# Supplementary material for: Characterization of circulating breast cancer cells with tumorigenic and metastatic capacity
Source: EMBO Mol Med. 2020 Jul 15;12(9):e11908. doi: 10.15252/emmm.201911908 (PMC7507517; doi:10.15252/emmm.201911908)
Supplement: Supplementary file 1 — Appendix [file EMMM-12-e11908-s001.docx]

**Appendix**

**Table of contents**

1. Appendix Supplementary Material and Methods
2. Appendix Figures S1-S3 and Legends
3. Appendix Table S1
4. **Appendix Supplementary Material and Methods:**

**Treatment and disease progression of the BCa patient giving rise to CTC-ITB-01**

Upon diagnosis, the patient received 18 cycles of palliative chemotherapy with paclitaxel, followed by the second generation aromatase inhibitor Letrozole combined with antiresorptive therapy (Denosumab). Letrozole was continued for 1.5 years until additional metastases were detected in the spleen and liver. Then, endocrine therapy was switched to Tamoxifen. Denosumab was continued through all treatment switches. Shortly afterwards, prior to a therapy change to Eribulin we received the blood sample leading to the establishment of the CTC line. At this time point, a strongly dispersing adenocarcinoma of the vagina had been diagnosed as breast cancer metastasis and classified as ER/PR positive and ERBB2 negative after biopsy. Eribulin was administered briefly before changing therapy to Doxorubicin, due to low tolerance towards Eribulin. The patient died of her progressive disease shortly afterwards.

**Isolation and cultivation of CTC-ITB-01**

Following initial *ex vivo* growth, CTC-ITB-01 was cultured in various culture media to determine optimal growth conditions. These were RPMI 1640 and DMEM containing 10% FCS, 1 % penicillin-streptomycin and 1% L-glutamine. Cells were grown in these media and RPMI *complete* in parallel for three weeks. Judged by morphology, growth pattern, and cell density following three weeks of culture in the respective medium, RPMI *complete* medium proved to be the most suitable medium for the newly established cell line, while standard RPMI and DMEM medium led to lower proliferation rates and cell density (data not shown). RPMI *complete* medium contains additional FGF2, EGF, and insulin previously established by our group as important factors for the establishment of cell lines from disseminated tumor cells (DTCs) in bone marrow of patients with breast cancer and other solid epithelial tumors [1-3].

**Western blot analysis**

Cells were harvested in urea lysis buffer (9.8 M Urea, 15 mM EDTA, 30 mM Tris) and homogenized by ultrasonic treatment using UP50H (Hielscher). Protein concentration was measured using the Pierce BCA Protein Assay Kit (Pierce) according to the instruction manual and samples were stored at -80°C. All samples were generated in biological triplicates. Proteins were separated by size with the Novex XCell Sure-Lock mini system (Invitrogen) or the Protean II xi cell (Bio-Rad) using a Laemmli buffer system and 10 % polyacrylamide separation gels. Prestained peqGOLD Protein-Marker V (VWR) was used as a molecular size standard. For Western Blot analysis 20 µg of total protein per sample (EpCAM: 40 µg of total protein per sample) was applied. The primary antibodies were utilized according to the instruction manual of the supplier using appropriate dilutions (Table EV4). The appropriate secondary antibodies conjugated with horseradish peroxidase (DakoCytomation) according to the species of the primary antibody were used at dilutions from 1:500 to 1:10.000 depending on the signal intensity. A detailed overview of the antibodies and dilutions can be gained from Table EV4. Protein bands were visualized using SignalFire™Plus ECL reagent (Cell Signaling Technology) and X-ray films (CEA) according to the instruction manual. Prior to re-use membranes were stripped with stripping buffer to remove bound antibodies (62.4 mM Tris, 69.4 mM SDS, 99.8 mM  2-mercaptoethanol, adjusted to pH 9.5 using HCl, 0.01% DTT, H_2_O dest. ad 1 l).

**EPISPOT assay**

The fluoro-EPISPOT assay was carried out as previously described [4] and the following antibodies were applied: For the K19-EPISPOT, the anti-K19 Ks19.1 (6 μg/ml) and the Alexa555-conjugated anti-CK19 Ks19.2 (3 μg/ml) mAbs (Progen Biotechnik GMBH) were used. For the vascular endothelial growth factor (VEGF)-EPISPOT, the anti-VEGF Avastin (7 μg/ml, Roche) and the biotin-conjugated anti-VEGF (4 μg/ml, Ray Biotech Inc) mAbs were used. For the EGFR-EPISPOT, the anti-EGFR Vectibix (1.5 μg/ml, Amgen) and the Alexa488-conjugated anti-EGFR (2 μg/ml, AF231, R&D system) mAbs were used. For the FGF2-EPISPOT, the anti-FGF2 M38 (6 μg/ml) and the biotin-conjugated FGF2 P18 (0.9 μg/ml) mAbs (Peprotech) were used. For the osteoprotegerin (OPG)-EPISPOT, the anti-OPG (6 μg/ml, mab8051, R&D systems) and the biotin-conjugated OPG (6 μg/ml, Baf 805, R&D systems) mAbs (R&D systems) were used. Biotin-labeled antibodies were detected by streptavidin conjugated to Alexa555 (Invitrogen). Single fluorescent immunospots were observed under an ELISPOT reader (C.T.L ImmunoSpot).

**References**

1. Pantel, K., et al., *Establishment of micrometastatic carcinoma cell lines: a novel source of tumor cell vaccines.* J Natl Cancer Inst, 1995. **87**(15): p. 1162-8.

2. Putz, E., et al., *Phenotypic characteristics of cell lines derived from disseminated cancer cells in bone marrow of patients with solid epithelial tumors: establishment of working models for human micrometastases.* Cancer Res, 1999. **59**(1): p. 241-8.

3. Bartkowiak, K., et al., *Disseminated Tumor Cells Persist in the Bone Marrow of Breast Cancer Patients through Sustained Activation of the Unfolded Protein Response.* Cancer Research, 2015. **75**(24): p. 5367-5377.

4. Alix-Panabieres, C., et al., *Full-length cytokeratin-19 is released by human tumor cells: a potential role in metastatic progression of breast cancer.* Breast Cancer Research, 2009. **11**(3).

1. **Appendix Figures S1-S3 and Legends**

**Appendix Figure S1 – ERBB2 status of CTC-ITB-01 cells:**

**A** *FISH* analysis using fluorescent *ERBB2* (green) and *CEP17* (*centromere 17,* orange) probes. Only two ERBB2-specific signals were detected, negating *ERBB2* amplification. The grey scale bars correspond to 50 µm.

**B** ICC staining of CTC-ITB-01 for ERBB2 (orange), and DAPI (blue). The grey scale bars correspond to 50 µm. SKBR-3, as strongly ERBB2 expressing and MCF-7 cells as ERBB2- reference cell lines are depicted.

**Appendix Figure S2 – Secretome analysis of CTC-ITB-01 with functional fluoro-EPISPOT assay:** Assessment of proteins secreted by CTC-ITB-01 including K19, VEGF, OPG, EGFR and FGF2. Secretion was evaluated in comparison to the appropriate cell line controls, including CTC-MCC-41, 11B (laryngeal squamous cell carcinoma) and NBTII (bladder tumor). K19 and VEGF were released and secreted (+), respectively while other proteins were not (-). Pos. Ctrl.: Cell lines secreting the appropriate proteins, Neg. Ctrl. No cells added.

**

**

**Appendix Figure S3 – CTC-ITB-01 cell sizes:** Distribution of CTC-ITB-01 cell sizes in both the adherent and the non-adherent cell fraction. Both distinct phenotypes display large variances in cell size, ranging from 44.06 - 2712.2 μm² in the adherent and 99.11- 4228.88 μm² for the non-adherent fraction.

**Appendix Table S1: Frequently mutated genes selected based on the COSMIC database, and genes involved in hereditary cancer predispositions syndromes.**

| **Genes** |
| --- |
| *ACVR1B* |
| *AKT1* |
| *AKT2* |
| *AKT3* |
| *ALDH1A1* |
| *APC* |
| *ATM* |
| *ATR* |
| *AXL* |
| *BAP1* |
| *BARD1* |
| *BCAR1* |
| *BCAR3* |
| *BCL2* |
| *BMI1* |
| *BMP7* |
| *BMPR1A* |
| *BRAF* |
| *BRCA1* |
| *BRCA2* |
| *BRIP1* |
| *CAV1* |
| *CBFB* |
| *CCL2* |
| *CCL5* |
| *CCND1* |
| *CD24* |
| *CD274* |
| *CD44* |
| *CD47* |
| *CDH1* |
| *CDH2* |
| *CDK4* |
| *CDK4* |
| *CDK6* |
| *CDKN1A* |
| *CDKN1B* |
| *CDKN2A* |
| *CEACAM5* |
| *CFTR* |
| *CHEK1* |
| *CHEK2* |
| *CR1* |
| *CREBBP* |
| *CSF1R* |
| *CTGF* |
| *CTLA4* |
| *CTNNA1* |
| *CTNNB1* |
| *CXCL12* |
| *DCC* |
| *DNMT1* |
| *EGFR* |
| *ELF5* |
| *ENG* |
| *EPCAM* |
| *ERBB2* |
| *ERBB3* |
| *ERBB4* |
| *ERCC4* |
| *ESR1* |
| *ESR2* |
| *ETFA* |
| *EZH2* |
| *FAM175A* |
| *FAN1* |
| *FANCA* |
| *FANCB* |
| *FANCC* |
| *FANCD2* |
| *FANCE* |
| *FANCF* |
| *FANCG* |
| *FANCI* |
| *FANCL* |
| *FANCM* |
| *FAT1* |
| *FGF2* |
| *FGFR1* |
| *FGFR2* |
| *FGFR3* |
| *FH* |
| *FOXA1* |
| *FOXA2* |
| *GALNT12* |
| *GATA3* |
| *GREM1* |
| *HDAC1* |
| *HERC1* |
| *HIF1A* |
| *HLA-A* |
| *HLA-B* |
| *HLA-C* |
| *HLA-E* |
| *HLA-F* |
| *HLA-G* |
| *HNF1A* |
| *HOXB13* |
| *HPSE* |
| *HRAS* |
| *IDH1* |
| *IDH2* |
| *IDO1* |
| *IFNG* |
| *IGF1R* |
| *IL6* |
| *ITGAV* |
| *JAK1* |
| *JAK2* |
| *JAK3* |
| *KIT* |
| *KRAS* |
| *MAP2K1* |
| *MAP2K4* |
| *MAP3K1* |
| *MAP3K6* |
| *MAPK1* |
| *MAPK3* |
| *MAX* |
| *MEN1* |
| *MET* |
| *MKI67* |
| *MLH1* |
| *MLH3* |
| *MRE11A* |
| *MSH2* |
| *MSH3* |
| *MSH6* |
| *MSR1* |
| *mTOR* |
| *MUC1* |
| *MUTYH* |
| *MYC* |
| *MYC* |
| *NANOG* |
| *NBN* |
| *NCOR2* |
| *NF1* |
| *NF2* |
| *NOTCH1* |
| *NOTCH3* |
| *NRAS* |
| *NTHL1* |
| *PALB2* |
| *PALLD* |
| *PAX2* |
| *PDCD1* |
| *PDGFRA* |
| *PDGFRB* |
| *PIK3CA* |
| *PIK3CG* |
| *PMS1* |
| *PMS2* |
| *POLD1* |
| *POLE* |
| *POU5F1* |
| *PPM1D* |
| *PPM1l* |
| *PROM1* |
| *PRSS1* |
| *PTEN* |
| *RAC1* |
| *RAD50* |
| *RAD50* |
| *RAD51B* |
| *RAD51C* |
| *RAD51D* |
| *RB1* |
| *RET* |
| *RINT1* |
| *RNASEL* |
| *RNF43* |
| *ROS1* |
| *RPS20* |
| *RUNX1* |
| *RUNX2* |
| *RUNX3* |
| *SCRIB* |
| *SDHA* |
| *SDHAF2* |
| *SDHB* |
| *SDHC* |
| *SDHD* |
| *SEPT9* |
| *SLX4* |
| *SMAD2* |
| *SMAD4* |
| *SNAI1* |
| *SNAI2* |
| *SOX2* |
| *SPINK1* |
| *SRC* |
| *STAT3* |
| *STK11* |
| *TAP1* |
| *TAP2* |
| *TBX3* |
| *TGFB1* |
| *TGFBR2* |
| *TMEM127* |
| *TNFSF11* |
| *TP53* |
| *TRERF11* |
| *TRRAP* |
| *TSC1* |
| *TSC2* |
| *TWIST1* |
| *TWIST2* |
| *TYK2* |
| *UBE2T* |
| *VEGFA* |
| *VHL* |
| *VHL* |
| *VIM* |
| *WNT1* |
| *WT1* |
| *XBP1* |
| *ZEB1* |
| *ZEB2* |
