# Supplementary material for: Characterization of circulating breast cancer cells with tumorigenic and metastatic capacity
Source: EMBO Mol Med. 2020 Jul 15;12(9):e11908. doi: 10.15252/emmm.201911908 (PMC7507517; doi:10.15252/emmm.201911908)
Supplement: Supplementary file 2 — Expanded View Figures PDF [file EMMM-12-e11908-s002.pdf]

Expanded View Figures

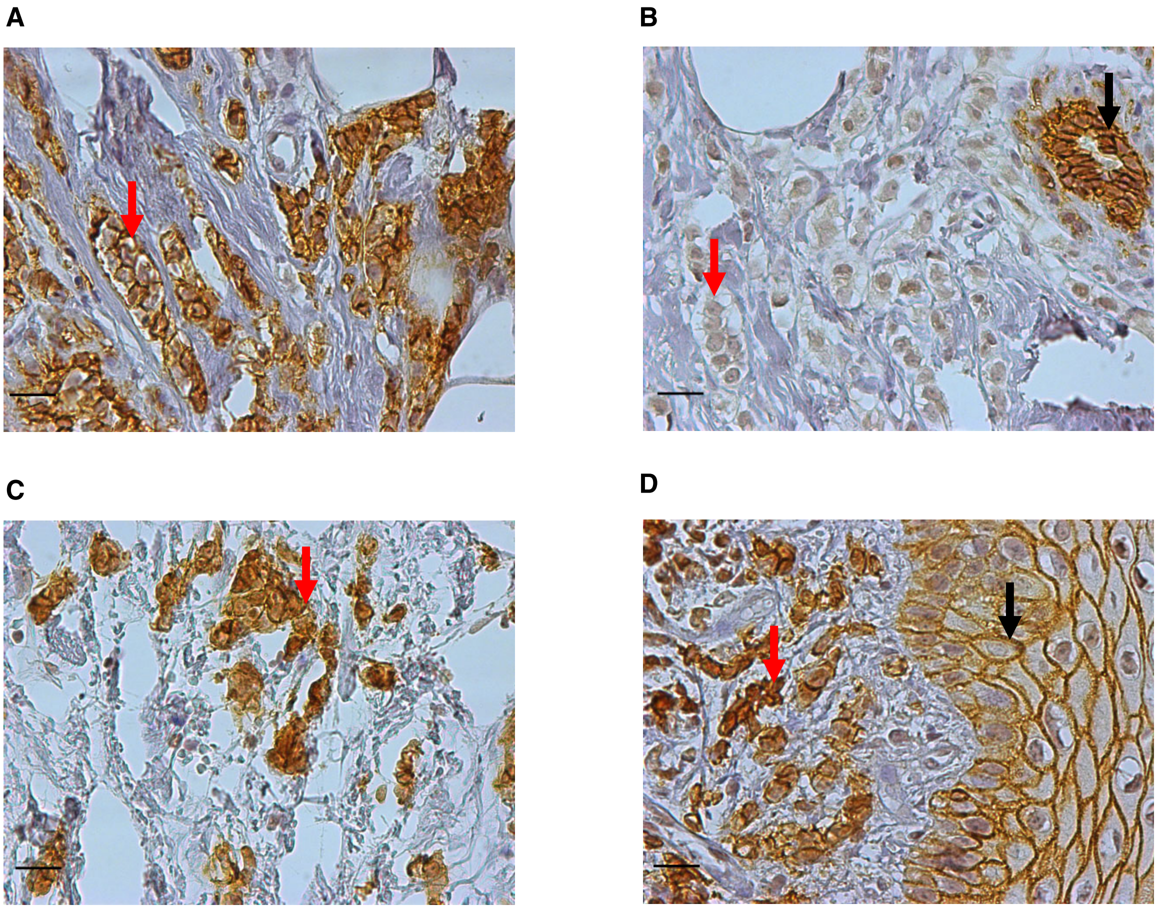

Figure EV1. E-cadherin re-immunostaining of biopsies taken from the primary tumors and from the vaginal metastasis.

Formalin-fixed paraffin-embedded (FFPE) tissue of both primary tumors and of the vaginal metastasis was analyzed by immunohistochemistry for E-cadherin protein expression (brown). Nuclear visualization was performed using Mayer's hemalum solution. Figures show exemplary IHC images of E-cadherin staining. The scale bars correspond to 20 µm.

- A Area of the primary left tumor (primarily diagnosed as lobular carcinoma) with strongly E-cadherin-positive tumor cells (red arrow).
- B Area of the primary lobular left tumor with negative or very weakly E-cadherin-positive tumor cells (red arrow). The black arrow shows strong E-cadherin staining in normal mammary ductal epithelium.
- C Strongly E-cadherin-positive tumor cells (red arrow) of the primary ductal (right) tumor.
- D Strongly E-cadherin-positive tumor cells (red arrow) of the vaginal metastasis. E-cadherin staining in normal vulvar squamous epithelium is shown by a black arrow.

Source data are available online for this figure.

© 2020 The Authors

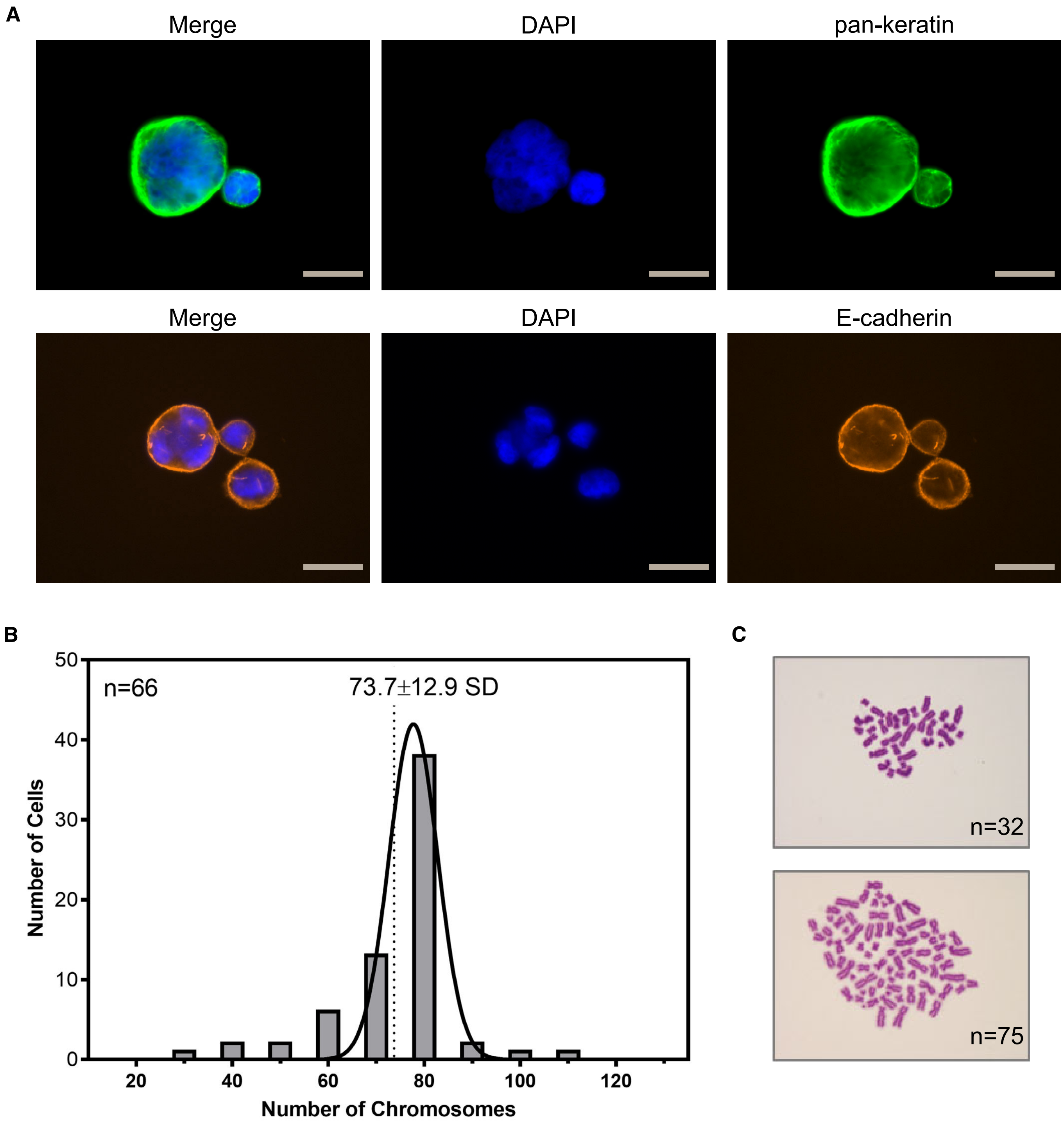

Figure EV2. CTC-ITB-01 nuclei and karyotype.

A Representative ICC images of large CTC-ITB-01 cells containing multiple or lobed nuclei. Cells were stained with pan-keratin (green) or E-cadherin (orange). Nuclear visualization was performed with DAPI (blue). Gray scale bars represent 20 μm.

B Histogram showing the chromosome distribution across 66 measured CTC-ITB-01 cells. A mean chromosome count of 73.7 (SD = 12.9) was calculated, representing triploidy.

C Representative bright field images of Giemsa staining used for karyotyping (purple). Examples of 32 (upper image) or 75 chromosomes (lower image).

Source data are available online for this figure.

 © 2020 The Authors

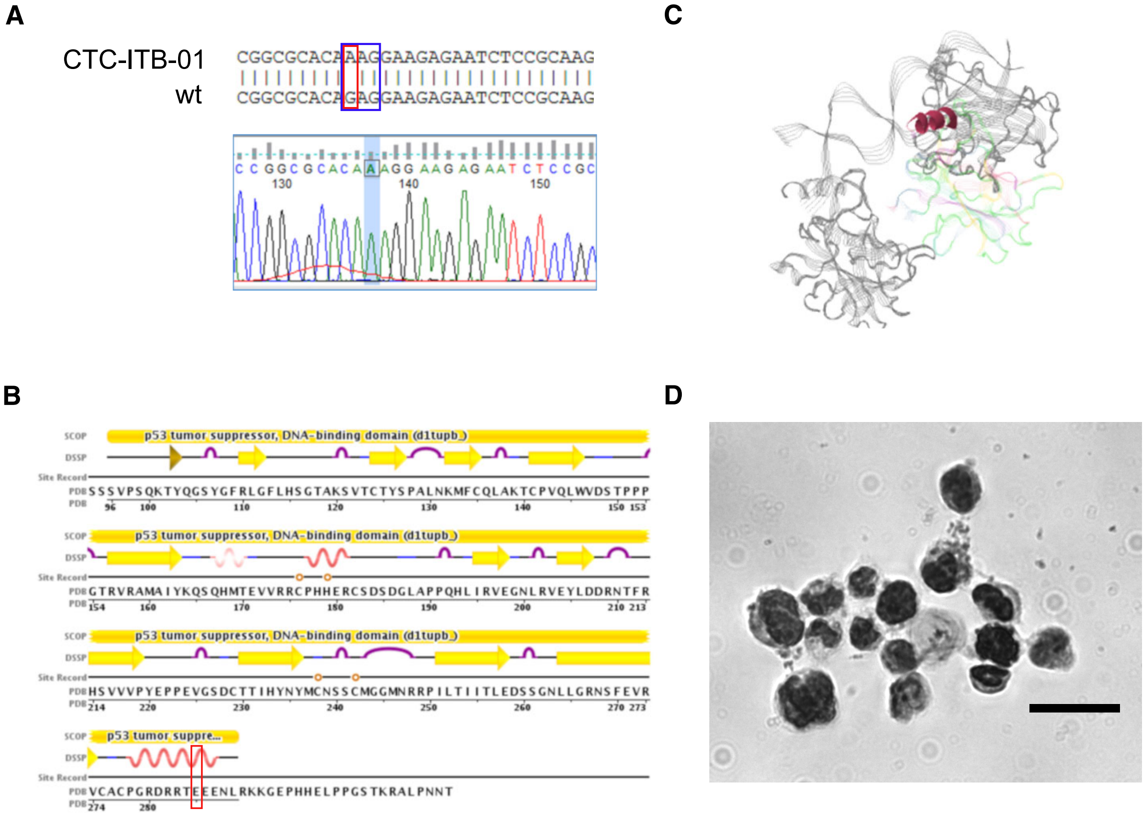

Figure EV3. TP53 analysis in CTC-ITB-01 cells.

A Red square indicating point mutation from guanine (G) to adenine (A) localized on exon 8 of the *TP53* gene sequence. Wild-type (wt) sequence and gene sequence (Finch TV) shown for visualization. Blue square indicates the affected amino acid sequence.

B Localization of the E285K mutation in the secondary protein structure of TP53 and integration into the functional domains of the protein (https://www.rcsb.org/pdb/explore/remediatedSequence.do?structureId=1TUP&bionumber=1). Red square marks altered region with amino acid exchange from E (glutamic acid) to K (lysine).

C Localization of the detected mutation within the quaternary structure of TP53. The E to K exchange affects a helical structure within the DNA binding domain of the protein.

D Strong accumulation of TP53 in the nucleus of CTC-ITB-01 cells, assessed via ICC staining. Scale bar corresponds to 20 µm.

Source data are available online for this figure.

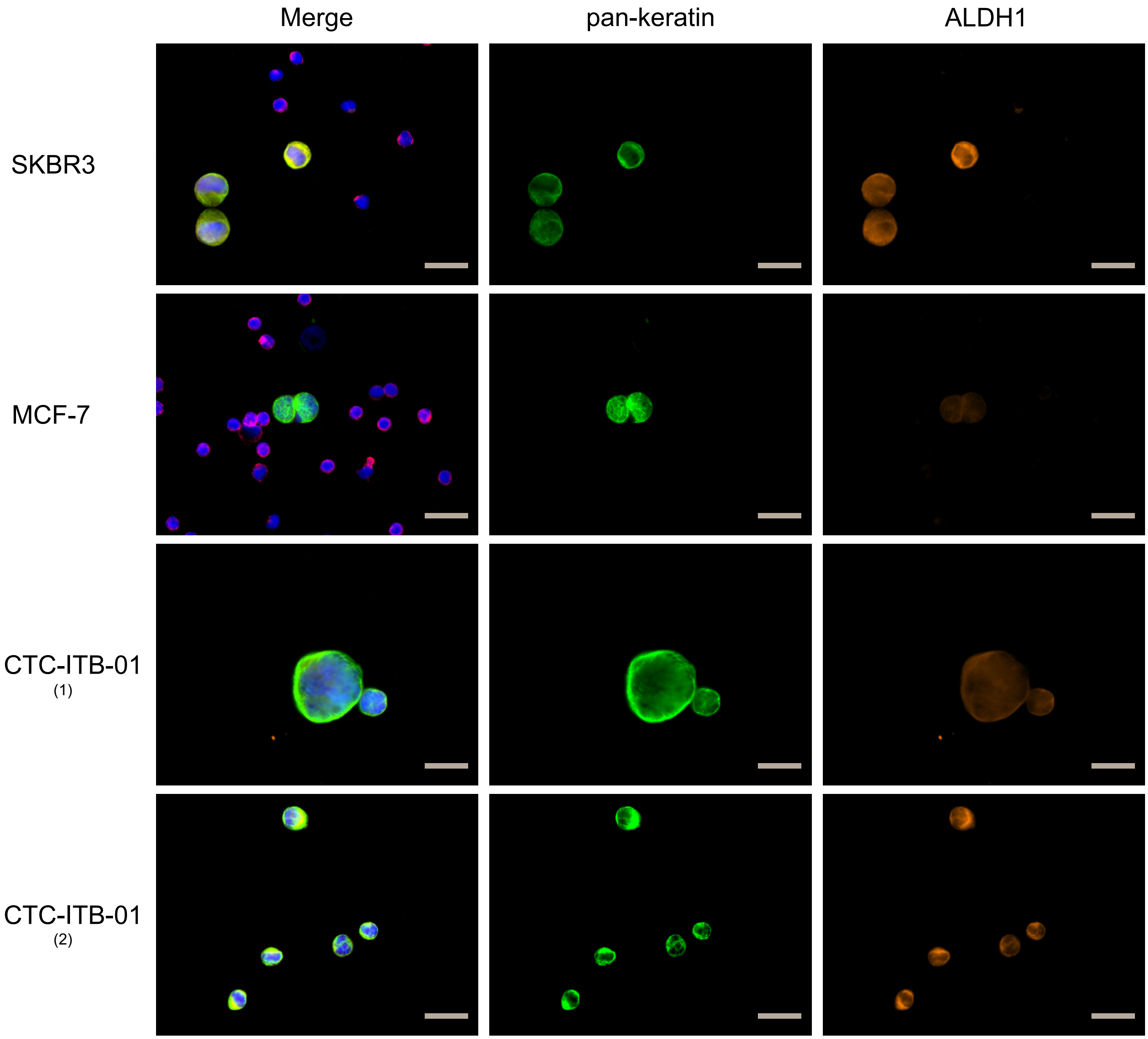

Figure EV4. ALDH1 status of CTC-ITB-01 cells.

ICC staining of CTC-ITB-01 for pan-keratin (green), ALDH1 (orange), and DAPI (blue). The gray scale bars correspond to 20 μm. Two representative panels are shown for CTC-ITB-01, one with weaker (1) and the other stronger (2) ALHD1 expressing cells. SKBR-3, as strongly ALDH1 expressing and MCF-7 cells as ALDH1-negative reference cell lines, spiked into blood from healthy donors are depicted. Leukocytes are visible on reference cell line slides and stained with CD45 (red).

Source data are available online for this figure.

 © 2020 The Authors

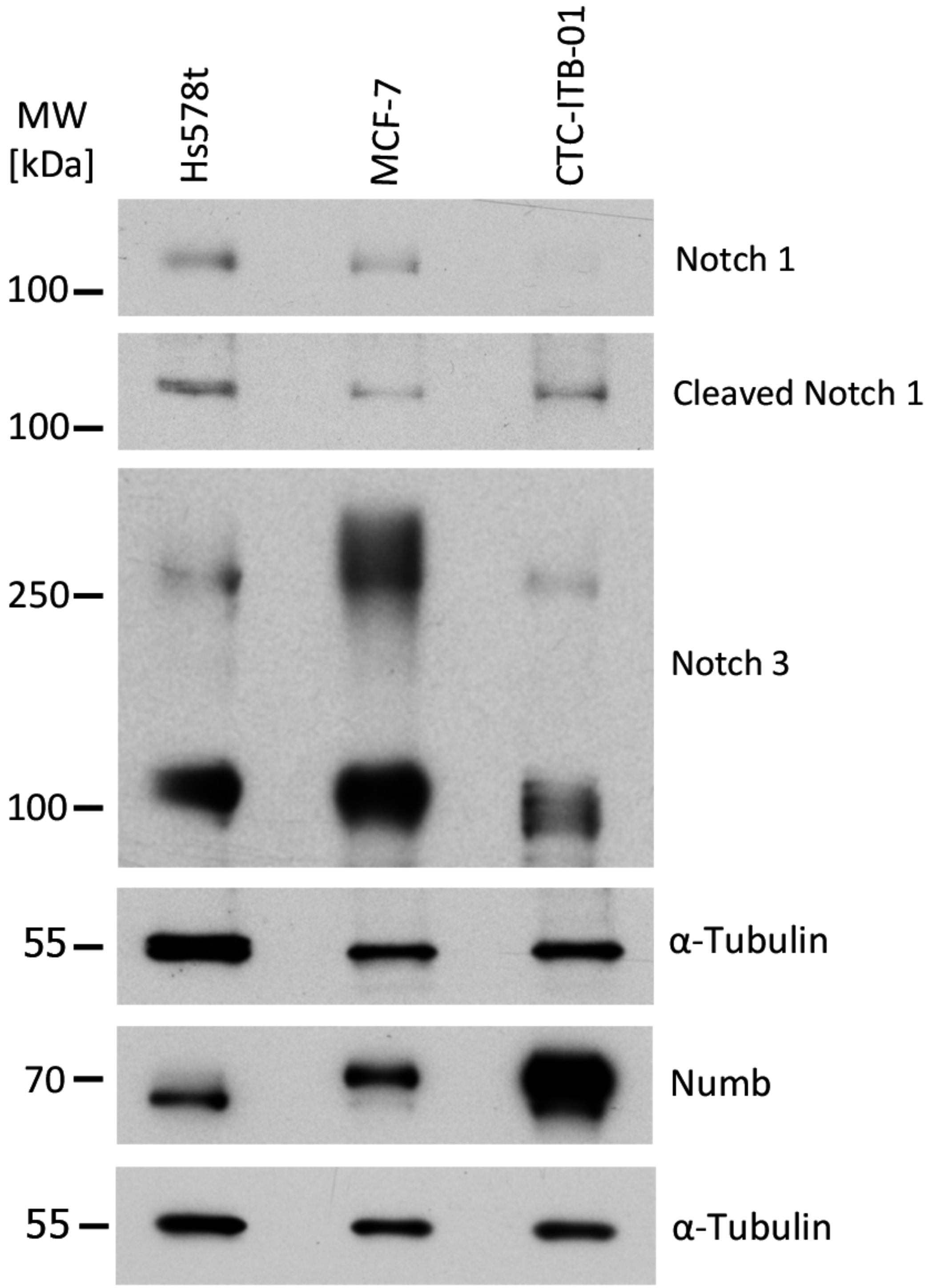

Figure EV5. CTC-ITB-01 phenotype in culture.

Western blot analysis for selected proteins, including NUMB, NOTCH1, NOTCH3, Cleaved NOTCH, and α-tubulin (as a loading control). CTC-ITB-01 was compared to more mesenchymal ER^- Hs578t and epithelial ER^+ MCF-7 breast cancer cell lines.

Source data are available online for this figure.

© 2020 The Authors
