# Supplementary material for: Characterization of circulating breast cancer cells with tumorigenic and metastatic capacity
Source: EMBO Mol Med. 2020 Jul 15;12(9):e11908. doi: 10.15252/emmm.201911908 (PMC7507517; doi:10.15252/emmm.201911908)
Supplement: Supplementary file 3 — Table EV1 [file EMMM-12-e11908-s003.docx]

**Table EV1 – Histological and receptor expression information of both primary breast tumors**

|  | left primary breast tumor | right primary breast tumor |
| --- | --- | --- |
| **ILC / IDC:** | ILC | IDC |
| **Tumor grade** | G1 | G1 |
| **ER/PR/HER2 status:** | positive/ positive/ negative | positive/ positive/ negative |
| **E-cadherin expression:** | n/a | clear membrane staining |
| **specifics on ER expression:** | moderate expression in over 80% of nuclei (IRS 8) | strong expression in over 80% of nuclei (IRS 12) |
| **specifics on PR expression:** | strong expression in over 80% of nuclei (IRS 12) | Progesterone receptor:  strong expression in over 80% of nuclei (IRS 12) |
| **Ki67-index [%]:** | 5% | 5% |
