# Supplementary material for: Characterization of circulating breast cancer cells with tumorigenic and metastatic capacity
Source: EMBO Mol Med. 2020 Jul 15;12(9):e11908. doi: 10.15252/emmm.201911908 (PMC7507517; doi:10.15252/emmm.201911908)
Supplement: Supplementary file 4 — Table EV2 [file EMMM-12-e11908-s004.docx]

Table EV 2: **List of additional, probably pathogenic variants identified by whole-exome sequencing in a panel of 219 cancer-associated genes**

| **Gene Name** | **CTC-ITB-01** | **Vaginal metastasis** | **Left primary tumor** | **Right primary tumor** |
| --- | --- | --- | --- | --- |
| ***ATM*** | c.6067G>A, p.G2023R (47%) | c.6067G>A, p.G2023R (50%) | c.6067G>A, p.G2023R (40%) | c.6067G>A, p.G2023R (42%) |
| ***CDH1*** | c.1204G>A; p.D402N (84%) | - | - | - |
| ***CDKN1A*** | c.251G>A; p.R84Q (39%) | c.251G>A; p.R84Q (34%) | c.251G>A; p.R84Q (44%) | c.251G>A; p.R84Q (46%) |
| ***FGF2*** | c.439G>A; p.E147K (47%) | c.439G>A; p.E147K (33%) | c.439G>A; p.E147K (50%) | c.439G>A; p.E147K (31%) |
| ***IDH2*** | - | c.1054A>C; p.T352P (44%) | - | c.1054A>C; p.T352P (39%) |
| ***MAP3K1*** | c.2782delT; p.S928Lfs*9 (41%) | c.2782delT; p.S928Lfs*9 (15%) | c.2782delT; p.S928Lfs*9 (22%) | c.2782delT; p.S928Lfs*9 (16%) |
| ***MAP3K6*** | c.2837C>T; p.P946L (30%) | c.2837C>T; p.P946L (40%) | c.2837C>T; p.P946L (30%) | c.2837C>T; p.P946L (45%) |
| ***POLD1*** | - | - | - | c.1932C>G;p.D644E (13%) |
| ***RNASEL*** | c.793G>T; p.E265* (53%) | c.793G>T; p.E265* (39%) | c.793G>T; p.E265* (46%) | c.793G>T; p.E265* (44%) |
|  | c.175G>A; p.G59S (46%) | c.175G>A; p.G59S (41%) | c.175G>A; p.G59S (40%) | c.175G>A; p.G59S (46%) |
| ***SCRIB*** | c.3995C>T; p.P1332L (37%) | c.3995C>T; p.P1332L (45%) | c.3995C>T; p.P1332L (23%) | c.3995C>T; p.P1332L (39%) |

All four samples were analyzed for rare (MAF <1%), functionally relevant variants with an allele frequency of 10% and higher in at least one of the samples. 219 cancer-associated genes curated from the COSMIC, HGMD and OMIM databases were analysed. Identified variants were classified by different *in silico* predictions tools regarding their functional relevance. Gene symbol were used as approved by the HGNC, and location of mutations on cDNA and protein (one letter code) level, and allele frequency is shown. del, deletion; ins, insertion; *, stop codon; fs, frameshift.
