# Supplementary material for: Characterization of circulating breast cancer cells with tumorigenic and metastatic capacity
Source: EMBO Mol Med. 2020 Jul 15;12(9):e11908. doi: 10.15252/emmm.201911908 (PMC7507517; doi:10.15252/emmm.201911908)
Supplement: Supplementary file 5 — Table EV3 [file EMMM-12-e11908-s005.docx]

**Table EV3: LOH in CTC-ITB-01:** Chromosomal regions affected by loss of heterozygosity (LOH) found exclusively in CTC-ITB-01 calculated from whole-exome sequencing data of this cell line. Start and end points as well as size of the 15 largest regions are indicated.

| **Chromosome** | **Start** | **End** | **Size** |
| --- | --- | --- | --- |
| **6** | 63921456 | 136597449 | 72675994 |
| **18** | 14542979 | 77894844 | 63351866 |
| **12** | 9585631 | 50745791 | 41160161 |
| **4** | 54373464 | 94316763 | 39943300 |
| **16** | 55854412 | 90160948 | 34306537 |
| **6** | 136599842 | 167590541 | 30990700 |
| **17** | 21319302 | 44248837 | 22929536 |
| **22** | 24325047 | 45723947 | 21398901 |
| **8** | 7720995 | 27369334 | 19648340 |
| **17** | 13905 | 18391123 | 18377219 |
| **4** | 7735311 | 25394078 | 17658768 |
| **6** | 46135984 | 63257354 | 17121371 |
| **18** | 48476 | 14183720 | 14135245 |
| **17** | 44344970 | 57648129 | 13303160 |
| **12** | 50745791 | 62104219 | 11358429 |
