# Supplementary material for: Characterization of circulating breast cancer cells with tumorigenic and metastatic capacity
Source: EMBO Mol Med. 2020 Jul 15;12(9):e11908. doi: 10.15252/emmm.201911908 (PMC7507517; doi:10.15252/emmm.201911908)
Supplement: Supplementary file 6 — Table EV4 [file EMMM-12-e11908-s006.docx]

**Table EV4: Primary antibody used for Western blot analyses**

| **Primary antibody** | **Company** | **Clone** | **Clonality** | **Dilution** | **Species** |
| --- | --- | --- | --- | --- | --- |
| anti-EGFR | Cell Signaling Technology, Danvers, USA | D38B1 | monoclonal | 1:1000 | Rabbit |
| anti-N-Cadherin | Cell Signaling Technology, Danvers, USA | D4R1H | monoclonal | 1:10000 | Rabbit |
| anti-CD44 | Cell Signaling Technology, Danvers, USA | - | polyclonal | 1:5000 | Rabbit |
| anti-α-Tubulin | Cell Signaling Technology, Danvers, USA | 11H10 | monoclonal | 1:20000 | Rabbit |
| anti-estrogen receptor alpha | Abcam, Cambridge, United Kingdom | SP1 | monoclonal | 1:1000 | Rabbit |
| anti-CD24 | Abcam, Cambridge, United Kingdom | EPR3006(N) | monoclonal | 1:4000 | Rabbit |
| anti-ErbB2 | Leica Biosystems (Novocastra) | CB11 | monoclonal | 1:10000 | Mouse |
| anti-EpCAM | Leica Biosystems (Novocastra) | VU-1D9 | monoclonal | 1:500 | Mouse |
| anti-CK 18 | Progen Biotechnik, Heidelberg, Germany | Ks18.04 | monoclonal | 1:10000 | Mouse |
| anti-CK 8 | Progen Biotechnik, Heidelberg, Germany | Ks8.7 | monoclonal | 1:5000 | Mouse |
| anti-CK 19 | Antibodies-online, Atlanta, USA | BA-17 | monoclonal | 1:100000 | Mouse |
| anti-E-Cadherin | Epitomics, Burlingame, USA | EP700Y | monoclonal | 1:50000 | Rabbit |
| anti-Vimentin | BD Pharmingen, Erembodegem, Belgium | RV202 | monoclonal | 1:200000 | Mouse |
| anti-Notch 3 | Cell Signaling Technology, Danvers, USA | D11B8 | monoclonal | 1:1000 | Rabbit |
| anti-Numb | Cell Signaling Technology, Danvers, USA | C29G11 | monoclonal | 1:1000 | Rabbit |
| anti-TWIST1 | Cell Signaling Technology, Danvers, USA | - | polyclonal | 1:1000 | Rabbit |
| anti-cleaved Notch1 | Cell Signaling Technology, Danvers, USA | D3B8 | monoclonal | 1:1000 | Rabbit |
| anti-Notch 1 | Cell Signaling Technology, Danvers, USA | D6F11 | monoclonal | 1:1000 | Rabbit |
| anti-Snail | Cell Signaling Technology, Danvers, USA | C15D3 | monoclonal | 1:1000 | Rabbit |
| anti-SLUG | Santa Cruz Biotechnology, Dallas, USA | A7 | monoclonal | 1:1000 | Mouse |
| anti-ALDH1A1 | Santa Cruz Biotechnology, Santa Cruz, USA | H8 | monoclonal | 1:1000 | Mouse |
| anti-ER-alpha | Santa Cruz Biotechnology, Santa Cruz, USA | D12 | monoclonal | 1:200 | Mouse |
| anti-FOXM1 | Cell Signaling Technology, Danvers, USA | D12D5 | monoclonal | 1:500 | Rabbit |
| anti-Bcl-2 | Santa Cruz Biotechnology, Santa Cruz, USA | C-2 | monoclonal | 1:200 | Mouse |
| anti-PR | Abcam, Cambridge, United Kingdom | - | polyclonal | 1:1000 | Rabbit |
| anti-Id1 | Santa Cruz Biotechnology, Santa Cruz, USA | C-20 | monoclonal | 1:200 | Rabbit |
| anti-actin | Santa Cruz Biotechnology, Santa Cruz, USA | C-4 | monoclonal | 1:200 | Mouse |
