# Supplementary material for: Characterization of circulating breast cancer cells with tumorigenic and metastatic capacity
Source: EMBO Mol Med. 2020 Jul 15;12(9):e11908. doi: 10.15252/emmm.201911908 (PMC7507517; doi:10.15252/emmm.201911908)
Supplement: Supplementary file 7 — Source Data for Expanded View and Appendix [file EMMM-12-e11908-s012.zip › SourceDataForFigureEV5.pdf.pdf]

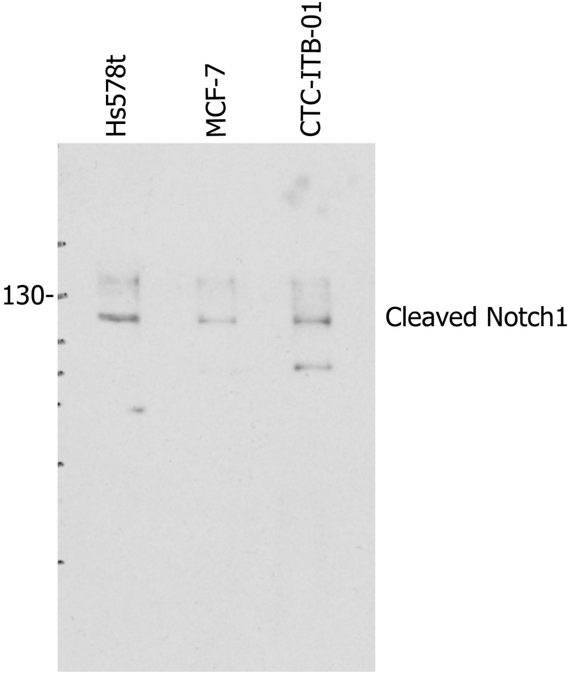

Hs578t
MCF-7
CTC-ITB-01
130-
Cleaved Notch1

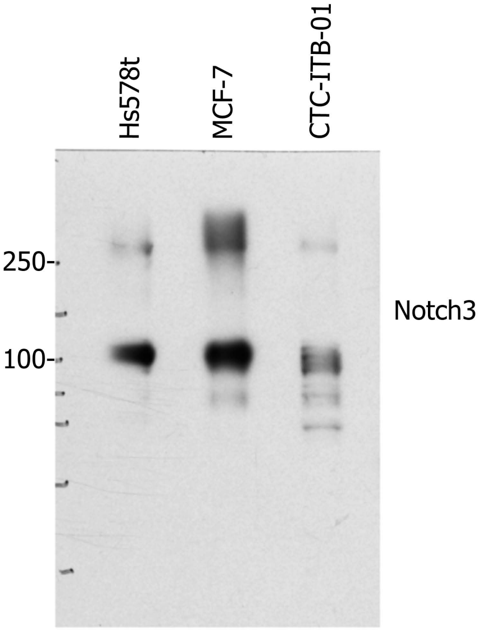
Hs578t
MCF-7
CTC-ITB-01
250-
100-
Notch3

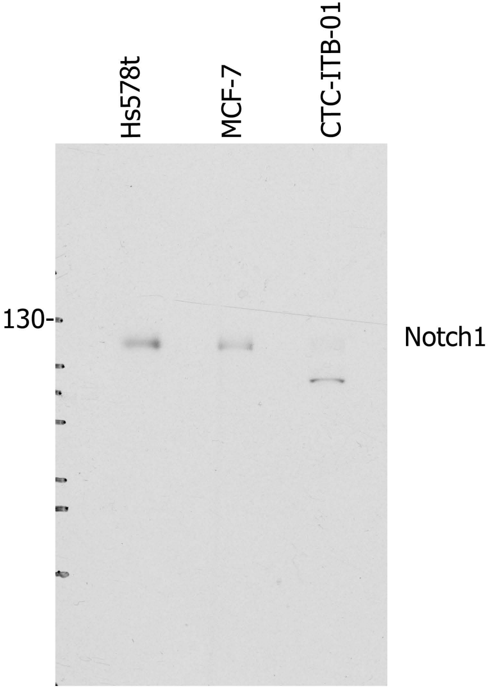
Hs578t
MCF-7
CTC-ITB-01
130-
Notch1

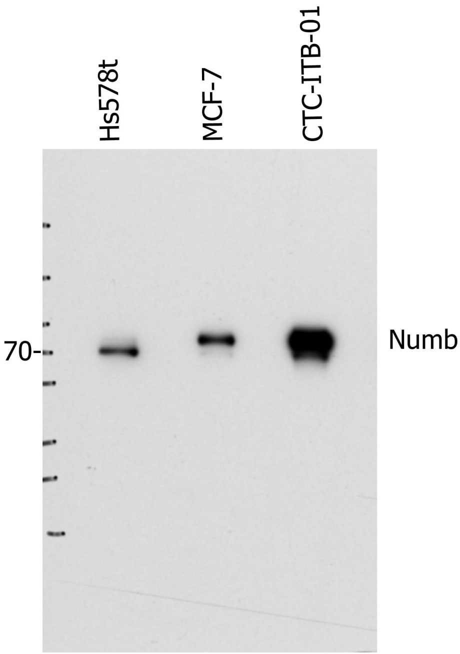
Hs578t
MCF-7
CTC-ITB-01
70-
Numb

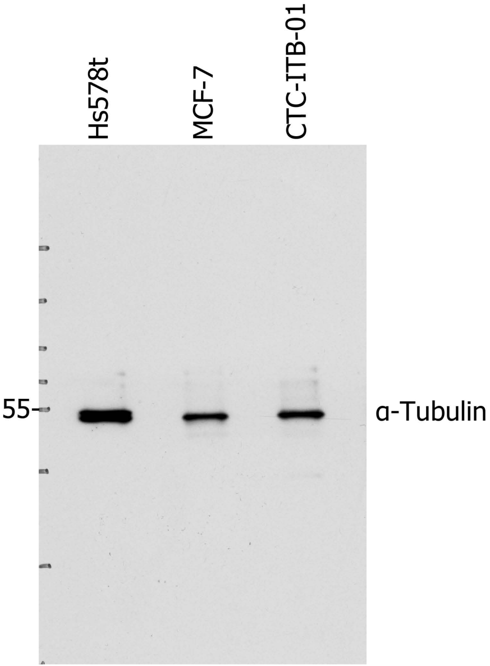

Hs578t
MCF-7
CTC-ITB-01
55-
α-Tubulin

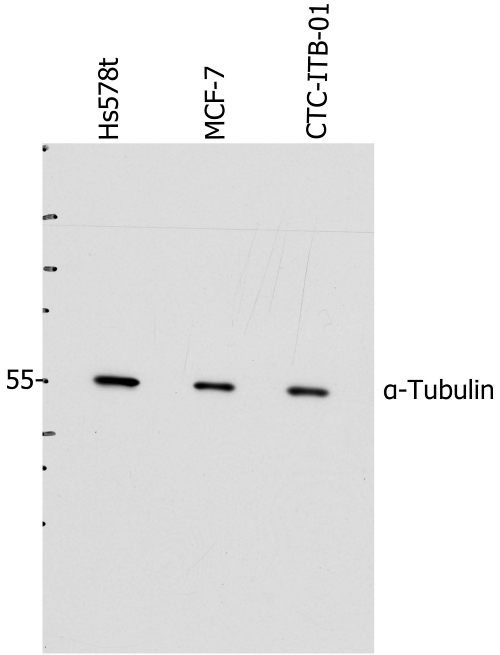
Hs578t
MCF-7
CTC-ITB-01
55-
α-Tubulin
