# Supplementary figures and images for: Characterization of circulating breast cancer cells with tumorigenic and metastatic capacity
Source: EMBO Mol Med. 2020 Jul 15;12(9):e11908. doi: 10.15252/emmm.201911908 (PMC7507517; doi:10.15252/emmm.201911908)

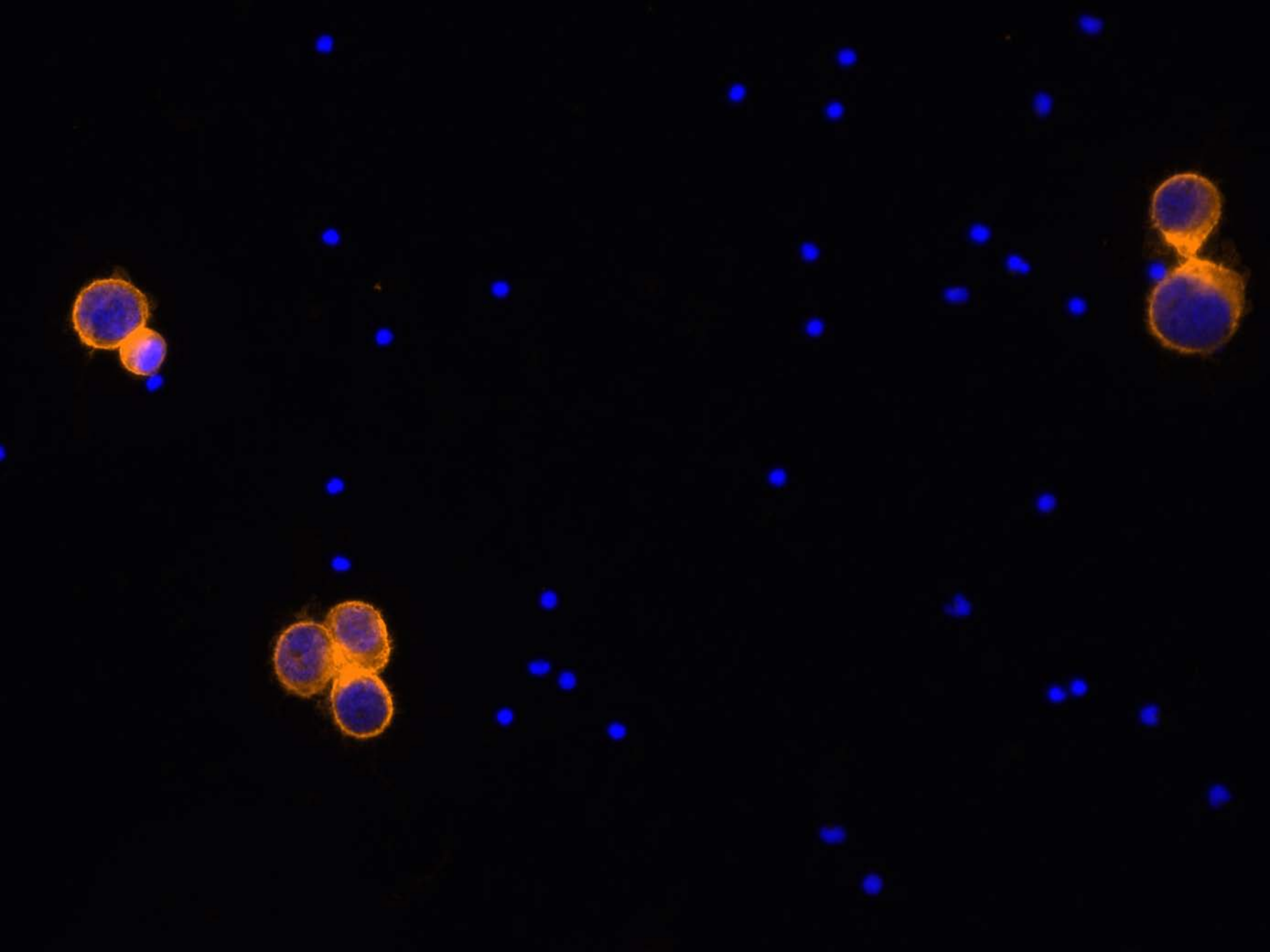



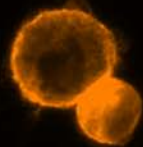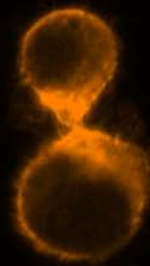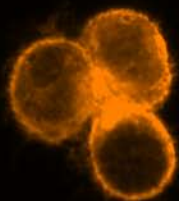

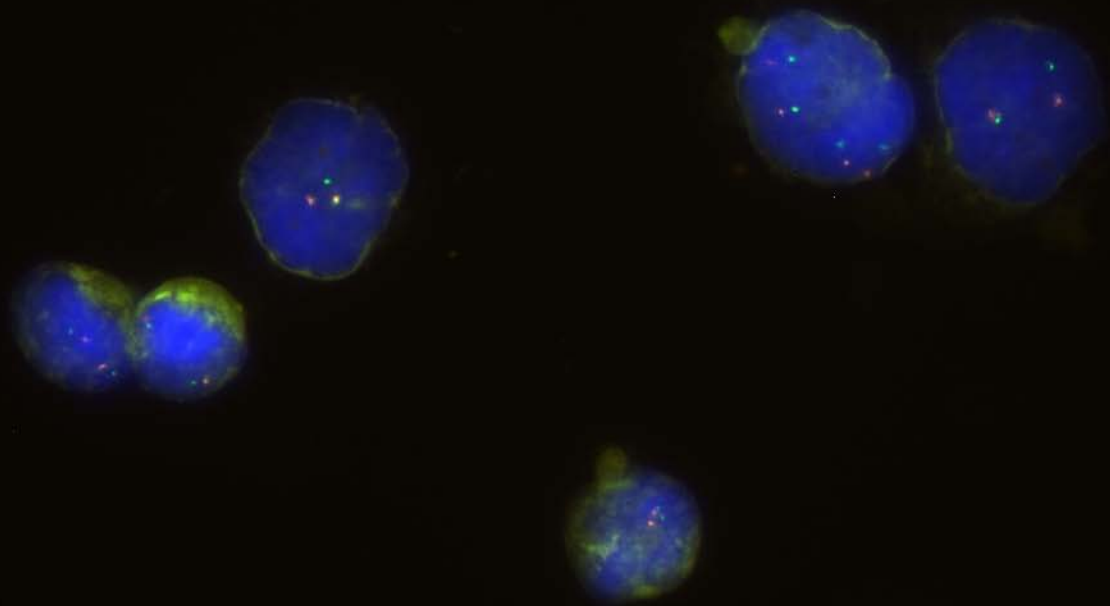







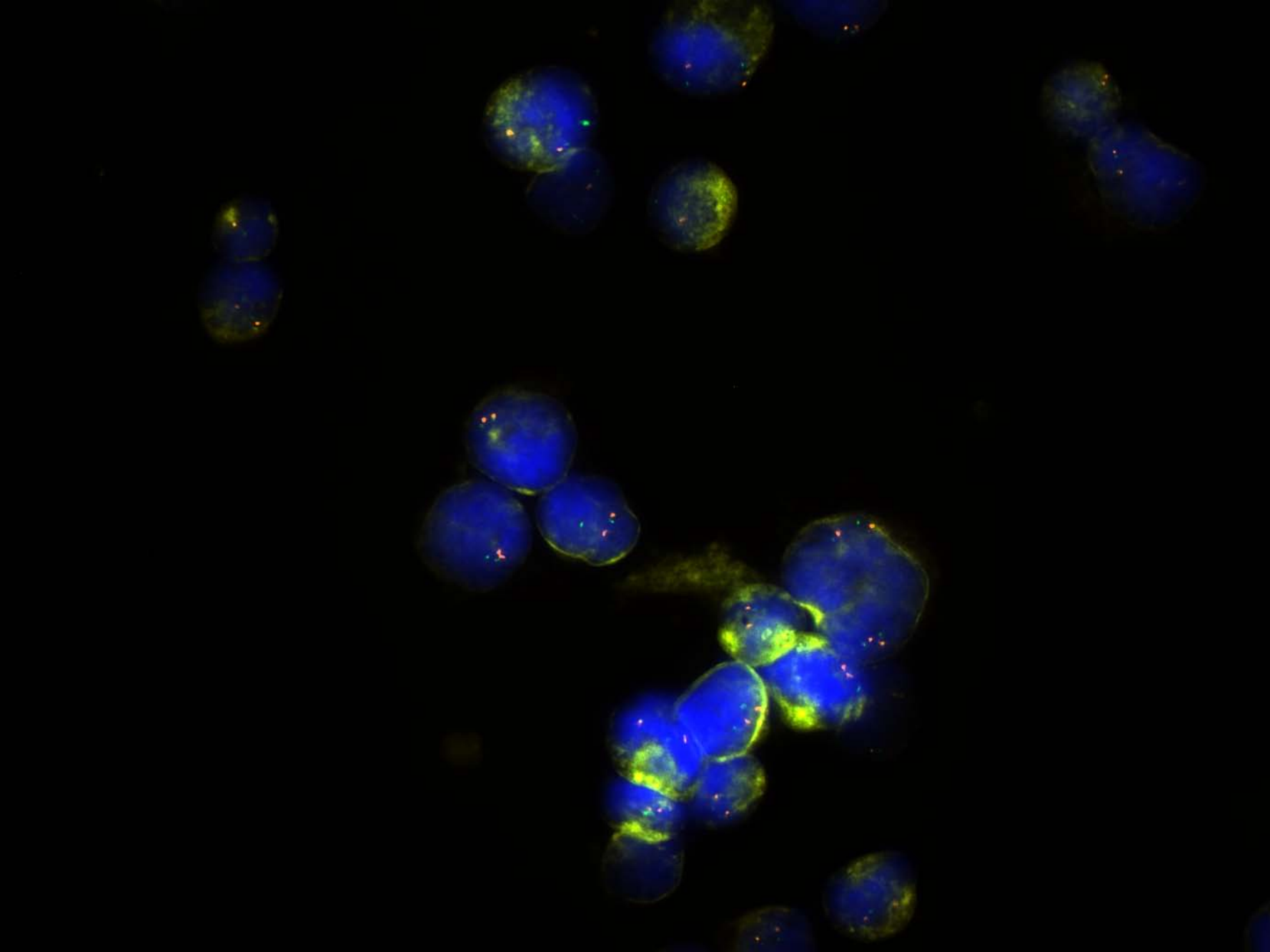

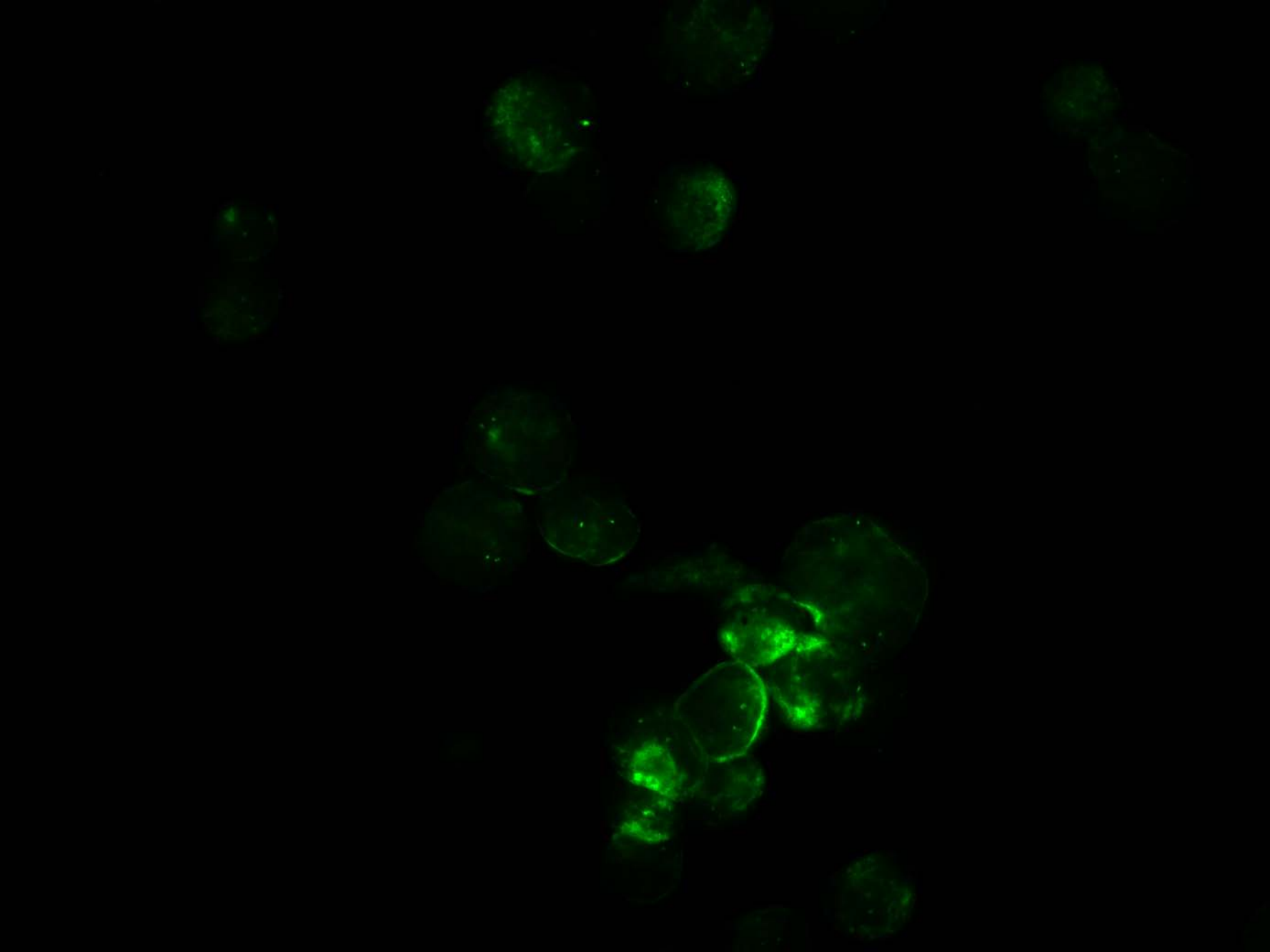



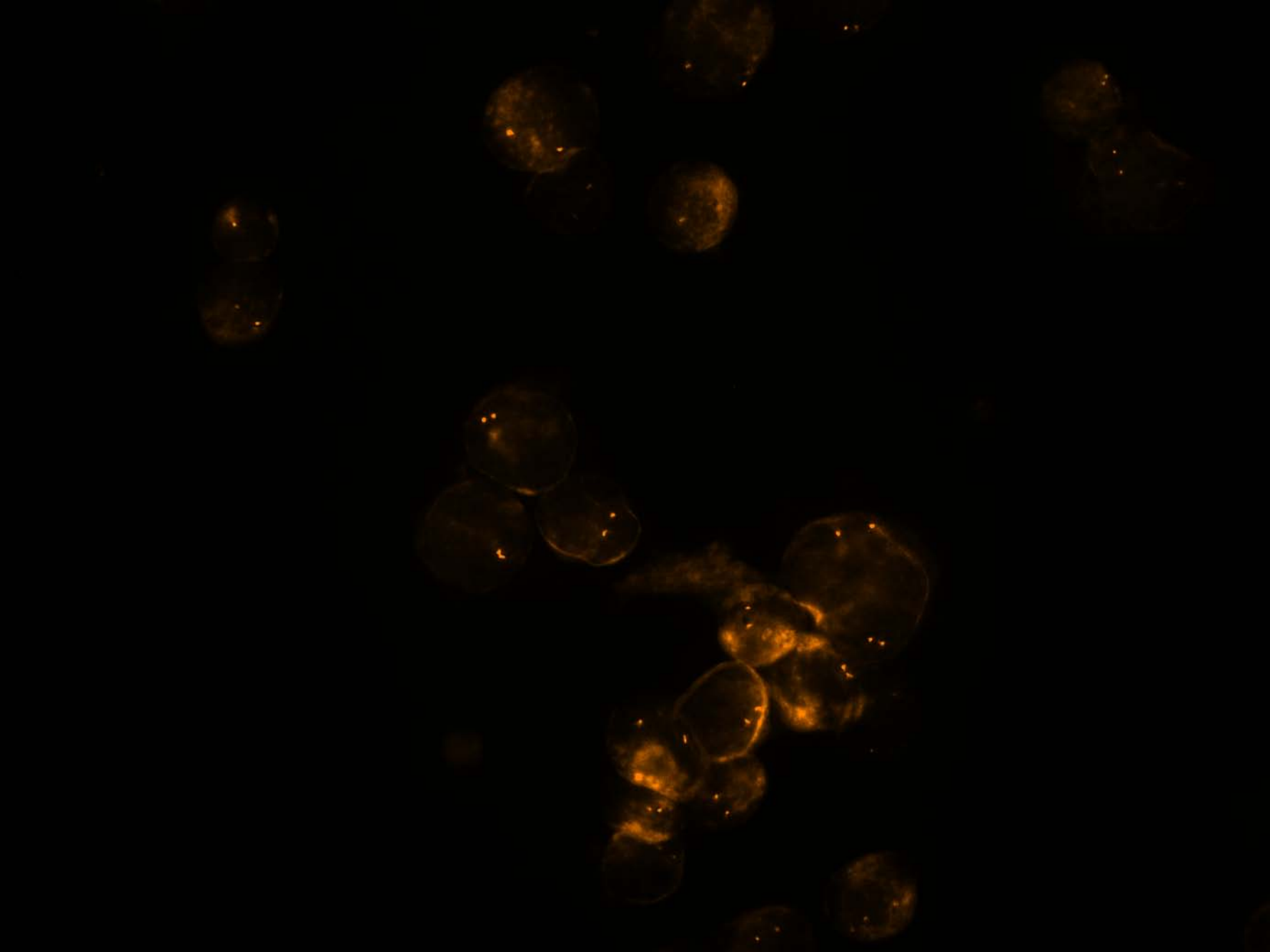

Supplement: Supplementary file 7 — Source Data for Expanded View and Appendix [file EMMM-12-e11908-s012.zip › SourceDataForAppendixFigure1.pdf.pdf]

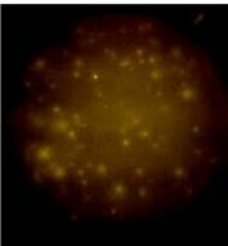

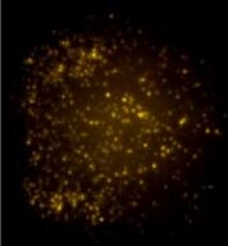



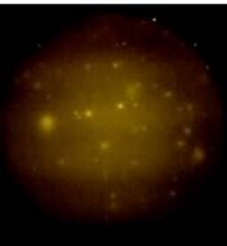



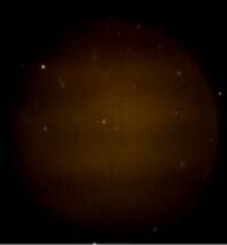





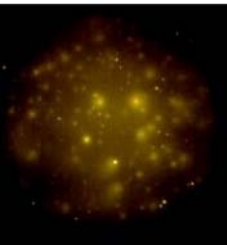



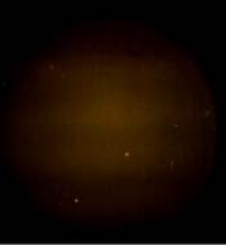





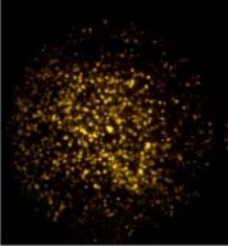

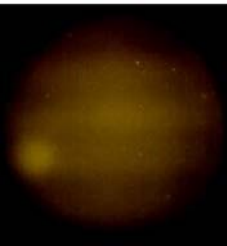



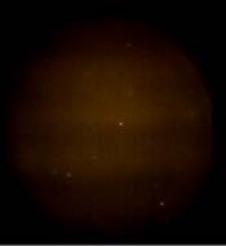



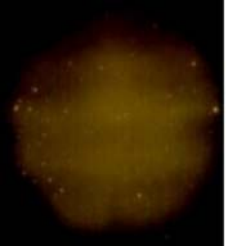

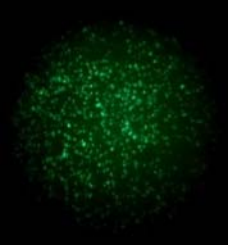

Supplement: Supplementary file 7 — Source Data for Expanded View and Appendix [file EMMM-12-e11908-s012.zip › SourceDataForAppendixFigure2.pdf.pdf]

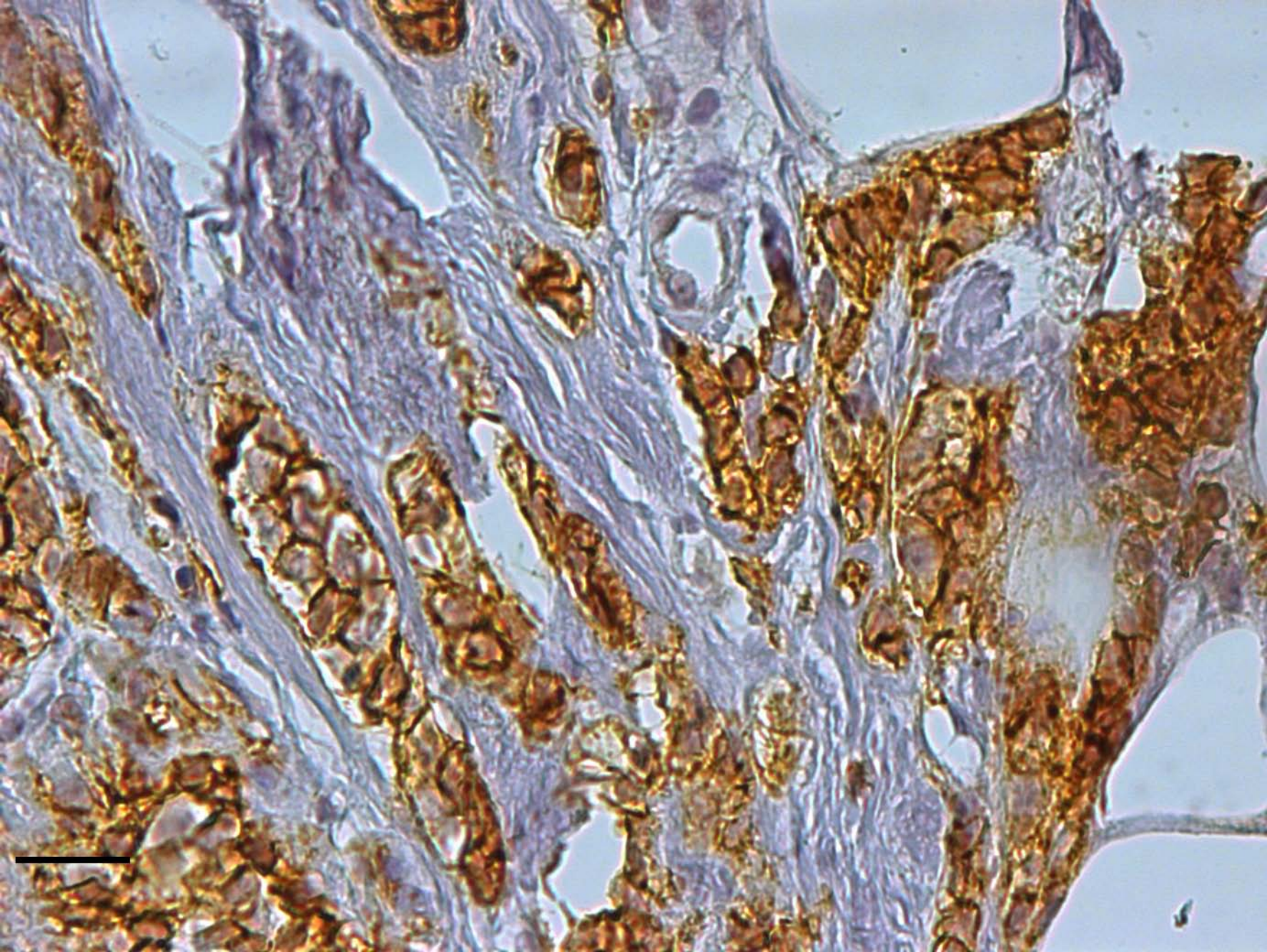

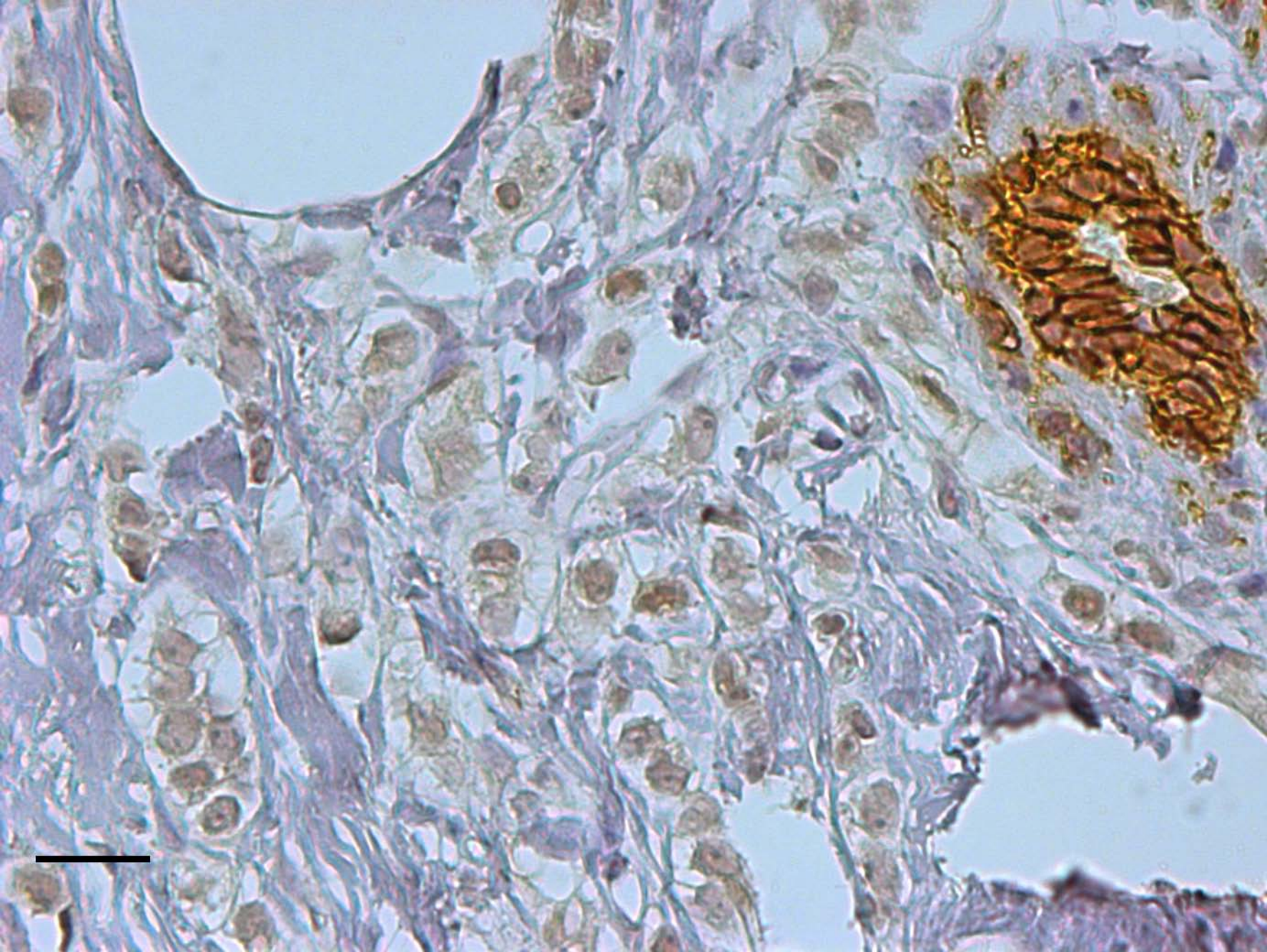

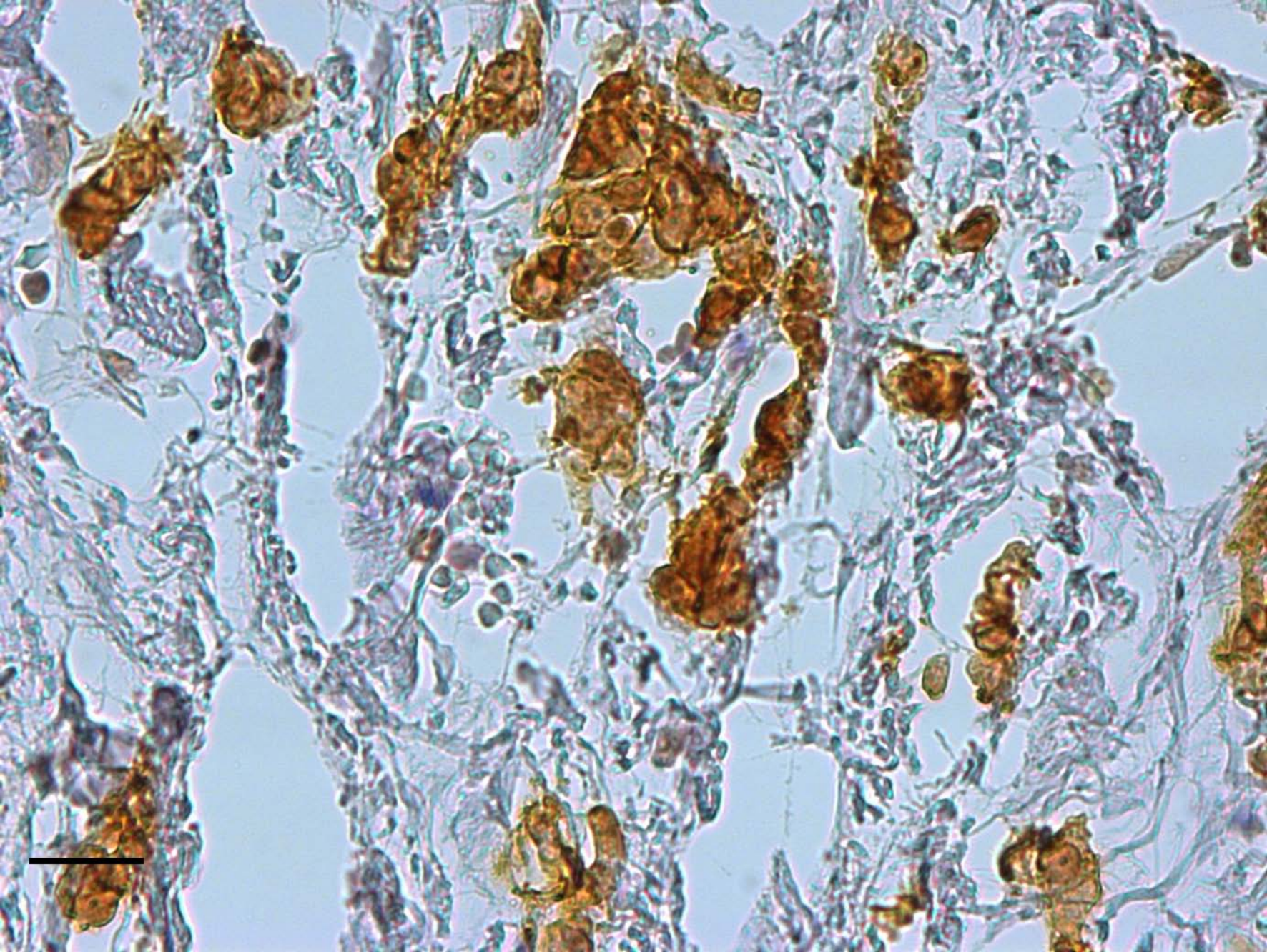

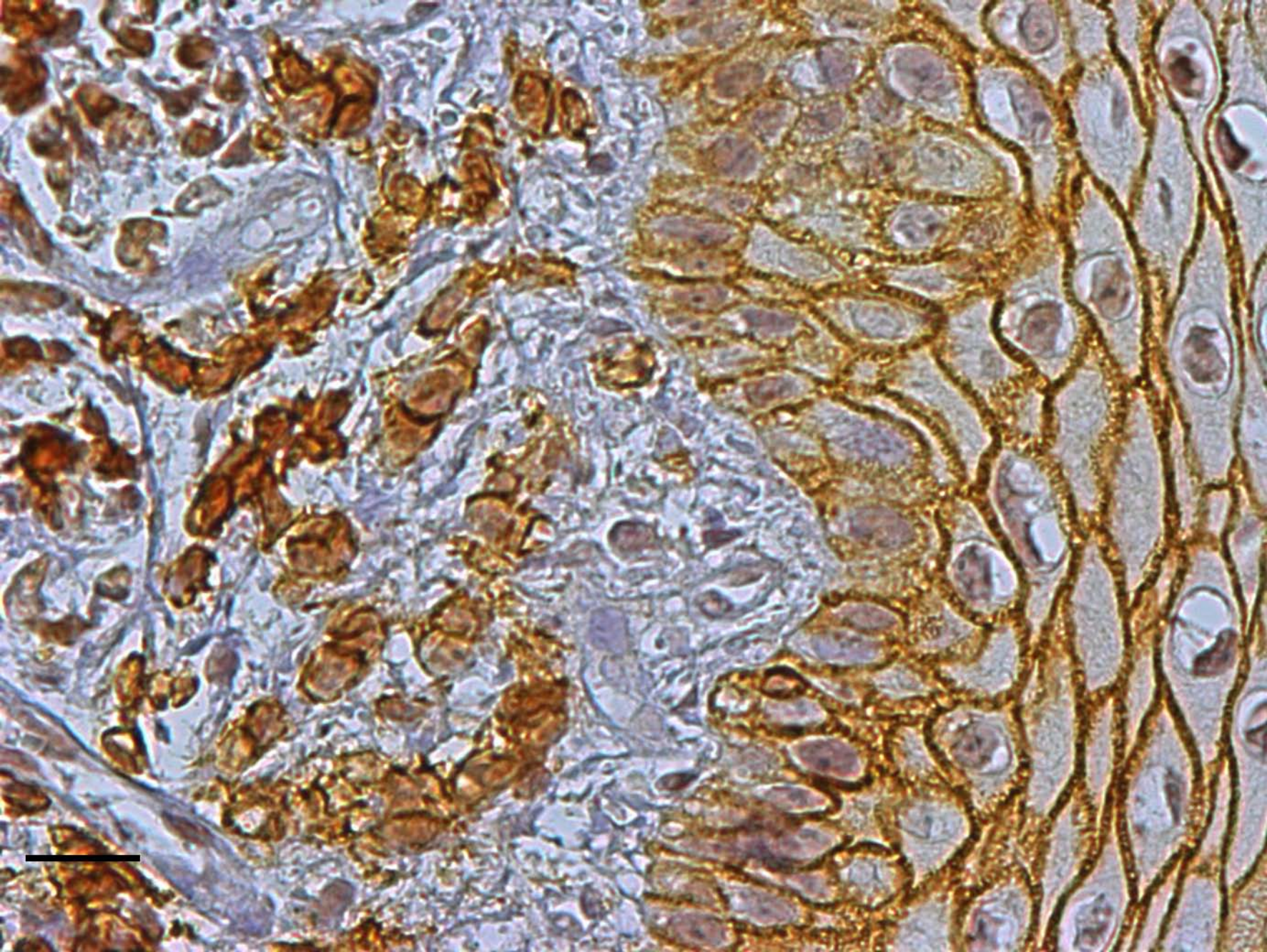

Supplement: Supplementary file 7 — Source Data for Expanded View and Appendix [file EMMM-12-e11908-s012.zip › SourceDataForFigureEV1.pdf.pdf]

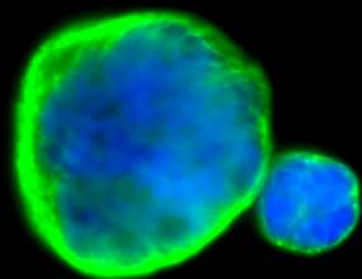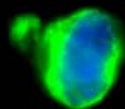

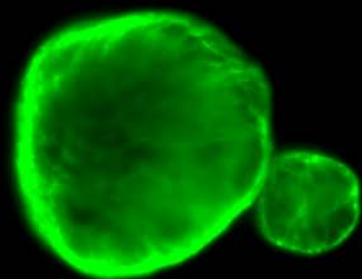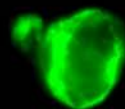



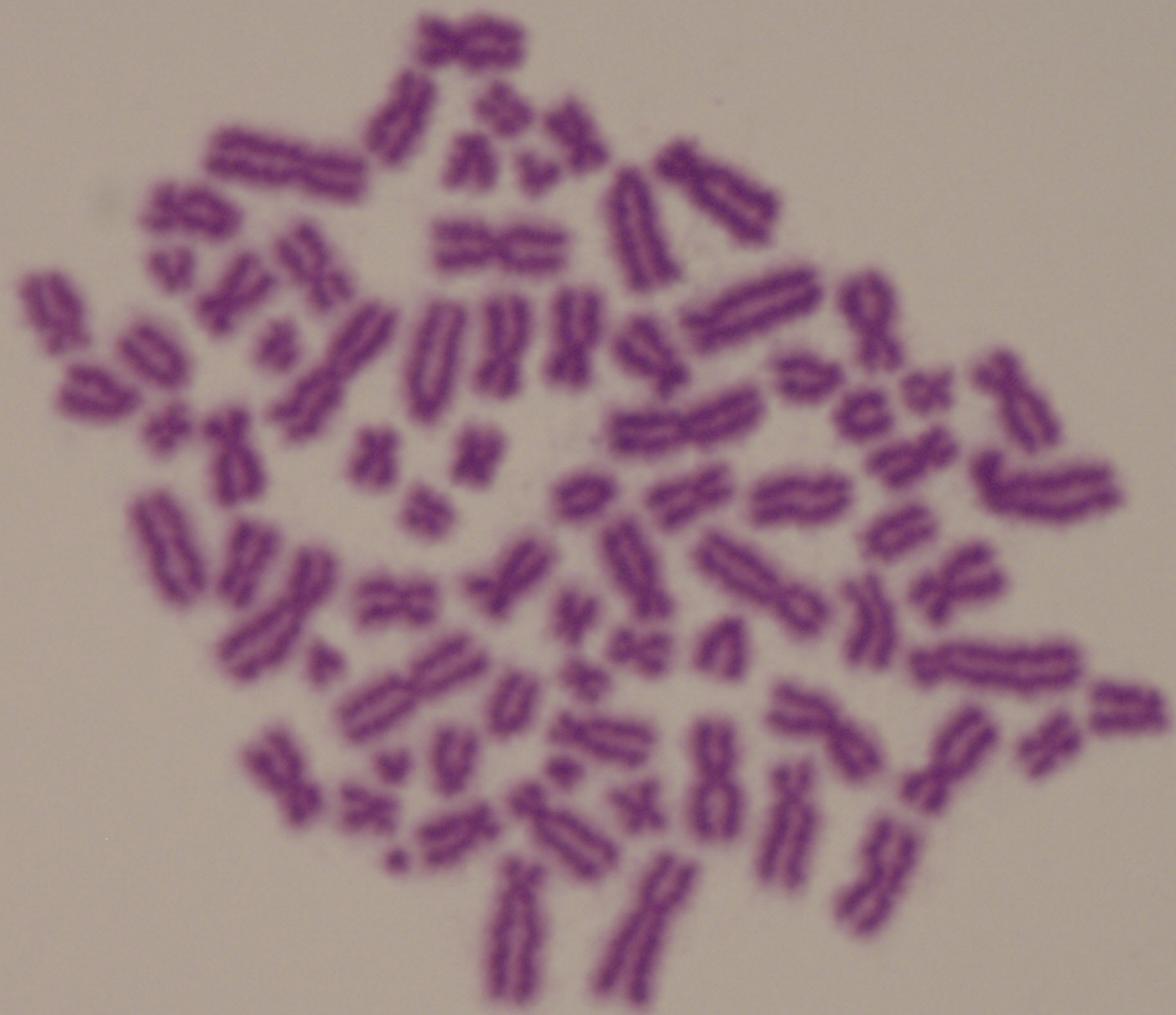

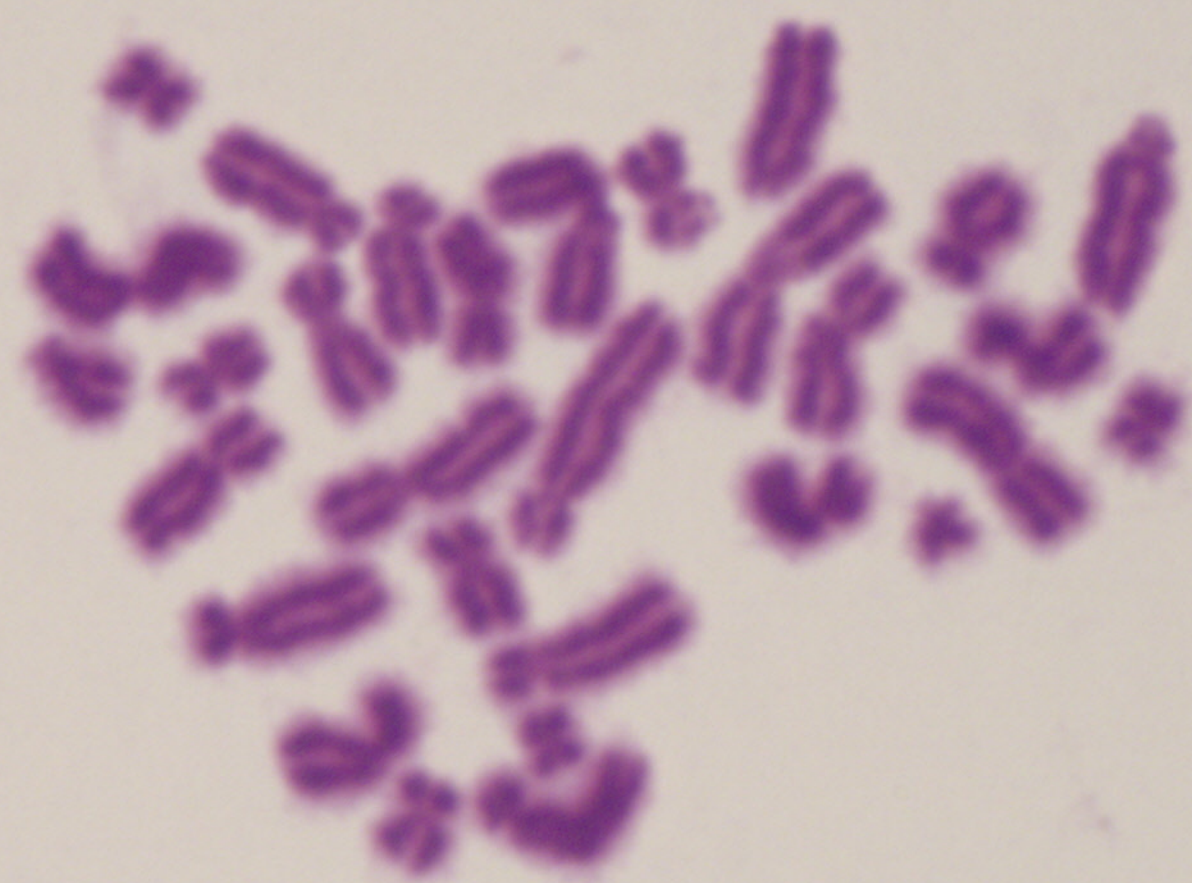

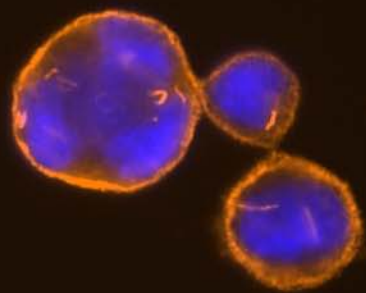



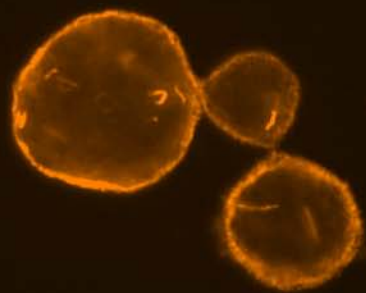

Supplement: Supplementary file 7 — Source Data for Expanded View and Appendix [file EMMM-12-e11908-s012.zip › SourceDataForFigureEV2.pdf.pdf]

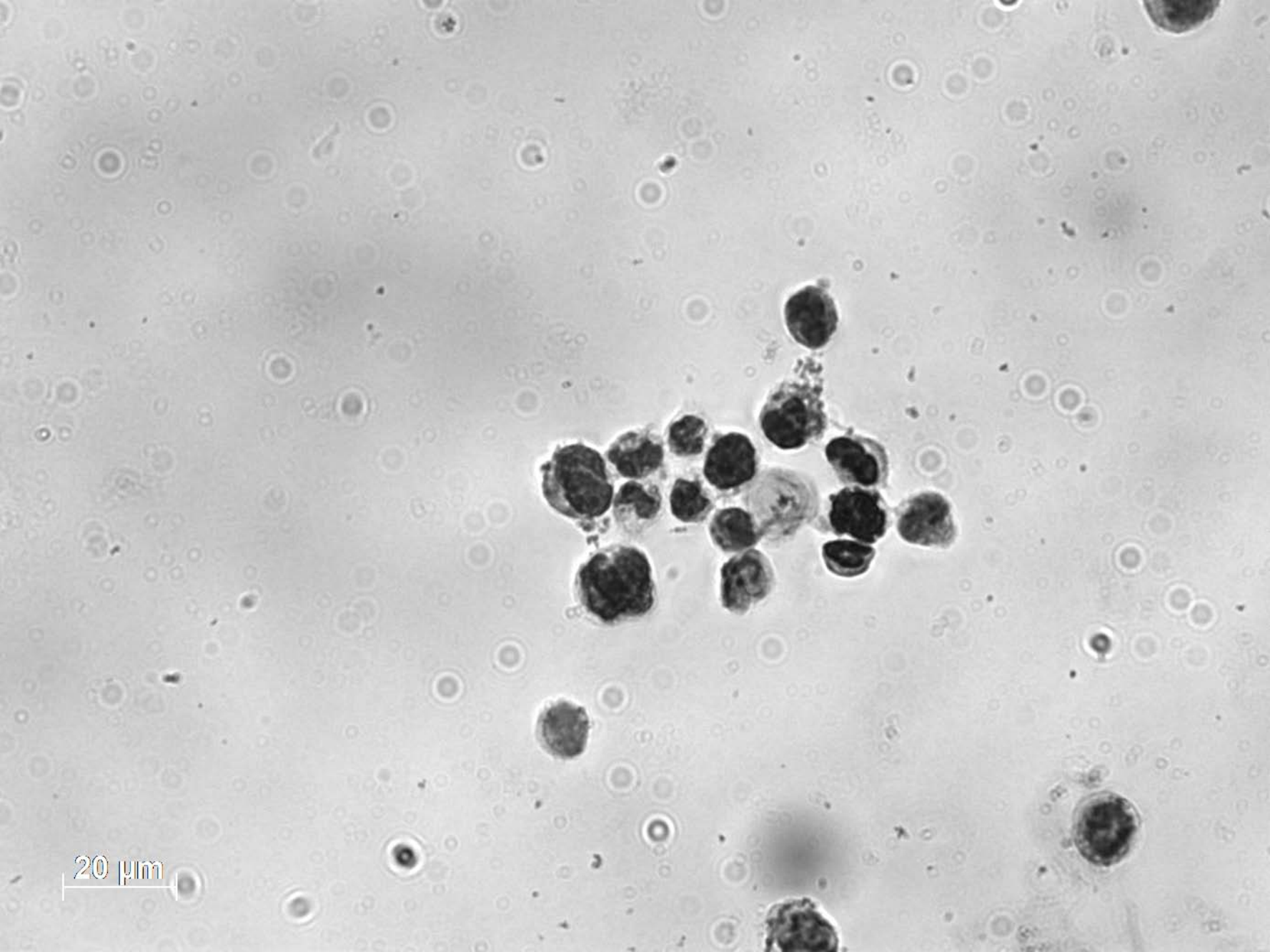

20  $\mu\text{m}$

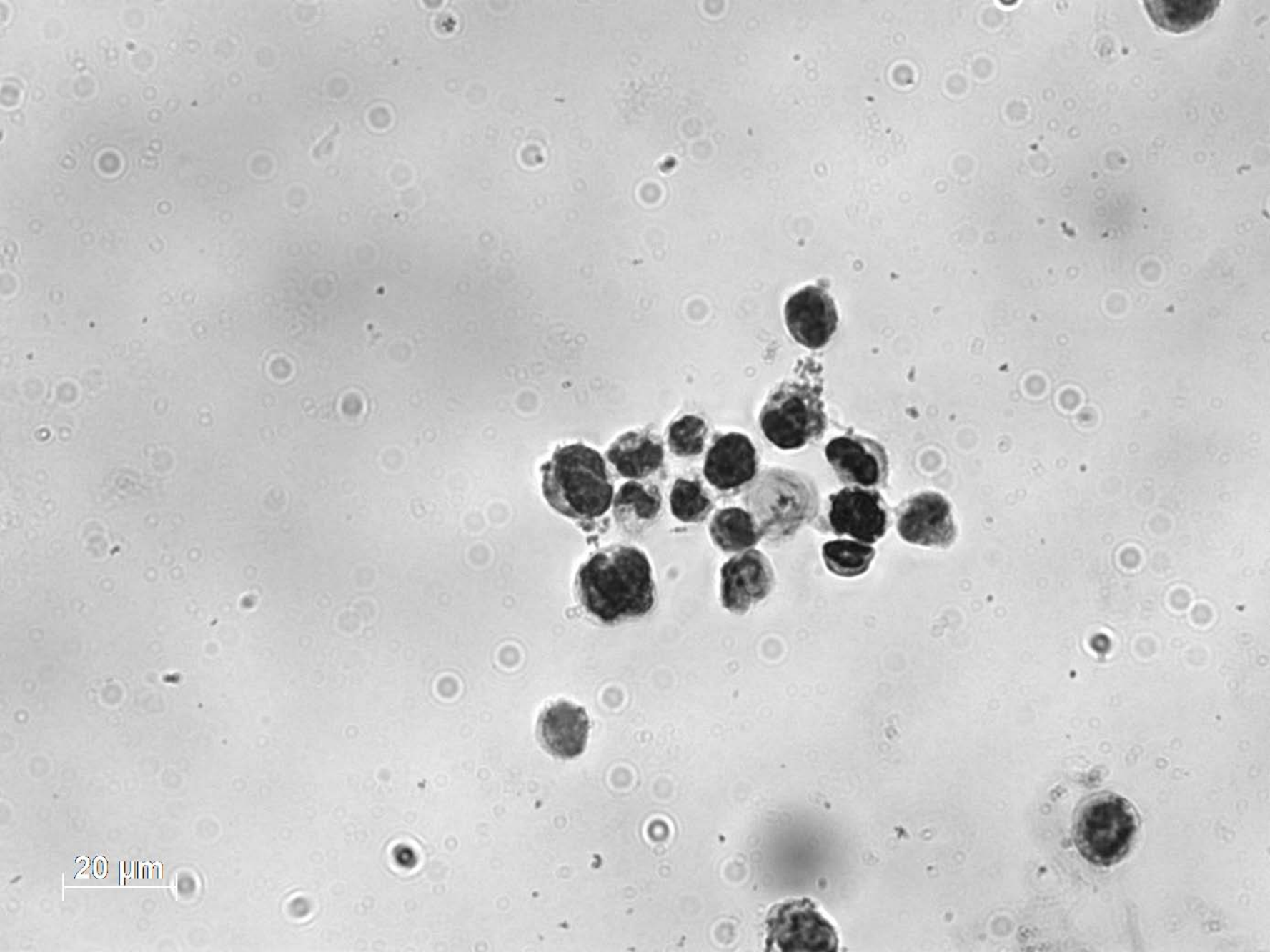

20  $\mu\text{m}$

Supplement: Supplementary file 7 — Source Data for Expanded View and Appendix [file EMMM-12-e11908-s012.zip › SourceDataForFigureEV3D.pdf.pdf]

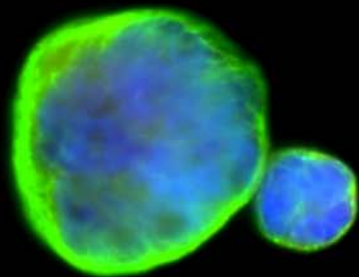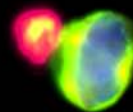

124,03 Pixel

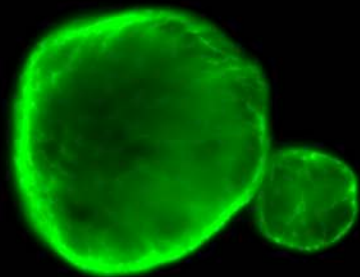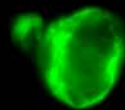

124,03 Pixel

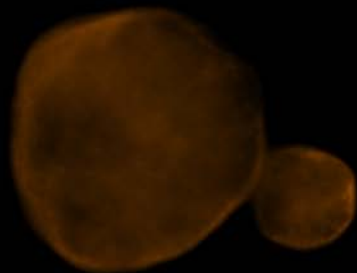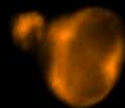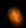

124,03 Pixel

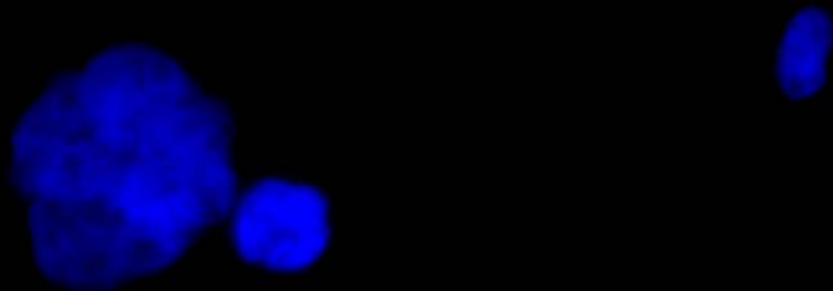

124,03 Pixel

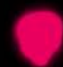

124,03 Pixel

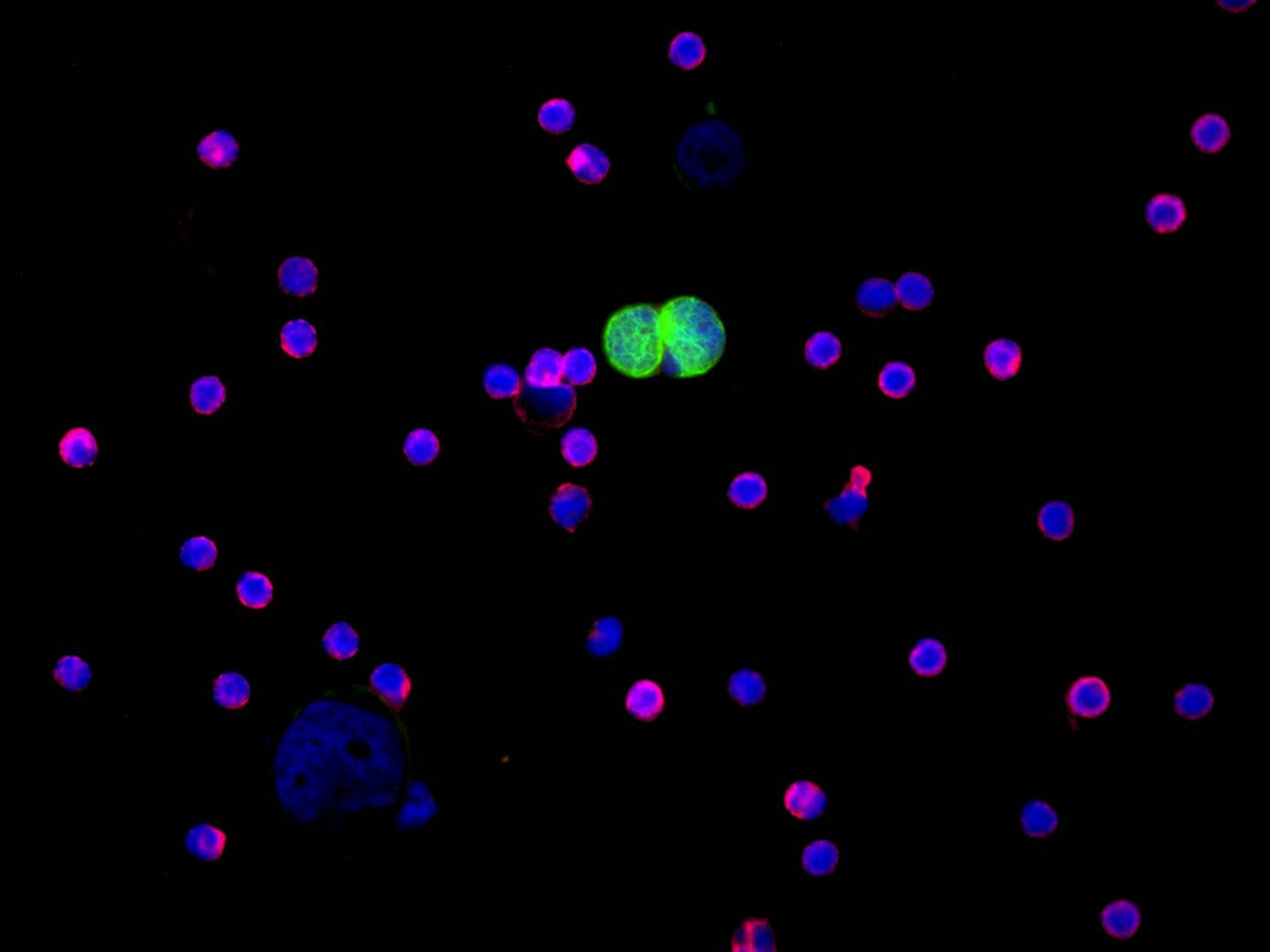

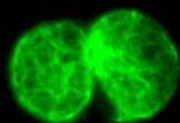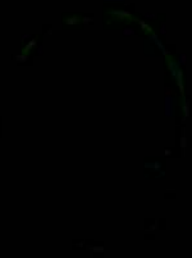





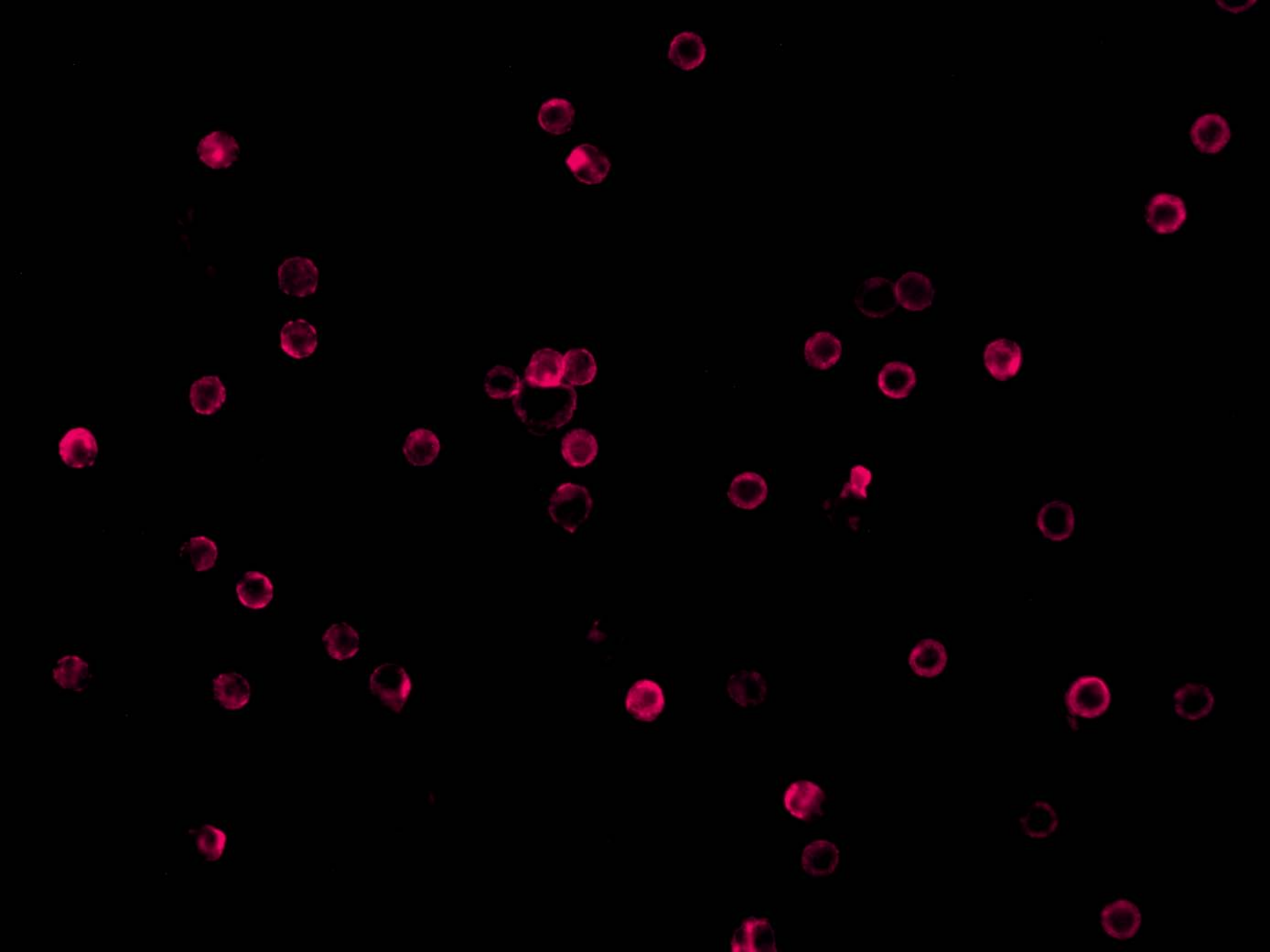

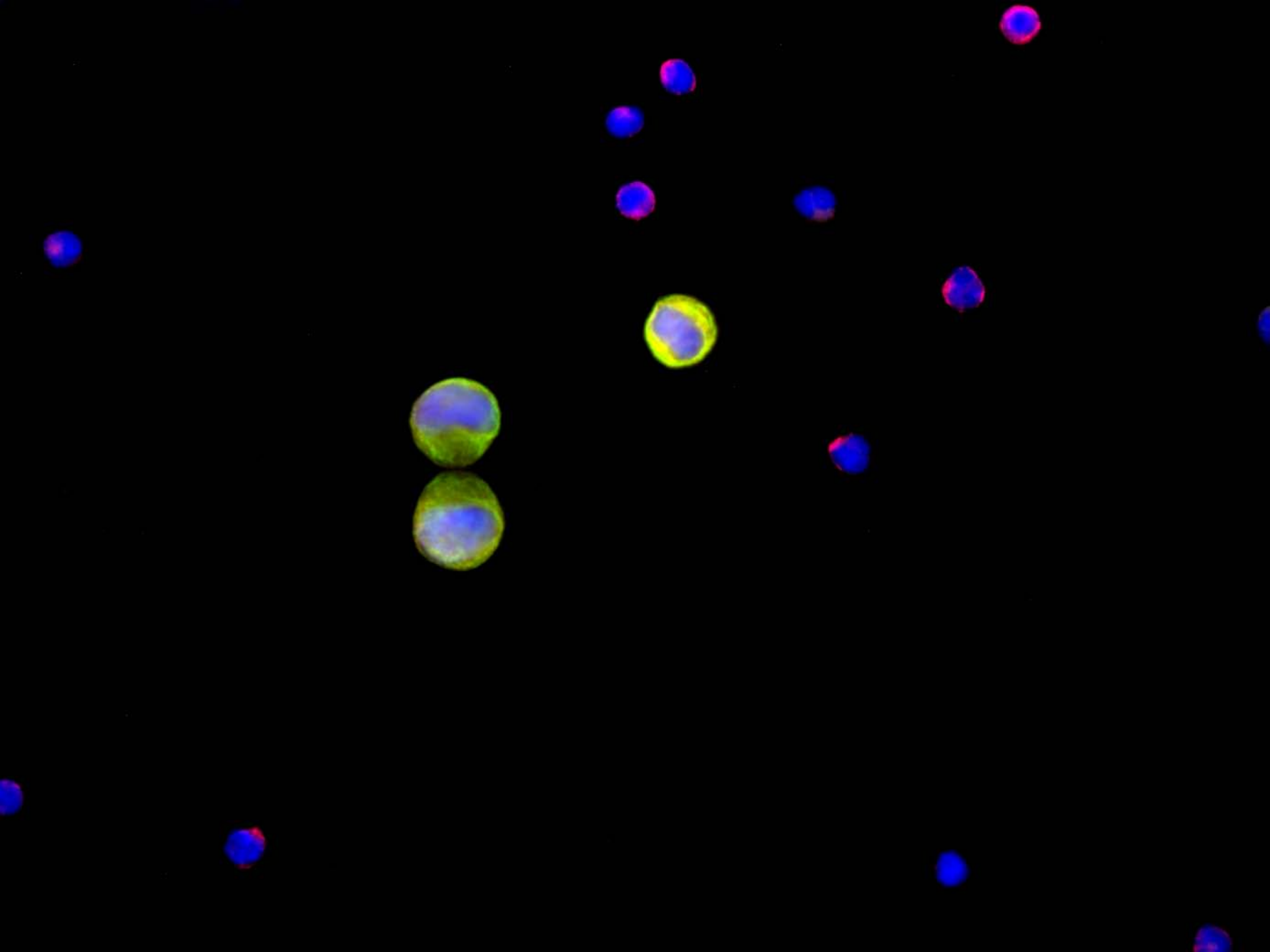

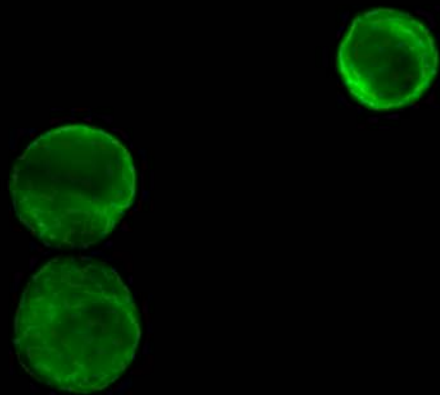

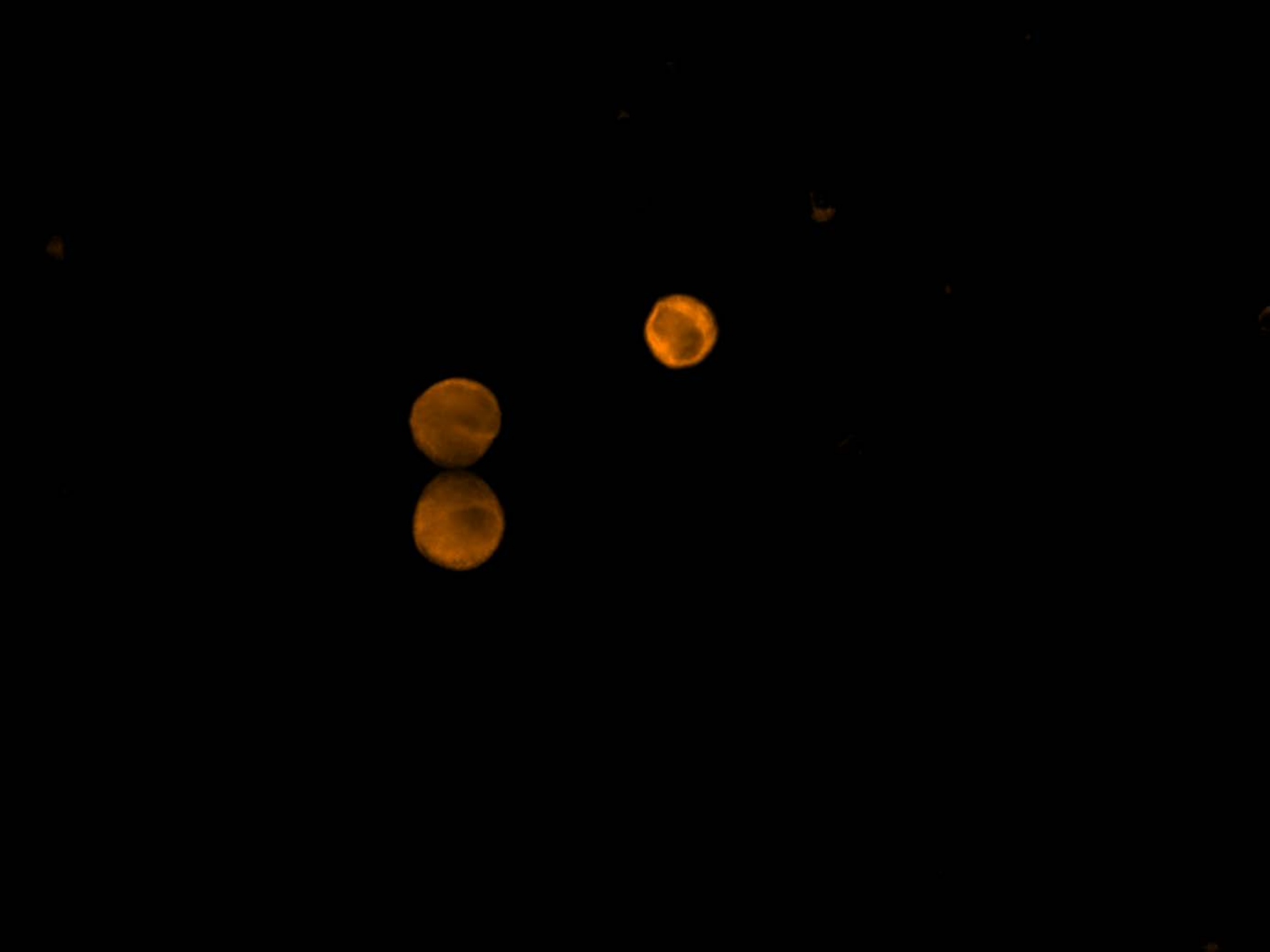

Supplement: Supplementary file 7 — Source Data for Expanded View and Appendix [file EMMM-12-e11908-s012.zip › SourceDataForFigureEV4.pdf.pdf]

20  $\mu$ m

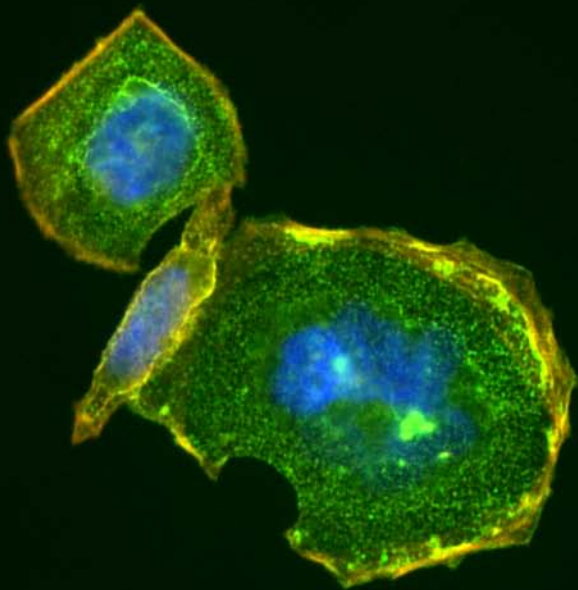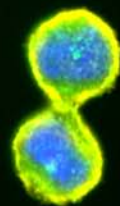

20  $\mu$ m

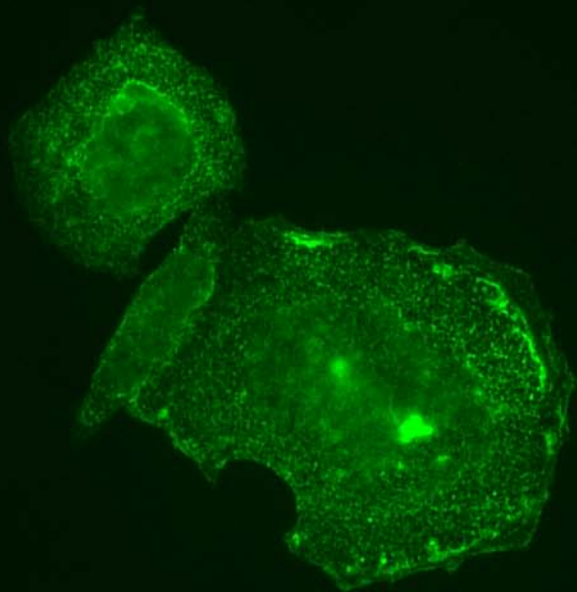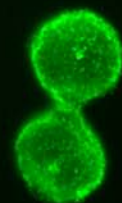

20  $\mu$ m

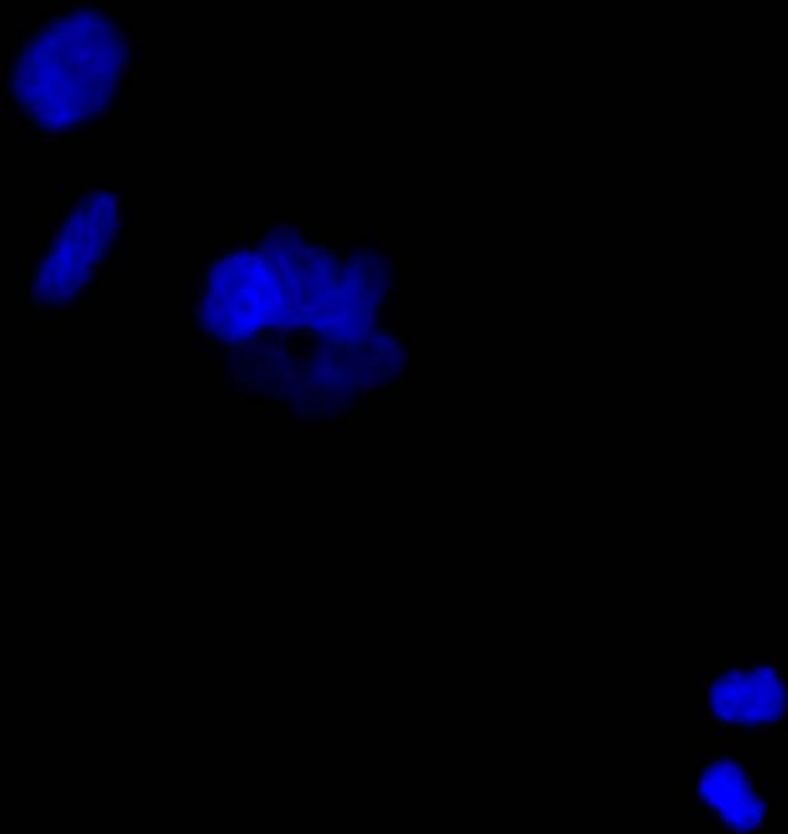

20  $\mu$ m

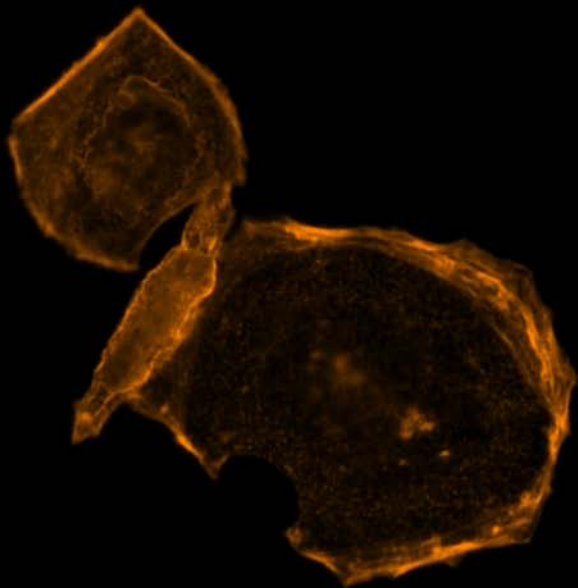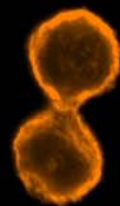

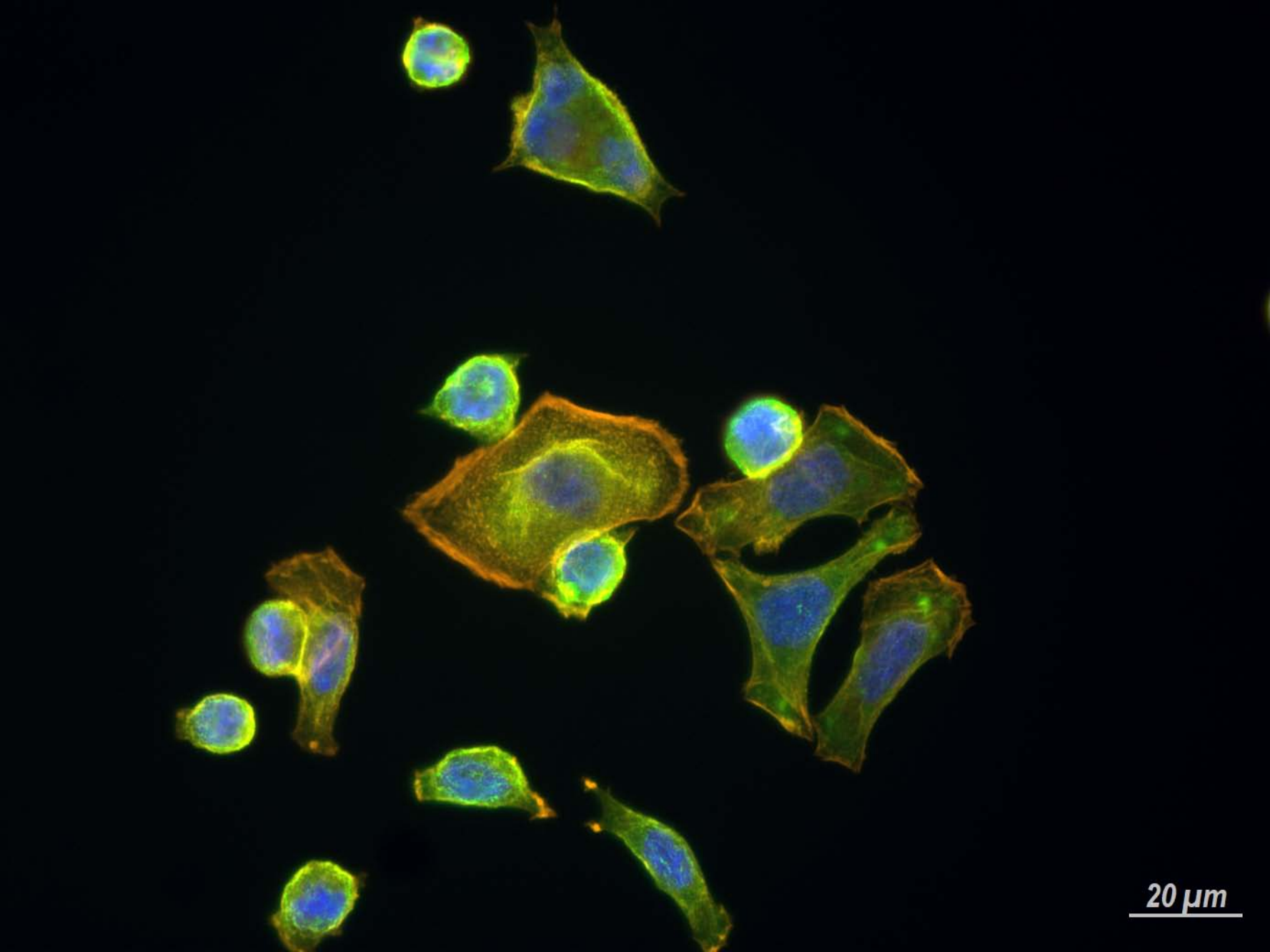

20  $\mu$ m

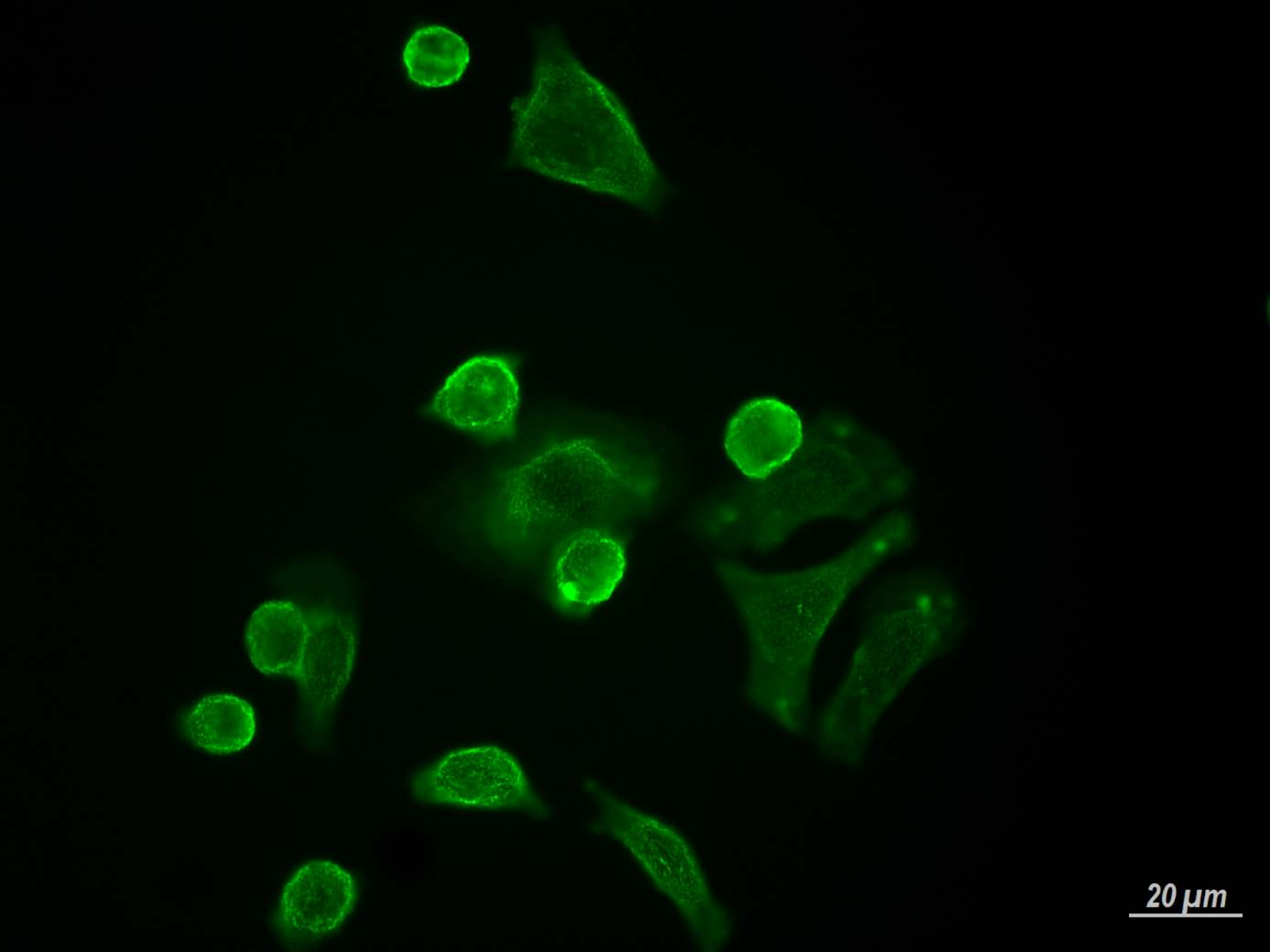

20  $\mu$ m

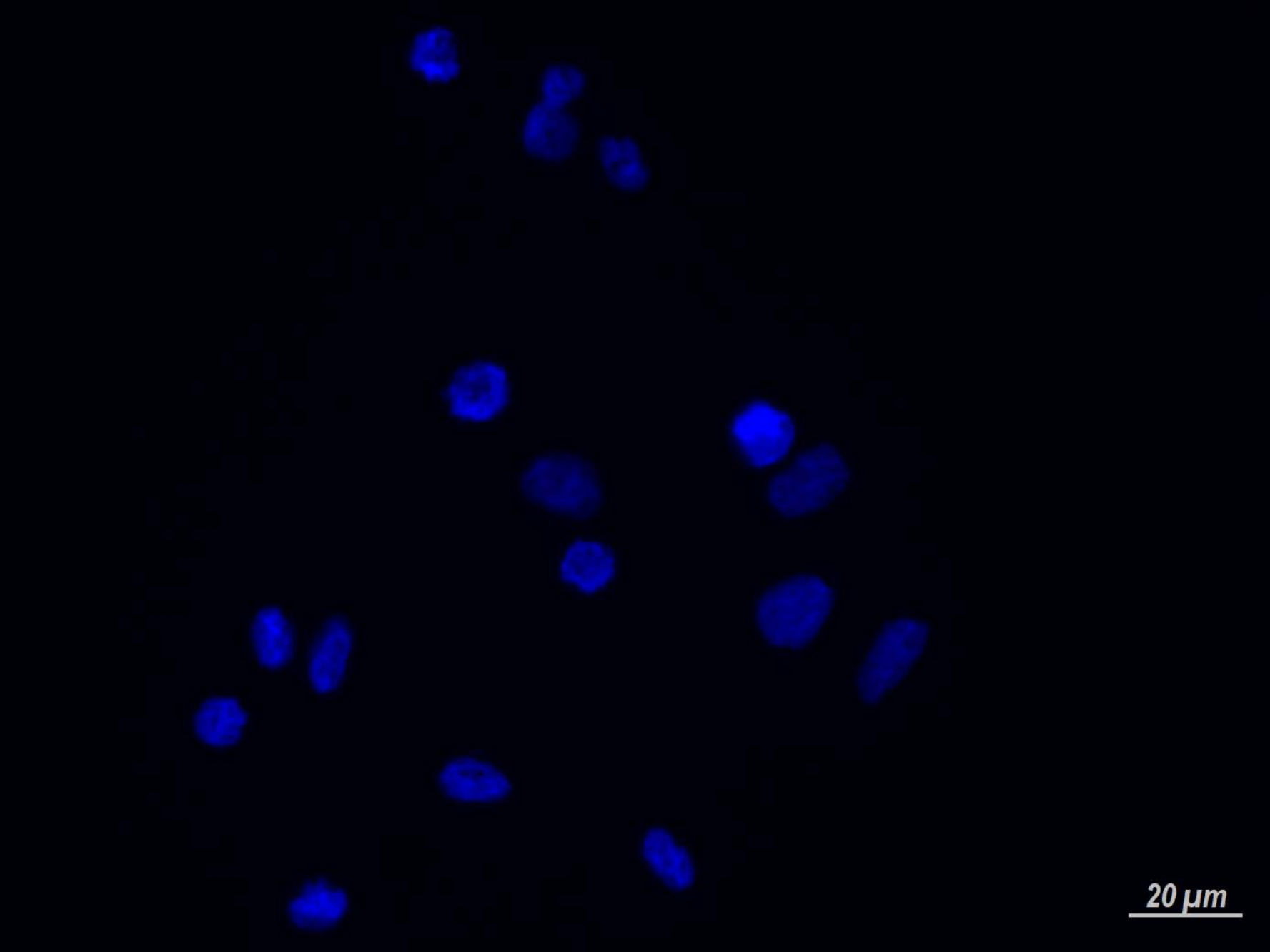

20  $\mu$ m

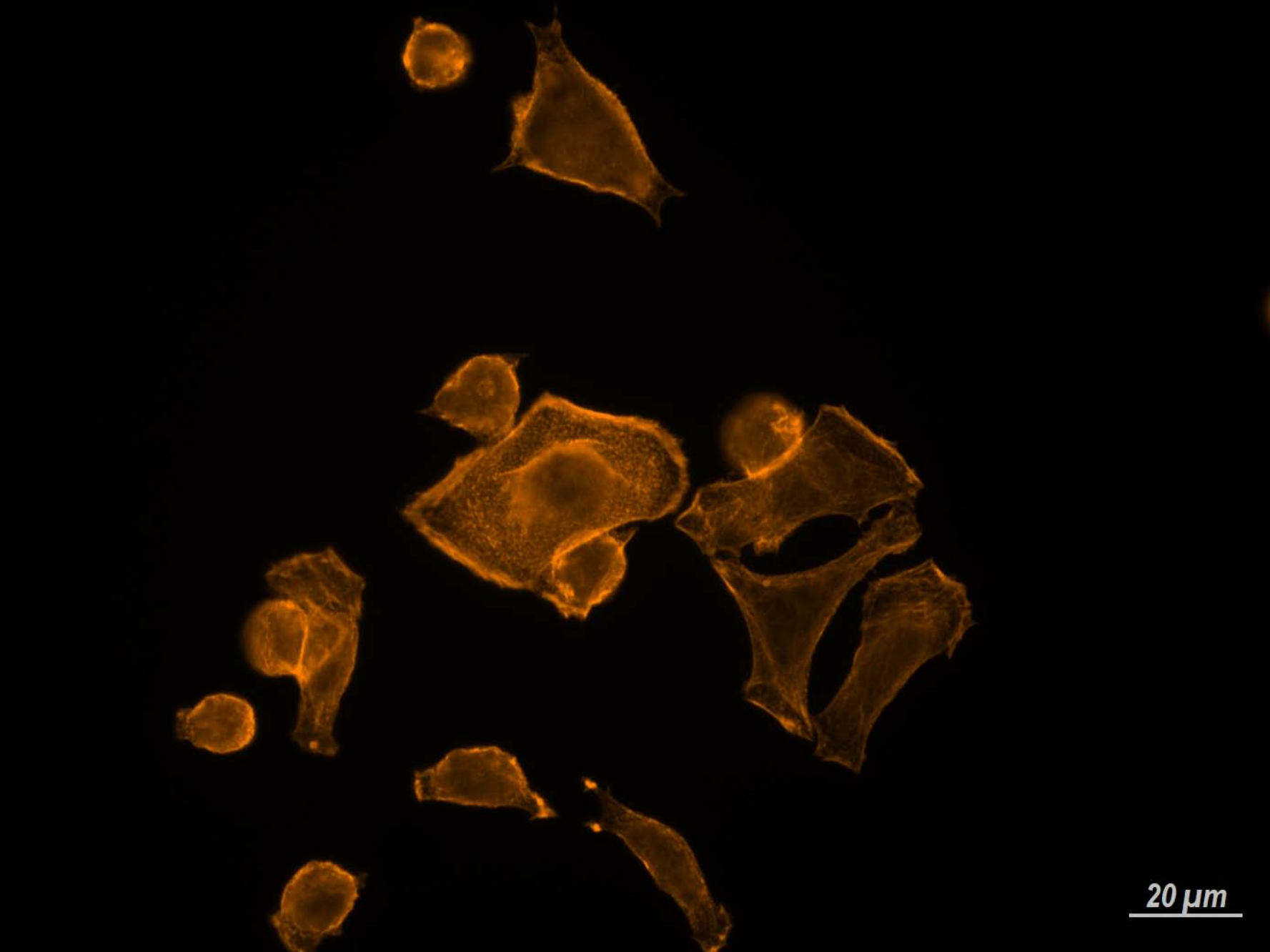

20  $\mu\text{m}$

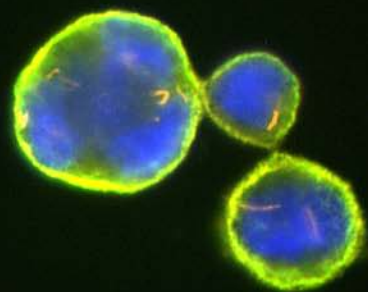

20  $\mu$ m

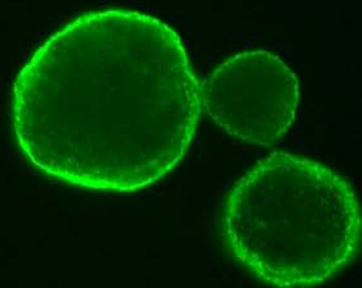

20  $\mu$ m

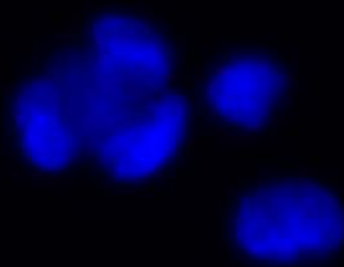

20  $\mu$ m

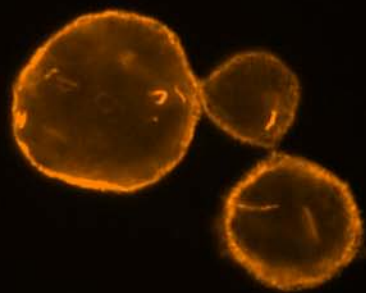

20  $\mu$ m

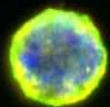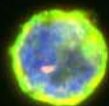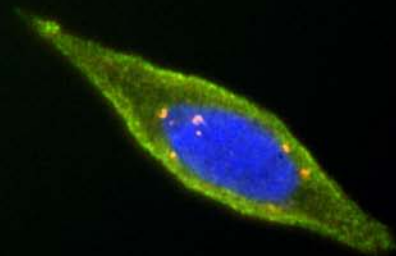

20  $\mu m$

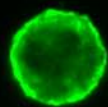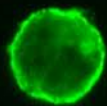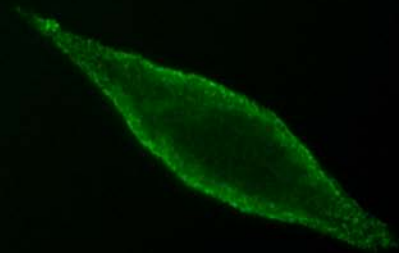

20  $\mu m$

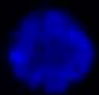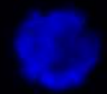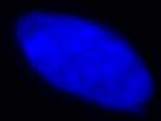

20  $\mu m$

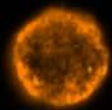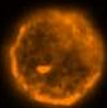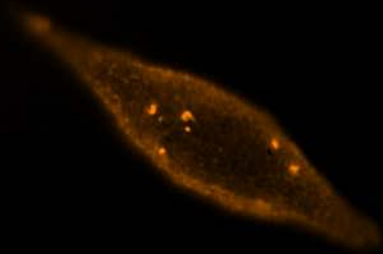

20  $\mu m$

Supplement: Supplementary file 11 — Source Data for Figure 5 [file EMMM-12-e11908-s009.pdf]

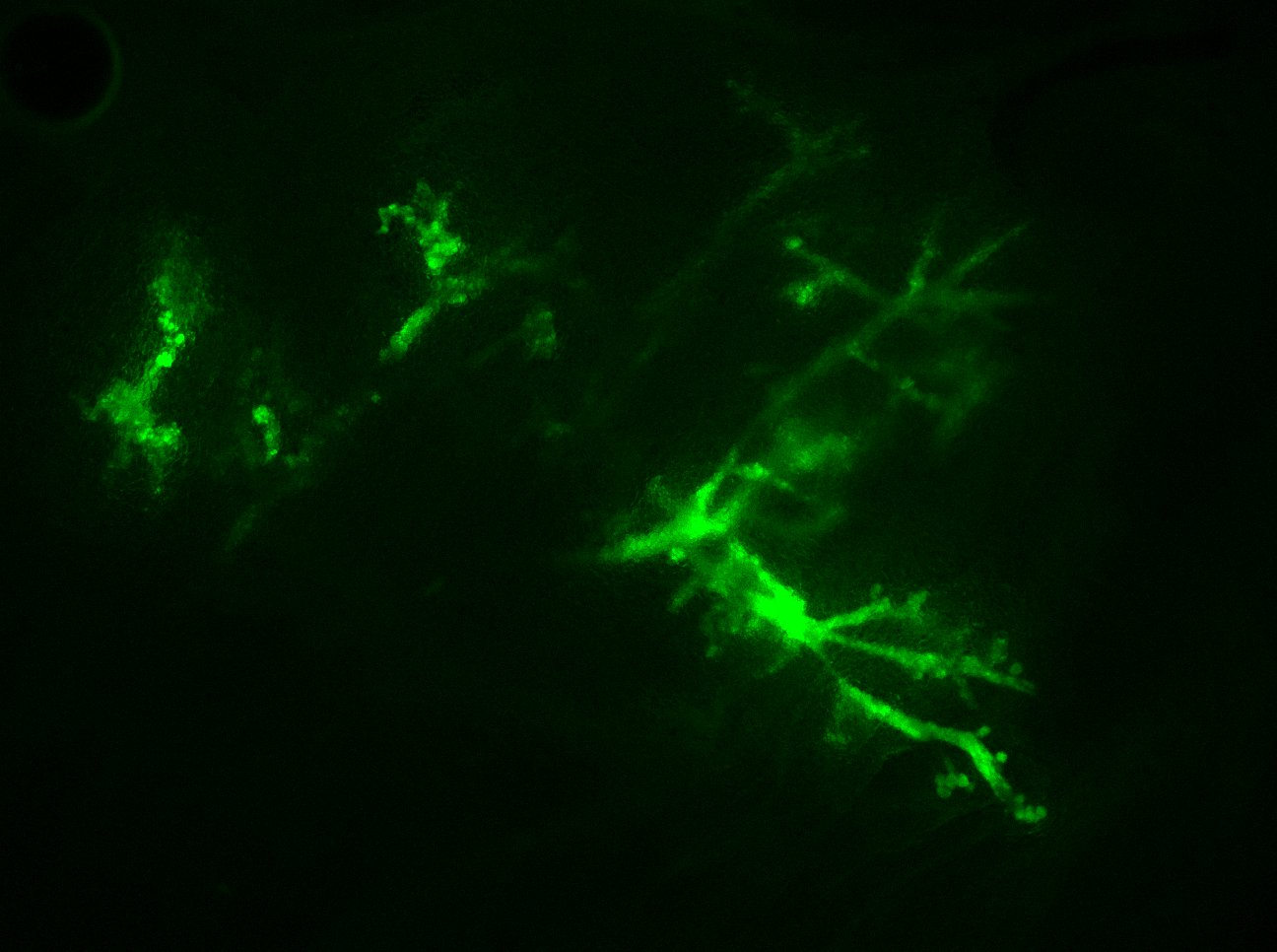

Supplement: Supplementary file 12 — Source Data for Figure 6 [file EMMM-12-e11908-s010.zip › _Source Data Part_2/Fig.6D/Mammary gland of CTC-ITB-01_MIND xenograft.jpg]

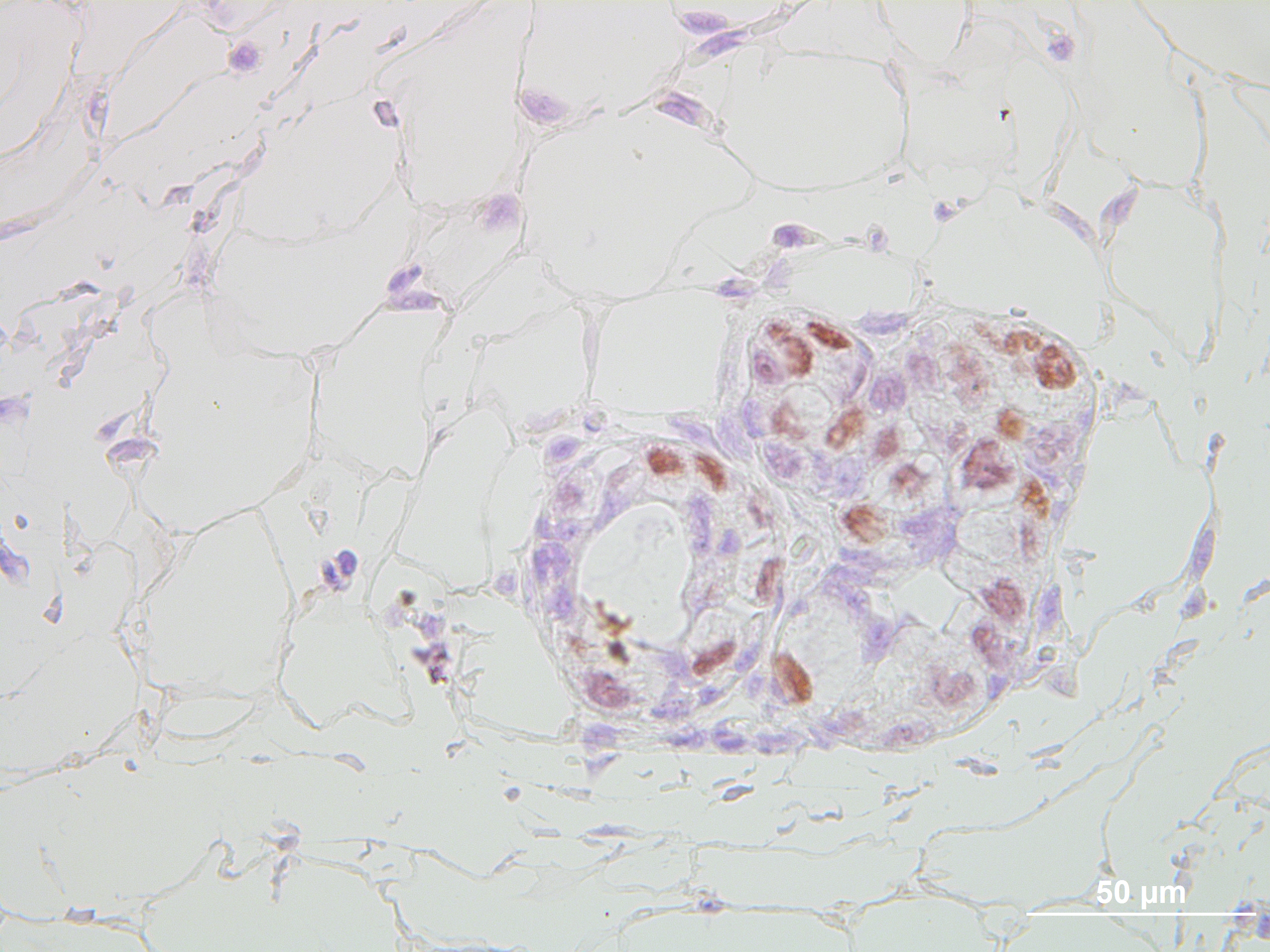

Supplement: Supplementary file 12 — Source Data for Figure 6 [file EMMM-12-e11908-s010.zip › _Source Data Part_2/Fig.6E/C463 ER image 1.jpg]

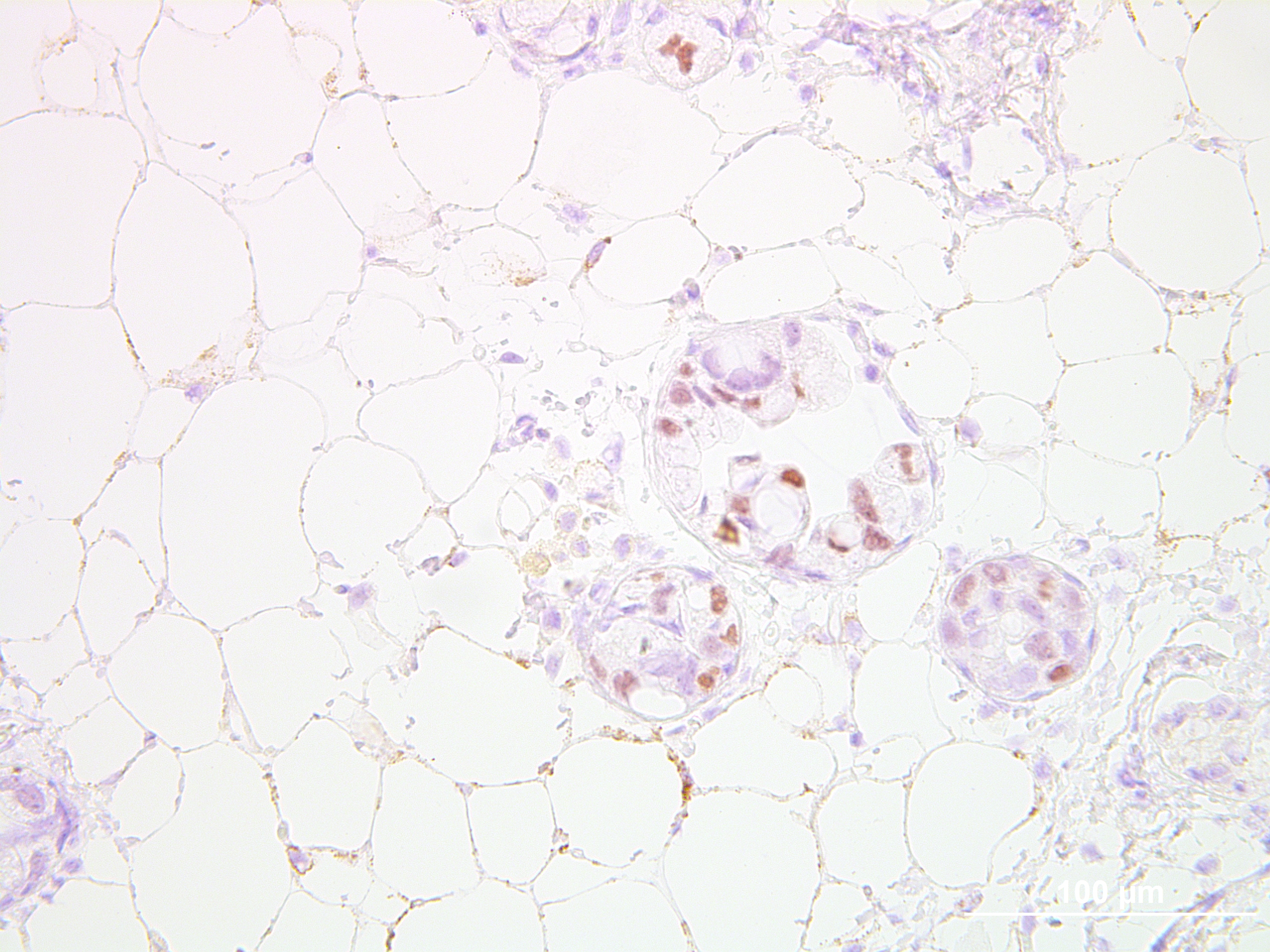

Supplement: Supplementary file 12 — Source Data for Figure 6 [file EMMM-12-e11908-s010.zip › _Source Data Part_2/Fig.6E/C463 ER image 2.jpg]

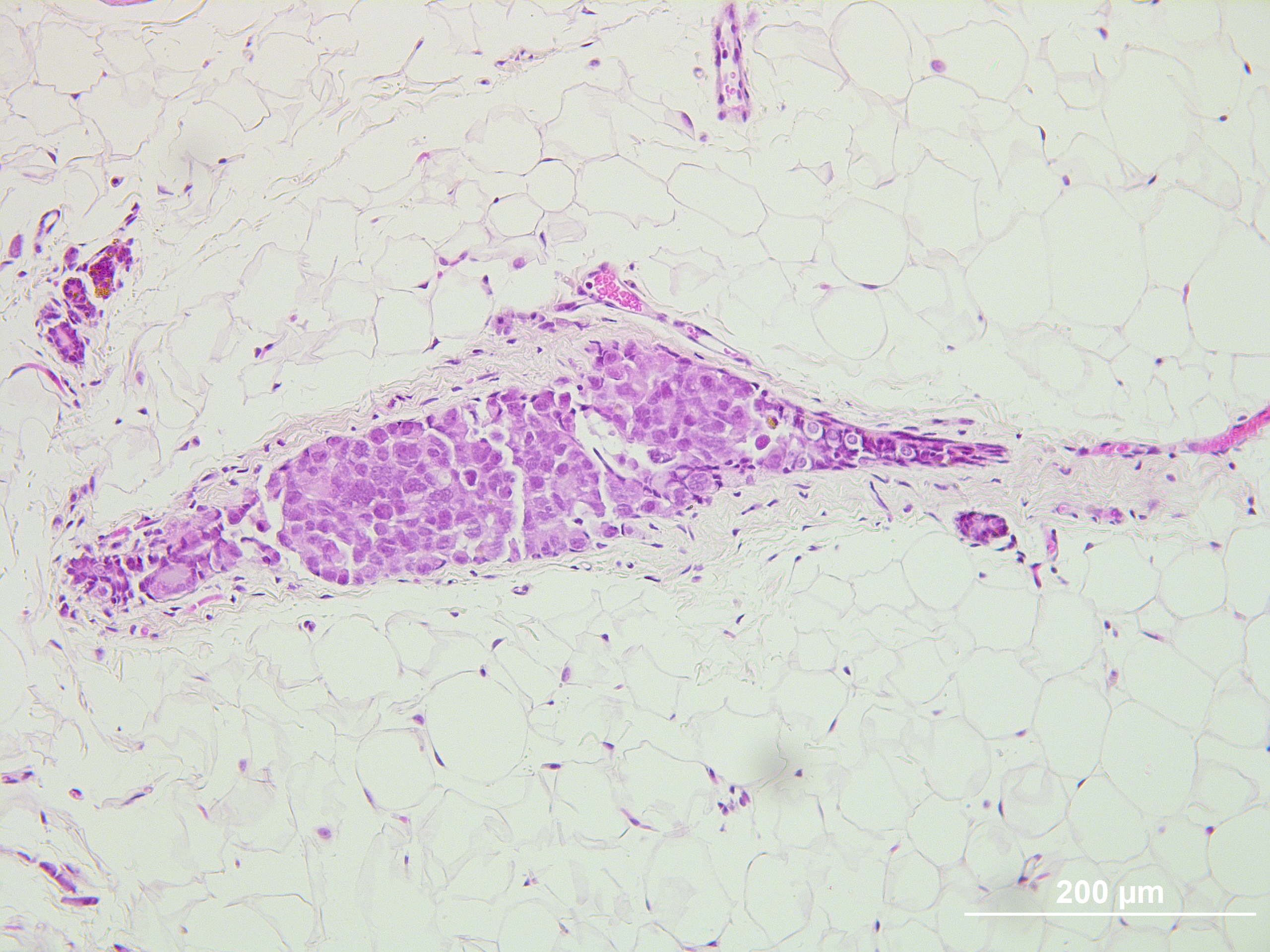

Supplement: Supplementary file 12 — Source Data for Figure 6 [file EMMM-12-e11908-s010.zip › SourceDataForFigure6_1.zip/Fig.6C/H&E_1.jpg]

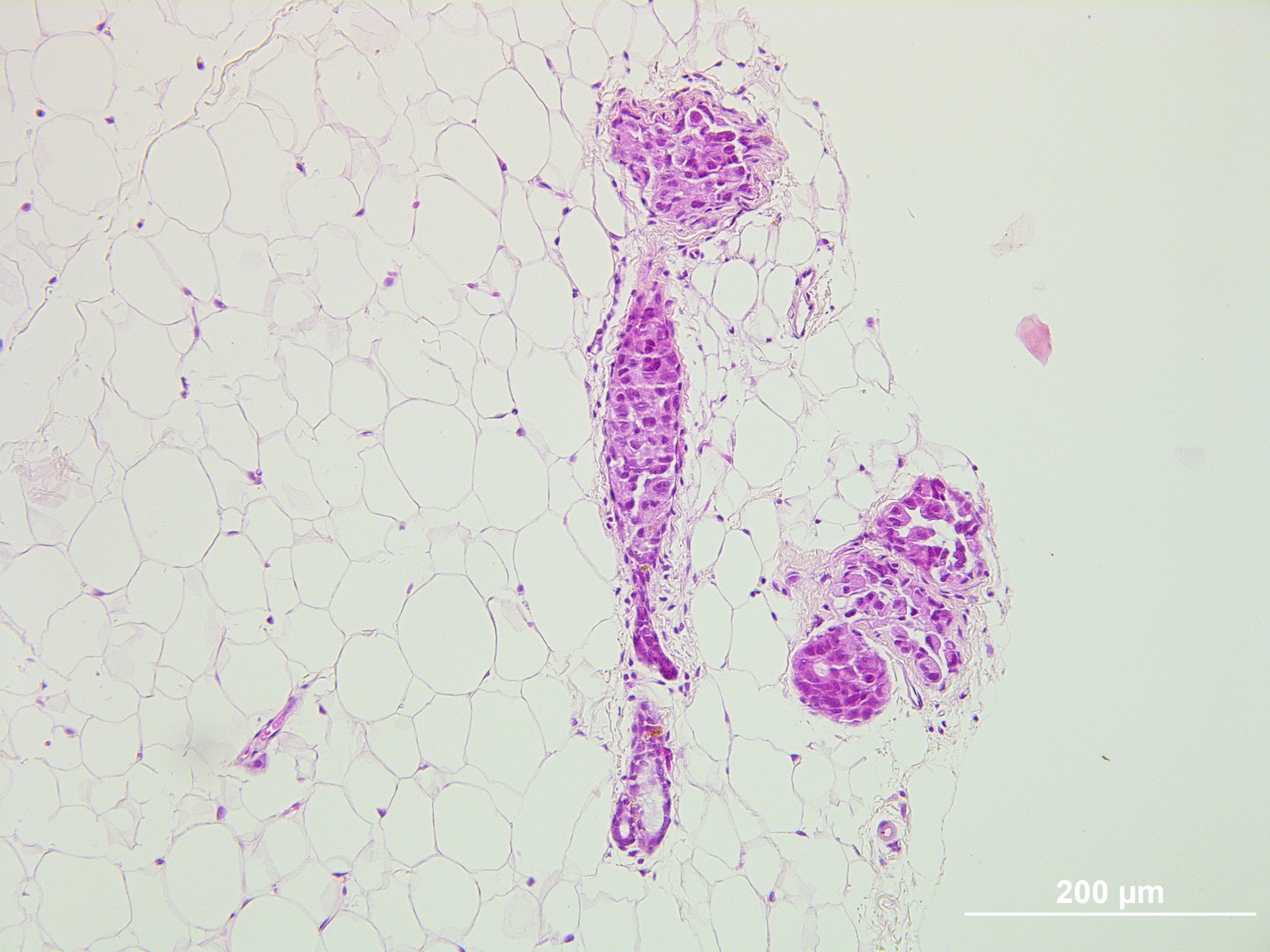

Supplement: Supplementary file 12 — Source Data for Figure 6 [file EMMM-12-e11908-s010.zip › SourceDataForFigure6_1.zip/Fig.6C/H&E_2.jpg]

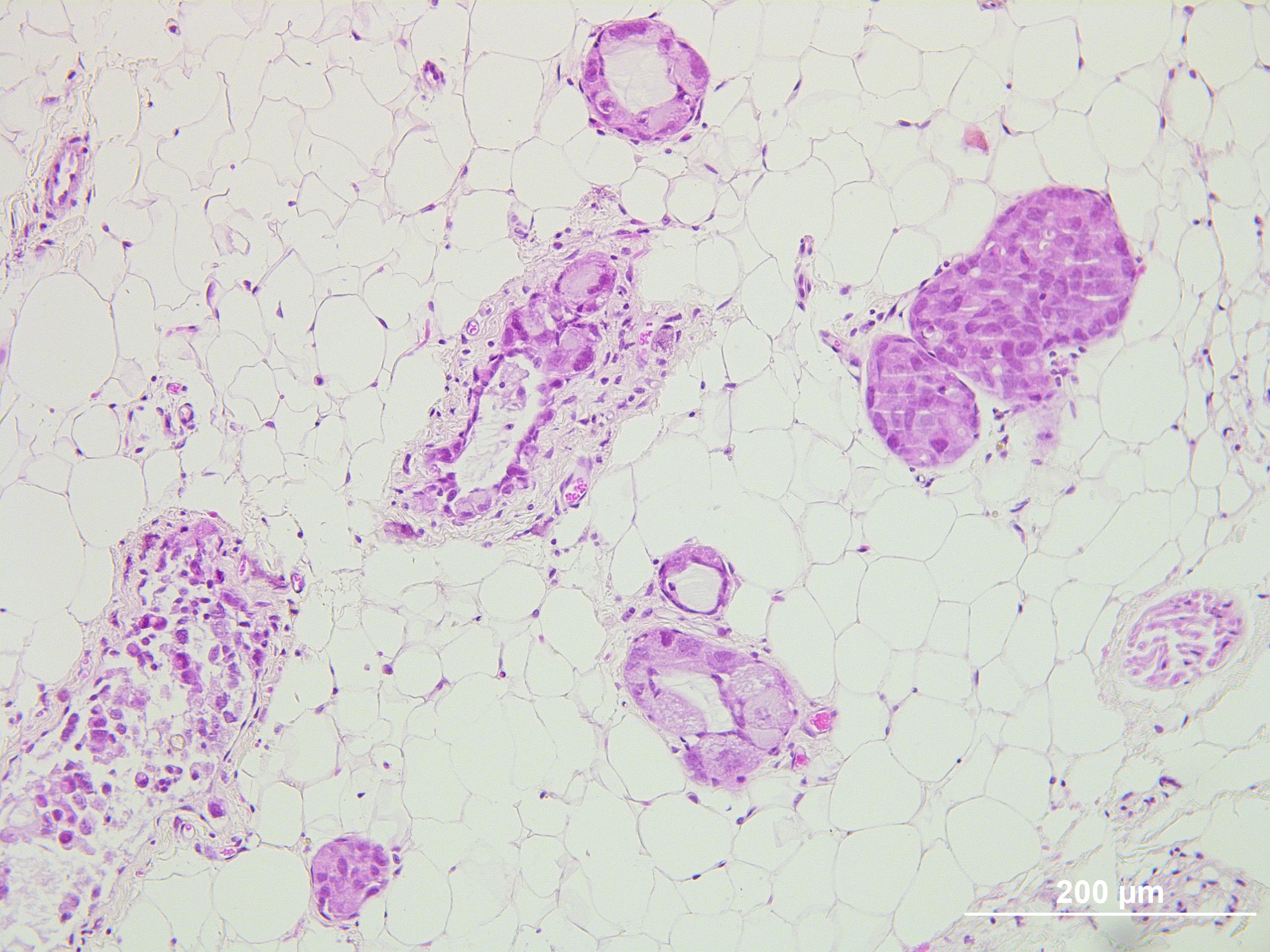

Supplement: Supplementary file 12 — Source Data for Figure 6 [file EMMM-12-e11908-s010.zip › SourceDataForFigure6_1.zip/Fig.6C/H&E_3.jpg]

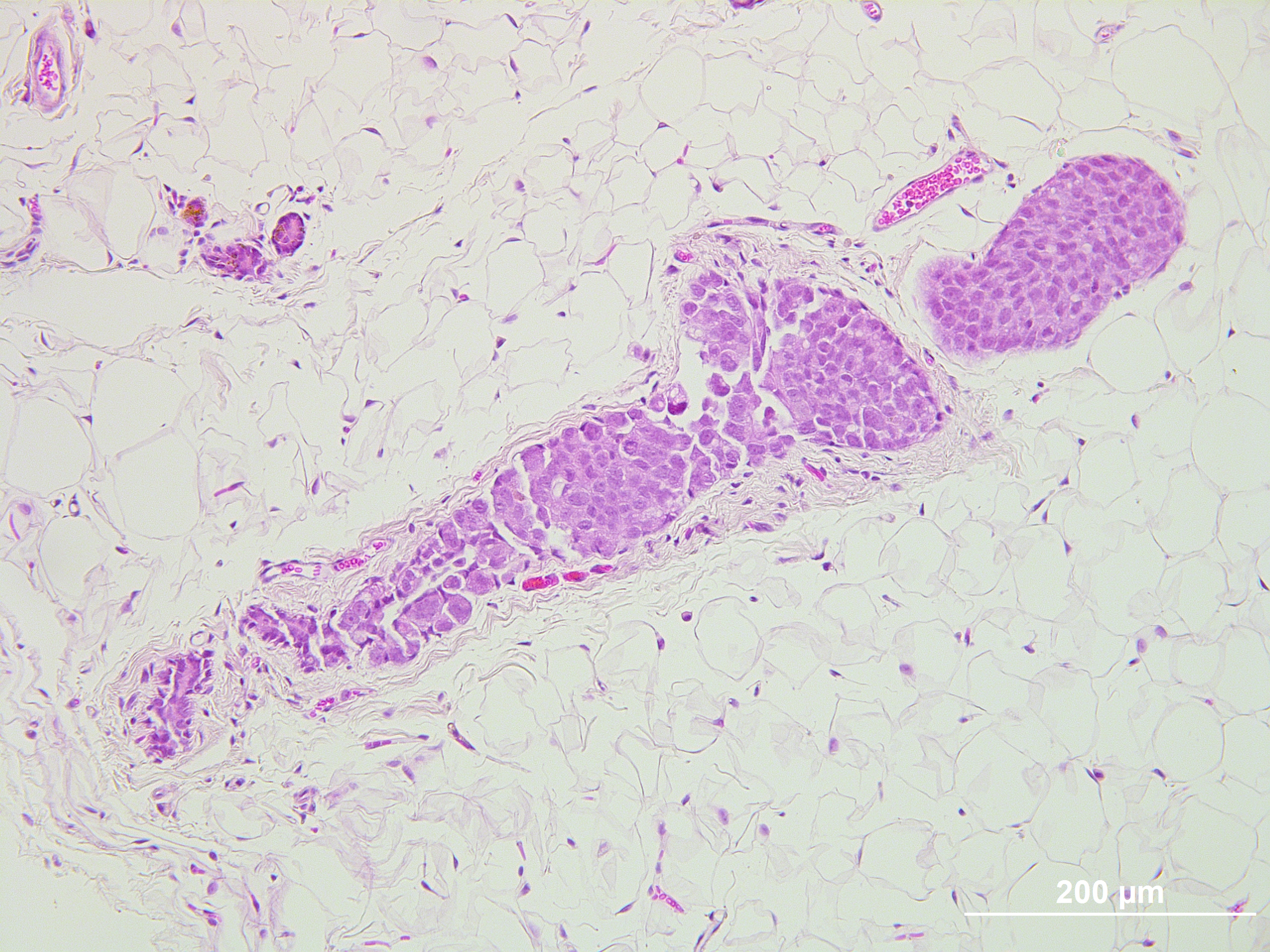

Supplement: Supplementary file 12 — Source Data for Figure 6 [file EMMM-12-e11908-s010.zip › SourceDataForFigure6_1.zip/Fig.6C/H&E_4.jpg]

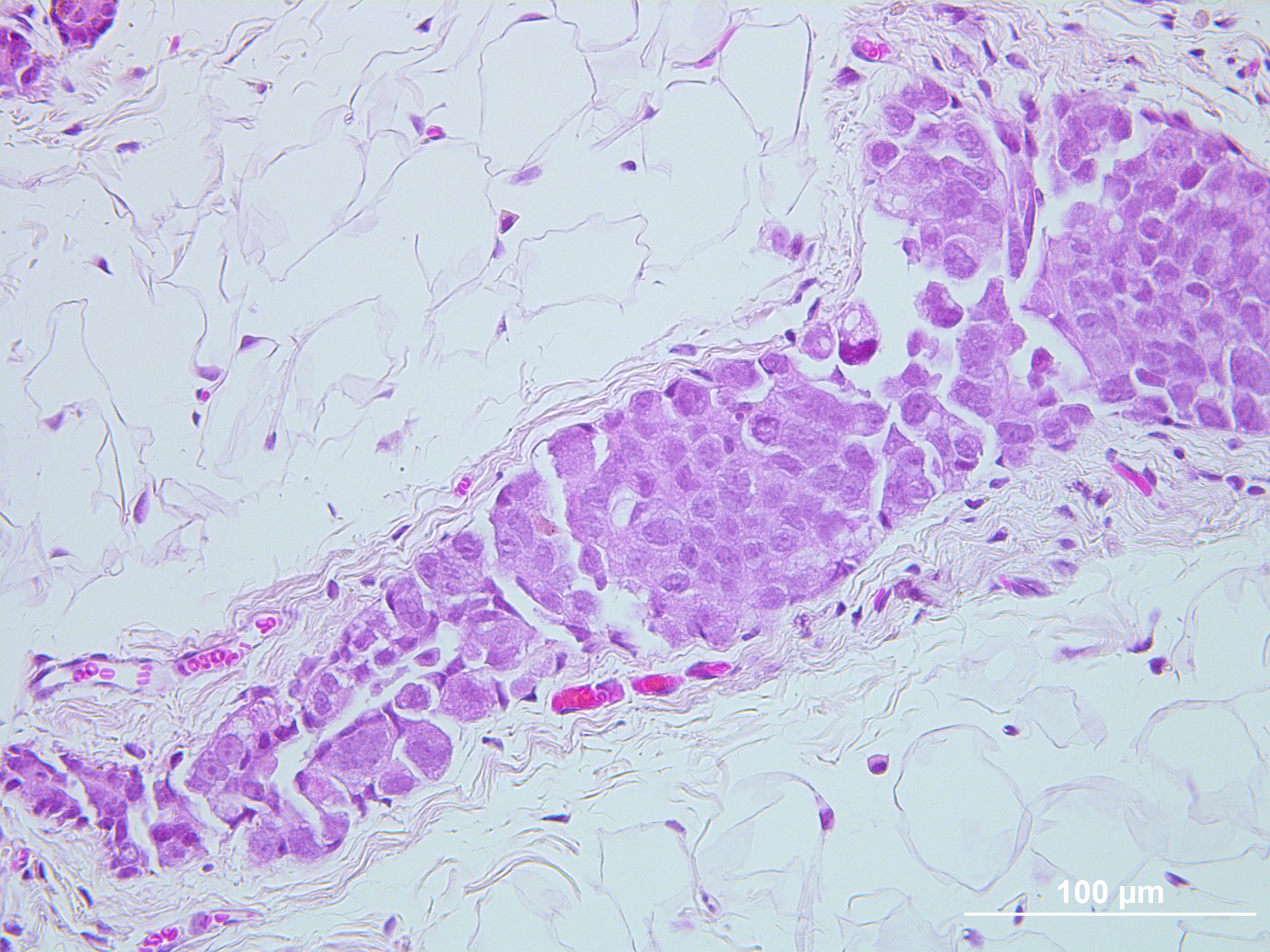

Supplement: Supplementary file 12 — Source Data for Figure 6 [file EMMM-12-e11908-s010.zip › SourceDataForFigure6_1.zip/Fig.6C/H&E_5.jpg]

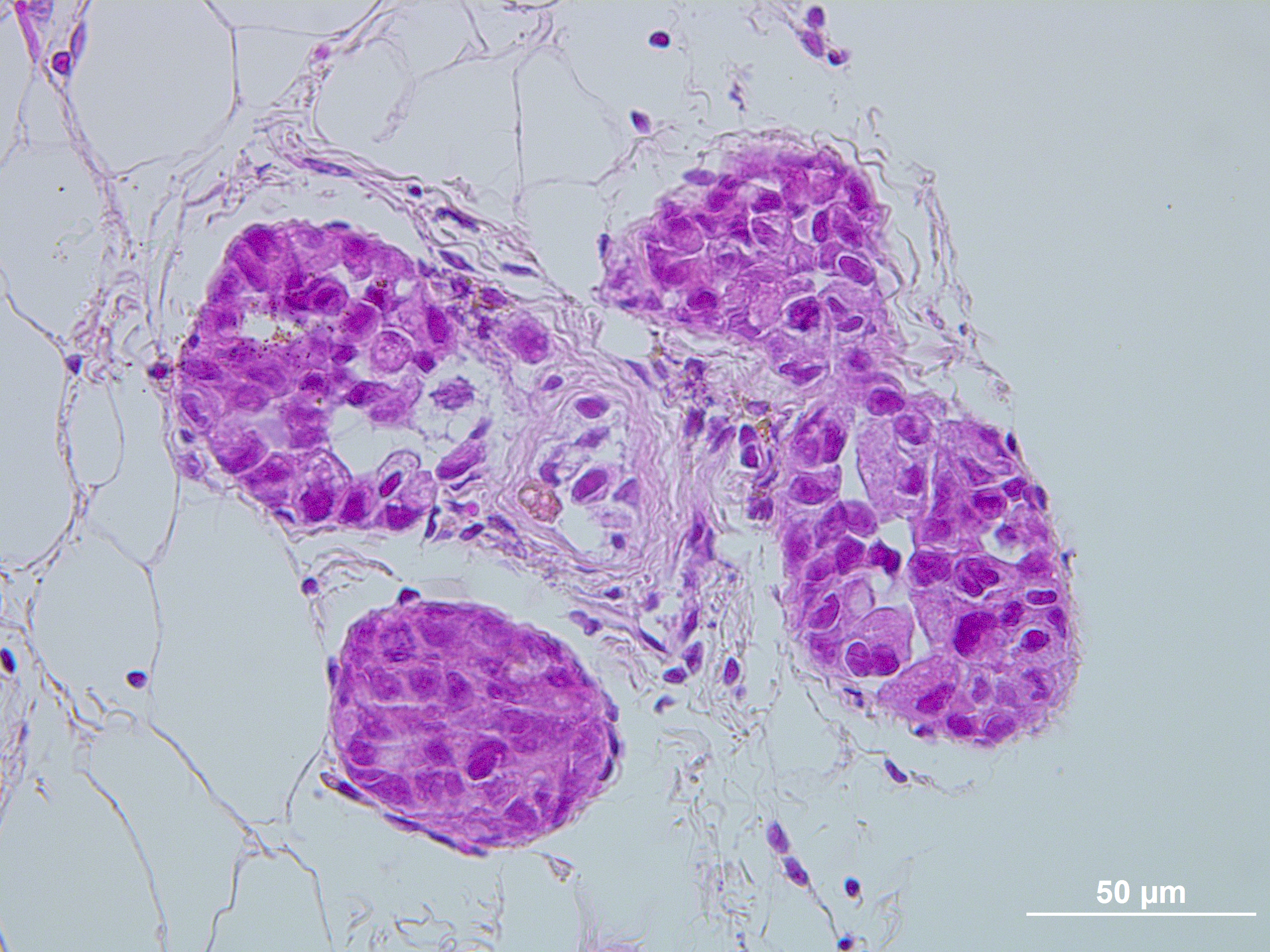

Supplement: Supplementary file 12 — Source Data for Figure 6 [file EMMM-12-e11908-s010.zip › SourceDataForFigure6_1.zip/Fig.6C/H&E_6.jpg]
