# Supplementary material for: Characterization of circulating breast cancer cells with tumorigenic and metastatic capacity
Source: EMBO Mol Med. 2020 Jul 15;12(9):e11908. doi: 10.15252/emmm.201911908 (PMC7507517; doi:10.15252/emmm.201911908)
Supplement: Supplementary file 10 — Source Data for Figure 4 [file EMMM-12-e11908-s008.zip › RE-EMM-2019-11908_SourceDataForFigure4A_B.pdf]

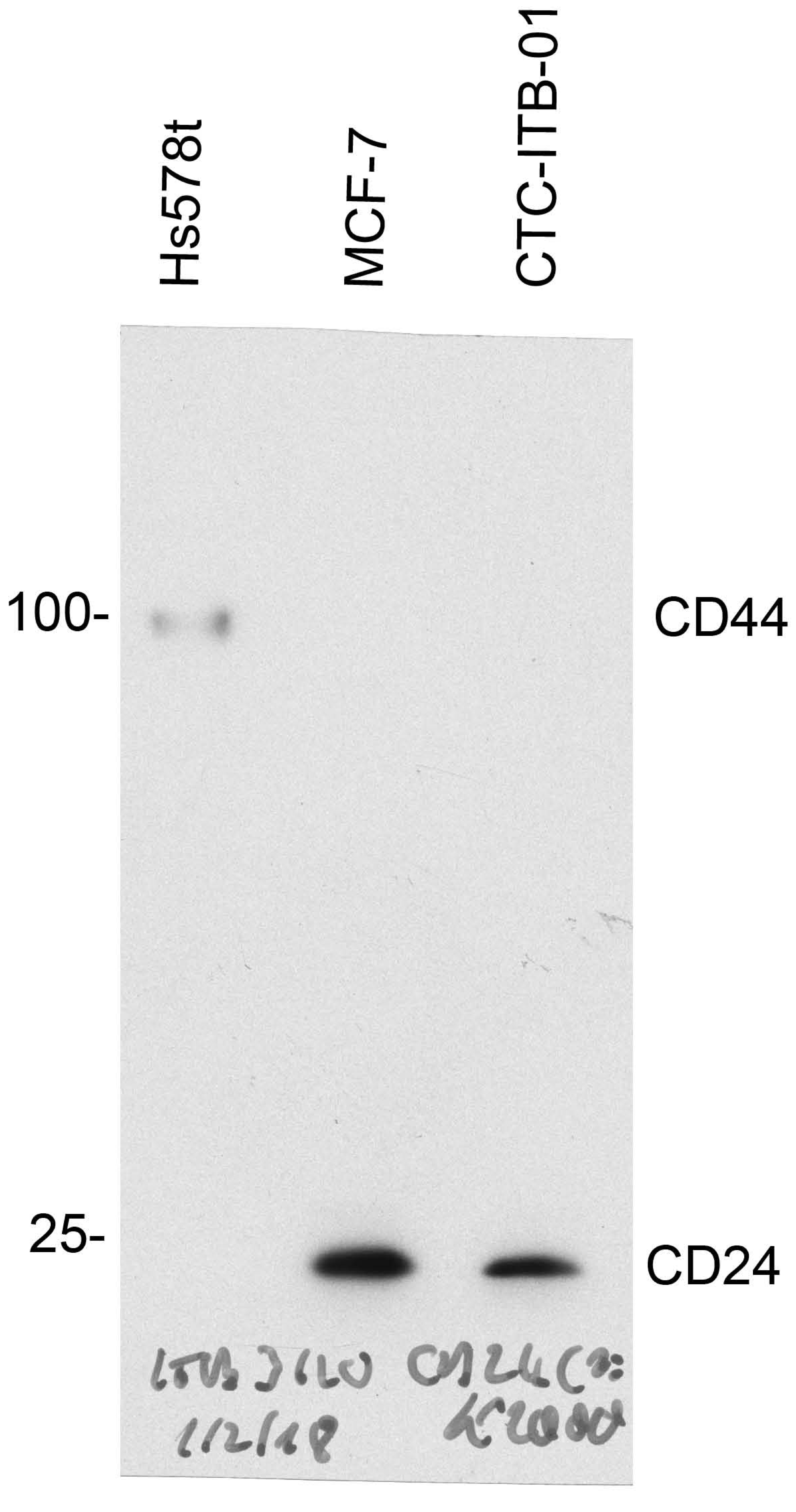
Hs578t
MCF-7
CTC-ITB-01
100-
25-
CD44
CD24

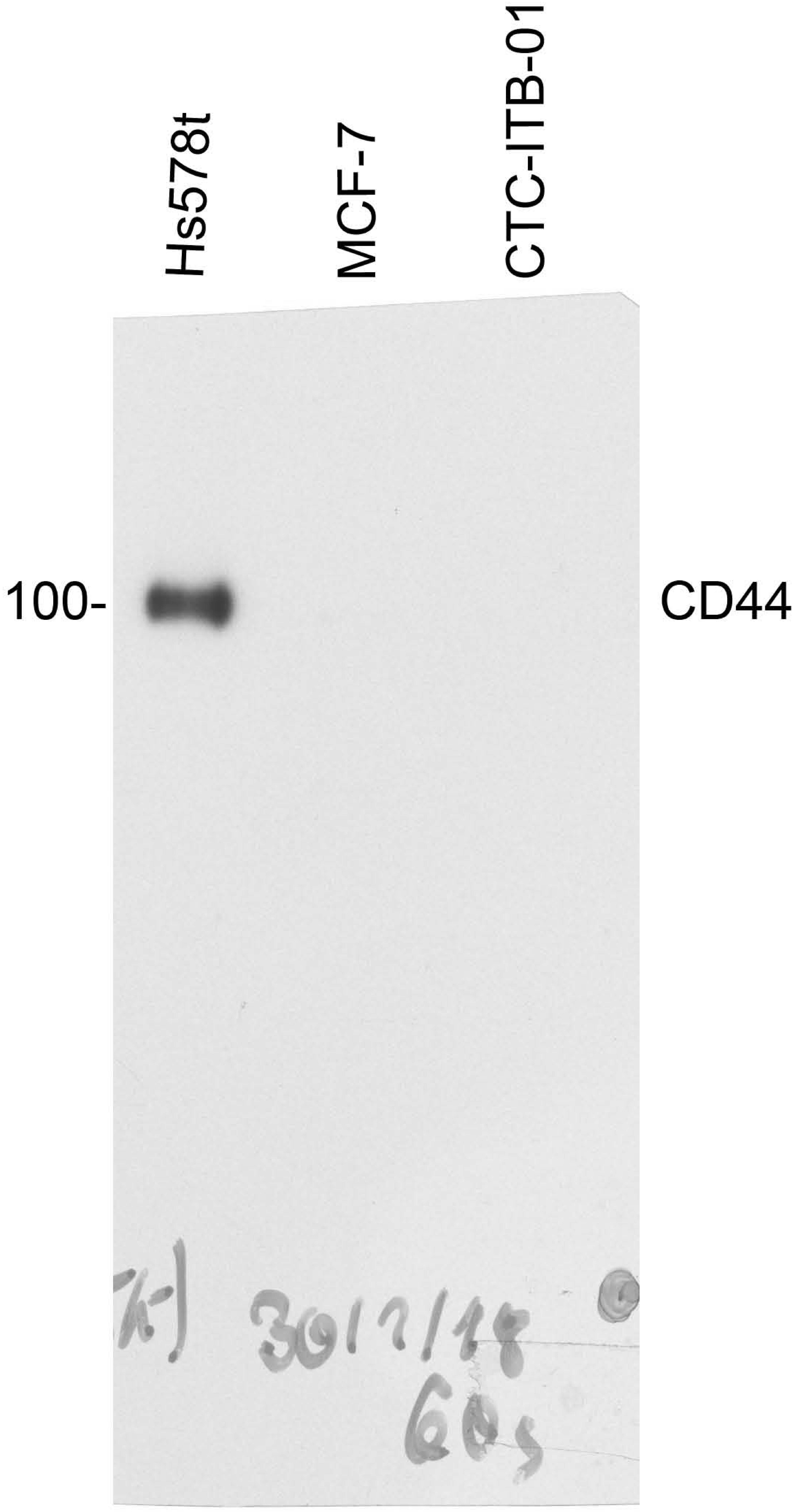

Hs578t
MCF-7
CTC-ITB-01
100-
CD44

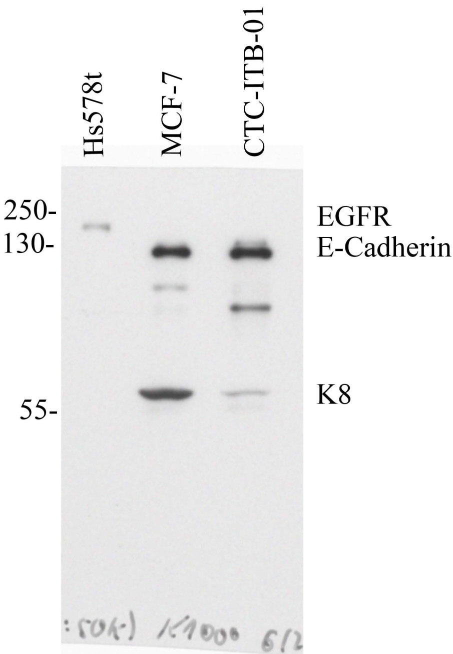
Hs578t
MCF-7
CTC-ITB-01
250-
130-
55-
EGFR
E-Cadherin
K8

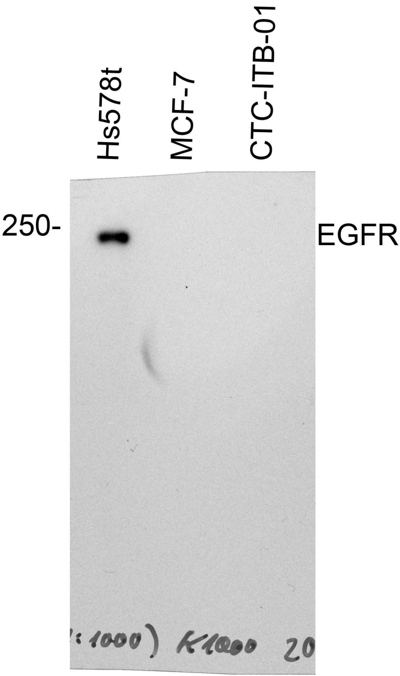
Hs578t
MCF-7
CTC-ITB-01
250-
EGFR

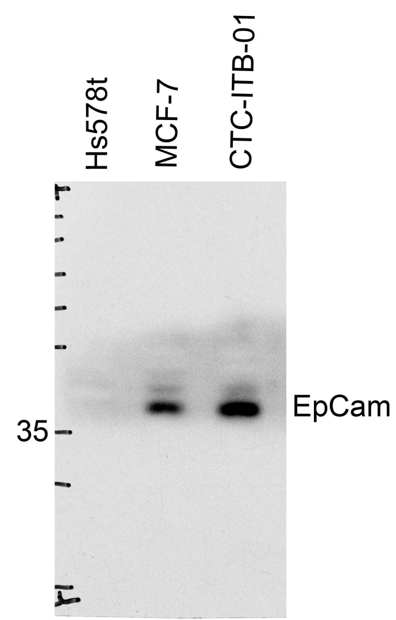

Hs578t
MCF-7
CTC-ITB-01
EpCam
35

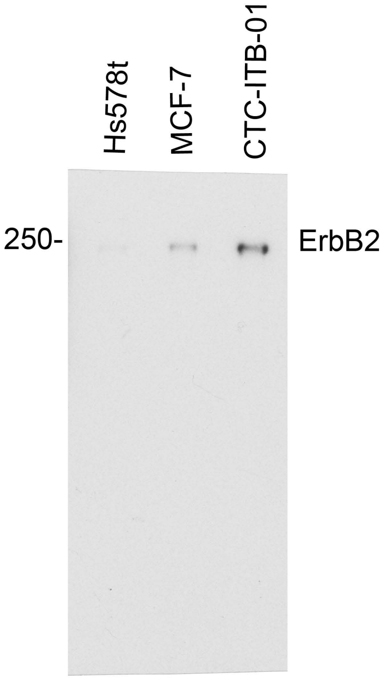
Hs578t
MCF-7
CTC-ITB-01
250-
ErbB2

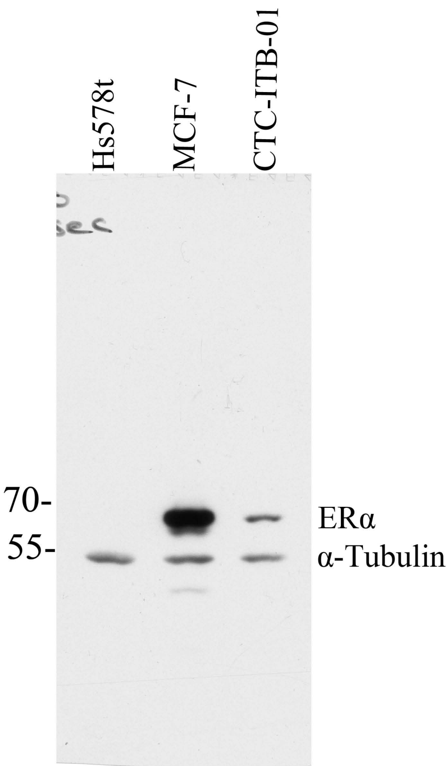
Hs578t
MCF-7
CTC-ITB-01
70-
55-
ERα
α-Tubulin

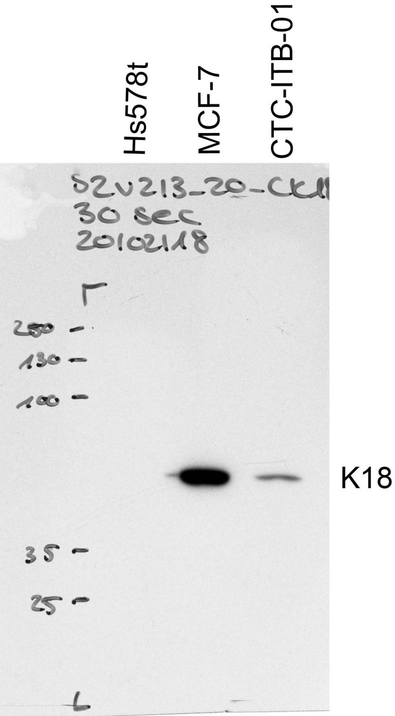

Hs578t
MCF-7
CTC-ITB-01
30 sec
250
130
100
35
25
K18

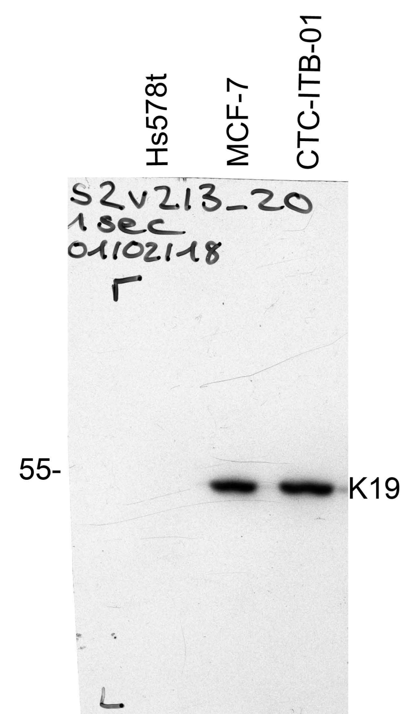
Hs578t
MCF-7
CTC-ITB-01
S2v213_20
1sec
01/02/18
55-
K19

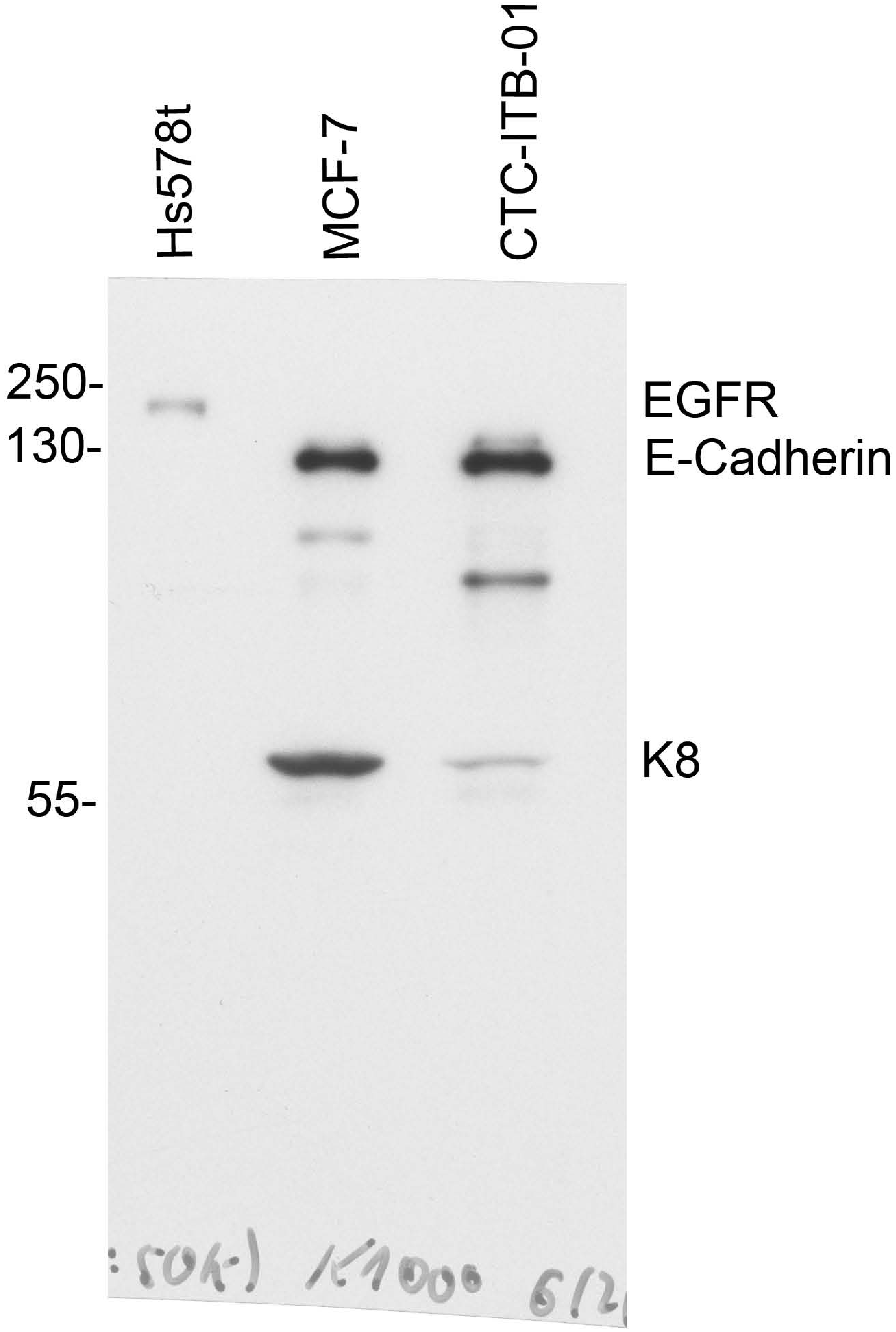
Hs578t
MCF-7
CTC-ITB-01
250-
130-
55-
EGFR
E-Cadherin
K8

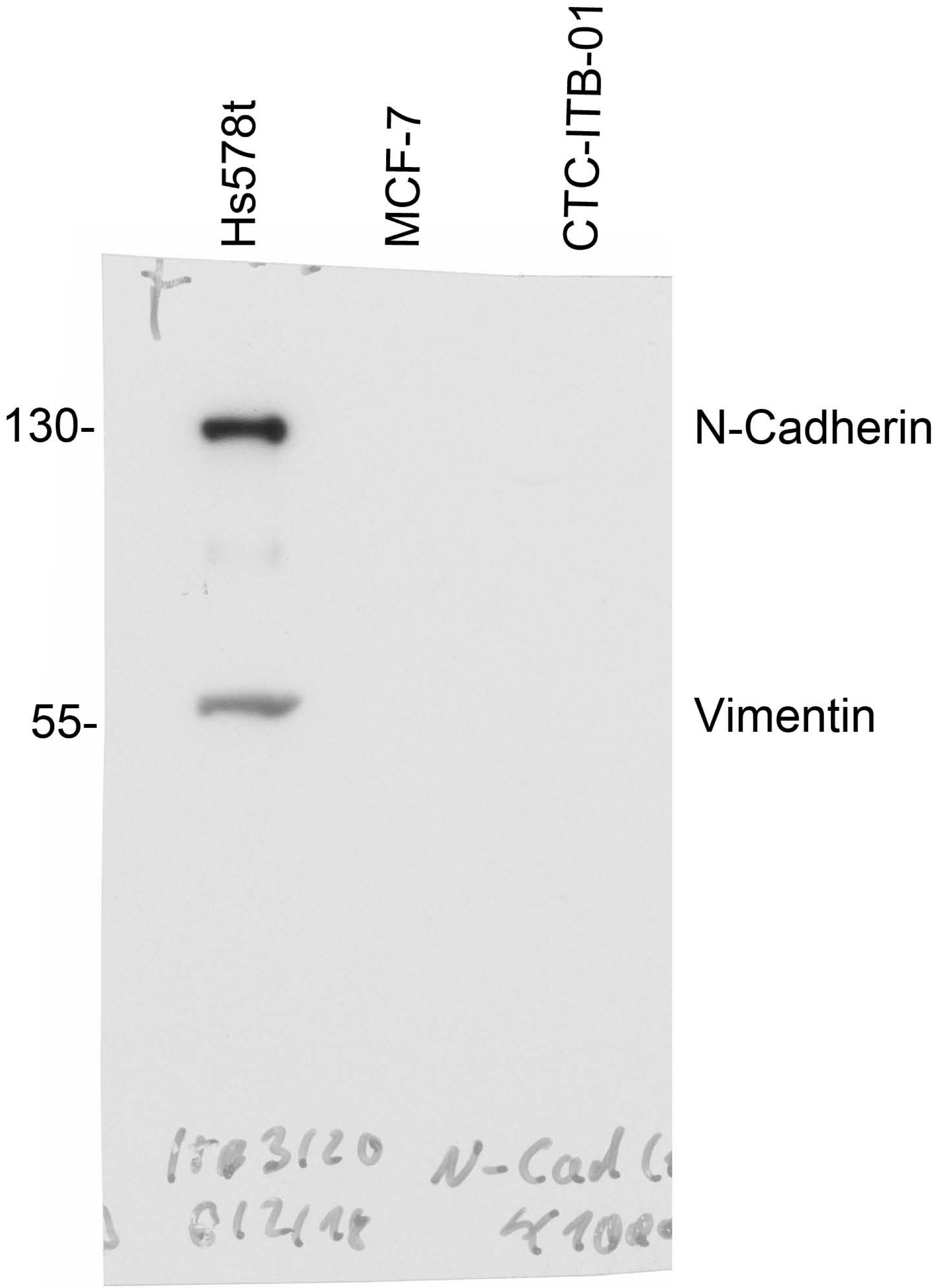
Hs578t
MCF-7
CTC-ITB-01
130-
55-
N-Cadherin
Vimentin

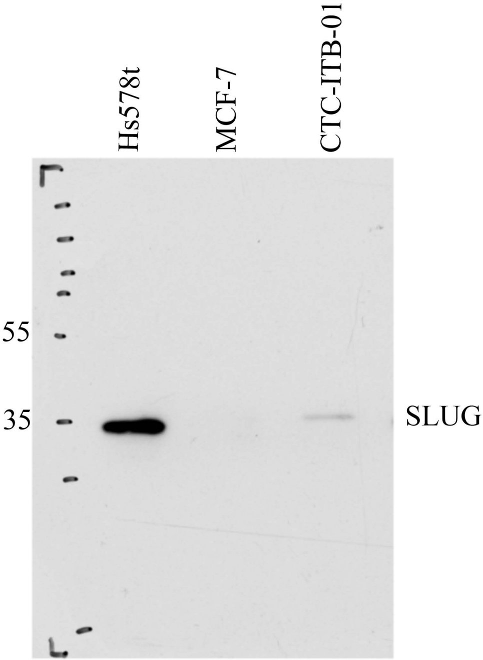

Hs578t
MCF-7
CTC-ITB-01
55
35
SLUG

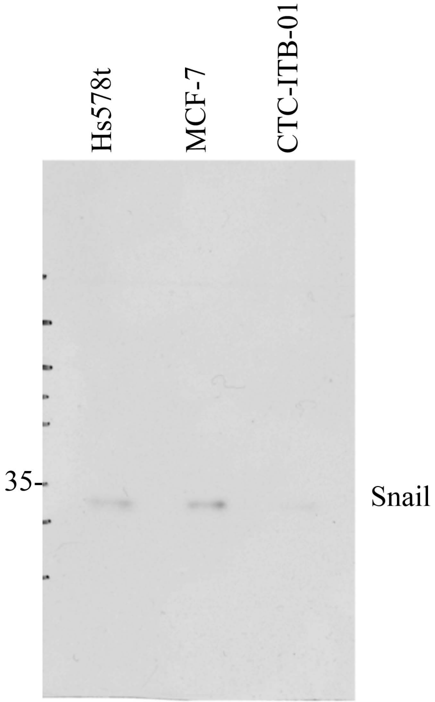
Hs578t
MCF-7
CTC-ITB-01
35-
Snail

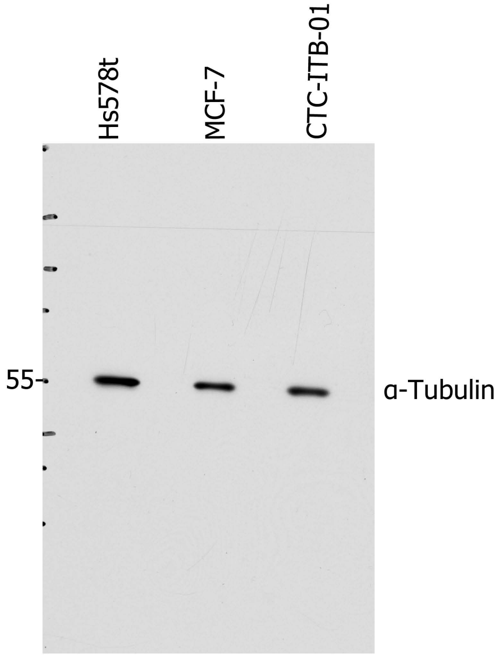

Hs578t
MCF-7
CTC-ITB-01
55-
α-Tubulin

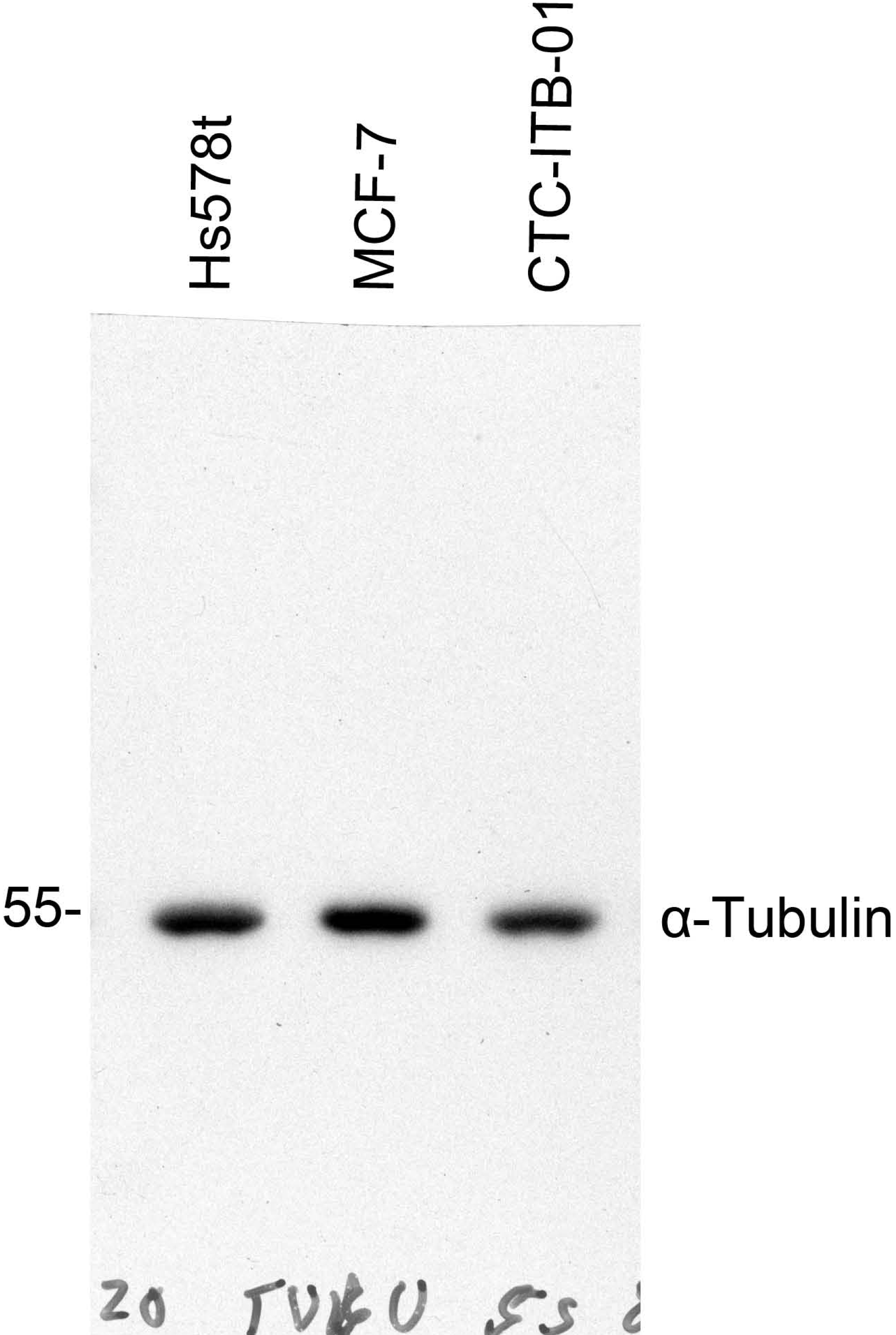
Hs578t
MCF-7
CTC-ITB-01
55-
α-Tubulin

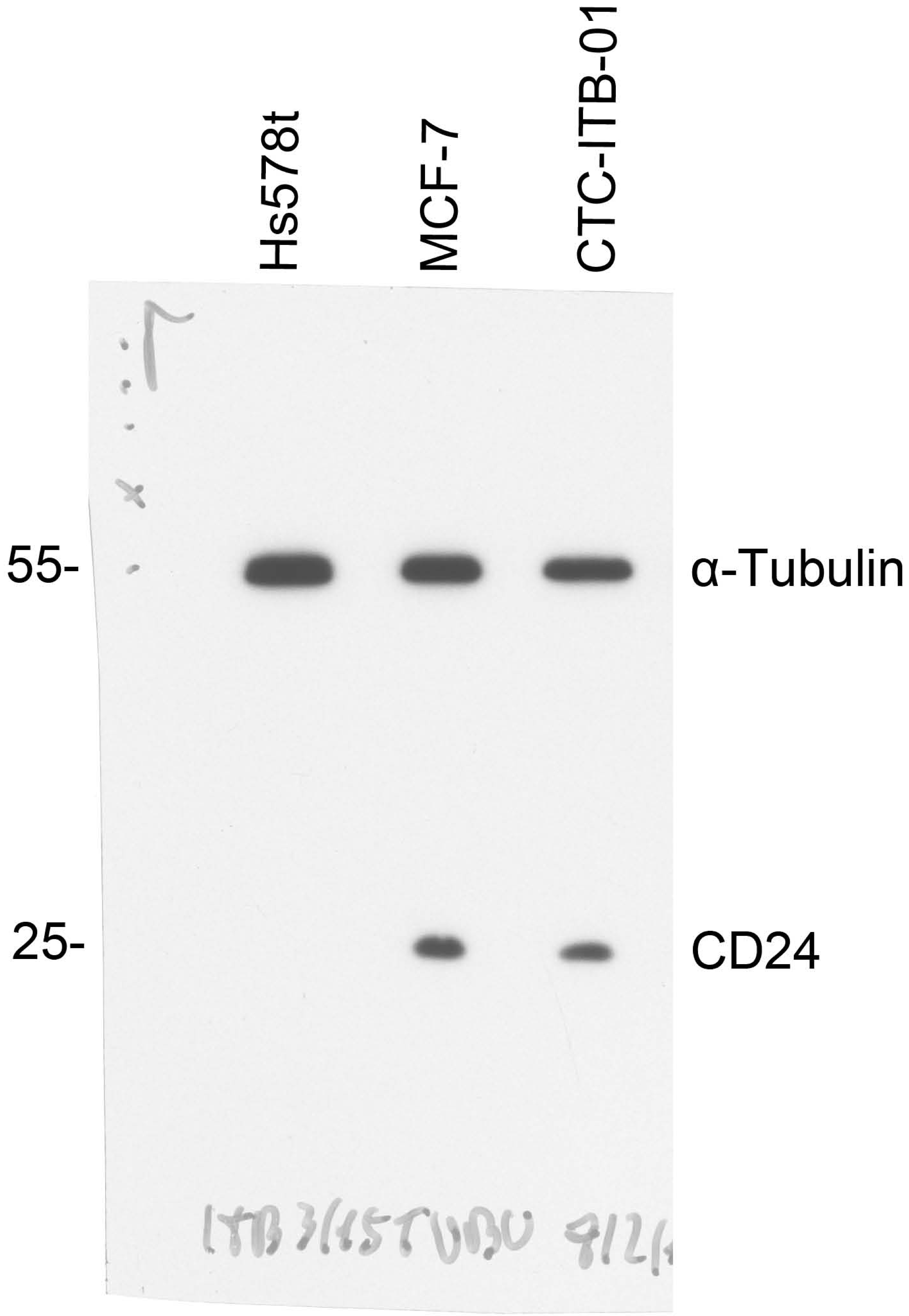
Hs578t
MCF-7
CTC-ITB-01
55-
α-Tubulin
25-
CD24

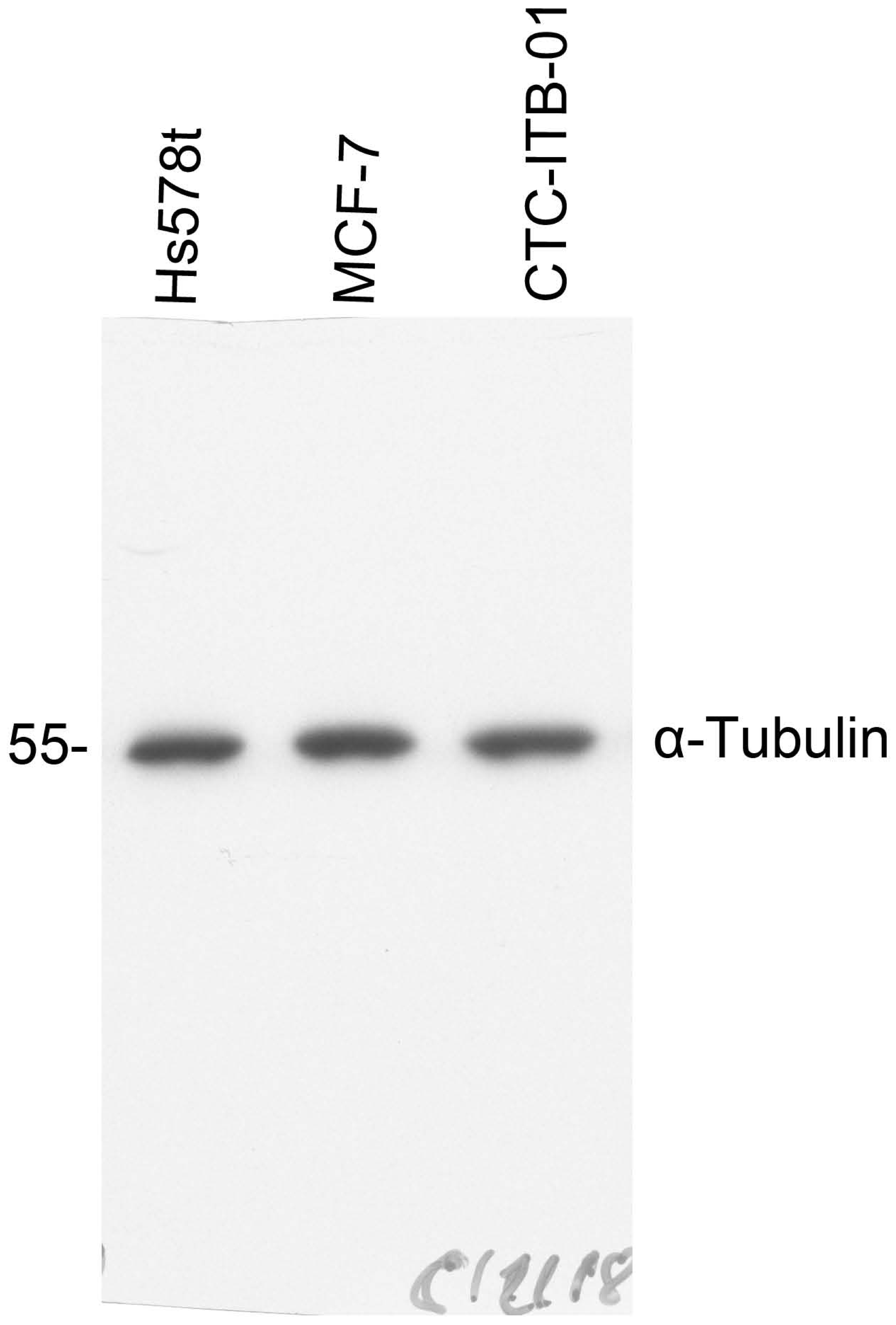
Hs578t
MCF-7
CTC-ITB-01
55-
α-Tubulin

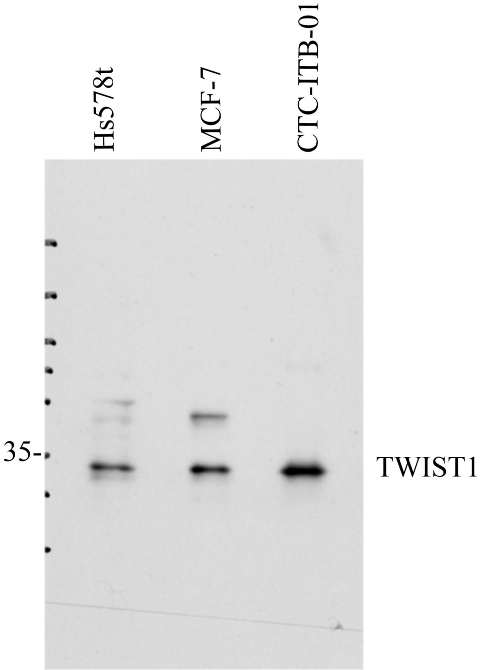
Hs578t
MCF-7
CTC-ITB-01
35-
TWIST1

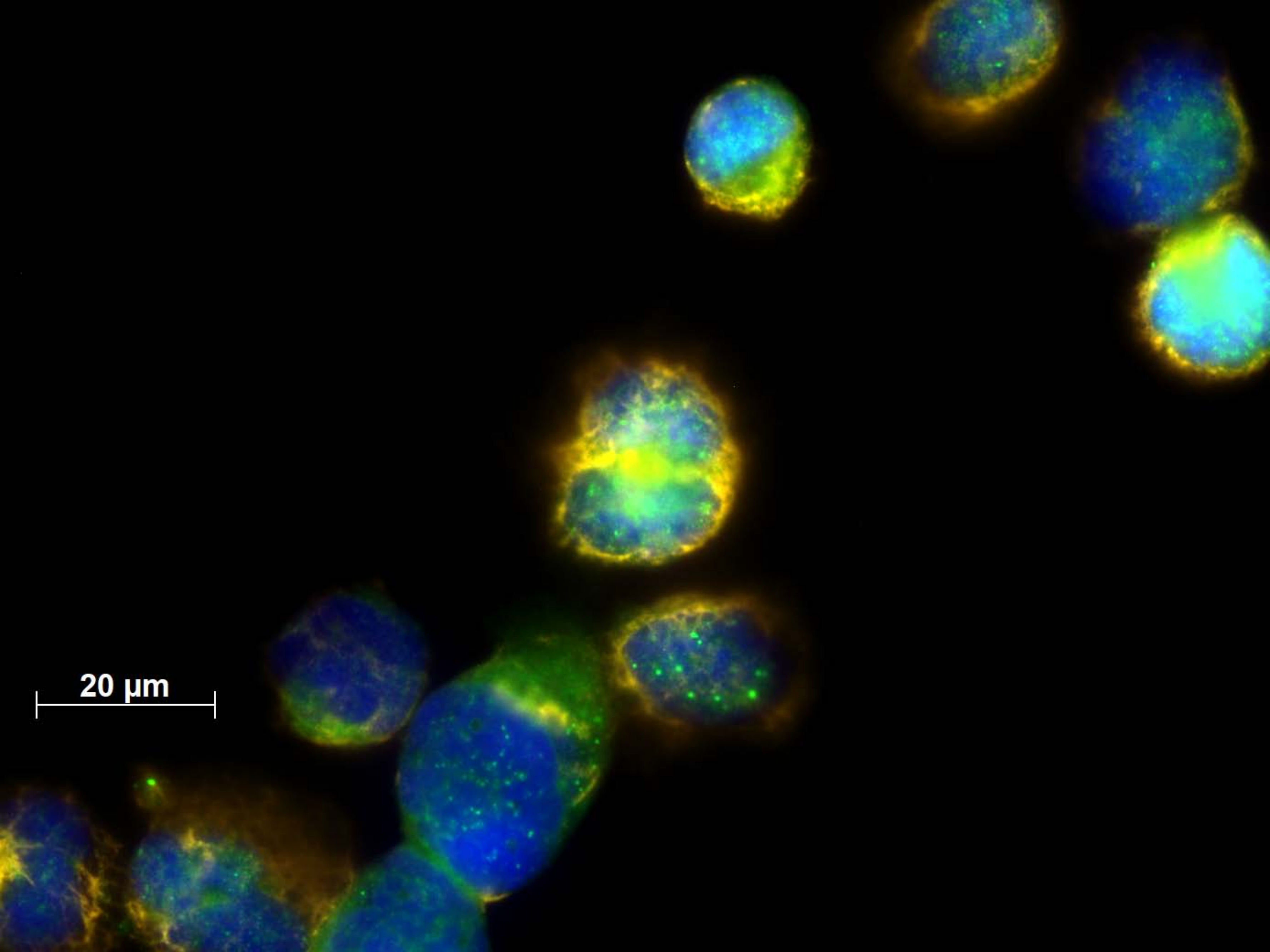
20 µm

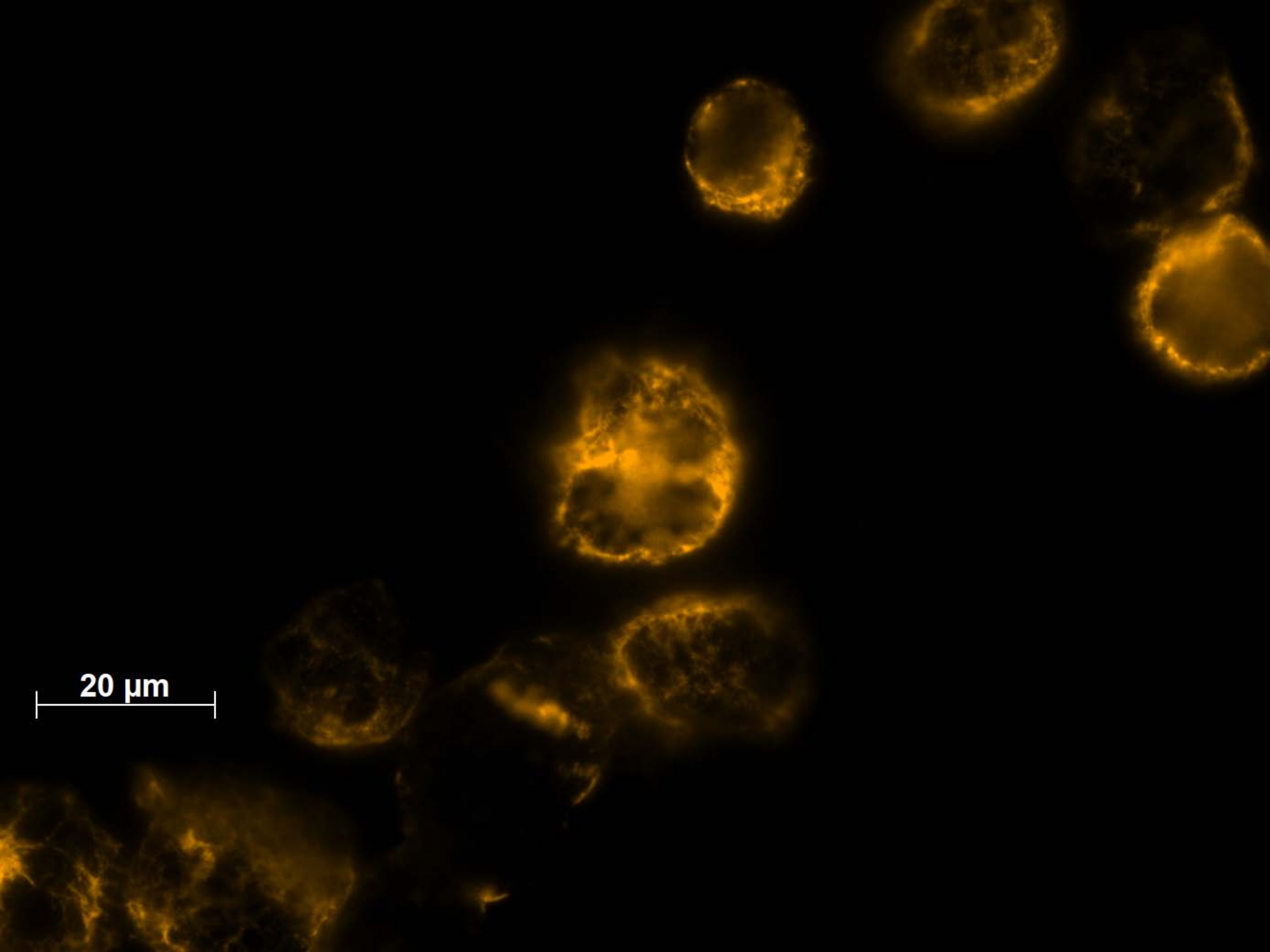
20 µm

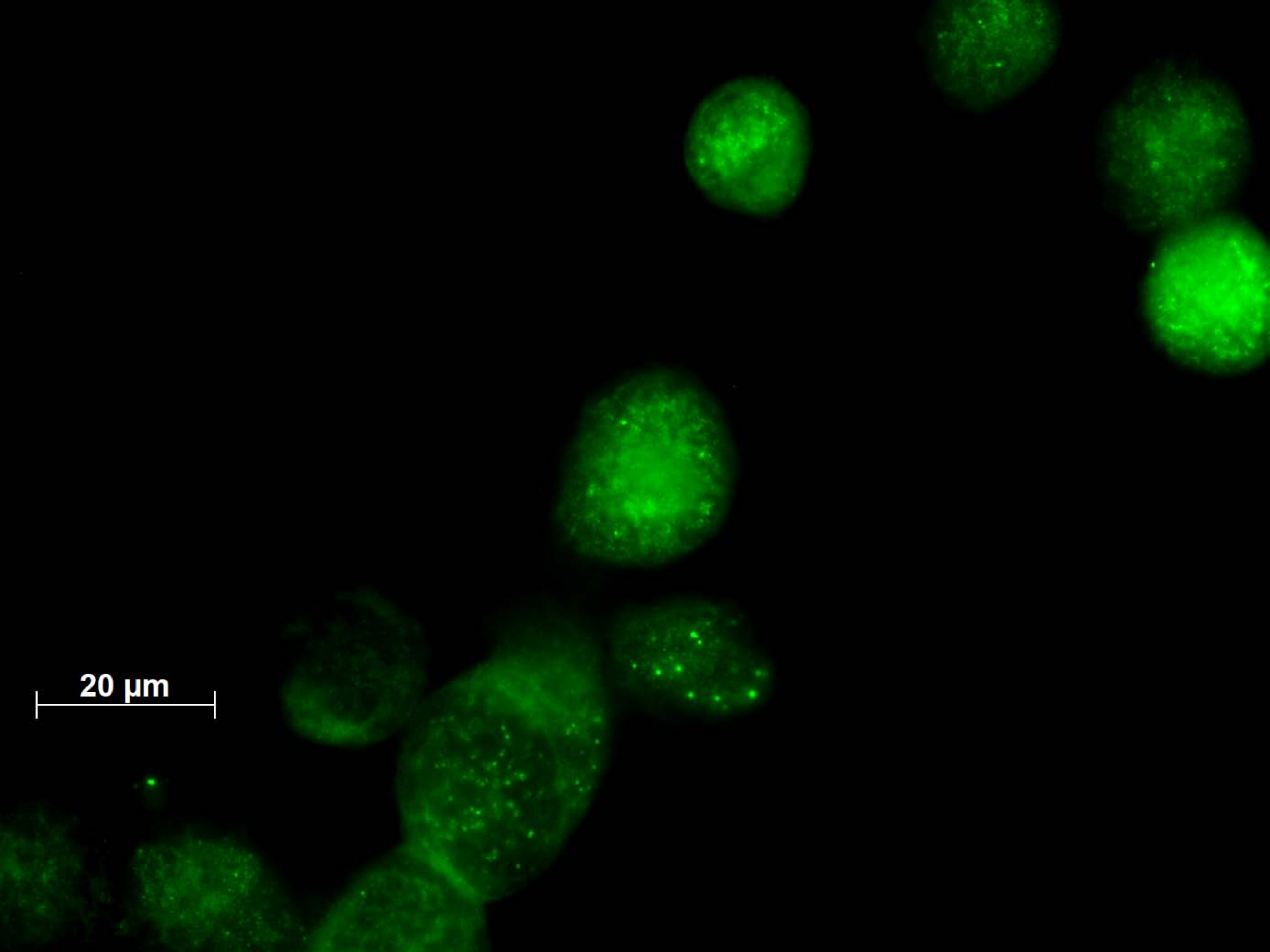
20 µm

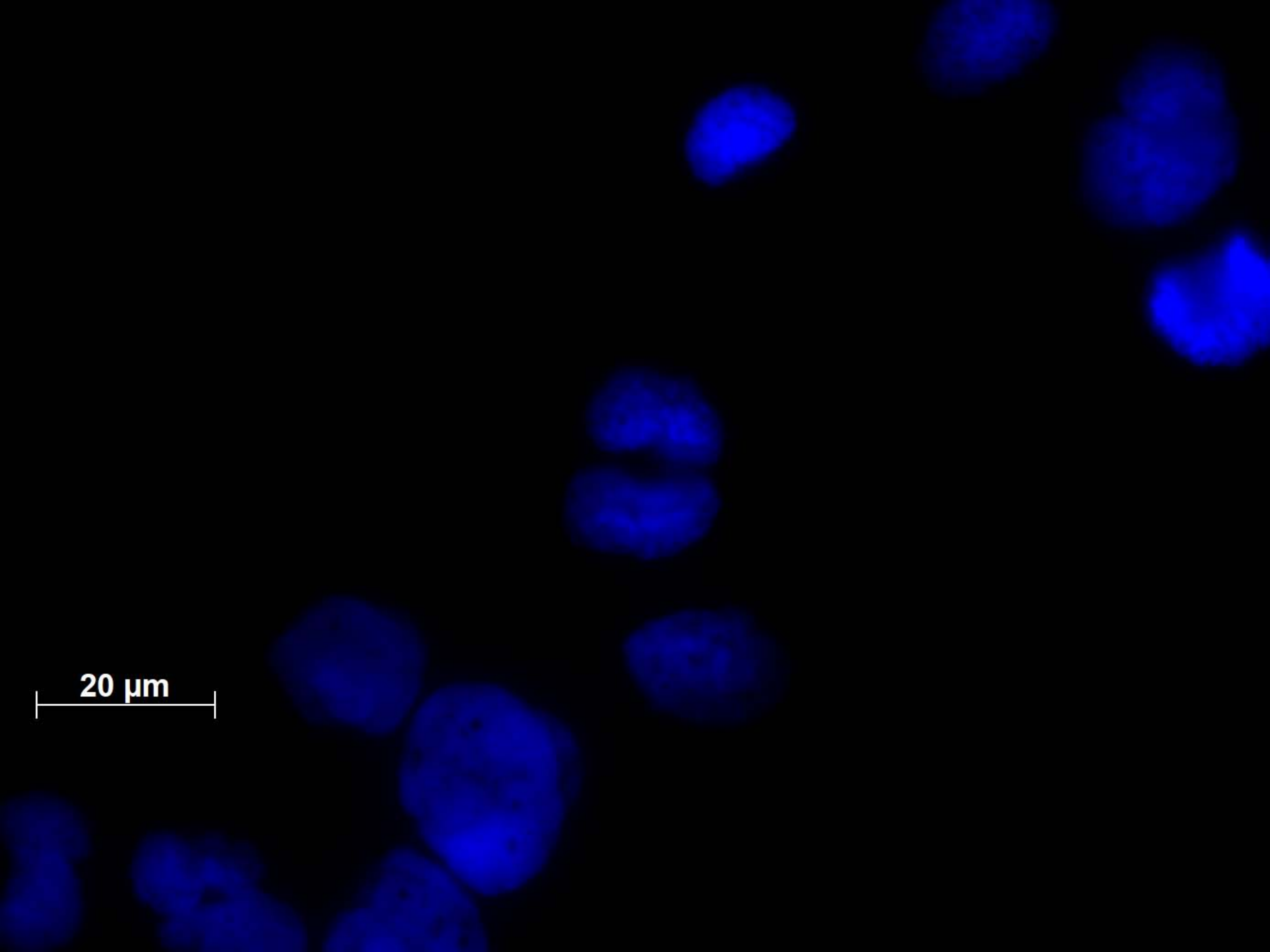
20 µm

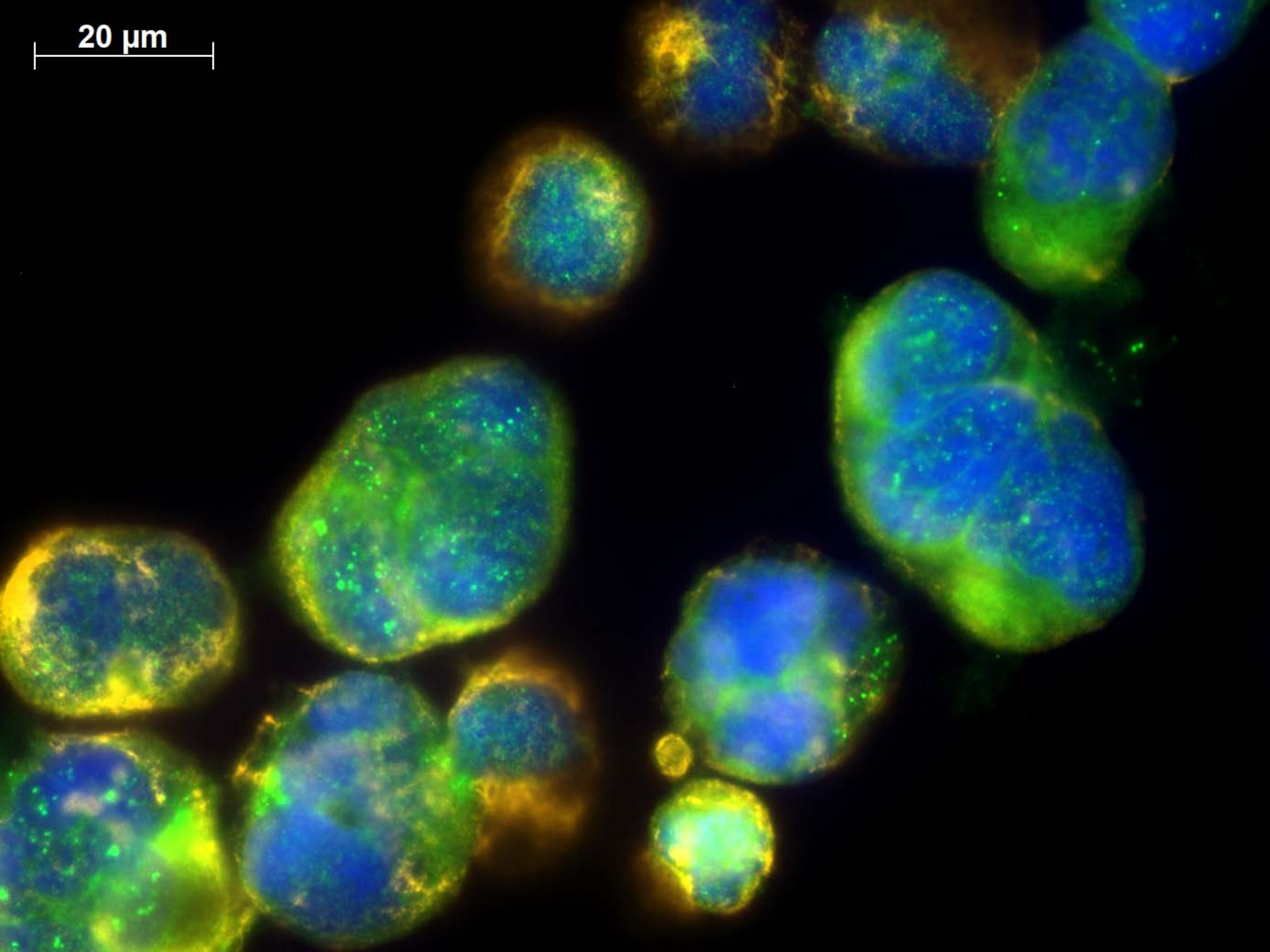
20 µm

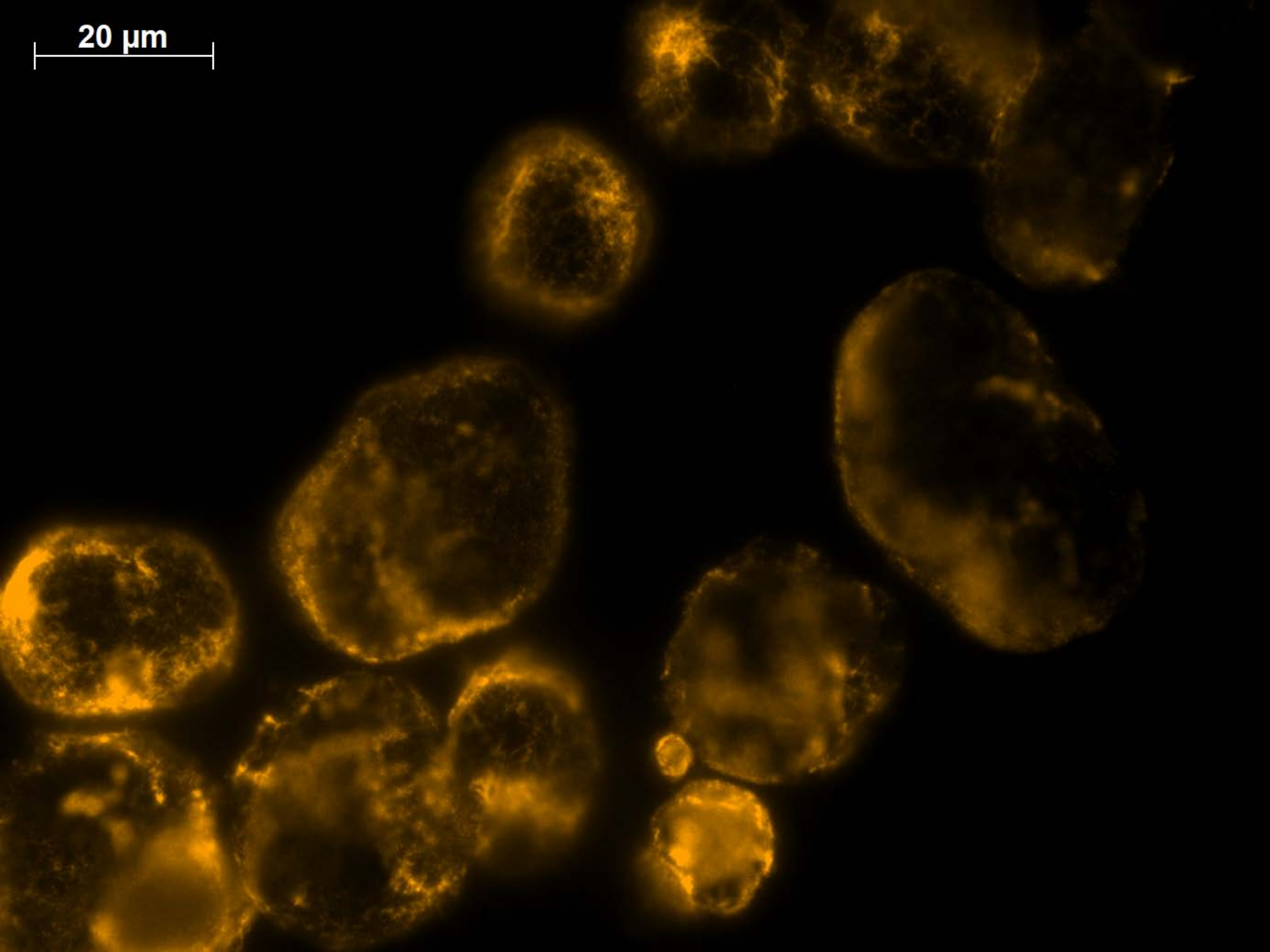
20 µm

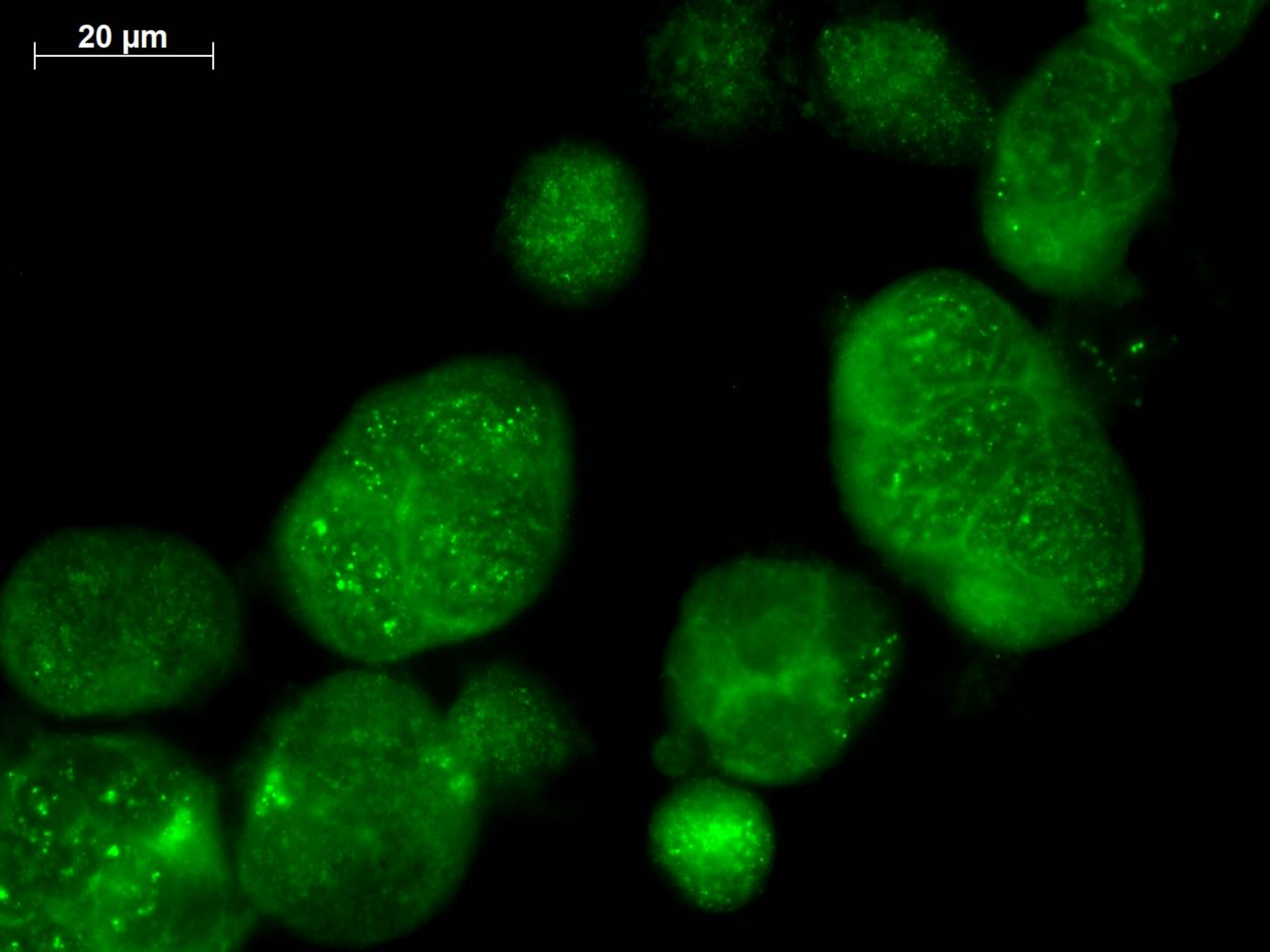
20 µm

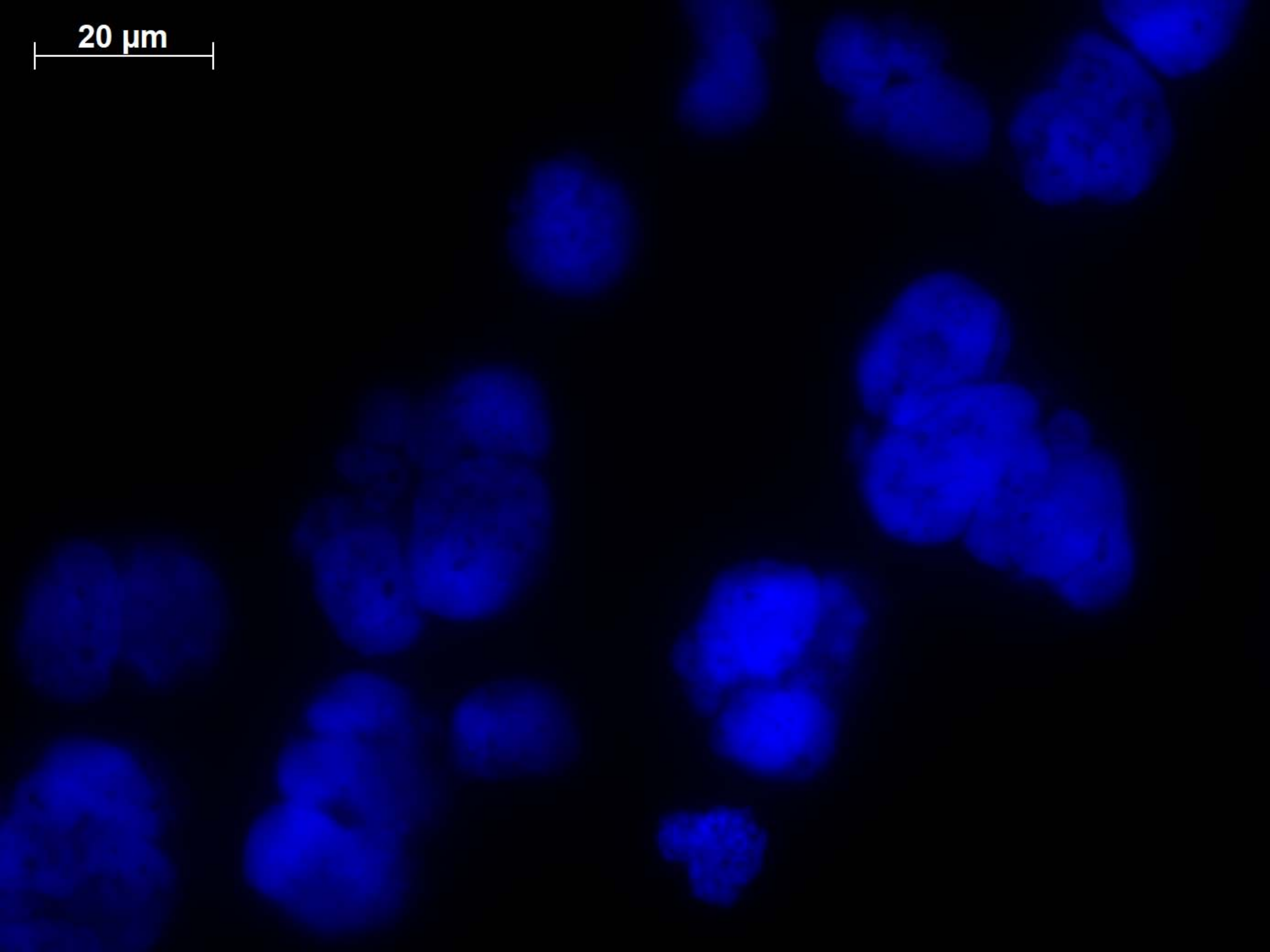
20 µm

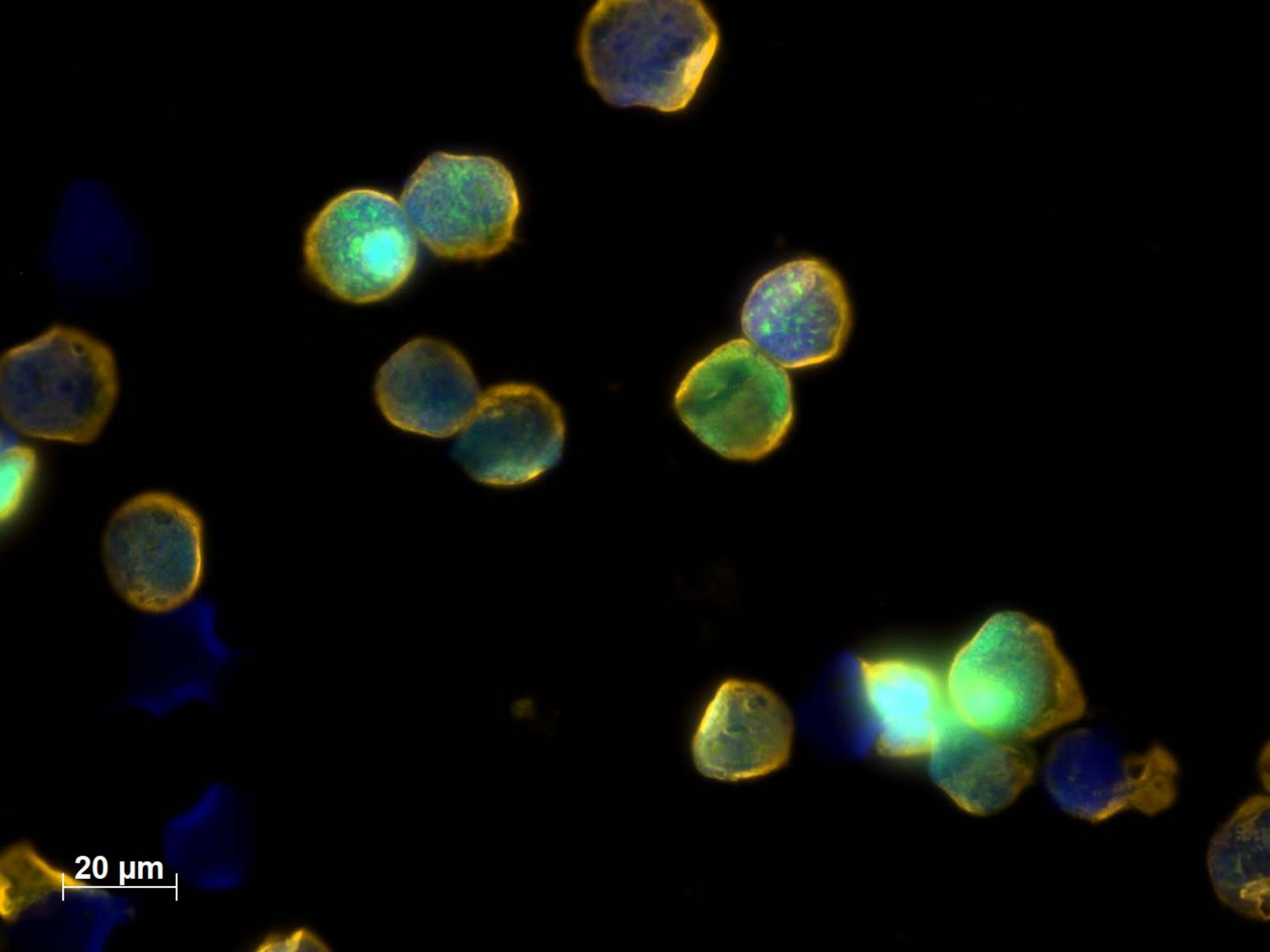
20 µm

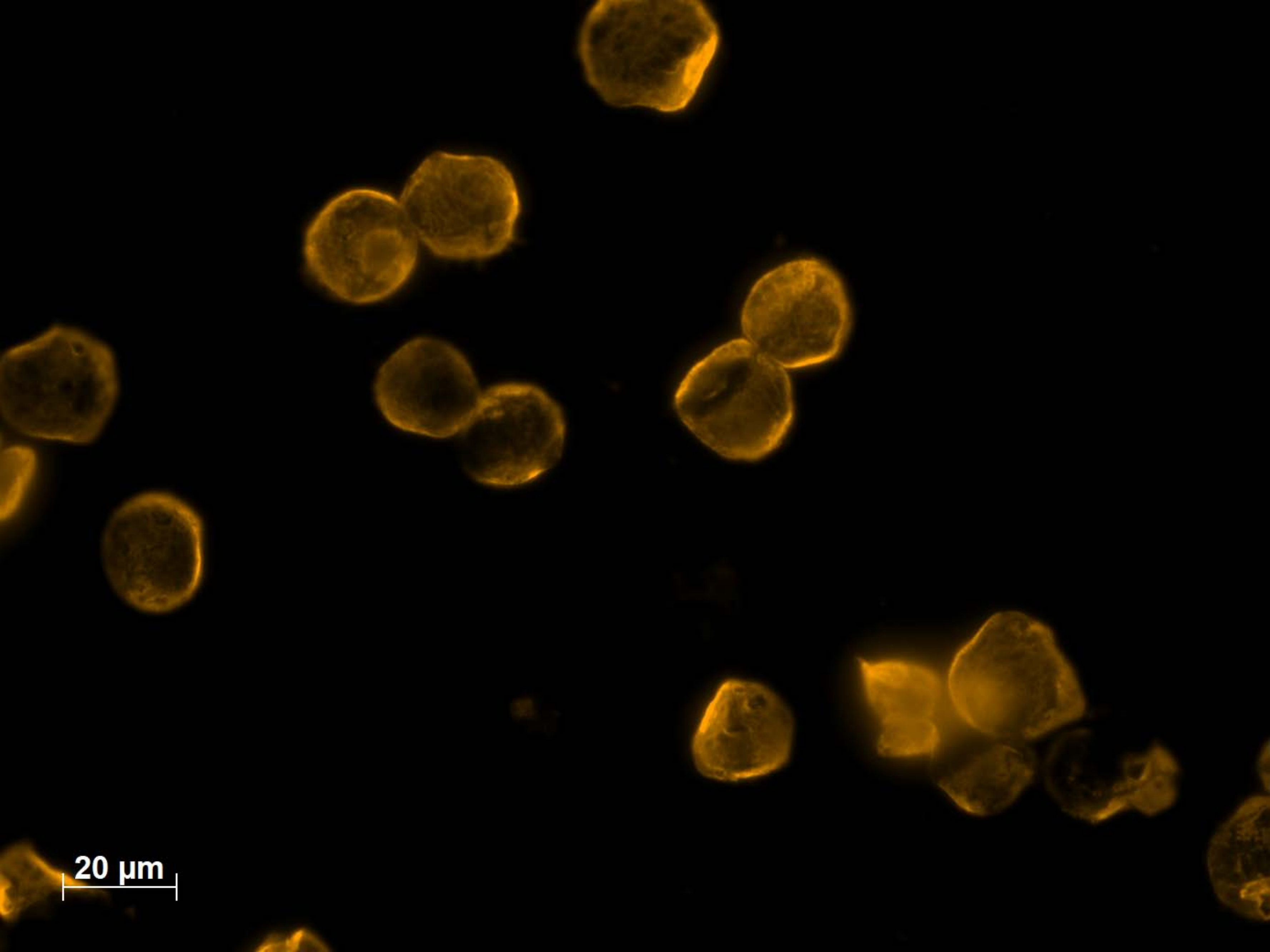
20 µm

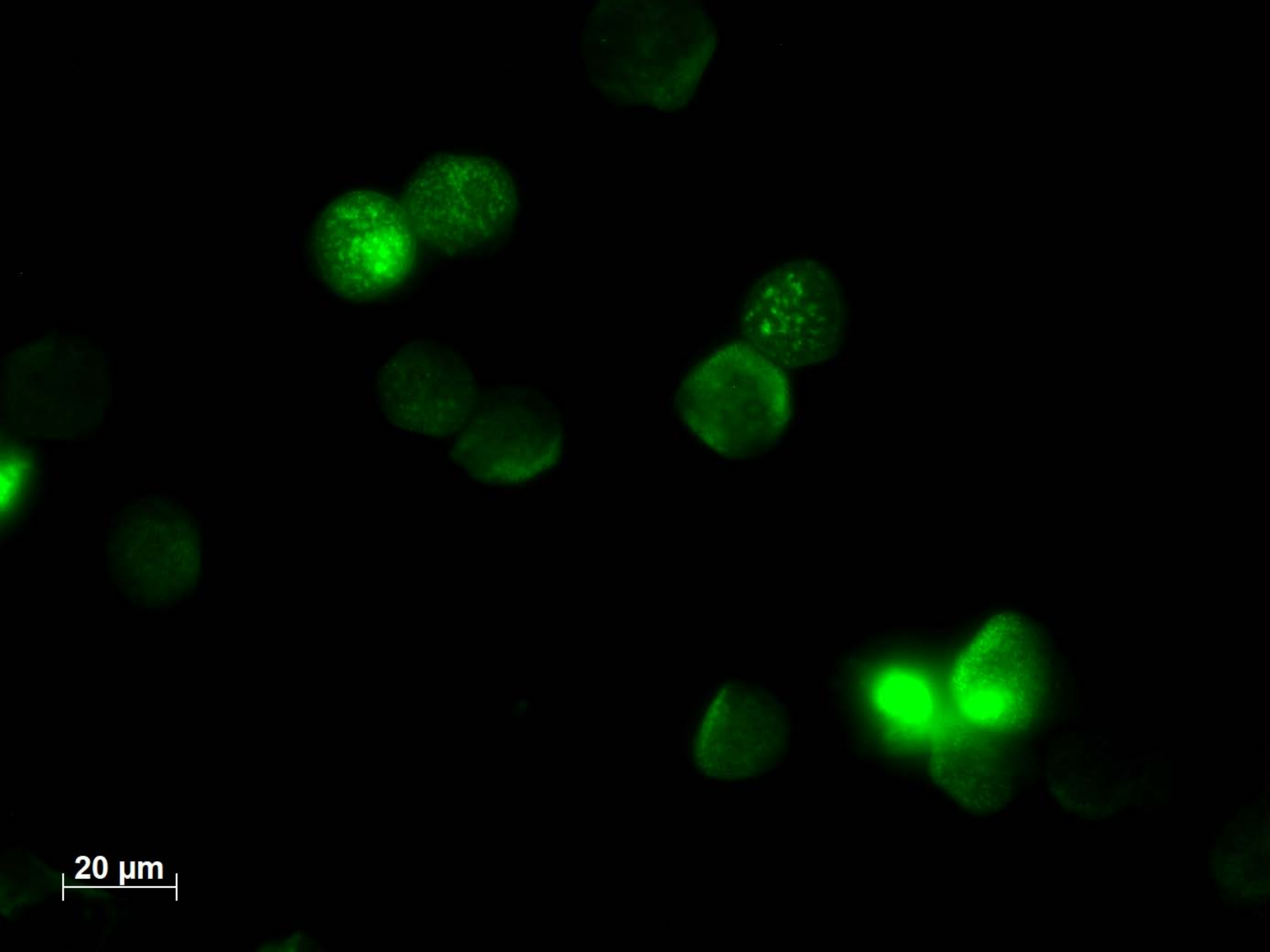
20 µm

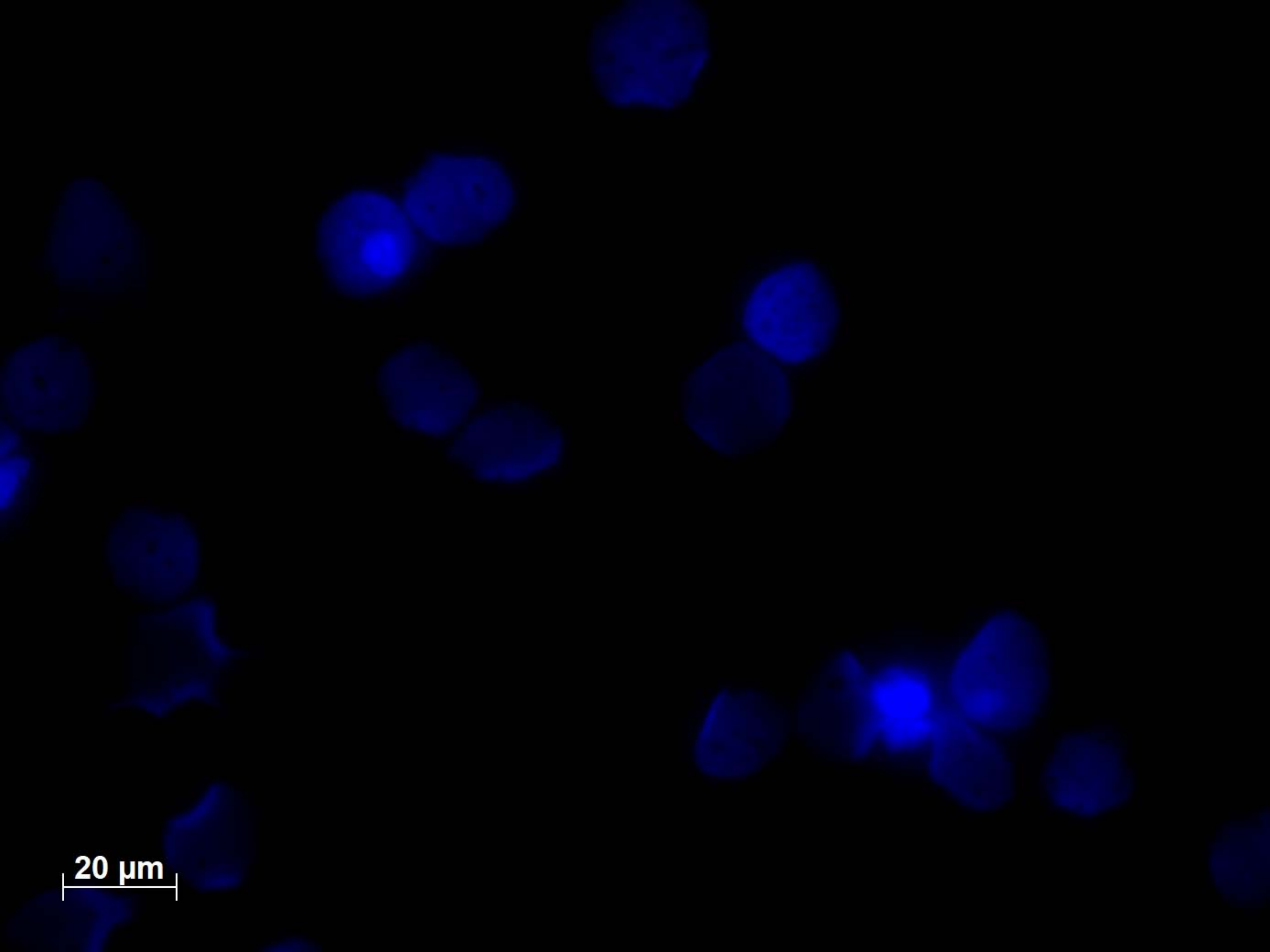
20 µm
