# Supplementary material for: Characterization of circulating breast cancer cells with tumorigenic and metastatic capacity
Source: EMBO Mol Med. 2020 Jul 15;12(9):e11908. doi: 10.15252/emmm.201911908 (PMC7507517; doi:10.15252/emmm.201911908)
Supplement: Supplementary file 13 — Source Data for Figure 7 [file EMMM-12-e11908-s011.zip › EMM-2019-11908_SourceDataForFigure7A-C.pdf.pdf]

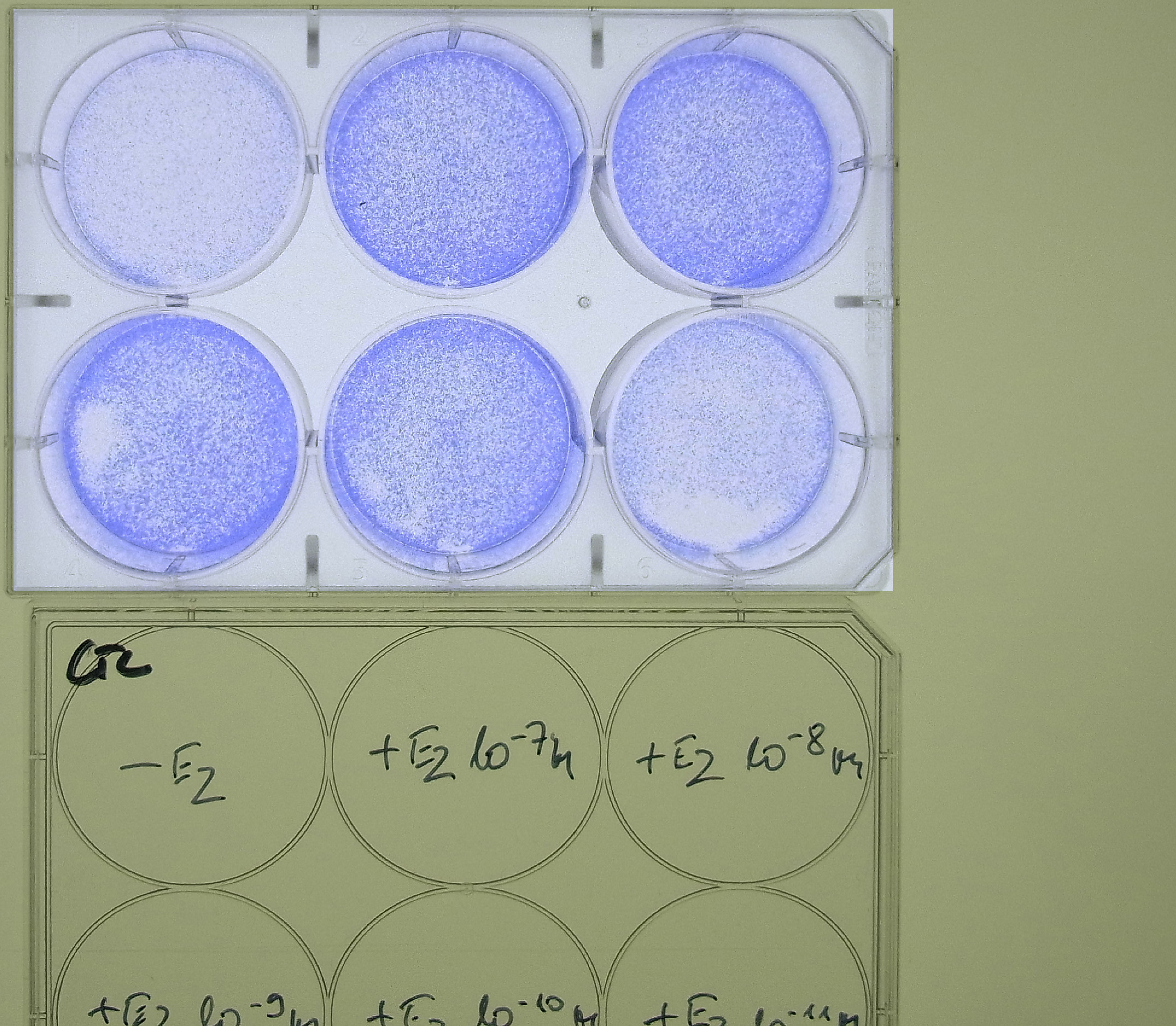
−E2
+E2 10-7 M
+E2 10-8 M

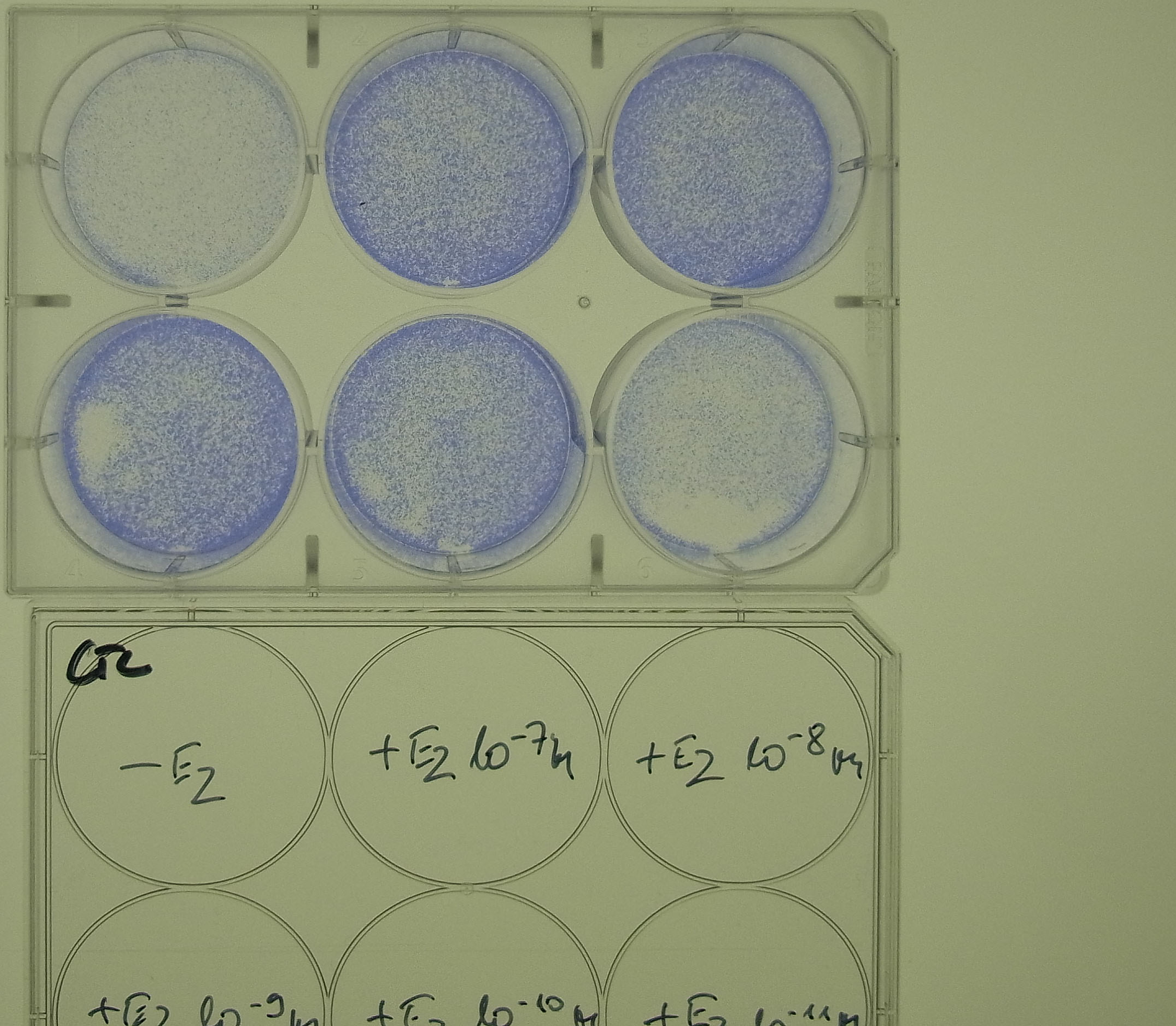
−E2
+E2 10-7 M
+E2 10-8 M
+E2 10-9 M
+E2 10-10 M
+E2 10-11 M

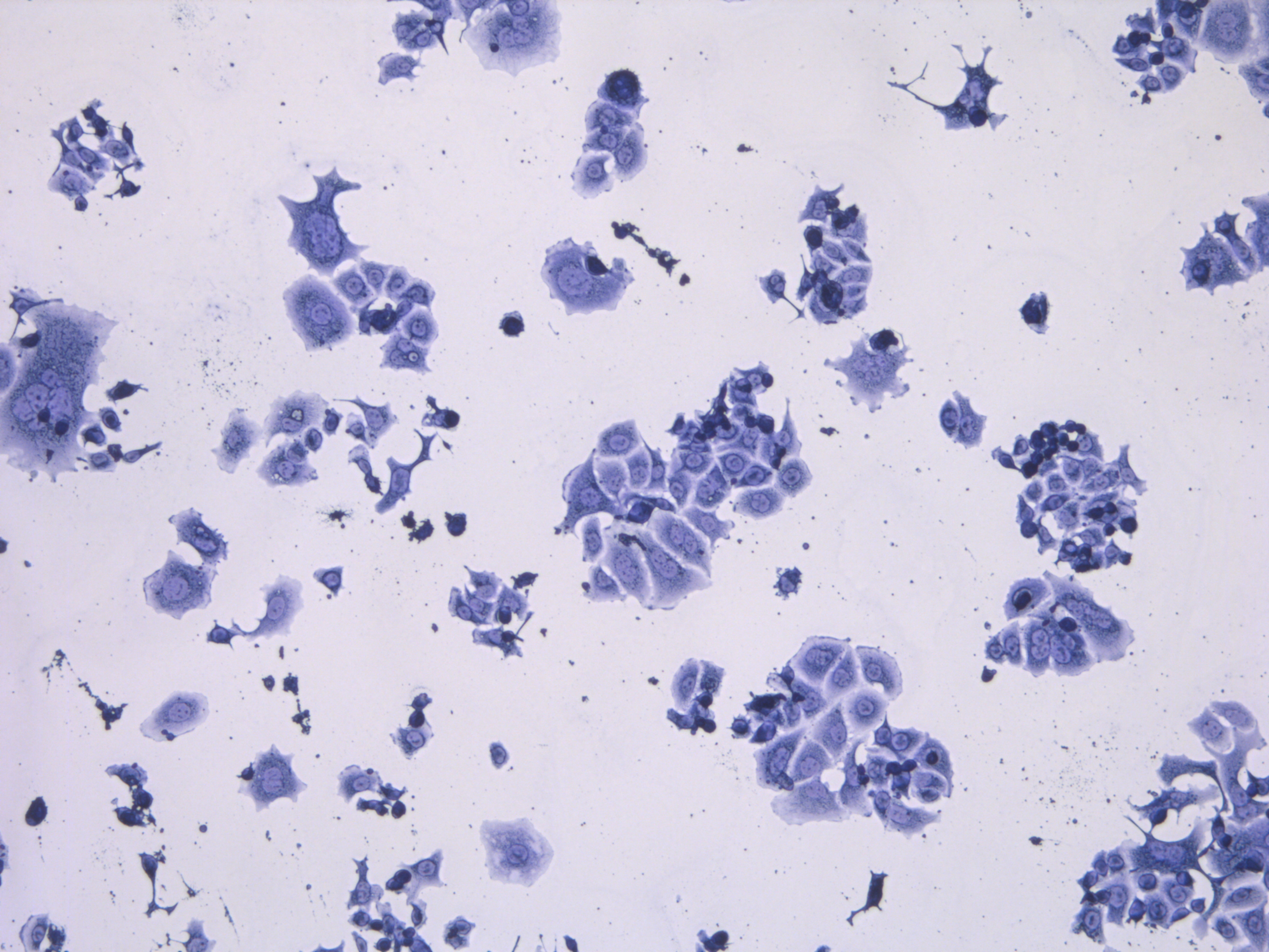

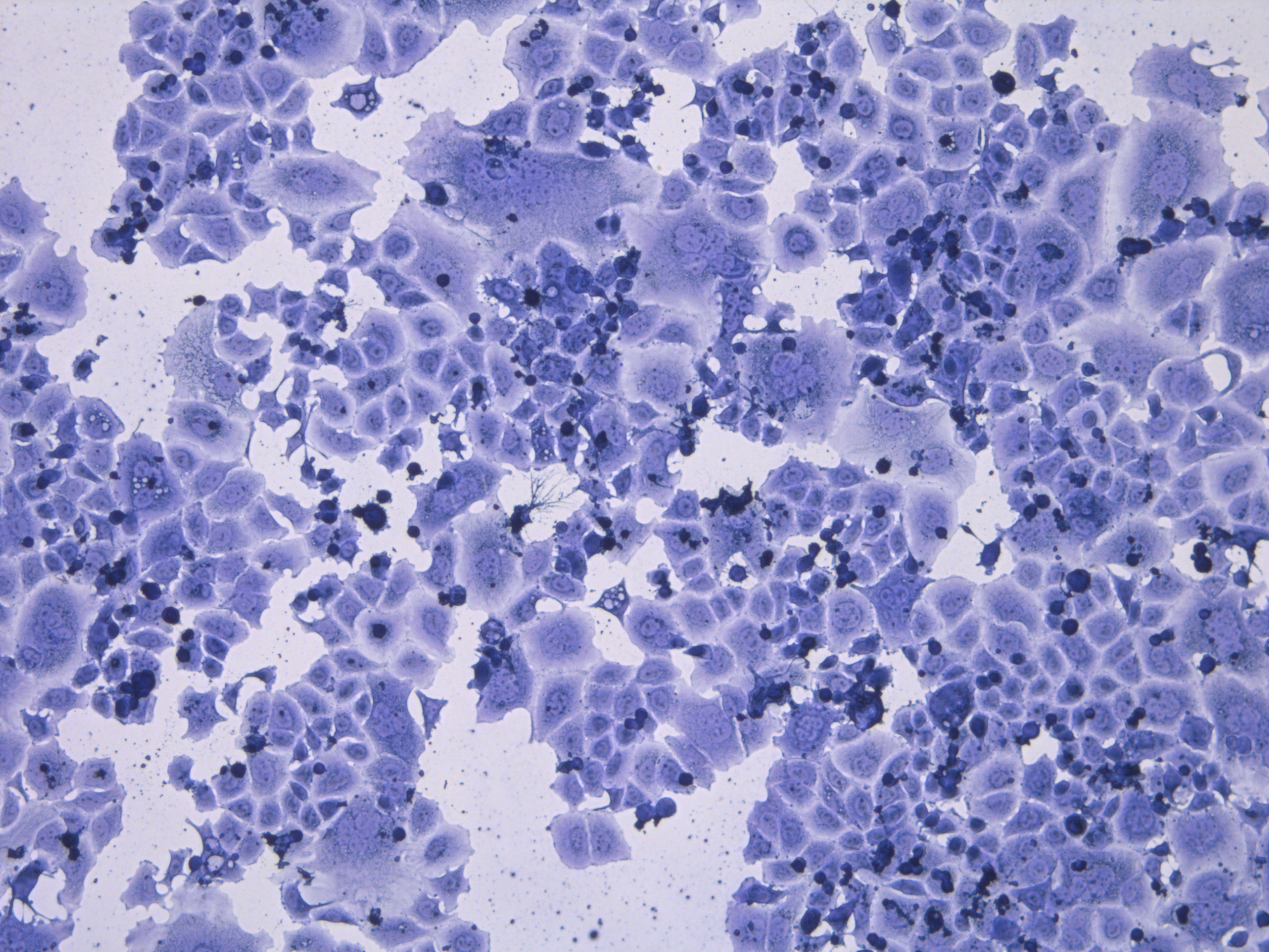

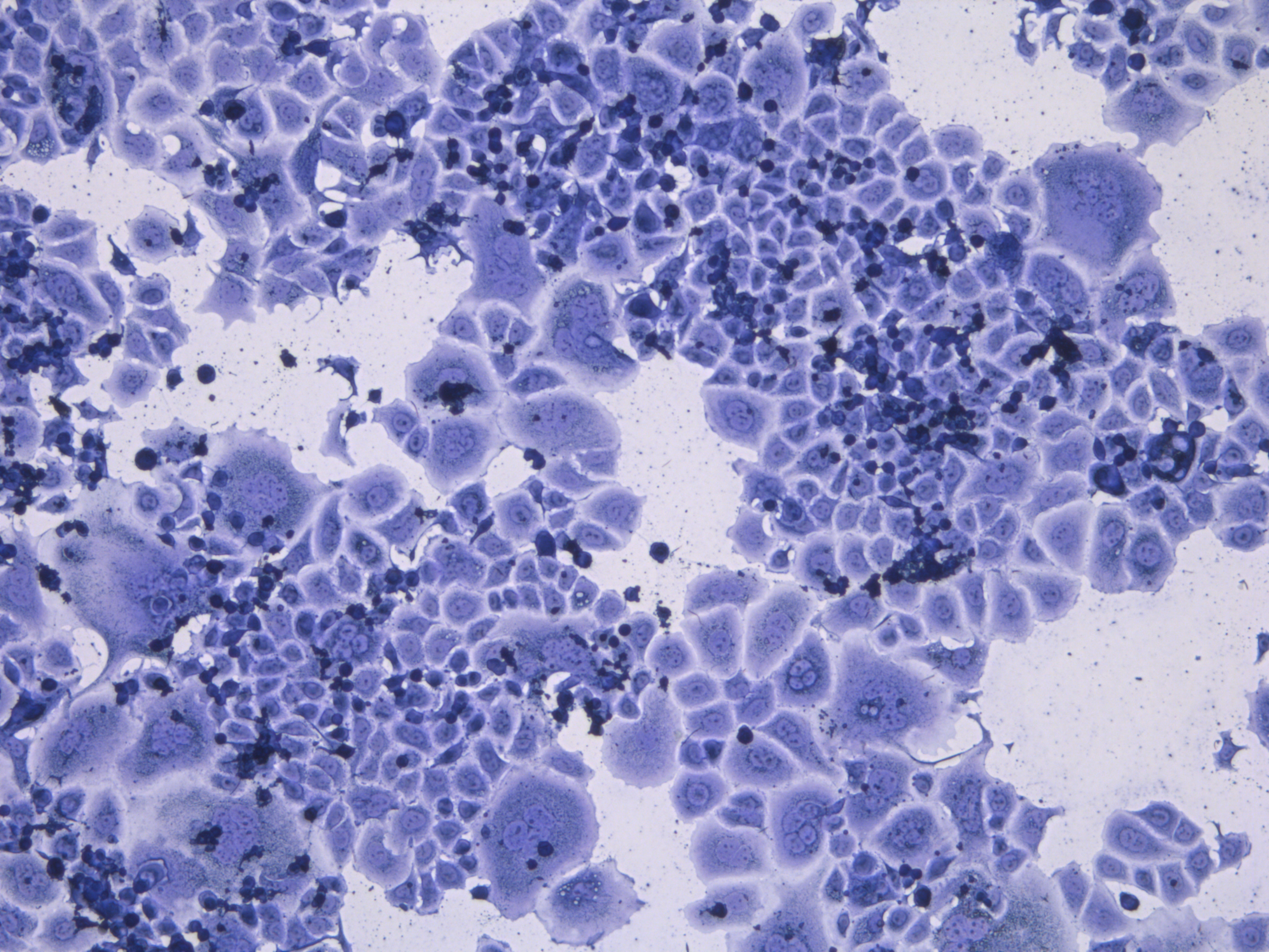

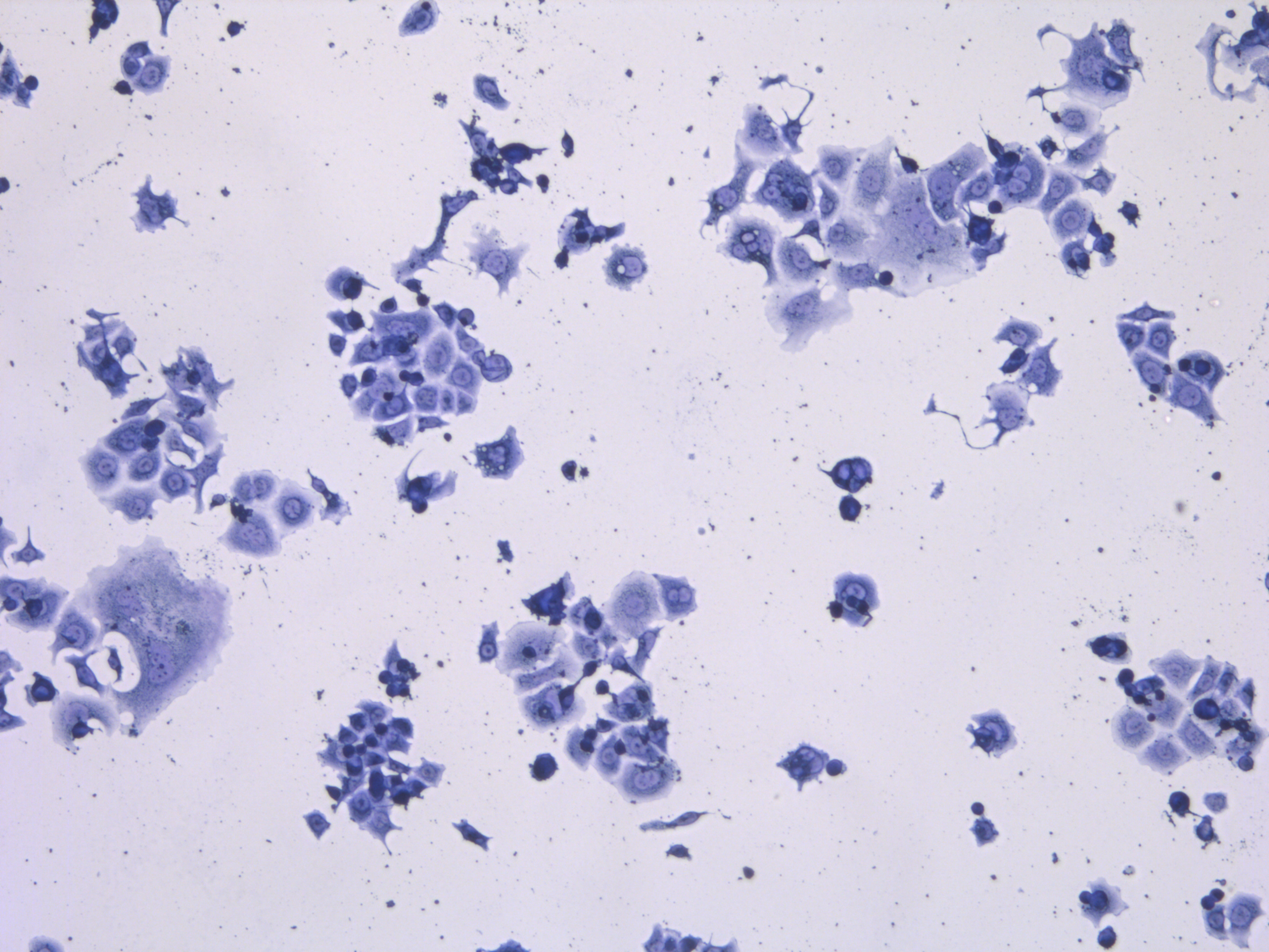

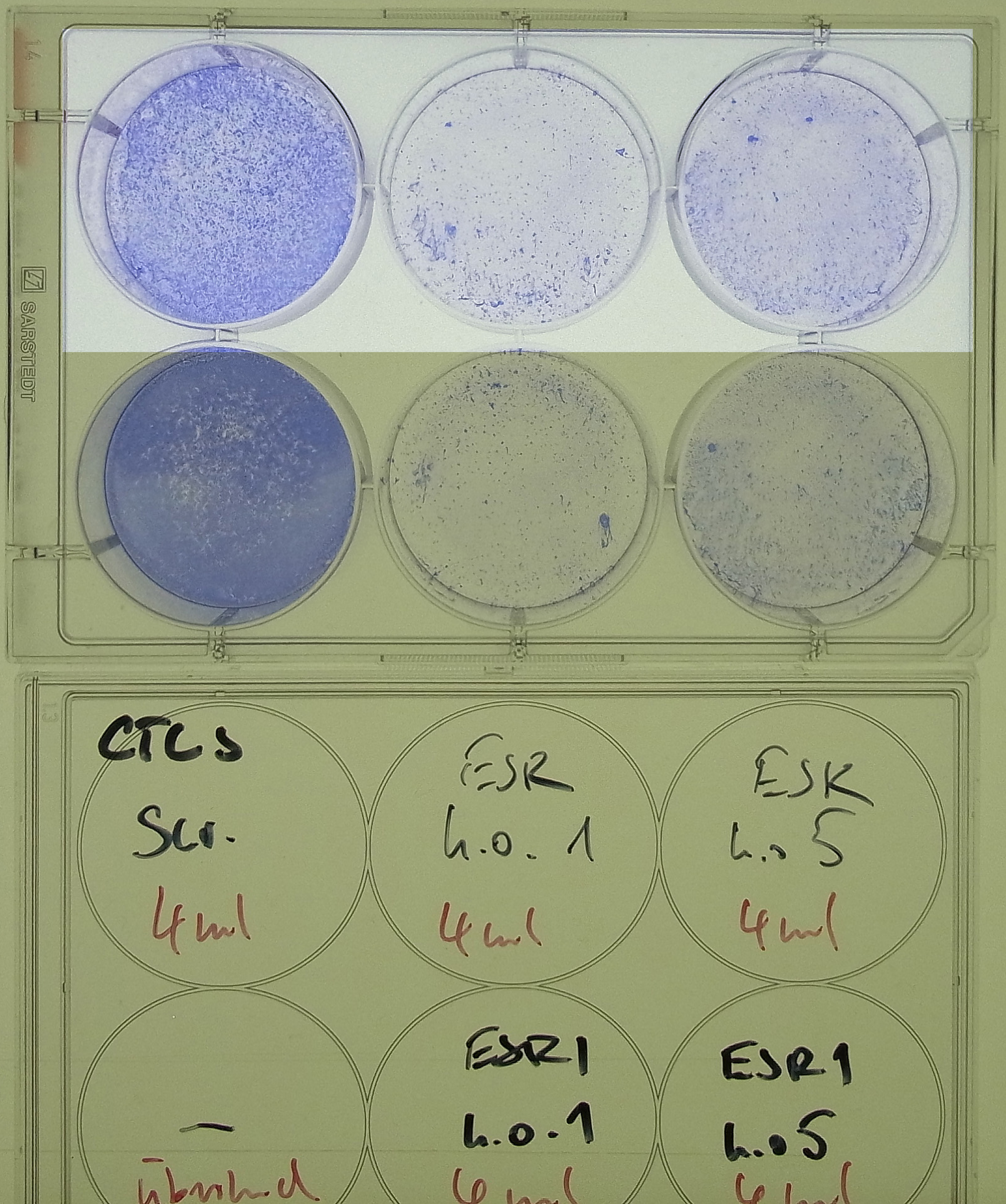
SARSTEDT
CTCs
Scr.
4ml
ESR
h.o. 1
4ml
h.o 5
4ml
ESR1
h.o.1
ESR1
h.o5

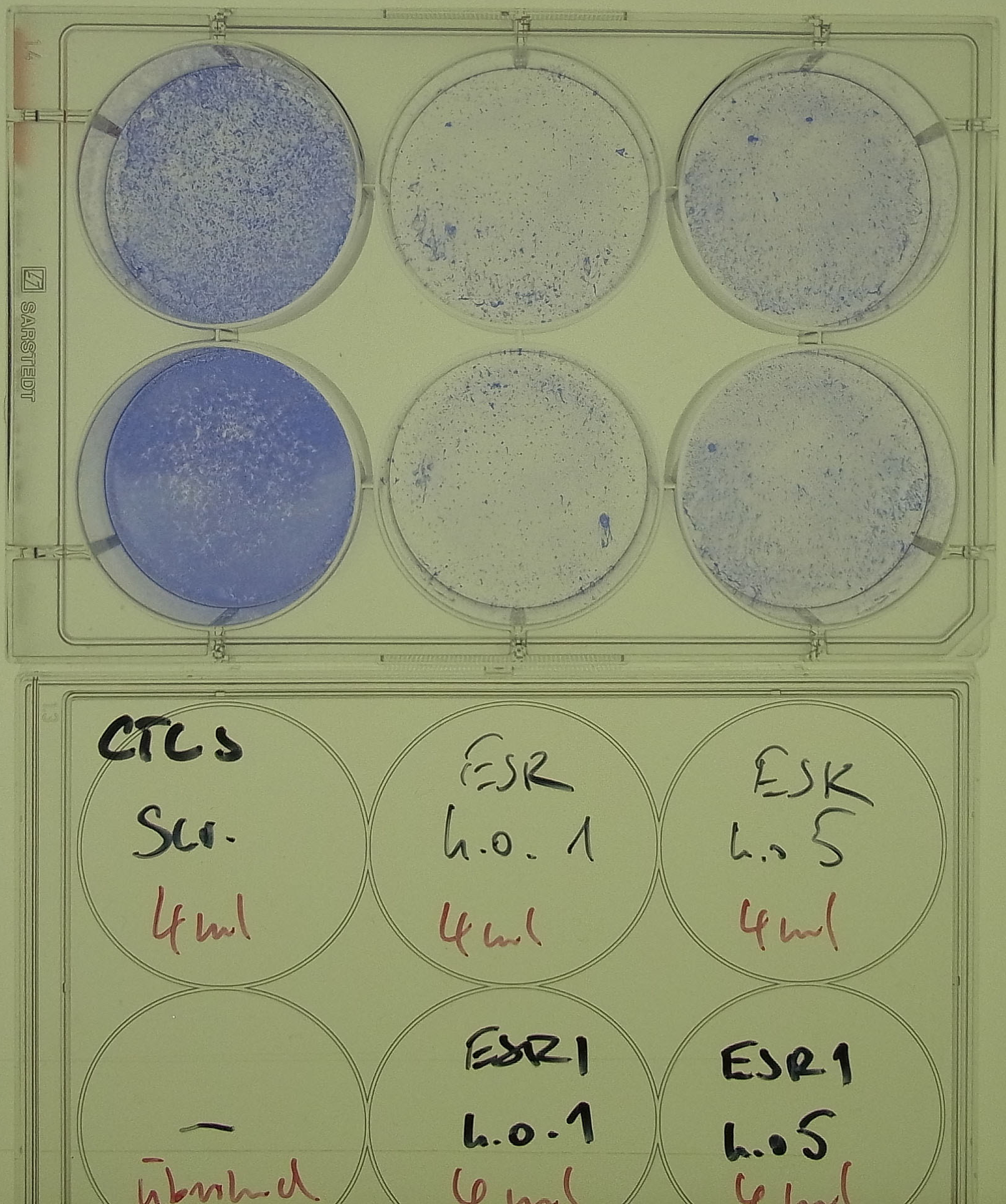

SARSTEDT
CTCs
Scr.
4ml
ESR
k.o. 1
4ml
ESR
k.o 5
4ml
—
ESR1
k.o.1
ESR1
k.o5

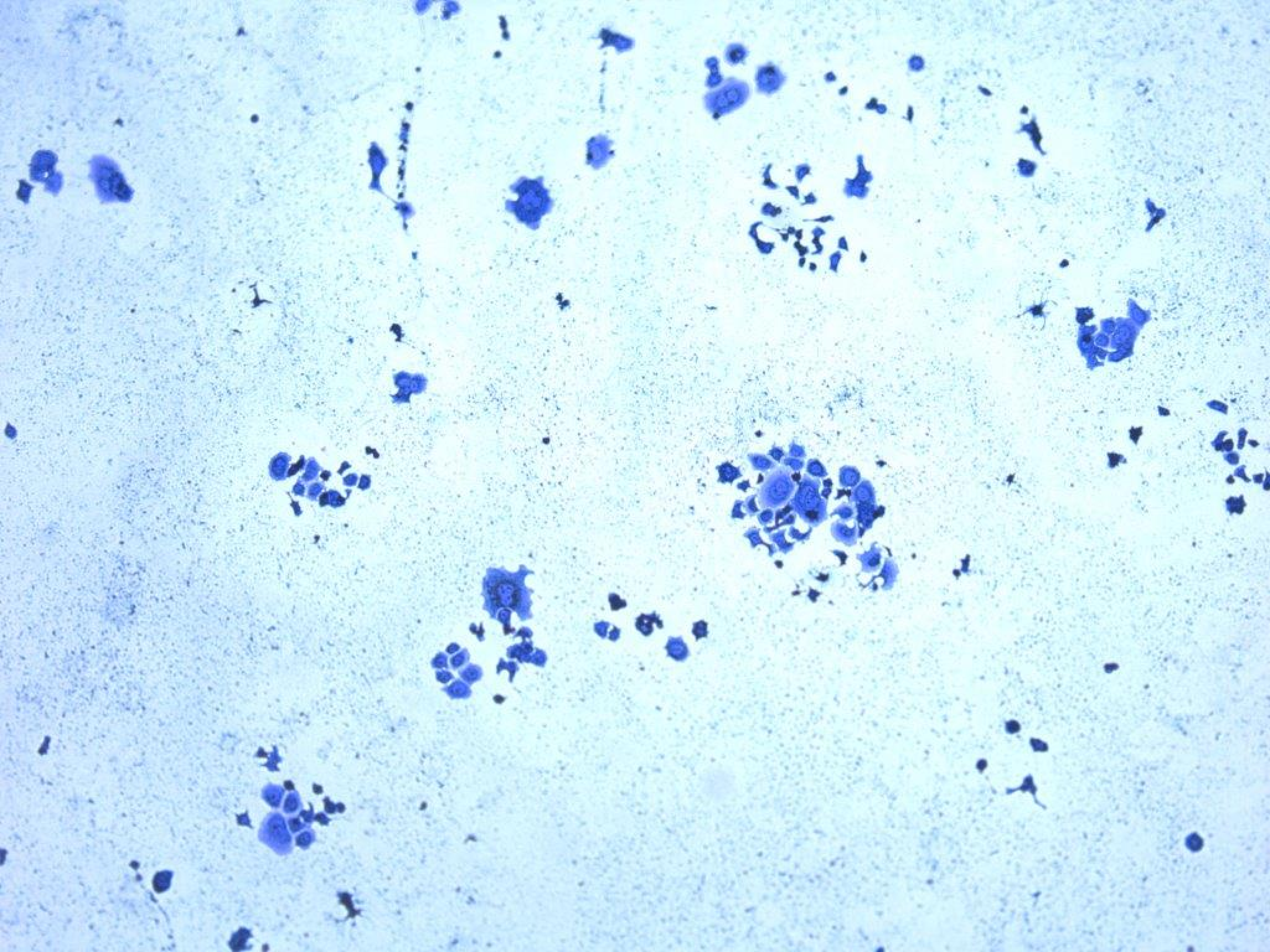

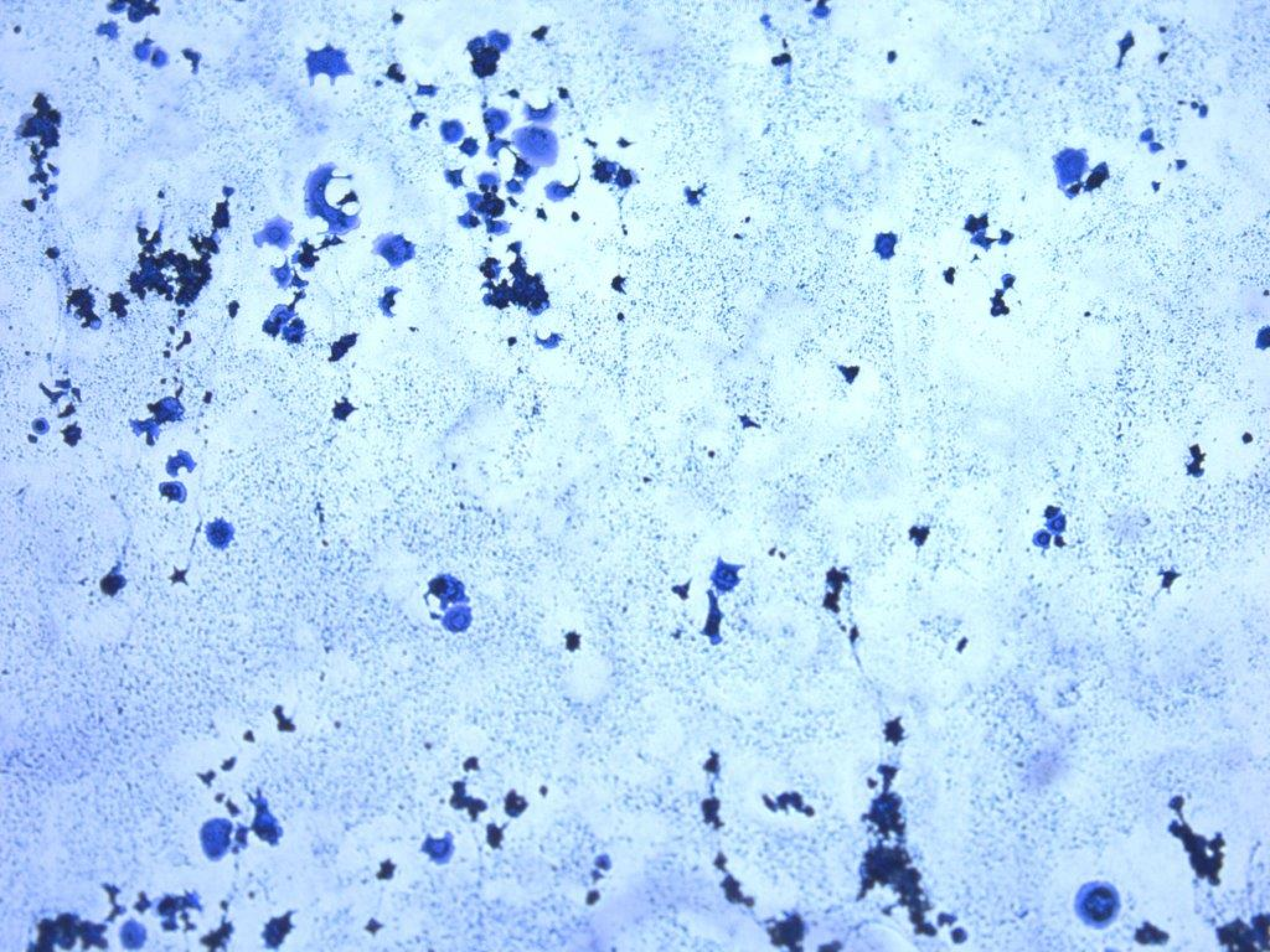

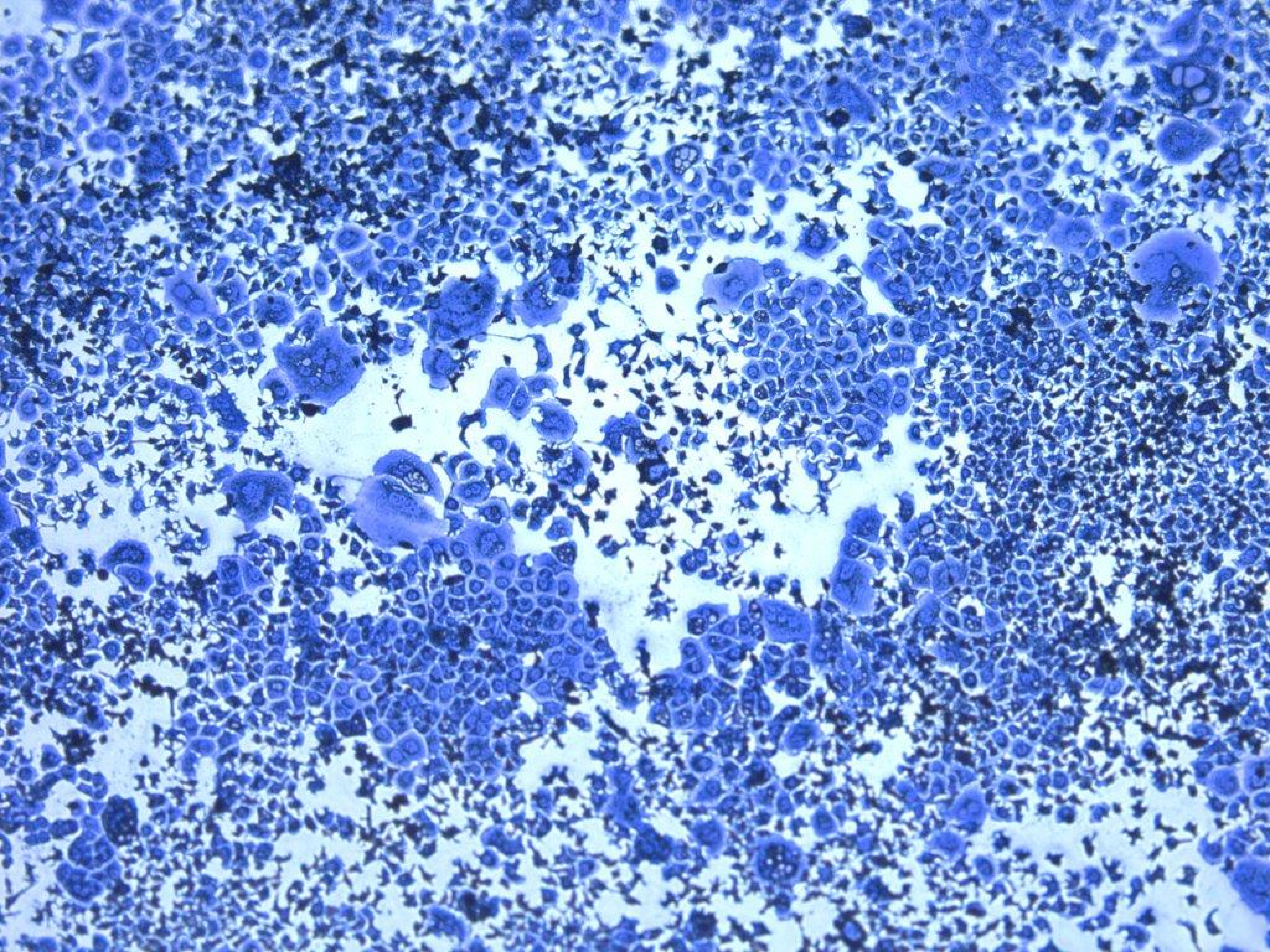

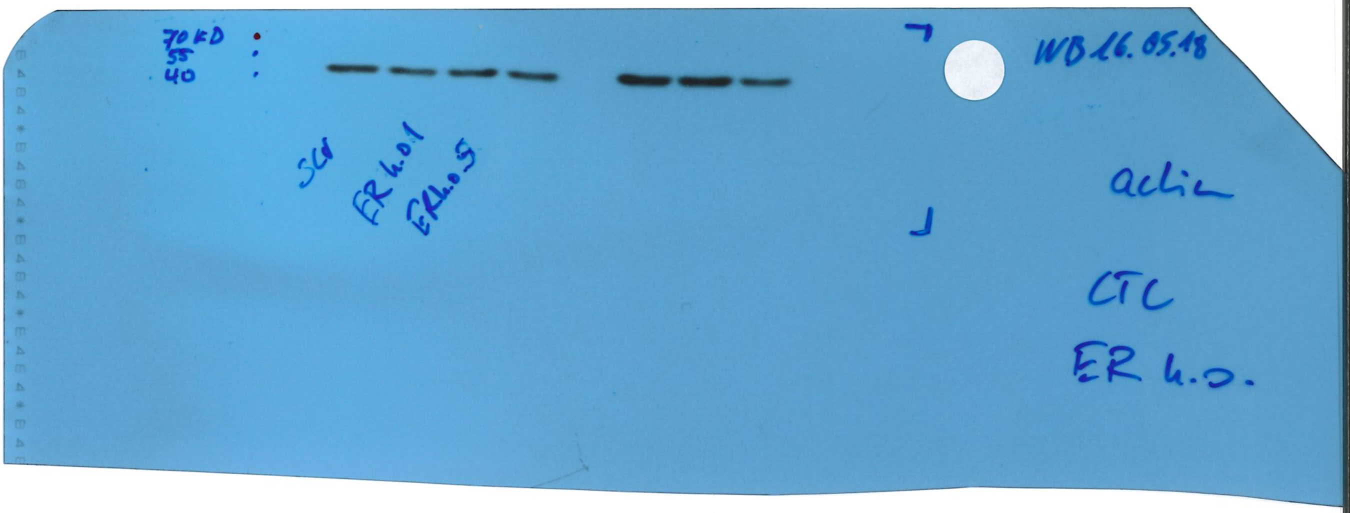
70 KD
55
40
Scr
ER h.0.1
ER h.0.5
WB 16.05.18
actin
CTC
ER h.o.

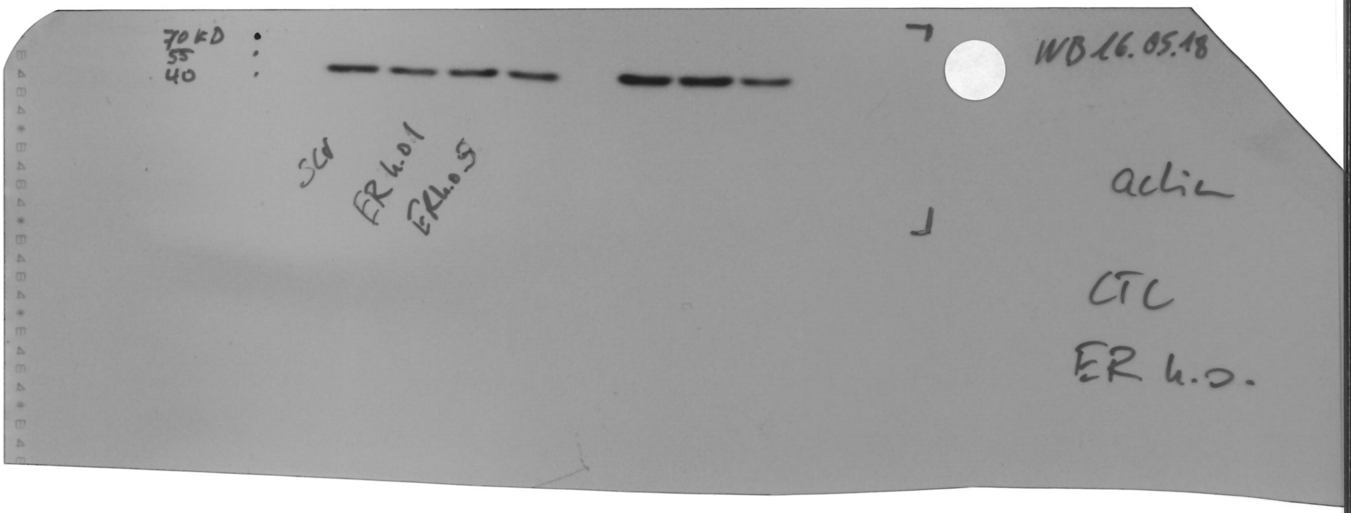
70 kD
55
40
Scr
ER k.o.1
ER k.o.5
WB 16.05.18
actin
CTC
ER k.o.

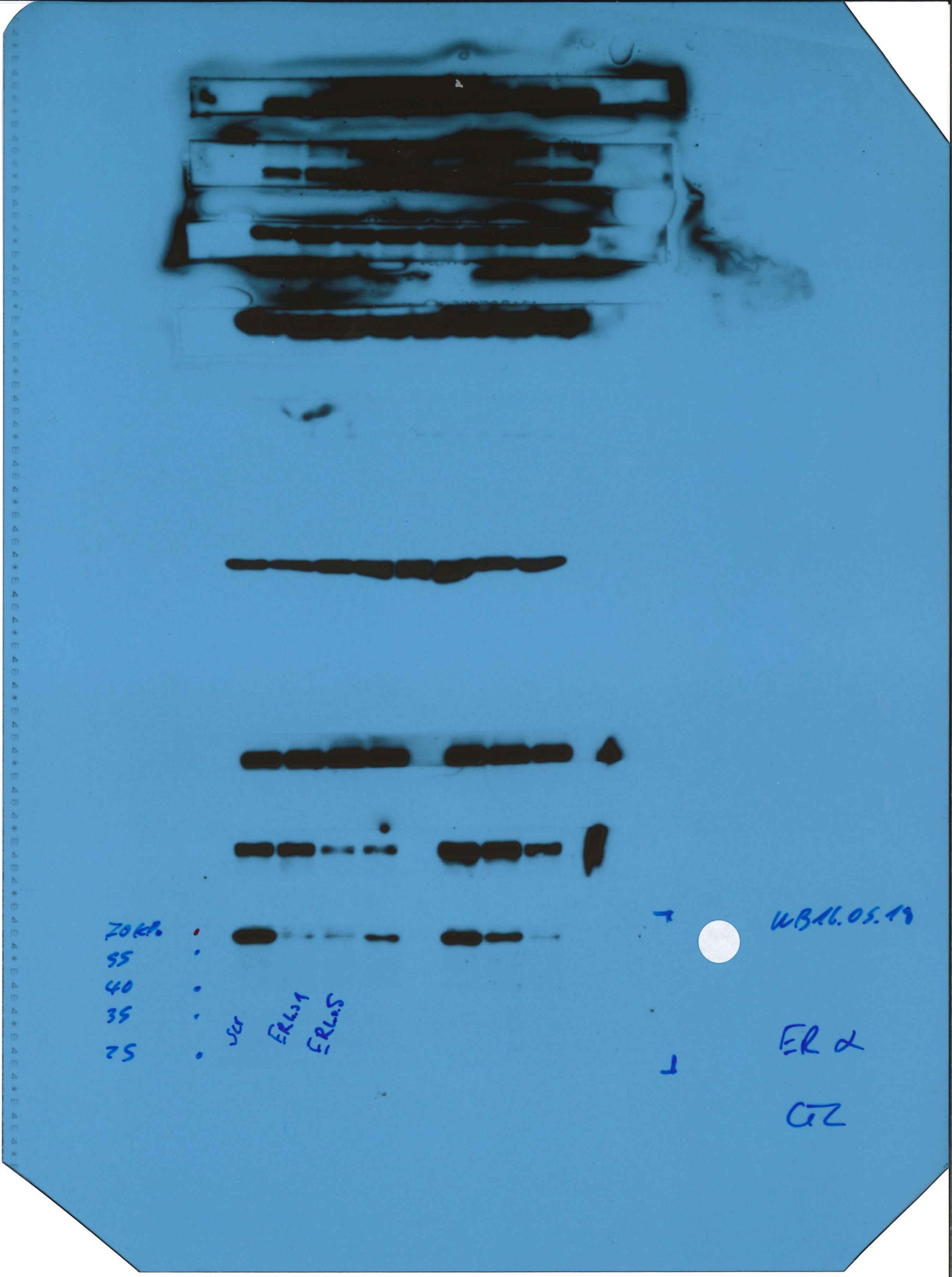
70 kDa
55
40
35
25
scr
WB 16.05.19
ER α
GZ

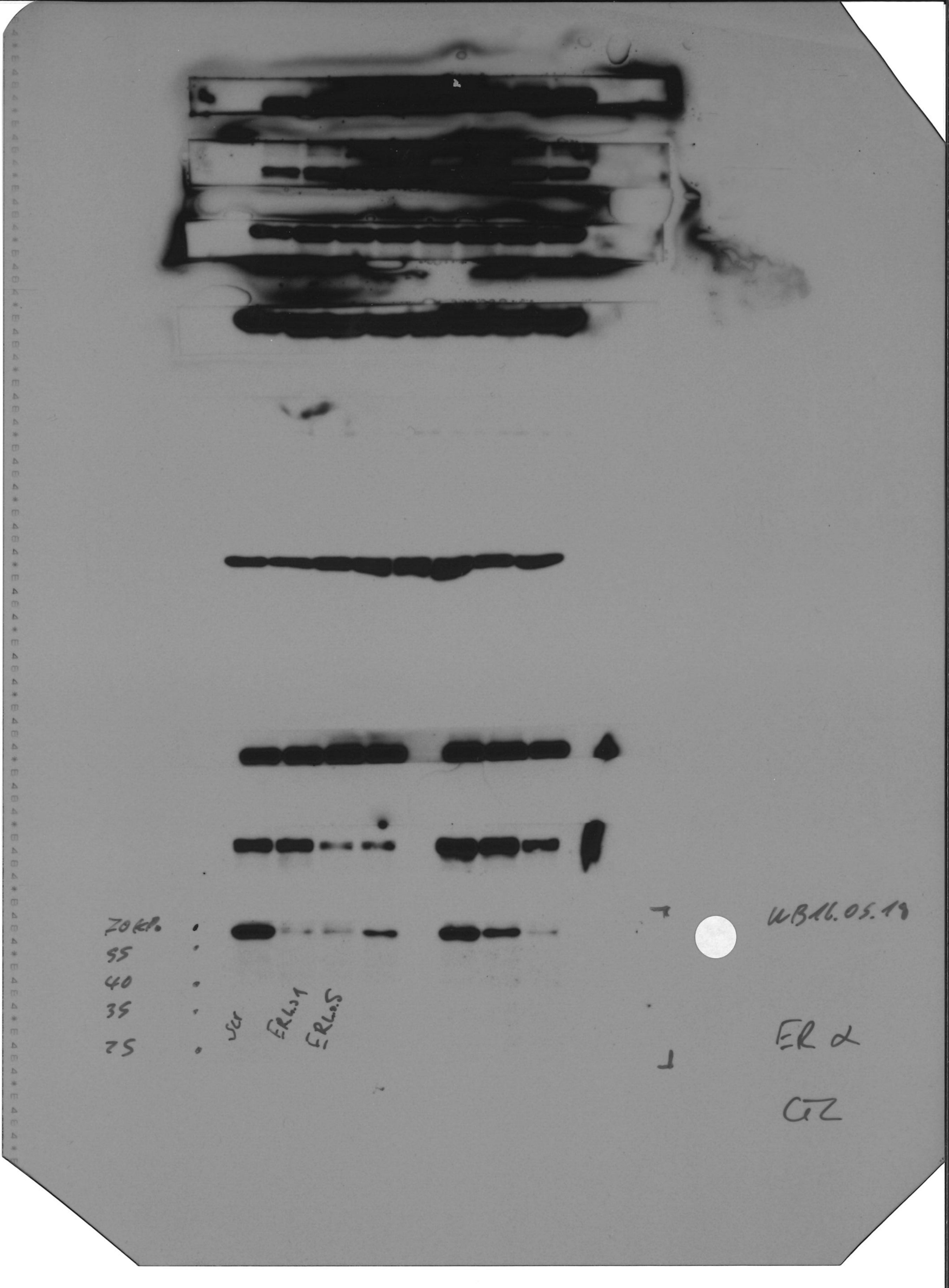
70kDa
55
40
35
25
scr
ERL0.1
ERL0.5
WB16.05.18
ER α
GZ

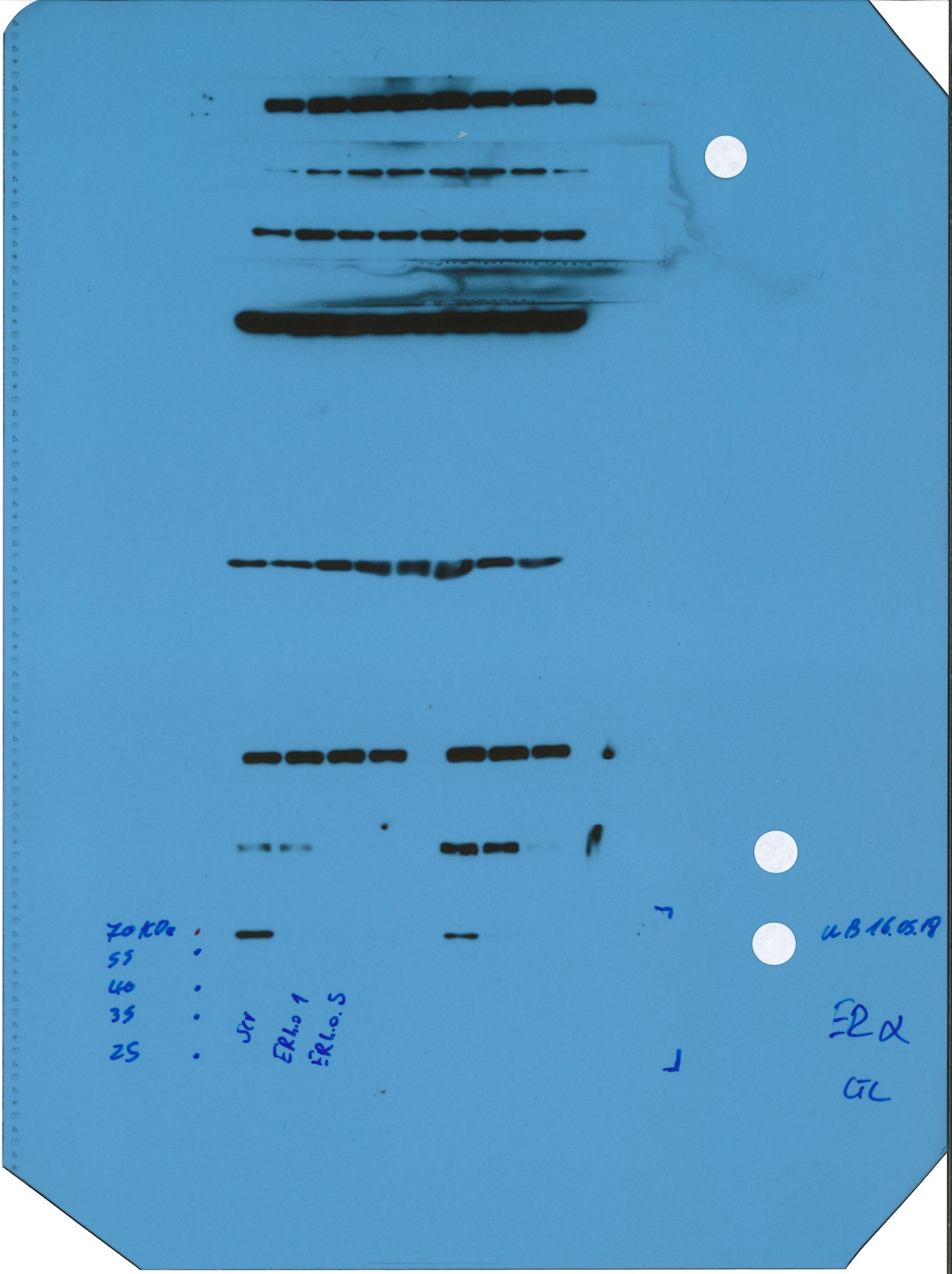
70 KDa
55
40
35
25
Scr
ERLo 1
ERLo.5
uB 16.05.19
ERα
GL

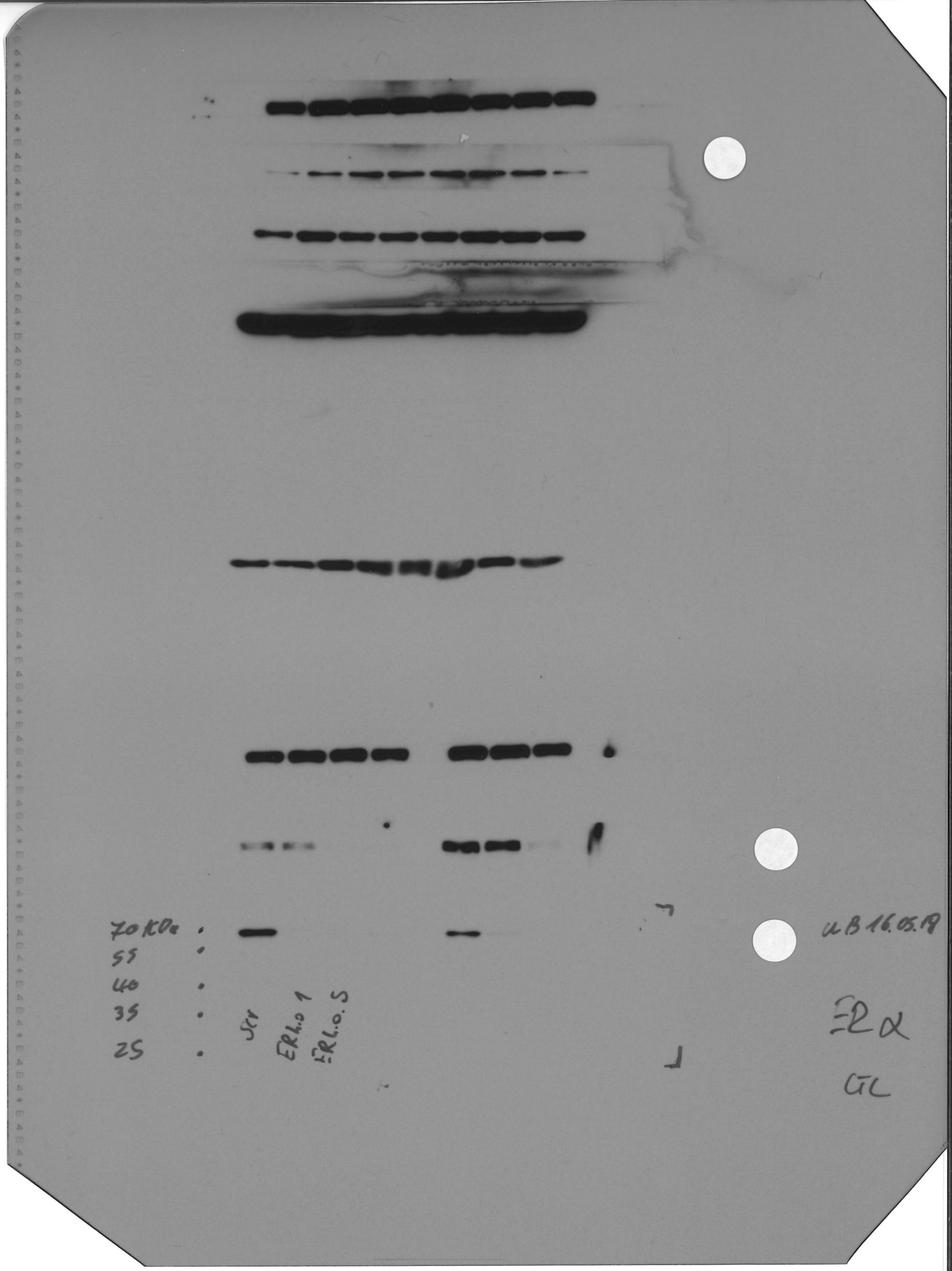
70 kDa
55
40
35
25
scr
ERL.o 1
ERL.o.5
uB 16.05.19
ERα
GL

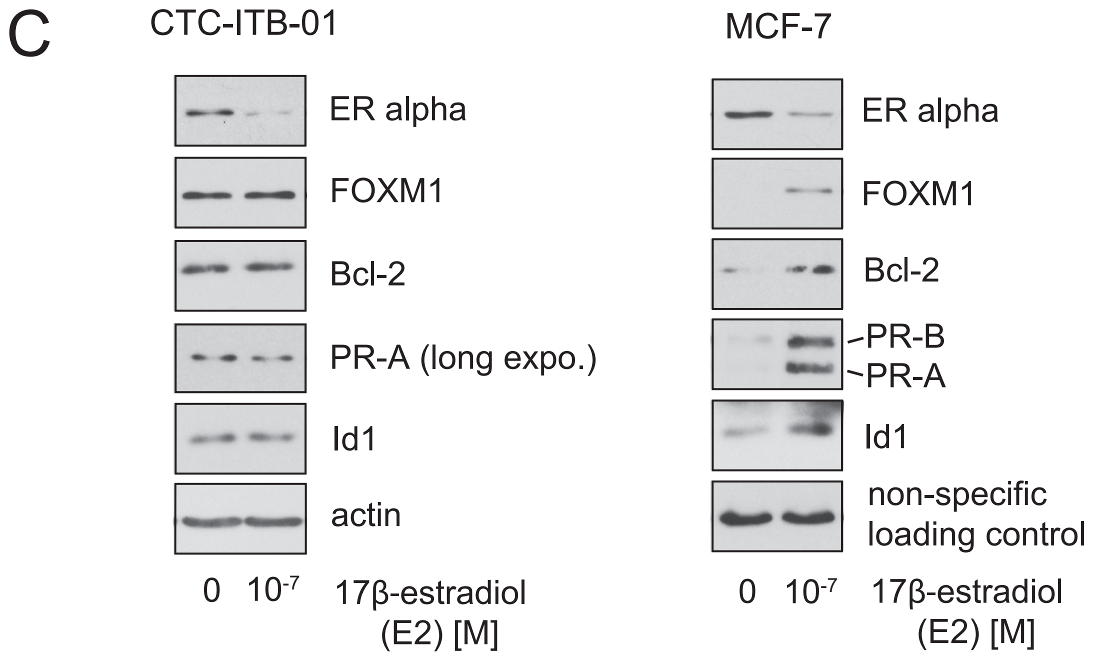

C
CTC-ITB-01
ER alpha
FOXM1
Bcl-2
PR-A (long expo.)
Id1
actin
0 10-7 17β-estradiol (E2) [M]
MCF-7
ER alpha
FOXM1
Bcl-2
PR-B
PR-A
Id1
non-specific loading control
0 10-7 17β-estradiol (E2) [M]

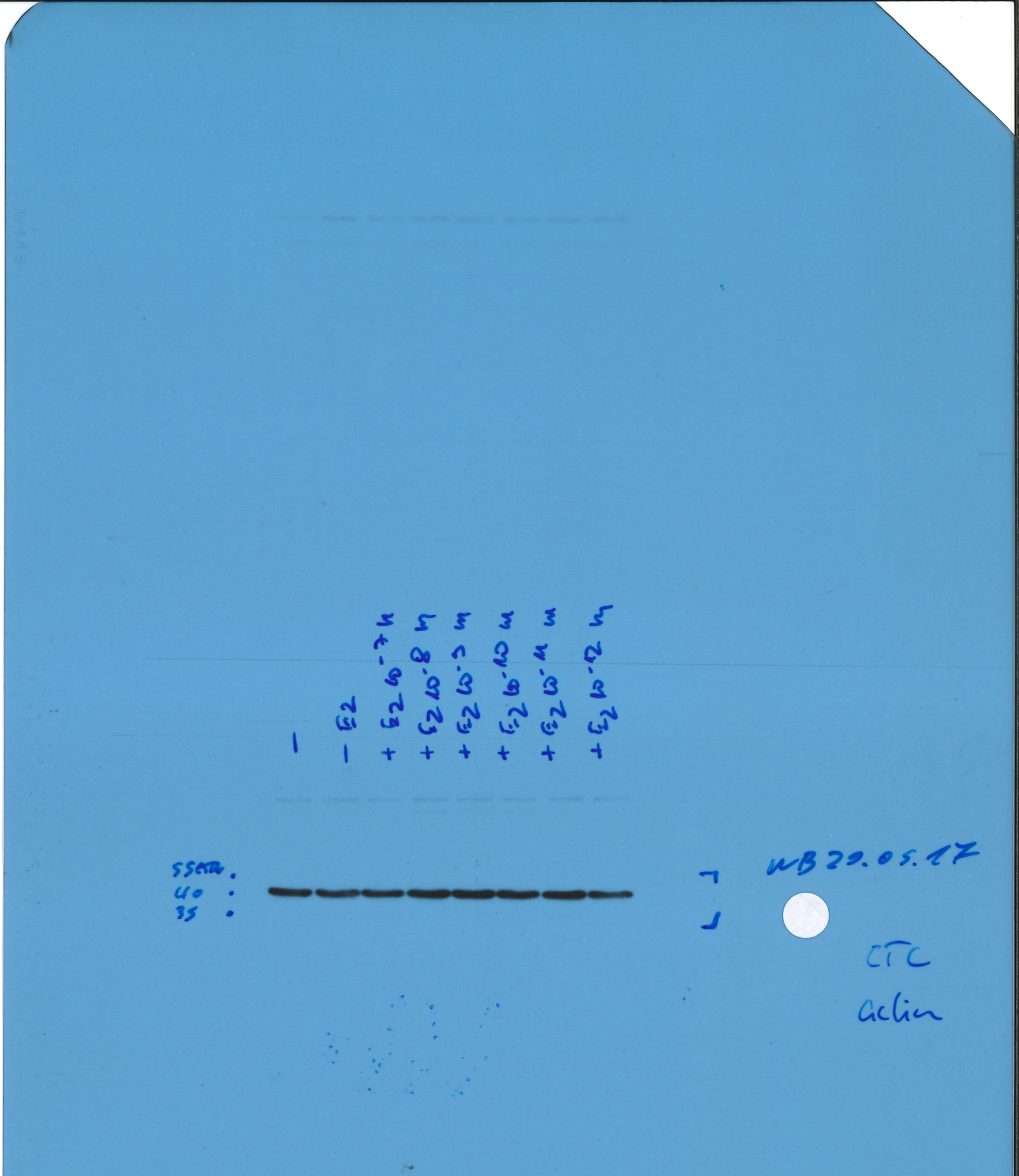
55kDa
40
35
WB 29.05.17
CTC
actin

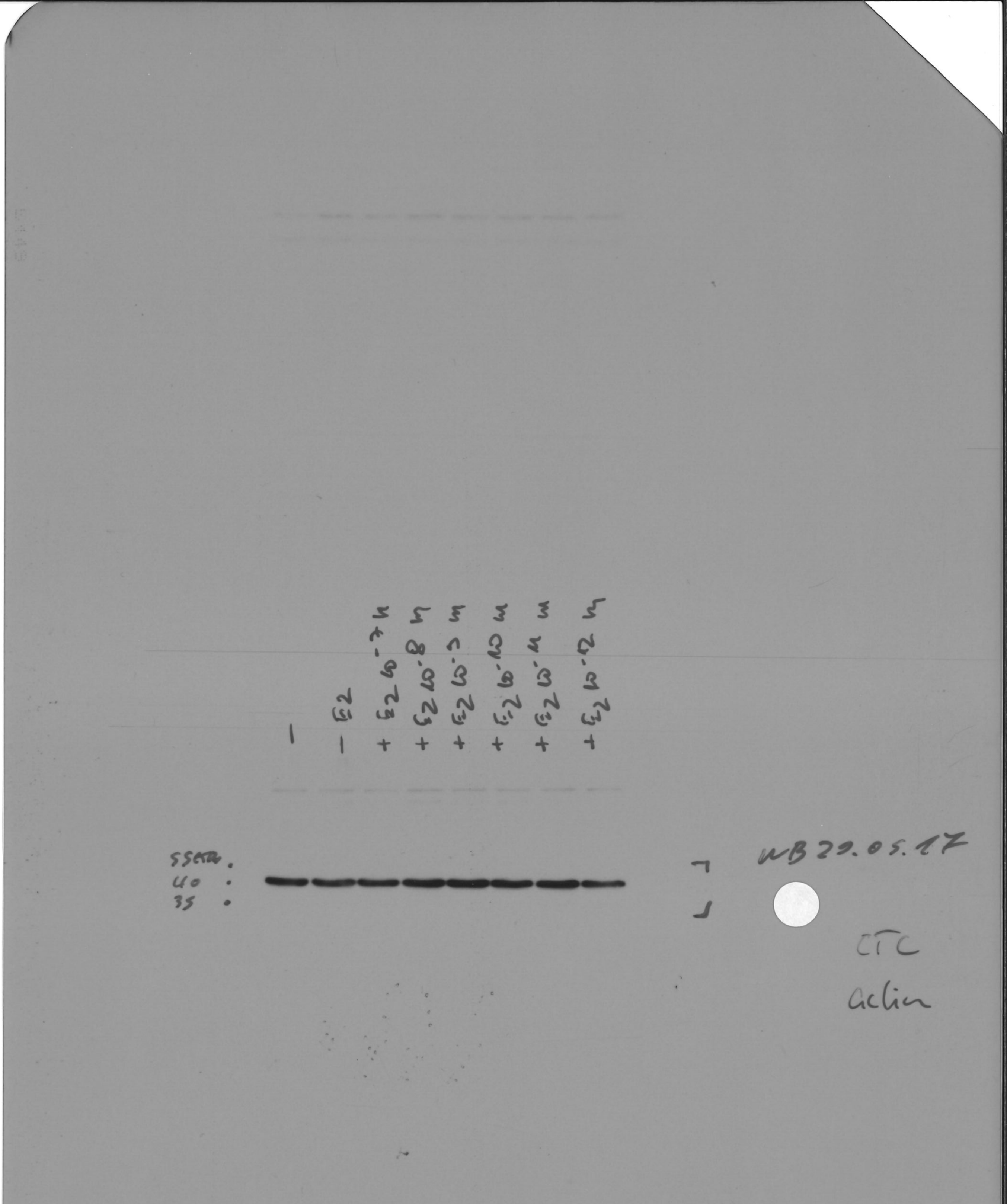

WB 29.05.17
CTC
actin
40
35

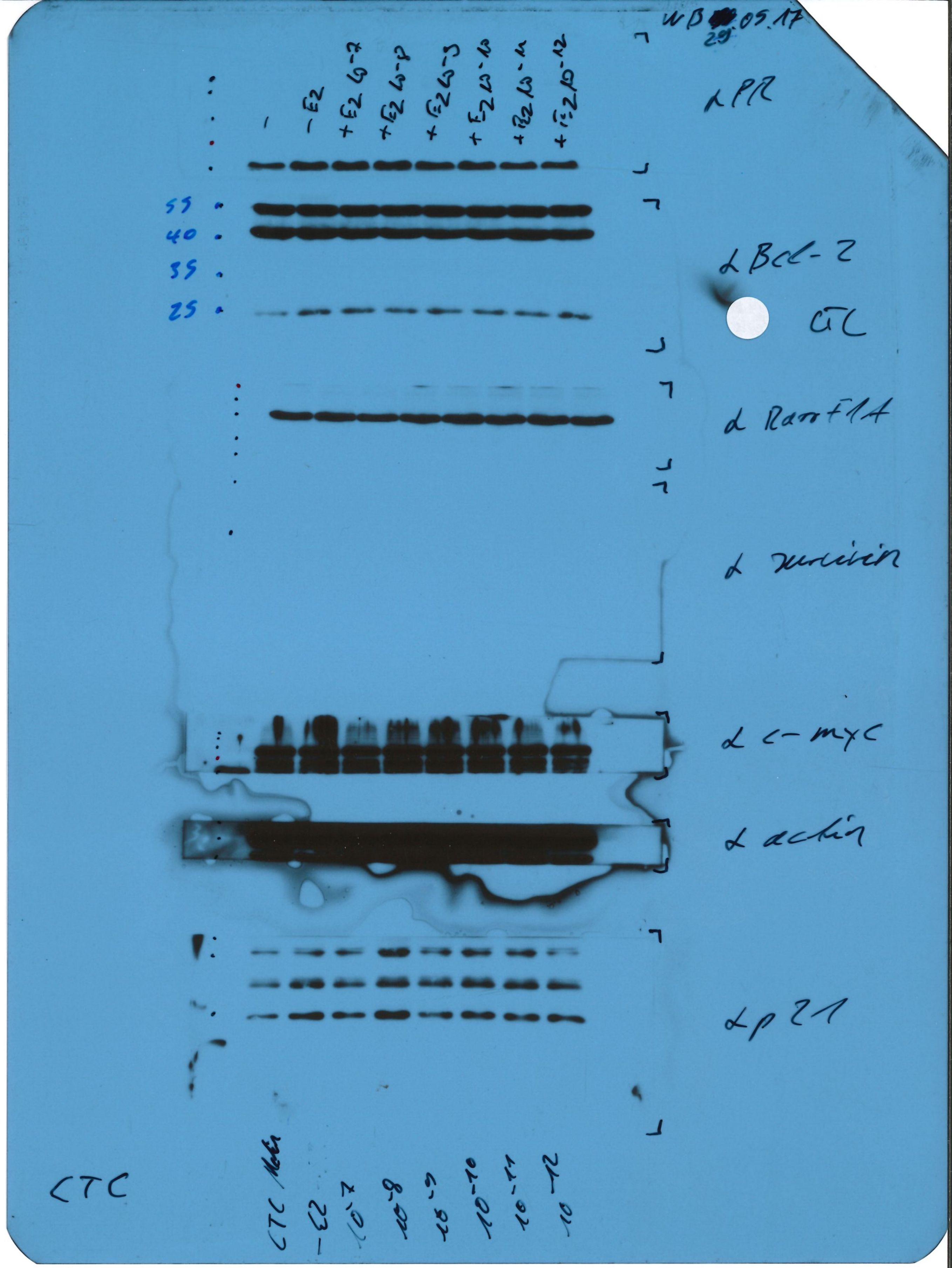
WB 29.05.17
αPR
–
–E2
+E2 10-7
+E2 10-8
+E2 10-9
+E2 10-10
+E2 10-11
+E2 10-12
55
40
35
25
αBcl-2
CTC
α RassF1A
α survivin
α c-myc
α actin
α p21
CTC Marker
–E2
10-7
10-8
10-9
10-10
10-11
10-12
CTC

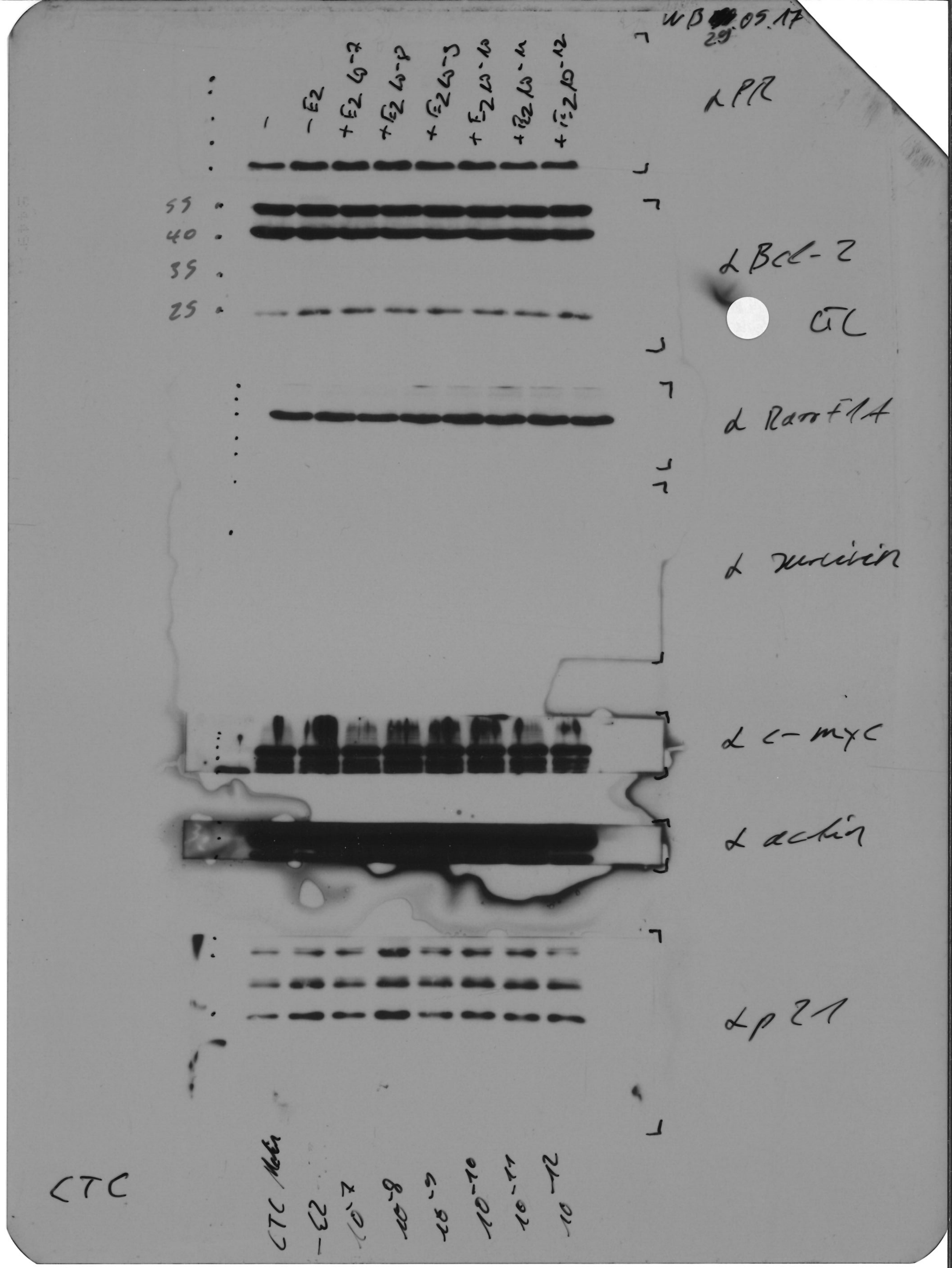

WB 29.05.17
αPR
55
40
35
25
αBcl-2
CTC
α c-myc
α actin
α p21
CTC

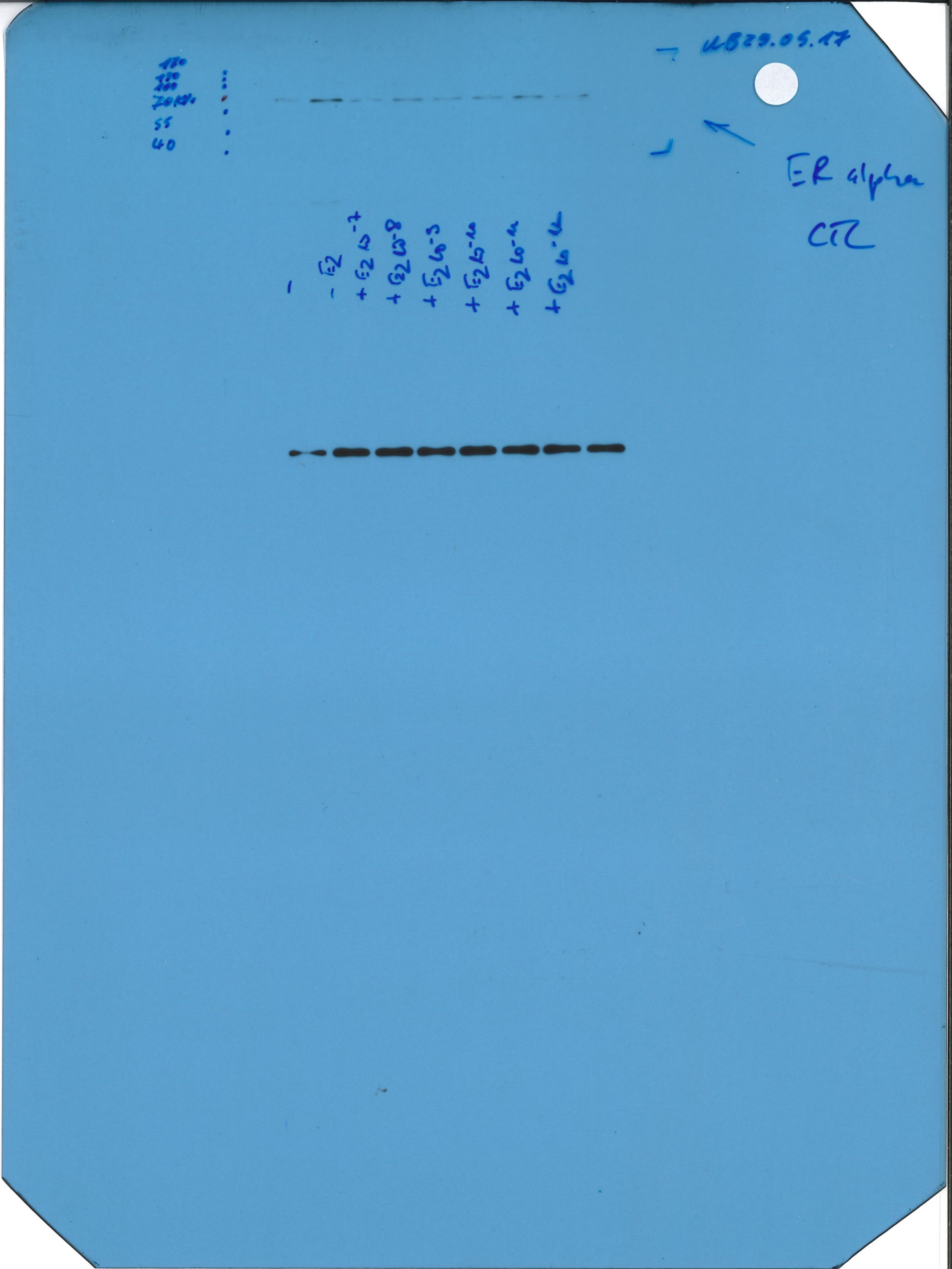

WB 29.05.17
180
130
100
70 kDa
55
40
ER alpha
CTL

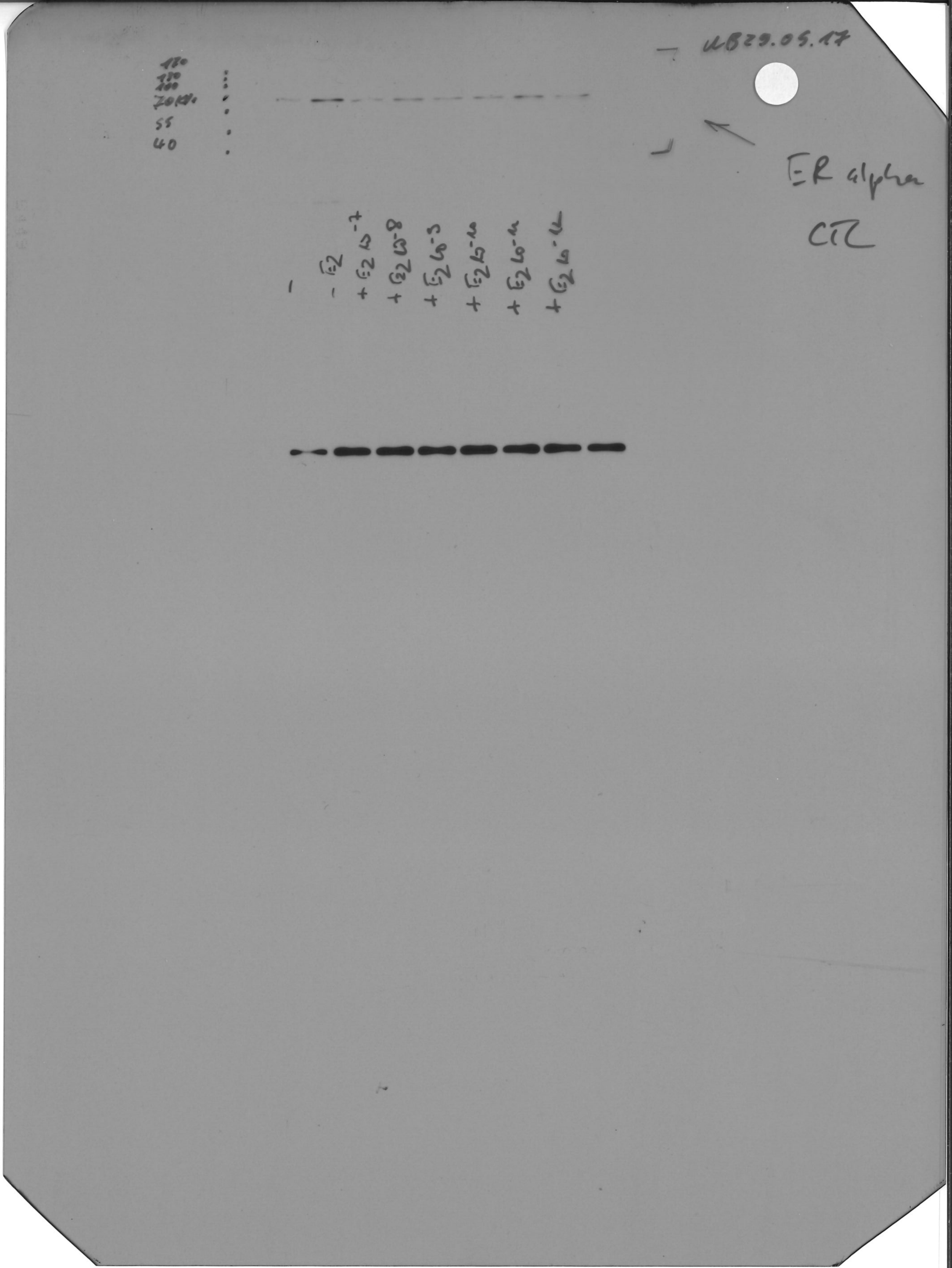
55
40
ER alpha
CTL

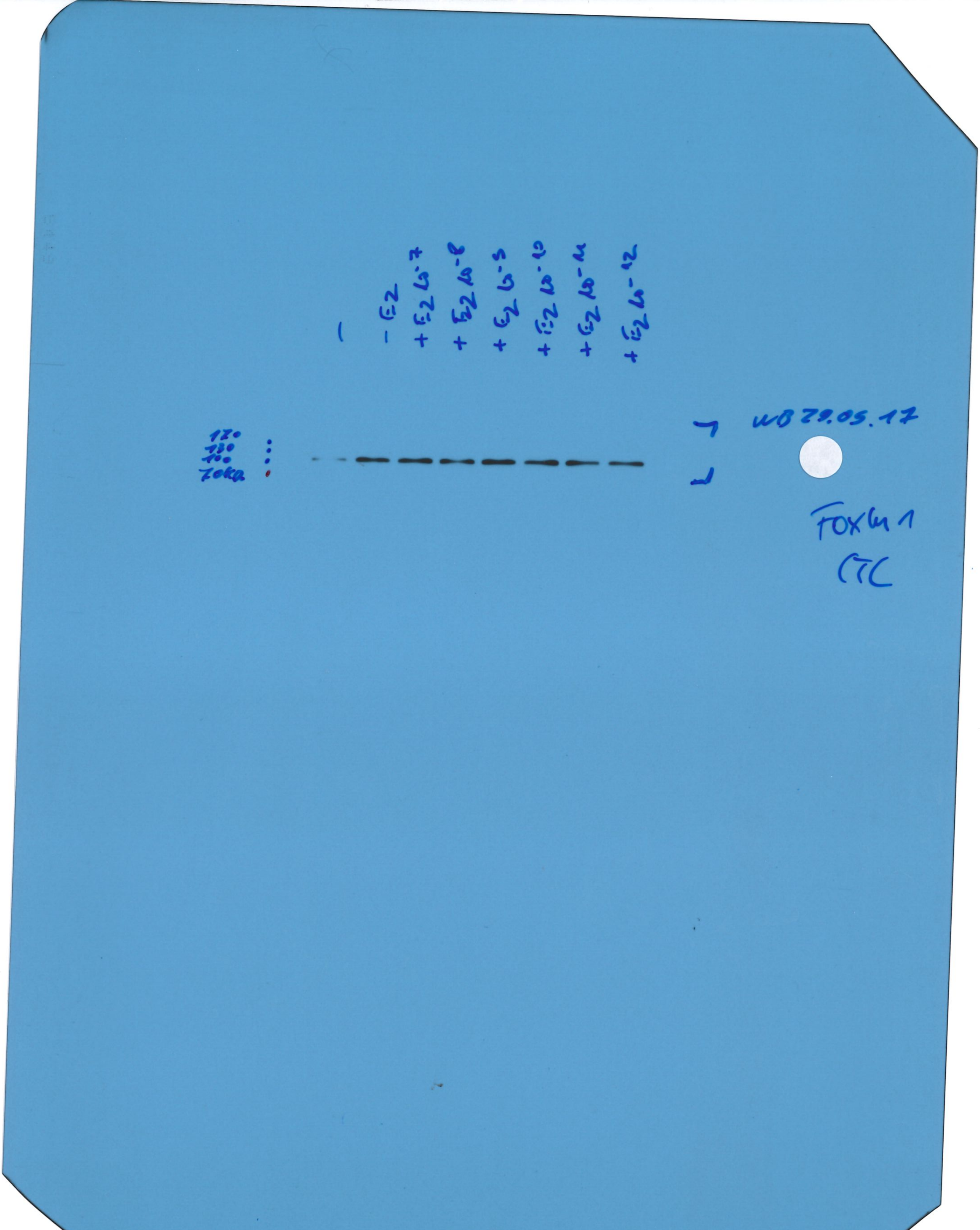

-
- E2
+ E2 10-7
+ E2 10-8
+ E2 10-9
+ E2 10-10
+ E2 10-11
+ E2 10-12
WB 29.05.17
FoxM1
CTC

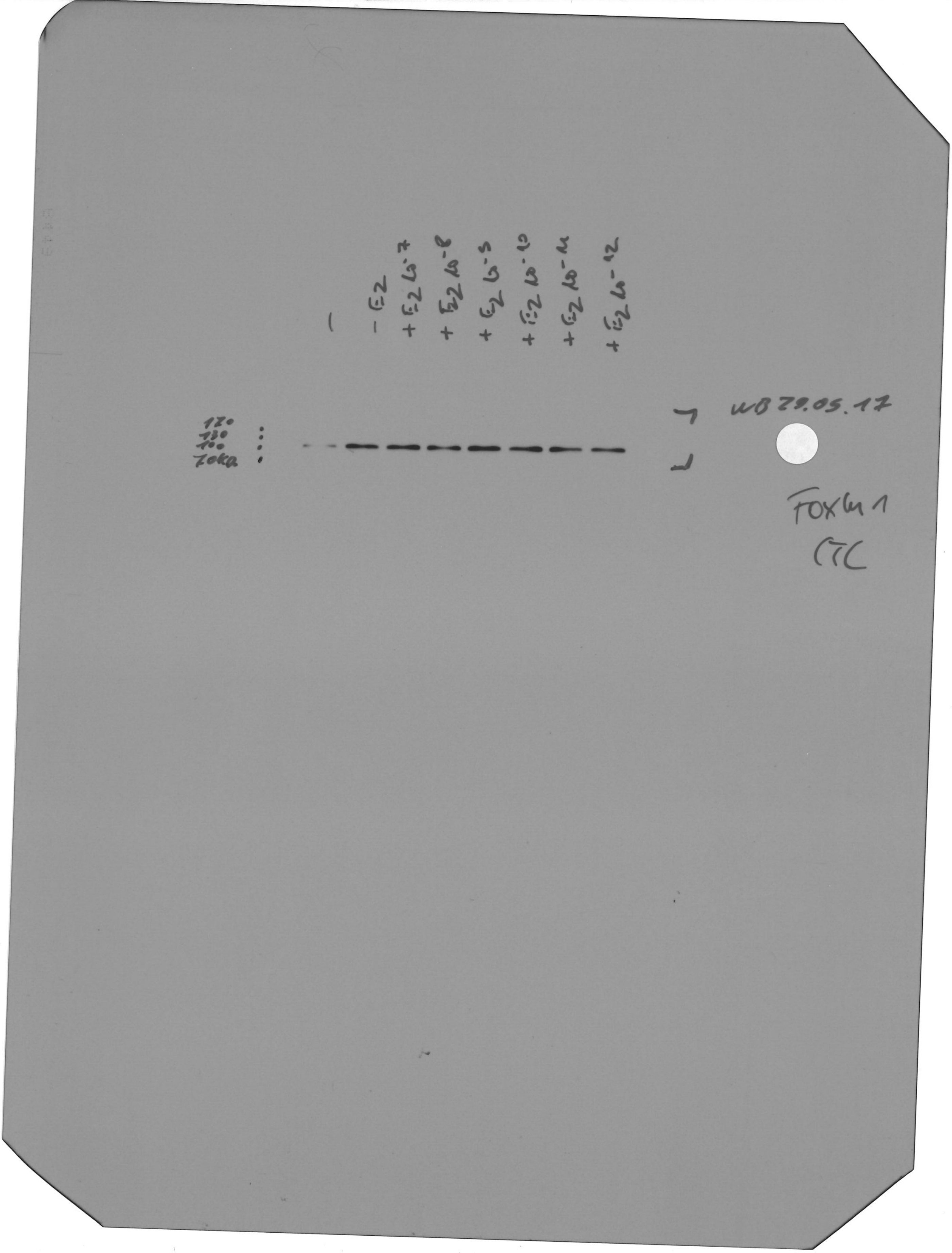
WB 29.05.17
FoxM1
CTC

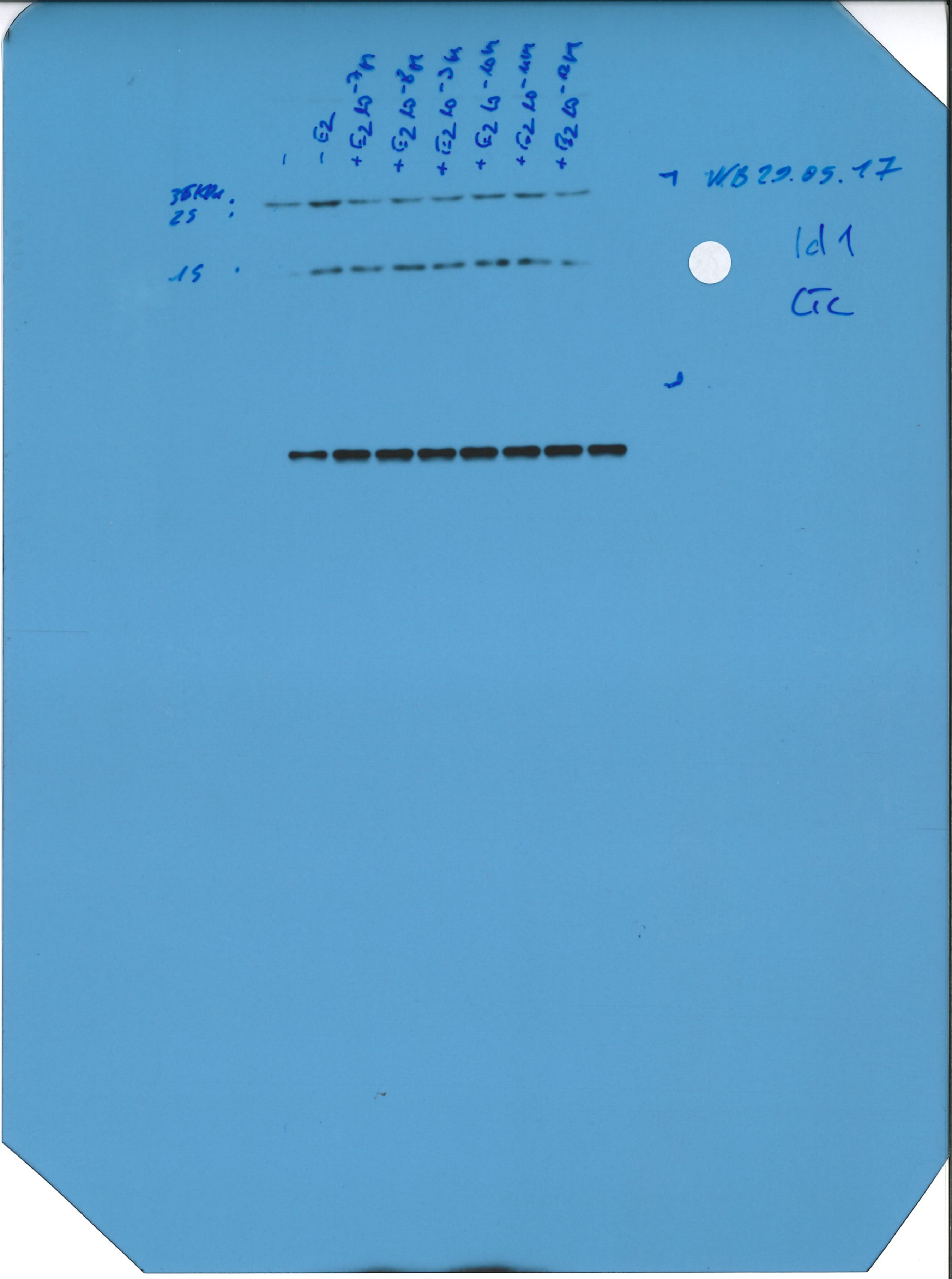
35 kDa
25
15
WB 29.05.17
Id1

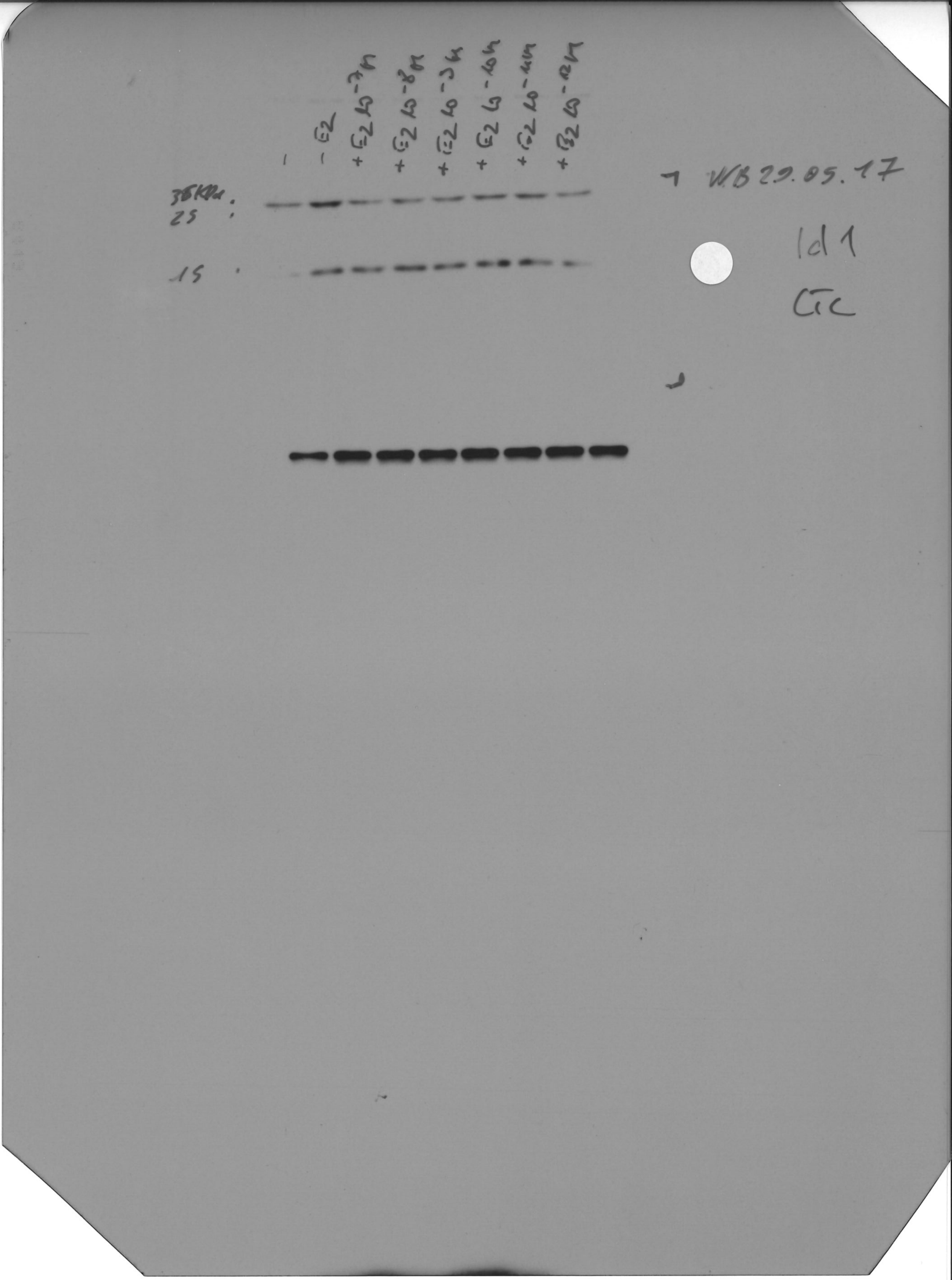
-
- E2
+ E2 10^{-7} M
+ E2 10^{-8} M
+ E2 10^{-9} M
+ E2 10^{-10} M
+ E2 10^{-11} M
+ E2 10^{-12} M
35 kDa
25
15
WB 29.05.17
Id1
GC

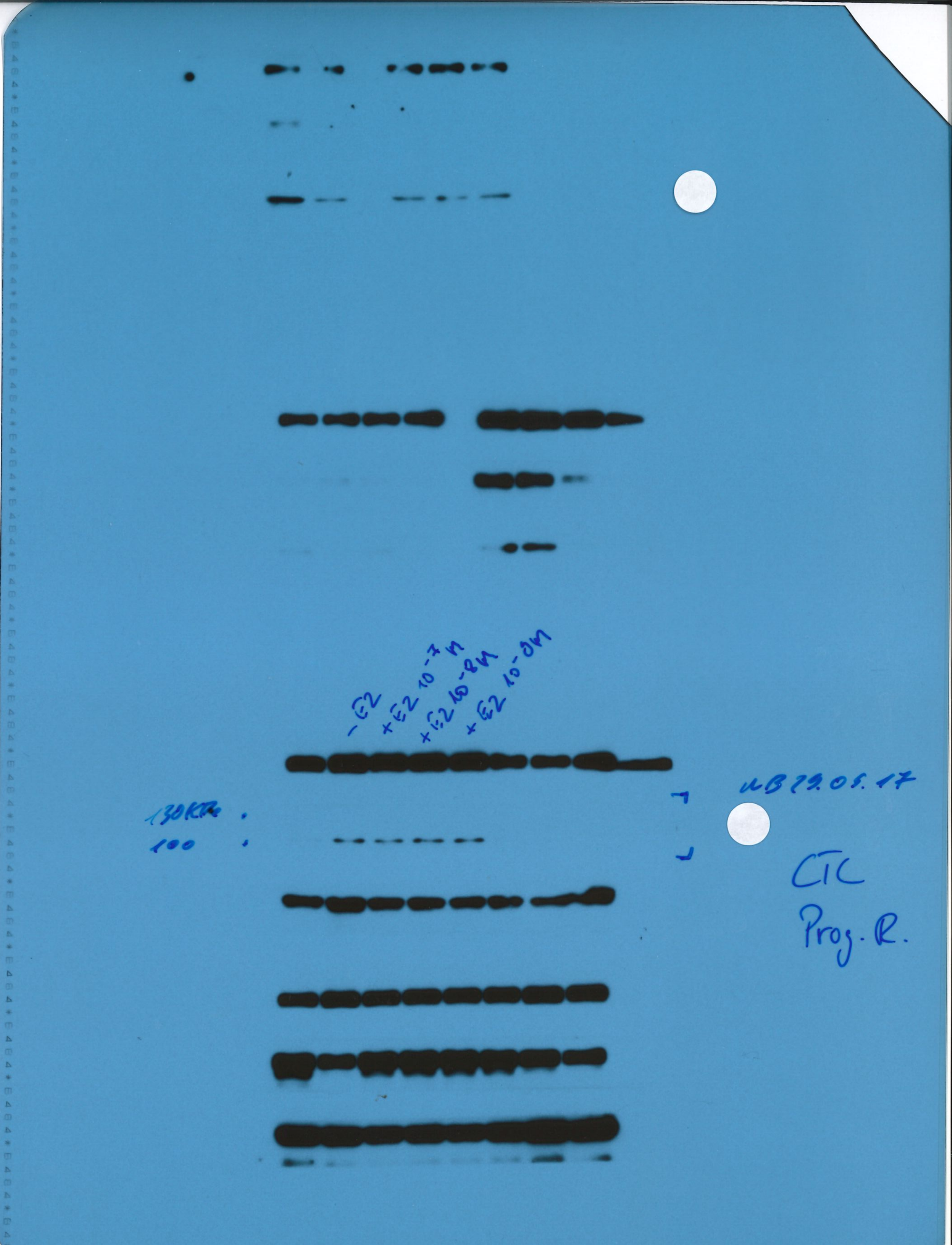

– E2
+ E2 10-7 M
+ E2 10-8 M
+ E2 10-9 M
130 kDa
100
uB 29.05.17
CTC
Prog. R.

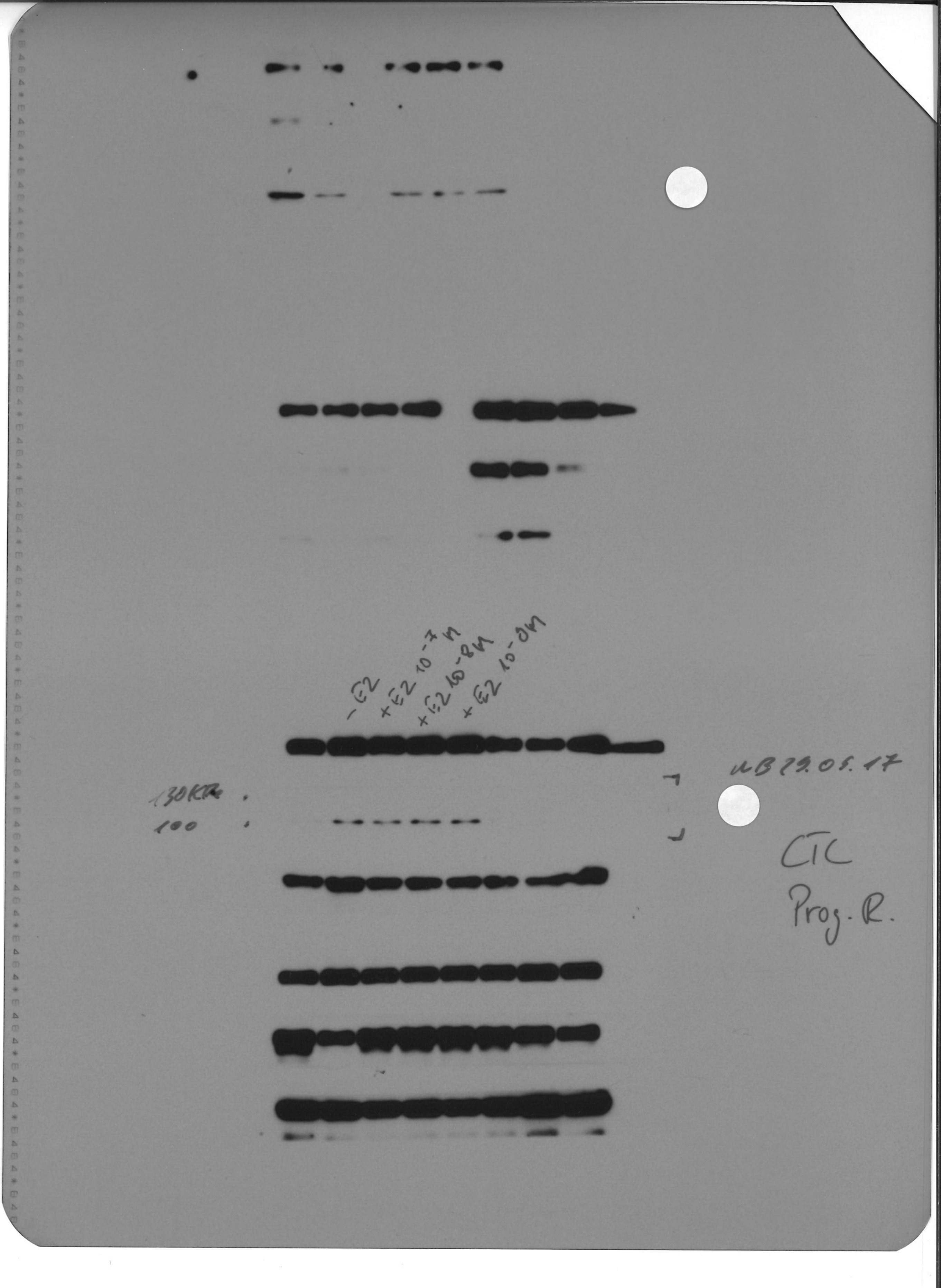
– E2
+ E2 10-7 M
+ E2 10-8M
+ E2 10-9M
130 kDa
100
uB 29.05.17
CTC
Prog. R.

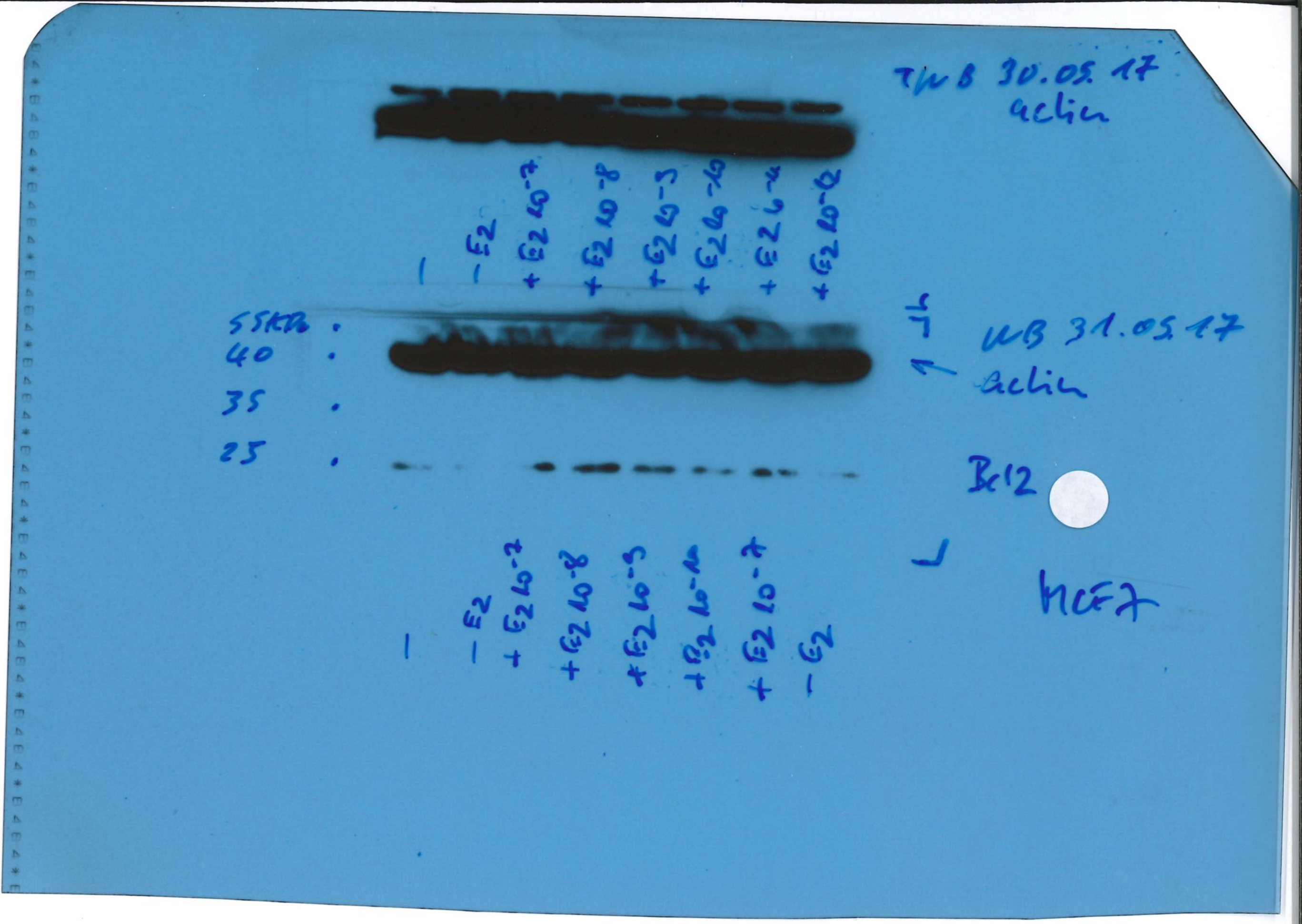
WB 30.05.17
actin
WB 31.05.17
actin
55kDa
40
35
25
Bcl2
MCF7

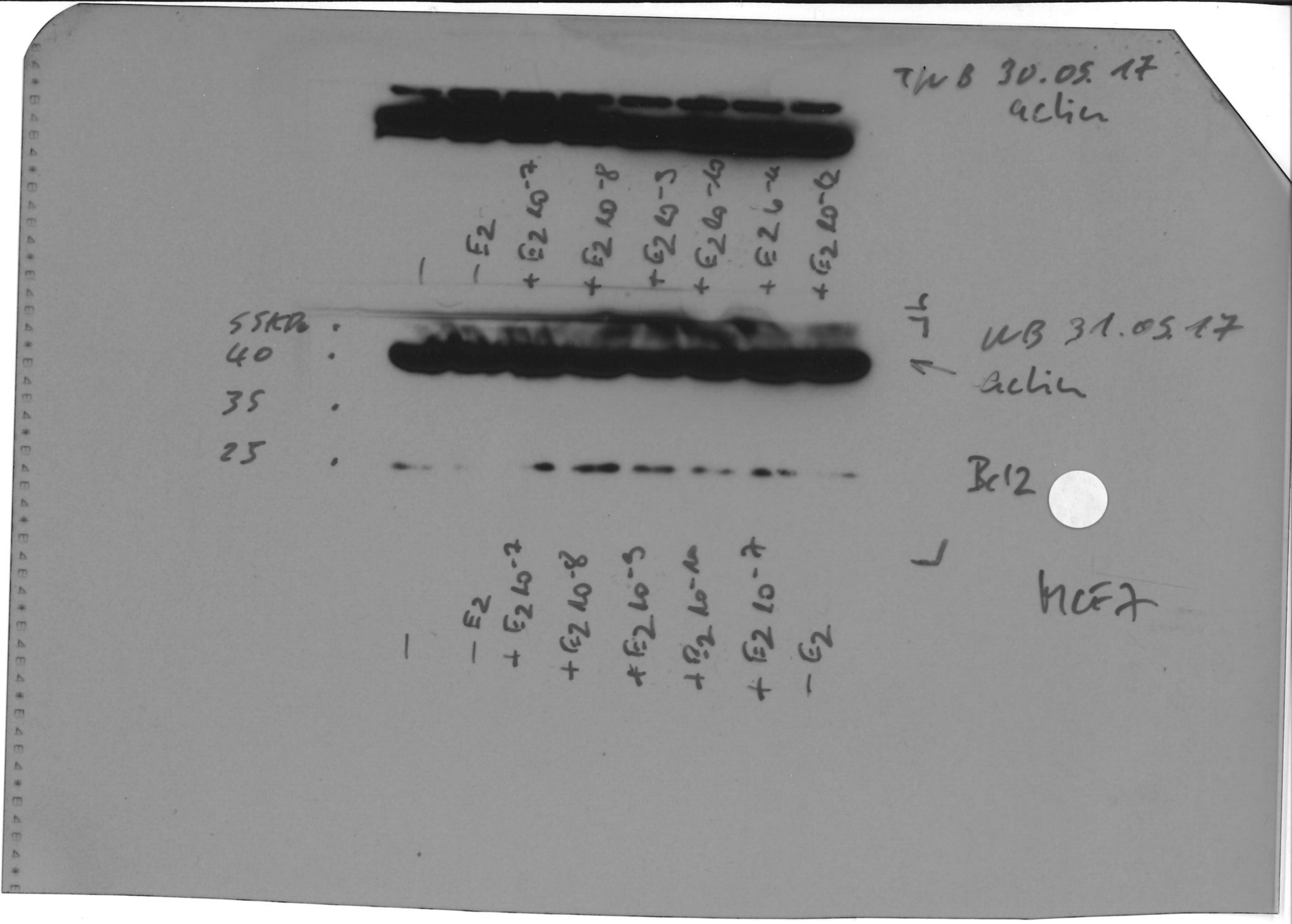
WB 30.05.17
actin
55kDa
40
35
25
WB 31.05.17
actin
Bcl2
MCF7

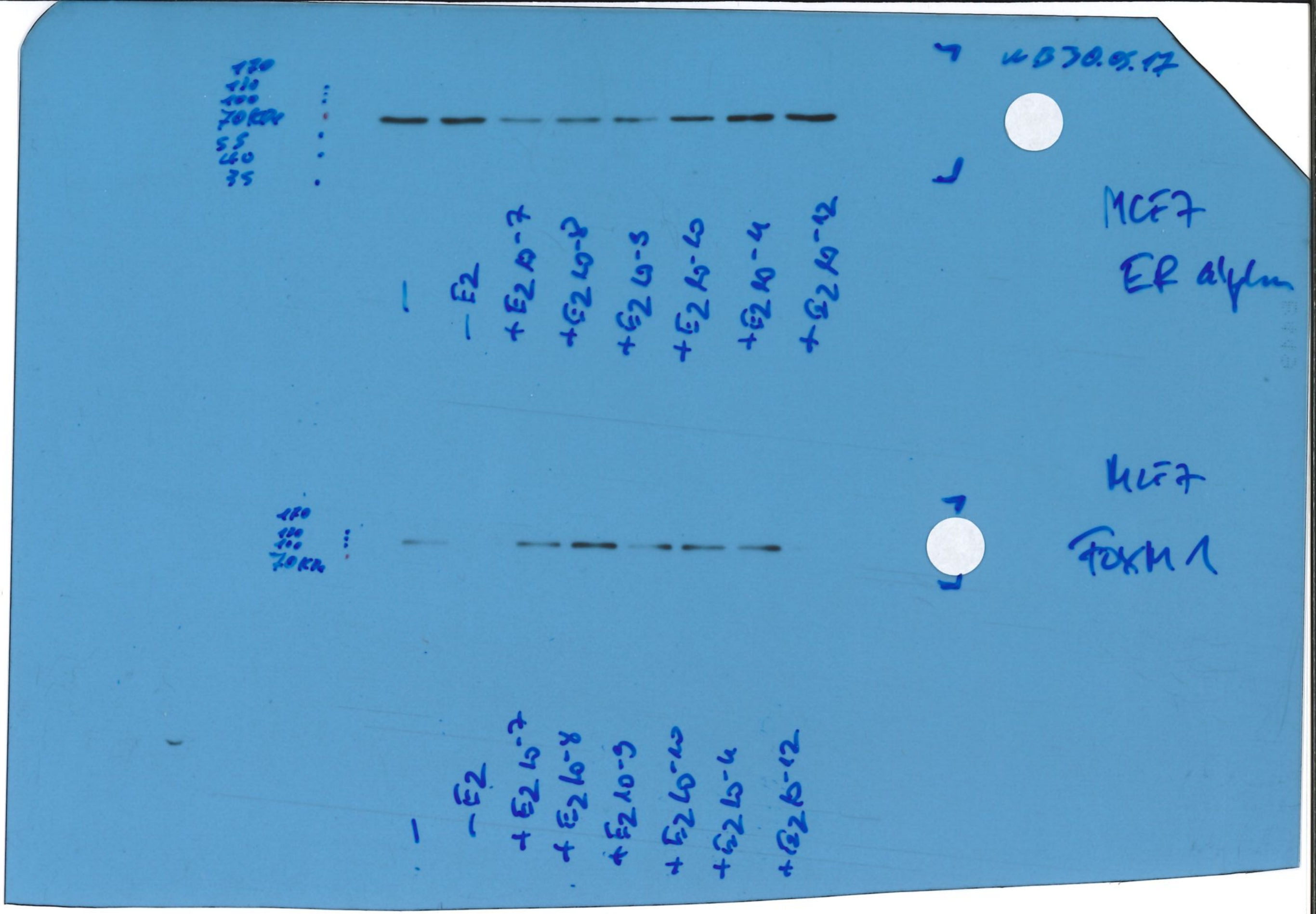

MCF7
MCF7
FoxM1
70 kDa

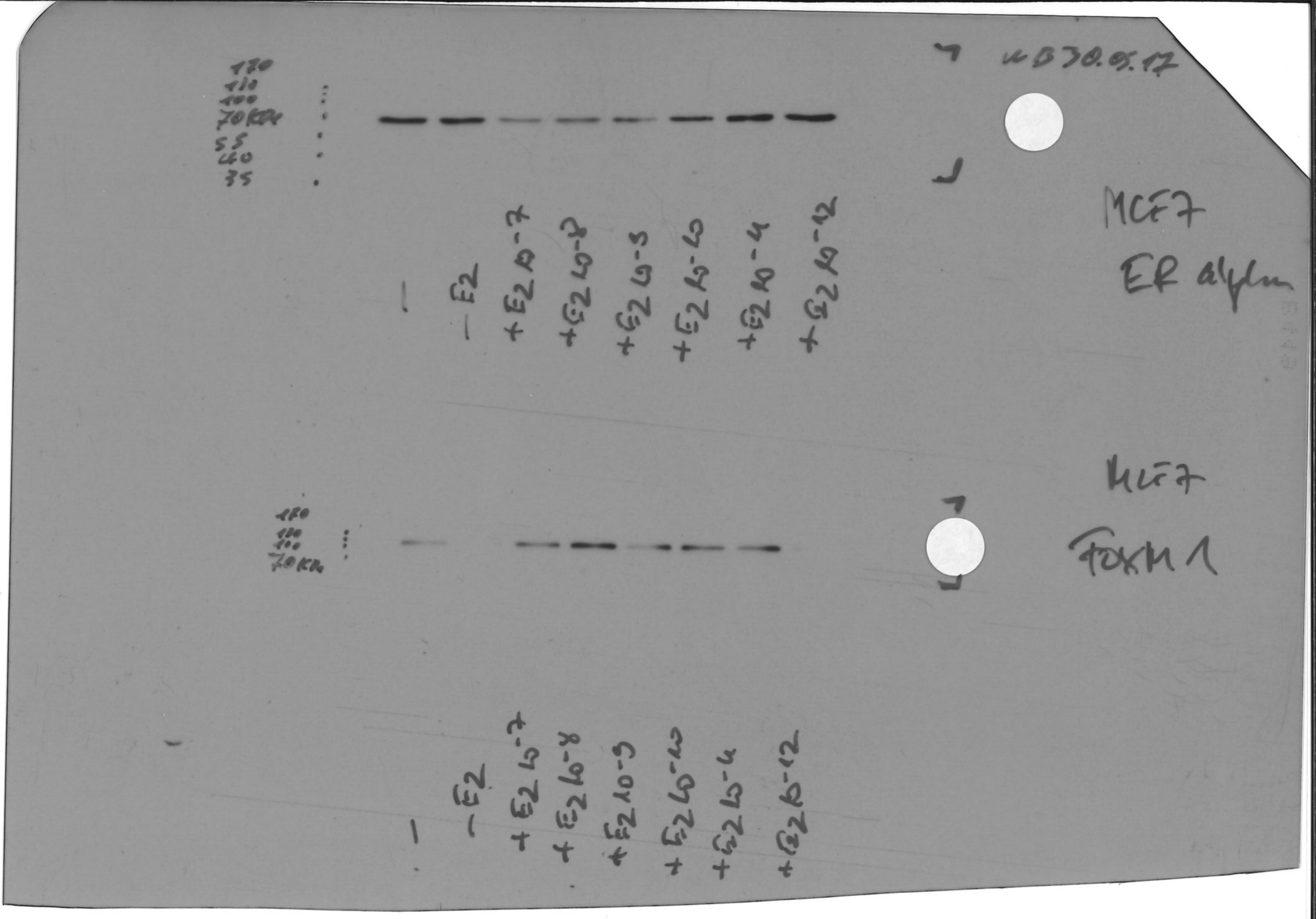
WB 30.05.17
170
130
100
70 kDa
55
40
35
MCF7
MCF7
FoxM1

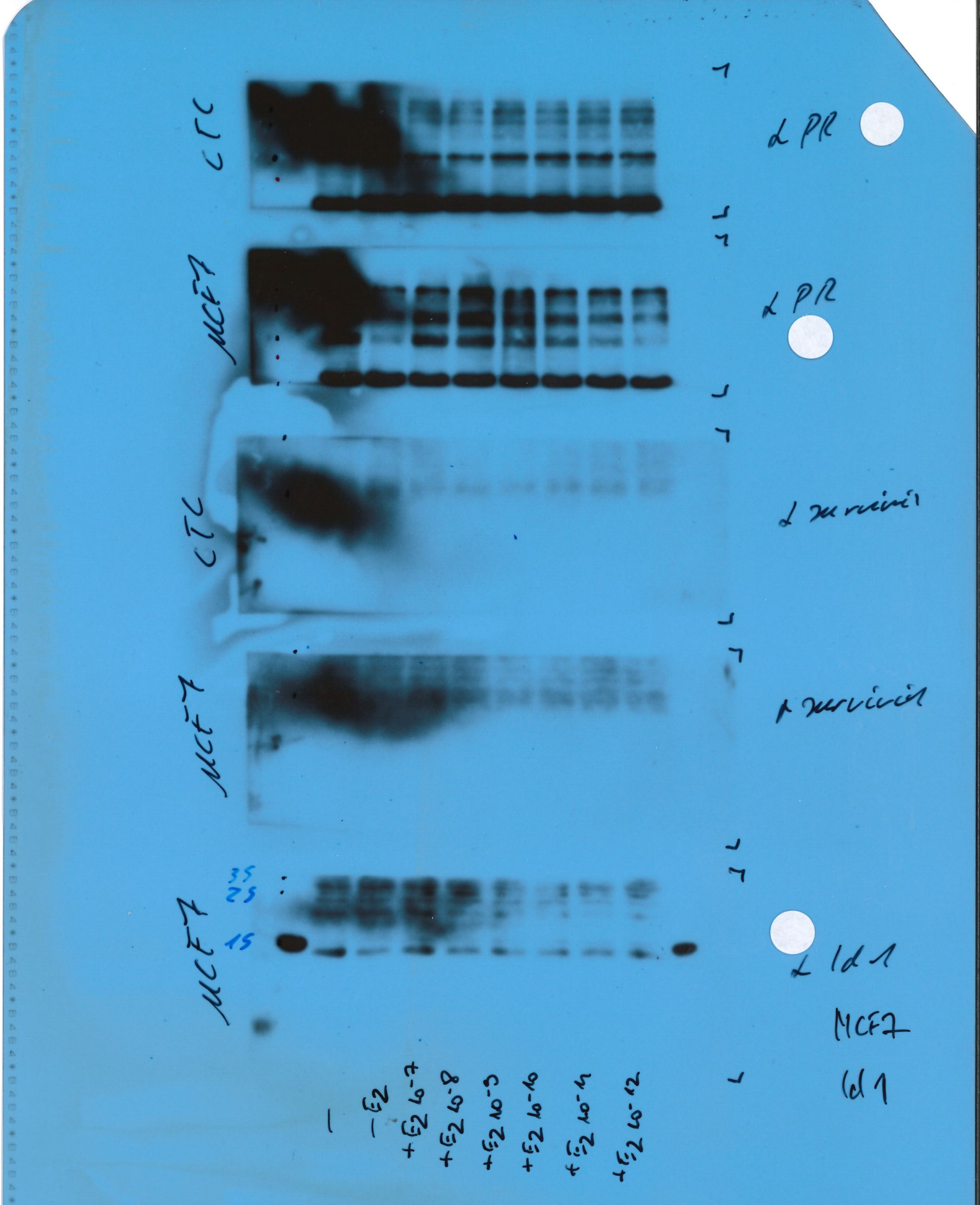
α PR
α PR
α survivin
α survivin
α Id1
MCF7
Id1
CTC
MCF7
CTC
MCF7
MCF7
35
25
15

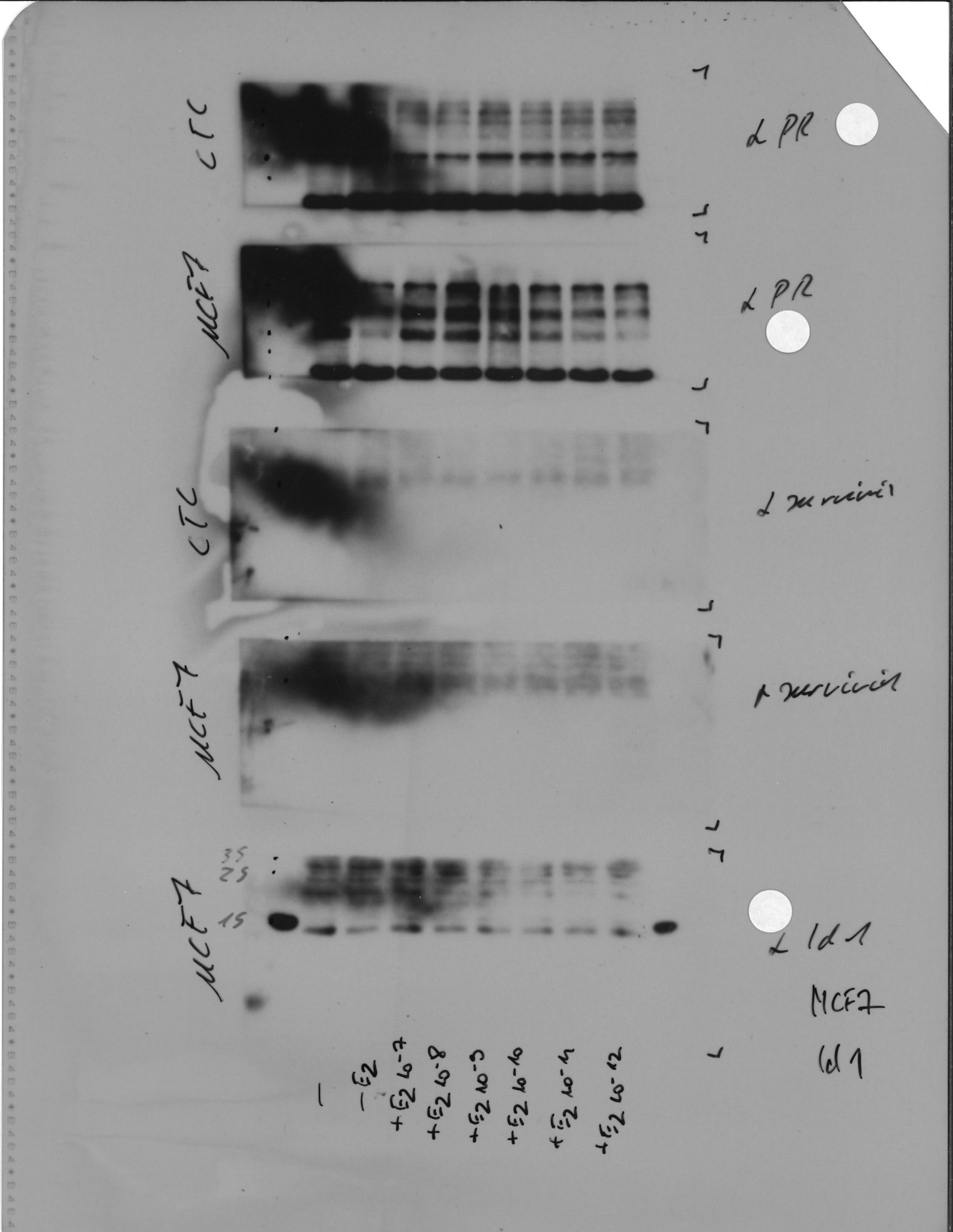
CTC
α PR
MCF7
α PR
CTC
α survivin
MCF7
α survivin
35
25
15
MCF7
α Id1
MCF7
Id1
+E2 10-7
+E2 10-8
+E2 10-10
+E2 10-12

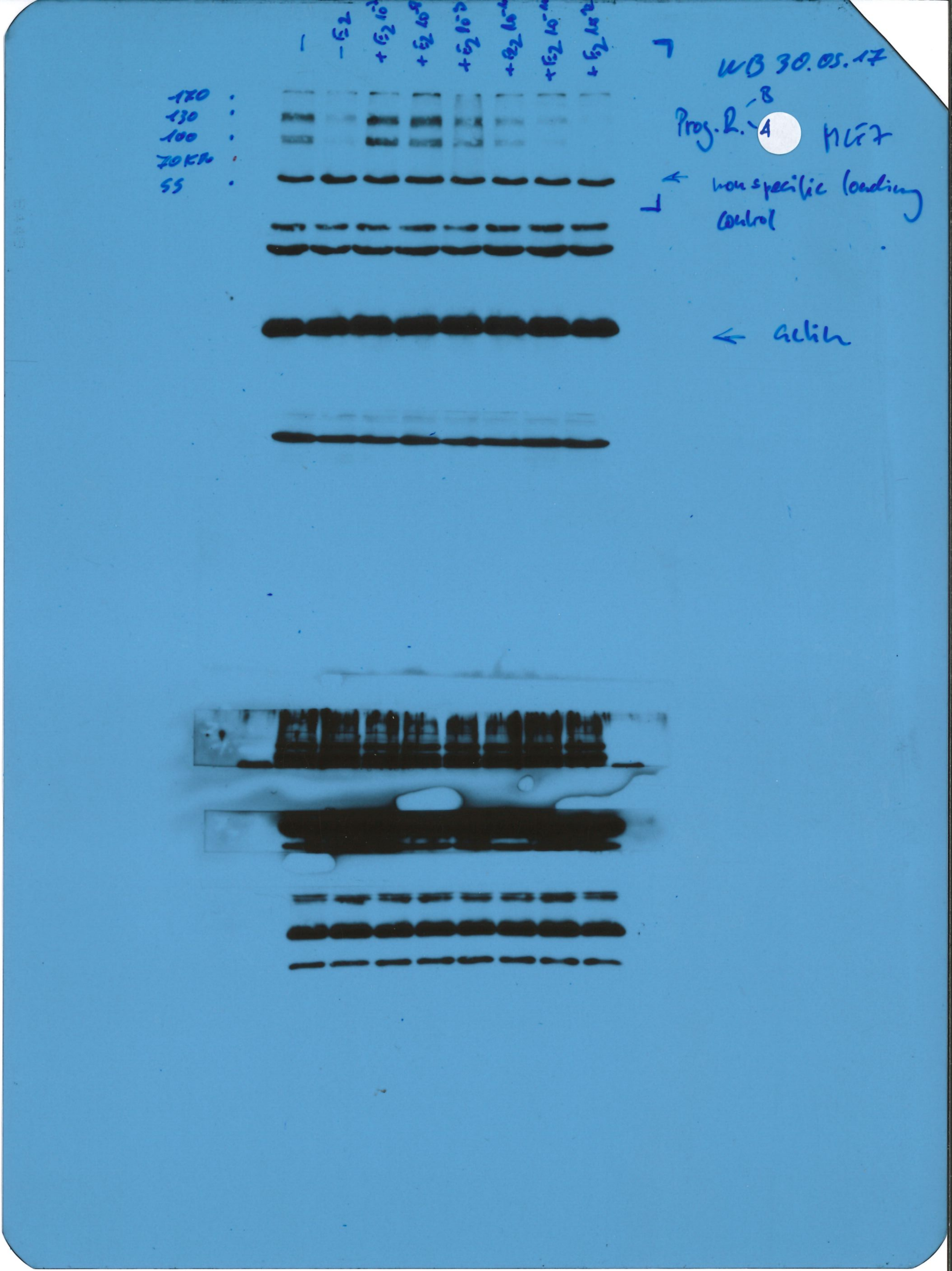

WB 30.05.17
Prog. R. B A
MCF7
170
130
100
70 kDa
55
non specific loading control
actin

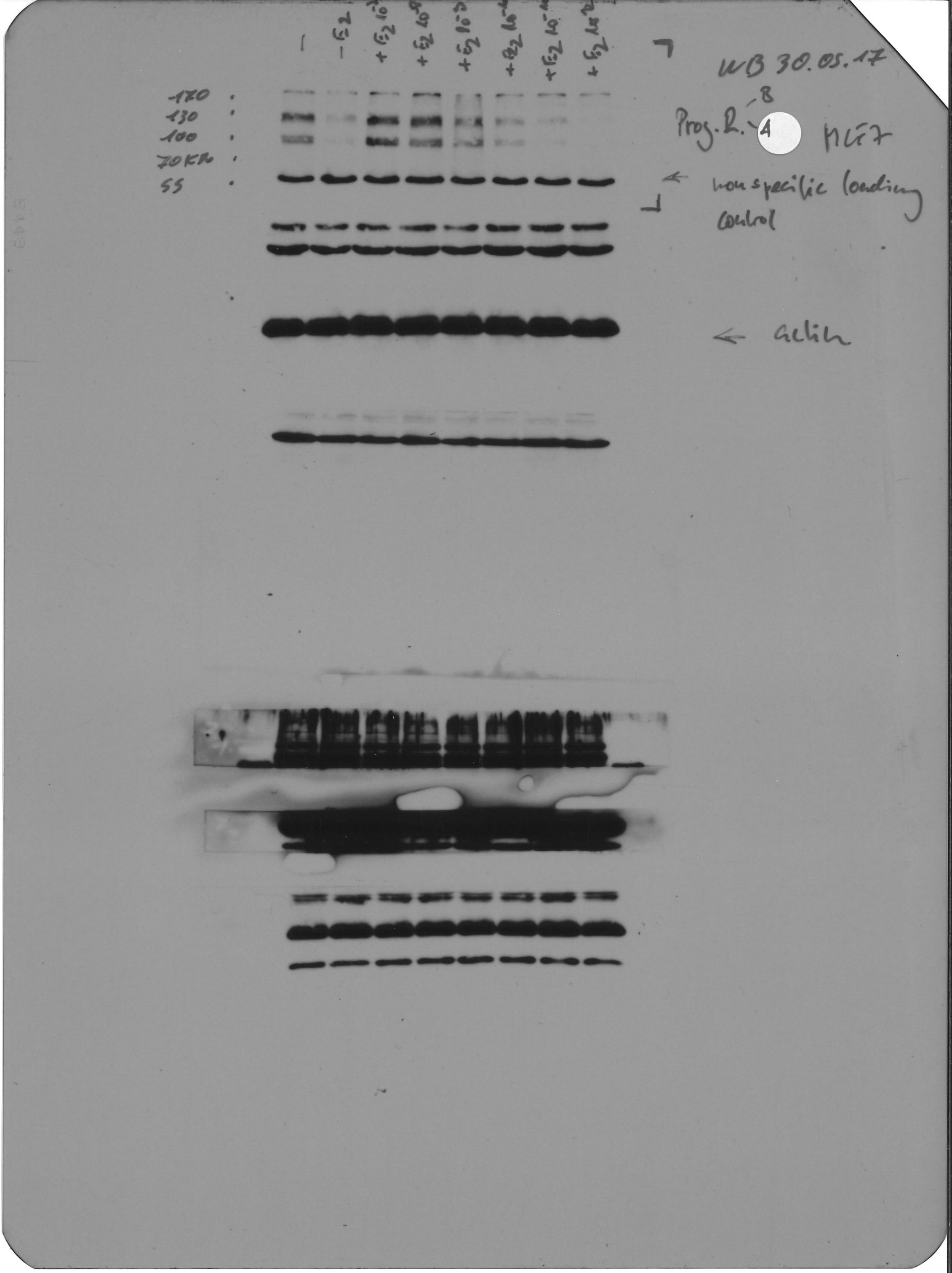
WB 30.05.17
Prog. R. -B -A
MCF7
170
130
100
70 KDa
55
non specific loading control
actin
