# Supplementary material for: Characterization of circulating breast cancer cells with tumorigenic and metastatic capacity
Source: EMBO Mol Med. 2020 Jul 15;12(9):e11908. doi: 10.15252/emmm.201911908 (PMC7507517; doi:10.15252/emmm.201911908)
Supplement: Supplementary file 9 — Source Data for Figure 1 [file EMMM-12-e11908-s007.pdf]

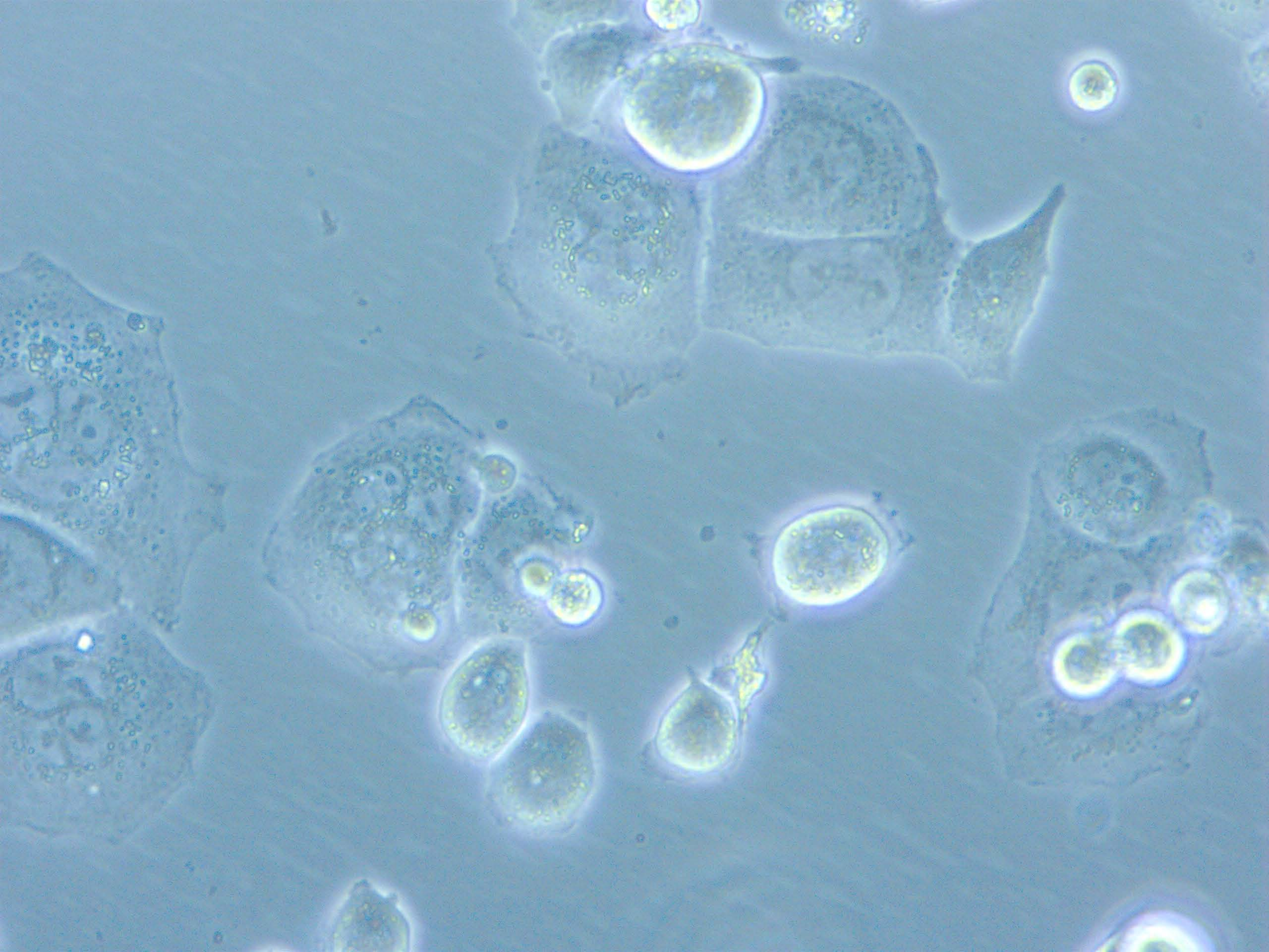

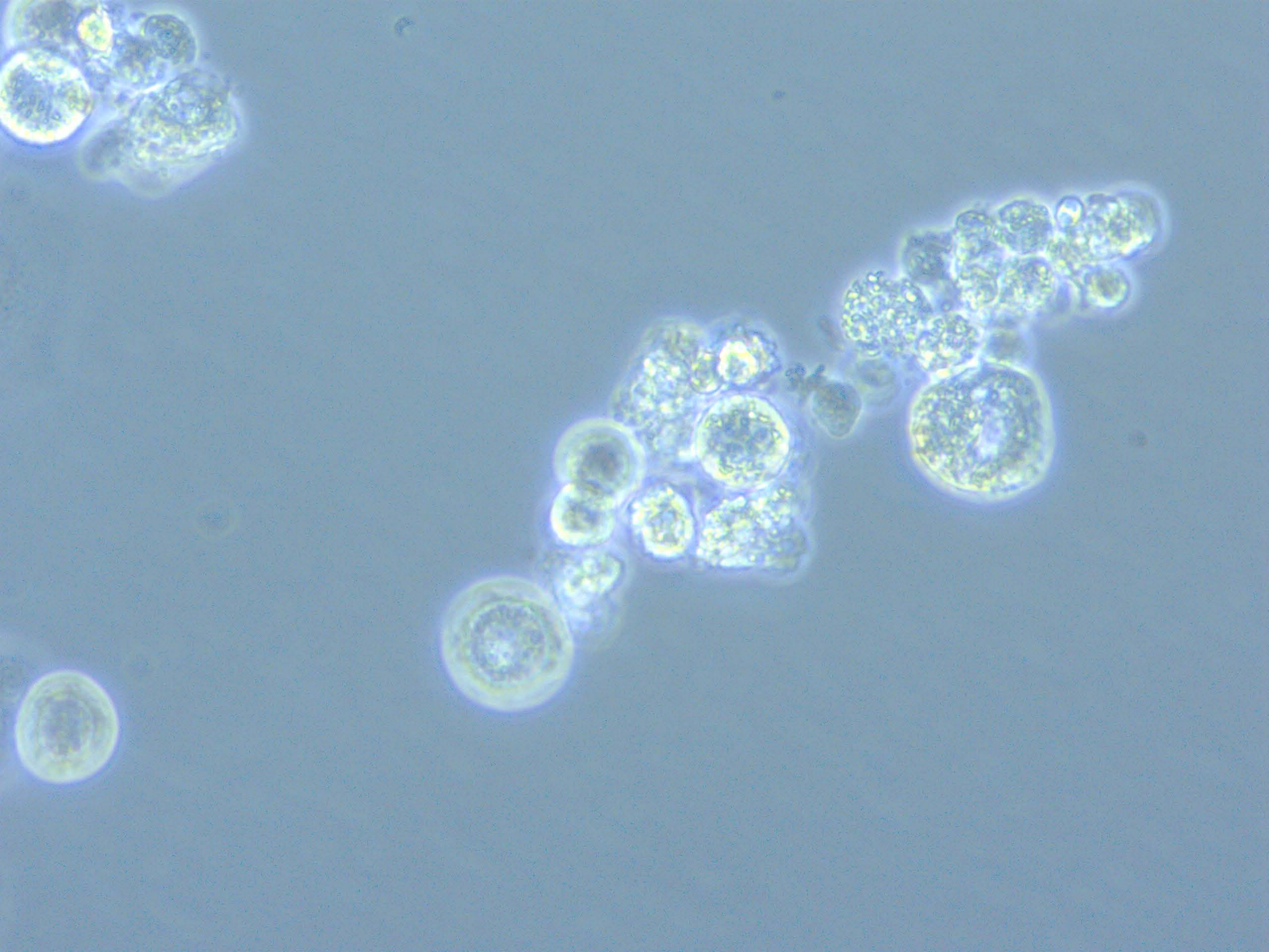

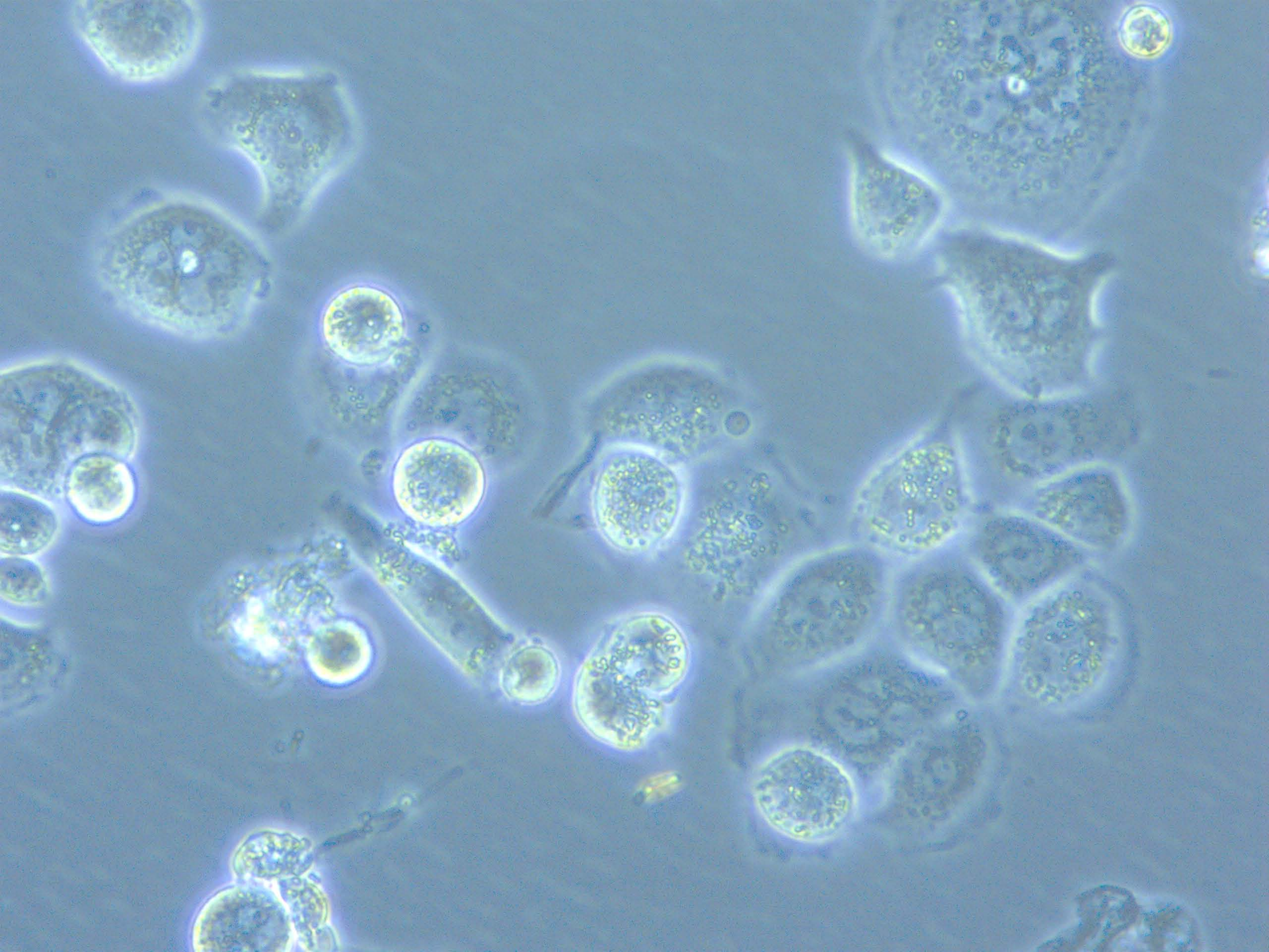

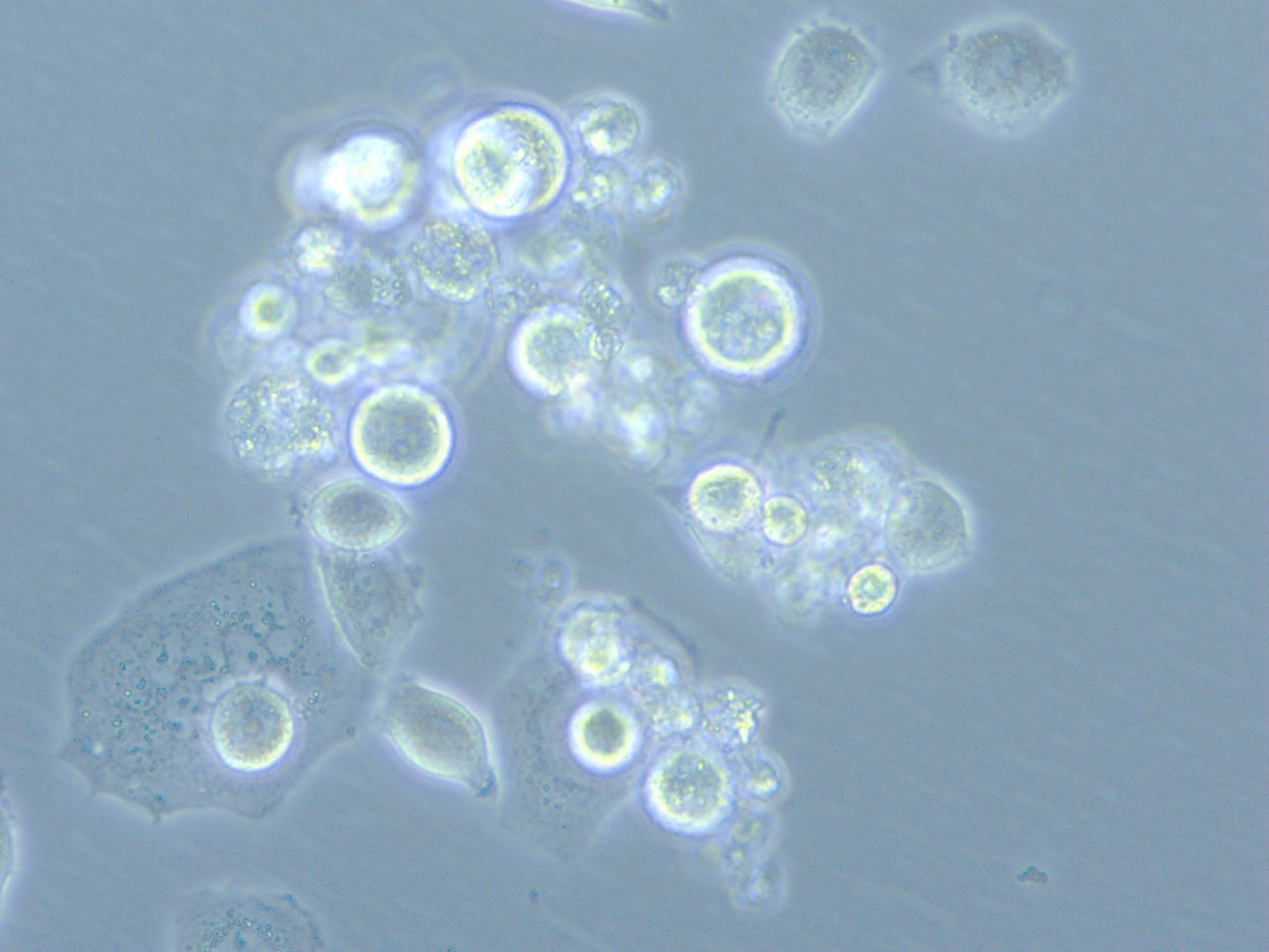

ÜBERPRÜFUNG DER FORSCHUNGSPROBE DRUCKEN

Druckdatum:02/06/2020 16:41

Proben-ID: Gyn14.208 **Kassetten-ID:**01327106 **Patienten-ID:**

Ereignis	Aufnahme	DAPI/CK-PE	CK-PE	DAPI	CD45-APC	HER-2/neu
4001	32	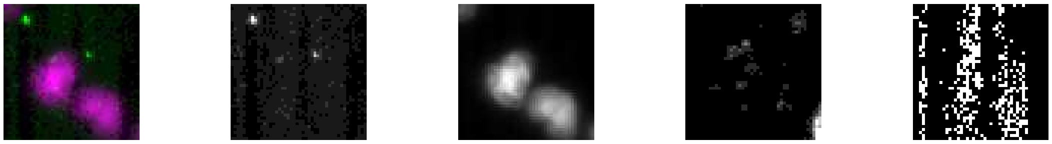				
4002	32	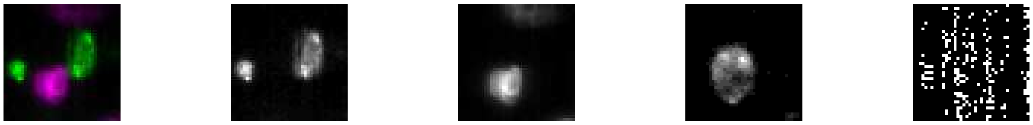				
4003	32	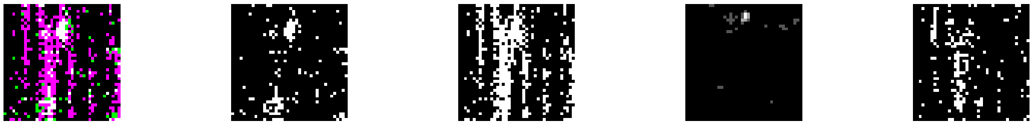				
4004	32	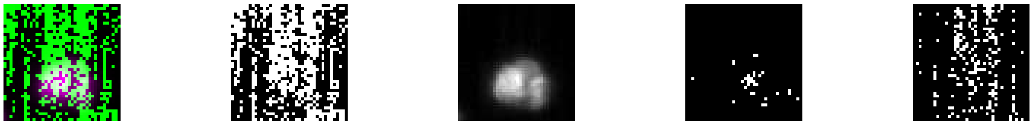				
4005	32	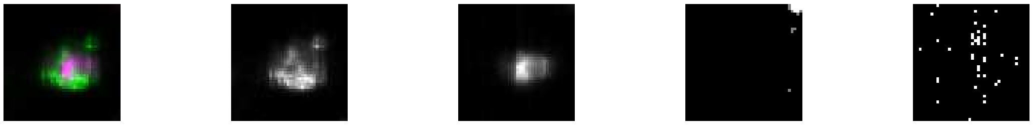				
4006	32	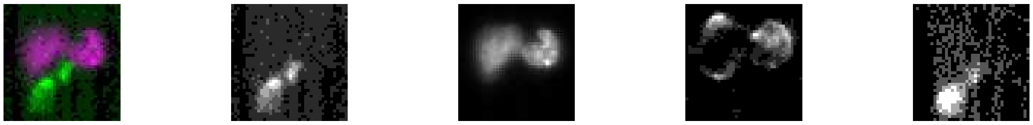				
4007	32	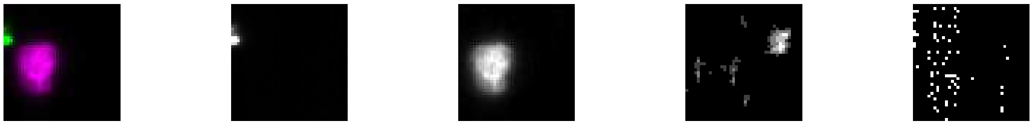				
4008	32	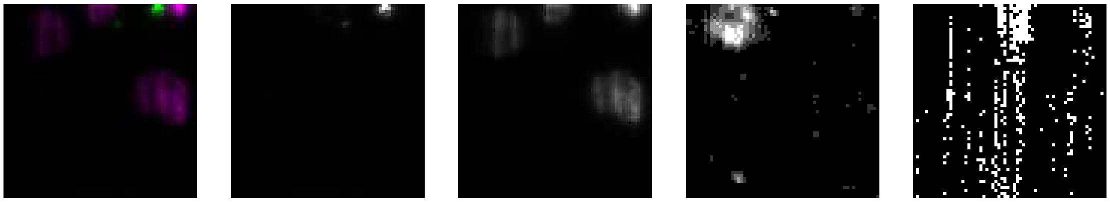				
4009	32	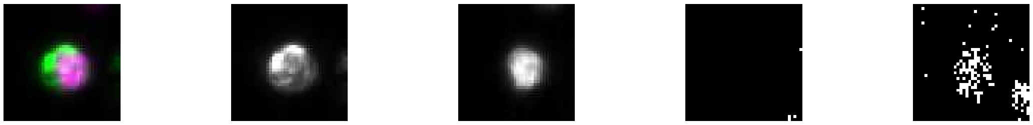				
4010	32	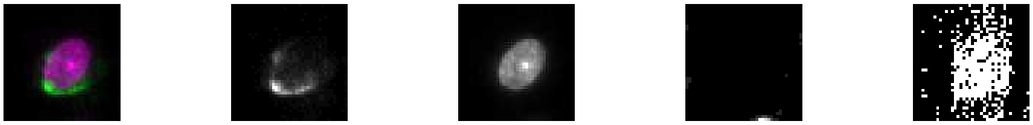				
4011	32	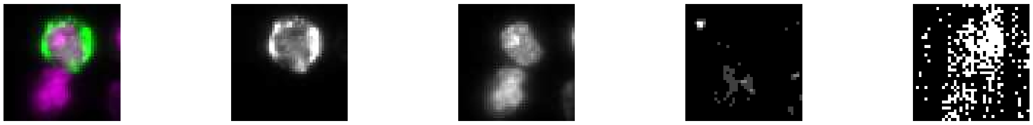				
4012	32	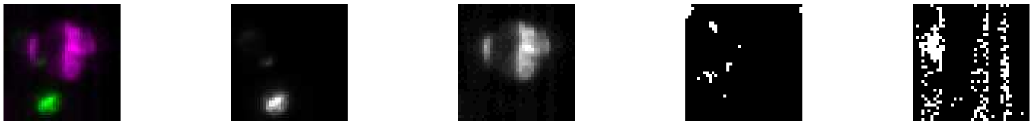				

Ereignis	Aufnahme	DAPI/CK-PE	CK-PE	DAPI	CD45-APC	HER-2/neu
4013	32	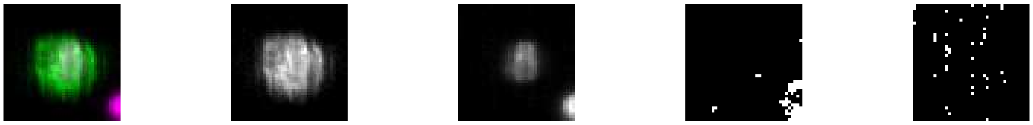				
4014	32	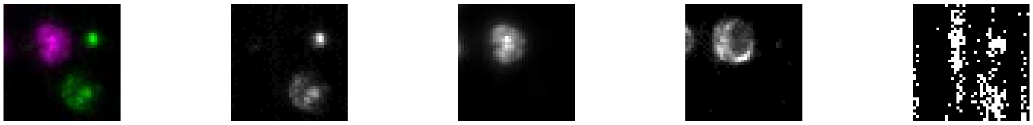				
4015	32	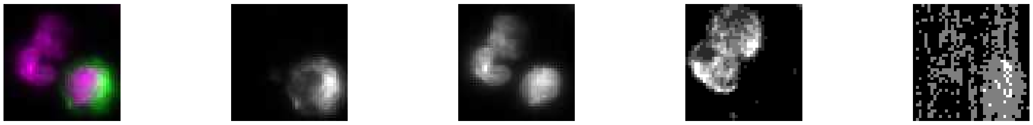				
4016	31	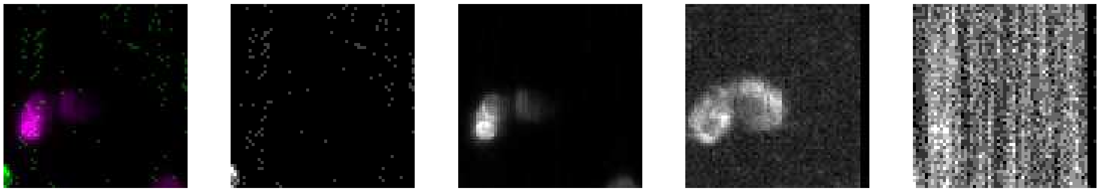				
4017	32	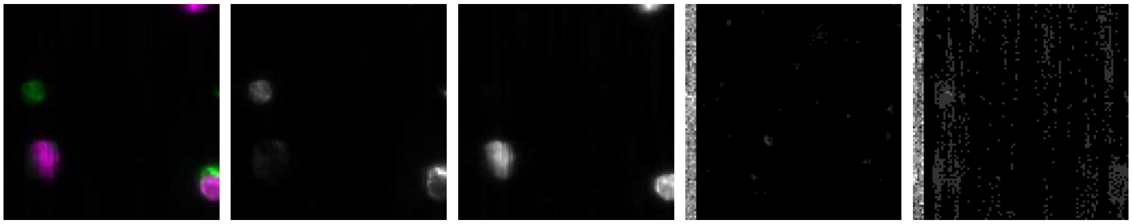				
4018	33	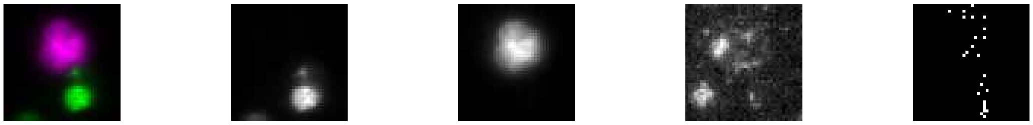				
4019	33	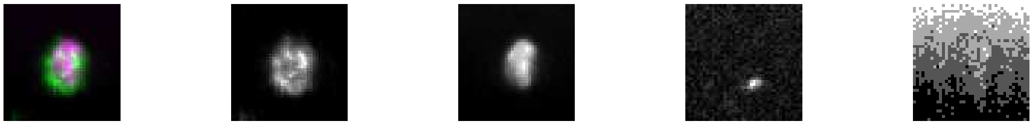				
4020	33	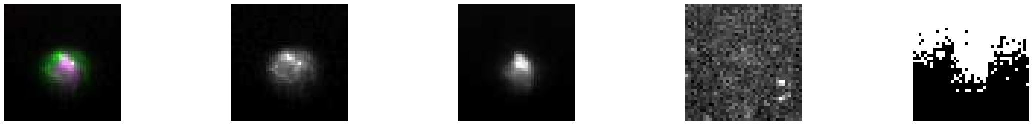				
4021	33	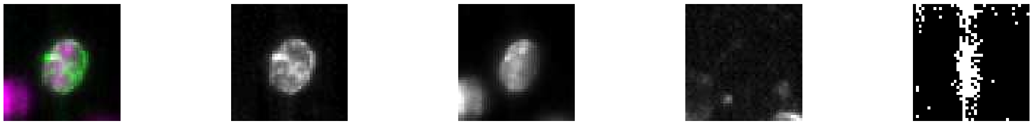				
4022	33	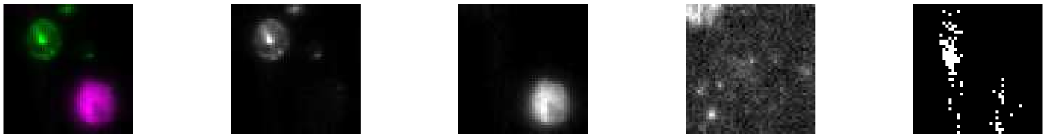				
4023	33	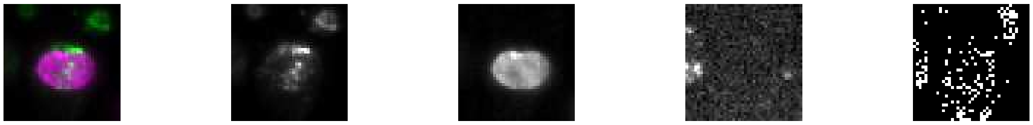				
4024	33	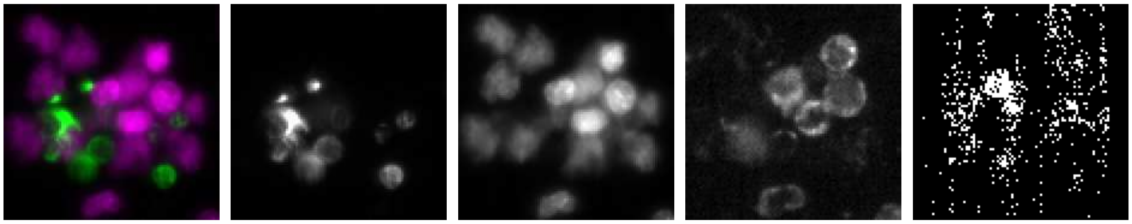				

Ereignis	Aufnahme	DAPI/CK-PE	CK-PE	DAPI	CD45-APC	HER-2/neu
4025	33	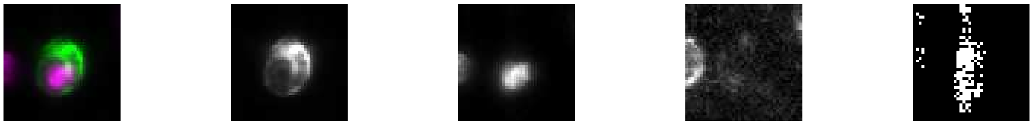				
4026	33	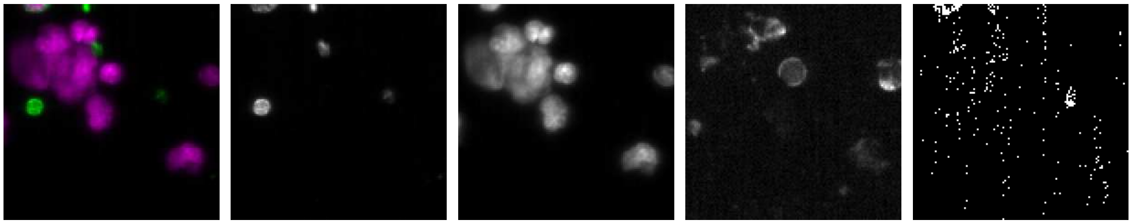				
4027	33	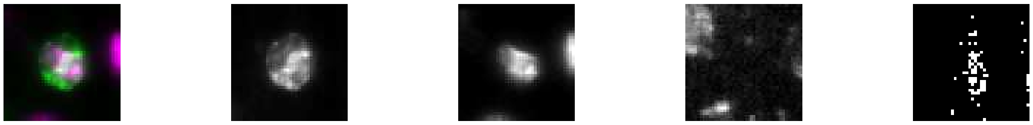				
4028	33	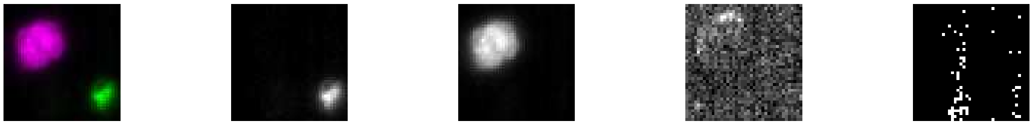				
4029	33	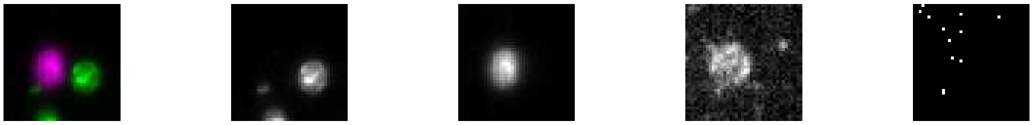				
4030	33	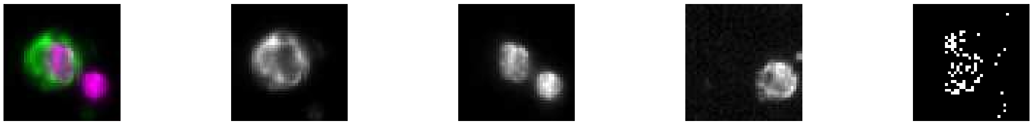				
4031	33	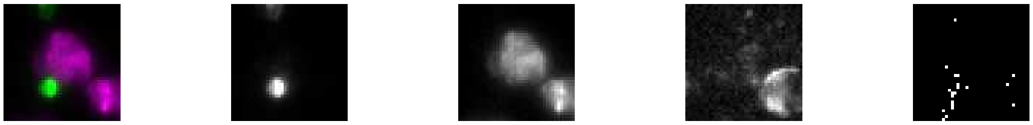				
4032	33	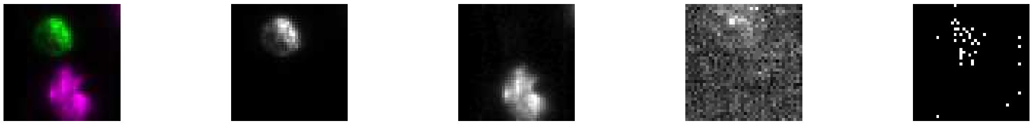				
4033	33	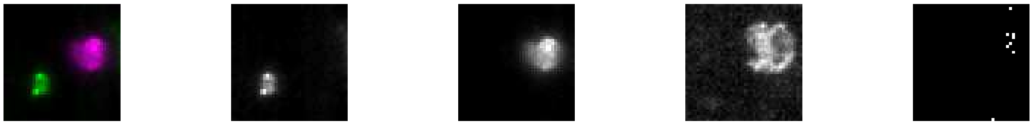				
4034	33	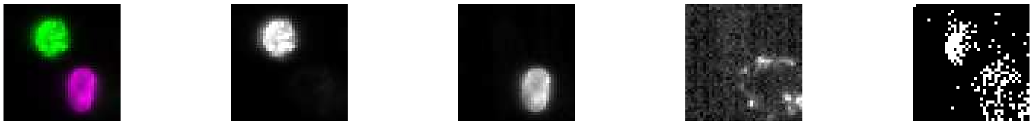				
4035	33	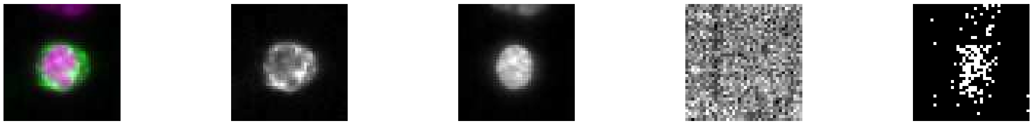				
4036	33	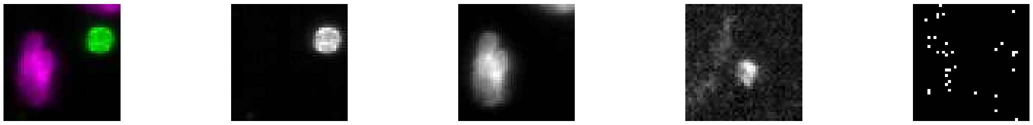				
4037	33	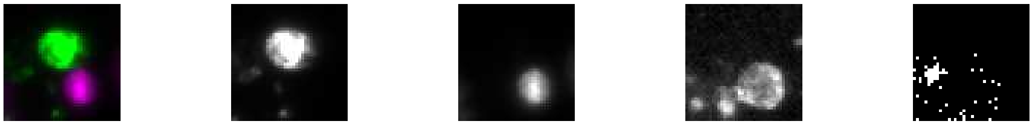				

Ereignis	Aufnahme	DAPI/CK-PE	CK-PE	DAPI	CD45-APC	HER-2/neu
4038	33	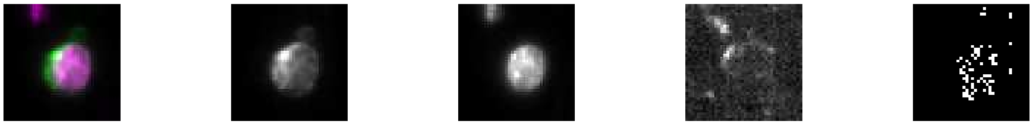				
4039	33	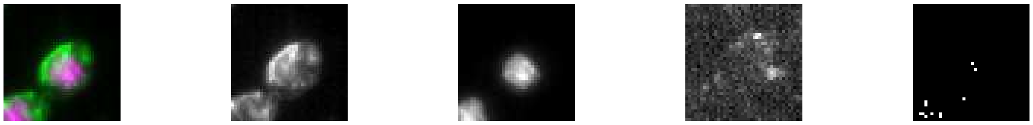				
4040	33	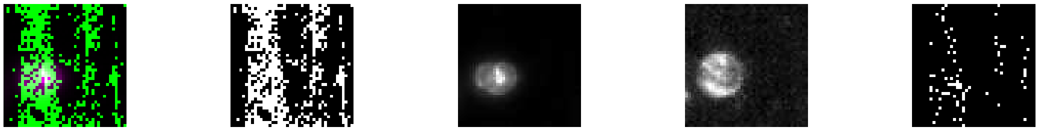				
4041	33	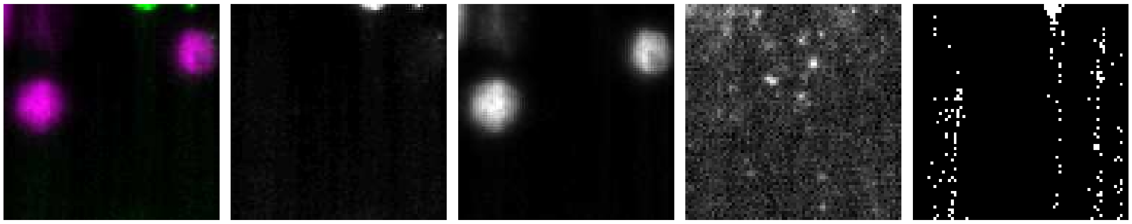				
4042	33	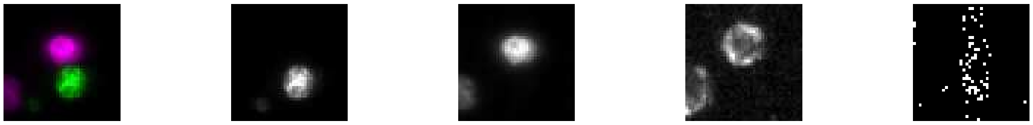				
4043	33	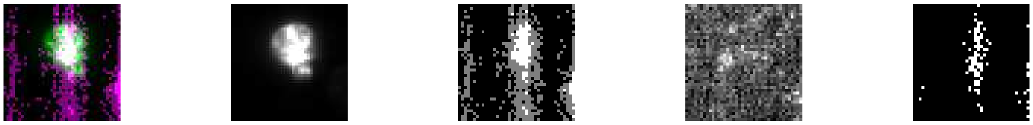				
4044	33	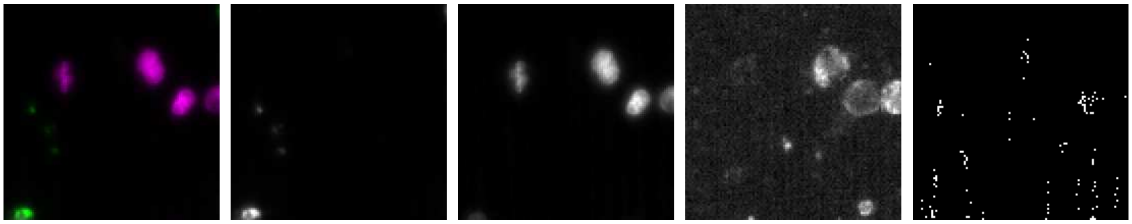				
4045	33	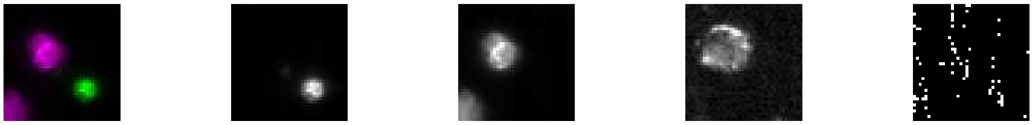				
4046	33	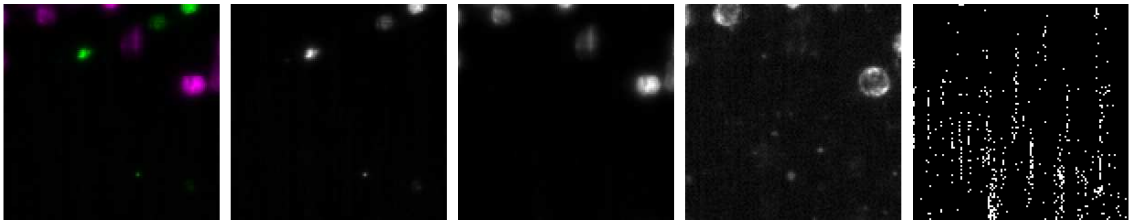				
4047	33	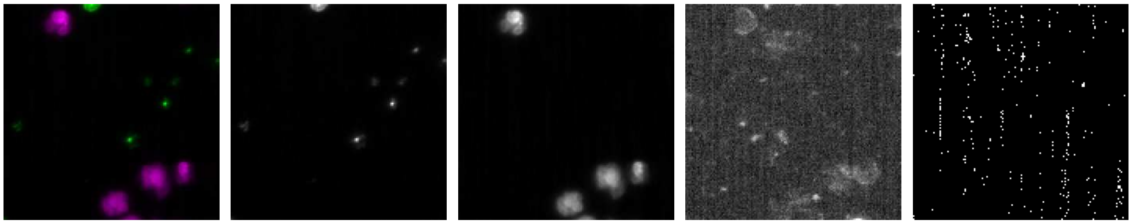				
4048	33	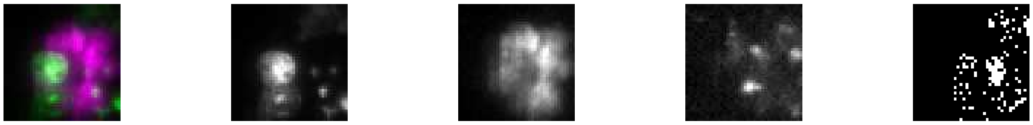				

Ereignis	Aufnahme	DAPI/CK-PE	CK-PE	DAPI	CD45-APC	HER-2/neu
4049	33	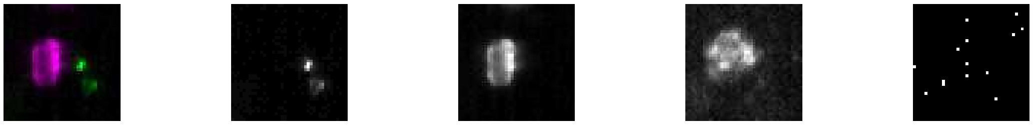				
4050	33	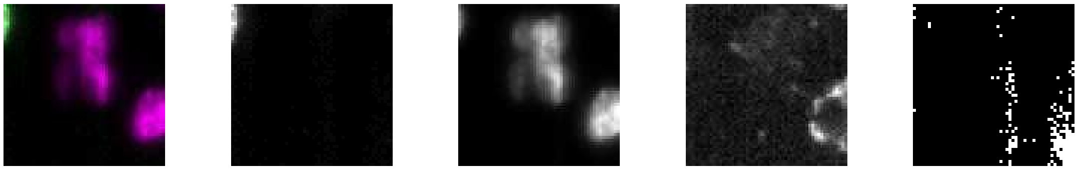				
4051	33	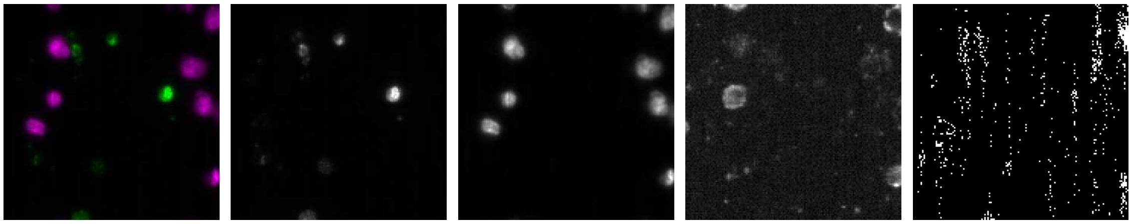				
4052	33	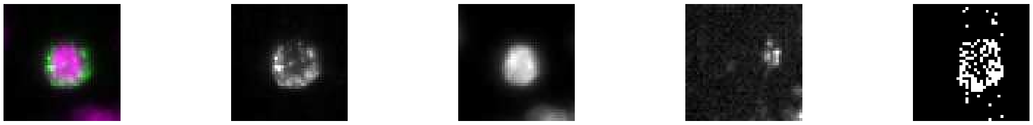				
4053	33	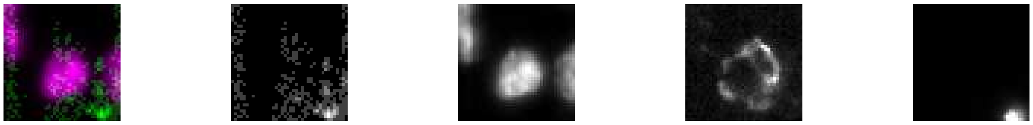				
4054	33	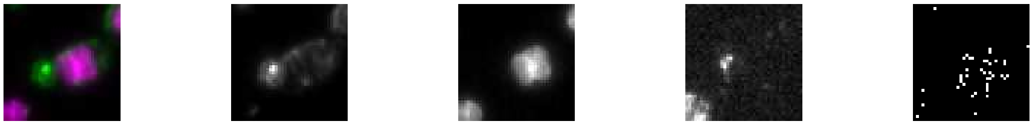				
4055	33	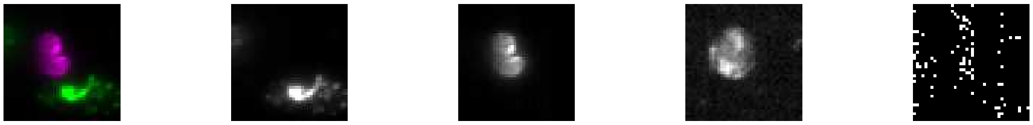				
4056	33	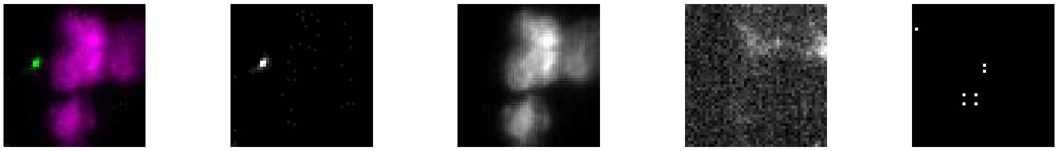				
4057	33	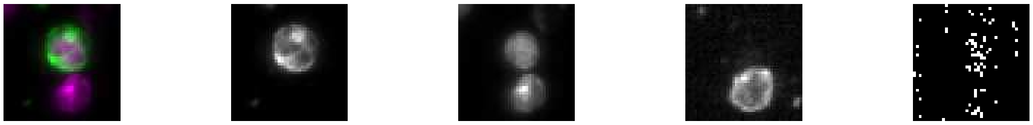				
4058	33	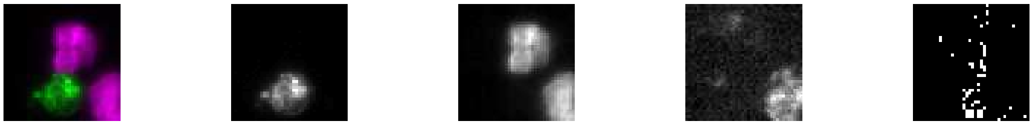				
4059	33	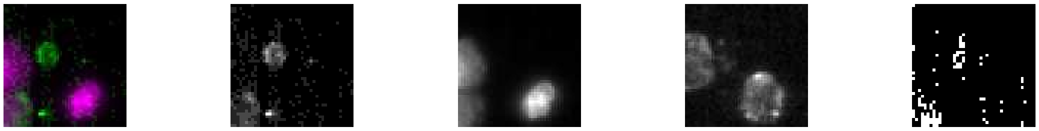				
4060	33	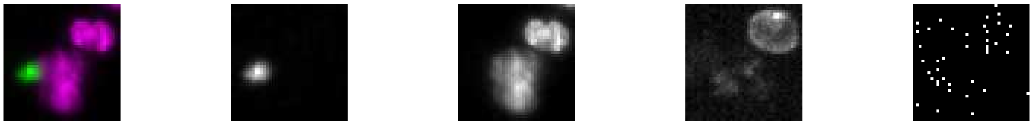				
4061	33	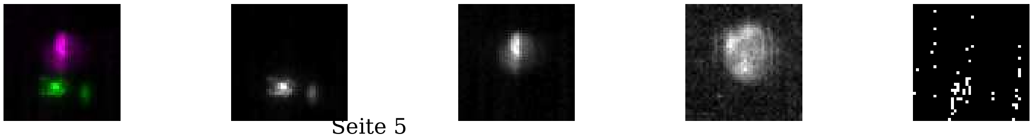				

Ereignis	Aufnahme	DAPI/CK-PE	CK-PE	DAPI	CD45-APC	HER-2/neu
4062	33	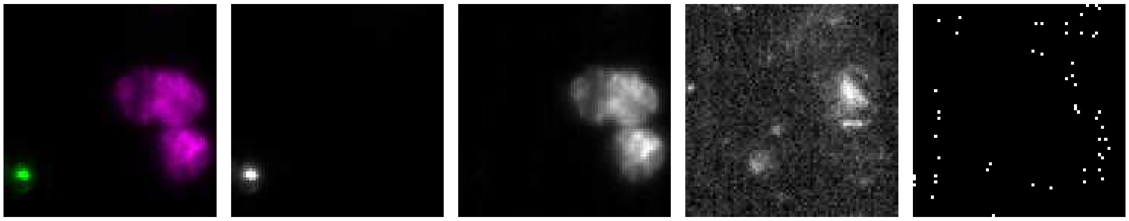				
4063	33	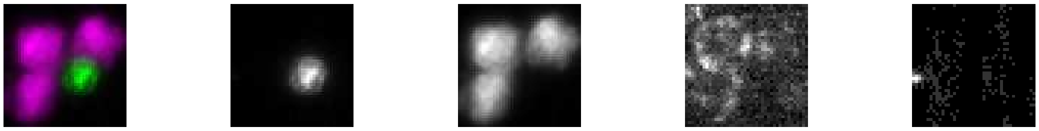				
4064	33	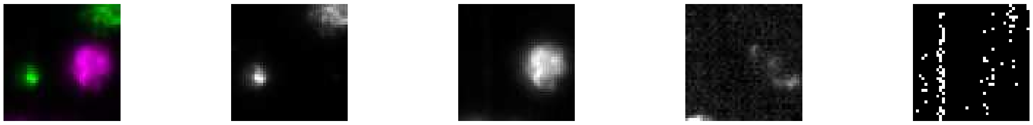				
4065	33	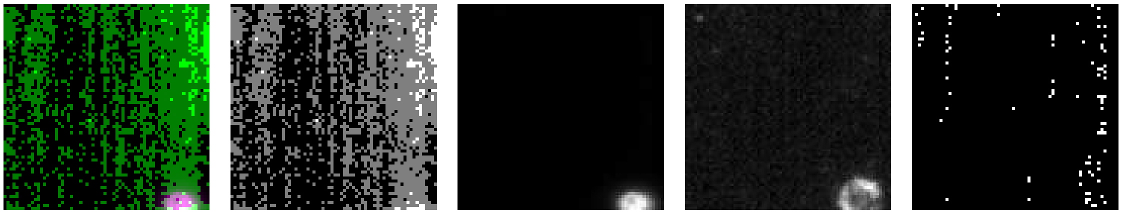				
4066	33	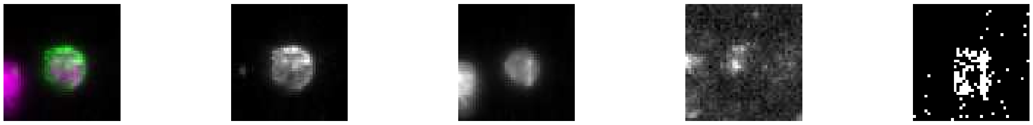				
4067	33	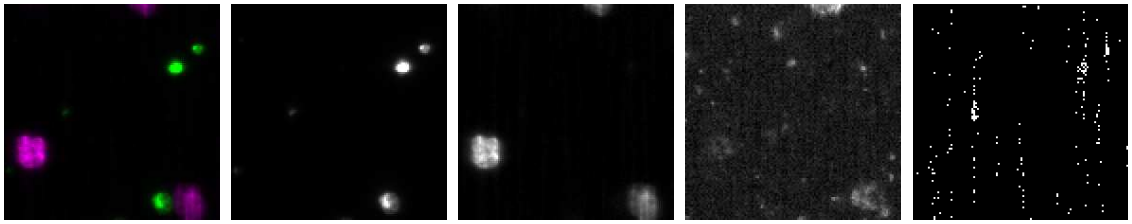				
4068	33	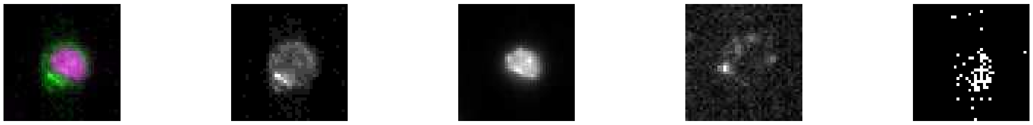				
4069	33	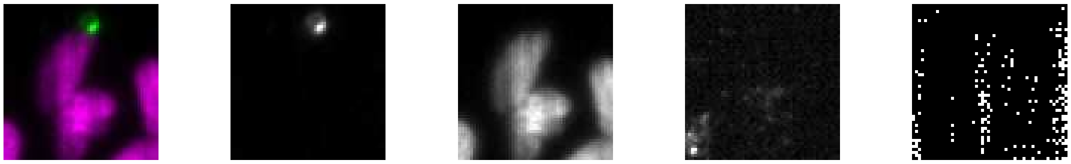				
4070	33	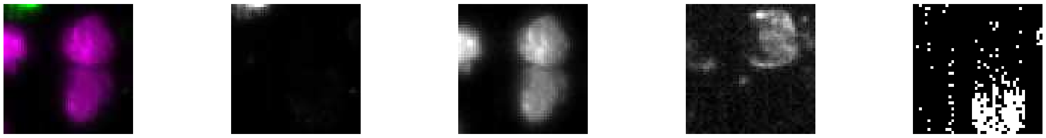				
4071	33	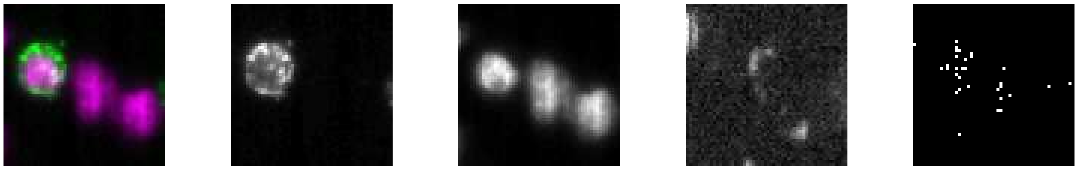				
4072	33	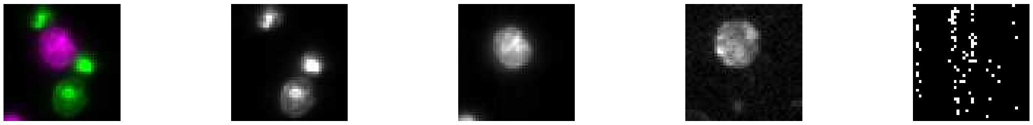				

Ereignis	Aufnahme	DAPI/CK-PE	CK-PE	DAPI	CD45-APC	HER-2/neu
4073	33	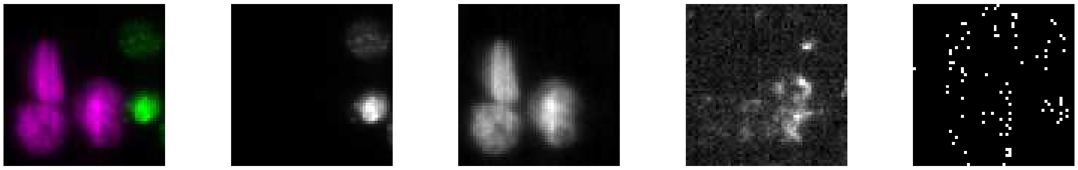				
4074	33	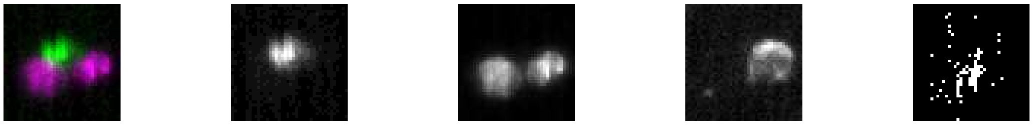				
4075	33	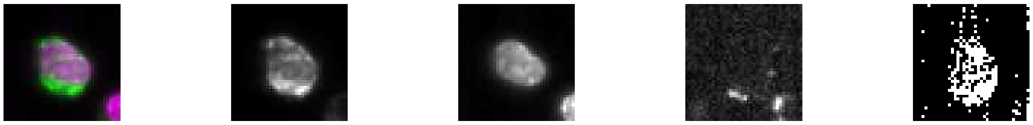				
4076	33	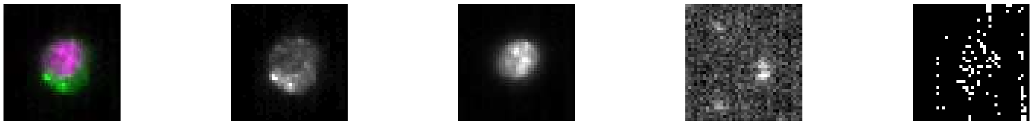				
4077	33	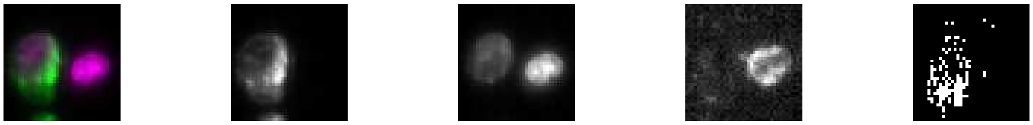				
4078	33	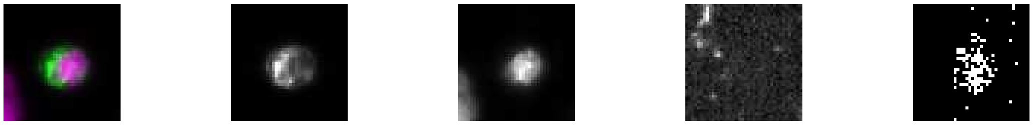				
4079	33	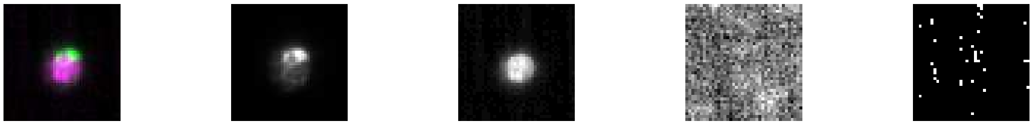				
4080	33	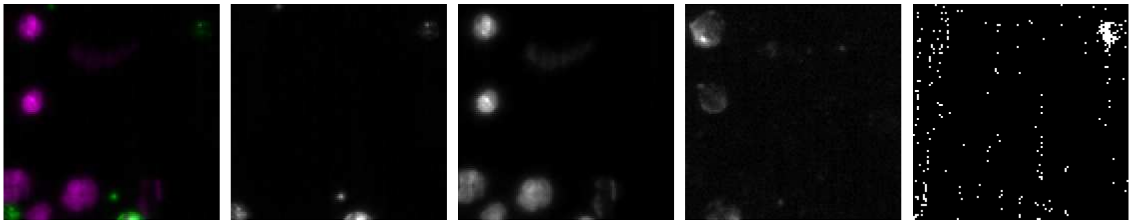				
4081	33	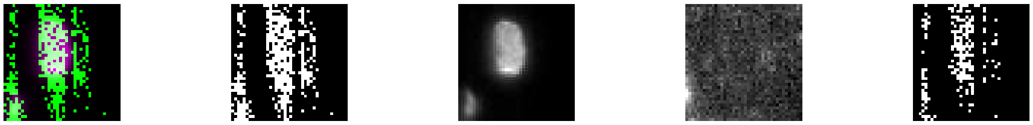				
4082	33	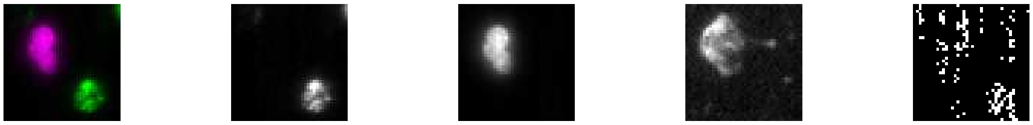				
4083	33	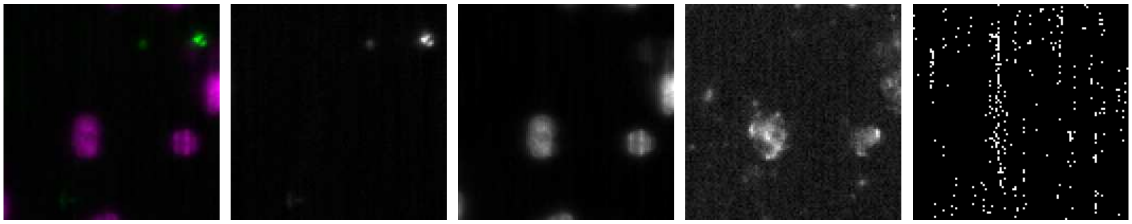				
4084	33	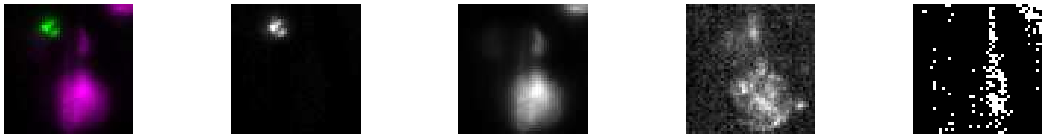				

Proben-ID: Gyn14.208 **Kassetten-ID:**01327106 **Patienten-ID:**

Ereignis	Aufnahme	DAPI/CK-PE	CK-PE	DAPI	CD45-APC	HER-2/neu
4085	33	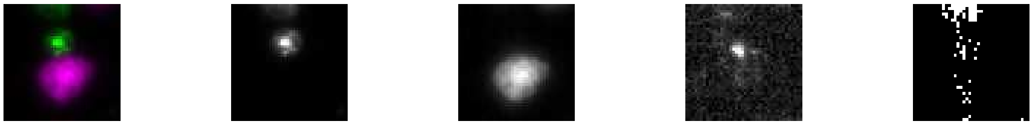				
4086	33	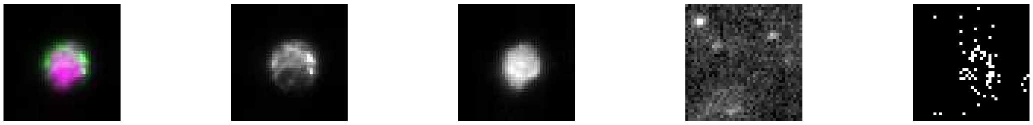				
4087	33	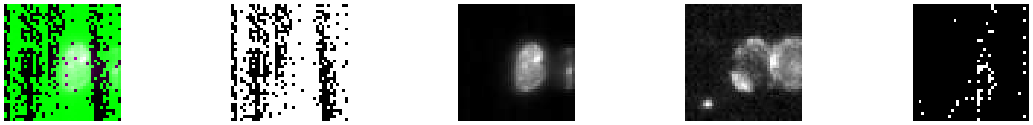				
4088	33	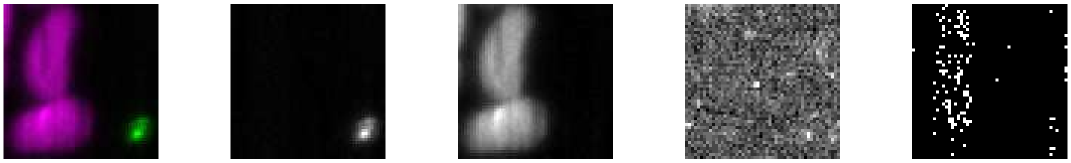				
4089	33	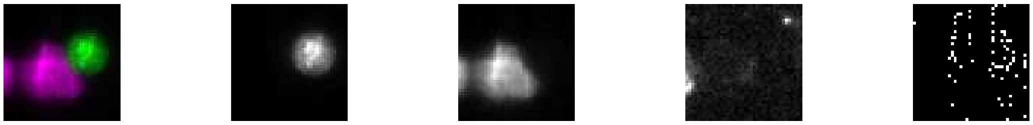				
4090	33	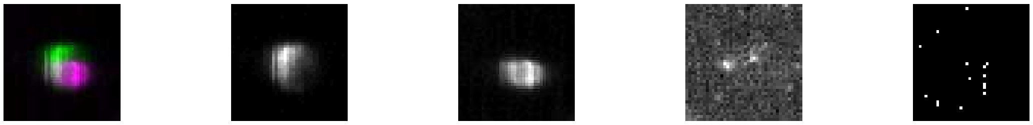				
4091	33	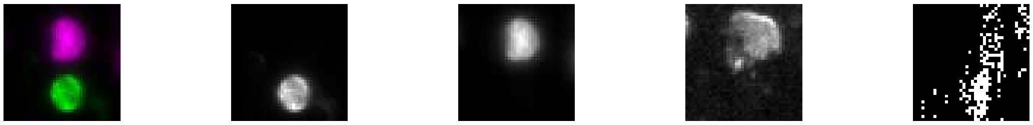				
4092	33	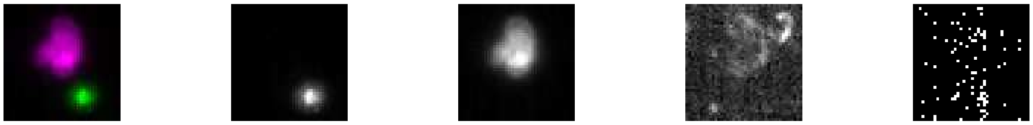				
4093	33	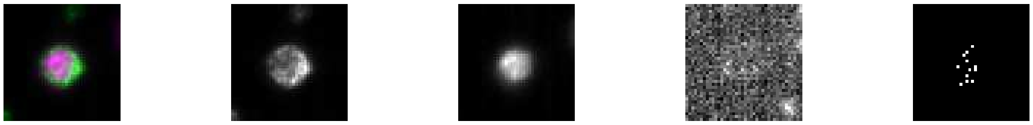				
4094	33	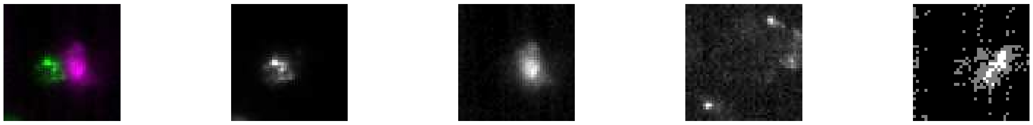				
4095	33	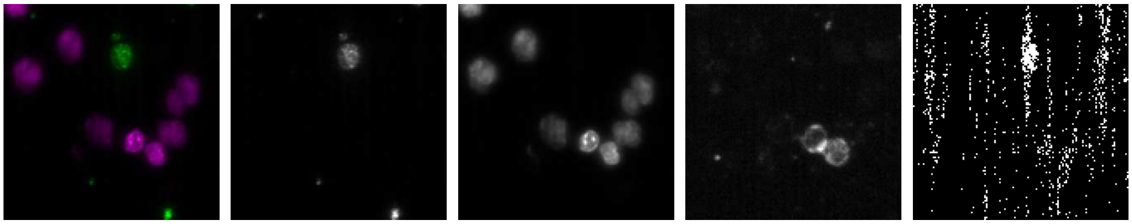				
4096	33	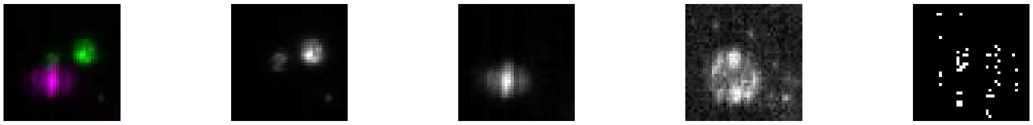				

Proben-ID: Gyn14.208 **Kassetten-ID:**01327106 **Patienten-ID:**

Ereignis	Aufnahme	DAPI/CK-PE	CK-PE	DAPI	CD45-APC	HER-2/neu
4097	33	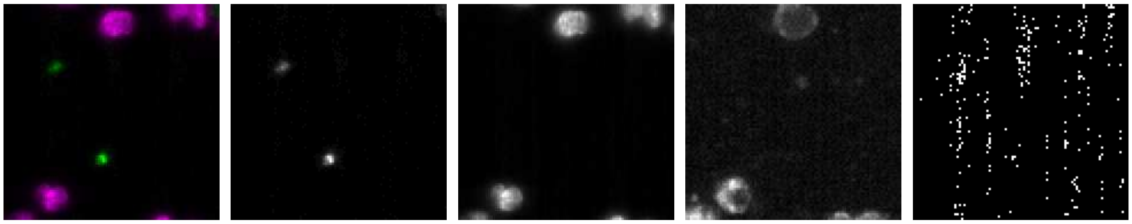				
4098	33	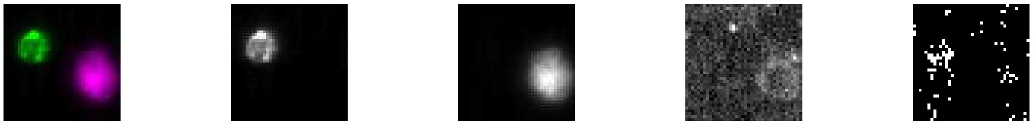				
4099	33	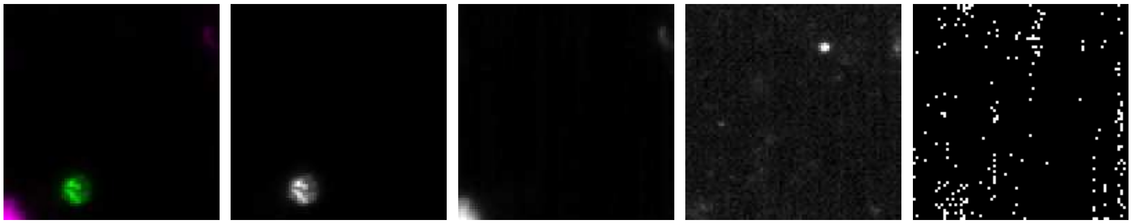				
4100	33	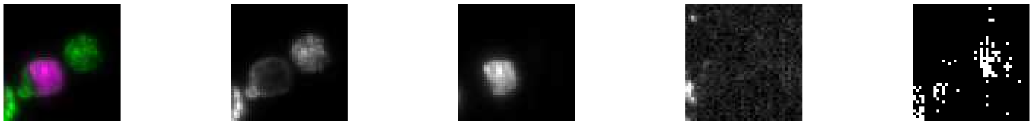				
4101	33	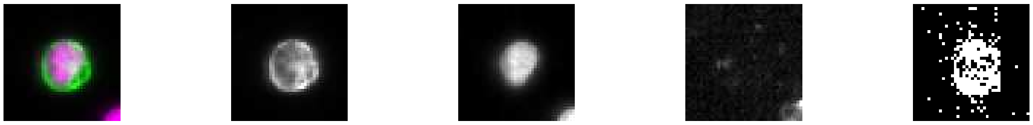				
4102	33	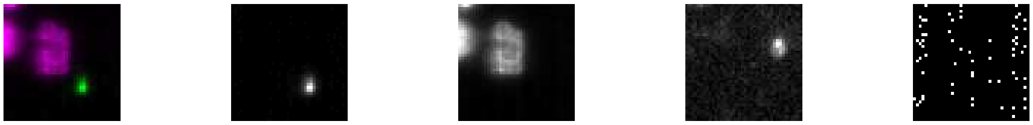				
4103	33	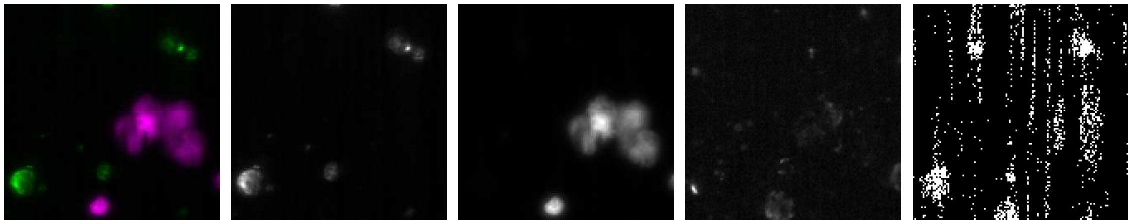				
4104	33	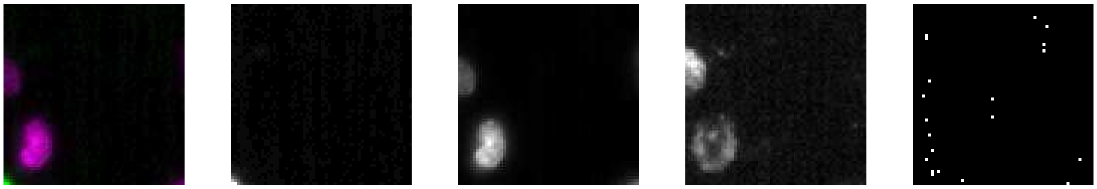				
4105	33	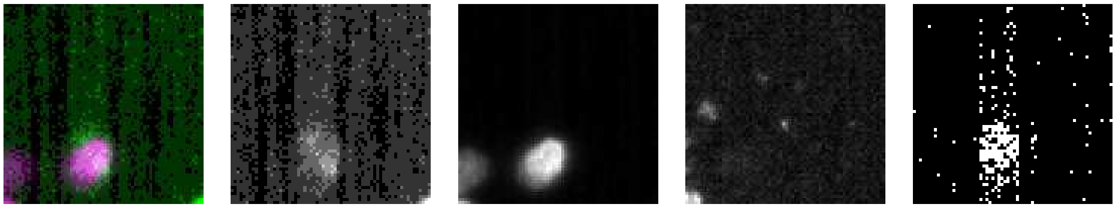				
4106	33	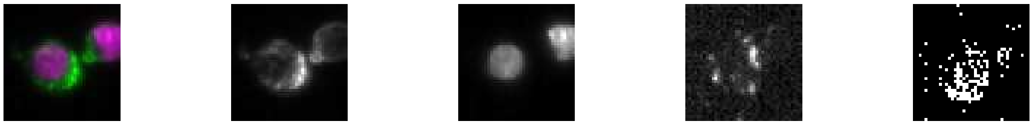				

Proben-ID: Gyn14.208 **Kassetten-ID:** 01327106 **Patienten-ID:**

Ereignis	Aufnahme	DAPI/CK-PE	CK-PE	DAPI	CD45-APC	HER-2/neu
4107	33	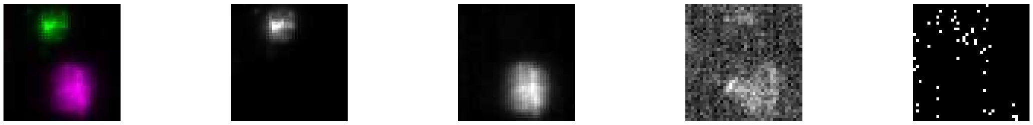				
4108	33	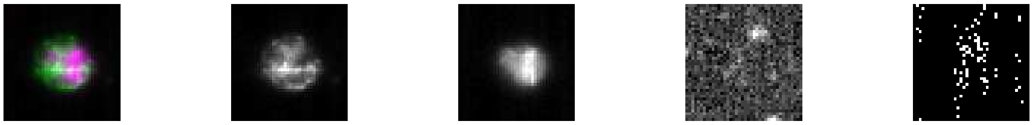				
4109	33	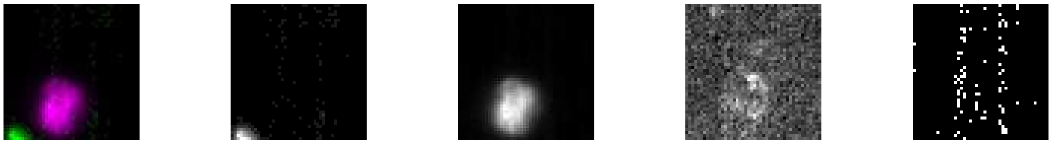				
4110	33	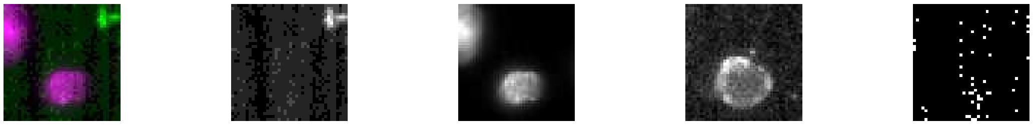				
4111	33	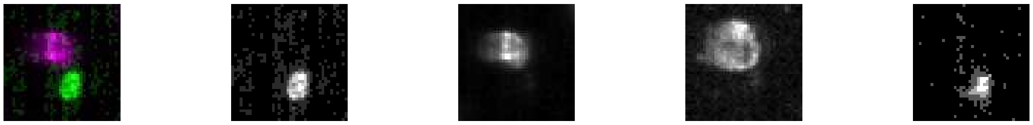				
4112	33	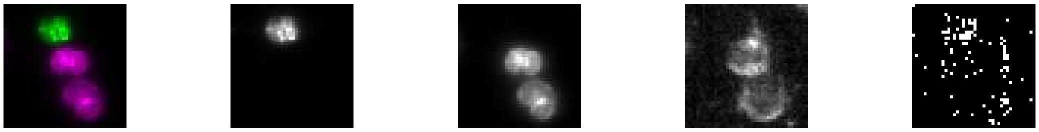				
4113	33	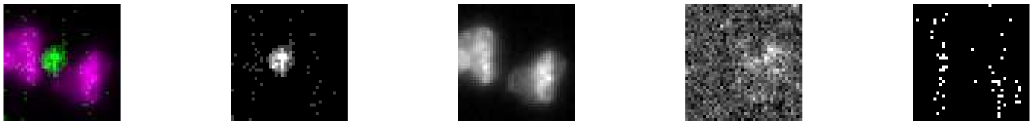				
4114	33	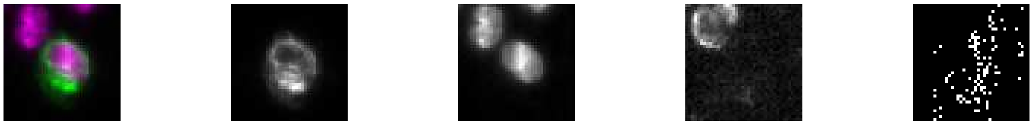				
4115	33	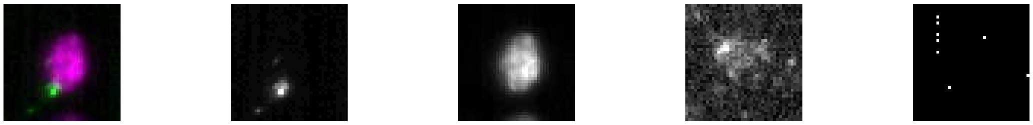				
4116	33	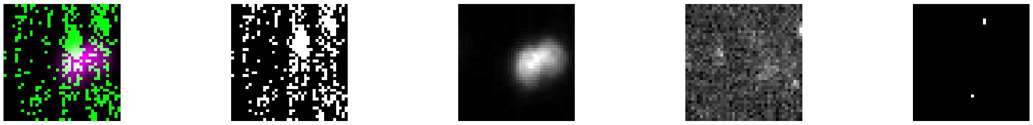				
4117	33	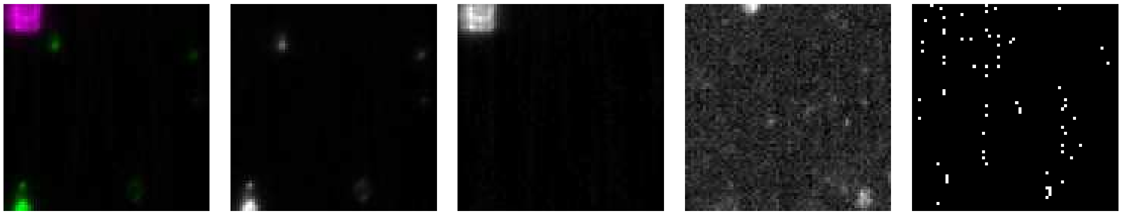				
4118	33	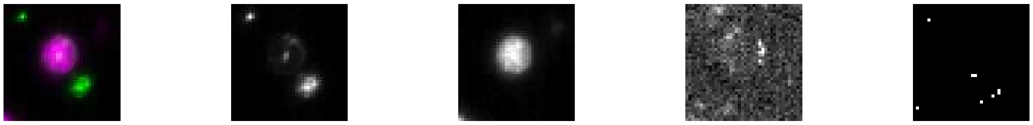				
4119	33	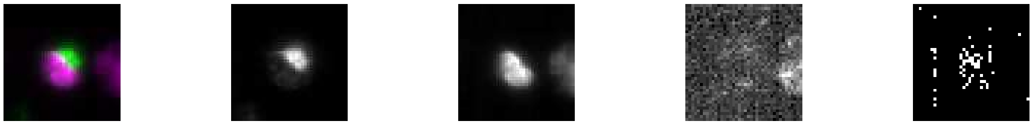				

Proben-ID: Gyn14.208 **Kassetten-ID:**01327106 **Patienten-ID:**

Ereignis	Aufnahme	DAPI/CK-PE	CK-PE	DAPI	CD45-APC	HER-2/neu
4120	33	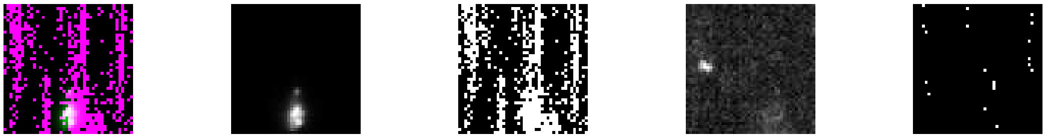				
4121	33	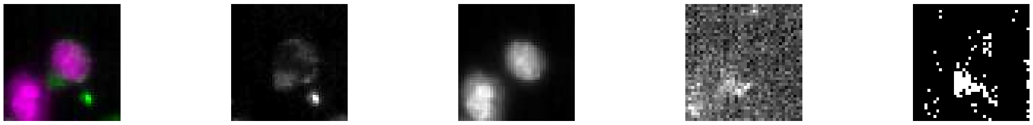				
4122	33	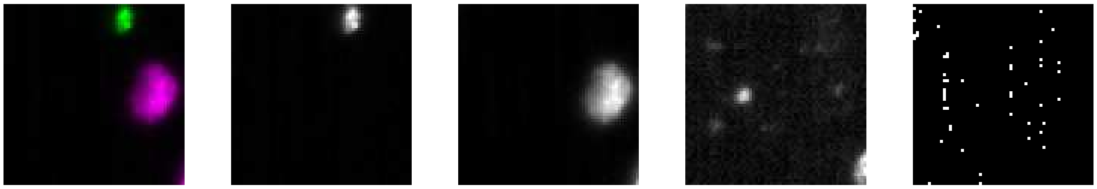				
4123	33	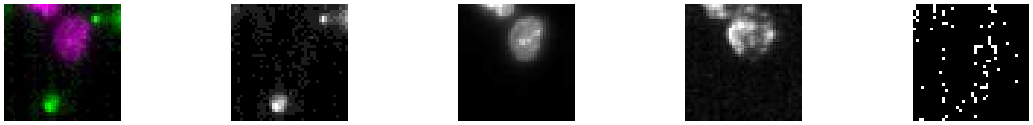				
4124	33	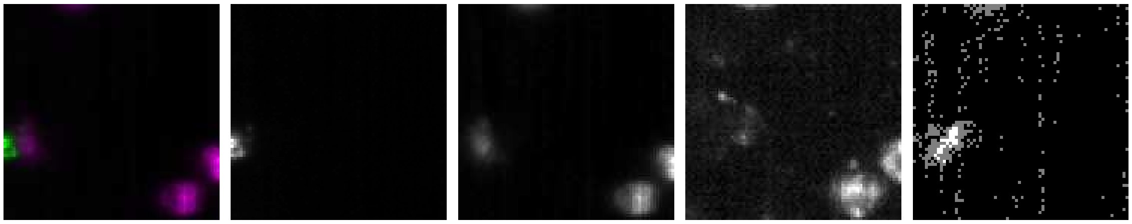				
4125	33	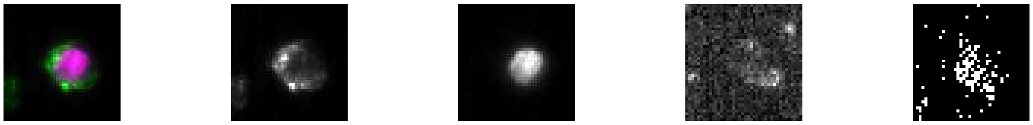				
4126	33	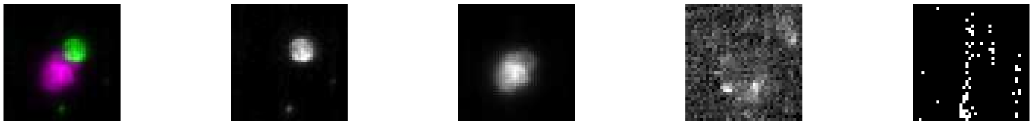				
4127	33	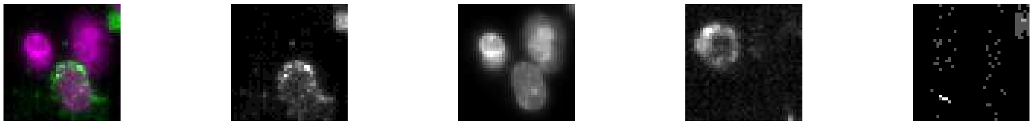				
4128	33	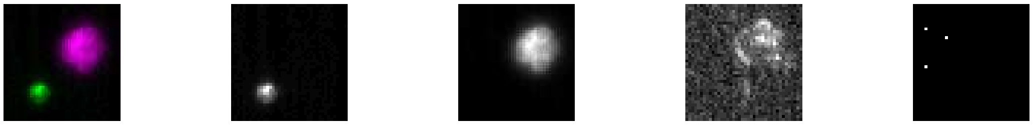				
4129	33	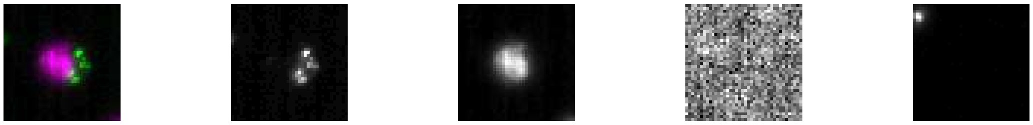				
4130	32	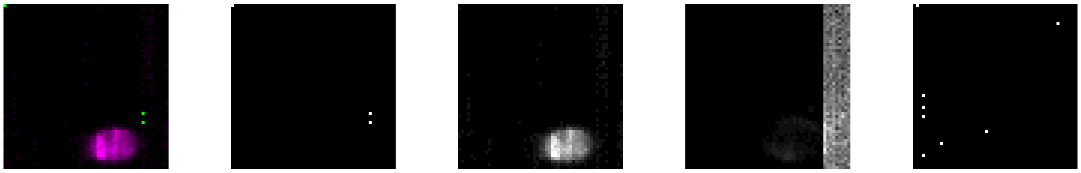				

Proben-ID: Gyn14.208 **Kassetten-ID:**01327106 **Druckdatum:**02/06/2020 16:41
Patienten-ID:

Ereignis	Aufnahme	DAPI/CK-PE	CK-PE	DAPI	CD45-APC	HER-2/neu
4131	32	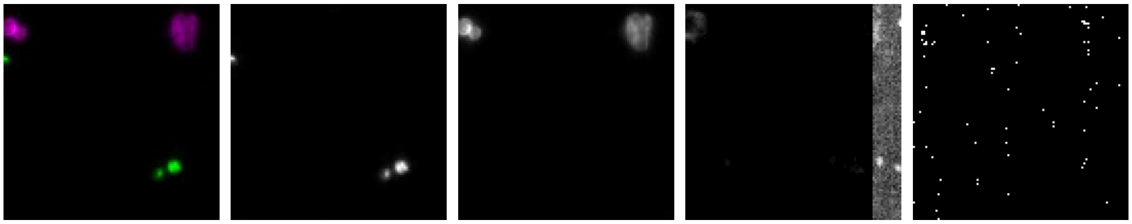				
4132	32	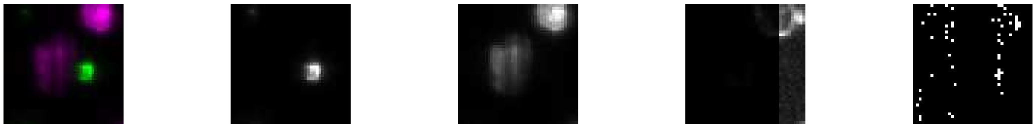				
4133	33	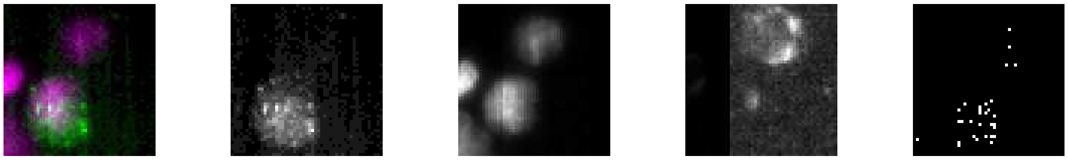				
4134	34	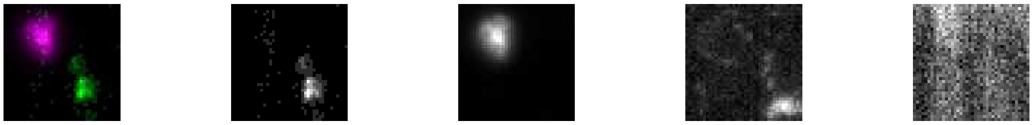				
4135	34	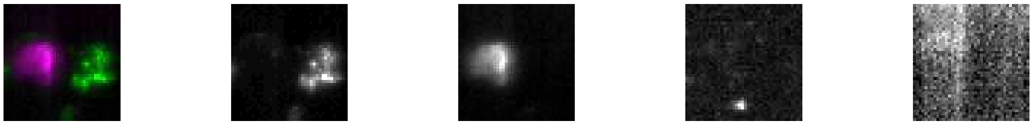				
4136	34	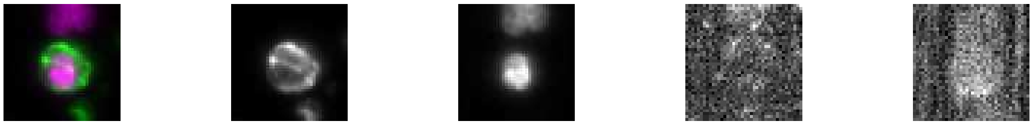				
4137	34	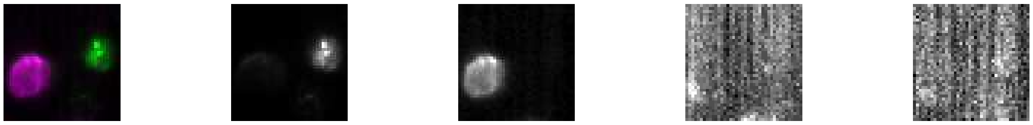				
4138	34	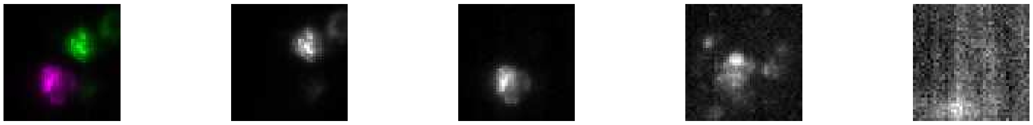				
4139	34	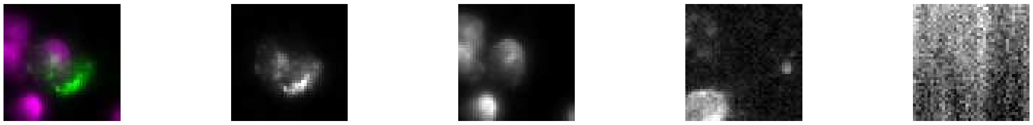				
4140	34	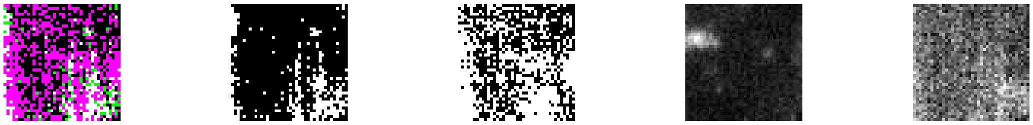				
4141	34	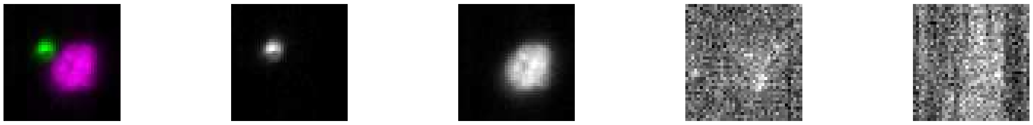				
4142	34	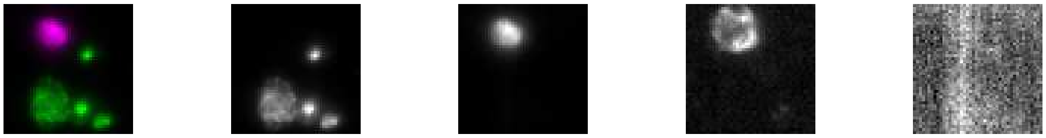				
4143	34	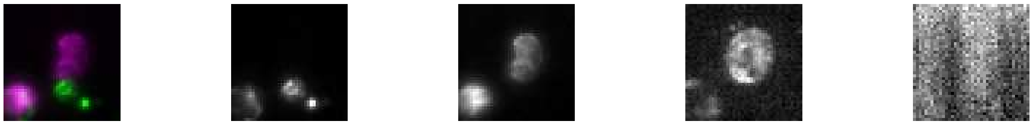				

Ereignis	Aufnahme	DAPI/CK-PE	CK-PE	DAPI	CD45-APC	HER-2/neu
4144	34	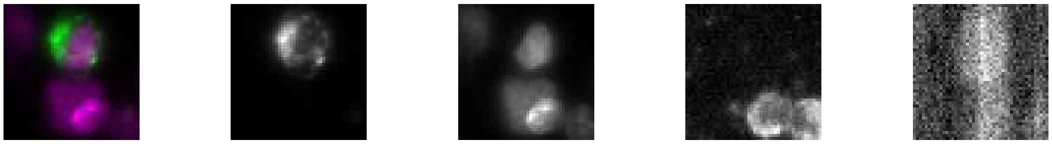				
4145	34	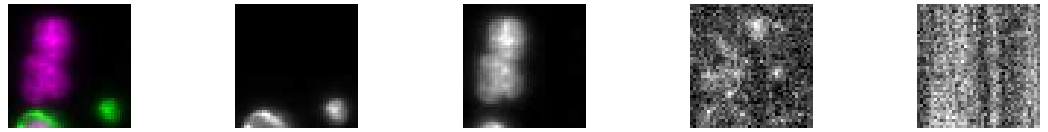				
4146	34	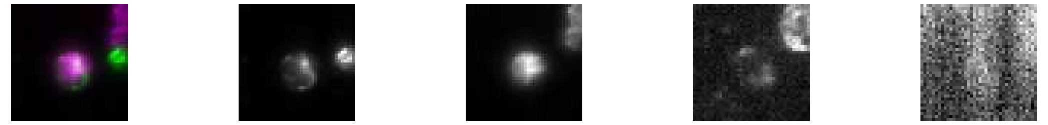				
4147	34	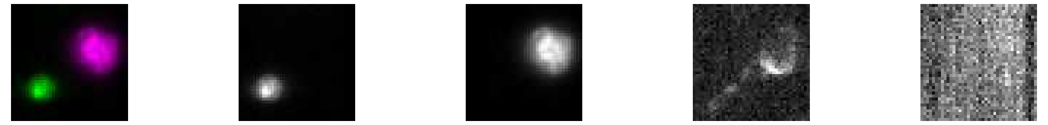				
4148	34	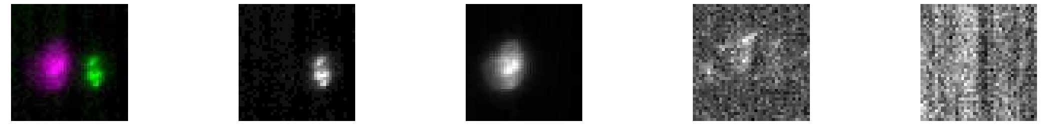				
4149	34	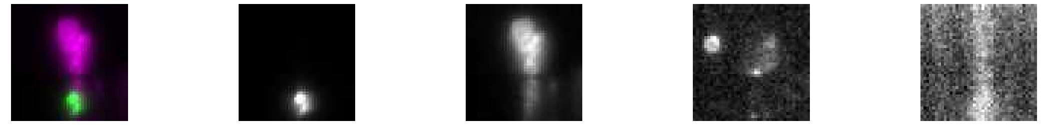				
4150	34	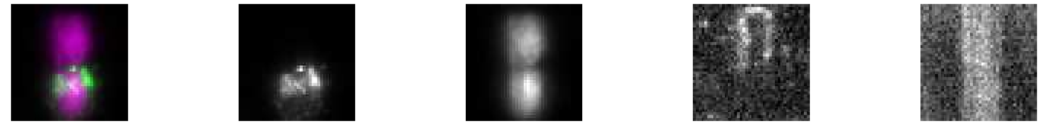				
4151	34	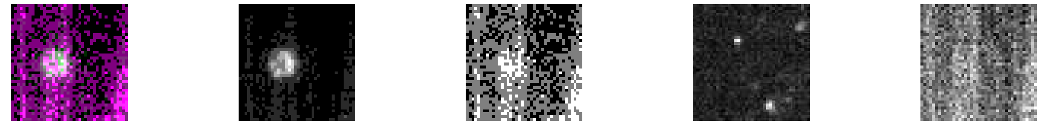				
4152	34	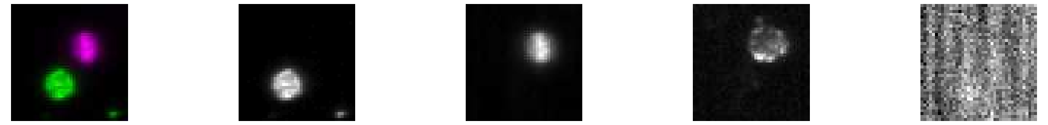				
4153	34	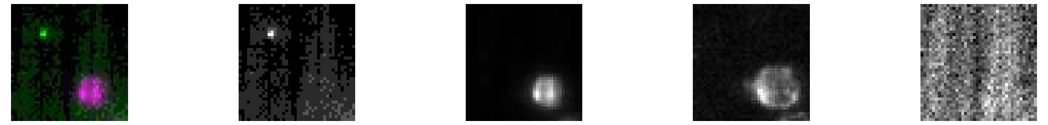				
4154	34	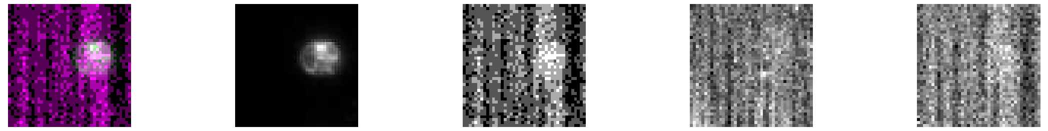				
4155	34	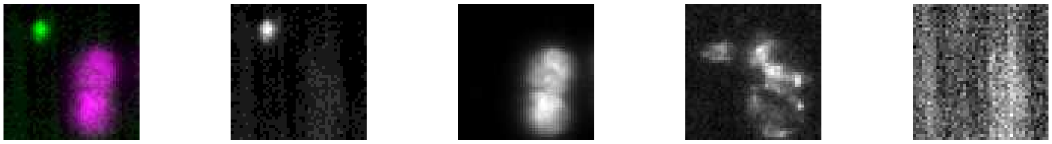				
4156	34	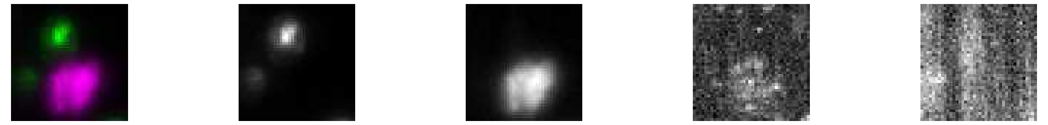				

Ereignis	Aufnahme	DAPI/CK-PE	CK-PE	DAPI	CD45-APC	HER-2/neu
4157	34	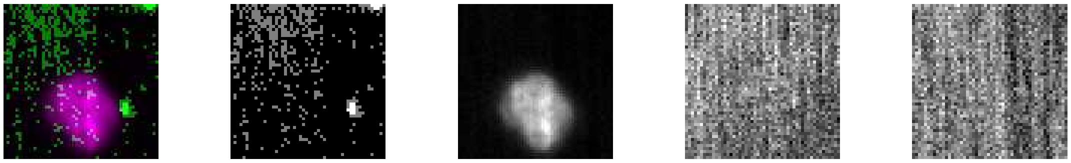				
4158	34	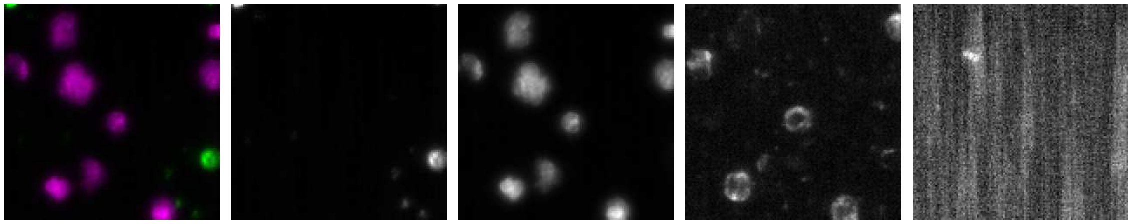				
4159	34	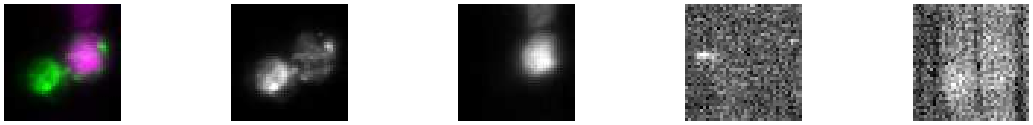				
4160	34	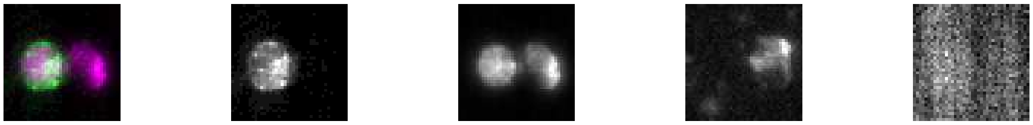				
4161	34	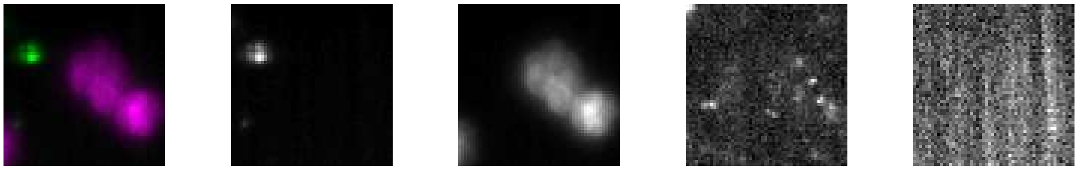				
4162	34	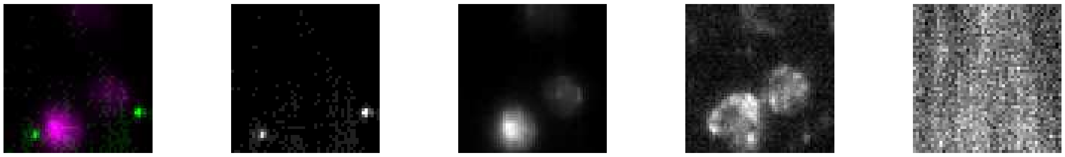				
4163	34	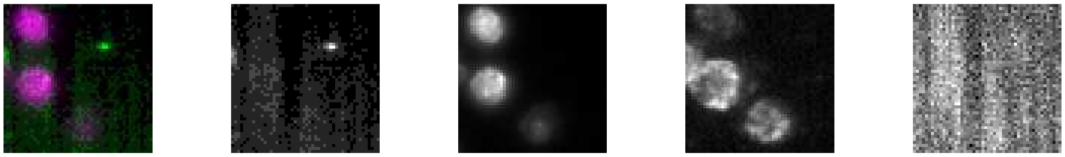				
4164	34	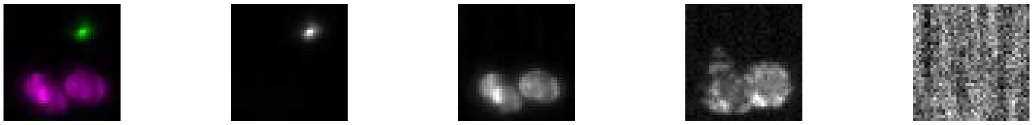				
4165	34	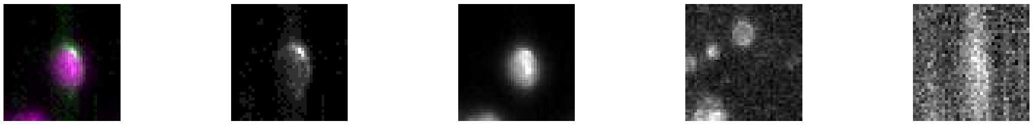				
4166	34	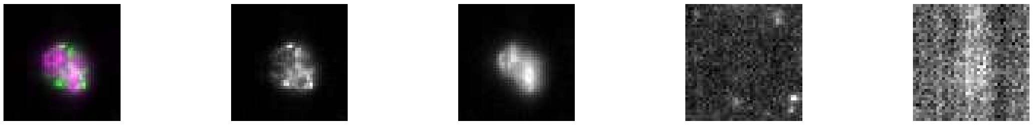				
4167	34	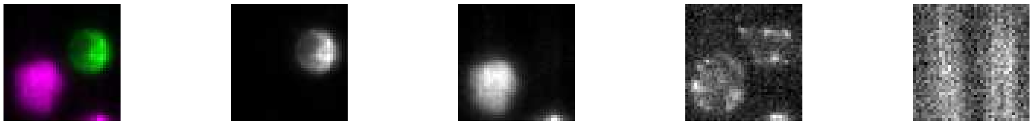				
4168	34	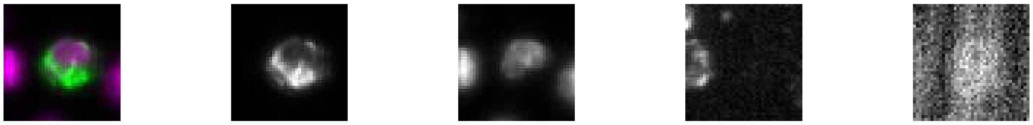				

Proben-ID: Gyn14.208 **Kassetten-ID:**01327106 **Patienten-ID:**

Ereignis	Aufnahme	DAPI/CK-PE	CK-PE	DAPI	CD45-APC	HER-2/neu
4169	34	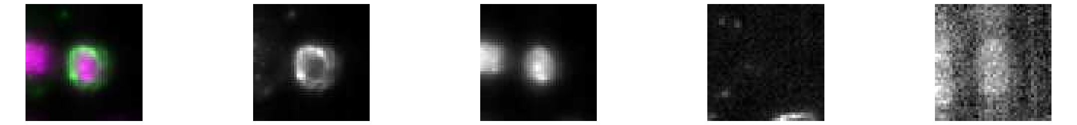				
4170	34	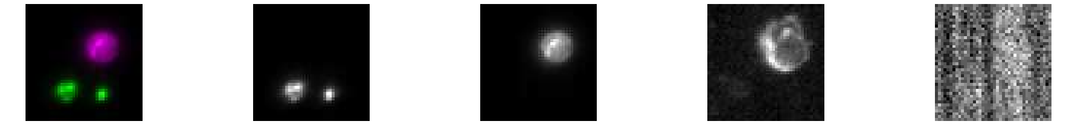				
4171	34	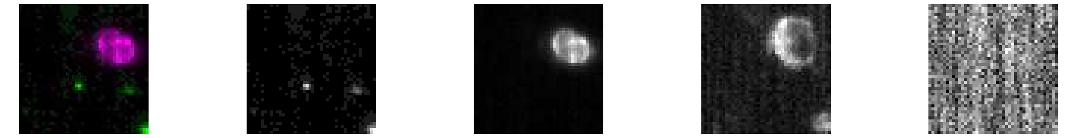				
4172	34	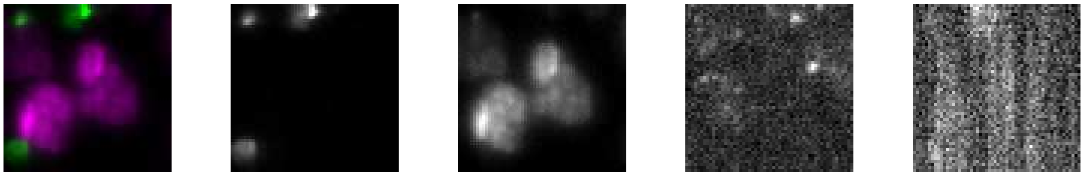				
4173	34	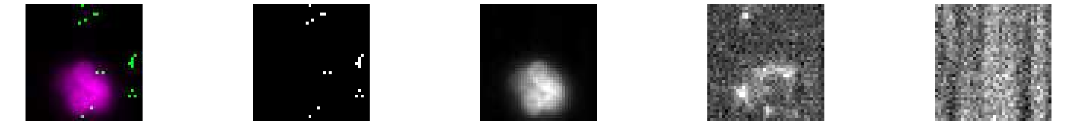				
4174	34	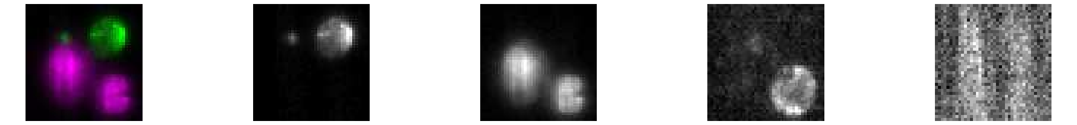				
4175	34	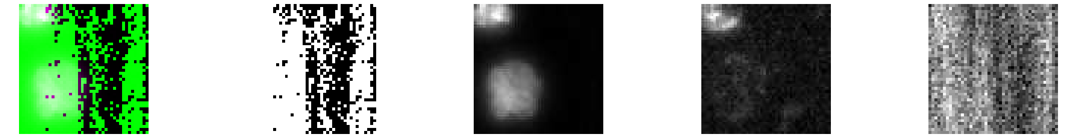				
4176	34	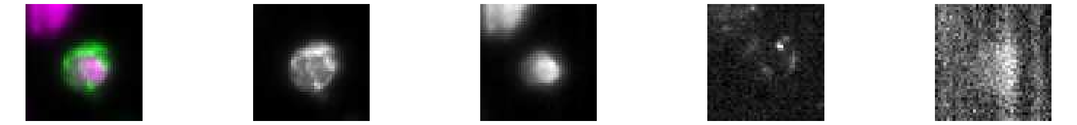				
4177	34	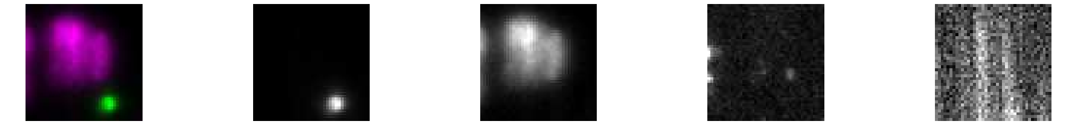				
4178	34	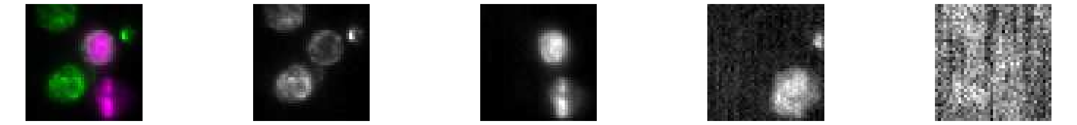				
4179	34	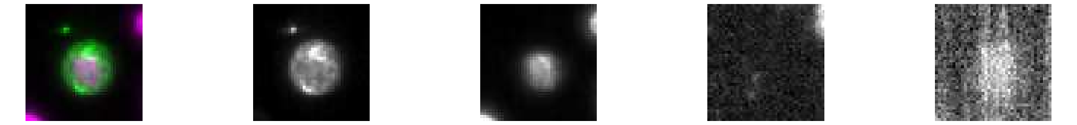				
4180	34	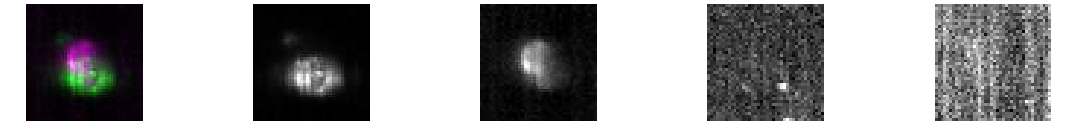				
4181	34	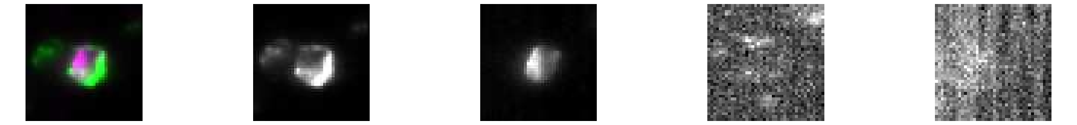				

Ereignis	Aufnahme	DAPI/CK-PE	CK-PE	DAPI	CD45-APC	HER-2/neu
4182	34	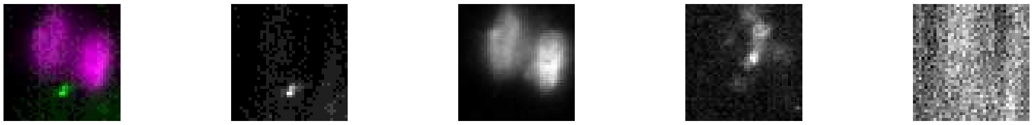				
4183	34	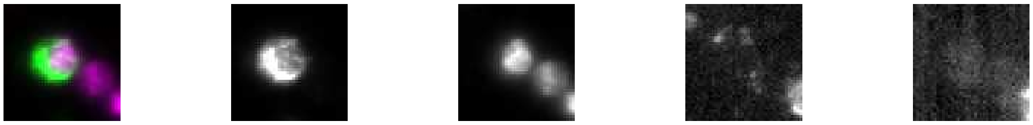				
4184	34	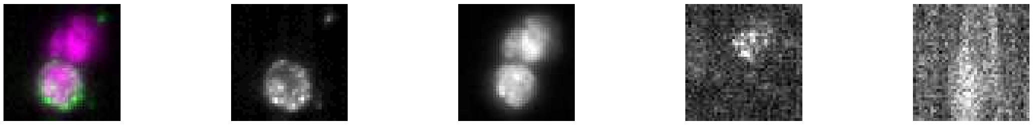				
4185	34	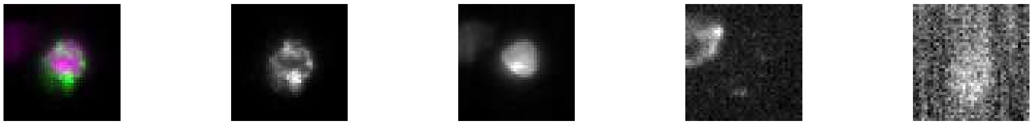				
4186	34	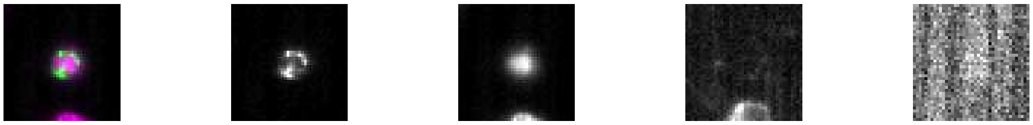				
4187	34	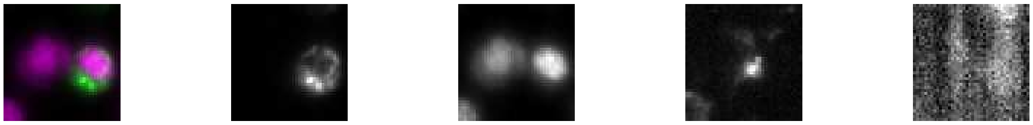				
4188	34	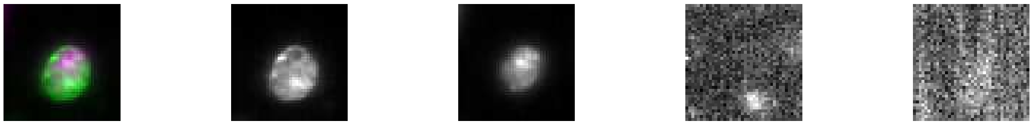				
4189	34	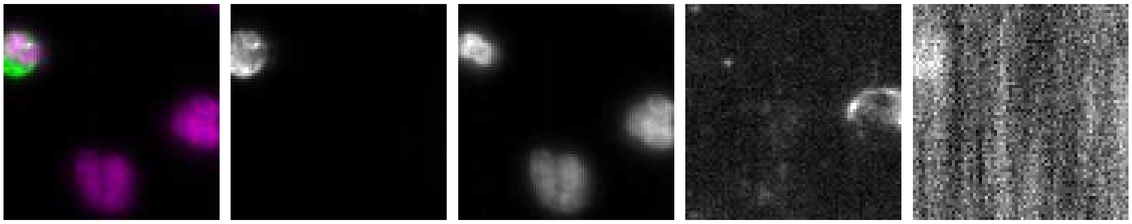				
4190	34	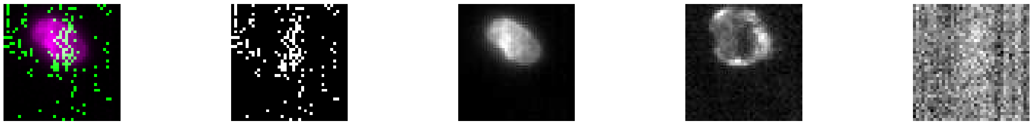				
4191	34	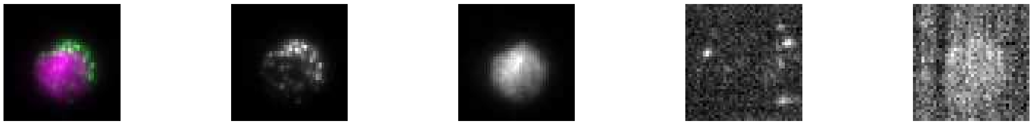				
4192	34	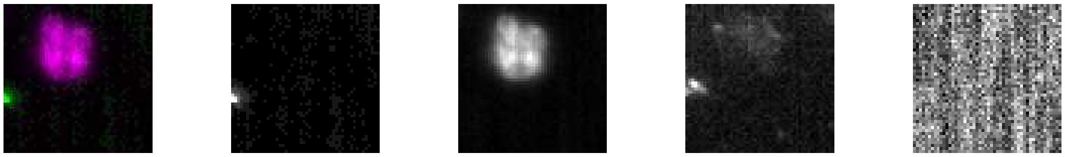				
4193	34	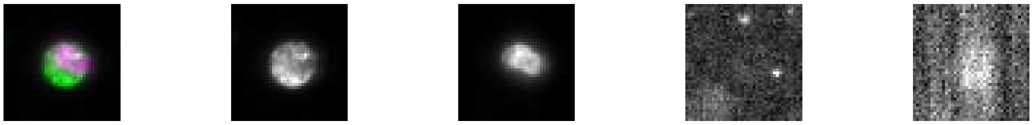				
4194	34	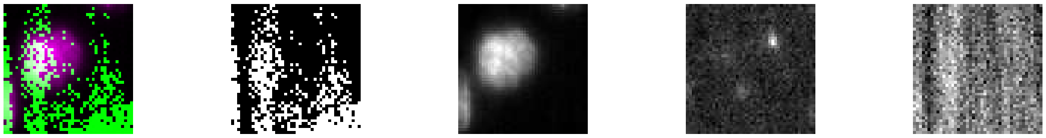				

**Proben-ID:** Gyn14.208		**Kassetten-ID:** 01327106		**Druckdatum:** 02/06/2020 16:41
				**Patienten-ID:**

Ereignis	Aufnahme	DAPI/CK-PE	CK-PE	DAPI	CD45-APC	HER-2/neu
4195	34	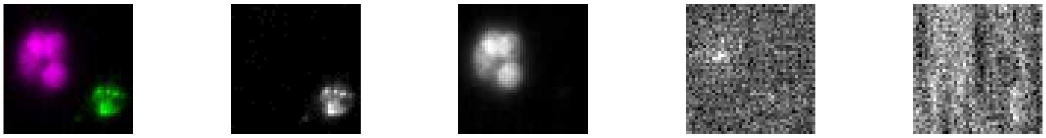				
4196	34	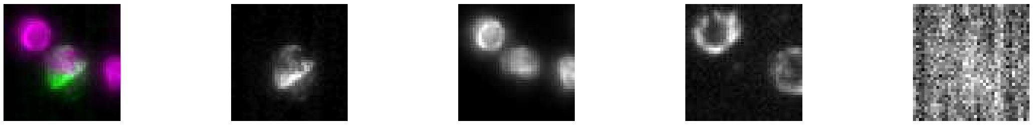				
4197	34	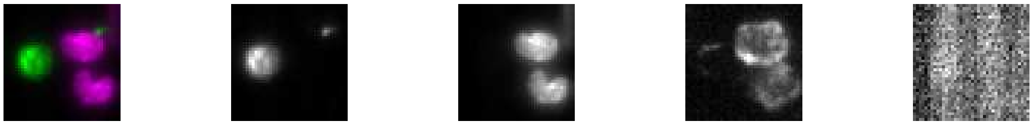				
4198	34	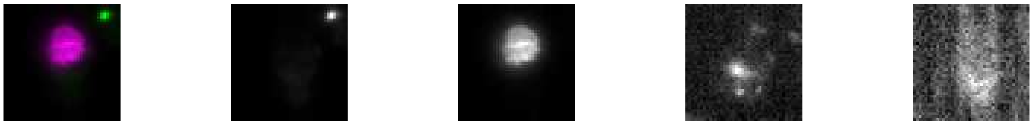				
4199	34	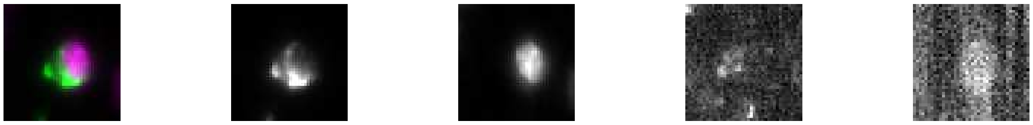				
4200	34	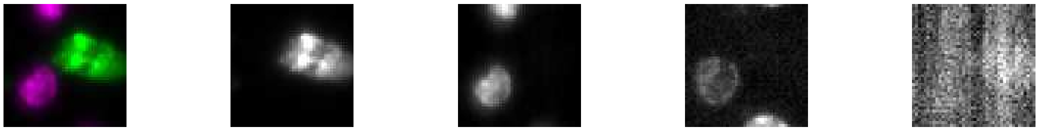				
4201	34	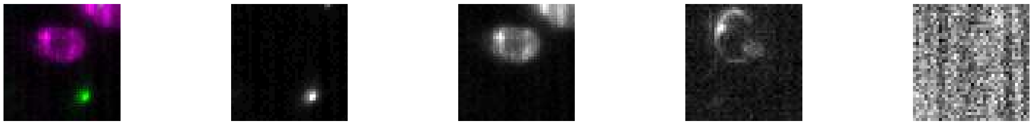				
4202	34	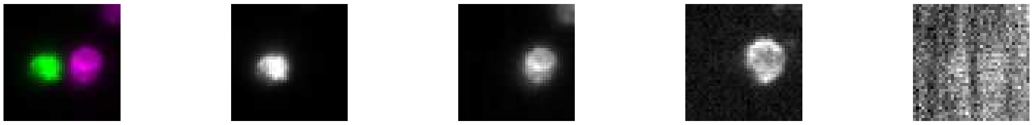				
4203	34	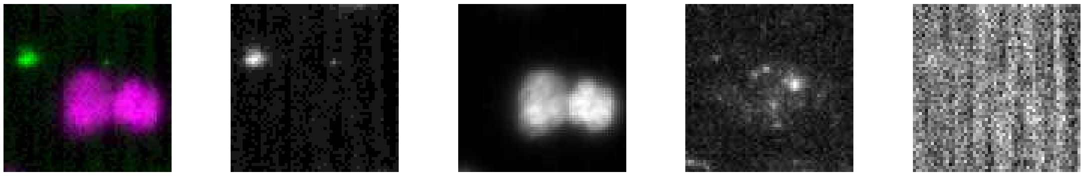				
4204	34	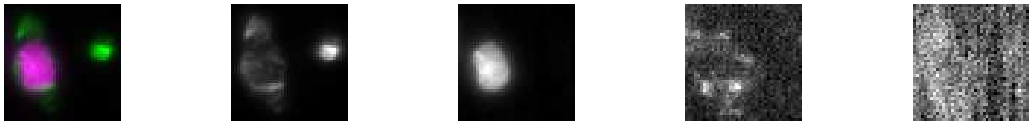				
4205	34	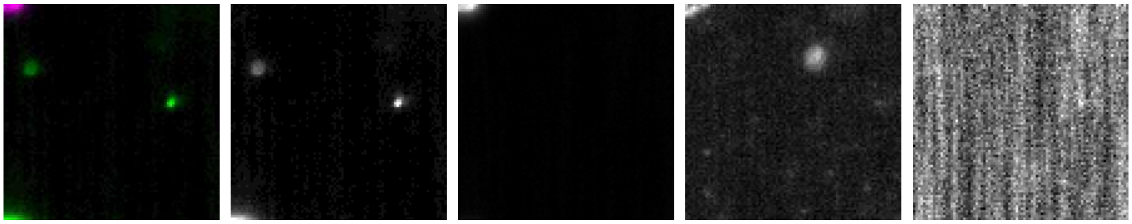				
4206	34	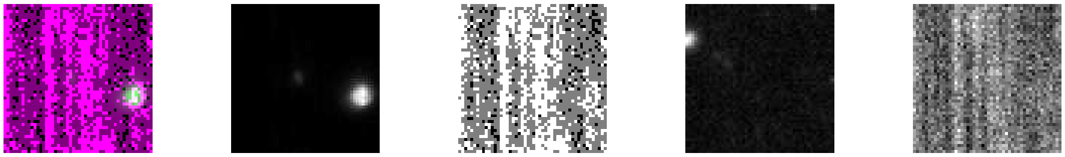				

Ereignis	Aufnahme	DAPI/CK-PE	CK-PE	DAPI	CD45-APC	HER-2/neu
4207	34	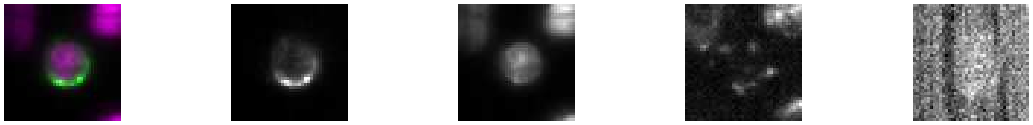				
4208	34	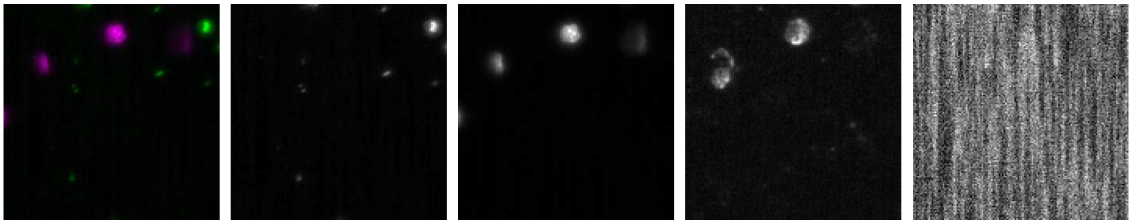				
4209	34	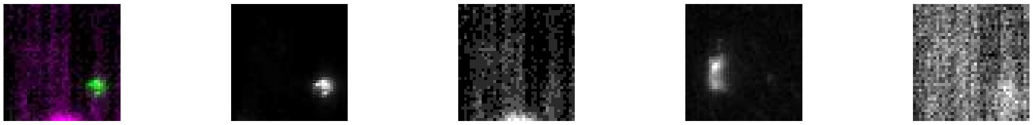				
4210	34	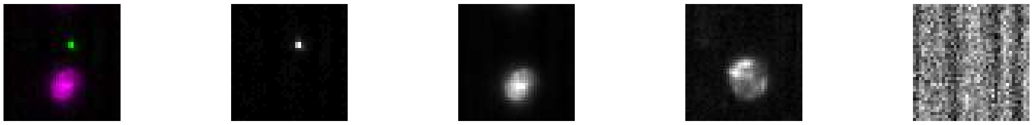				
4211	34	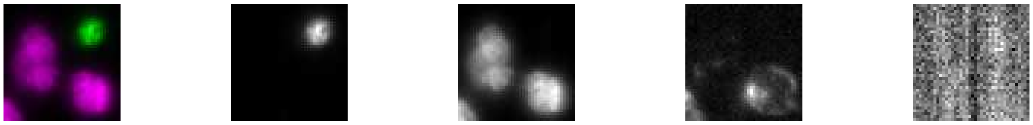				
4212	34	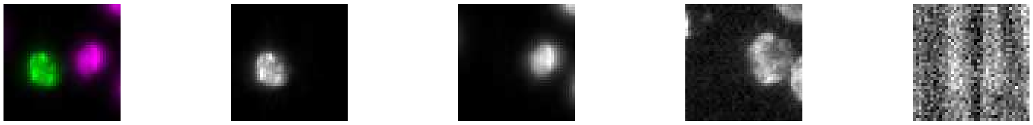				
4213	34	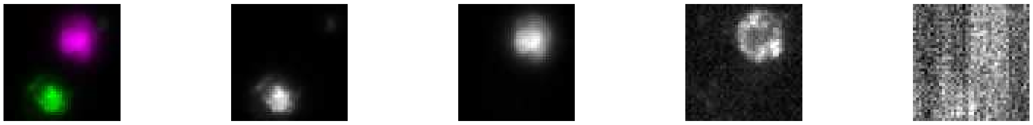				
4214	34	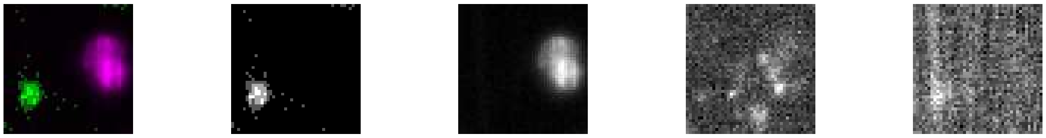				
4215	34	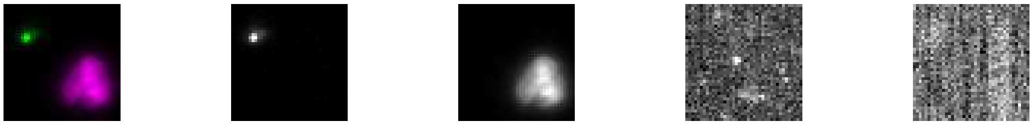				
4216	34	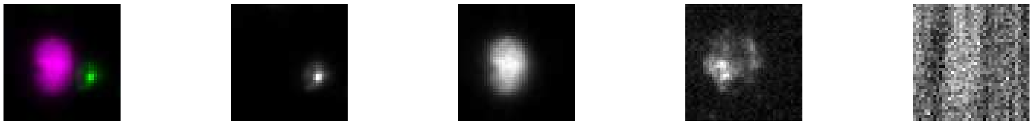				
4217	34	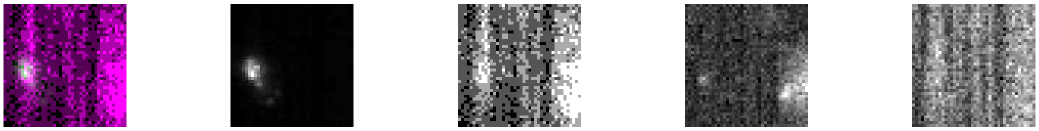				
4218	34	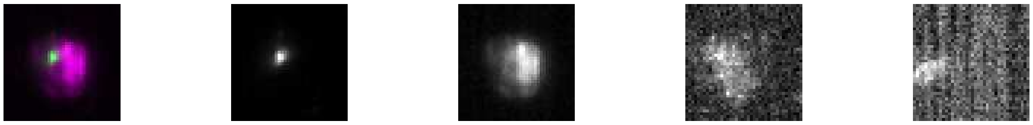				
4219	34	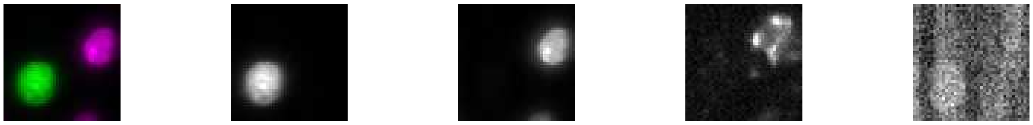				

Druckdatum:02/06/2020 16:41

Proben-ID: Gyn14.208 **Kassetten-ID:**01327106 **Patienten-ID:**

Ereignis	Aufnahme	DAPI/CK-PE	CK-PE	DAPI	CD45-APC	HER-2/neu
4220	34	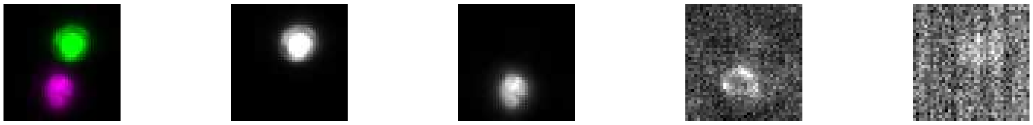				
4221	34	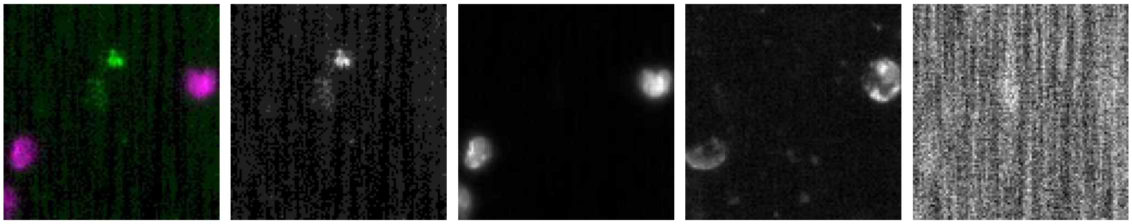				
4222	34	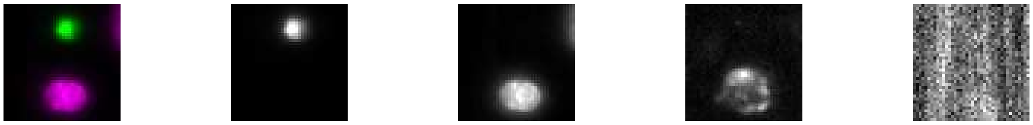				
4223	34	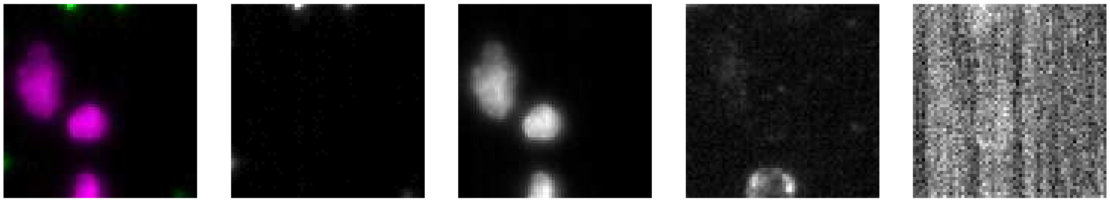				
4224	34	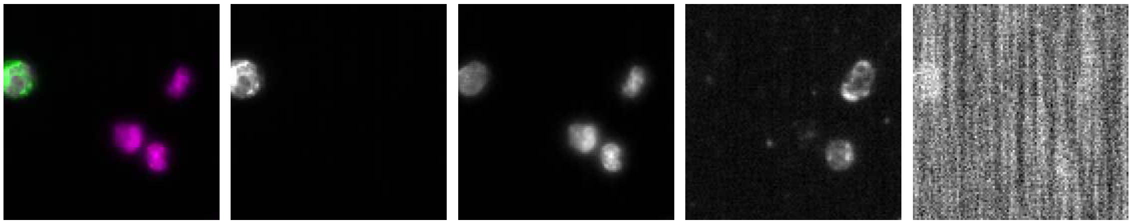				
4225	34	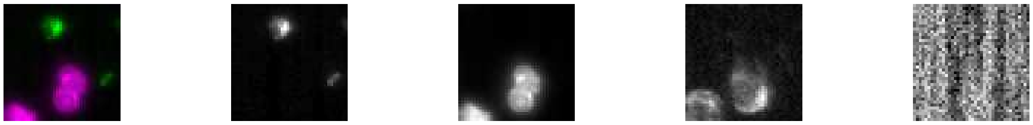				
4226	34	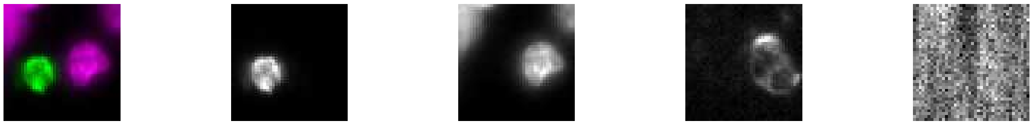				
4227	34	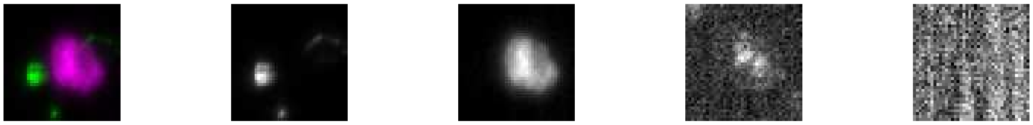				
4228	34	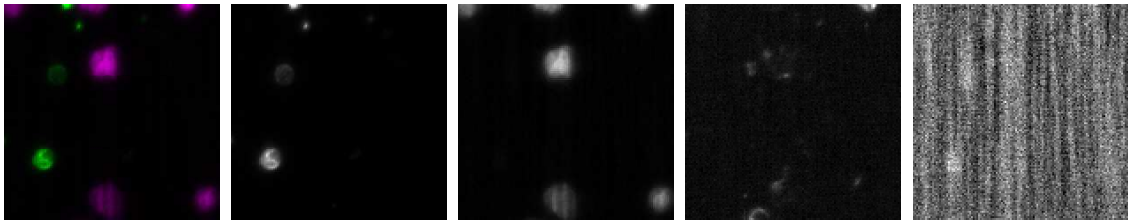				
4229	34	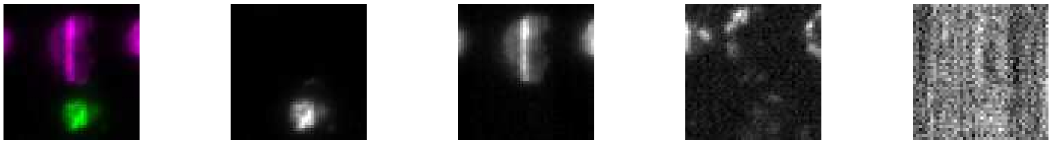				
4230	34	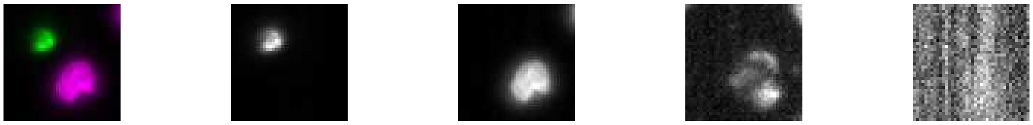				

Druckdatum: 02/06/2020 16:41

Proben-ID: Gyn14.208 **Kassetten-ID:** 01327106 **Patienten-ID:**

Ereignis	Aufnahme	DAPI/CK-PE	CK-PE	DAPI	CD45-APC	HER-2/neu
4231	34	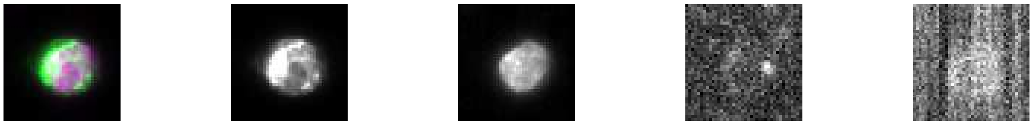				
4232	34	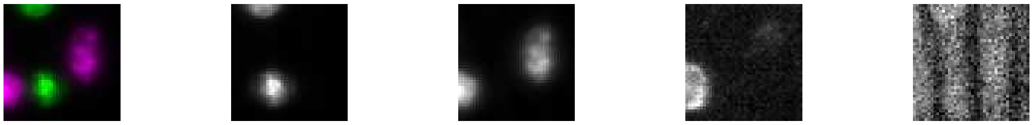				
4233	34	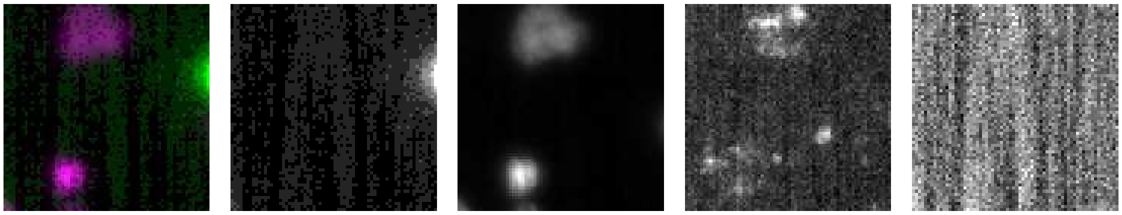				
4234	34	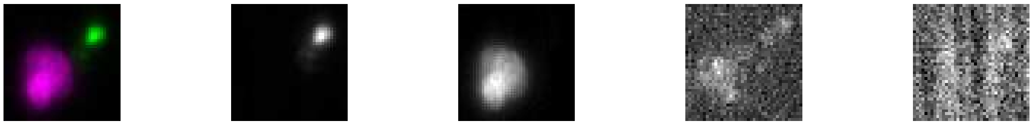				
4235	34	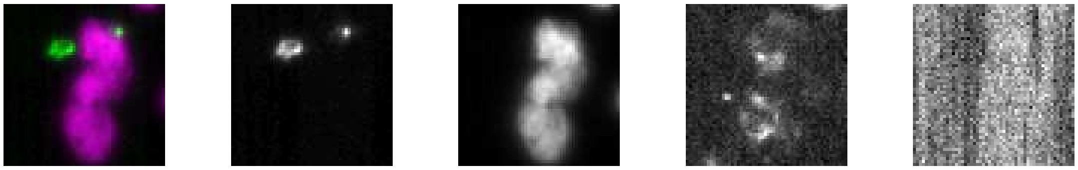				
4236	34	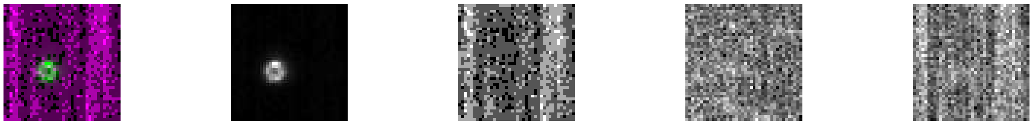				
4237	34	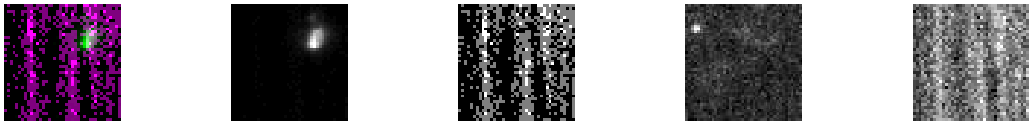				
4238	34	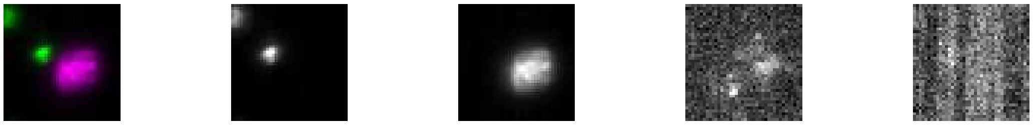				
4239	34	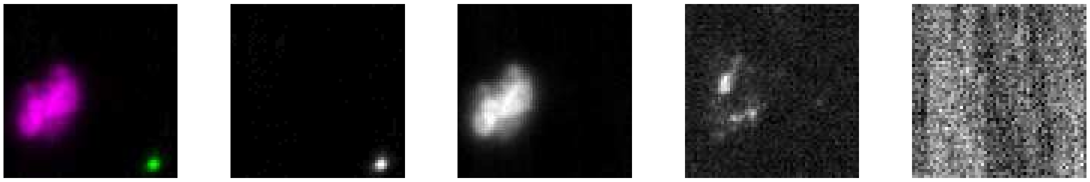				
4240	34	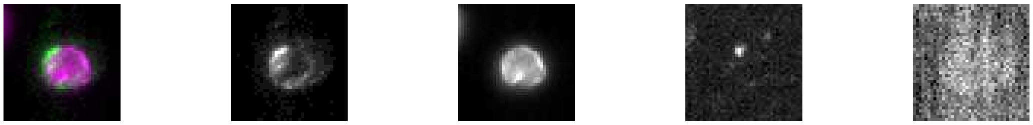				
4241	34	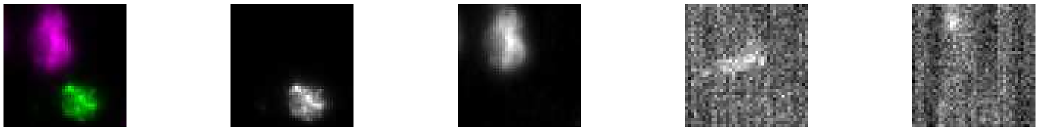				
4242	34	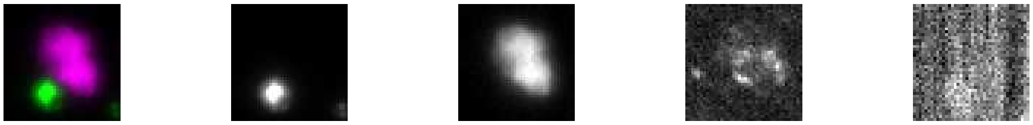				

Druckdatum: 02/06/2020 16:41

Proben-ID: Gyn14.208 **Kassetten-ID:** 01327106 **Patienten-ID:**

Ereignis	Aufnahme	DAPI/CK-PE	CK-PE	DAPI	CD45-APC	HER-2/neu
4243	33	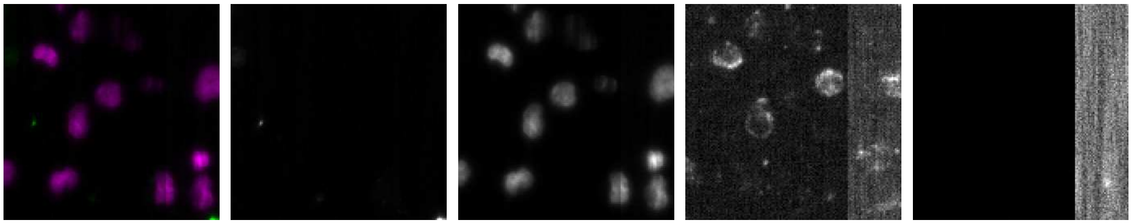				
4244	33	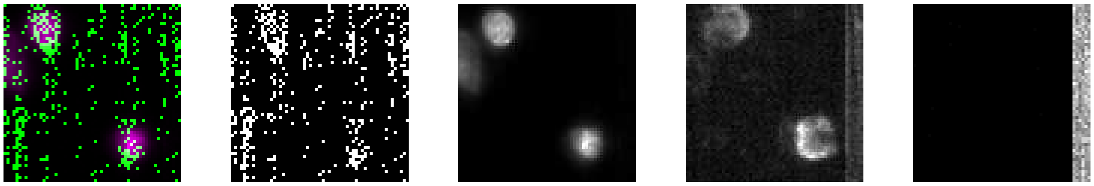				
4245	33	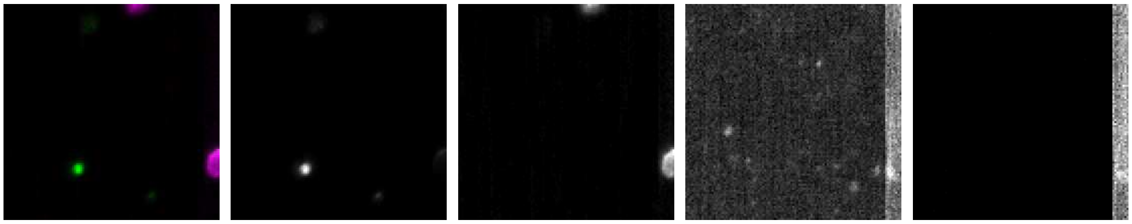				
4246	35	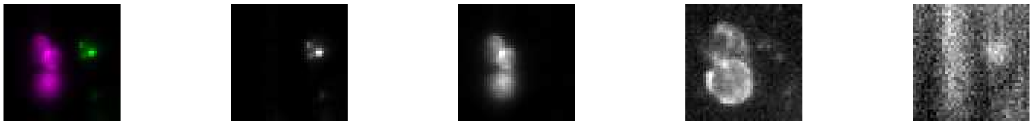				
4247	35	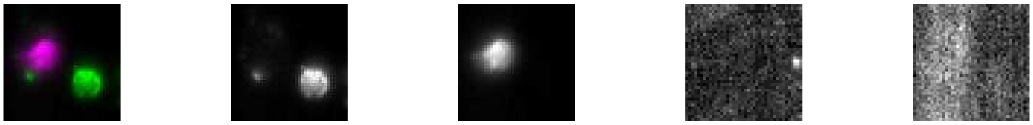				
4248	35	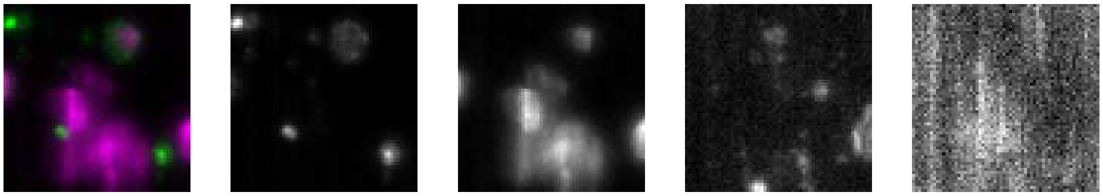				
4249	35	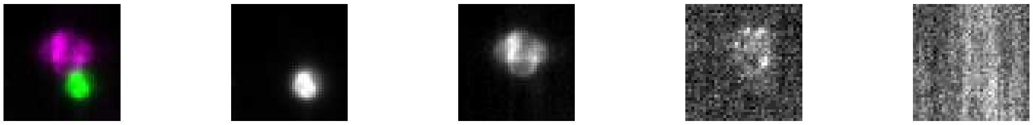				
4250	35	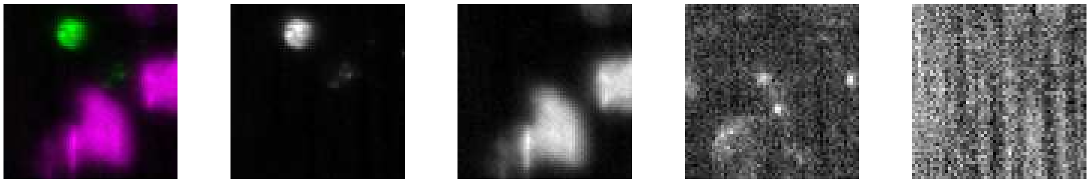				
4251	35	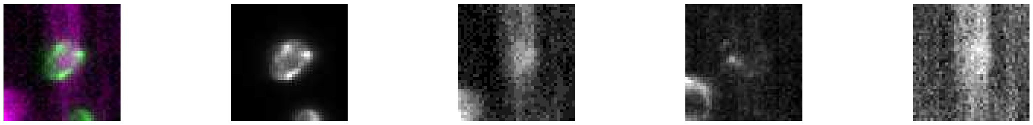				
4252	35	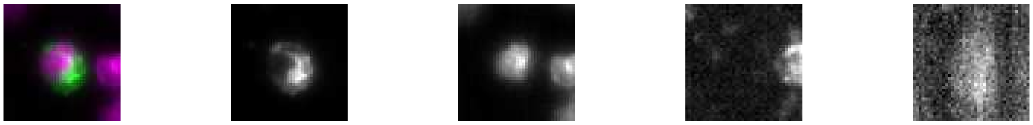				
4253	35	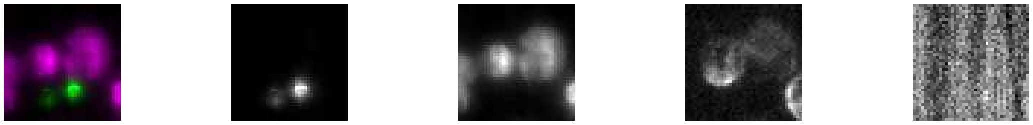				

Ereignis	Aufnahme	DAPI/CK-PE	CK-PE	DAPI	CD45-APC	HER-2/neu
4254	35	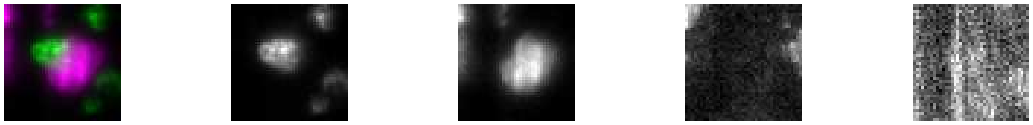				
4255	35	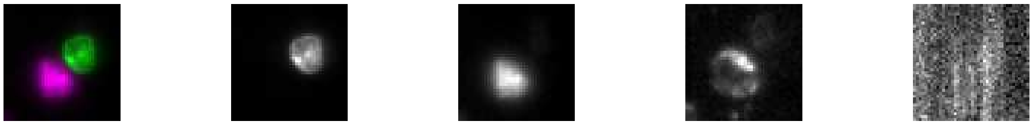				
4256	35	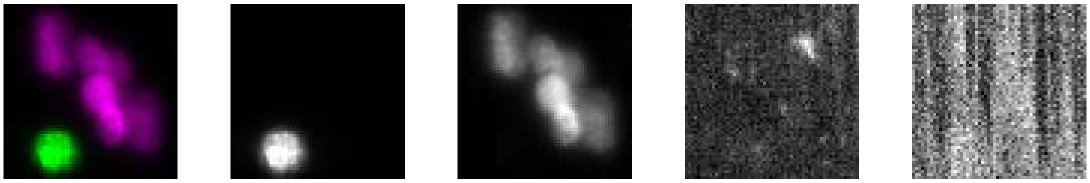				
4257	35	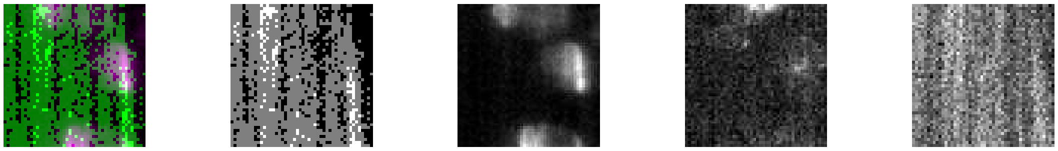				
4258	35	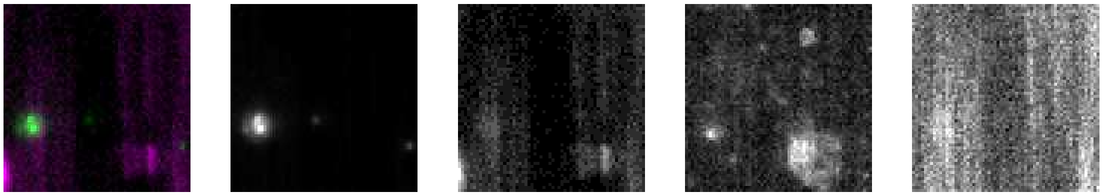				
4259	35	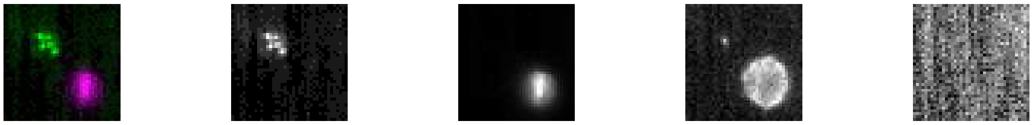				
4260	35	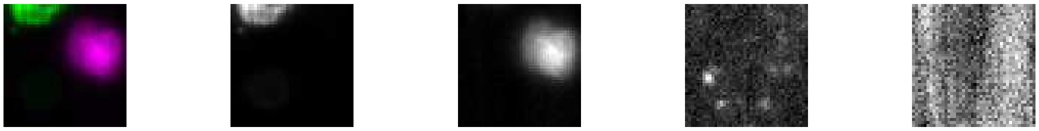				
4261	35	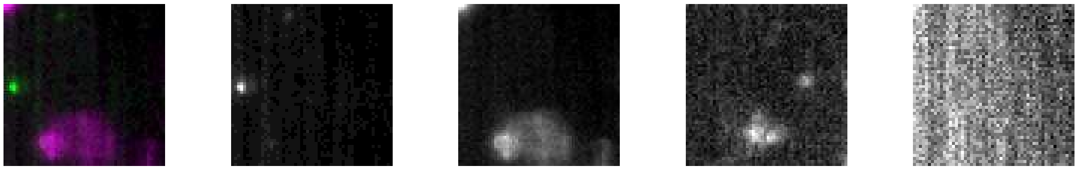				
4262	35	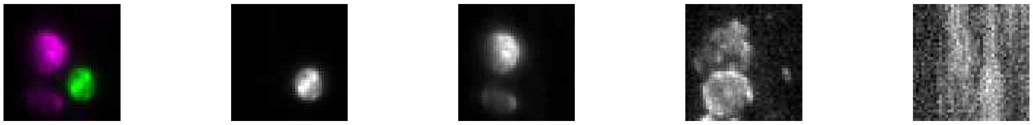				
4263	34	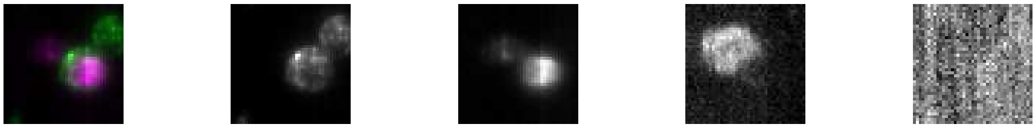				
4264	34	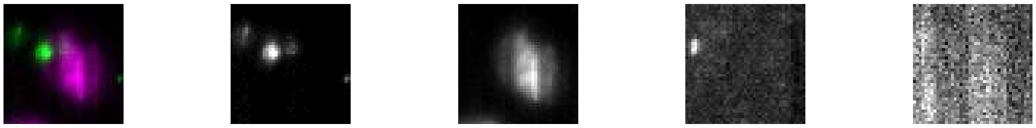				
4265	34	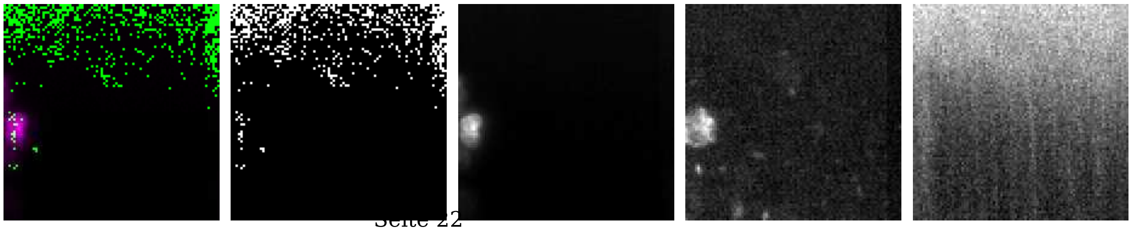				

Ereignis	Aufnahme	DAPI/CK-PE	CK-PE	DAPI	CD45-APC	HER-2/neu
4266	36	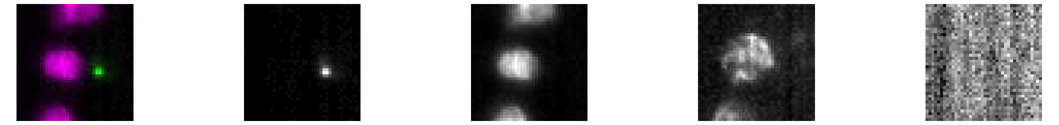				
4267	36	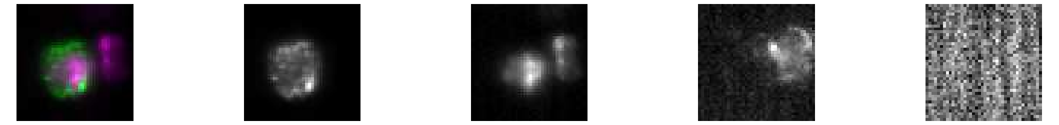				
4268	36	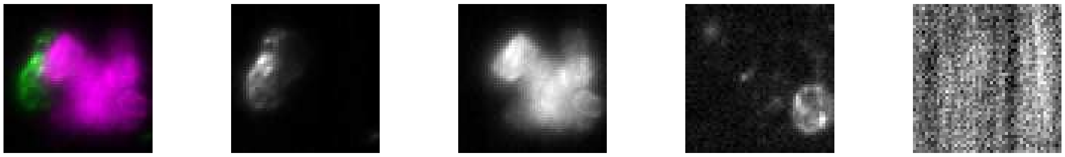				
4269	36	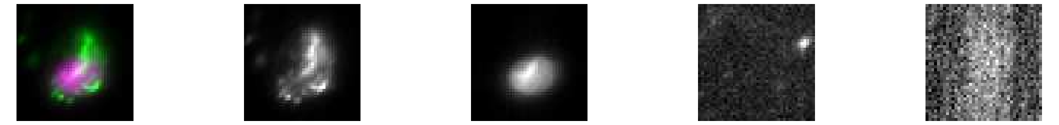				
4270	36	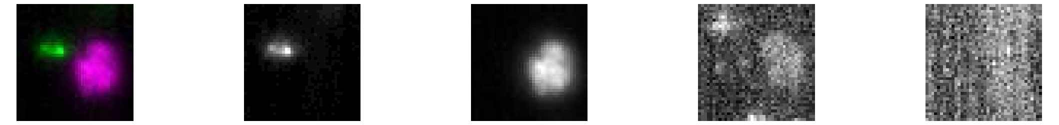				
4271	36	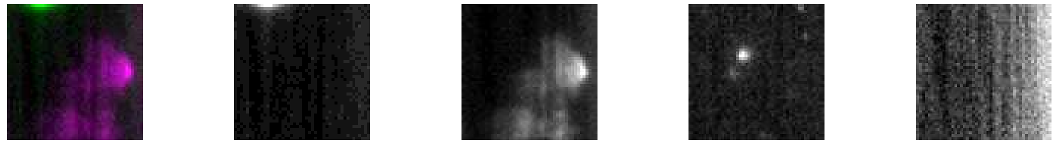				
4272	36	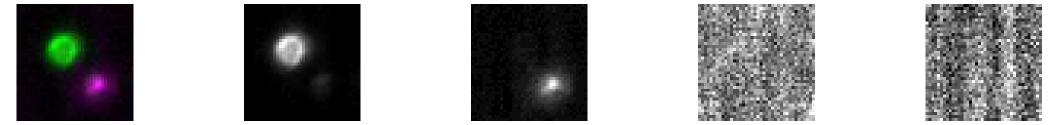				
4273	36	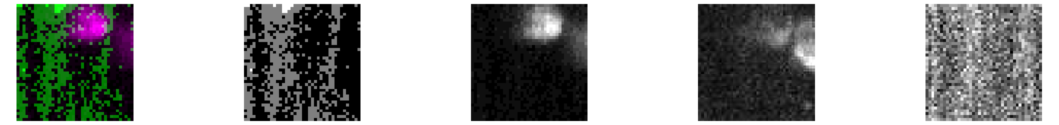				
4274	36	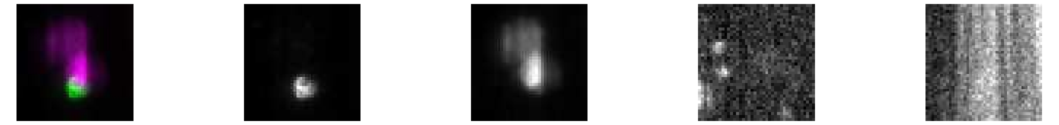				
4275	36	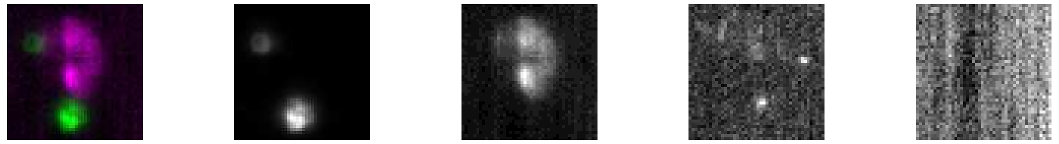				
4276	36	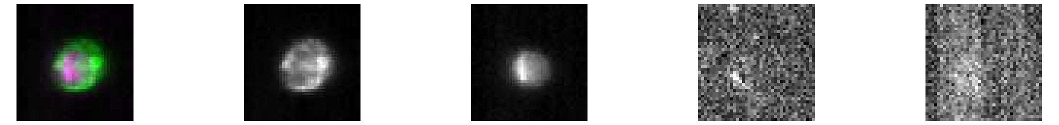				
4277	36	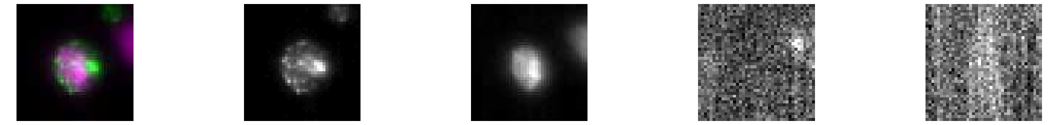				
4278	36	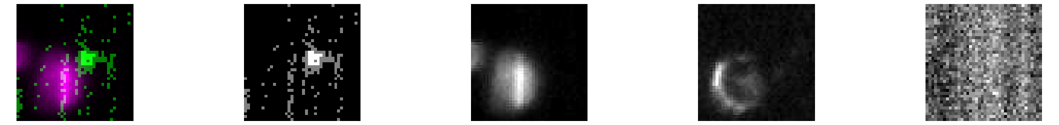				

Ereignis	Aufnahme	DAPI/CK-PE	CK-PE	DAPI	CD45-APC	HER-2/neu
4279	36	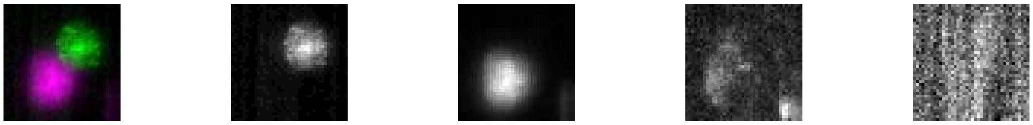				
4280	36	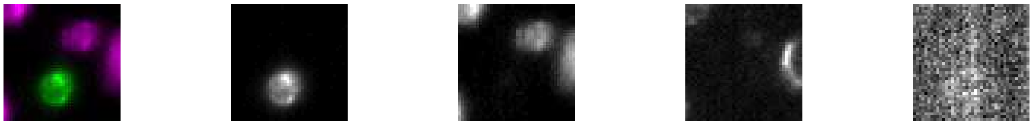				
4281	36	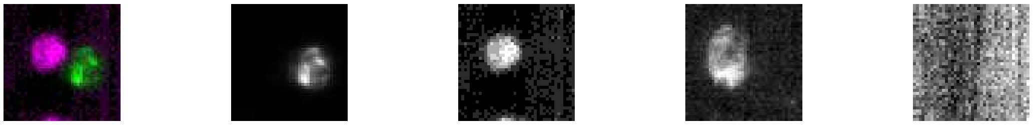				
4282	36	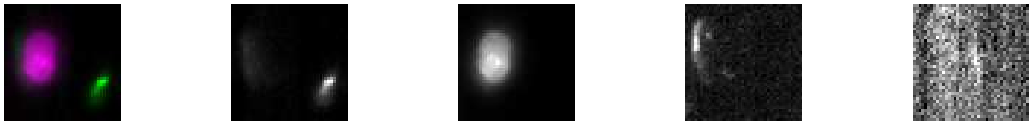				
4283	36	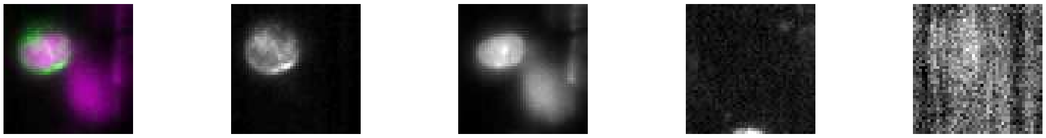				
4284	36	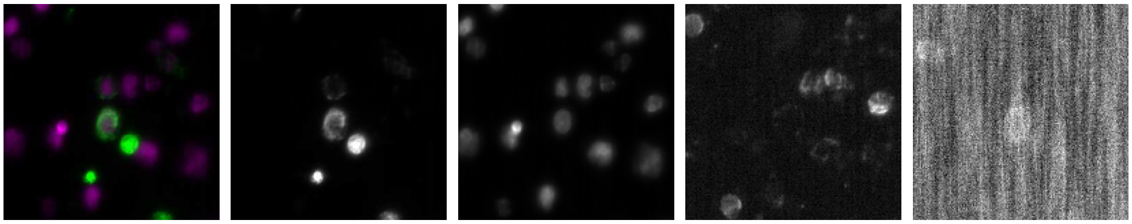				
4285	36	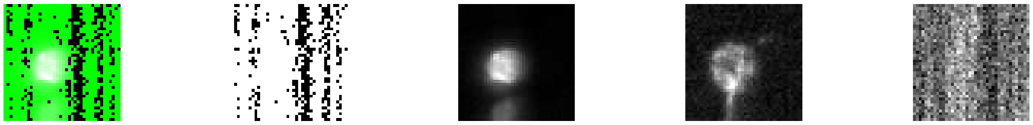				
4286	36	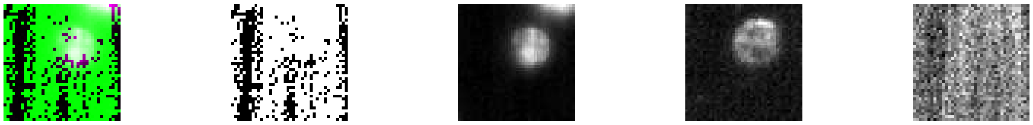				
4287	36	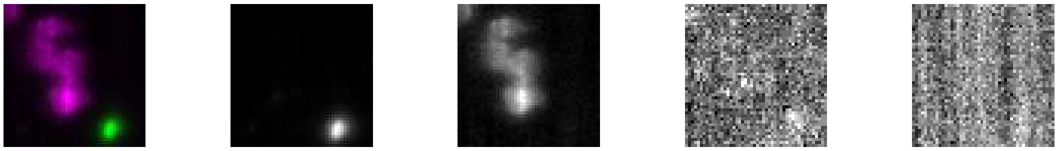				
4288	36	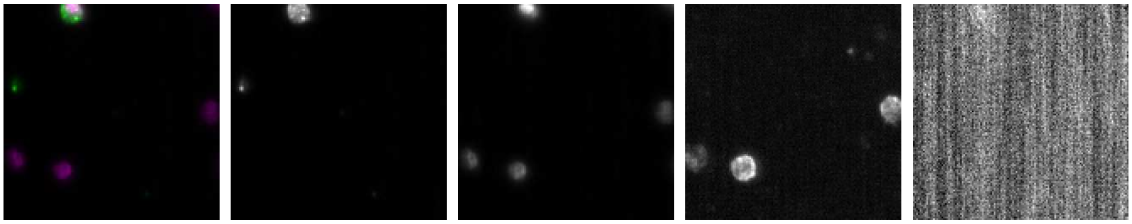				
4289	36	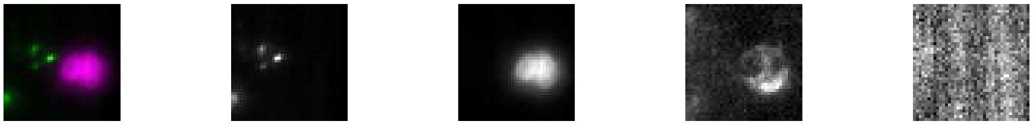				
4290	36	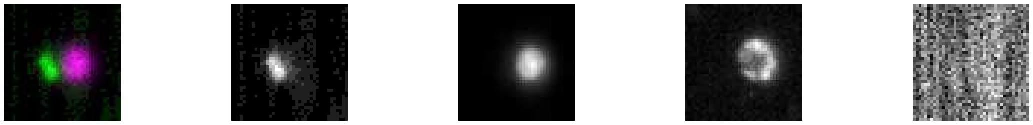				

Ereignis	Aufnahme	DAPI/CK-PE	CK-PE	DAPI	CD45-APC	HER-2/neu
4291	36	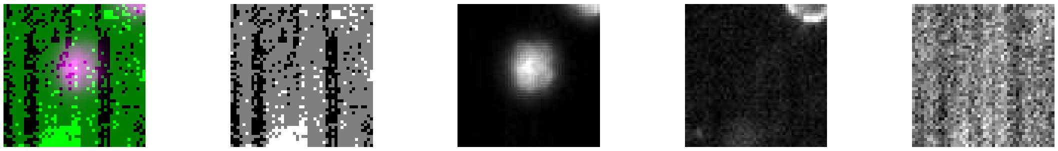				
4292	36	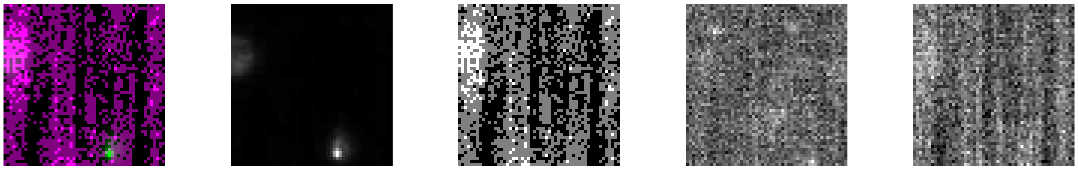				
4293	36	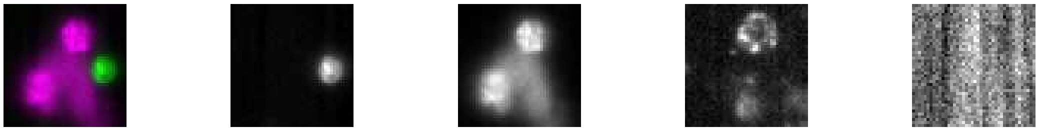				
4294	36	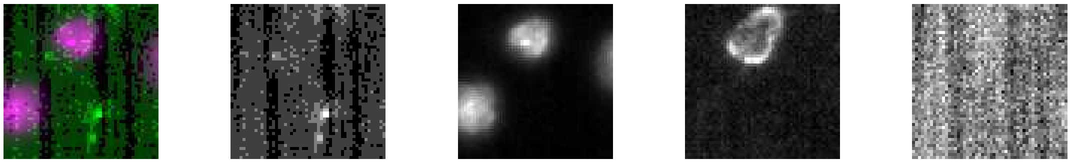				
4295	36	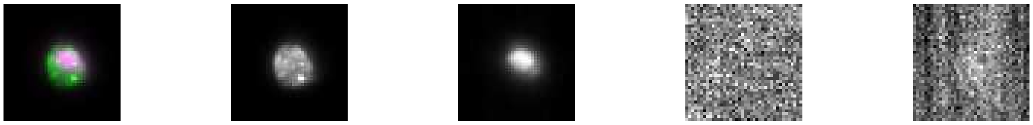				
4296	36	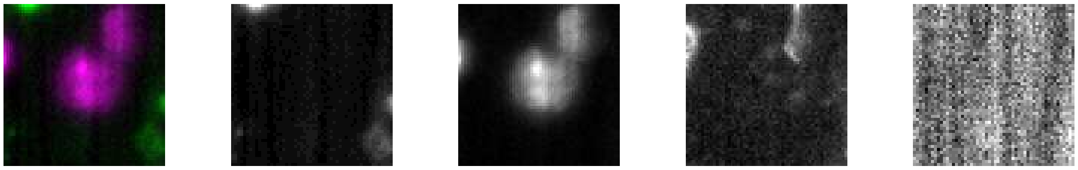				
4297	36	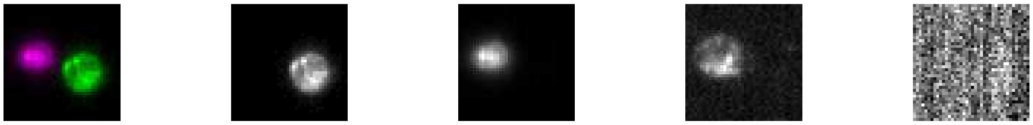				
4298	36	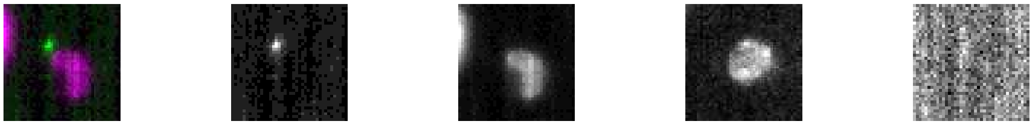				
4299	36	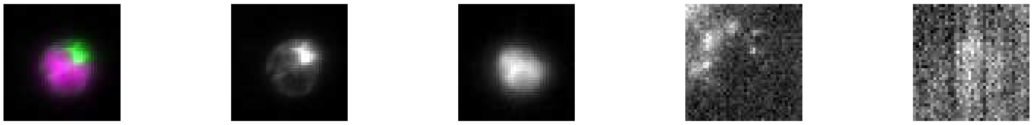				
4300	36	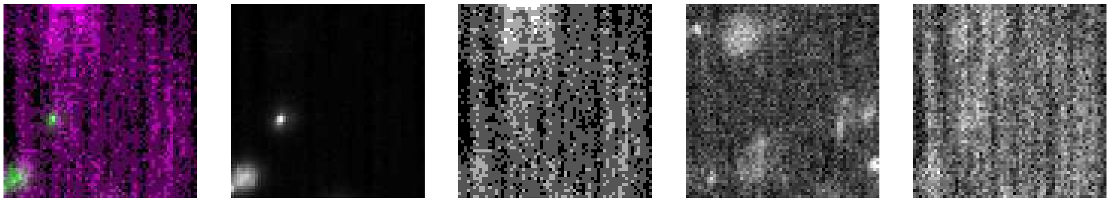				
4301	36	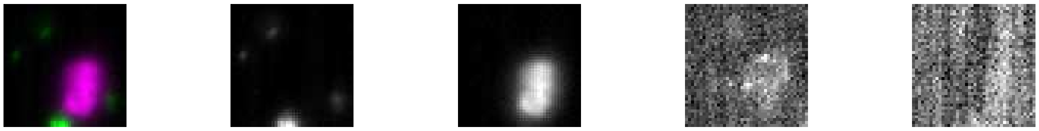				
4302	36	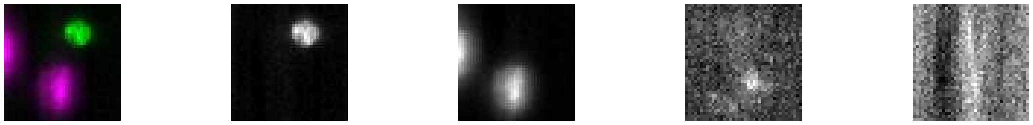				

Proben-ID: Gyn14.208 **Kassetten-ID:** 01327106 **Patienten-ID:**

Ereignis	Aufnahme	DAPI/CK-PE	CK-PE	DAPI	CD45-APC	HER-2/neu
4303	36	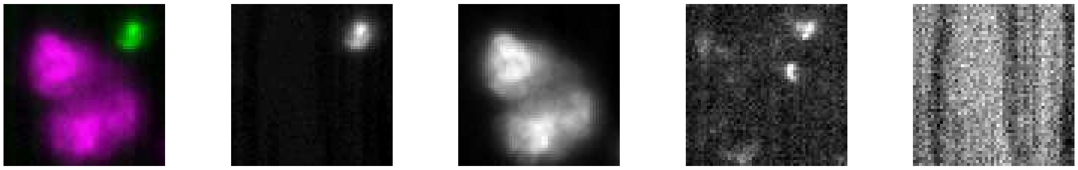				
4304	36	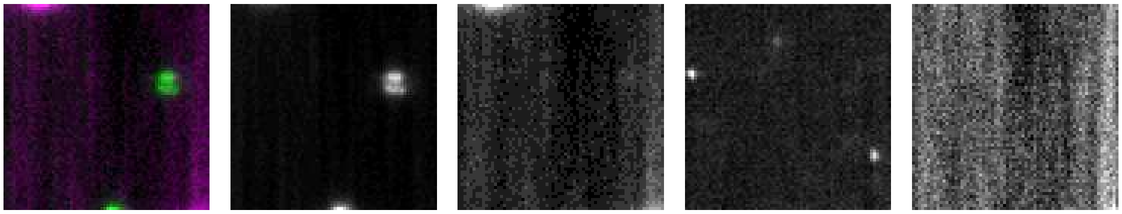				
4305	36	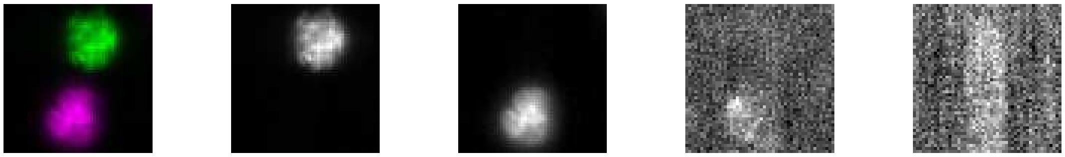				
4306	36	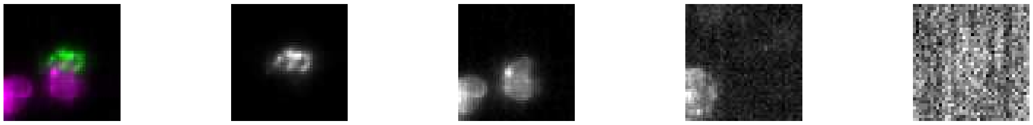				
4307	36	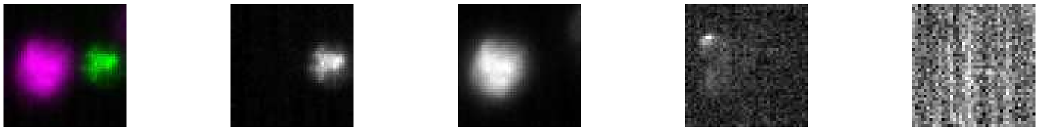				
4308	36	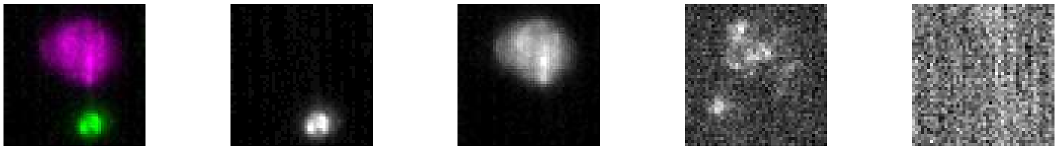				
4309	36	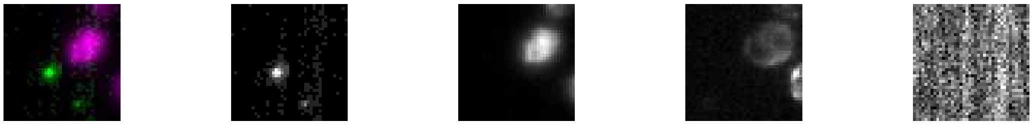				
4310	36	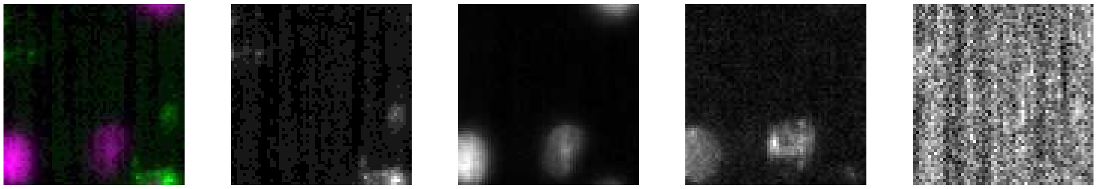				
4311	36	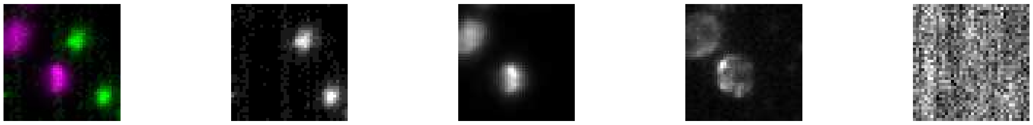				
4312	36	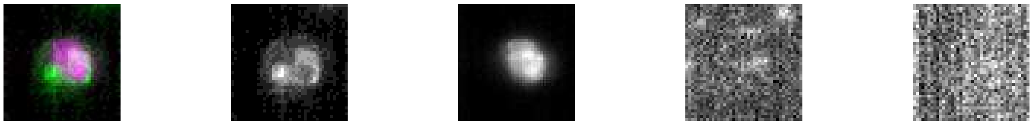				
4313	36	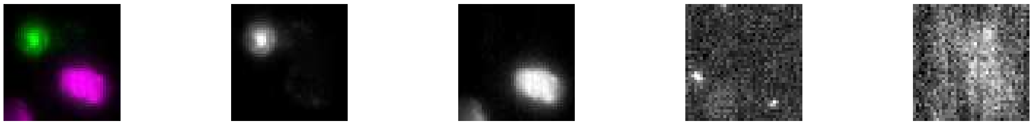				

Ereignis	Aufnahme	DAPI/CK-PE	CK-PE	DAPI	CD45-APC	HER-2/neu
4314	36	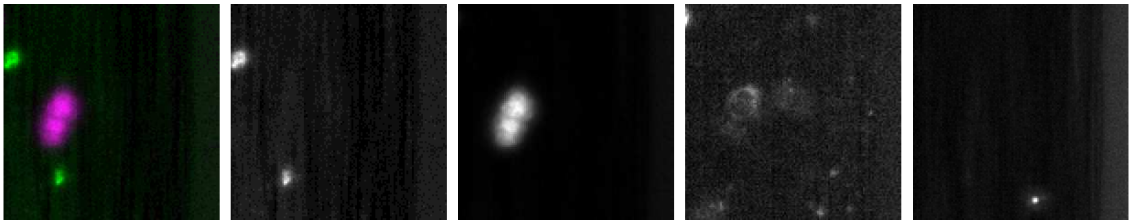				
4315	36	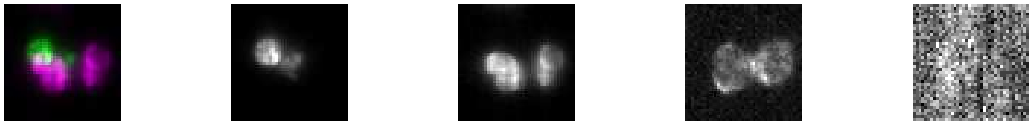				
4316	36	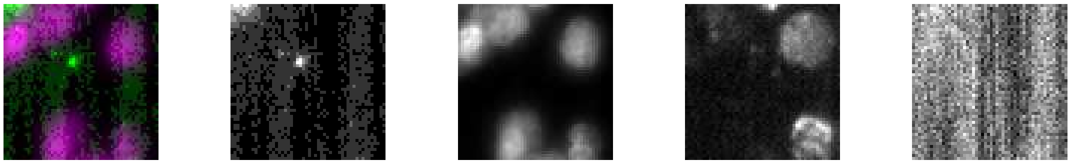				
4317	36	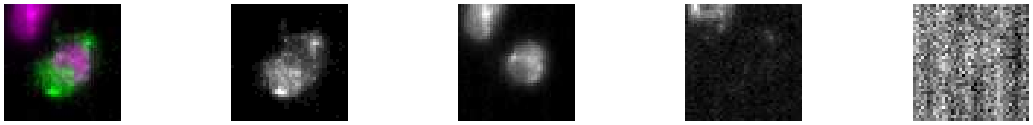				
4318	36	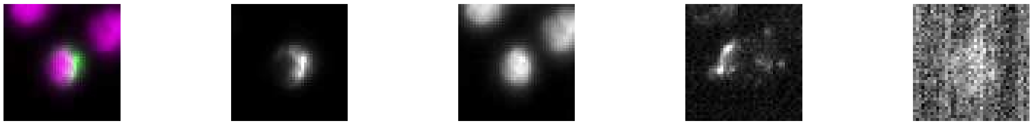				
4319	36	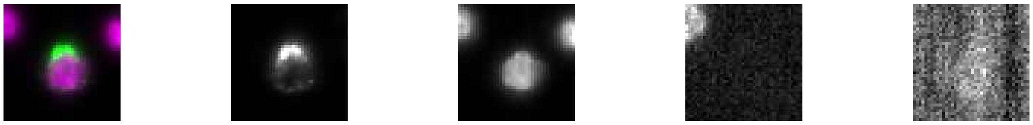				
4320	35	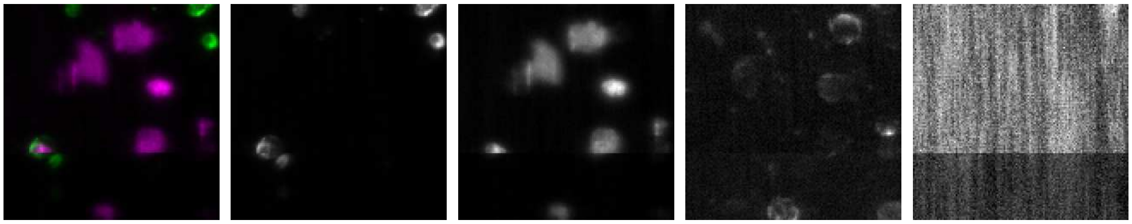				
4321	37	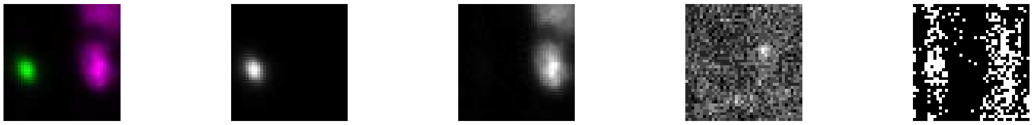				
4322	37	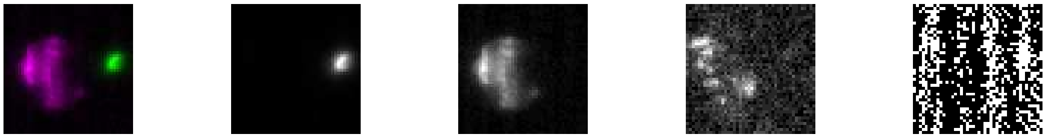				
4323	37	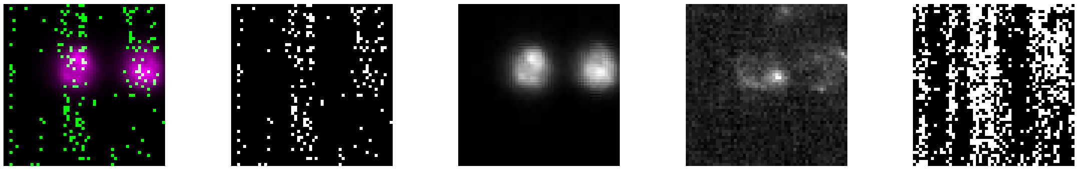				
4324	37	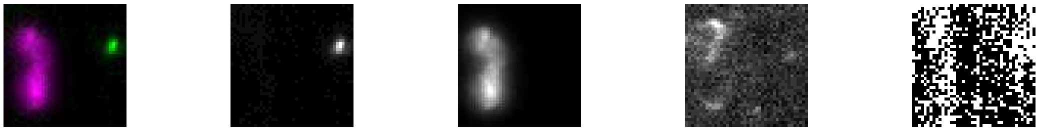				

Ereignis	Aufnahme	DAPI/CK-PE	CK-PE	DAPI	CD45-APC	HER-2/neu
4325	37	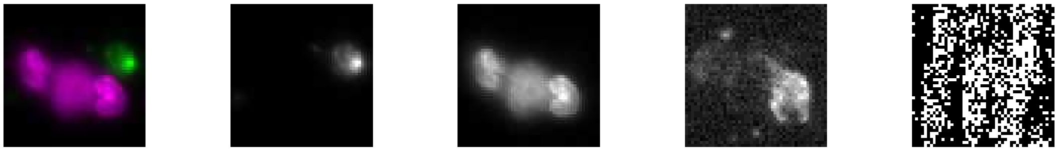				
4326	37	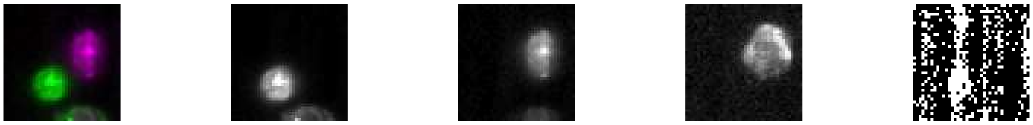				
4327	37	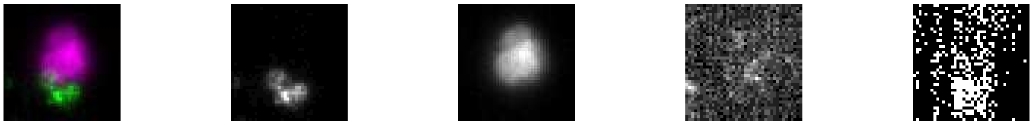				
4328	37	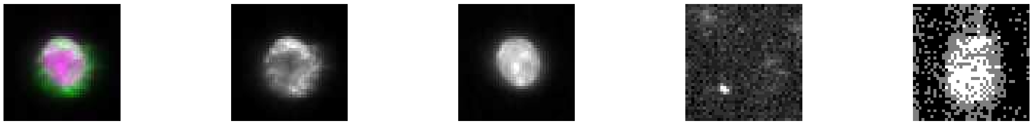				
4329	37	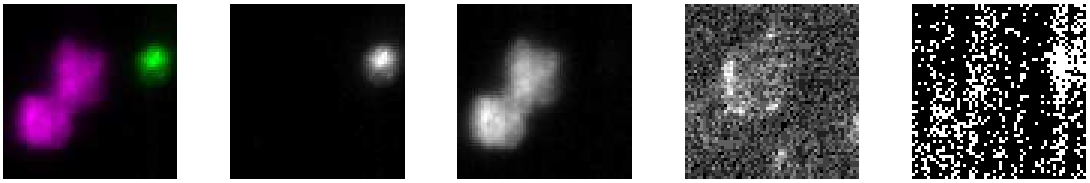				
4330	37	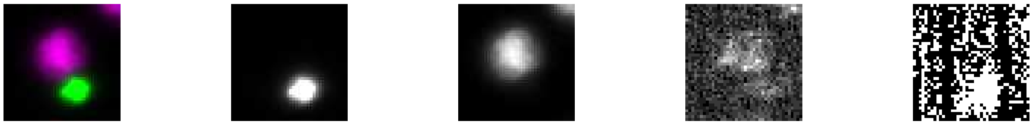				
4331	37	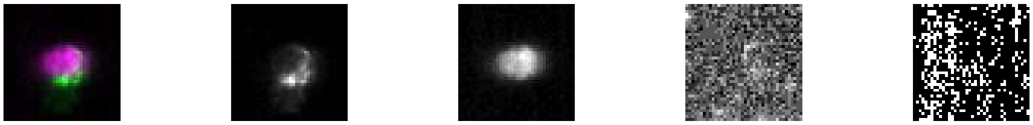				
4332	37	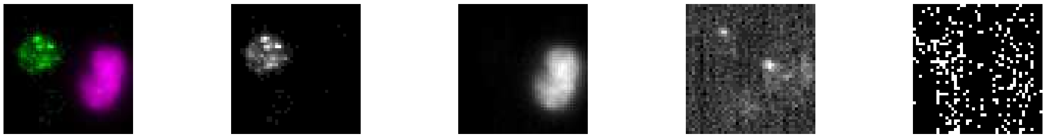				
4333	37	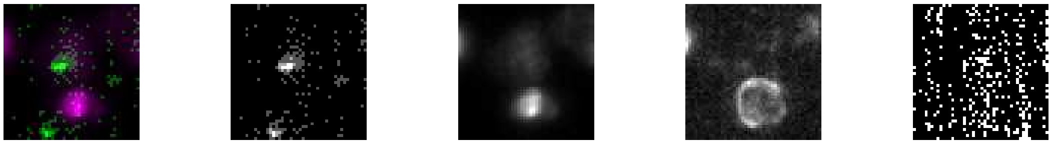				
4334	37	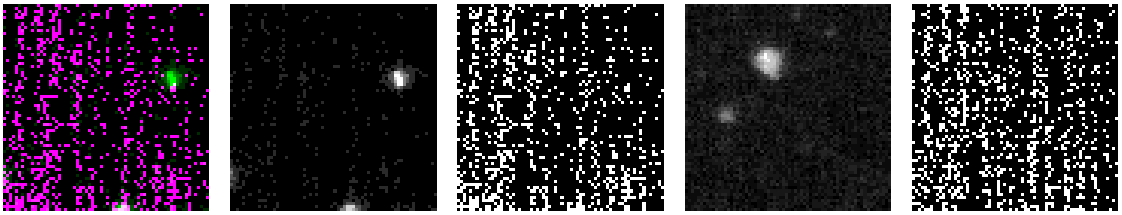				
4335	37	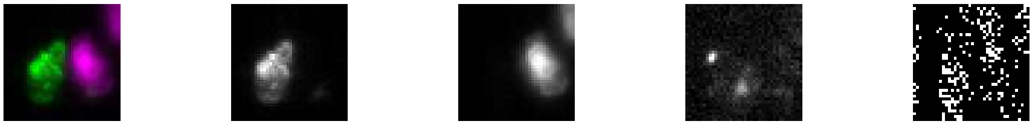				
4336	37	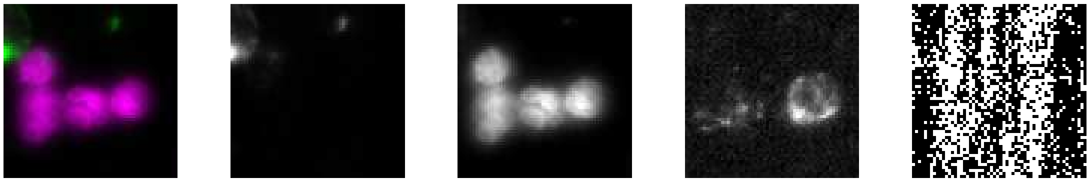				

Ereignis	Aufnahme	DAPI/CK-PE	CK-PE	DAPI	CD45-APC	HER-2/neu
4337	37	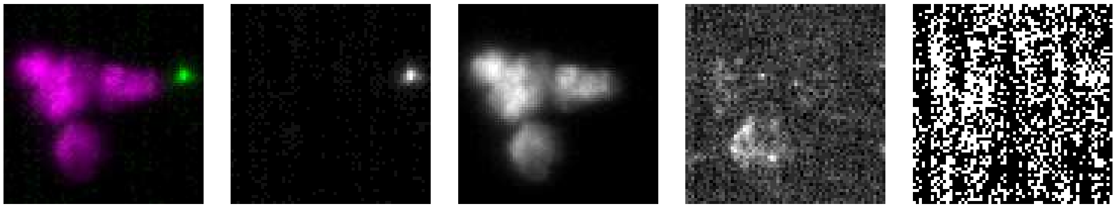				
4338	37	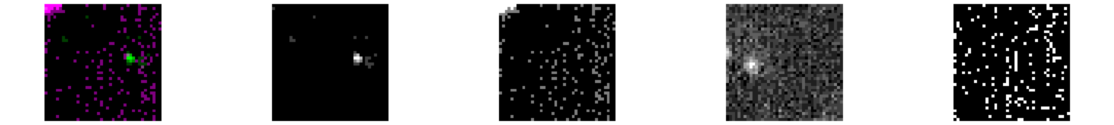				
4339	37	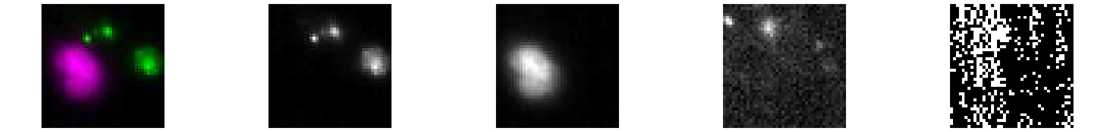				
4340	37	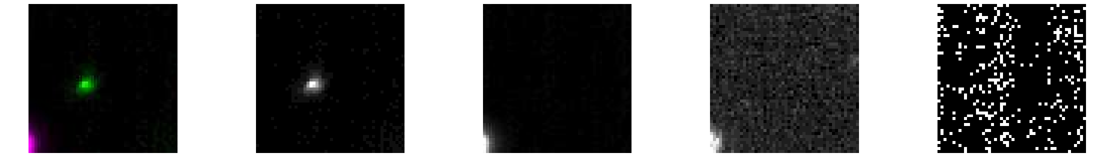				
4341	37	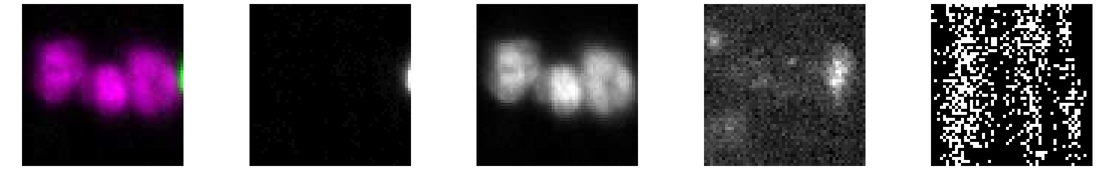				
4342	37	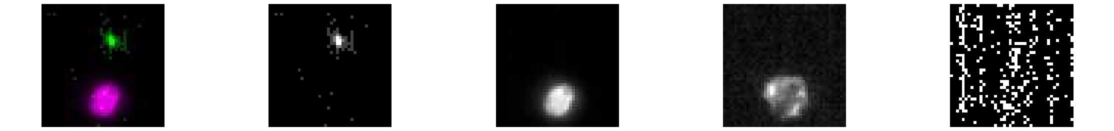				
4343	37	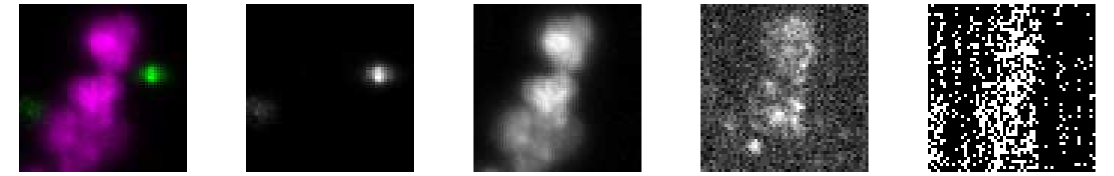				
4344	37	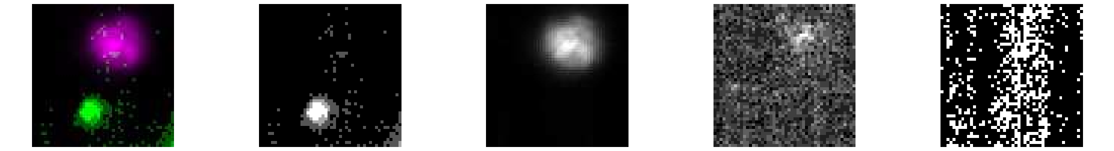				
4345	37	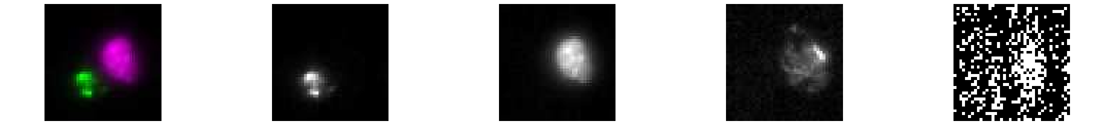				
4346	37	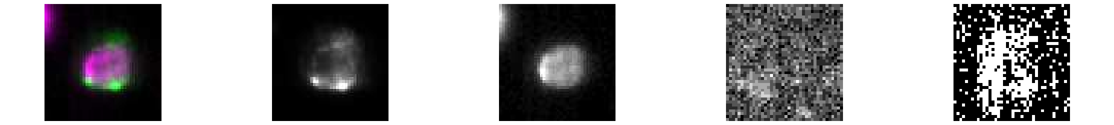				
4347	37	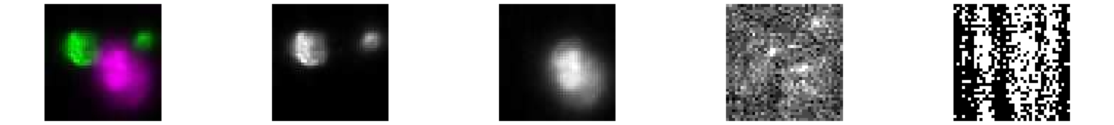				
4348	37	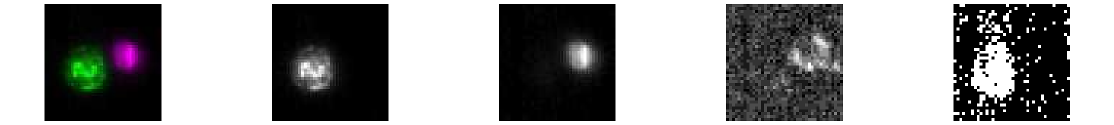				

Ereignis	Aufnahme	DAPI/CK-PE	CK-PE	DAPI	CD45-APC	HER-2/neu
4349	37	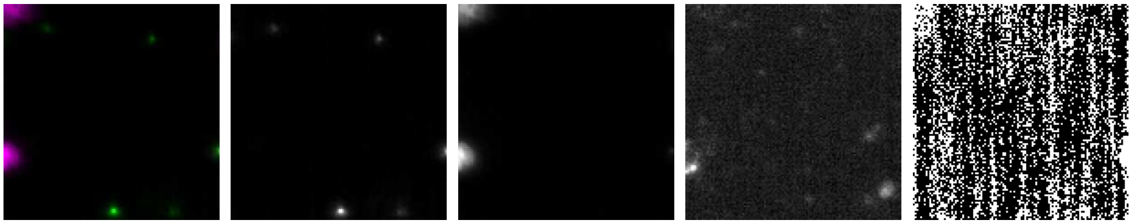				
4350	37	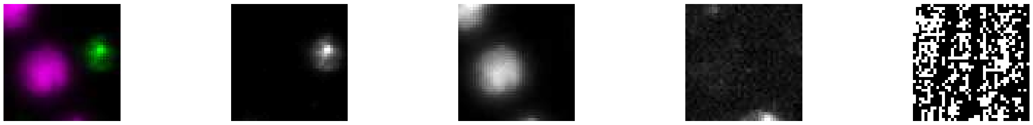				
4351	37	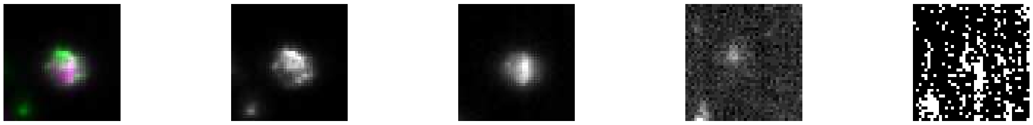				
4352	37	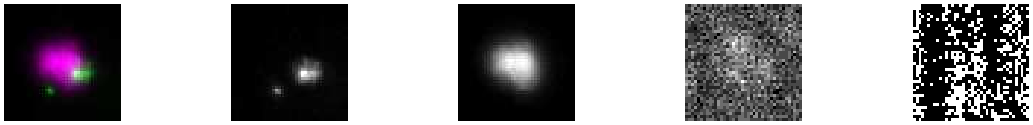				
4353	37	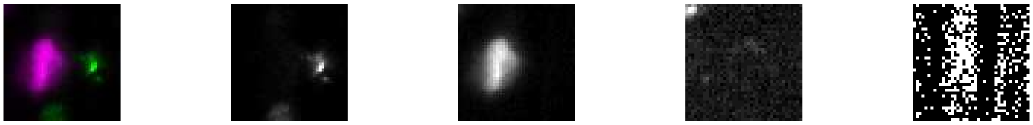				
4354	37	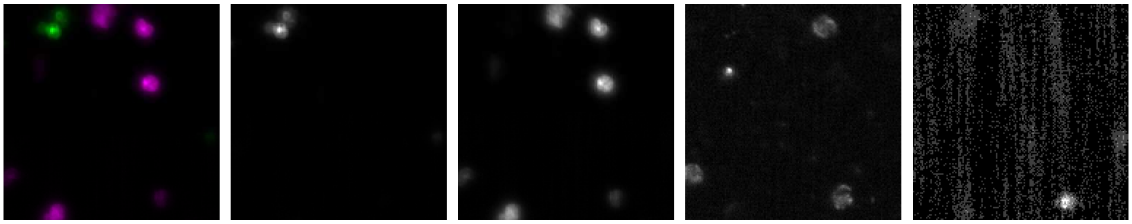				
4355	37	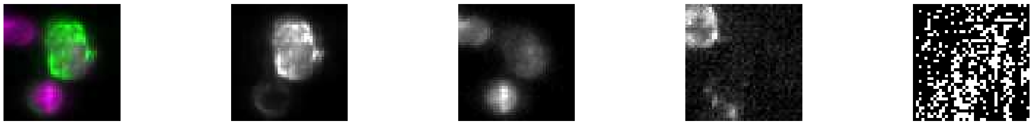				
4356	37	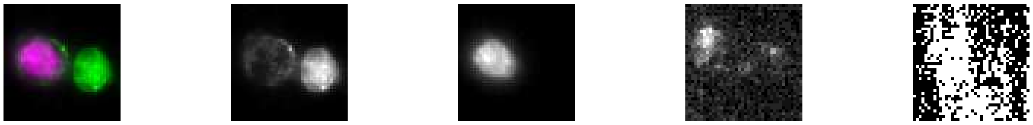				
4357	37	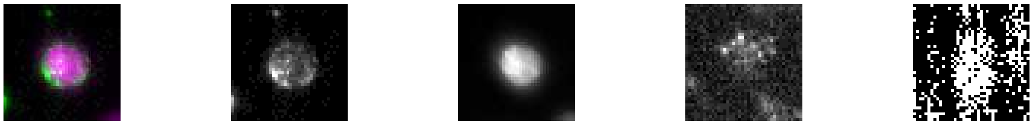				
4358	37	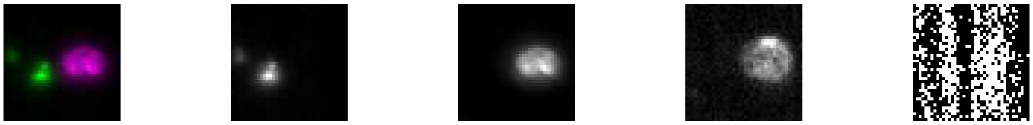				
4359	37	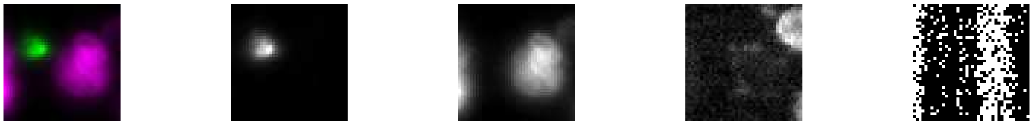				
4360	37	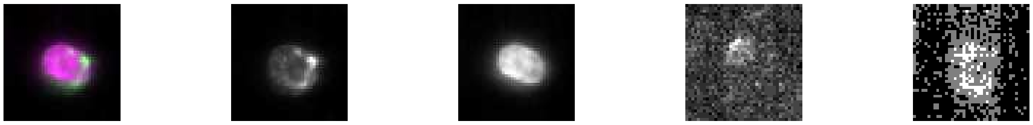				

Ereignis	Aufnahme	DAPI/CK-PE	CK-PE	DAPI	CD45-APC	HER-2/neu
4361	37	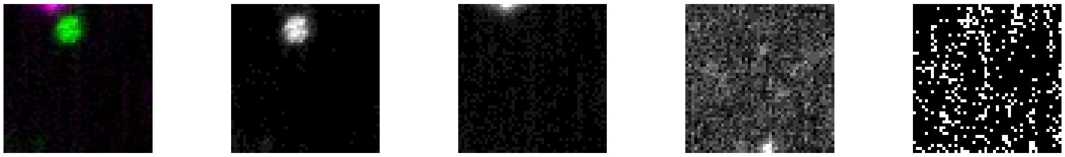				
4362	37	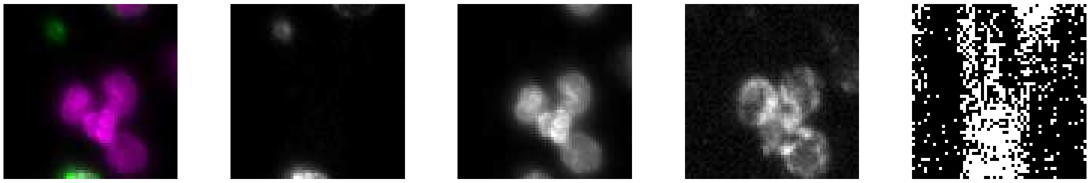				
4363	37	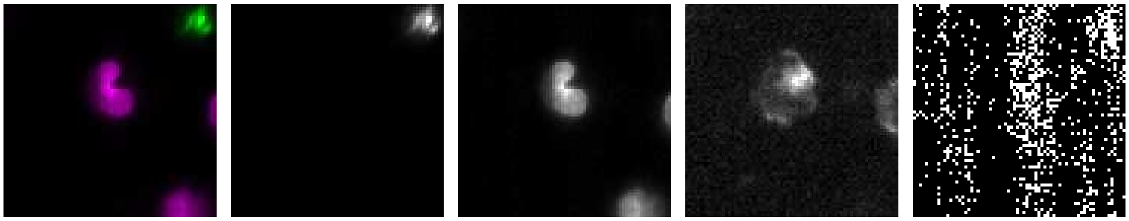				
4364	37	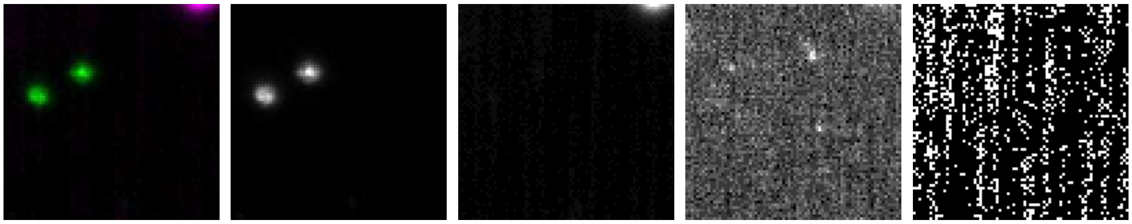				
4365	37	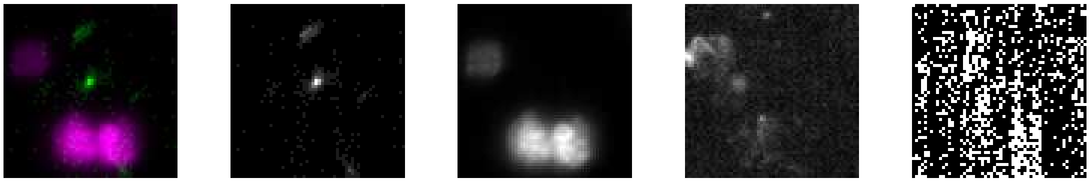				
4366	37	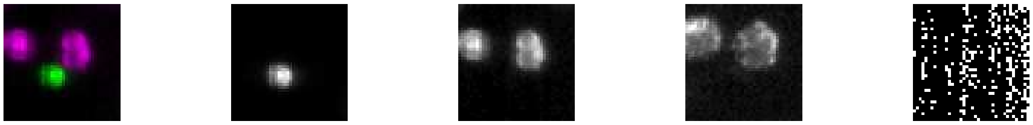				
4367	37	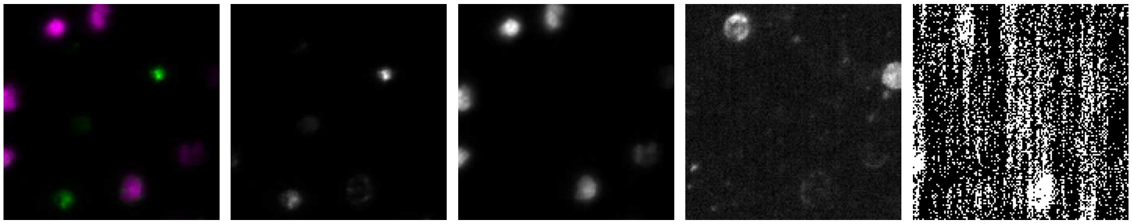				
4368	37	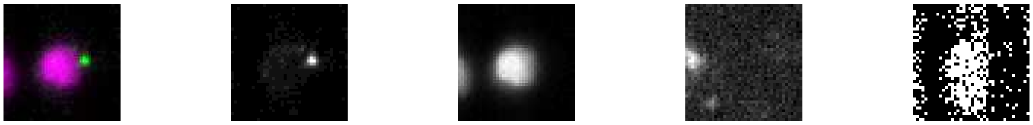				
4369	37	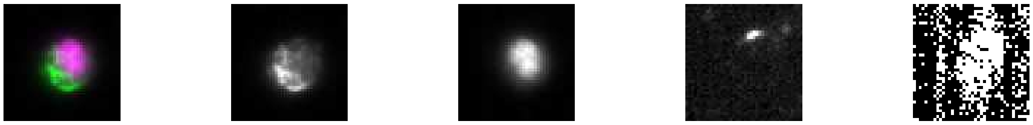				
4370	37	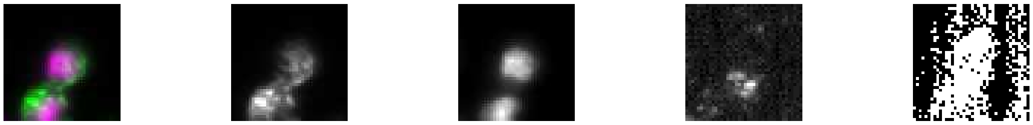				

Ereignis	Aufnahme	DAPI/CK-PE	CK-PE	DAPI	CD45-APC	HER-2/neu
4371	37	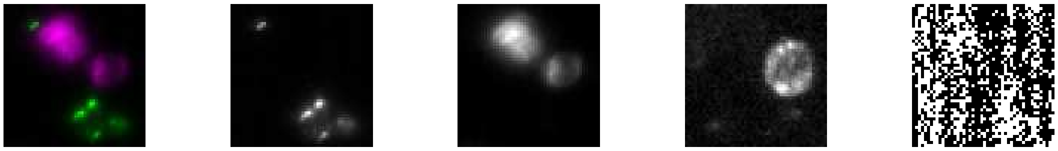				
4372	37	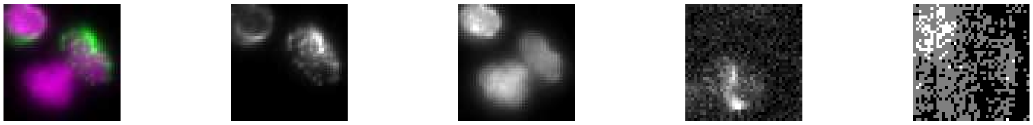				
4373	37	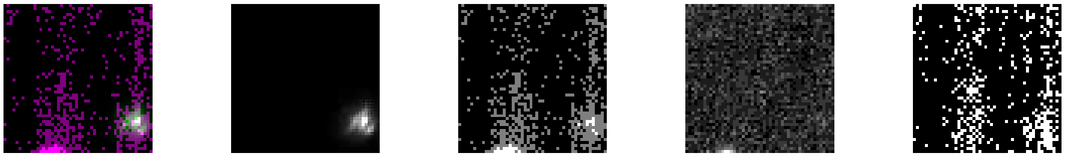				
4374	37	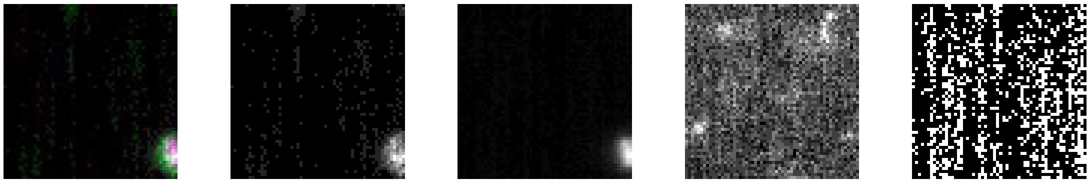				
4375	37	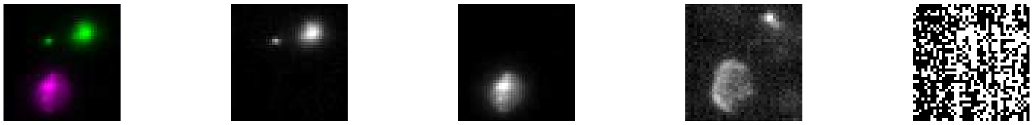				
4376	37	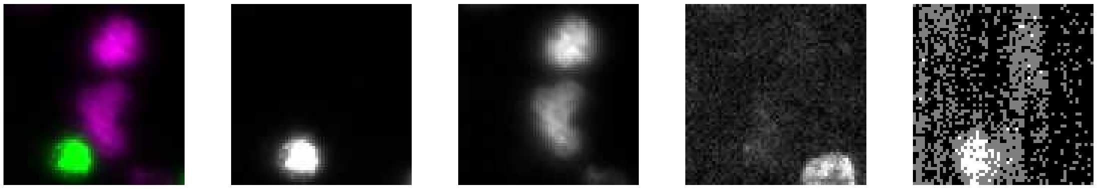				
4377	37	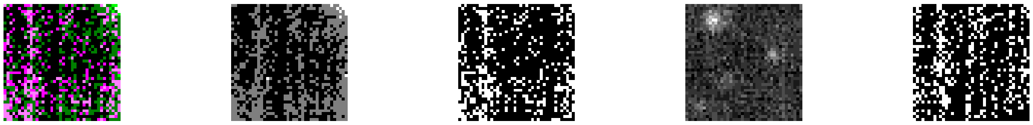				
4378	37	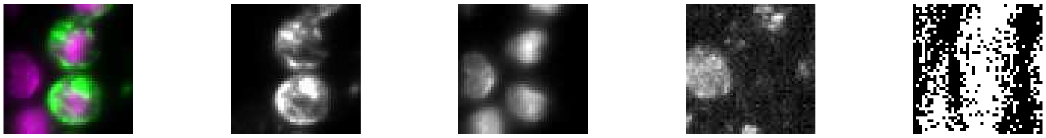				
4379	37	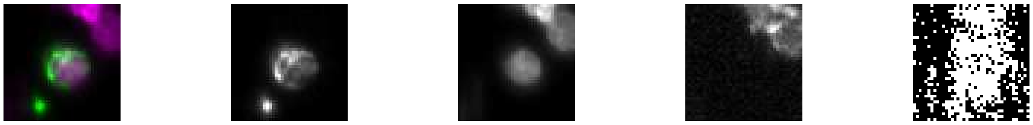				
4380	37	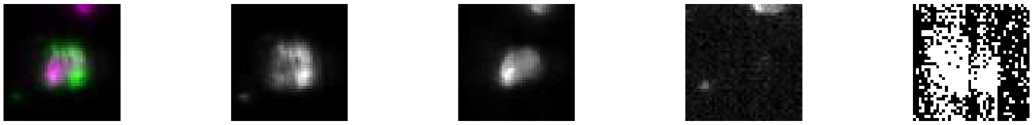				
4381	37	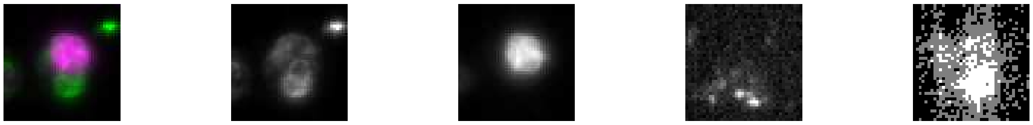				
4382	37	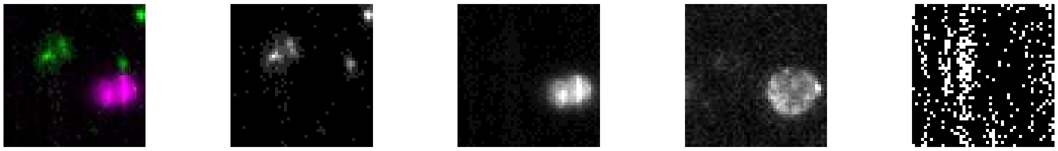				

Proben-ID: Gyn14.208 **Kassetten-ID:**01327106 **Patienten-ID:**

Ereignis	Aufnahme	DAPI/CK-PE	CK-PE	DAPI	CD45-APC	HER-2/neu
4383	37	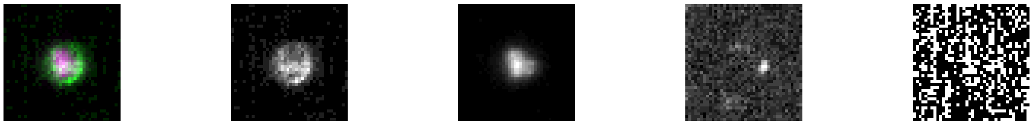				
4384	37	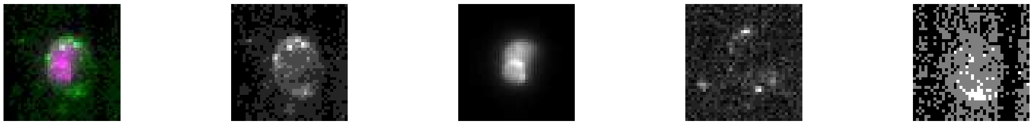				
4385	37	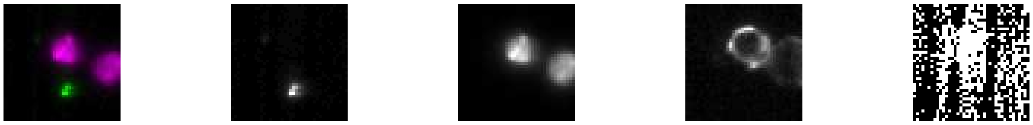				
4386	37	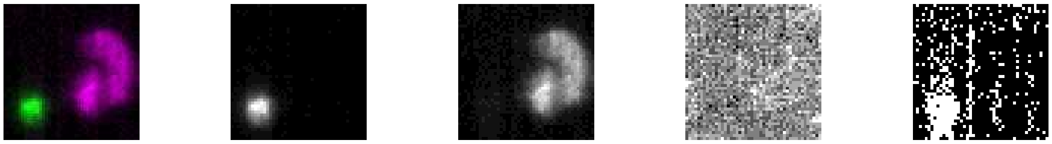				
4387	37	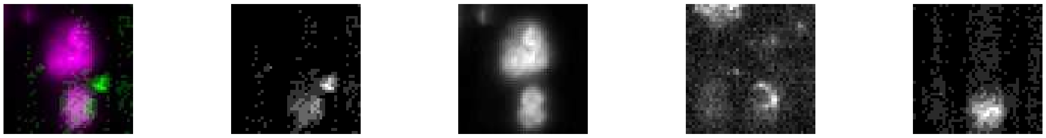				
4388	37	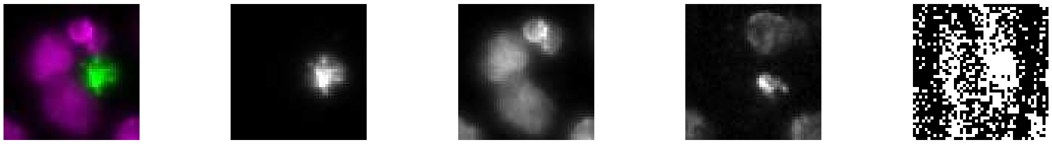				
4389	37	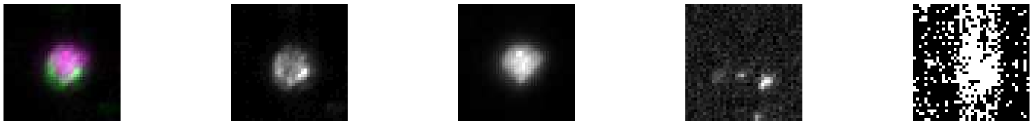				
4390	37	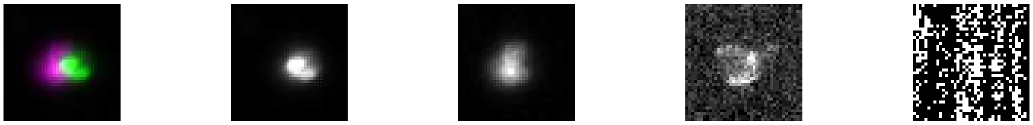				
4391	37	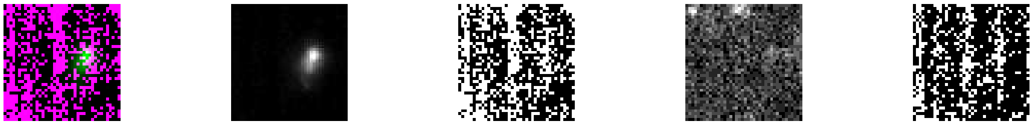				
4392	37	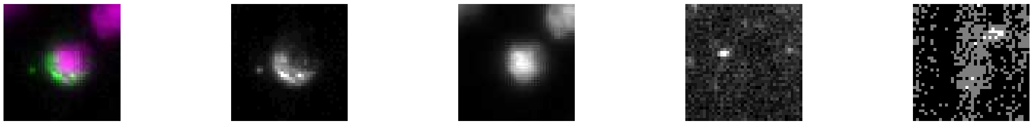				
4393	37	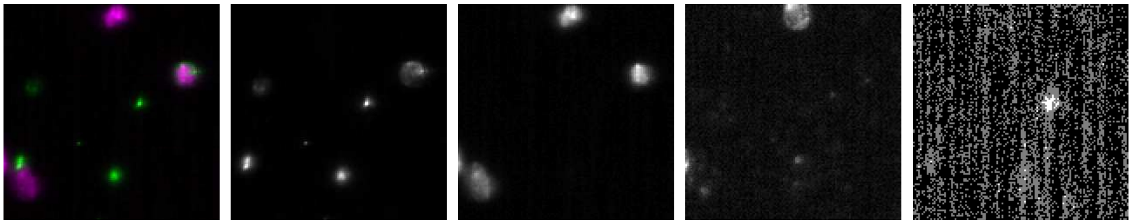				
4394	37	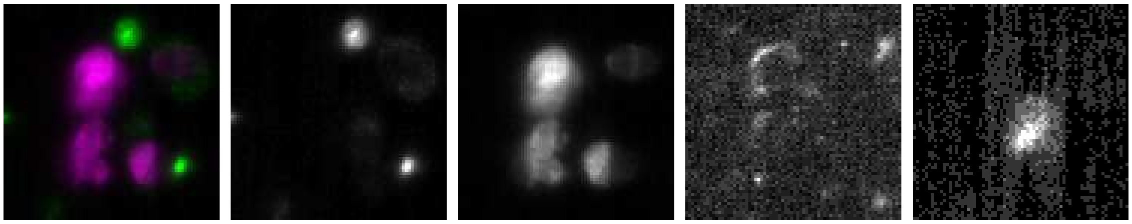				

Ereignis	Aufnahme	DAPI/CK-PE	CK-PE	DAPI	CD45-APC	HER-2/neu
4395	37	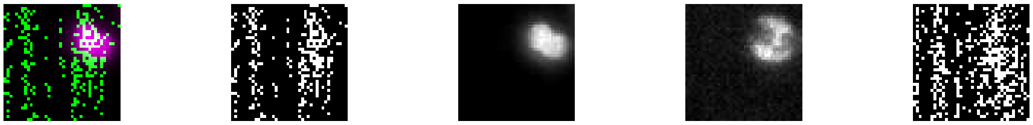				
4396	37	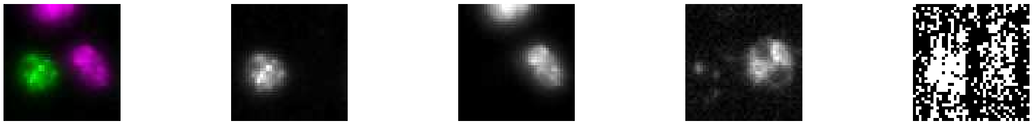				
4397	37	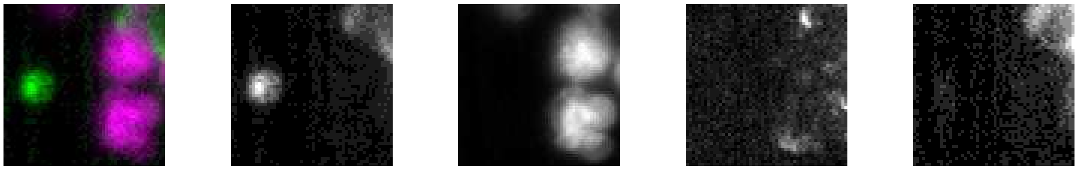				
4398	37	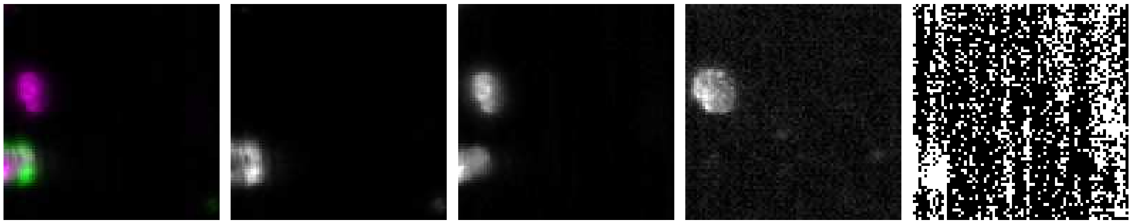				
4399	37	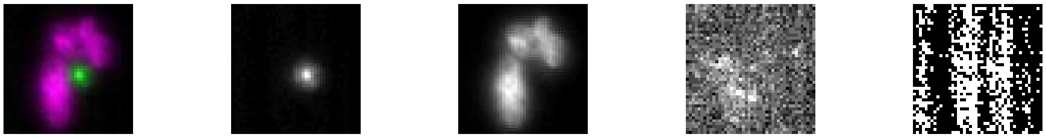				
4400	37	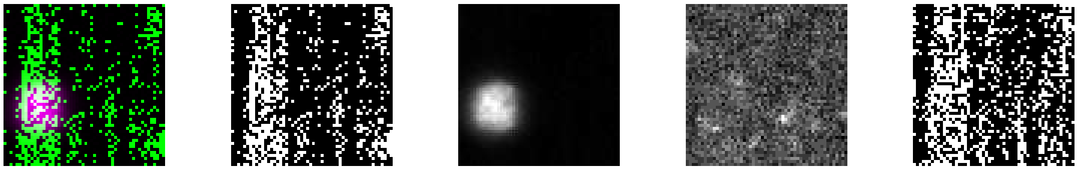				
4401	37	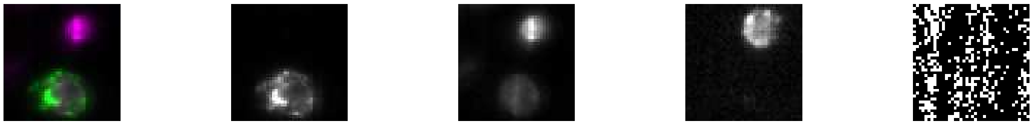				
4402	37	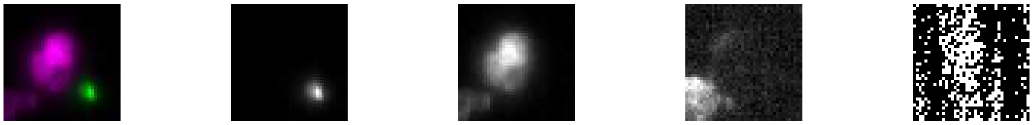				
4403	37	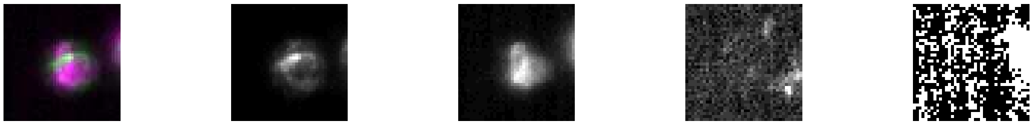				
4404	37	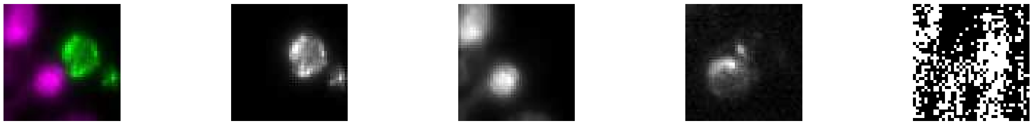				
4405	37	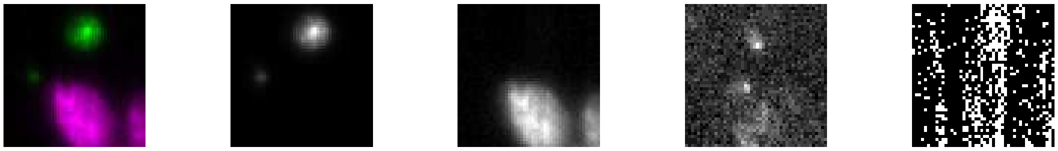				
4406	37	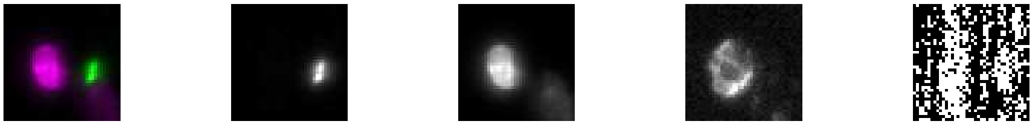				

Ereignis	Aufnahme	DAPI/CK-PE	CK-PE	DAPI	CD45-APC	HER-2/neu
4407	37	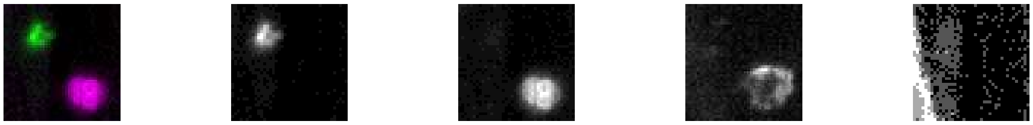				
4408	37	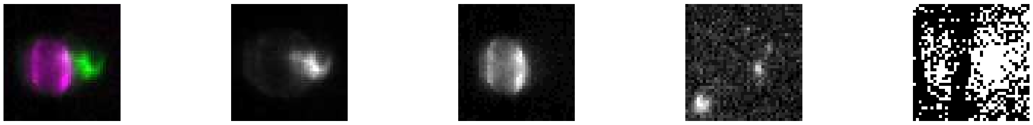				
4409	37	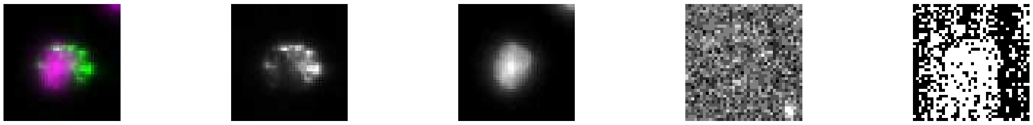				
4410	37	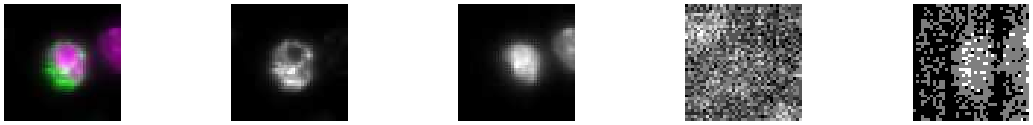				
4411	37	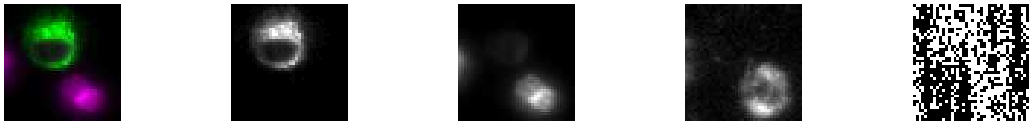				
4412	37	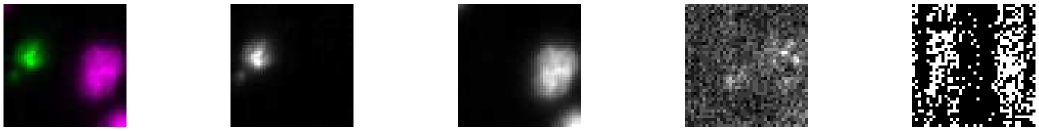				
4413	37	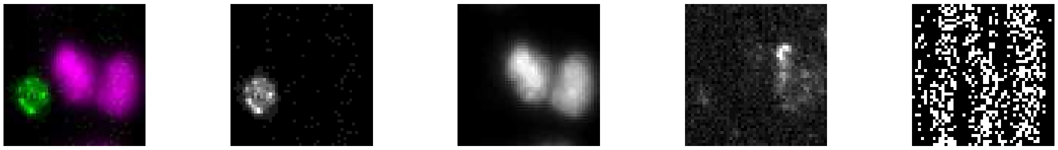				
4414	37	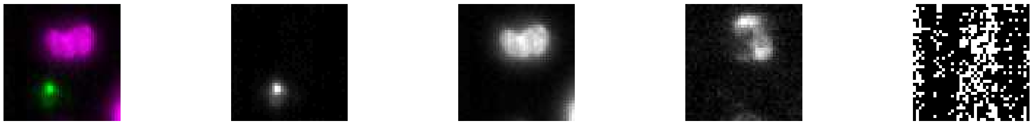				
4415	37	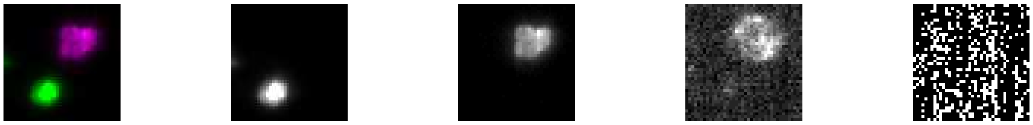				
4416	37	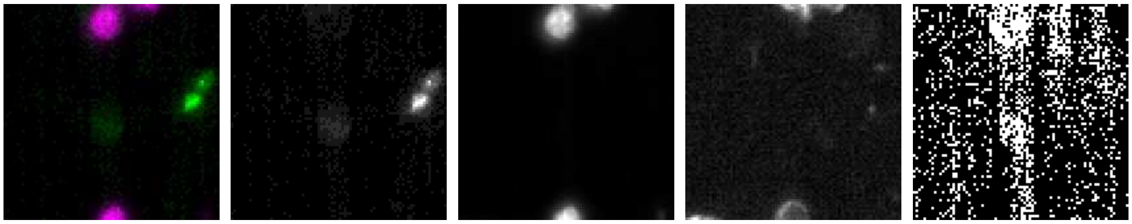				
4417	37	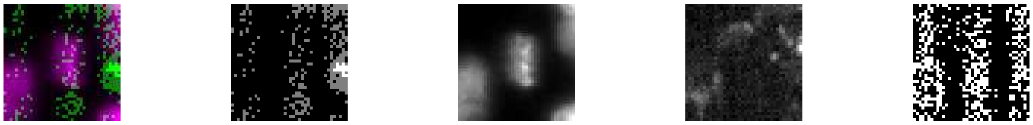				
4418	37	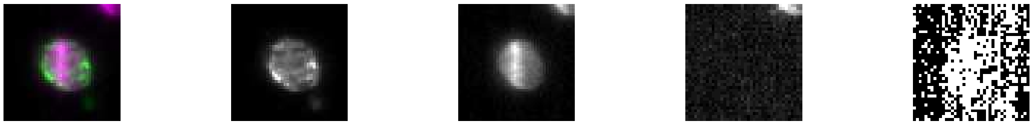				
4419	37	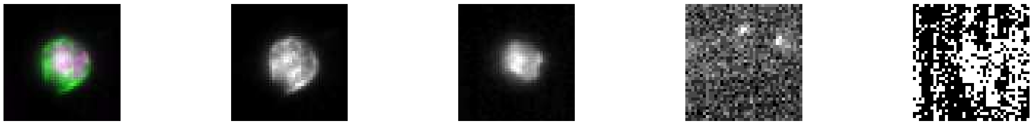				

Ereignis	Aufnahme	DAPI/CK-PE	CK-PE	DAPI	CD45-APC	HER-2/neu
4420	37	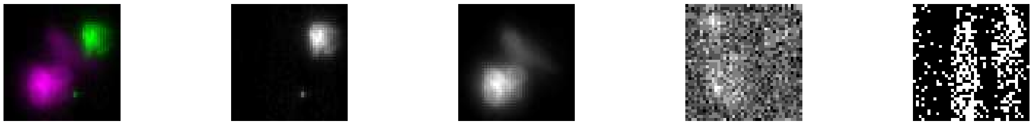				
4421	37	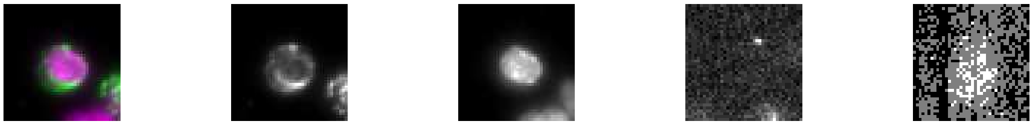				
4422	37	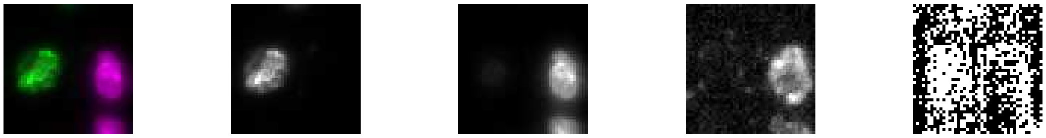				
4423	37	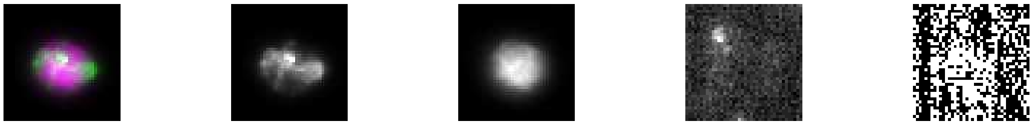				
4424	37	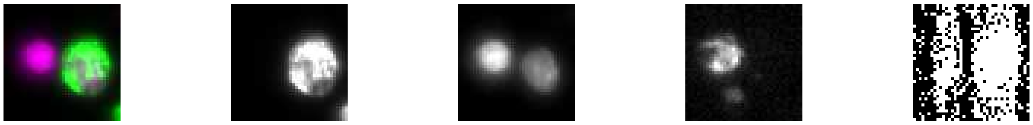				
4425	37	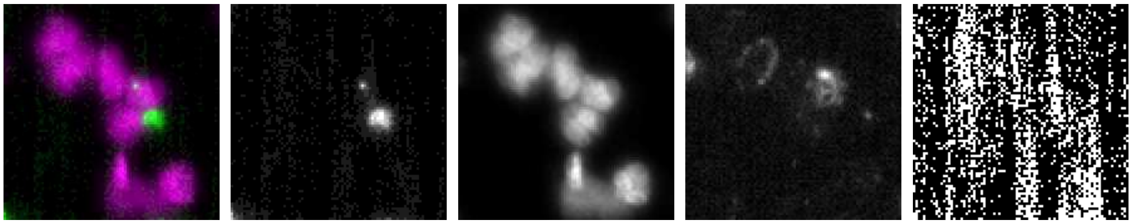				
4426	37	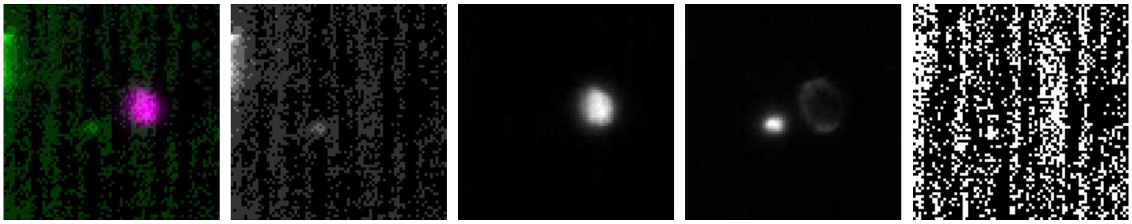				
4427	37	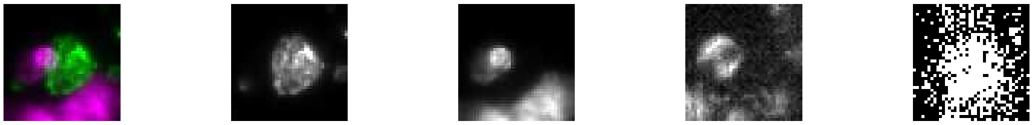				
4428	37	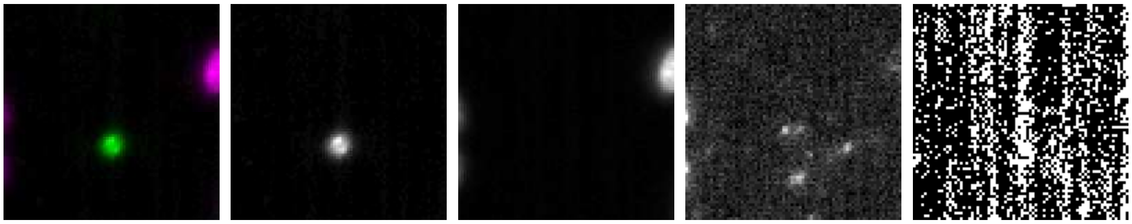				
4429	37	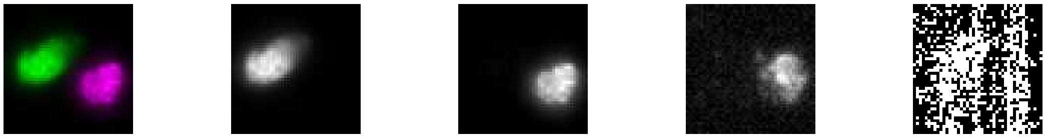				
4430	37	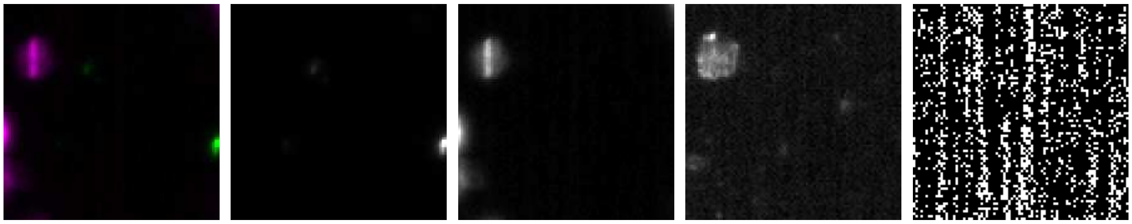				

Ereignis	Aufnahme	DAPI/CK-PE	CK-PE	DAPI	CD45-APC	HER-2/neu
4431	37	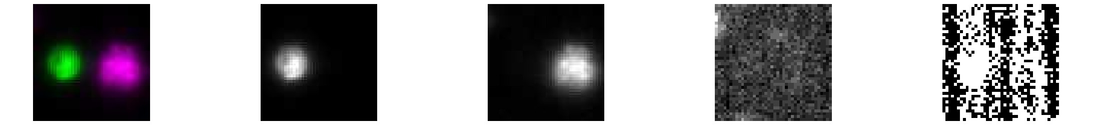				
4432	37	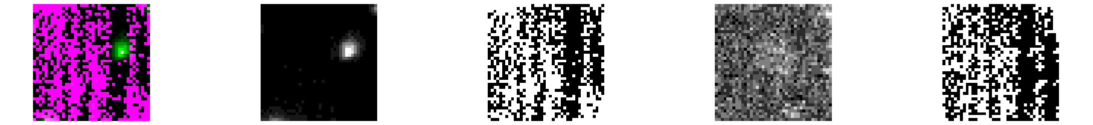				
4433	37	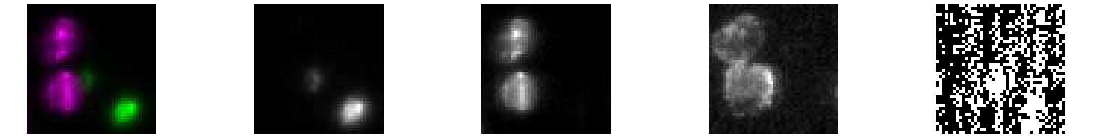				
4434	37	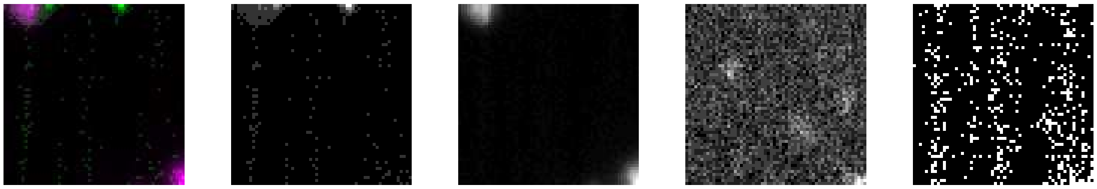				
4435	37	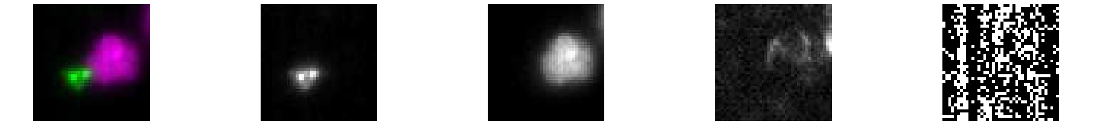				
4436	37	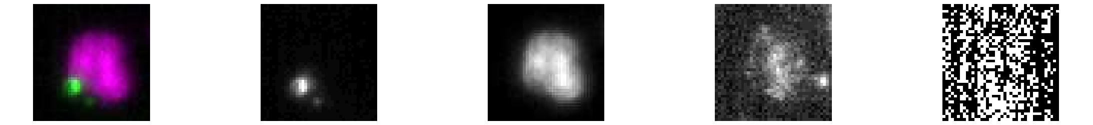				
4437	37	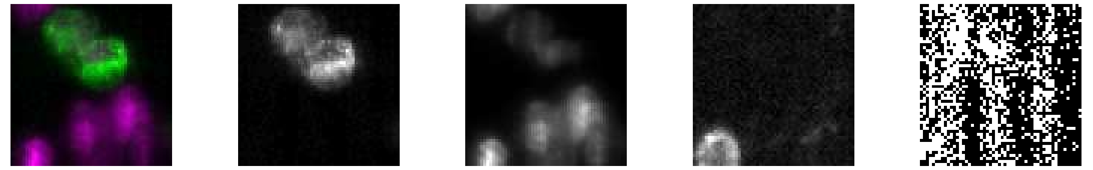				
4438	37	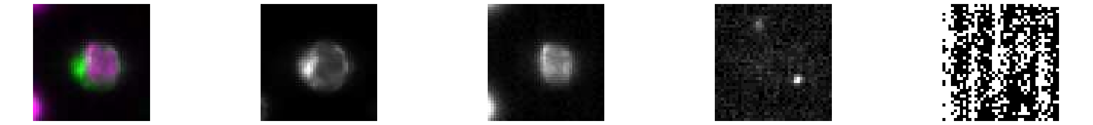				
4439	37	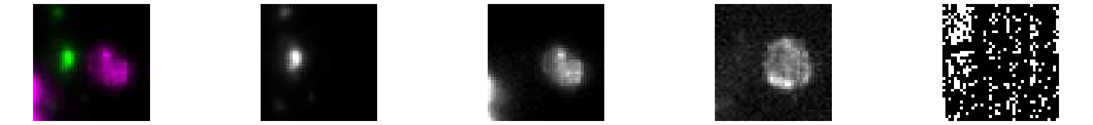				
4440	37	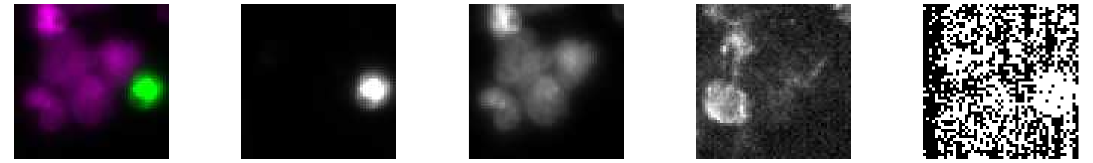				
4441	37	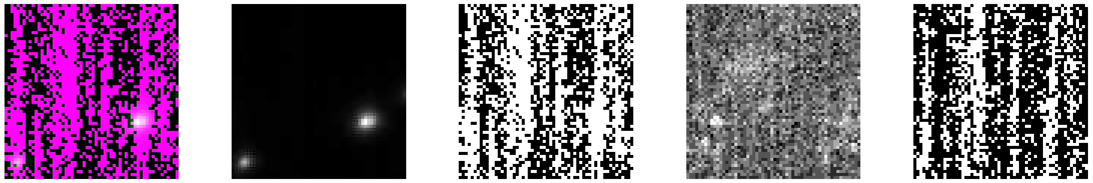				
4442	37	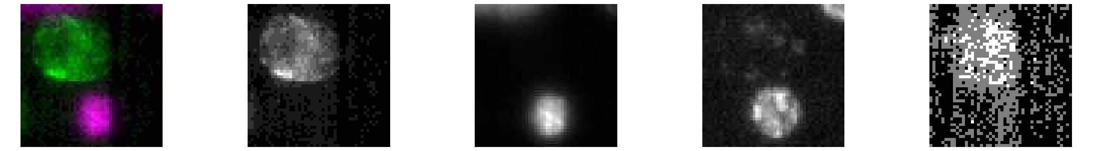				

Ereignis	Aufnahme	DAPI/CK-PE	CK-PE	DAPI	CD45-APC	HER-2/neu
4443	37	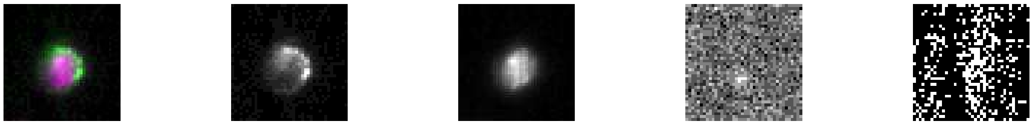				
4444	37	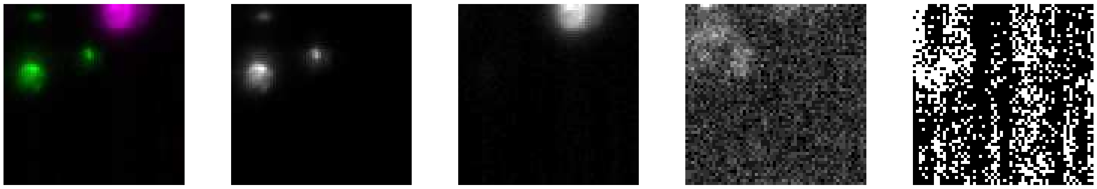				
4445	37	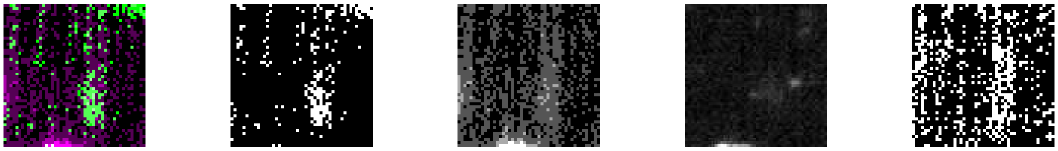				
4446	37	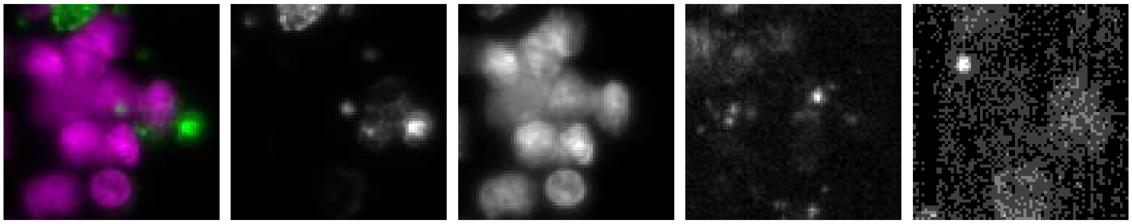				
4447	37	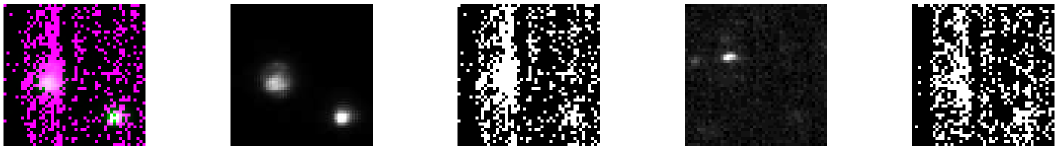				
4448	37	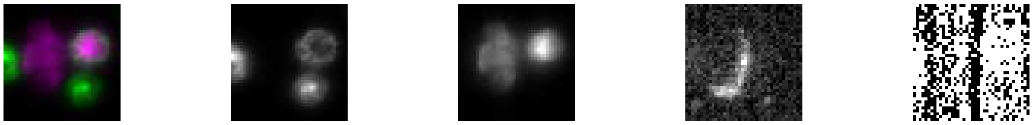				
4449	37	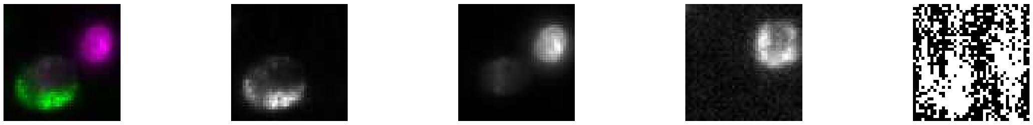				
4450	37	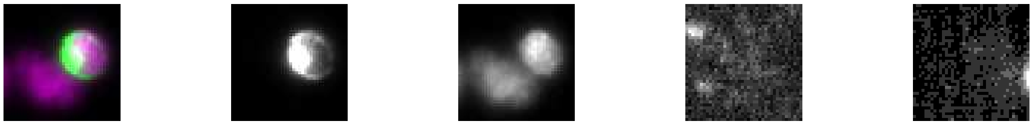				
4451	37	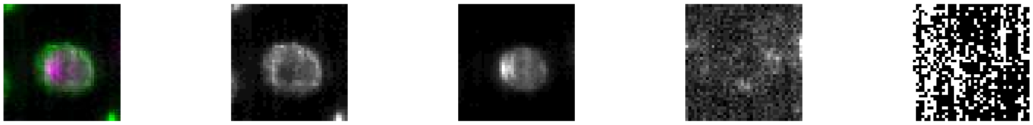				
4452	37	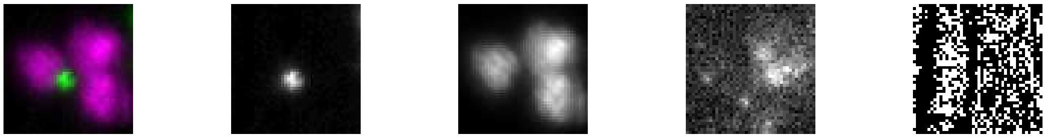				
4453	37	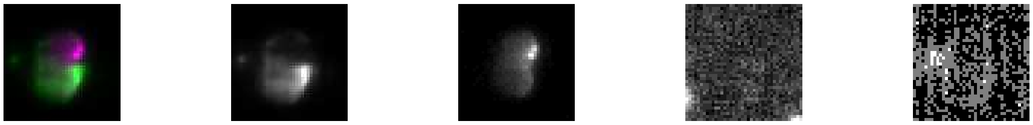				
4454	37	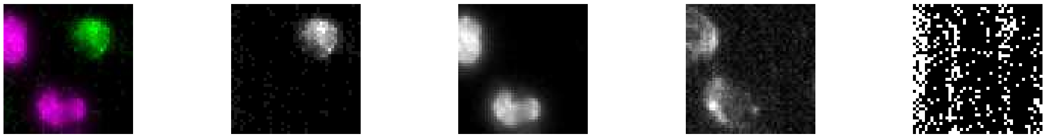				

Ereignis	Aufnahme	DAPI/CK-PE	CK-PE	DAPI	CD45-APC	HER-2/neu
4455	37	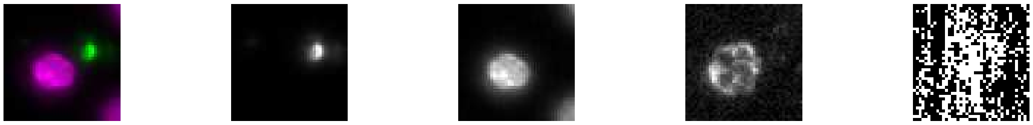				
4456	37	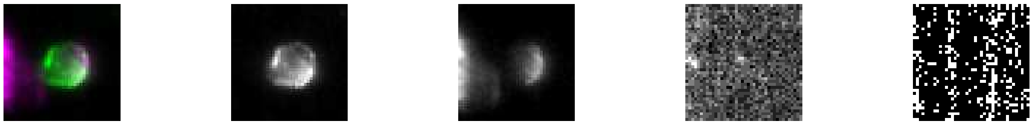				
4457	37	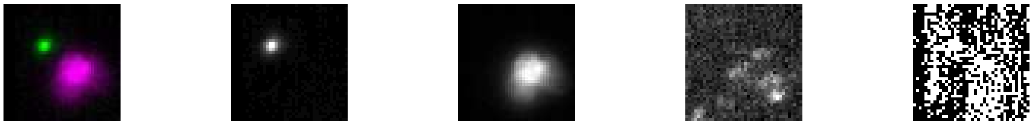				
4458	37	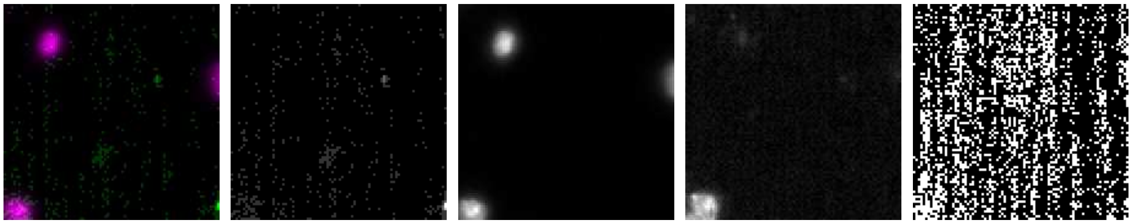				
4459	37	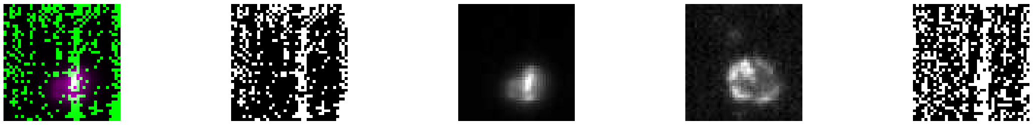				
4460	37	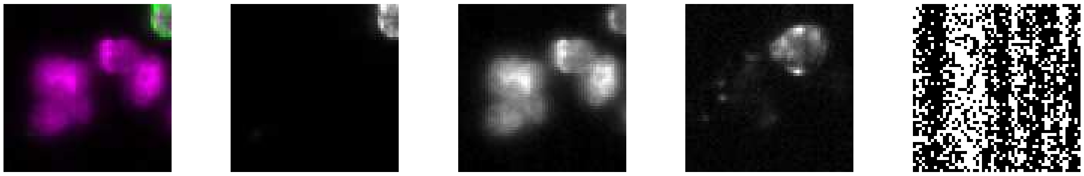				
4461	37	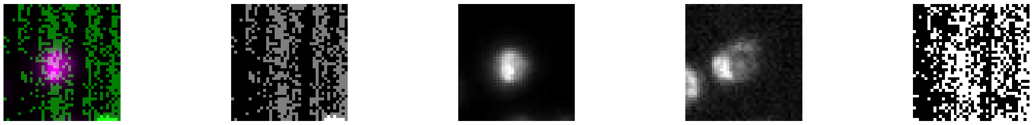				
4462	37	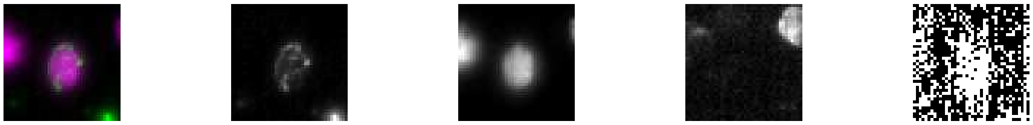				
4463	37	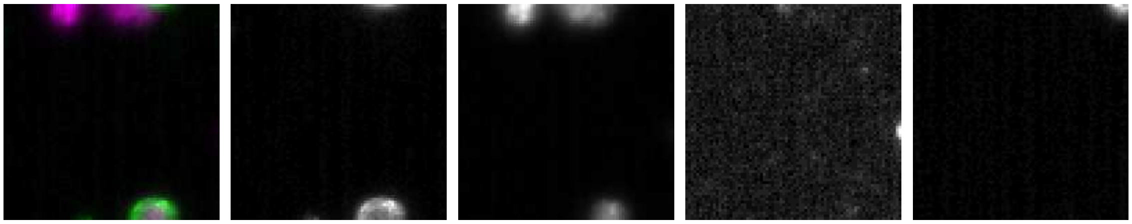				
4464	37	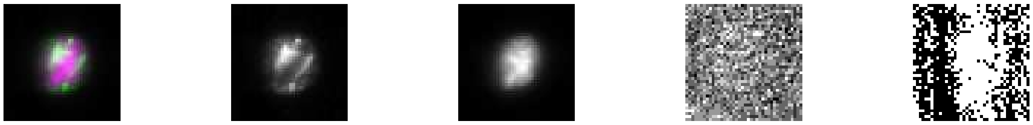				
4465	37	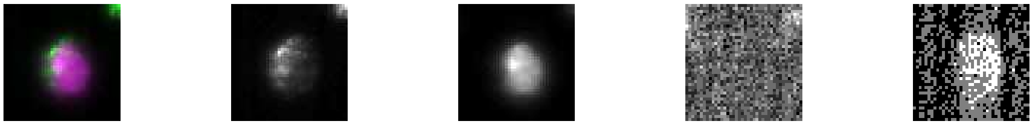				
4466	37	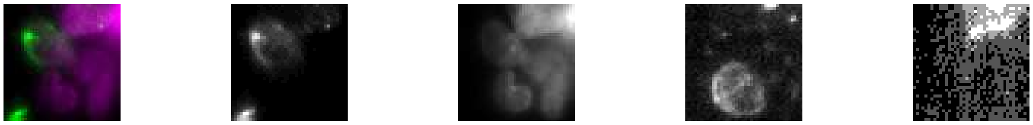				

Ereignis	Aufnahme	DAPI/CK-PE	CK-PE	DAPI	CD45-APC	HER-2/neu
4467	37	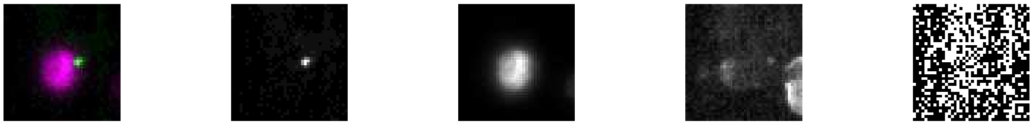				
4468	37	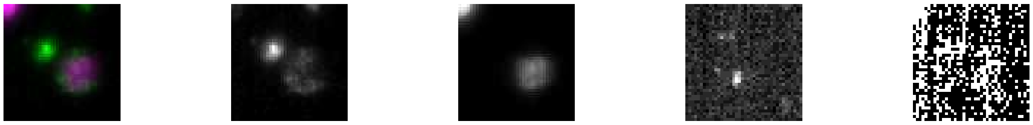				
4469	37	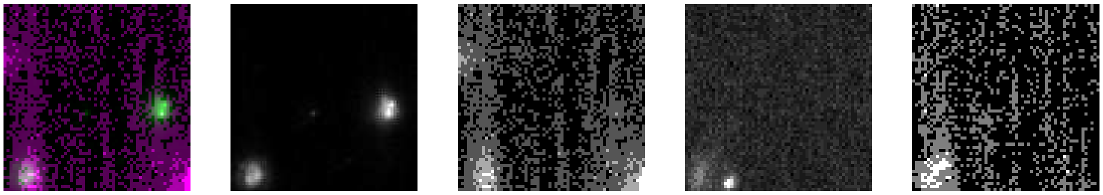				
4470	37	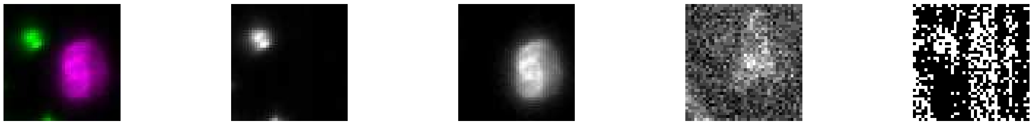				
4471	37	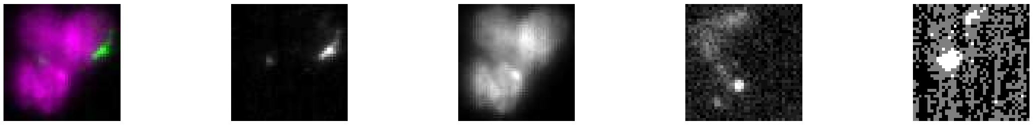				
4472	37	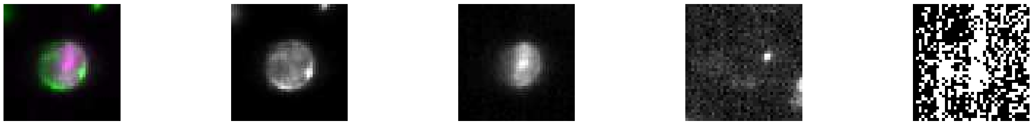				
4473	37	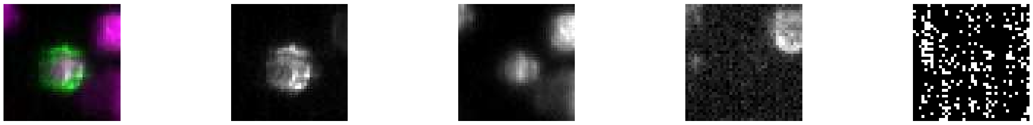				
4474	37	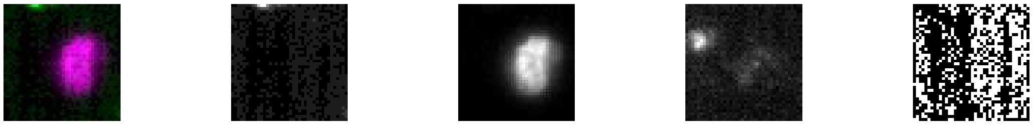				
4475	37	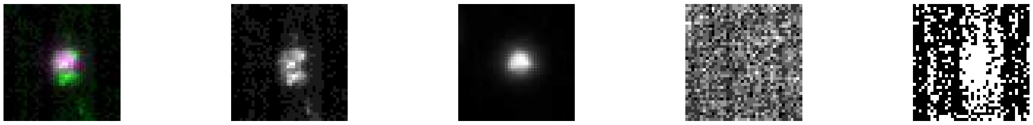				
4476	37	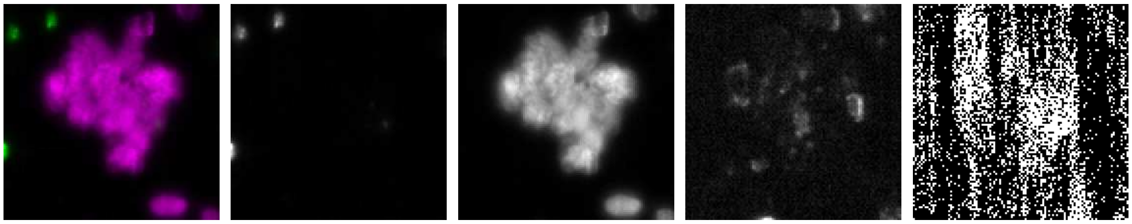				
4477	37	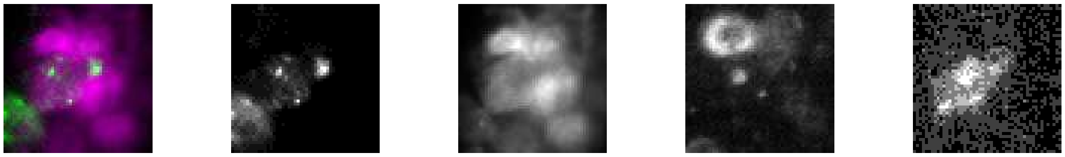				
4478	37	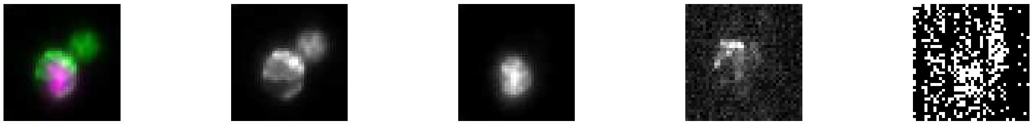				

Ereignis	Aufnahme	DAPI/CK-PE	CK-PE	DAPI	CD45-APC	HER-2/neu
4479	37	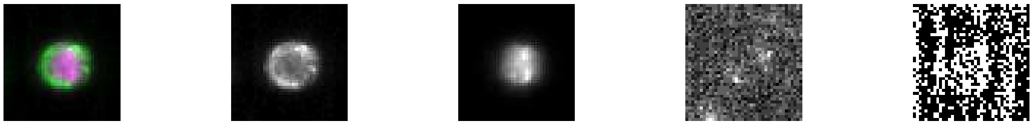				
4480	37	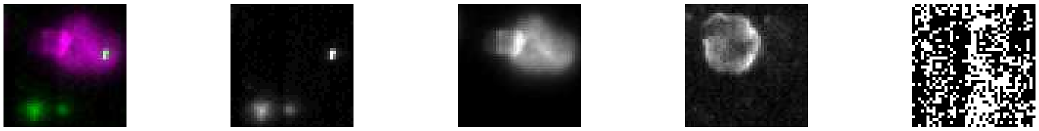				
4481	37	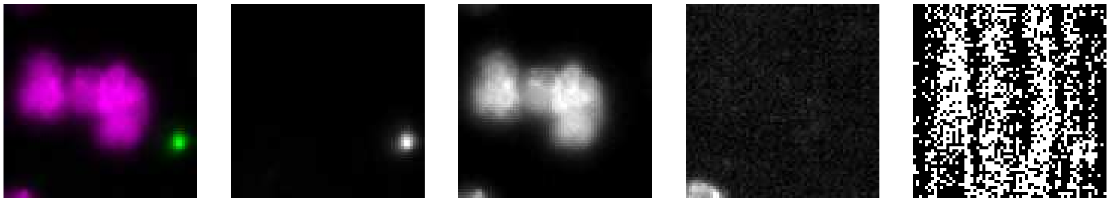				
4482	37	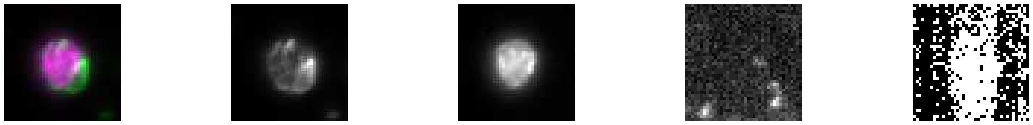				
4483	37	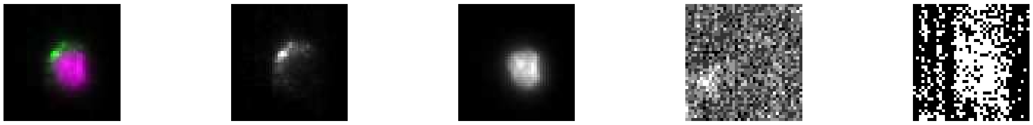				
4484	37	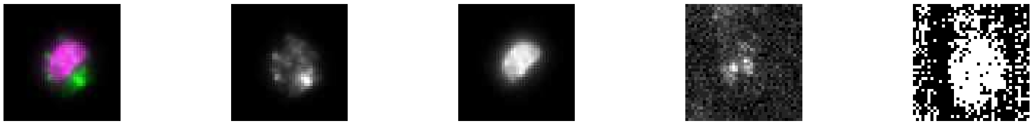				
4485	37	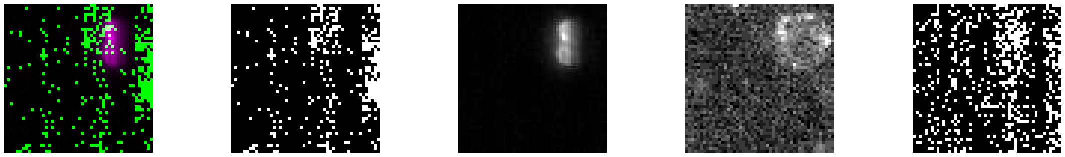				
4486	37	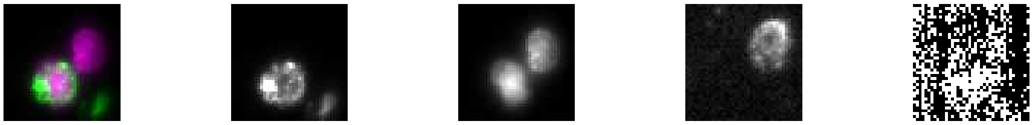				
4487	37	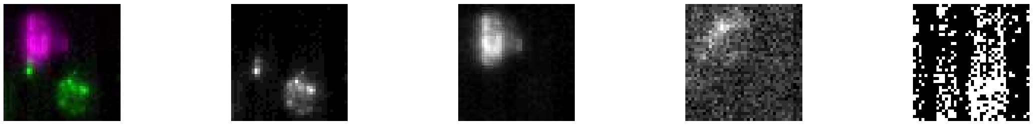				
4488	37	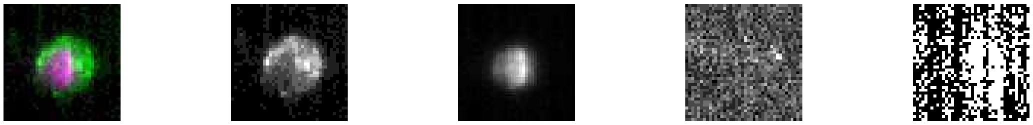				
4489	37	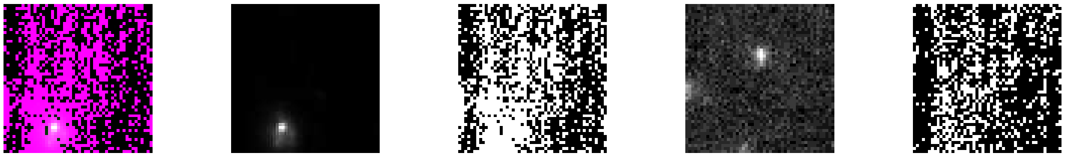				
4490	37	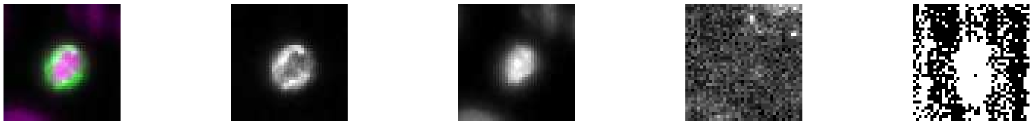				
4491	37	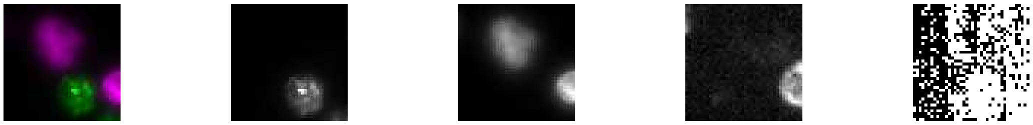				

Ereignis	Aufnahme	DAPI/CK-PE	CK-PE	DAPI	CD45-APC	HER-2/neu
4492	37	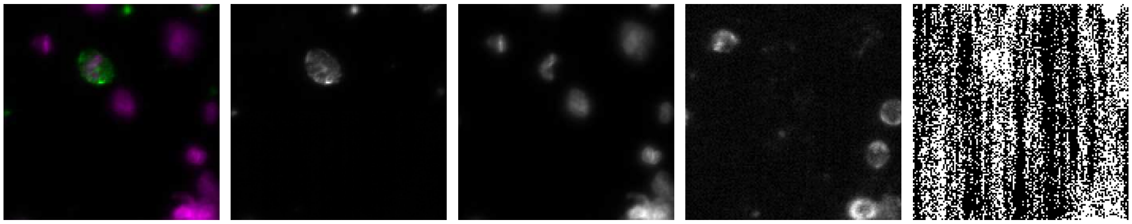				
4493	37	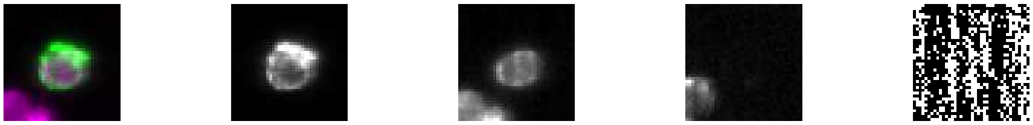				
4494	37	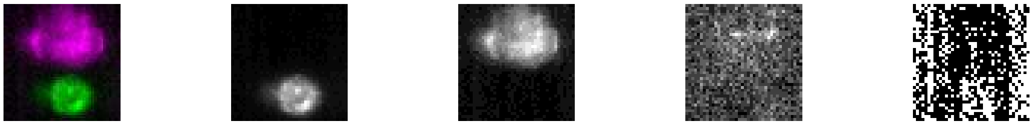				
4495	37	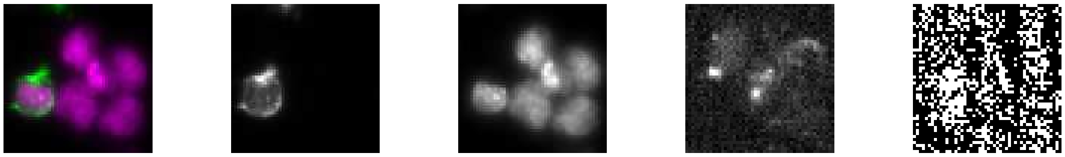				
4496	37	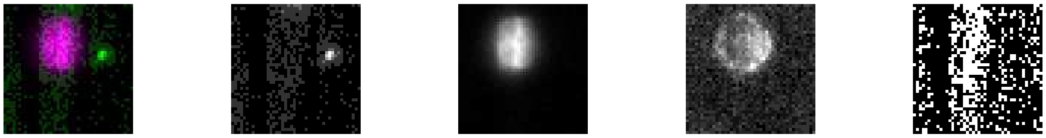				
4497	37	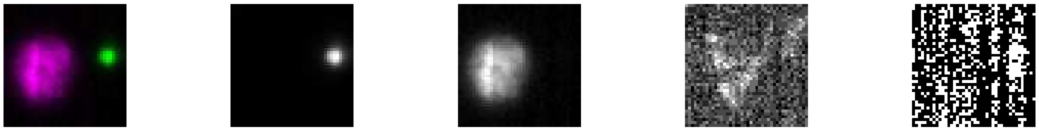				
4498	37	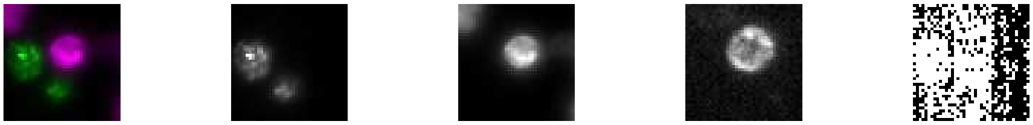				
4499	37	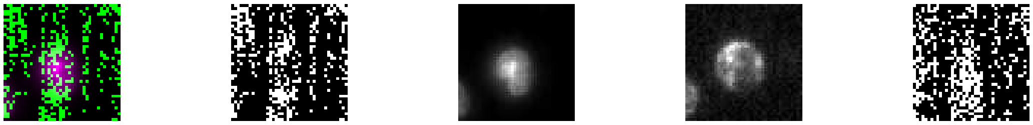				
4500	37	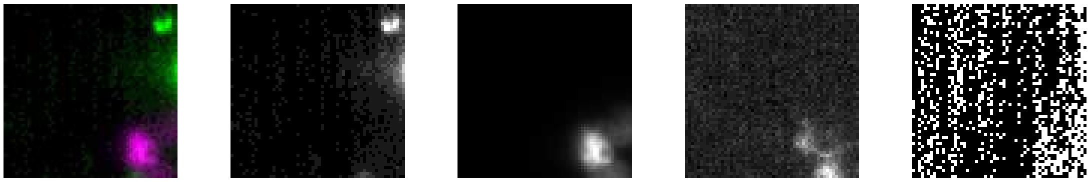				
4501	37	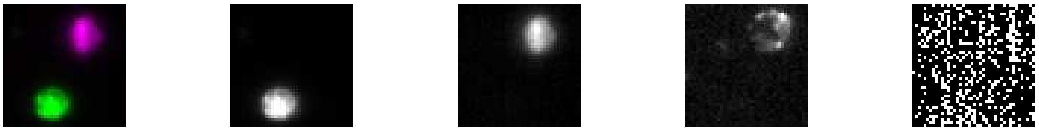				
4502	37	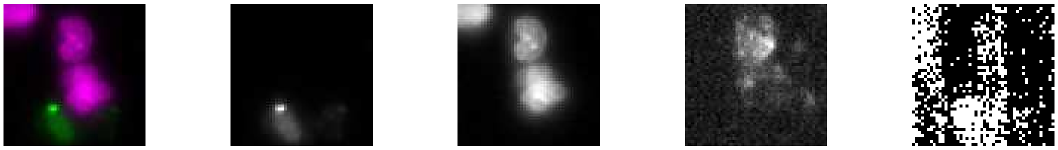				
4503	37	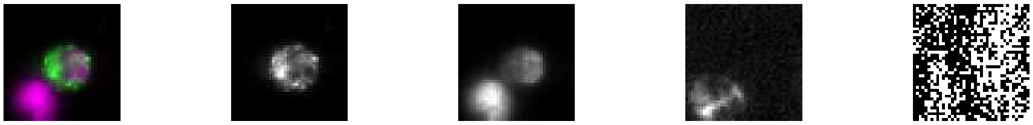				

Druckdatum:02/06/2020 16:41

Proben-ID: Gyn14.208 **Kassetten-ID:**01327106 **Patienten-ID:**

Ereignis	Aufnahme	DAPI/CK-PE	CK-PE	DAPI	CD45-APC	HER-2/neu
4504	37	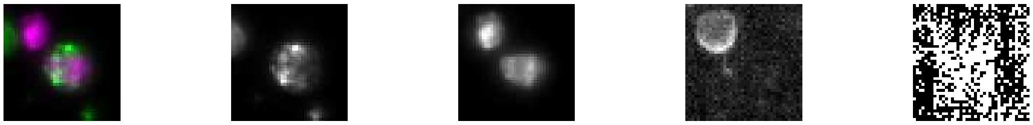				
4505	37	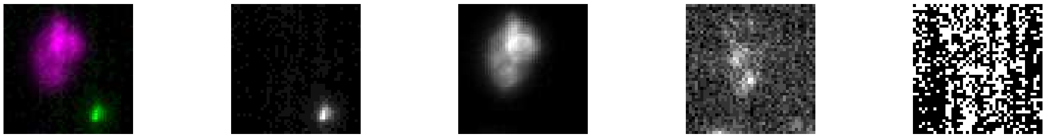				
4506	37	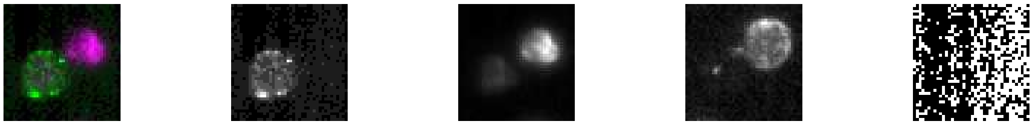				
4507	37	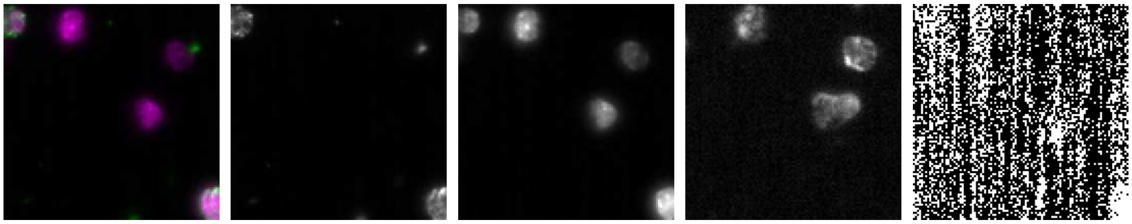				
4508	37	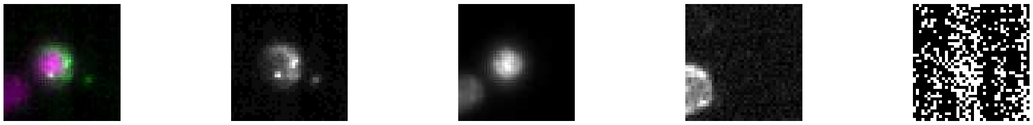				
4509	37	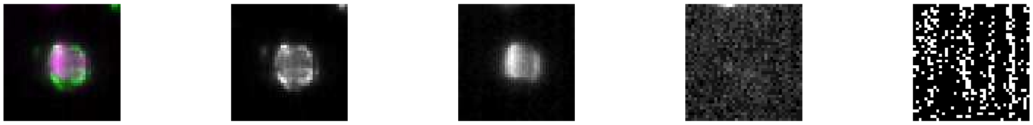				
4510	37	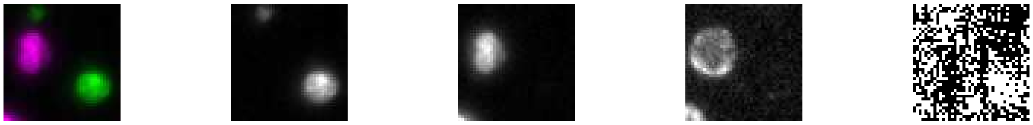				
4511	37	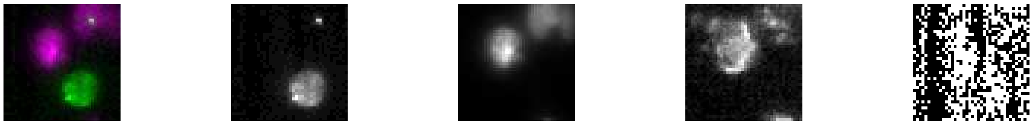				
4512	37	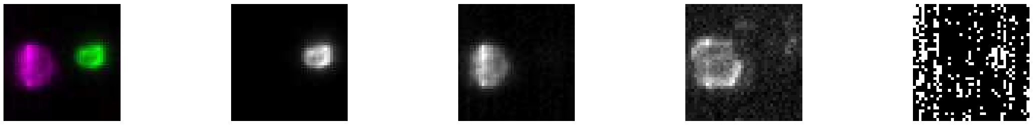				
4513	37	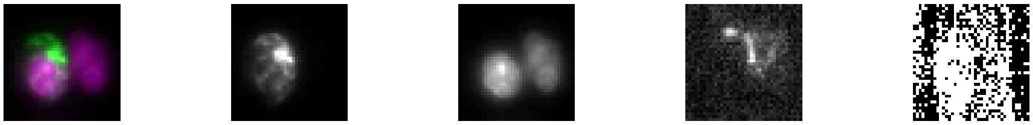				
4514	36	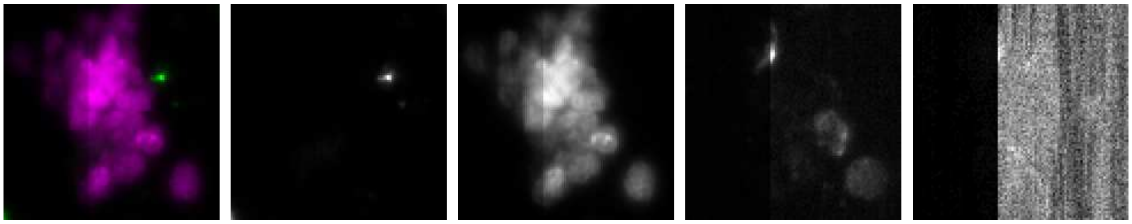				
4515	37	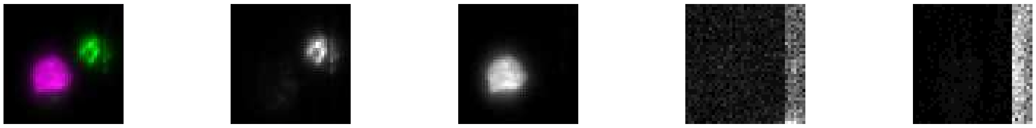				

Ereignis	Aufnahme	DAPI/CK-PE	CK-PE	DAPI	CD45-APC	HER-2/neu
4516	37	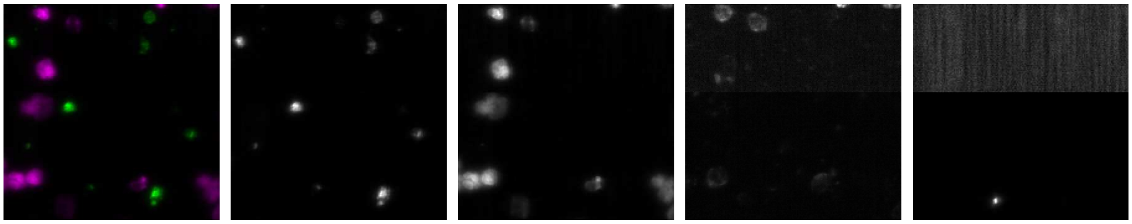				
4517	36	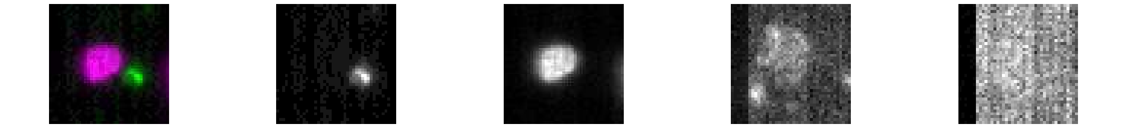				
4518	34	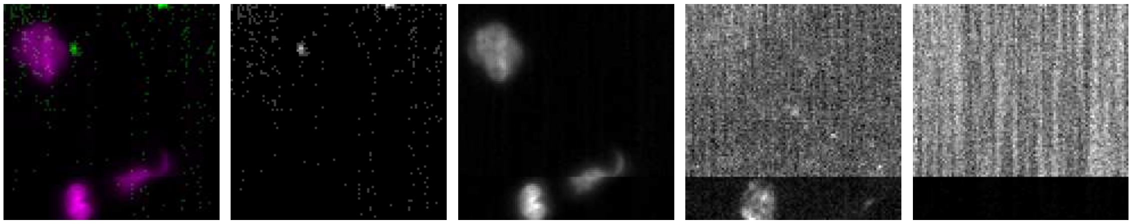				
4519	37	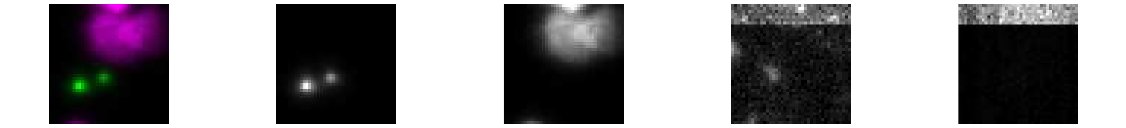				
4520	38	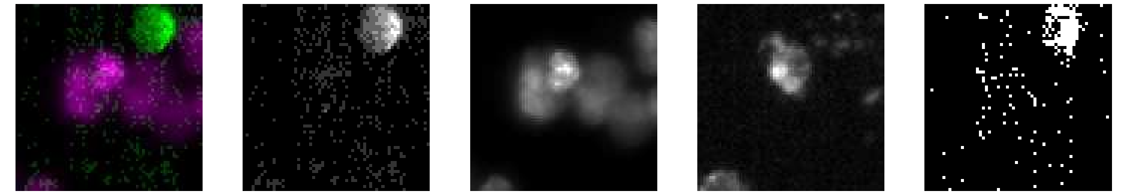				
4521	38	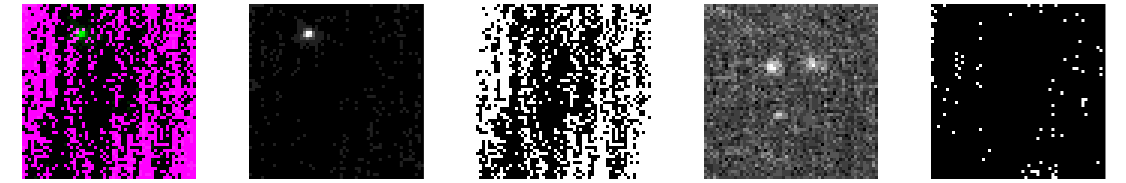				
4522	38	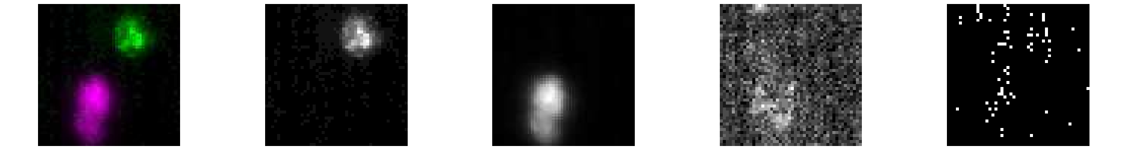				
4523	38	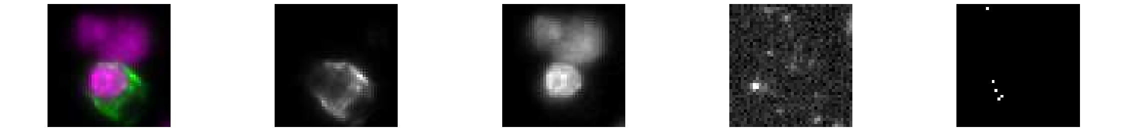				
4524	38	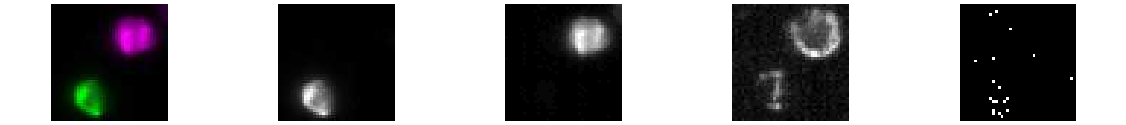				
4525	38	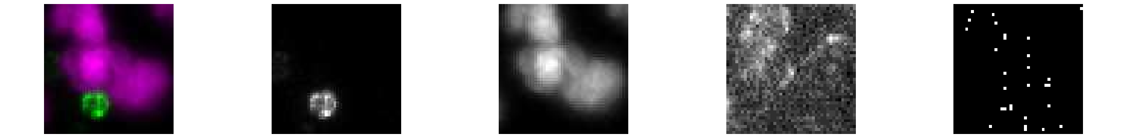				
4526	38	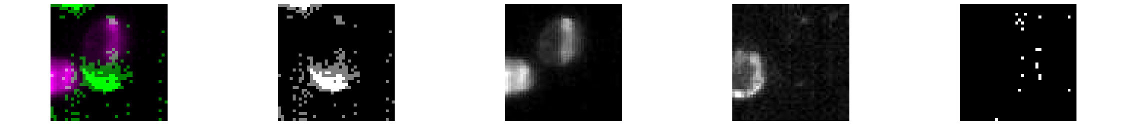				

Ereignis	Aufnahme	DAPI/CK-PE	CK-PE	DAPI	CD45-APC	HER-2/neu
4527	38	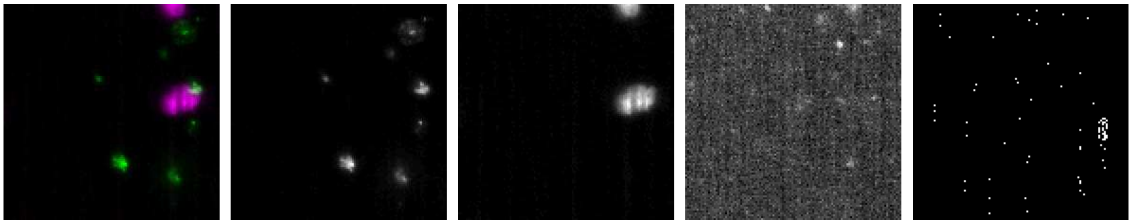				
4528	38	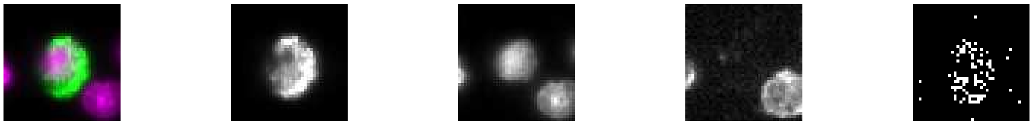				
4529	38	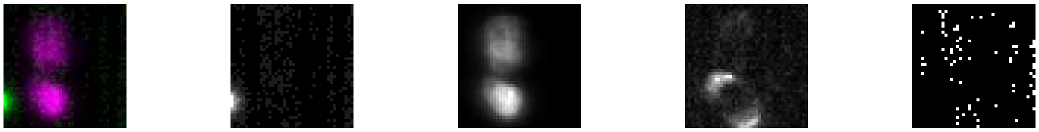				
4530	38	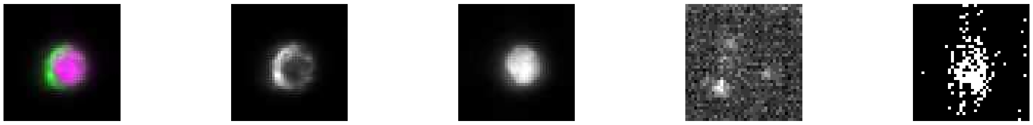				
4531	38	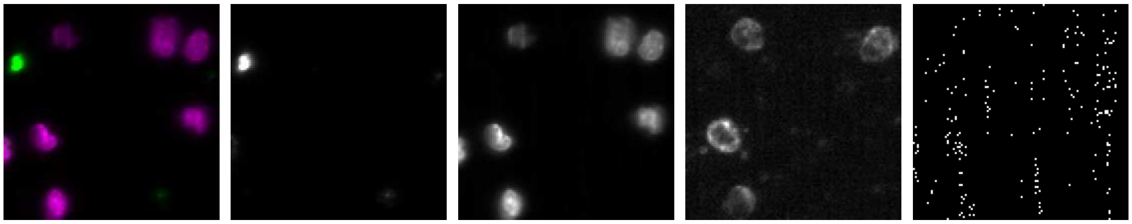				
4532	38	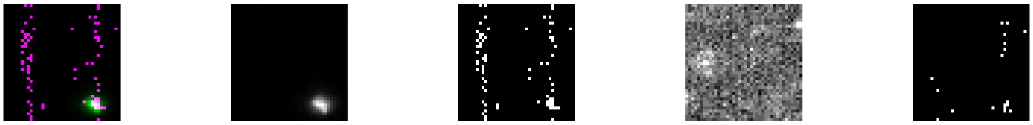				
4533	38	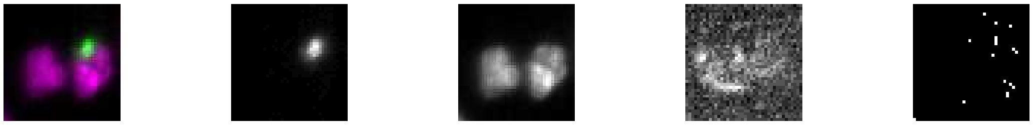				
4534	38	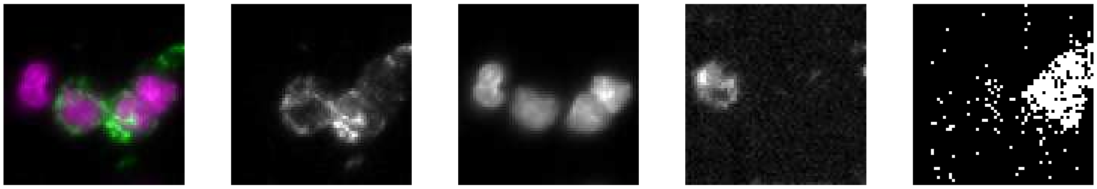				
4535	38	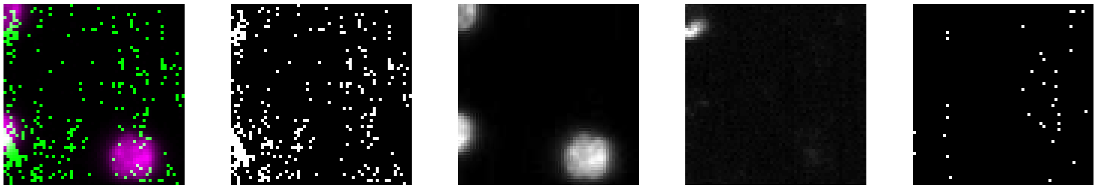				
4536	38	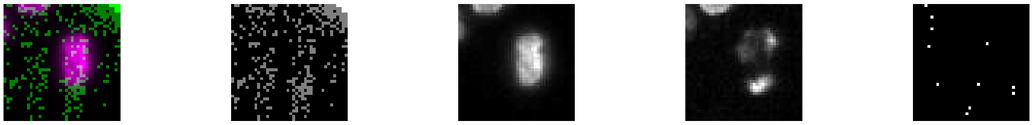				
4537	38	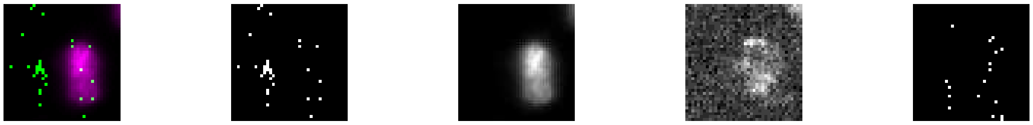				

Ereignis	Aufnahme	DAPI/CK-PE	CK-PE	DAPI	CD45-APC	HER-2/neu
4538	38	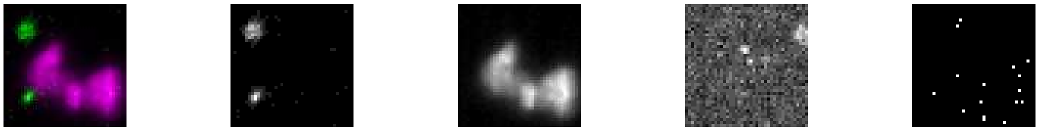				
4539	38	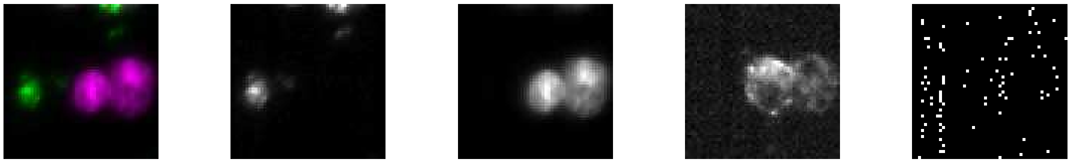				
4540	38	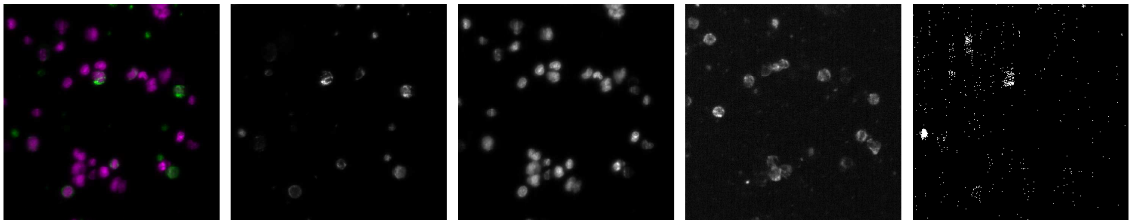				
4541	38	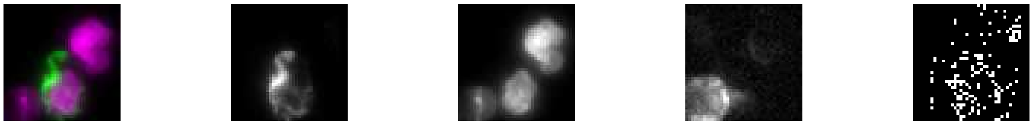				
4542	38	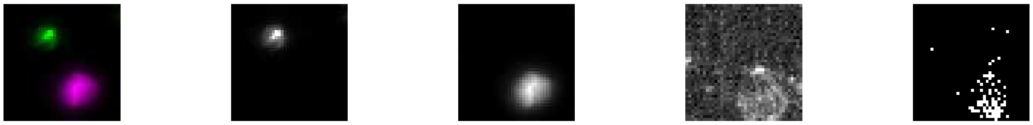				
4543	38	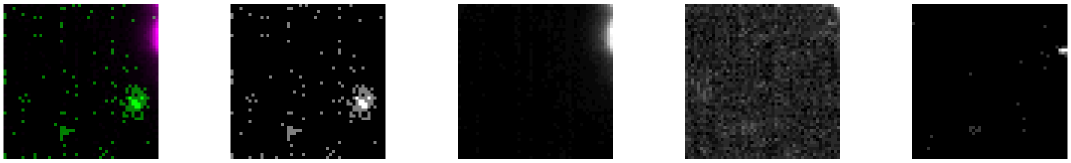				
4544	38	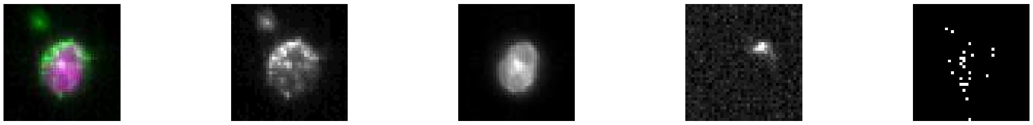				
4545	38	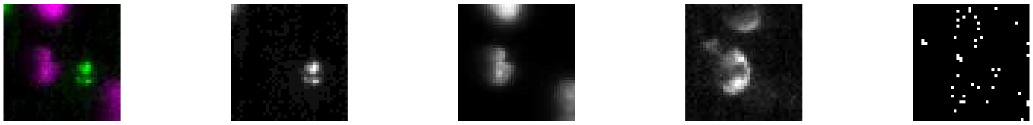				
4546	38	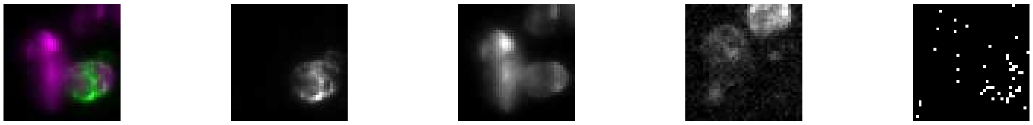				
4547	38	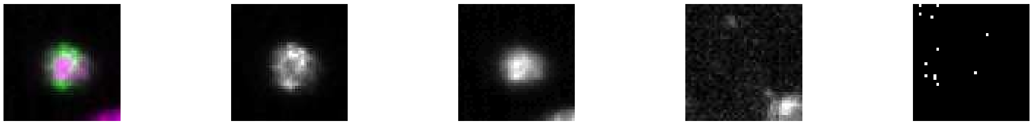				
4548	38	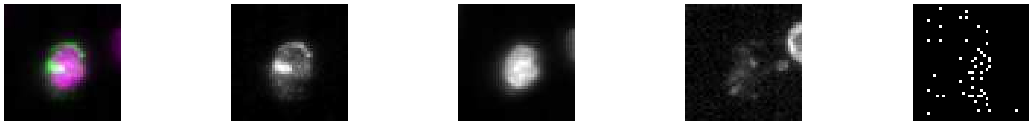				
4549	38	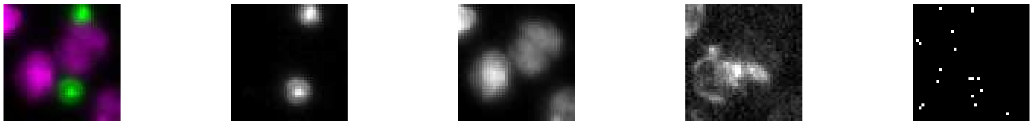				
4550	38	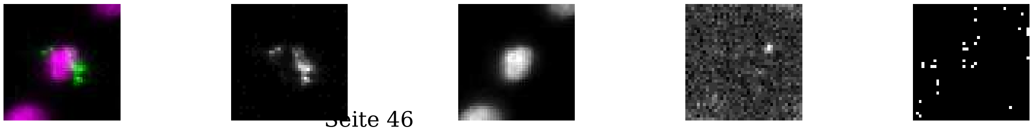				

Ereignis	Aufnahme	DAPI/CK-PE	CK-PE	DAPI	CD45-APC	HER-2/neu
4551	38	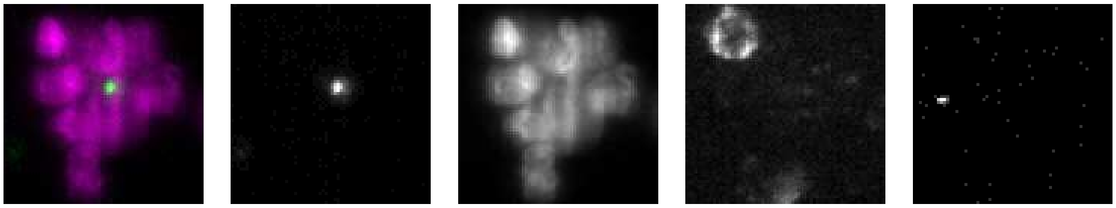				
4552	38	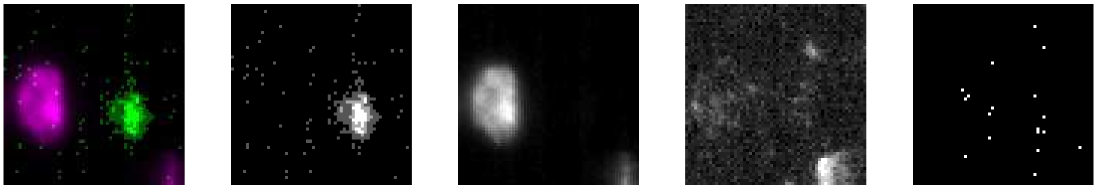				
4553	38	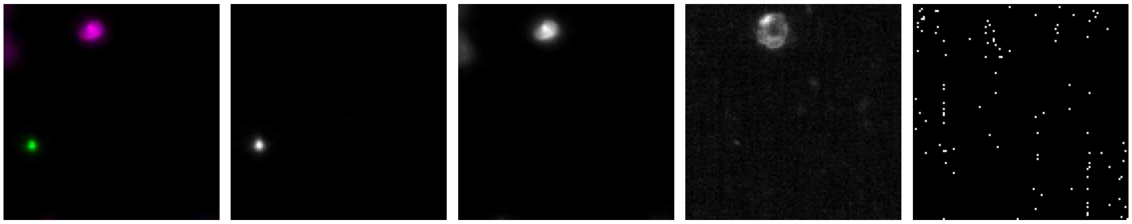				
4554	38	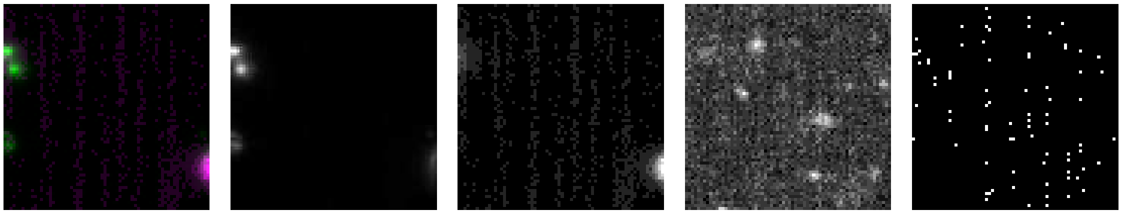				
4555	38	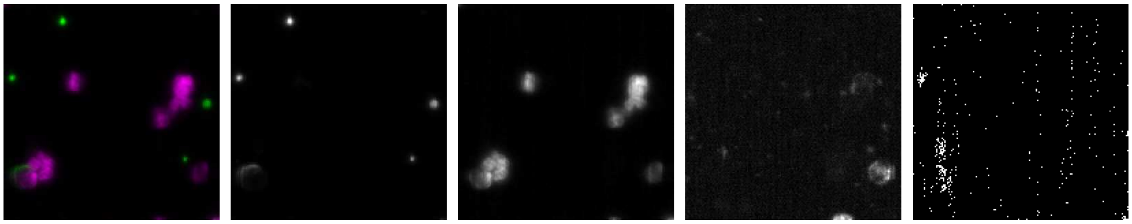				
4556	38	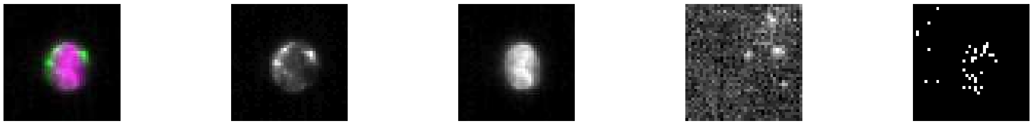				
4557	38	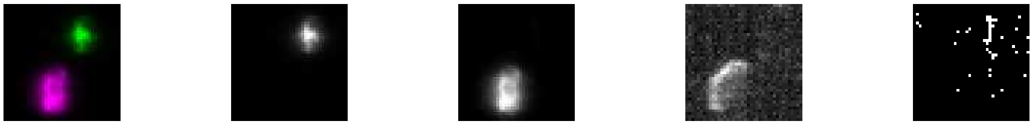				
4558	38	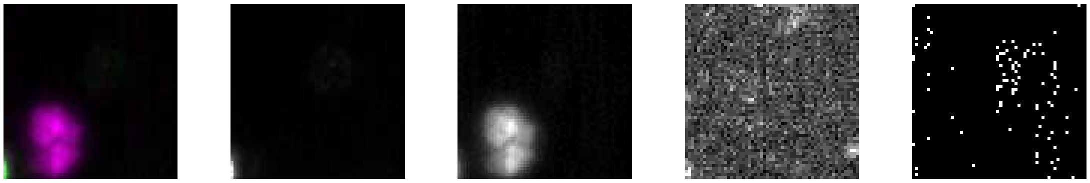				
4559	38	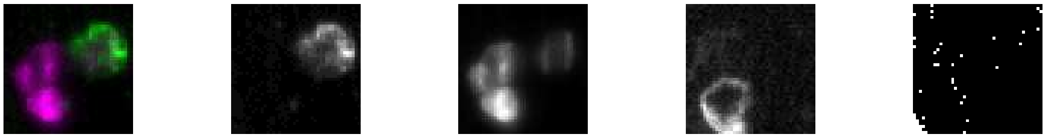				
4560	38	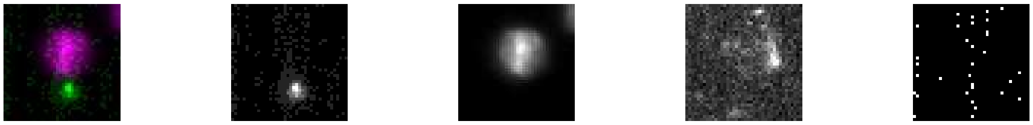				

Ereignis	Aufnahme	DAPI/CK-PE	CK-PE	DAPI	CD45-APC	HER-2/neu
4561	38	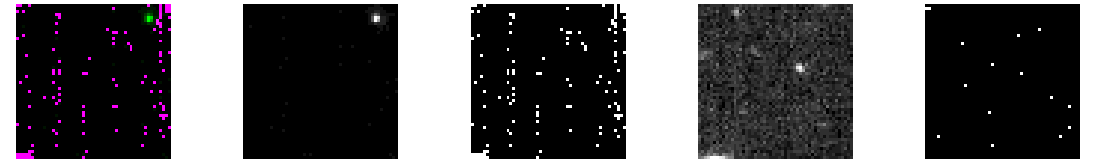				
4562	38	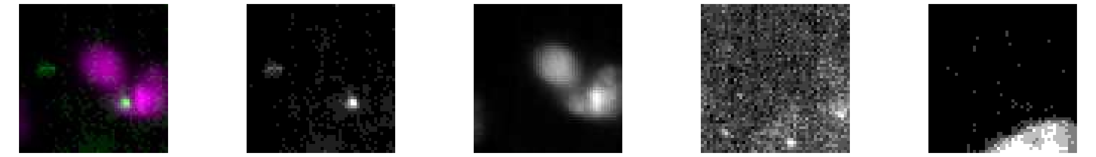				
4563	38	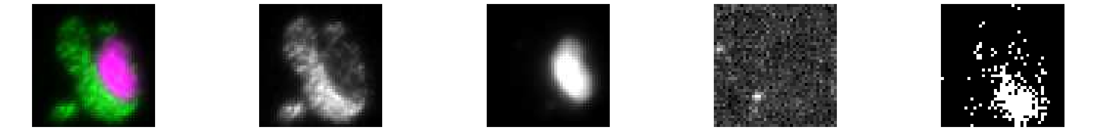				
4564	38	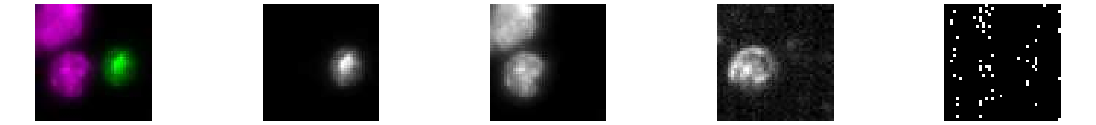				
4565	38	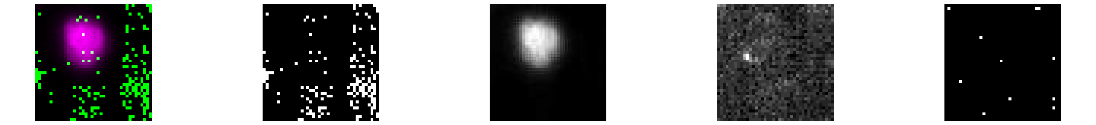				
4566	38	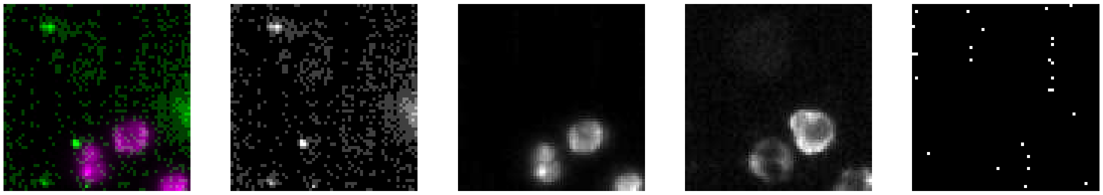				
4567	38	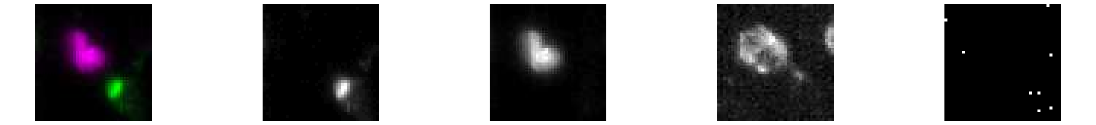				
4568	38	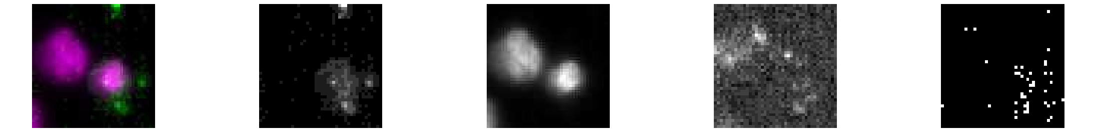				
4569	38	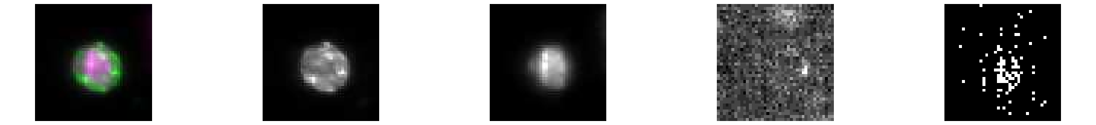				
4570	38	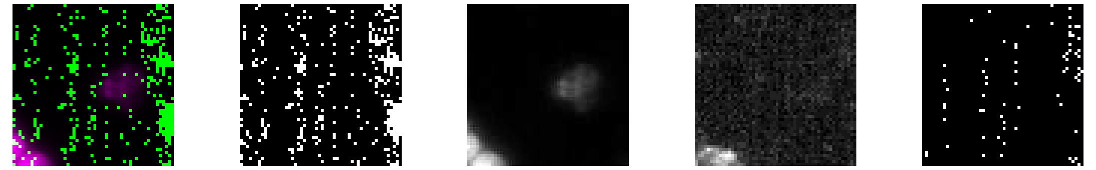				
4571	38	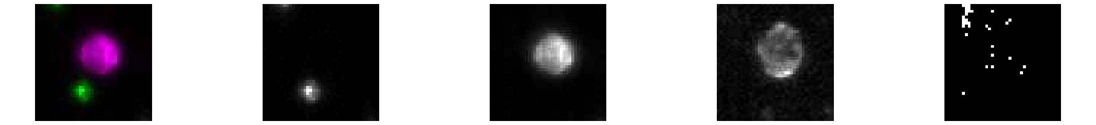				
4572	38	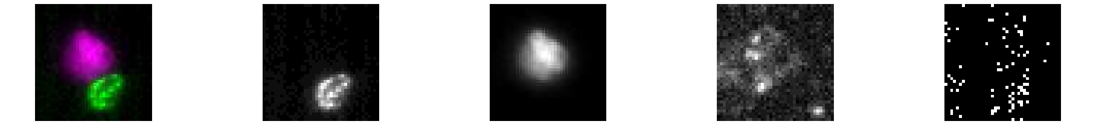				

Ereignis	Aufnahme	DAPI/CK-PE	CK-PE	DAPI	CD45-APC	HER-2/neu
4573	38	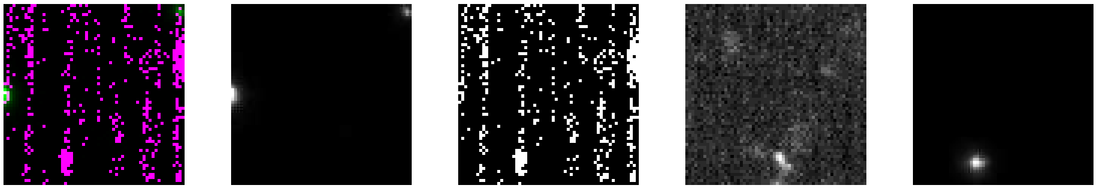				
4574	38	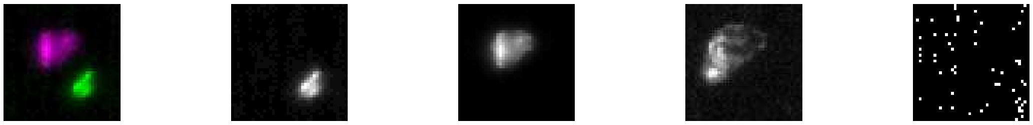				
4575	38	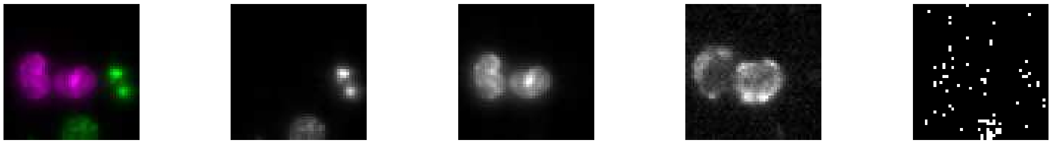				
4576	38	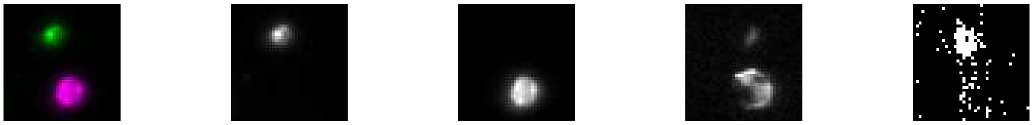				
4577	38	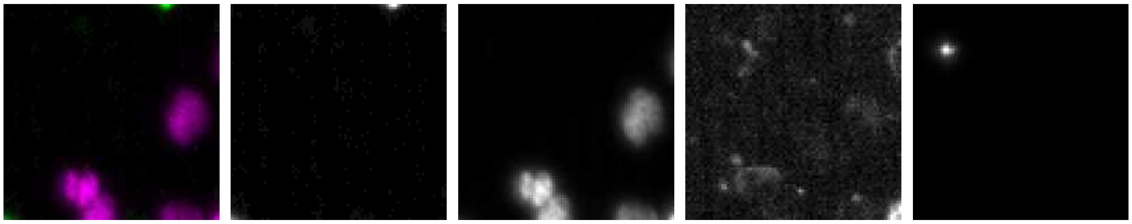				
4578	38	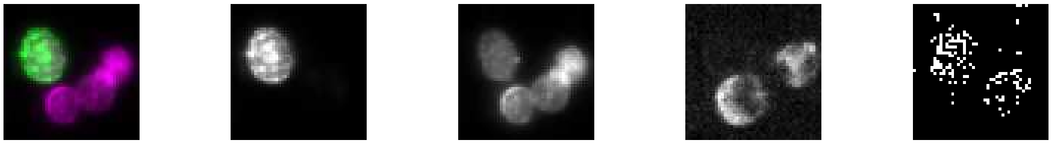				
4579	38	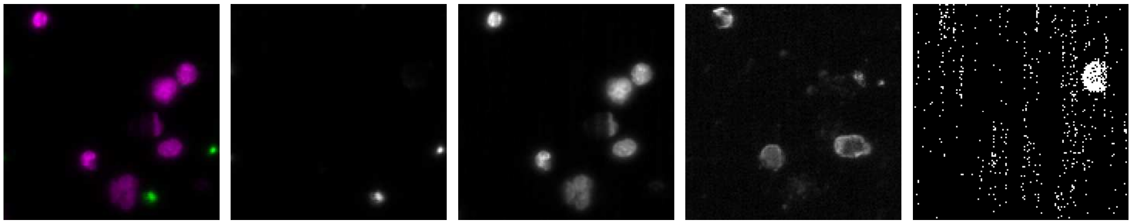				
4580	38	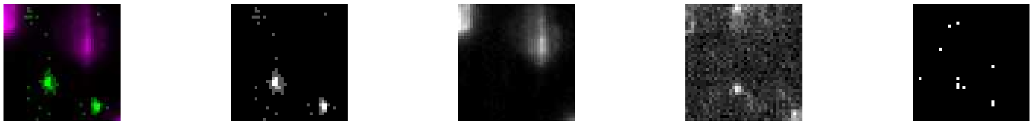				
4581	38	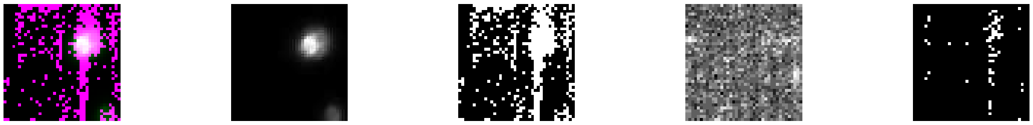				
4582	38	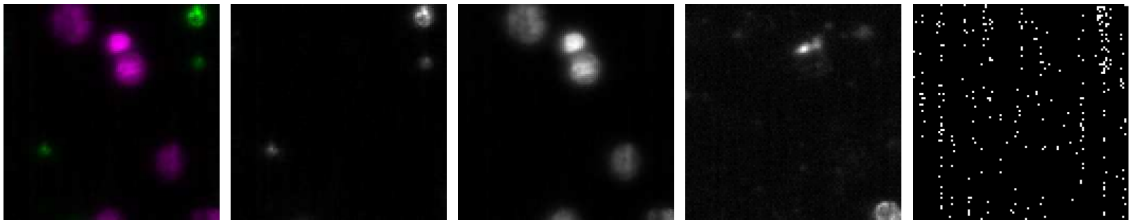				
4583	38	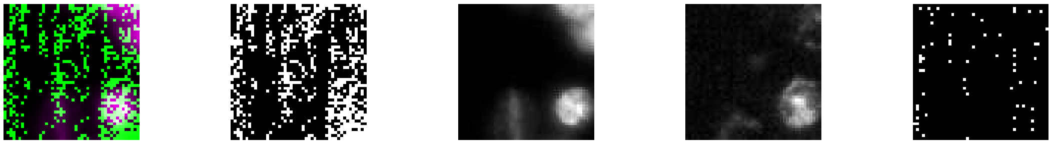				

Ereignis	Aufnahme	DAPI/CK-PE	CK-PE	DAPI	CD45-APC	HER-2/neu
4584	38	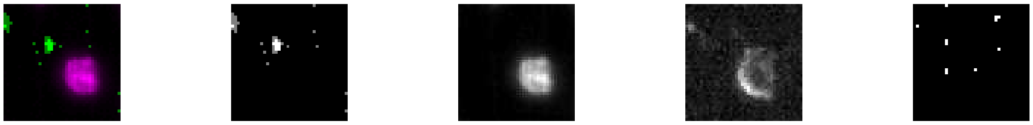				
4585	38	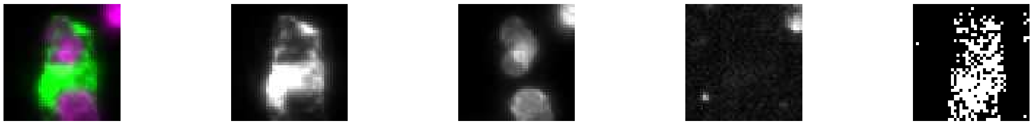				
4586	38	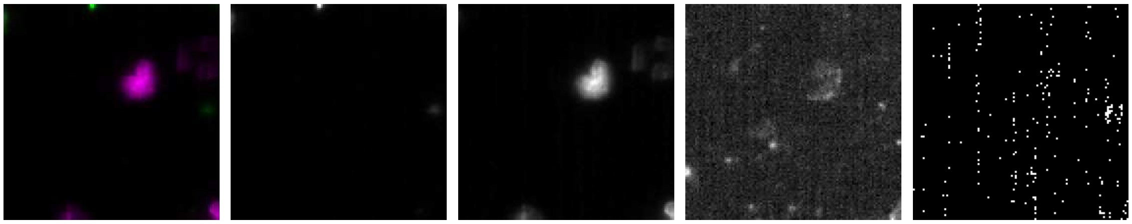				
4587	38	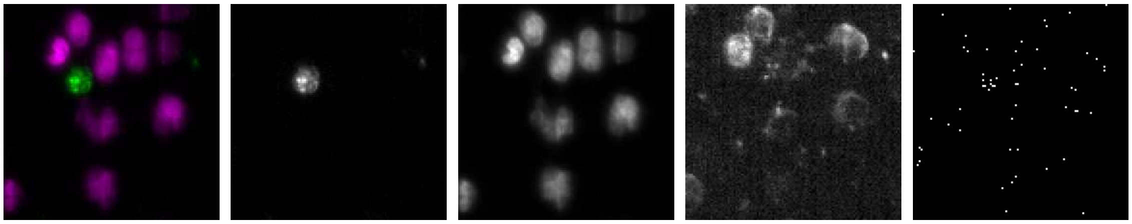				
4588	38	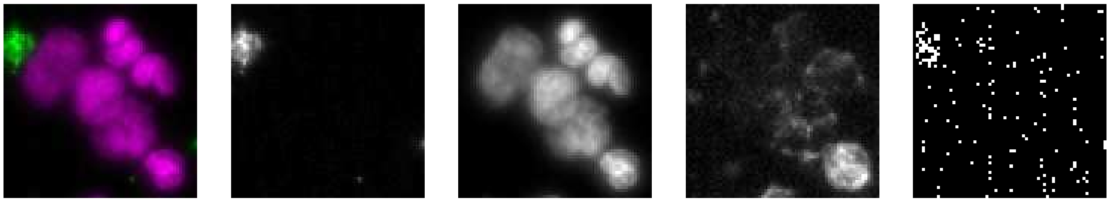				
4589	38	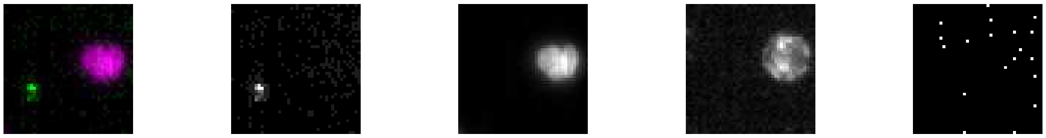				
4590	38	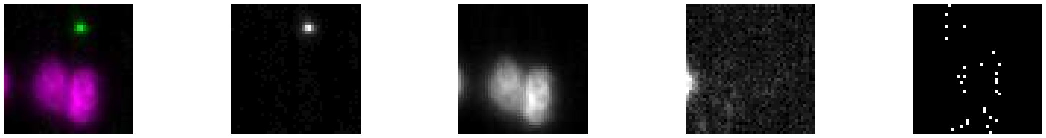				
4591	38	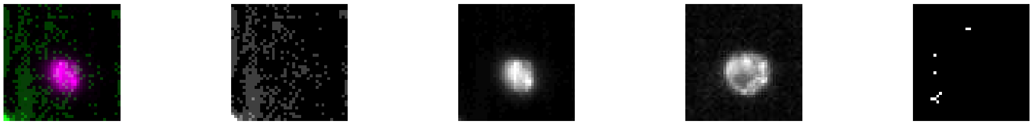				
4592	38	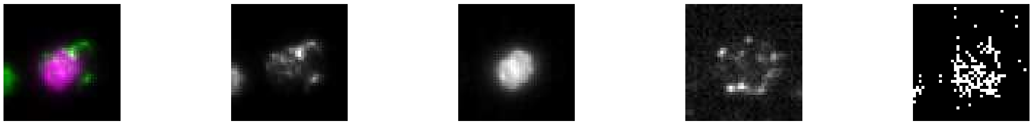				
4593	38	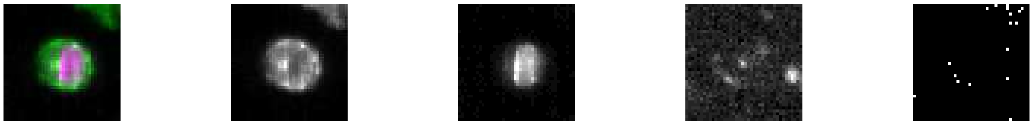				
4594	38	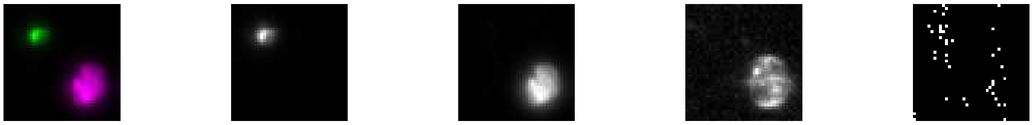				
4595	38	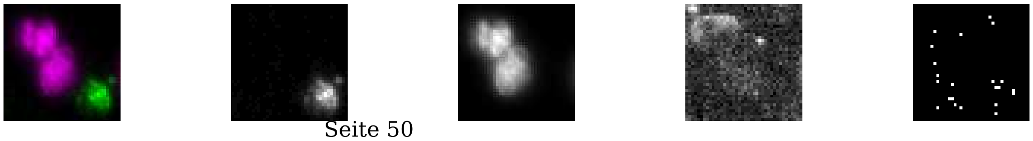				

Ereignis	Aufnahme	DAPI/CK-PE	CK-PE	DAPI	CD45-APC	HER-2/neu
4596	38	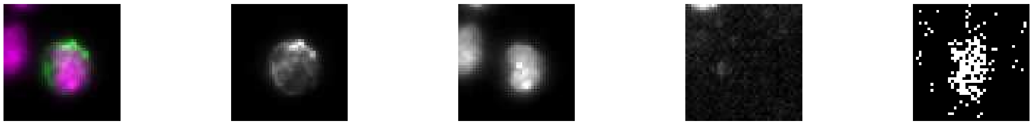				
4597	38	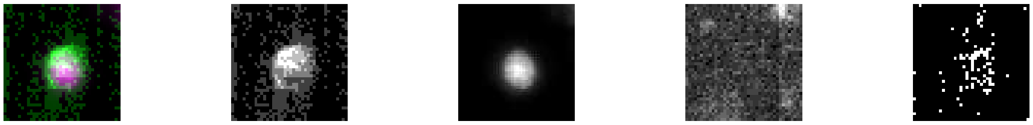				
4598	38	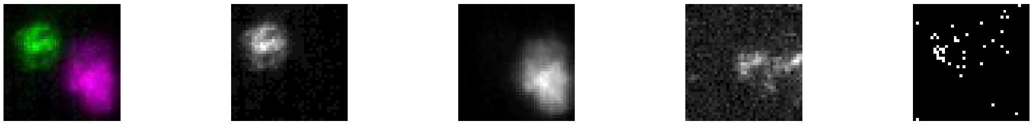				
4599	38	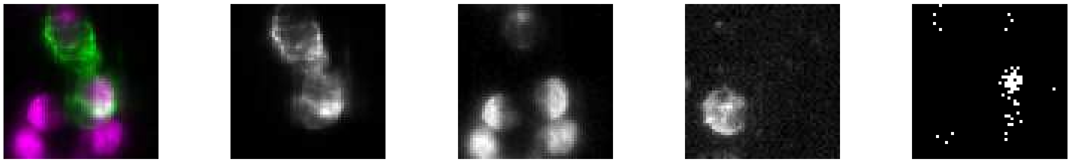				
4600	38	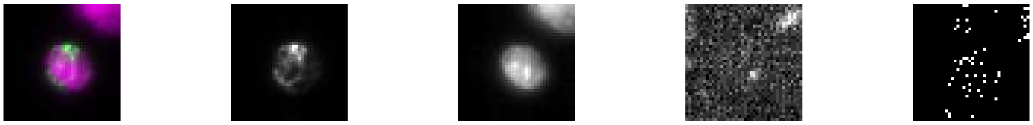				
4601	38	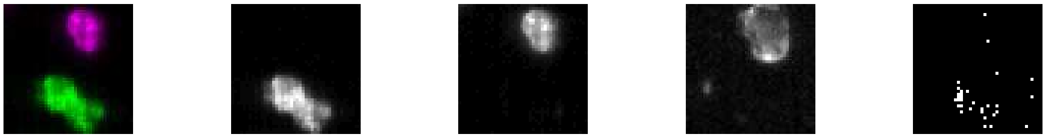				
4602	38	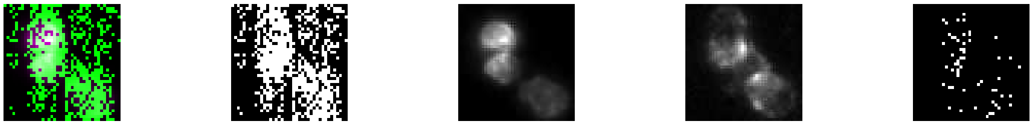				
4603	38	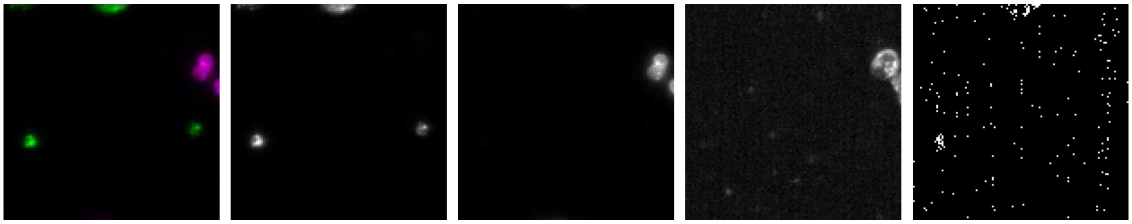				
4604	38	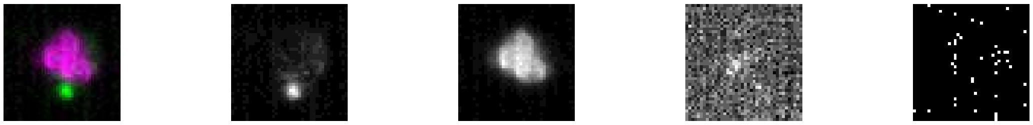				
4605	38	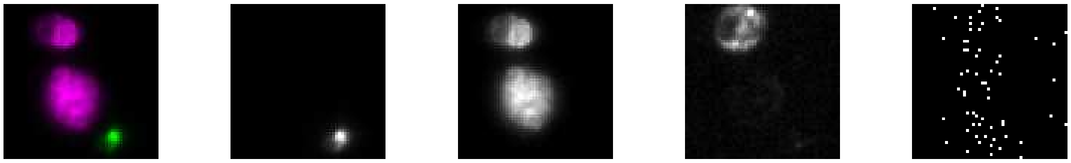				
4606	38	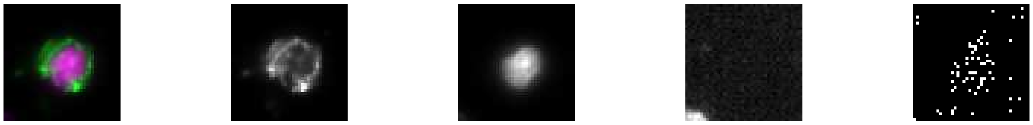				
4607	38	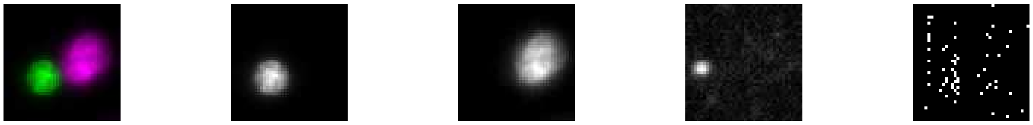				

Druckdatum: 02/06/2020 16:41

Proben-ID: Gyn14.208 **Kassetten-ID:** 01327106 **Patienten-ID:**

Ereignis	Aufnahme	DAPI/CK-PE	CK-PE	DAPI	CD45-APC	HER-2/neu
4608	38	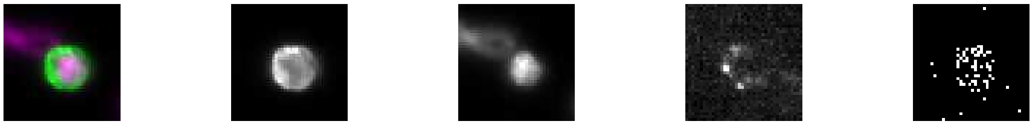				
4609	38	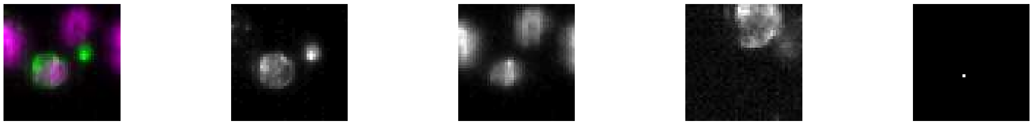				
4610	38	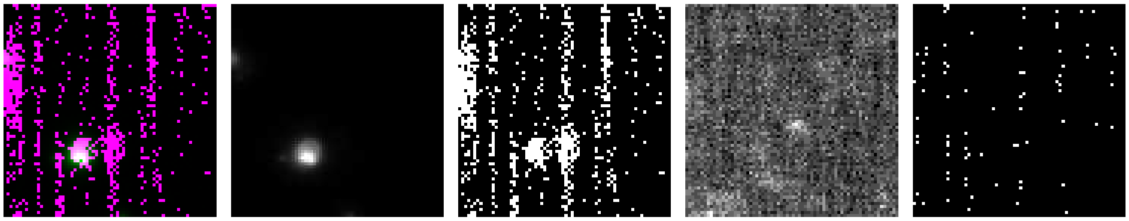				
4611	38	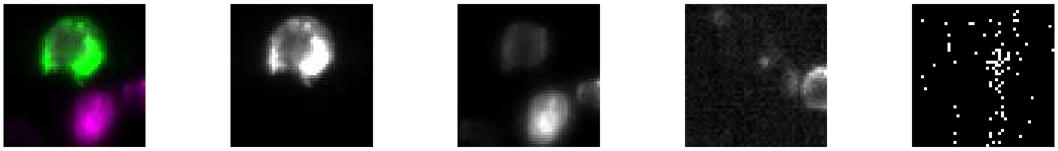				
4612	38	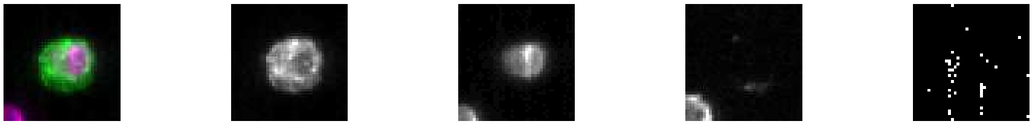				
4613	38	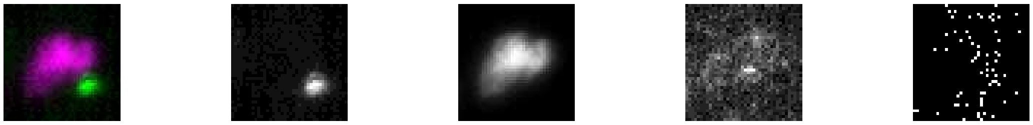				
4614	38	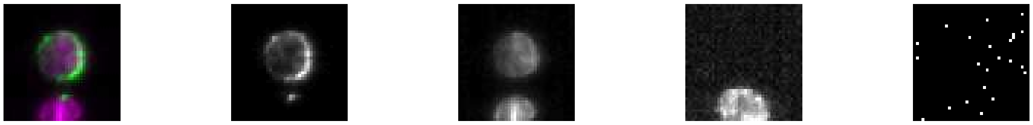				
4615	38	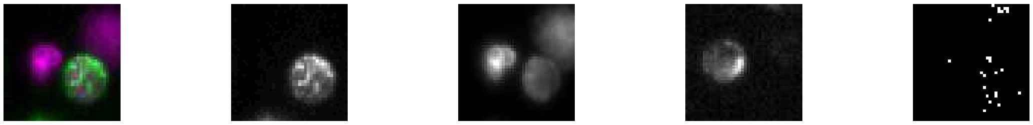				
4616	38	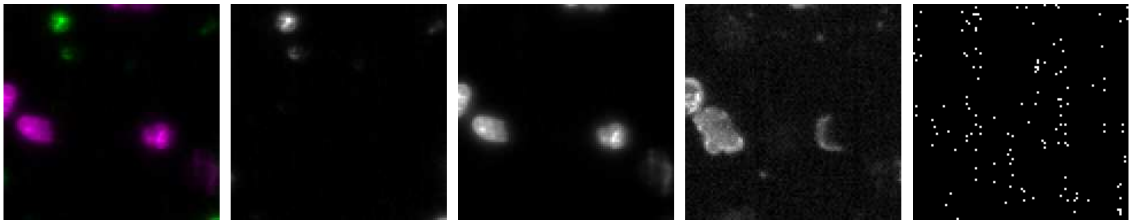				
4617	38	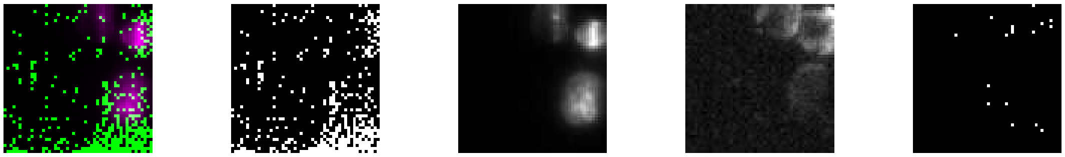				
4618	38	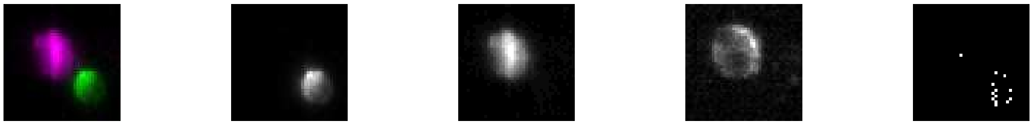				
4619	38	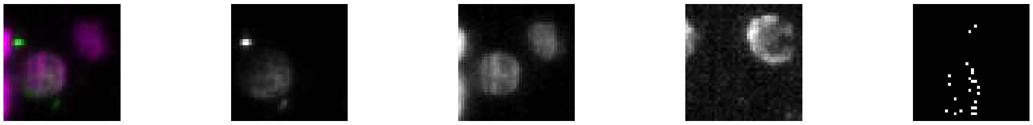				

Ereignis	Aufnahme	DAPI/CK-PE	CK-PE	DAPI	CD45-APC	HER-2/neu
4620	38	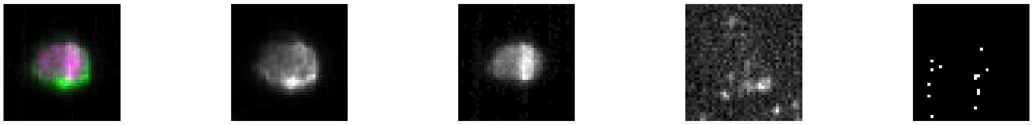				
4621	38	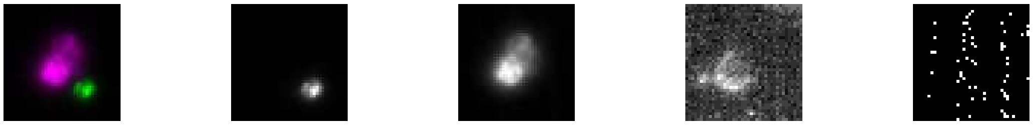				
4622	38	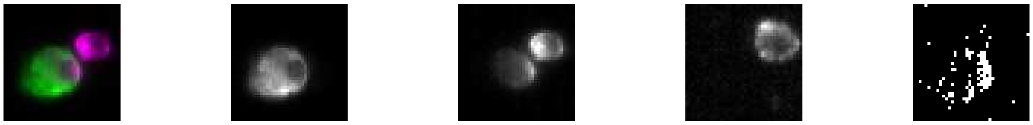				
4623	38	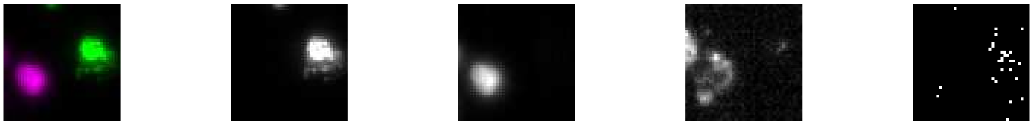				
4624	38	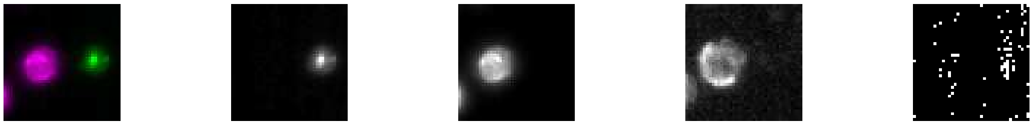				
4625	38	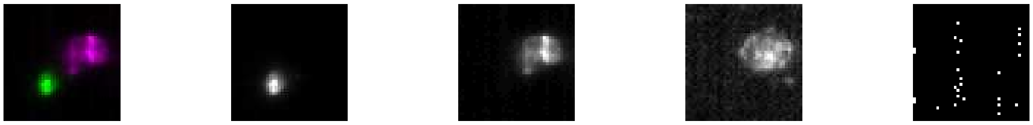				
4626	38	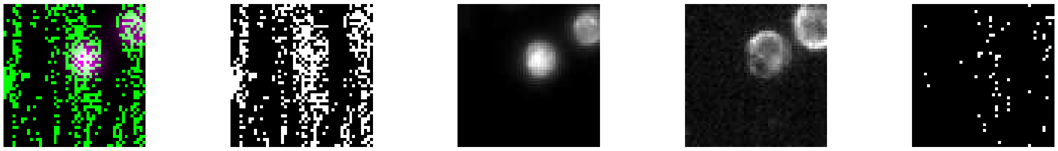				
4627	38	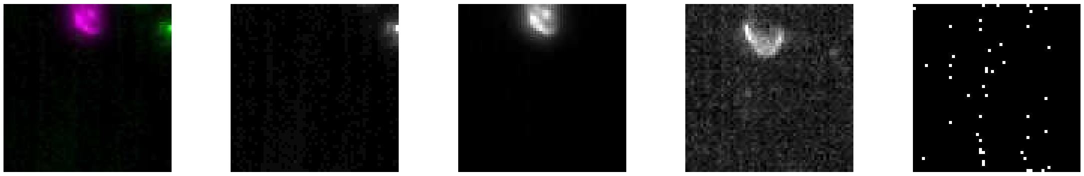				
4628	38	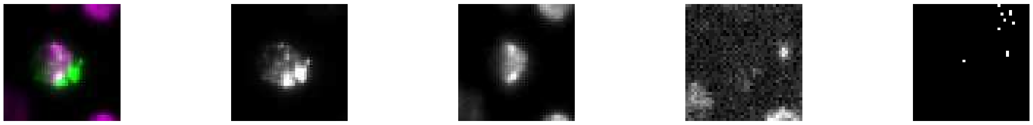				
4629	38	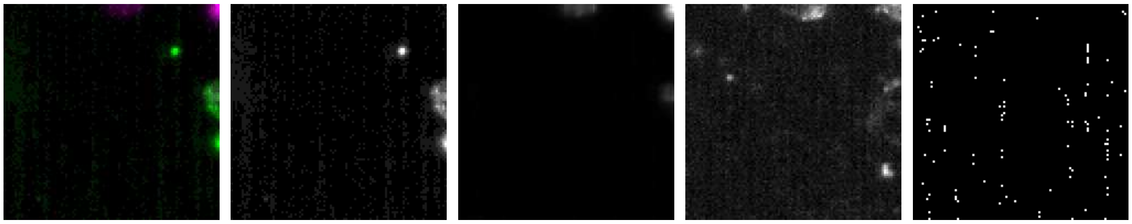				
4630	38	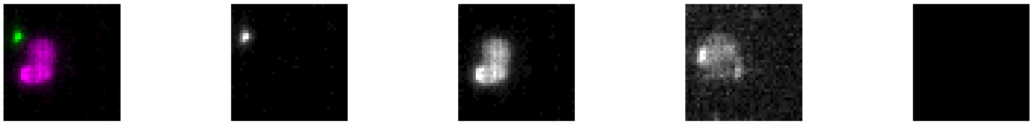				
4631	38	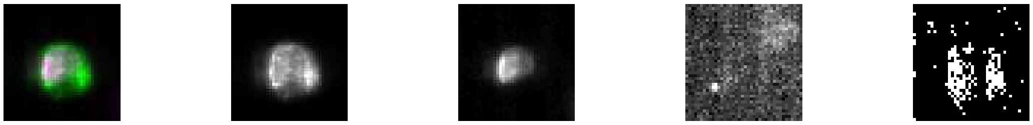				

Ereignis	Aufnahme	DAPI/CK-PE · CK-PE · DAPI · CD45-APC · HER-2/neu
4632	38	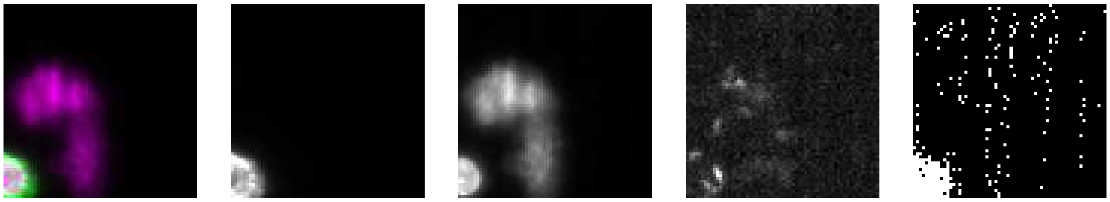
4633	38	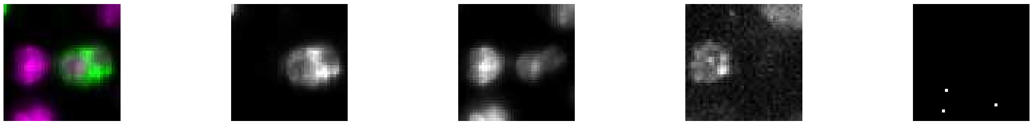
4634	38	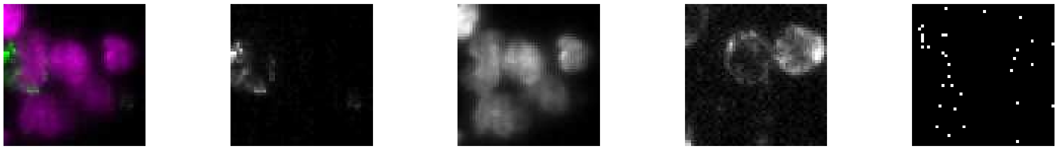
4635	38	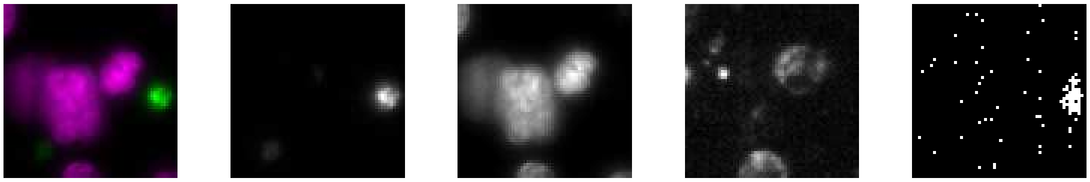
4636	38	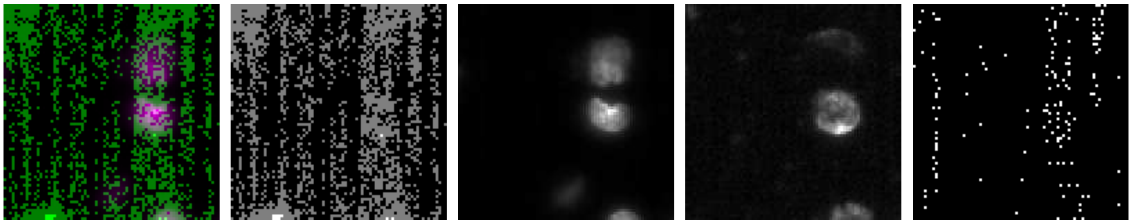
4637	38	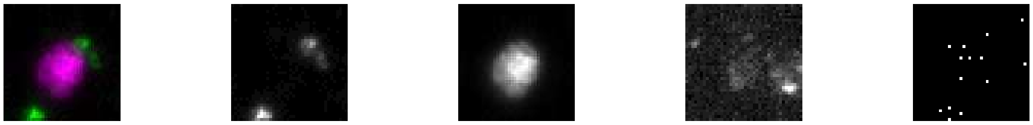
4638	38	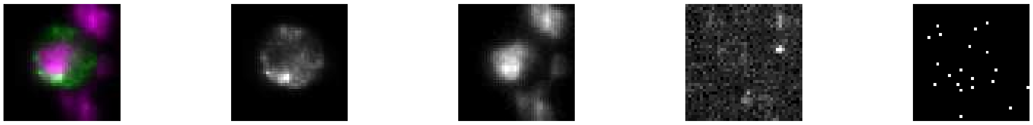
4639	38	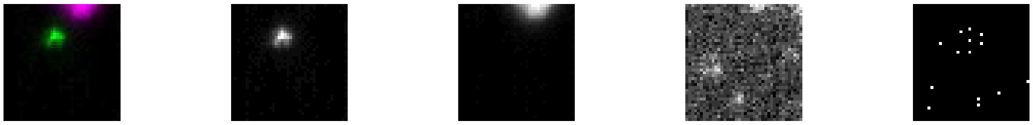
4640	38	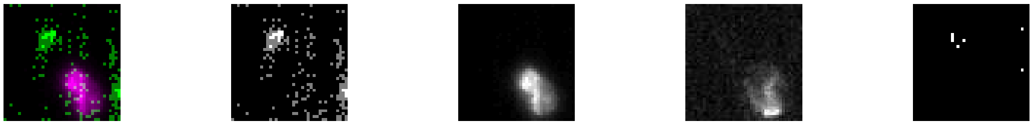
4641	38	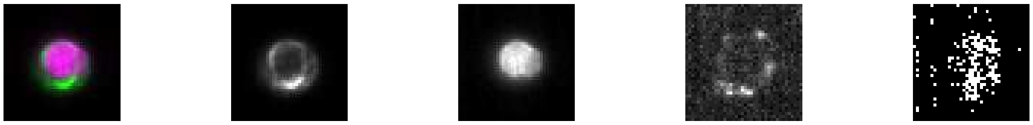
4642	38	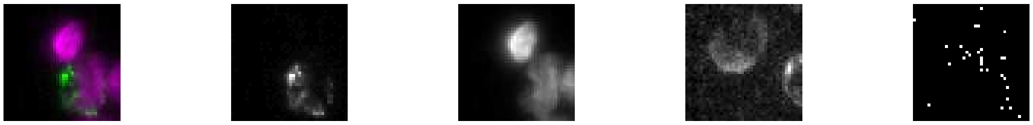
4643	38	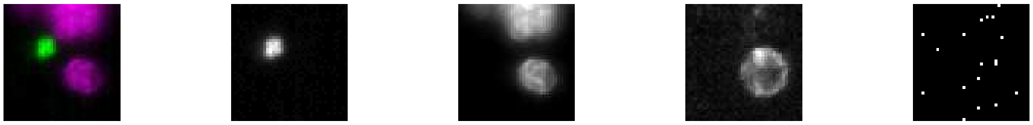

Ereignis	Aufnahme	DAPI/CK-PE	CK-PE	DAPI	CD45-APC	HER-2/neu
4644	38	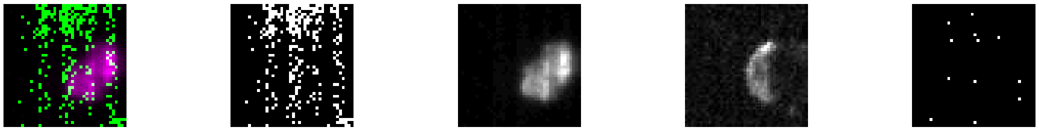				
4645	38	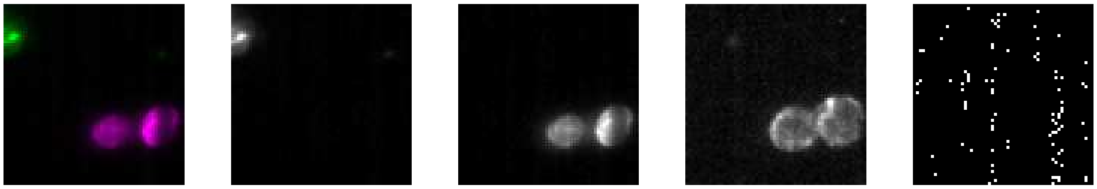				
4646	38	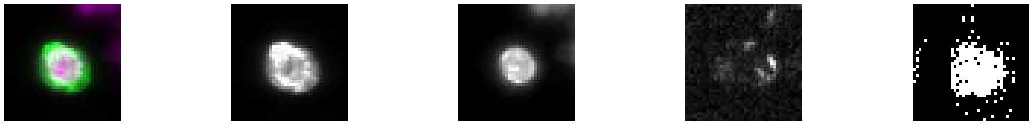				
4647	38	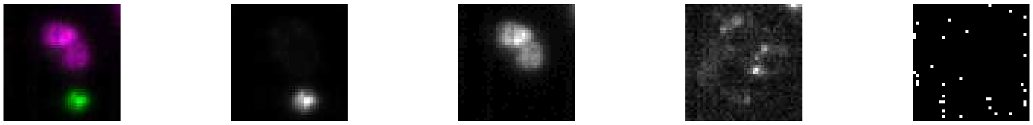				
4648	38	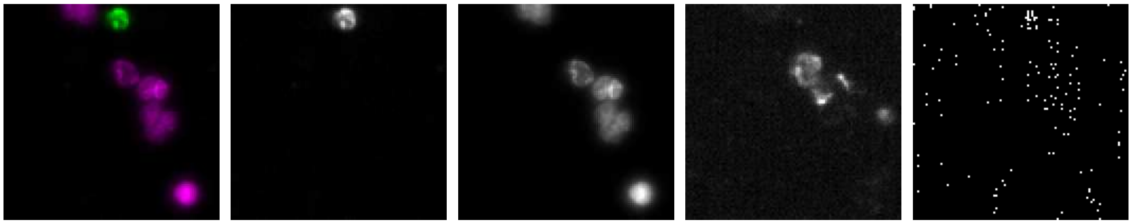				
4649	38	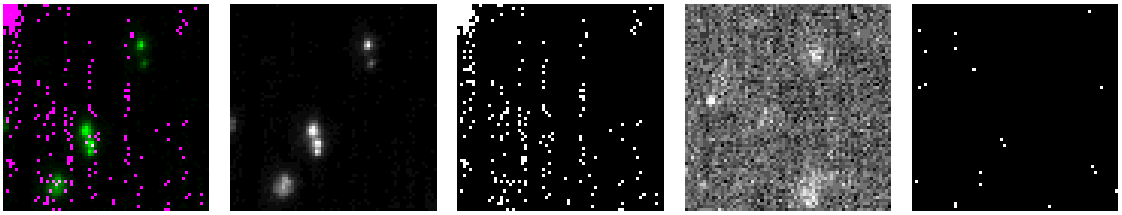				
4650	38	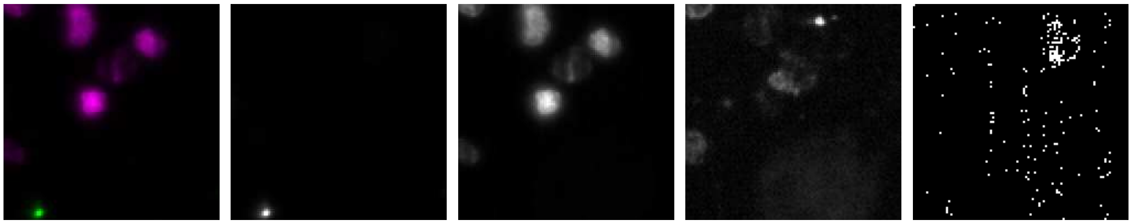				
4651	38	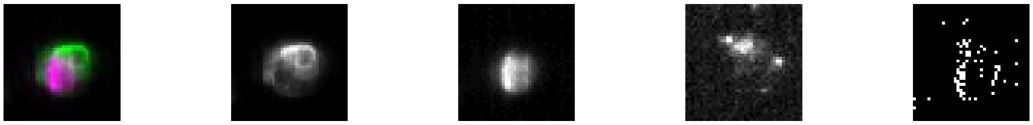				
4652	38	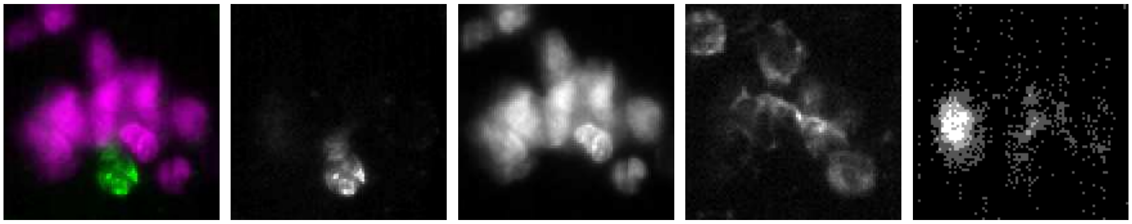				
4653	38	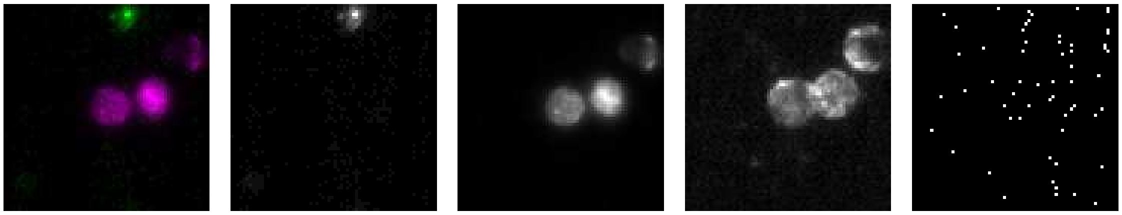				

Proben-ID: Gyn14.208 **Kassetten-ID:** 01327106 **Patienten-ID:**

Ereignis	Aufnahme	DAPI/CK-PE	CK-PE	DAPI	CD45-APC	HER-2/neu
4654	38	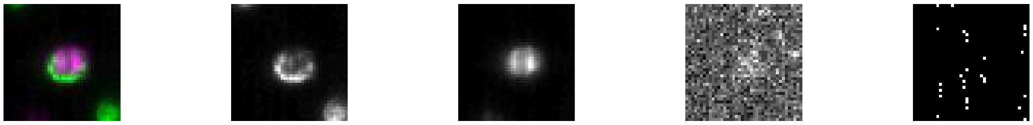				
4655	38	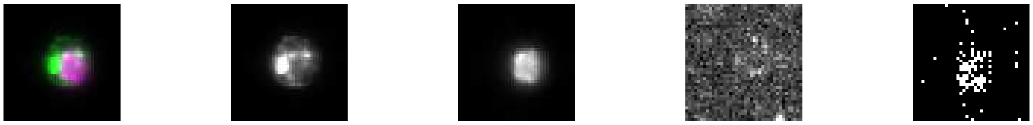				
4656	38	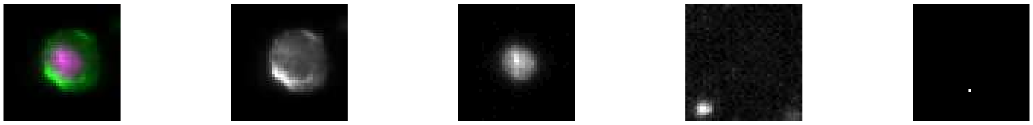				
4657	38	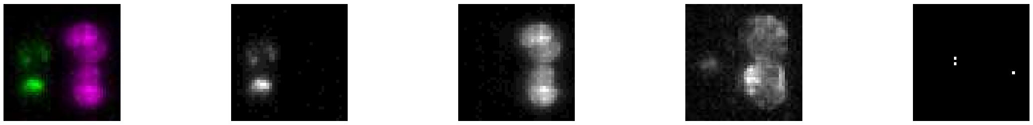				
4658	38	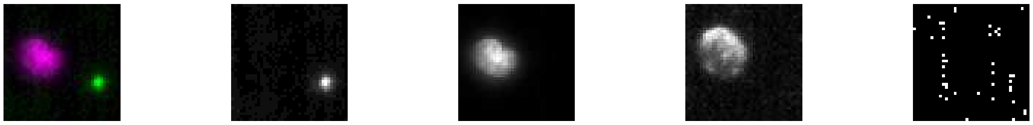				
4659	38	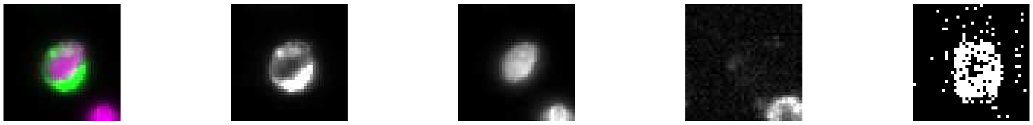				
4660	38	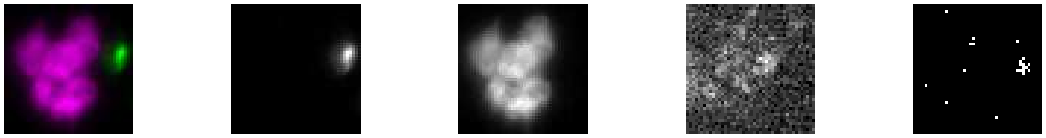				
4661	38	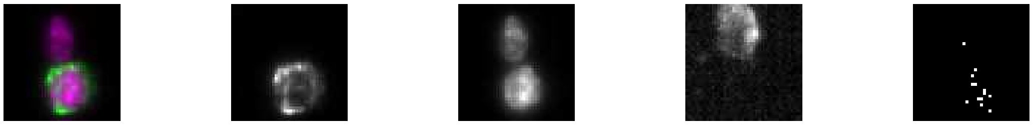				
4662	38	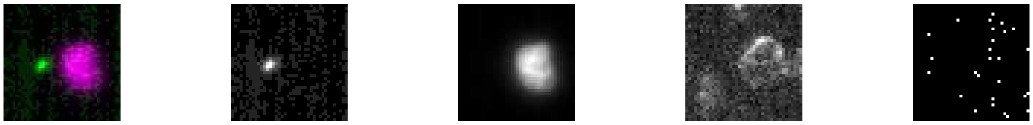				
4663	38	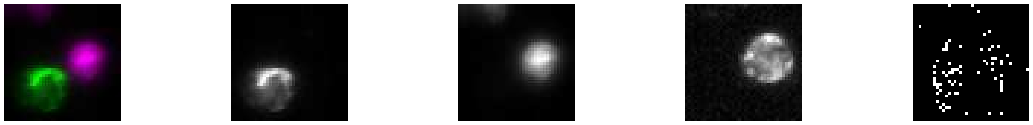				
4664	38	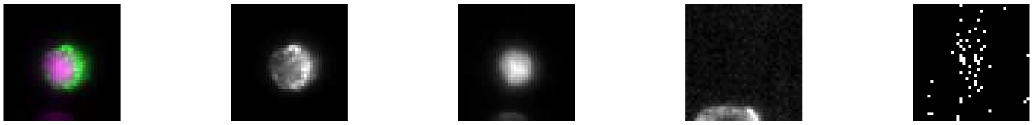				
4665	38	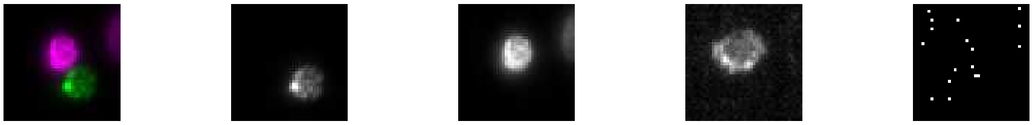				
4666	38	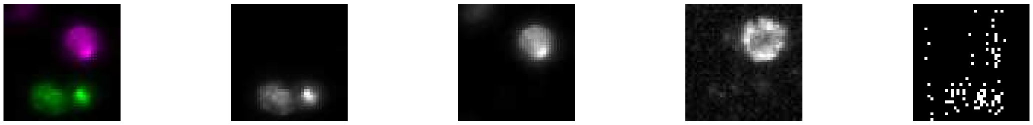				
4667	38	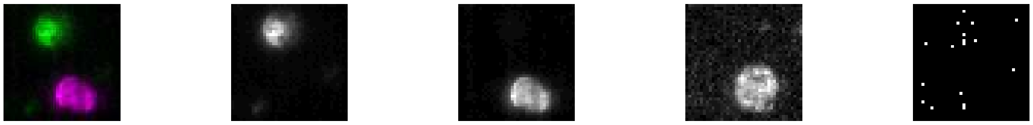				

Ereignis	Aufnahme	DAPI/CK-PE	CK-PE	DAPI	CD45-APC	HER-2/neu
4668	38	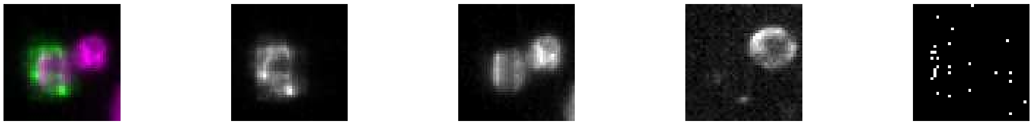				
4669	38	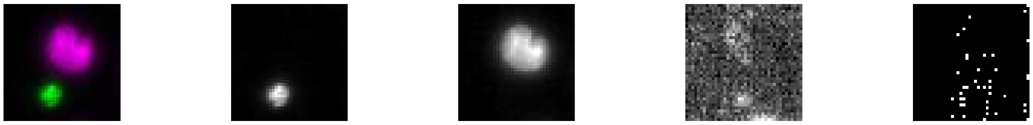				
4670	38	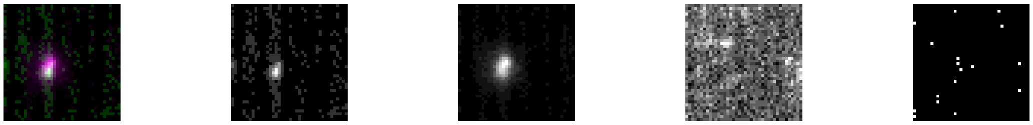				
4671	38	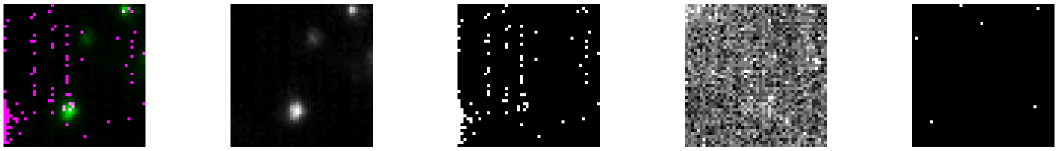				
4672	38	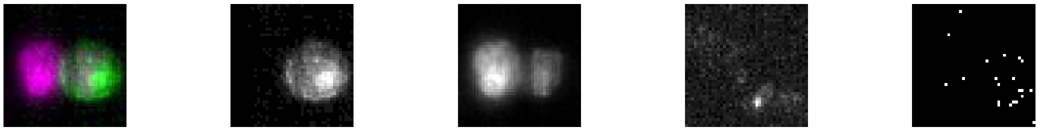				
4673	38	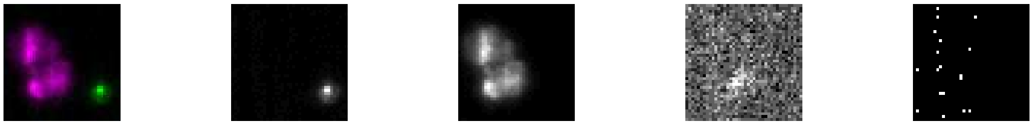				
4674	38	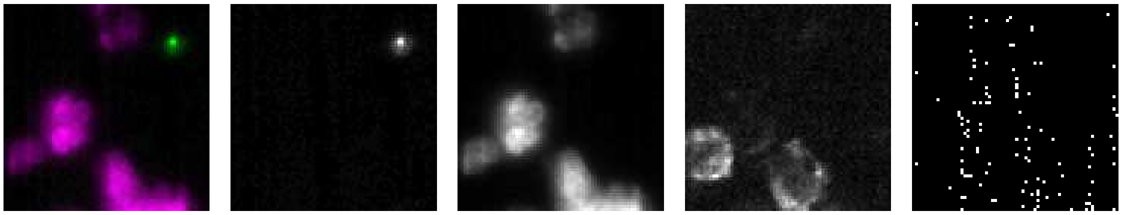				
4675	38	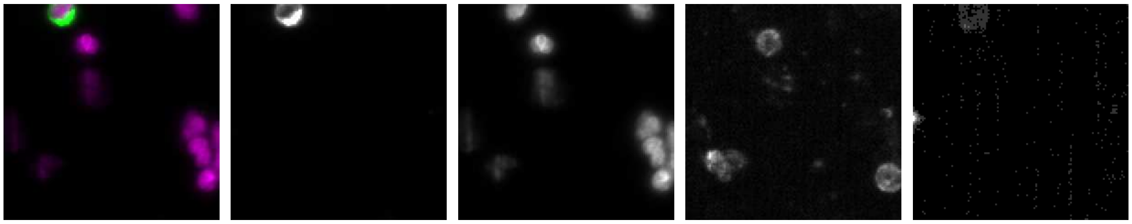				
4676	38	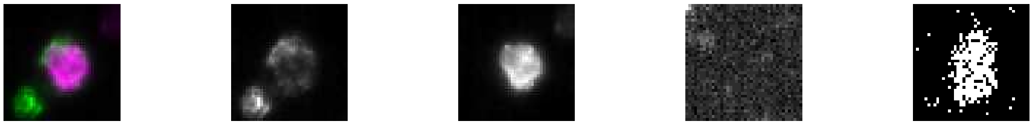				
4677	38	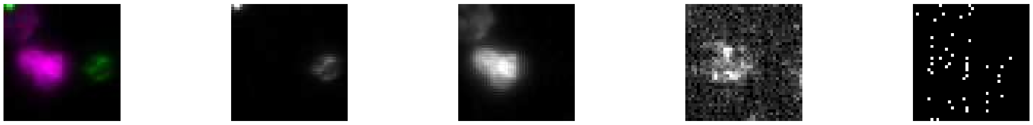				
4678	38	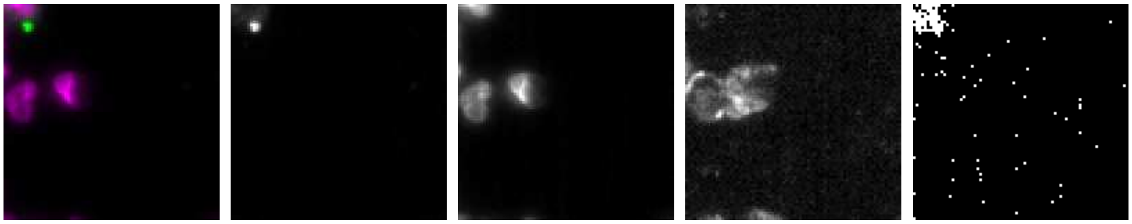				

Ereignis	Aufnahme	DAPI/CK-PE	CK-PE	DAPI	CD45-APC	HER-2/neu
4679	38	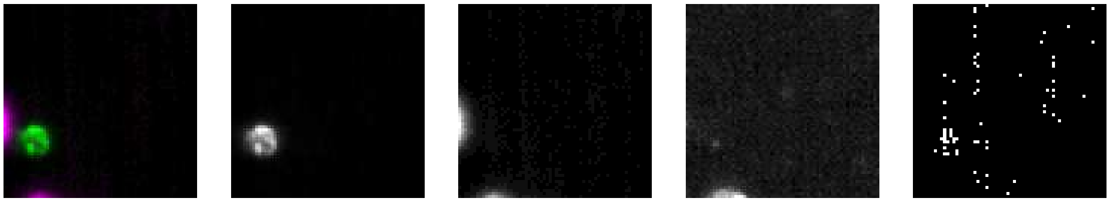				
4680	38	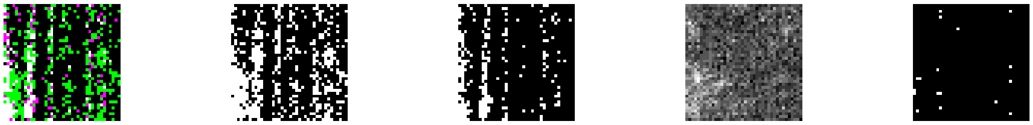				
4681	38	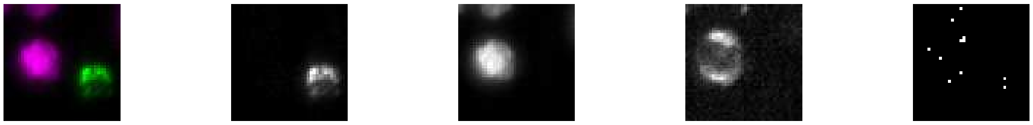				
4682	38	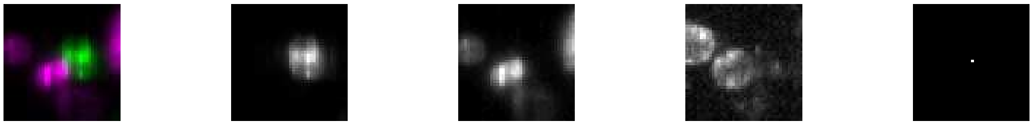				
4683	38	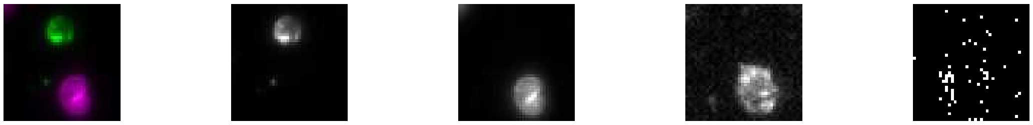				
4684	38	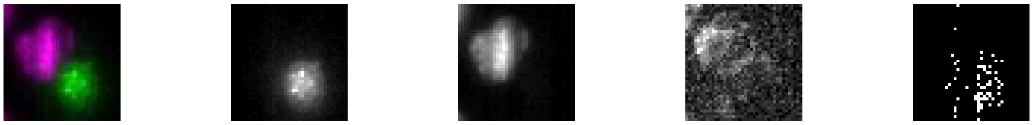				
4685	38	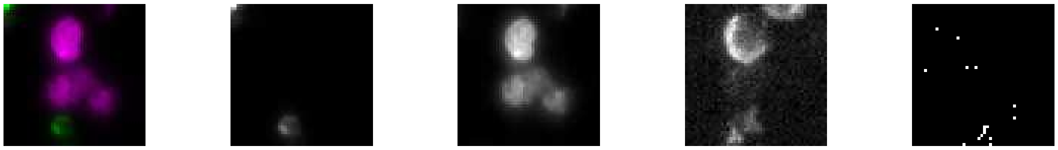				
4686	38	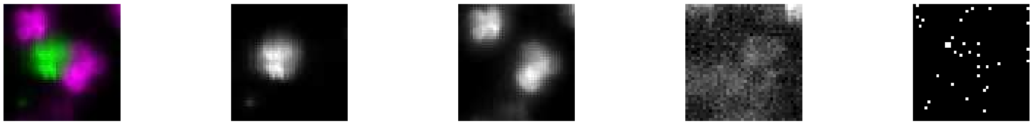				
4687	38	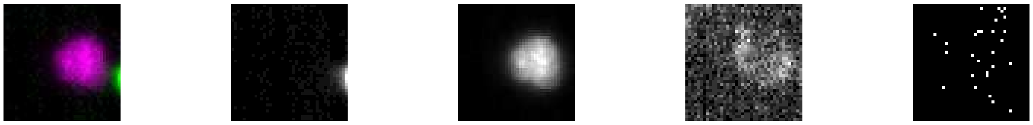				
4688	38	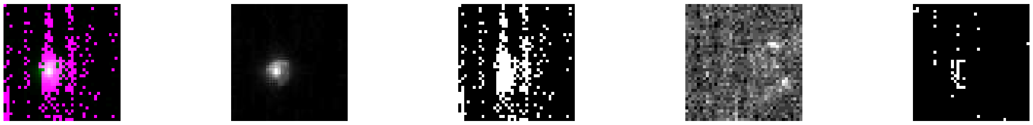				
4689	38	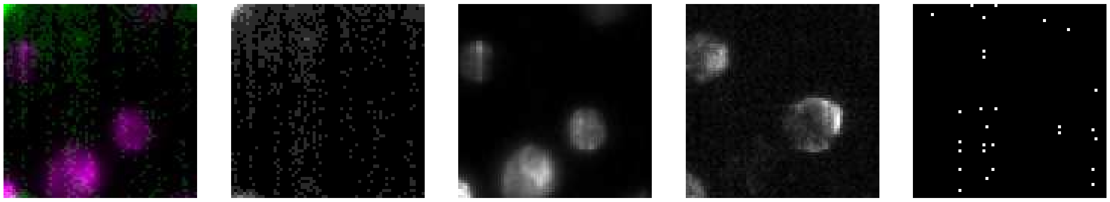				
4690	38	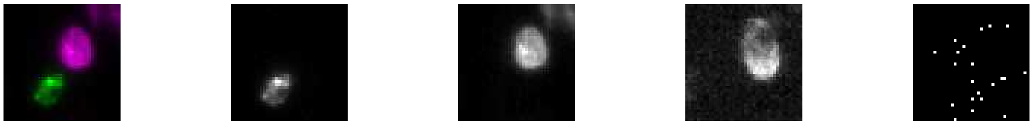				
4691	38	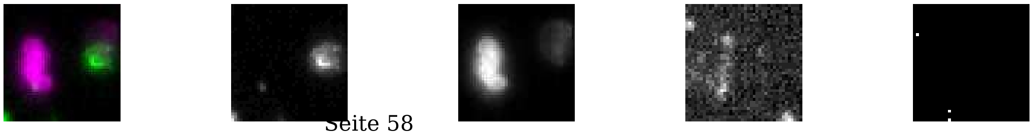				

Ereignis	Aufnahme	DAPI/CK-PE	CK-PE	DAPI	CD45-APC	HER-2/neu
4692	38	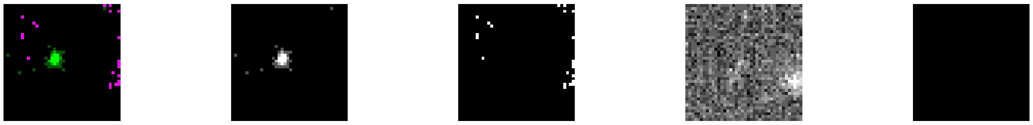				
4693	38	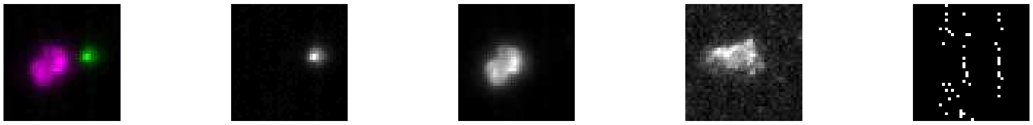				
4694	38	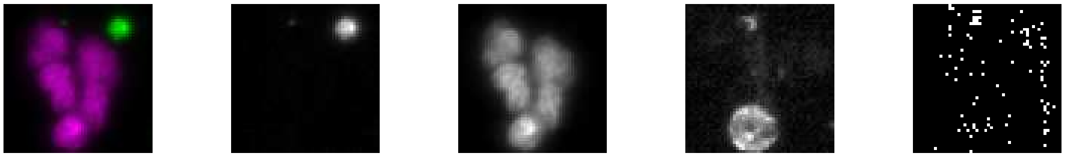				
4695	38	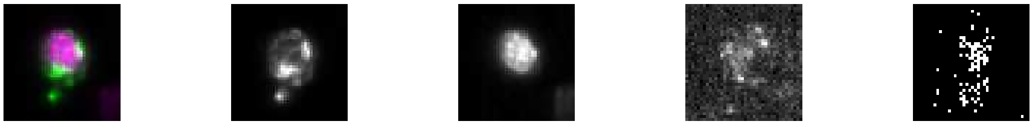				
4696	38	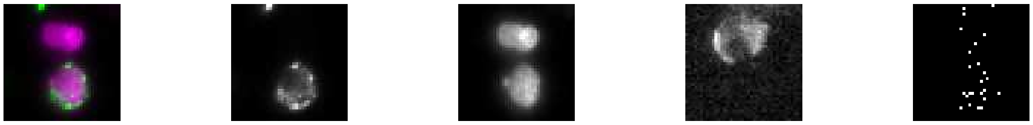				
4697	38	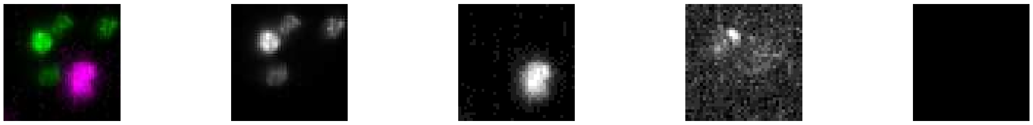				
4698	38	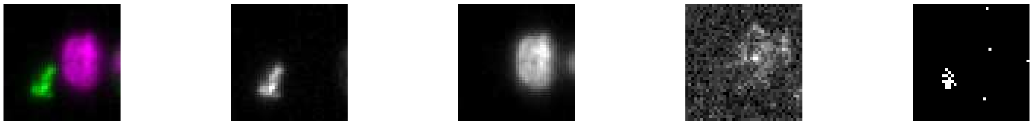				
4699	38	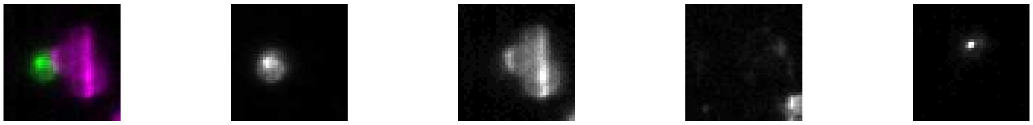				
4700	38	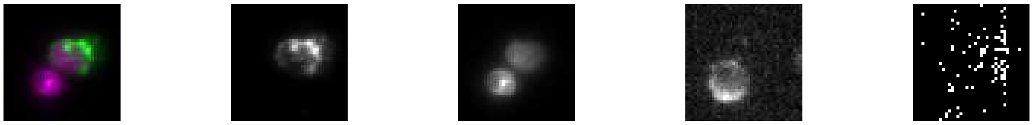				
4701	38	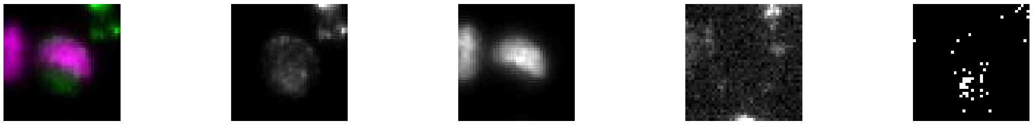				
4702	38	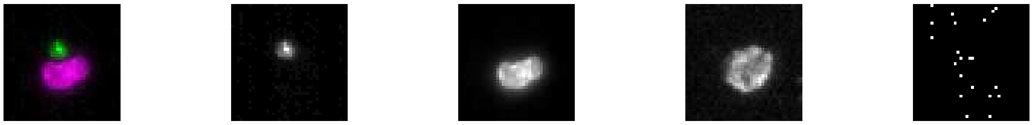				
4703	38	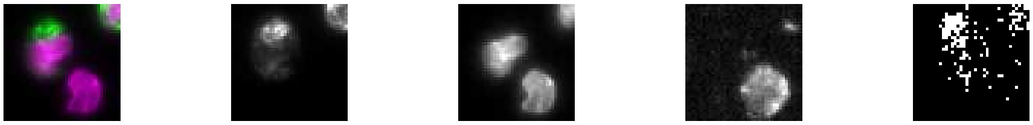				
4704	38	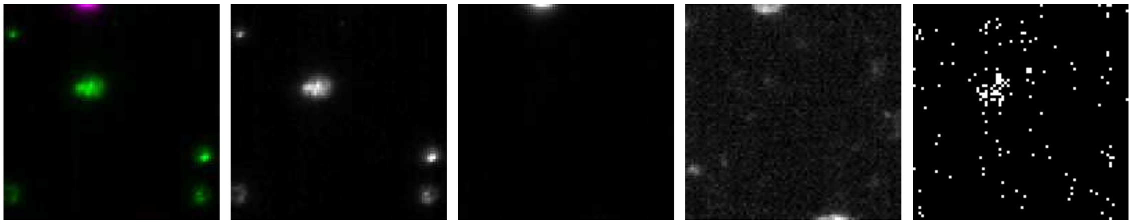				

Ereignis	Aufnahme	DAPI/CK-PE	CK-PE	DAPI	CD45-APC	HER-2/neu
4705	38	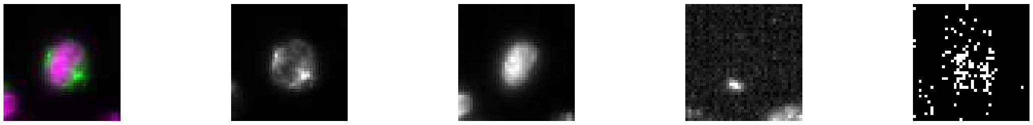				
4706	38	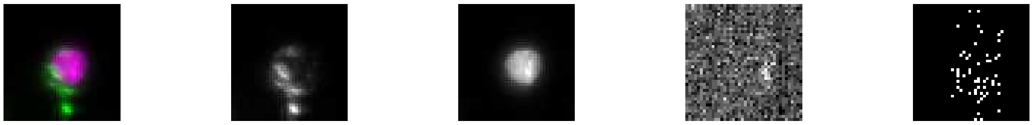				
4707	38	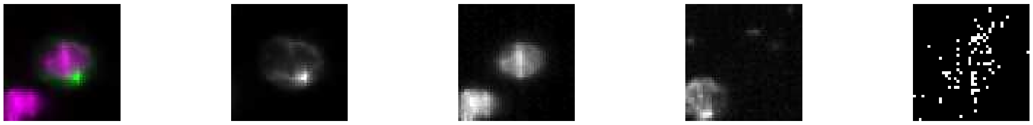				
4708	38	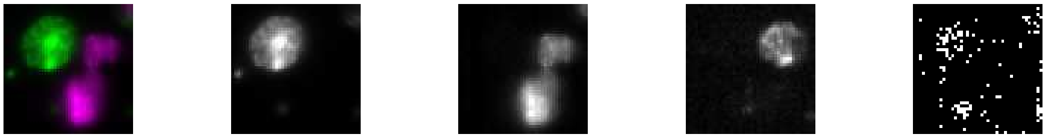				
4709	38	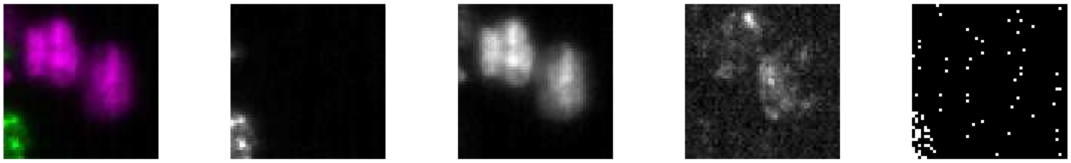				
4710	38	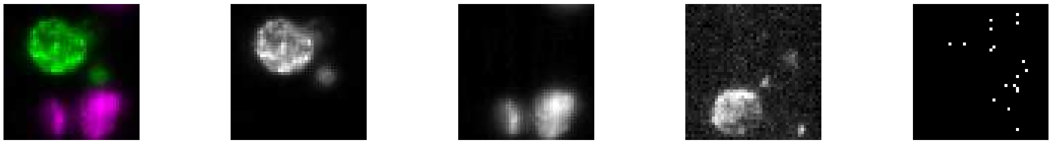				
4711	38	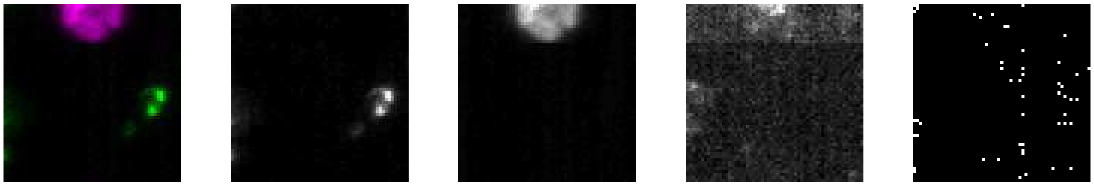				
4712	38	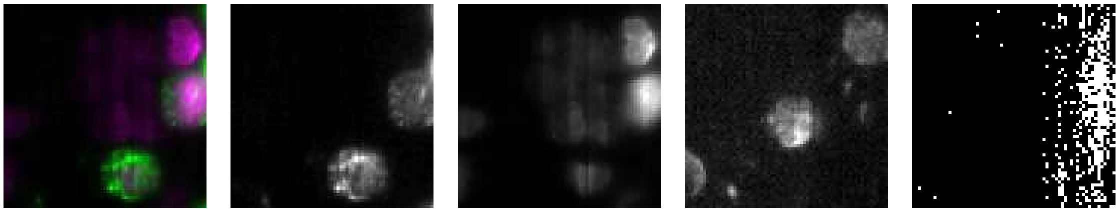				
4713	33	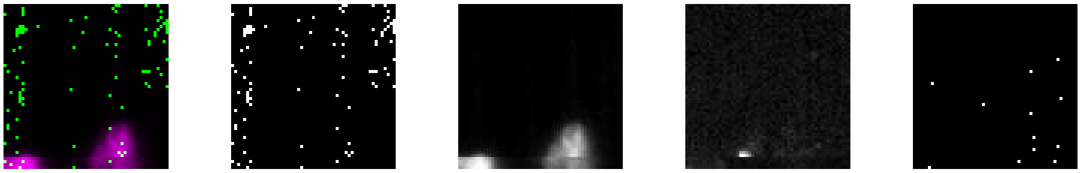				
4714	38	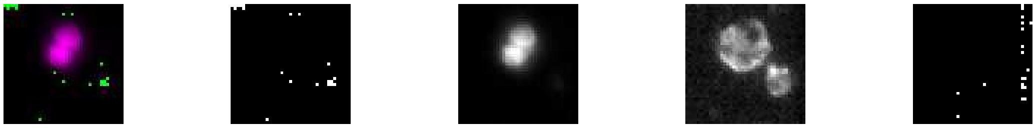				
4715	38	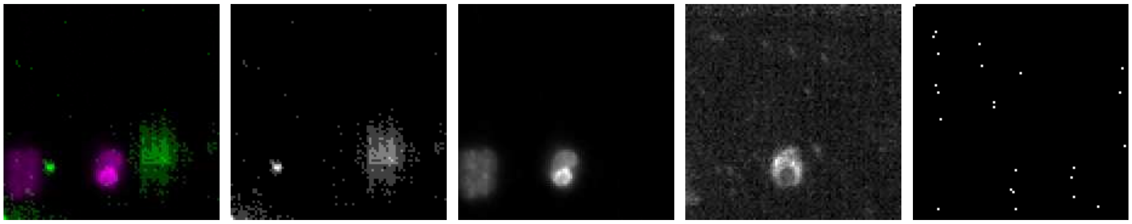				

Ereignis	Aufnahme	DAPI/CK-PE	CK-PE	DAPI	CD45-APC	HER-2/neu
4716	38	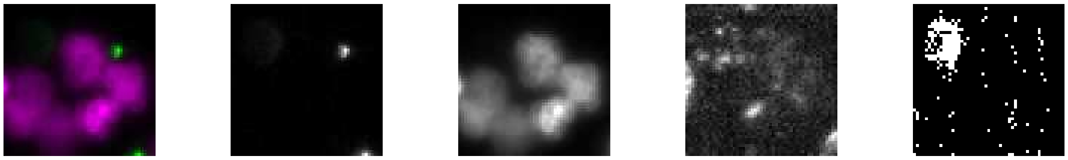				
4717	39	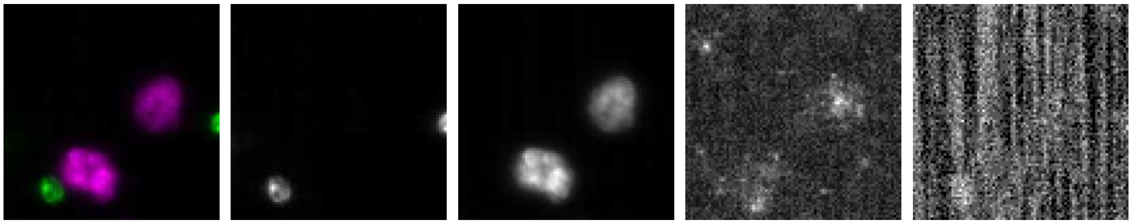				
4718	39	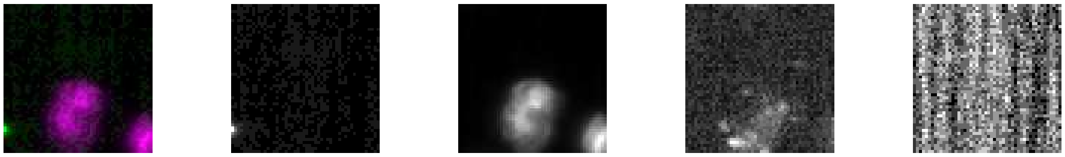				
4719	39	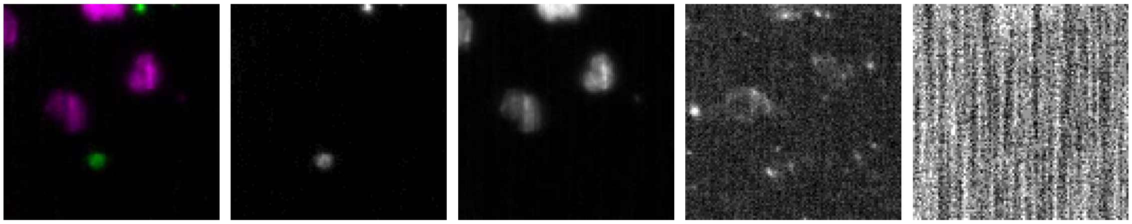				
4720	39	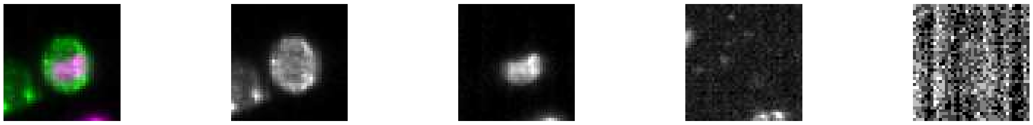				
4721	39	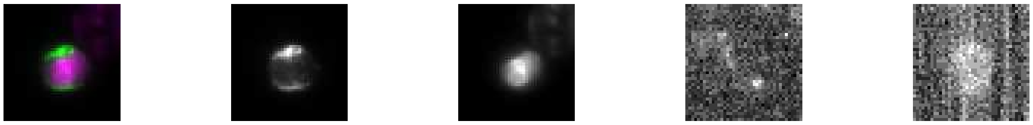				
4722	39	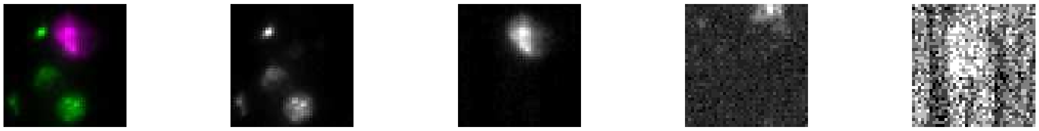				
4723	39	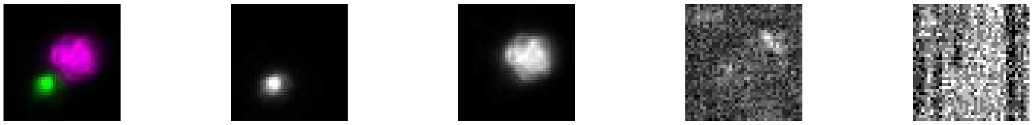				
4724	39	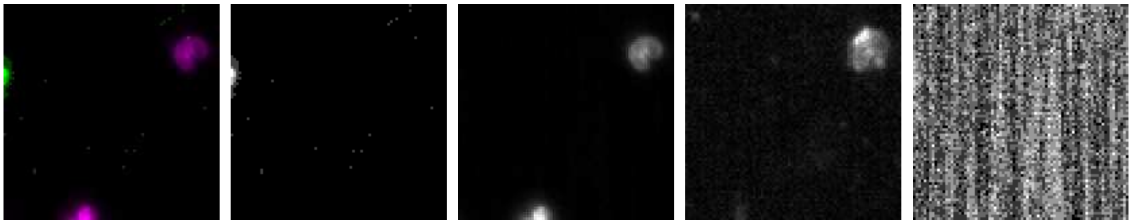				
4725	39	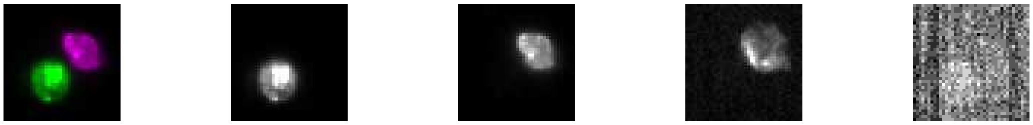				
4726	39	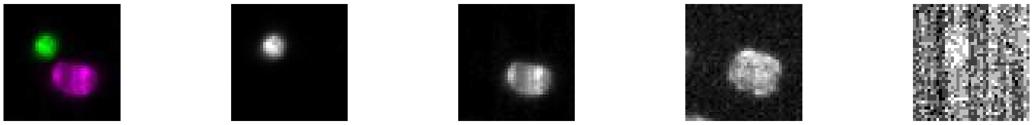				

Ereignis	Aufnahme	DAPI/CK-PE	CK-PE	DAPI	CD45-APC	HER-2/neu
4727	39	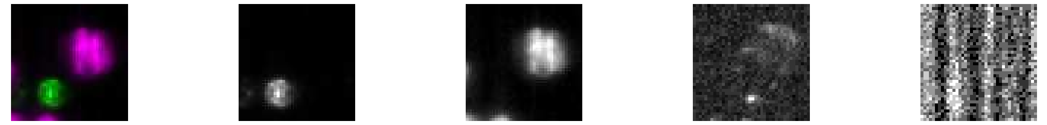				
4728	39	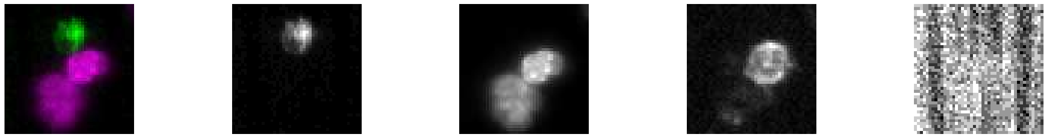				
4729	39	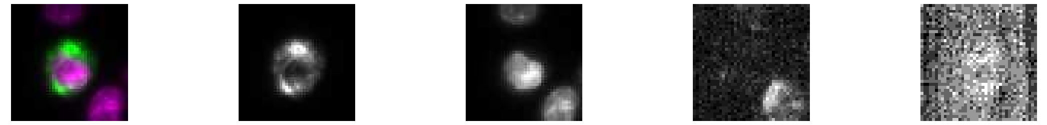				
4730	39	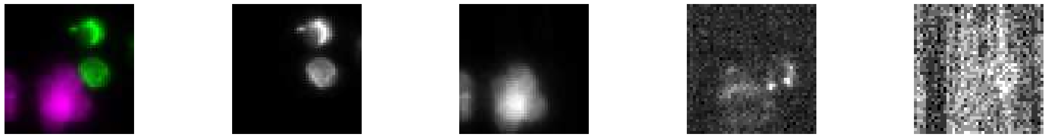				
4731	39	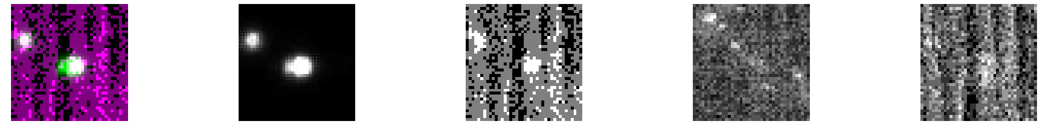				
4732	39	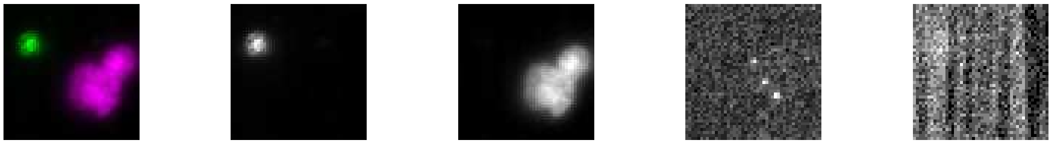				
4733	39	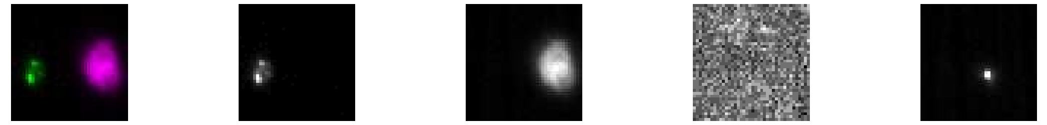				
4734	39	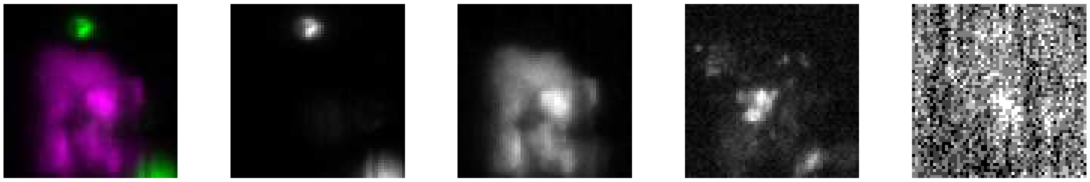				
4735	39	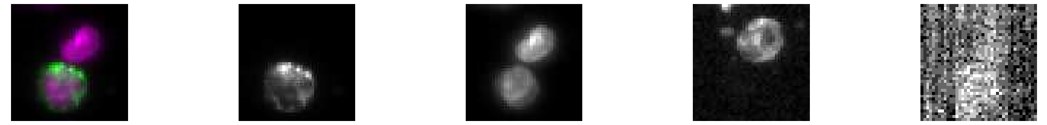				
4736	39	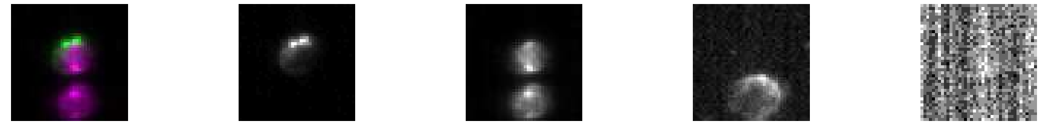				
4737	39	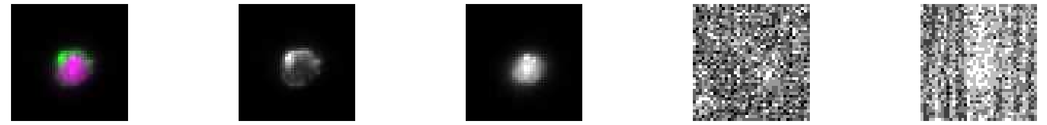				
4738	39	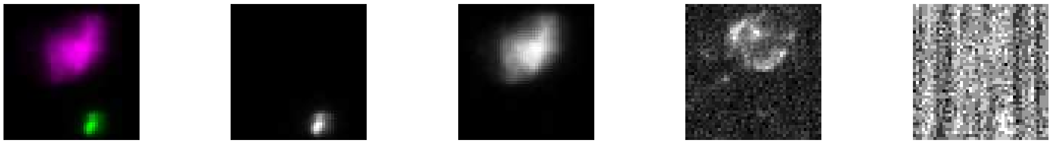				
4739	39	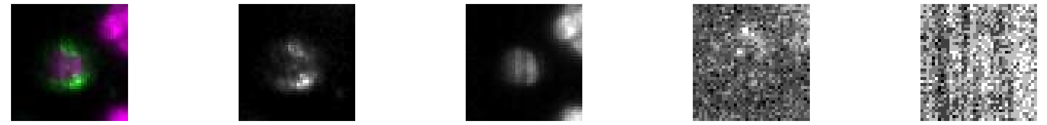				

Ereignis	Aufnahme	DAPI/CK-PE	CK-PE	DAPI	CD45-APC	HER-2/neu
4740	39	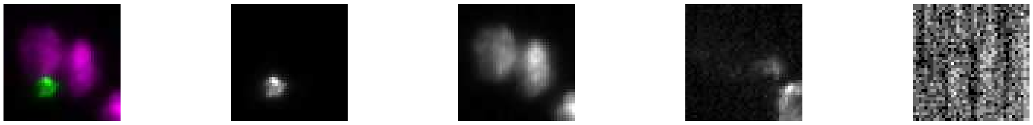				
4741	39	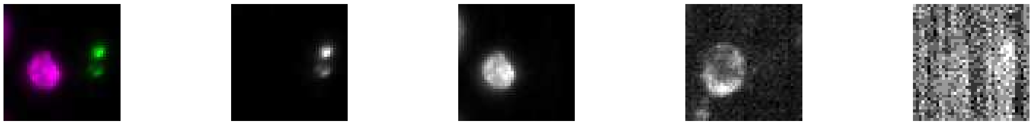				
4742	39	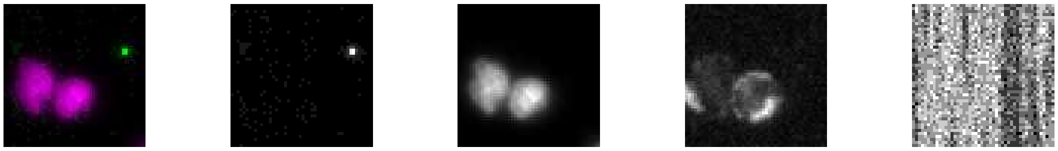				
4743	39	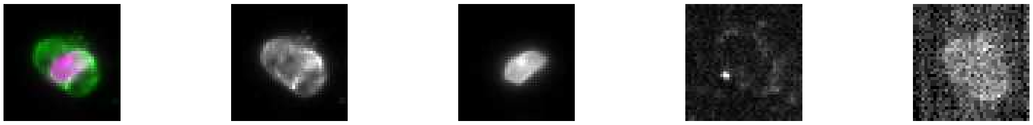				
4744	39	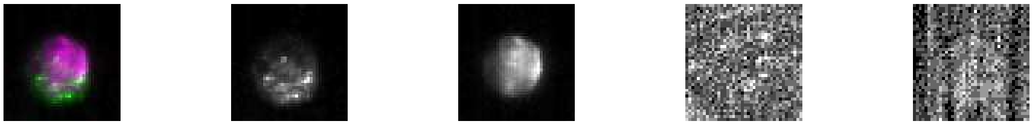				
4745	39	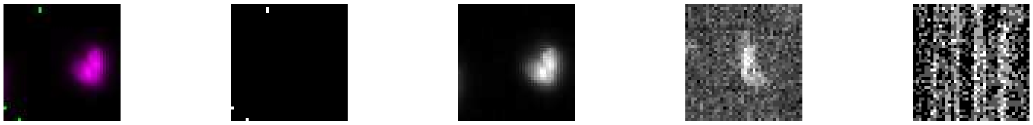				
4746	39	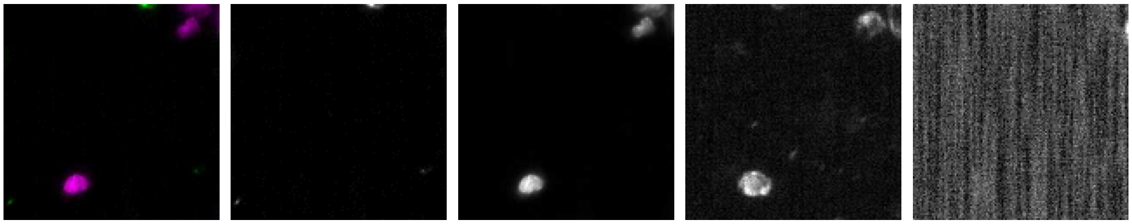				
4747	39	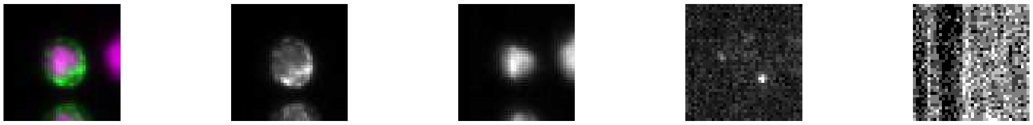				
4748	39	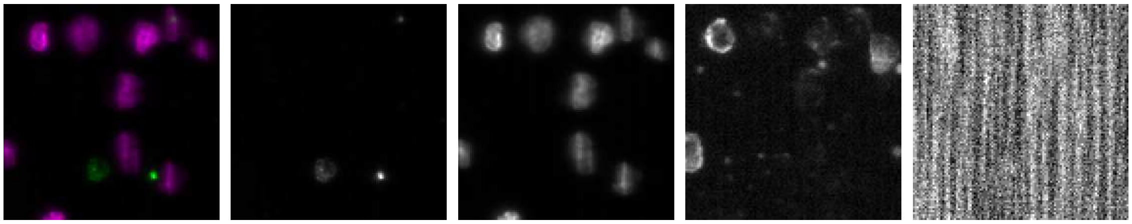				
4749	39	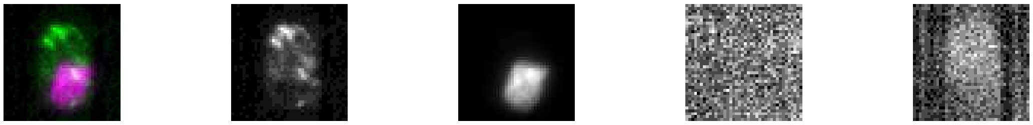				
4750	39	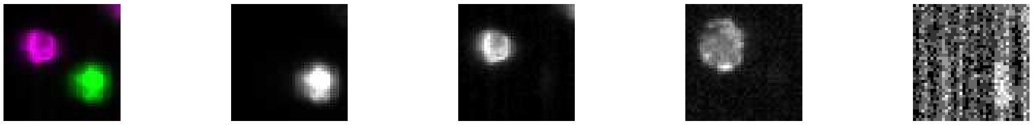				
4751	39	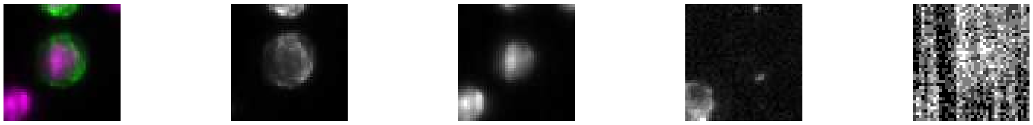				

Ereignis	Aufnahme	DAPI/CK-PE	CK-PE	DAPI	CD45-APC	HER-2/neu
4752	39	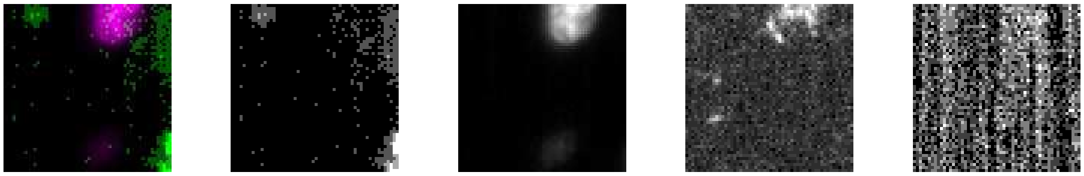				
4753	39	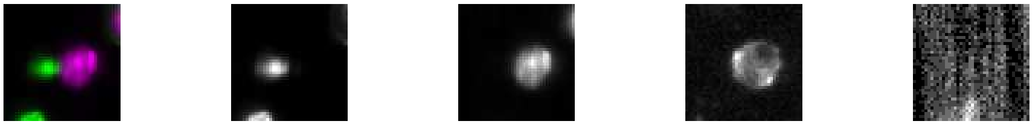				
4754	39	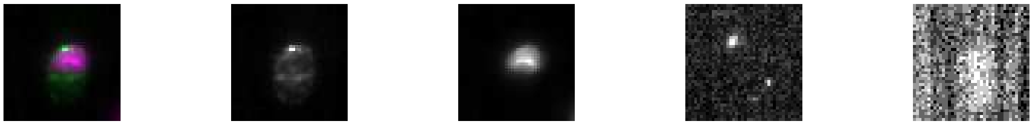				
4755	39	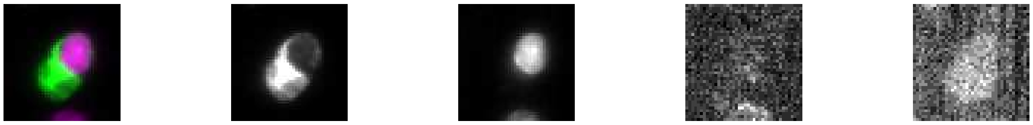				
4756	39	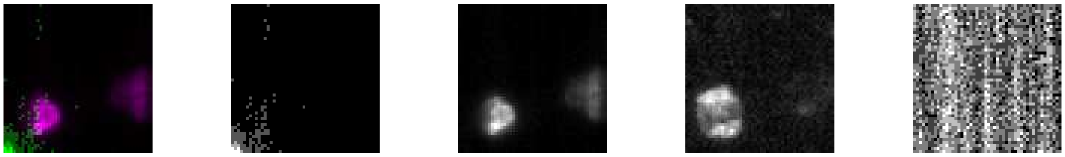				
4757	39	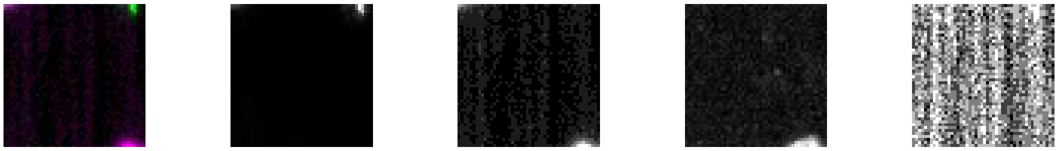				
4758	39	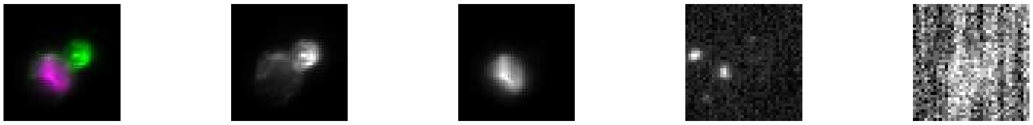				
4759	39	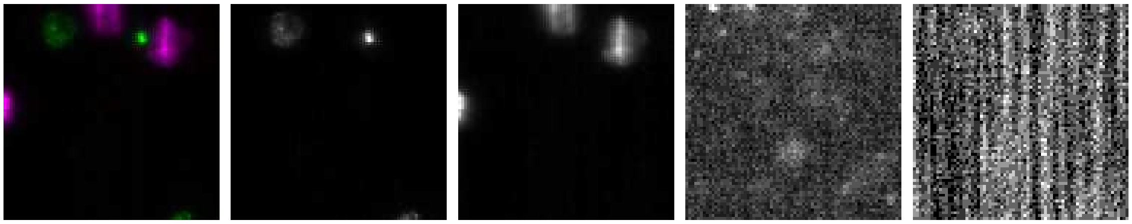				
4760	39	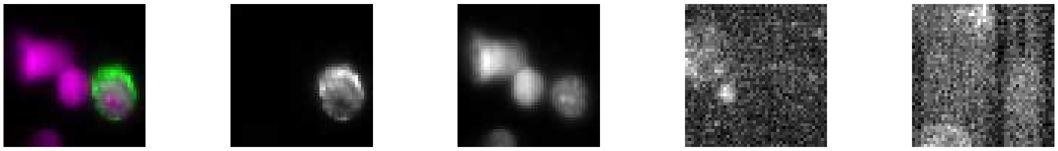				
4761	39	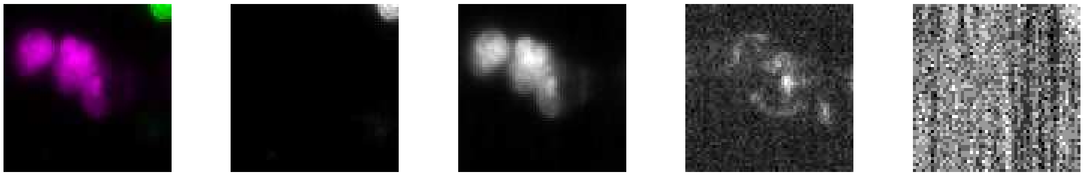				
4762	39	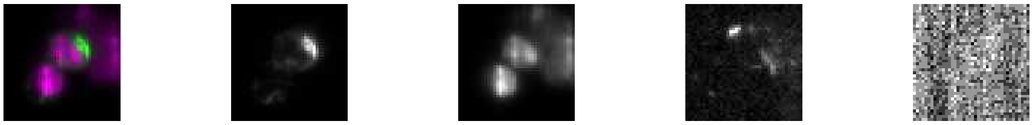				
4763	39	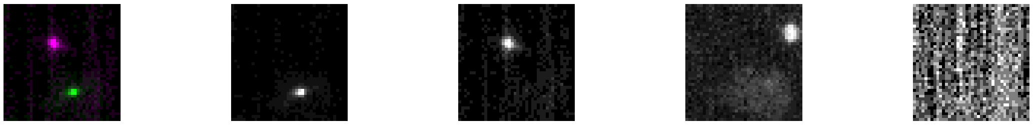				

Ereignis	Aufnahme	DAPI/CK-PE	CK-PE	DAPI	CD45-APC	HER-2/neu
4764	39	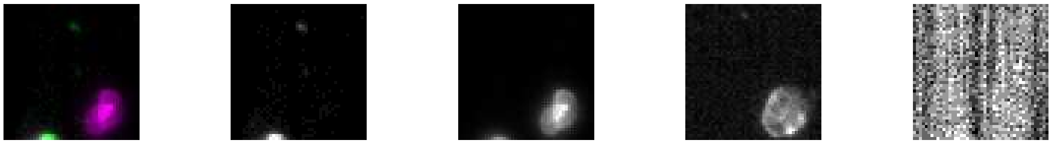				
4765	39	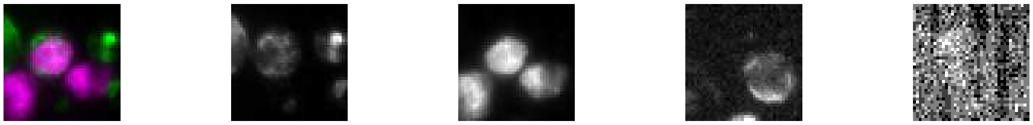				
4766	39	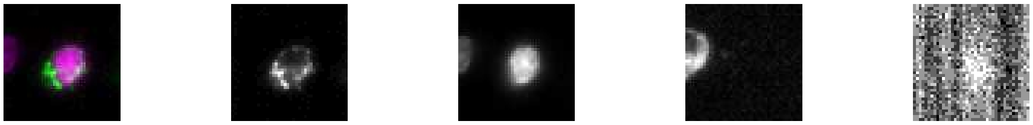				
4767	39	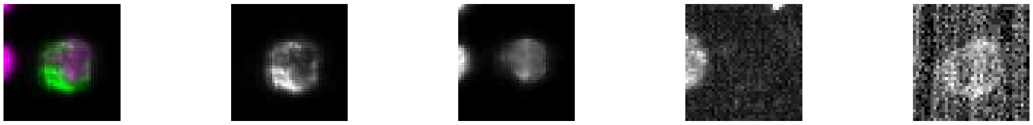				
4768	39	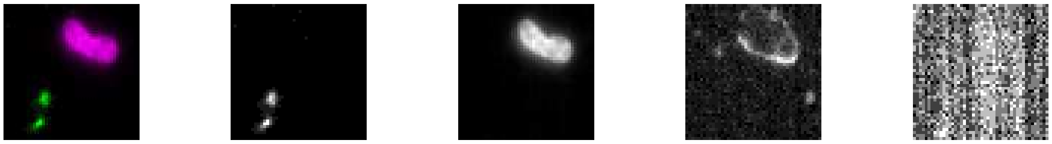				
4769	39	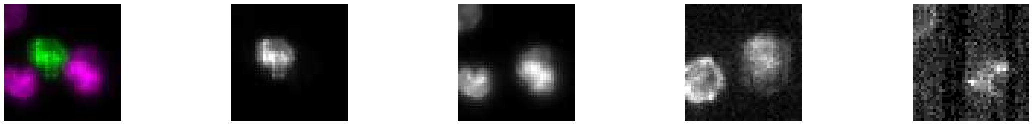				
4770	39	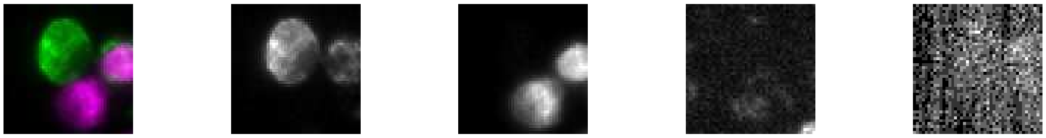				
4771	39	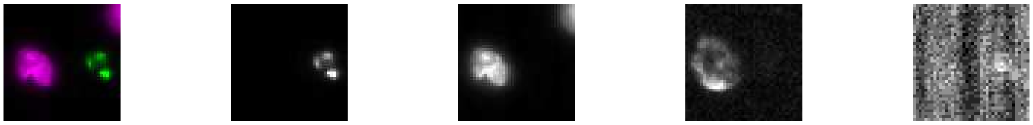				
4772	39	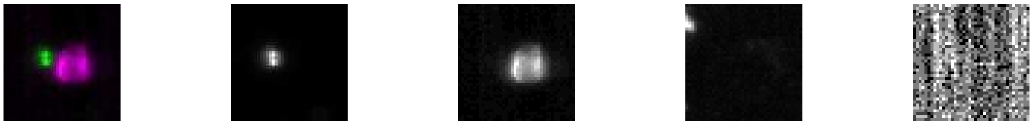				
4773	39	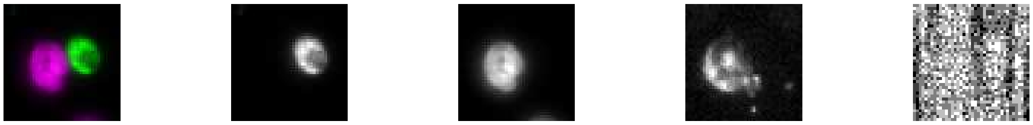				
4774	39	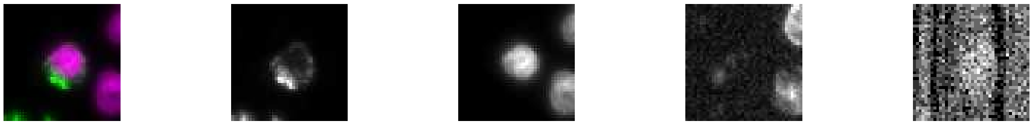				
4775	39	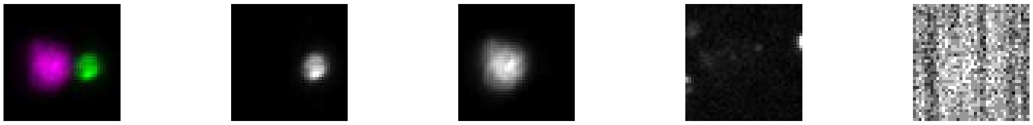				
4776	39	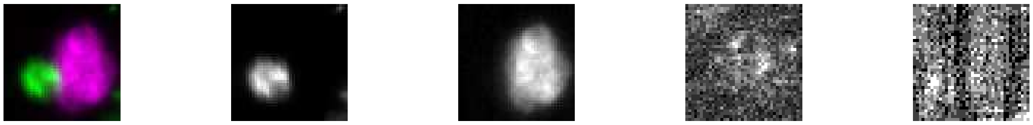				

Proben-ID: Gyn14.208 **Kassetten-ID:** 01327106 **Patienten-ID:**

Ereignis	Aufnahme	DAPI/CK-PE	CK-PE	DAPI	CD45-APC	HER-2/neu
4777	39	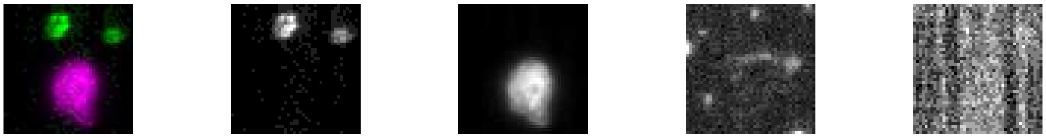				
4778	39	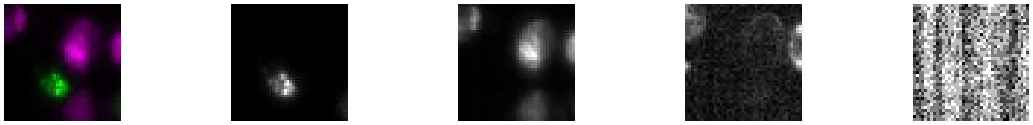				
4779	39	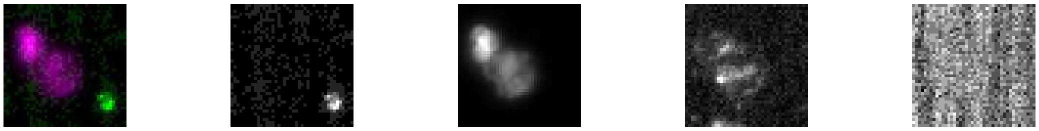				
4780	39	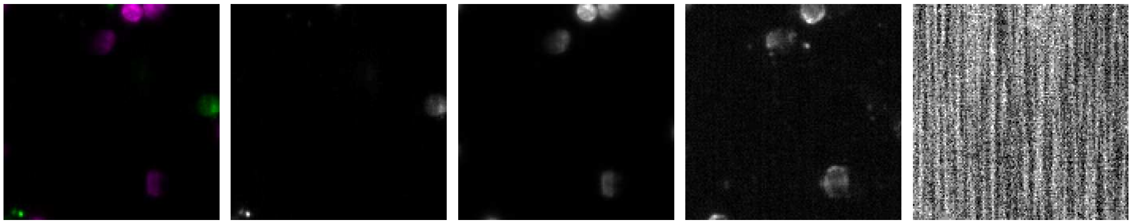				
4781	39	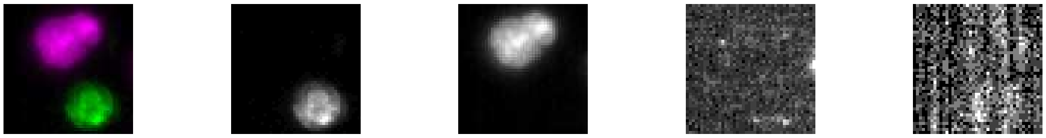				
4782	39	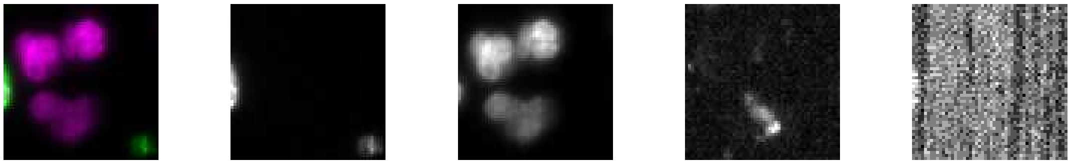				
4783	39	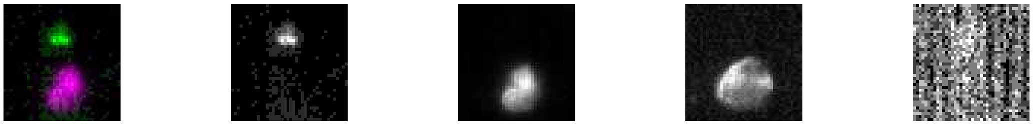				
4784	39	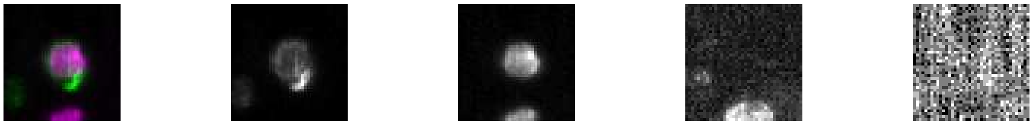				
4785	39	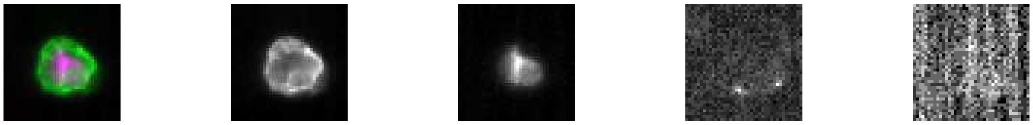				
4786	39	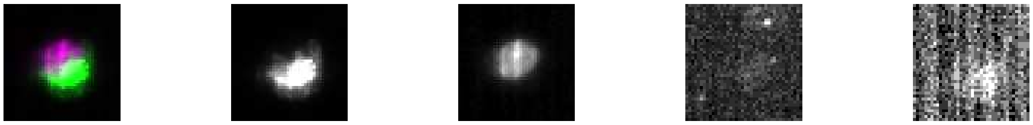				
4787	39	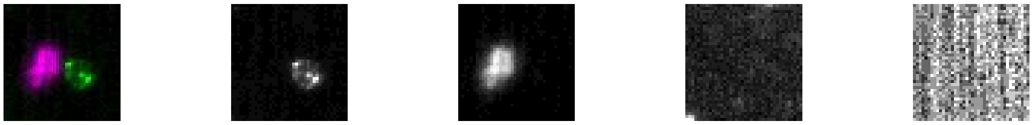				
4788	39	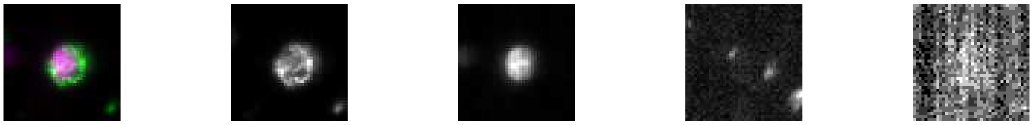				

Proben-ID: Gyn14.208 **Kassetten-ID:** 01327106 **Patienten-ID:**

Ereignis	Aufnahme	DAPI/CK-PE	CK-PE	DAPI	CD45-APC	HER-2/neu
4789	39	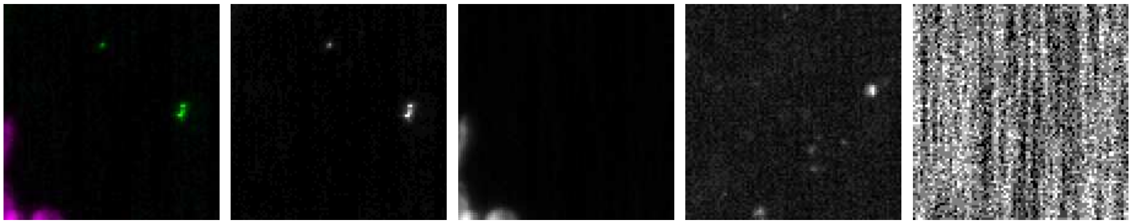				
4790	39	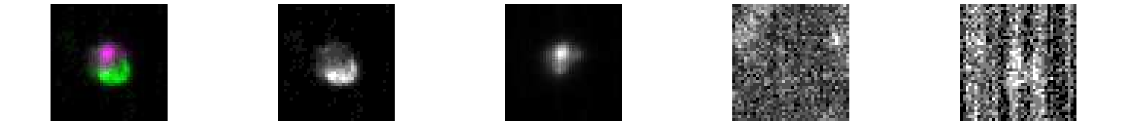				
4791	39	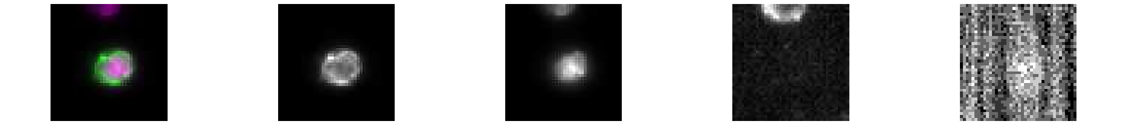				
4792	39	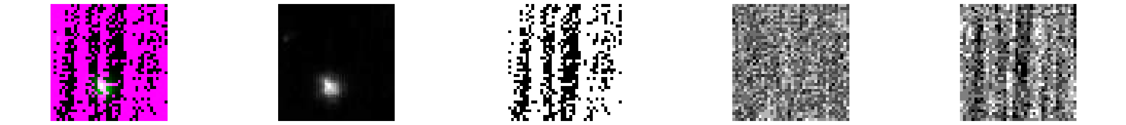				
4793	39	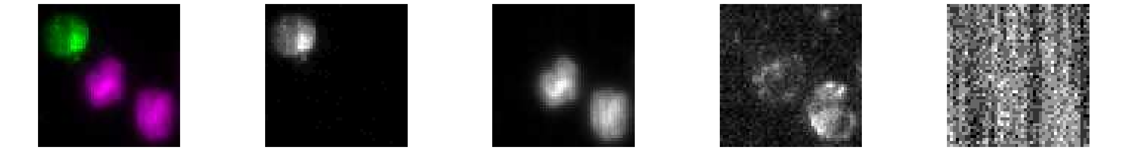				
4794	39	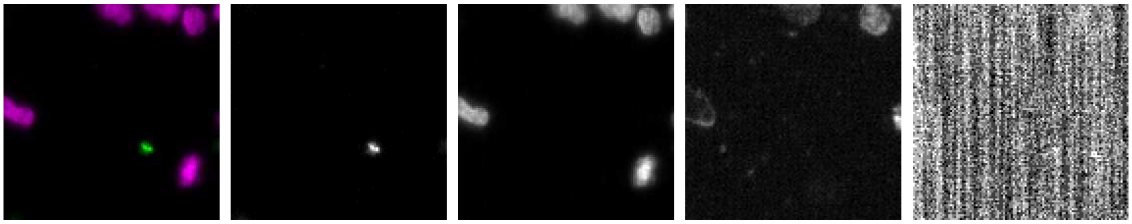				
4795	39	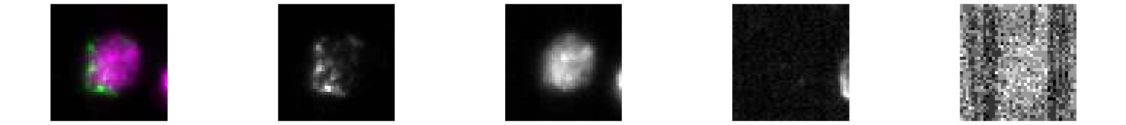				
4796	39	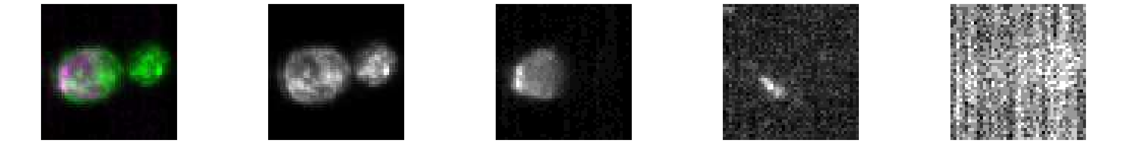				
4797	39	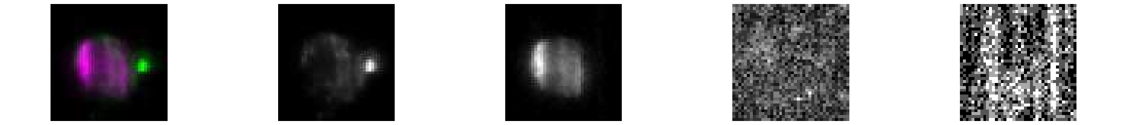				
4798	39	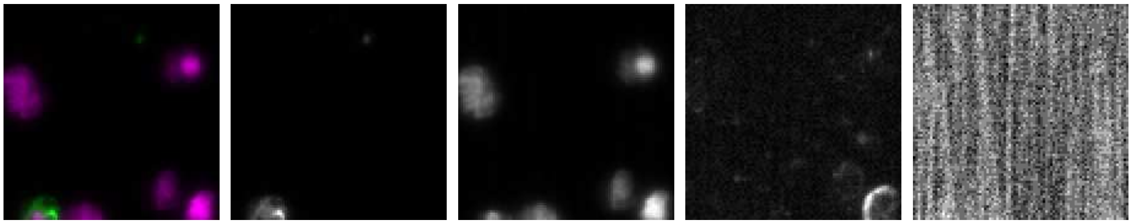				
4799	39	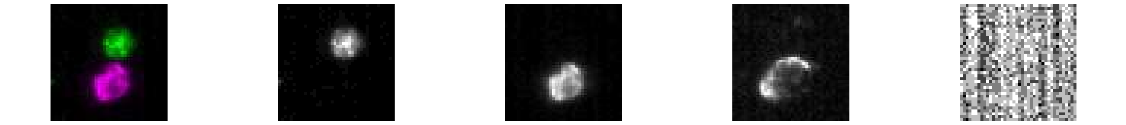				

Ereignis	Aufnahme	DAPI/CK-PE	CK-PE	DAPI	CD45-APC	HER-2/neu
4800	39	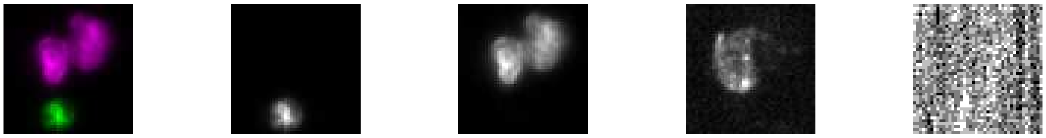				
4801	39	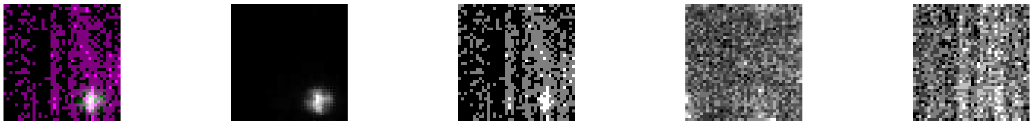				
4802	39	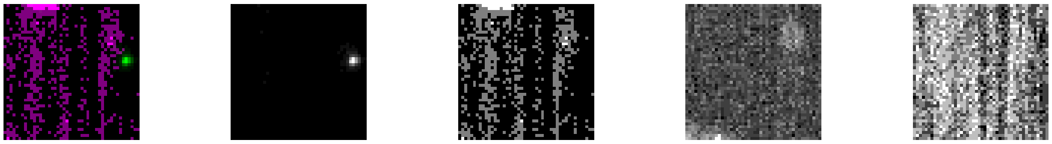				
4803	39	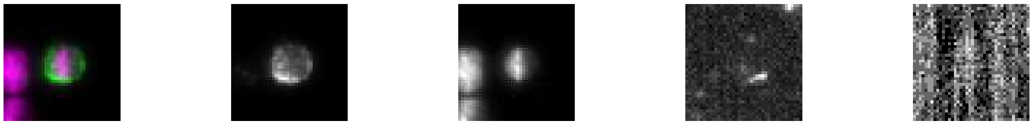				
4804	39	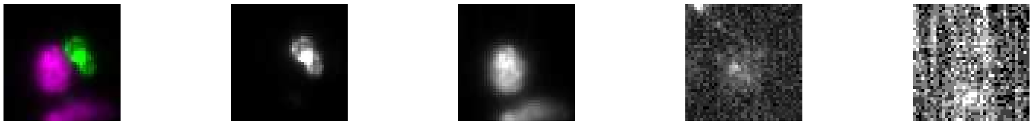				
4805	39	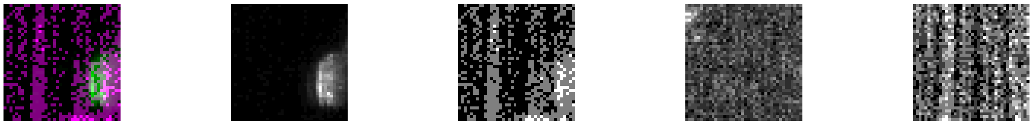				
4806	39	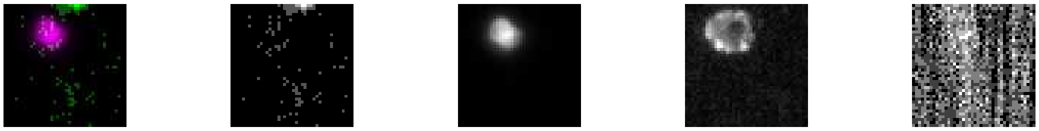				
4807	39	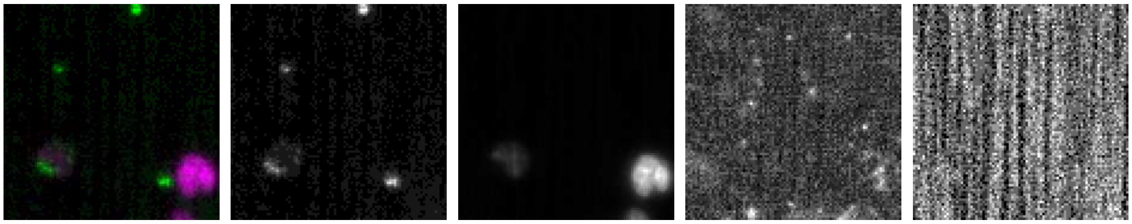				
4808	39	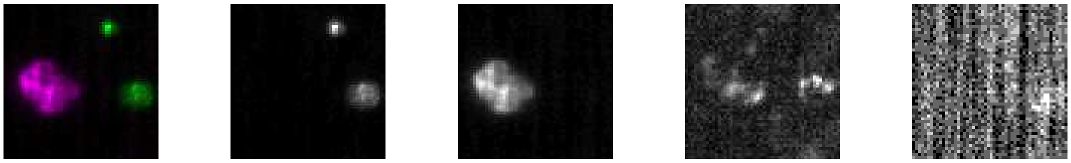				
4809	39	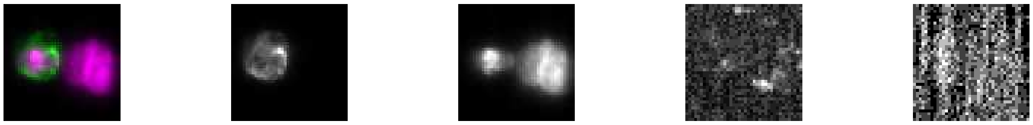				
4810	39	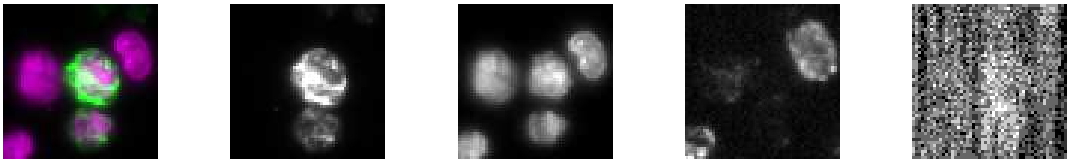				
4811	39	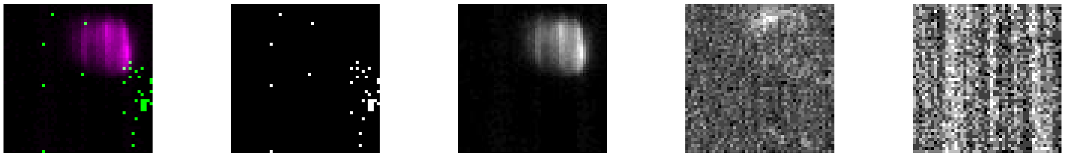				

Ereignis	Aufnahme	DAPI/CK-PE	CK-PE	DAPI	CD45-APC	HER-2/neu
4812	39	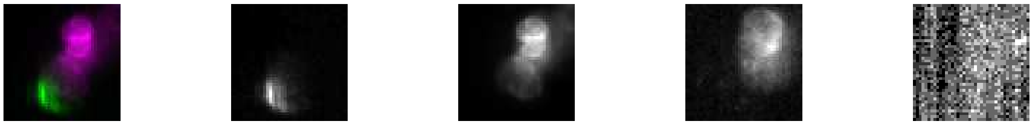				
4813	39	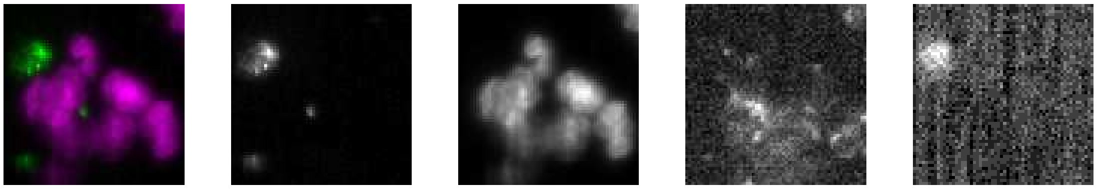				
4814	39	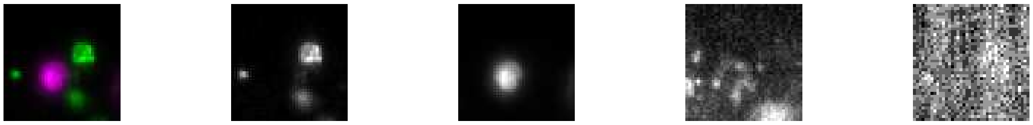				
4815	39	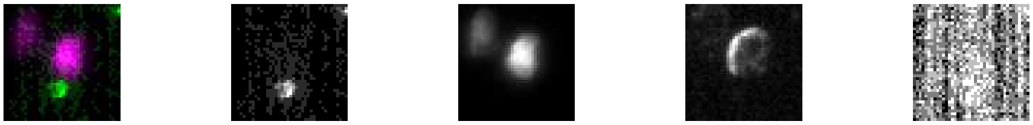				
4816	39	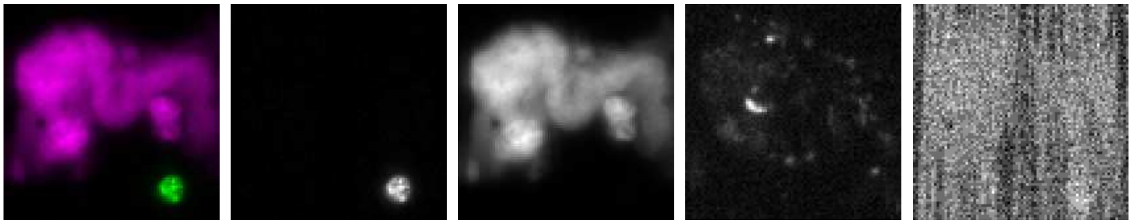				
4817	39	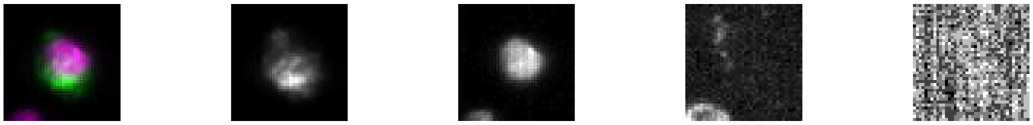				
4818	39	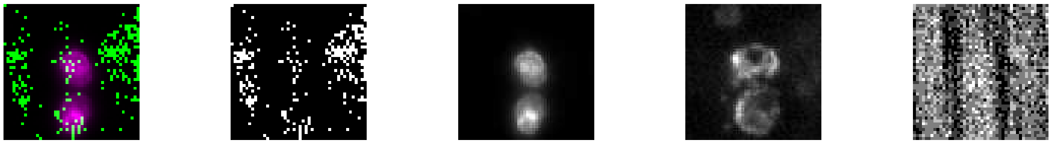				
4819	39	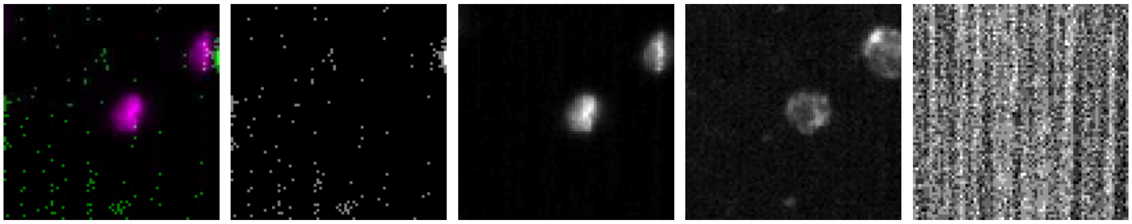				
4820	39	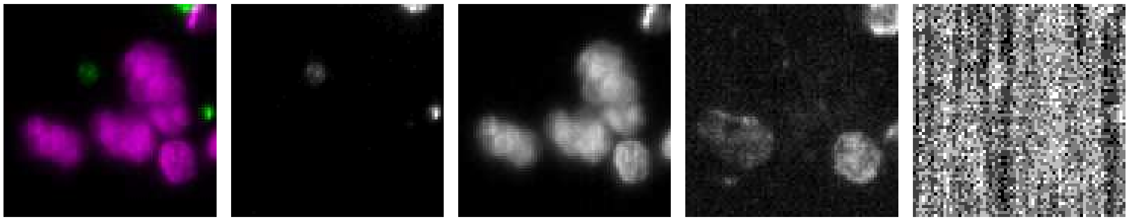				
4821	39	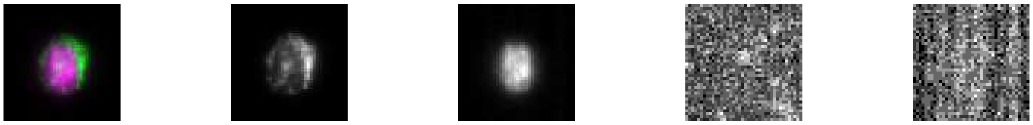				
4822	39	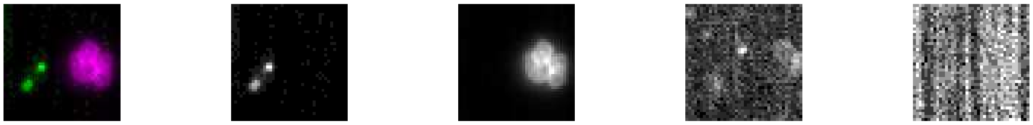				

Ereignis	Aufnahme	DAPI/CK-PE	CK-PE	DAPI	CD45-APC	HER-2/neu
4823	39	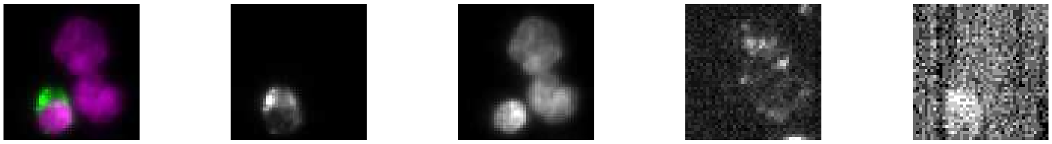				
4824	39	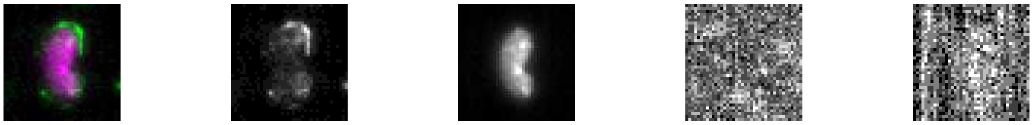				
4825	39	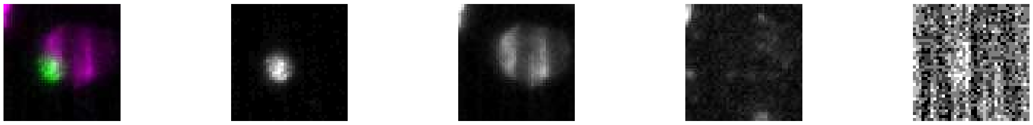				
4826	39	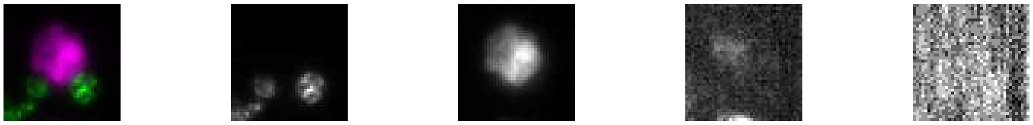				
4827	39	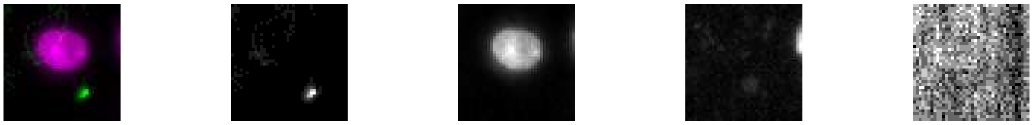				
4828	39	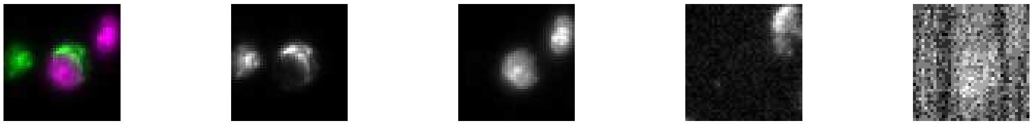				
4829	39	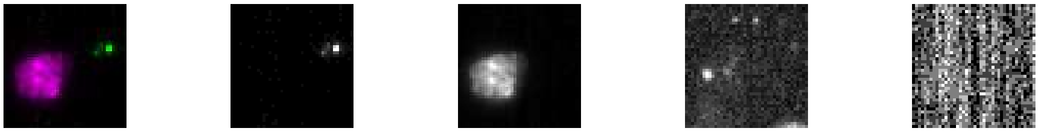				
4830	39	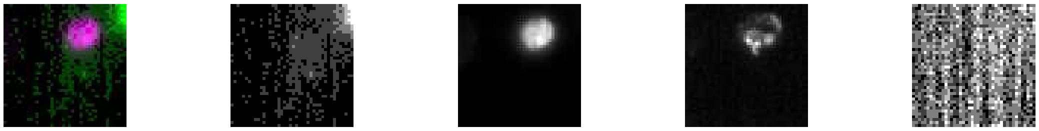				
4831	39	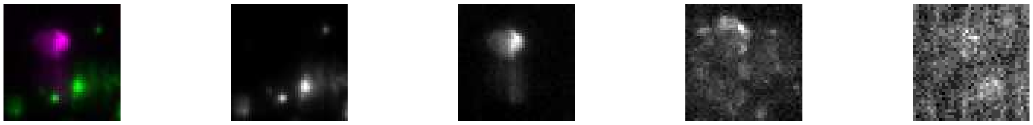				
4832	39	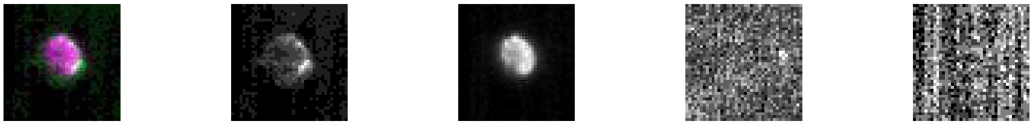				
4833	39	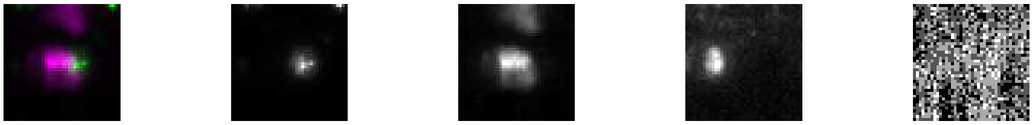				
4834	39	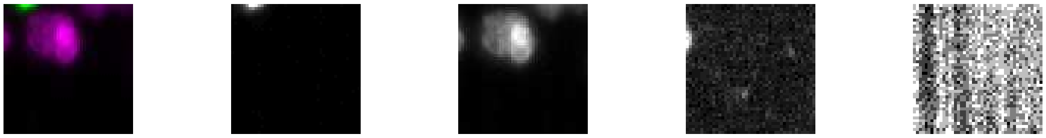				
4835	39	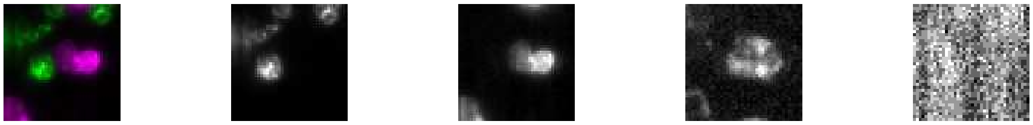				
4836	39	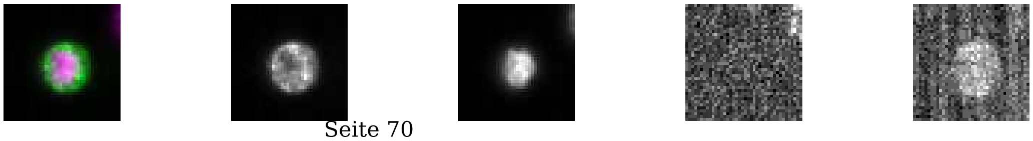				

Ereignis	Aufnahme	DAPI/CK-PE	CK-PE	DAPI	CD45-APC	HER-2/neu
4837	39	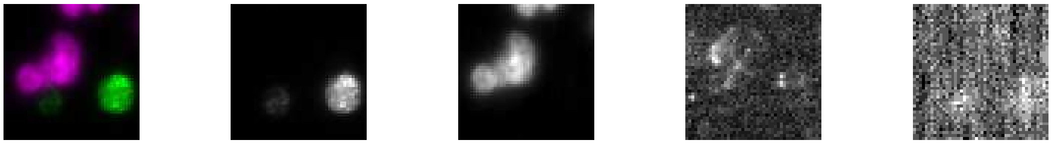				
4838	39	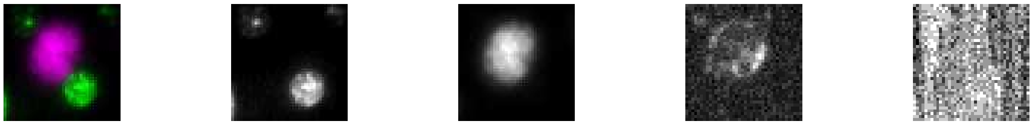				
4839	39	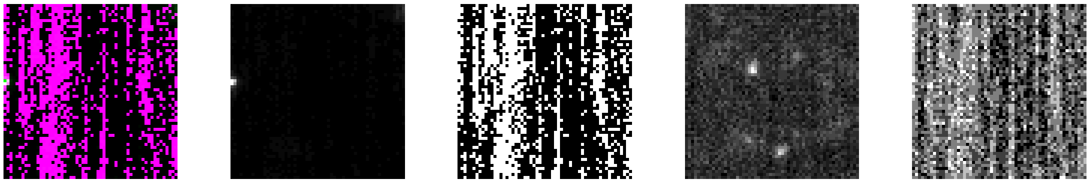				
4840	39	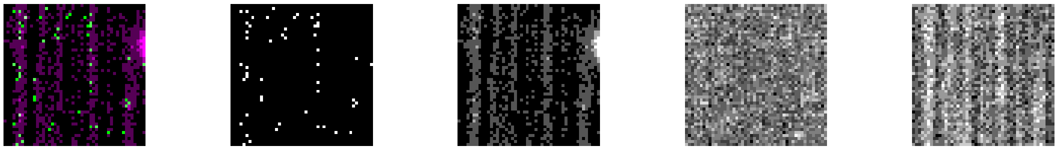				
4841	39	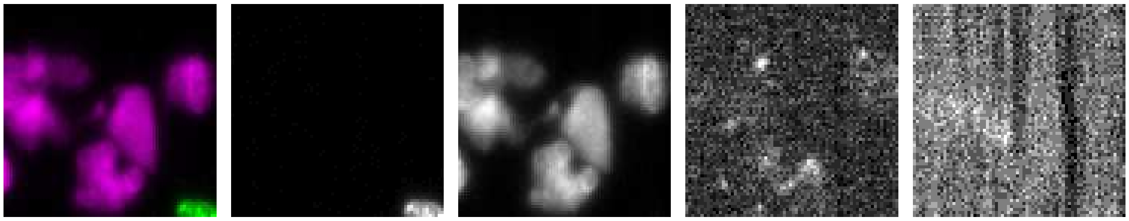				
4842	39	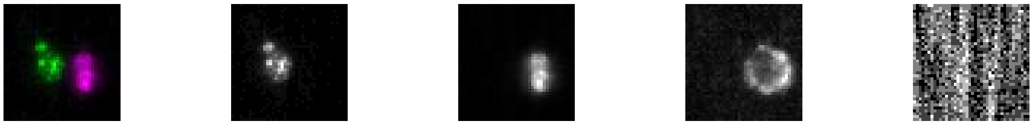				
4843	39	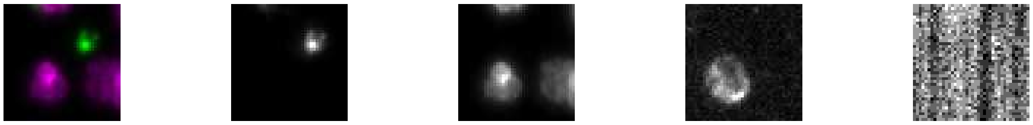				
4844	39	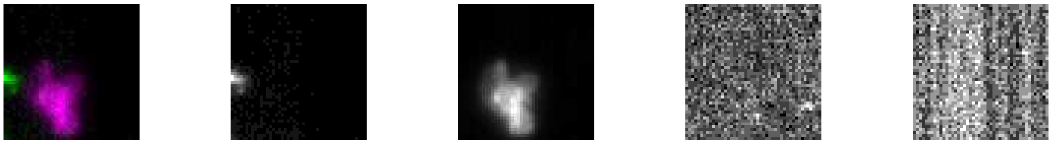				
4845	39	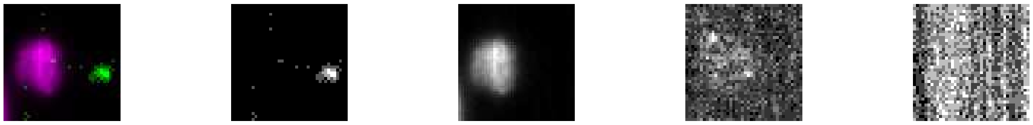				
4846	39	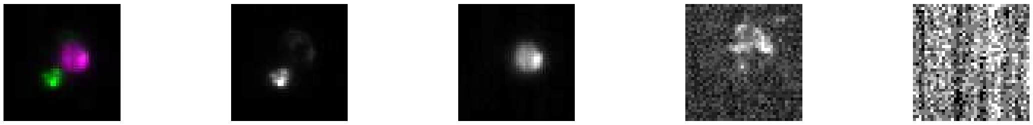				
4847	39	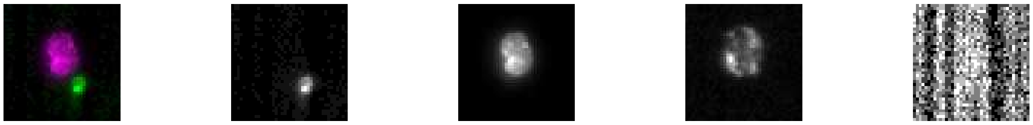				

Ereignis	Aufnahme	DAPI/CK-PE	CK-PE	DAPI	CD45-APC	HER-2/neu
4848	32	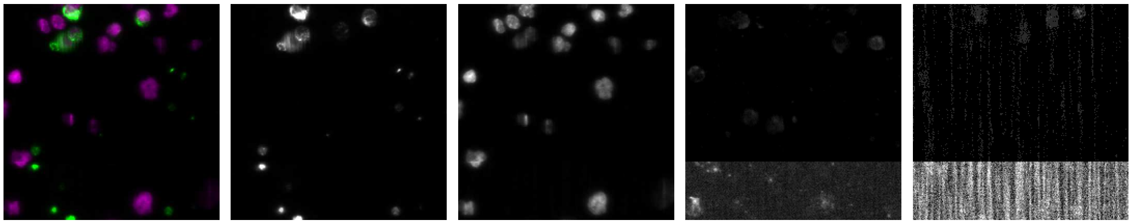				
4849	39	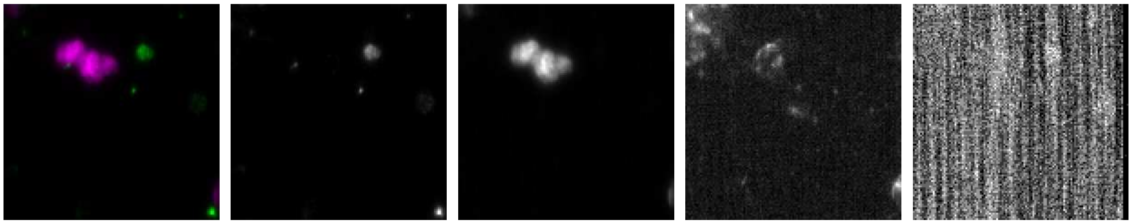				
4850	39	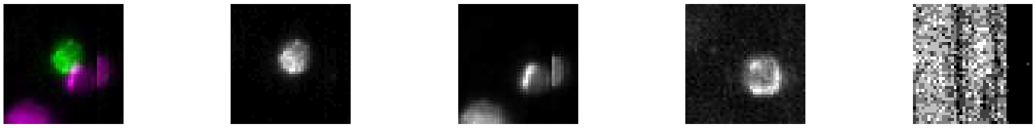				
4851	39	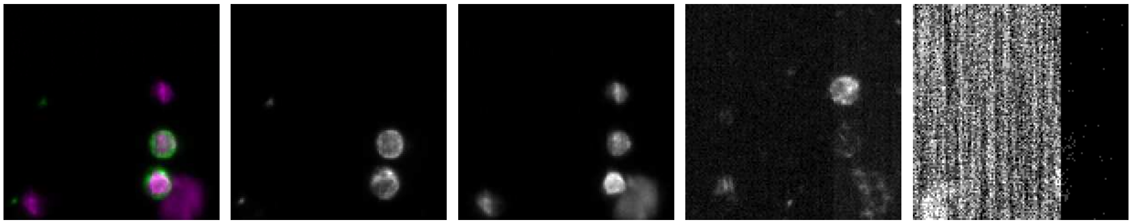				
4852	39	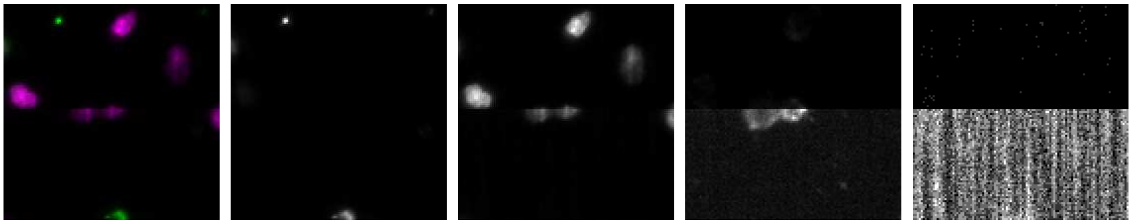				
4853	39	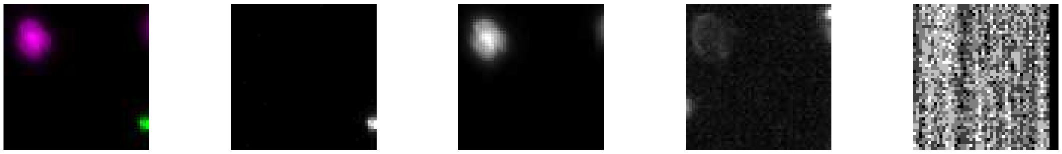				
4854	39	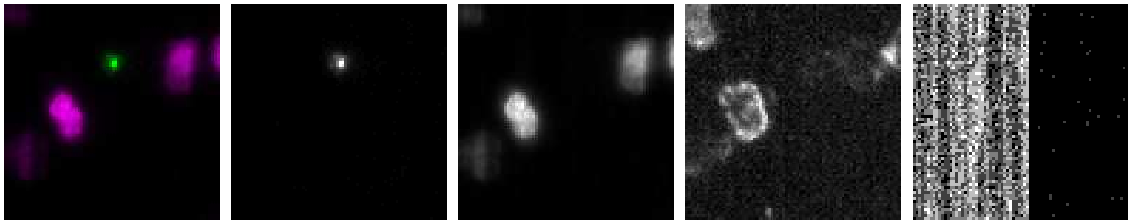				
4855	32	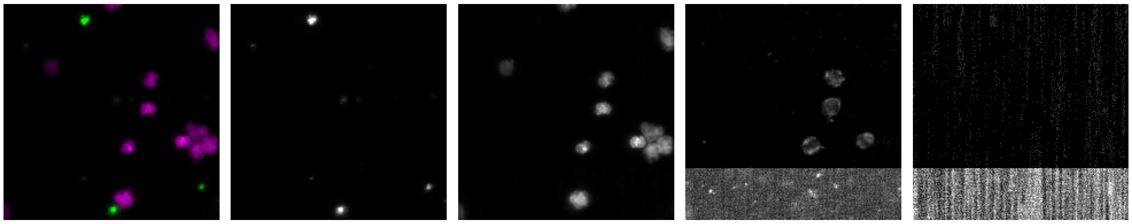				

Ereignis	Aufnahme	DAPI/CK-PE	CK-PE	DAPI	CD45-APC	HER-2/neu
4856	32	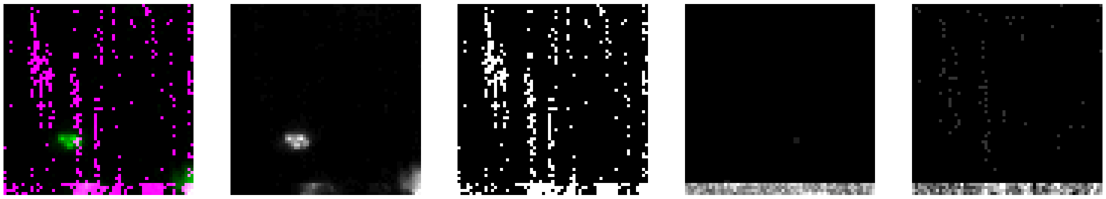				
4857	32	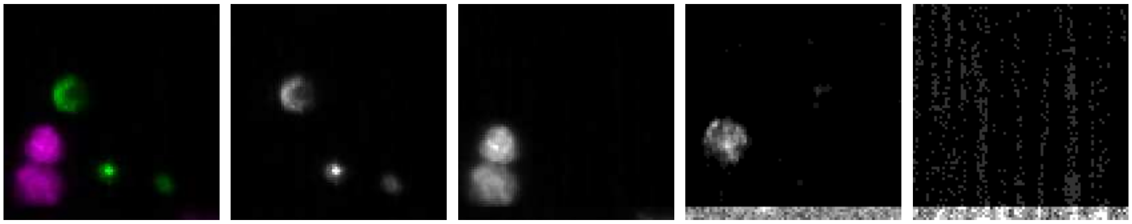				
4858	39	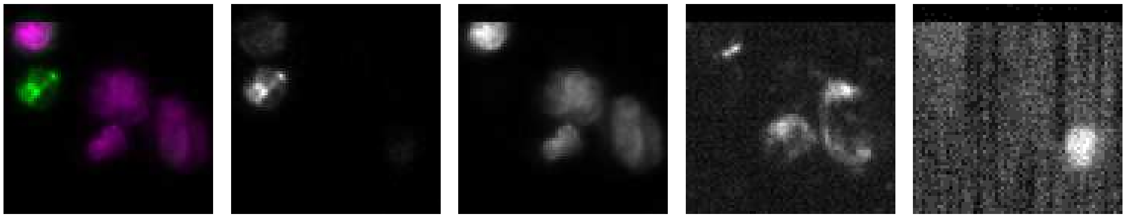				
4859	40	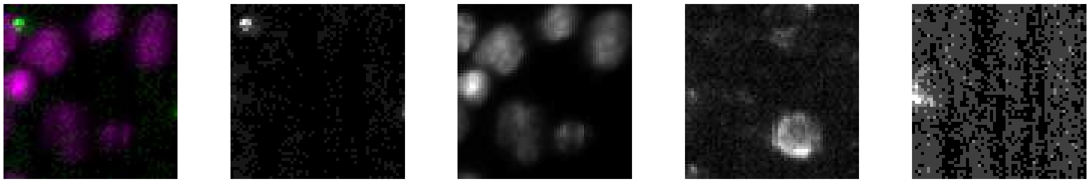				
4860	40	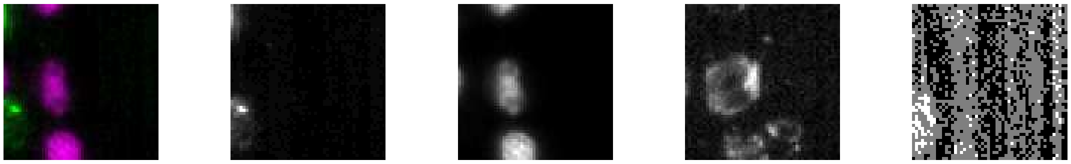				
4861	40	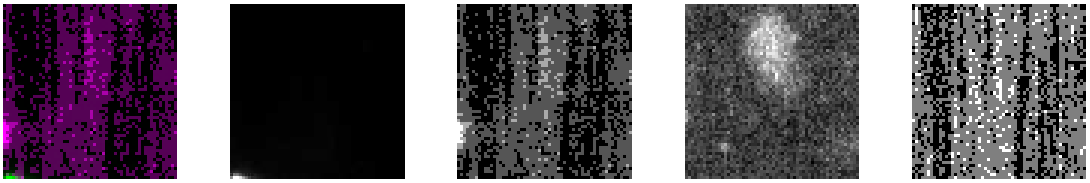				
4862	40	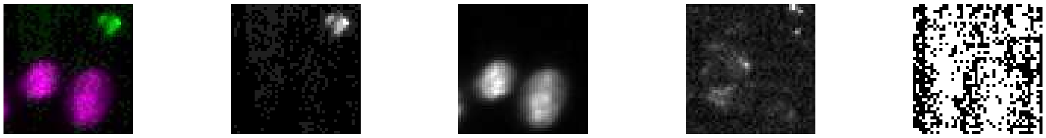				
4863	40	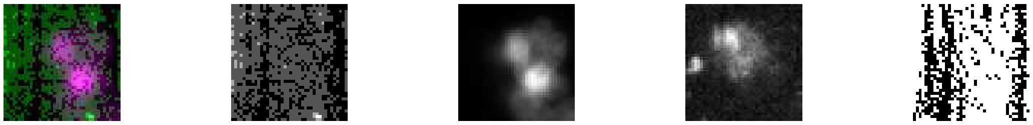				
4864	40	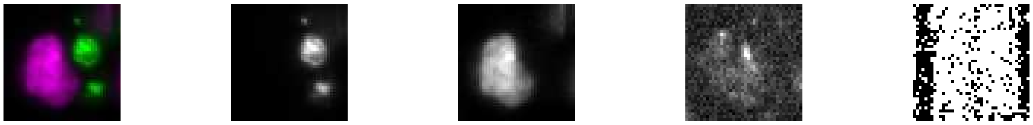				
4865	40	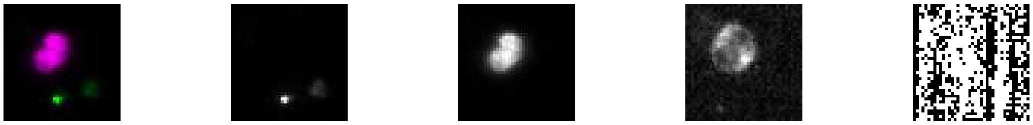				
4866	40	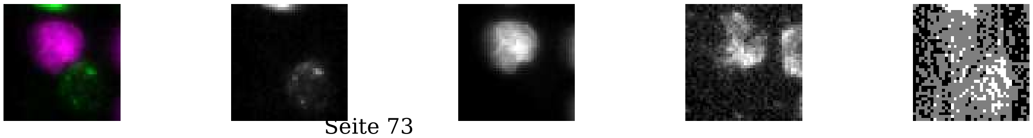				

Ereignis	Aufnahme	DAPI/CK-PE	CK-PE	DAPI	CD45-APC	HER-2/neu
4867	40	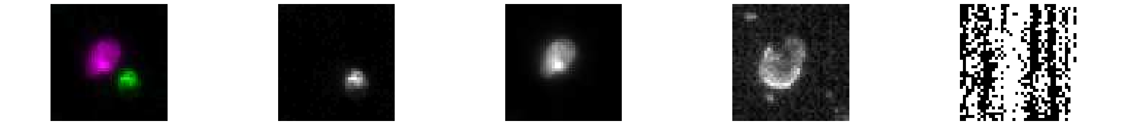				
4868	40	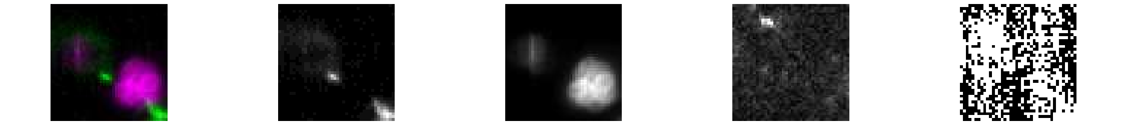				
4869	40	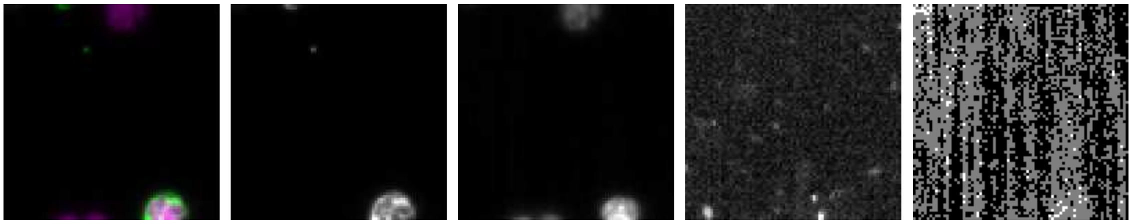				
4870	40	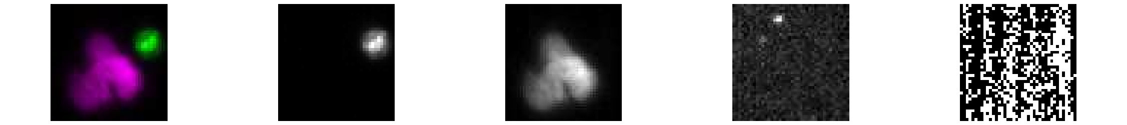				
4871	40	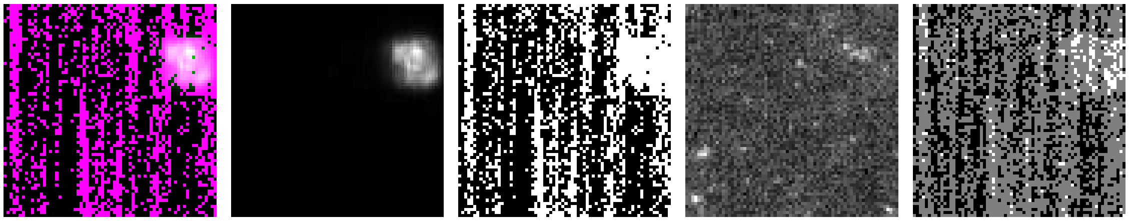				
4872	40	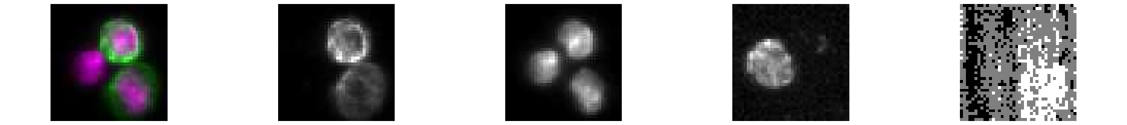				
4873	40	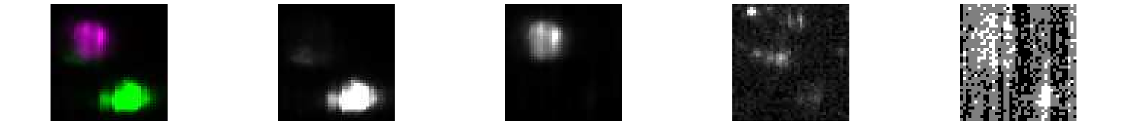				
4874	40	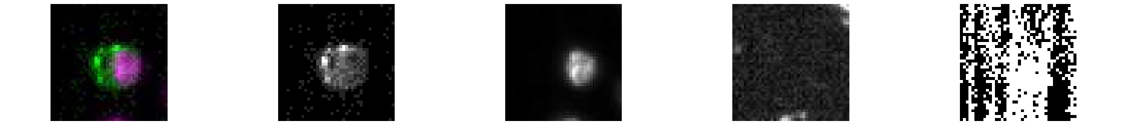				
4875	40	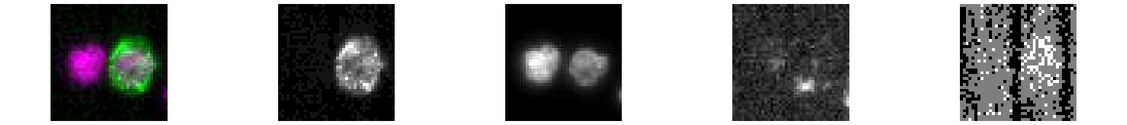				
4876	40	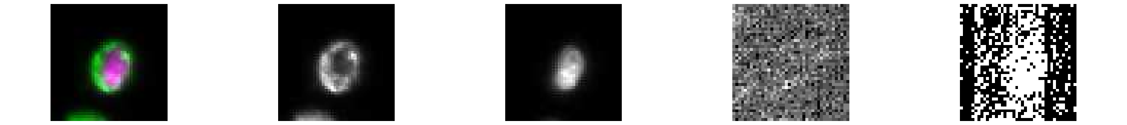				
4877	40	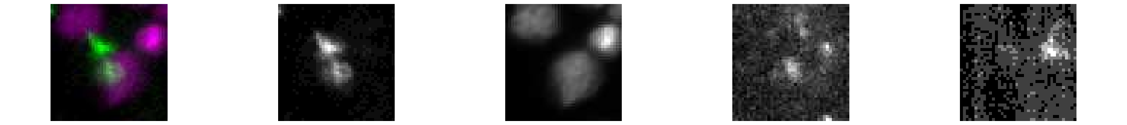				
4878	40	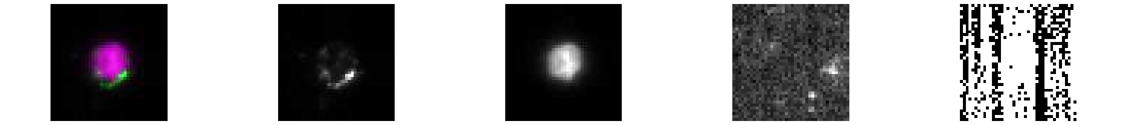				

Ereignis	Aufnahme	DAPI/CK-PE	CK-PE	DAPI	CD45-APC	HER-2/neu
4879	40	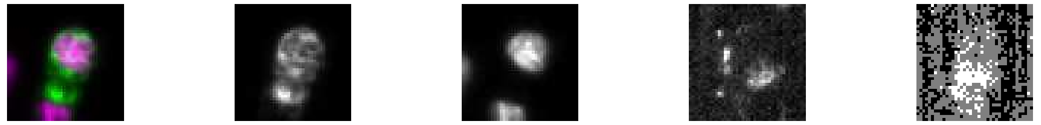				
4880	40	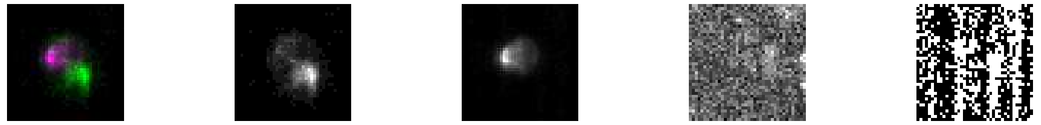				
4881	40	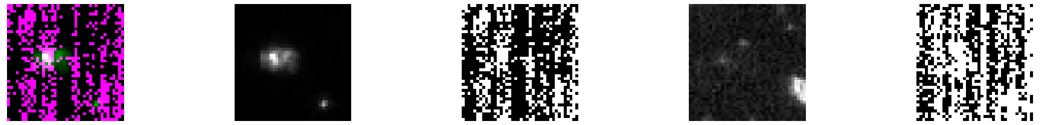				
4882	40	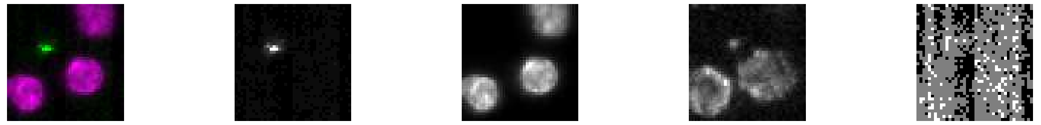				
4883	40	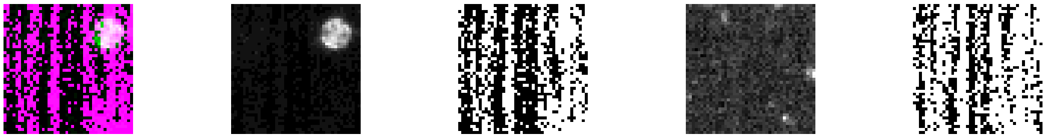				
4884	40	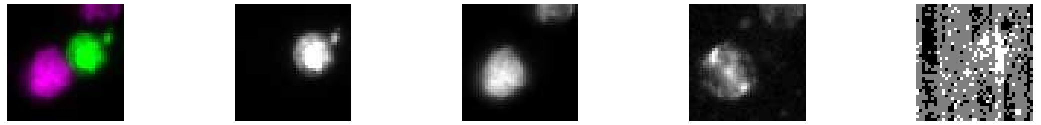				
4885	40	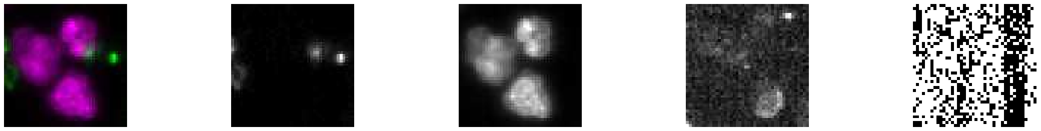				
4886	40	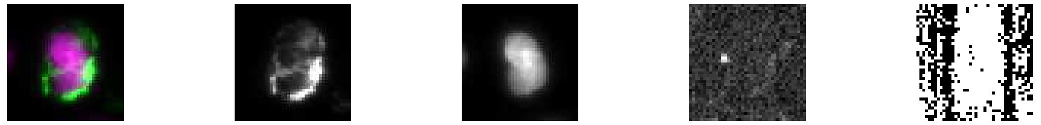				
4887	40	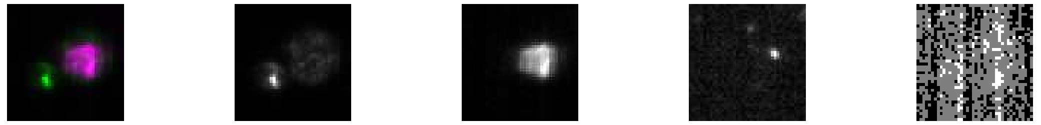				
4888	40	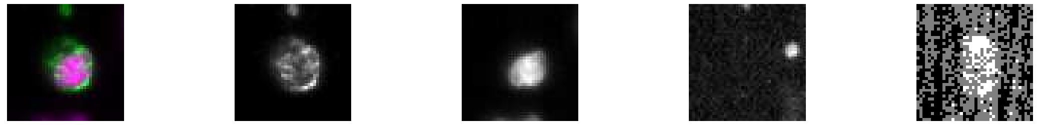				
4889	40	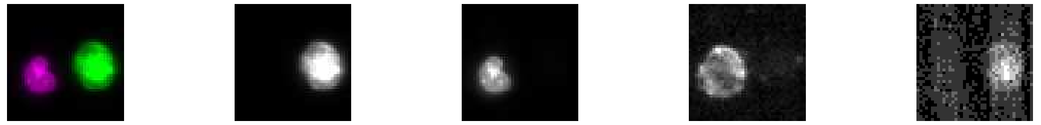				
4890	40	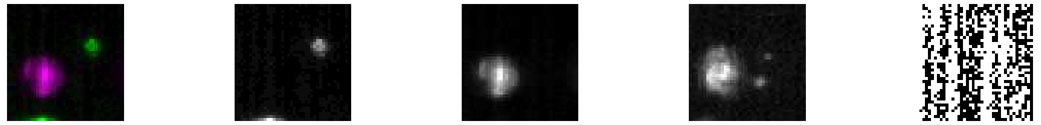				
4891	40	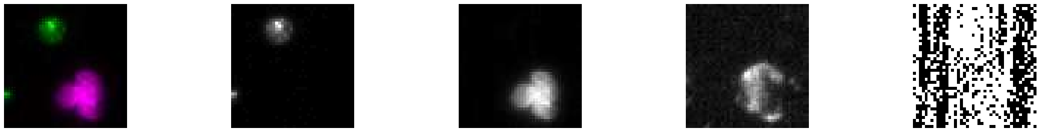				
4892	40	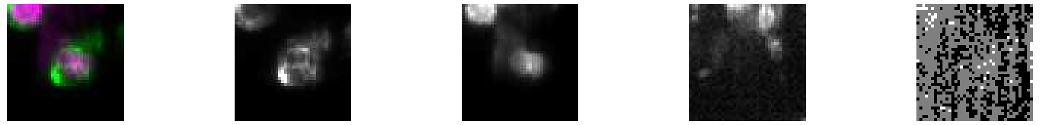				

Proben-ID: Gyn14.208 **Kassetten-ID:**01327106 **Patienten-ID:**

Ereignis	Aufnahme	DAPI/CK-PE	CK-PE	DAPI	CD45-APC	HER-2/neu
4893	40	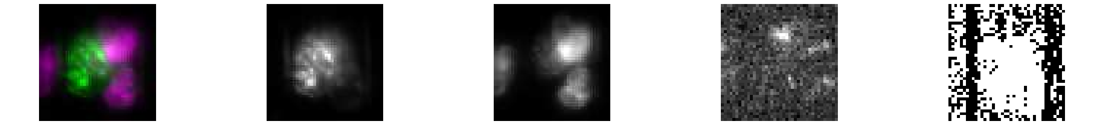				
4894	40	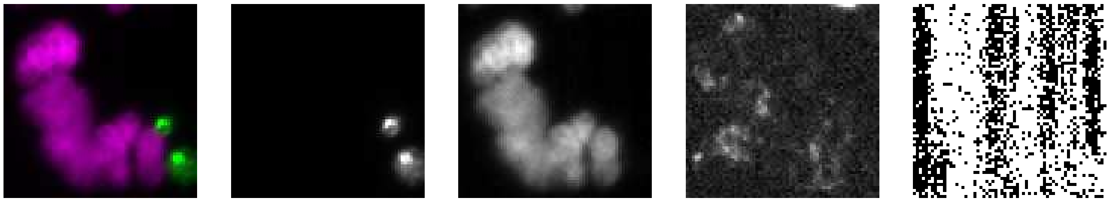				
4895	40	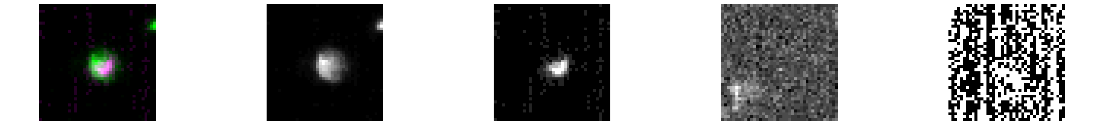				
4896	40	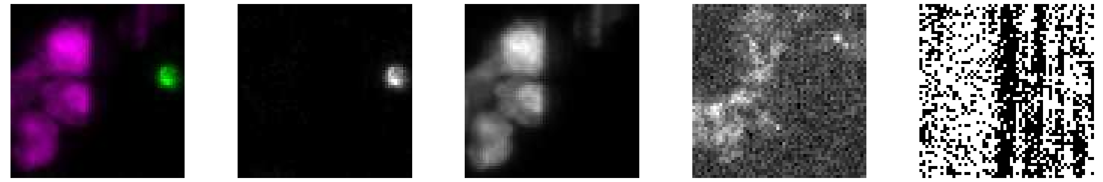				
4897	40	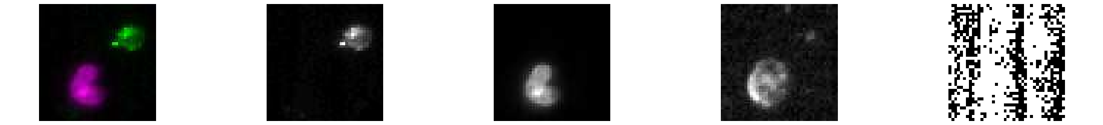				
4898	40	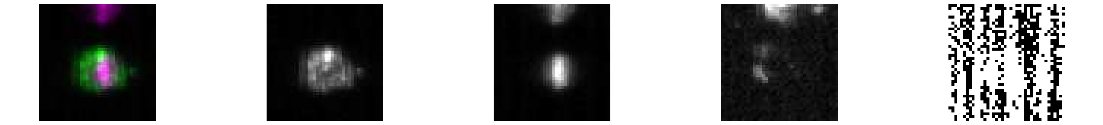				
4899	40	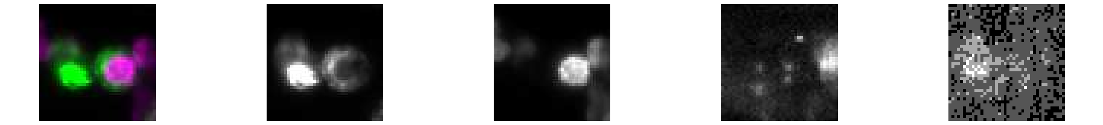				
4900	40	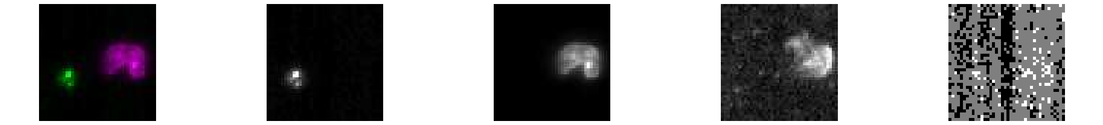				
4901	40	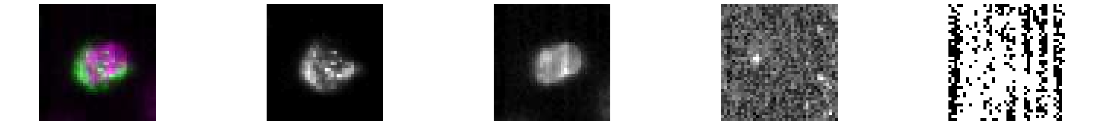				
4902	40	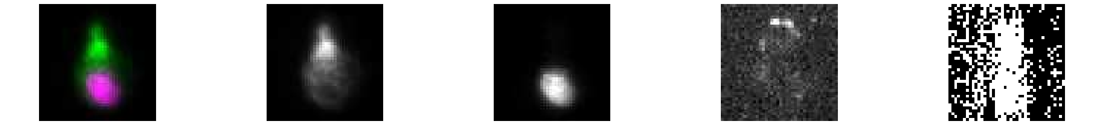				
4903	40	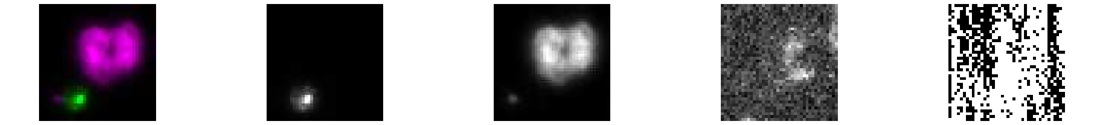				
4904	40	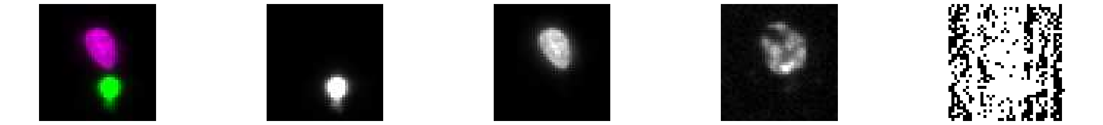				
4905	40	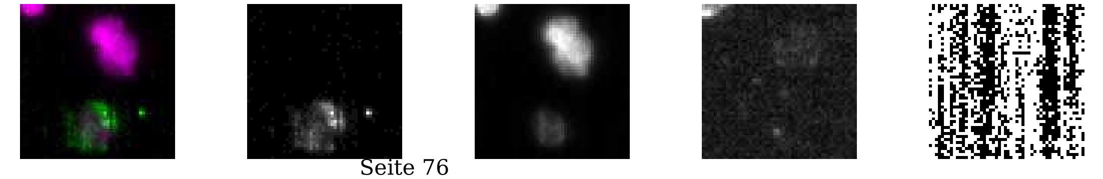				

Ereignis	Aufnahme	DAPI/CK-PE	CK-PE	DAPI	CD45-APC	HER-2/neu
4906	40	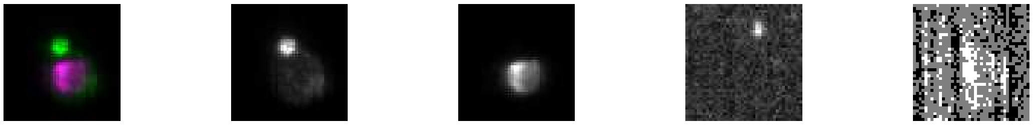				
4907	40	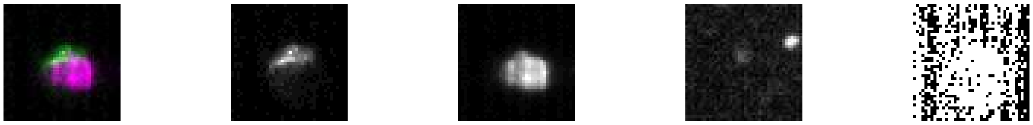				
4908	40	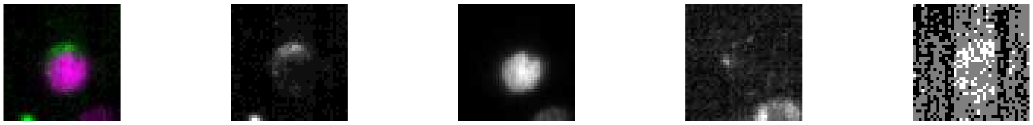				
4909	40	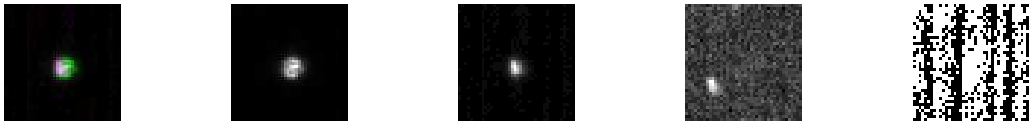				
4910	40	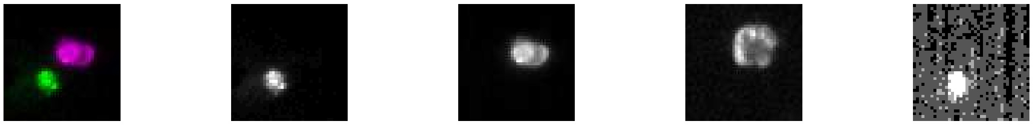				
4911	40	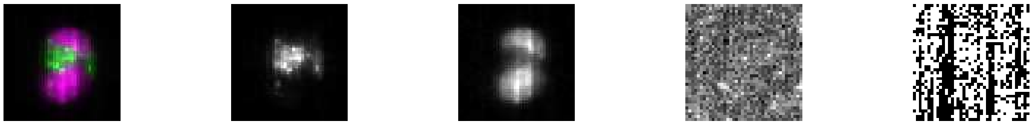				
4912	40	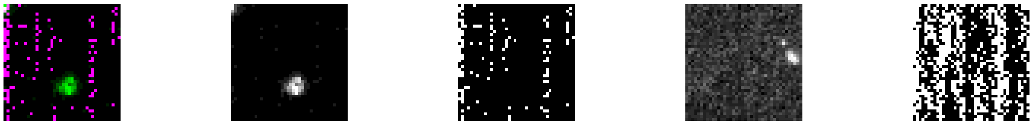				
4913	40	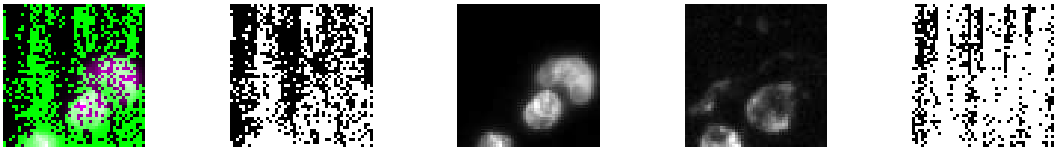				
4914	40	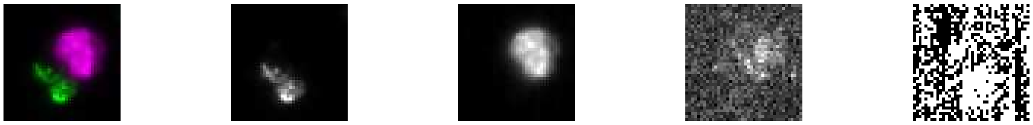				
4915	40	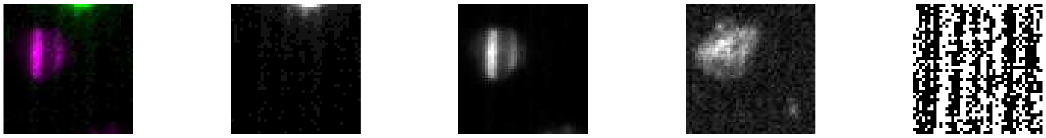				
4916	40	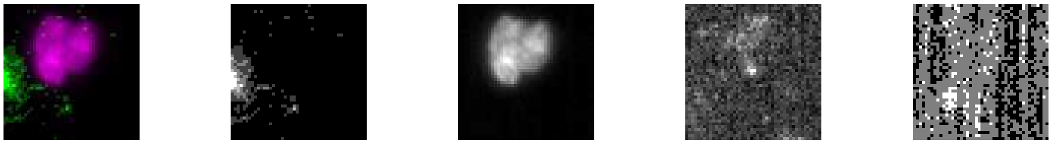				
4917	40	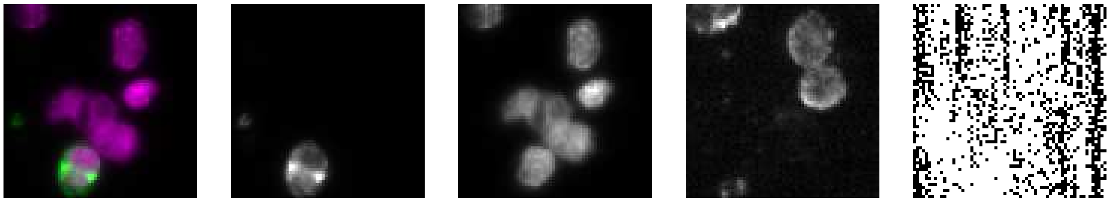				
4918	40	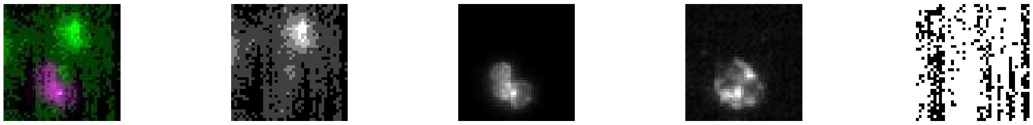				

Ereignis	Aufnahme	DAPI/CK-PE	CK-PE	DAPI	CD45-APC	HER-2/neu
4919	40	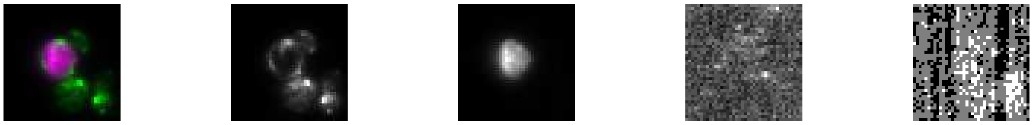				
4920	40	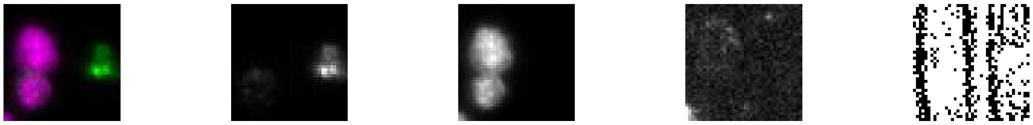				
4921	40	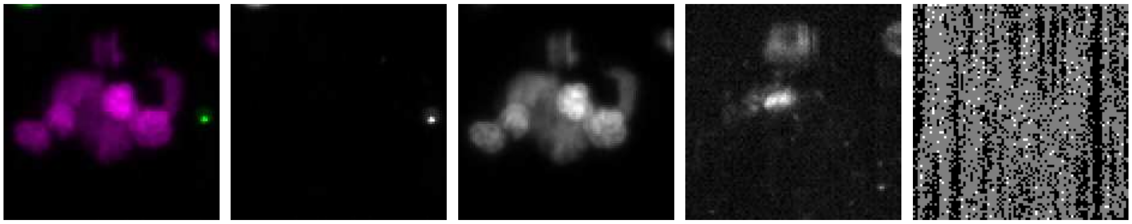				
4922	40	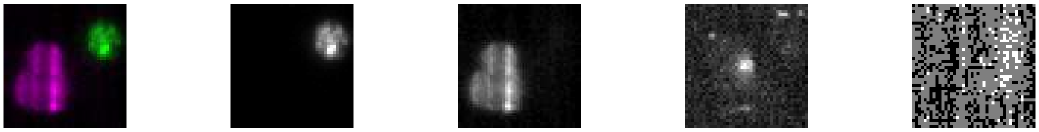				
4923	40	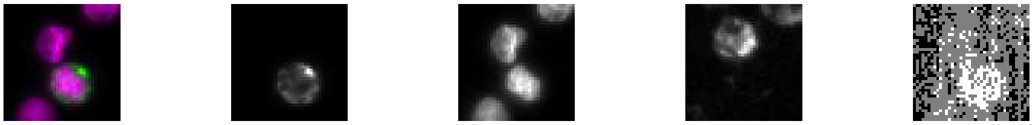				
4924	40	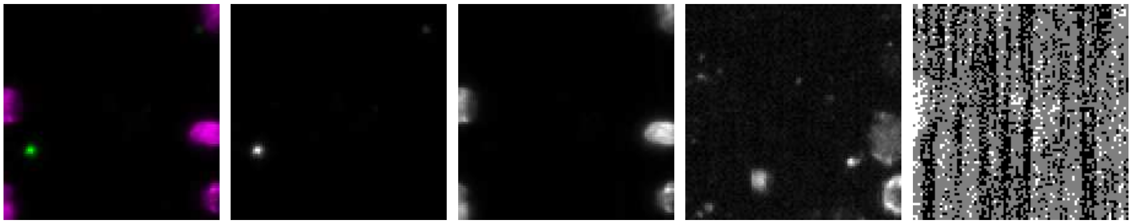				
4925	40	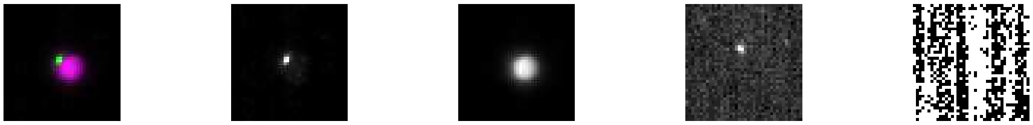				
4926	40	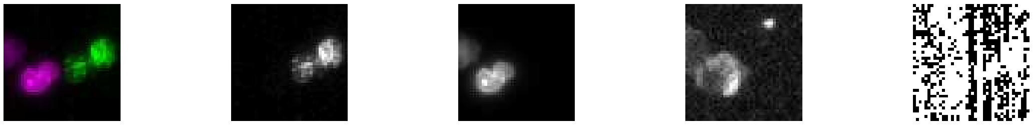				
4927	40	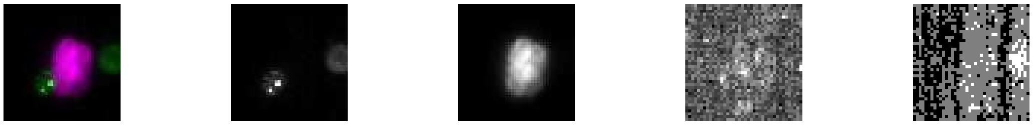				
4928	40	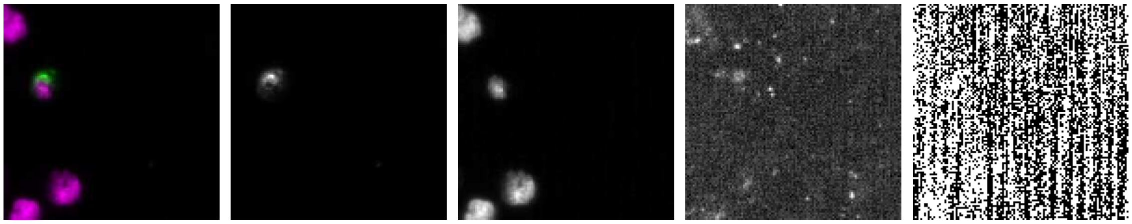				
4929	40	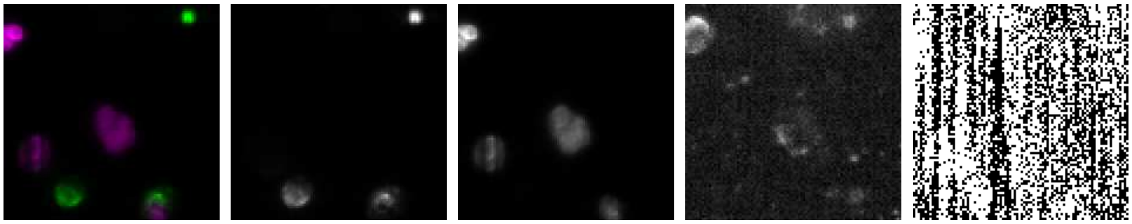				

Ereignis	Aufnahme	DAPI/CK-PE	CK-PE	DAPI	CD45-APC	HER-2/neu
4930	40	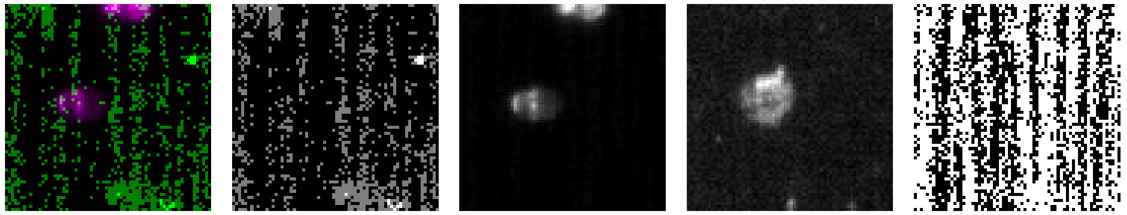				
4931	40	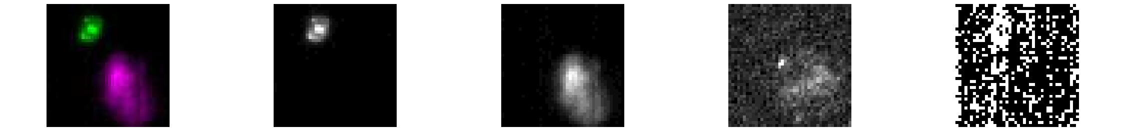				
4932	40	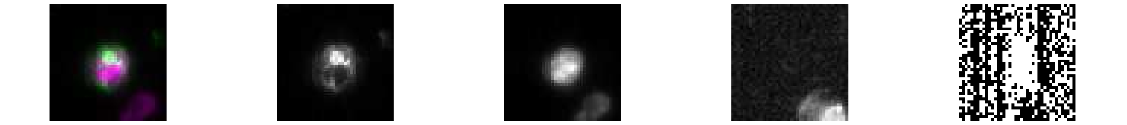				
4933	40	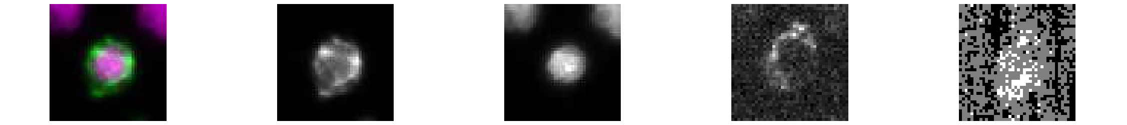				
4934	40	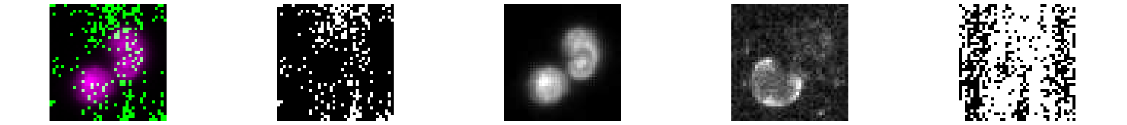				
4935	40	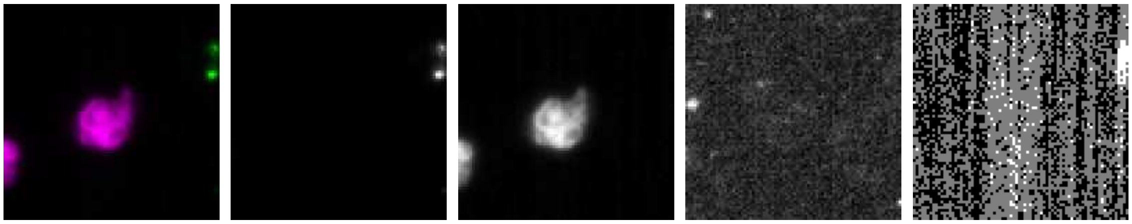				
4936	40	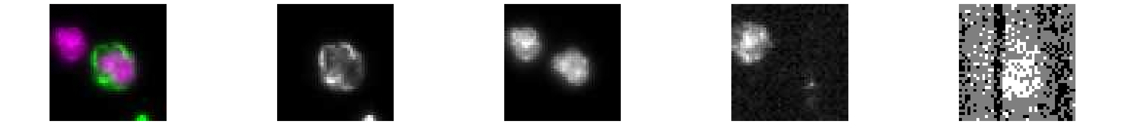				
4937	40	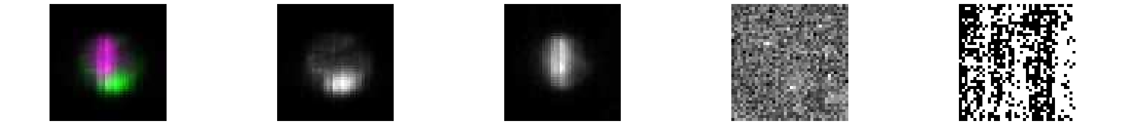				
4938	40	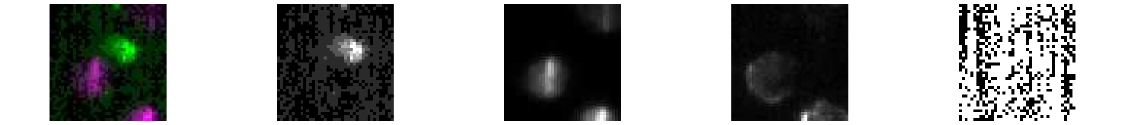				
4939	40	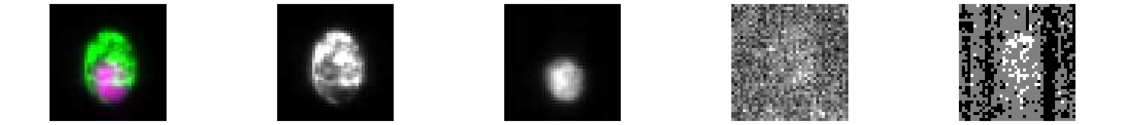				
4940	40	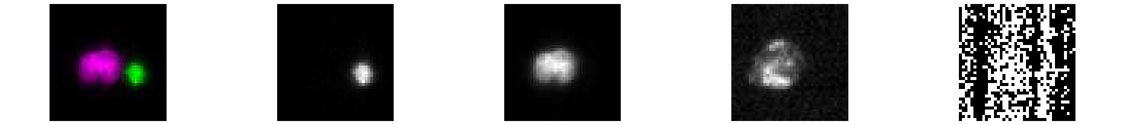				
4941	40	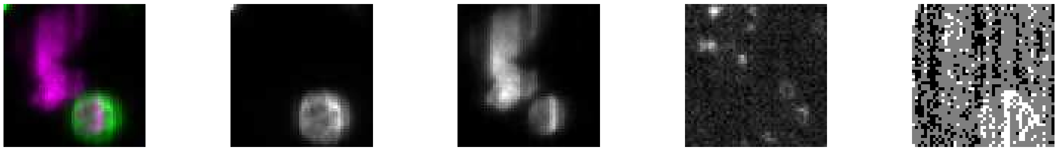				

Ereignis	Aufnahme	DAPI/CK-PE	CK-PE	DAPI	CD45-APC	HER-2/neu
4942	40	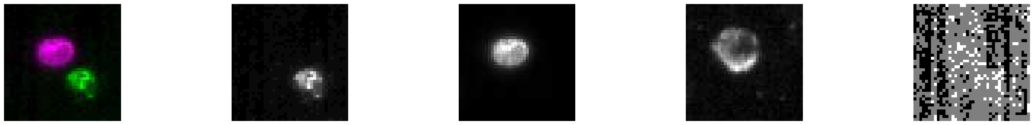				
4943	40	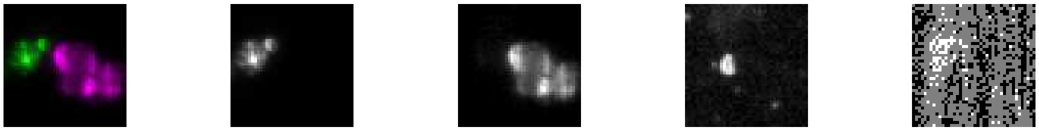				
4944	40	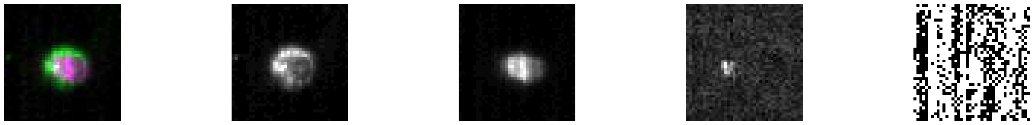				
4945	40	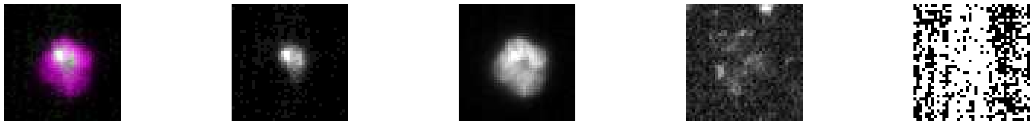				
4946	40	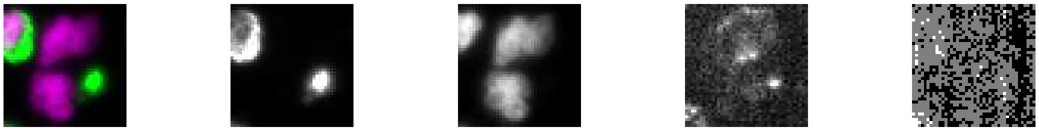				
4947	40	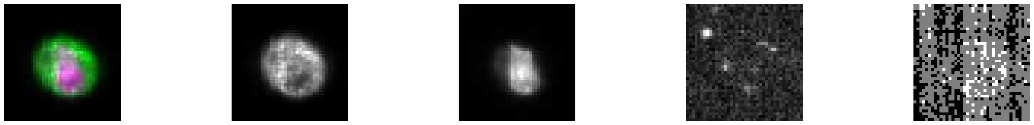				
4948	40	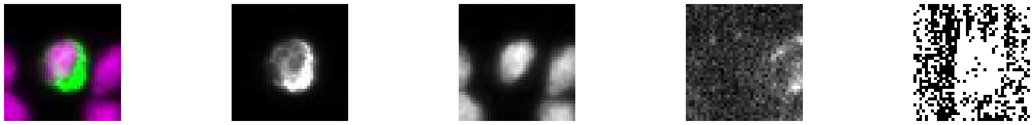				
4949	40	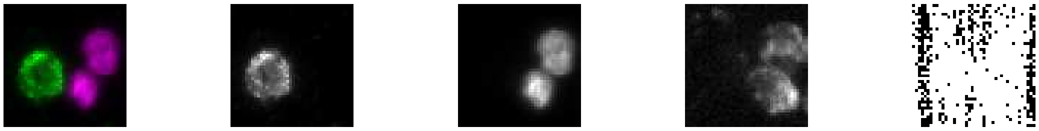				
4950	40	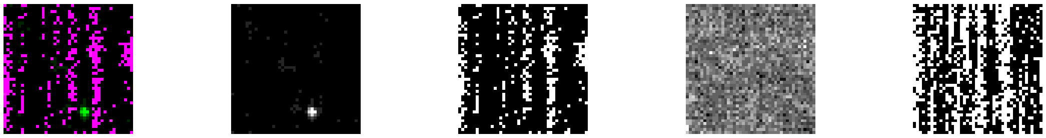				
4951	40	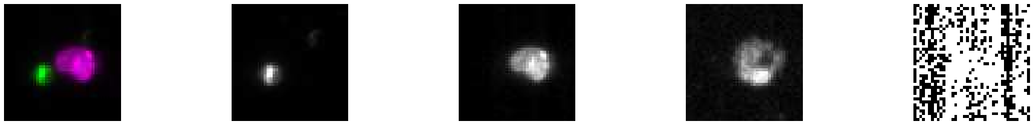				
4952	40	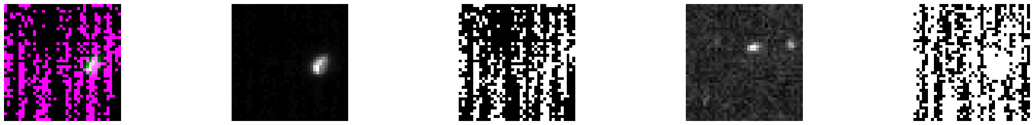				
4953	40	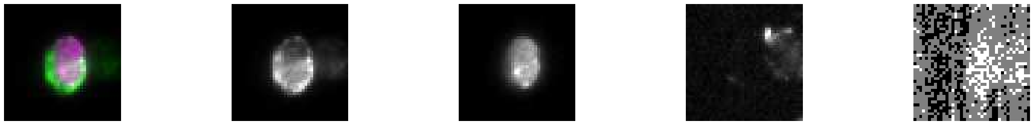				
4954	40	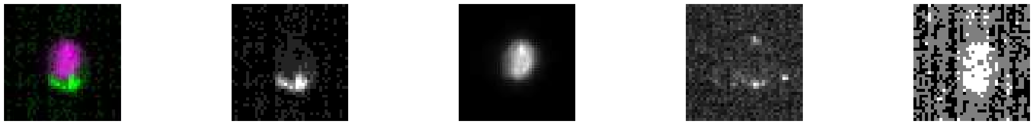				
4955	40	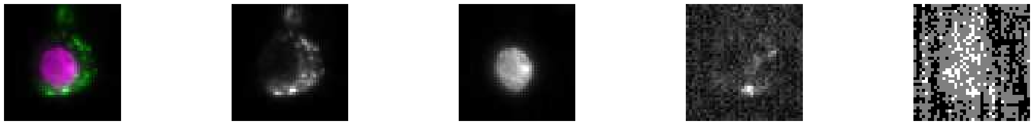				

Ereignis	Aufnahme	DAPI/CK-PE	CK-PE	DAPI	CD45-APC	HER-2/neu
4956	40	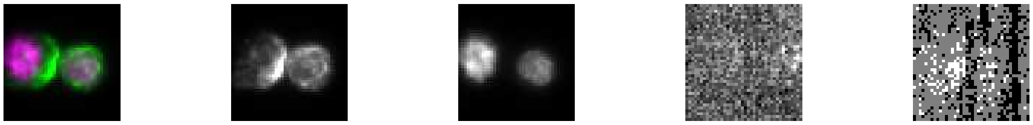				
4957	40	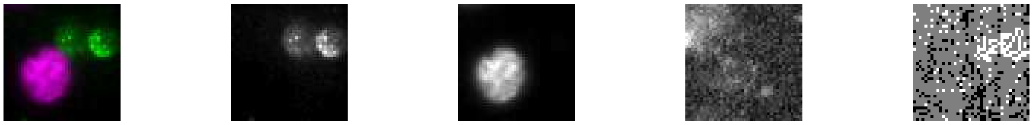				
4958	40	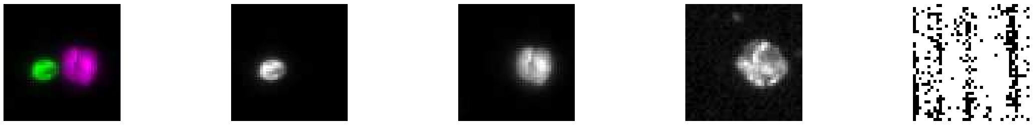				
4959	40	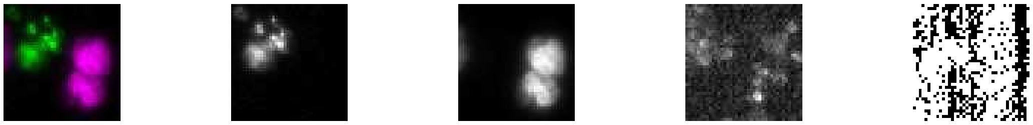				
4960	40	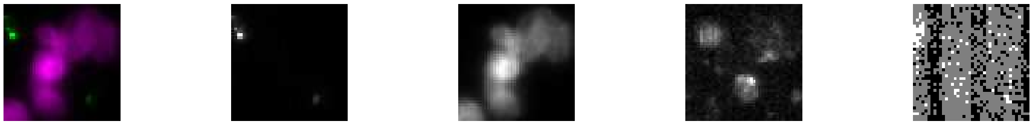				
4961	40	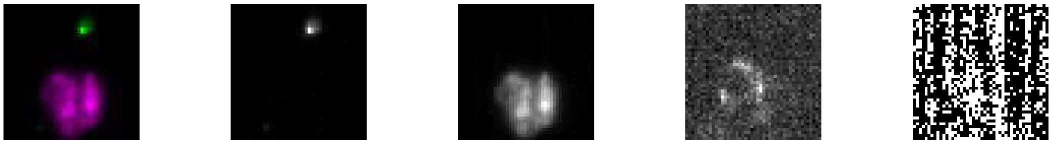				
4962	40	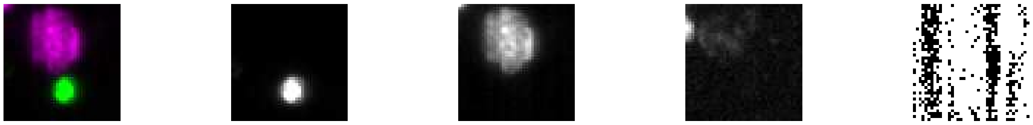				
4963	40	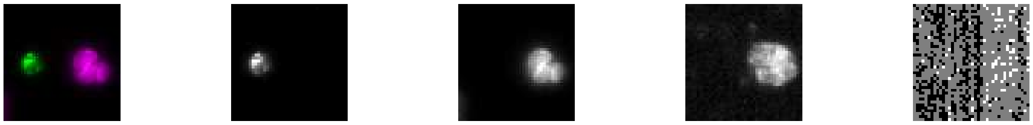				
4964	40	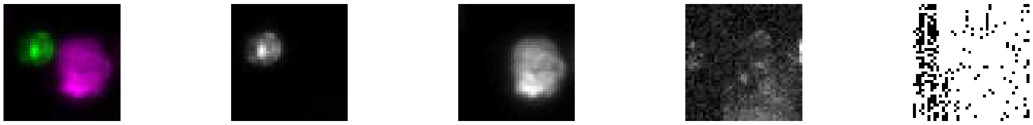				
4965	40	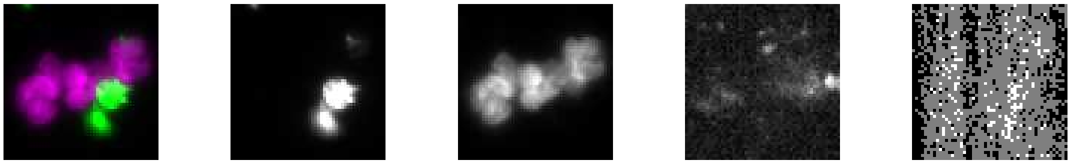				
4966	40	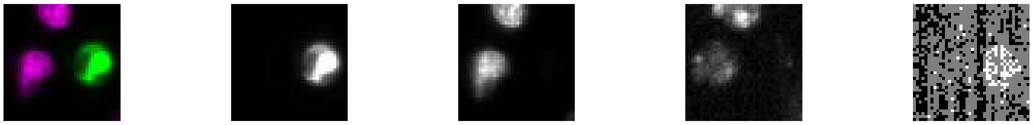				
4967	40	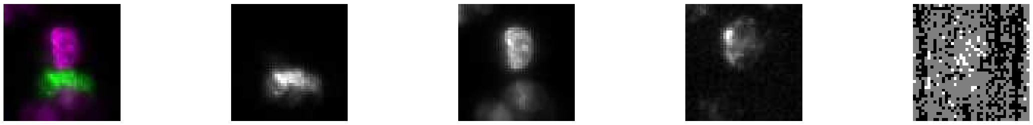				
4968	40	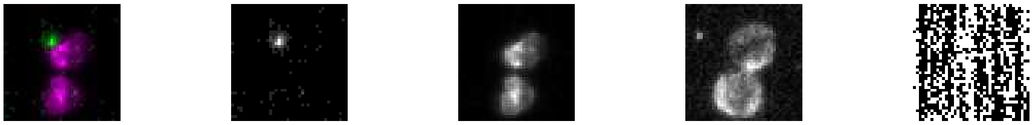				

Ereignis	Aufnahme	DAPI/CK-PE	CK-PE	DAPI	CD45-APC	HER-2/neu
4969	40	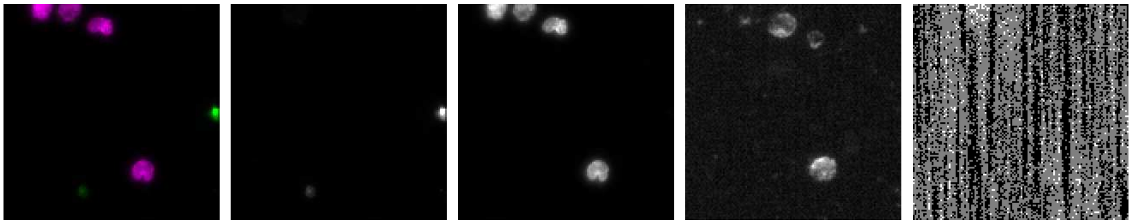				
4970	40	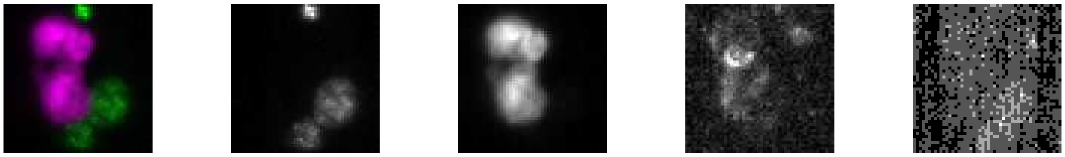				
4971	40	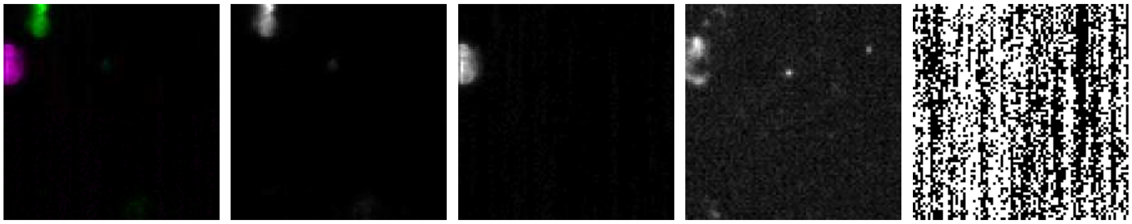				
4972	40	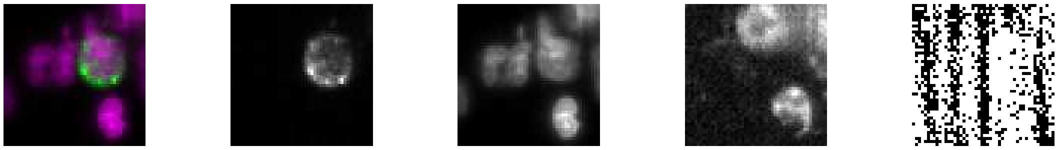				
4973	40	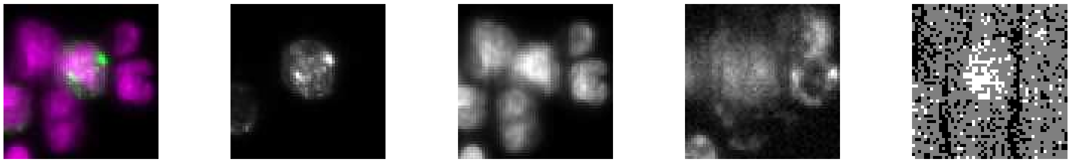				
4974	40	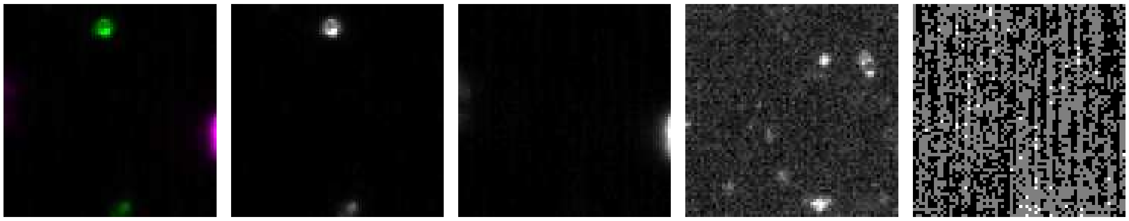				
4975	40	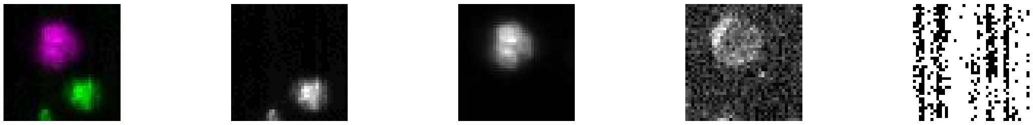				
4976	40	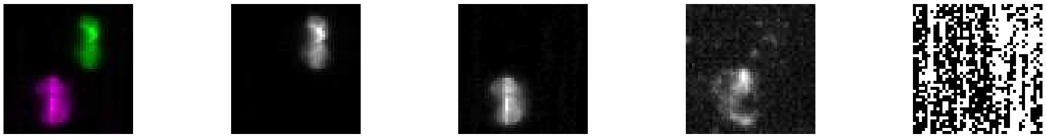				
4977	40	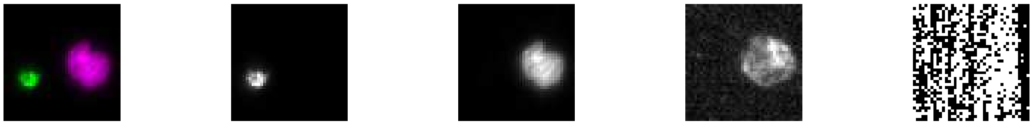				
4978	40	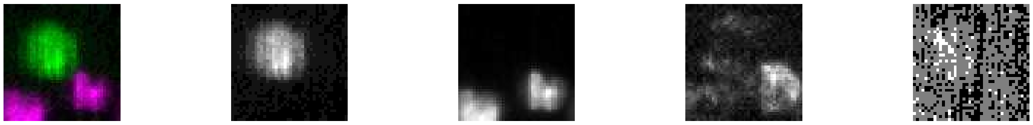				
4979	40	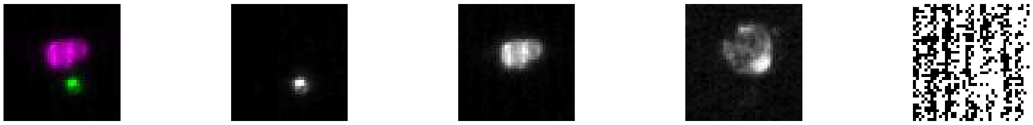				

Ereignis	Aufnahme	DAPI/CK-PE	CK-PE	DAPI	CD45-APC	HER-2/neu
4980	40	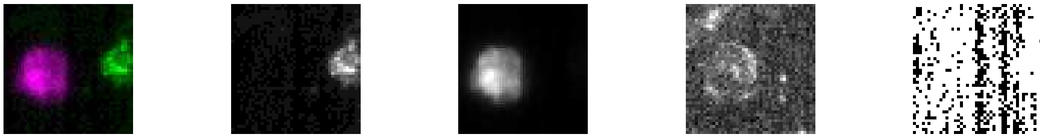				
4981	40	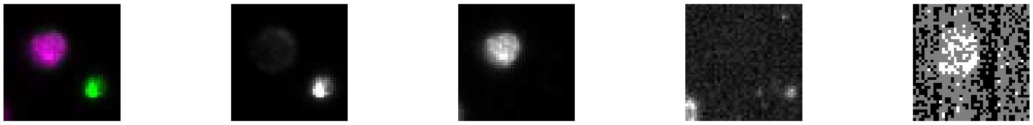				
4982	40	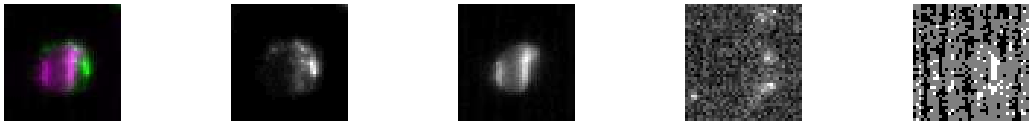				
4983	40	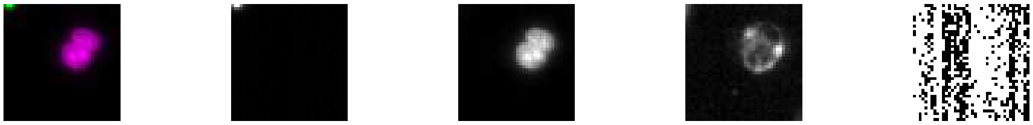				
4984	40	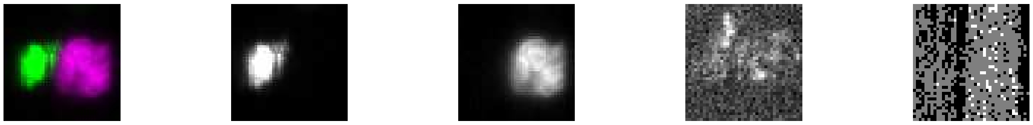				
4985	40	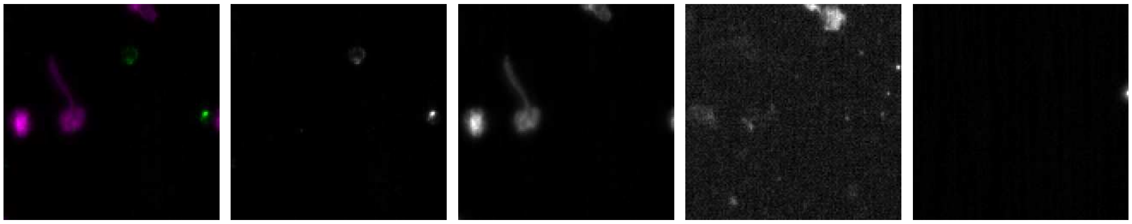				
4986	40	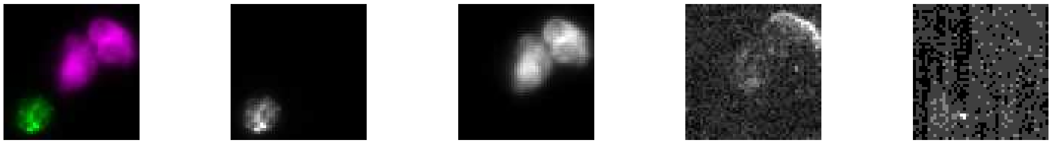				
4987	40	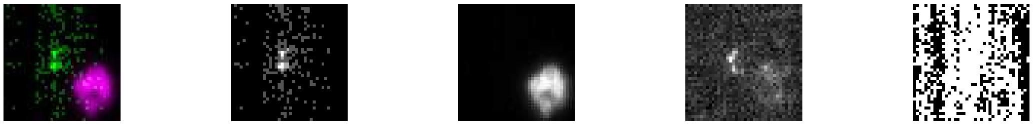				
4988	40	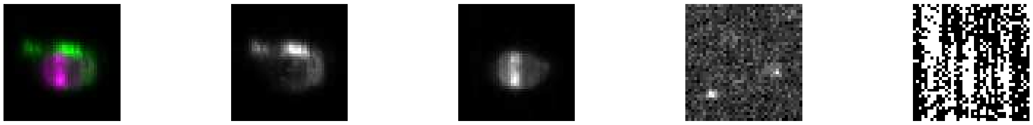				
4989	40	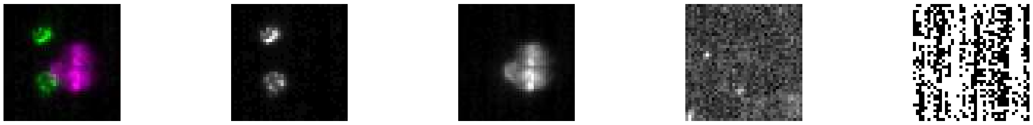				
4990	40	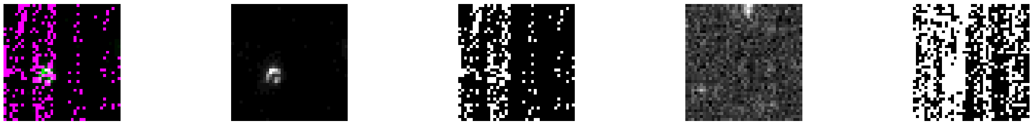				
4991	40	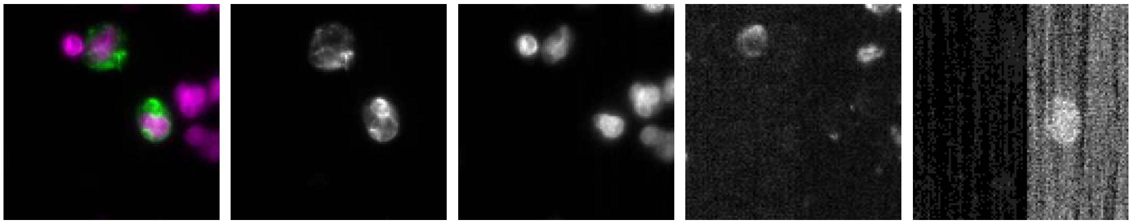				

Druckdatum:02/06/2020 16:41

Proben-ID: Gyn14.208 **Kassetten-ID:**01327106 **Patienten-ID:**

Ereignis	Aufnahme	DAPI/CK-PE	CK-PE	DAPI	CD45-APC	HER-2/neu
4992	40	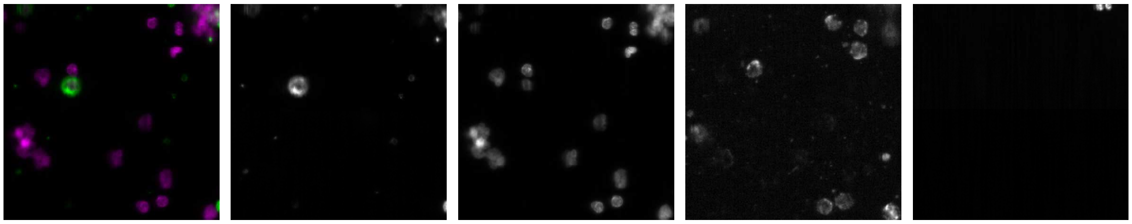				
4993	40	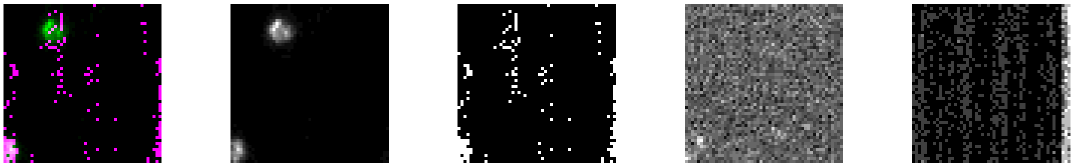				
4994	40	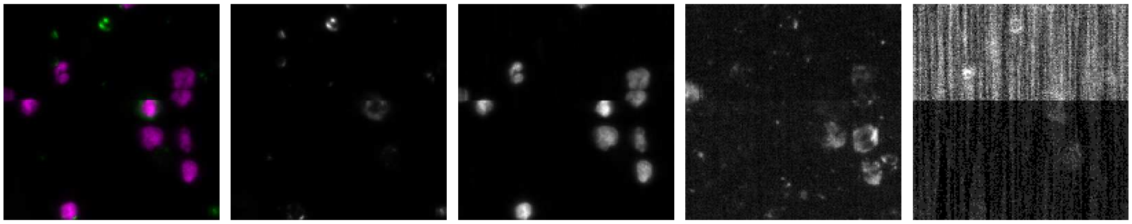				
4995	31	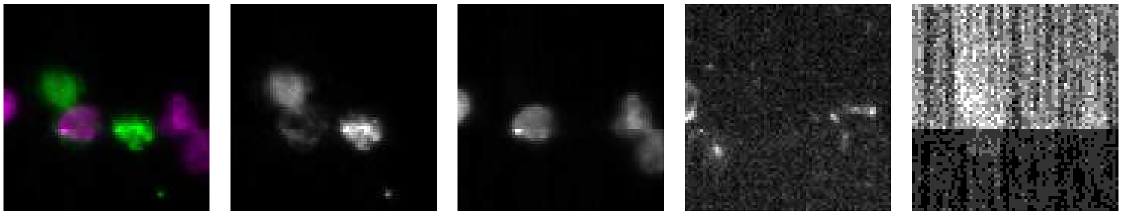				
4996	40	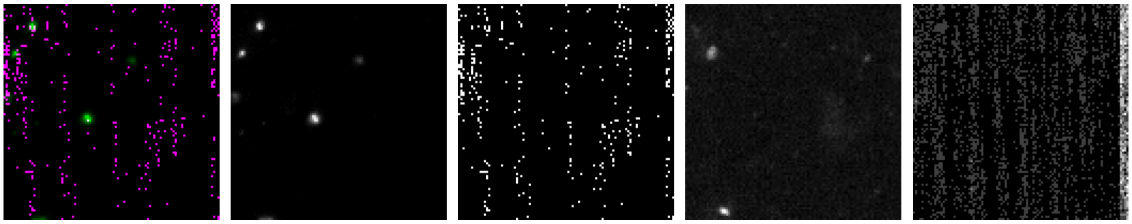				
4997	31	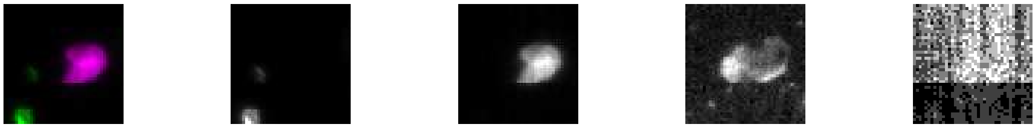				
4998	40	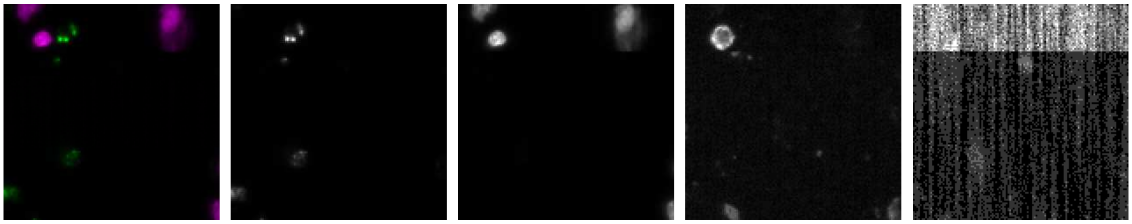				
4999	40	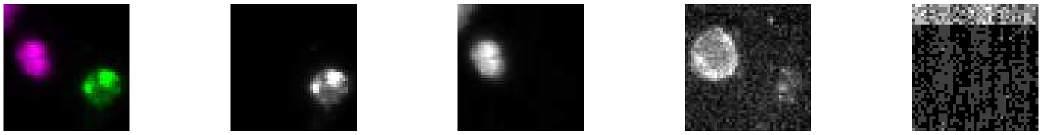				
5000	41	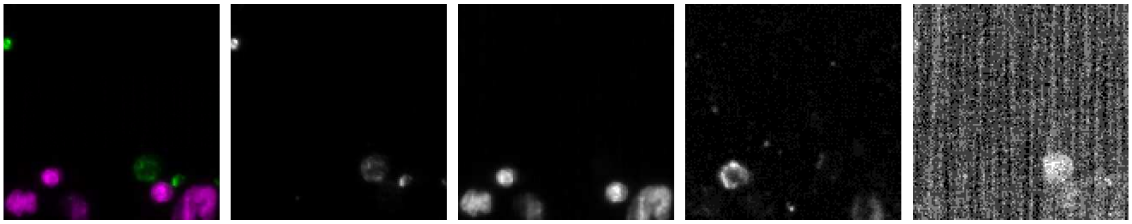				

Ereignis	Aufnahme	DAPI/CK-PE	CK-PE	DAPI	CD45-APC	HER-2/neu
5001	41	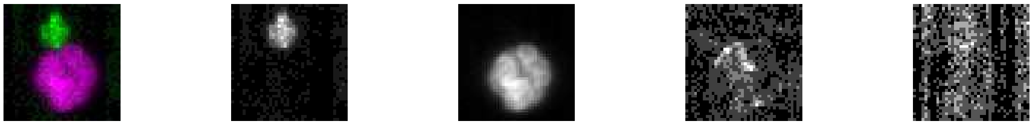				
5002	41	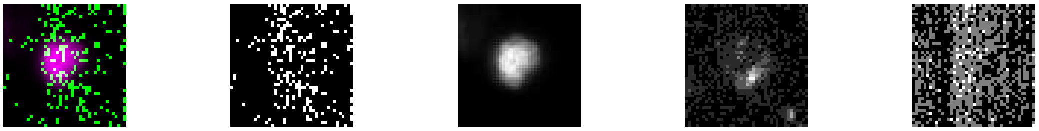				
5003	41	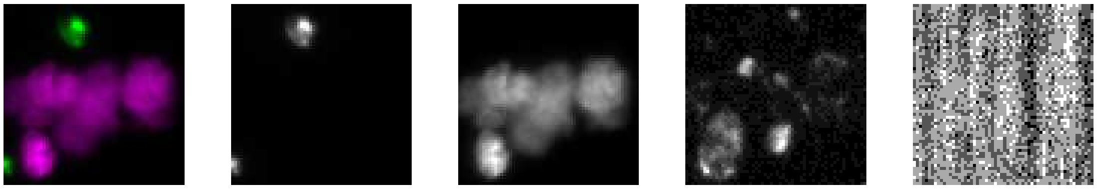				
5004	41	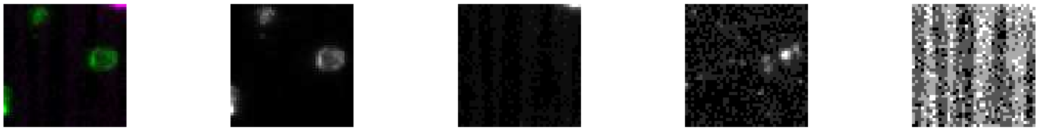				
5005	41	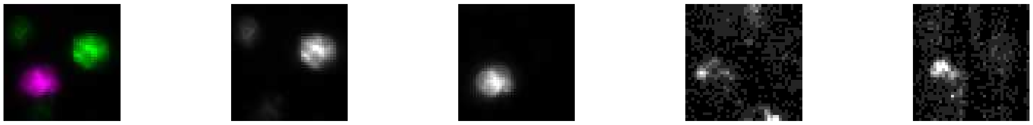				
5006	41	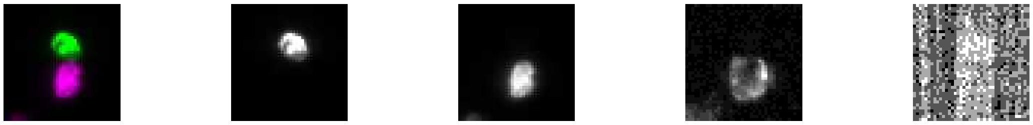				
5007	41	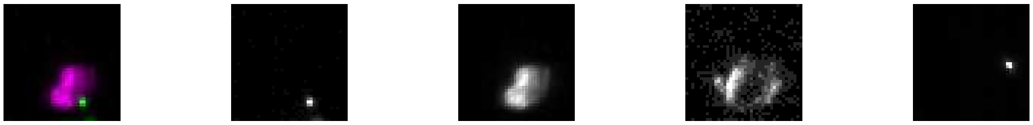				
5008	41	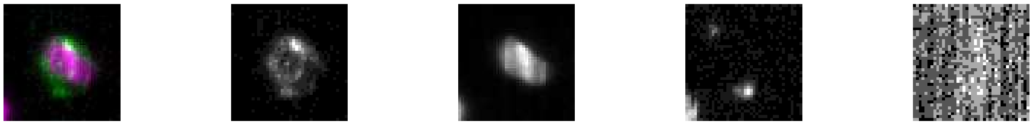				
5009	41	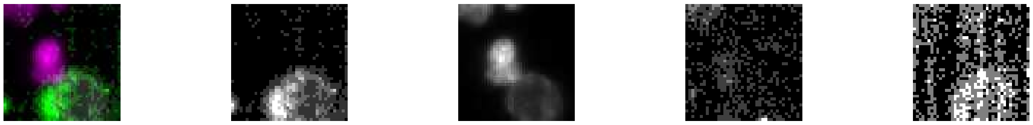				
5010	41	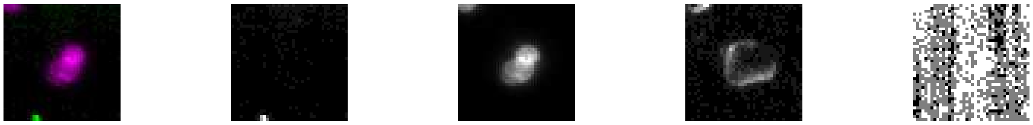				
5011	41	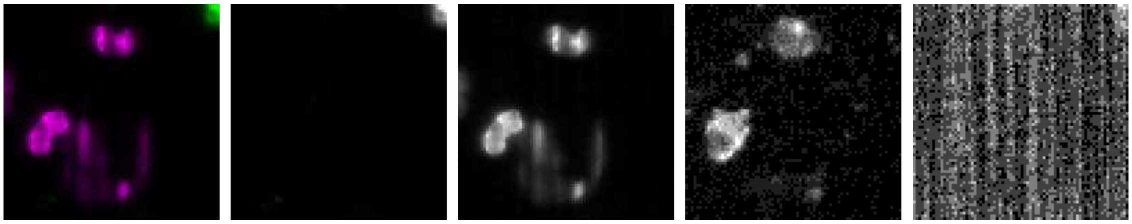				
5012	41	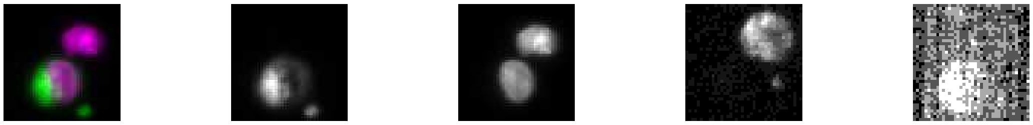				
5013	41	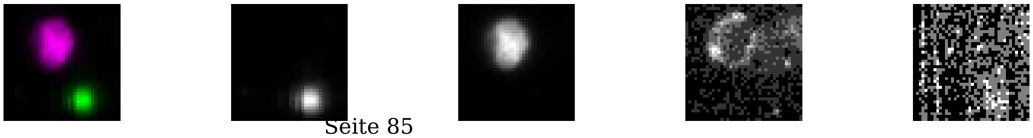				

Ereignis	Aufnahme	DAPI/CK-PE	CK-PE	DAPI	CD45-APC	HER-2/neu
5014	41	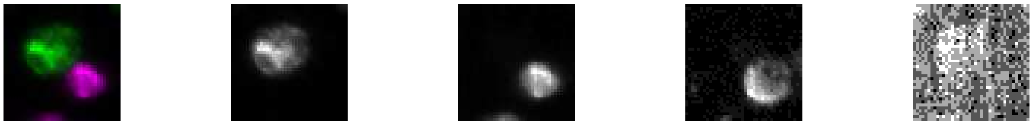				
5015	41	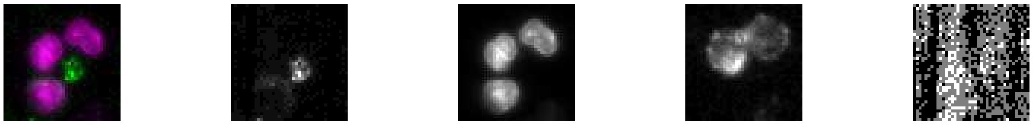				
5016	41	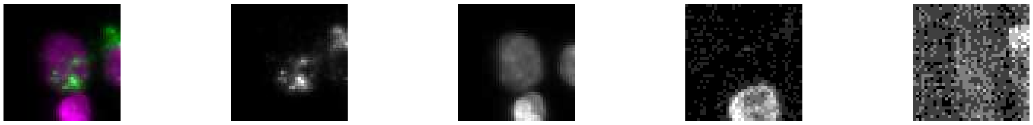				
5017	41	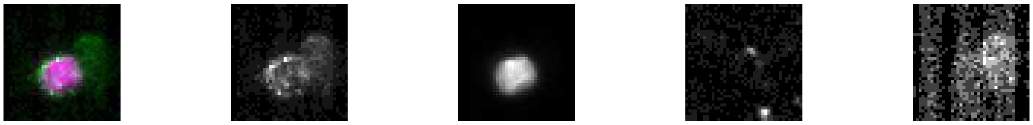				
5018	41	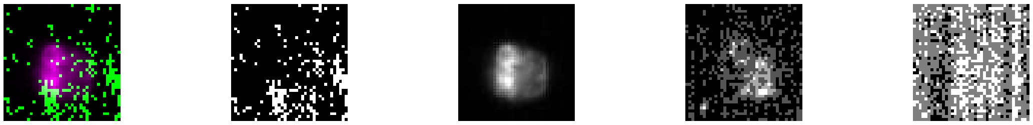				
5019	41	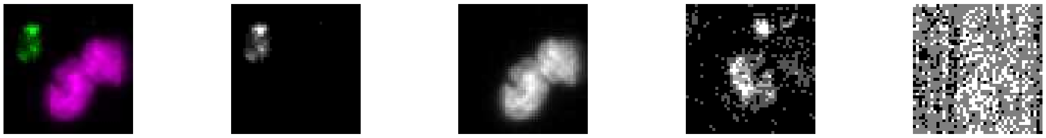				
5020	41	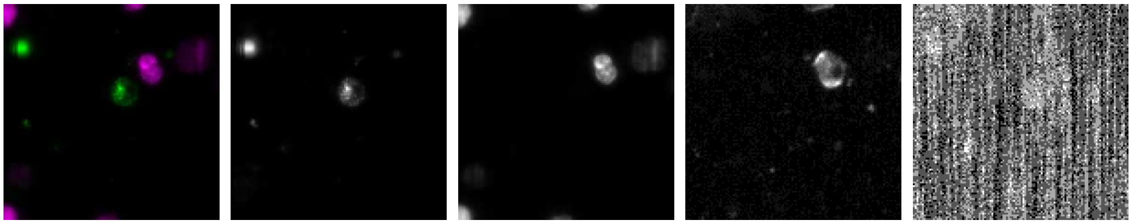				
5021	41	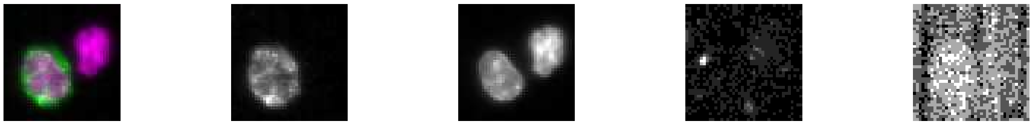				
5022	41	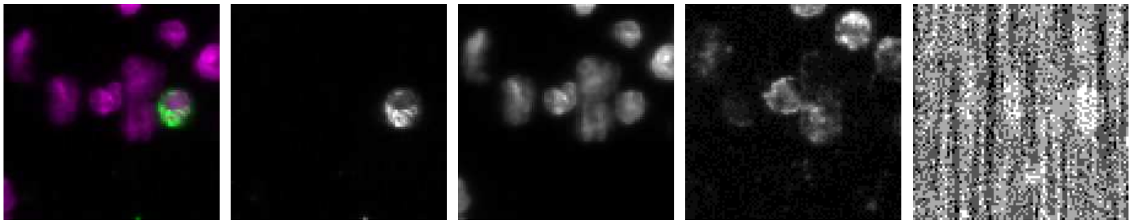				
5023	41	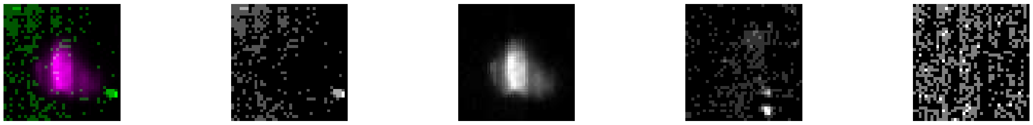				
5024	41	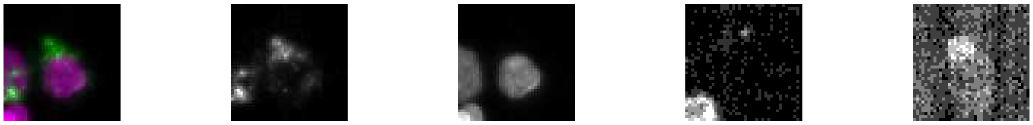				
5025	41	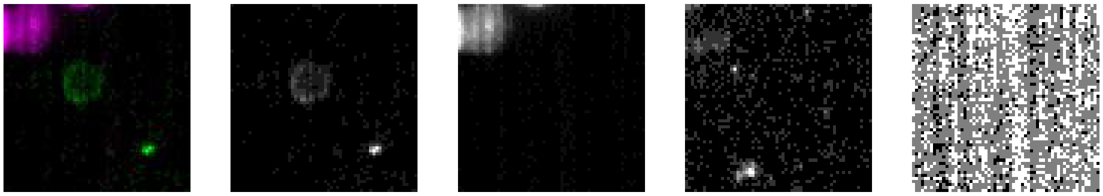				

Ereignis	Aufnahme	DAPI/CK-PE	CK-PE	DAPI	CD45-APC	HER-2/neu
5026	41	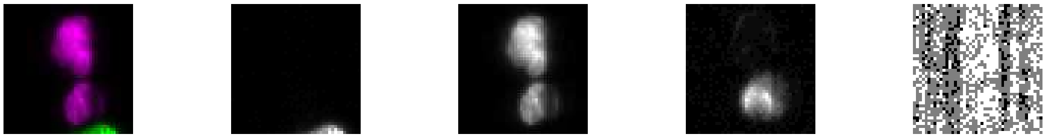				
5027	41	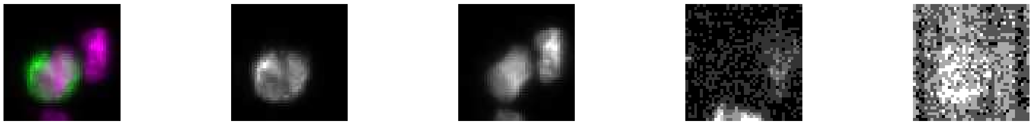				
5028	41	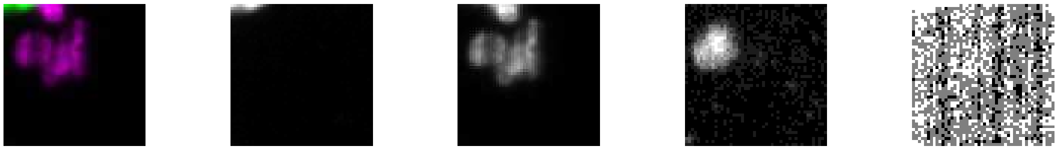				
5029	41	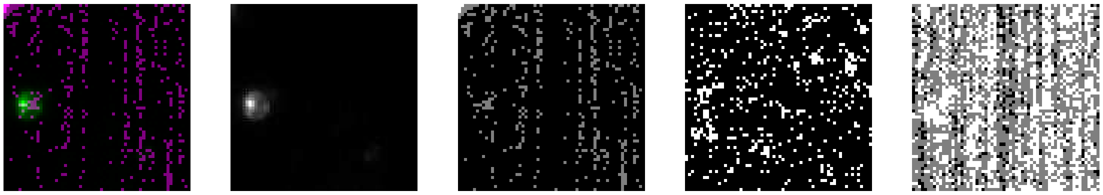				
5030	41	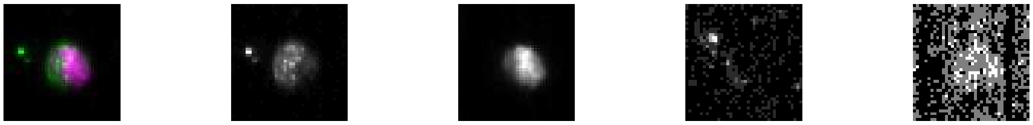				
5031	41	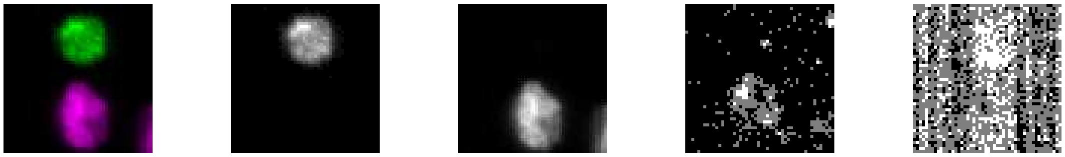				
5032	41	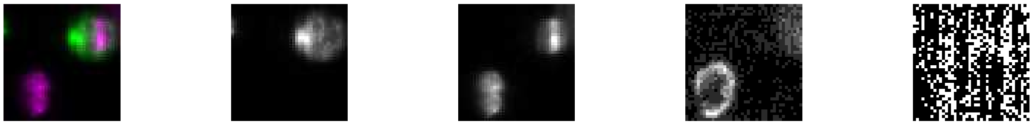				
5033	41	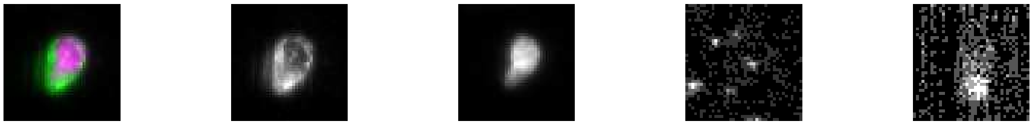				
5034	41	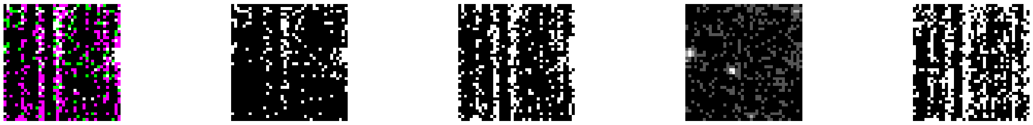				
5035	41	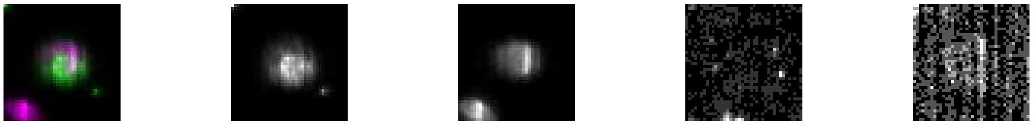				
5036	41	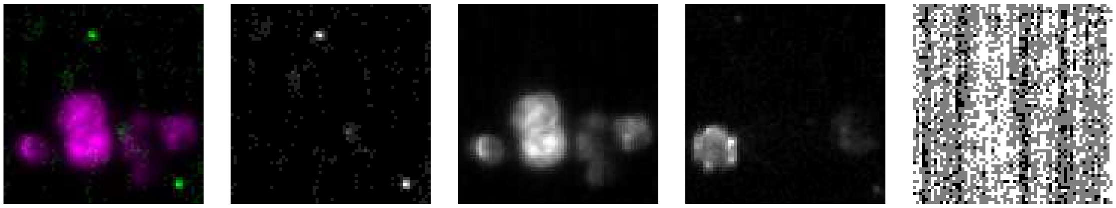				
5037	41	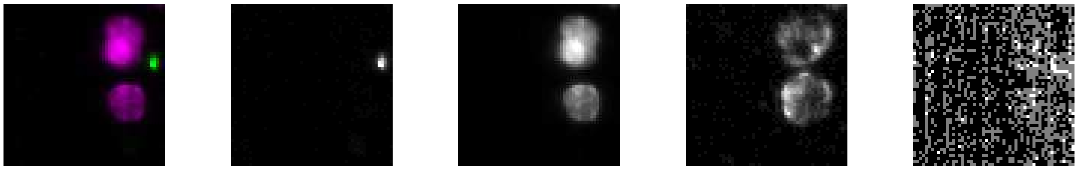				

Proben-ID: Gyn14.208 **Kassetten-ID:**01327106 **Patienten-ID:**

Ereignis	Aufnahme	DAPI/CK-PE	CK-PE	DAPI	CD45-APC	HER-2/neu
5038	41	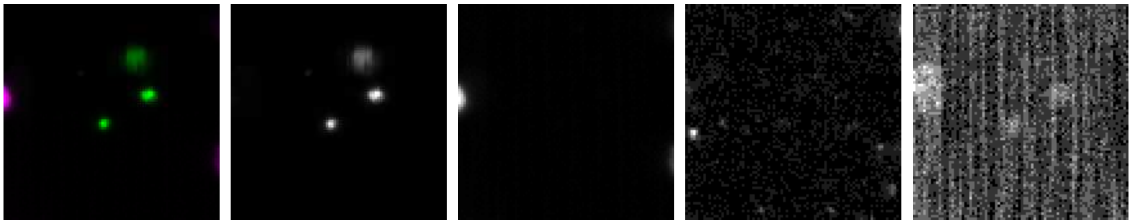				
5039	41	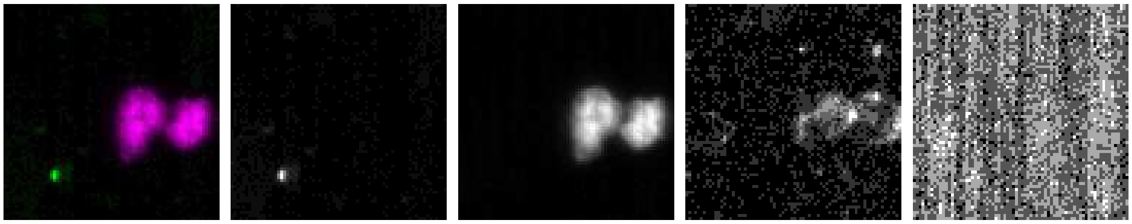				
5040	41	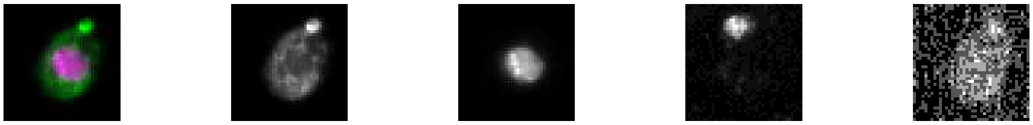				
5041	41	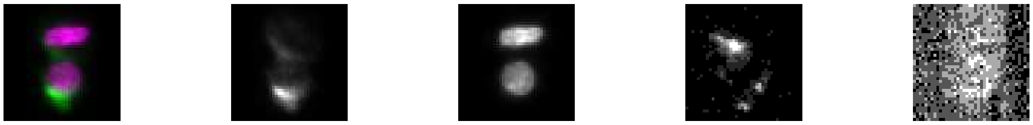				
5042	41	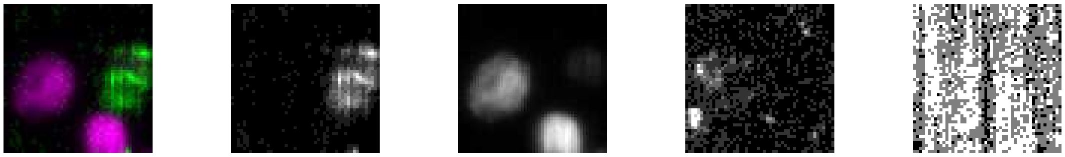				
5043	41	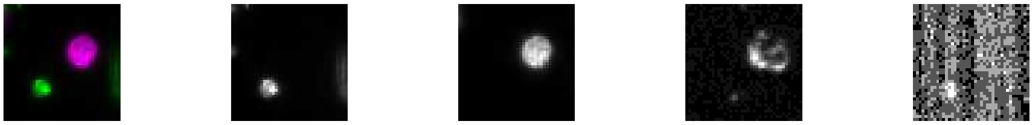				
5044	41	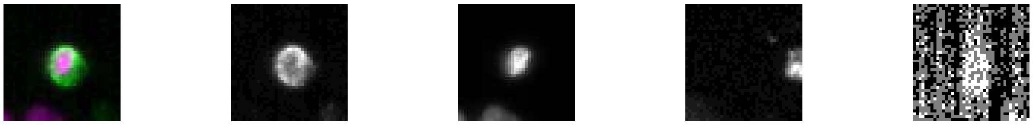				
5045	41	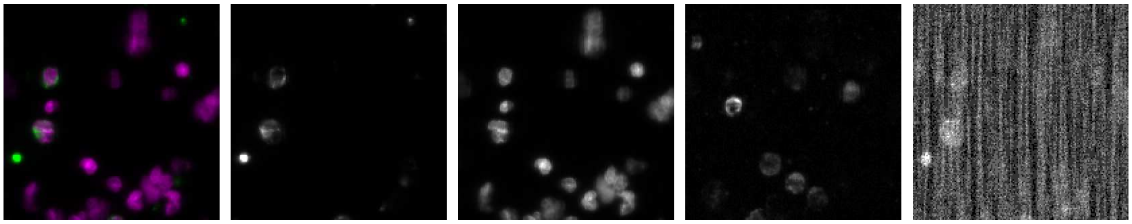				
5046	41	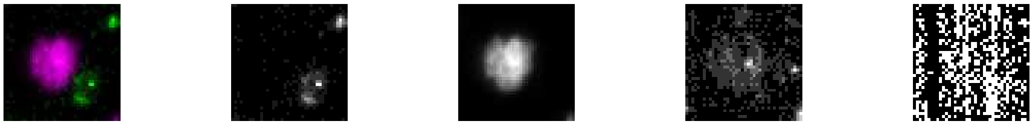				
5047	41	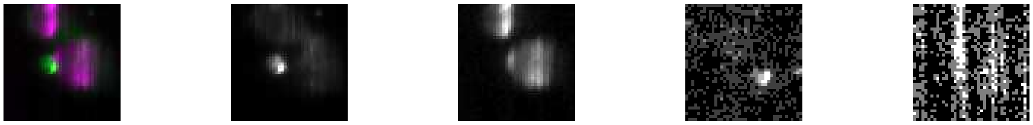				
5048	41	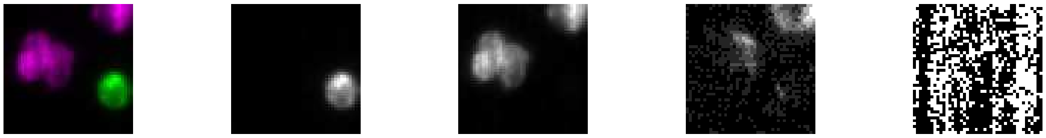				

Ereignis	Aufnahme	DAPI/CK-PE	CK-PE	DAPI	CD45-APC	HER-2/neu
5049	41	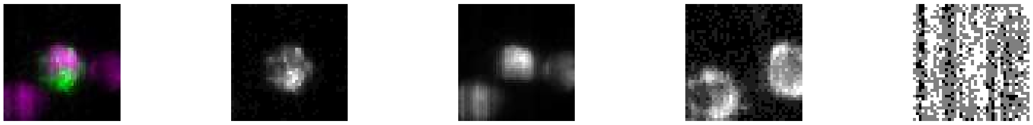				
5050	41	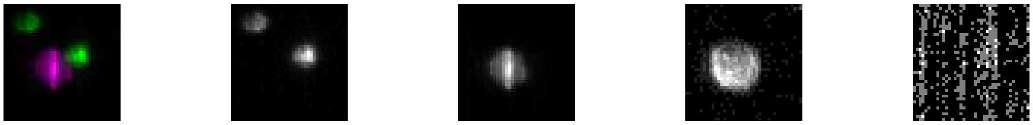				
5051	41	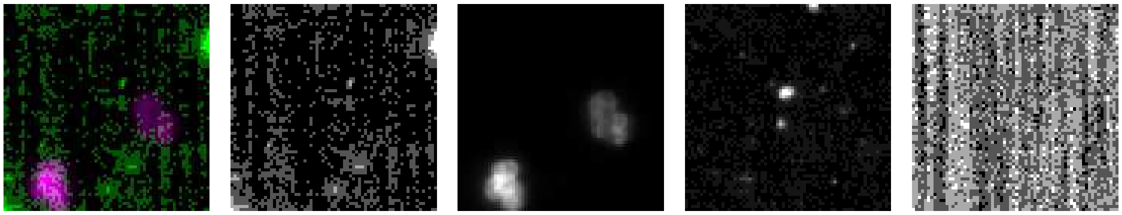				
5052	41	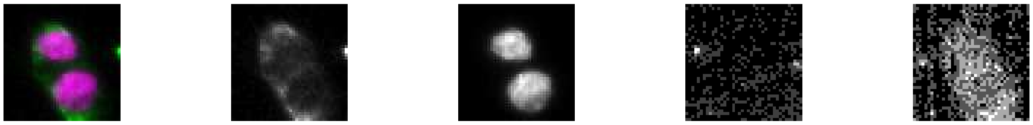				
5053	41	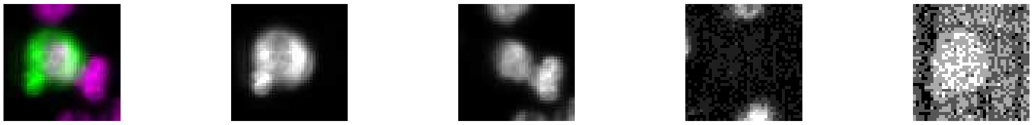				
5054	41	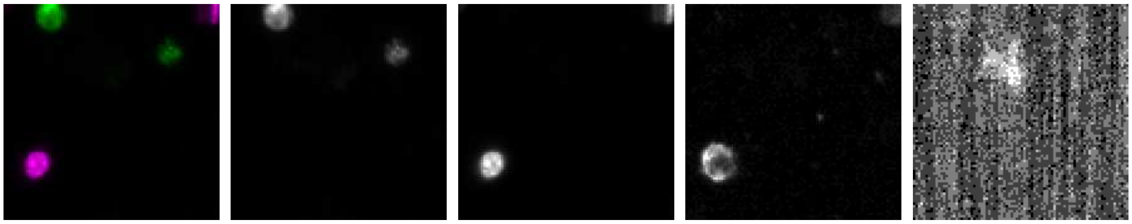				
5055	41	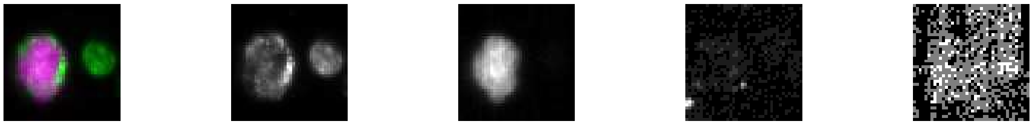				
5056	41	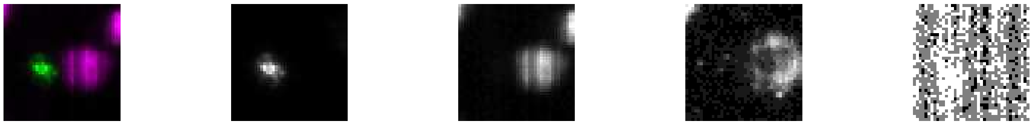				
5057	41	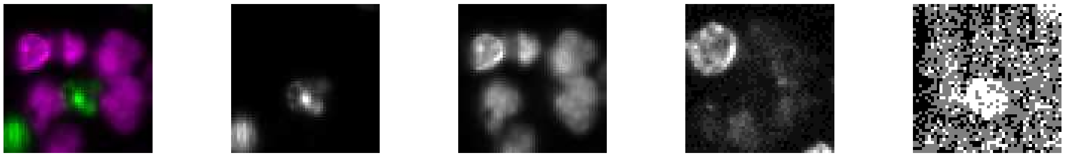				
5058	41	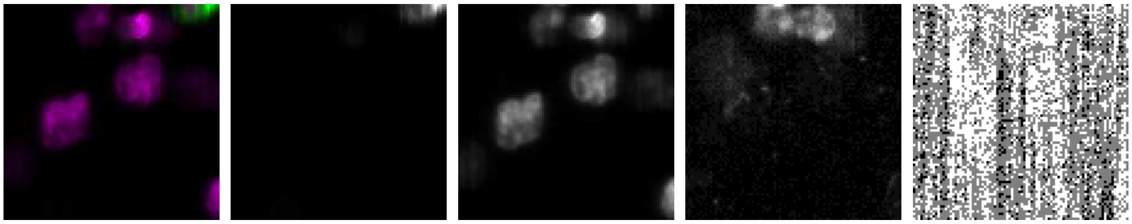				
5059	41	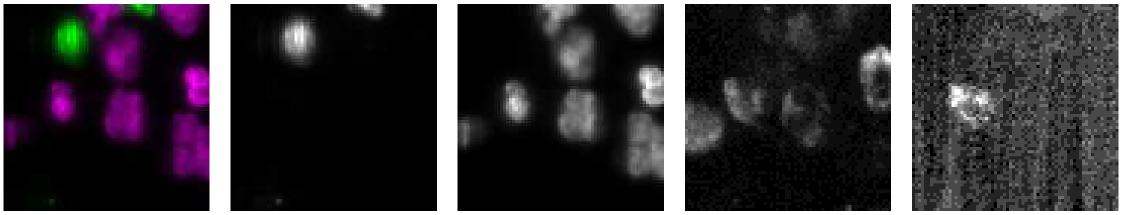				

Ereignis	Aufnahme	DAPI/CK-PE	CK-PE	DAPI	CD45-APC	HER-2/neu
5060	41	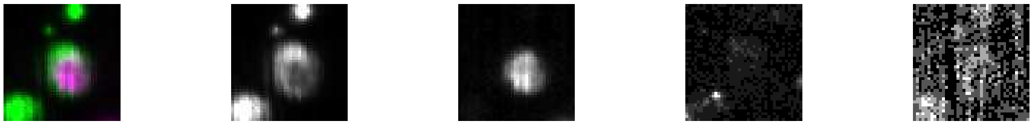				
5061	41	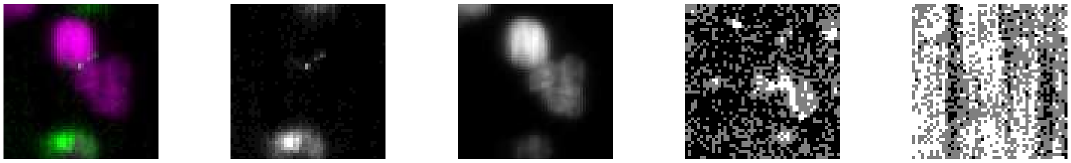				
5062	41	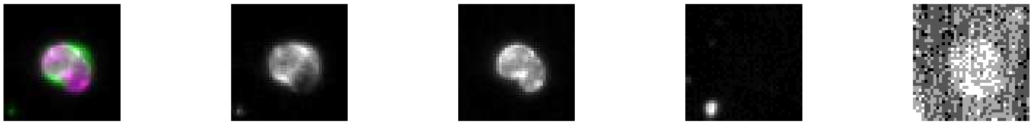				
5063	41	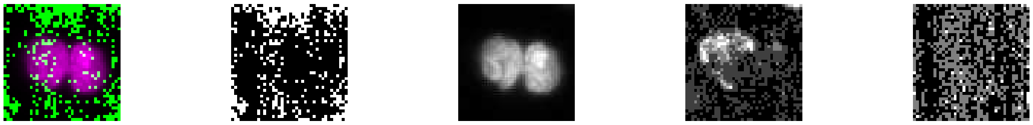				
5064	41	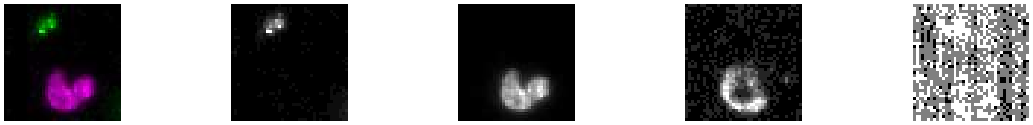				
5065	41	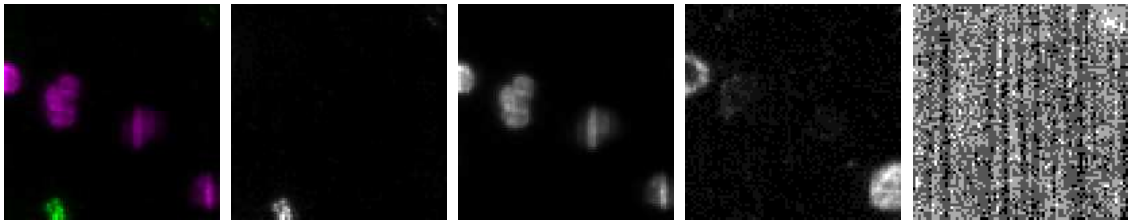				
5066	41	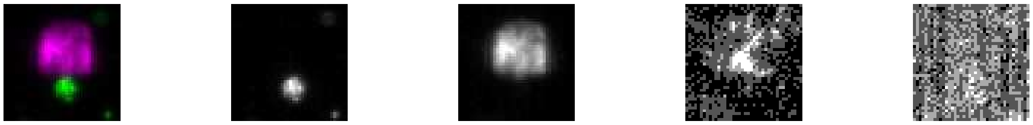				
5067	41	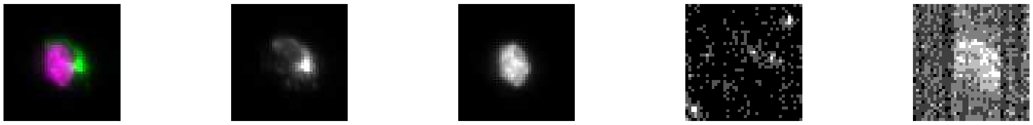				
5068	41	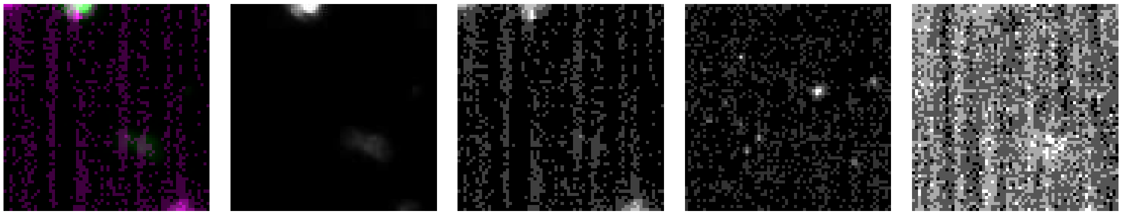				
5069	41	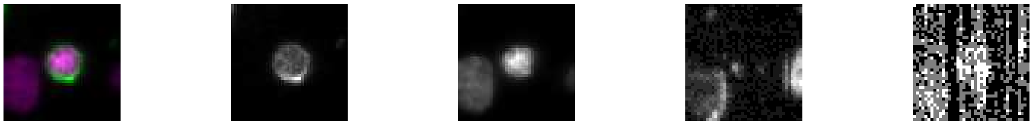				
5070	41	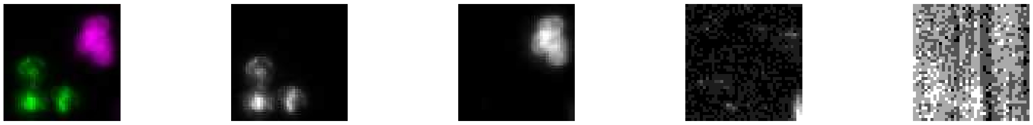				
5071	41	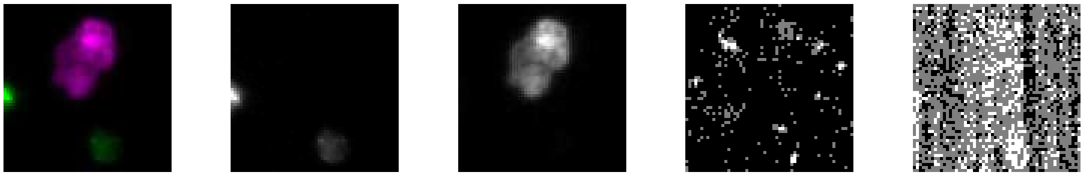				

Ereignis	Aufnahme	DAPI/CK-PE	CK-PE	DAPI	CD45-APC	HER-2/neu
5072	41	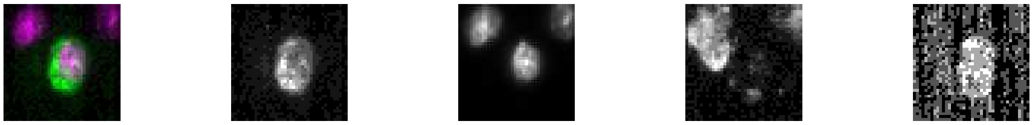				
5073	41	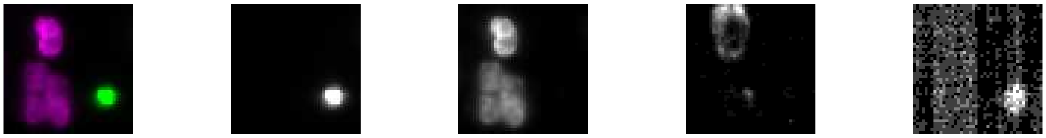				
5074	41	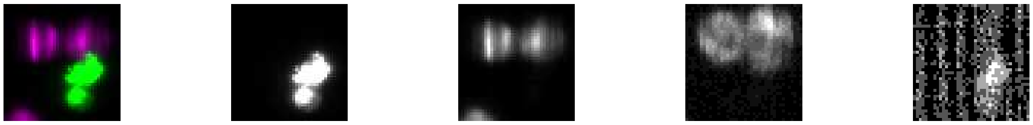				
5075	41	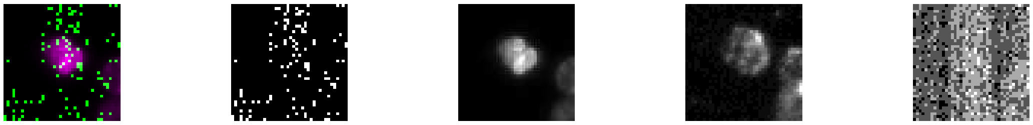				
5076	41	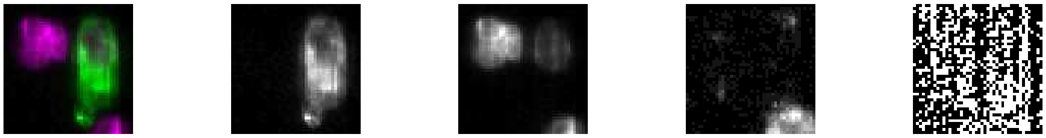				
5077	41	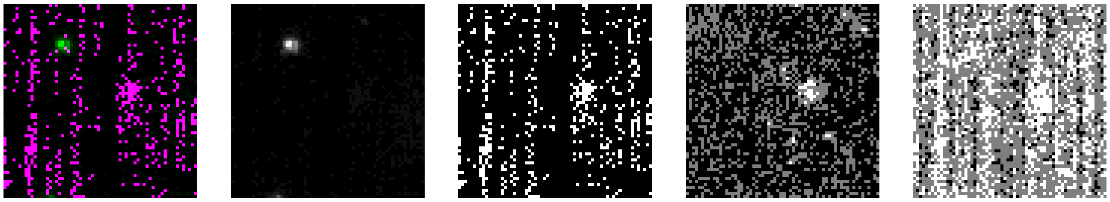				
5078	41	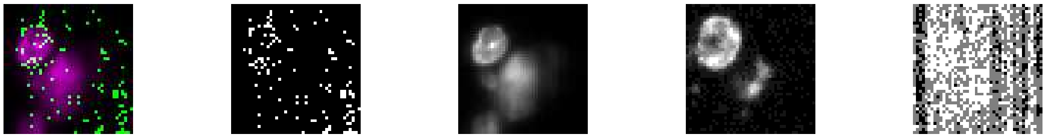				
5079	41	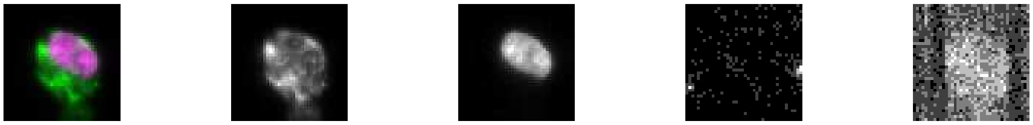				
5080	41	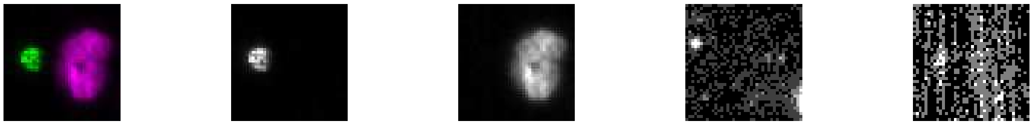				
5081	41	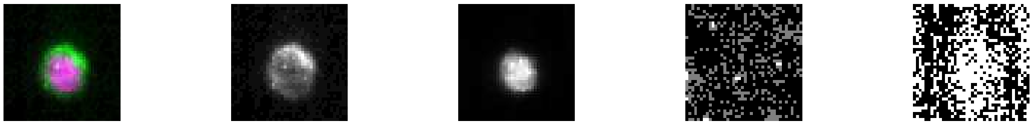				
5082	41	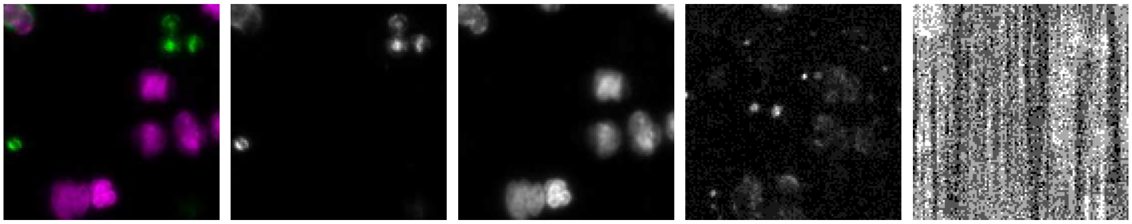				
5083	41	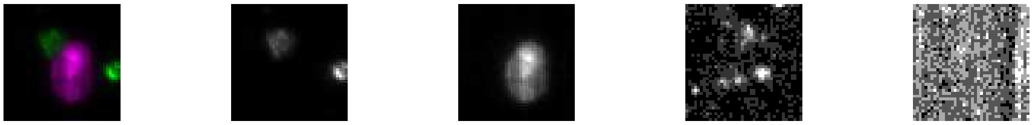				

Ereignis	Aufnahme	DAPI/CK-PE	CK-PE	DAPI	CD45-APC	HER-2/neu
5084	41	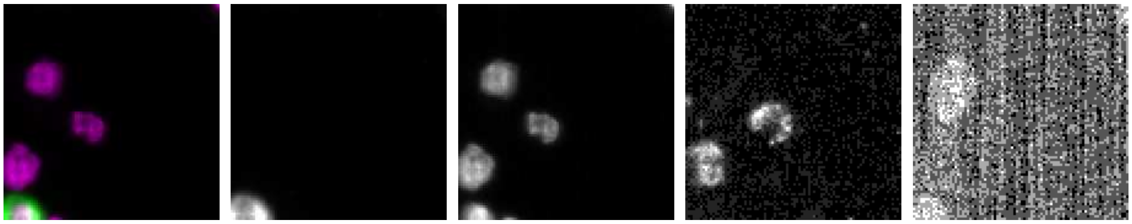				
5085	41	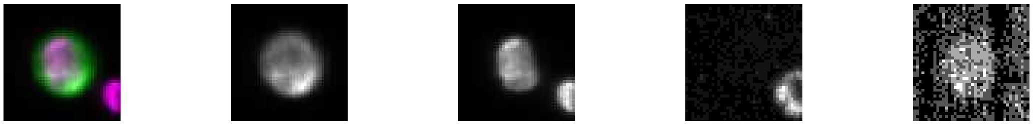				
5086	41	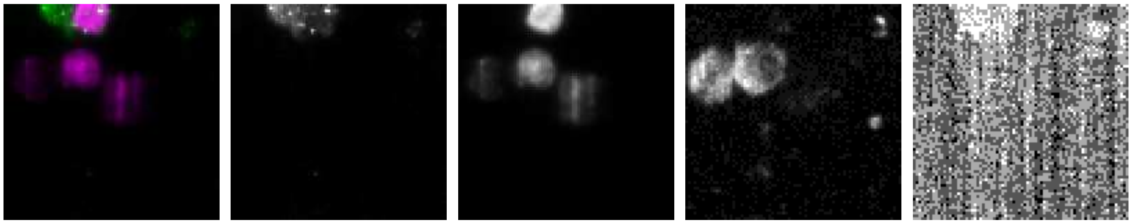				
5087	41	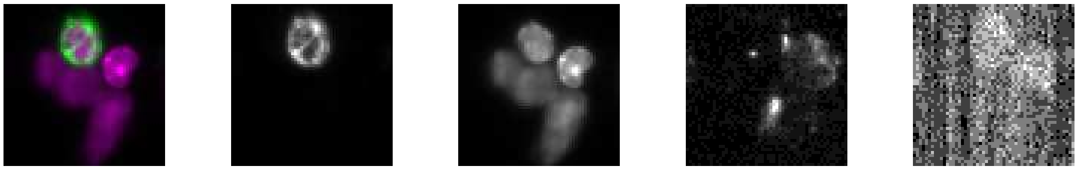				
5088	41	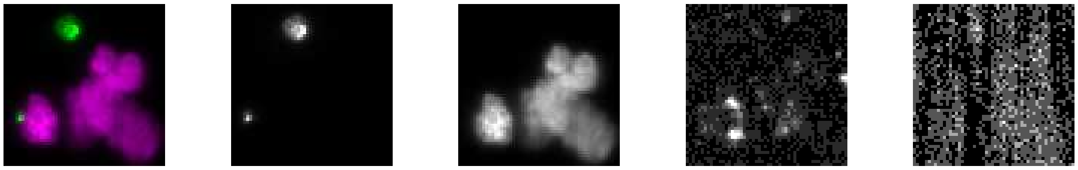				
5089	41	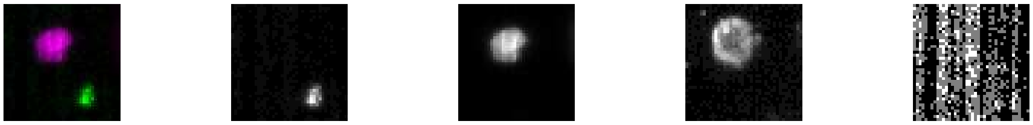				
5090	41	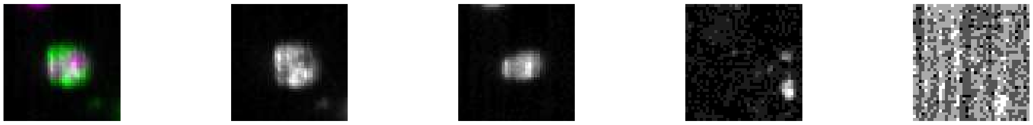				
5091	41	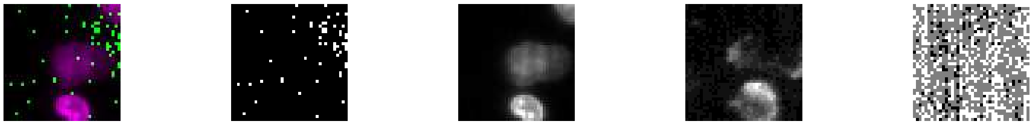				
5092	41	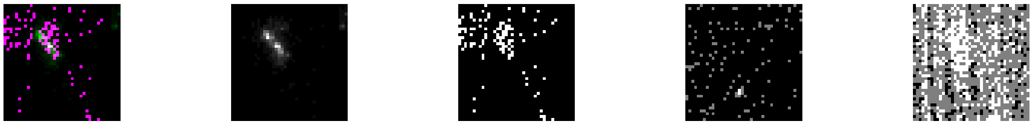				
5093	41	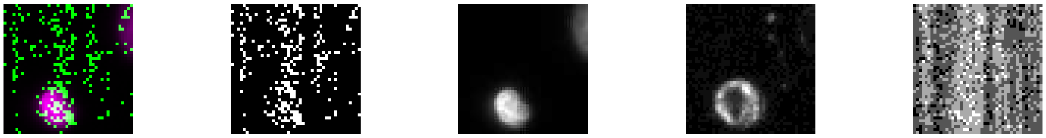				
5094	41	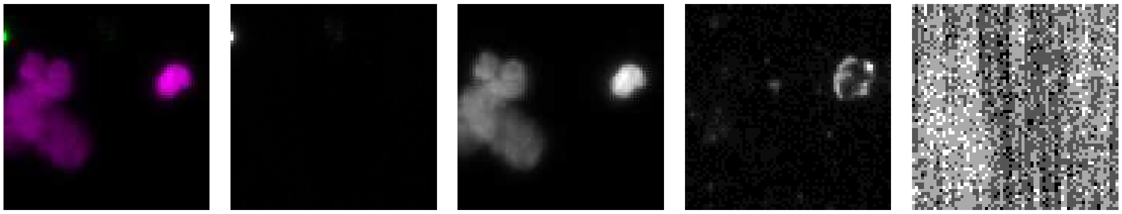				

Ereignis	Aufnahme	DAPI/CK-PE	CK-PE	DAPI	CD45-APC	HER-2/neu
5095	41	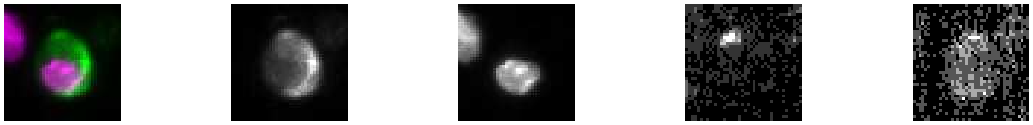				
5096	41	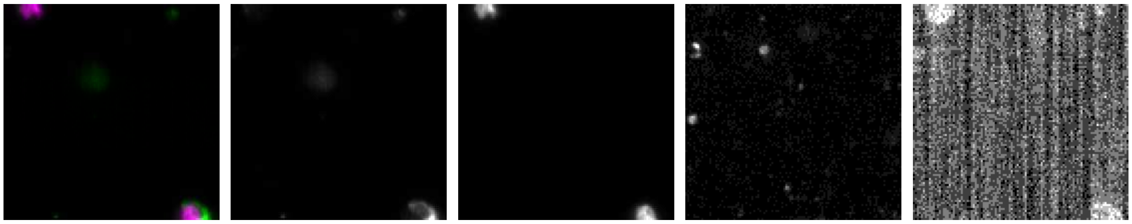				
5097	41	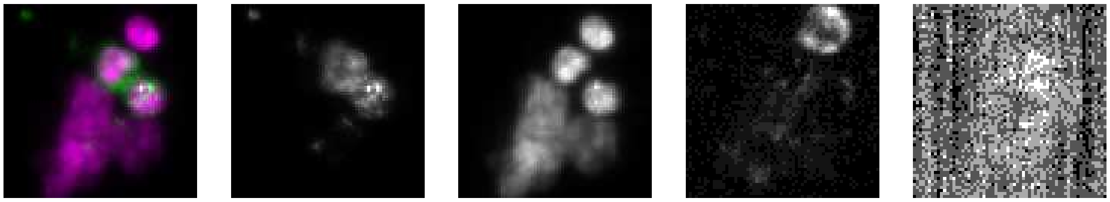				
5098	41	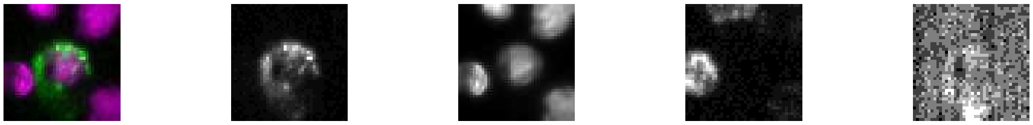				
5099	41	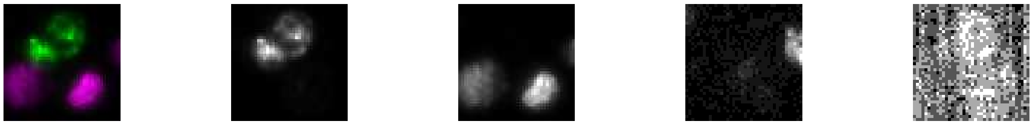				
5100	41	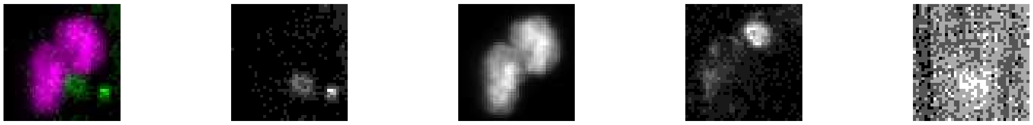				
5101	41	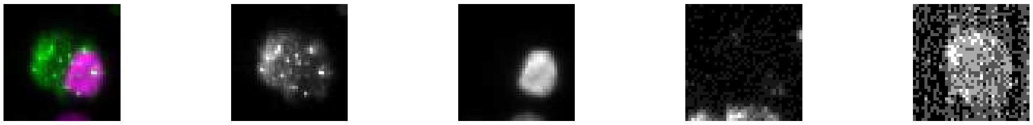				
5102	41	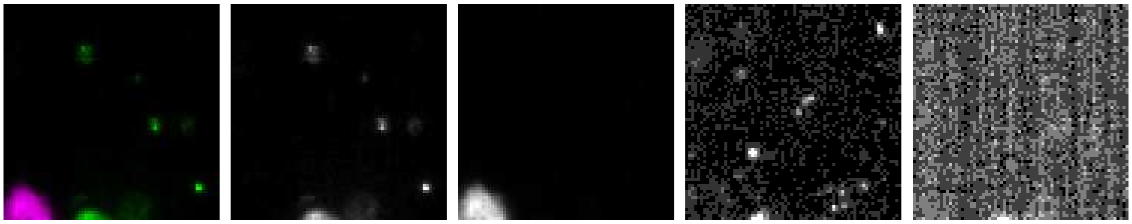				
5103	41	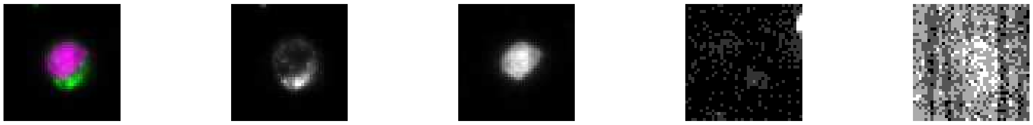				
5104	41	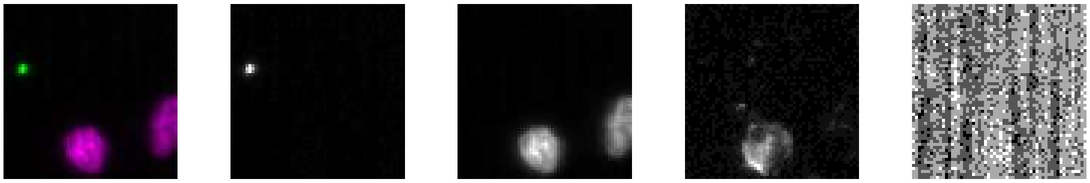				
5105	41	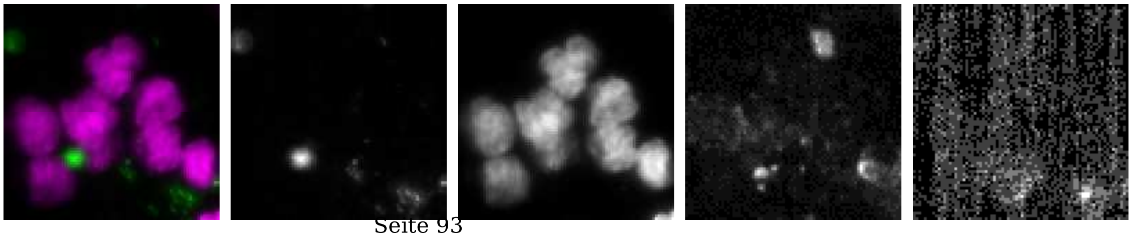				

Ereignis	Aufnahme	DAPI/CK-PE	CK-PE	DAPI	CD45-APC	HER-2/neu
5106	41	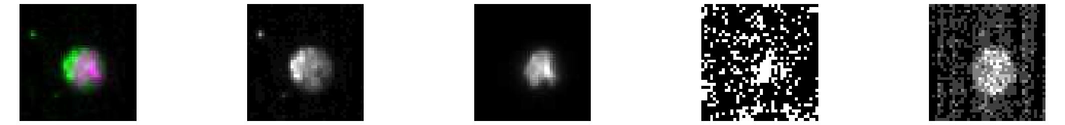				
5107	41	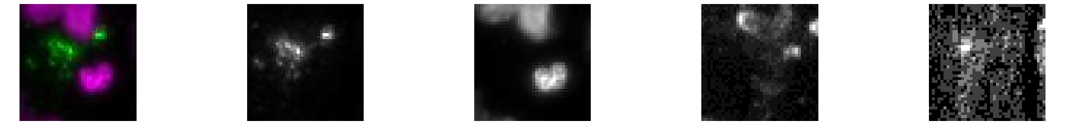				
5108	41	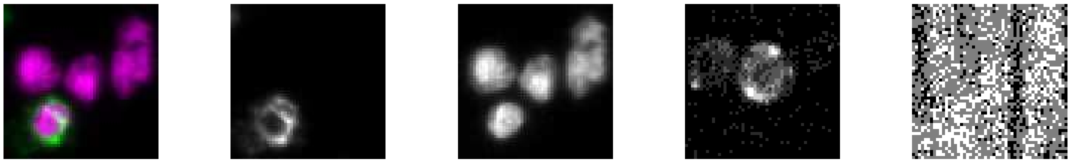				
5109	41	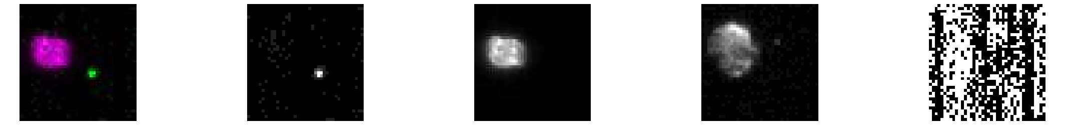				
5110	41	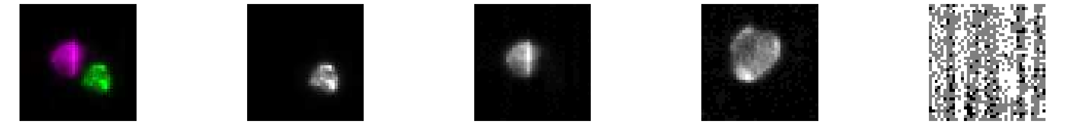				
5111	41	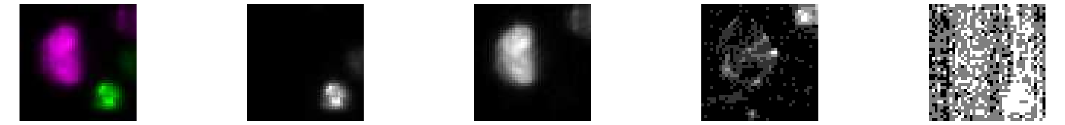				
5112	41	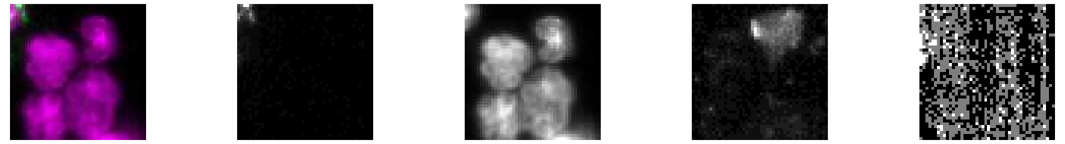				
5113	41	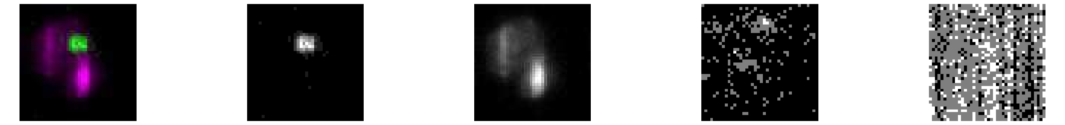				
5114	41	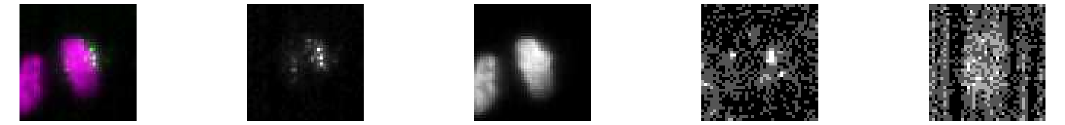				
5115	41	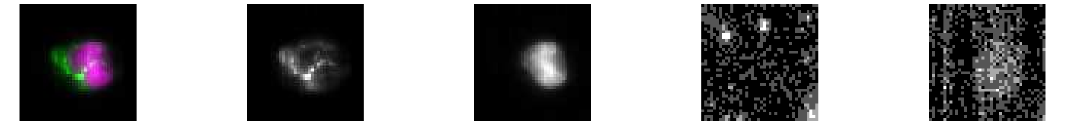				
5116	41	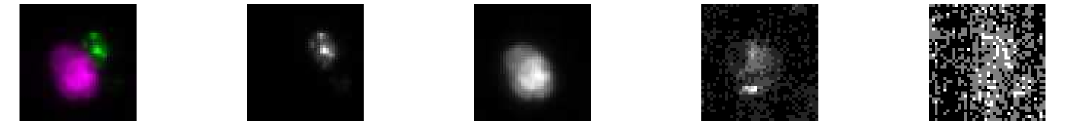				
5117	41	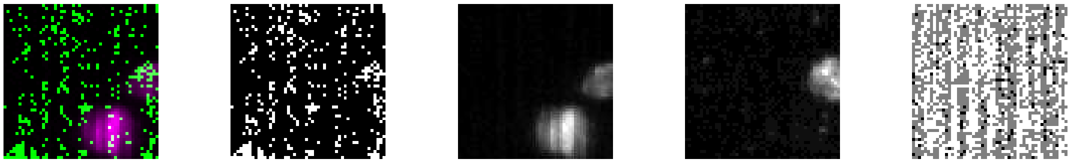				
5118	41	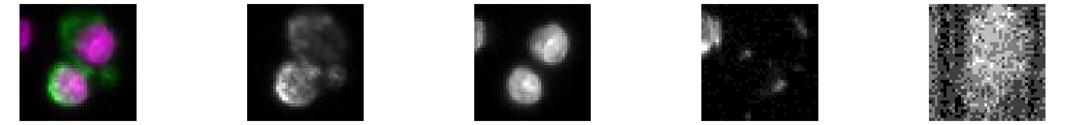				

Ereignis	Aufnahme	DAPI/CK-PE	CK-PE	DAPI	CD45-APC	HER-2/neu
5119	41	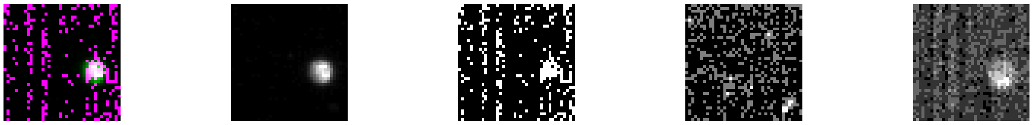				
5120	41	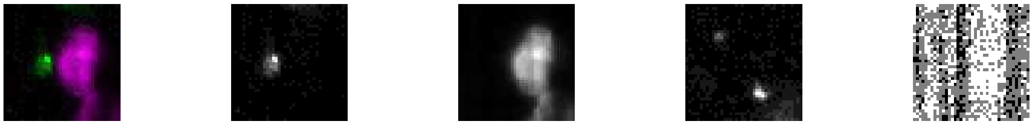				
5121	41	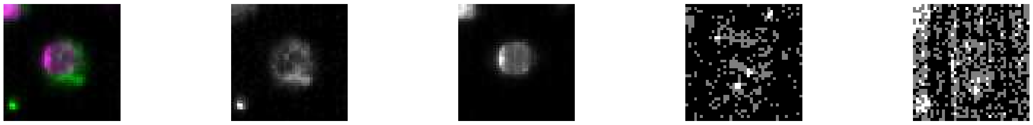				
5122	41	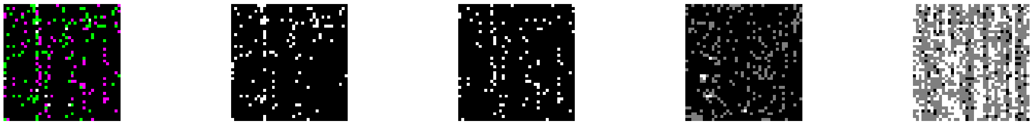				
5123	41	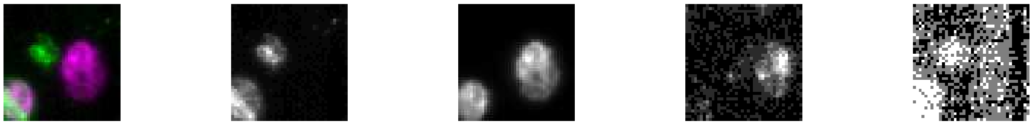				
5124	41	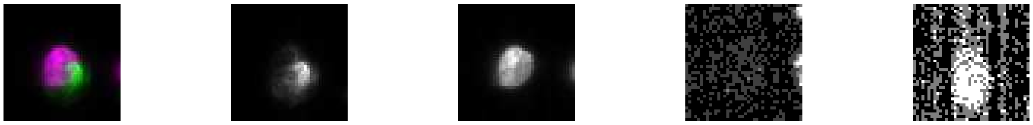				
5125	41	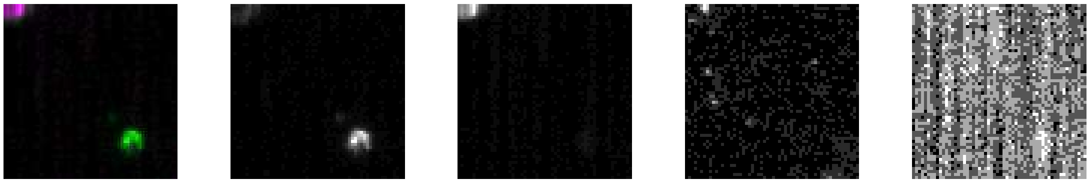				
5126	41	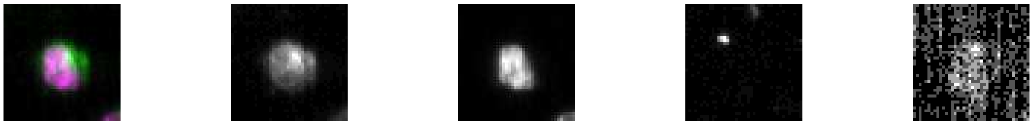				
5127	41	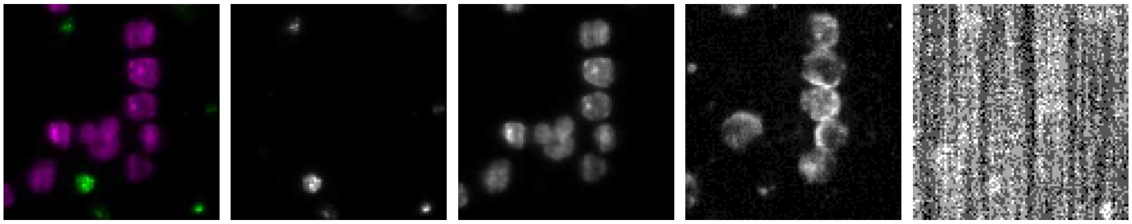				
5128	41	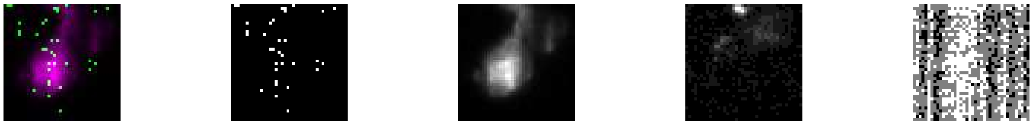				
5129	41	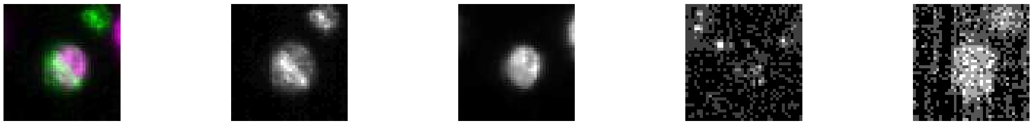				
5130	41	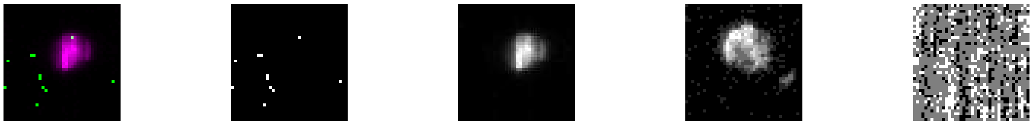				
5131	41	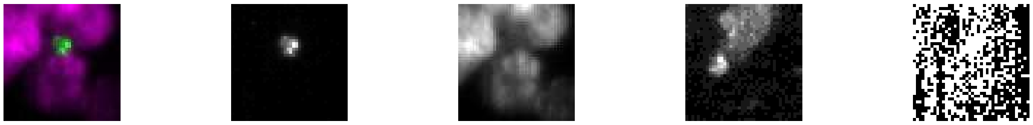				

Ereignis	Aufnahme	DAPI/CK-PE	CK-PE	DAPI	CD45-APC	HER-2/neu
5132	41	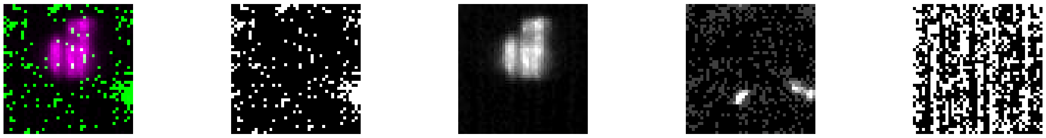				
5133	41	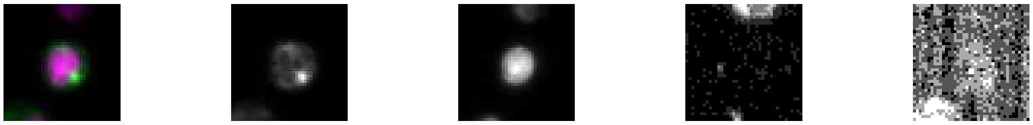				
5134	41	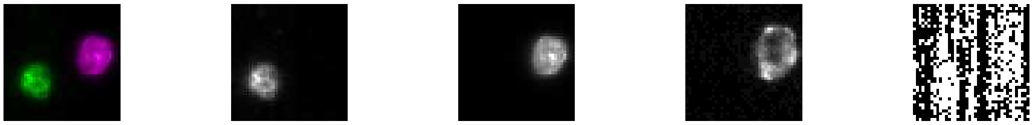				
5135	41	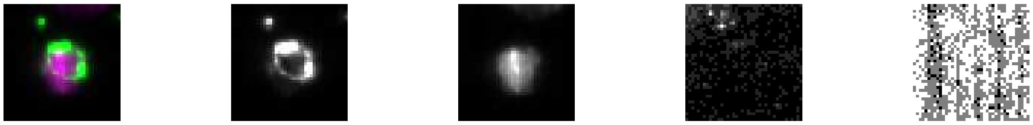				
5136	41	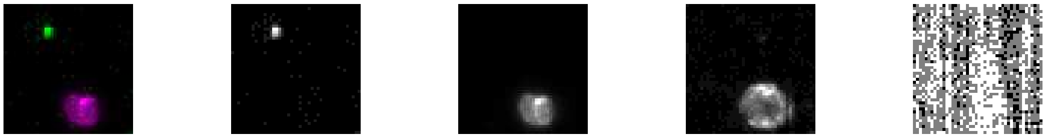				
5137	41	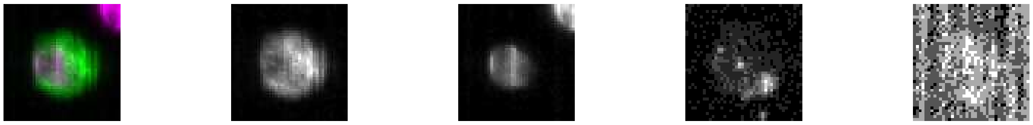				
5138	41	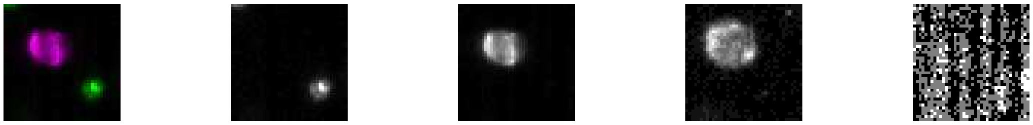				
5139	41	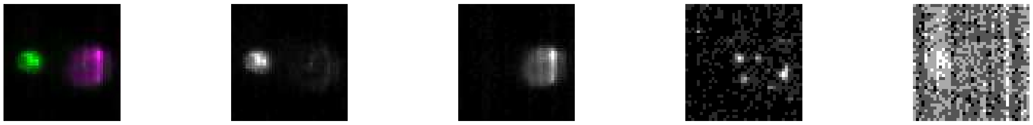				
5140	41	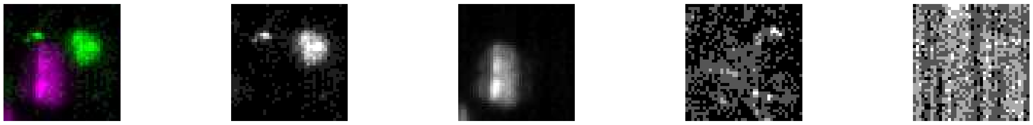				
5141	41	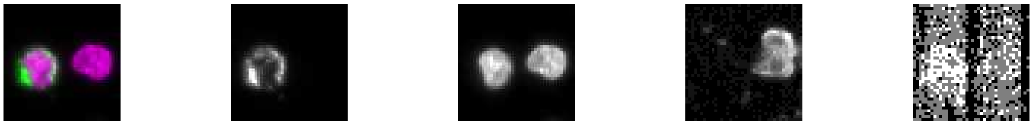				
5142	41	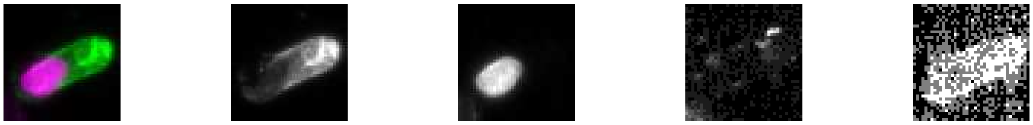				
5143	41	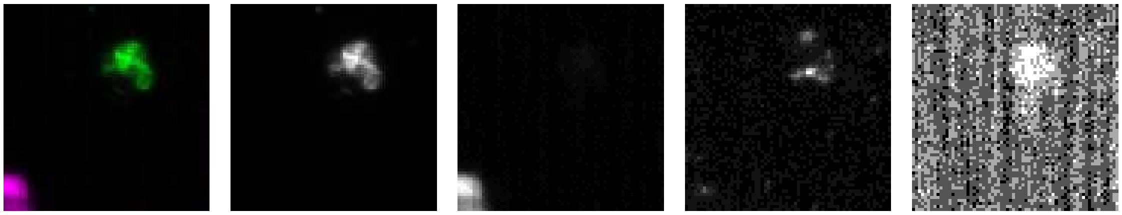				

Druckdatum:02/06/2020 16:41

Proben-ID: Gyn14.208 **Kassetten-ID:**01327106 **Patienten-ID:**

Ereignis	Aufnahme	DAPI/CK-PE	CK-PE	DAPI	CD45-APC	HER-2/neu
5144	41	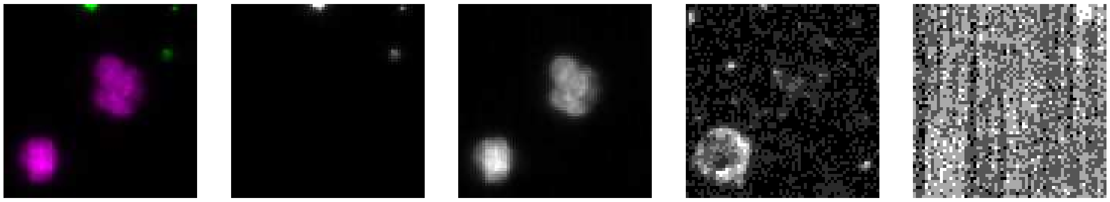				
5145	41	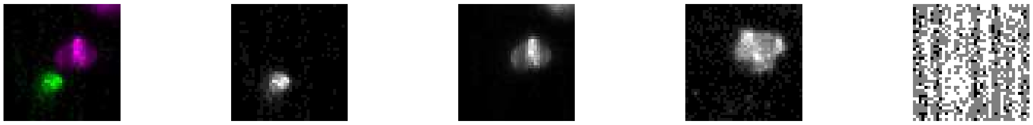				
5146	41	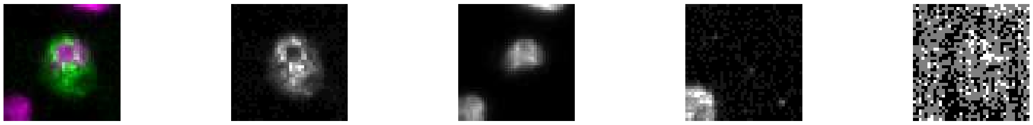				
5147	41	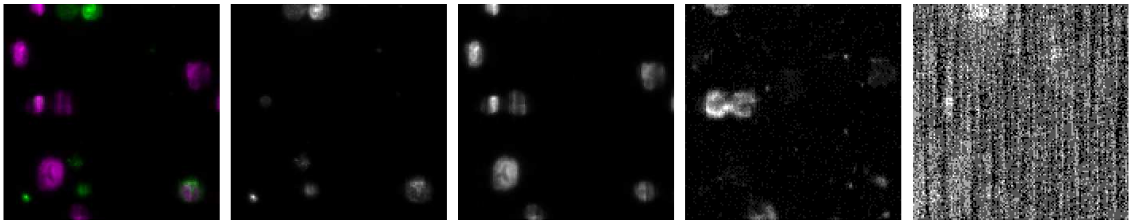				
5148	41	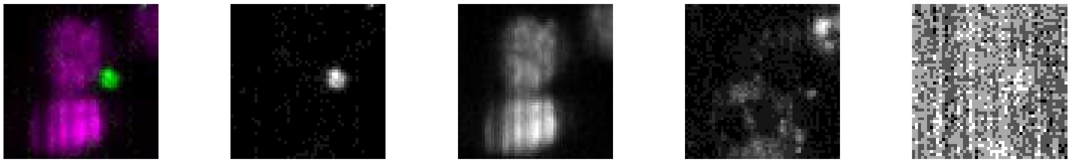				
5149	41	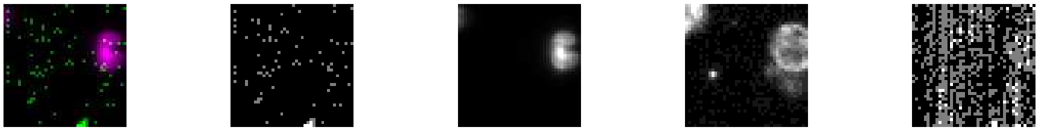				
5150	41	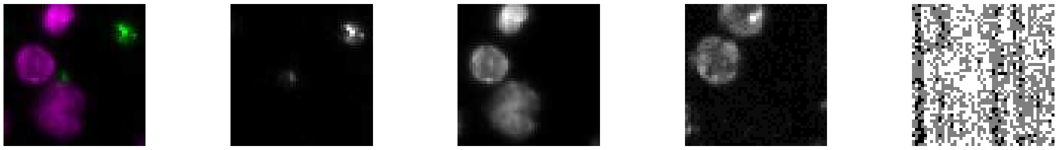				
5151	41	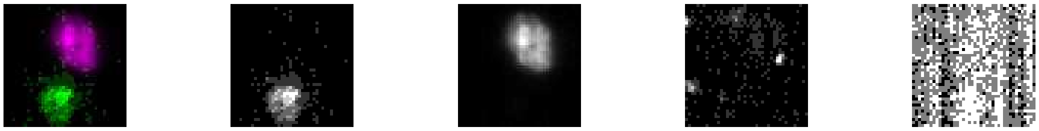				
5152	41	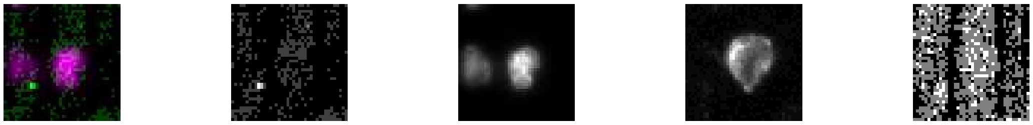				
5153	41	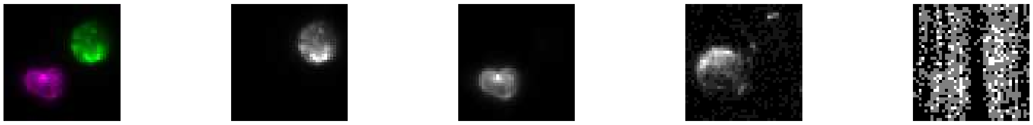				
5154	41	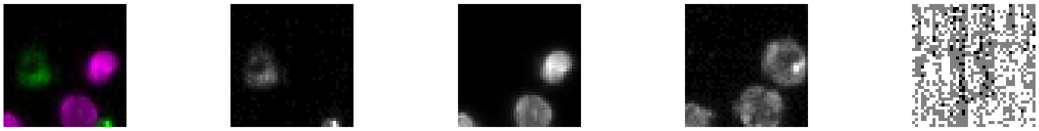				
5155	41	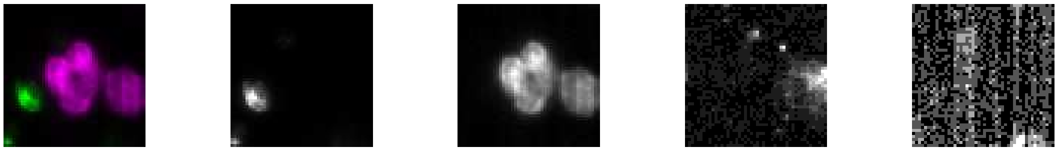				

Ereignis	Aufnahme	DAPI/CK-PE	CK-PE	DAPI	CD45-APC	HER-2/neu
5156	41	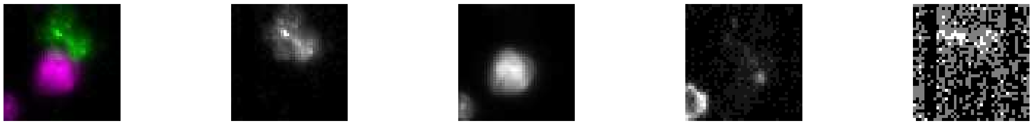				
5157	41	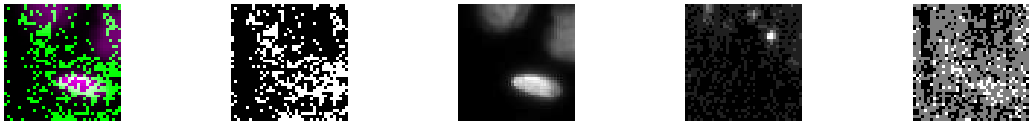				
5158	41	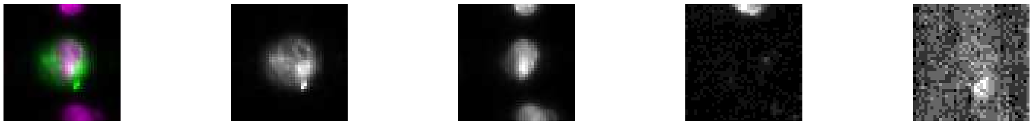				
5159	41	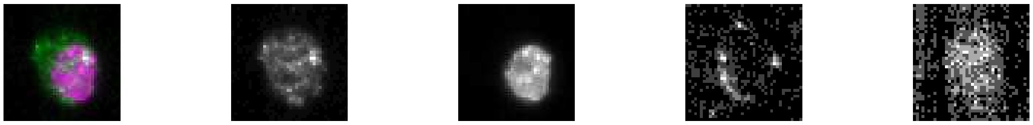				
5160	41	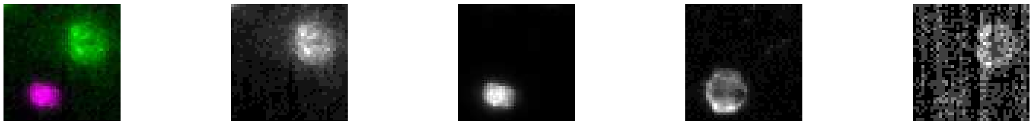				
5161	41	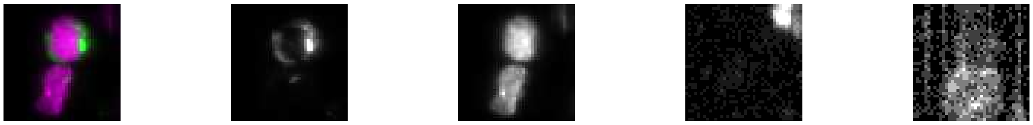				
5162	41	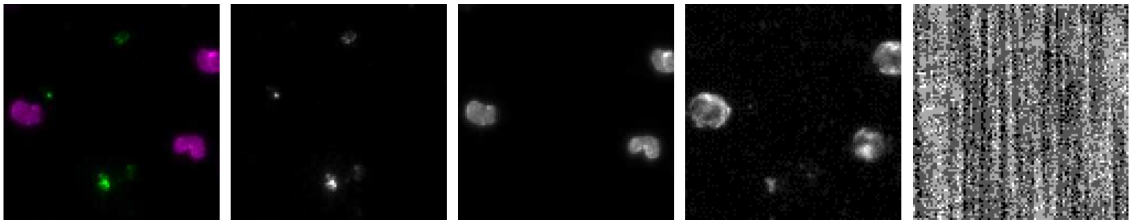				
5163	41	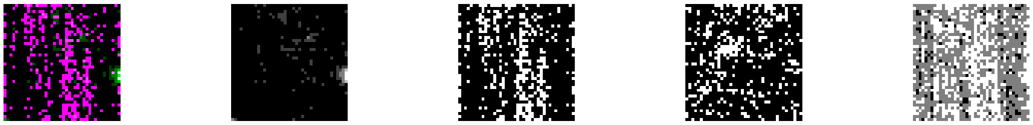				
5164	41	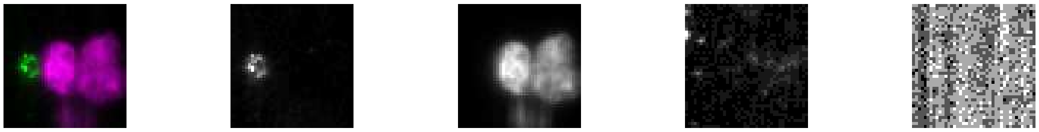				
5165	41	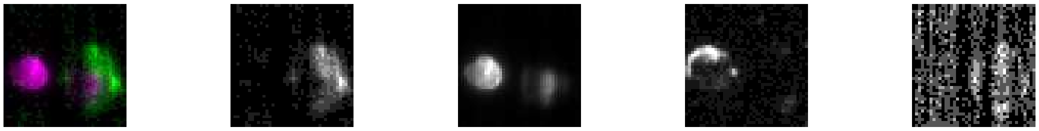				
5166	41	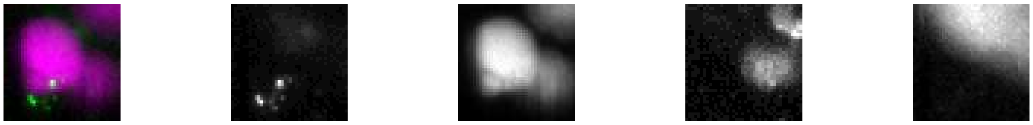				
5167	41	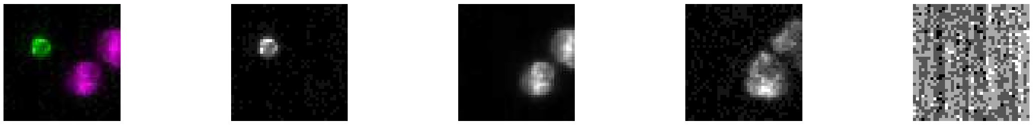				
5168	41	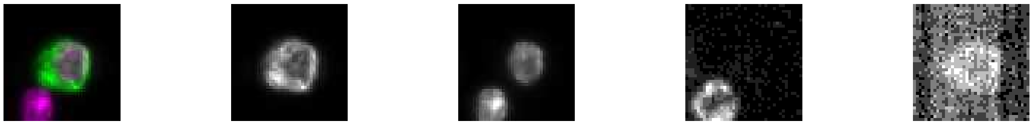				

Proben-ID: Gyn14.208 **Kassetten-ID:** 01327106 **Patienten-ID:**

Ereignis	Aufnahme	DAPI/CK-PE	CK-PE	DAPI	CD45-APC	HER-2/neu
5169	41	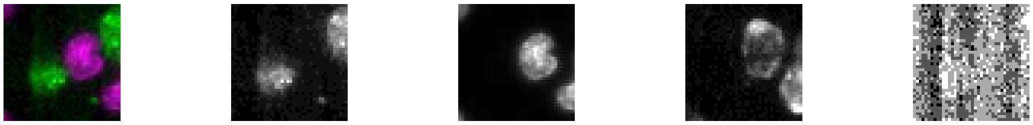				
5170	41	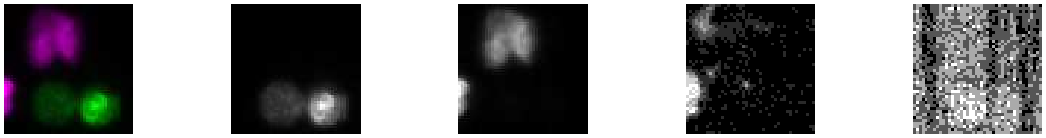				
5171	41	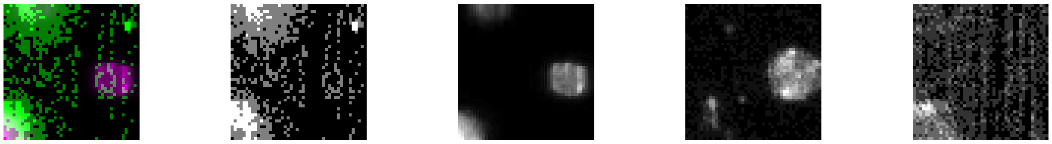				
5172	41	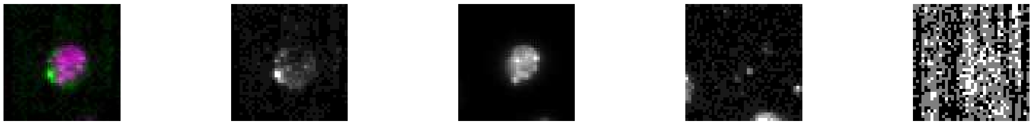				
5173	41	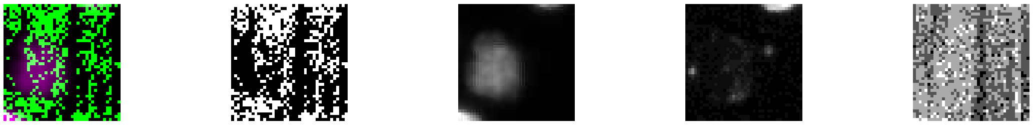				
5174	41	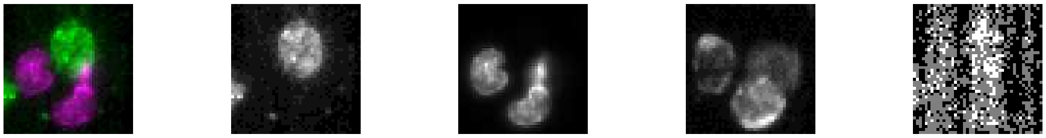				
5175	40	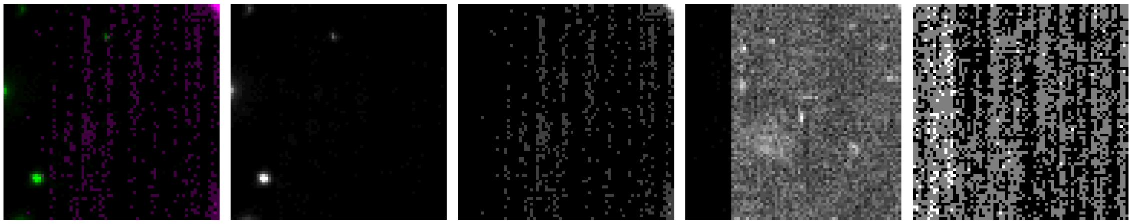				
5176	30	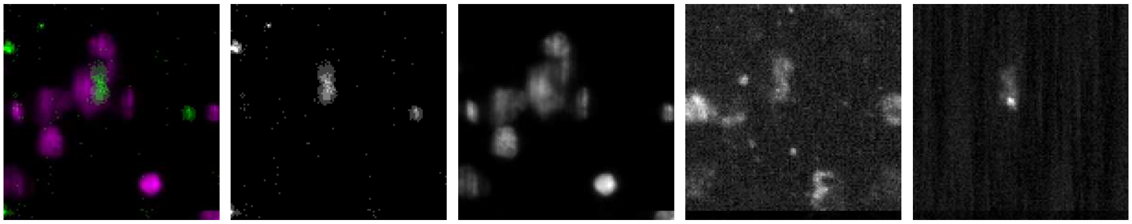				
5177	30	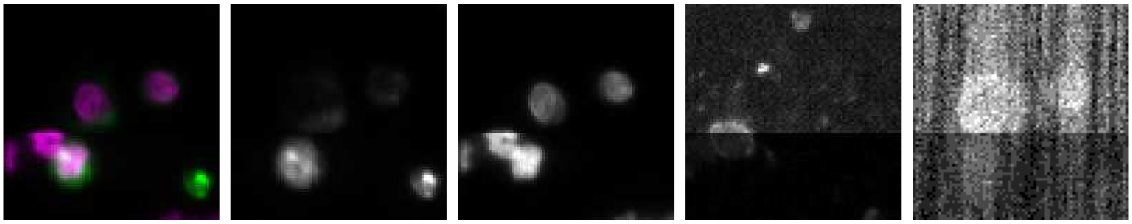				
5178	30	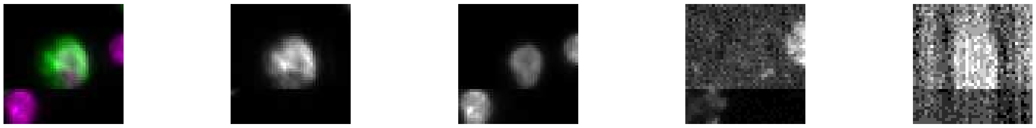				
5179	30	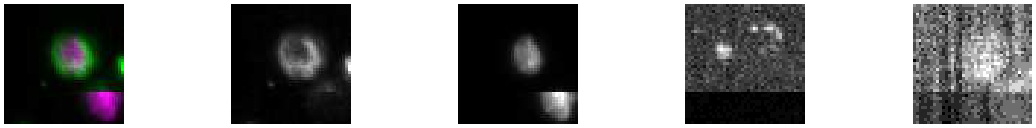				

Ereignis	Aufnahme	DAPI/CK-PE	CK-PE	DAPI	CD45-APC	HER-2/neu
5180	41	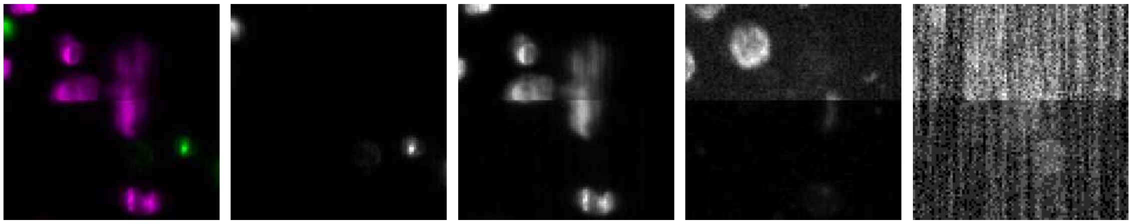				
5181	41	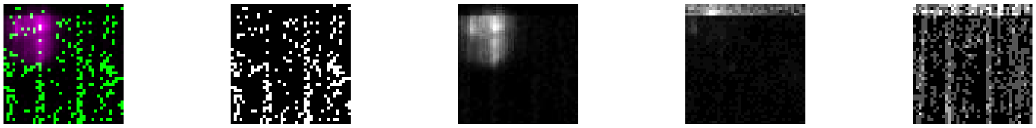				
5182	41	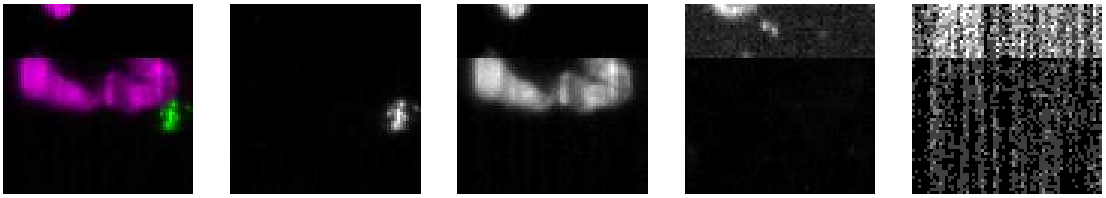				
5183	40	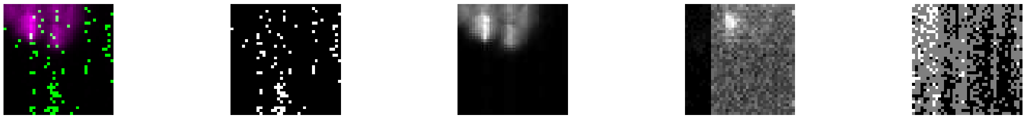				
5184	41	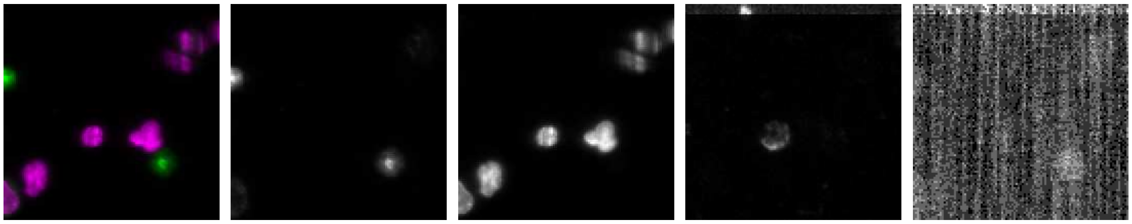				
5185	41	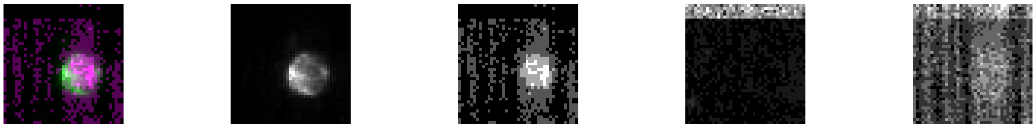				
5186	42	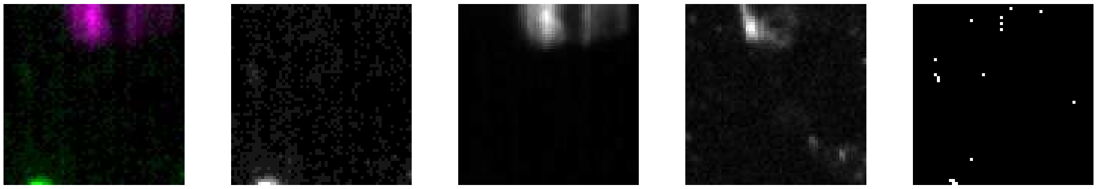				
5187	42	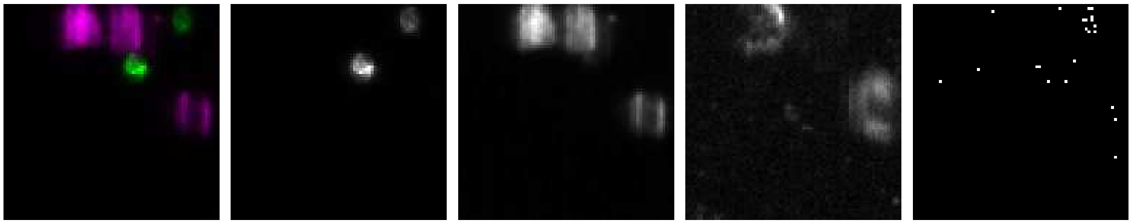				
5188	42	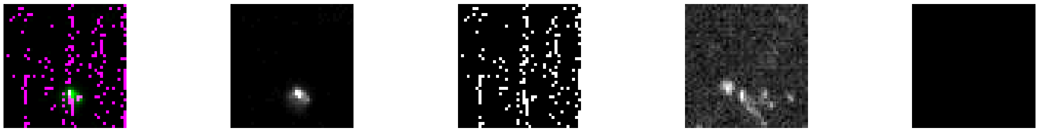				
5189	42	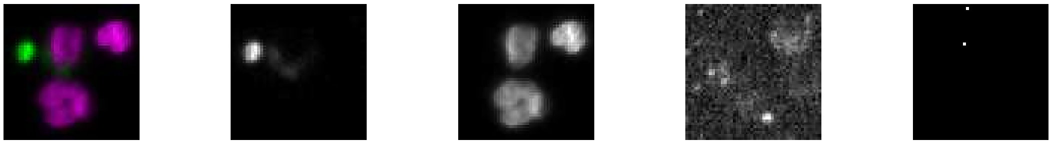				

Ereignis	Aufnahme	DAPI/CK-PE	CK-PE	DAPI	CD45-APC	HER-2/neu
5190	42	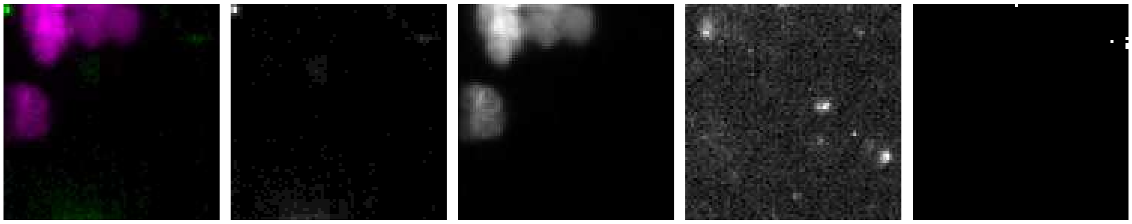				
5191	42	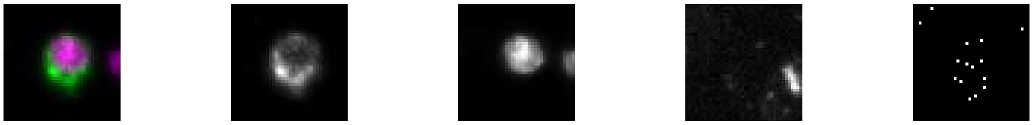				
5192	42	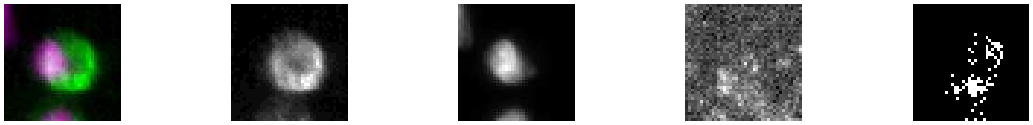				
5193	42	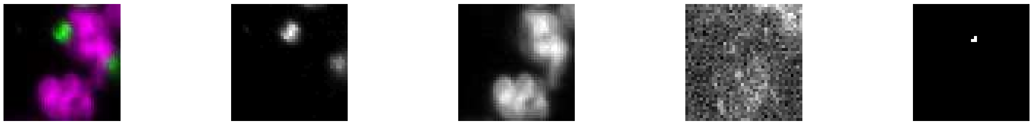				
5194	42	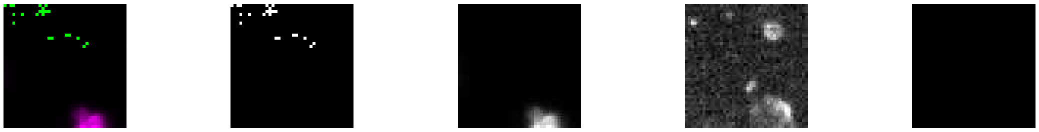				
5195	42	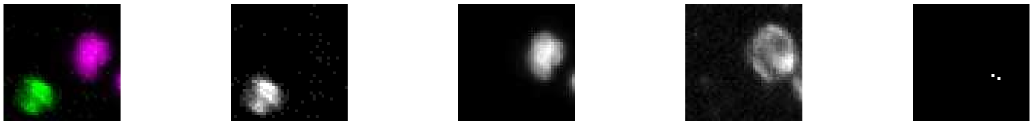				
5196	42	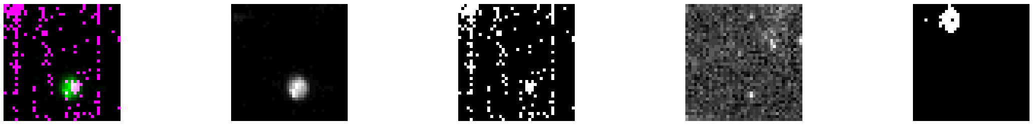				
5197	42	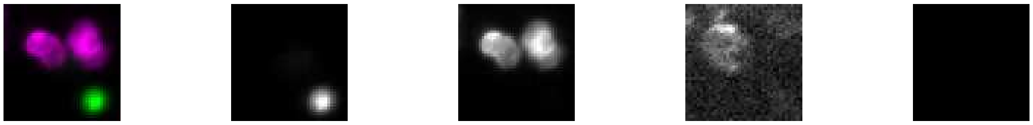				
5198	42	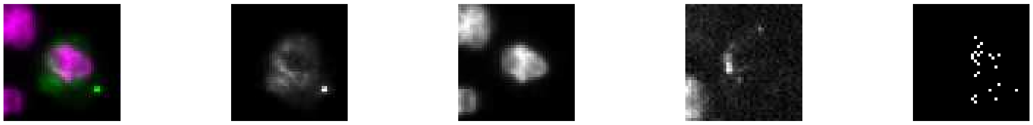				
5199	42	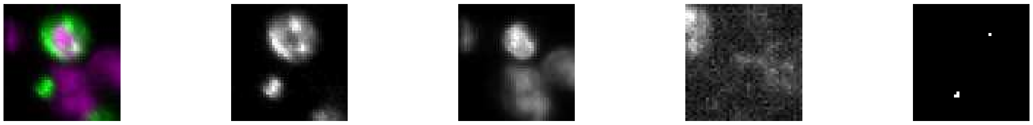				
5200	42	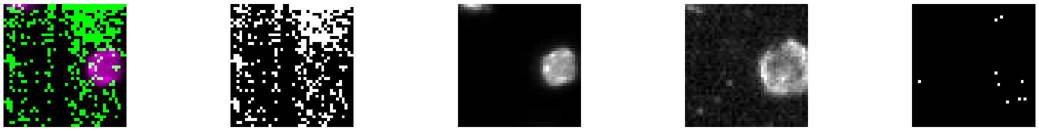				
5201	42	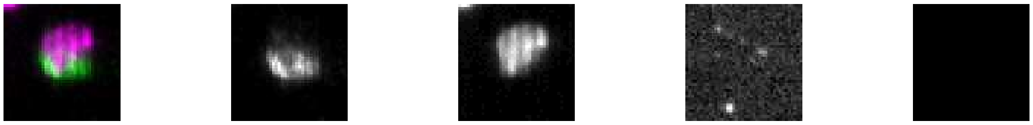				
5202	42	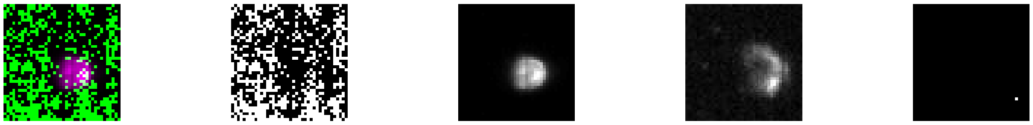				

Ereignis	Aufnahme	DAPI/CK-PE	CK-PE	DAPI	CD45-APC	HER-2/neu
5203	42	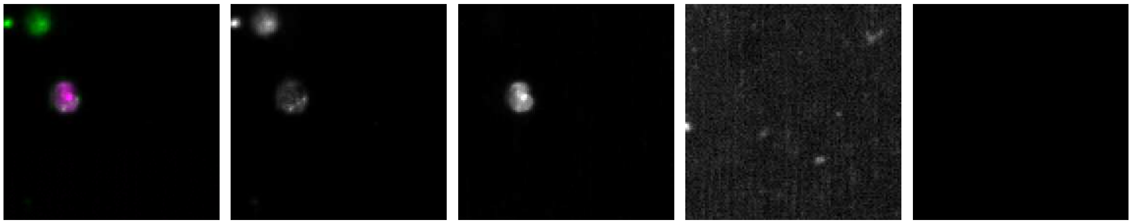				
5204	42	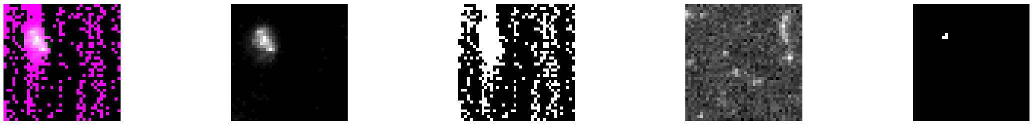				
5205	42	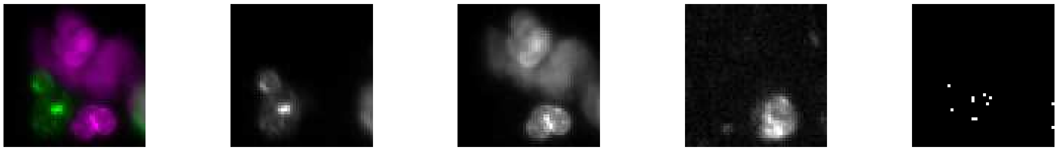				
5206	42	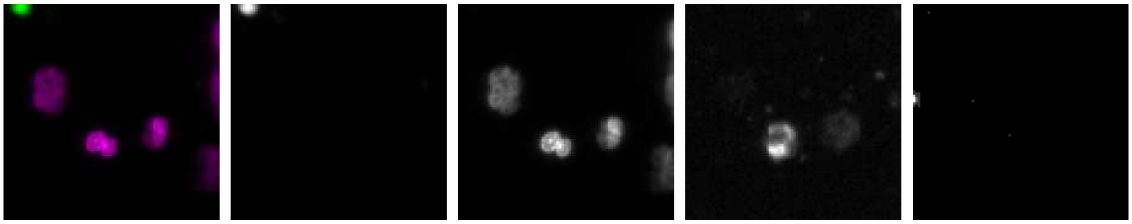				
5207	42	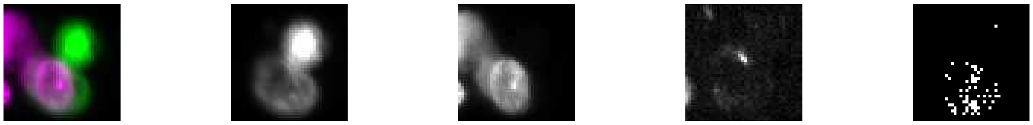				
5208	42	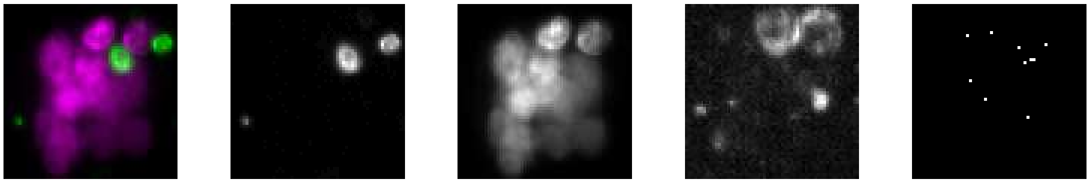				
5209	42	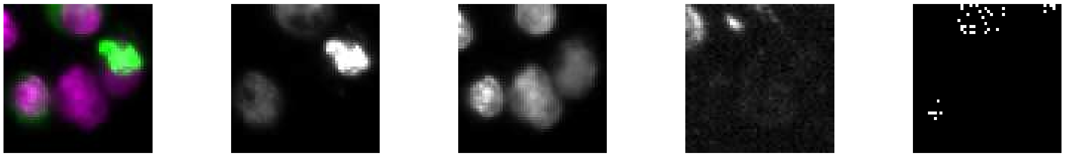				
5210	42	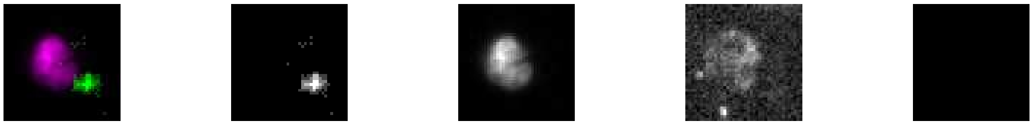				
5211	42	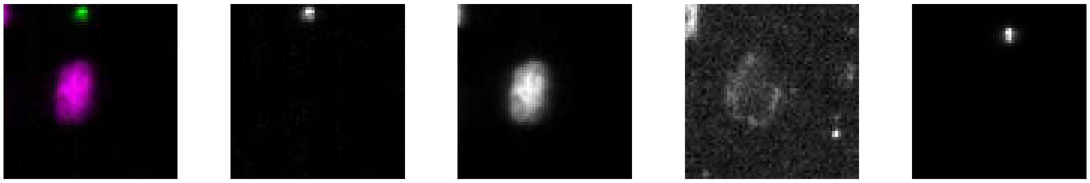				
5212	42	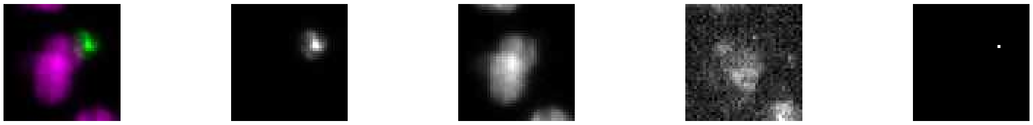				
5213	42	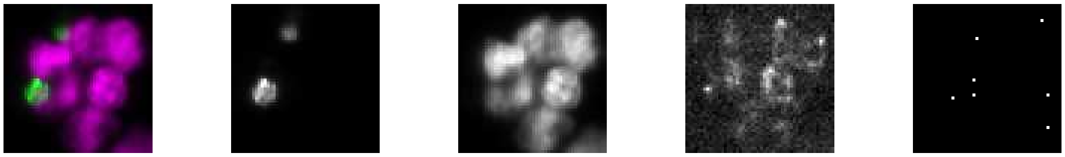				

Ereignis	Aufnahme	DAPI/CK-PE	CK-PE	DAPI	CD45-APC	HER-2/neu
5214	42	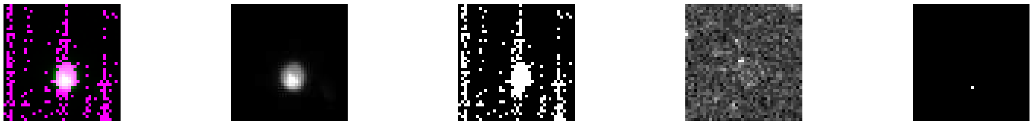				
5215	42	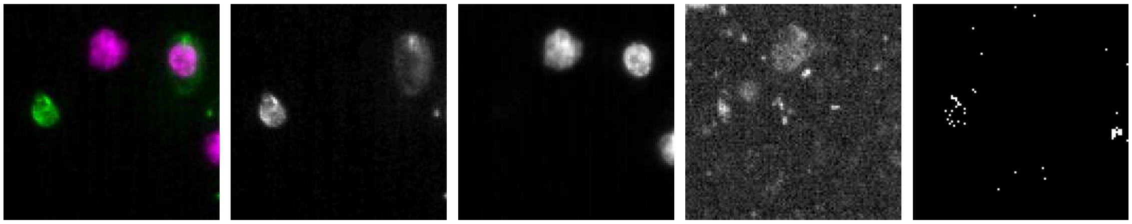				
5216	42	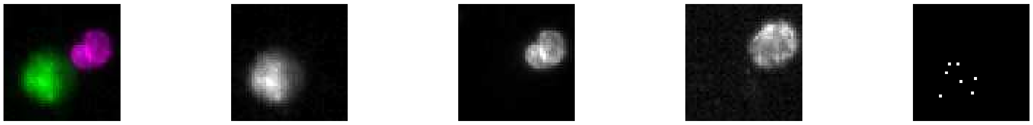				
5217	42	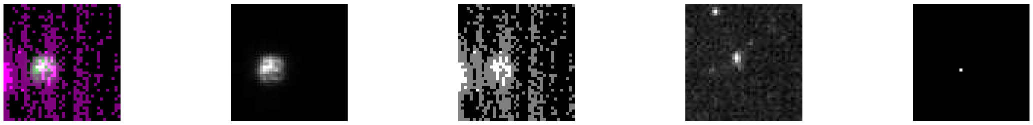				
5218	42	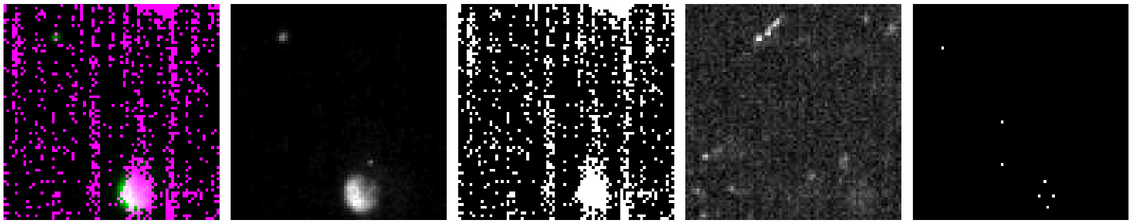				
5219	42	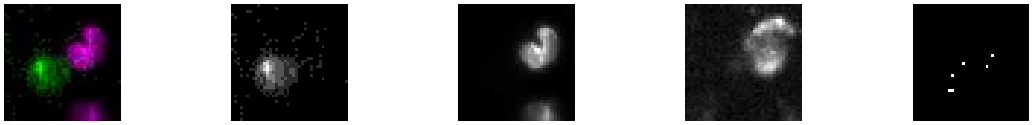				
5220	42	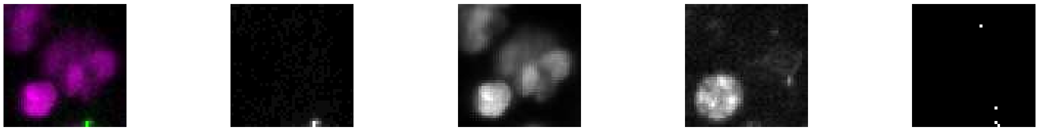				
5221	42	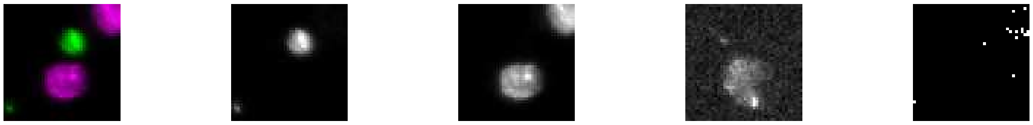				
5222	42	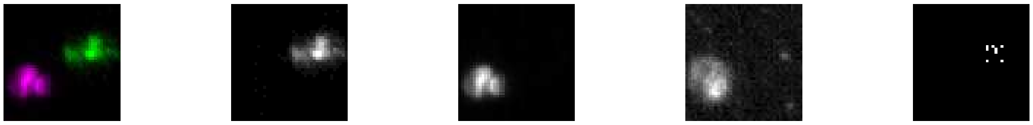				
5223	42	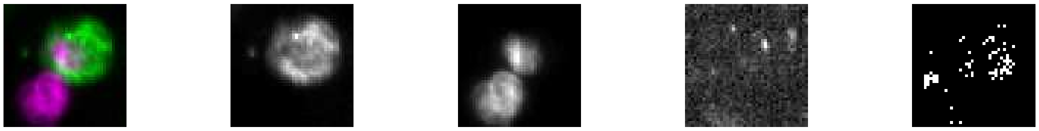				
5224	42	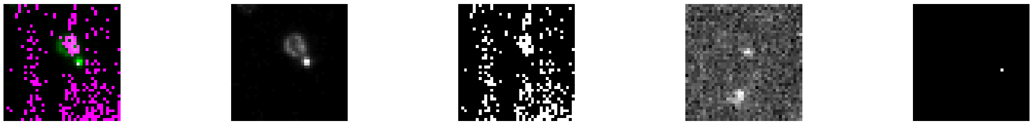				
5225	42	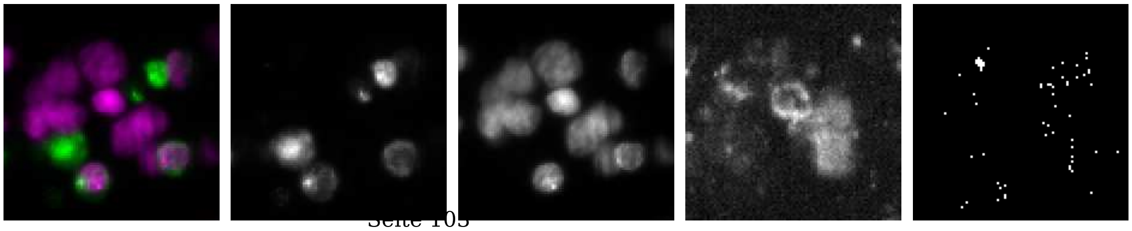				

Ereignis	Aufnahme	DAPI/CK-PE	CK-PE	DAPI	CD45-APC	HER-2/neu
5226	42	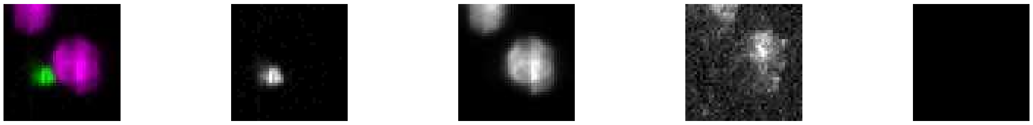				
5227	42	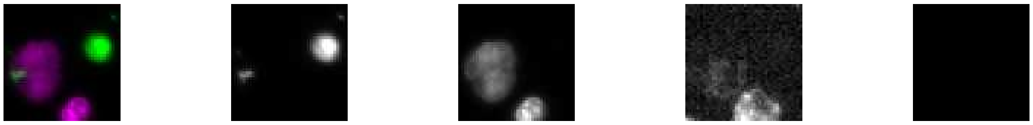				
5228	42	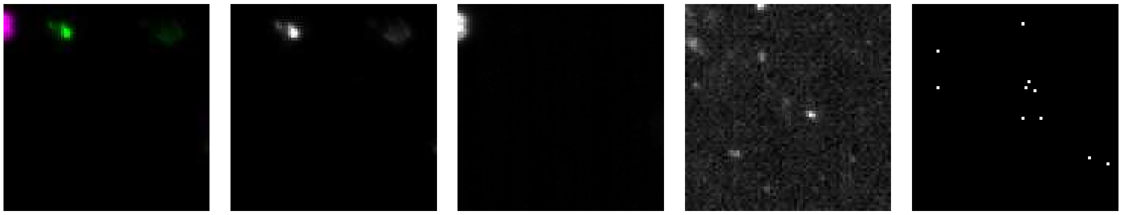				
5229	42	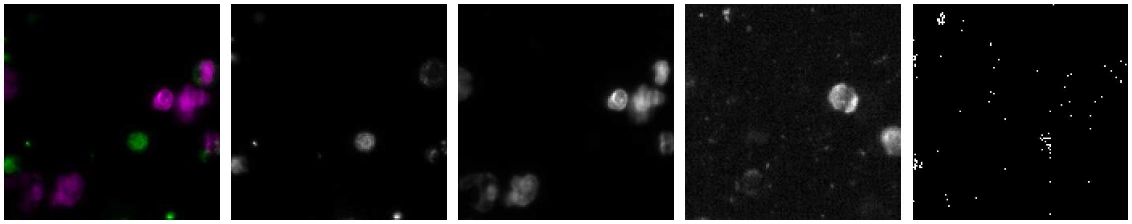				
5230	42	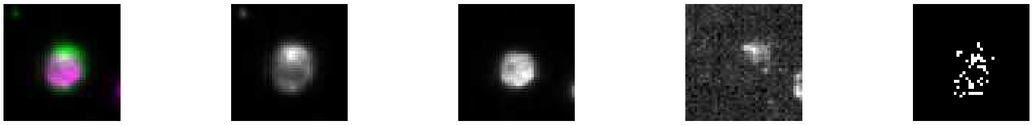				
5231	42	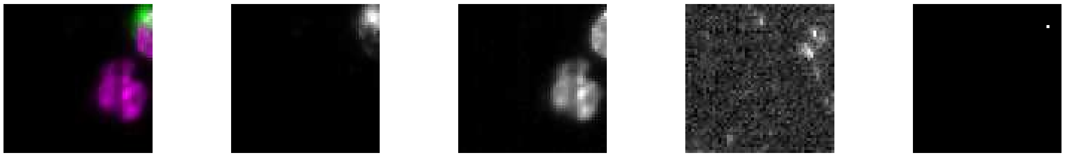				
5232	42	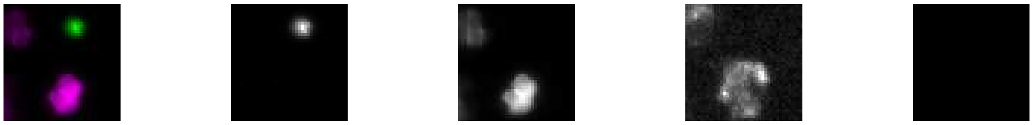				
5233	42	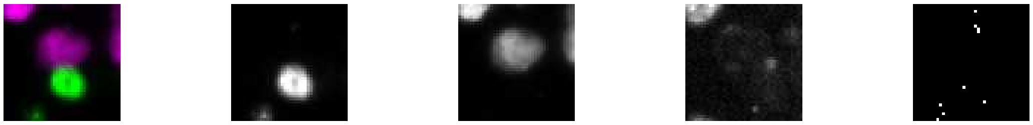				
5234	42	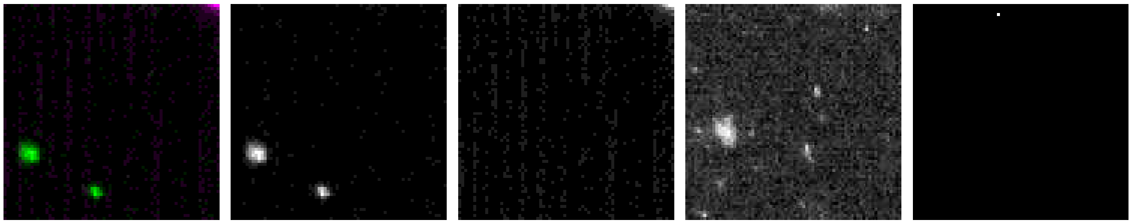				
5235	42	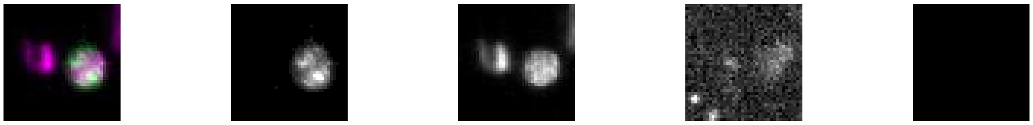				
5236	42	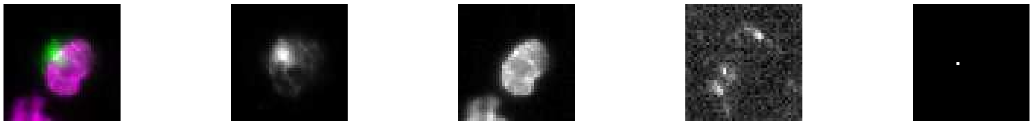				

Ereignis	Aufnahme	DAPI/CK-PE	CK-PE	DAPI	CD45-APC	HER-2/neu
5237	42	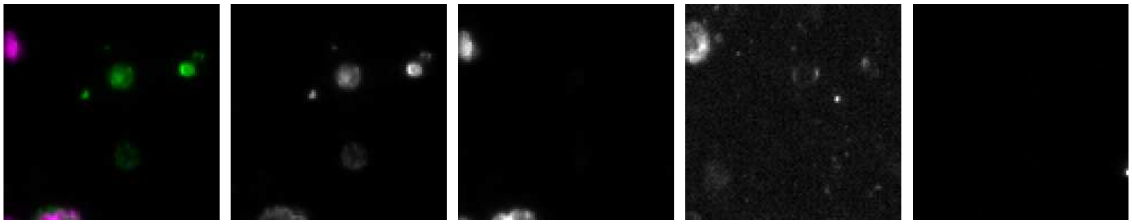				
5238	42	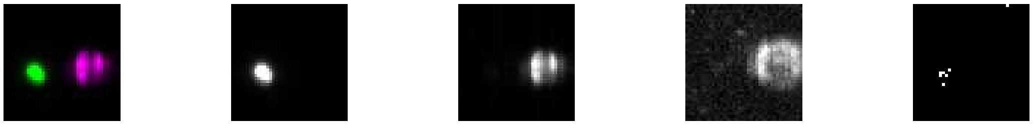				
5239	42	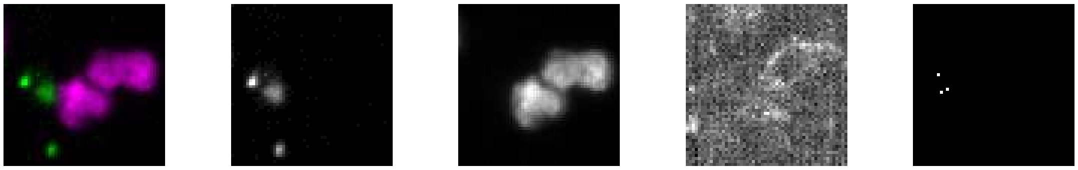				
5240	42	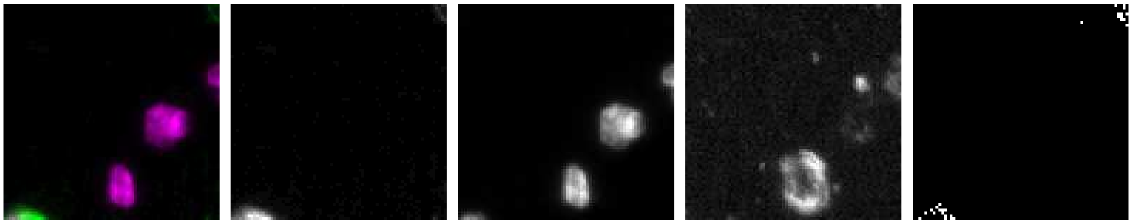				
5241	42	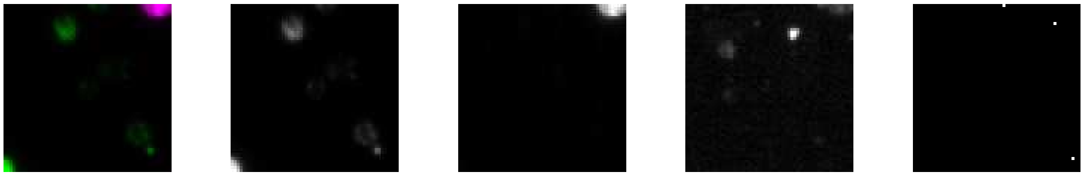				
5242	42	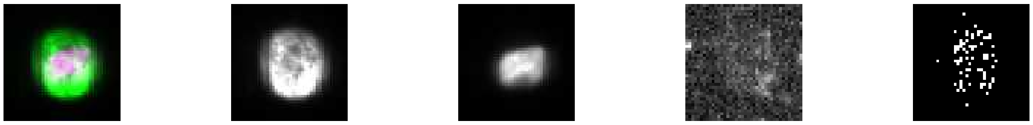				
5243	42	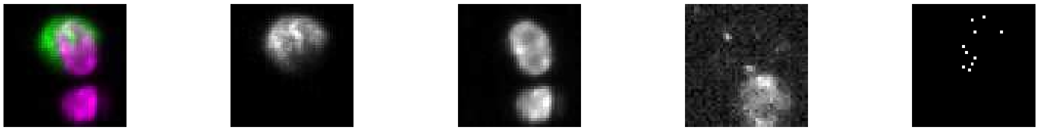				
5244	42	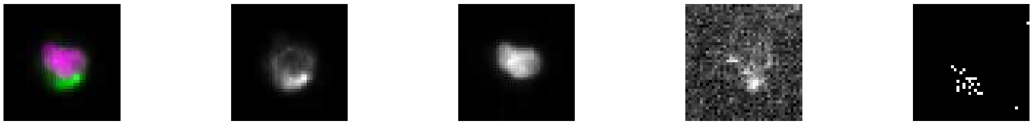				
5245	42	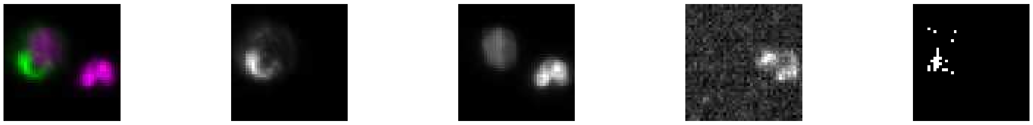				
5246	42	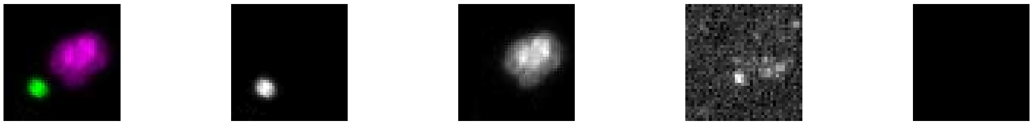				
5247	42	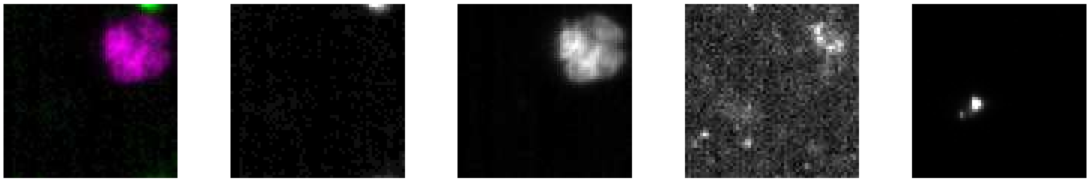				

Ereignis	Aufnahme	DAPI/CK-PE	CK-PE	DAPI	CD45-APC	HER-2/neu
5248	42	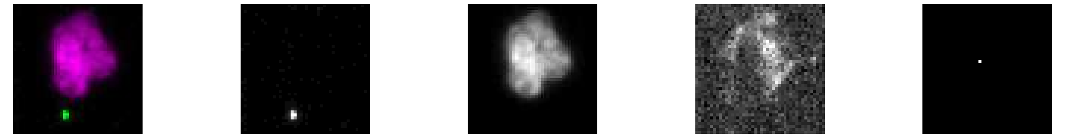				
5249	42	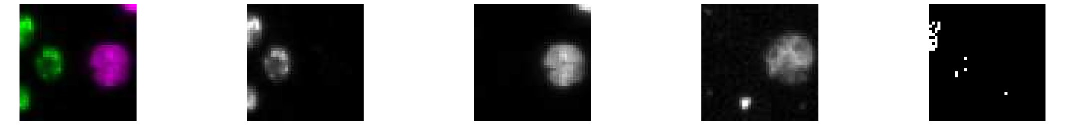				
5250	42	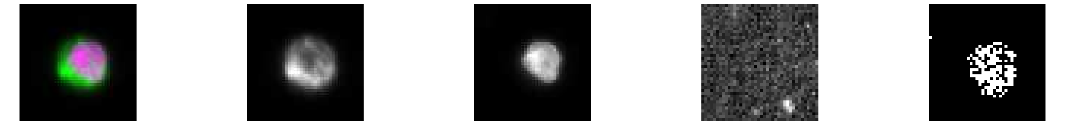				
5251	42	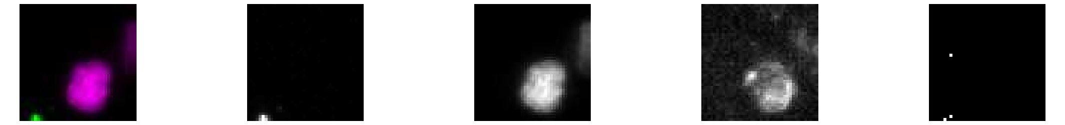				
5252	42	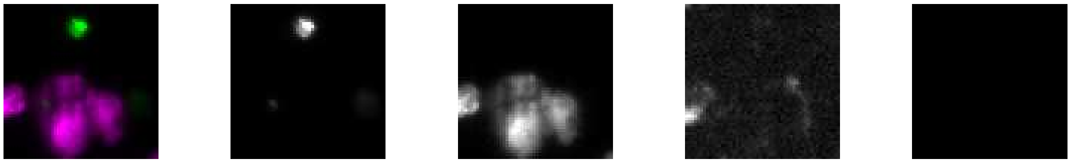				
5253	42	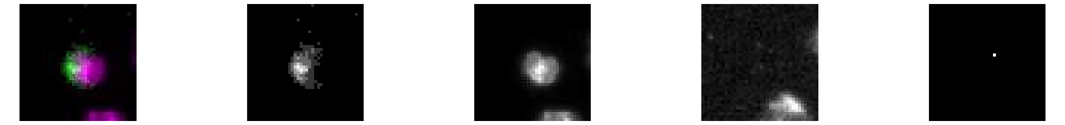				
5254	42	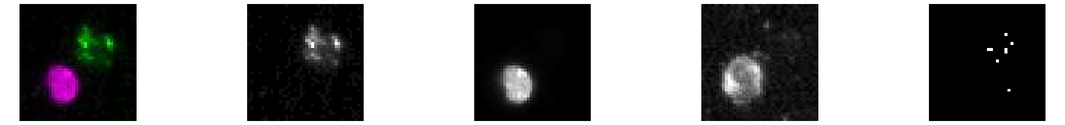				
5255	42	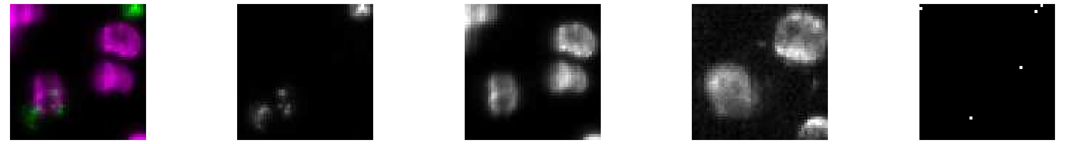				
5256	42	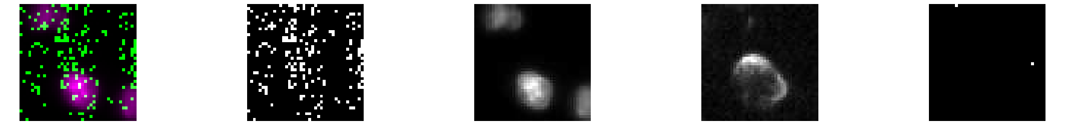				
5257	42	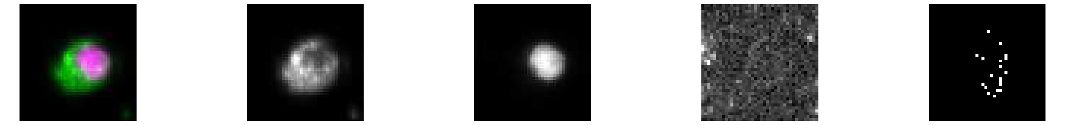				
5258	42	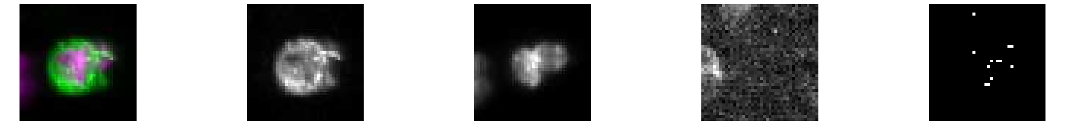				
5259	42	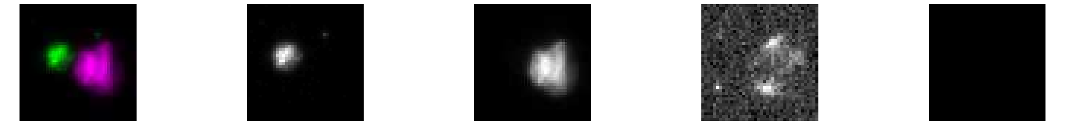				
5260	42	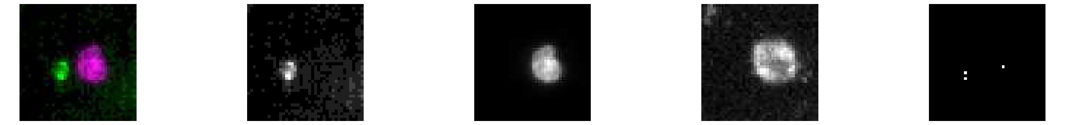				

Ereignis	Aufnahme	DAPI/CK-PE	CK-PE	DAPI	CD45-APC	HER-2/neu
5261	42	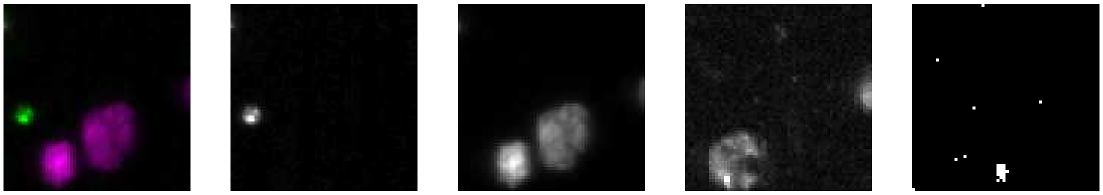				
5262	42	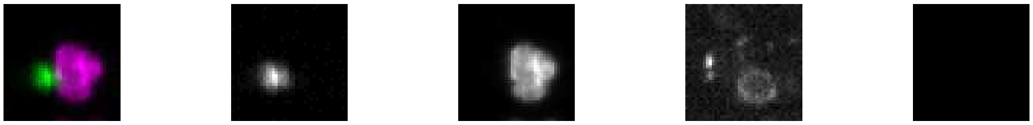				
5263	42	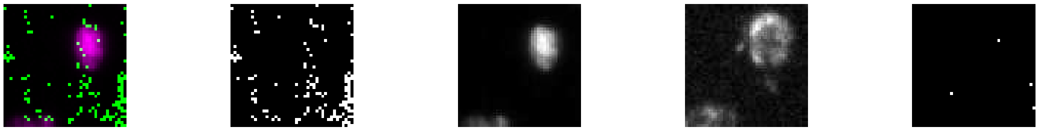				
5264	42	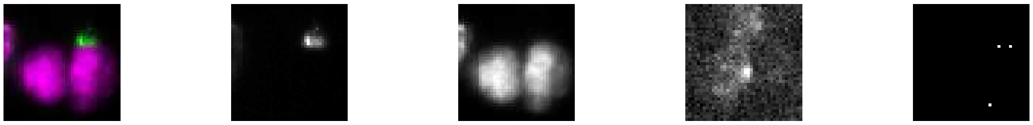				
5265	42	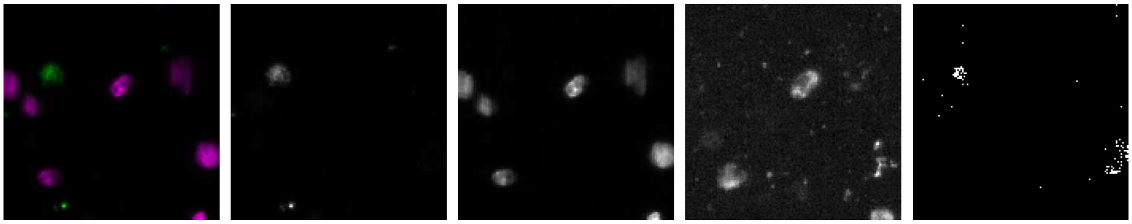				
5266	42	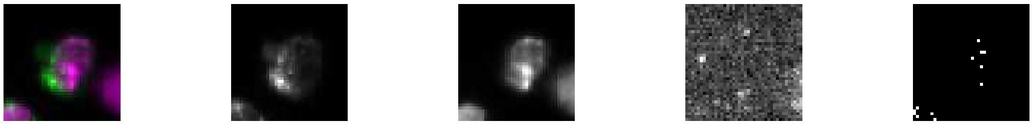				
5267	42	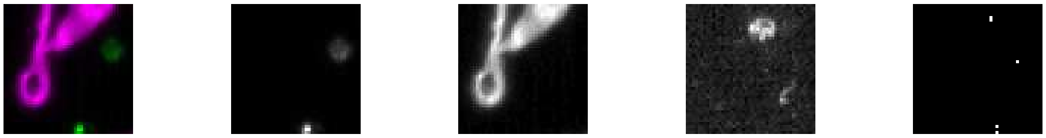				
5268	42	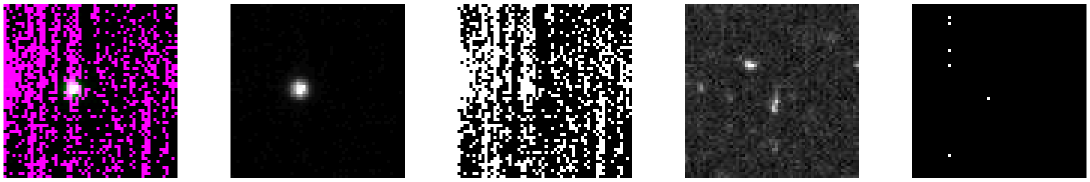				
5269	42	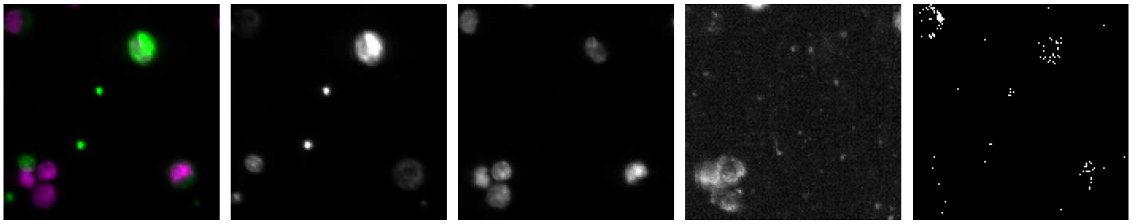				
5270	42	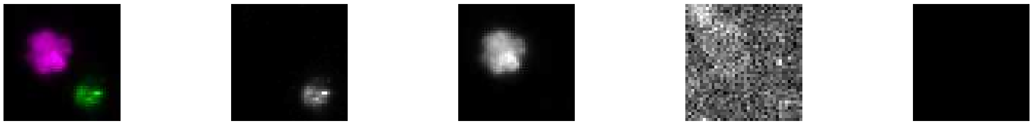				
5271	42	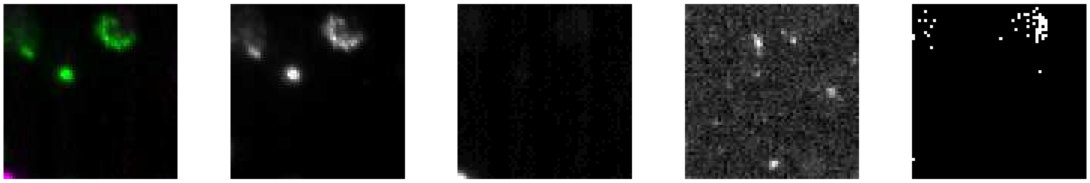				

Ereignis	Aufnahme	DAPI/CK-PE	CK-PE	DAPI	CD45-APC	HER-2/neu
5272	42	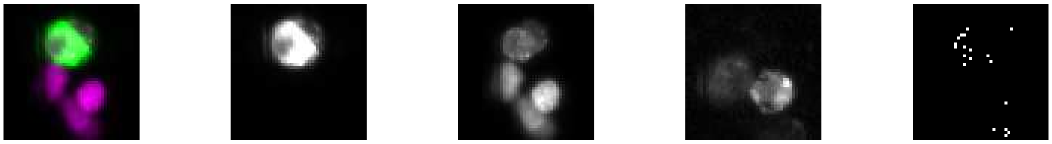				
5273	42	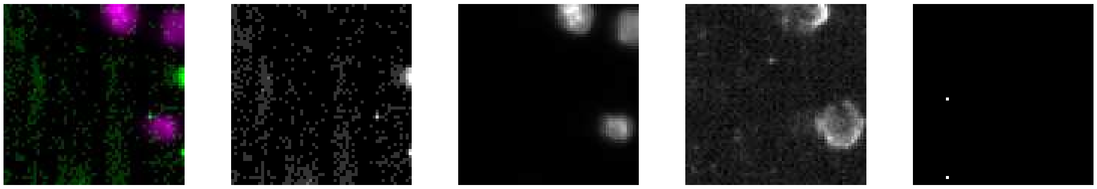				
5274	42	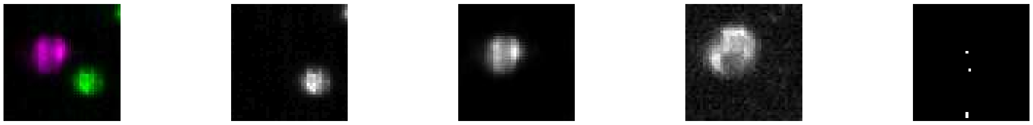				
5275	42	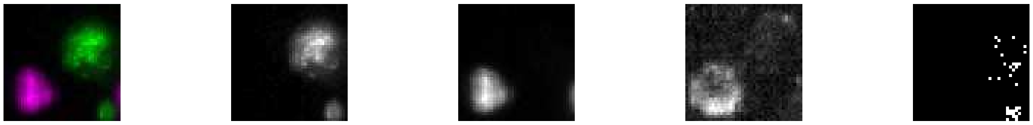				
5276	42	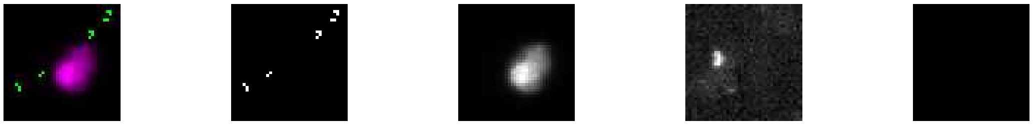				
5277	42	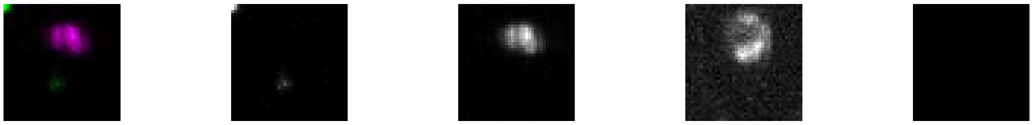				
5278	42	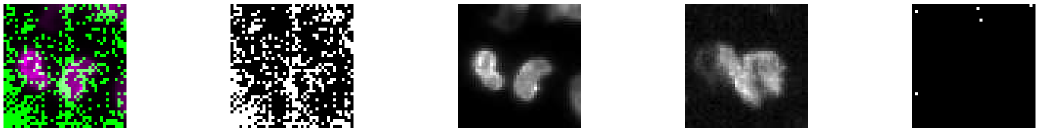				
5279	42	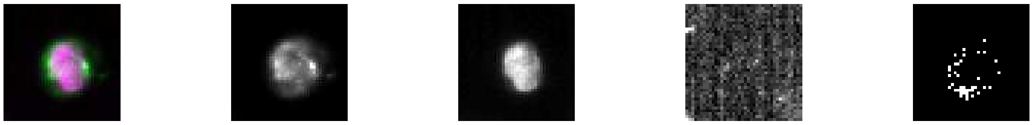				
5280	42	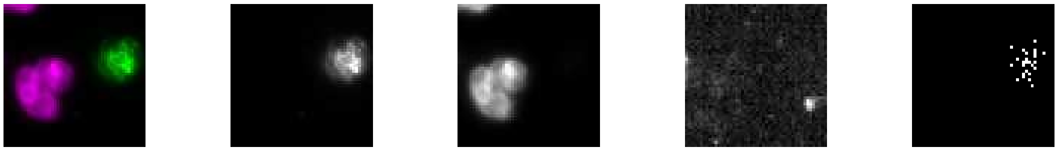				
5281	42	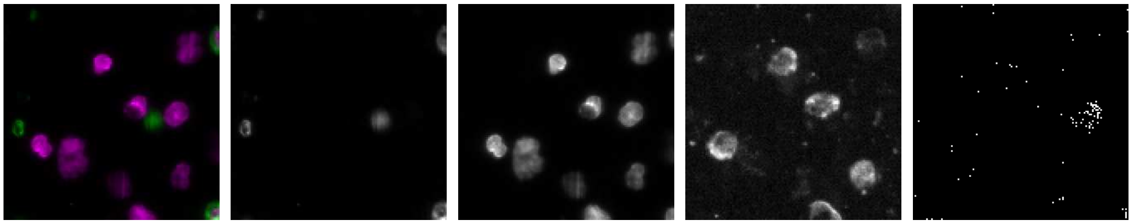				
5282	42	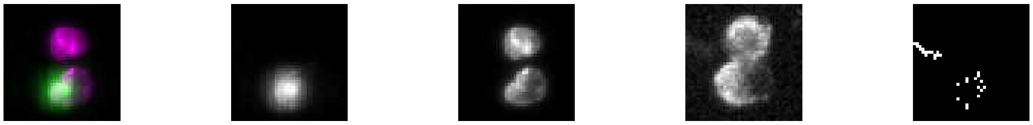				
5283	42	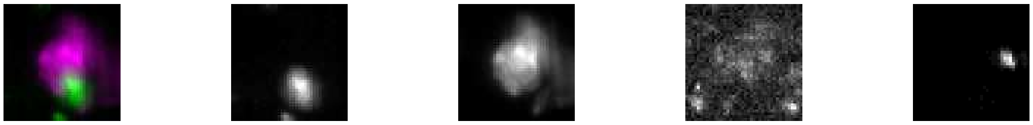				

Ereignis	Aufnahme	DAPI/CK-PE	CK-PE	DAPI	CD45-APC	HER-2/neu
5284	42	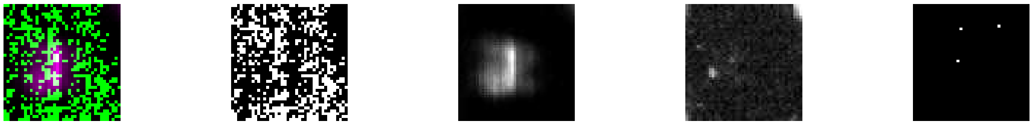				
5285	42	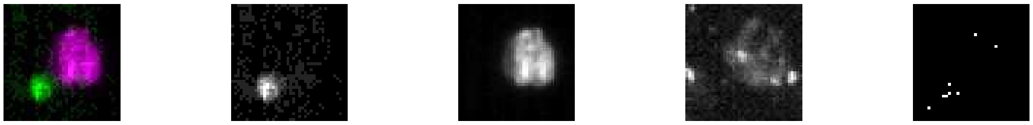				
5286	42	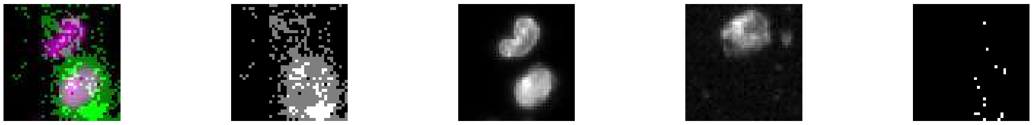				
5287	42	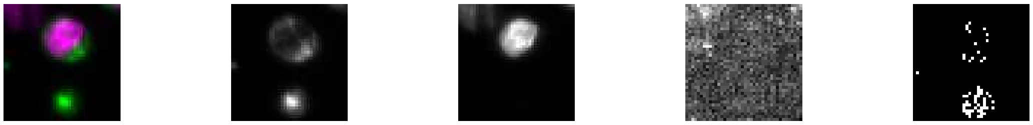				
5288	42	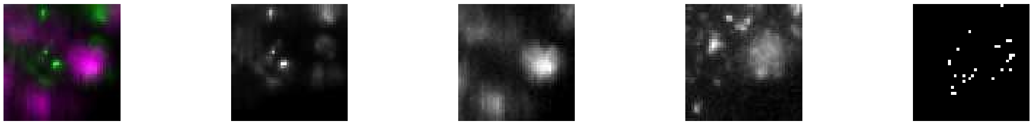				
5289	42	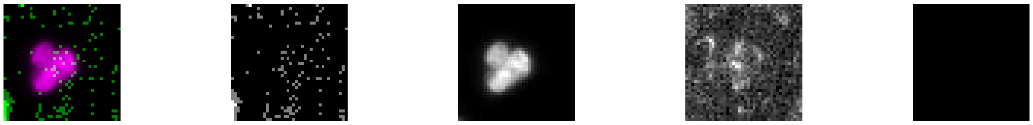				
5290	42	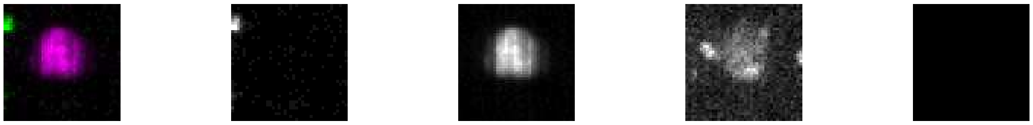				
5291	42	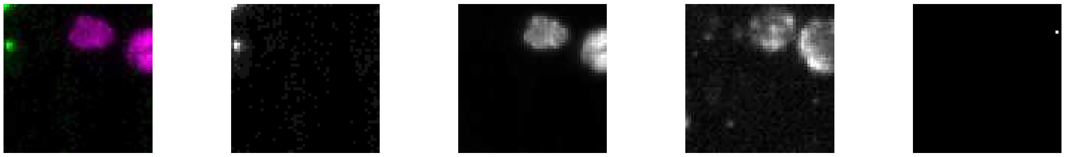				
5292	42	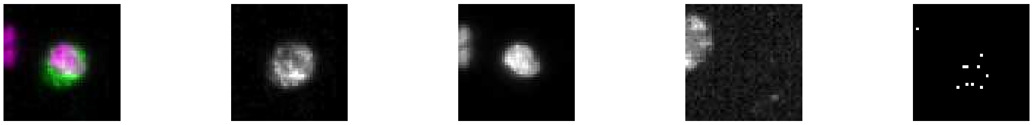				
5293	42	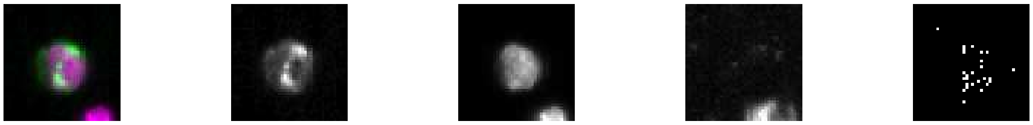				
5294	42	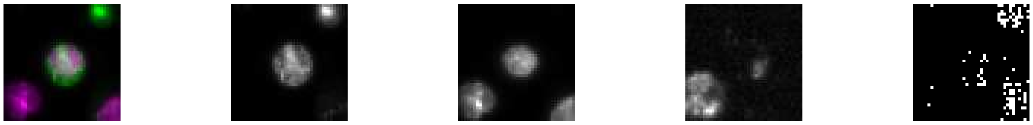				
5295	42	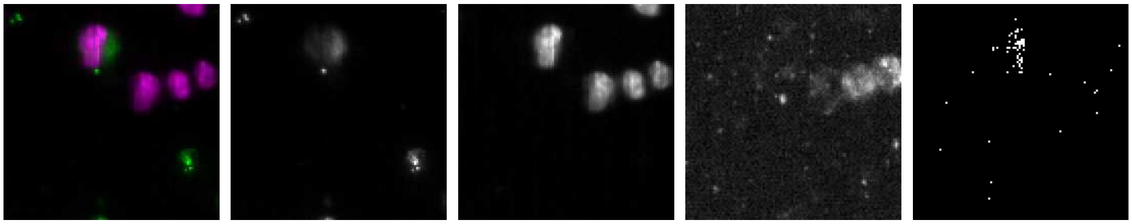				
5296	42	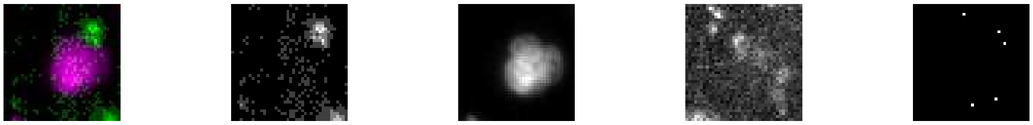				

Ereignis	Aufnahme	DAPI/CK-PE	CK-PE	DAPI	CD45-APC	HER-2/neu
5297	42	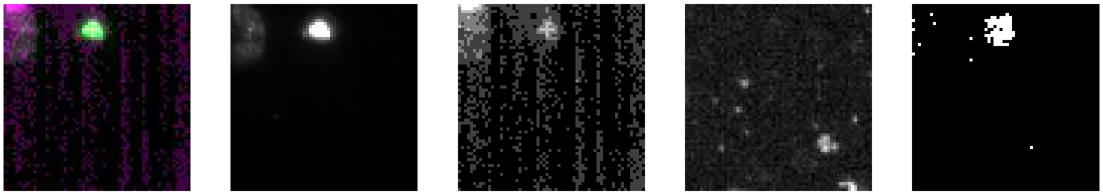				
5298	42	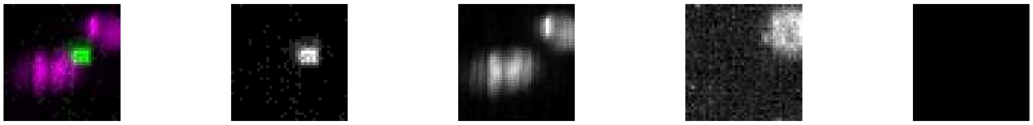				
5299	42	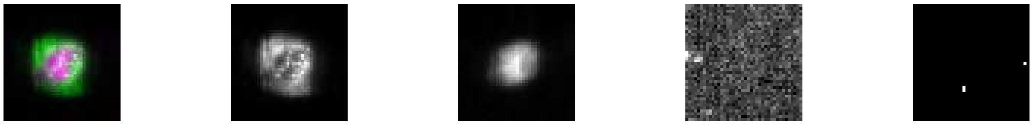				
5300	42	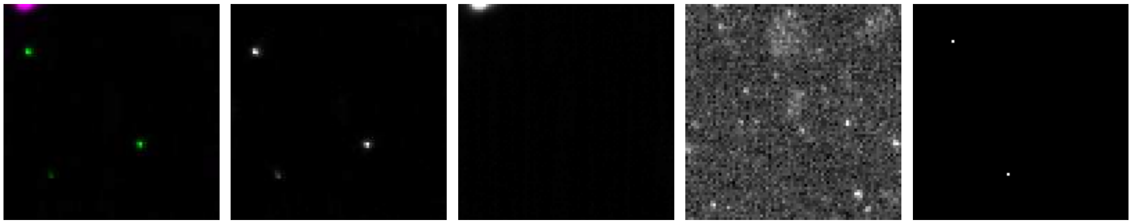				
5301	42	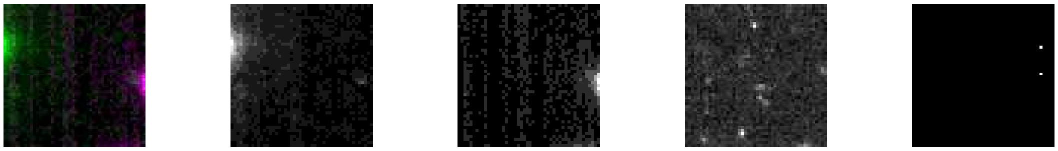				
5302	42	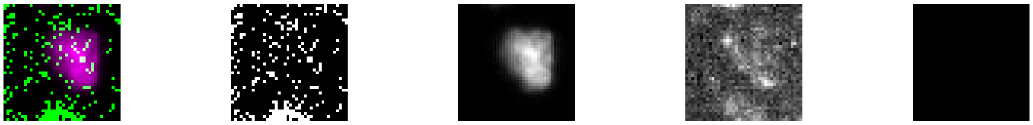				
5303	42	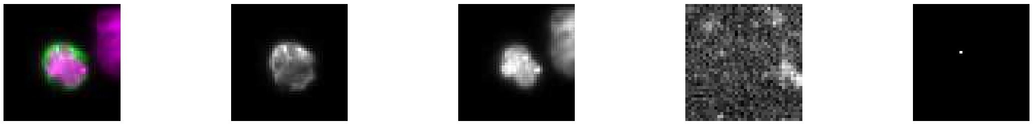				
5304	42	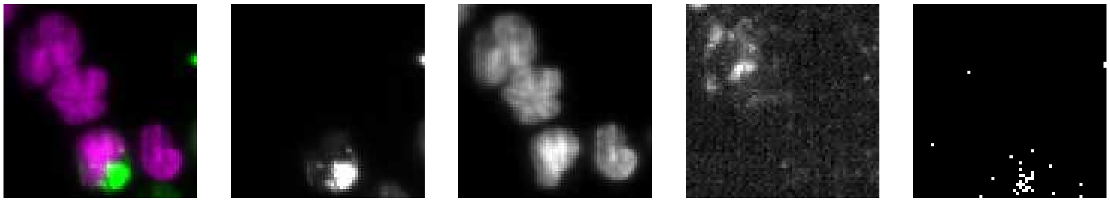				
5305	42	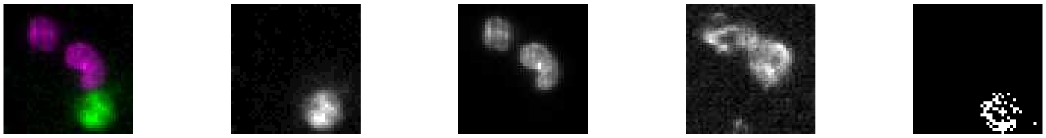				
5306	42	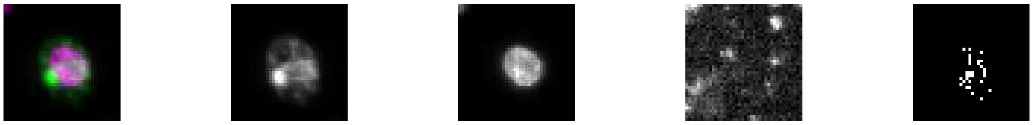				
5307	42	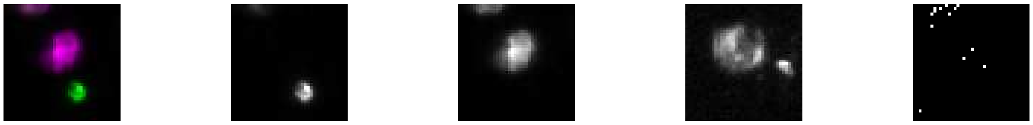				
5308	42	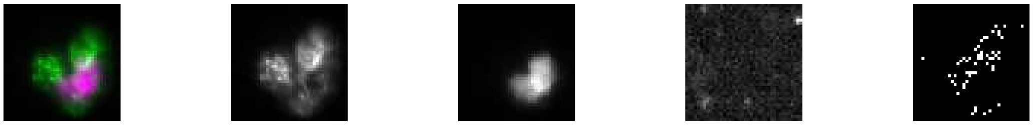				

Ereignis	Aufnahme	DAPI/CK-PE	CK-PE	DAPI	CD45-APC	HER-2/neu
5309	42	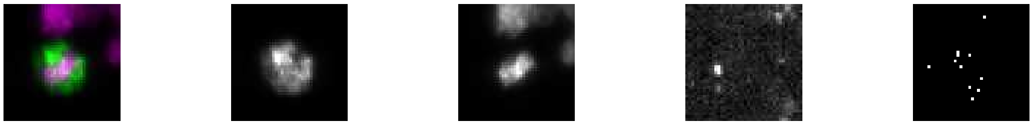				
5310	42	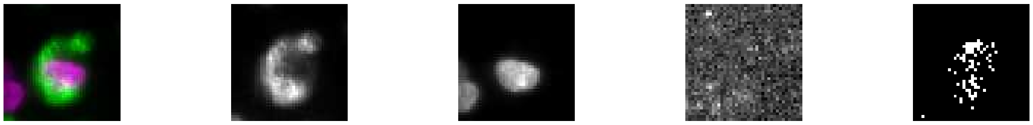				
5311	42	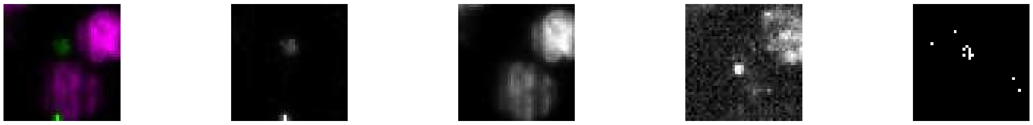				
5312	42	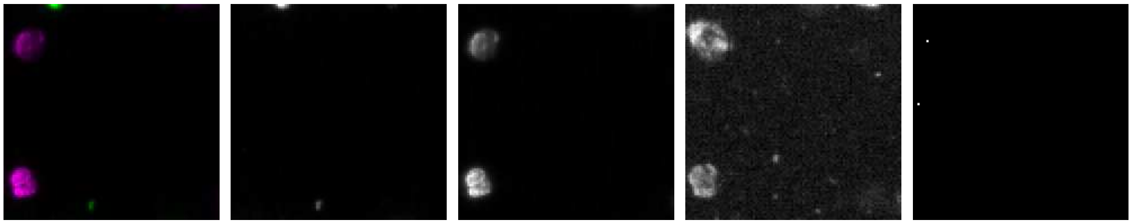				
5313	42	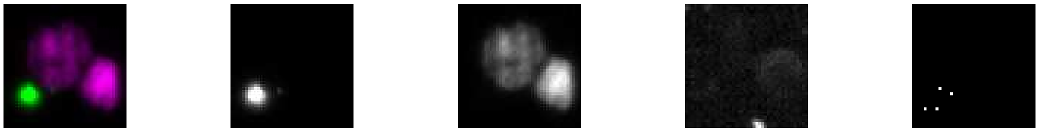				
5314	42	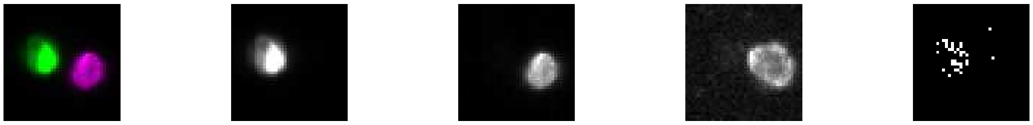				
5315	42	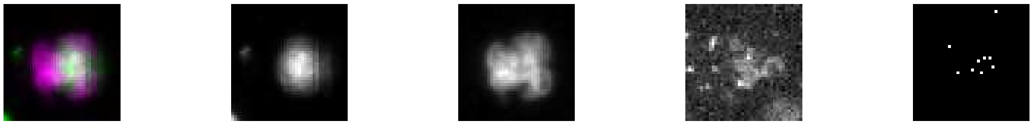				
5316	42	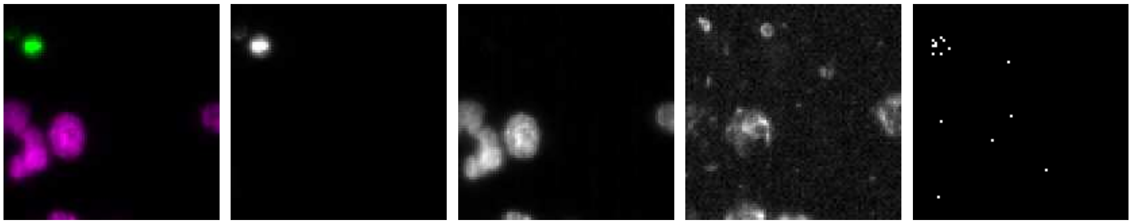				
5317	42	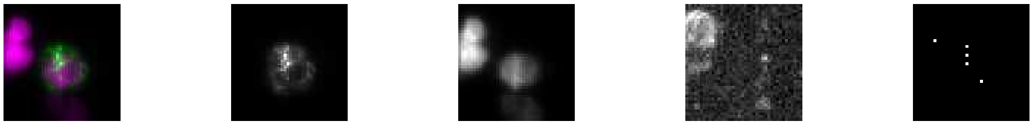				
5318	42	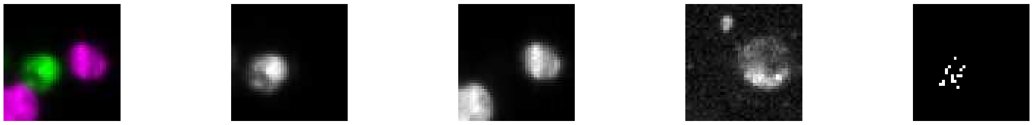				
5319	42	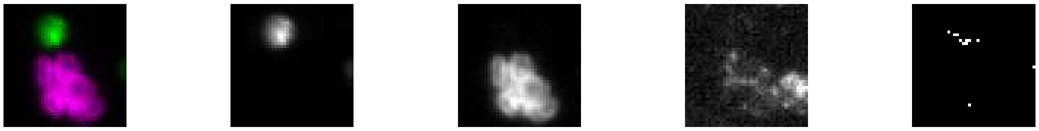				
5320	42	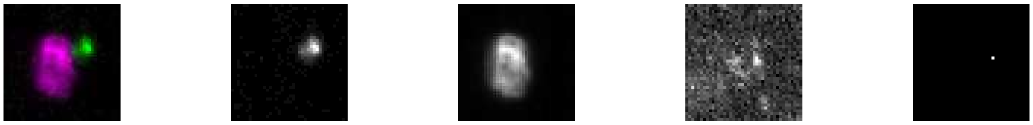				

Ereignis	Aufnahme	DAPI/CK-PE	CK-PE	DAPI	CD45-APC	HER-2/neu
5321	42	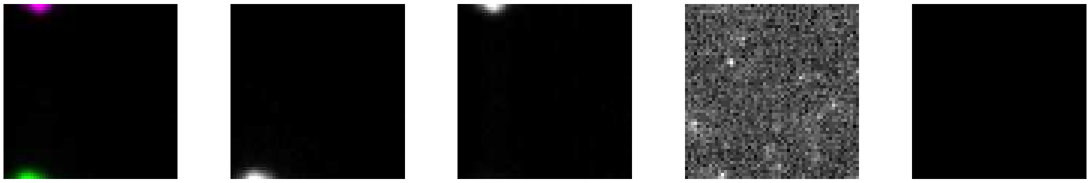				
5322	42	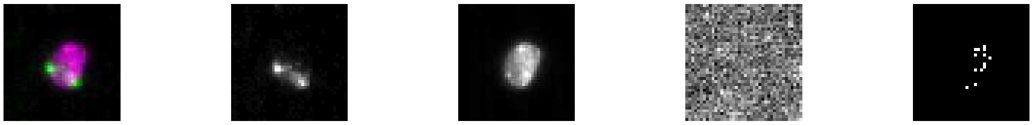				
5323	42	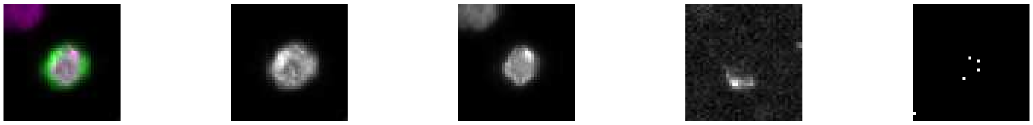				
5324	42	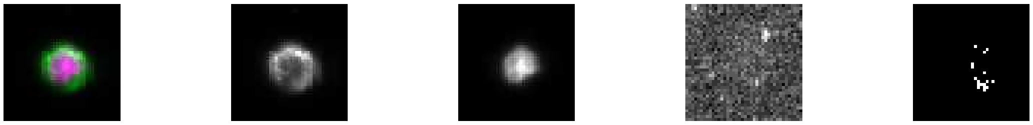				
5325	42	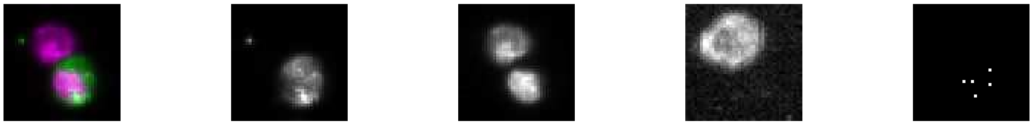				
5326	42	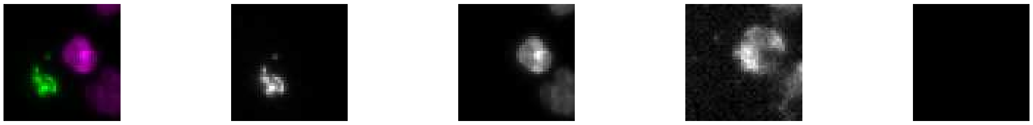				
5327	42	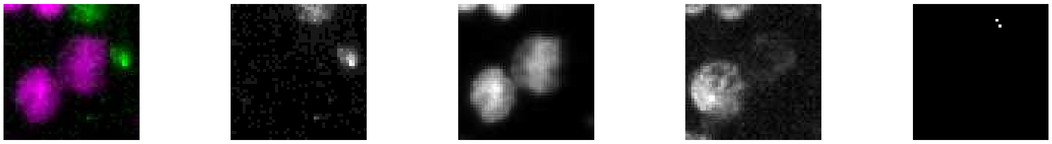				
5328	42	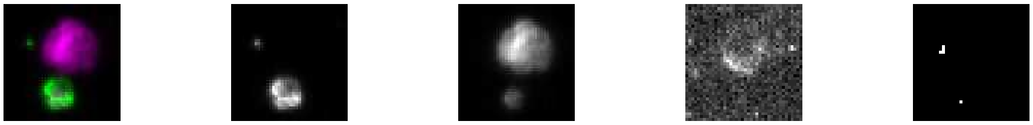				
5329	42	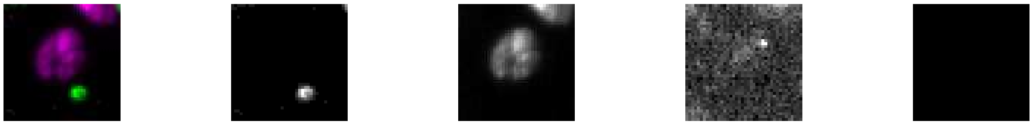				
5330	42	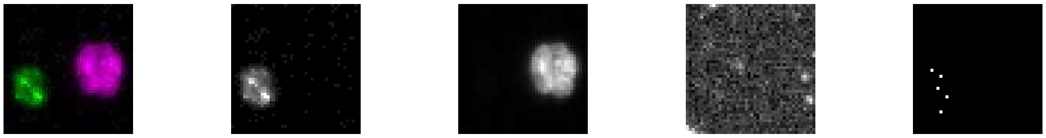				
5331	42	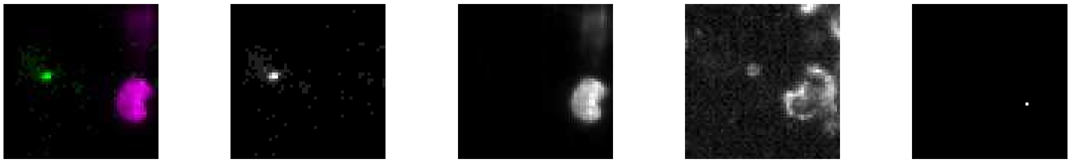				
5332	42	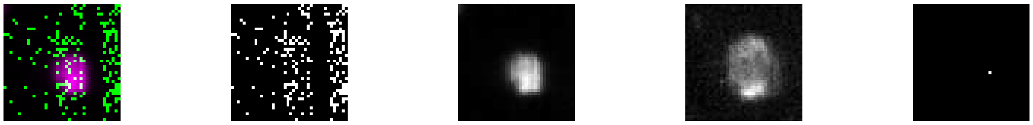				
5333	42	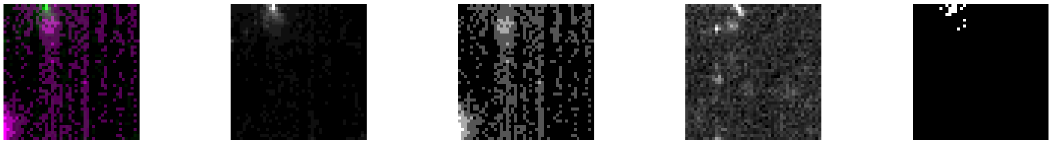				

Ereignis	Aufnahme	DAPI/CK-PE	CK-PE	DAPI	CD45-APC	HER-2/neu
5334	42	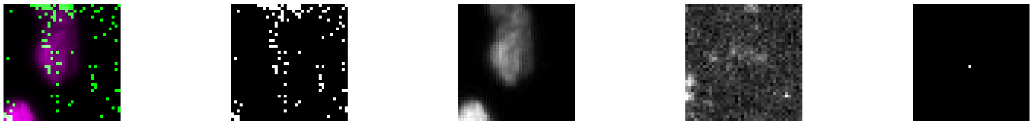				
5335	42	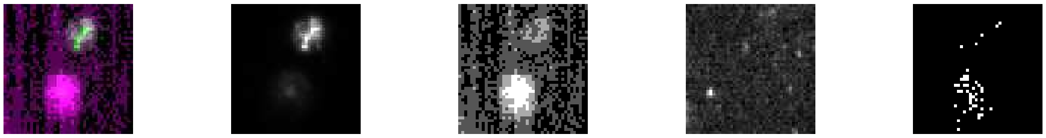				
5336	42	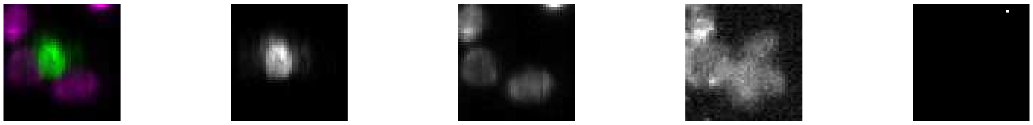				
5337	42	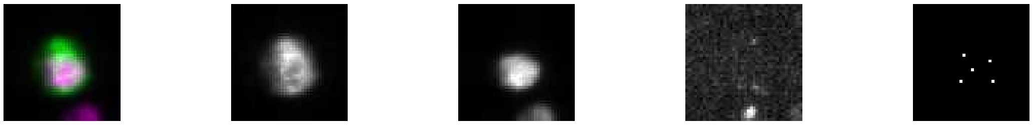				
5338	42	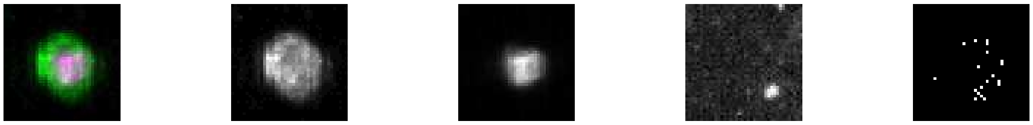				
5339	42	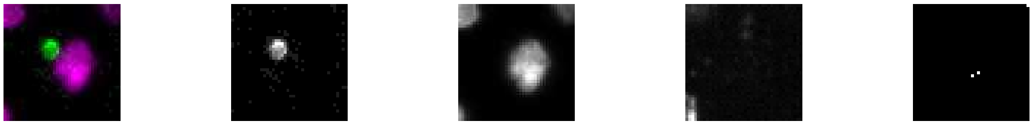				
5340	42	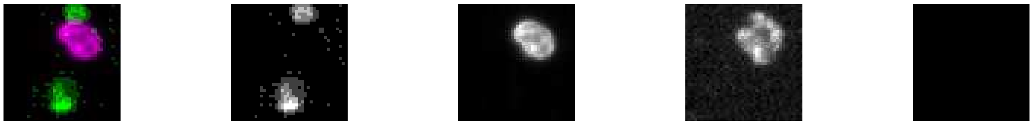				
5341	42	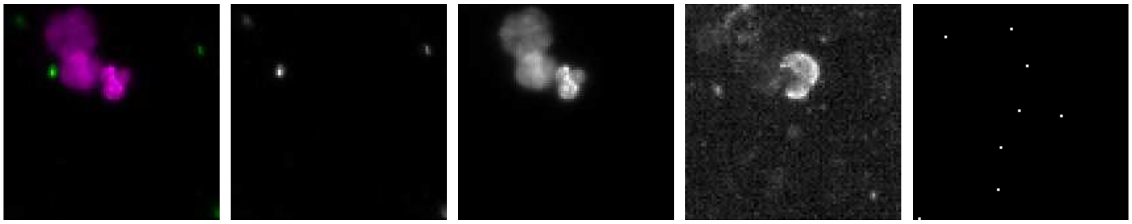				
5342	42	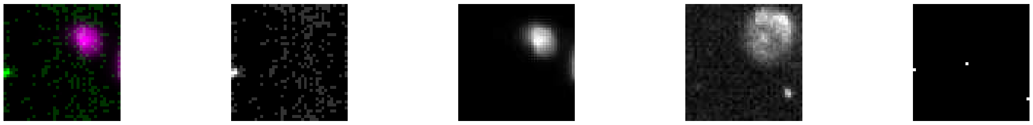				
5343	42	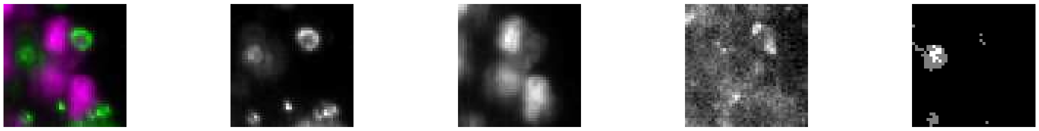				
5344	42	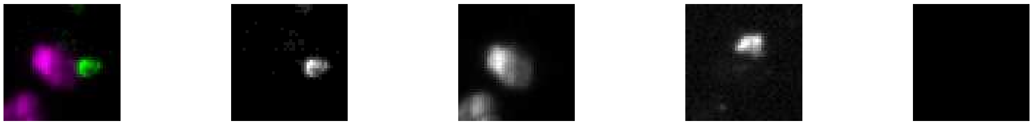				
5345	42	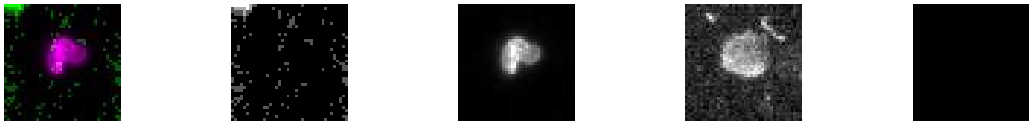				
5346	42	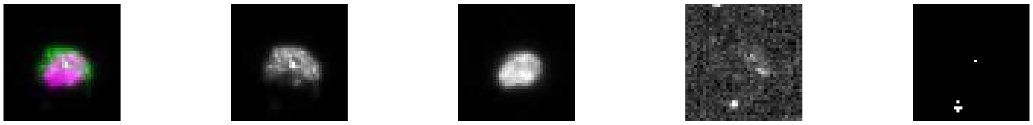				

Ereignis	Aufnahme	DAPI/CK-PE	CK-PE	DAPI	CD45-APC	HER-2/neu
5347	42	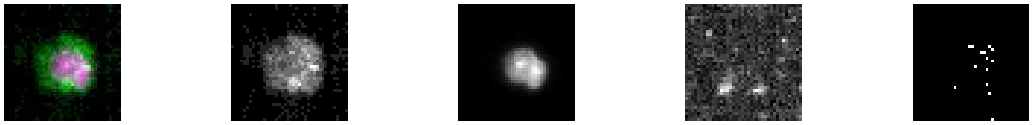				
5348	42	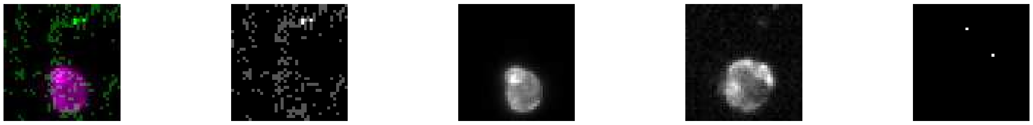				
5349	42	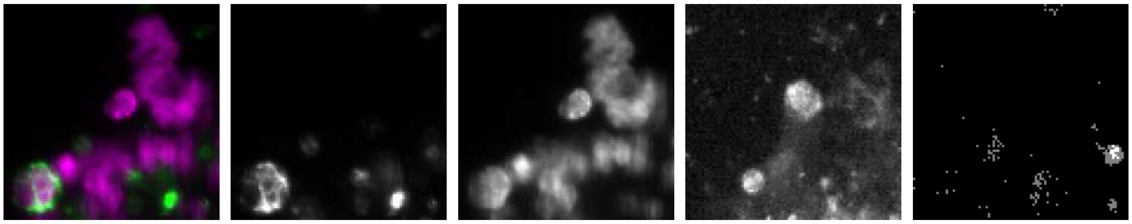				
5350	42	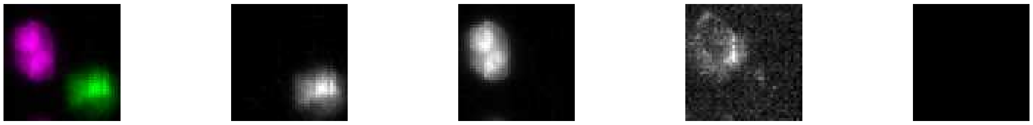				
5351	42	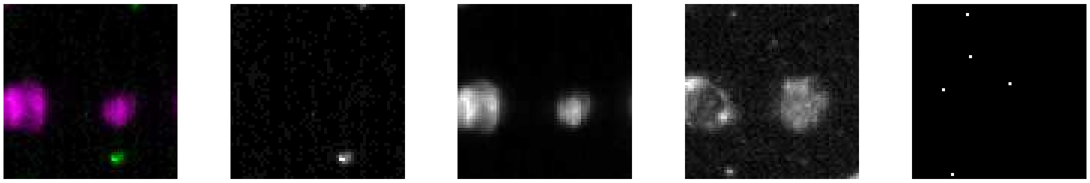				
5352	42	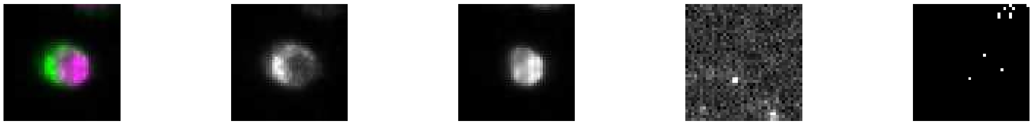				
5353	42	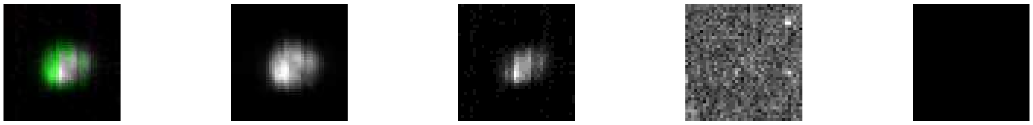				
5354	42	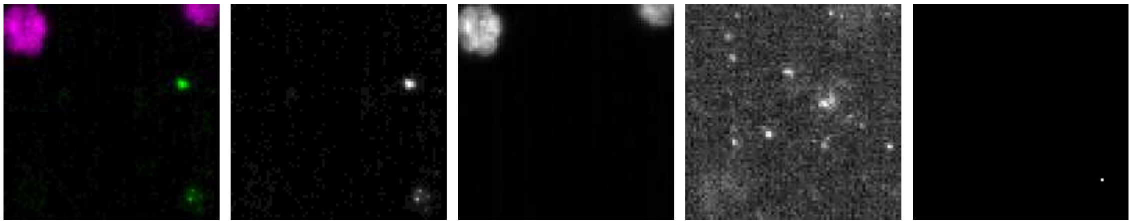				
5355	42	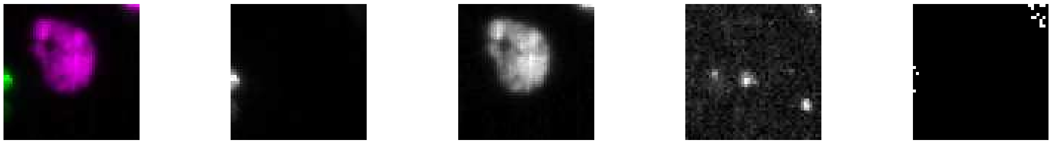				
5356	42	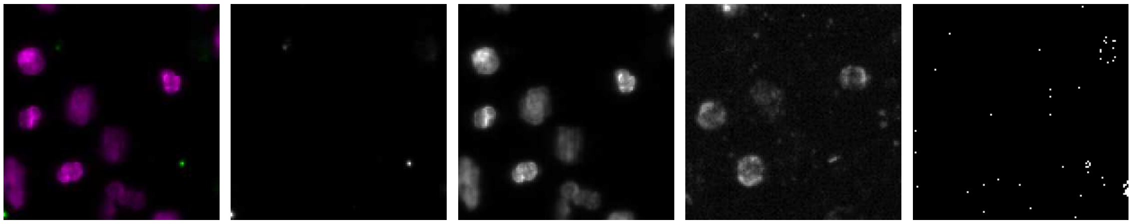				
5357	42	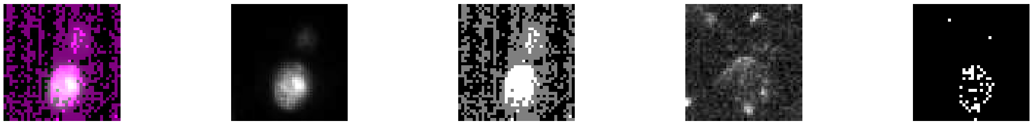				

Ereignis	Aufnahme	DAPI/CK-PE	CK-PE	DAPI	CD45-APC	HER-2/neu
5358	42	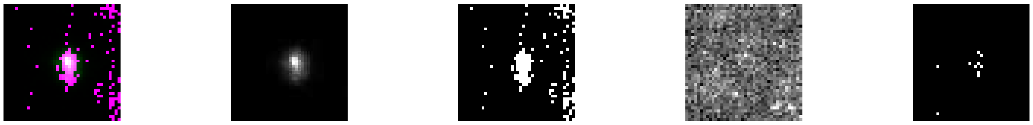				
5359	42	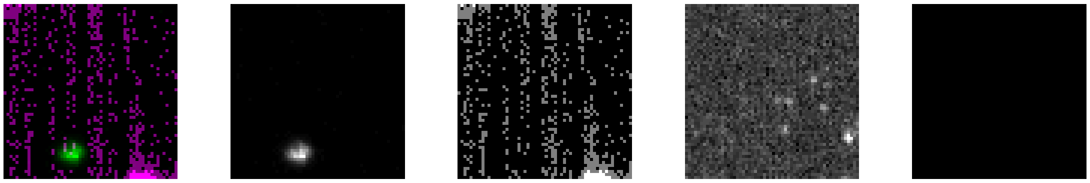				
5360	42	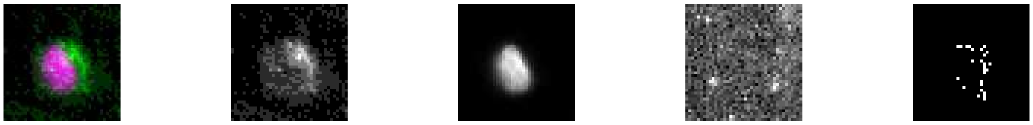				
5361	42	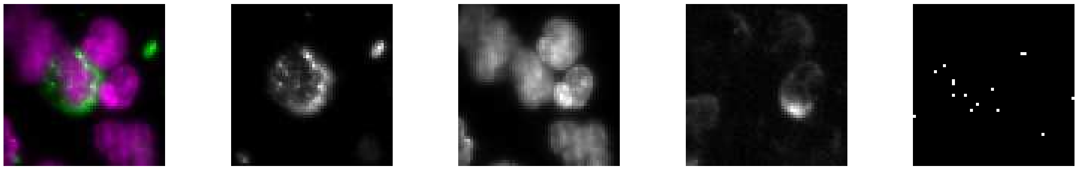				
5362	42	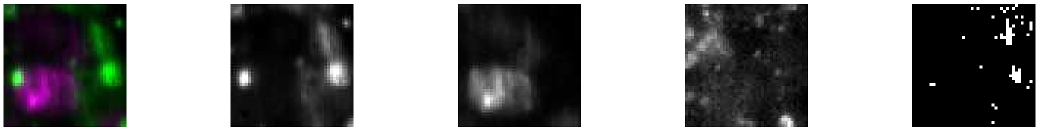				
5363	42	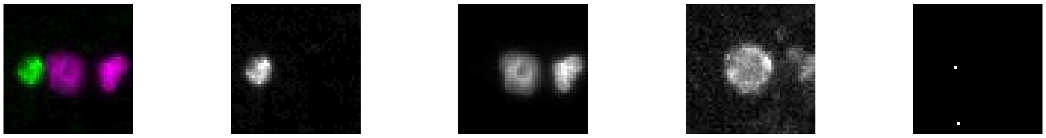				
5364	42	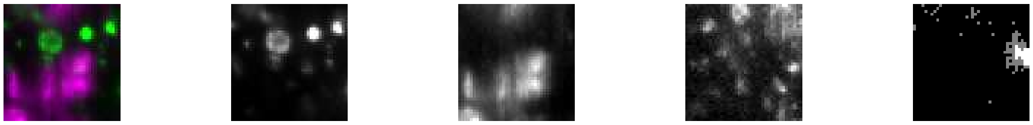				
5365	42	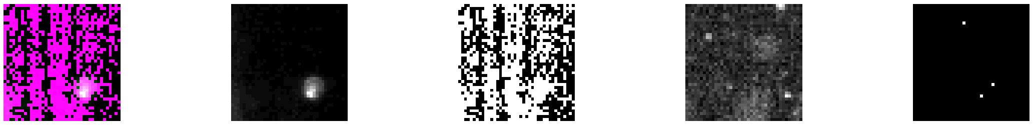				
5366	42	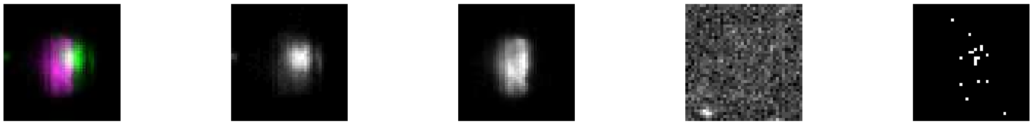				
5367	42	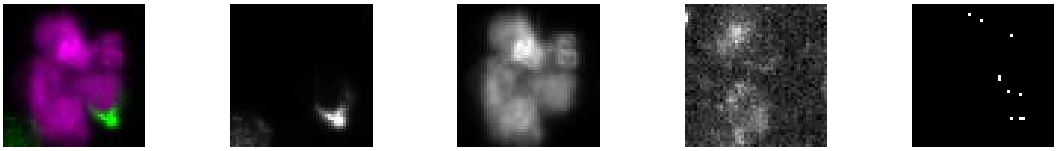				
5368	42	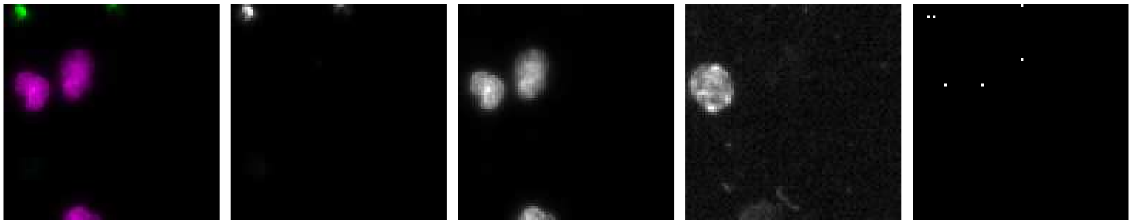				
5369	42	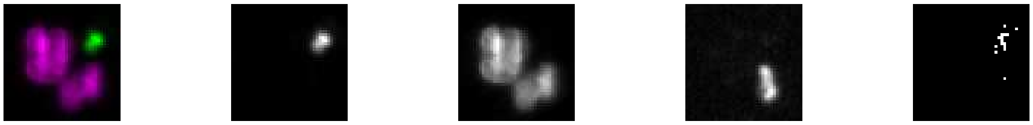				

Ereignis	Aufnahme	DAPI/CK-PE	CK-PE	DAPI	CD45-APC	HER-2/neu
5370	42	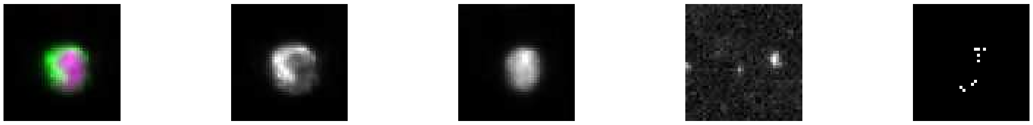				
5371	42	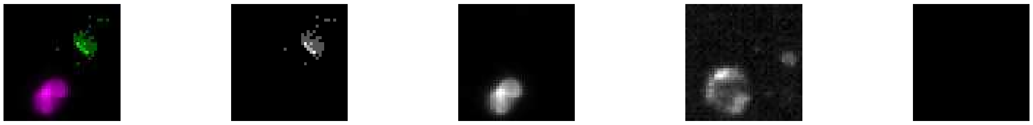				
5372	42	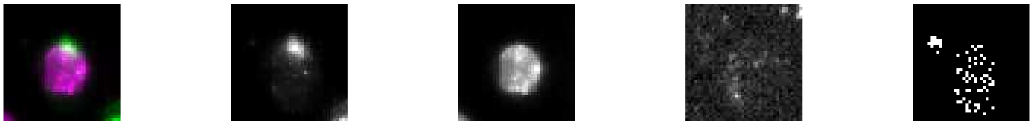				
5373	42	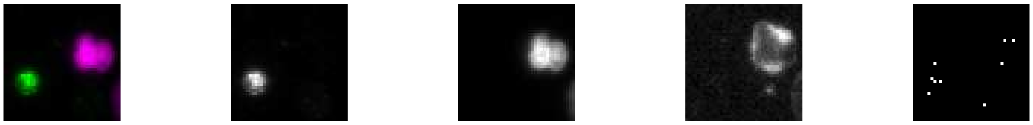				
5374	42	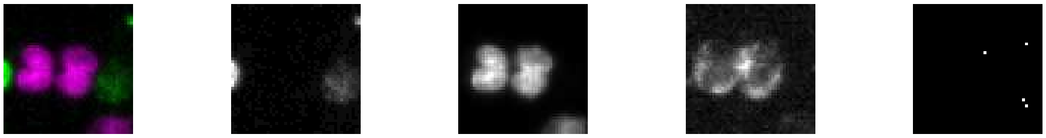				
5375	42	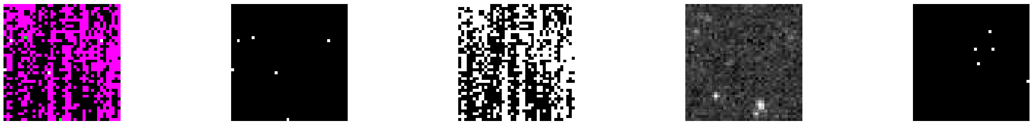				
5376	42	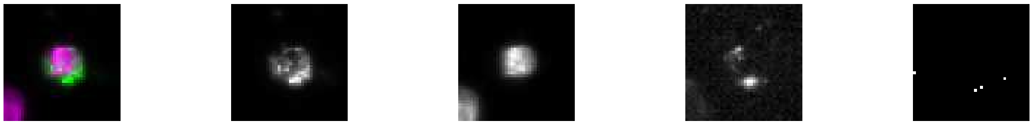				
5377	42	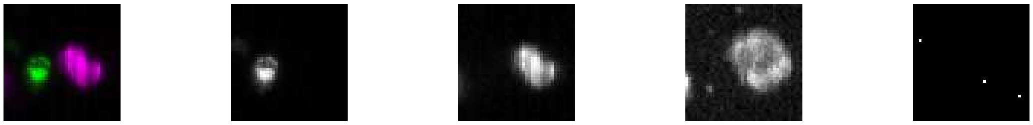				
5378	42	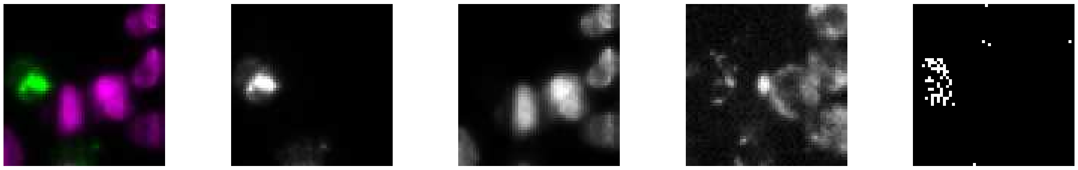				
5379	42	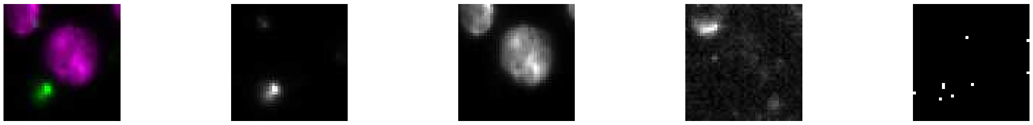				
5380	42	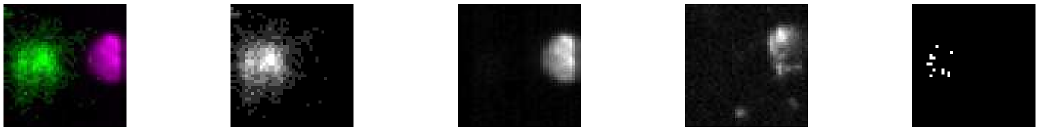				
5381	42	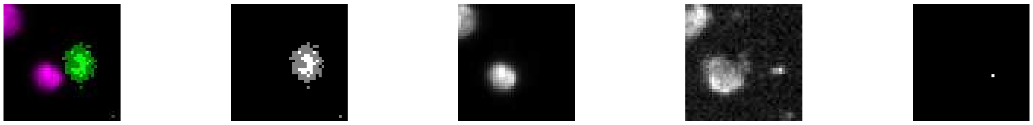				
5382	42	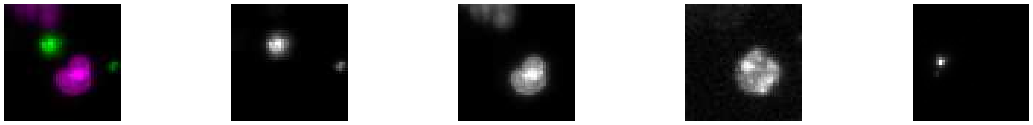				

Ereignis	Aufnahme	DAPI/CK-PE	CK-PE	DAPI	CD45-APC	HER-2/neu
5383	42	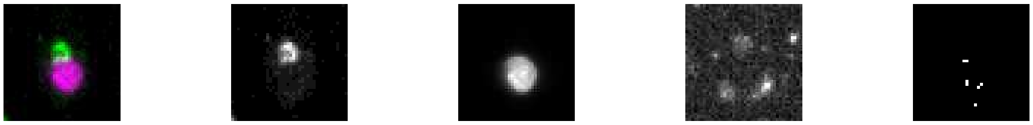				
5384	42	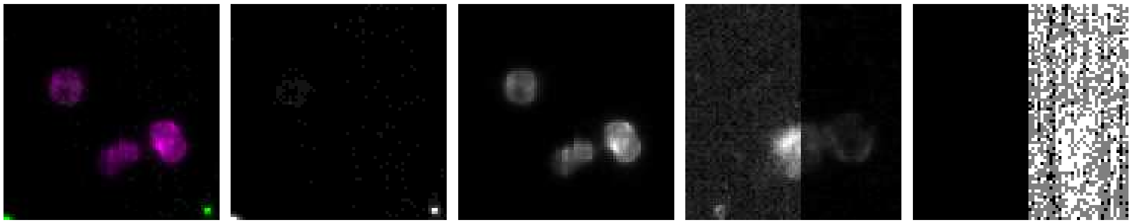				
5385	41	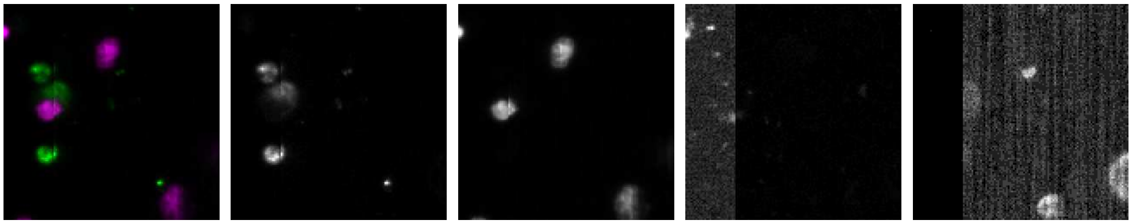				
5386	42	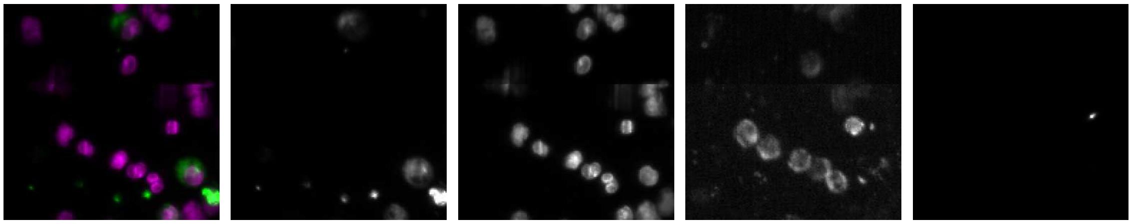				
5387	42	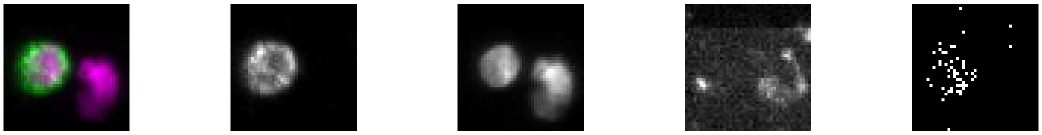				
5388	41	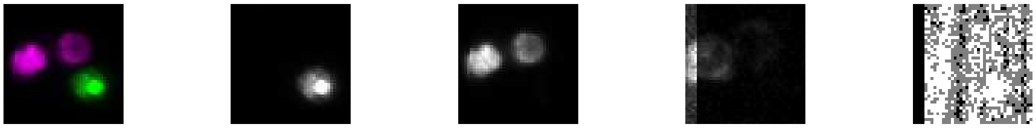				
5389	29	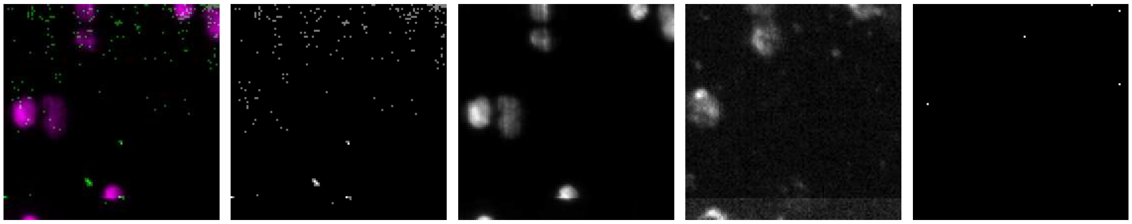				
5390	29	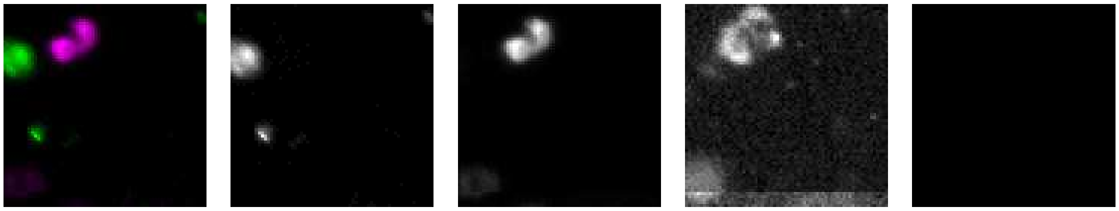				
5391	41	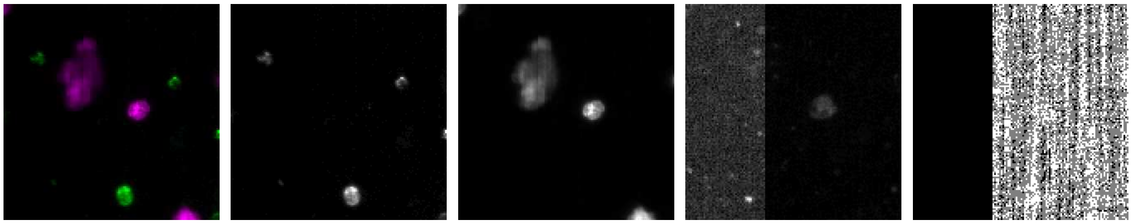				

Ereignis	Aufnahme	DAPI/CK-PE	CK-PE	DAPI	CD45-APC	HER-2/neu
5392	41	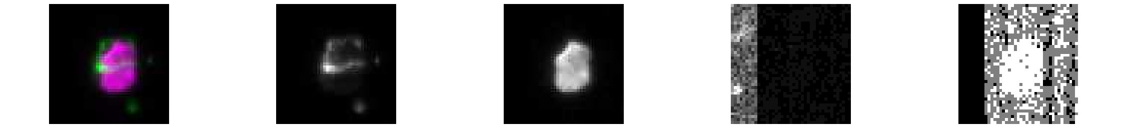				
5393	42	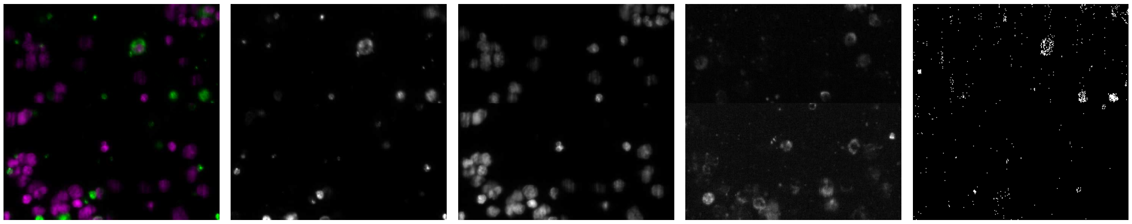				
5394	42	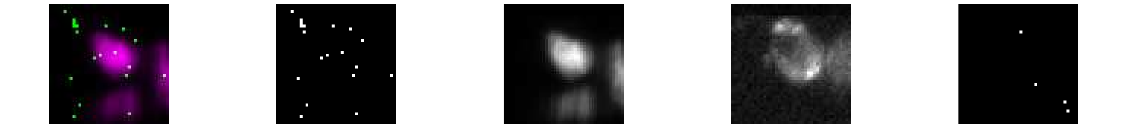				
5395	43	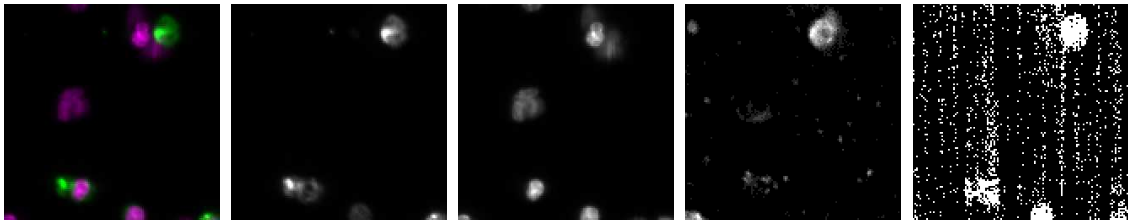				
5396	43	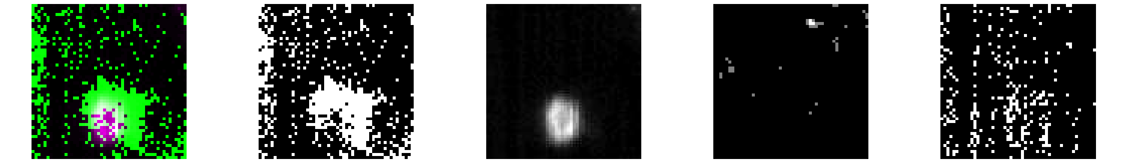				
5397	43	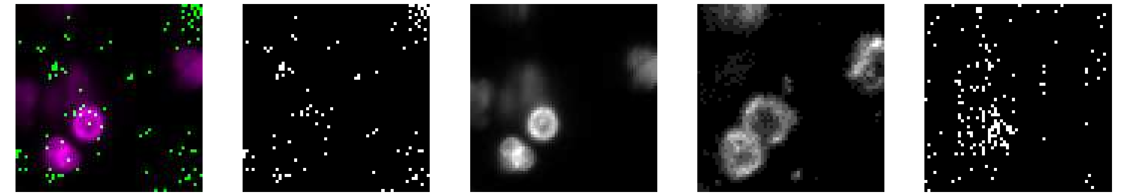				
5398	43	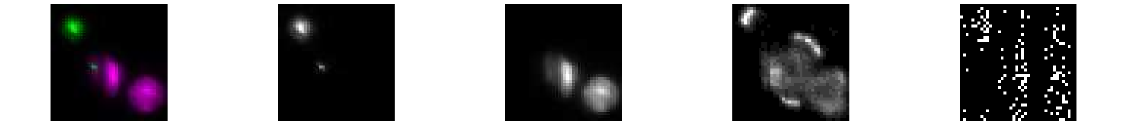				
5399	43	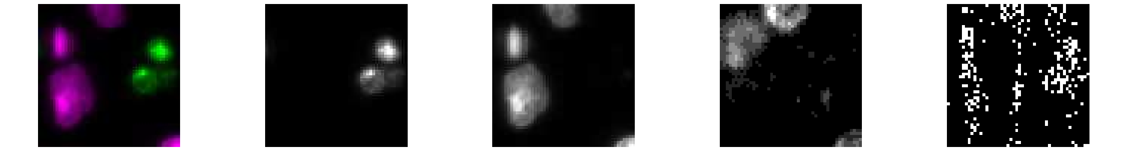				
5400	43	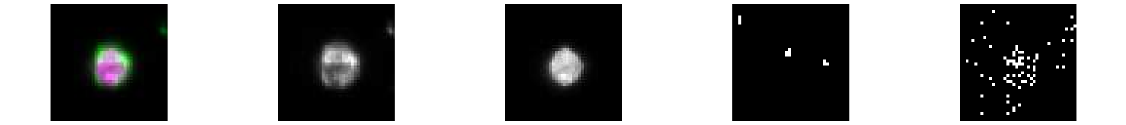				
5401	43	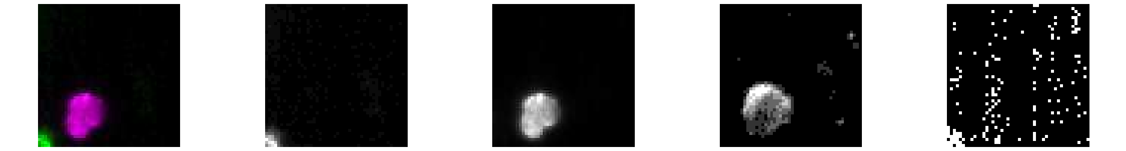				
5402	43	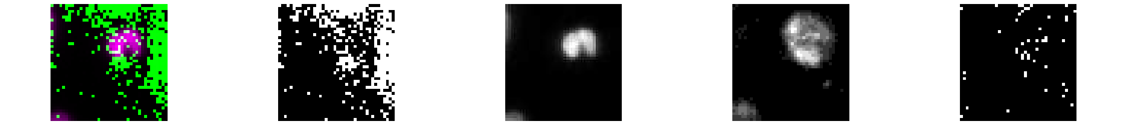				

Proben-ID: Gyn14.208 **Kassetten-ID:** 01327106 **Druckdatum:** 02/06/2020 16:41 **Patienten-ID:**

Ereignis	Aufnahme	DAPI/CK-PE	CK-PE	DAPI	CD45-APC	HER-2/neu
5403	43	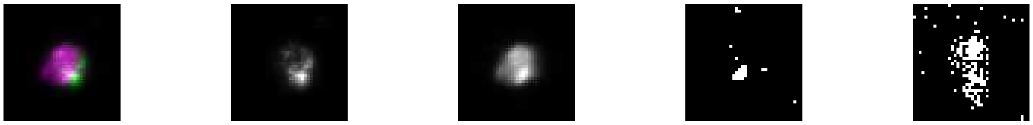				
5404	43	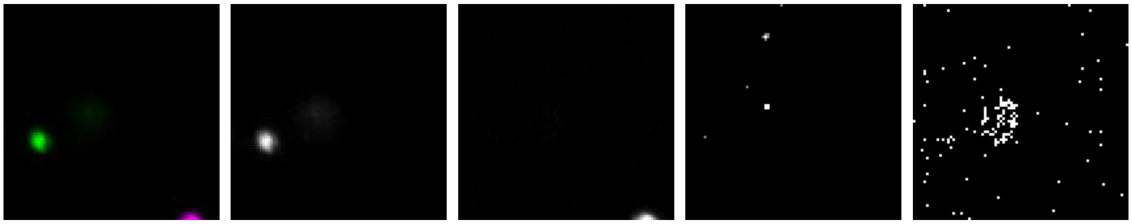				
5405	43	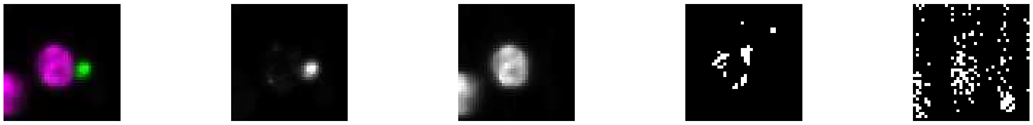				
5406	43	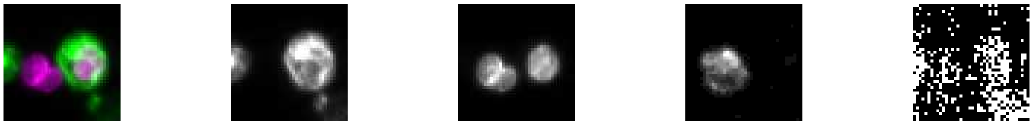				
5407	43	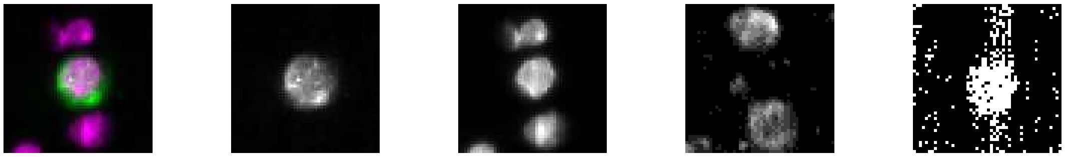				
5408	43	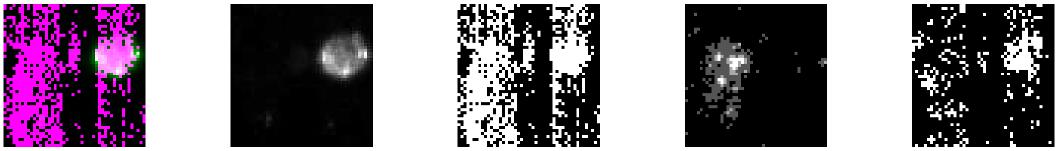				
5409	43	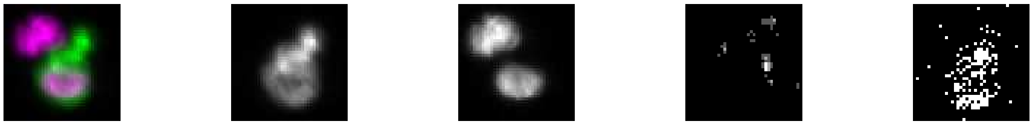				
5410	43	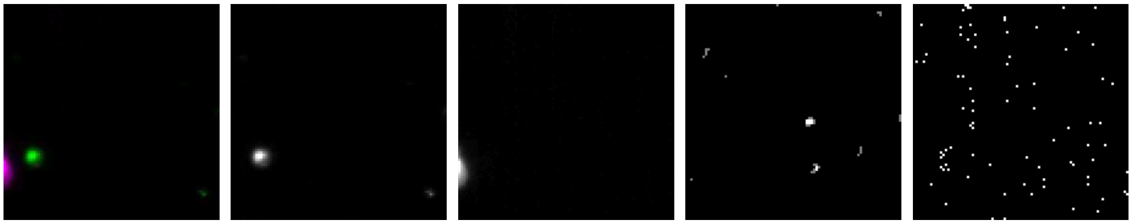				
5411	43	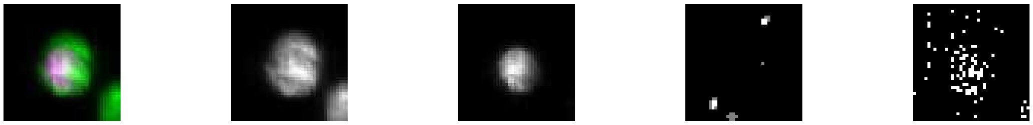				
5412	43	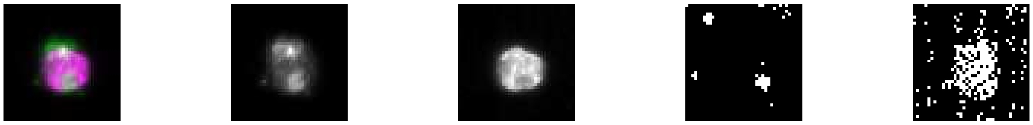				
5413	43	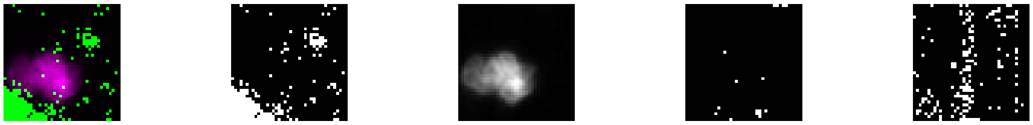				
5414	43	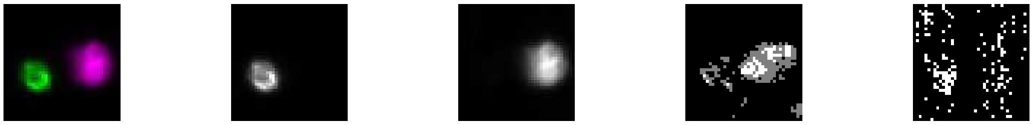				

Ereignis	Aufnahme	DAPI/CK-PE	CK-PE	DAPI	CD45-APC	HER-2/neu
5415	43	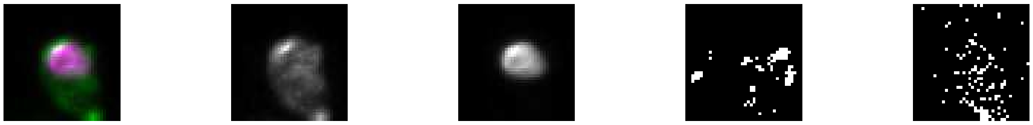				
5416	43	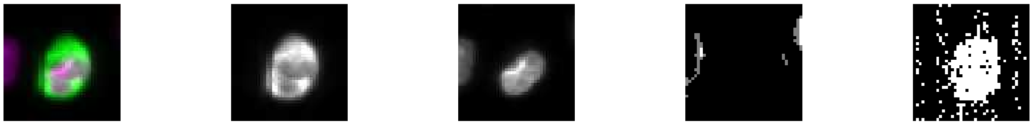				
5417	43	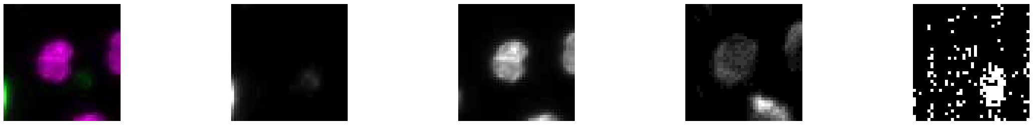				
5418	43	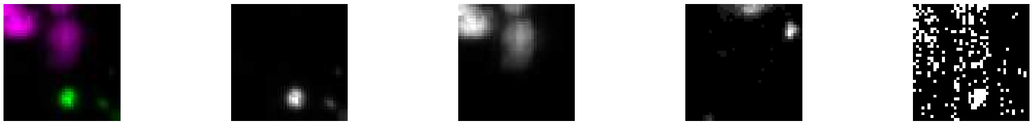				
5419	43	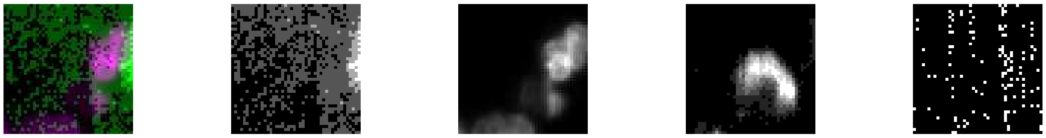				
5420	43	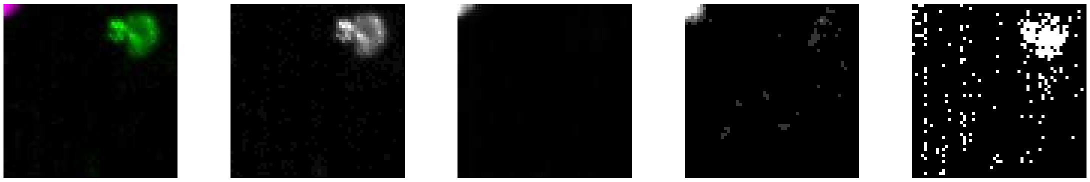				
5421	43	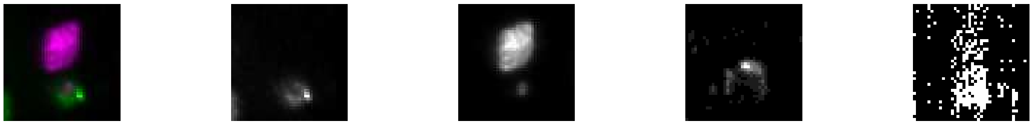				
5422	43	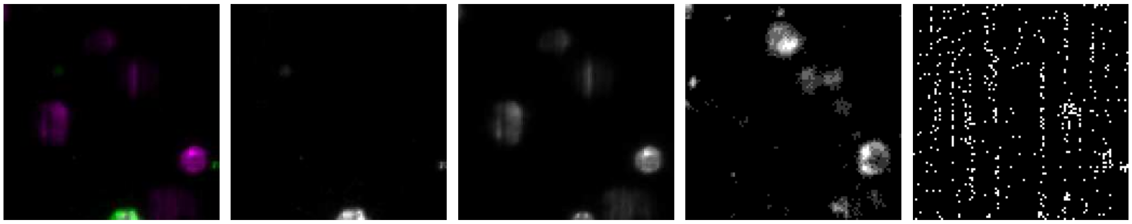				
5423	43	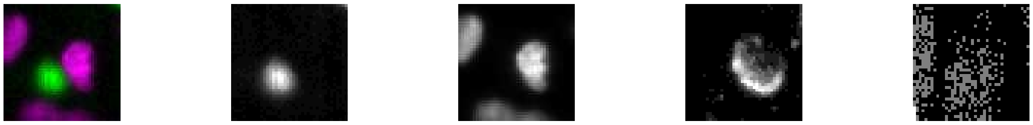				
5424	43	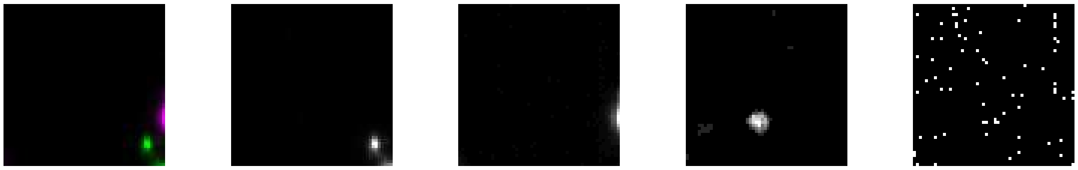				
5425	43	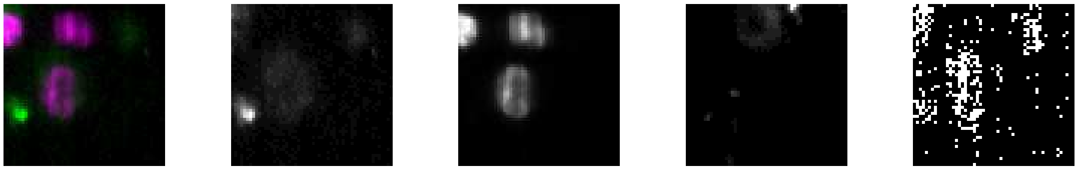				
5426	43	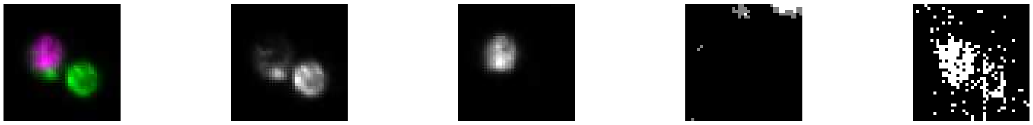				

Ereignis	Aufnahme	DAPI/CK-PE	CK-PE	DAPI	CD45-APC	HER-2/neu
5427	43	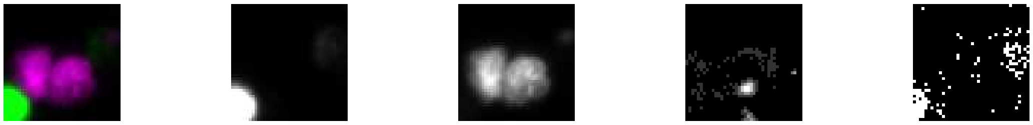				
5428	43	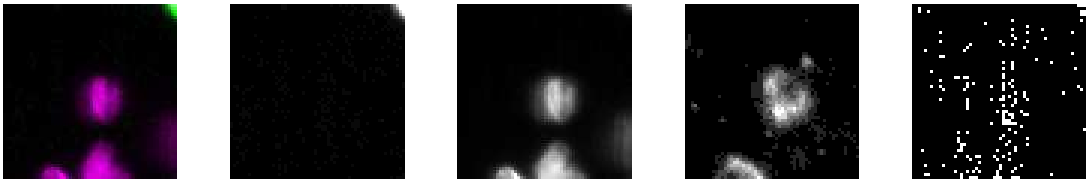				
5429	43	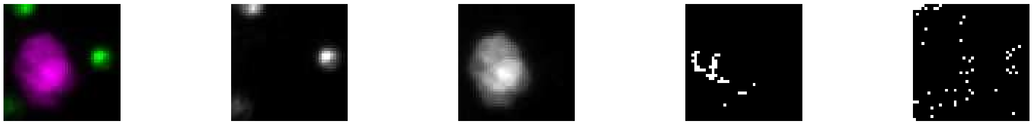				
5430	43	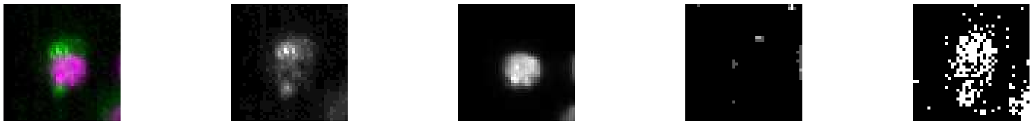				
5431	43	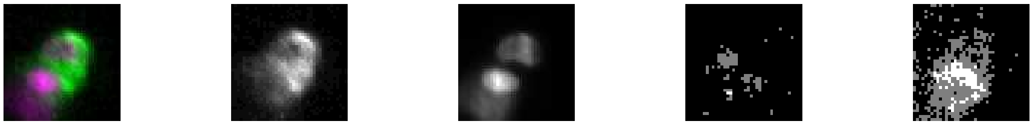				
5432	43	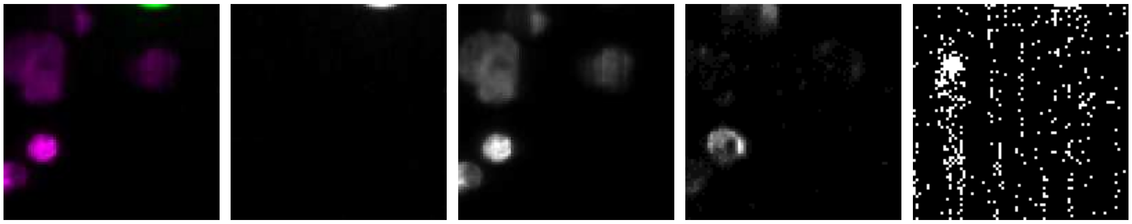				
5433	43	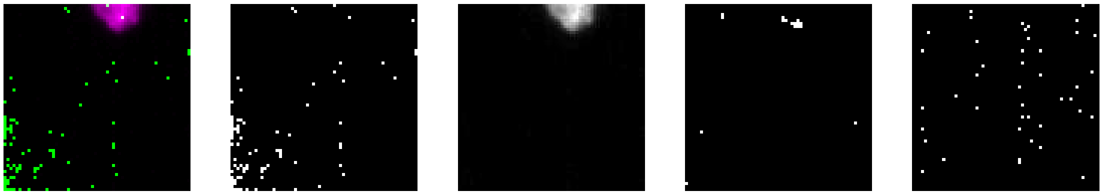				
5434	43	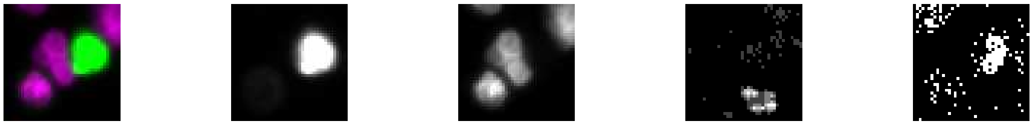				
5435	43	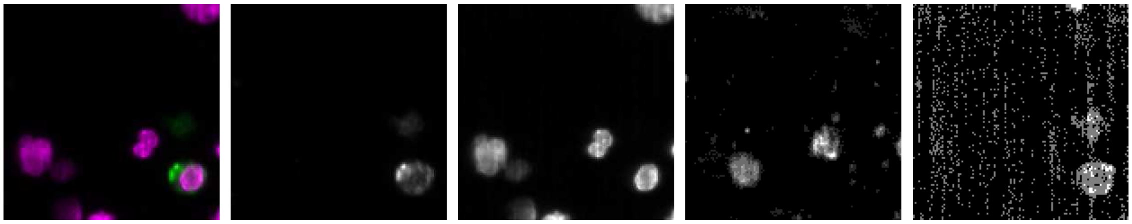				
5436	43	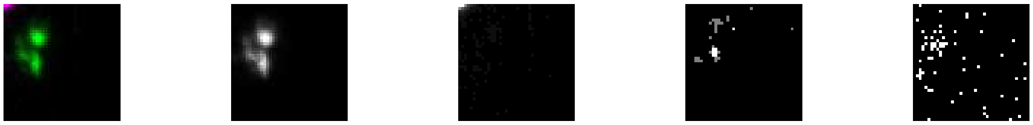				
5437	43	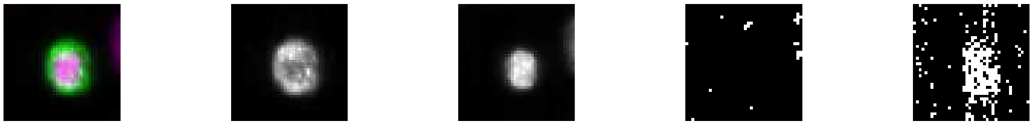				

Ereignis	Aufnahme	DAPI/CK-PE	CK-PE	DAPI	CD45-APC	HER-2/neu
5438	43	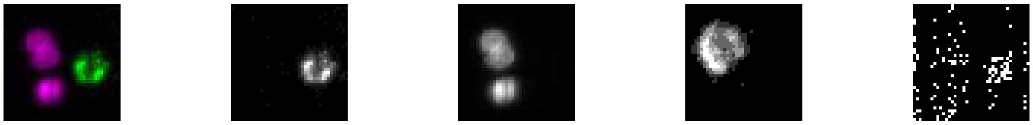				
5439	43	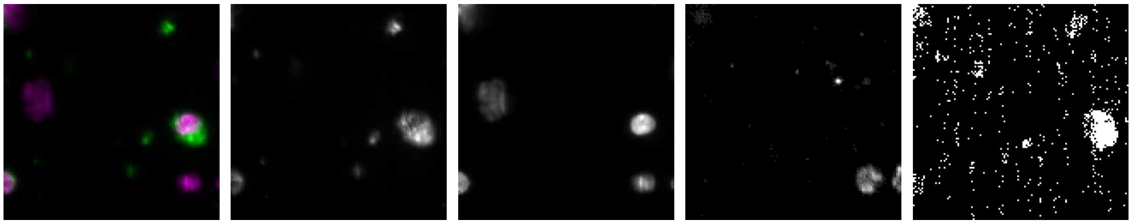				
5440	43	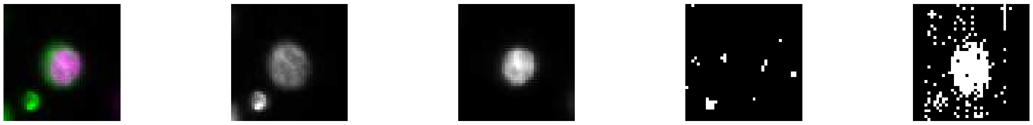				
5441	43	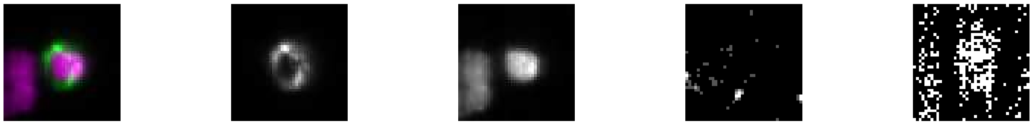				
5442	43	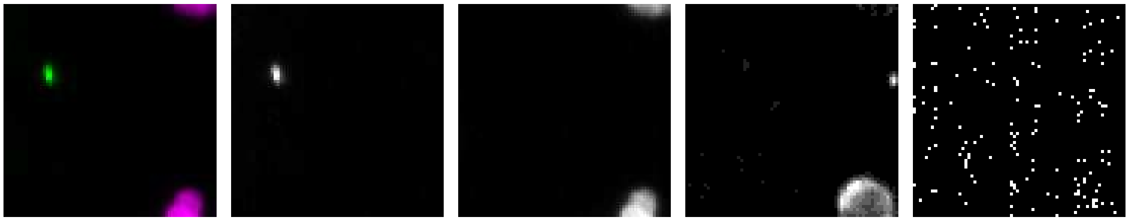				
5443	43	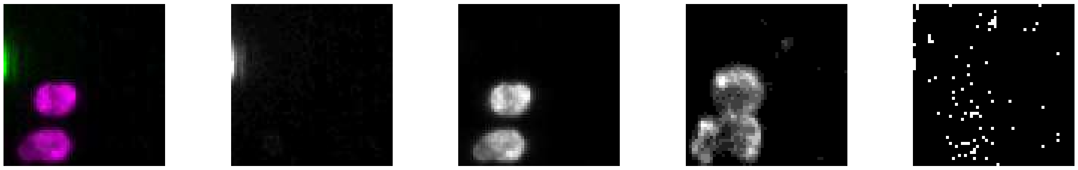				
5444	43	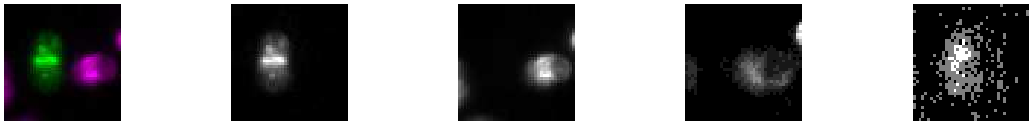				
5445	43	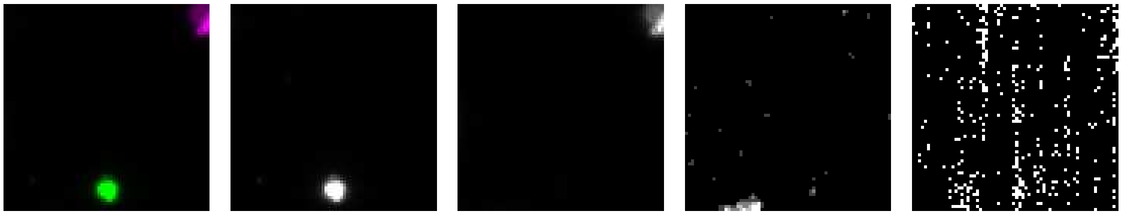				
5446	43	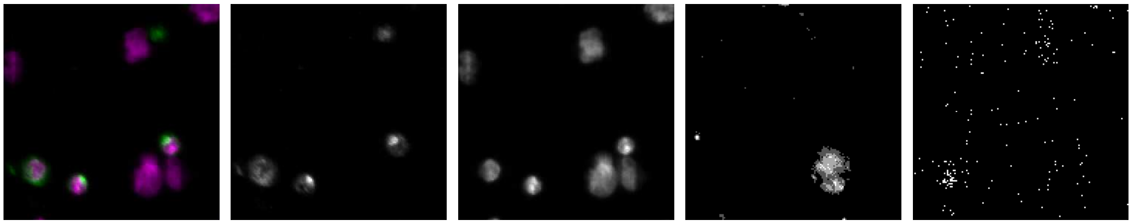				
5447	43	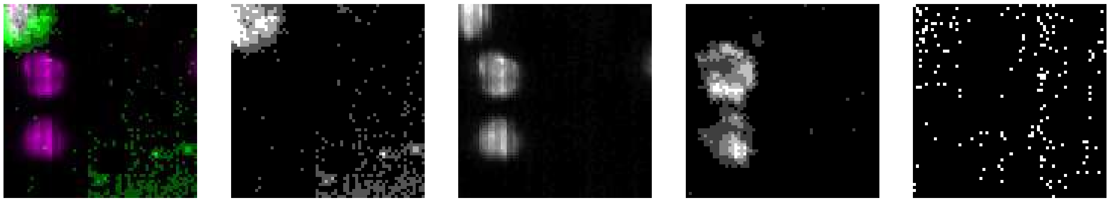				

Ereignis	Aufnahme	DAPI/CK-PE	CK-PE	DAPI	CD45-APC	HER-2/neu
5448	43	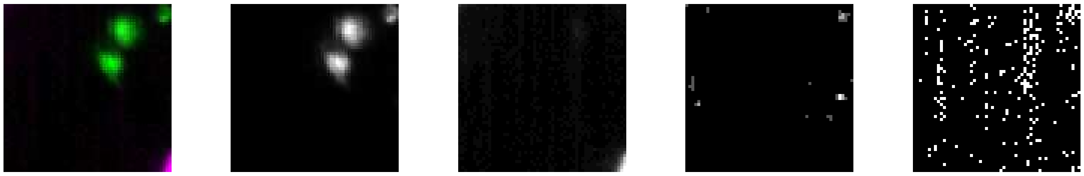				
5449	43	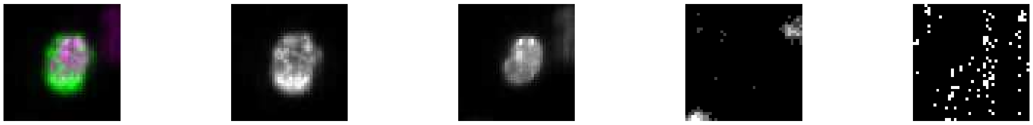				
5450	43	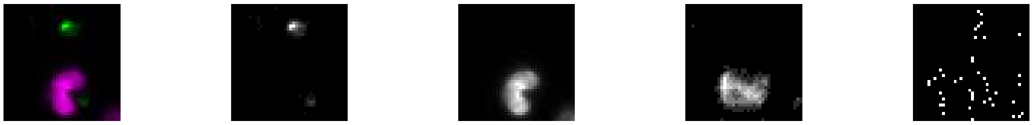				
5451	43	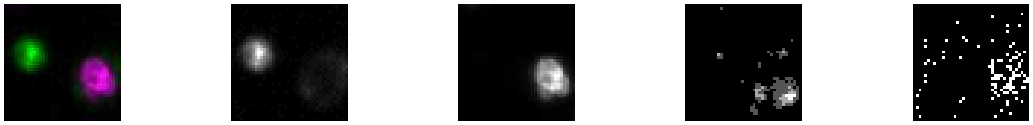				
5452	43	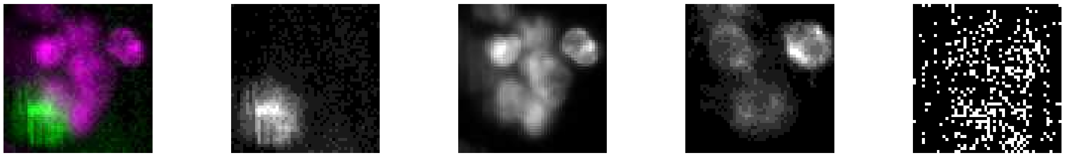				
5453	43	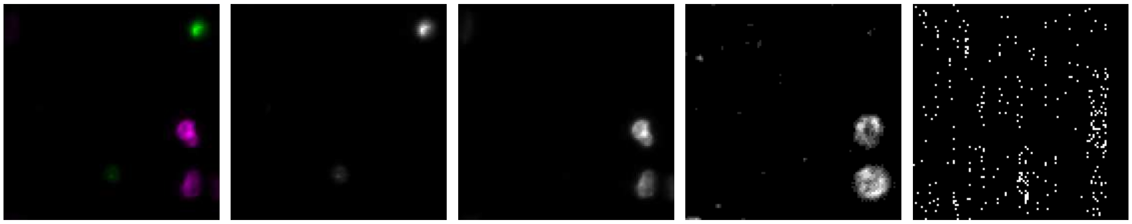				
5454	43	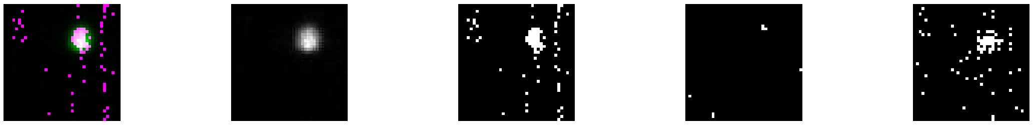				
5455	43	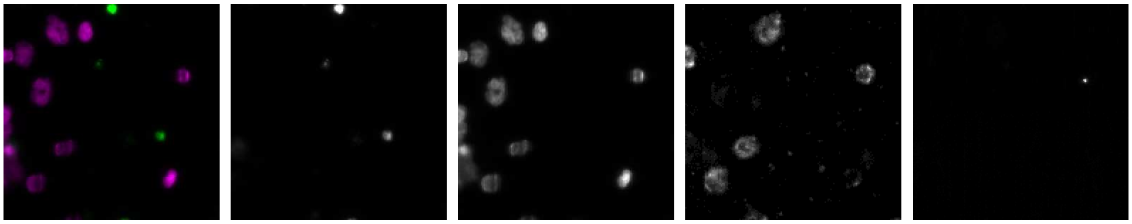				
5456	43	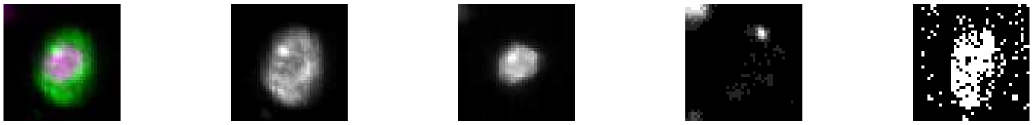				
5457	43	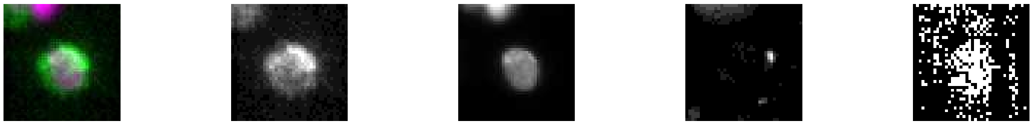				
5458	43	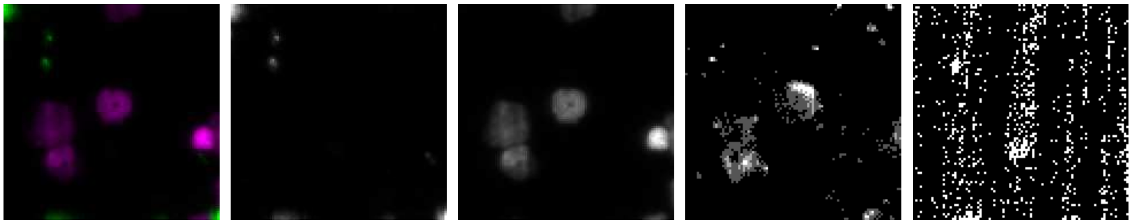				

Ereignis	Aufnahme	DAPI/CK-PE	CK-PE	DAPI	CD45-APC	HER-2/neu
5459	43	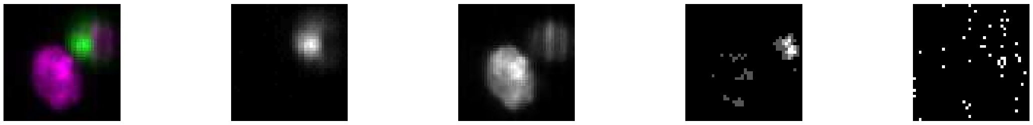				
5460	43	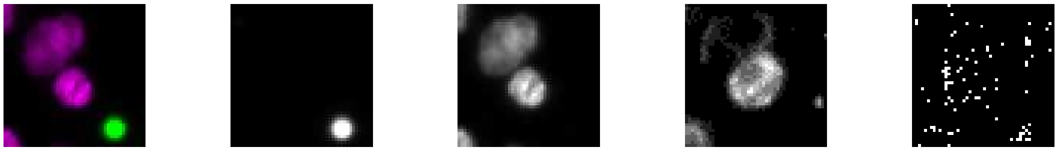				
5461	43	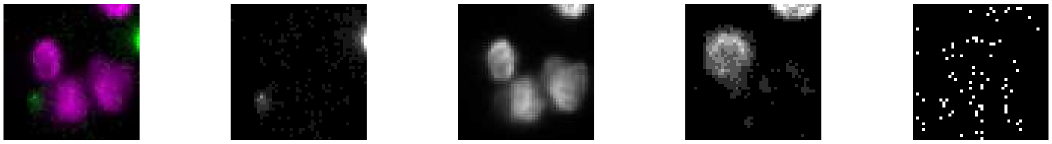				
5462	43	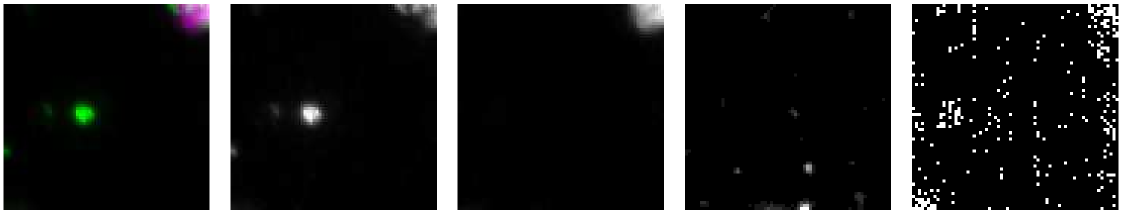				
5463	43	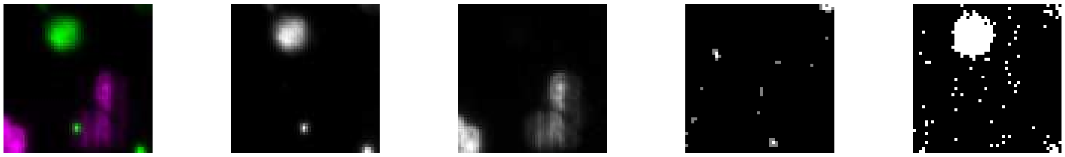				
5464	43	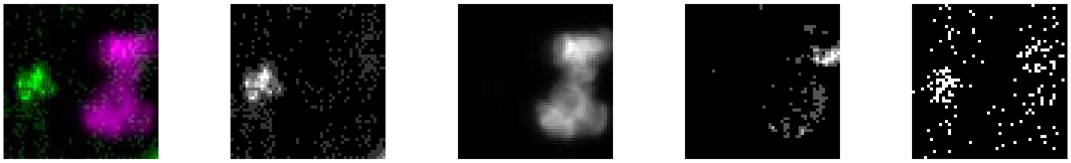				
5465	43	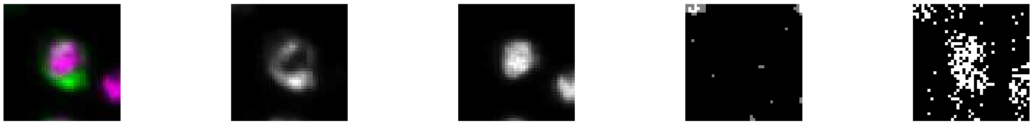				
5466	43	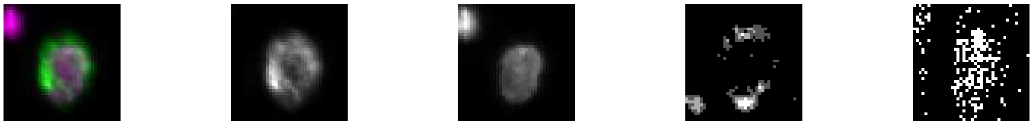				
5467	43	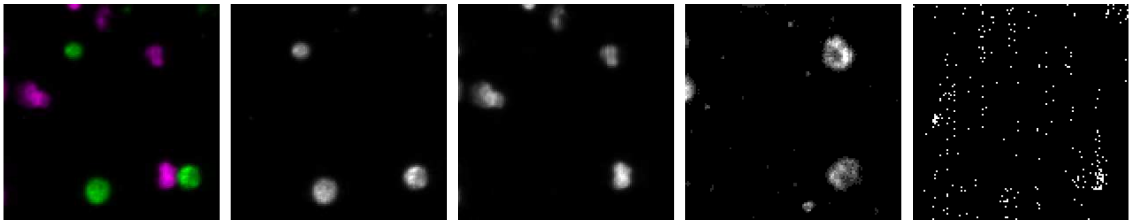				
5468	43	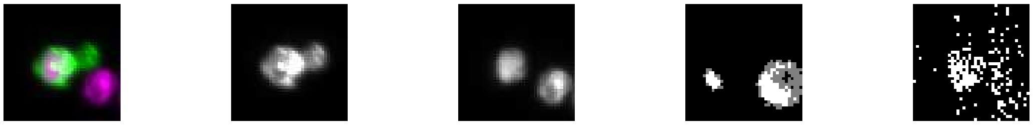				
5469	43	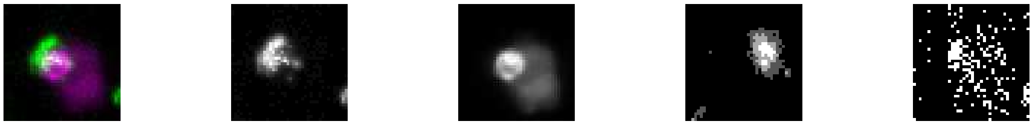				

Ereignis	Aufnahme	DAPI/CK-PE	CK-PE	DAPI	CD45-APC	HER-2/neu
5470	43	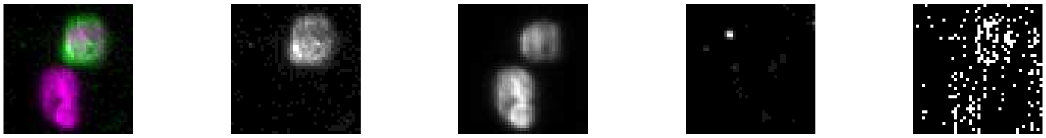				
5471	43	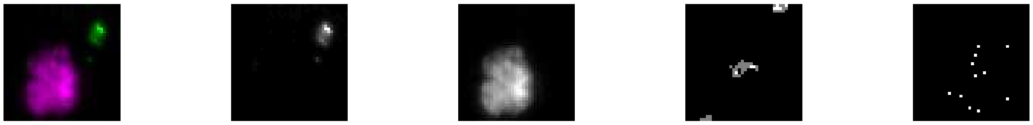				
5472	43	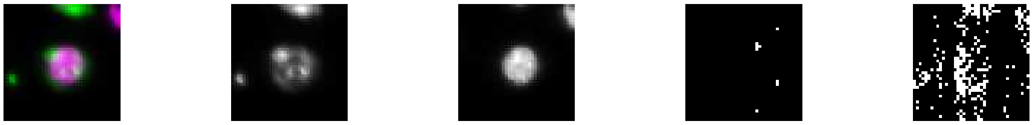				
5473	43	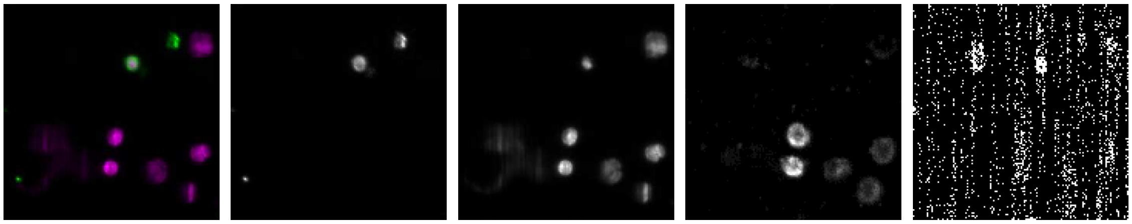				
5474	43	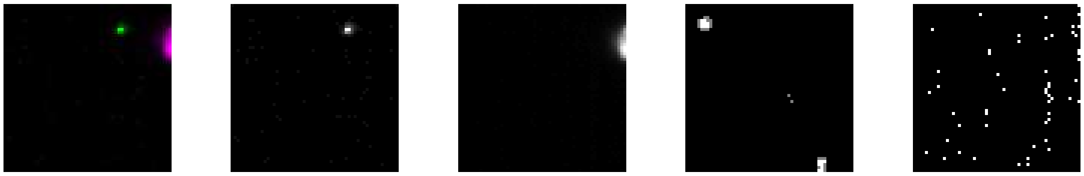				
5475	43	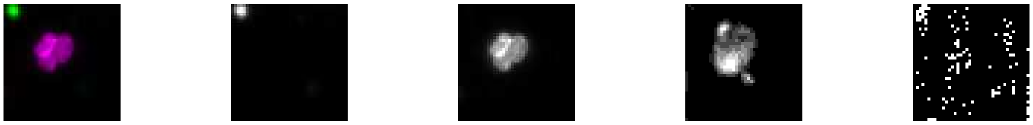				
5476	43	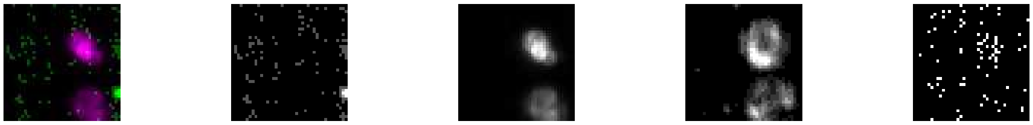				
5477	43	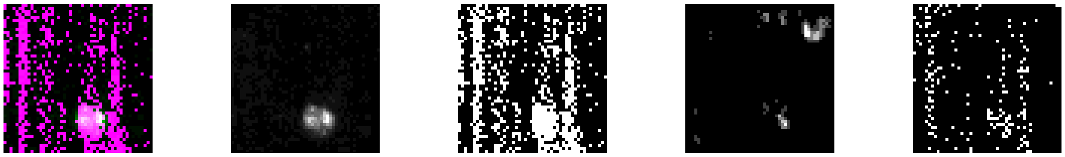				
5478	43	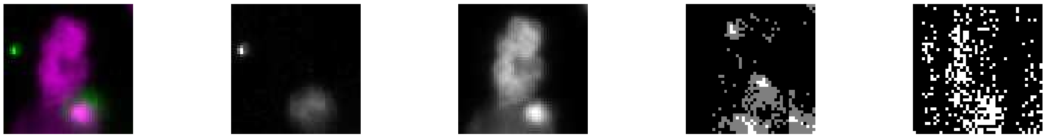				
5479	43	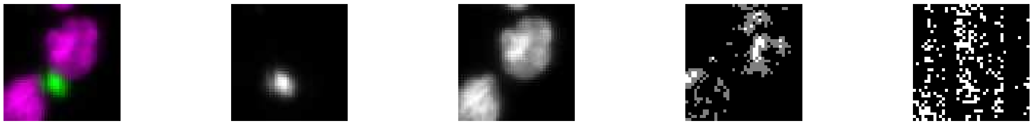				
5480	43	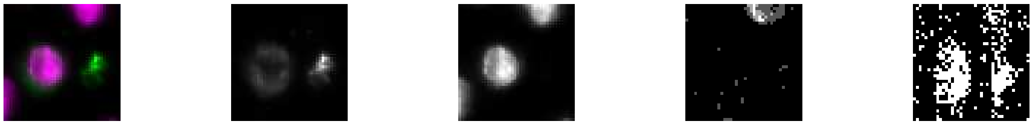				
5481	43	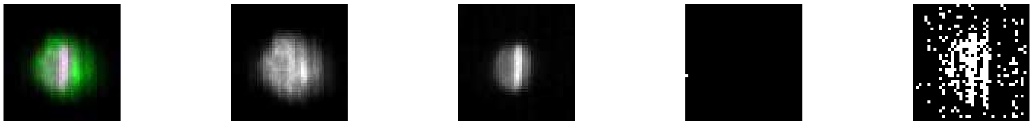				

Ereignis	Aufnahme	DAPI/CK-PE	CK-PE	DAPI	CD45-APC	HER-2/neu
5482	43	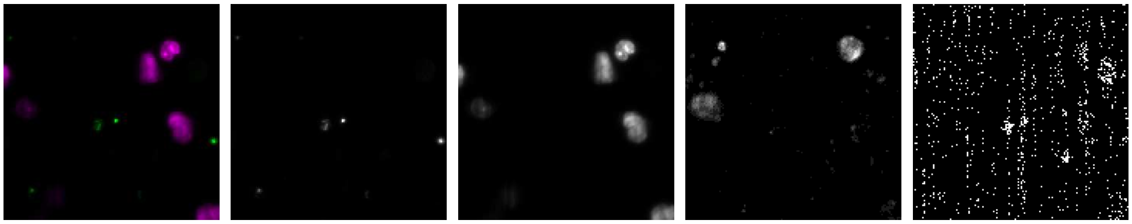				
5483	43	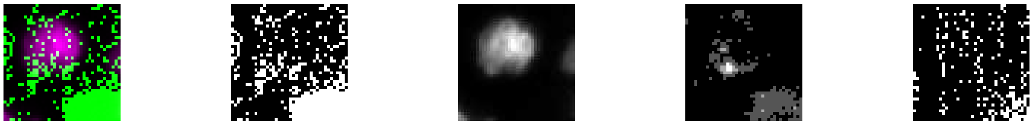				
5484	43	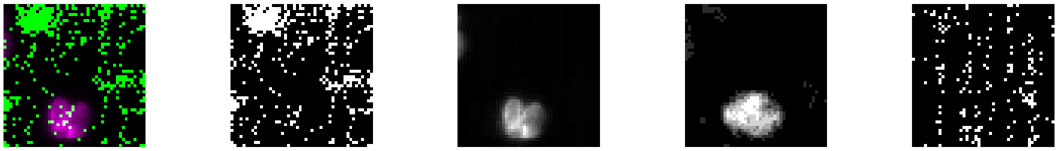				
5485	43	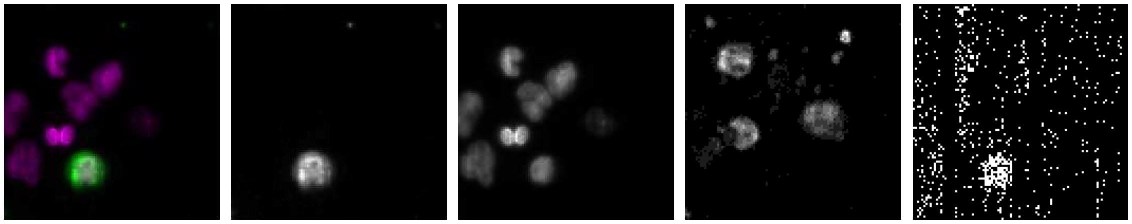				
5486	43	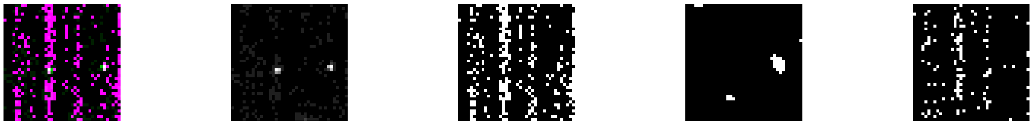				
5487	43	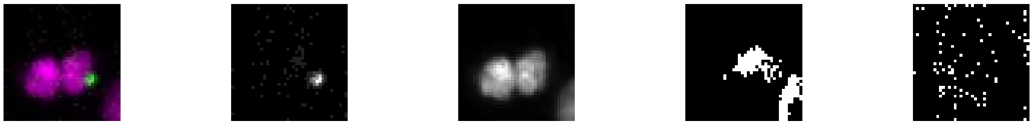				
5488	43	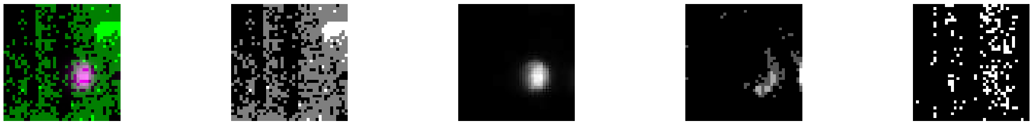				
5489	43	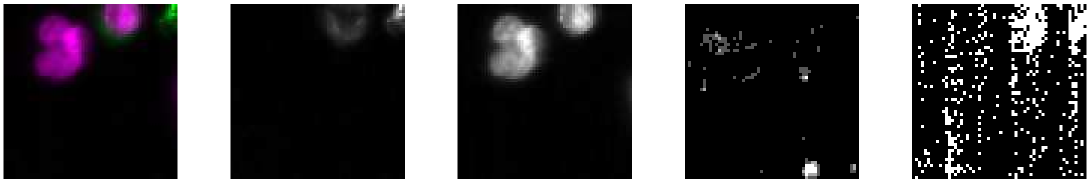				
5490	43	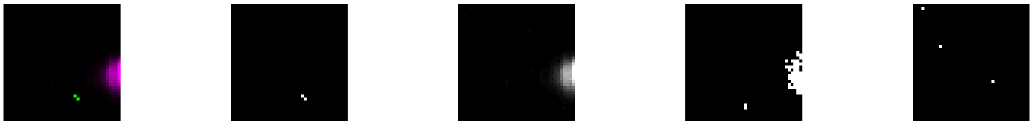				
5491	43	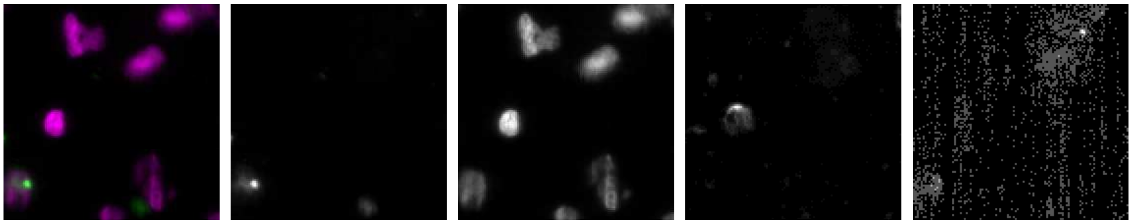				
5492	43	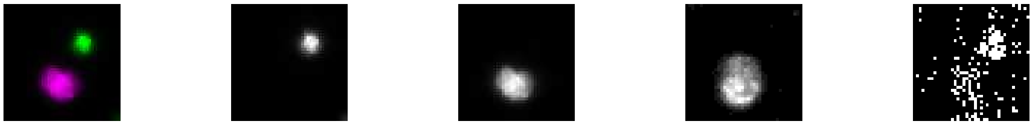				

Druckdatum: 02/06/2020 16:41

Proben-ID: Gyn14.208 **Kassetten-ID:** 01327106 **Patienten-ID:**

Ereignis	Aufnahme	DAPI/CK-PE	CK-PE	DAPI	CD45-APC	HER-2/neu
5493	43	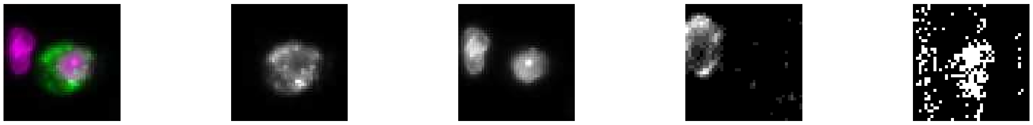				
5494	43	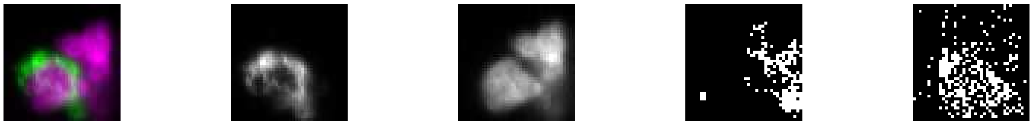				
5495	43	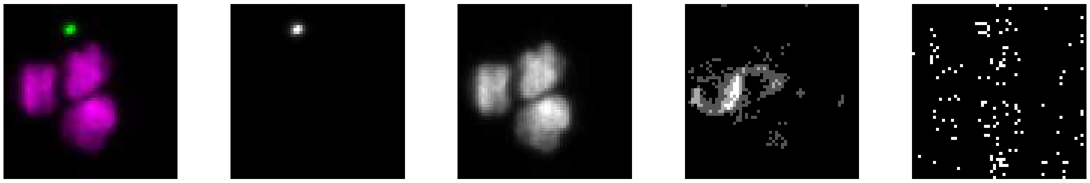				
5496	43	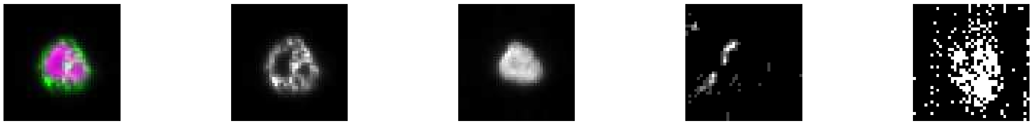				
5497	43	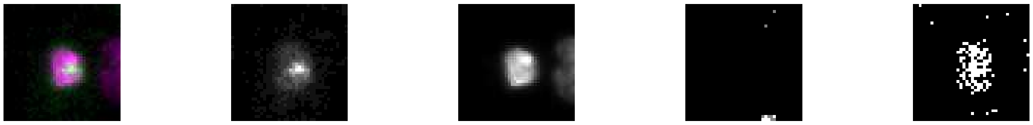				
5498	43	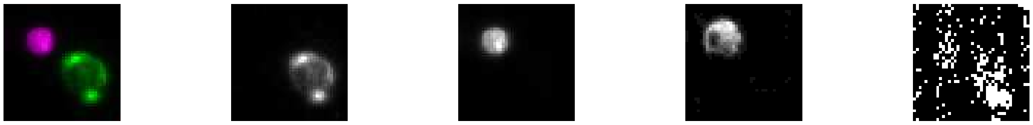				
5499	43	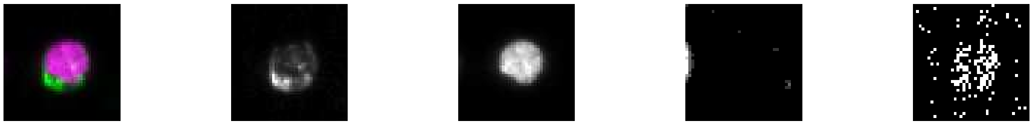				
5500	43	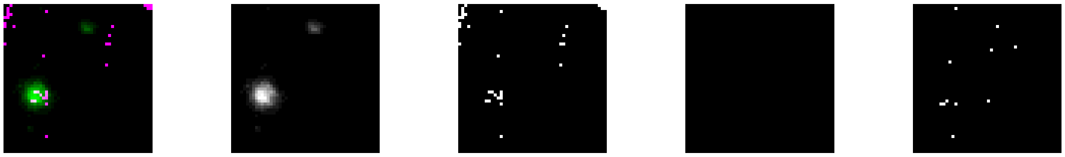				
5501	43	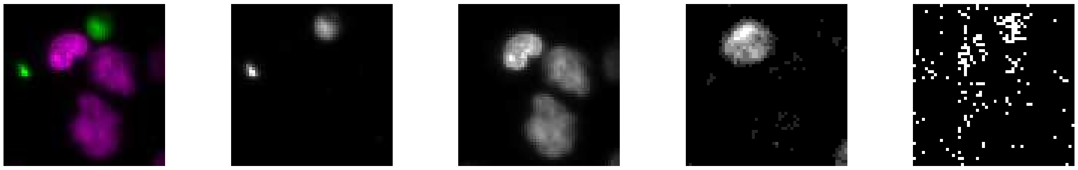				
5502	43	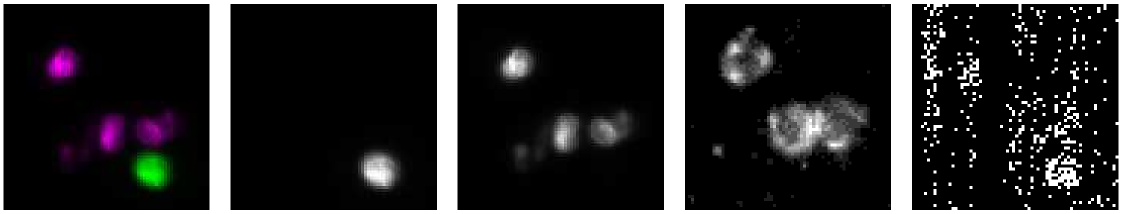				
5503	43	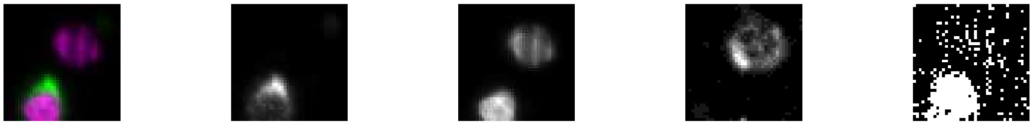				

Ereignis	Aufnahme	DAPI/CK-PE	CK-PE	DAPI	CD45-APC	HER-2/neu
5504	43	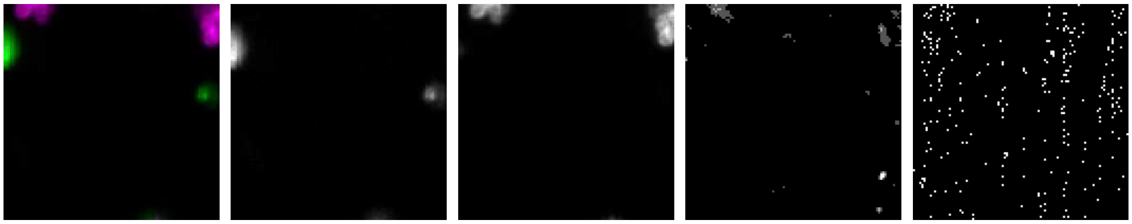				
5505	43	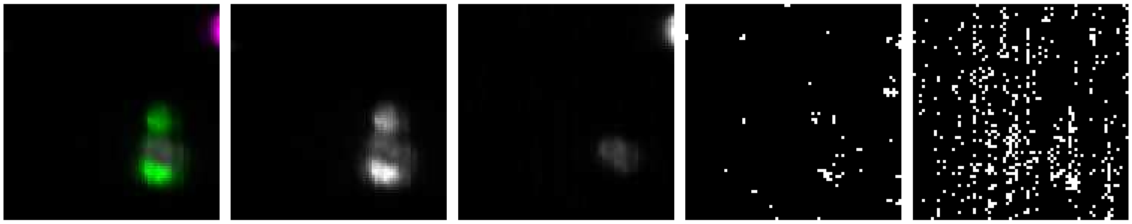				
5506	43	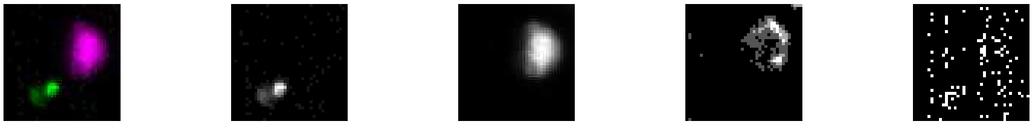				
5507	43	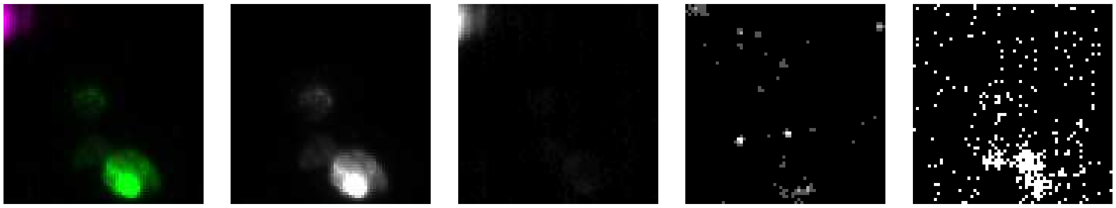				
5508	43	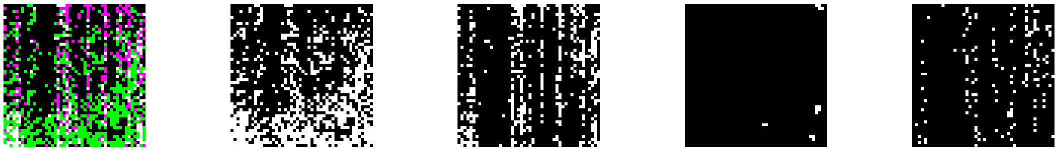				
5509	43	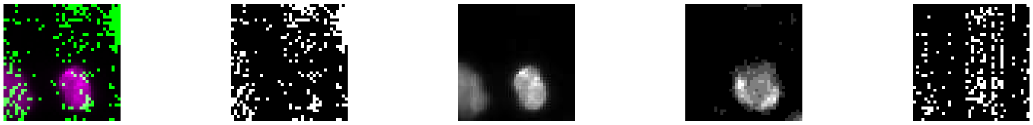				
5510	43	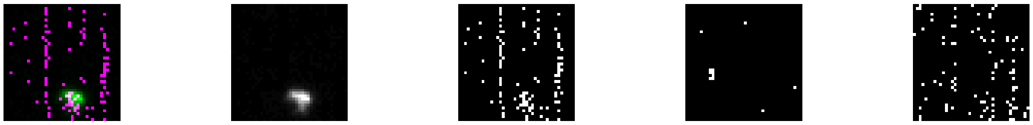				
5511	43	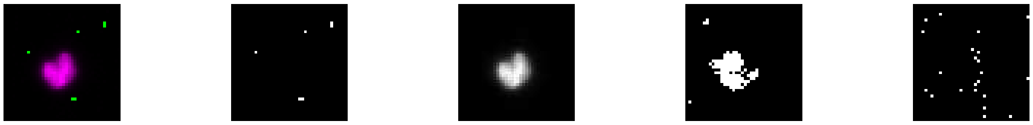				
5512	43	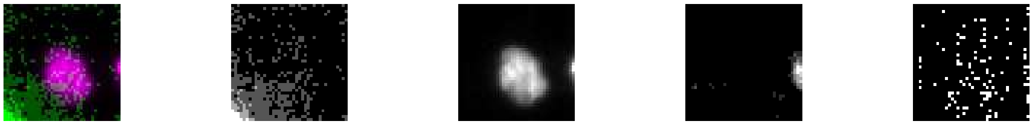				
5513	43	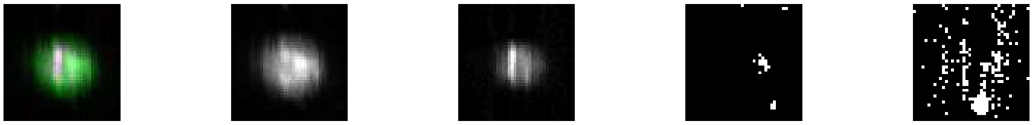				
5514	43	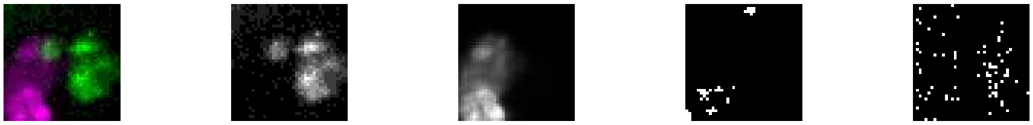				

Ereignis	Aufnahme	DAPI/CK-PE	CK-PE	DAPI	CD45-APC	HER-2/neu
5515	43	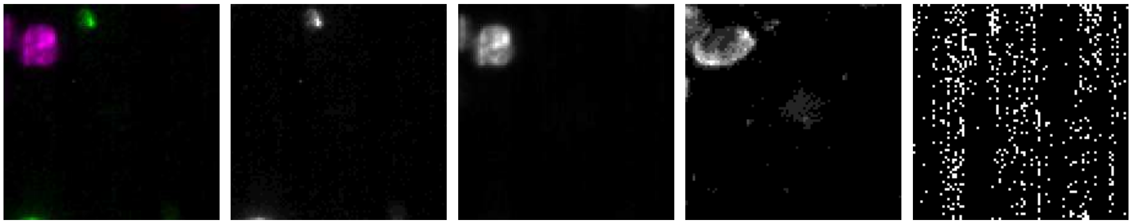				
5516	43	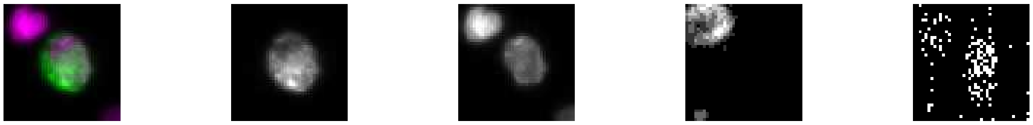				
5517	43	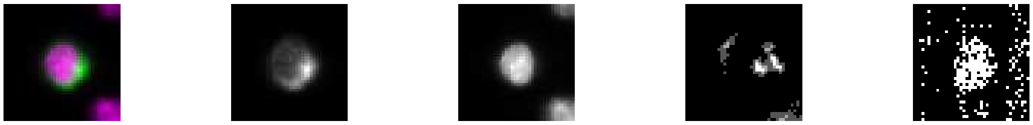				
5518	43	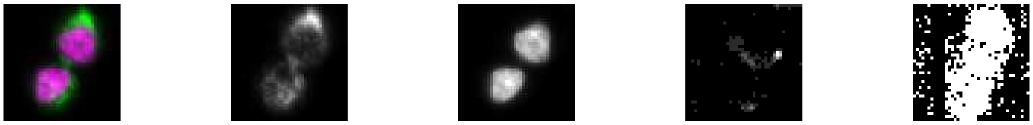				
5519	43	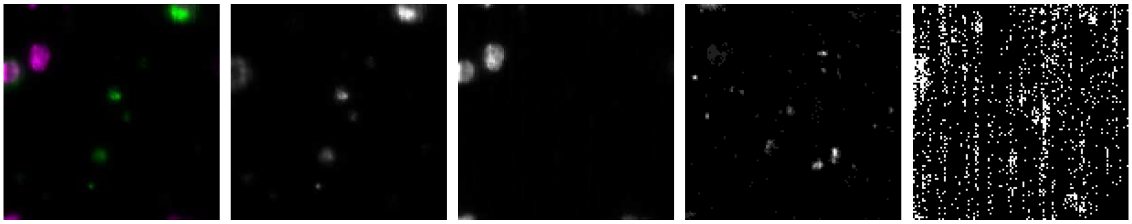				
5520	43	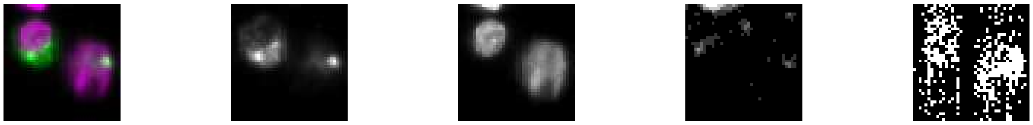				
5521	43	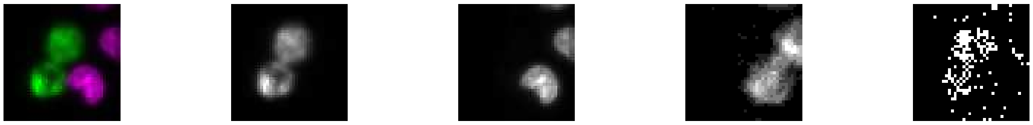				
5522	43	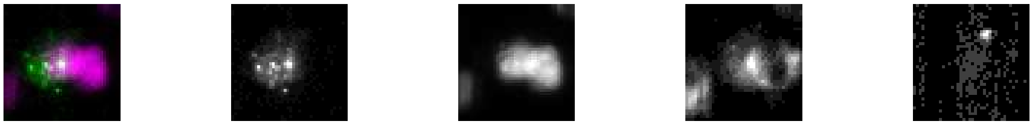				
5523	43	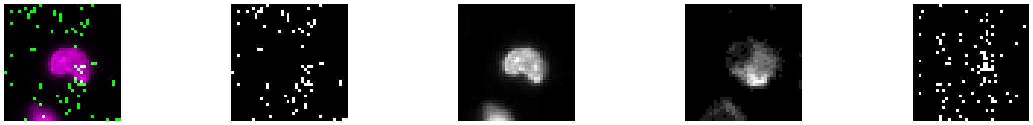				
5524	43	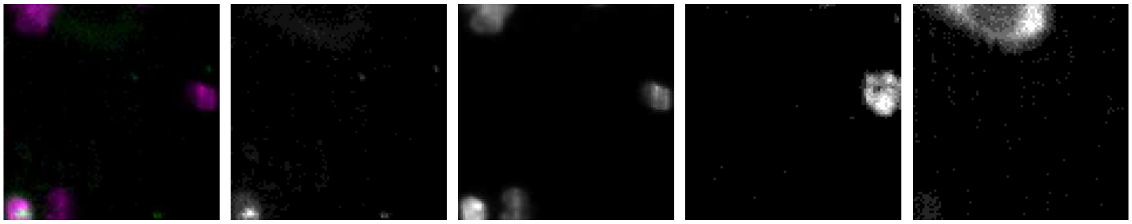				
5525	43	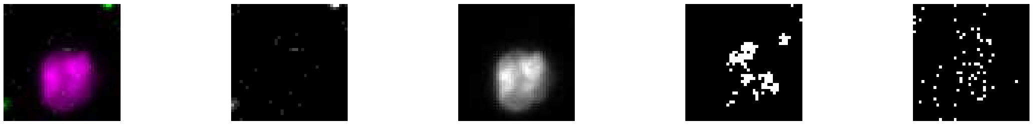				

Ereignis	Aufnahme	DAPI/CK-PE	CK-PE	DAPI	CD45-APC	HER-2/neu
5526	43	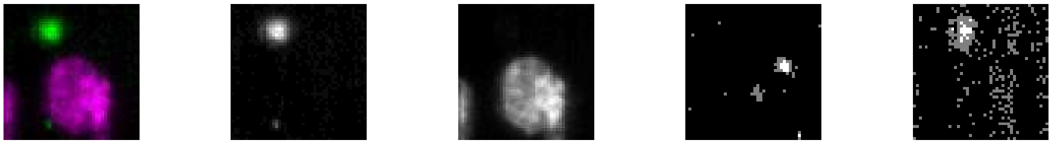				
5527	43	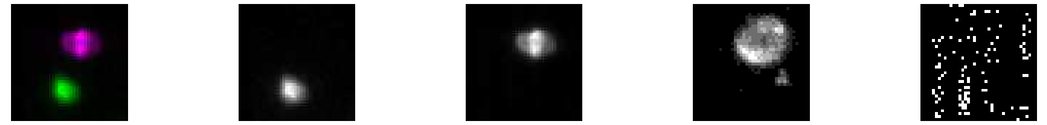				
5528	43	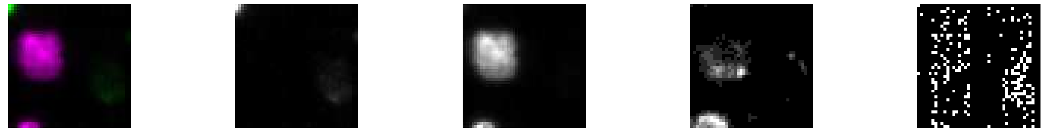				
5529	43	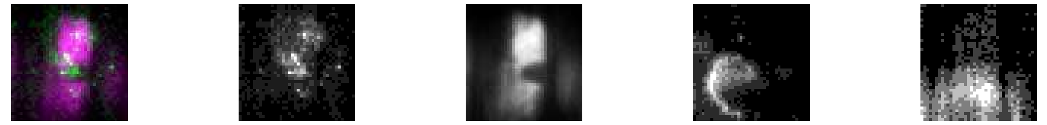				
5530	43	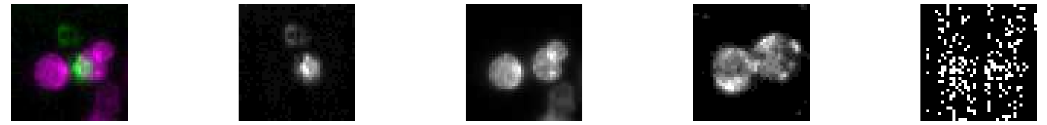				
5531	43	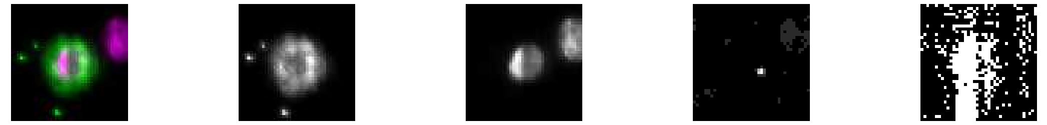				
5532	43	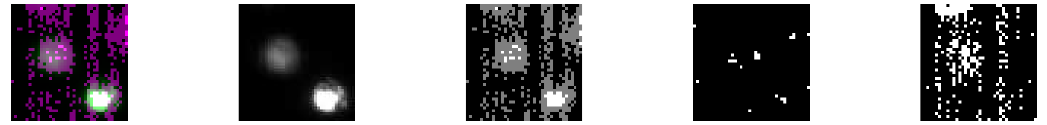				
5533	43	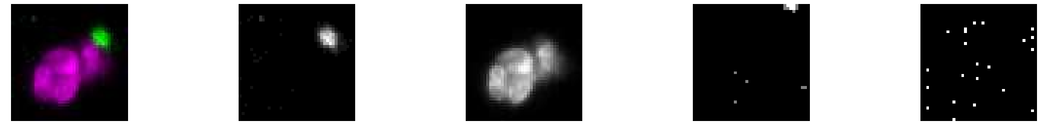				
5534	43	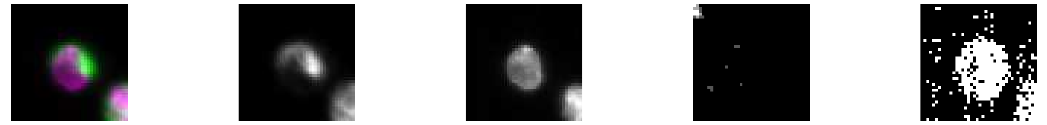				
5535	43	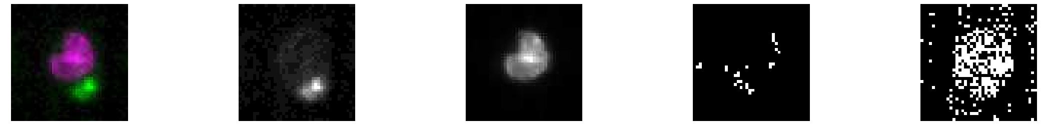				
5536	43	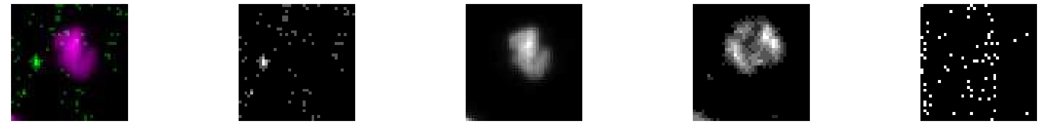				
5537	43	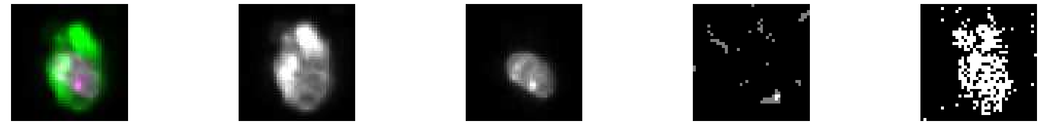				
5538	43	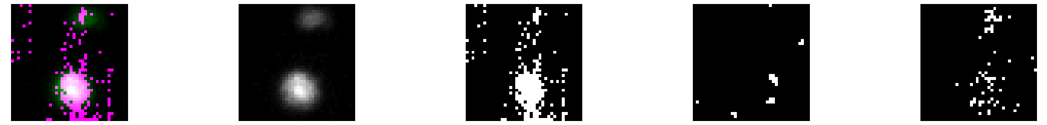				
5539	43	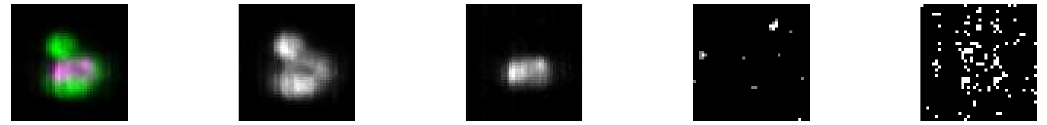				

Ereignis	Aufnahme	DAPI/CK-PE	CK-PE	DAPI	CD45-APC	HER-2/neu
5540	43	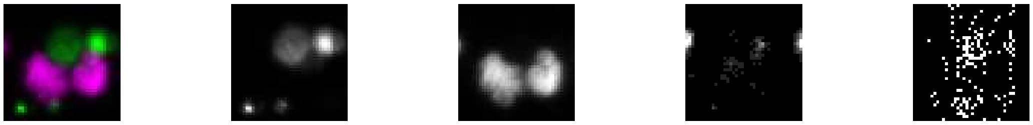				
5541	43	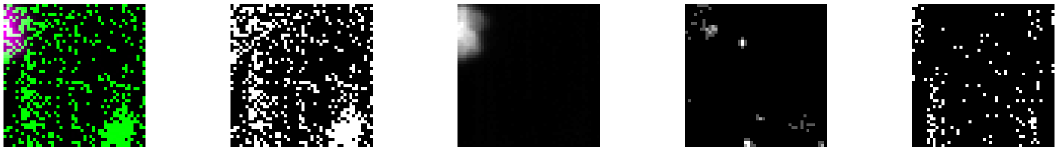				
5542	43	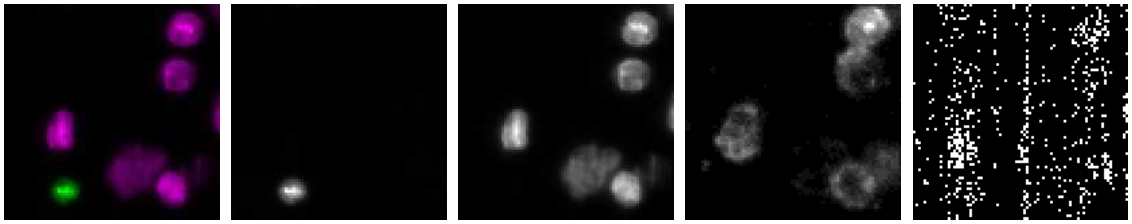				
5543	43	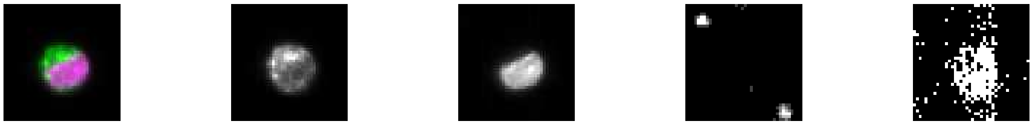				
5544	43	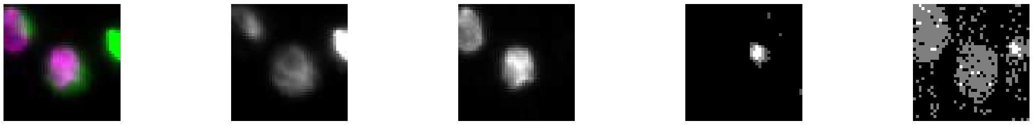				
5545	43	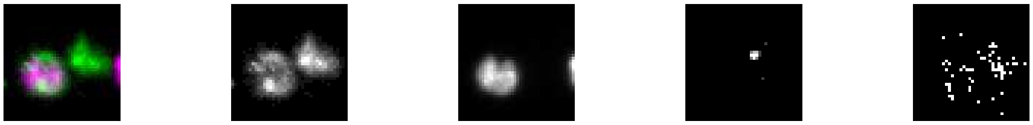				
5546	43	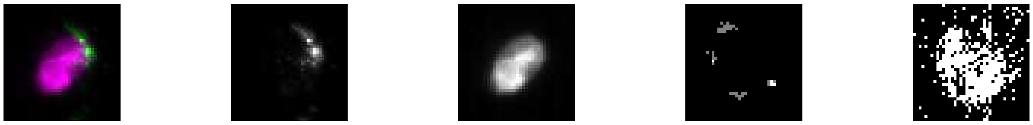				
5547	43	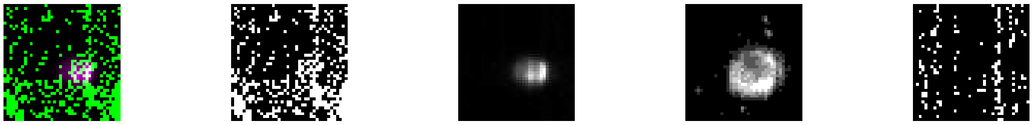				
5548	43	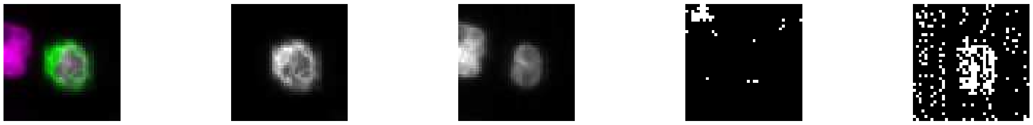				
5549	43	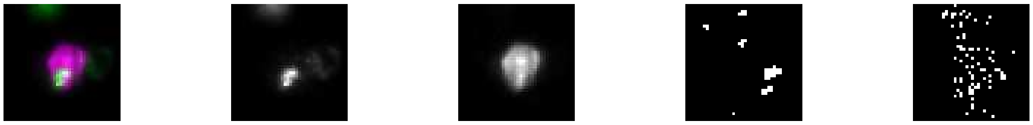				
5550	43	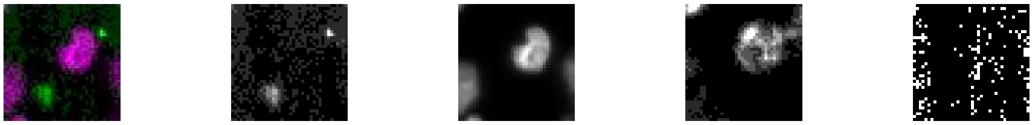				
5551	43	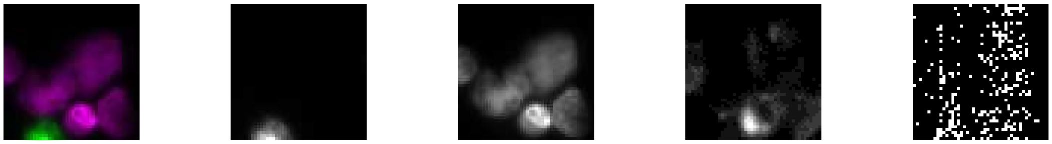				

Druckdatum:02/06/2020 16:41

Proben-ID: Gyn14.208 **Kassetten-ID:**01327106 **Patienten-ID:**

Ereignis	Aufnahme	DAPI/CK-PE	CK-PE	DAPI	CD45-APC	HER-2/neu
5552	43	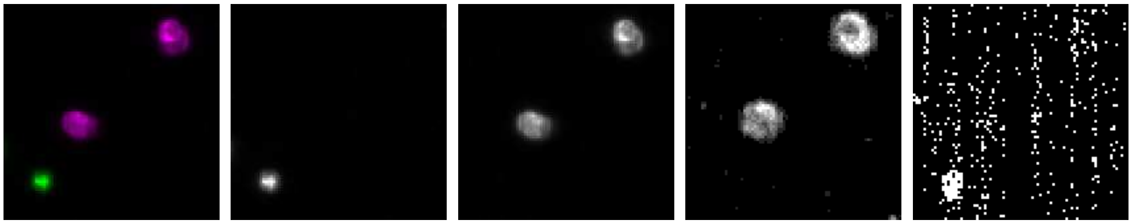				
5553	43	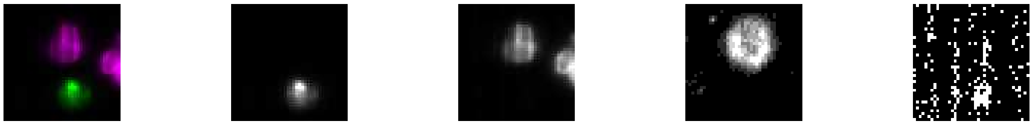				
5554	43	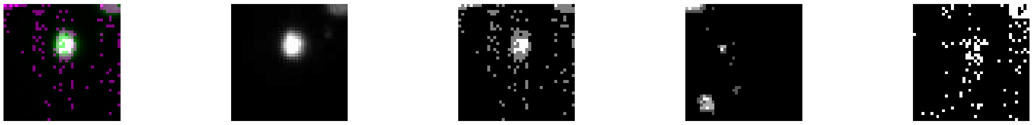				
5555	43	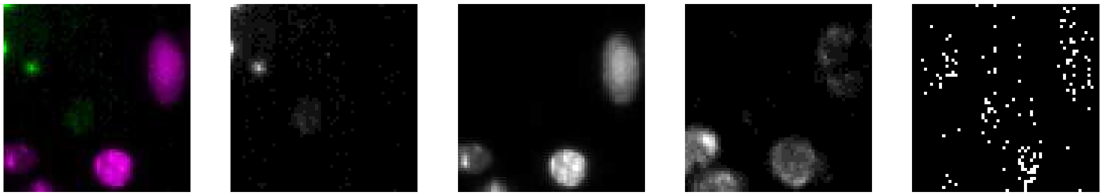				
5556	43	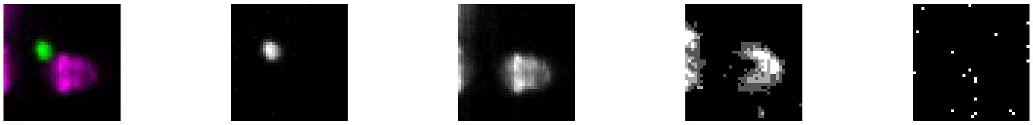				
5557	43	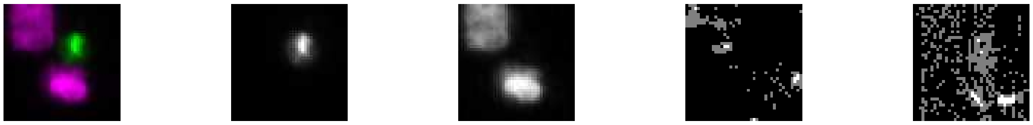				
5558	43	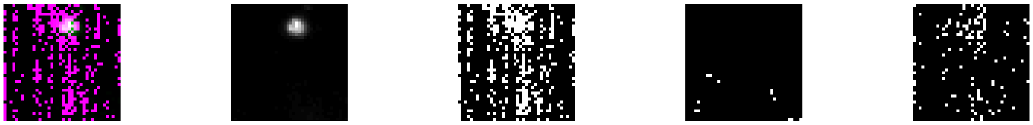				
5559	43	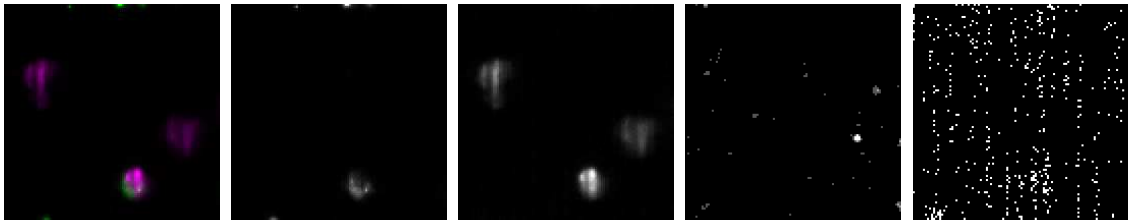				
5560	43	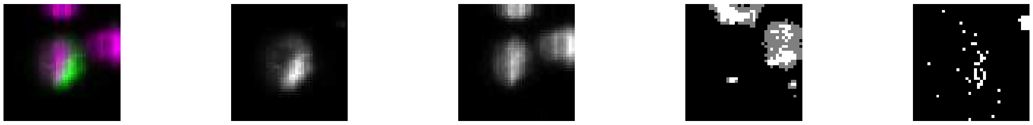				
5561	43	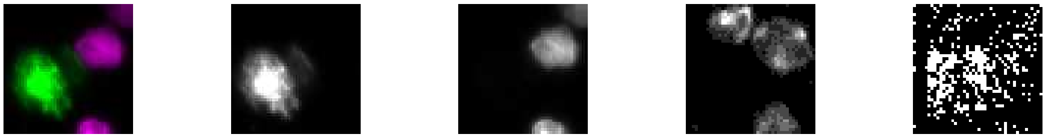				
5562	43	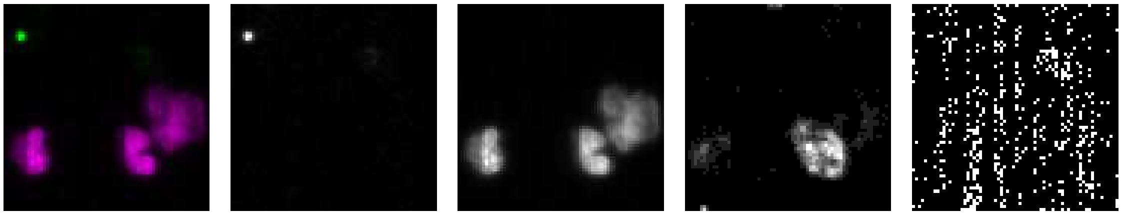				

Ereignis	Aufnahme	DAPI/CK-PE	CK-PE	DAPI	CD45-APC	HER-2/neu
5563	43	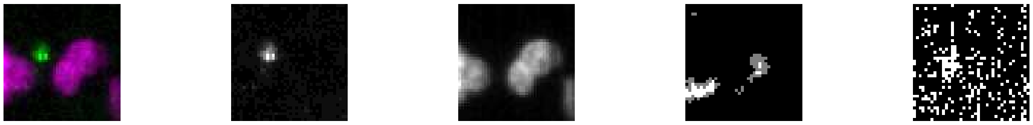				
5564	43	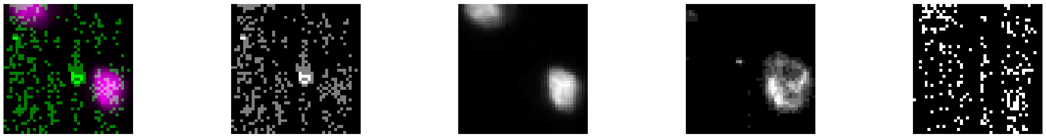				
5565	43	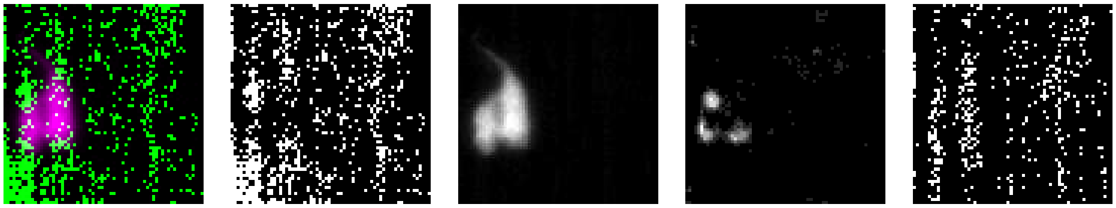				
5566	43	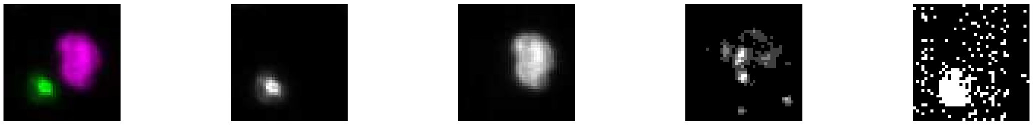				
5567	43	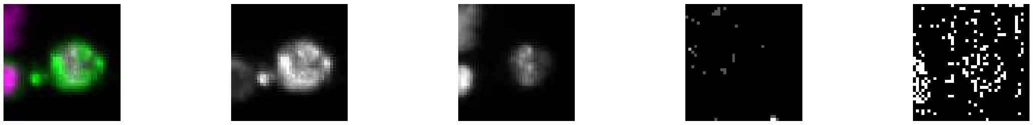				
5568	43	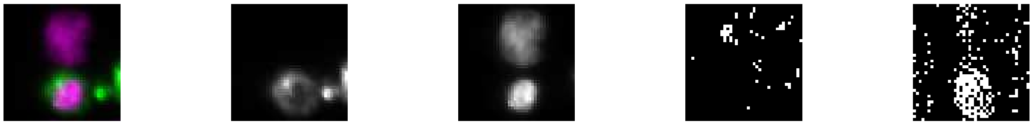				
5569	43	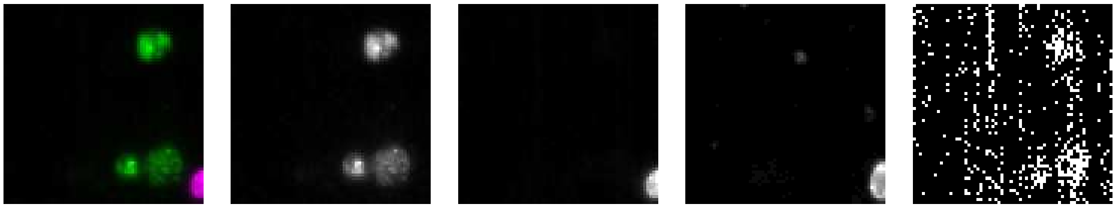				
5570	43	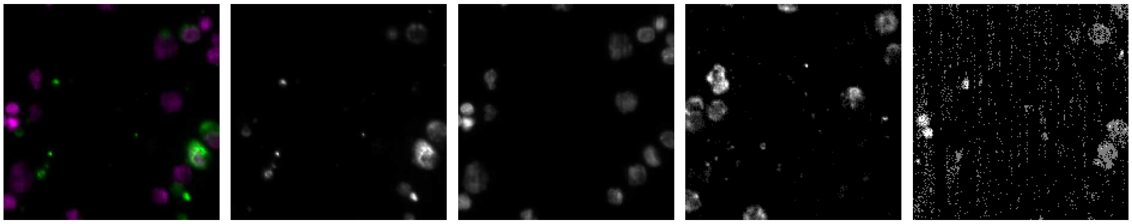				
5571	43	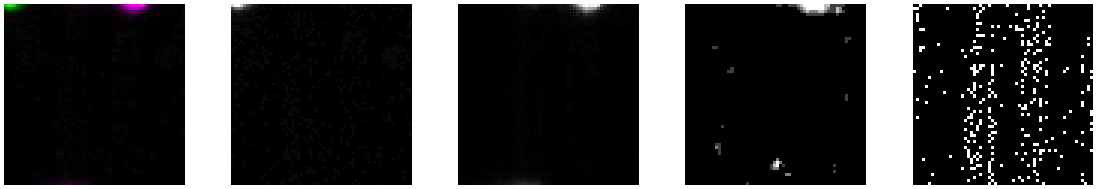				
5572	43	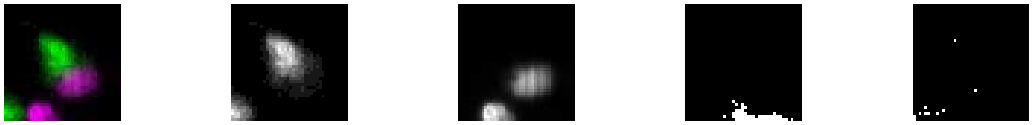				
5573	43	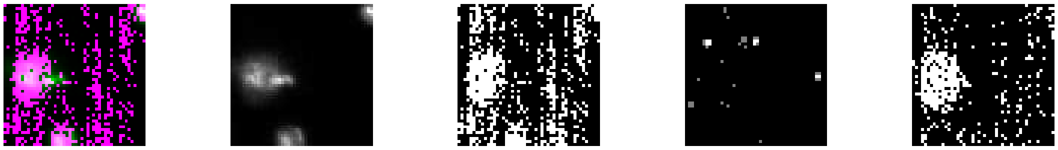				

Ereignis	Aufnahme	DAPI/CK-PE	CK-PE	DAPI	CD45-APC	HER-2/neu
5574	43	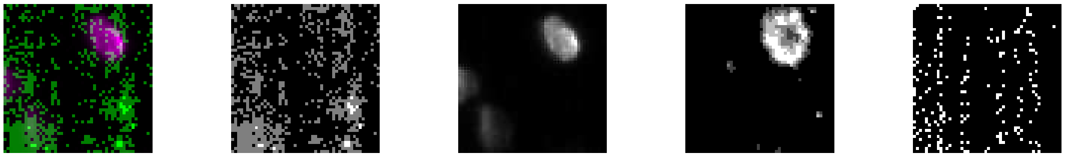				
5575	43	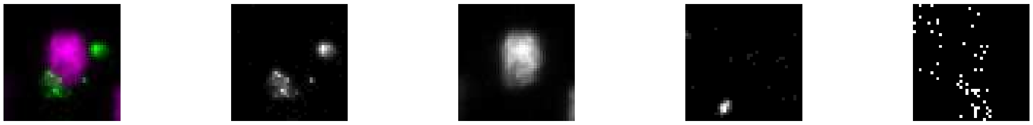				
5576	43	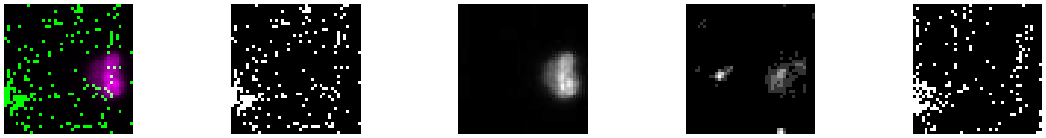				
5577	43	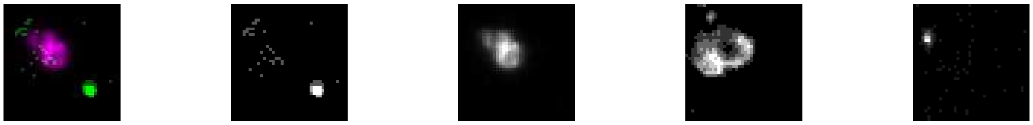				
5578	43	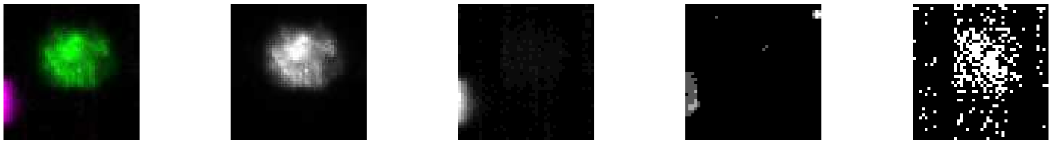				
5579	43	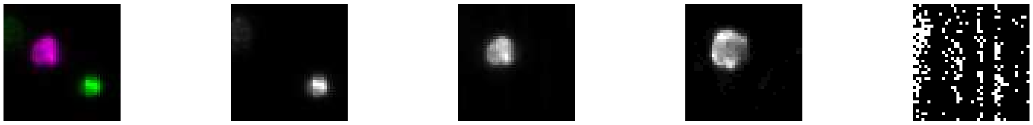				
5580	43	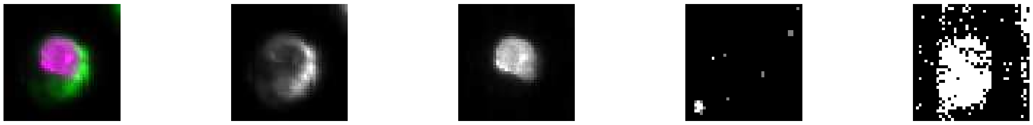				
5581	43	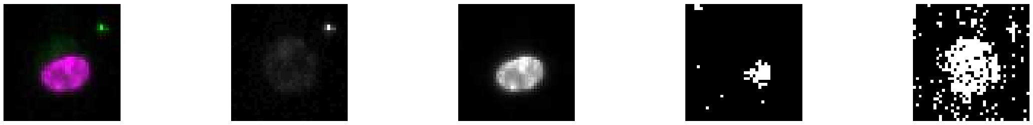				
5582	43	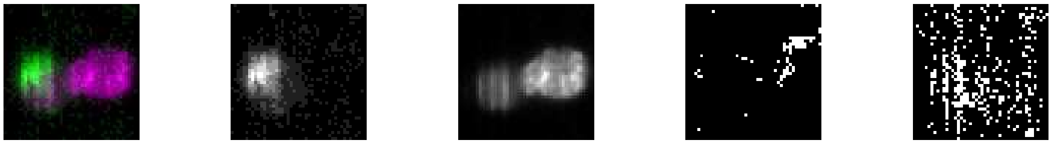				
5583	43	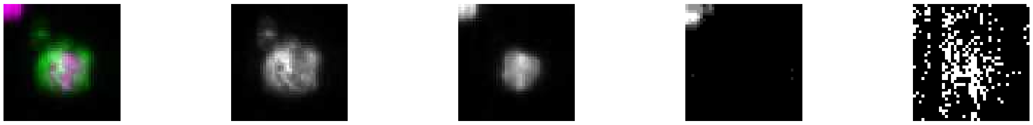				
5584	43	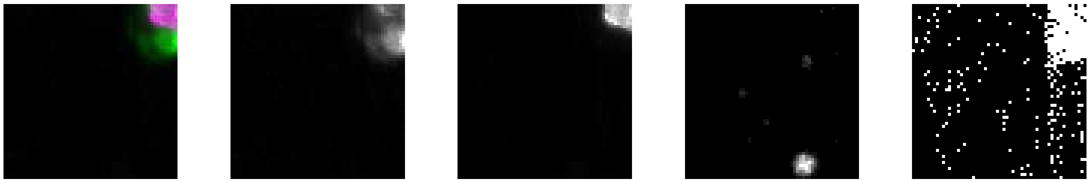				
5585	43	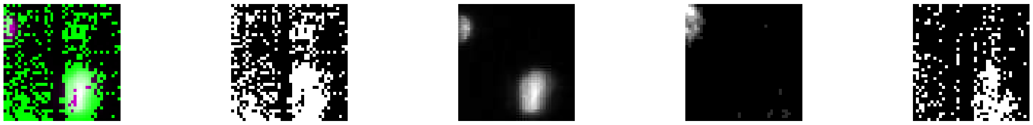				
5586	43	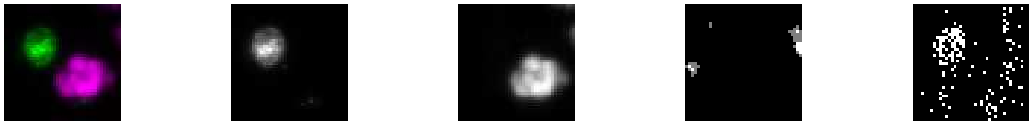				

Ereignis	Aufnahme	DAPI/CK-PE	CK-PE	DAPI	CD45-APC	HER-2/neu
5587	43	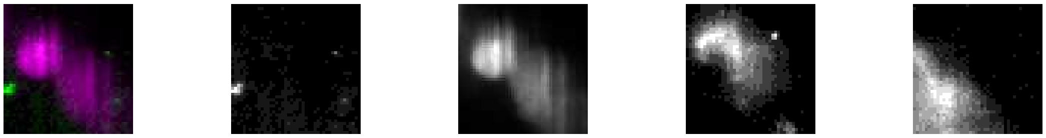				
5588	43	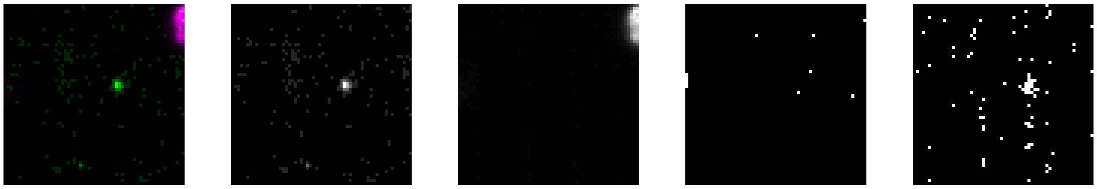				
5589	43	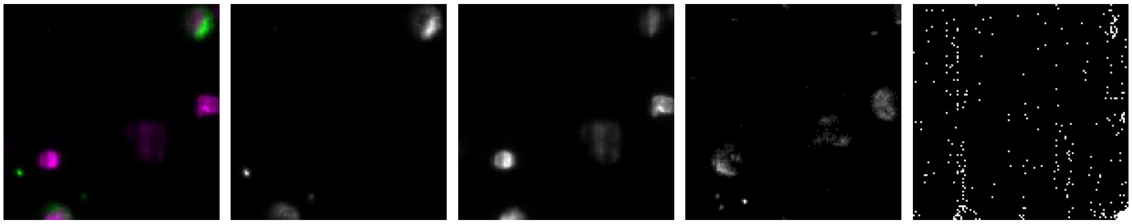				
5590	43	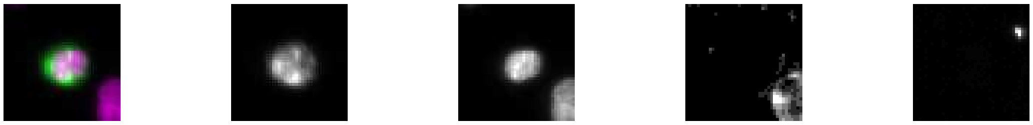				
5591	43	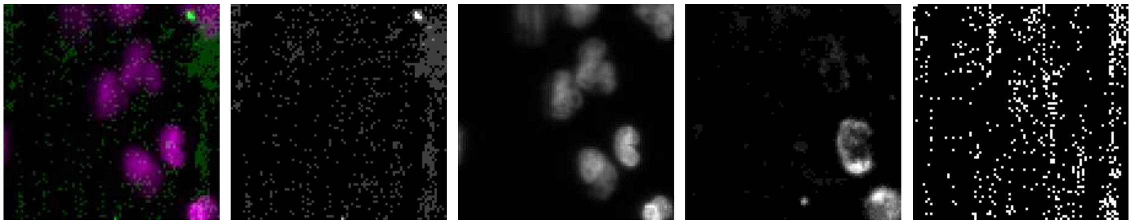				
5592	43	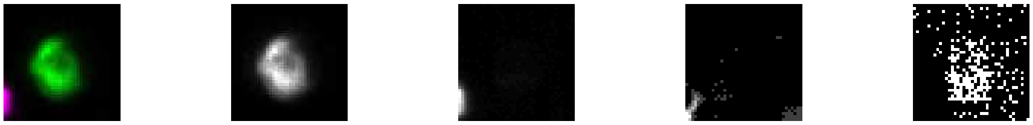				
5593	43	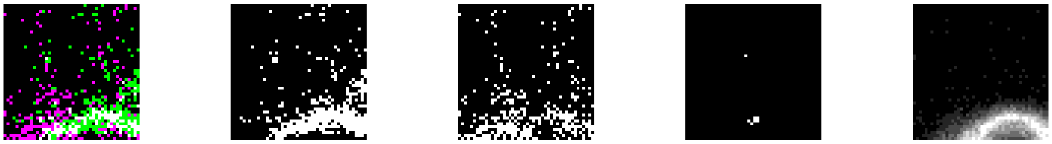				
5594	43	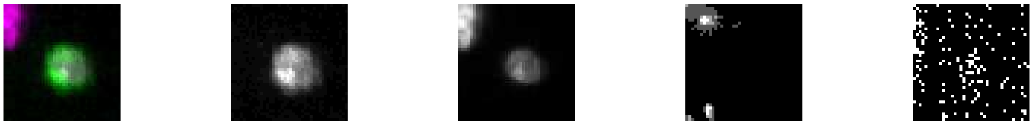				
5595	43	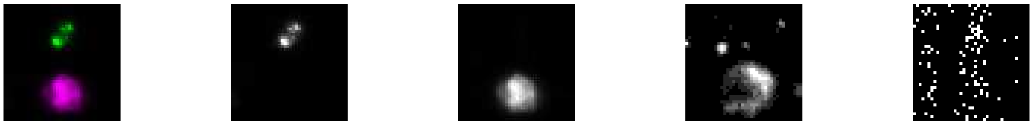				
5596	43	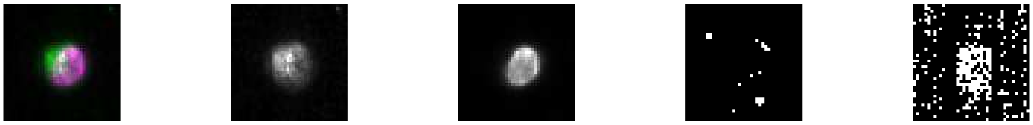				
5597	43	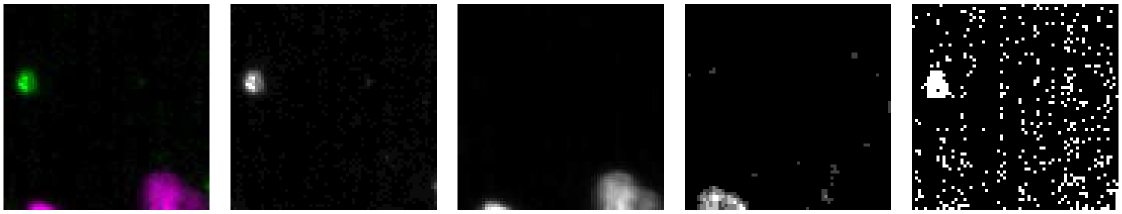				

Druckdatum: 02/06/2020 16:41

Proben-ID: Gyn14.208 **Kassetten-ID:** 01327106 **Patienten-ID:**

Ereignis	Aufnahme	DAPI/CK-PE	CK-PE	DAPI	CD45-APC	HER-2/neu
5598	43	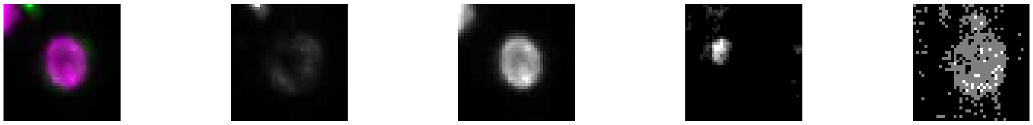				
5599	43	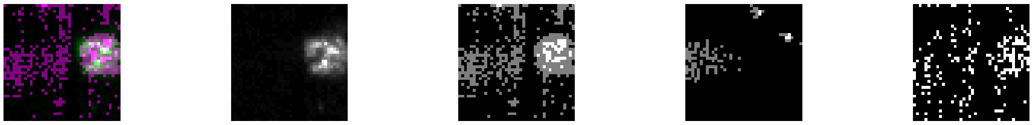				
5600	43	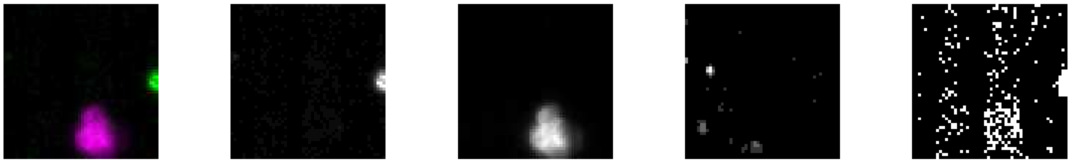				
5601	43	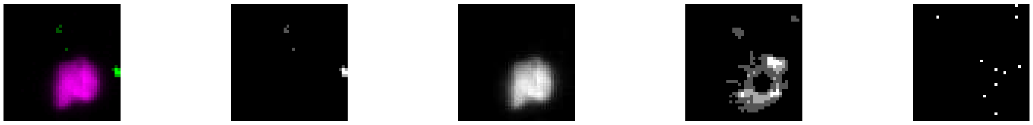				
5602	43	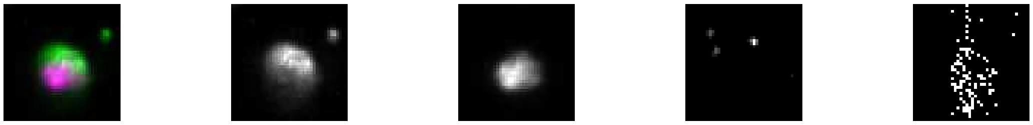				
5603	43	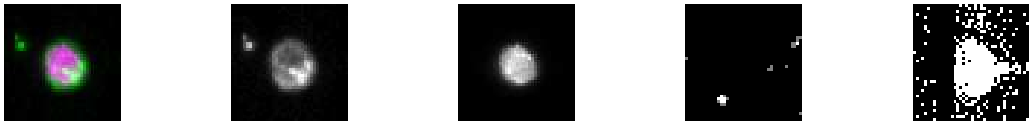				
5604	43	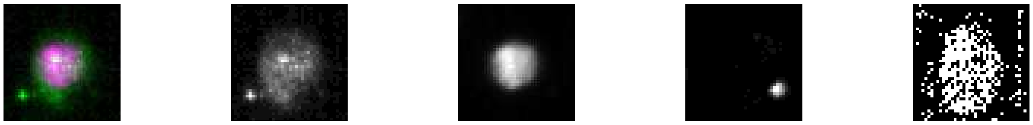				
5605	43	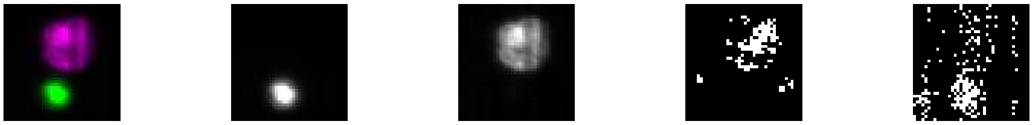				
5606	43	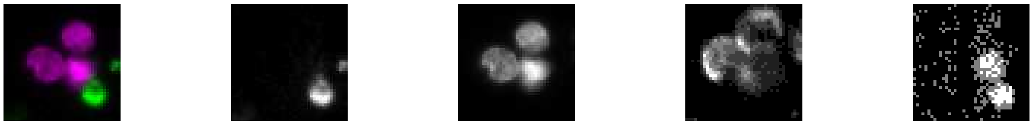				
5607	43	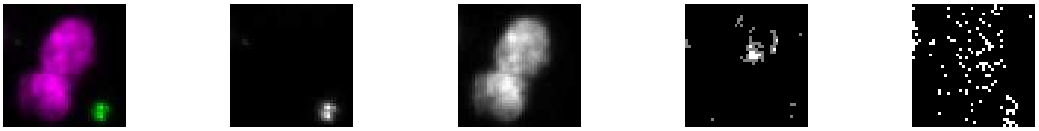				
5608	43	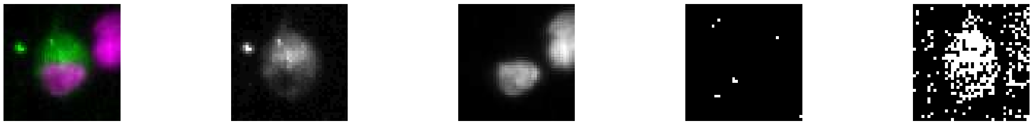				
5609	43	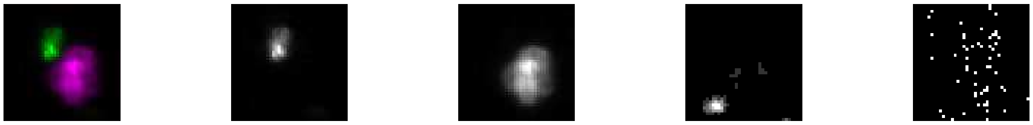				
5610	43	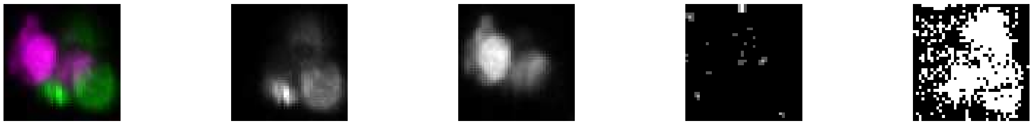				

Ereignis	Aufnahme	DAPI/CK-PE	CK-PE	DAPI	CD45-APC	HER-2/neu
5611	43	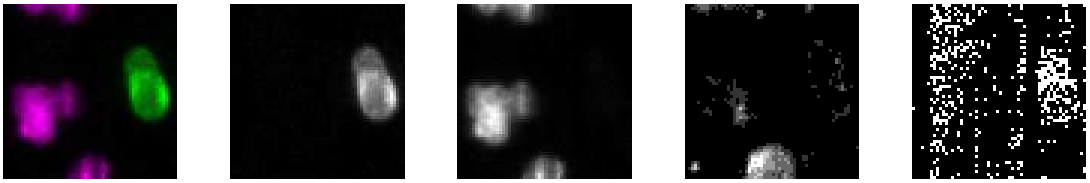				
5612	43	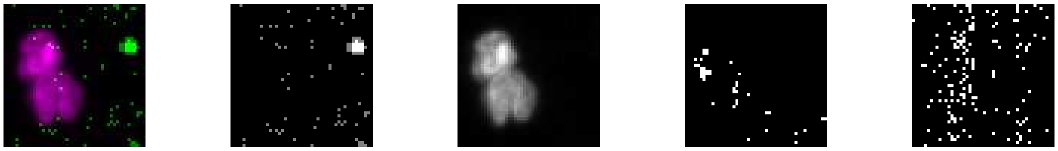				
5613	43	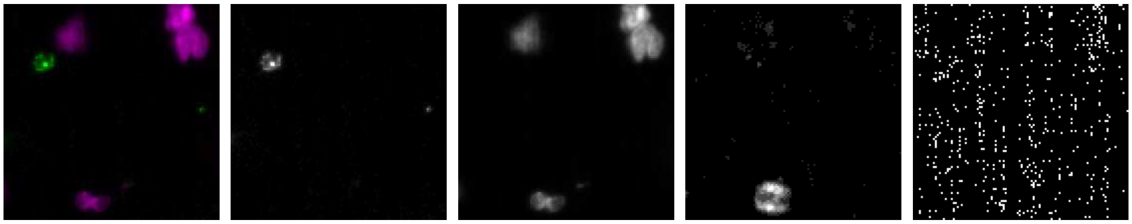				
5614	43	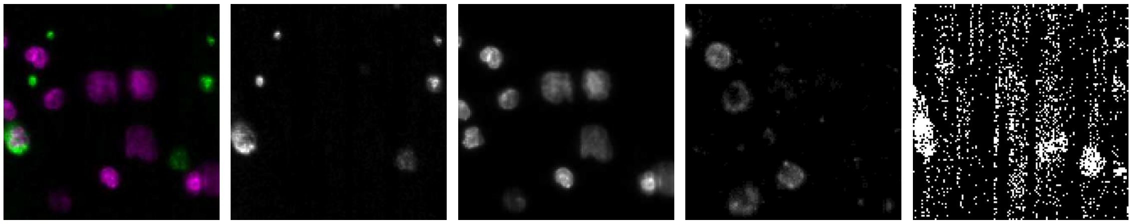				
5615	43	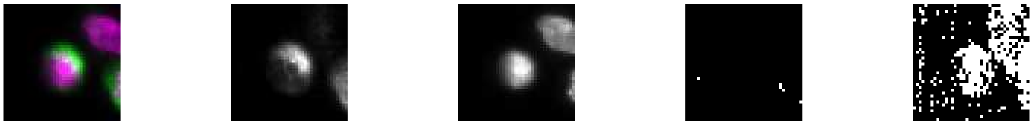				
5616	43	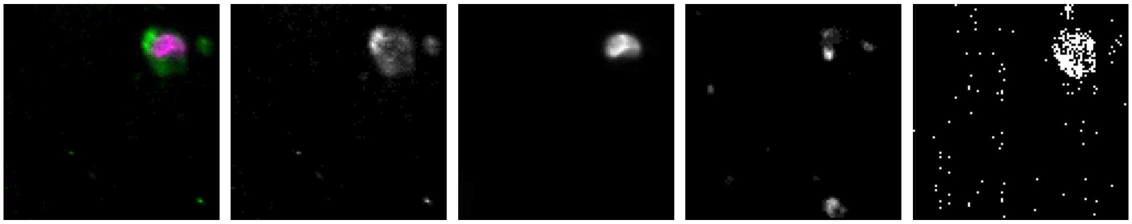				
5617	43	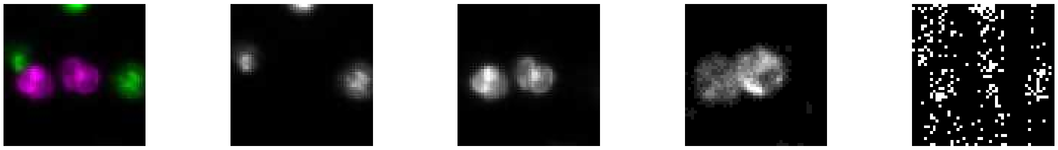				
5618	43	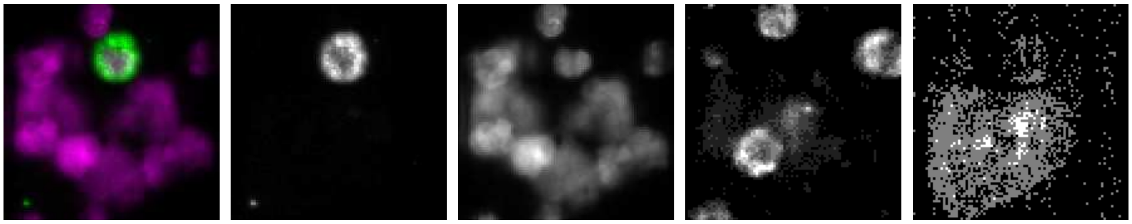				
5619	43	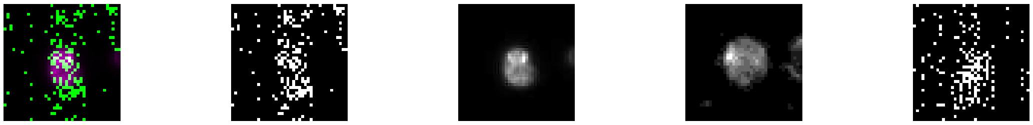				

Ereignis	Aufnahme	DAPI/CK-PE	CK-PE	DAPI	CD45-APC	HER-2/neu
5620	43	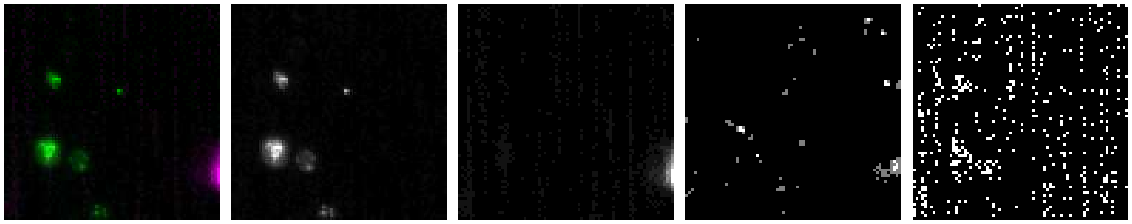				
5621	43	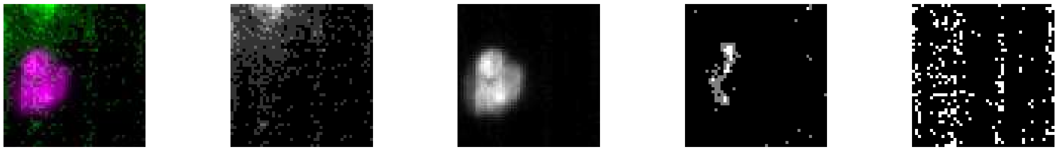				
5622	43	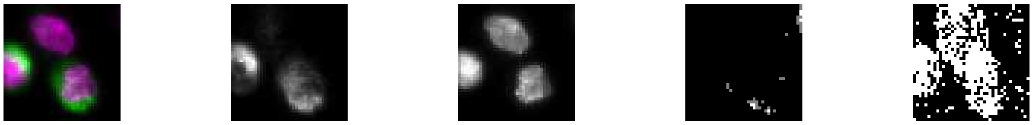				
5623	43	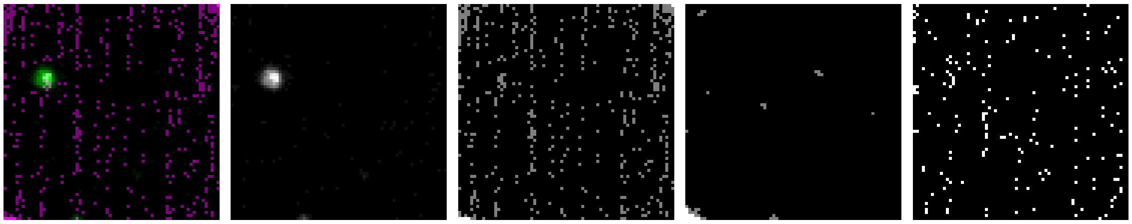				
5624	43	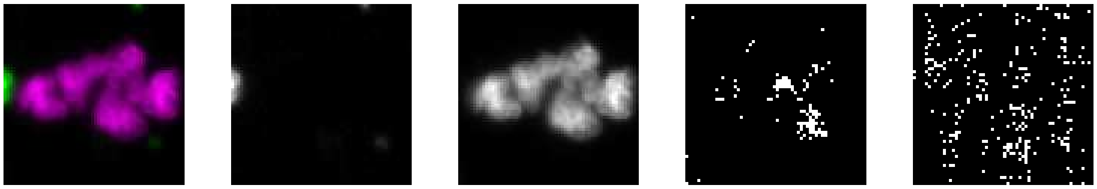				
5625	43	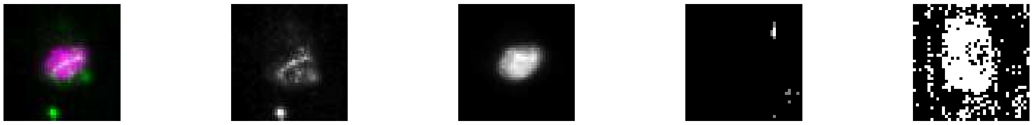				
5626	43	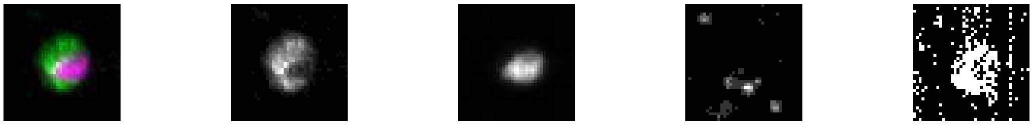				
5627	43	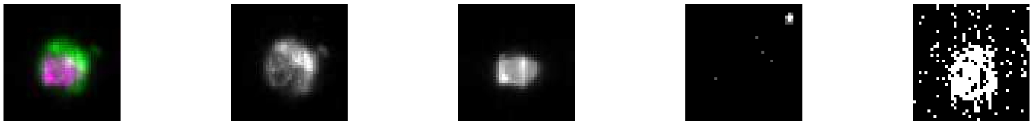				
5628	43	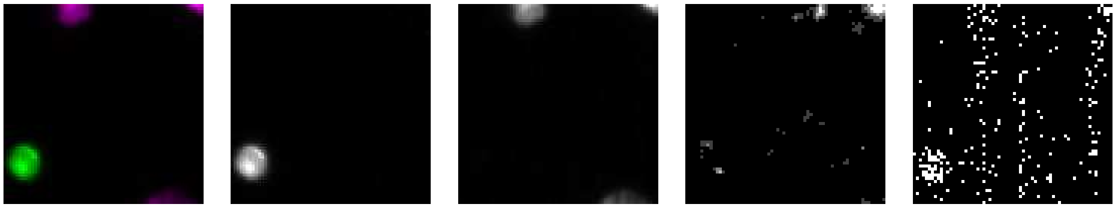				
5629	43	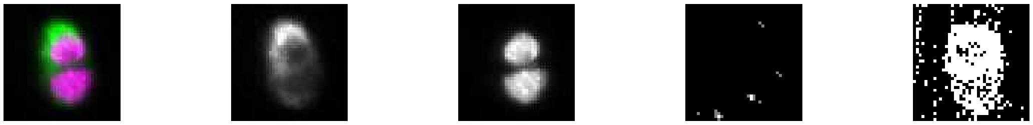				
5630	43	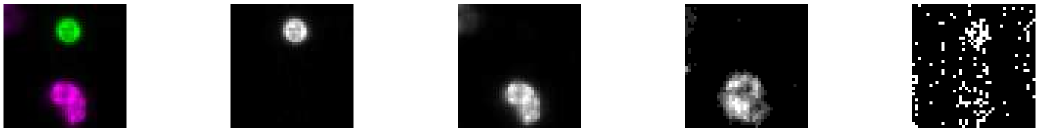				

Ereignis	Aufnahme	DAPI/CK-PE	CK-PE	DAPI	CD45-APC	HER-2/neu
5631	43	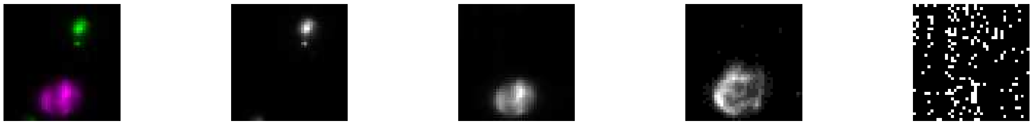				
5632	43	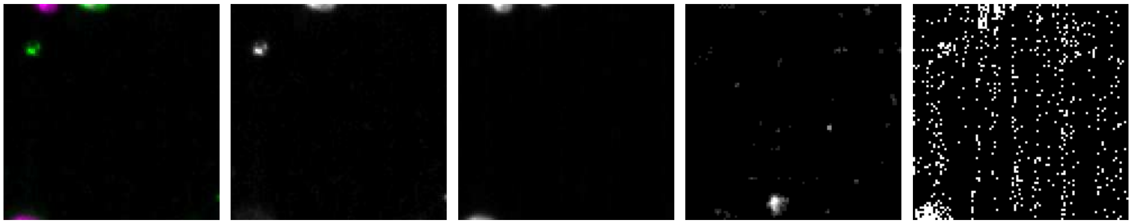				
5633	43	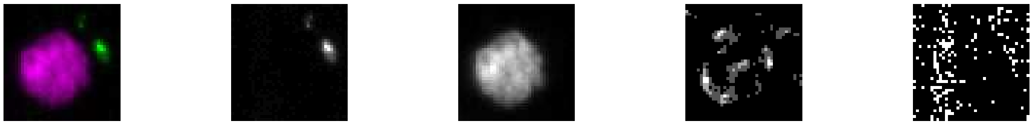				
5634	43	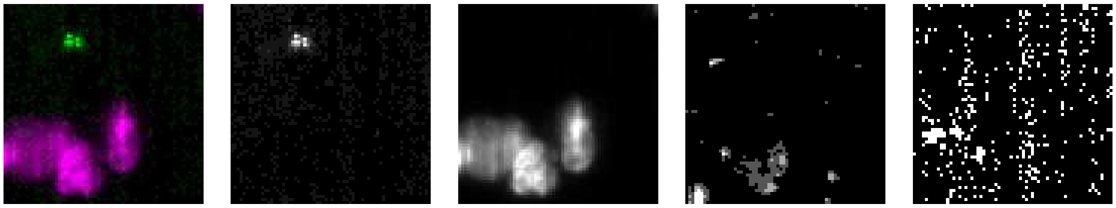				
5635	43	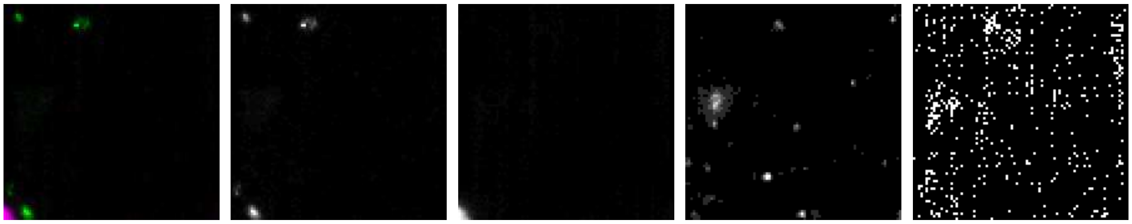				
5636	43	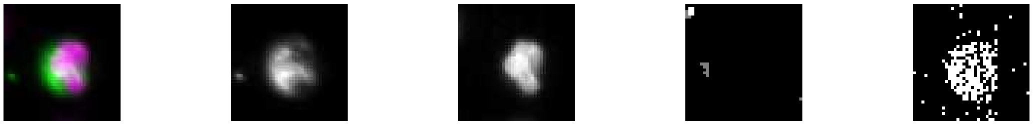				
5637	43	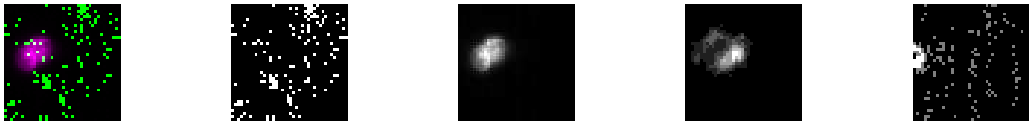				
5638	43	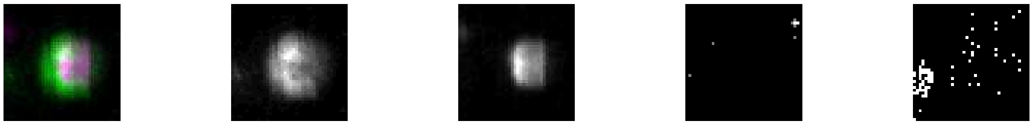				
5639	43	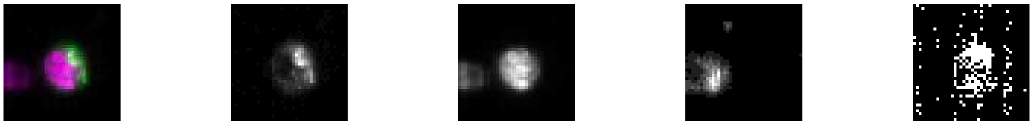				
5640	43	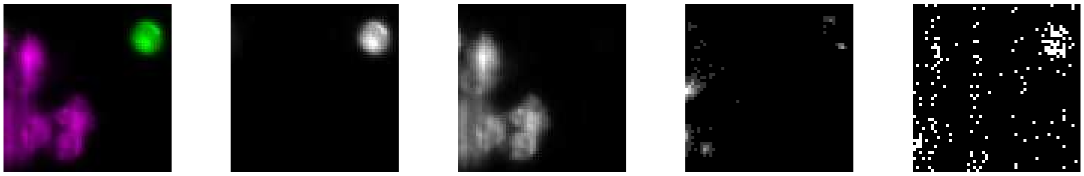				
5641	43	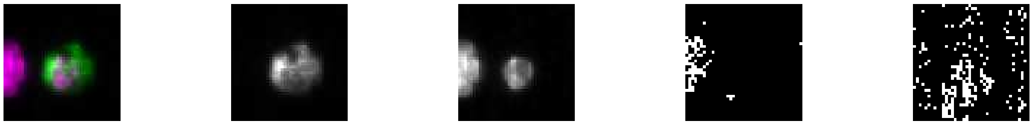				

Ereignis	Aufnahme	DAPI/CK-PE	CK-PE	DAPI	CD45-APC	HER-2/neu
5642	43	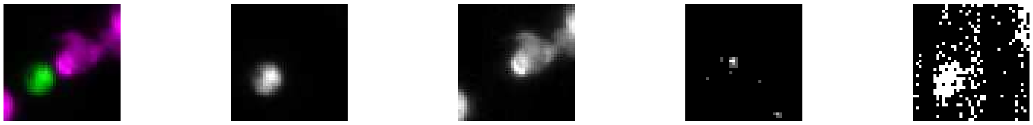				
5643	43	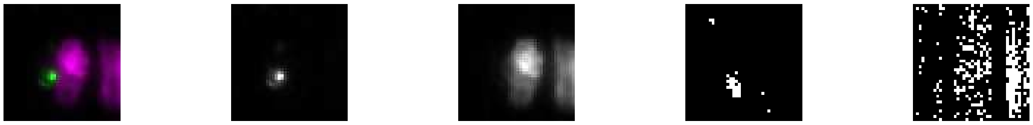				
5644	43	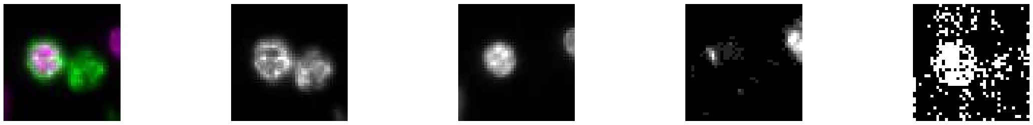				
5645	43	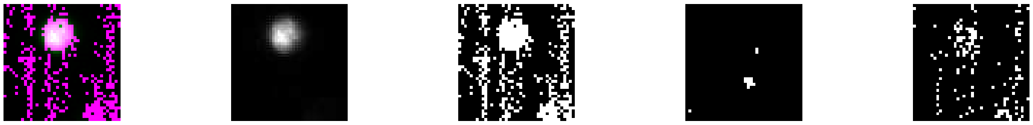				
5646	42	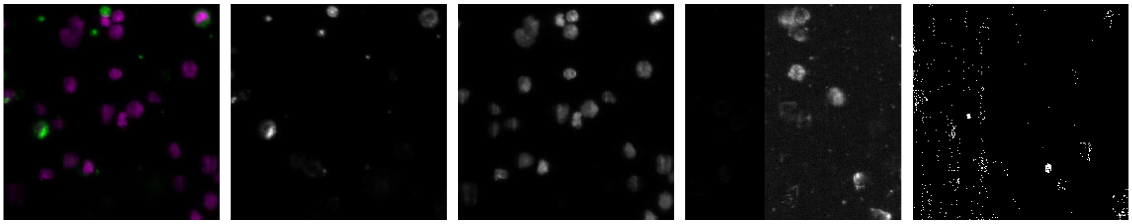				
5647	42	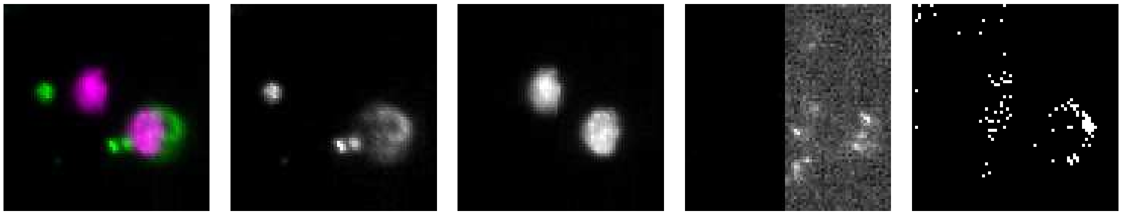				
5648	28	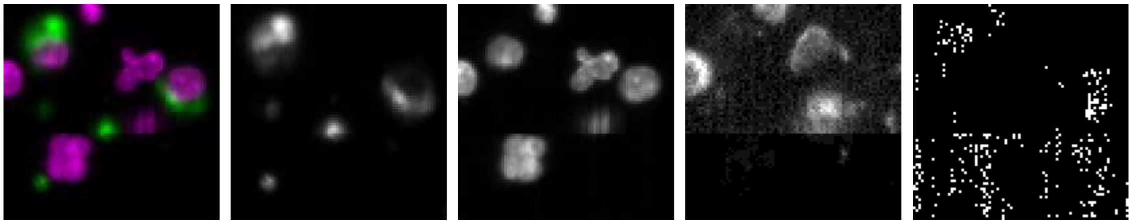				
5649	28	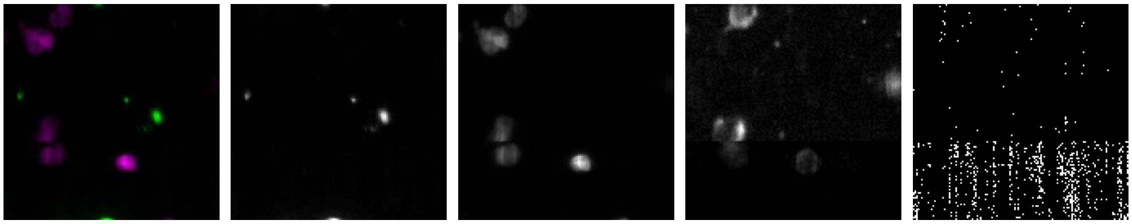				
5650	28	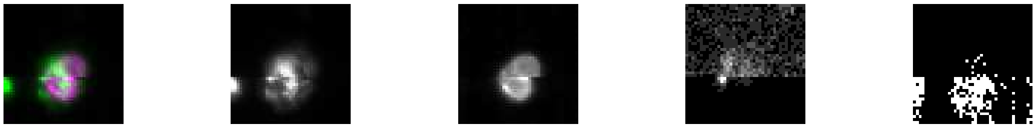				
5651	42	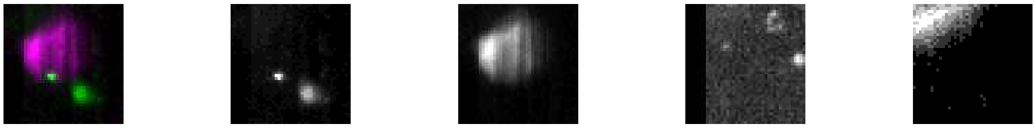				

Ereignis	Aufnahme	DAPI/CK-PE	CK-PE	DAPI	CD45-APC	HER-2/neu
5652	43	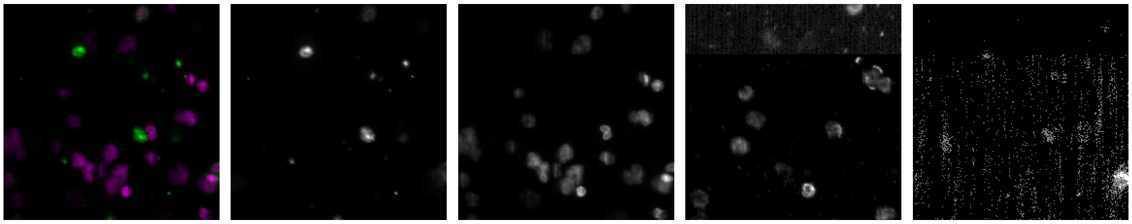				
5653	28	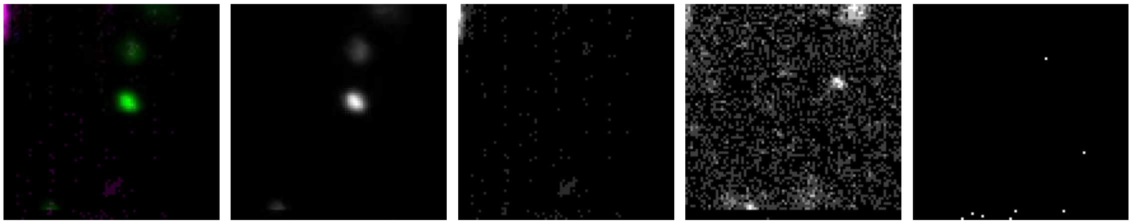				
5654	43	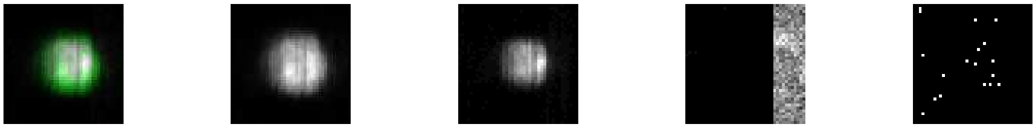				
5655	43	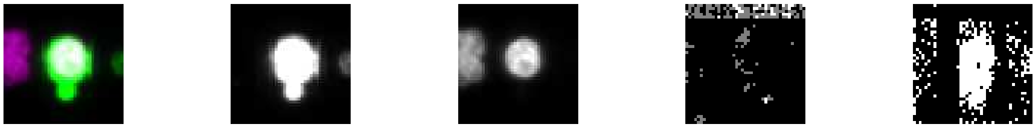				
5656	43	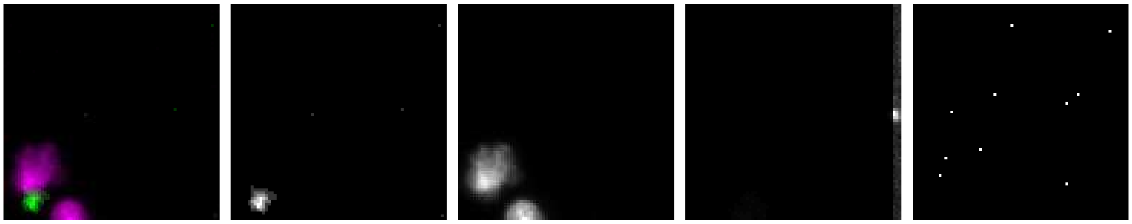				
5657	43	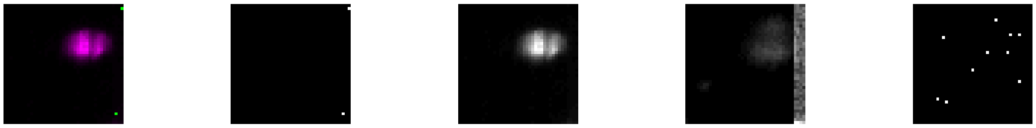				
5658	28	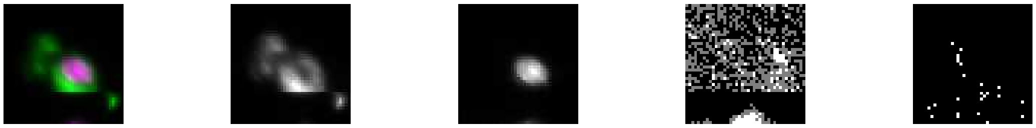				
5659	28	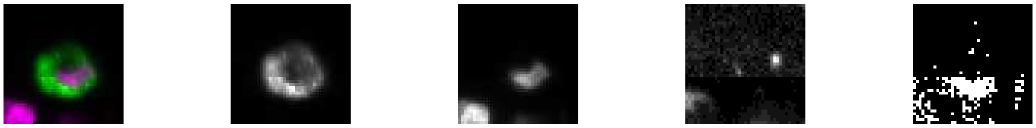				
5660	28	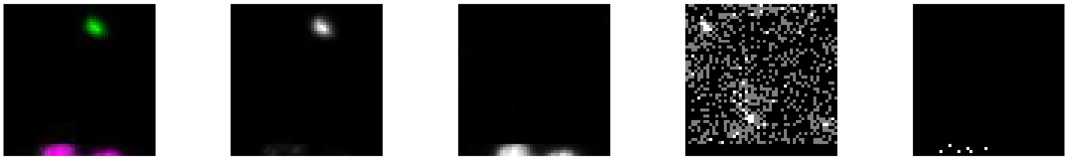				
5661	28	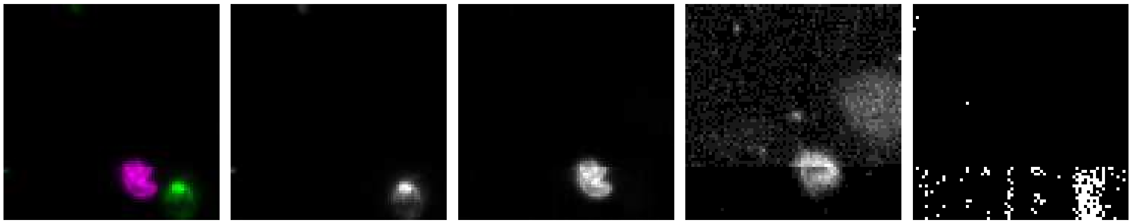				

Ereignis	Aufnahme	DAPI/CK-PE	CK-PE	DAPI	CD45-APC	HER-2/neu
5662	44	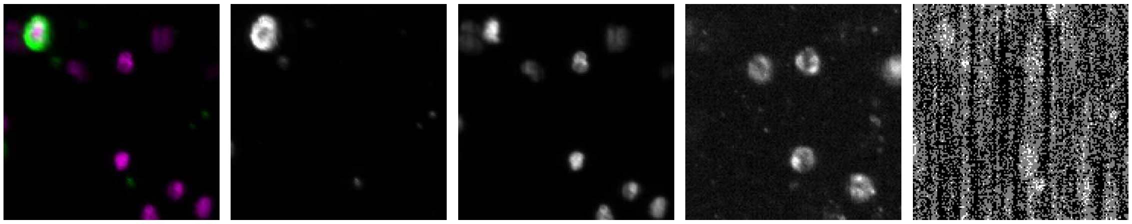				
5663	44	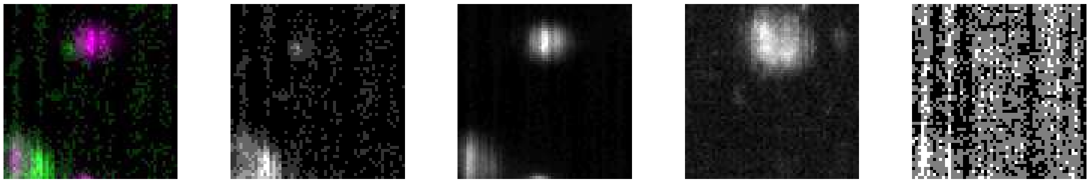				
5664	44	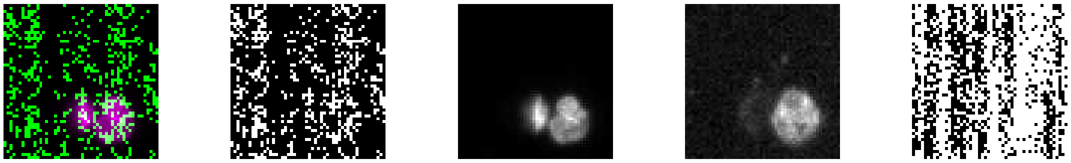				
5665	44	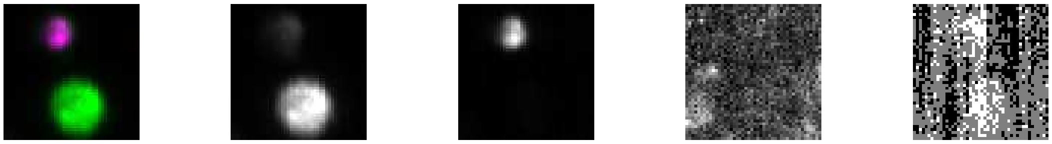				
5666	44	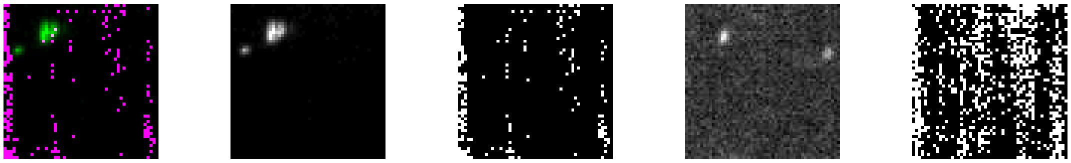				
5667	44	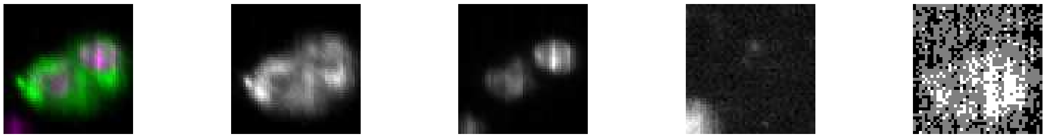				
5668	44	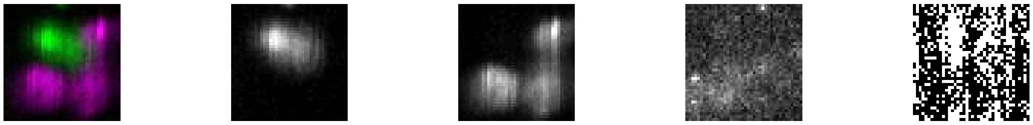				
5669	44	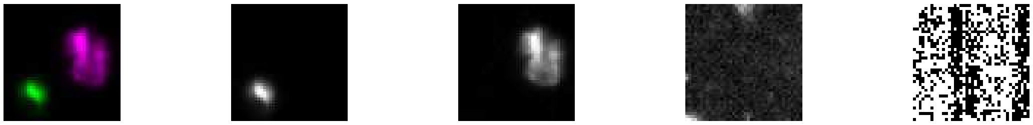				
5670	44	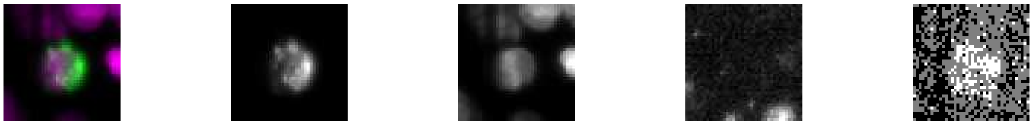				
5671	44	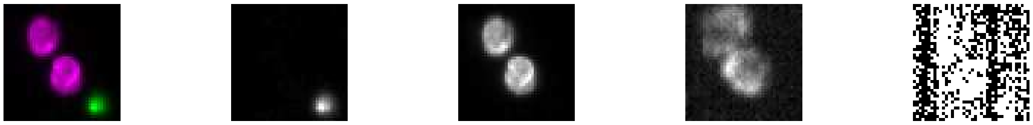				
5672	44	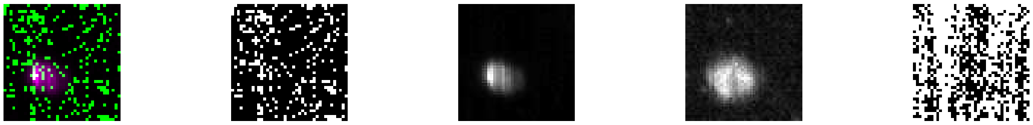				
5673	44	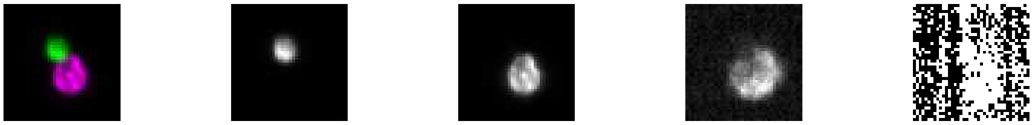				

Ereignis	Aufnahme	DAPI/CK-PE	CK-PE	DAPI	CD45-APC	HER-2/neu
5674	44	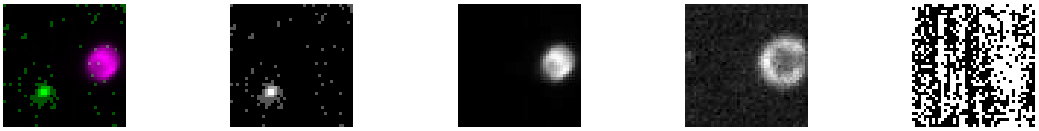				
5675	44	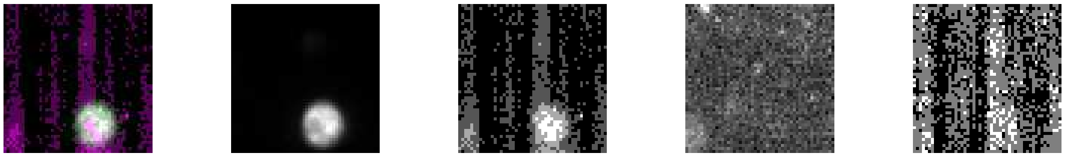				
5676	44	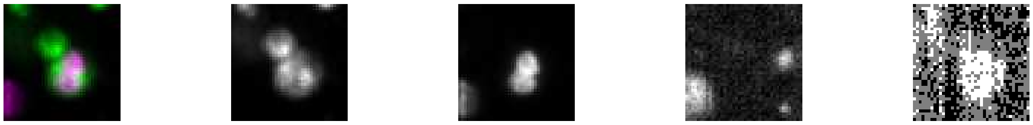				
5677	44	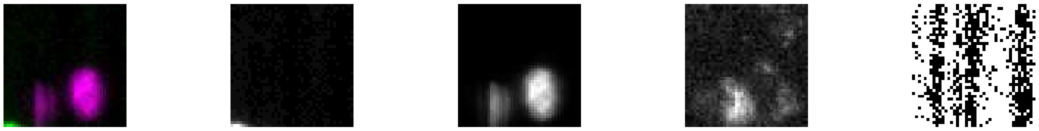				
5678	44	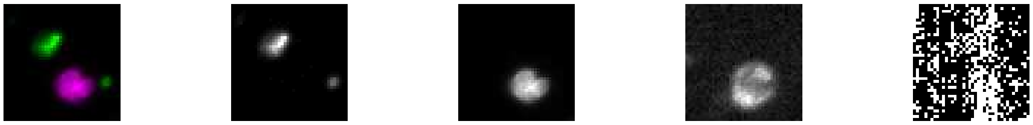				
5679	44	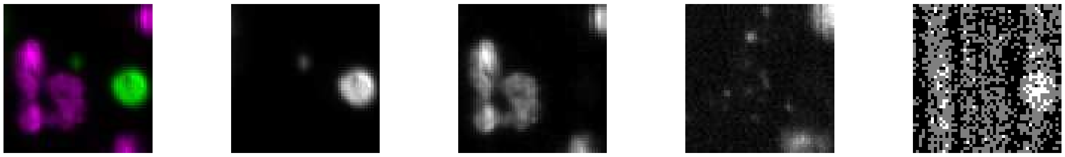				
5680	44	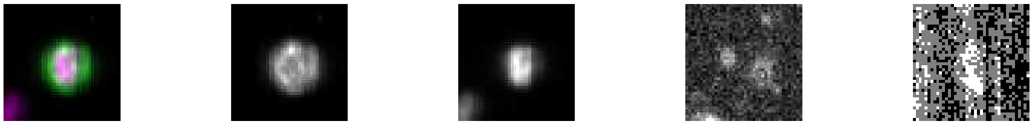				
5681	44	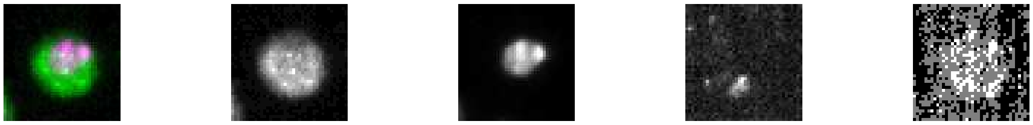				
5682	44	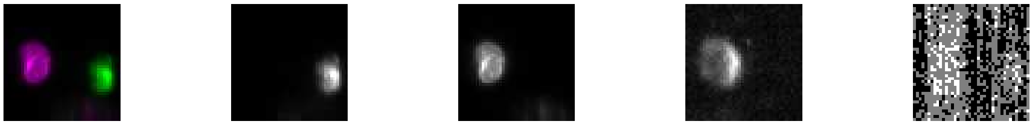				
5683	44	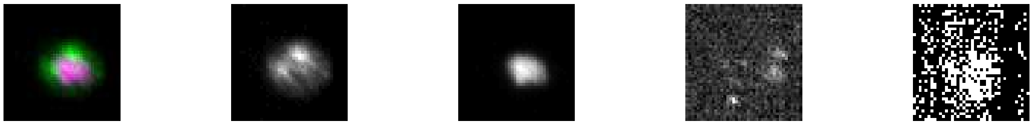				
5684	44	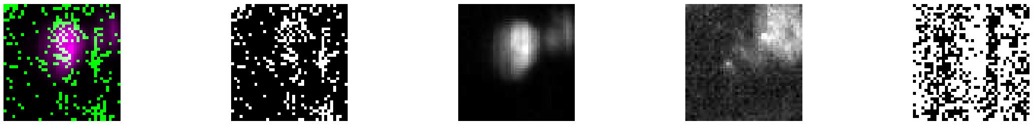				
5685	44	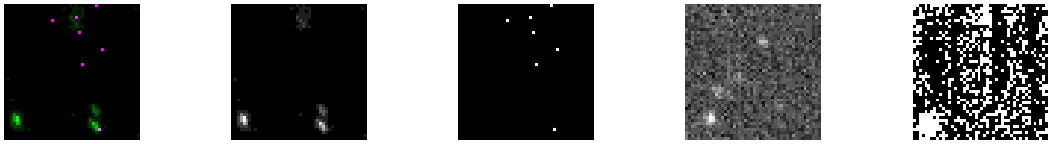				

Ereignis	Aufnahme	DAPI/CK-PE	CK-PE	DAPI	CD45-APC	HER-2/neu
5686	44	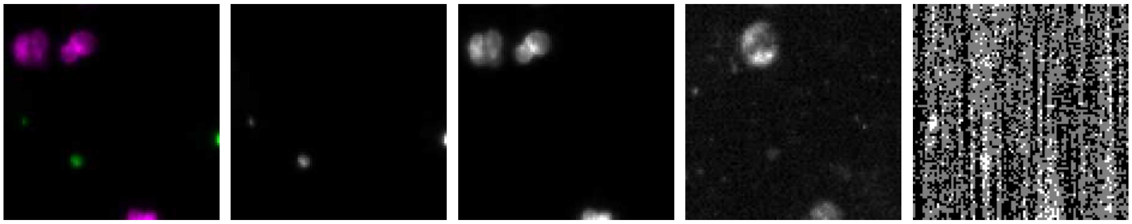				
5687	44	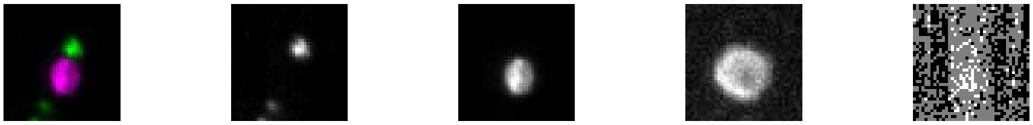				
5688	44	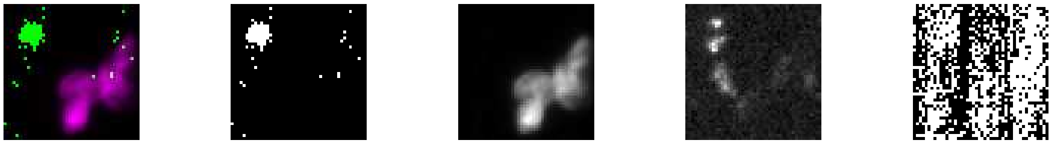				
5689	44	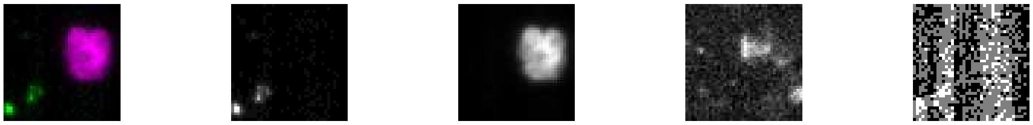				
5690	44	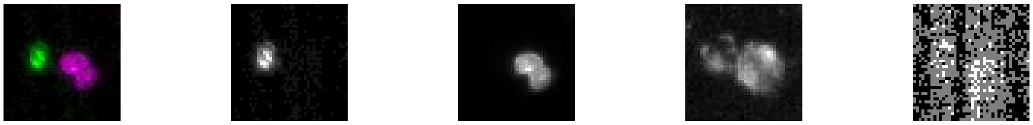				
5691	44	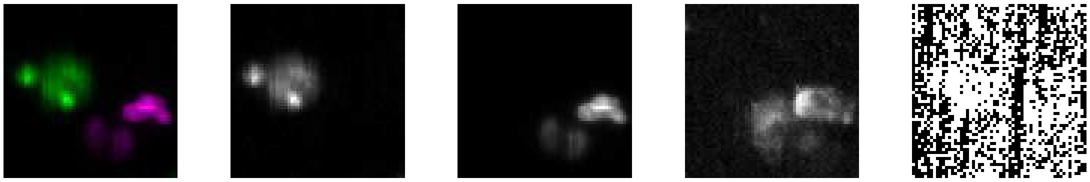				
5692	44	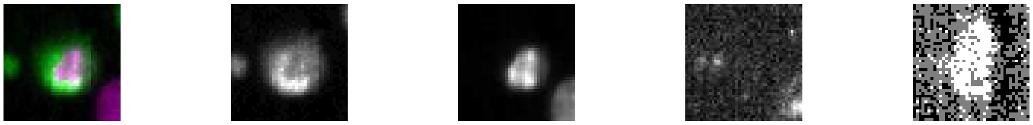				
5693	44	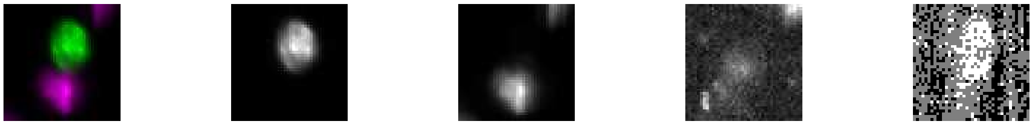				
5694	44	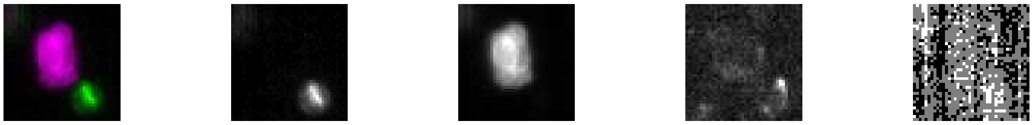				
5695	44	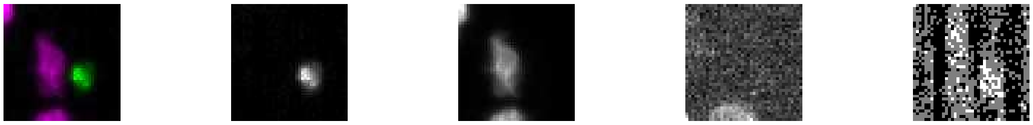				
5696	44	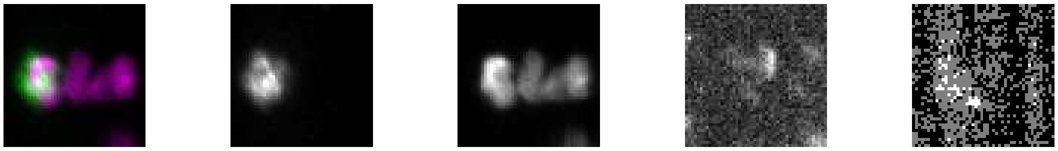				
5697	44	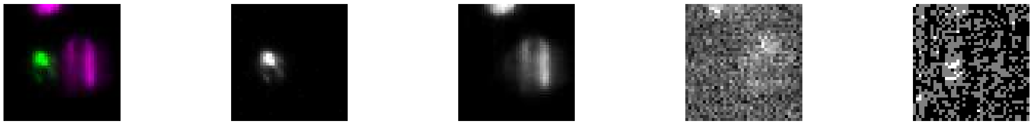				

Ereignis	Aufnahme	DAPI/CK-PE	CK-PE	DAPI	CD45-APC	HER-2/neu
5698	44	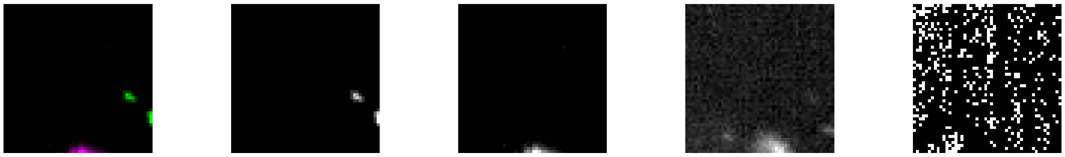				
5699	44	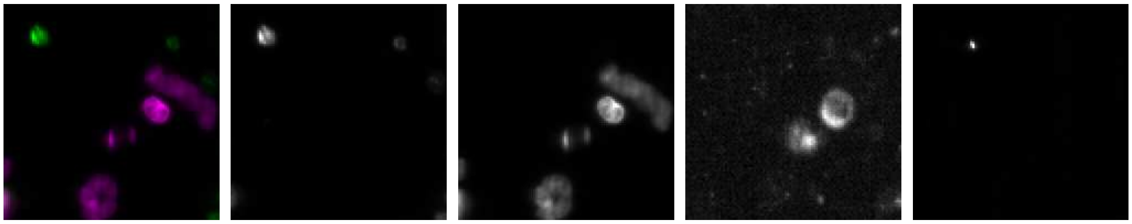				
5700	44	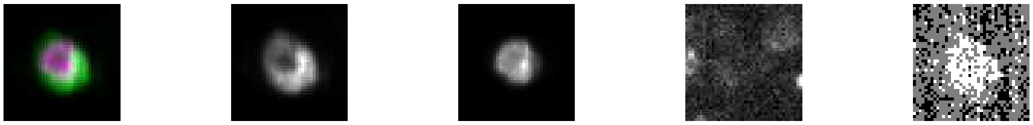				
5701	44	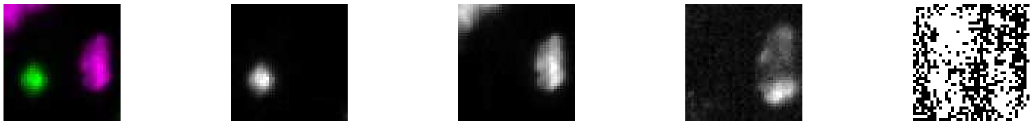				
5702	44	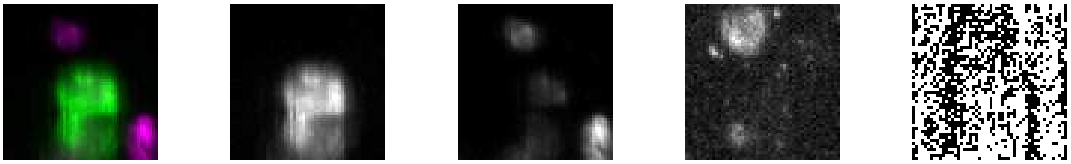				
5703	44	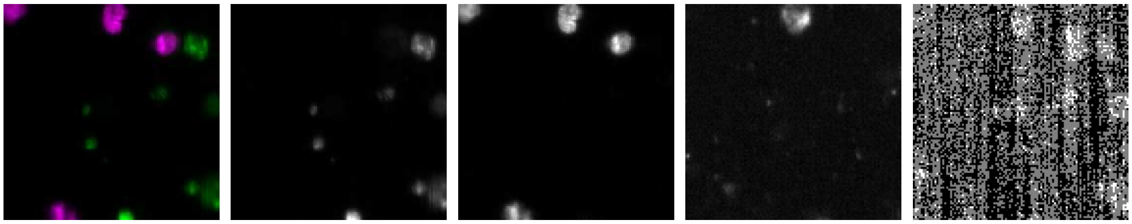				
5704	44	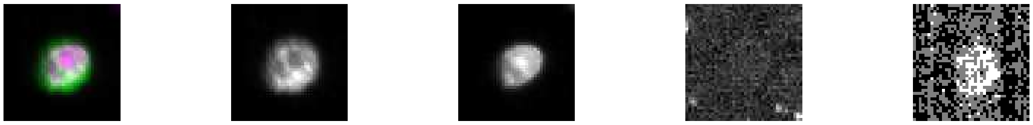				
5705	44	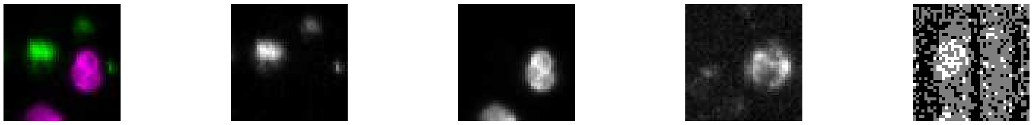				
5706	44	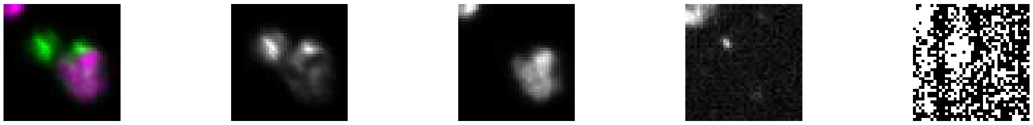				
5707	44	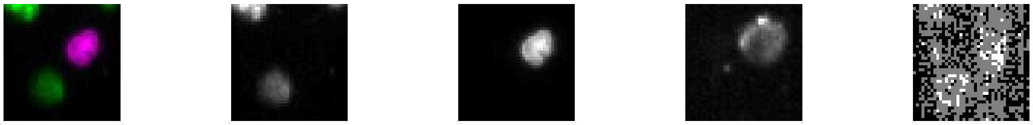				
5708	44	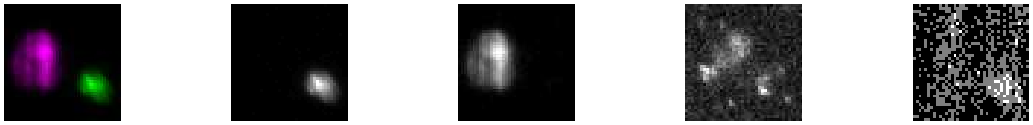				
5709	44	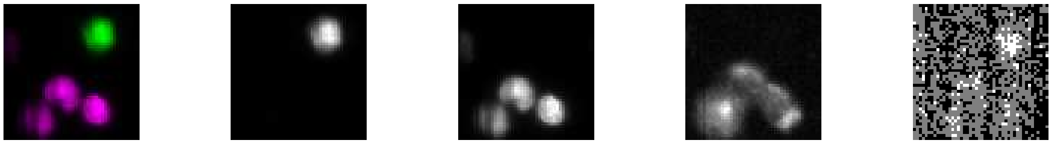				

Ereignis	Aufnahme	DAPI/CK-PE	CK-PE	DAPI	CD45-APC	HER-2/neu
5710	44	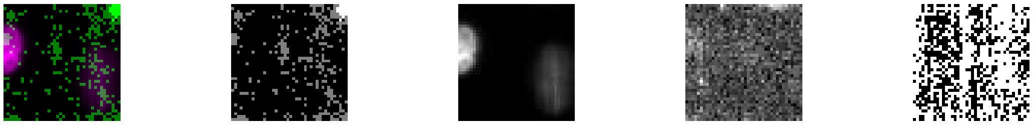				
5711	44	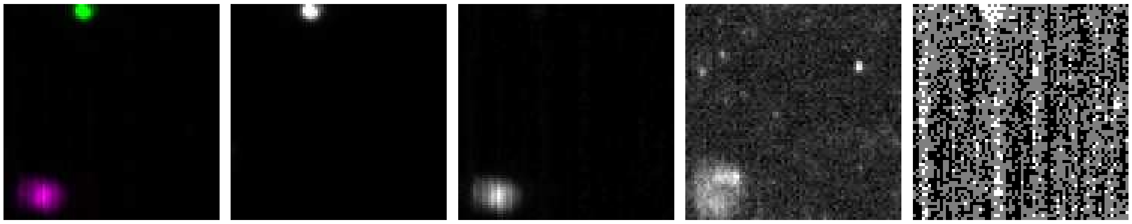				
5712	44	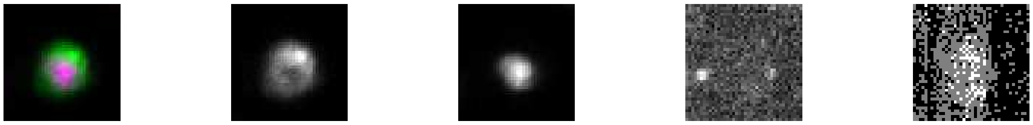				
5713	44	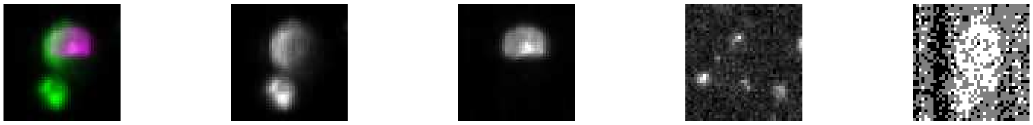				
5714	44	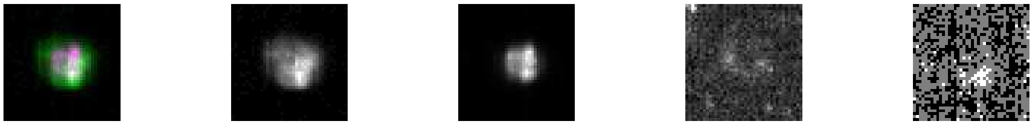				
5715	44	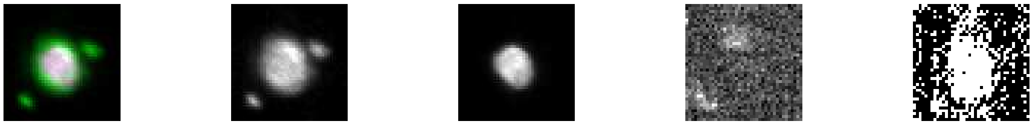				
5716	44	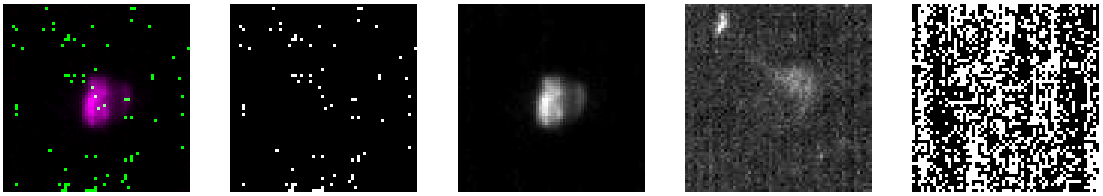				
5717	44	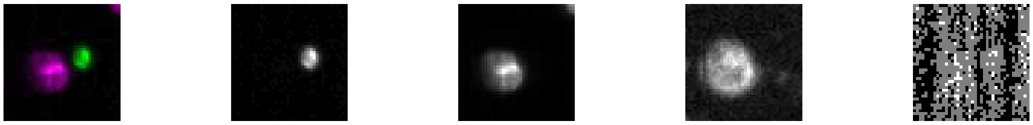				
5718	44	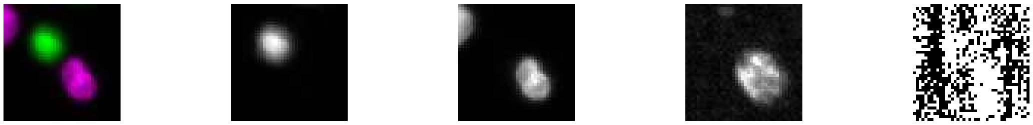				
5719	44	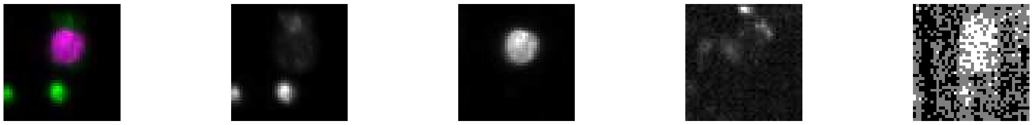				
5720	44	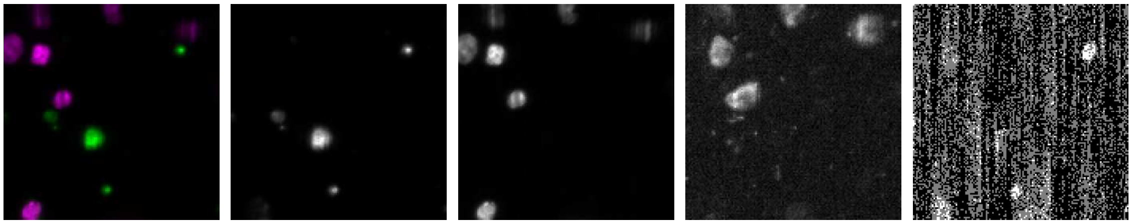				
5721	44	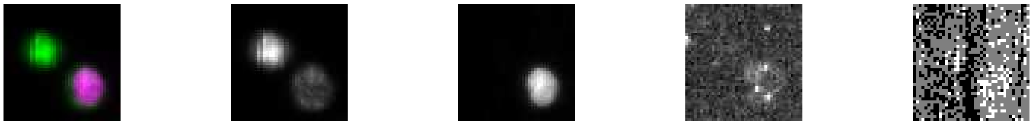				

Ereignis	Aufnahme	DAPI/CK-PE	CK-PE	DAPI	CD45-APC	HER-2/neu
5722	44	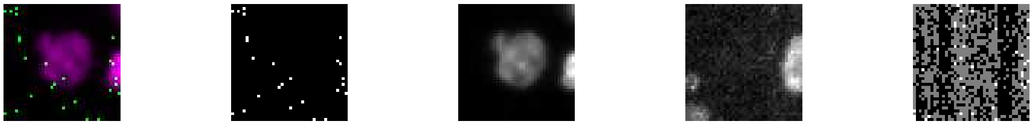				
5723	44	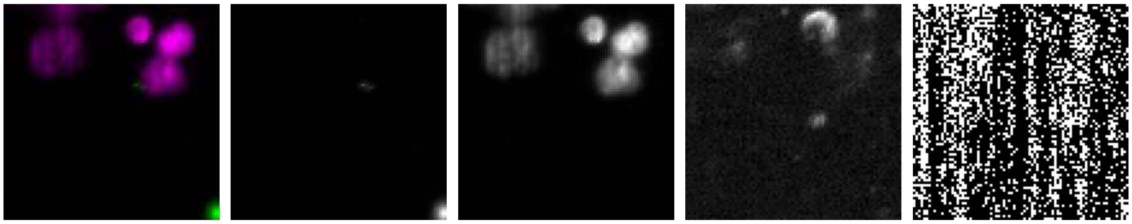				
5724	44	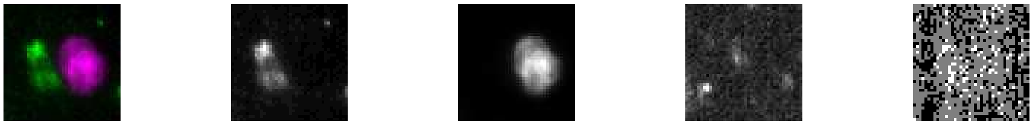				
5725	44	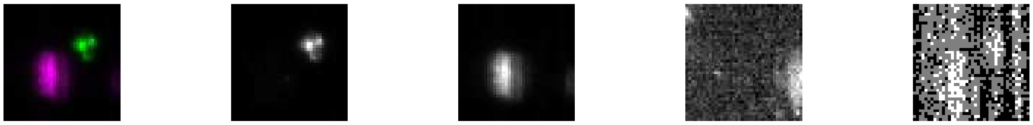				
5726	44	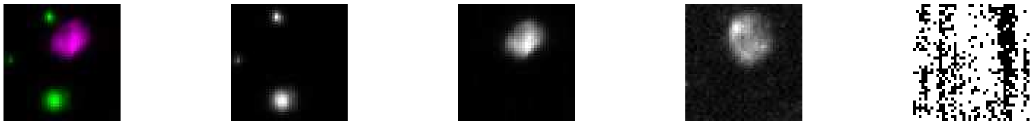				
5727	44	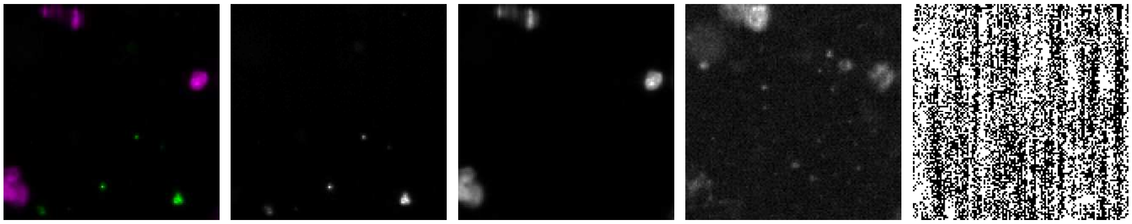				
5728	44	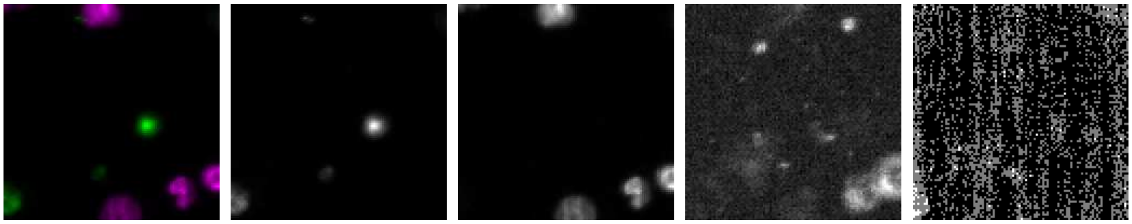				
5729	44	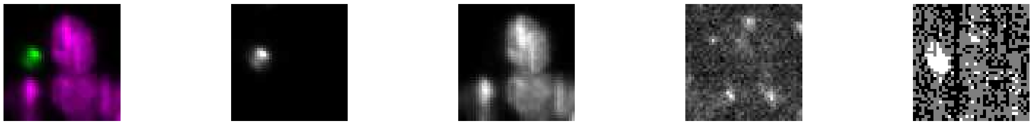				
5730	44	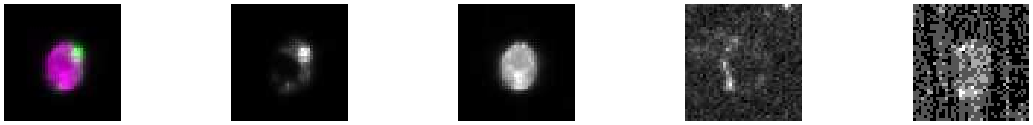				
5731	44	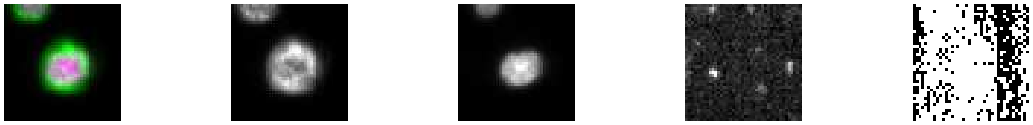				
5732	44	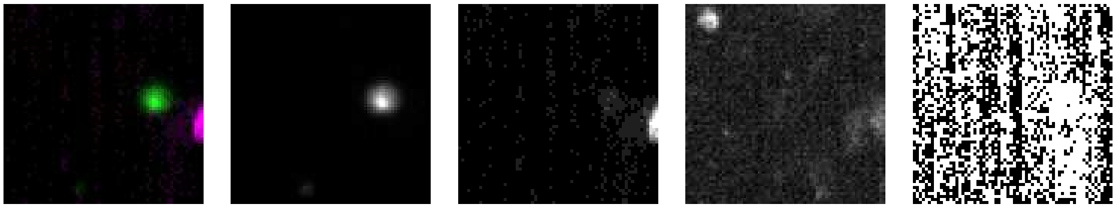				

Ereignis	Aufnahme	DAPI/CK-PE	CK-PE	DAPI	CD45-APC	HER-2/neu
5733	44	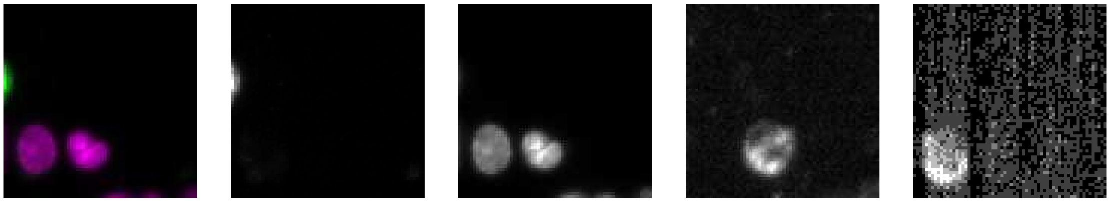				
5734	44	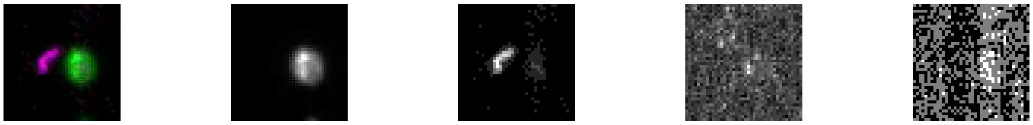				
5735	44	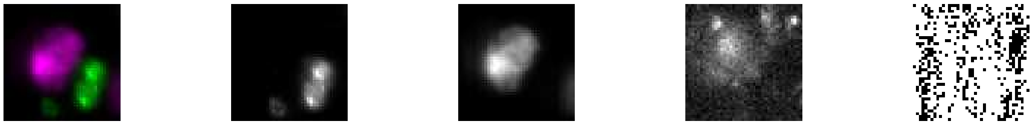				
5736	44	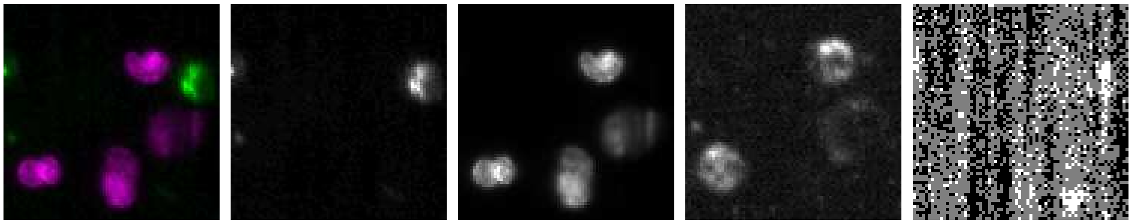				
5737	44	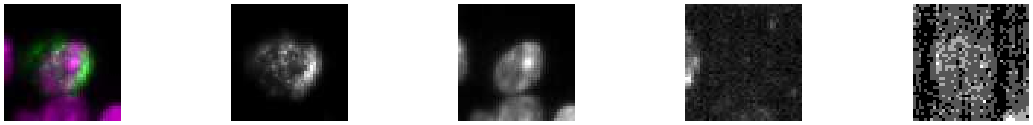				
5738	44	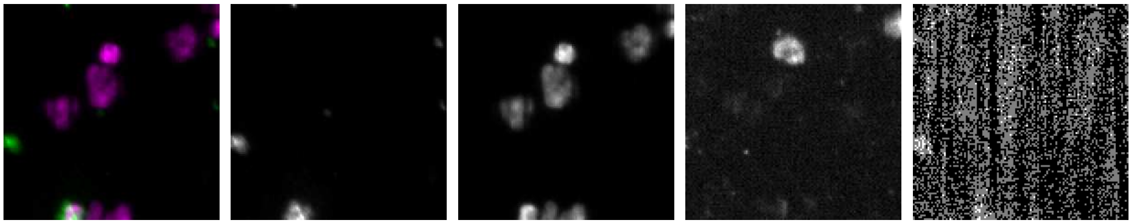				
5739	44	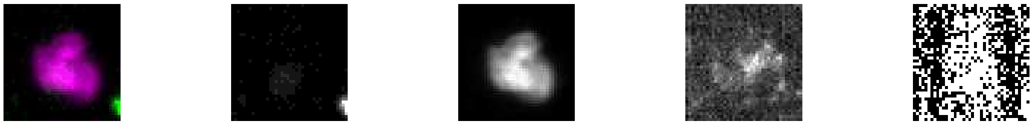				
5740	44	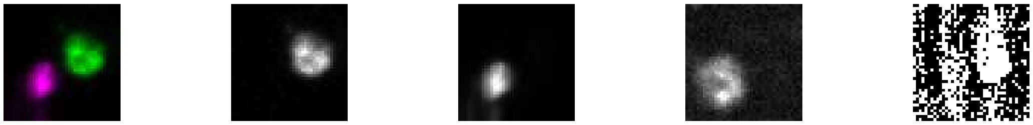				
5741	44	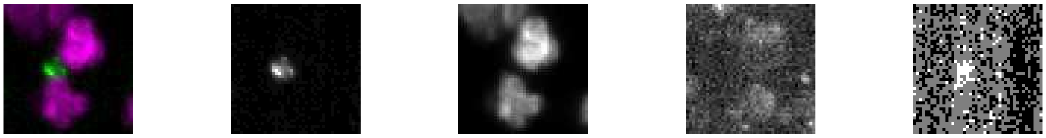				
5742	44	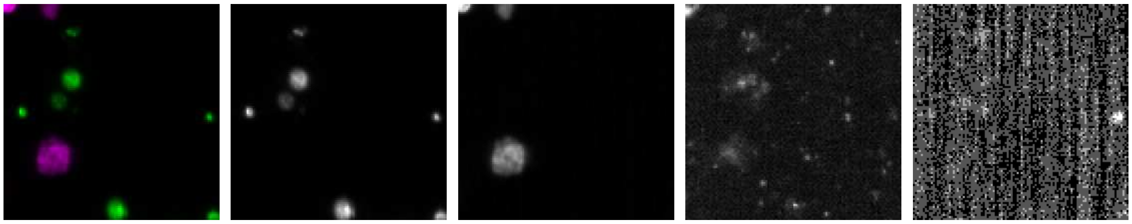				
5743	44	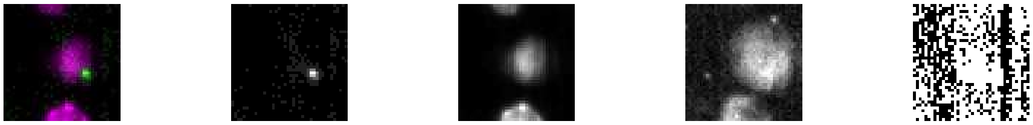				

Proben-ID: Gyn14.208 **Kassetten-ID:**01327106 **Patienten-ID:**

Ereignis	Aufnahme	DAPI/CK-PE	CK-PE	DAPI	CD45-APC	HER-2/neu
5744	44	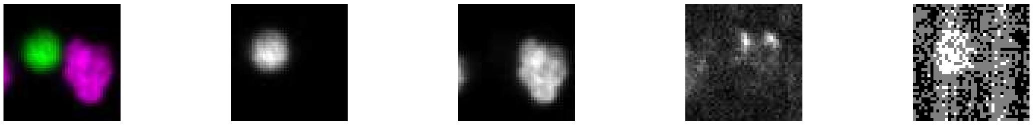				
5745	44	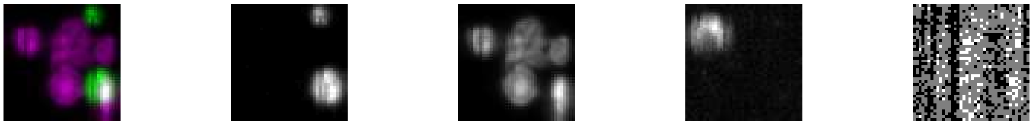				
5746	44	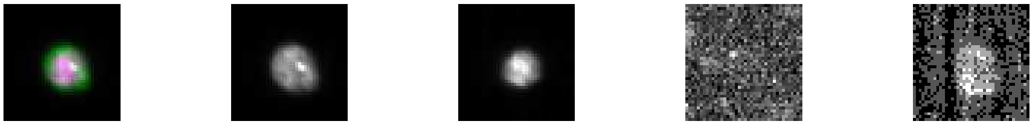				
5747	44	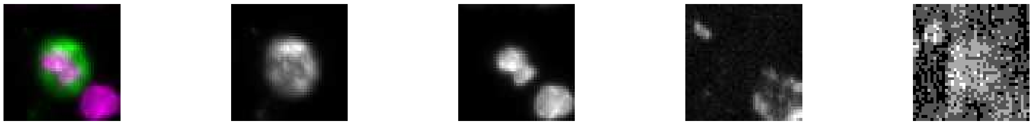				
5748	44	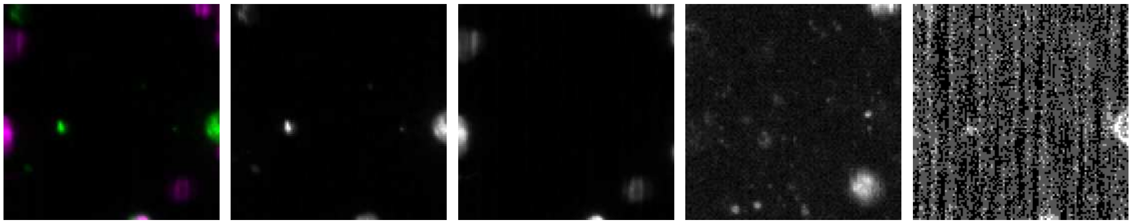				
5749	44	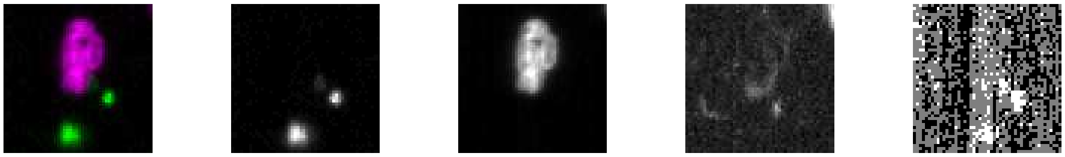				
5750	44	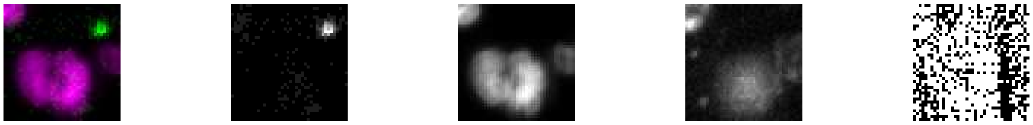				
5751	44	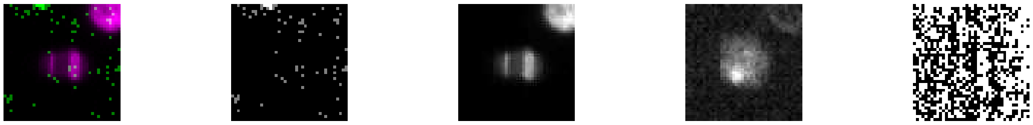				
5752	44	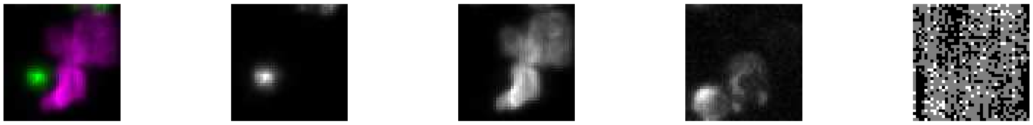				
5753	44	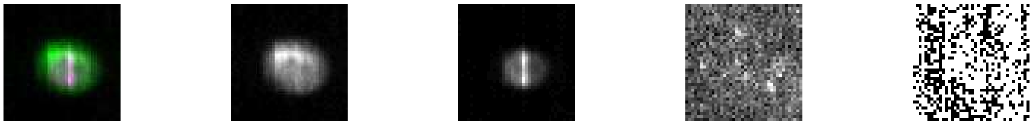				
5754	44	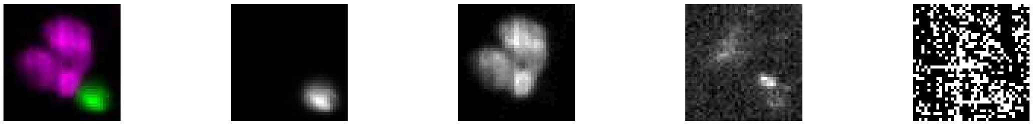				
5755	44	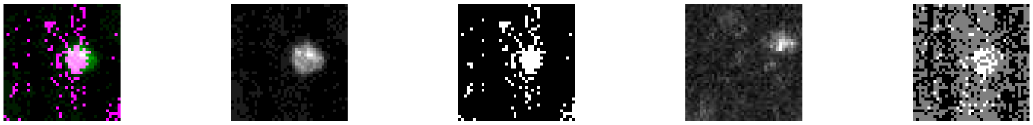				
5756	44	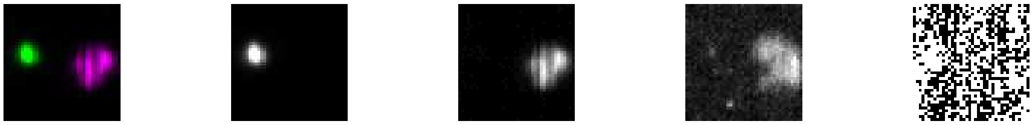				

Ereignis	Aufnahme	DAPI/CK-PE	CK-PE	DAPI	CD45-APC	HER-2/neu
5757	44	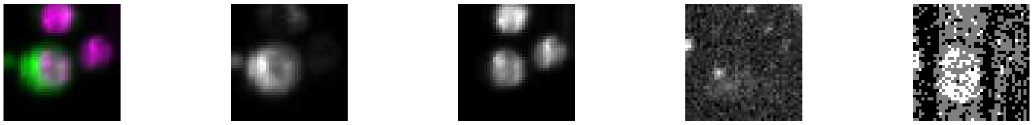				
5758	44	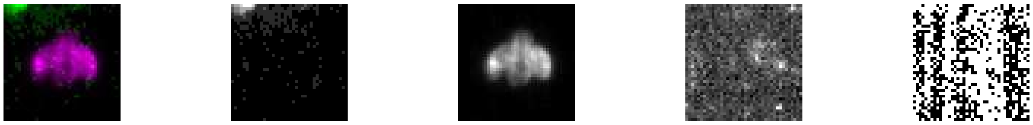				
5759	44	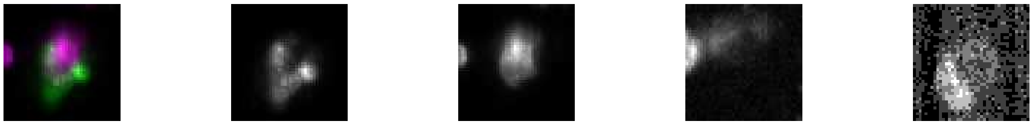				
5760	44	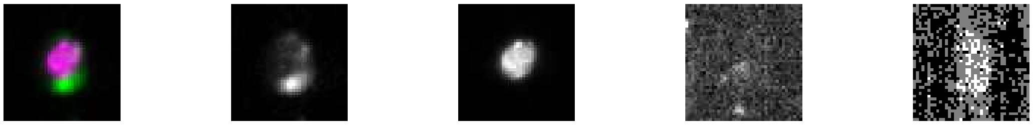				
5761	44	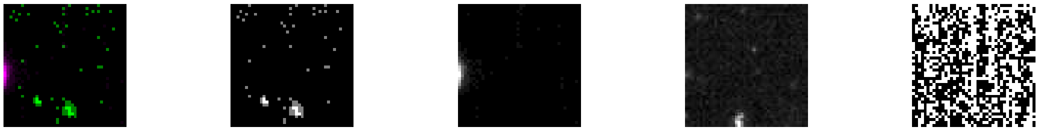				
5762	44	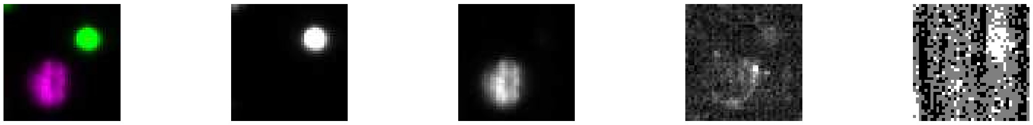				
5763	44	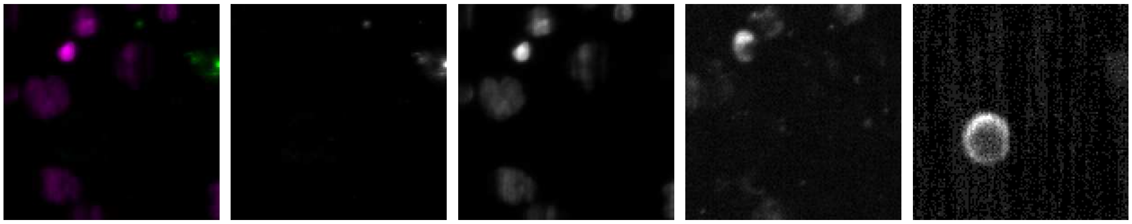				
5764	44	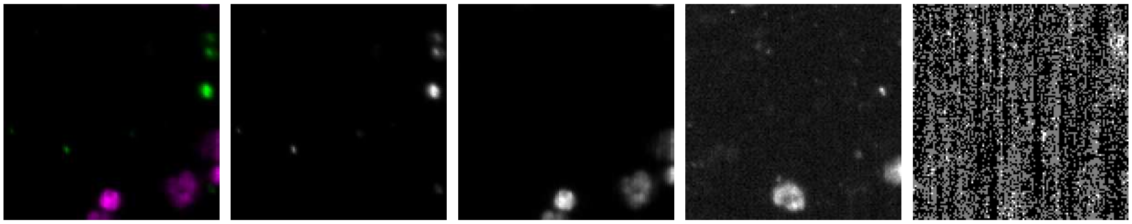				
5765	44	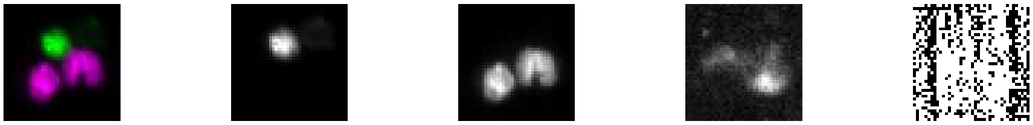				
5766	44	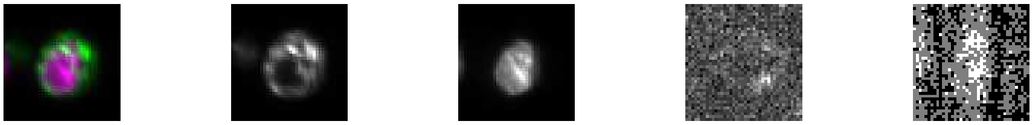				
5767	44	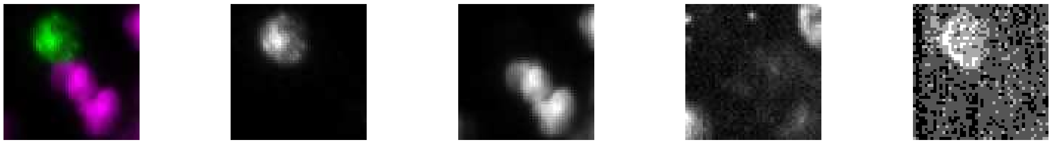				
5768	44	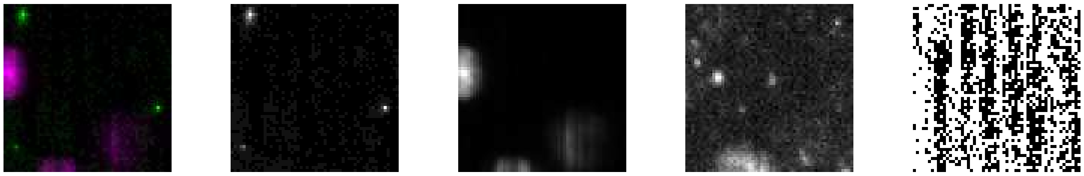				

Ereignis	Aufnahme	DAPI/CK-PE	CK-PE	DAPI	CD45-APC	HER-2/neu
5769	44	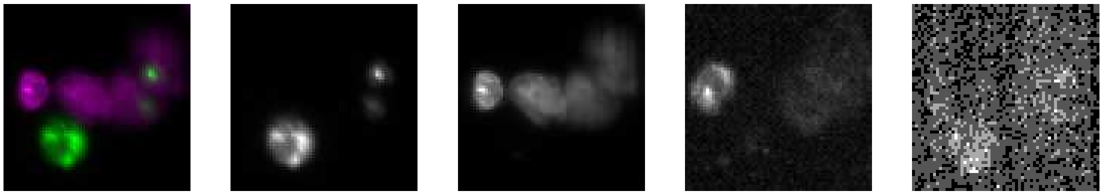				
5770	44	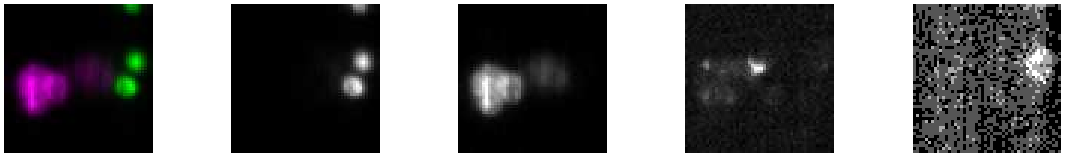				
5771	44	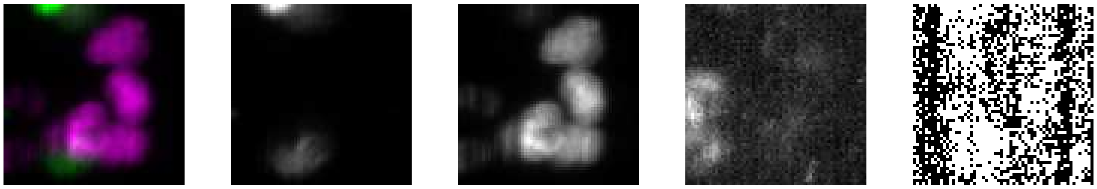				
5772	44	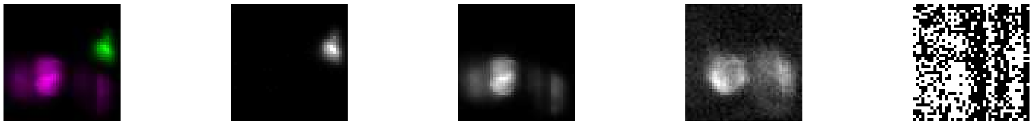				
5773	44	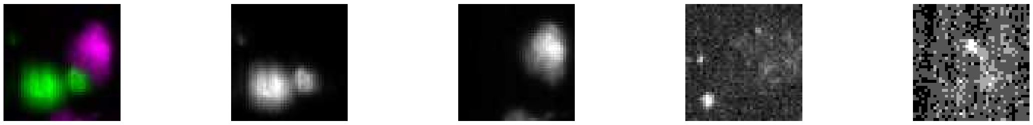				
5774	44	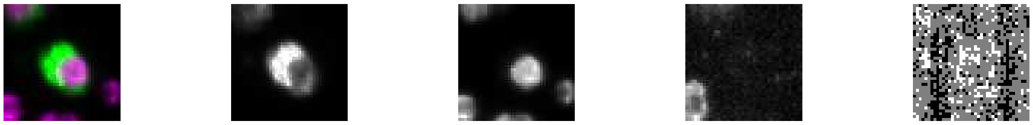				
5775	44	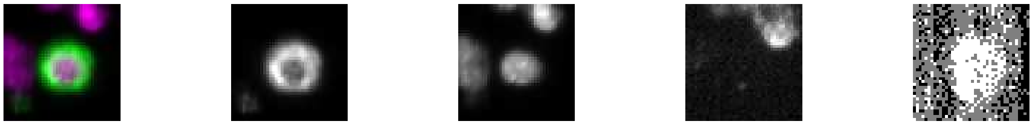				
5776	44	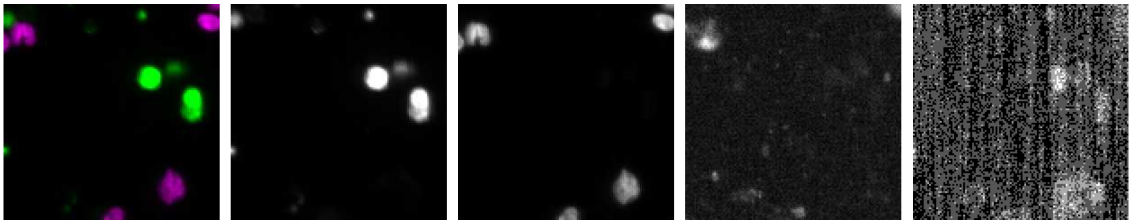				
5777	44	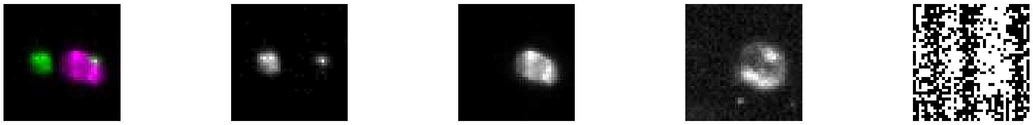				
5778	44	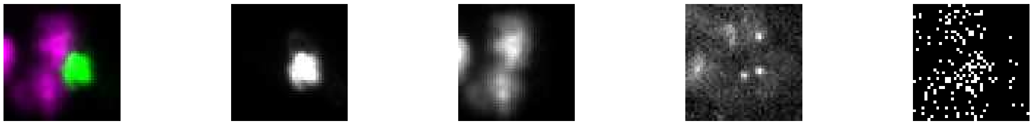				
5779	44	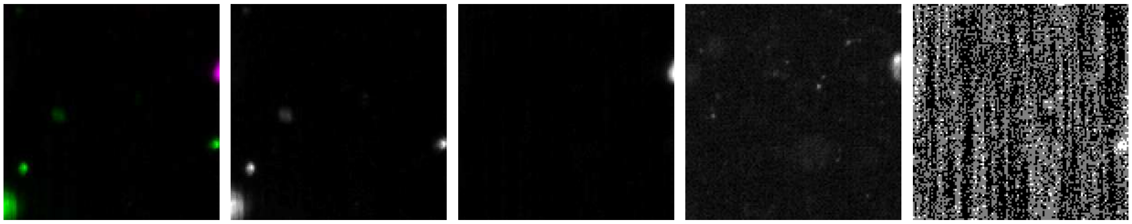				

Proben-ID: Gyn14.208 **Kassetten-ID:**01327106 **Patienten-ID:**

Ereignis	Aufnahme	DAPI/CK-PE	CK-PE	DAPI	CD45-APC	HER-2/neu
5780	44	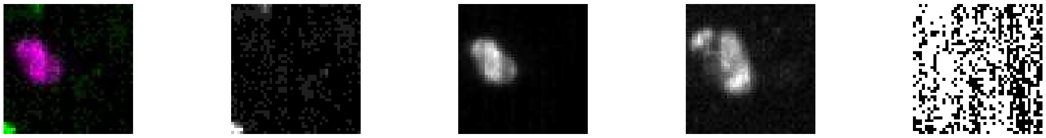				
5781	44	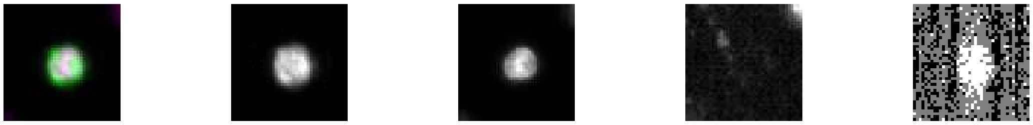				
5782	44	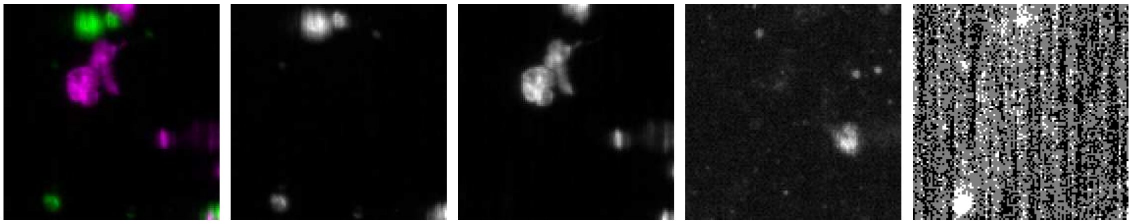				
5783	44	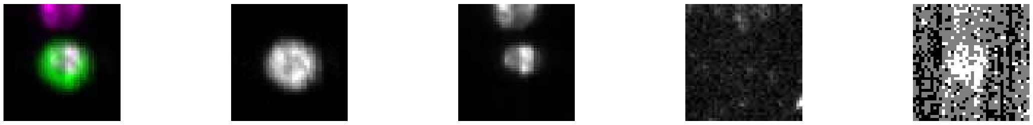				
5784	44	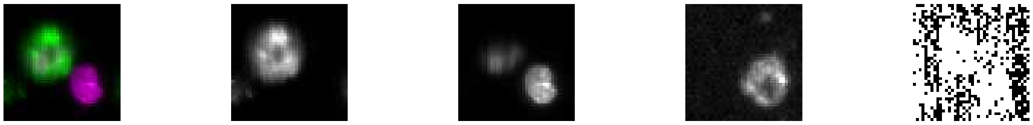				
5785	44	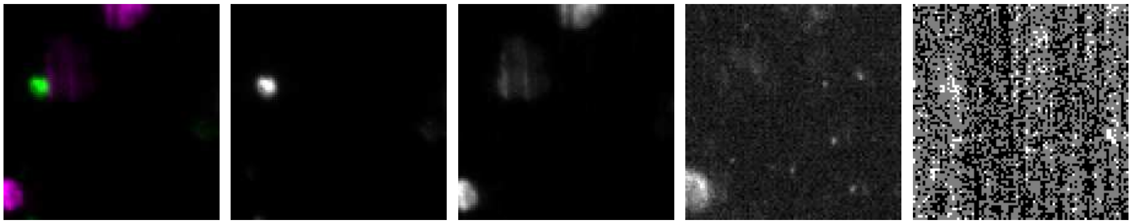				
5786	44	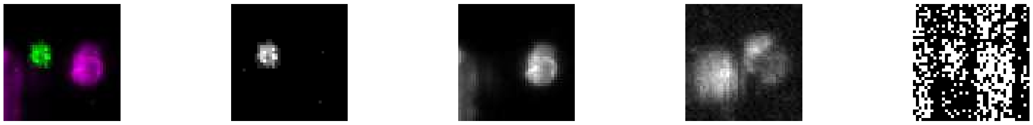				
5787	44	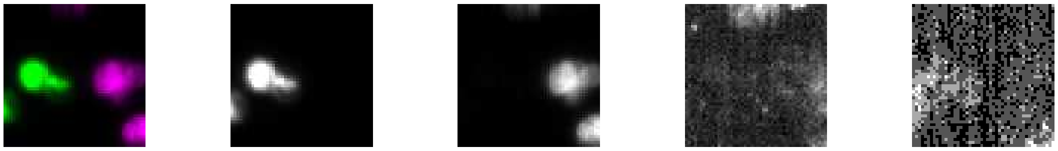				
5788	44	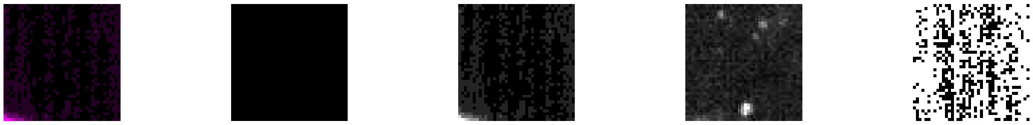				
5789	44	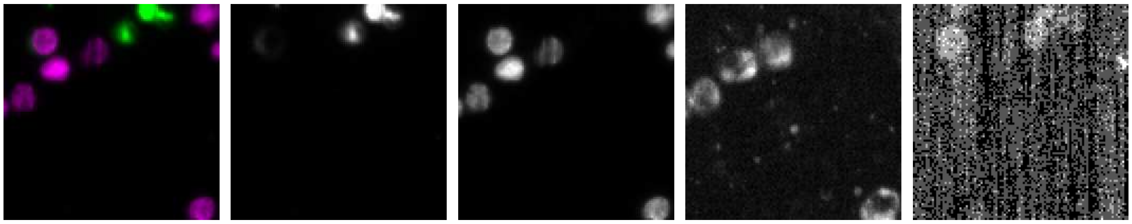				
5790	44	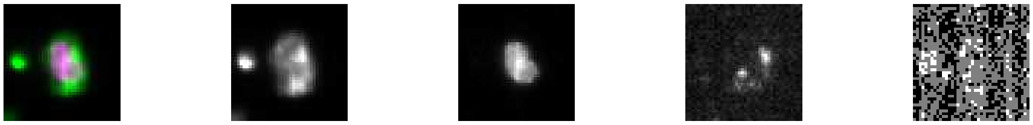				

Ereignis	Aufnahme	DAPI/CK-PE	CK-PE	DAPI	CD45-APC	HER-2/neu
5791	44	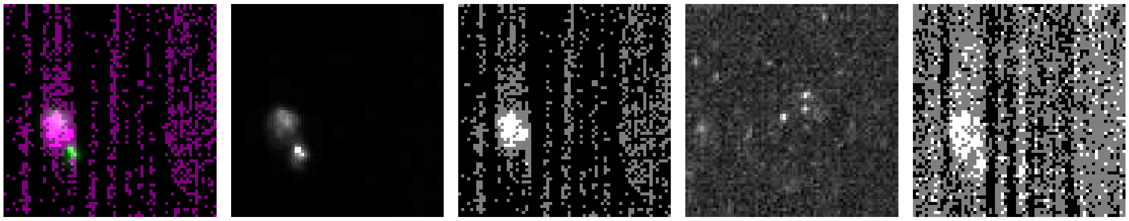				
5792	44	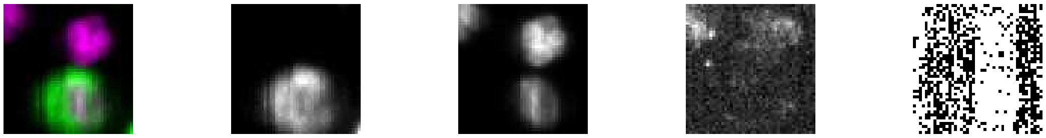				
5793	44	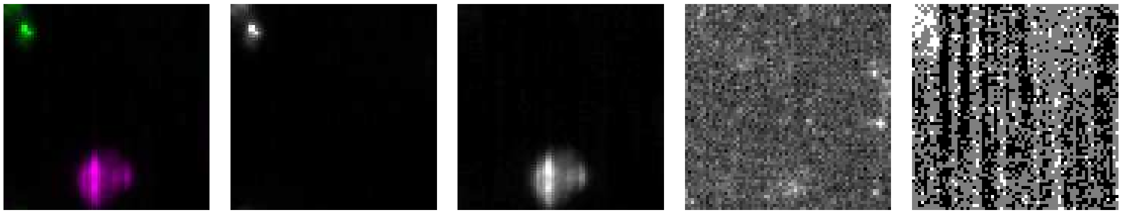				
5794	44	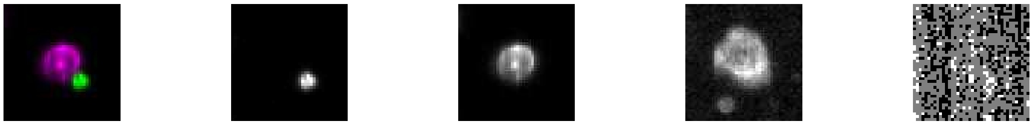				
5795	44	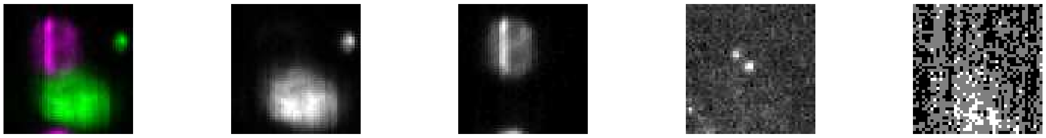				
5796	44	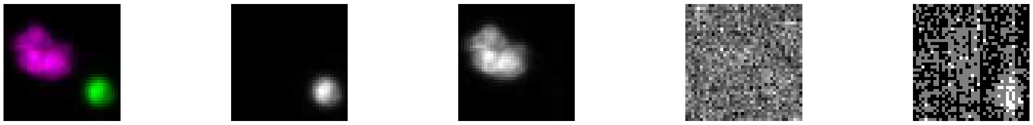				
5797	44	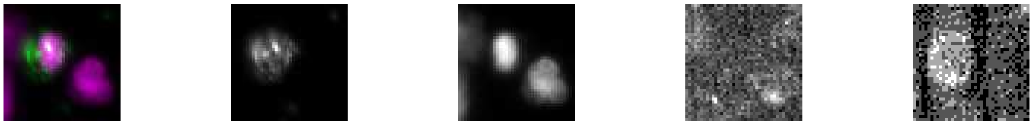				
5798	44	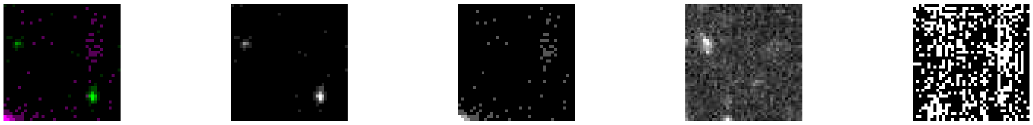				
5799	44	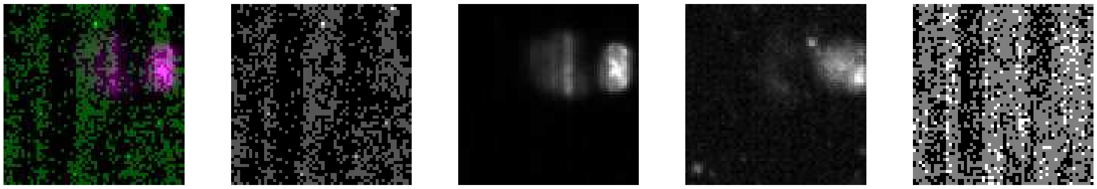				
5800	44	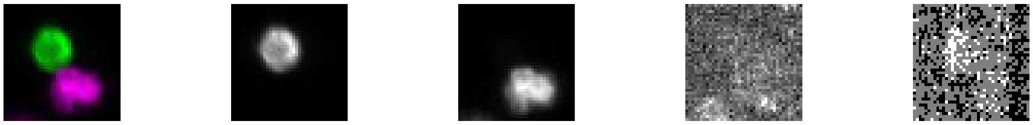				
5801	44	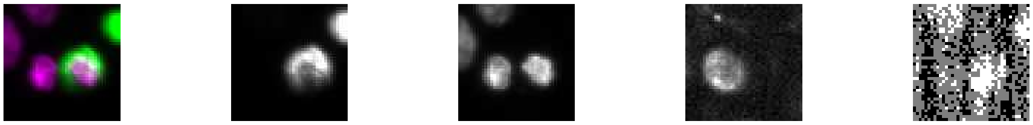				
5802	44	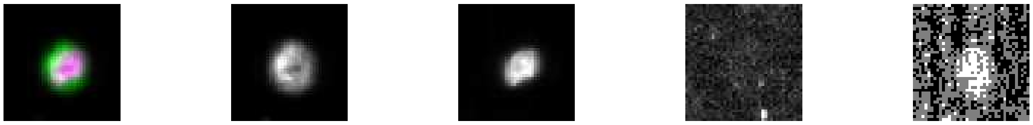				

Ereignis	Aufnahme	DAPI/CK-PE	CK-PE	DAPI	CD45-APC	HER-2/neu
5803	44	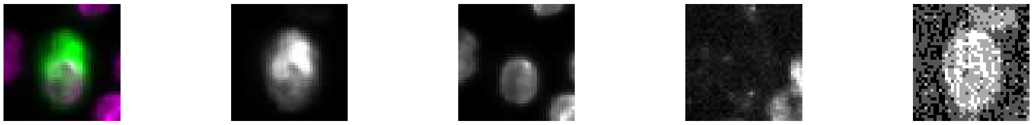				
5804	44	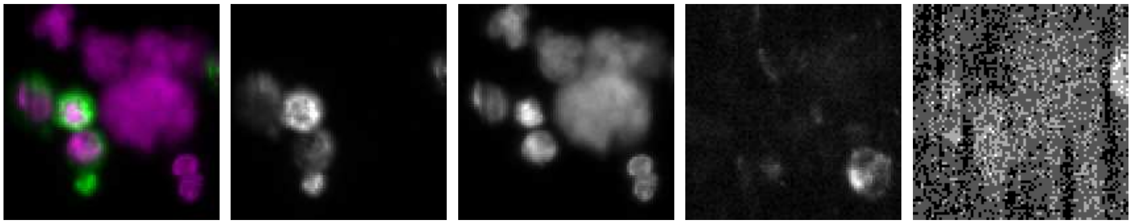				
5805	44	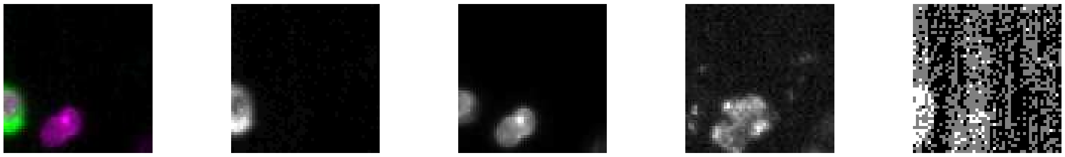				
5806	44	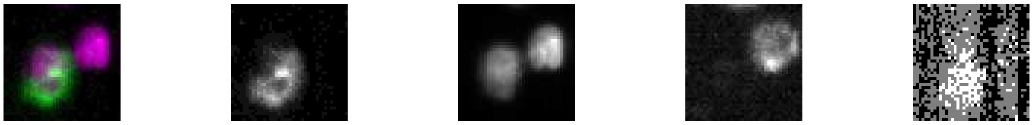				
5807	44	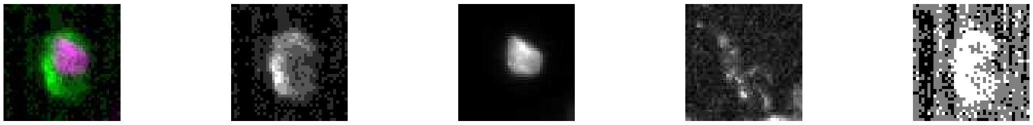				
5808	44	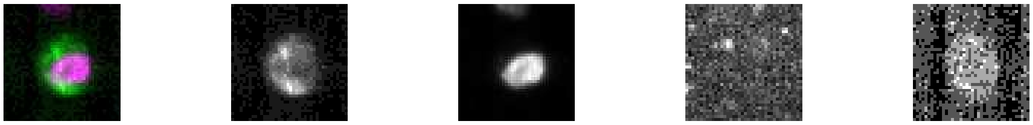				
5809	44	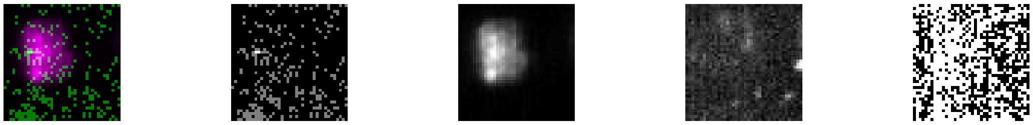				
5810	44	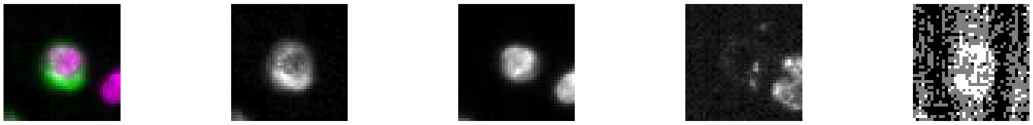				
5811	44	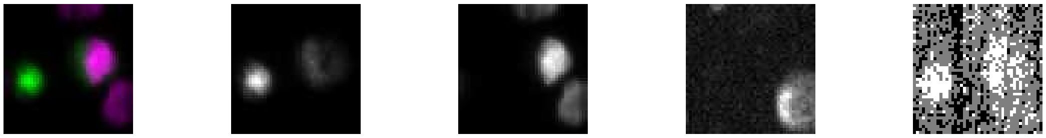				
5812	44	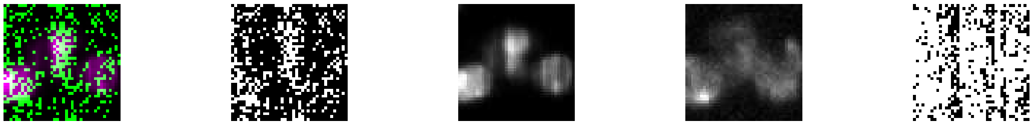				
5813	44	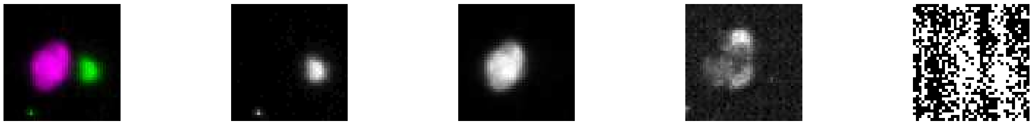				
5814	44	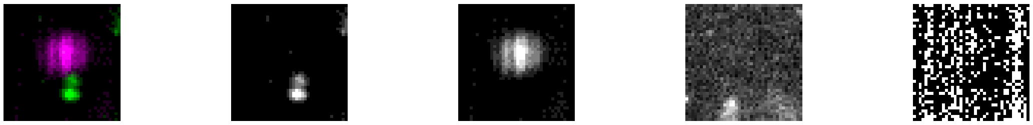				
5815	44	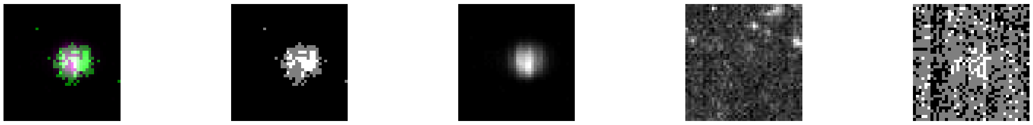				

Ereignis	Aufnahme	DAPI/CK-PE	CK-PE	DAPI	CD45-APC	HER-2/neu
5816	44	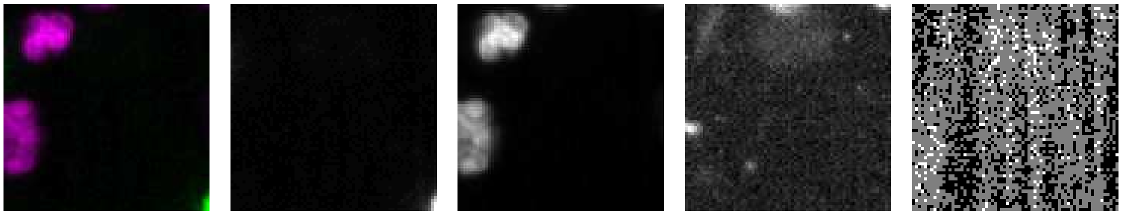				
5817	44	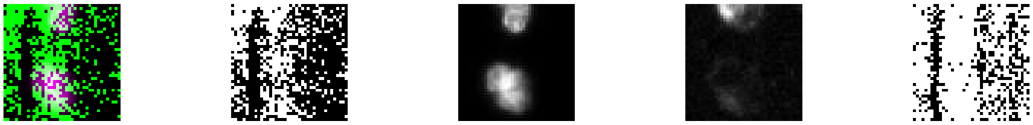				
5818	44	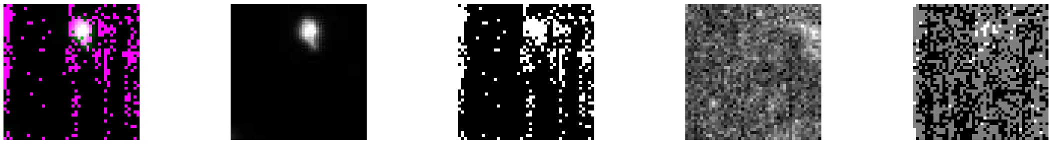				
5819	44	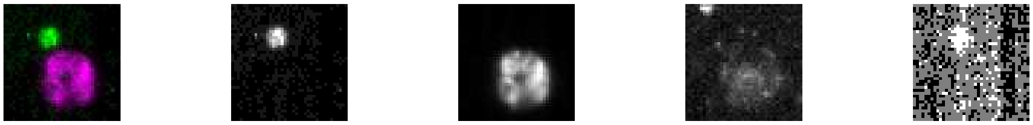				
5820	44	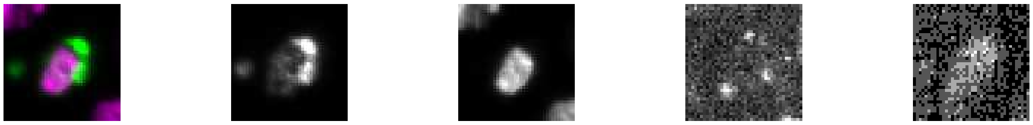				
5821	44	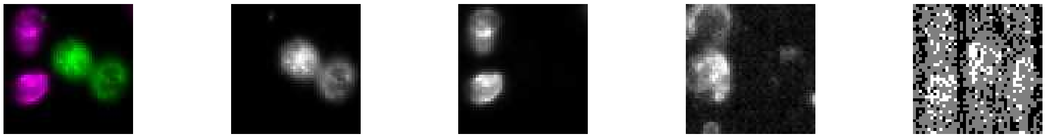				
5822	44	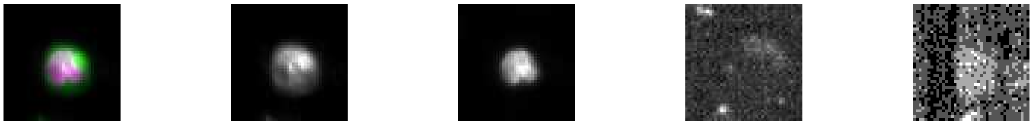				
5823	44	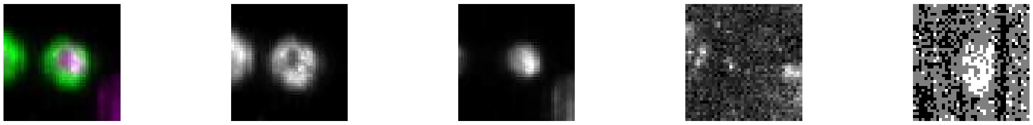				
5824	44	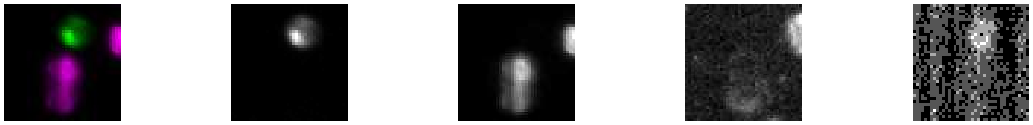				
5825	44	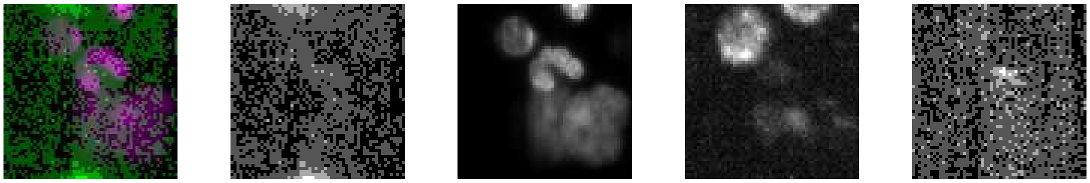				
5826	44	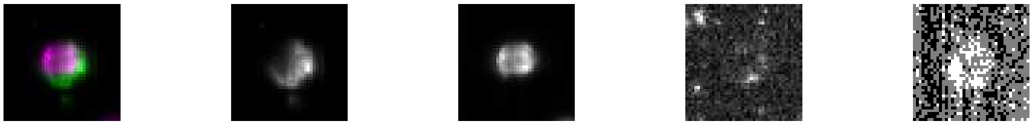				
5827	44	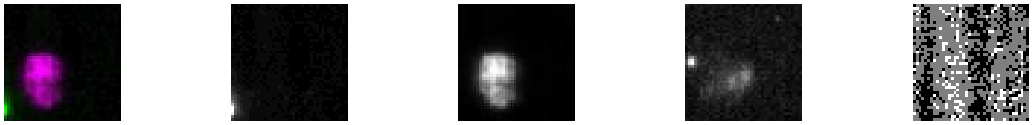				

Ereignis	Aufnahme	DAPI/CK-PE	CK-PE	DAPI	CD45-APC	HER-2/neu
5828	44	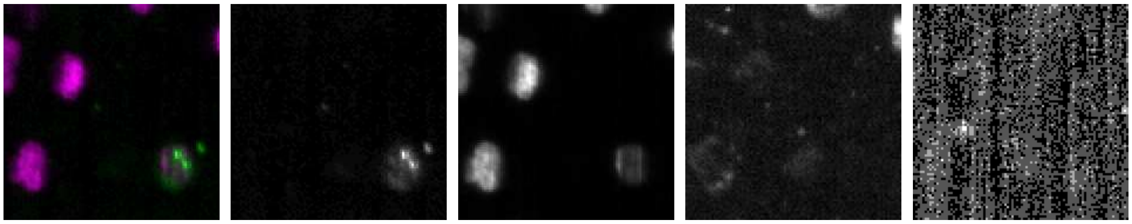				
5829	44	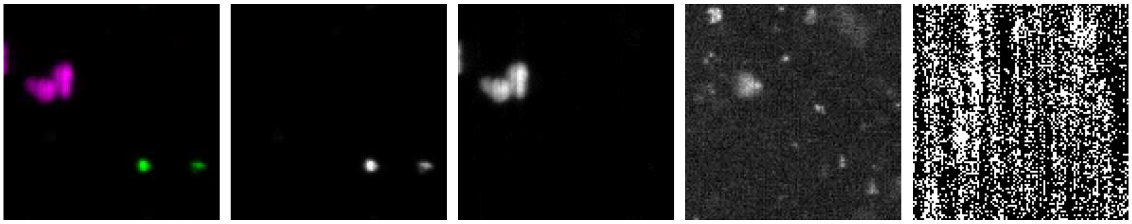				
5830	44	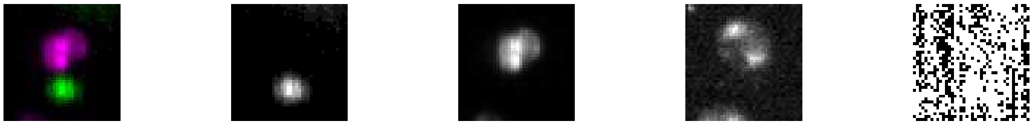				
5831	44	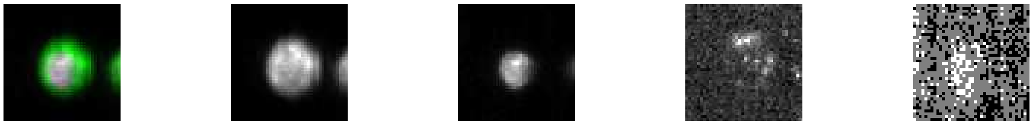				
5832	44	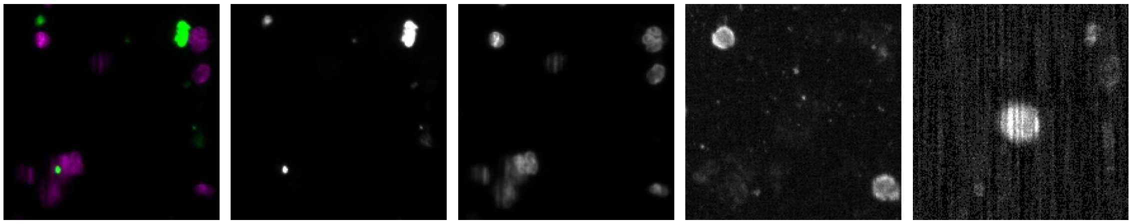				
5833	44	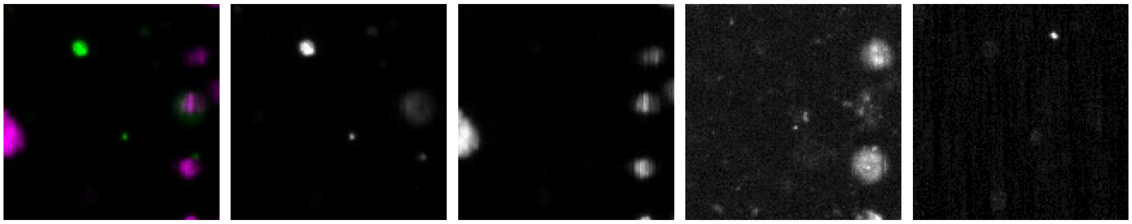				
5834	44	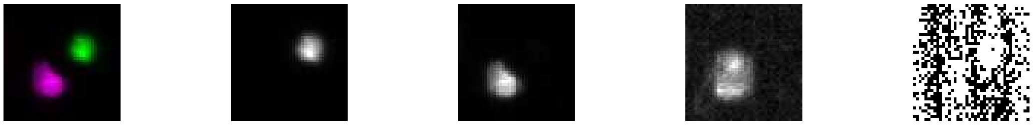				
5835	44	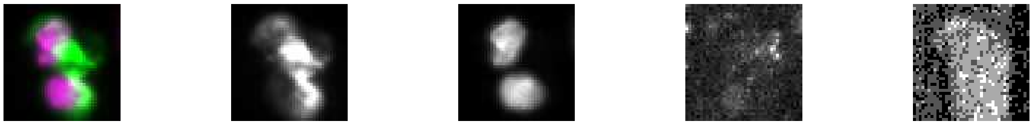				
5836	44	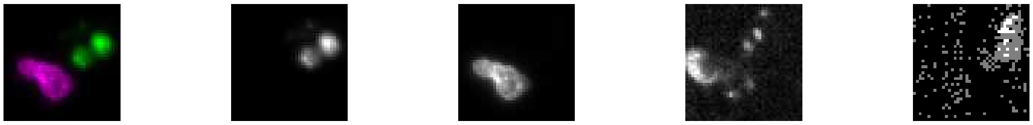				
5837	44	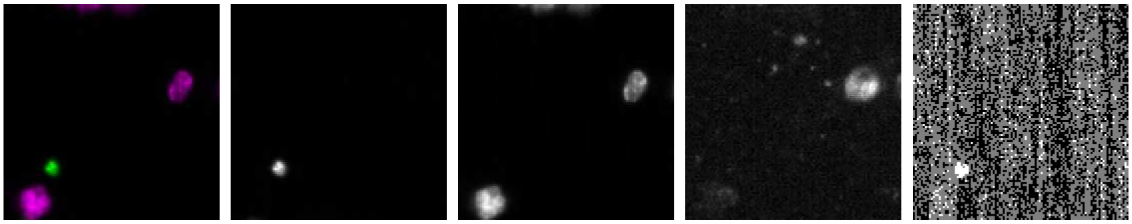				

Druckdatum:02/06/2020 16:41

Proben-ID:Gyn14.208 Kassetten-ID:01327106 Patienten-ID:

Ereignis	Aufnahme	DAPI/CK-PE	CK-PE	DAPI	CD45-APC	HER-2/neu
5838	44	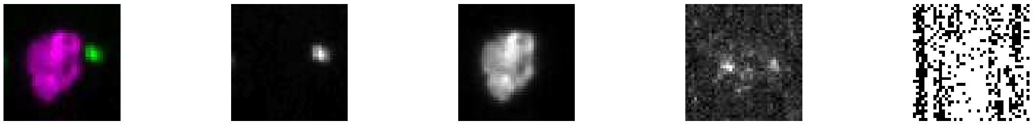				
5839	44	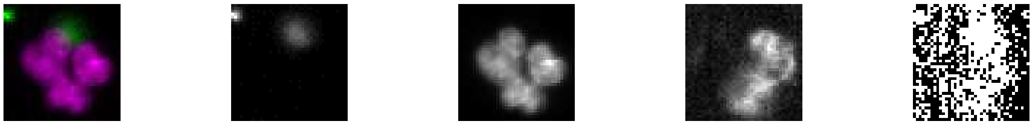				
5840	44	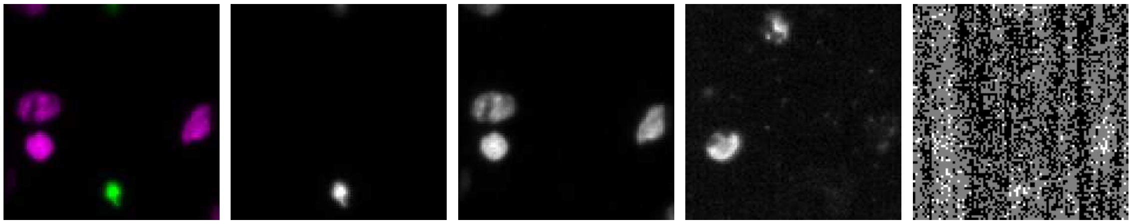				
5841	44	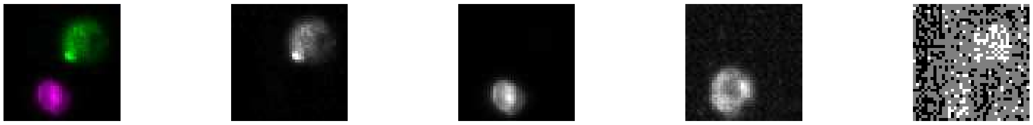				
5842	44	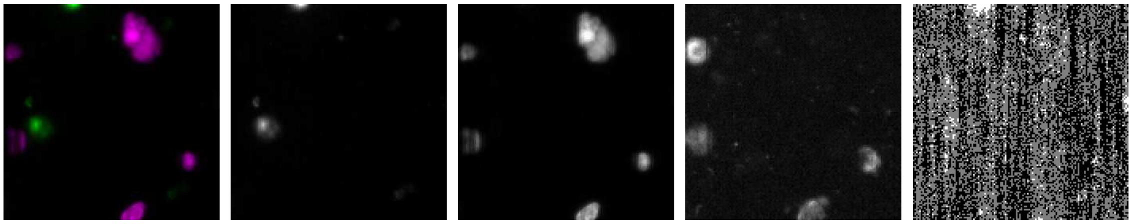				
5843	44	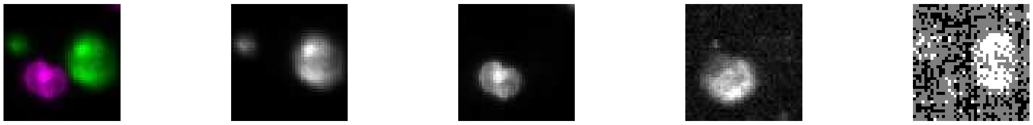				
5844	44	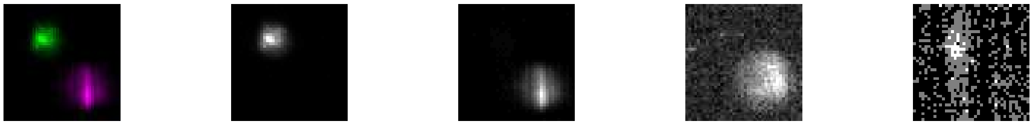				
5845	44	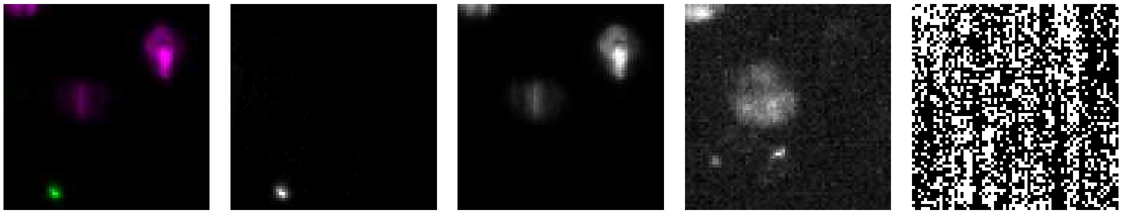				
5846	44	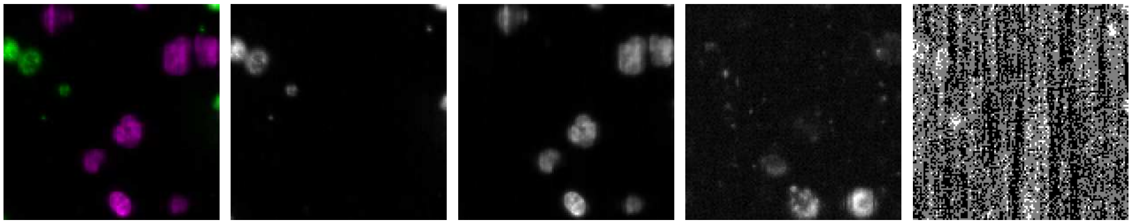				
5847	44	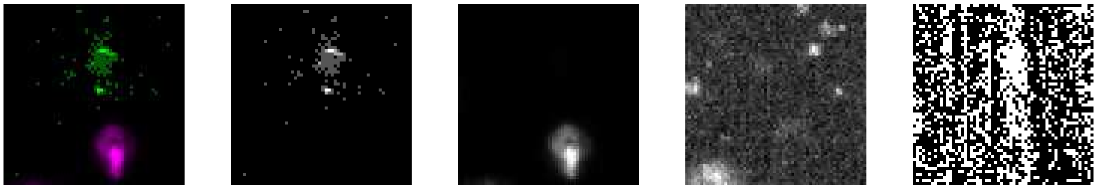				

Ereignis	Aufnahme	DAPI/CK-PE	CK-PE	DAPI	CD45-APC	HER-2/neu
5848	44	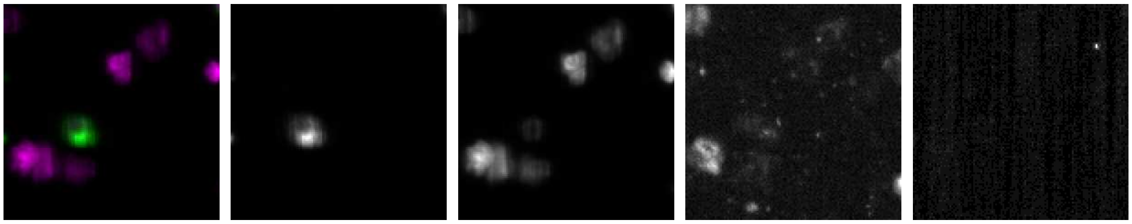				
5849	44	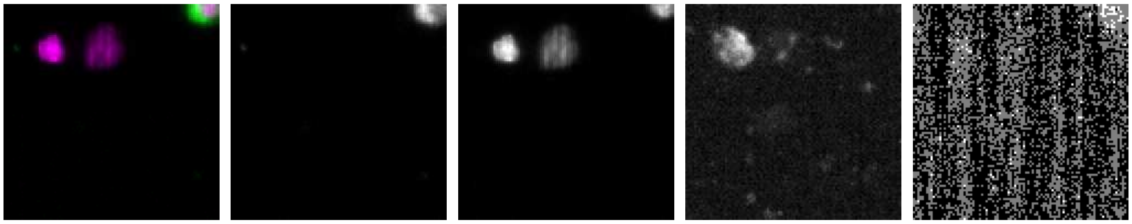				
5850	44	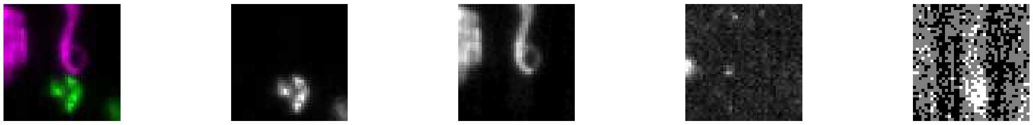				
5851	44	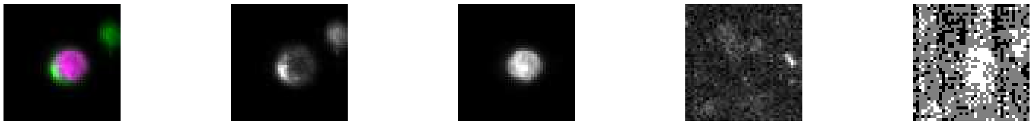				
5852	44	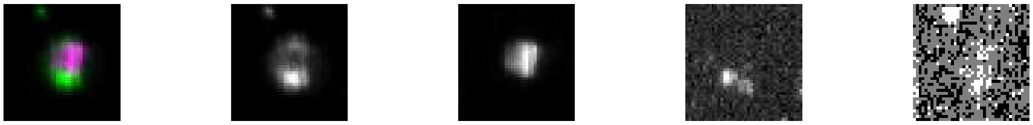				
5853	44	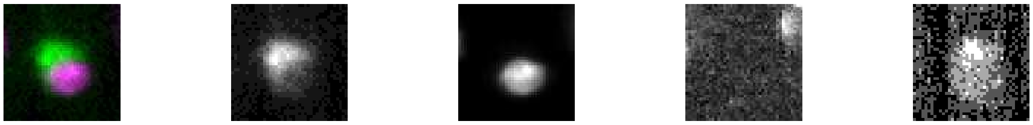				
5854	44	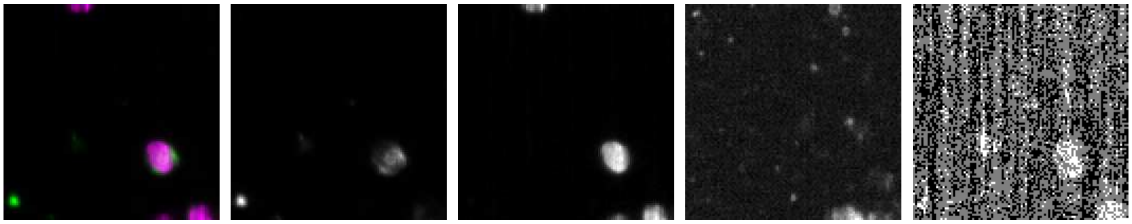				
5855	44	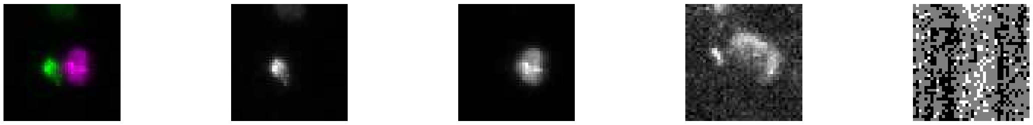				
5856	44	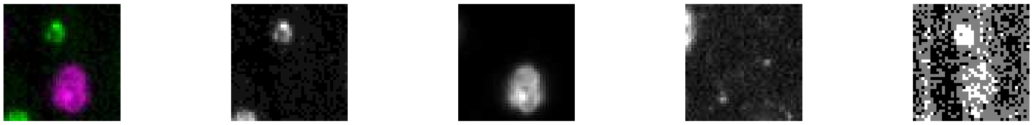				
5857	44	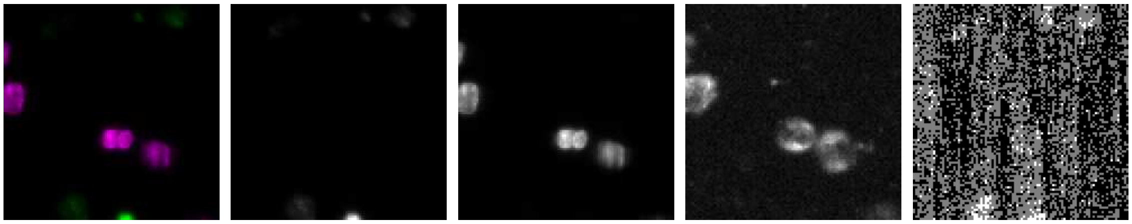				
5858	44	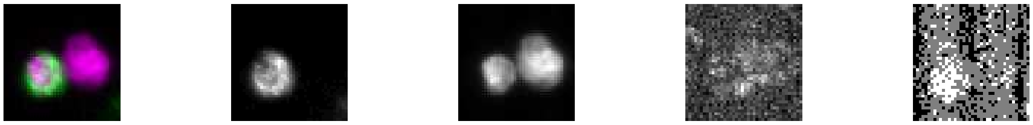				

Ereignis	Aufnahme	DAPI/CK-PE	CK-PE	DAPI	CD45-APC	HER-2/neu
5859	44	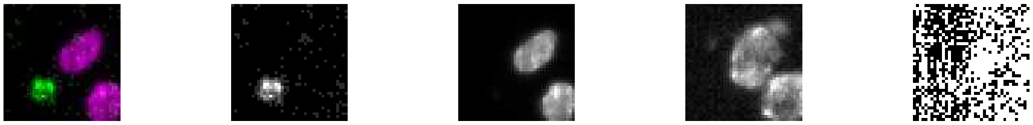				
5860	44	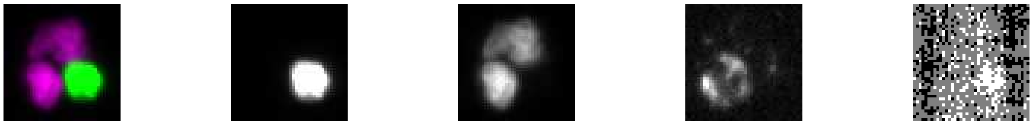				
5861	44	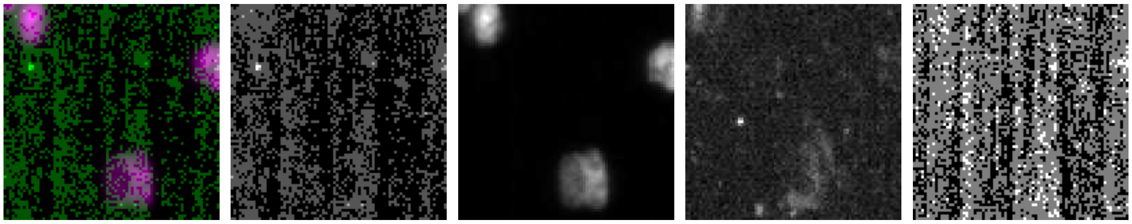				
5862	44	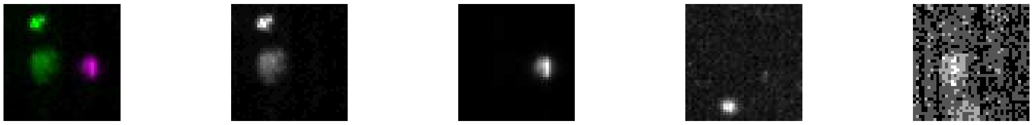				
5863	44	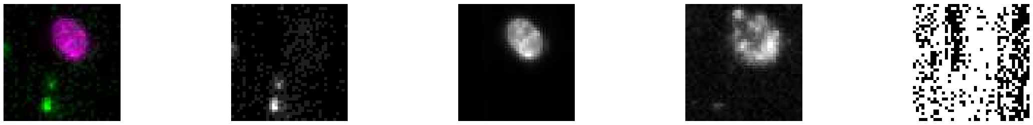				
5864	44	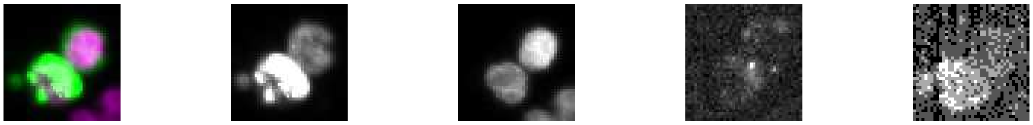				
5865	44	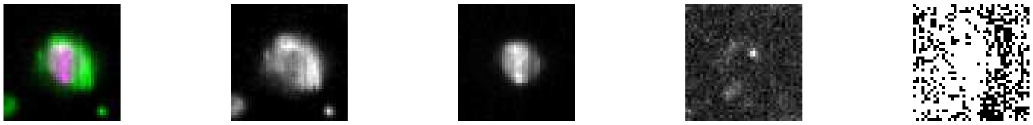				
5866	44	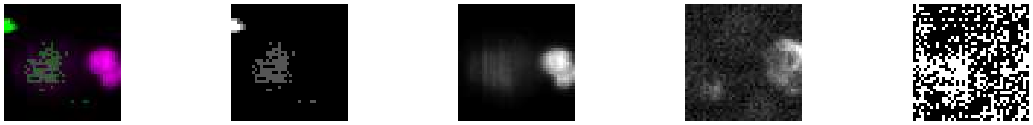				
5867	44	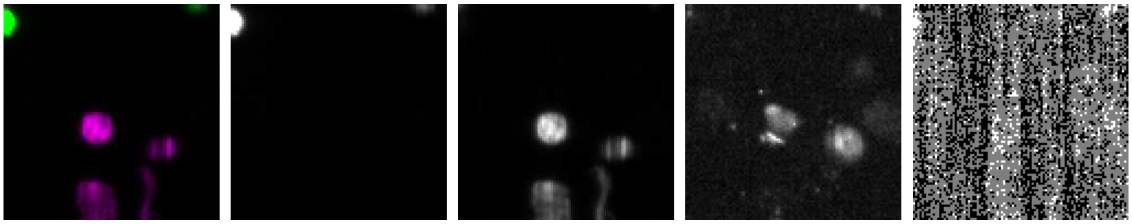				
5868	44	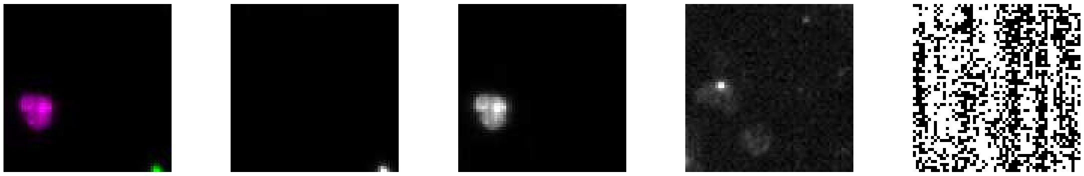				
5869	44	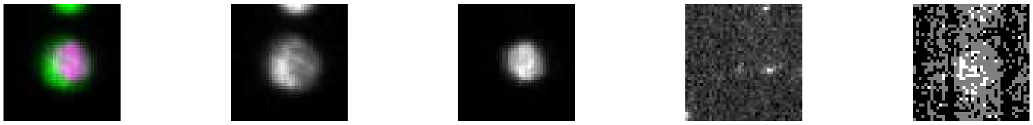				
5870	44	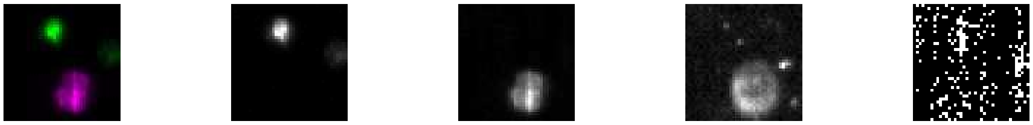				

Ereignis	Aufnahme	DAPI/CK-PE	CK-PE	DAPI	CD45-APC	HER-2/neu
5871	44	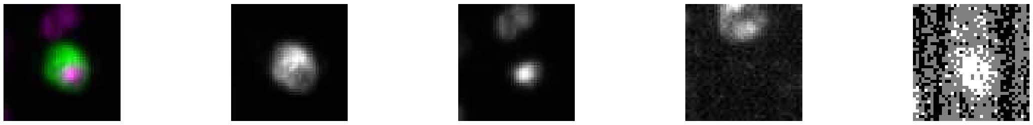				
5872	44	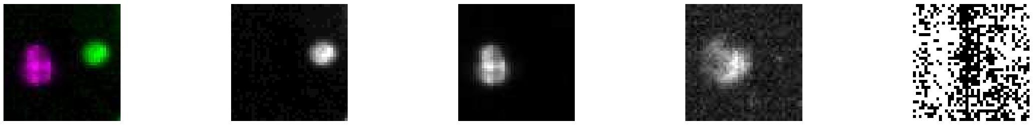				
5873	44	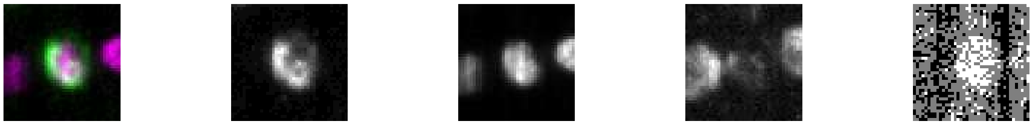				
5874	44	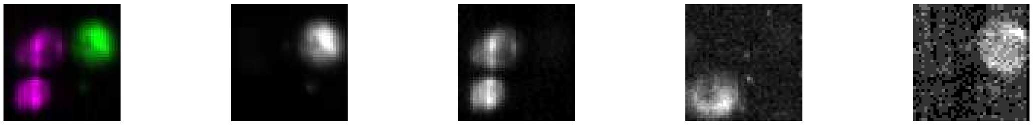				
5875	44	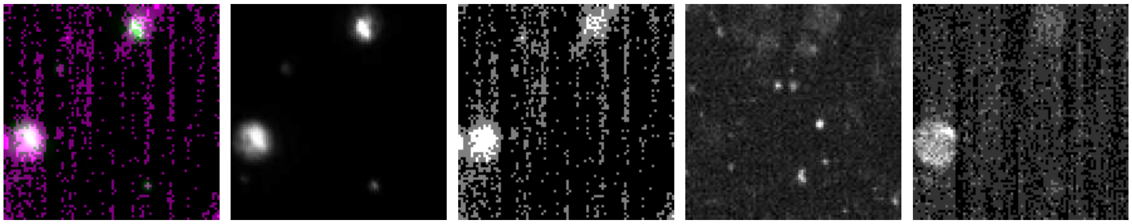				
5876	44	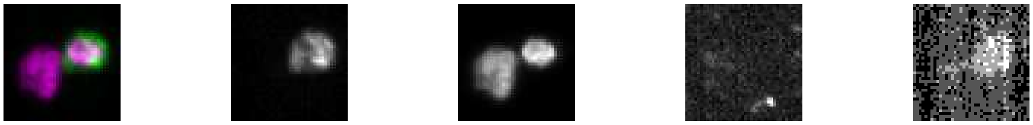				
5877	44	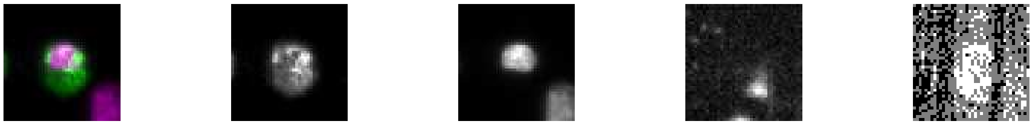				
5878	44	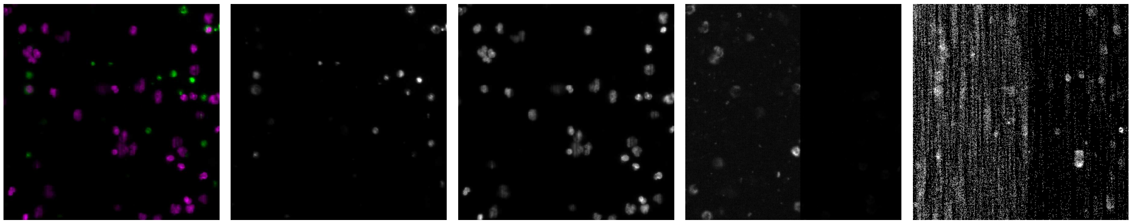				
5879	43	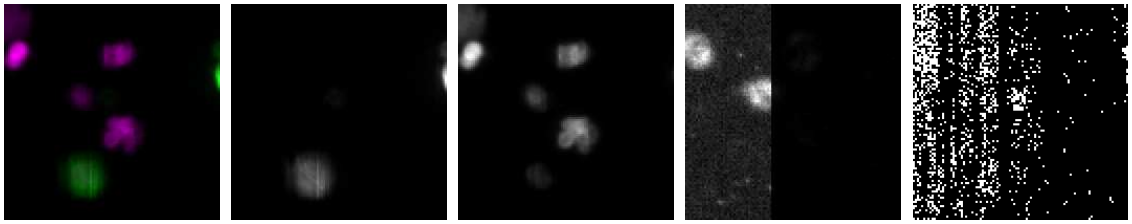				
5880	27	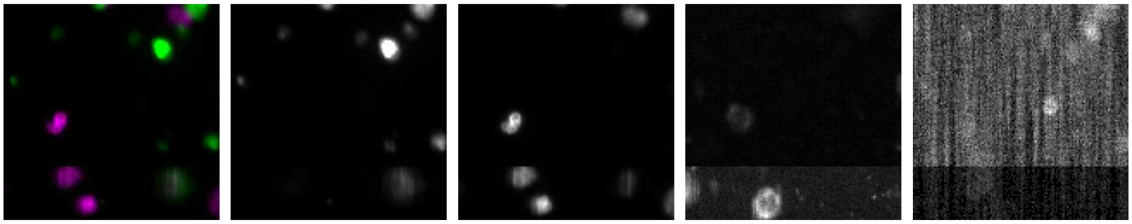				

Proben-ID: Gyn14.208 **Kassetten-ID:**01327106 **Patienten-ID:**

Ereignis	Aufnahme	DAPI/CK-PE	CK-PE	DAPI	CD45-APC	HER-2/neu
5881	27	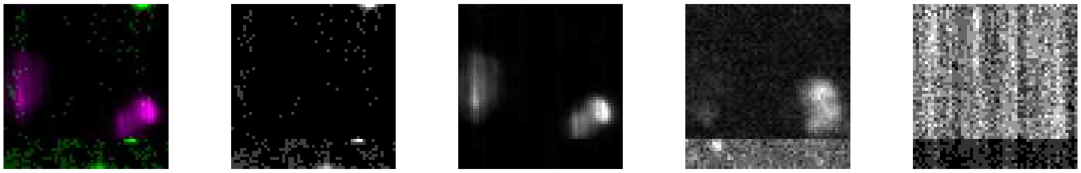				
5882	27	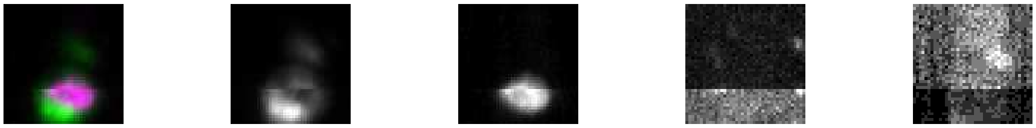				
5883	44	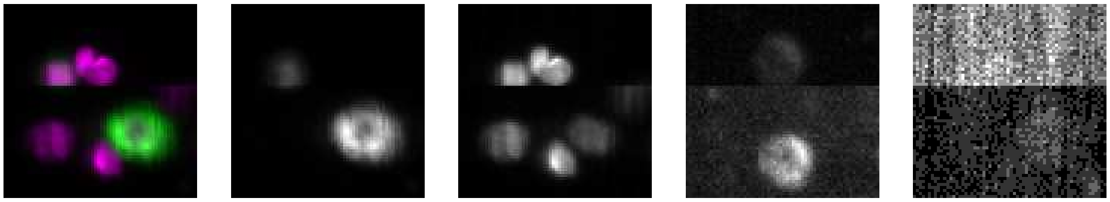				
5884	44	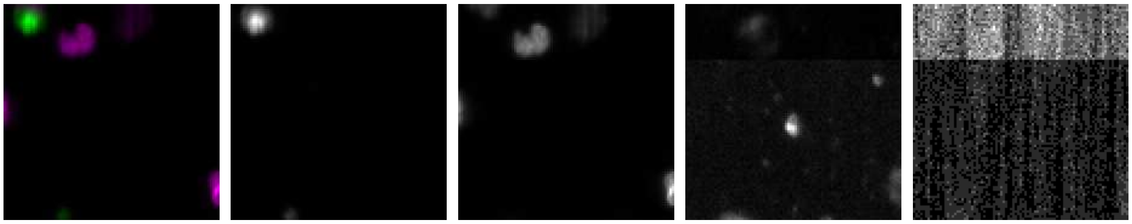				
5885	44	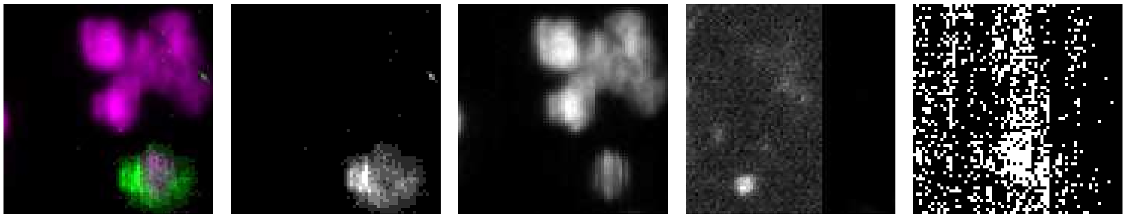				
5886	27	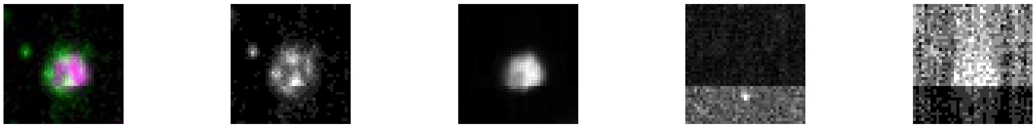				
5887	27	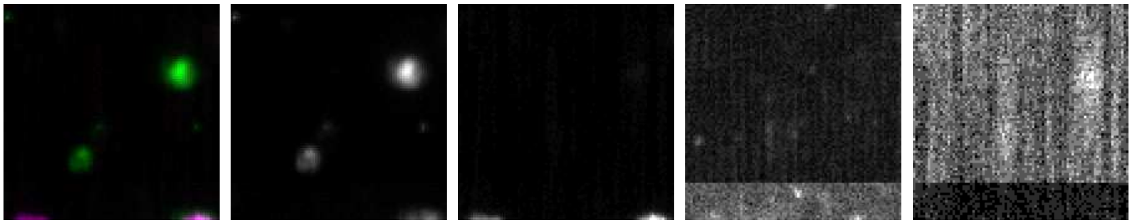				
5888	44	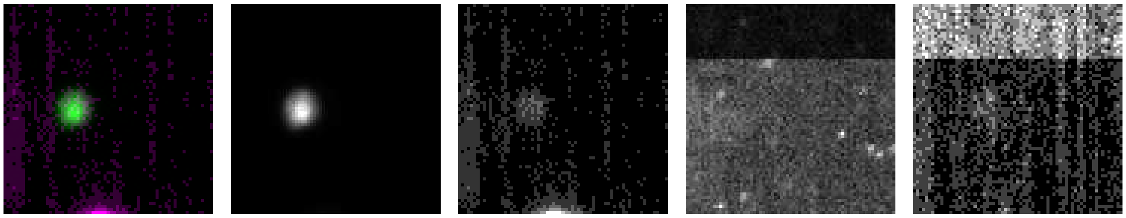				
5889	27	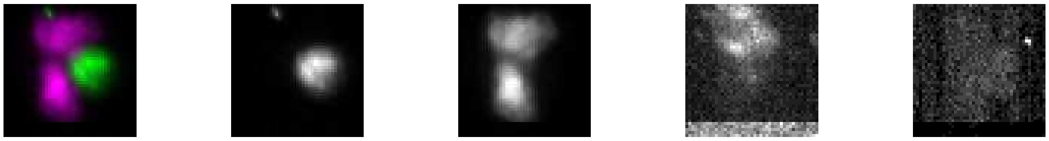				
5890	27	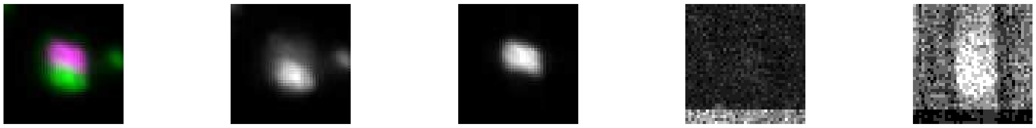				

Ereignis	Aufnahme	DAPI/CK-PE	CK-PE	DAPI	CD45-APC	HER-2/neu
5891	44	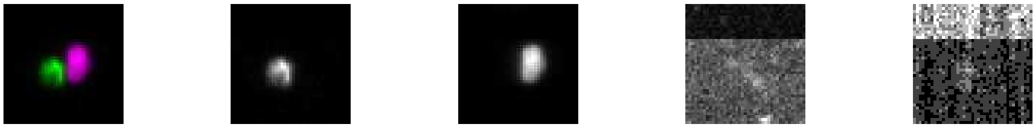				
5892	45	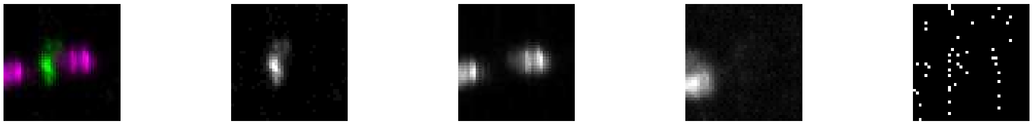				
5893	45	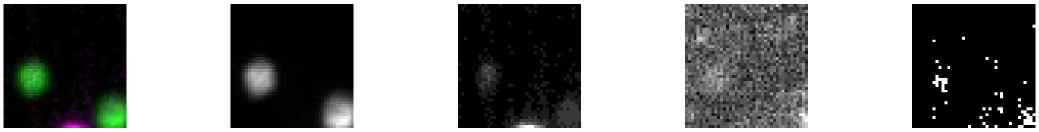				
5894	45	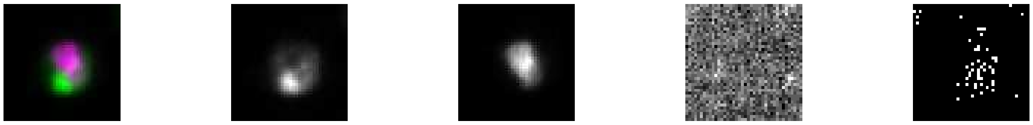				
5895	45	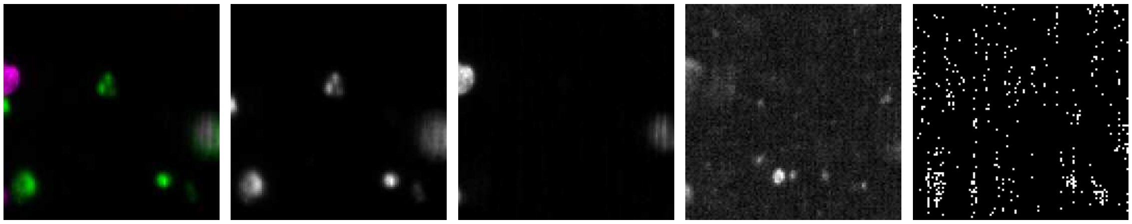				
5896	45	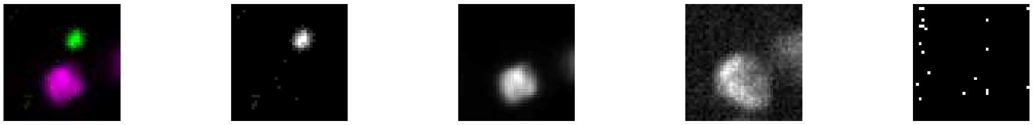				
5897	45	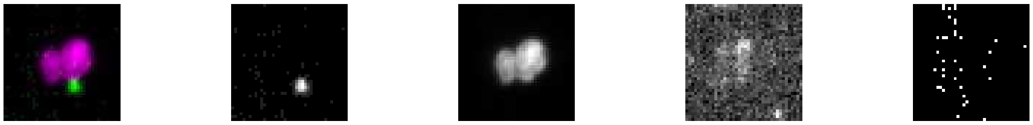				
5898	45	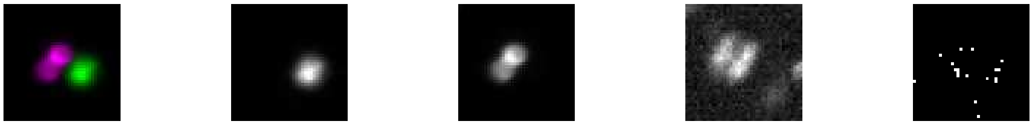				
5899	45	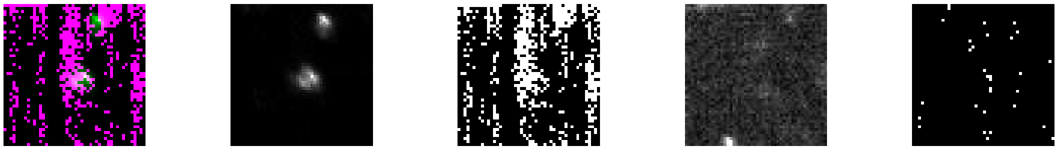				
5900	45	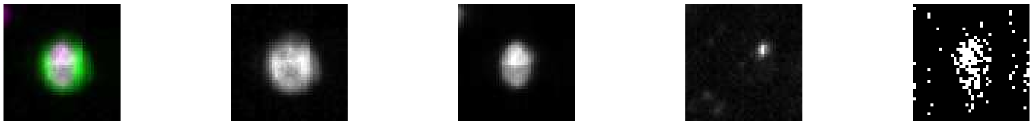				
5901	45	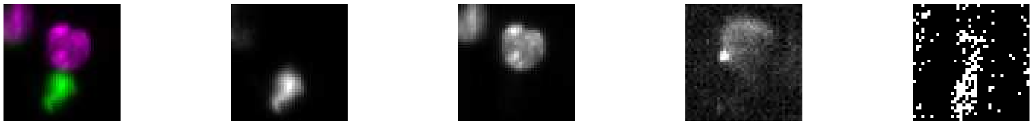				
5902	45	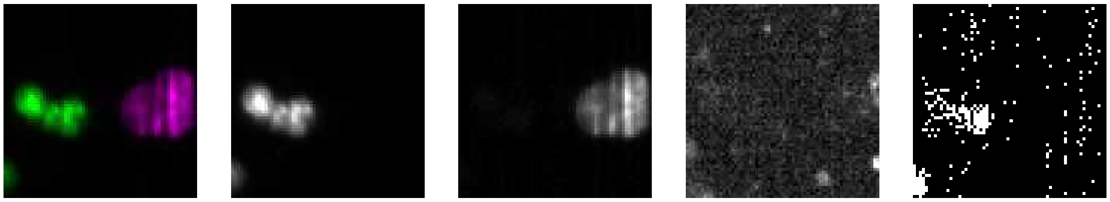				

Ereignis	Aufnahme	DAPI/CK-PE	CK-PE	DAPI	CD45-APC	HER-2/neu
5903	45	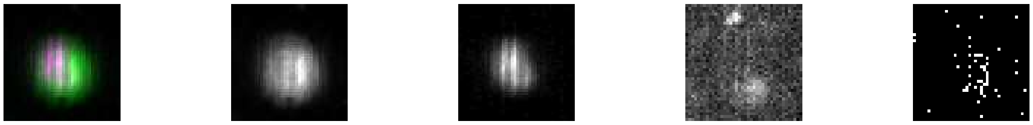				
5904	45	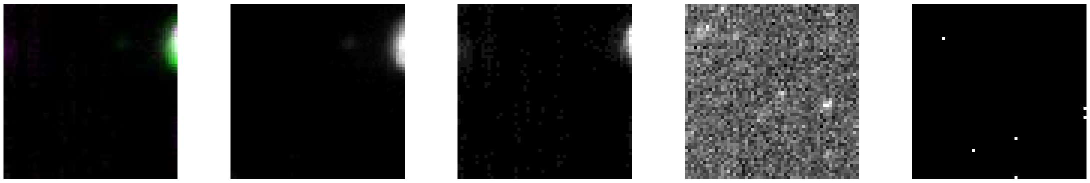				
5905	45	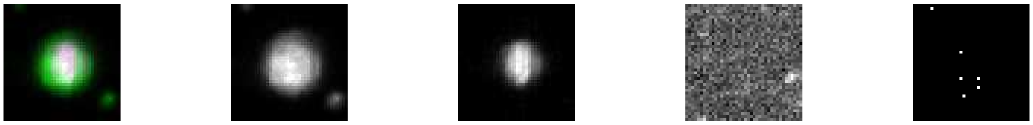				
5906	45	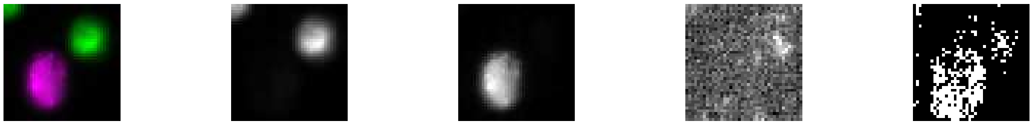				
5907	45	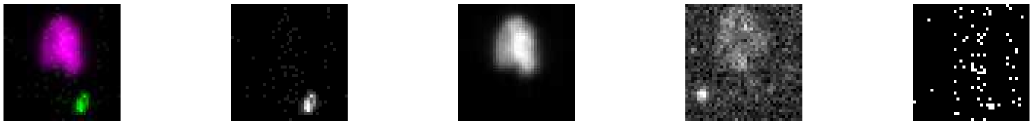				
5908	45	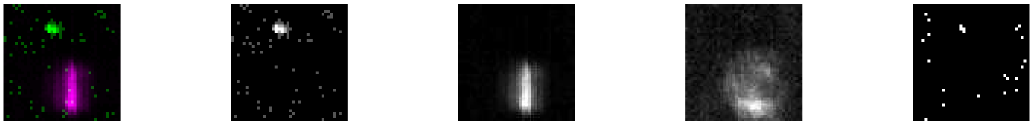				
5909	45	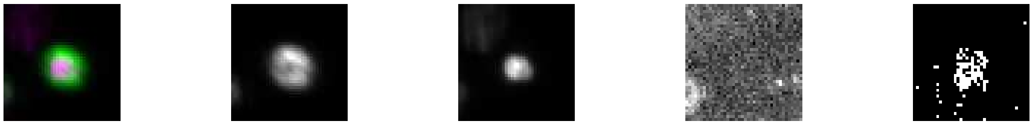				
5910	45	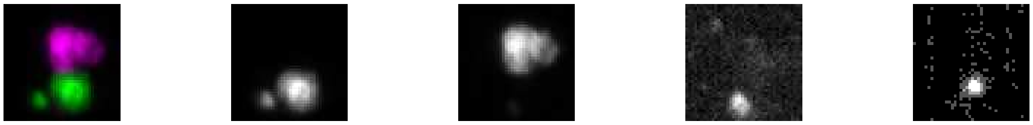				
5911	45	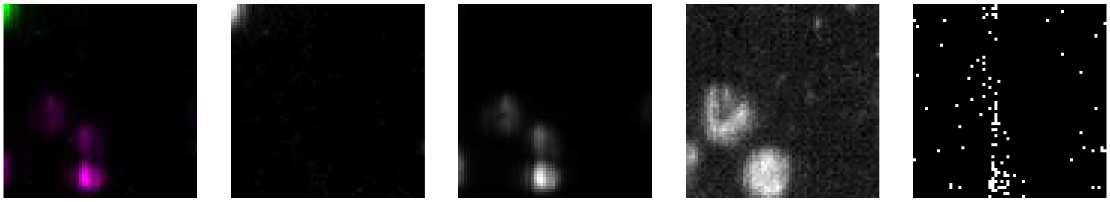				
5912	45	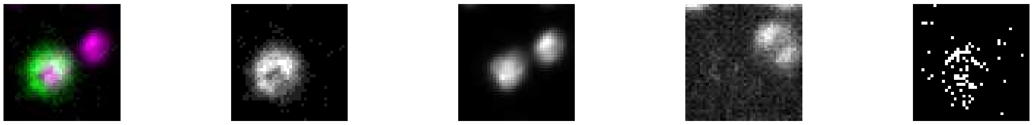				
5913	45	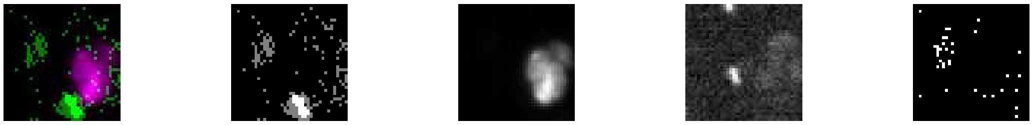				
5914	45	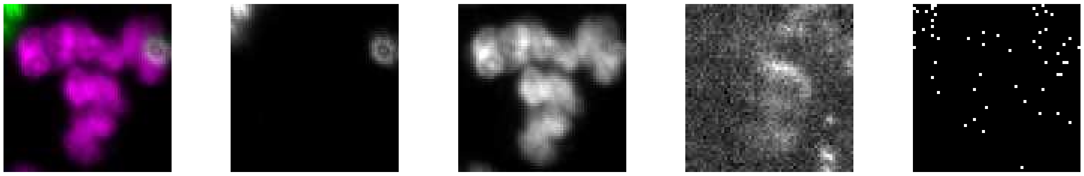				

Druckdatum:02/06/2020 16:41

Proben-ID: Gyn14.208 **Kassetten-ID:**01327106 **Patienten-ID:**

Ereignis	Aufnahme	DAPI/CK-PE	CK-PE	DAPI	CD45-APC	HER-2/neu
5915	45	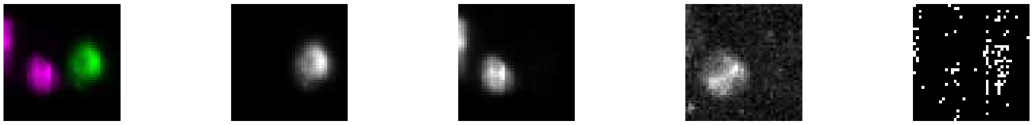				
5916	45	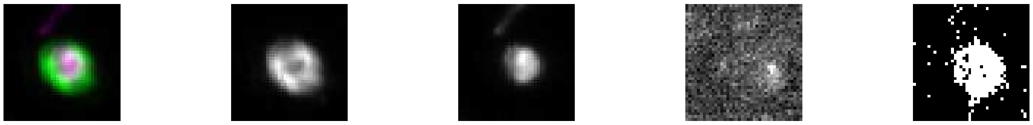				
5917	45	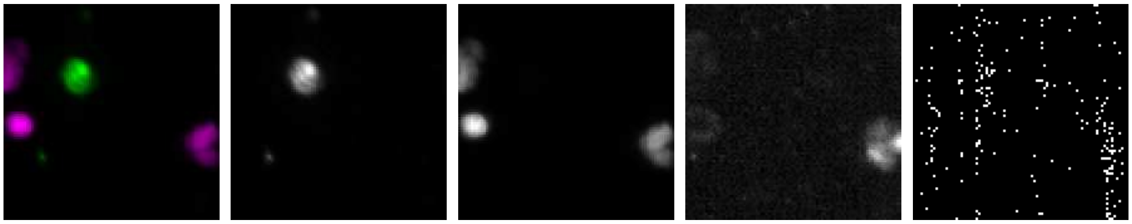				
5918	45	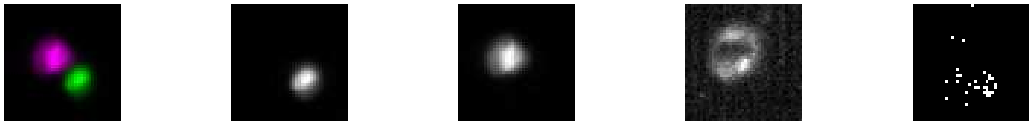				
5919	45	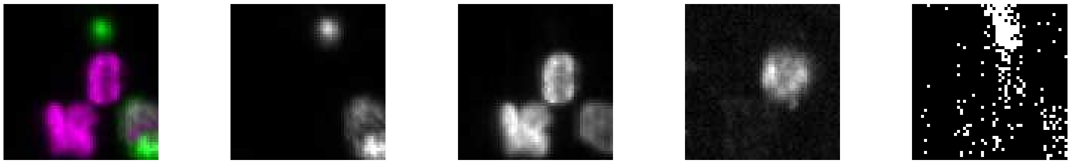				
5920	45	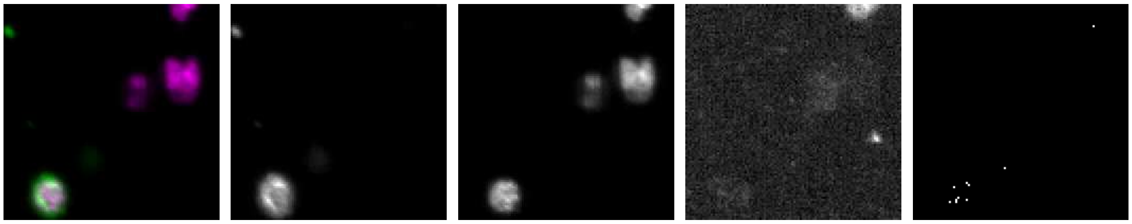				
5921	45	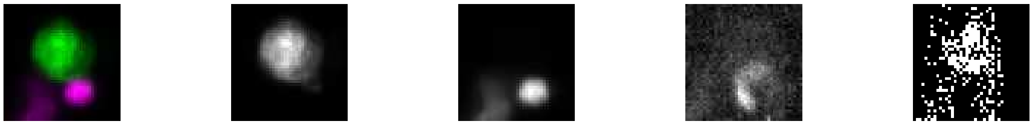				
5922	45	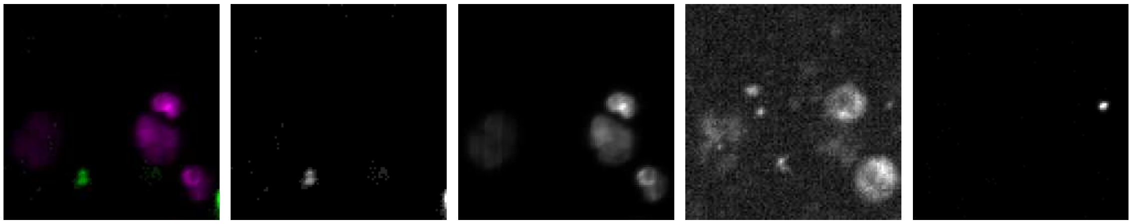				
5923	45	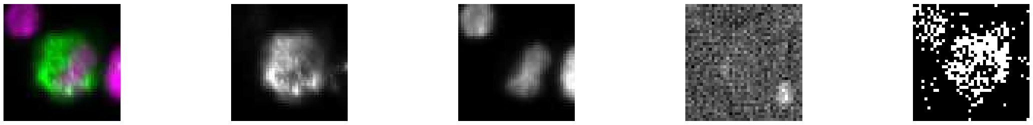				
5924	45	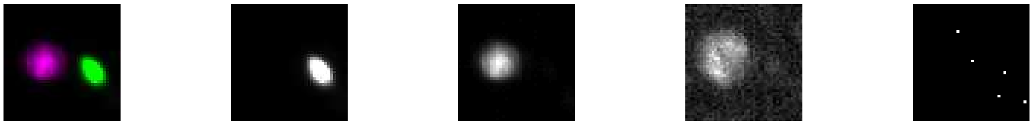				
5925	45	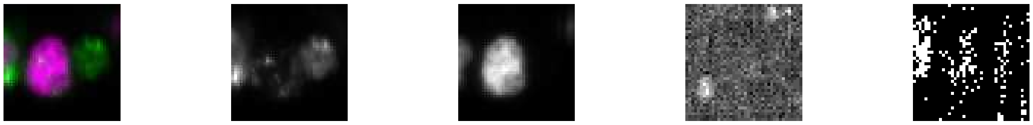				

Ereignis	Aufnahme	DAPI/CK-PE	CK-PE	DAPI	CD45-APC	HER-2/neu
5926	45	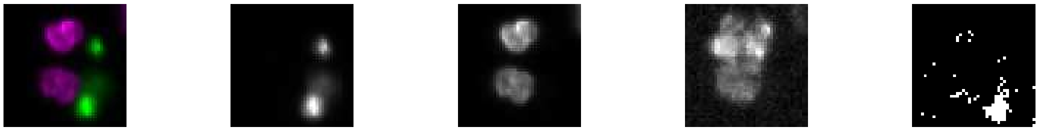				
5927	45	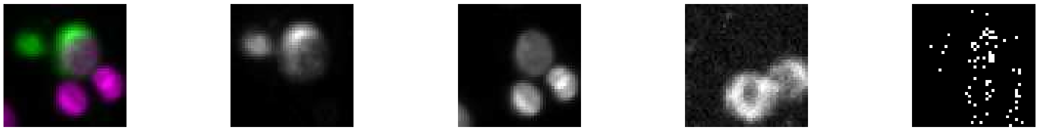				
5928	45	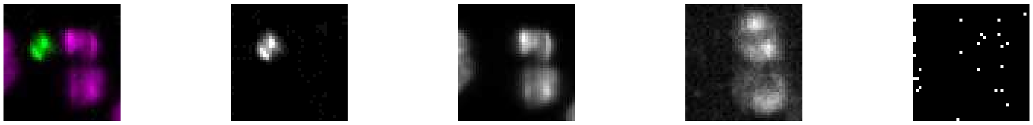				
5929	45	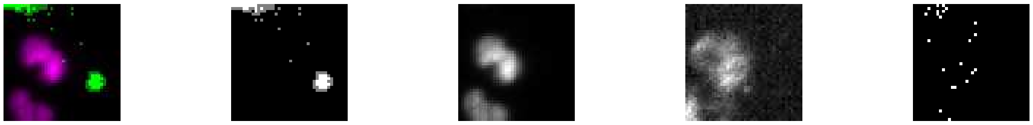				
5930	45	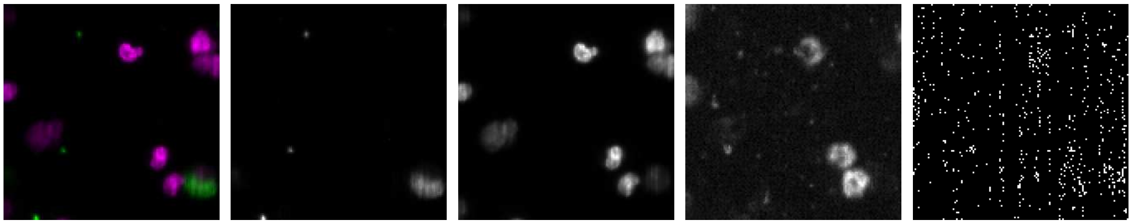				
5931	45	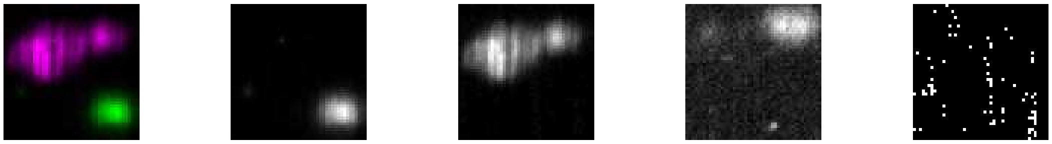				
5932	45	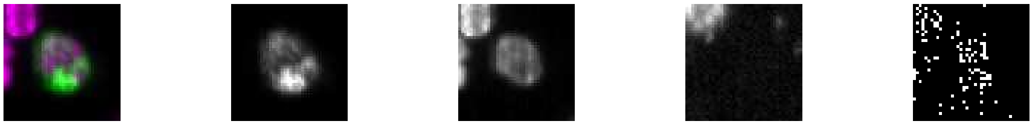				
5933	45	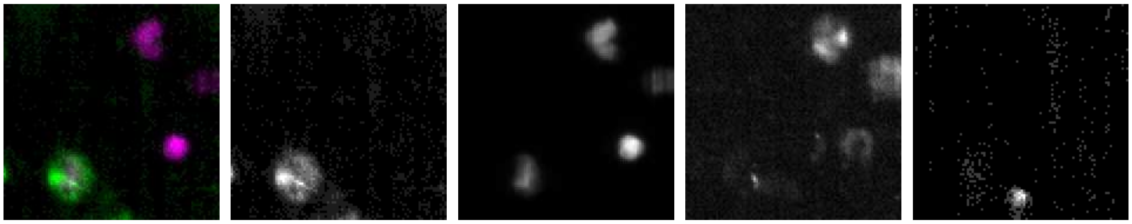				
5934	45	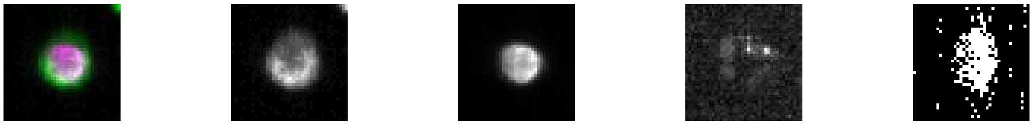				
5935	45	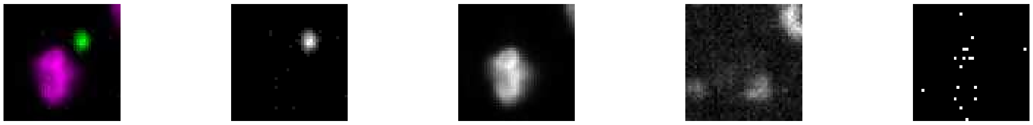				
5936	45	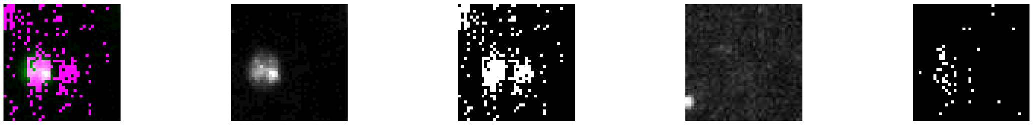				
5937	45	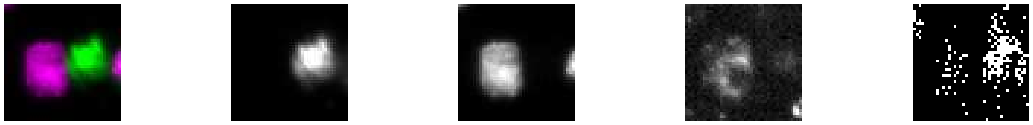				

Ereignis	Aufnahme	DAPI/CK-PE	CK-PE	DAPI	CD45-APC	HER-2/neu
5938	45	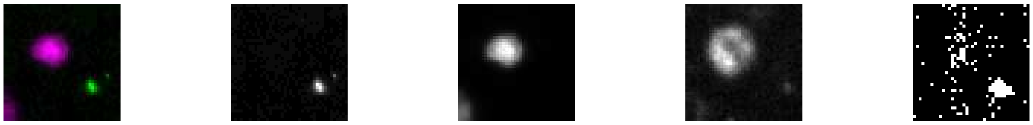				
5939	45	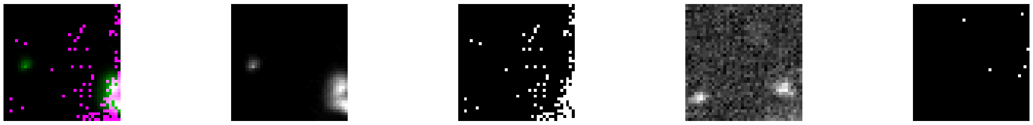				
5940	45	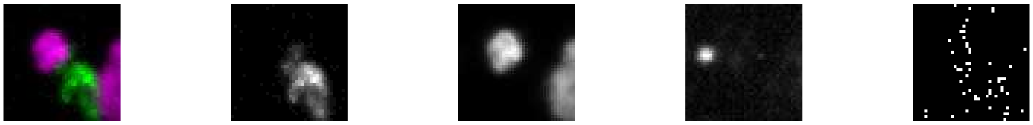				
5941	45	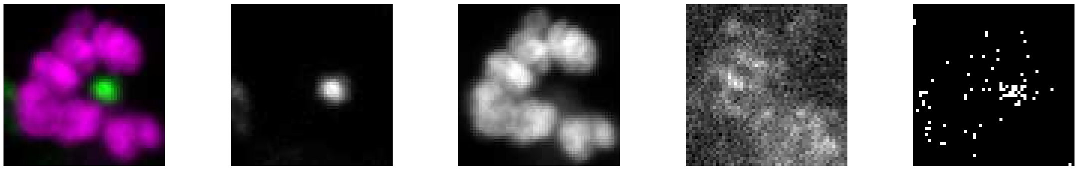				
5942	45	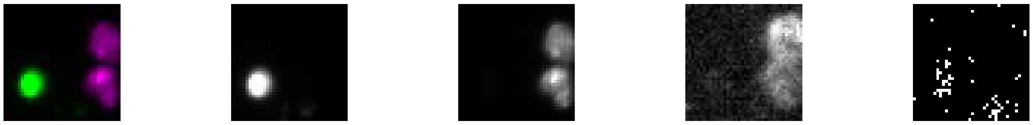				
5943	45	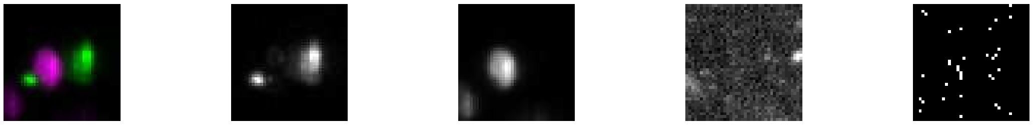				
5944	45	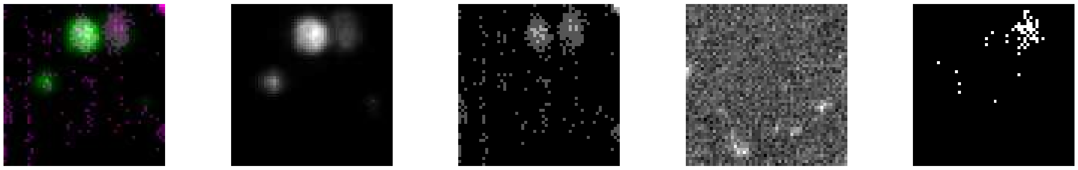				
5945	45	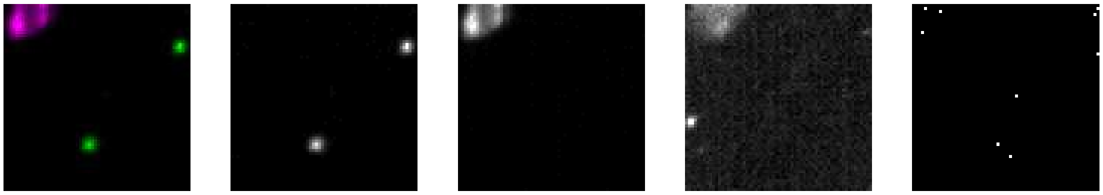				
5946	45	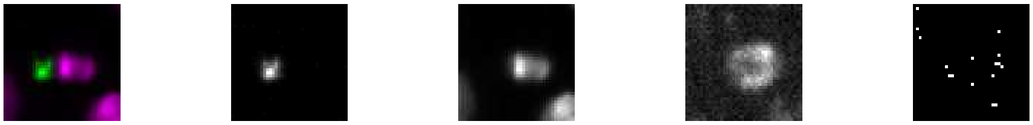				
5947	45	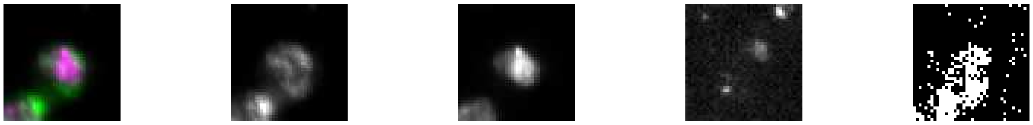				
5948	45	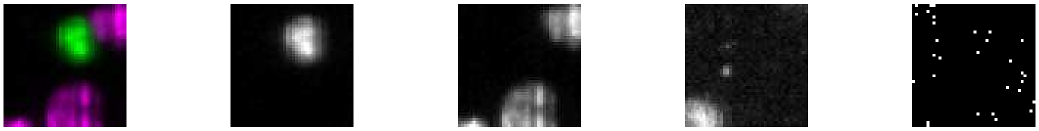				
5949	45	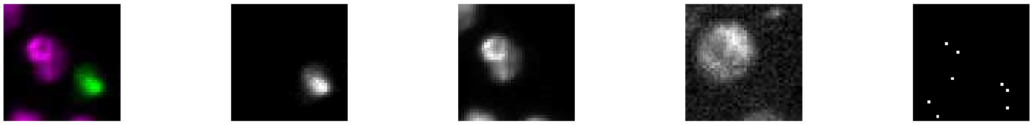				
5950	45	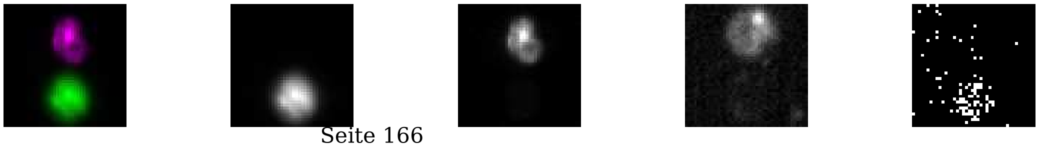				

Druckdatum:02/06/2020 16:41
Proben-ID:Gyn14.208 Kassetten-ID:01327106 Patienten-ID:

Ereignis	Aufnahme	DAPI/CK-PE	CK-PE	DAPI	CD45-APC	HER-2/neu
5951	45	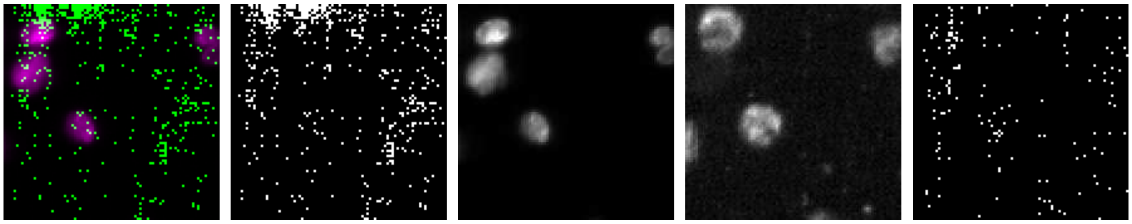				
5952	45	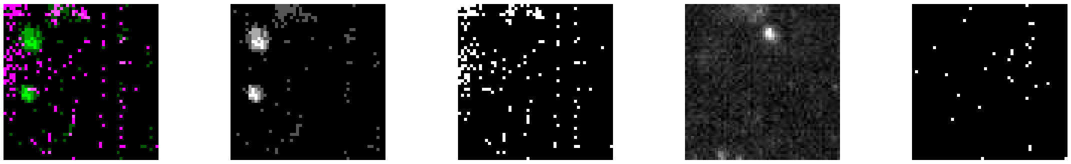				
5953	45	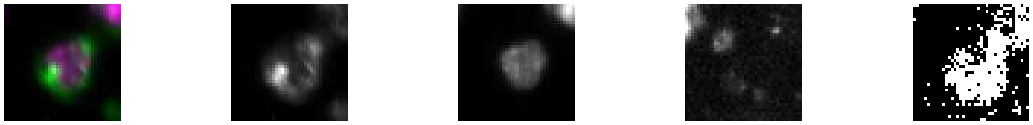				
5954	45	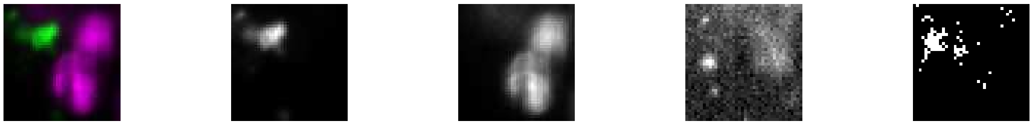				
5955	45	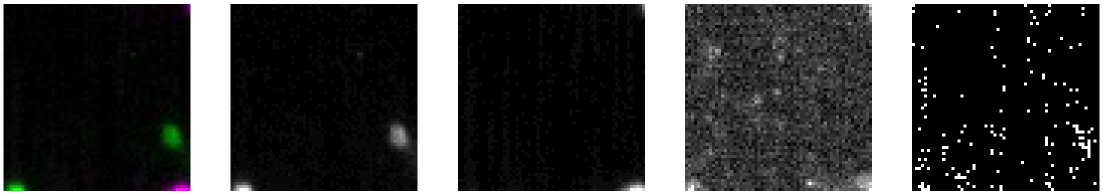				
5956	45	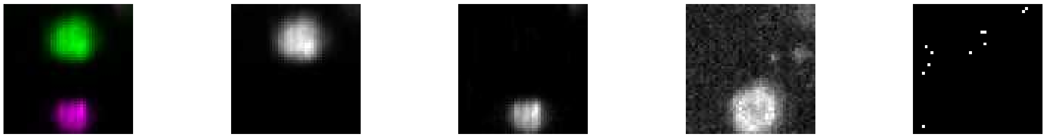				
5957	45	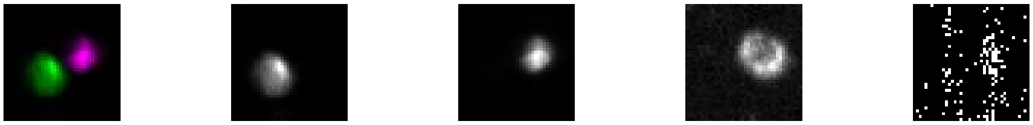				
5958	45	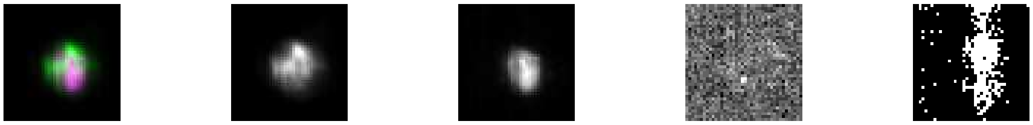				
5959	45	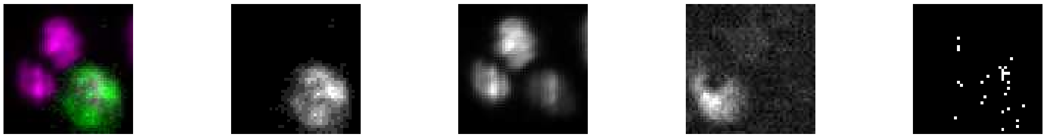				
5960	45	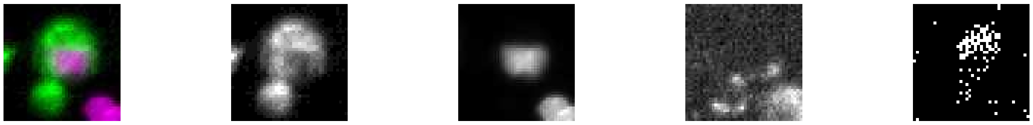				
5961	45	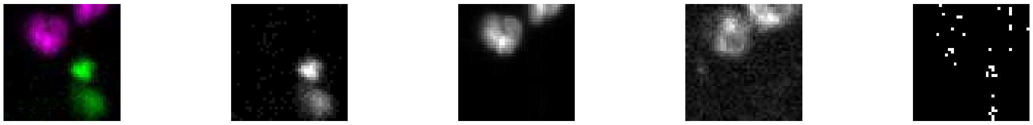				
5962	45	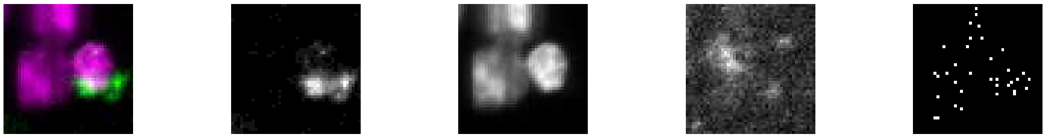				

Ereignis	Aufnahme	DAPI/CK-PE	CK-PE	DAPI	CD45-APC	HER-2/neu
5963	45	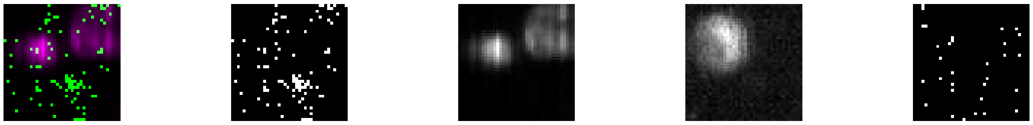				
5964	45	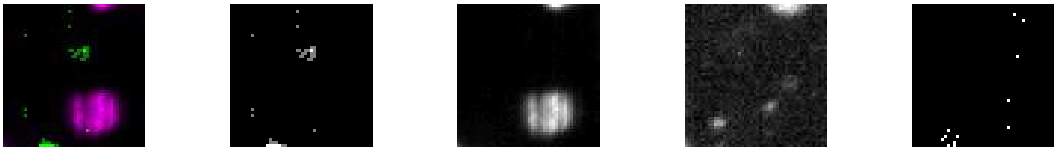				
5965	45	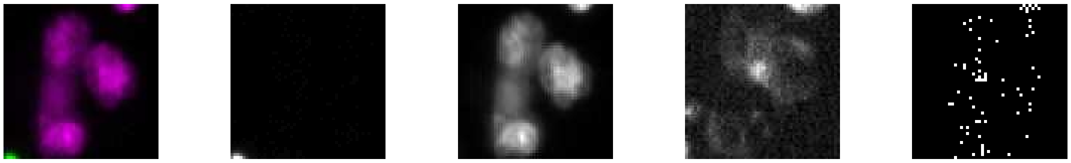				
5966	45	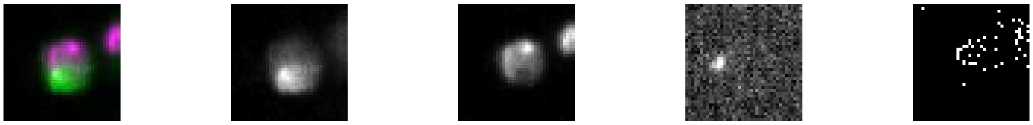				
5967	45	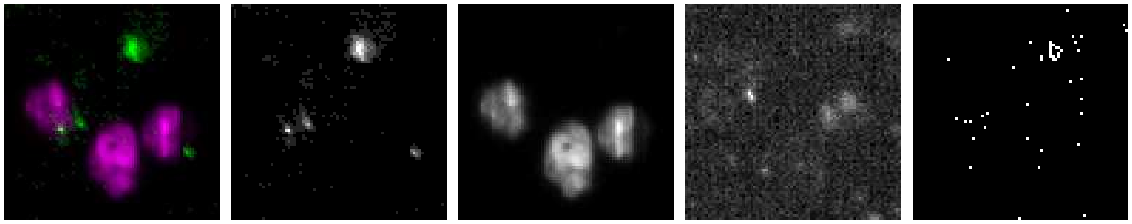				
5968	45	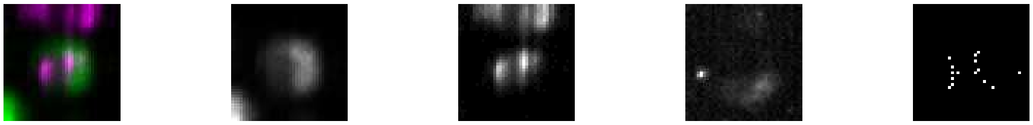				
5969	45	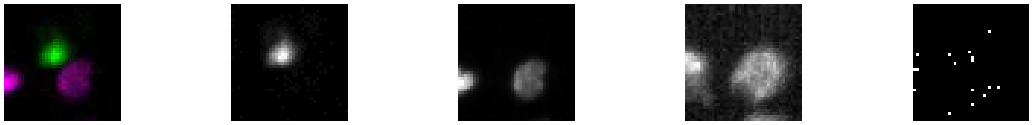				
5970	45	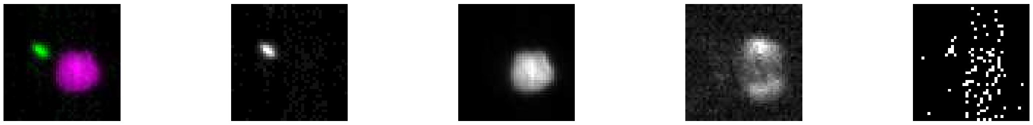				
5971	45	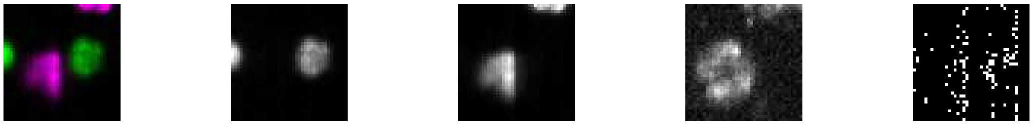				
5972	45	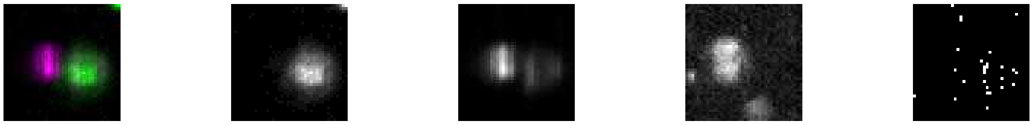				
5973	45	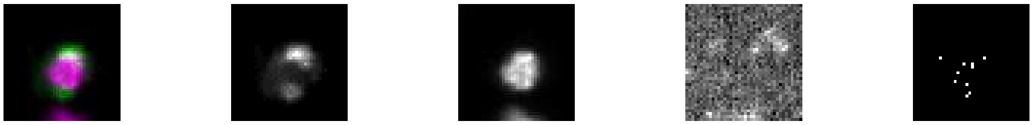				
5974	45	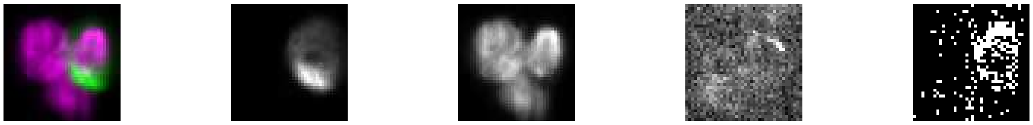				
5975	45	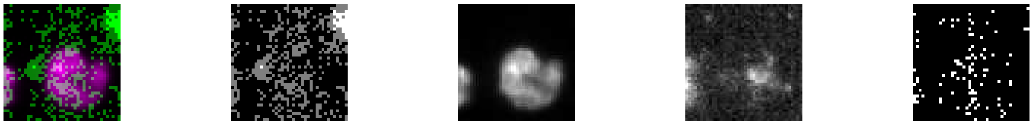				

Druckdatum:02/06/2020 16:41

Proben-ID: Gyn14.208 **Kassetten-ID:**01327106 **Patienten-ID:**

Ereignis	Aufnahme	DAPI/CK-PE	CK-PE	DAPI	CD45-APC	HER-2/neu
5976	45	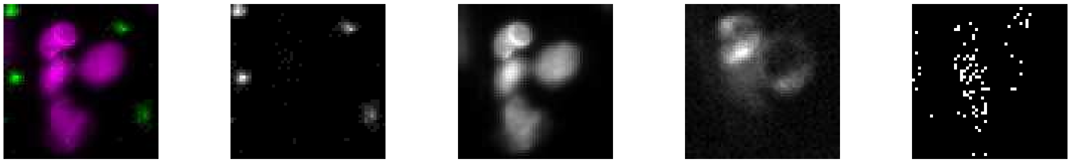				
5977	45	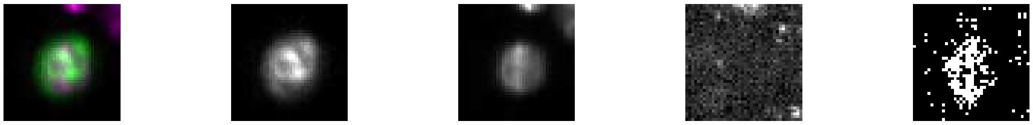				
5978	45	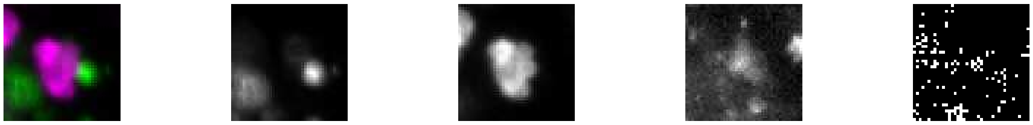				
5979	45	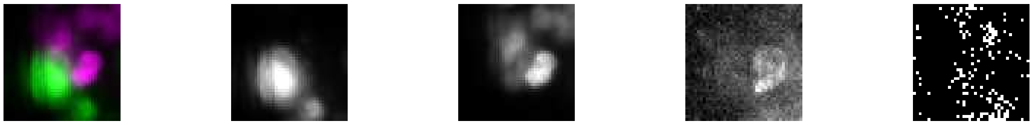				
5980	45	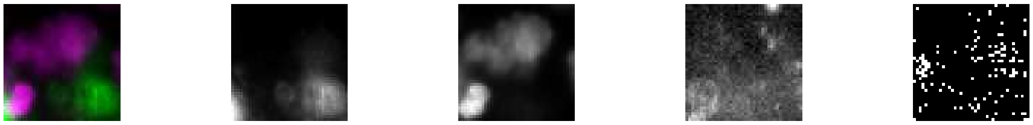				
5981	45	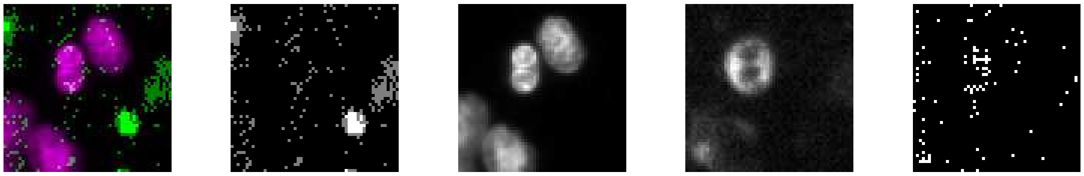				
5982	45	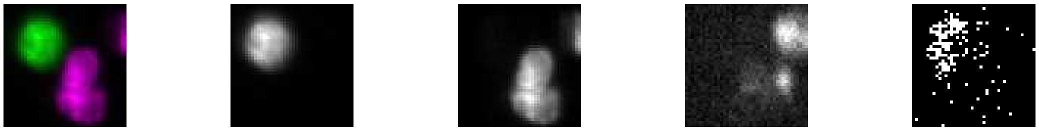				
5983	45	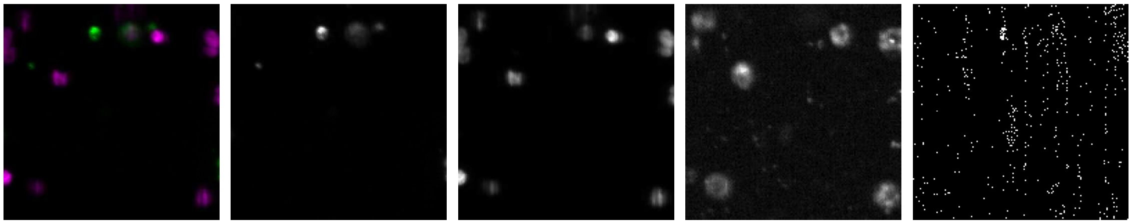				
5984	45	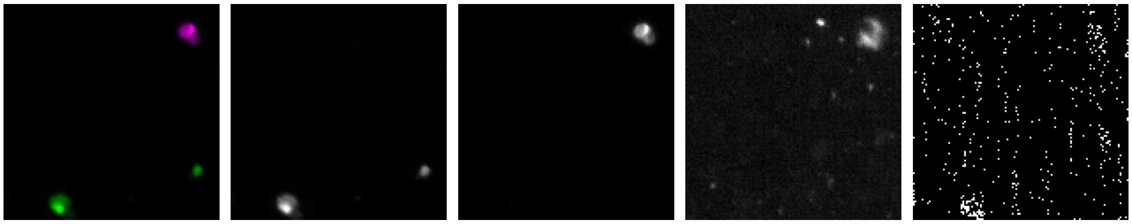				
5985	45	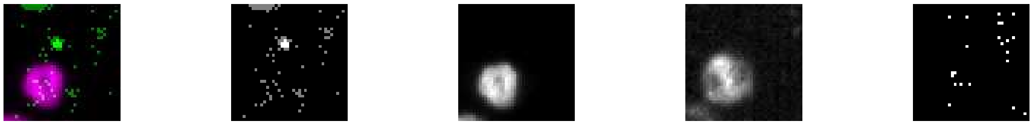				
5986	45	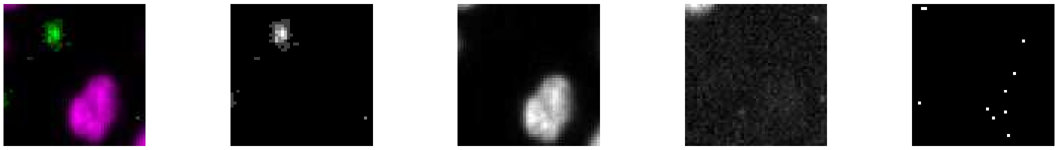				

Ereignis	Aufnahme	DAPI/CK-PE	CK-PE	DAPI	CD45-APC	HER-2/neu
5987	45	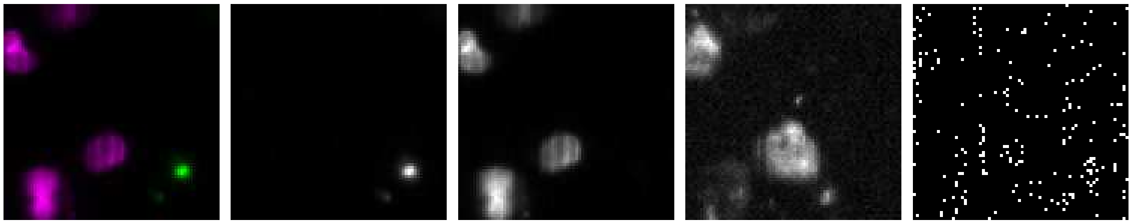				
5988	45	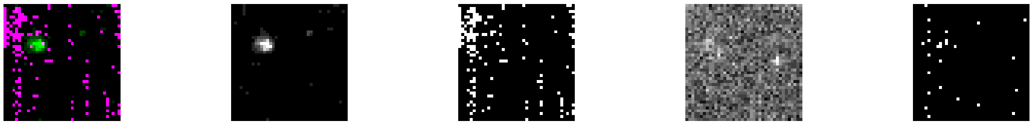				
5989	45	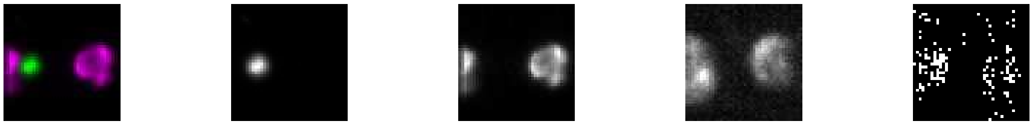				
5990	45	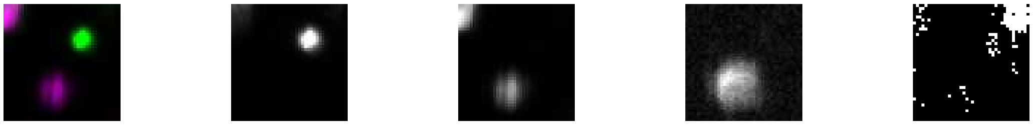				
5991	45	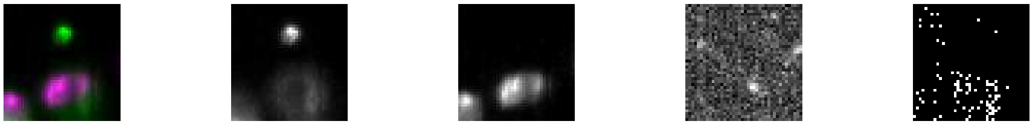				
5992	45	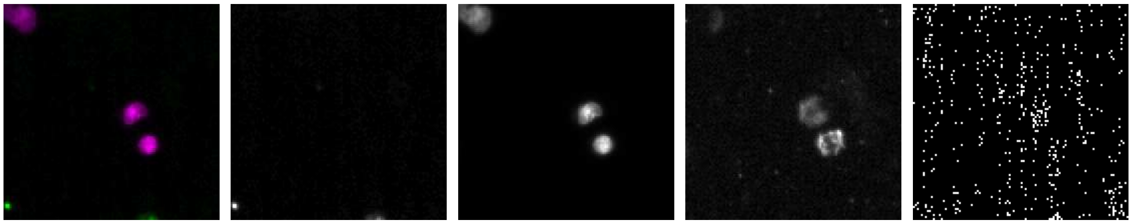				
5993	45	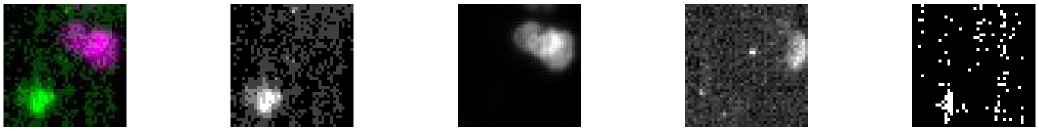				
5994	45	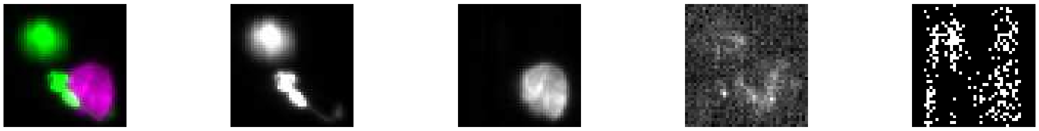				
5995	45	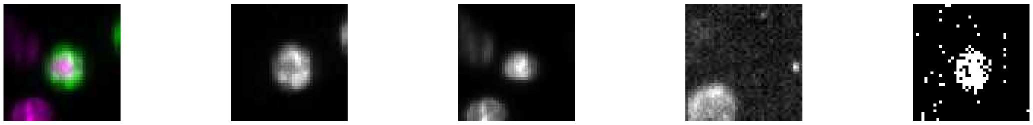				
5996	45	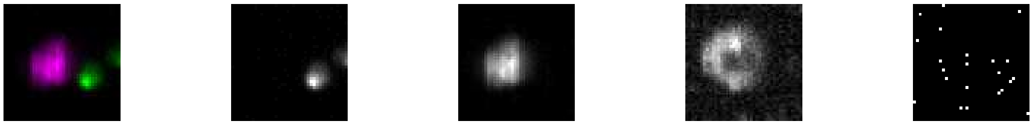				
5997	45	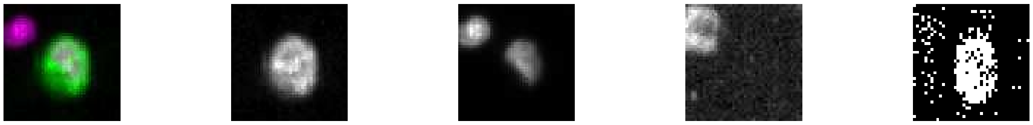				
5998	45	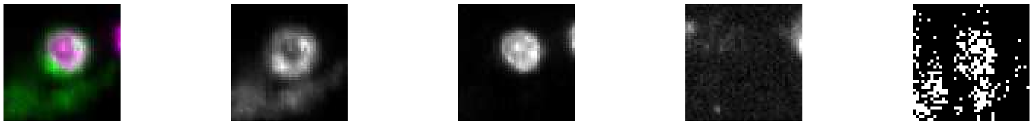				

Ereignis	Aufnahme	DAPI/CK-PE	CK-PE	DAPI	CD45-APC	HER-2/neu
5999	45	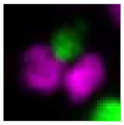	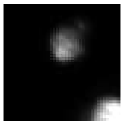	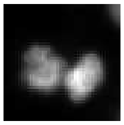	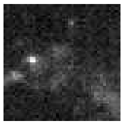	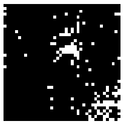
6000	45	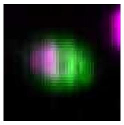	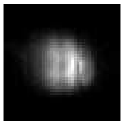	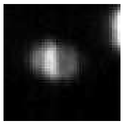	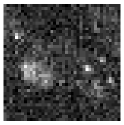	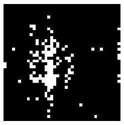

ÜBERPRÜFUNG DER FORSCHUNGSPROBE DRUCKEN

Druckdatum:02/06/2020 17:00

Proben-ID: Gyn14.208 **Kassetten-ID:**01327106 **Patienten-ID:**

Ereignis	Aufnahme	DAPI/CK-PE	CK-PE	DAPI	CD45-APC	HER-2/neu
12001	80	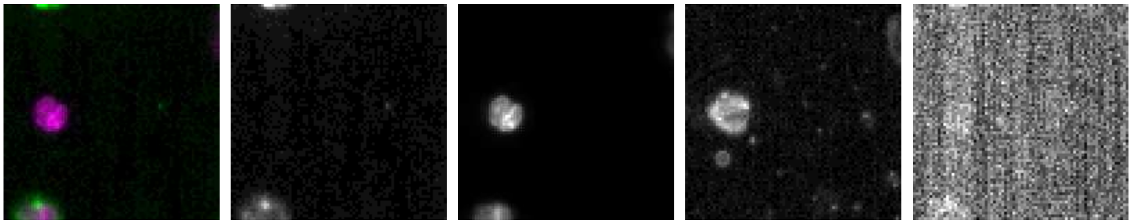				
12002	80	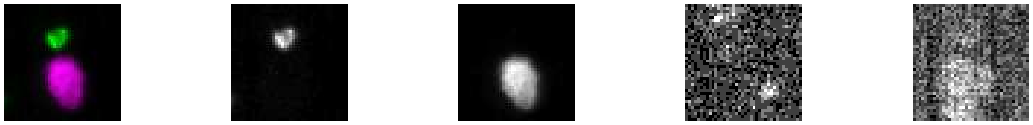				
12003	80	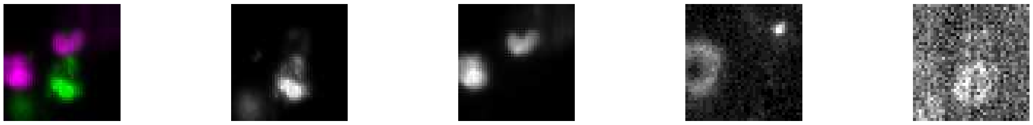				
12004	80	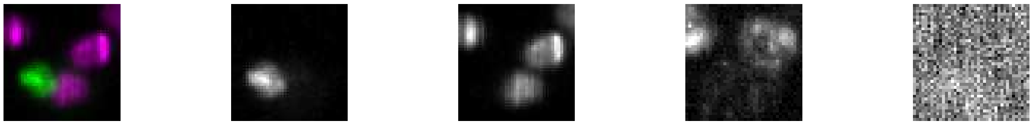				
12005	80	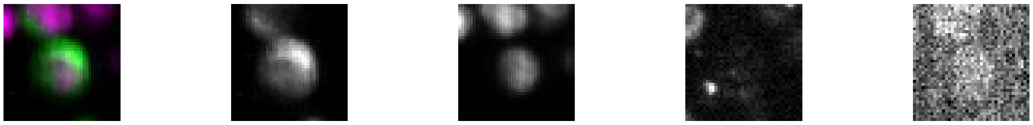				
12006	80	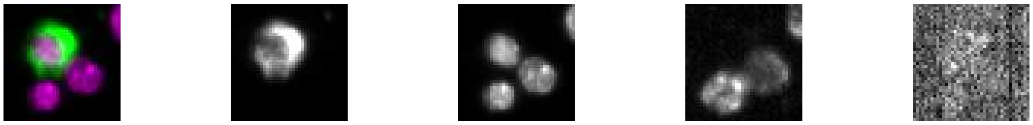				
12007	80	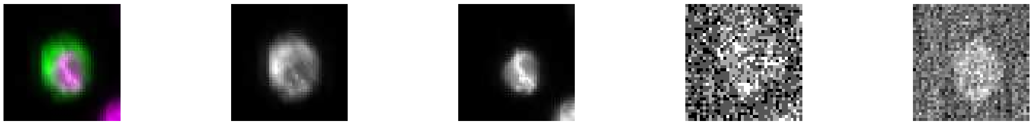				
12008	80	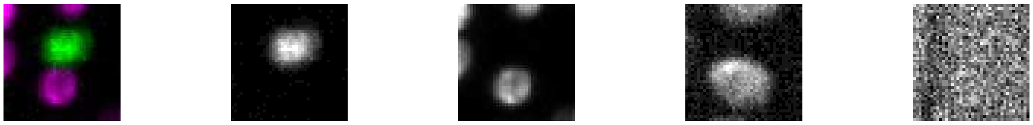				
12009	80	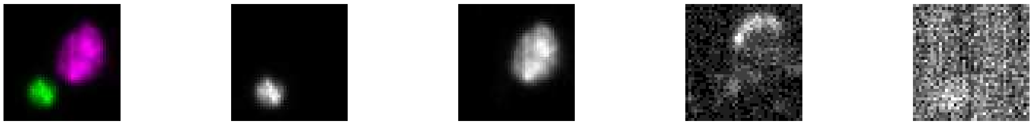				
12010	80	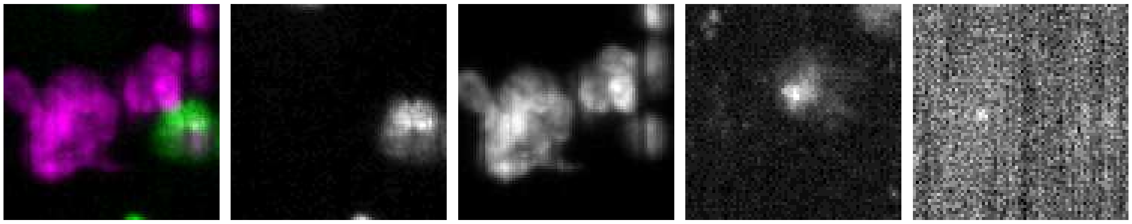				
12011	80	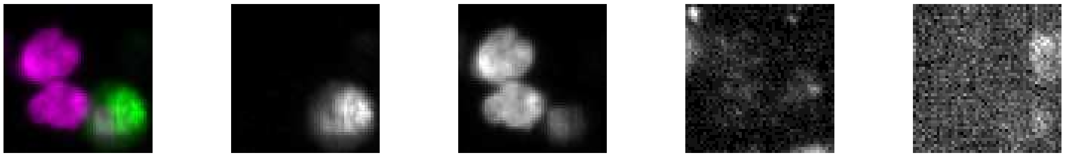				

Proben-ID: Gyn14.208 **Kassetten-ID:**01327106 **Patienten-ID:**

Ereignis	Aufnahme	DAPI/CK-PE	CK-PE	DAPI	CD45-APC	HER-2/neu
12012	80	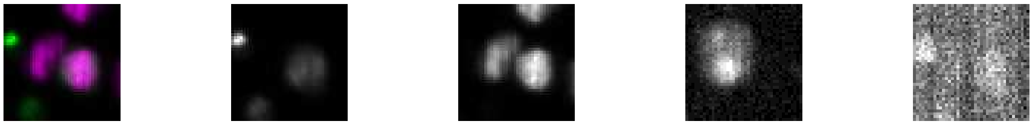				
12013	80	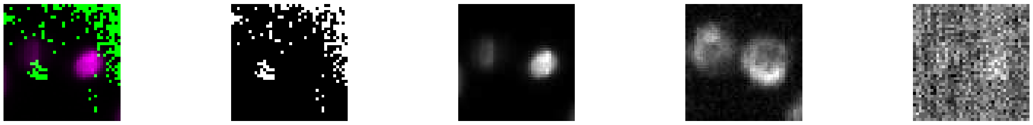				
12014	80	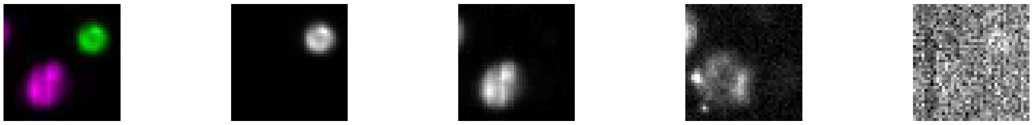				
12015	80	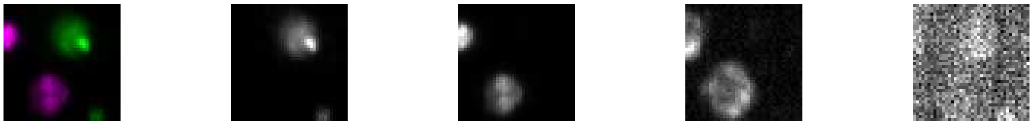				
12016	80	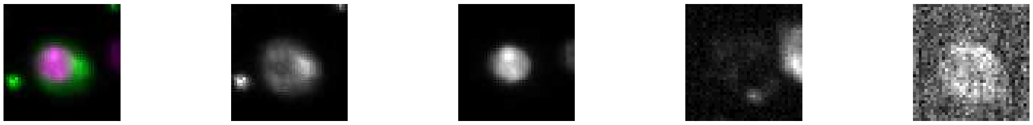				
12017	80	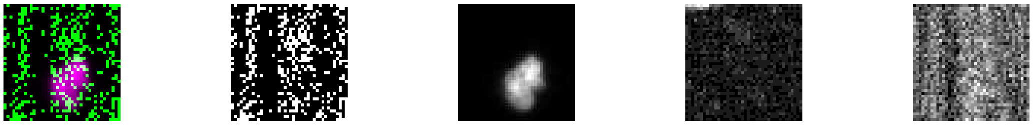				
12018	80	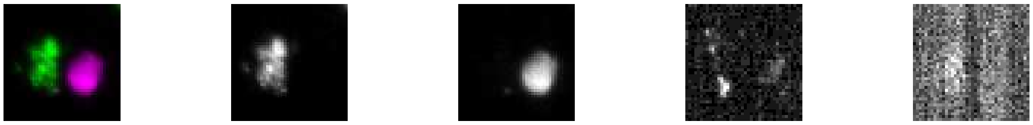				
12019	80	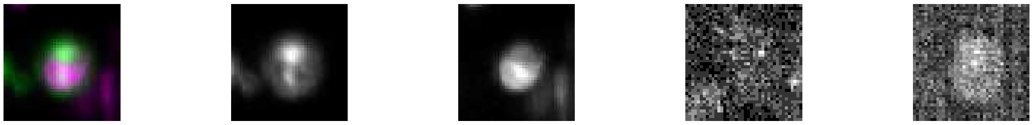				
12020	80	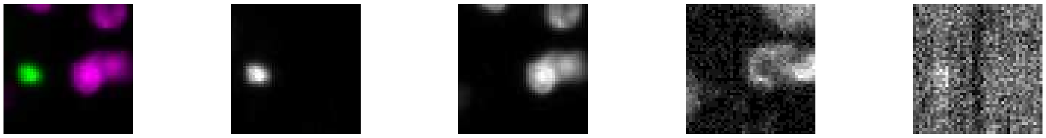				
12021	80	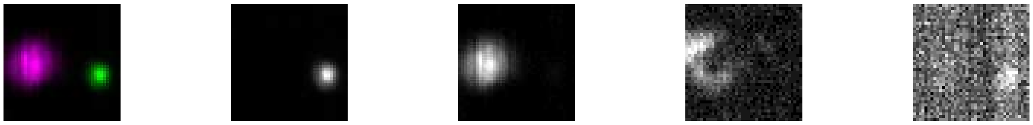				
12022	80	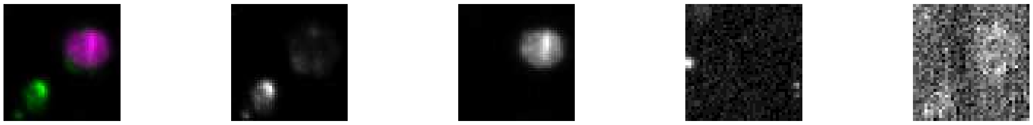				
12023	80	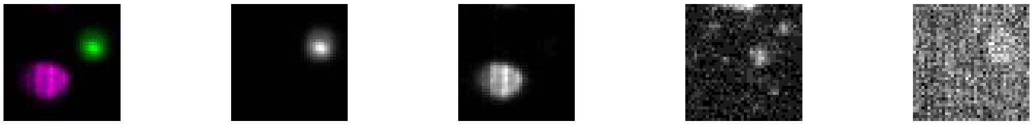				
12024	80	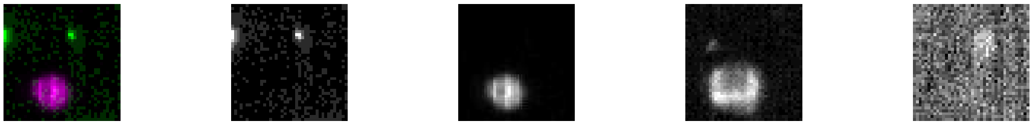				
12025	80	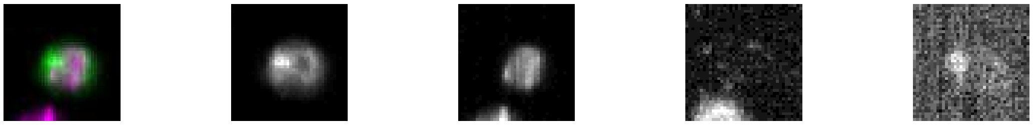				

Proben-ID: Gyn14.208 **Kassetten-ID:** 01327106 **Patienten-ID:**

Ereignis	Aufnahme	DAPI/CK-PE	CK-PE	DAPI	CD45-APC	HER-2/neu
12026	80	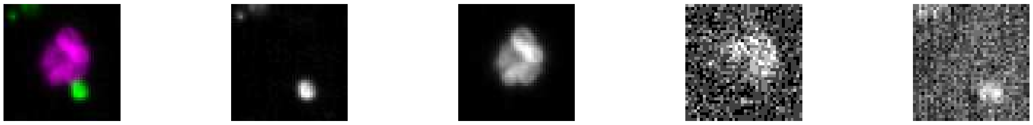				
12027	80	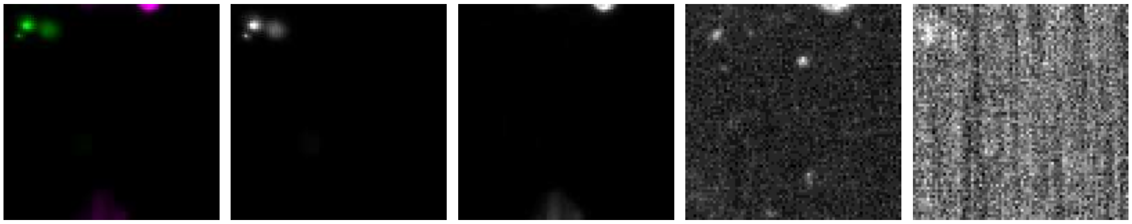				
12028	80	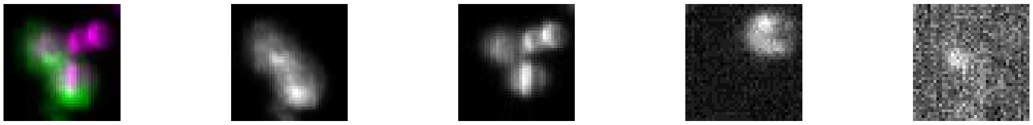				
12029	80	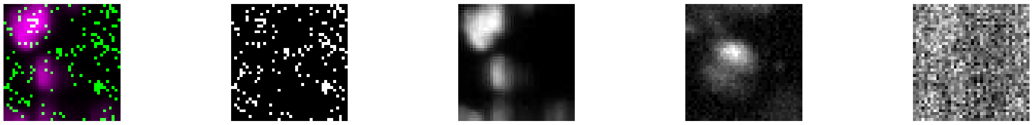				
12030	80	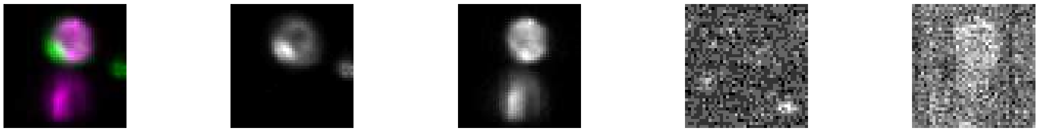				
12031	80	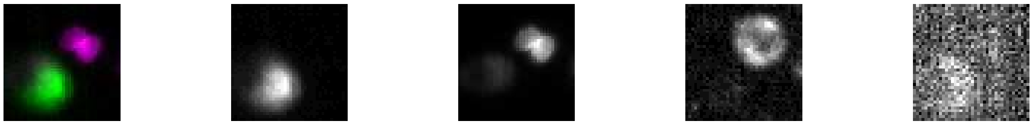				
12032	61	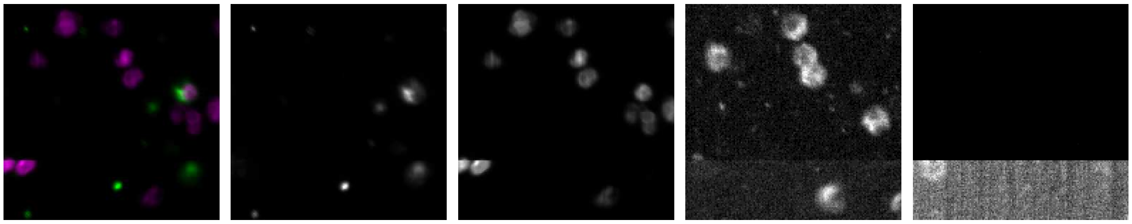				
12033	80	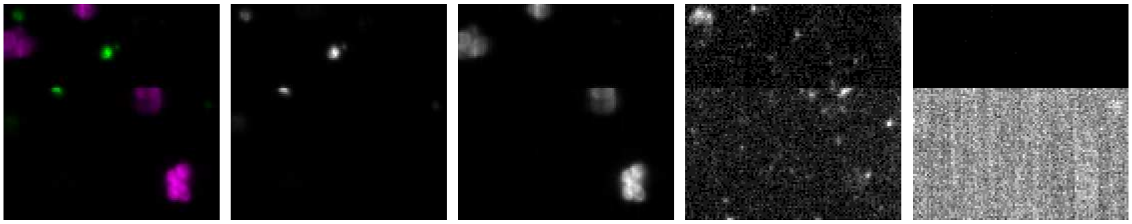				
12034	61	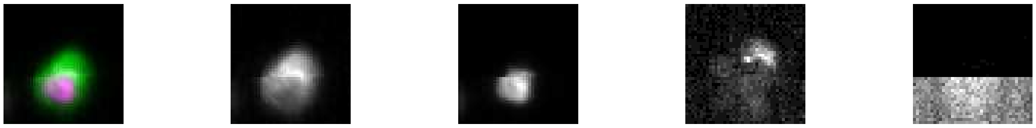				
12035	80	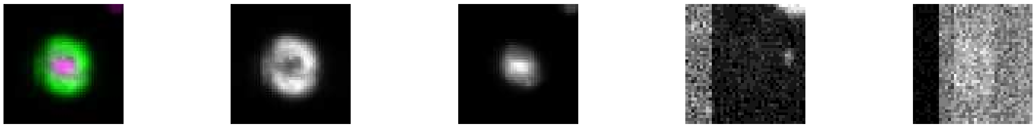				
12036	61	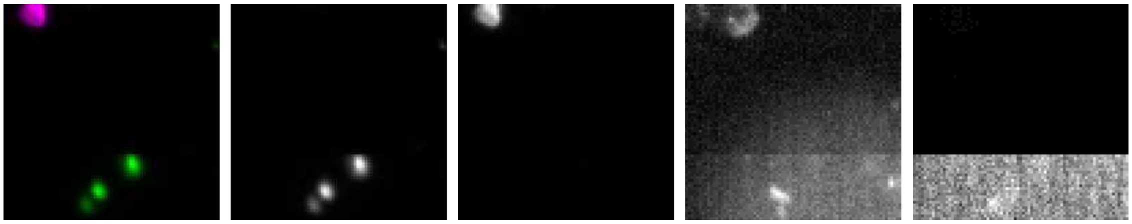				

Druckdatum: 02/06/2020 17:00

Proben-ID: Gyn14.208 **Kassetten-ID:** 01327106 **Patienten-ID:**

Ereignis	Aufnahme	DAPI/CK-PE	CK-PE	DAPI	CD45-APC	HER-2/neu
12037	61	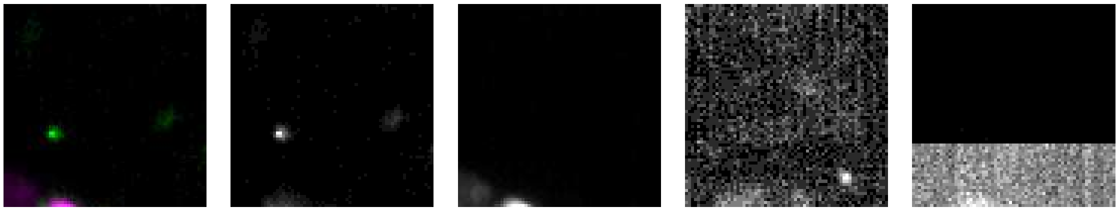				
12038	81	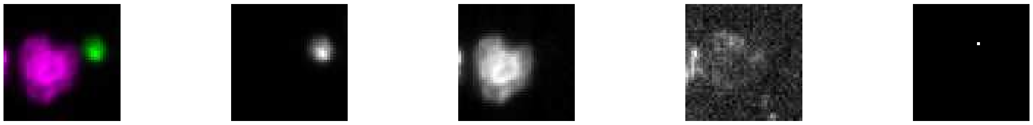				
12039	81	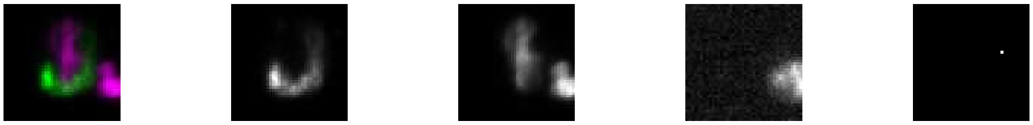				
12040	81	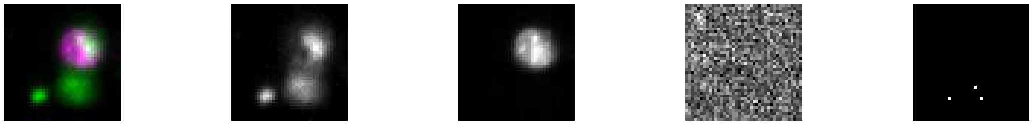				
12041	81	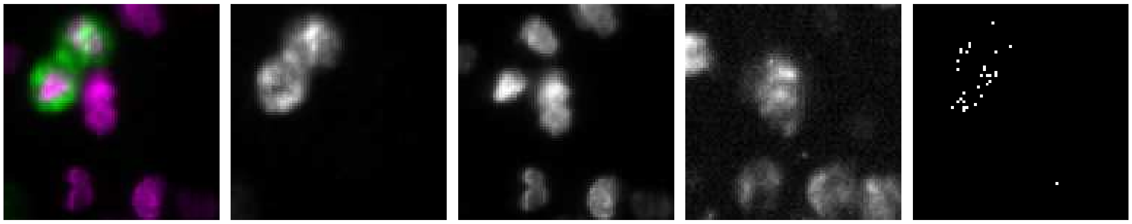				
12042	81	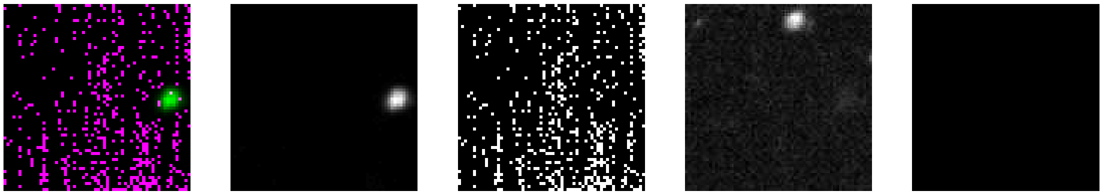				
12043	81	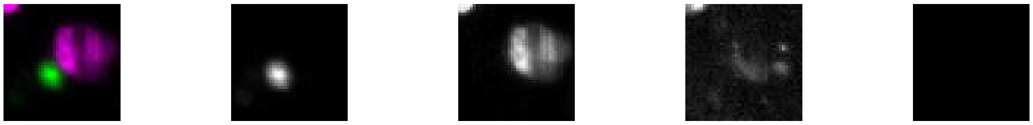				
12044	81	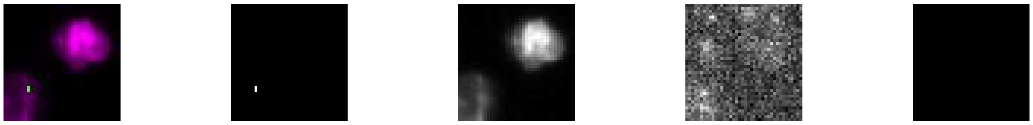				
12045	81	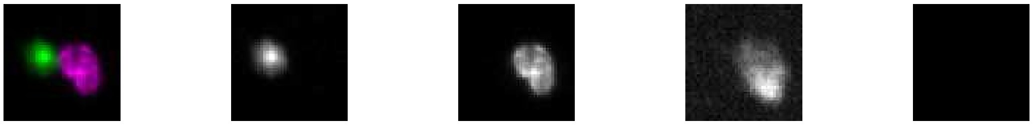				
12046	81	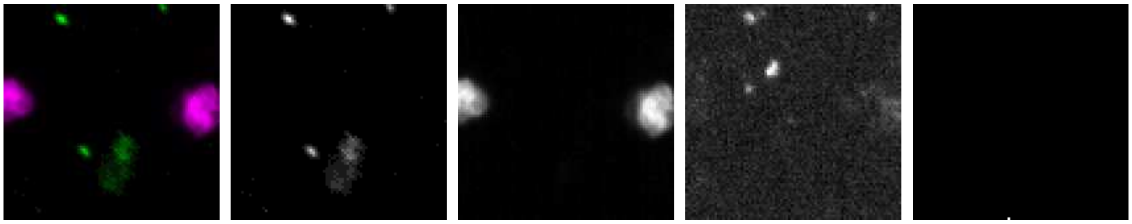				
12047	81	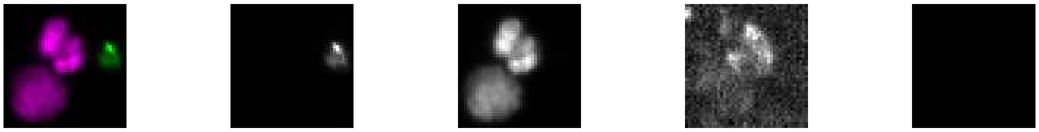				

Ereignis	Aufnahme	DAPI/CK-PE	CK-PE	DAPI	CD45-APC	HER-2/neu
12048	81	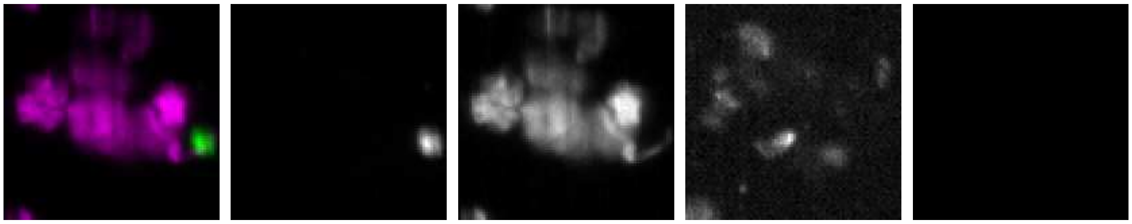				
12049	81	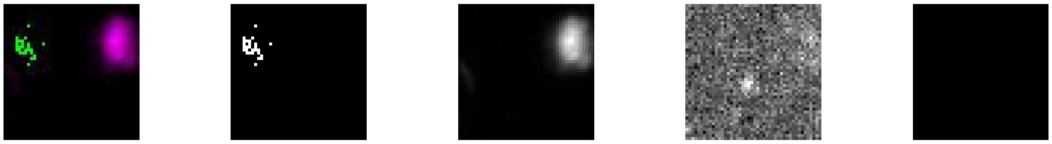				
12050	81	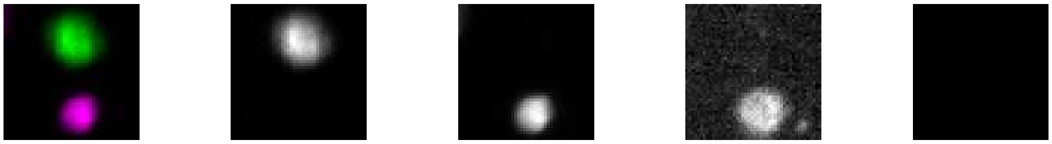				
12051	81	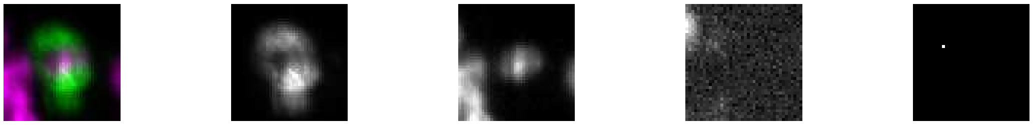				
12052	81	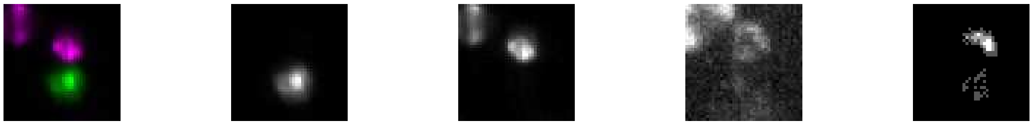				
12053	81	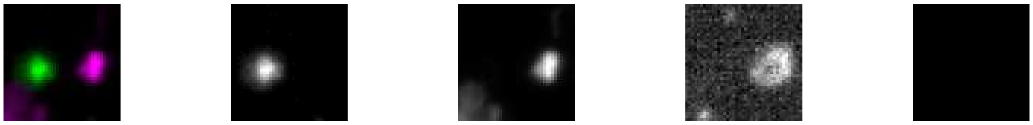				
12054	81	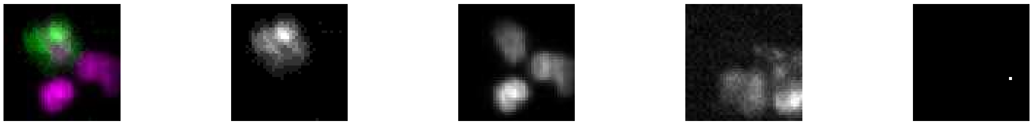				
12055	81	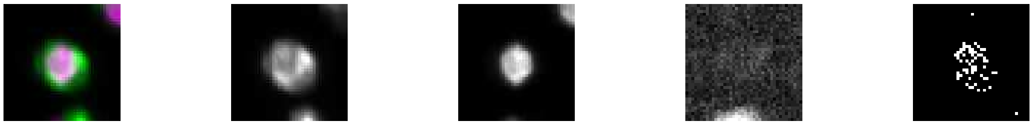				
12056	81	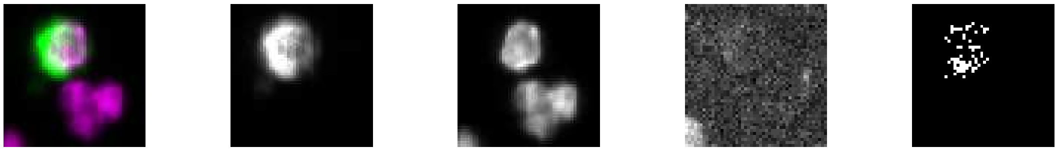				
12057	81	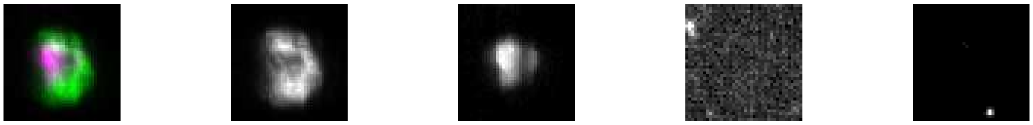				
12058	81	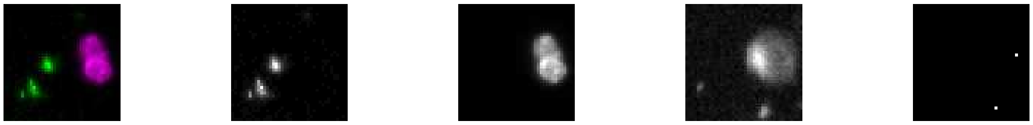				
12059	81	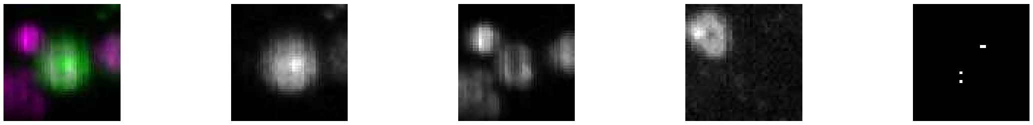				
12060	81	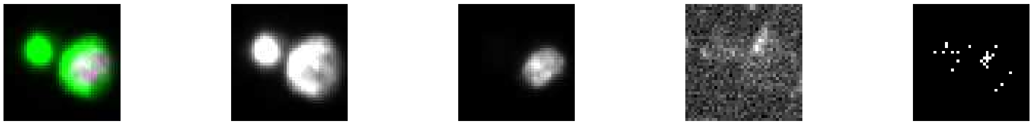				

Druckdatum:02/06/2020 17:00

Proben-ID: Gyn14.208 **Kassetten-ID:**01327106 **Patienten-ID:**

Ereignis	Aufnahme	DAPI/CK-PE	CK-PE	DAPI	CD45-APC	HER-2/neu
12061	81	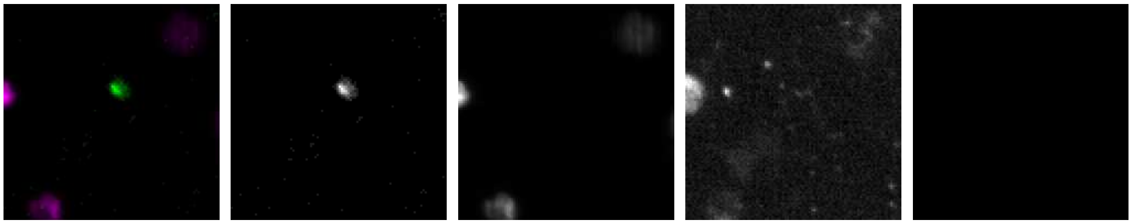				
12062	81	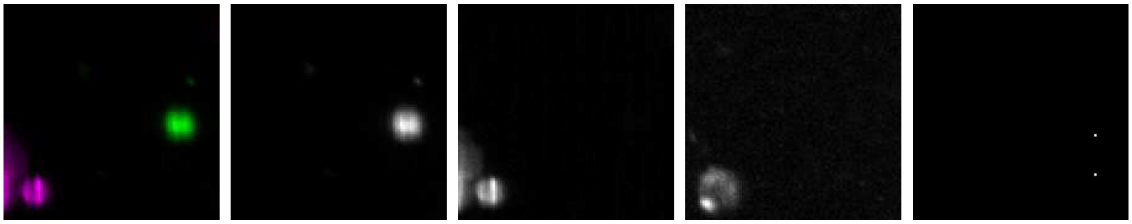				
12063	81	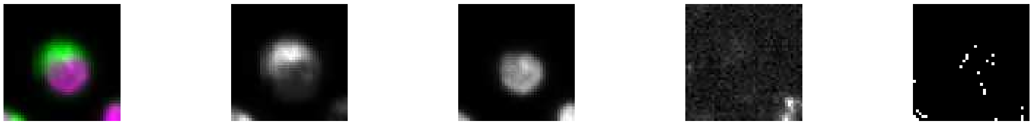				
12064	81	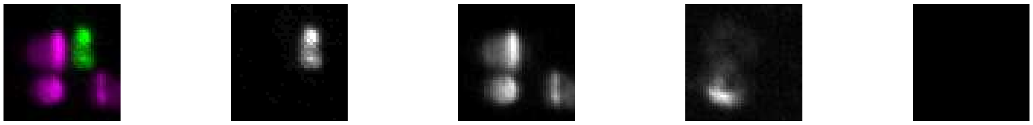				
12065	81	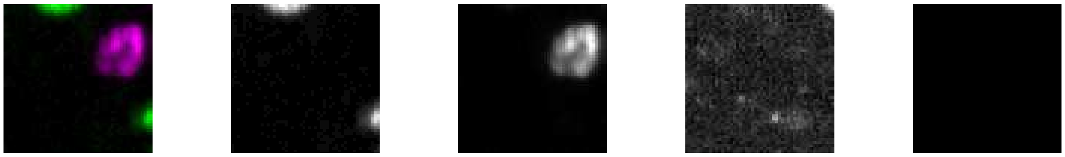				
12066	81	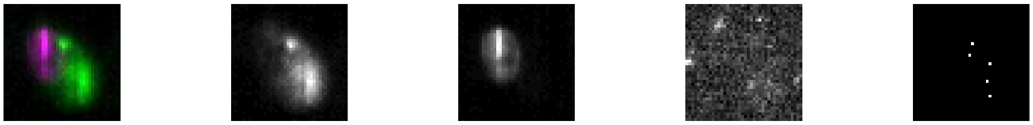				
12067	81	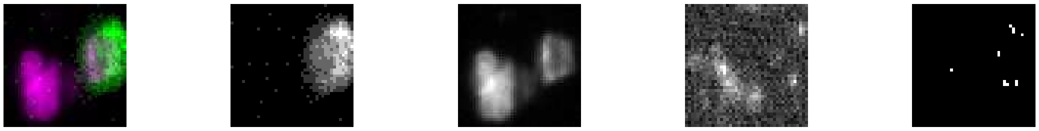				
12068	81	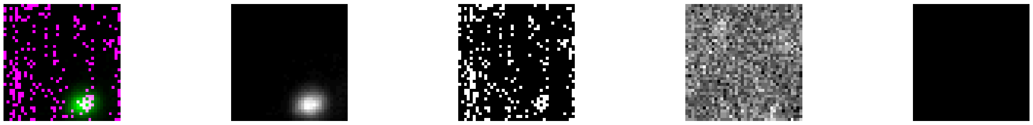				
12069	81	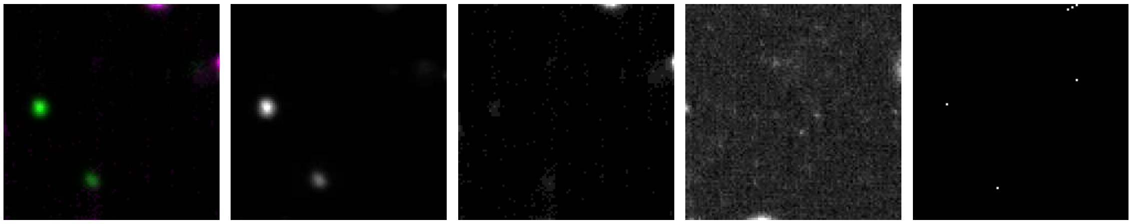				
12070	81	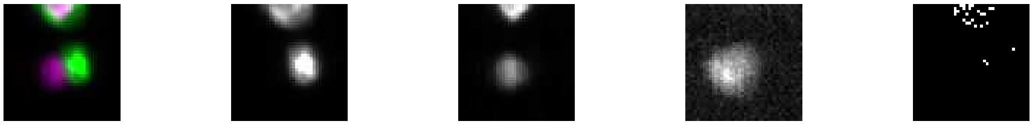				
12071	81	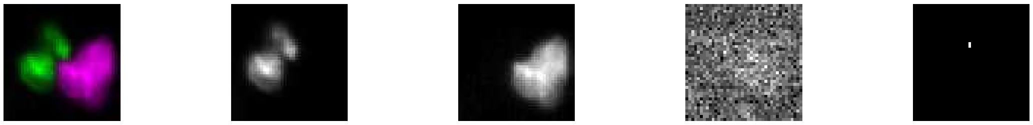				

Ereignis	Aufnahme	DAPI/CK-PE	CK-PE	DAPI	CD45-APC	HER-2/neu
12072	81	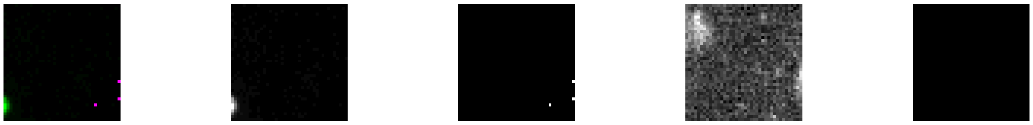				
12073	81	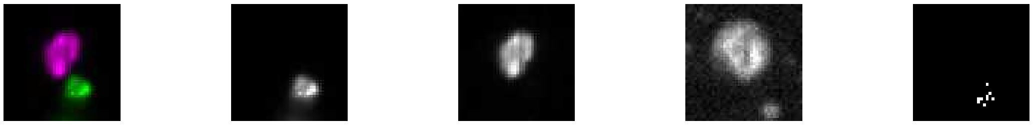				
12074	81	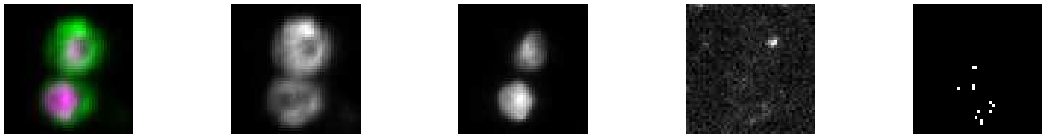				
12075	81	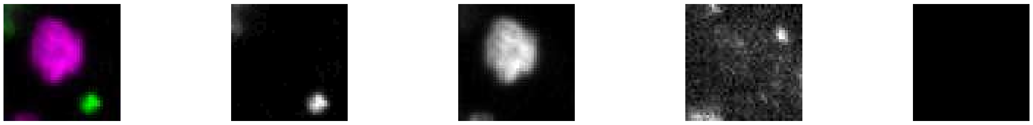				
12076	81	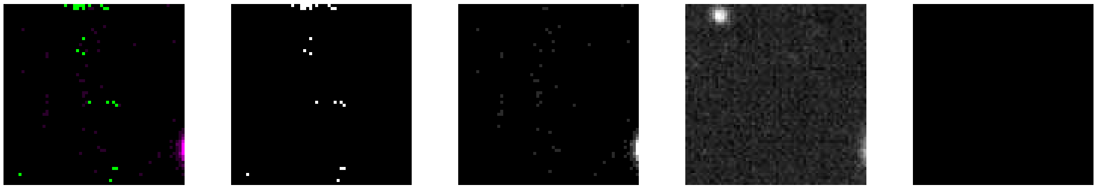				
12077	81	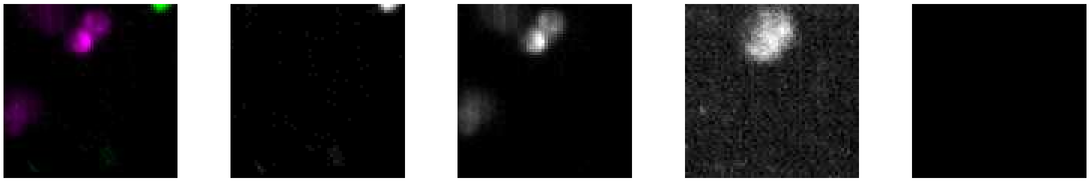				
12078	81	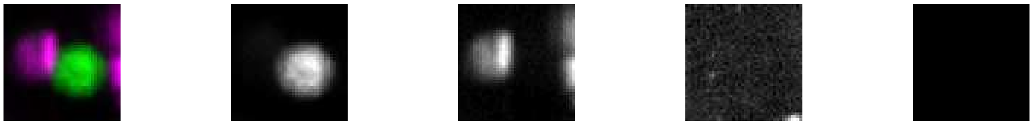				
12079	81	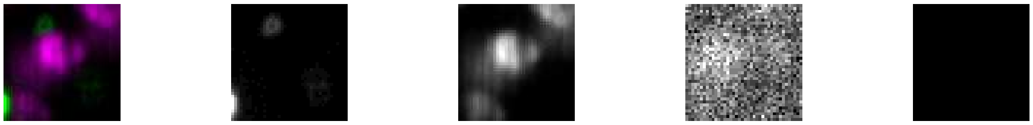				
12080	81	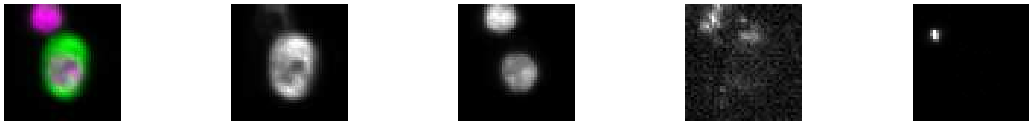				
12081	81	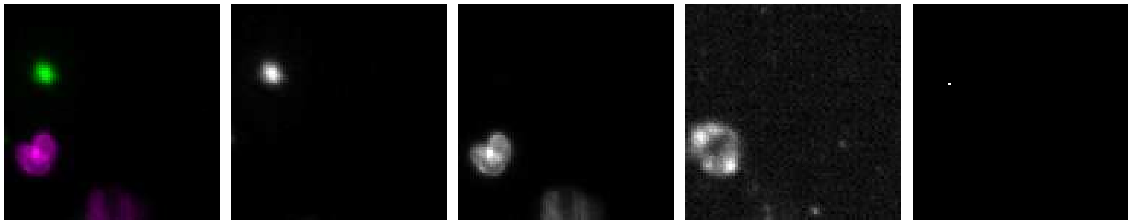				
12082	81	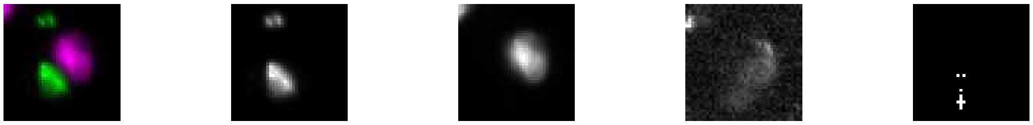				
12083	81	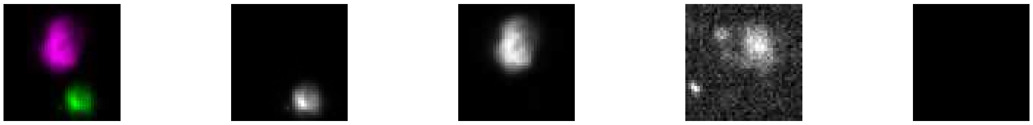				

Ereignis	Aufnahme	DAPI/CK-PE, CK-PE, DAPI, CD45-APC, HER-2/neu
12084	81	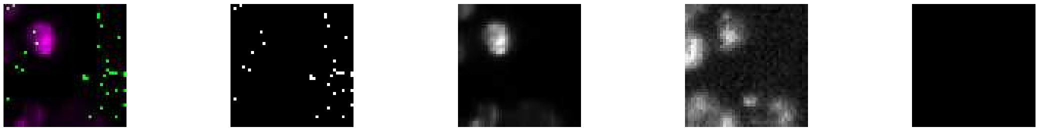
12085	81	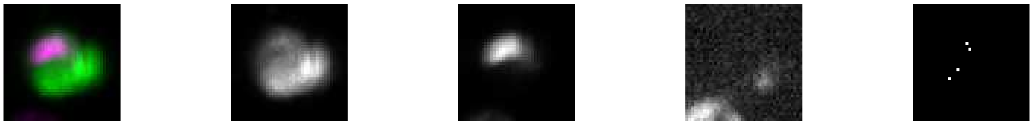
12086	81	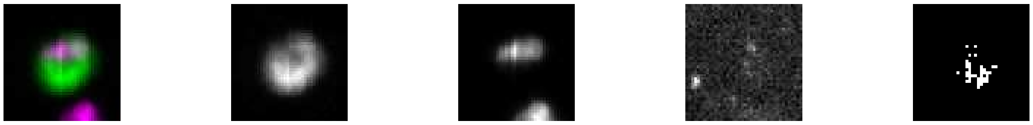
12087	81	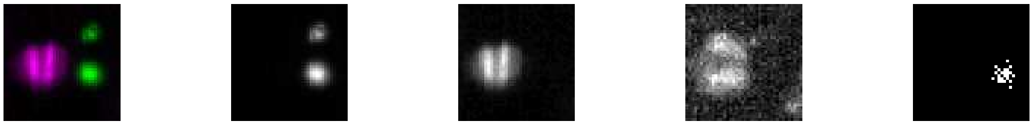
12088	81	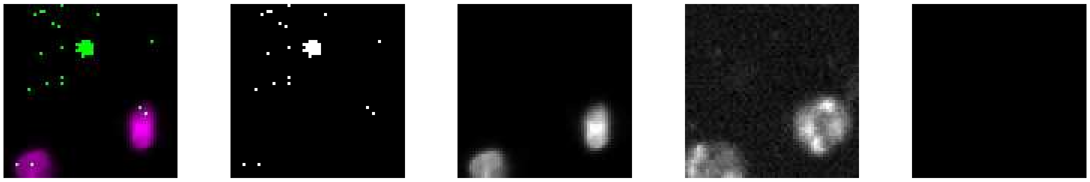
12089	81	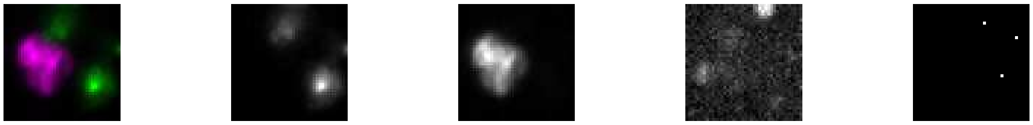
12090	81	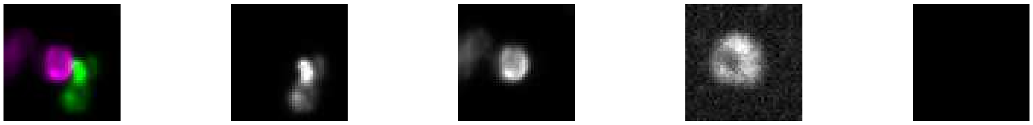
12091	81	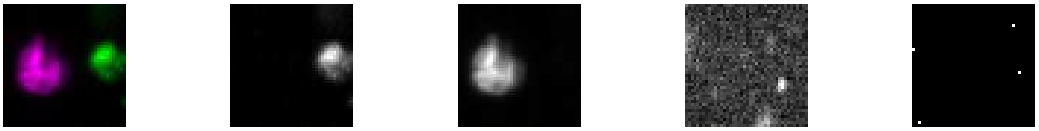
12092	81	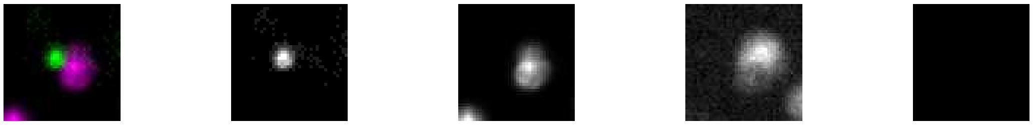
12093	81	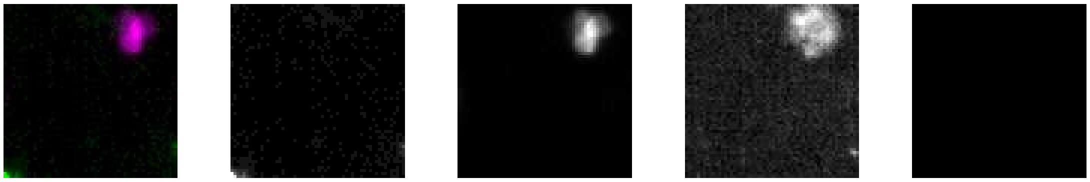
12094	81	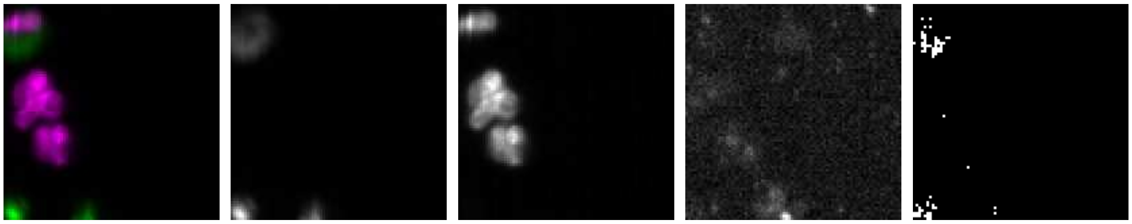
12095	81	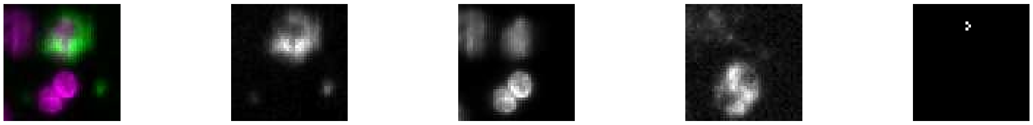

Ereignis	Aufnahme	DAPI/CK-PE	CK-PE	DAPI	CD45-APC	HER-2/neu
12096	81	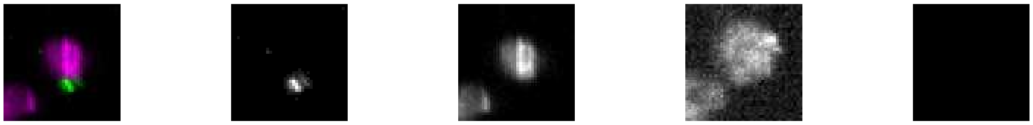				
12097	81	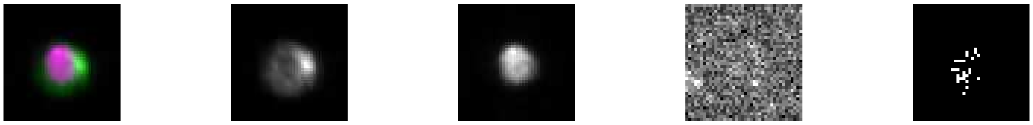				
12098	81	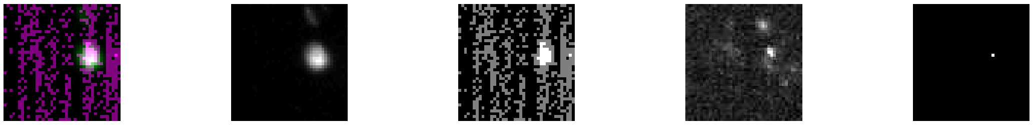				
12099	81	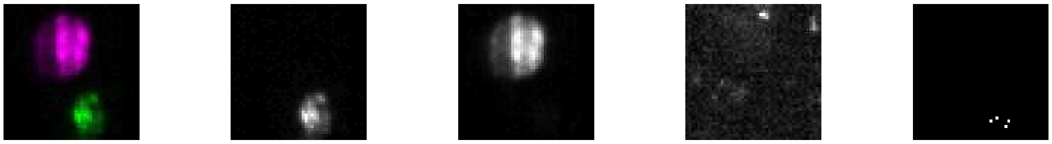				
12100	81	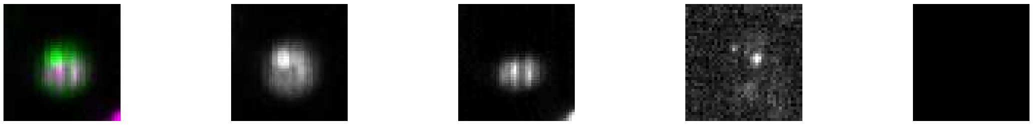				
12101	81	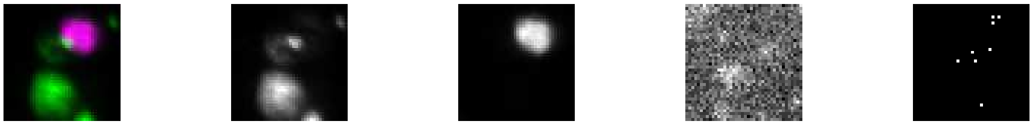				
12102	81	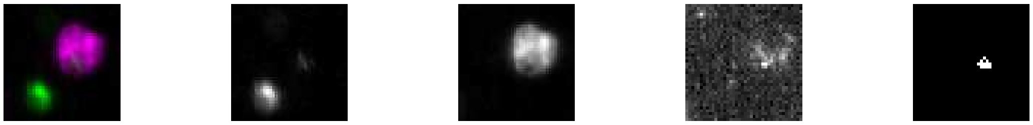				
12103	81	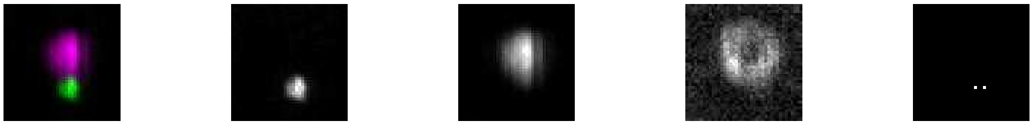				
12104	81	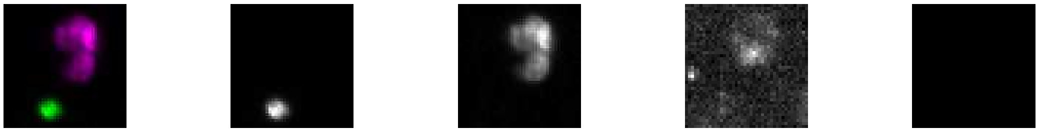				
12105	81	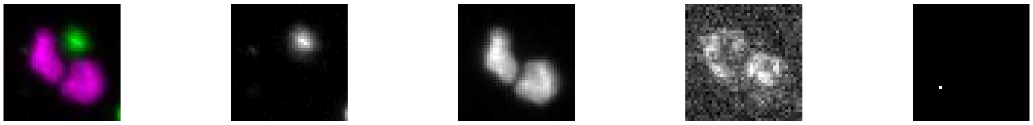				
12106	81	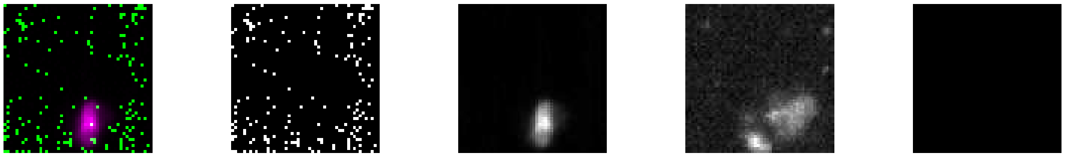				
12107	81	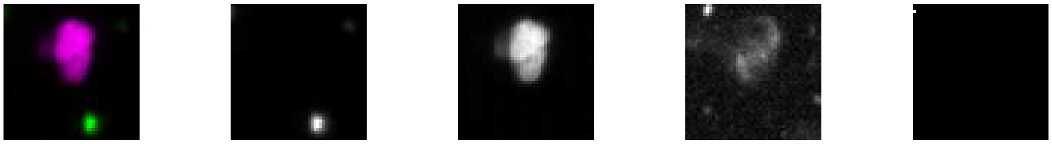				
12108	81	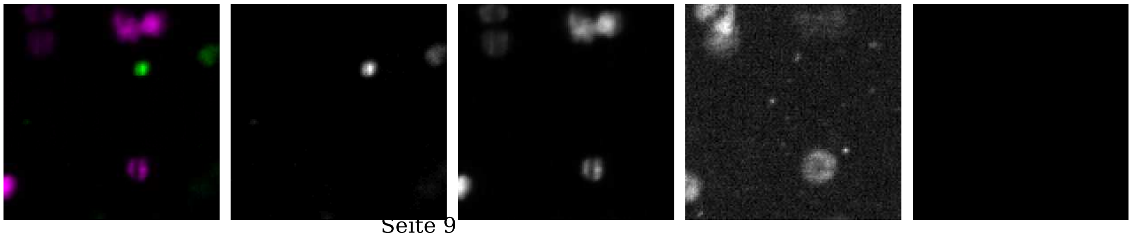				

Ereignis	Aufnahme	DAPI/CK-PE	CK-PE	DAPI	CD45-APC	HER-2/neu
12109	81	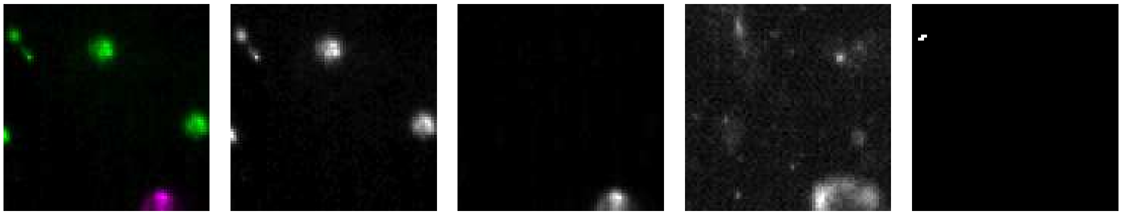				
12110	81	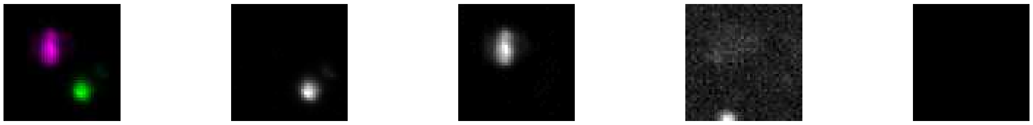				
12111	81	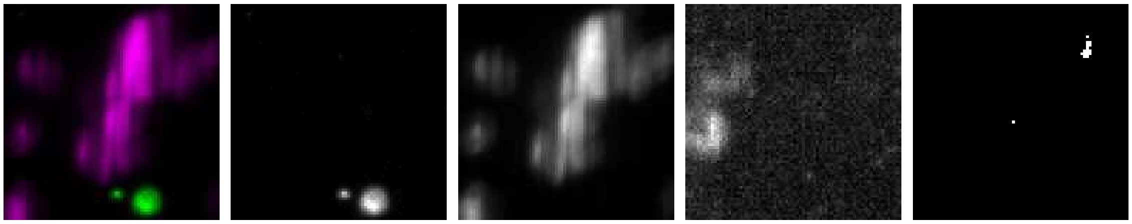				
12112	81	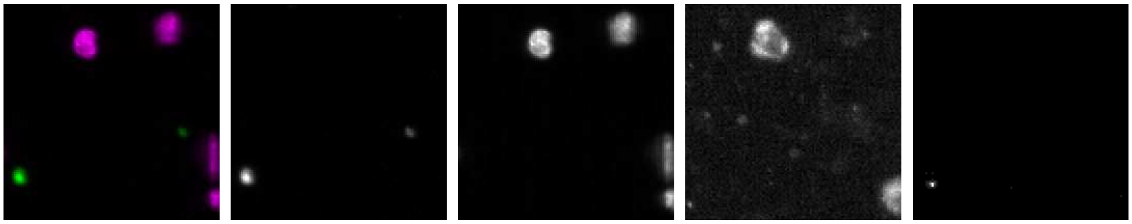				
12113	81	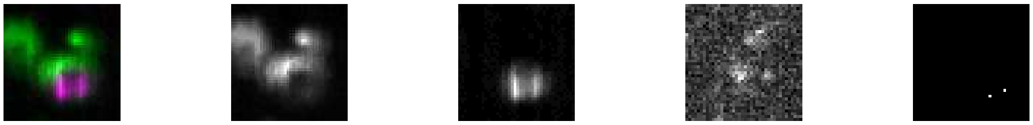				
12114	81	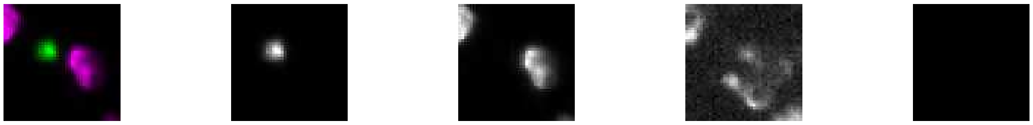				
12115	81	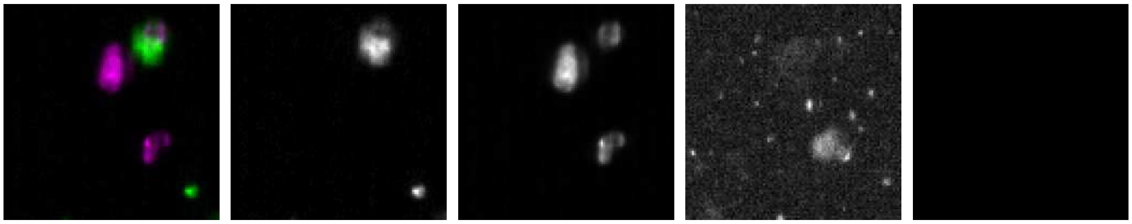				
12116	81	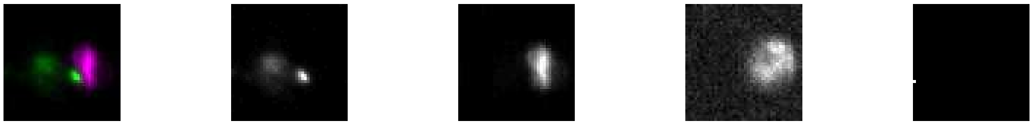				
12117	81	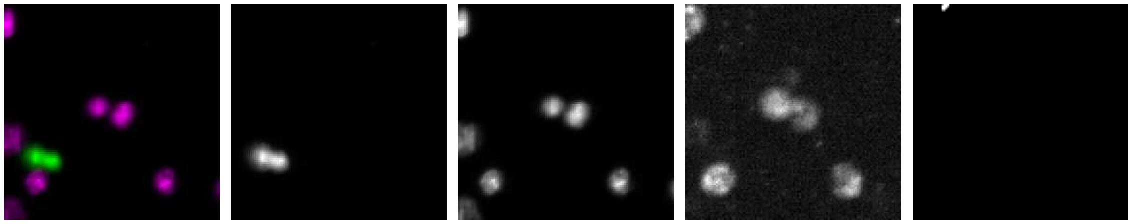				
12118	81	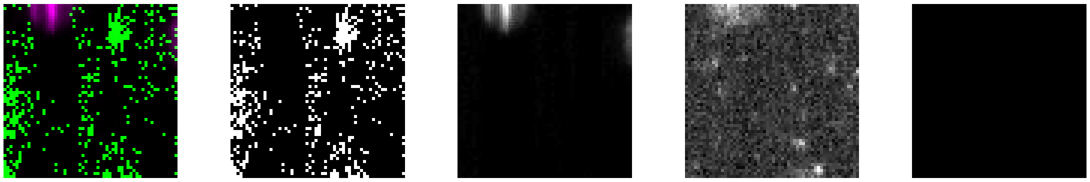				

Druckdatum: 02/06/2020 17:00

Proben-ID: Gyn14.208 **Kassetten-ID:** 01327106 **Patienten-ID:**

Ereignis	Aufnahme	DAPI/CK-PE	CK-PE	DAPI	CD45-APC	HER-2/neu
12119	81	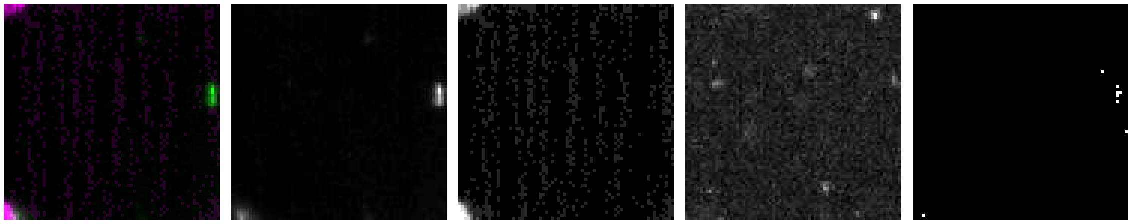				
12120	81	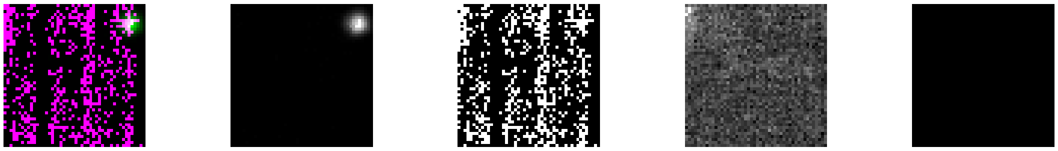				
12121	81	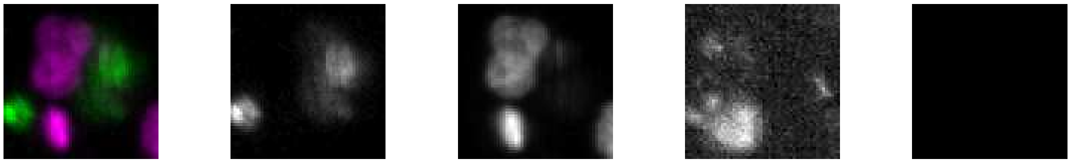				
12122	81	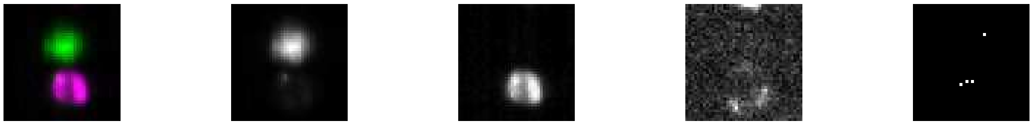				
12123	81	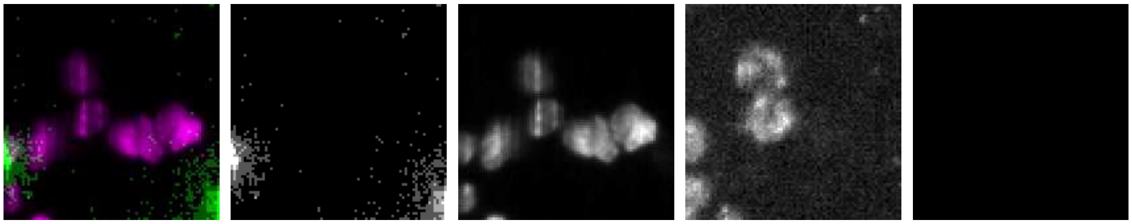				
12124	81	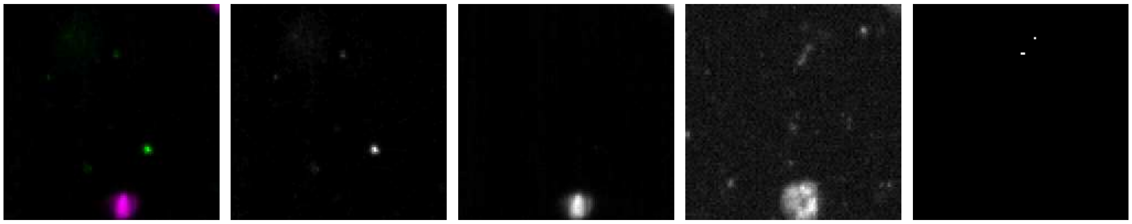				
12125	81	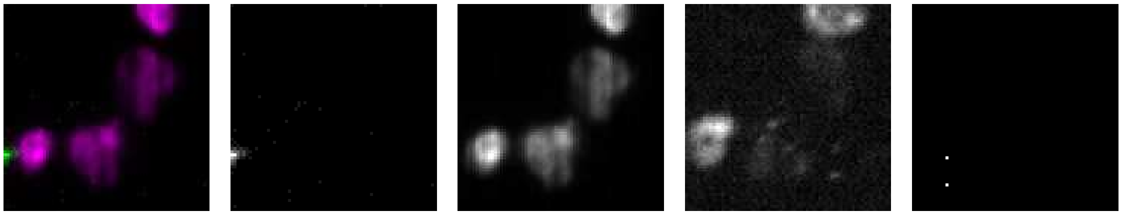				
12126	81	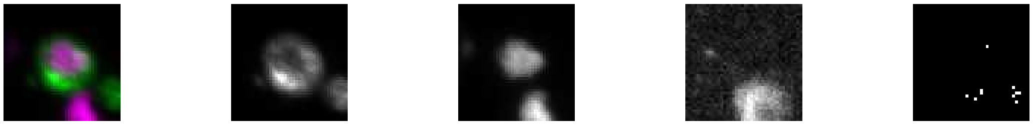				
12127	81	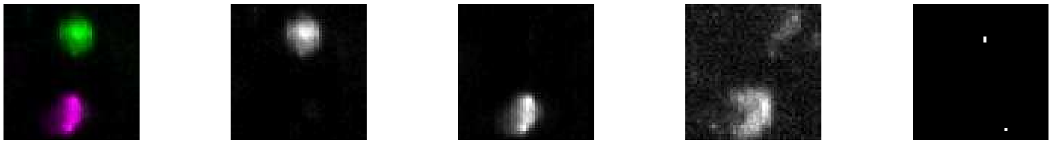				
12128	81	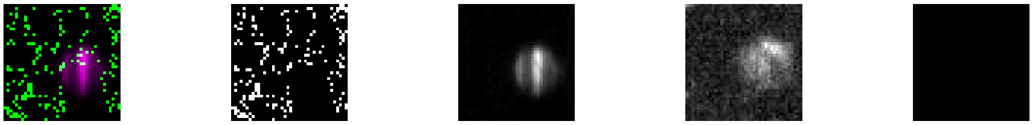				

Ereignis	Aufnahme	DAPI/CK-PE	CK-PE	DAPI	CD45-APC	HER-2/neu
12129	81	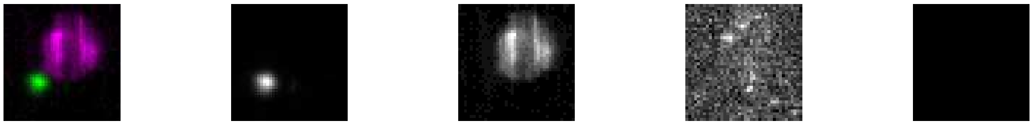				
12130	81	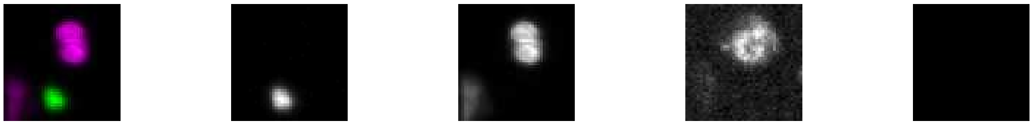				
12131	81	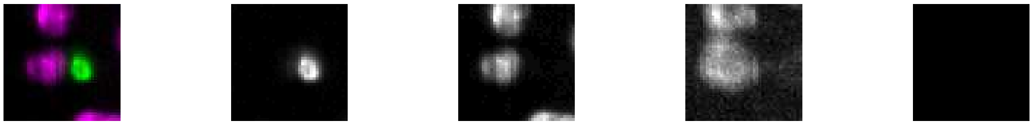				
12132	81	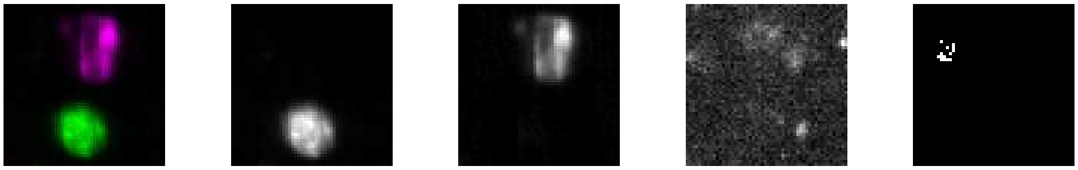				
12133	81	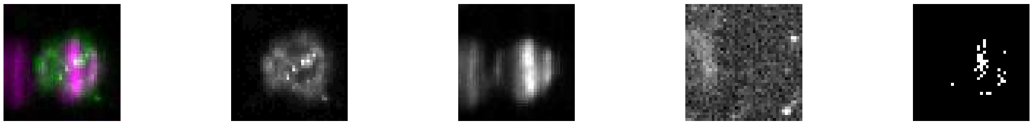				
12134	81	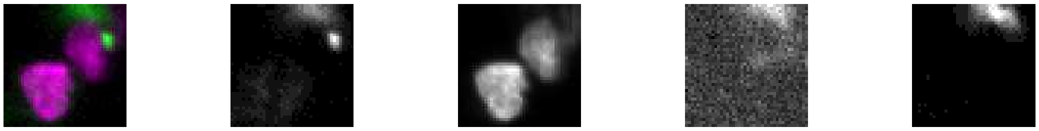				
12135	81	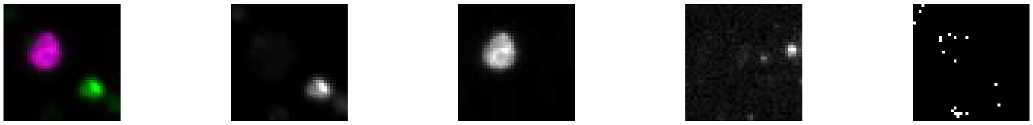				
12136	81	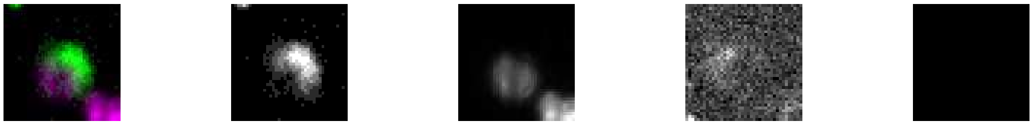				
12137	81	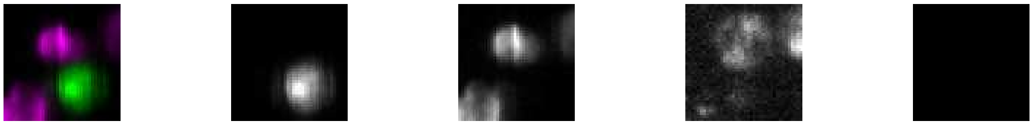				
12138	81	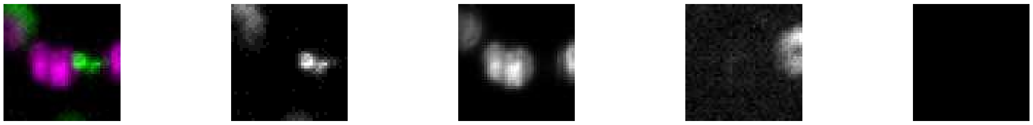				
12139	81	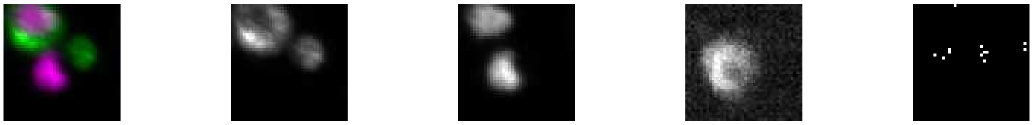				
12140	81	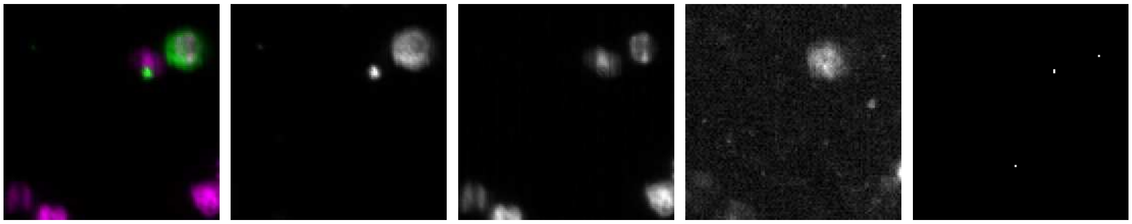				
12141	81	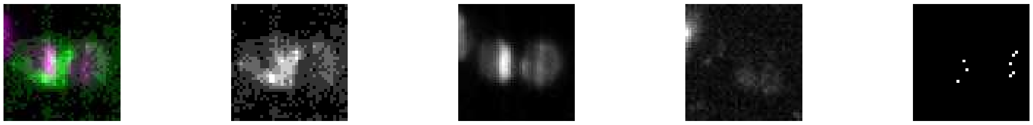				

Ereignis	Aufnahme	DAPI/CK-PE	CK-PE	DAPI	CD45-APC	HER-2/neu
12142	81	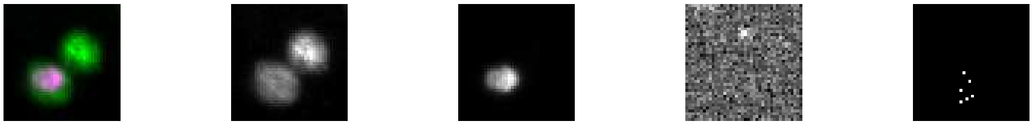				
12143	81	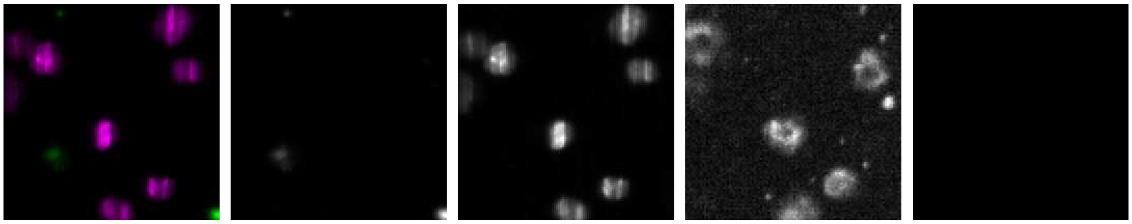				
12144	81	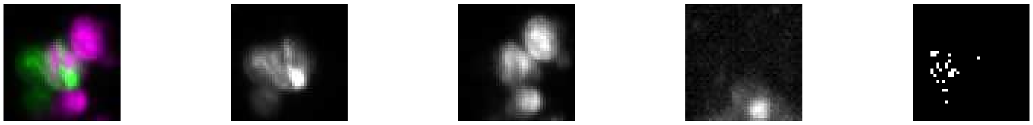				
12145	81	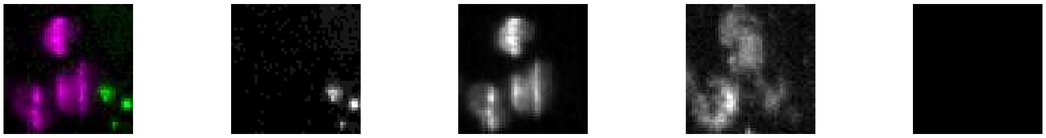				
12146	81	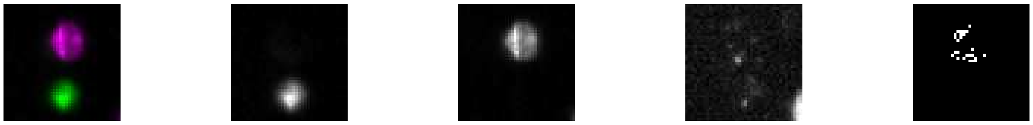				
12147	81	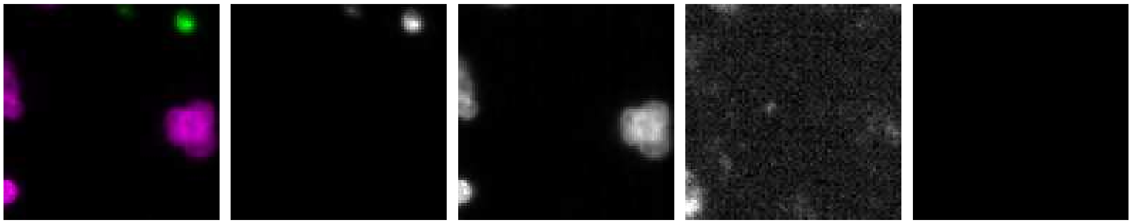				
12148	81	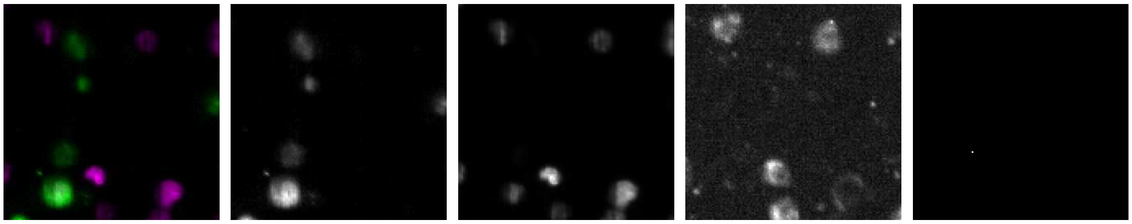				
12149	81	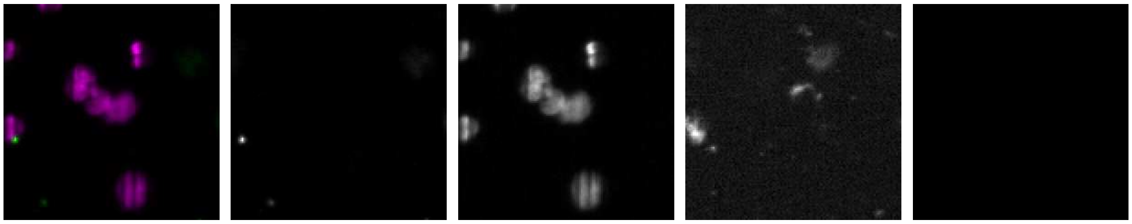				
12150	81	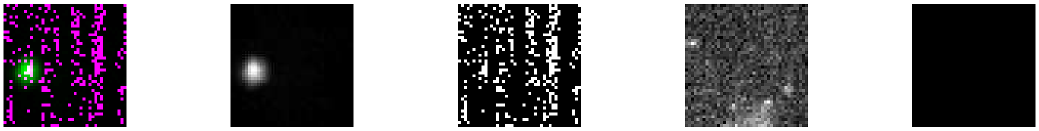				
12151	81	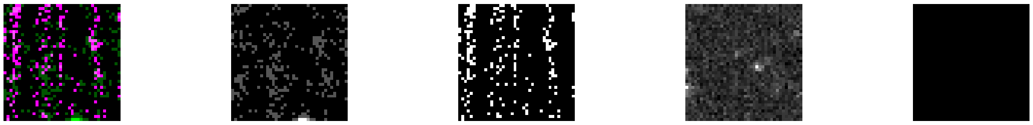				

Ereignis	Aufnahme	DAPI/CK-PE	CK-PE	DAPI	CD45-APC	HER-2/neu
12152	81	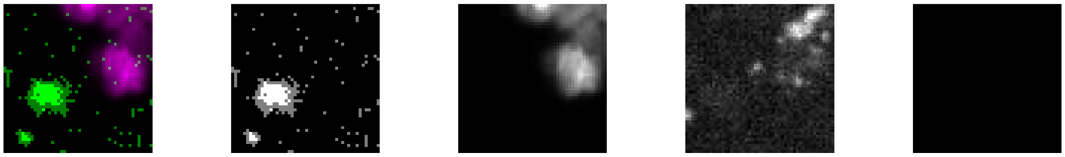				
12153	81	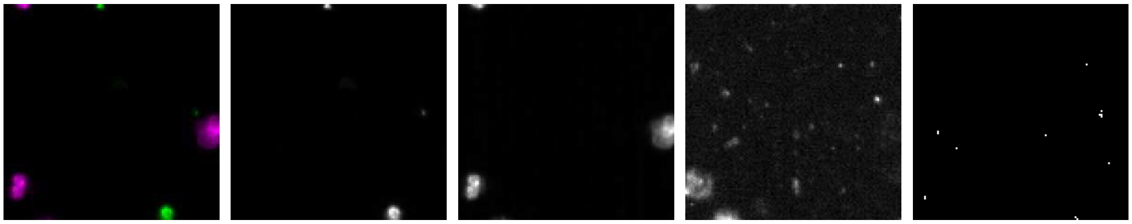				
12154	81	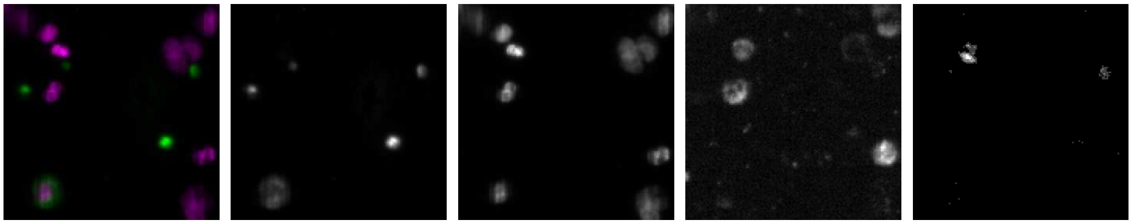				
12155	81	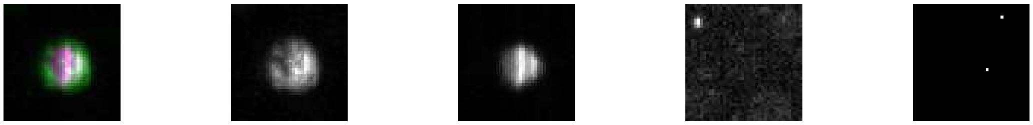				
12156	81	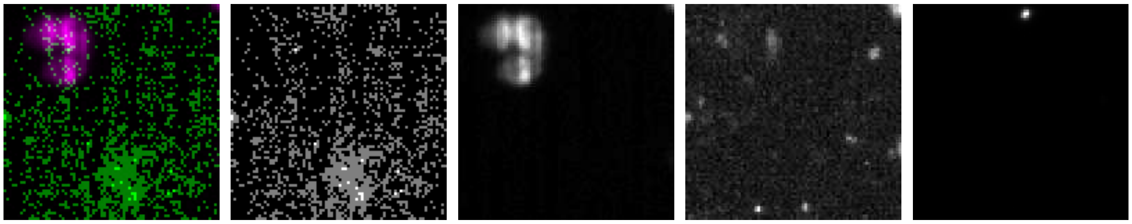				
12157	81	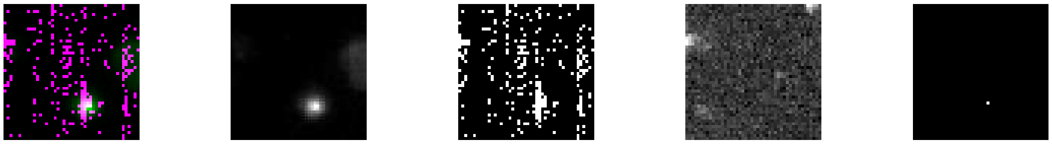				
12158	81	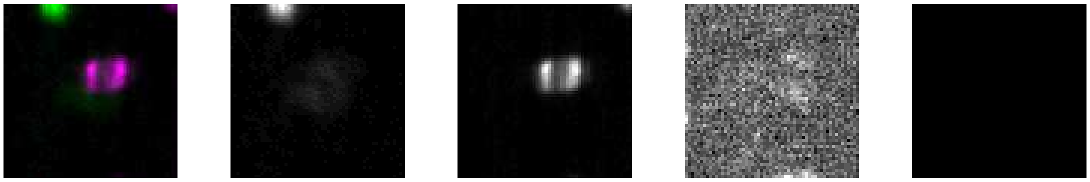				
12159	81	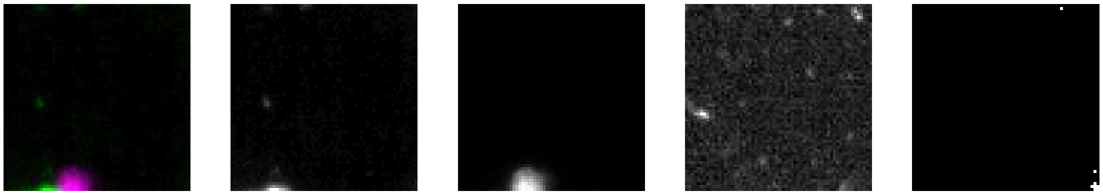				
12160	81	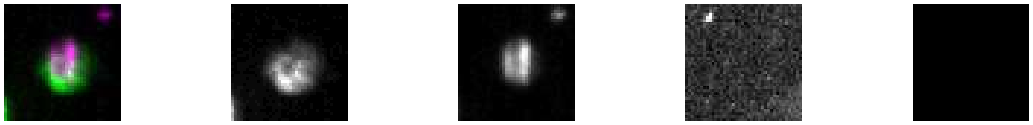				
12161	81	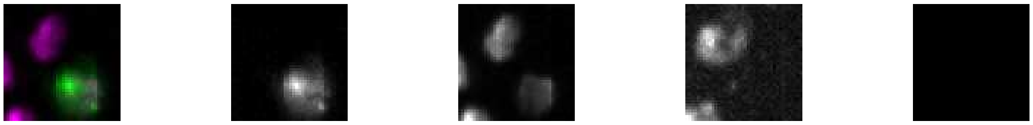				

Proben-ID: Gyn14.208 **Kassetten-ID:** 01327106 **Patienten-ID:**

Ereignis	Aufnahme	DAPI/CK-PE	CK-PE	DAPI	CD45-APC	HER-2/neu
12162	81	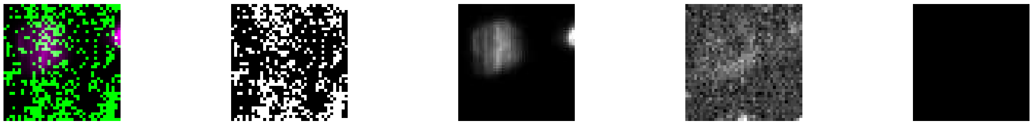				
12163	81	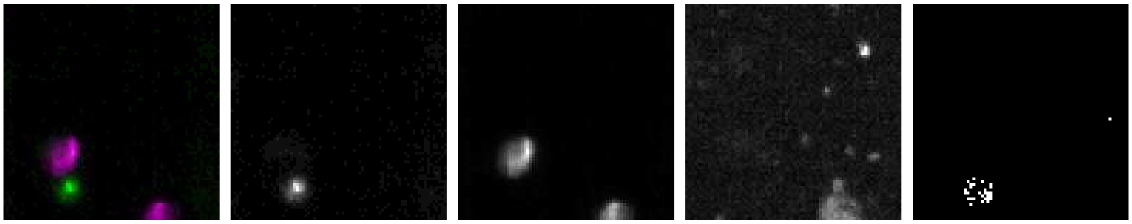				
12164	81	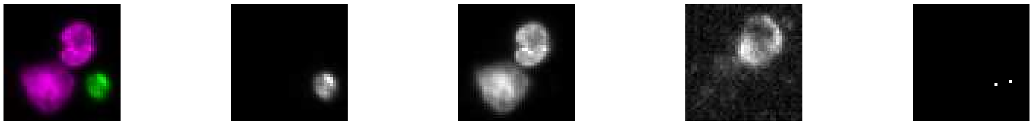				
12165	81	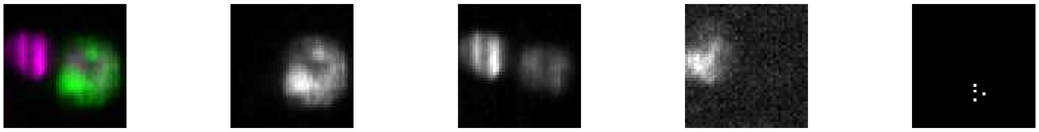				
12166	81	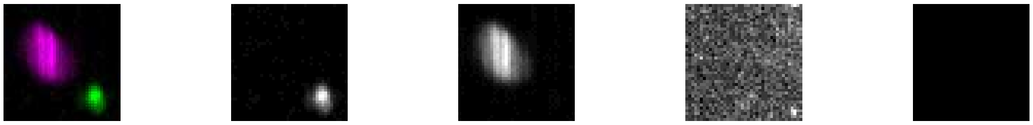				
12167	81	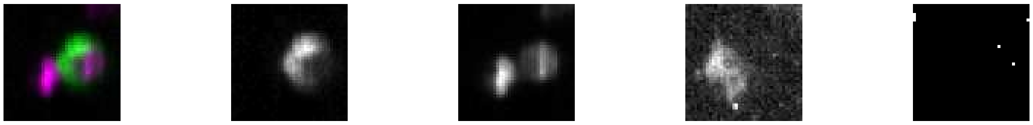				
12168	81	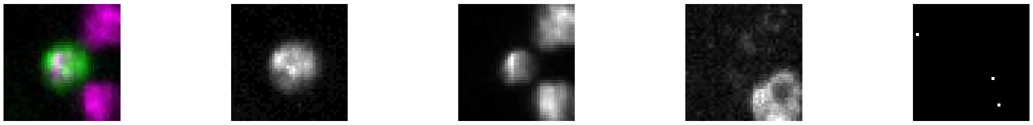				
12169	81	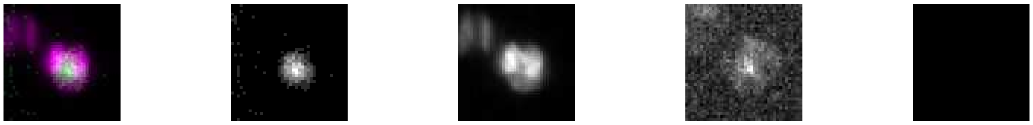				
12170	81	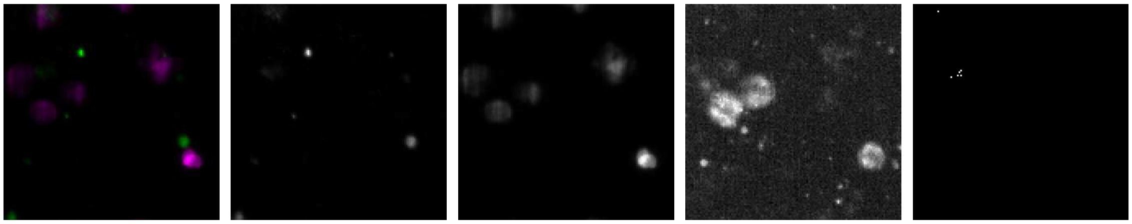				
12171	81	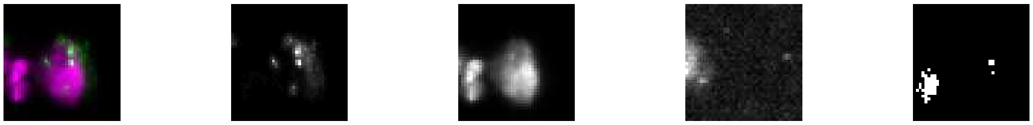				
12172	81	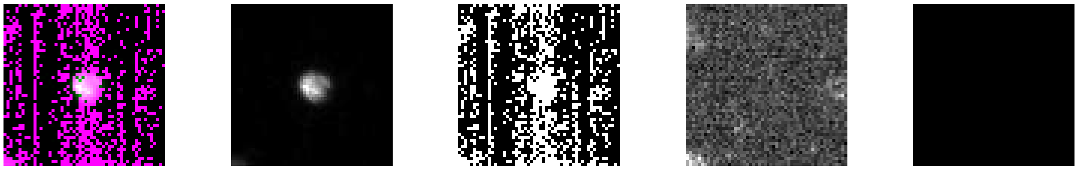				

Ereignis	Aufnahme	DAPI/CK-PE	CK-PE	DAPI	CD45-APC	HER-2/neu
12173	81	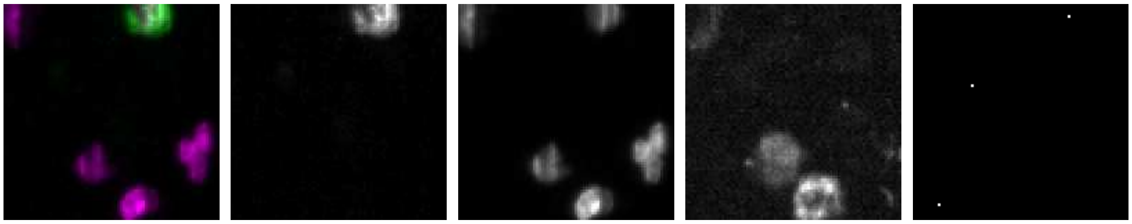				
12174	81	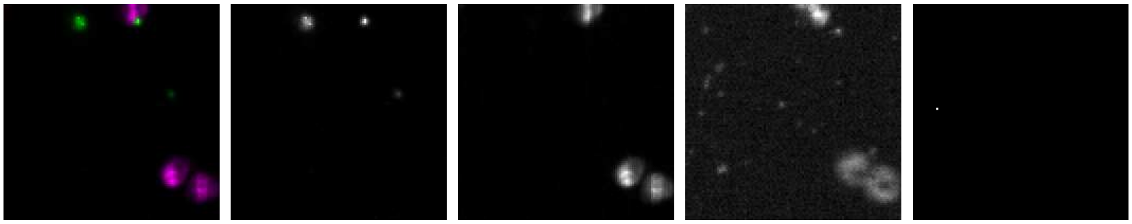				
12175	81	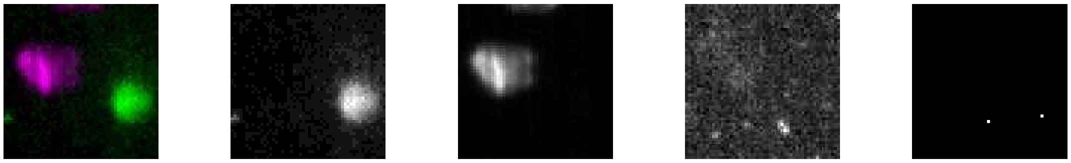				
12176	81	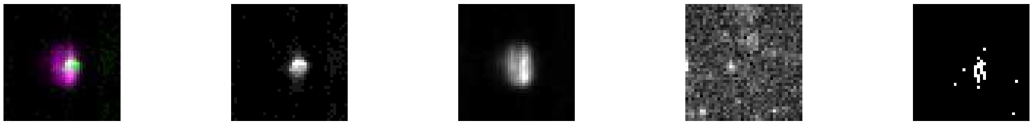				
12177	81	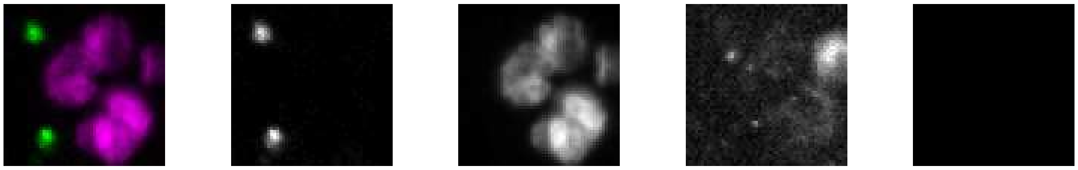				
12178	81	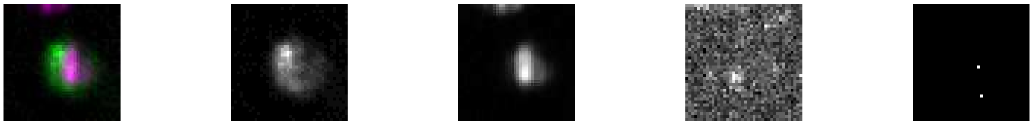				
12179	81	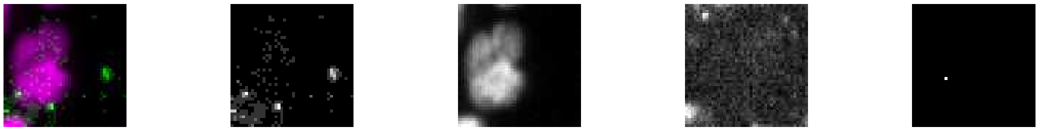				
12180	81	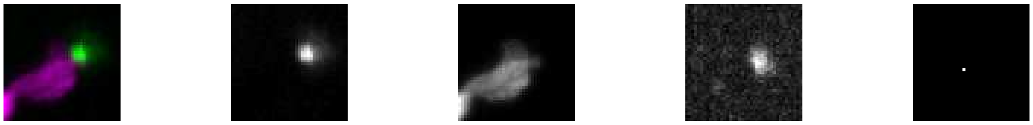				
12181	81	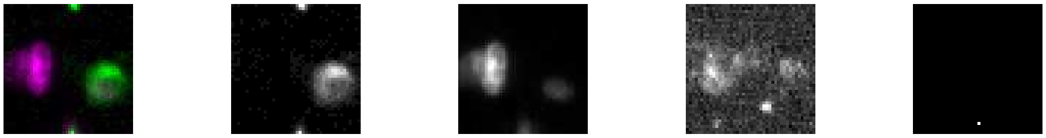				
12182	81	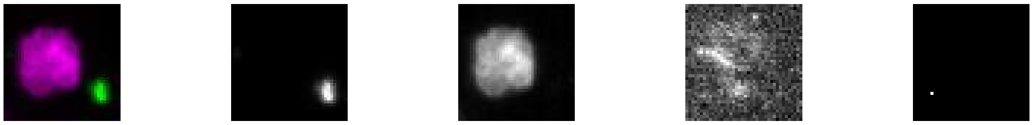				
12183	81	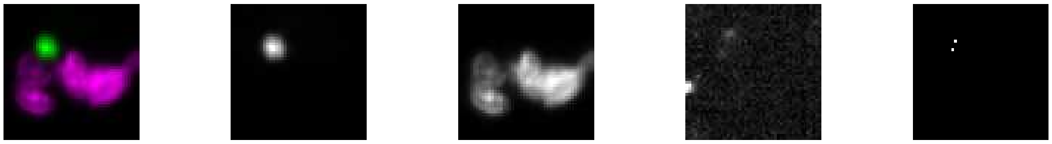				

Druckdatum: 02/06/2020 17:00

Proben-ID: Gyn14.208 **Kassetten-ID:** 01327106 **Patienten-ID:**

Ereignis	Aufnahme	DAPI/CK-PE	CK-PE	DAPI	CD45-APC	HER-2/neu
12184	81	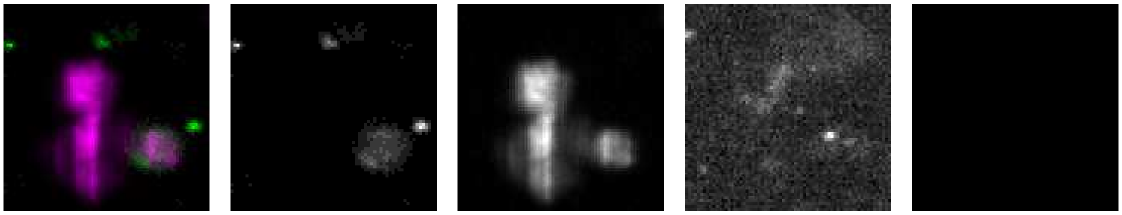				
12185	81	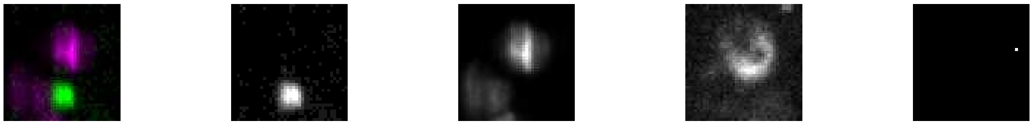				
12186	81	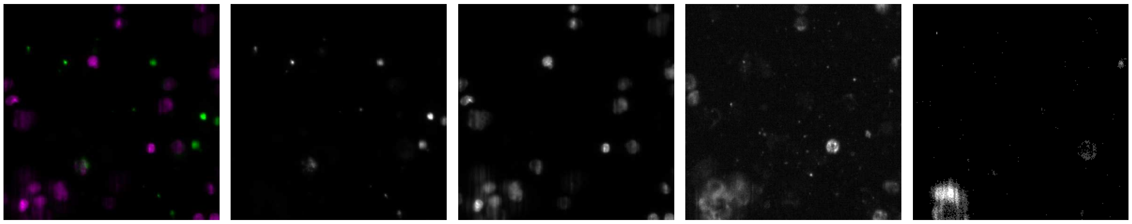				
12187	81	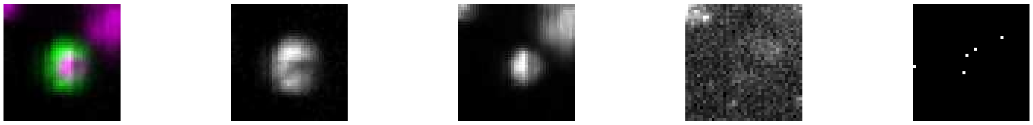				
12188	81	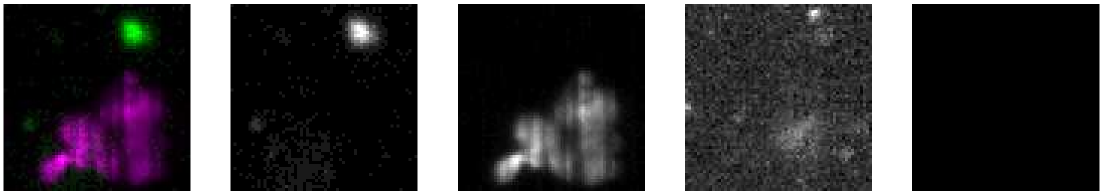				
12189	81	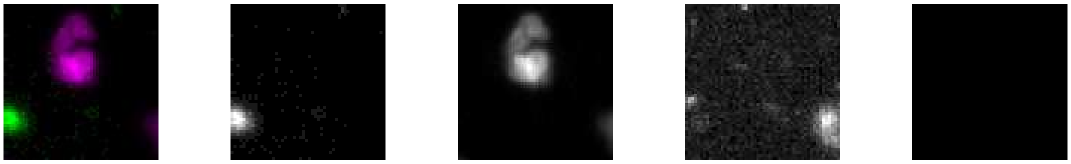				
12190	81	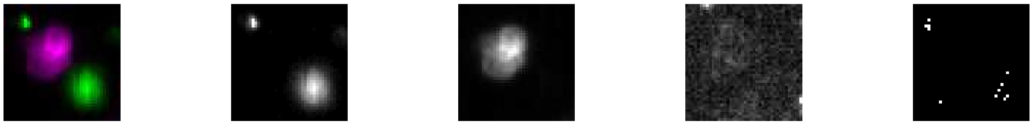				
12191	81	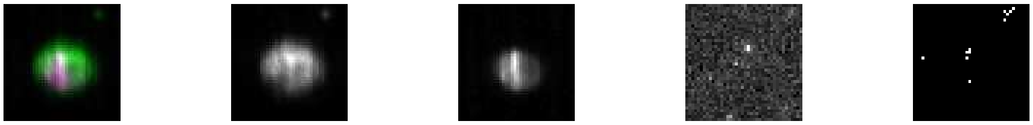				
12192	81	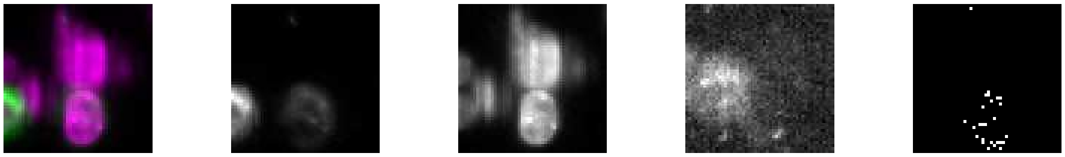				
12193	81	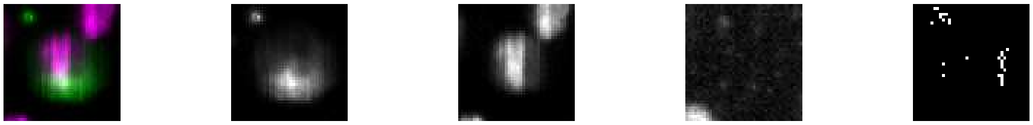				
12194	81	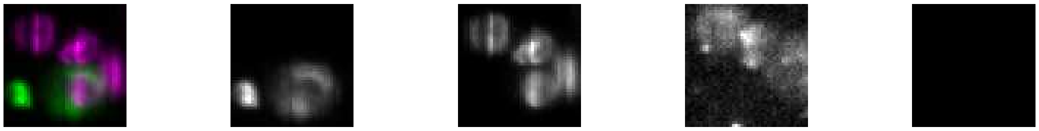				

Ereignis	Aufnahme	DAPI/CK-PE	CK-PE	DAPI	CD45-APC	HER-2/neu
12195	81	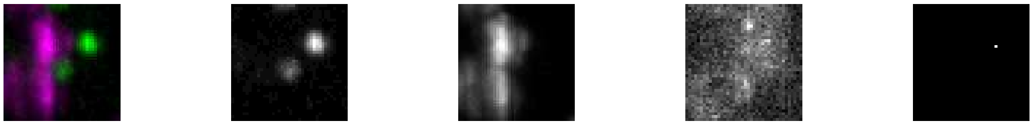				
12196	81	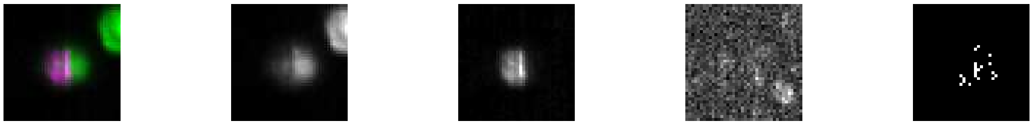				
12197	81	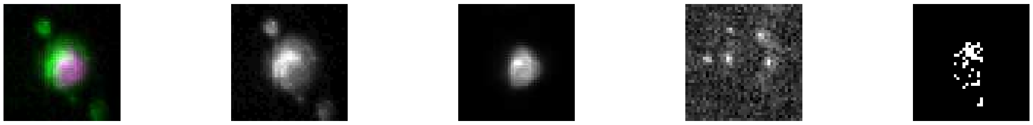				
12198	81	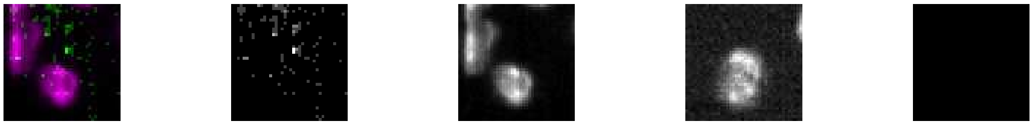				
12199	81	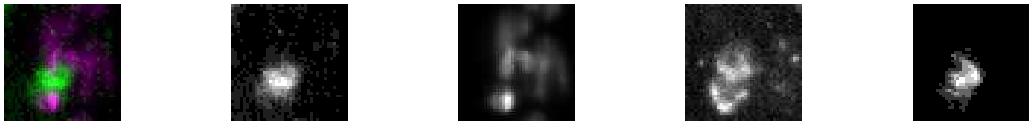				
12200	81	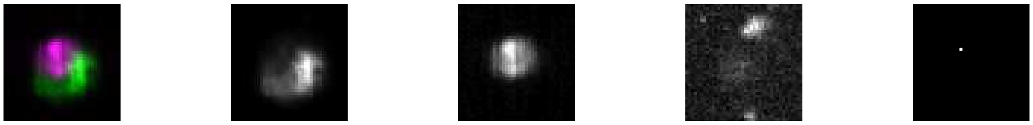				
12201	81	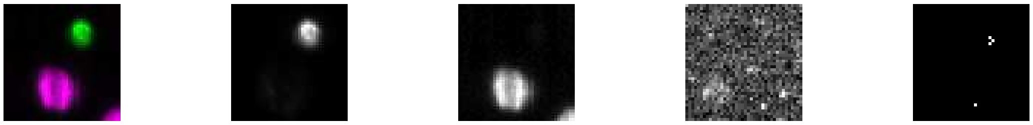				
12202	81	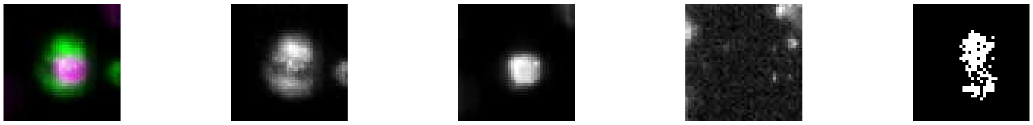				
12203	81	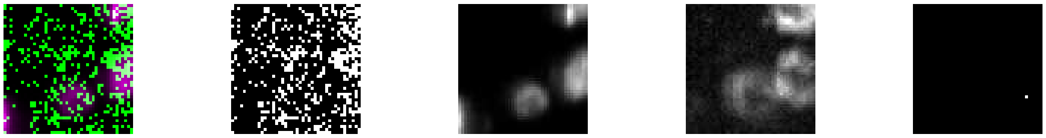				
12204	81	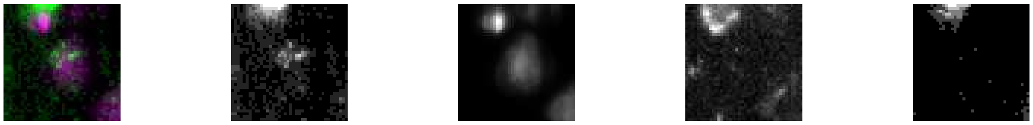				
12205	81	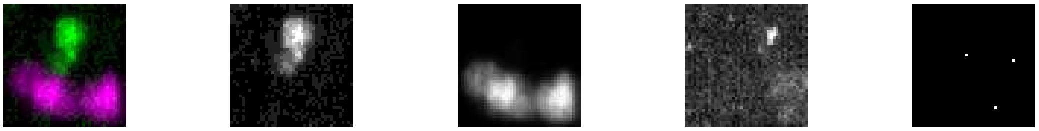				
12206	81	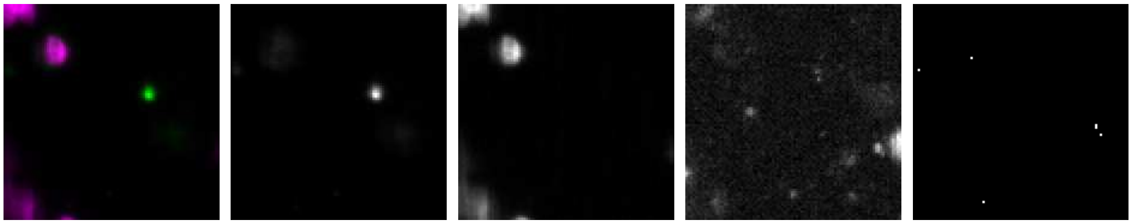				

Ereignis	Aufnahme	DAPI/CK-PE	CK-PE	DAPI	CD45-APC	HER-2/neu
12207	81	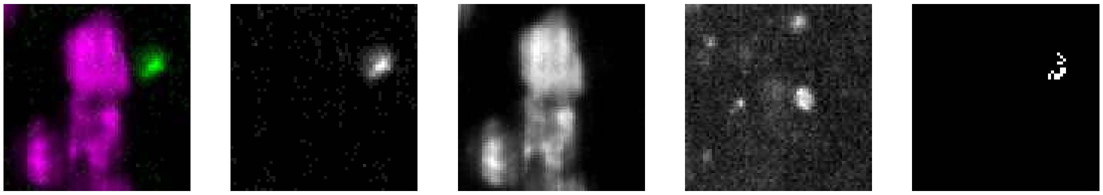				
12208	81	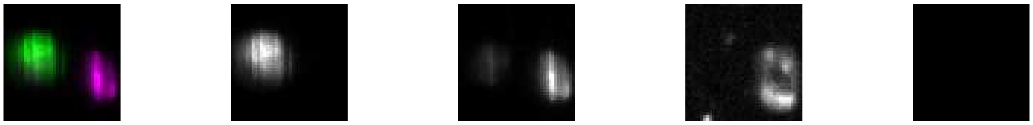				
12209	81	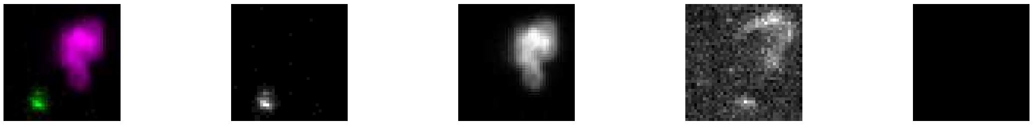				
12210	81	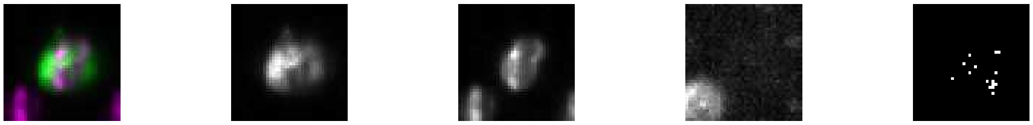				
12211	81	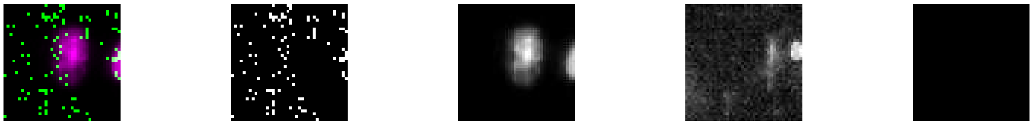				
12212	81	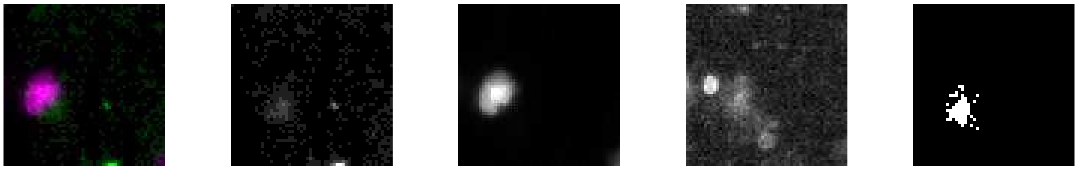				
12213	81	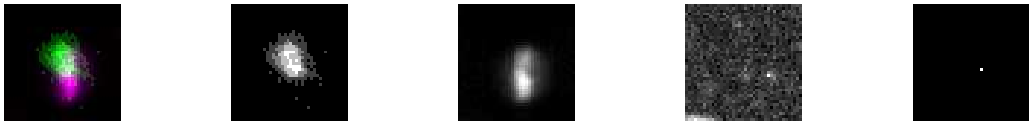				
12214	81	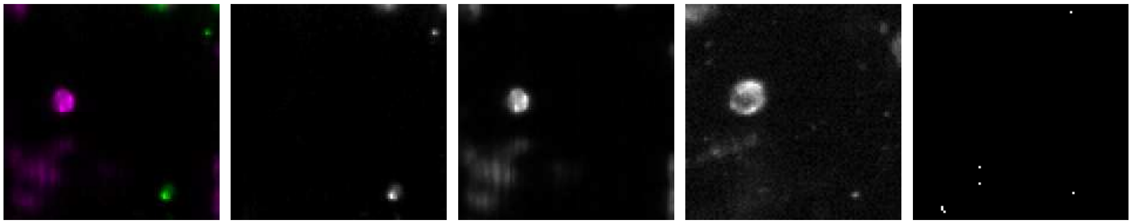				
12215	81	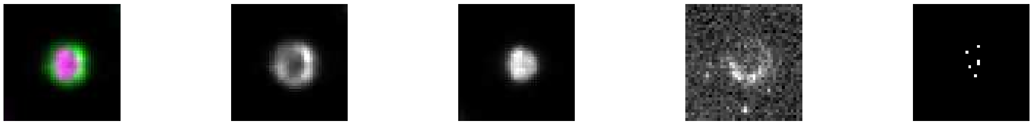				
12216	81	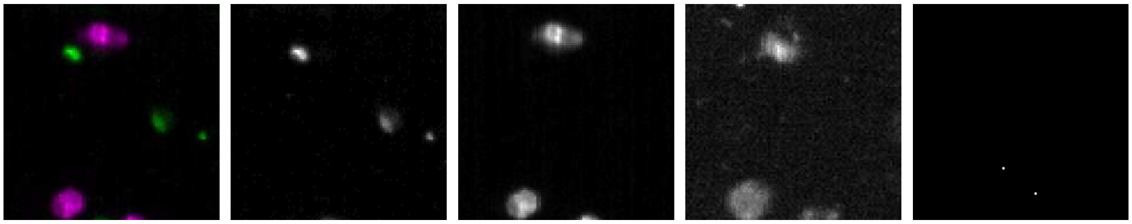				
12217	81	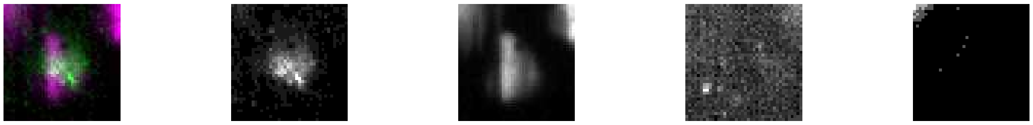				

Ereignis	Aufnahme	DAPI/CK-PE	CK-PE	DAPI	CD45-APC	HER-2/neu
12218	81	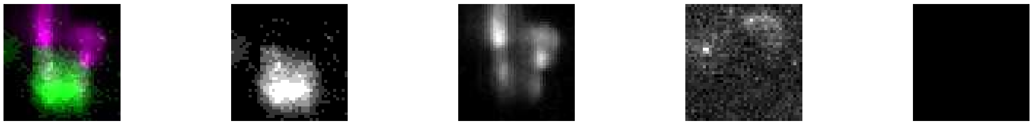				
12219	81	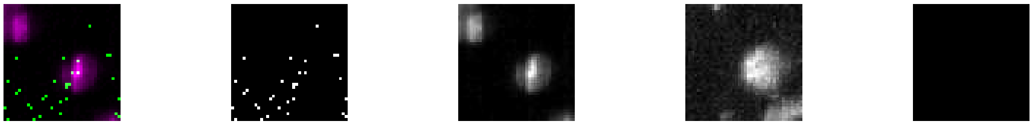				
12220	81	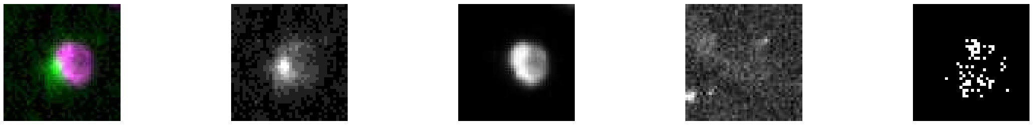				
12221	81	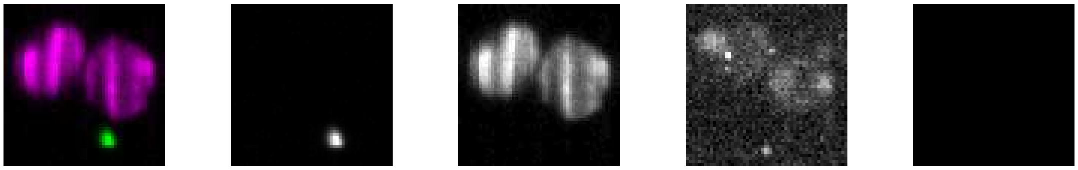				
12222	81	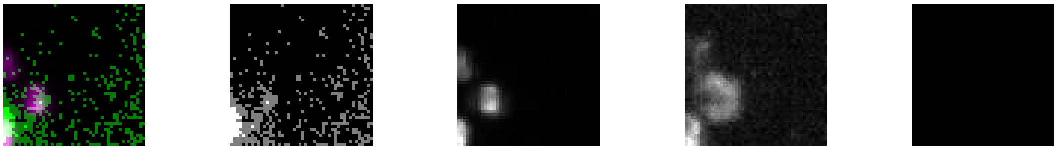				
12223	81	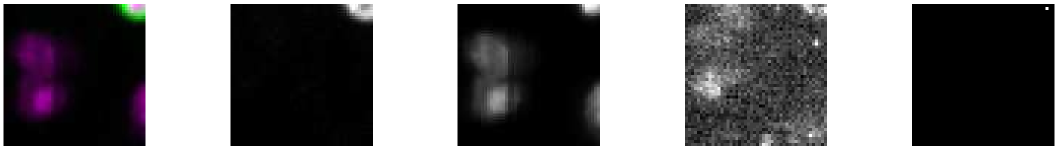				
12224	81	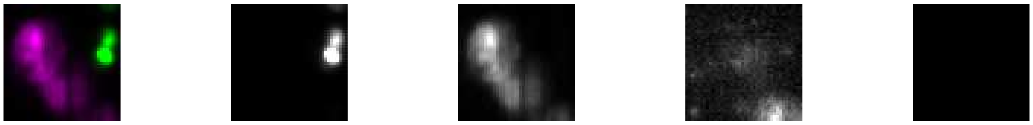				
12225	81	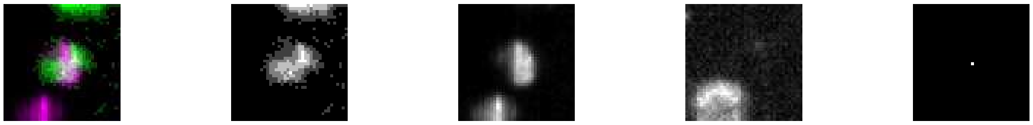				
12226	81	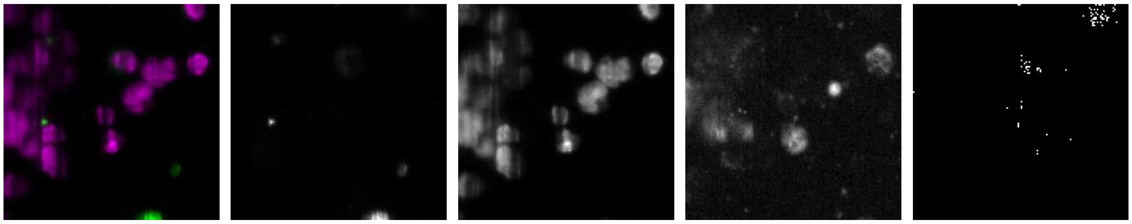				
12227	81	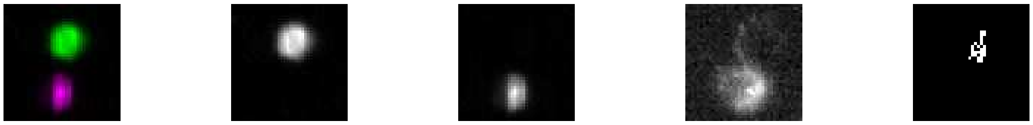				
12228	81	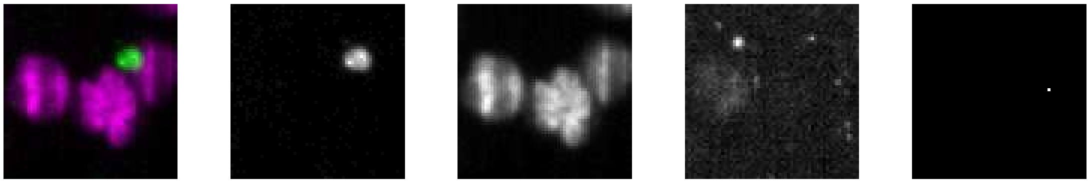				
12229	81	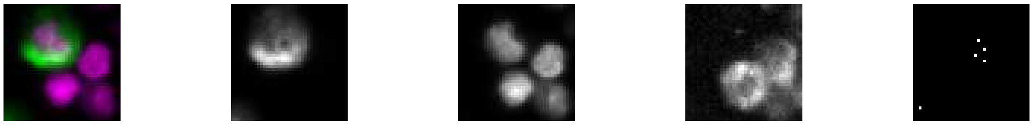				

Ereignis	Aufnahme	DAPI/CK-PE	CK-PE	DAPI	CD45-APC	HER-2/neu
12230	81	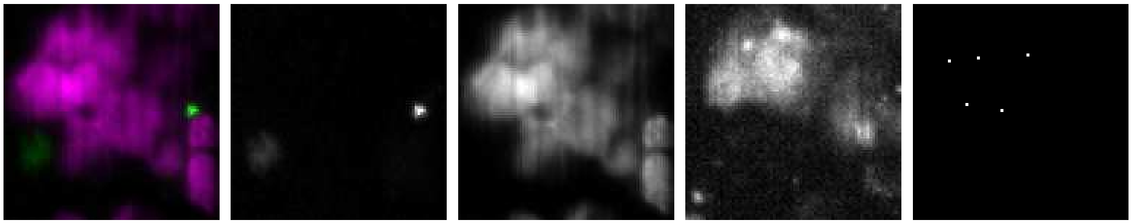				
12231	81	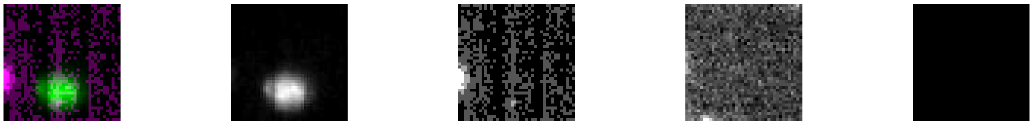				
12232	81	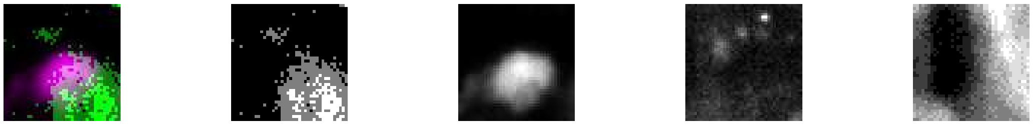				
12233	81	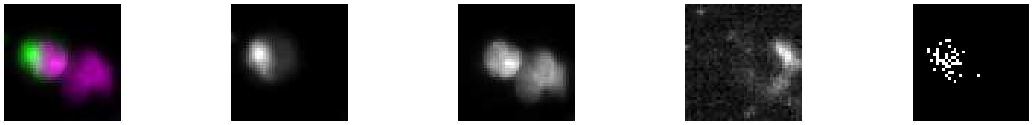				
12234	81	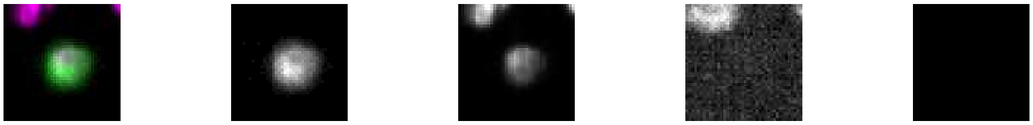				
12235	80	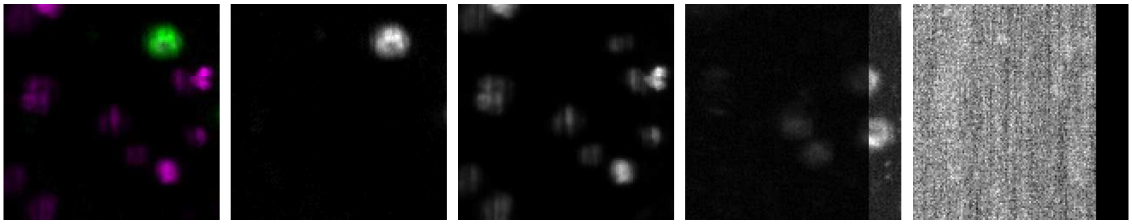				
12236	60	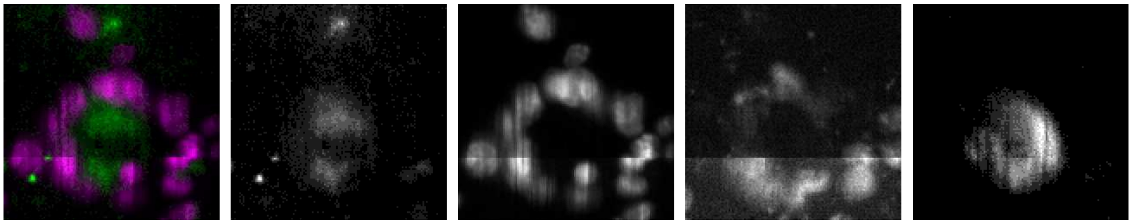				
12237	80	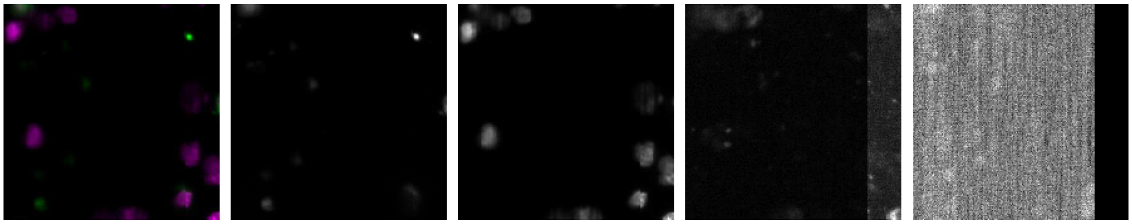				
12238	81	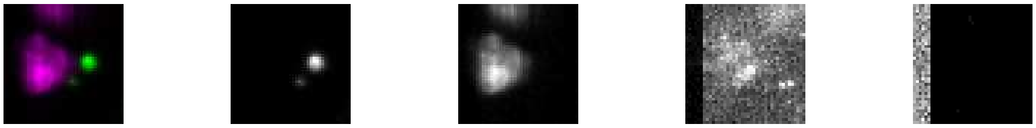				
12239	81	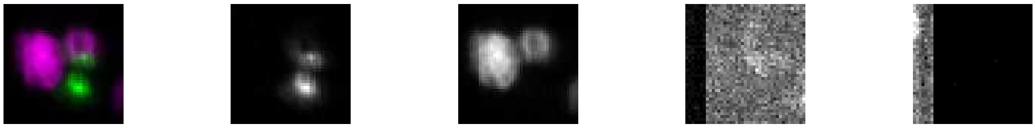				

Ereignis	Aufnahme	DAPI/CK-PE	CK-PE	DAPI	CD45-APC	HER-2/neu
12240	80	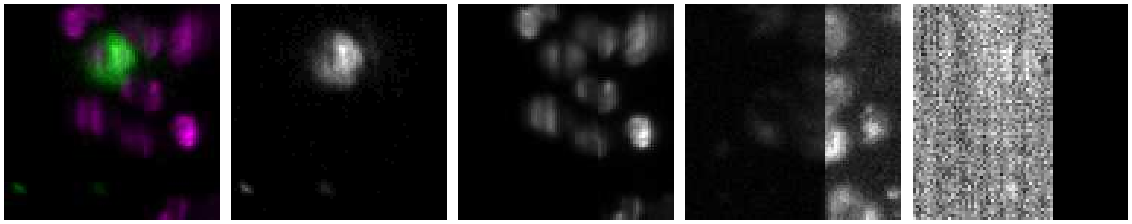				
12241	81	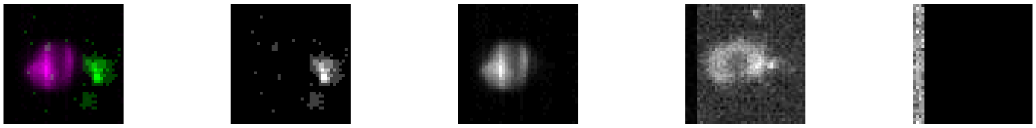				
12242	81	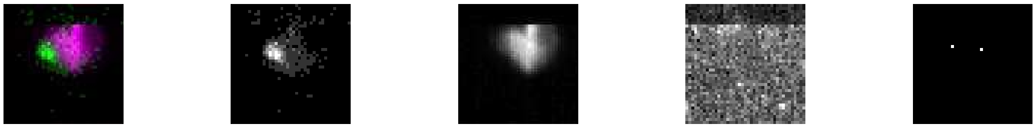				
12243	80	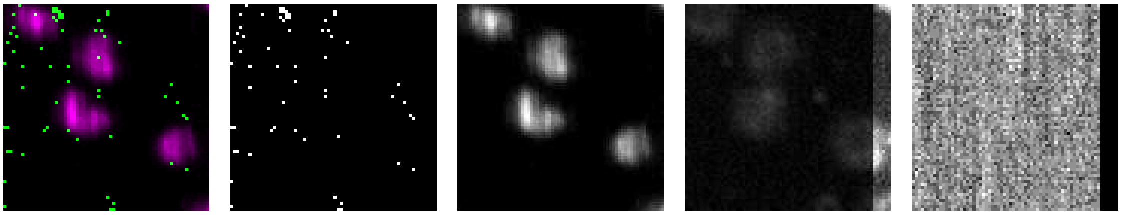				
12244	60	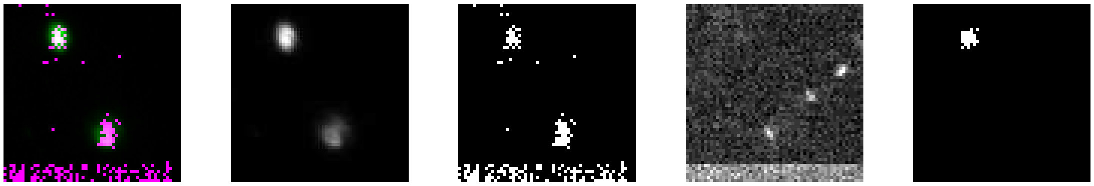				
12245	81	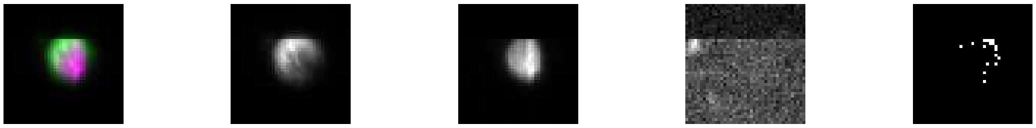				
12246	60	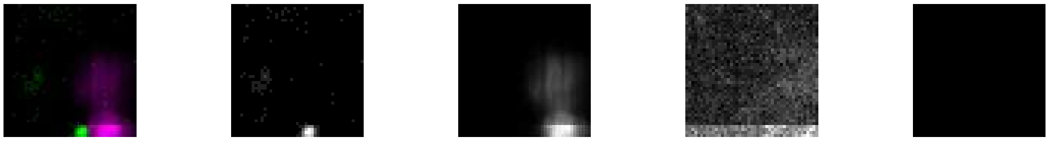				
12247	60	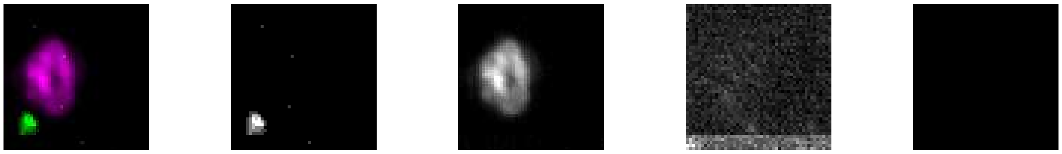				
12248	60	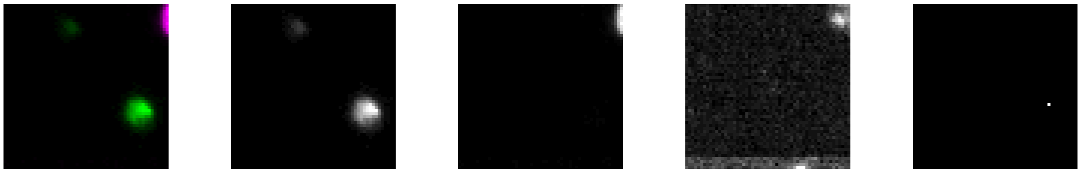				
12249	60	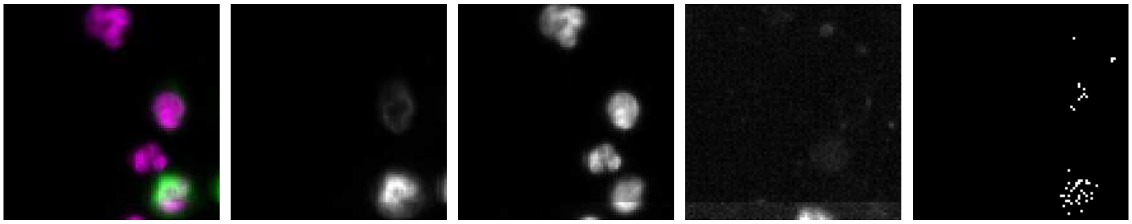				

Ereignis	Aufnahme	DAPI/CK-PE	CK-PE	DAPI	CD45-APC	HER-2/neu
12250	82	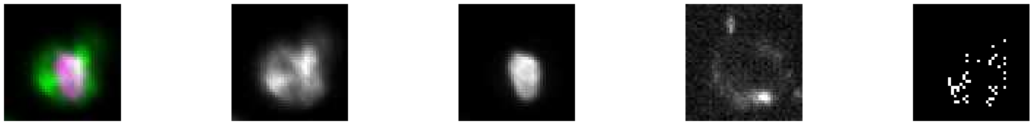				
12251	82	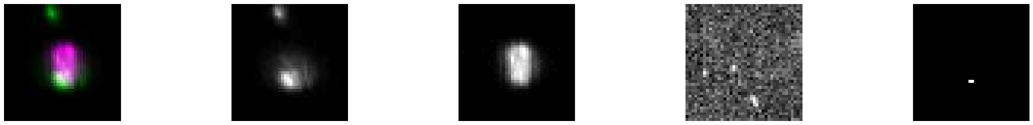				
12252	82	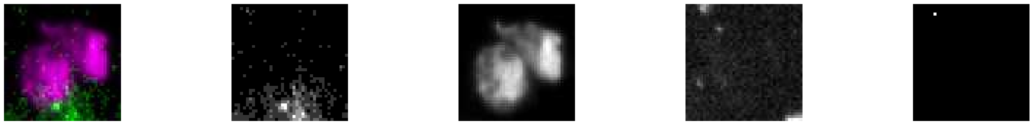				
12253	82	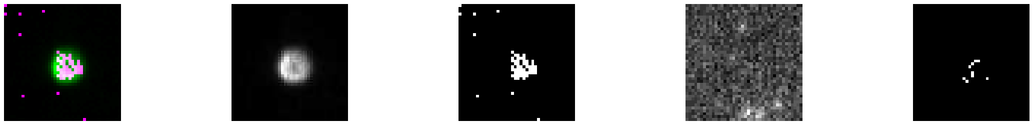				
12254	82	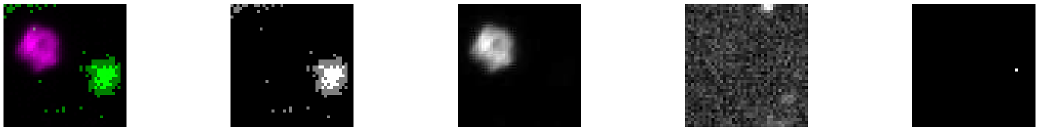				
12255	82	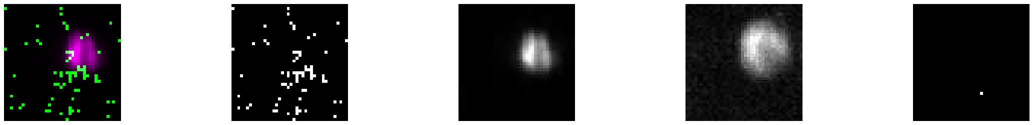				
12256	82	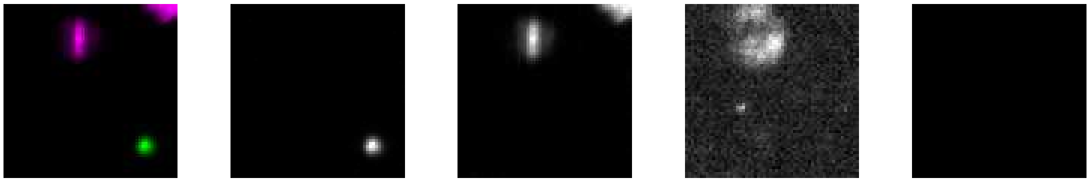				
12257	82	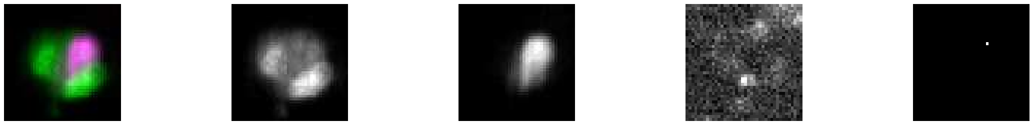				
12258	82	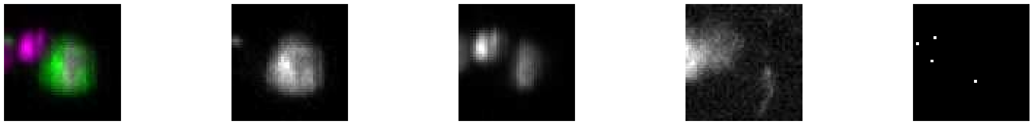				
12259	82	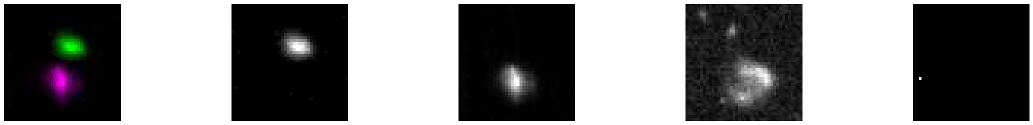				
12260	82	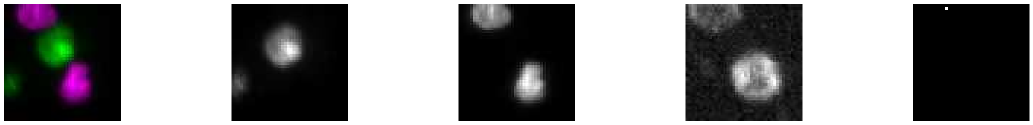				
12261	82	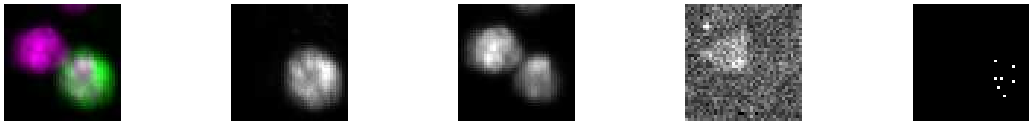				
12262	82	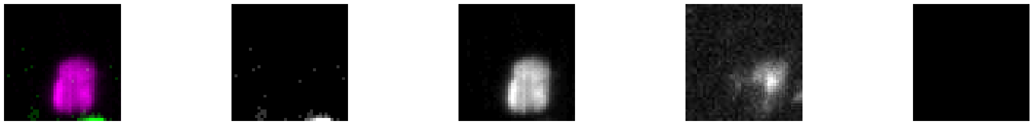				

Proben-ID: Gyn14.208 **Kassetten-ID:**01327106 **Patienten-ID:**

Ereignis	Aufnahme	DAPI/CK-PE	CK-PE	DAPI	CD45-APC	HER-2/neu
12263	82	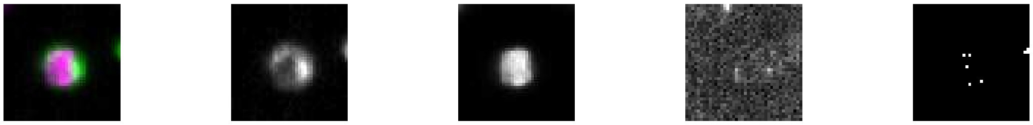				
12264	82	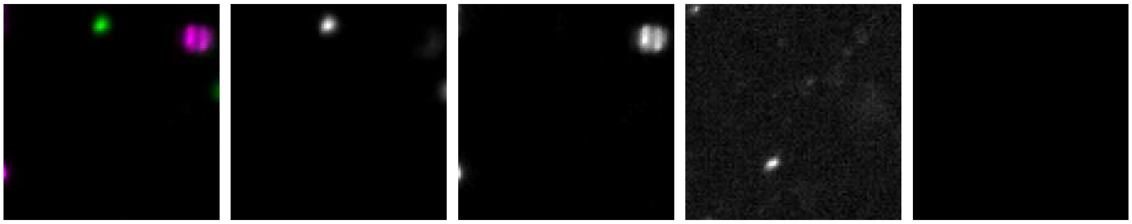				
12265	82	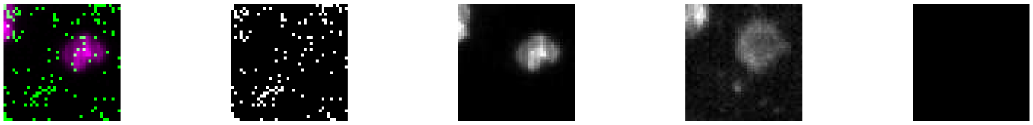				
12266	82	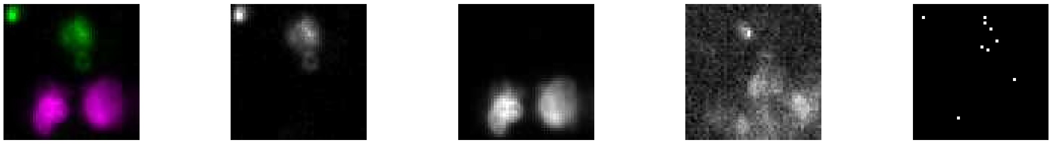				
12267	82	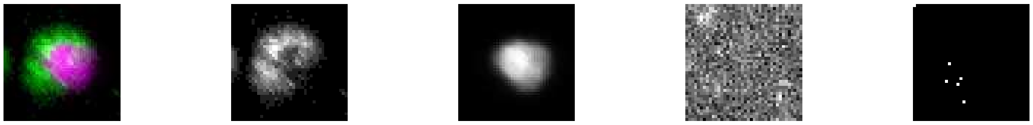				
12268	82	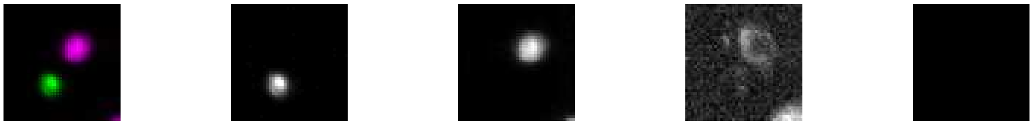				
12269	82	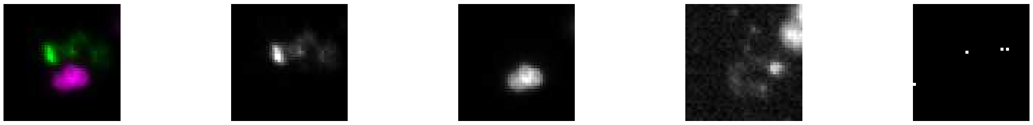				
12270	82	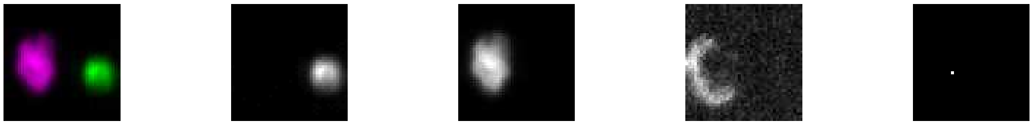				
12271	82	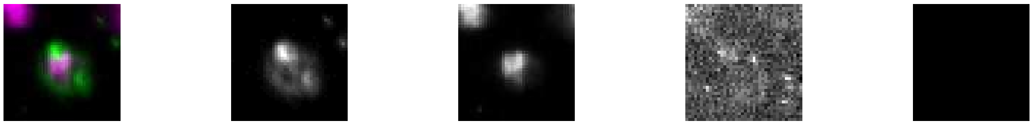				
12272	82	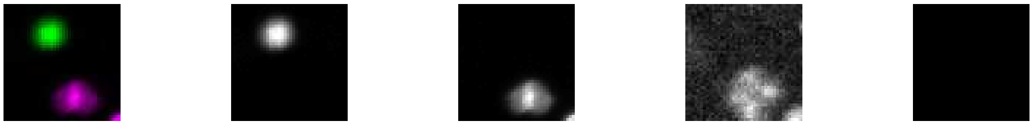				
12273	82	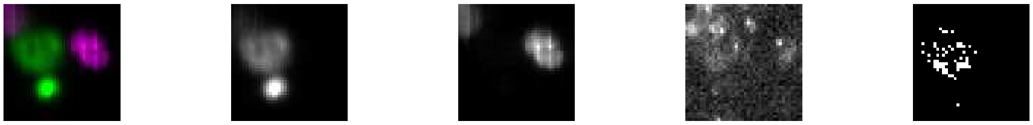				
12274	82	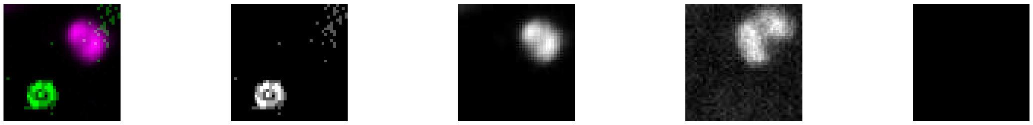				
12275	82	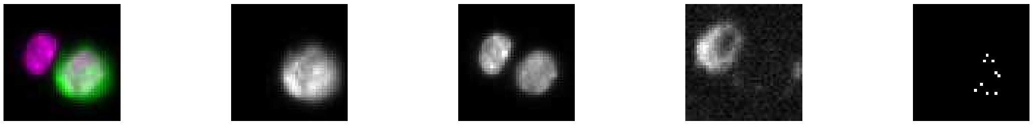				

Ereignis	Aufnahme	DAPI/CK-PE	CK-PE	DAPI	CD45-APC	HER-2/neu
12276	82	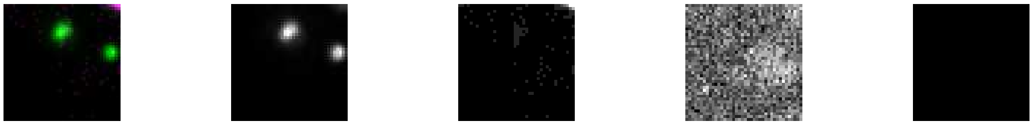				
12277	82	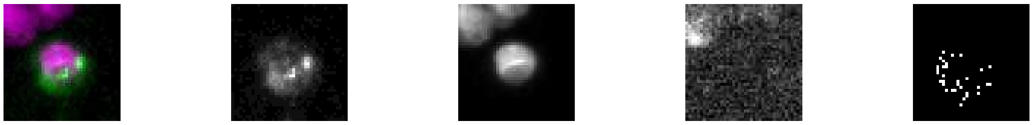				
12278	82	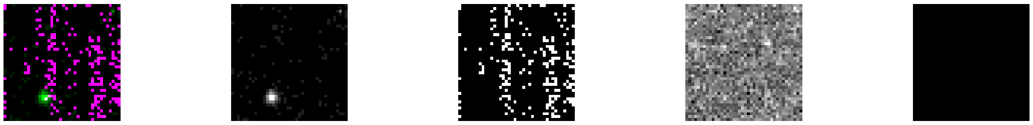				
12279	82	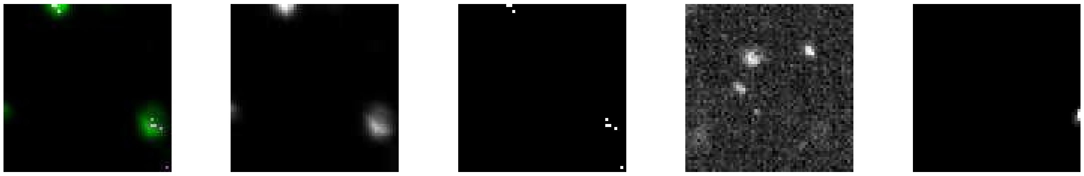				
12280	82	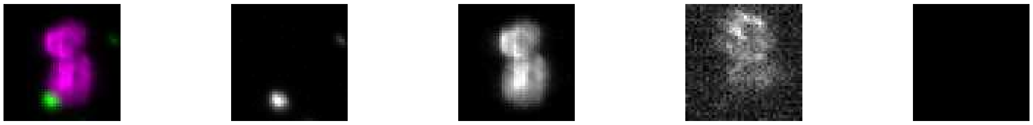				
12281	82	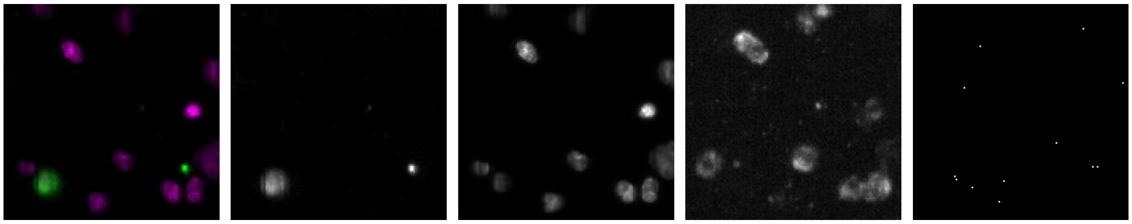				
12282	82	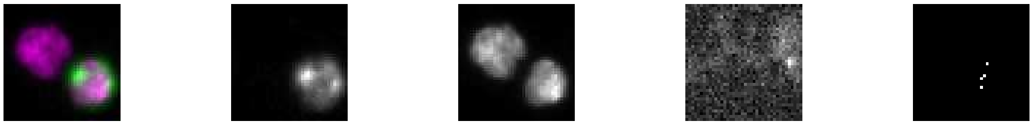				
12283	82	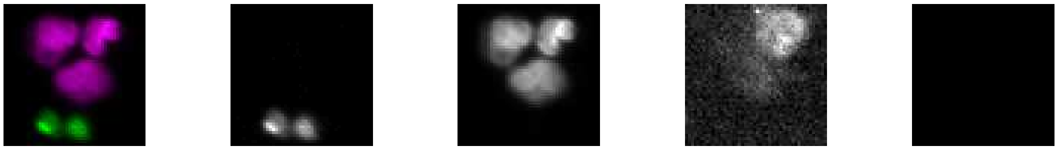				
12284	82	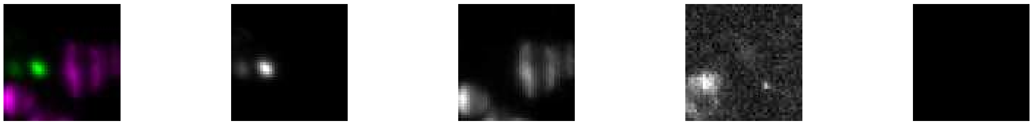				
12285	82	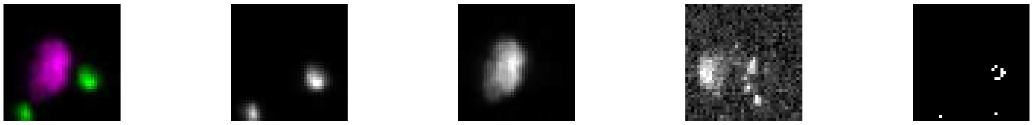				
12286	82	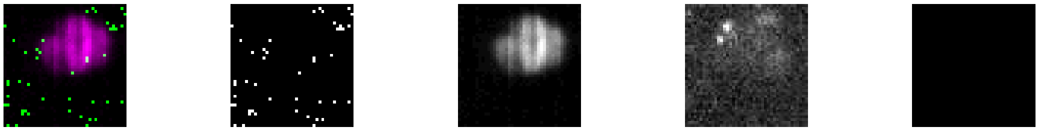				
12287	82	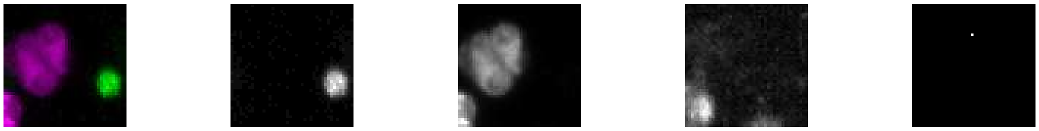				

Ereignis	Aufnahme	DAPI/CK-PE	CK-PE	DAPI	CD45-APC	HER-2/neu
12288	82	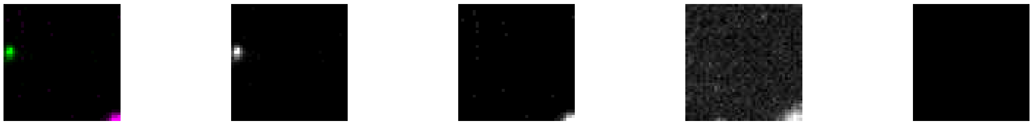				
12289	82	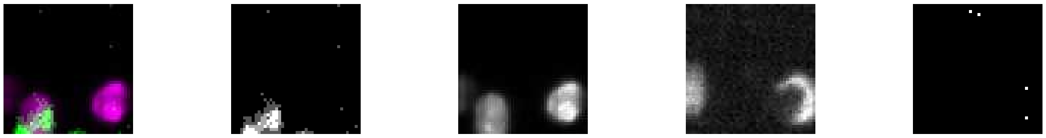				
12290	82	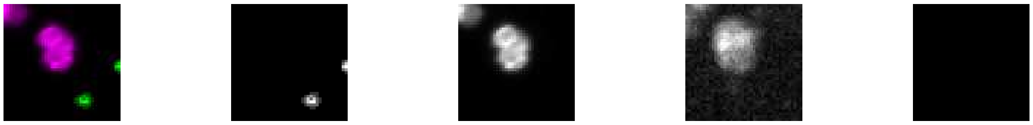				
12291	82	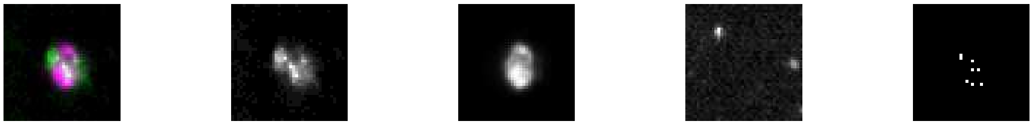				
12292	82	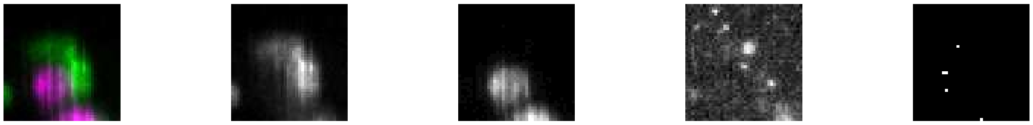				
12293	82	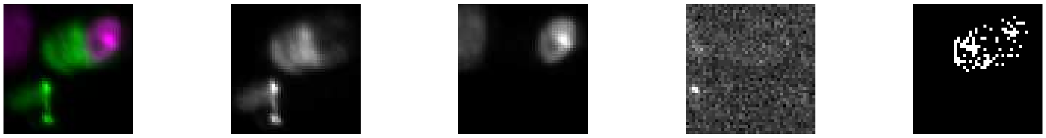				
12294	82	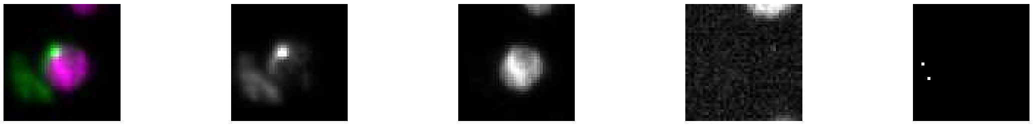				
12295	82	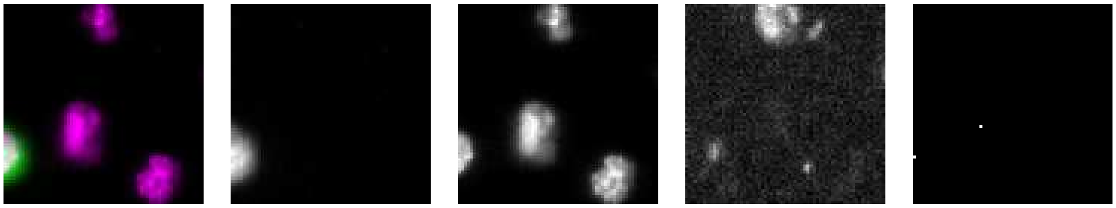				
12296	82	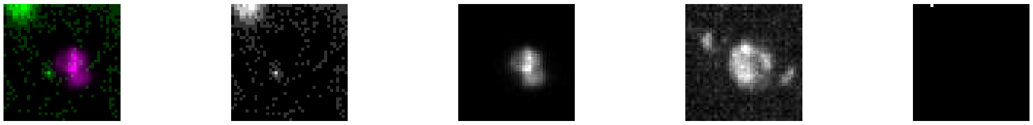				
12297	82	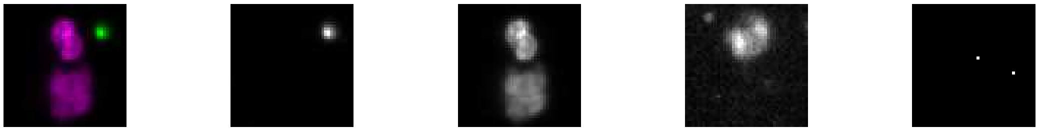				
12298	82	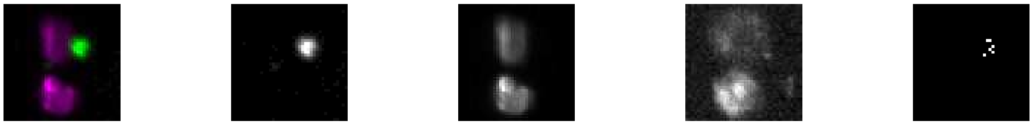				
12299	82	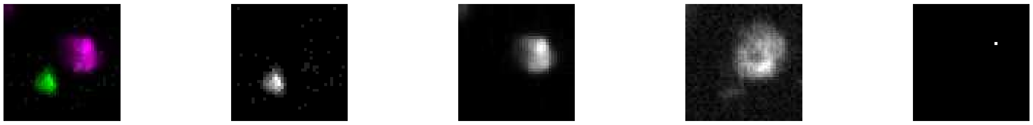				

Ereignis	Aufnahme	DAPI/CK-PE	CK-PE	DAPI	CD45-APC	HER-2/neu
12300	82	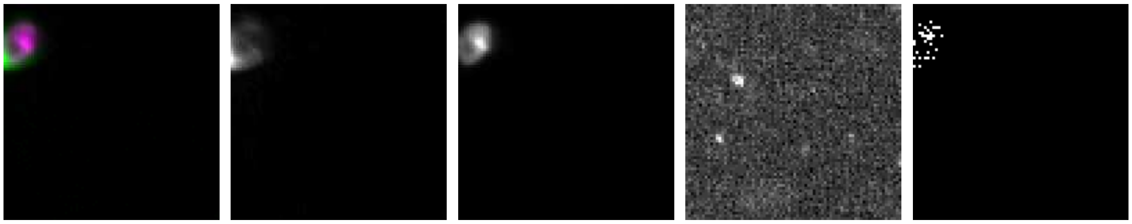				
12301	82	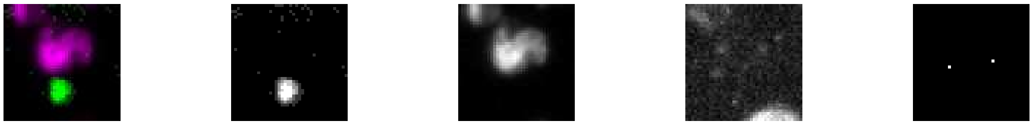				
12302	82	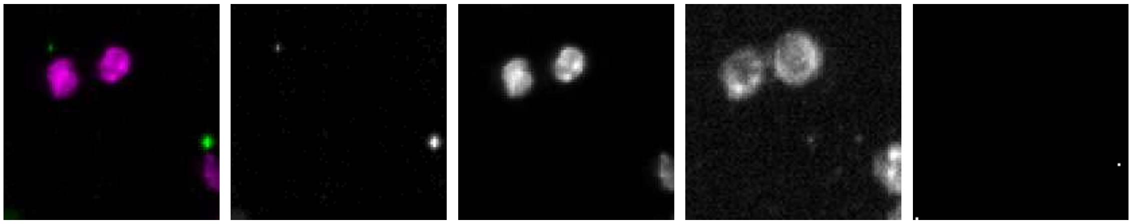				
12303	82	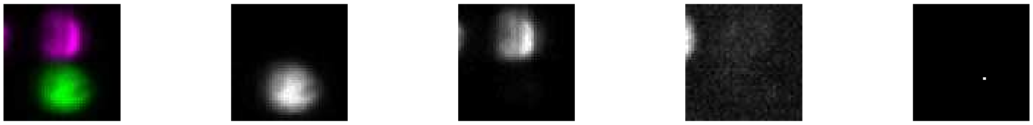				
12304	82	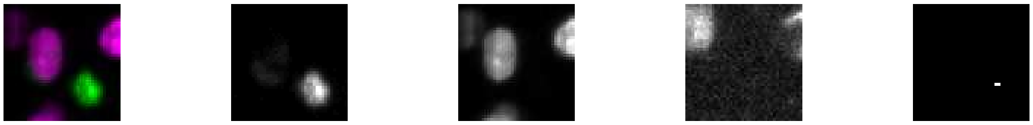				
12305	82	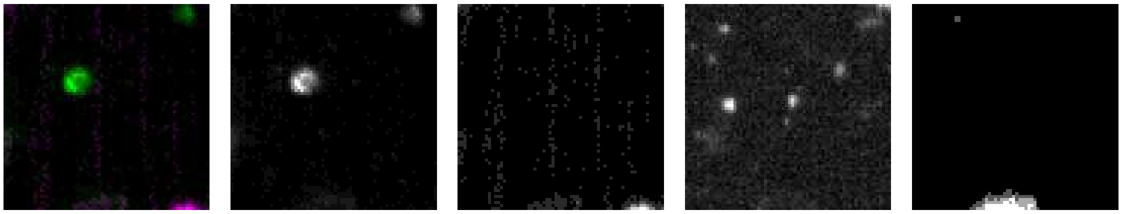				
12306	82	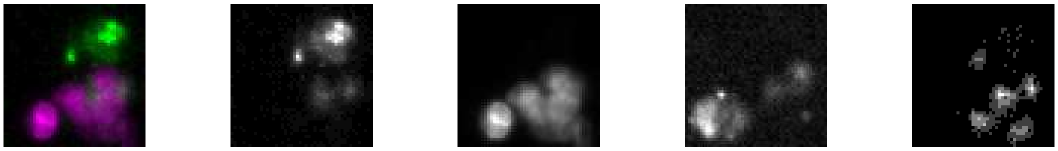				
12307	82	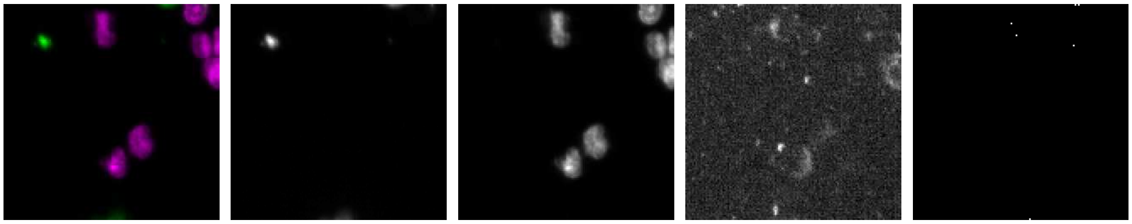				
12308	82	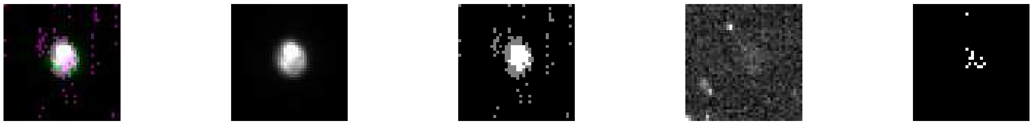				
12309	82	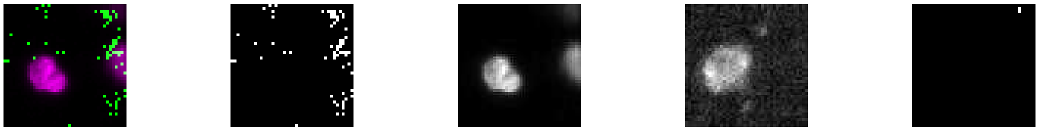				
12310	82	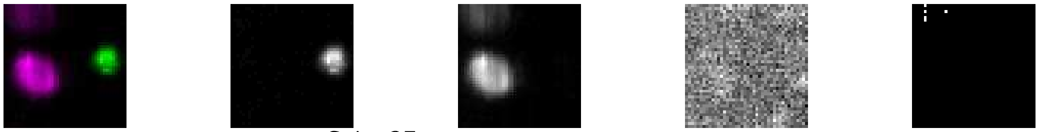				

Ereignis	Aufnahme	DAPI/CK-PE	CK-PE	DAPI	CD45-APC	HER-2/neu
12311	82	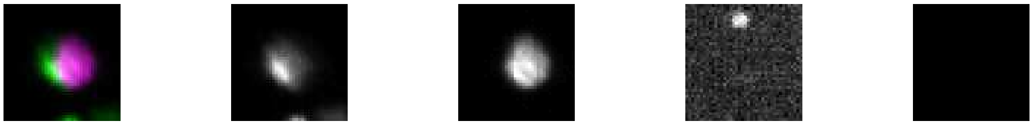				
12312	82	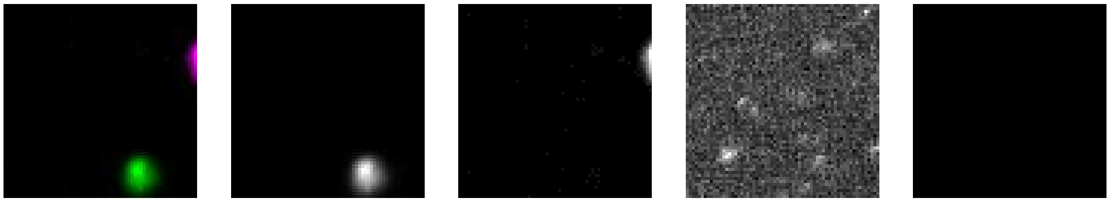				
12313	82	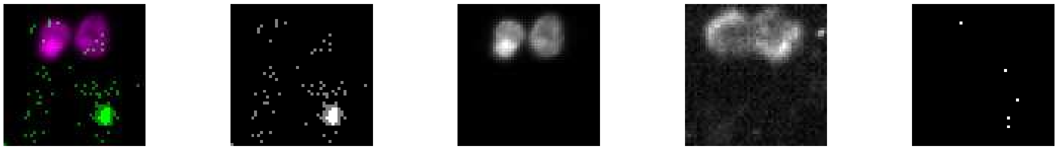				
12314	82	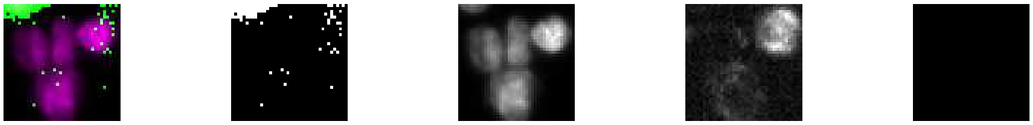				
12315	82	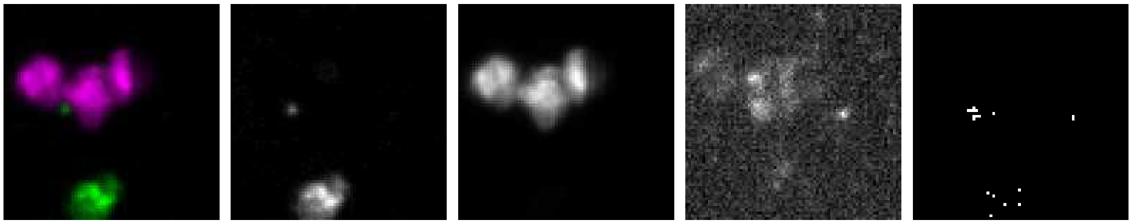				
12316	82	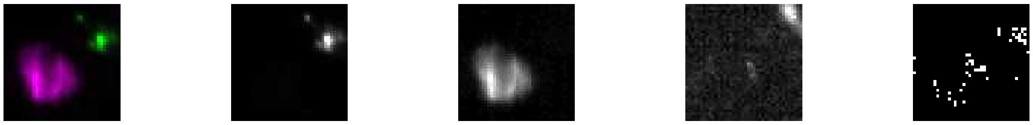				
12317	82	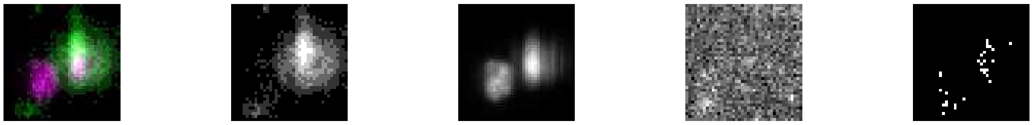				
12318	82	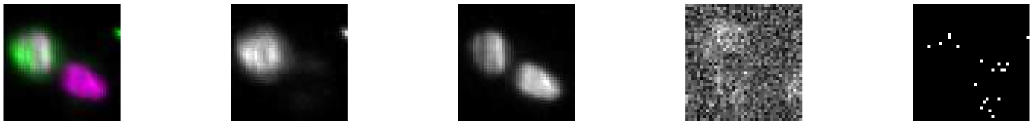				
12319	82	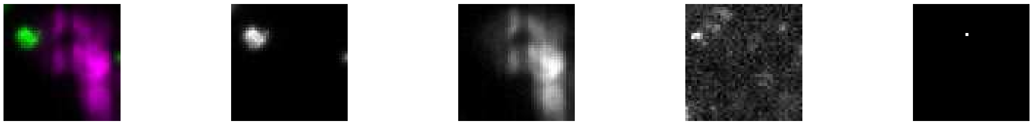				
12320	82	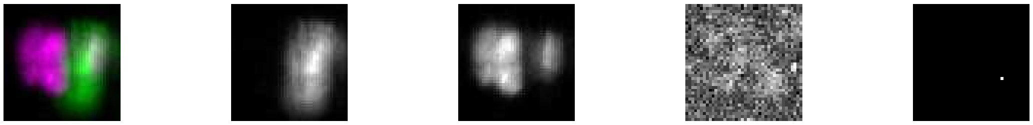				
12321	82	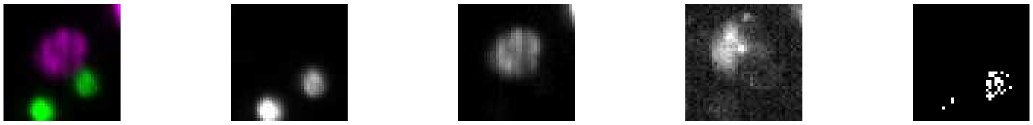				
12322	82	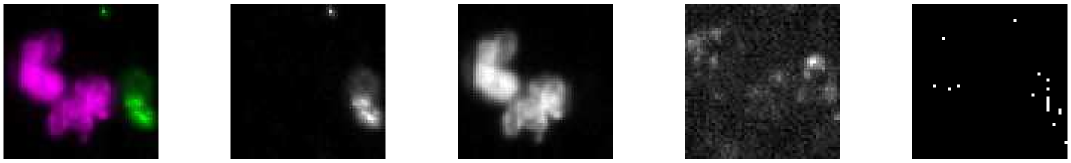				

Ereignis	Aufnahme	DAPI/CK-PE	CK-PE	DAPI	CD45-APC	HER-2/neu
12323	82	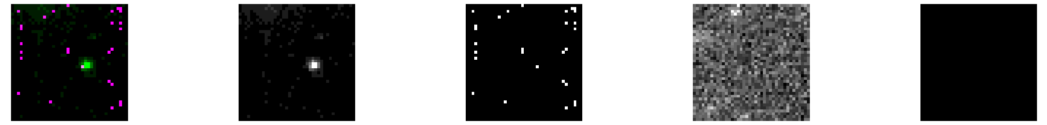				
12324	82	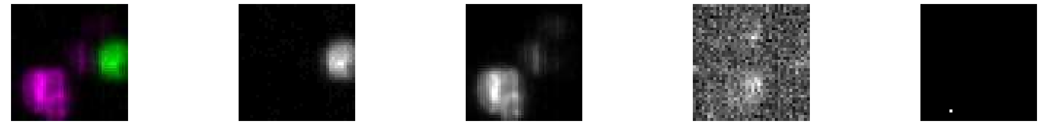				
12325	82	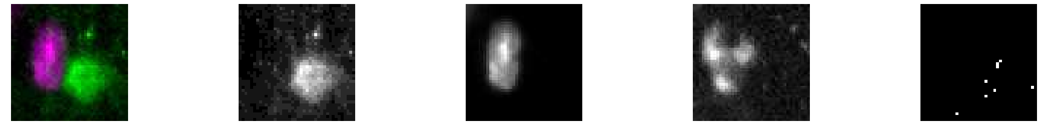				
12326	82	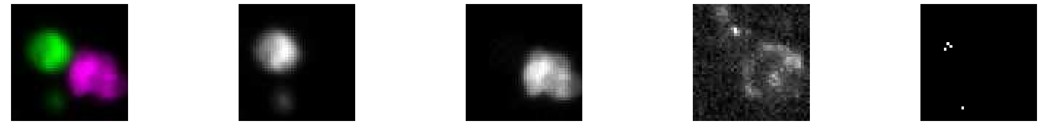				
12327	82	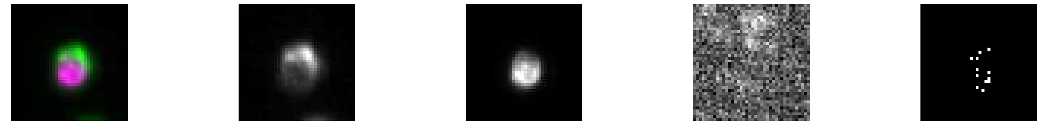				
12328	82	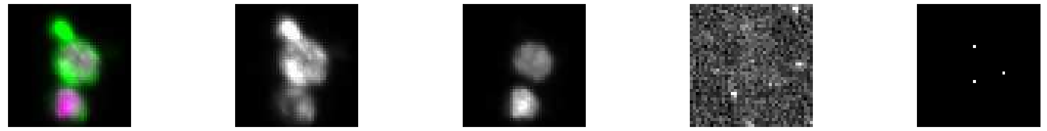				
12329	82	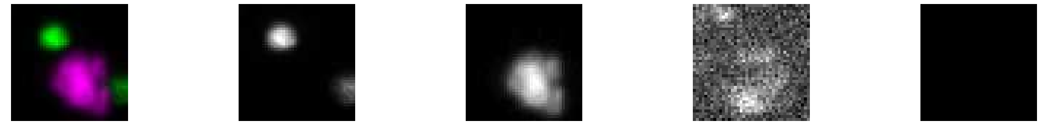				
12330	82	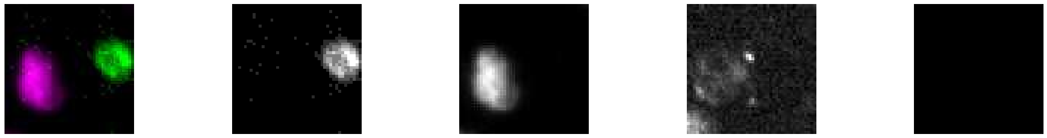				
12331	82	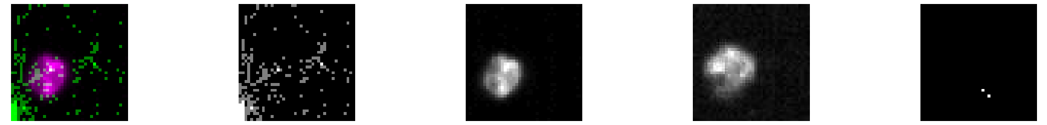				
12332	82	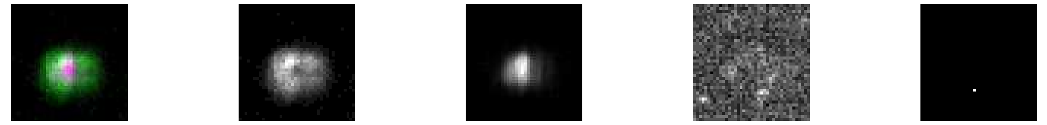				
12333	82	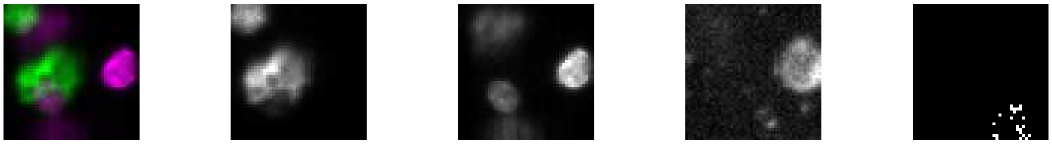				
12334	82	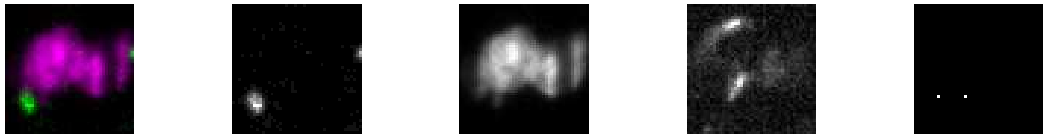				
12335	82	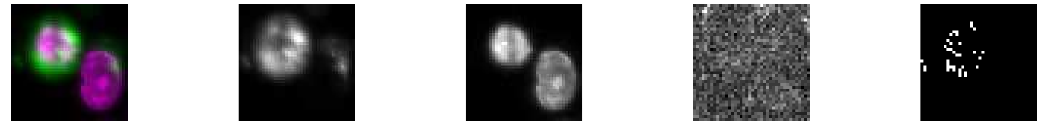				

Druckdatum: 02/06/2020 17:00

Proben-ID: Gyn14.208 **Kassetten-ID:** 01327106 **Patienten-ID:**

Ereignis	Aufnahme	DAPI/CK-PE	CK-PE	DAPI	CD45-APC	HER-2/neu
12336	82	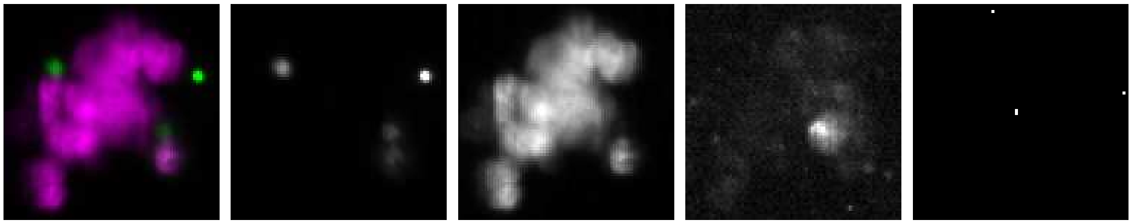				
12337	82	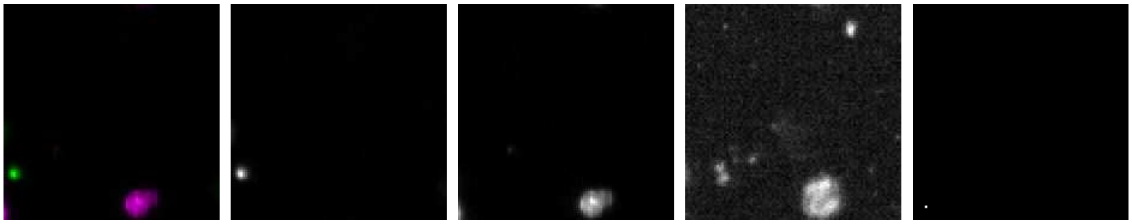				
12338	82	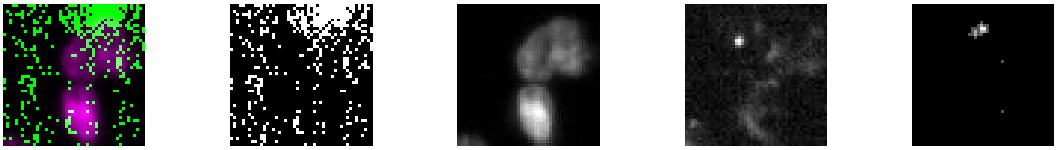				
12339	82	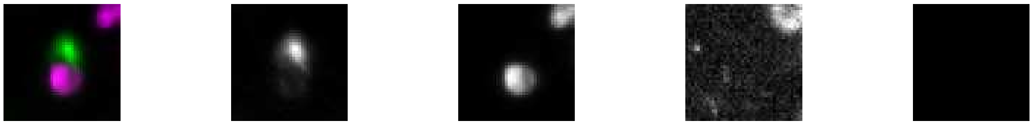				
12340	82	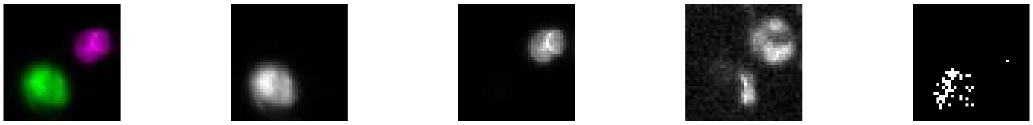				
12341	82	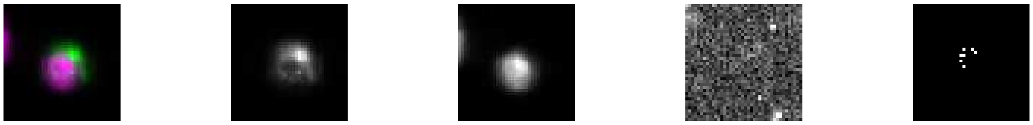				
12342	82	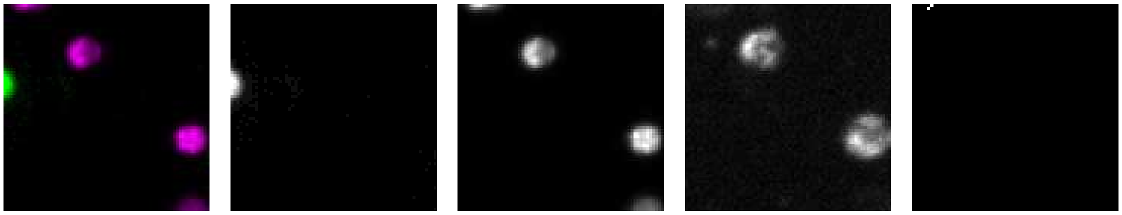				
12343	82	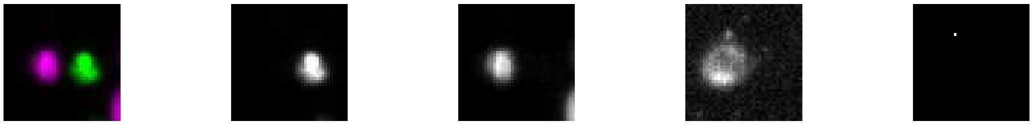				
12344	82	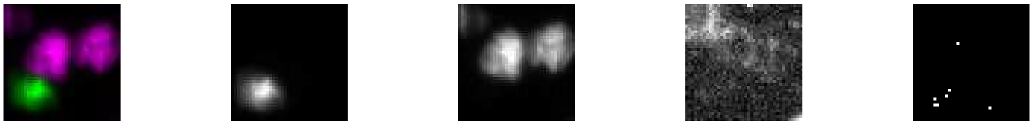				
12345	82	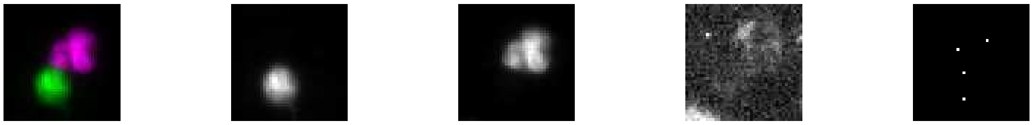				
12346	82	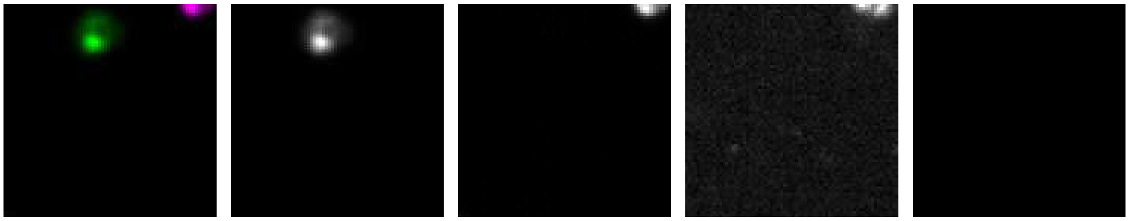				

Ereignis	Aufnahme	DAPI/CK-PE	CK-PE	DAPI	CD45-APC	HER-2/neu
12347	82	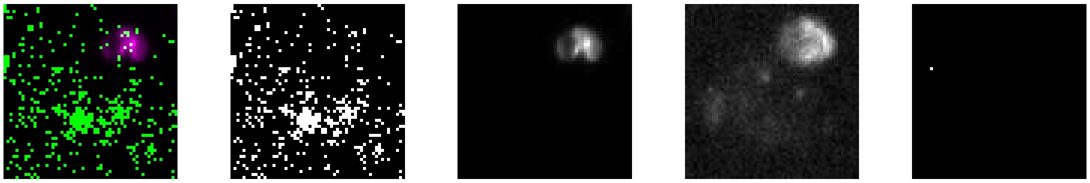				
12348	82	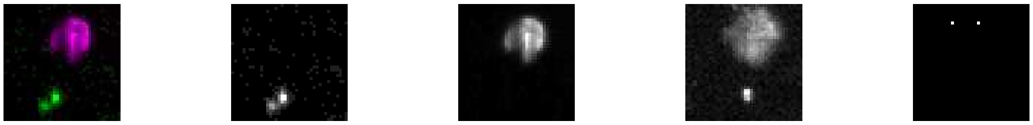				
12349	82	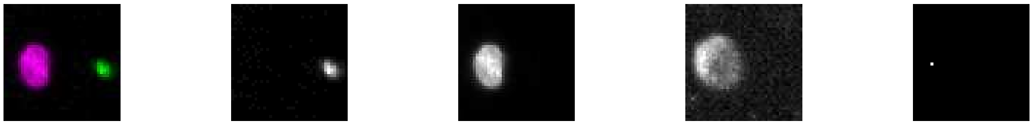				
12350	82	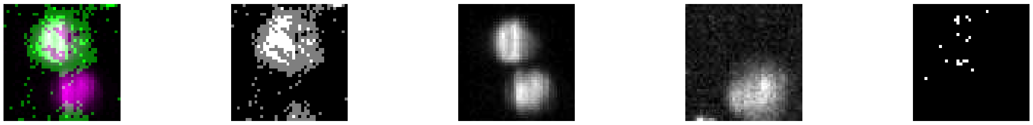				
12351	82	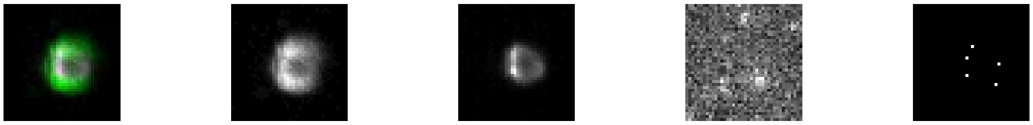				
12352	82	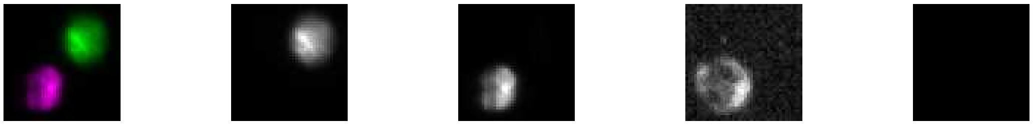				
12353	82	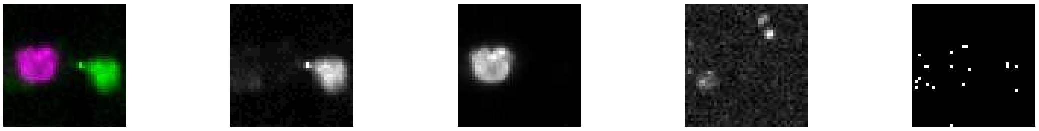				
12354	82	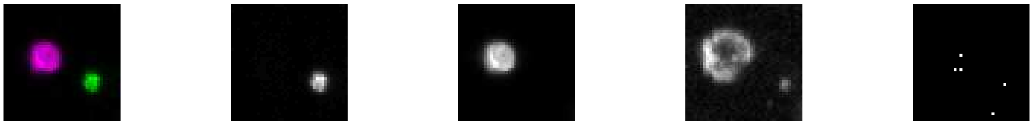				
12355	82	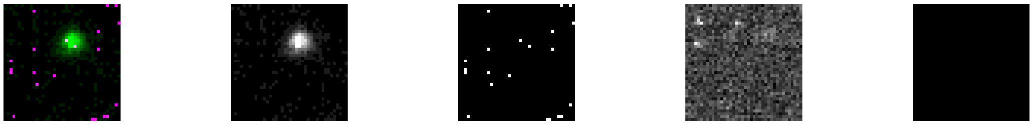				
12356	82	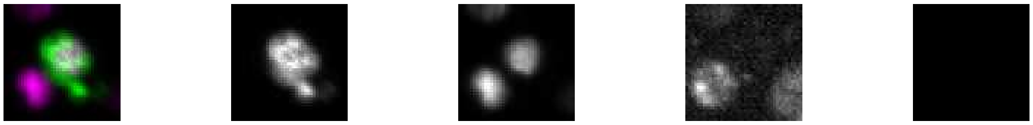				
12357	82	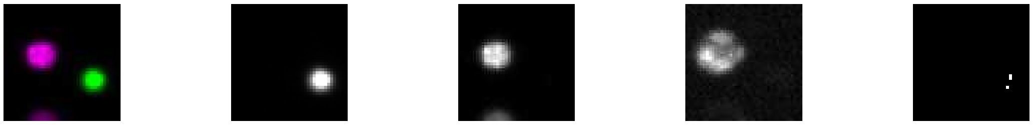				
12358	82	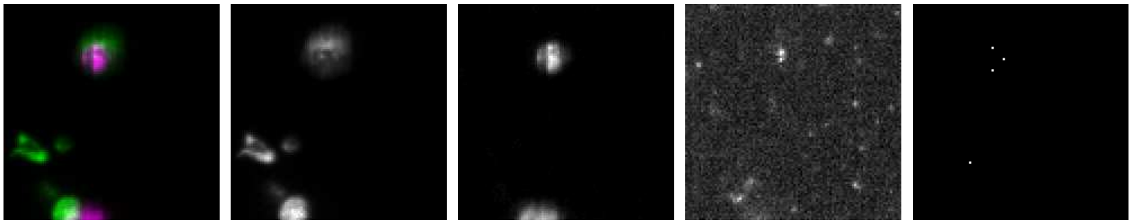				
12359	82	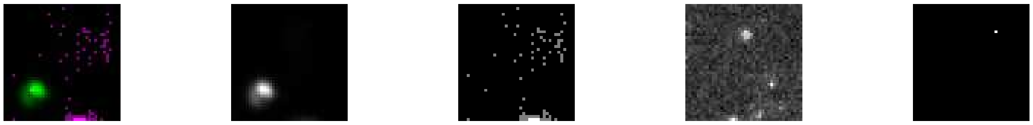				

Proben-ID: Gyn14.208 **Kassetten-ID:**01327106 **Druckdatum:**02/06/2020 17:00
Patienten-ID:

Ereignis	Aufnahme	DAPI/CK-PE	CK-PE	DAPI	CD45-APC	HER-2/neu
12360	82	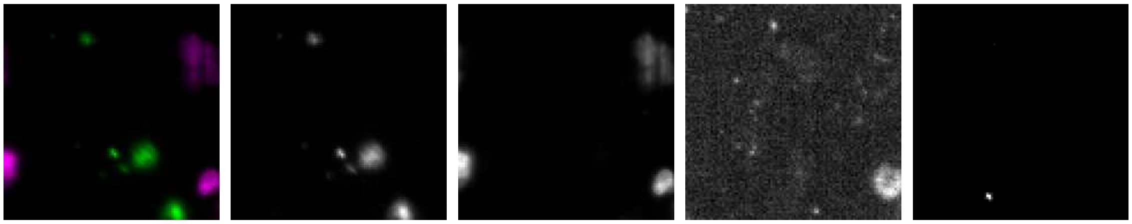				
12361	82	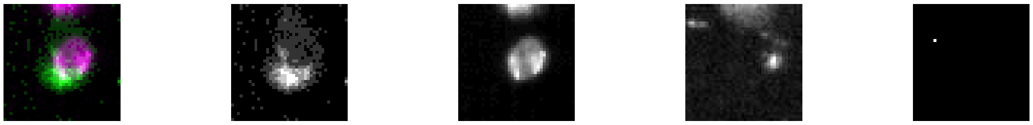				
12362	82	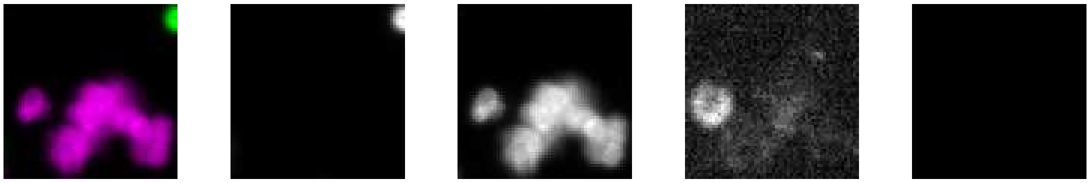				
12363	82	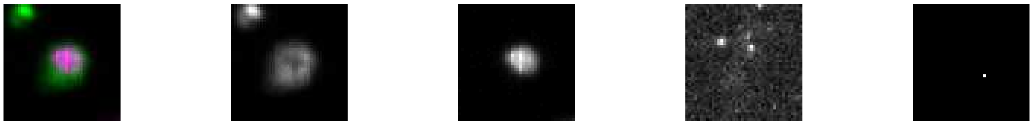				
12364	82	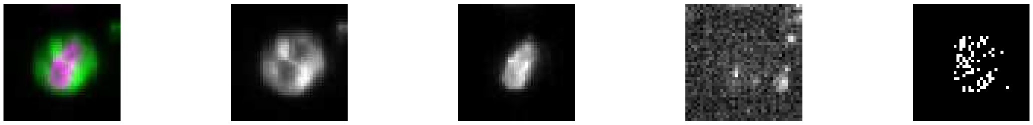				
12365	82	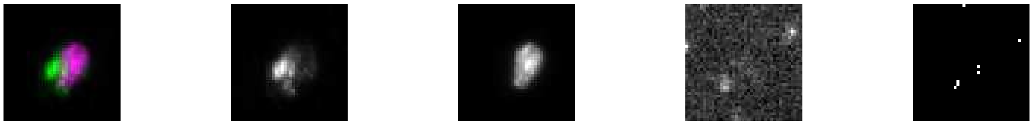				
12366	82	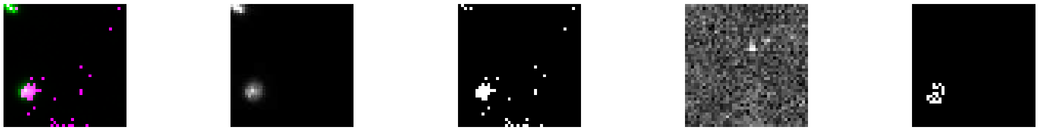				
12367	82	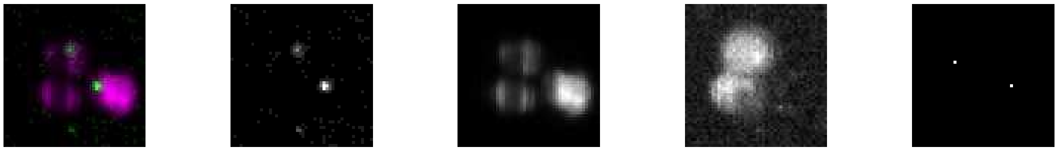				
12368	82	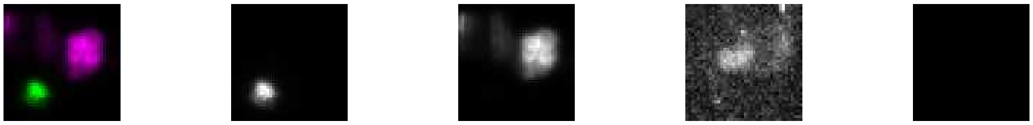				
12369	82	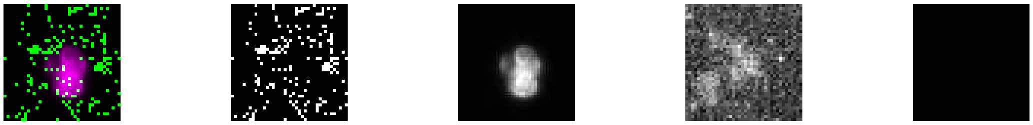				
12370	82	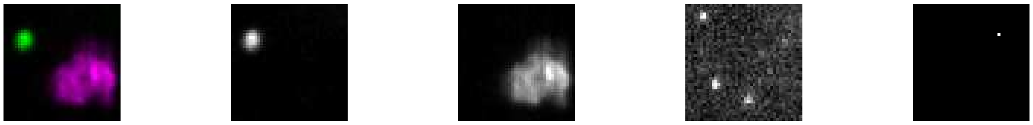				
12371	82	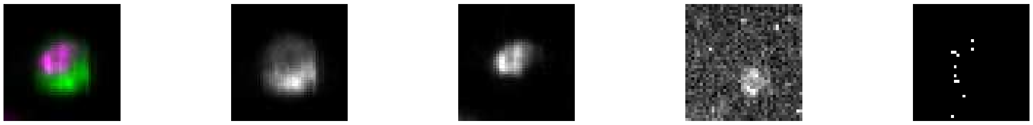				

Ereignis	Aufnahme	DAPI/CK-PE	CK-PE	DAPI	CD45-APC	HER-2/neu
12372	82	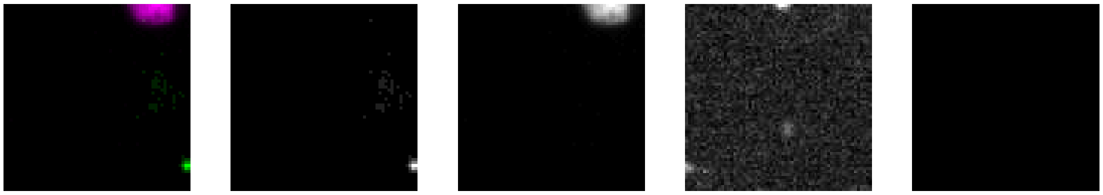				
12373	82	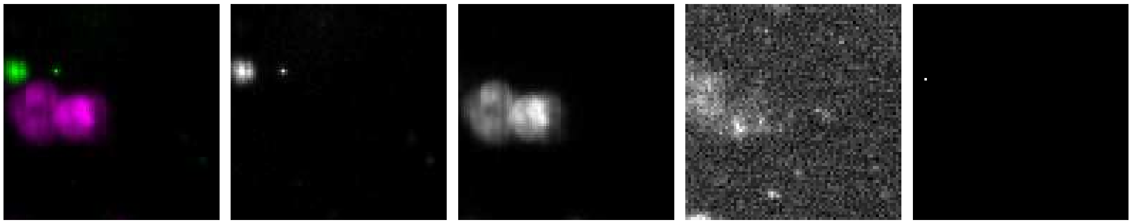				
12374	82	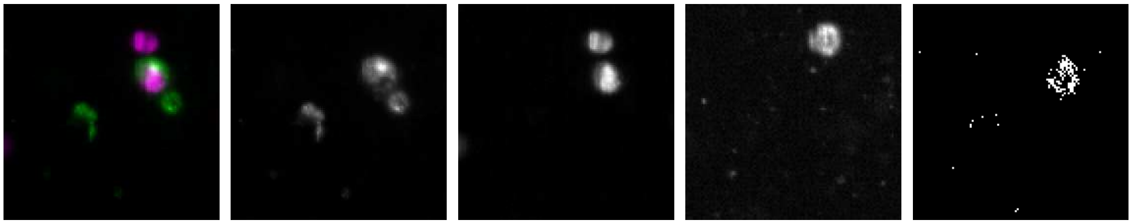				
12375	82	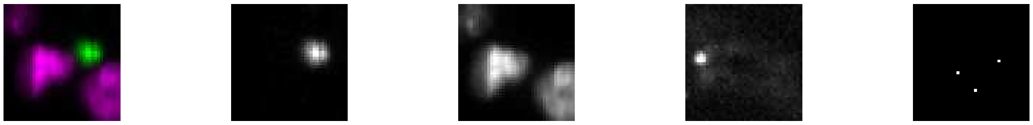				
12376	82	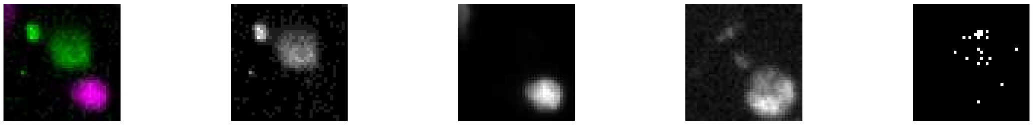				
12377	82	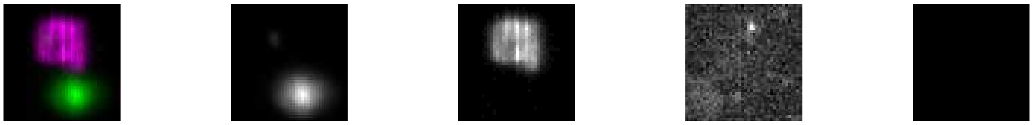				
12378	82	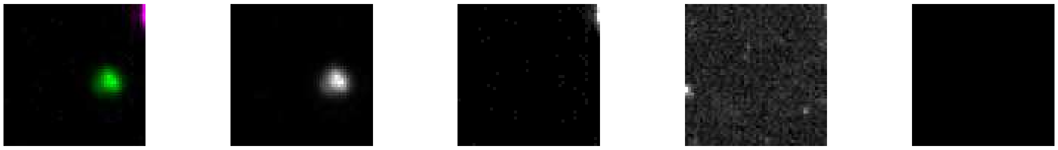				
12379	82	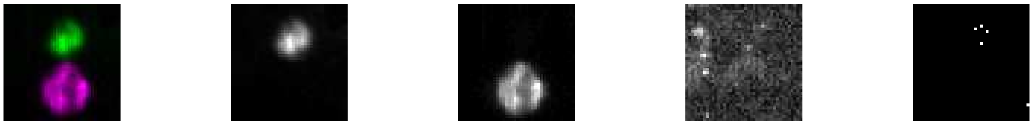				
12380	82	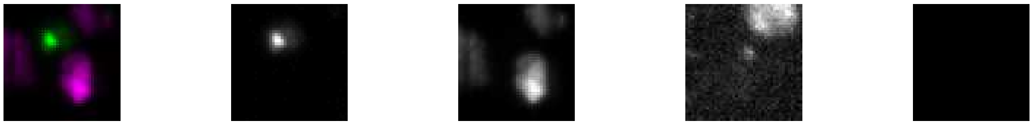				
12381	82	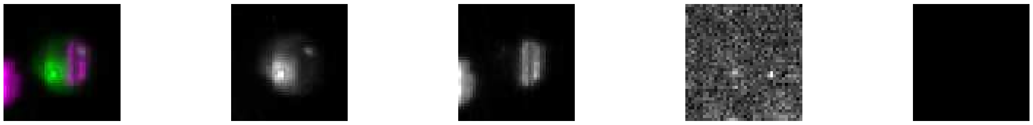				
12382	82	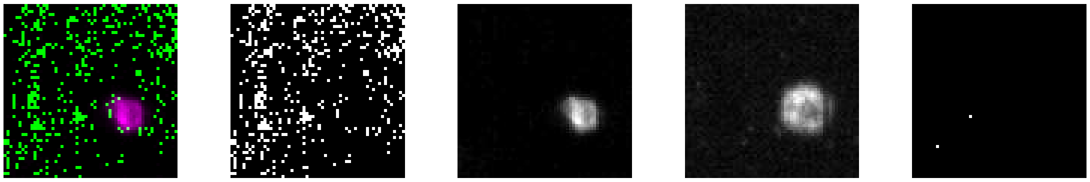				

Ereignis	Aufnahme	DAPI/CK-PE	CK-PE	DAPI	CD45-APC	HER-2/neu
12383	82	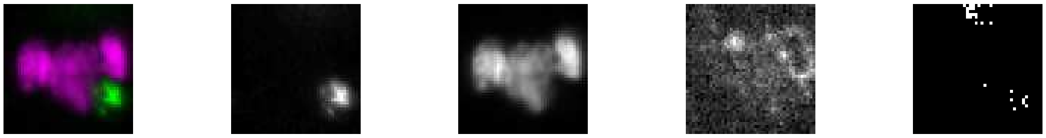				
12384	82	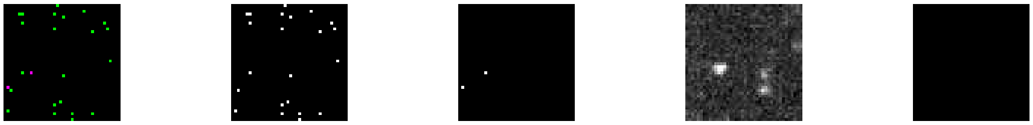				
12385	82	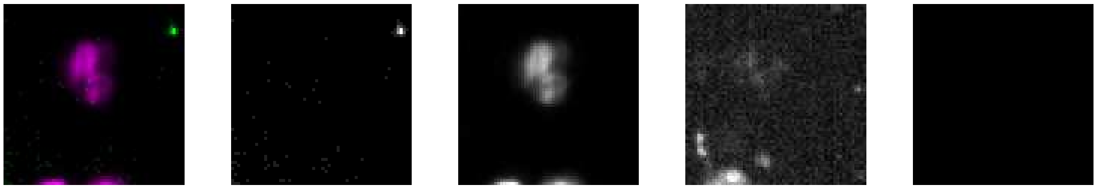				
12386	82	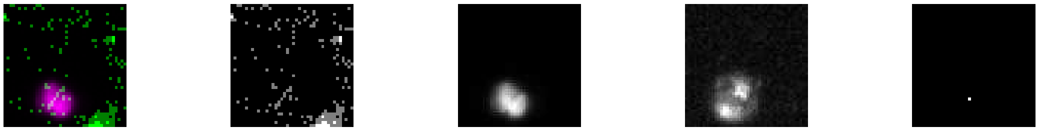				
12387	82	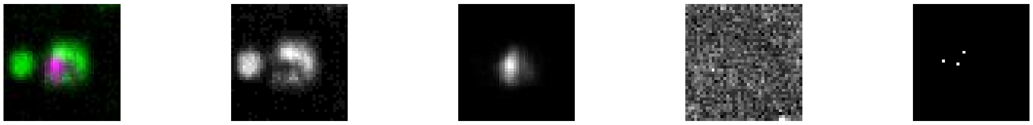				
12388	82	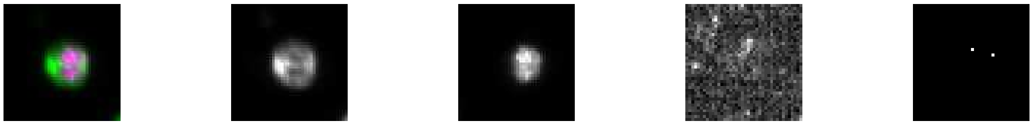				
12389	82	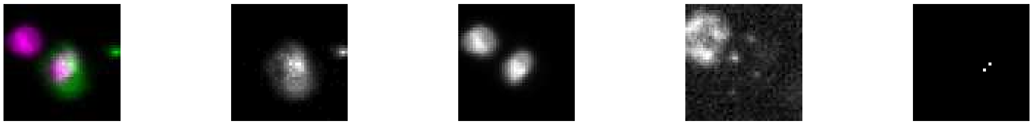				
12390	82	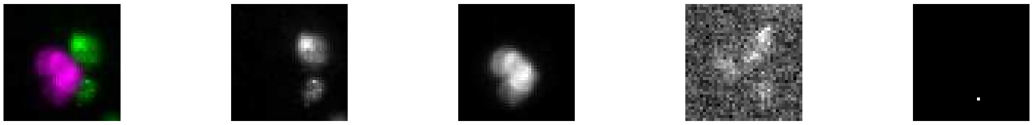				
12391	82	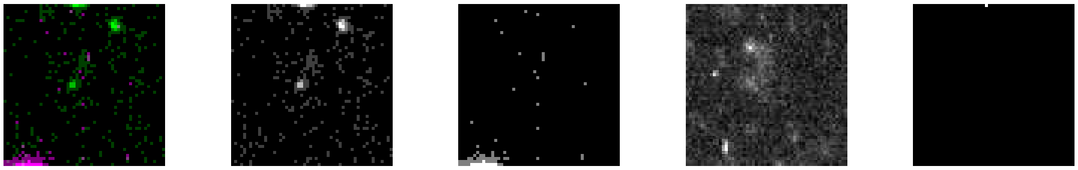				
12392	82	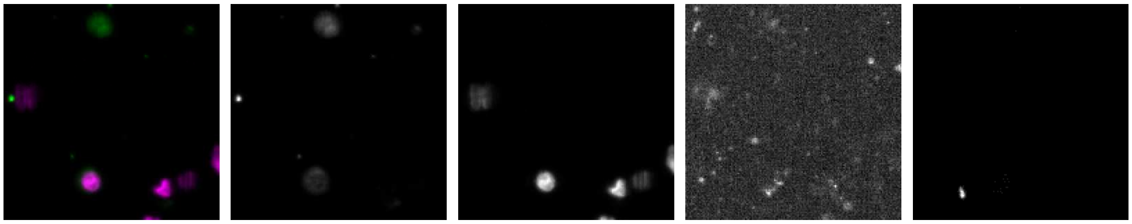				
12393	82	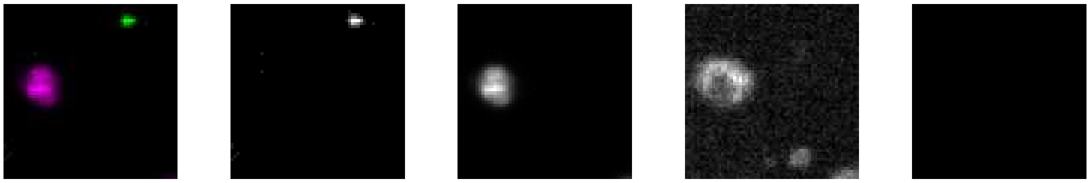				
12394	82	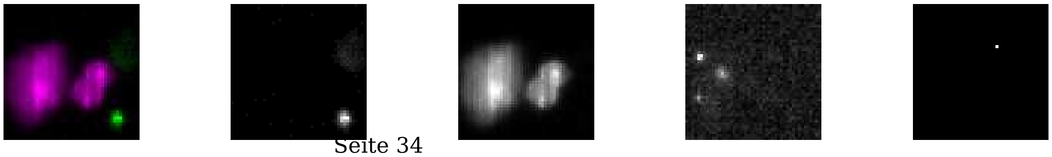				

Ereignis	Aufnahme	DAPI/CK-PE	CK-PE	DAPI	CD45-APC	HER-2/neu
12395	82					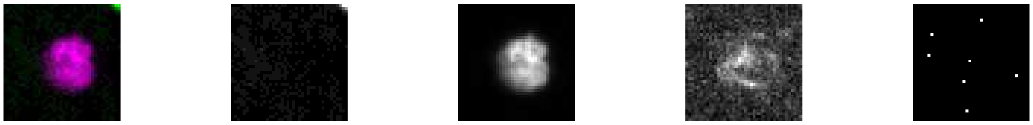
12396	82					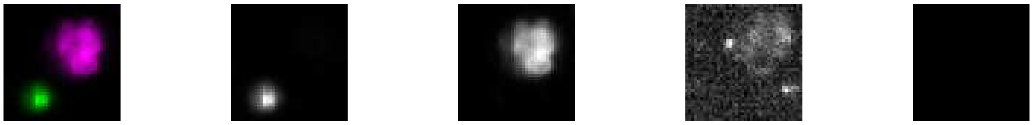
12397	82					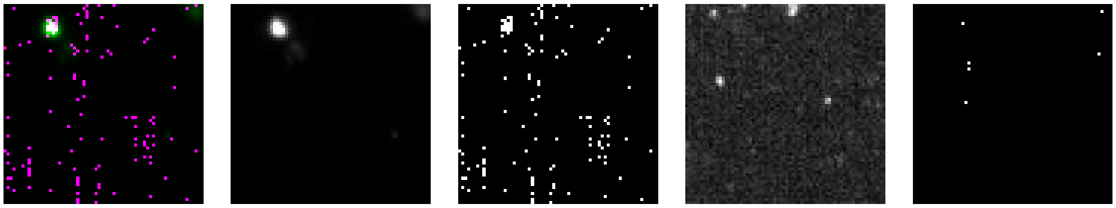
12398	82					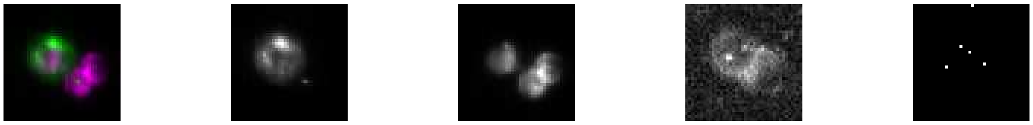
12399	82					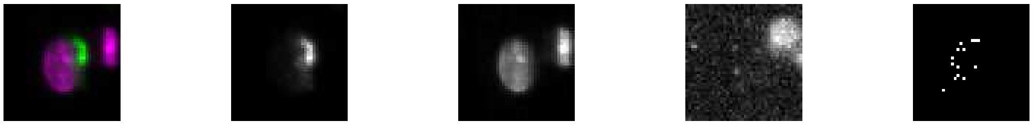
12400	82					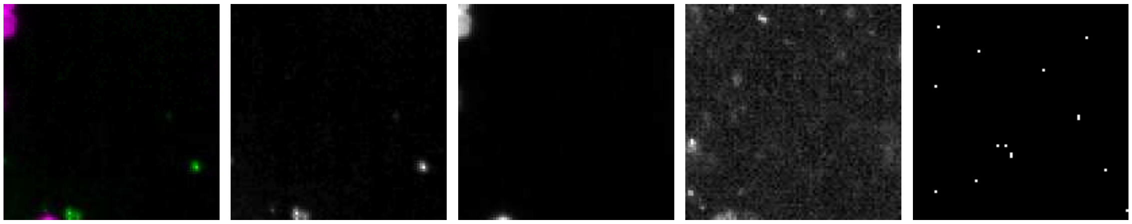
12401	82					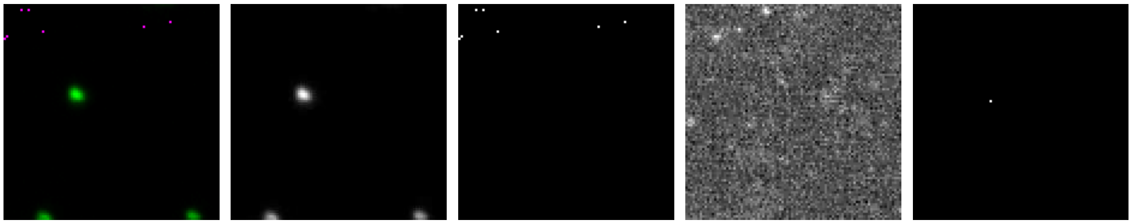
12402	82					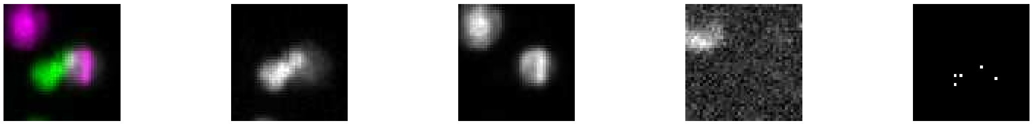
12403	82					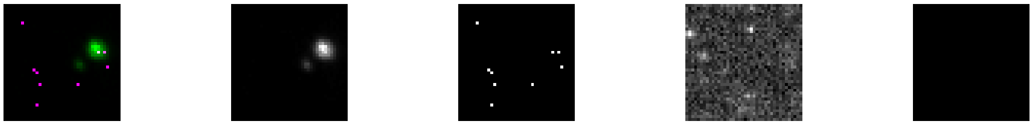
12404	82					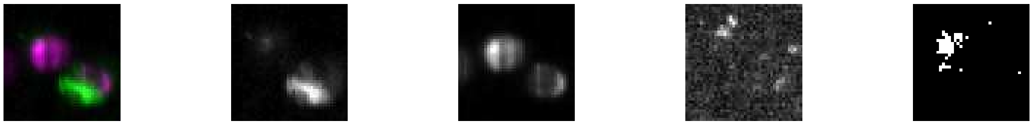
12405	82					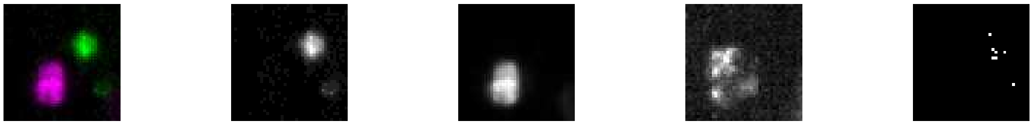
12406	82					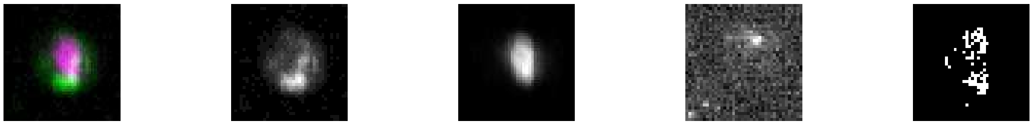

Ereignis	Aufnahme	DAPI/CK-PE	CK-PE	DAPI	CD45-APC	HER-2/neu
12407	82	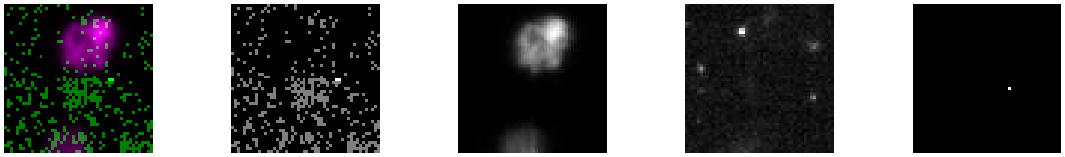				
12408	82	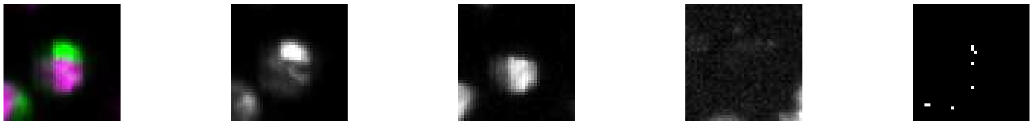				
12409	82	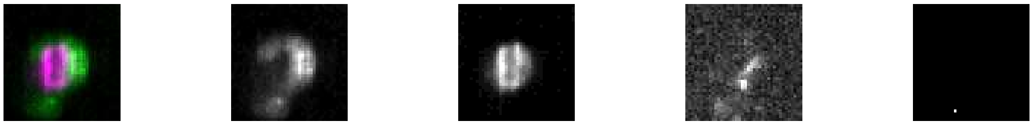				
12410	82	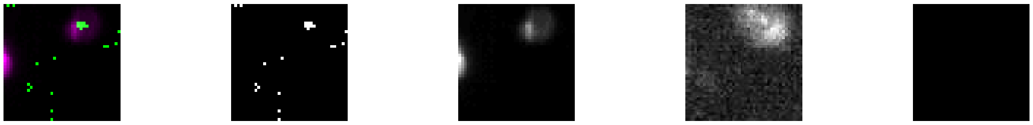				
12411	82	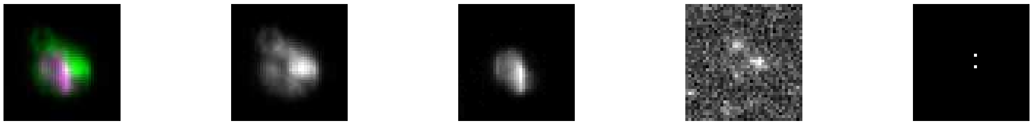				
12412	82	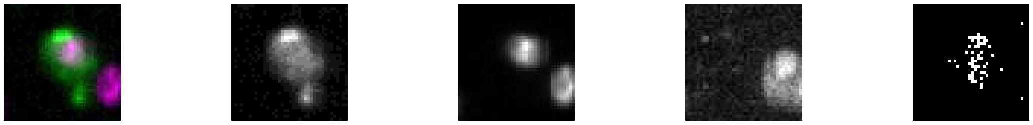				
12413	82	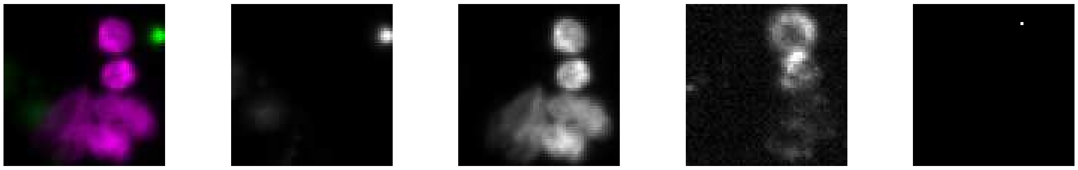				
12414	82	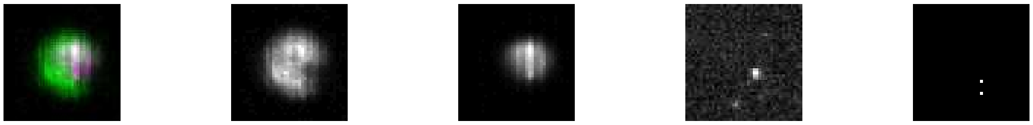				
12415	82	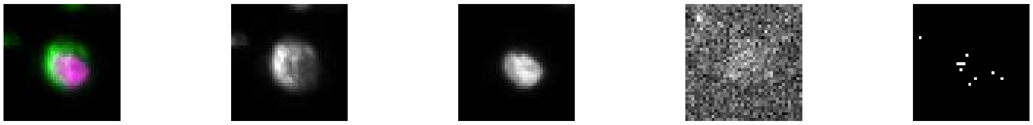				
12416	82	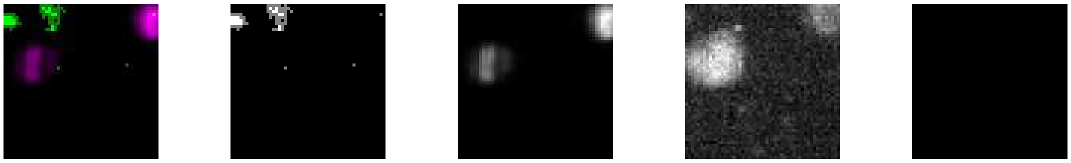				
12417	82	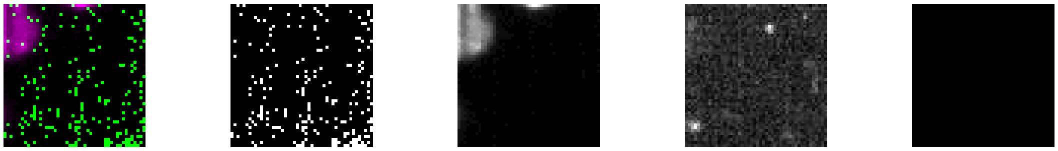				
12418	82	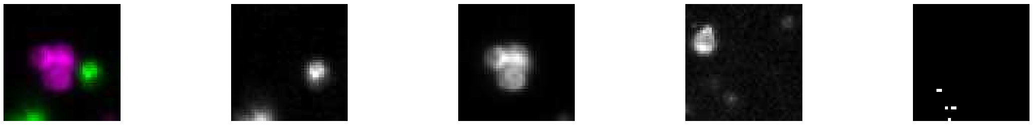				
12419	82	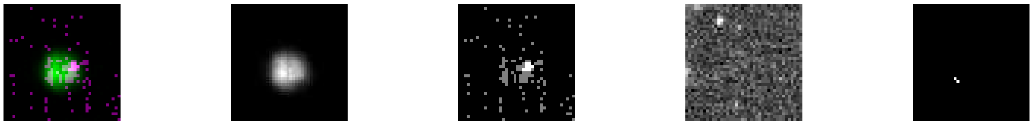				

Ereignis	Aufnahme	DAPI/CK-PE	CK-PE	DAPI	CD45-APC	HER-2/neu
12420	82	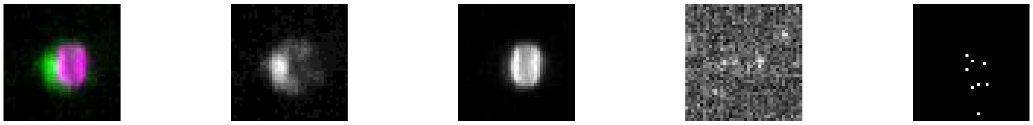				
12421	82	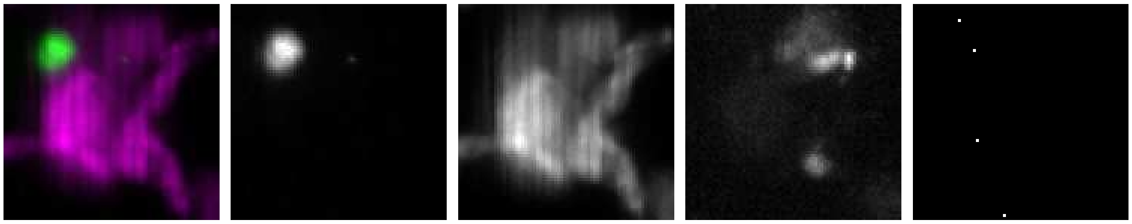				
12422	82	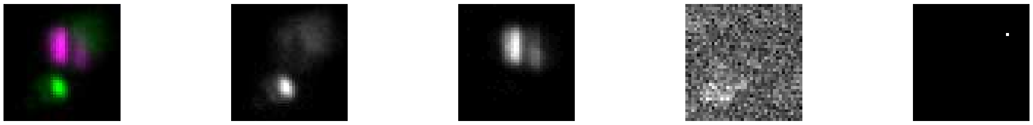				
12423	82	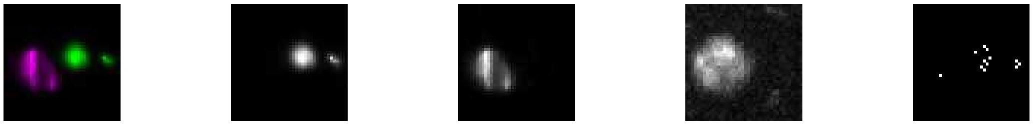				
12424	82	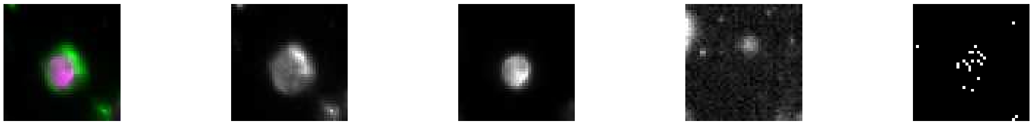				
12425	82	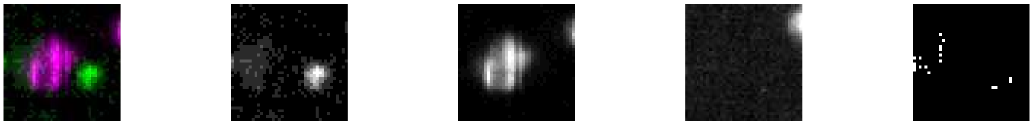				
12426	82	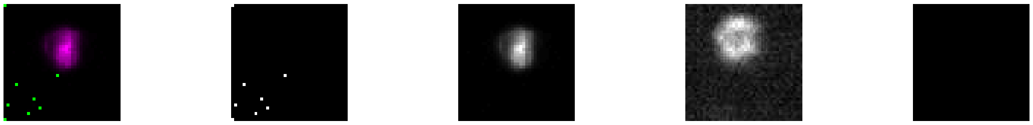				
12427	82	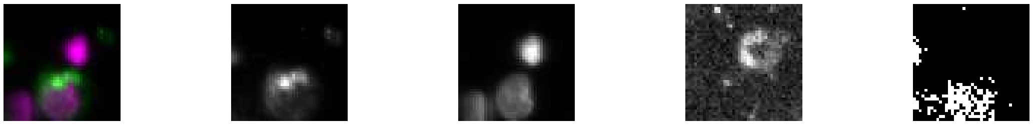				
12428	82	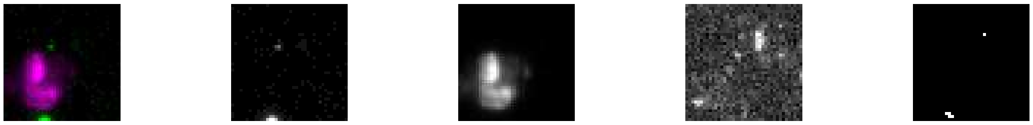				
12429	82	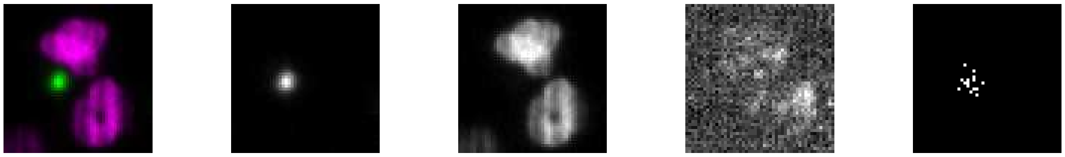				
12430	82	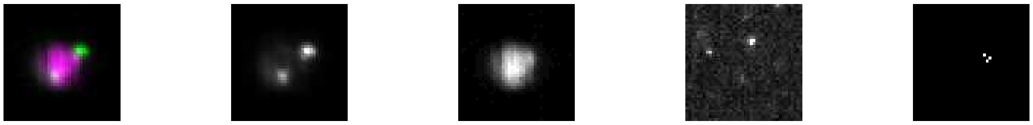				
12431	82	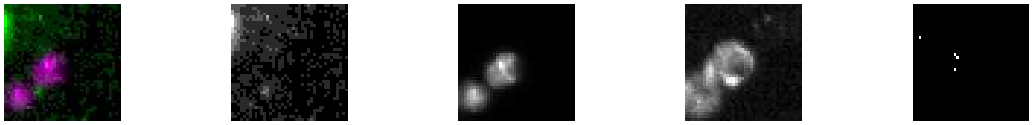				
12432	82	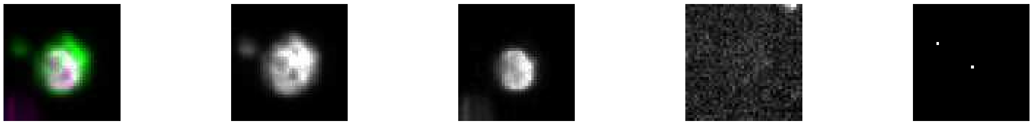				

Ereignis	Aufnahme	DAPI/CK-PE	CK-PE	DAPI	CD45-APC	HER-2/neu
12433	82	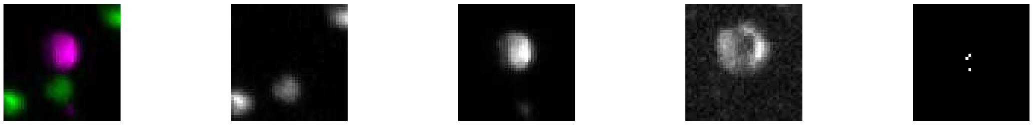				
12434	82	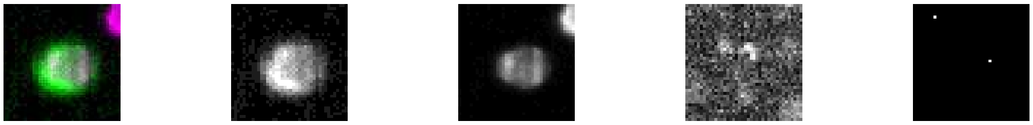				
12435	82	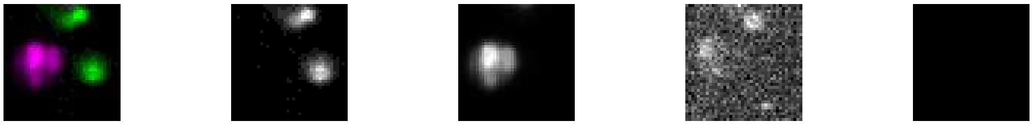				
12436	82	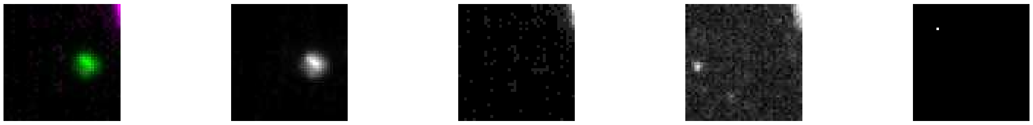				
12437	82	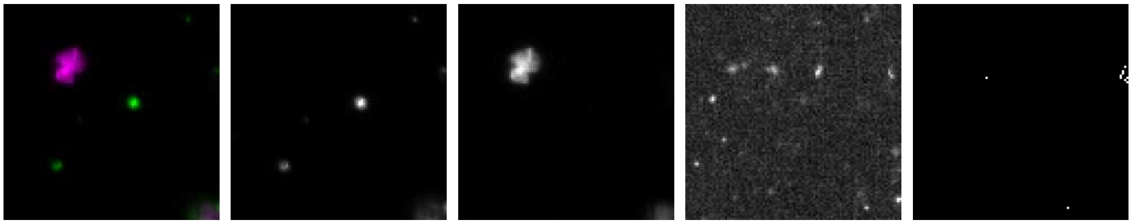				
12438	82	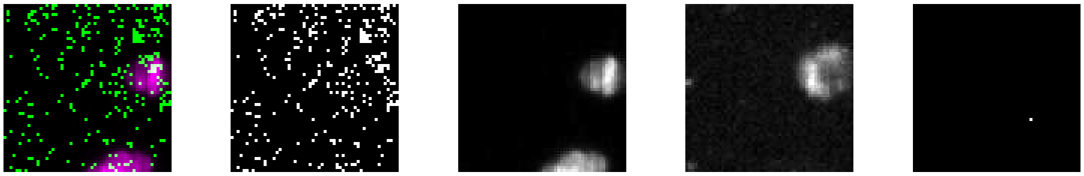				
12439	82	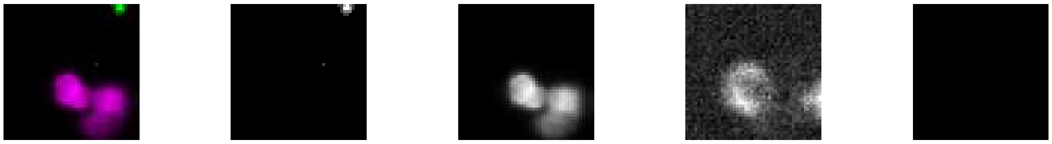				
12440	82	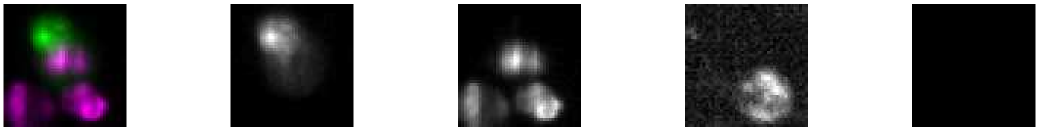				
12441	82	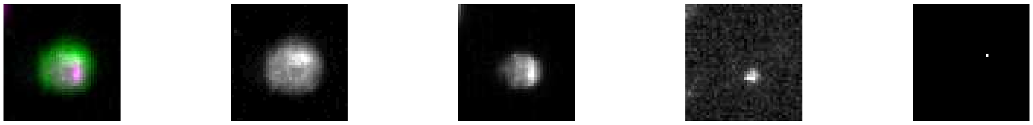				
12442	82	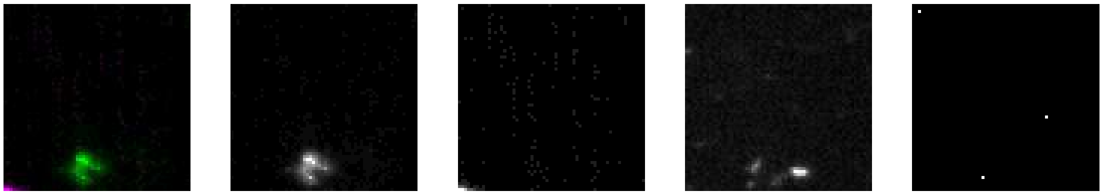				
12443	82	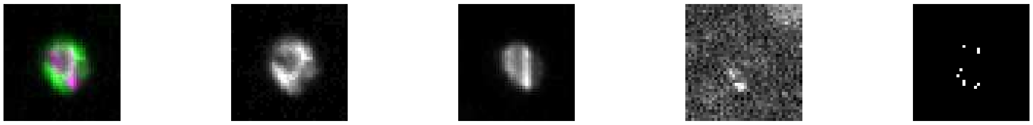				
12444	82	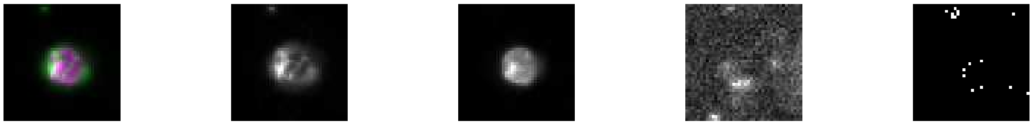				

Ereignis	Aufnahme	DAPI/CK-PE	CK-PE	DAPI	CD45-APC	HER-2/neu
12445	82	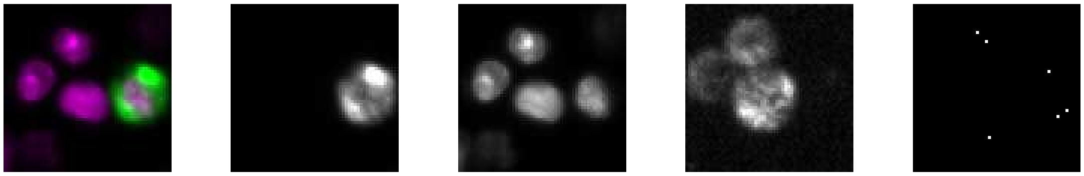				
12446	82	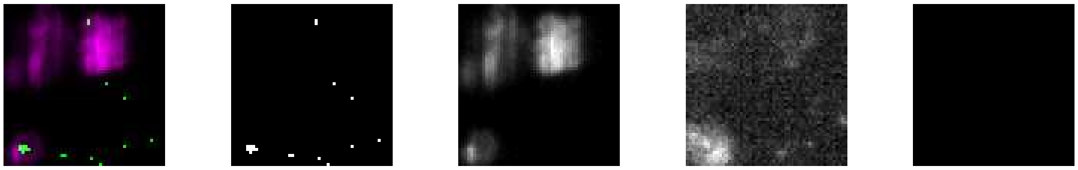				
12447	82	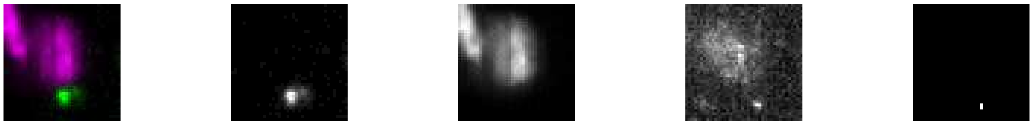				
12448	82	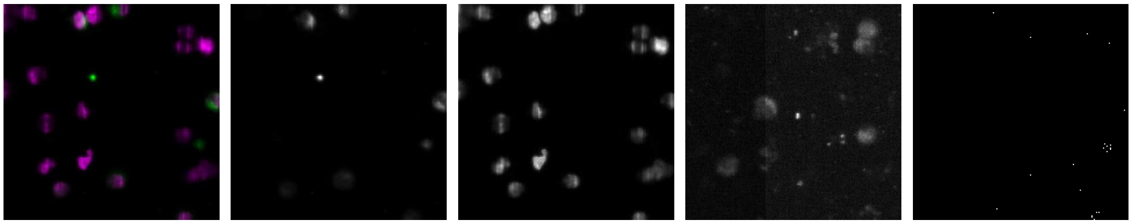				
12449	81	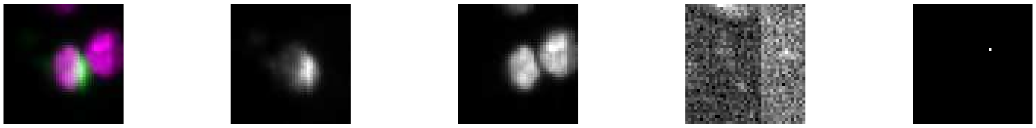				
12450	82	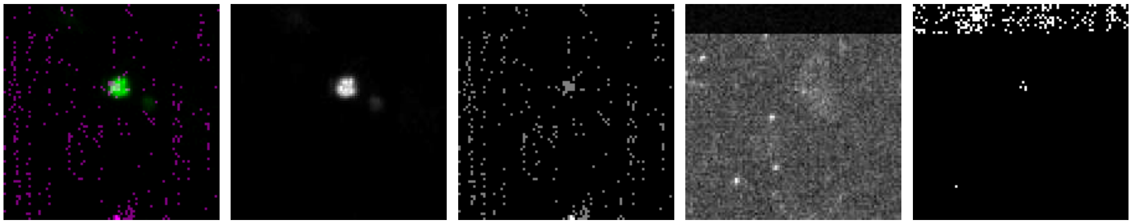				
12451	82	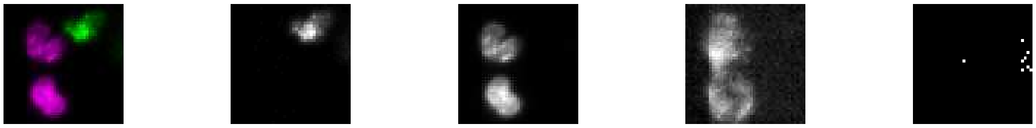				
12452	81	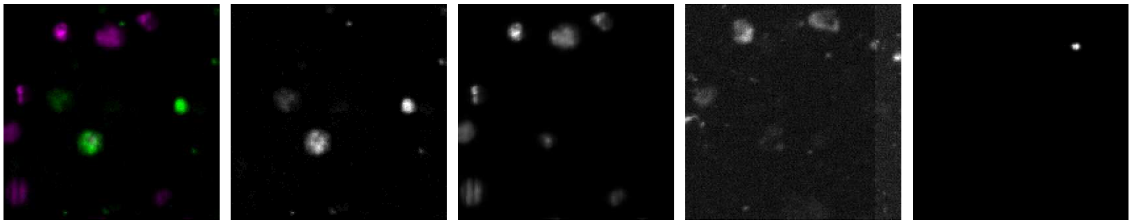				
12453	81	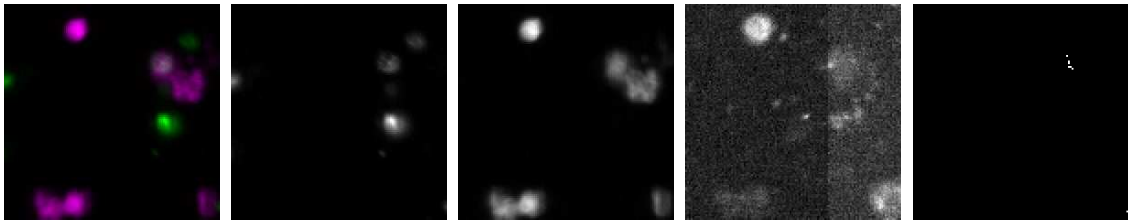				

Ereignis	Aufnahme	DAPI/CK-PE	CK-PE	DAPI	CD45-APC	HER-2/neu
12454	82	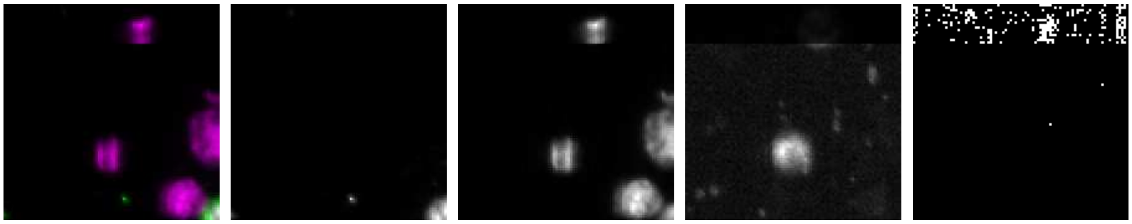				
12455	82	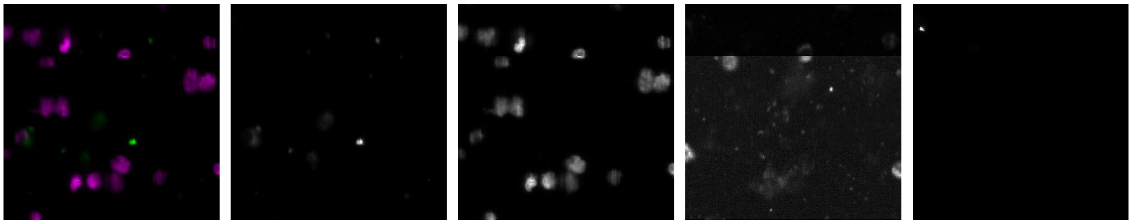				
12456	59	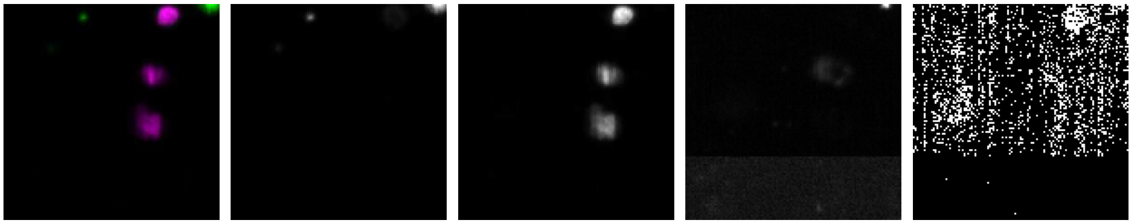				
12457	82	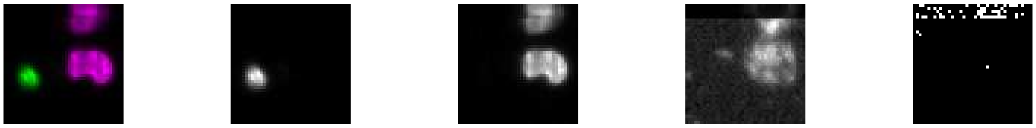				
12458	81	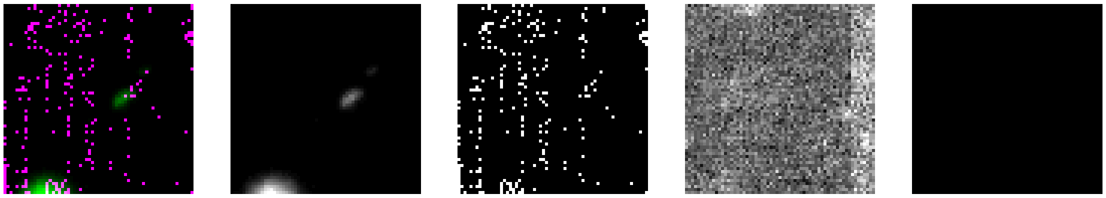				
12459	83	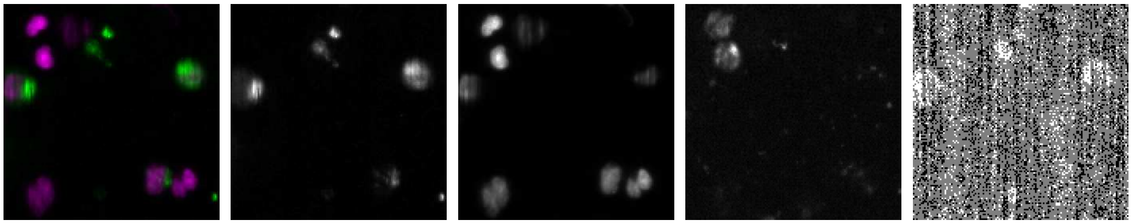				
12460	83	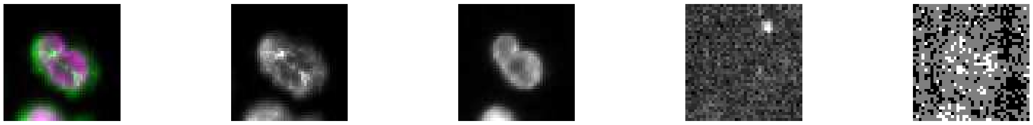				
12461	83	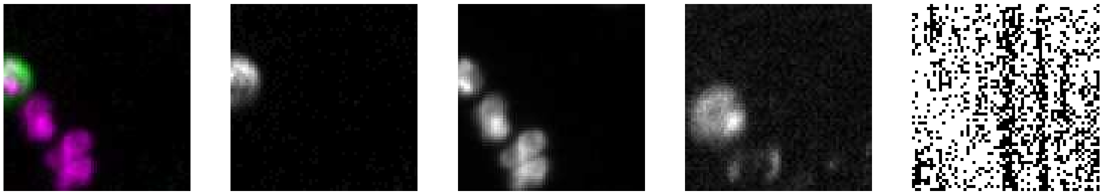				
12462	83	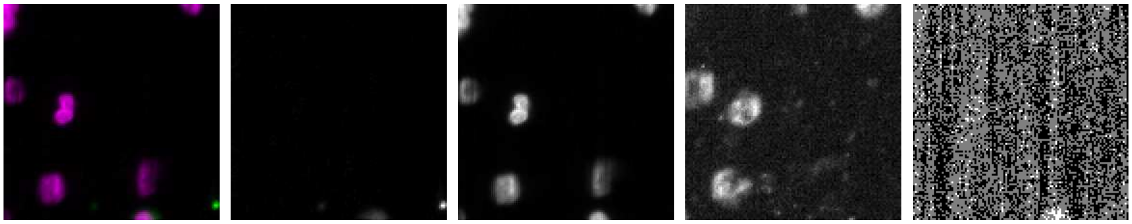				

Ereignis	Aufnahme	DAPI/CK-PE	CK-PE	DAPI	CD45-APC	HER-2/neu
12463	83	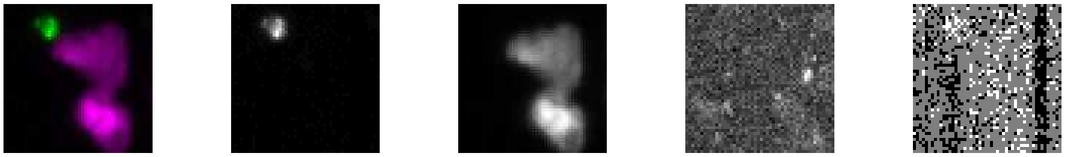				
12464	83	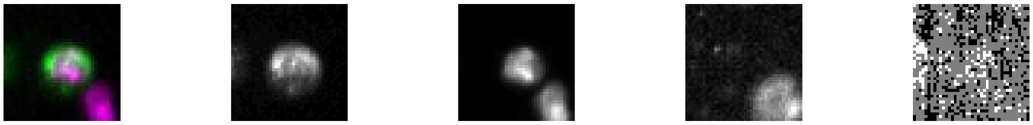				
12465	83	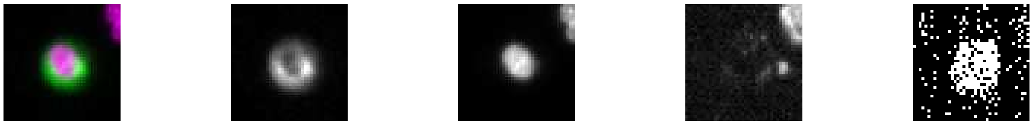				
12466	83	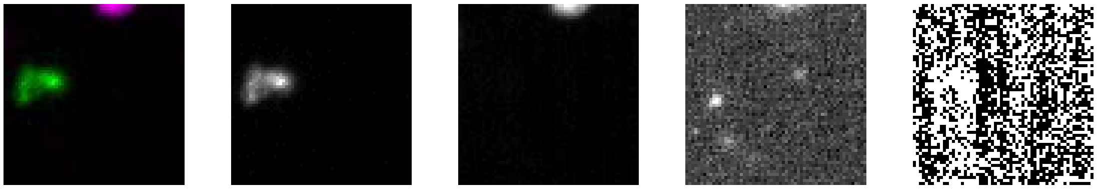				
12467	83	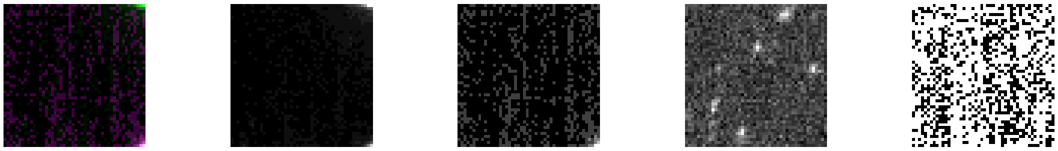				
12468	83	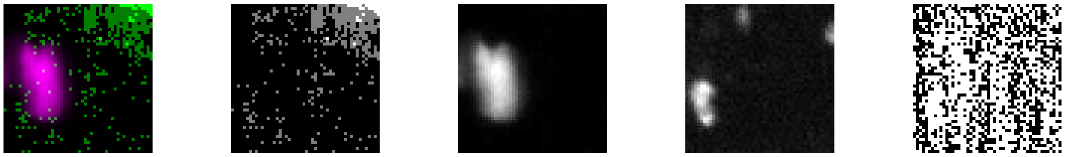				
12469	83	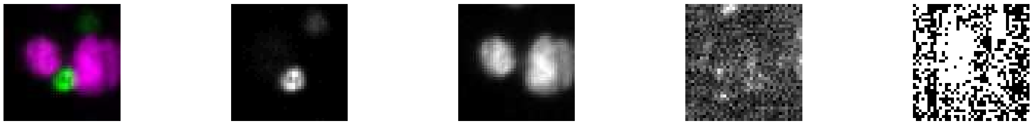				
12470	83	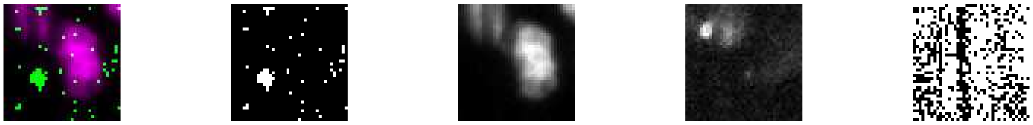				
12471	83	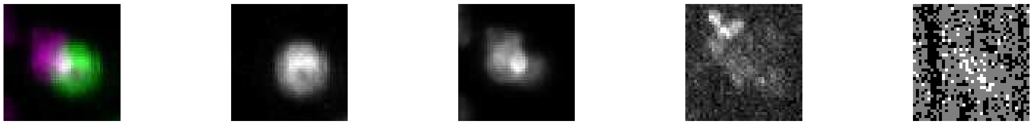				
12472	83	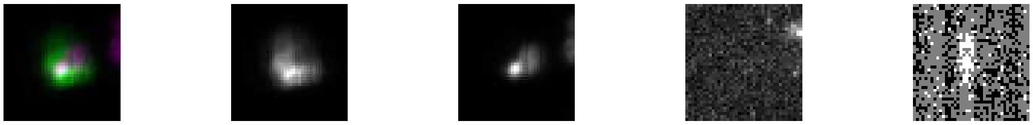				
12473	83	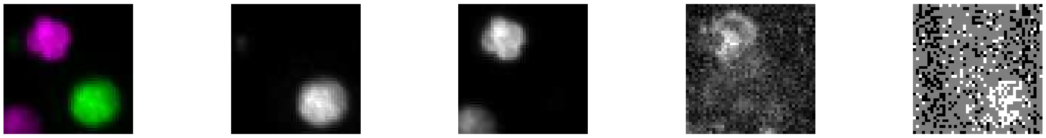				
12474	83	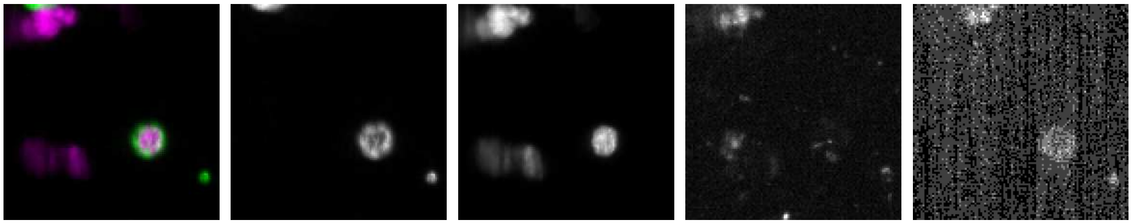				

Ereignis	Aufnahme	DAPI/CK-PE	CK-PE	DAPI	CD45-APC	HER-2/neu
12475	83	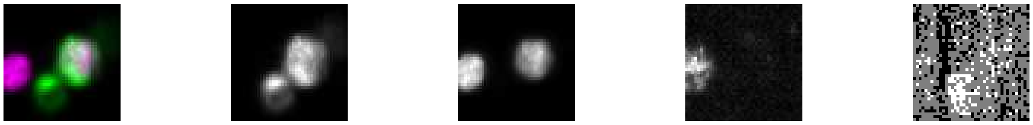				
12476	83	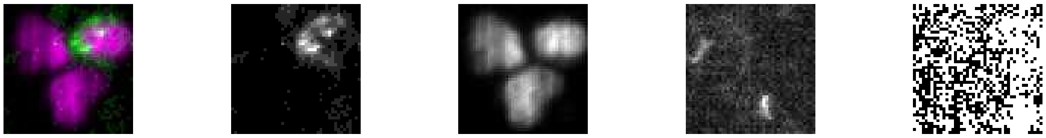				
12477	83	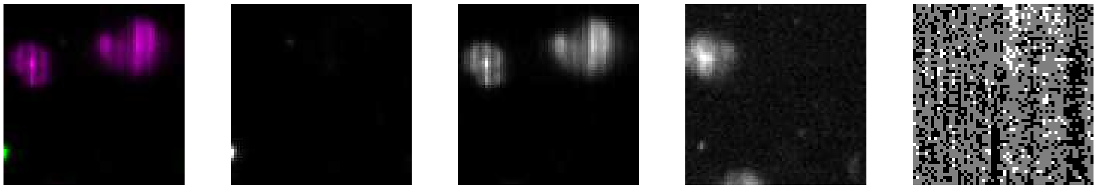				
12478	83	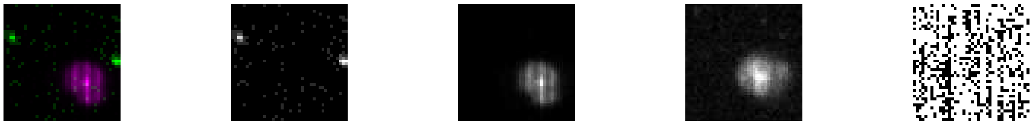				
12479	83	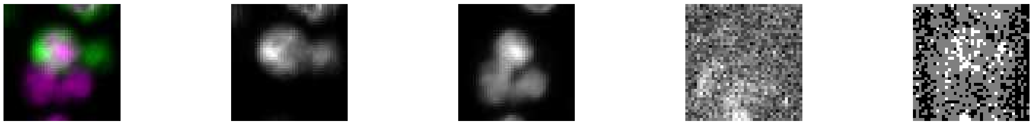				
12480	83	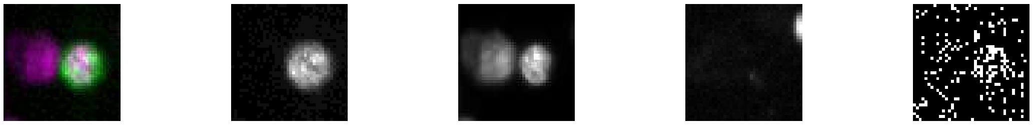				
12481	83	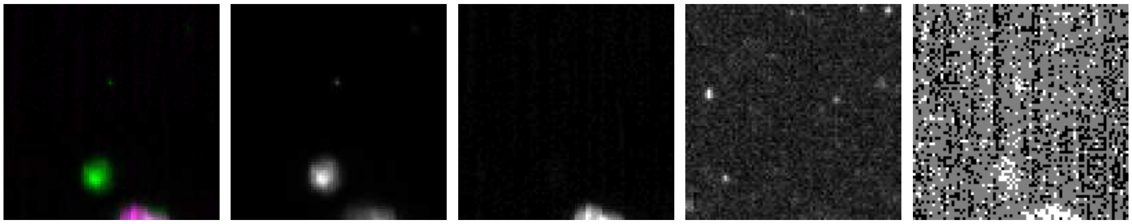				
12482	83	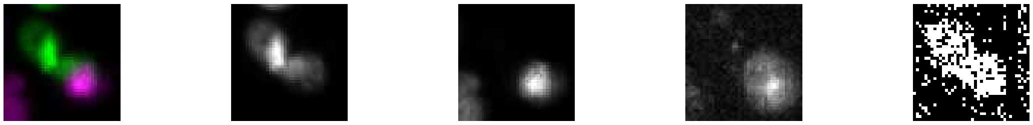				
12483	83	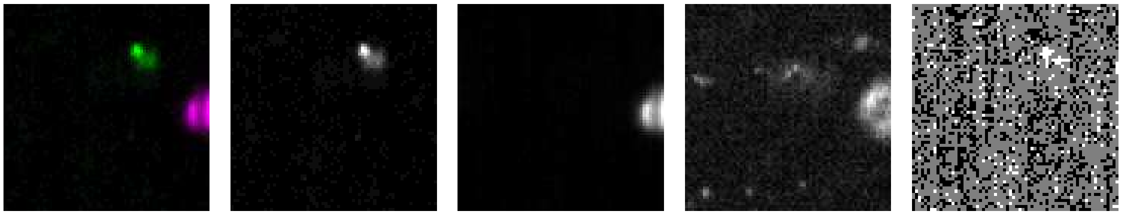				
12484	83	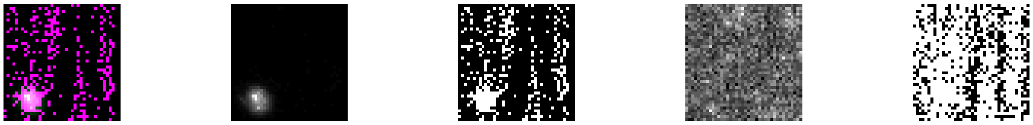				
12485	83	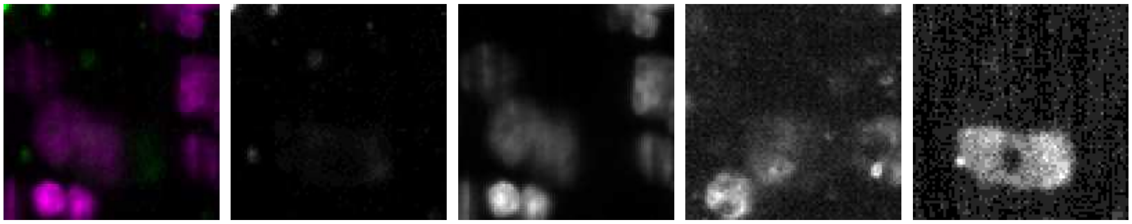				

Ereignis	Aufnahme	DAPI/CK-PE	CK-PE	DAPI	CD45-APC	HER-2/neu
12486	83	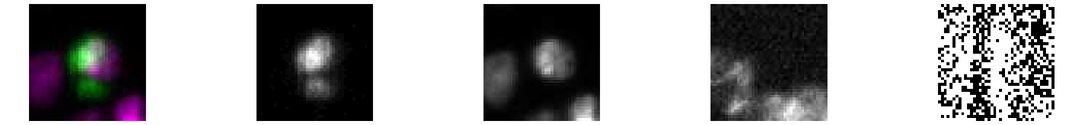				
12487	83	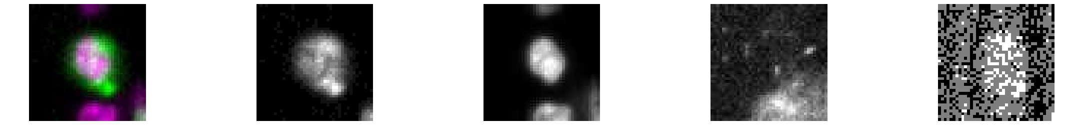				
12488	83	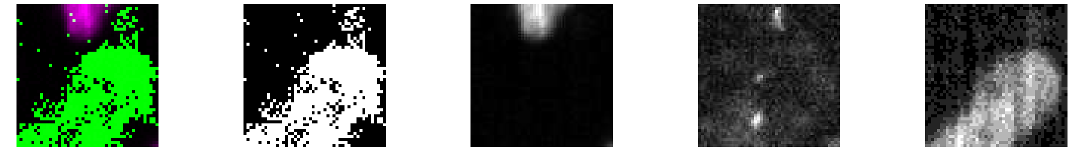				
12489	83	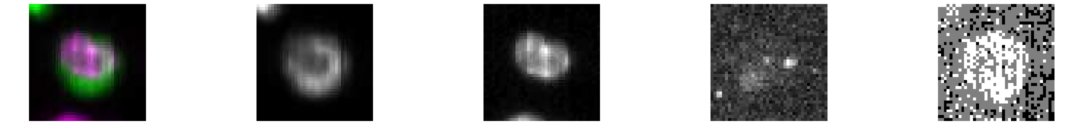				
12490	83	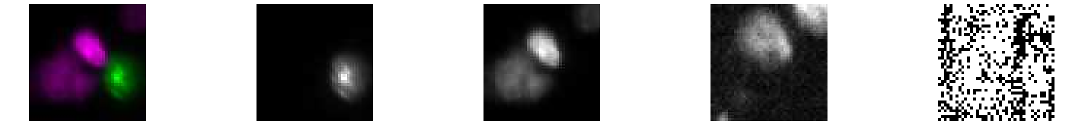				
12491	83	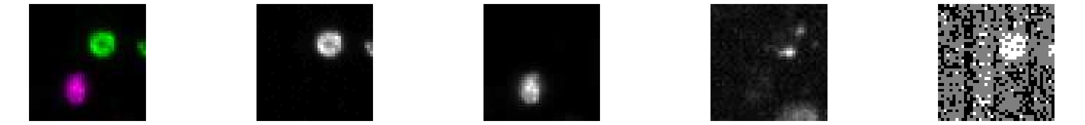				
12492	83	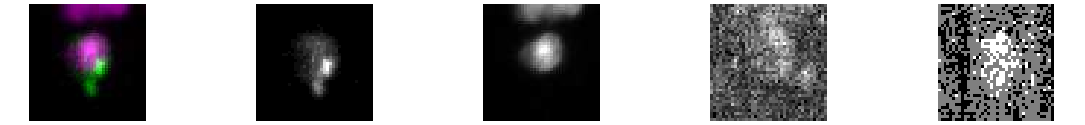				
12493	83	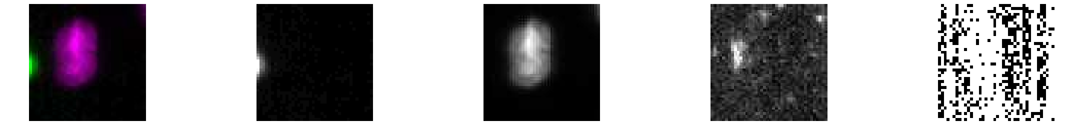				
12494	83	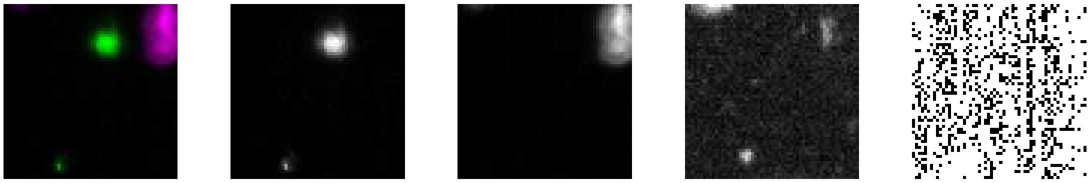				
12495	83	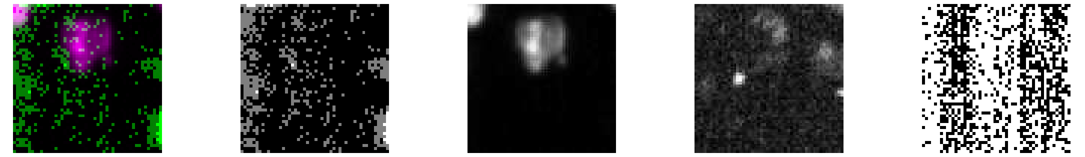				
12496	83	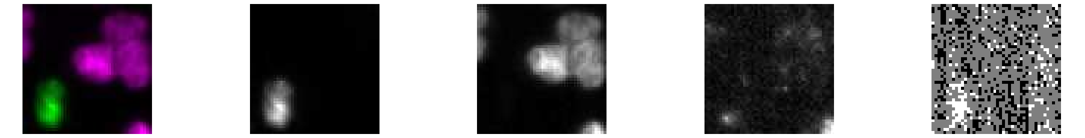				
12497	83	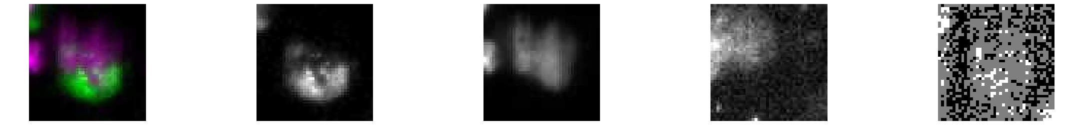				
12498	83	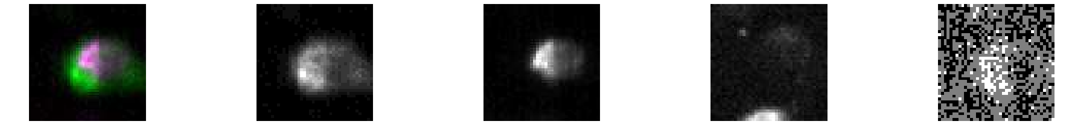				

Ereignis	Aufnahme	DAPI/CK-PE	CK-PE	DAPI	CD45-APC	HER-2/neu
12499	83	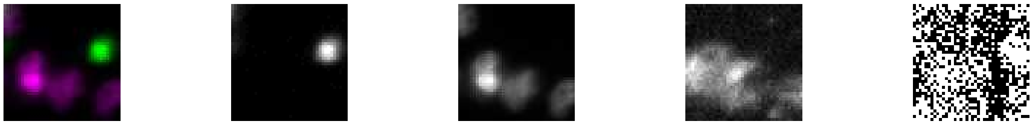				
12500	83	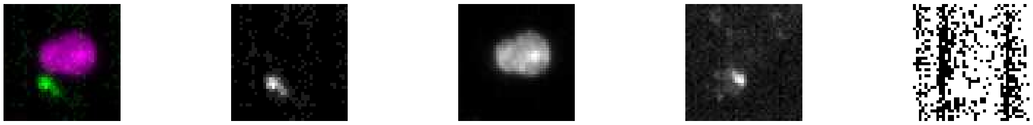				
12501	83	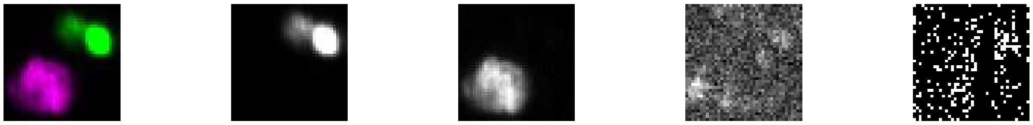				
12502	83	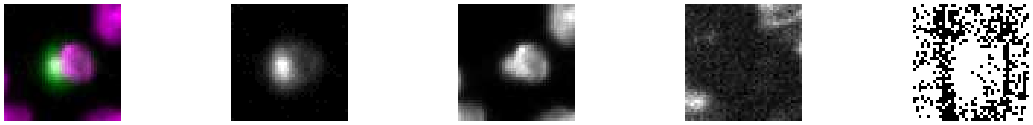				
12503	83	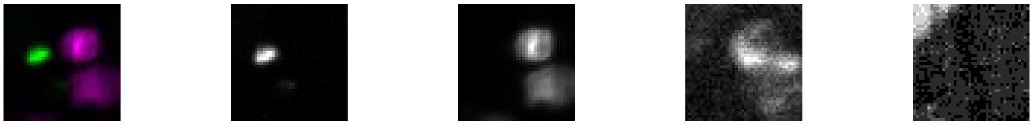				
12504	83	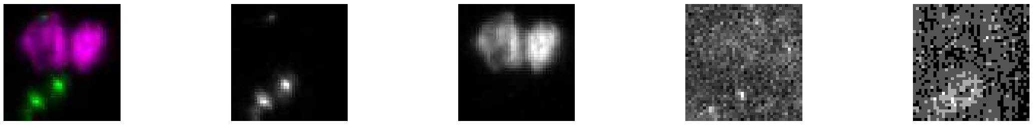				
12505	83	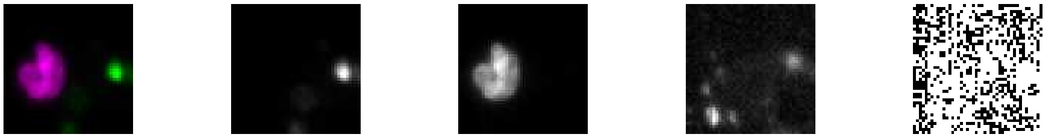				
12506	83	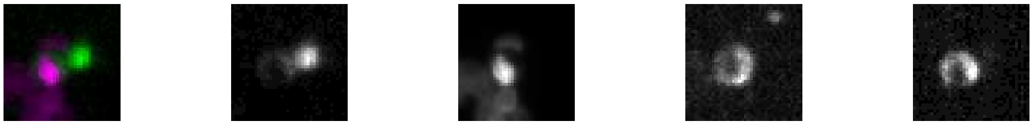				
12507	83	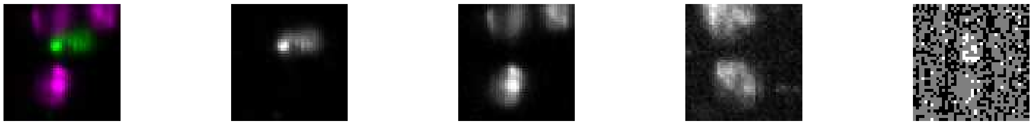				
12508	83	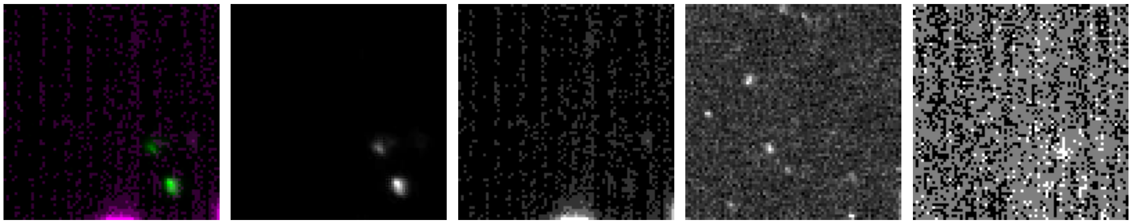				
12509	83	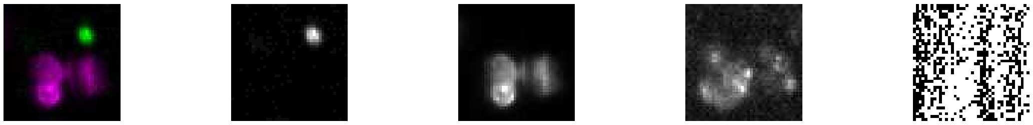				
12510	83	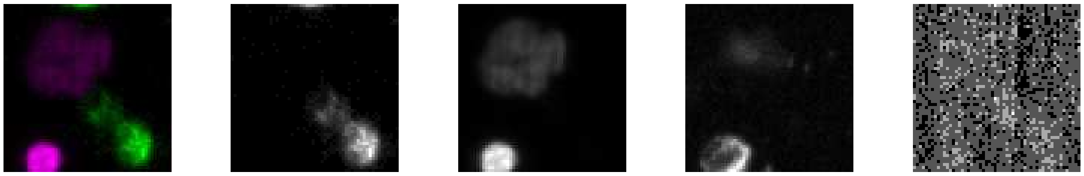				
12511	83	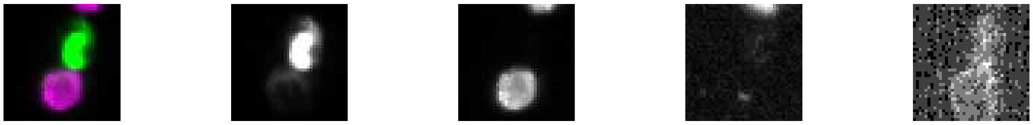				

Ereignis	Aufnahme	DAPI/CK-PE	CK-PE	DAPI	CD45-APC	HER-2/neu
12512	83	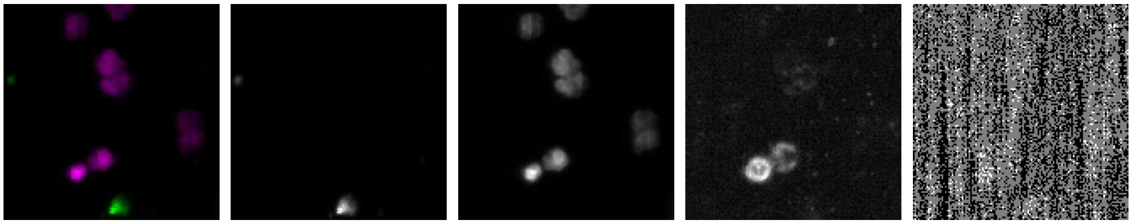				
12513	83	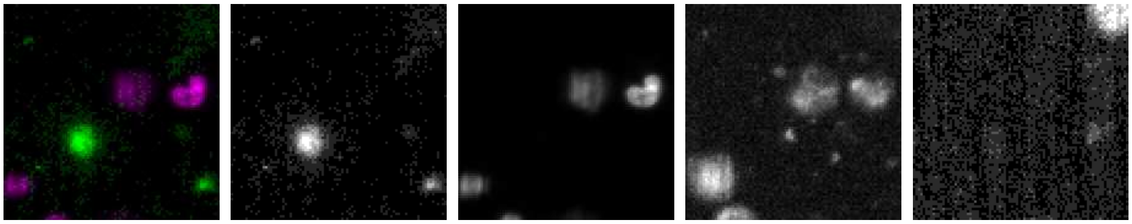				
12514	83	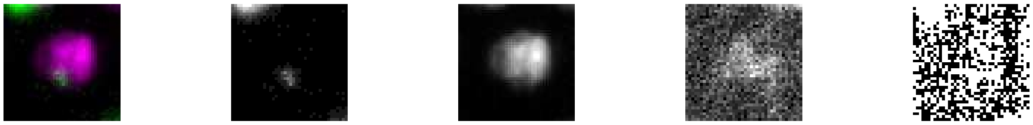				
12515	83	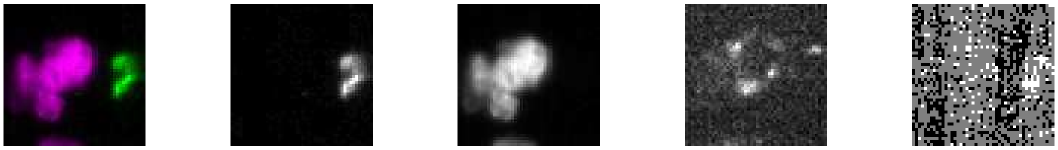				
12516	83	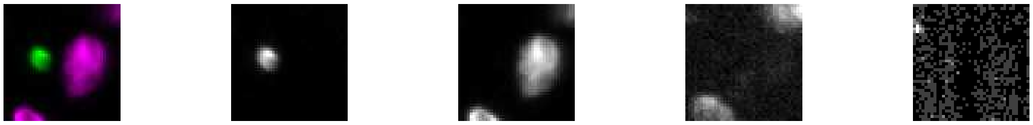				
12517	83	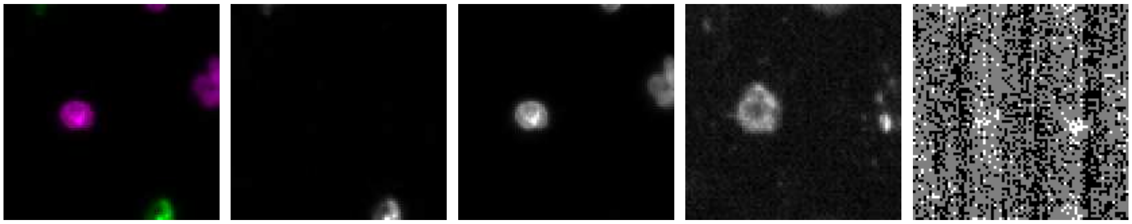				
12518	83	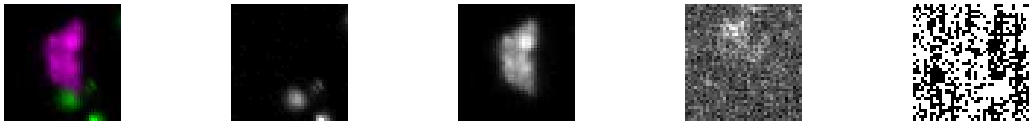				
12519	83	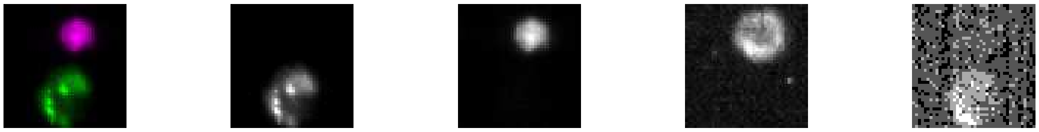				
12520	83	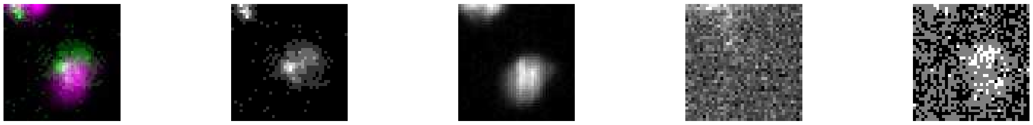				
12521	83	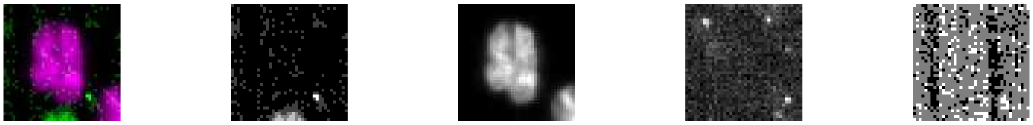				
12522	83	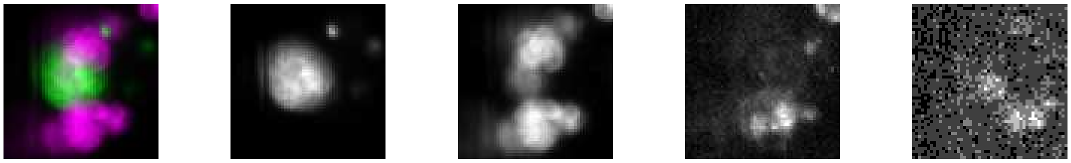				

Ereignis	Aufnahme	DAPI/CK-PE	CK-PE	DAPI	CD45-APC	HER-2/neu
12523	83	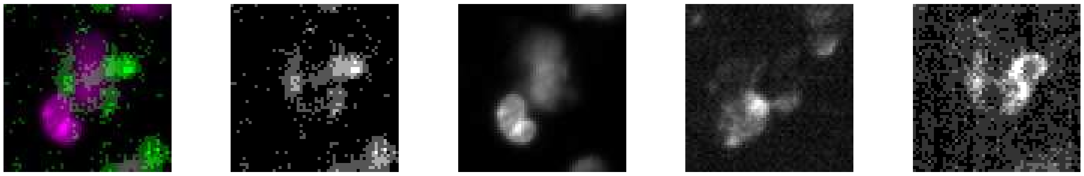				
12524	83	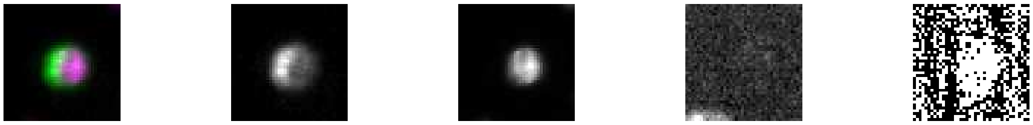				
12525	83	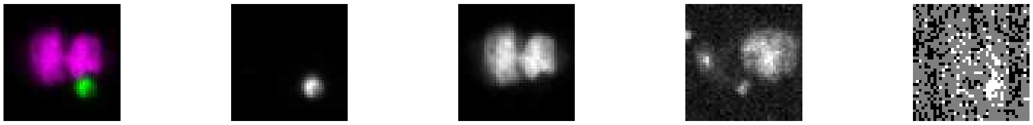				
12526	83	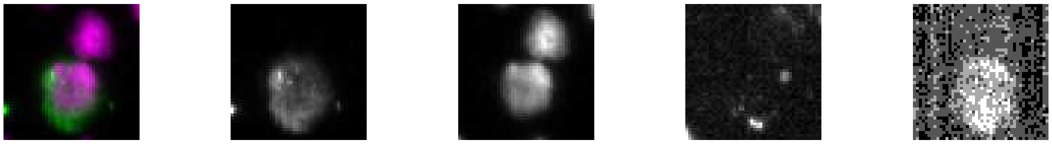				
12527	83	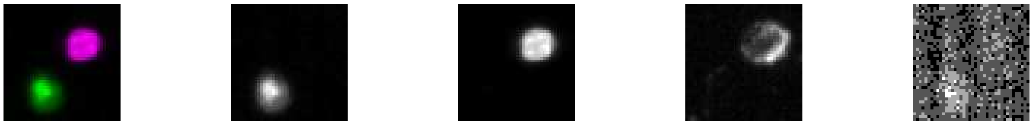				
12528	83	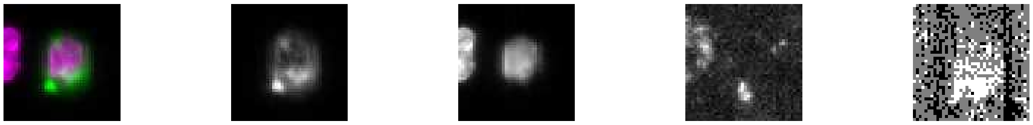				
12529	83	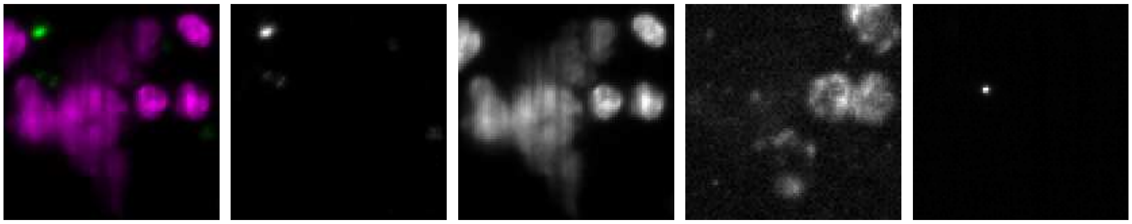				
12530	83	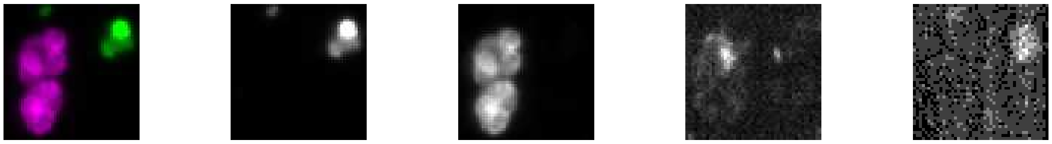				
12531	83	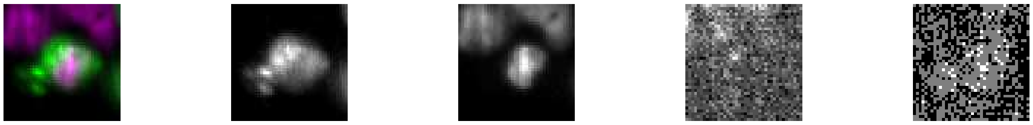				
12532	83	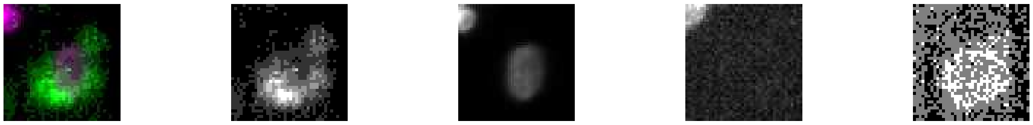				
12533	83	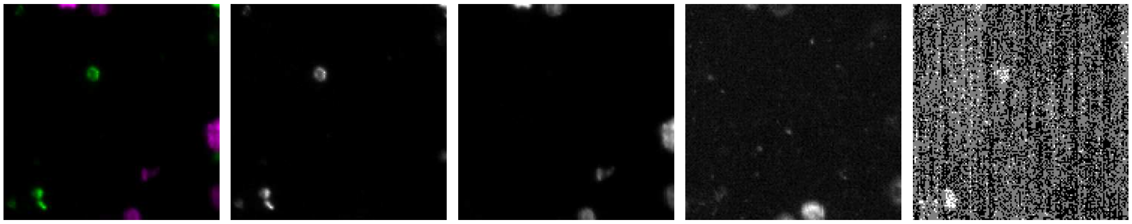				

Ereignis	Aufnahme	DAPI/CK-PE	CK-PE	DAPI	CD45-APC	HER-2/neu
12534	83	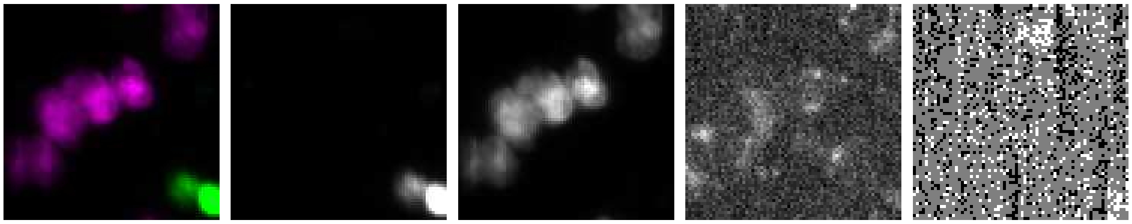				
12535	83	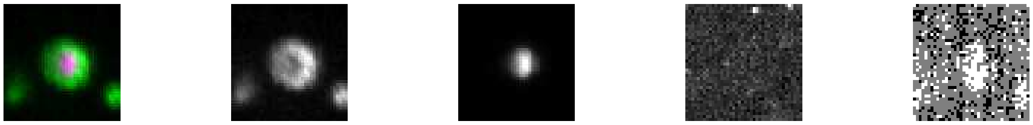				
12536	83	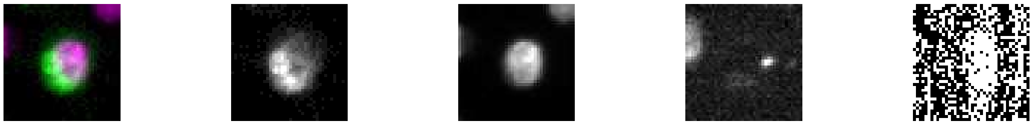				
12537	83	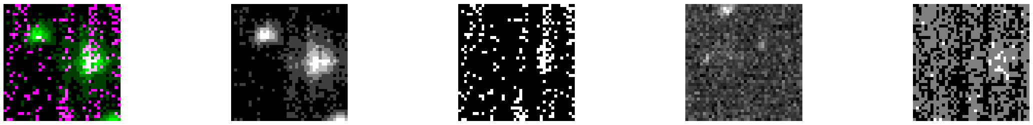				
12538	83	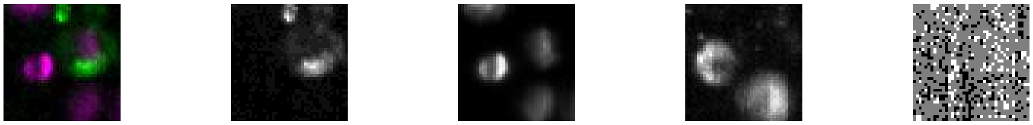				
12539	83	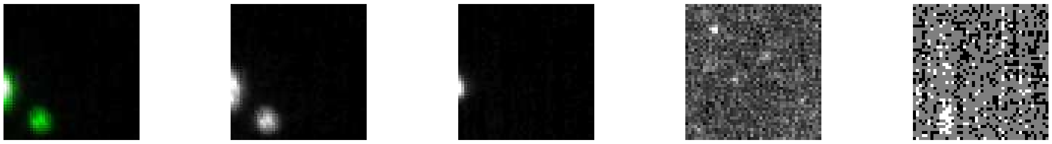				
12540	83	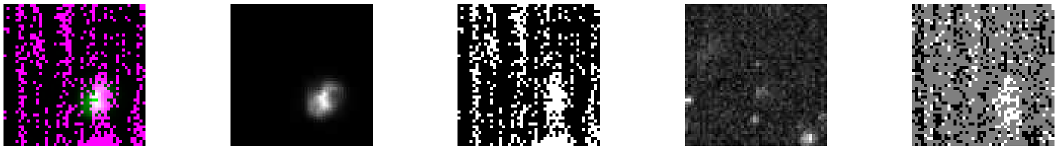				
12541	83	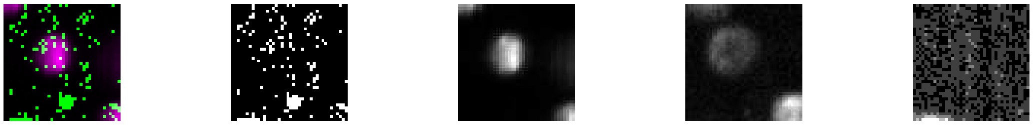				
12542	83	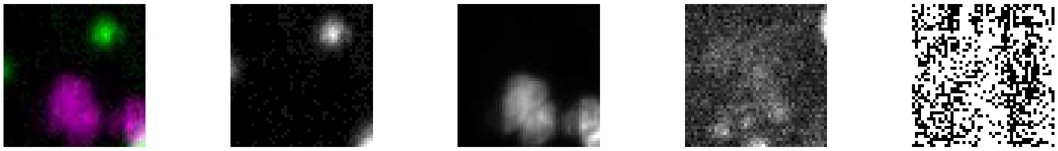				
12543	83	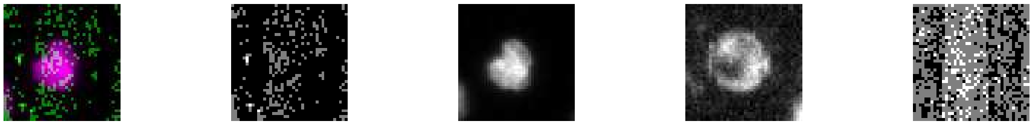				
12544	83	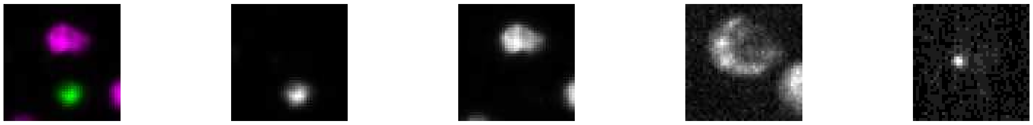				
12545	83	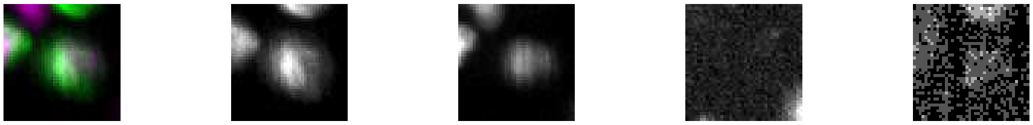				

Ereignis	Aufnahme	DAPI/CK-PE	CK-PE	DAPI	CD45-APC	HER-2/neu
12546	83	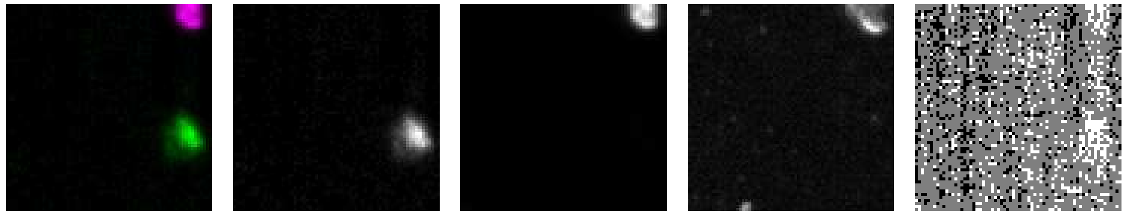				
12547	83	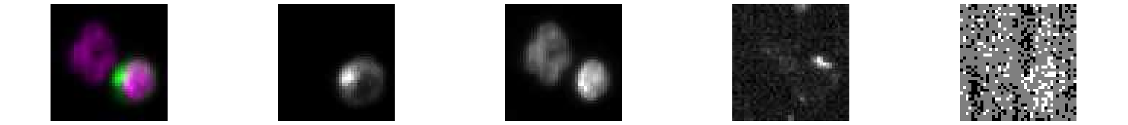				
12548	83	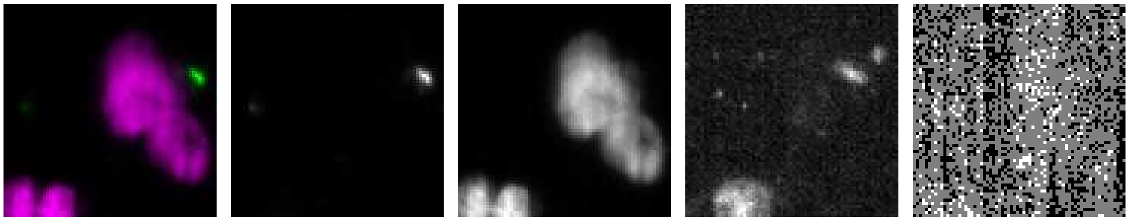				
12549	83	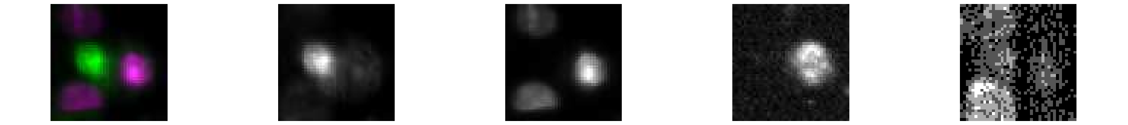				
12550	83	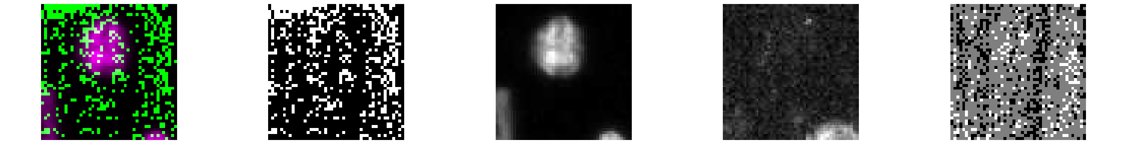				
12551	83	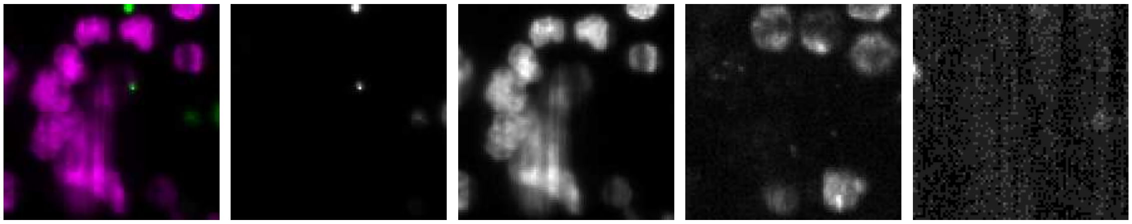				
12552	83	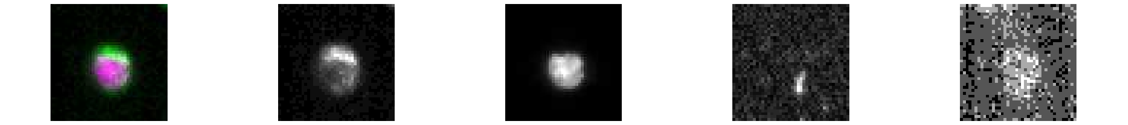				
12553	83	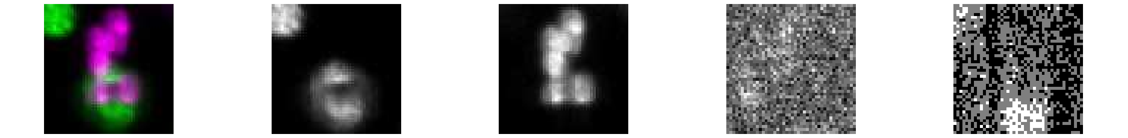				
12554	83	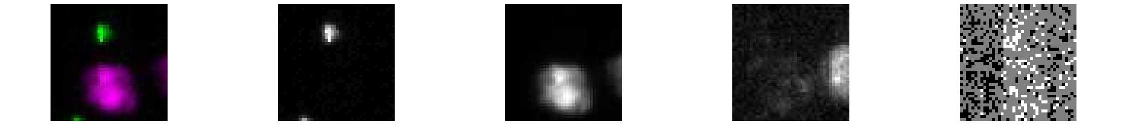				
12555	83	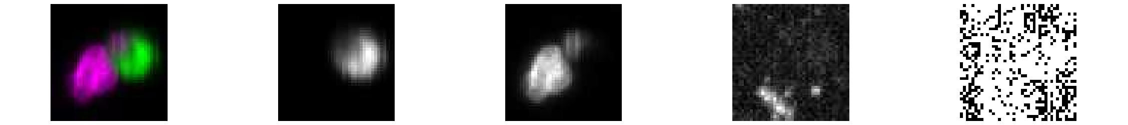				
12556	83	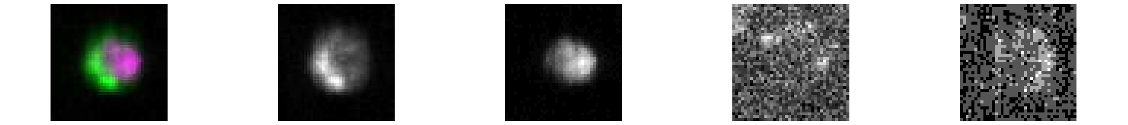				

Ereignis	Aufnahme	DAPI/CK-PE	CK-PE	DAPI	CD45-APC	HER-2/neu
12557	83	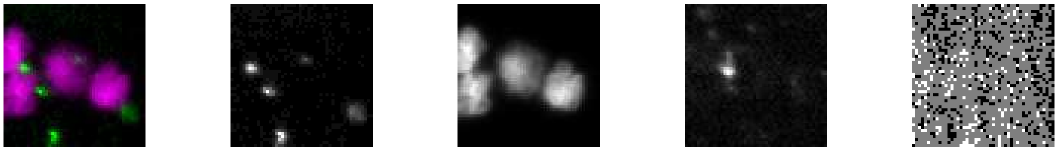				
12558	83	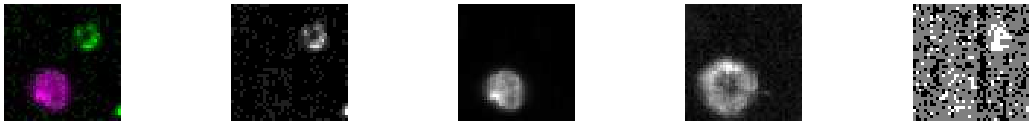				
12559	83	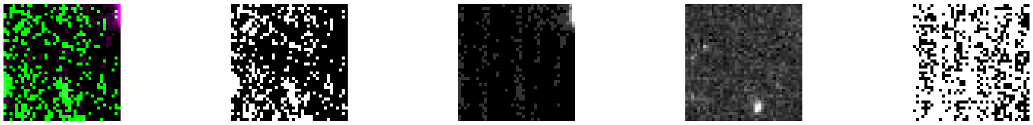				
12560	83	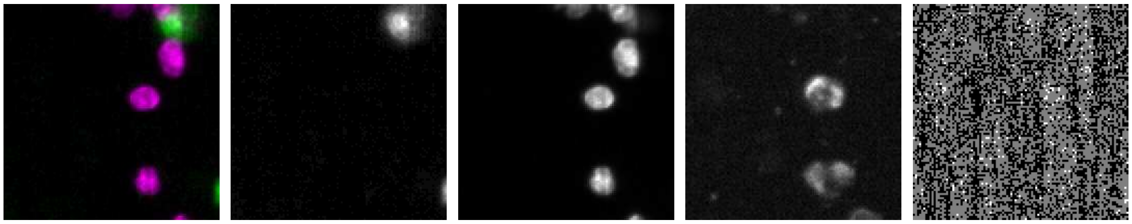				
12561	83	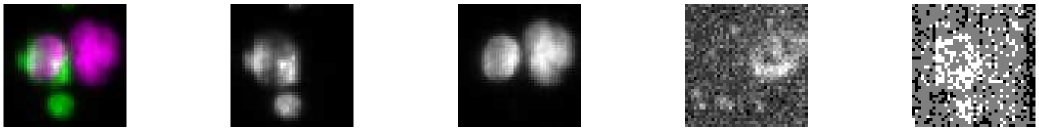				
12562	83	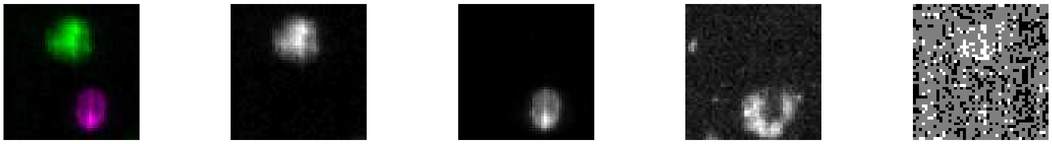				
12563	83	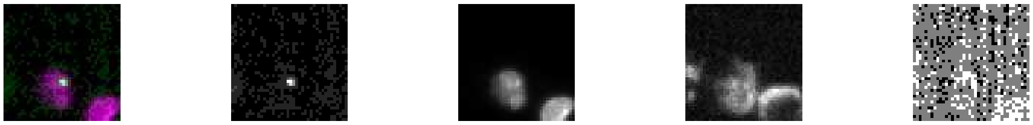				
12564	83	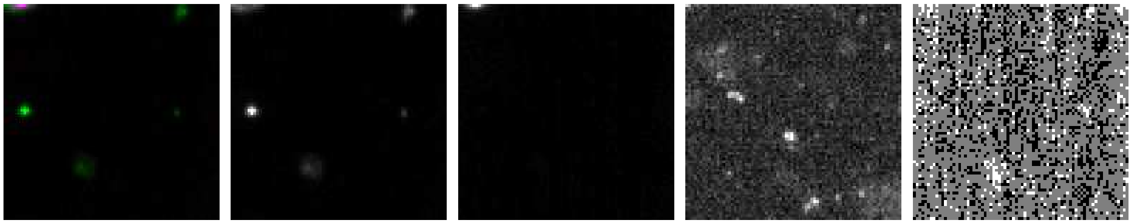				
12565	83	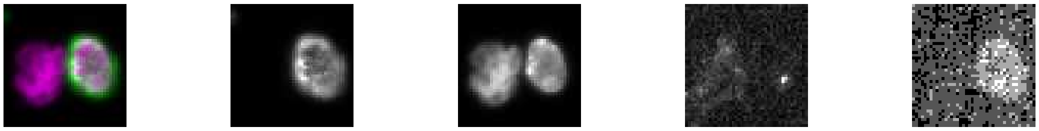				
12566	83	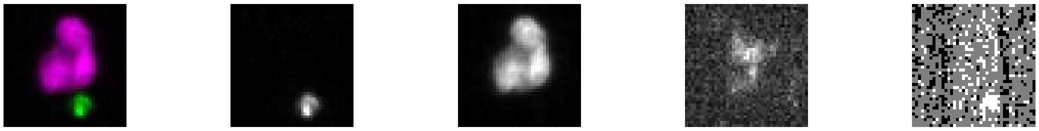				
12567	83	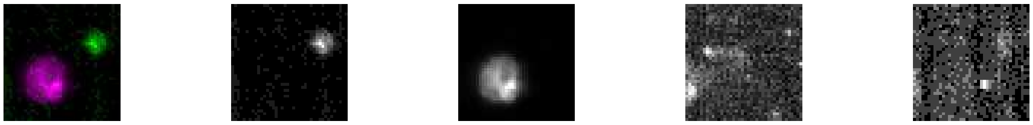				

Ereignis	Aufnahme	DAPI/CK-PE	CK-PE	DAPI	CD45-APC	HER-2/neu
12568	83	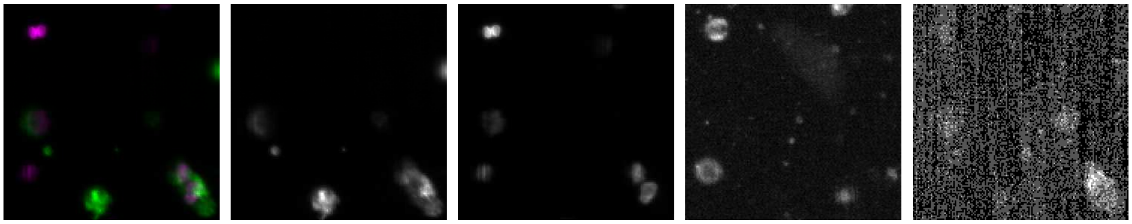				
12569	83	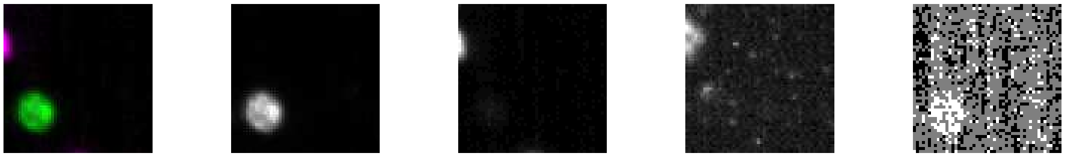				
12570	83	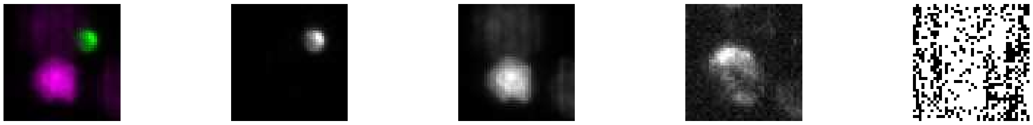				
12571	83	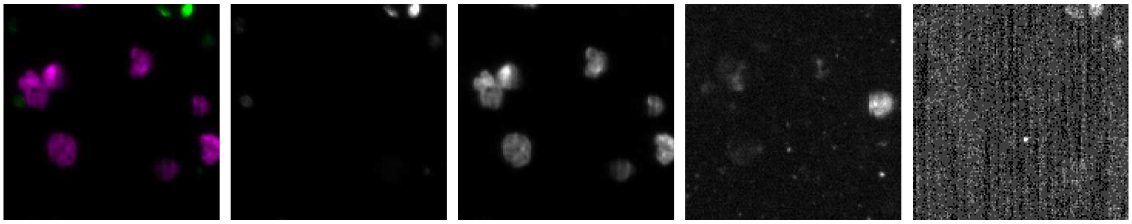				
12572	83	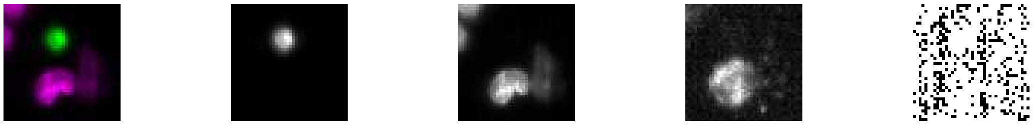				
12573	83	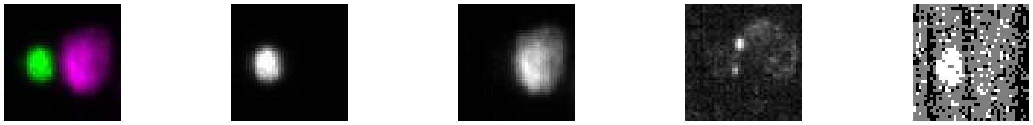				
12574	83	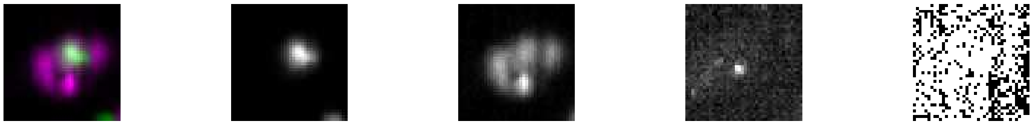				
12575	83	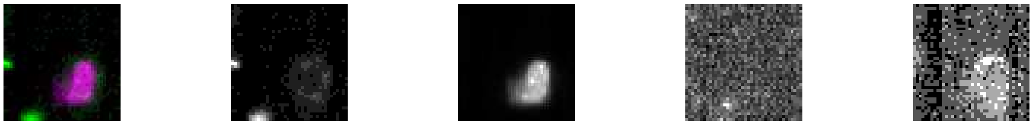				
12576	83	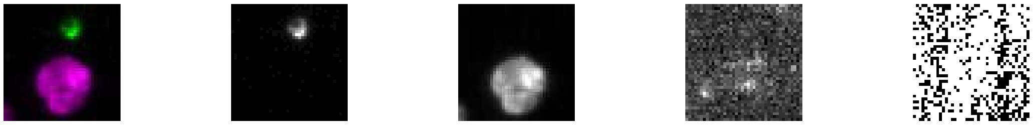				
12577	83	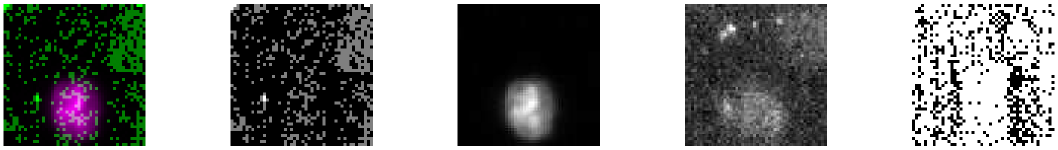				
12578	83	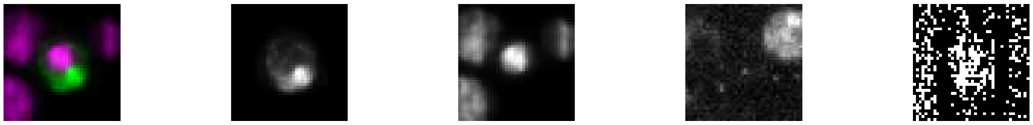				

Ereignis	Aufnahme	DAPI/CK-PE	CK-PE	DAPI	CD45-APC	HER-2/neu
12579	83	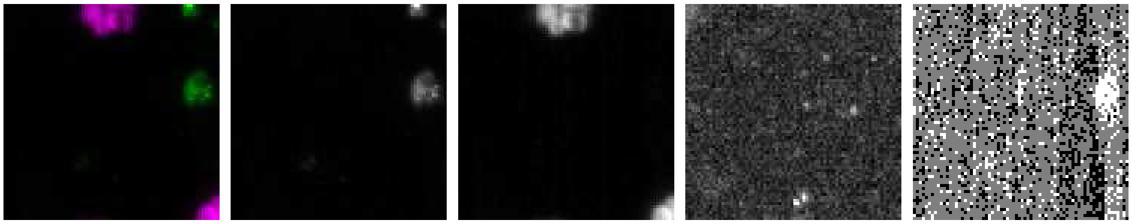				
12580	83	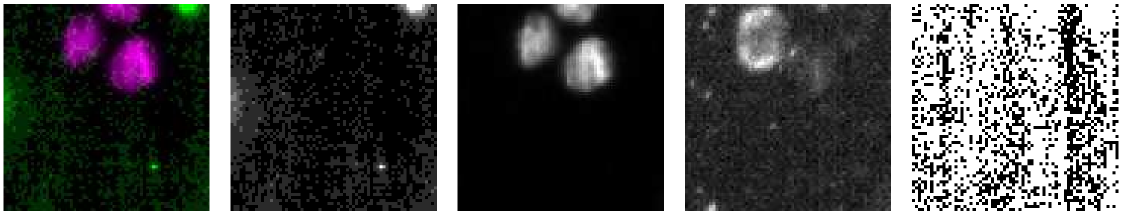				
12581	83	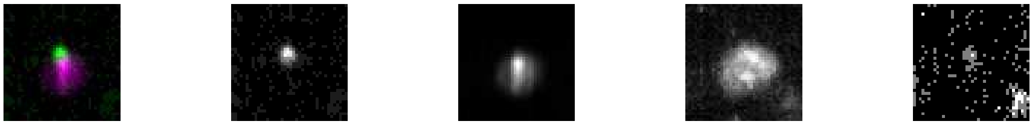				
12582	83	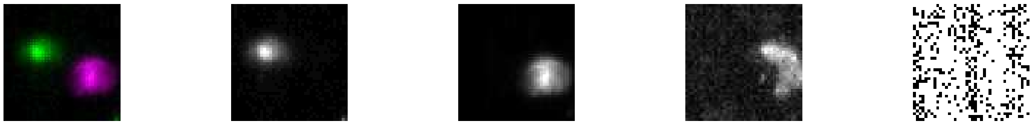				
12583	83	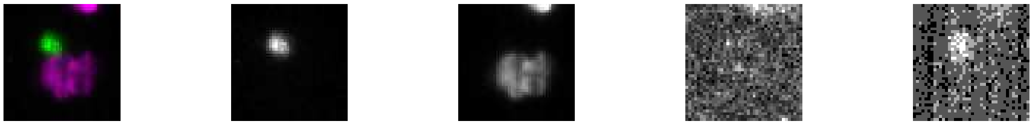				
12584	83	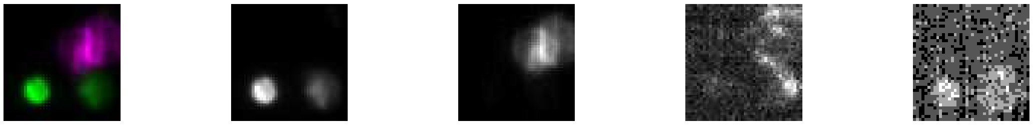				
12585	83	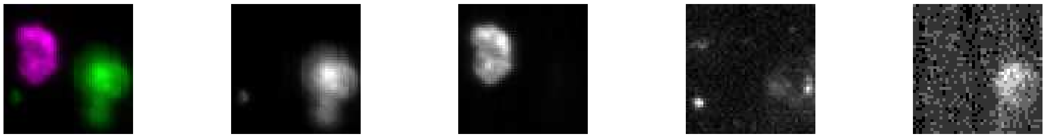				
12586	83	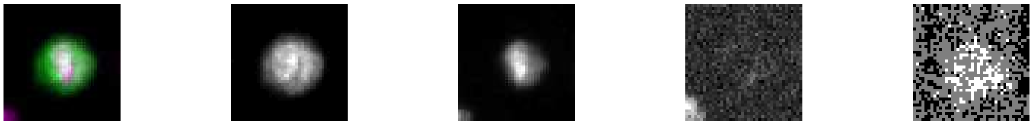				
12587	83	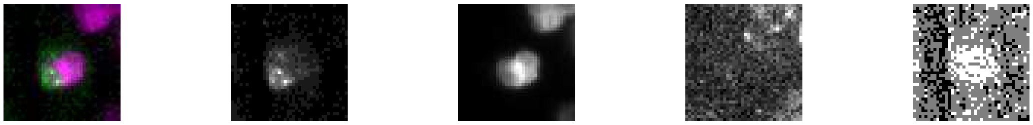				
12588	83	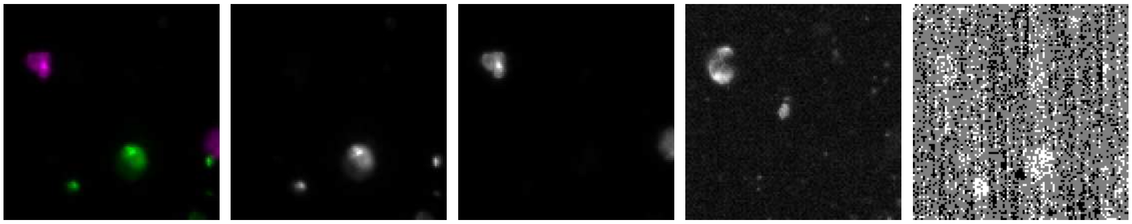				
12589	83	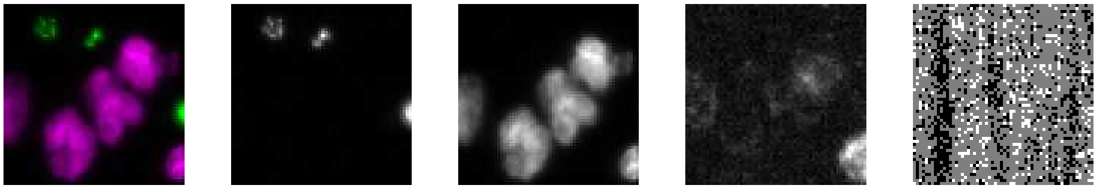				

Ereignis	Aufnahme	DAPI/CK-PE	CK-PE	DAPI	CD45-APC	HER-2/neu
12590	83	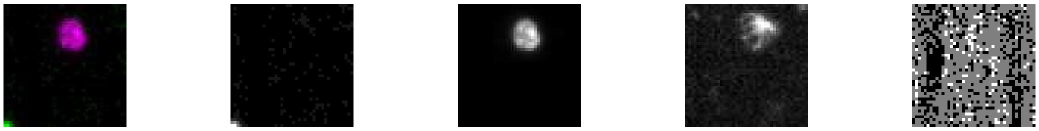				
12591	83	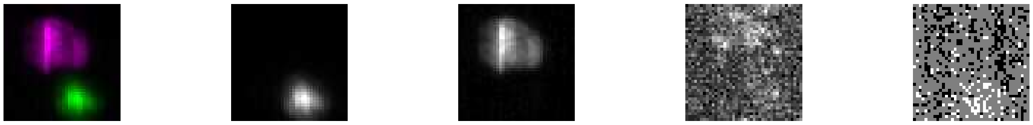				
12592	83	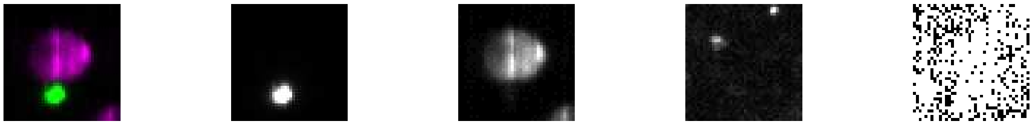				
12593	83	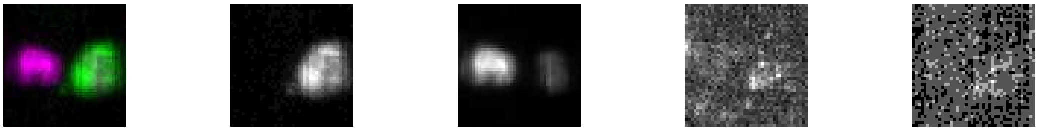				
12594	83	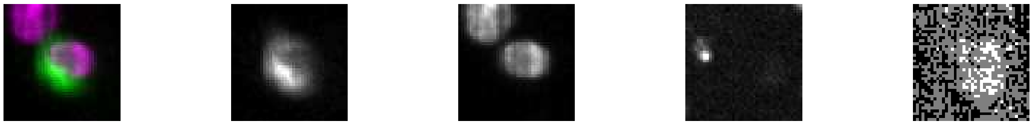				
12595	83	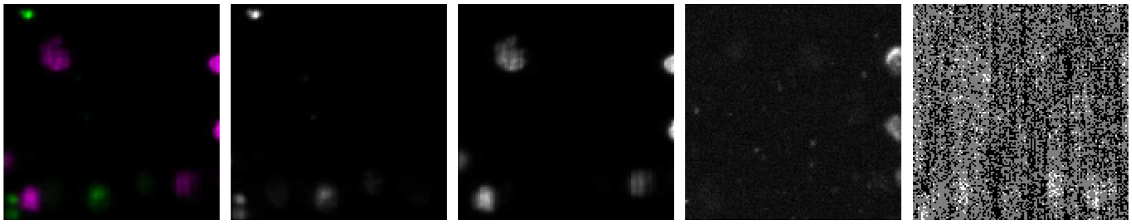				
12596	83	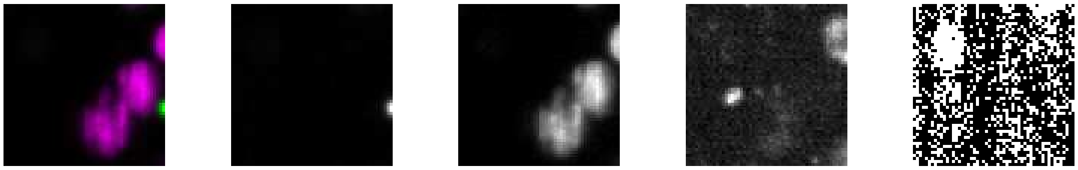				
12597	83	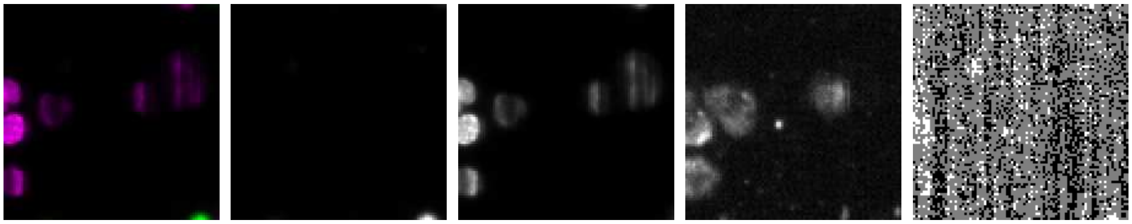				
12598	83	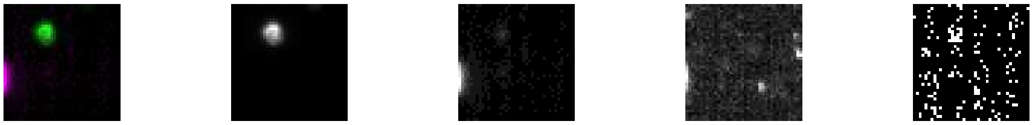				
12599	83	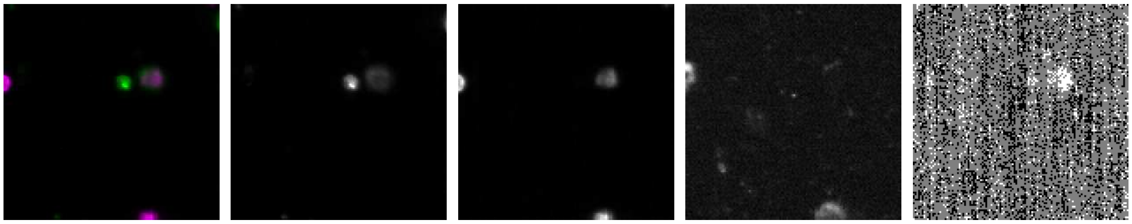				
12600	83	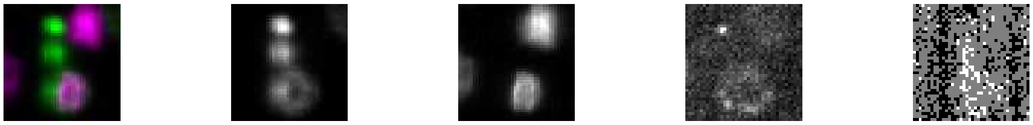				

Druckdatum: 02/06/2020 17:00

Proben-ID: Gyn14.208 **Kassetten-ID:** 01327106 **Patienten-ID:**

Ereignis	Aufnahme	DAPI/CK-PE	CK-PE	DAPI	CD45-APC	HER-2/neu
12601	83	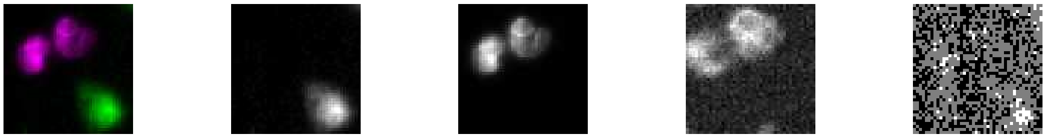				
12602	83	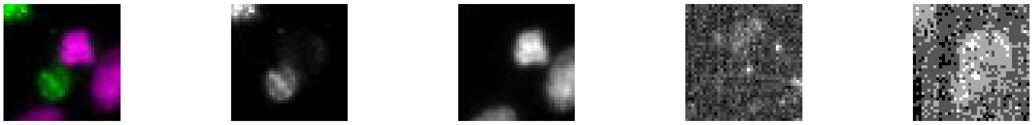				
12603	83	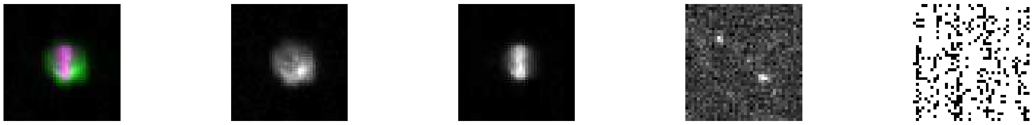				
12604	83	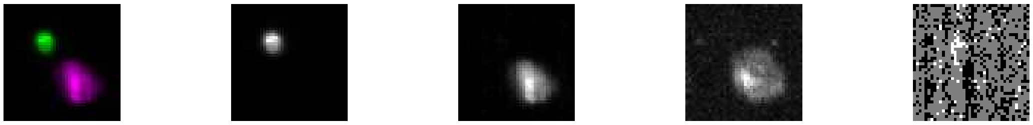				
12605	83	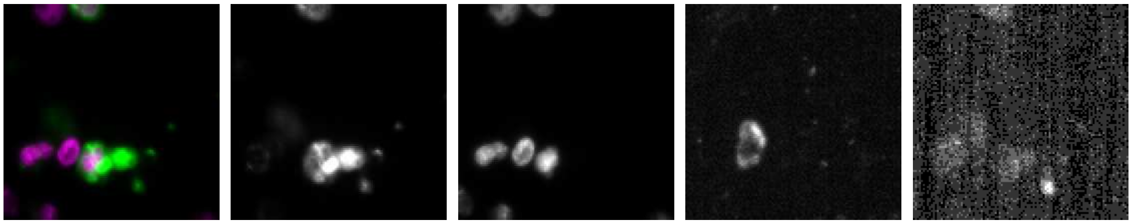				
12606	83	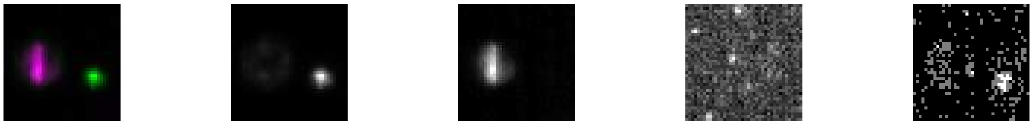				
12607	83	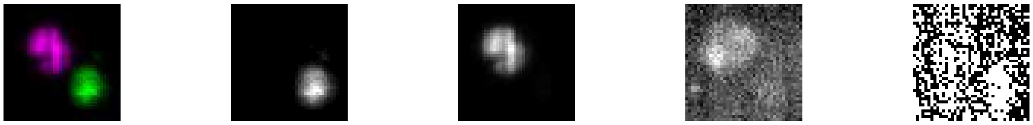				
12608	83	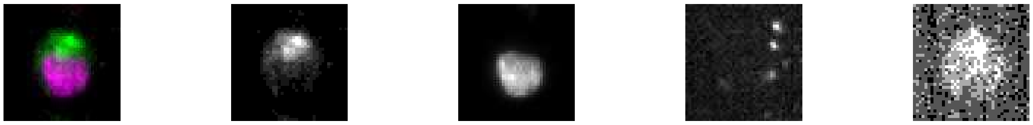				
12609	83	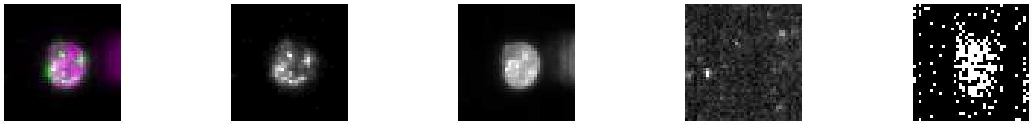				
12610	83	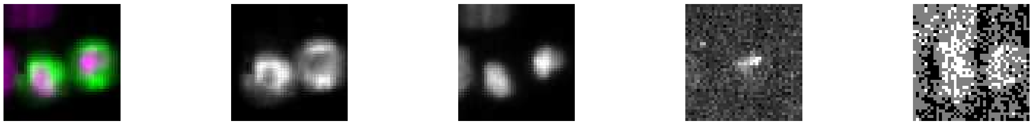				
12611	83	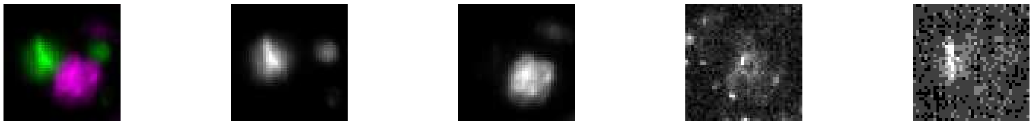				
12612	83	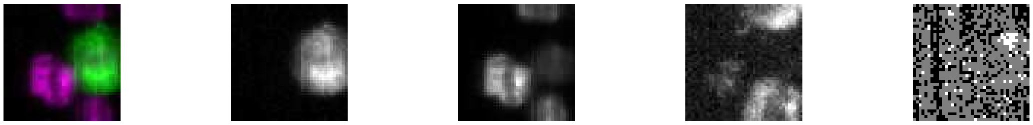				
12613	83	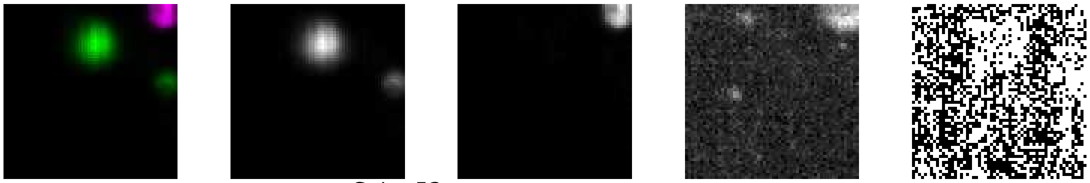				

Ereignis	Aufnahme	DAPI/CK-PE	CK-PE	DAPI	CD45-APC	HER-2/neu
12614	83	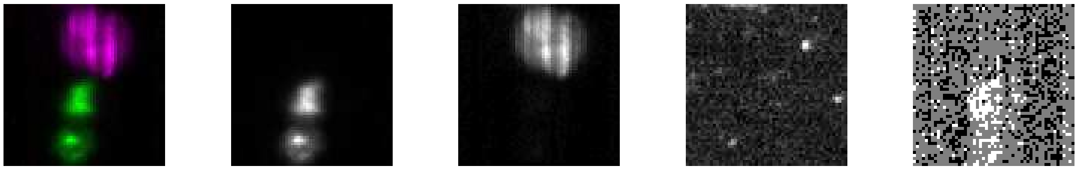				
12615	83	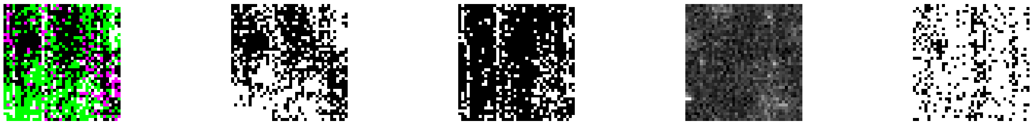				
12616	83	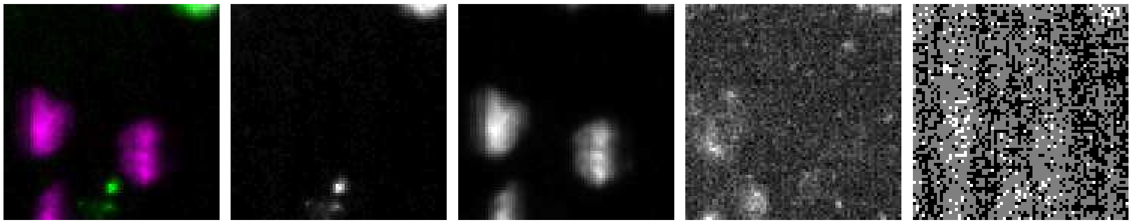				
12617	83	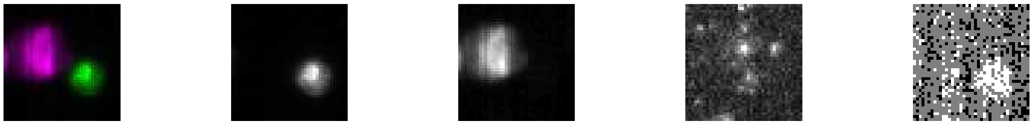				
12618	83	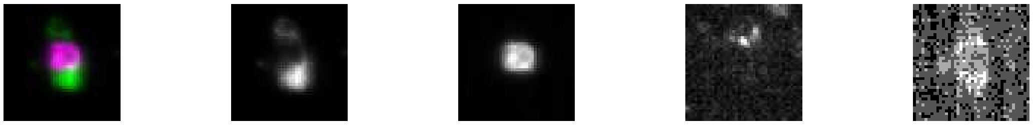				
12619	83	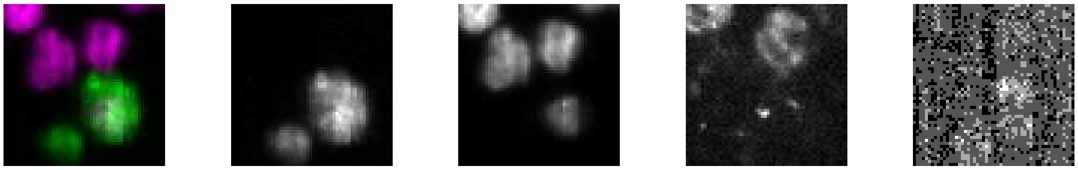				
12620	83	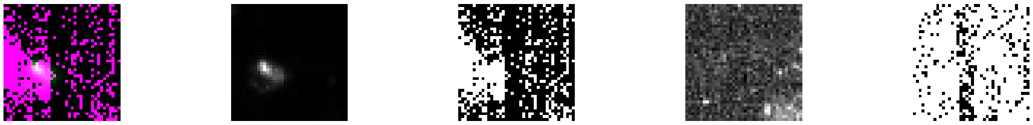				
12621	83	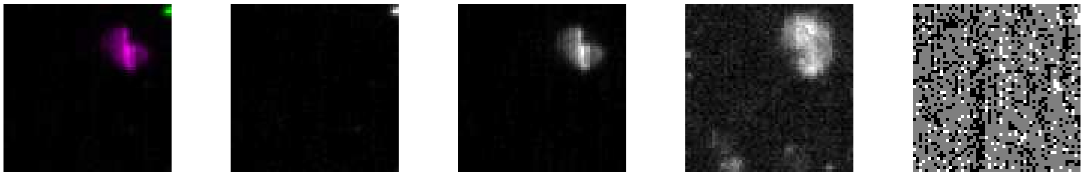				
12622	83	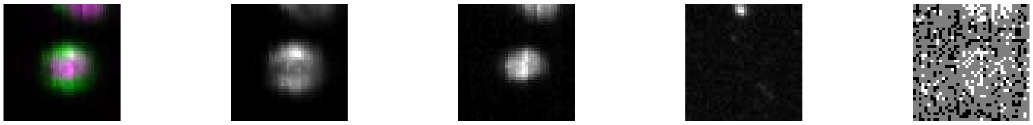				
12623	83	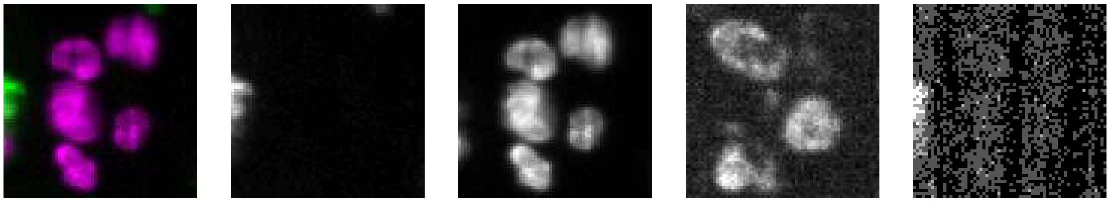				
12624	83	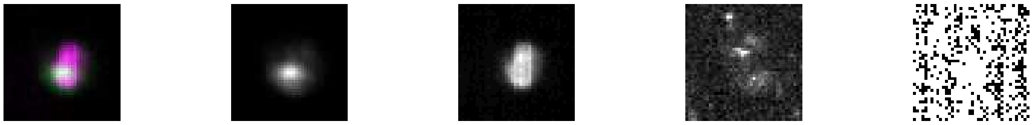				

Ereignis	Aufnahme	DAPI/CK-PE	CK-PE	DAPI	CD45-APC	HER-2/neu
12625	83	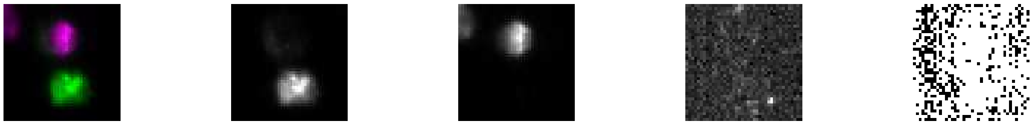				
12626	83	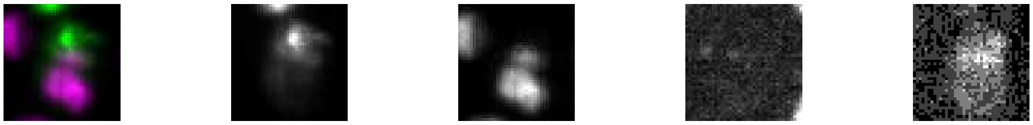				
12627	83	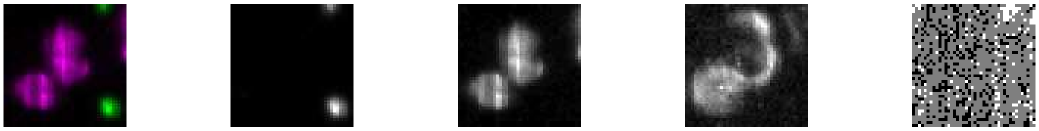				
12628	83	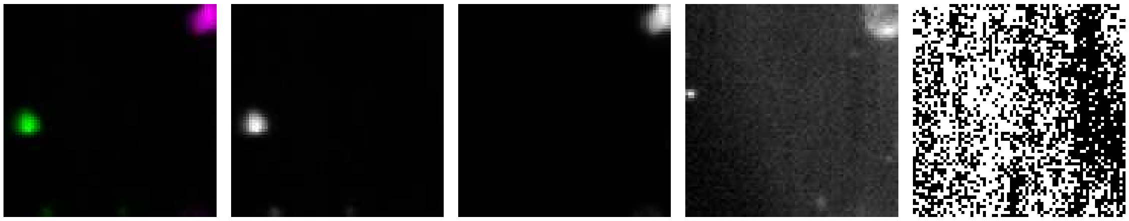				
12629	83	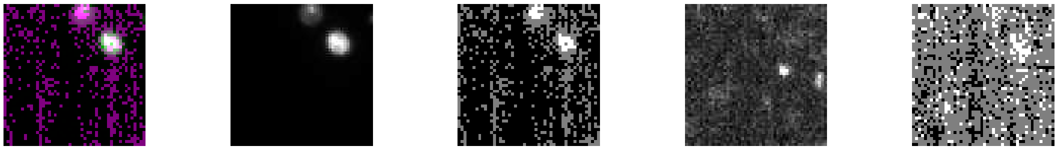				
12630	83	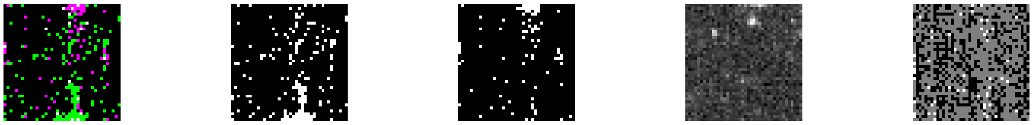				
12631	83	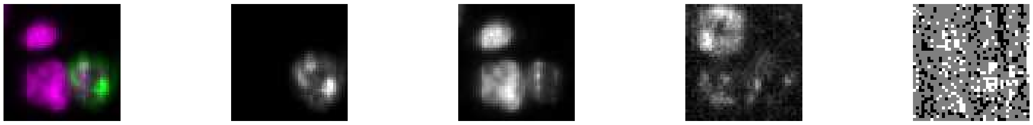				
12632	83	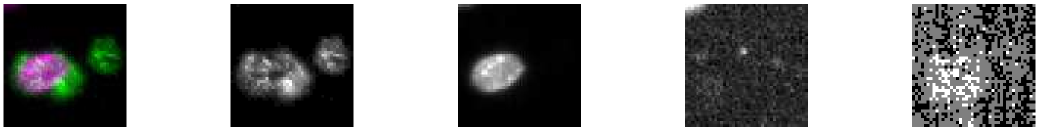				
12633	83	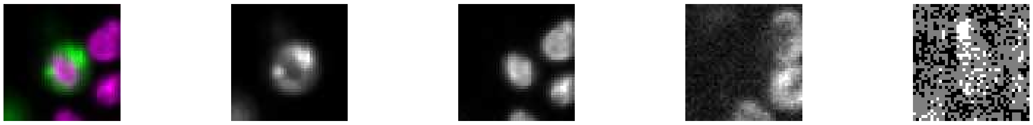				
12634	83	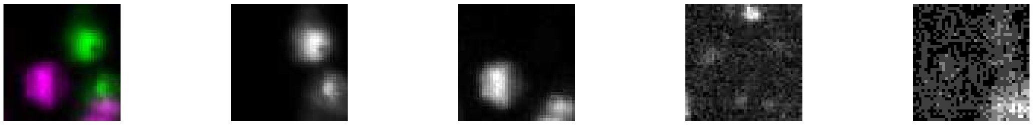				
12635	83	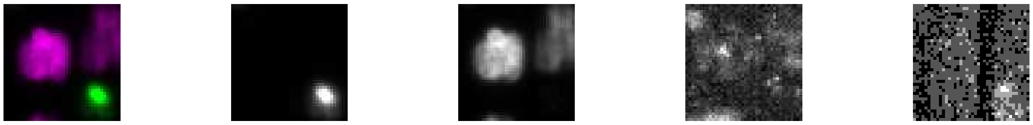				
12636	83	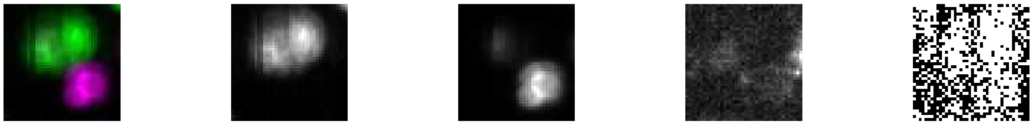				

Ereignis	Aufnahme	DAPI/CK-PE	CK-PE	DAPI	CD45-APC	HER-2/neu
12637	83	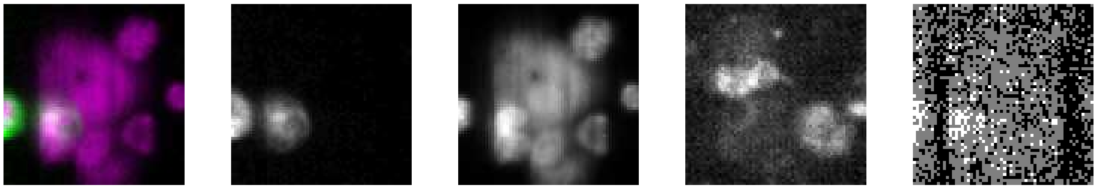				
12638	83	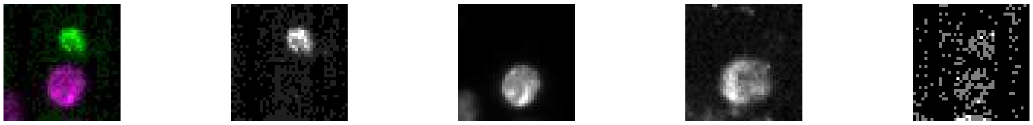				
12639	83	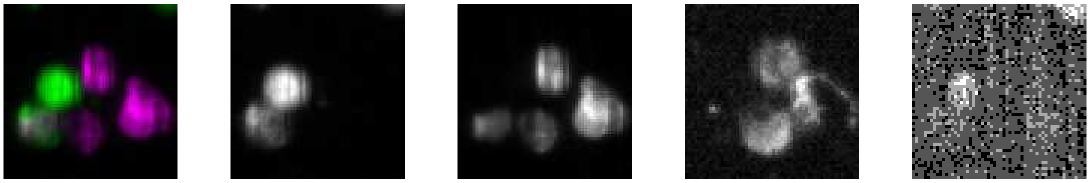				
12640	83	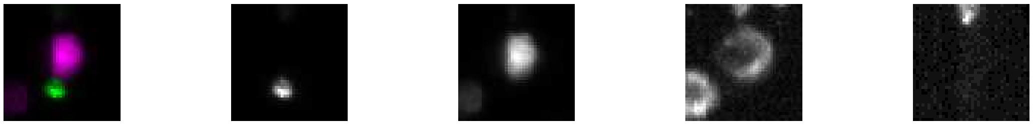				
12641	83	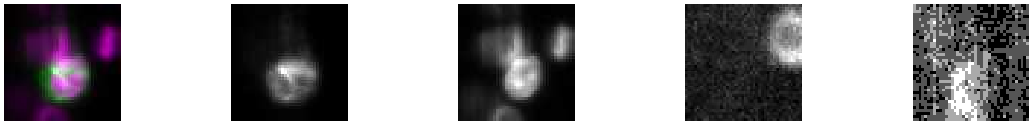				
12642	83	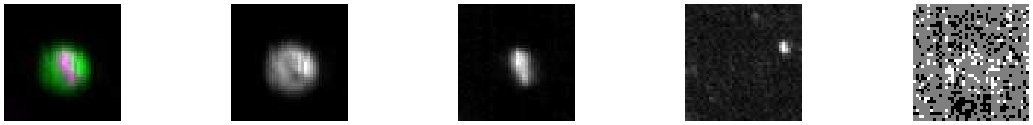				
12643	83	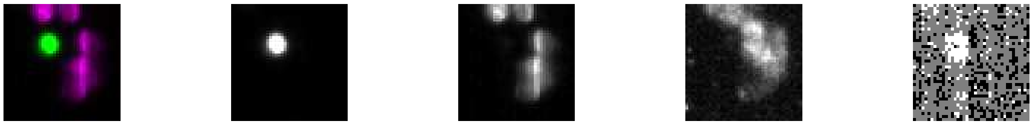				
12644	83	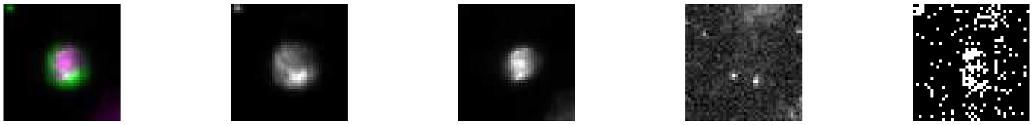				
12645	83	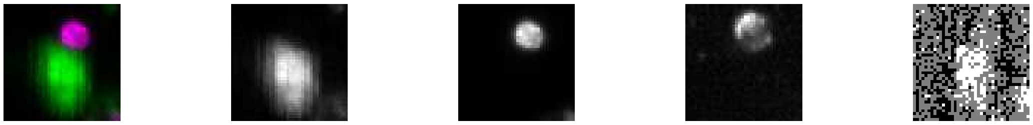				
12646	83	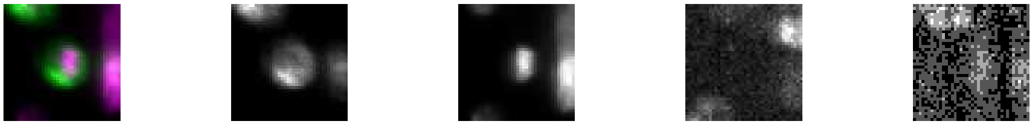				
12647	83	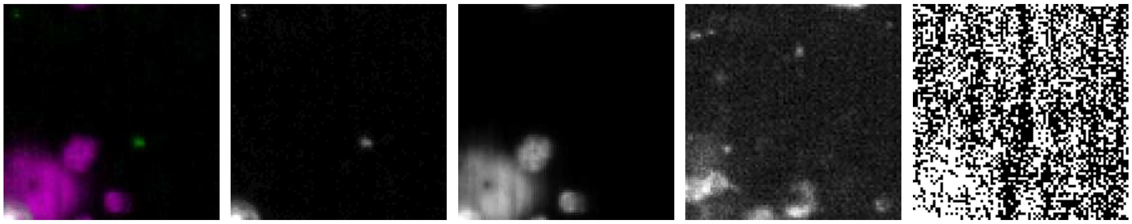				
12648	83	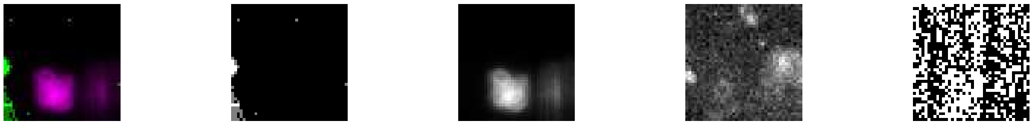				

Ereignis	Aufnahme	DAPI/CK-PE	CK-PE	DAPI	CD45-APC	HER-2/neu
12649	83	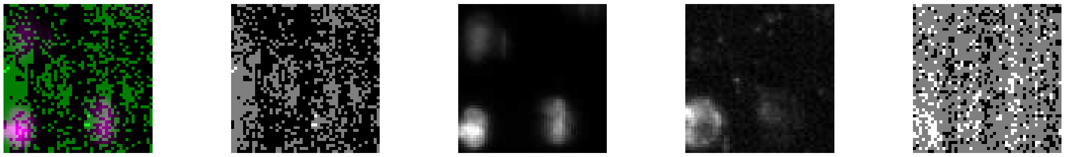				
12650	83	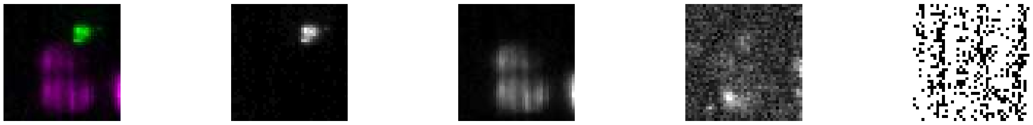				
12651	83	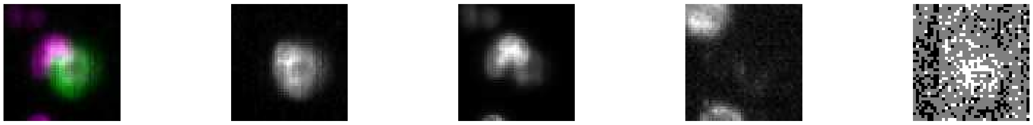				
12652	83	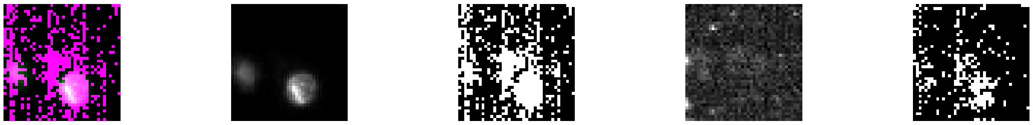				
12653	83	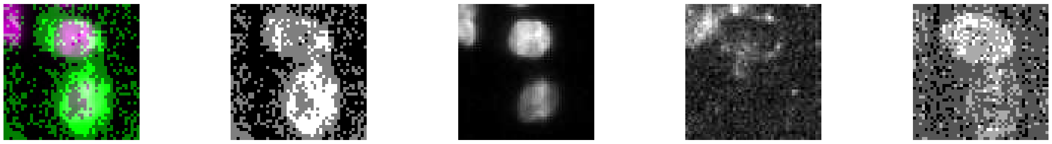				
12654	83	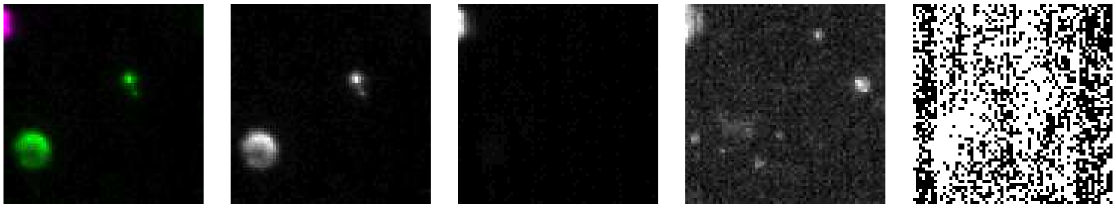				
12655	83	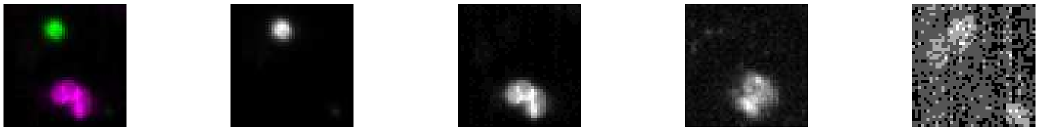				
12656	83	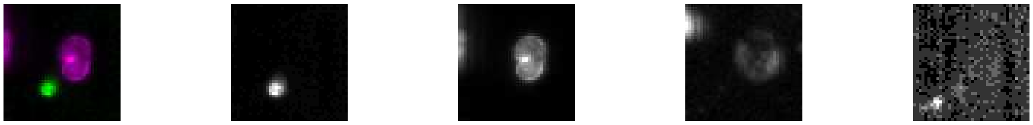				
12657	83	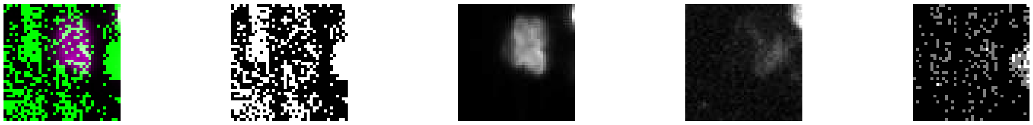				
12658	83	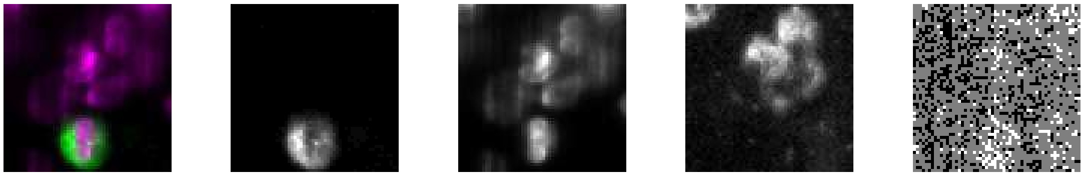				
12659	83	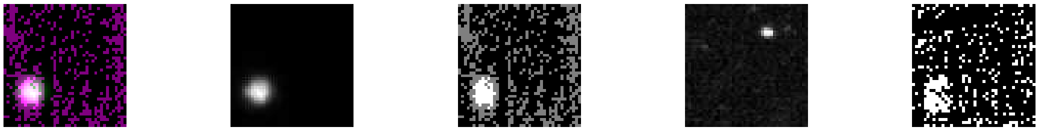				
12660	83	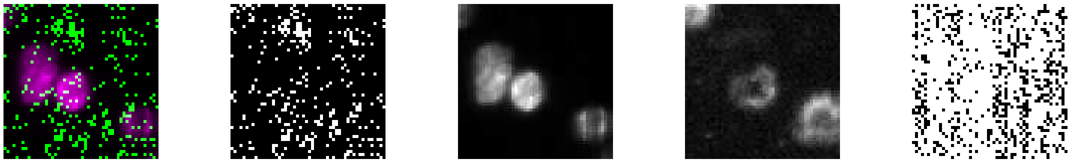				

Ereignis	Aufnahme	DAPI/CK-PE	CK-PE	DAPI	CD45-APC	HER-2/neu
12661	83	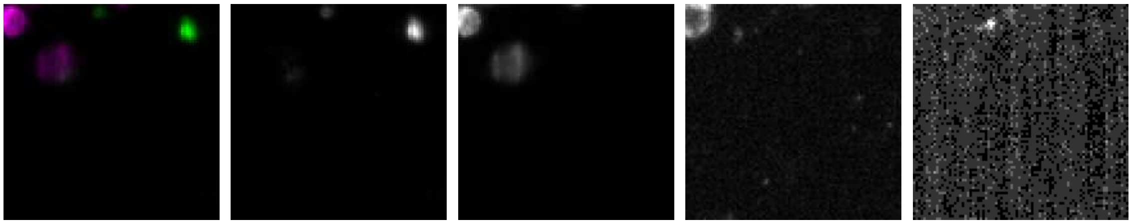				
12662	83	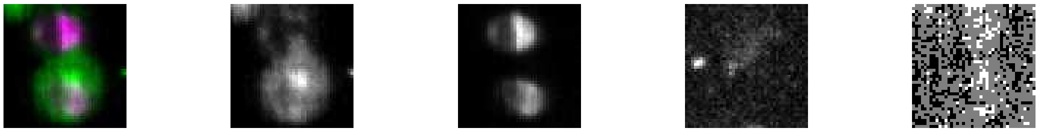				
12663	83	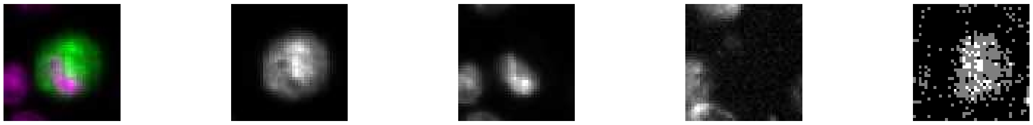				
12664	83	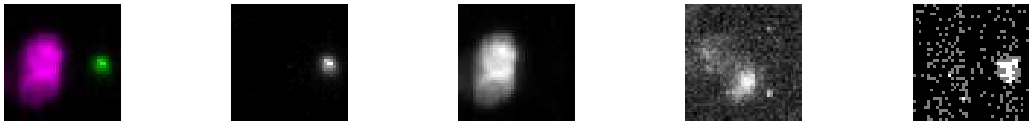				
12665	83	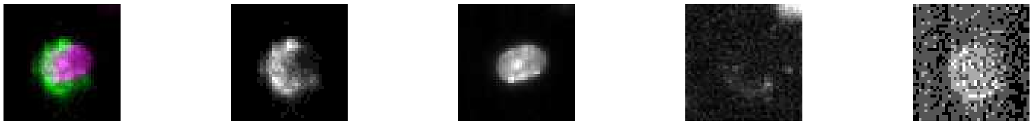				
12666	83	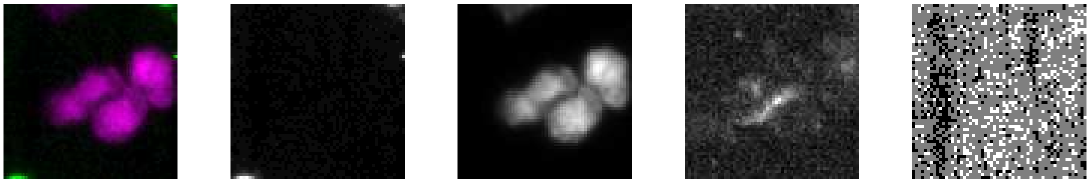				
12667	83	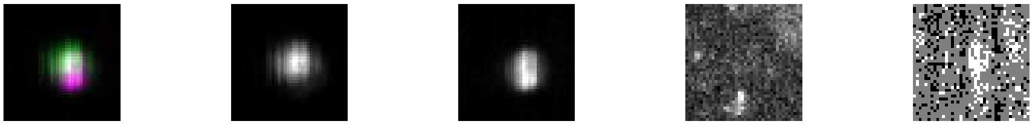				
12668	83	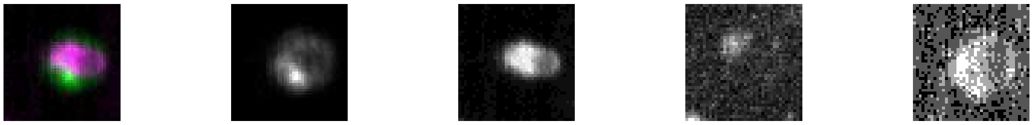				
12669	83	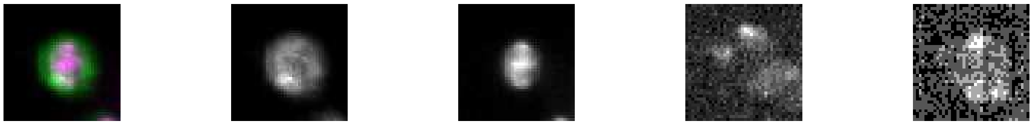				
12670	83	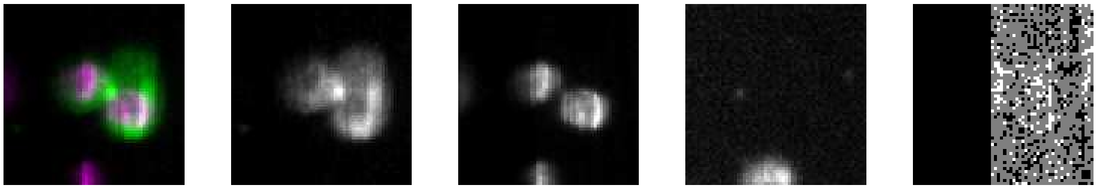				
12671	83	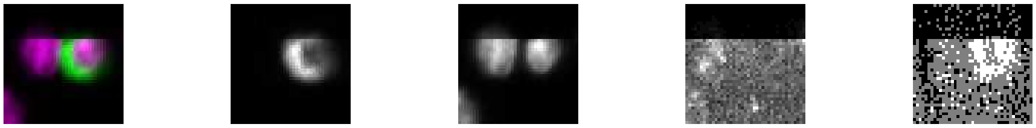				

Ereignis	Aufnahme	DAPI/CK-PE	CK-PE	DAPI	CD45-APC	HER-2/neu
12672	82	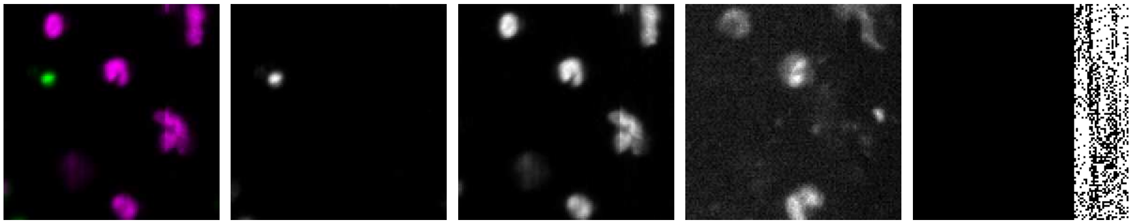				
12673	82	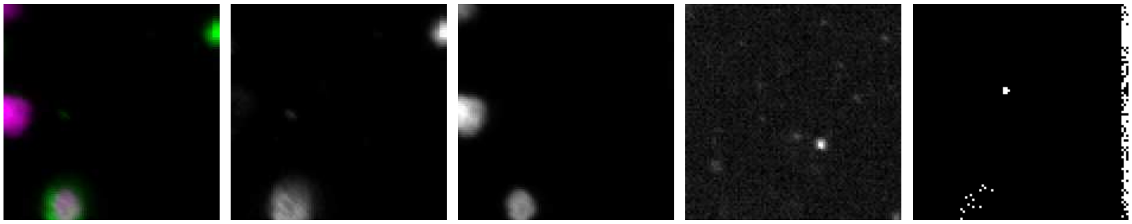				
12674	83	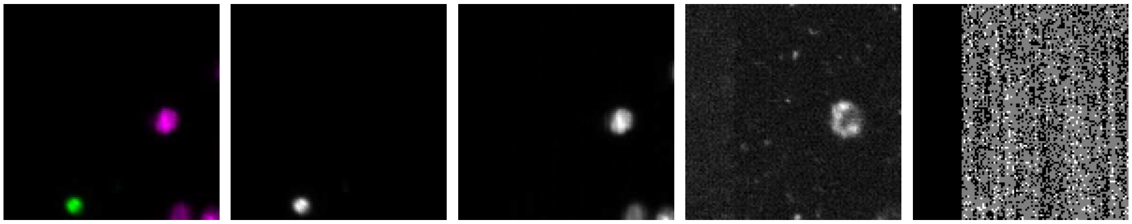				
12675	58	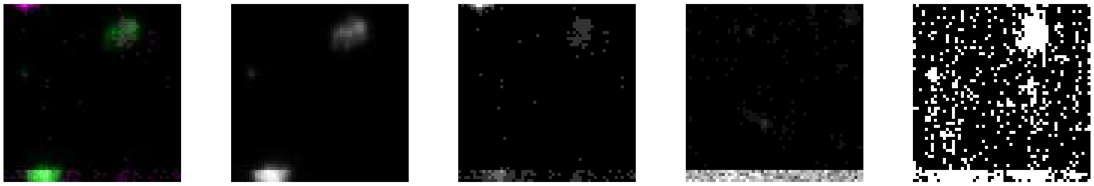				
12676	58	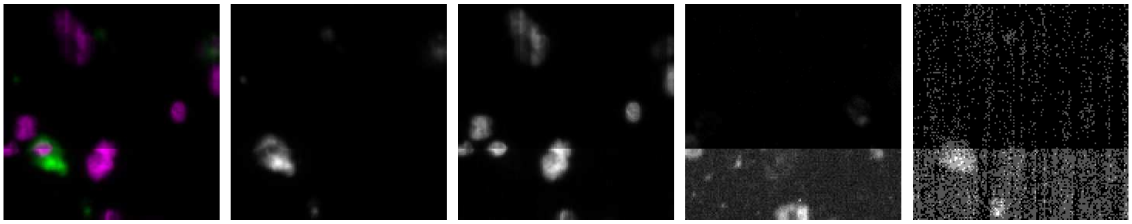				
12677	58	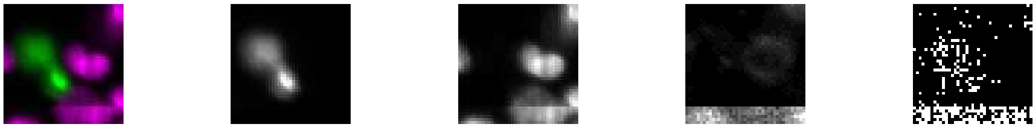				
12678	58	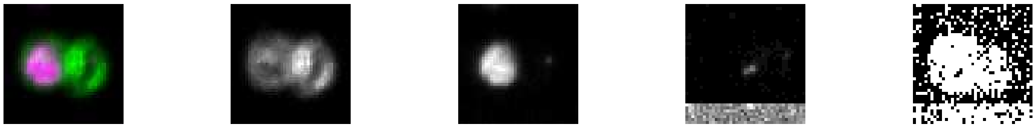				
12679	83	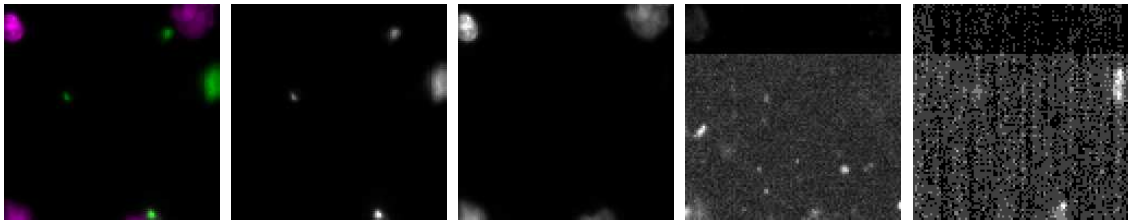				
12680	83	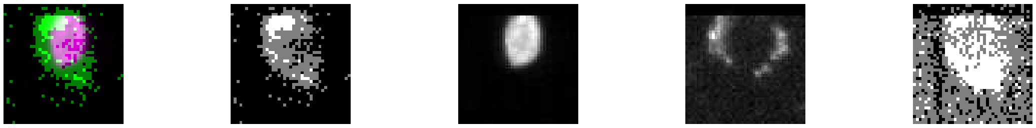				

Ereignis	Aufnahme	DAPI/CK-PE	CK-PE	DAPI	CD45-APC	HER-2/neu
12681	84	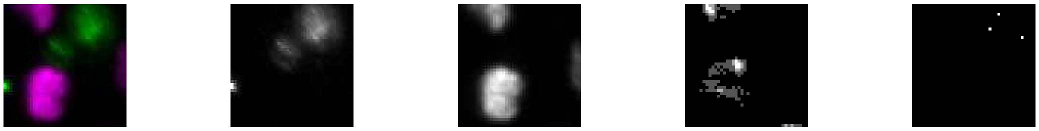				
12682	84	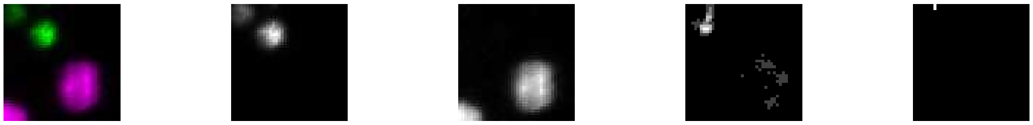				
12683	84	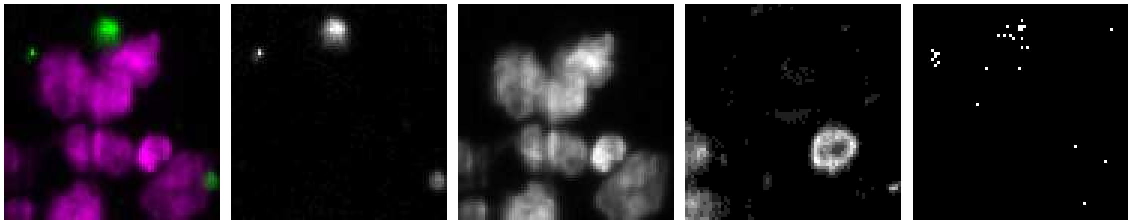				
12684	84	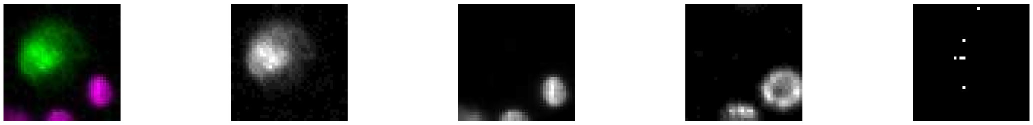				
12685	84	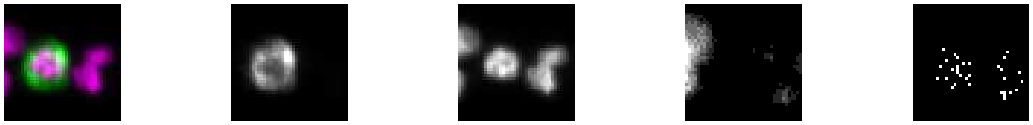				
12686	84	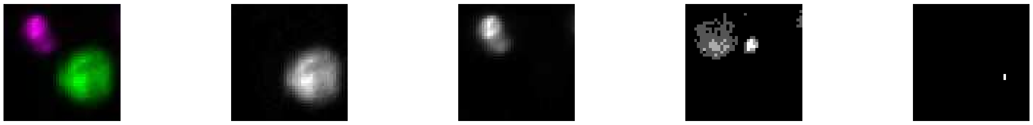				
12687	84	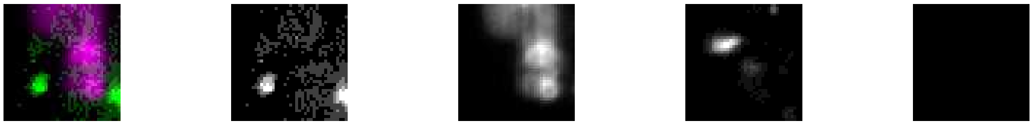				
12688	84	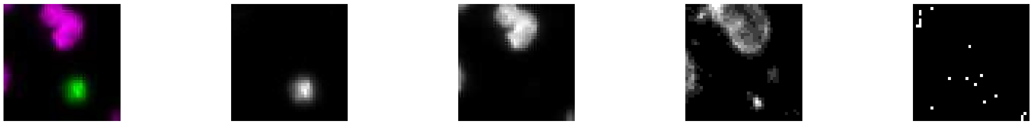				
12689	84	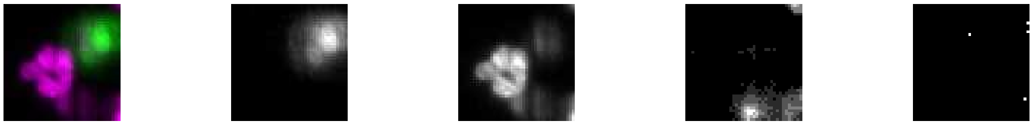				
12690	84	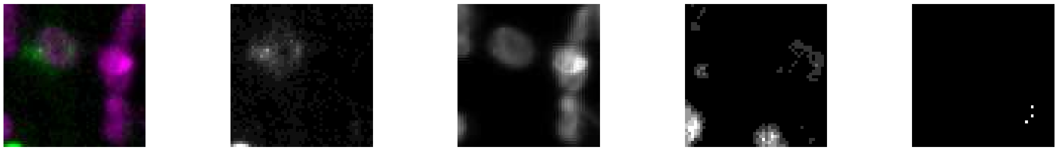				
12691	84	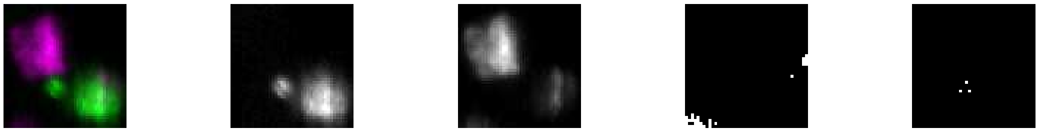				
12692	84	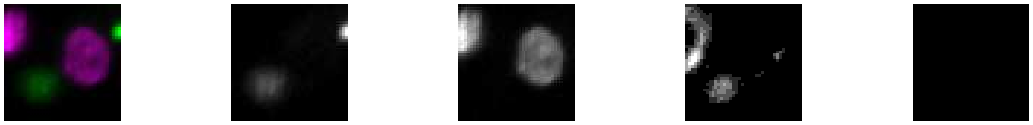				

Ereignis	Aufnahme	DAPI/CK-PE	CK-PE	DAPI	CD45-APC	HER-2/neu
12693	84	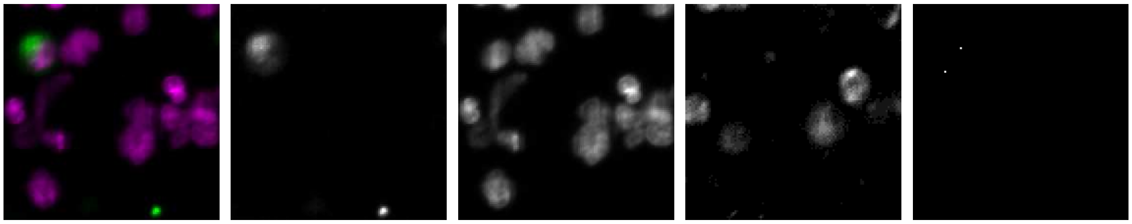				
12694	84	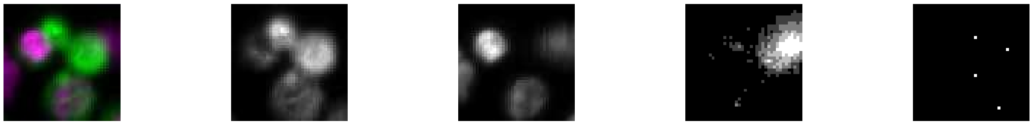				
12695	84	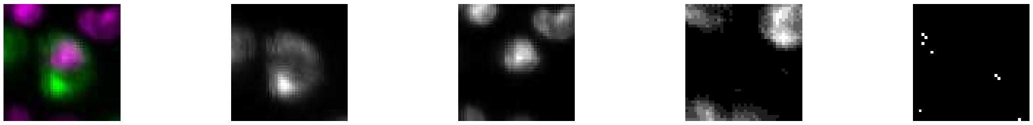				
12696	84	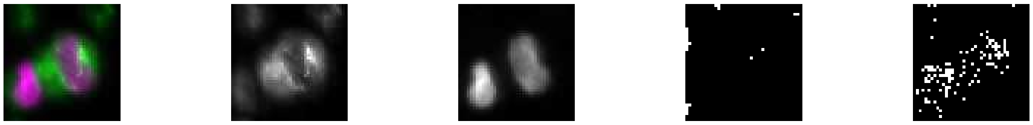				
12697	84	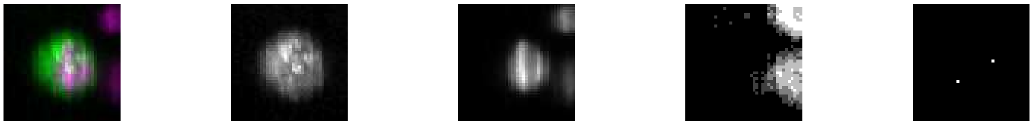				
12698	84	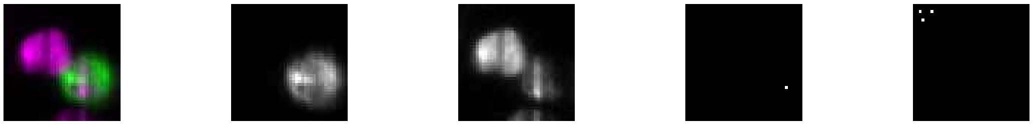				
12699	84	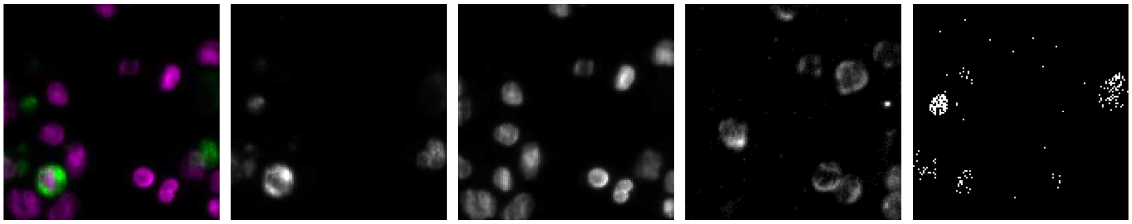				
12700	84	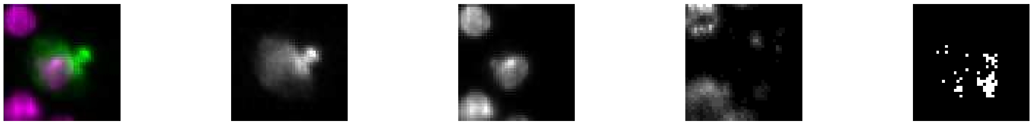				
12701	84	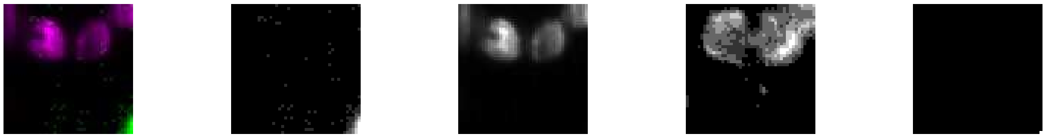				
12702	84	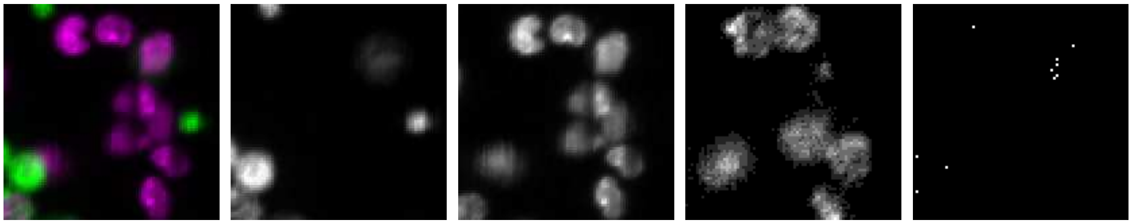				
12703	84	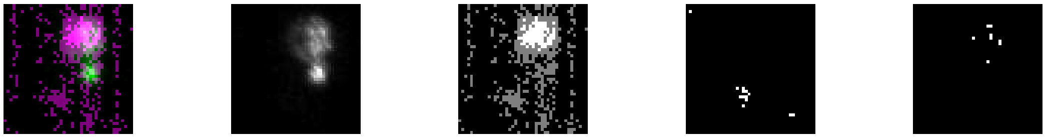				

Ereignis	Aufnahme	DAPI/CK-PE	CK-PE	DAPI	CD45-APC	HER-2/neu
12704	84	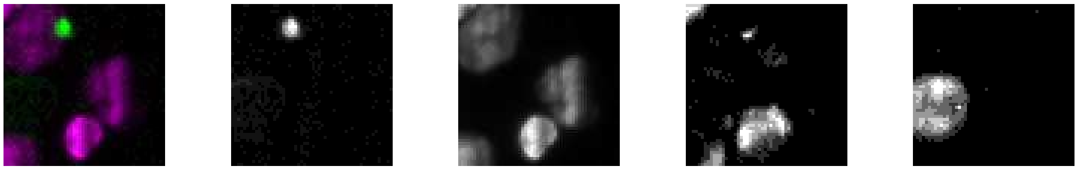				
12705	84	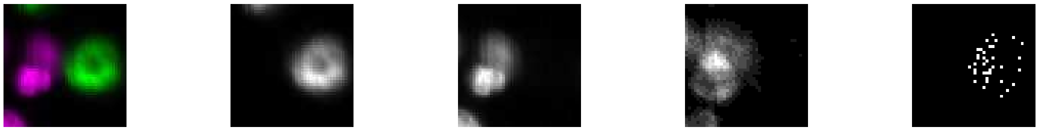				
12706	84	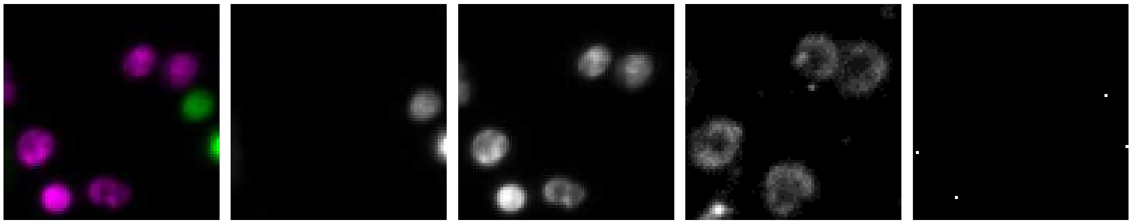				
12707	84	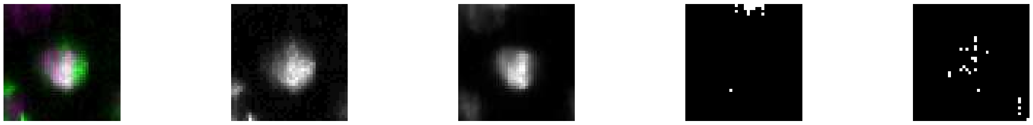				
12708	84	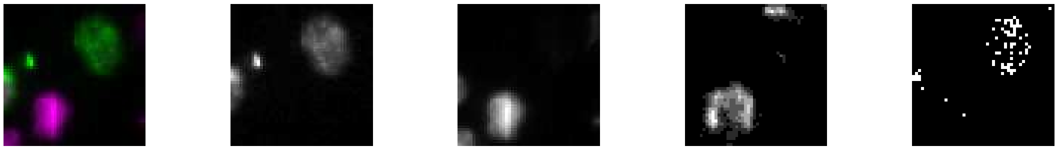				
12709	84	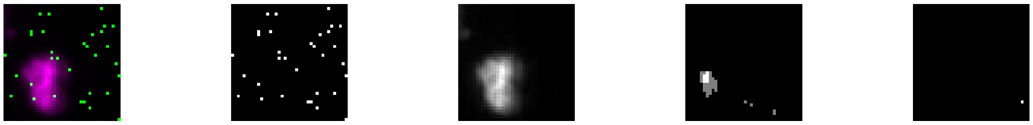				
12710	84	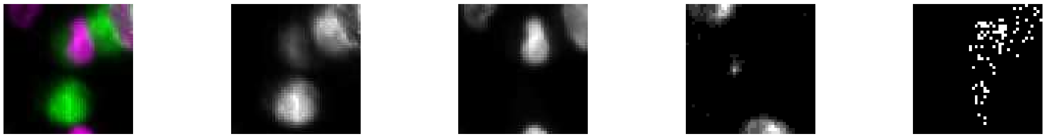				
12711	84	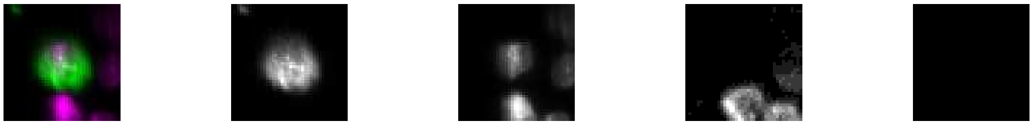				
12712	84	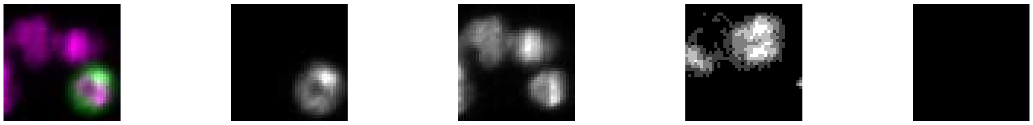				
12713	84	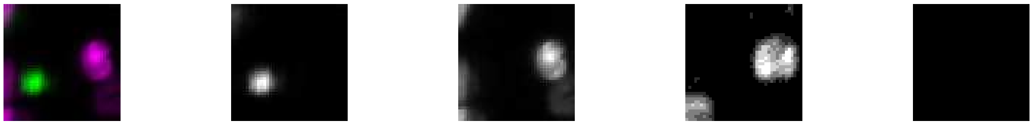				
12714	84	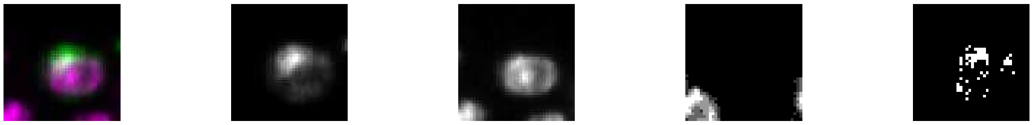				
12715	84	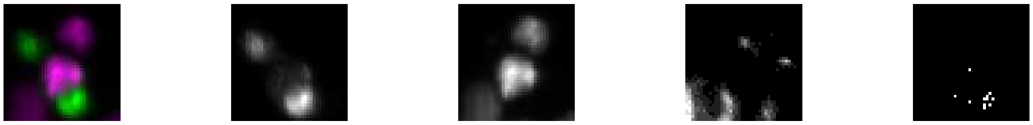				

Ereignis	Aufnahme	DAPI/CK-PE	CK-PE	DAPI	CD45-APC	HER-2/neu
12716	84	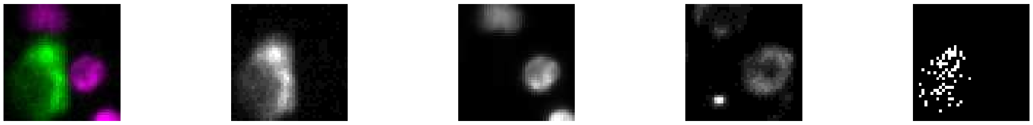				
12717	84	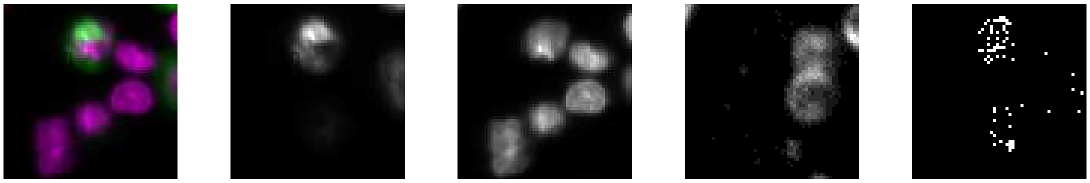				
12718	84	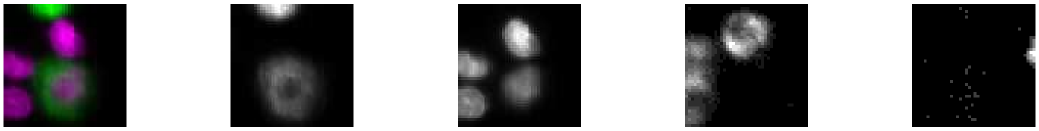				
12719	84	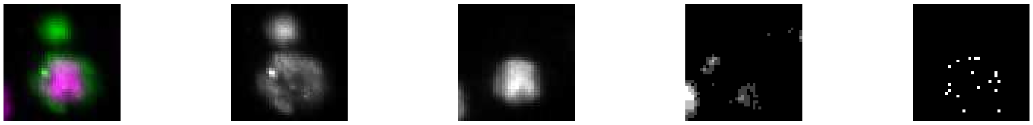				
12720	84	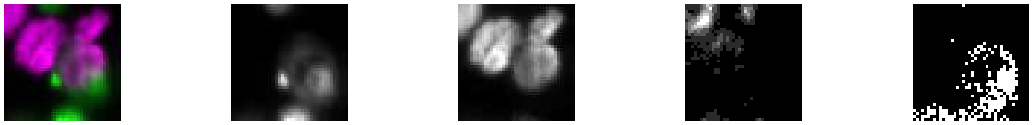				
12721	84	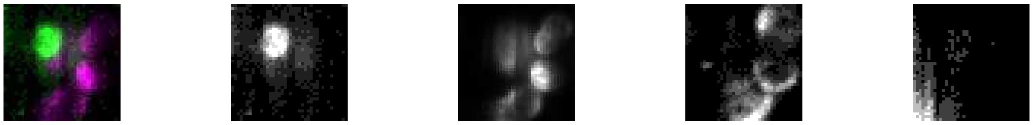				
12722	84	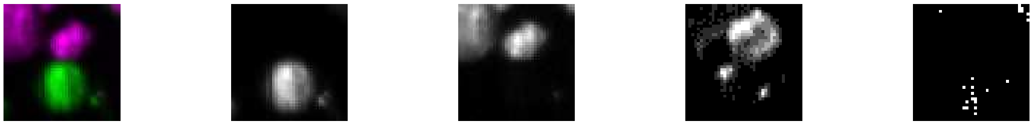				
12723	84	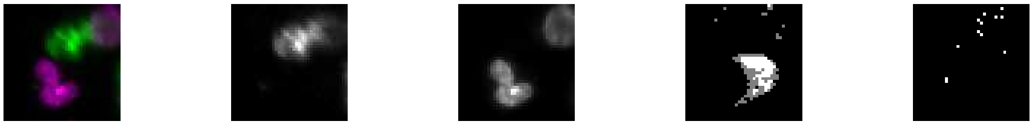				
12724	84	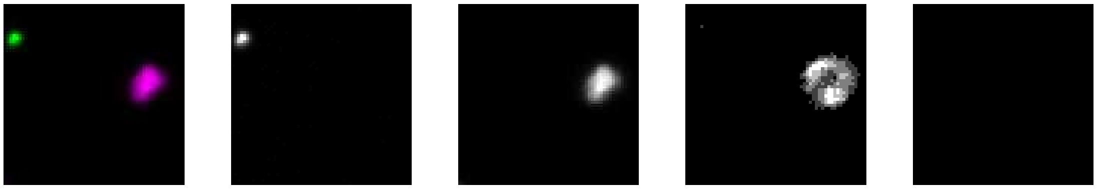				
12725	84	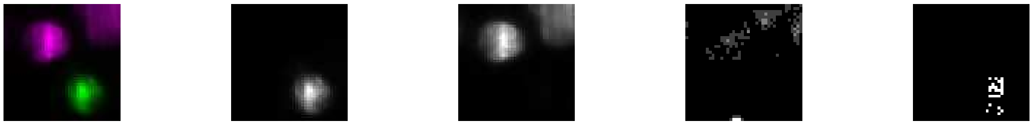				
12726	84	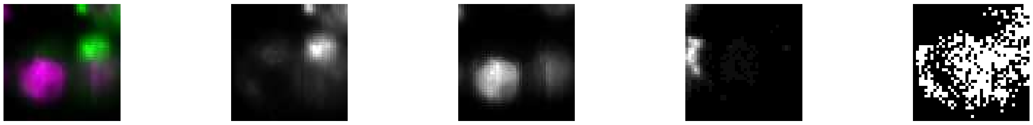				
12727	84	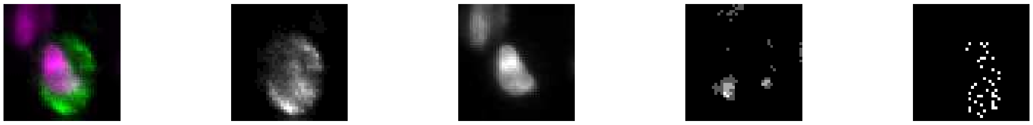				
12728	84	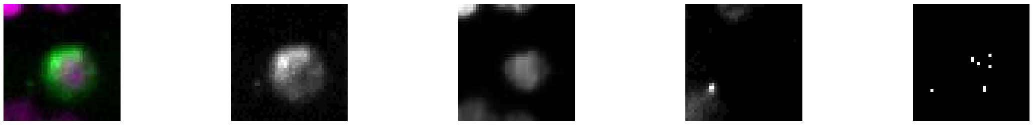				

Ereignis	Aufnahme	DAPI/CK-PE	CK-PE	DAPI	CD45-APC	HER-2/neu
12729	84	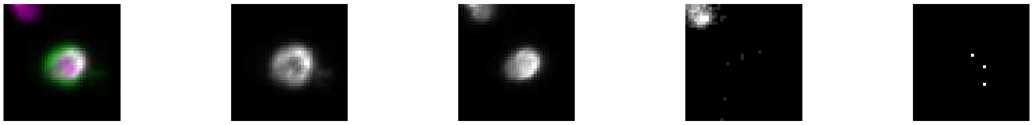				
12730	84	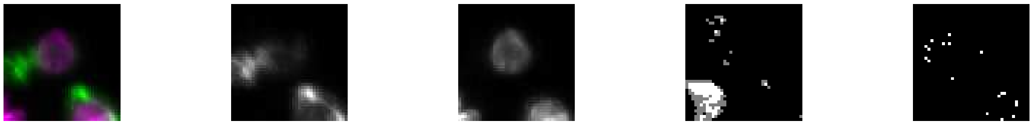				
12731	84	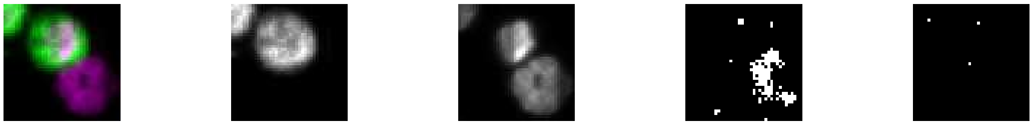				
12732	84	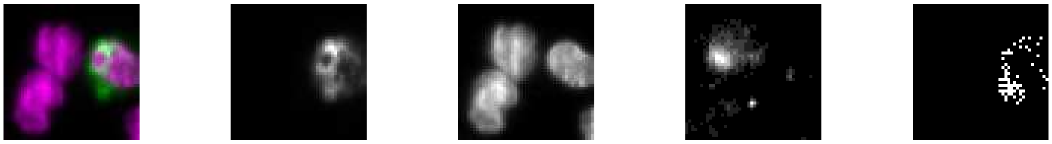				
12733	84	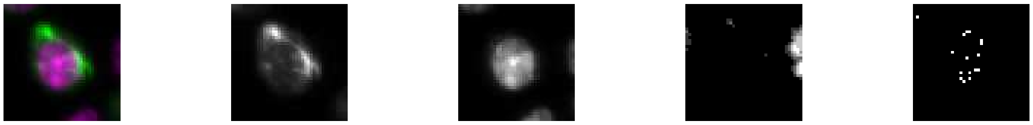				
12734	84	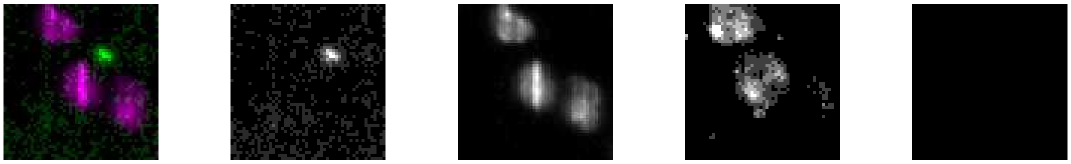				
12735	84	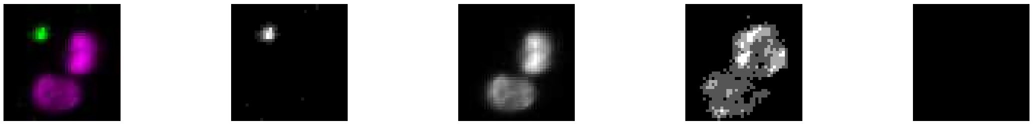				
12736	84	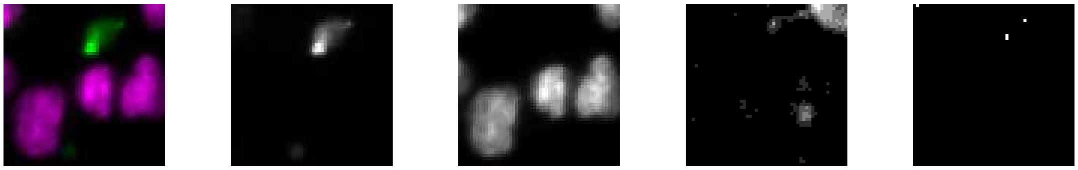				
12737	84	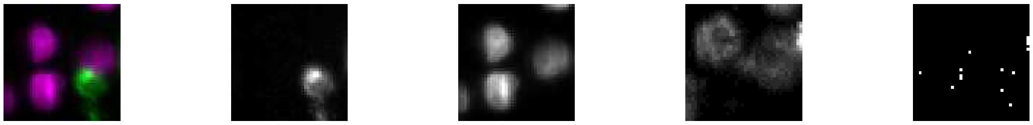				
12738	84	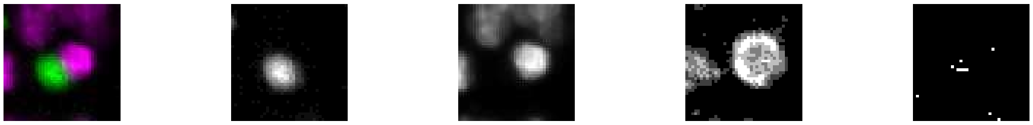				
12739	84	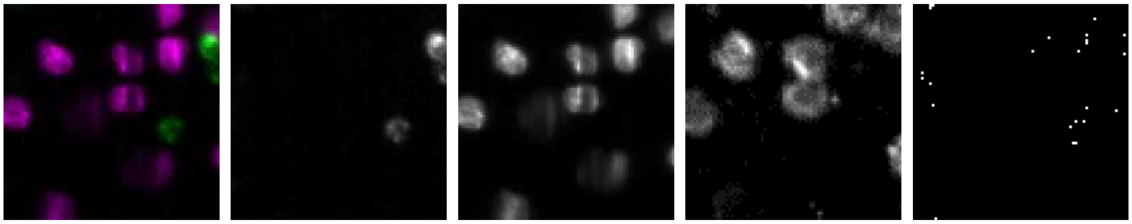				
12740	84	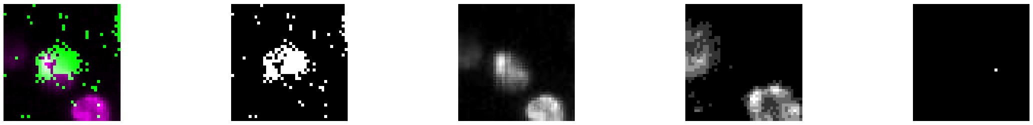				

Ereignis	Aufnahme	DAPI/CK-PE	CK-PE	DAPI	CD45-APC	HER-2/neu
12741	84	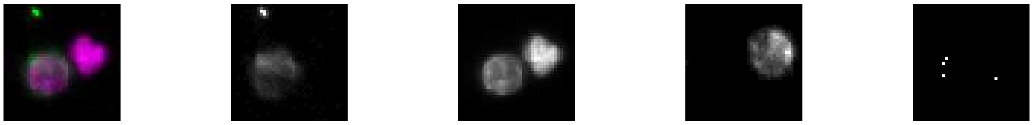				
12742	84	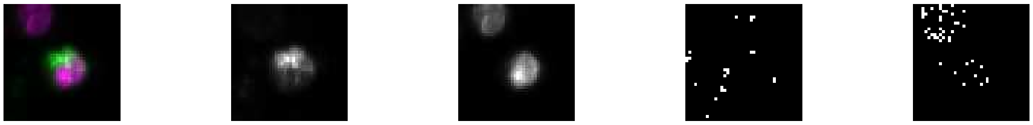				
12743	84	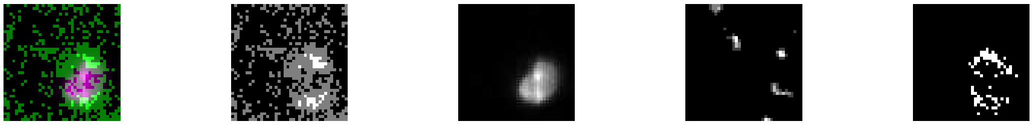				
12744	84	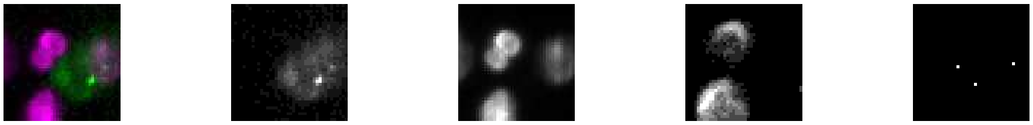				
12745	84	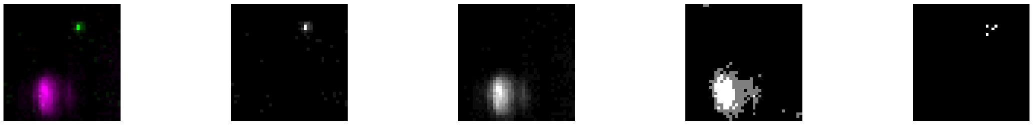				
12746	84	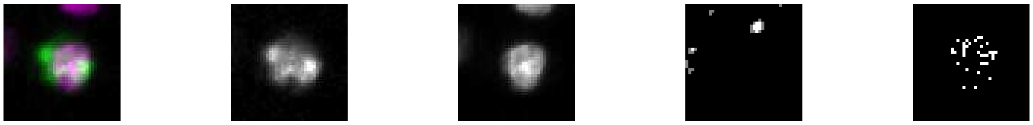				
12747	84	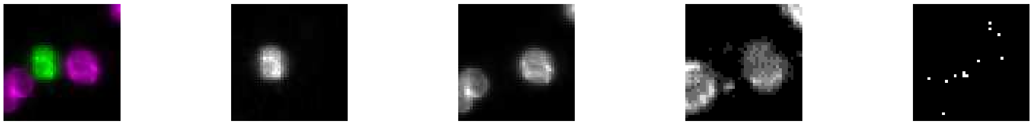				
12748	84	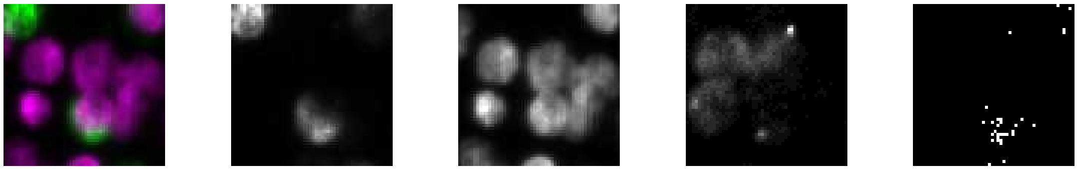				
12749	84	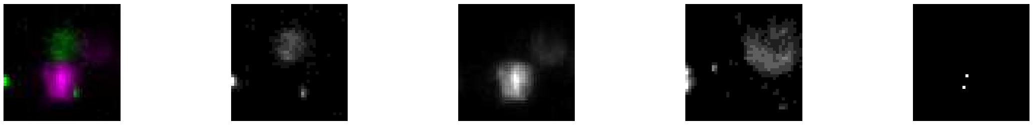				
12750	84	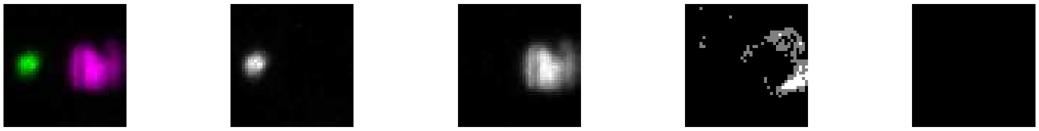				
12751	84	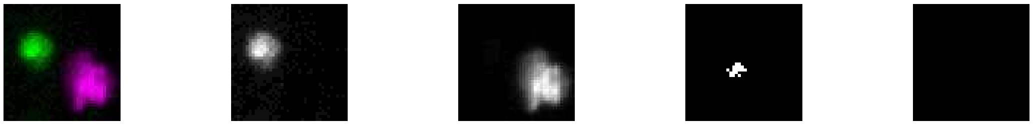				
12752	84	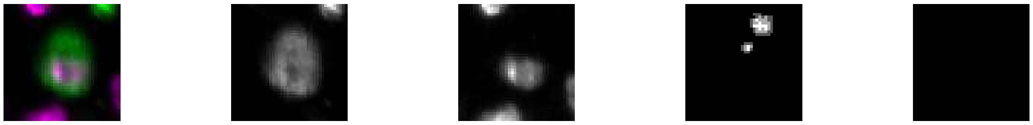				
12753	84	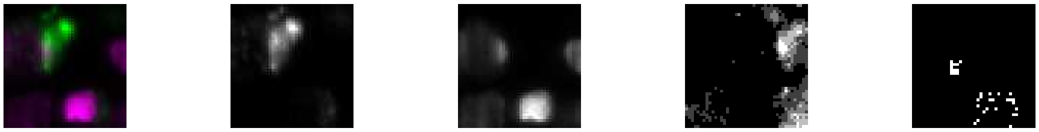				

Ereignis	Aufnahme	DAPI/CK-PE	CK-PE	DAPI	CD45-APC	HER-2/neu
12754	84	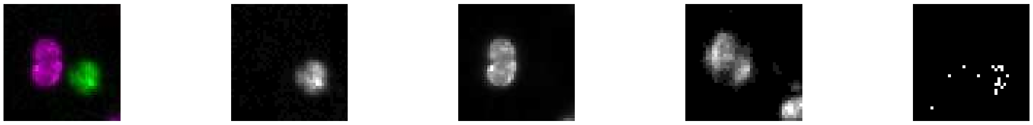				
12755	84	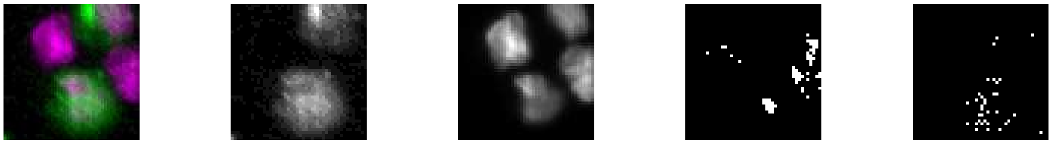				
12756	84	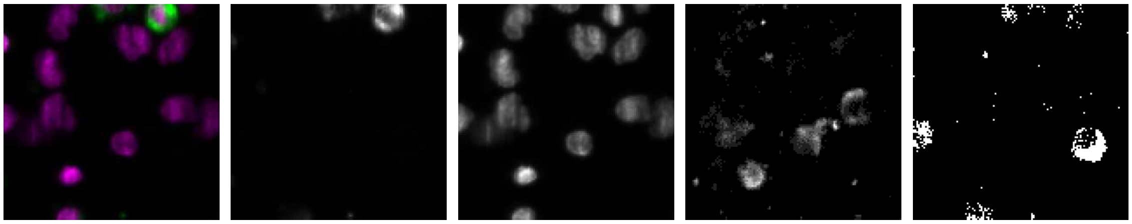				
12757	84	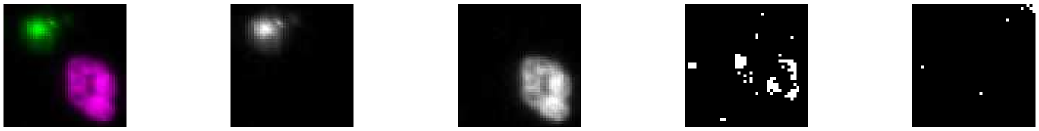				
12758	84	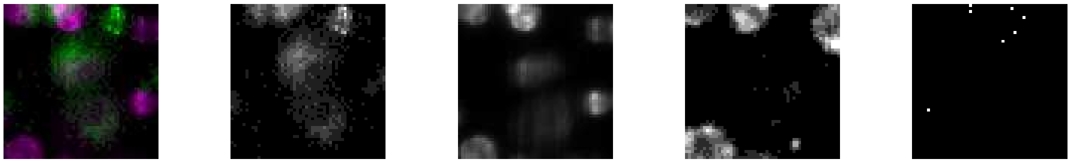				
12759	84	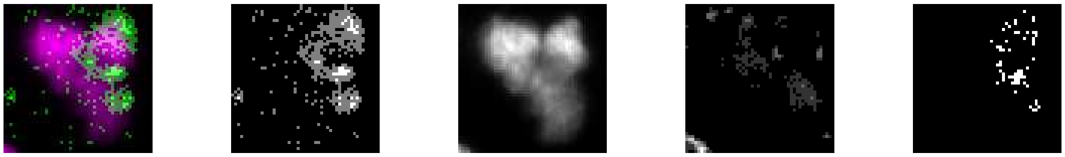				
12760	84	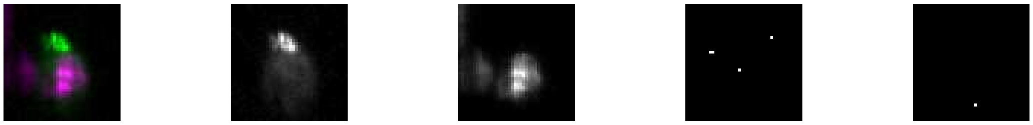				
12761	84	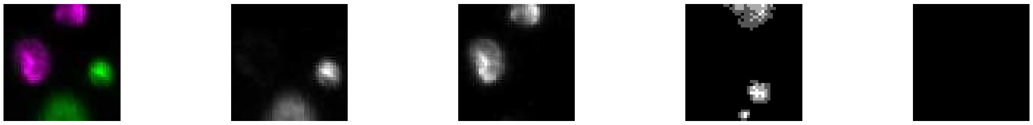				
12762	84	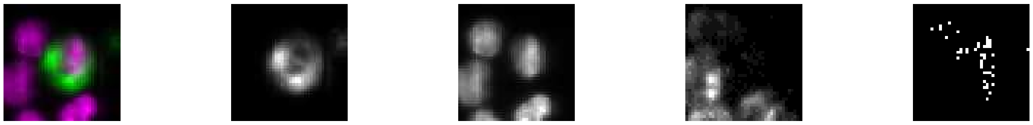				
12763	84	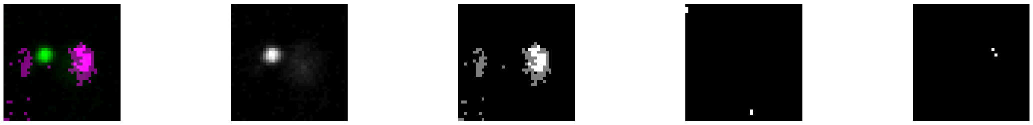				
12764	84	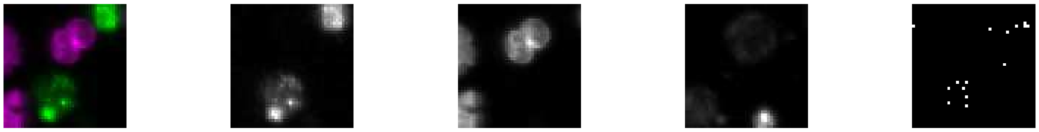				
12765	84	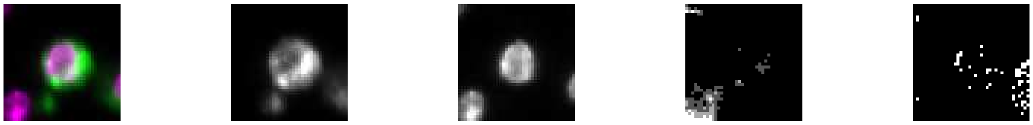				

Ereignis	Aufnahme	DAPI/CK-PE	CK-PE	DAPI	CD45-APC	HER-2/neu
12766	84	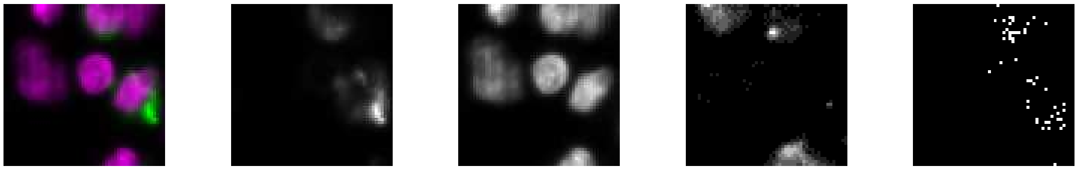				
12767	84	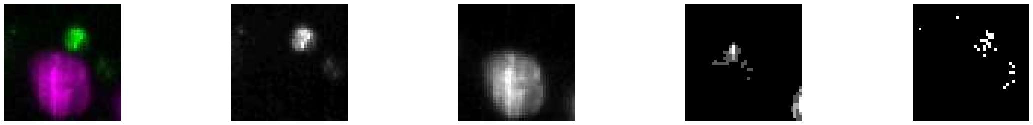				
12768	84	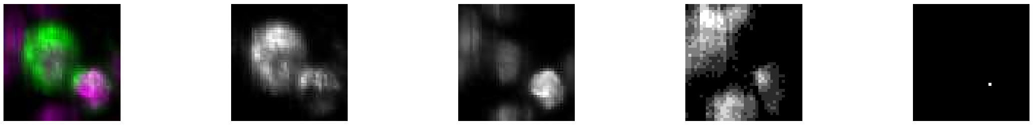				
12769	84	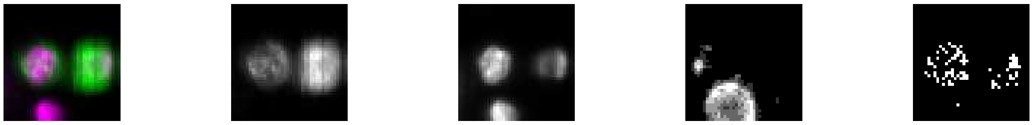				
12770	84	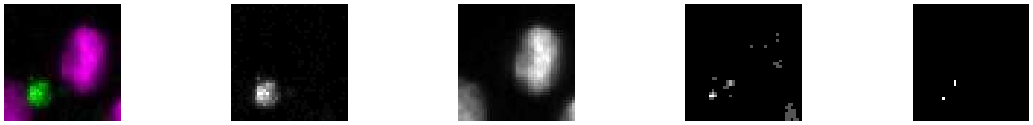				
12771	84	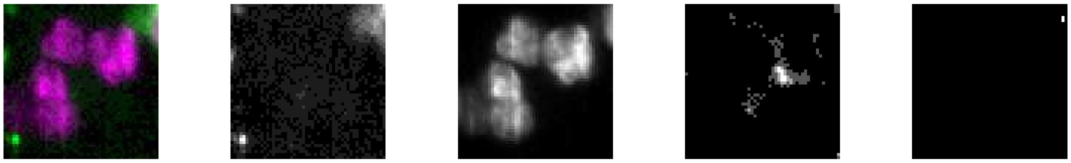				
12772	84	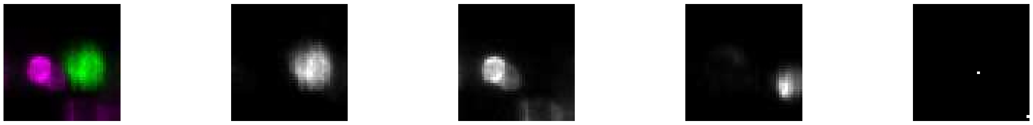				
12773	84	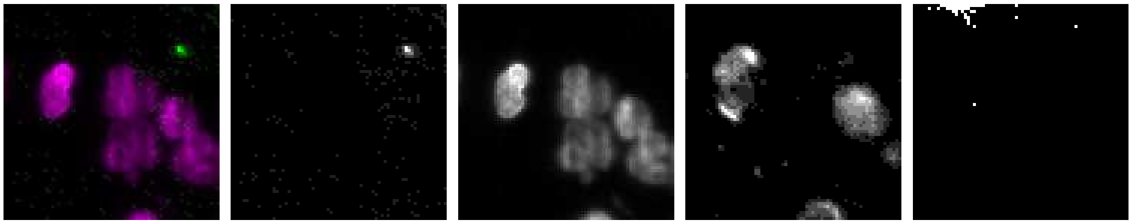				
12774	84	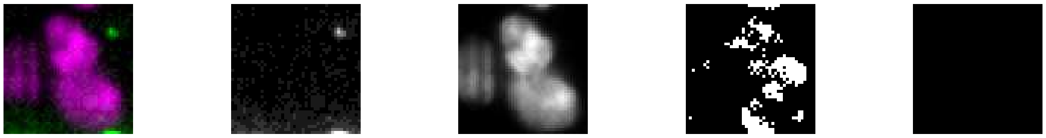				
12775	84	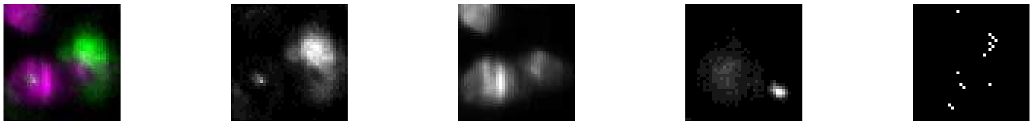				
12776	84	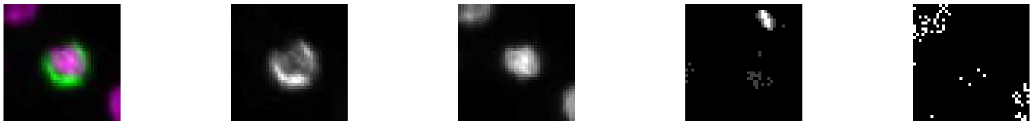				
12777	84	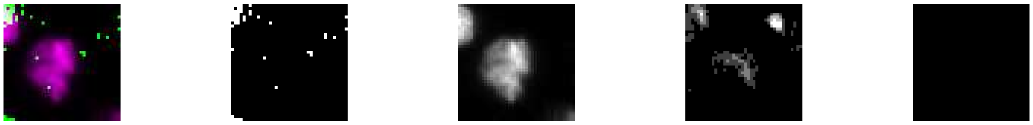				

Ereignis	Aufnahme	DAPI/CK-PE	CK-PE	DAPI	CD45-APC	HER-2/neu
12778	84	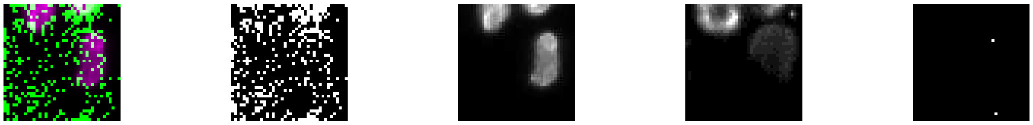				
12779	84	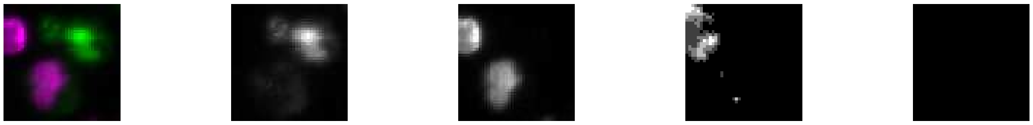				
12780	84	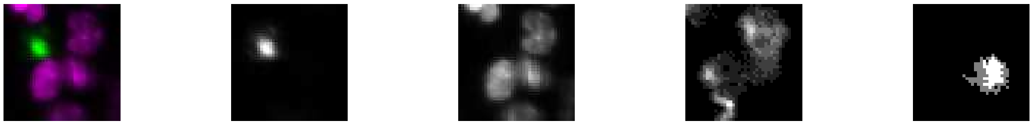				
12781	84	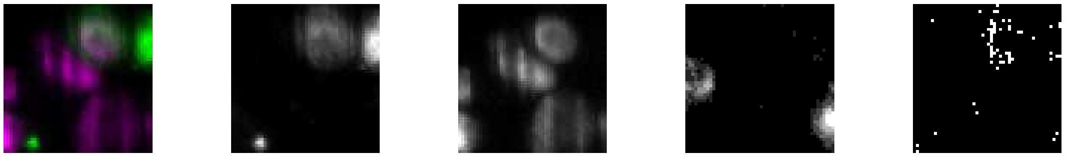				
12782	84	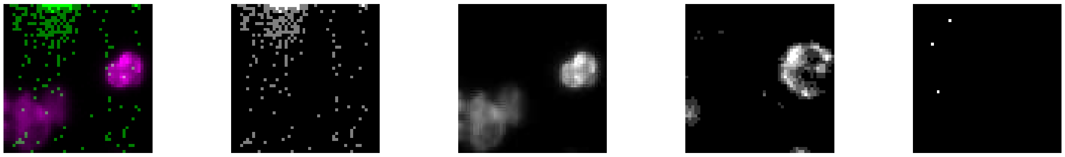				
12783	84	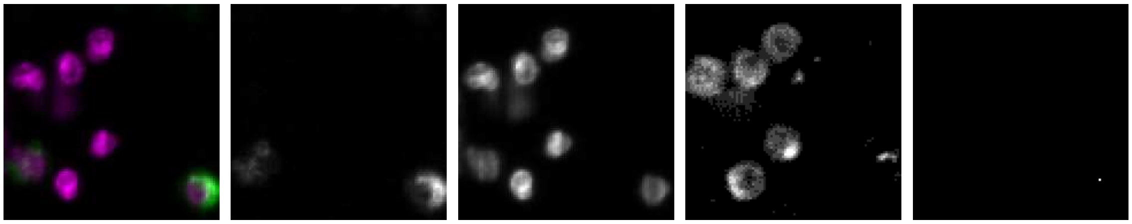				
12784	84	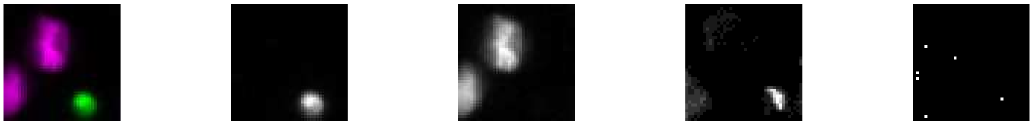				
12785	84	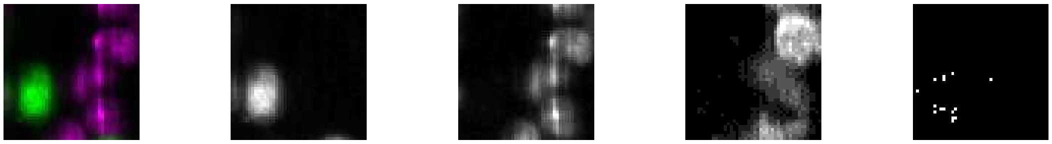				
12786	84	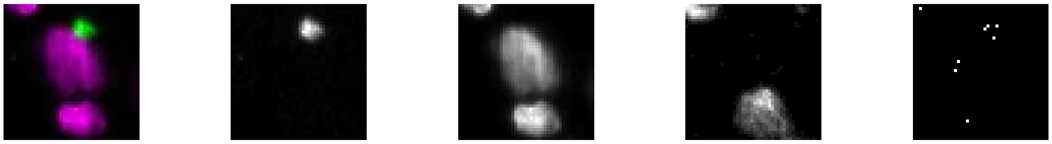				
12787	84	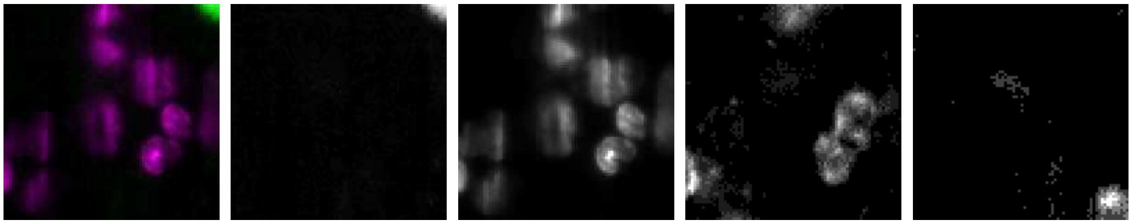				
12788	84	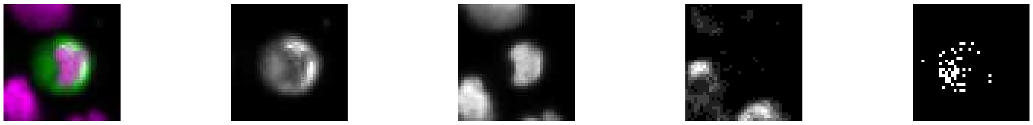				

Ereignis	Aufnahme	DAPI/CK-PE	CK-PE	DAPI	CD45-APC	HER-2/neu
12789	84	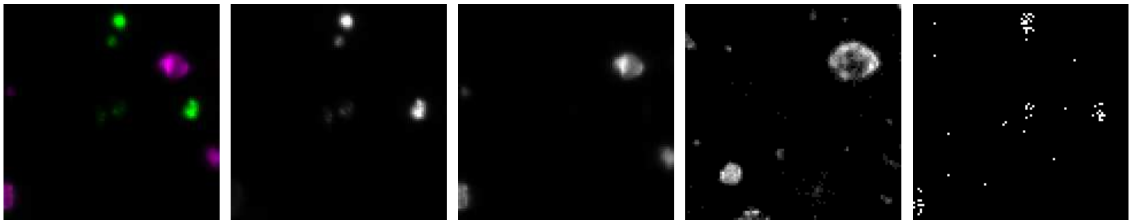				
12790	84	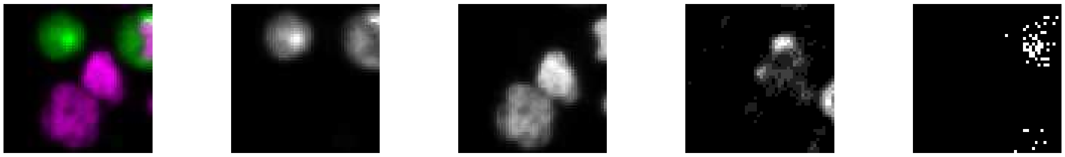				
12791	84	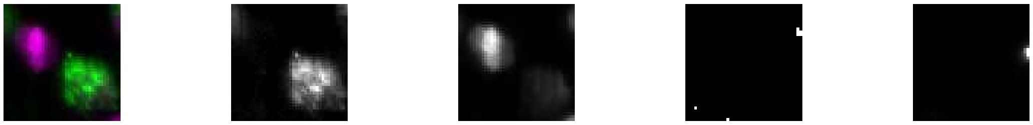				
12792	84	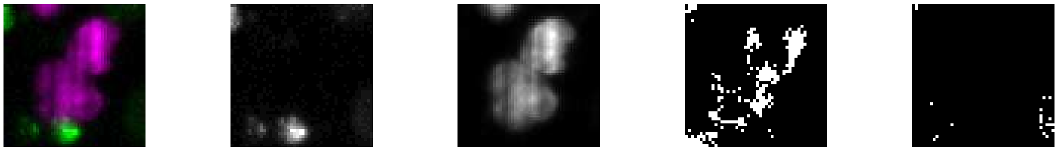				
12793	84	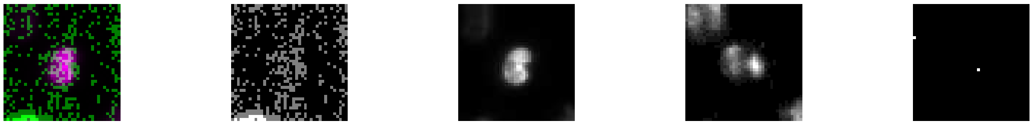				
12794	84	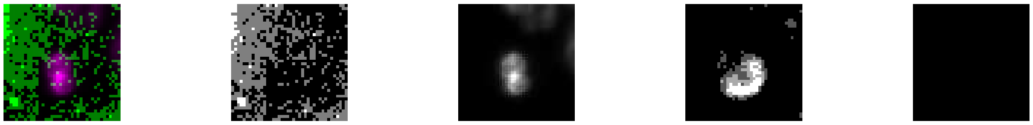				
12795	84	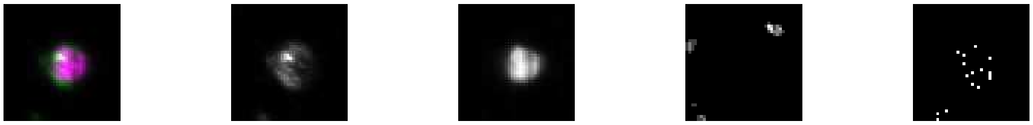				
12796	84	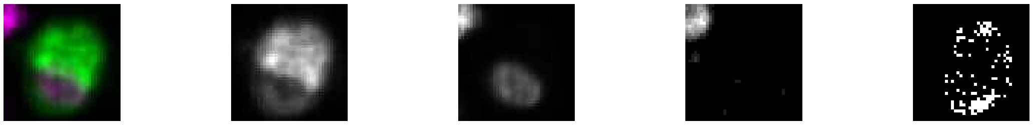				
12797	84	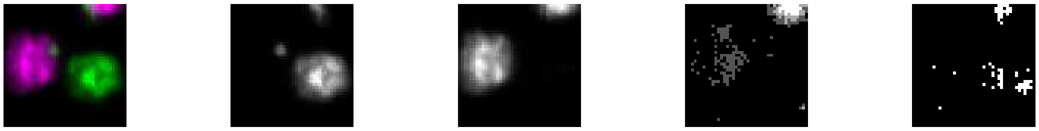				
12798	84	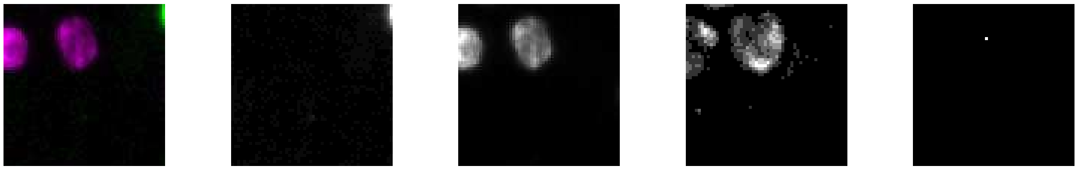				
12799	84	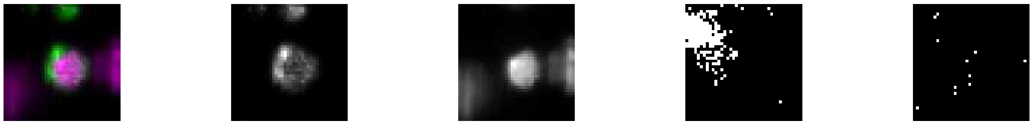				
12800	84	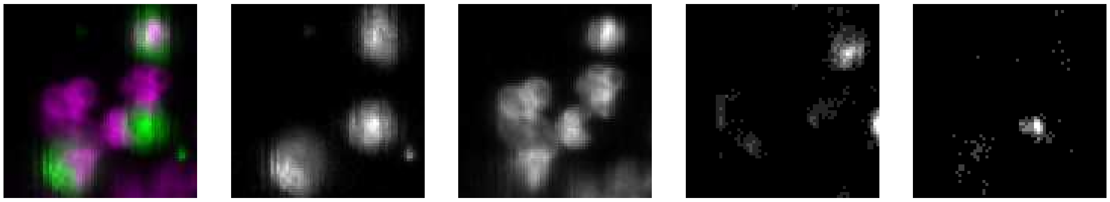				

Ereignis	Aufnahme	DAPI/CK-PE	CK-PE	DAPI	CD45-APC	HER-2/neu
12801	84	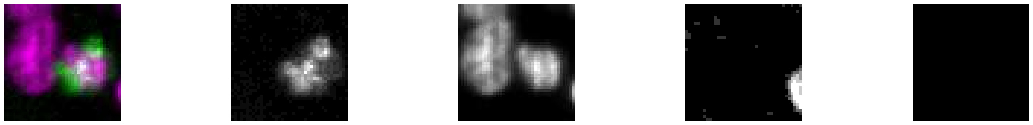				
12802	84	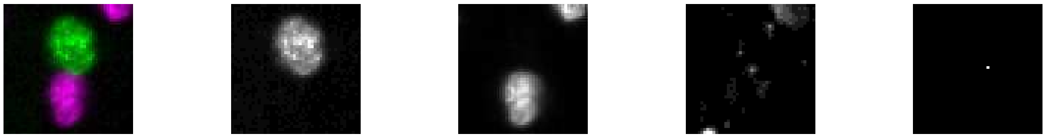				
12803	84	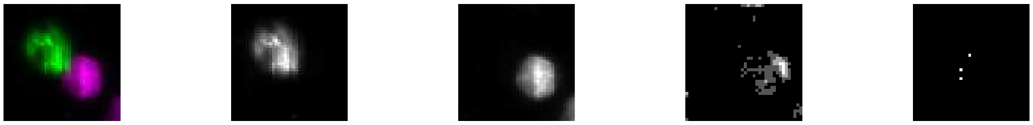				
12804	84	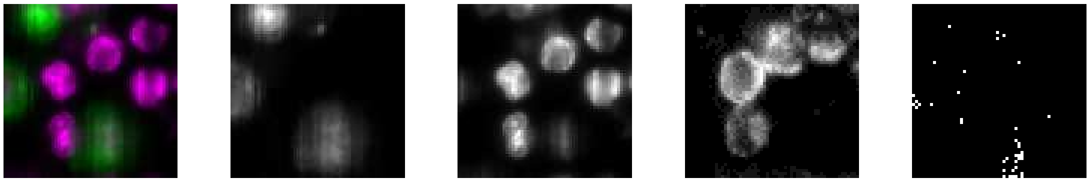				
12805	84	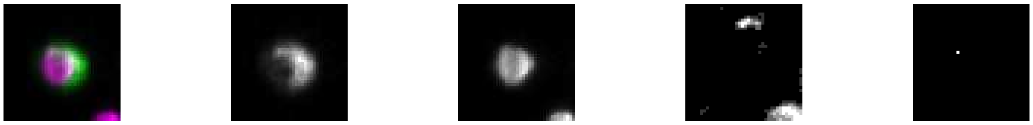				
12806	84	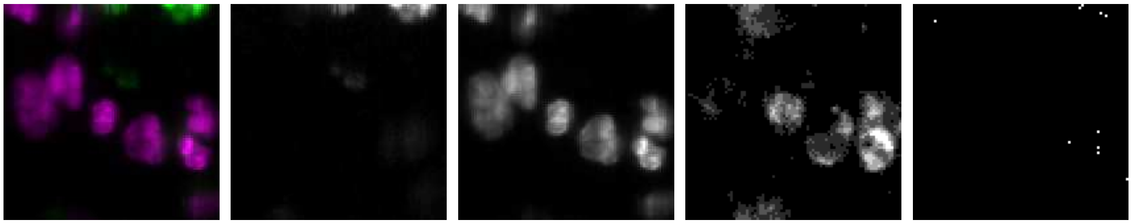				
12807	84	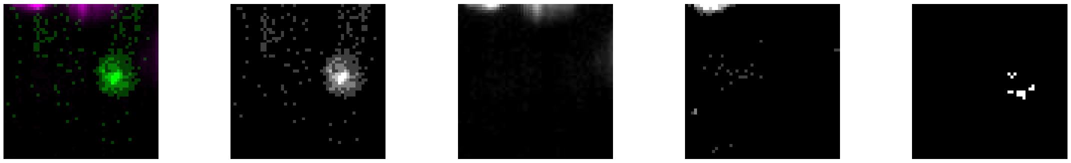				
12808	84	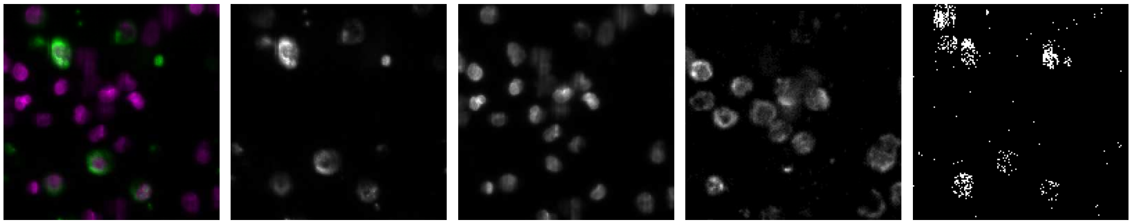				
12809	84	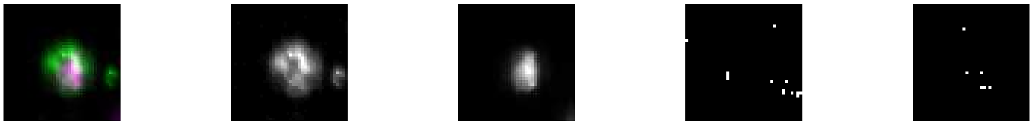				
12810	84	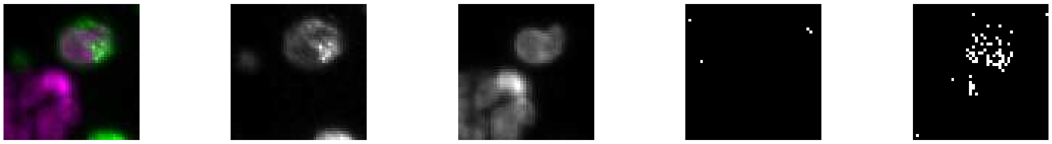				
12811	84	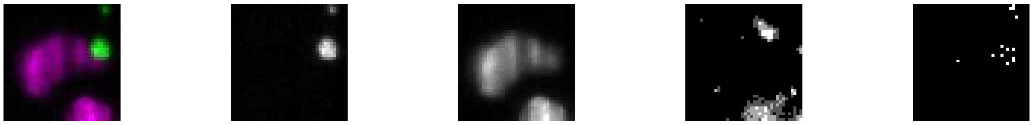				

Ereignis	Aufnahme	DAPI/CK-PE	CK-PE	DAPI	CD45-APC	HER-2/neu
12812	84	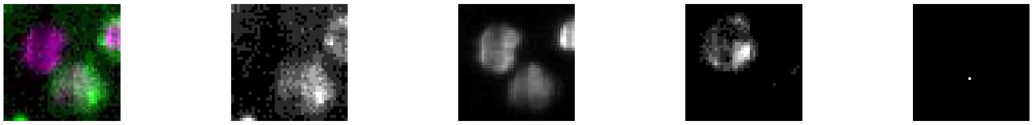				
12813	84	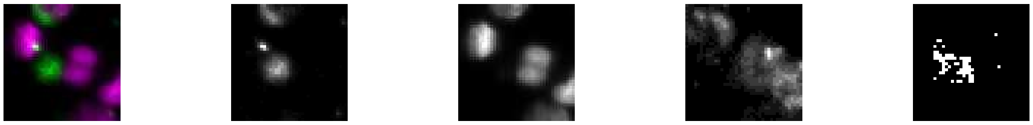				
12814	84	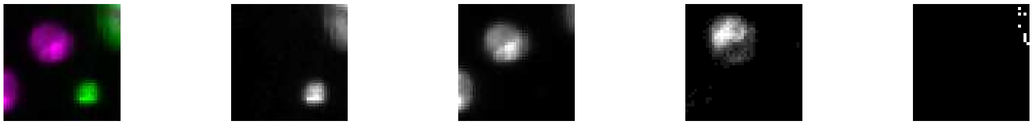				
12815	84	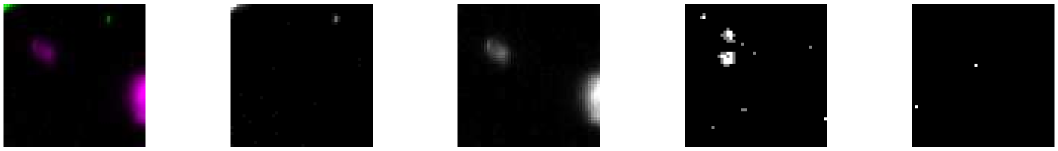				
12816	84	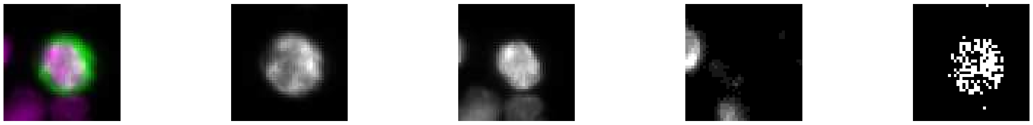				
12817	84	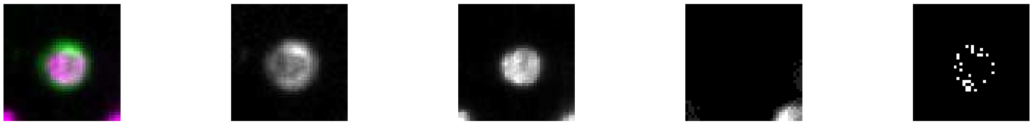				
12818	84	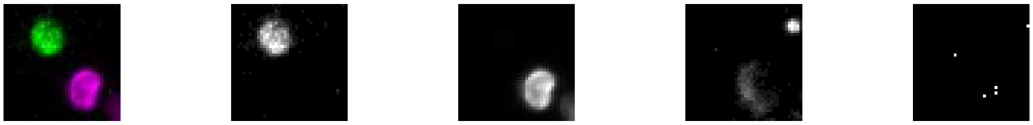				
12819	84	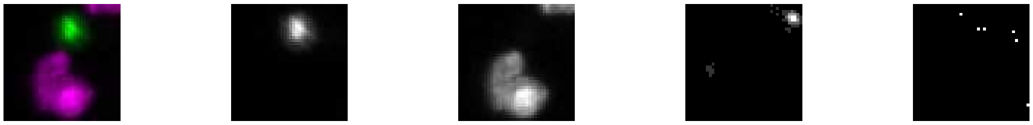				
12820	84	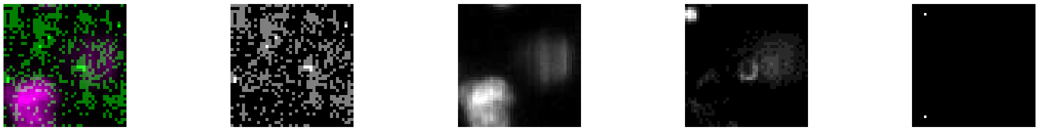				
12821	84	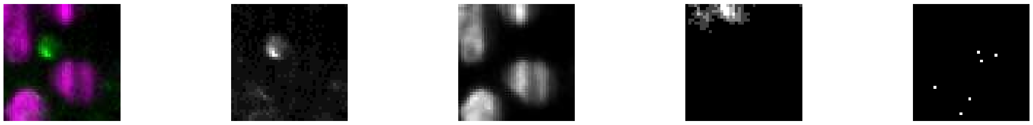				
12822	84	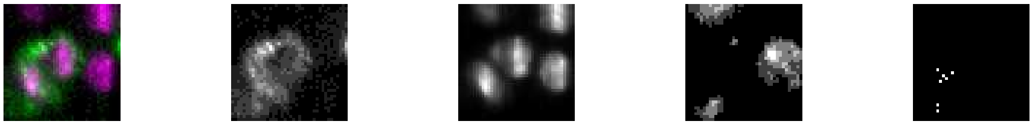				
12823	84	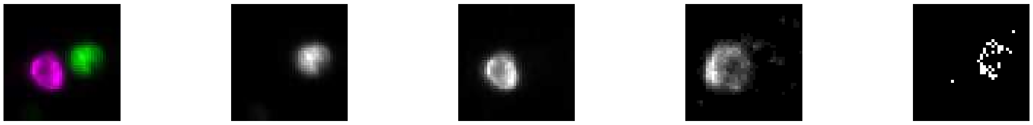				
12824	84	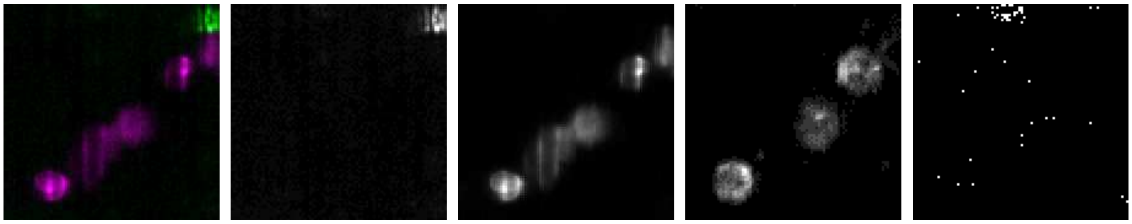				

Druckdatum: 02/06/2020 17:00

Proben-ID: Gyn14.208 **Kassetten-ID:** 01327106 **Patienten-ID:**

Ereignis	Aufnahme	DAPI/CK-PE	CK-PE	DAPI	CD45-APC	HER-2/neu
12825	84	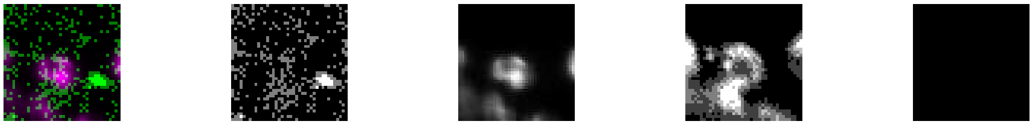				
12826	84	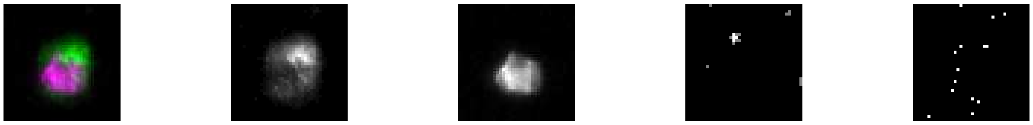				
12827	84	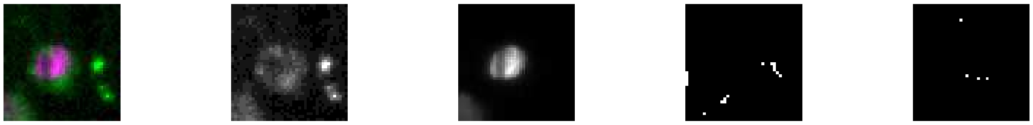				
12828	84	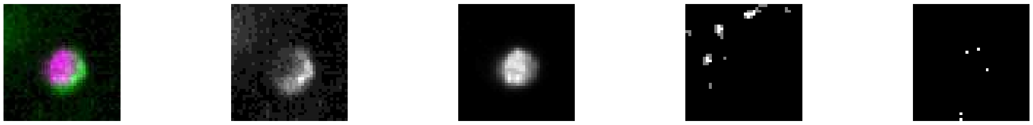				
12829	84	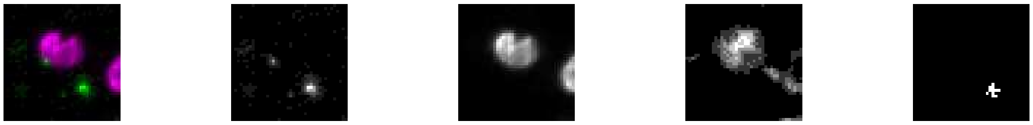				
12830	84	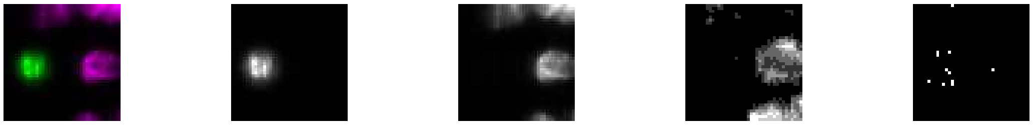				
12831	84	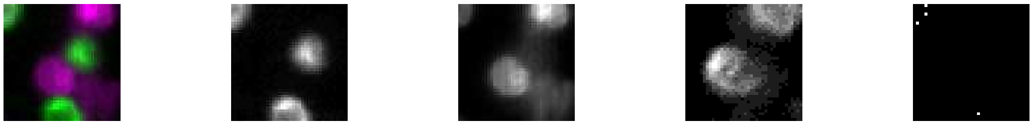				
12832	84	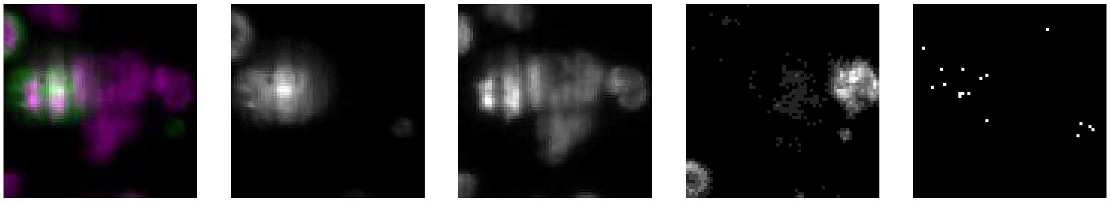				
12833	84	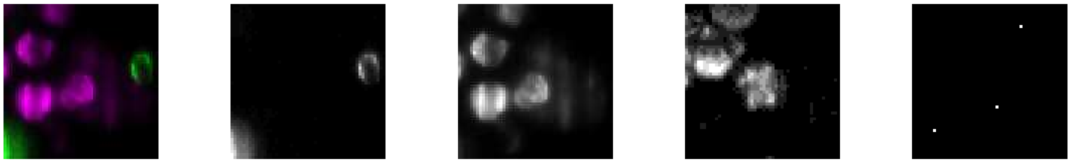				
12834	84	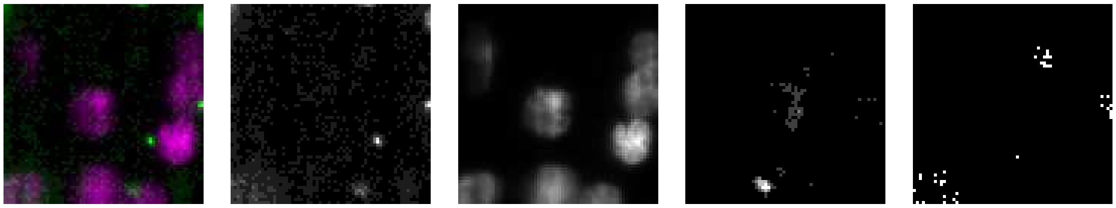				
12835	84	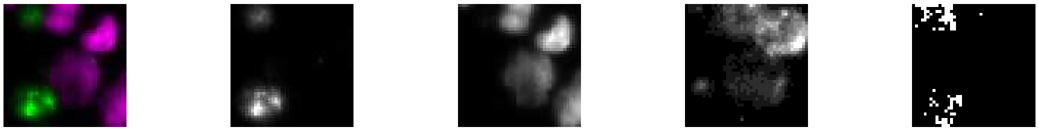				
12836	84	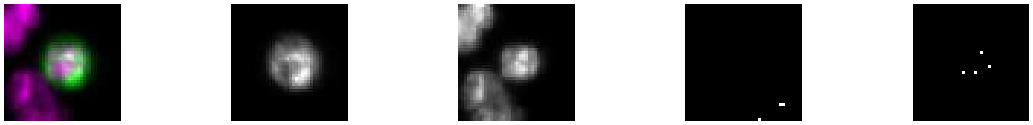				

Ereignis	Aufnahme	DAPI/CK-PE	CK-PE	DAPI	CD45-APC	HER-2/neu
12837	84	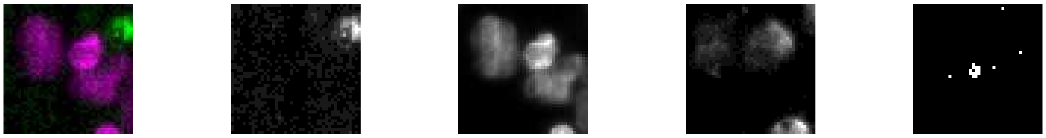				
12838	84	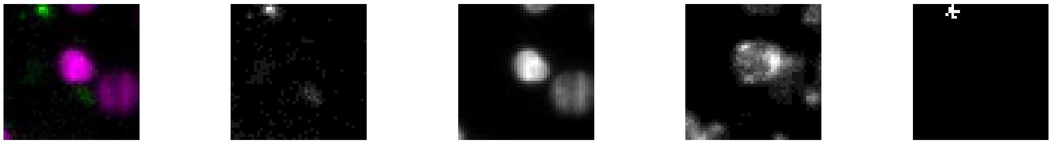				
12839	84	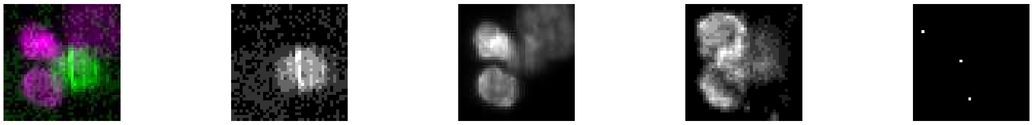				
12840	84	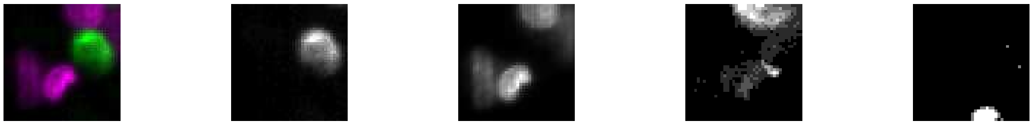				
12841	84	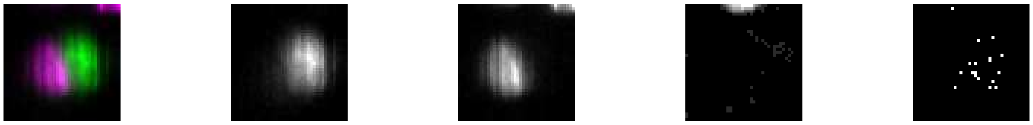				
12842	84	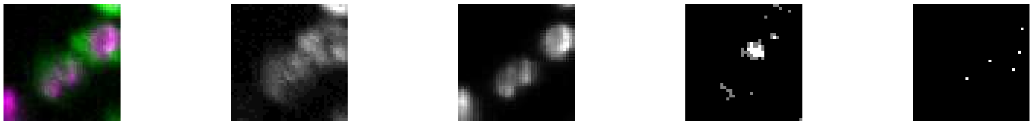				
12843	84	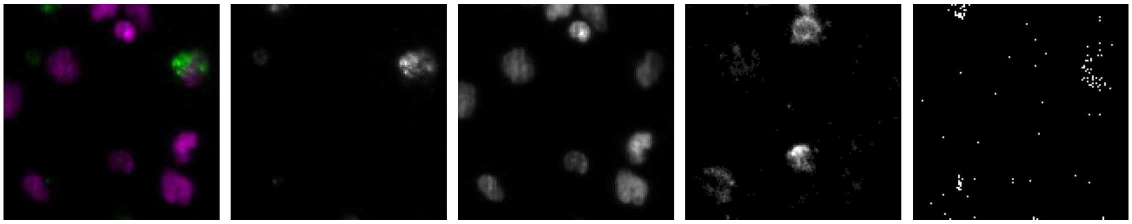				
12844	84	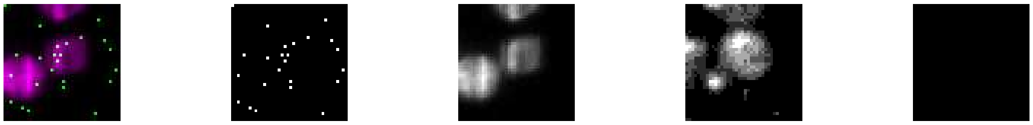				
12845	84	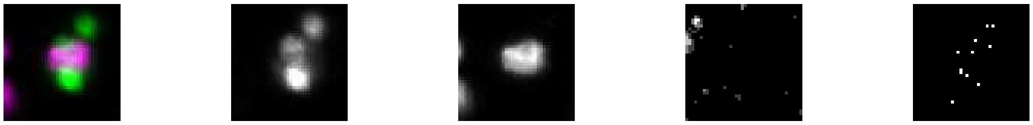				
12846	84	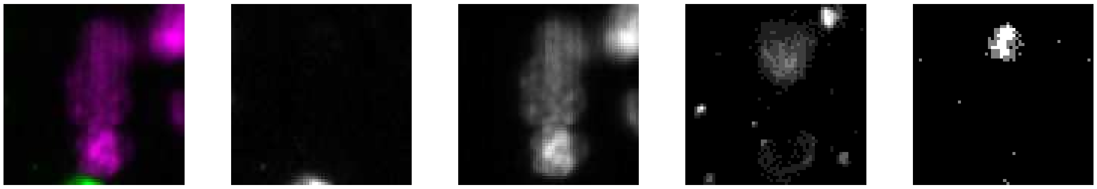				
12847	84	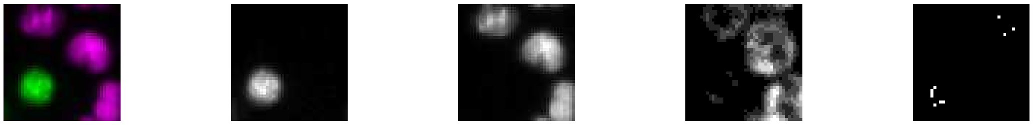				
12848	84	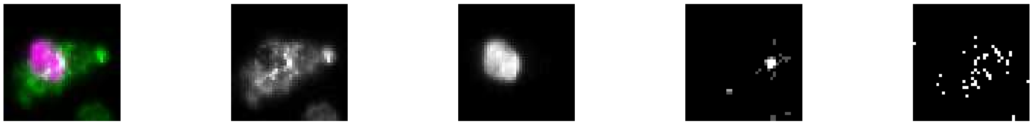				

Ereignis	Aufnahme	DAPI/CK-PE	CK-PE	DAPI	CD45-APC	HER-2/neu
12849	84	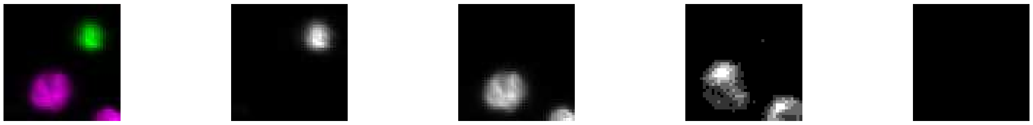				
12850	84	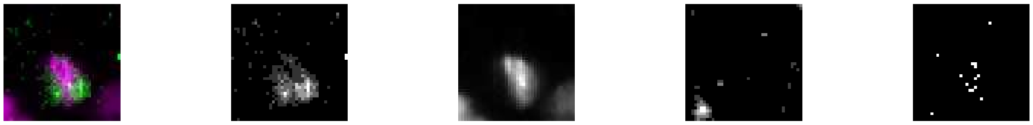				
12851	84	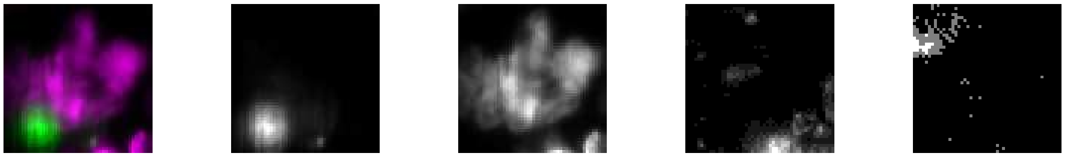				
12852	84	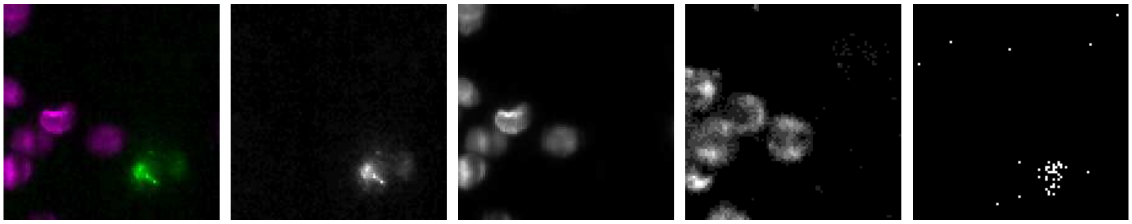				
12853	84	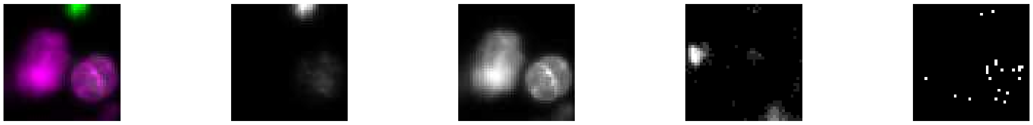				
12854	84	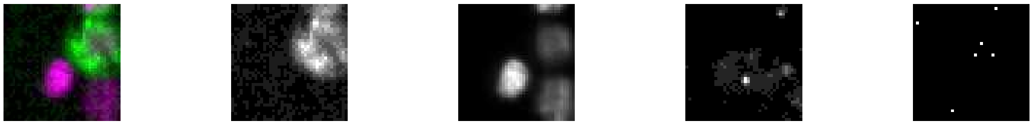				
12855	84	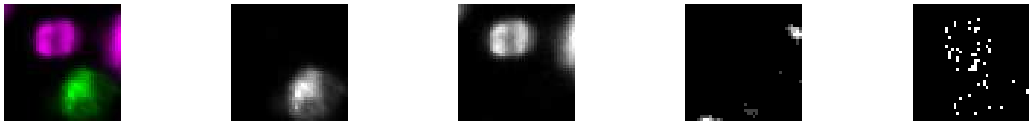				
12856	84	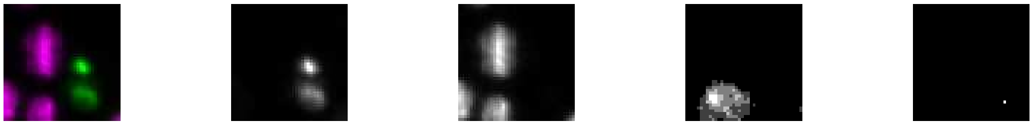				
12857	84	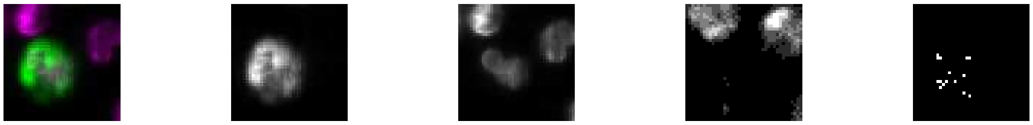				
12858	84	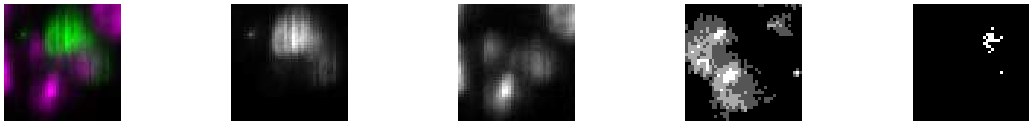				
12859	84	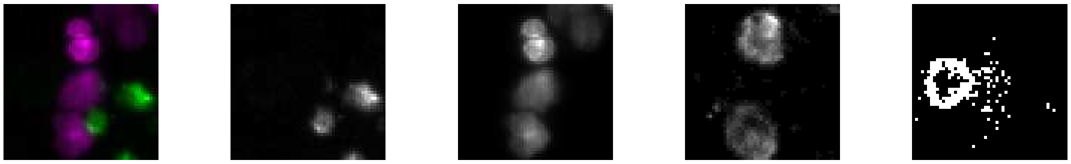				
12860	84	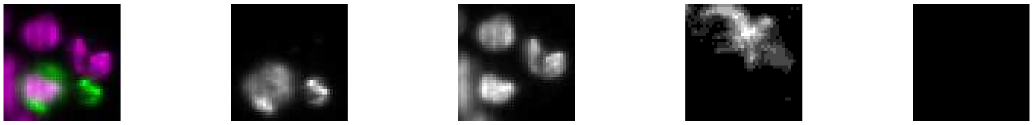				
12861	84	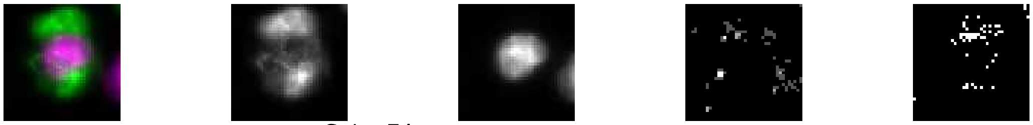				

Ereignis	Aufnahme	DAPI/CK-PE	CK-PE	DAPI	CD45-APC	HER-2/neu
12862	84	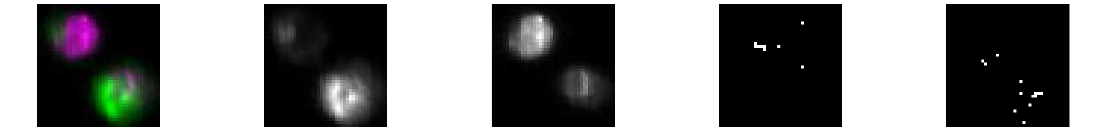				
12863	84	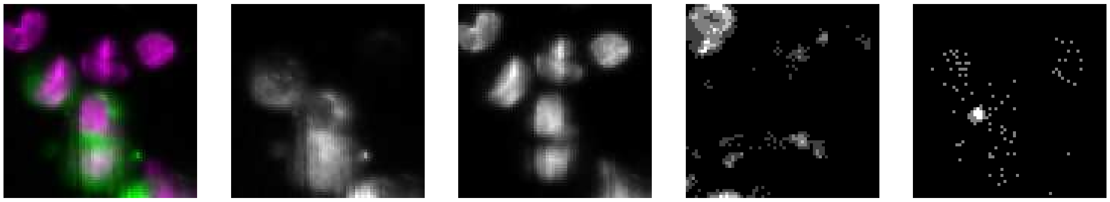				
12864	84	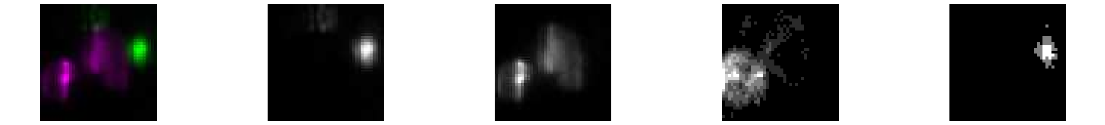				
12865	84	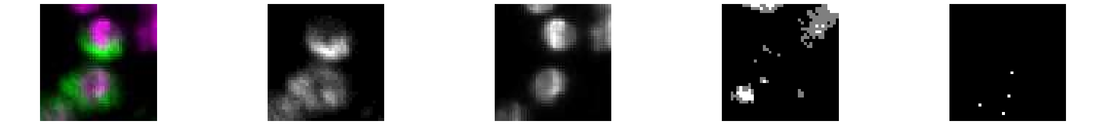				
12866	84	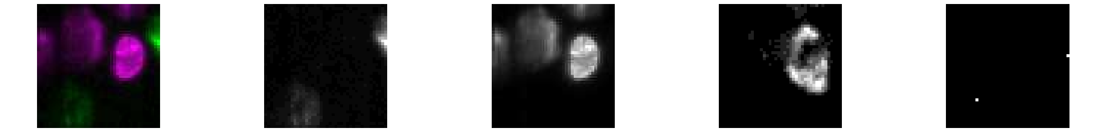				
12867	84	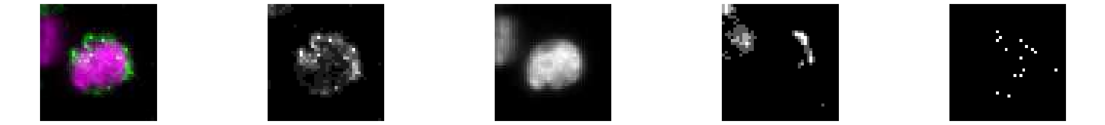				
12868	84	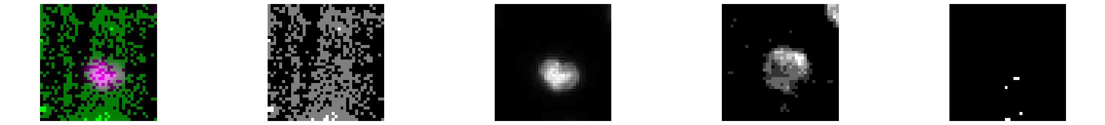				
12869	84	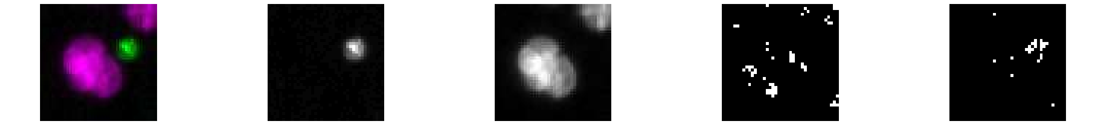				
12870	84	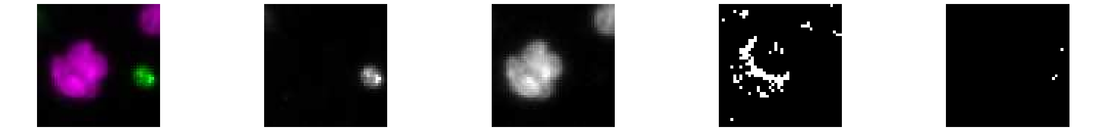				
12871	84	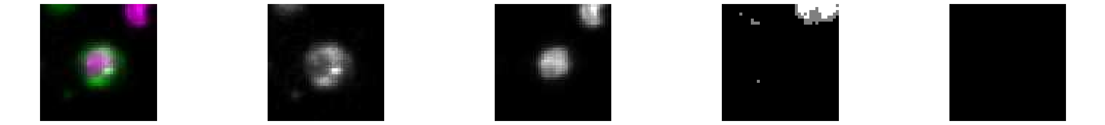				
12872	84	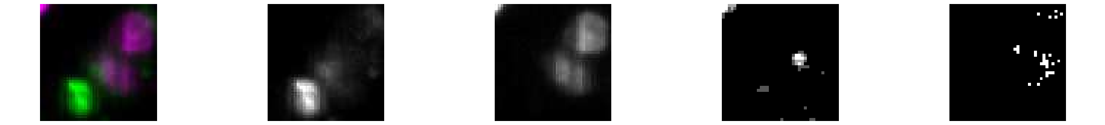				
12873	84	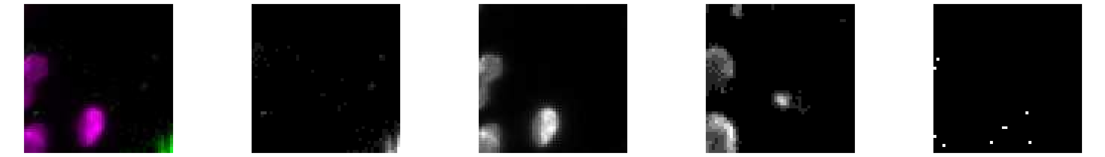				
12874	84	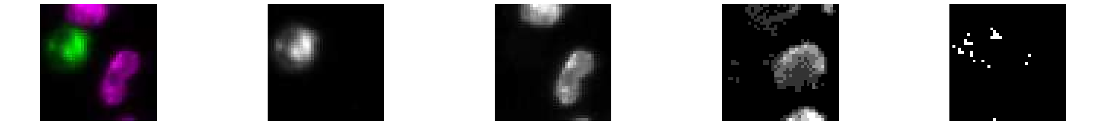				

Ereignis	Aufnahme	DAPI/CK-PE	CK-PE	DAPI	CD45-APC	HER-2/neu
12875	84	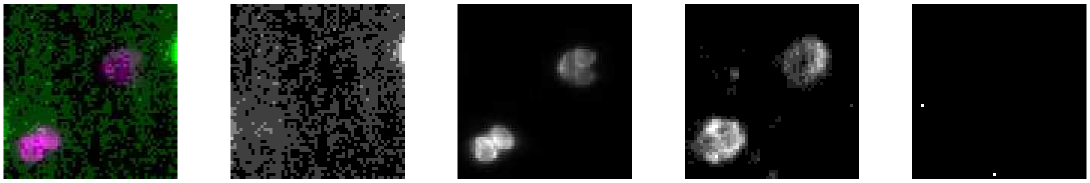				
12876	84	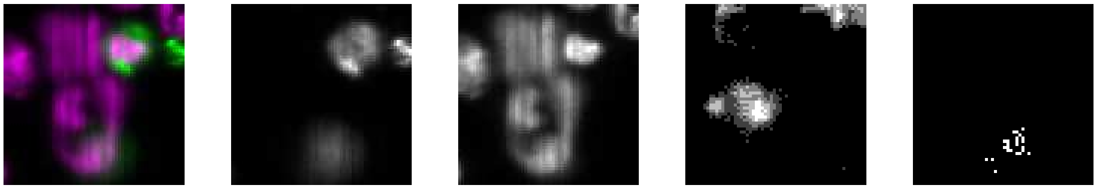				
12877	84	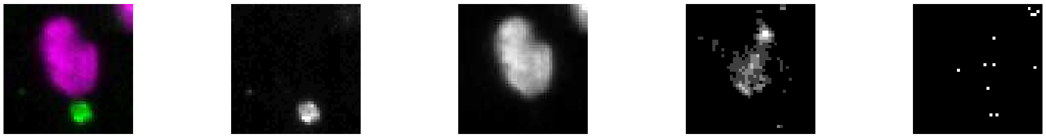				
12878	84	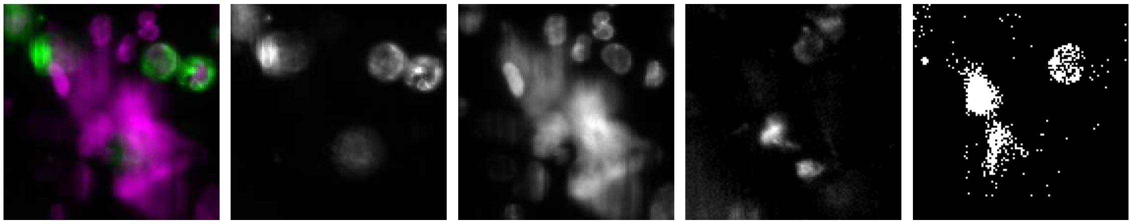				
12879	84	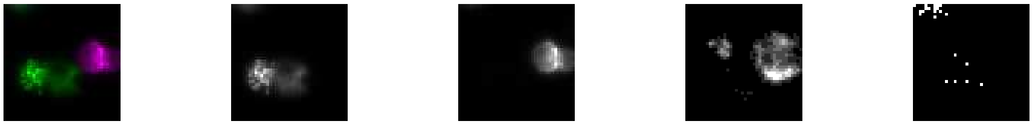				
12880	84	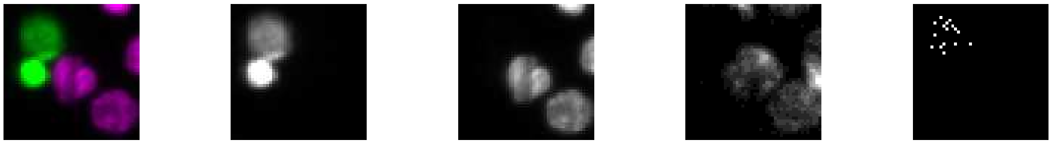				
12881	84	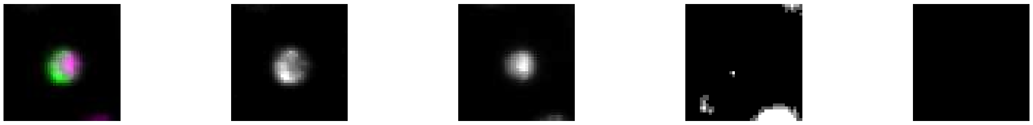				
12882	84	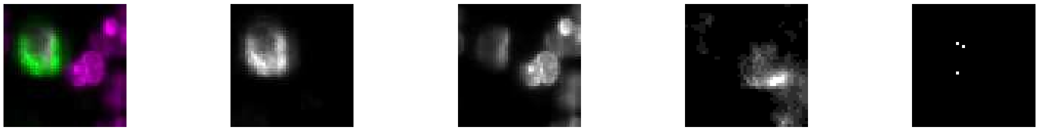				
12883	84	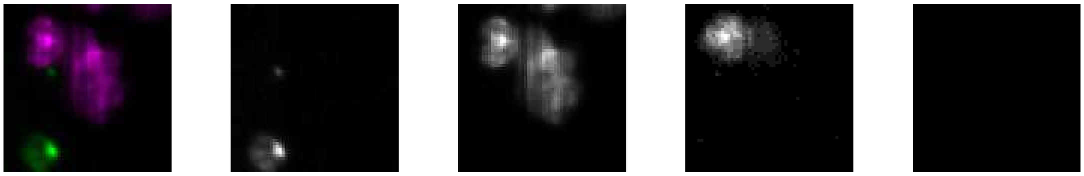				
12884	84	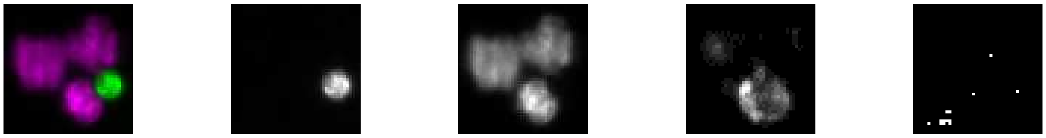				
12885	84	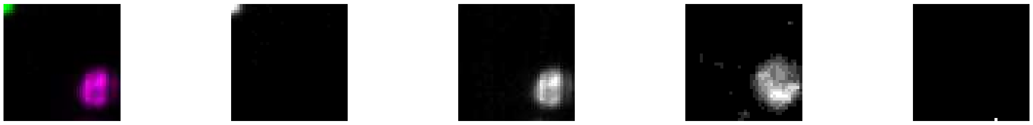				

Ereignis	Aufnahme	DAPI/CK-PE	CK-PE	DAPI	CD45-APC	HER-2/neu
12886	84	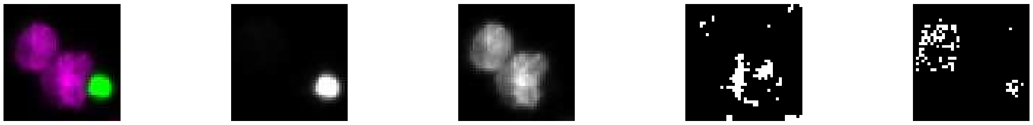				
12887	84	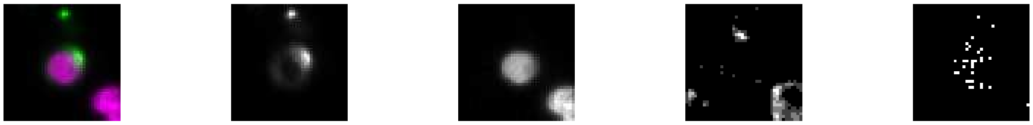				
12888	84	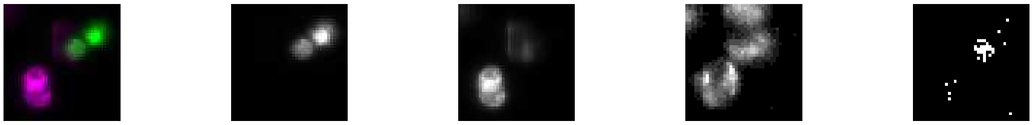				
12889	84	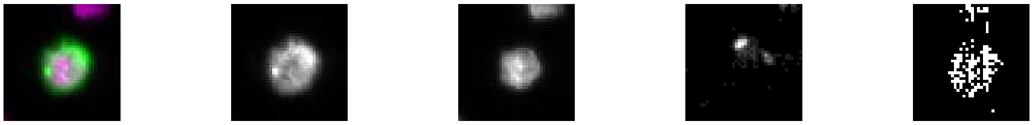				
12890	84	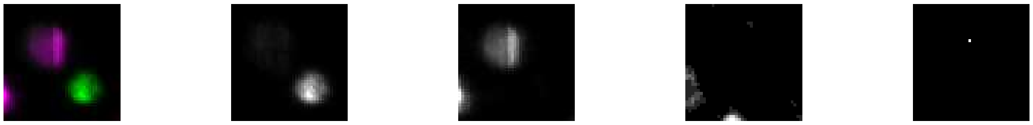				
12891	84	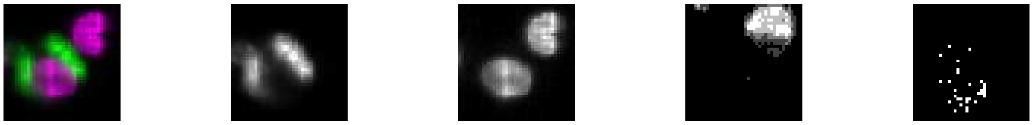				
12892	84	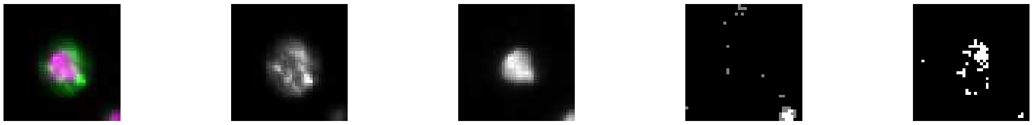				
12893	84	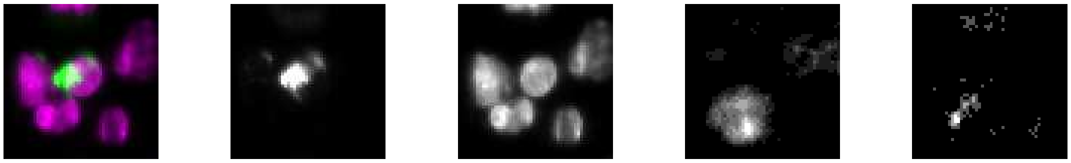				
12894	84	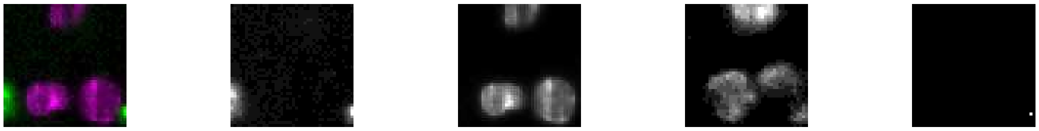				
12895	84	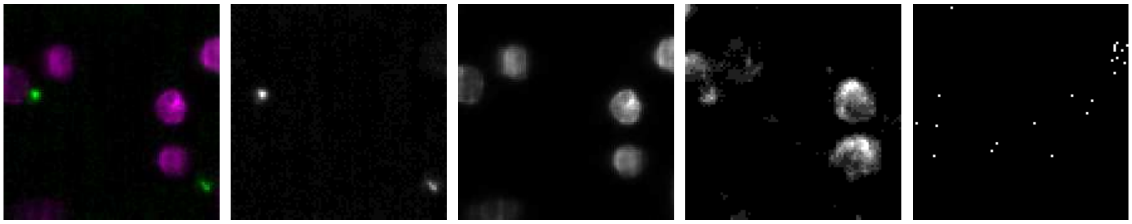				
12896	84	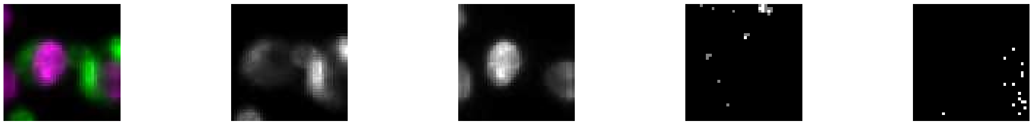				
12897	84	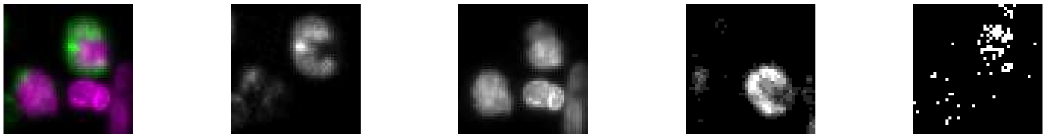				
12898	84	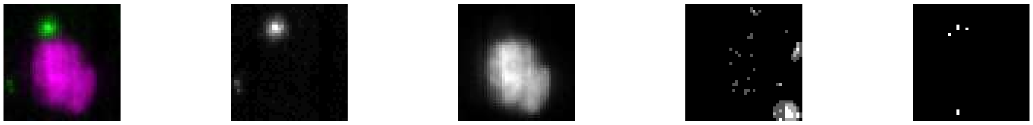				

Ereignis	Aufnahme	DAPI/CK-PE	CK-PE	DAPI	CD45-APC	HER-2/neu
12899	84	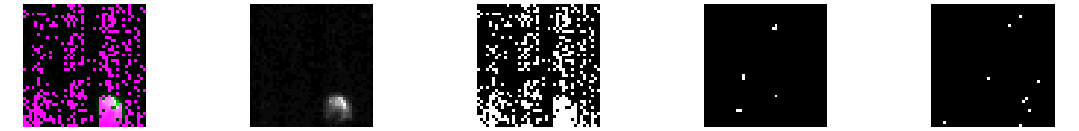				
12900	84	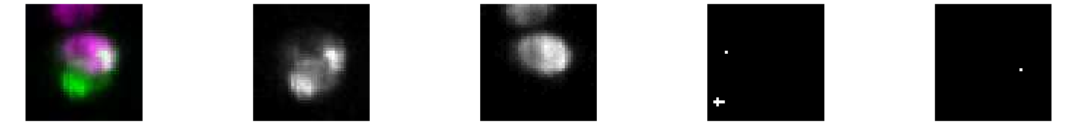				
12901	84	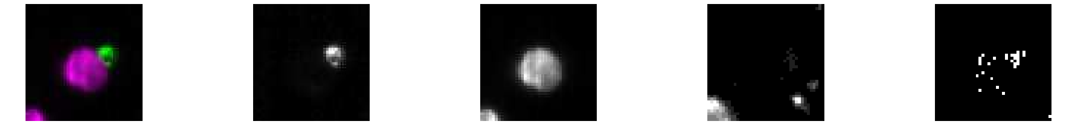				
12902	84	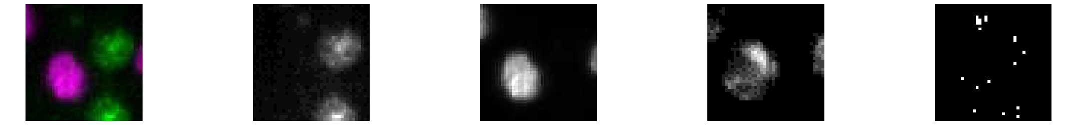				
12903	84	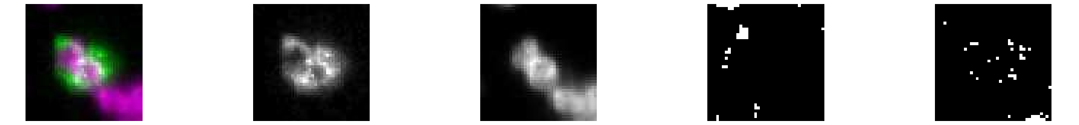				
12904	84	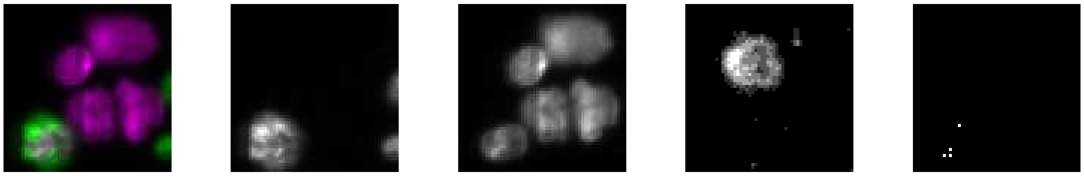				
12905	84	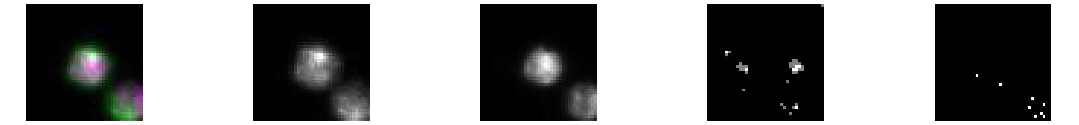				
12906	84	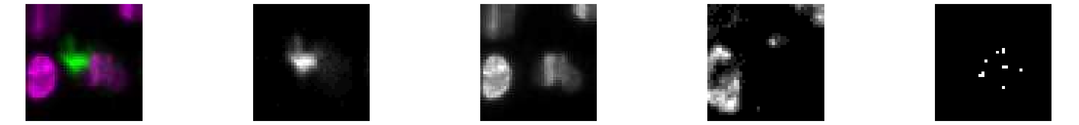				
12907	84	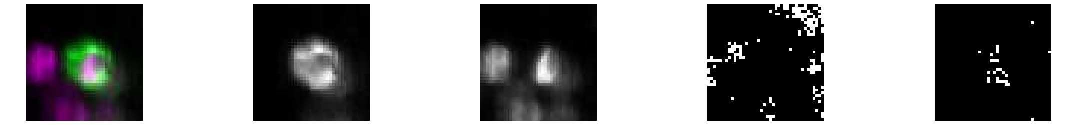				
12908	84	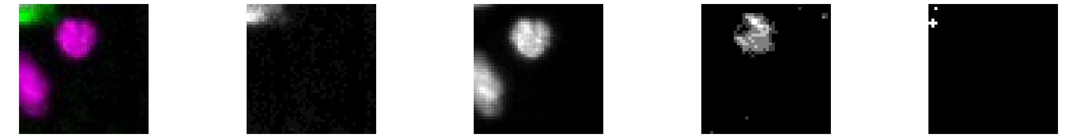				
12909	84	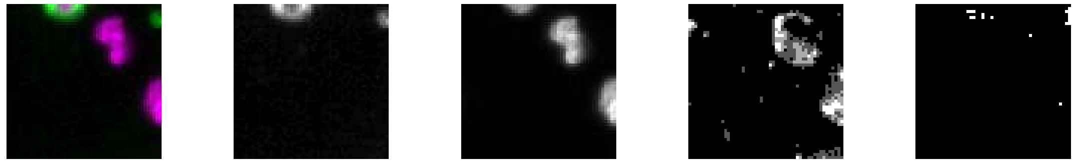				
12910	84	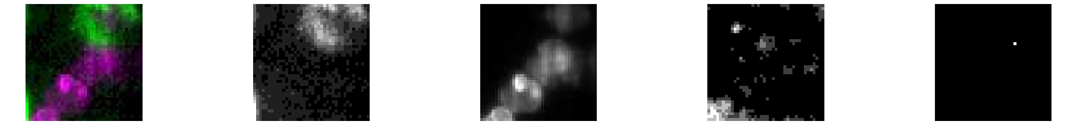				
12911	84	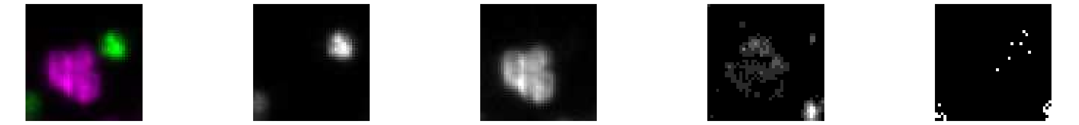				

Ereignis	Aufnahme	DAPI/CK-PE	CK-PE	DAPI	CD45-APC	HER-2/neu
12912	84	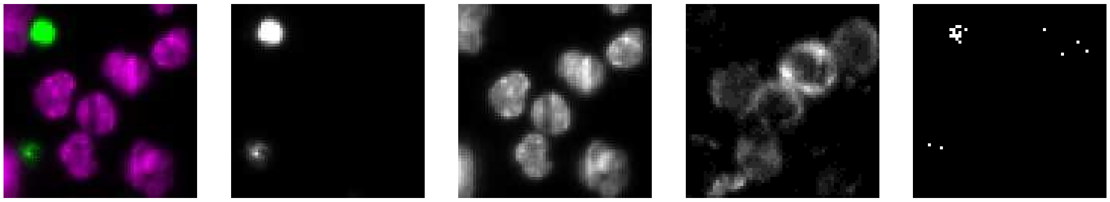				
12913	84	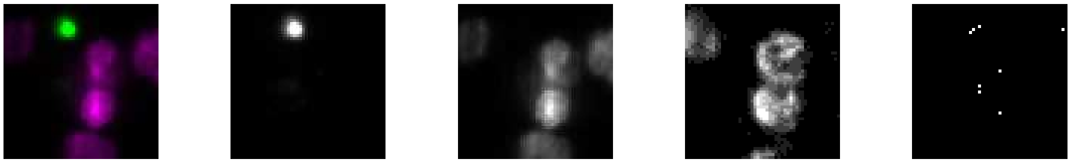				
12914	84	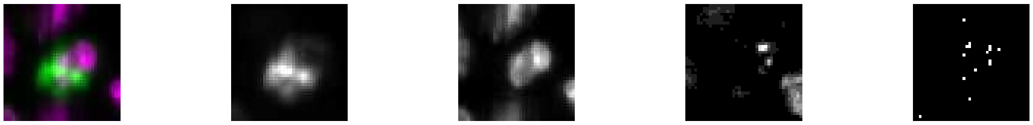				
12915	84	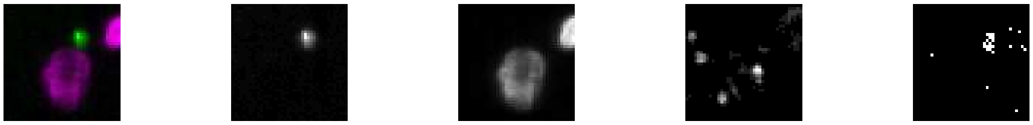				
12916	84	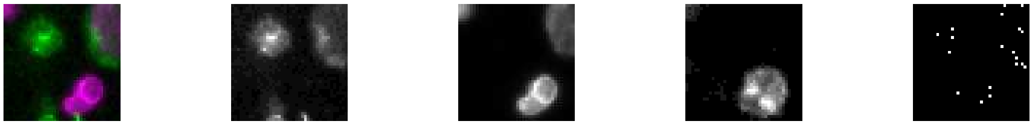				
12917	84	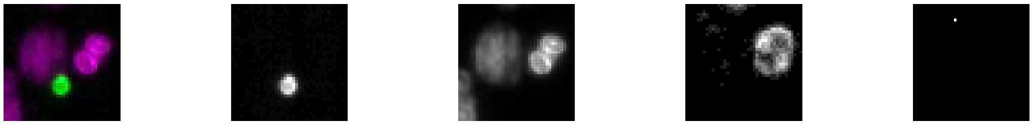				
12918	84	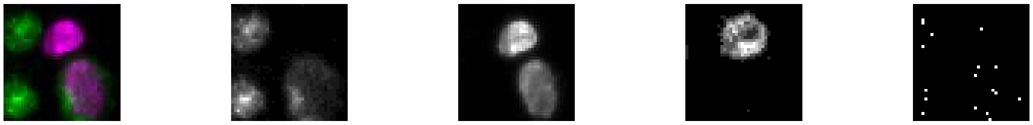				
12919	84	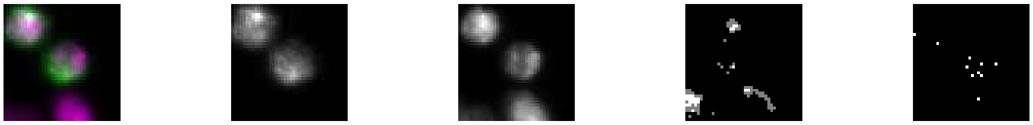				
12920	84	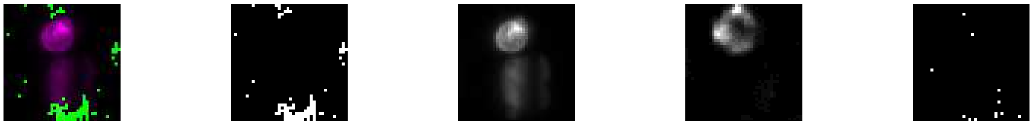				
12921	84	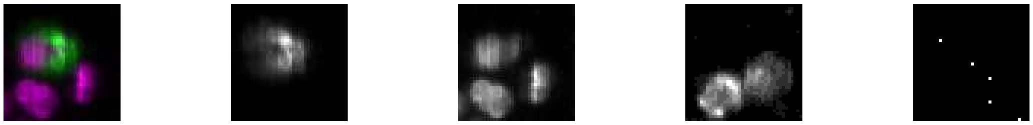				
12922	84	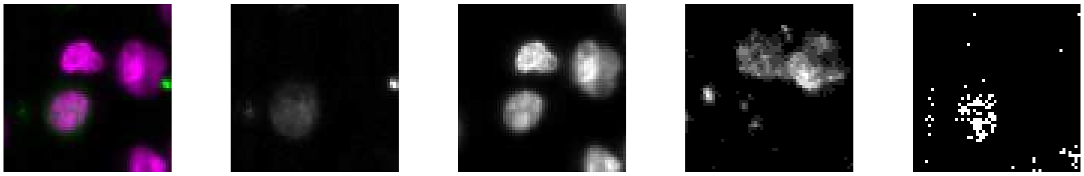				
12923	84	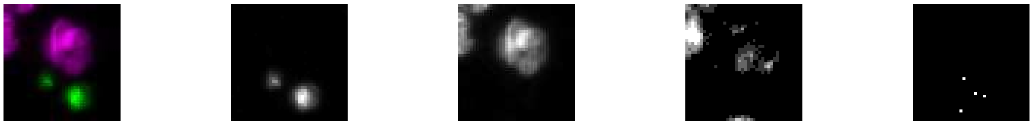				

Ereignis	Aufnahme	DAPI/CK-PE	CK-PE	DAPI	CD45-APC	HER-2/neu
12924	84	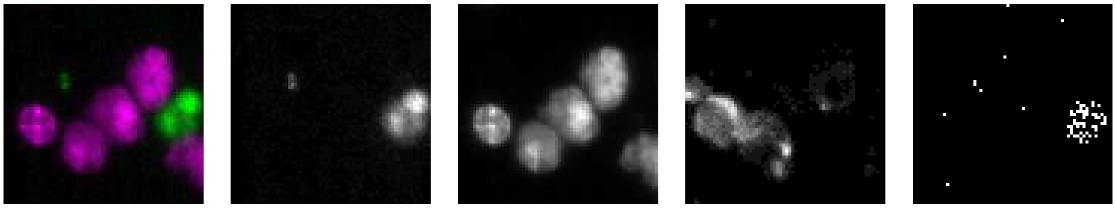				
12925	84	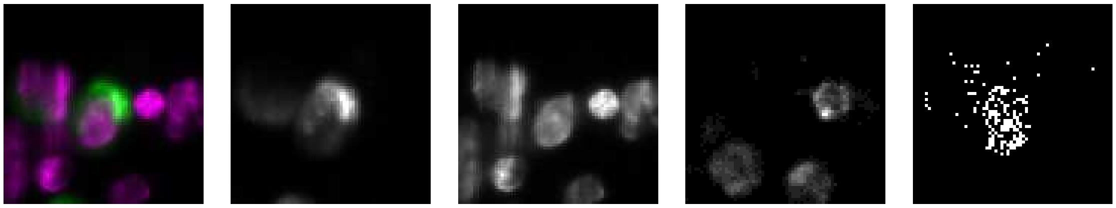				
12926	84	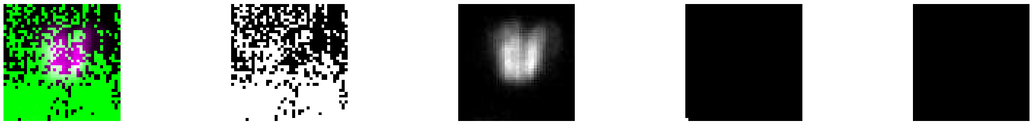				
12927	84	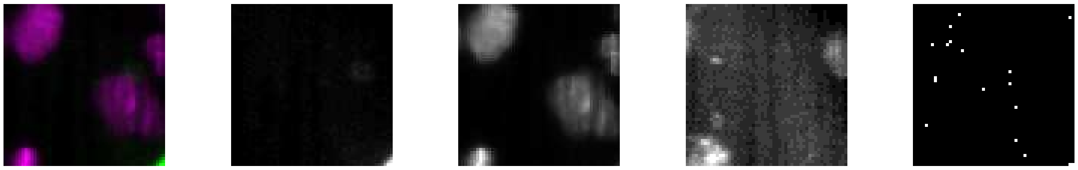				
12928	84	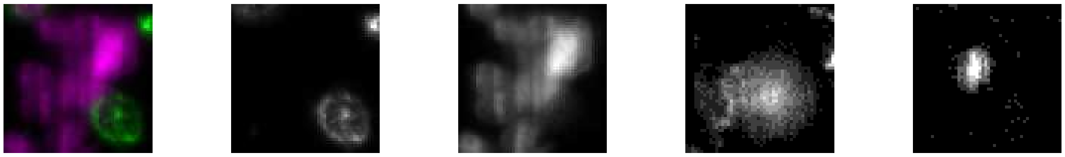				
12929	84	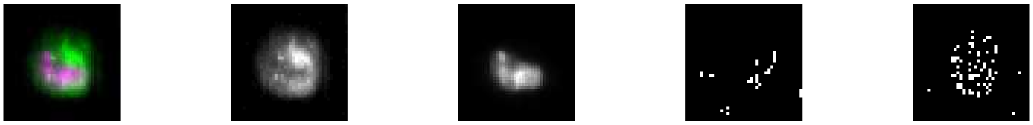				
12930	84	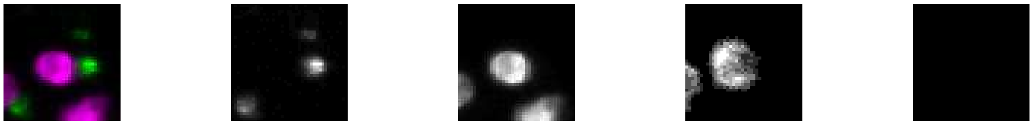				
12931	84	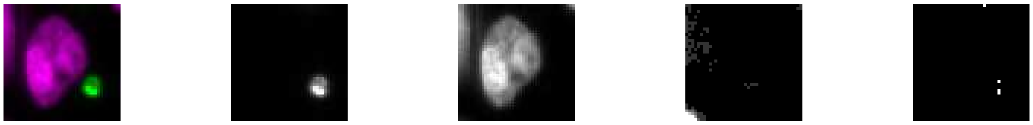				
12932	84	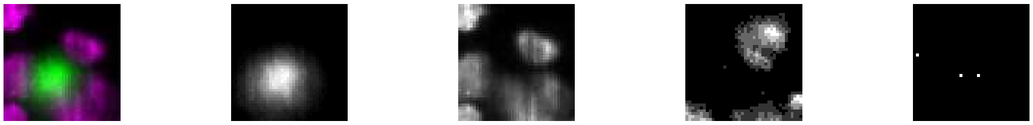				
12933	84	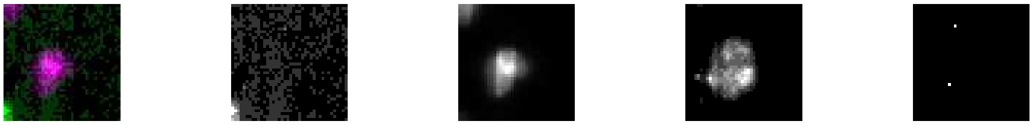				
12934	84	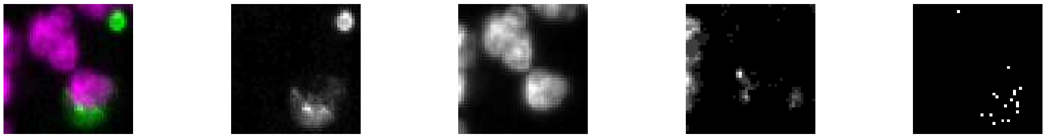				
12935	84	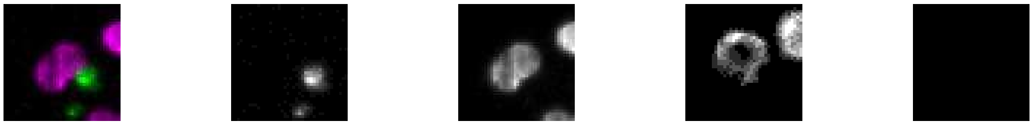				

Ereignis	Aufnahme	DAPI/CK-PE	CK-PE	DAPI	CD45-APC	HER-2/neu
12936	84	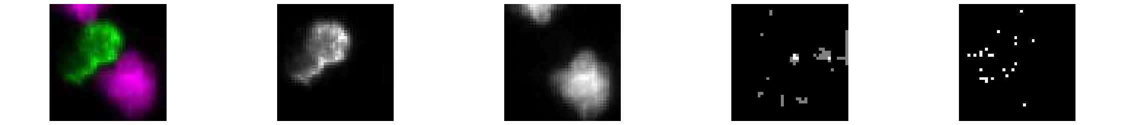				
12937	84	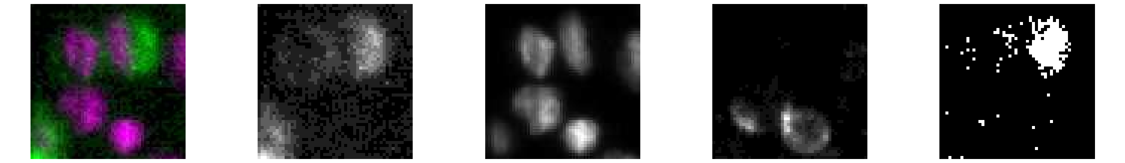				
12938	84	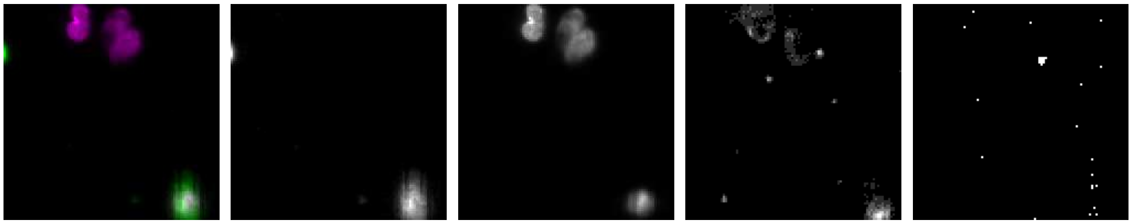				
12939	84	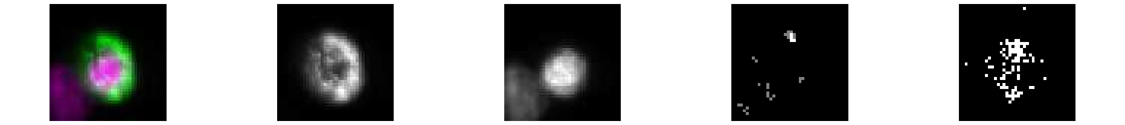				
12940	84	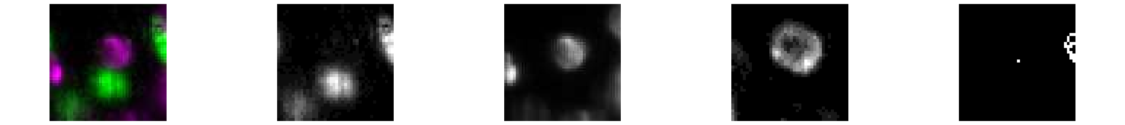				
12941	84	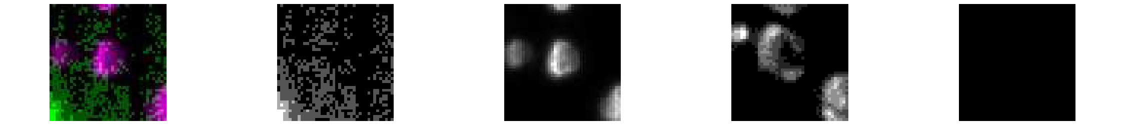				
12942	84	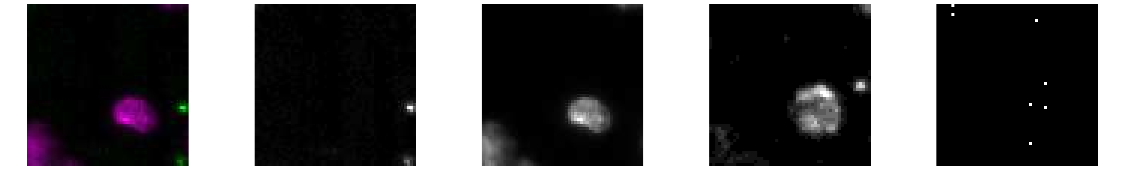				
12943	84	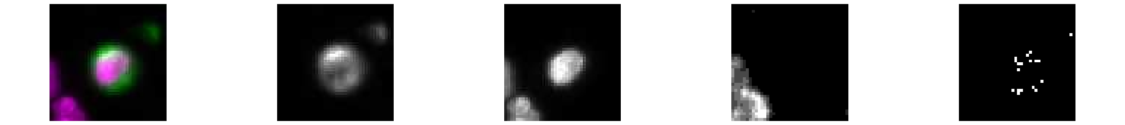				
12944	84	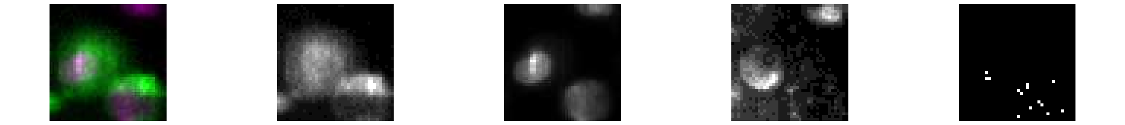				
12945	84	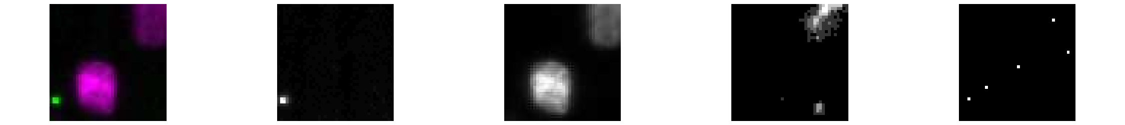				
12946	84	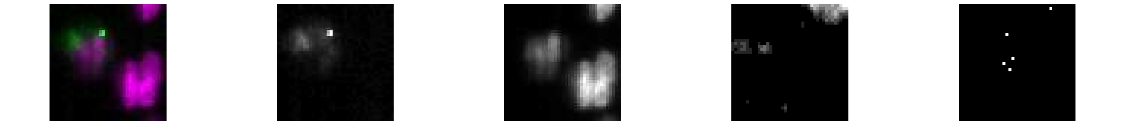				
12947	84	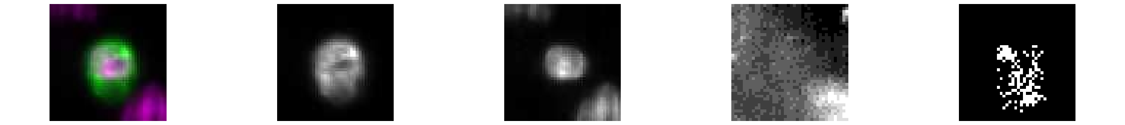				
12948	84	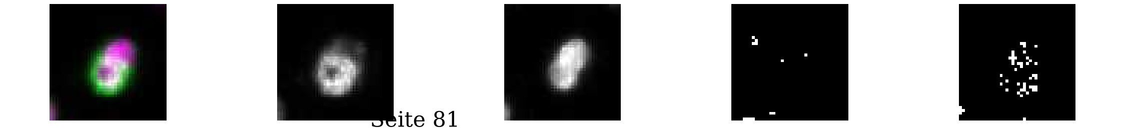				

Ereignis	Aufnahme	DAPI/CK-PE	CK-PE	DAPI	CD45-APC	HER-2/neu
12949	84	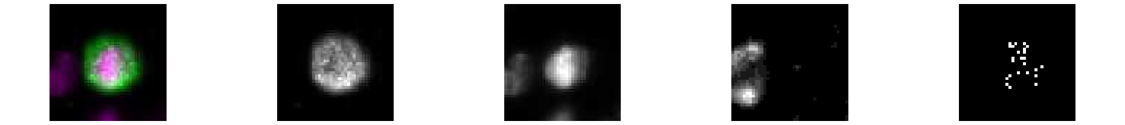				
12950	84	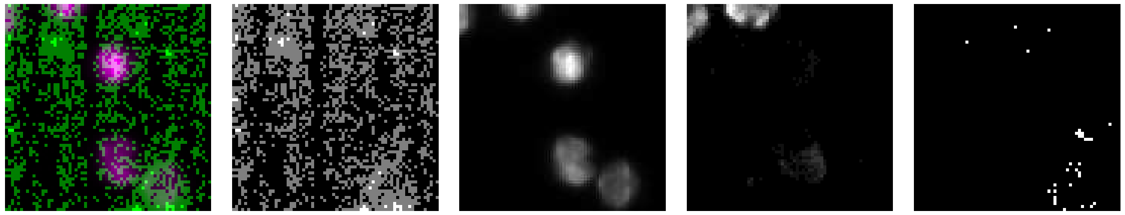				
12951	84	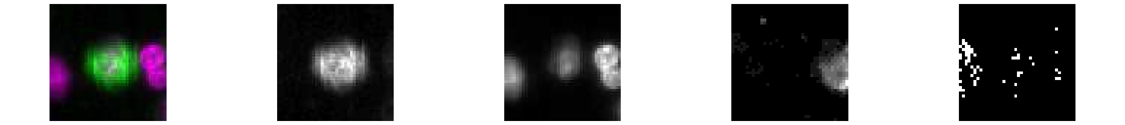				
12952	84	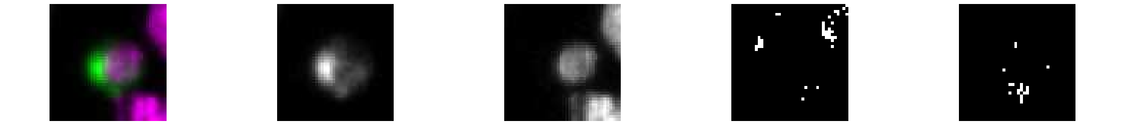				
12953	84	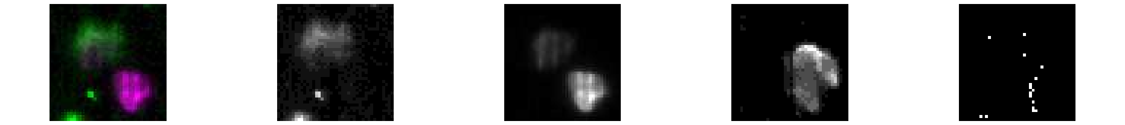				
12954	84	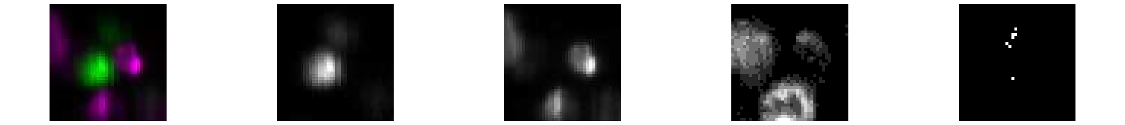				
12955	84	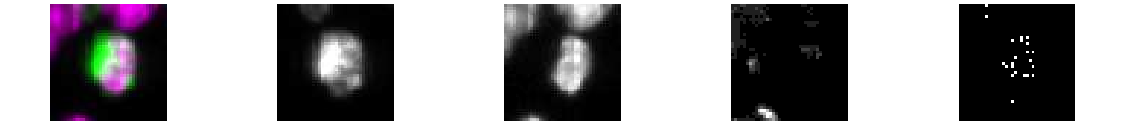				
12956	84	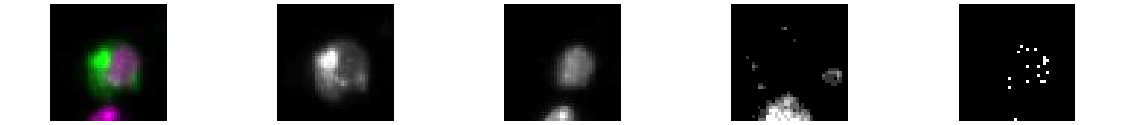				
12957	84	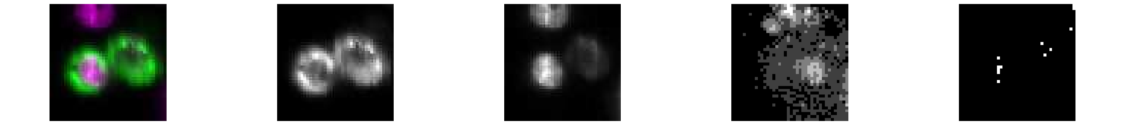				
12958	84	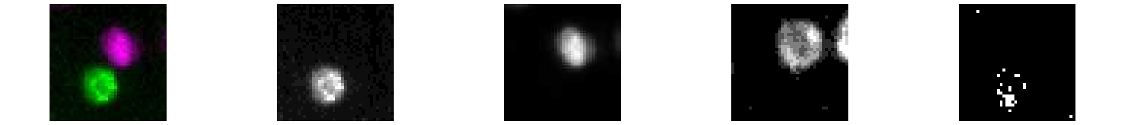				
12959	84	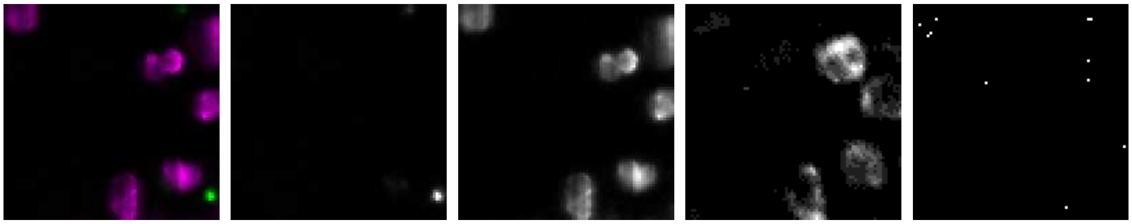				
12960	84	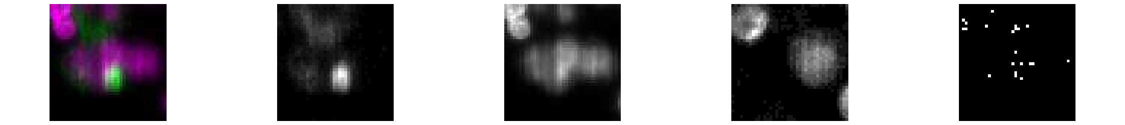				

Ereignis	Aufnahme	DAPI/CK-PE	CK-PE	DAPI	CD45-APC	HER-2/neu
12961	84	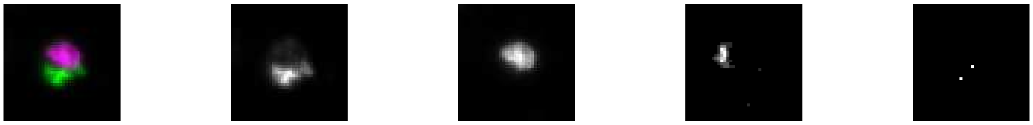				
12962	84	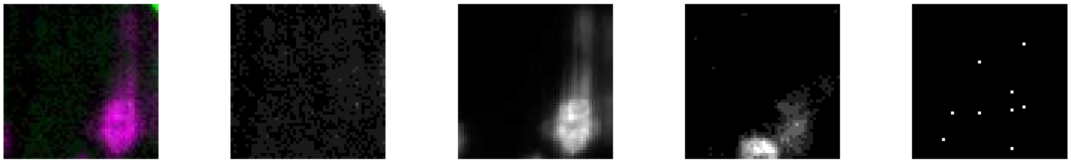				
12963	84	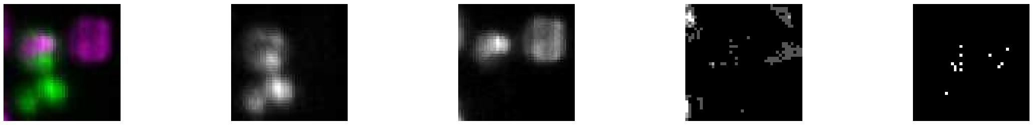				
12964	84	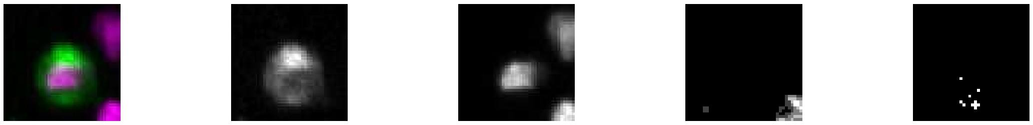				
12965	84	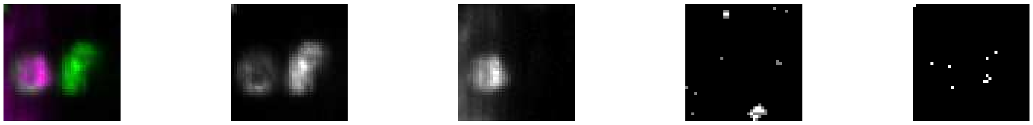				
12966	84	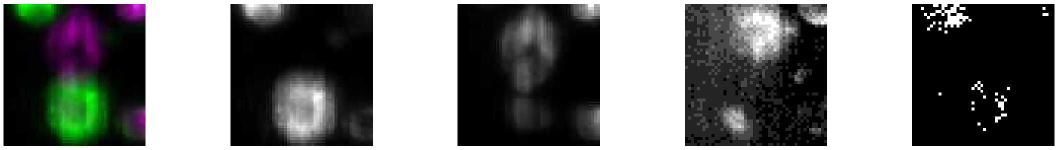				
12967	84	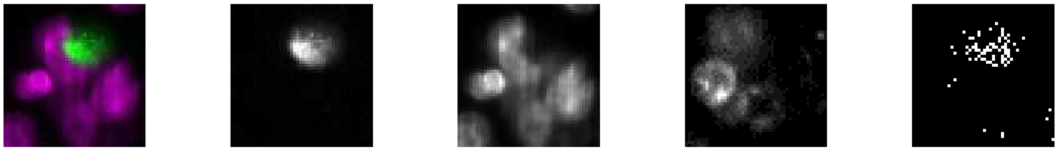				
12968	84	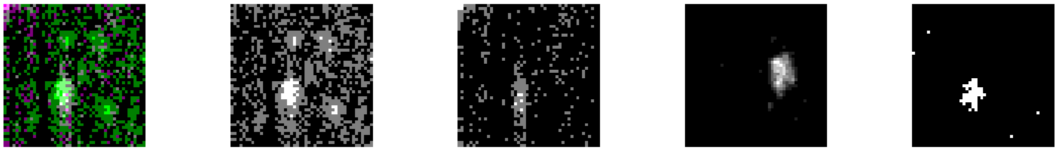				
12969	84	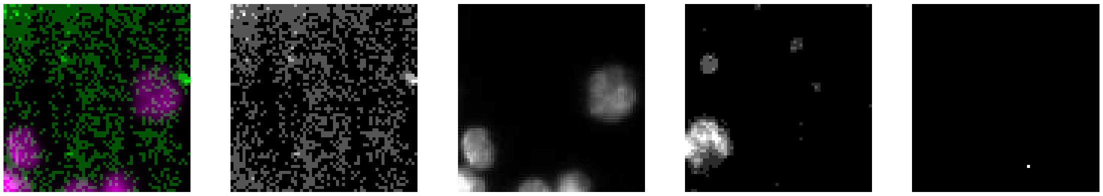				
12970	84	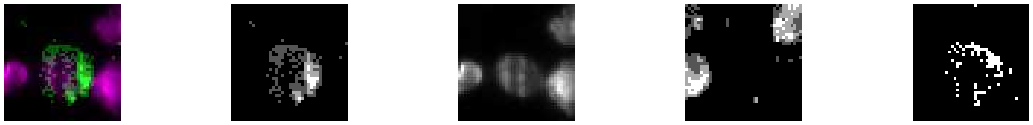				
12971	84	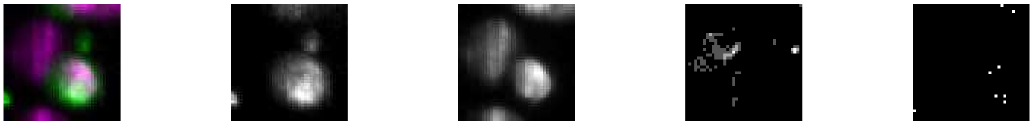				
12972	84	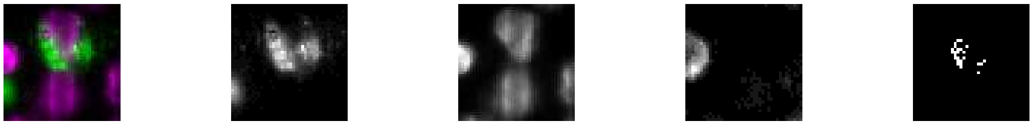				

Ereignis	Aufnahme	DAPI/CK-PE	CK-PE	DAPI	CD45-APC	HER-2/neu
12973	84	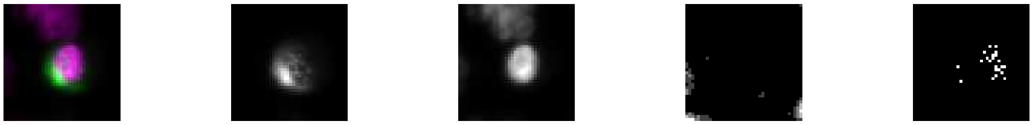				
12974	84	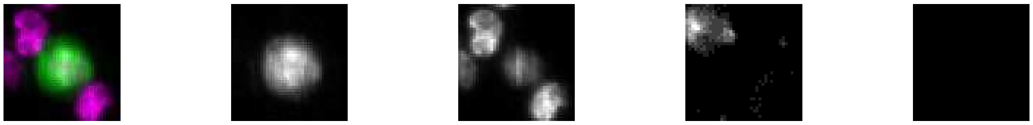				
12975	84	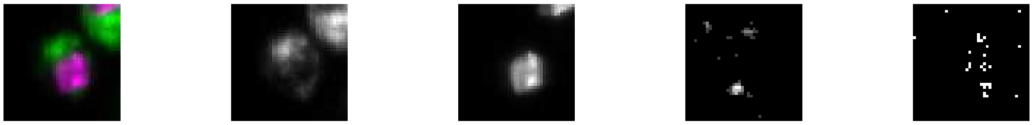				
12976	84	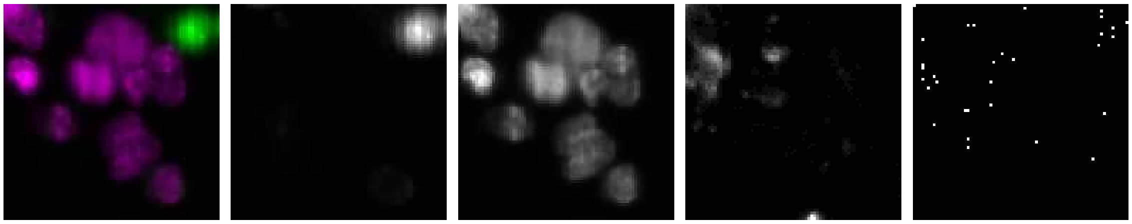				
12977	84	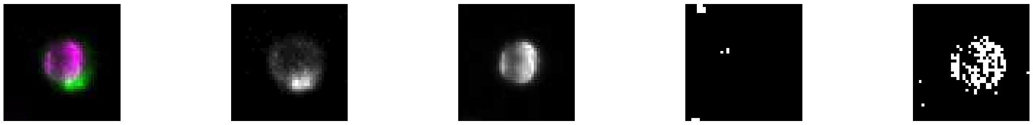				
12978	84	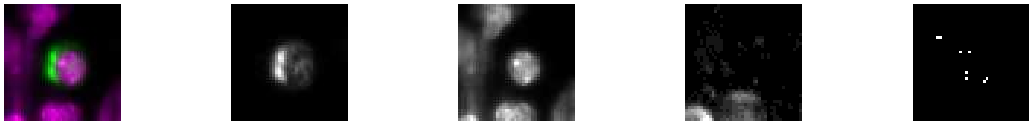				
12979	84	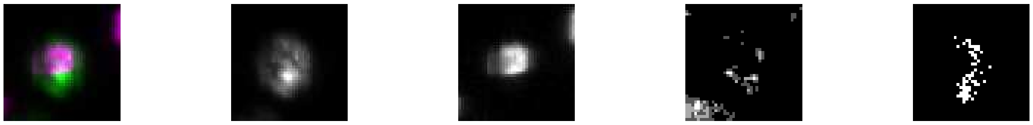				
12980	84	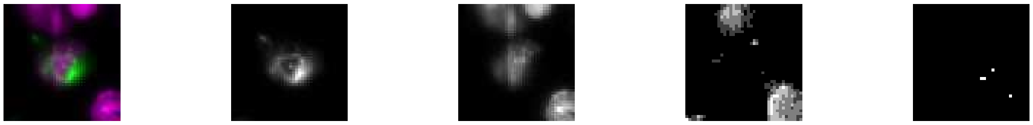				
12981	84	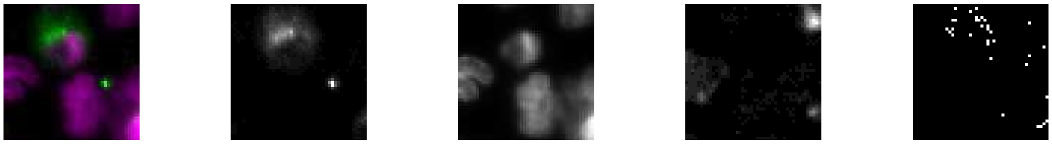				
12982	84	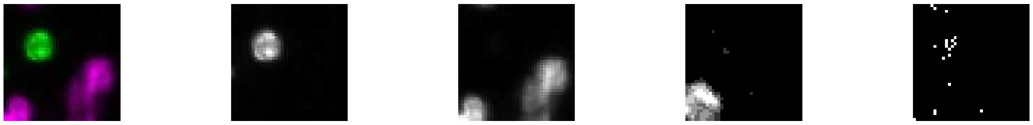				
12983	84	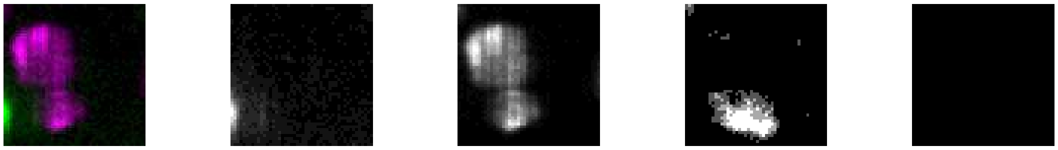				
12984	84	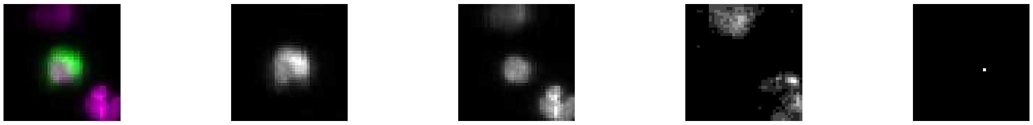				

Ereignis	Aufnahme	DAPI/CK-PE	CK-PE	DAPI	CD45-APC	HER-2/neu
12985	84	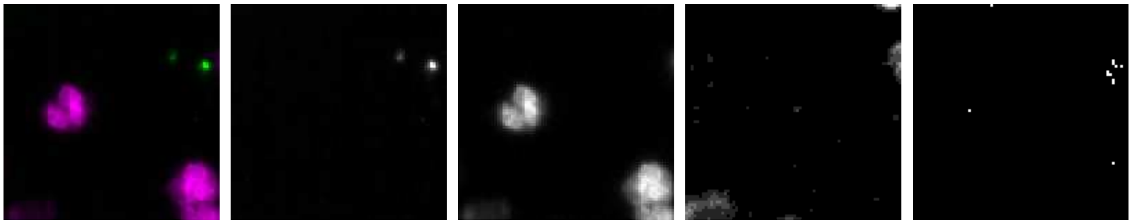				
12986	84	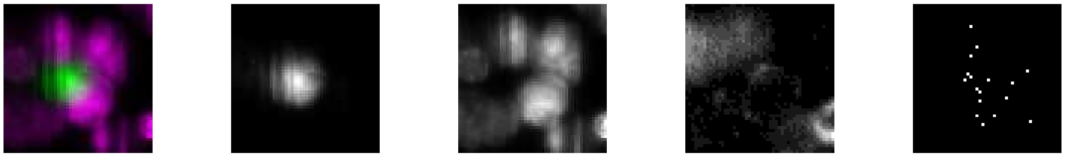				
12987	84	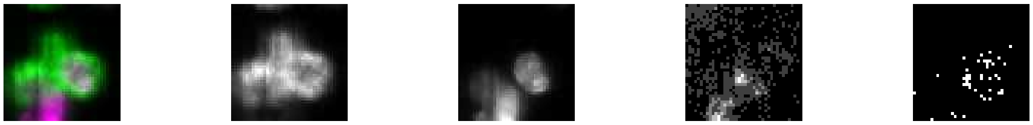				
12988	84	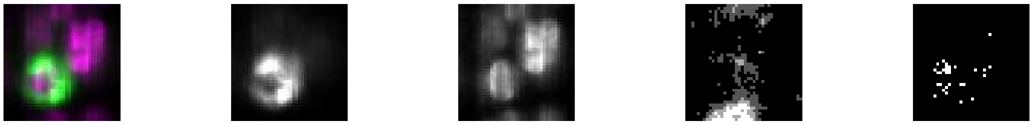				
12989	84	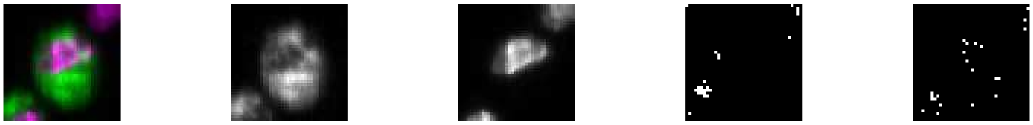				
12990	84	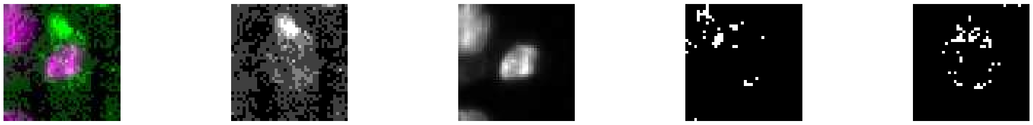				
12991	84	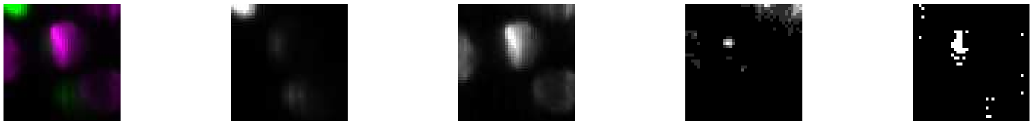				
12992	84	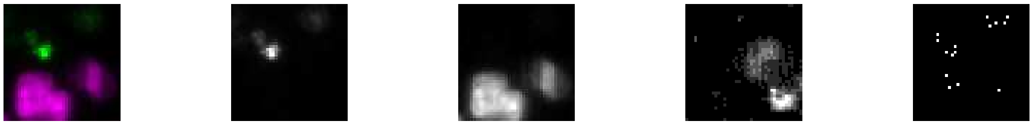				
12993	84	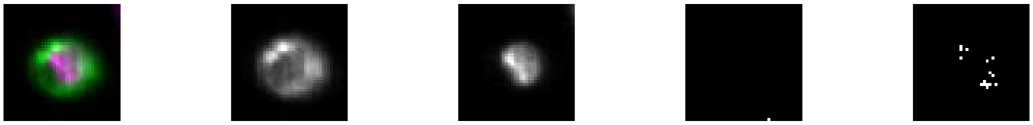				
12994	84	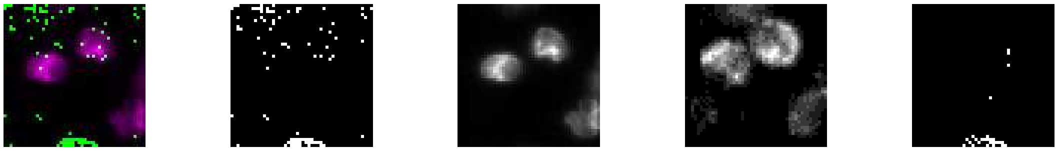				
12995	84	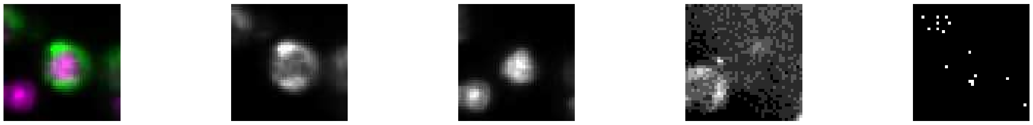				
12996	84	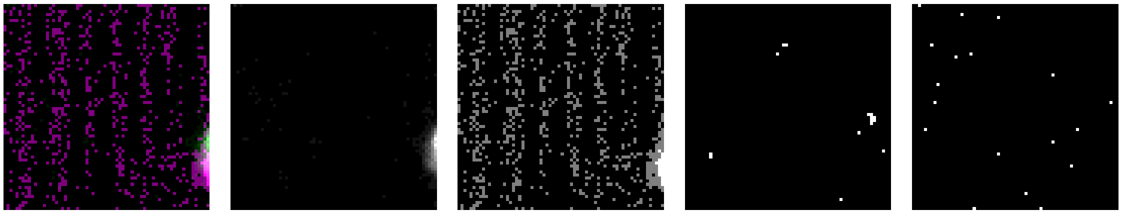				

Druckdatum:02/06/2020 17:00

Proben-ID: Gyn14.208 **Kassetten-ID:**01327106 **Patienten-ID:**

Ereignis	Aufnahme	DAPI/CK-PE	CK-PE	DAPI	CD45-APC	HER-2/neu
12997	84	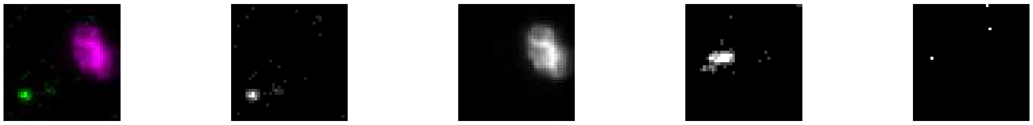				
12998	84	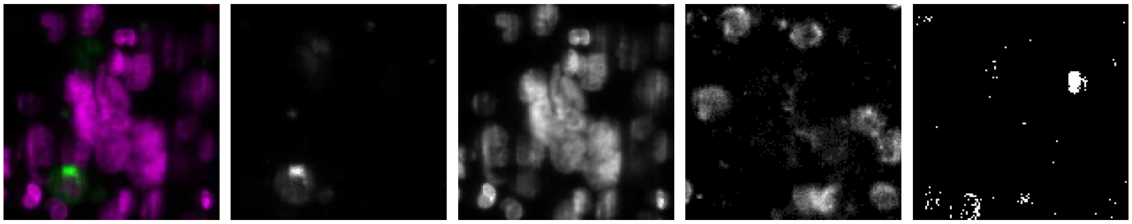				
12999	84	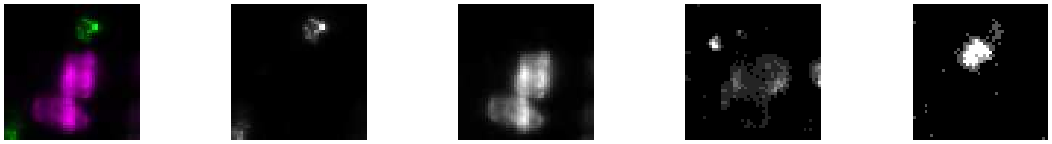				
13000	84	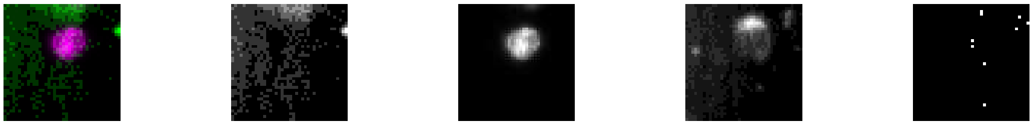				
13001	84	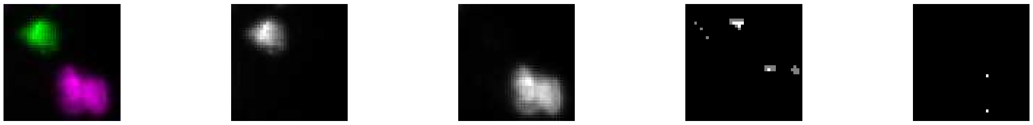				
13002	84	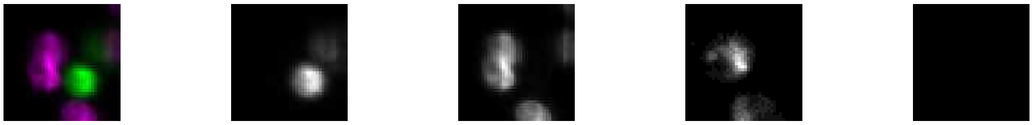				
13003	84	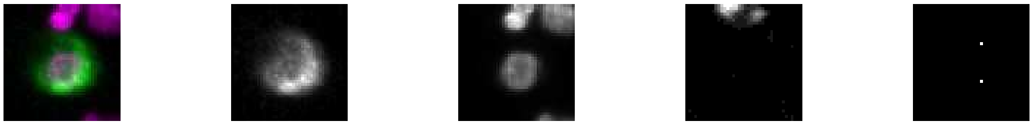				
13004	84	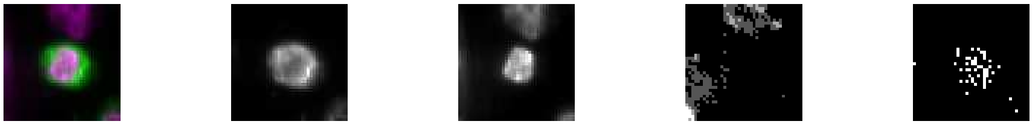				
13005	84	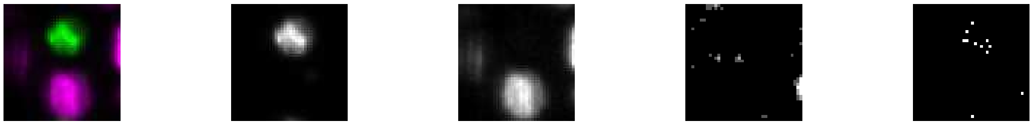				
13006	84	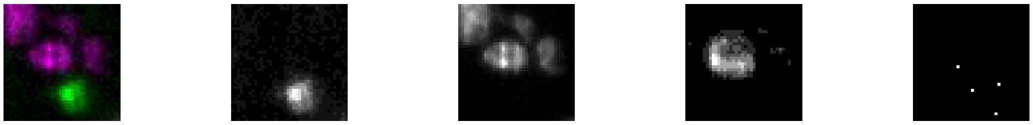				
13007	84	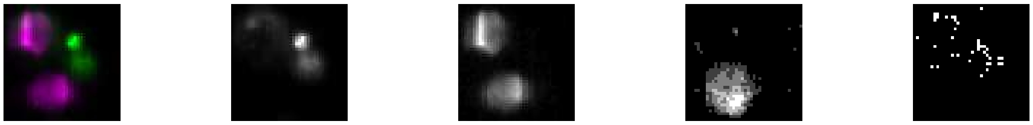				
13008	84	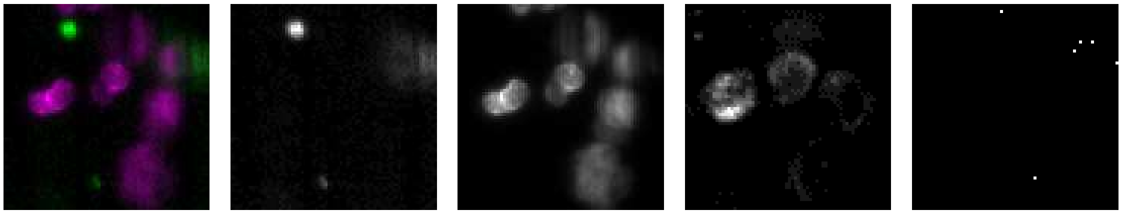				

Ereignis	Aufnahme	DAPI/CK-PE	CK-PE	DAPI	CD45-APC	HER-2/neu
13009	84	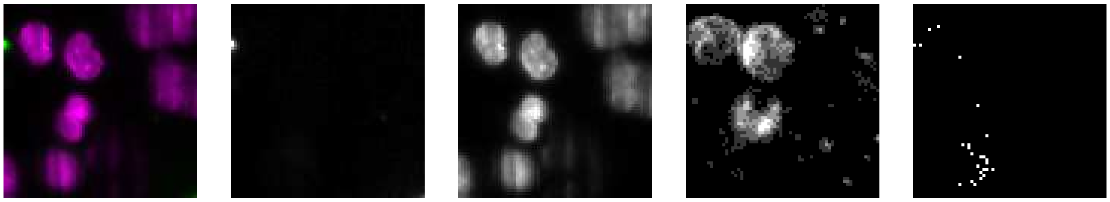				
13010	84	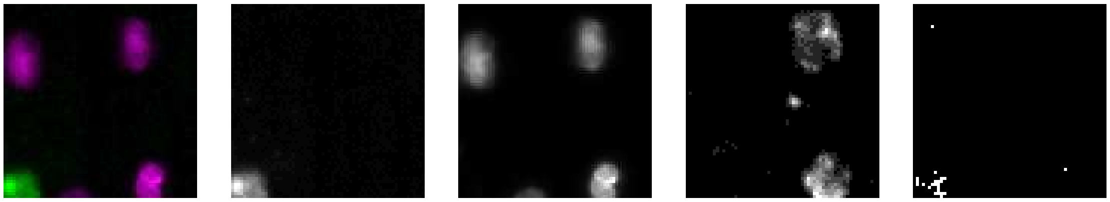				
13011	84	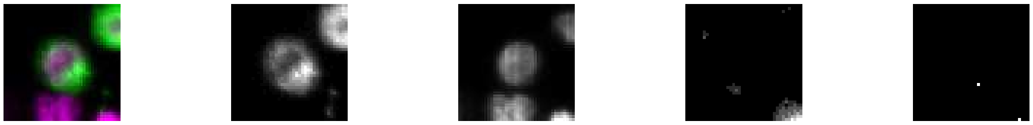				
13012	84	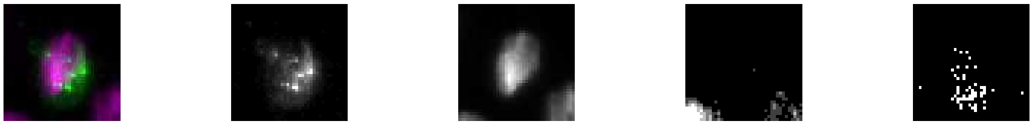				
13013	84	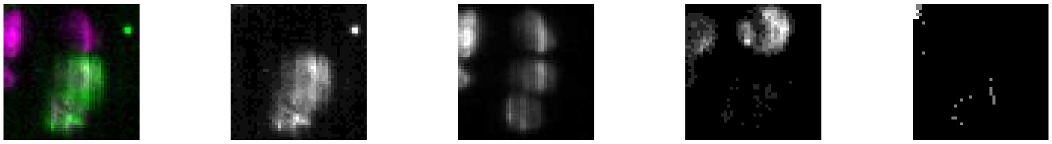				
13014	84	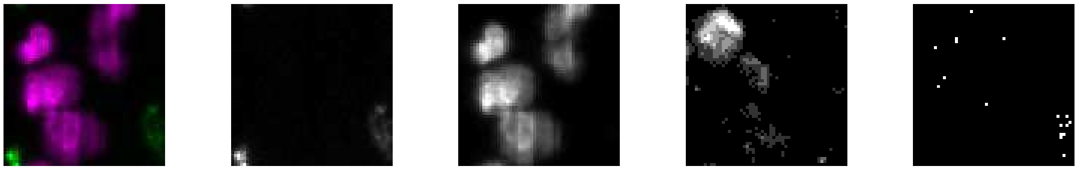				
13015	84	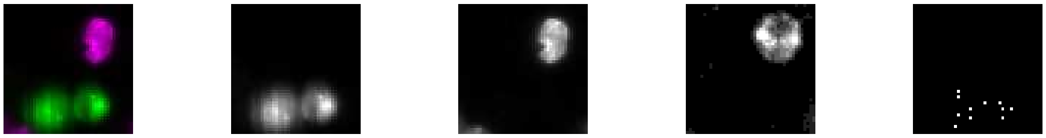				
13016	84	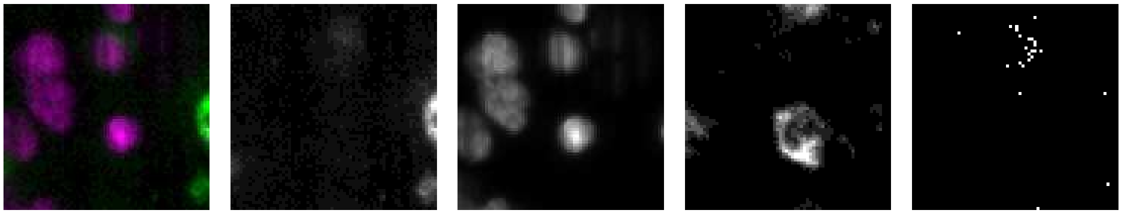				
13017	84	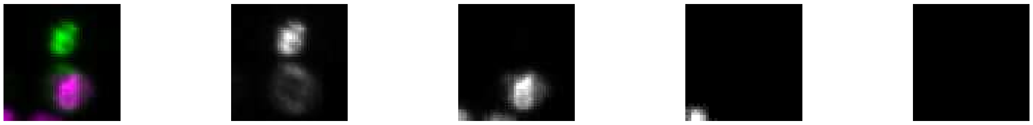				
13018	84	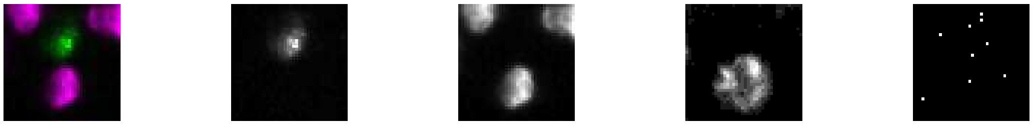				
13019	84	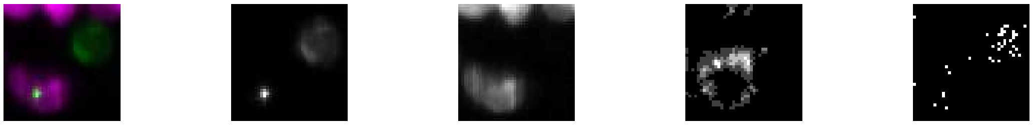				

Druckdatum: 02/06/2020 17:00

Proben-ID: Gyn14.208 **Kassetten-ID:** 01327106 **Patienten-ID:**

Ereignis	Aufnahme	DAPI/CK-PE	CK-PE	DAPI	CD45-APC	HER-2/neu
13020	84	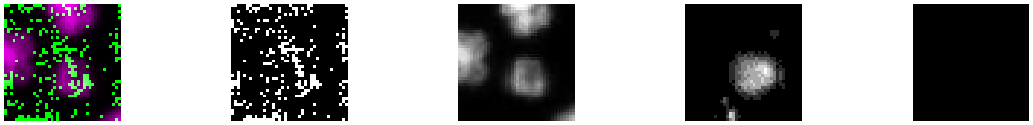				
13021	84	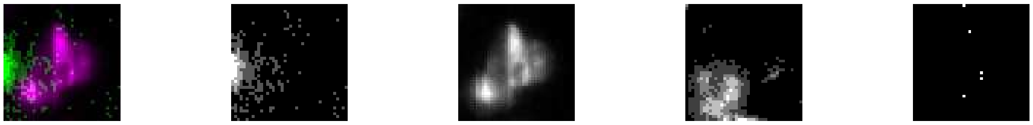				
13022	84	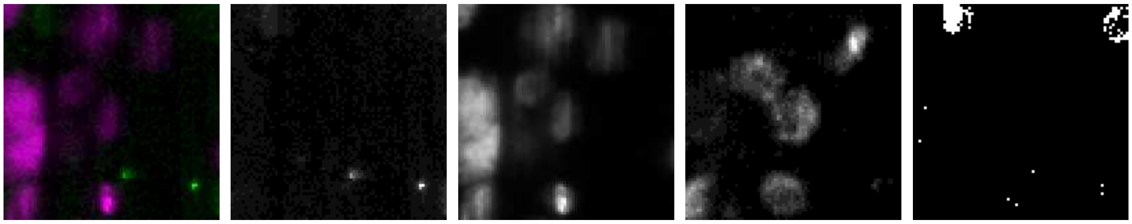				
13023	84	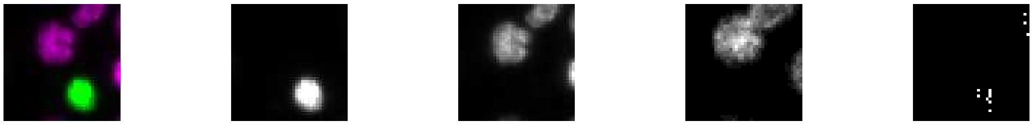				
13024	84	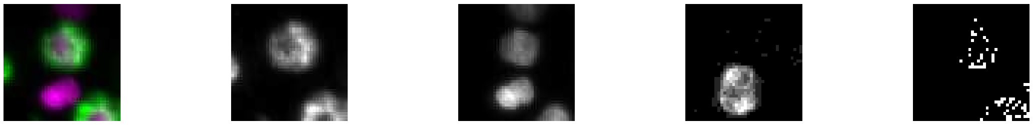				
13025	84	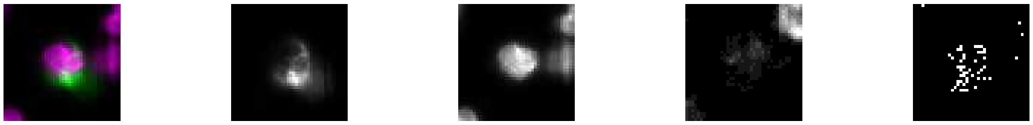				
13026	84	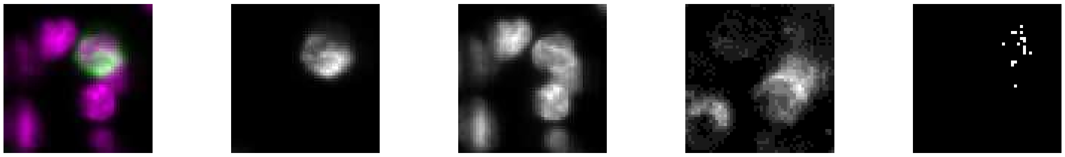				
13027	84	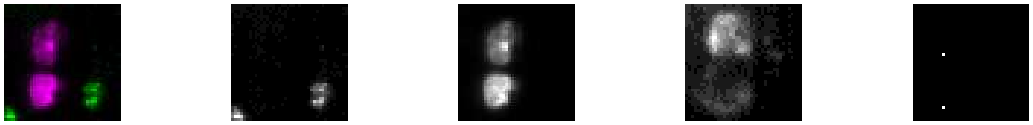				
13028	84	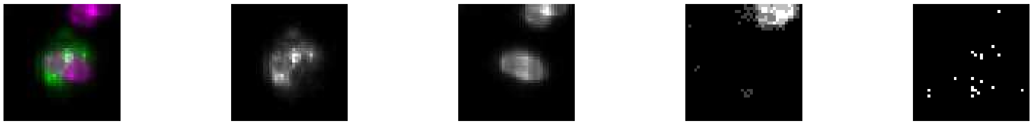				
13029	84	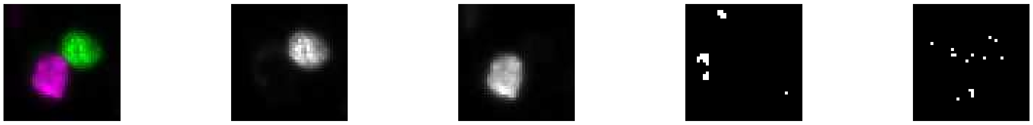				
13030	84	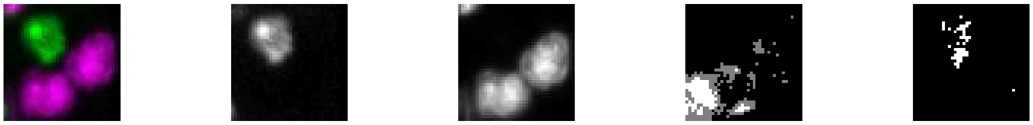				
13031	84	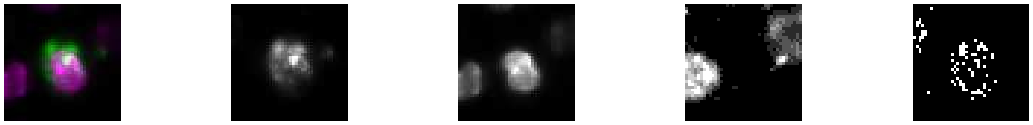				
13032	84	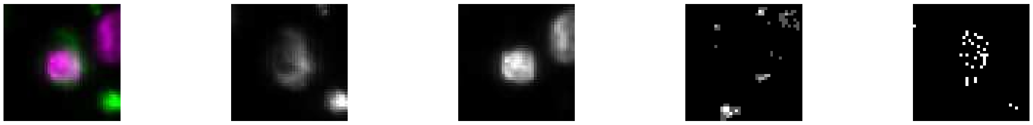				

Ereignis	Aufnahme	DAPI/CK-PE	CK-PE	DAPI	CD45-APC	HER-2/neu
13033	84	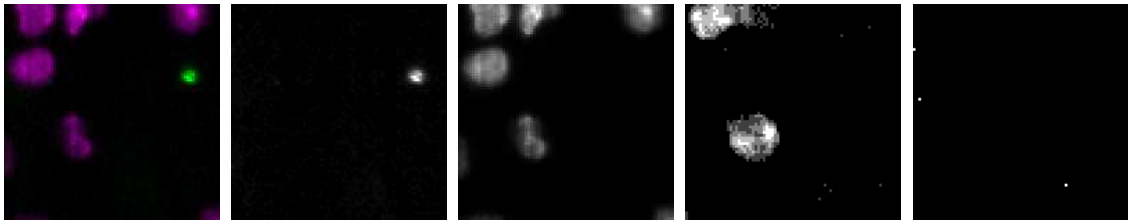				
13034	84	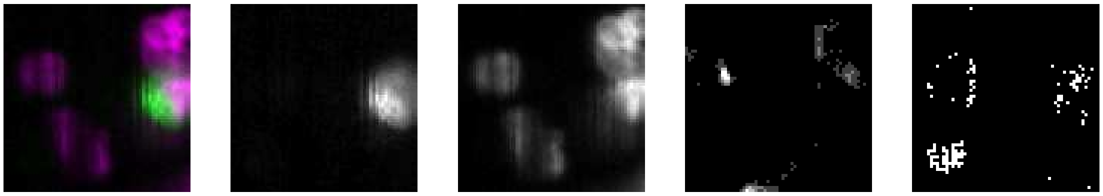				
13035	84	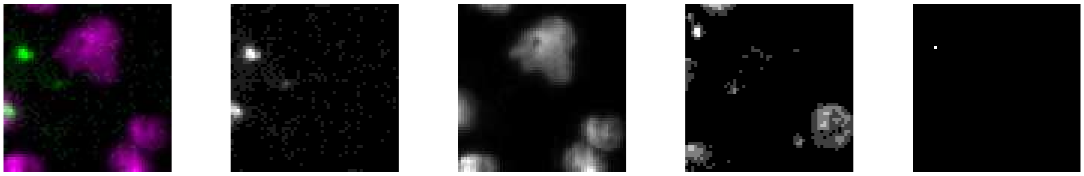				
13036	84	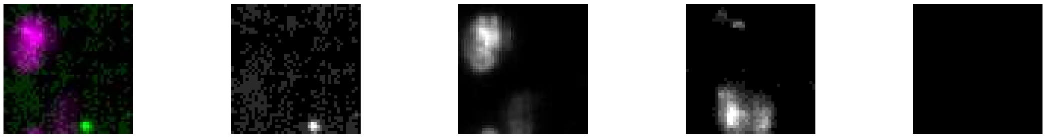				
13037	84	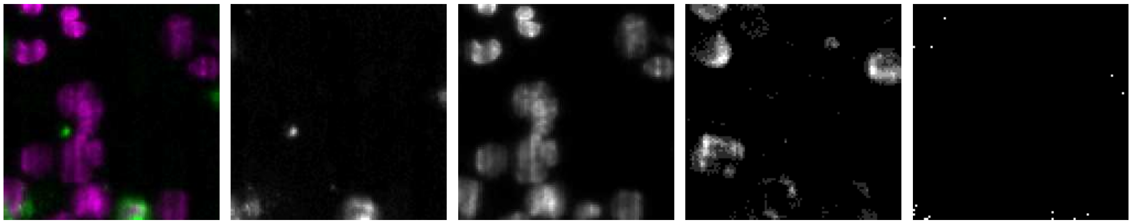				
13038	84	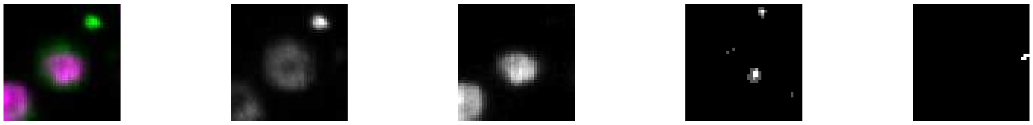				
13039	84	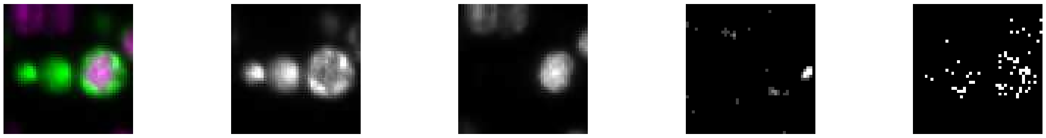				
13040	84	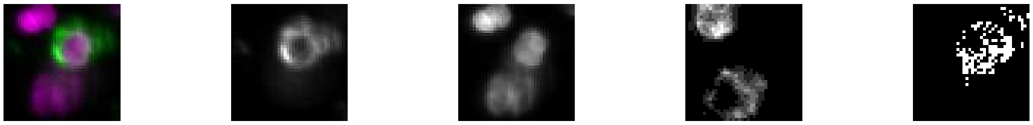				
13041	84	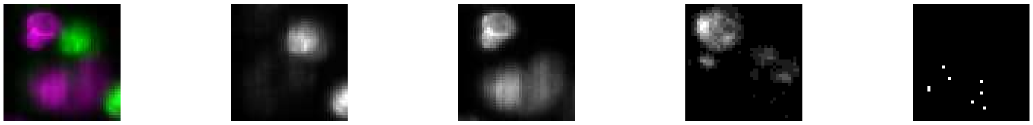				
13042	84	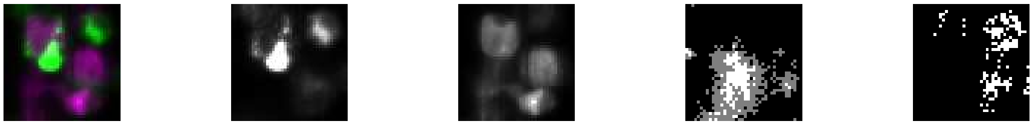				
13043	84	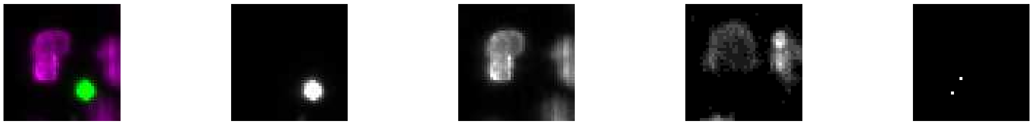				

Druckdatum:02/06/2020 17:00

Proben-ID: Gyn14.208 **Kassetten-ID:**01327106 **Patienten-ID:**

Ereignis	Aufnahme	DAPI/CK-PE, CK-PE, DAPI, CD45-APC, HER-2/neu
13044	84	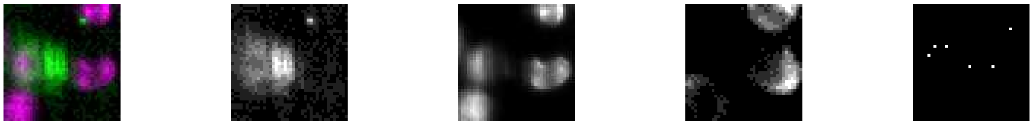
13045	84	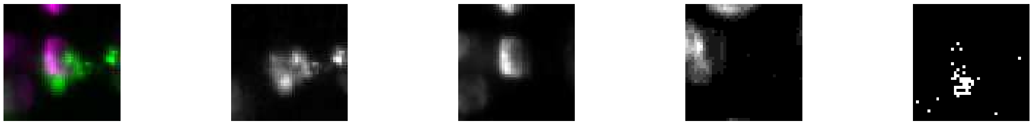
13046	84	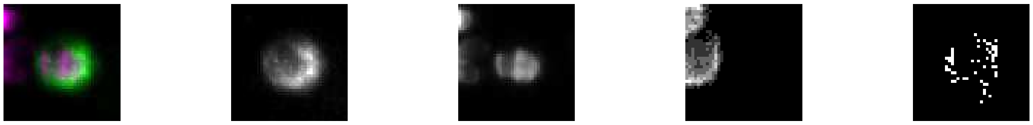
13047	84	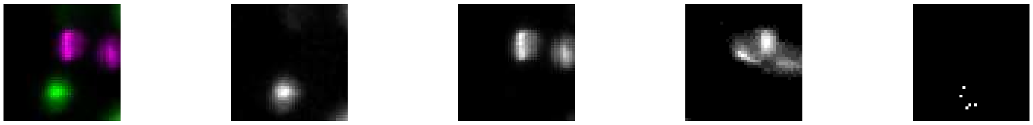
13048	84	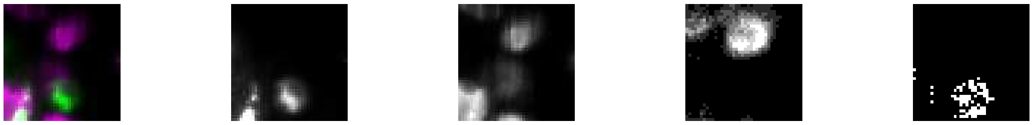
13049	84	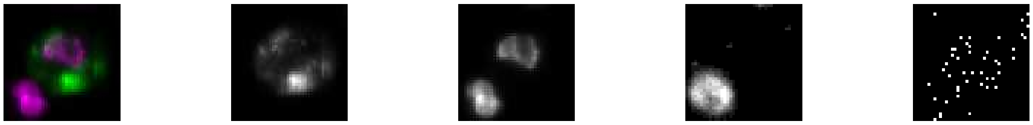
13050	84	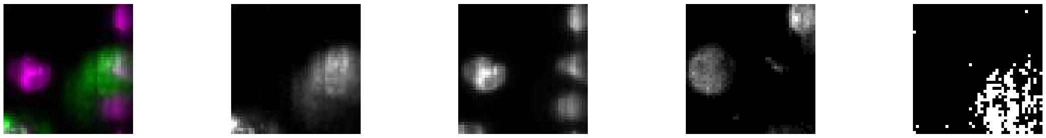
13051	84	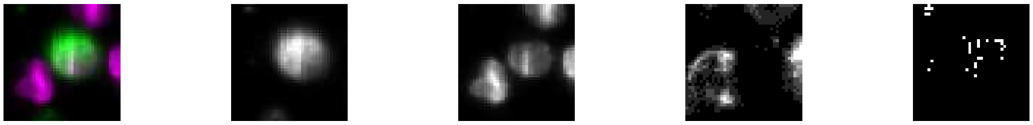
13052	84	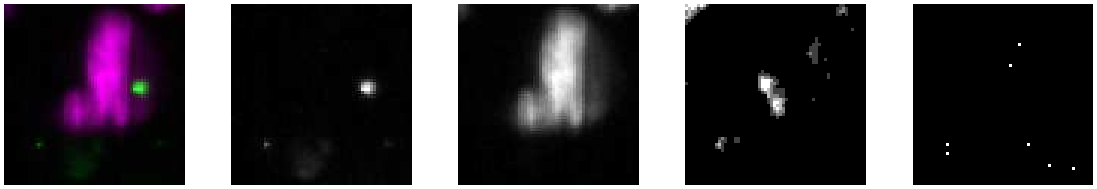
13053	84	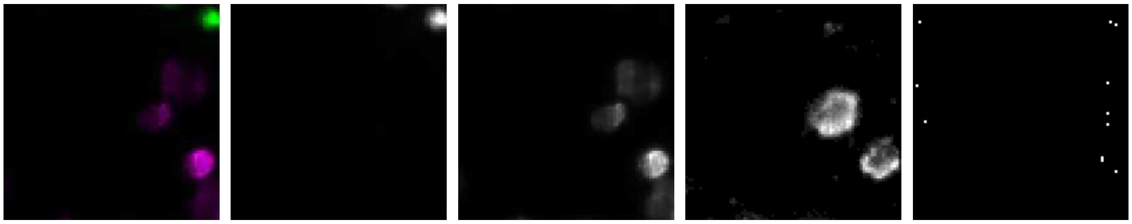
13054	84	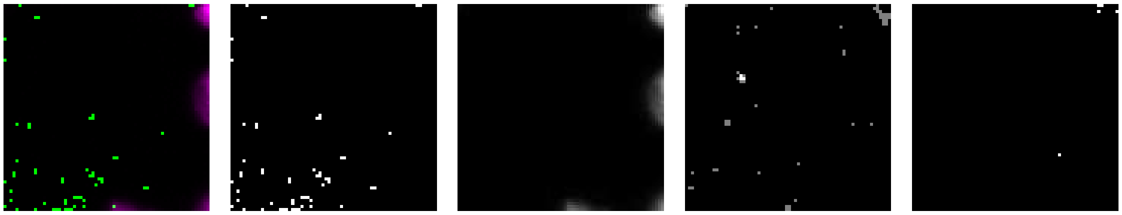
13055	84	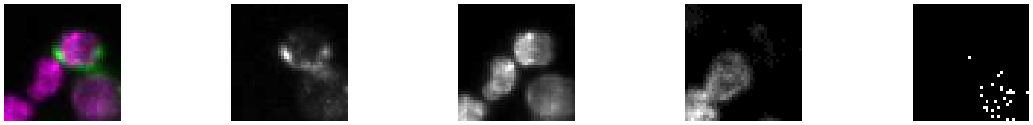

Ereignis	Aufnahme	DAPI/CK-PE	CK-PE	DAPI	CD45-APC	HER-2/neu
13056	84	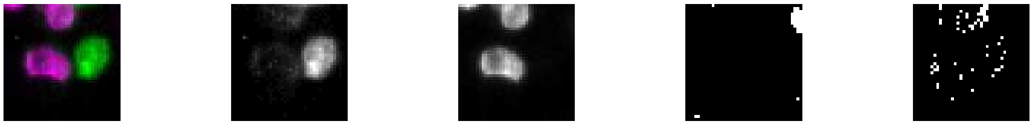				
13057	84	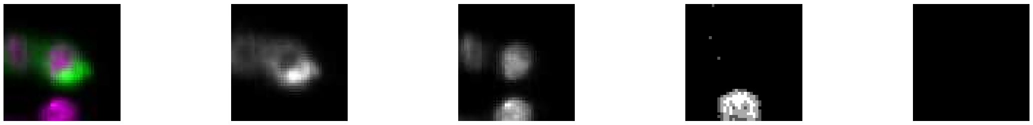				
13058	84	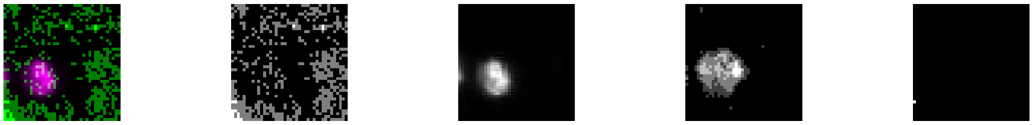				
13059	84	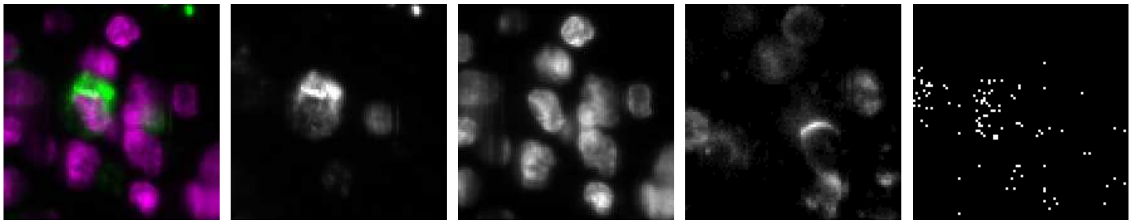				
13060	84	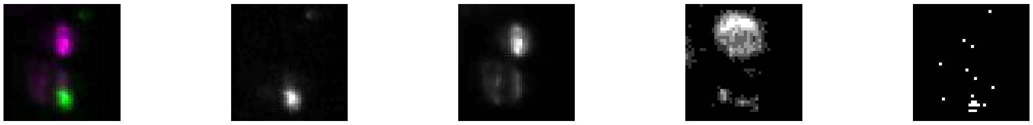				
13061	84	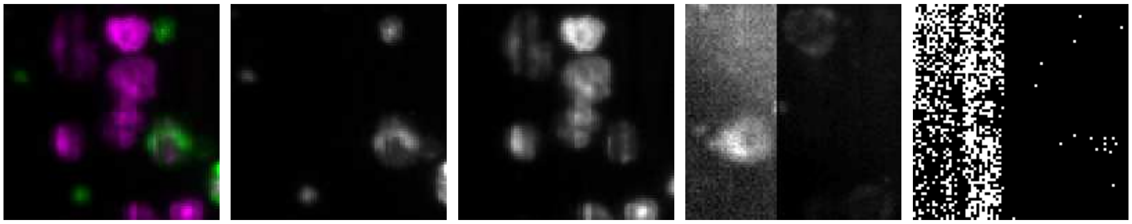				
13062	83	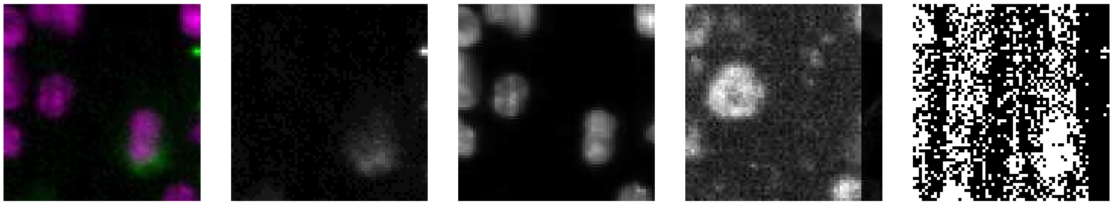				
13063	57	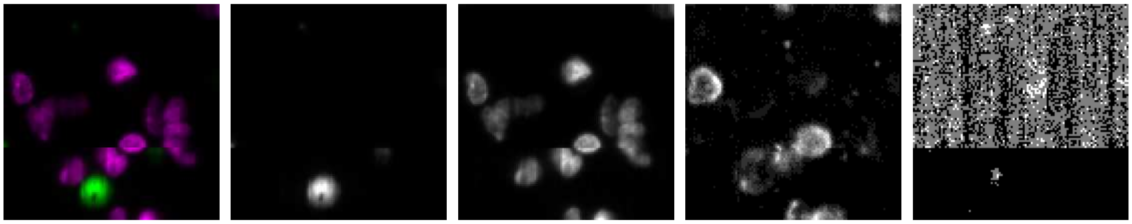				
13064	83	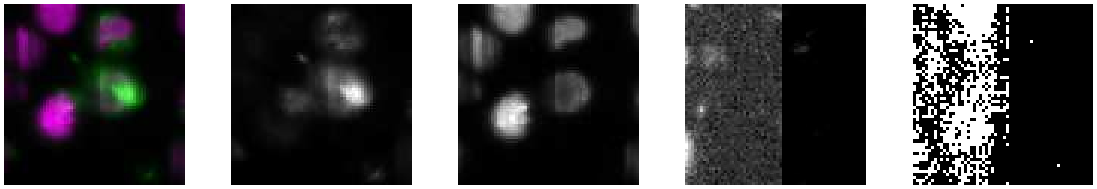				
13065	57	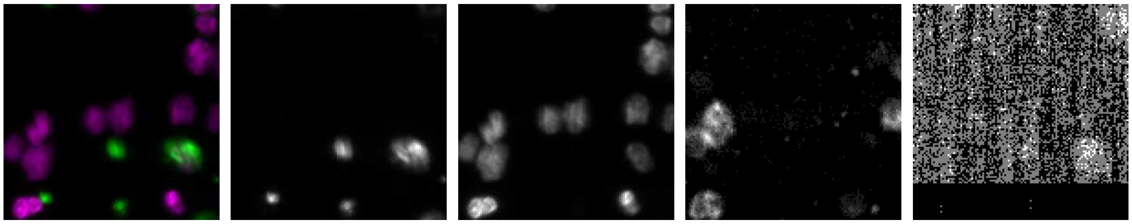				

Ereignis	Aufnahme	DAPI/CK-PE	CK-PE	DAPI	CD45-APC	HER-2/neu
13066	83	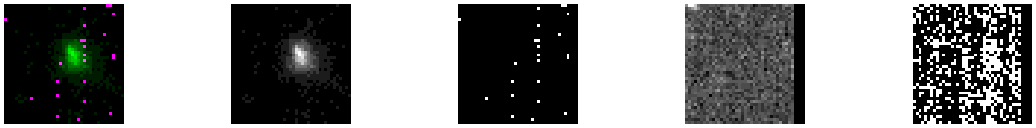				
13067	84	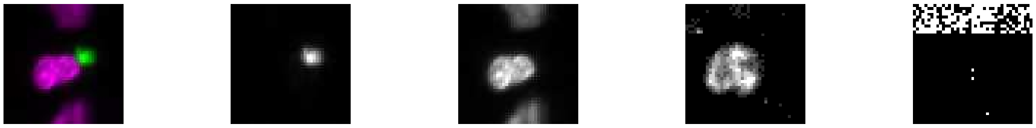				
13068	57	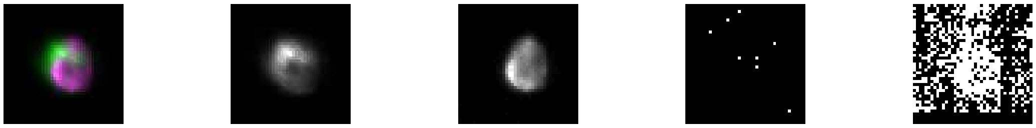				
13069	57	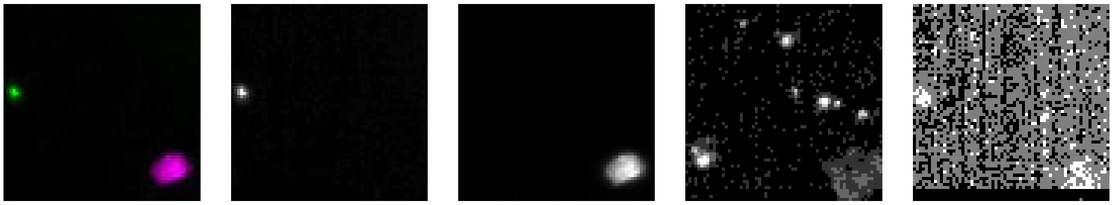				
13070	85	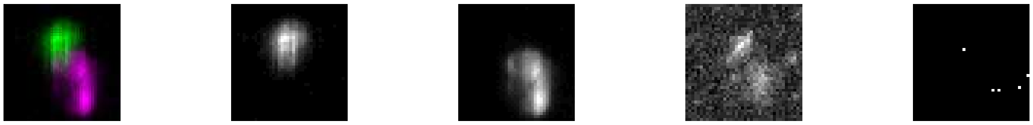				
13071	85	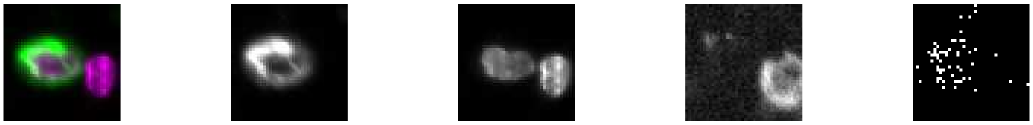				
13072	85	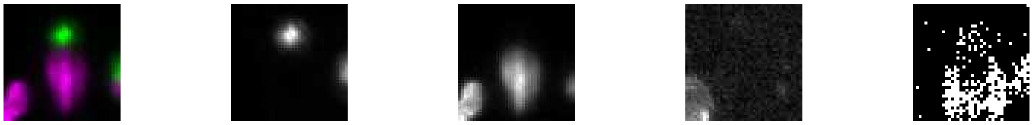				
13073	85	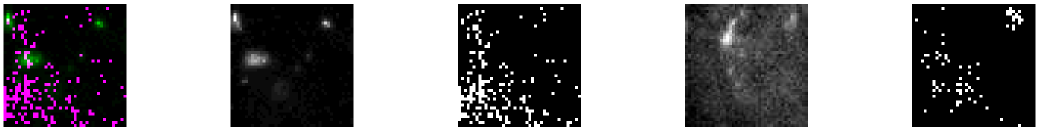				
13074	85	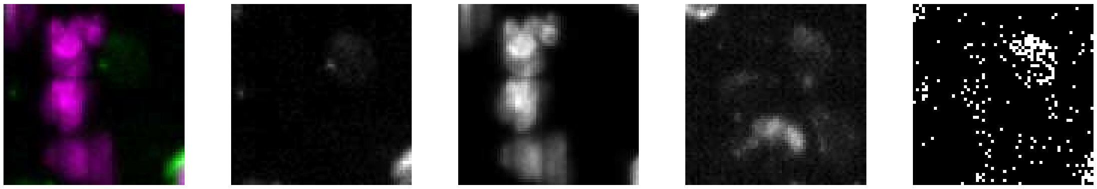				
13075	85	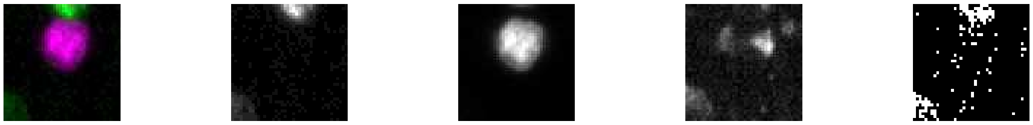				
13076	85	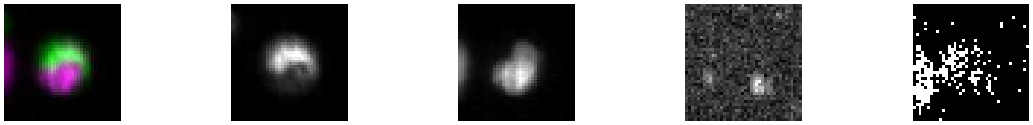				
13077	85	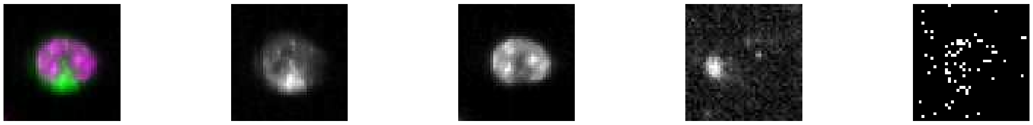				
13078	85	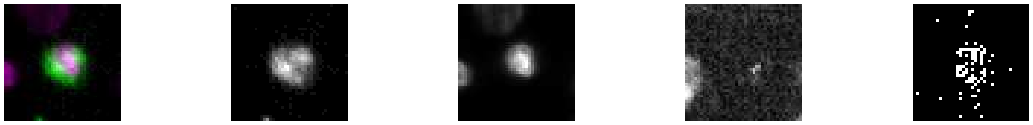				

Ereignis	Aufnahme	DAPI/CK-PE	CK-PE	DAPI	CD45-APC	HER-2/neu
13079	85	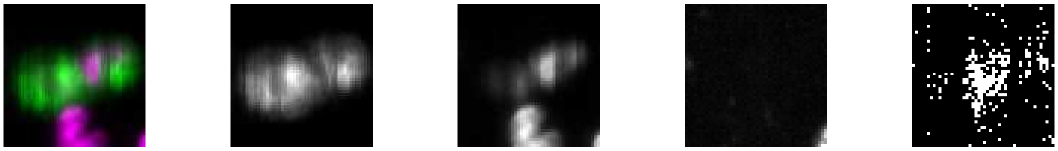				
13080	85	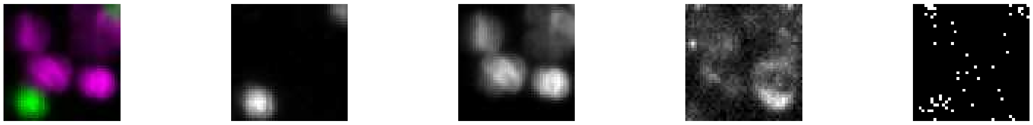				
13081	85	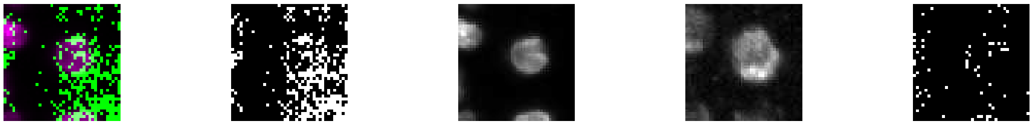				
13082	85	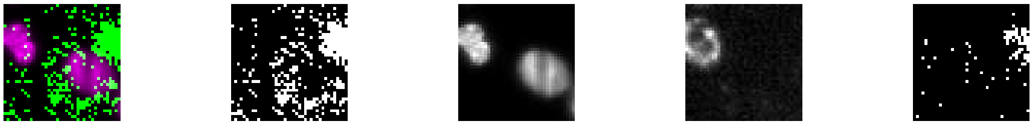				
13083	85	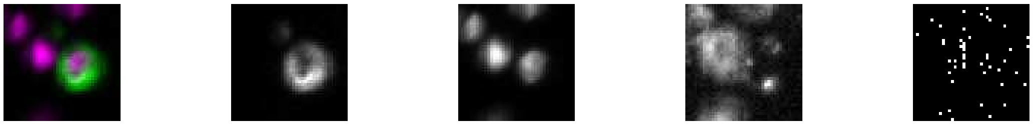				
13084	85	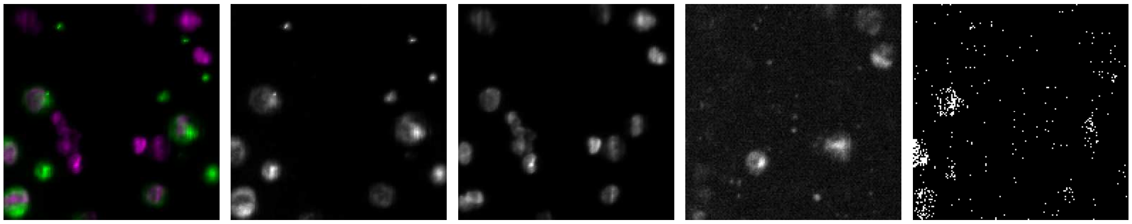				
13085	85	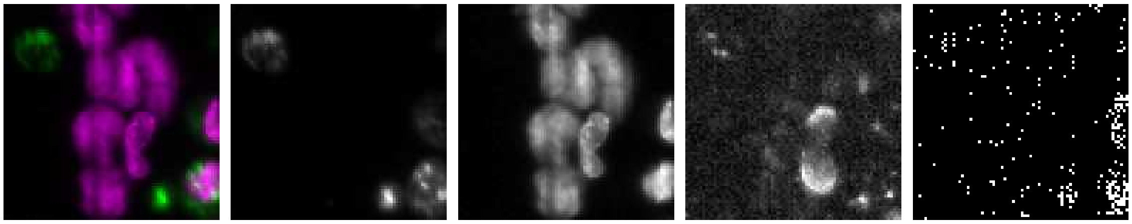				
13086	85	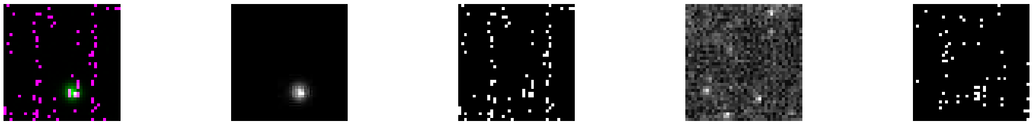				
13087	85	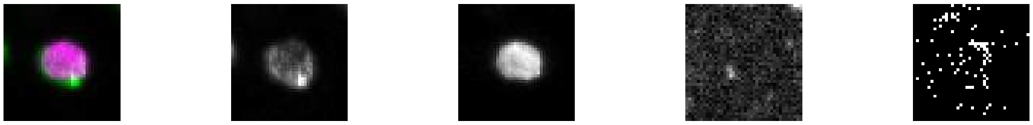				
13088	85	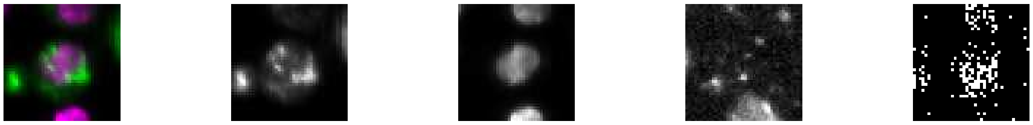				
13089	85	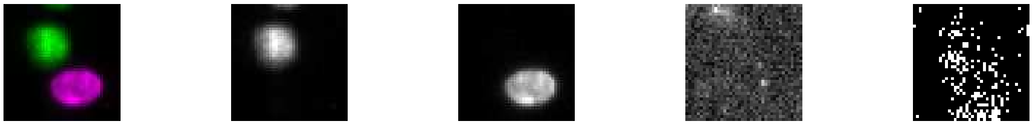				

Ereignis	Aufnahme	DAPI/CK-PE	CK-PE	DAPI	CD45-APC	HER-2/neu
13090	85	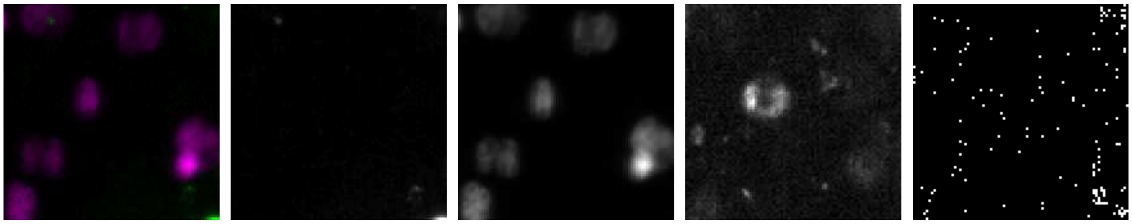				
13091	85	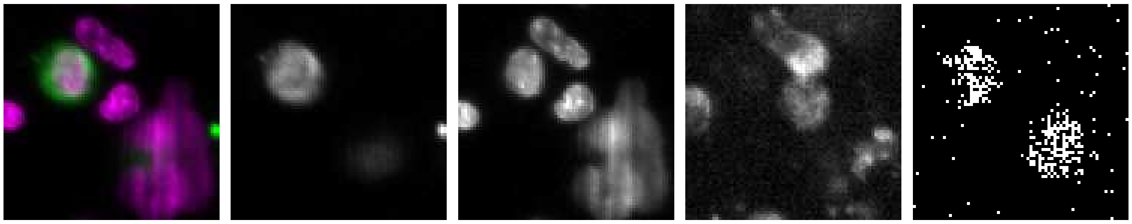				
13092	85	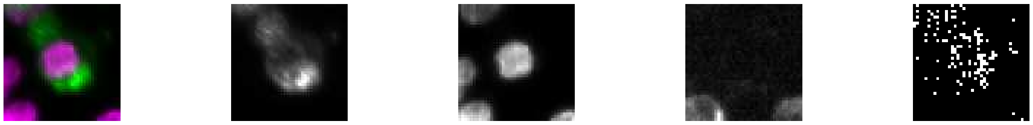				
13093	85	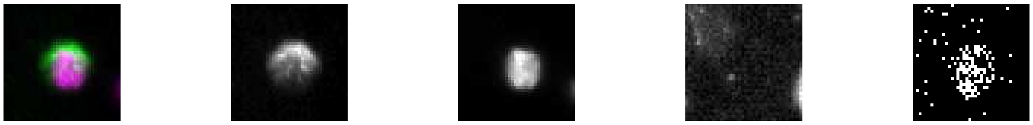				
13094	85	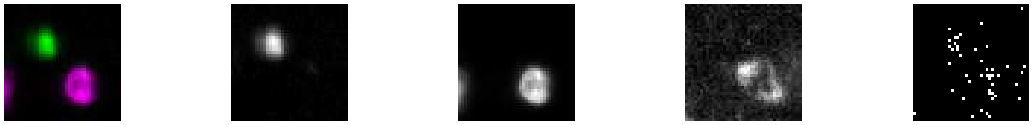				
13095	85	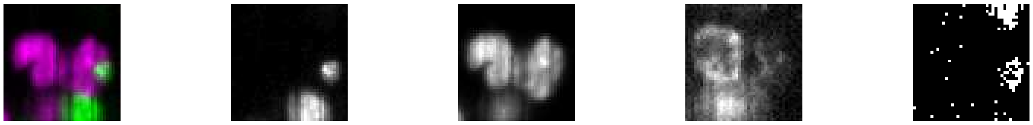				
13096	85	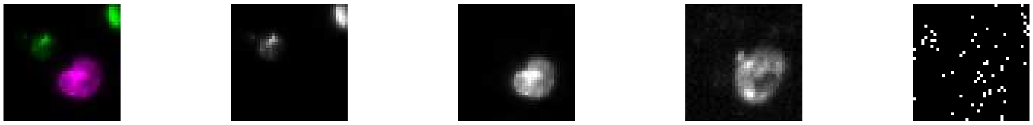				
13097	85	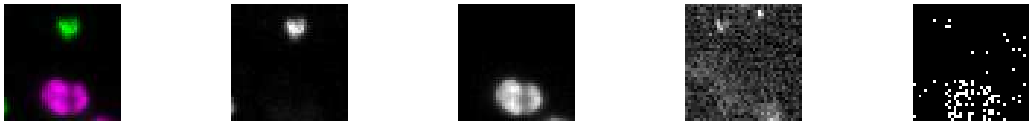				
13098	85	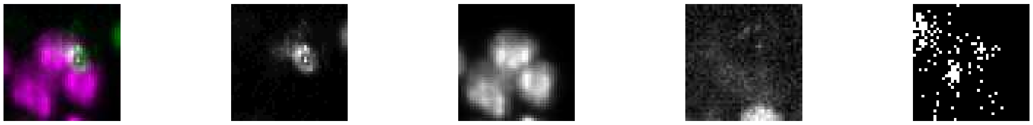				
13099	85	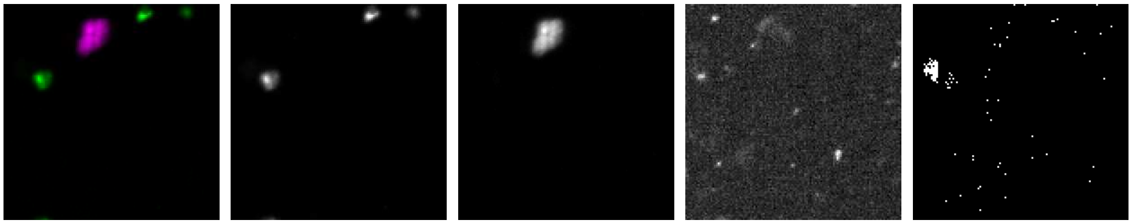				
13100	85	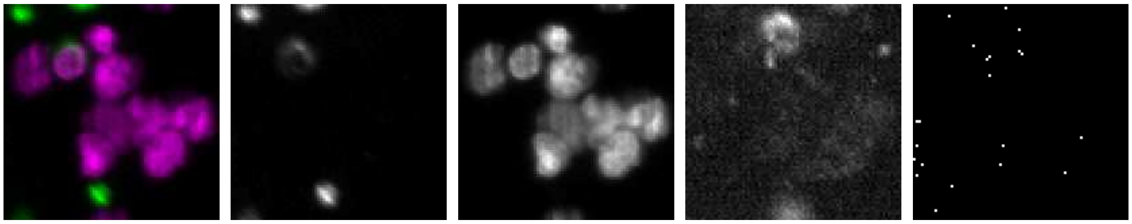				

Ereignis	Aufnahme	DAPI/CK-PE	CK-PE	DAPI	CD45-APC	HER-2/neu
13101	85	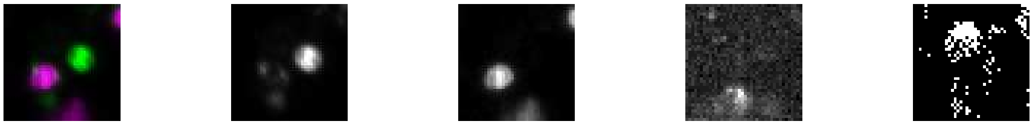				
13102	85	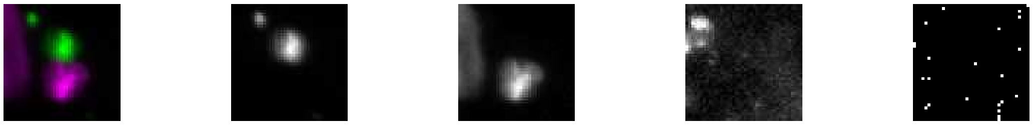				
13103	85	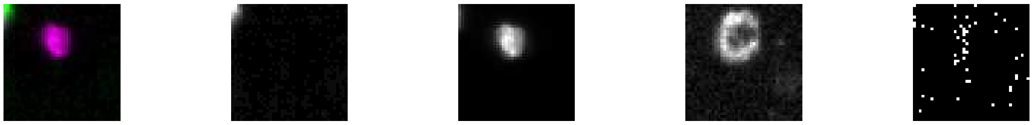				
13104	85	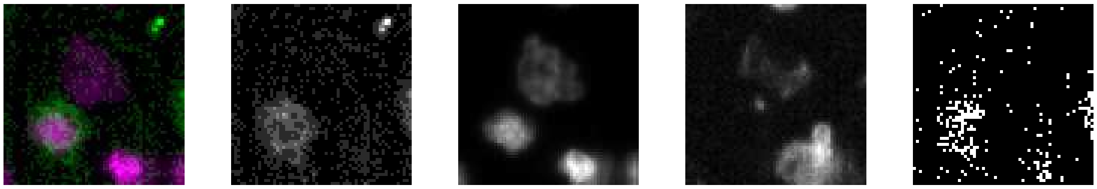				
13105	85	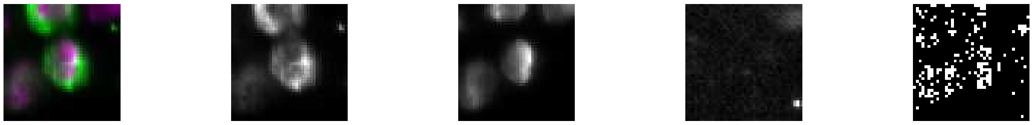				
13106	85	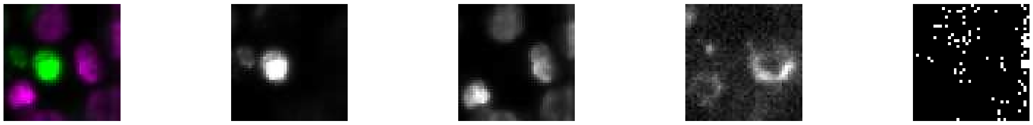				
13107	85	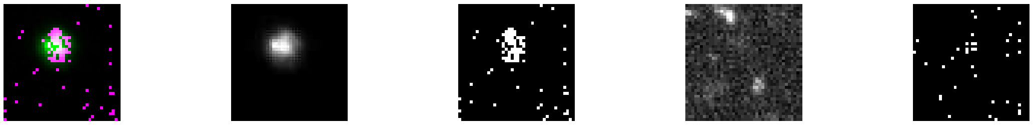				
13108	85	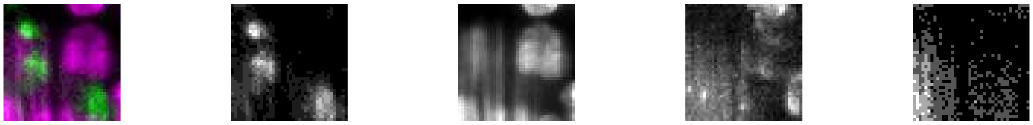				
13109	85	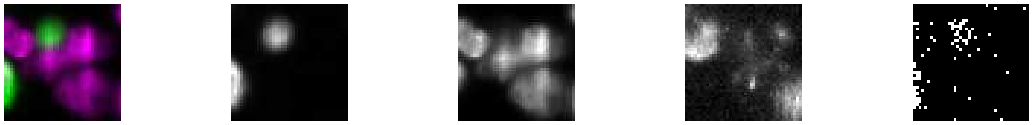				
13110	85	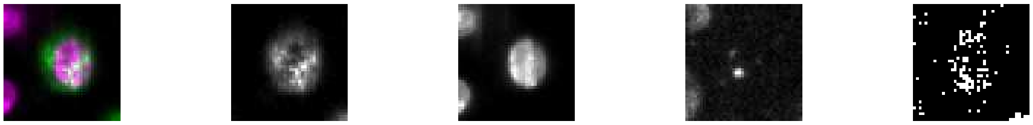				
13111	85	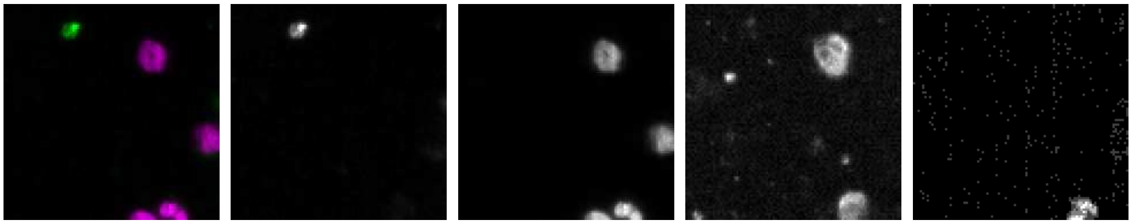				
13112	85	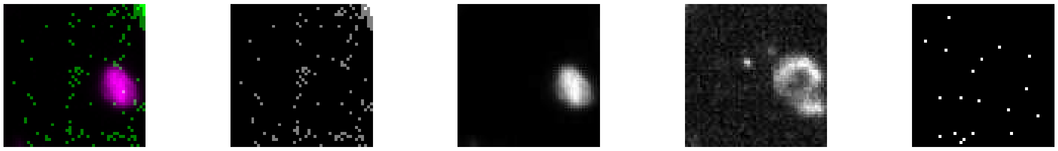				

Ereignis	Aufnahme	DAPI/CK-PE	CK-PE	DAPI	CD45-APC	HER-2/neu
13113	85	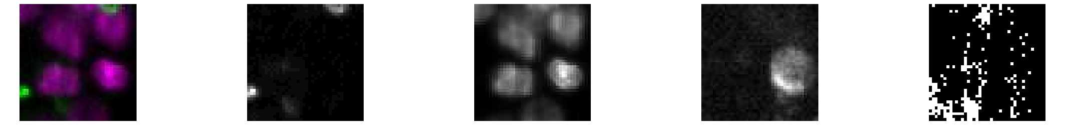				
13114	85	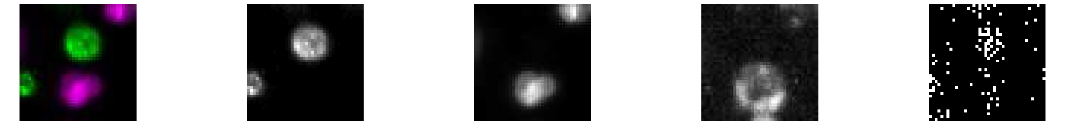				
13115	85	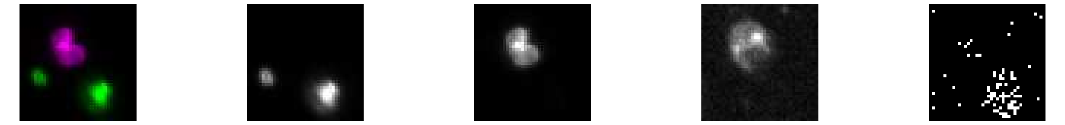				
13116	85	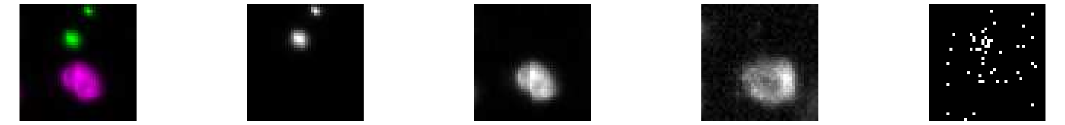				
13117	85	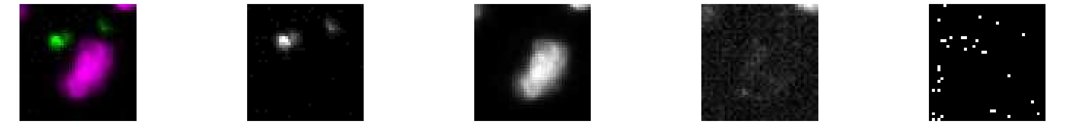				
13118	85	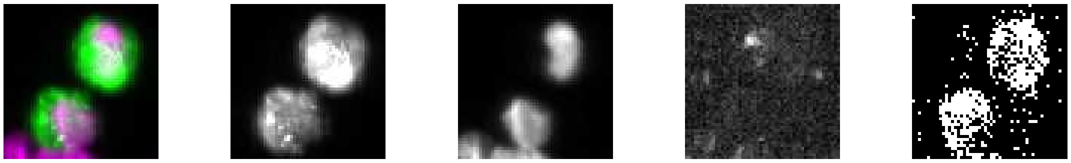				
13119	85	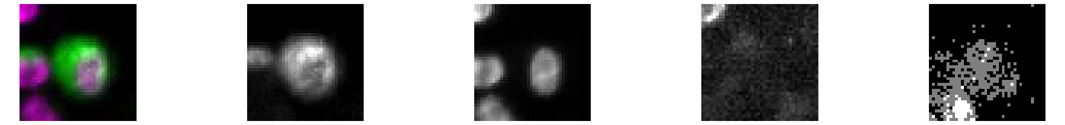				
13120	85	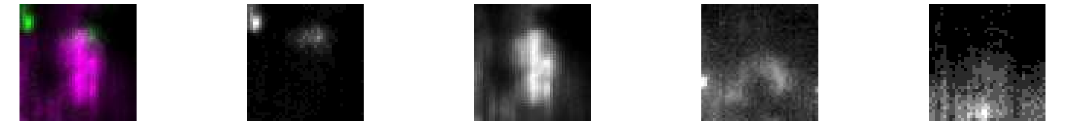				
13121	85	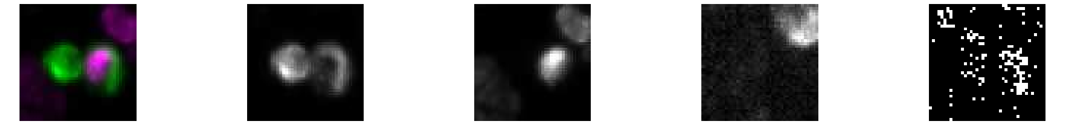				
13122	85	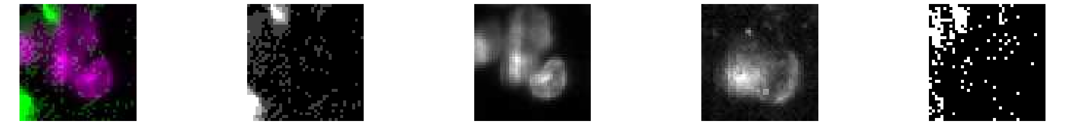				
13123	85	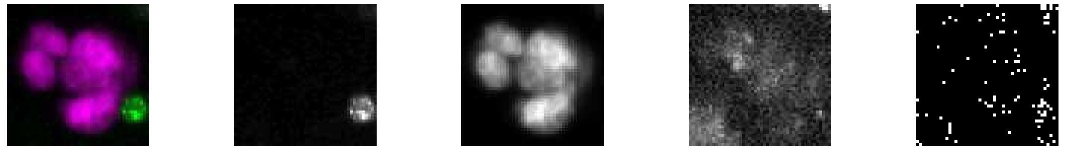				
13124	85	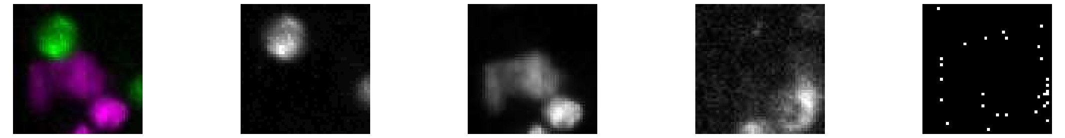				
13125	85	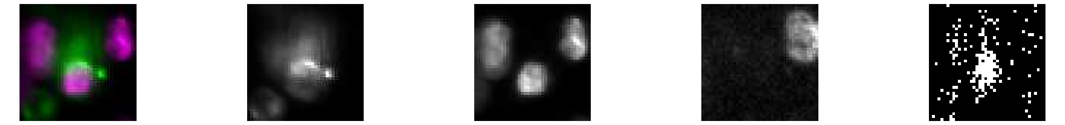				

Druckdatum:02/06/2020 17:00

Proben-ID: Gyn14.208 **Kassetten-ID:**01327106 **Patienten-ID:**

Ereignis	Aufnahme	DAPI/CK-PE	CK-PE	DAPI	CD45-APC	HER-2/neu
13126	85	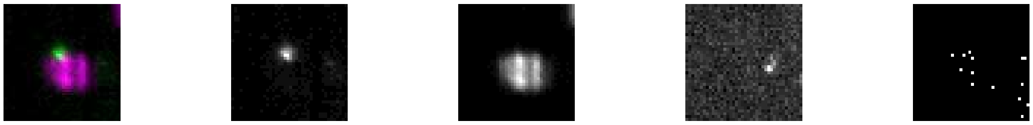				
13127	85	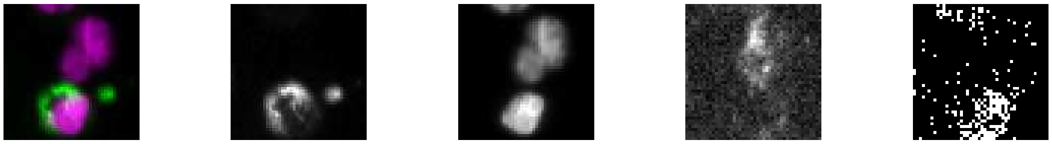				
13128	85	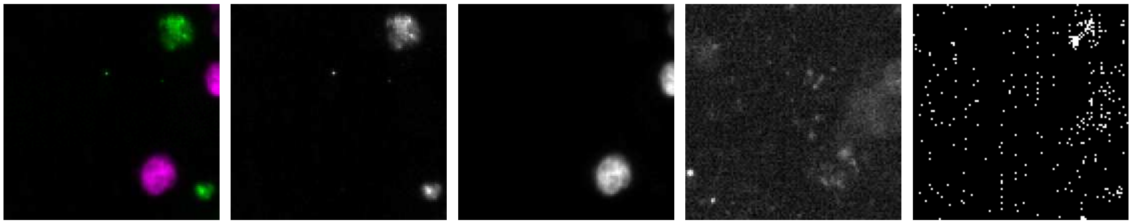				
13129	85	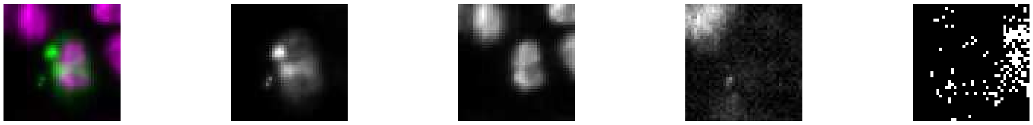				
13130	85	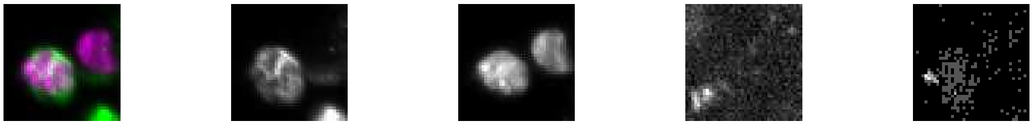				
13131	85	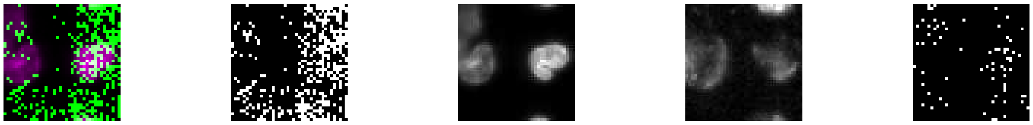				
13132	85	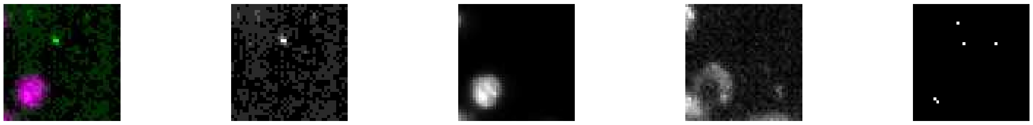				
13133	85	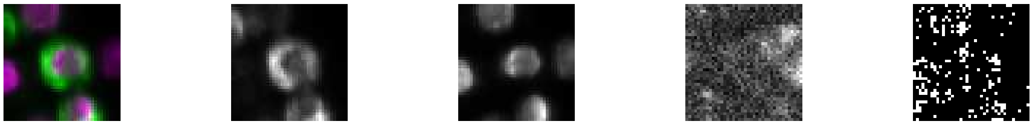				
13134	85	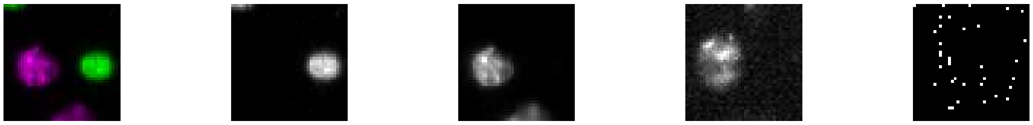				
13135	85	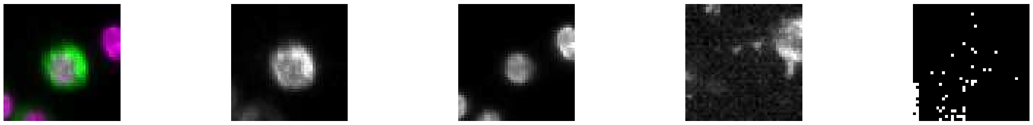				
13136	85	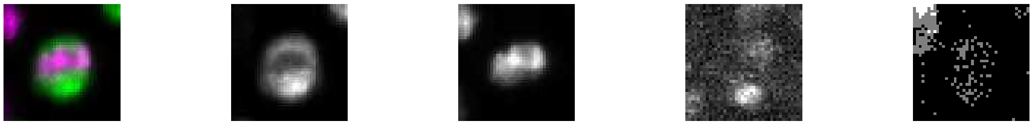				
13137	85	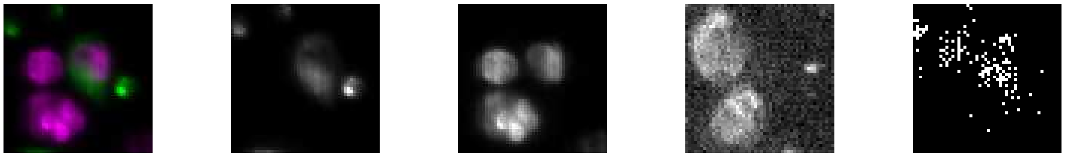				
13138	85	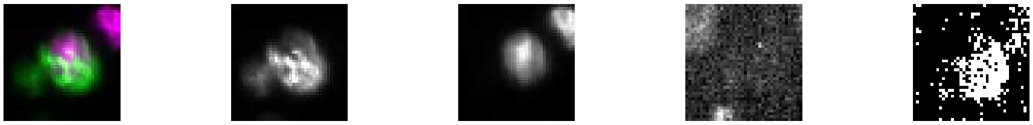				

Ereignis	Aufnahme	DAPI/CK-PE	CK-PE	DAPI	CD45-APC	HER-2/neu
13139	85	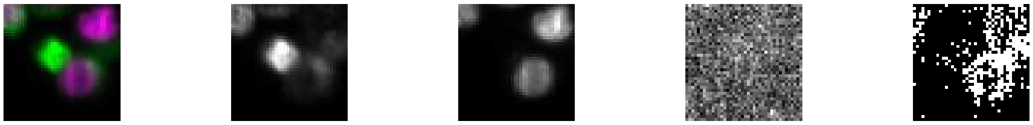				
13140	85	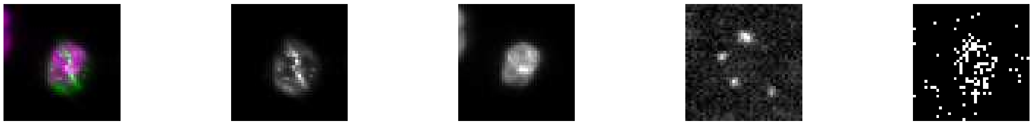				
13141	85	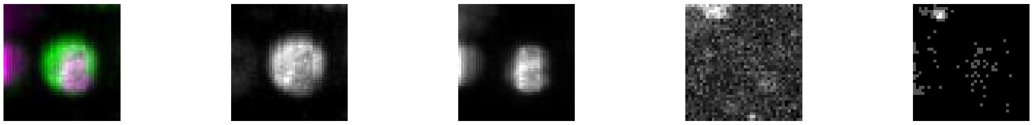				
13142	85	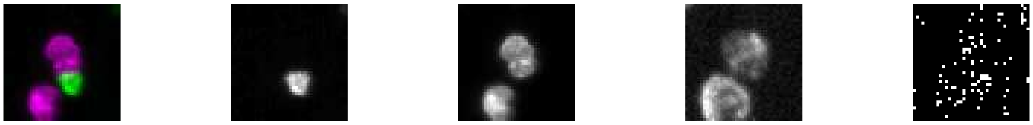				
13143	85	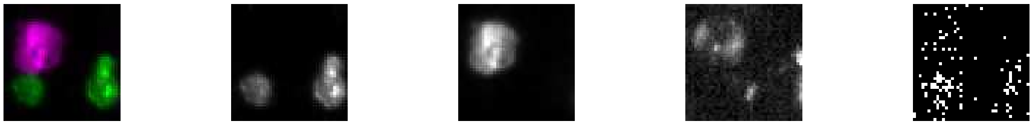				
13144	85	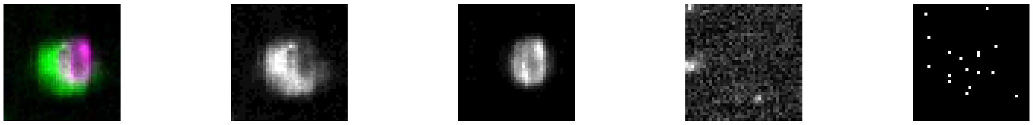				
13145	85	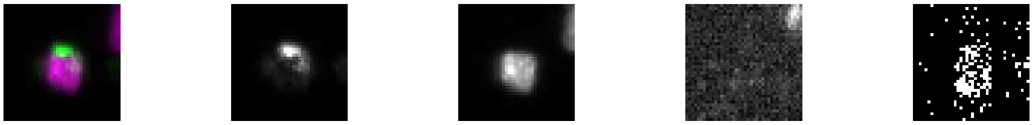				
13146	85	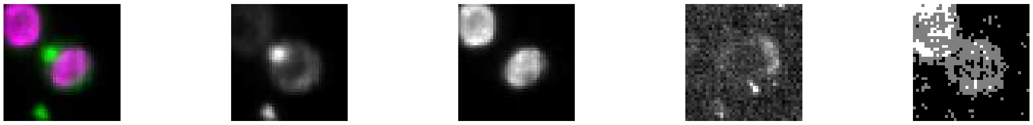				
13147	85	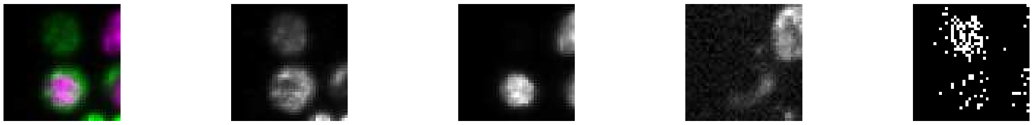				
13148	85	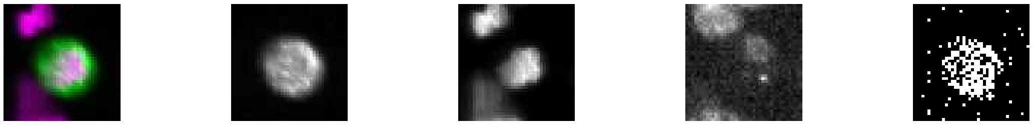				
13149	85	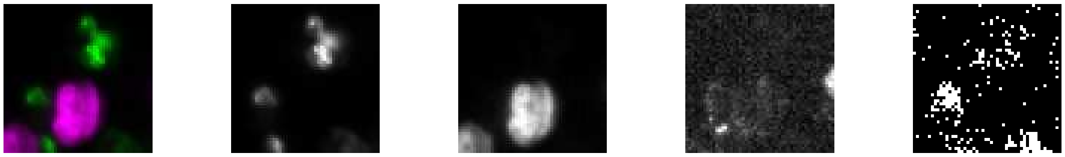				
13150	85	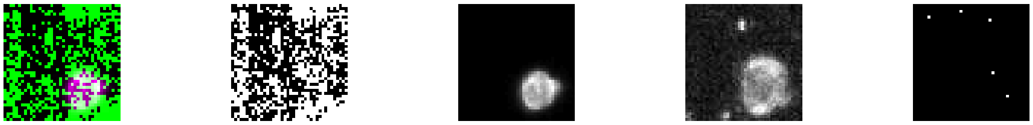				
13151	85	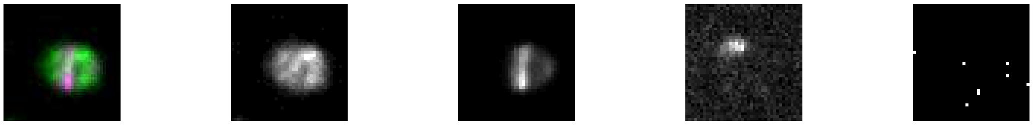				
13152	85	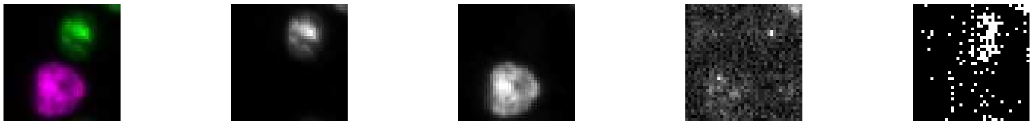				

Ereignis	Aufnahme	DAPI/CK-PE	CK-PE	DAPI	CD45-APC	HER-2/neu
13153	85	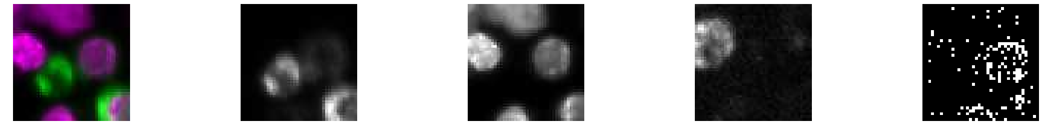				
13154	85	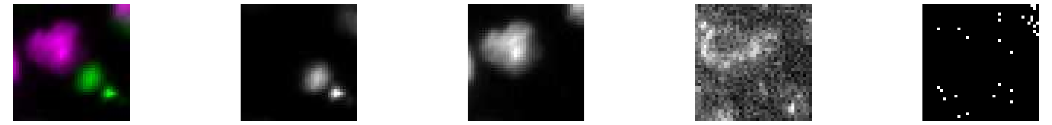				
13155	85	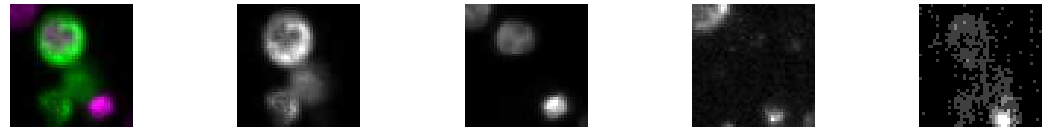				
13156	85	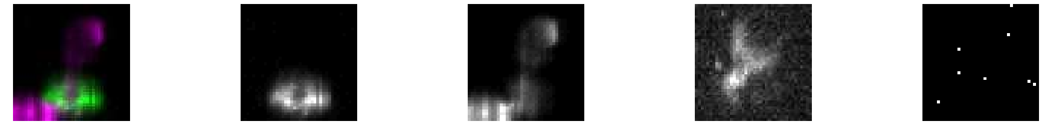				
13157	85	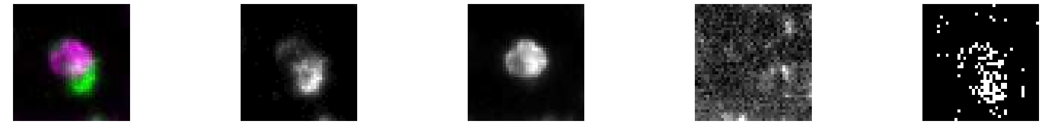				
13158	85	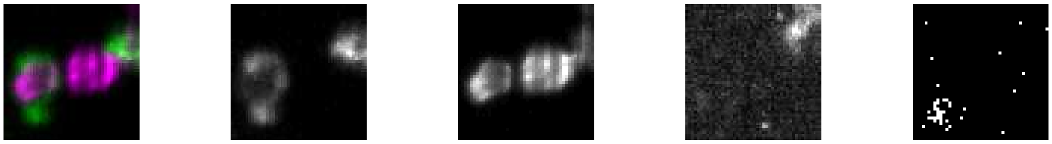				
13159	85	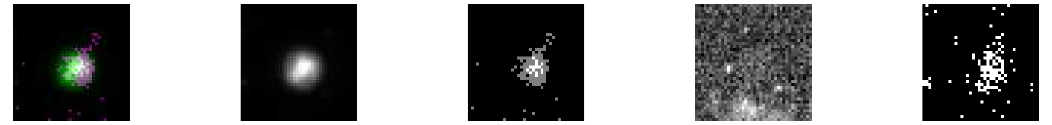				
13160	85	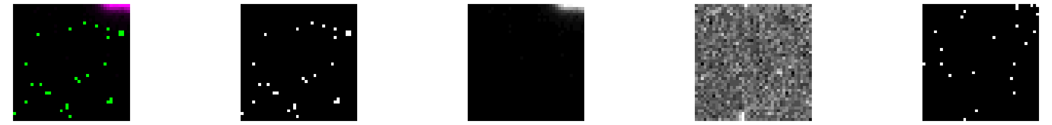				
13161	85	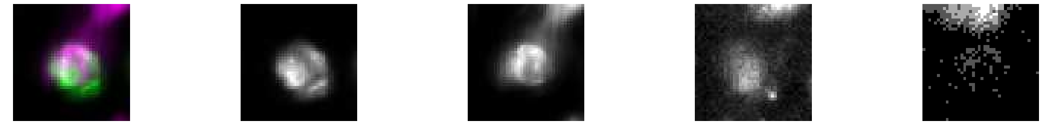				
13162	85	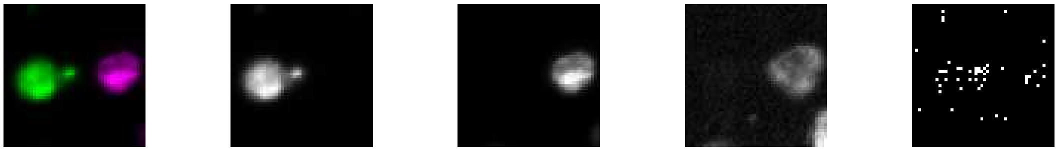				
13163	85	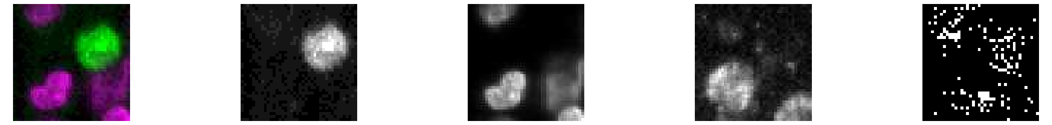				
13164	85	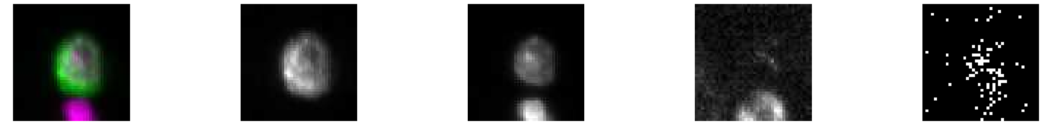				
13165	85	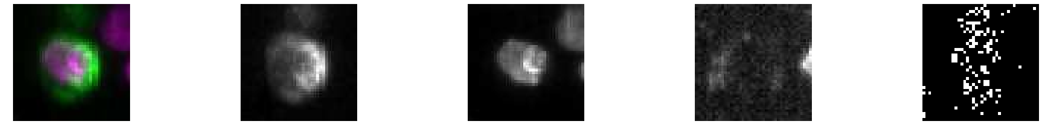				
13166	85	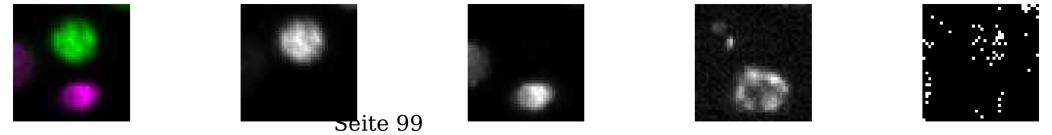				

Ereignis	Aufnahme	DAPI/CK-PE	CK-PE	DAPI	CD45-APC	HER-2/neu
13167	85	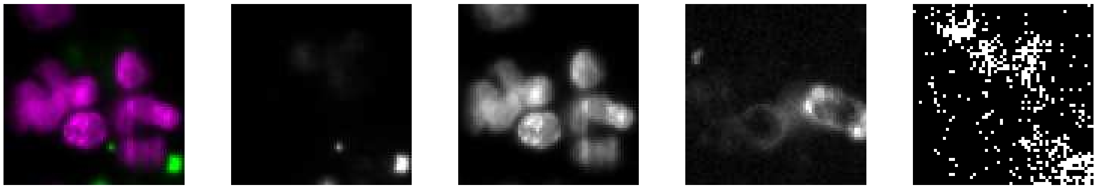				
13168	85	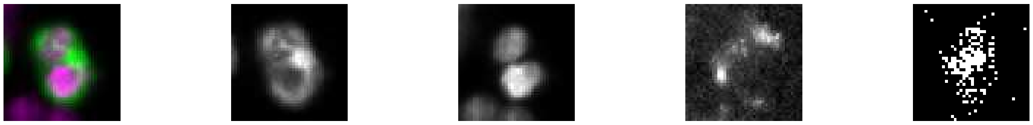				
13169	85	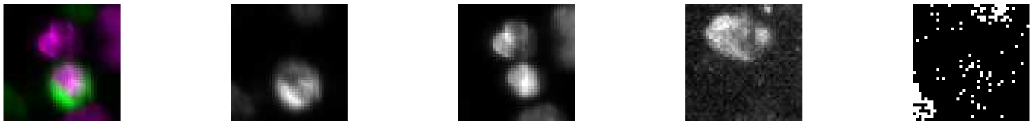				
13170	85	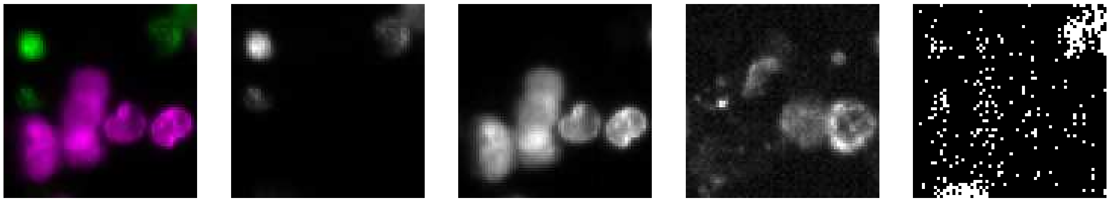				
13171	85	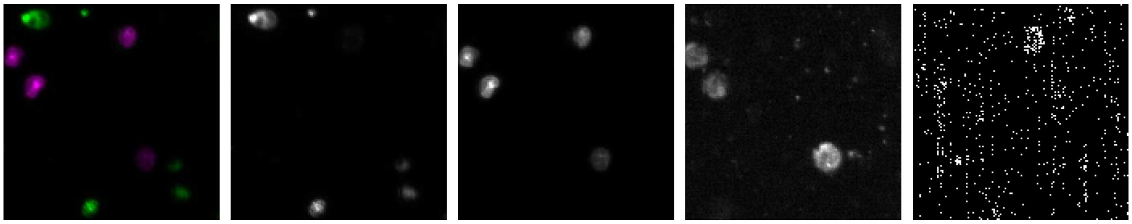				
13172	85	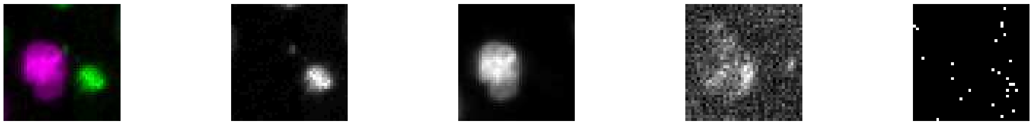				
13173	85	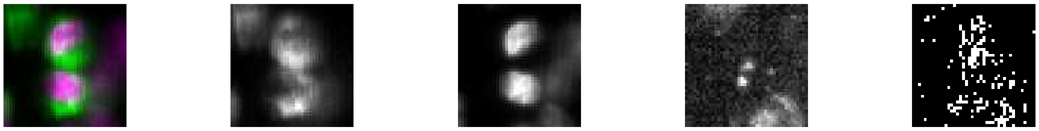				
13174	85	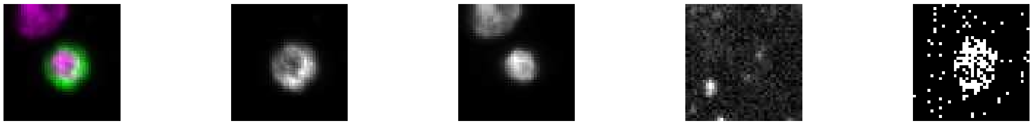				
13175	85	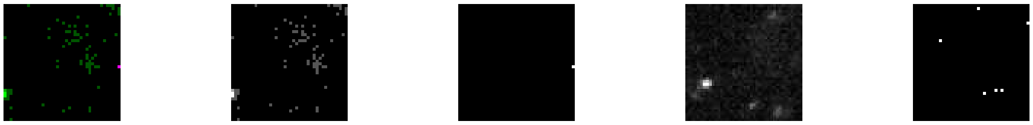				
13176	85	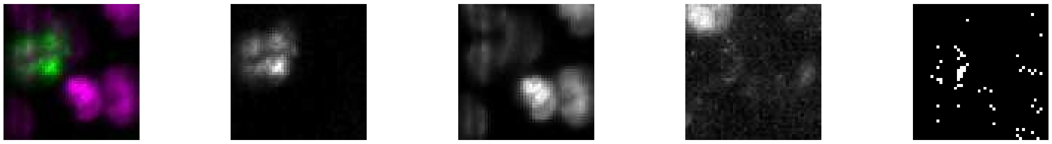				
13177	85	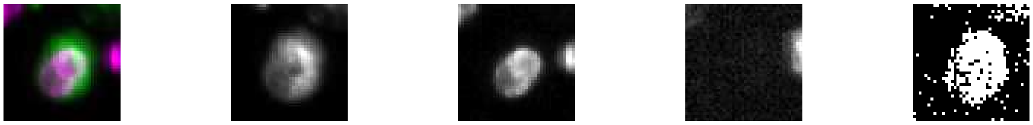				
13178	85	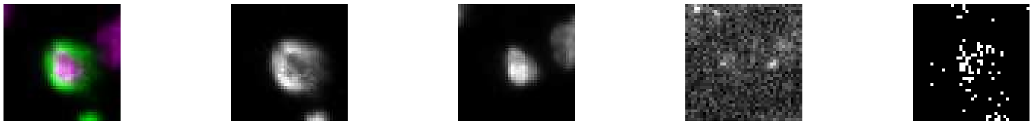				

Ereignis	Aufnahme	DAPI/CK-PE	CK-PE	DAPI	CD45-APC	HER-2/neu
13179	85	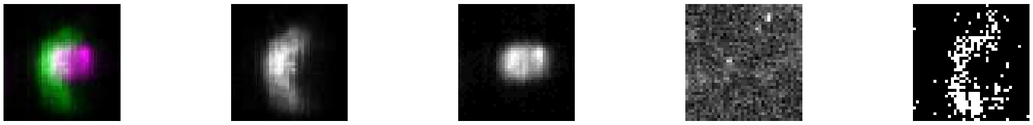				
13180	85	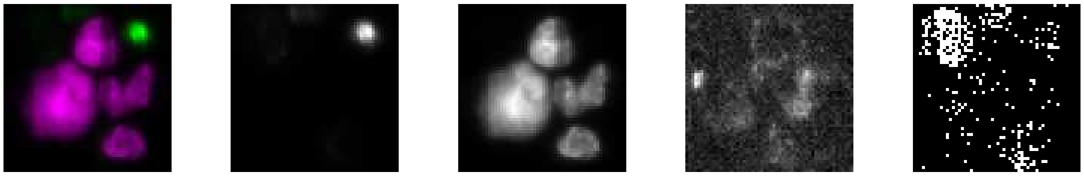				
13181	85	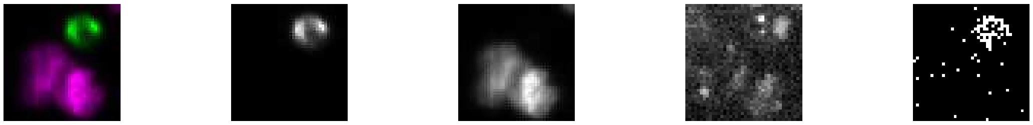				
13182	85	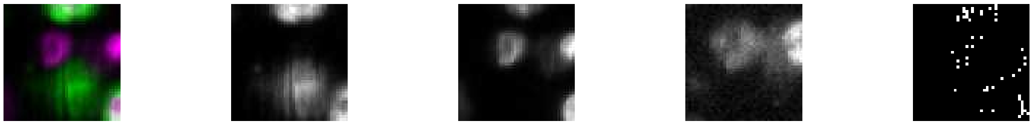				
13183	85	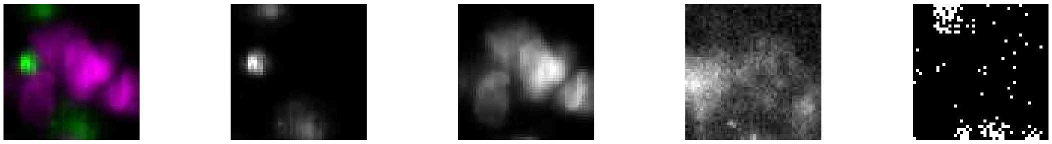				
13184	85	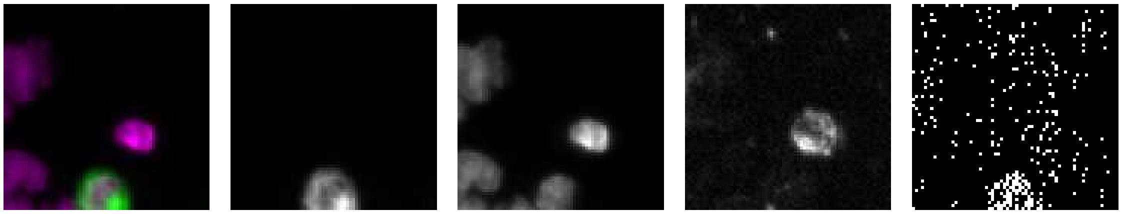				
13185	85	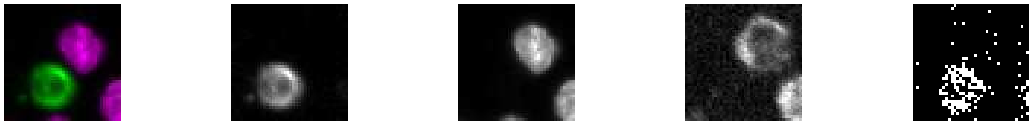				
13186	85	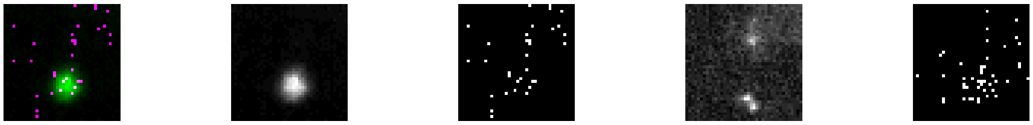				
13187	85	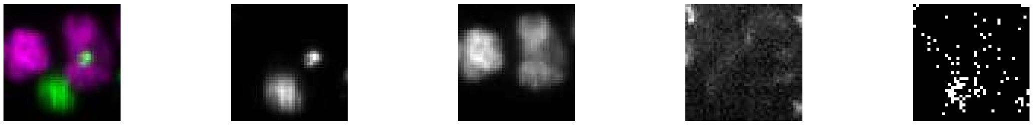				
13188	85	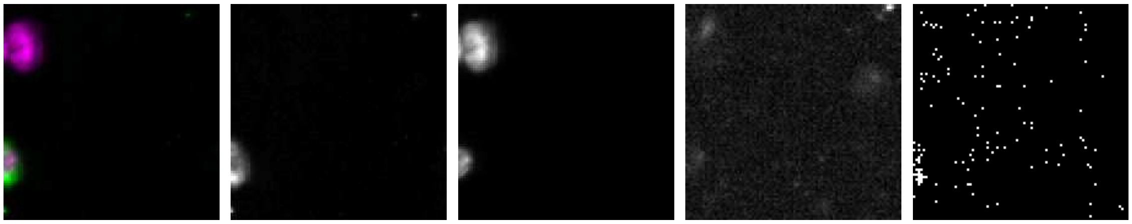				
13189	85	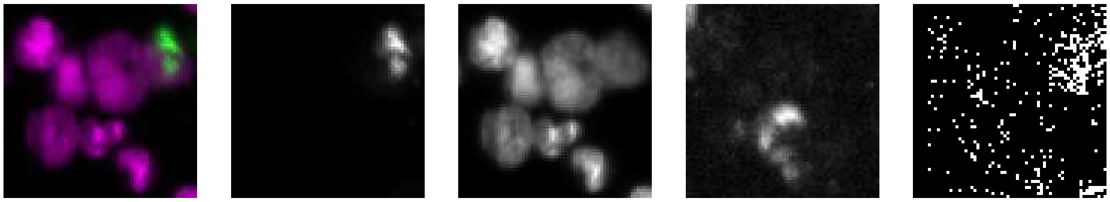				

Ereignis	Aufnahme	DAPI/CK-PE	CK-PE	DAPI	CD45-APC	HER-2/neu
13190	85	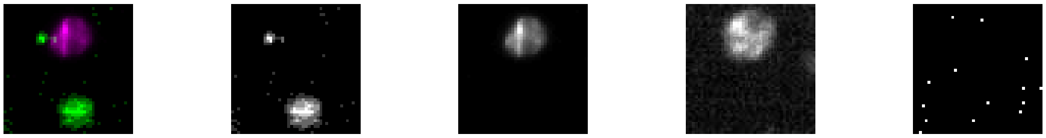				
13191	85	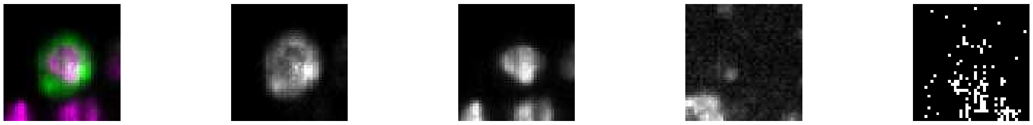				
13192	85	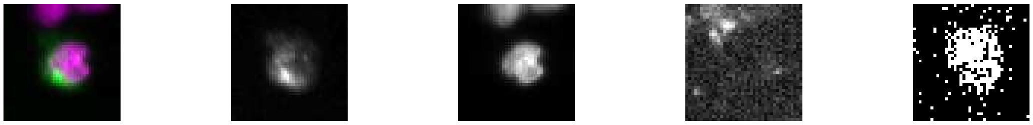				
13193	85	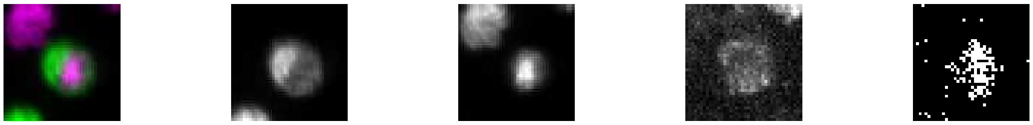				
13194	85	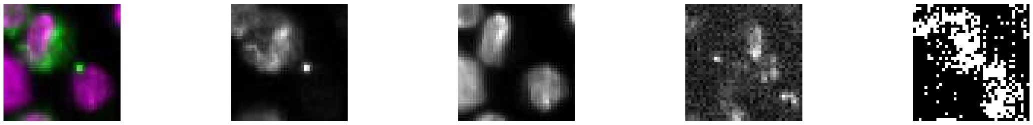				
13195	85	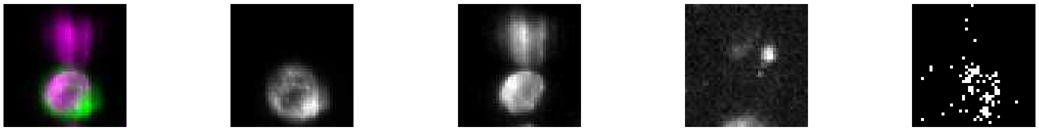				
13196	85	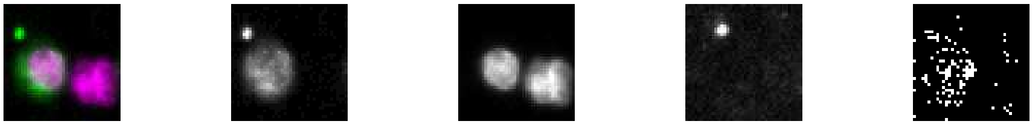				
13197	85	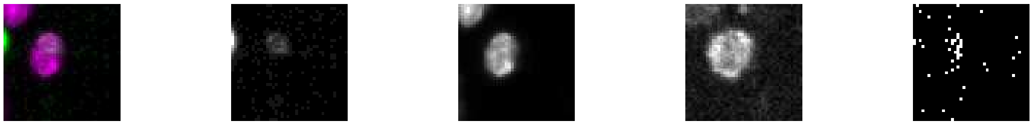				
13198	85	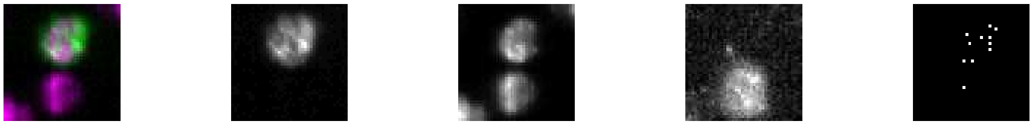				
13199	85	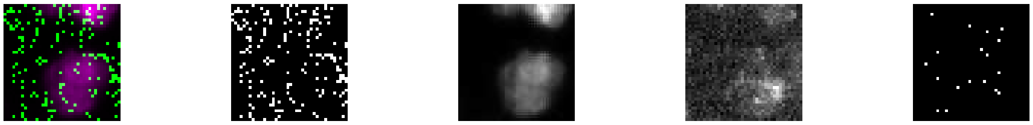				
13200	85	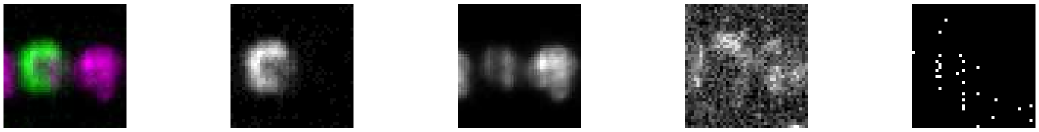				
13201	85	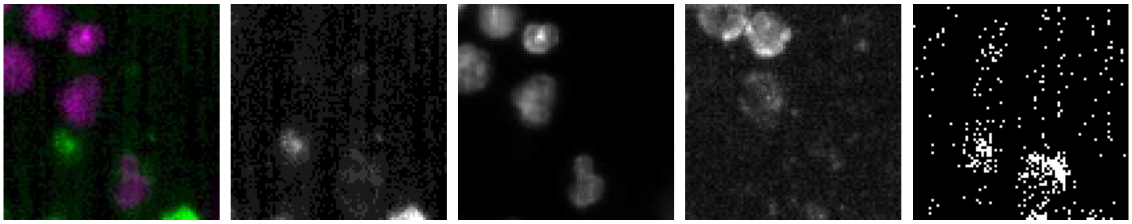				
13202	85	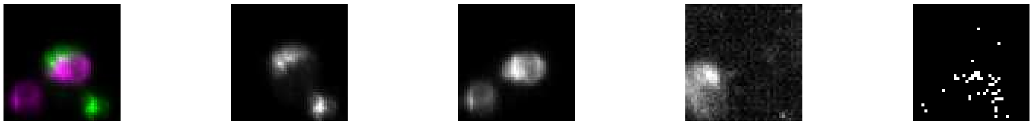				

Ereignis	Aufnahme	DAPI/CK-PE	CK-PE	DAPI	CD45-APC	HER-2/neu
13203	85	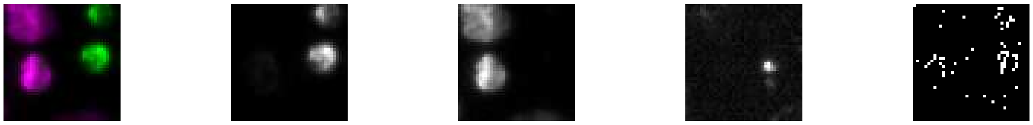				
13204	85	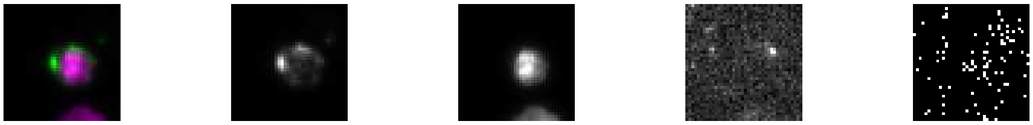				
13205	85	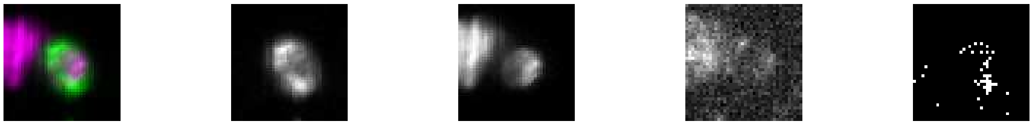				
13206	85	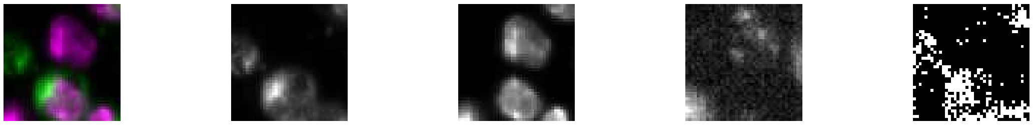				
13207	85	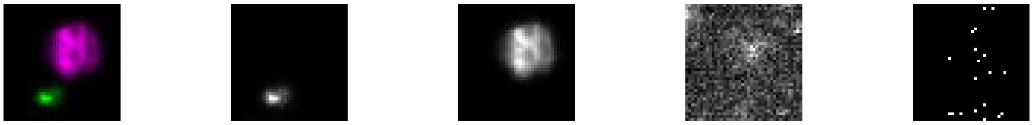				
13208	85	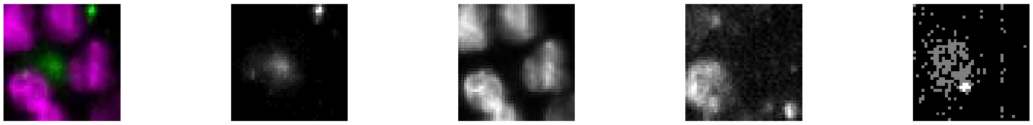				
13209	85	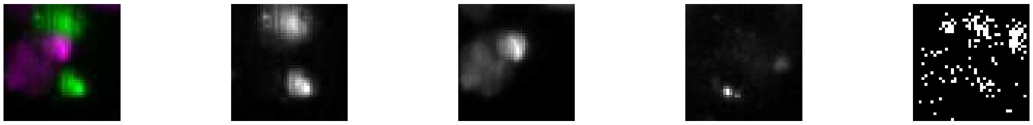				
13210	85	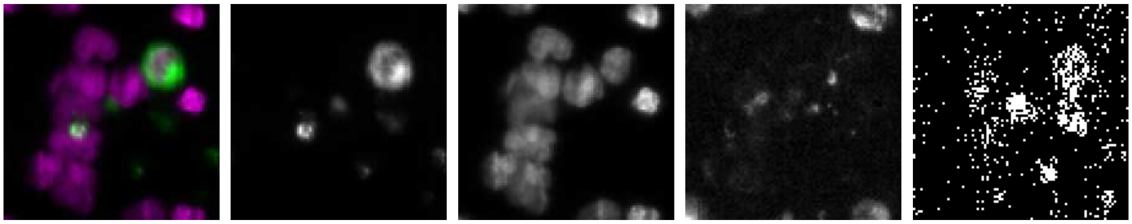				
13211	85	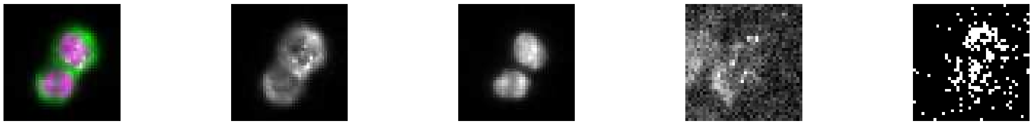				
13212	85	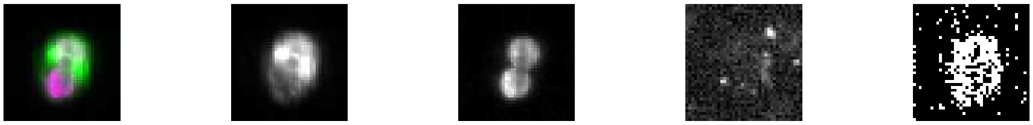				
13213	85	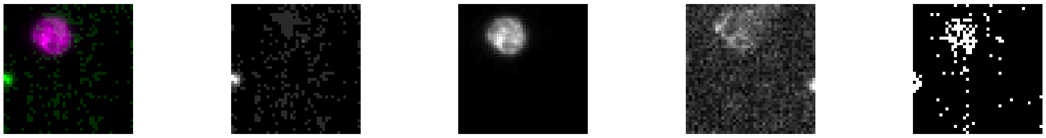				
13214	85	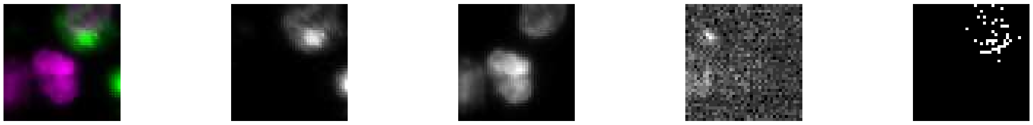				
13215	85	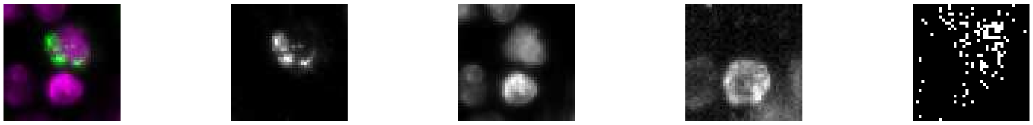				

Ereignis	Aufnahme	DAPI/CK-PE	CK-PE	DAPI	CD45-APC	HER-2/neu
13216	85	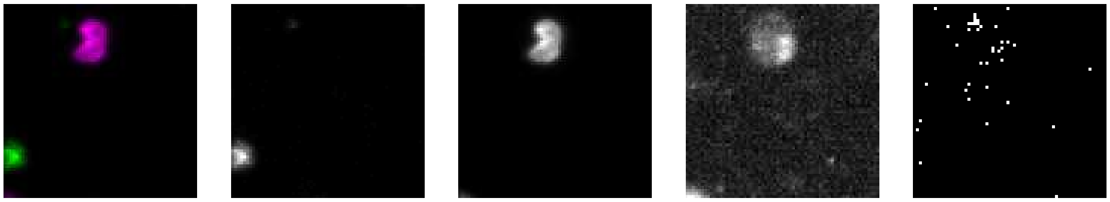				
13217	85	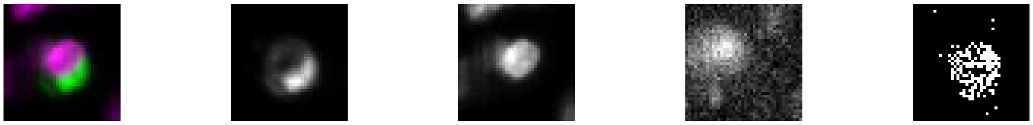				
13218	85	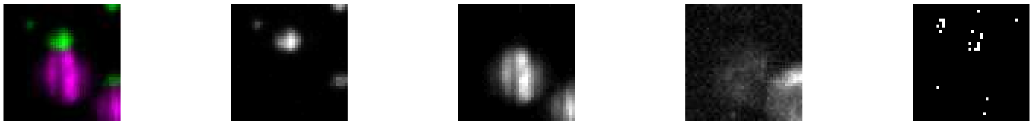				
13219	85	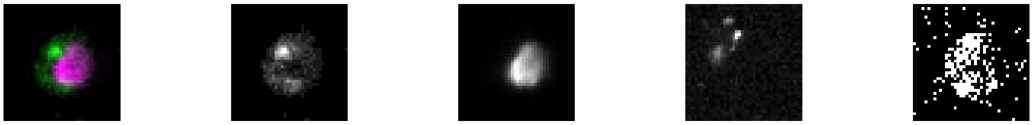				
13220	85	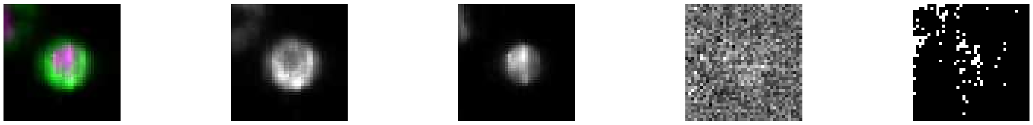				
13221	85	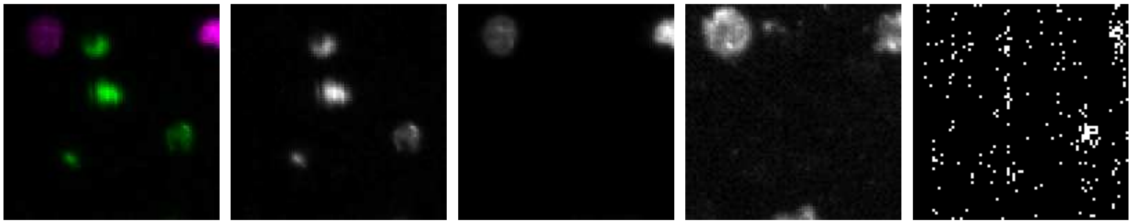				
13222	85	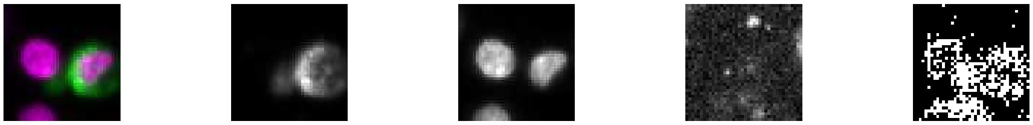				
13223	85	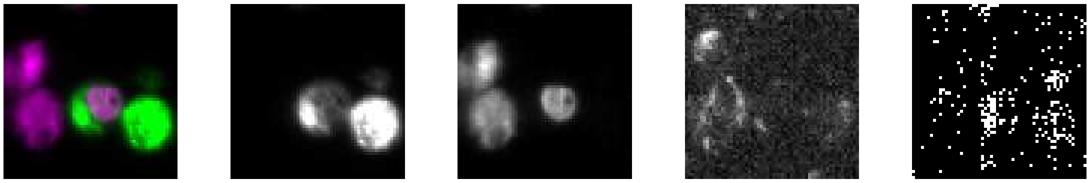				
13224	85	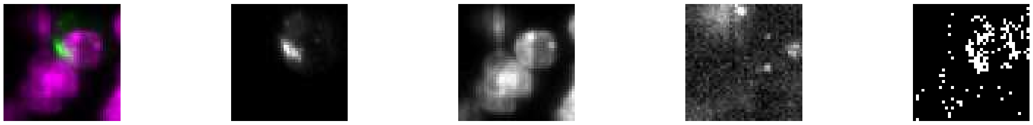				
13225	85	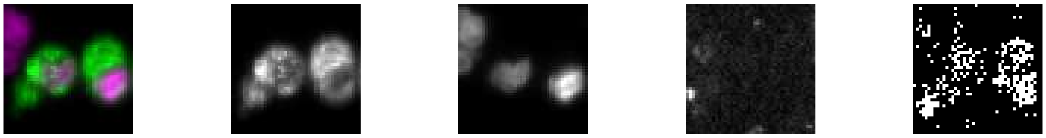				
13226	85	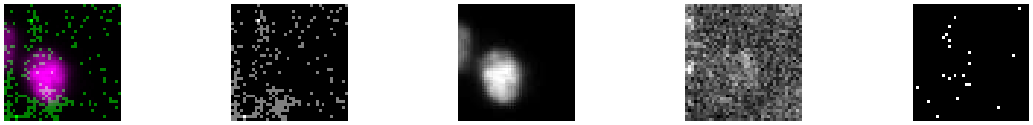				
13227	85	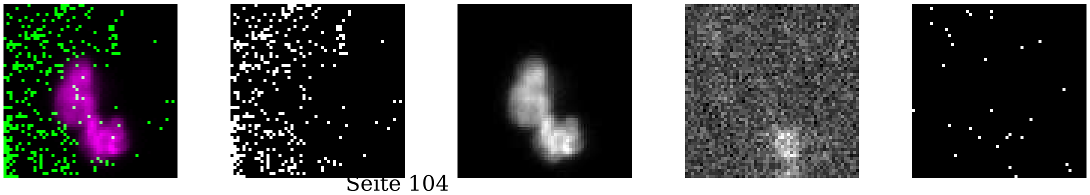				

Ereignis	Aufnahme	DAPI/CK-PE	CK-PE	DAPI	CD45-APC	HER-2/neu
13228	85	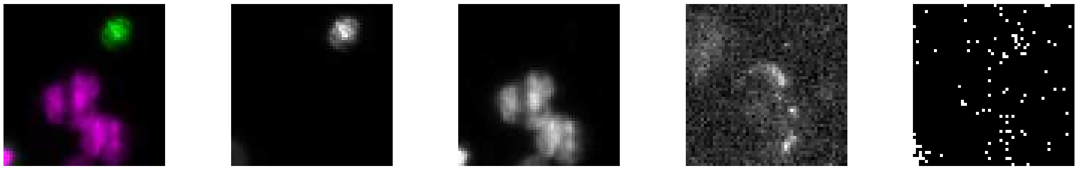				
13229	85	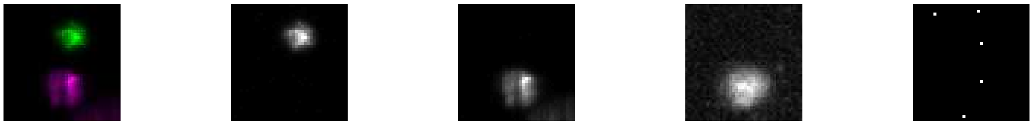				
13230	85	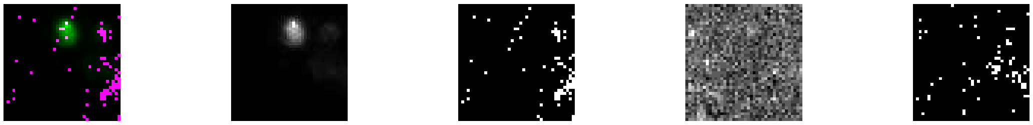				
13231	85	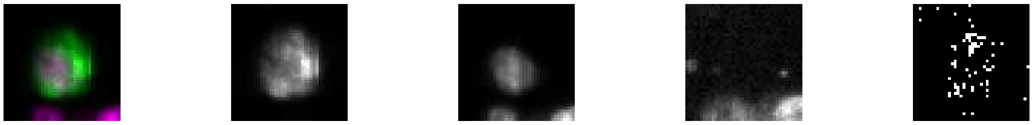				
13232	85	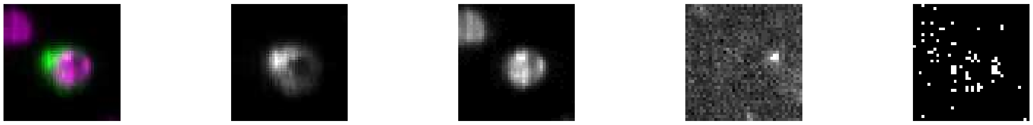				
13233	85	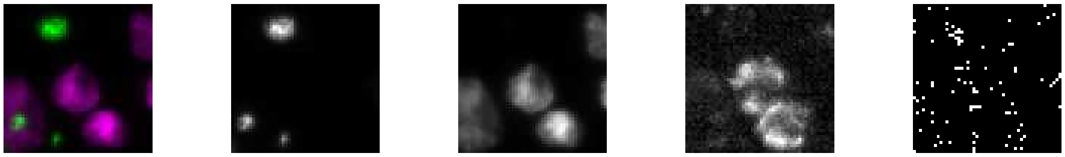				
13234	85	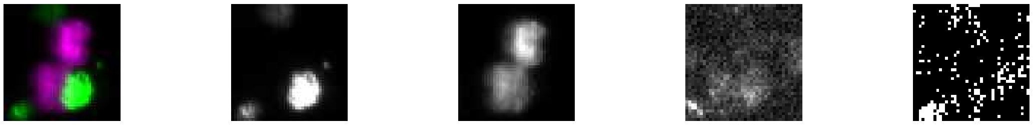				
13235	85	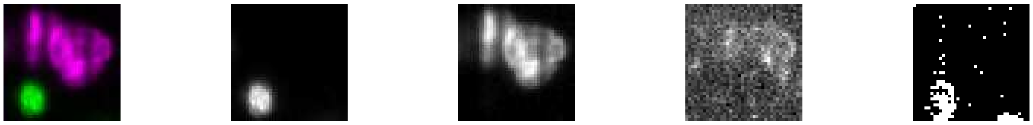				
13236	85	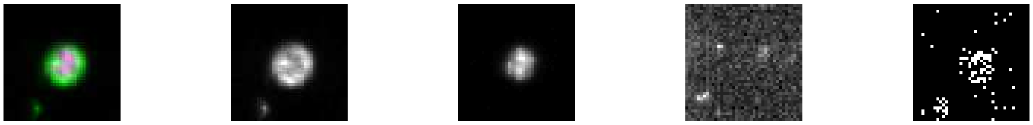				
13237	85	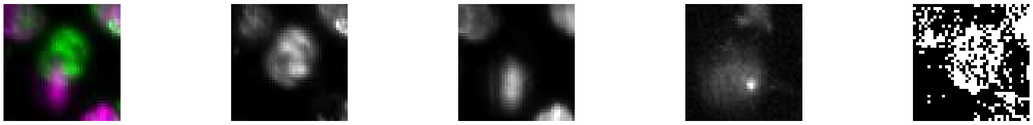				
13238	85	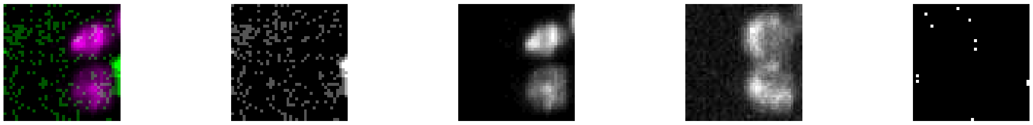				
13239	85	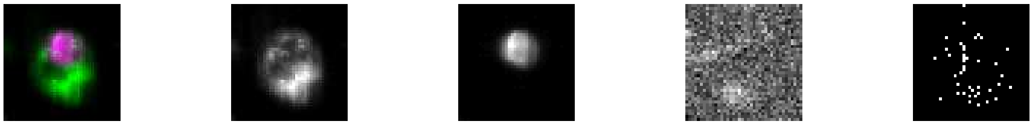				
13240	85	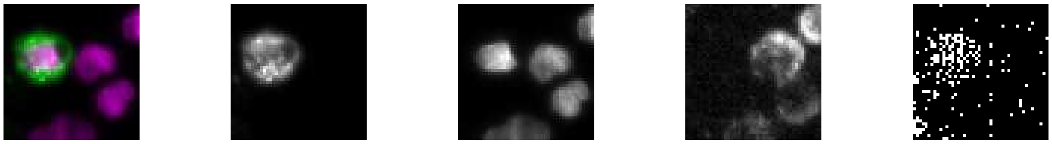				

Ereignis	Aufnahme	DAPI/CK-PE	CK-PE	DAPI	CD45-APC	HER-2/neu
13241	85	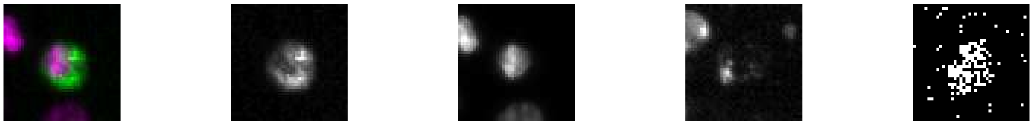				
13242	85	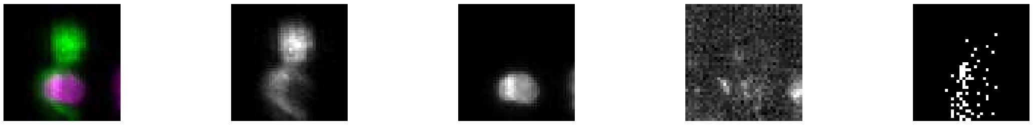				
13243	85	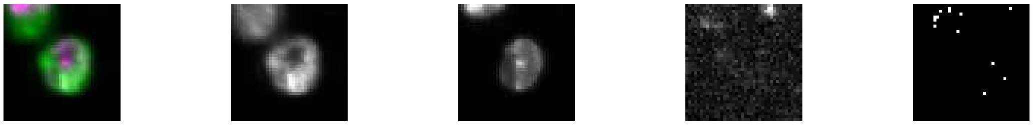				
13244	85	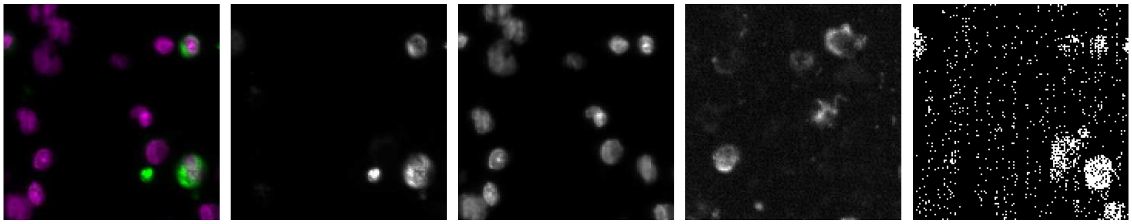				
13245	85	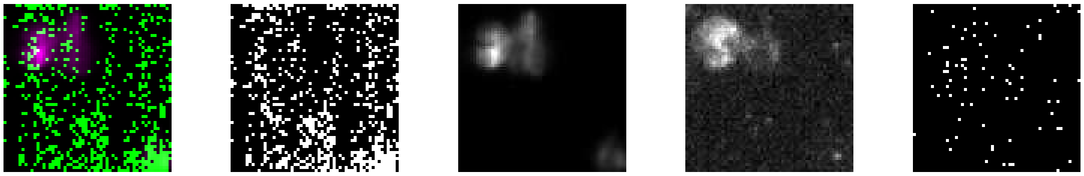				
13246	85	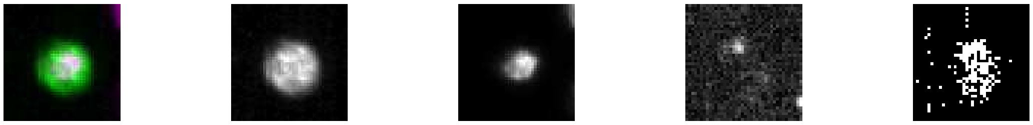				
13247	85	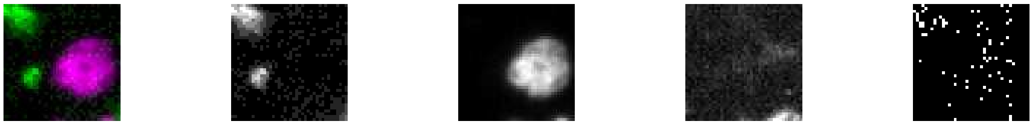				
13248	85	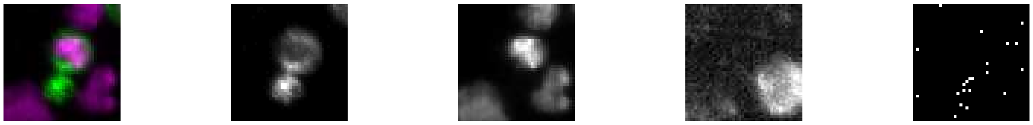				
13249	85	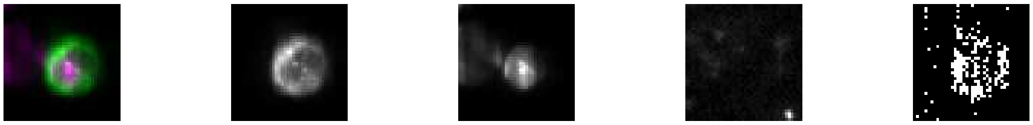				
13250	85	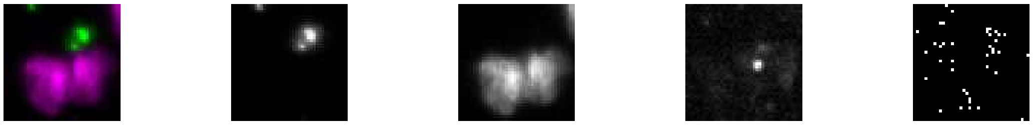				
13251	85	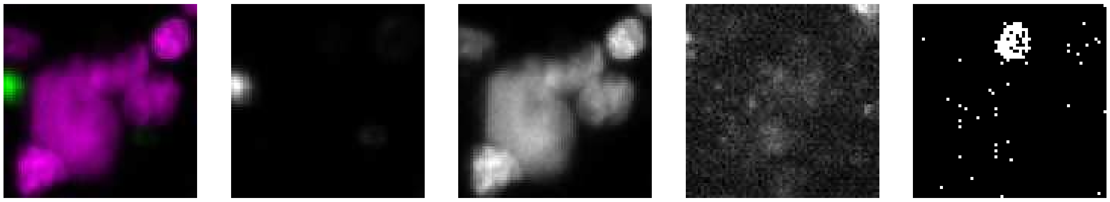				
13252	85	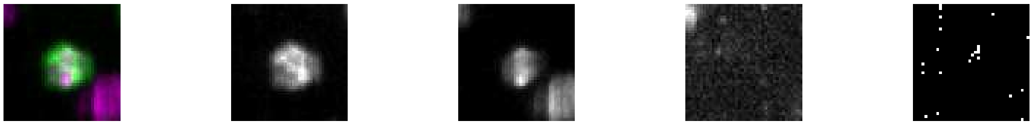				

Ereignis	Aufnahme	DAPI/CK-PE	CK-PE	DAPI	CD45-APC	HER-2/neu
13253	85	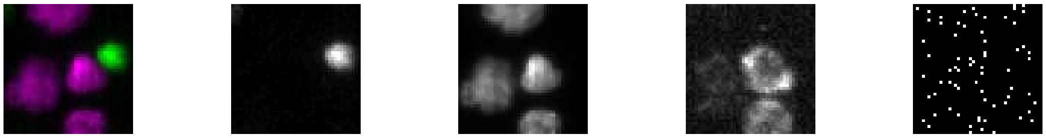				
13254	85	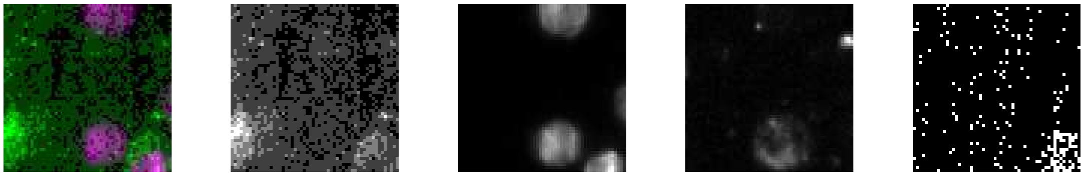				
13255	85	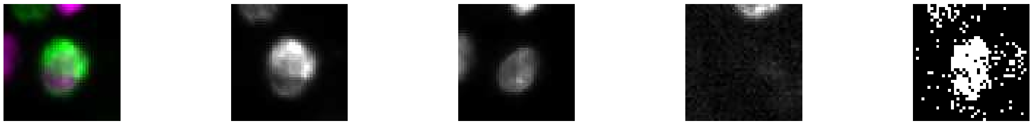				
13256	85	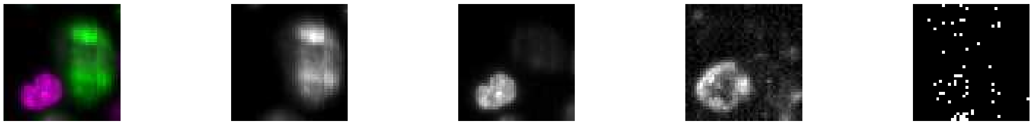				
13257	85	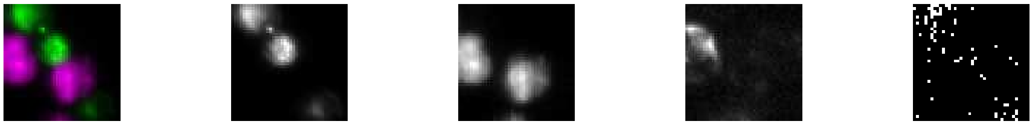				
13258	85	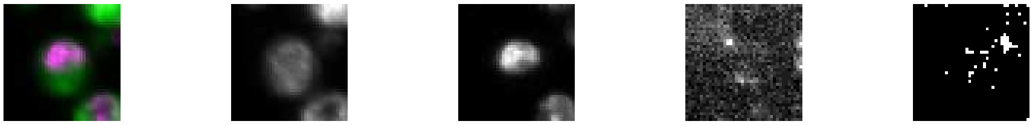				
13259	85	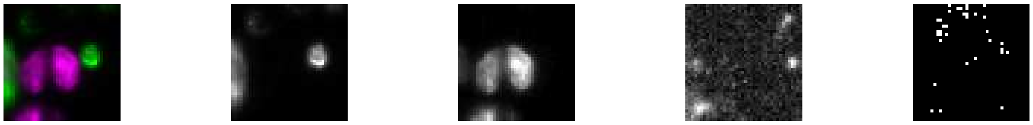				
13260	85	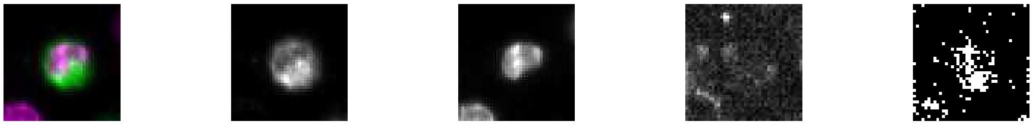				
13261	85	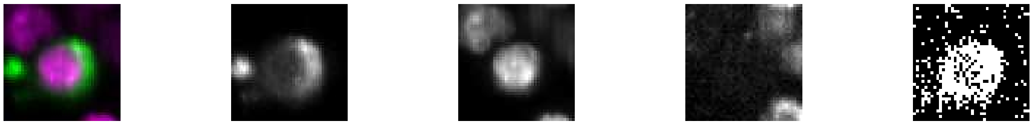				
13262	85	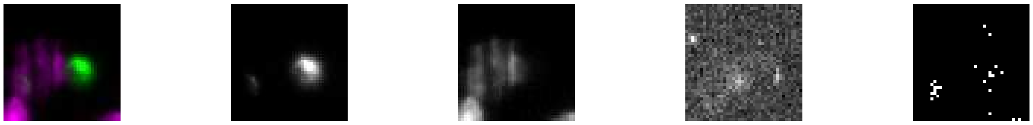				
13263	85	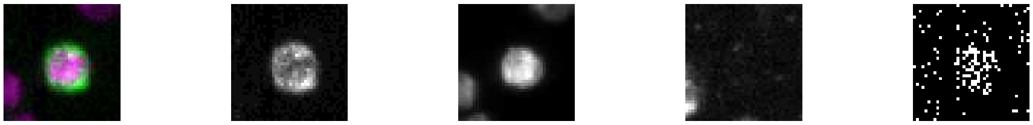				
13264	85	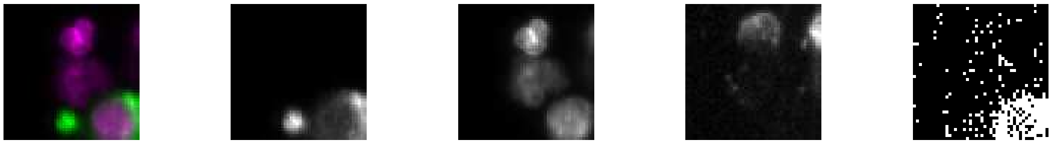				
13265	85	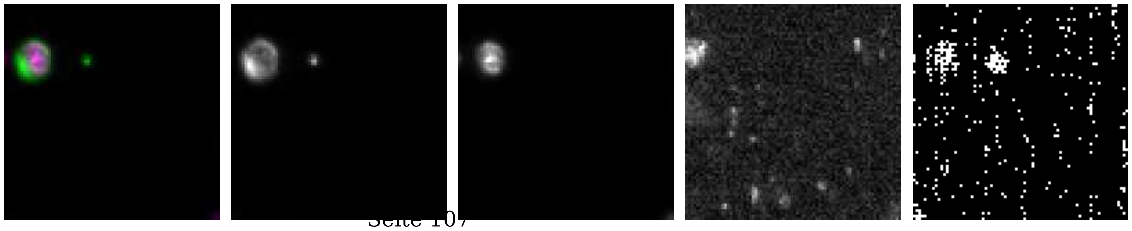				

Proben-ID: Gyn14.208 **Kassetten-ID:** 01327106 **Patienten-ID:**

Ereignis	Aufnahme	DAPI/CK-PE	CK-PE	DAPI	CD45-APC	HER-2/neu
13266	85	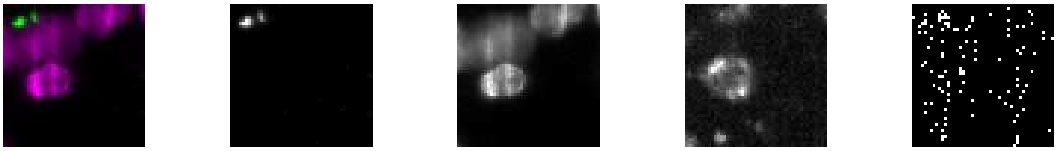				
13267	85	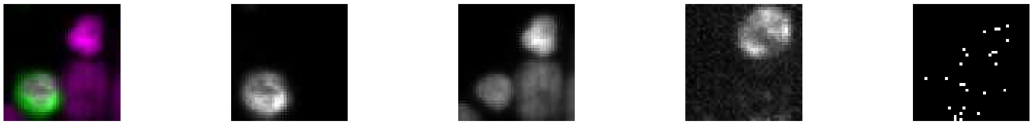				
13268	85	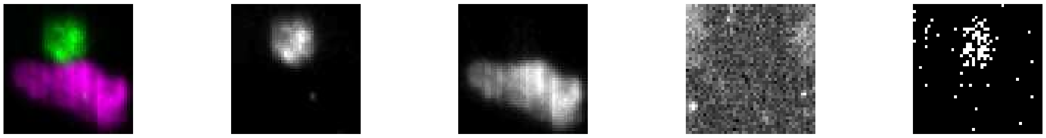				
13269	85	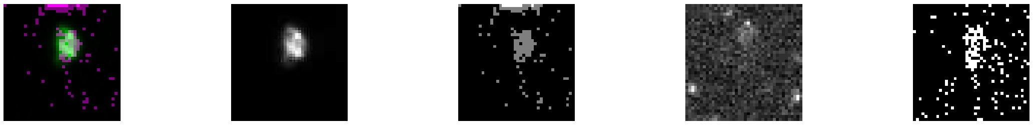				
13270	85	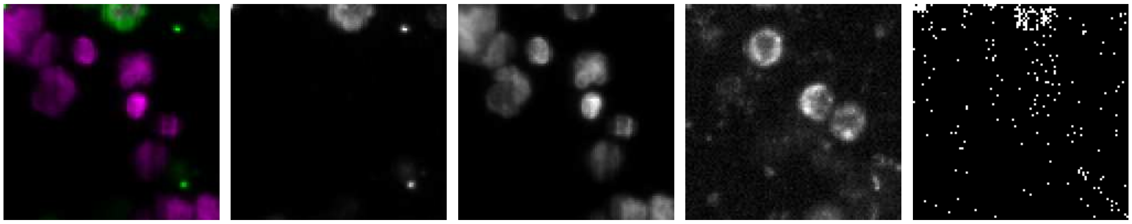				
13271	85	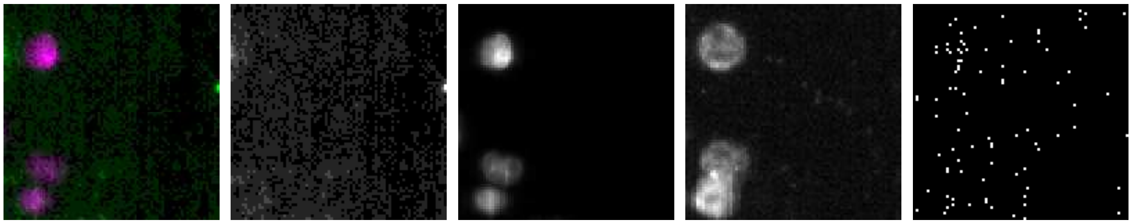				
13272	85	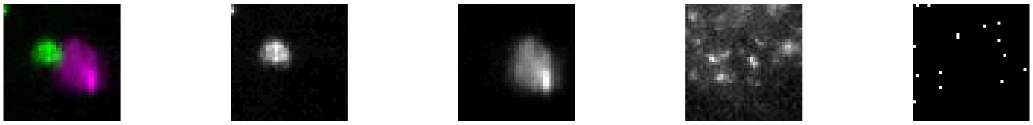				
13273	85	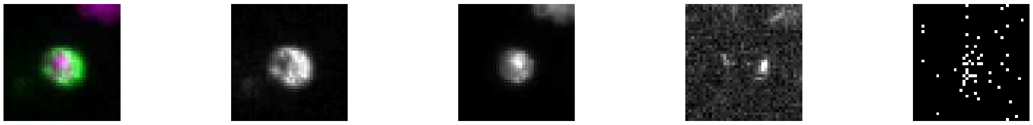				
13274	85	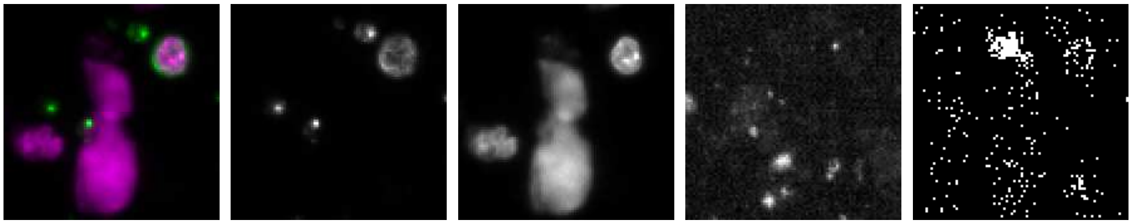				
13275	85	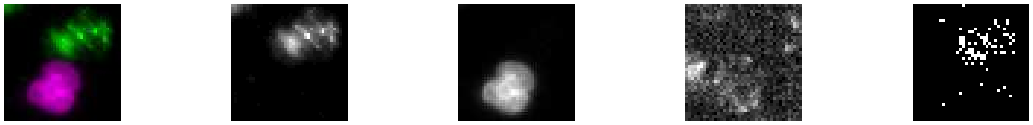				
13276	85	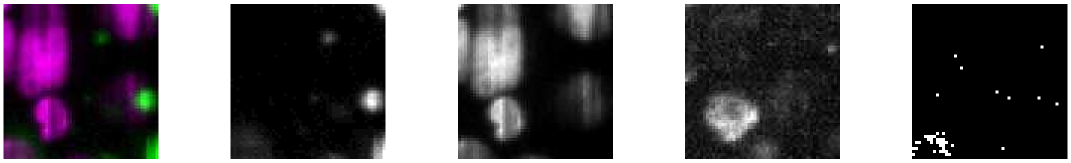				

Ereignis	Aufnahme	DAPI/CK-PE	CK-PE	DAPI	CD45-APC	HER-2/neu
13277	85	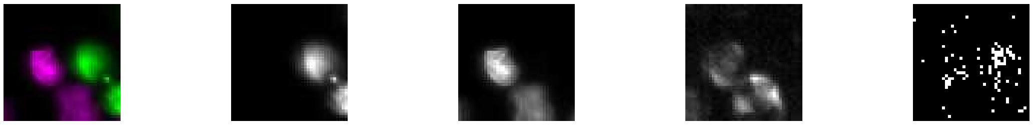				
13278	85	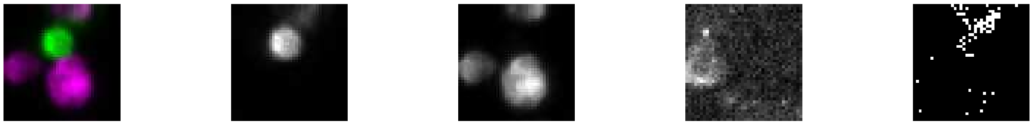				
13279	85	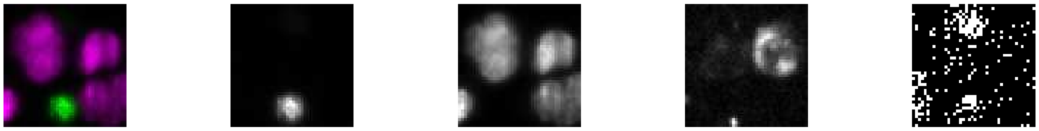				
13280	85	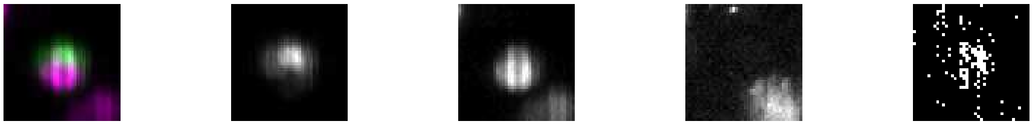				
13281	85	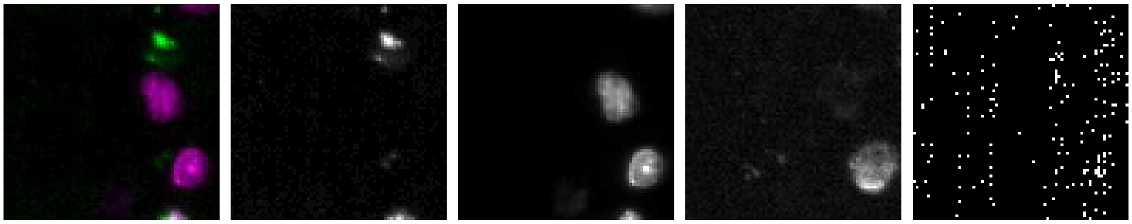				
13282	85	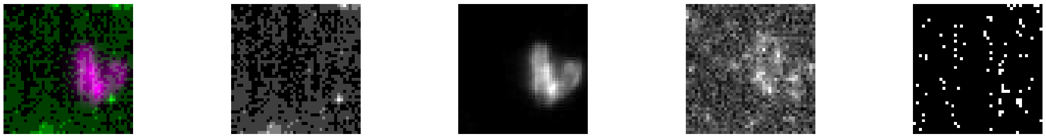				
13283	85	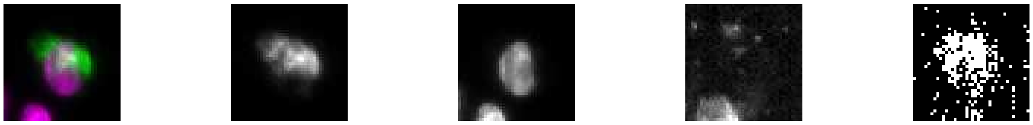				
13284	85	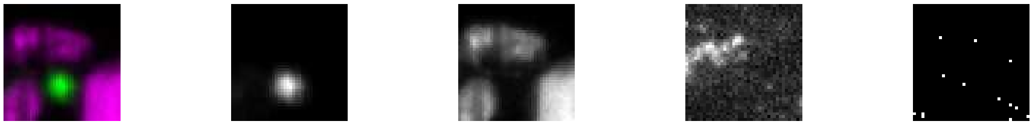				
13285	85	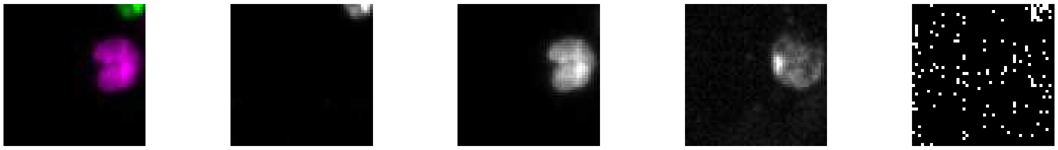				
13286	85	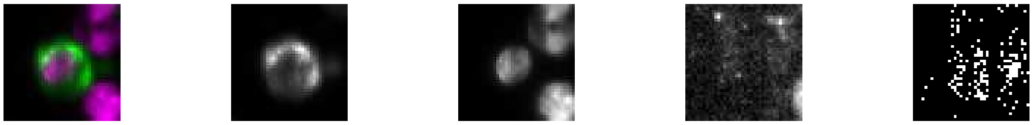				
13287	85	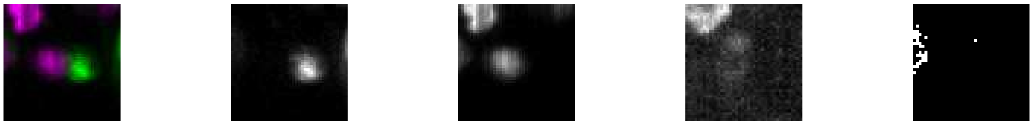				
13288	85	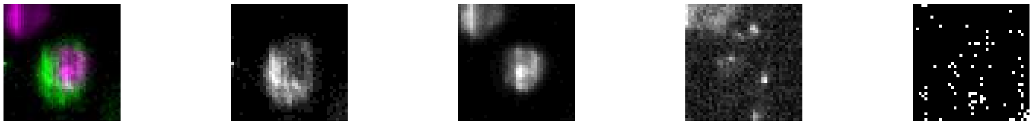				
13289	85	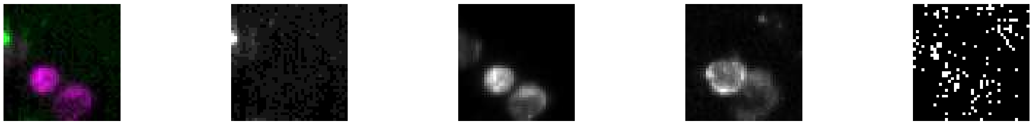				

Ereignis	Aufnahme	DAPI/CK-PE	CK-PE	DAPI	CD45-APC	HER-2/neu
13290	85	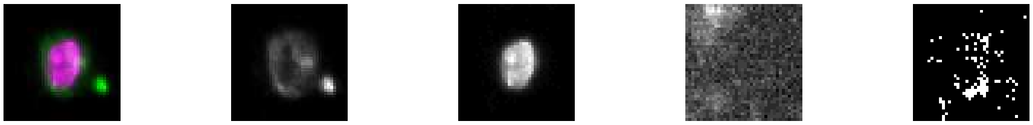				
13291	85	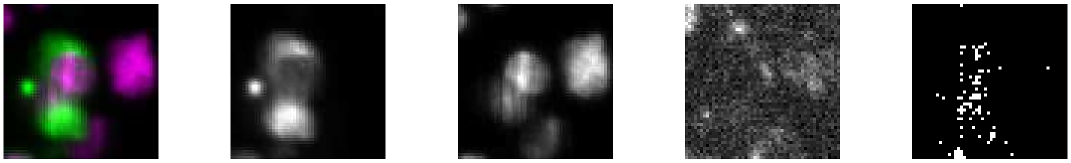				
13292	85	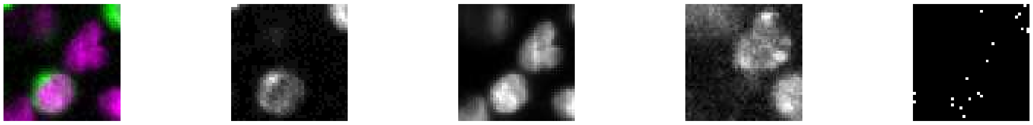				
13293	85	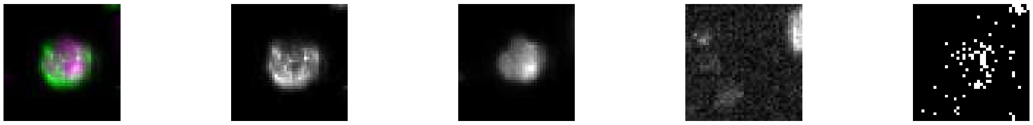				
13294	85	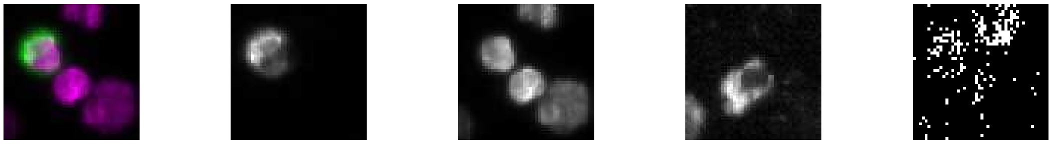				
13295	85	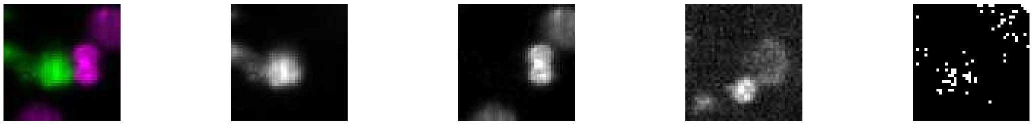				
13296	85	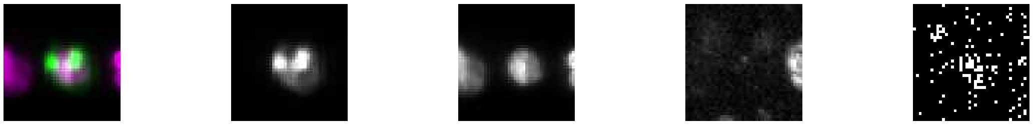				
13297	85	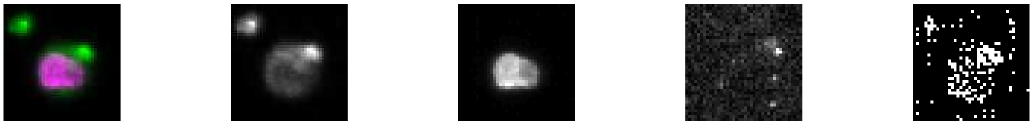				
13298	85	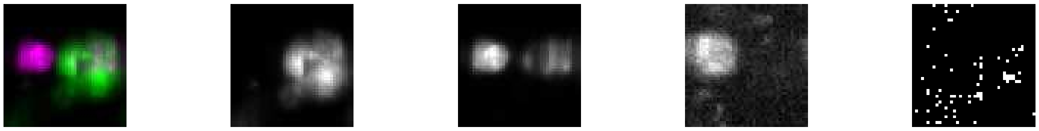				
13299	85	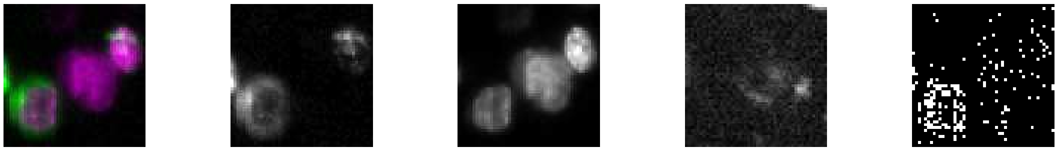				
13300	85	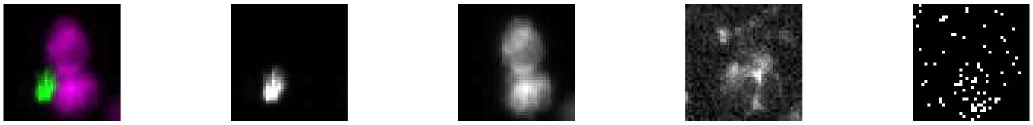				
13301	85	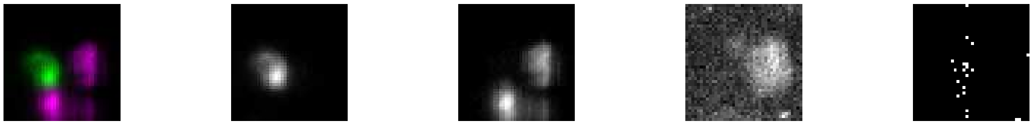				
13302	85	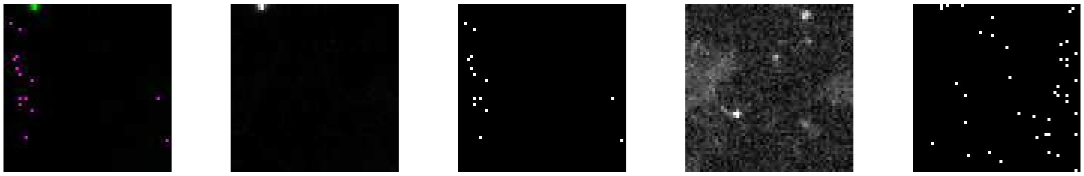				

Ereignis	Aufnahme	DAPI/CK-PE	CK-PE	DAPI	CD45-APC	HER-2/neu
13303	85	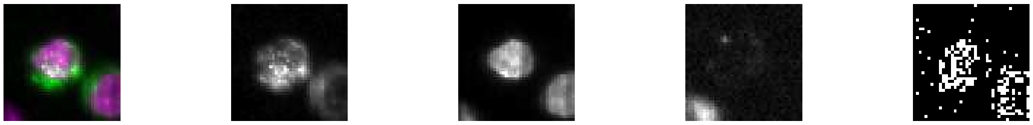				
13304	85	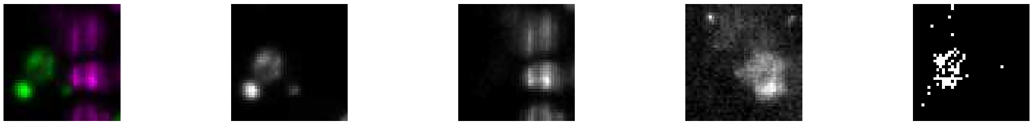				
13305	85	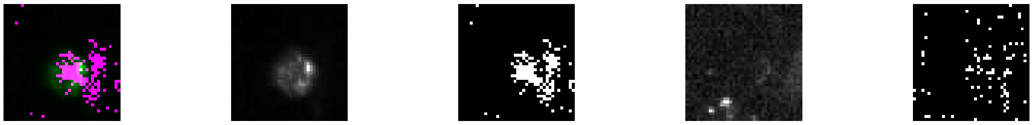				
13306	85	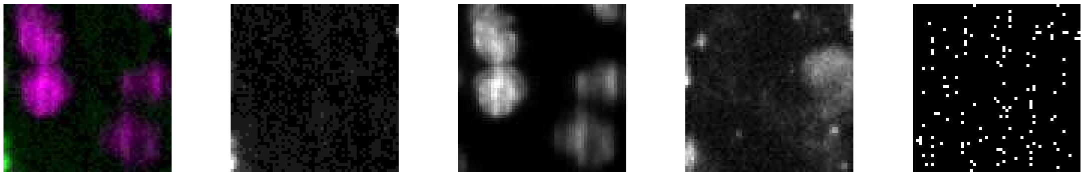				
13307	85	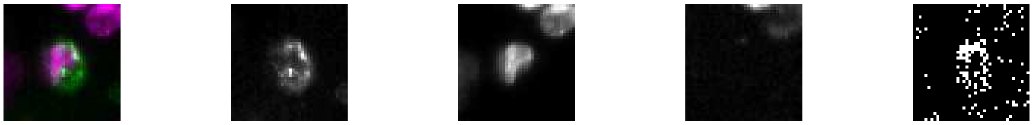				
13308	85	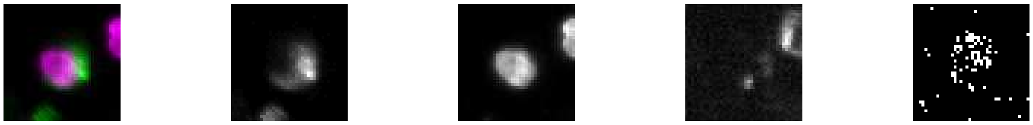				
13309	85	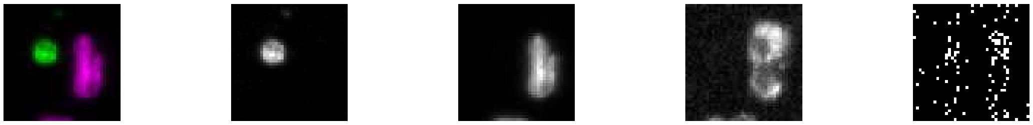				
13310	85	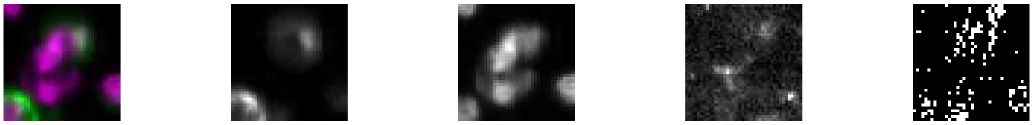				
13311	85	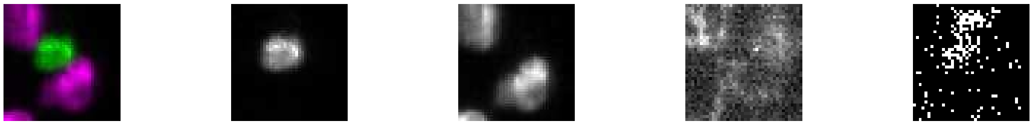				
13312	85	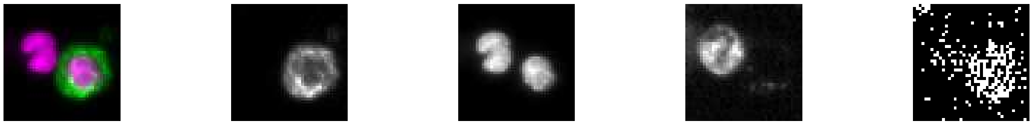				
13313	85	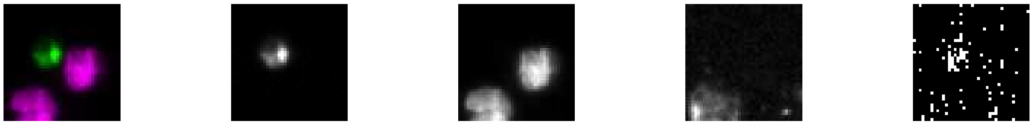				
13314	85	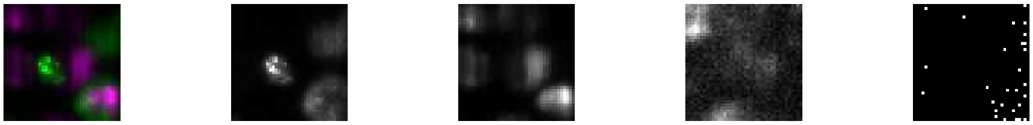				
13315	85	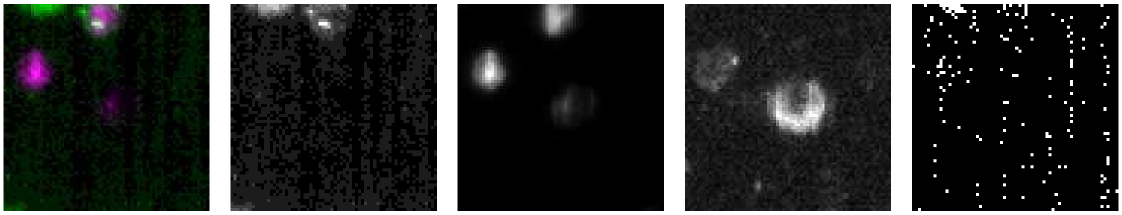				

Proben-ID: Gyn14.208 **Kassetten-ID:**01327106 **Patienten-ID:**

Ereignis	Aufnahme	DAPI/CK-PE	CK-PE	DAPI	CD45-APC	HER-2/neu
13316	85	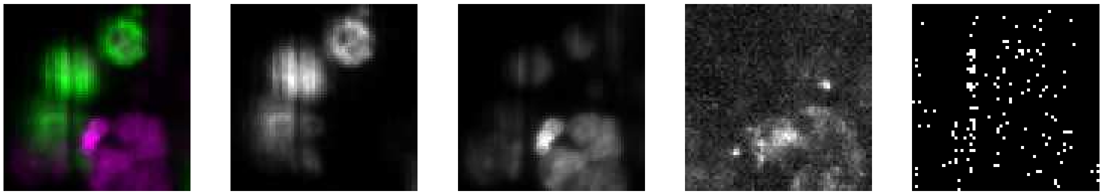				
13317	85	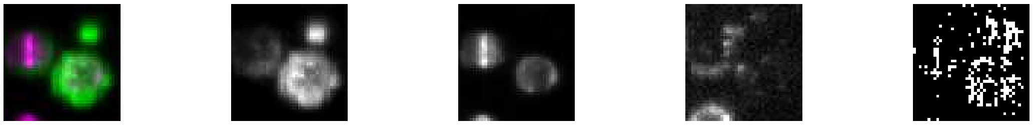				
13318	85	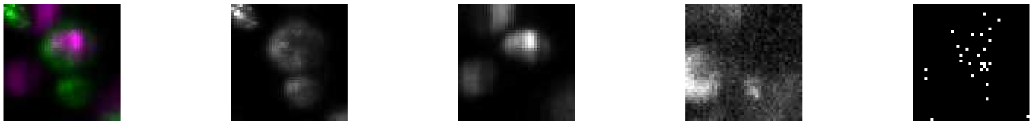				
13319	85	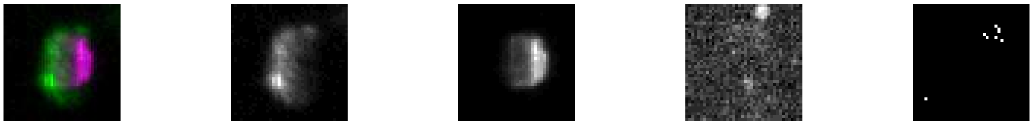				
13320	85	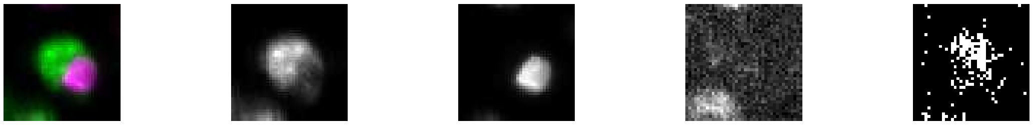				
13321	85	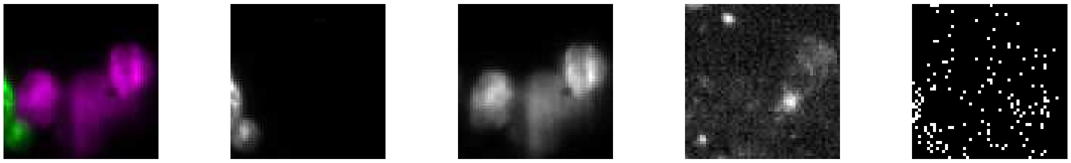				
13322	85	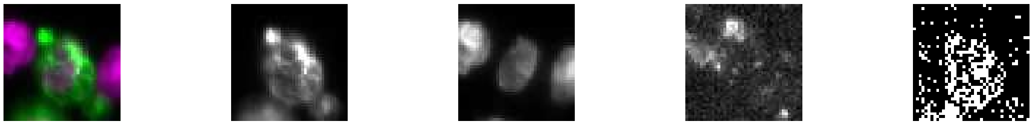				
13323	85	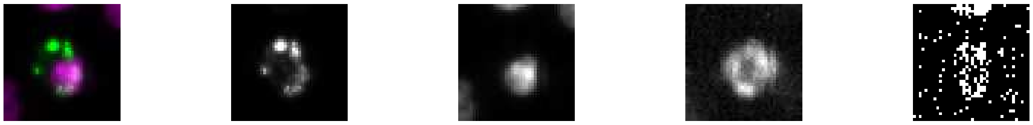				
13324	85	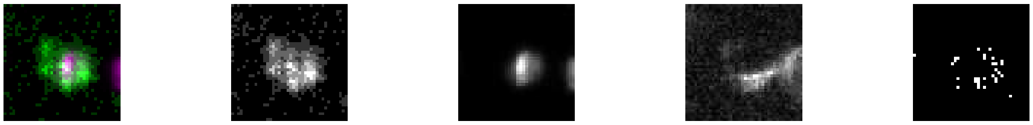				
13325	85	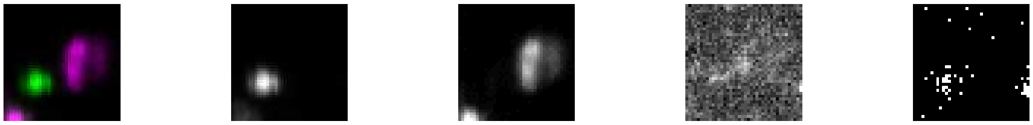				
13326	85	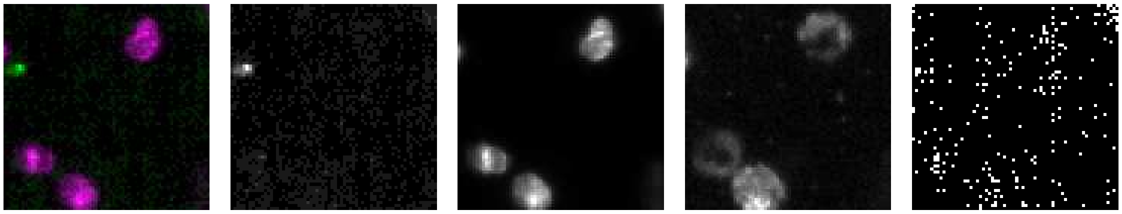				
13327	85	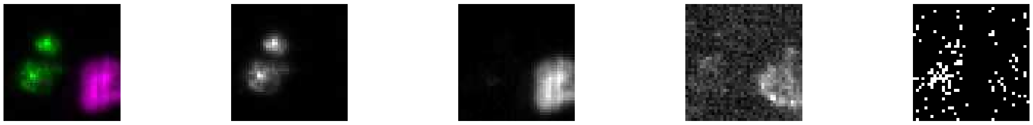				

Ereignis	Aufnahme	DAPI/CK-PE	CK-PE	DAPI	CD45-APC	HER-2/neu
13328	85	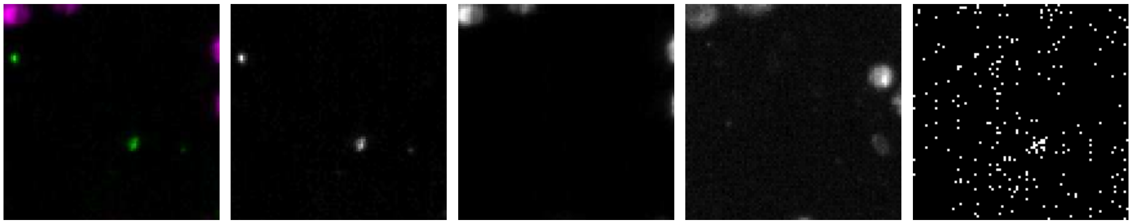				
13329	85	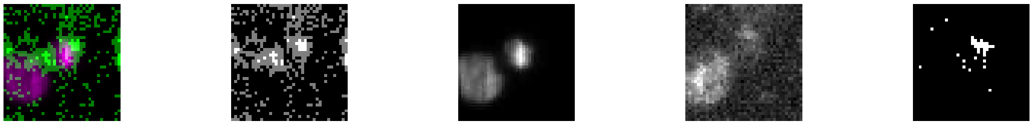				
13330	85	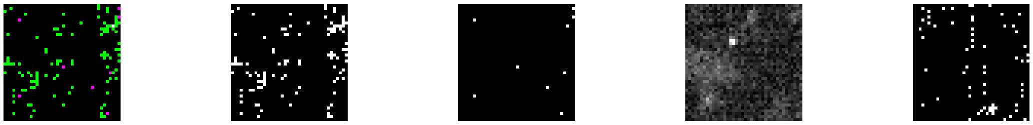				
13331	85	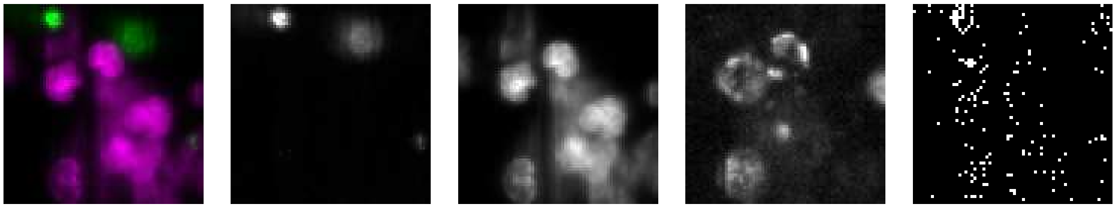				
13332	85	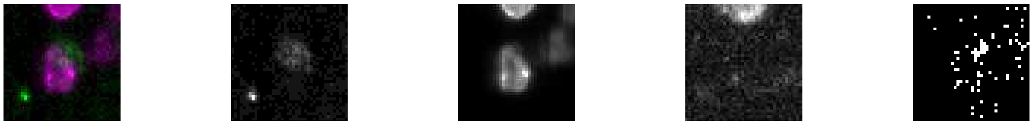				
13333	85	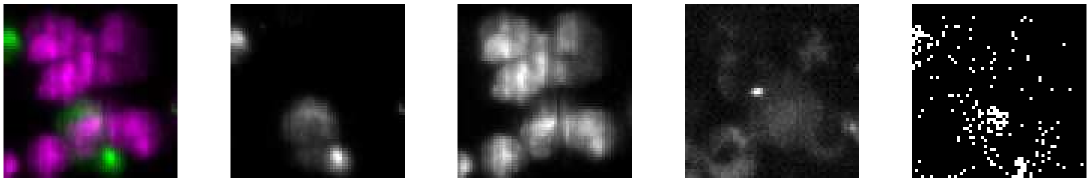				
13334	85	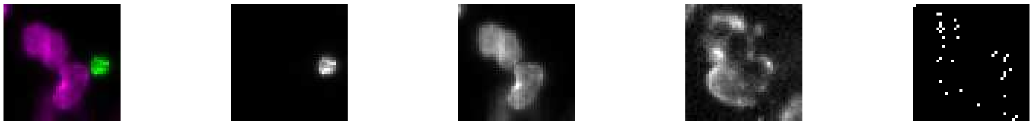				
13335	85	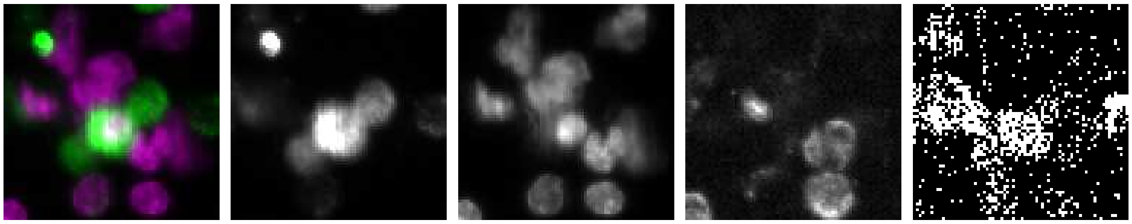				
13336	85	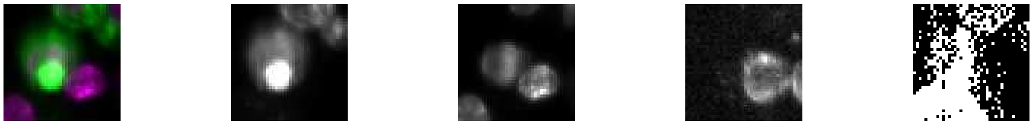				
13337	85	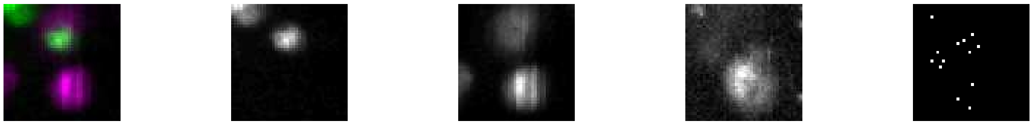				
13338	85	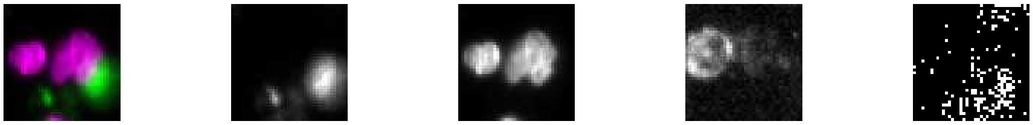				

Ereignis	Aufnahme	DAPI/CK-PE	CK-PE	DAPI	CD45-APC	HER-2/neu
13339	85	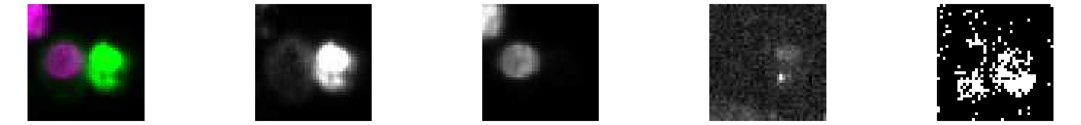				
13340	85	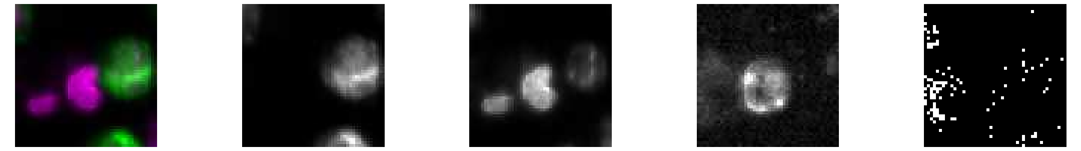				
13341	85	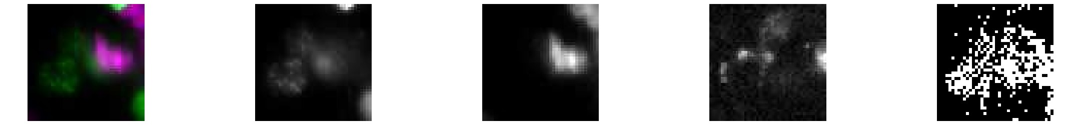				
13342	85	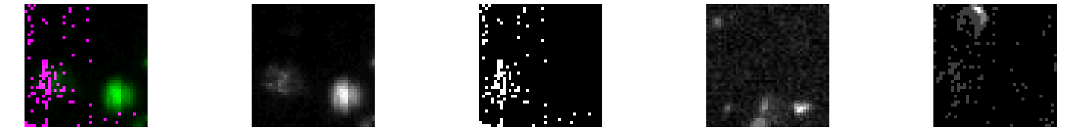				
13343	85	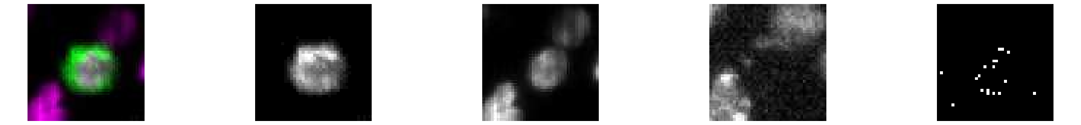				
13344	85	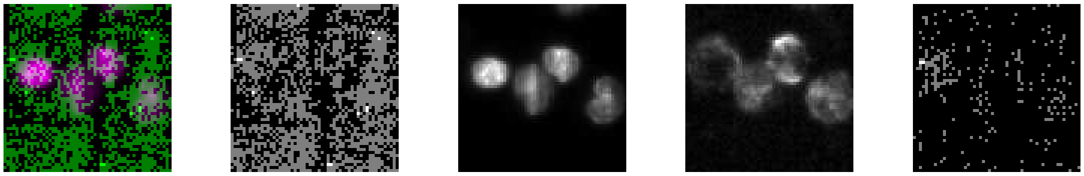				
13345	85	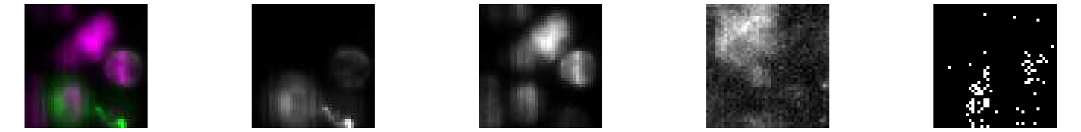				
13346	85	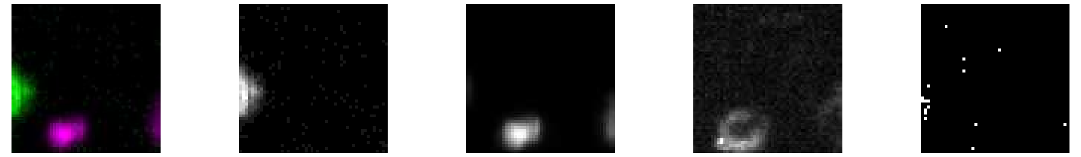				
13347	85	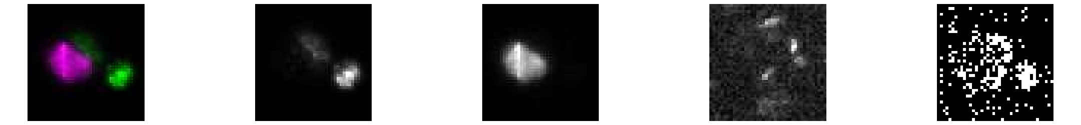				
13348	85	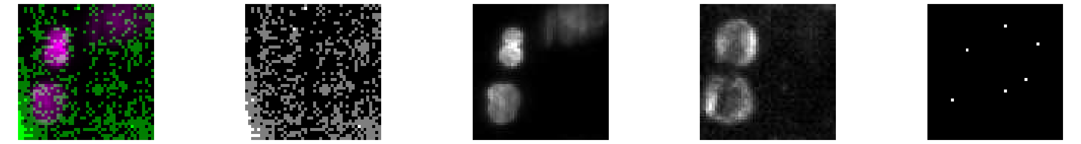				
13349	85	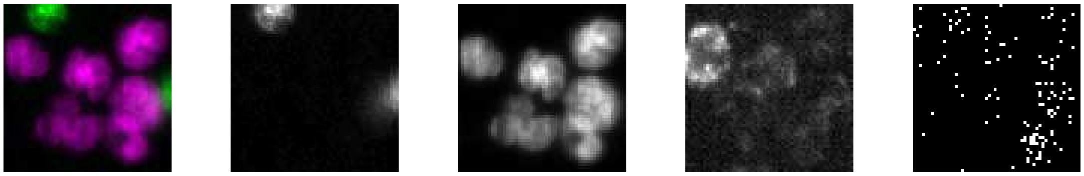				
13350	85	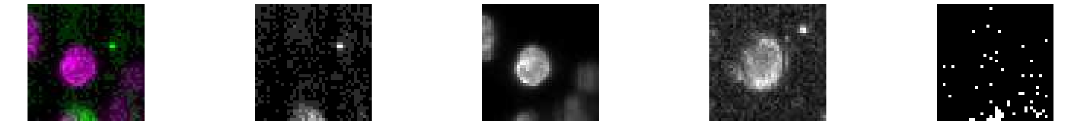				

Druckdatum: 02/06/2020 17:00

Proben-ID: Gyn14.208 **Kassetten-ID:** 01327106 **Patienten-ID:**

Ereignis	Aufnahme	DAPI/CK-PE	CK-PE	DAPI	CD45-APC	HER-2/neu
13351	85	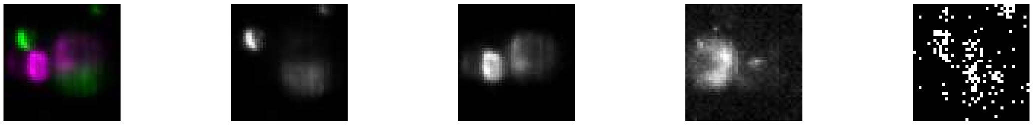				
13352	85	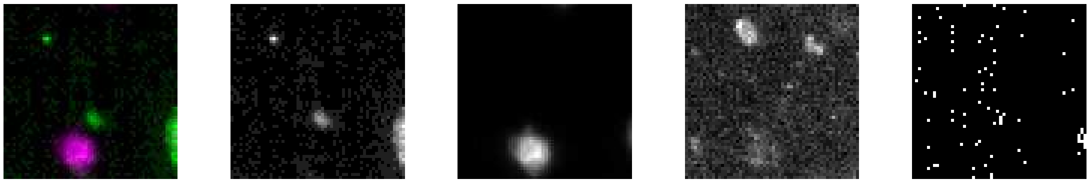				
13353	85	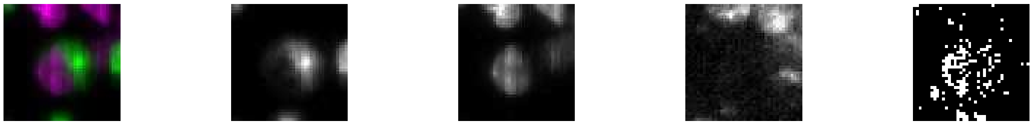				
13354	85	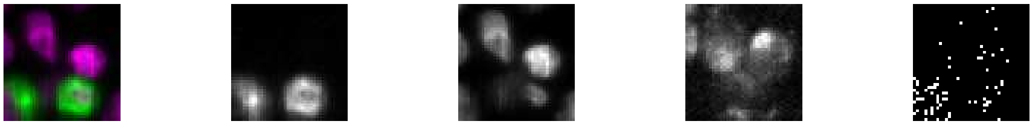				
13355	85	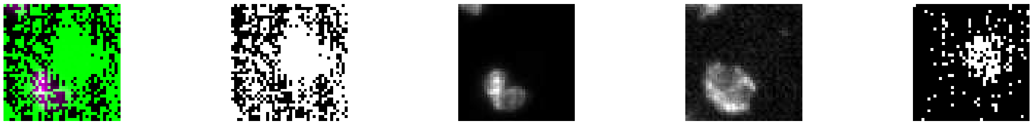				
13356	85	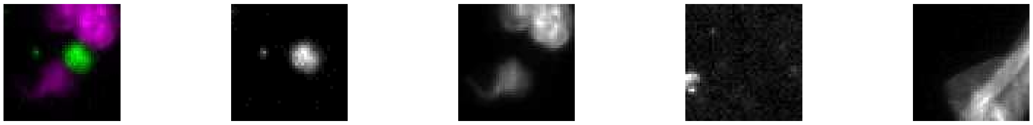				
13357	85	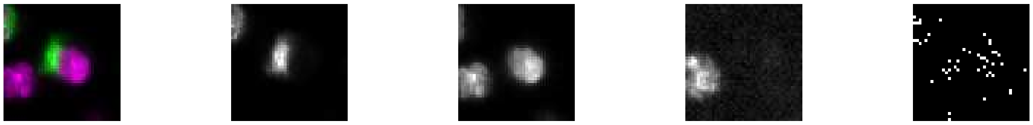				
13358	85	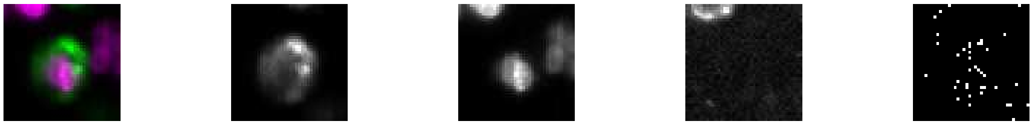				
13359	85	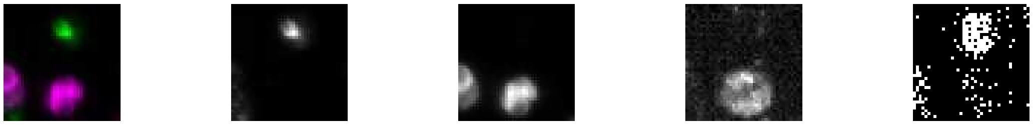				
13360	85	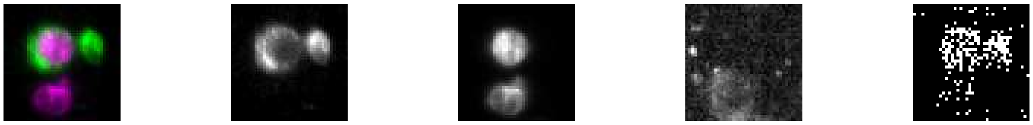				
13361	85	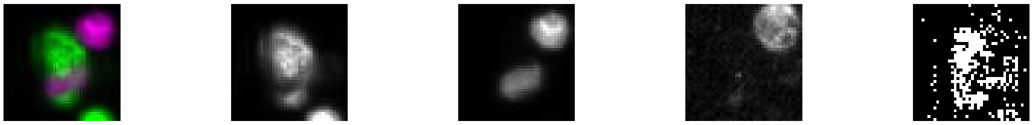				
13362	85	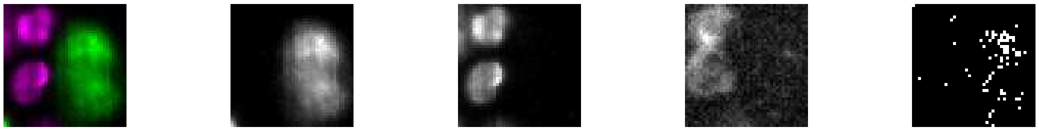				
13363	85	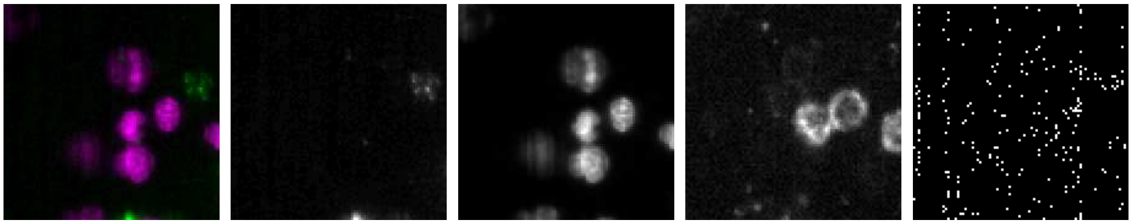				

Proben-ID: Gyn14.208 **Kassetten-ID:** 01327106 **Druckdatum:** 02/06/2020 17:00 **Patienten-ID:**

Ereignis	Aufnahme	DAPI/CK-PE	CK-PE	DAPI	CD45-APC	HER-2/neu
13364	85	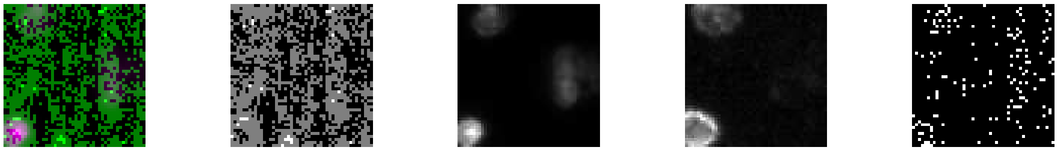				
13365	85	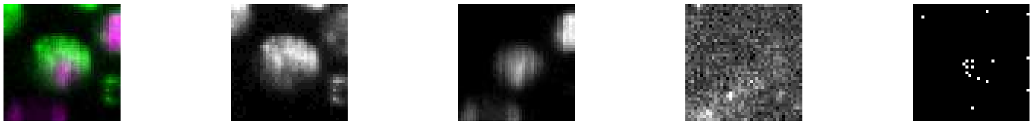				
13366	85	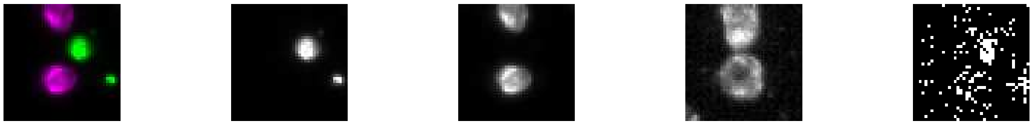				
13367	85	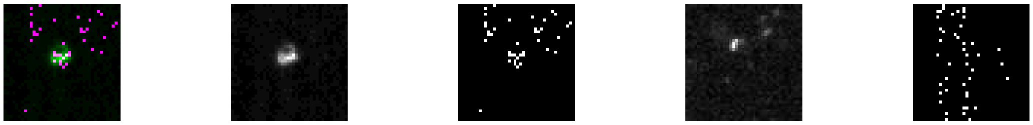				
13368	85	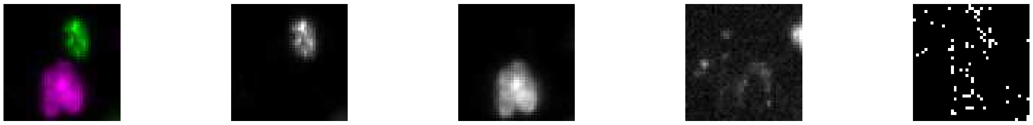				
13369	85	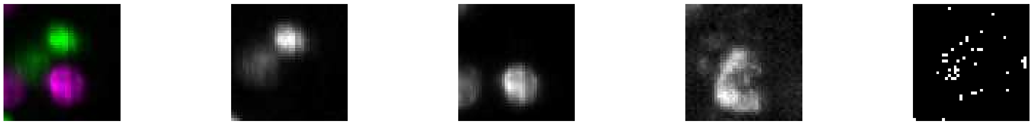				
13370	85	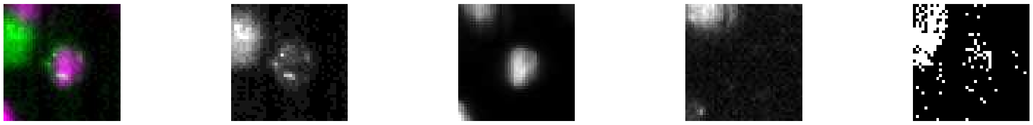				
13371	85	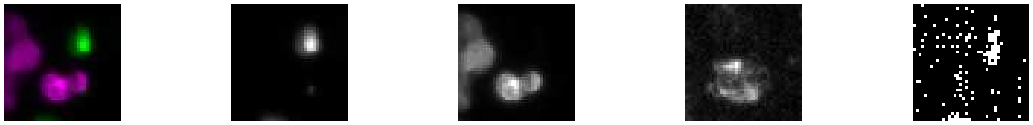				
13372	85	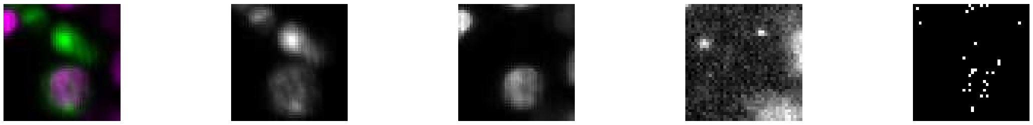				
13373	85	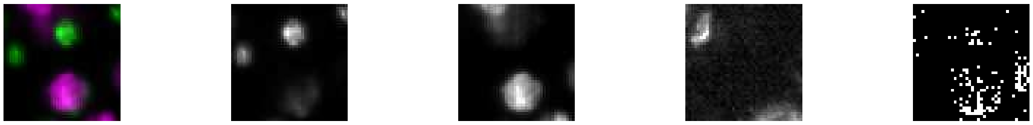				
13374	85	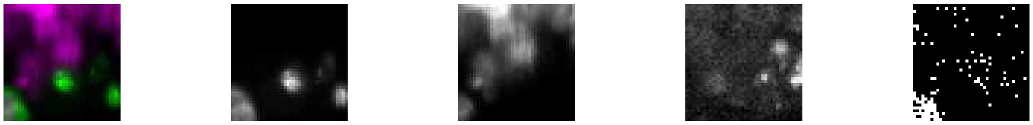				
13375	85	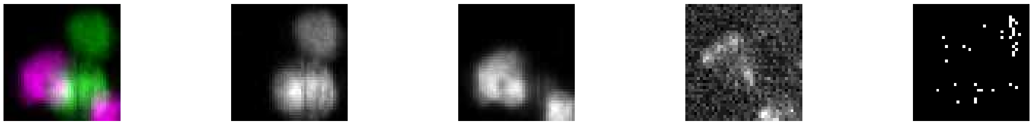				
13376	85	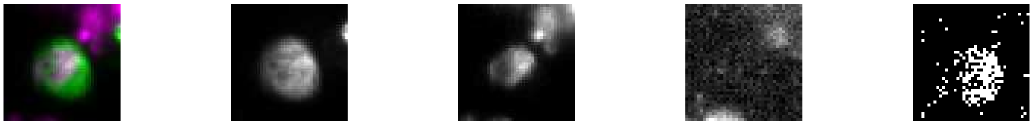				
13377	85	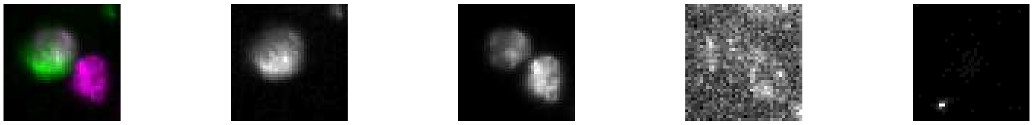				

Ereignis	Aufnahme	DAPI/CK-PE	CK-PE	DAPI	CD45-APC	HER-2/neu
13378	85	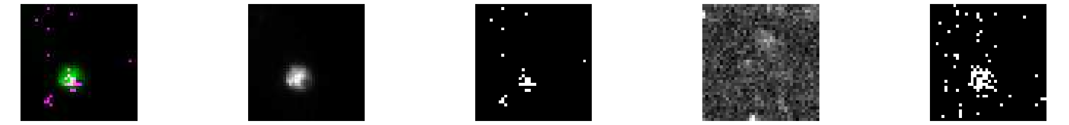				
13379	85	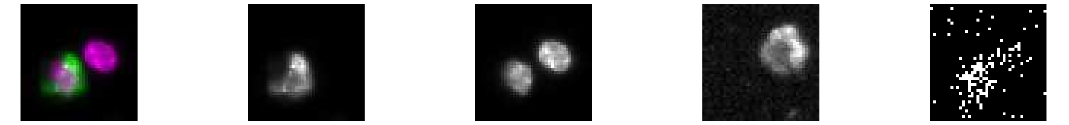				
13380	85	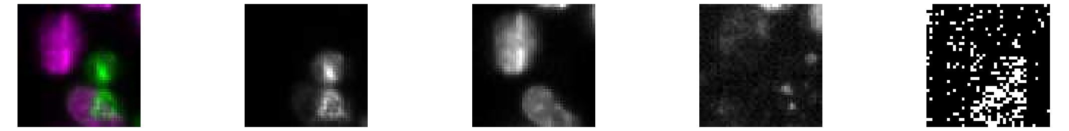				
13381	85	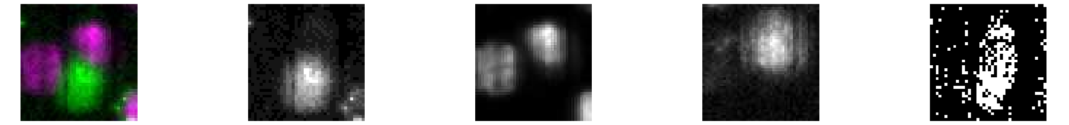				
13382	85	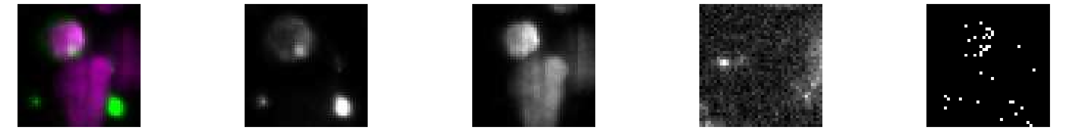				
13383	85	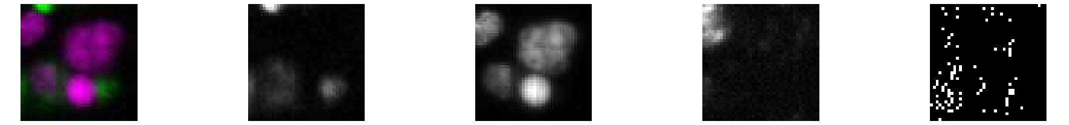				
13384	85	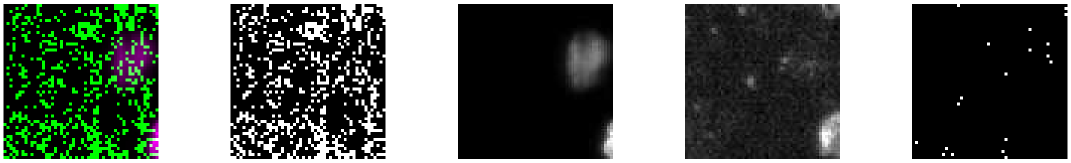				
13385	85	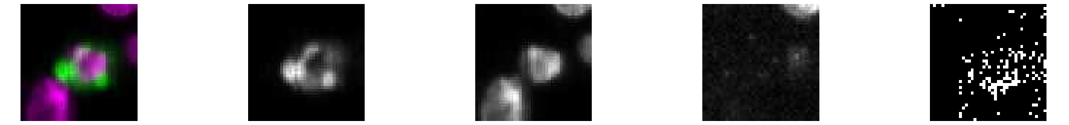				
13386	85	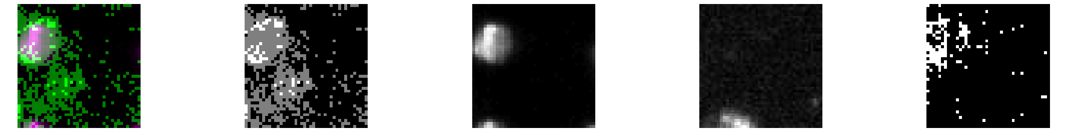				
13387	85	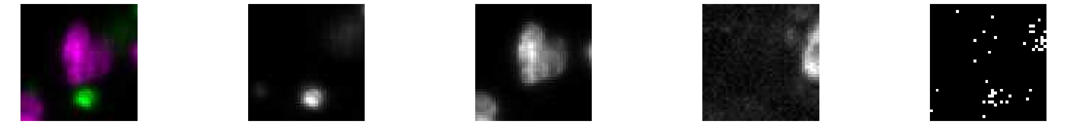				
13388	85	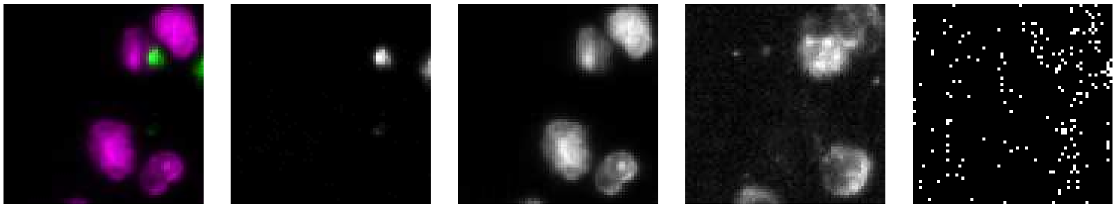				
13389	85	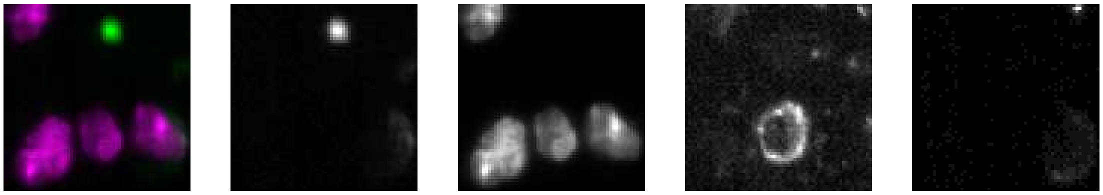				

Proben-ID: Gyn14.208 **Kassetten-ID:** 01327106 **Patienten-ID:**

Ereignis	Aufnahme	DAPI/CK-PE	CK-PE	DAPI	CD45-APC	HER-2/neu
13390	85	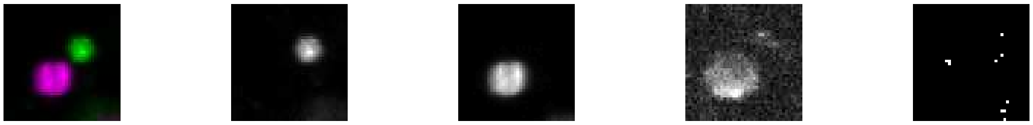				
13391	85	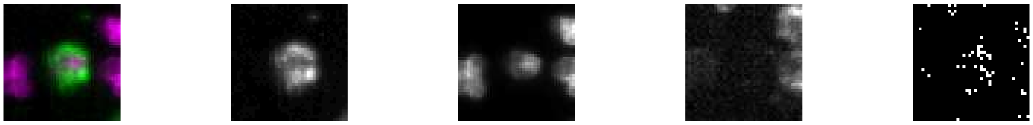				
13392	85	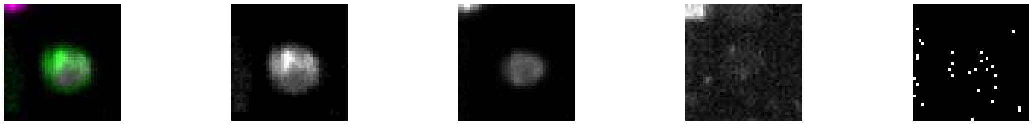				
13393	85	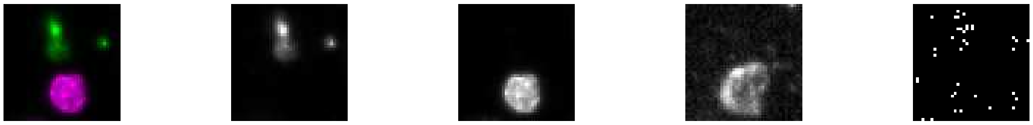				
13394	85	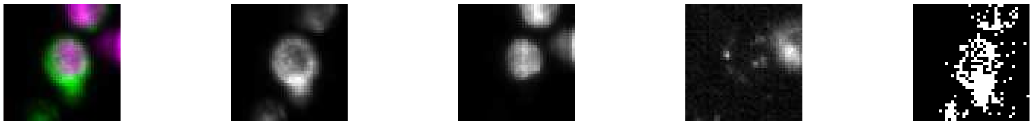				
13395	85	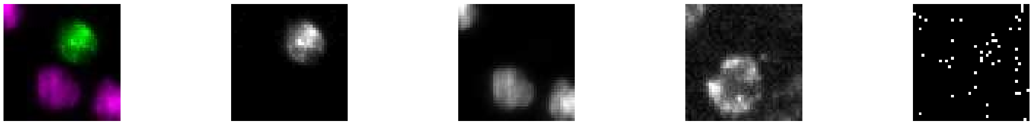				
13396	85	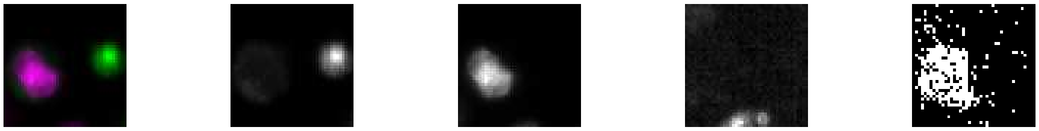				
13397	85	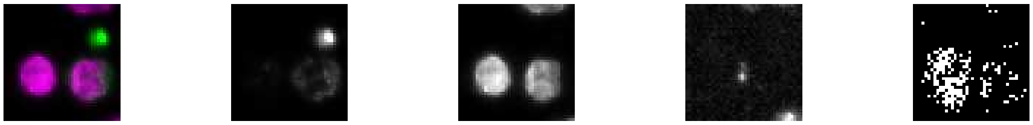				
13398	85	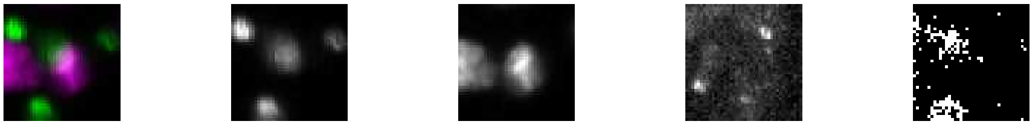				
13399	85	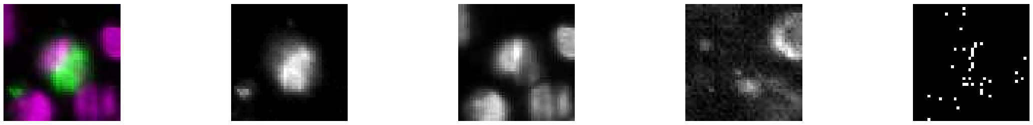				
13400	85	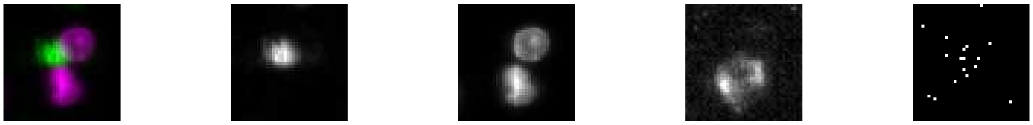				
13401	85	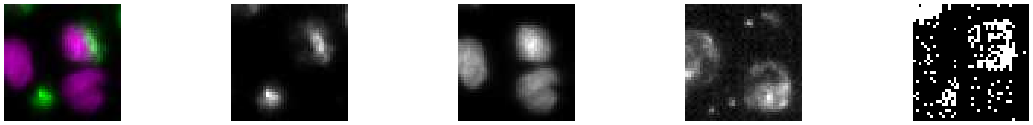				
13402	85	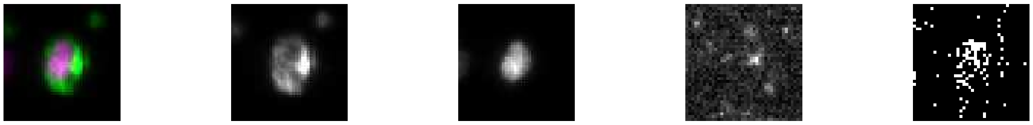				
13403	85	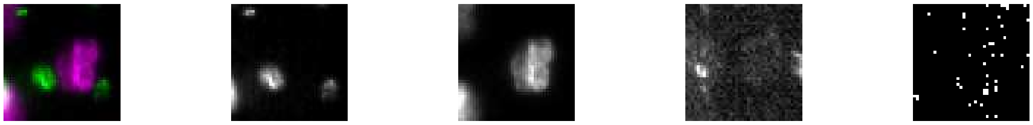				

Ereignis	Aufnahme	DAPI/CK-PE	CK-PE	DAPI	CD45-APC	HER-2/neu
13404	85	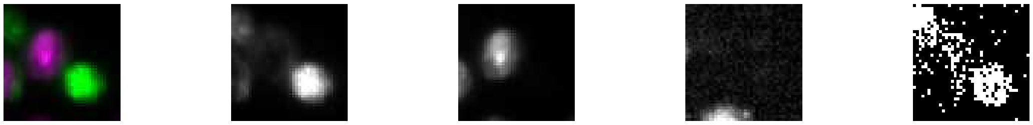				
13405	85	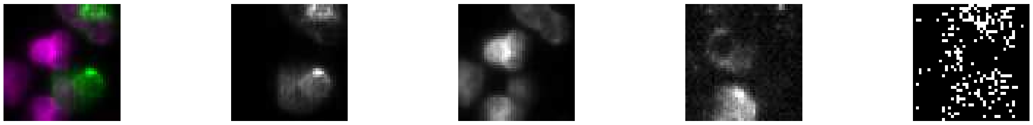				
13406	85	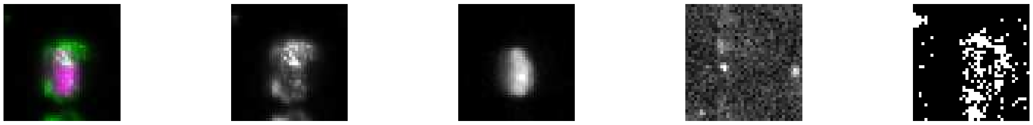				
13407	85	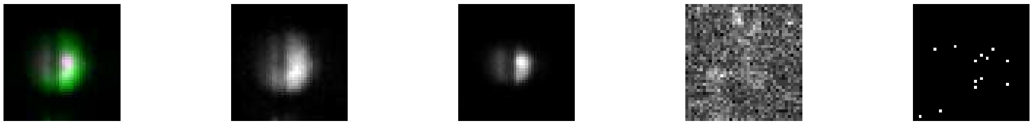				
13408	85	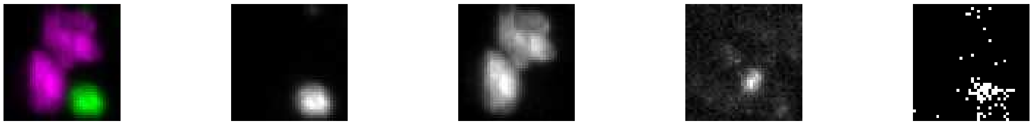				
13409	85	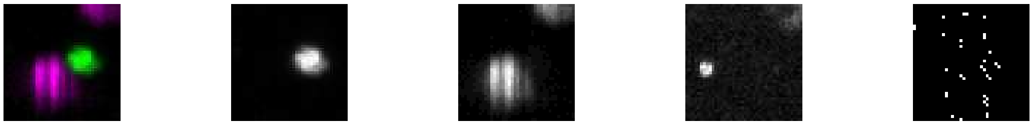				
13410	85	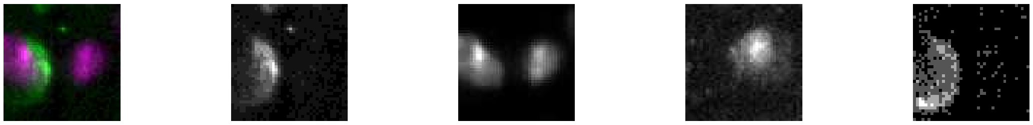				
13411	85	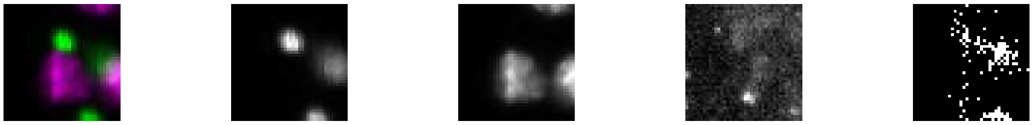				
13412	85	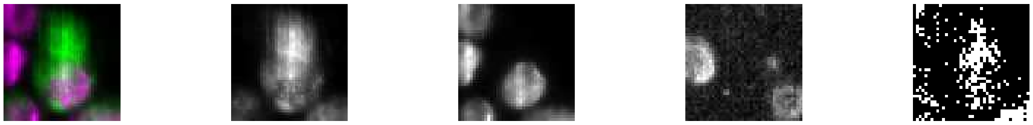				
13413	85	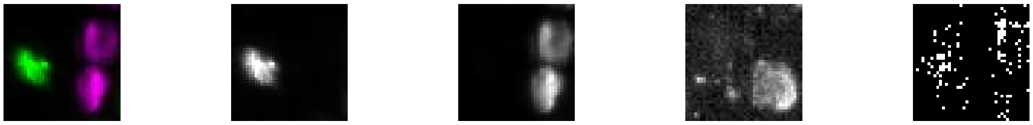				
13414	85	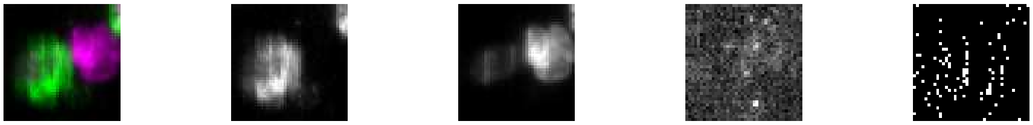				
13415	85	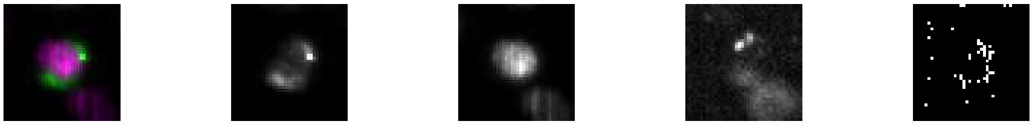				
13416	85	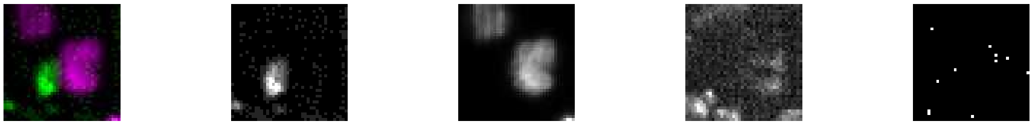				
13417	85	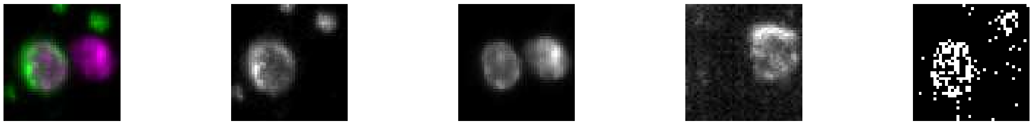				

Ereignis	Aufnahme	DAPI/CK-PE	CK-PE	DAPI	CD45-APC	HER-2/neu
13418	85	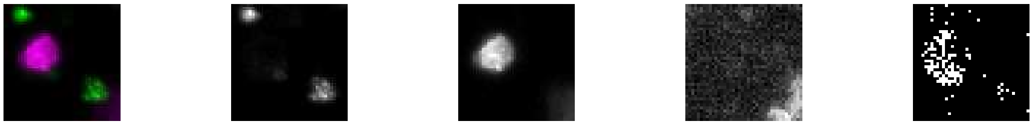				
13419	85	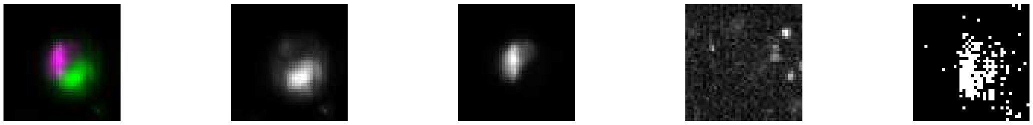				
13420	85	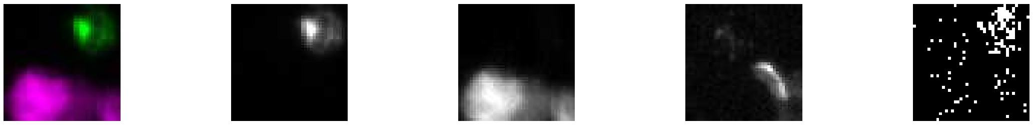				
13421	85	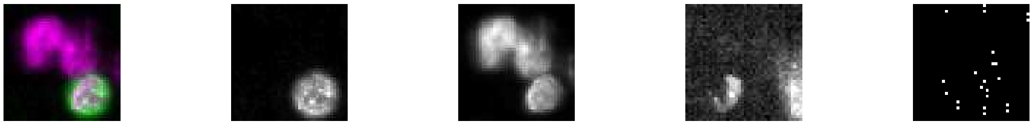				
13422	85	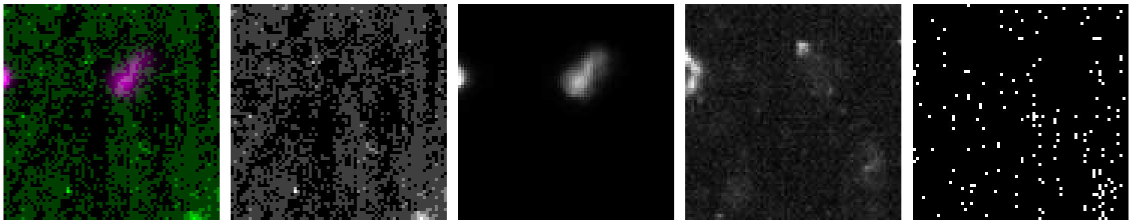				
13423	85	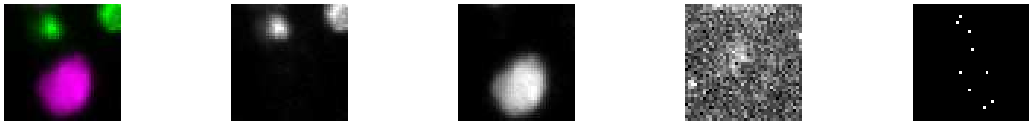				
13424	85	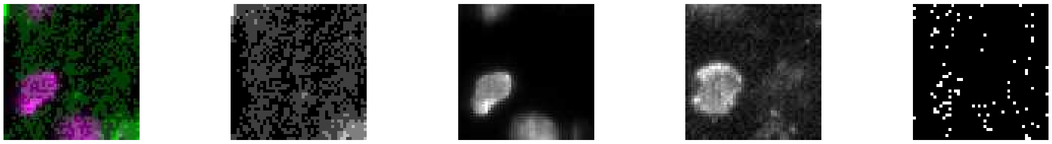				
13425	85	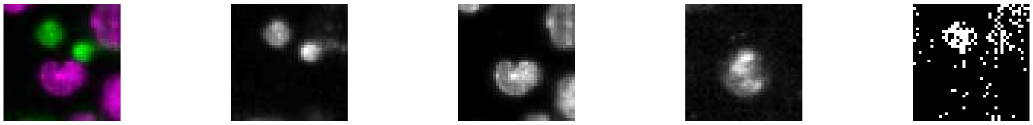				
13426	85	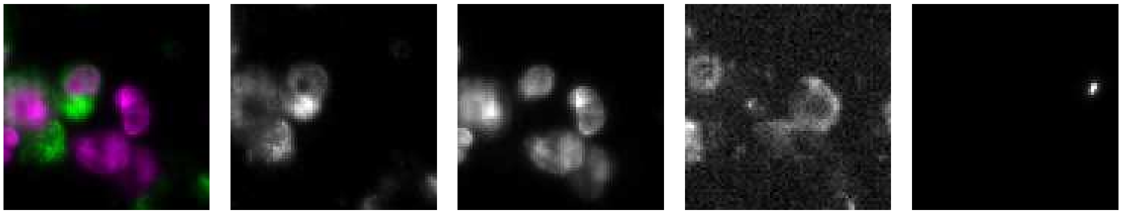				
13427	85	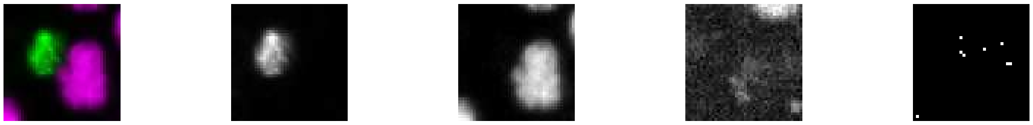				
13428	85	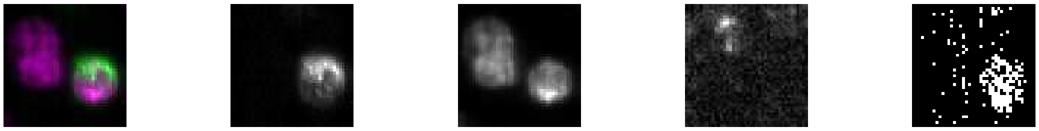				
13429	85	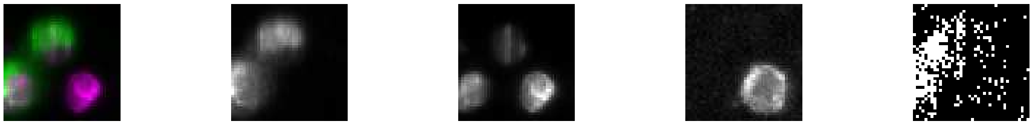				

Proben-ID: Gyn14.208 **Kassetten-ID:** 01327106 **Patienten-ID:**

Ereignis	Aufnahme	DAPI/CK-PE	CK-PE	DAPI	CD45-APC	HER-2/neu
13430	85	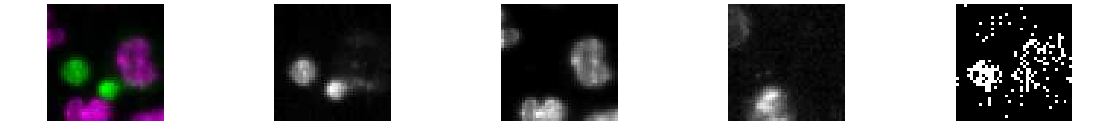				
13431	85	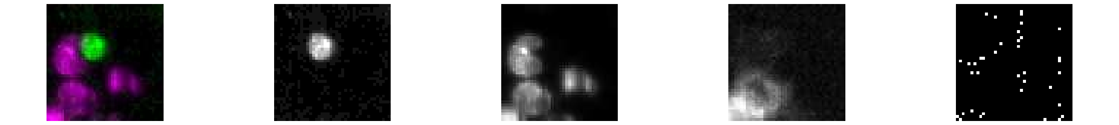				
13432	85	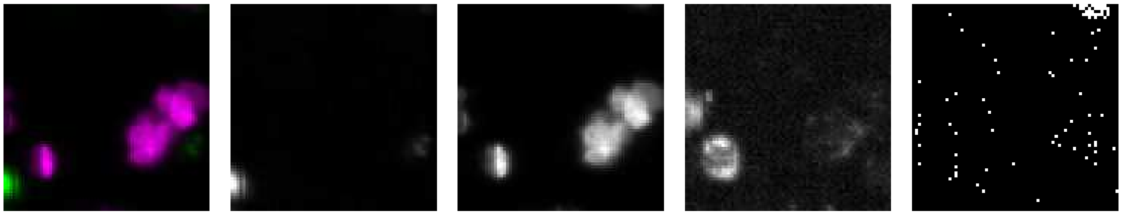				
13433	85	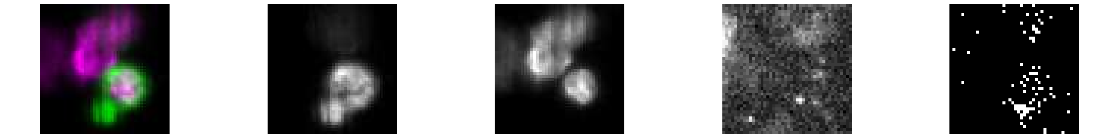				
13434	85	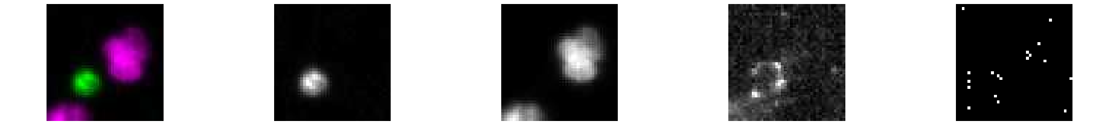				
13435	85	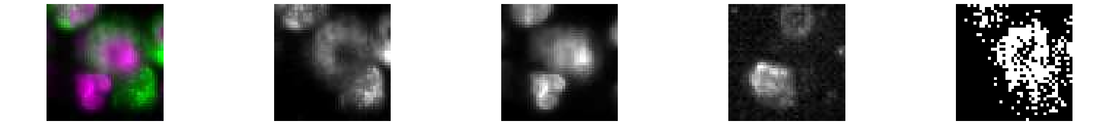				
13436	85	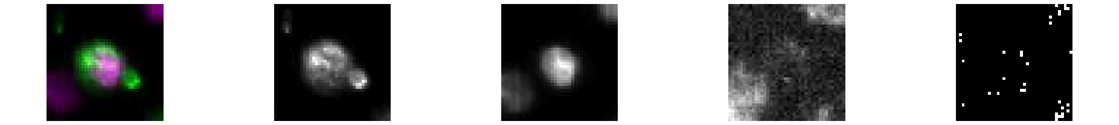				
13437	85	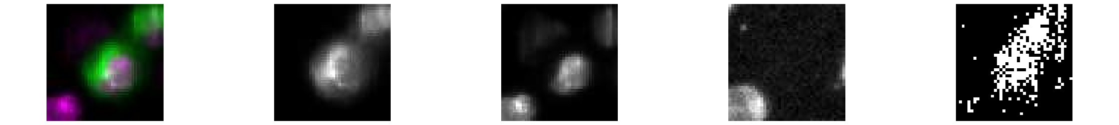				
13438	85	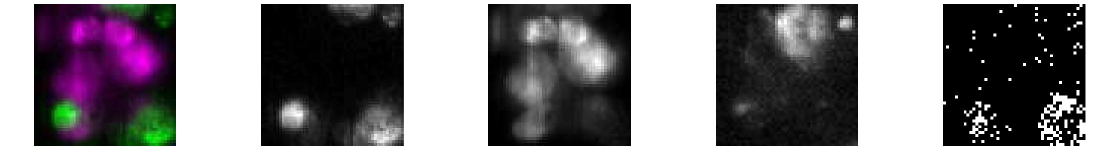				
13439	85	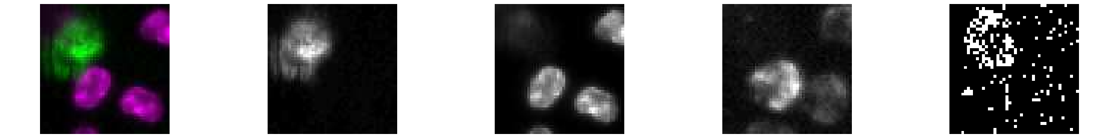				
13440	85	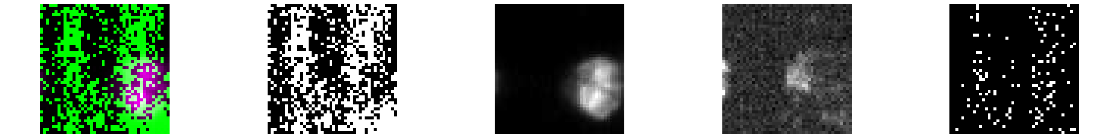				
13441	85	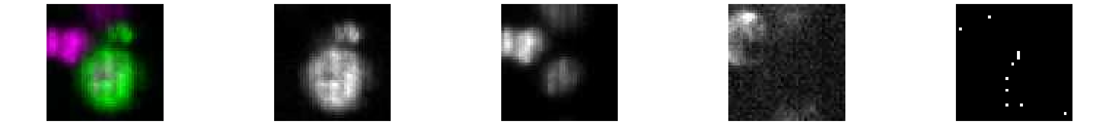				
13442	85	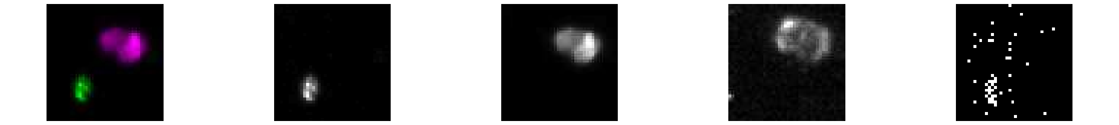				

Ereignis	Aufnahme	DAPI/CK-PE	CK-PE	DAPI	CD45-APC	HER-2/neu
13443	85	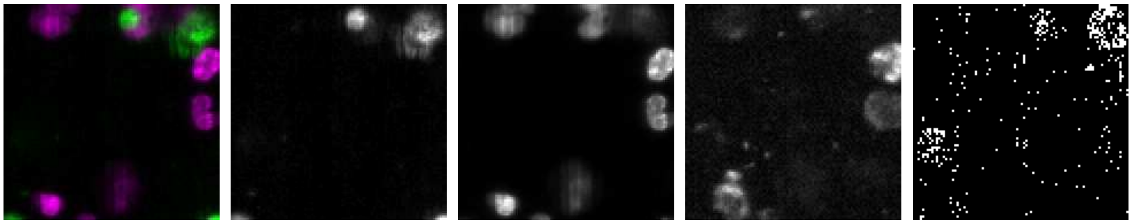				
13444	85	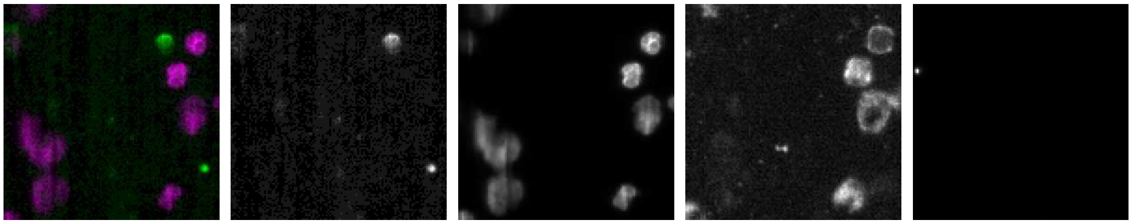				
13445	85	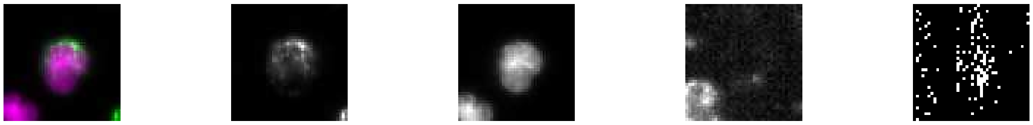				
13446	85	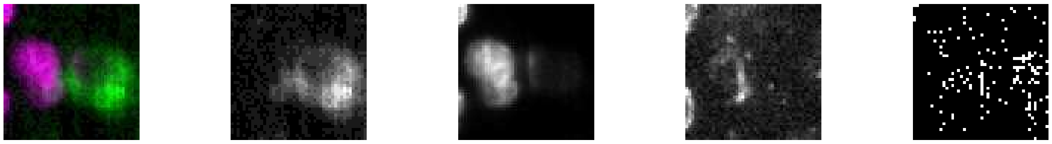				
13447	85	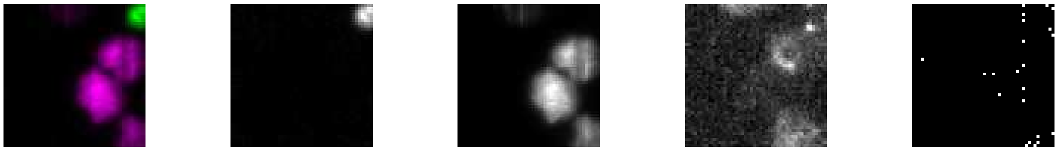				
13448	85	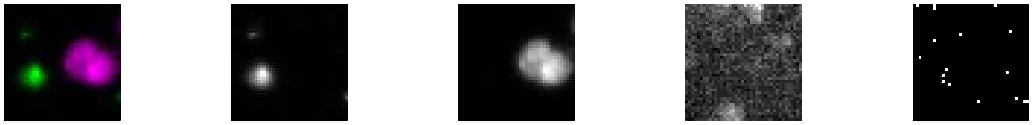				
13449	85	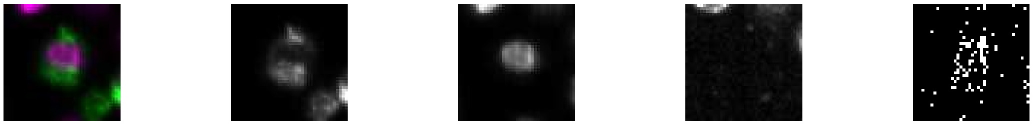				
13450	85	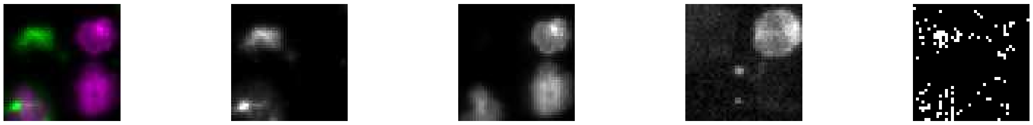				
13451	85	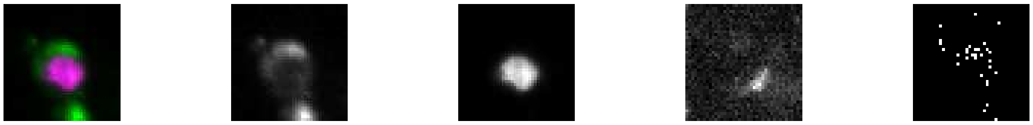				
13452	85	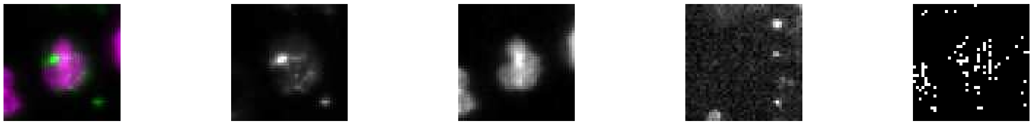				
13453	85	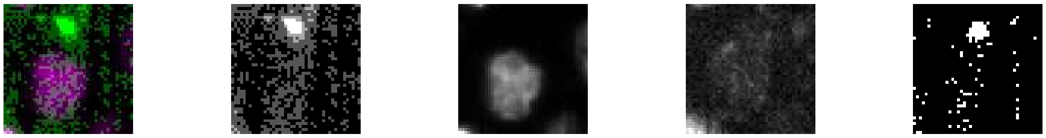				
13454	85	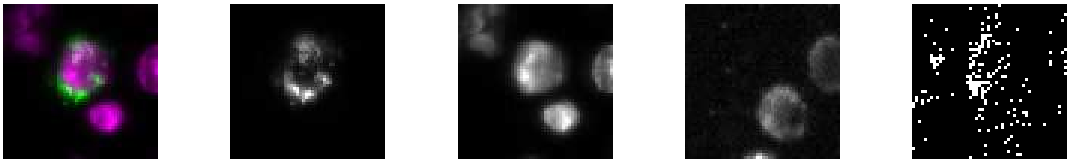				

Ereignis	Aufnahme	DAPI/CK-PE	CK-PE	DAPI	CD45-APC	HER-2/neu
13455	85	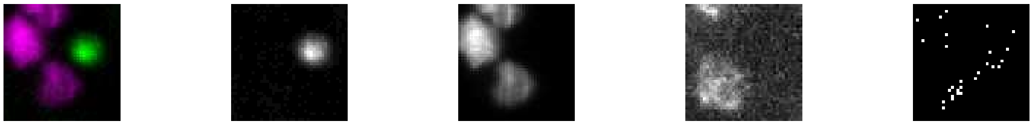				
13456	85	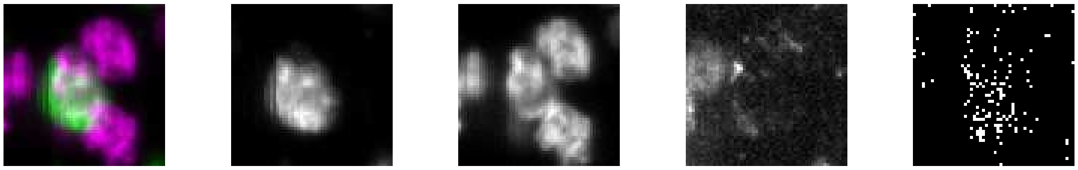				
13457	85	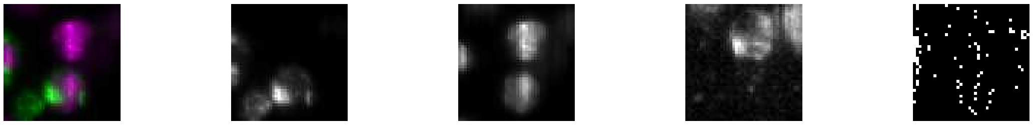				
13458	85	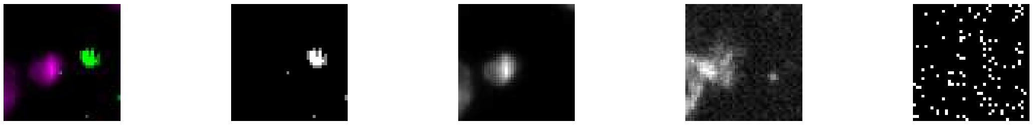				
13459	85	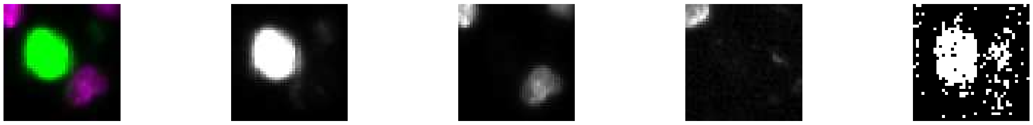				
13460	85	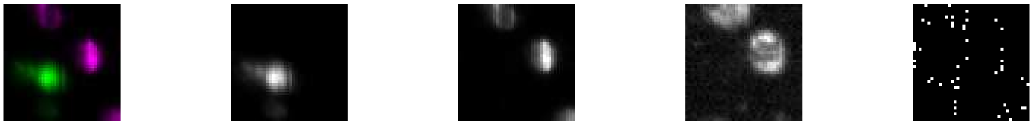				
13461	84	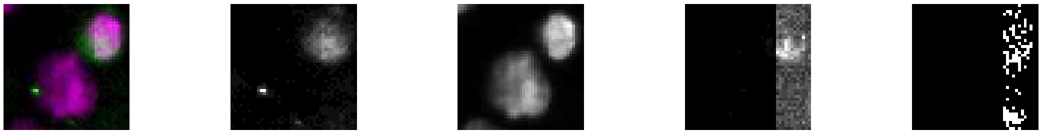				
13462	85	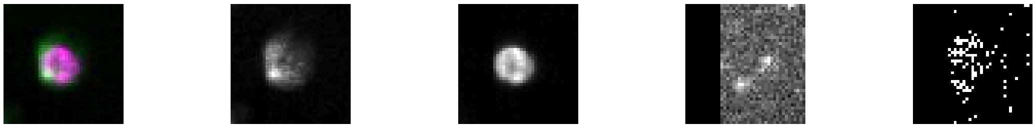				
13463	85	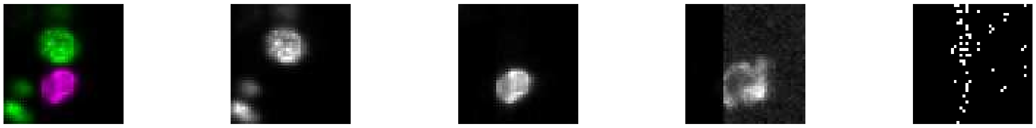				
13464	85	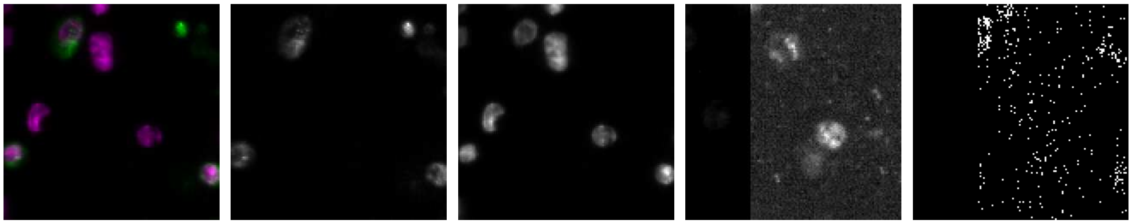				
13465	84	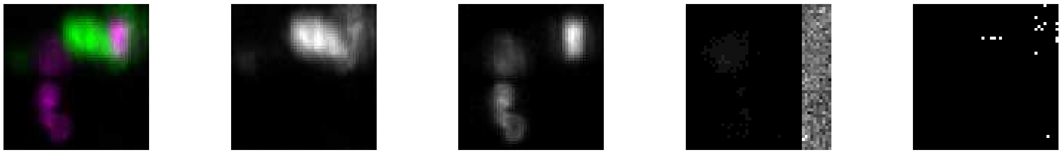				
13466	84	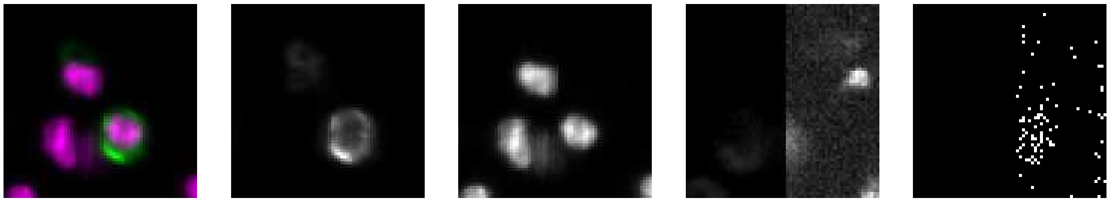				

Ereignis	Aufnahme	DAPI/CK-PE	CK-PE	DAPI	CD45-APC	HER-2/neu
13467	85	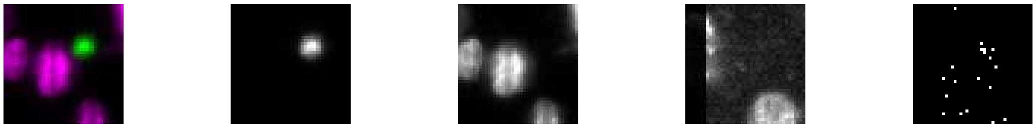				
13468	56	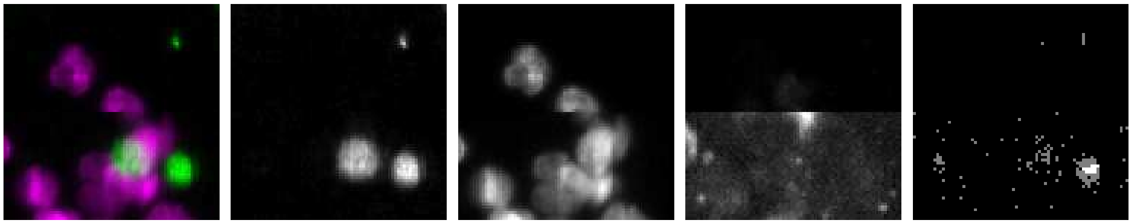				
13469	56	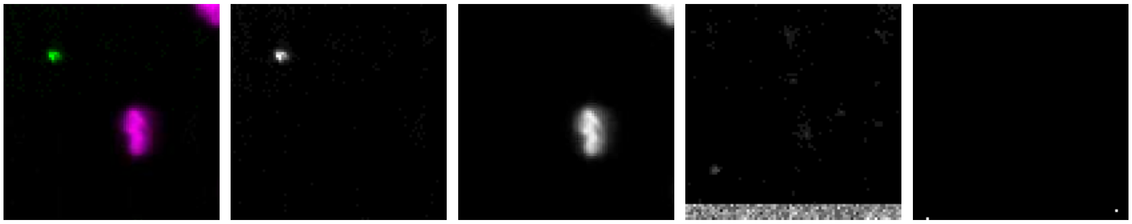				
13470	85	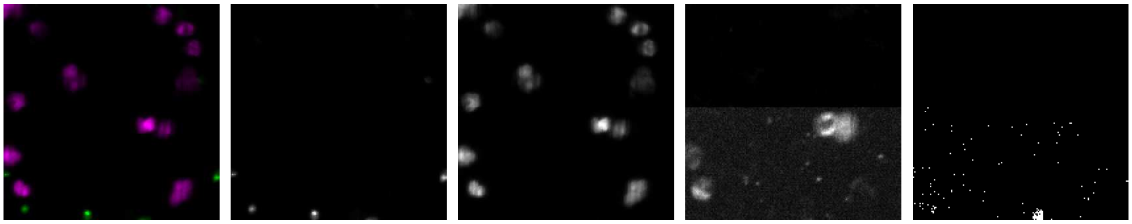				
13471	85	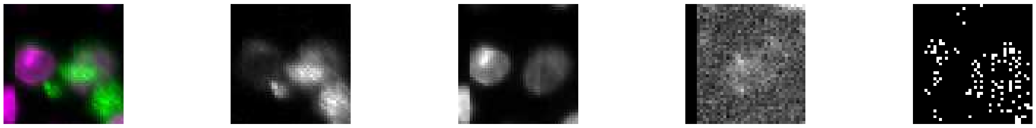				
13472	85	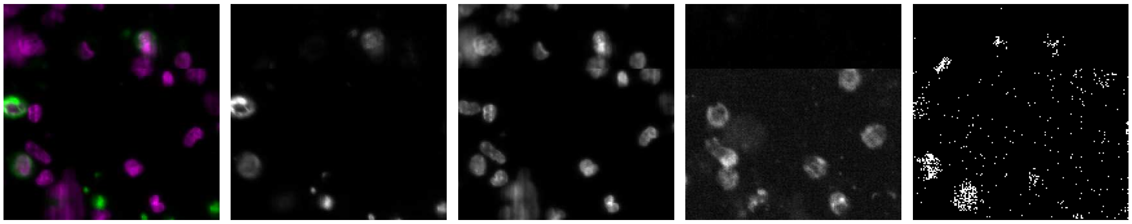				
13473	56	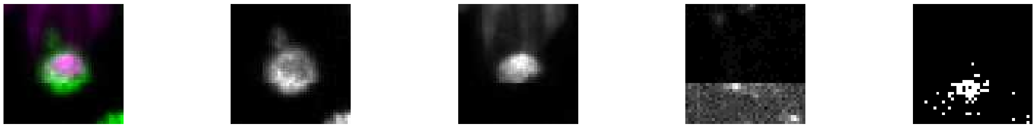				
13474	56	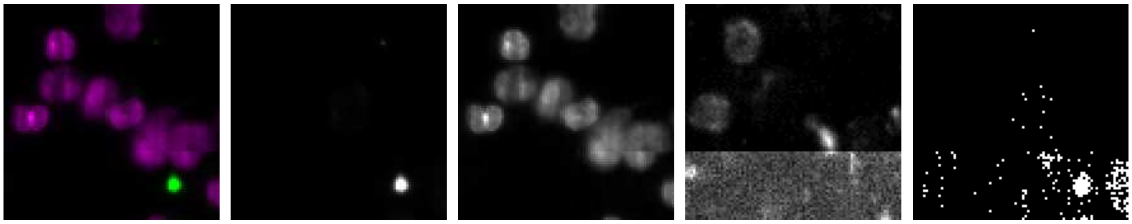				
13475	85	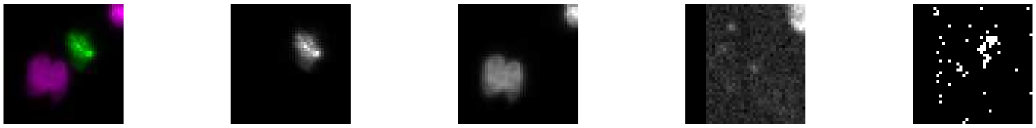				
13476	86	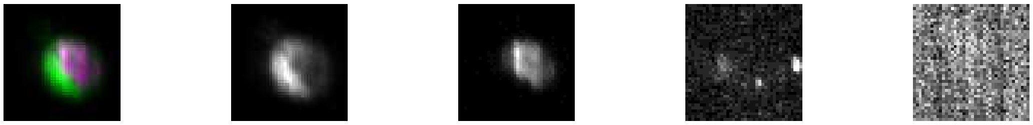				

Ereignis	Aufnahme	DAPI/CK-PE	CK-PE	DAPI	CD45-APC	HER-2/neu
13477	86	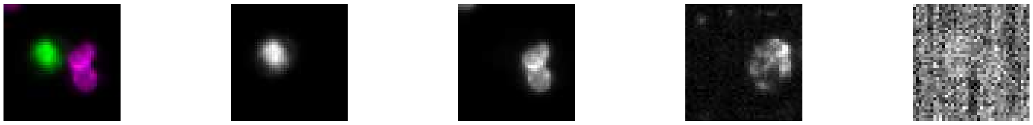				
13478	86	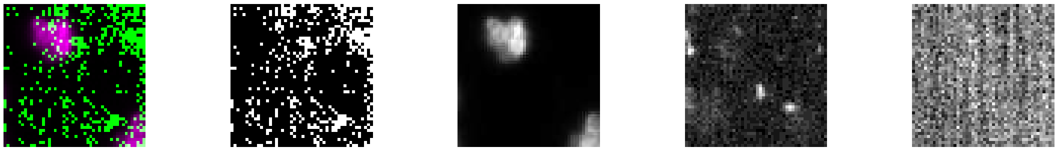				
13479	86	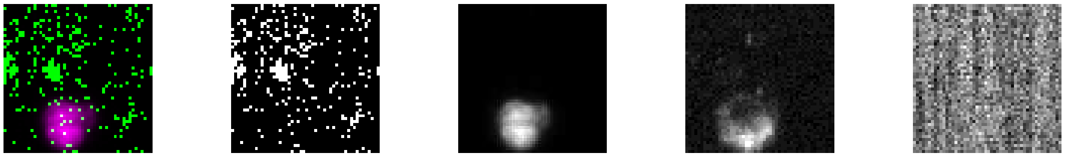				
13480	86	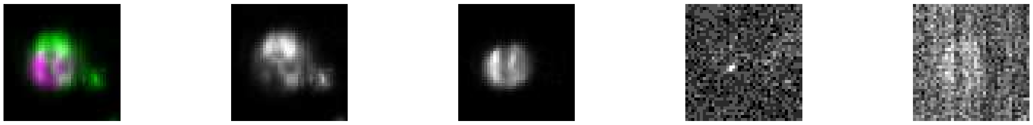				
13481	86	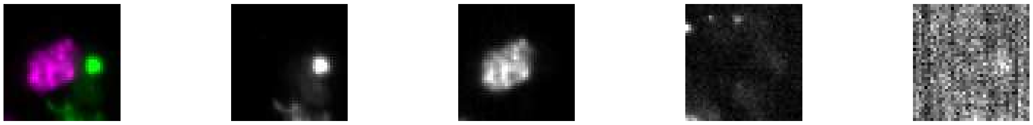				
13482	86	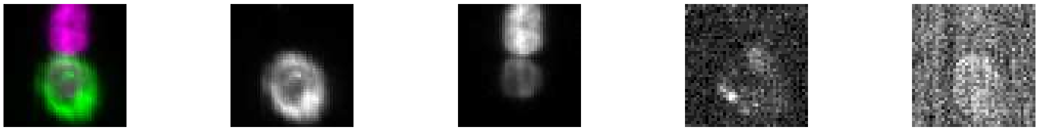				
13483	86	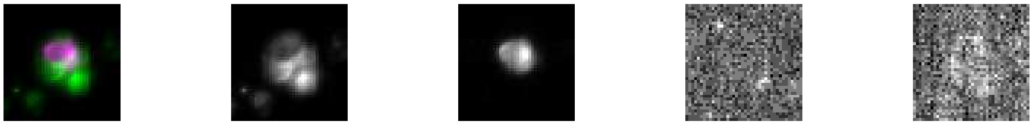				
13484	86	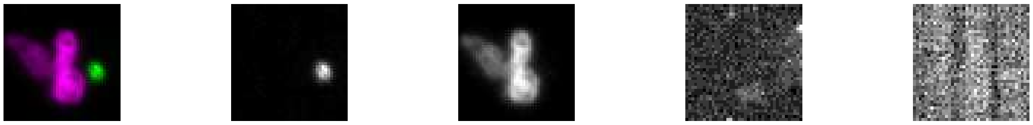				
13485	86	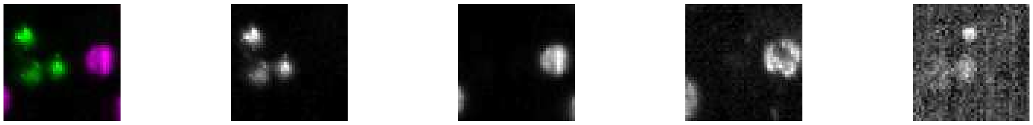				
13486	86	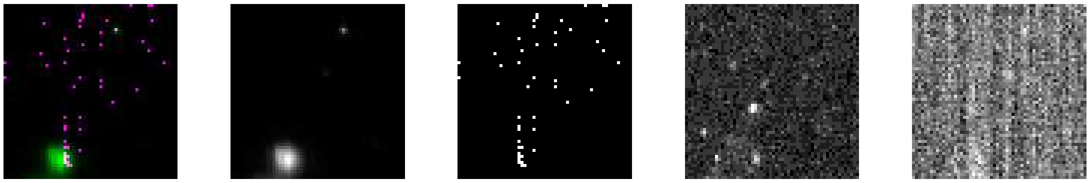				
13487	86	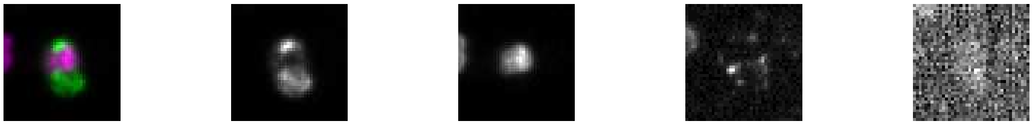				
13488	86	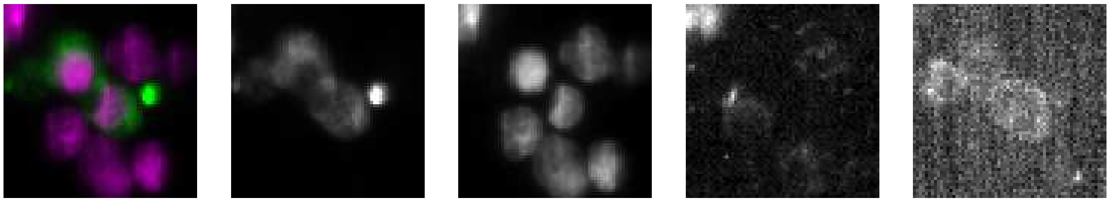				

Ereignis	Aufnahme	DAPI/CK-PE	CK-PE	DAPI	CD45-APC	HER-2/neu
13489	86	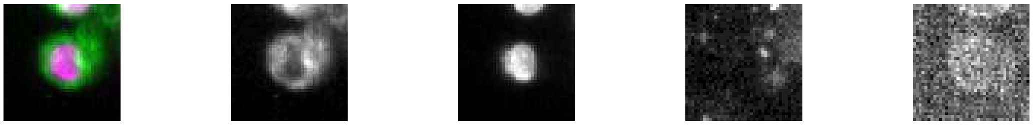				
13490	86	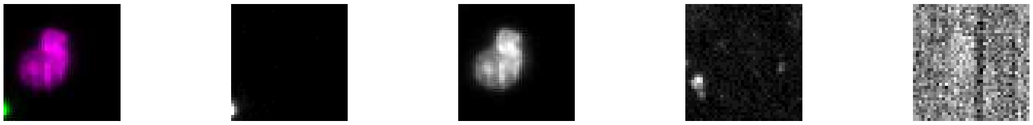				
13491	86	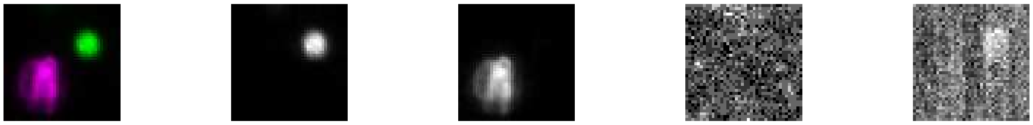				
13492	86	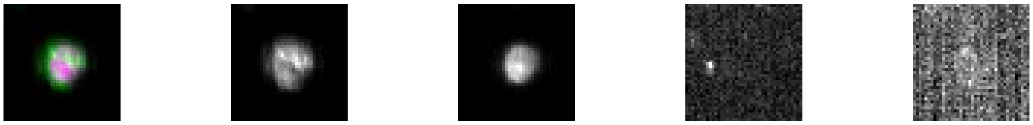				
13493	86	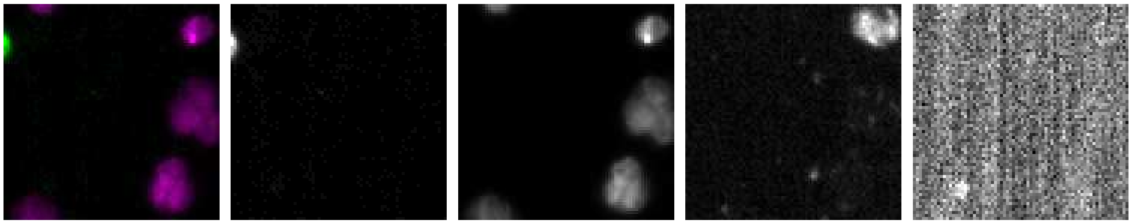				
13494	86	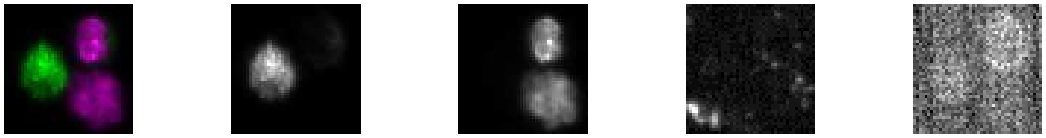				
13495	86	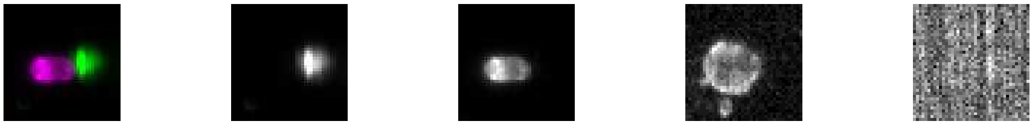				
13496	86	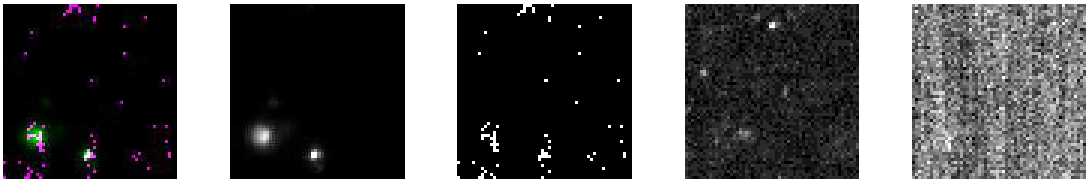				
13497	86	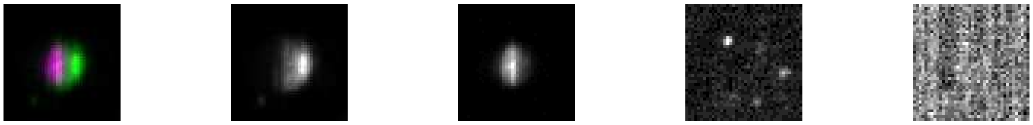				
13498	86	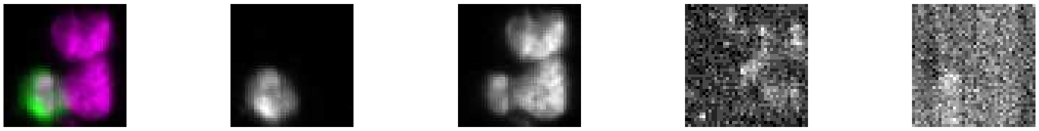				
13499	86	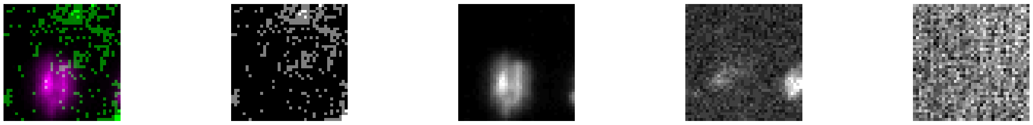				
13500	86	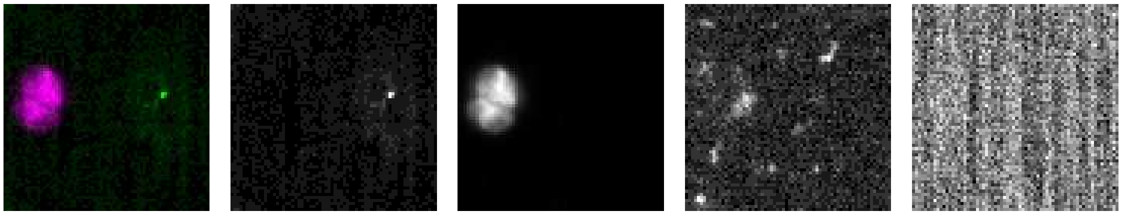				

Ereignis	Aufnahme	DAPI/CK-PE	CK-PE	DAPI	CD45-APC	HER-2/neu
13501	86	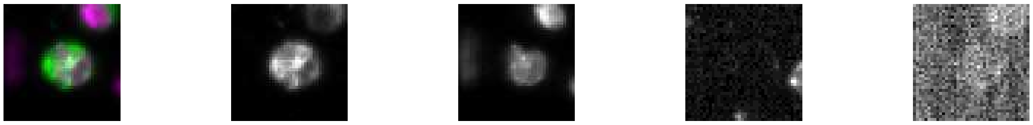				
13502	86	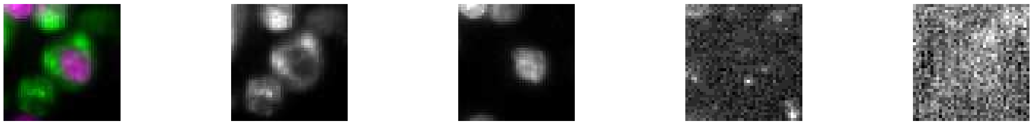				
13503	86	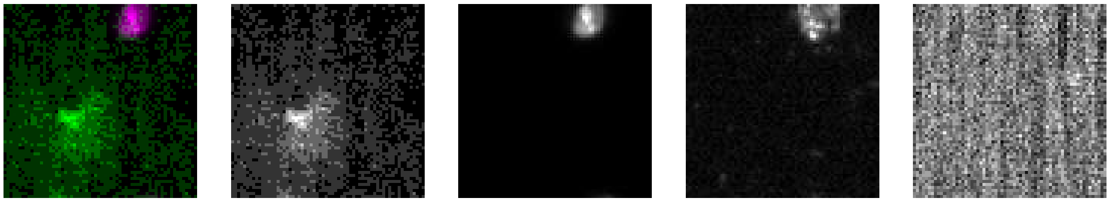				
13504	86	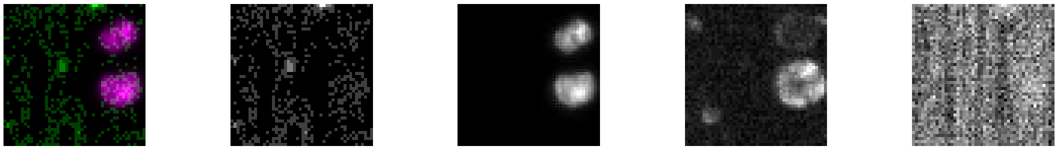				
13505	86	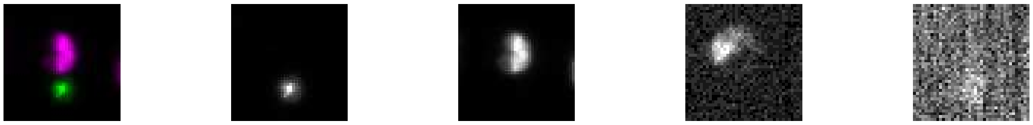				
13506	86	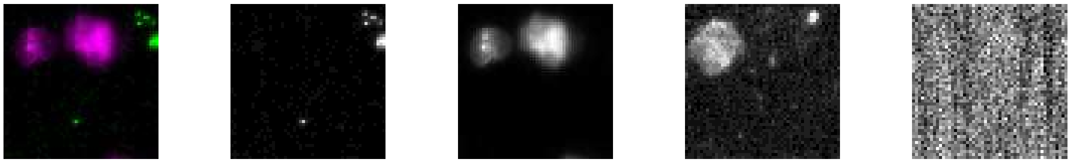				
13507	86	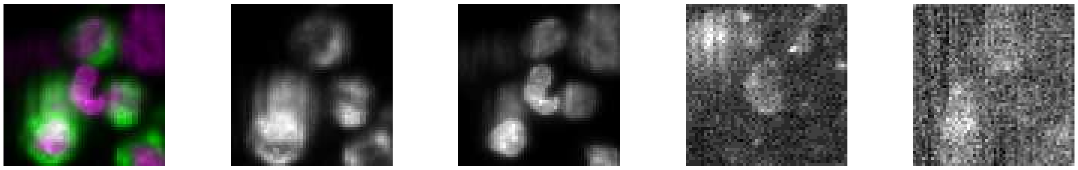				
13508	86	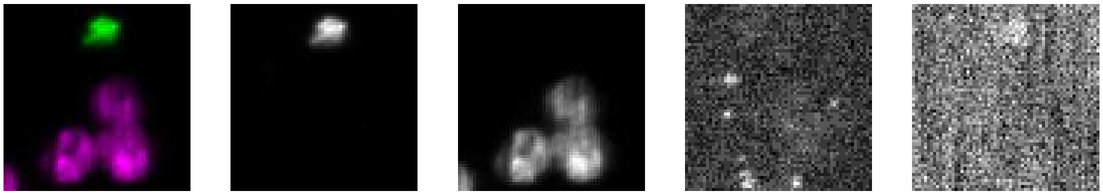				
13509	86	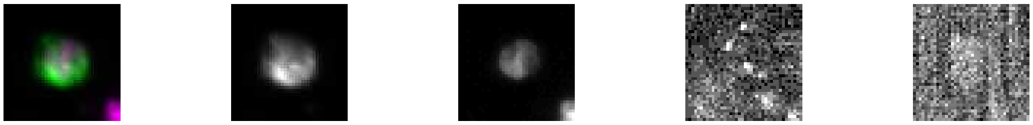				
13510	86	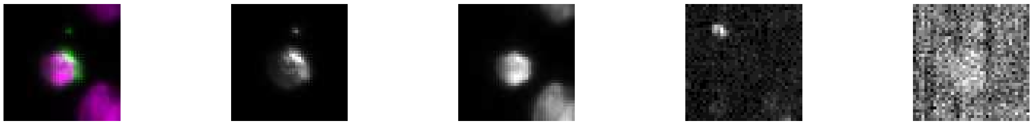				
13511	86	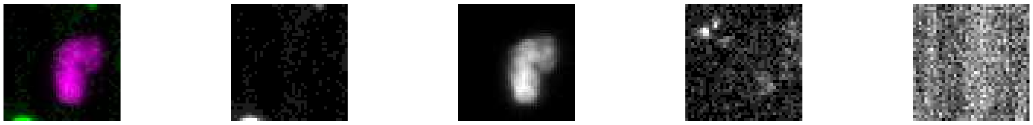				
13512	86	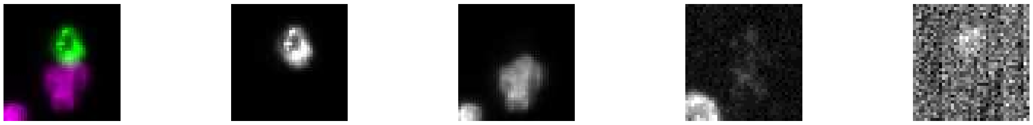				

Proben-ID: Gyn14.208 **Kassetten-ID:** 01327106 **Patienten-ID:**

Ereignis	Aufnahme	DAPI/CK-PE	CK-PE	DAPI	CD45-APC	HER-2/neu
13513	86	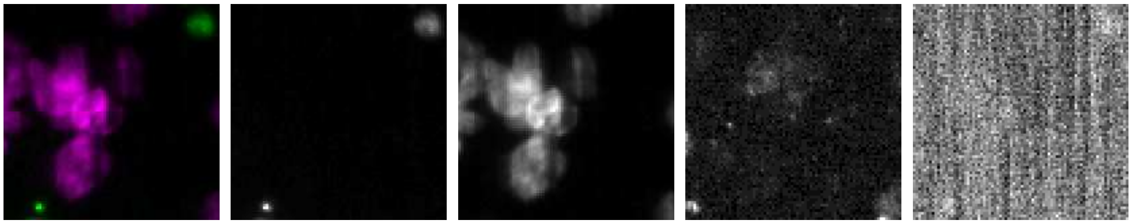				
13514	86	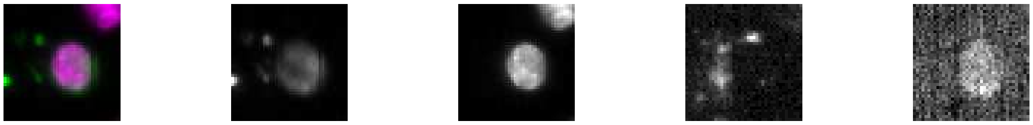				
13515	86	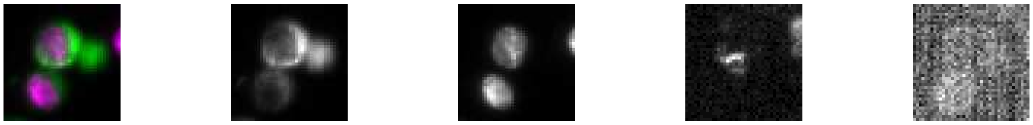				
13516	86	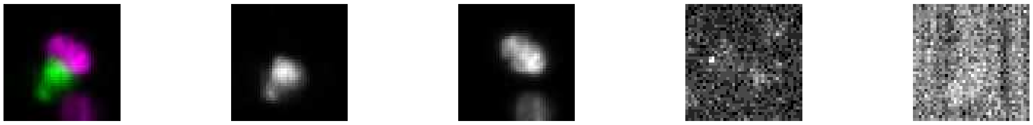				
13517	86	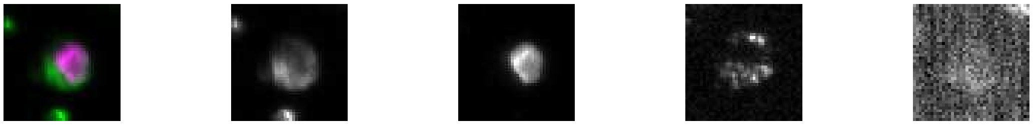				
13518	86	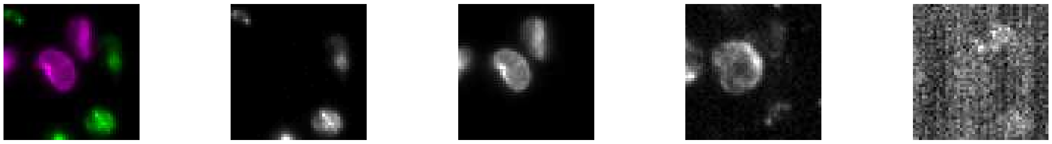				
13519	86	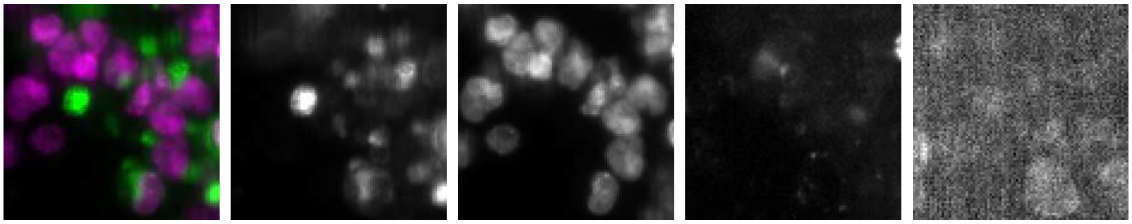				
13520	86	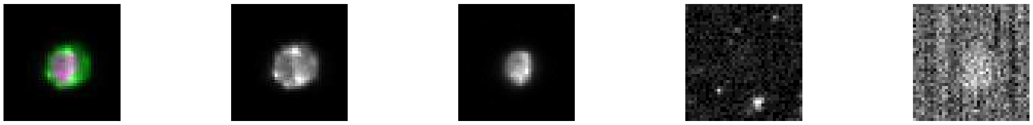				
13521	86	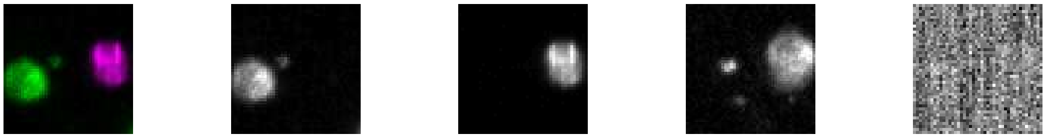				
13522	86	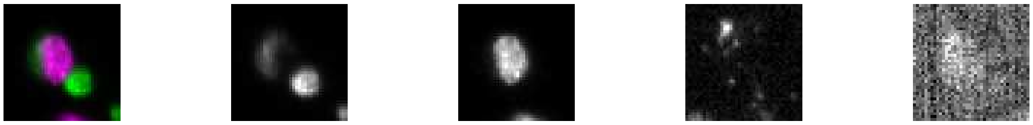				
13523	86	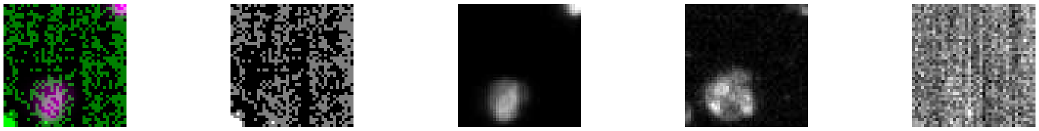				
13524	86	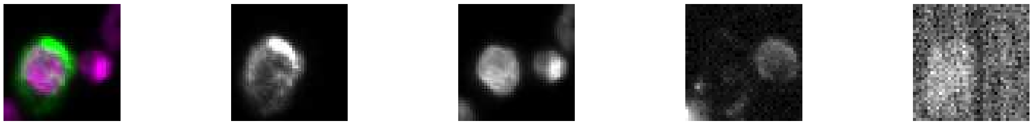				

Ereignis	Aufnahme	DAPI/CK-PE	CK-PE	DAPI	CD45-APC	HER-2/neu
13525	86	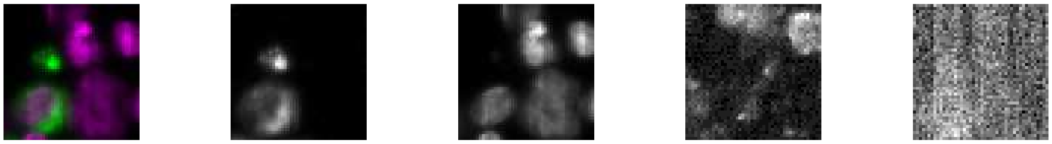				
13526	86	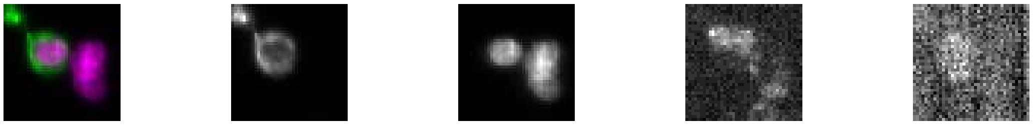				
13527	86	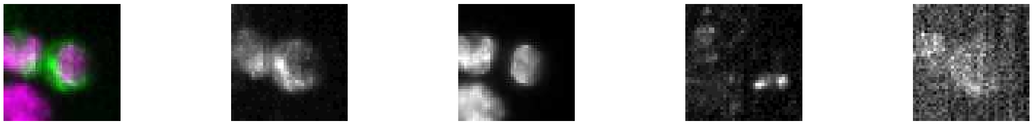				
13528	86	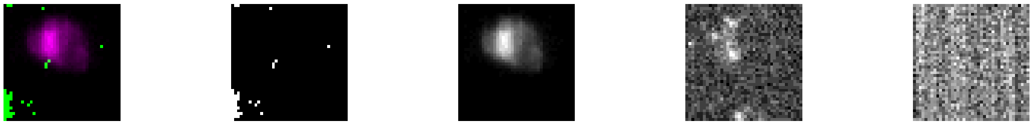				
13529	86	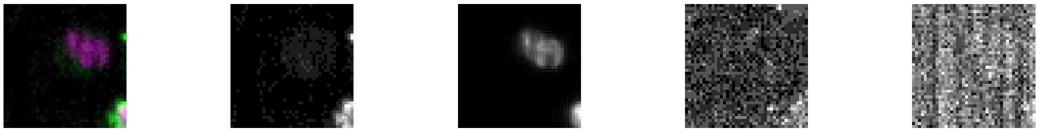				
13530	86	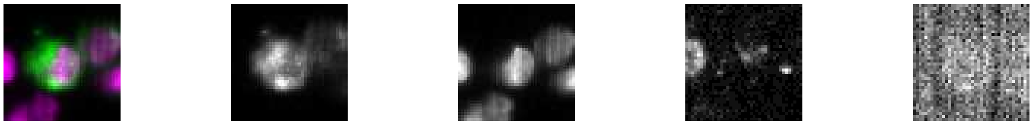				
13531	86	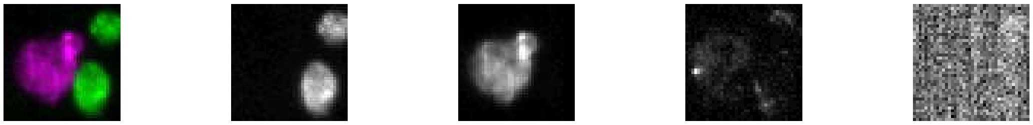				
13532	86	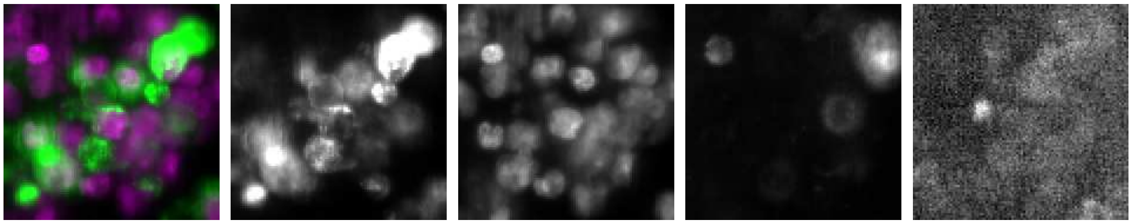				
13533	86	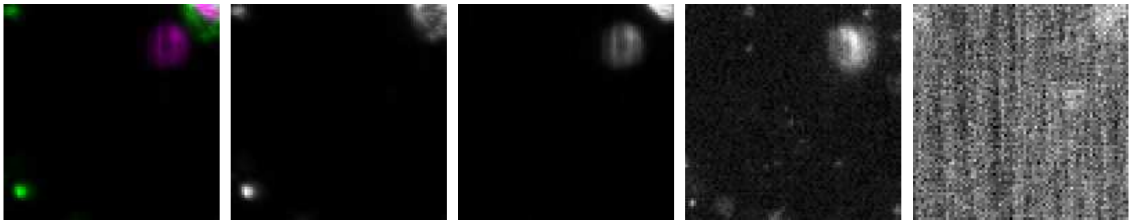				
13534	86	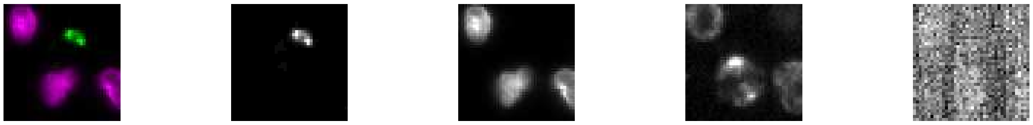				
13535	86	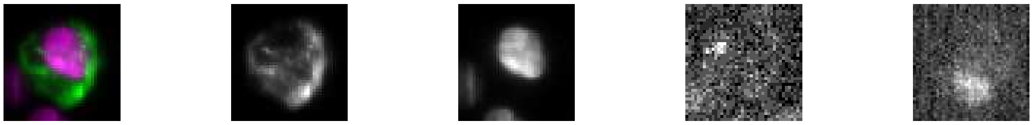				
13536	86	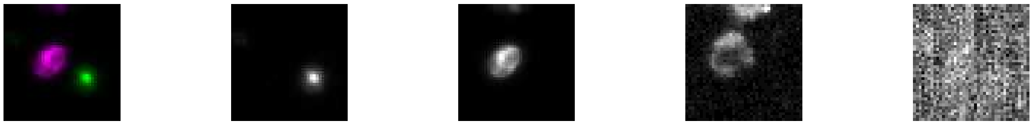				

Ereignis	Aufnahme	DAPI/CK-PE	CK-PE	DAPI	CD45-APC	HER-2/neu
13537	86	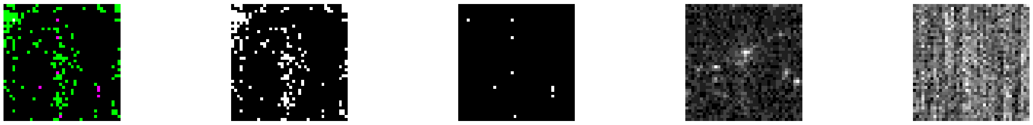				
13538	86	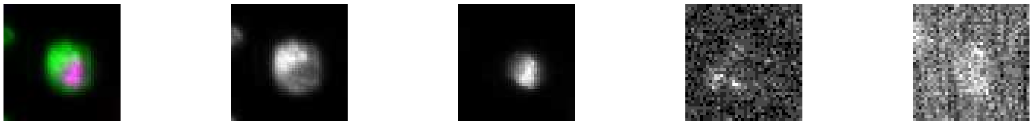				
13539	86	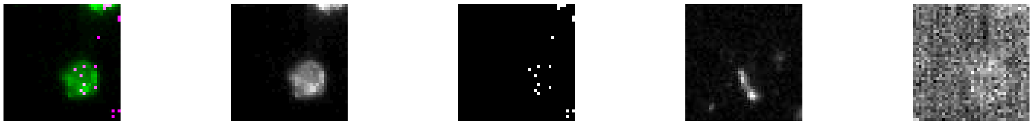				
13540	86	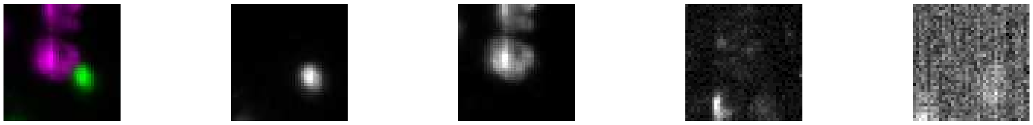				
13541	86	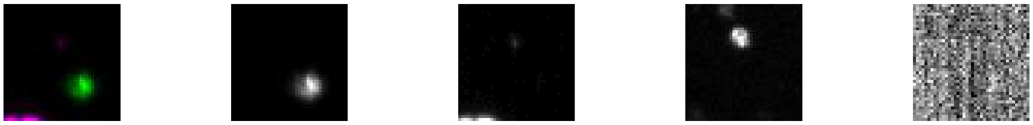				
13542	86	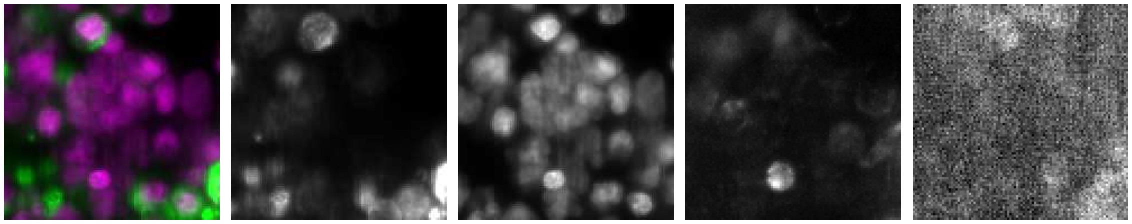				
13543	86	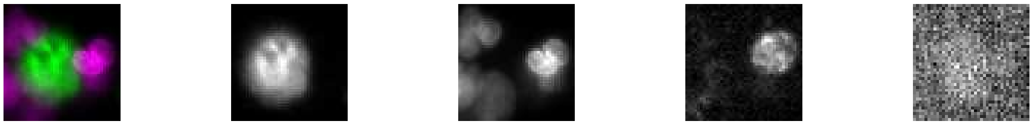				
13544	86	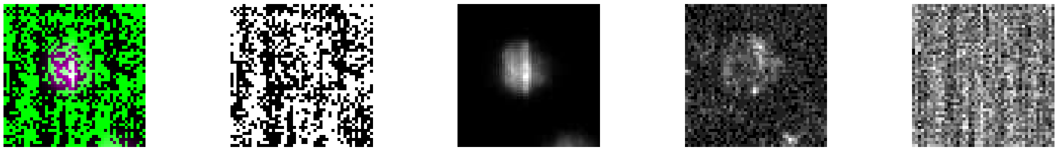				
13545	86	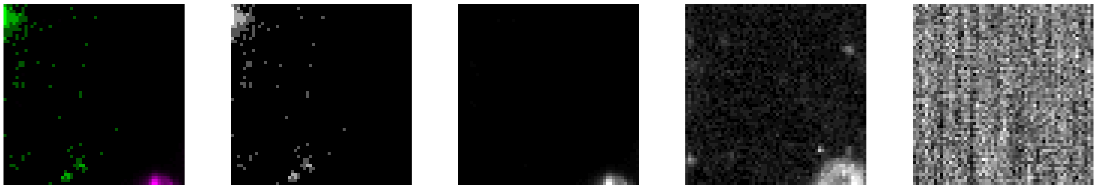				
13546	86	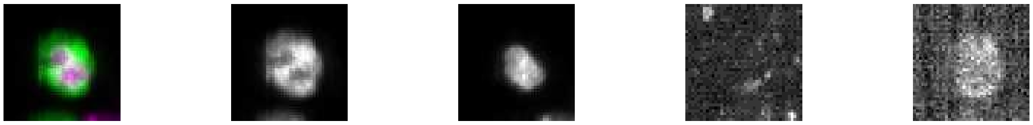				
13547	86	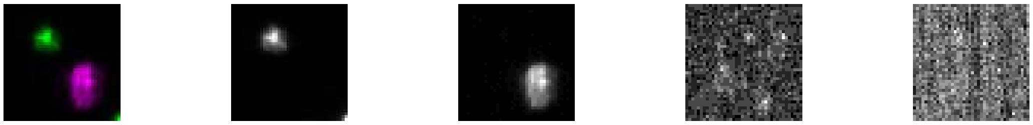				
13548	86	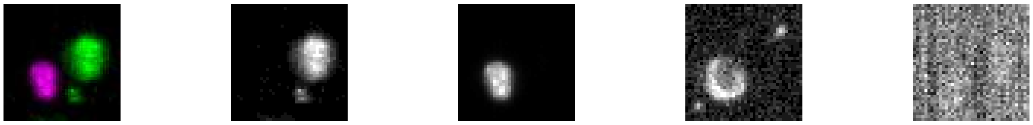				

Ereignis	Aufnahme	DAPI/CK-PE	CK-PE	DAPI	CD45-APC	HER-2/neu
13549	86	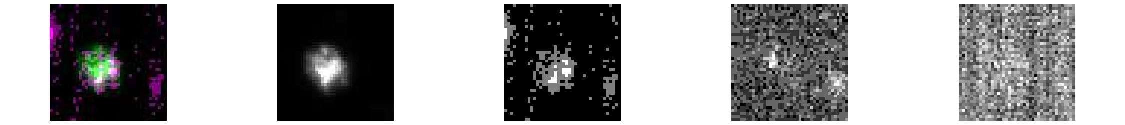				
13550	86	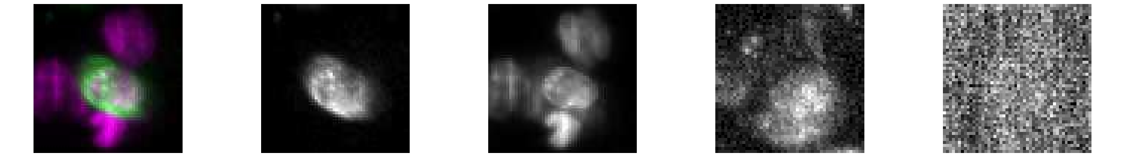				
13551	86	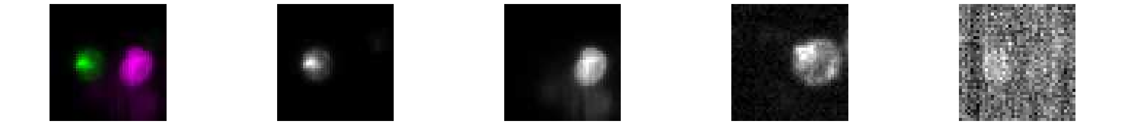				
13552	86	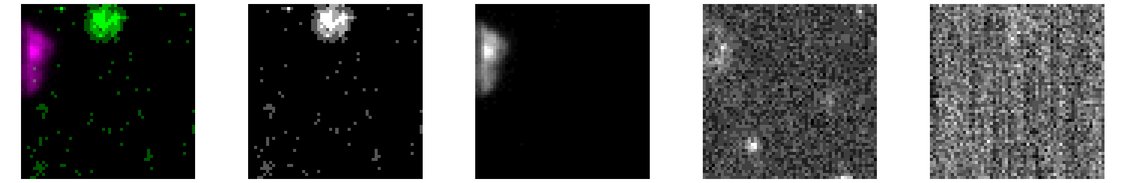				
13553	86	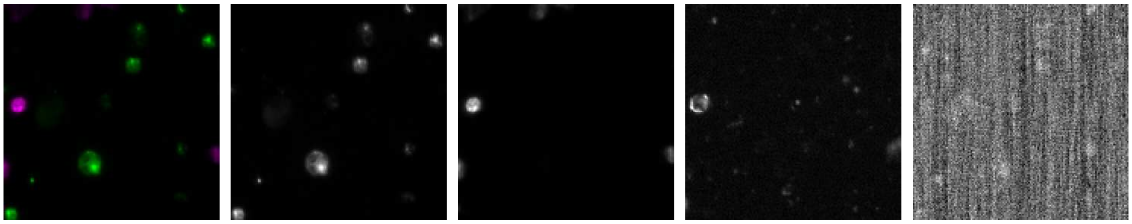				
13554	86	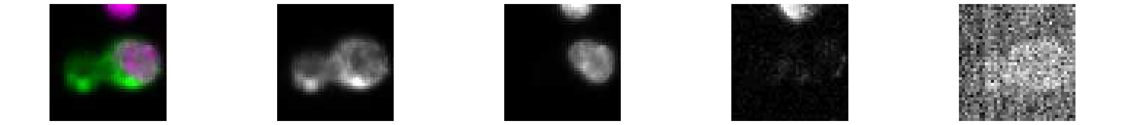				
13555	86	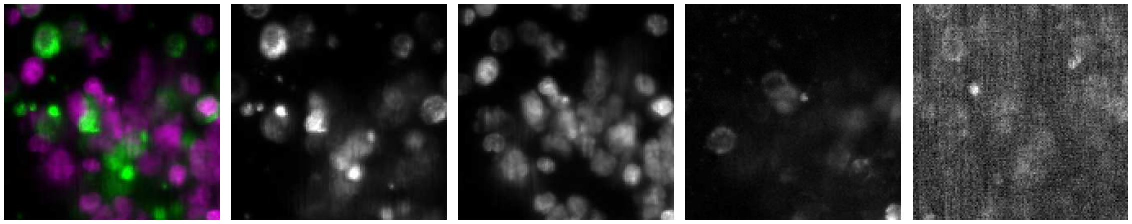				
13556	86	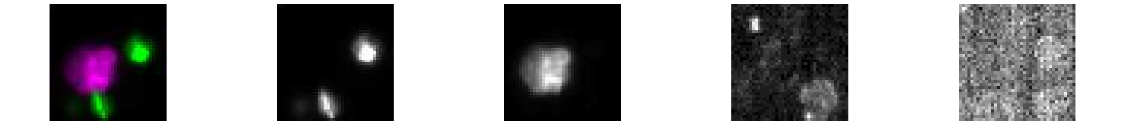				
13557	86	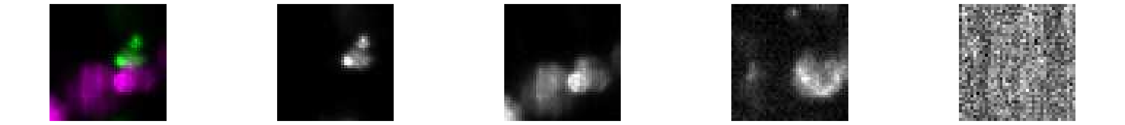				
13558	86	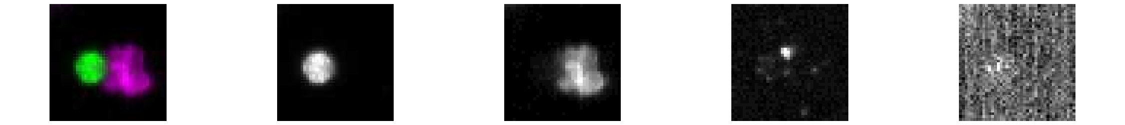				
13559	86	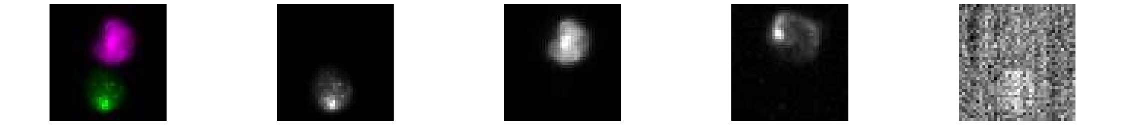				
13560	86	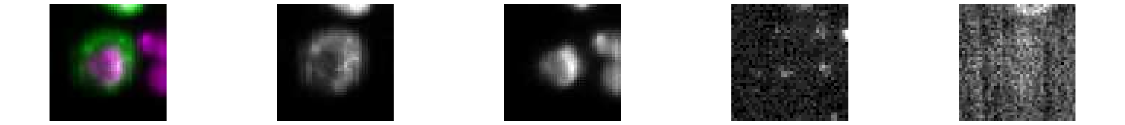				

Ereignis	Aufnahme	DAPI/CK-PE	CK-PE	DAPI	CD45-APC	HER-2/neu
13561	86	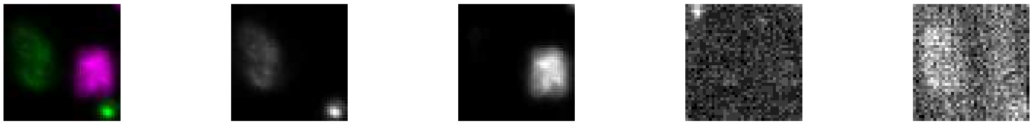				
13562	86	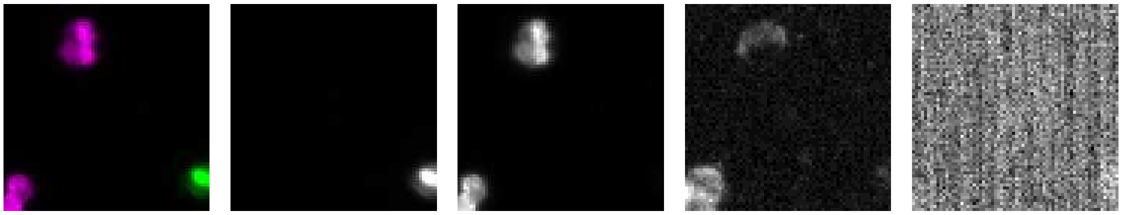				
13563	86	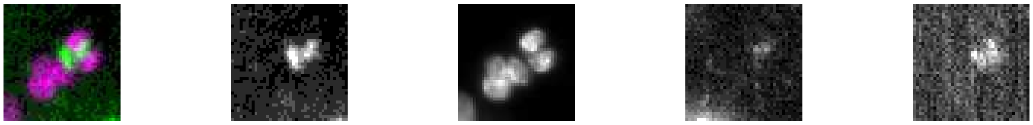				
13564	86	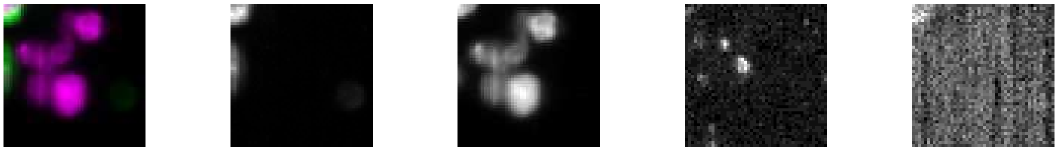				
13565	86	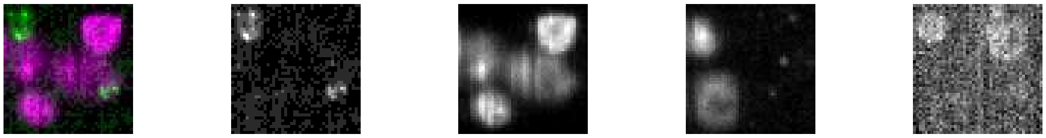				
13566	86	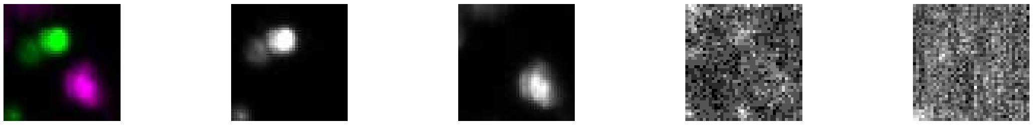				
13567	86	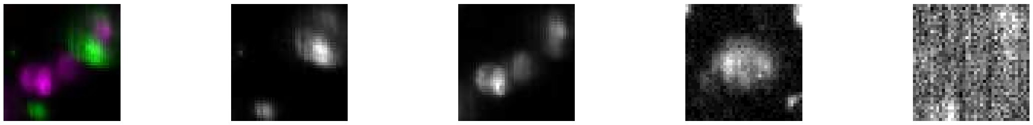				
13568	86	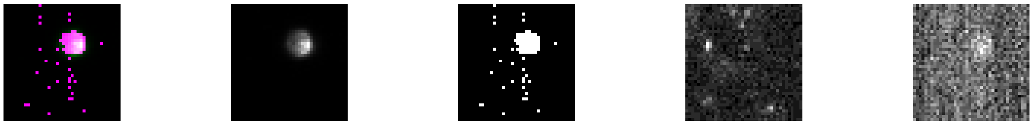				
13569	86	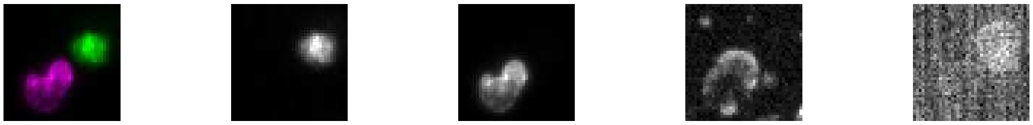				
13570	86	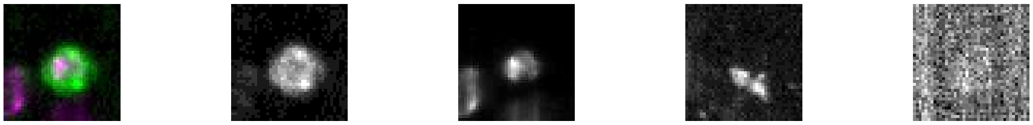				
13571	86	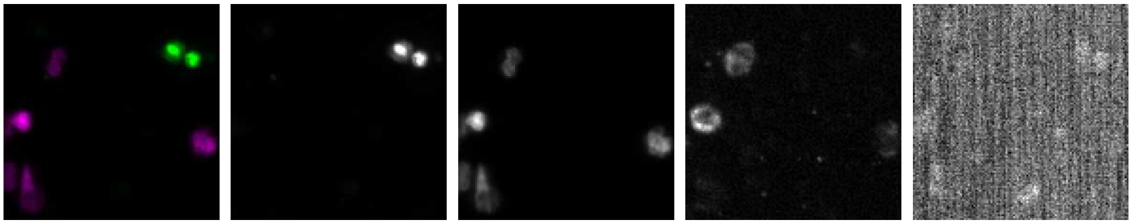				
13572	86	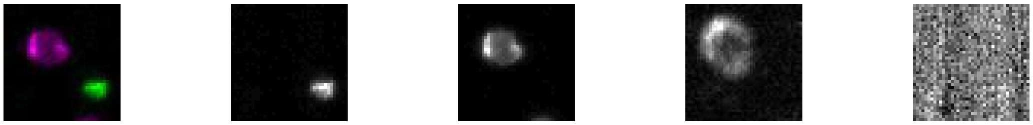				

Druckdatum: 02/06/2020 17:00

Proben-ID: Gyn14.208 **Kassetten-ID:** 01327106 **Patienten-ID:**

Ereignis	Aufnahme	DAPI/CK-PE	CK-PE	DAPI	CD45-APC	HER-2/neu
13573	86	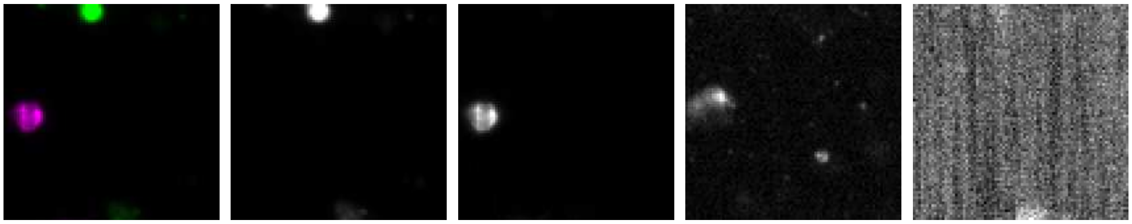				
13574	86	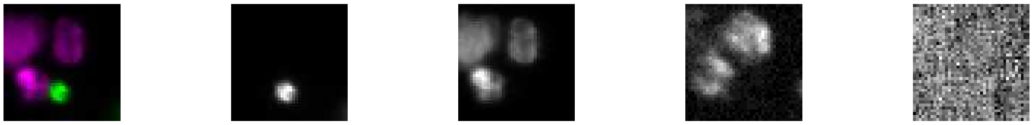				
13575	86	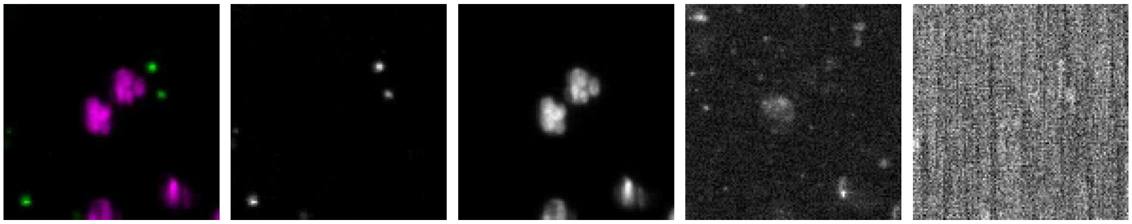				
13576	86	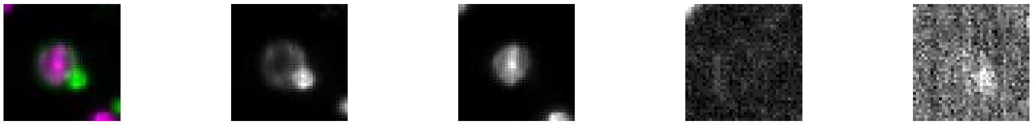				
13577	86	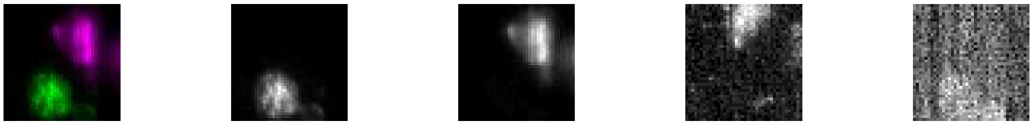				
13578	86	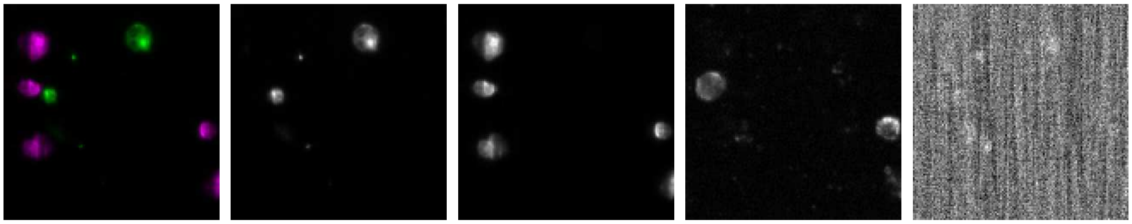				
13579	86	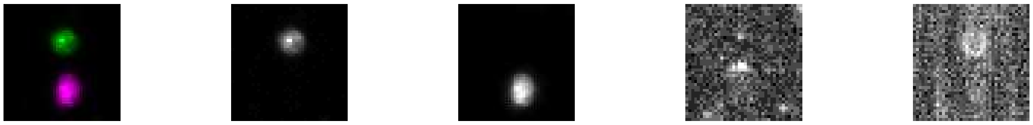				
13580	86	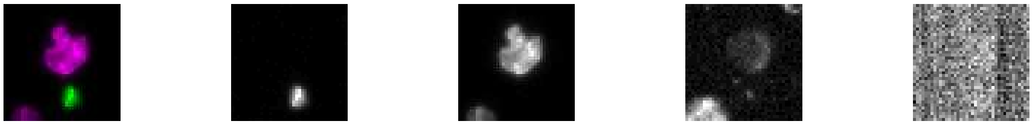				
13581	86	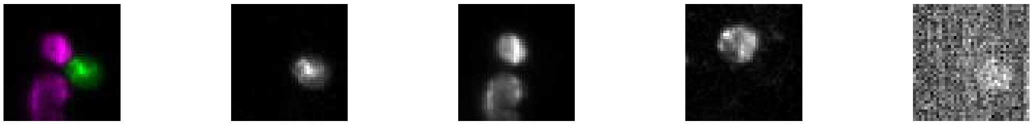				
13582	86	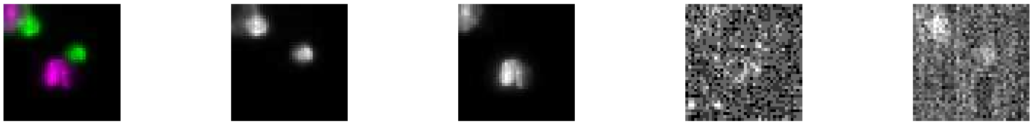				
13583	86	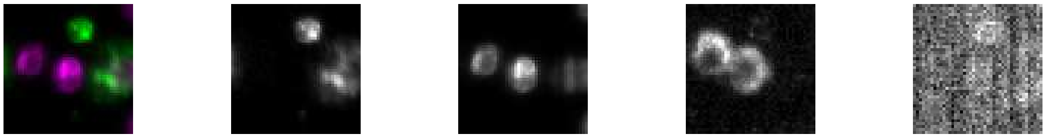				
13584	86	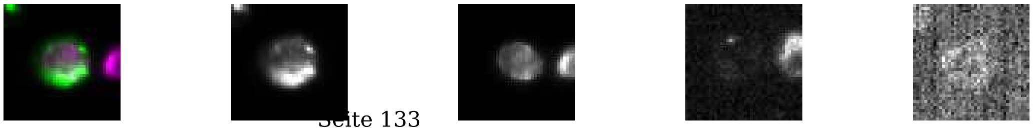				

Ereignis	Aufnahme	DAPI/CK-PE	CK-PE	DAPI	CD45-APC	HER-2/neu
13585	86	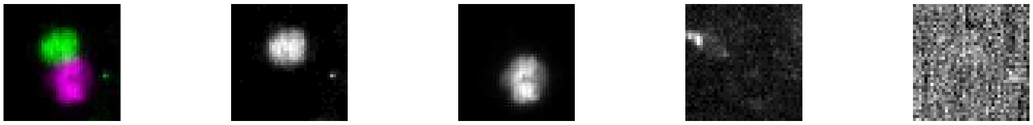				
13586	86	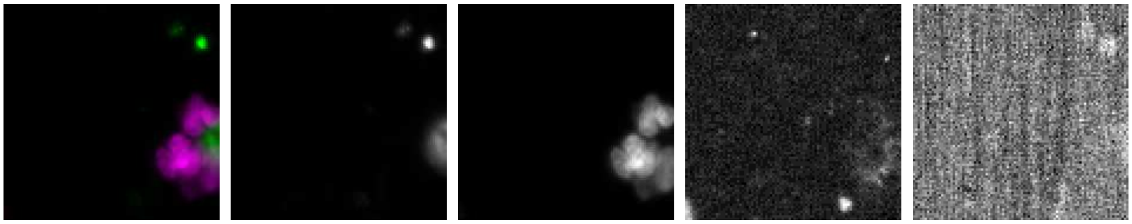				
13587	86	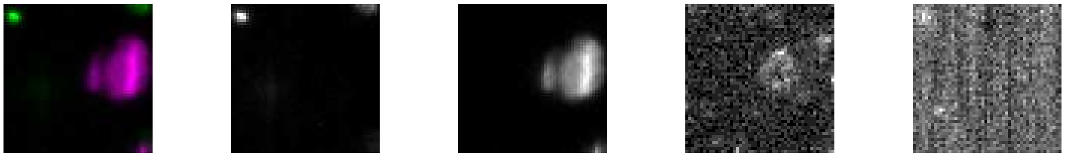				
13588	86	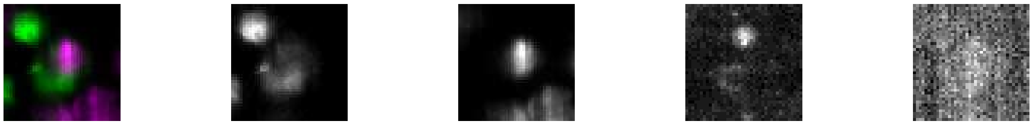				
13589	86	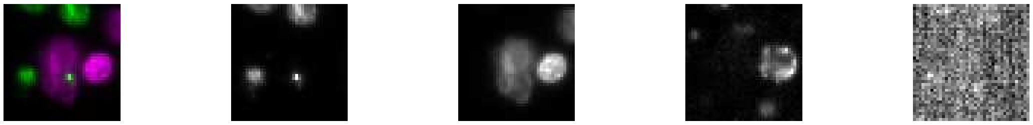				
13590	86	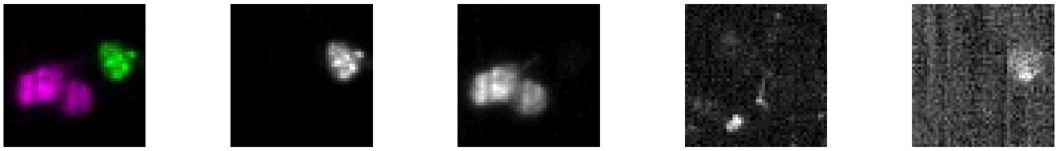				
13591	86	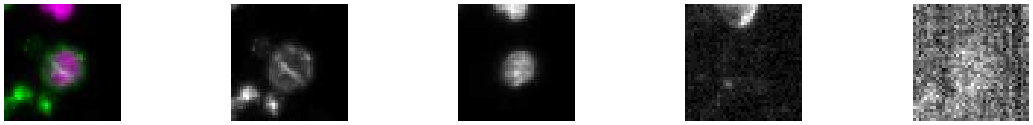				
13592	86	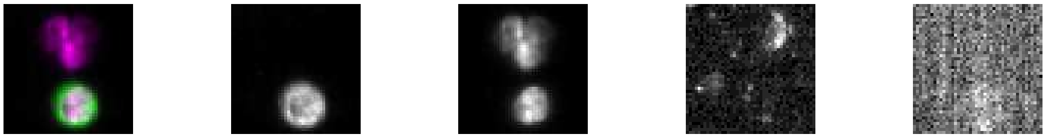				
13593	86	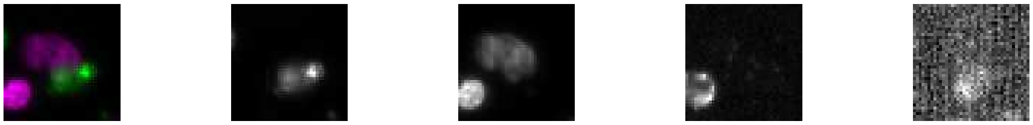				
13594	86	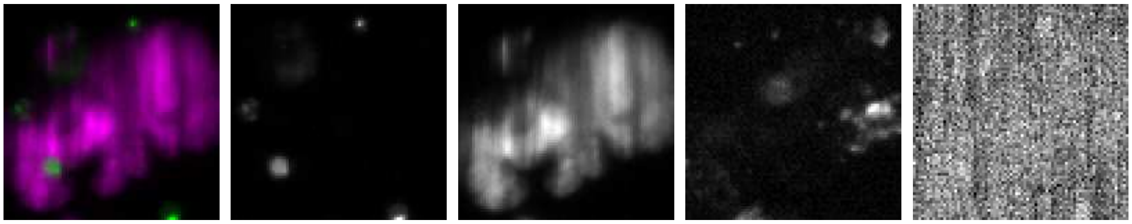				
13595	86	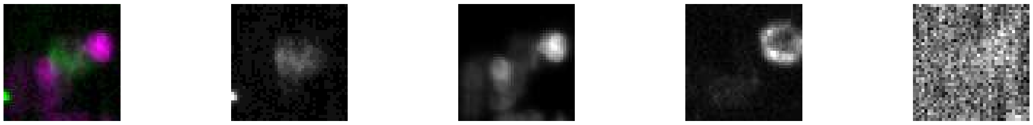				
13596	86	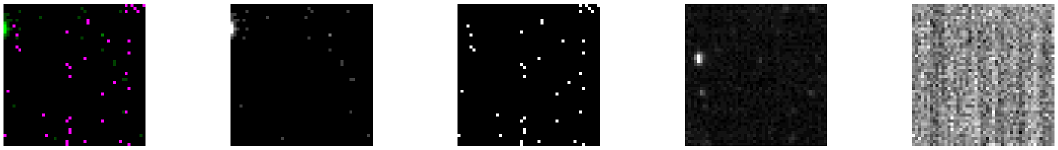				

Ereignis	Aufnahme	DAPI/CK-PE	CK-PE	DAPI	CD45-APC	HER-2/neu
13597	86	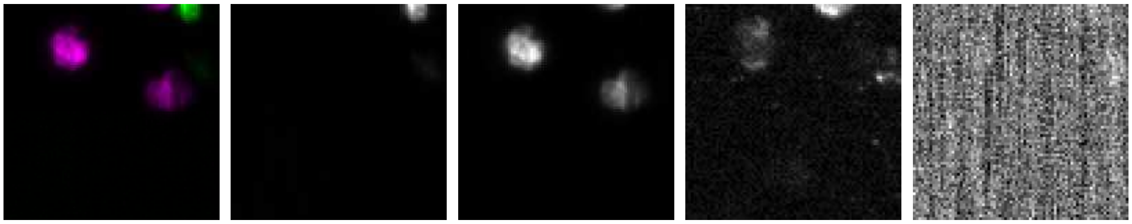				
13598	86	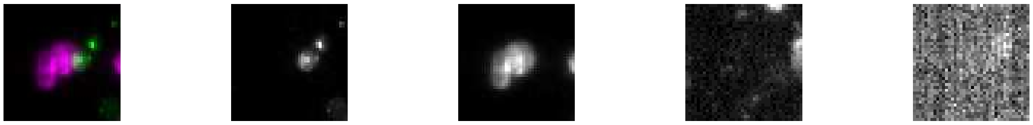				
13599	86	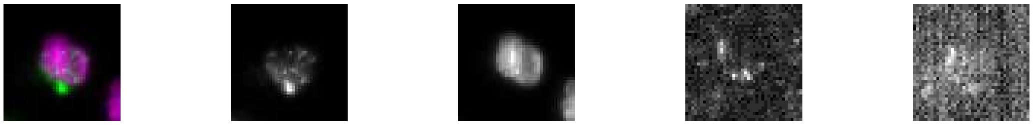				
13600	86	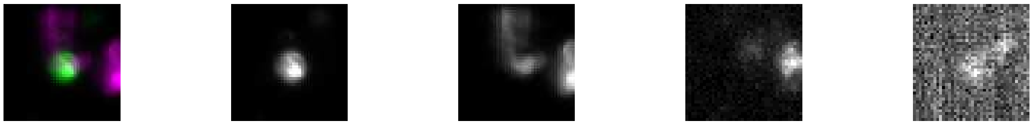				
13601	86	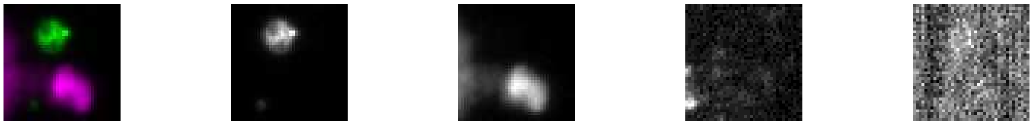				
13602	86	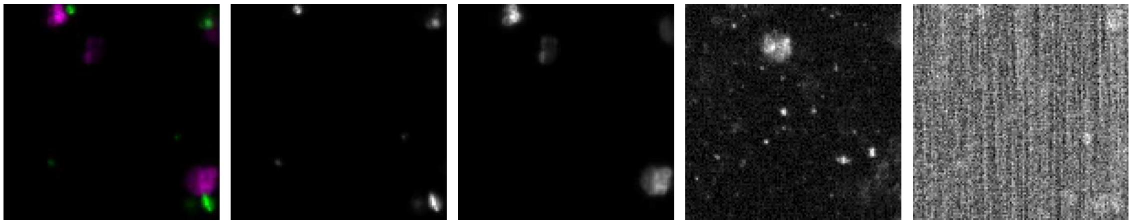				
13603	86	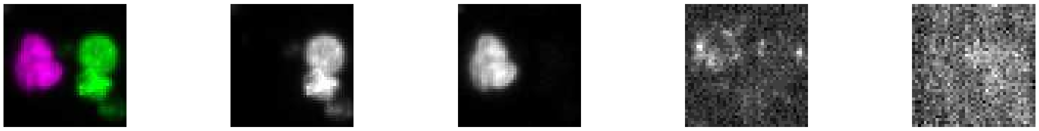				
13604	86	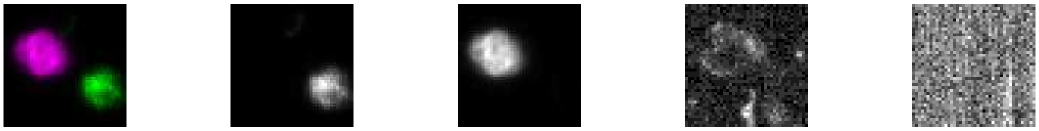				
13605	86	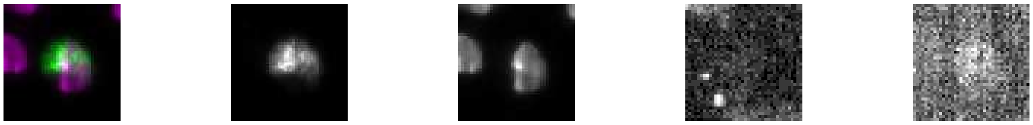				
13606	86	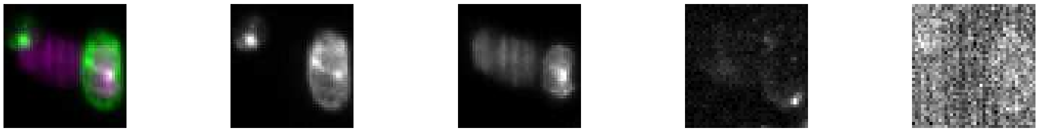				
13607	86	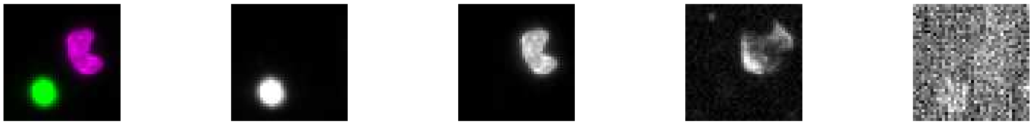				
13608	86	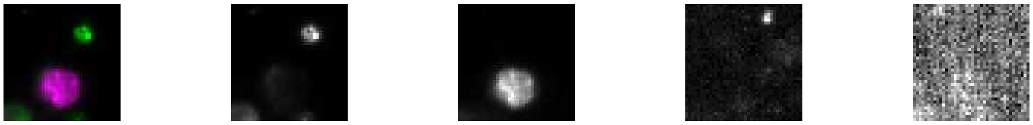				

Proben-ID: Gyn14.208 **Kassetten-ID:**01327106 **Patienten-ID:**

Ereignis	Aufnahme	DAPI/CK-PE	CK-PE	DAPI	CD45-APC	HER-2/neu
13609	86	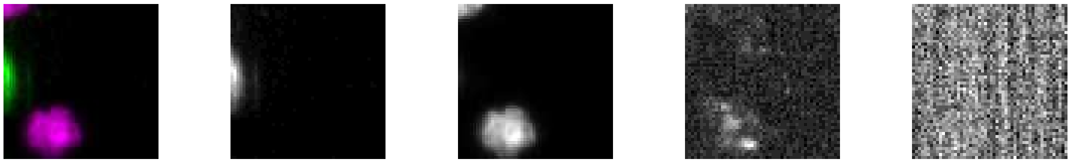				
13610	86	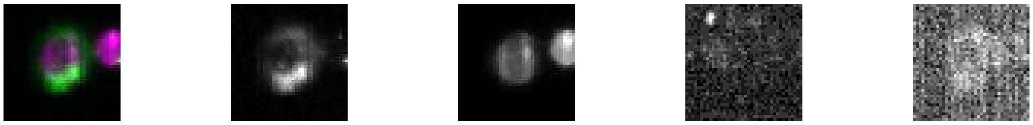				
13611	86	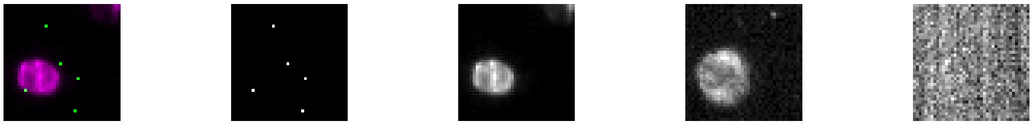				
13612	86	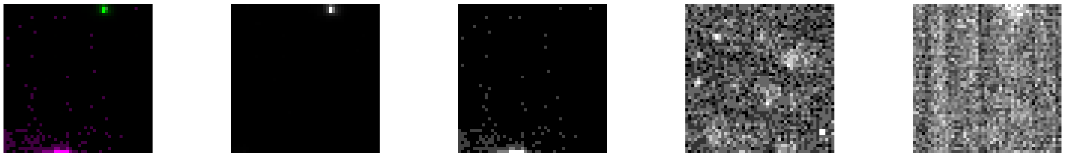				
13613	86	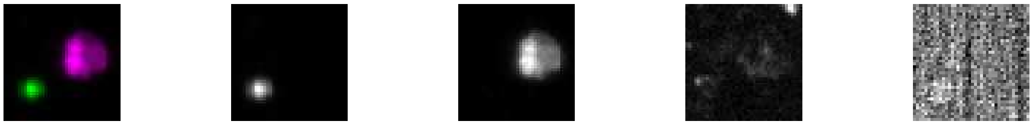				
13614	86	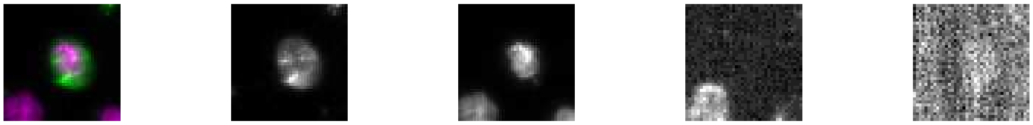				
13615	86	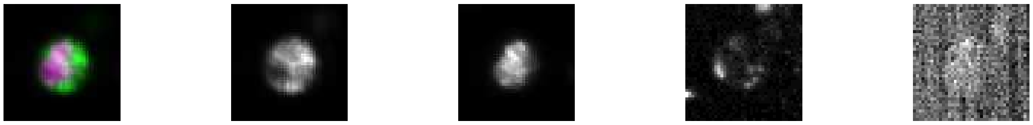				
13616	86	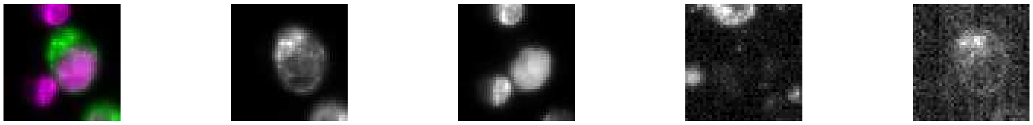				
13617	86	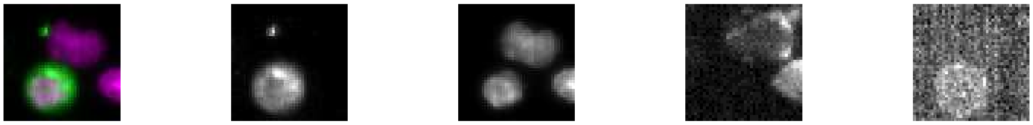				
13618	86	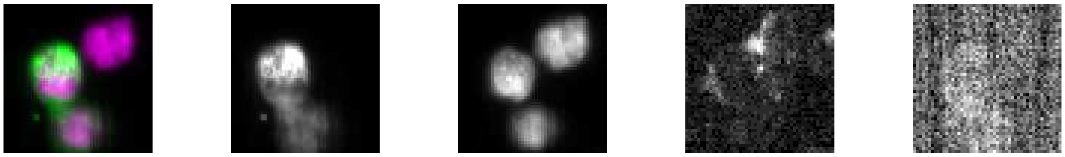				
13619	86	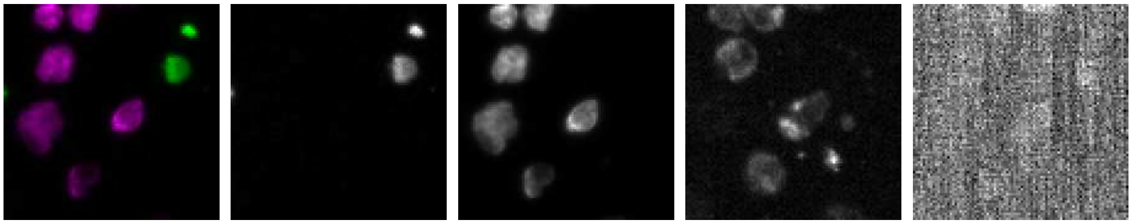				
13620	86	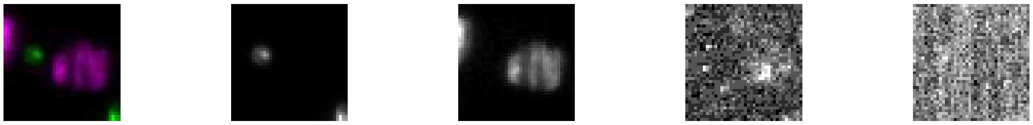				

Proben-ID: Gyn14.208 **Kassetten-ID:** 01327106 **Patienten-ID:**

Ereignis	Aufnahme	DAPI/CK-PE	CK-PE	DAPI	CD45-APC	HER-2/neu
13621	86	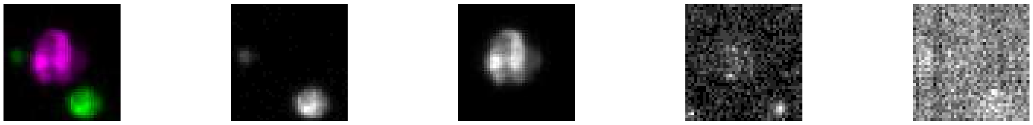				
13622	86	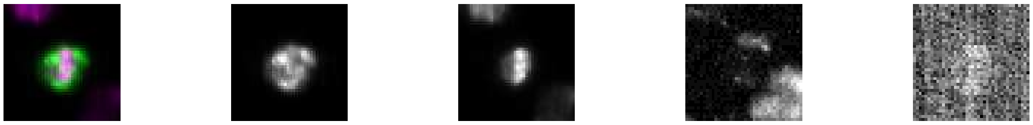				
13623	86	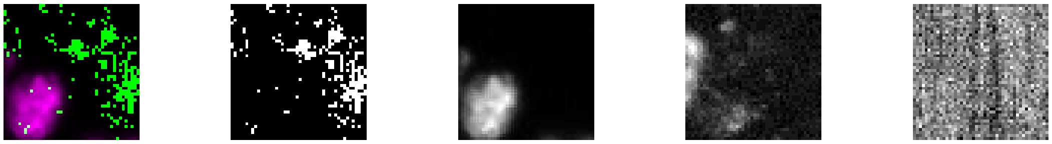				
13624	86	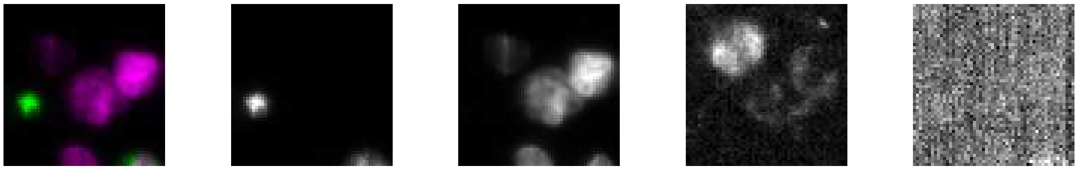				
13625	86	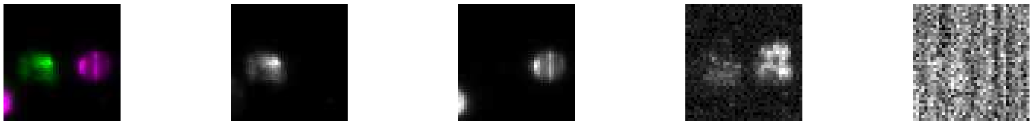				
13626	86	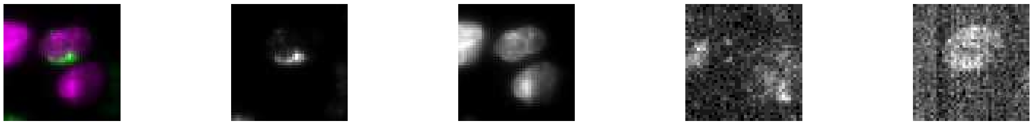				
13627	86	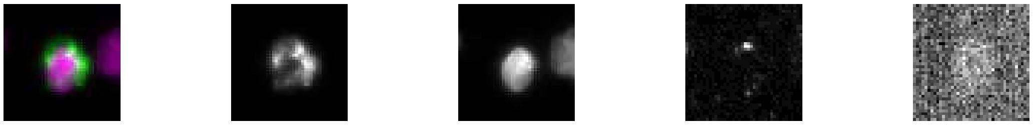				
13628	86	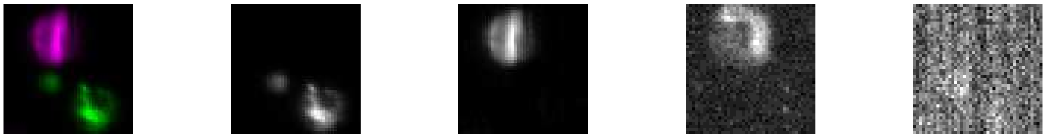				
13629	86	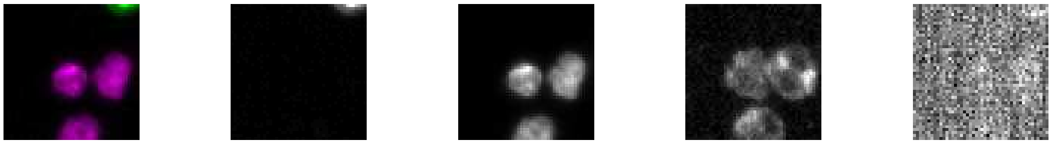				
13630	86	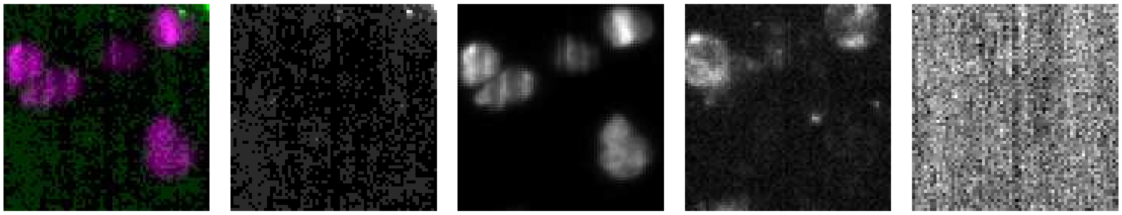				
13631	86	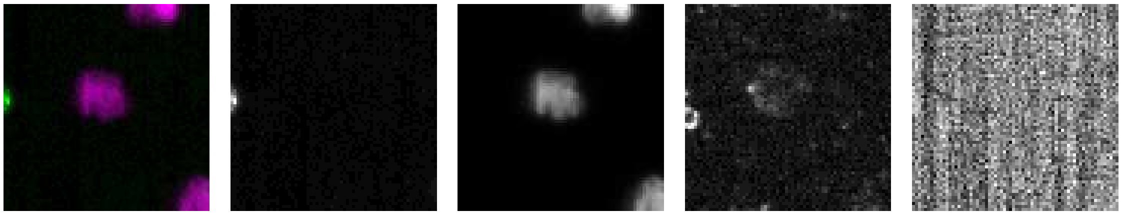				

Ereignis	Aufnahme	DAPI/CK-PE	CK-PE	DAPI	CD45-APC	HER-2/neu
13632	86	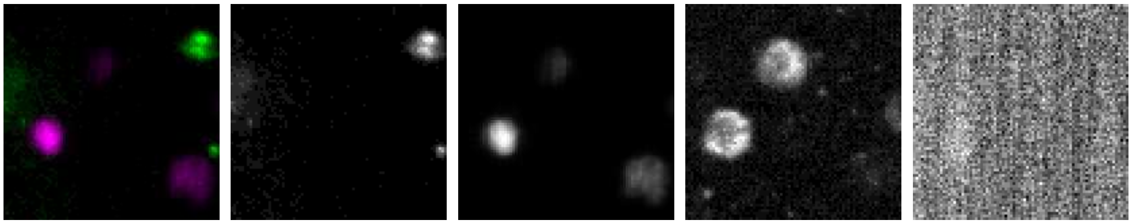				
13633	86	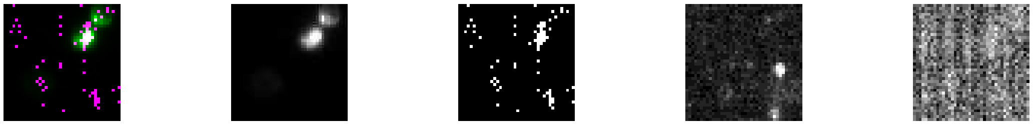				
13634	86	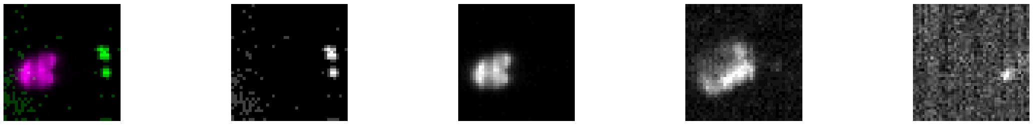				
13635	86	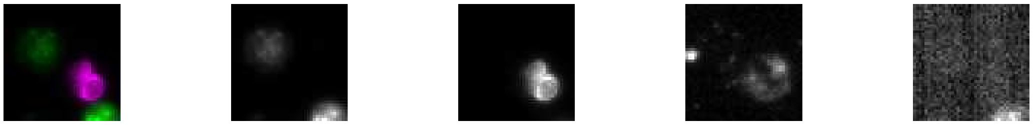				
13636	86	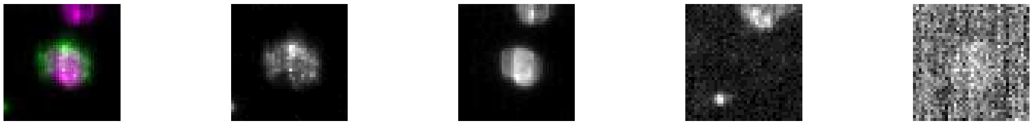				
13637	86	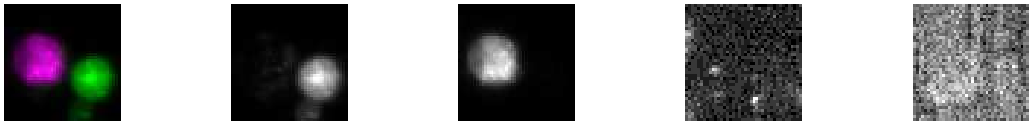				
13638	86	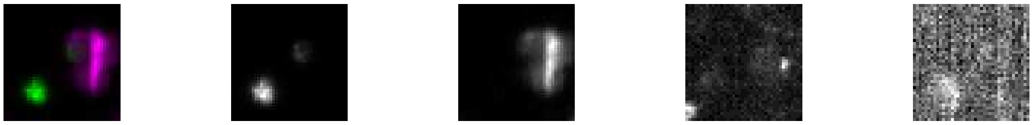				
13639	86	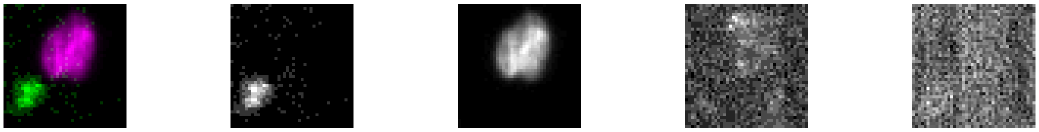				
13640	86	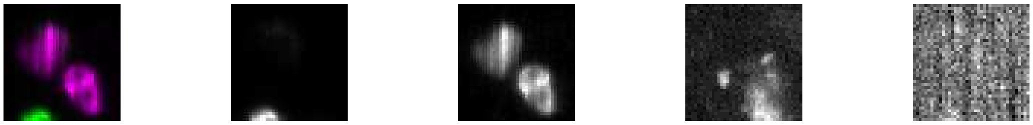				
13641	86	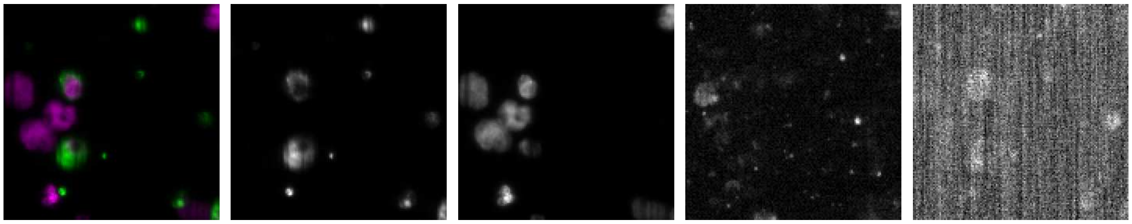				
13642	86	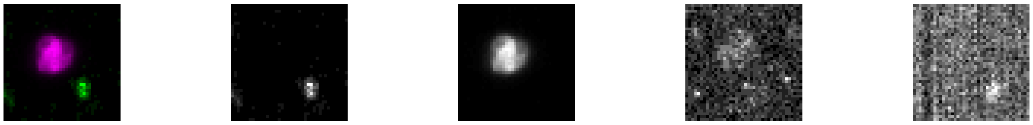				
13643	86	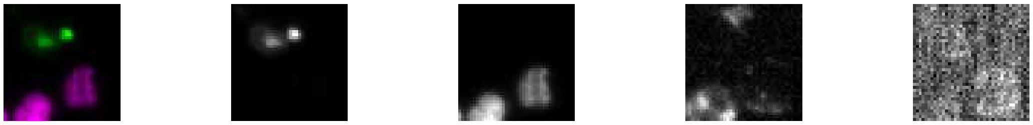				

Ereignis	Aufnahme	DAPI/CK-PE	CK-PE	DAPI	CD45-APC	HER-2/neu
13644	86	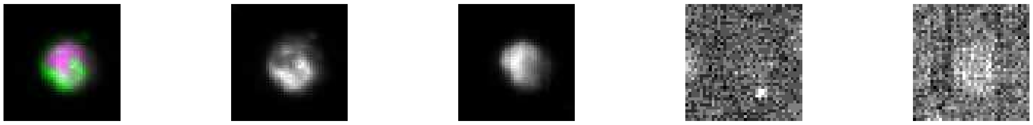				
13645	86	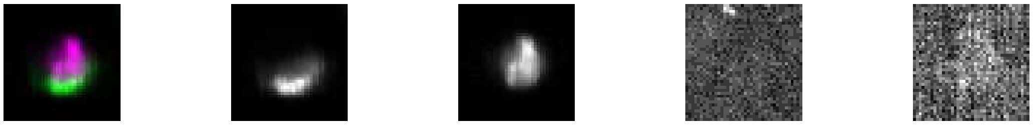				
13646	86	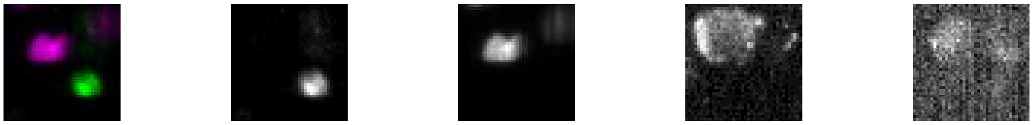				
13647	86	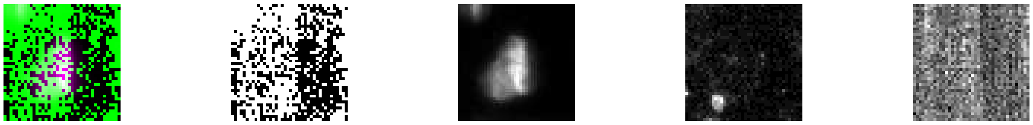				
13648	86	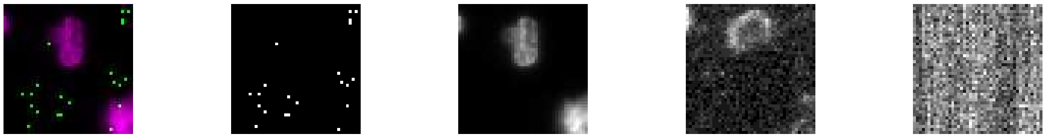				
13649	86	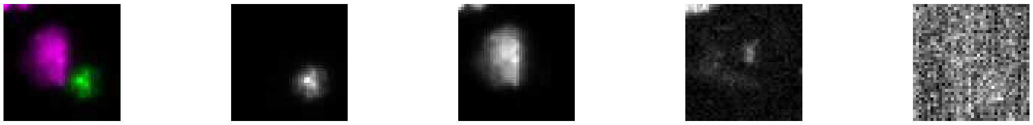				
13650	86	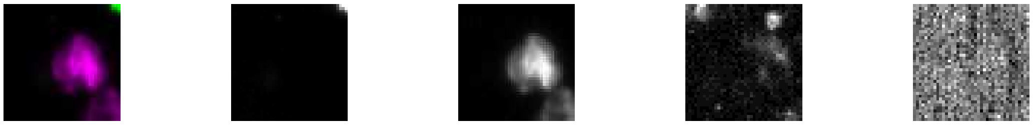				
13651	86	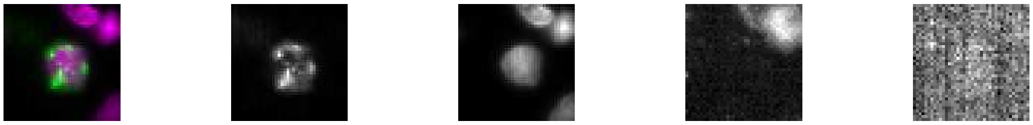				
13652	86	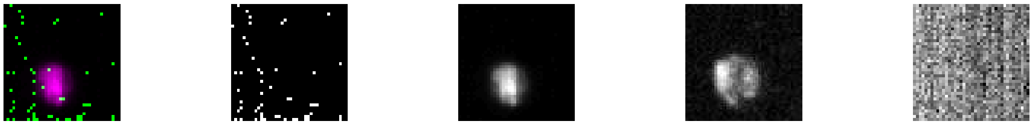				
13653	86	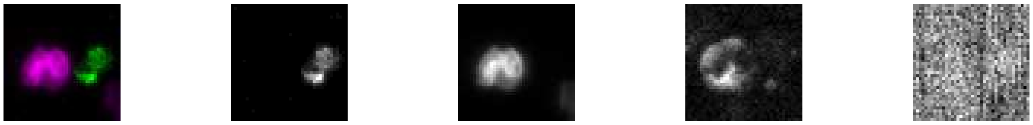				
13654	86	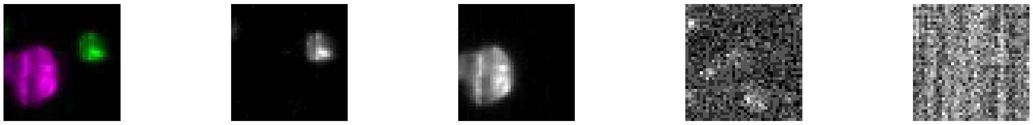				
13655	86	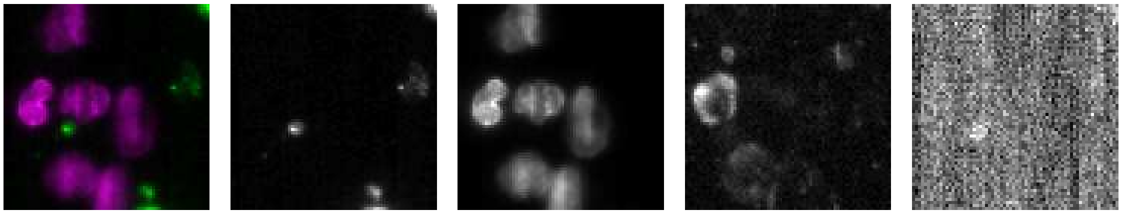				
13656	86	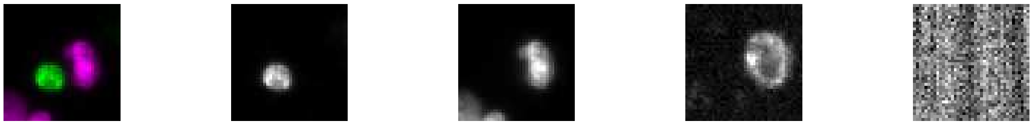				

Ereignis	Aufnahme	DAPI/CK-PE	CK-PE	DAPI	CD45-APC	HER-2/neu
13657	86	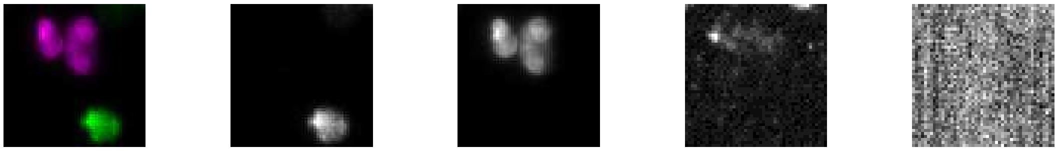				
13658	86	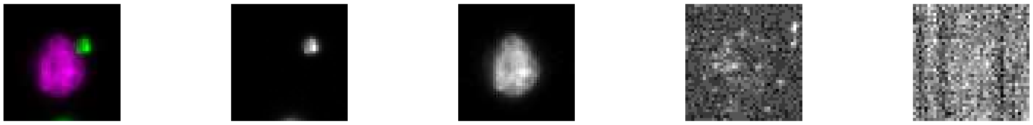				
13659	86	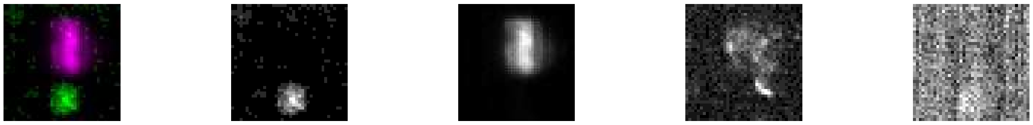				
13660	86	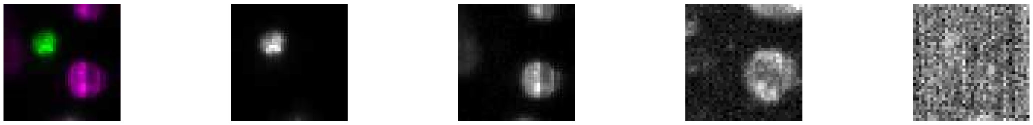				
13661	86	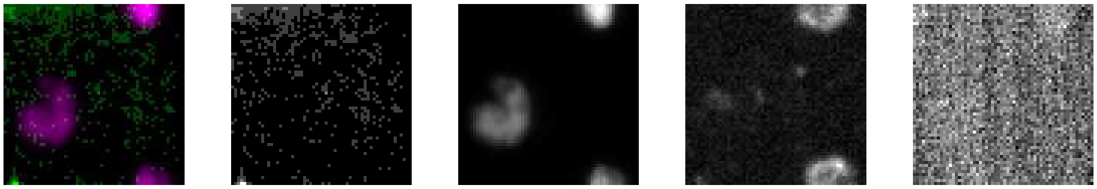				
13662	86	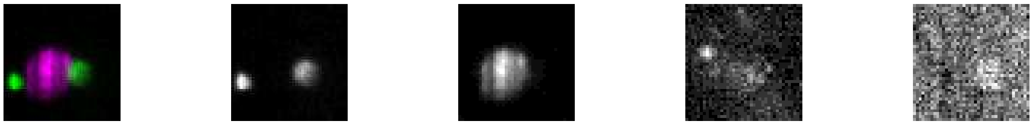				
13663	86	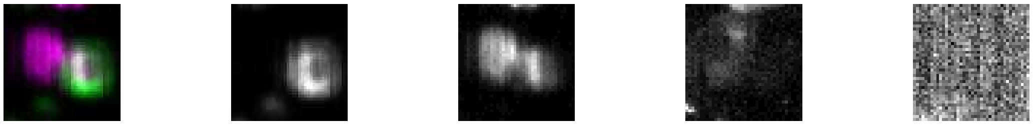				
13664	86	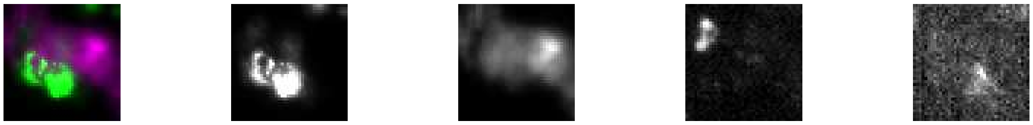				
13665	86	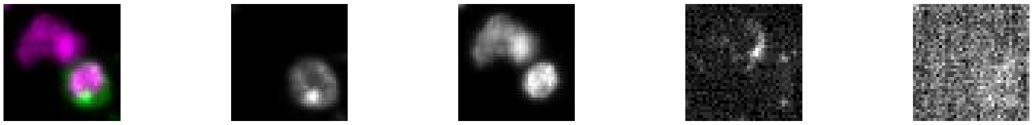				
13666	86	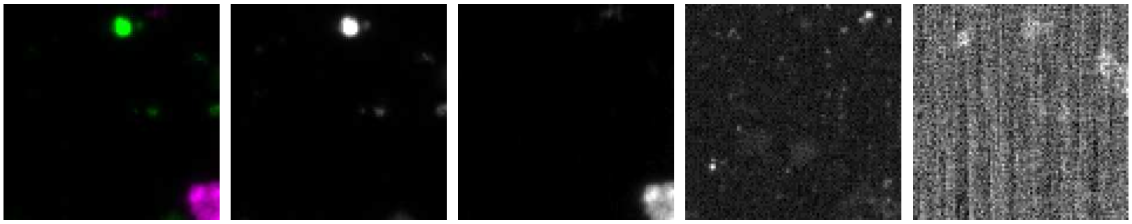				
13667	86	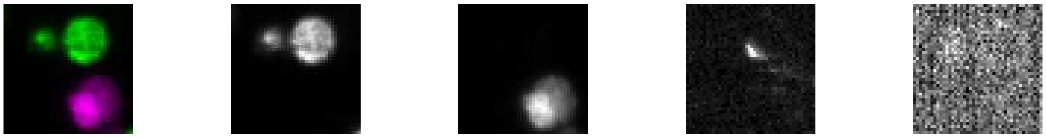				
13668	86	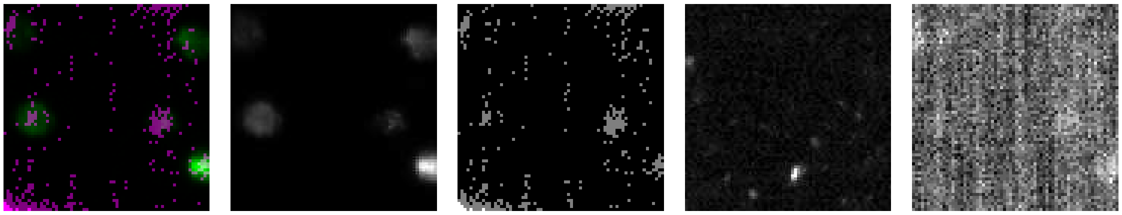				

Ereignis	Aufnahme	DAPI/CK-PE	CK-PE	DAPI	CD45-APC	HER-2/neu
13669	86	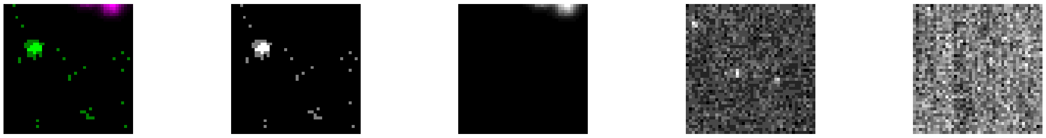				
13670	86	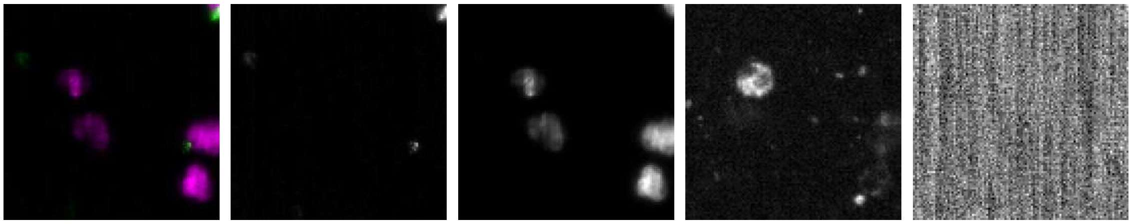				
13671	86	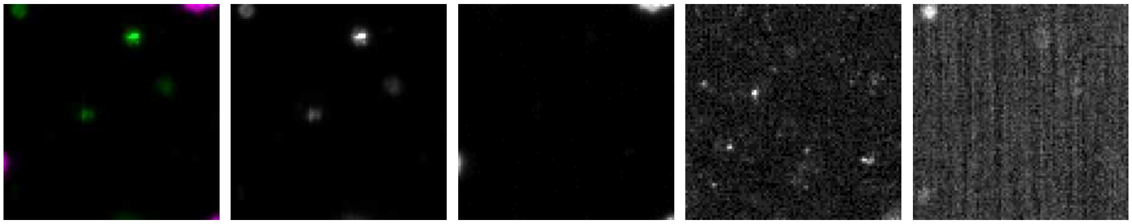				
13672	86	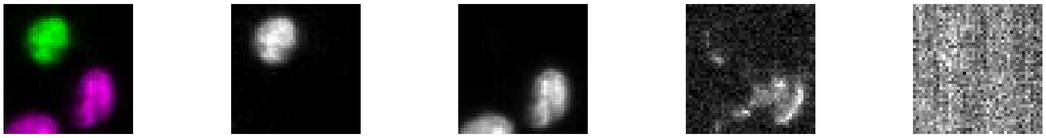				
13673	86	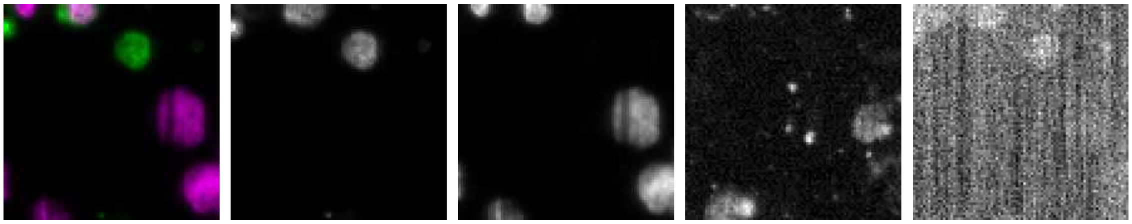				
13674	86	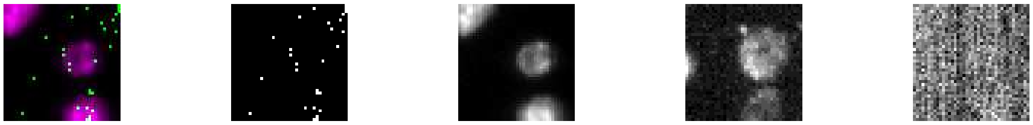				
13675	86	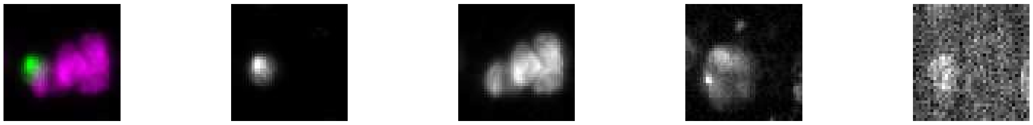				
13676	86	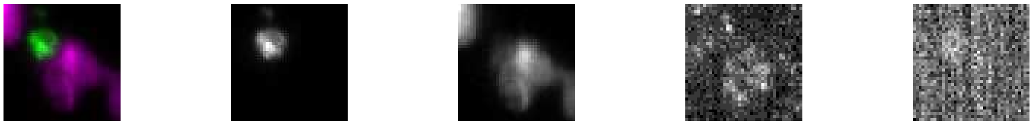				
13677	86	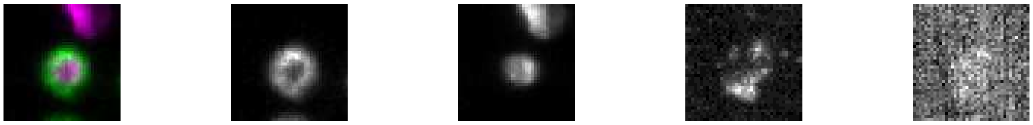				
13678	86	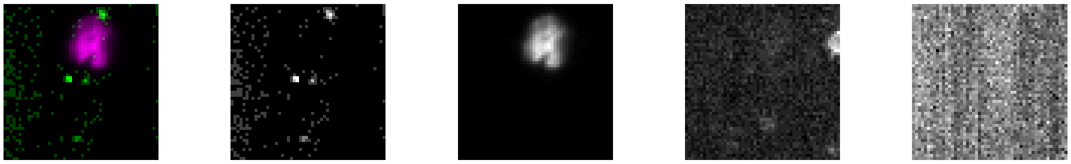				

Ereignis	Aufnahme	DAPI/CK-PE	CK-PE	DAPI	CD45-APC	HER-2/neu
13679	86	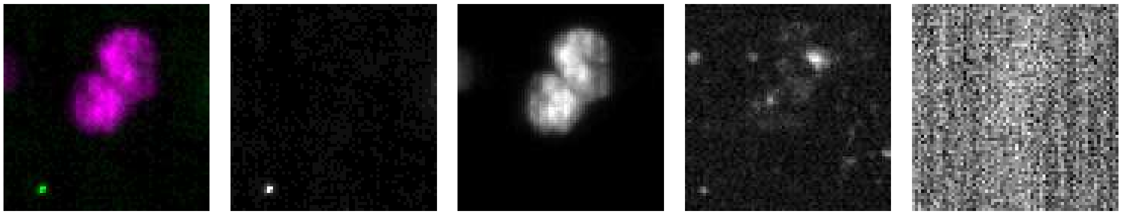				
13680	86	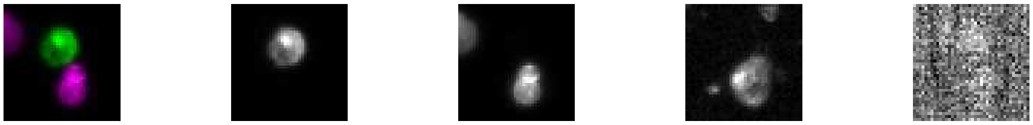				
13681	86	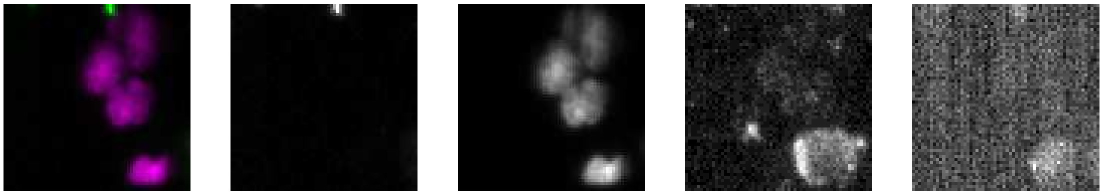				
13682	86	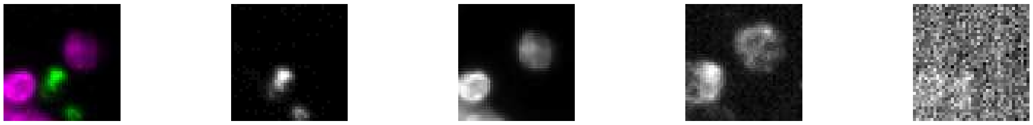				
13683	86	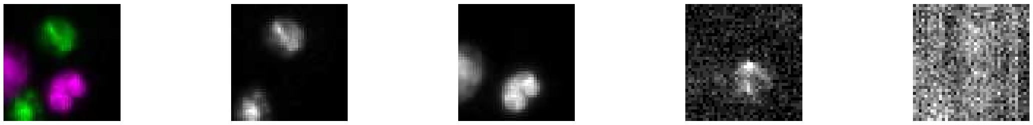				
13684	86	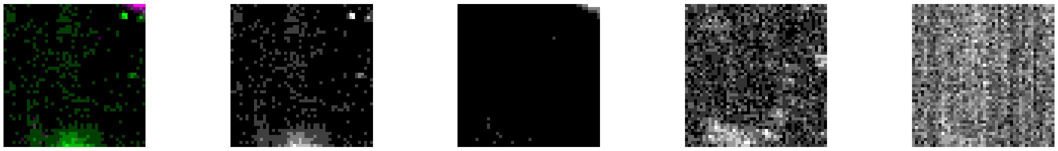				
13685	86	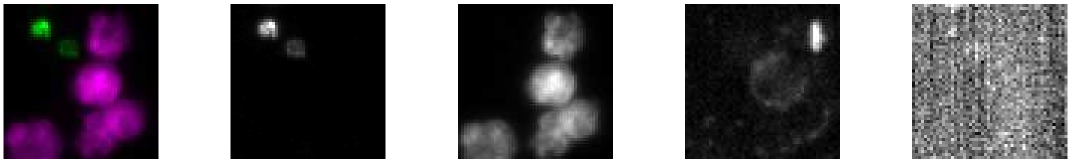				
13686	86	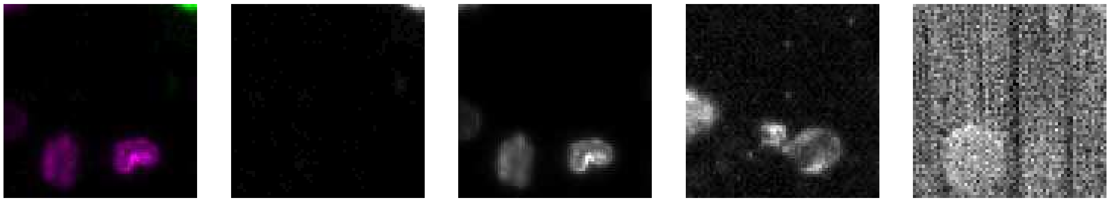				
13687	86	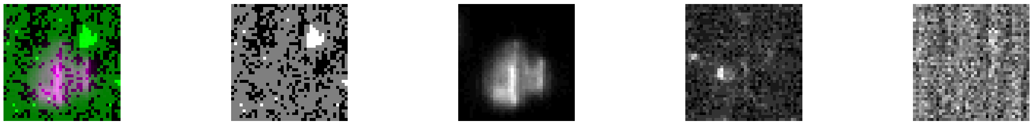				
13688	86	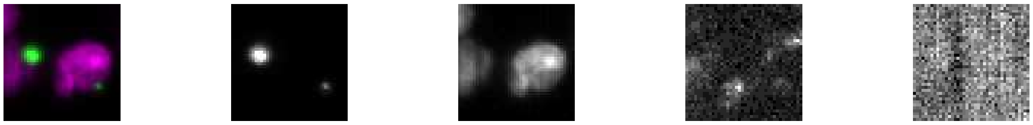				
13689	86	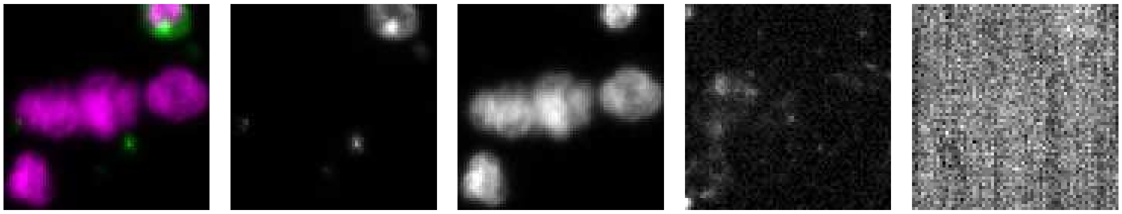				

Ereignis	Aufnahme	DAPI/CK-PE	CK-PE	DAPI	CD45-APC	HER-2/neu
13690	86					
13691	86					
13692	86					
13693	86					
13694	86					
13695	86					
13696	86					
13697	86					
13698	86					
13699	86					
13700	86					
13701	86					
13702	86					

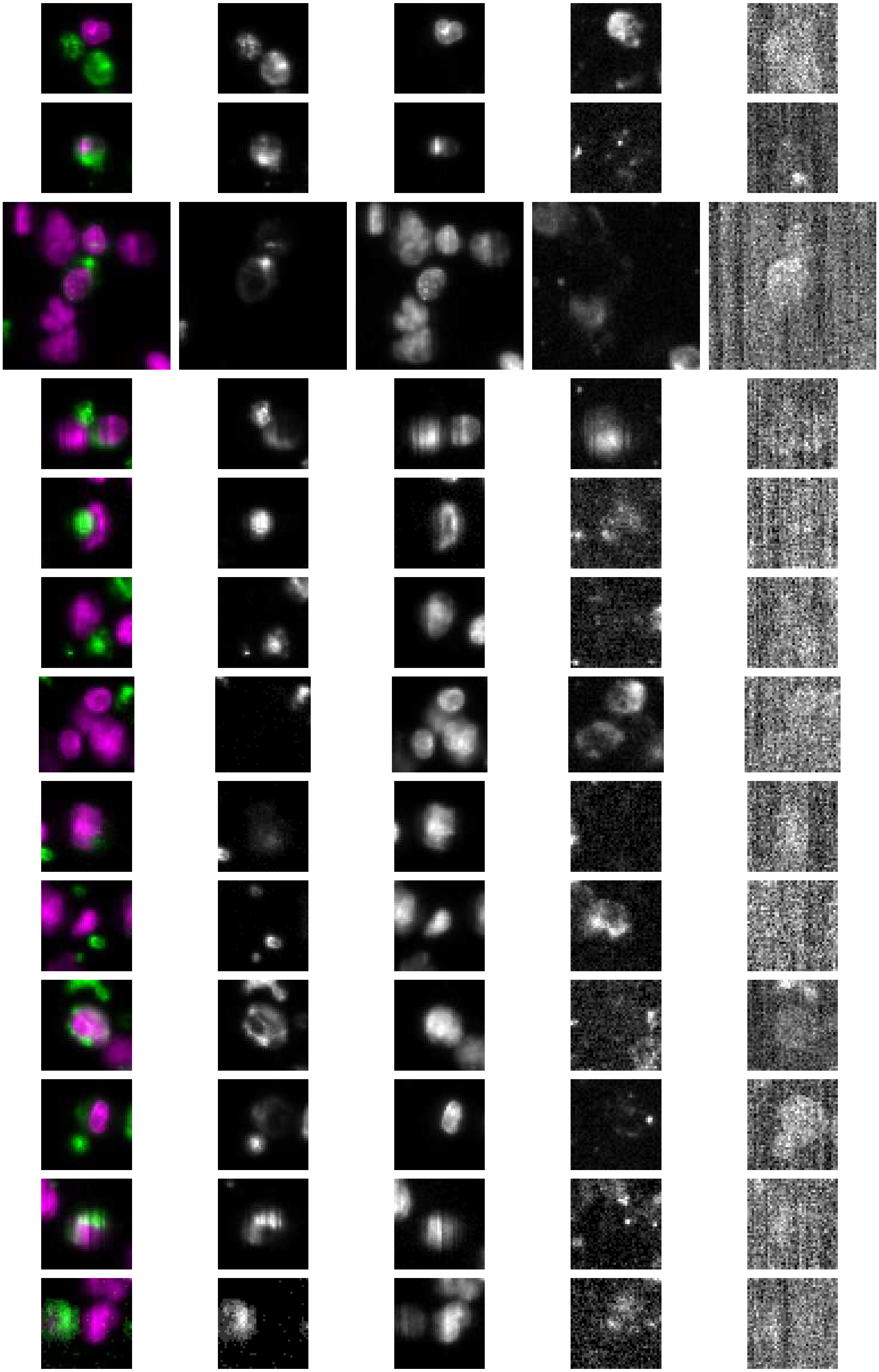

Proben-ID: Gyn14.208 **Kassetten-ID:** 01327106 **Druckdatum:** 02/06/2020 17:00 **Patienten-ID:**

Ereignis	Aufnahme	DAPI/CK-PE	CK-PE	DAPI	CD45-APC	HER-2/neu
13703	86	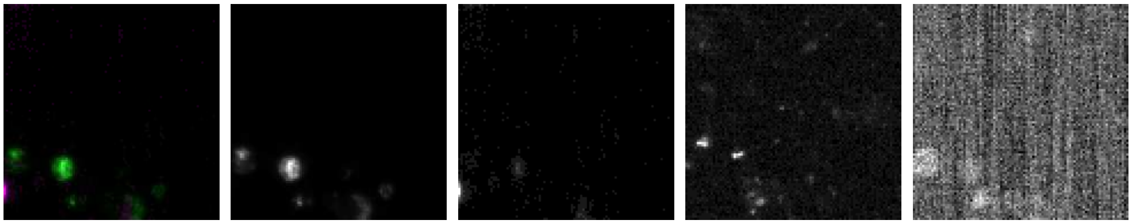				
13704	86	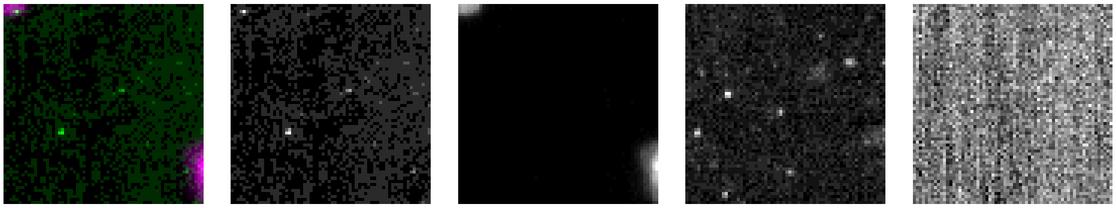				
13705	86	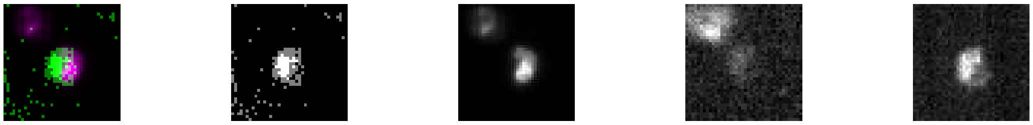				
13706	86	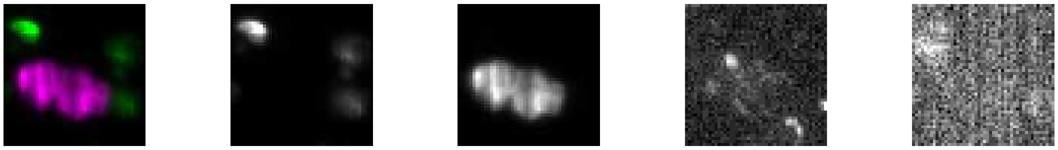				
13707	86	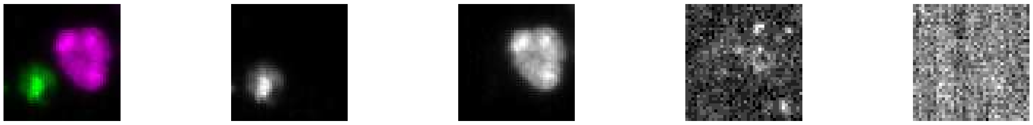				
13708	86	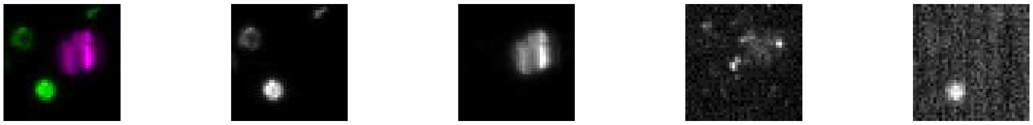				
13709	86	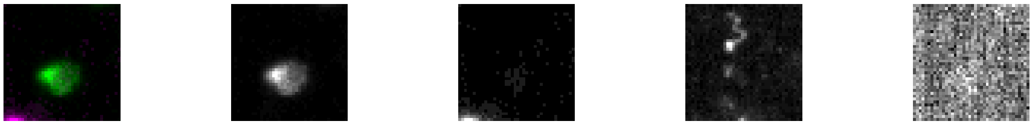				
13710	86	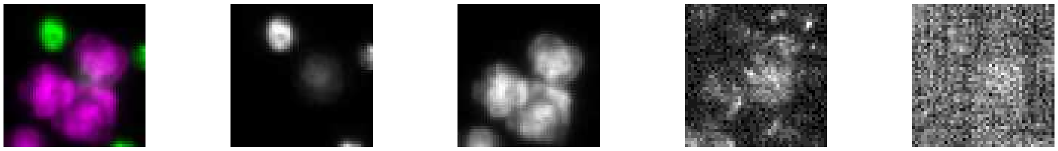				
13711	86	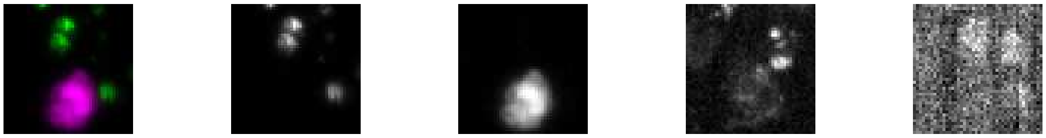				
13712	86	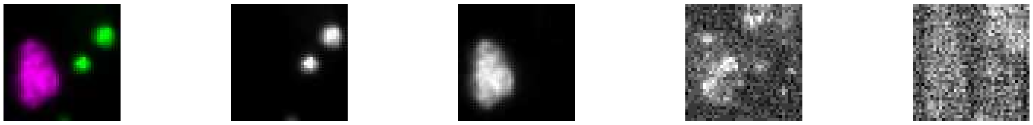				
13713	86	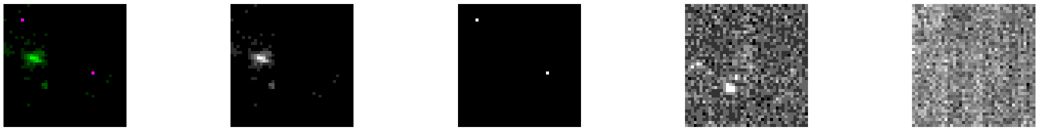				
13714	86	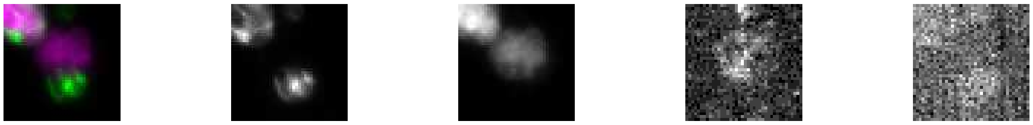				

Ereignis	Aufnahme	DAPI/CK-PE	CK-PE	DAPI	CD45-APC	HER-2/neu
13715	86	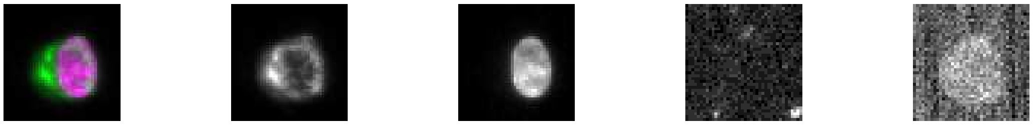				
13716	86	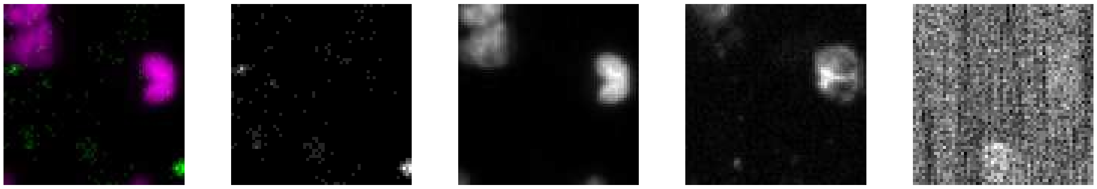				
13717	86	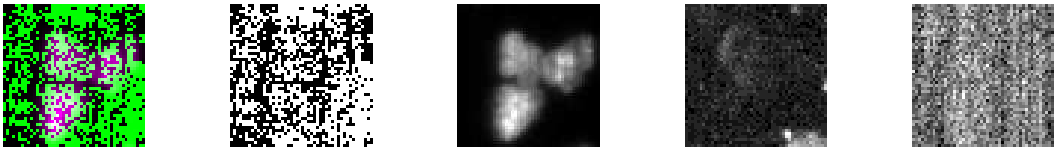				
13718	86	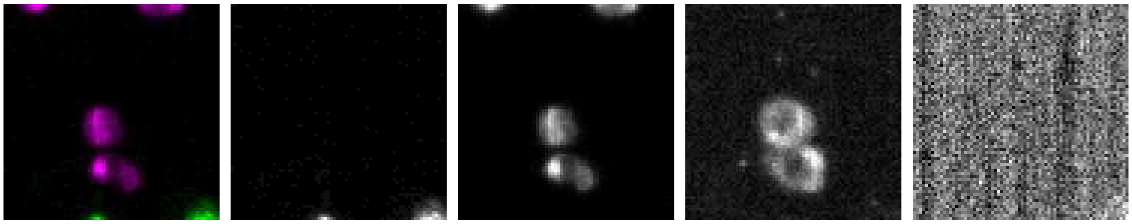				
13719	86	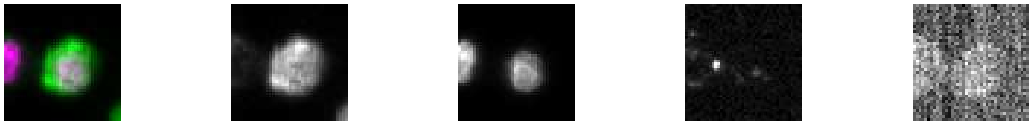				
13720	86	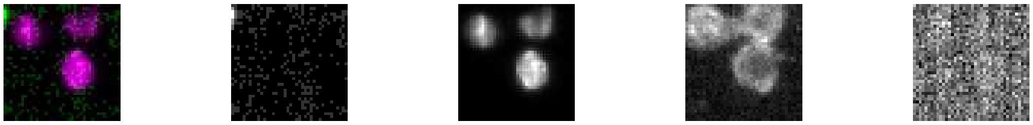				
13721	86	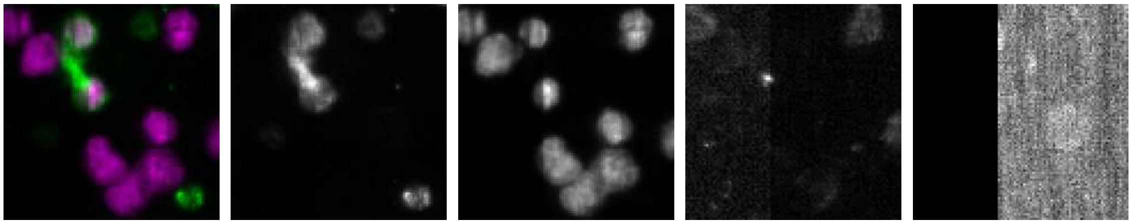				
13722	86	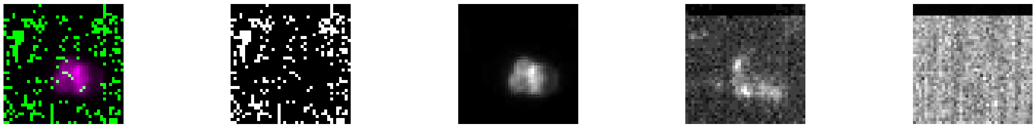				
13723	85	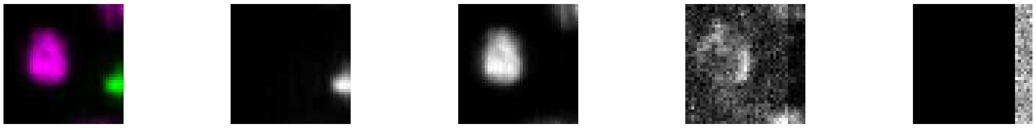				
13724	85	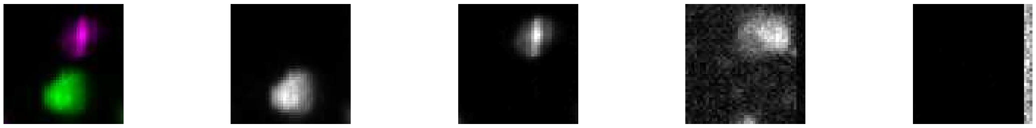				
13725	85	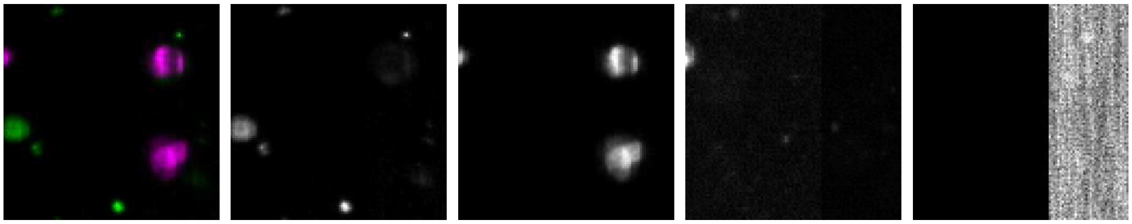				

Proben-ID: Gyn14.208 **Kassetten-ID:** 01327106 **Patienten-ID:**

Ereignis	Aufnahme	DAPI/CK-PE	CK-PE	DAPI	CD45-APC	HER-2/neu
13726	86	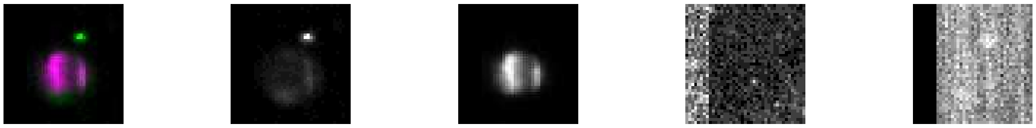				
13727	85	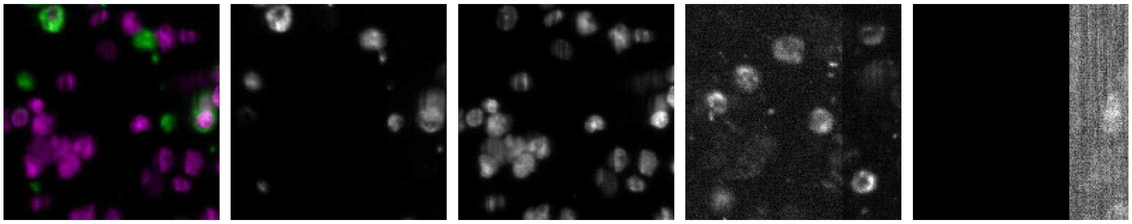				
13728	85	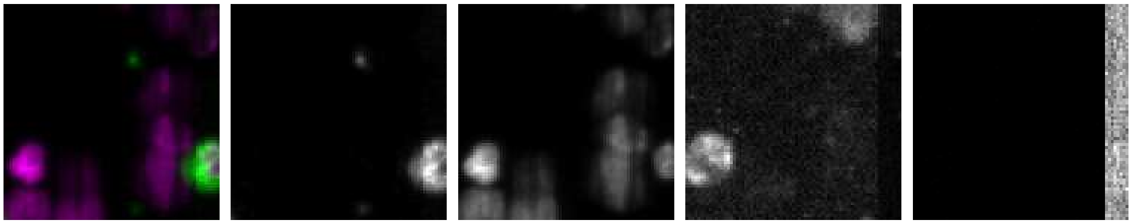				
13729	86	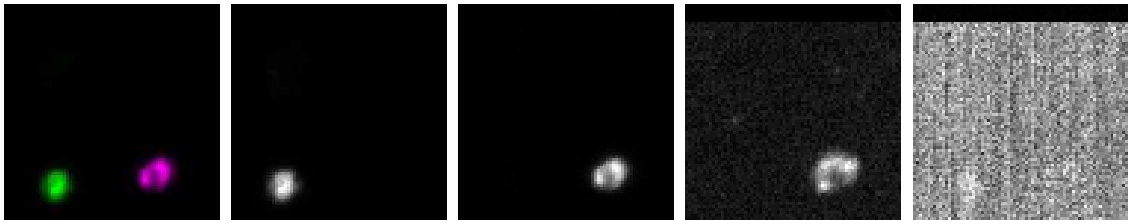				
13730	86	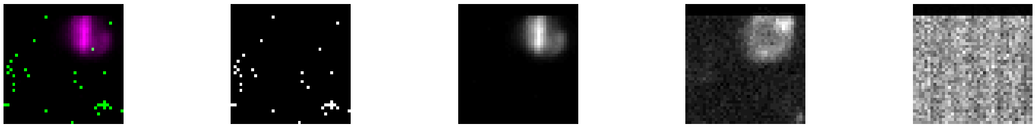				
13731	55	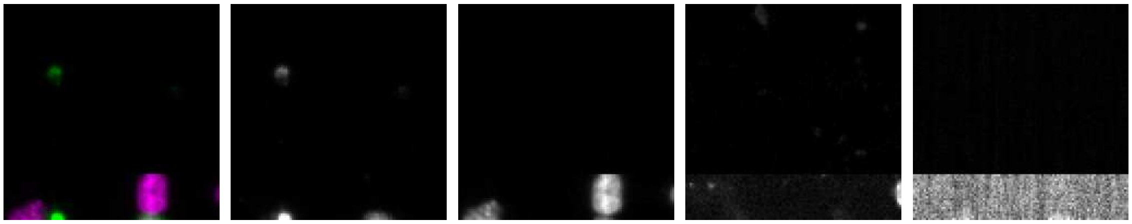				
13732	55	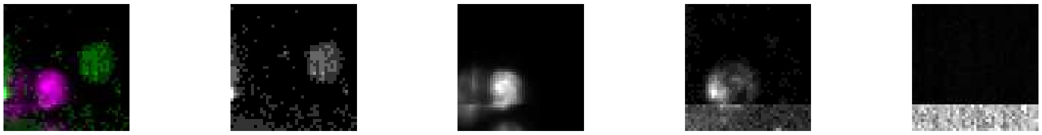				
13733	55	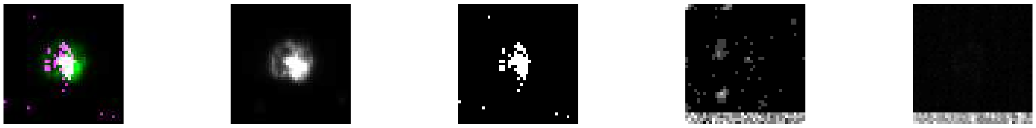				
13734	55	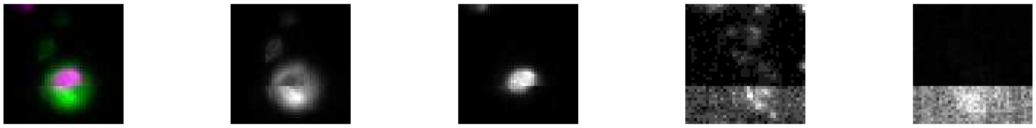				
13735	86	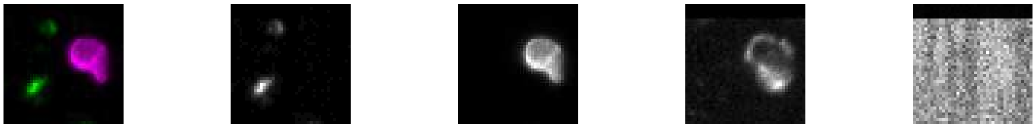				

Druckdatum: 02/06/2020 17:00

Proben-ID: Gyn14.208 **Kassetten-ID:** 01327106 **Patienten-ID:**

Ereignis	Aufnahme	DAPI/CK-PE	CK-PE	DAPI	CD45-APC	HER-2/neu
13736	86	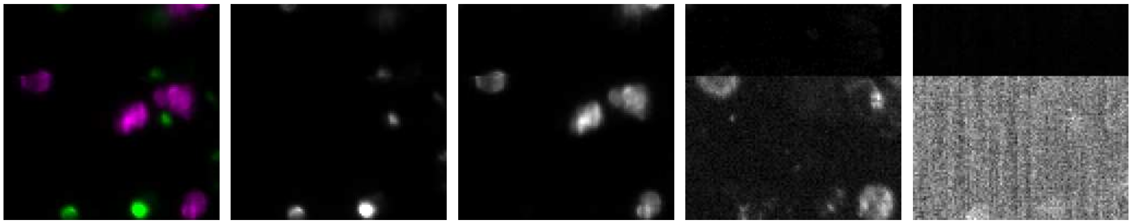				
13737	87	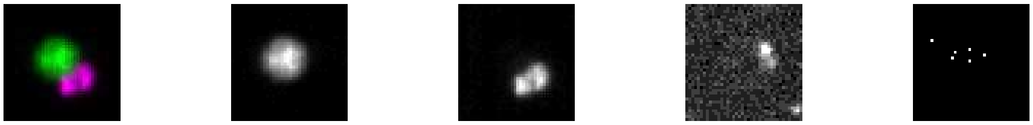				
13738	87	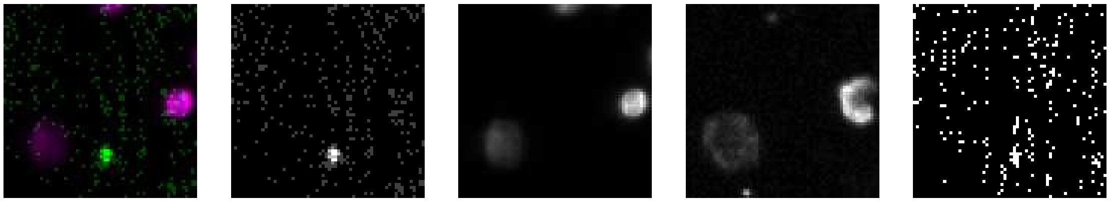				
13739	87	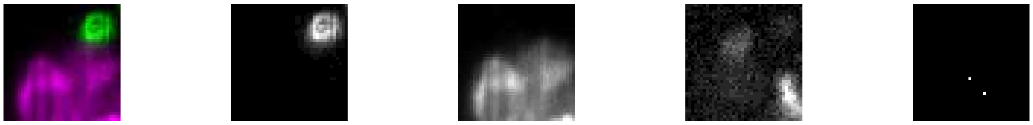				
13740	87	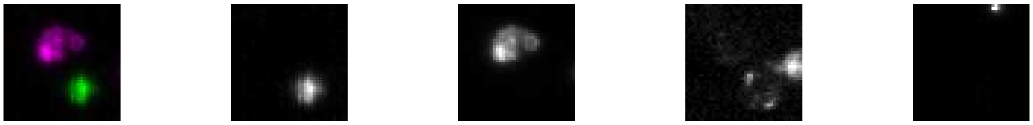				
13741	87	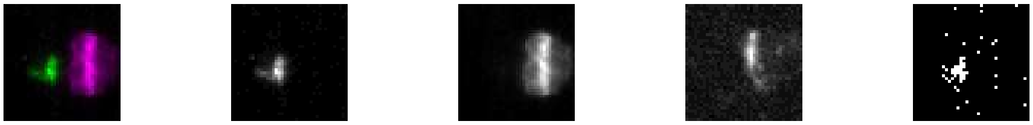				
13742	87	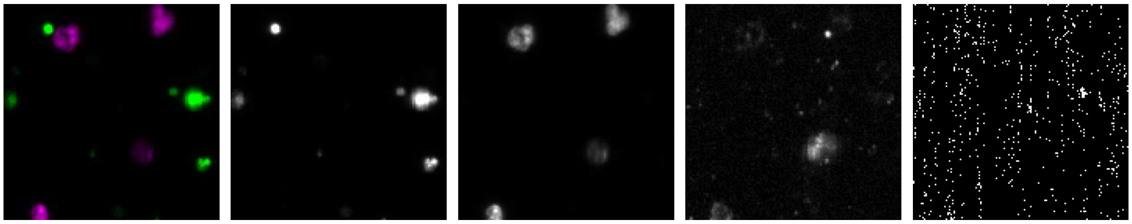				
13743	87	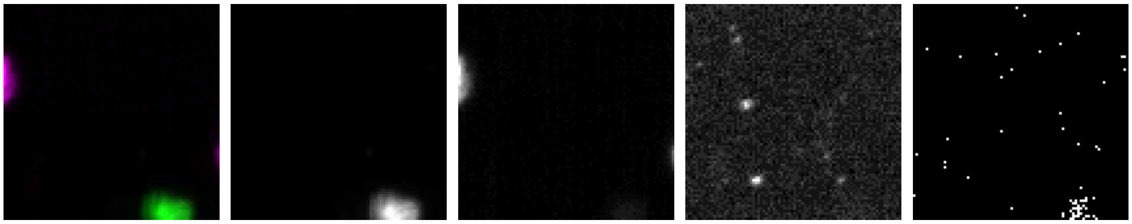				
13744	87	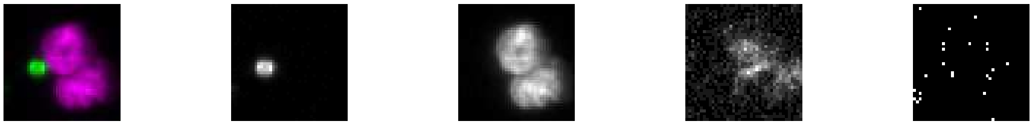				
13745	87	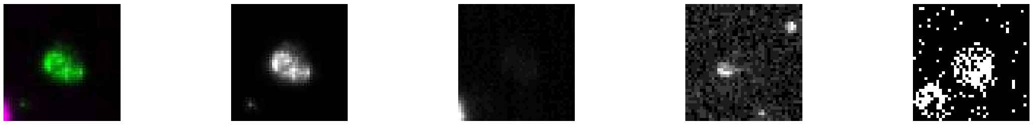				
13746	87	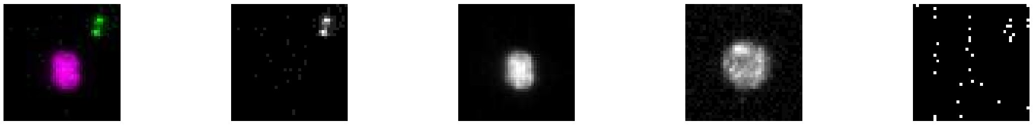				

Proben-ID: Gyn14.208 **Kassetten-ID:**01327106 **Patienten-ID:**

Ereignis	Aufnahme	DAPI/CK-PE	CK-PE	DAPI	CD45-APC	HER-2/neu
13747	87	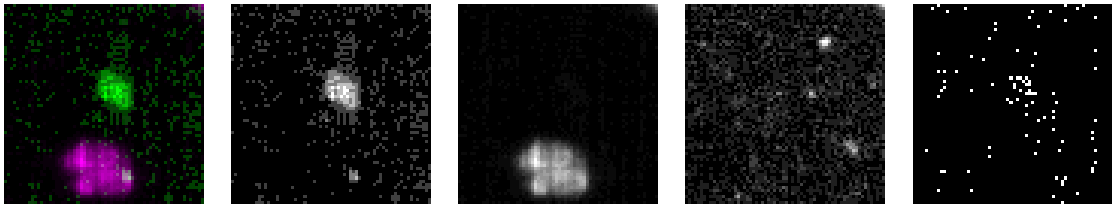				
13748	87	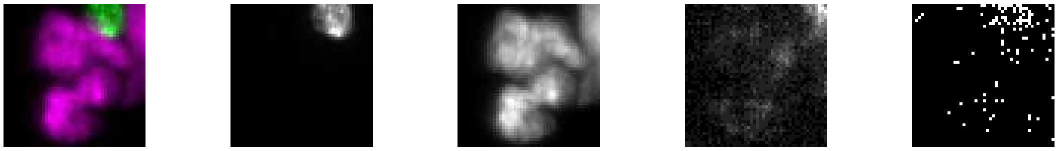				
13749	87	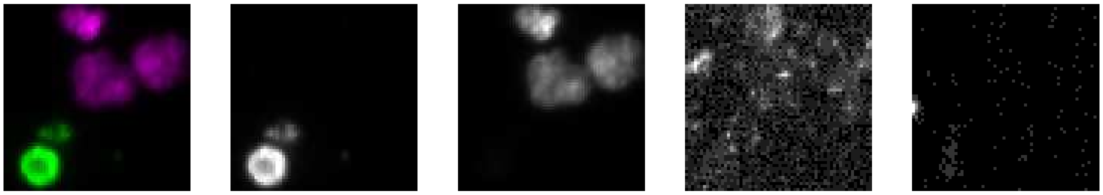				
13750	87	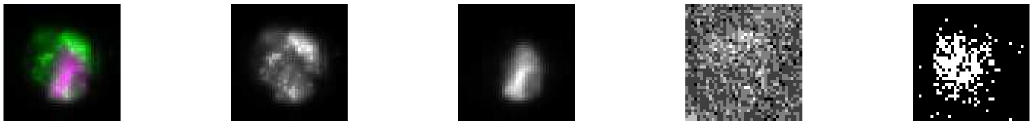				
13751	87	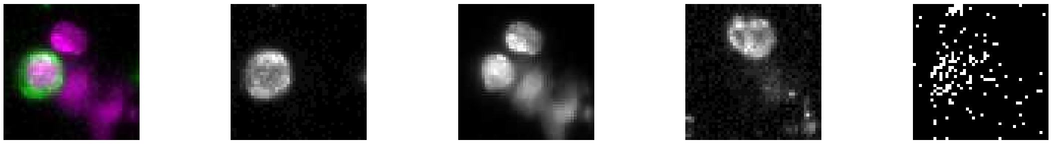				
13752	87	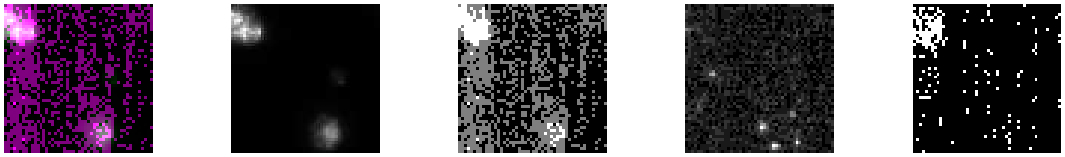				
13753	87	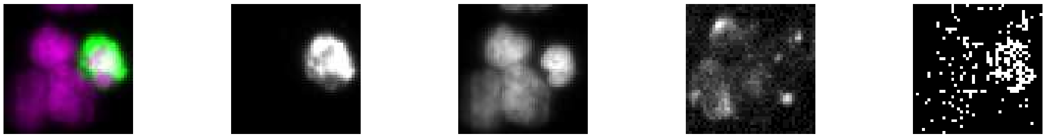				
13754	87	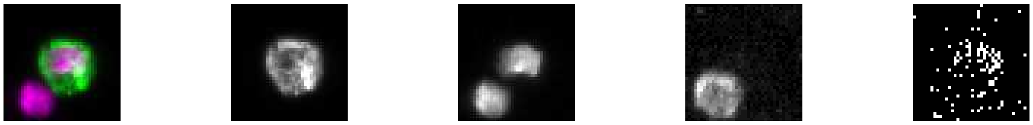				
13755	87	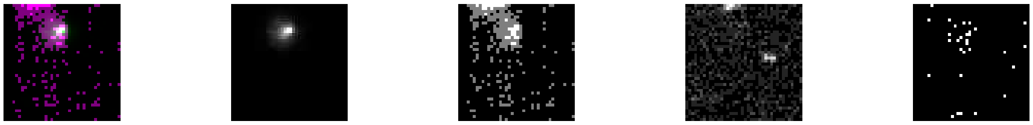				
13756	87	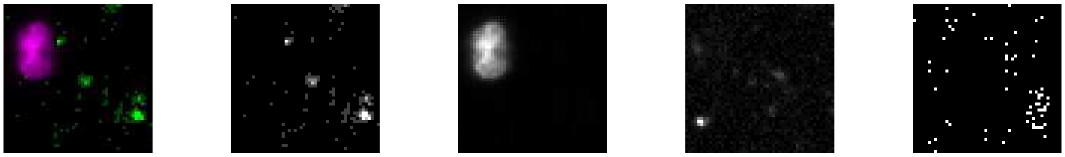				
13757	87	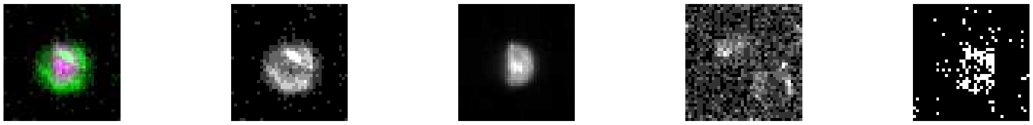				

Ereignis	Aufnahme	DAPI/CK-PE	CK-PE	DAPI	CD45-APC	HER-2/neu
13758	87	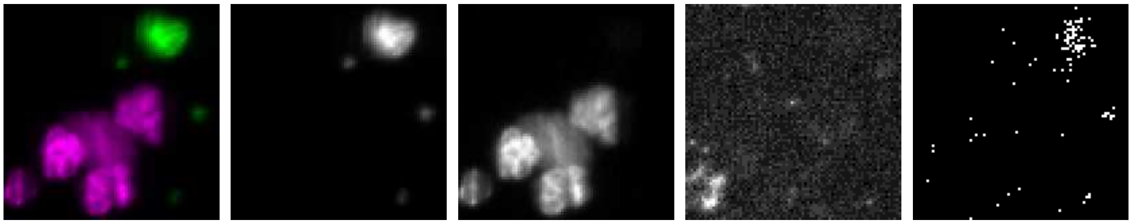				
13759	87	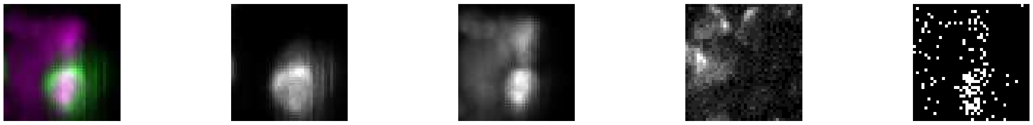				
13760	87	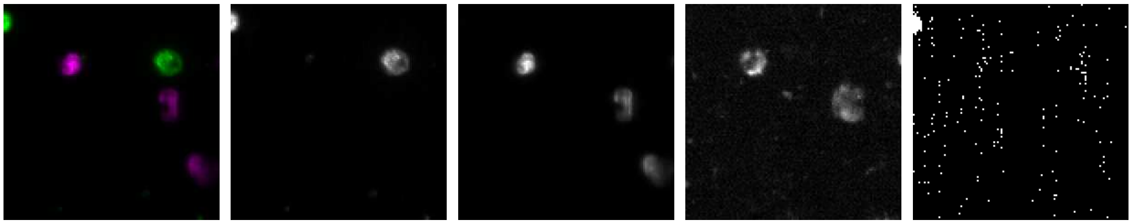				
13761	87	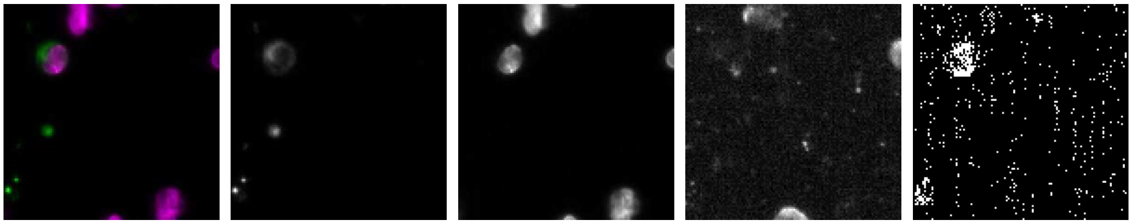				
13762	87	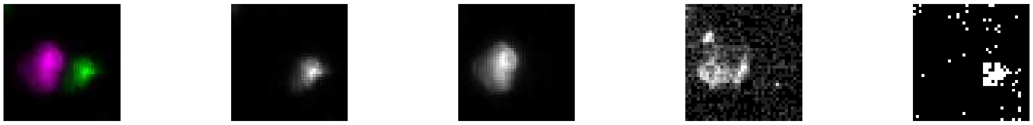				
13763	87	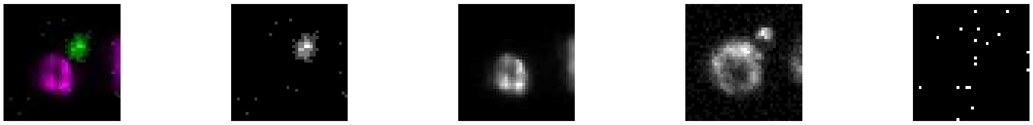				
13764	87	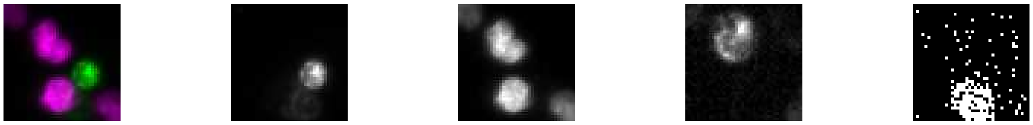				
13765	87	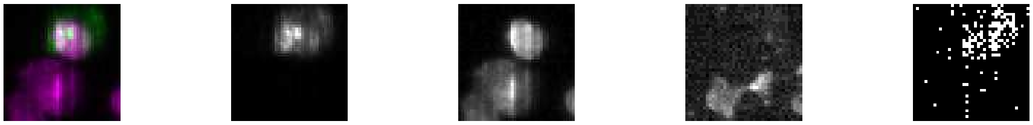				
13766	87	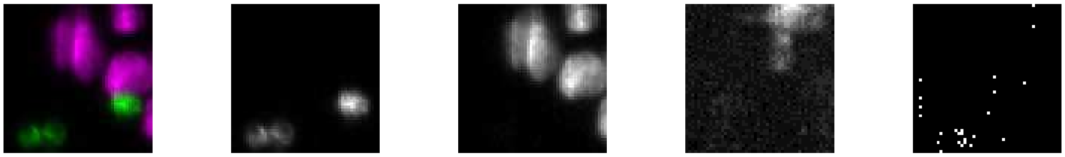				
13767	87	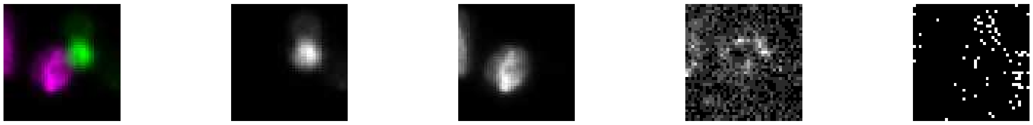				
13768	87	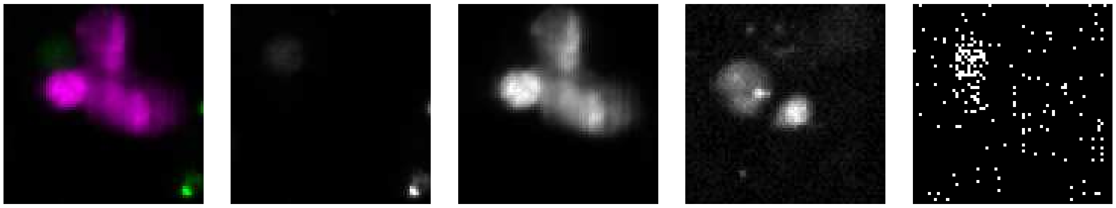				

Ereignis	Aufnahme	DAPI/CK-PE	CK-PE	DAPI	CD45-APC	HER-2/neu
13769	87	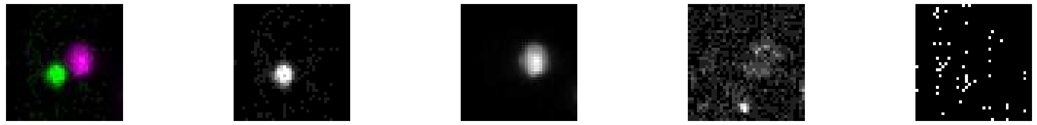				
13770	87	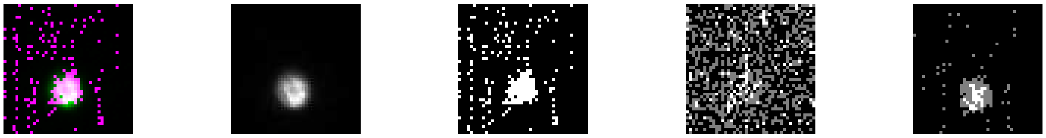				
13771	87	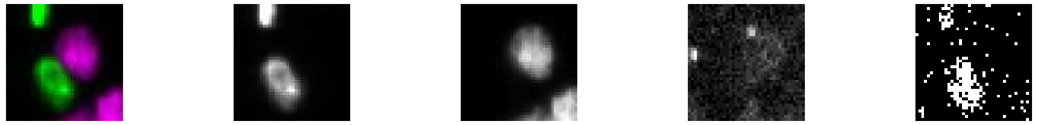				
13772	87	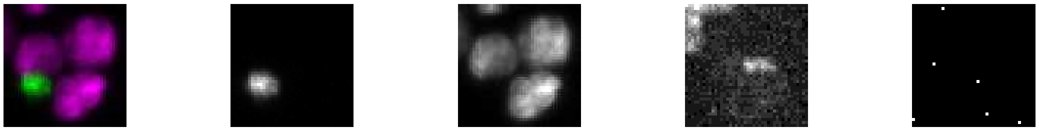				
13773	87	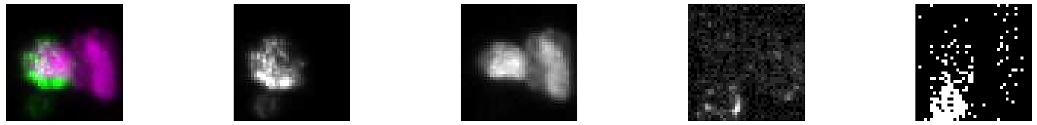				
13774	87	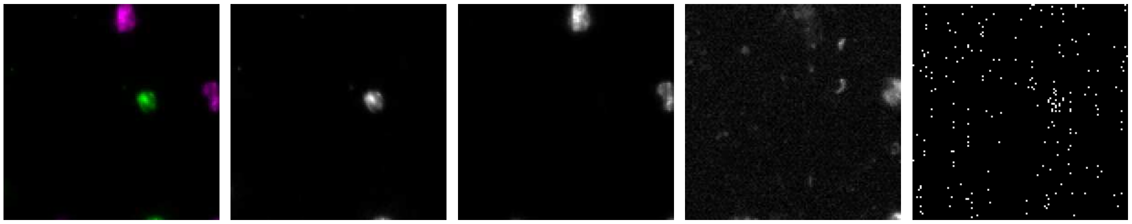				
13775	87	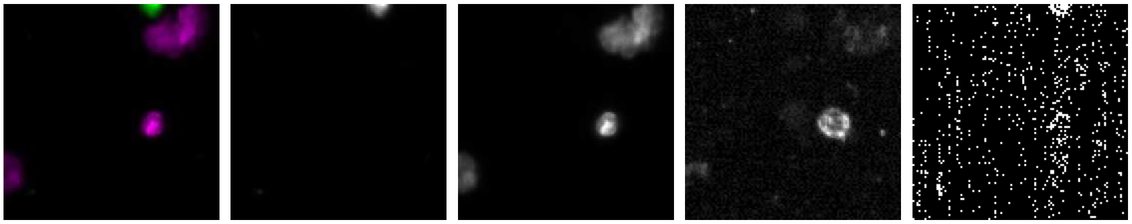				
13776	87	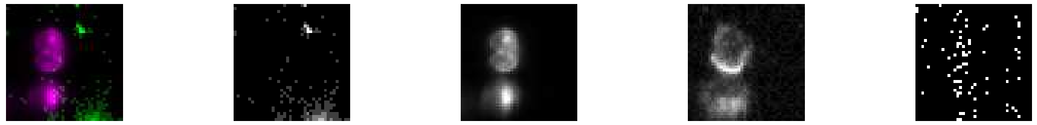				
13777	87	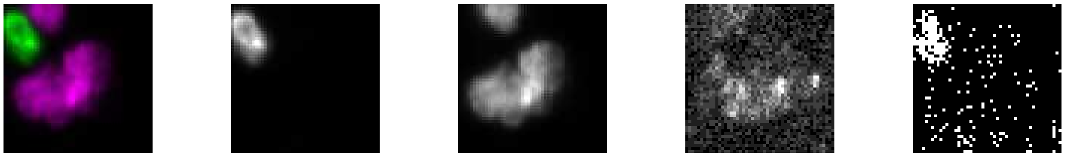				
13778	87	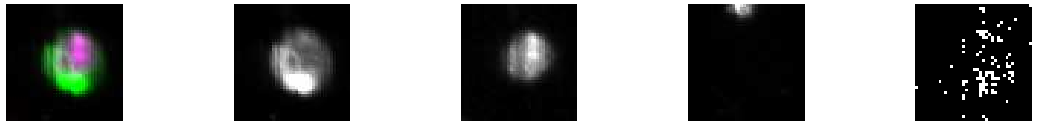				
13779	87	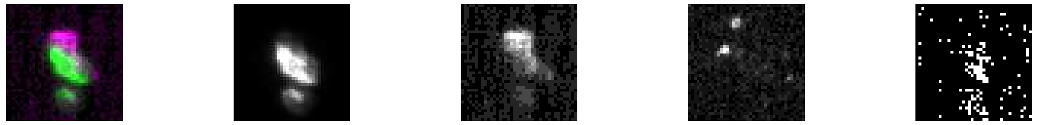				
13780	87	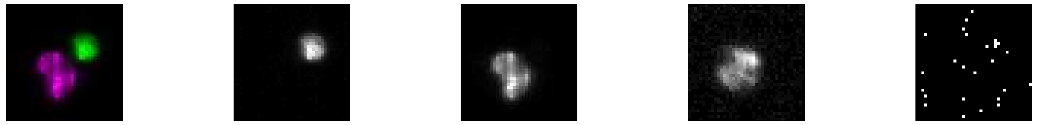				

Ereignis	Aufnahme	DAPI/CK-PE	CK-PE	DAPI	CD45-APC	HER-2/neu
13781	87	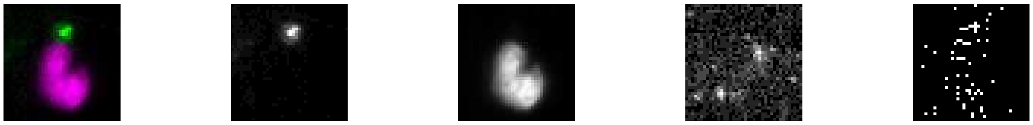				
13782	87	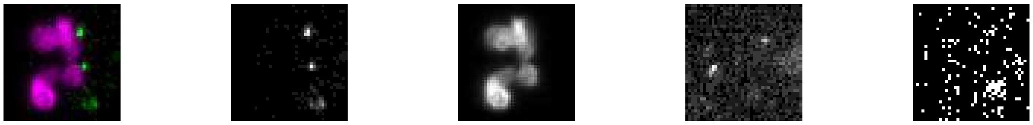				
13783	87	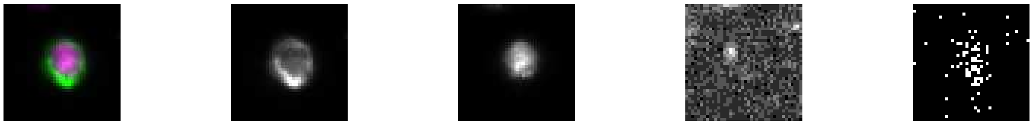				
13784	87	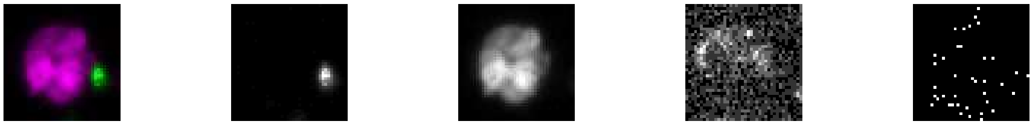				
13785	87	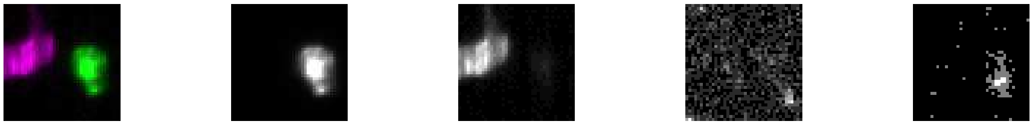				
13786	87	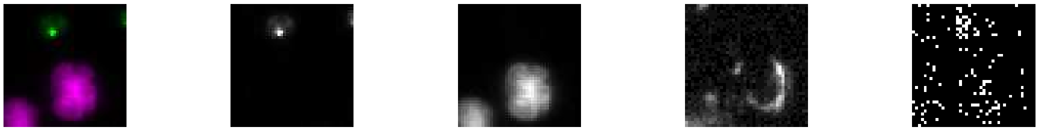				
13787	87	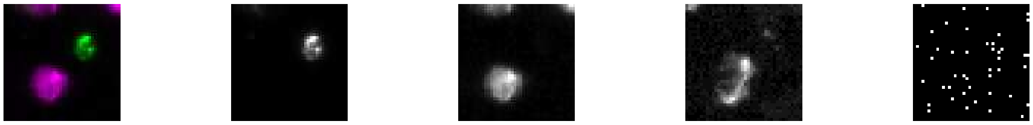				
13788	87	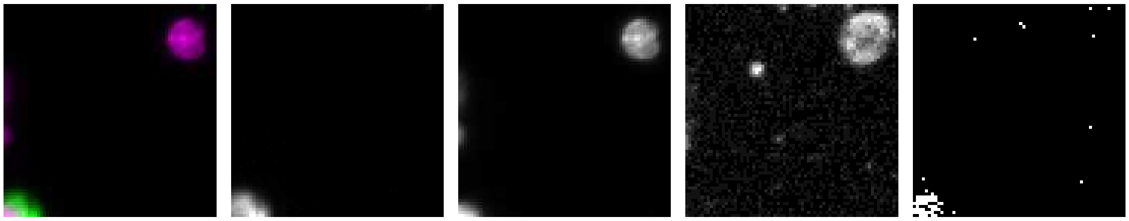				
13789	87	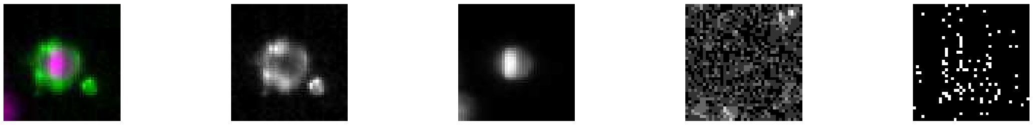				
13790	87	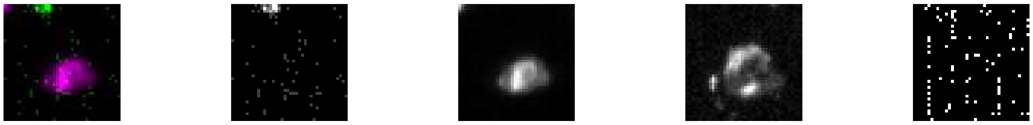				
13791	87	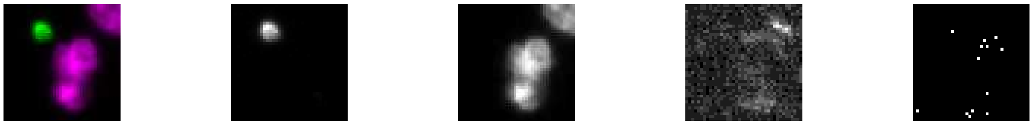				
13792	87	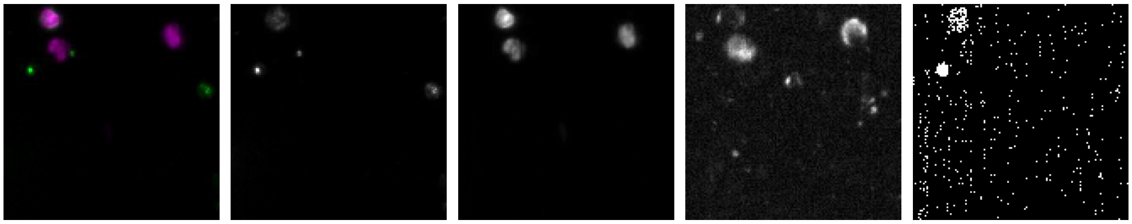				

Ereignis	Aufnahme	DAPI/CK-PE	CK-PE	DAPI	CD45-APC	HER-2/neu
13793	87	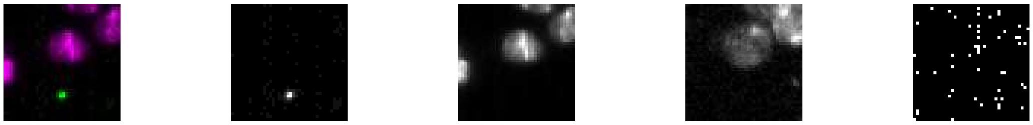				
13794	87	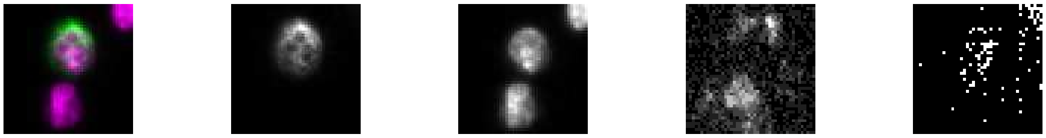				
13795	87	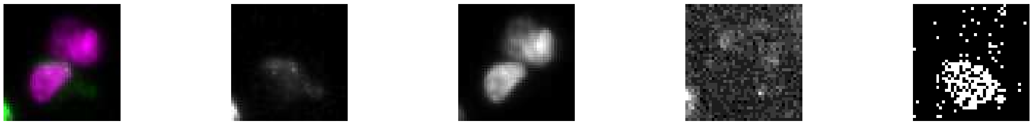				
13796	87	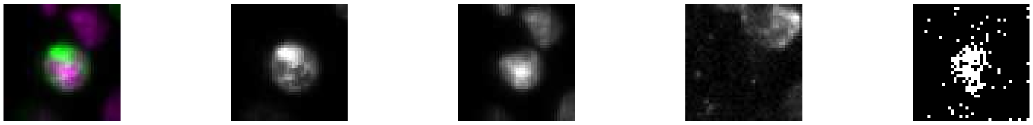				
13797	87	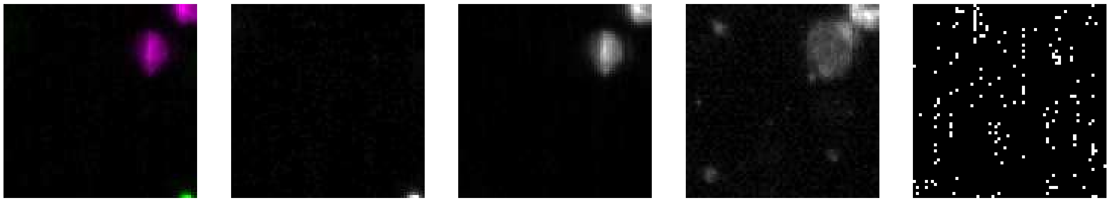				
13798	87	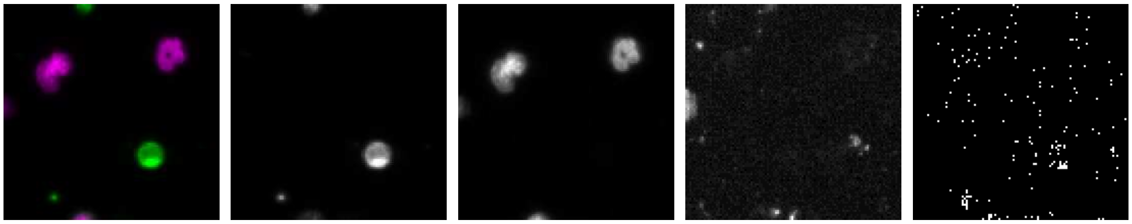				
13799	87	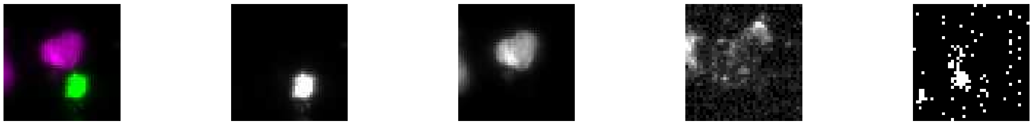				
13800	87	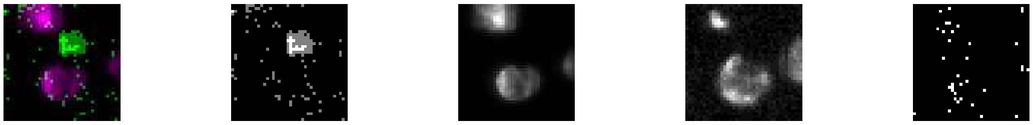				
13801	87	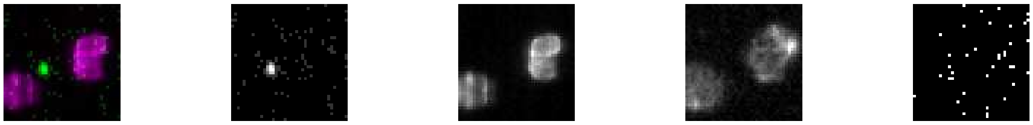				
13802	87	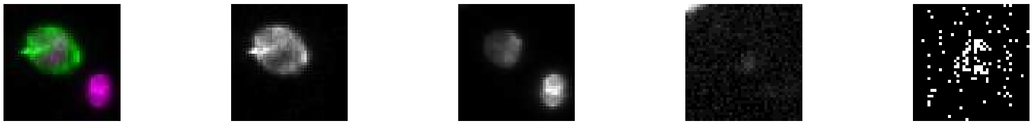				
13803	87	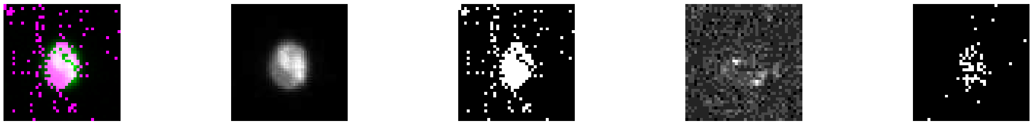				
13804	87	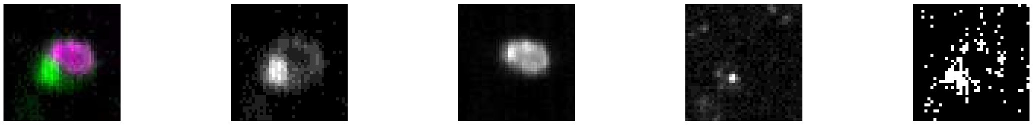				

Ereignis	Aufnahme	DAPI/CK-PE	CK-PE	DAPI	CD45-APC	HER-2/neu
13805	87	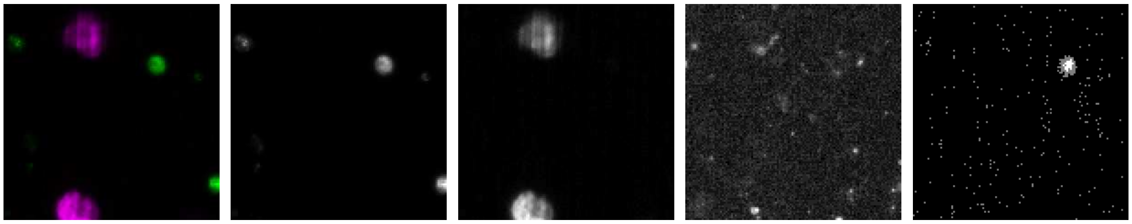				
13806	87	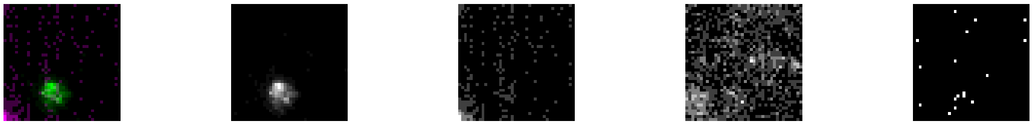				
13807	87	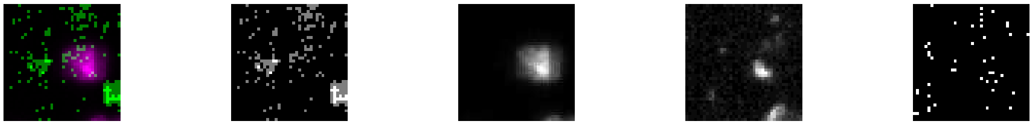				
13808	87	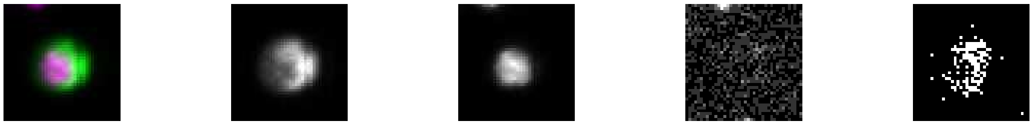				
13809	87	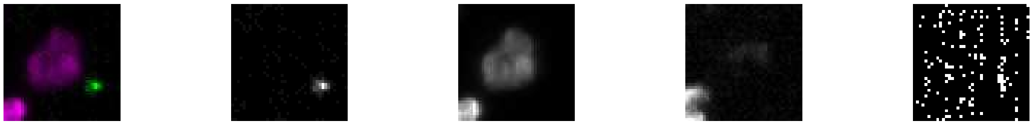				
13810	87	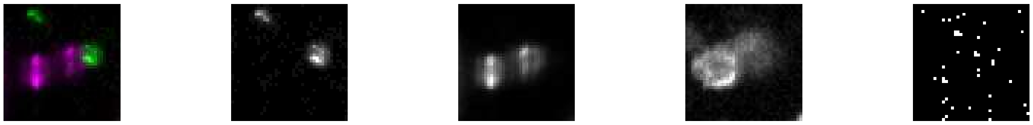				
13811	87	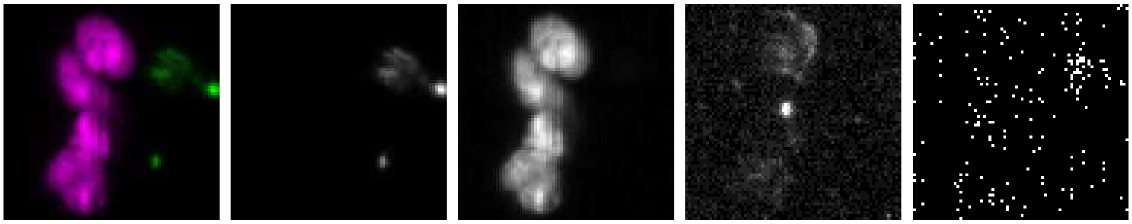				
13812	87	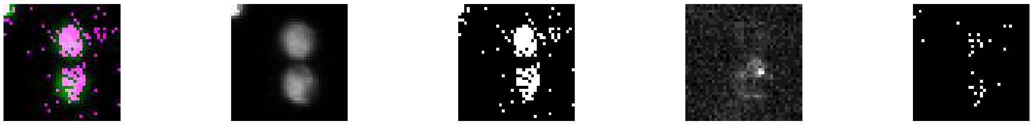				
13813	87	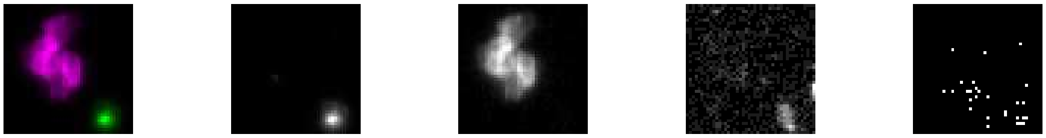				
13814	87	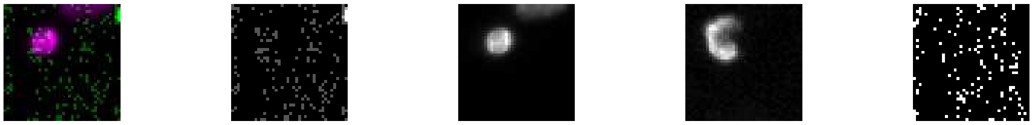				
13815	87	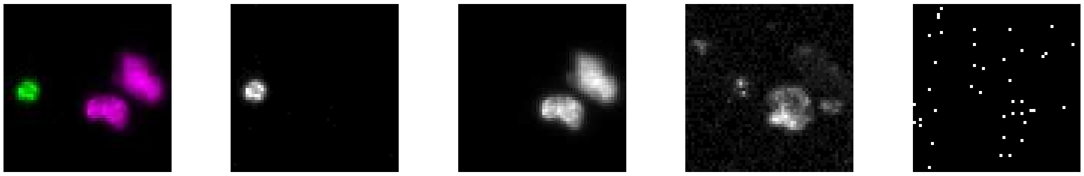				

Ereignis	Aufnahme	DAPI/CK-PE	CK-PE	DAPI	CD45-APC	HER-2/neu
13816	87	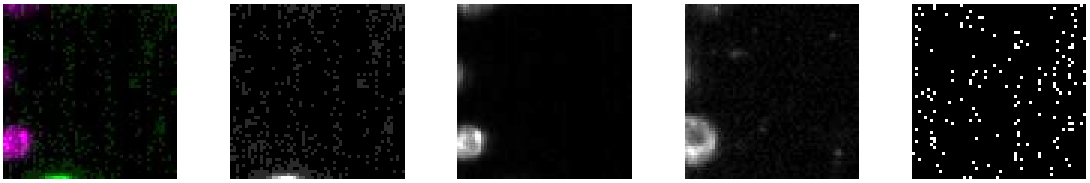				
13817	87	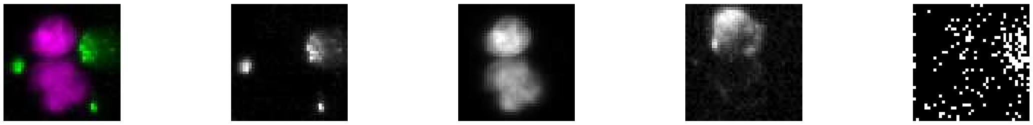				
13818	87	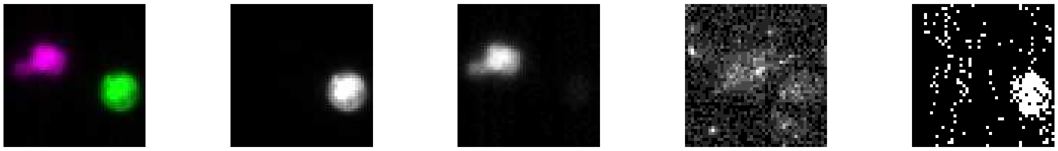				
13819	87	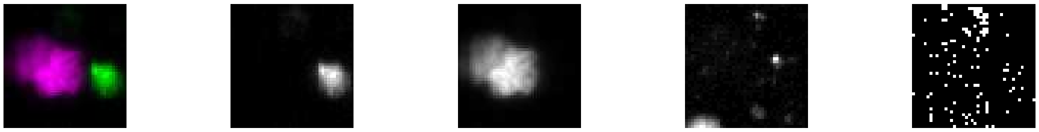				
13820	87	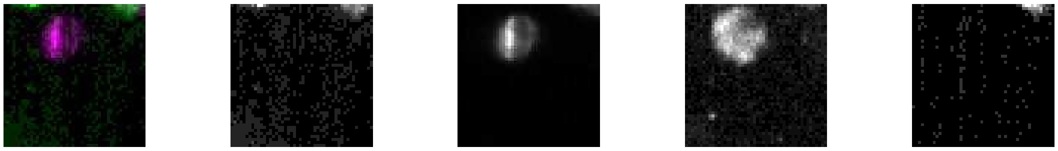				
13821	87	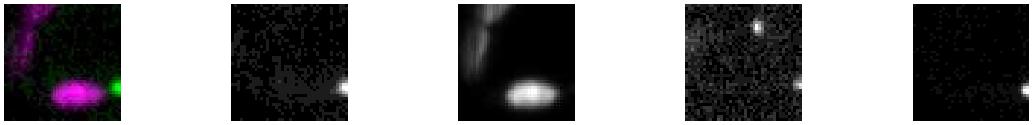				
13822	87	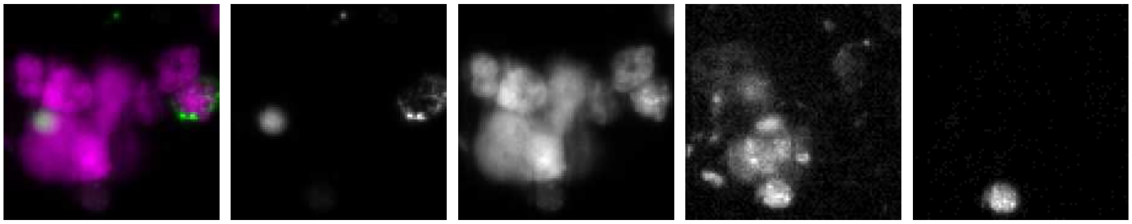				
13823	87	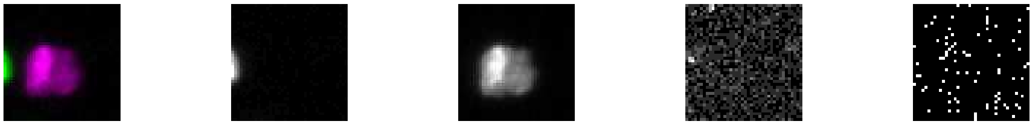				
13824	87	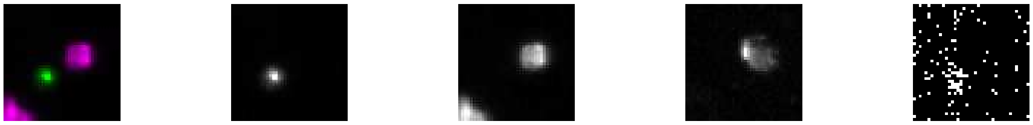				
13825	87	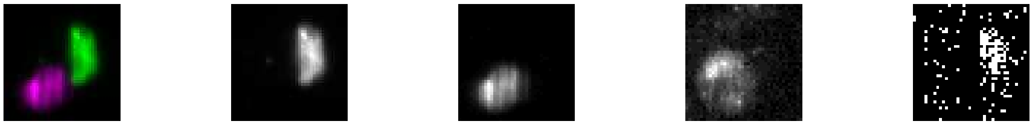				
13826	87	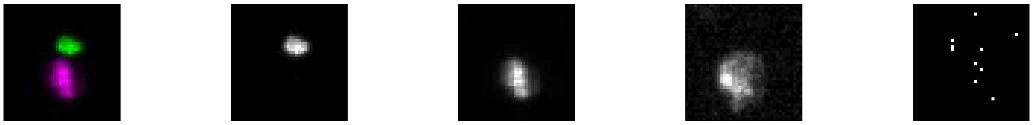				
13827	87	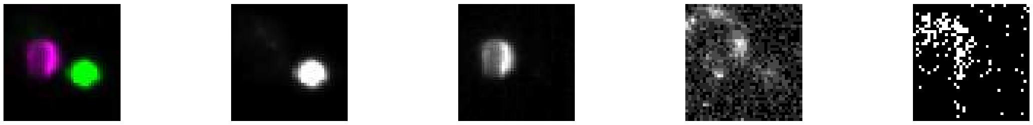				

Ereignis	Aufnahme	DAPI/CK-PE	CK-PE	DAPI	CD45-APC	HER-2/neu
13828	87	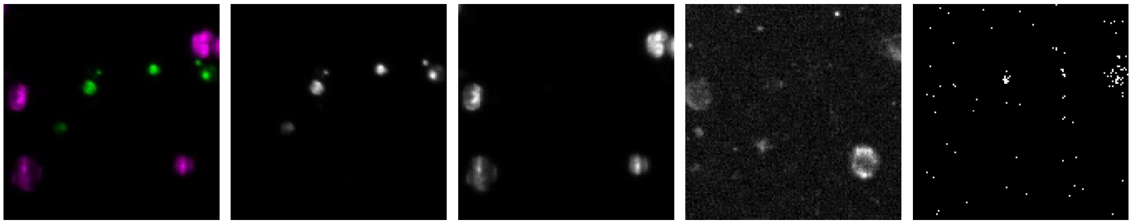				
13829	87	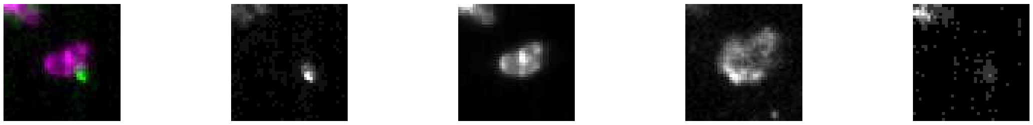				
13830	87	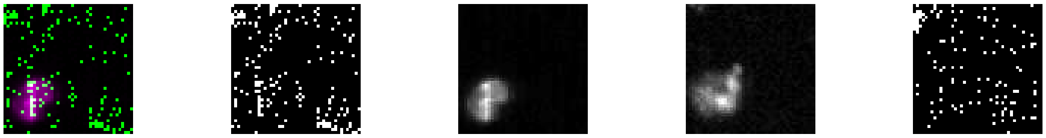				
13831	87	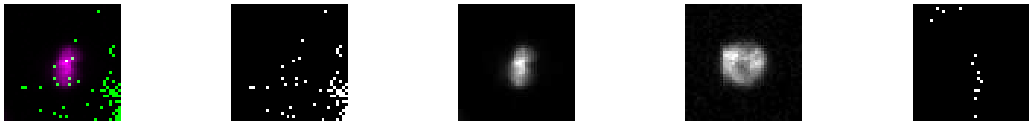				
13832	87	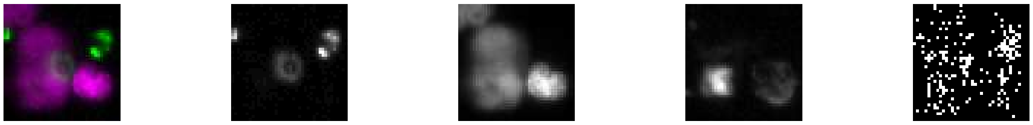				
13833	87	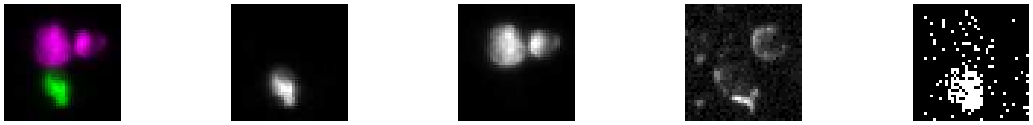				
13834	87	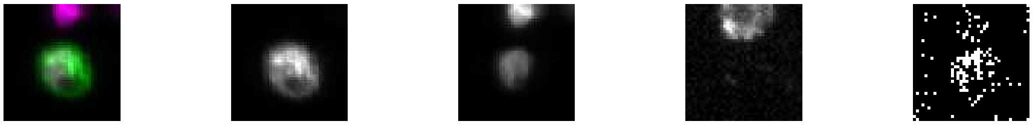				
13835	87	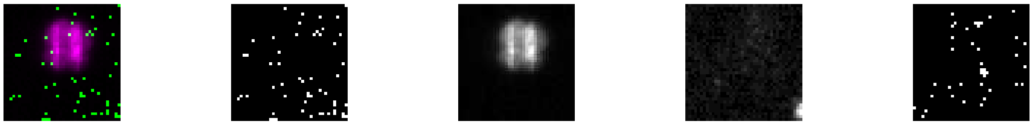				
13836	87	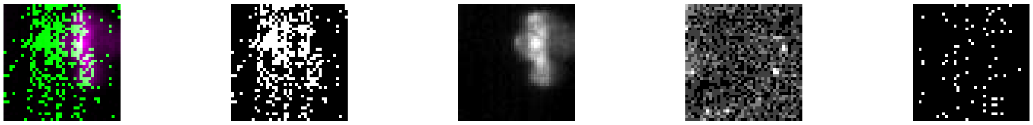				
13837	87	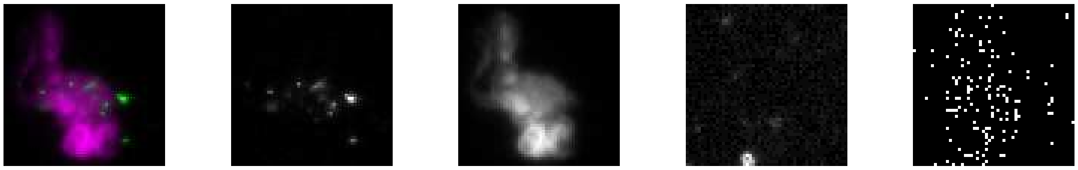				
13838	87	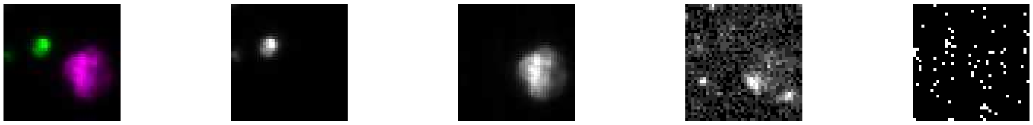				
13839	87	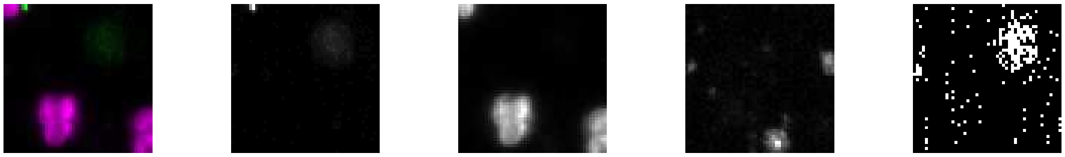				

Ereignis	Aufnahme	DAPI/CK-PE	CK-PE	DAPI	CD45-APC	HER-2/neu
13840	87	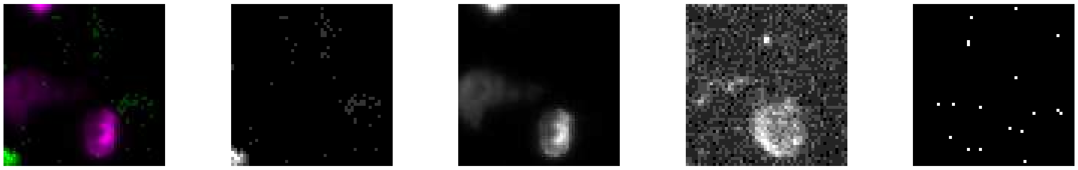				
13841	87	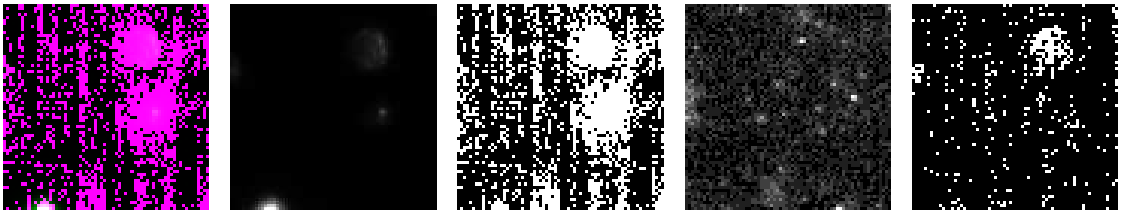				
13842	87	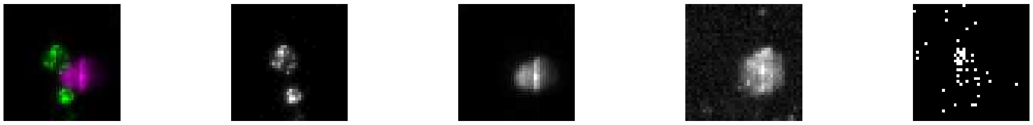				
13843	87	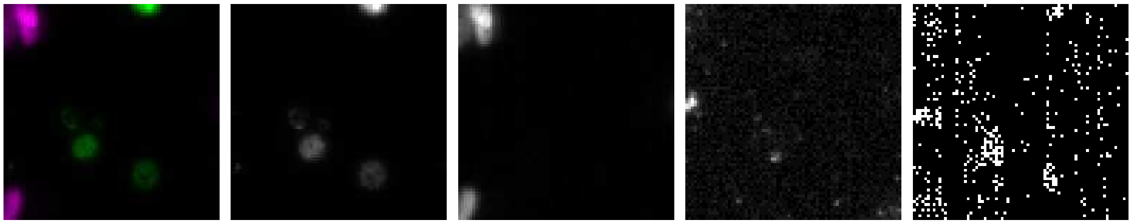				
13844	87	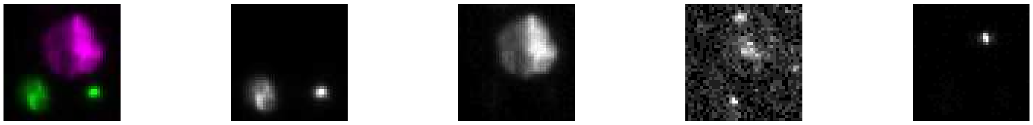				
13845	87	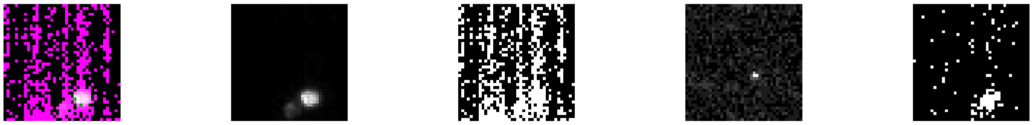				
13846	87	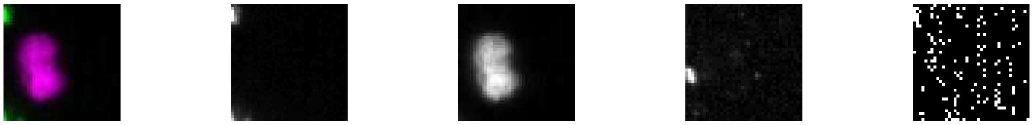				
13847	87	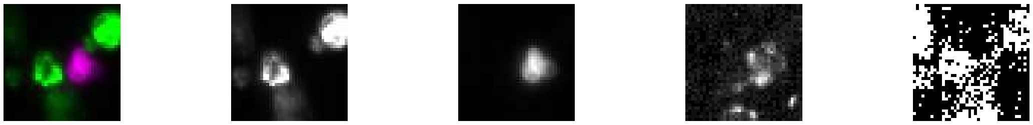				
13848	87	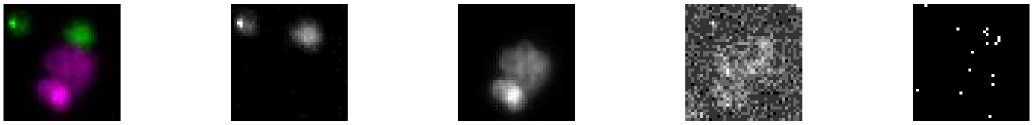				
13849	87	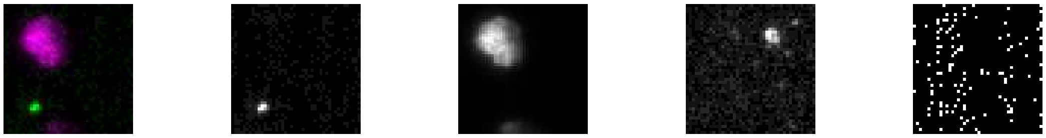				
13850	87	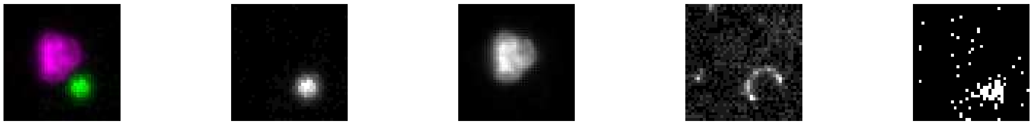				
13851	87	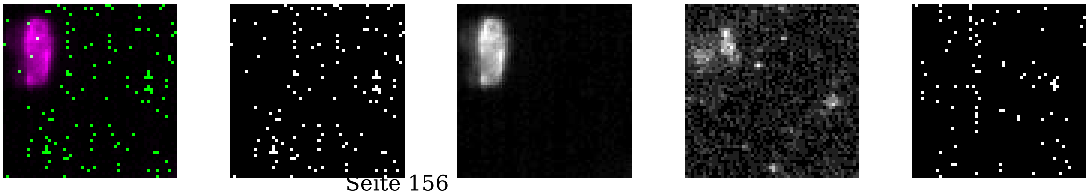				

Ereignis	Aufnahme	DAPI/CK-PE	CK-PE	DAPI	CD45-APC	HER-2/neu
13852	87	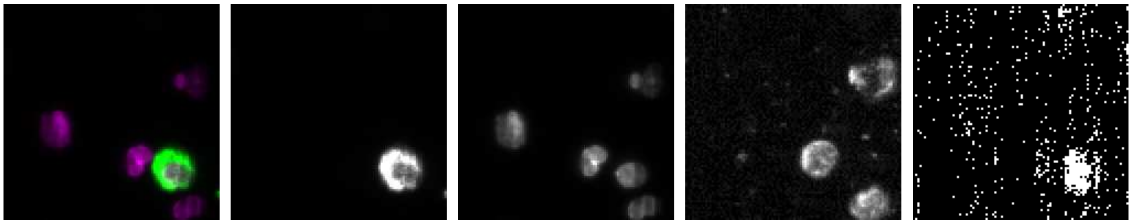				
13853	87	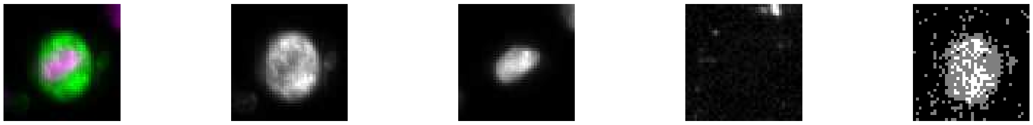				
13854	87	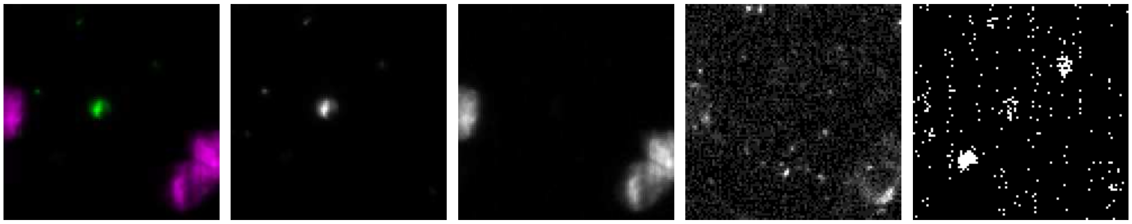				
13855	87	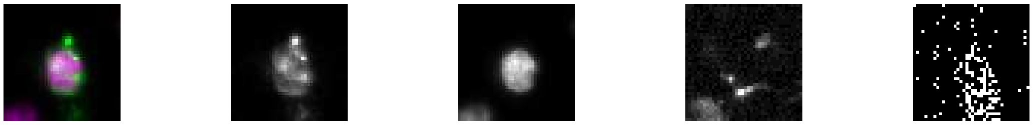				
13856	87	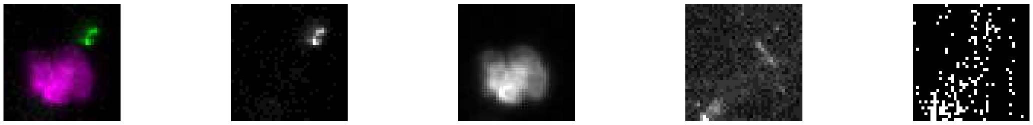				
13857	87	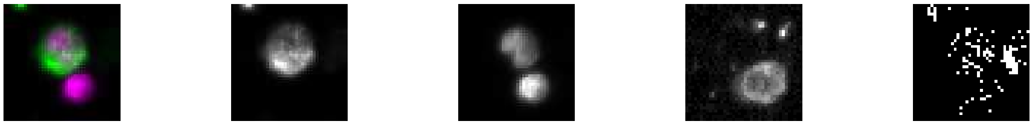				
13858	87	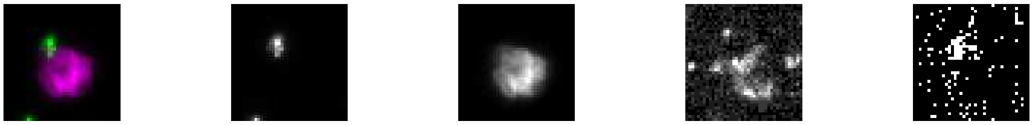				
13859	87	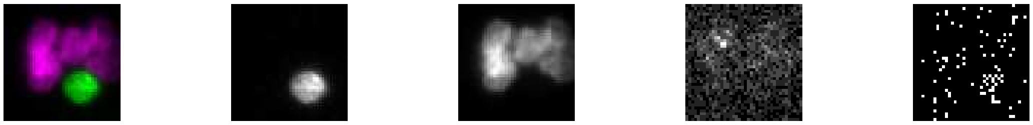				
13860	87	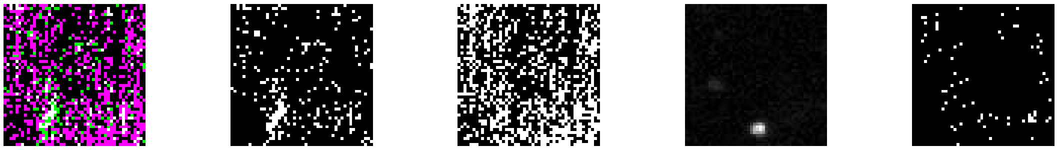				
13861	87	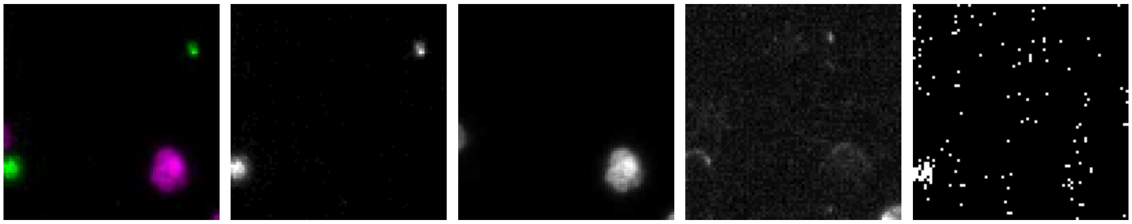				
13862	87	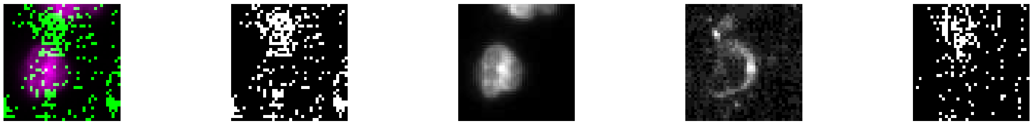				

Ereignis	Aufnahme	DAPI/CK-PE	CK-PE	DAPI	CD45-APC	HER-2/neu
13863	87	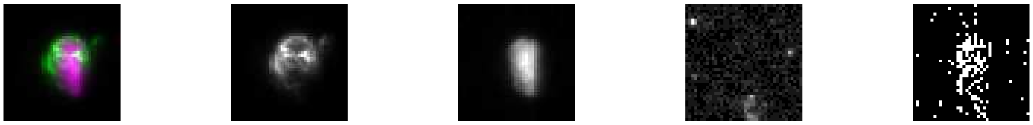				
13864	87	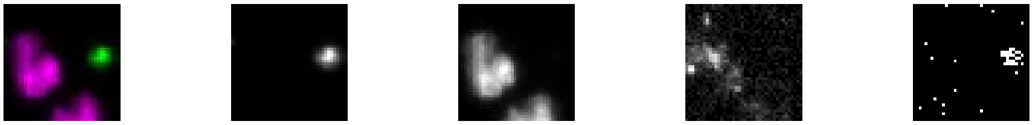				
13865	87	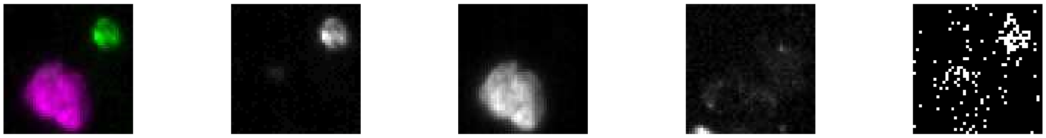				
13866	87	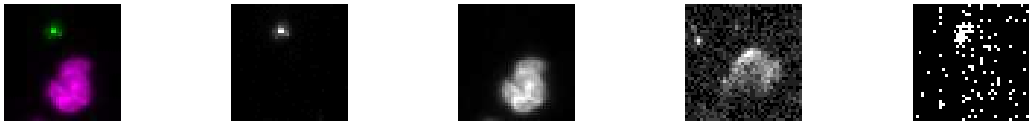				
13867	87	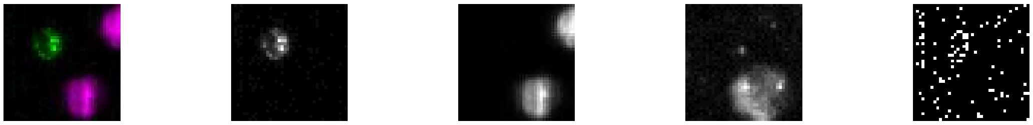				
13868	87	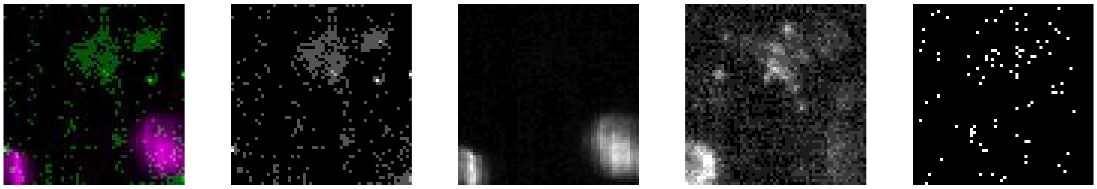				
13869	87	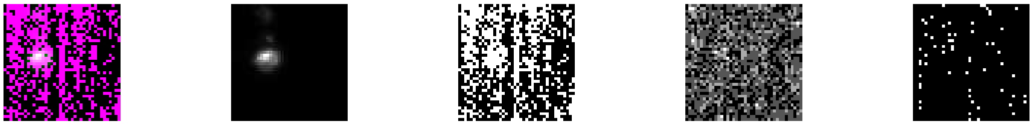				
13870	87	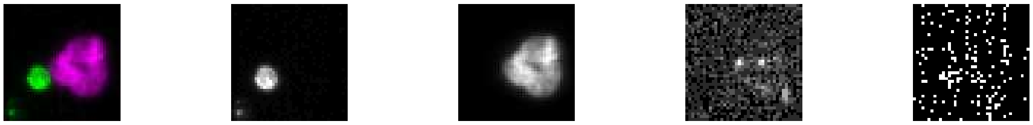				
13871	87	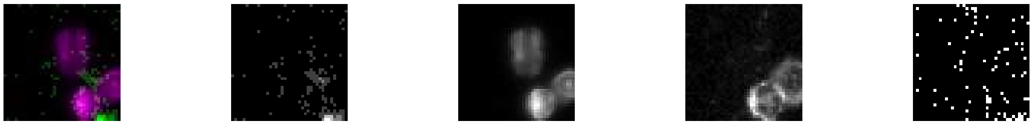				
13872	87	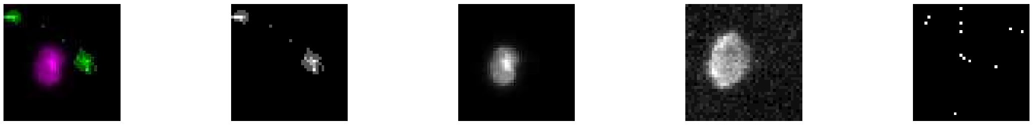				
13873	87	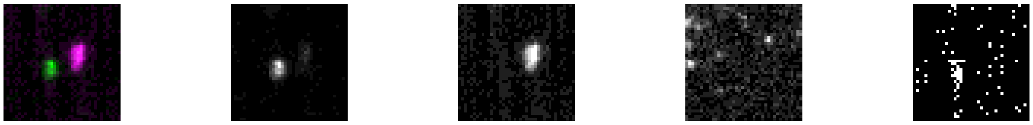				
13874	87	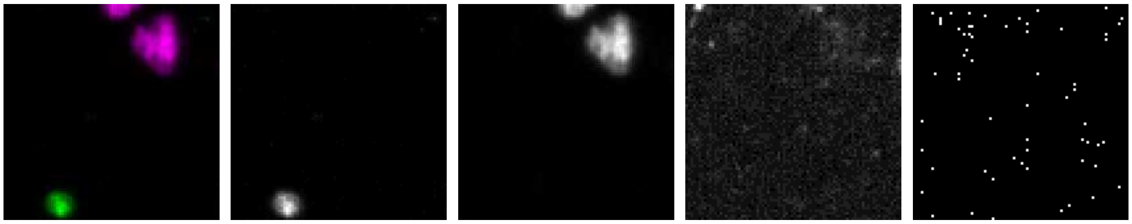				

Ereignis	Aufnahme	DAPI/CK-PE	CK-PE	DAPI	CD45-APC	HER-2/neu
13875	87	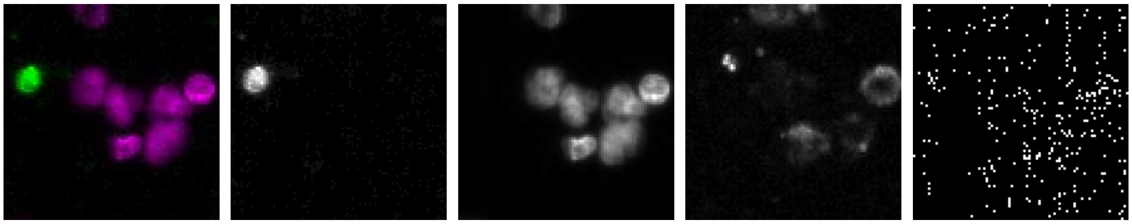				
13876	87	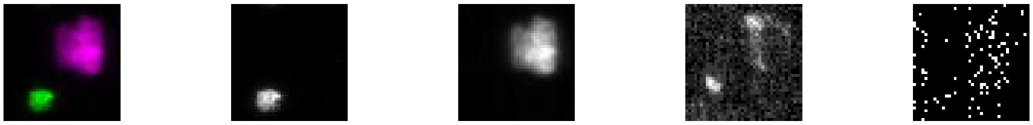				
13877	87	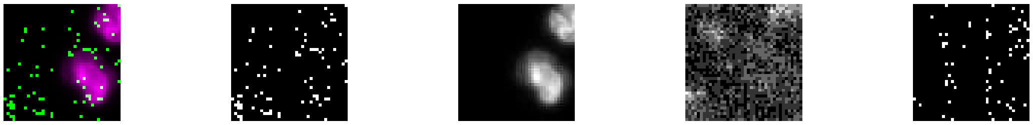				
13878	87	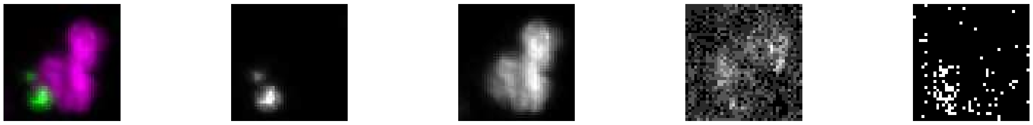				
13879	87	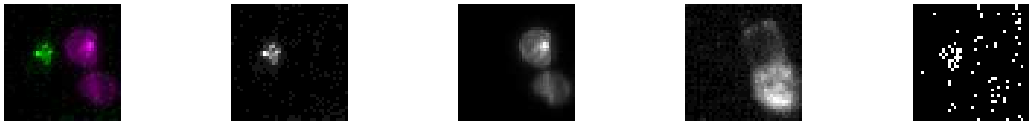				
13880	87	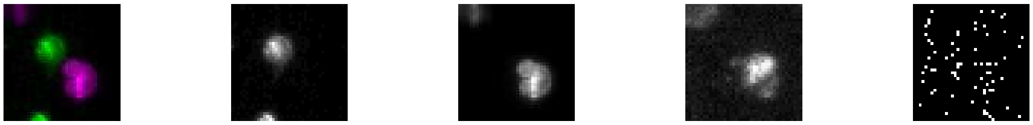				
13881	87	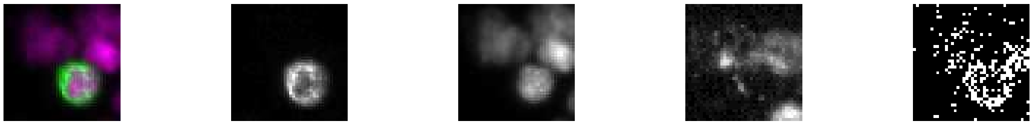				
13882	86	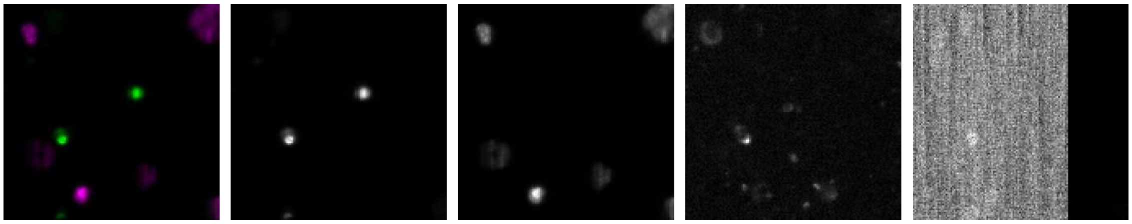				
13883	87	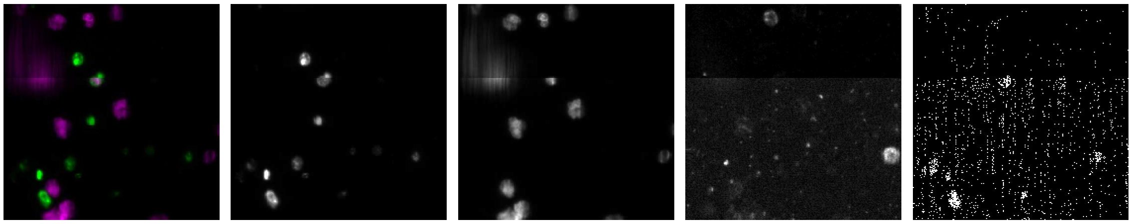				
13884	54	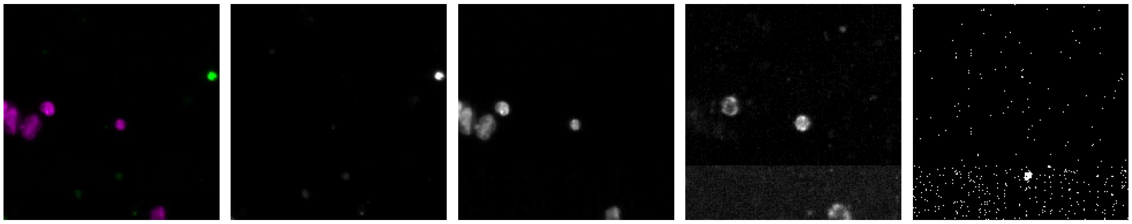				
13885	86	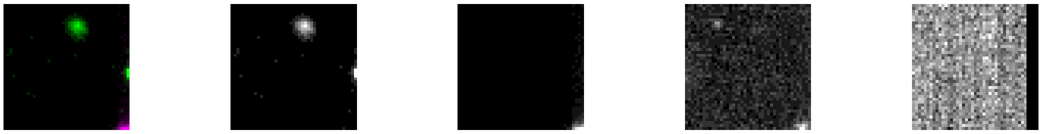				

Ereignis	Aufnahme	DAPI/CK-PE	CK-PE	DAPI	CD45-APC	HER-2/neu
13886	54	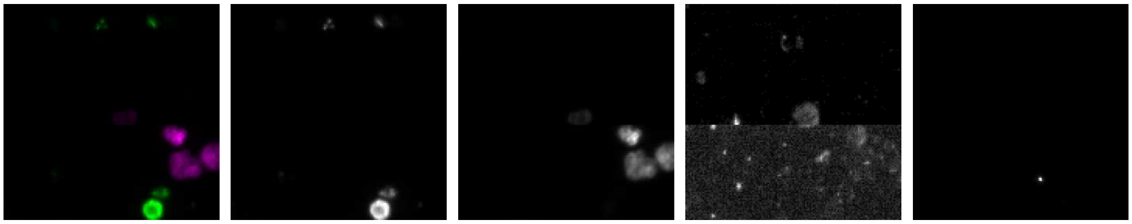				
13887	54	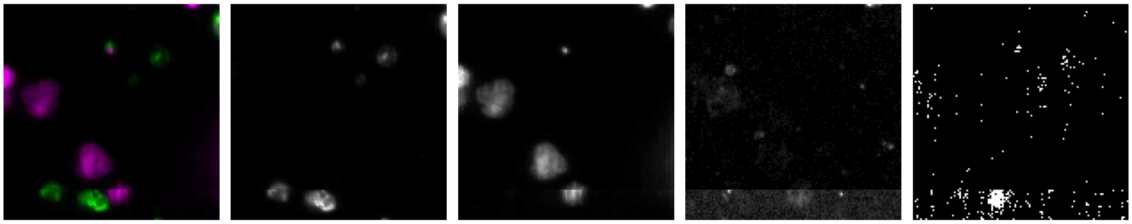				
13888	88	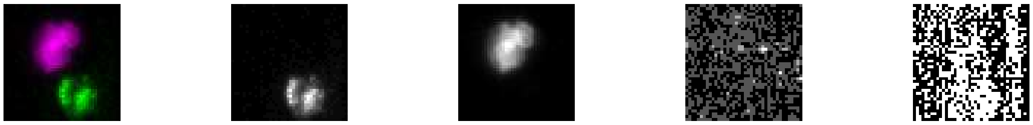				
13889	88	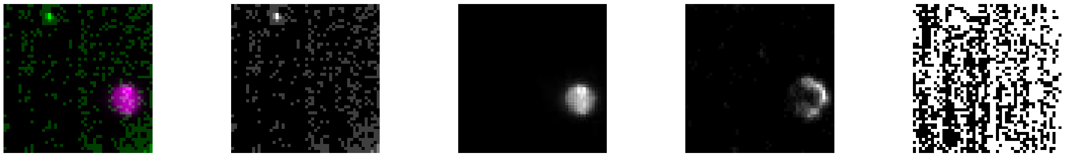				
13890	88	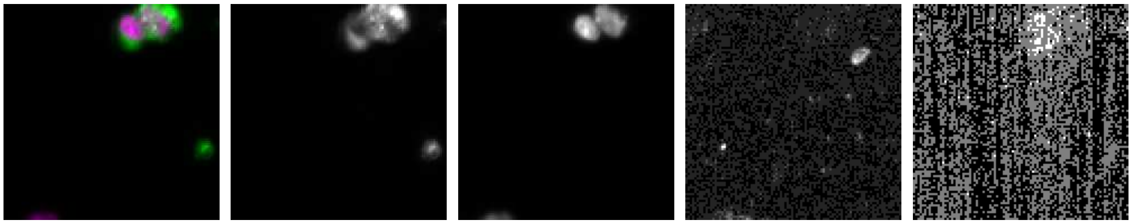				
13891	88	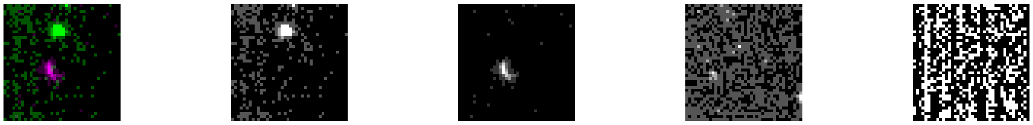				
13892	88	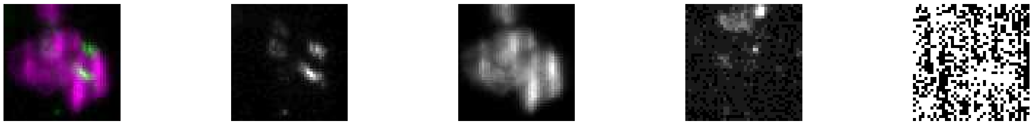				
13893	88	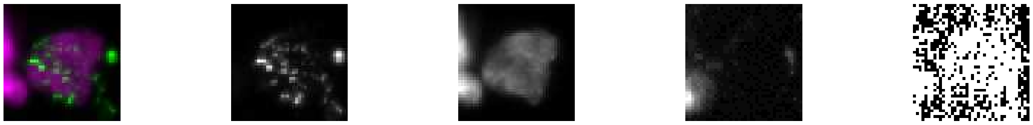				
13894	88	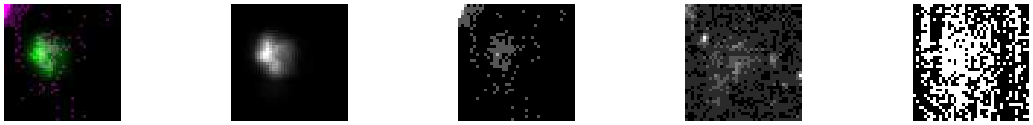				
13895	88	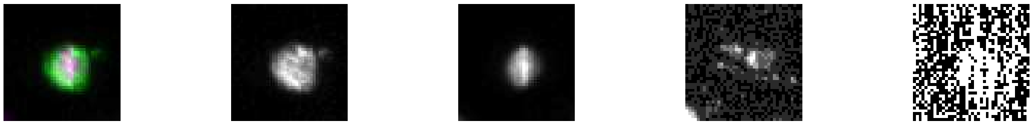				
13896	88	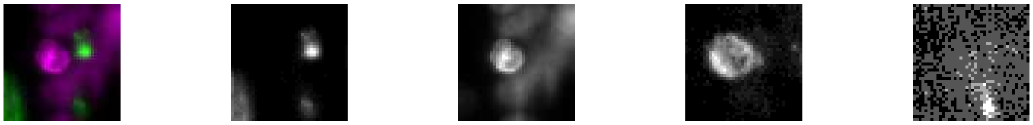				

Ereignis	Aufnahme	DAPI/CK-PE	CK-PE	DAPI	CD45-APC	HER-2/neu
13897	88	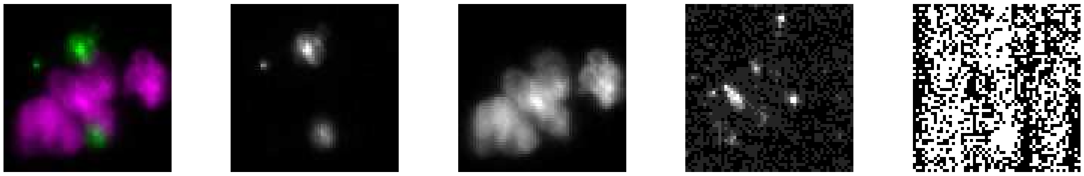				
13898	88	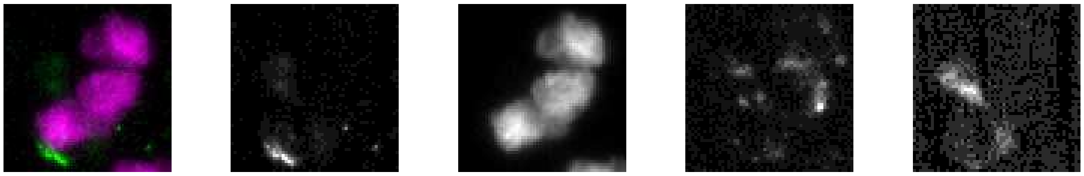				
13899	88	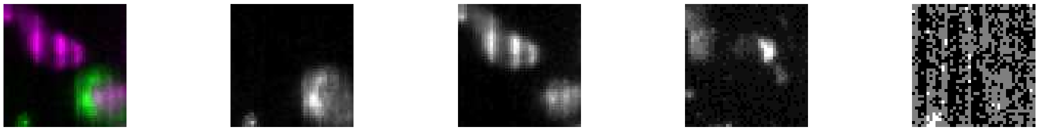				
13900	88	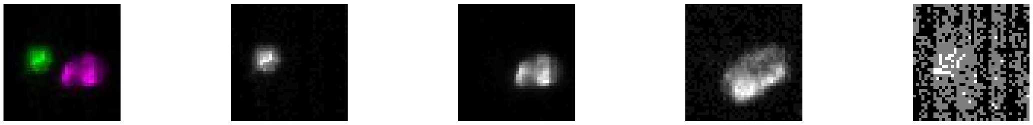				
13901	88	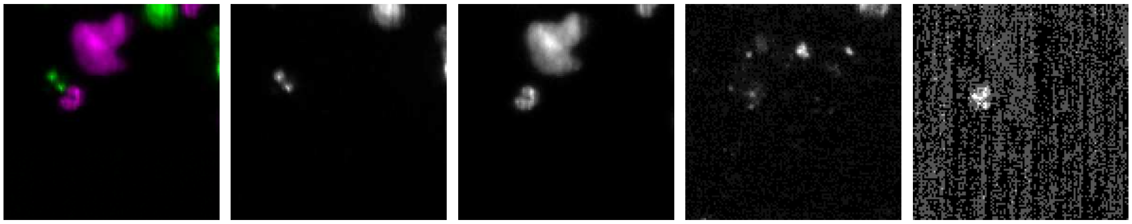				
13902	88	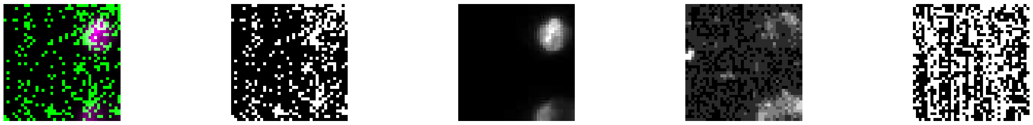				
13903	88	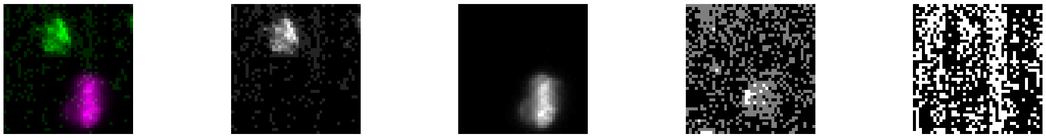				
13904	88	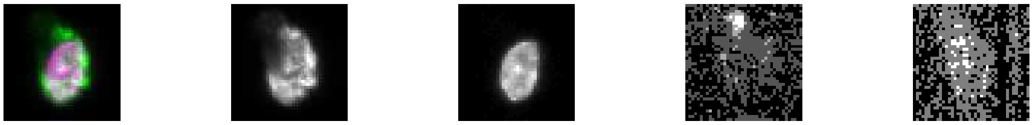				
13905	88	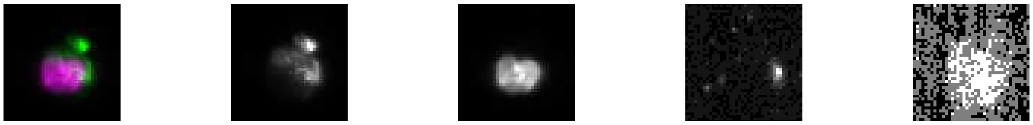				
13906	88	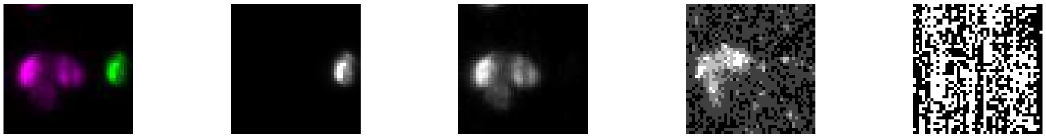				
13907	88	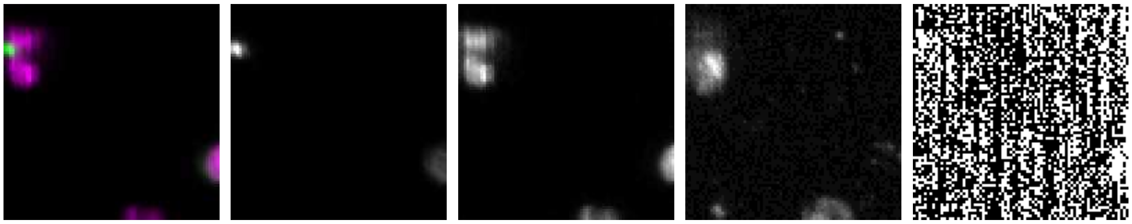				

Proben-ID: Gyn14.208 **Kassetten-ID:**01327106 **Patienten-ID:**

Ereignis	Aufnahme	DAPI/CK-PE	CK-PE	DAPI	CD45-APC	HER-2/neu
13908	88	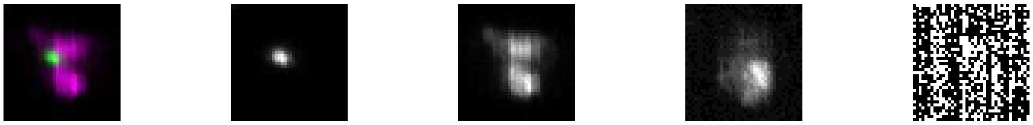				
13909	88	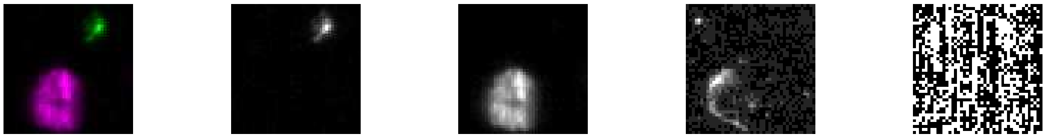				
13910	88	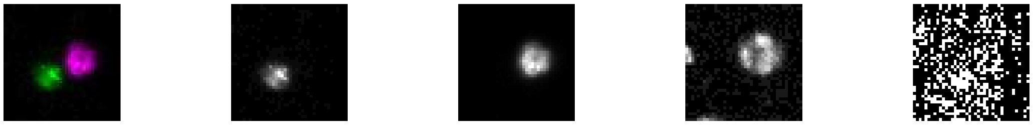				
13911	88	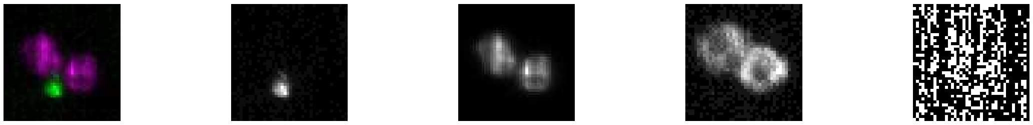				
13912	88	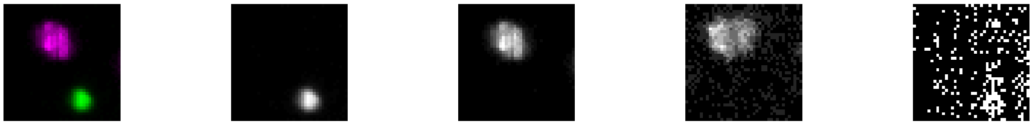				
13913	88	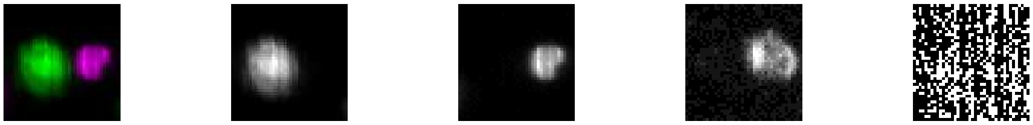				
13914	88	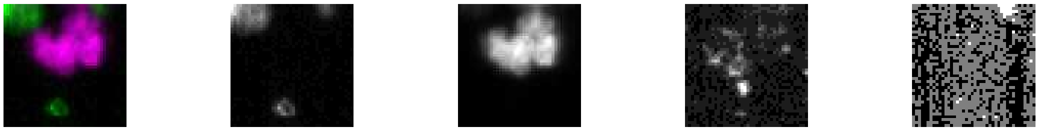				
13915	88	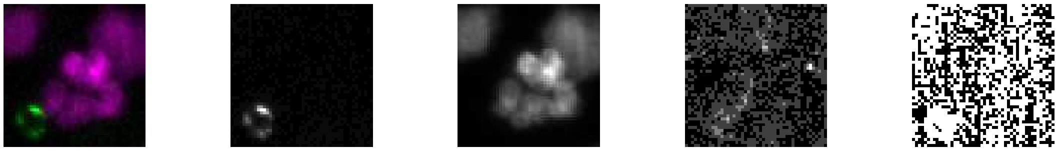				
13916	88	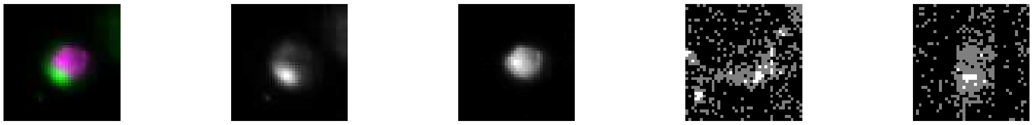				
13917	88	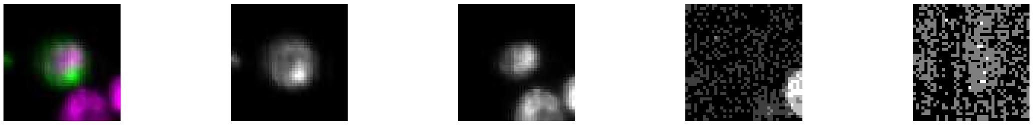				
13918	88	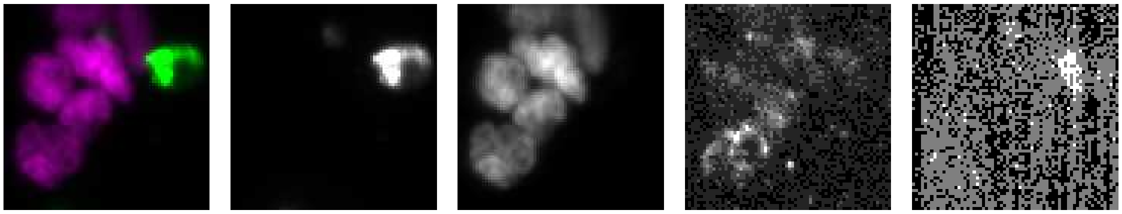				
13919	88	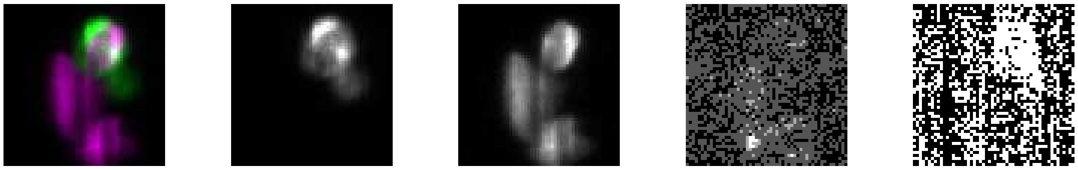				

Ereignis	Aufnahme	DAPI/CK-PE	CK-PE	DAPI	CD45-APC	HER-2/neu
13920	88	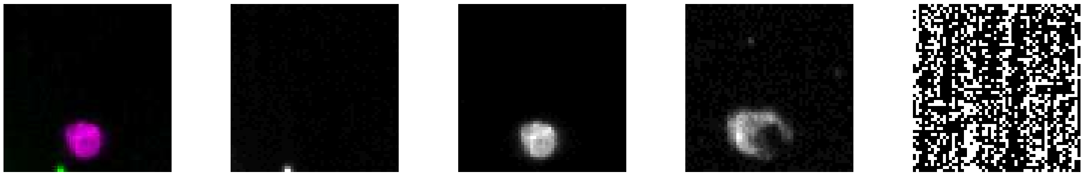				
13921	88	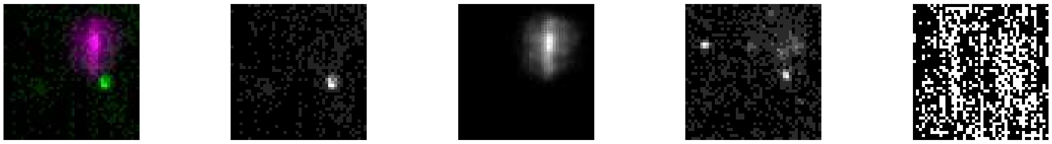				
13922	88	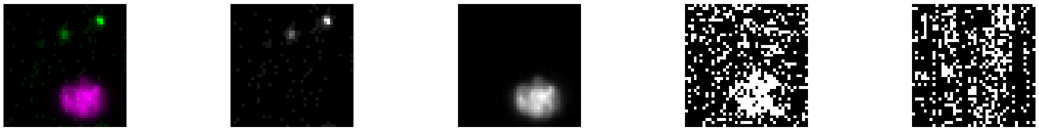				
13923	88	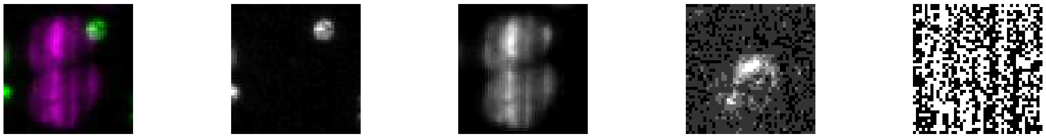				
13924	88	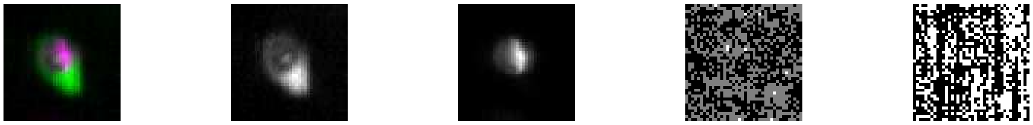				
13925	88	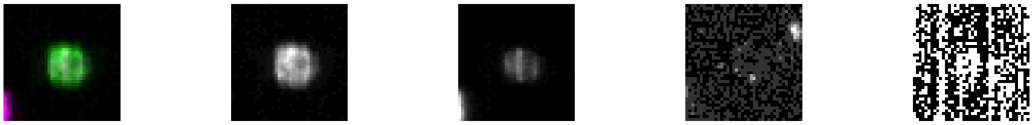				
13926	88	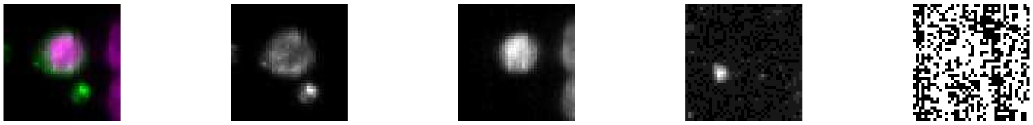				
13927	88	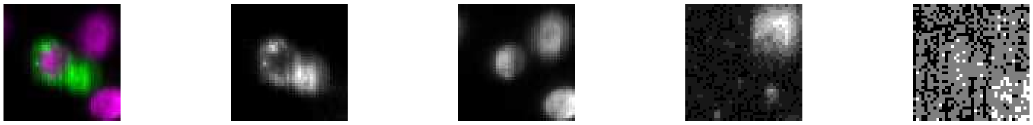				
13928	88	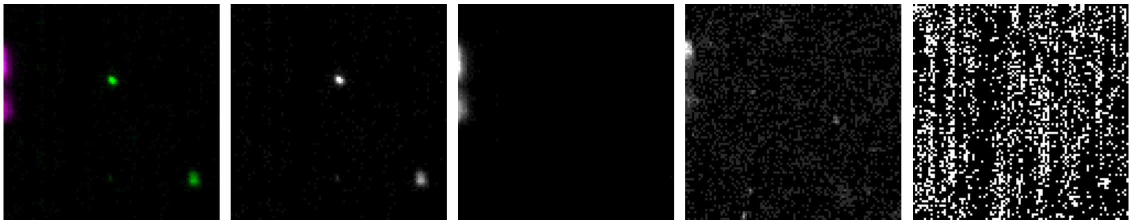				
13929	88	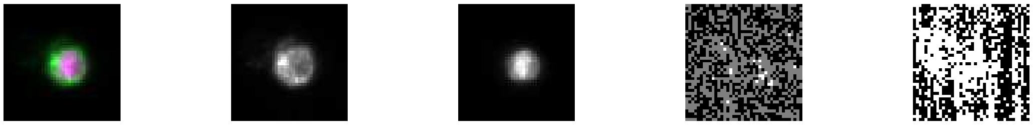				
13930	88	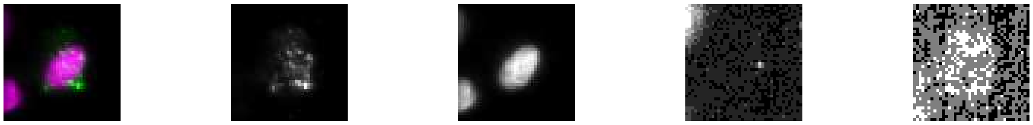				
13931	88	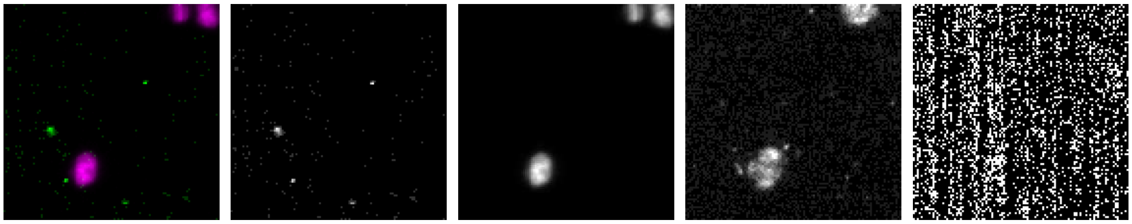				

Ereignis	Aufnahme	DAPI/CK-PE	CK-PE	DAPI	CD45-APC	HER-2/neu
13932	88	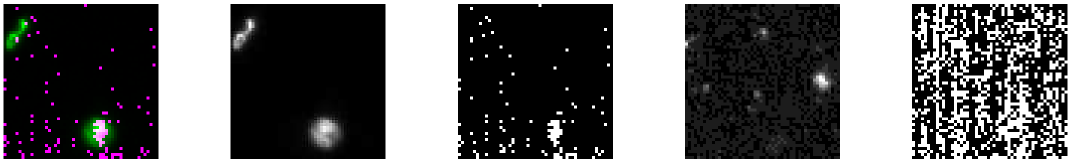				
13933	88	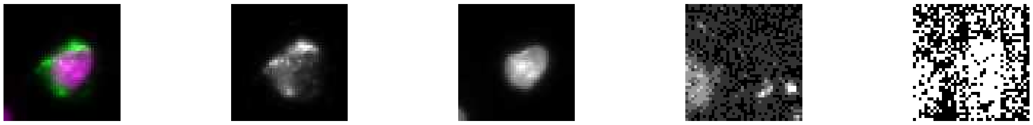				
13934	88	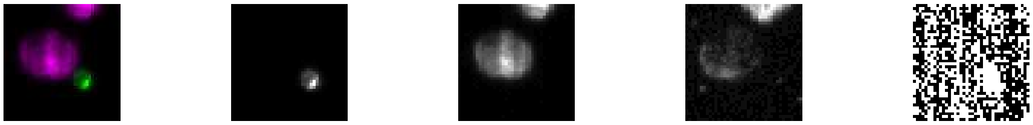				
13935	88	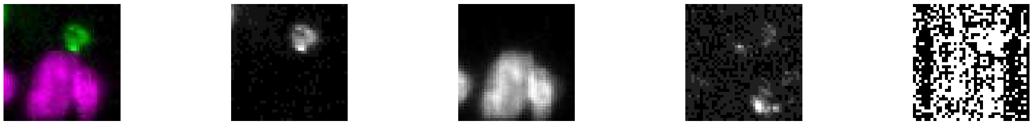				
13936	88	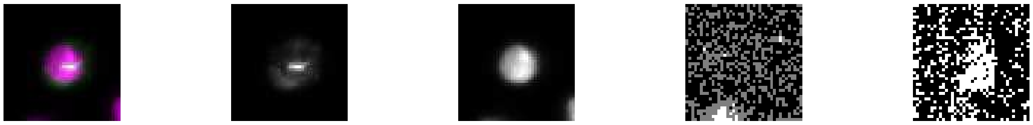				
13937	88	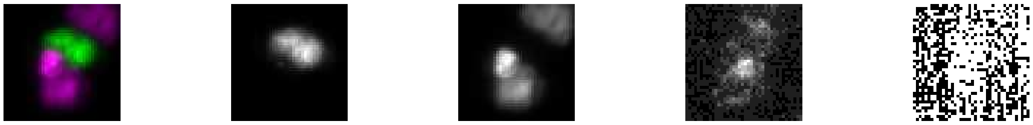				
13938	88	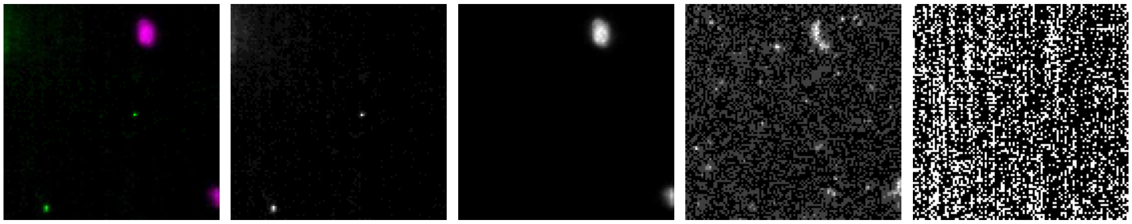				
13939	88	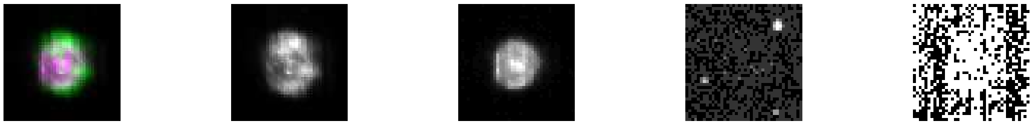				
13940	88	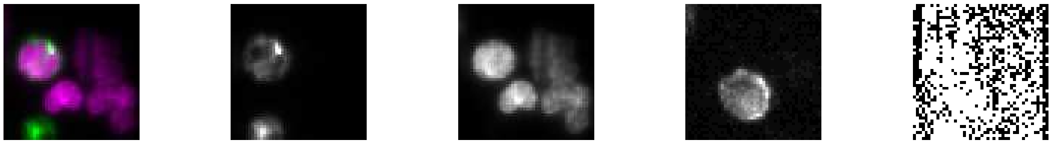				
13941	88	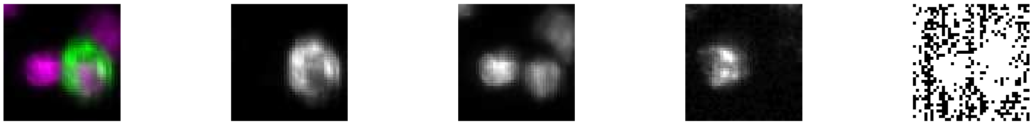				
13942	88	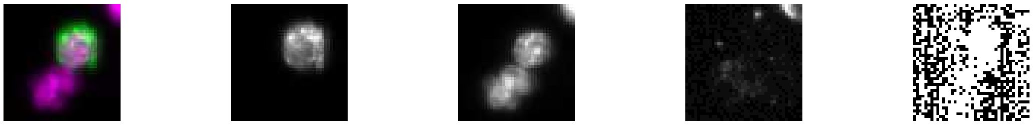				
13943	88	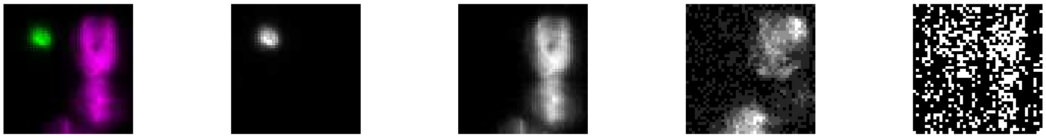				
13944	88	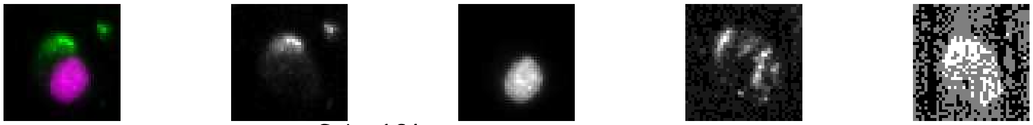				

Ereignis	Aufnahme	DAPI/CK-PE	CK-PE	DAPI	CD45-APC	HER-2/neu
13945	88	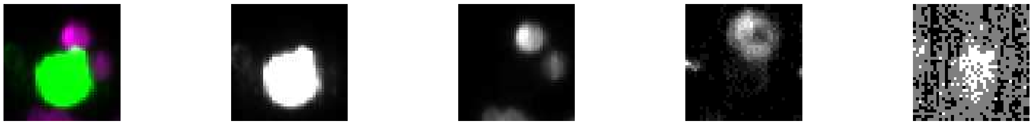				
13946	88	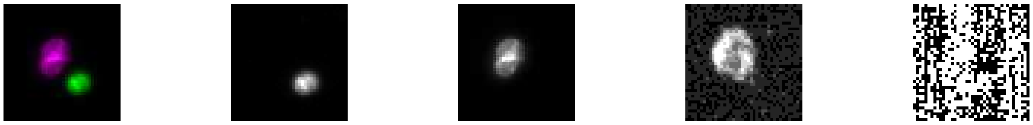				
13947	88	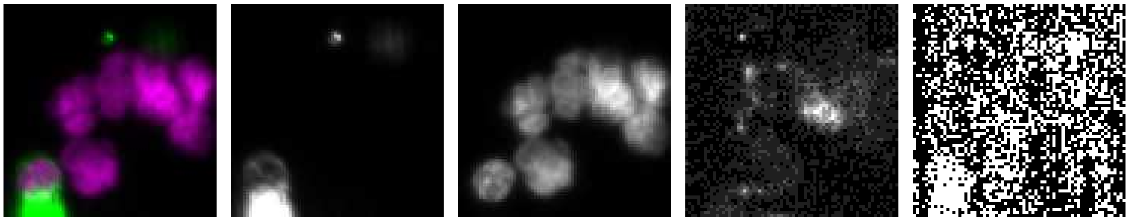				
13948	88	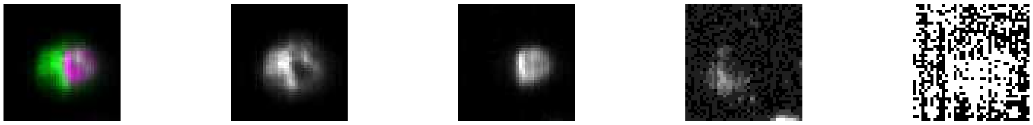				
13949	88	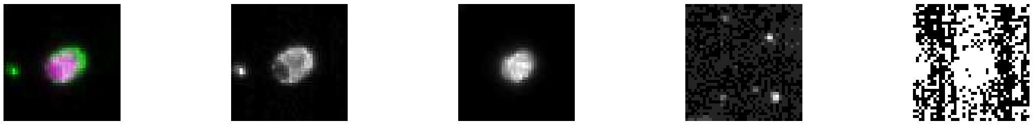				
13950	88	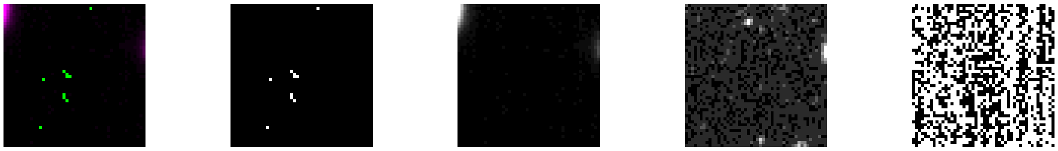				
13951	88	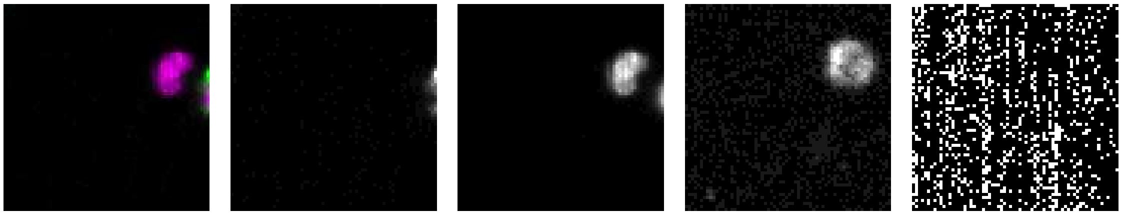				
13952	88	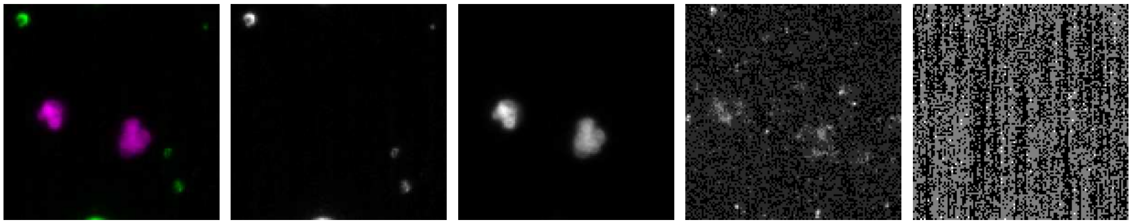				
13953	88	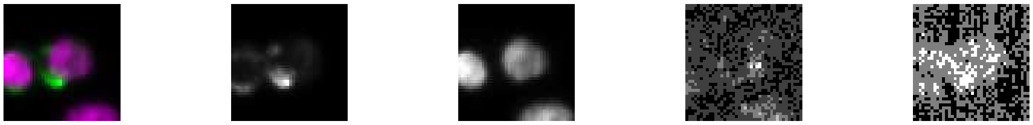				
13954	88	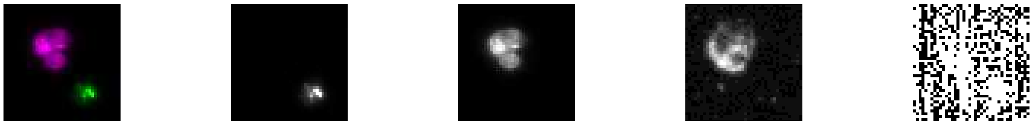				
13955	88	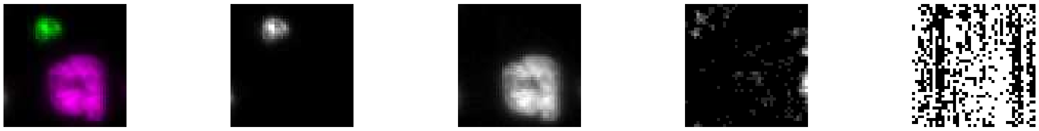				

Ereignis	Aufnahme	DAPI/CK-PE	CK-PE	DAPI	CD45-APC	HER-2/neu
13956	88	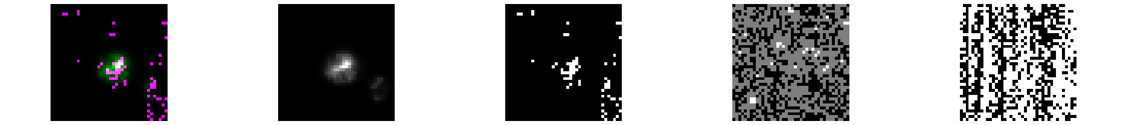				
13957	88	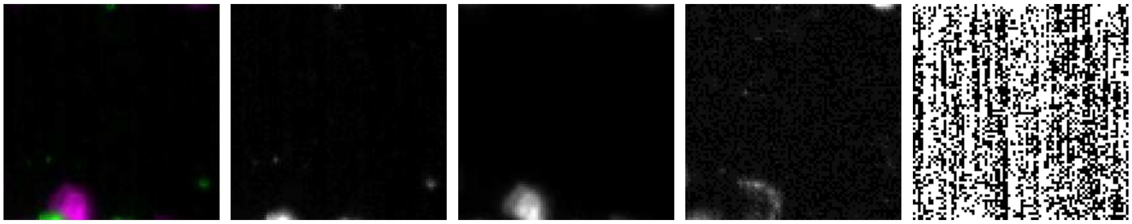				
13958	88	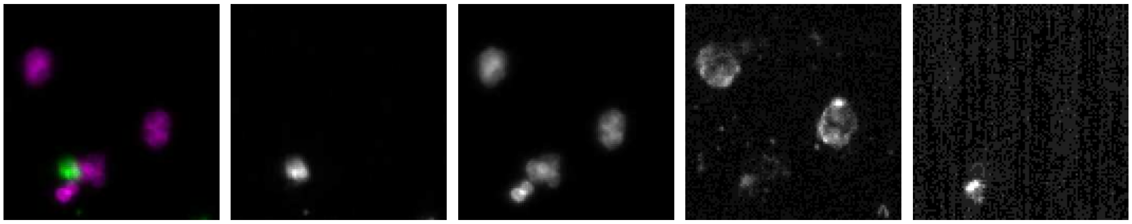				
13959	88	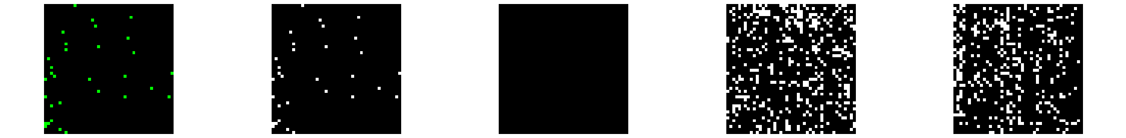				
13960	88	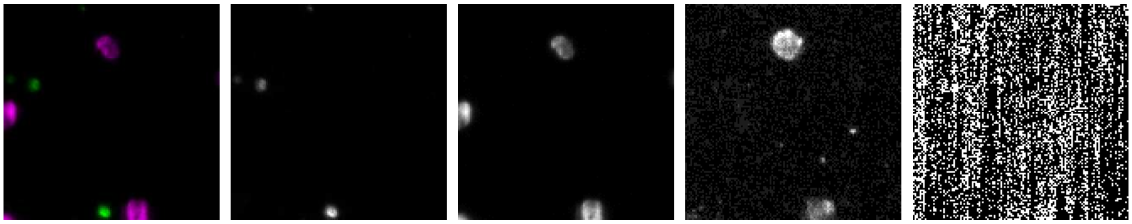				
13961	88	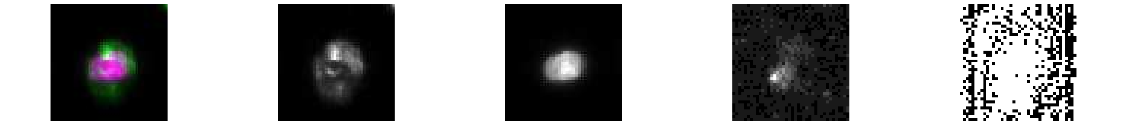				
13962	88	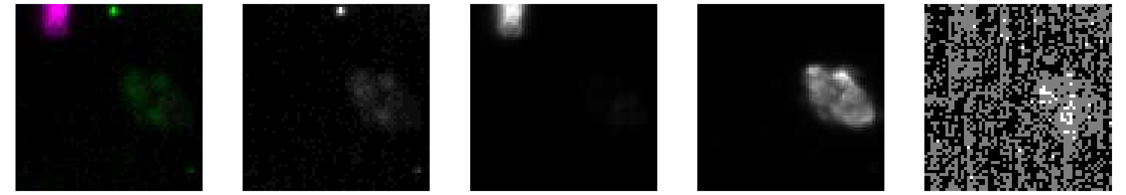				
13963	88	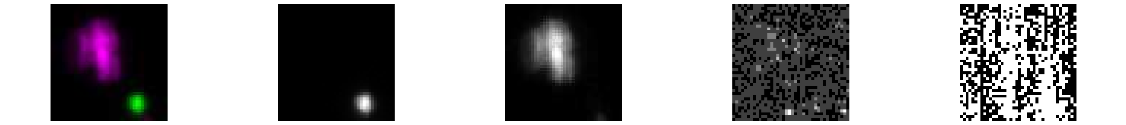				
13964	88	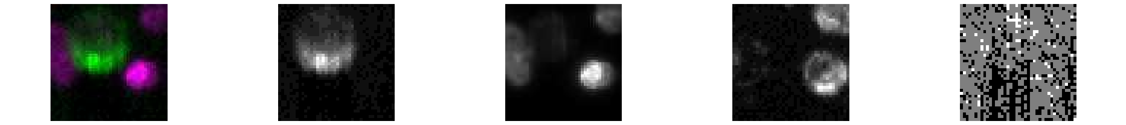				
13965	88	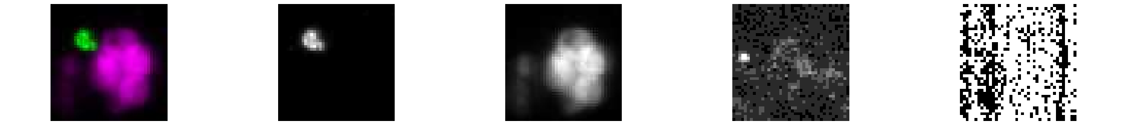				
13966	88	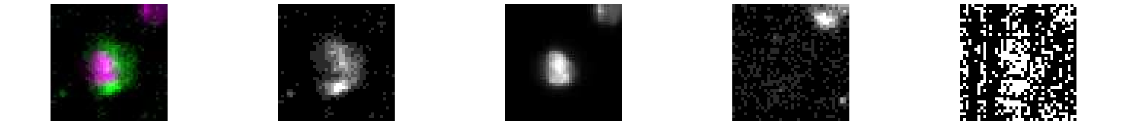				

Ereignis	Aufnahme	DAPI/CK-PE	CK-PE	DAPI	CD45-APC	HER-2/neu
13967	88	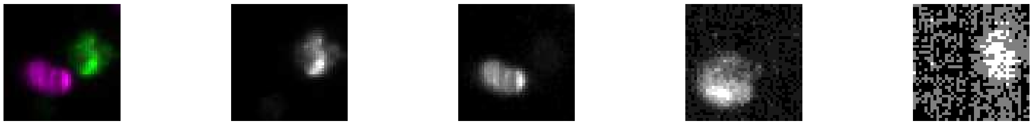				
13968	88	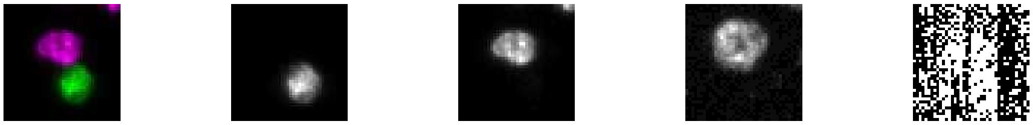				
13969	88	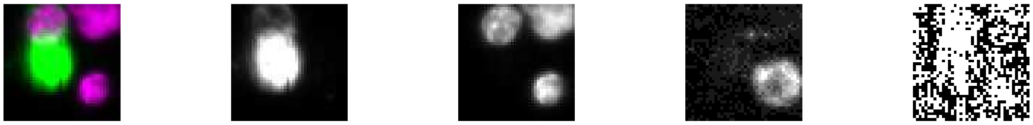				
13970	88	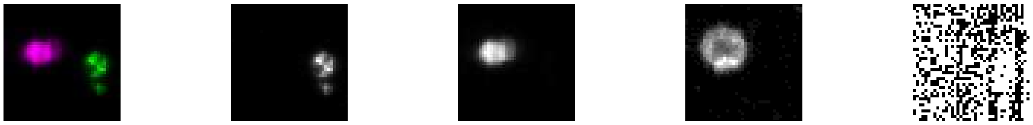				
13971	88	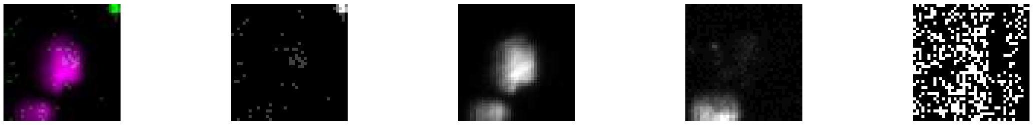				
13972	88	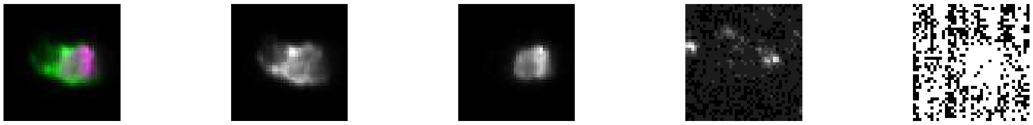				
13973	88	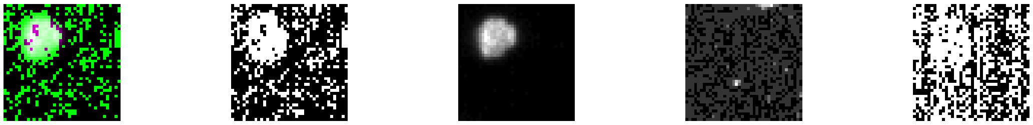				
13974	88	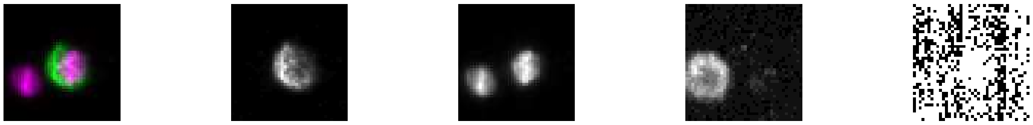				
13975	88	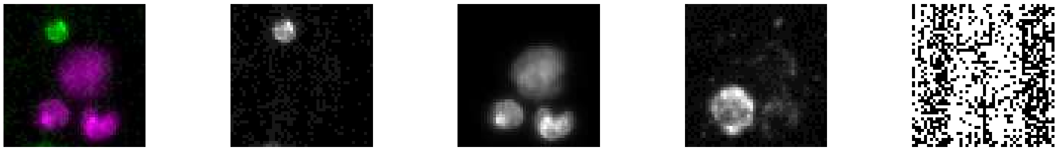				
13976	88	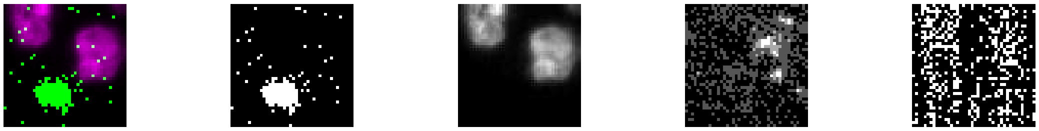				
13977	88	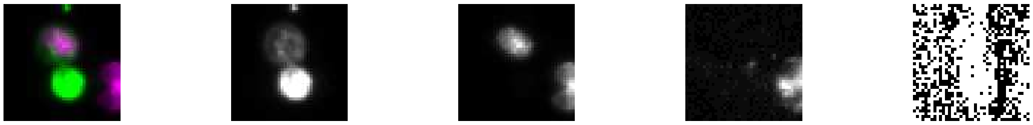				
13978	88	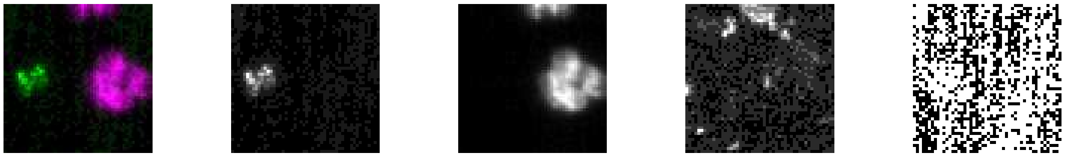				
13979	88	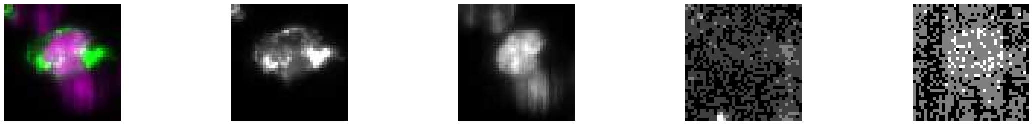				

Druckdatum: 02/06/2020 17:00

Proben-ID: Gyn14.208 **Kassetten-ID:** 01327106 **Patienten-ID:**

Ereignis	Aufnahme	DAPI/CK-PE	CK-PE	DAPI	CD45-APC	HER-2/neu
13980	88	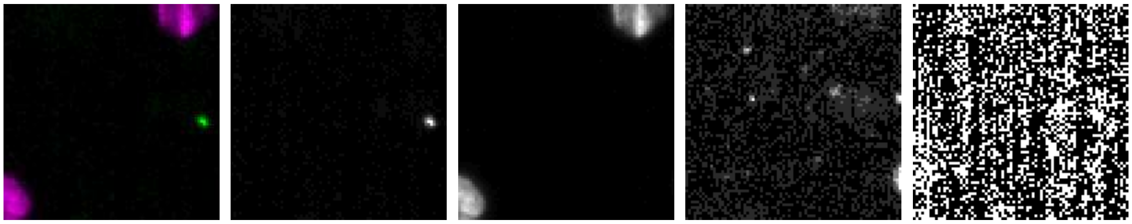				
13981	88	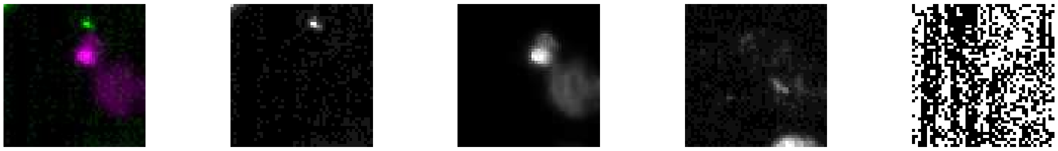				
13982	88	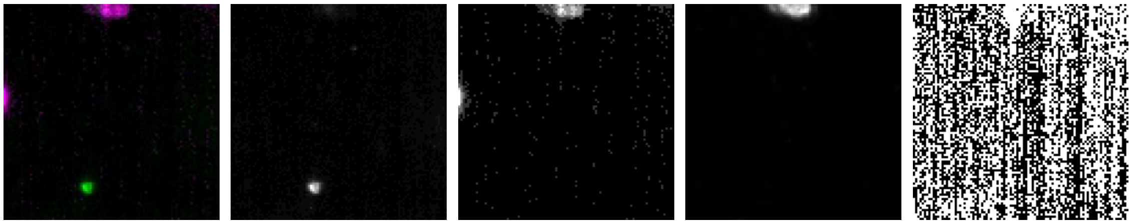				
13983	88	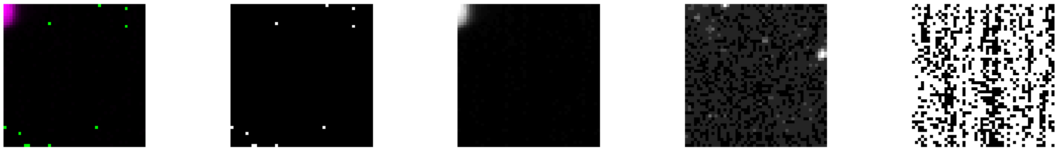				
13984	88	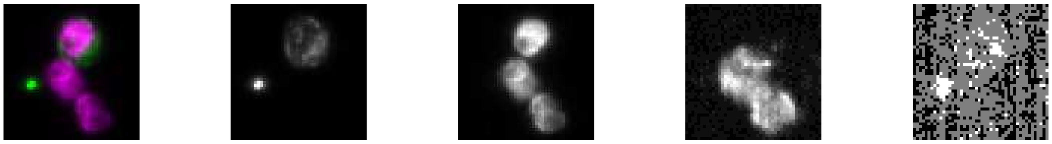				
13985	88	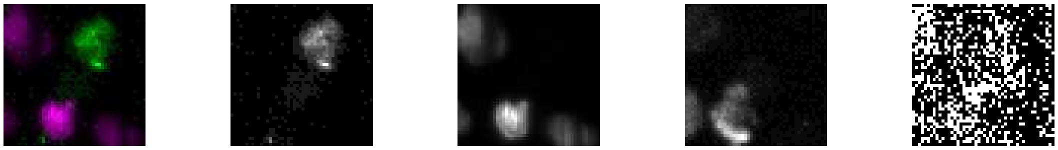				
13986	88	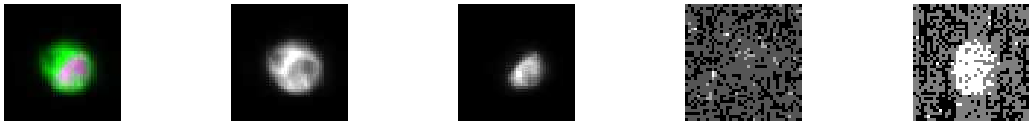				
13987	88	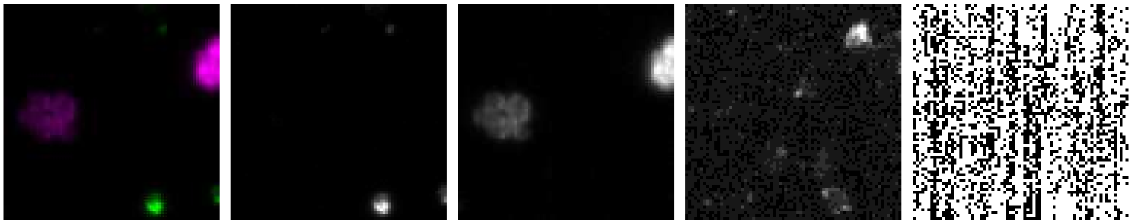				
13988	88	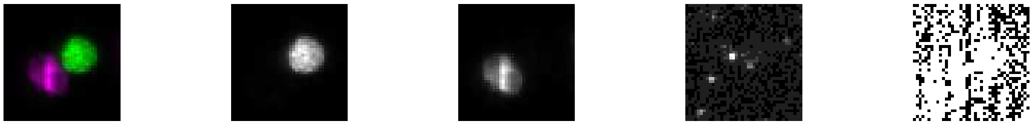				
13989	88	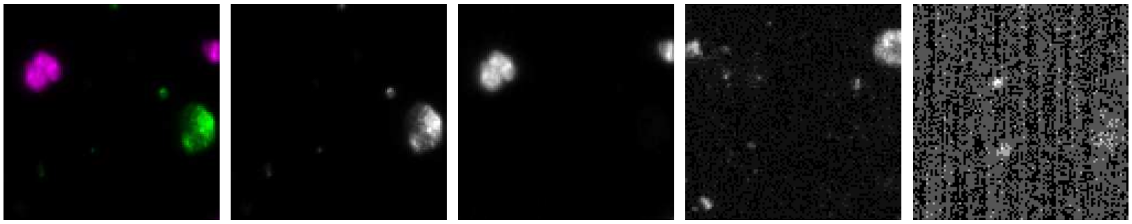				

Ereignis	Aufnahme	DAPI/CK-PE	CK-PE	DAPI	CD45-APC	HER-2/neu
13990	88	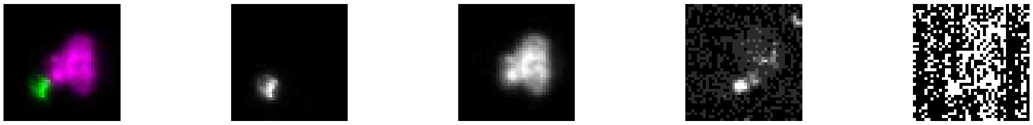				
13991	88	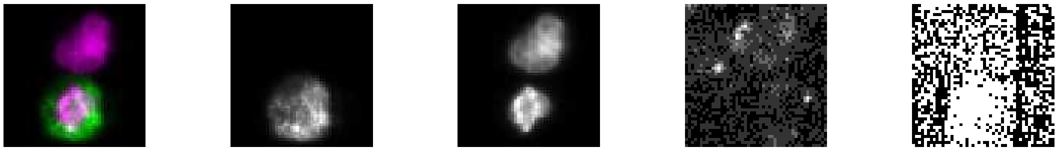				
13992	88	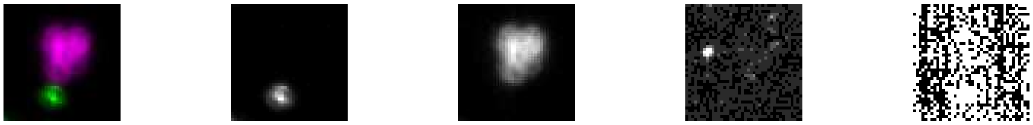				
13993	88	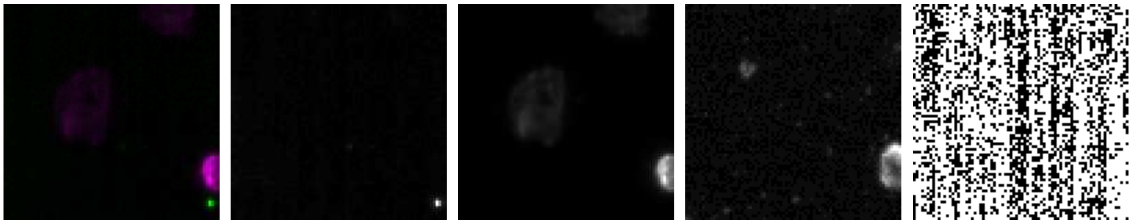				
13994	88	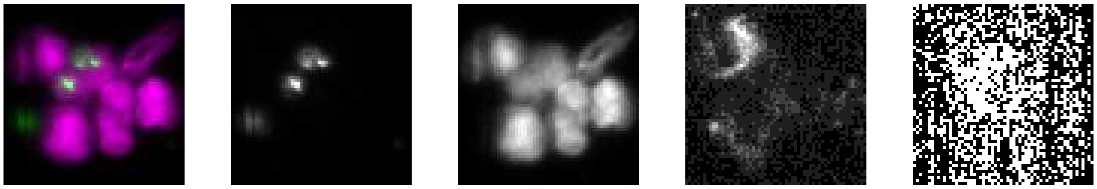				
13995	88	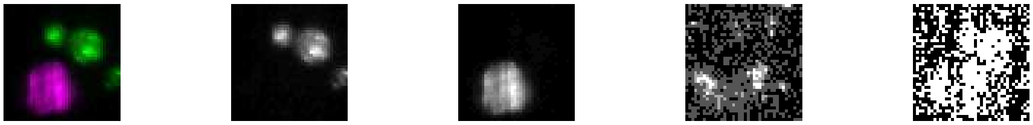				
13996	88	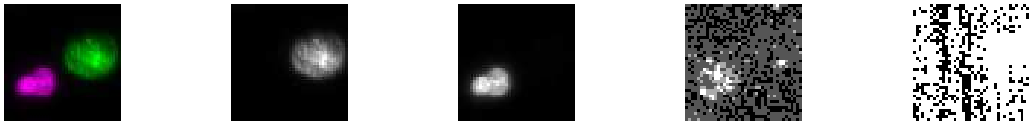				
13997	88	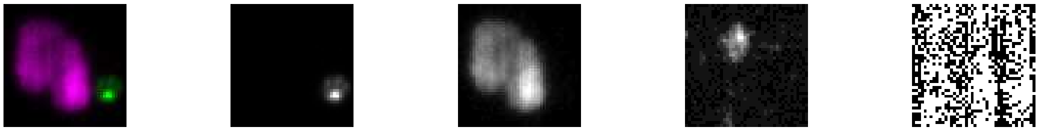				
13998	88	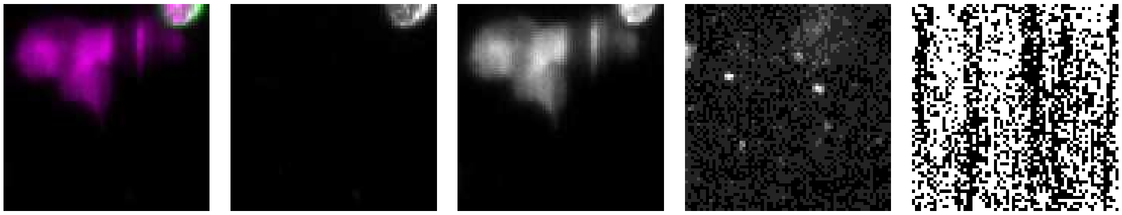				
13999	88	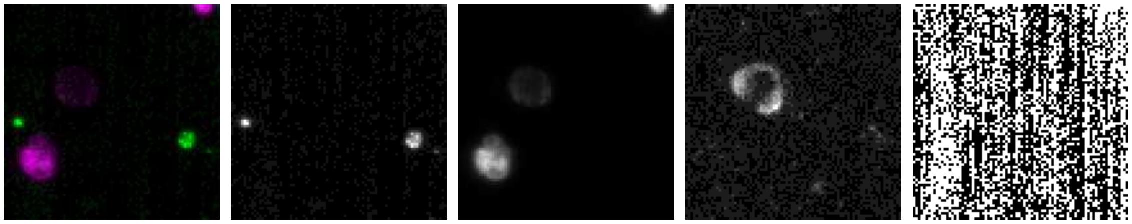				
14000	88	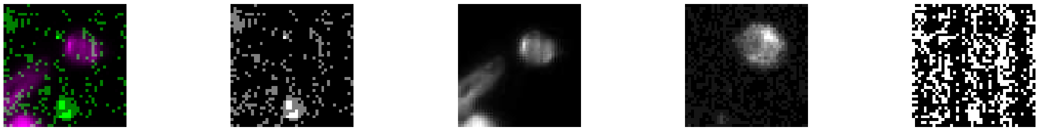				

ÜBERPRÜFUNG DER FORSCHUNGSPROBE DRUCKEN

Druckdatum: 02/06/2020 17:25

Proben-ID: Gyn14.208 **Kassetten-ID:** 01327106 **Patienten-ID:**

Ereignis	Aufnahme	DAPI/CK-PE	CK-PE	DAPI	CD45-APC	HER-2/neu
24001	135	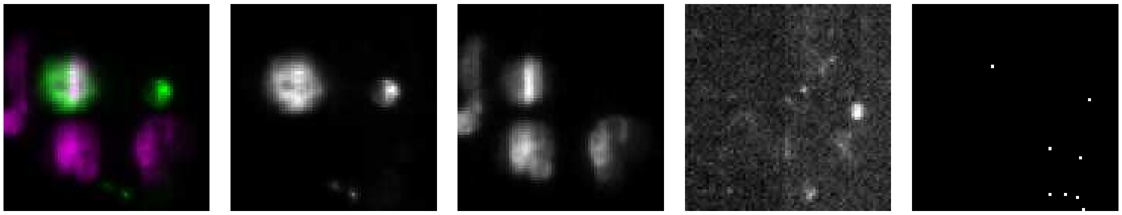				
24002	136	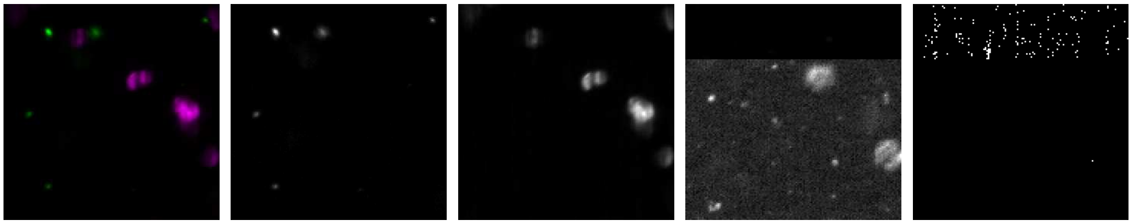				
24003	75	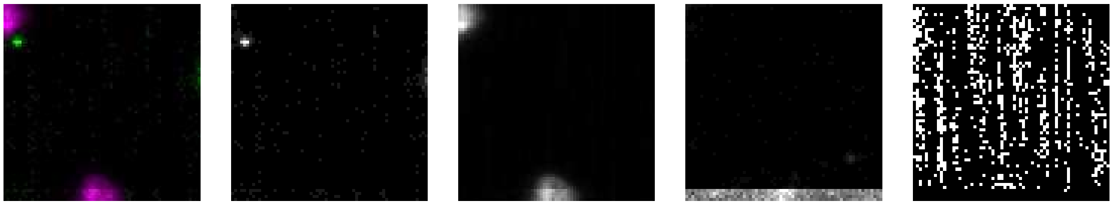				
24004	136	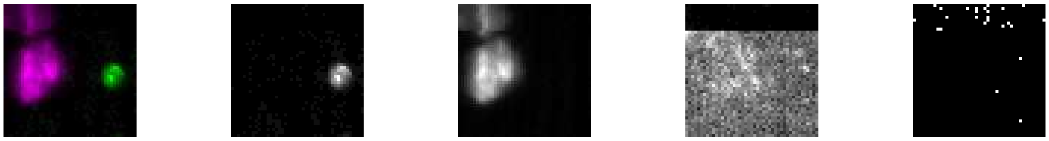				
24005	137	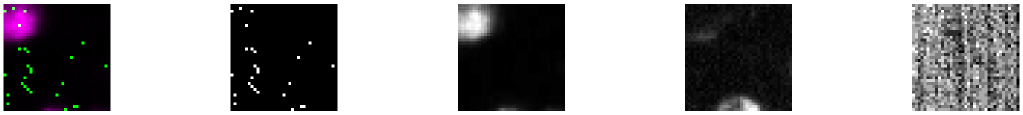				
24006	137	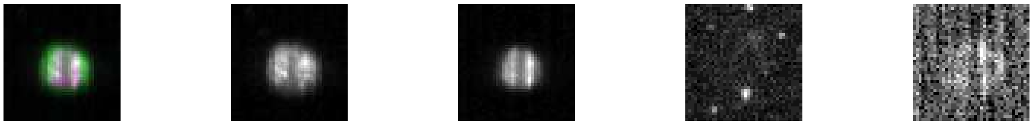				
24007	137	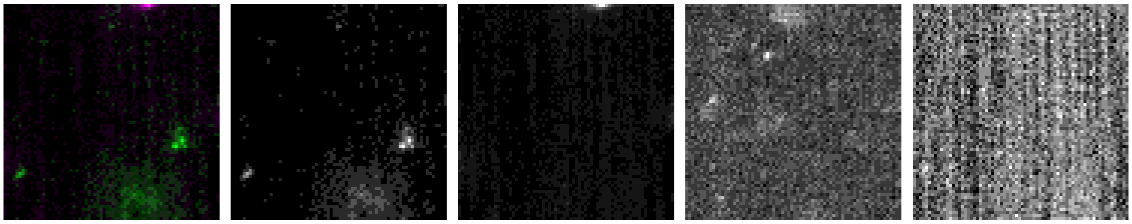				
24008	137	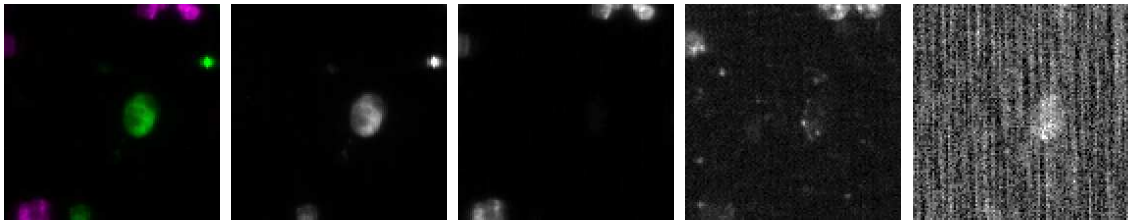				
24009	137	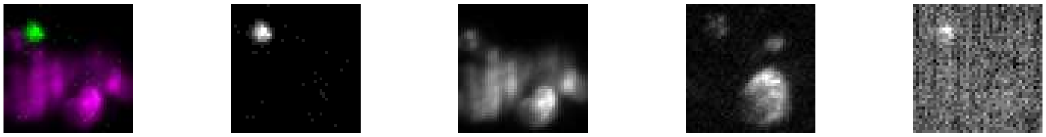				

Druckdatum:02/06/2020 17:25

Proben-ID: Gyn14.208 **Kassetten-ID:**01327106 **Patienten-ID:**

Ereignis	Aufnahme	DAPI/CK-PE	CK-PE	DAPI	CD45-APC	HER-2/neu
24010	137	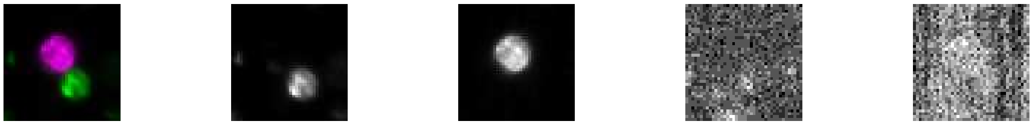				
24011	137	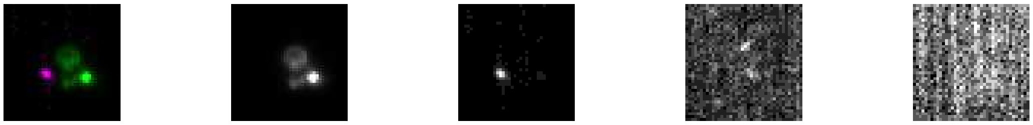				
24012	137	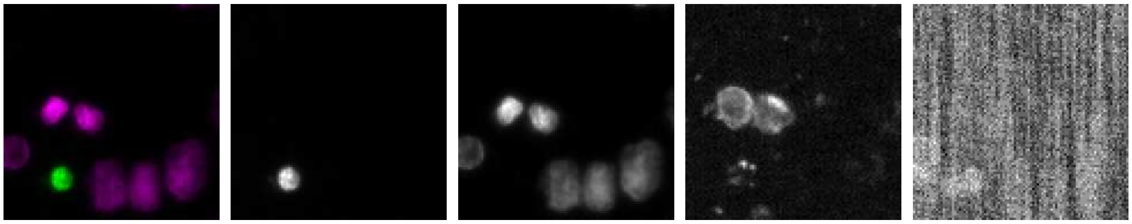				
24013	137	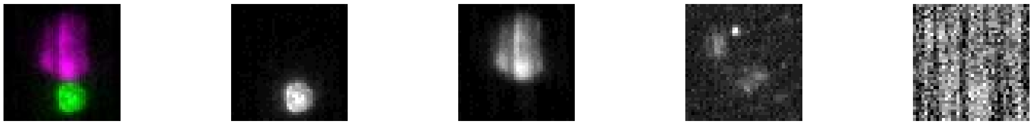				
24014	137	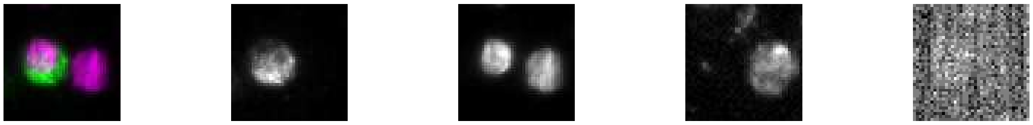				
24015	137	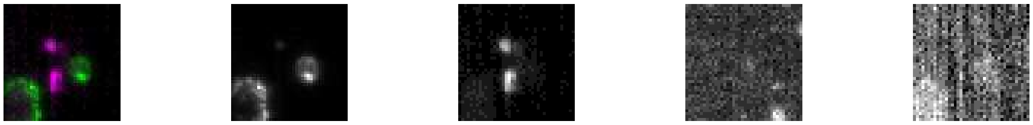				
24016	137	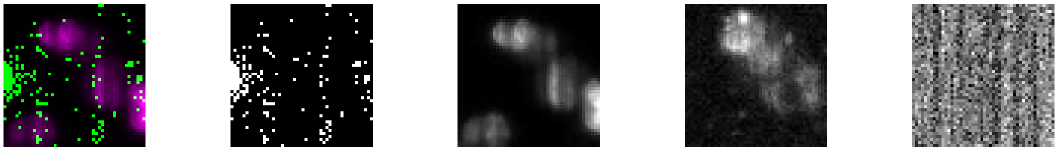				
24017	137	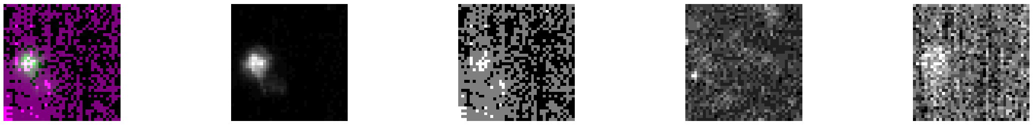				
24018	137	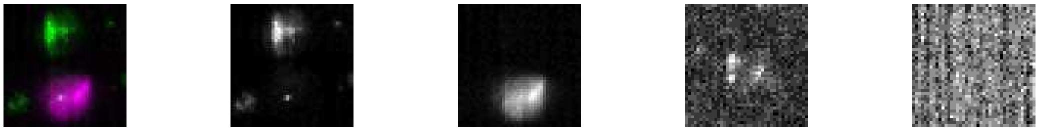				
24019	137	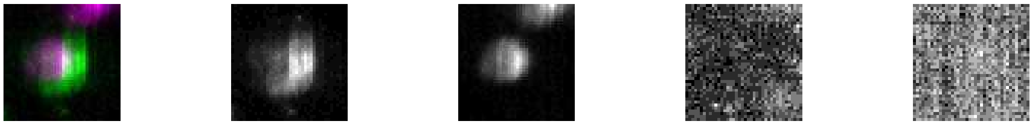				
24020	137	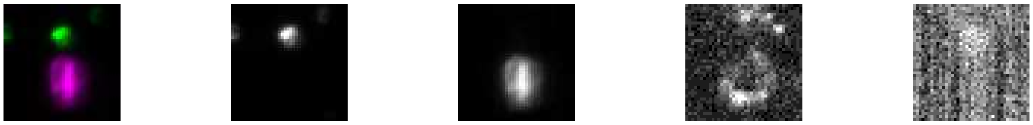				
24021	137	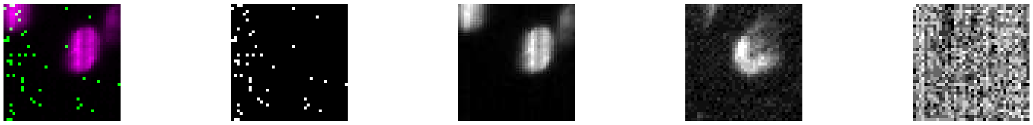				
24022	137	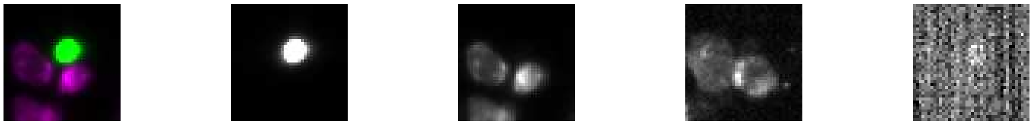				

Ereignis	Aufnahme	DAPI/CK-PE	CK-PE	DAPI	CD45-APC	HER-2/neu
24023	137	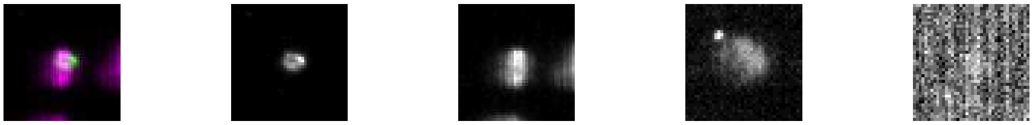				
24024	137	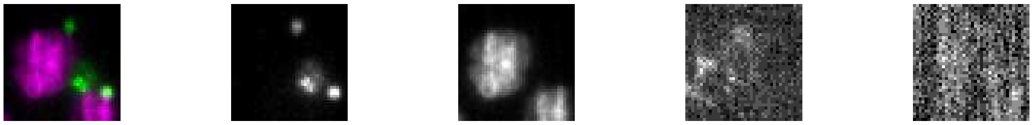				
24025	137	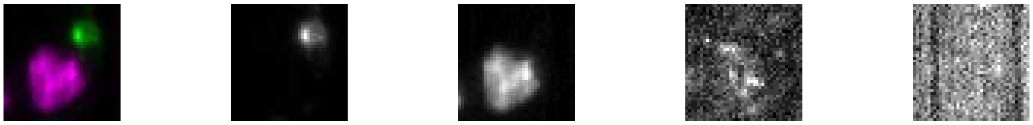				
24026	137	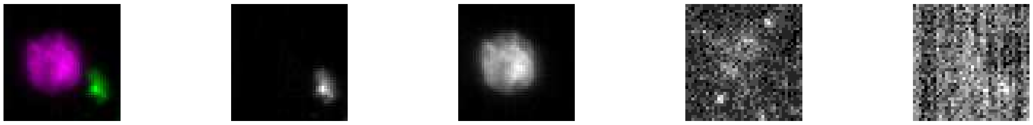				
24027	137	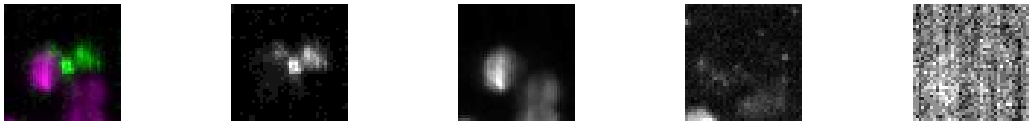				
24028	137	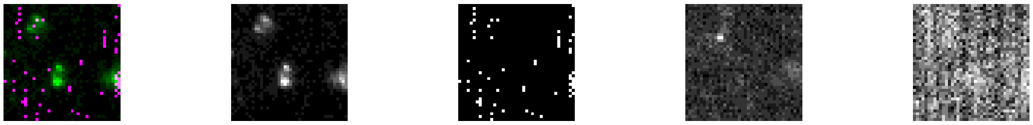				
24029	137	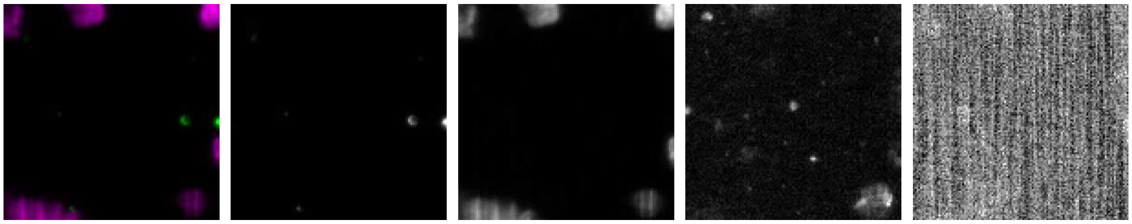				
24030	137	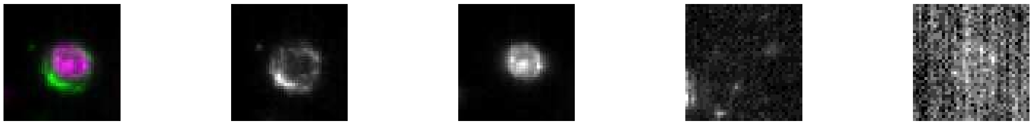				
24031	137	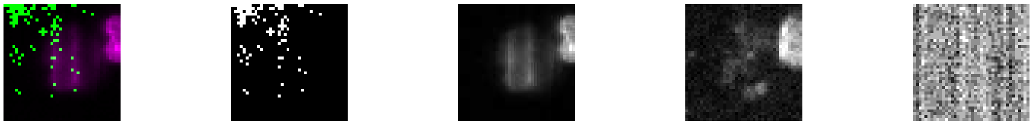				
24032	137	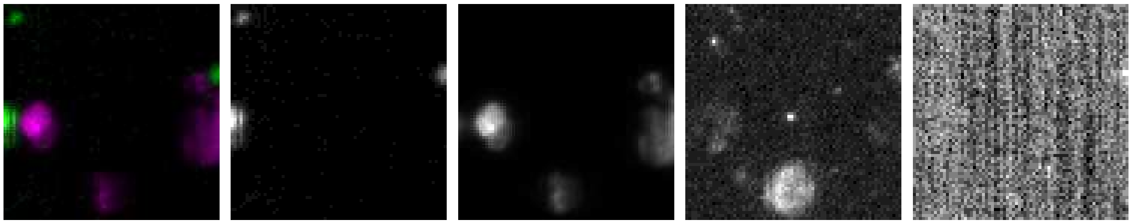				
24033	137	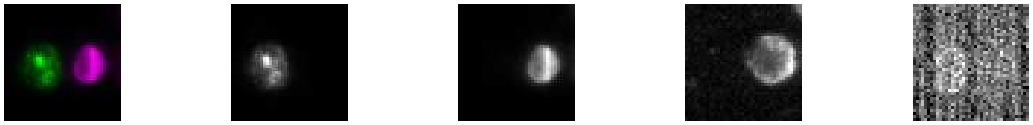				
24034	137	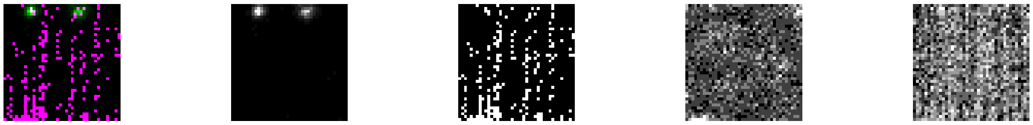				

Ereignis	Aufnahme	DAPI/CK-PE	CK-PE	DAPI	CD45-APC	HER-2/neu
24035	137	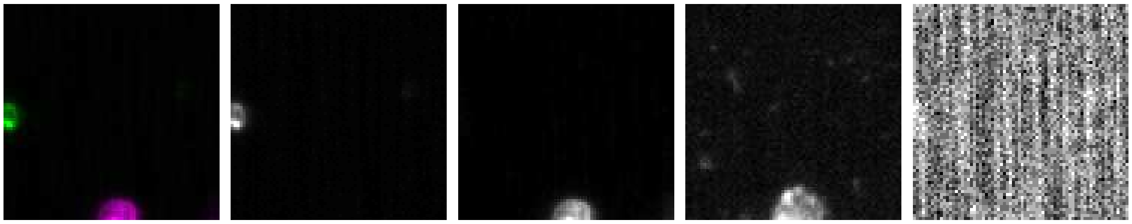				
24036	137	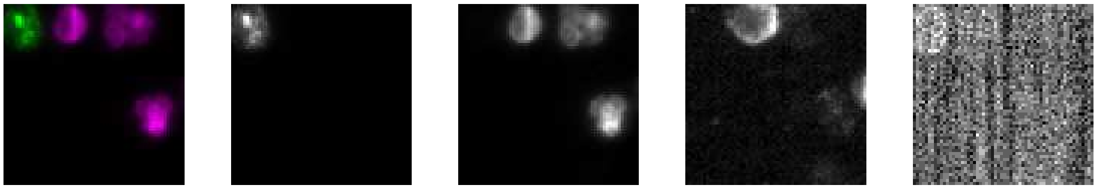				
24037	137	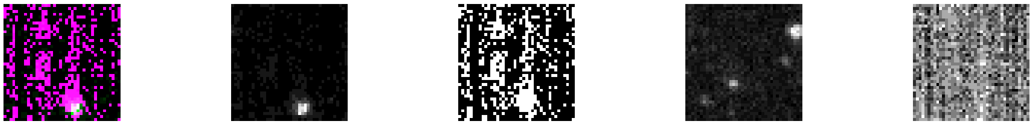				
24038	137	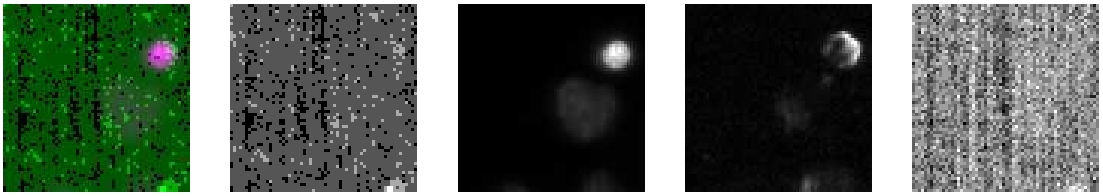				
24039	137	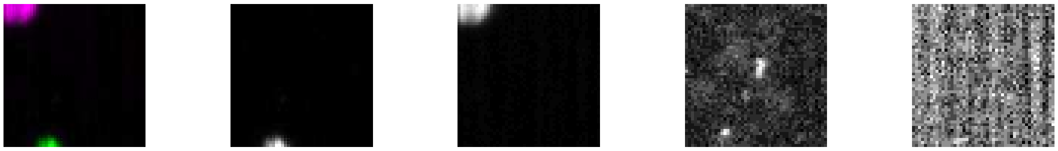				
24040	137	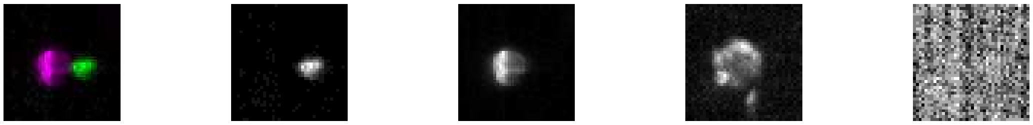				
24041	137	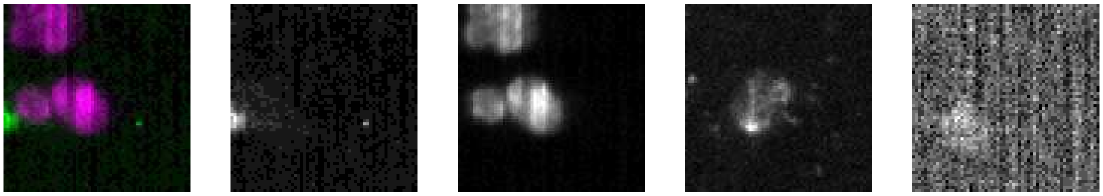				
24042	137	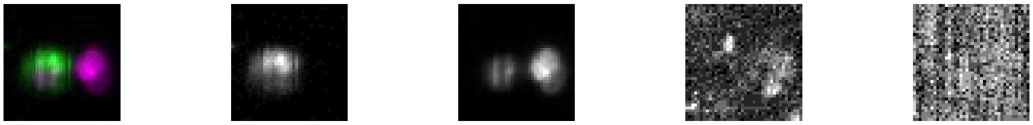				
24043	137	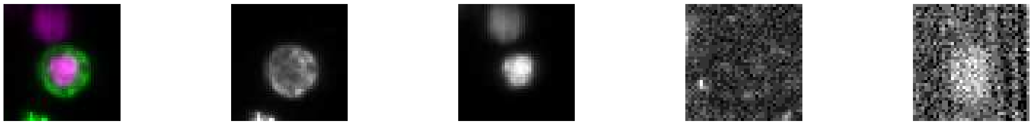				
24044	137	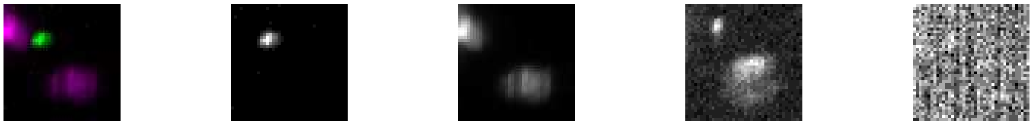				
24045	137	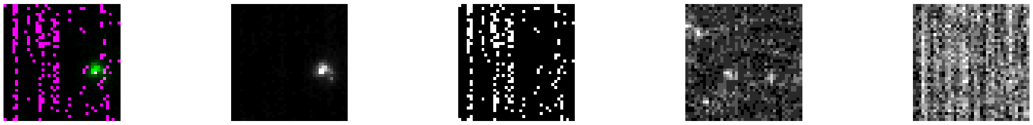				

Proben-ID: Gyn14.208 **Kassetten-ID:**01327106 **Druckdatum:**02/06/2020 17:25
Patienten-ID:

Ereignis	Aufnahme	DAPI/CK-PE	CK-PE	DAPI	CD45-APC	HER-2/neu
24046	137	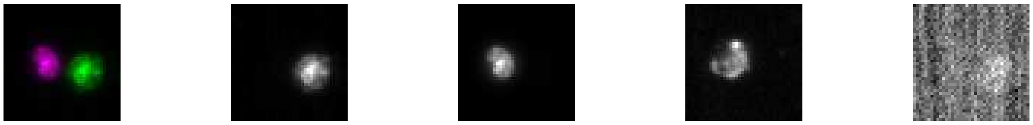				
24047	137	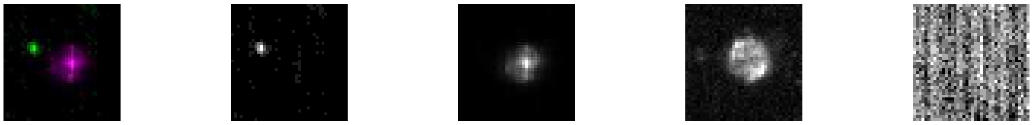				
24048	137	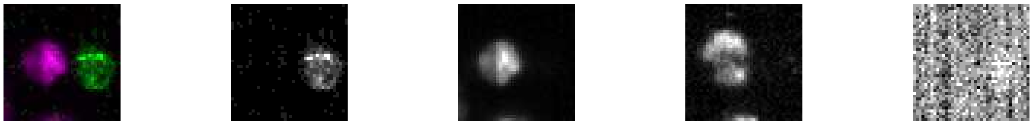				
24049	137	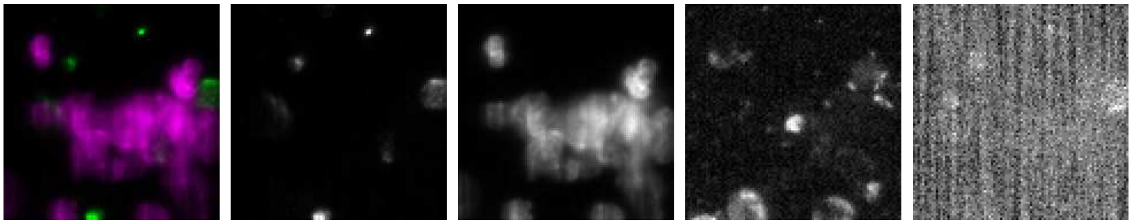				
24050	137	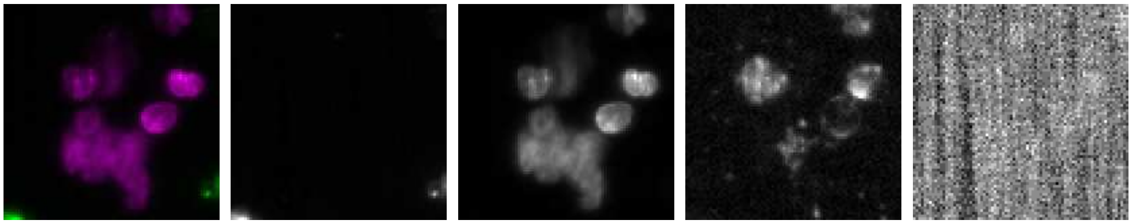				
24051	137	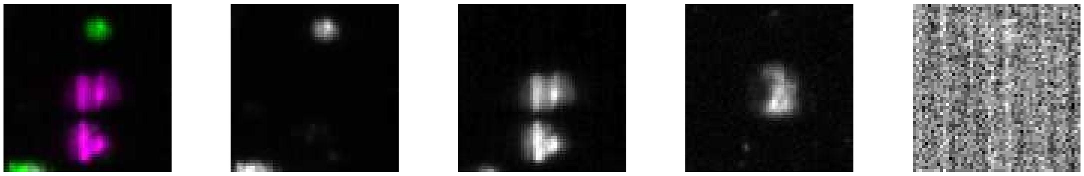				
24052	137	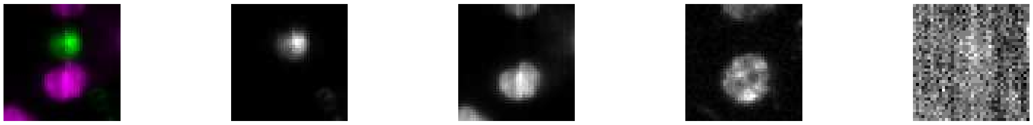				
24053	137	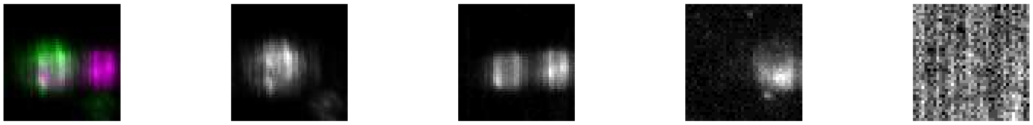				
24054	137	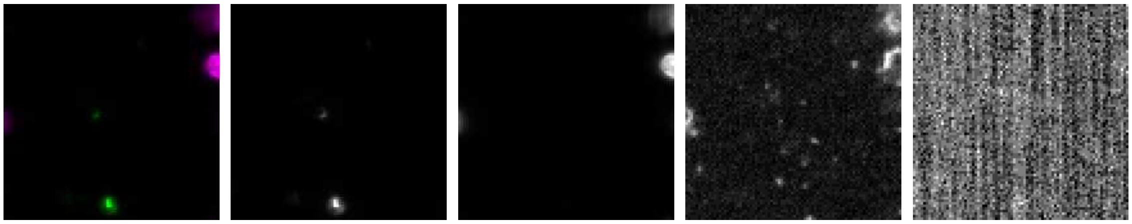				
24055	137	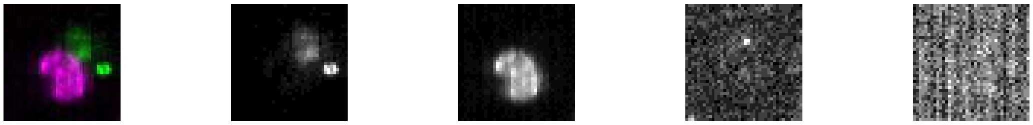				
24056	137	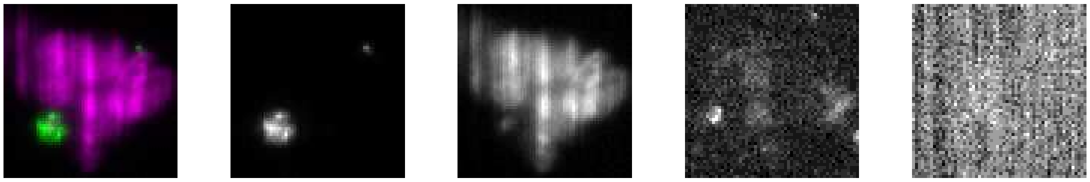				

Ereignis	Aufnahme	DAPI/CK-PE	CK-PE	DAPI	CD45-APC	HER-2/neu
24057	137	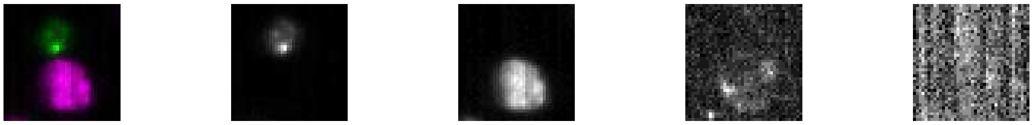				
24058	137	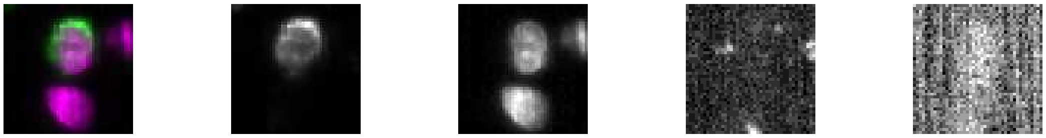				
24059	137	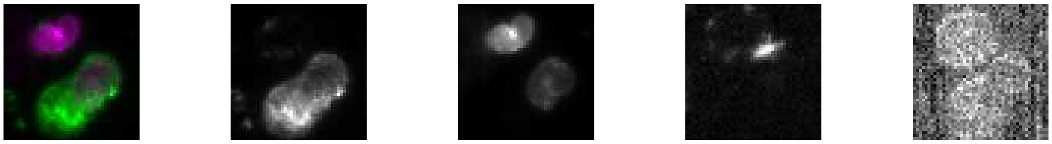				
24060	137	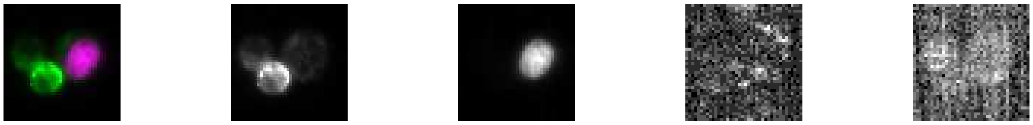				
24061	137	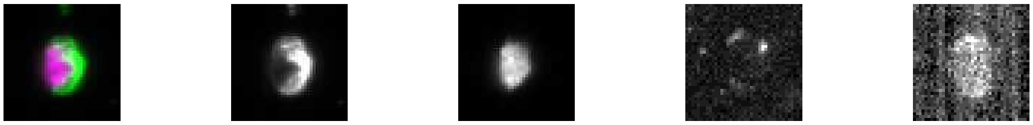				
24062	137	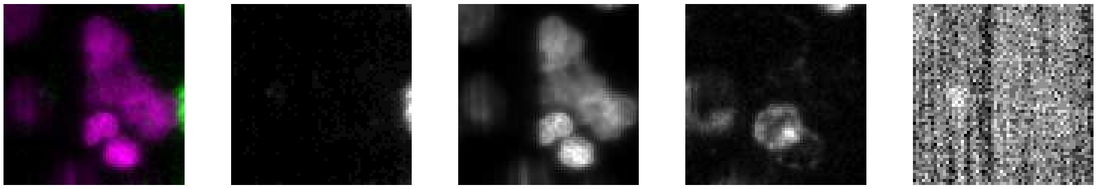				
24063	137	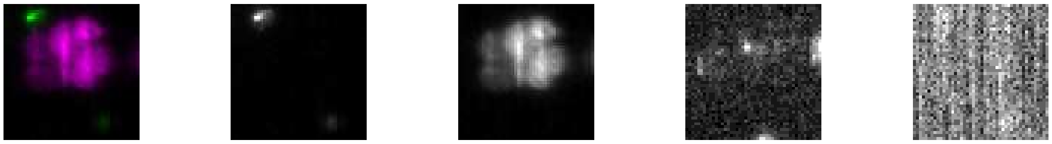				
24064	137	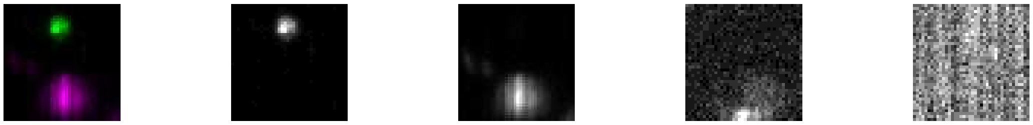				
24065	137	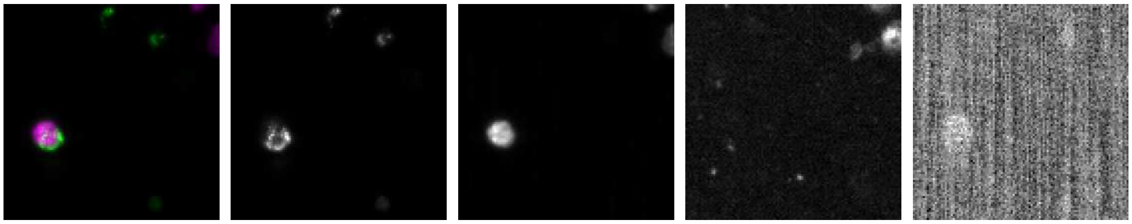				
24066	137	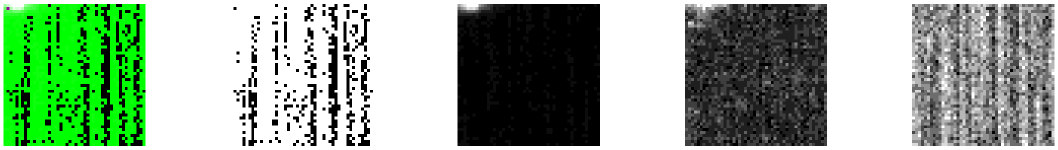				
24067	137	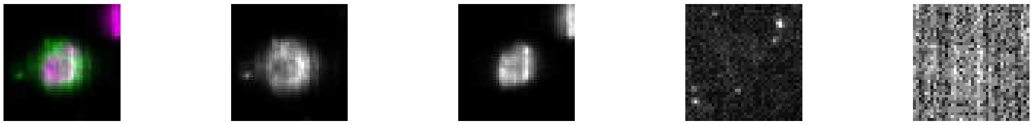				
24068	137	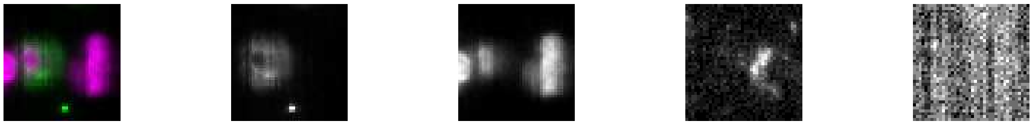				

Druckdatum: 02/06/2020 17:25

Proben-ID: Gyn14.208 **Kassetten-ID:** 01327106 **Patienten-ID:**

Ereignis	Aufnahme	DAPI/CK-PE	CK-PE	DAPI	CD45-APC	HER-2/neu
24069	137	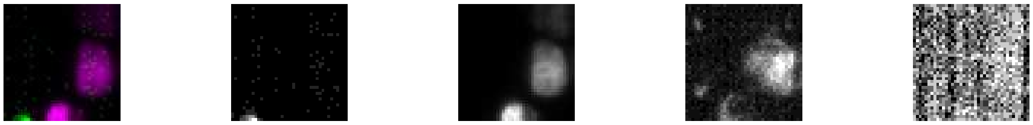				
24070	137	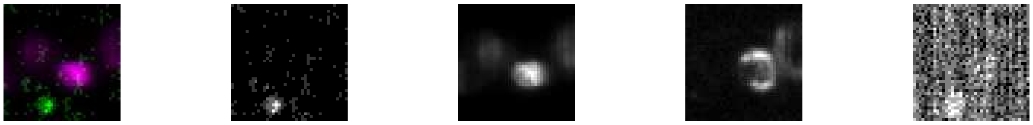				
24071	137	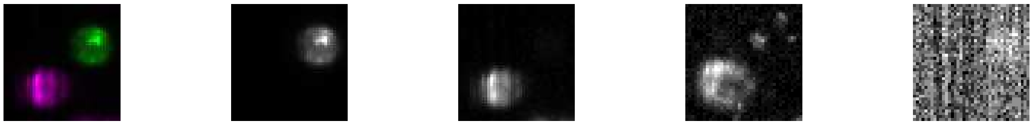				
24072	137	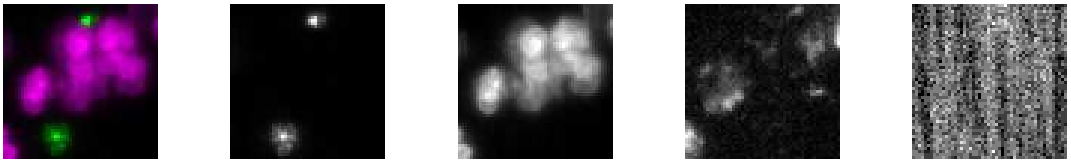				
24073	137	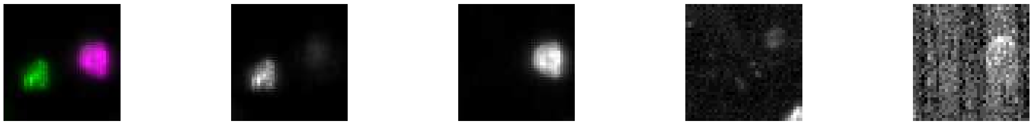				
24074	137	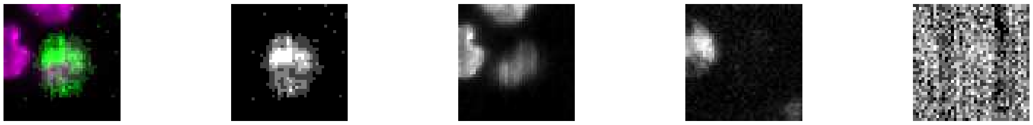				
24075	137	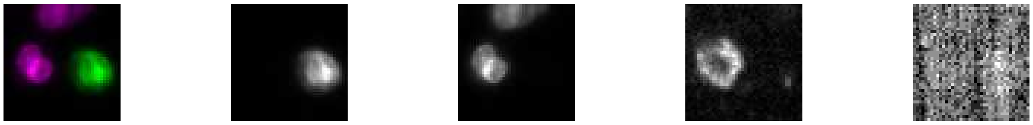				
24076	137	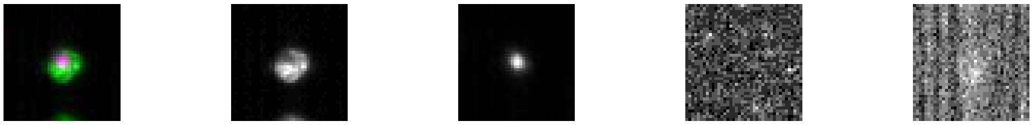				
24077	137	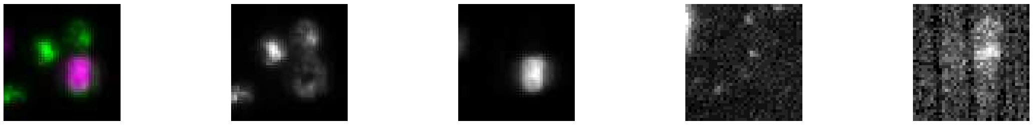				
24078	137	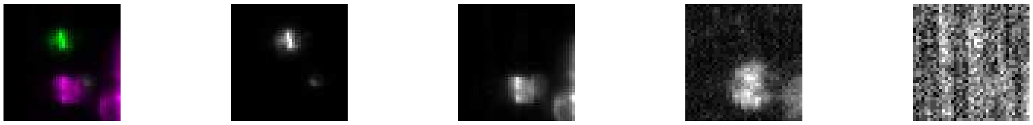				
24079	137	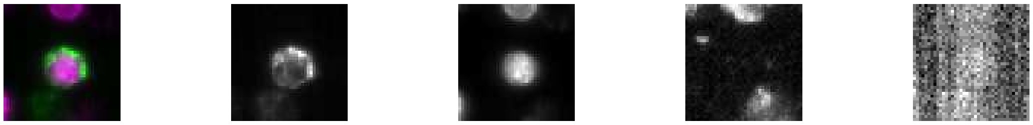				
24080	137	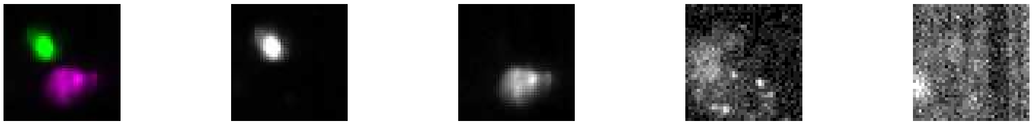				
24081	137	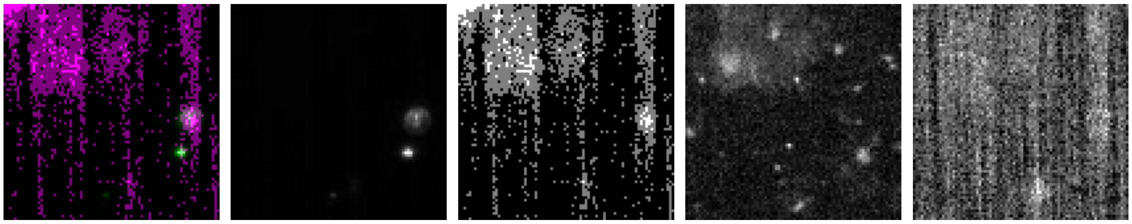				

Ereignis	Aufnahme	DAPI/CK-PE	CK-PE	DAPI	CD45-APC	HER-2/neu
24082	137	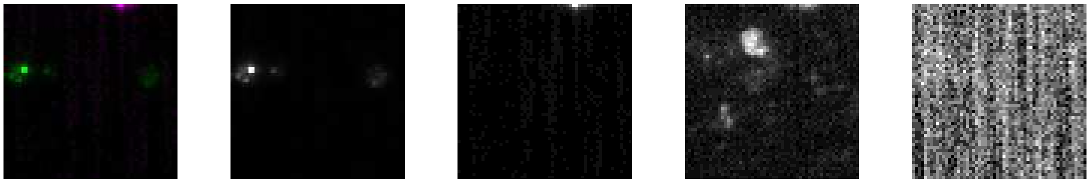				
24083	137	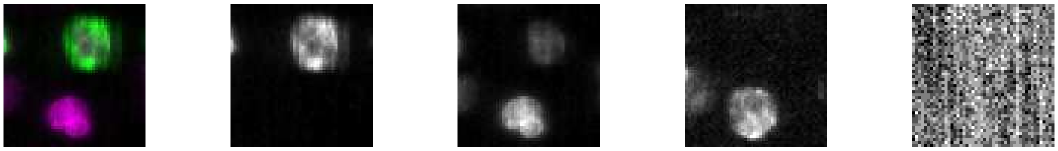				
24084	137	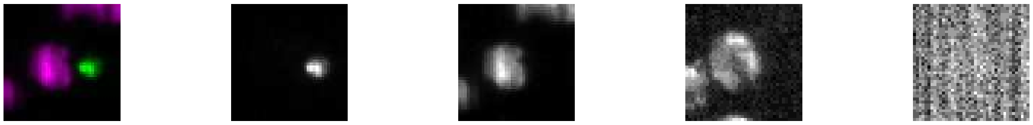				
24085	137	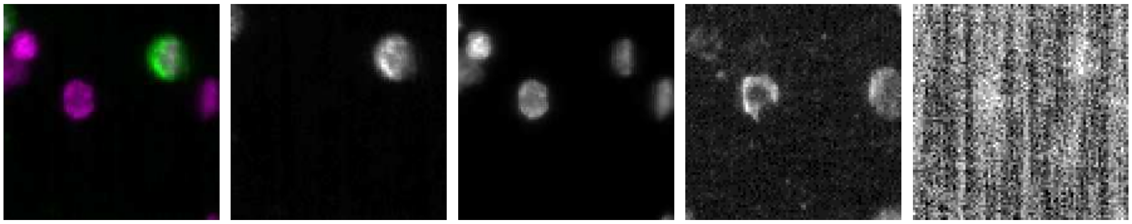				
24086	137	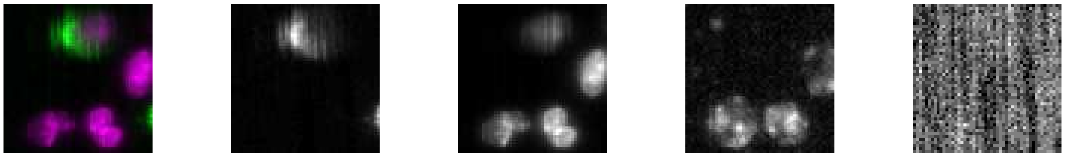				
24087	137	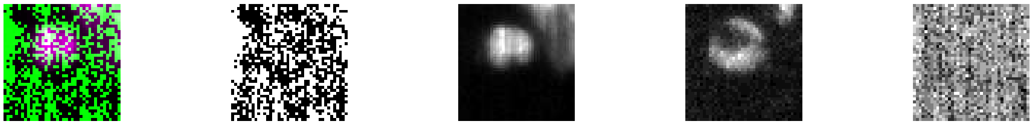				
24088	137	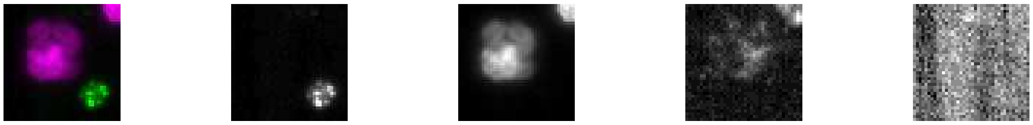				
24089	137	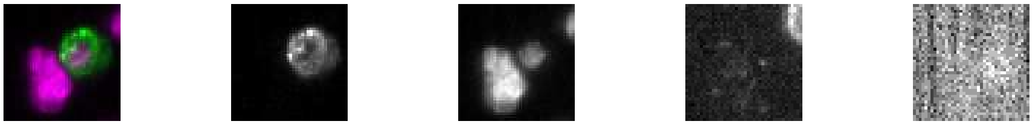				
24090	137	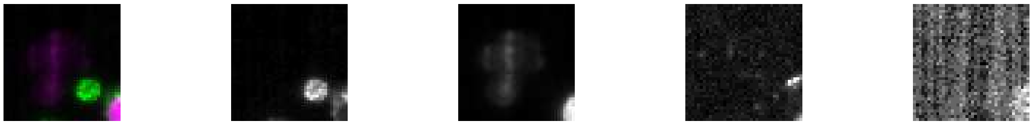				
24091	137	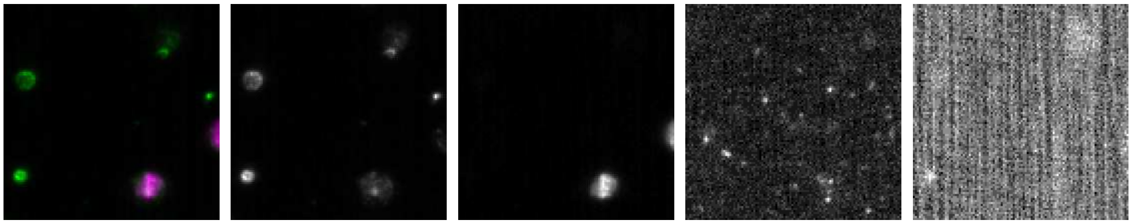				
24092	137	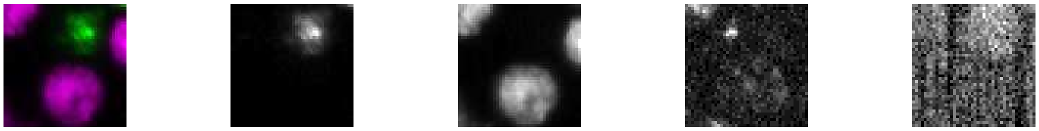				

Druckdatum:02/06/2020 17:25

Proben-ID:Gyn14.208 Kassetten-ID:01327106 Patienten-ID:

Ereignis	Aufnahme	DAPI/CK-PE	CK-PE	DAPI	CD45-APC	HER-2/neu
24093	137	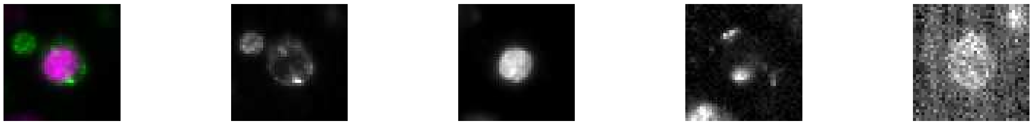				
24094	137	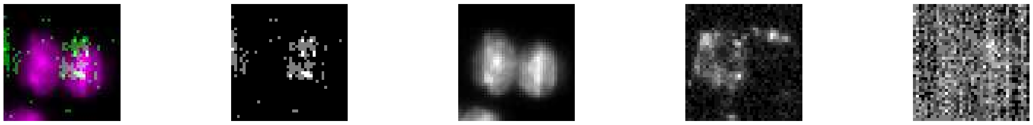				
24095	137	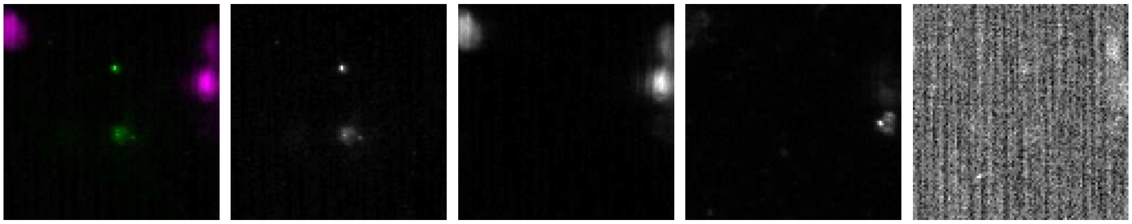				
24096	137	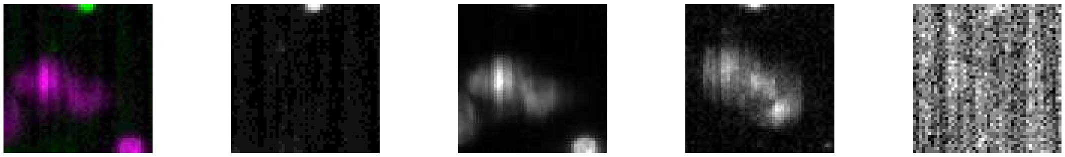				
24097	137	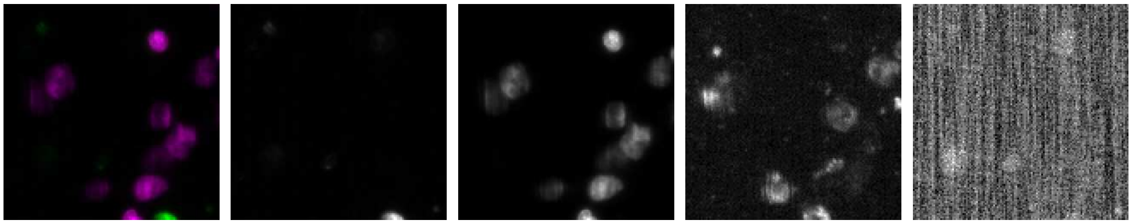				
24098	137	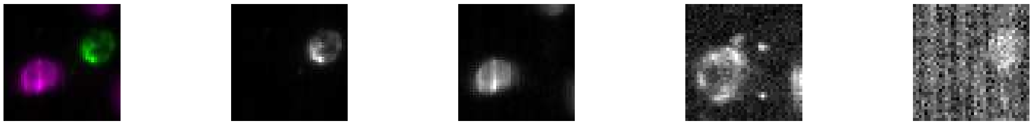				
24099	137	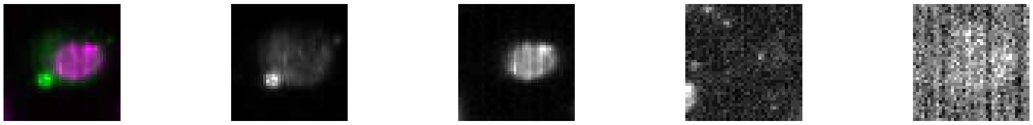				
24100	137	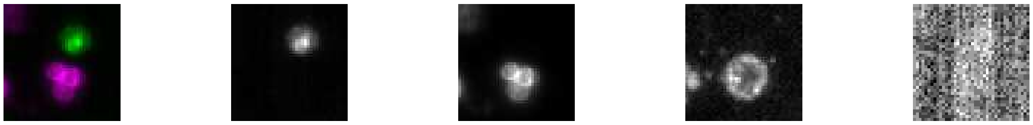				
24101	137	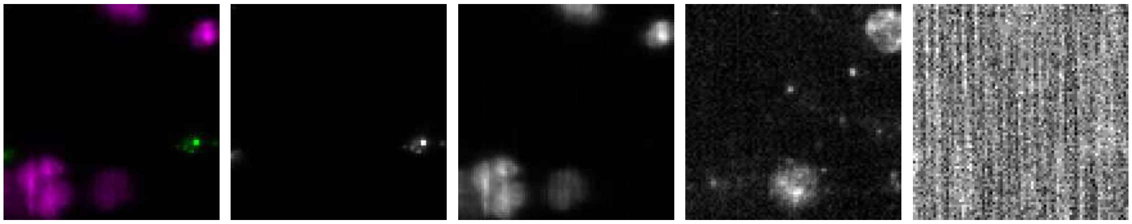				
24102	137	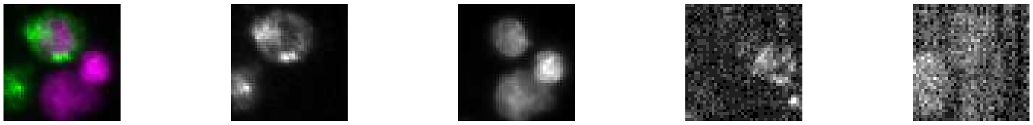				
24103	137	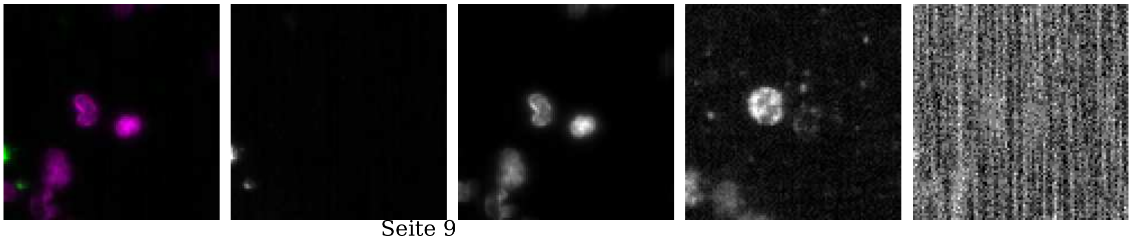				

Proben-ID: Gyn14.208 **Kassetten-ID:**01327106 **Druckdatum:**02/06/2020 17:25 **Patienten-ID:**

Ereignis	Aufnahme	DAPI/CK-PE	CK-PE	DAPI	CD45-APC	HER-2/neu
24104	137	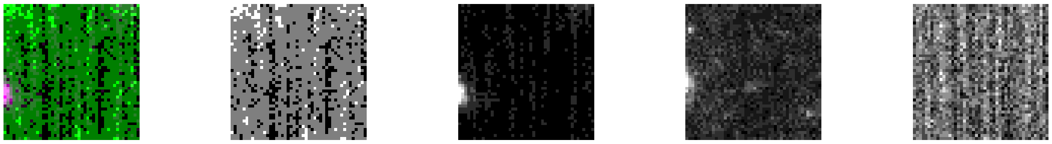				
24105	137	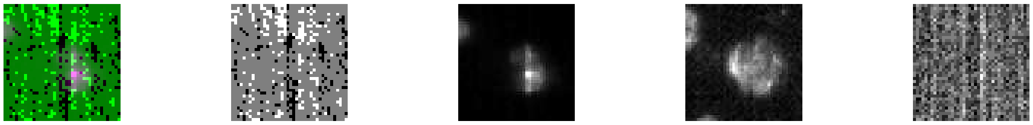				
24106	137	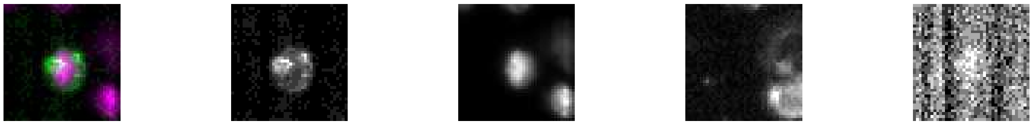				
24107	137	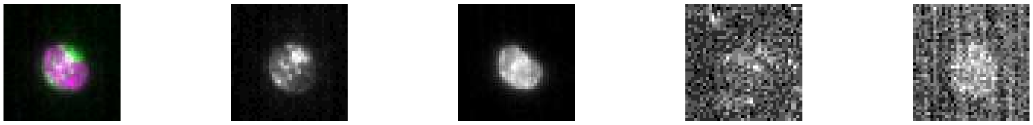				
24108	137	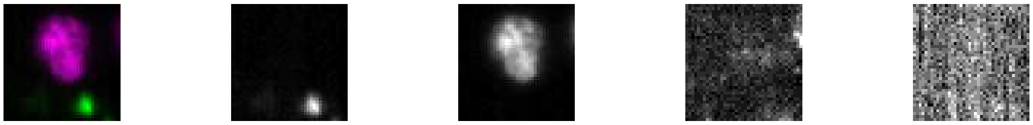				
24109	137	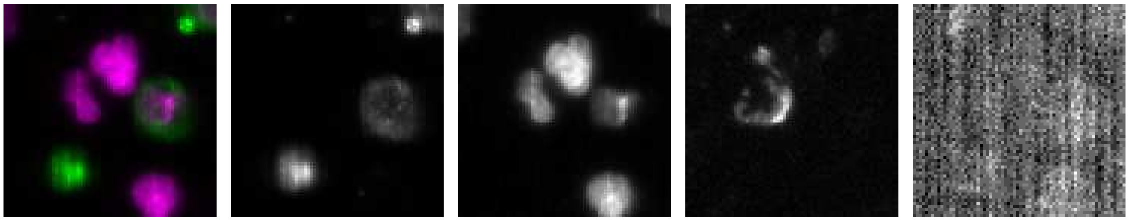				
24110	137	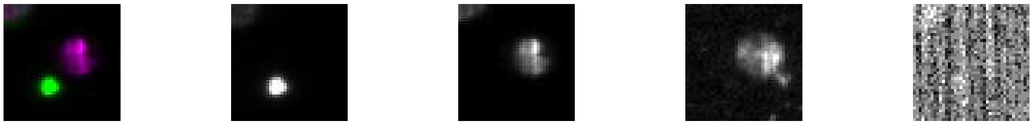				
24111	137	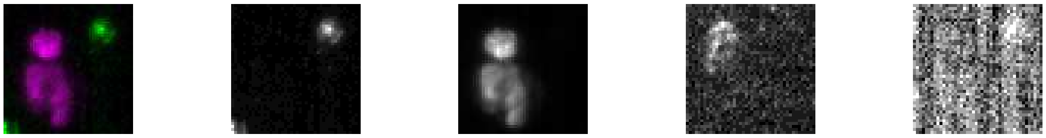				
24112	137	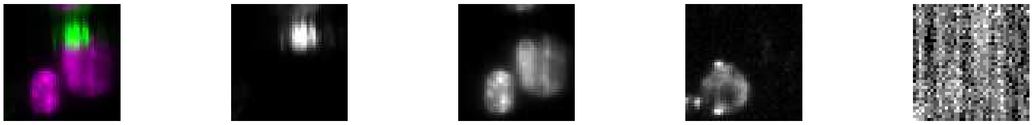				
24113	137	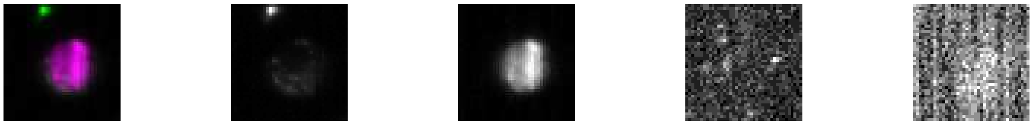				
24114	137	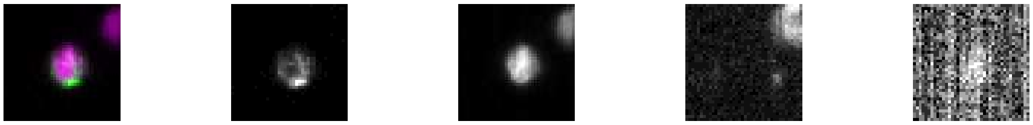				
24115	137	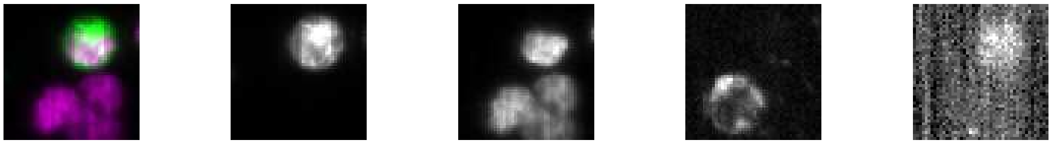				
24116	137	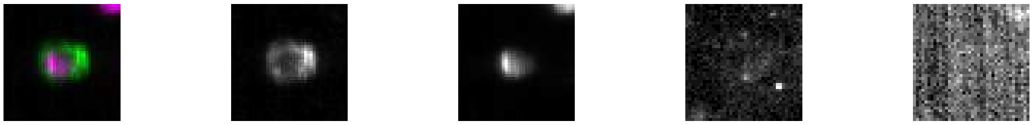				

Ereignis	Aufnahme	DAPI/CK-PE	CK-PE	DAPI	CD45-APC	HER-2/neu
24117	137	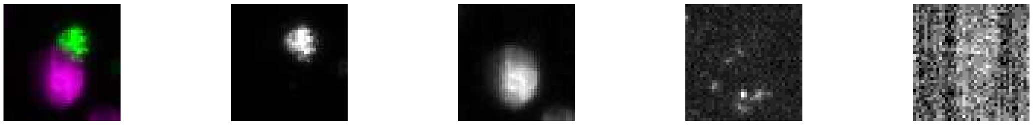				
24118	137	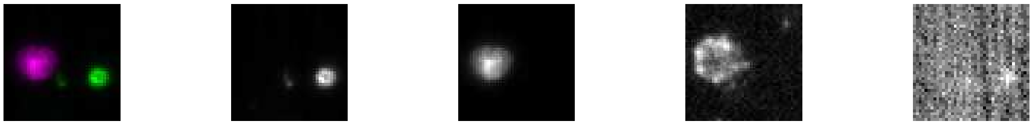				
24119	137	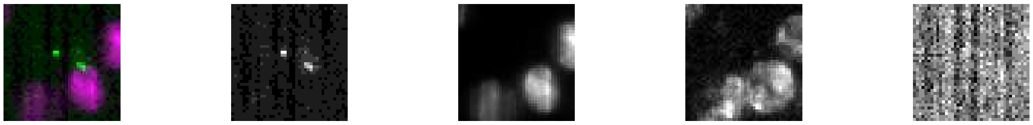				
24120	137	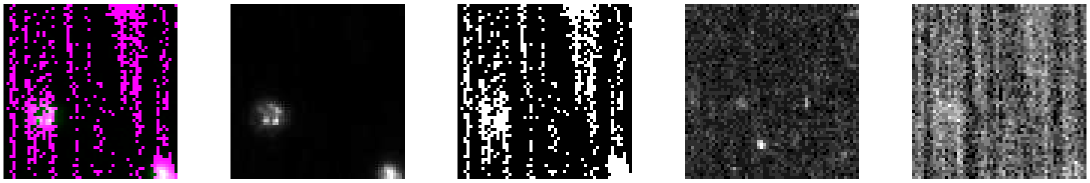				
24121	137	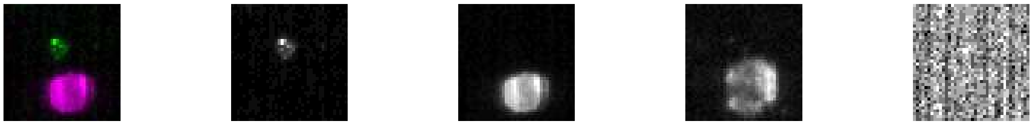				
24122	137	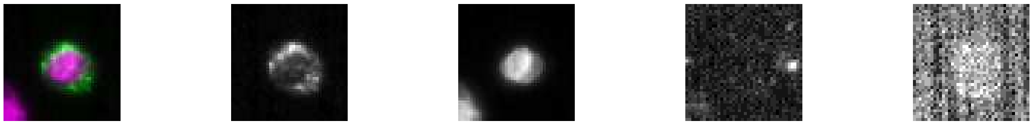				
24123	137	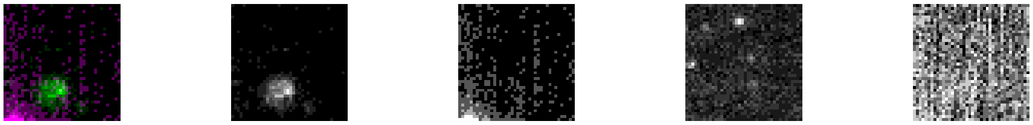				
24124	137	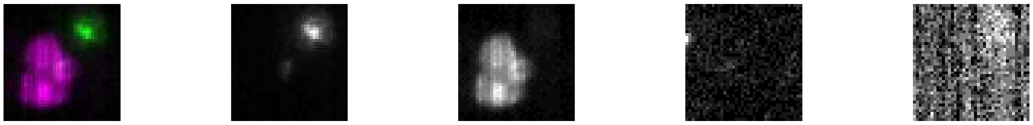				
24125	137	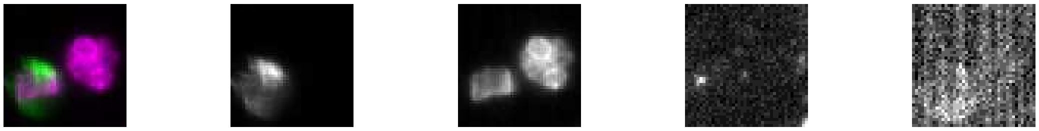				
24126	137	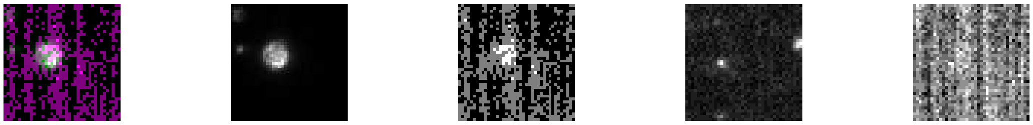				
24127	137	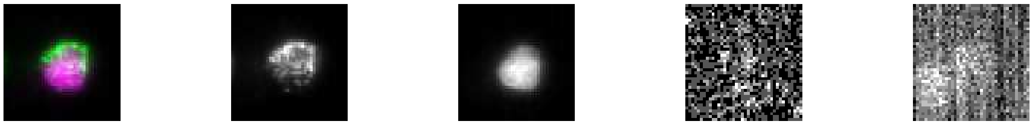				
24128	137	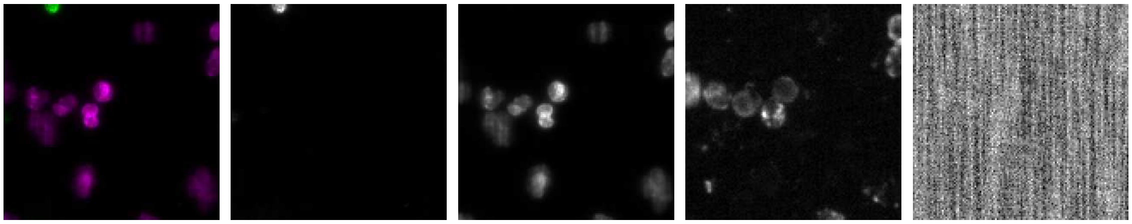				
24129	137	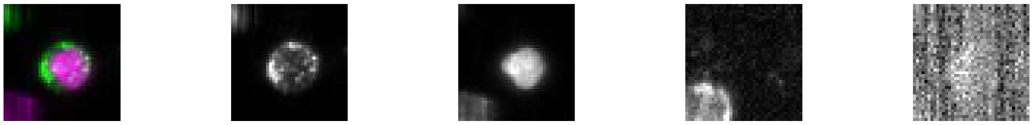				

Druckdatum: 02/06/2020 17:25

Proben-ID: Gyn14.208 **Kassetten-ID:** 01327106 **Patienten-ID:**

Ereignis	Aufnahme	DAPI/CK-PE / CK-PE / DAPI / CD45-APC / HER-2/neu
24130	137	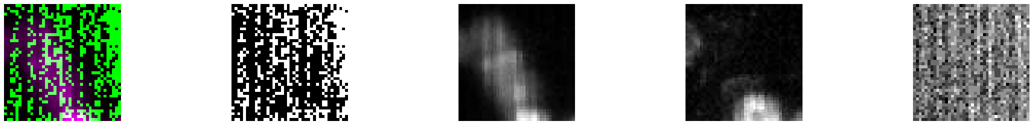
24131	137	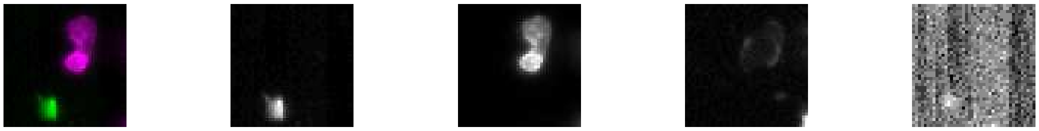
24132	137	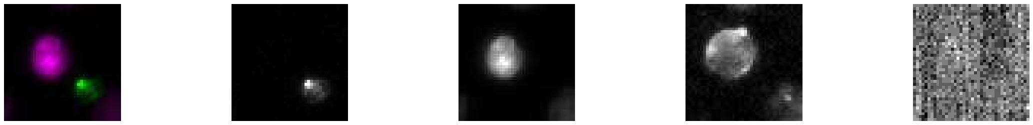
24133	137	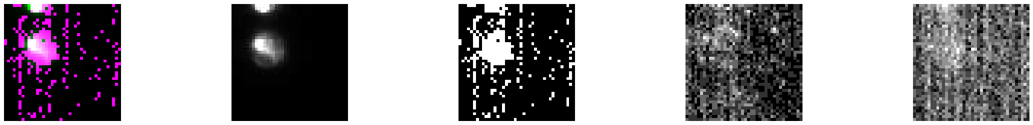
24134	137	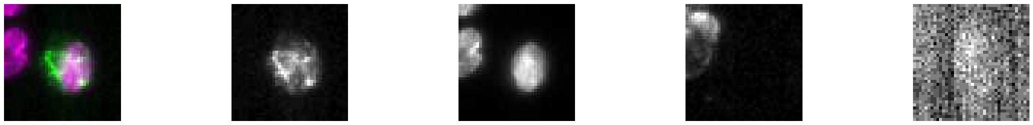
24135	137	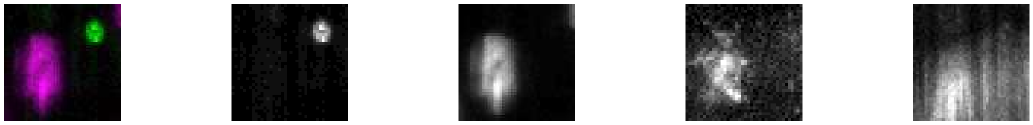
24136	137	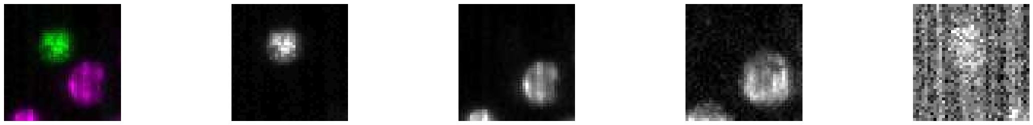
24137	137	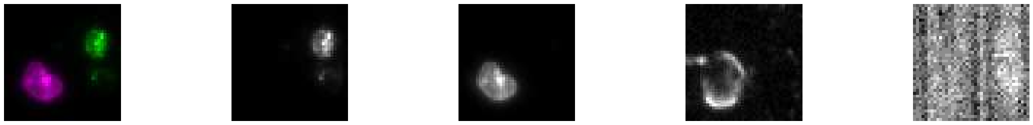
24138	137	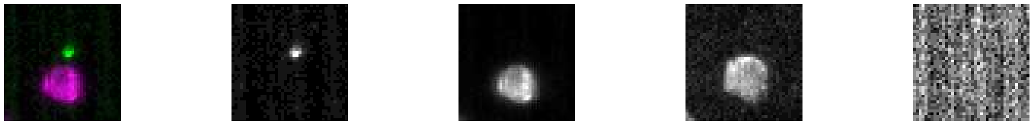
24139	137	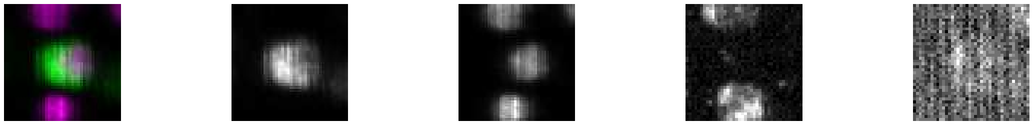
24140	137	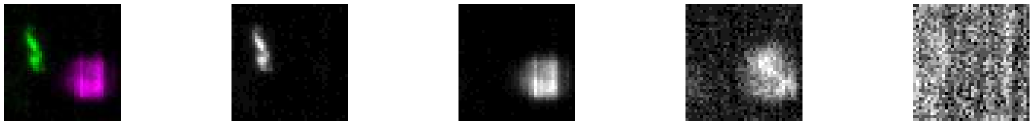
24141	137	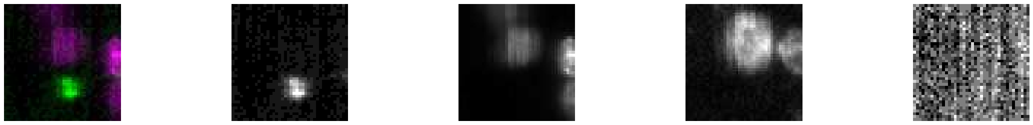
24142	137	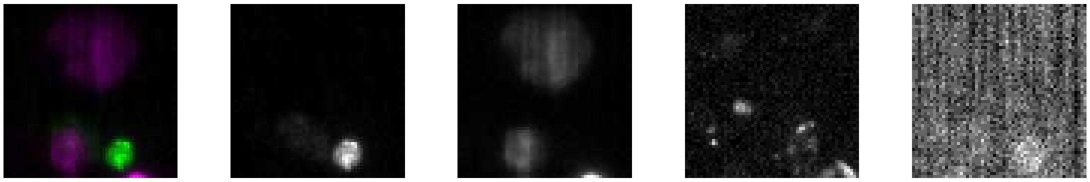

Proben-ID: Gyn14.208 **Kassetten-ID:** 01327106 **Patienten-ID:**

Ereignis	Aufnahme	DAPI/CK-PE	CK-PE	DAPI	CD45-APC	HER-2/neu
24143	137	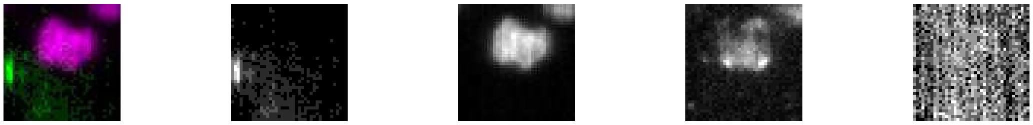				
24144	137	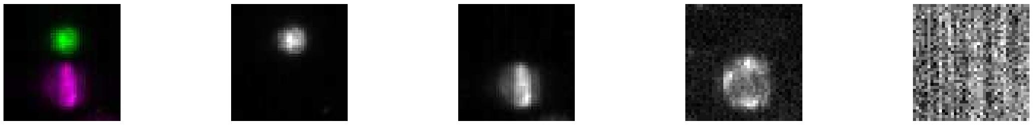				
24145	137	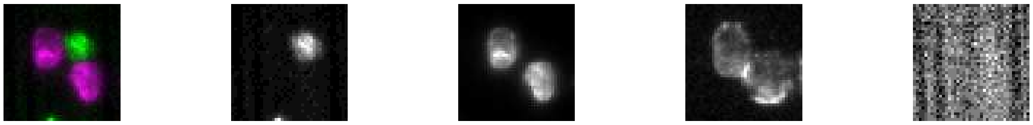				
24146	137	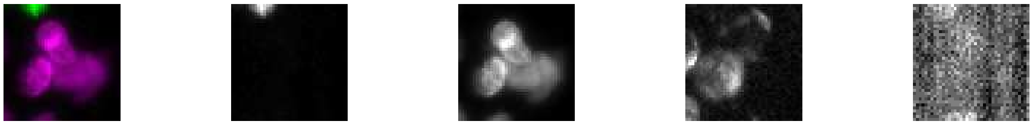				
24147	137	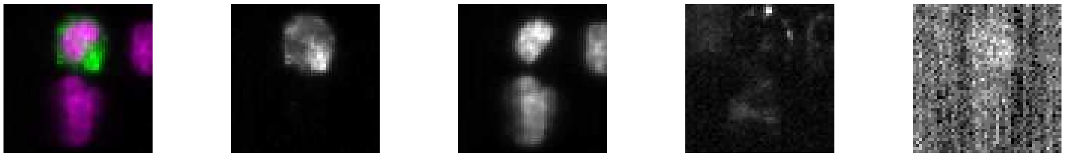				
24148	137	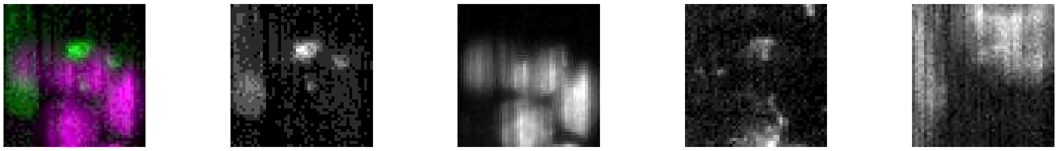				
24149	137	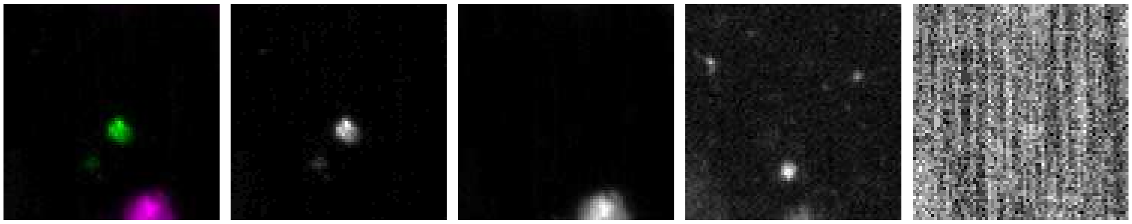				
24150	137	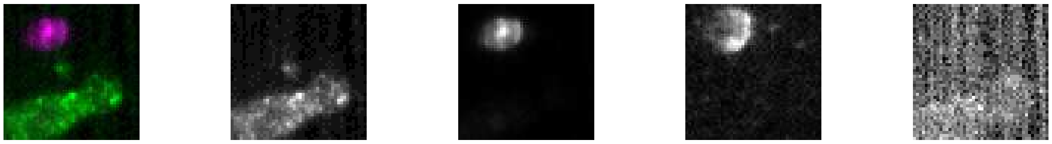				
24151	137	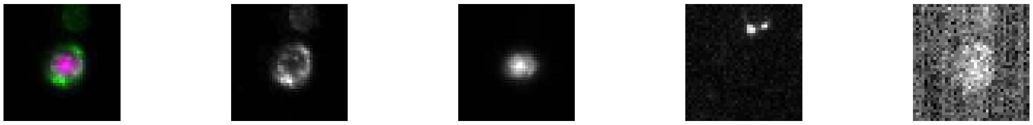				
24152	137	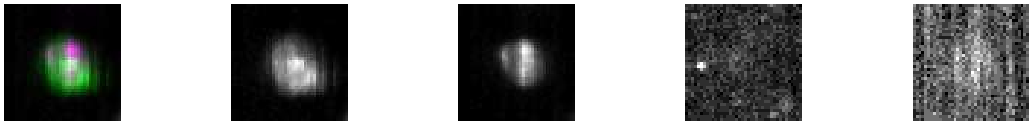				
24153	137	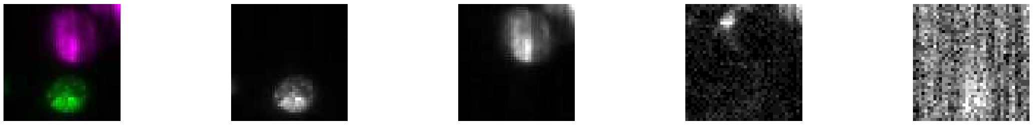				
24154	137	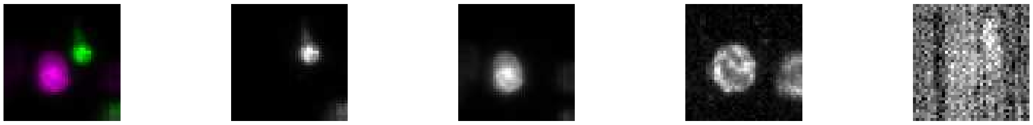				
24155	137	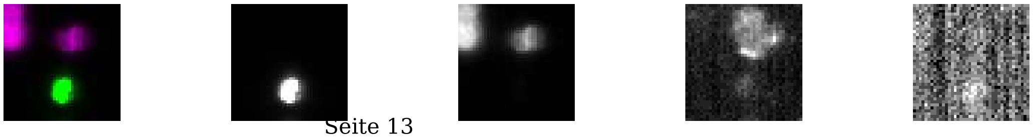				

Ereignis	Aufnahme	DAPI/CK-PE	CK-PE	DAPI	CD45-APC	HER-2/neu
24156	137	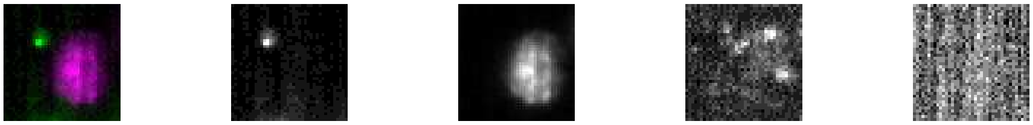				
24157	137	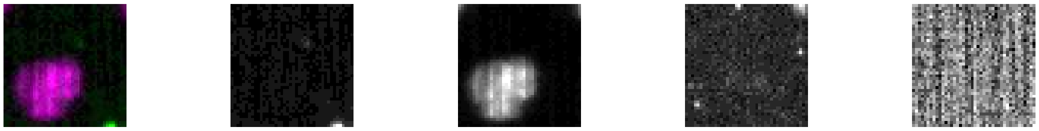				
24158	137	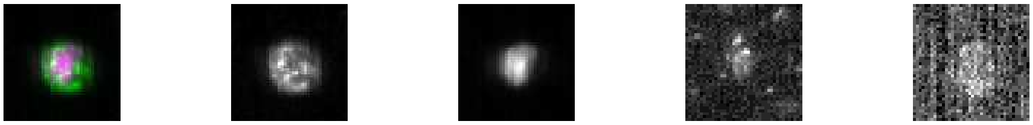				
24159	137	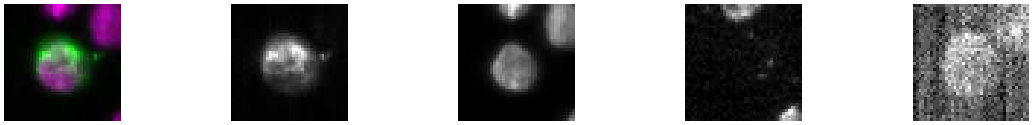				
24160	137	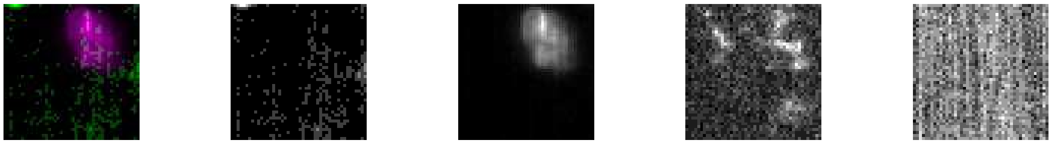				
24161	137	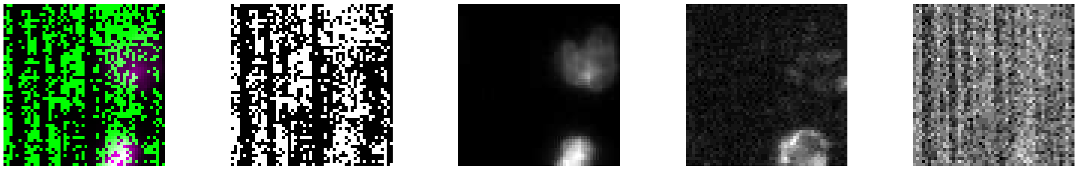				
24162	137	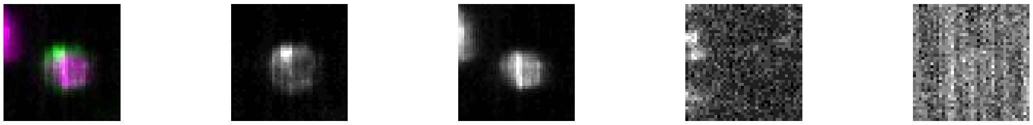				
24163	137	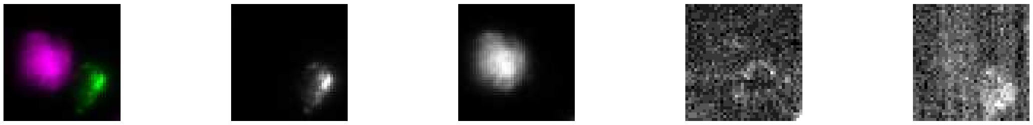				
24164	137	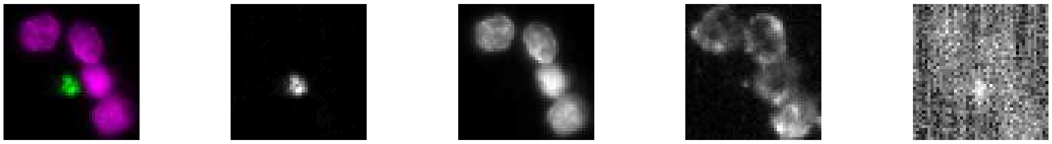				
24165	137	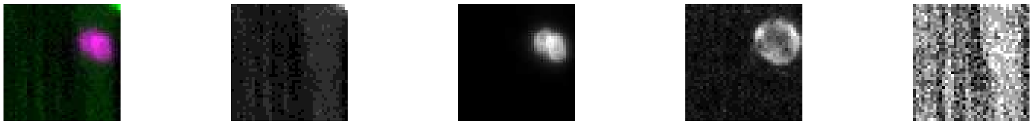				
24166	137	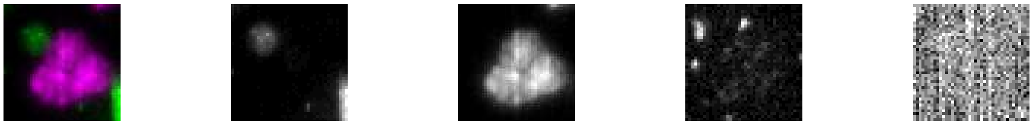				
24167	137	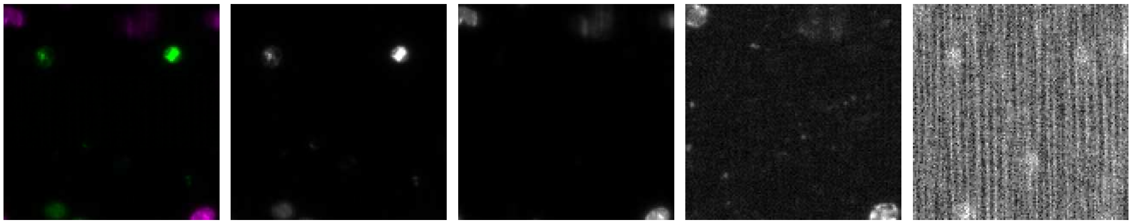				

Ereignis	Aufnahme	DAPI/CK-PE	CK-PE	DAPI	CD45-APC	HER-2/neu
24168	137	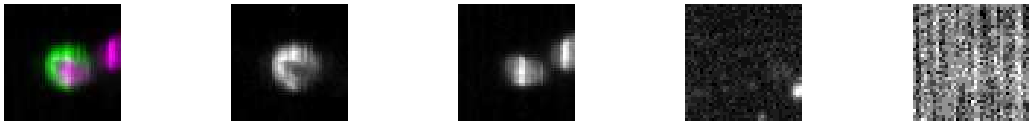				
24169	137	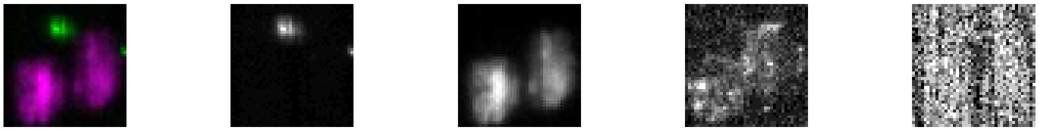				
24170	137	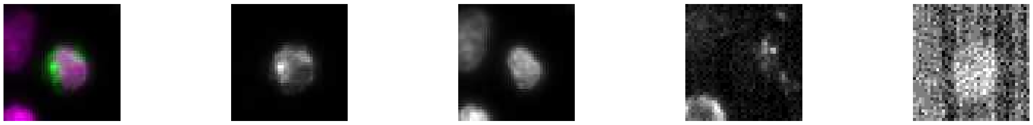				
24171	137	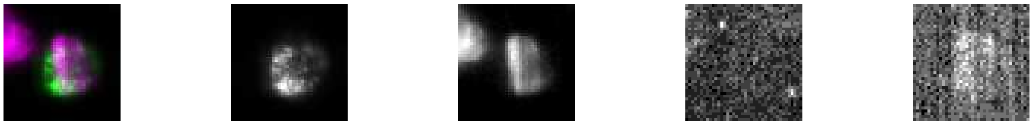				
24172	137	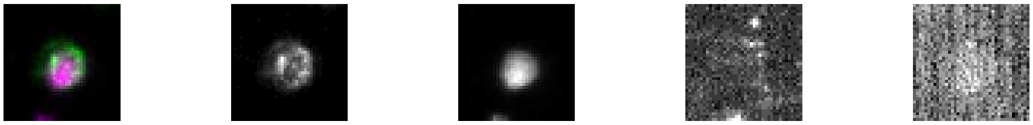				
24173	137	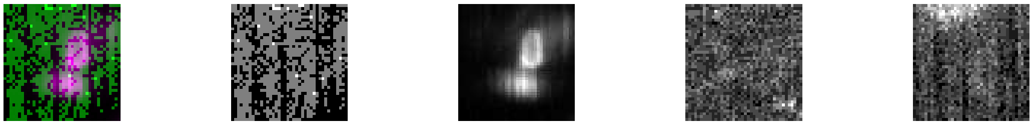				
24174	137	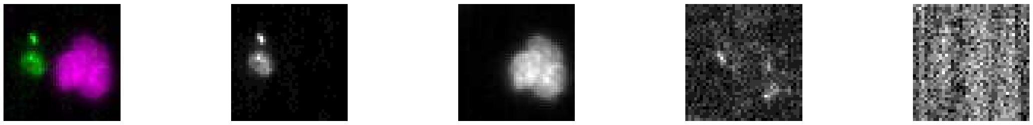				
24175	137	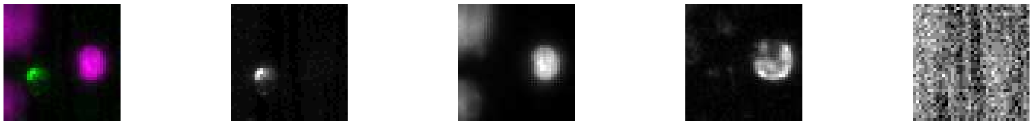				
24176	137	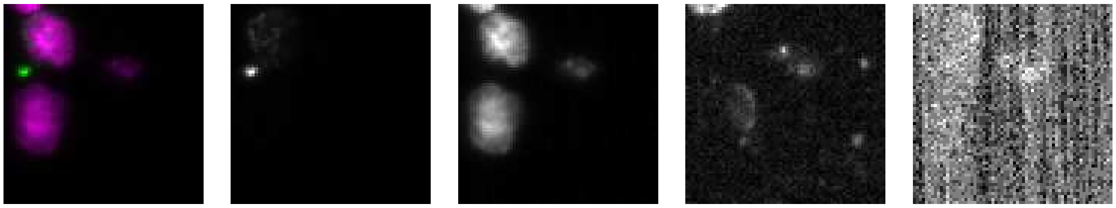				
24177	137	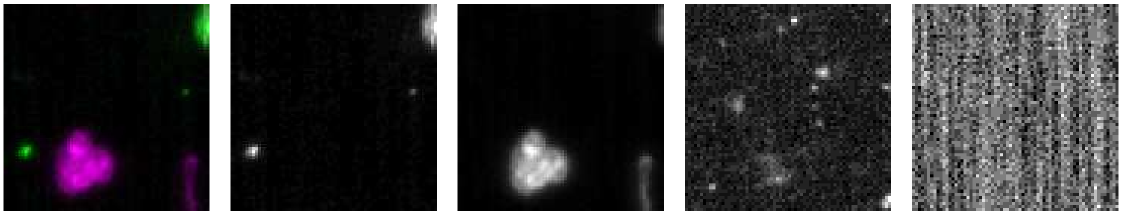				
24178	137	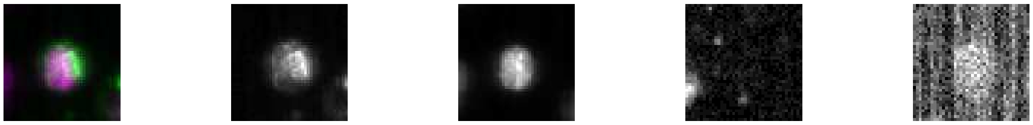				
24179	137	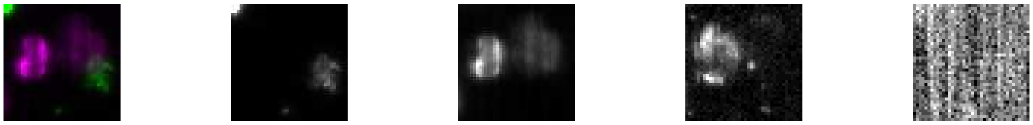				
24180	137	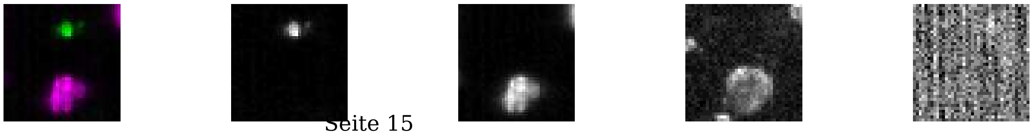				

Druckdatum:02/06/2020 17:25

Proben-ID: Gyn14.208 **Kassetten-ID:**01327106 **Patienten-ID:**

Ereignis	Aufnahme	DAPI/CK-PE	CK-PE	DAPI	CD45-APC	HER-2/neu
24181	137	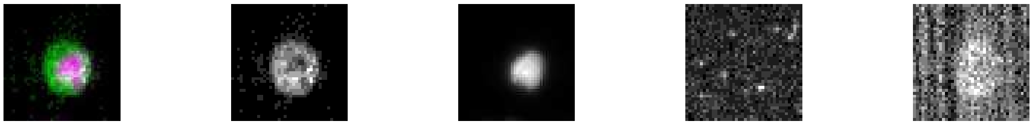				
24182	137	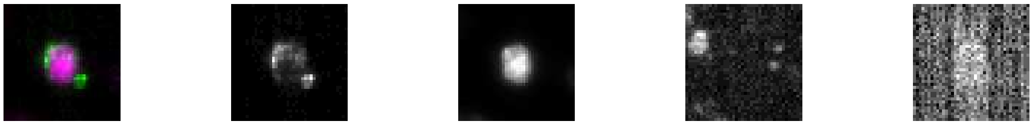				
24183	137	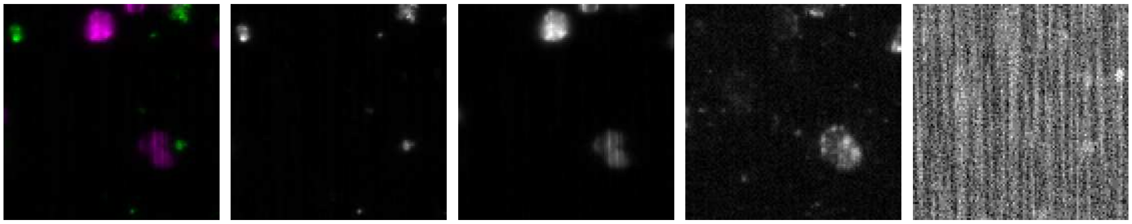				
24184	137	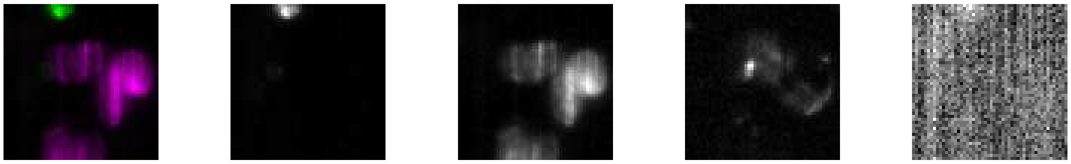				
24185	137	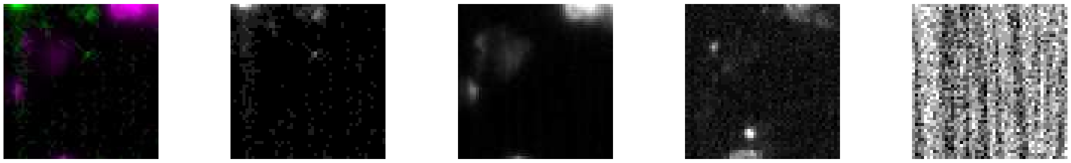				
24186	137	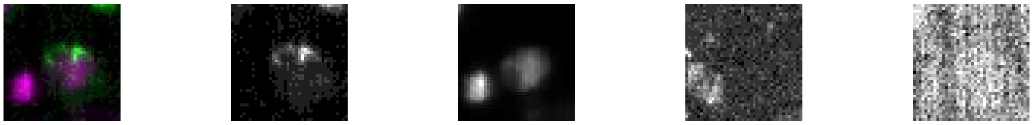				
24187	137	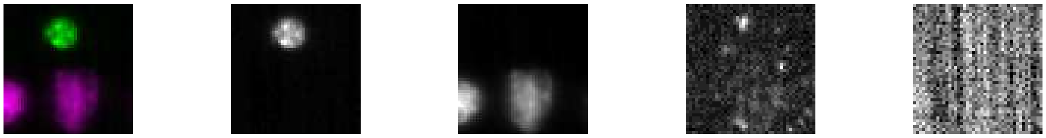				
24188	137	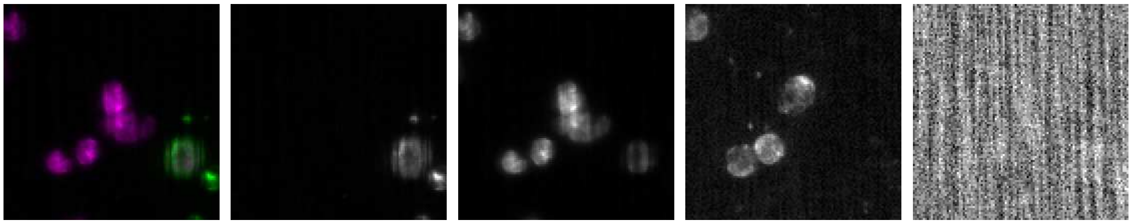				
24189	137	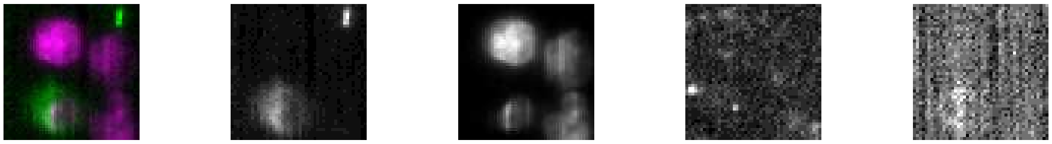				
24190	137	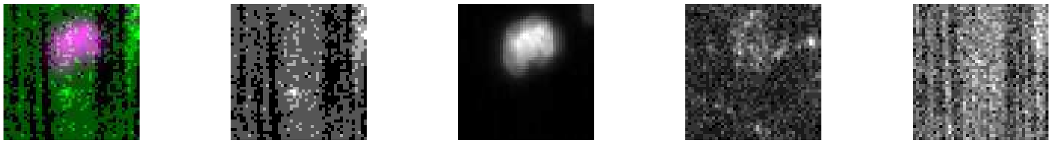				
24191	137	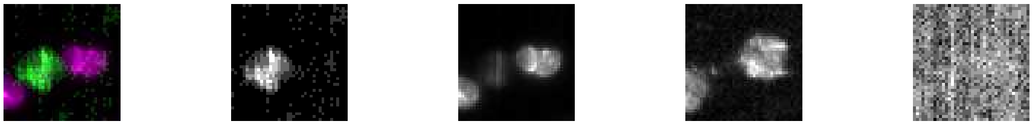				

Proben-ID: Gyn14.208 **Kassetten-ID:**01327106 **Patienten-ID:**

Ereignis	Aufnahme	DAPI/CK-PE	CK-PE	DAPI	CD45-APC	HER-2/neu
24192	137	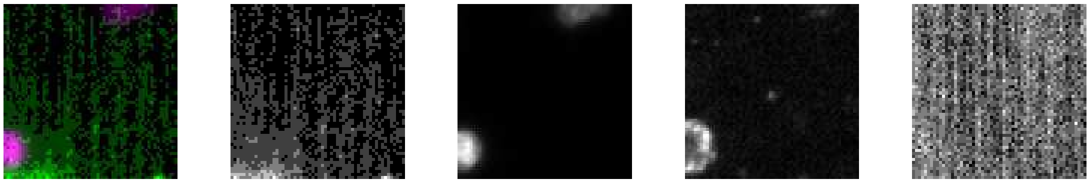				
24193	137	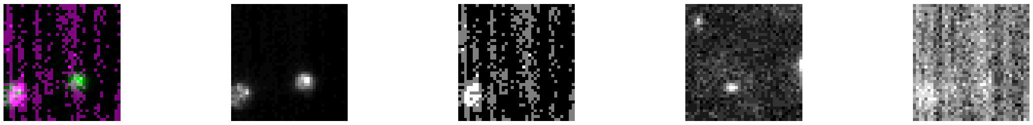				
24194	137	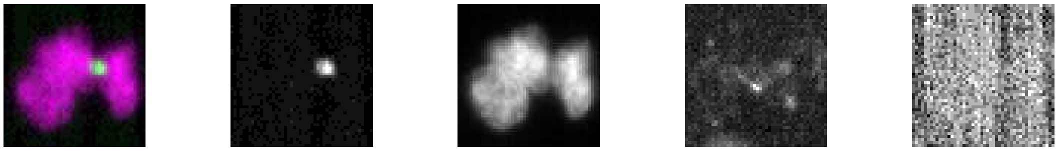				
24195	137	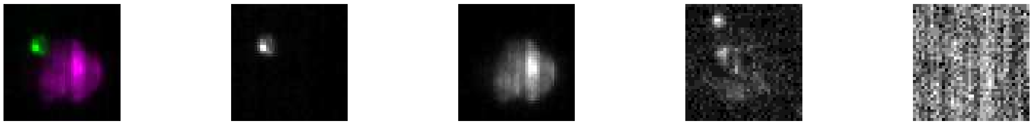				
24196	137	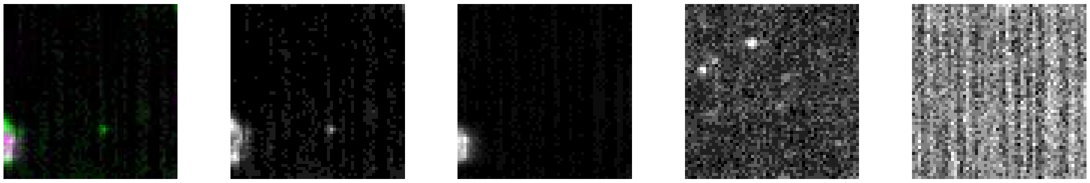				
24197	137	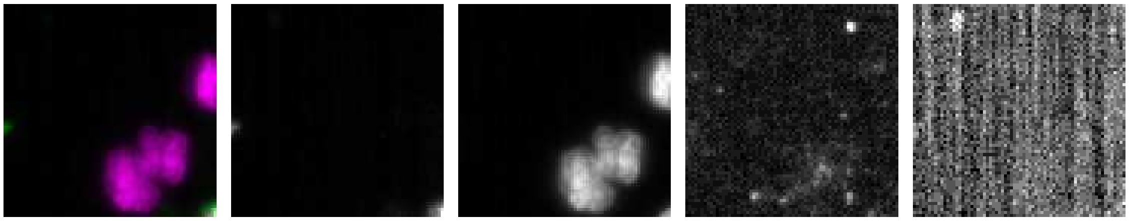				
24198	137	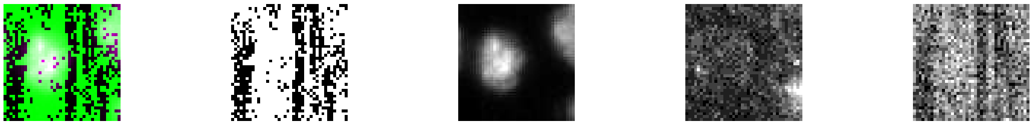				
24199	137	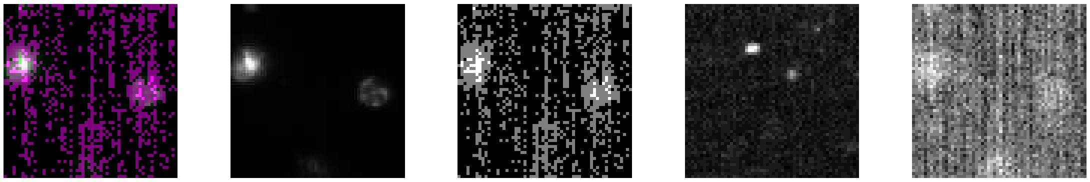				
24200	137	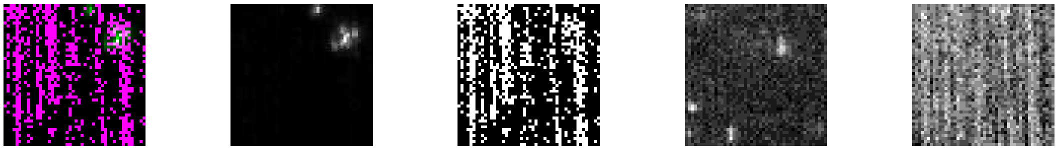				
24201	137	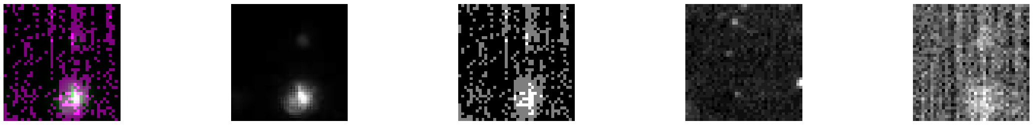				
24202	137	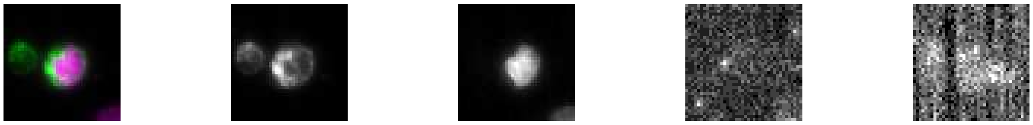				

Ereignis	Aufnahme	DAPI/CK-PE	CK-PE	DAPI	CD45-APC	HER-2/neu
24203	137	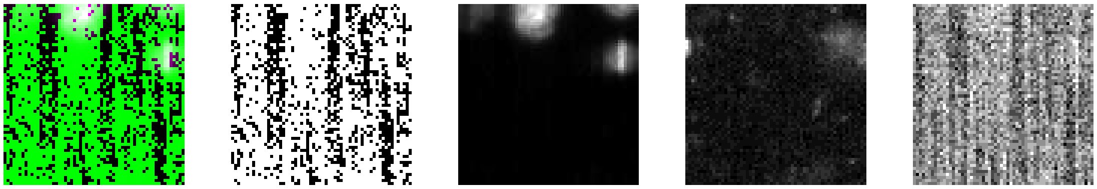				
24204	137	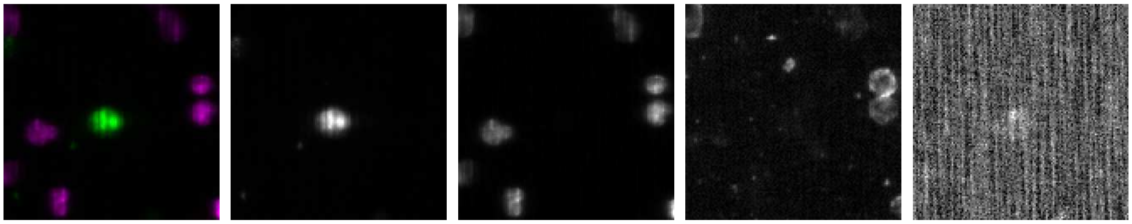				
24205	137	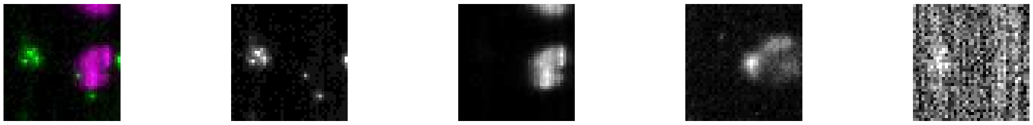				
24206	137	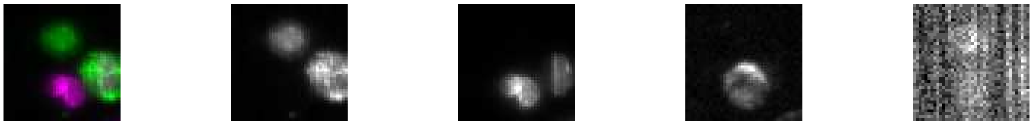				
24207	137	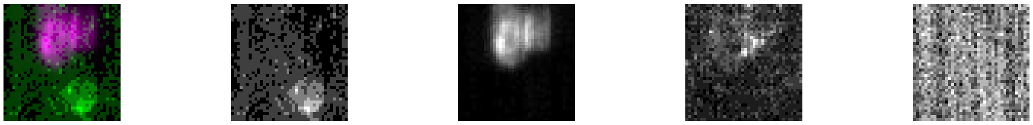				
24208	137	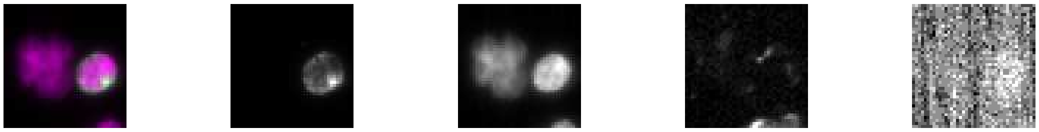				
24209	137	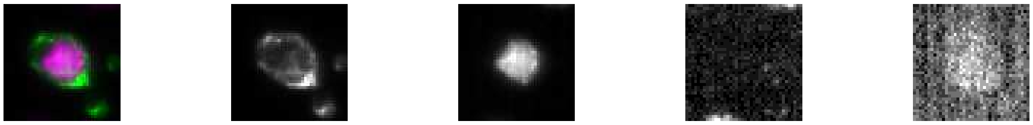				
24210	137	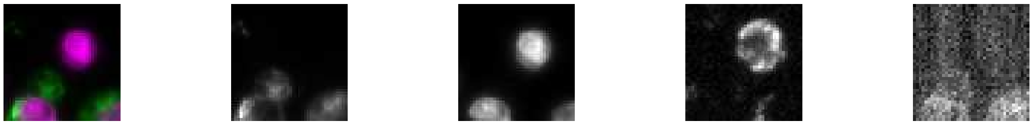				
24211	137	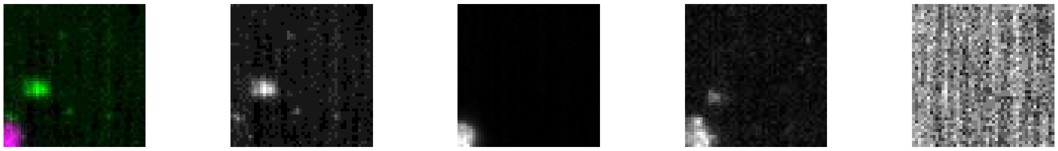				
24212	137	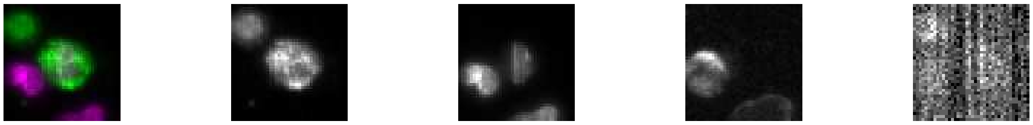				
24213	137	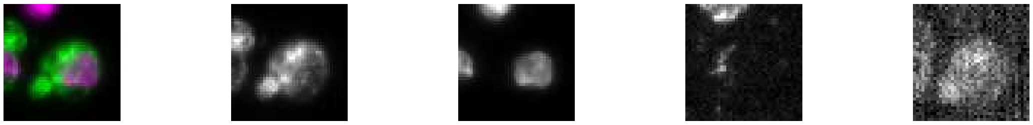				
24214	137	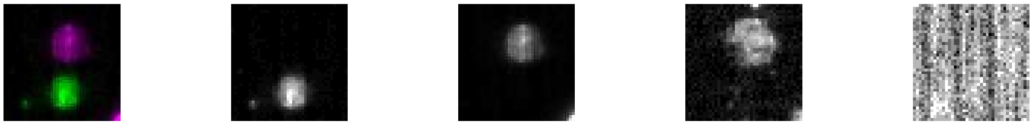				

Ereignis	Aufnahme	DAPI/CK-PE	CK-PE	DAPI	CD45-APC	HER-2/neu
24215	137	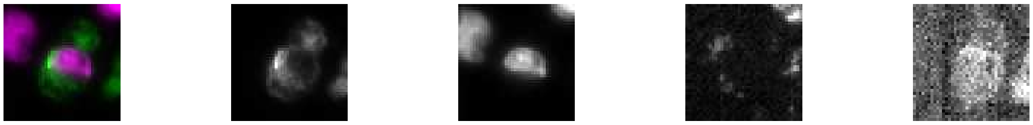				
24216	137	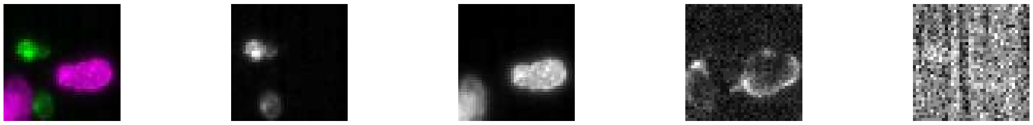				
24217	137	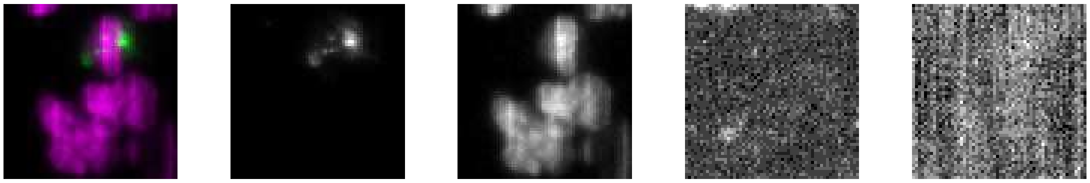				
24218	137	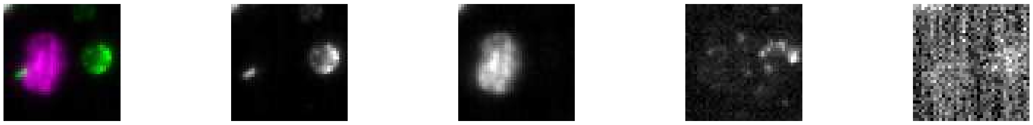				
24219	137	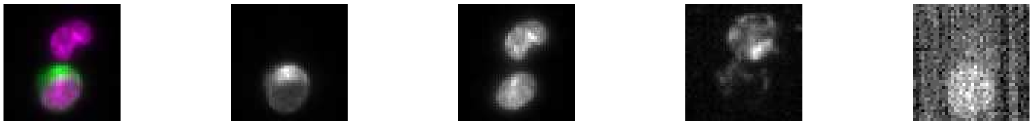				
24220	137	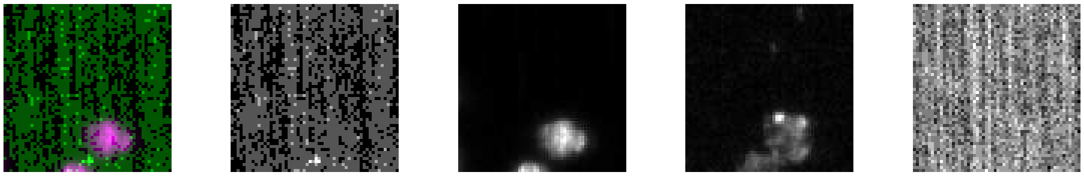				
24221	137	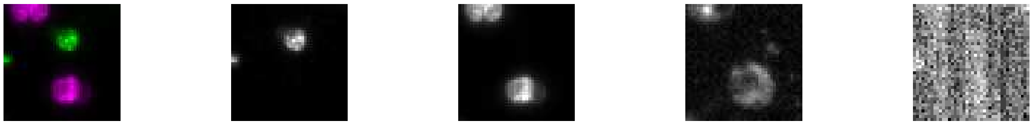				
24222	137	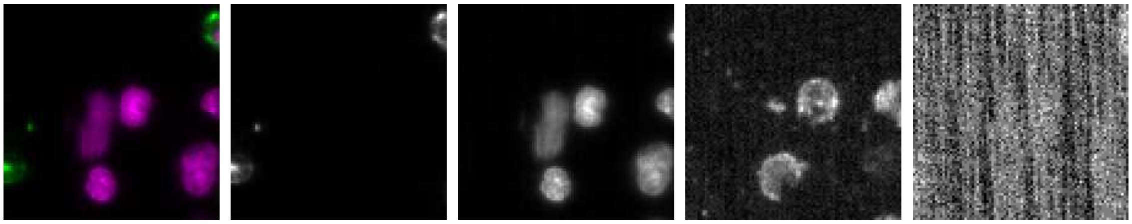				
24223	137	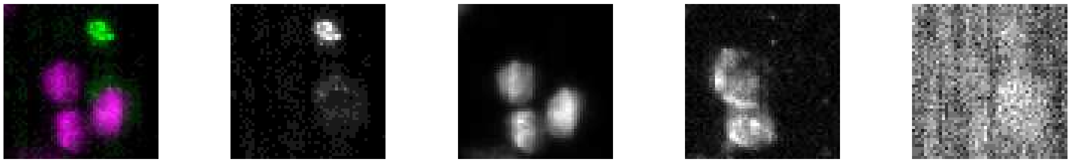				
24224	137	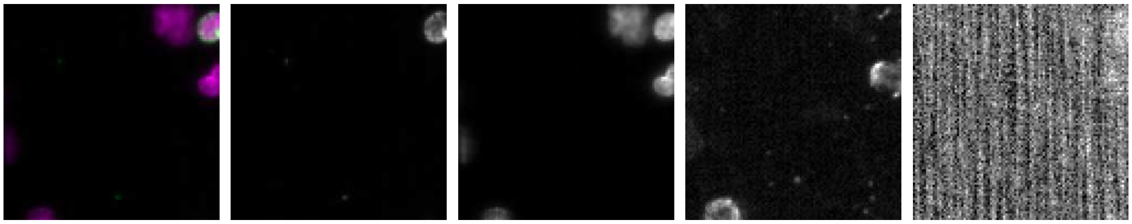				
24225	137	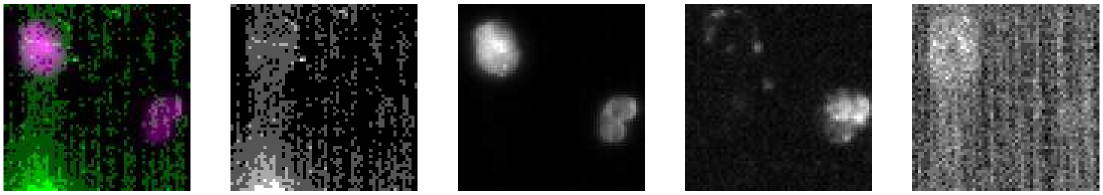				

Proben-ID: Gyn14.208 **Kassetten-ID:**01327106 **Patienten-ID:**

Ereignis	Aufnahme	DAPI/CK-PE	CK-PE	DAPI	CD45-APC	HER-2/neu
24226	137	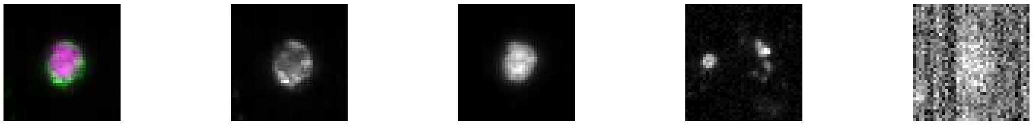				
24227	137	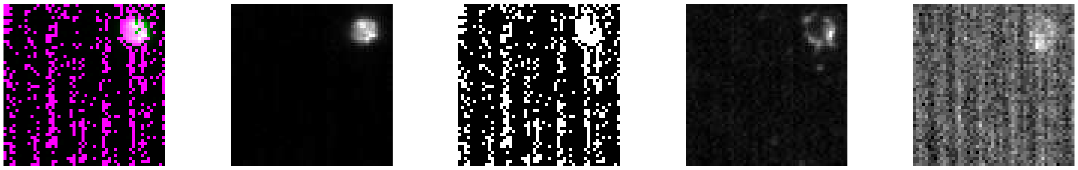				
24228	137	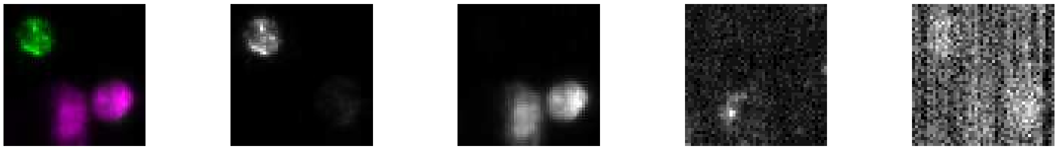				
24229	137	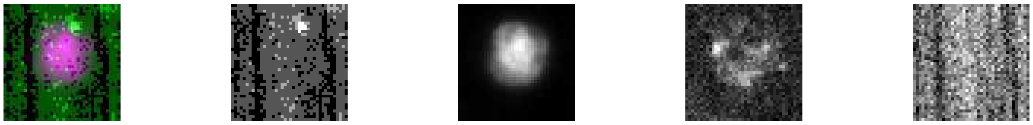				
24230	137	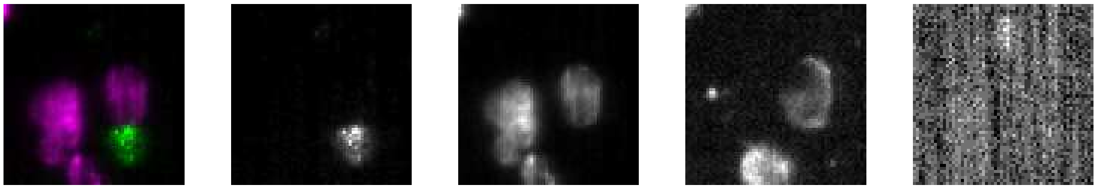				
24231	137	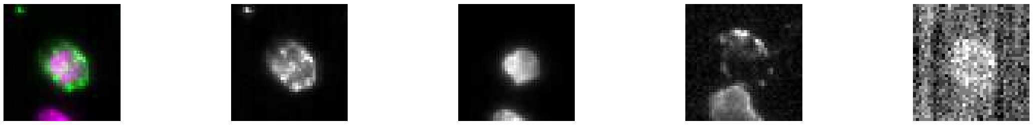				
24232	137	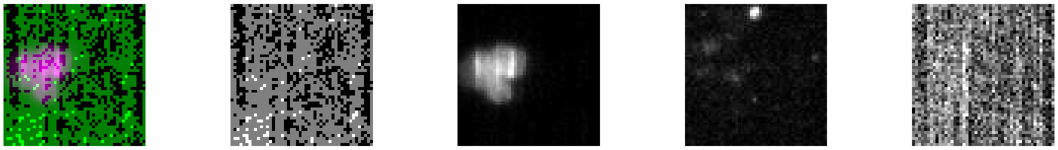				
24233	137	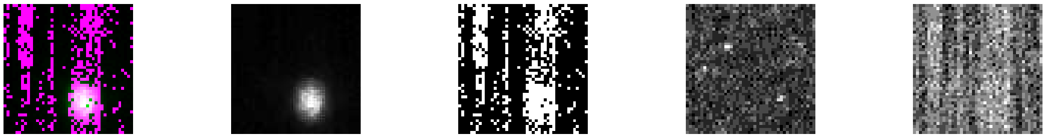				
24234	137	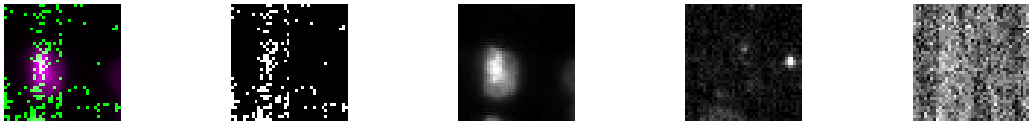				
24235	137	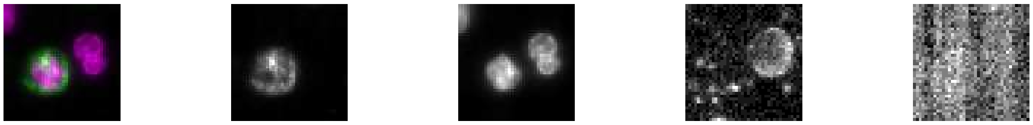				
24236	137	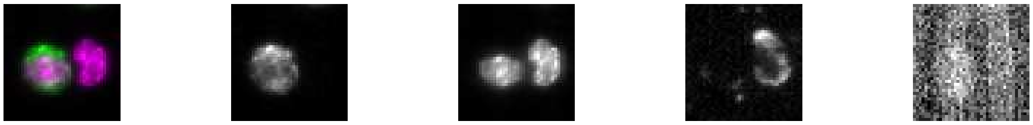				
24237	137	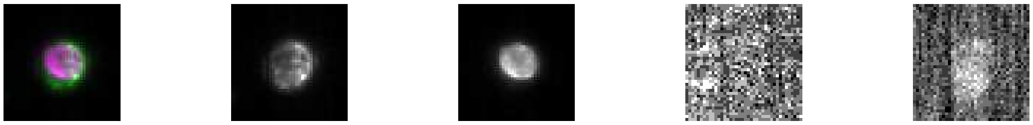				

Druckdatum:02/06/2020 17:25

Proben-ID: Gyn14.208 **Kassetten-ID:**01327106 **Patienten-ID:**

Ereignis	Aufnahme	DAPI/CK-PE	CK-PE	DAPI	CD45-APC	HER-2/neu
24238	136	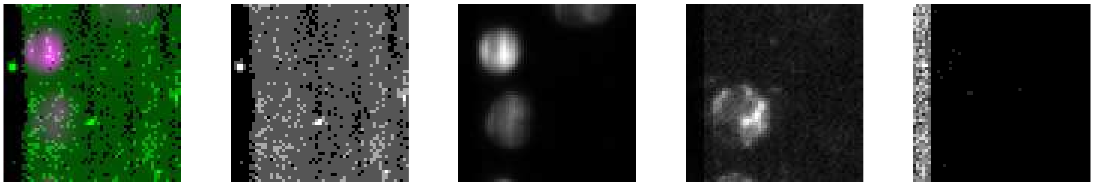				
24239	137	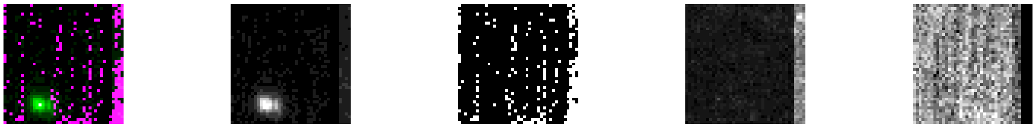				
24240	137	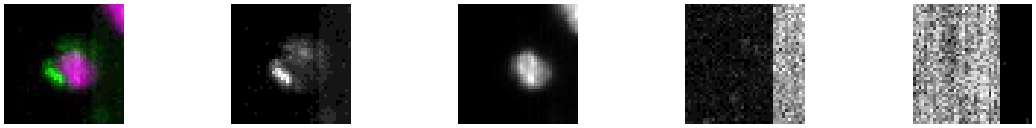				
24241	74	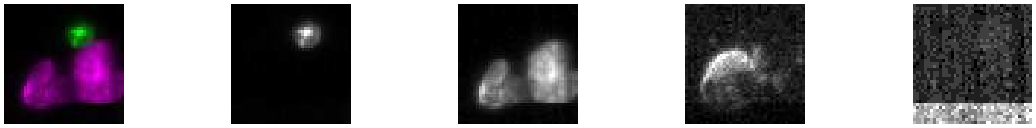				
24242	137	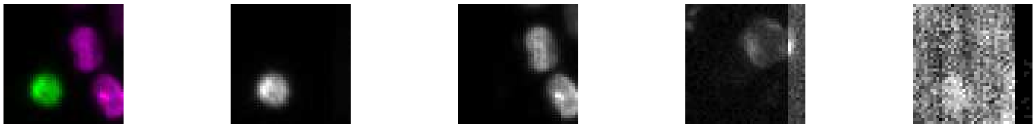				
24243	136	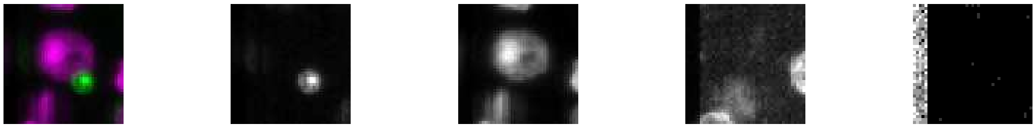				
24244	136	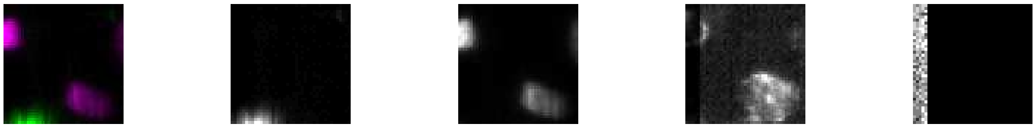				
24245	137	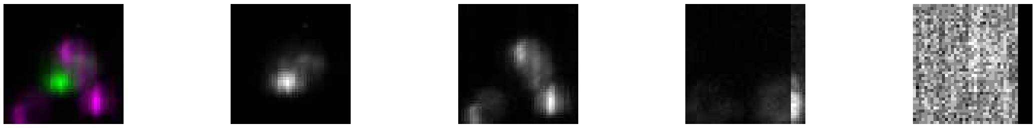				
24246	74	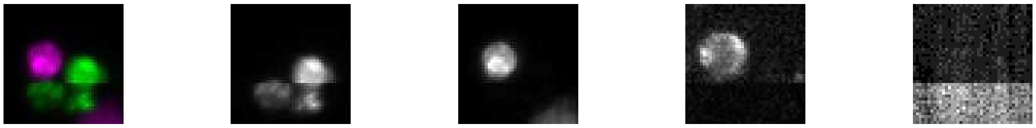				
24247	137	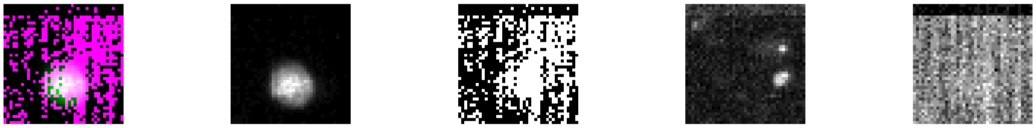				
24248	74	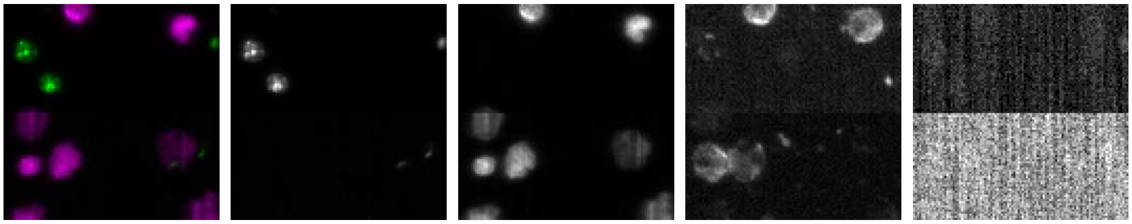				
24249	137	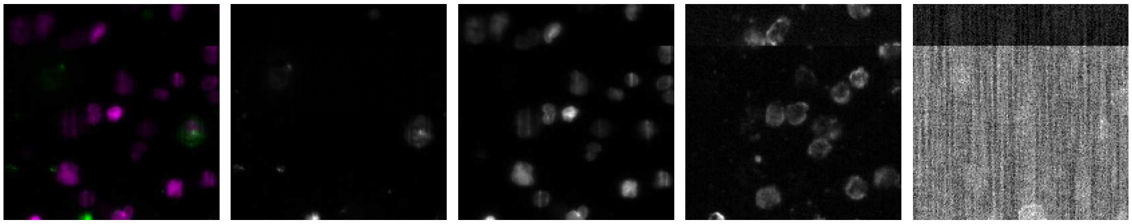				

Ereignis	Aufnahme	DAPI/CK-PE	CK-PE	DAPI	CD45-APC	HER-2/neu
24250	74	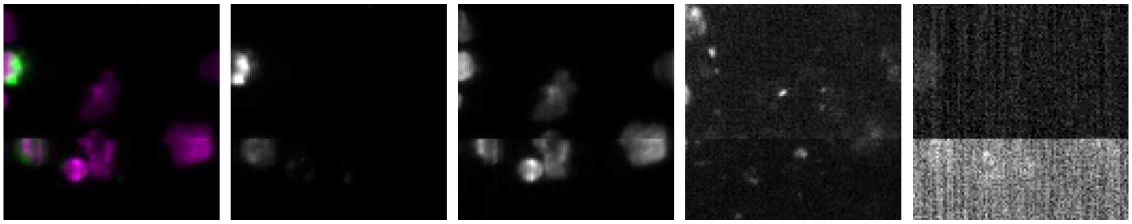				
24251	74	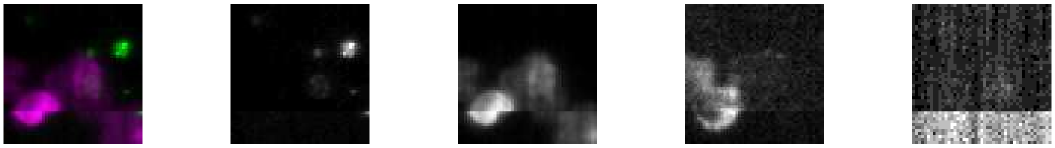				
24252	138	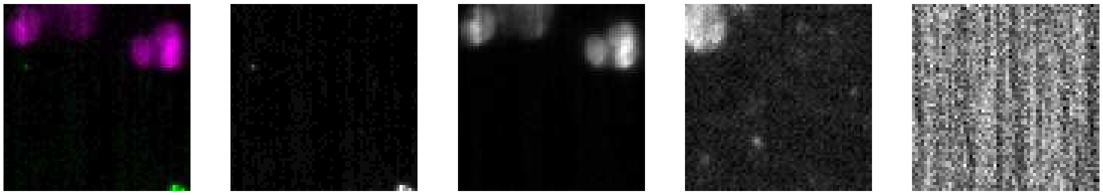				
24253	138	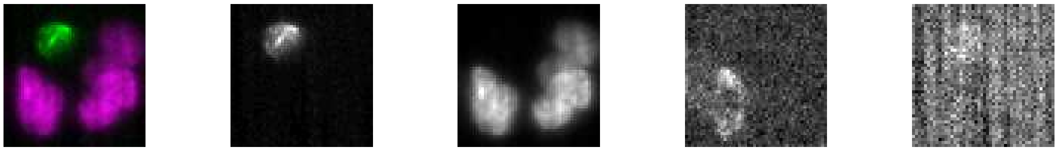				
24254	138	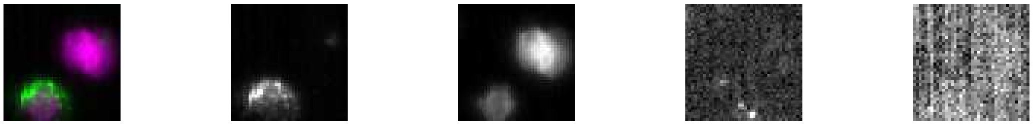				
24255	138	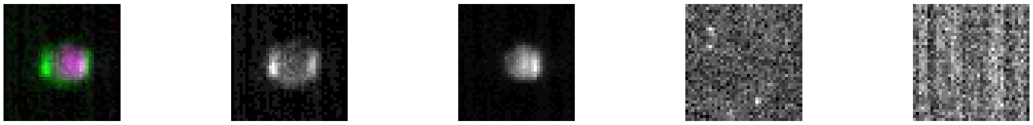				
24256	138	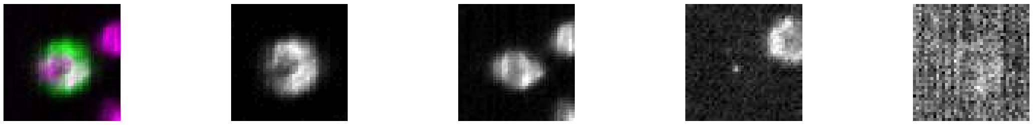				
24257	138	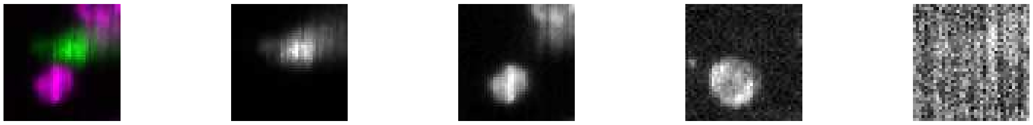				
24258	138	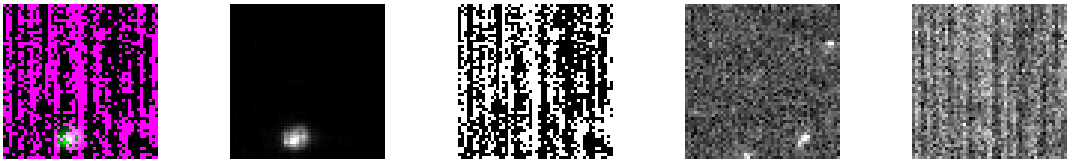				
24259	138	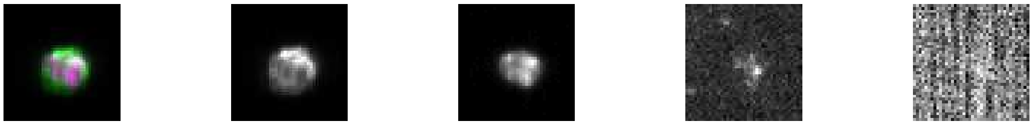				
24260	138	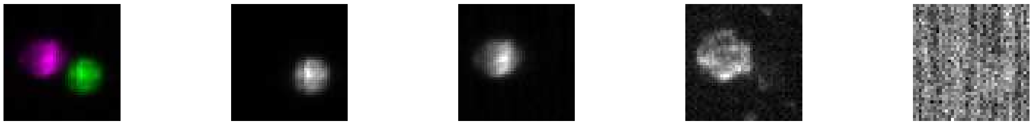				
24261	138	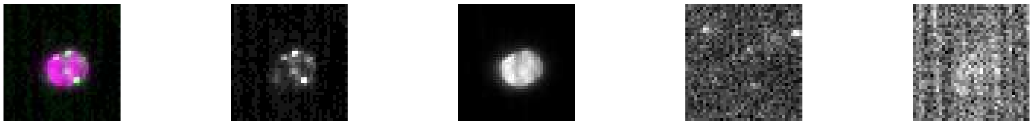				

Ereignis	Aufnahme	DAPI/CK-PE	CK-PE	DAPI	CD45-APC	HER-2/neu
24262	138	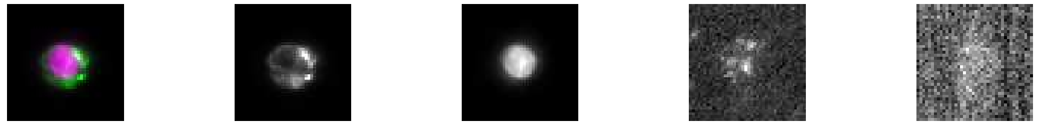				
24263	138	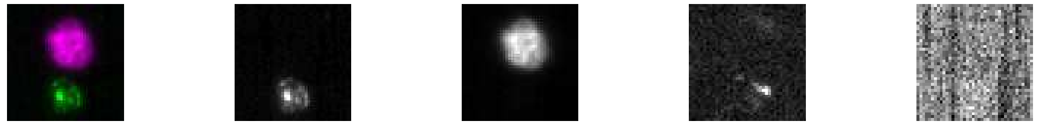				
24264	138	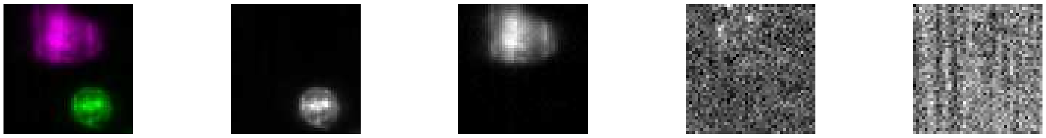				
24265	138	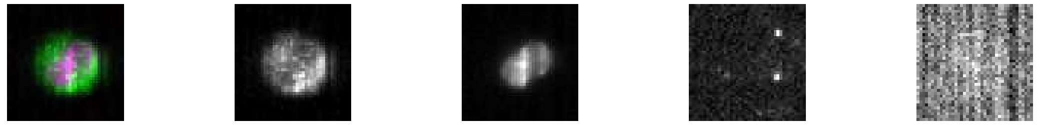				
24266	138	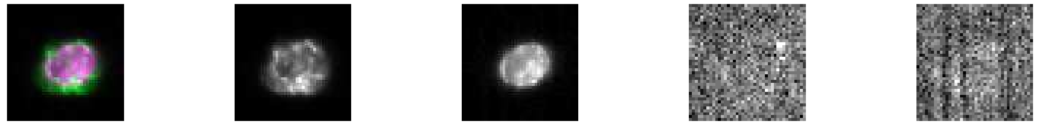				
24267	138	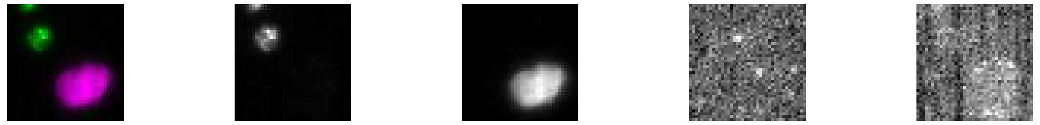				
24268	138	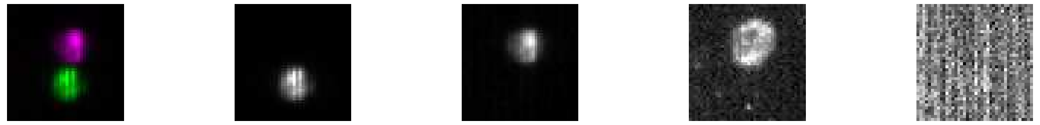				
24269	138	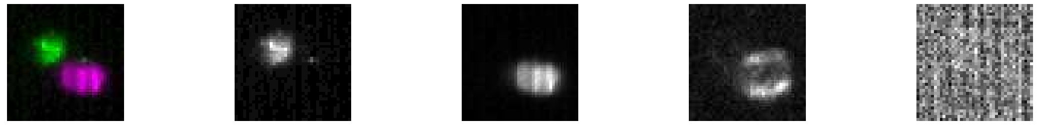				
24270	138	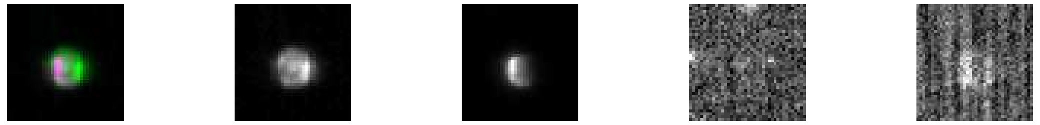				
24271	138	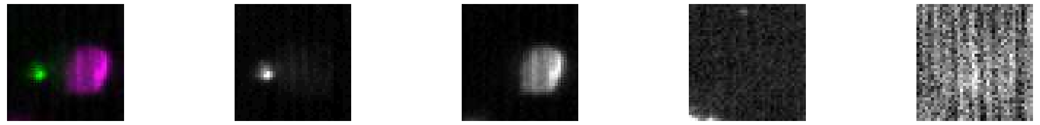				
24272	138	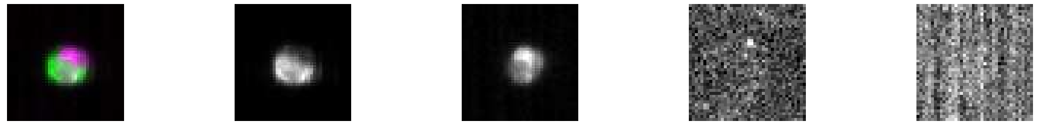				
24273	138	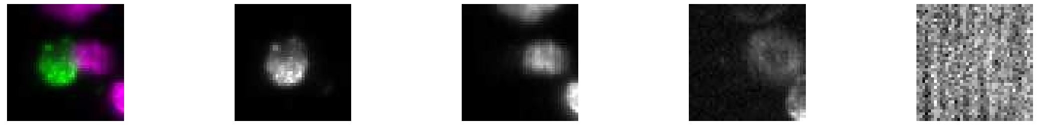				
24274	138	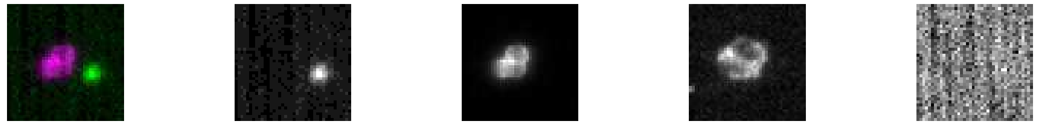				
24275	138	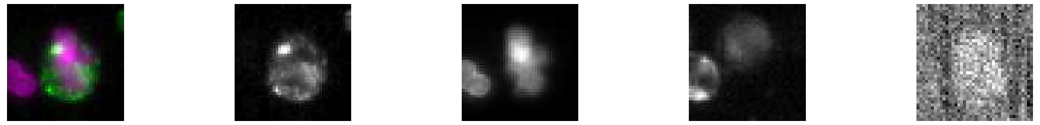				

Ereignis	Aufnahme	DAPI/CK-PE	CK-PE	DAPI	CD45-APC	HER-2/neu
24276	138	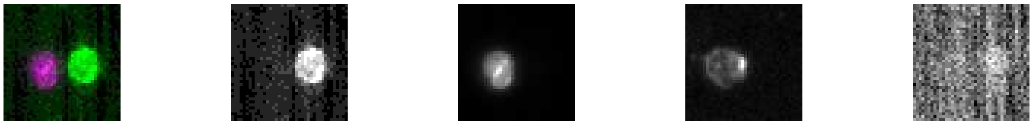				
24277	138	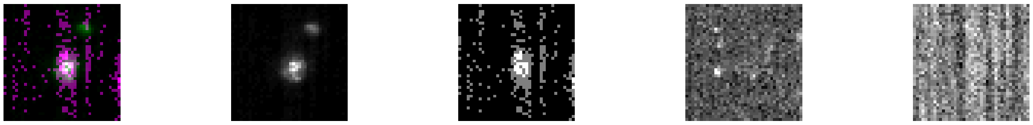				
24278	138	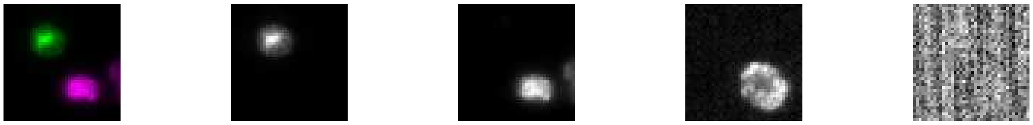				
24279	138	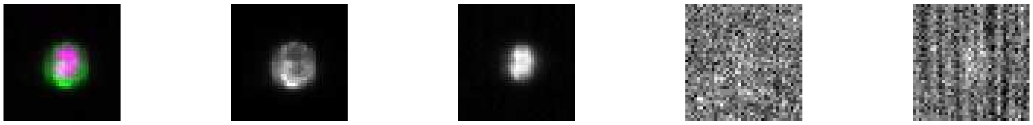				
24280	138	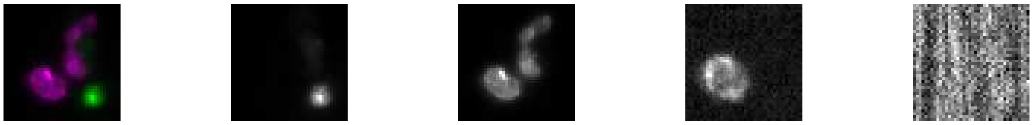				
24281	138	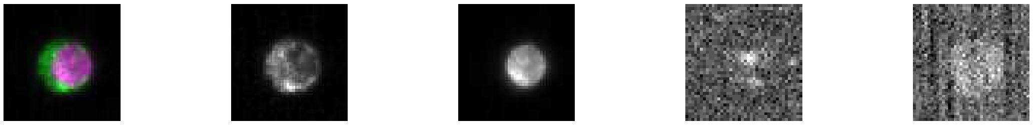				
24282	138	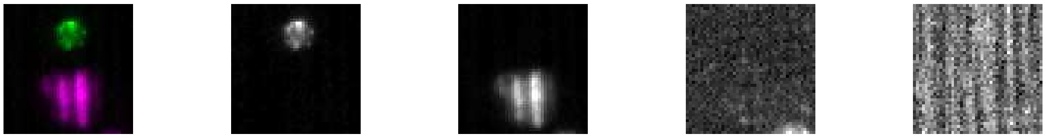				
24283	138	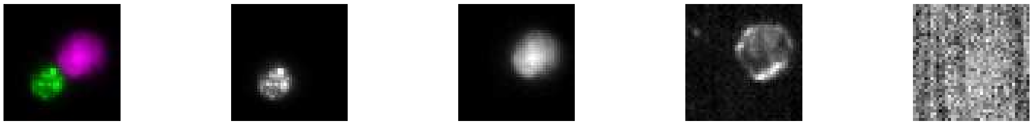				
24284	138	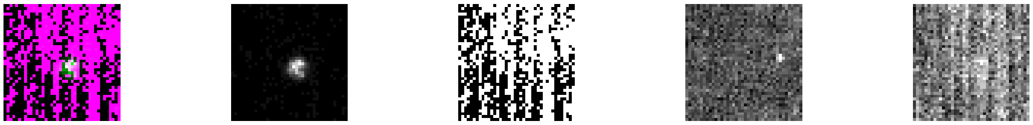				
24285	138	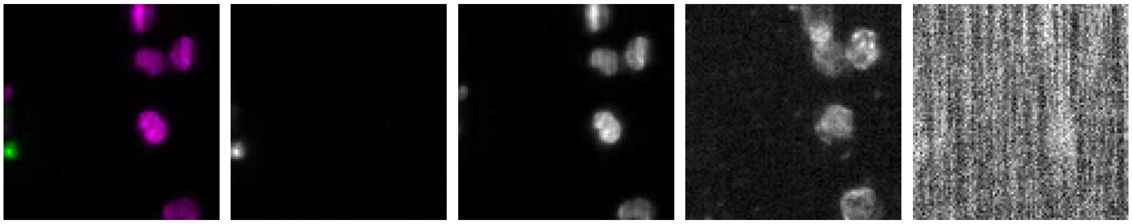				
24286	138	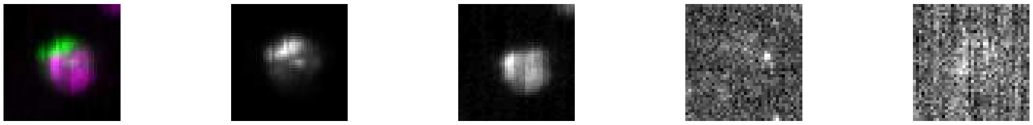				
24287	138	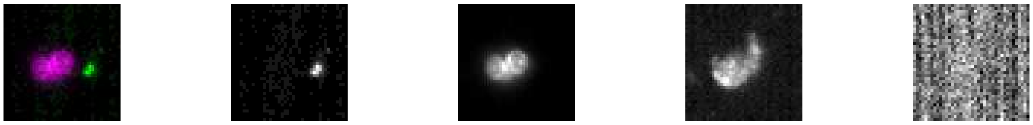				
24288	138	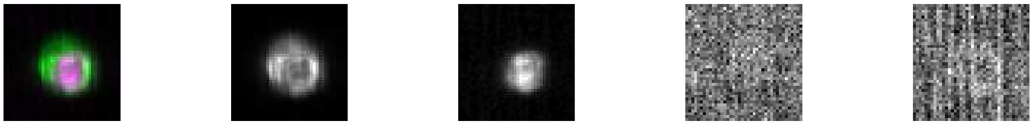				

Ereignis	Aufnahme	DAPI/CK-PE	CK-PE	DAPI	CD45-APC	HER-2/neu
24289	138	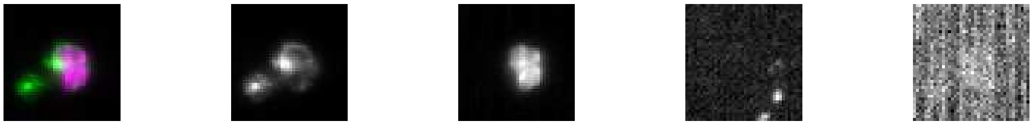				
24290	138	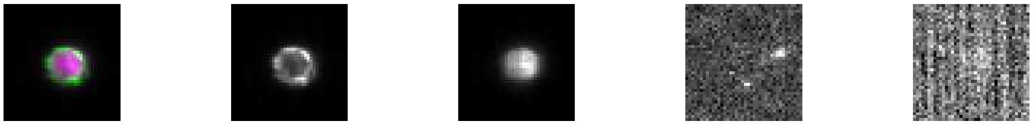				
24291	138	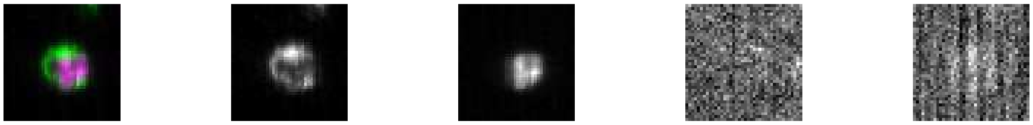				
24292	138	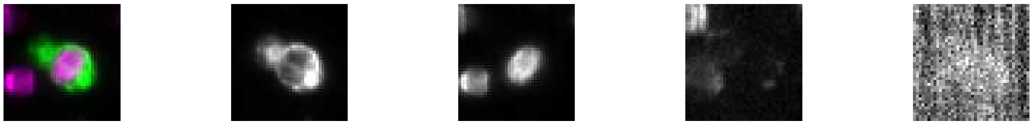				
24293	138	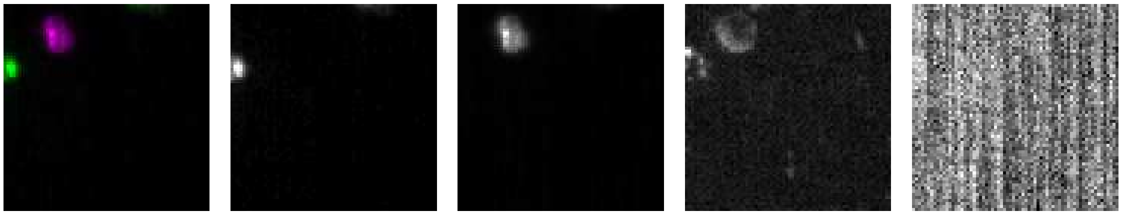				
24294	138	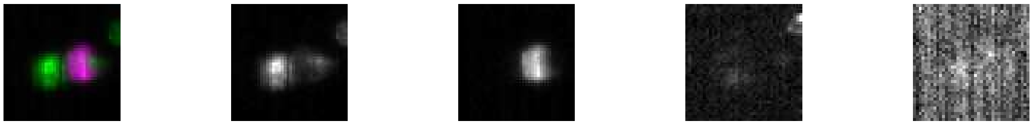				
24295	138	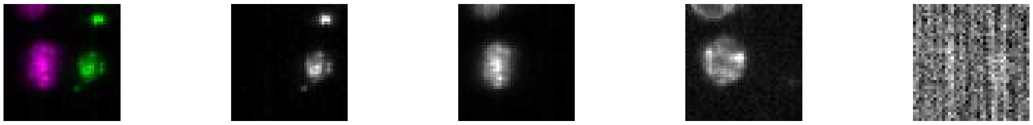				
24296	138	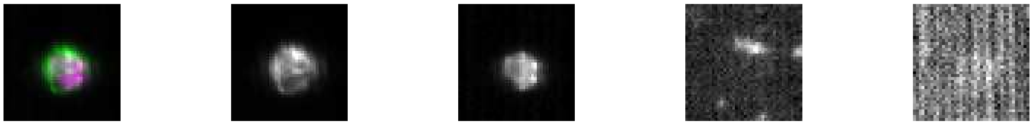				
24297	138	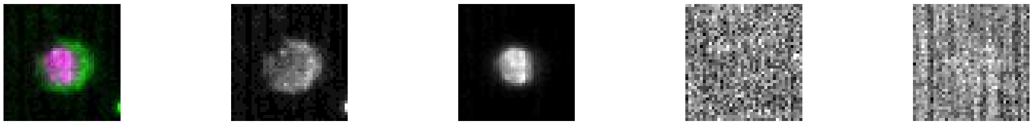				
24298	138	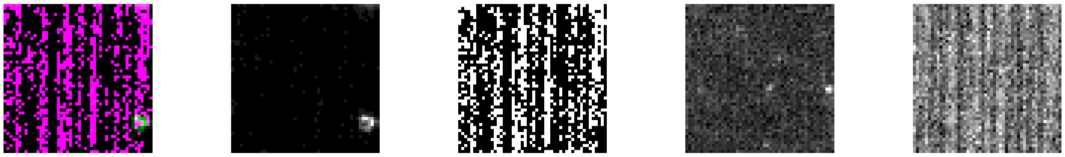				
24299	138	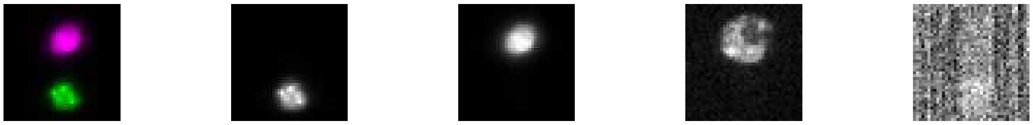				
24300	138	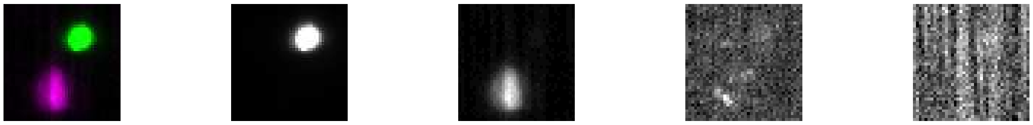				
24301	138	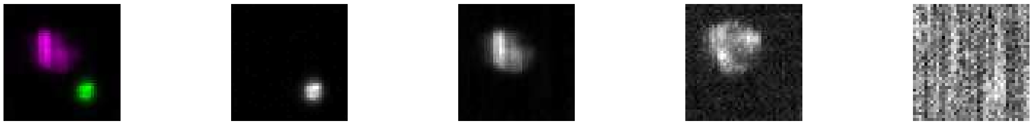				

Proben-ID: Gyn14.208 **Kassetten-ID:**01327106 **Patienten-ID:**

Ereignis	Aufnahme	DAPI/CK-PE	CK-PE	DAPI	CD45-APC	HER-2/neu
24302	138	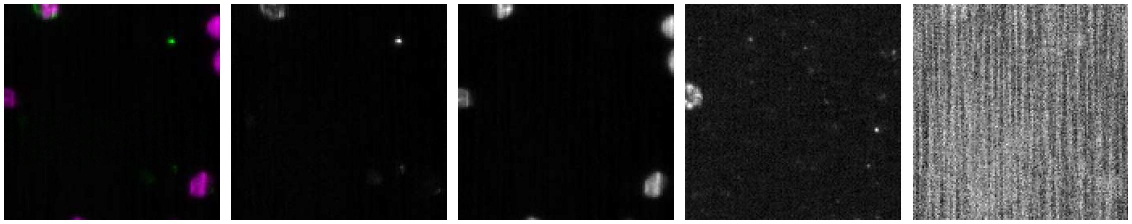				
24303	138	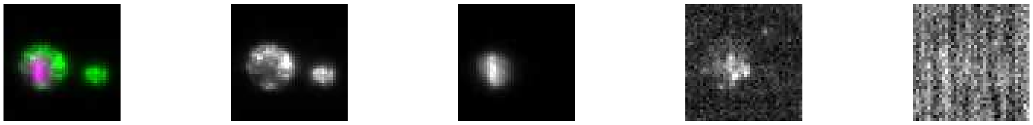				
24304	138	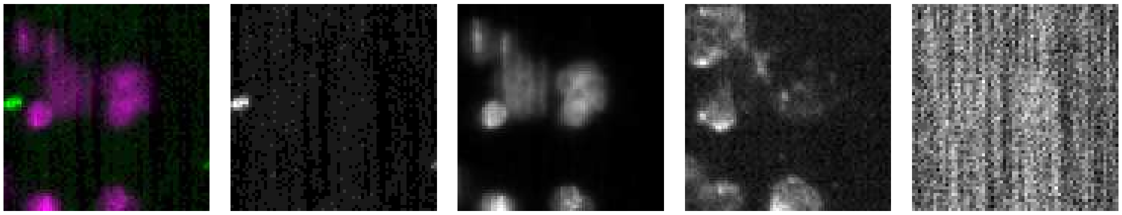				
24305	138	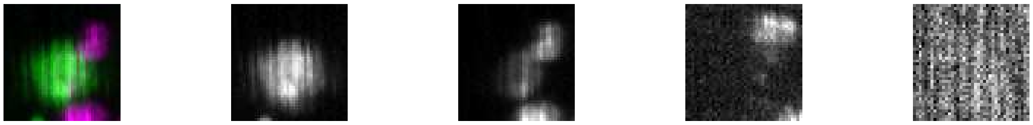				
24306	138	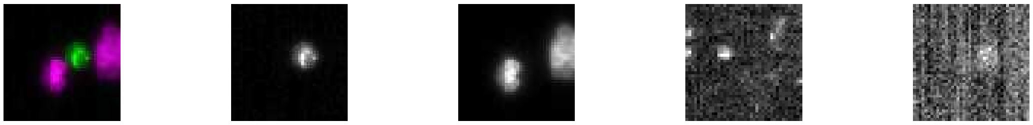				
24307	138	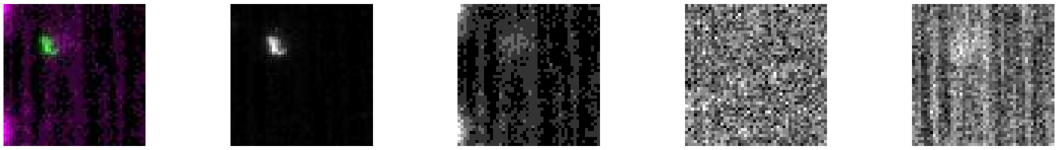				
24308	138	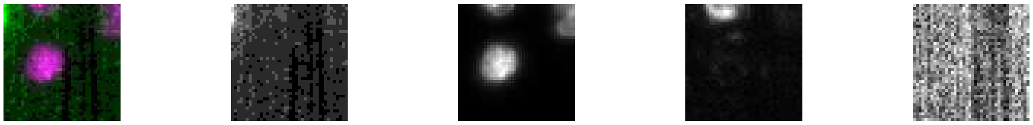				
24309	138	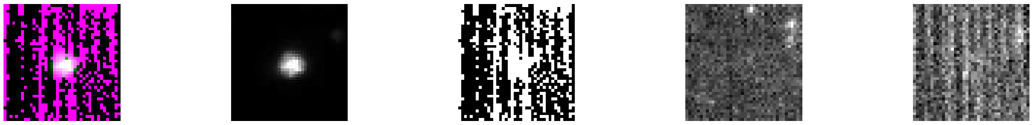				
24310	138	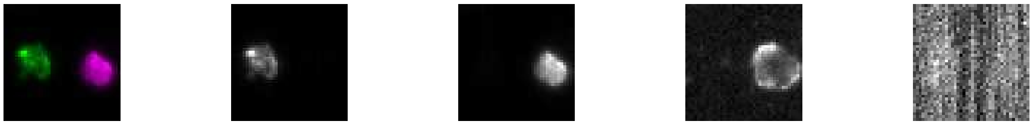				
24311	138	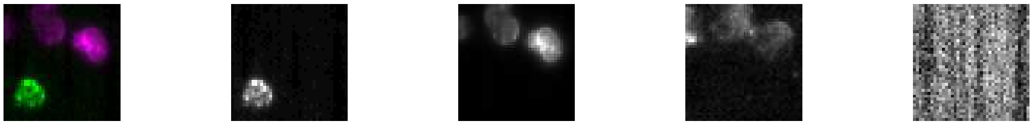				
24312	138	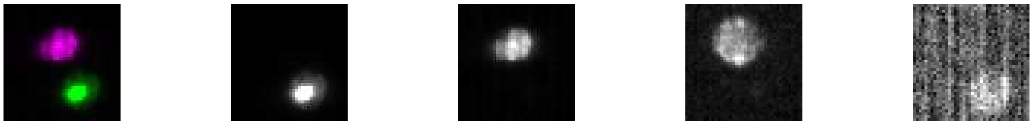				
24313	138	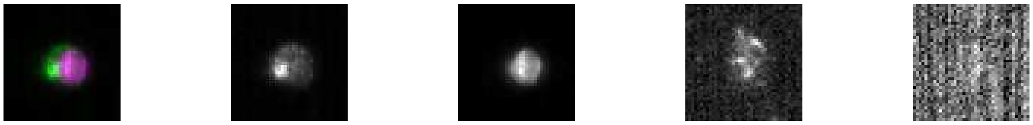				

Ereignis	Aufnahme	DAPI/CK-PE	CK-PE	DAPI	CD45-APC	HER-2/neu
24314	138	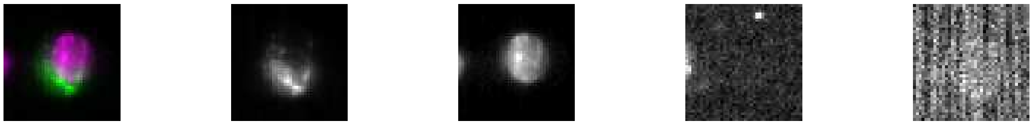				
24315	138	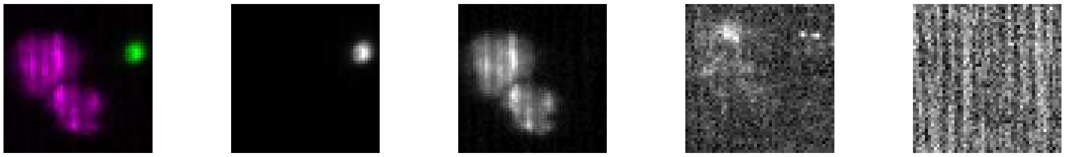				
24316	138	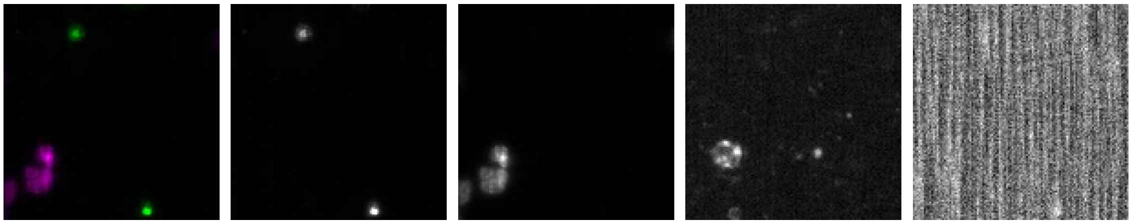				
24317	138	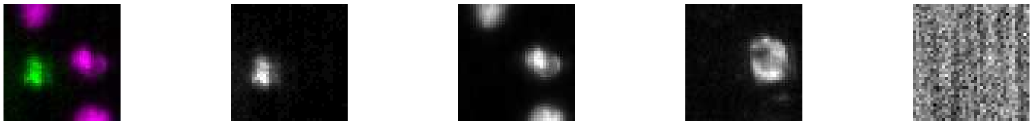				
24318	138	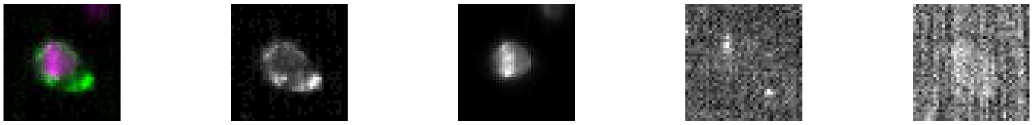				
24319	138	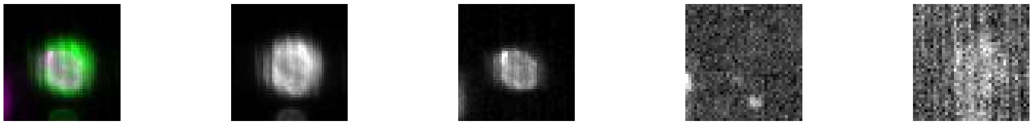				
24320	138	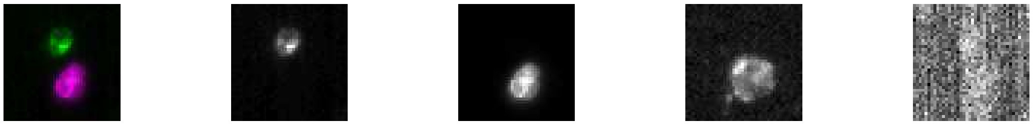				
24321	138	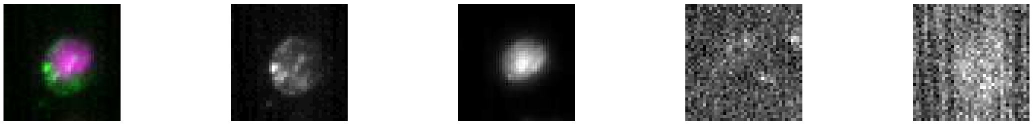				
24322	138	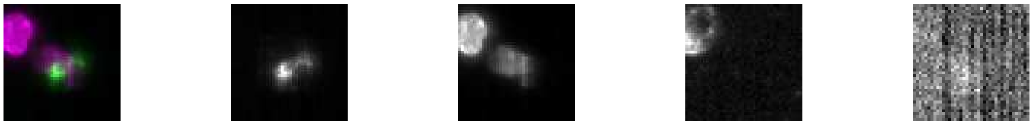				
24323	138	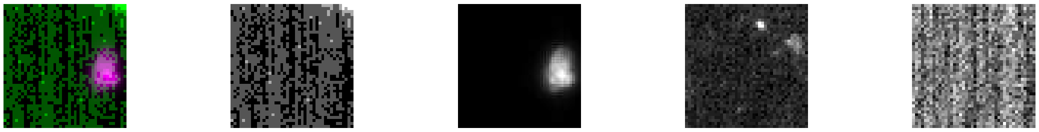				
24324	138	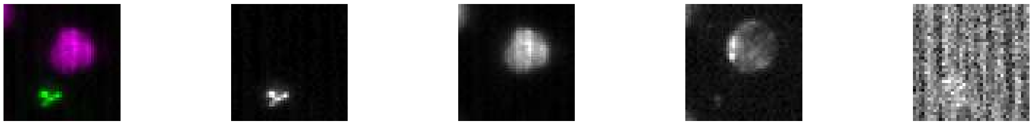				
24325	138	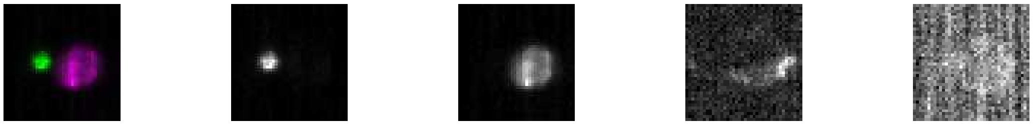				
24326	138	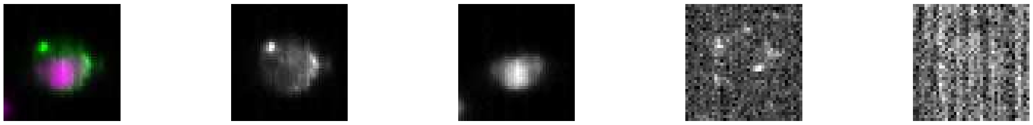				

Ereignis	Aufnahme	DAPI/CK-PE · CK-PE · DAPI · CD45-APC · HER-2/neu
24327	138	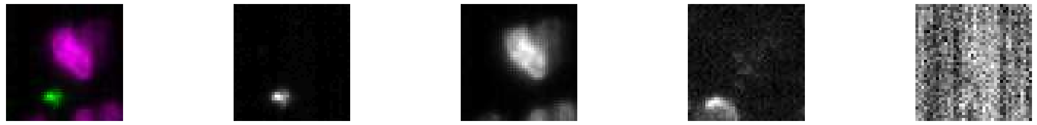
24328	138	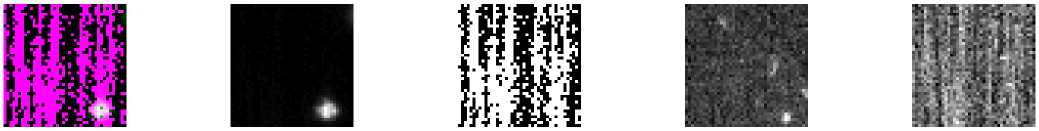
24329	138	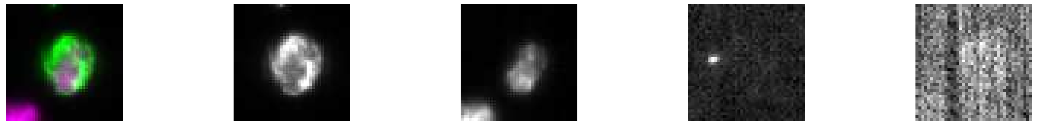
24330	138	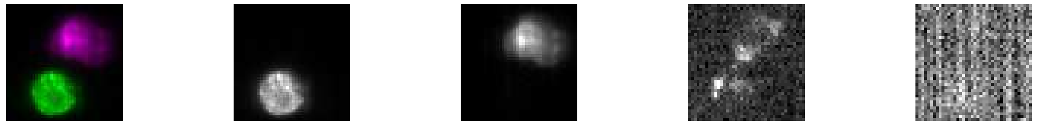
24331	138	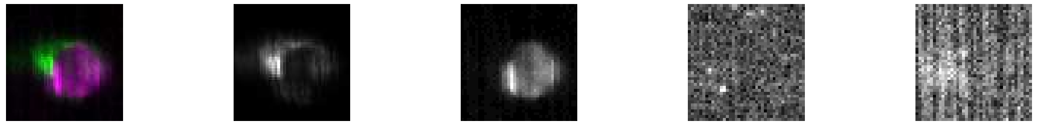
24332	138	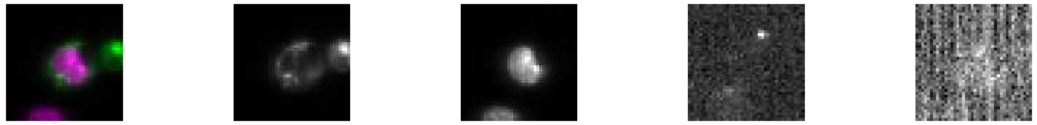
24333	138	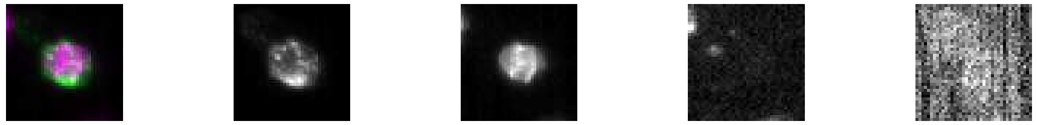
24334	138	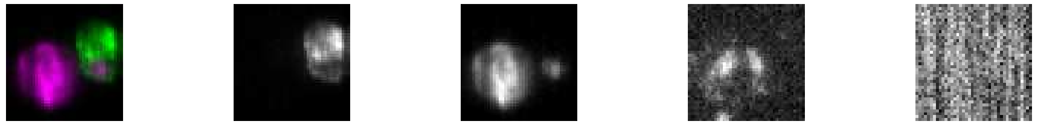
24335	138	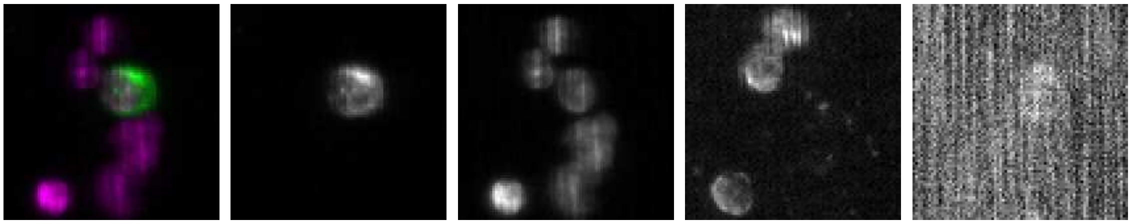
24336	138	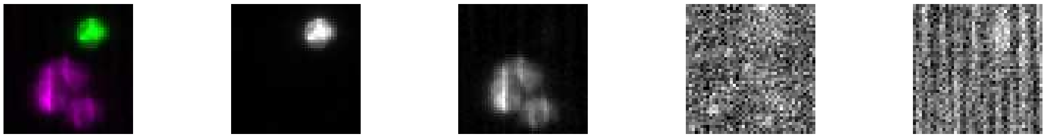
24337	138	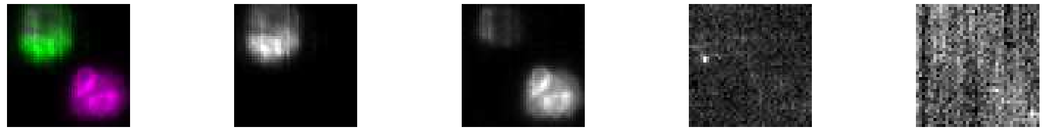
24338	138	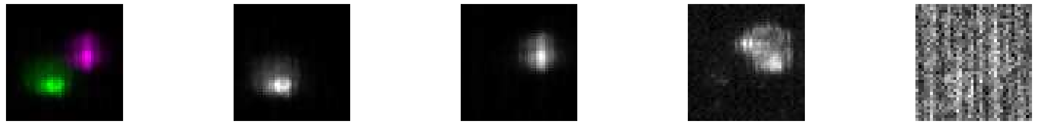
24339	138	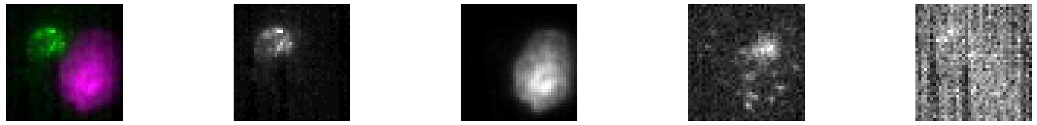

Ereignis	Aufnahme	DAPI/CK-PE	CK-PE	DAPI	CD45-APC	HER-2/neu
24340	138	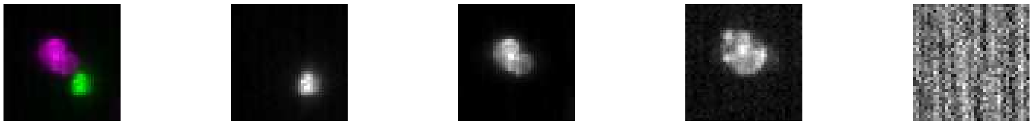				
24341	138	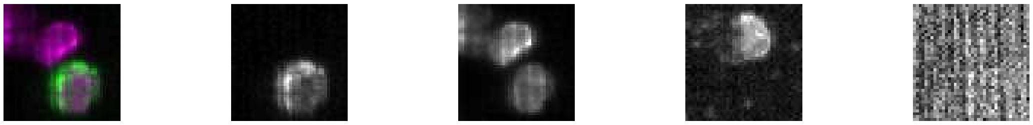				
24342	138	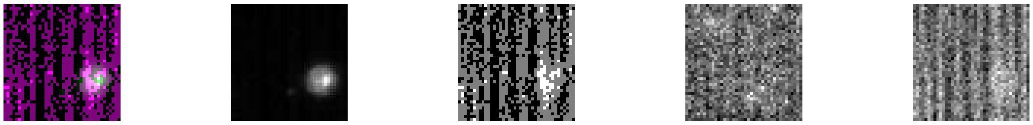				
24343	138	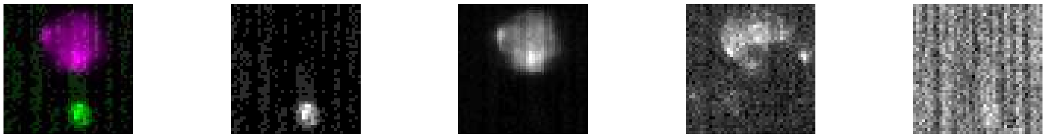				
24344	138	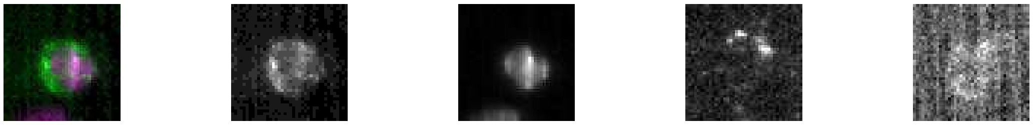				
24345	73	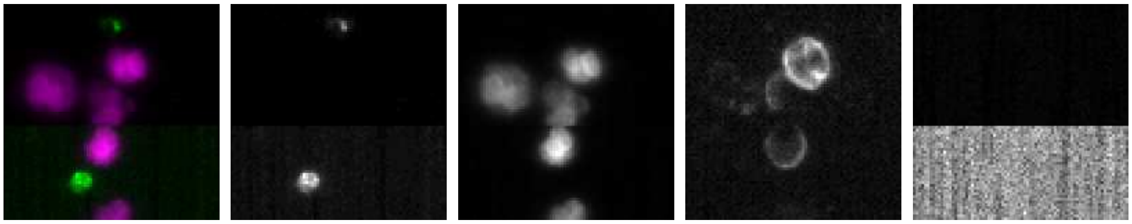				
24346	138	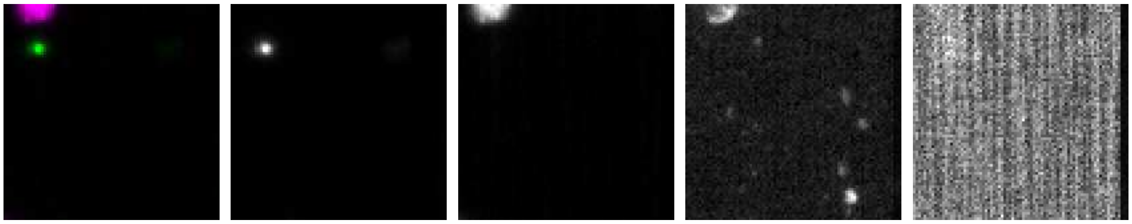				
24347	138	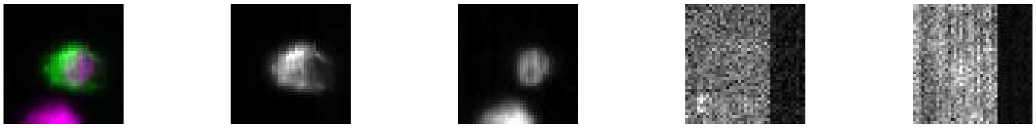				
24348	138	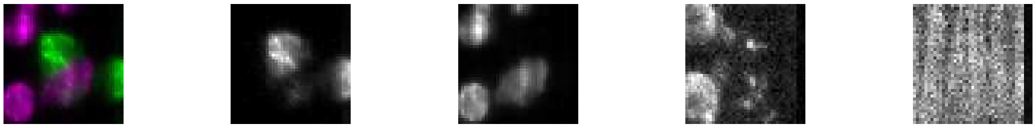				
24349	138	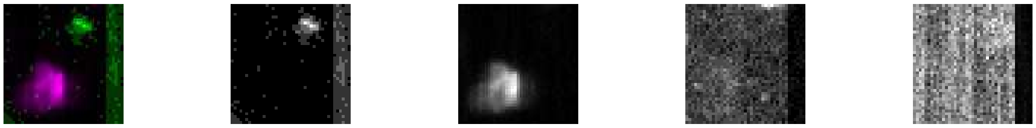				
24350	138	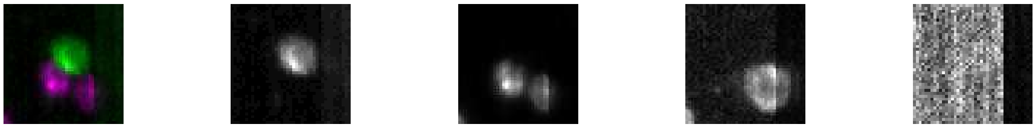				
24351	138	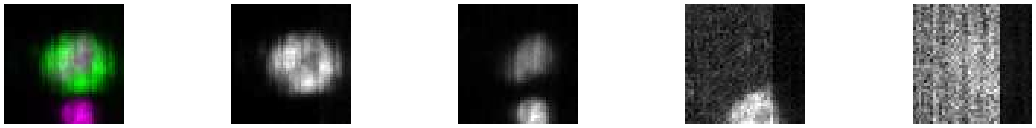				

Ereignis	Aufnahme	DAPI/CK-PE	CK-PE	DAPI	CD45-APC	HER-2/neu
24352	73	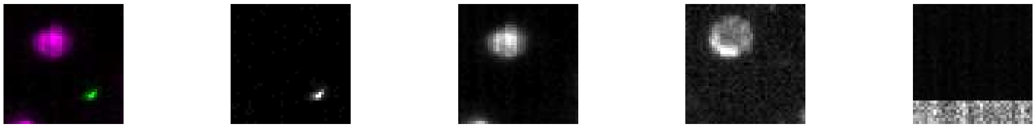				
24353	139	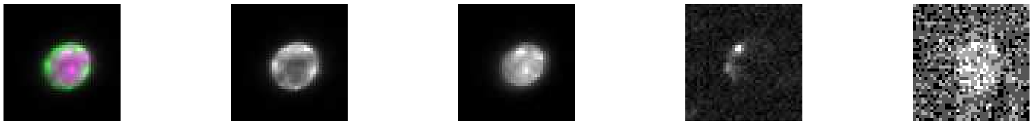				
24354	139	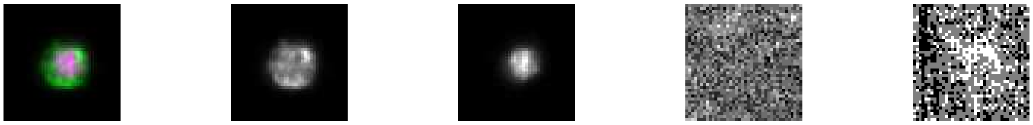				
24355	139	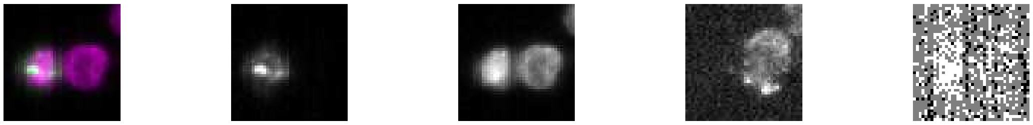				
24356	139	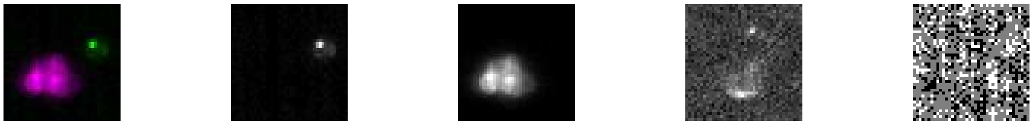				
24357	139	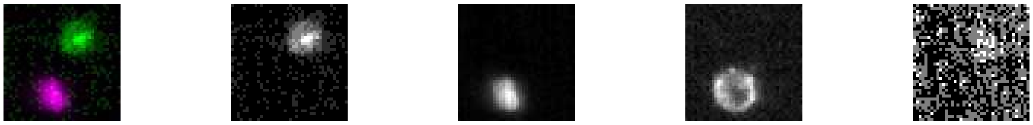				
24358	139	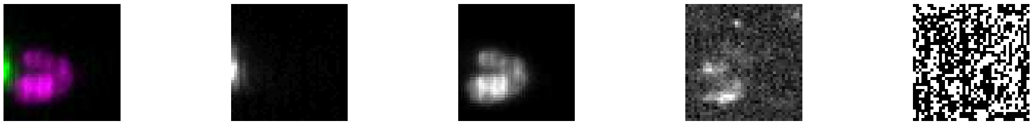				
24359	139	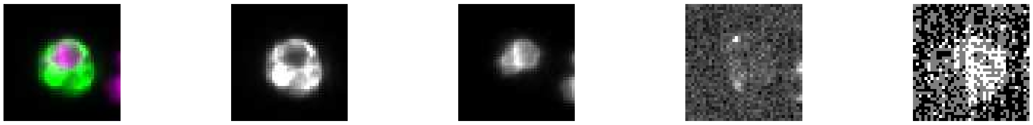				
24360	139	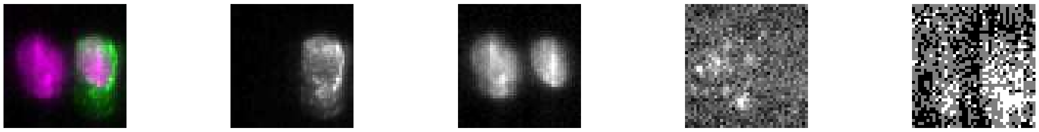				
24361	139	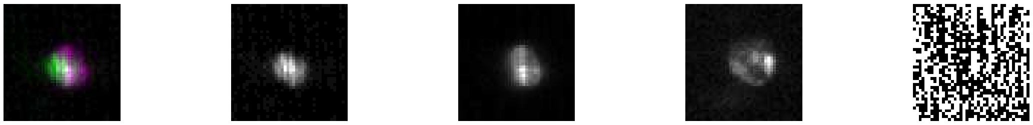				
24362	139	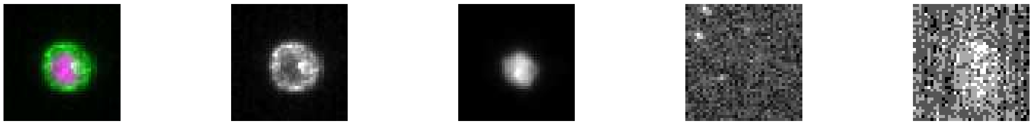				
24363	139	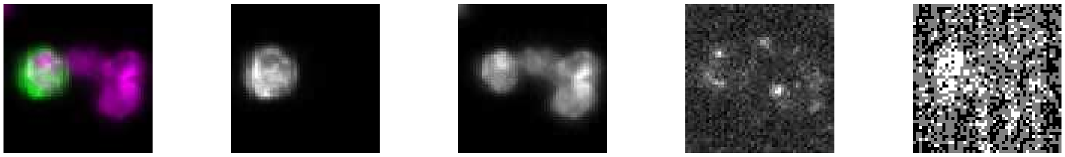				
24364	139	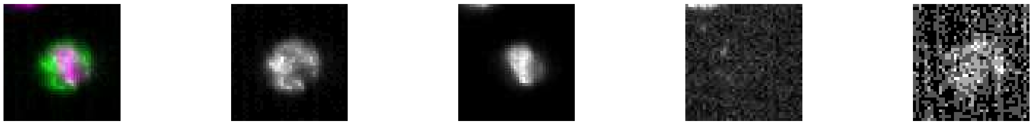				
24365	139	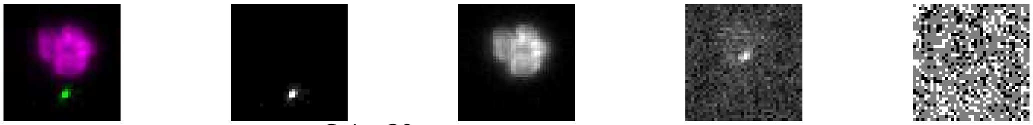				

Ereignis	Aufnahme	DAPI/CK-PE	CK-PE	DAPI	CD45-APC	HER-2/neu
24366	139	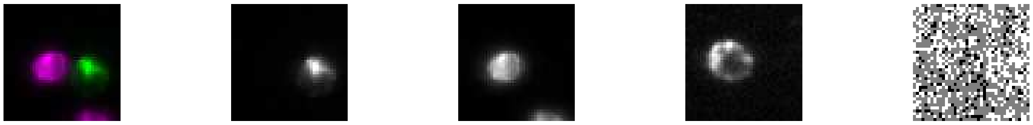				
24367	139	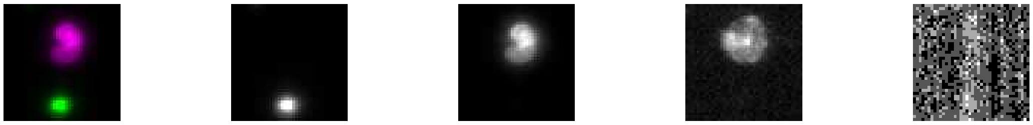				
24368	139	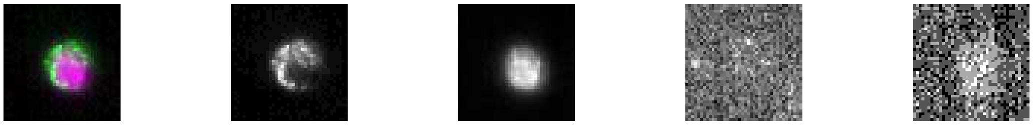				
24369	139	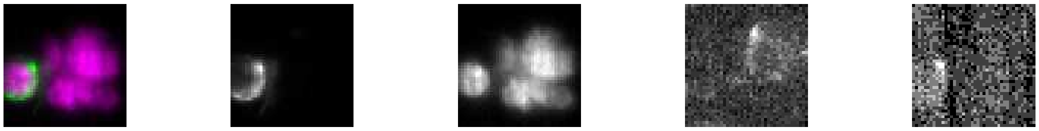				
24370	139	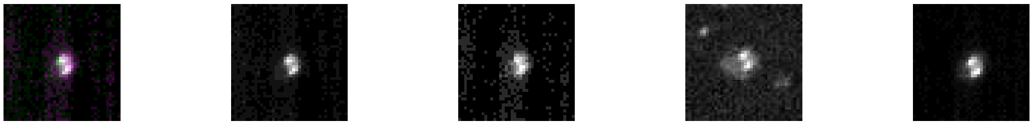				
24371	139	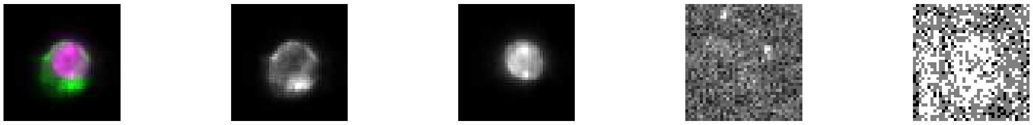				
24372	139	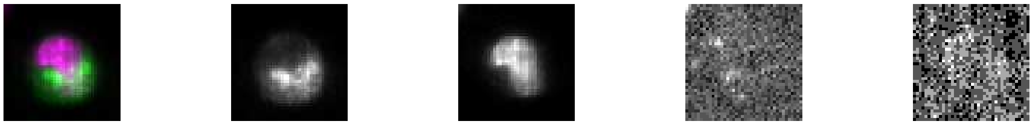				
24373	139	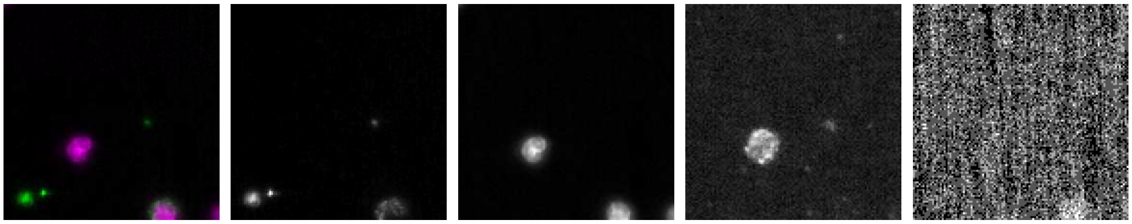				
24374	139	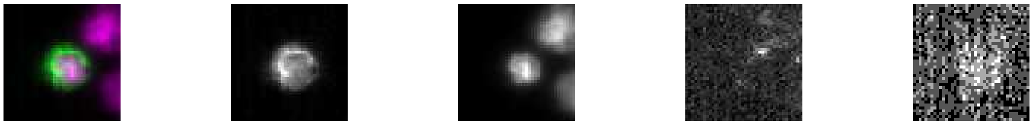				
24375	139	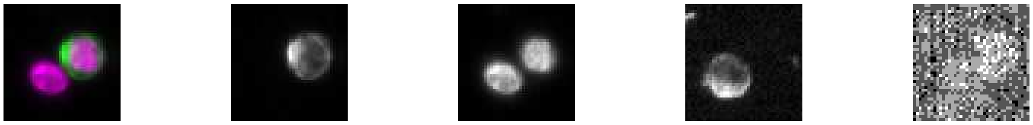				
24376	139	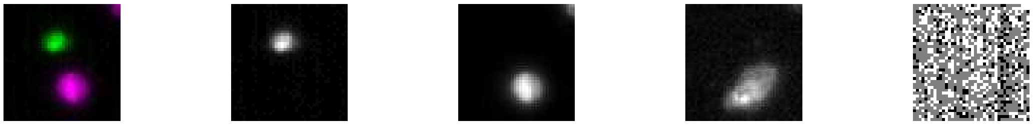				
24377	139	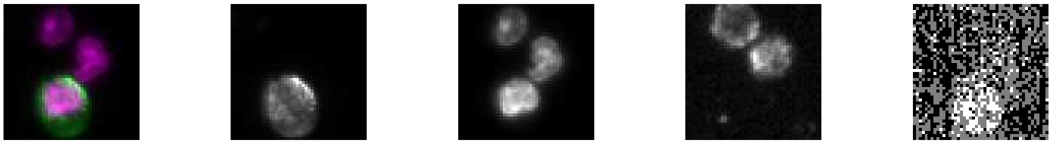				
24378	139	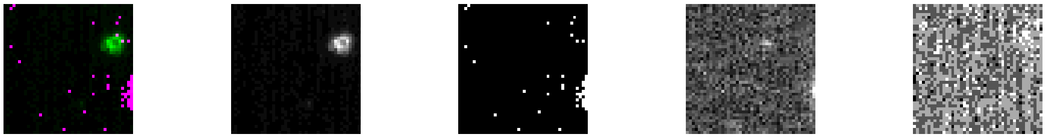				

Ereignis	Aufnahme	DAPI/CK-PE	CK-PE	DAPI	CD45-APC	HER-2/neu
24379	139	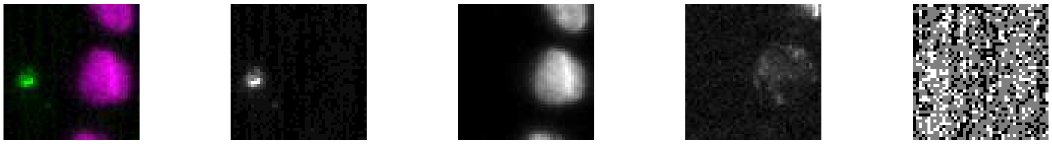				
24380	139	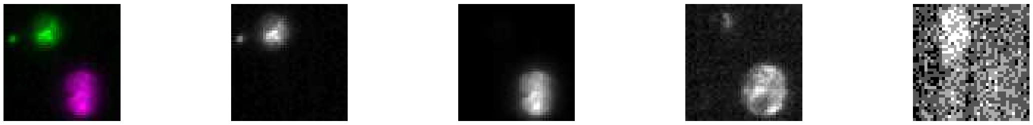				
24381	139	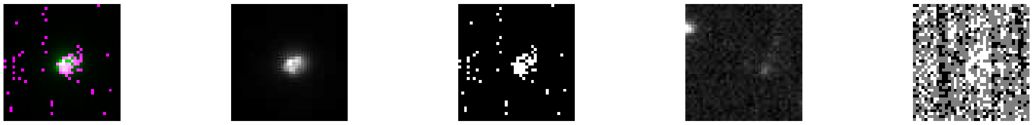				
24382	139	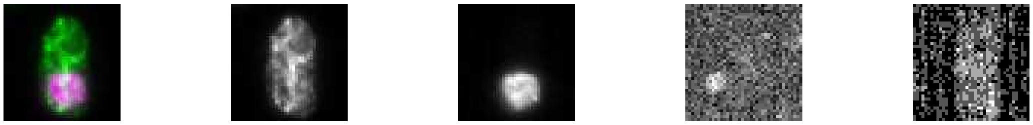				
24383	139	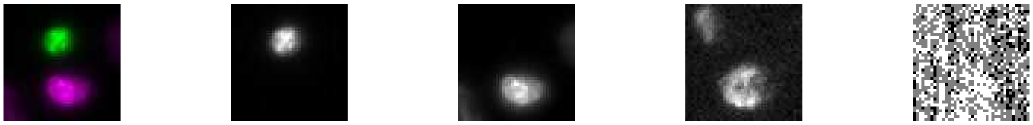				
24384	139	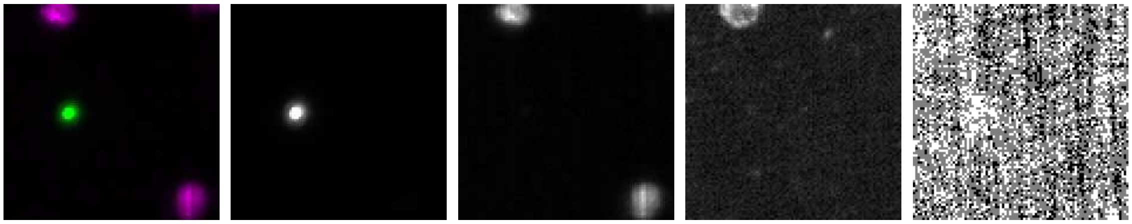				
24385	139	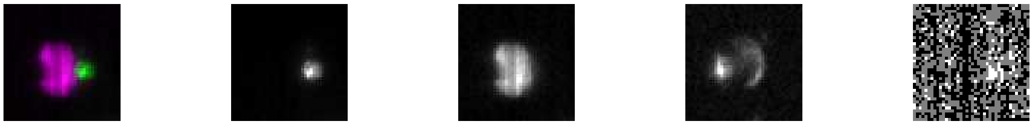				
24386	139	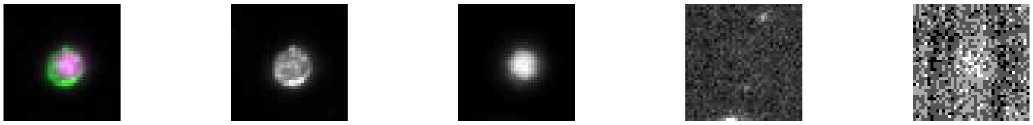				
24387	139	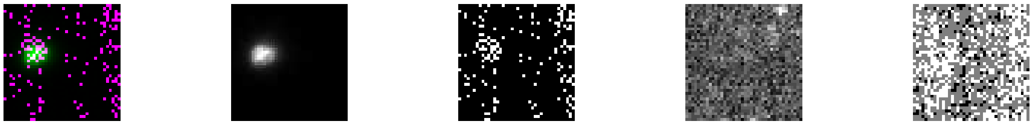				
24388	139	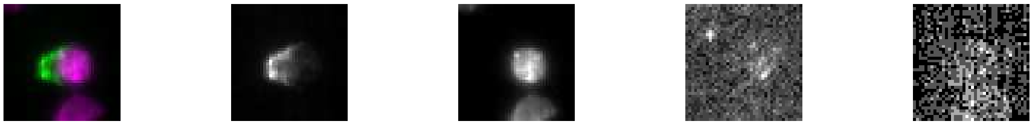				
24389	139	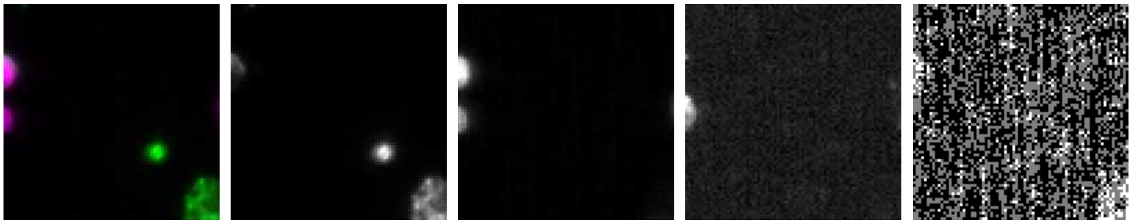				
24390	139	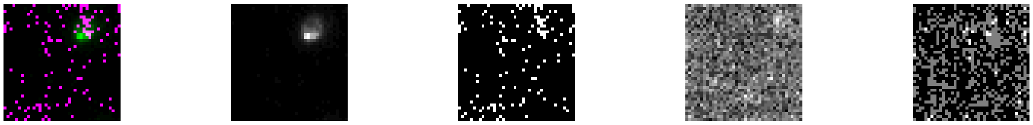				

Proben-ID: Gyn14.208 **Kassetten-ID:**01327106 **Patienten-ID:**

Ereignis	Aufnahme	DAPI/CK-PE	CK-PE	DAPI	CD45-APC	HER-2/neu
24391	139	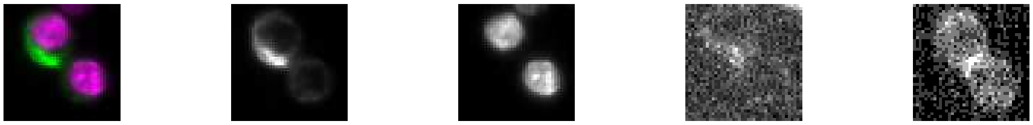				
24392	139	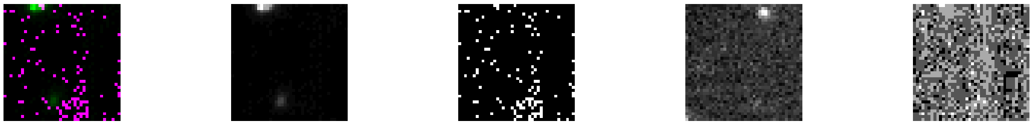				
24393	139	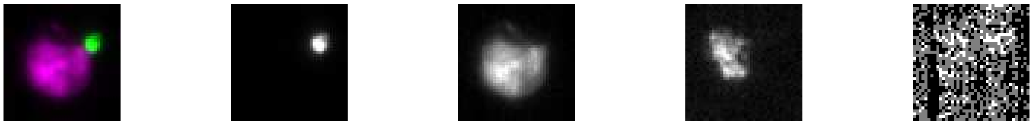				
24394	139	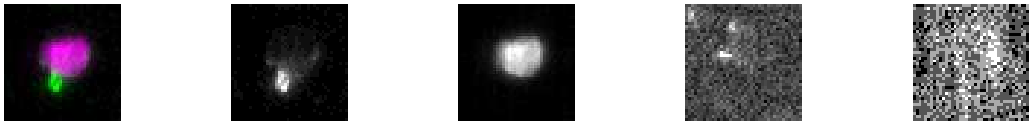				
24395	139	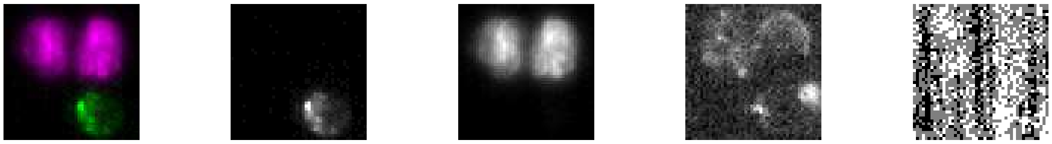				
24396	139	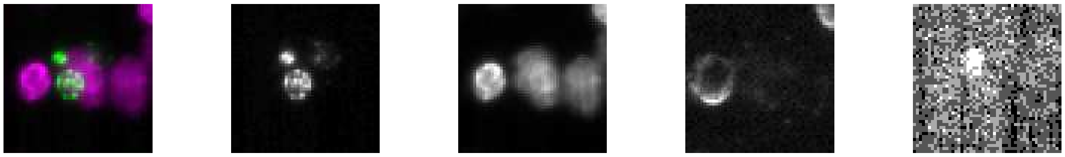				
24397	139	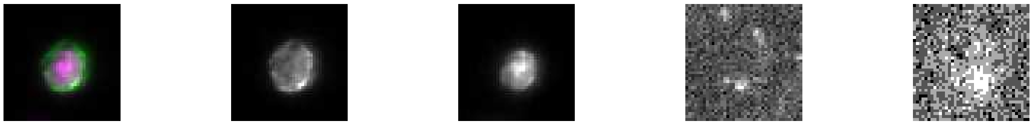				
24398	139	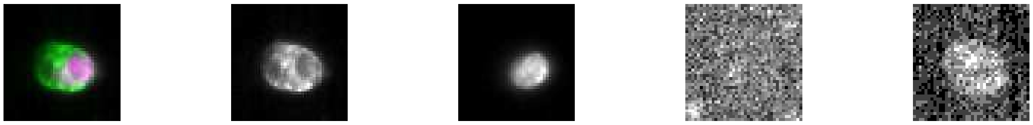				
24399	139	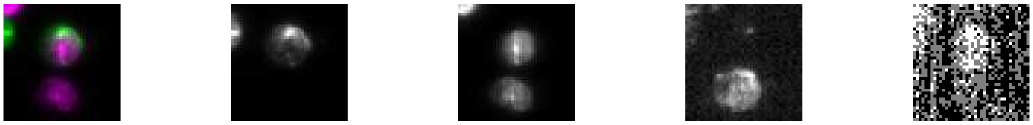				
24400	139	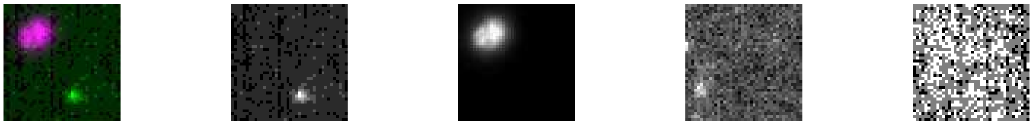				
24401	139	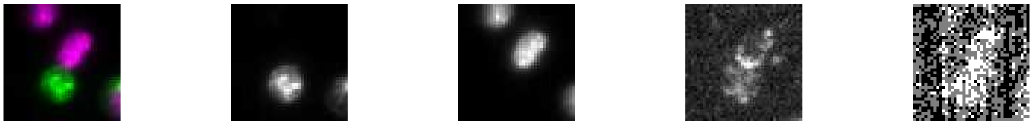				
24402	139	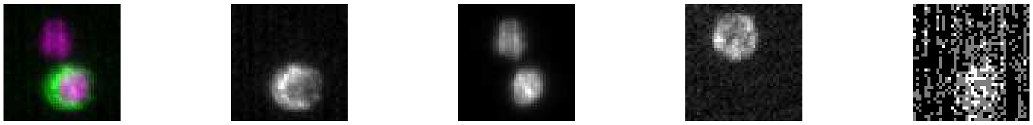				
24403	139	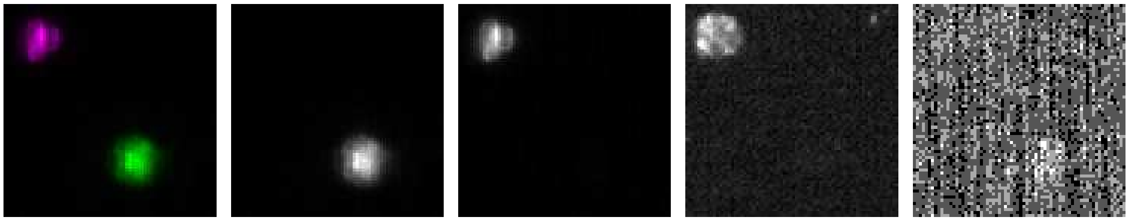				

Druckdatum: 02/06/2020 17:25

Proben-ID: Gyn14.208 **Kassetten-ID:** 01327106 **Patienten-ID:**

Ereignis	Aufnahme	DAPI/CK-PE	CK-PE	DAPI	CD45-APC	HER-2/neu
24404	139	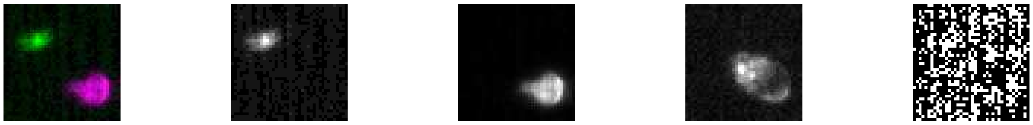				
24405	139	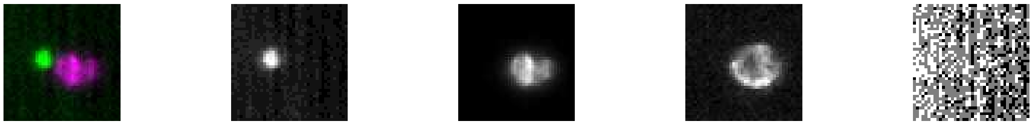				
24406	139	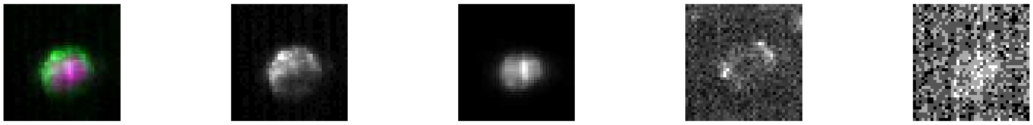				
24407	139	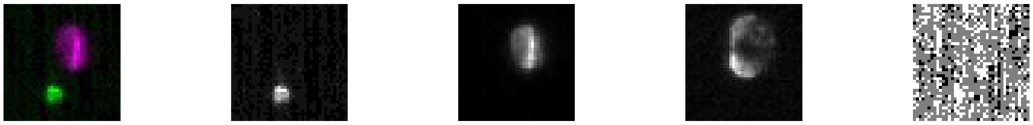				
24408	139	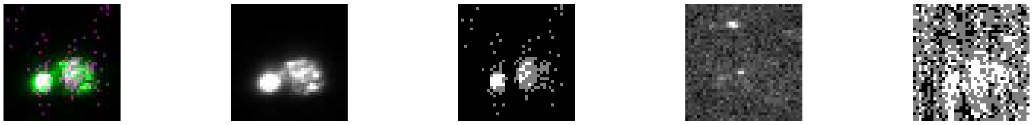				
24409	139	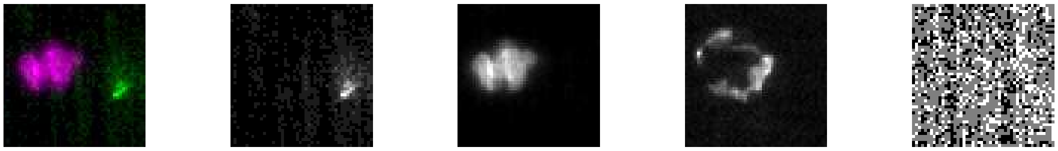				
24410	139	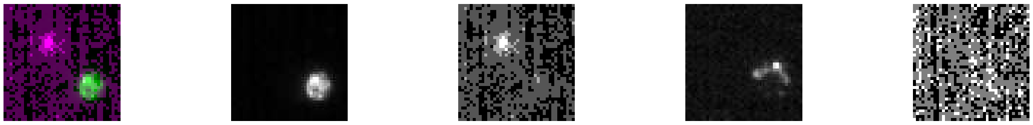				
24411	139	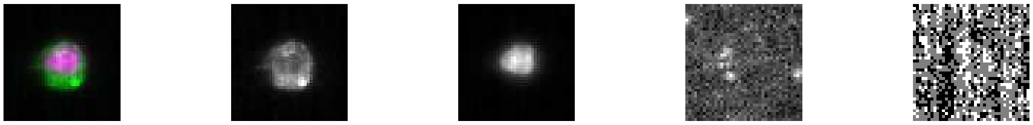				
24412	139	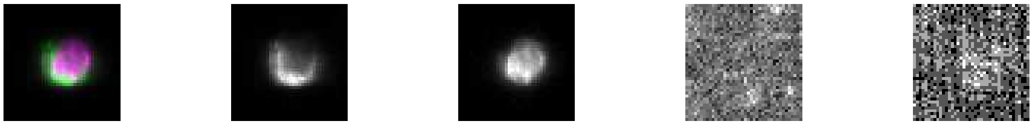				
24413	139	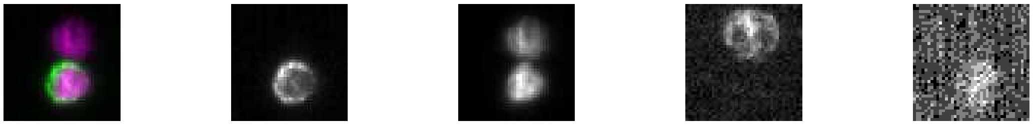				
24414	139	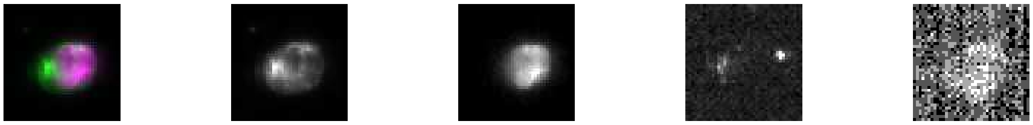				
24415	139	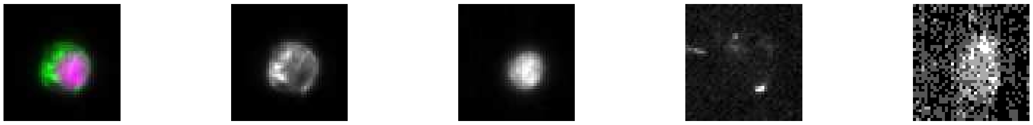				
24416	138	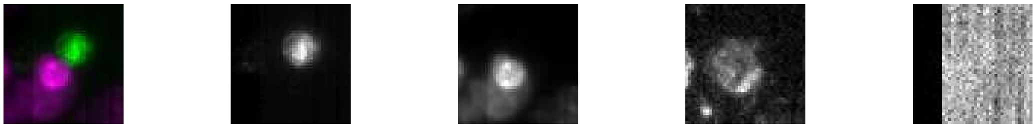				
24417	72	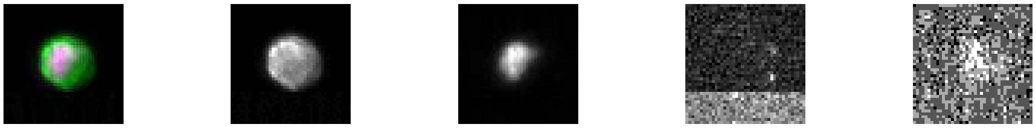				

Proben-ID: Gyn14.208 **Kassetten-ID:** 01327106 **Patienten-ID:**

Ereignis	Aufnahme	DAPI/CK-PE	CK-PE	DAPI	CD45-APC	HER-2/neu
24418	139	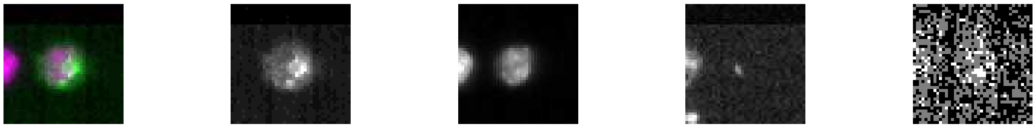				
24419	140	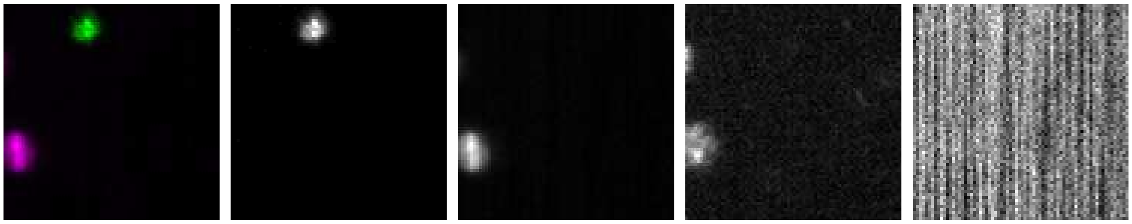				
24420	140	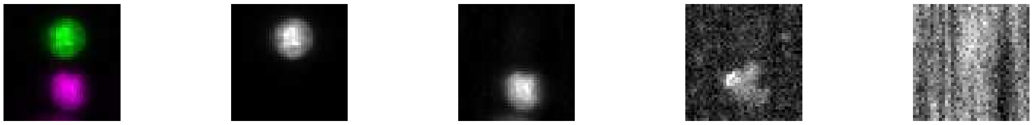				
24421	140	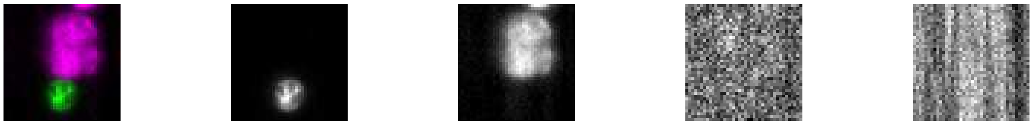				
24422	140	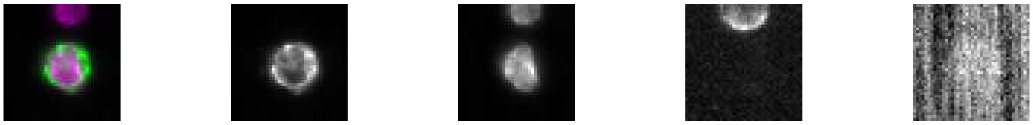				
24423	140	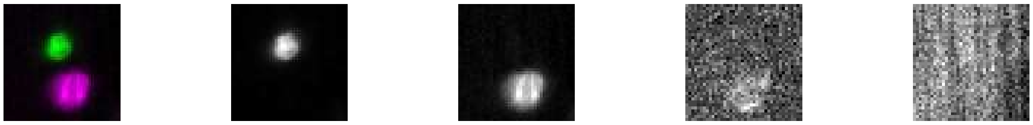				
24424	140	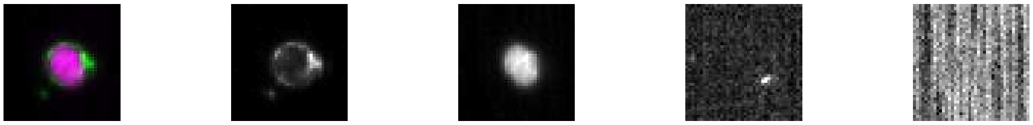				
24425	140	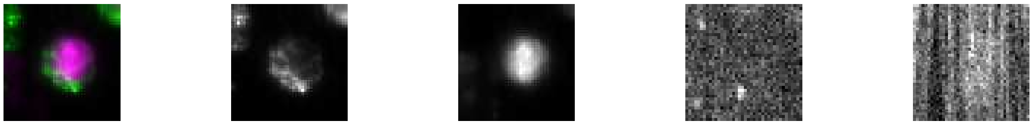				
24426	140	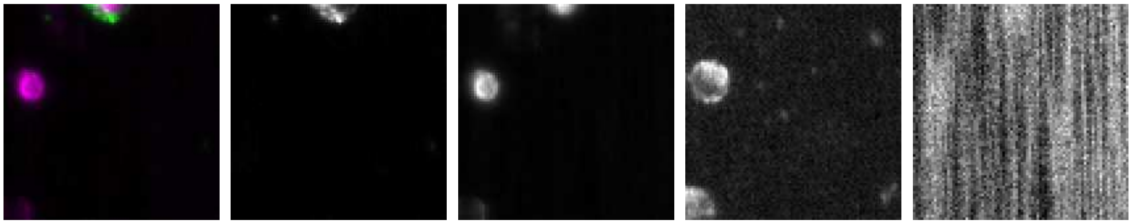				
24427	140	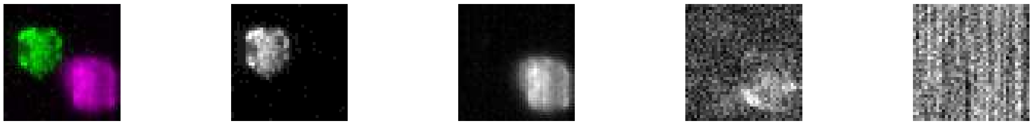				
24428	140	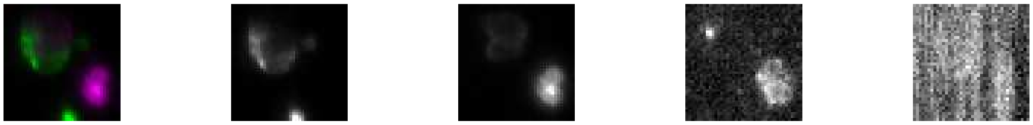				
24429	140	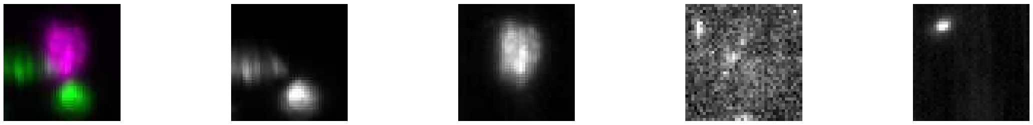				

Proben-ID: Gyn14.208 **Kassetten-ID:**01327106 **Patienten-ID:**

Ereignis	Aufnahme	DAPI/CK-PE	CK-PE	DAPI	CD45-APC	HER-2/neu
24430	140	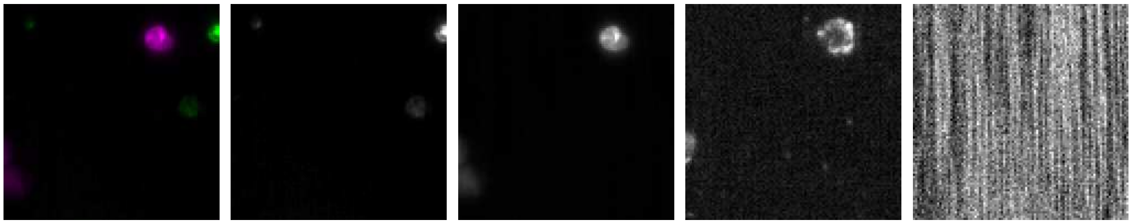				
24431	140	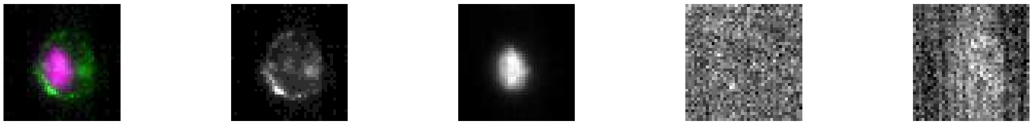				
24432	140	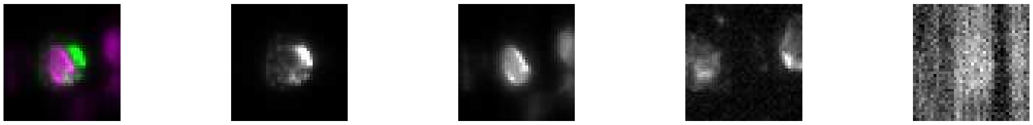				
24433	140	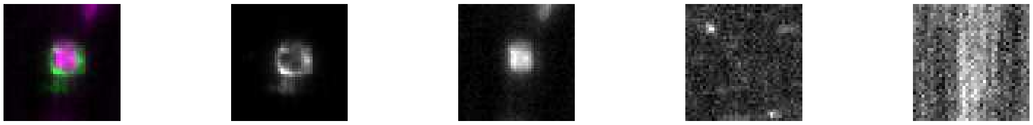				
24434	140	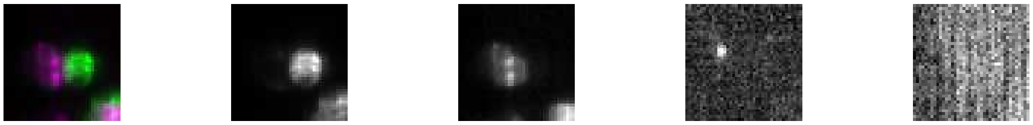				
24435	140	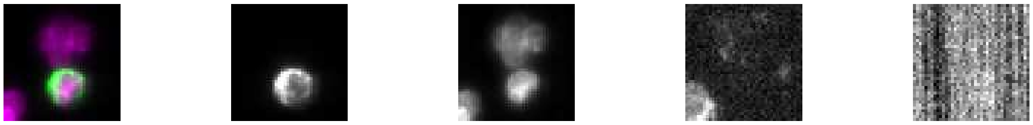				
24436	140	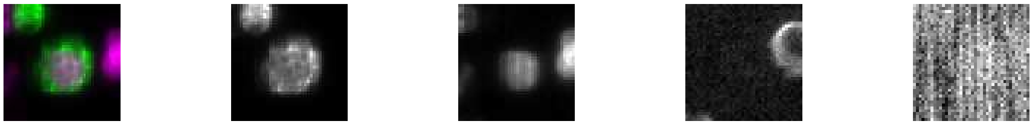				
24437	140	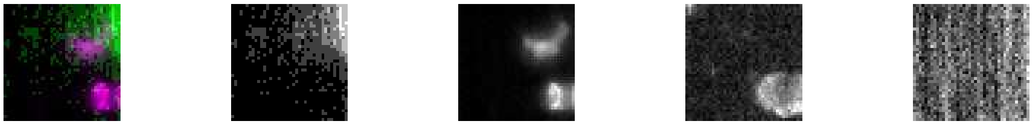				
24438	140	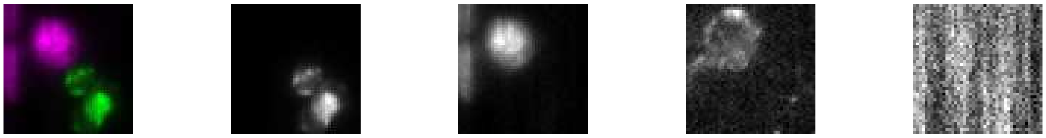				
24439	140	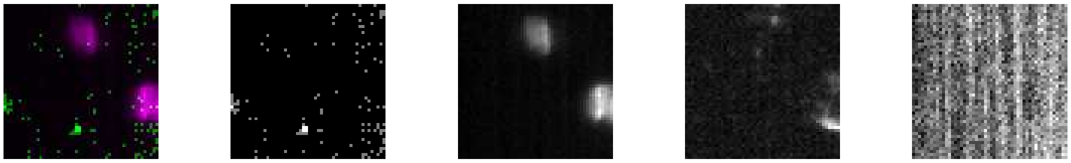				
24440	140	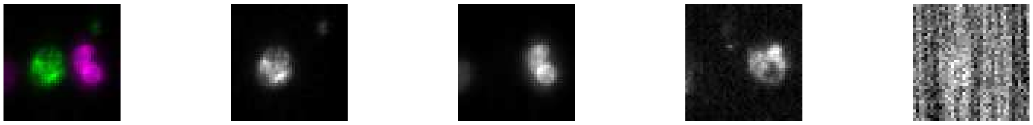				
24441	140	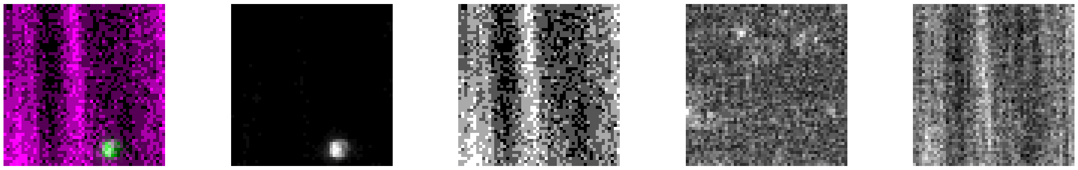				

Ereignis	Aufnahme	DAPI/CK-PE	CK-PE	DAPI	CD45-APC	HER-2/neu
24442	140	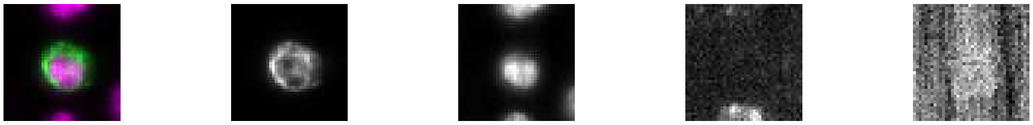				
24443	140	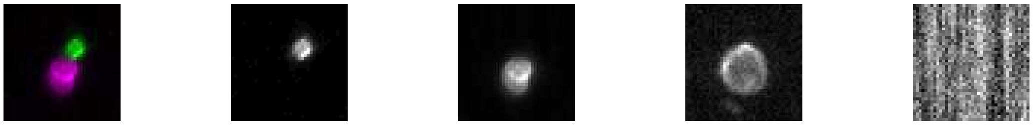				
24444	140	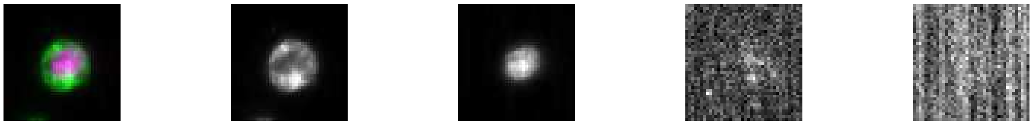				
24445	140	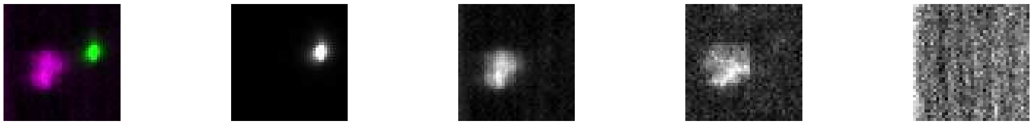				
24446	71	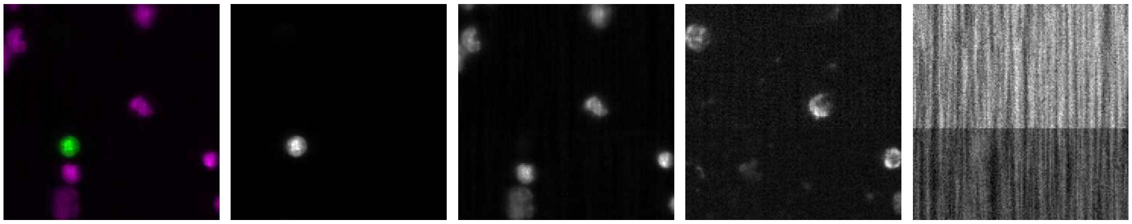				
24447	140	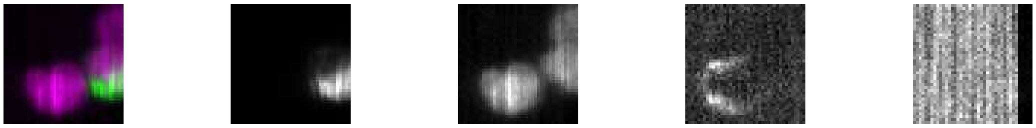				
24448	140	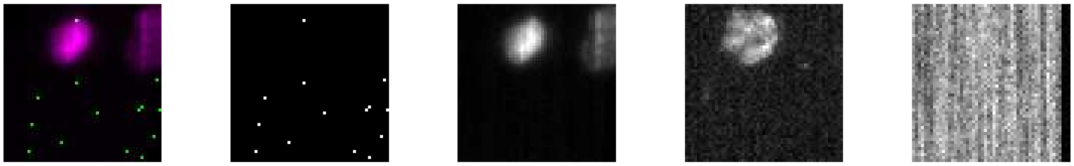				
24449	140	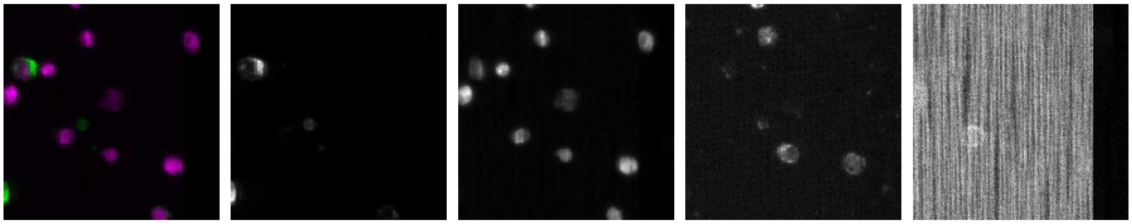				
24450	141	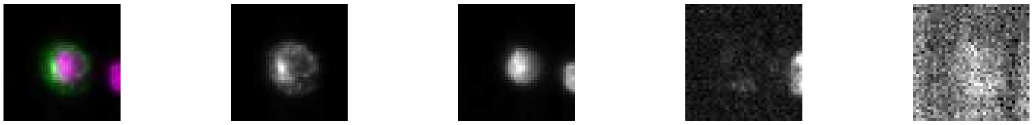				
24451	141	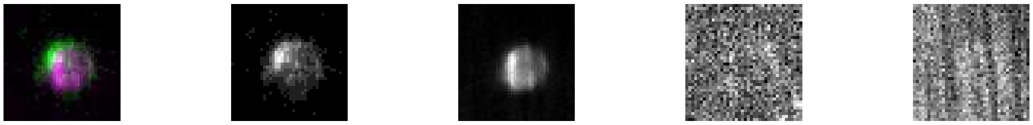				
24452	141	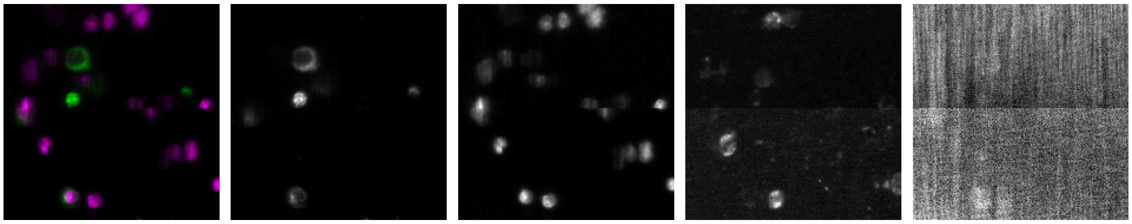				

Ereignis	Aufnahme	DAPI/CK-PE	CK-PE	DAPI	CD45-APC	HER-2/neu
24453	142	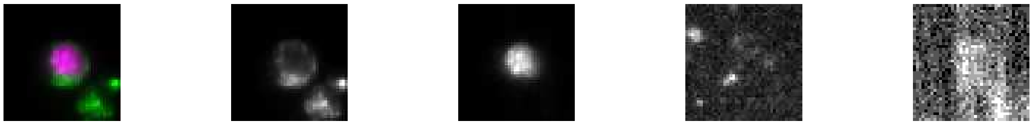				
24454	142	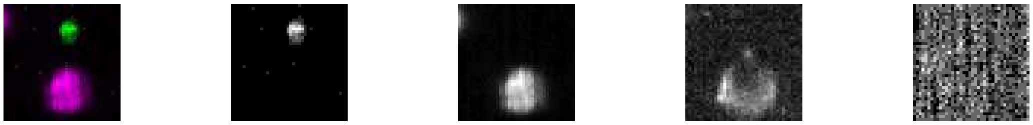				
24455	142	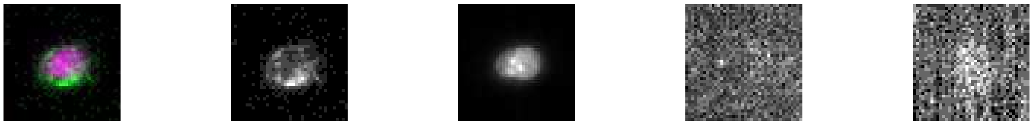				
24456	142	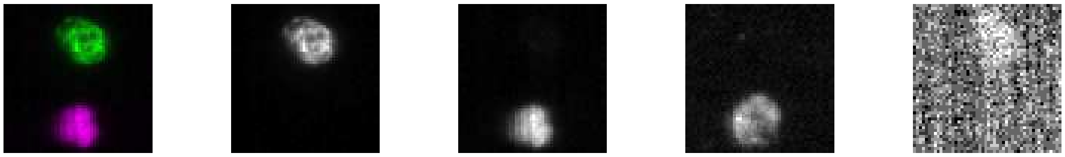				
24457	142	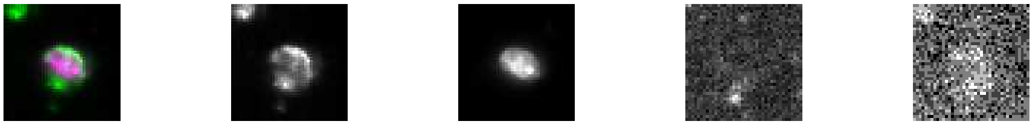				
24458	142	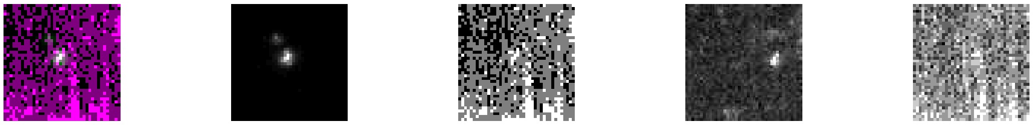				
24459	142	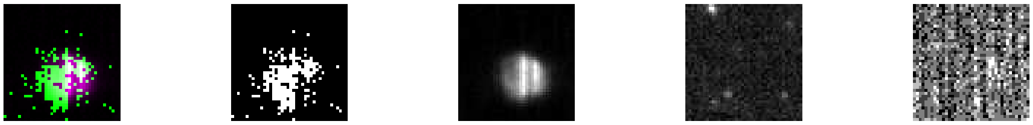				
24460	142	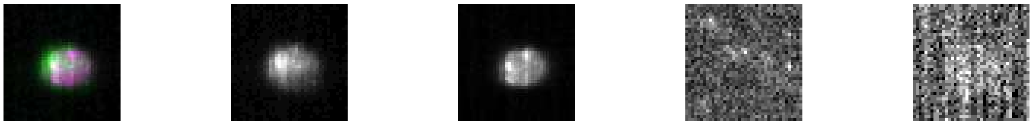				
24461	142	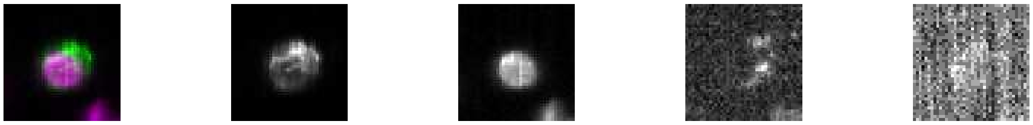				
24462	142	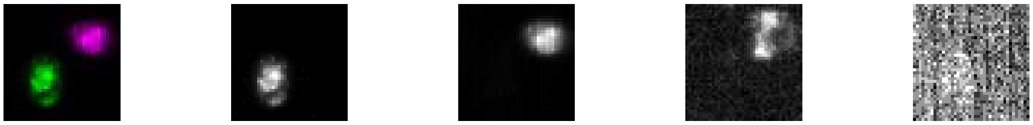				
24463	142	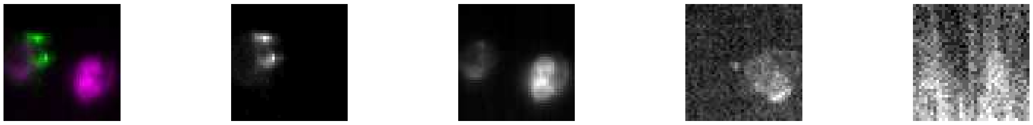				
24464	142	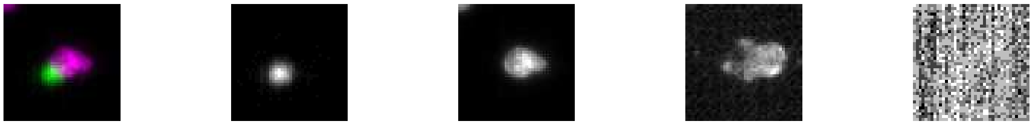				
24465	142	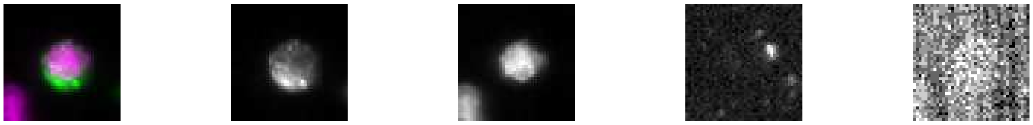				
24466	142	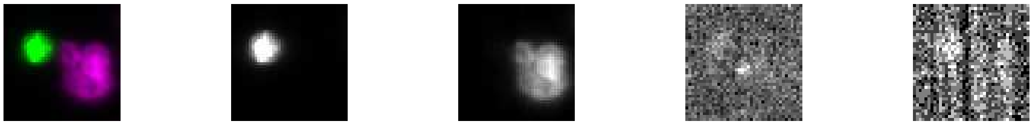				

Ereignis	Aufnahme	DAPI/CK-PE	CK-PE	DAPI	CD45-APC	HER-2/neu
24467	142	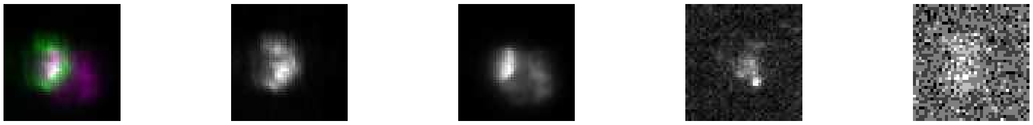				
24468	142	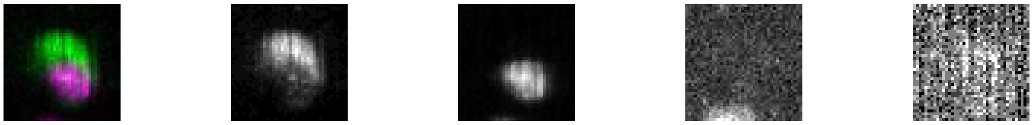				
24469	142	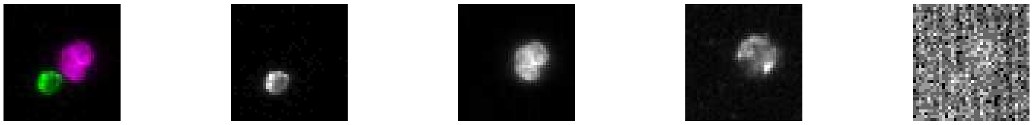				
24470	142	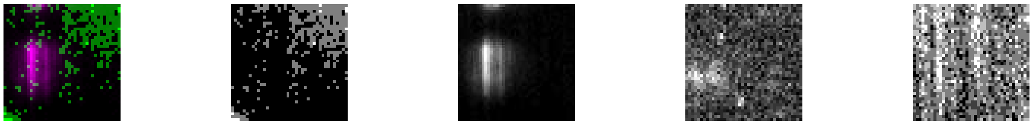				
24471	142	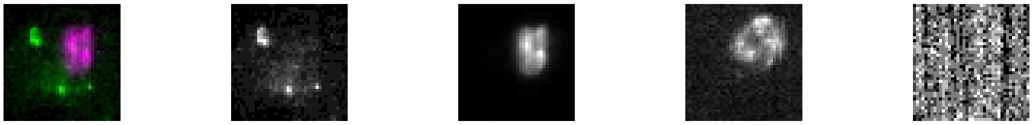				
24472	142	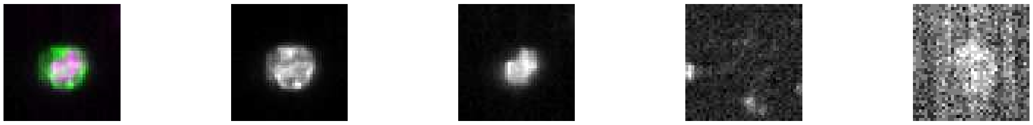				
24473	142	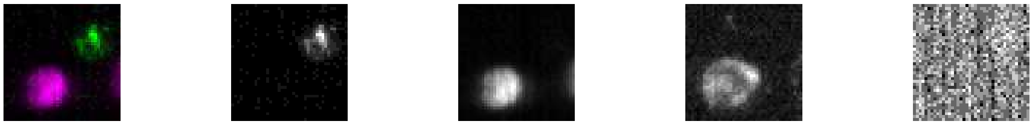				
24474	142	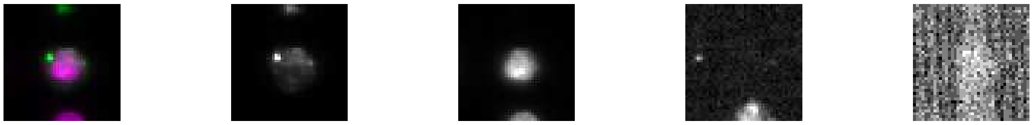				
24475	142	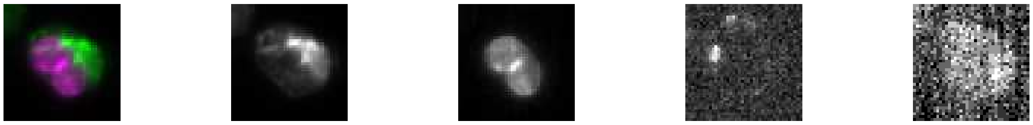				
24476	142	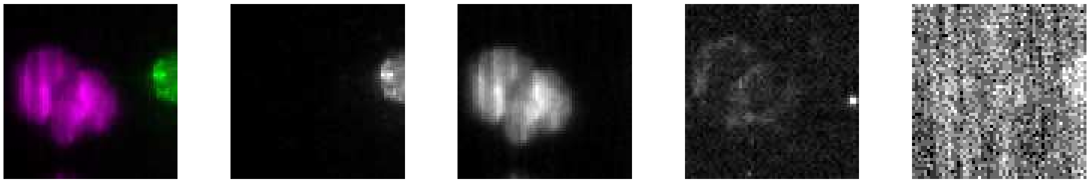				
24477	139	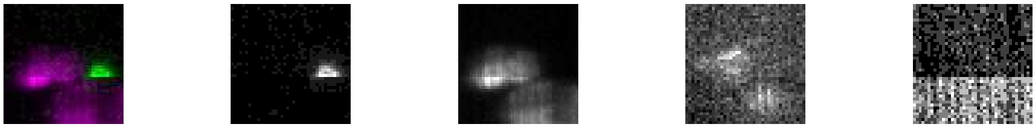				
24478	142	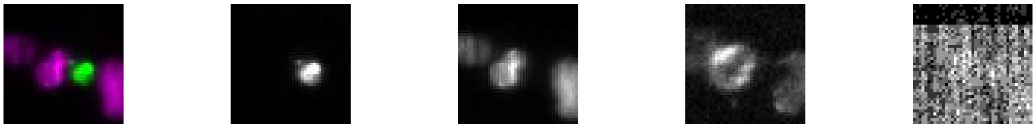				
24479	142	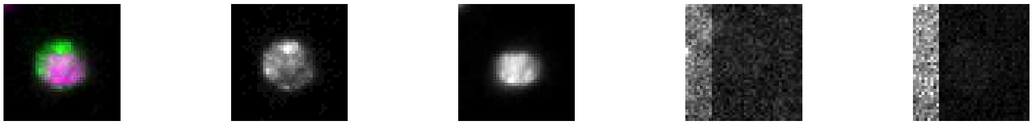				

Ereignis	Aufnahme	DAPI/CK-PE	CK-PE	DAPI	CD45-APC	HER-2/neu
24480	139	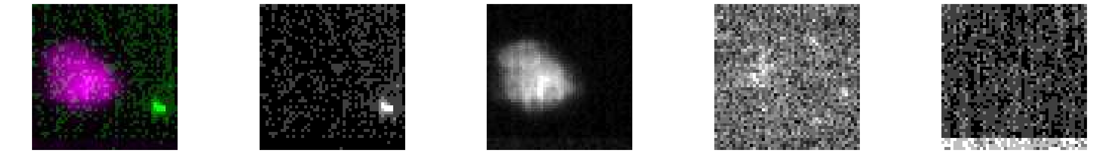				
24481	143	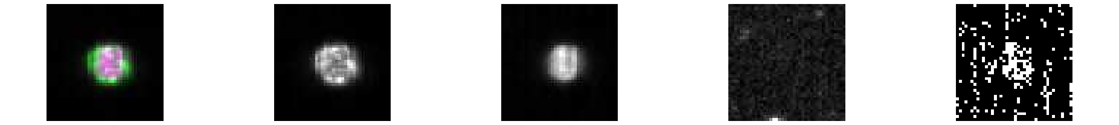				
24482	143	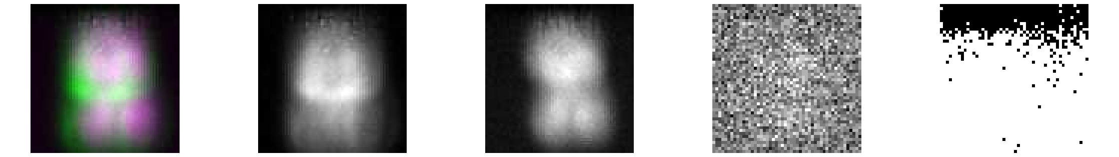				
24483	143	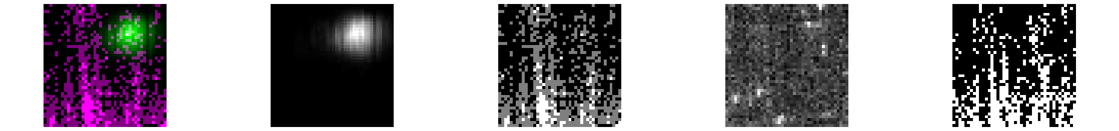				
24484	143	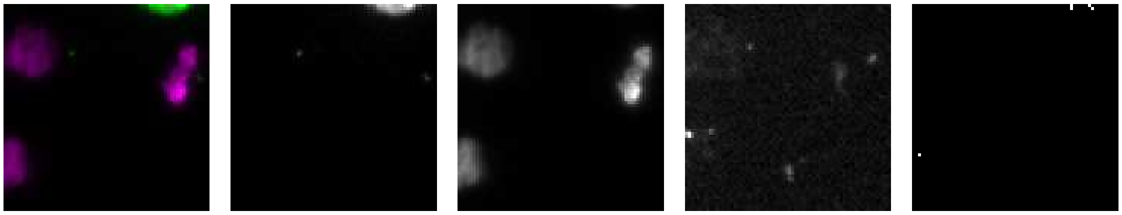				
24485	143	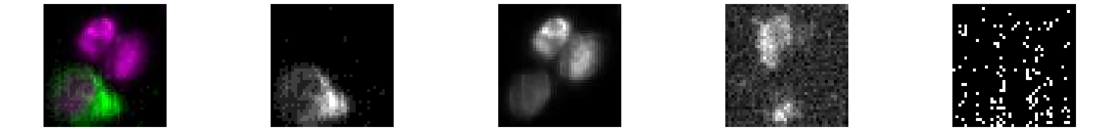				
24486	143	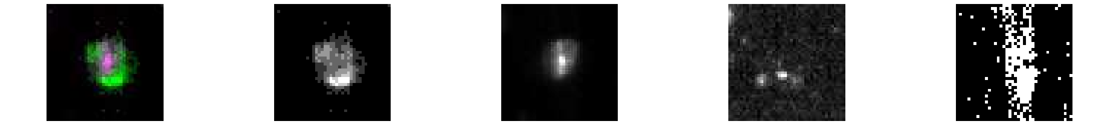				
24487	143	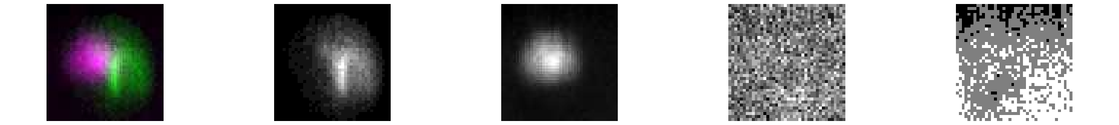				
24488	143	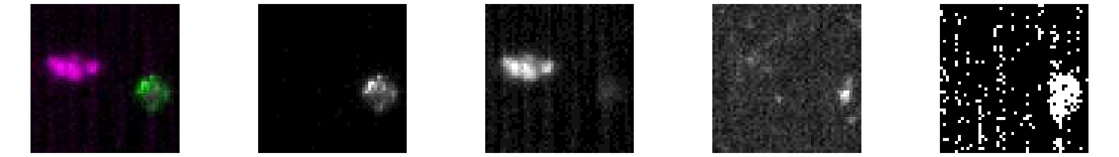				
24489	143	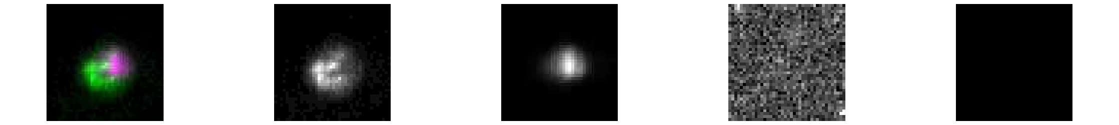				
24490	143	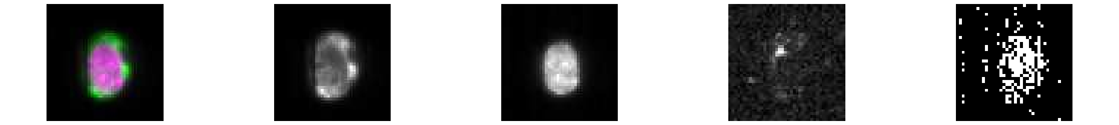				
24491	143	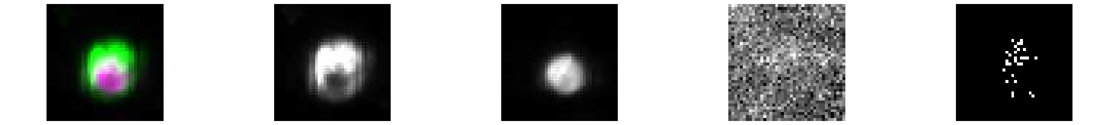				

Ereignis	Aufnahme	DAPI/CK-PE	CK-PE	DAPI	CD45-APC	HER-2/neu
24492	143					
24493	143					
24494	143					
24495	143					
24496	143					
24497	143					
24498	143					
24499	143					
24500	143					
24501	143					
24502	143					
24503	143					
24504	143					
24505	143					

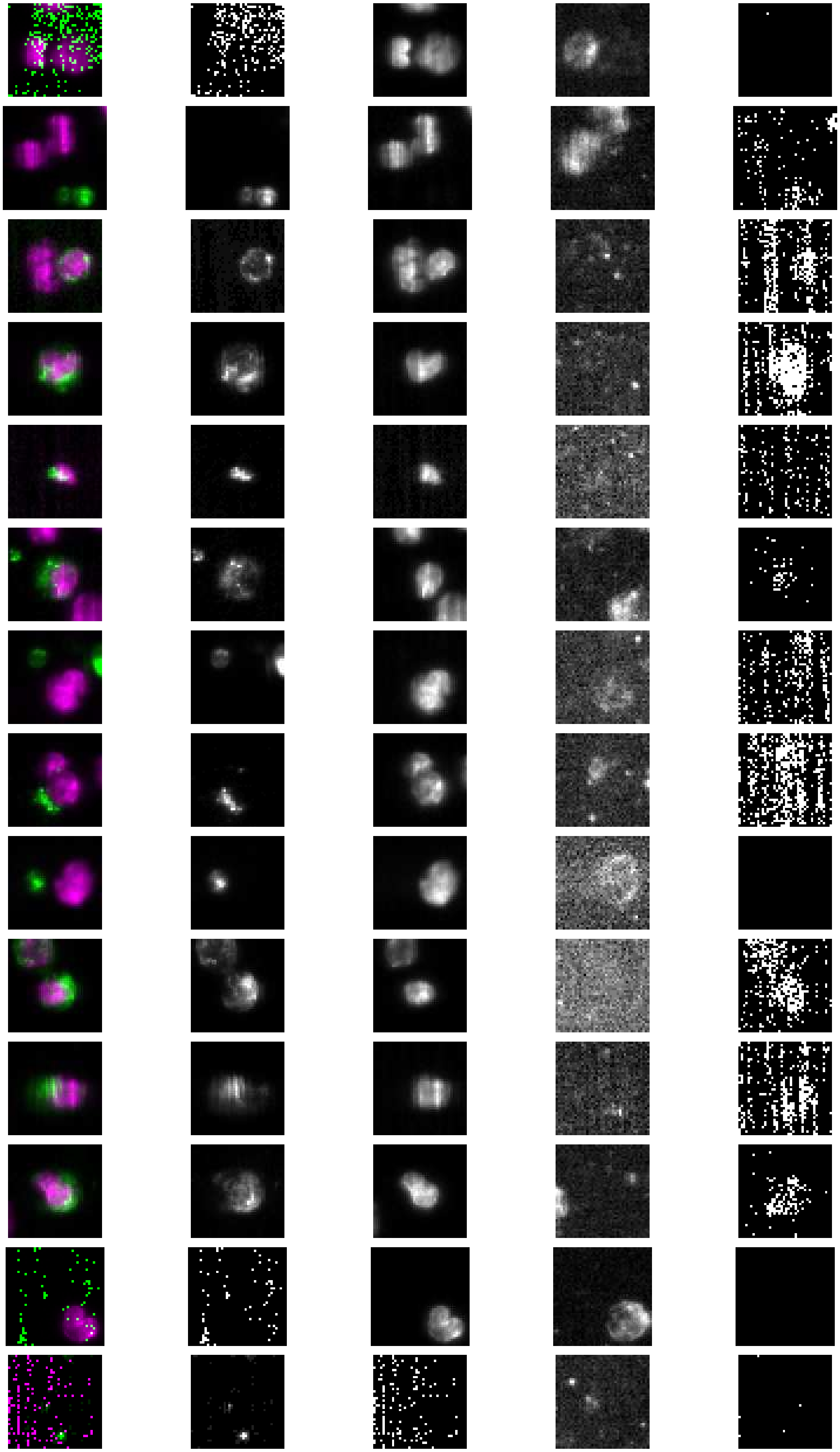

Druckdatum: 02/06/2020 17:25

Proben-ID: Gyn14.208 **Kassetten-ID:** 01327106 **Patienten-ID:**

Ereignis	Aufnahme	DAPI/CK-PE	CK-PE	DAPI	CD45-APC	HER-2/neu
24506	143	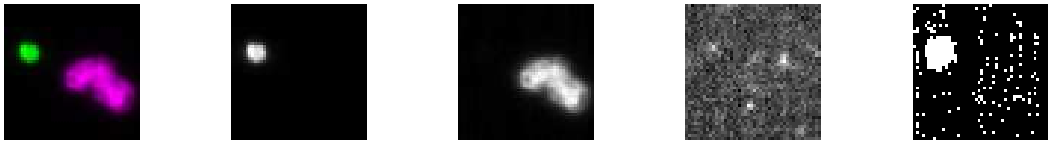				
24507	143	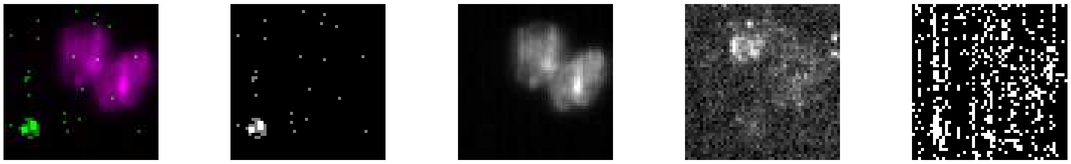				
24508	143	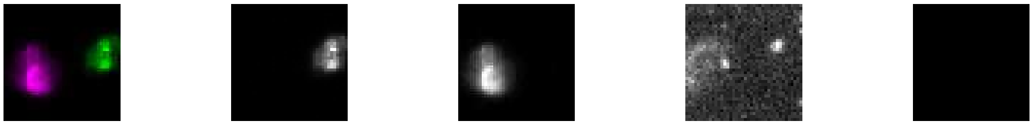				
24509	143	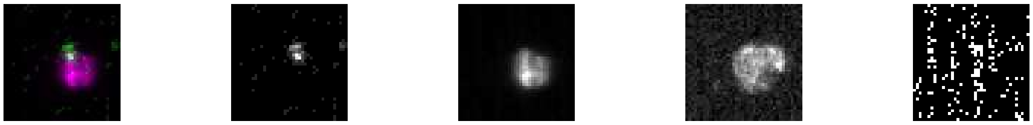				
24510	143	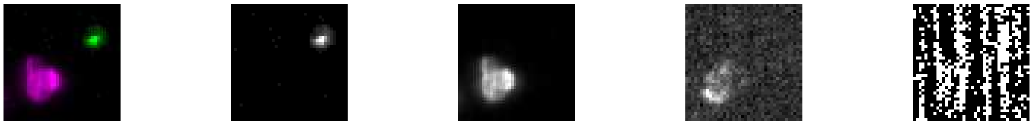				
24511	143	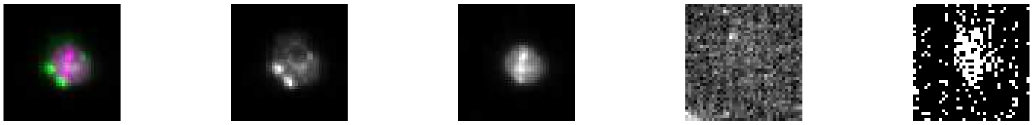				
24512	143	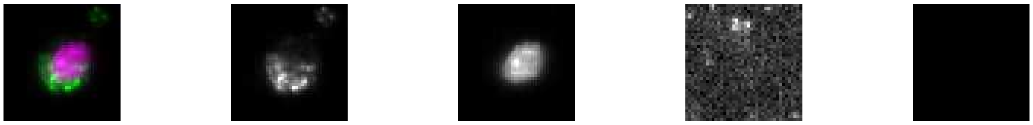				
24513	143	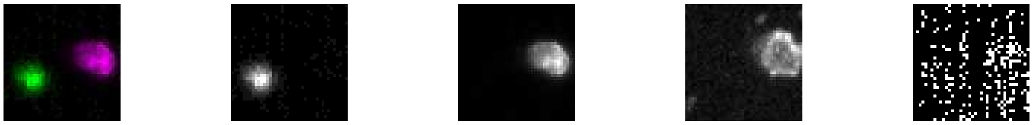				
24514	143	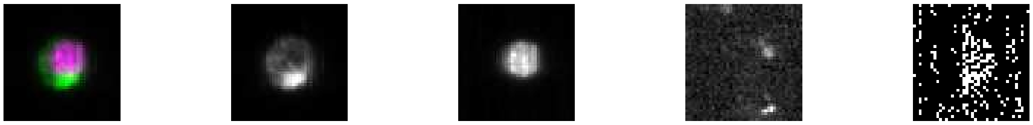				
24515	143	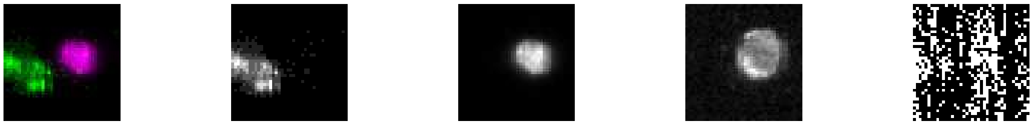				
24516	143	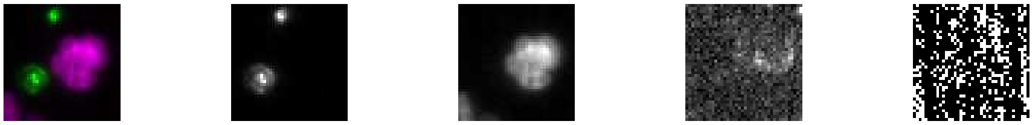				
24517	138	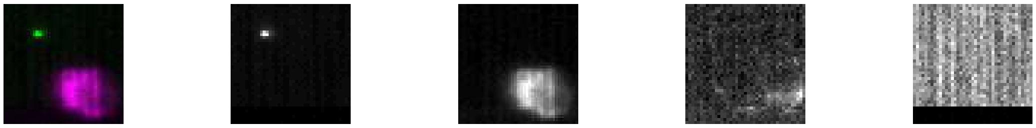				
24518	138	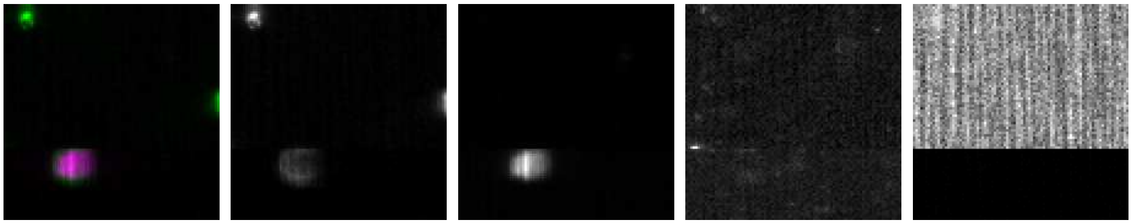				

Ereignis	Aufnahme	DAPI/CK-PE	CK-PE	DAPI	CD45-APC	HER-2/neu
24519	138	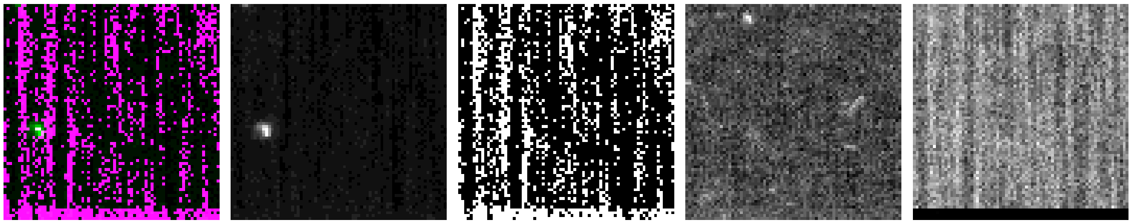				
24520	144	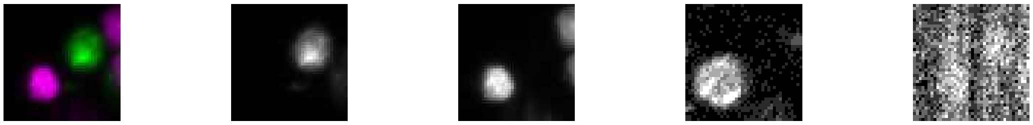				
24521	144	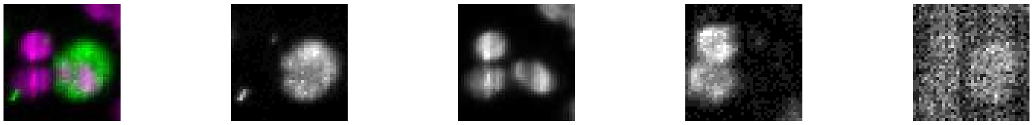				
24522	144	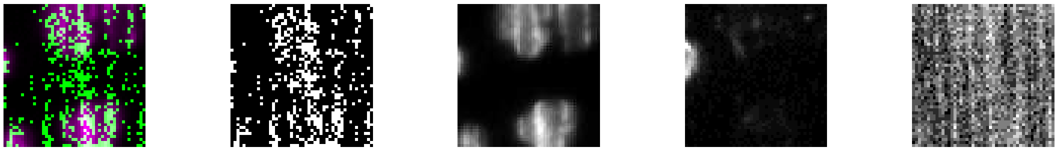				
24523	144	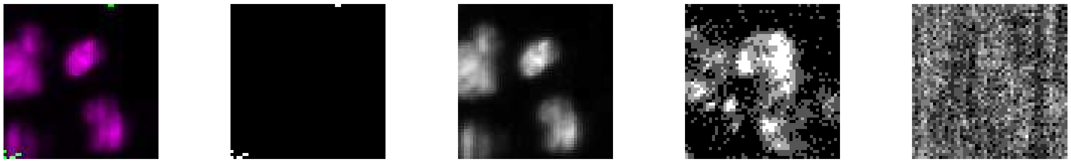				
24524	144	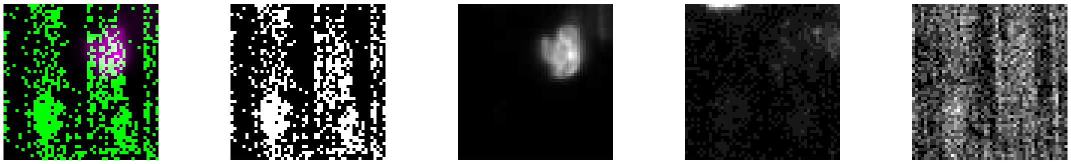				
24525	144	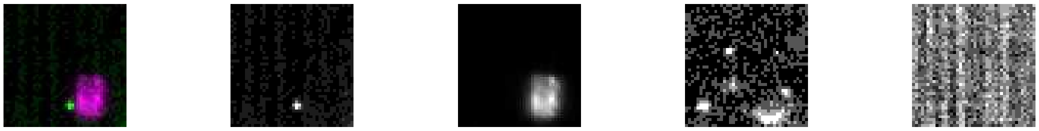				
24526	144	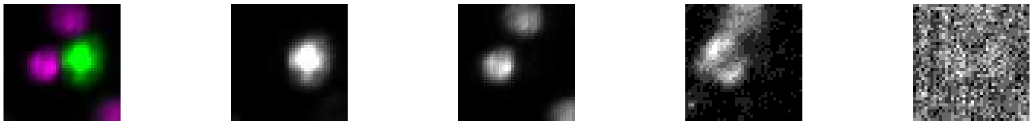				
24527	144	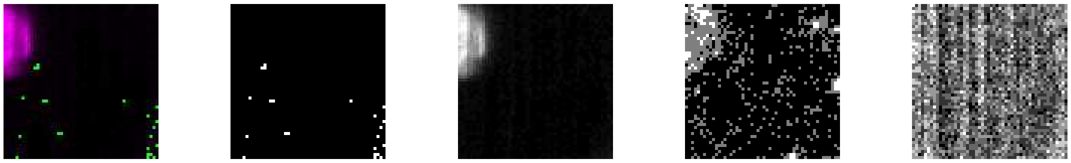				
24528	144	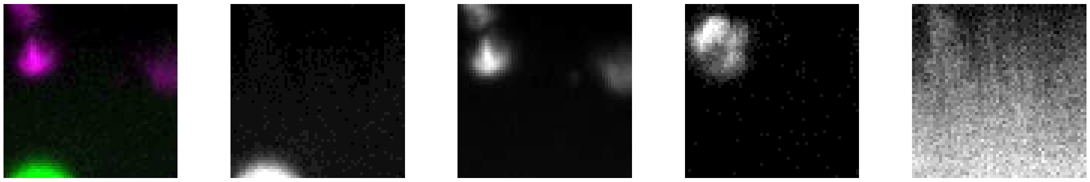				
24529	144	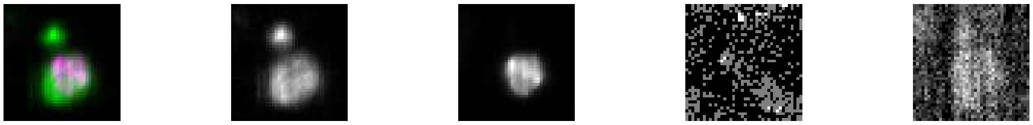				
24530	144	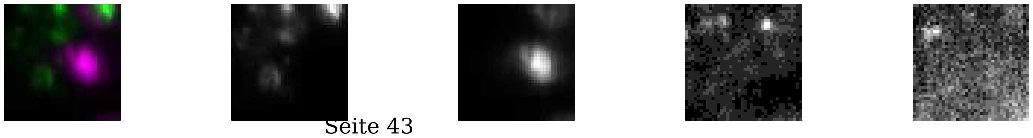				

Ereignis	Aufnahme	DAPI/CK-PE	CK-PE	DAPI	CD45-APC	HER-2/neu
24531	144	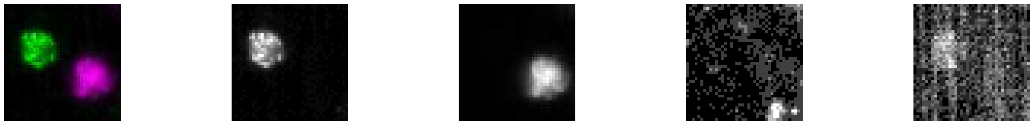				
24532	144	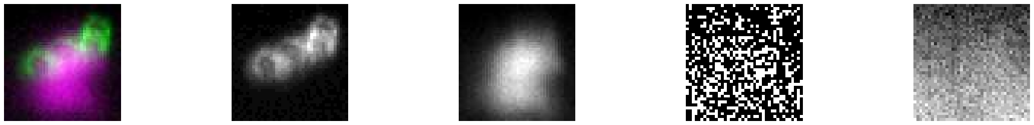				
24533	144	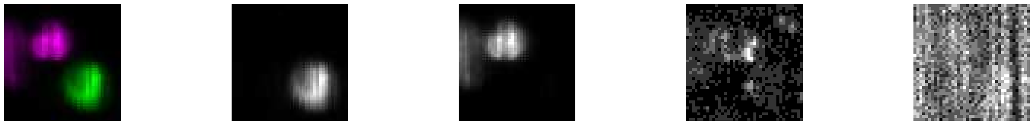				
24534	144	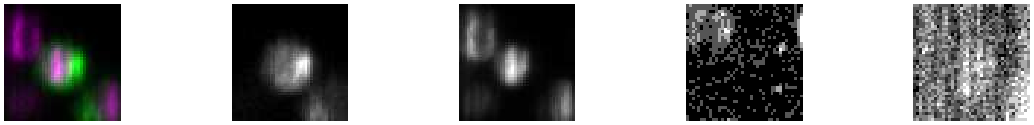				
24535	144	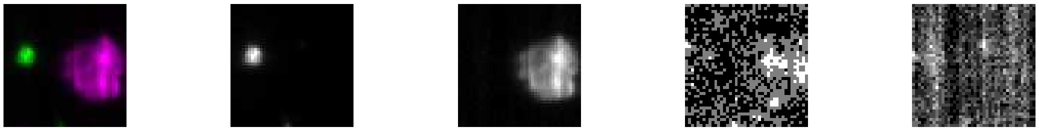				
24536	144	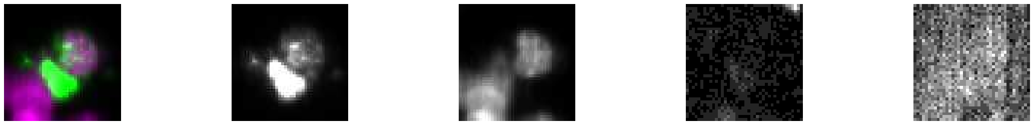				
24537	144	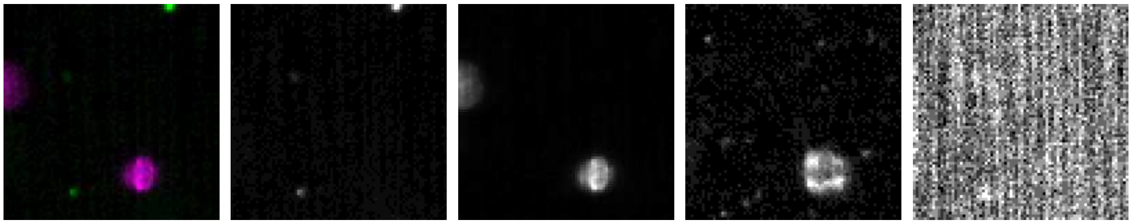				
24538	144	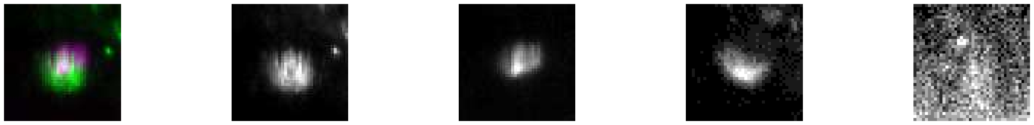				
24539	144	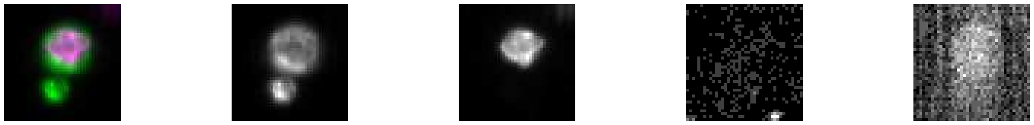				
24540	144	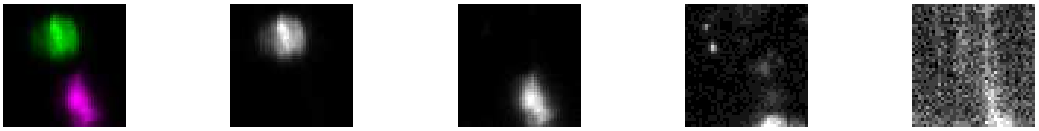				
24541	144	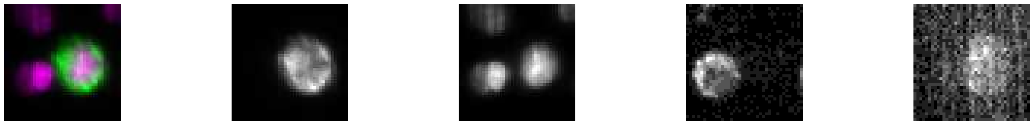				
24542	144	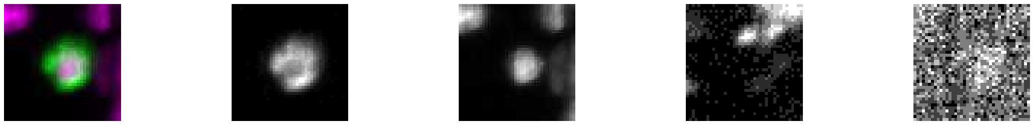				
24543	144	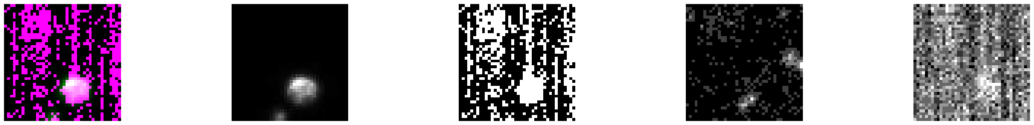				

Ereignis	Aufnahme	DAPI/CK-PE	CK-PE	DAPI	CD45-APC	HER-2/neu
24544	144	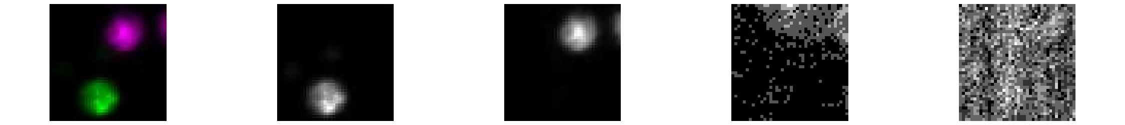				
24545	144	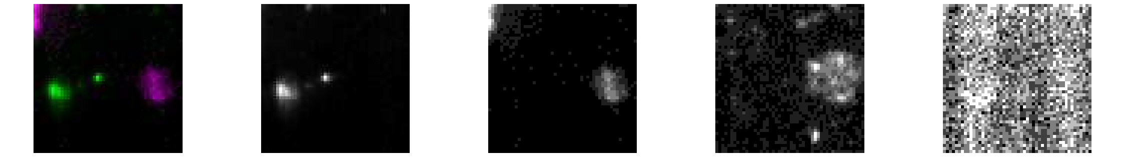				
24546	144	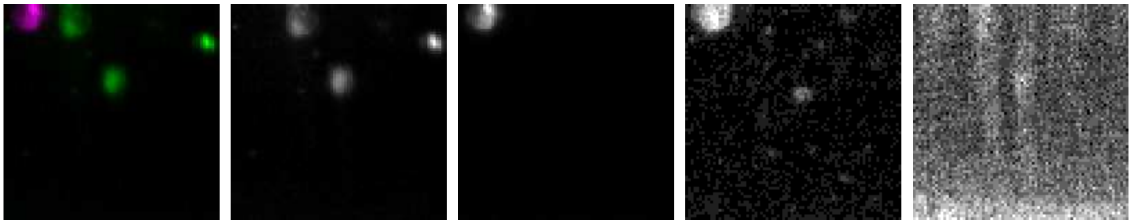				
24547	144	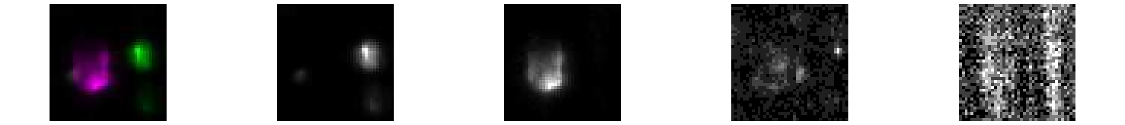				
24548	144	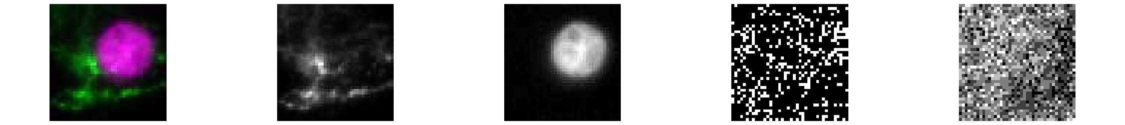				
24549	144	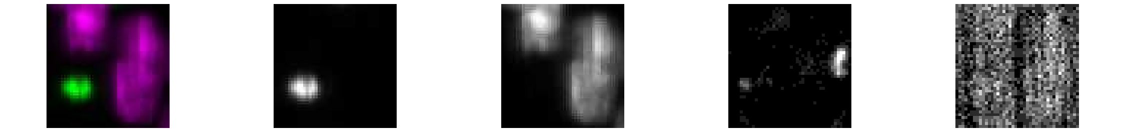				
24550	144	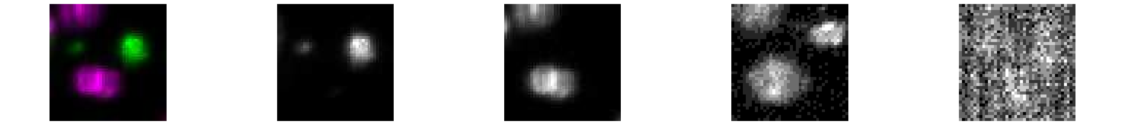				
24551	144	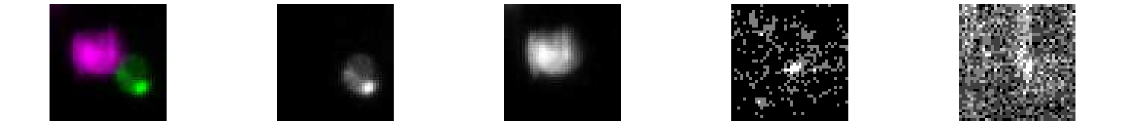				
24552	144	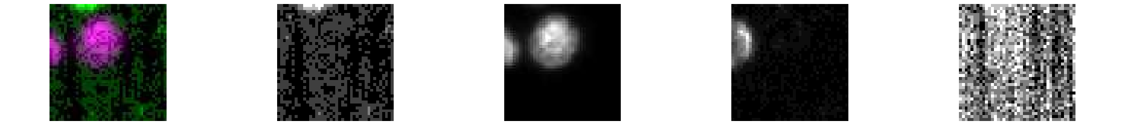				
24553	144	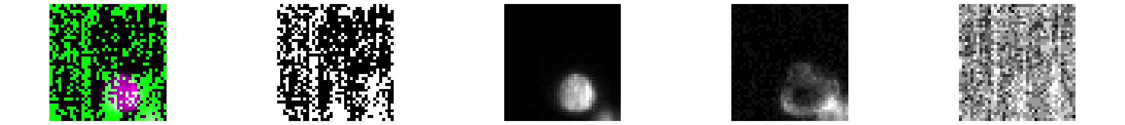				
24554	144	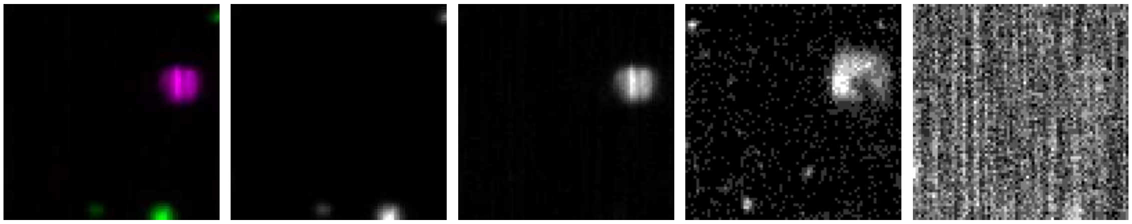				
24555	144	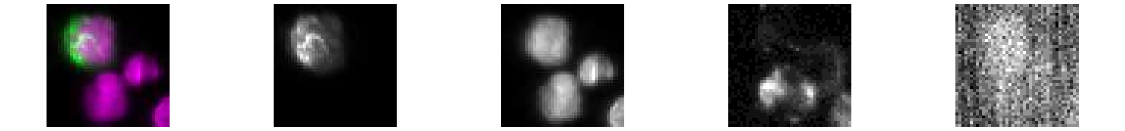				

Ereignis	Aufnahme	DAPI/CK-PE	CK-PE	DAPI	CD45-APC	HER-2/neu
24556	144	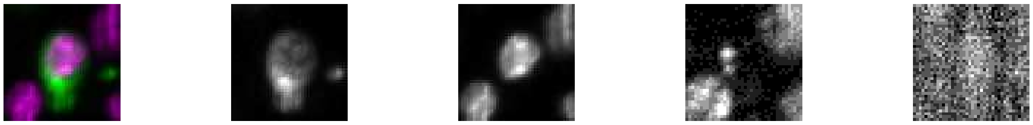				
24557	144	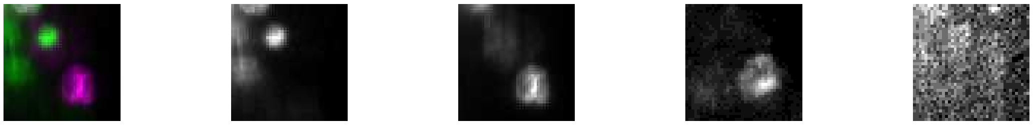				
24558	144	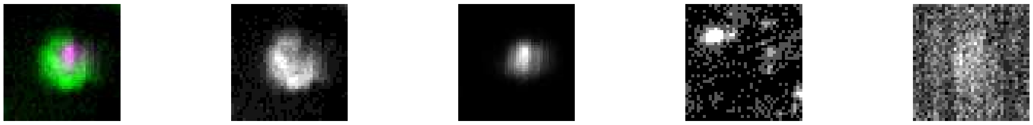				
24559	144	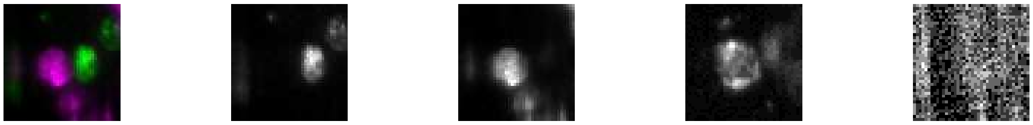				
24560	144	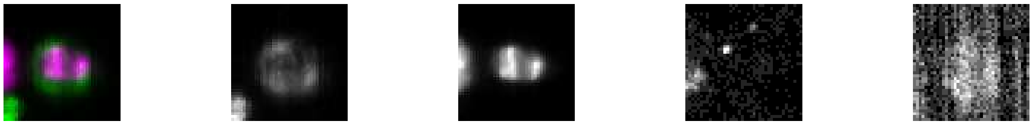				
24561	144	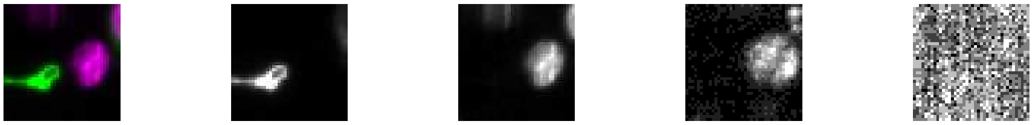				
24562	144	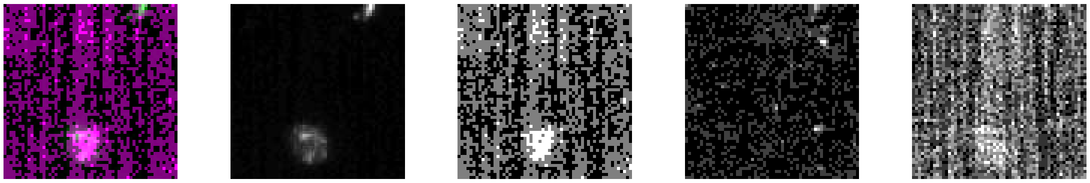				
24563	144	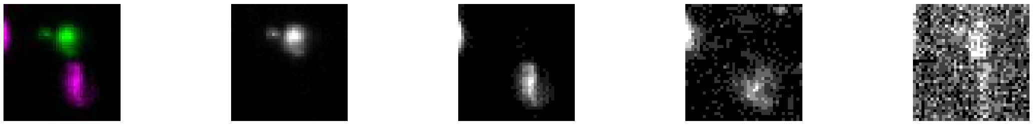				
24564	144	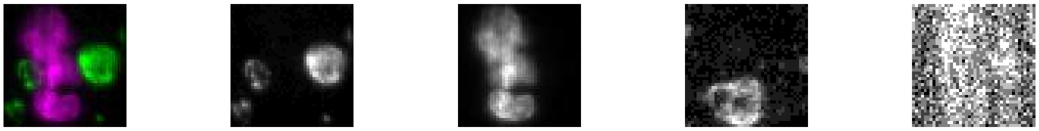				
24565	144	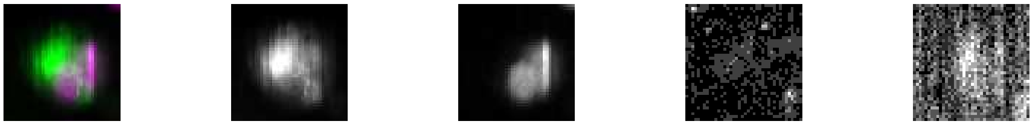				
24566	144	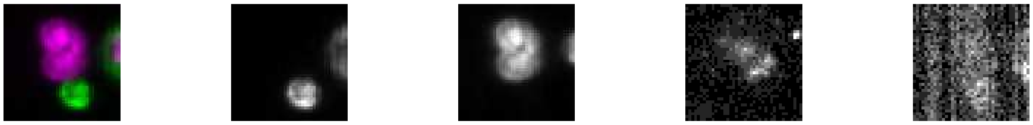				
24567	144	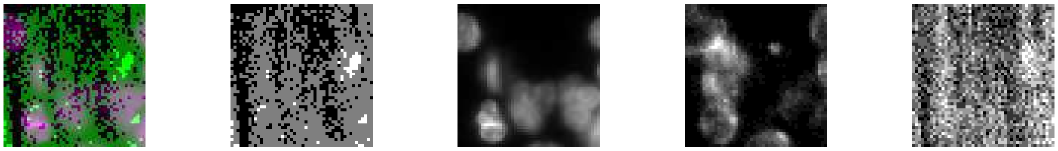				
24568	144	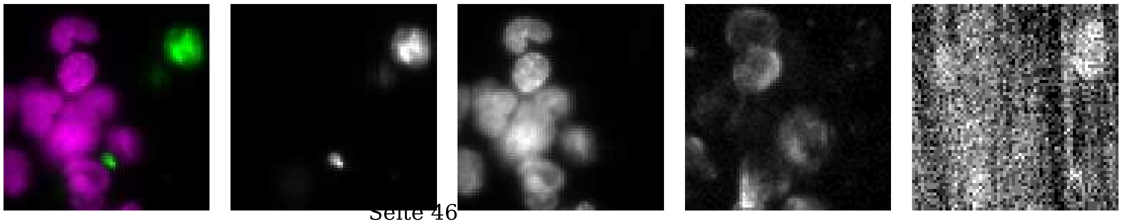				

Ereignis	Aufnahme	DAPI/CK-PE	CK-PE	DAPI	CD45-APC	HER-2/neu
24569	144	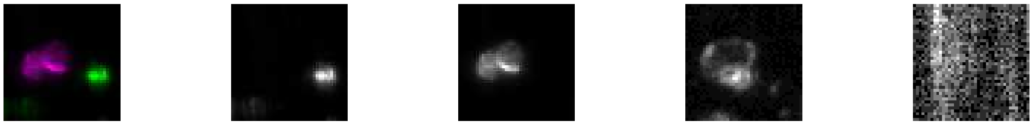				
24570	144	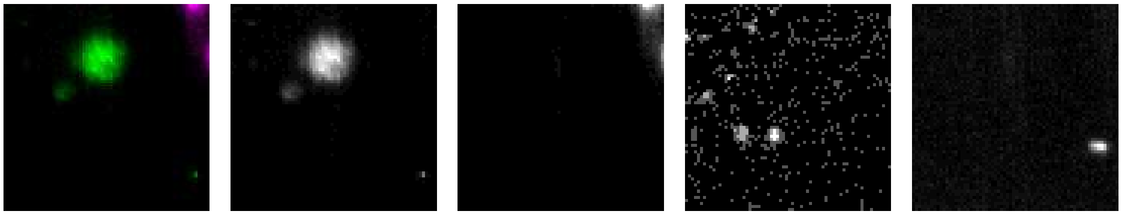				
24571	144	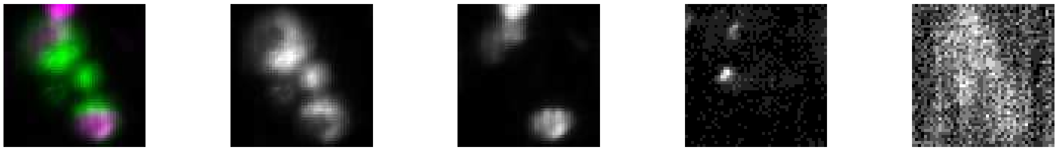				
24572	144	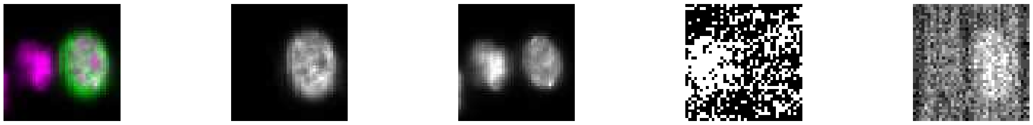				
24573	144	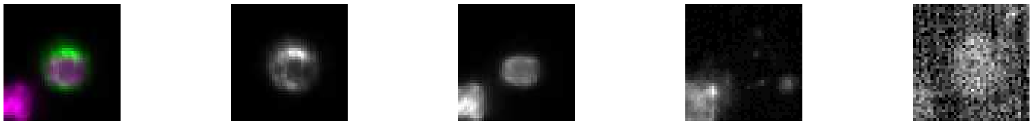				
24574	144	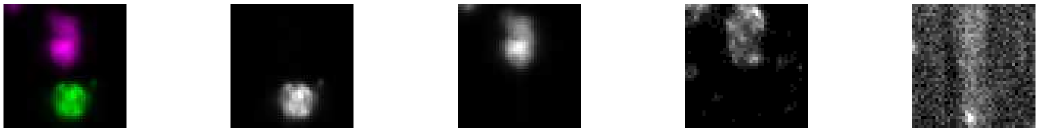				
24575	144	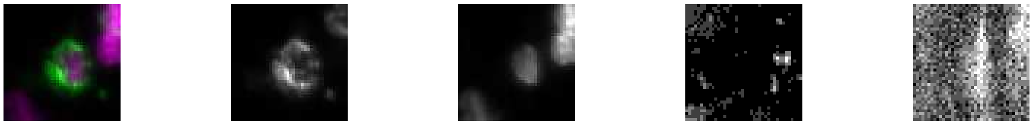				
24576	144	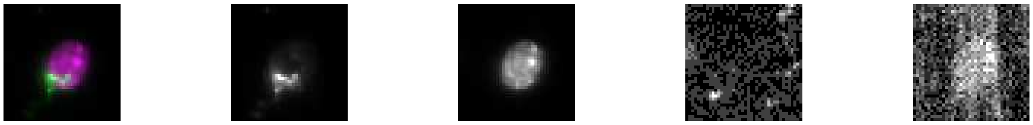				
24577	144	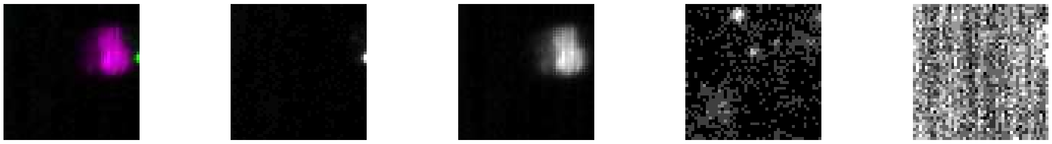				
24578	144	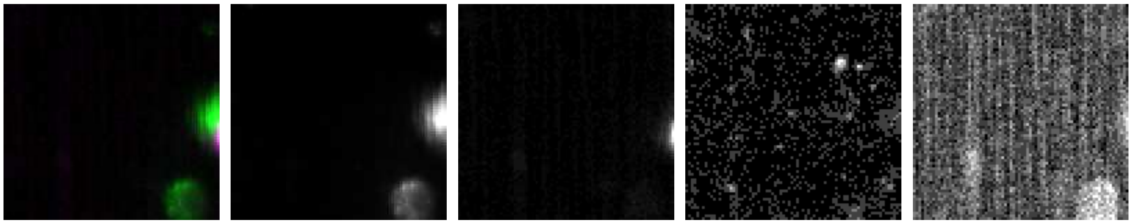				
24579	144	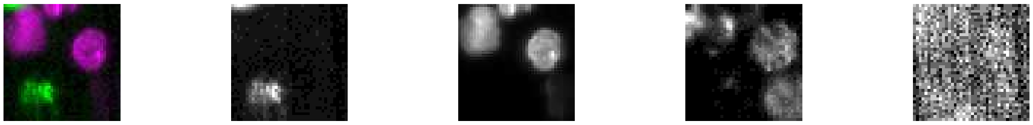				
24580	144	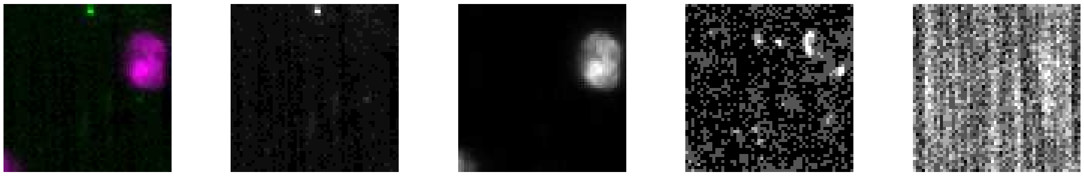				

Ereignis	Aufnahme	DAPI/CK-PE	CK-PE	DAPI	CD45-APC	HER-2/neu
24581	144	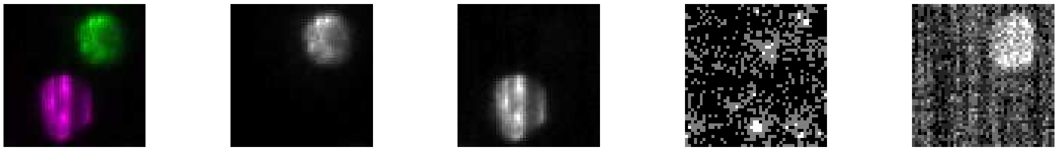				
24582	144	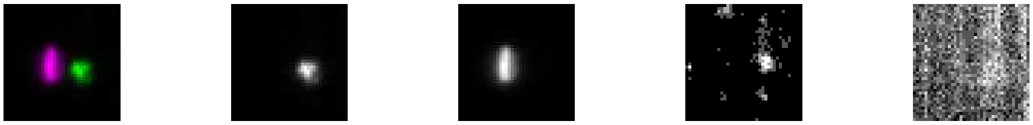				
24583	144	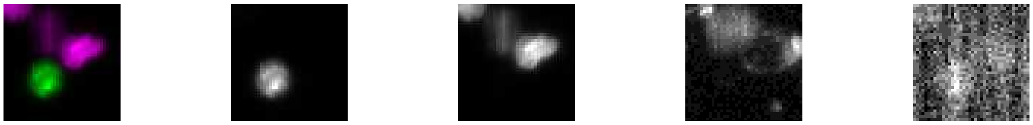				
24584	144	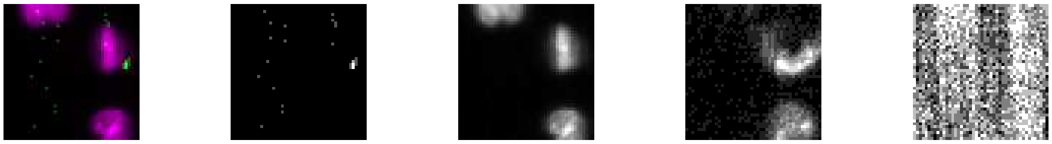				
24585	144	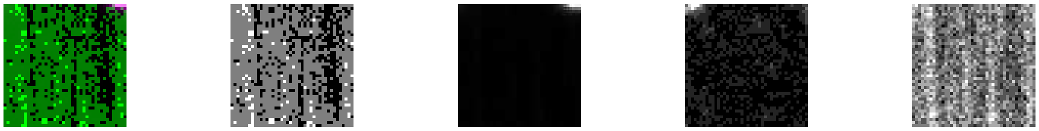				
24586	144	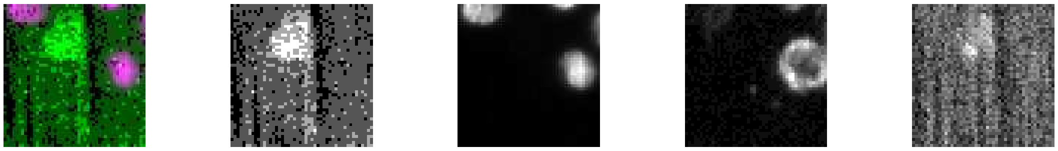				
24587	144	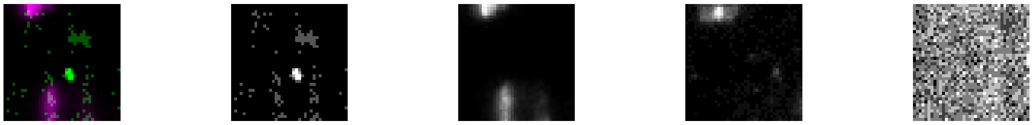				
24588	144	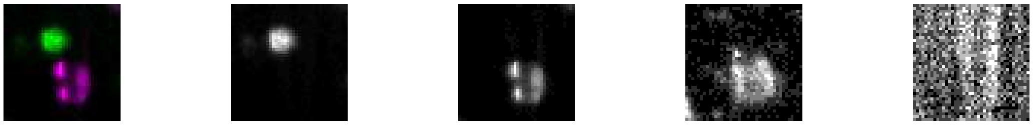				
24589	144	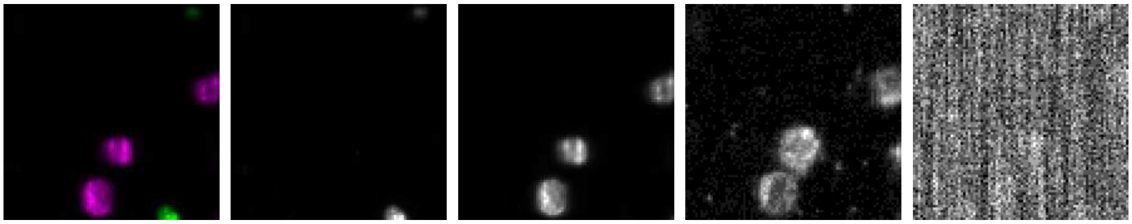				
24590	144	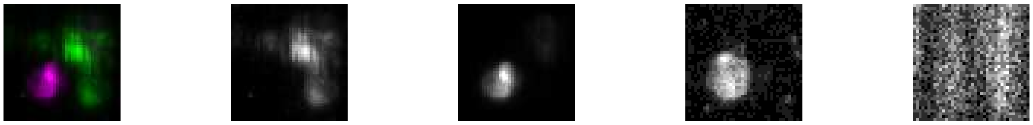				
24591	144	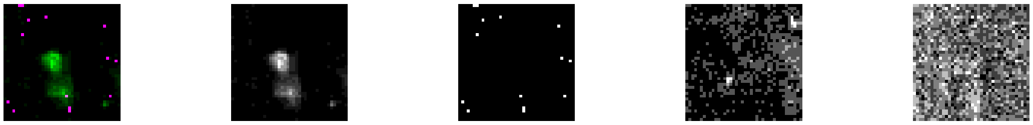				
24592	144	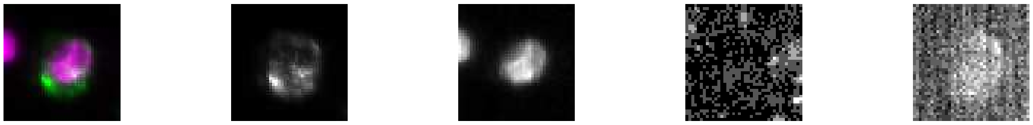				
24593	144	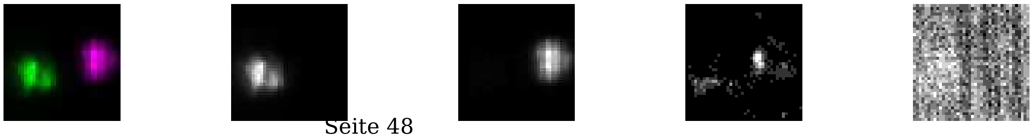				

Ereignis	Aufnahme	DAPI/CK-PE	CK-PE	DAPI	CD45-APC	HER-2/neu
24594	144	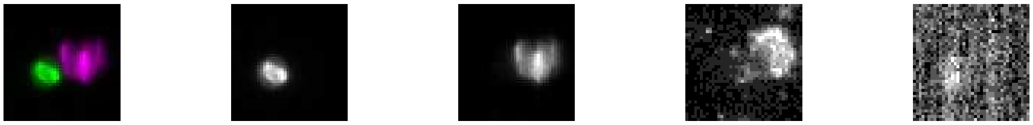				
24595	144	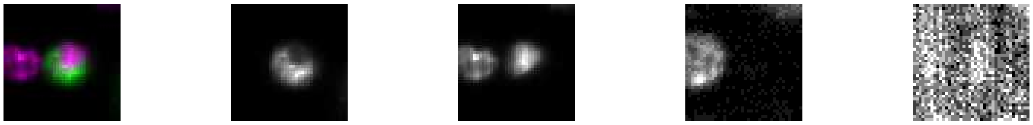				
24596	144	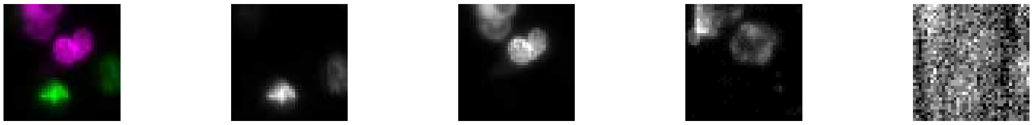				
24597	144	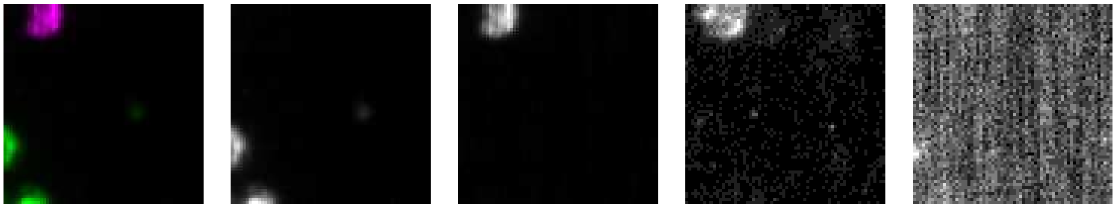				
24598	144	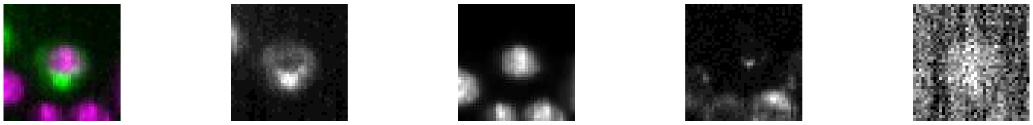				
24599	144	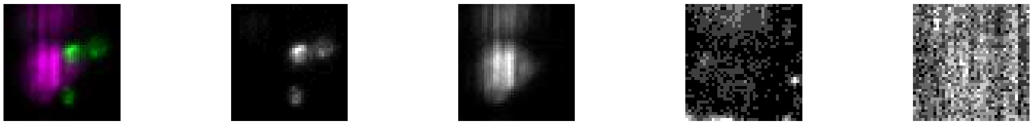				
24600	144	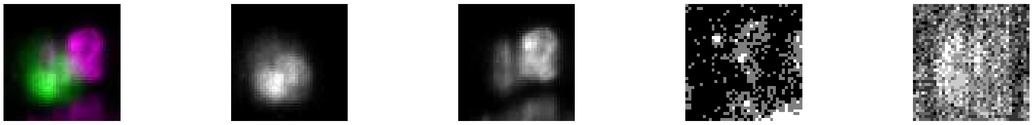				
24601	144	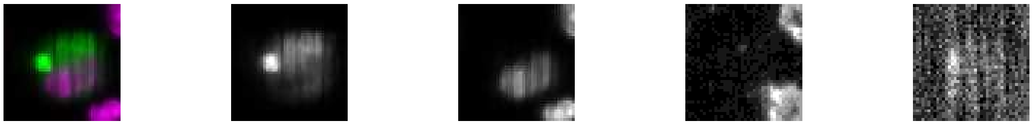				
24602	144	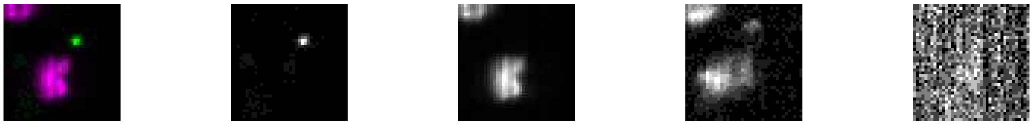				
24603	144	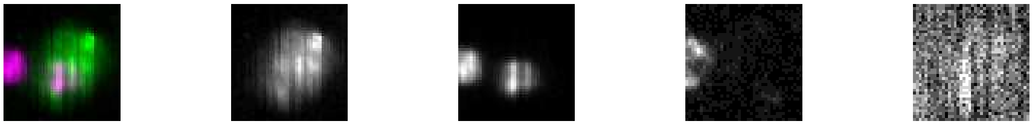				
24604	144	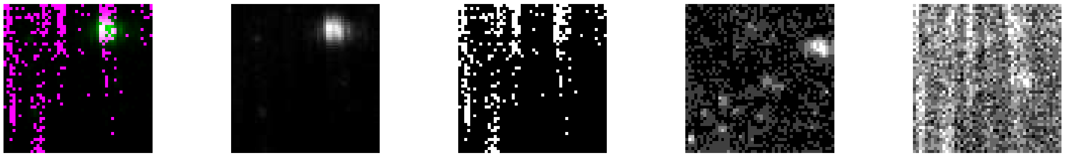				
24605	144	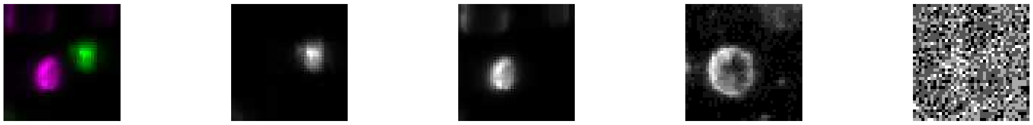				
24606	144	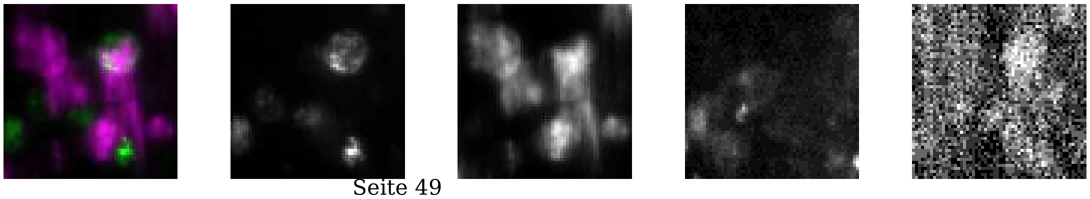				

Ereignis	Aufnahme	DAPI/CK-PE	CK-PE	DAPI	CD45-APC	HER-2/neu
24607	144	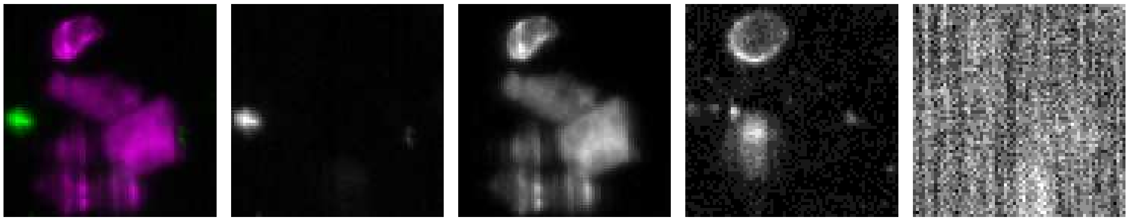				
24608	144	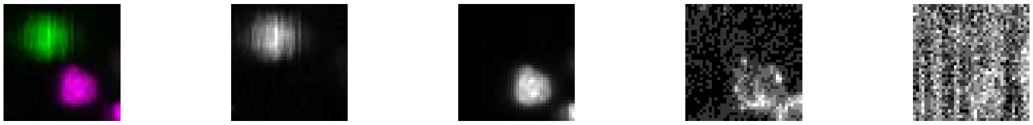				
24609	144	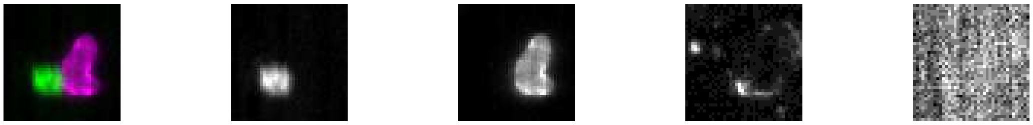				
24610	144	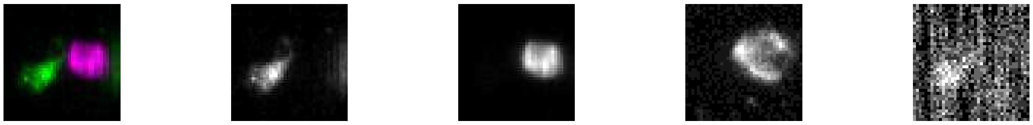				
24611	144	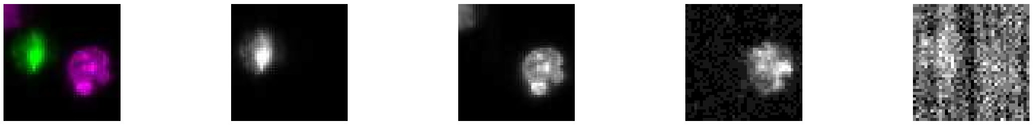				
24612	144	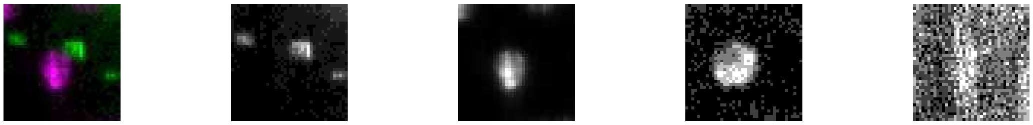				
24613	144	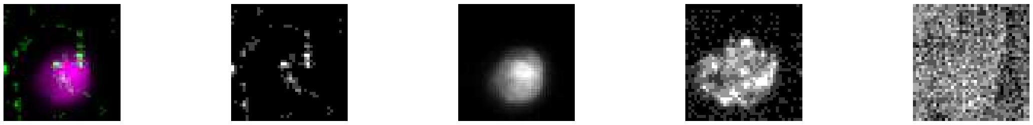				
24614	144	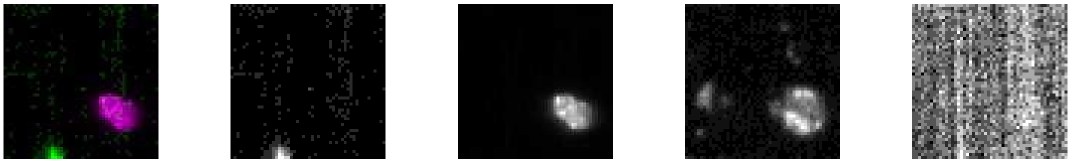				
24615	144	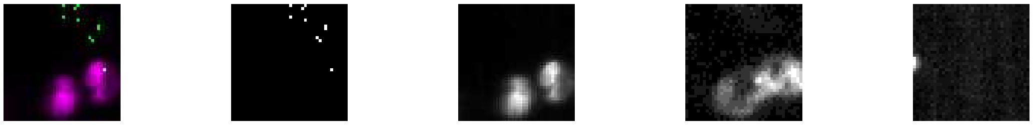				
24616	144	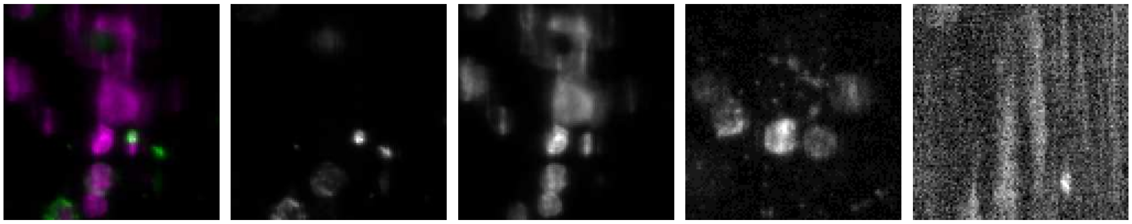				
24617	144	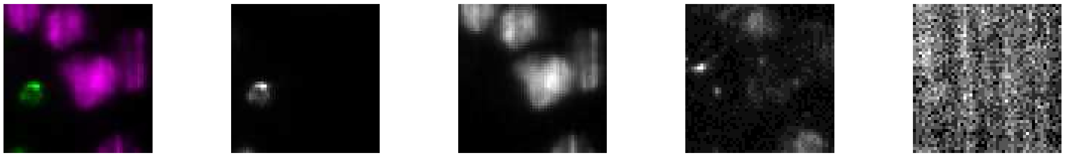				
24618	144	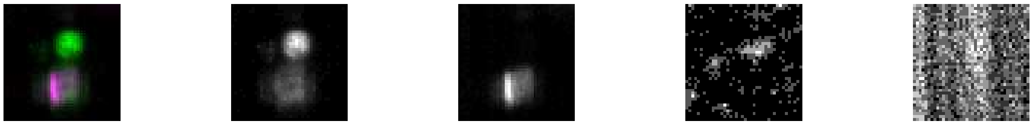				

Druckdatum: 02/06/2020 17:25

Proben-ID: Gyn14.208 **Kassetten-ID:** 01327106 **Patienten-ID:**

Ereignis	Aufnahme	DAPI/CK-PE	CK-PE	DAPI	CD45-APC	HER-2/neu
24619	144	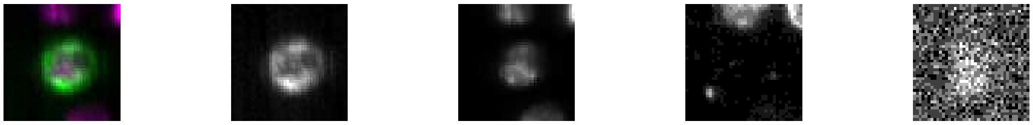				
24620	144	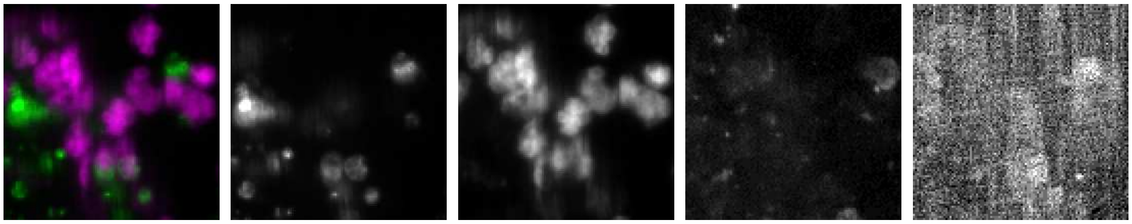				
24621	144	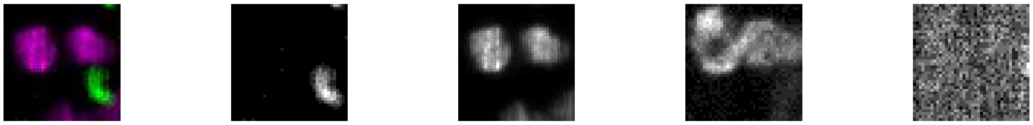				
24622	144	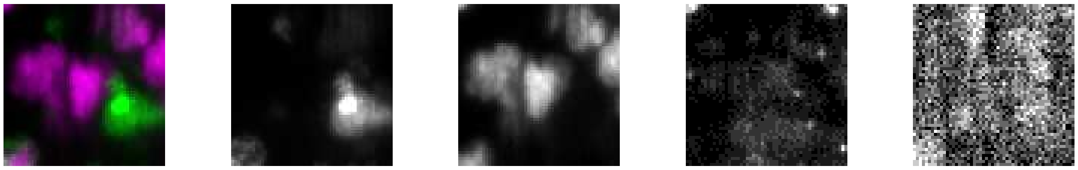				
24623	144	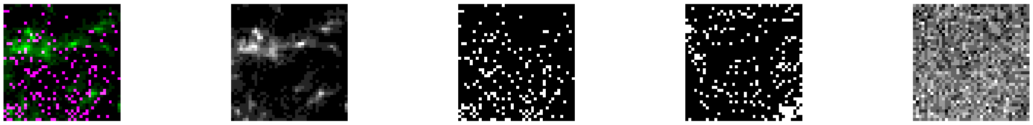				
24624	144	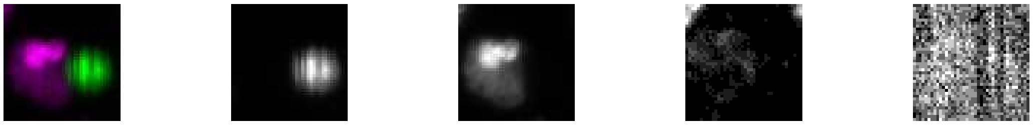				
24625	144	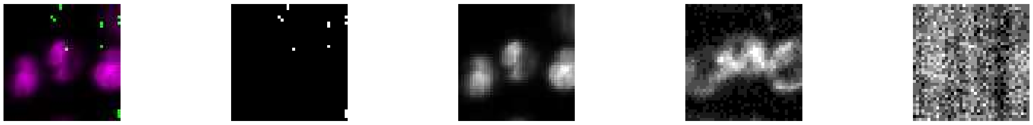				
24626	144	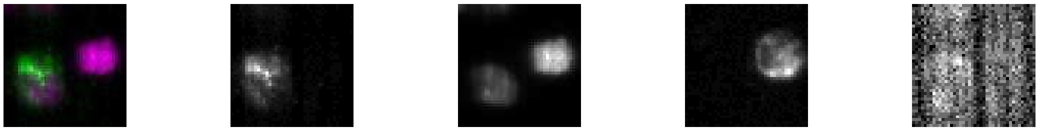				
24627	144	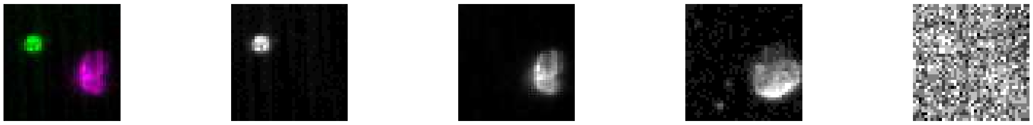				
24628	144	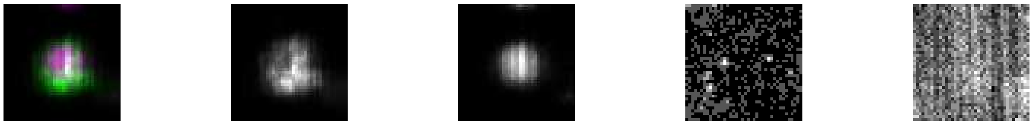				
24629	144	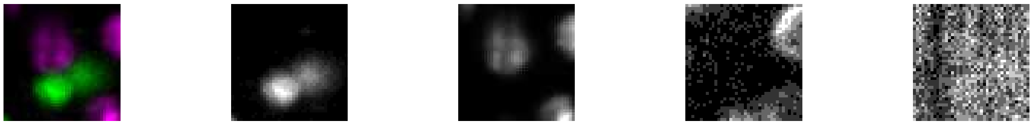				
24630	144	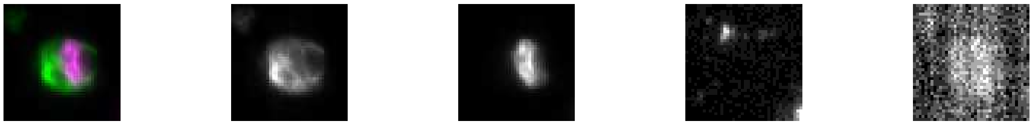				
24631	144	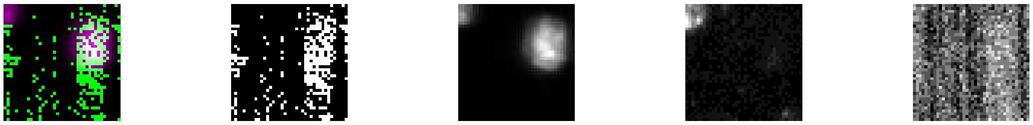				

Ereignis	Aufnahme	DAPI/CK-PE	CK-PE	DAPI	CD45-APC	HER-2/neu
24632	144	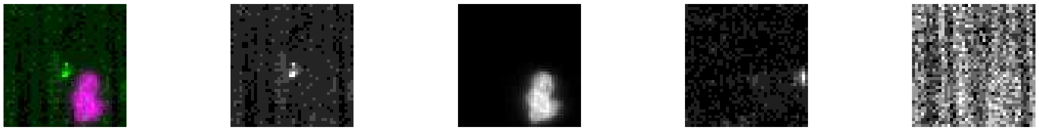				
24633	144	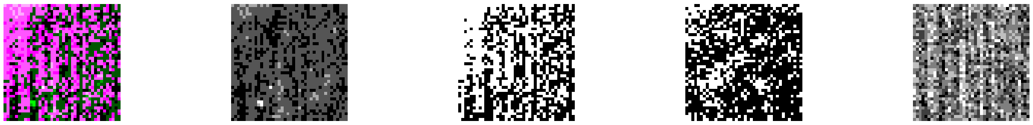				
24634	144	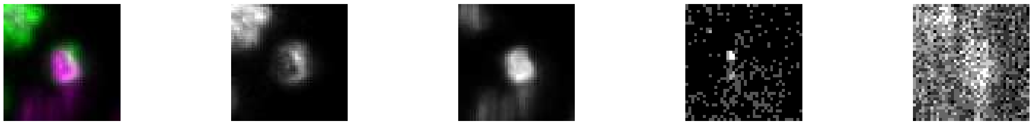				
24635	144	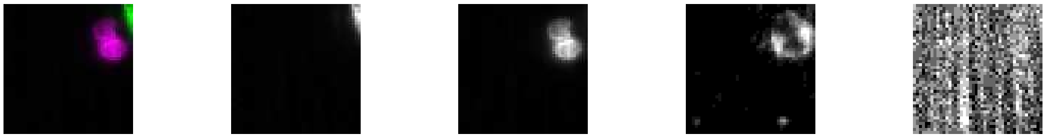				
24636	144	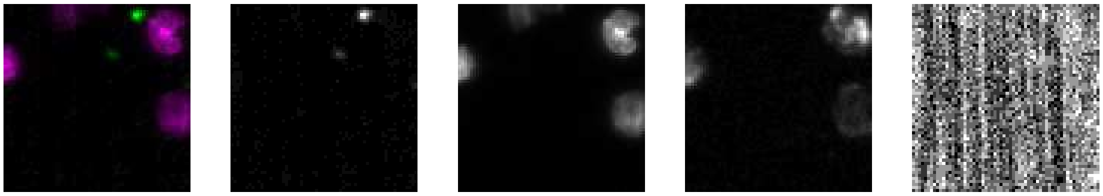				
24637	144	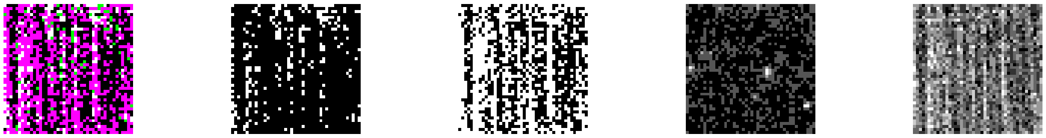				
24638	144	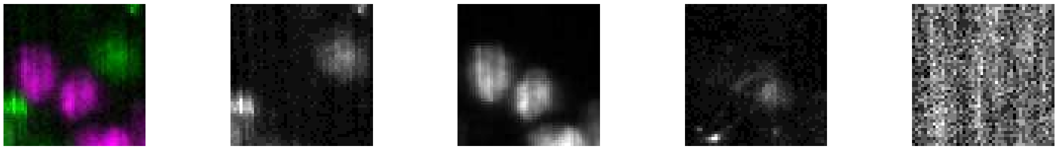				
24639	144	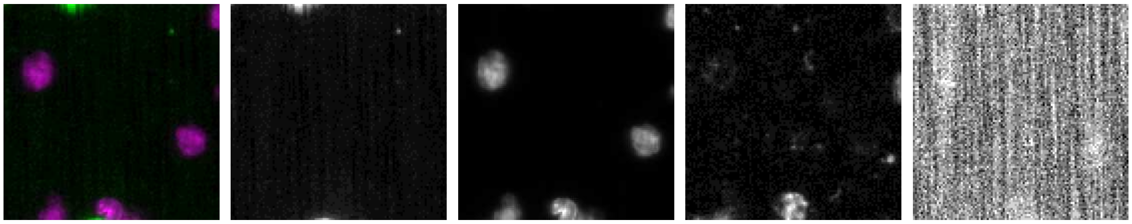				
24640	144	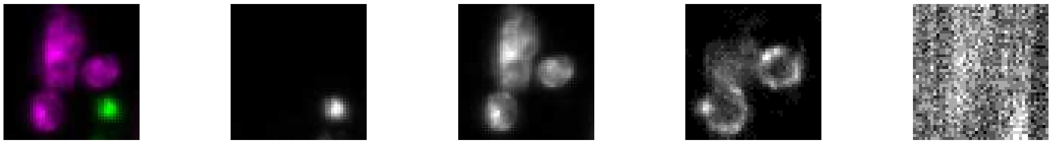				
24641	144	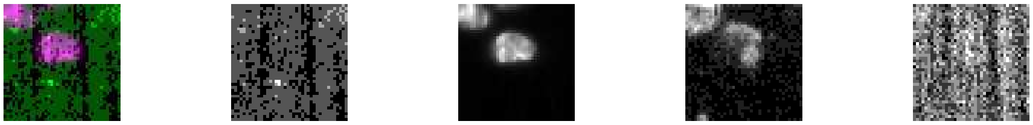				
24642	144	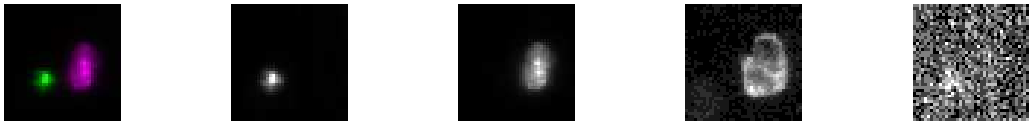				

Ereignis	Aufnahme	DAPI/CK-PE	CK-PE	DAPI	CD45-APC	HER-2/neu
24643	144	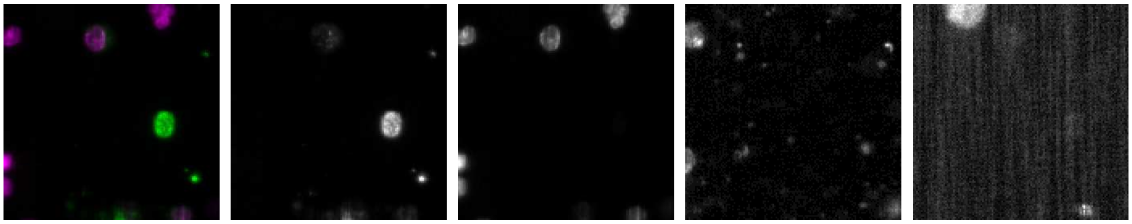				
24644	144	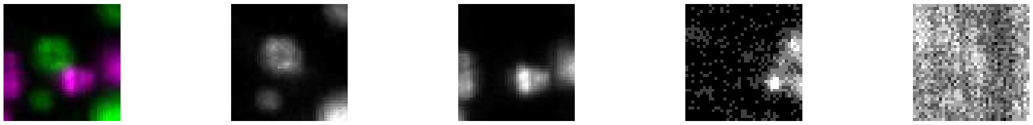				
24645	144	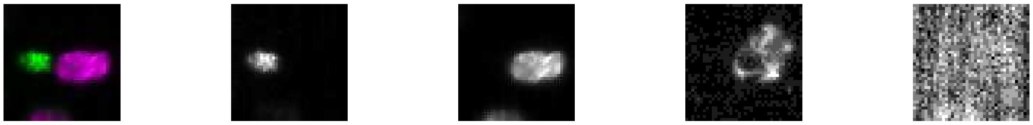				
24646	144	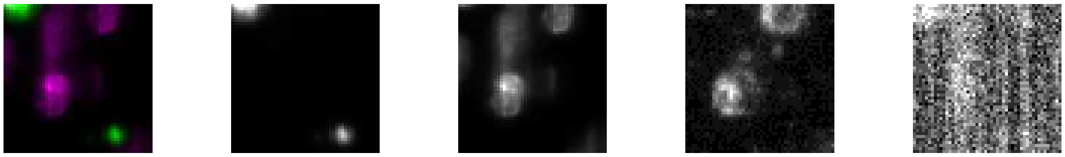				
24647	144	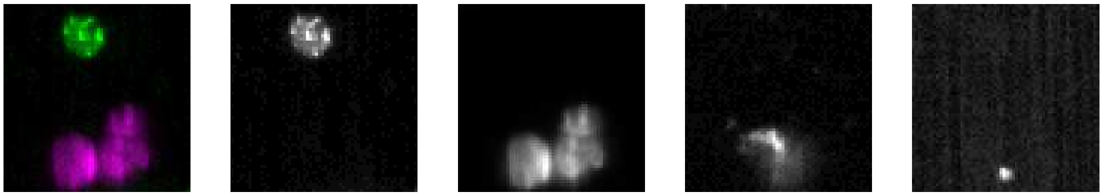				
24648	144	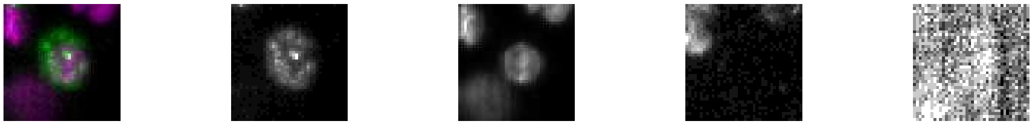				
24649	144	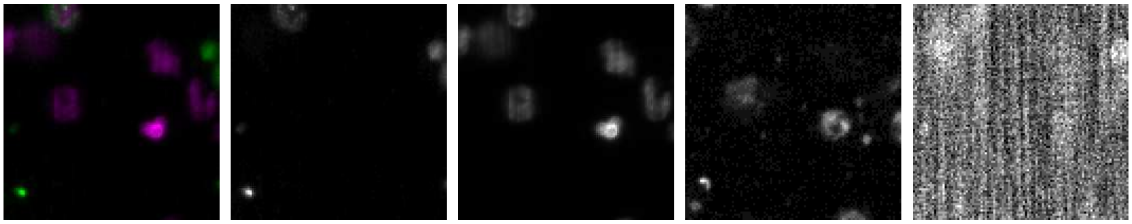				
24650	144	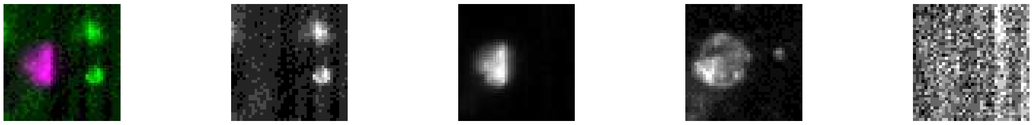				
24651	144	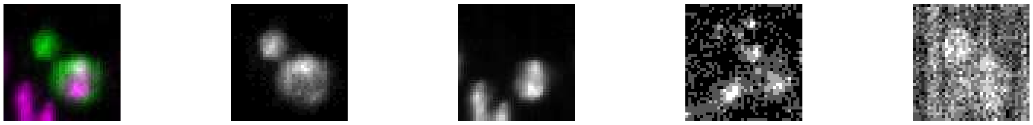				
24652	144	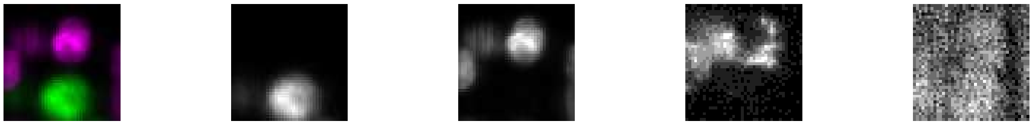				
24653	144	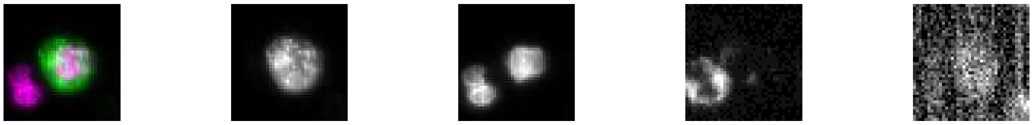				
24654	144	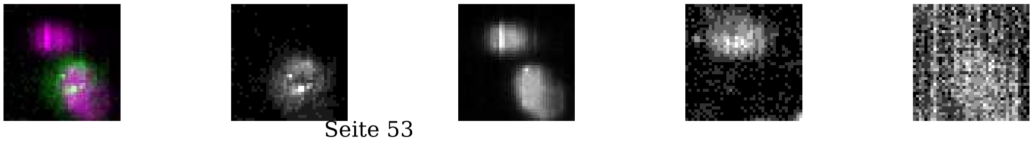				

Ereignis	Aufnahme	DAPI/CK-PE	CK-PE	DAPI	CD45-APC	HER-2/neu
24655	144	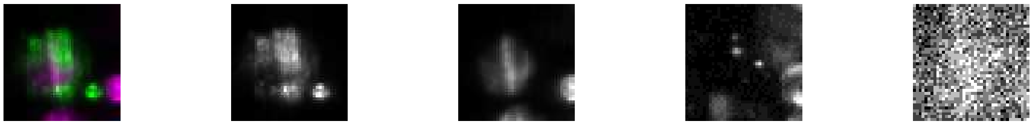				
24656	144	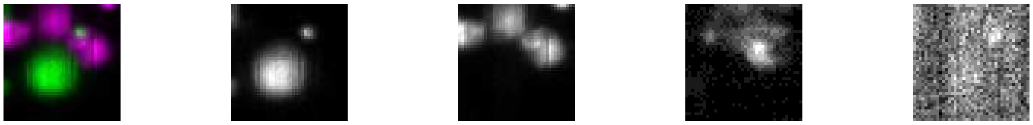				
24657	144	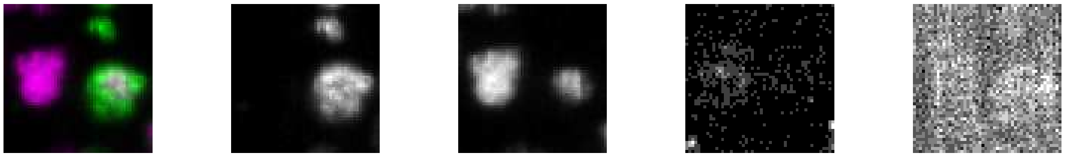				
24658	144	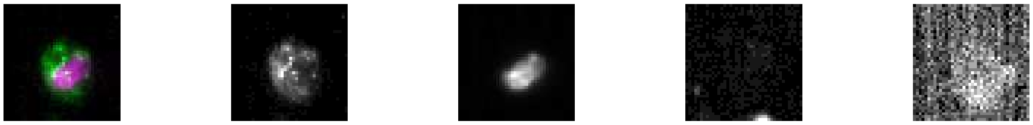				
24659	144	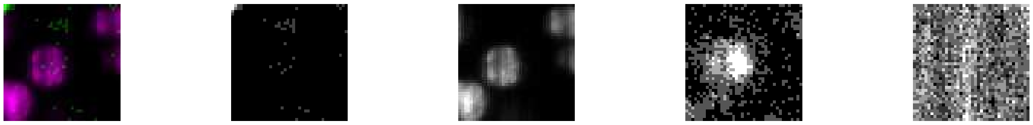				
24660	144	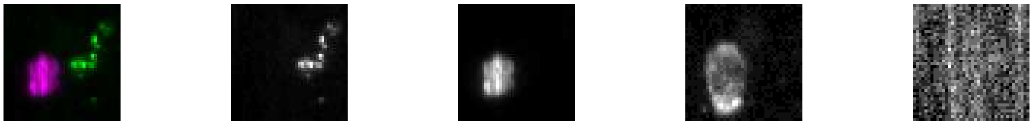				
24661	144	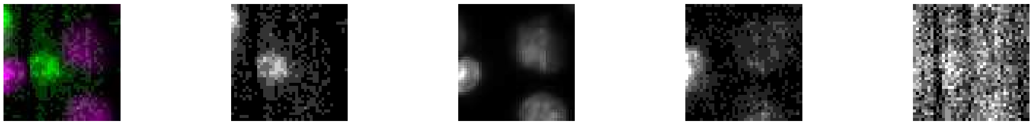				
24662	144	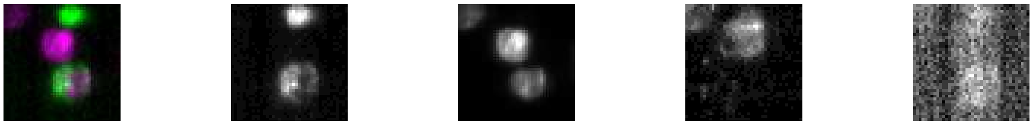				
24663	144	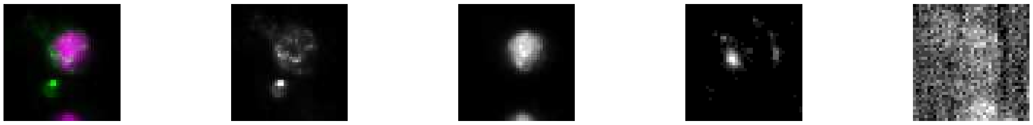				
24664	144	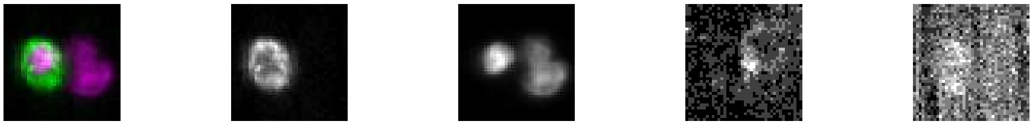				
24665	144	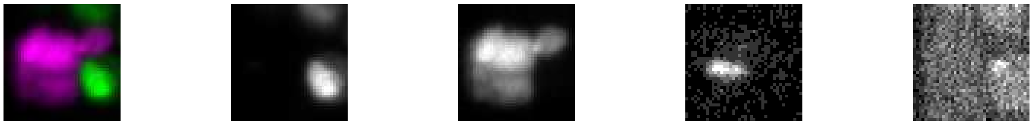				
24666	144	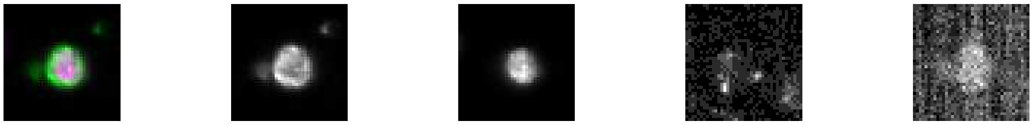				
24667	144	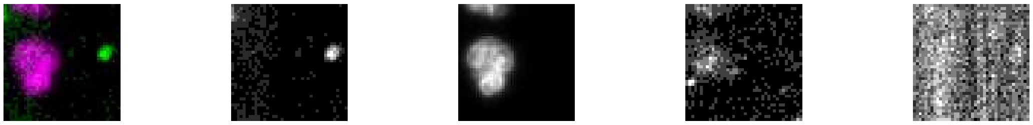				
24668	144	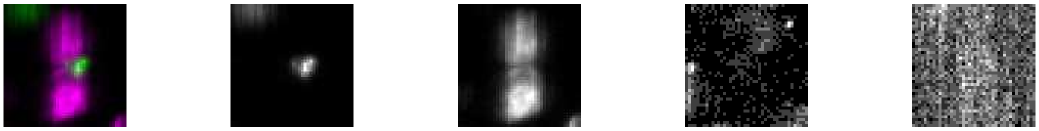				

Ereignis	Aufnahme	DAPI/CK-PE	CK-PE	DAPI	CD45-APC	HER-2/neu
24669	144	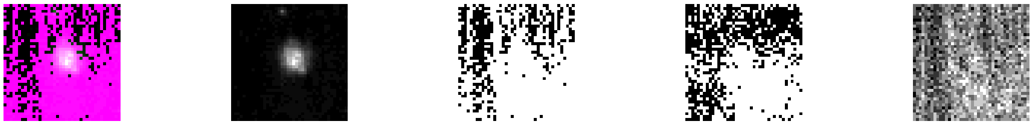				
24670	144	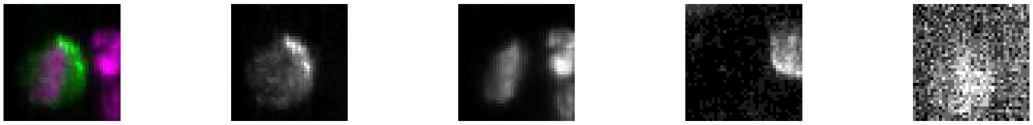				
24671	144	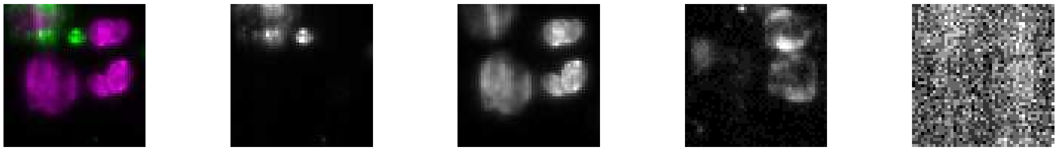				
24672	144	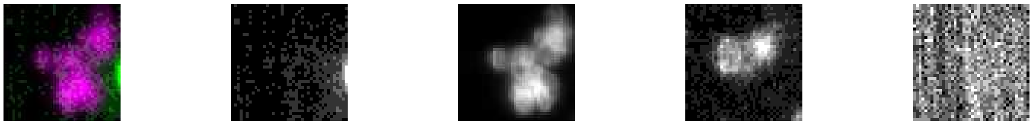				
24673	144	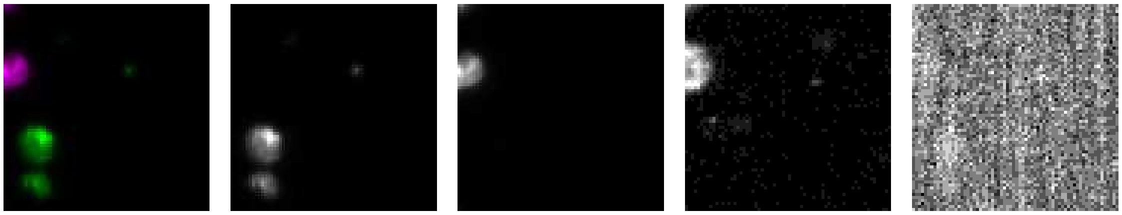				
24674	144	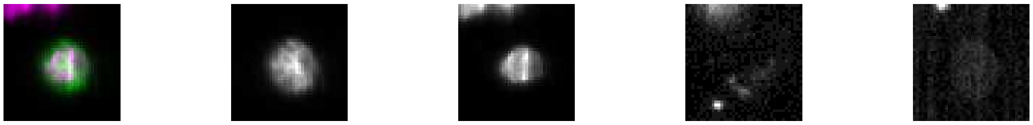				
24675	144	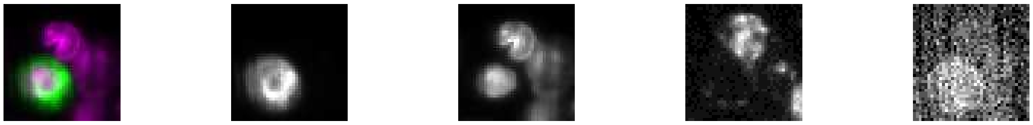				
24676	144	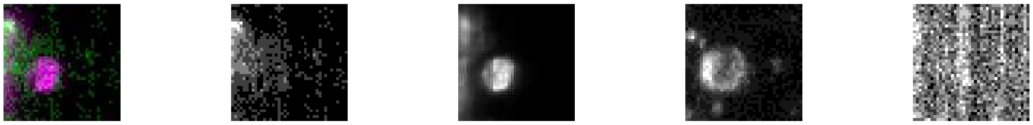				
24677	144	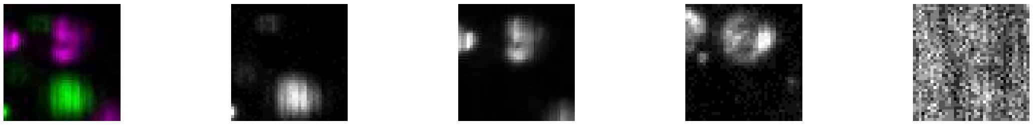				
24678	144	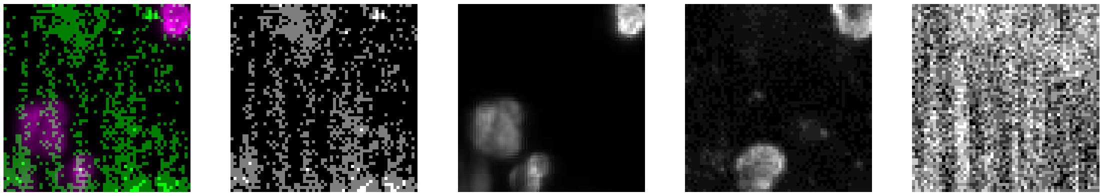				
24679	144	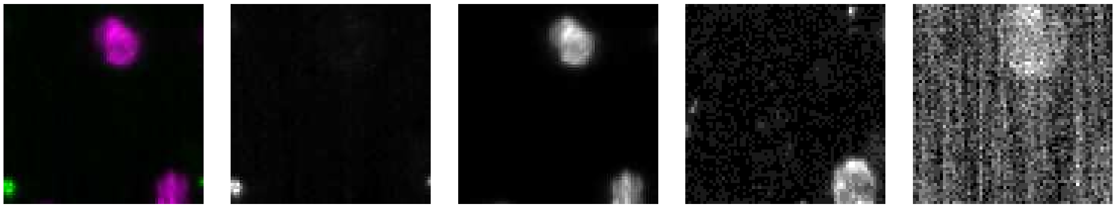				

Ereignis	Aufnahme	DAPI/CK-PE	CK-PE	DAPI	CD45-APC	HER-2/neu
24680	144	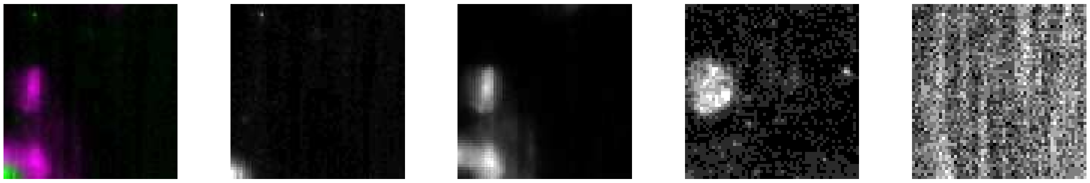				
24681	144	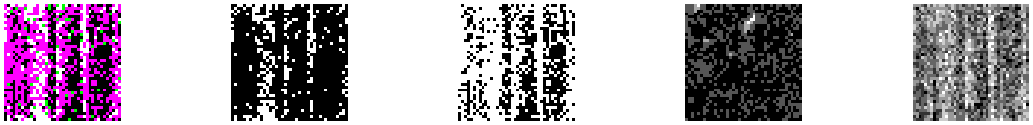				
24682	137	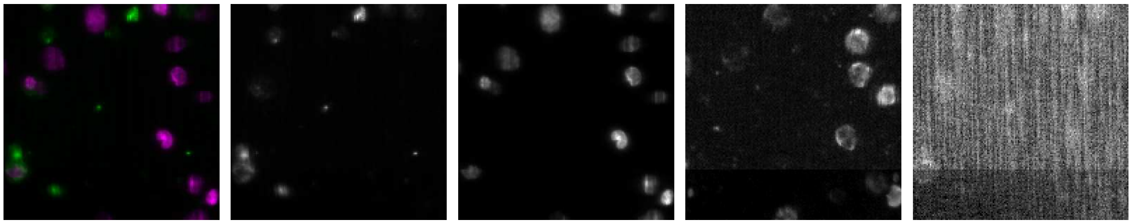				
24683	144	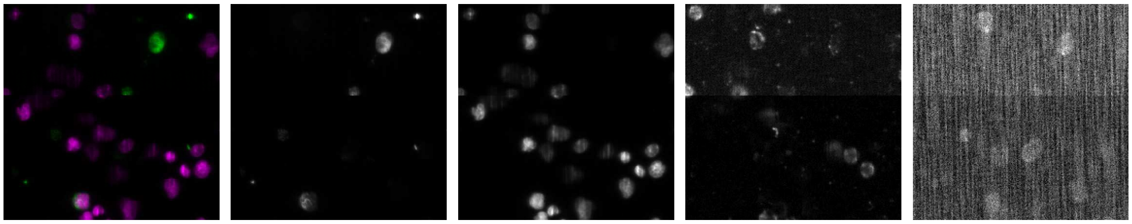				
24684	144	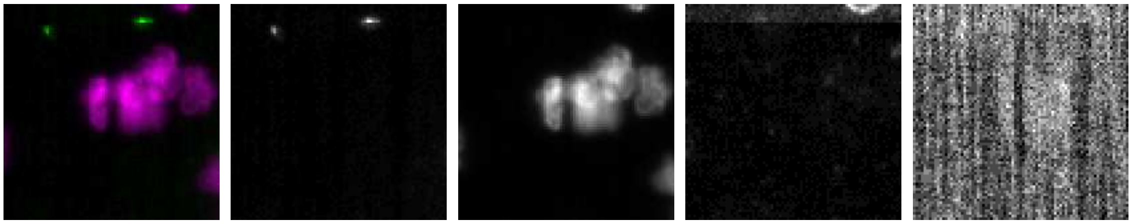				
24685	144	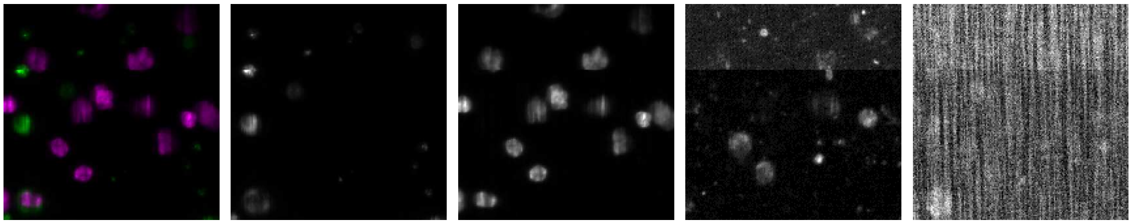				
24686	145	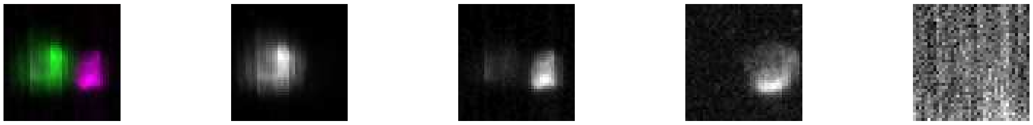				
24687	145	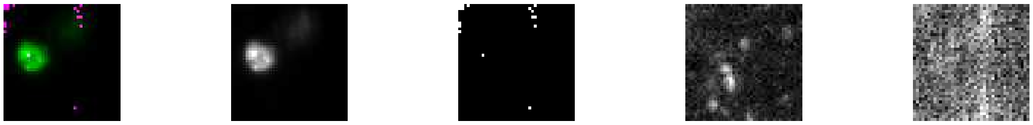				
24688	145	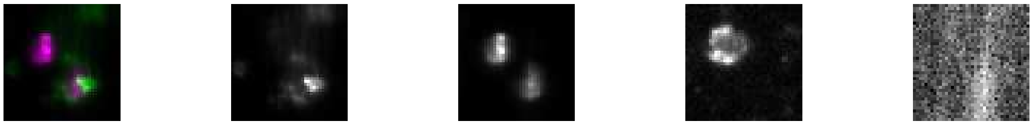				
24689	145	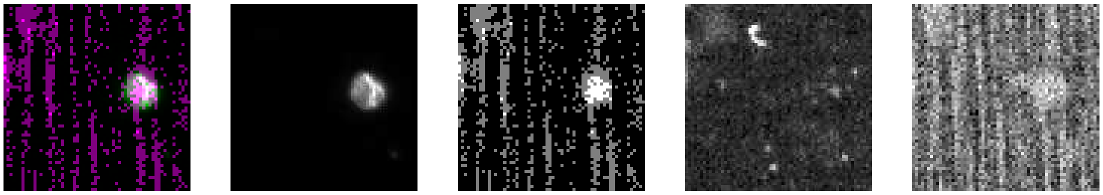				

Druckdatum: 02/06/2020 17:25

Proben-ID: Gyn14.208 **Kassetten-ID:** 01327106 **Patienten-ID:**

Ereignis	Aufnahme	DAPI/CK-PE	CK-PE	DAPI	CD45-APC	HER-2/neu
24690	145	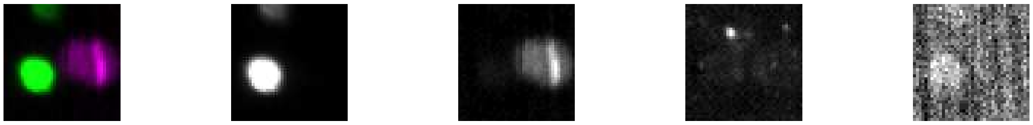				
24691	145	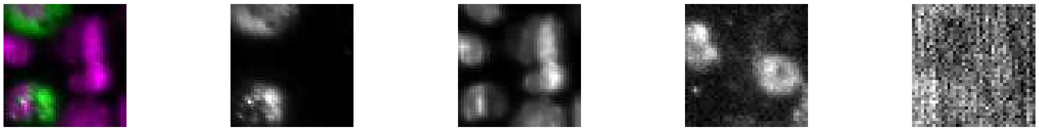				
24692	145	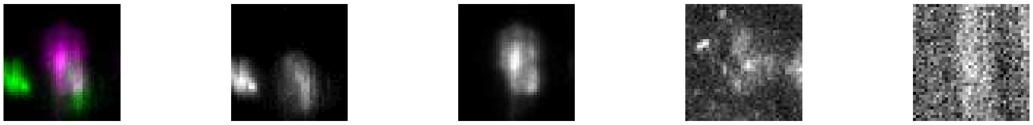				
24693	145	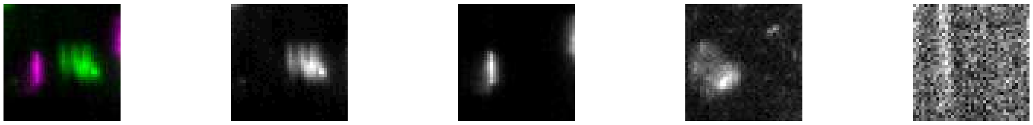				
24694	145	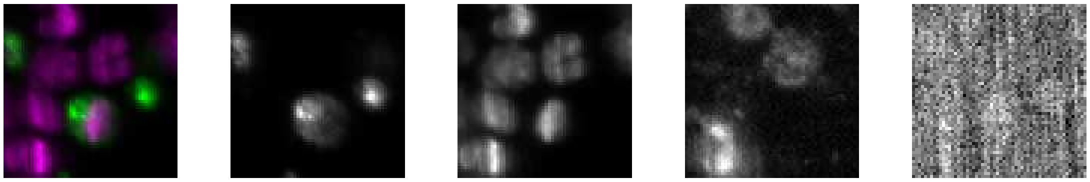				
24695	145	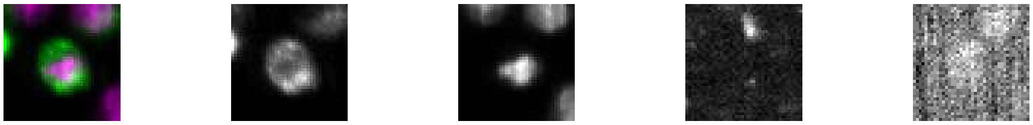				
24696	145	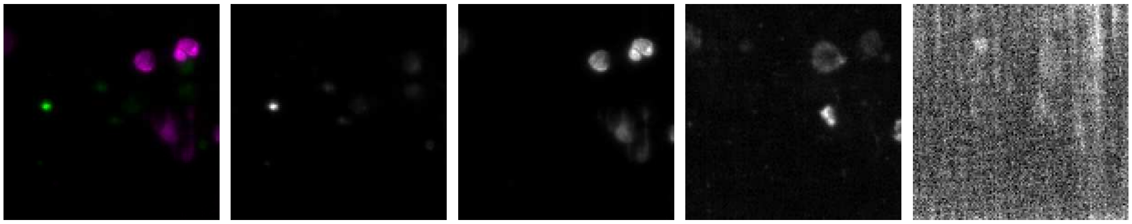				
24697	145	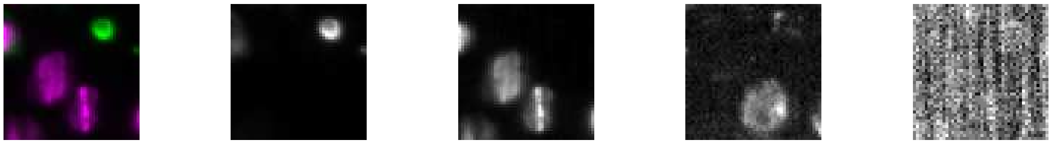				
24698	145	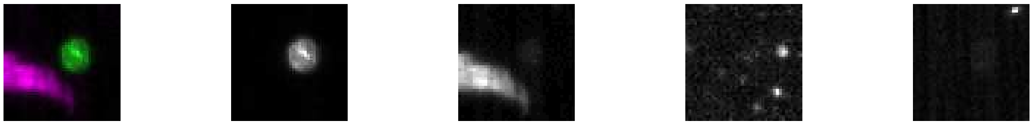				
24699	145	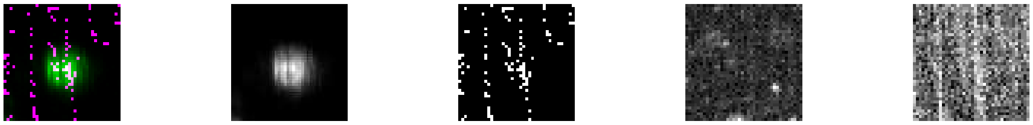				
24700	145	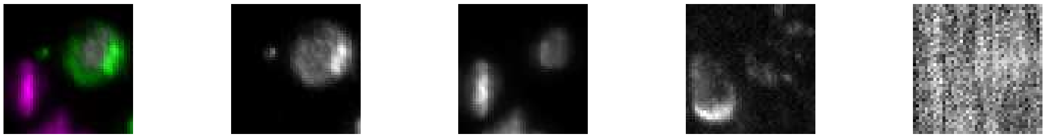				
24701	145	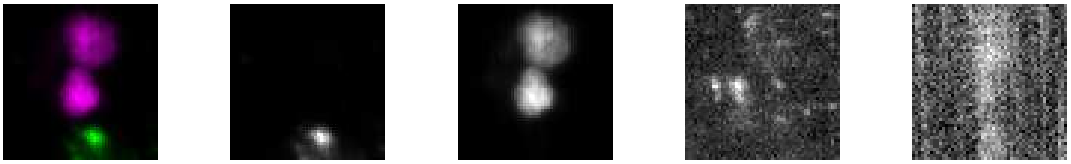				

Ereignis	Aufnahme	DAPI/CK-PE	CK-PE	DAPI	CD45-APC	HER-2/neu
24702	145	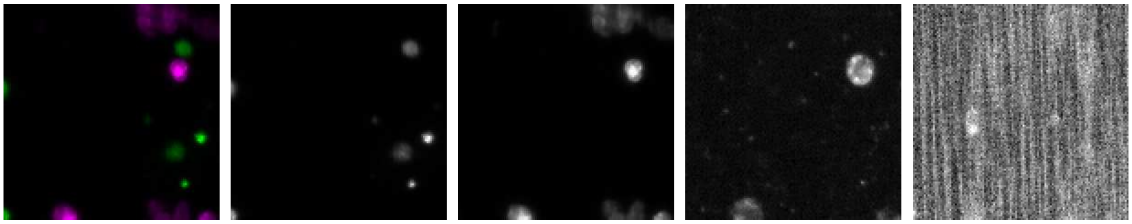				
24703	145	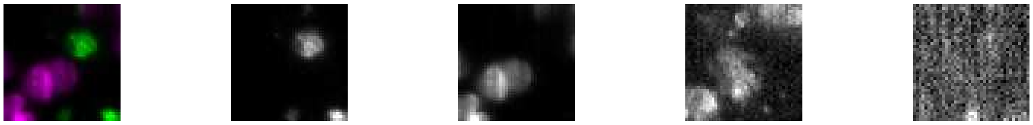				
24704	145	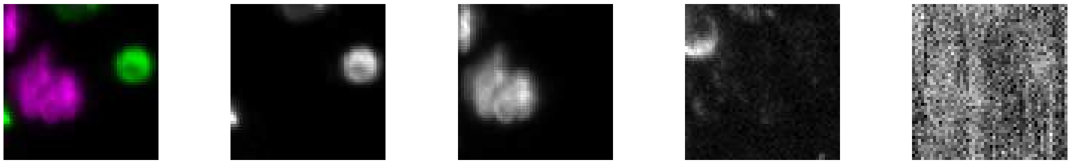				
24705	145	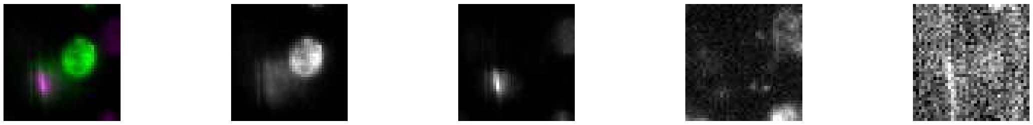				
24706	145	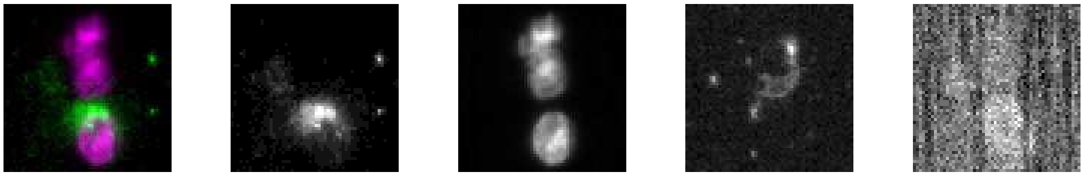				
24707	145	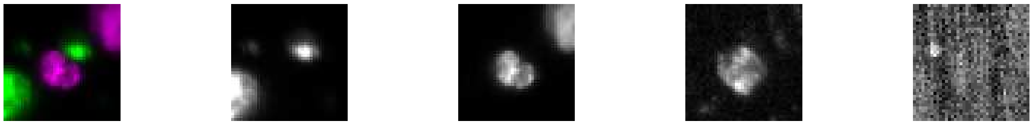				
24708	145	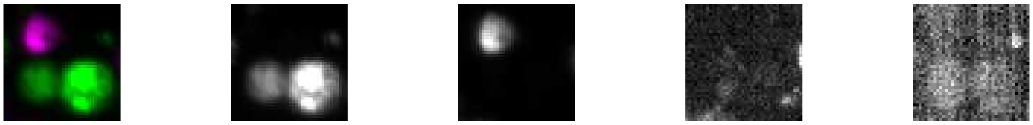				
24709	145	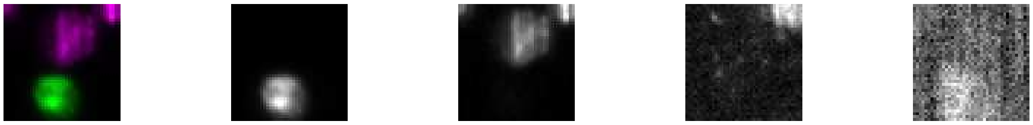				
24710	145	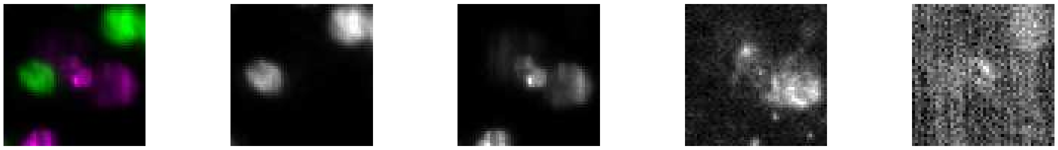				
24711	145	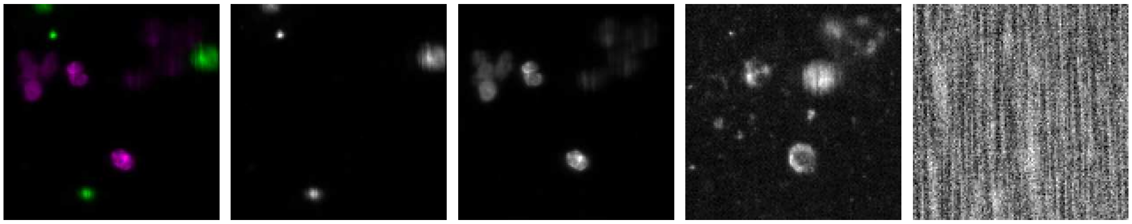				
24712	145	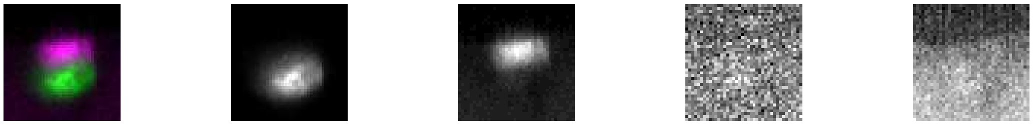				

Ereignis	Aufnahme	DAPI/CK-PE	CK-PE	DAPI	CD45-APC	HER-2/neu
24713	145	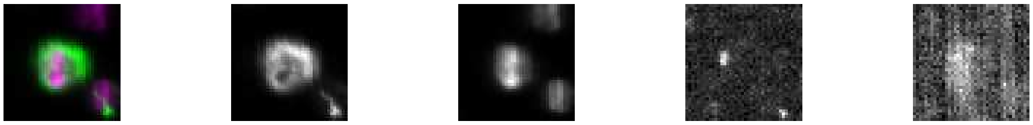				
24714	145	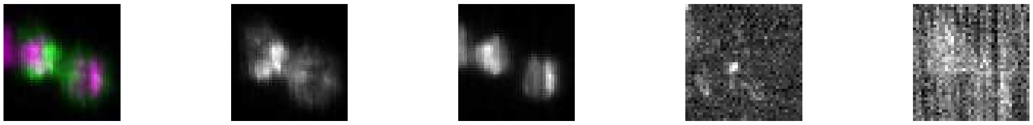				
24715	145	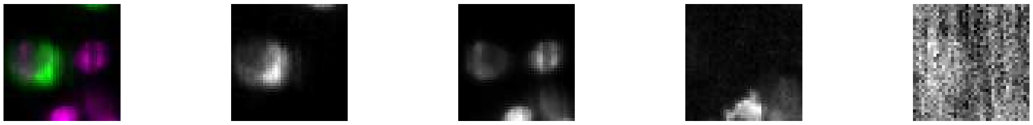				
24716	145	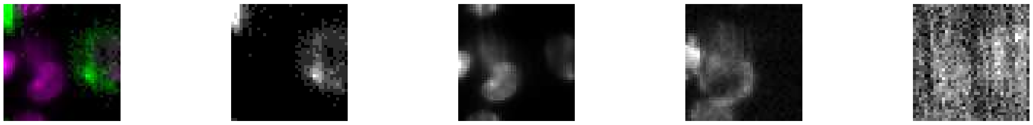				
24717	145	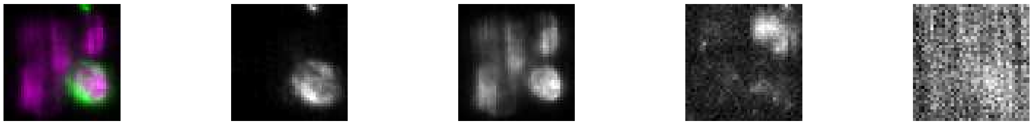				
24718	145	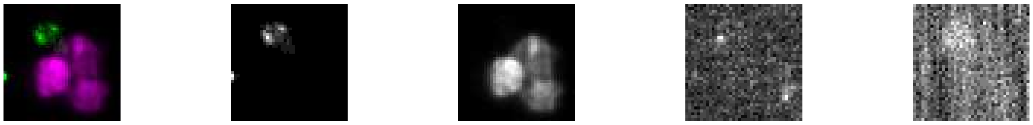				
24719	145	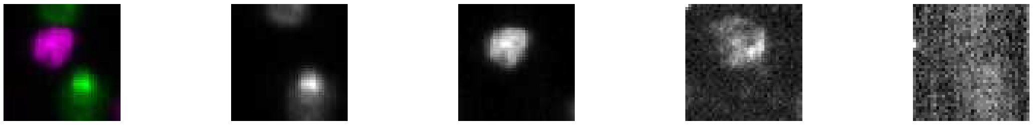				
24720	145	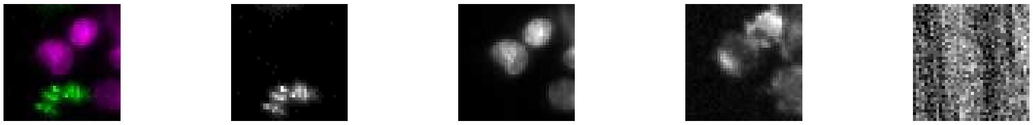				
24721	145	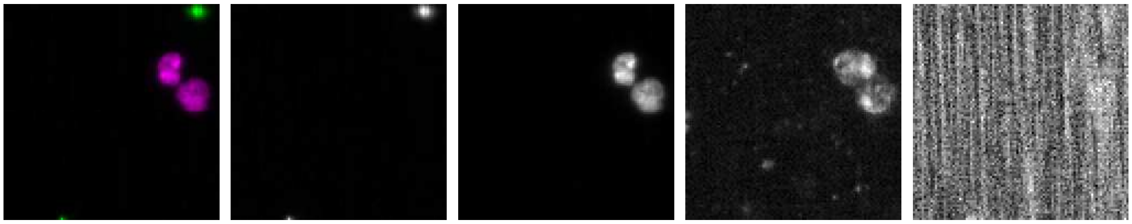				
24722	145	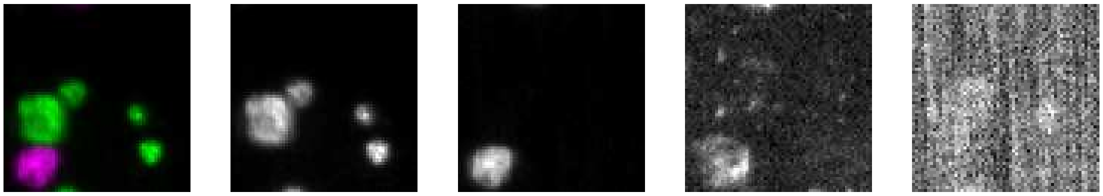				
24723	145	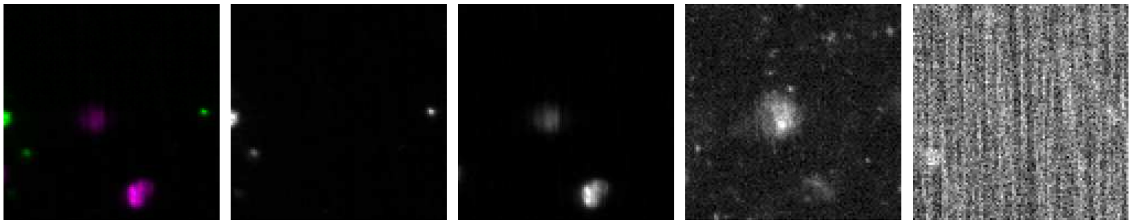				
24724	144	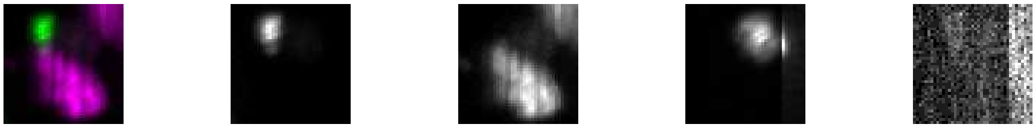				

Ereignis	Aufnahme	DAPI/CK-PE	CK-PE	DAPI	CD45-APC	HER-2/neu
24725	145	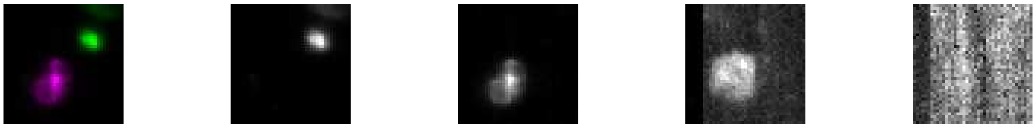				
24726	144	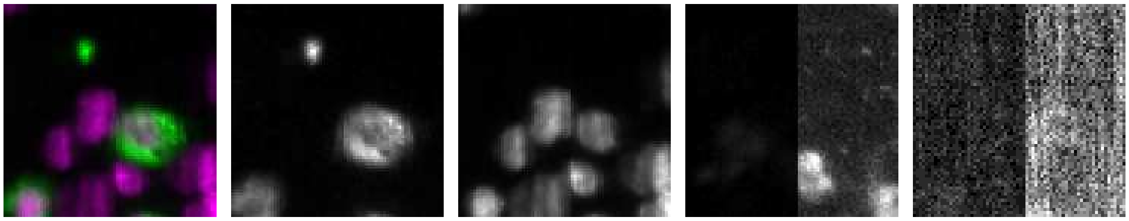				
24727	136	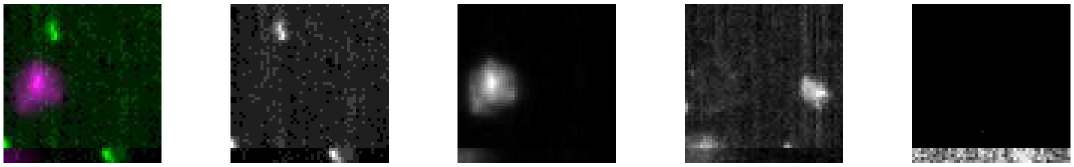				
24728	146	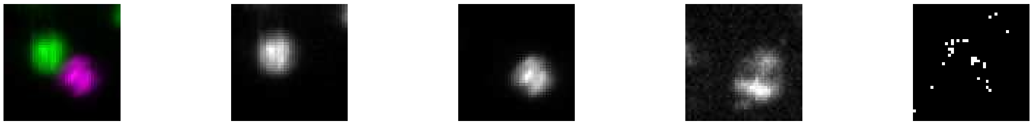				
24729	146	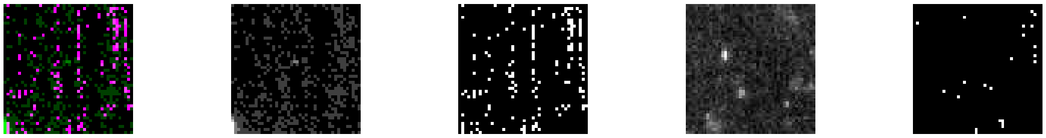				
24730	146	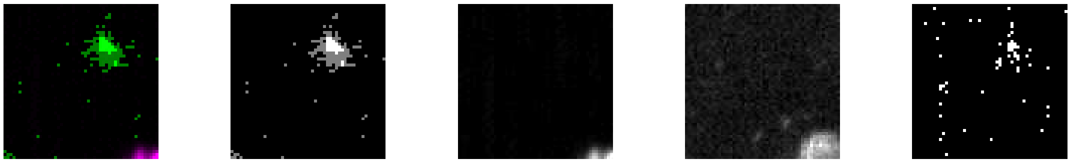				
24731	146	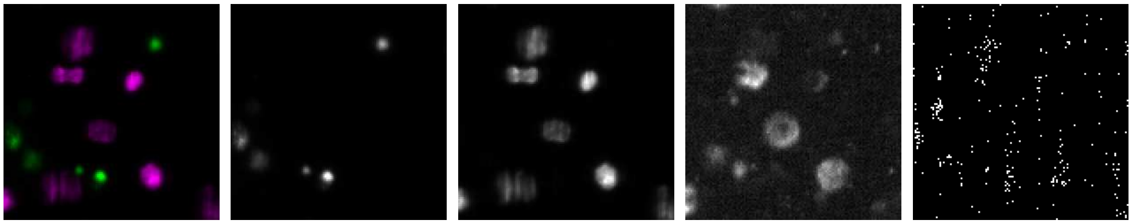				
24732	146	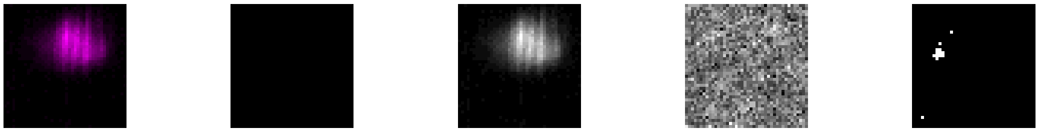				
24733	146	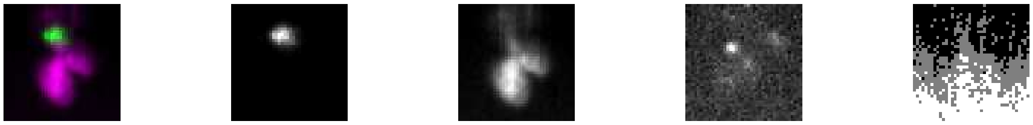				
24734	146	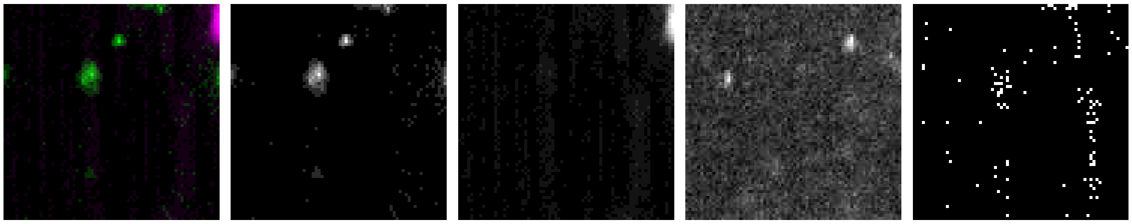				

Ereignis	Aufnahme	DAPI/CK-PE	CK-PE	DAPI	CD45-APC	HER-2/neu
24735	146	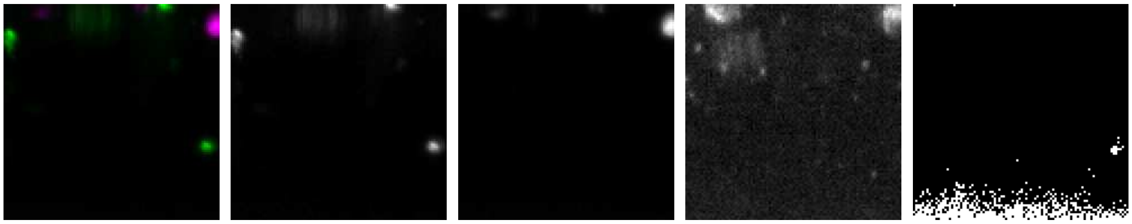				
24736	146	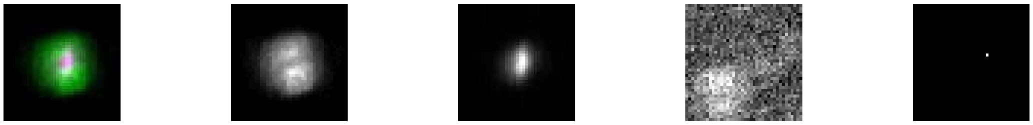				
24737	146	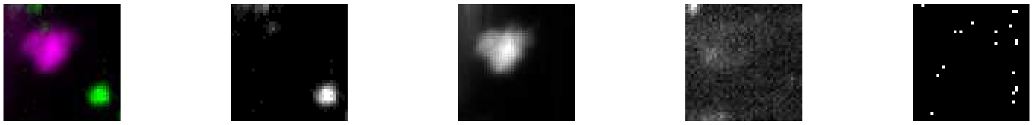				
24738	146	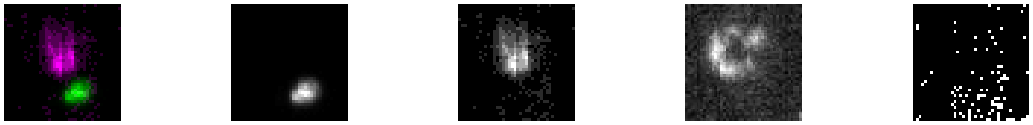				
24739	146	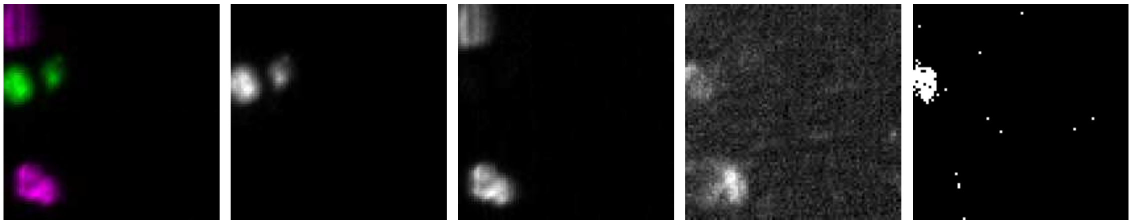				
24740	146	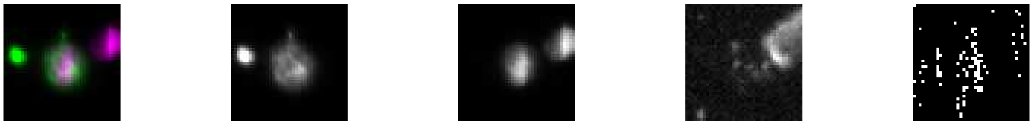				
24741	146	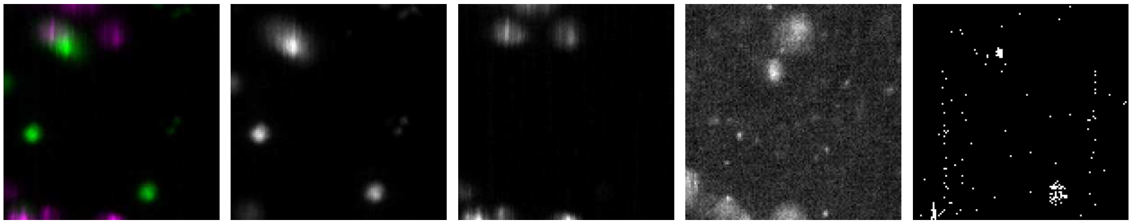				
24742	146	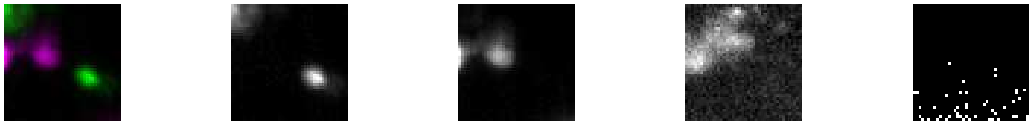				
24743	146	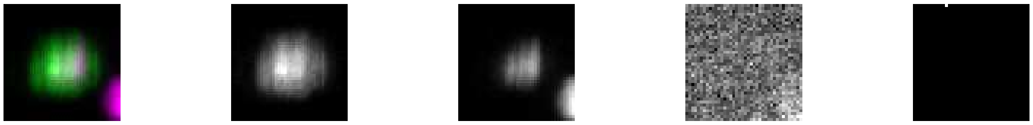				
24744	146	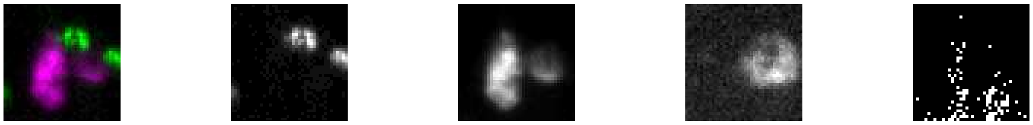				
24745	146	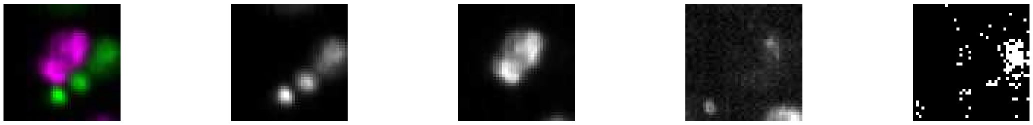				

Ereignis	Aufnahme	DAPI/CK-PE	CK-PE	DAPI	CD45-APC	HER-2/neu
24746	146	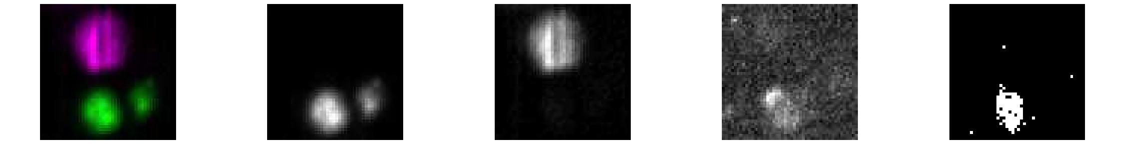				
24747	146	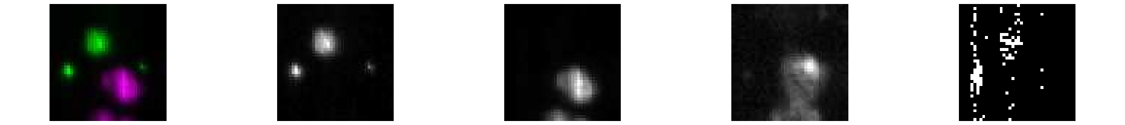				
24748	146	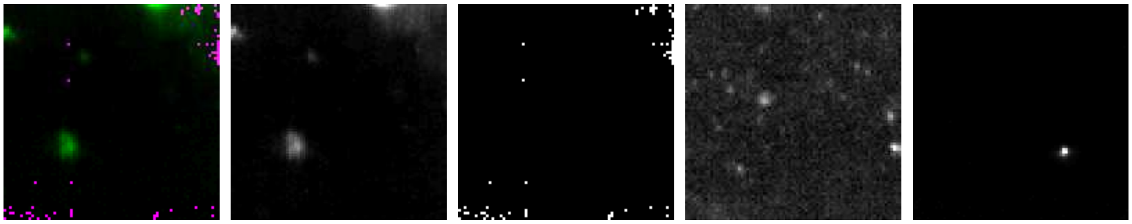				
24749	146	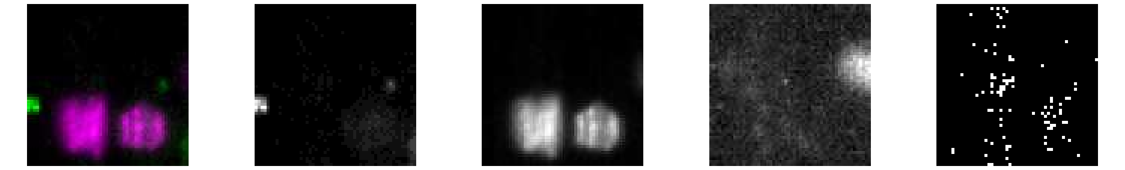				
24750	146	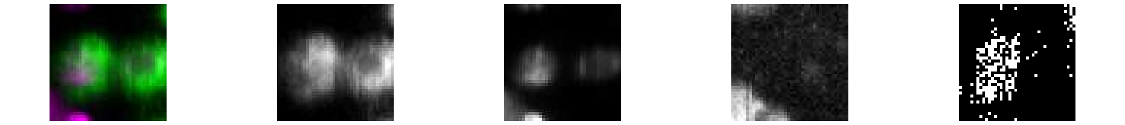				
24751	146	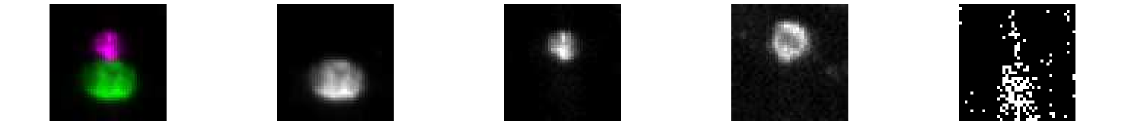				
24752	146	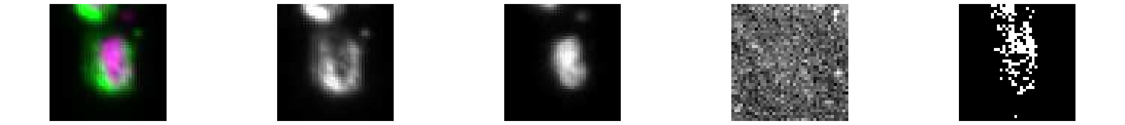				
24753	146	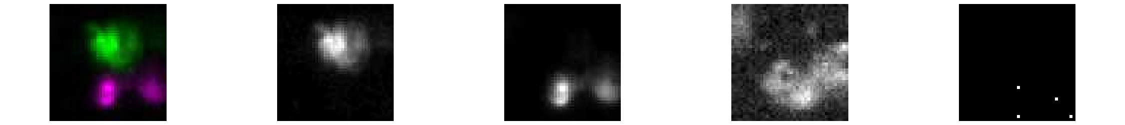				
24754	146	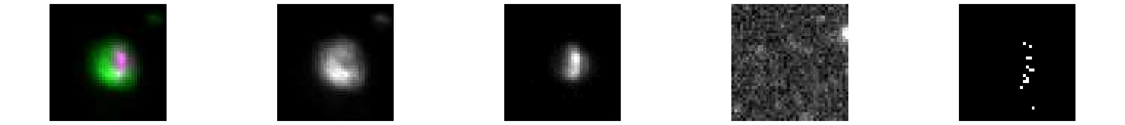				
24755	146	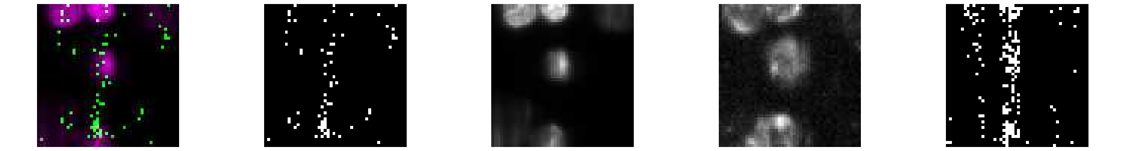				
24756	146	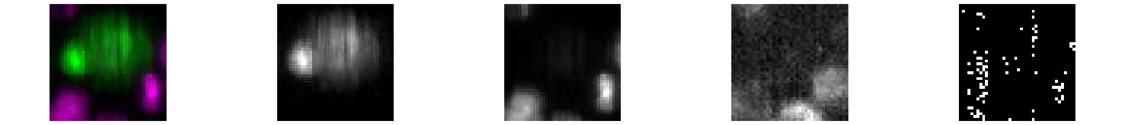				
24757	146	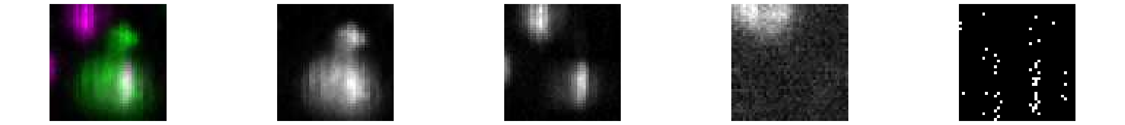				

Ereignis	Aufnahme	DAPI/CK-PE	CK-PE	DAPI	CD45-APC	HER-2/neu
24758	146	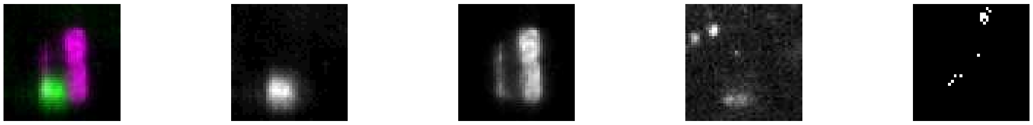				
24759	146	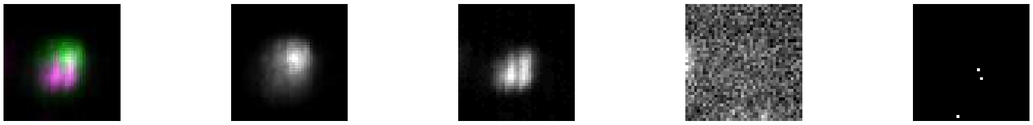				
24760	146	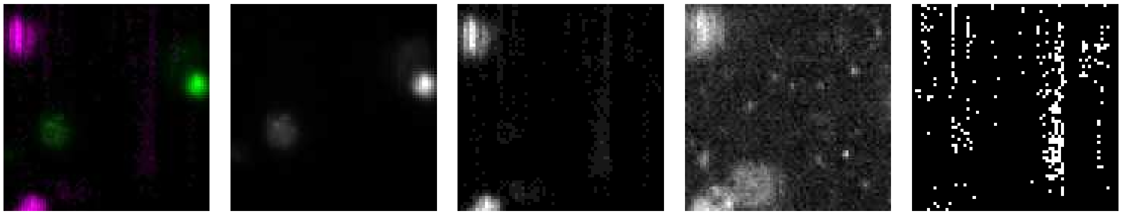				
24761	146	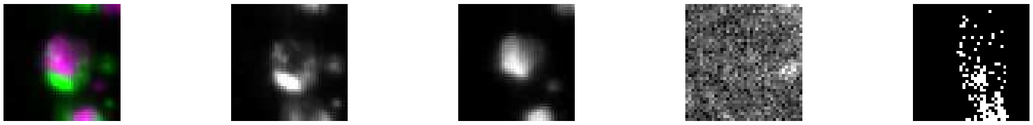				
24762	146	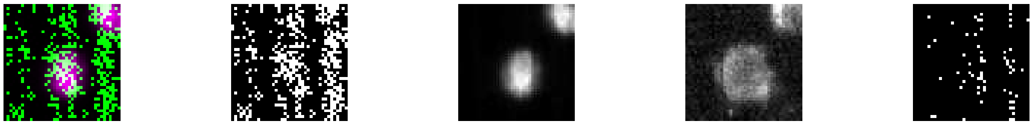				
24763	146	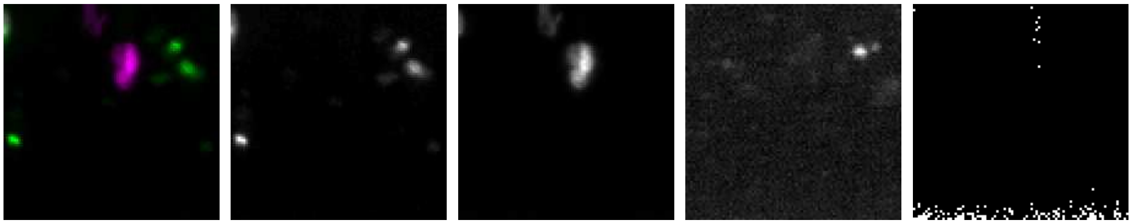				
24764	146	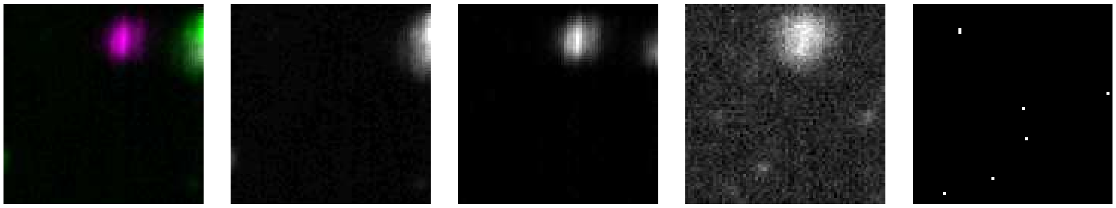				
24765	146	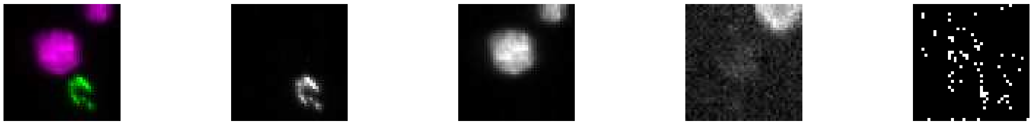				
24766	146	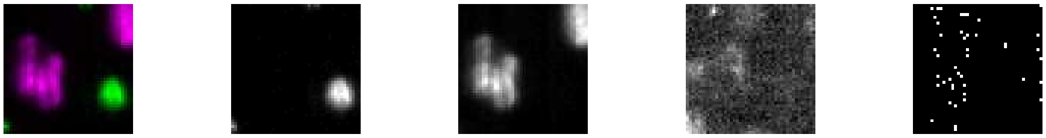				
24767	146	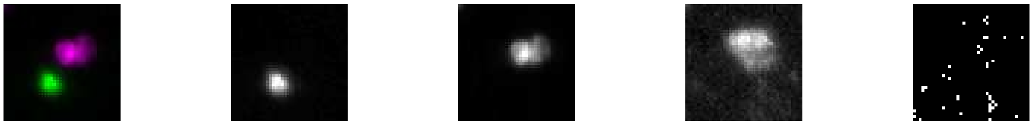				
24768	146	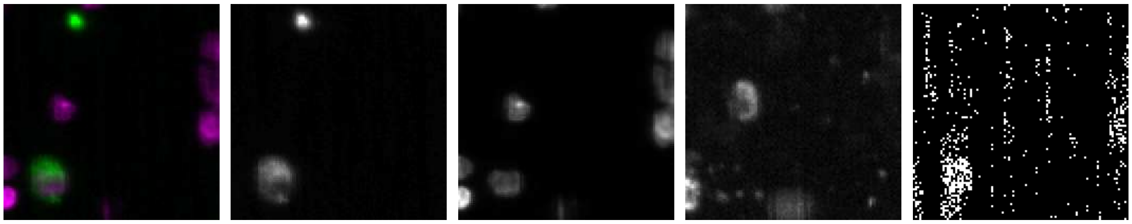				

Ereignis	Aufnahme	DAPI/CK-PE	CK-PE	DAPI	CD45-APC	HER-2/neu
24769	146	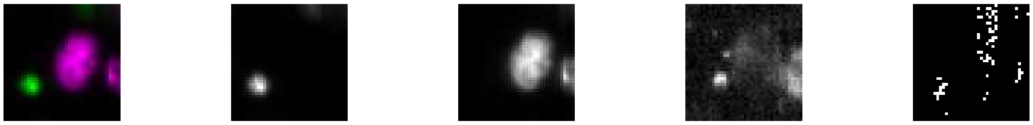				
24770	146	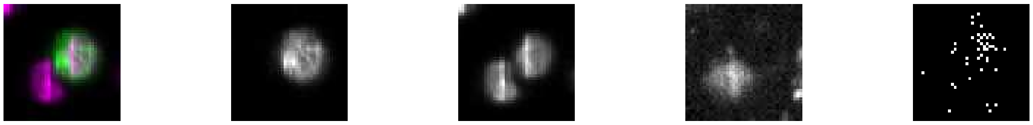				
24771	146	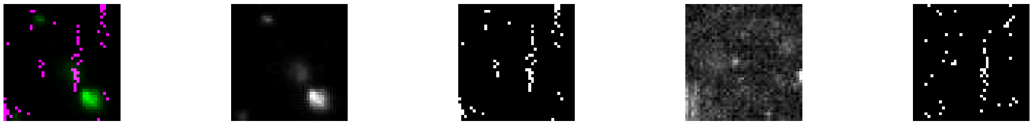				
24772	146	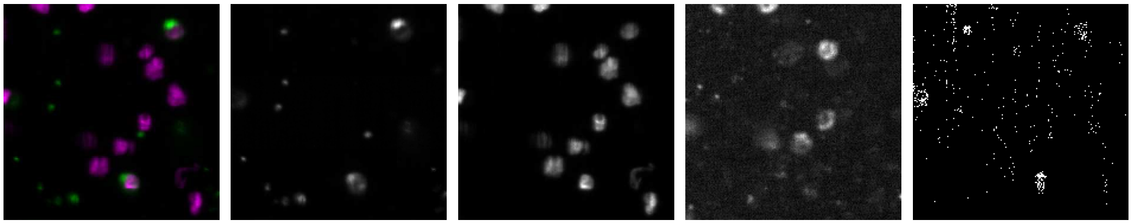				
24773	146	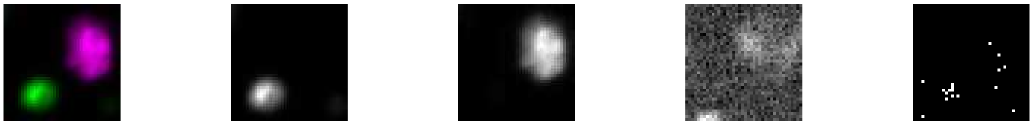				
24774	146	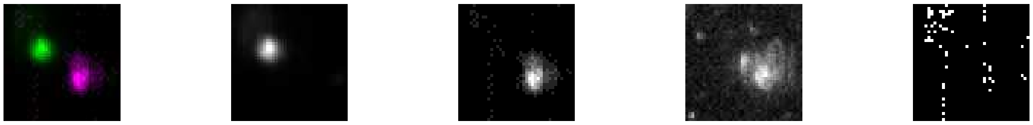				
24775	146	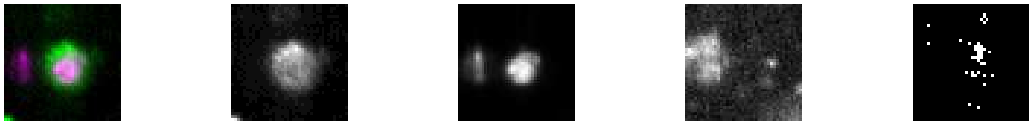				
24776	146	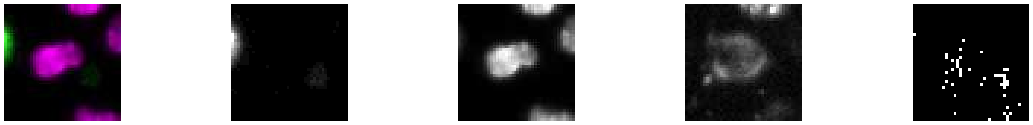				
24777	146	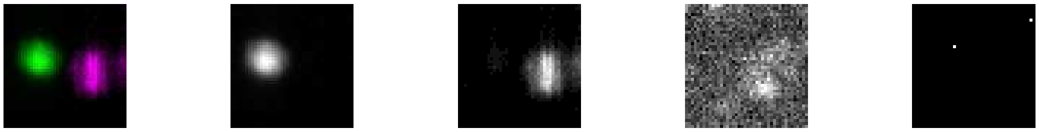				
24778	146	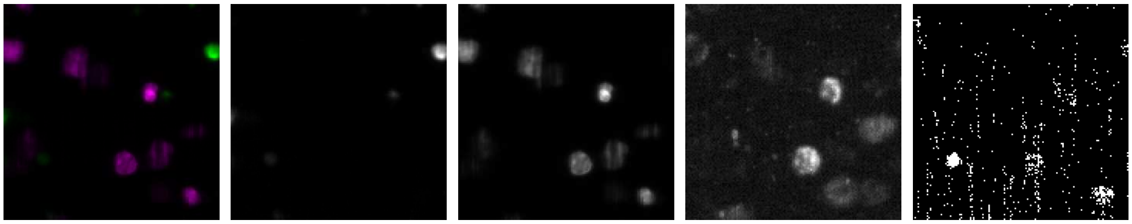				
24779	146	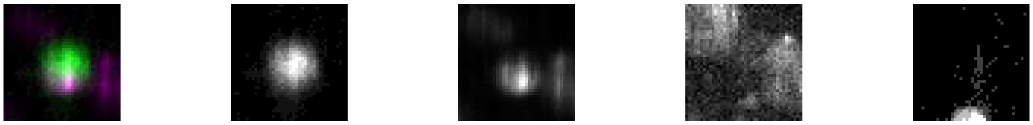				
24780	146	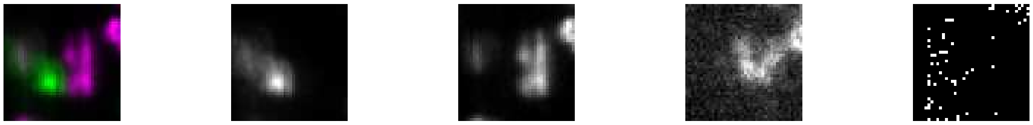				

Ereignis	Aufnahme	DAPI/CK-PE	CK-PE	DAPI	CD45-APC	HER-2/neu
24781	146	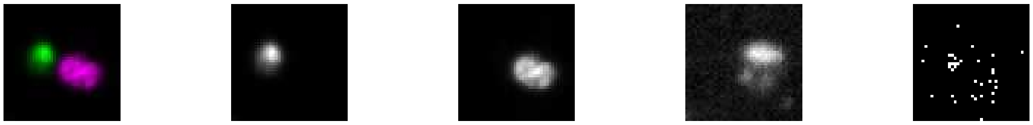				
24782	146	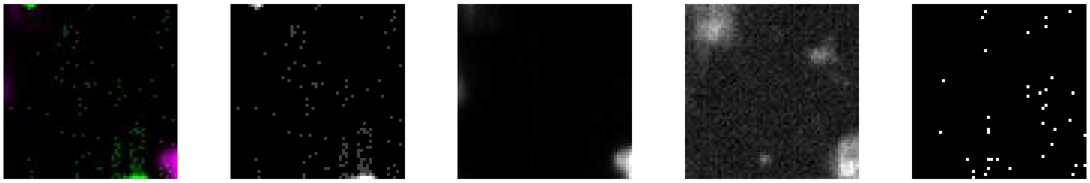				
24783	146	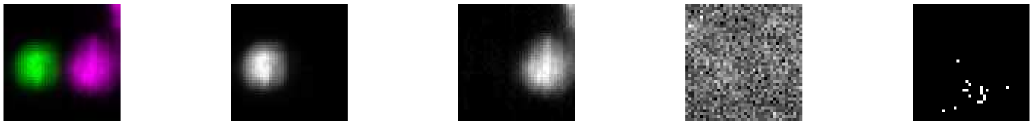				
24784	146	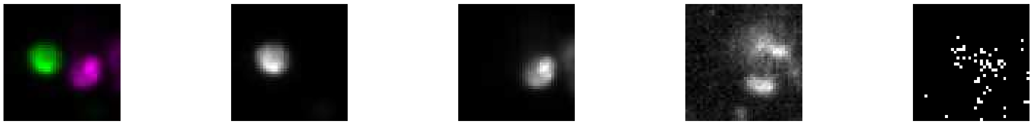				
24785	146	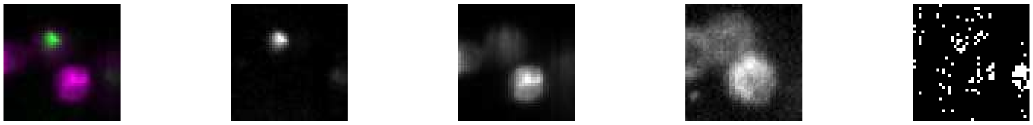				
24786	146	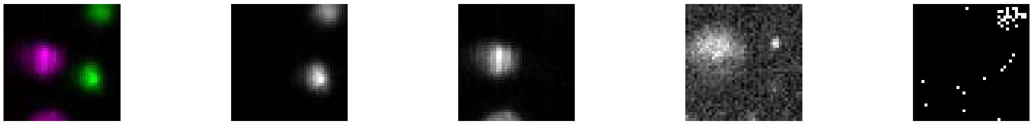				
24787	146	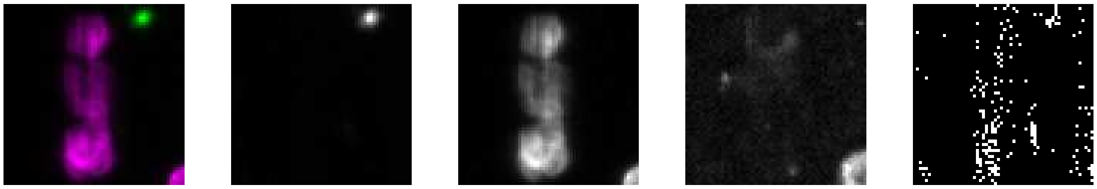				
24788	146	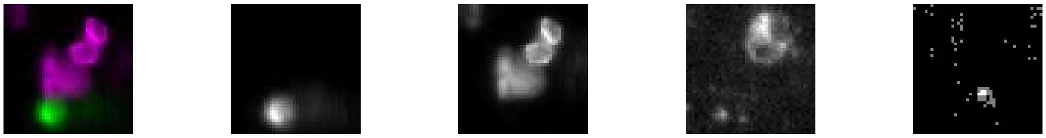				
24789	146	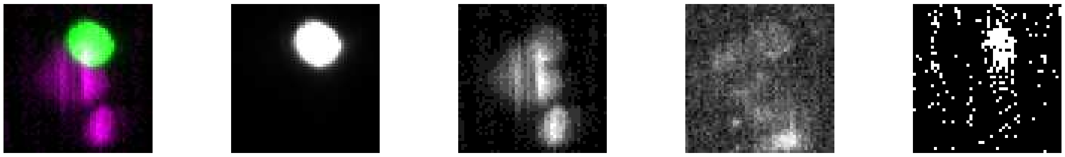				
24790	146	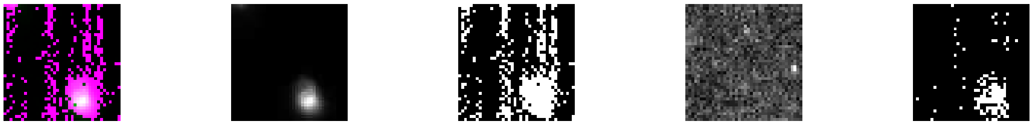				
24791	146	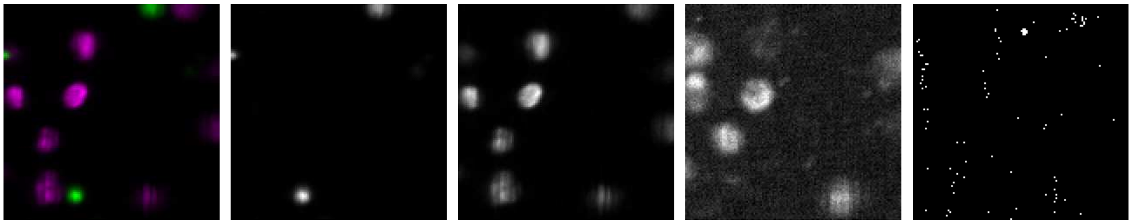				
24792	146	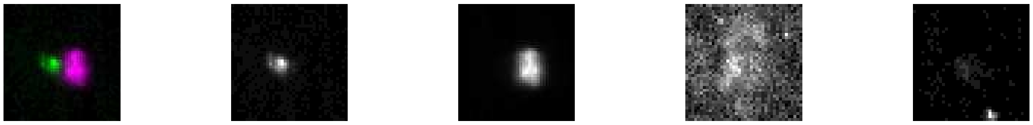				

Ereignis	Aufnahme	DAPI/CK-PE	CK-PE	DAPI	CD45-APC	HER-2/neu
24793	146	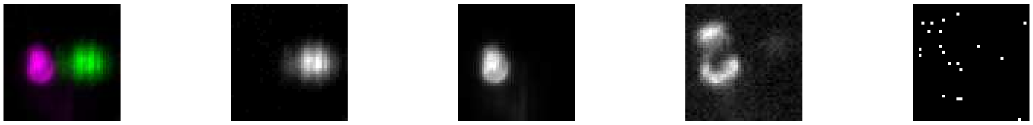				
24794	146	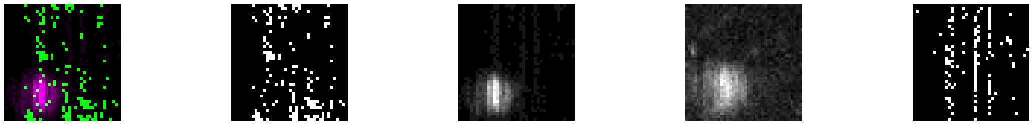				
24795	146	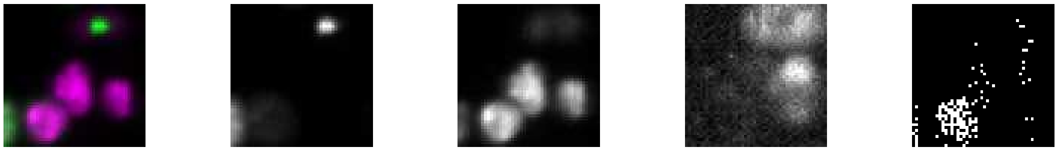				
24796	146	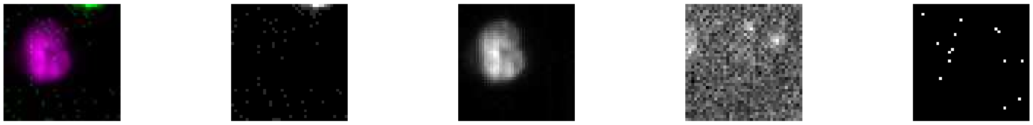				
24797	146	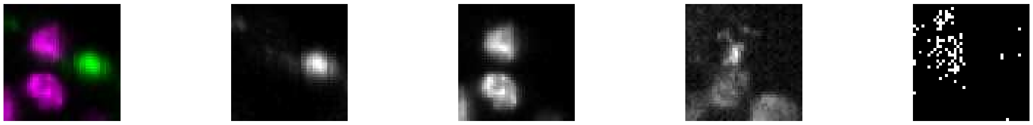				
24798	146	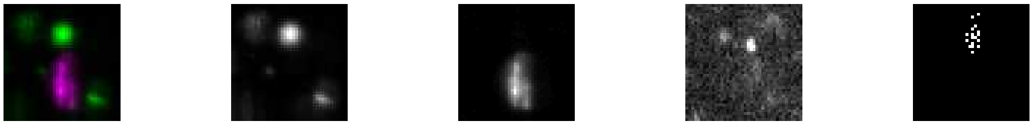				
24799	146	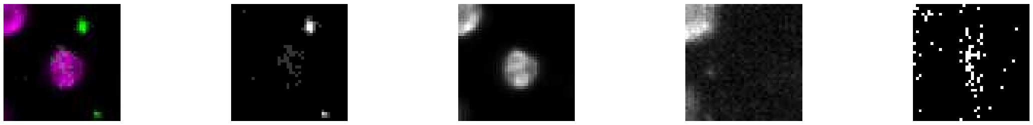				
24800	146	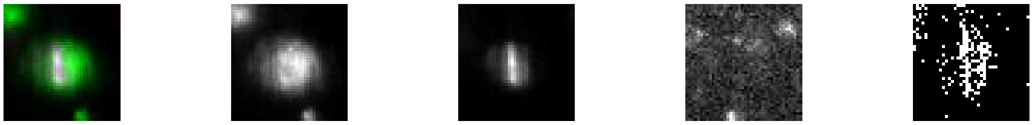				
24801	146	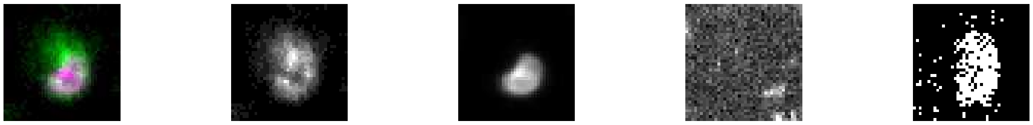				
24802	146	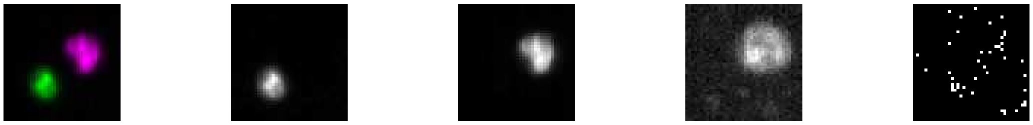				
24803	146	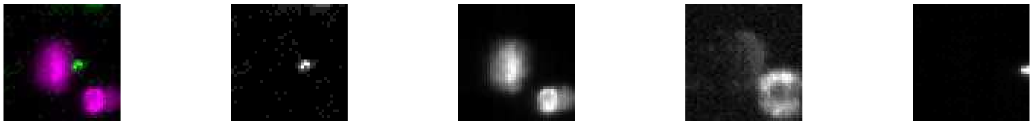				
24804	146	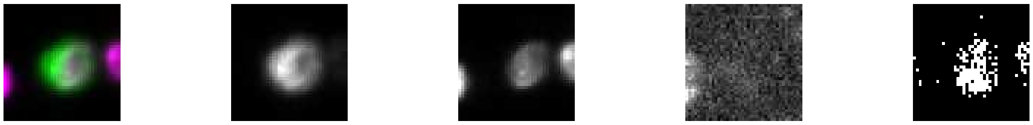				
24805	146	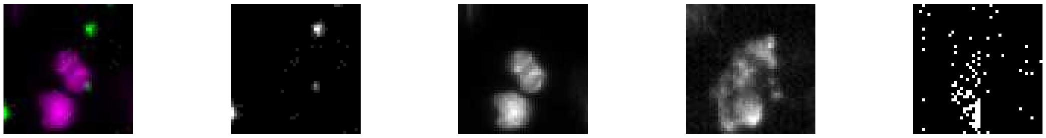				
24806	146	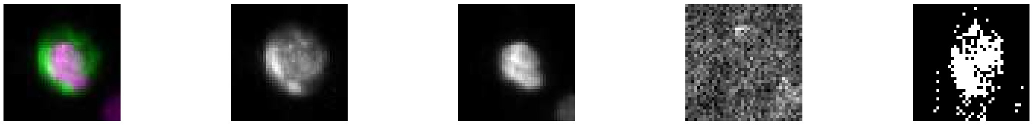				

Ereignis	Aufnahme	DAPI/CK-PE	CK-PE	DAPI	CD45-APC	HER-2/neu
24807	146	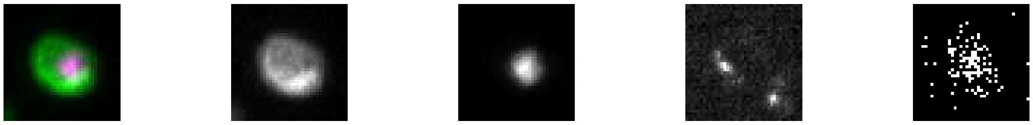				
24808	146	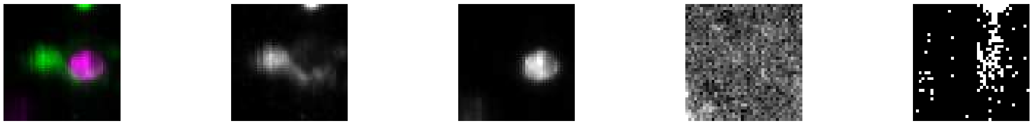				
24809	146	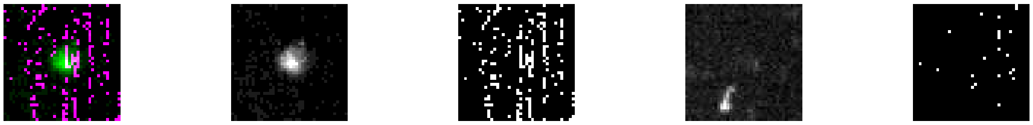				
24810	146	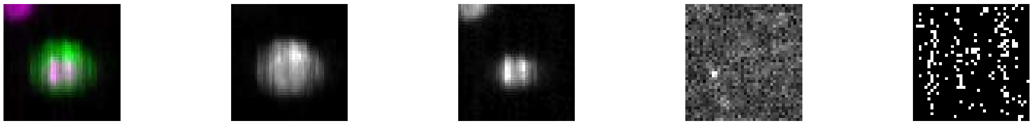				
24811	146	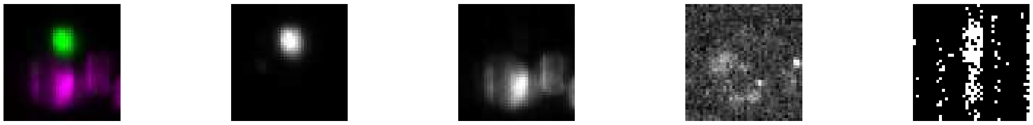				
24812	146	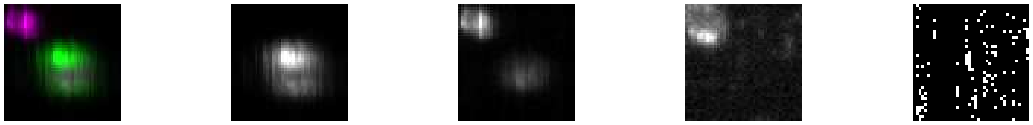				
24813	146	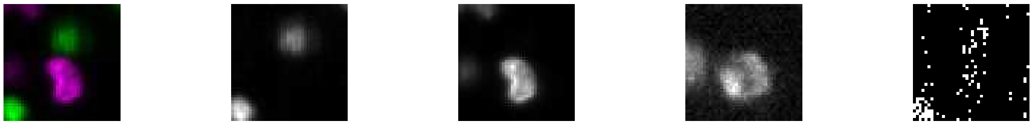				
24814	146	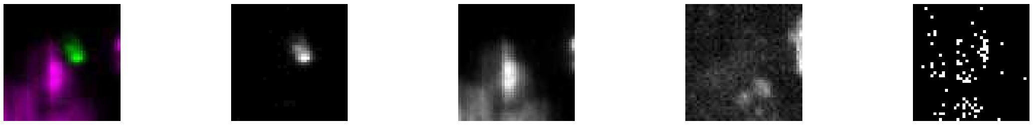				
24815	146	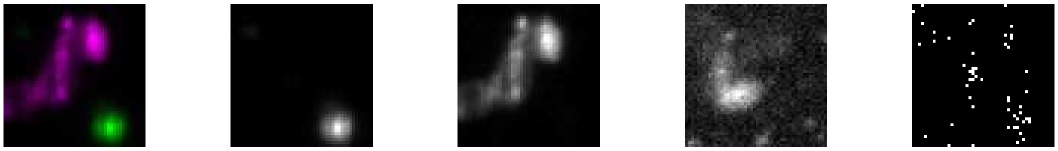				
24816	146	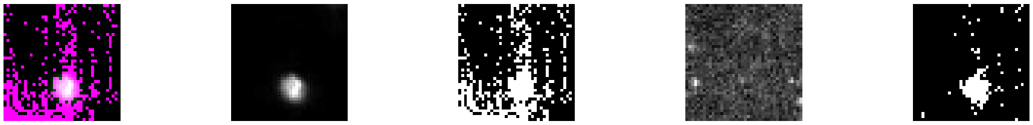				
24817	146	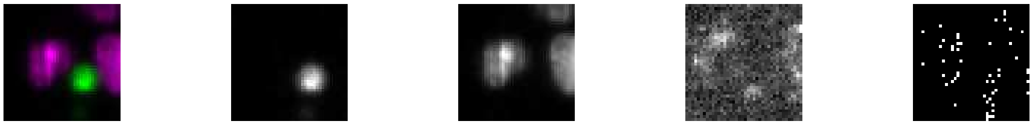				
24818	146	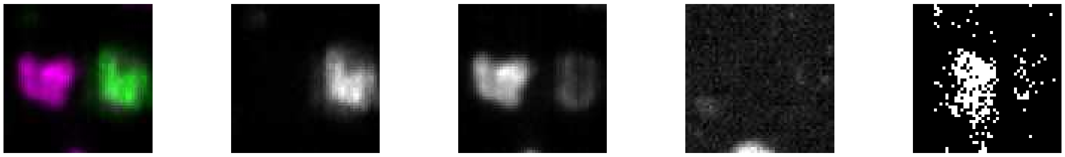				
24819	146	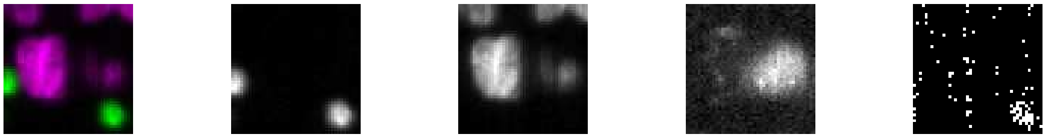				

Ereignis	Aufnahme	DAPI/CK-PE	CK-PE	DAPI	CD45-APC	HER-2/neu
24820	146	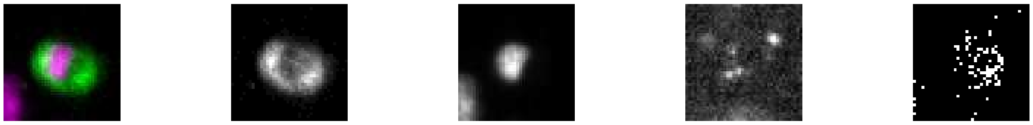				
24821	146	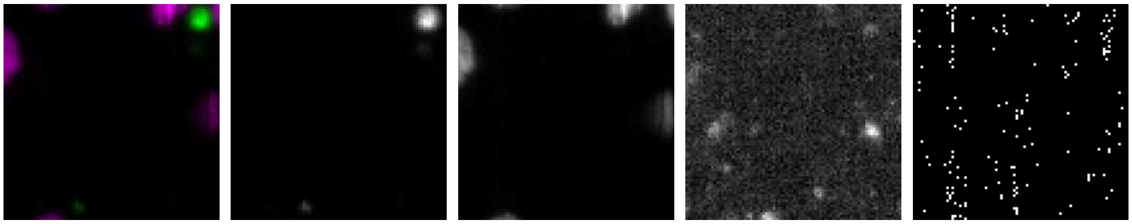				
24822	146	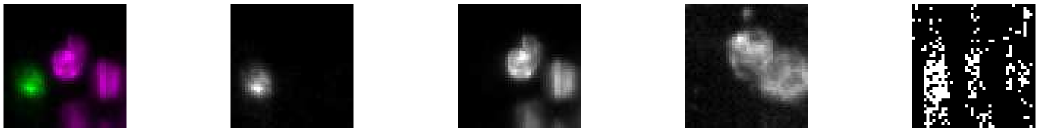				
24823	146	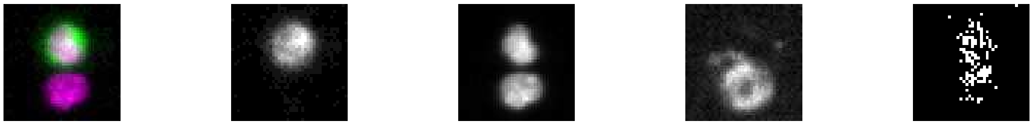				
24824	146	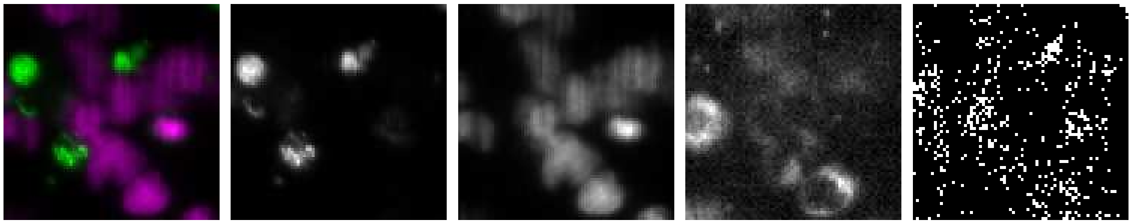				
24825	146	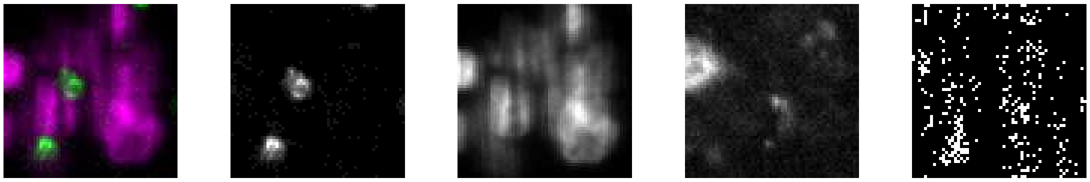				
24826	146	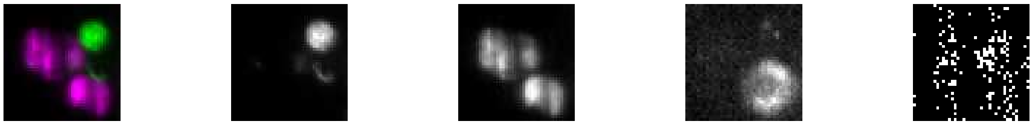				
24827	146	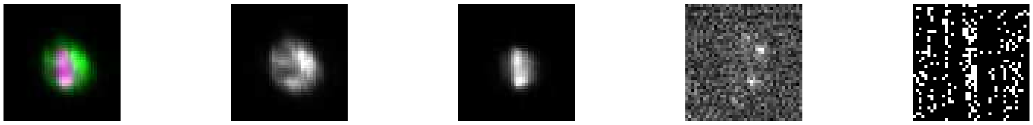				
24828	146	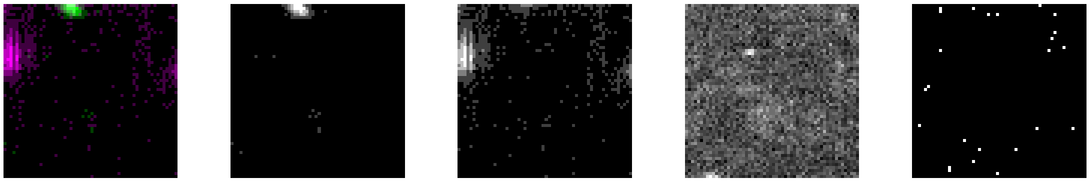				
24829	146	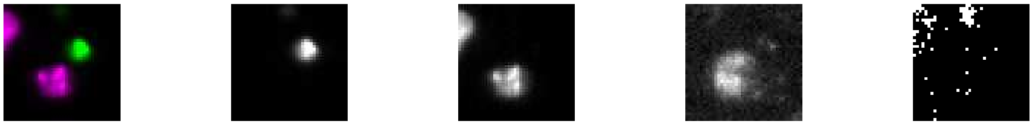				
24830	146	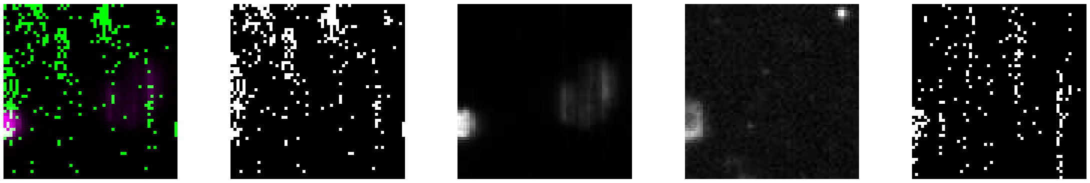				

Druckdatum: 02/06/2020 17:25
Proben-ID: Gyn14.208 **Kassetten-ID:** 01327106 **Patienten-ID:**

Ereignis	Aufnahme	DAPI/CK-PE	CK-PE	DAPI	CD45-APC	HER-2/neu
24831	146	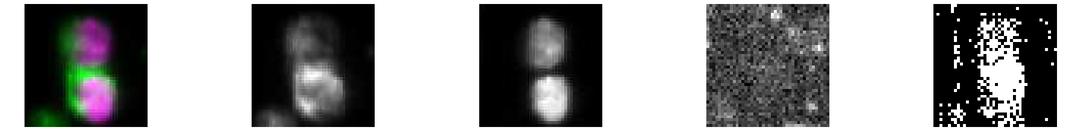				
24832	146	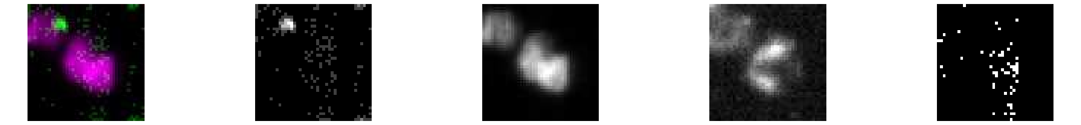				
24833	146	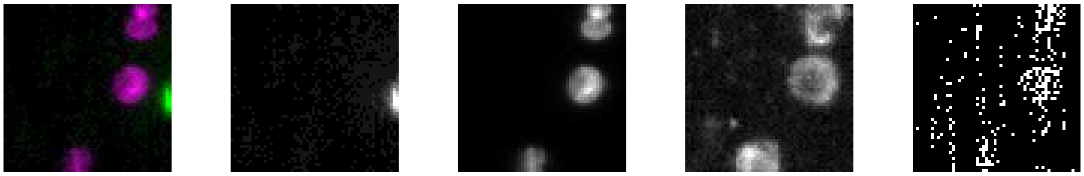				
24834	146	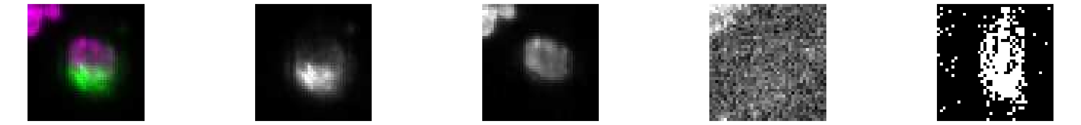				
24835	146	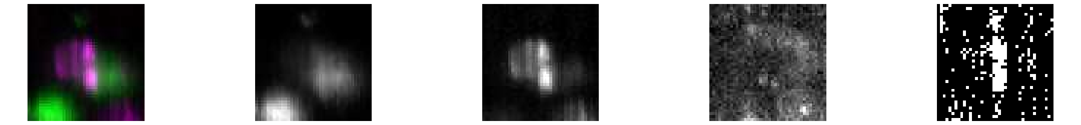				
24836	146	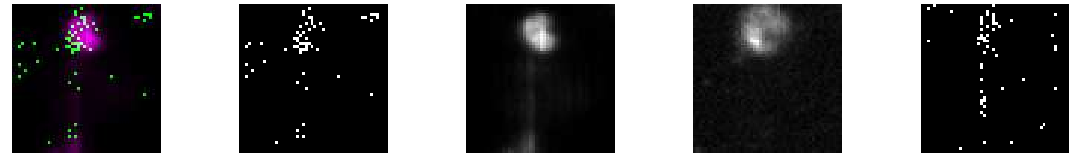				
24837	146	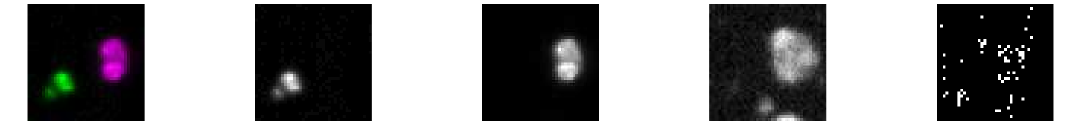				
24838	146	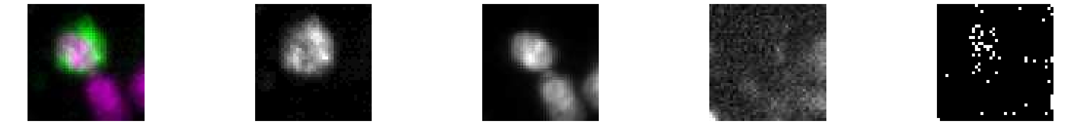				
24839	146	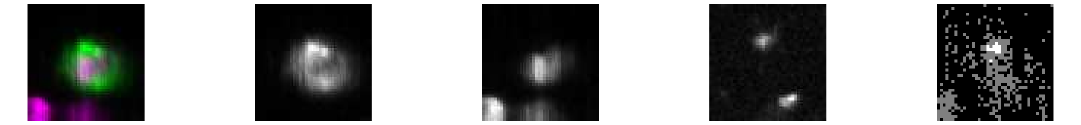				
24840	146	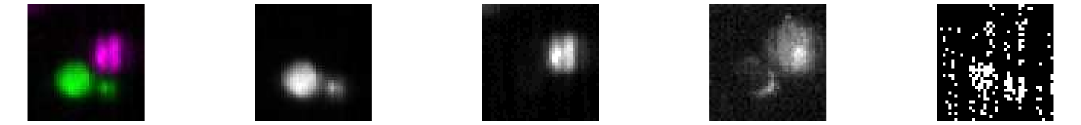				
24841	146	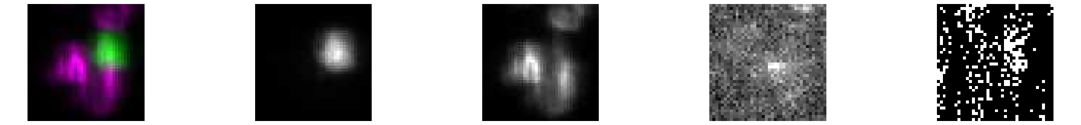				
24842	146	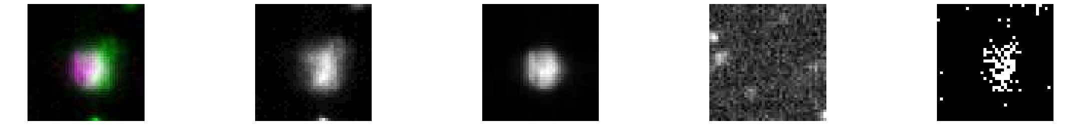				
24843	146	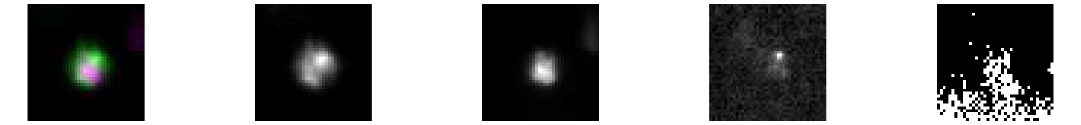				

Ereignis	Aufnahme	DAPI/CK-PE	CK-PE	DAPI	CD45-APC	HER-2/neu
24844	146	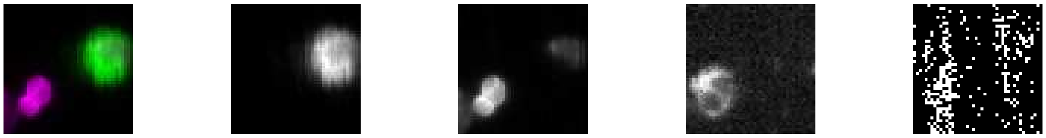				
24845	146	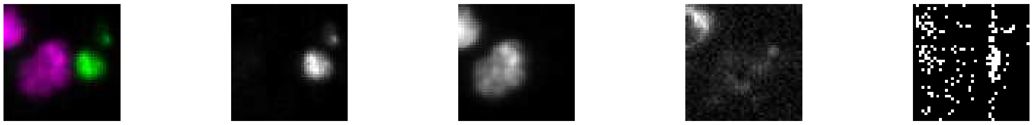				
24846	146	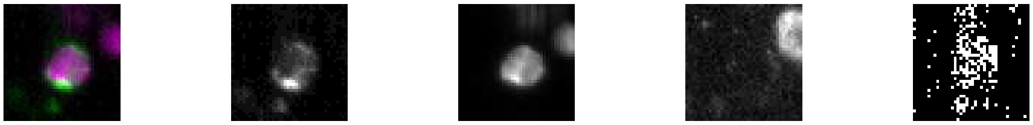				
24847	146	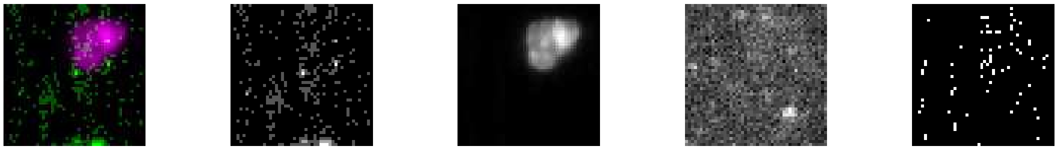				
24848	135	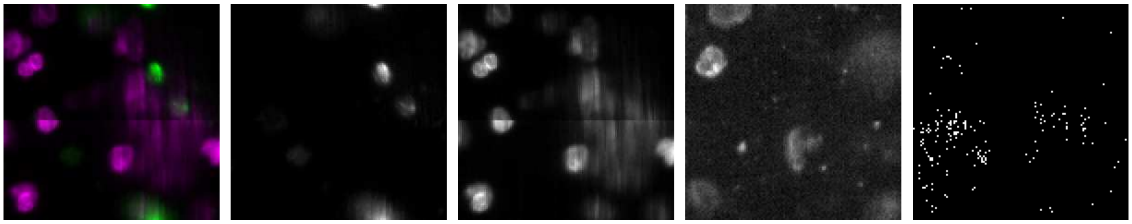				
24849	135	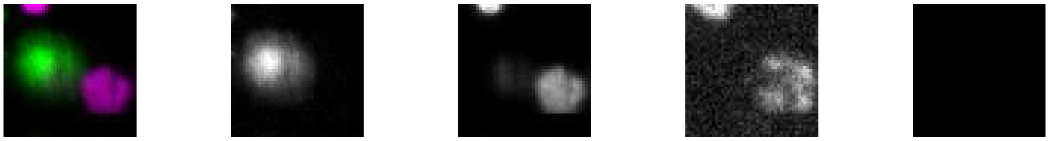				
24850	146	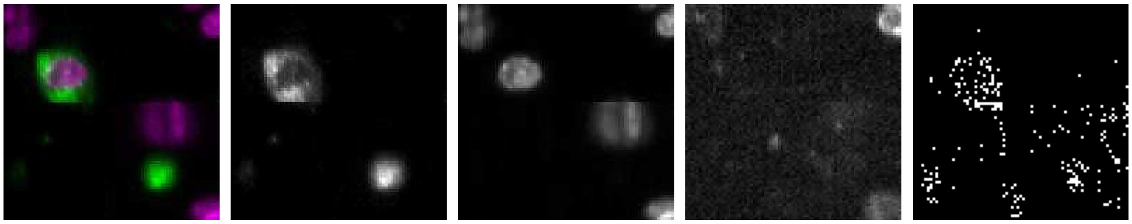				
24851	146	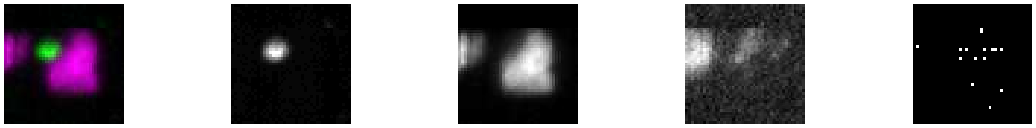				
24852	147	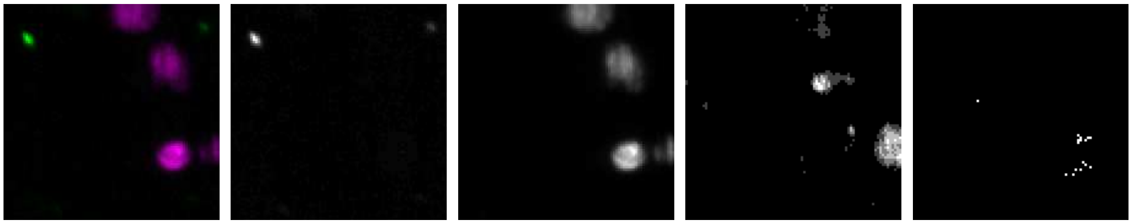				
24853	147	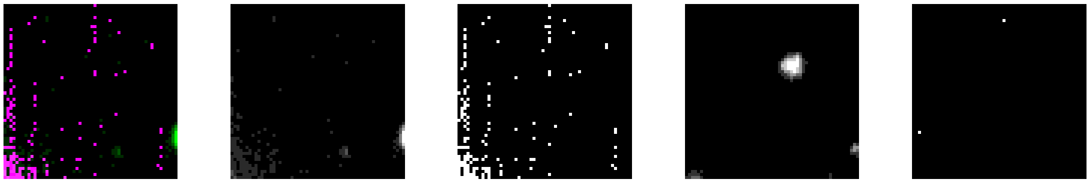				
24854	147	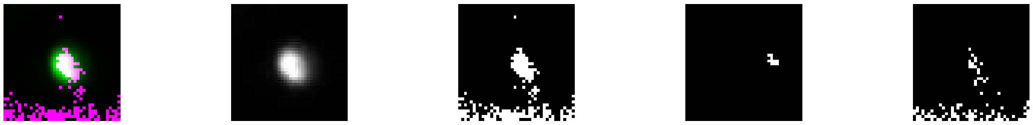				

Ereignis	Aufnahme	DAPI/CK-PE	CK-PE	DAPI	CD45-APC	HER-2/neu
24855	147	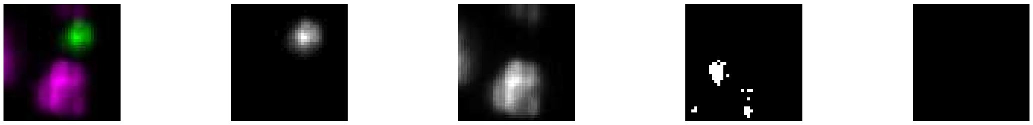				
24856	147	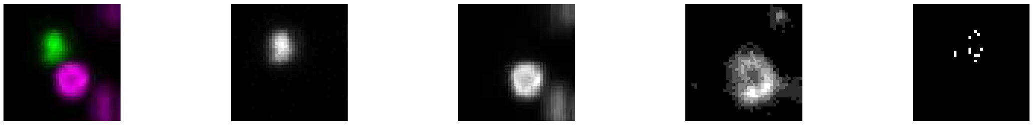				
24857	147	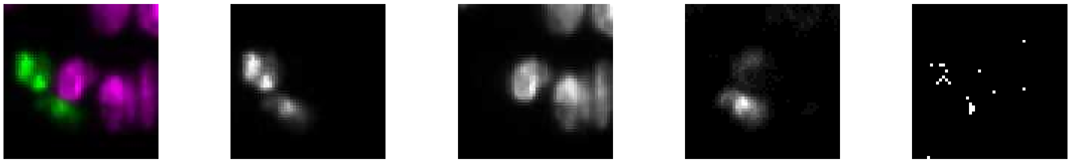				
24858	147	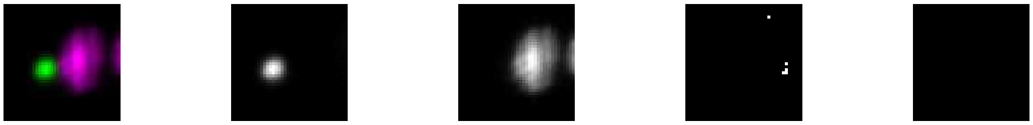				
24859	147	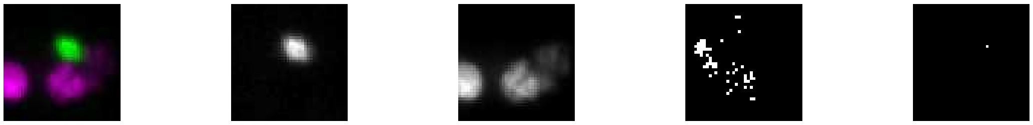				
24860	147	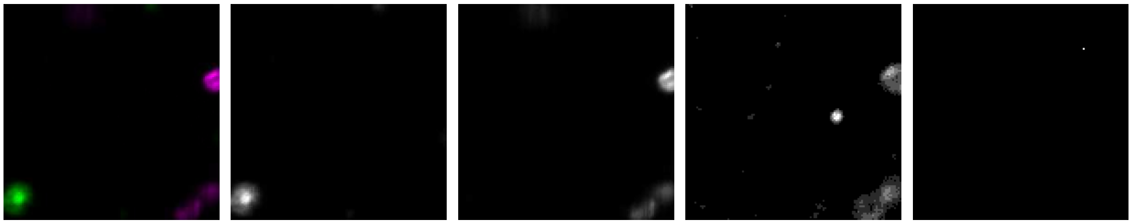				
24861	147	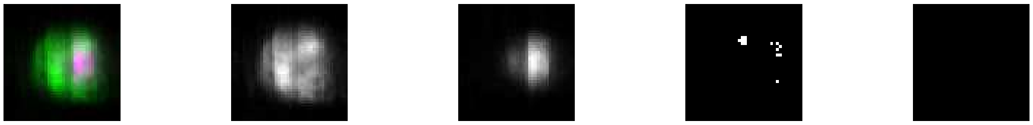				
24862	147	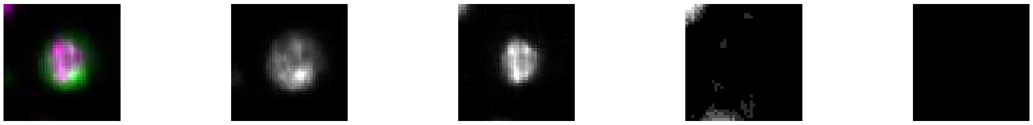				
24863	147	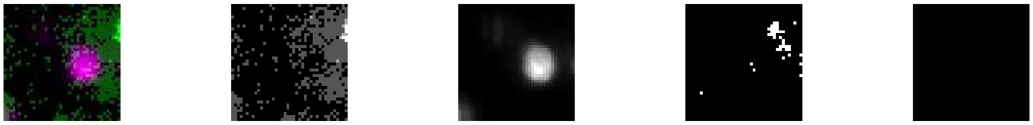				
24864	147	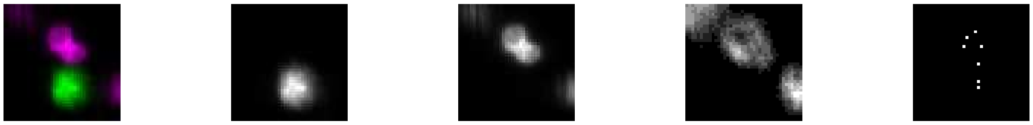				
24865	147	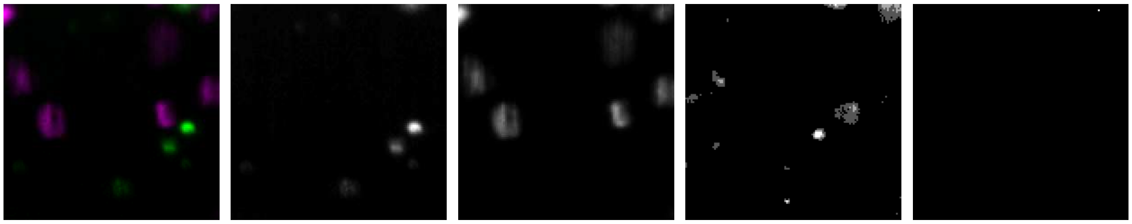				
24866	147	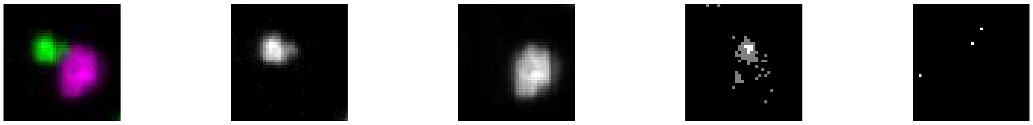				

Ereignis	Aufnahme	DAPI/CK-PE	CK-PE	DAPI	CD45-APC	HER-2/neu
24867	147	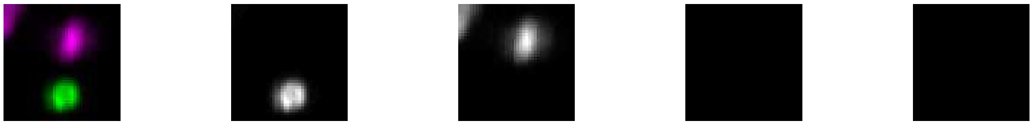				
24868	147	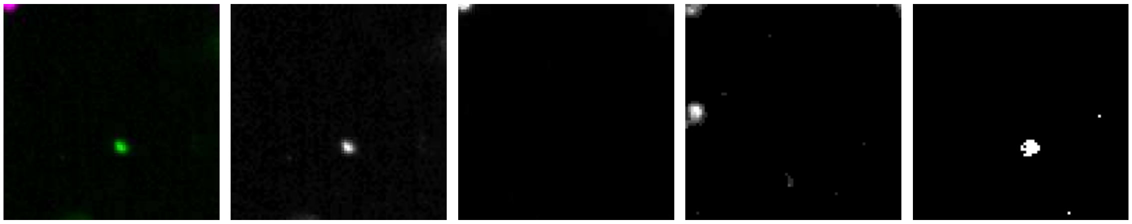				
24869	147	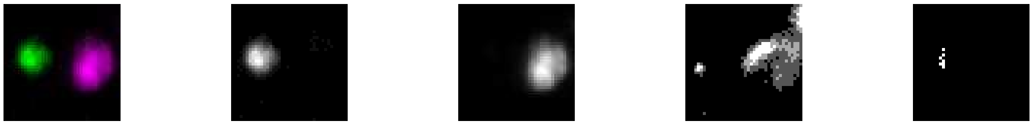				
24870	147	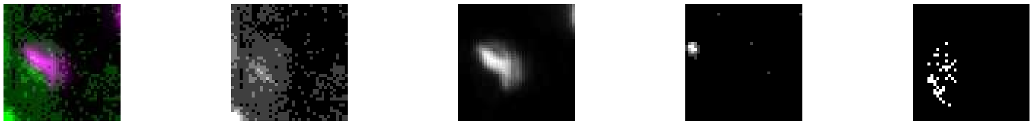				
24871	147	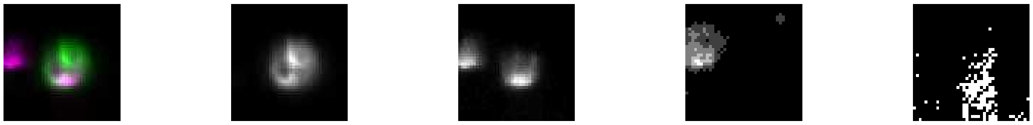				
24872	147	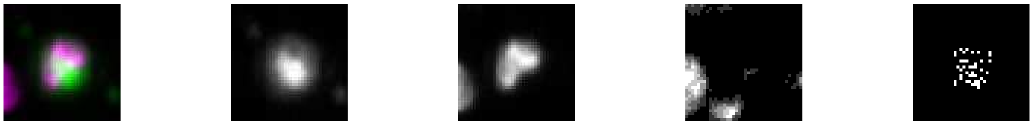				
24873	147	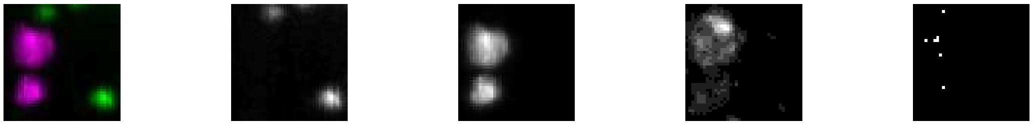				
24874	147	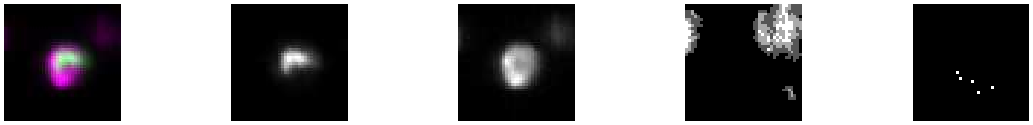				
24875	147	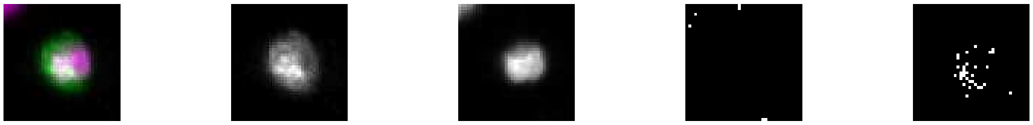				
24876	147	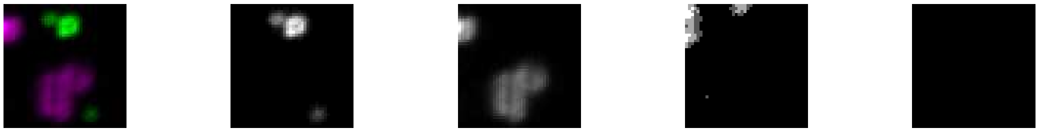				
24877	147	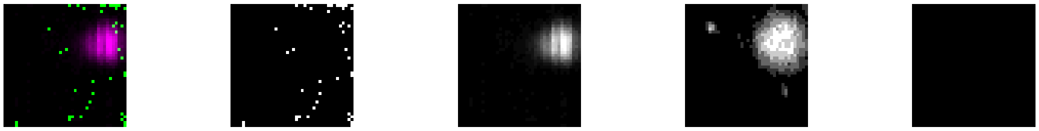				
24878	147	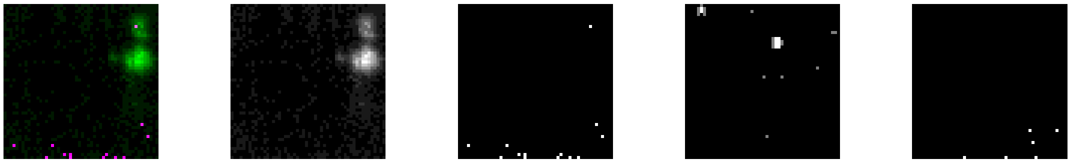				
24879	147	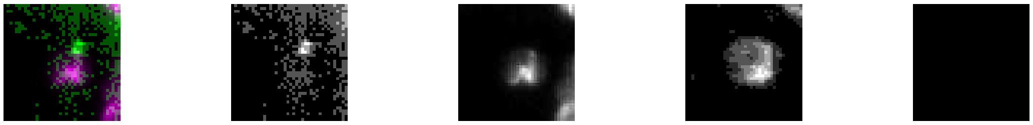				

Ereignis	Aufnahme	DAPI/CK-PE	CK-PE	DAPI	CD45-APC	HER-2/neu
24880	147	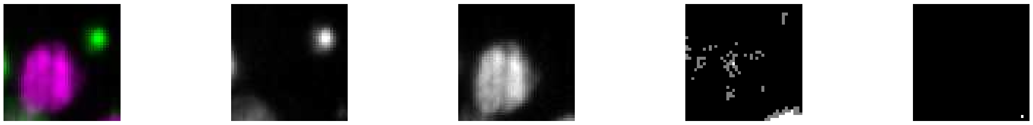				
24881	147	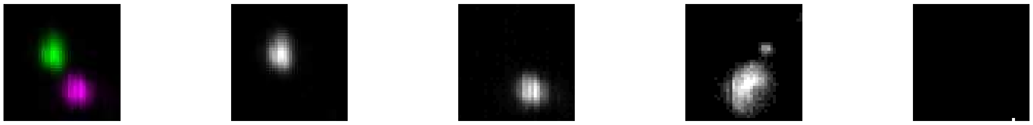				
24882	147	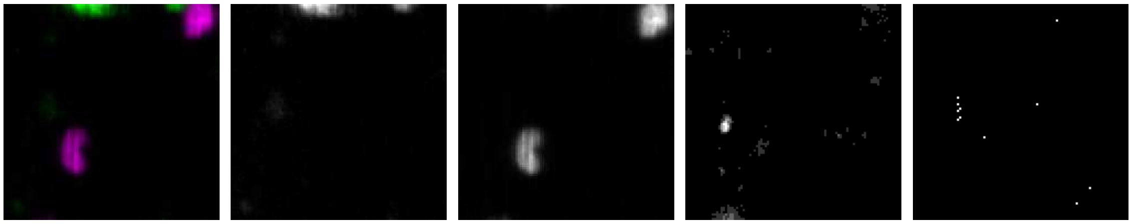				
24883	147	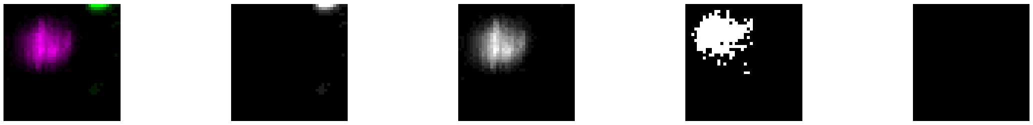				
24884	147	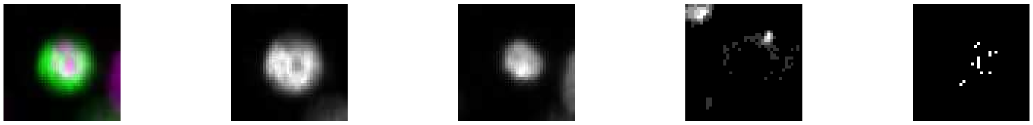				
24885	147	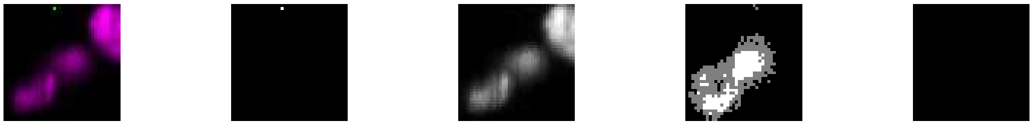				
24886	147	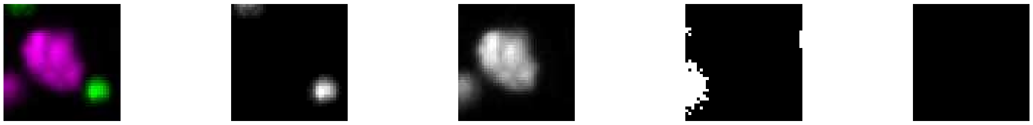				
24887	147	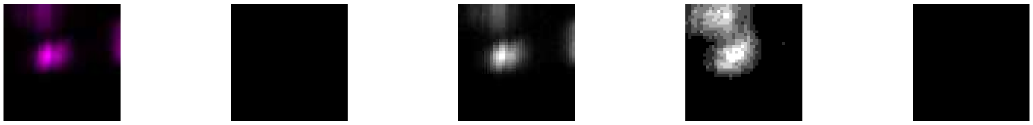				
24888	147	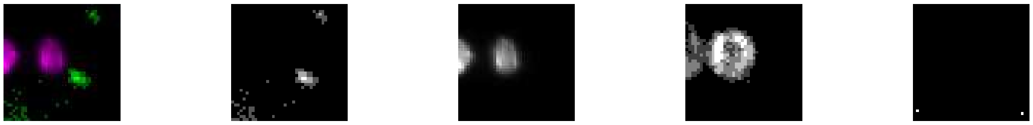				
24889	147	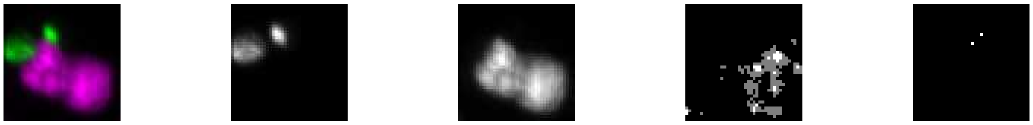				
24890	147	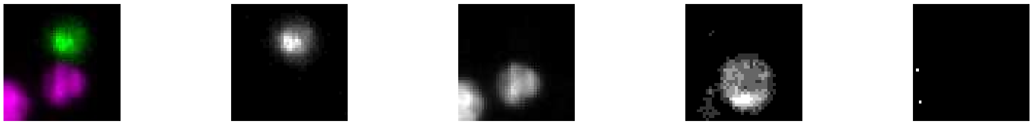				
24891	147	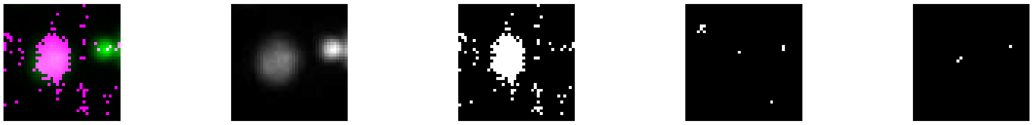				

Ereignis	Aufnahme	DAPI/CK-PE	CK-PE	DAPI	CD45-APC	HER-2/neu
24892	147	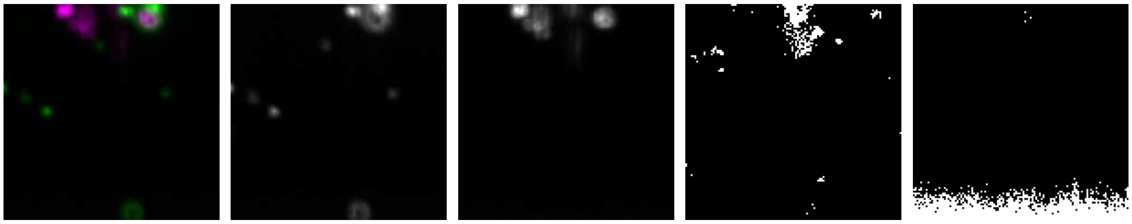				
24893	147	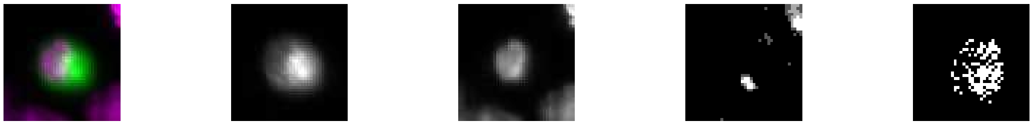				
24894	147	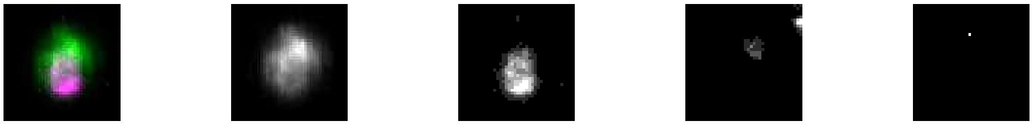				
24895	147	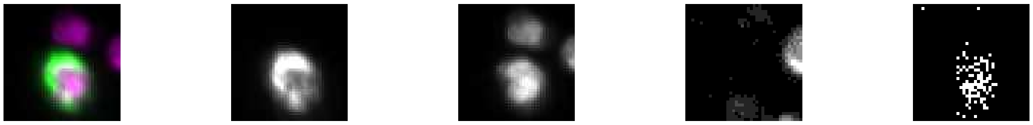				
24896	147	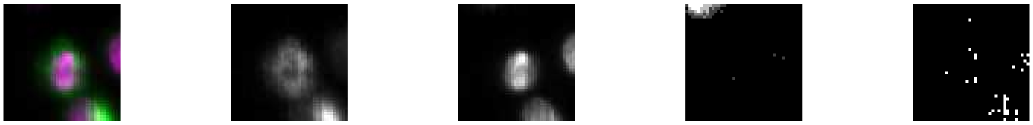				
24897	147	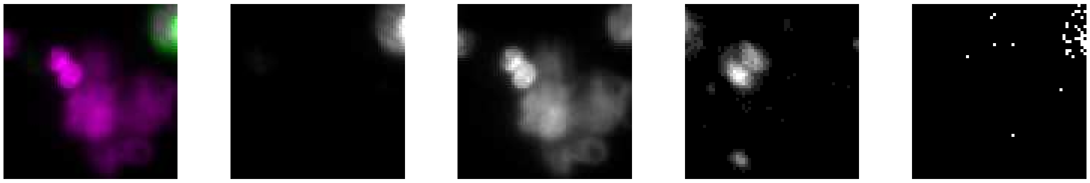				
24898	147	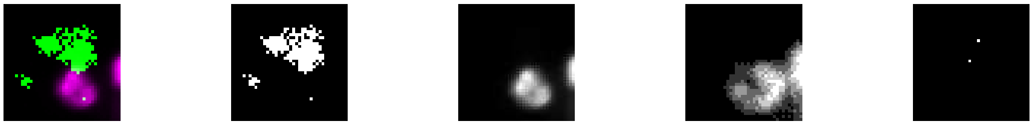				
24899	147	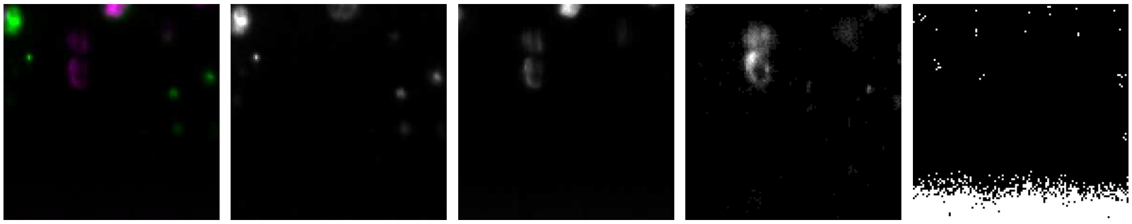				
24900	147	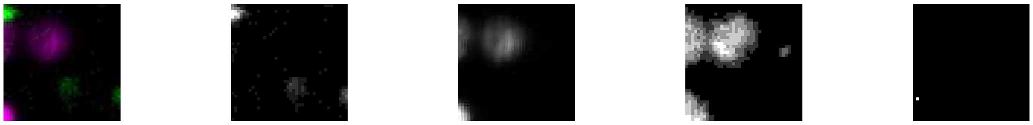				
24901	147	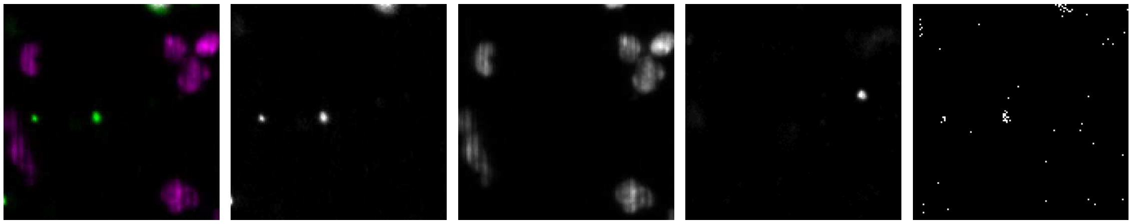				
24902	147	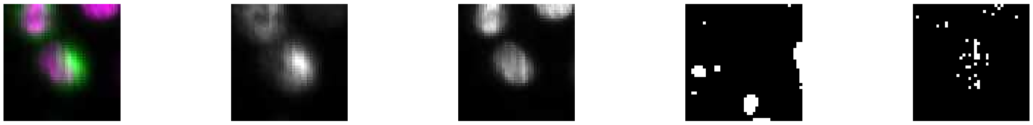				

Ereignis	Aufnahme	DAPI/CK-PE	CK-PE	DAPI	CD45-APC	HER-2/neu
24903	147	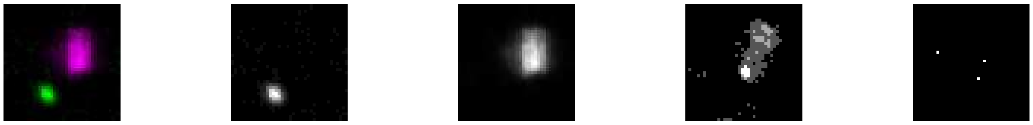				
24904	147	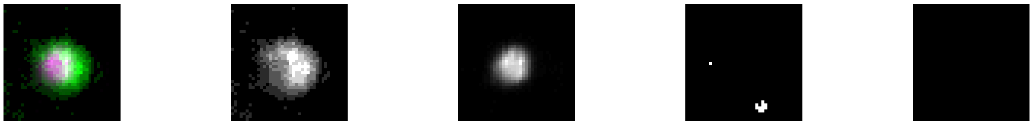				
24905	147	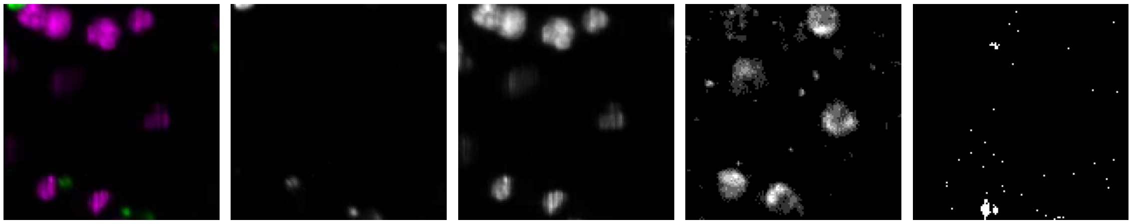				
24906	147	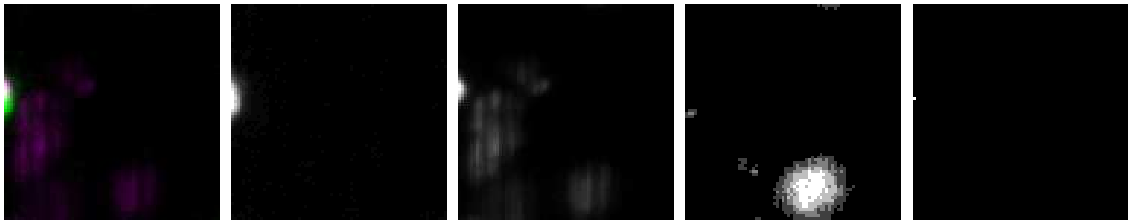				
24907	147	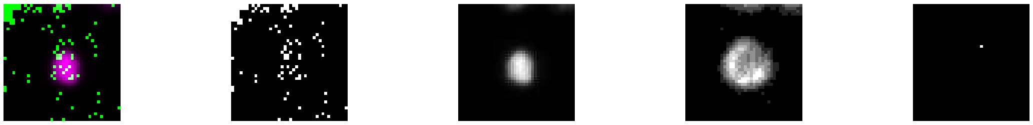				
24908	147	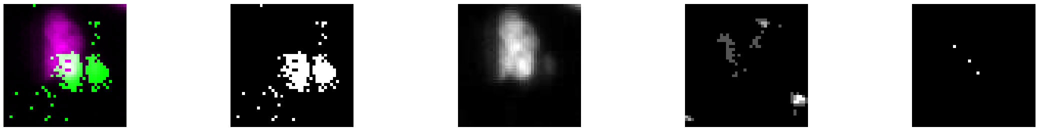				
24909	147	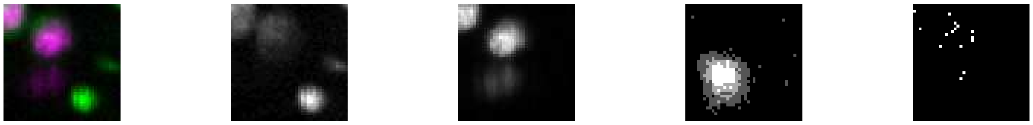				
24910	147	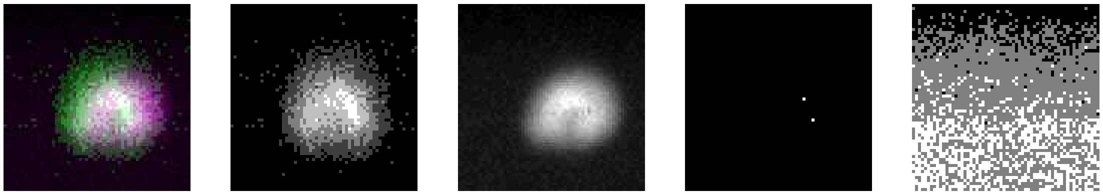				
24911	147	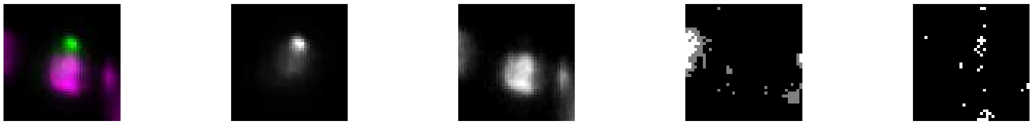				
24912	147	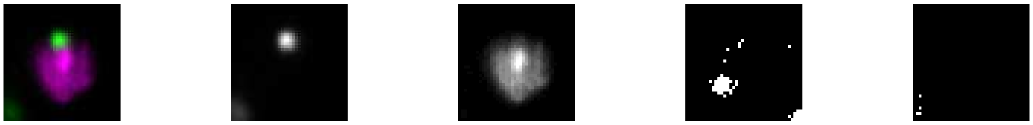				
24913	147	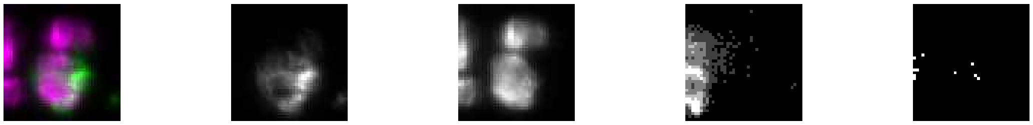				
24914	147	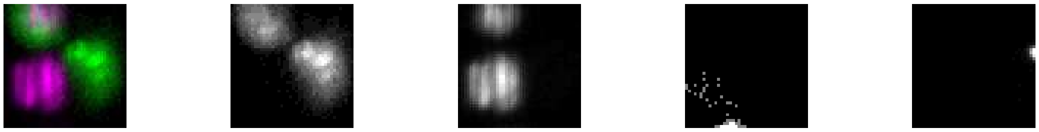				

Ereignis	Aufnahme	DAPI/CK-PE	CK-PE	DAPI	CD45-APC	HER-2/neu
24915	147	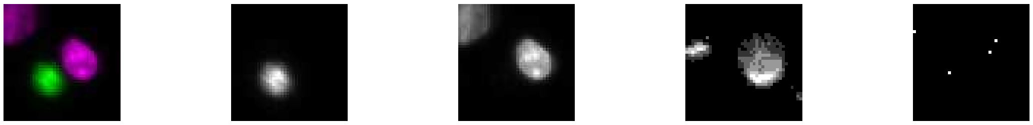				
24916	147	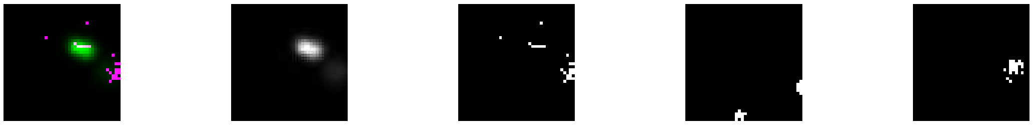				
24917	147	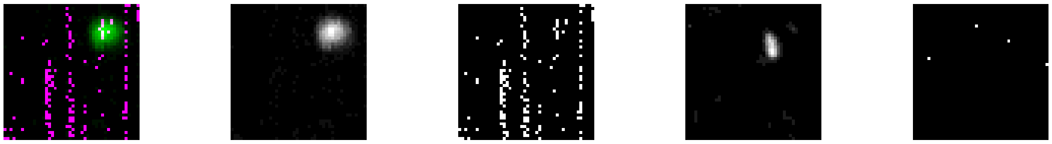				
24918	147	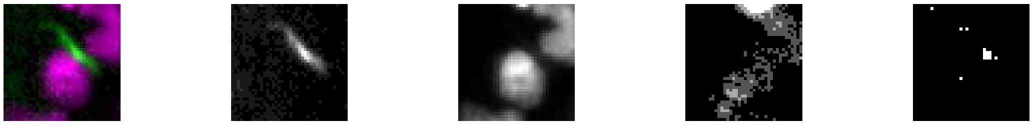				
24919	147	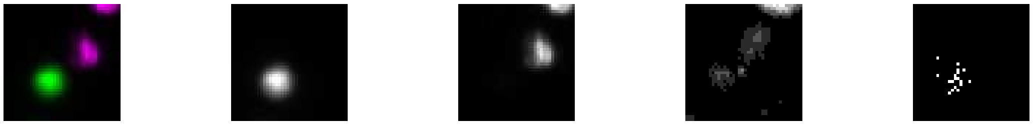				
24920	147	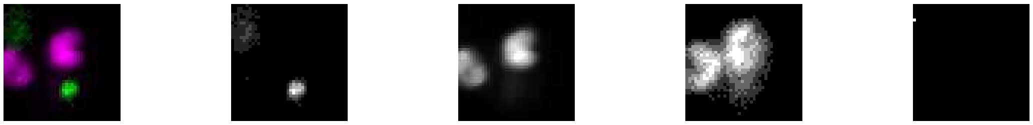				
24921	147	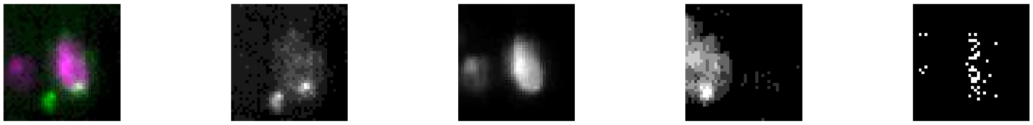				
24922	147	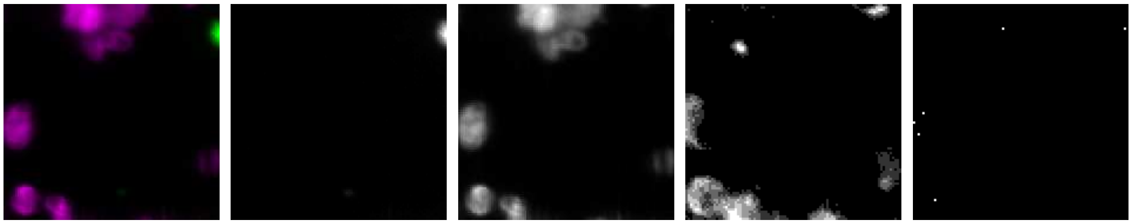				
24923	147	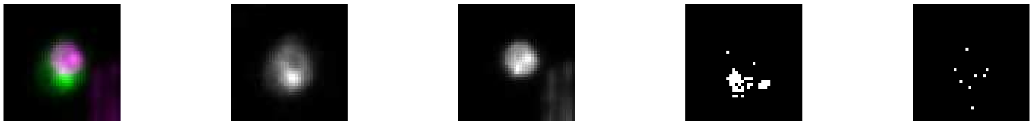				
24924	147	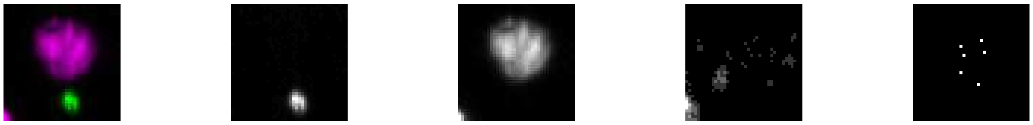				
24925	147	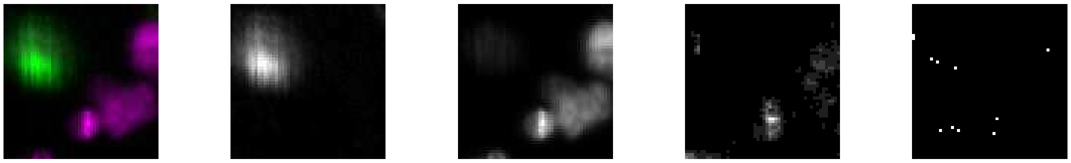				
24926	147	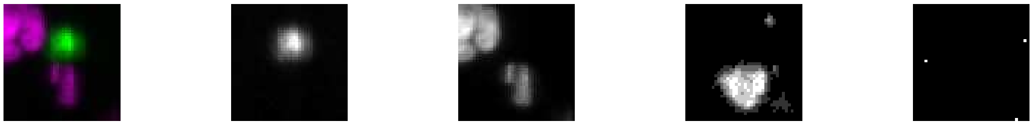				

Ereignis	Aufnahme	DAPI/CK-PE	CK-PE	DAPI	CD45-APC	HER-2/neu
24927	147	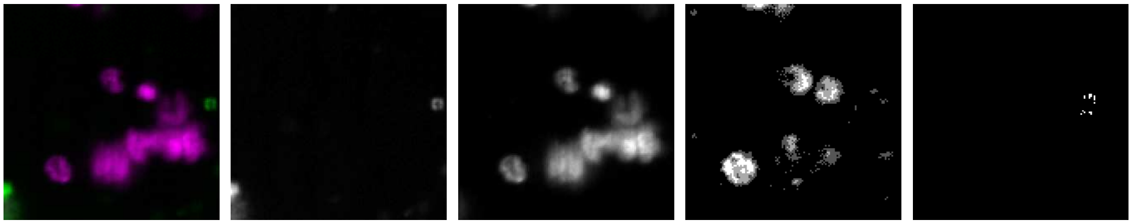				
24928	147	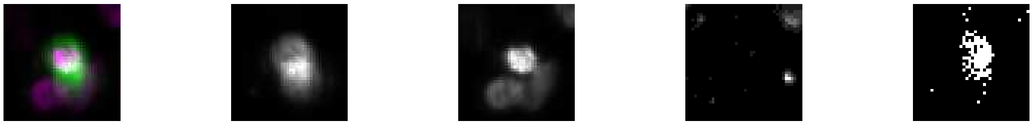				
24929	147	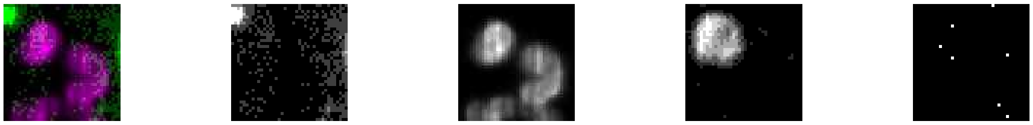				
24930	147	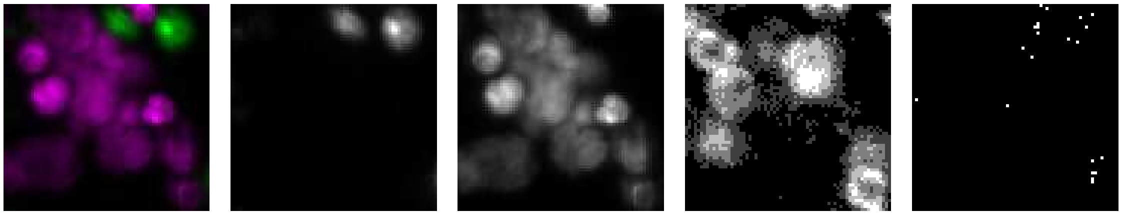				
24931	147	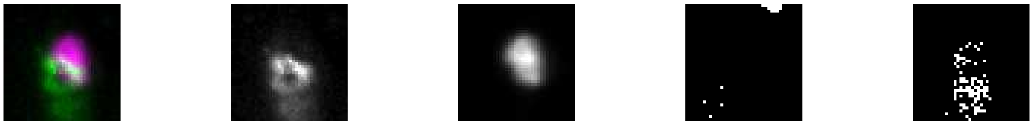				
24932	147	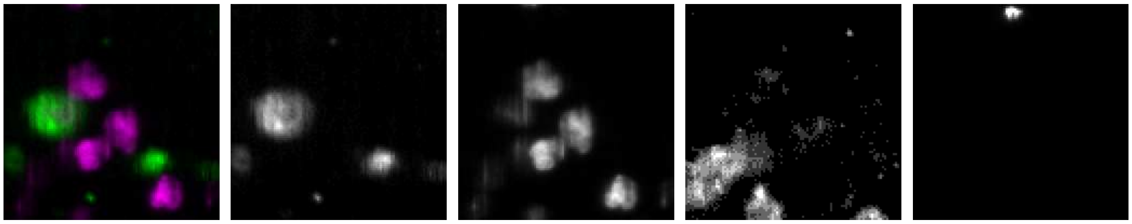				
24933	147	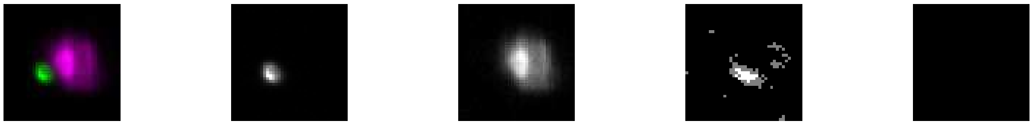				
24934	147	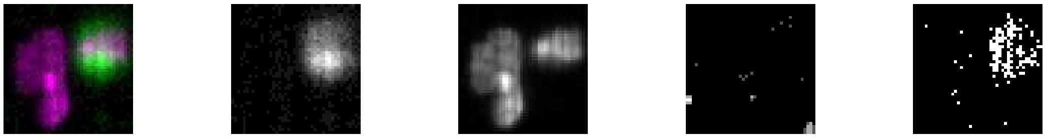				
24935	147	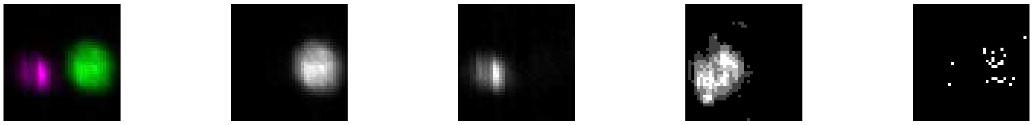				
24936	147	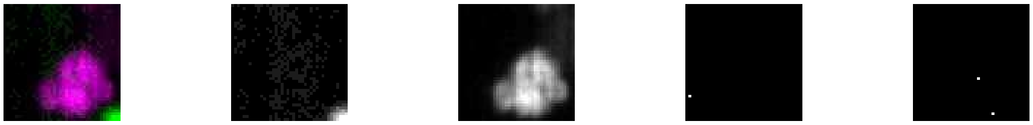				
24937	147	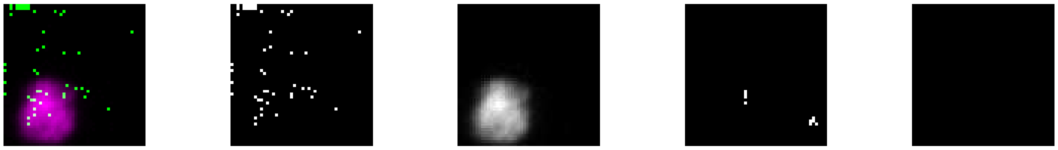				

Ereignis	Aufnahme	DAPI/CK-PE	CK-PE	DAPI	CD45-APC	HER-2/neu
24938	147	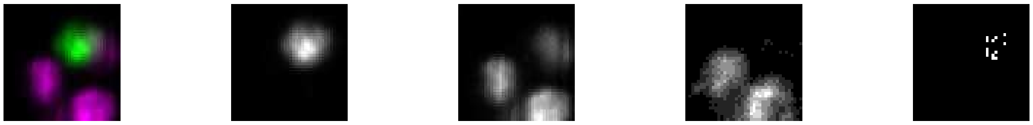				
24939	147	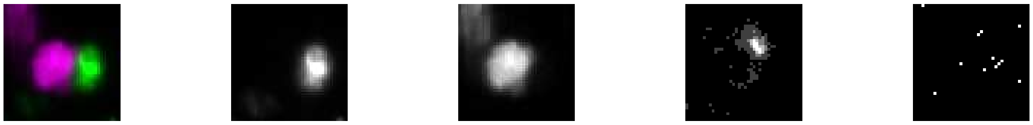				
24940	147	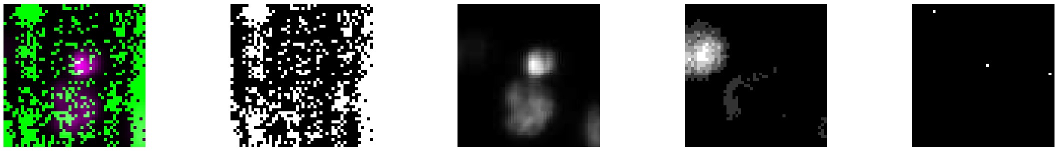				
24941	147	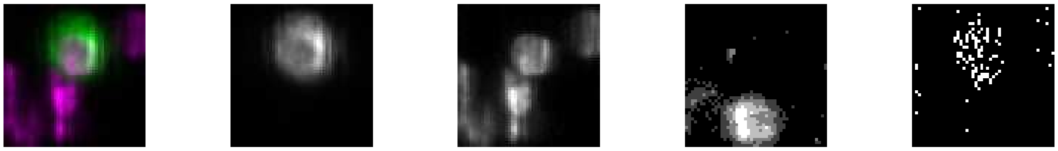				
24942	147	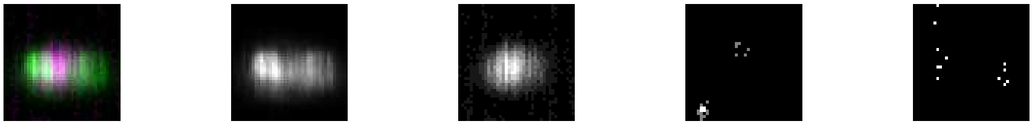				
24943	147	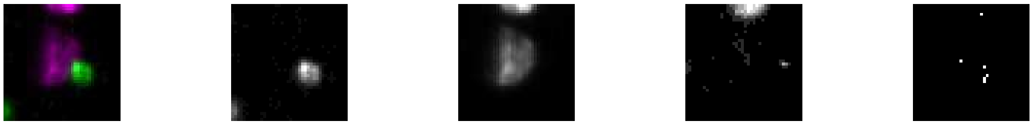				
24944	147	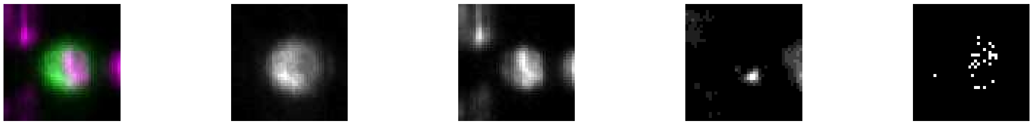				
24945	147	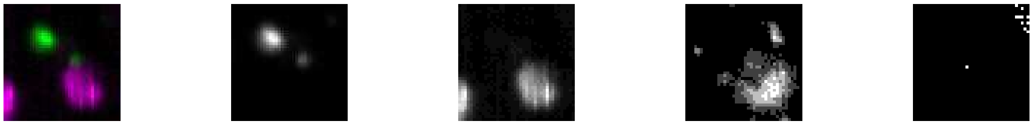				
24946	147	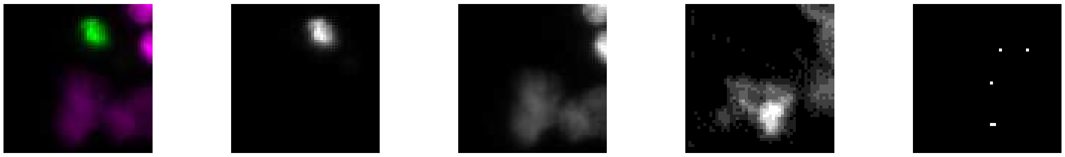				
24947	147	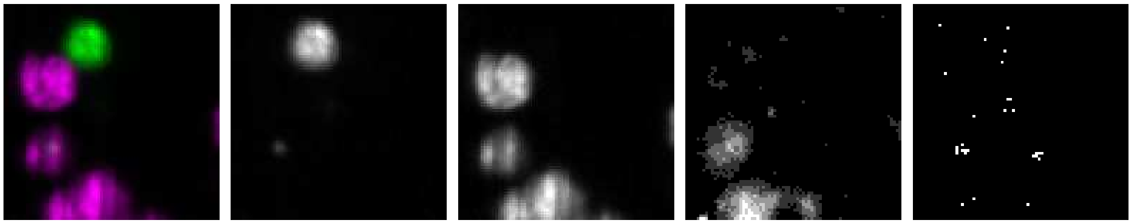				
24948	147	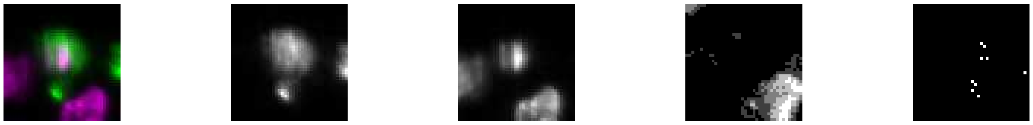				
24949	147	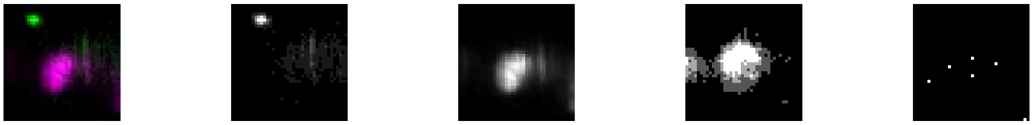				
24950	147	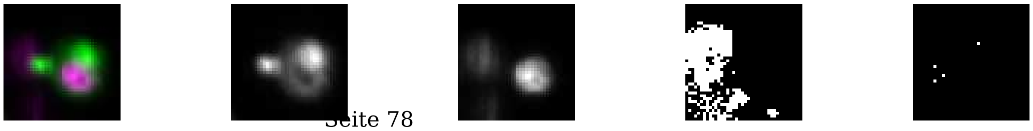				

Ereignis	Aufnahme	DAPI/CK-PE	CK-PE	DAPI	CD45-APC	HER-2/neu
24951	147	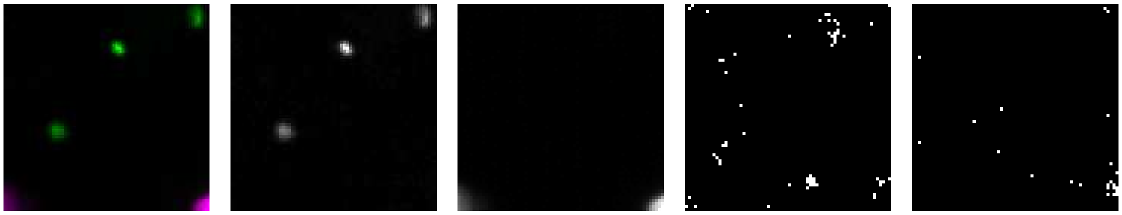				
24952	147	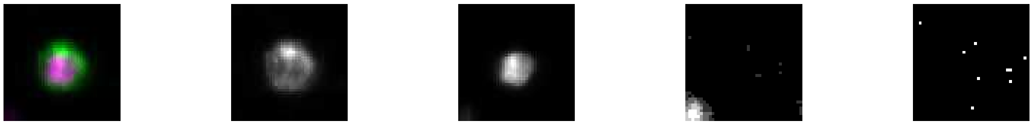				
24953	147	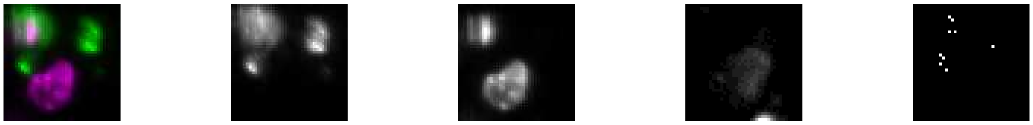				
24954	147	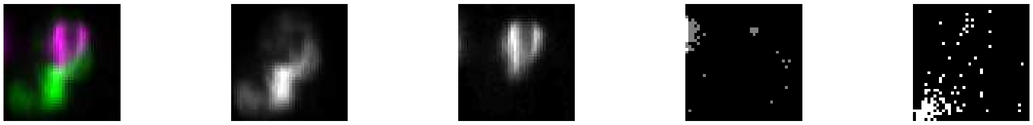				
24955	147	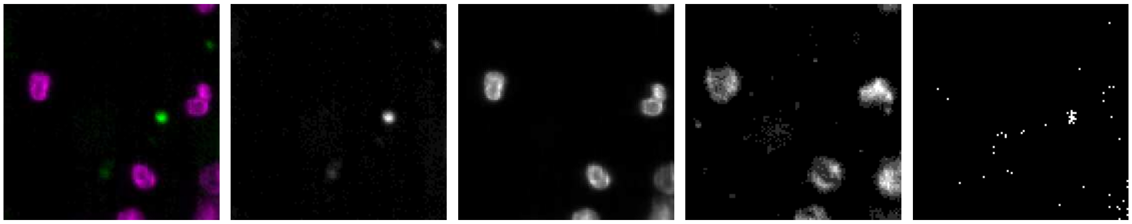				
24956	147	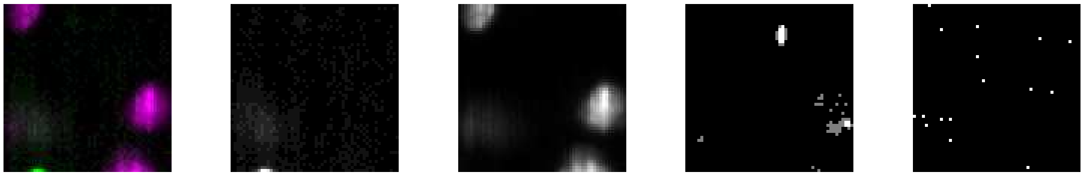				
24957	147	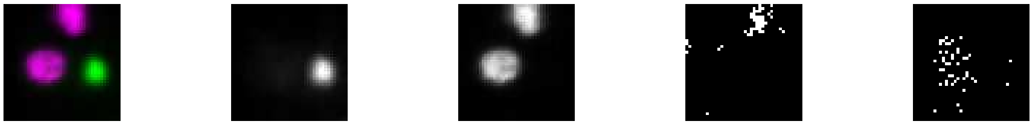				
24958	147	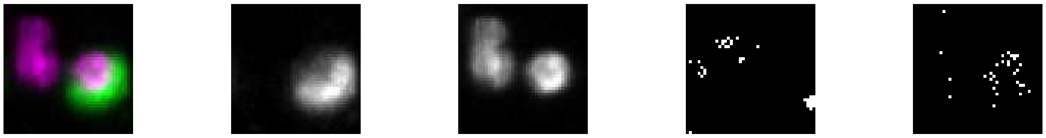				
24959	147	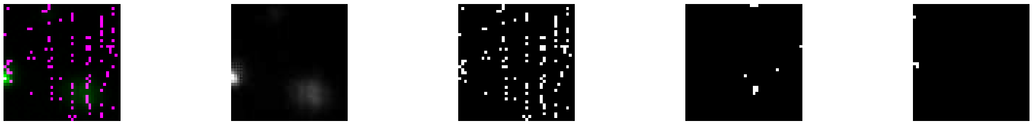				
24960	147	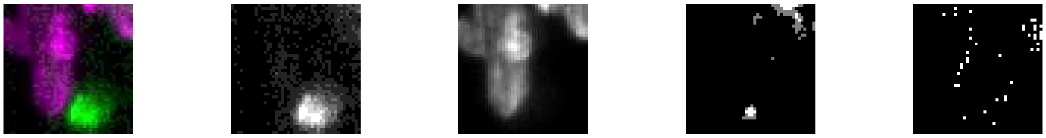				
24961	147	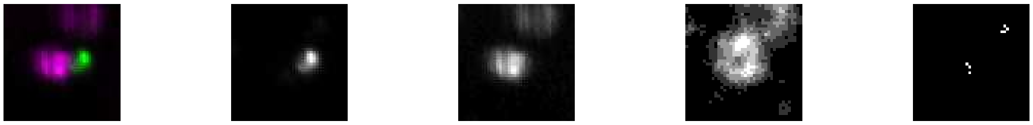				
24962	147	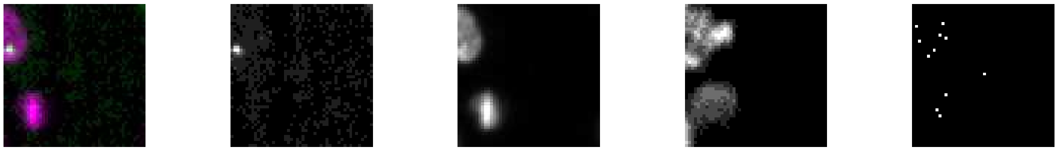				

Ereignis	Aufnahme	DAPI/CK-PE	CK-PE	DAPI	CD45-APC	HER-2/neu
24963	147	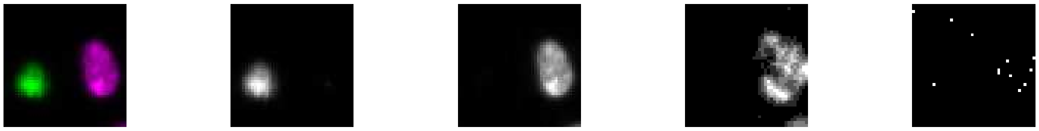				
24964	147	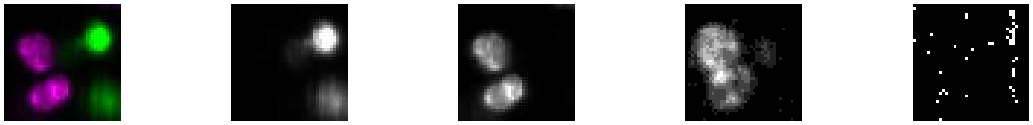				
24965	147	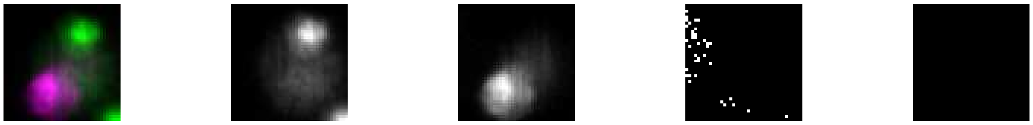				
24966	147	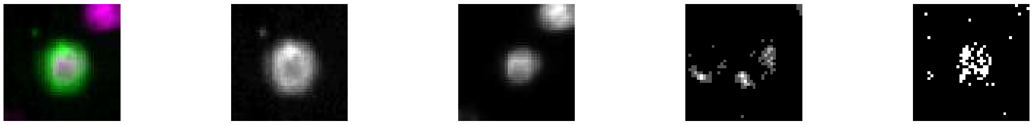				
24967	147	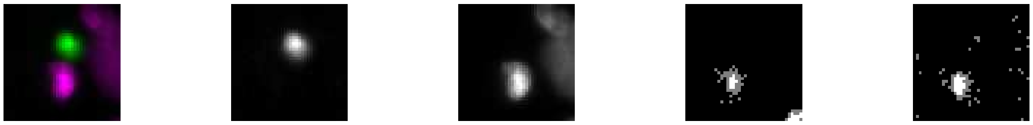				
24968	147	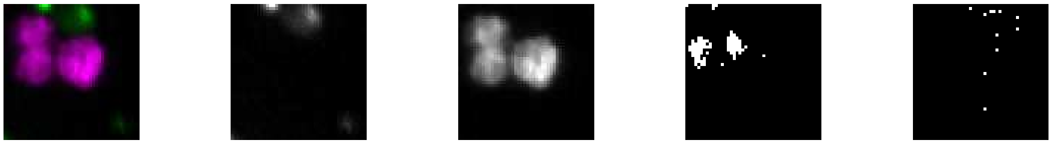				
24969	147	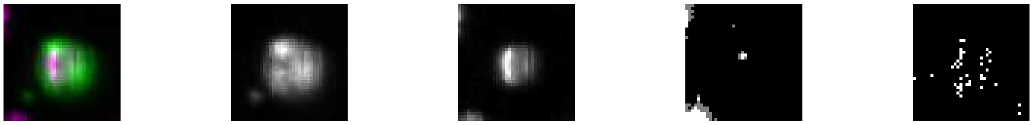				
24970	147	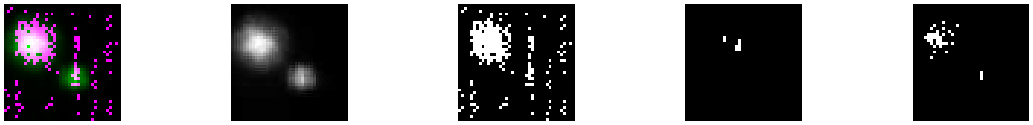				
24971	147	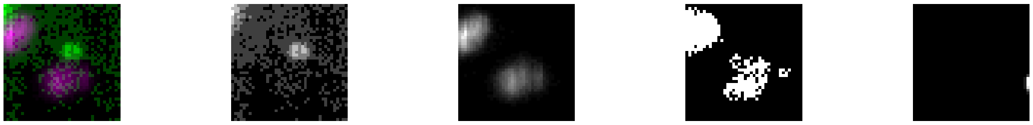				
24972	147	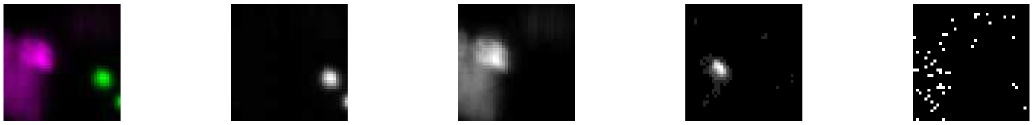				
24973	147	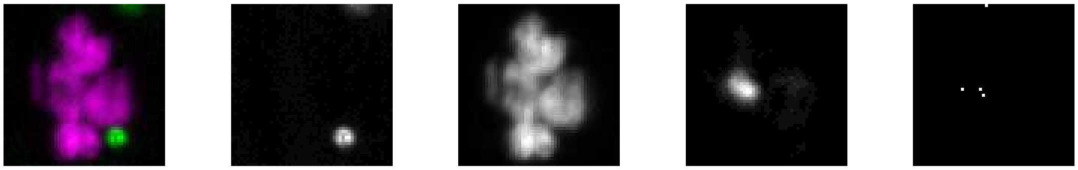				
24974	147	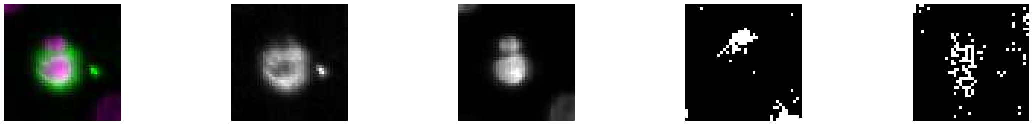				
24975	147	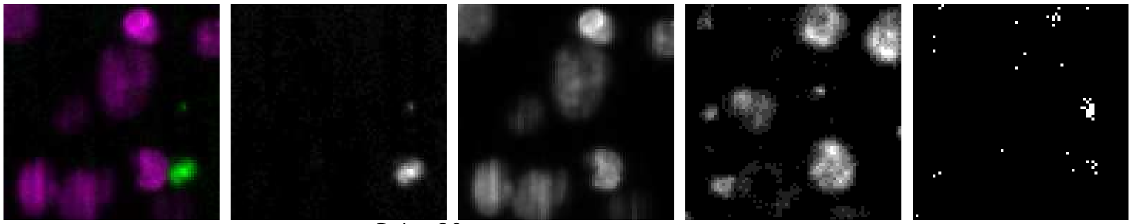				

Ereignis	Aufnahme	DAPI/CK-PE	CK-PE	DAPI	CD45-APC	HER-2/neu
24976	147	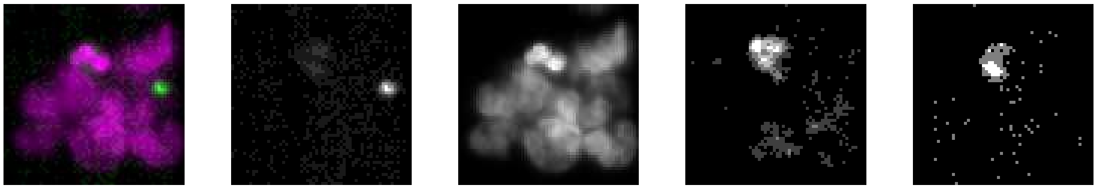				
24977	147	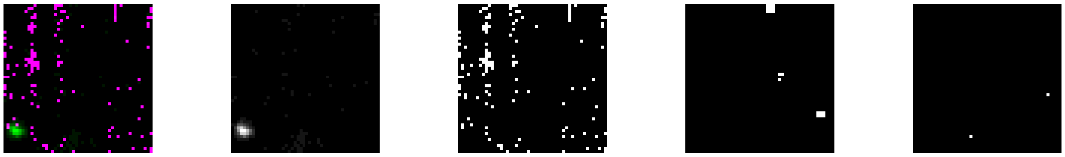				
24978	147	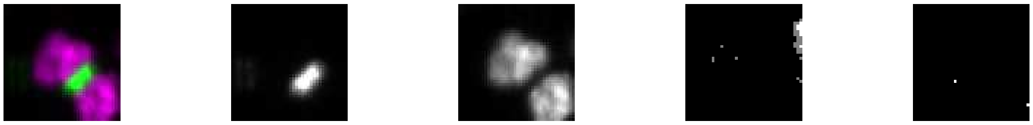				
24979	147	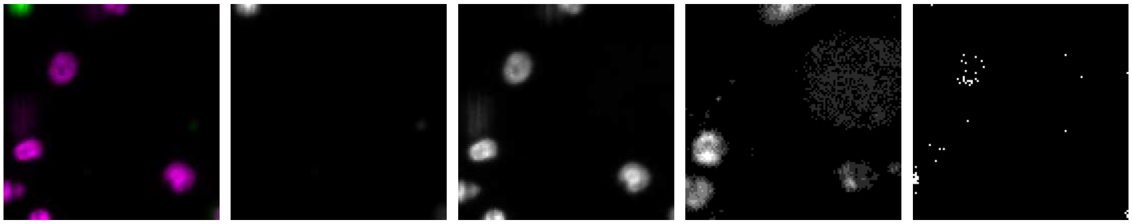				
24980	147	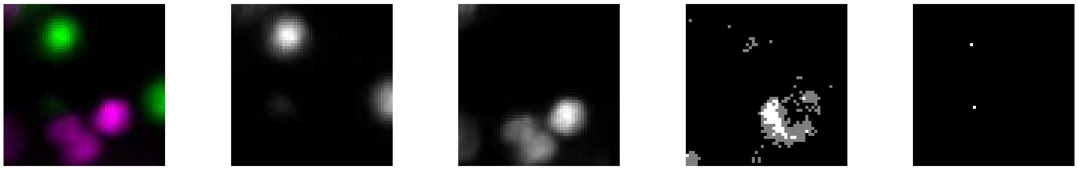				
24981	147	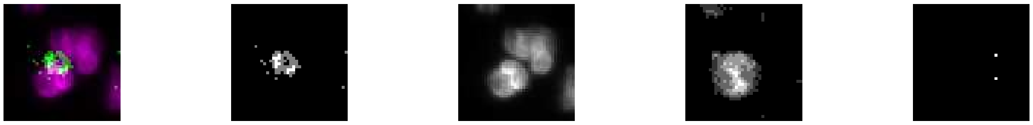				
24982	147	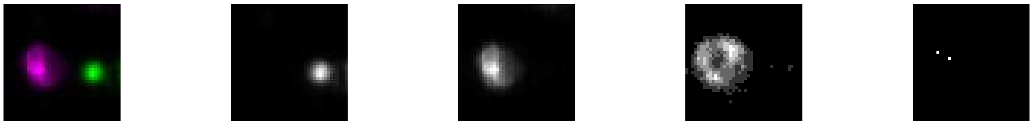				
24983	147	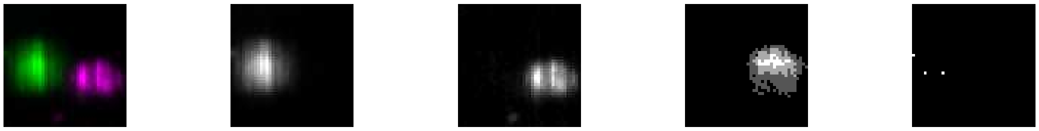				
24984	147	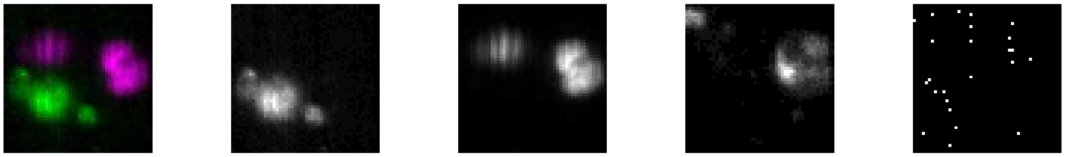				
24985	147	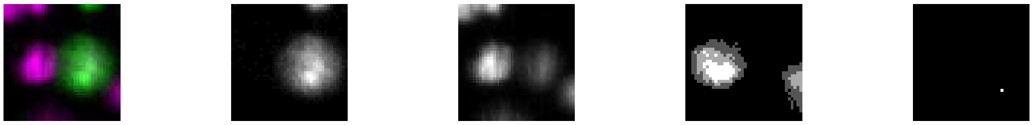				
24986	147	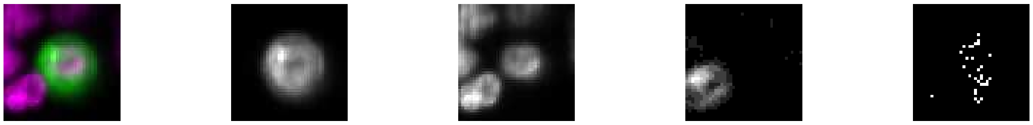				

Druckdatum:02/06/2020 17:25

Proben-ID: Gyn14.208 **Kassetten-ID:**01327106 **Patienten-ID:**

Ereignis	Aufnahme	DAPI/CK-PE	CK-PE	DAPI	CD45-APC	HER-2/neu
24987	147	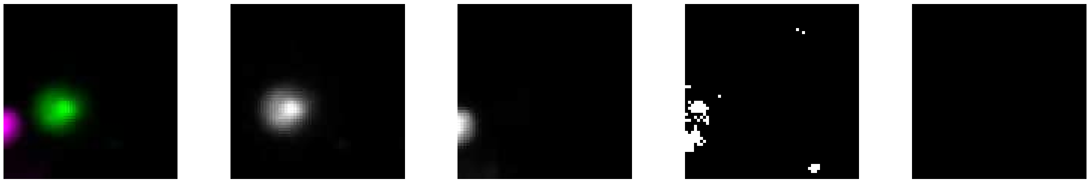				
24988	147	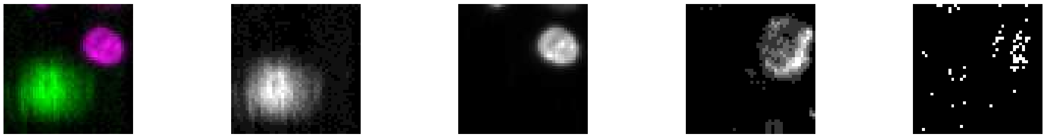				
24989	147	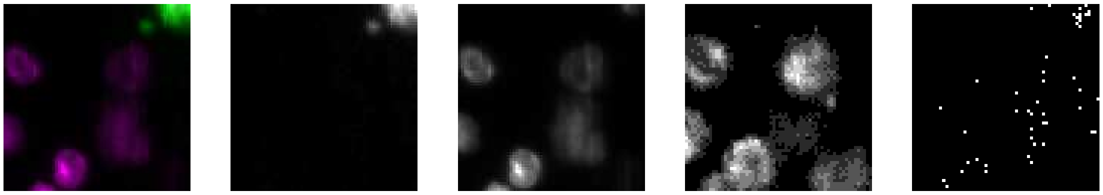				
24990	147	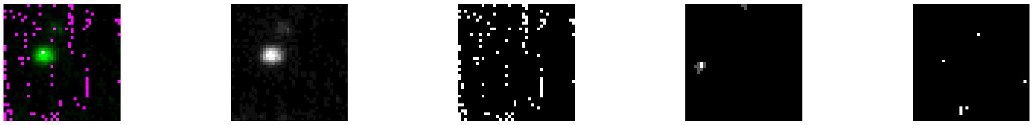				
24991	147	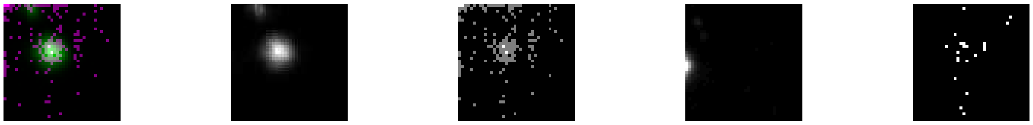				
24992	147	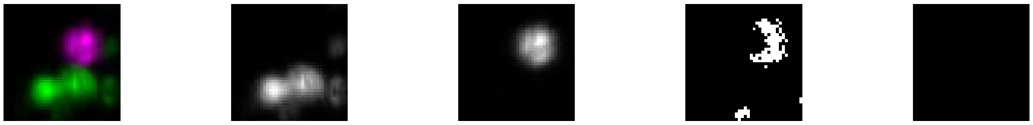				
24993	147	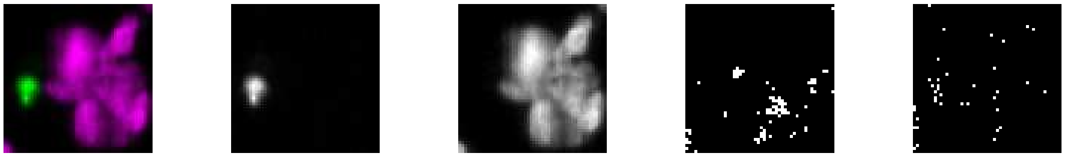				
24994	147	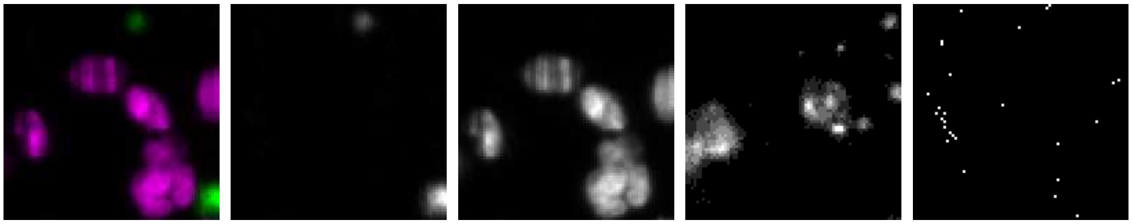				
24995	147	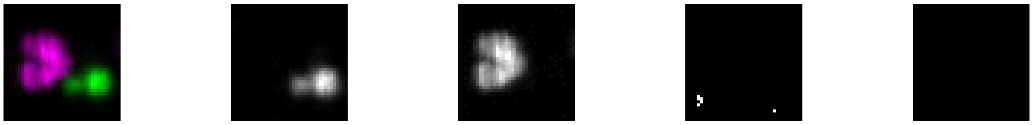				
24996	147	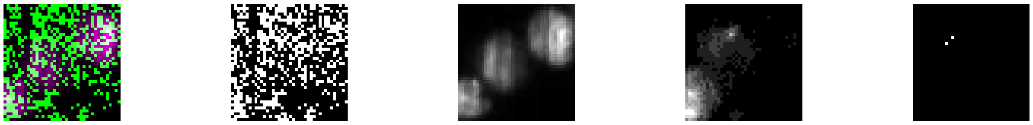				
24997	147	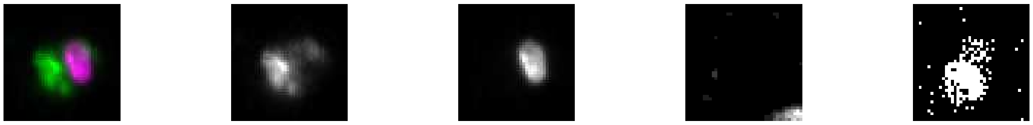				
24998	147	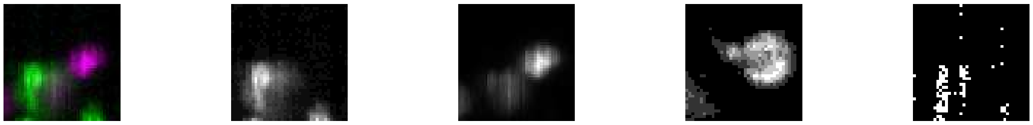				

Ereignis	Aufnahme	DAPI/CK-PE	CK-PE	DAPI	CD45-APC	HER-2/neu
24999	147	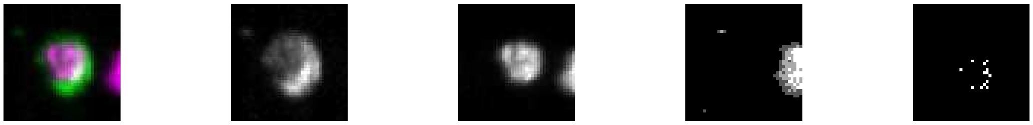				
25000	147	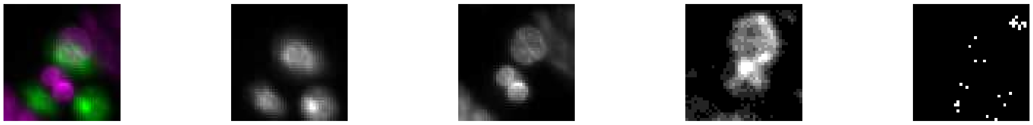				
25001	147	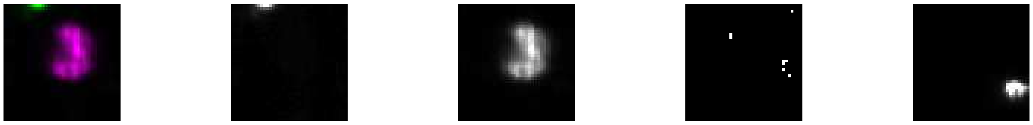				
25002	147	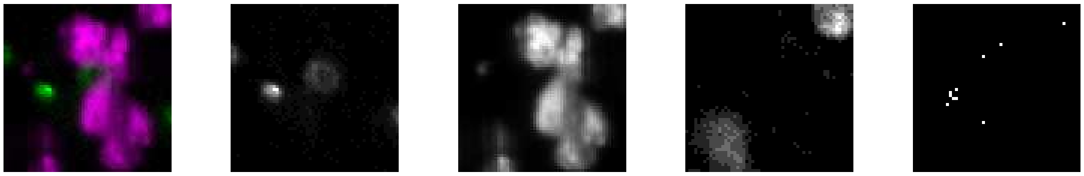				
25003	147	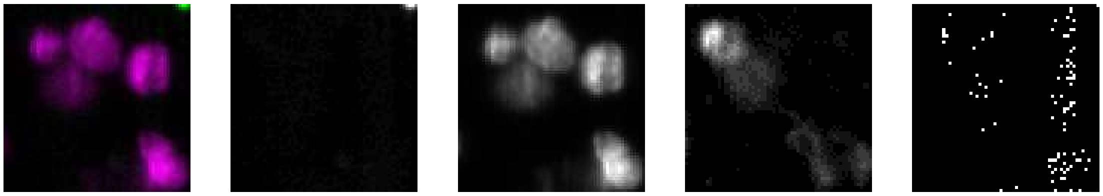				
25004	147	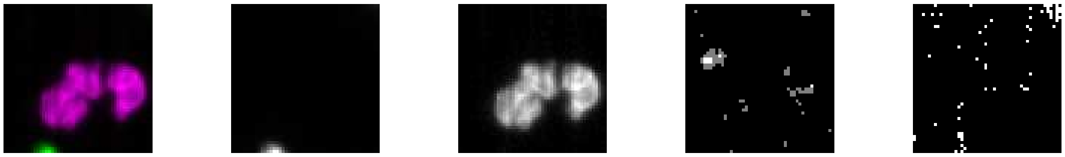				
25005	147	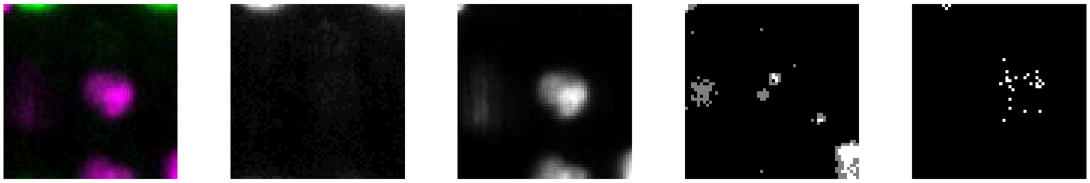				
25006	147	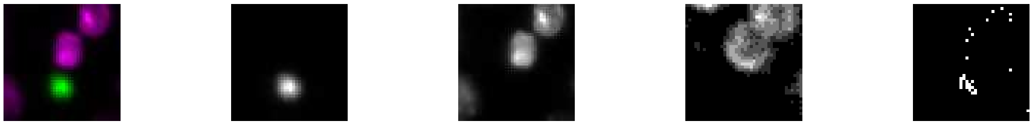				
25007	147	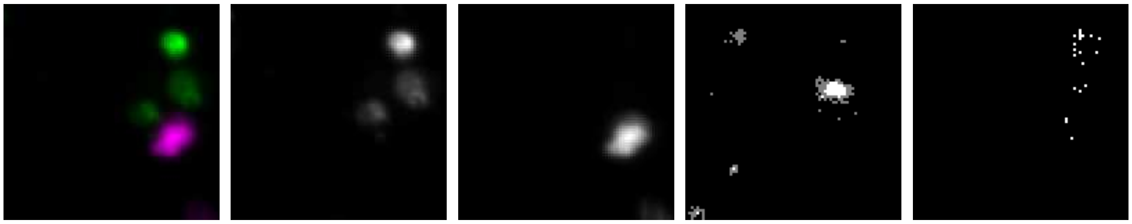				
25008	147	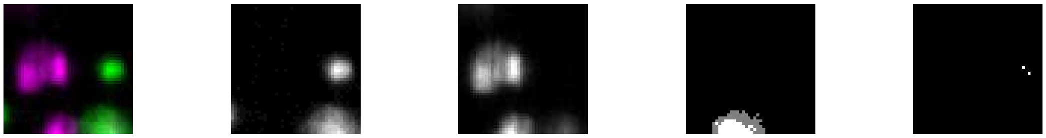				
25009	147	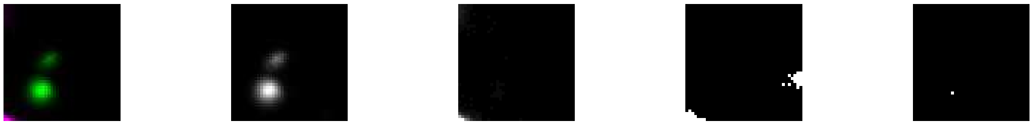				

Ereignis	Aufnahme	DAPI/CK-PE	CK-PE	DAPI	CD45-APC	HER-2/neu
25010	147	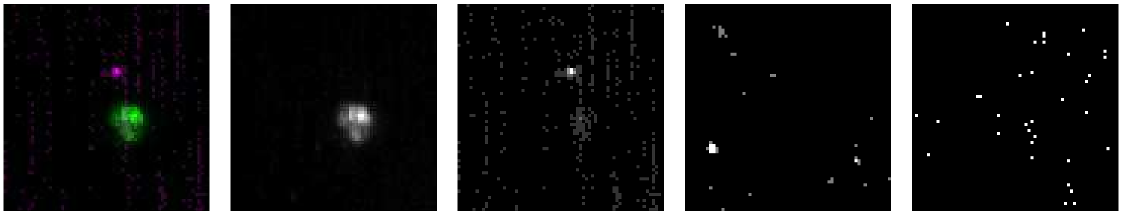				
25011	147	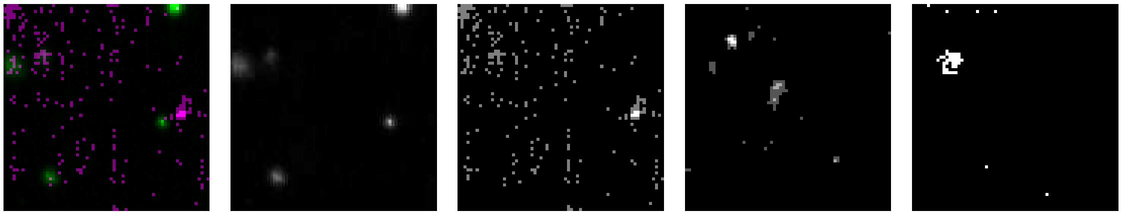				
25012	146	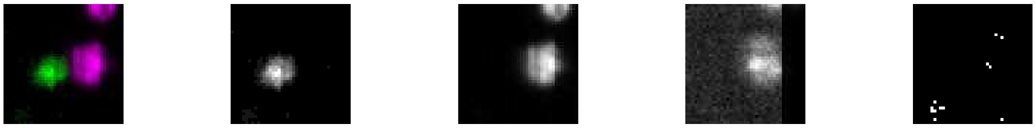				
25013	146	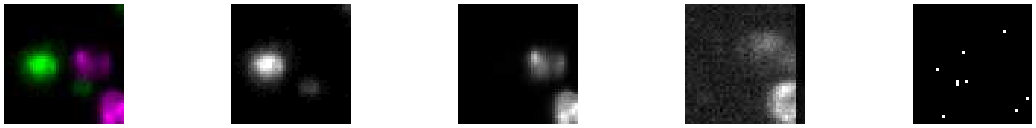				
25014	134	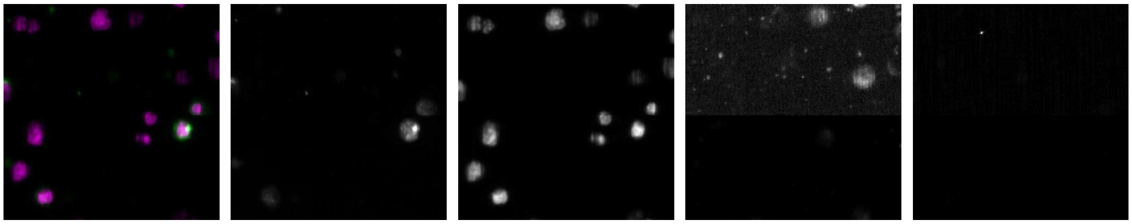				
25015	134	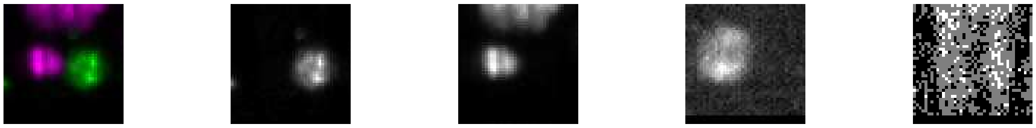				
25016	147	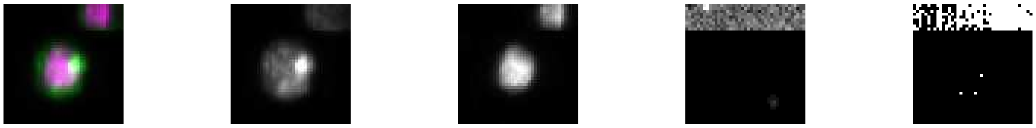				
25017	134	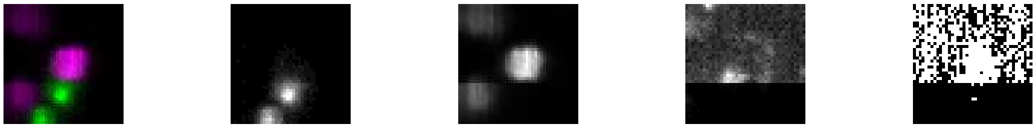				
25018	148	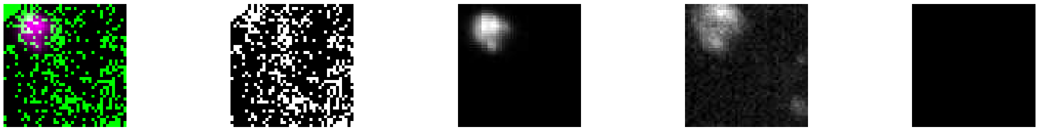				
25019	148	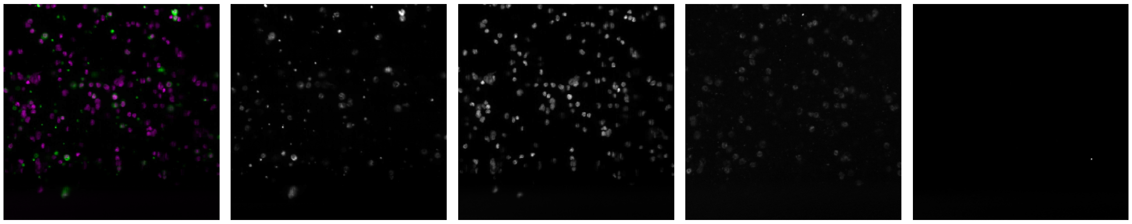				
25020	148	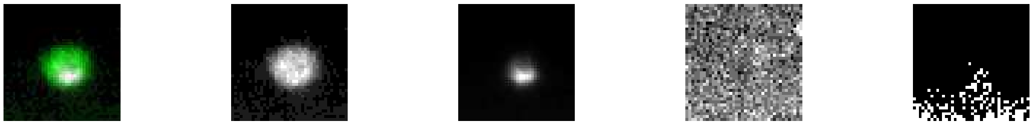				

Ereignis	Aufnahme	DAPI/CK-PE	CK-PE	DAPI	CD45-APC	HER-2/neu
25021	148	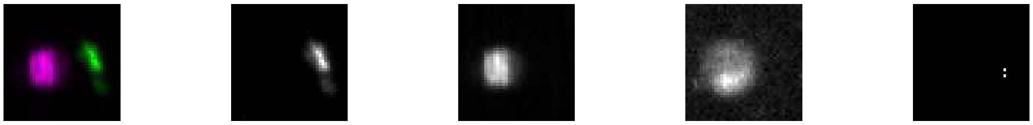				
25022	148	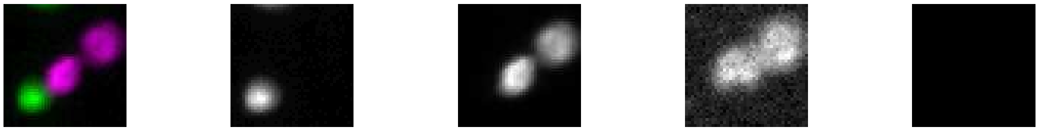				
25023	148	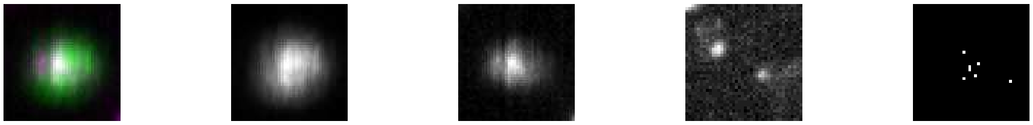				
25024	148	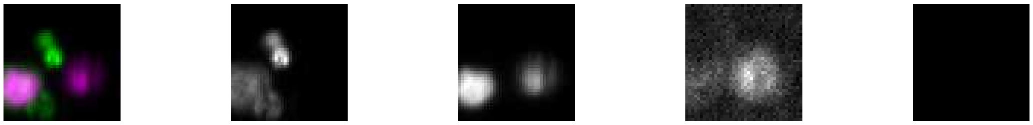				
25025	148	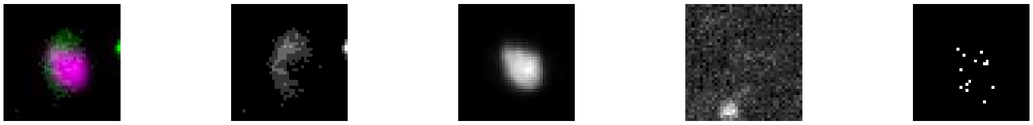				
25026	148	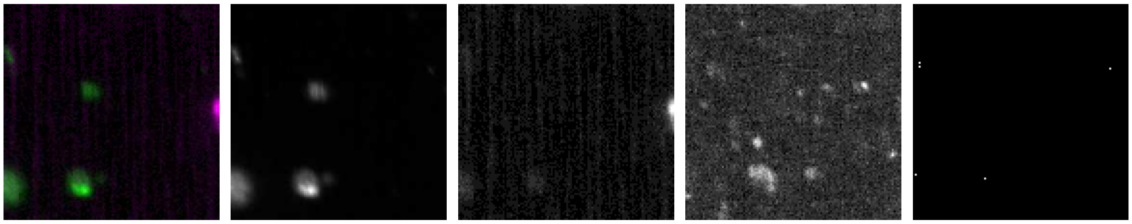				
25027	148	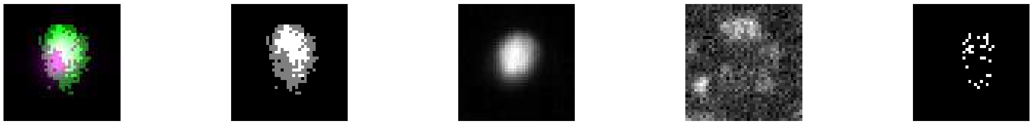				
25028	148	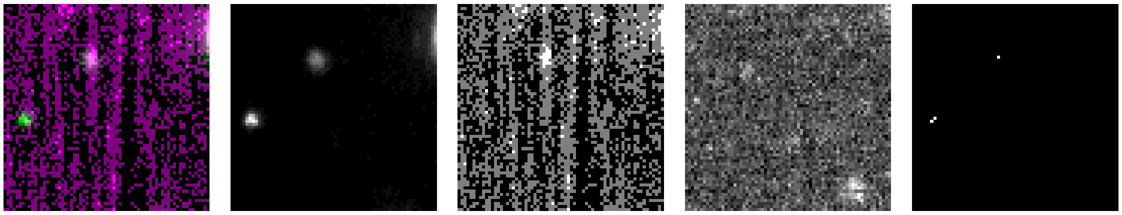				
25029	148	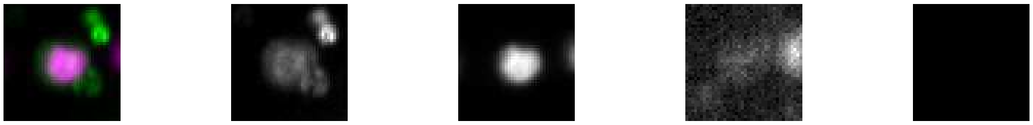				
25030	148	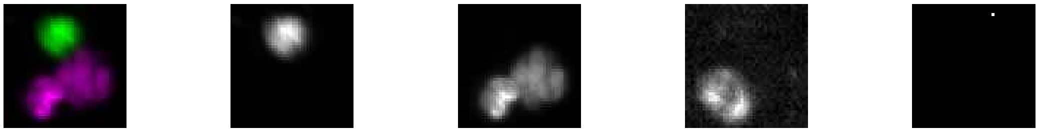				
25031	148	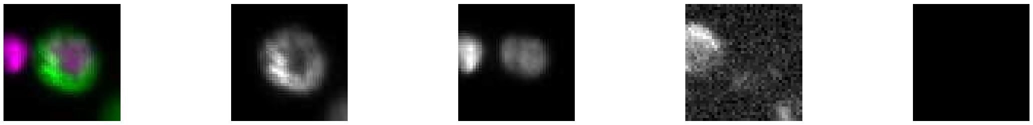				
25032	148	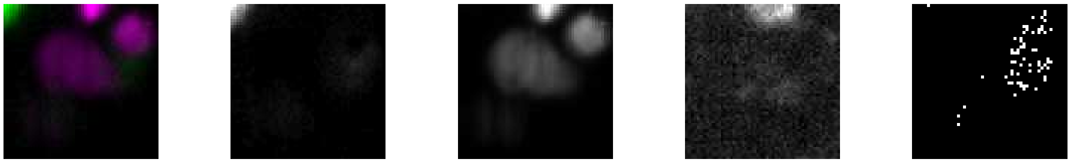				

Ereignis	Aufnahme	DAPI/CK-PE	CK-PE	DAPI	CD45-APC	HER-2/neu
25033	148	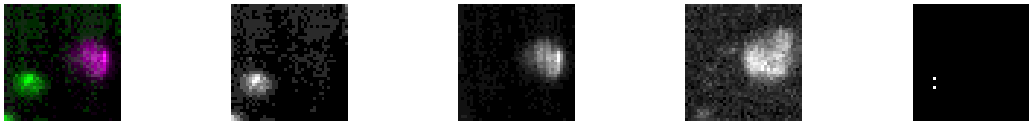				
25034	148	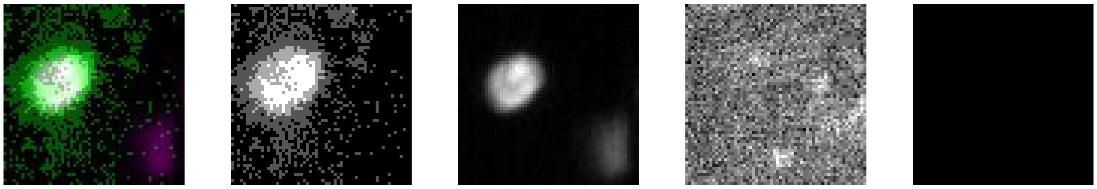				
25035	148	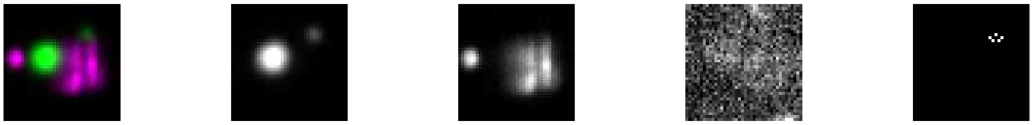				
25036	148	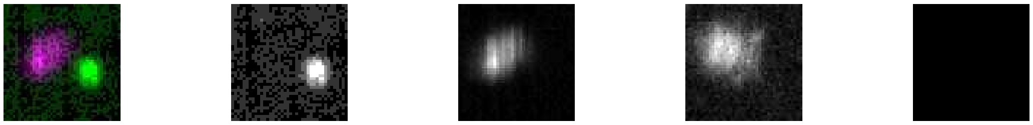				
25037	148	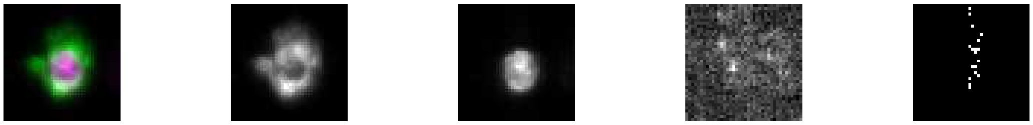				
25038	148	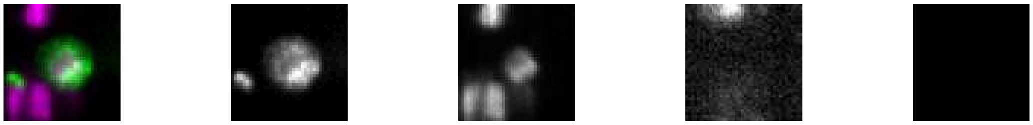				
25039	148	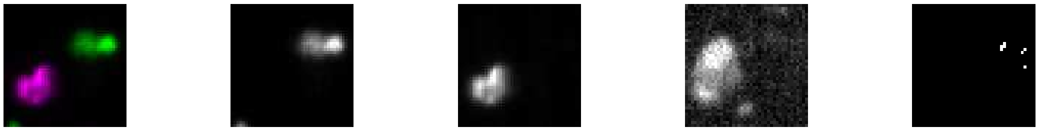				
25040	148	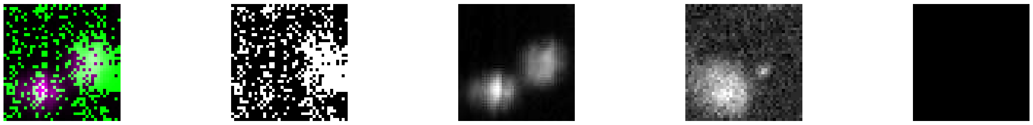				
25041	148	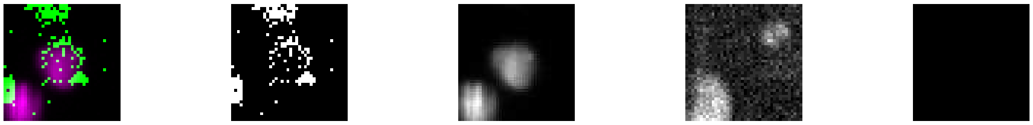				
25042	148	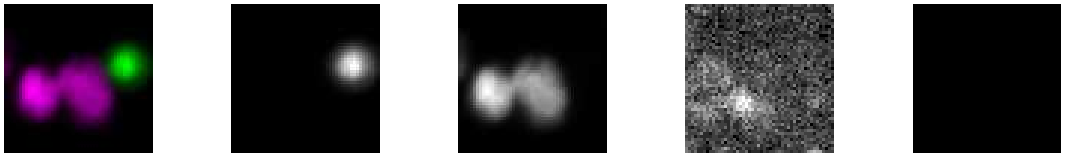				
25043	148	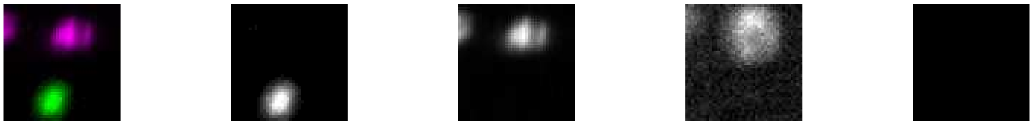				
25044	148	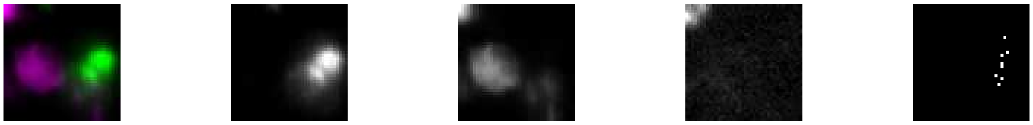				
25045	148	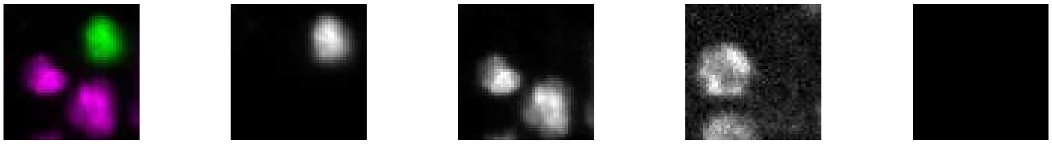				

Ereignis	Aufnahme	DAPI/CK-PE	CK-PE	DAPI	CD45-APC	HER-2/neu
25046	148	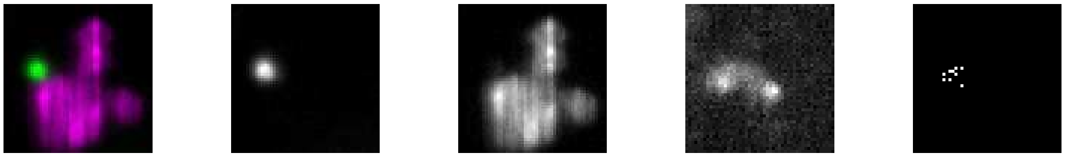				
25047	148	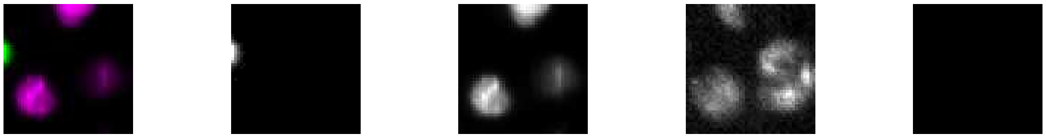				
25048	148	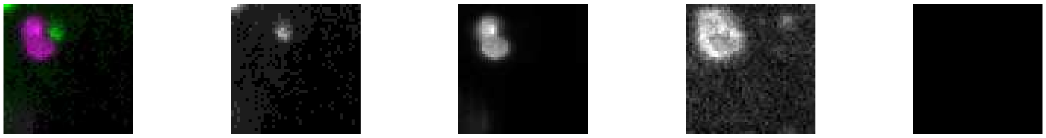				
25049	148	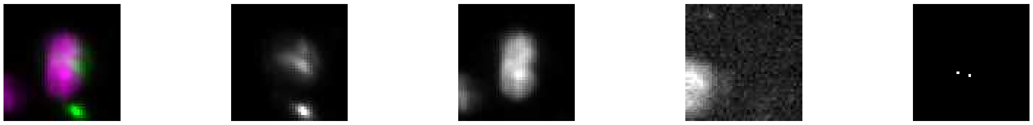				
25050	148	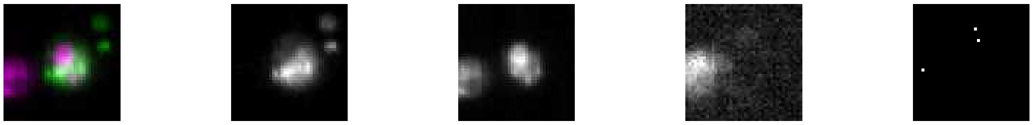				
25051	148	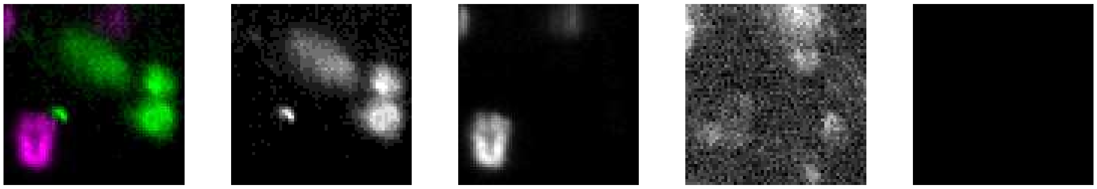				
25052	148	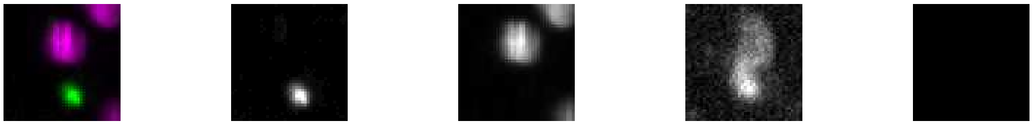				
25053	148	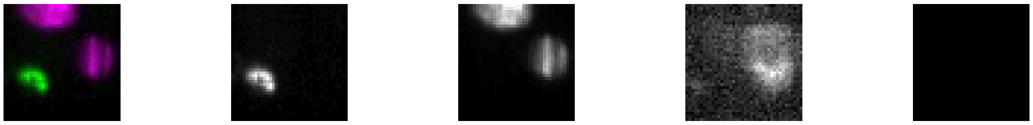				
25054	148	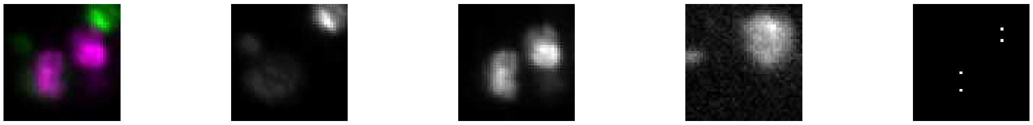				
25055	148	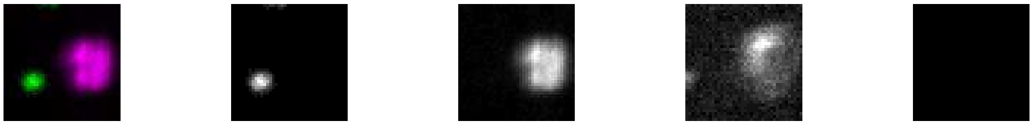				
25056	148	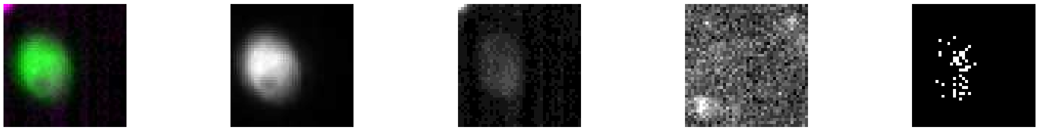				
25057	148	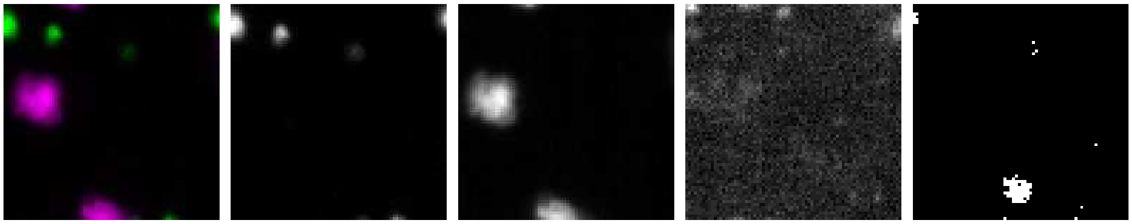				

Ereignis	Aufnahme	DAPI/CK-PE	CK-PE	DAPI	CD45-APC	HER-2/neu
25058	148	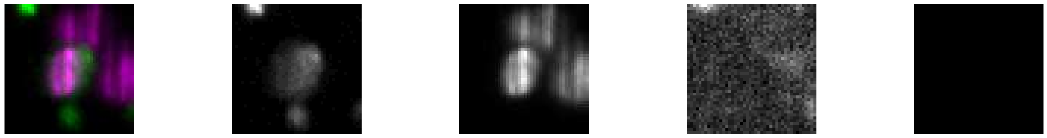				
25059	148	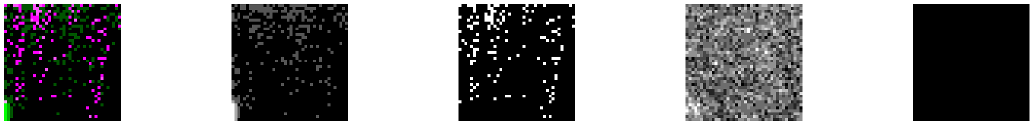				
25060	148	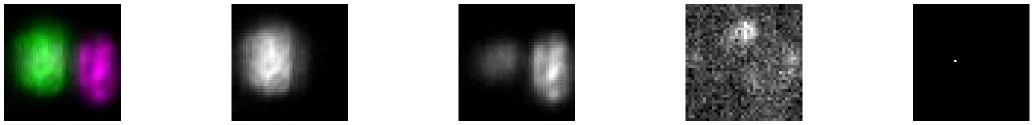				
25061	148	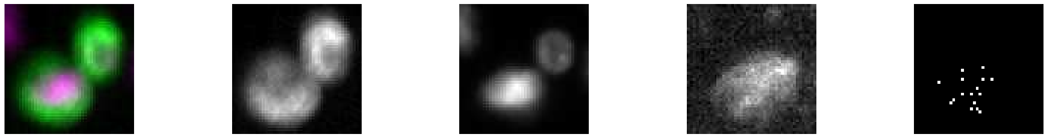				
25062	148	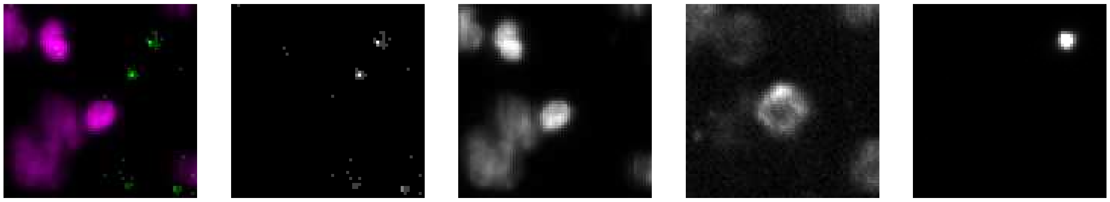				
25063	148	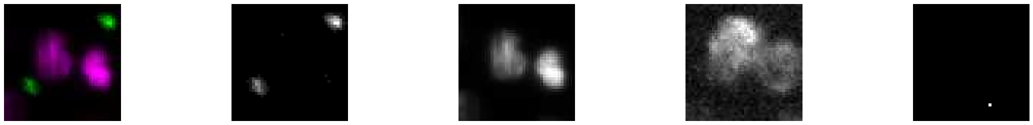				
25064	148	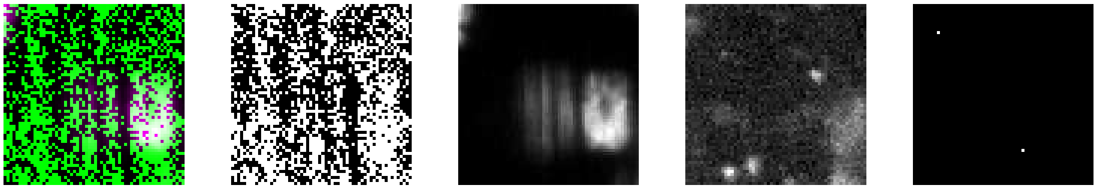				
25065	148	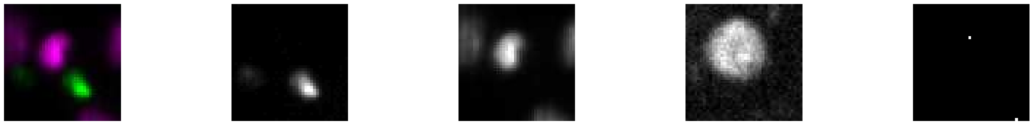				
25066	148	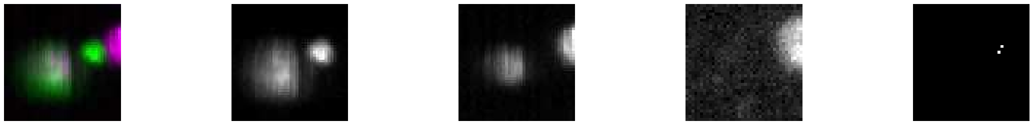				
25067	148	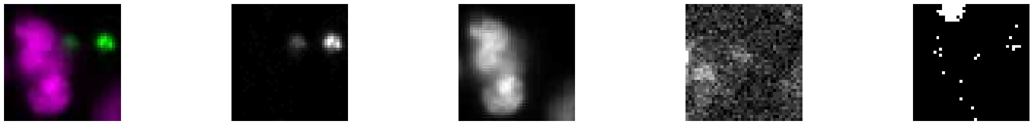				
25068	148	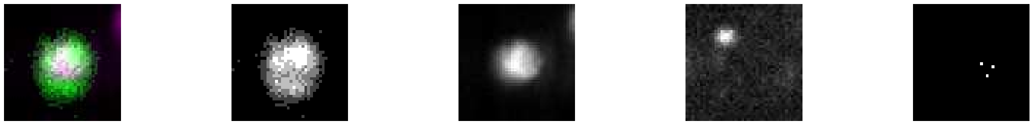				
25069	148	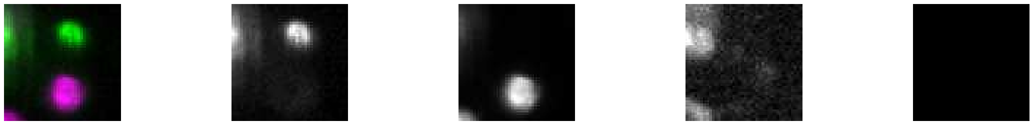				
25070	148	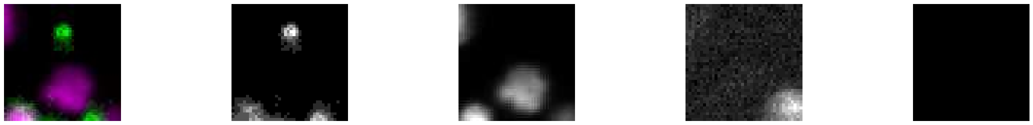				

Ereignis	Aufnahme	DAPI/CK-PE	CK-PE	DAPI	CD45-APC	HER-2/neu
25071	148	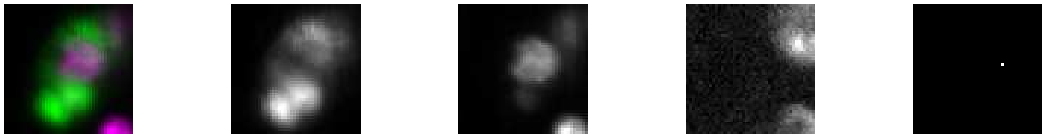				
25072	148	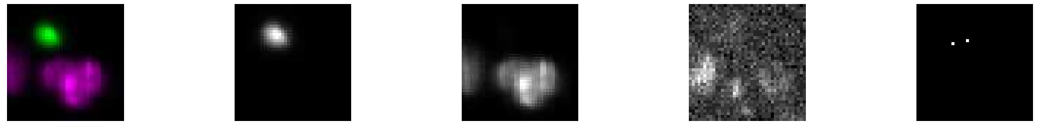				
25073	148	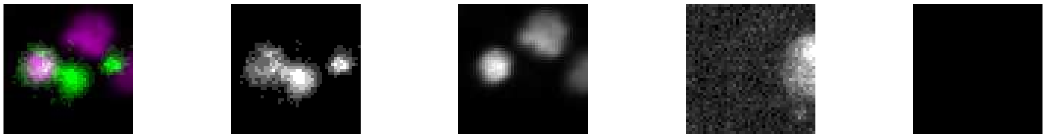				
25074	148	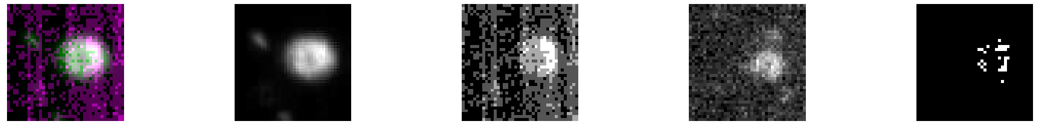				
25075	148	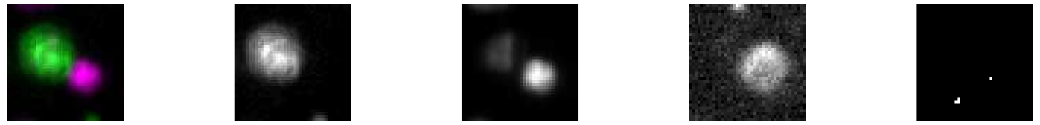				
25076	148	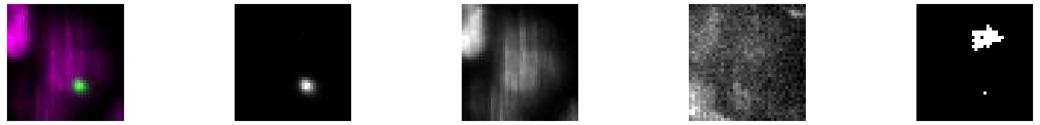				
25077	148	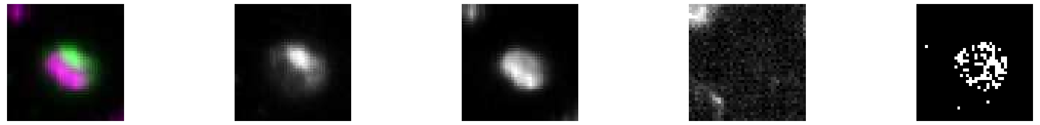				
25078	148	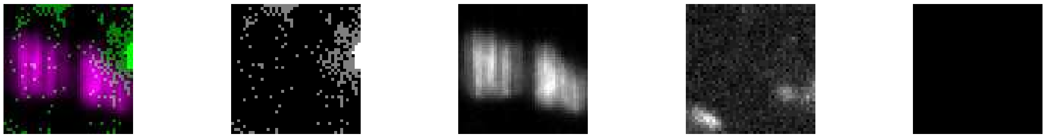				
25079	148	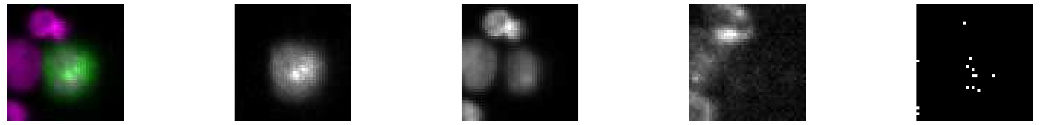				
25080	148	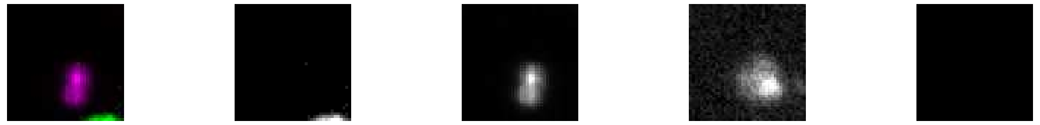				
25081	148	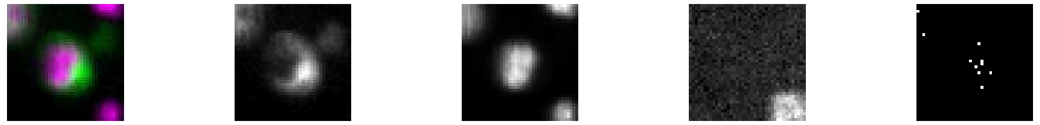				
25082	148	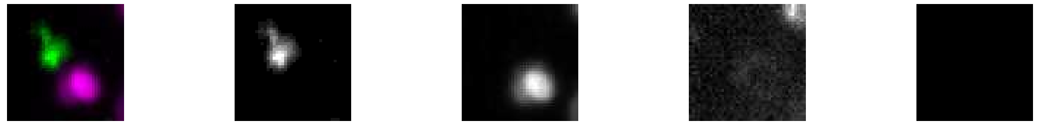				
25083	148	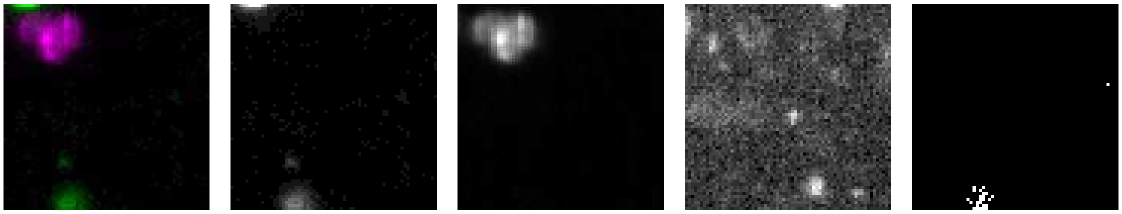				

Ereignis	Aufnahme	DAPI/CK-PE	CK-PE	DAPI	CD45-APC	HER-2/neu
25084	148	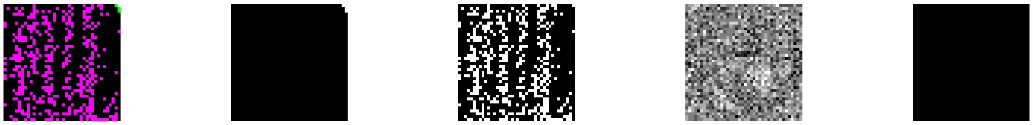				
25085	148	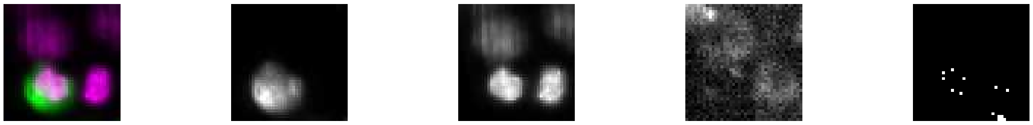				
25086	148	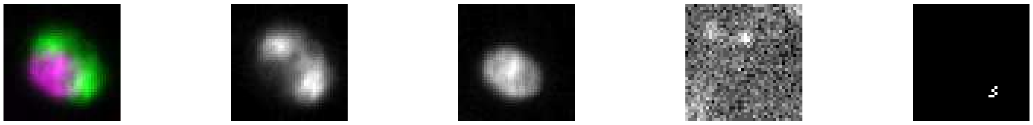				
25087	148	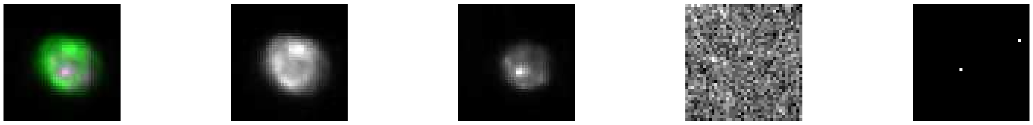				
25088	148	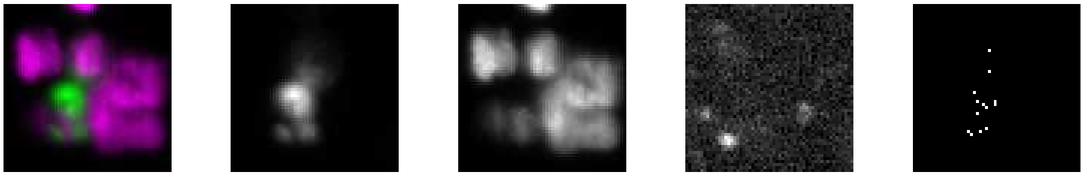				
25089	148	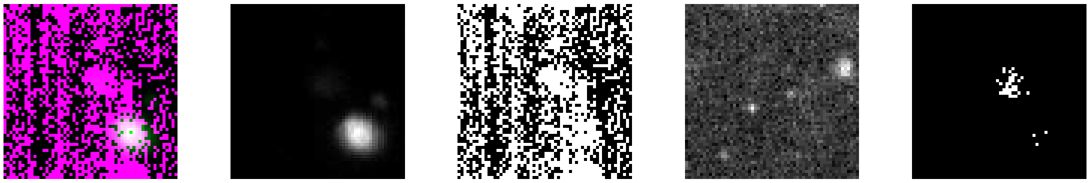				
25090	148	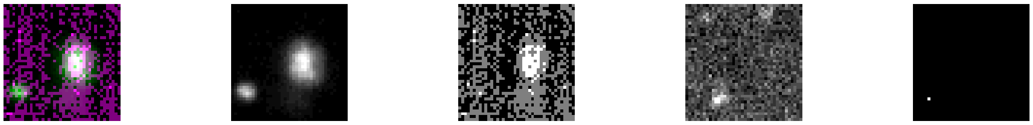				
25091	148	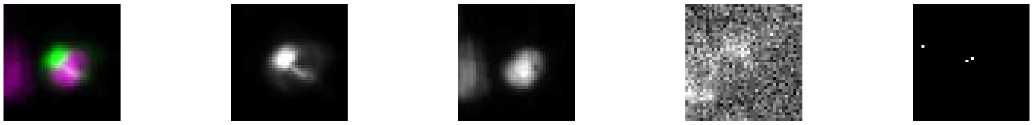				
25092	148	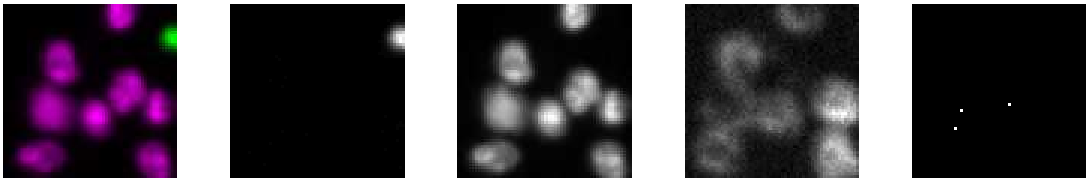				
25093	148	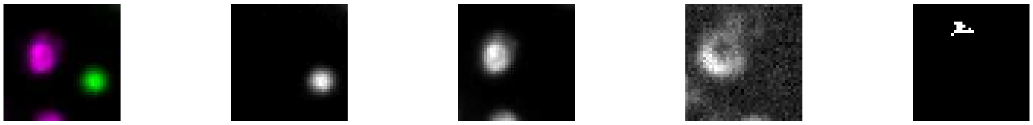				
25094	148	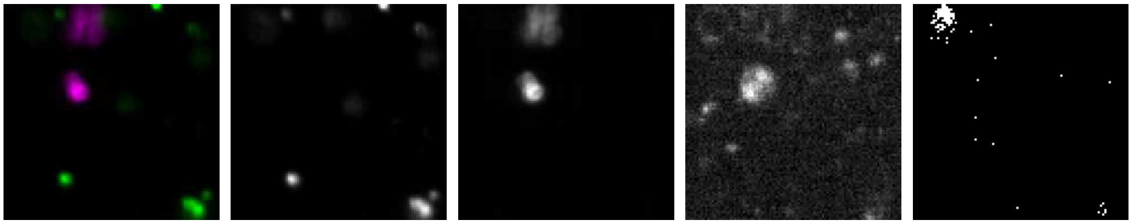				
25095	148	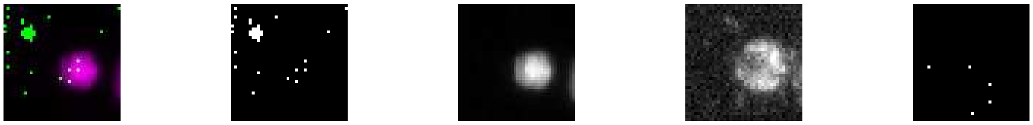				

Ereignis	Aufnahme	DAPI/CK-PE	CK-PE	DAPI	CD45-APC	HER-2/neu
25096	148	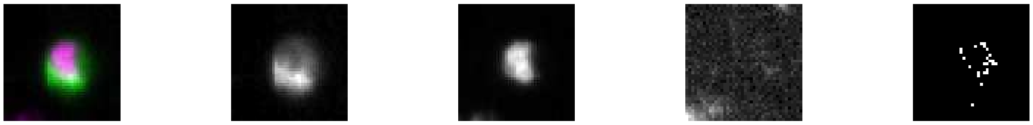				
25097	148	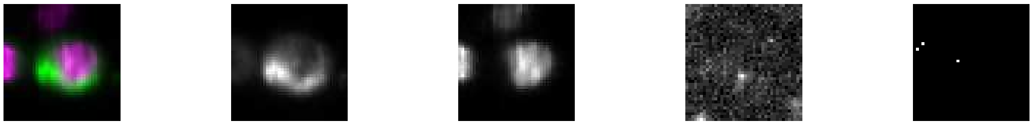				
25098	148	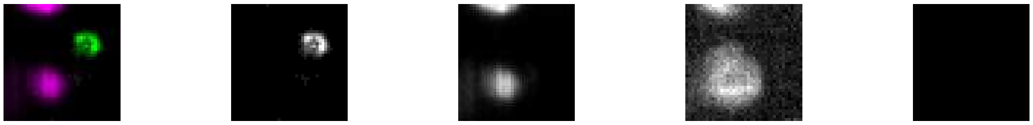				
25099	148	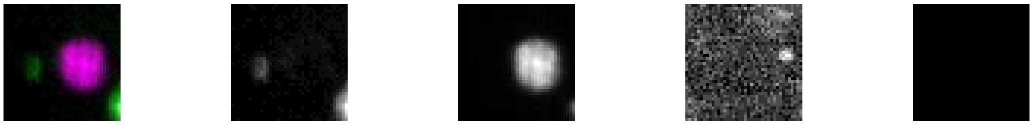				
25100	148	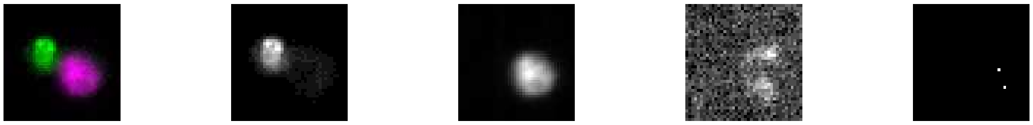				
25101	148	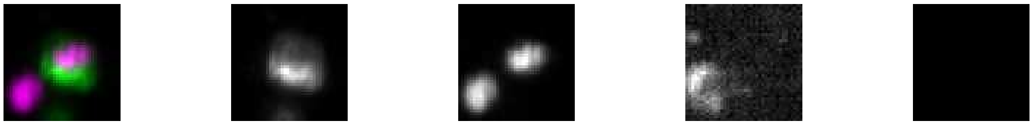				
25102	148	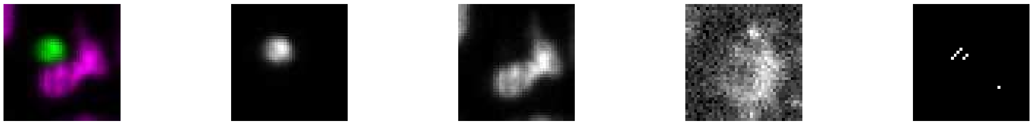				
25103	148	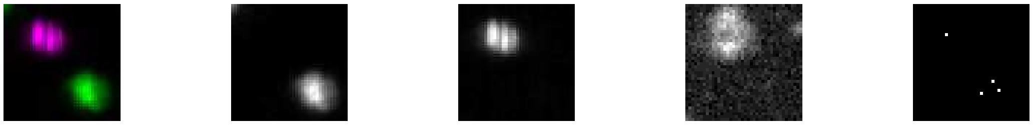				
25104	148	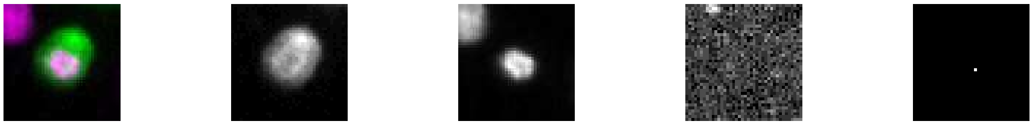				
25105	148	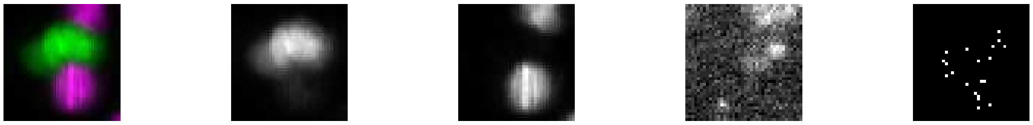				
25106	147	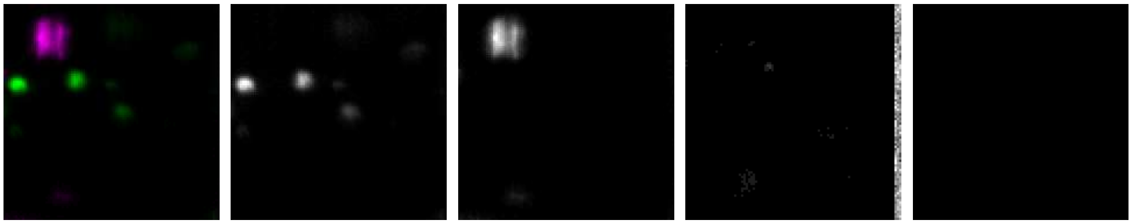				
25107	147	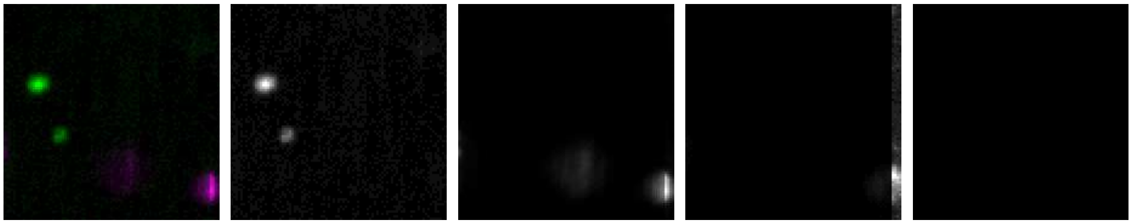				

Druckdatum: 02/06/2020 17:25

Proben-ID: Gyn14.208 **Kassetten-ID:** 01327106 **Patienten-ID:**

Ereignis	Aufnahme	DAPI/CK-PE	CK-PE	DAPI	CD45-APC	HER-2/neu
25108	147	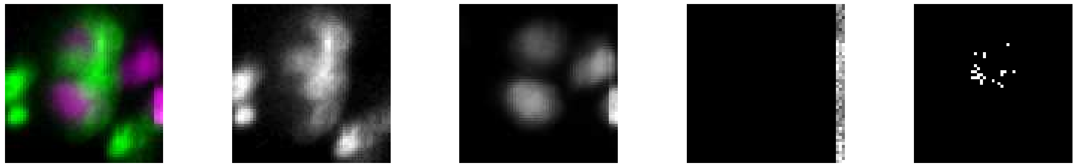				
25109	148	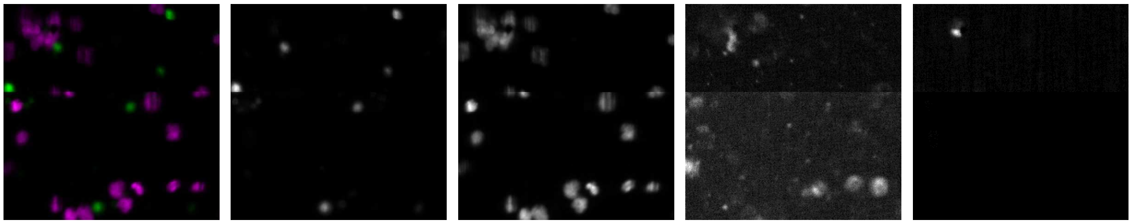				
25110	133	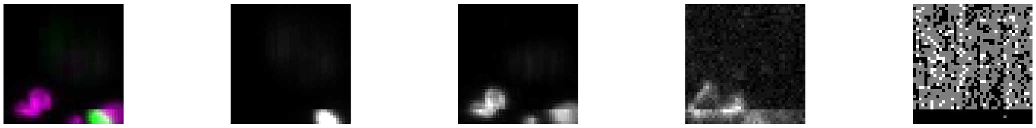				
25111	133	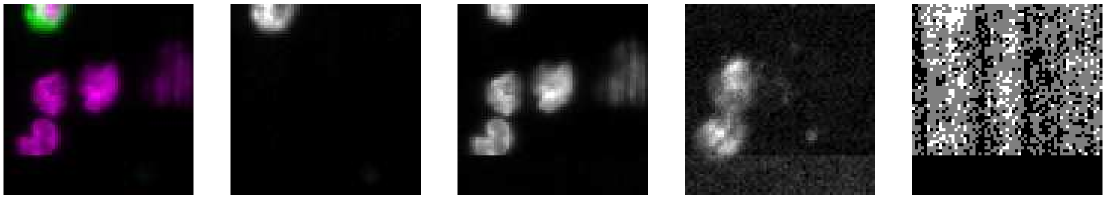				
25112	133	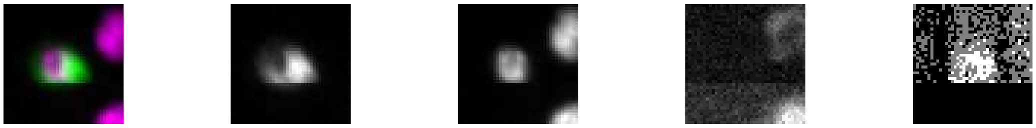				
25113	133	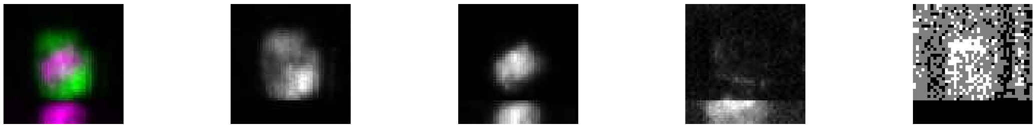				
25114	133	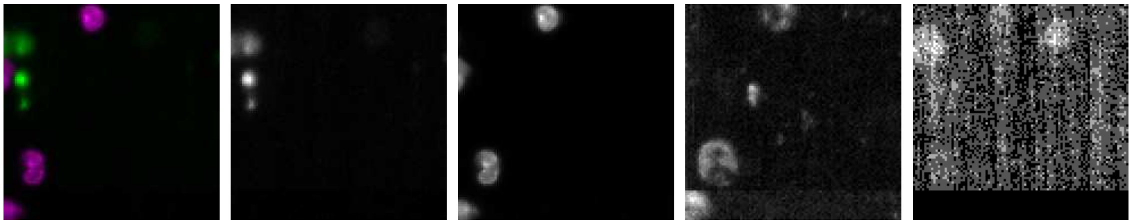				
25115	133	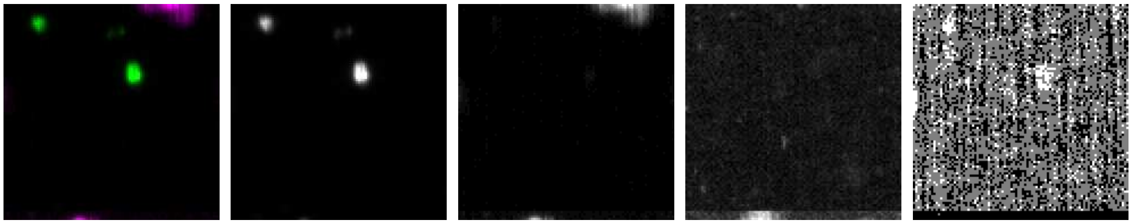				
25116	149	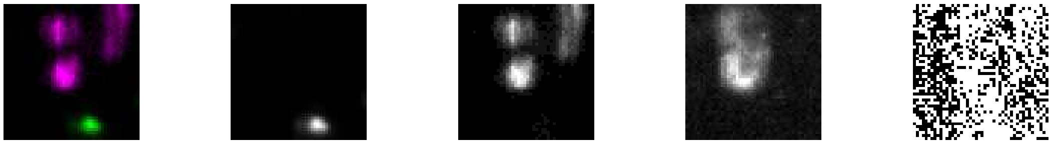				
25117	149	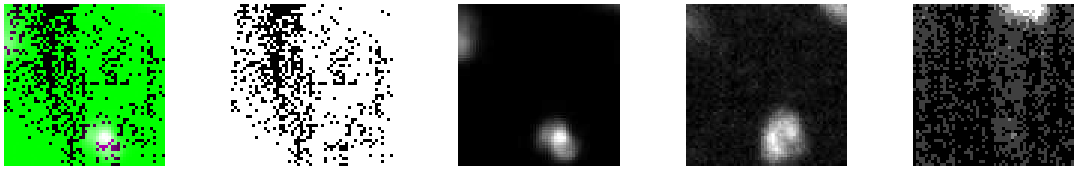				

Ereignis	Aufnahme	DAPI/CK-PE	CK-PE	DAPI	CD45-APC	HER-2/neu
25118	149	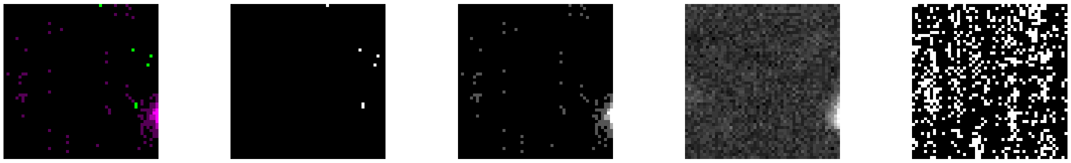				
25119	149	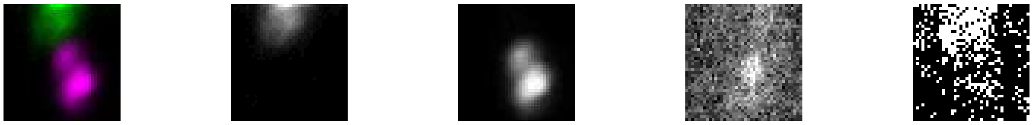				
25120	149	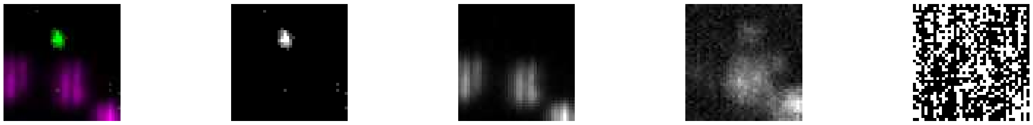				
25121	149	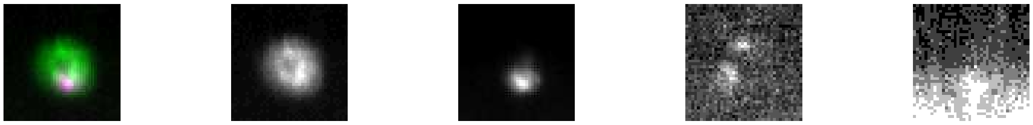				
25122	149	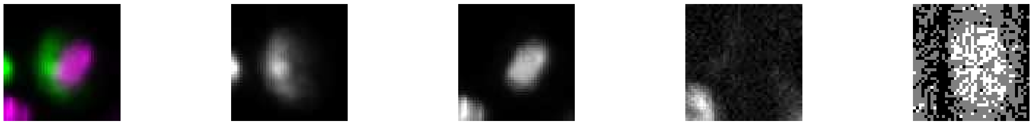				
25123	149	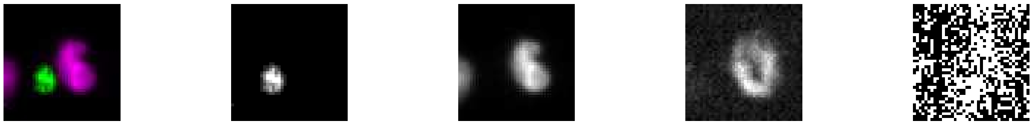				
25124	149	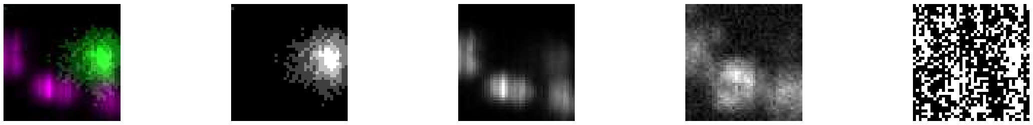				
25125	149	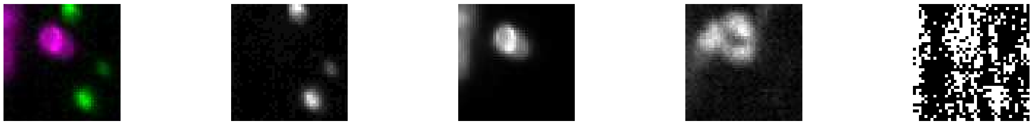				
25126	149	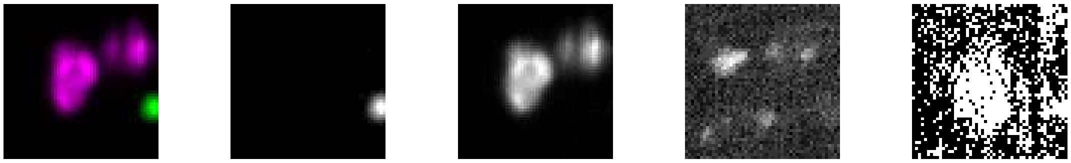				
25127	149	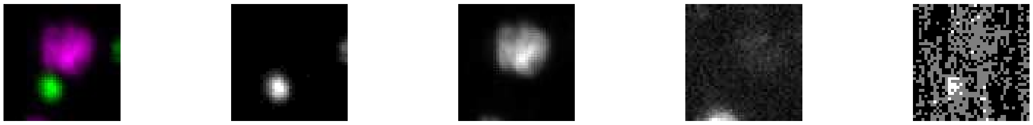				
25128	149	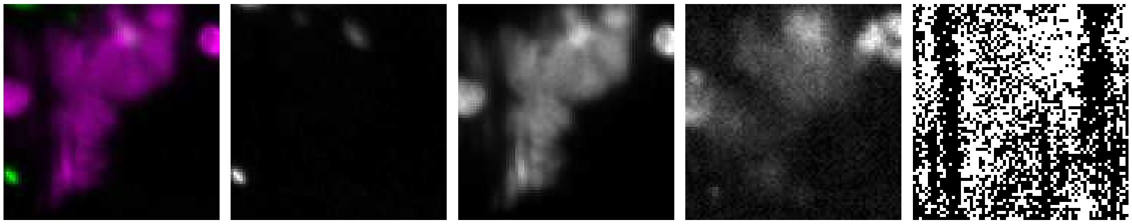				
25129	149	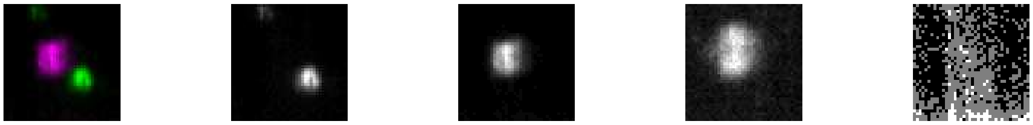				
25130	149	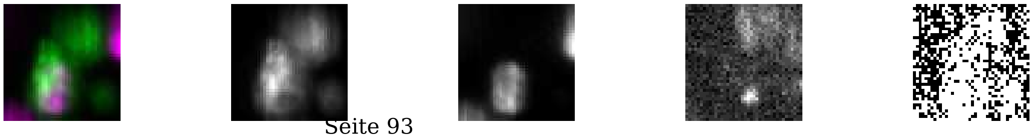				

Ereignis	Aufnahme	DAPI/CK-PE	CK-PE	DAPI	CD45-APC	HER-2/neu
25131	149	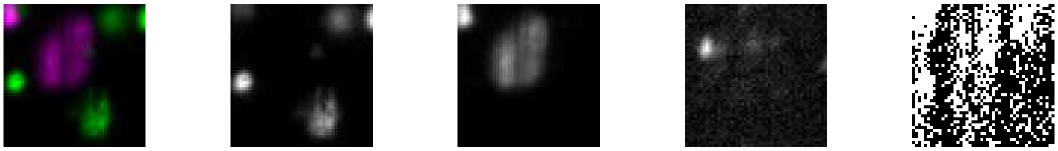				
25132	149	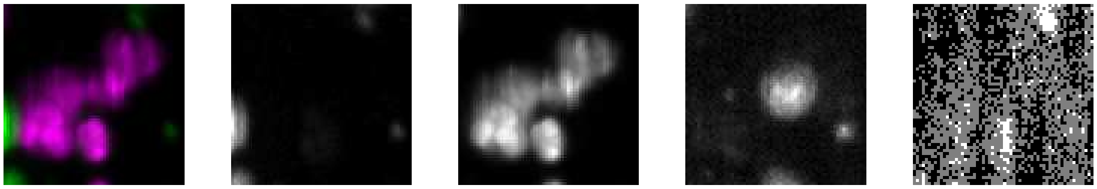				
25133	149	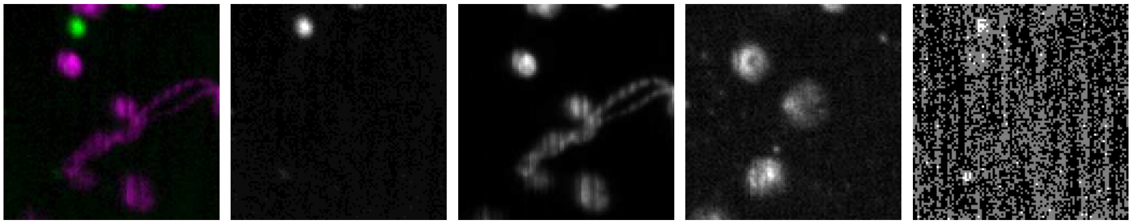				
25134	149	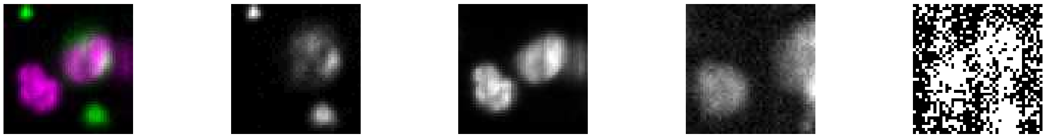				
25135	149	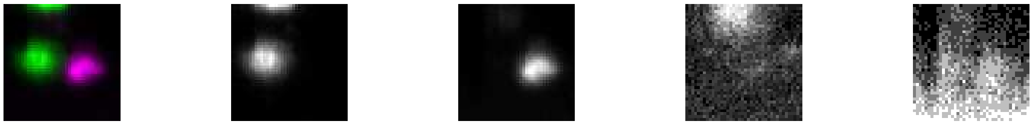				
25136	149	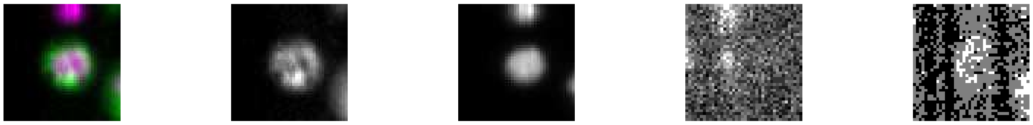				
25137	149	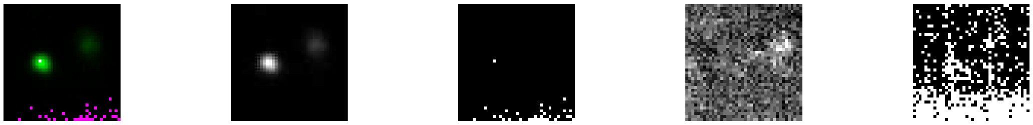				
25138	149	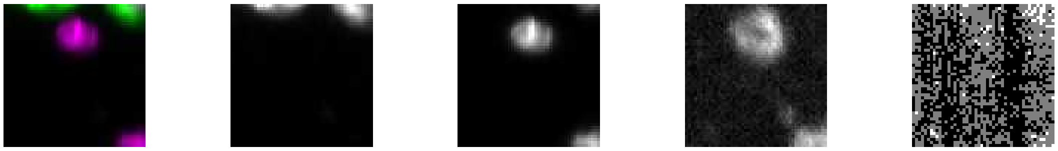				
25139	149	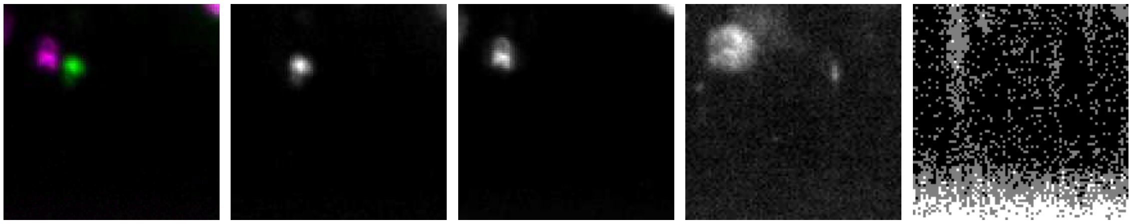				
25140	149	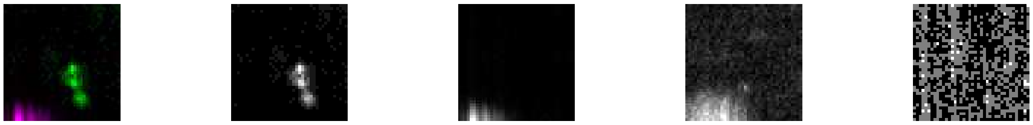				
25141	149	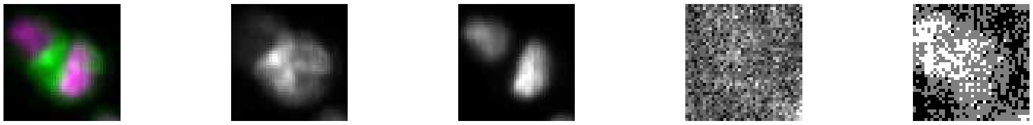				

Ereignis	Aufnahme	DAPI/CK-PE	CK-PE	DAPI	CD45-APC	HER-2/neu
25142	149	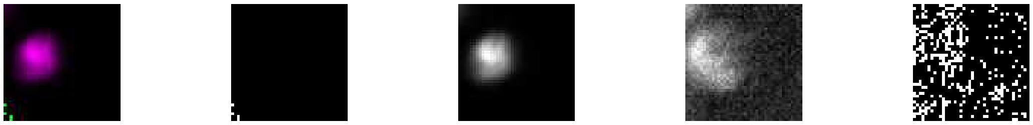				
25143	149	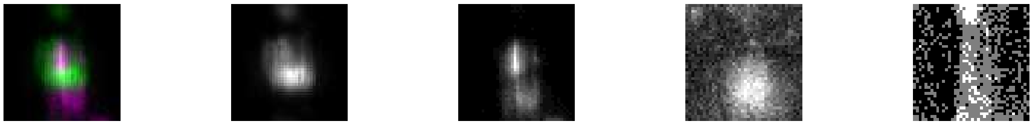				
25144	149	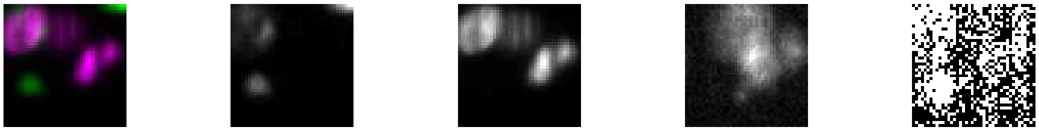				
25145	149	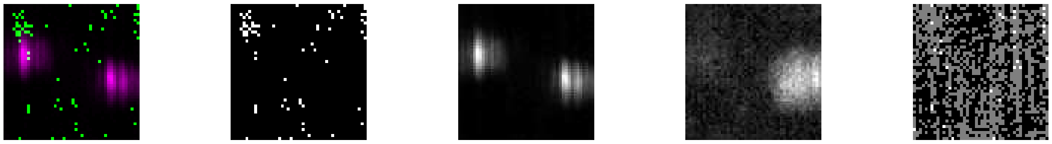				
25146	149	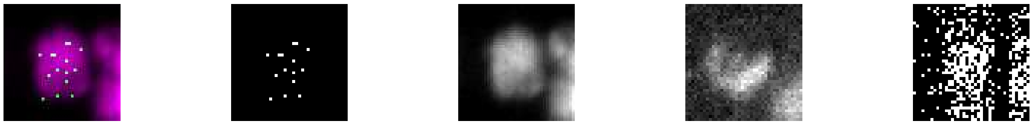				
25147	149	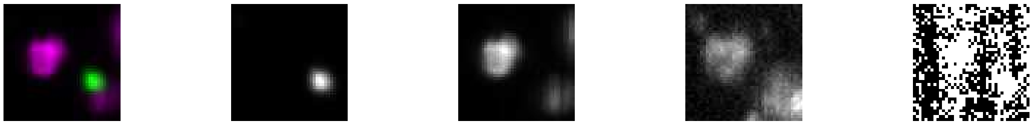				
25148	149	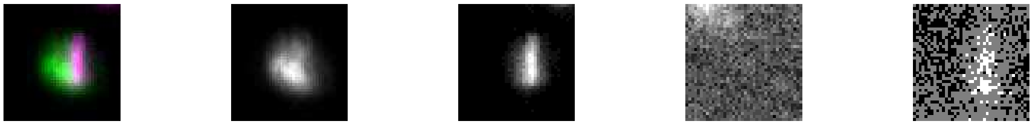				
25149	149	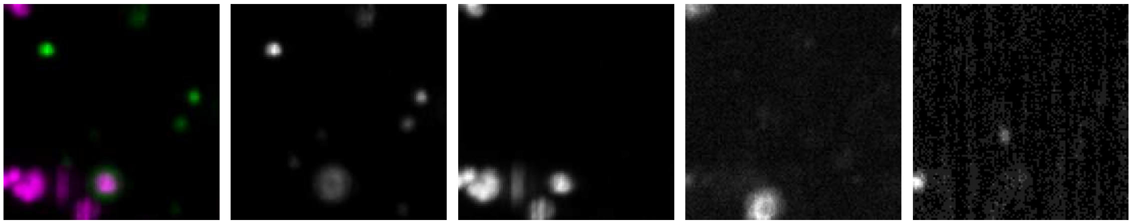				
25150	149	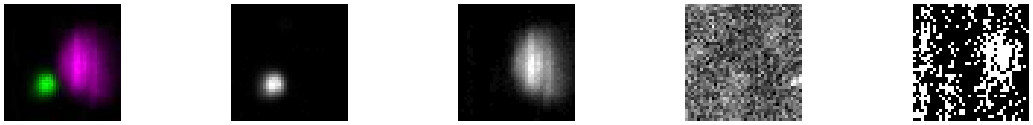				
25151	149	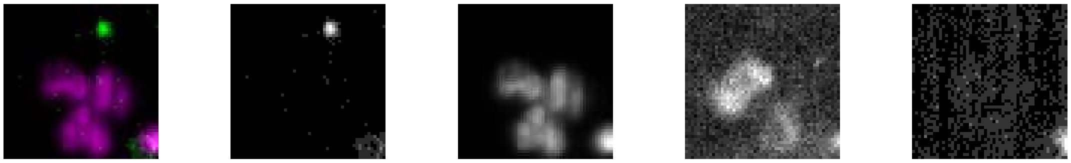				
25152	149	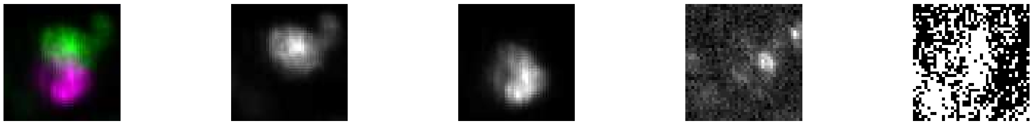				
25153	149	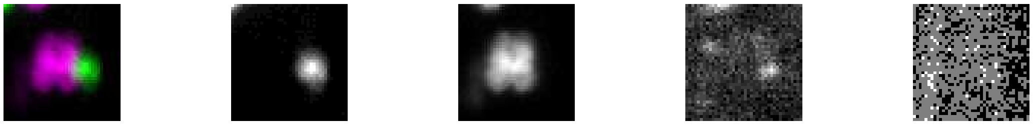				
25154	149	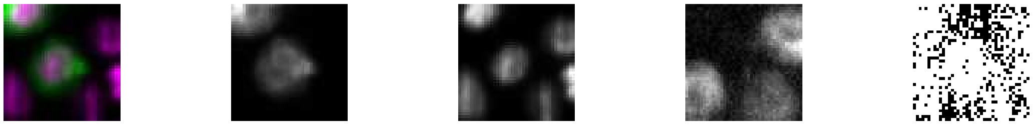				

Ereignis	Aufnahme	DAPI/CK-PE	CK-PE	DAPI	CD45-APC	HER-2/neu
25155	149	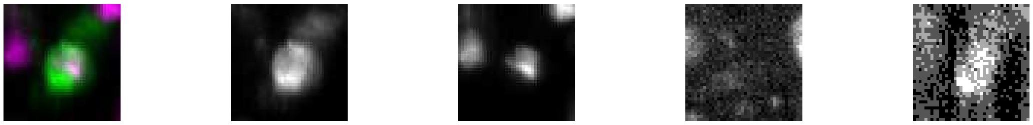				
25156	149	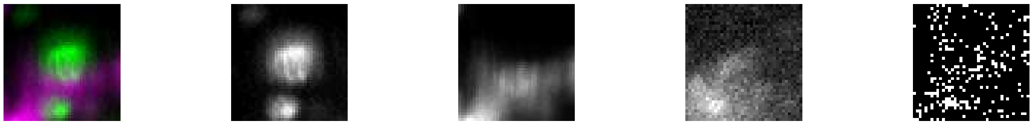				
25157	149	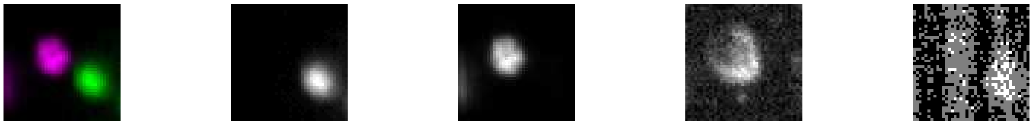				
25158	149	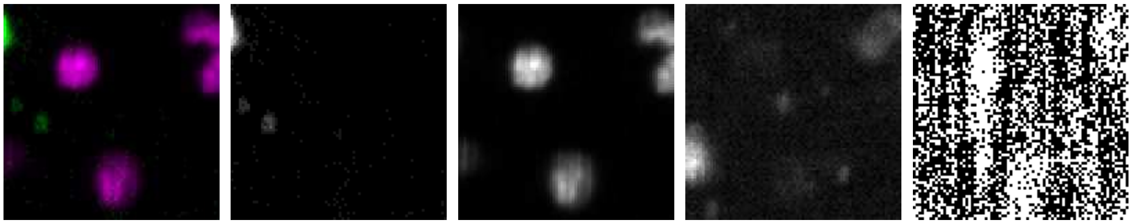				
25159	149	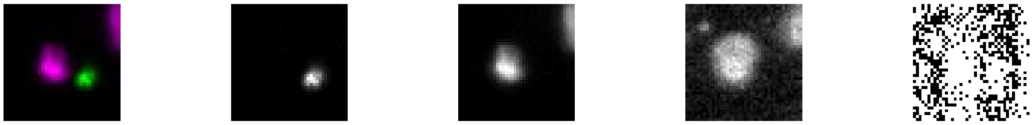				
25160	149	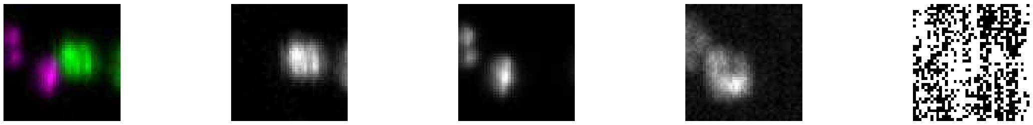				
25161	149	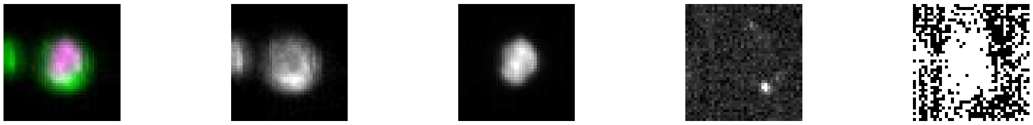				
25162	149	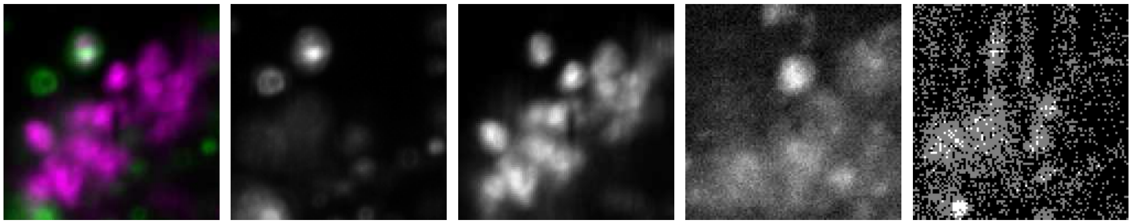				
25163	149	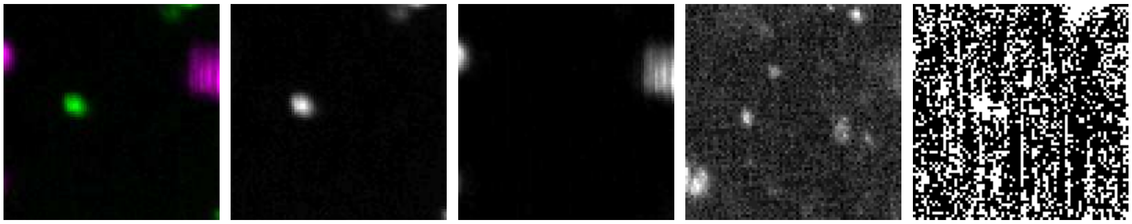				
25164	149	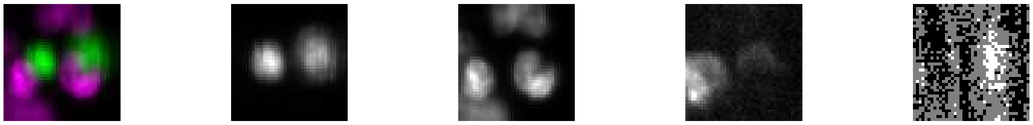				
25165	149	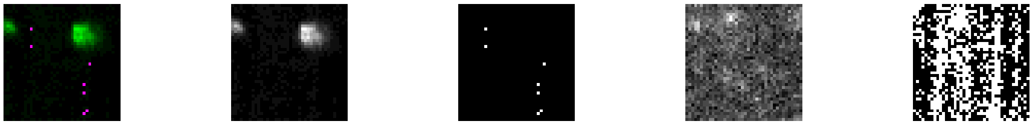				
25166	149	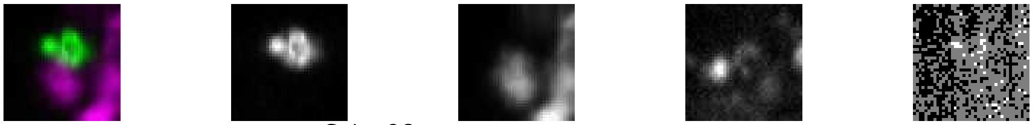				

Ereignis	Aufnahme	DAPI/CK-PE	CK-PE	DAPI	CD45-APC	HER-2/neu
25167	149	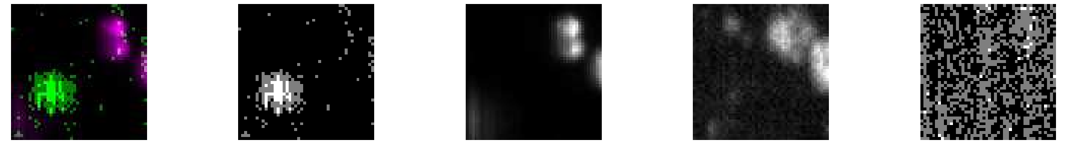				
25168	149	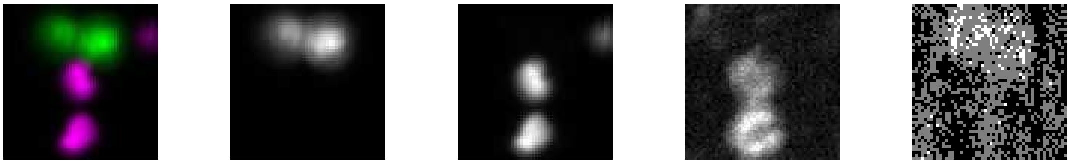				
25169	149	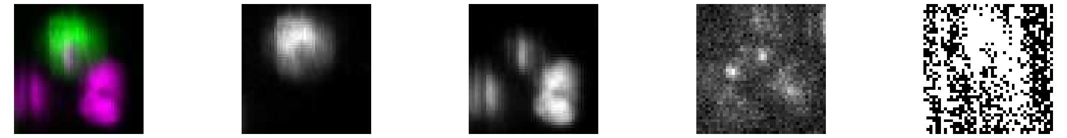				
25170	149	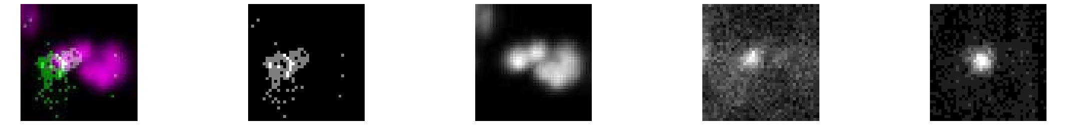				
25171	149	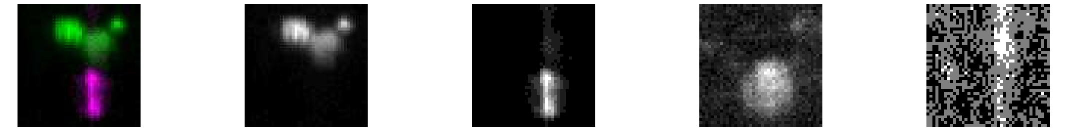				
25172	149	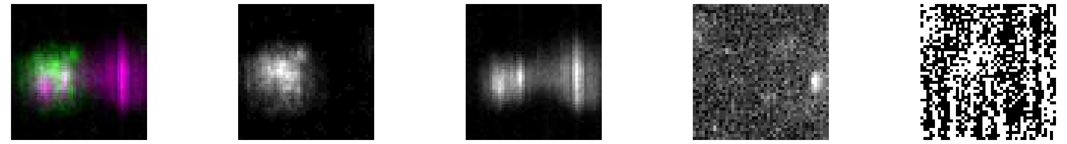				
25173	149	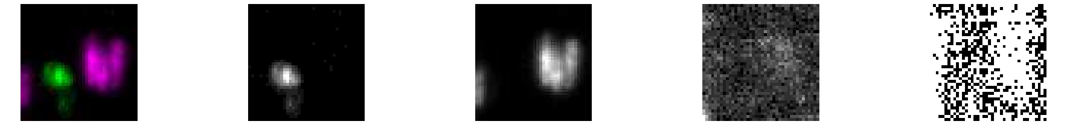				
25174	149	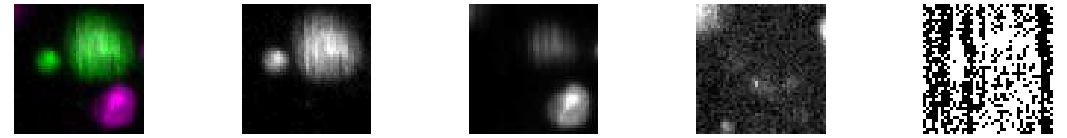				
25175	149	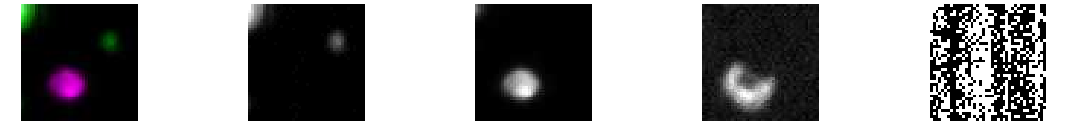				
25176	149	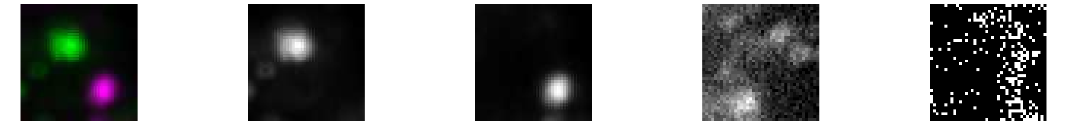				
25177	149	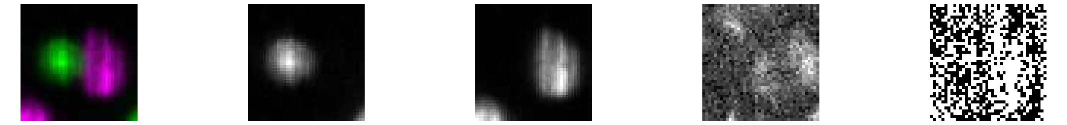				
25178	149	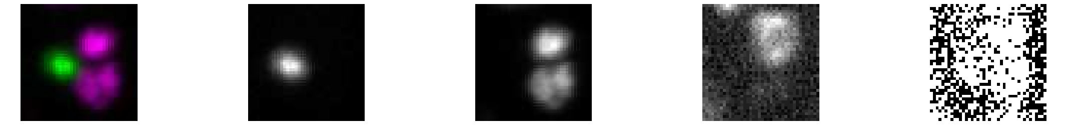				
25179	149	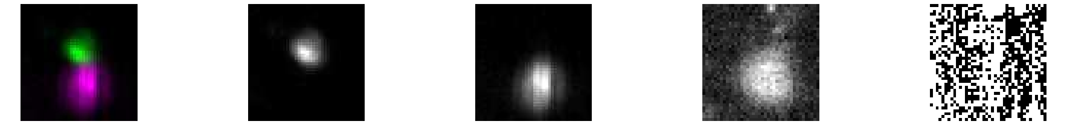				

Ereignis	Aufnahme	DAPI/CK-PE	CK-PE	DAPI	CD45-APC	HER-2/neu
25180	149	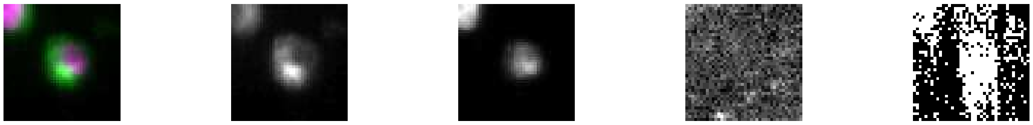				
25181	149	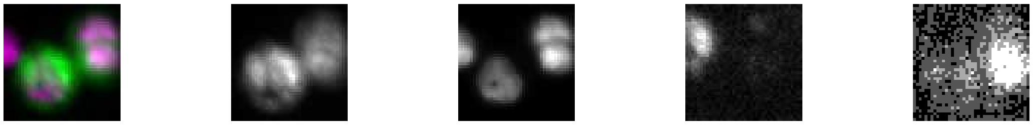				
25182	149	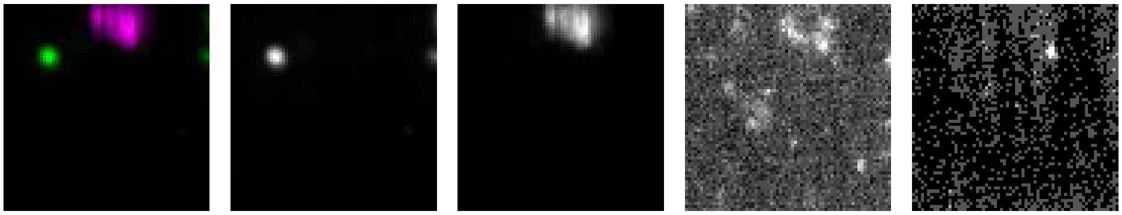				
25183	149	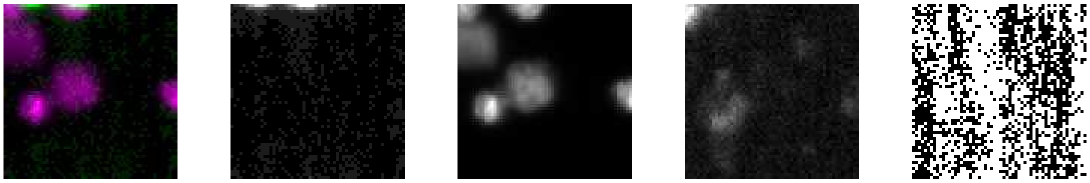				
25184	149	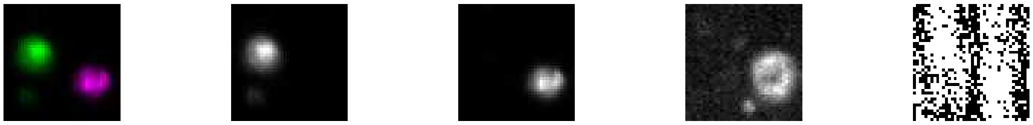				
25185	149	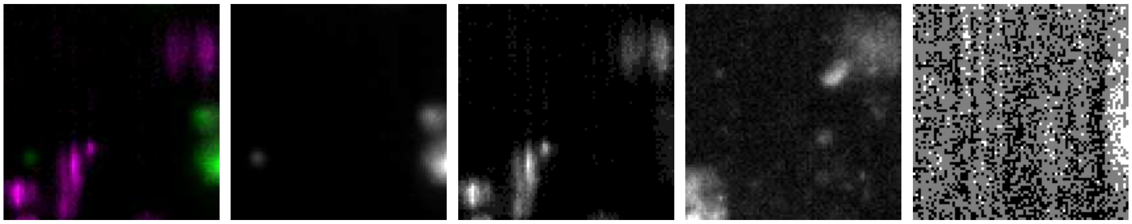				
25186	149	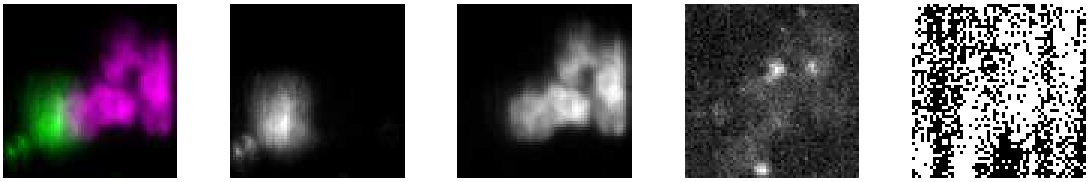				
25187	149	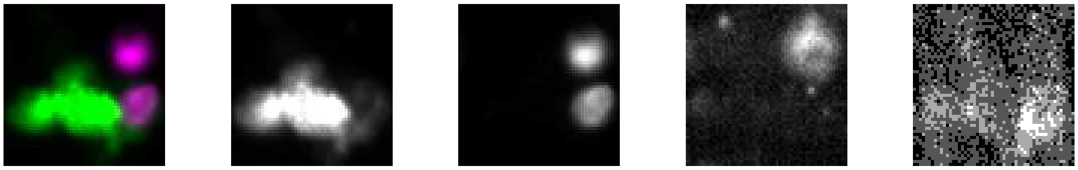				
25188	149	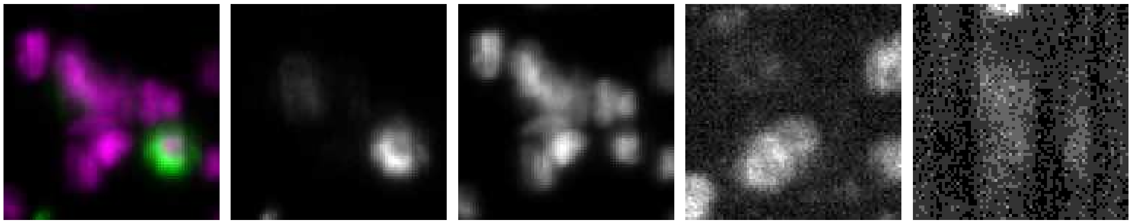				
25189	149	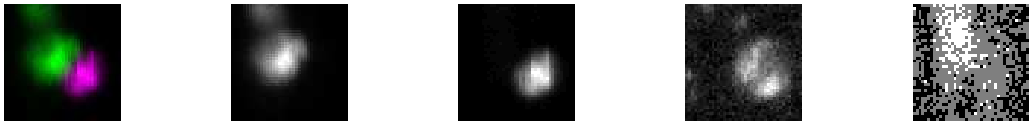				

Druckdatum: 02/06/2020 17:25

Proben-ID: Gyn14.208 **Kassetten-ID:** 01327106 **Patienten-ID:**

Ereignis	Aufnahme	DAPI/CK-PE	CK-PE	DAPI	CD45-APC	HER-2/neu
25190	149	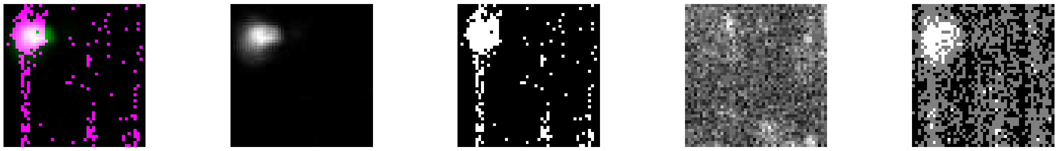				
25191	149	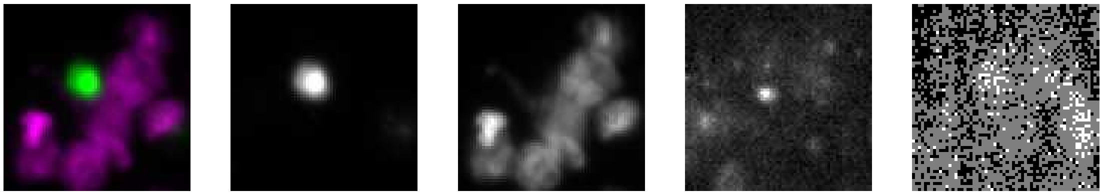				
25192	149	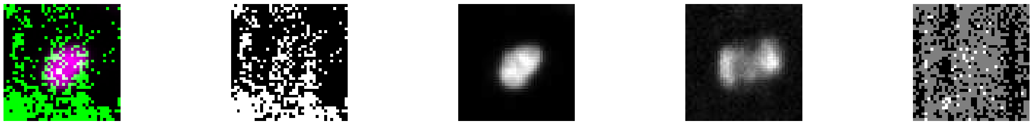				
25193	149	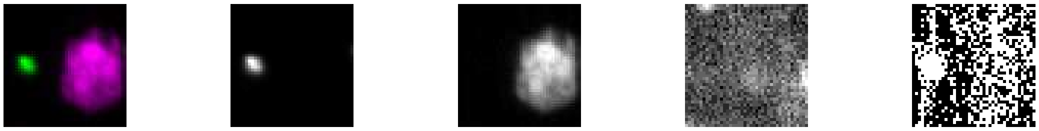				
25194	149	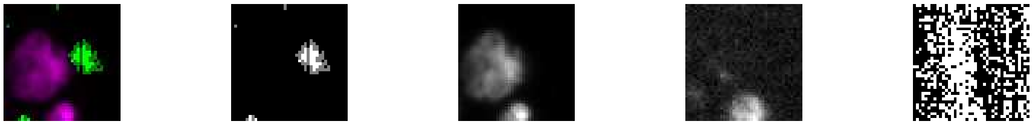				
25195	149	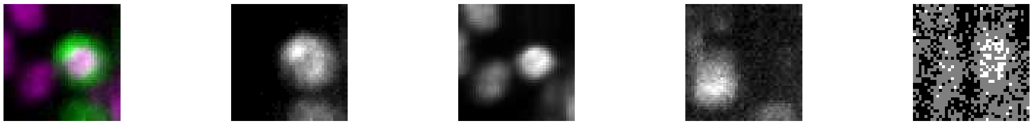				
25196	149	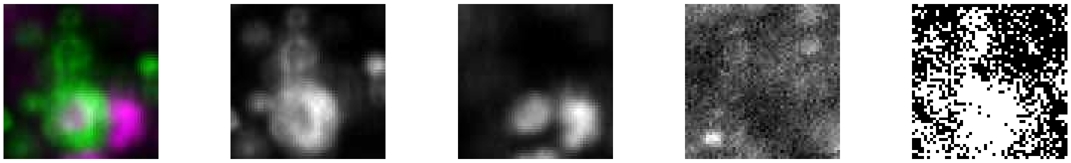				
25197	149	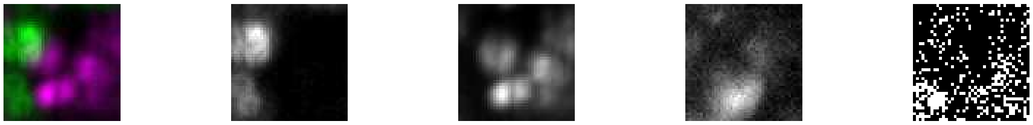				
25198	149	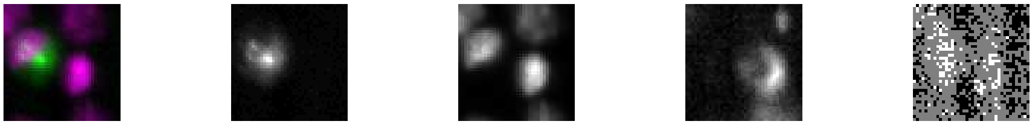				
25199	149	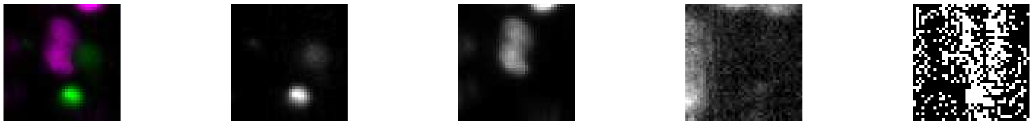				
25200	149	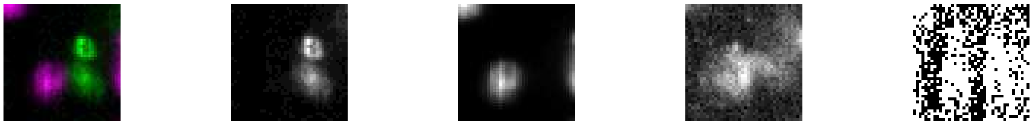				
25201	149	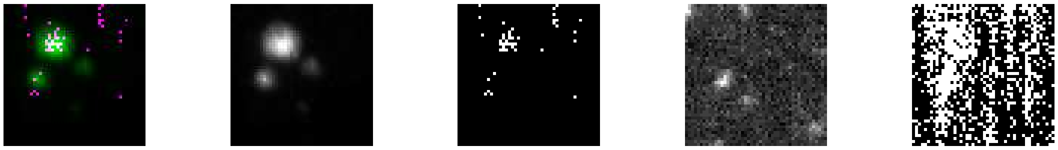				

Ereignis	Aufnahme	DAPI/CK-PE	CK-PE	DAPI	CD45-APC	HER-2/neu
25202	149	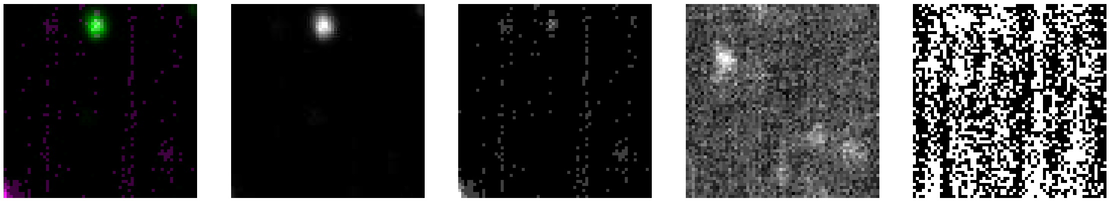				
25203	149	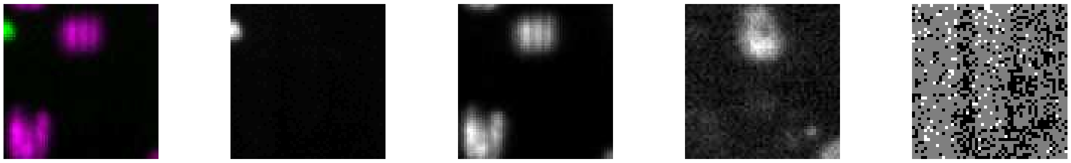				
25204	149	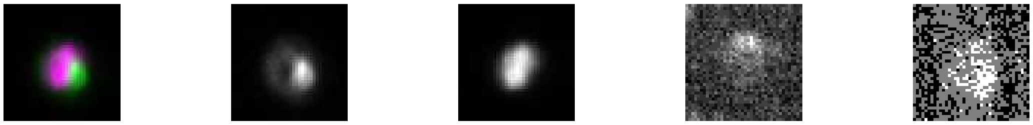				
25205	149	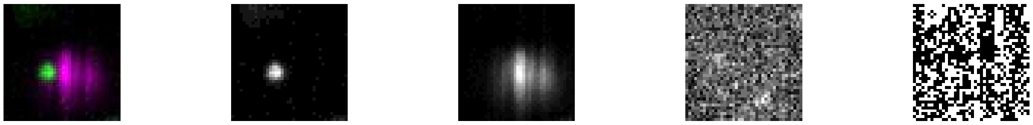				
25206	149	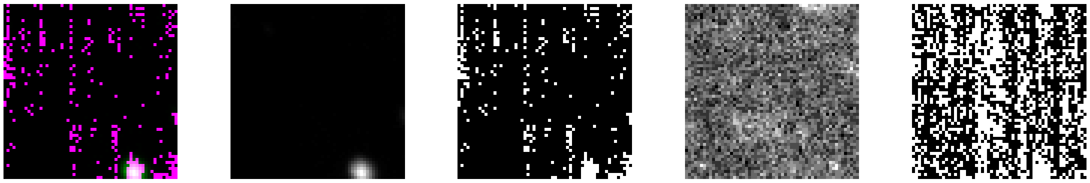				
25207	149	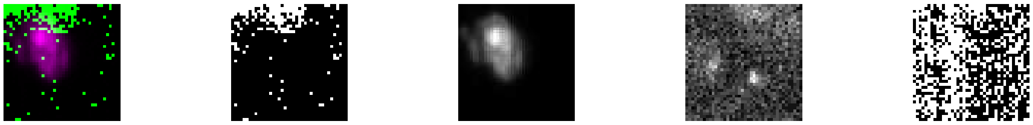				
25208	149	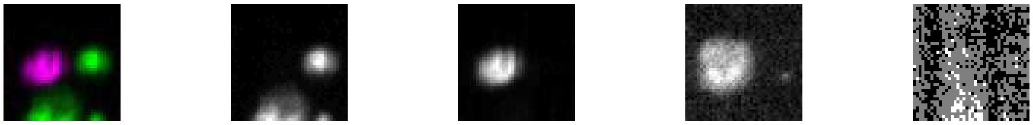				
25209	149	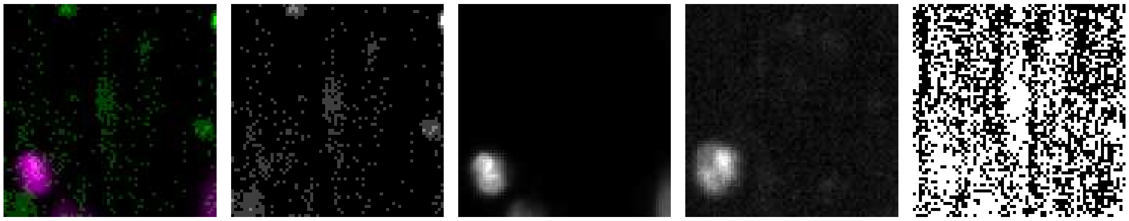				
25210	149	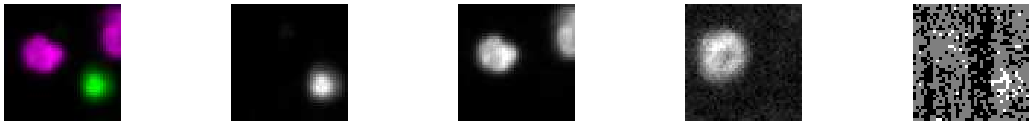				
25211	149	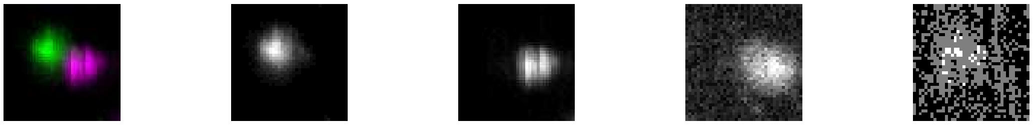				
25212	149	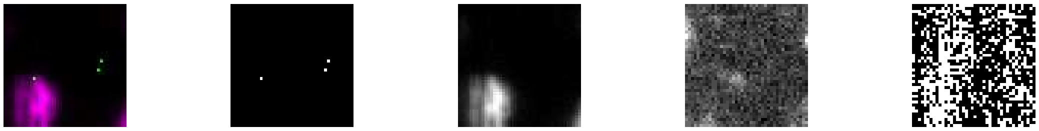				
25213	149	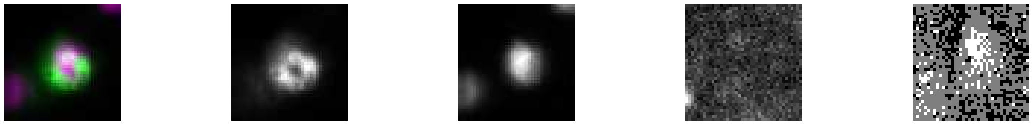				

Ereignis	Aufnahme	DAPI/CK-PE	CK-PE	DAPI	CD45-APC	HER-2/neu
25214	149	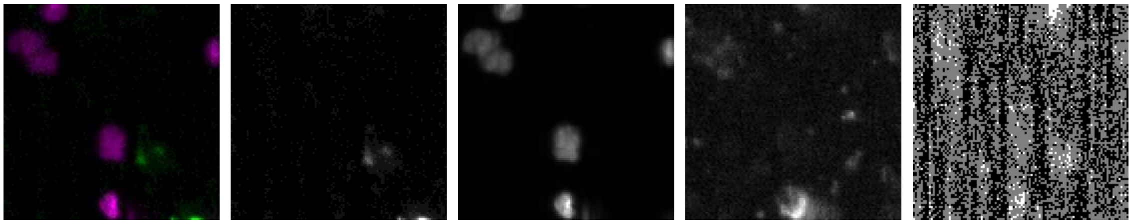				
25215	149	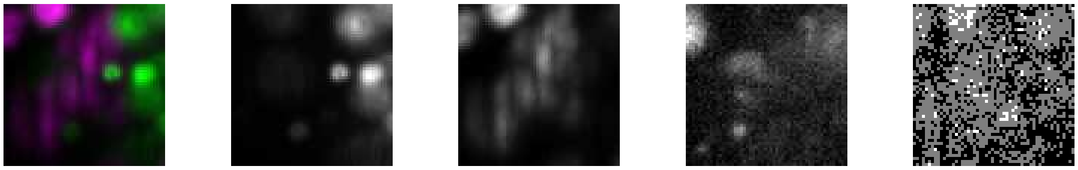				
25216	149	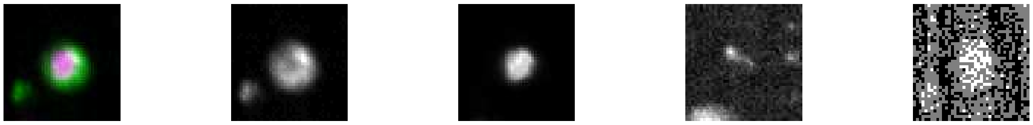				
25217	149	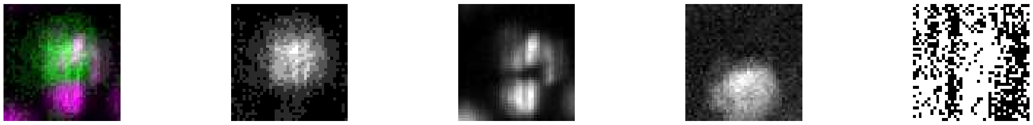				
25218	149	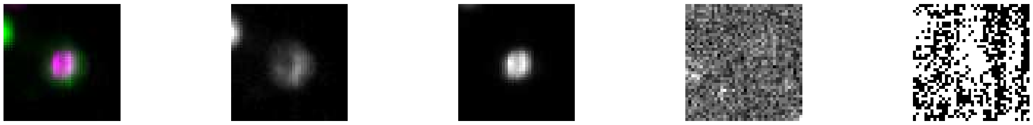				
25219	149	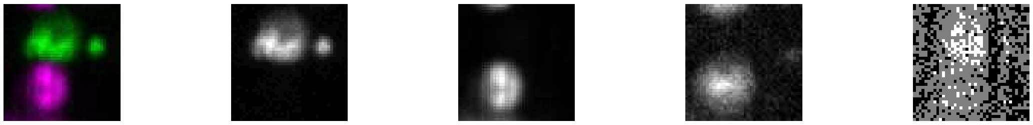				
25220	149	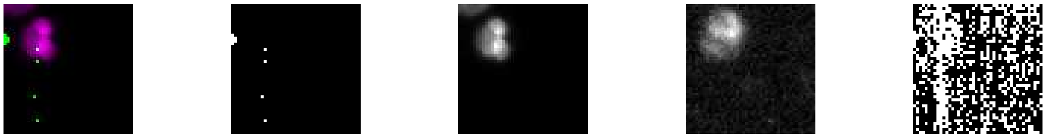				
25221	149	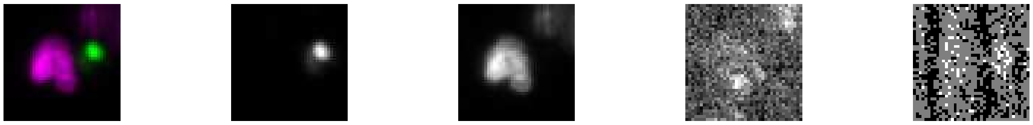				
25222	149	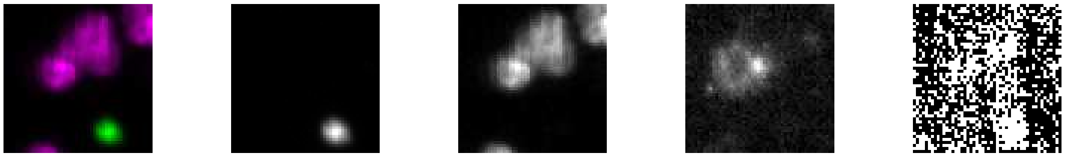				
25223	149	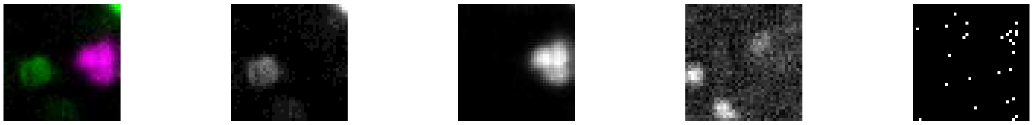				
25224	149	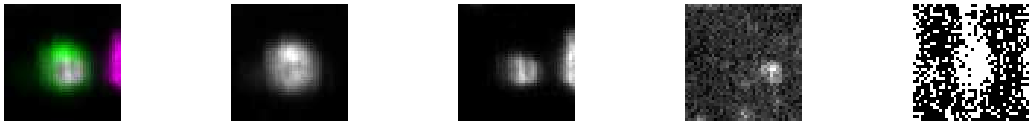				
25225	149	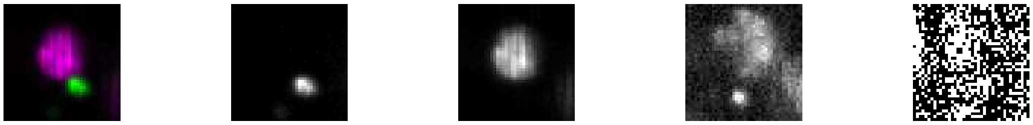				

Ereignis	Aufnahme	DAPI/CK-PE	CK-PE	DAPI	CD45-APC	HER-2/neu
25226	149	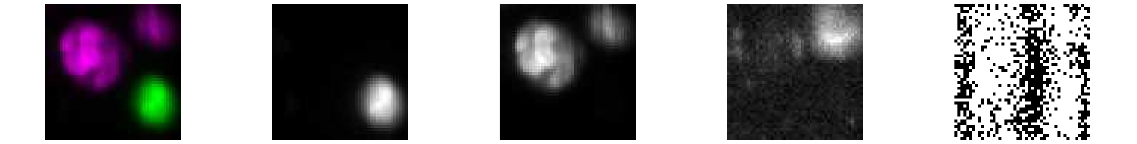				
25227	149	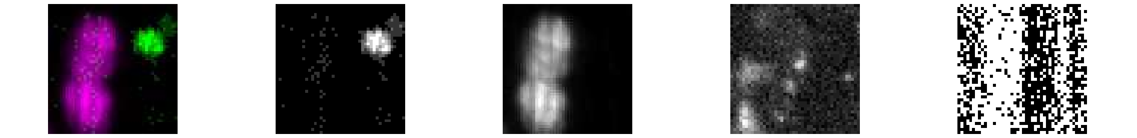				
25228	149	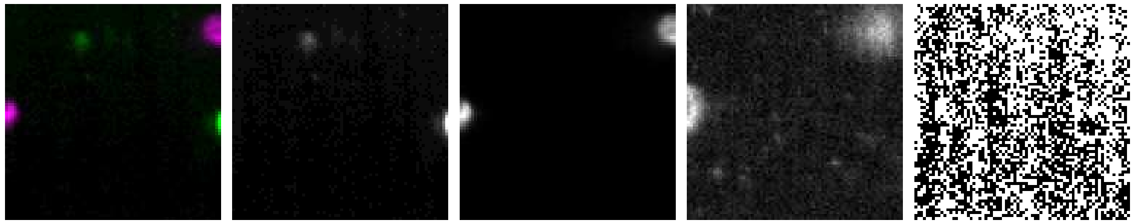				
25229	149	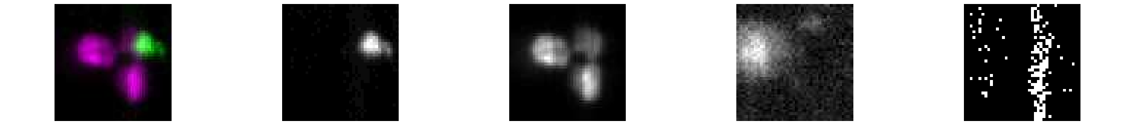				
25230	149	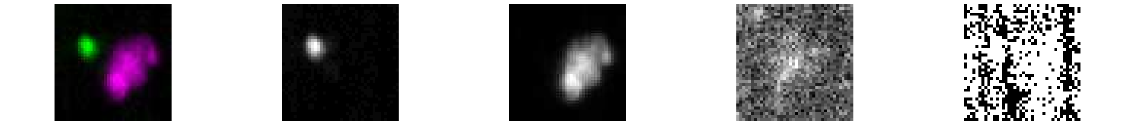				
25231	149	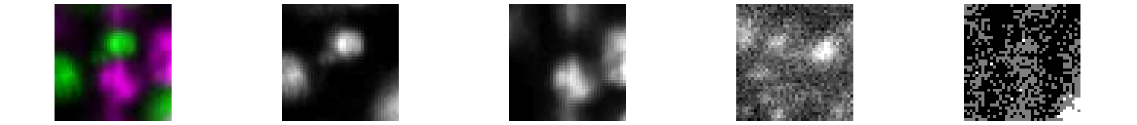				
25232	149	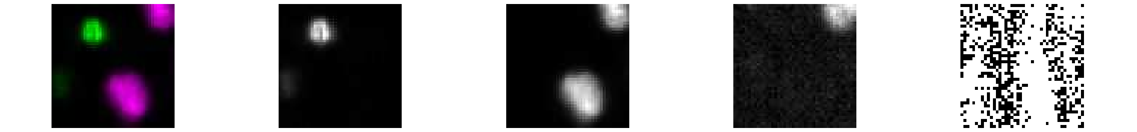				
25233	149	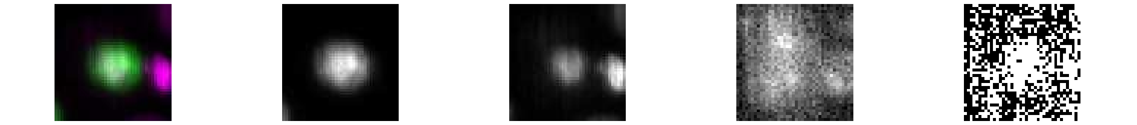				
25234	149	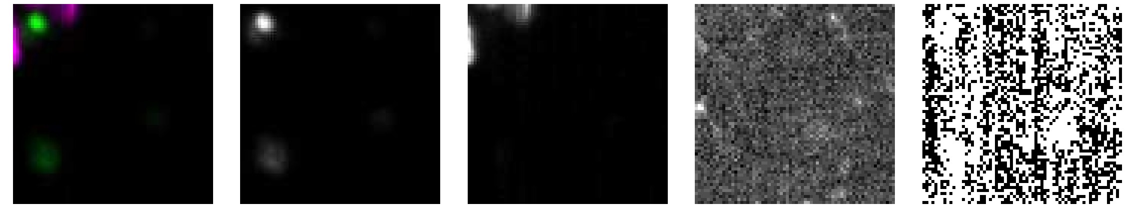				
25235	149	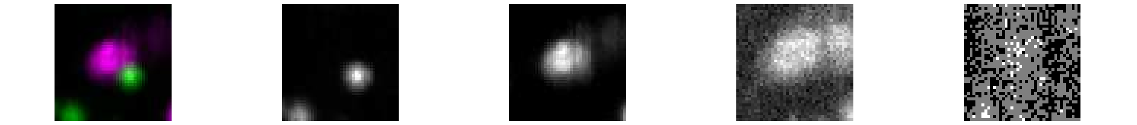				
25236	149	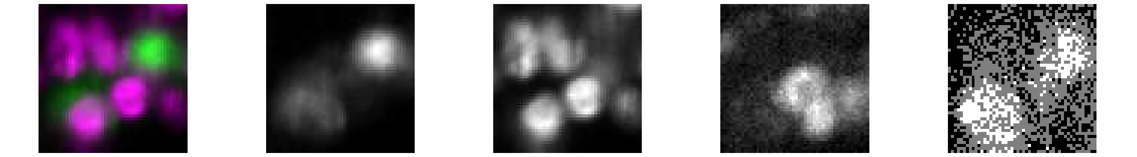				
25237	149	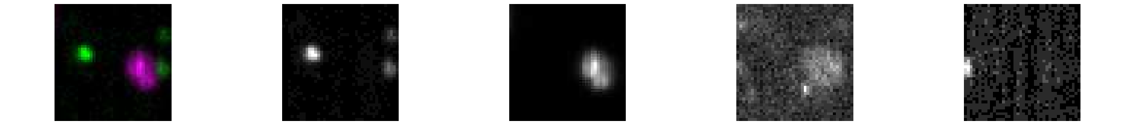				

Ereignis	Aufnahme	DAPI/CK-PE	CK-PE	DAPI	CD45-APC	HER-2/neu
25238	149	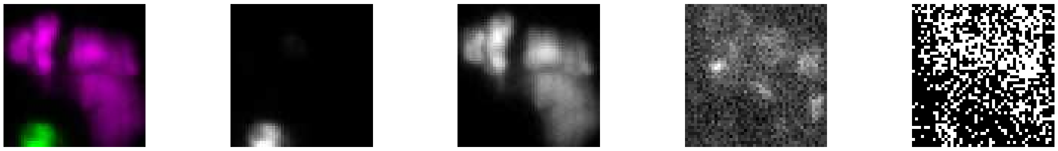				
25239	149	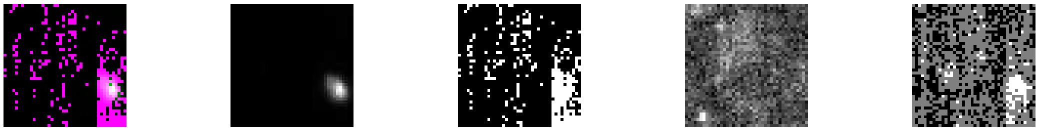				
25240	149	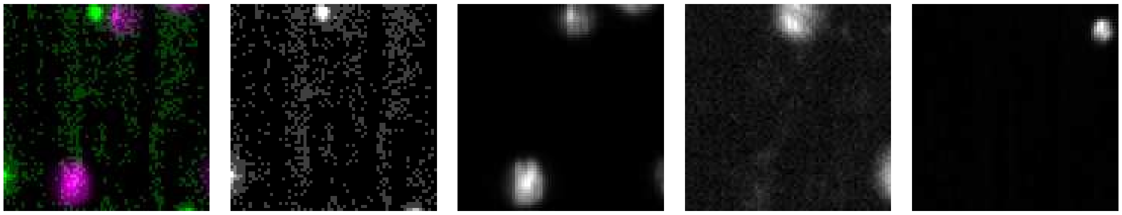				
25241	149	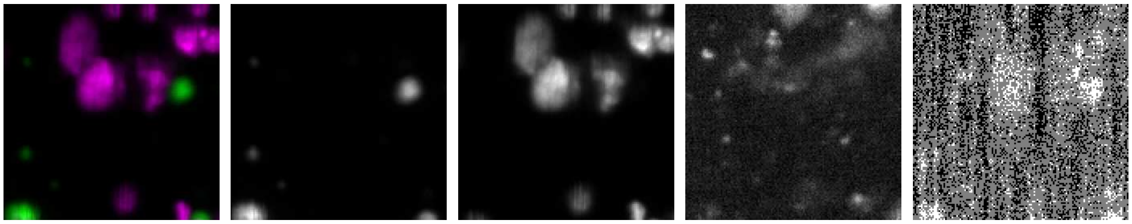				
25242	149	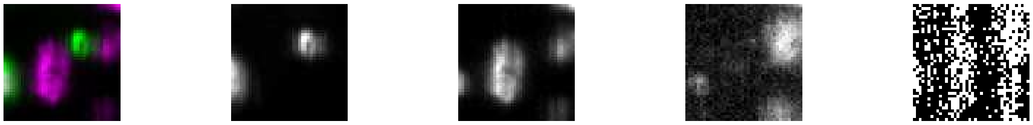				
25243	149	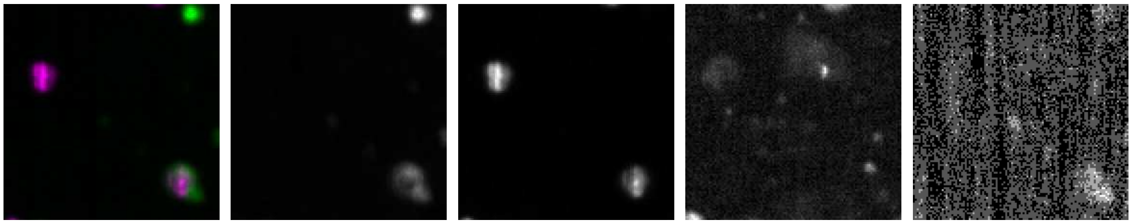				
25244	149	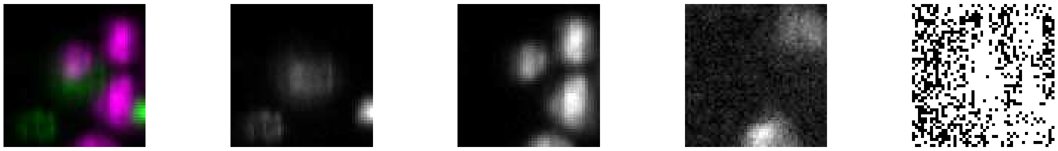				
25245	149	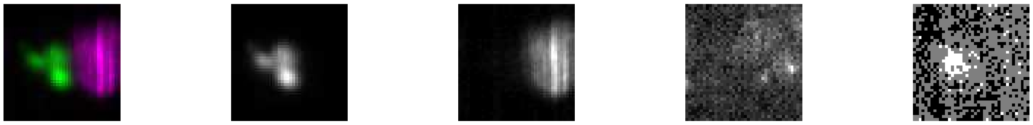				
25246	149	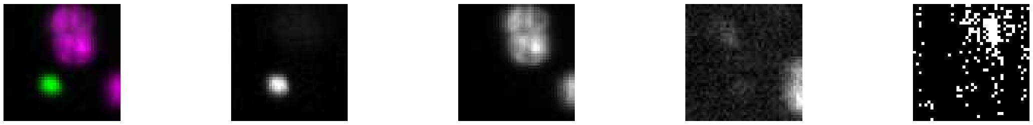				
25247	149	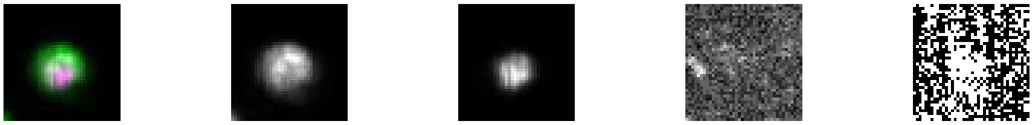				
25248	149	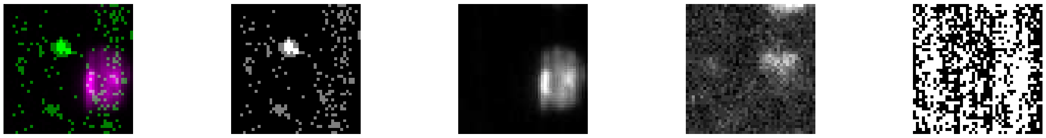				

Ereignis	Aufnahme	DAPI/CK-PE	CK-PE	DAPI	CD45-APC	HER-2/neu
25249	149	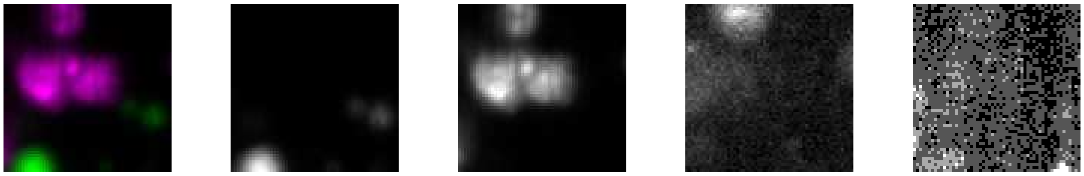				
25250	149	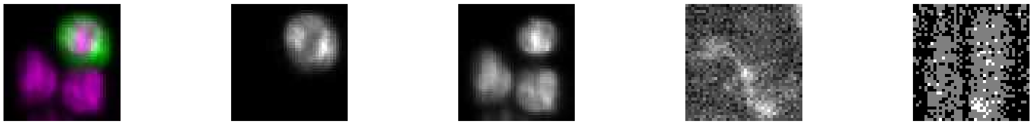				
25251	149	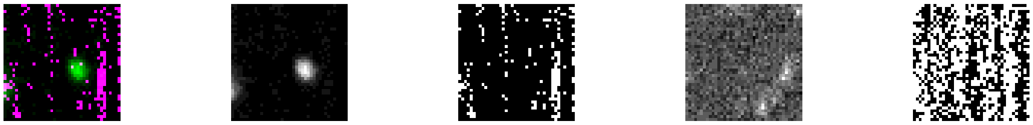				
25252	149	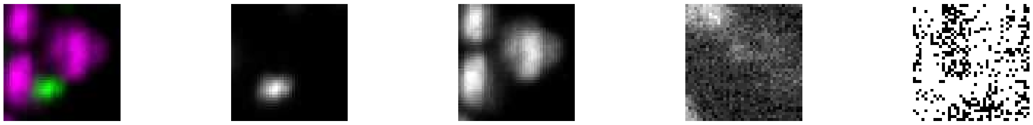				
25253	149	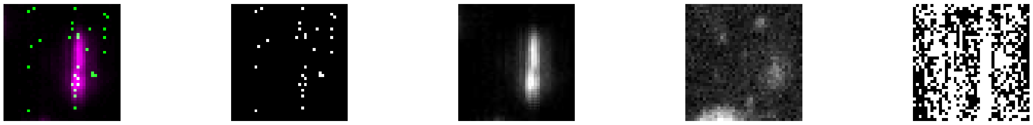				
25254	149	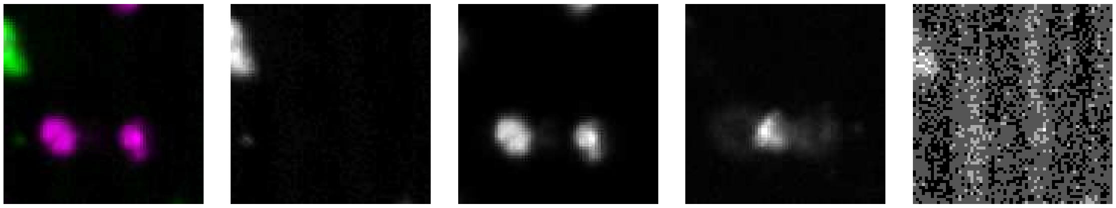				
25255	149	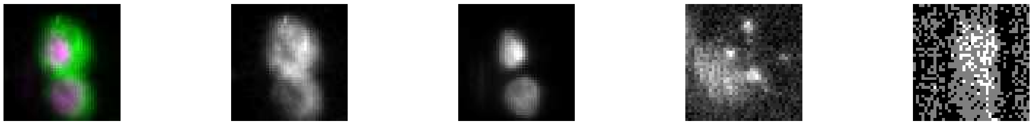				
25256	149	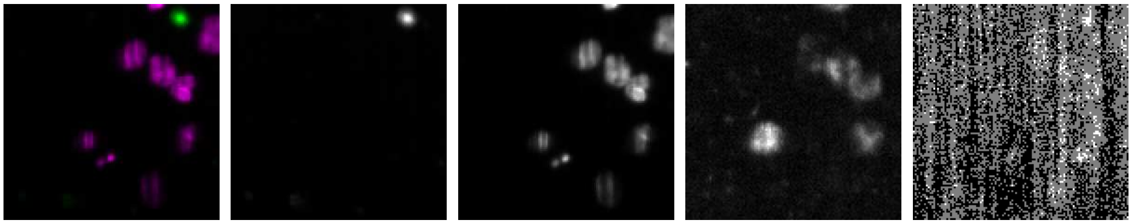				
25257	149	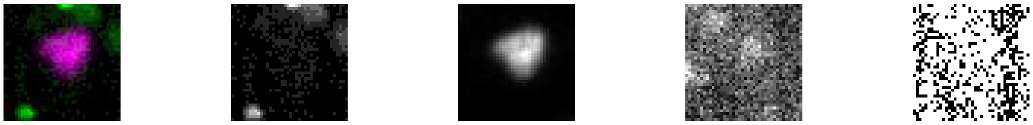				
25258	149	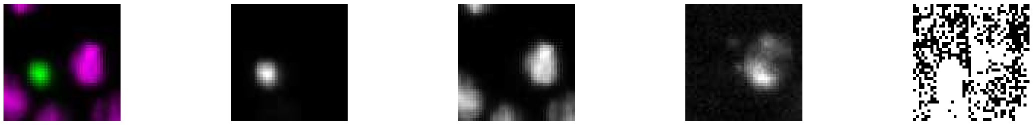				
25259	149	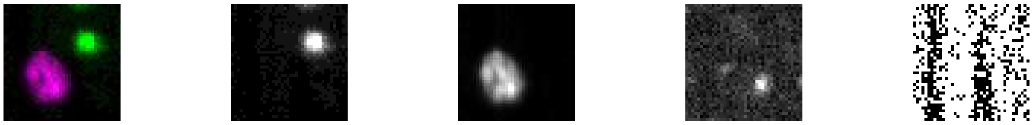				
25260	149	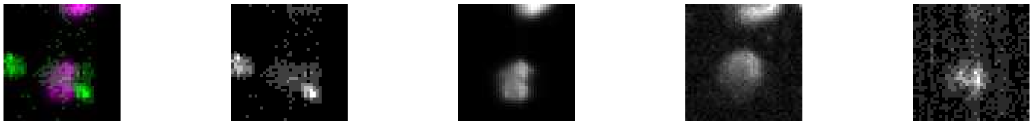				

Ereignis	Aufnahme	DAPI/CK-PE	CK-PE	DAPI	CD45-APC	HER-2/neu
25261	149	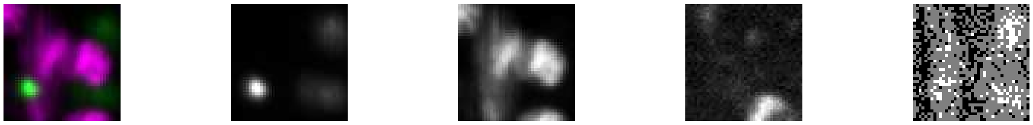				
25262	149	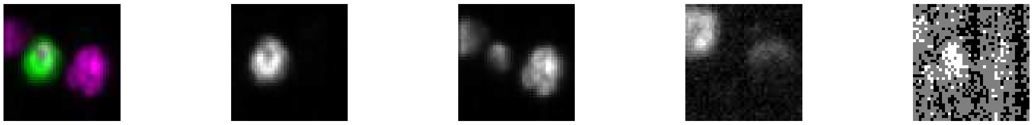				
25263	149	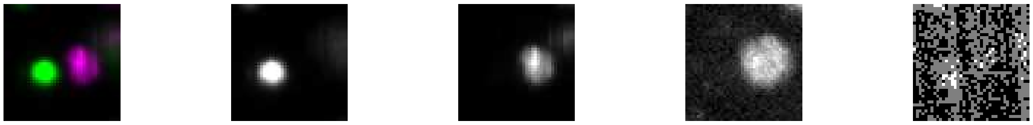				
25264	149	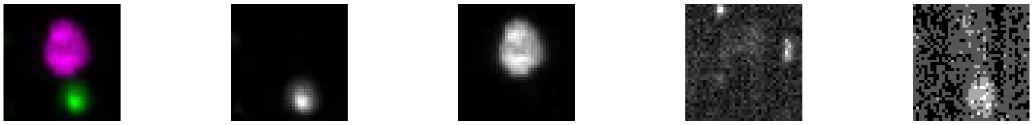				
25265	149	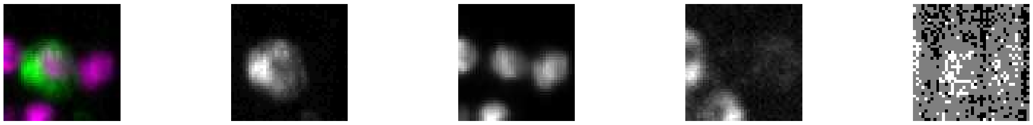				
25266	149	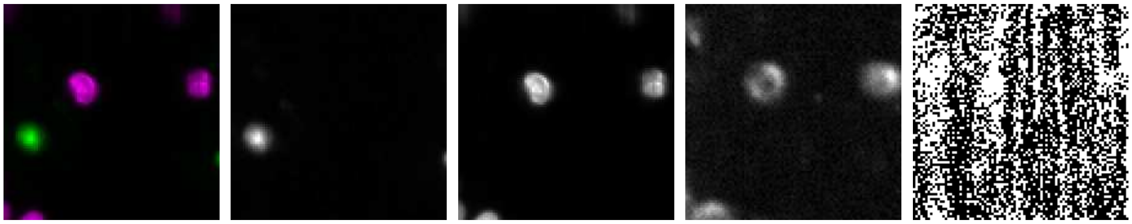				
25267	149	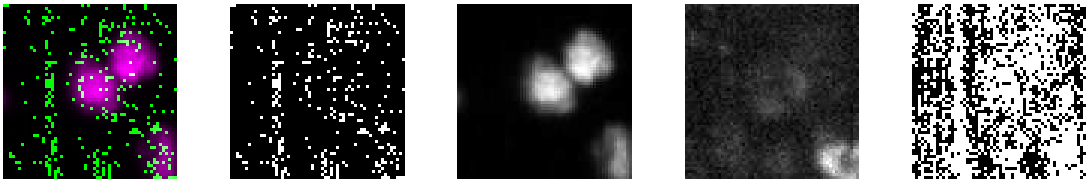				
25268	149	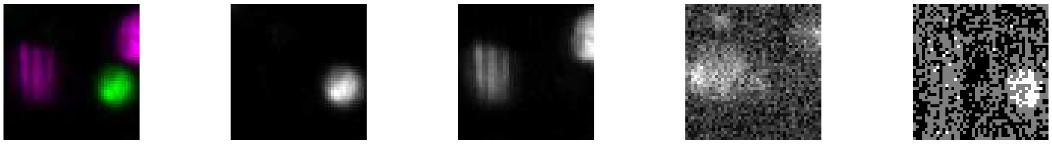				
25269	149	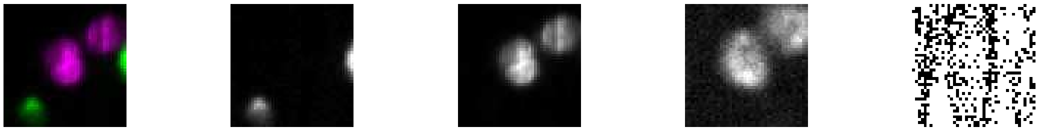				
25270	149	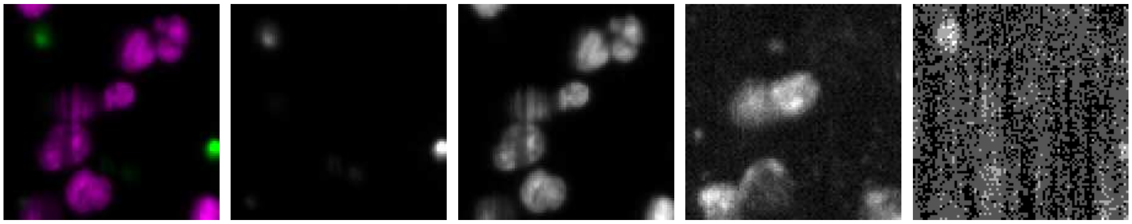				
25271	149	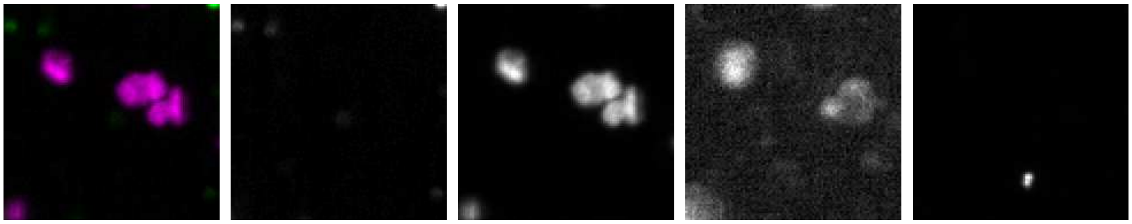				

Ereignis	Aufnahme	DAPI/CK-PE	CK-PE	DAPI	CD45-APC	HER-2/neu
25272	149	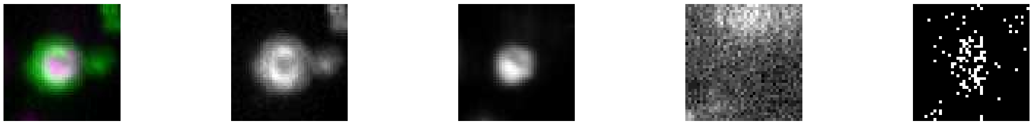				
25273	149	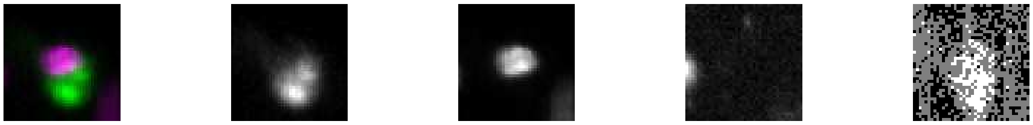				
25274	149	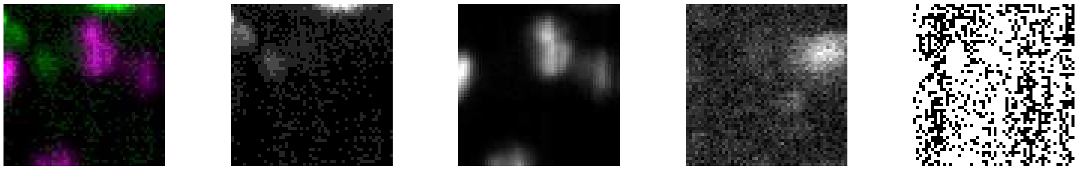				
25275	149	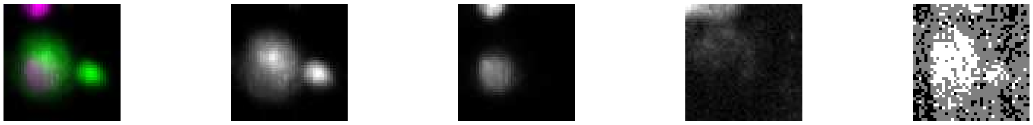				
25276	149	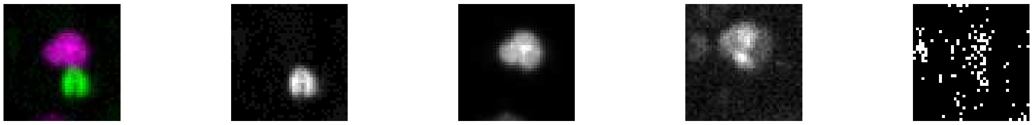				
25277	149	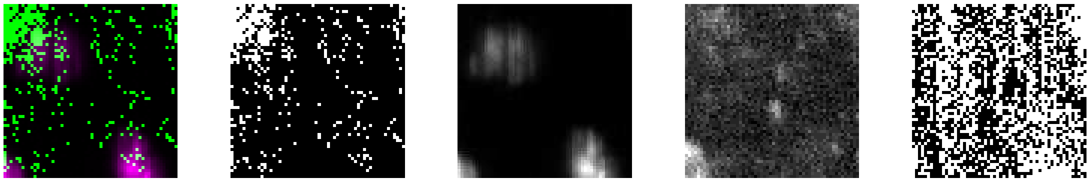				
25278	149	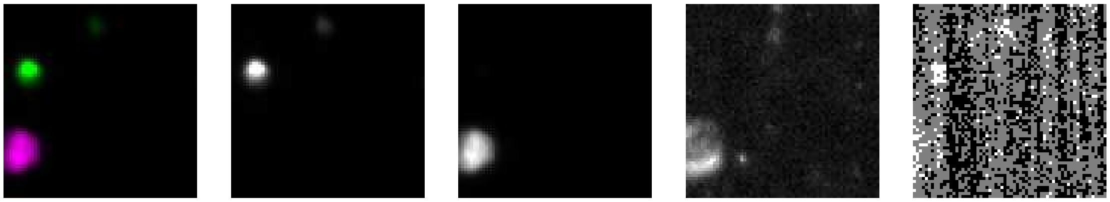				
25279	149	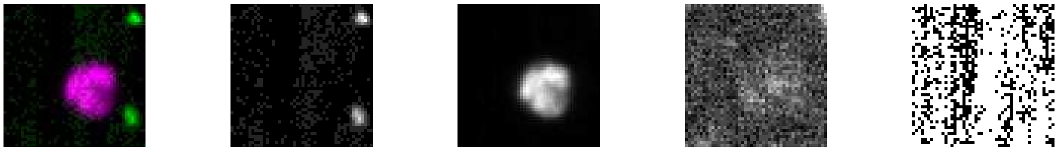				
25280	149	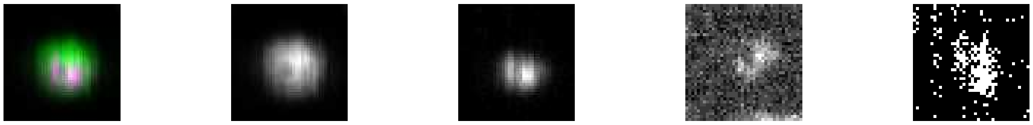				
25281	149	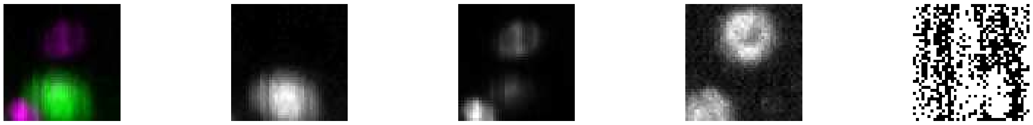				
25282	149	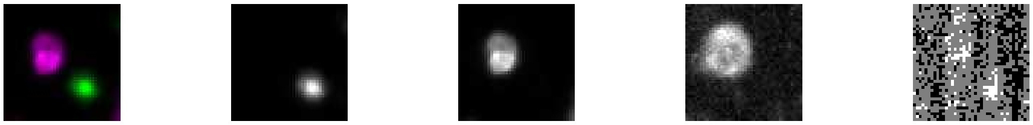				
25283	149	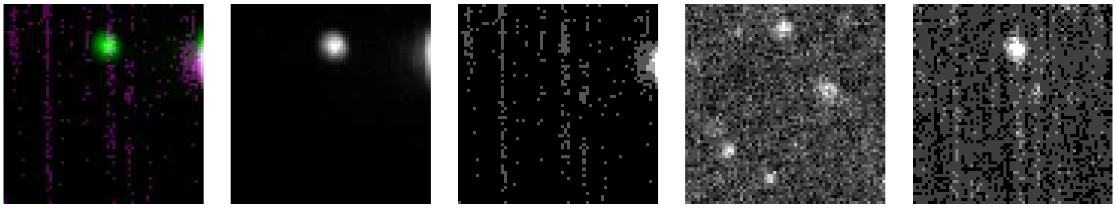				

Ereignis	Aufnahme	DAPI/CK-PE	CK-PE	DAPI	CD45-APC	HER-2/neu
25284	149	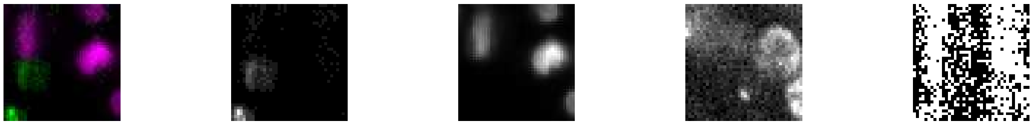				
25285	149	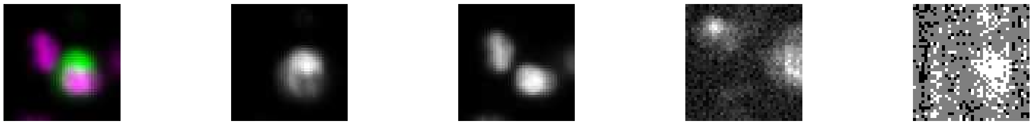				
25286	149	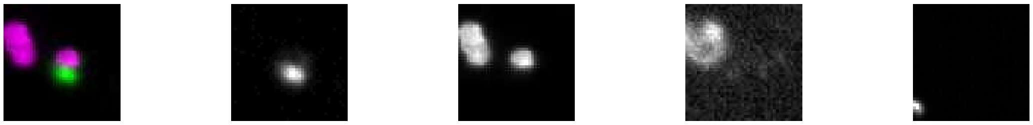				
25287	149	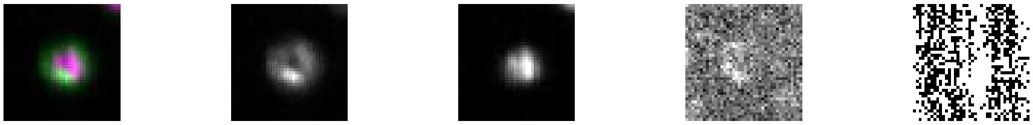				
25288	149	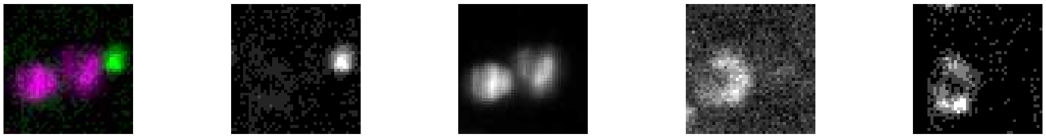				
25289	149	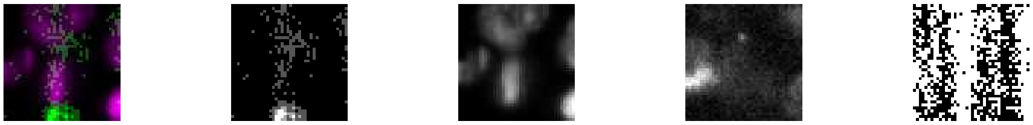				
25290	149	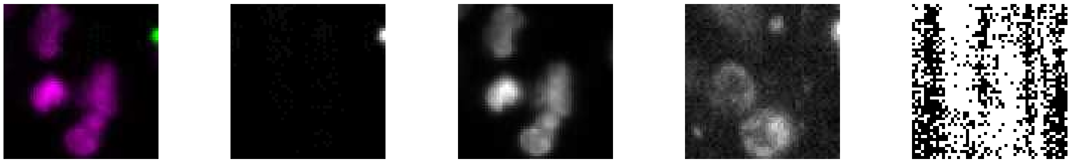				
25291	149	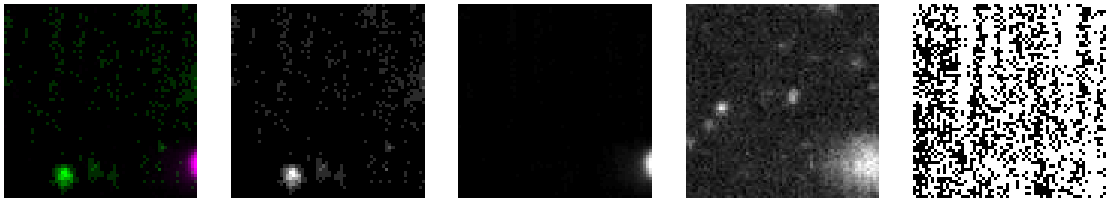				
25292	149	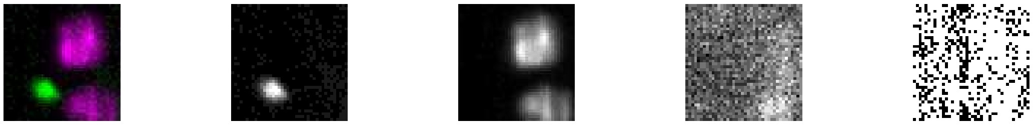				
25293	149	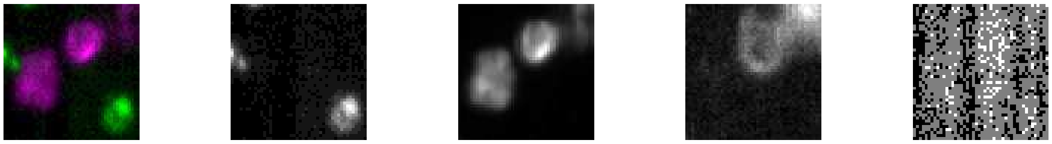				
25294	149	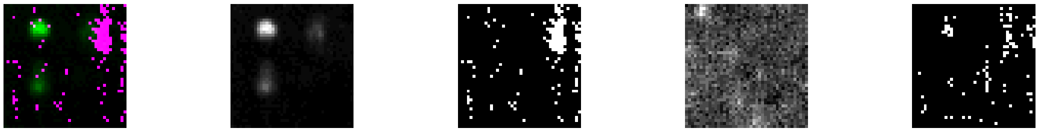				
25295	148	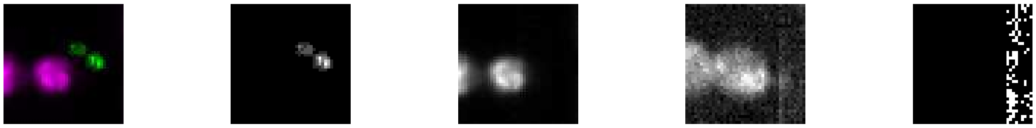				

Proben-ID: Gyn14.208 **Kassetten-ID:**01327106 **Patienten-ID:**

Ereignis	Aufnahme	DAPI/CK-PE	CK-PE	DAPI	CD45-APC	HER-2/neu
25296	148	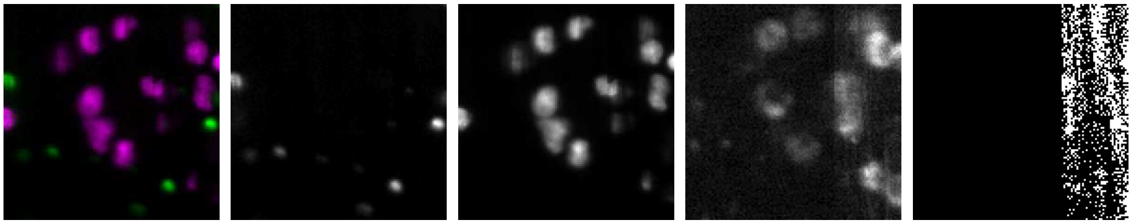				
25297	149	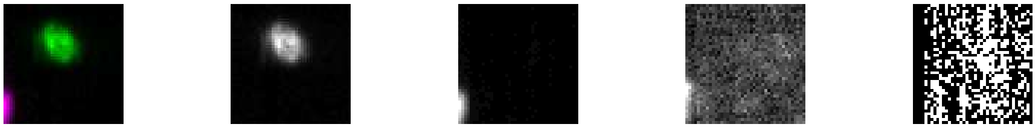				
25298	132	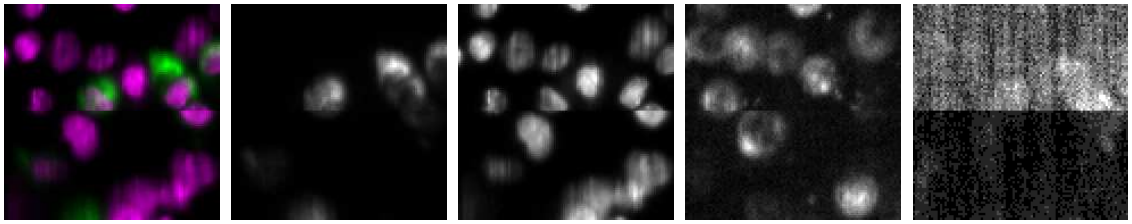				
25299	132	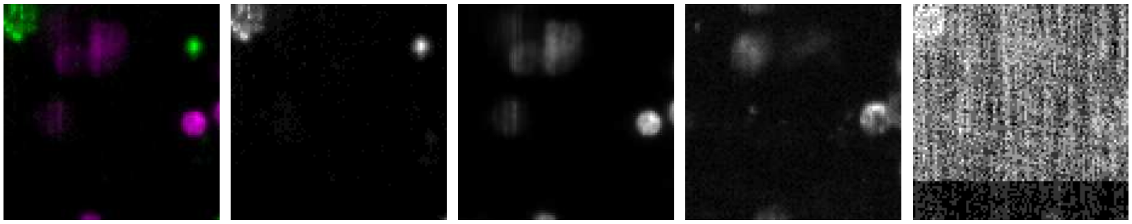				
25300	132	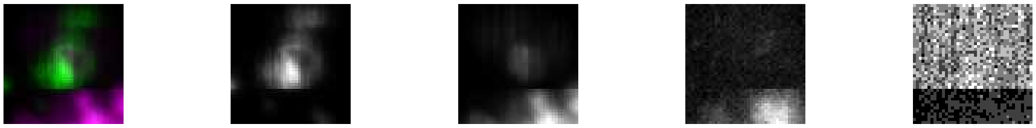				
25301	149	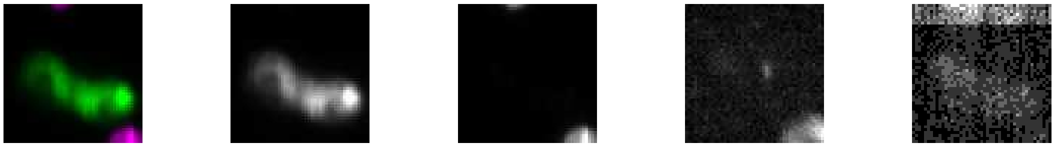				
25302	149	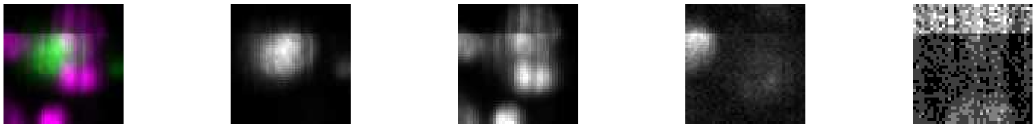				
25303	132	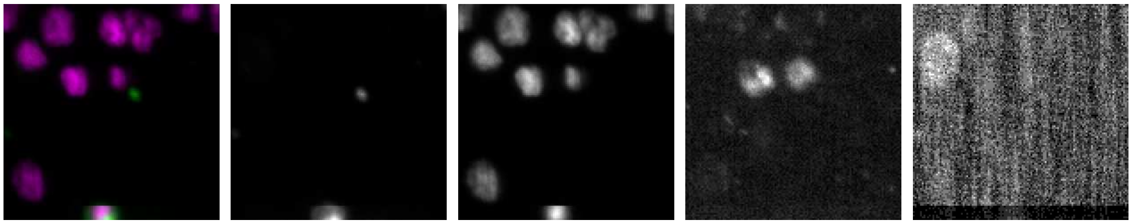				
25304	150	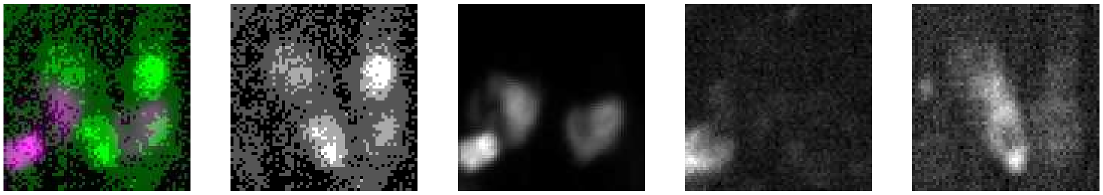				
25305	150	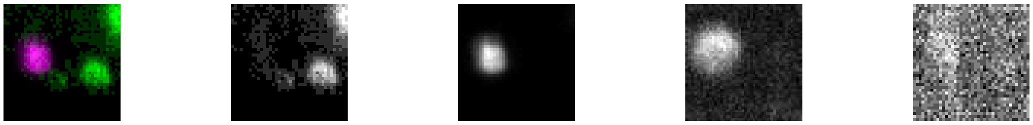				

Ereignis	Aufnahme	DAPI/CK-PE	CK-PE	DAPI	CD45-APC	HER-2/neu
25306	150	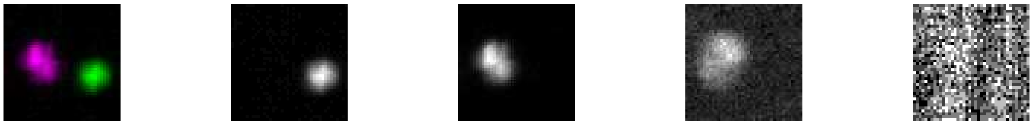				
25307	150	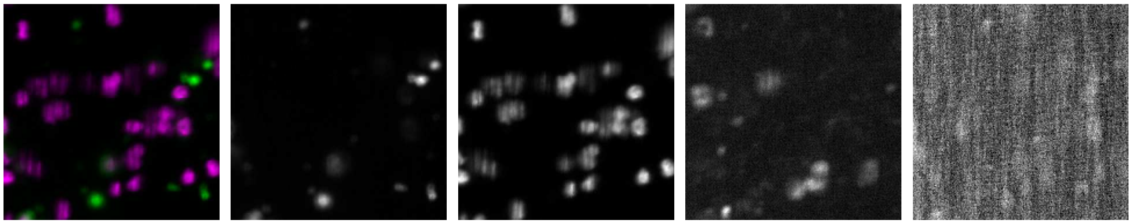				
25308	150	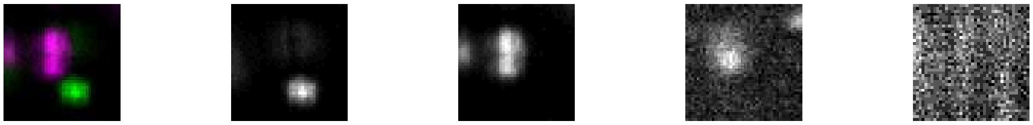				
25309	150	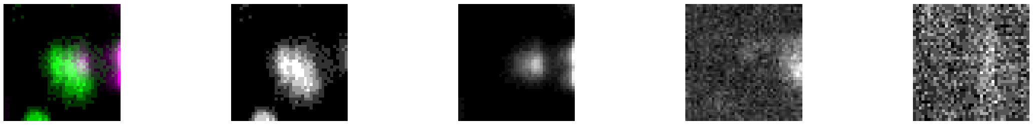				
25310	150	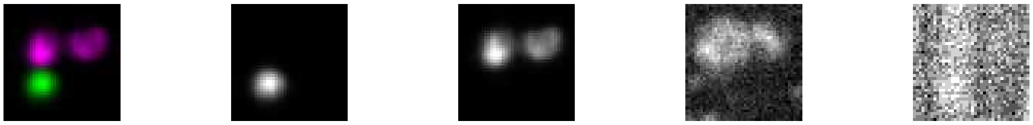				
25311	150	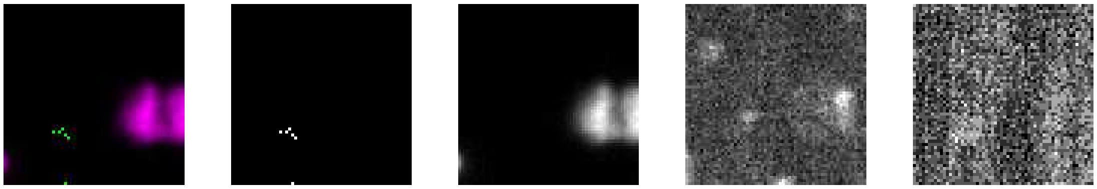				
25312	150	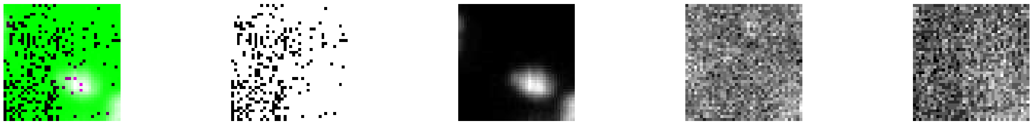				
25313	150	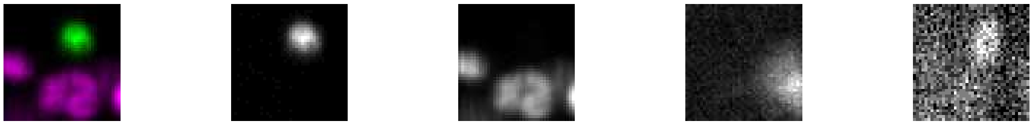				
25314	150	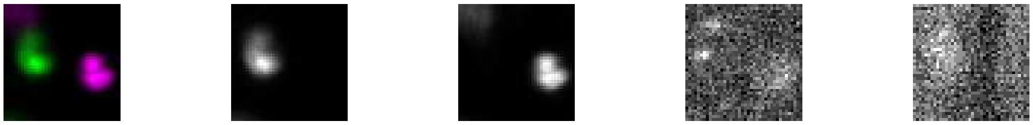				
25315	150	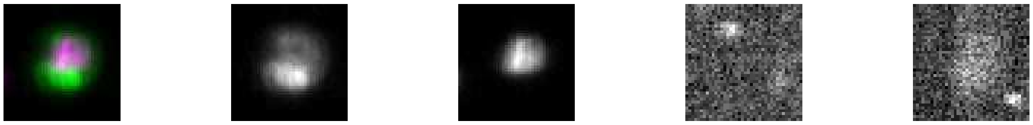				
25316	150	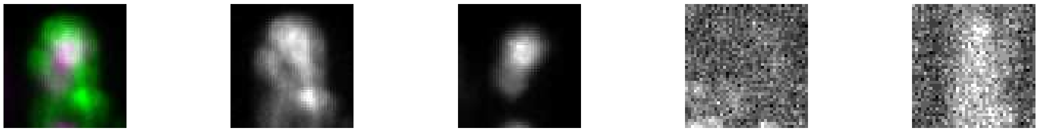				
25317	150	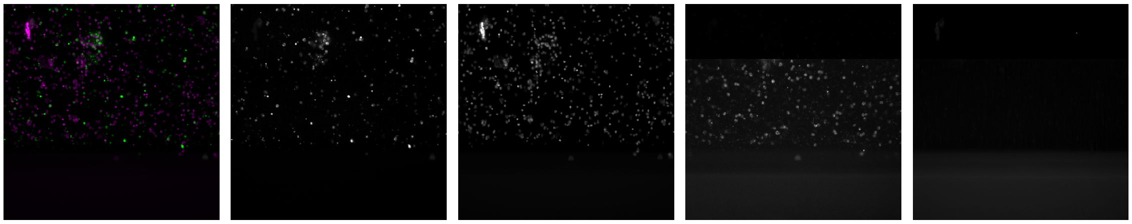				

Ereignis	Aufnahme	DAPI/CK-PE	CK-PE	DAPI	CD45-APC	HER-2/neu
25318	150	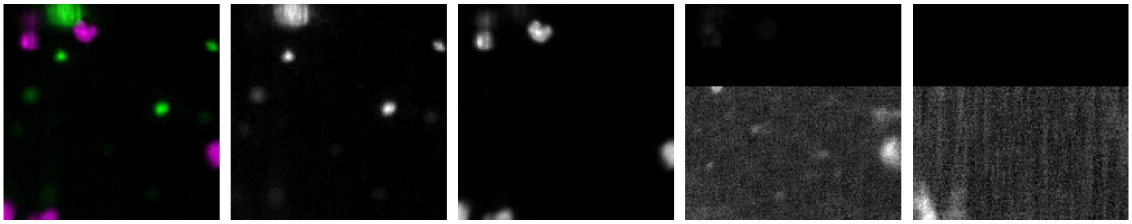				
25319	131	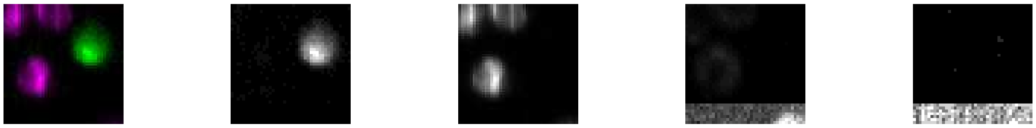				
25320	149	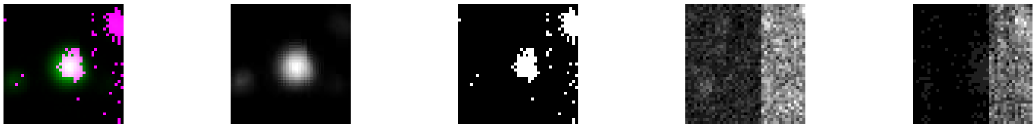				
25321	151	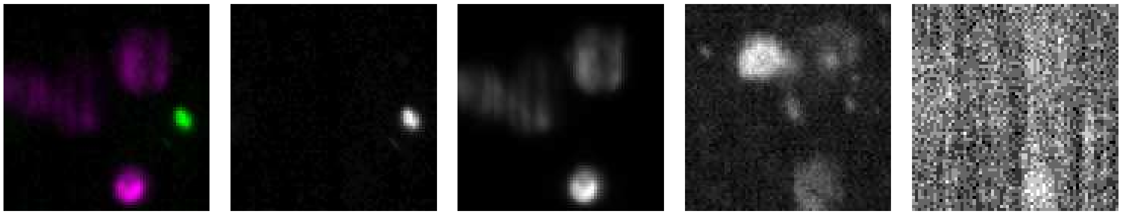				
25322	151	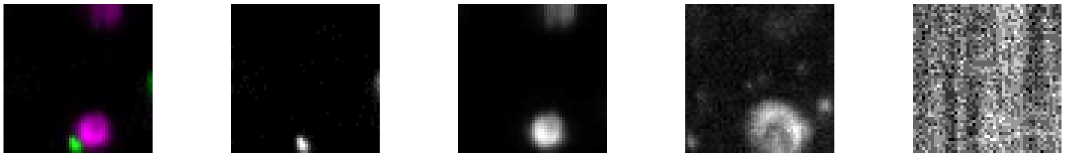				
25323	151	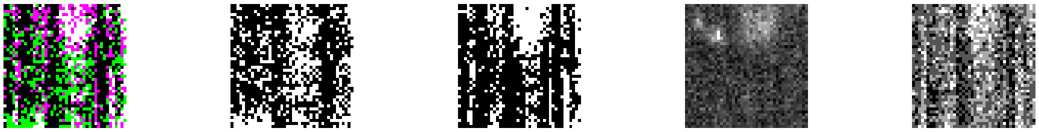				
25324	151	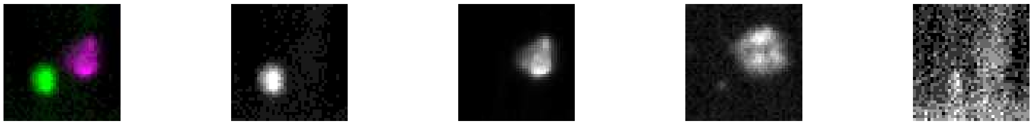				
25325	151	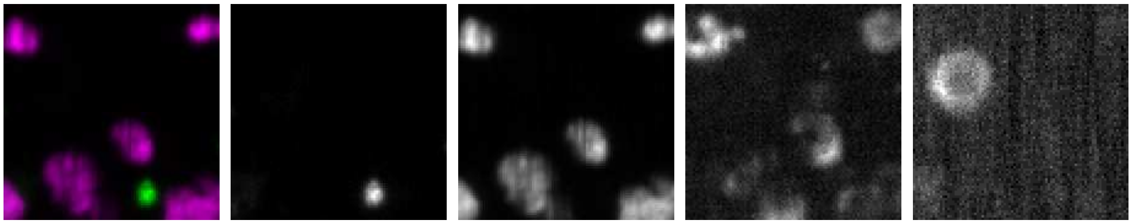				
25326	151	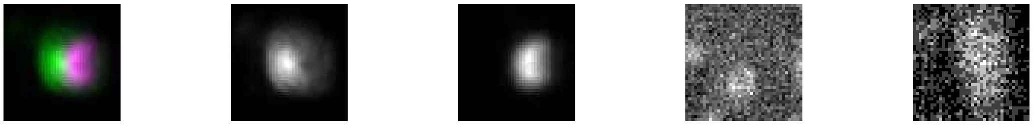				
25327	151	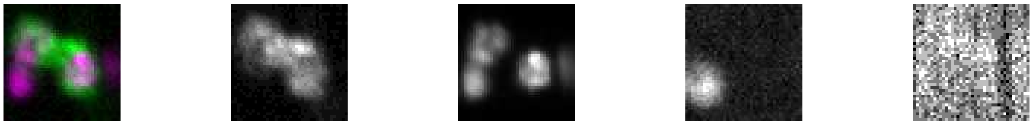				
25328	151	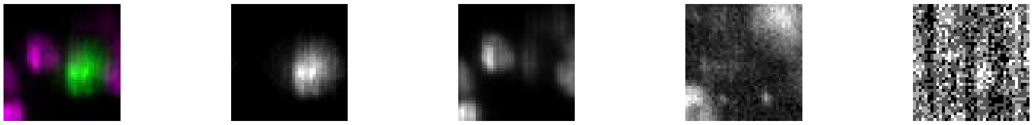				

Ereignis	Aufnahme	DAPI/CK-PE	CK-PE	DAPI	CD45-APC	HER-2/neu
25329	151	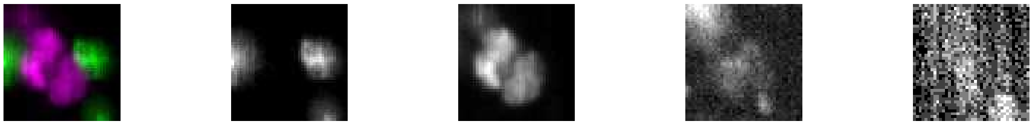				
25330	151	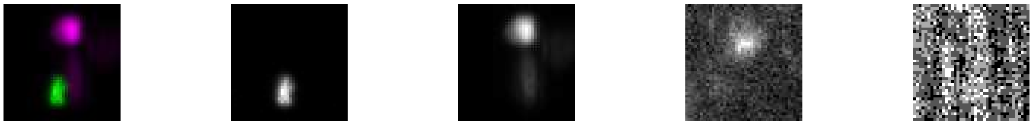				
25331	151	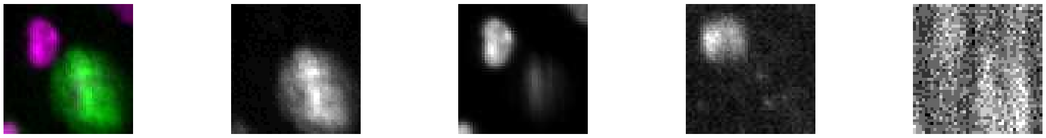				
25332	151	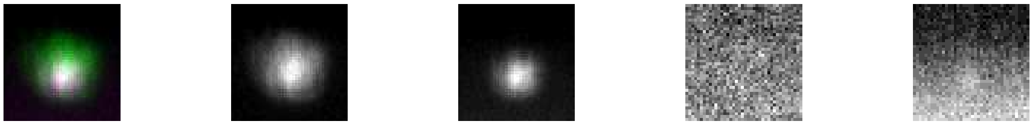				
25333	151	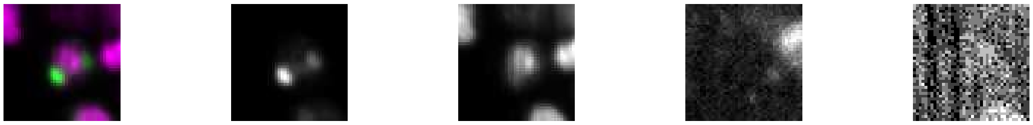				
25334	151	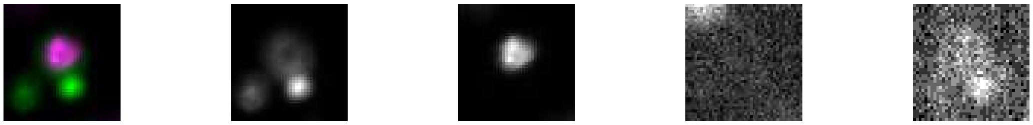				
25335	151	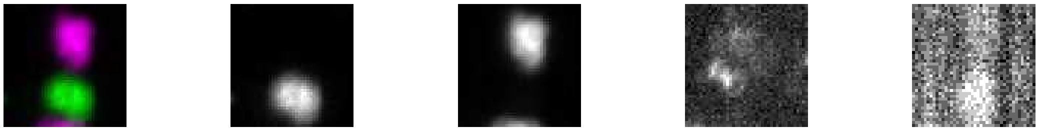				
25336	151	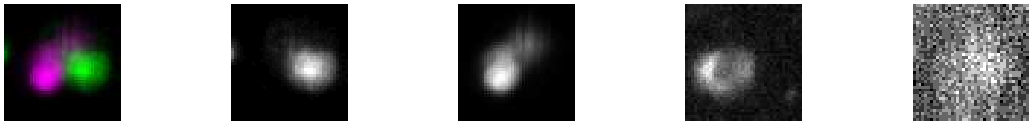				
25337	151	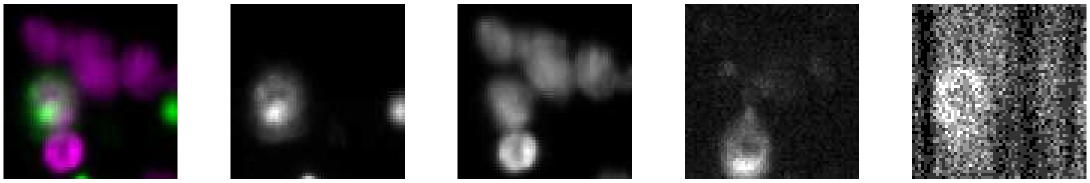				
25338	151	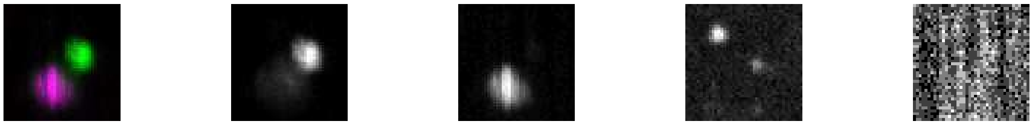				
25339	151	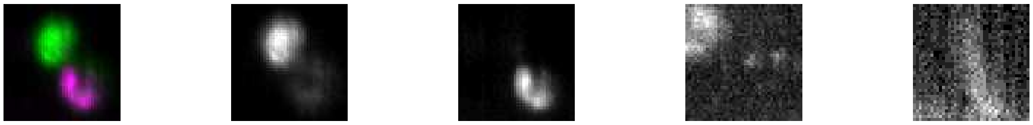				
25340	151	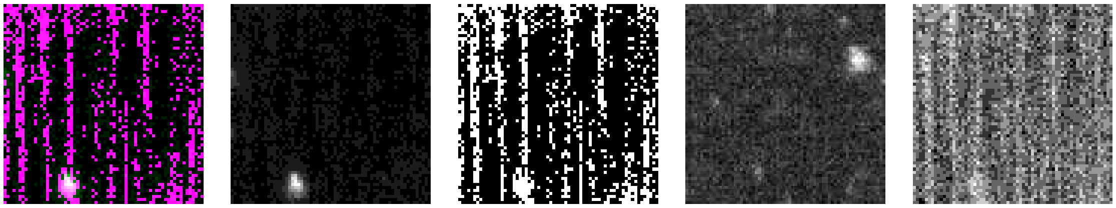				
25341	151	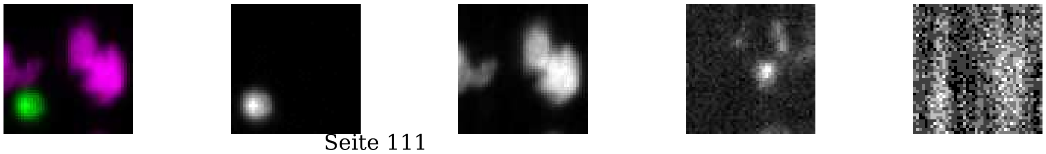				

Ereignis	Aufnahme	DAPI/CK-PE	CK-PE	DAPI	CD45-APC	HER-2/neu
25342	151	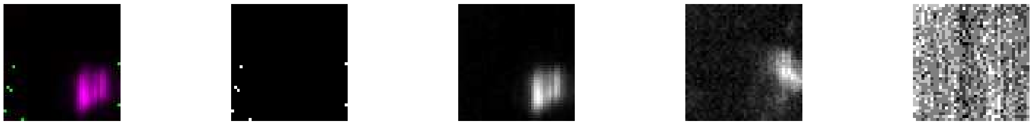				
25343	151	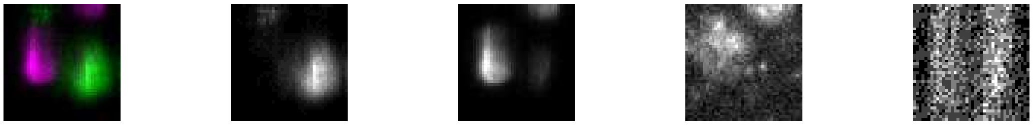				
25344	151	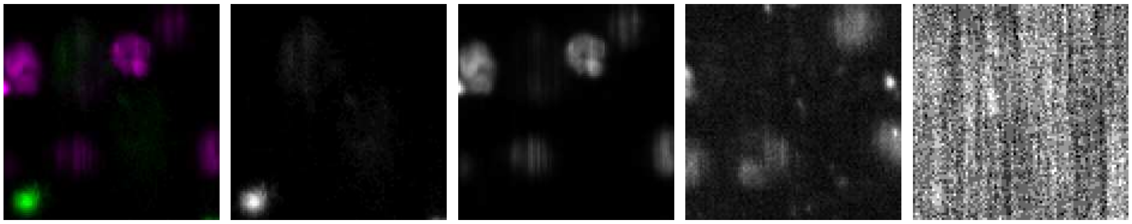				
25345	151	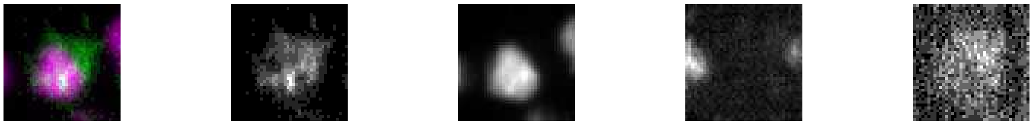				
25346	151	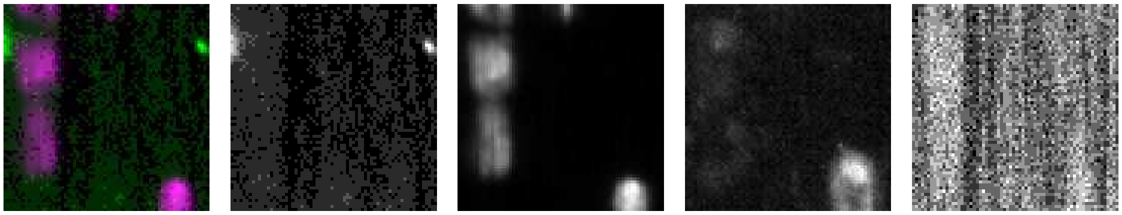				
25347	151	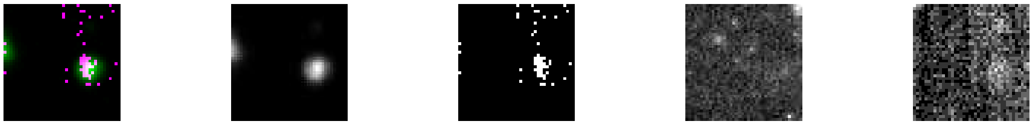				
25348	151	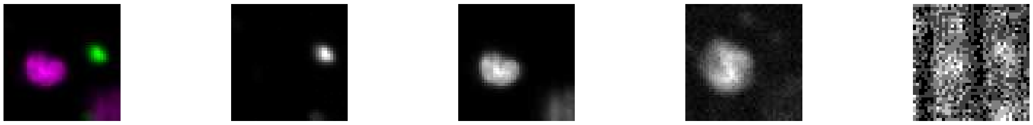				
25349	151	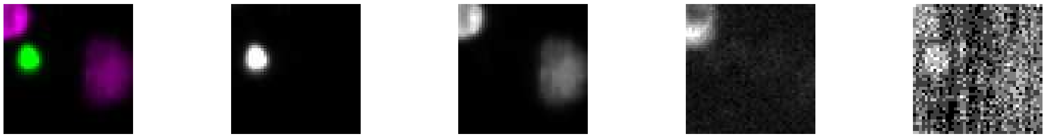				
25350	151	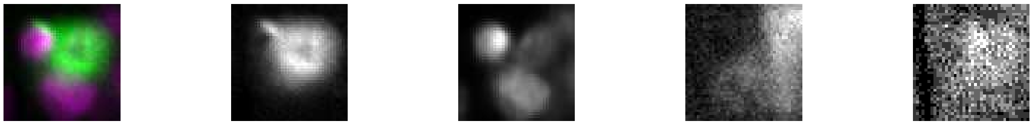				
25351	151	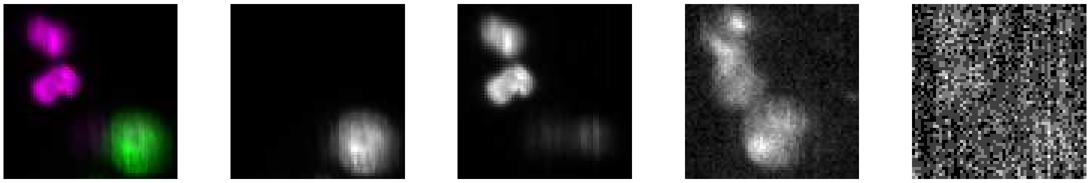				
25352	151	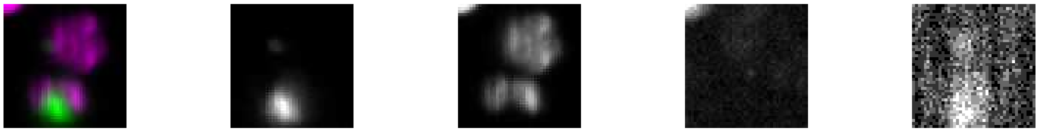				

Ereignis	Aufnahme	DAPI/CK-PE	CK-PE	DAPI	CD45-APC	HER-2/neu
25353	151	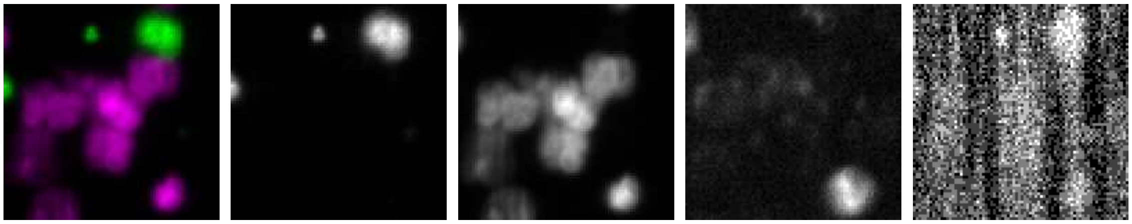				
25354	151	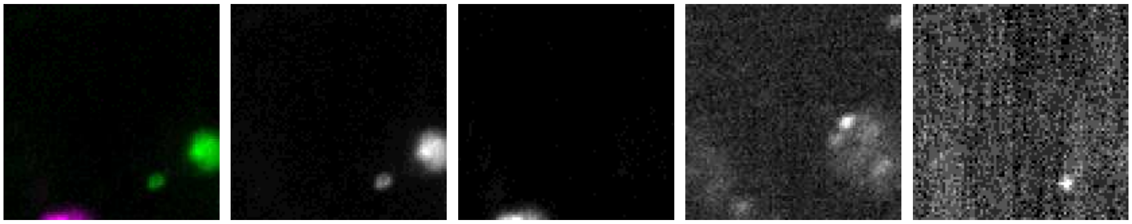				
25355	151	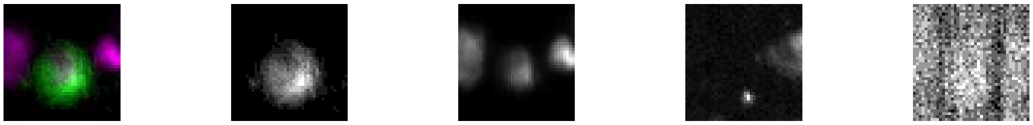				
25356	151	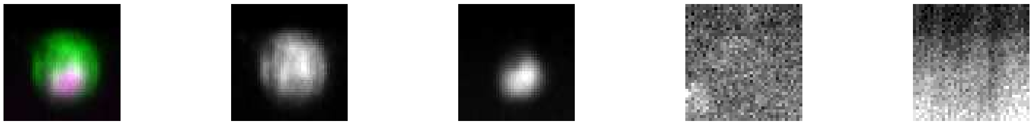				
25357	151	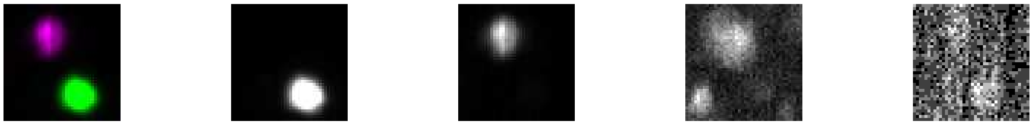				
25358	151	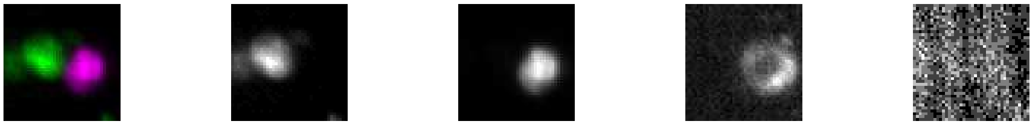				
25359	151	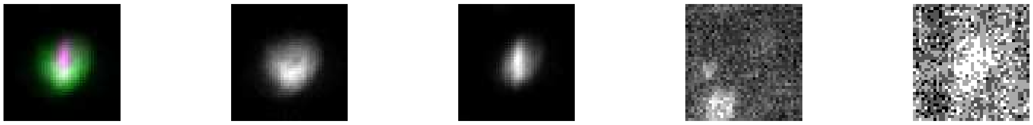				
25360	151	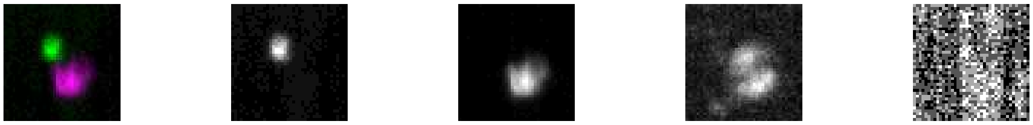				
25361	151	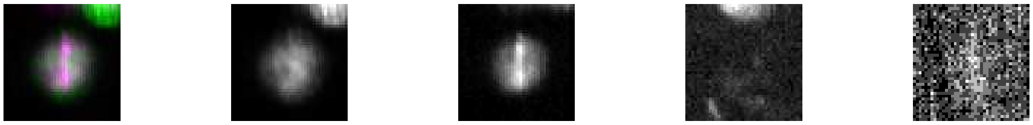				
25362	151	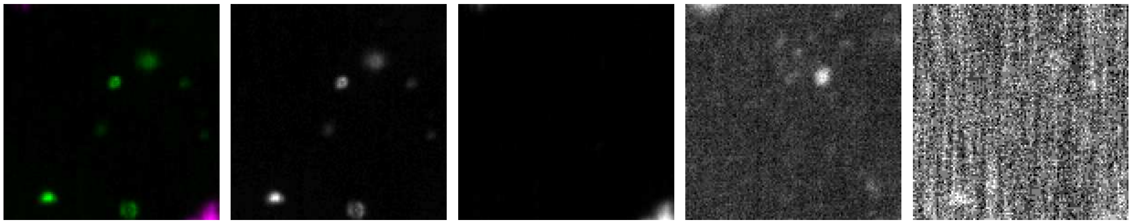				
25363	151	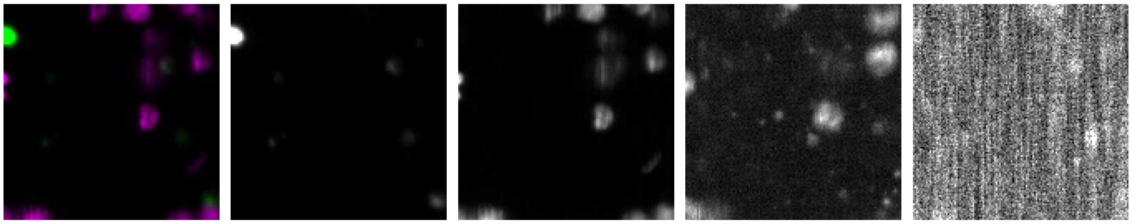				

Druckdatum:02/06/2020 17:25

Proben-ID: Gyn14.208 **Kassetten-ID:**01327106 **Patienten-ID:**

Ereignis	Aufnahme	DAPI/CK-PE	CK-PE	DAPI	CD45-APC	HER-2/neu
25364	151	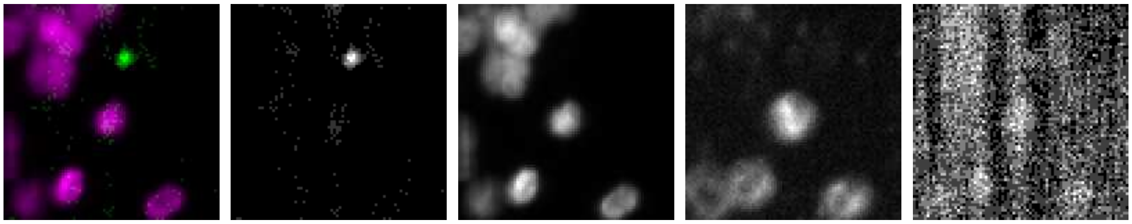				
25365	151	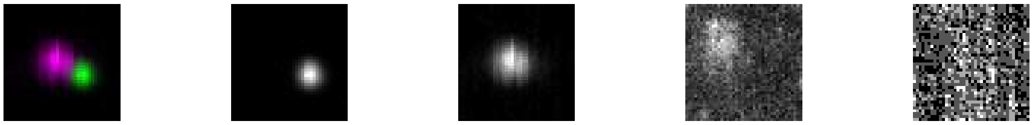				
25366	151	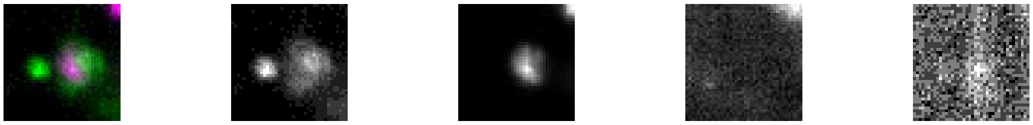				
25367	151	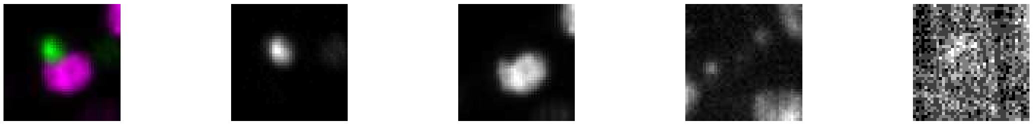				
25368	151	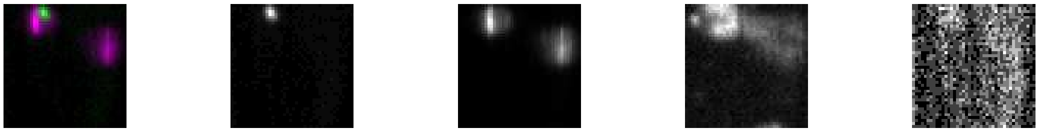				
25369	151	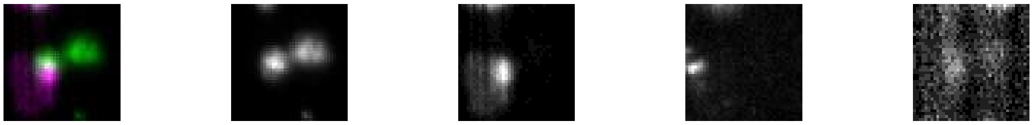				
25370	151	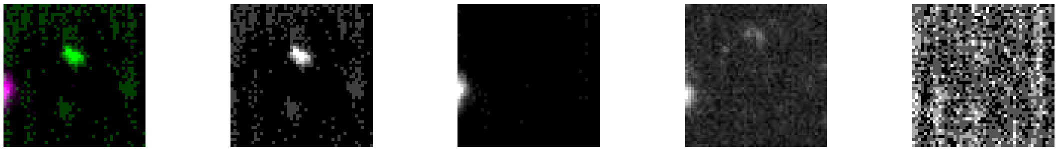				
25371	151	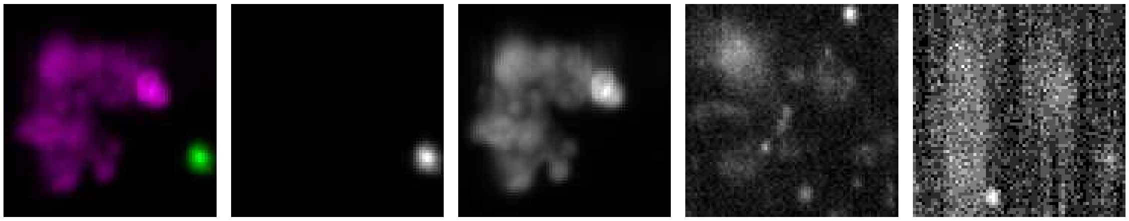				
25372	151	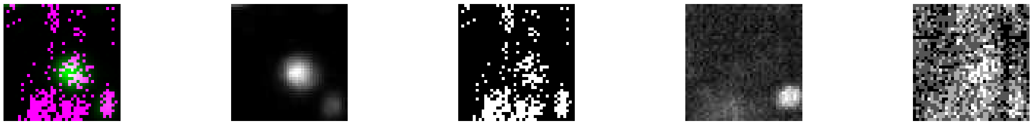				
25373	151	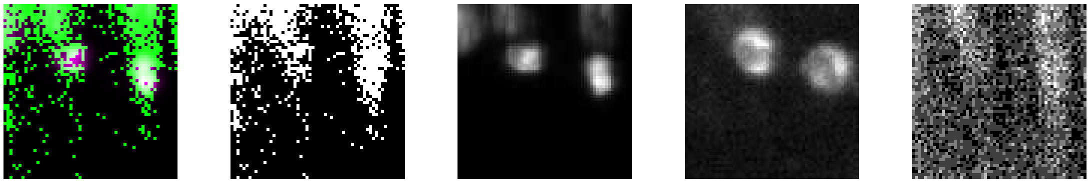				
25374	151	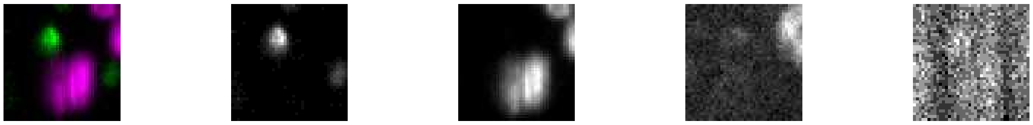				
25375	151	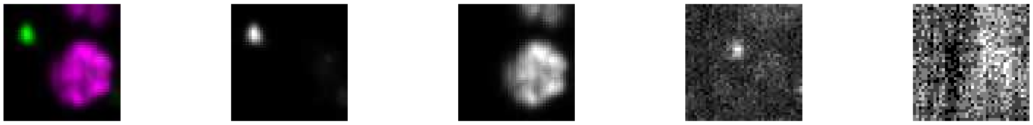				

Ereignis	Aufnahme	DAPI/CK-PE	CK-PE	DAPI	CD45-APC	HER-2/neu
25376	151	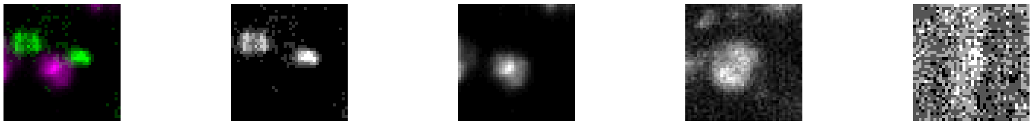				
25377	151	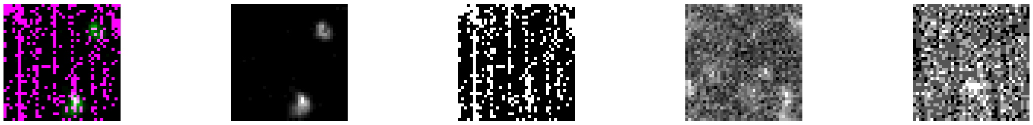				
25378	151	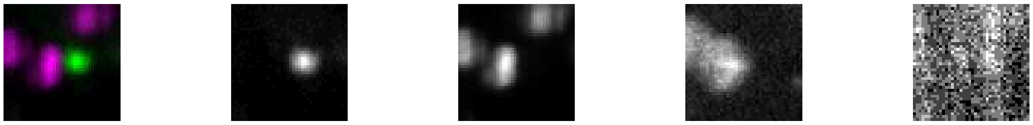				
25379	151	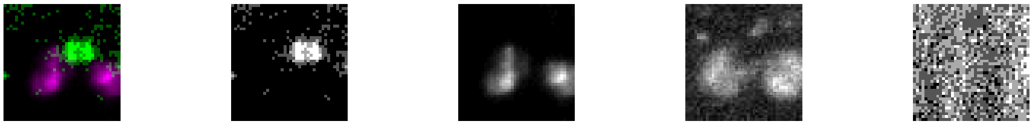				
25380	151	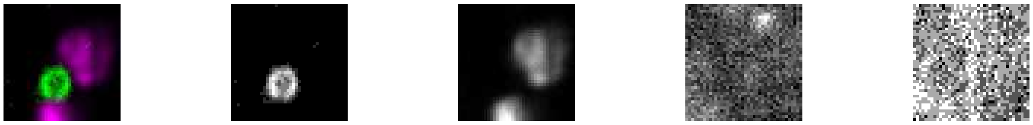				
25381	151	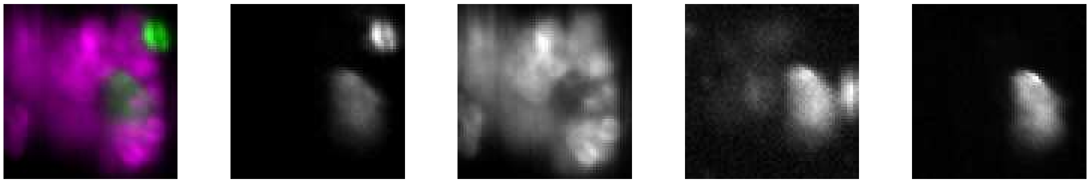				
25382	151	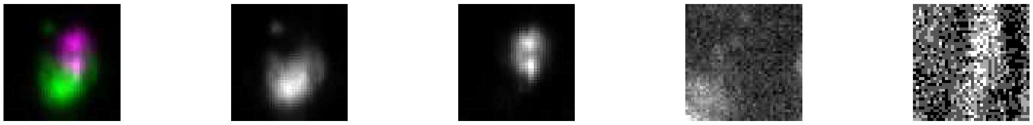				
25383	151	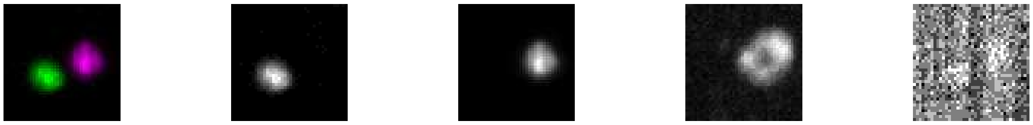				
25384	151	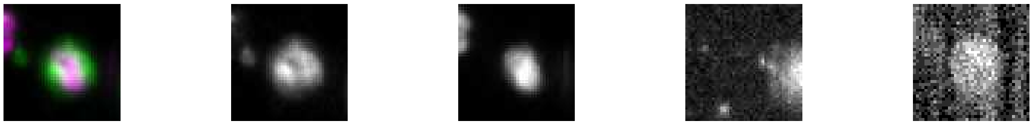				
25385	151	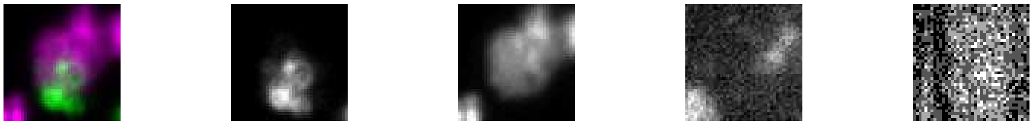				
25386	151	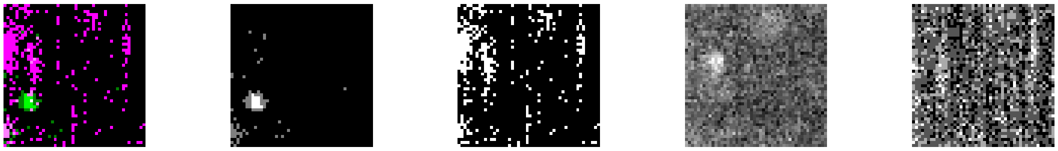				
25387	151	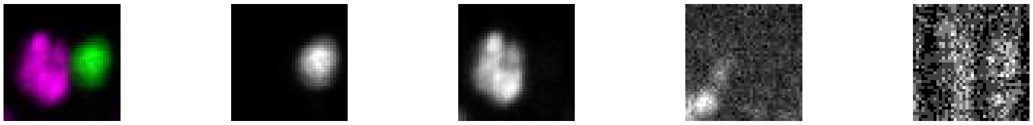				
25388	151	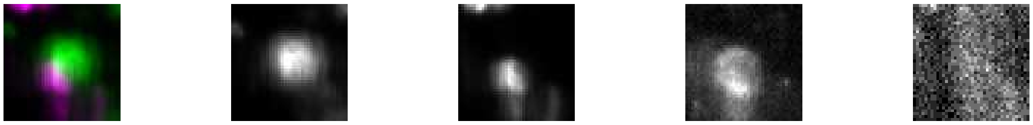				

Proben-ID: Gyn14.208 **Kassetten-ID:**01327106 **Patienten-ID:**

Ereignis	Aufnahme	DAPI/CK-PE	CK-PE	DAPI	CD45-APC	HER-2/neu
25389	151	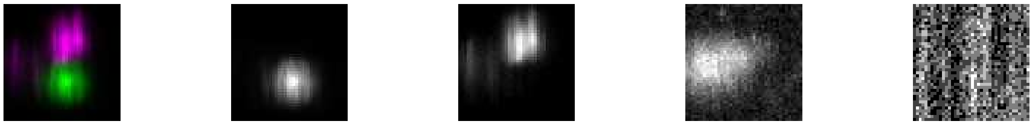				
25390	151	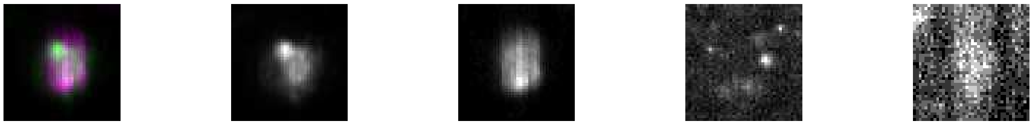				
25391	151	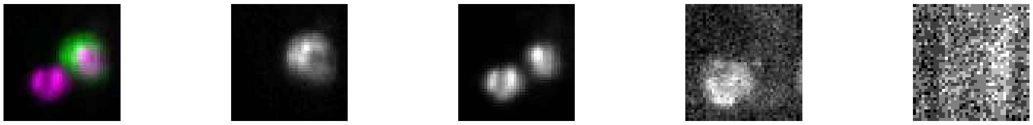				
25392	151	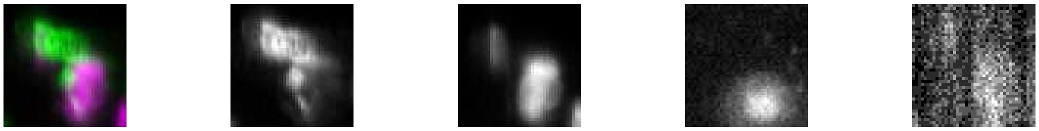				
25393	151	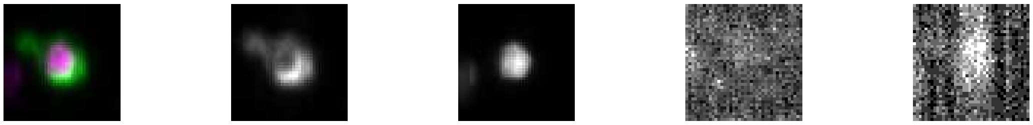				
25394	151	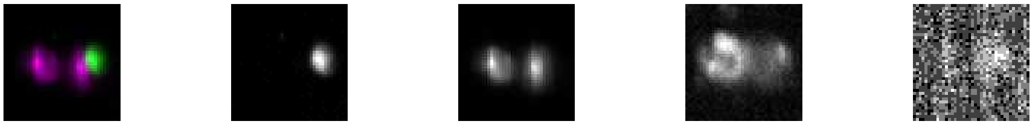				
25395	151	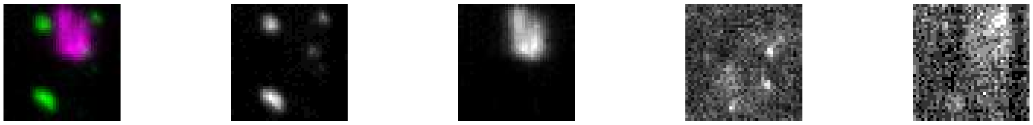				
25396	151	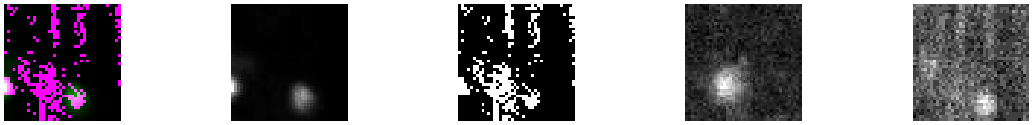				
25397	151	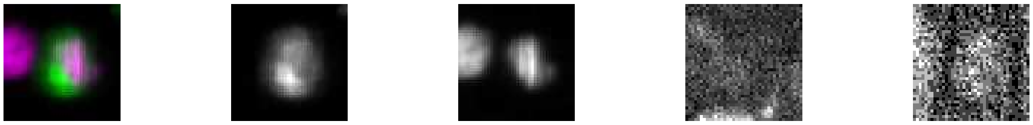				
25398	151	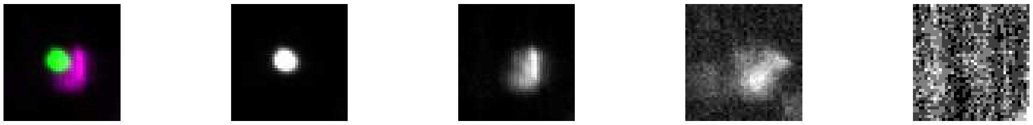				
25399	151	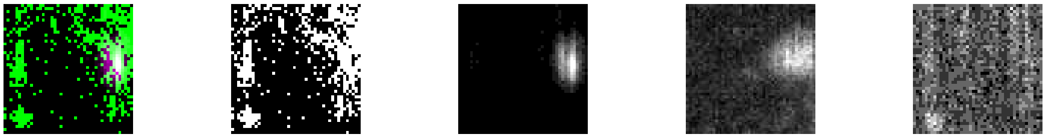				
25400	151	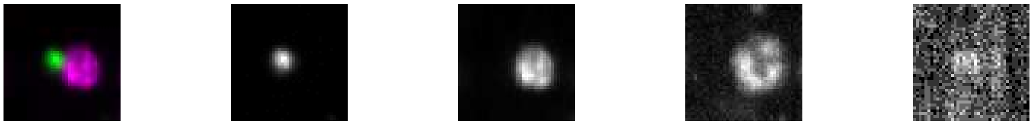				
25401	151	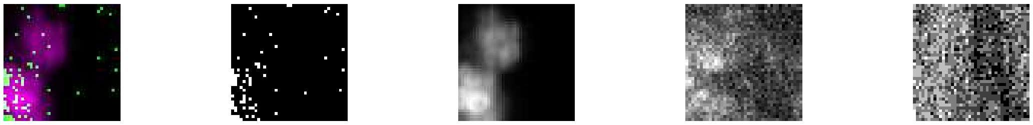				
25402	151	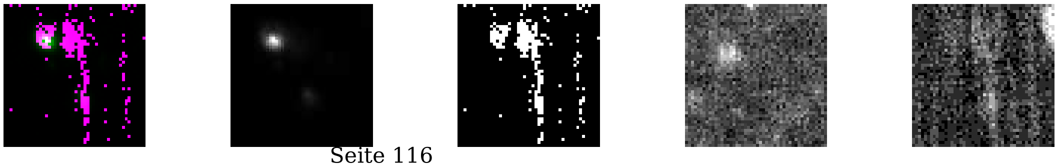				

Ereignis	Aufnahme	DAPI/CK-PE	CK-PE	DAPI	CD45-APC	HER-2/neu
25403	151	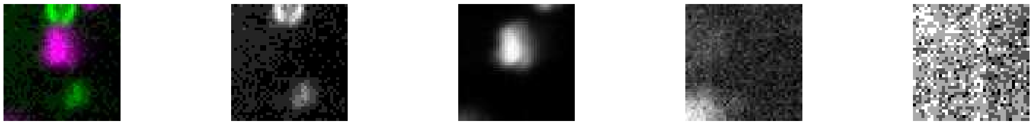				
25404	151	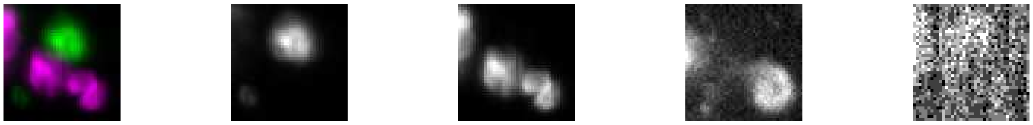				
25405	151	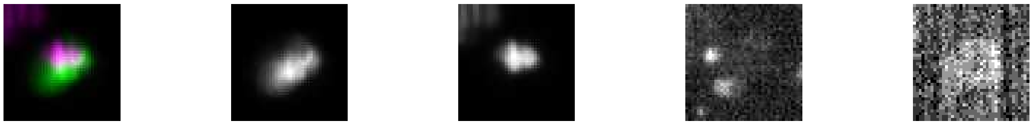				
25406	151	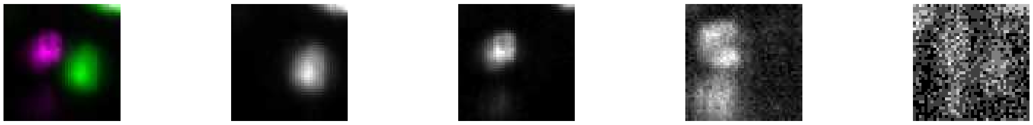				
25407	151	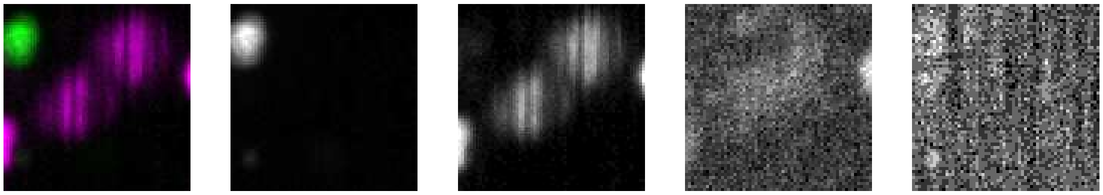				
25408	151	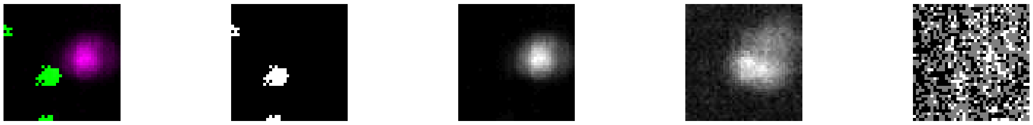				
25409	151	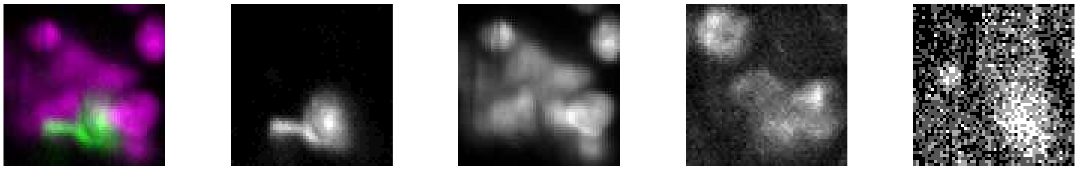				
25410	151	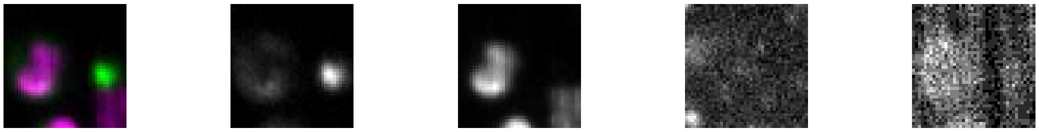				
25411	151	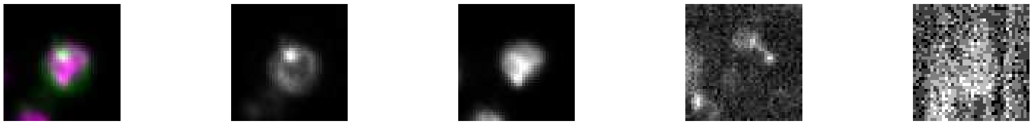				
25412	151	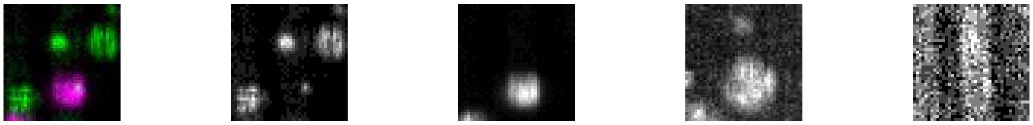				
25413	151	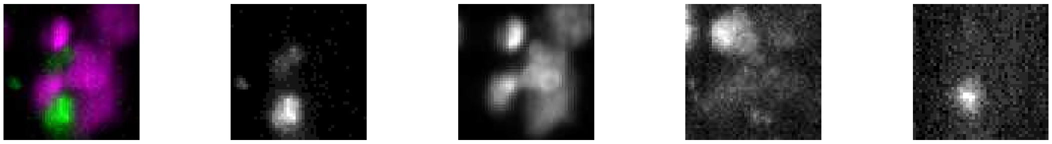				
25414	151	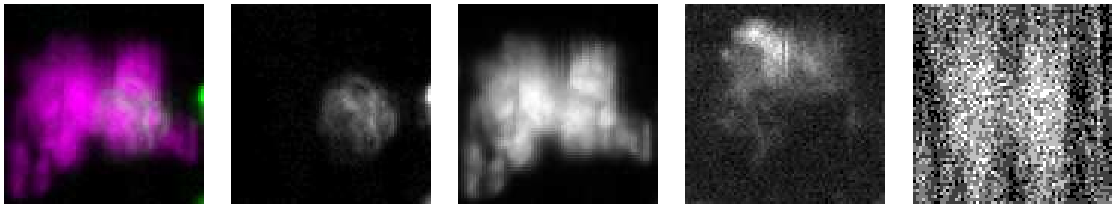				

Ereignis	Aufnahme	DAPI/CK-PE	CK-PE	DAPI	CD45-APC	HER-2/neu
25415	151	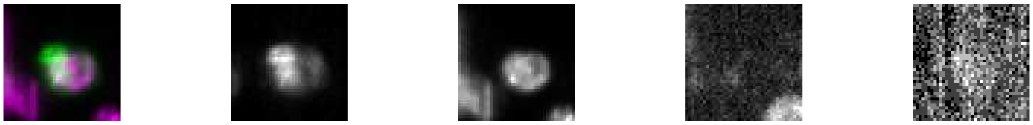				
25416	151	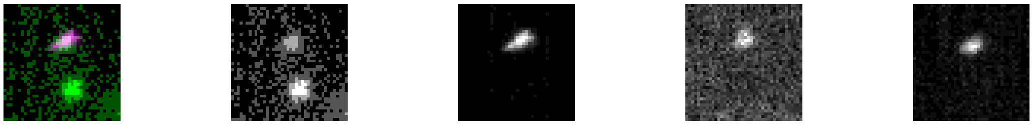				
25417	151	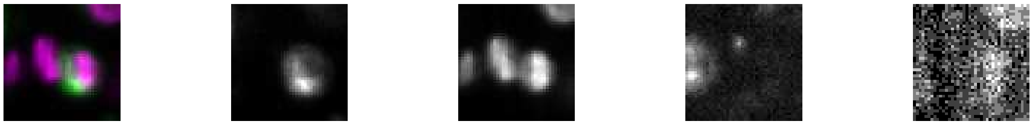				
25418	151	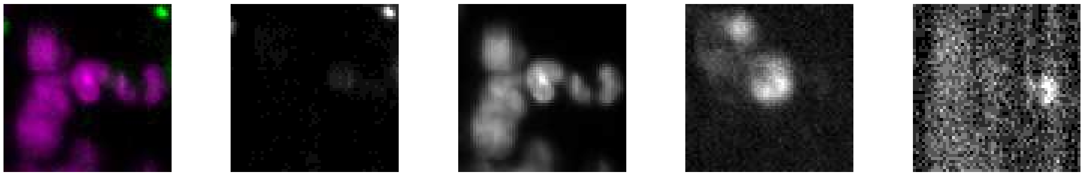				
25419	151	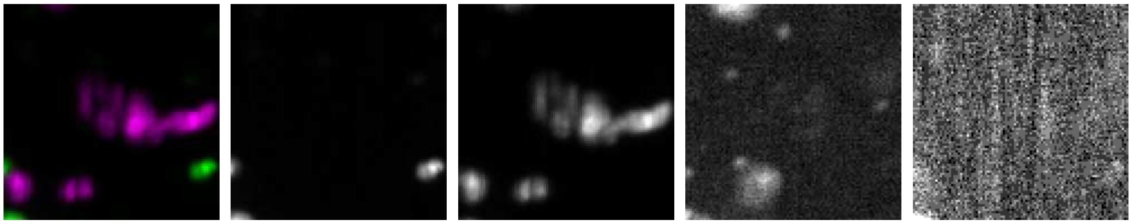				
25420	151	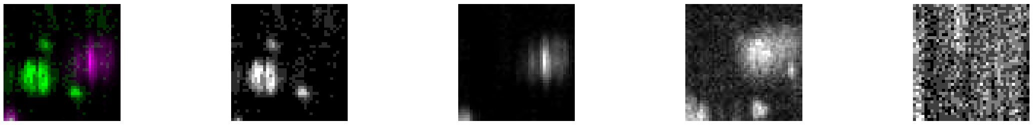				
25421	151	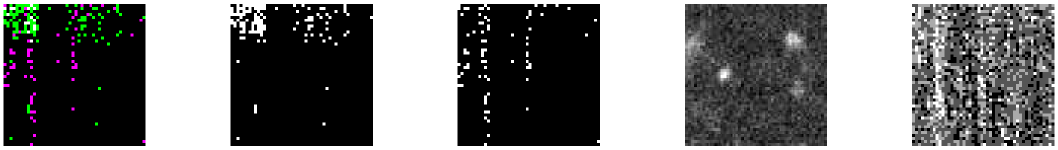				
25422	151	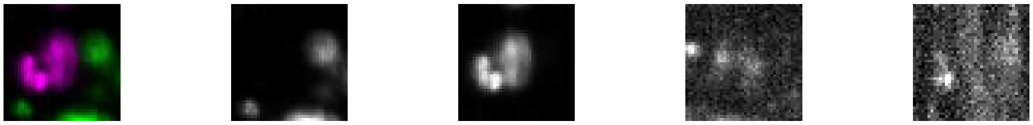				
25423	151	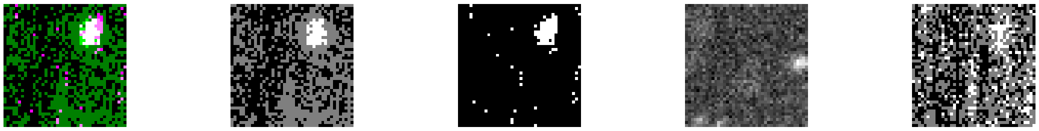				
25424	151	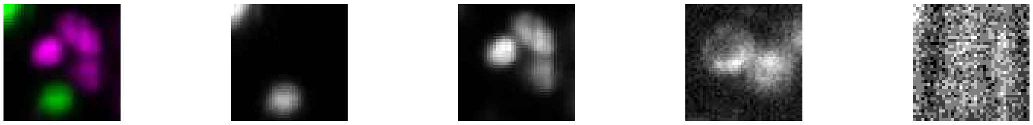				
25425	151	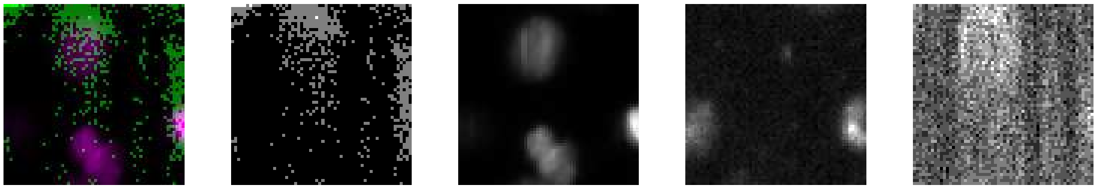				
25426	151	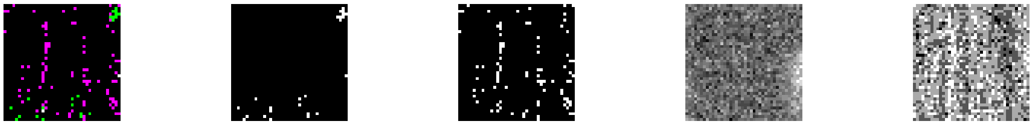				

Druckdatum:02/06/2020 17:25

Proben-ID: Gyn14.208 **Kassetten-ID:**01327106 **Patienten-ID:**

Ereignis	Aufnahme	DAPI/CK-PE	CK-PE	DAPI	CD45-APC	HER-2/neu
25427	151	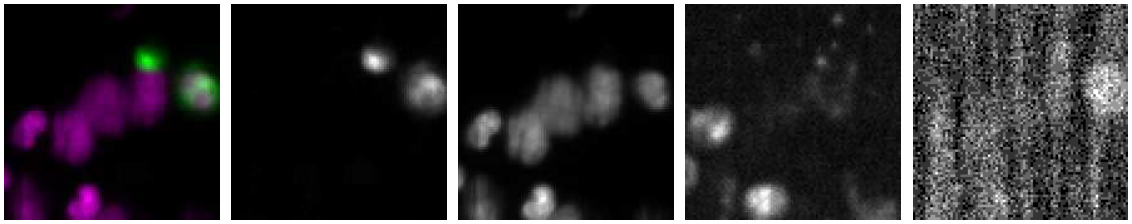				
25428	151	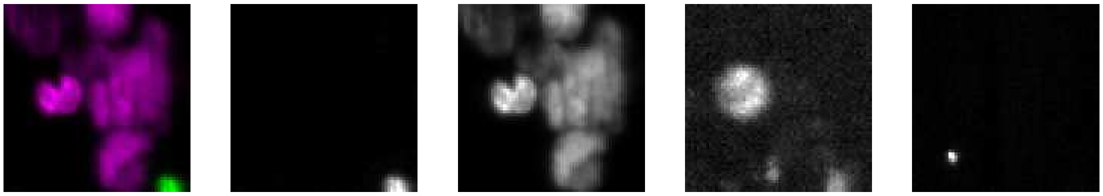				
25429	151	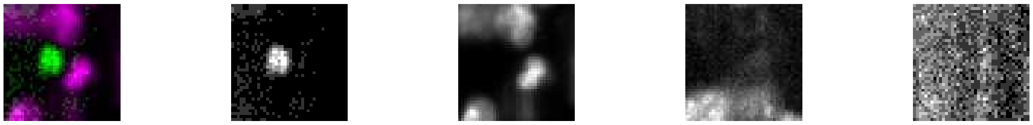				
25430	151	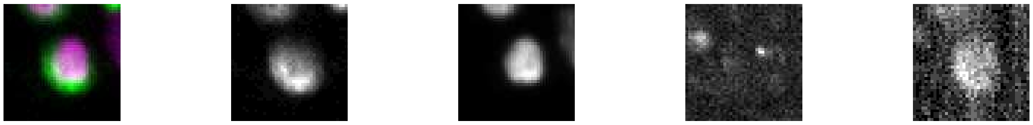				
25431	151	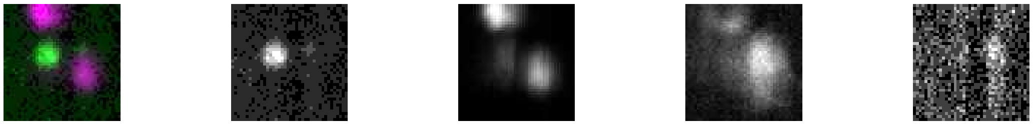				
25432	151	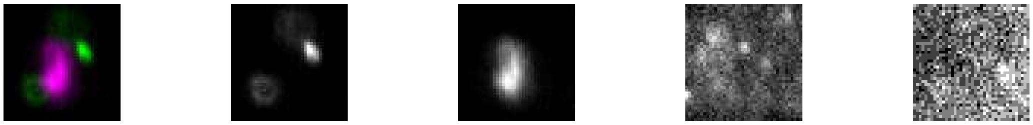				
25433	151	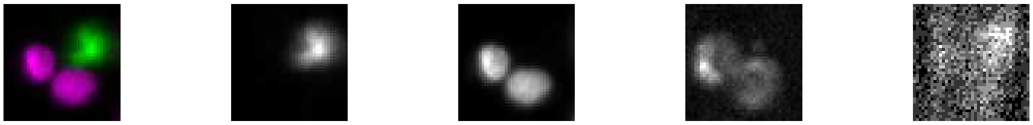				
25434	151	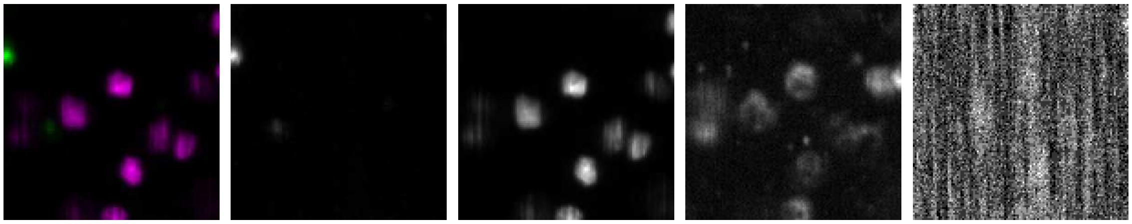				
25435	151	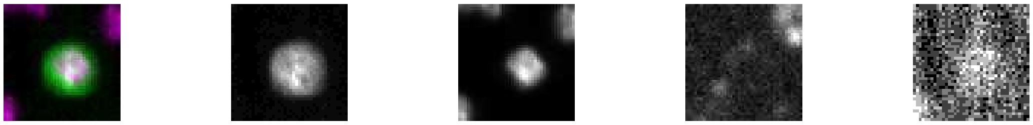				
25436	151	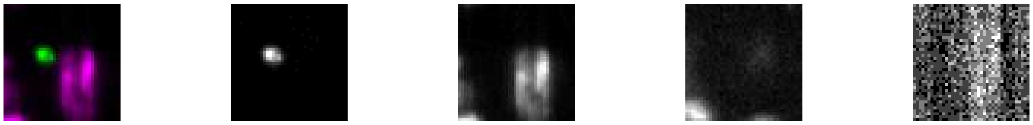				
25437	151	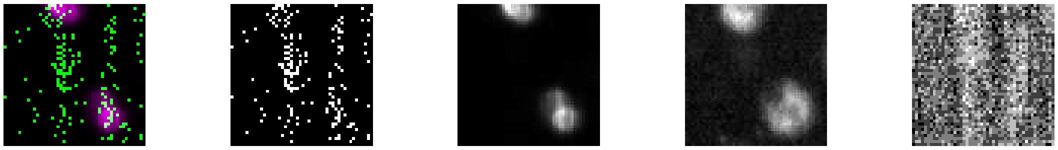				
25438	151	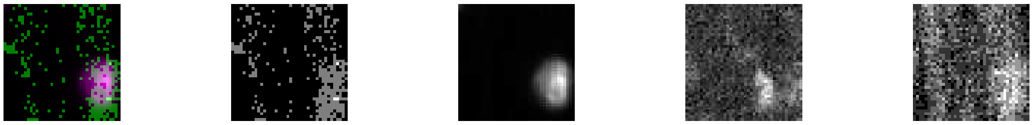				

Ereignis	Aufnahme	DAPI/CK-PE	CK-PE	DAPI	CD45-APC	HER-2/neu
25439	151	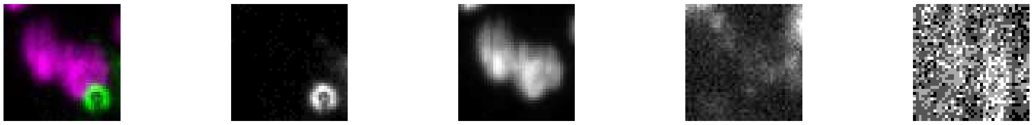				
25440	151	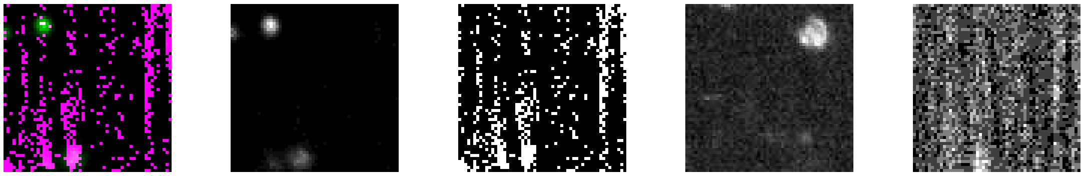				
25441	151	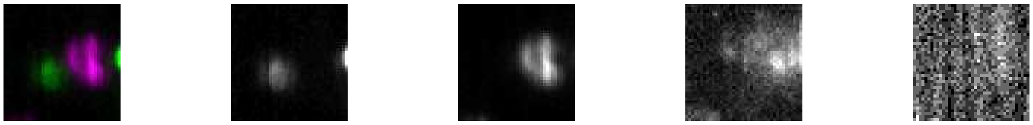				
25442	151	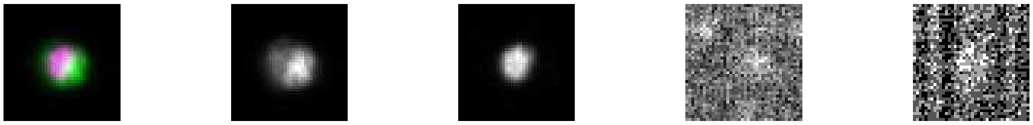				
25443	151	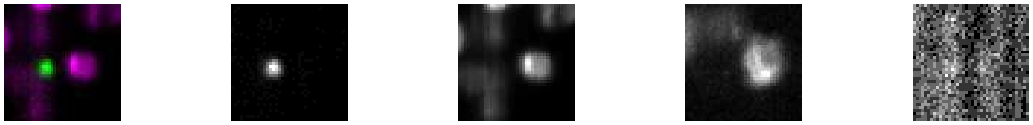				
25444	151	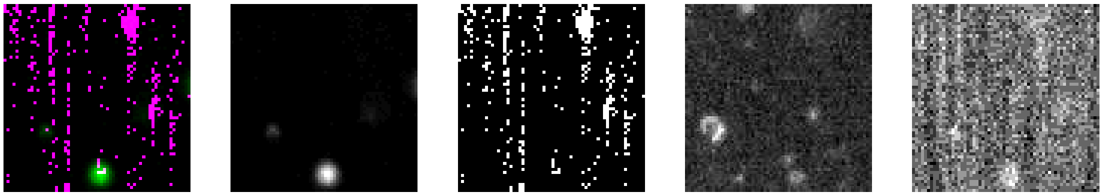				
25445	151	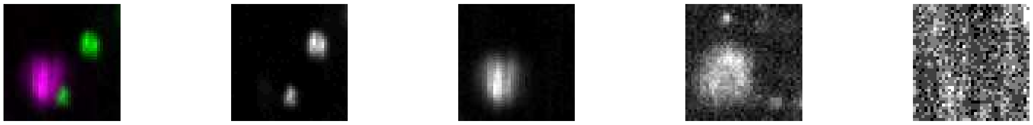				
25446	151	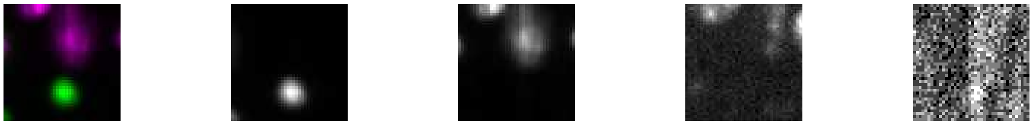				
25447	151	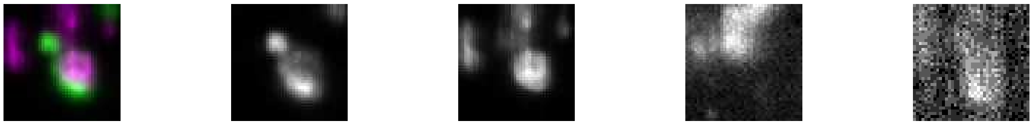				
25448	151	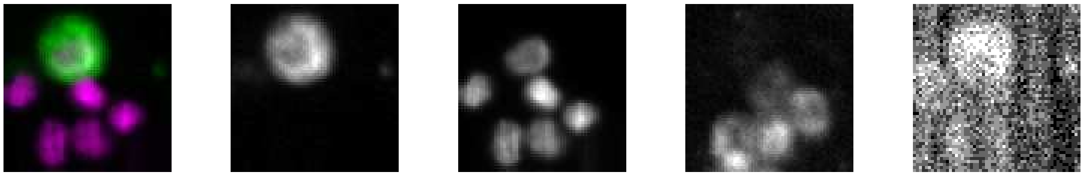				
25449	151	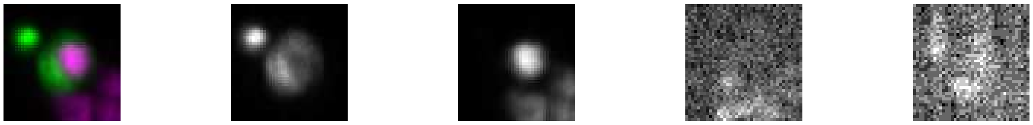				
25450	151	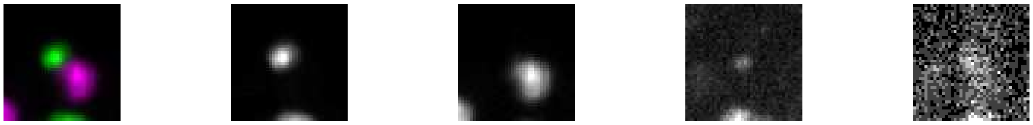				
25451	151	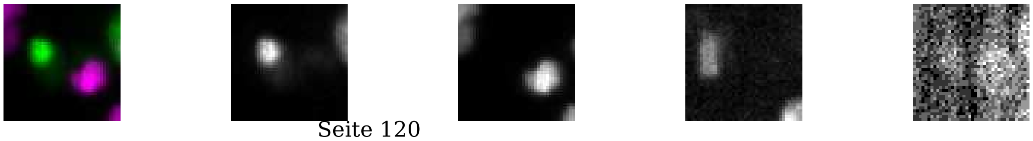				

Ereignis	Aufnahme	DAPI/CK-PE	CK-PE	DAPI	CD45-APC	HER-2/neu
25452	151	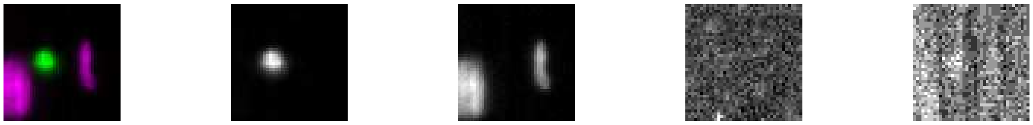				
25453	151	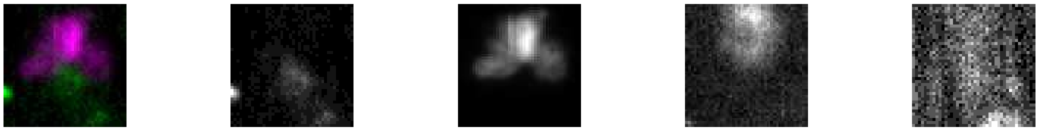				
25454	151	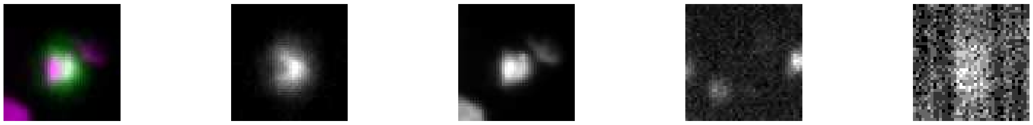				
25455	151	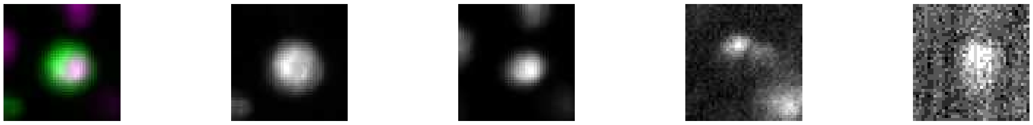				
25456	151	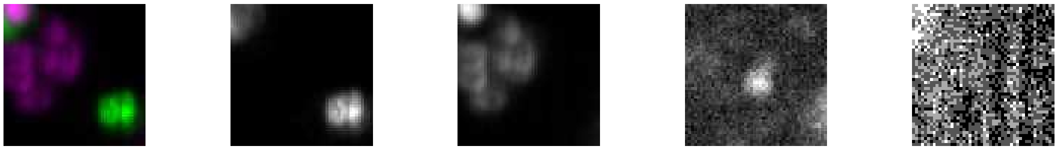				
25457	151	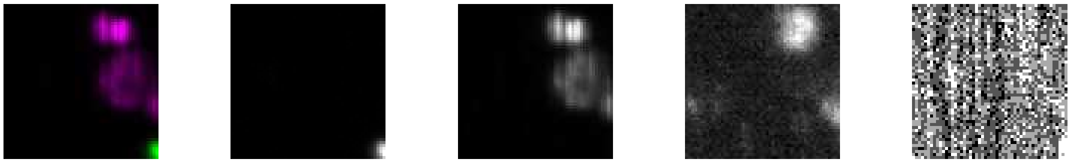				
25458	151	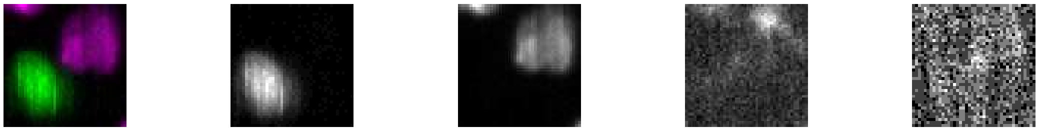				
25459	151	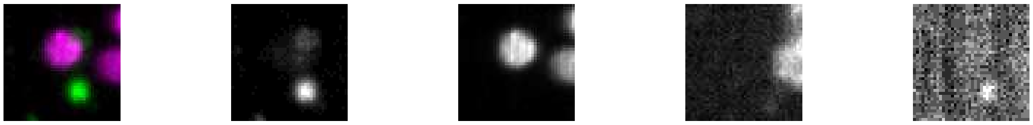				
25460	151	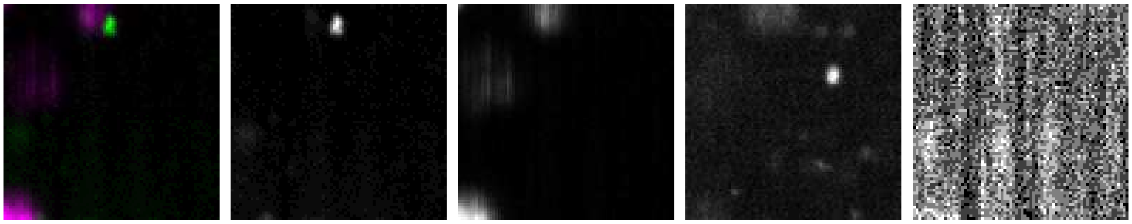				
25461	151	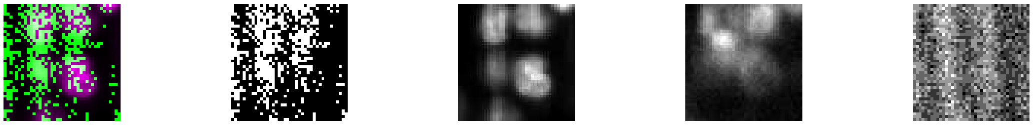				
25462	151	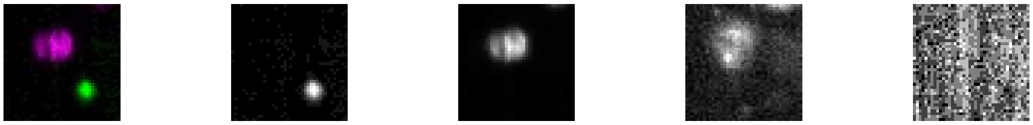				
25463	151	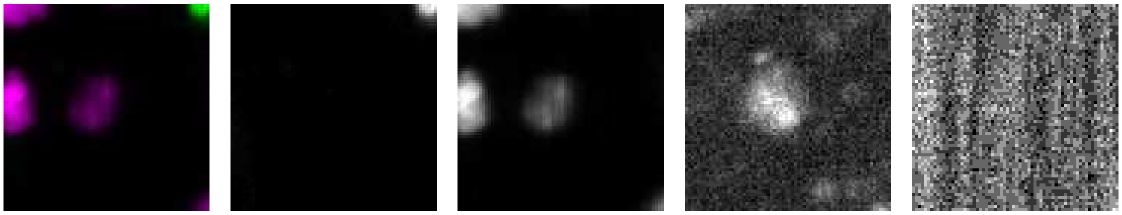				

Ereignis	Aufnahme	DAPI/CK-PE	CK-PE	DAPI	CD45-APC	HER-2/neu
25464	151	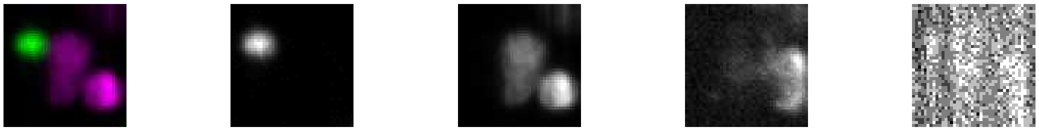				
25465	151	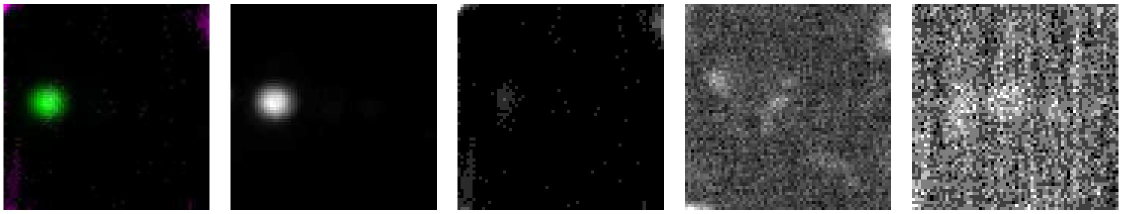				
25466	151	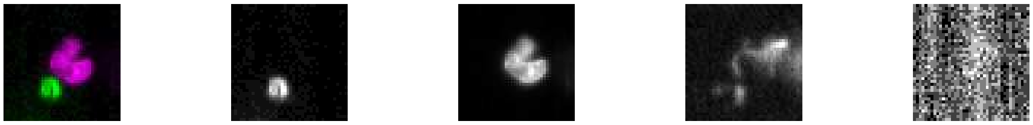				
25467	151	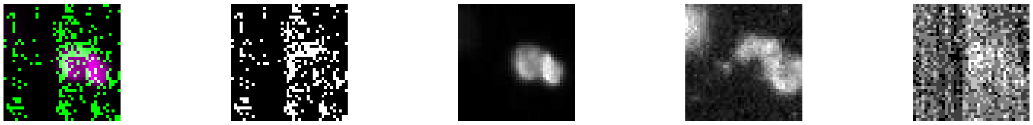				
25468	151	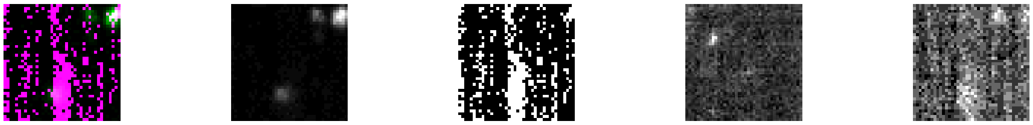				
25469	151	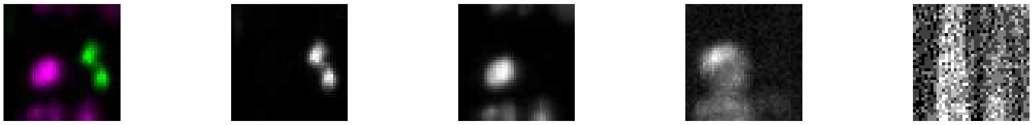				
25470	151	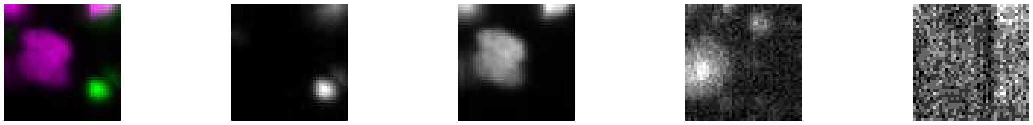				
25471	151	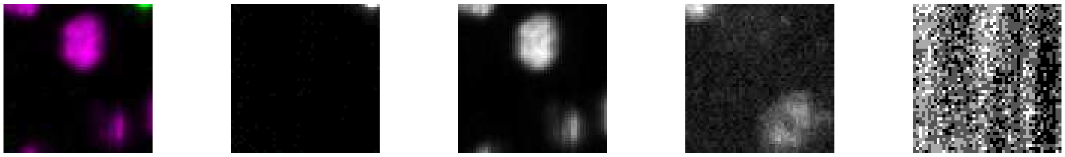				
25472	151	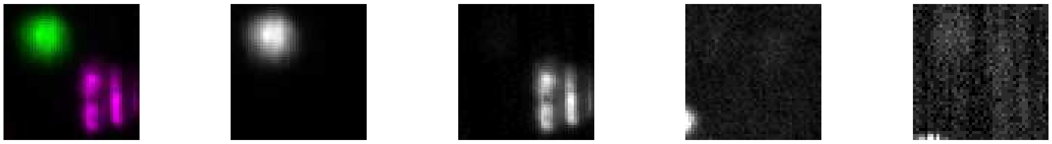				
25473	151	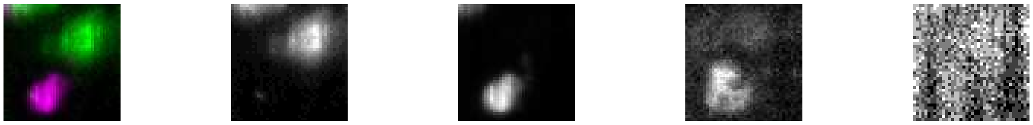				
25474	151	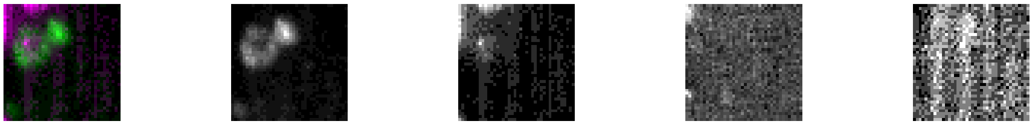				
25475	151	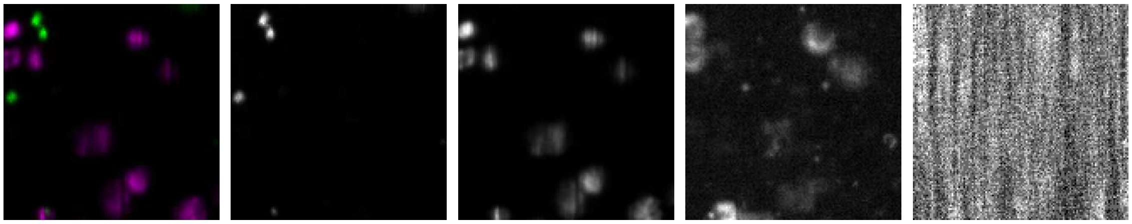				

Ereignis	Aufnahme	DAPI/CK-PE	CK-PE	DAPI	CD45-APC	HER-2/neu
25476	151	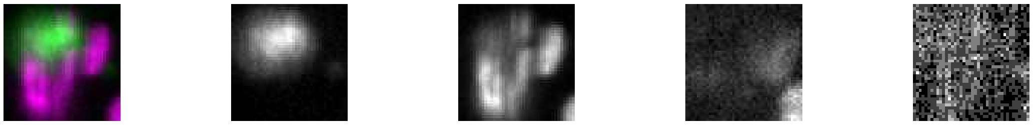				
25477	151	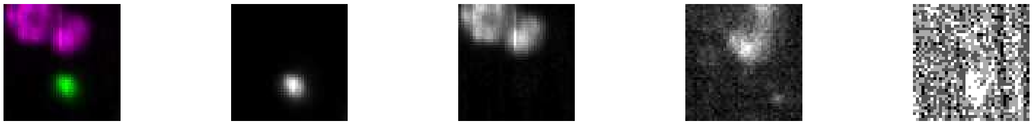				
25478	151	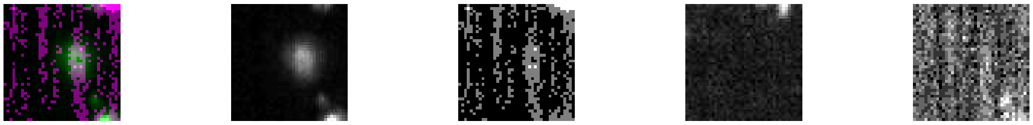				
25479	151	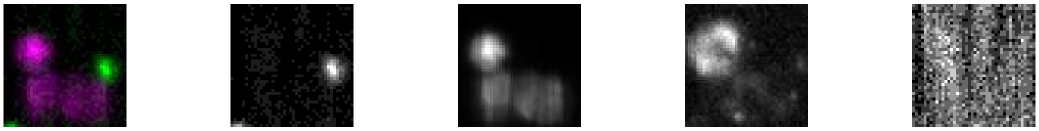				
25480	151	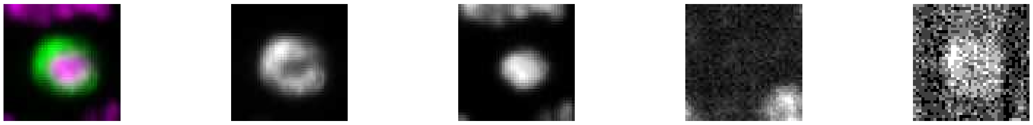				
25481	151	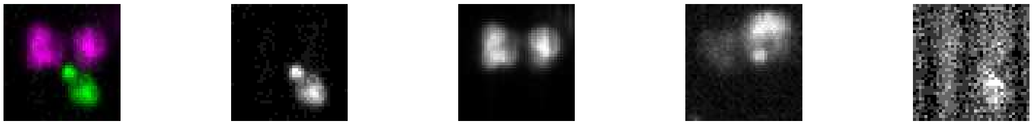				
25482	151	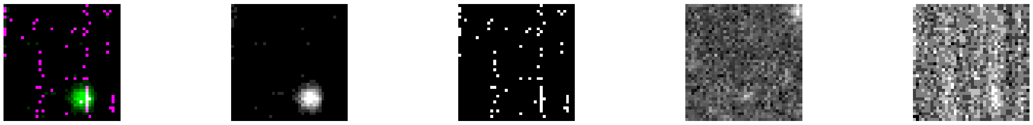				
25483	151	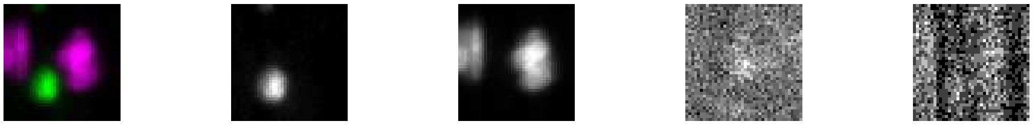				
25484	151	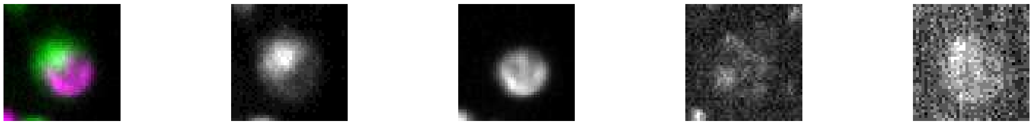				
25485	151	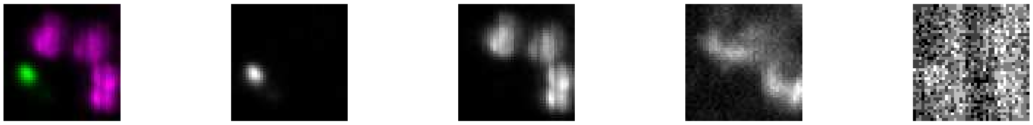				
25486	151	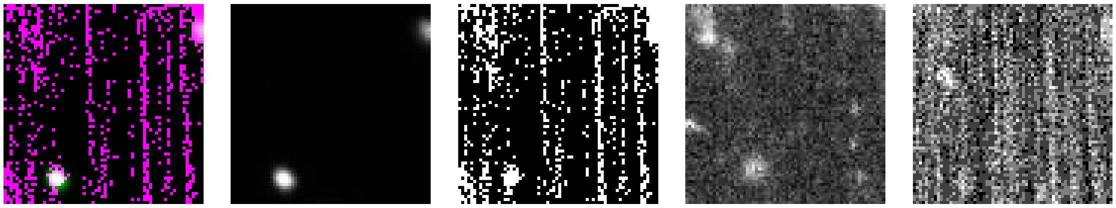				
25487	151	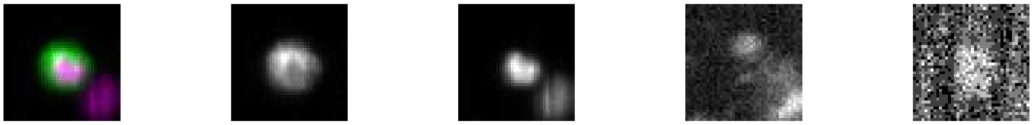				
25488	151	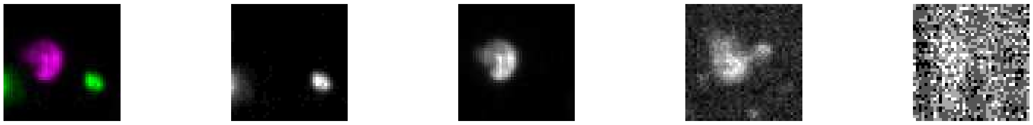				

Druckdatum:02/06/2020 17:25

Proben-ID: Gyn14.208 **Kassetten-ID:**01327106 **Patienten-ID:**

Ereignis	Aufnahme	DAPI/CK-PE	CK-PE	DAPI	CD45-APC	HER-2/neu
25489	151	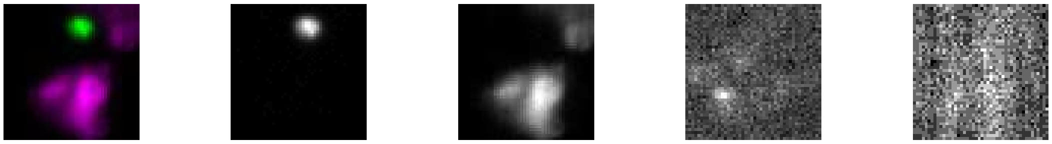				
25490	151	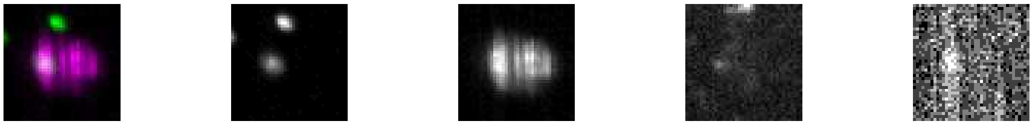				
25491	151	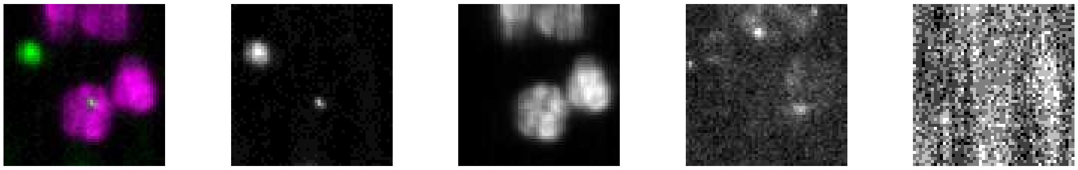				
25492	151	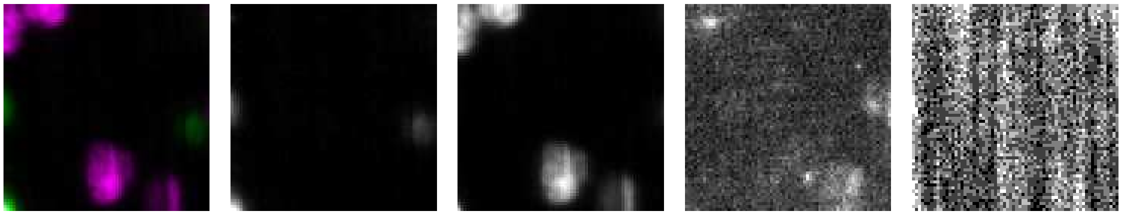				
25493	151	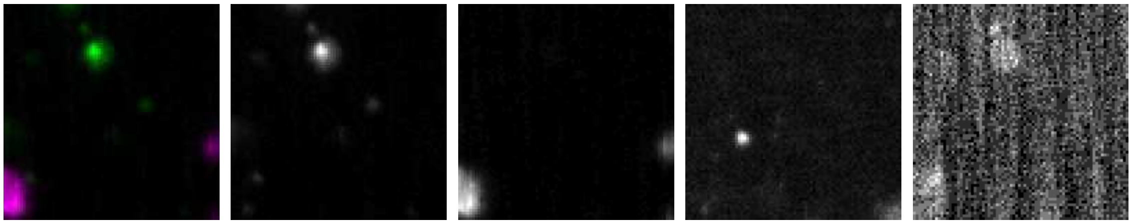				
25494	151	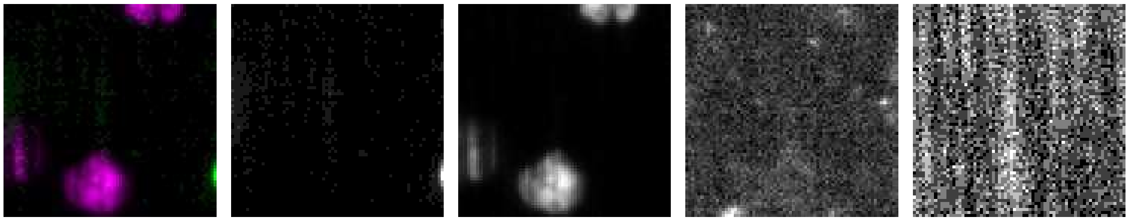				
25495	151	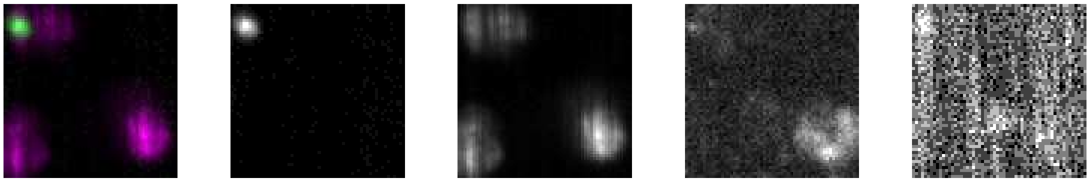				
25496	151	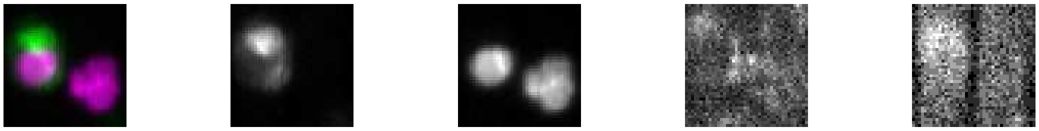				
25497	151	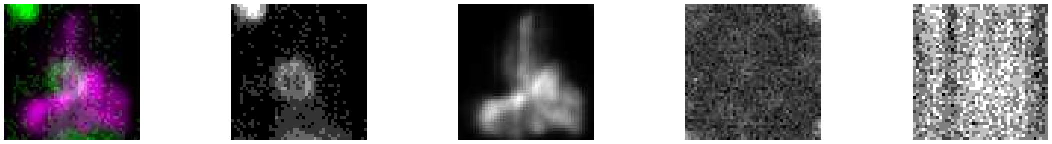				
25498	151	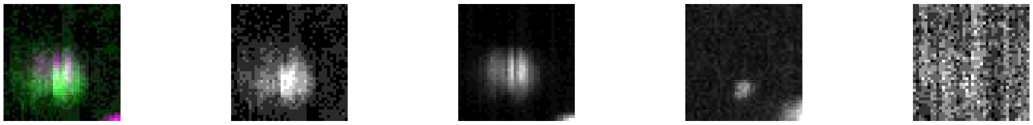				

Ereignis	Aufnahme	DAPI/CK-PE	CK-PE	DAPI	CD45-APC	HER-2/neu
25499	151	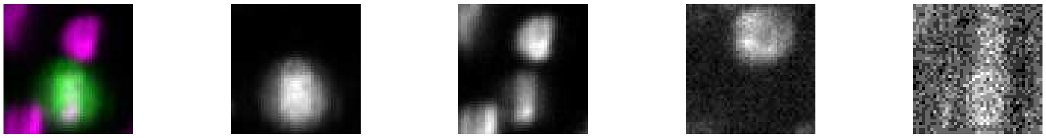				
25500	151	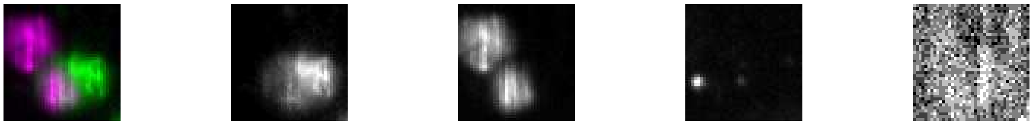				
25501	151	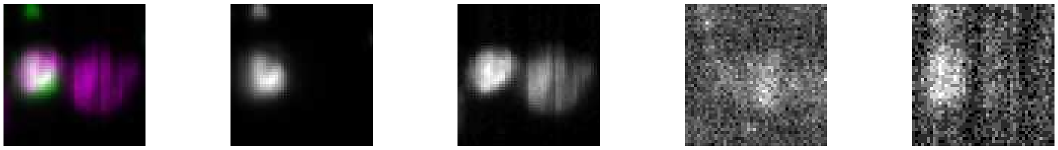				
25502	151	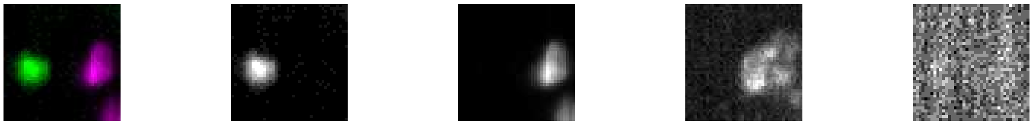				
25503	130	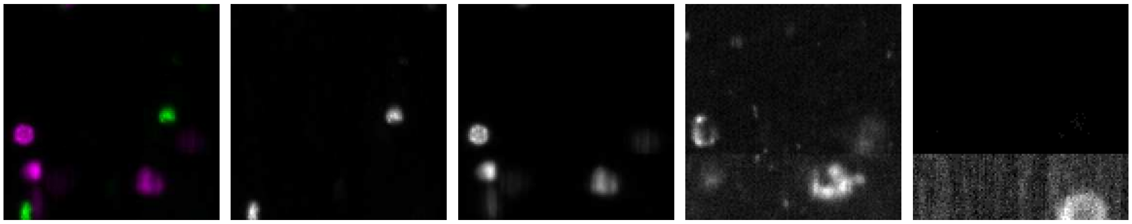				
25504	130	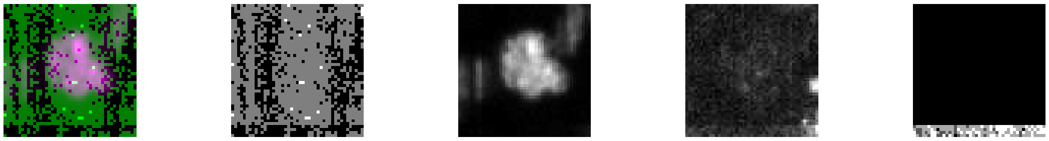				
25505	151	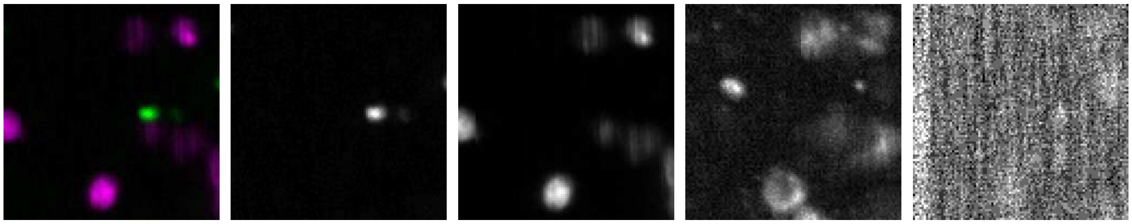				
25506	130	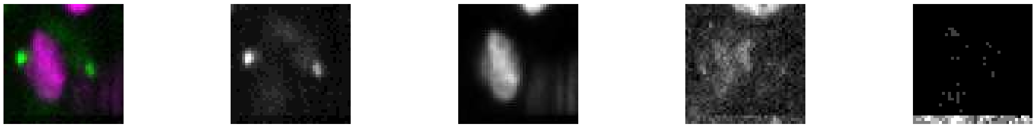				
25507	151	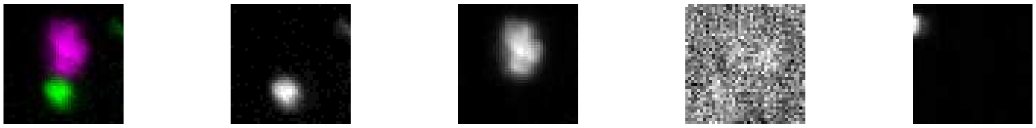				
25508	130	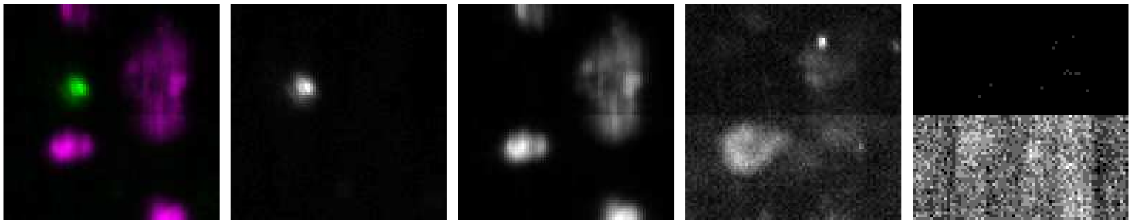				
25509	130	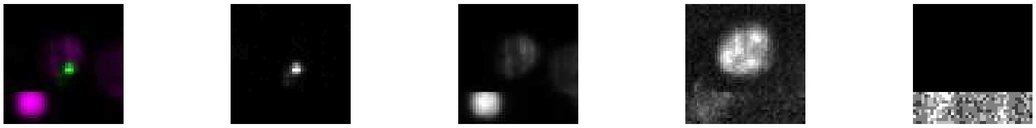				

Druckdatum:02/06/2020 17:25

Proben-ID: Gyn14.208 **Kassetten-ID:**01327106 **Patienten-ID:**

Ereignis	Aufnahme	DAPI/CK-PE	CK-PE	DAPI	CD45-APC	HER-2/neu
25510	151	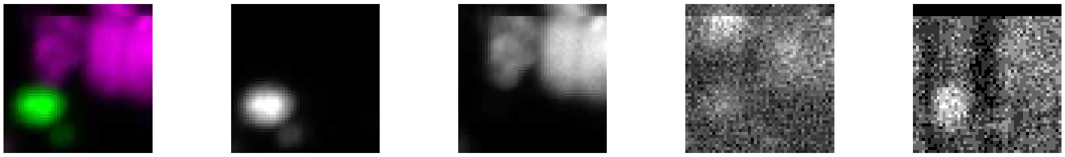				
25511	130	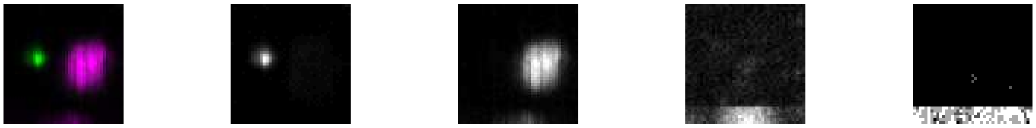				
25512	130	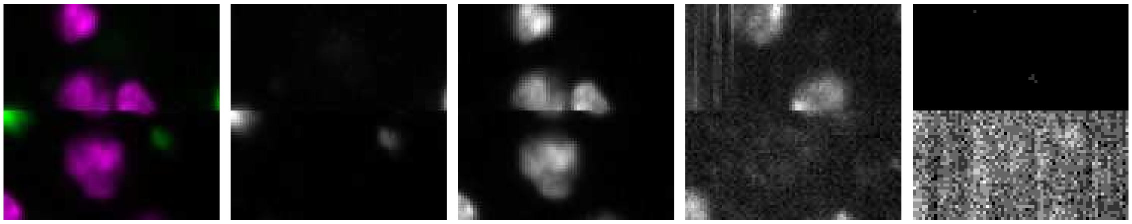				
25513	152	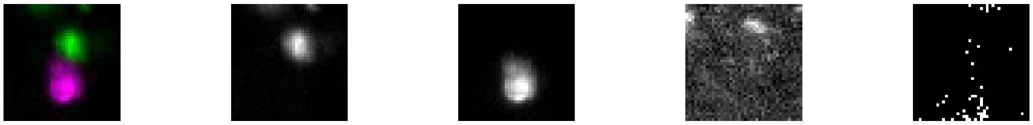				
25514	152	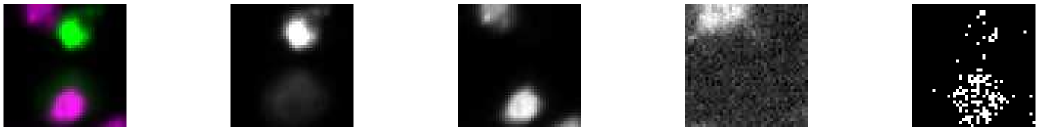				
25515	152	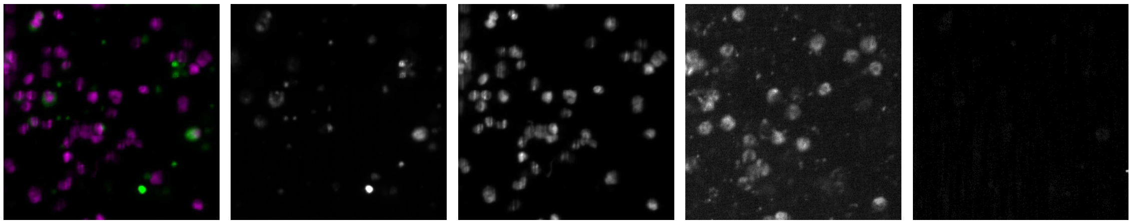				
25516	152	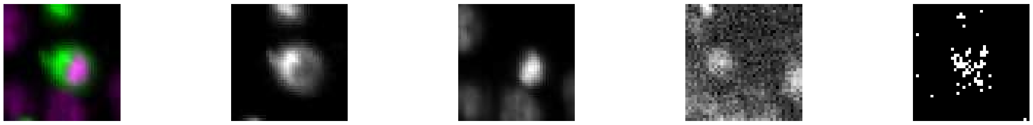				
25517	152	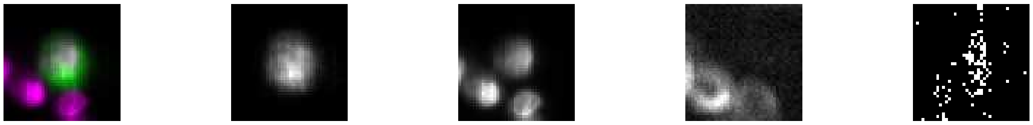				
25518	152	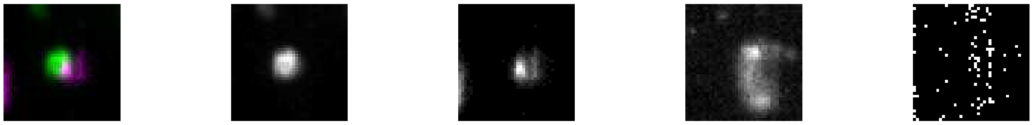				
25519	152	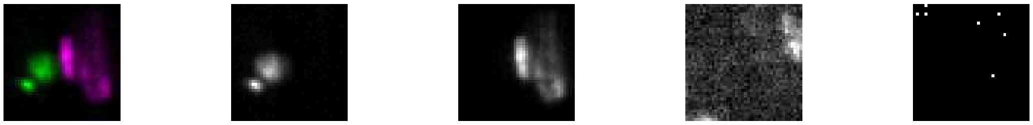				
25520	152	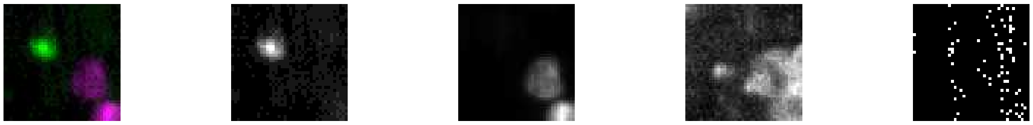				
25521	152	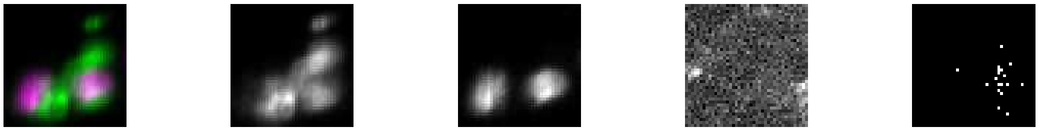				

Ereignis	Aufnahme	DAPI/CK-PE	CK-PE	DAPI	CD45-APC	HER-2/neu
25522	152	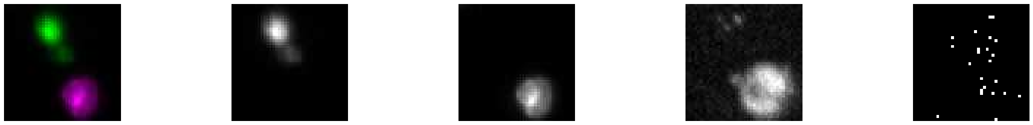				
25523	152	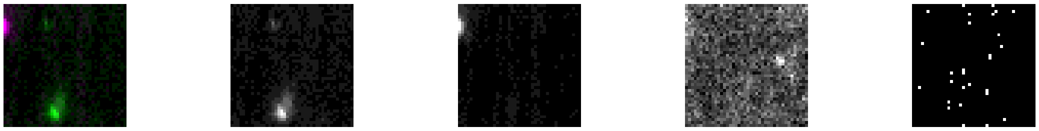				
25524	152	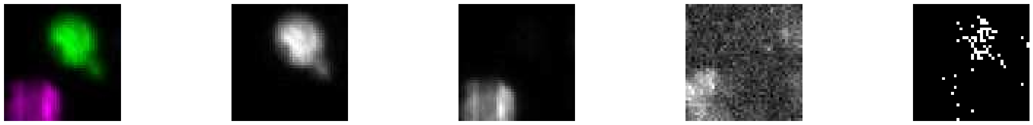				
25525	152	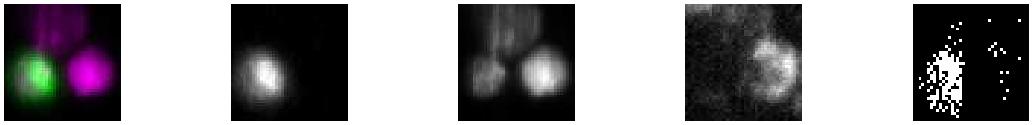				
25526	152	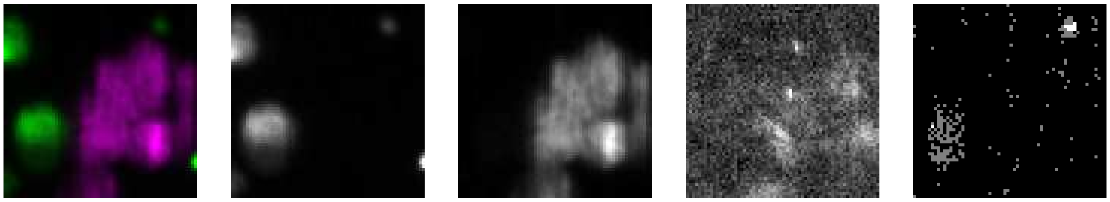				
25527	152	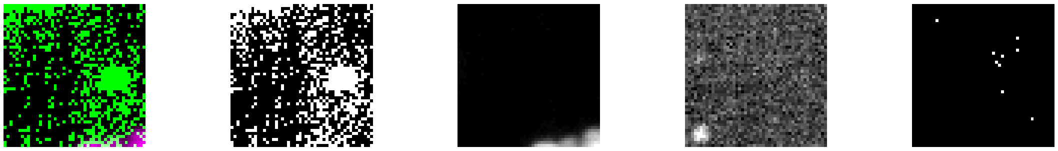				
25528	152	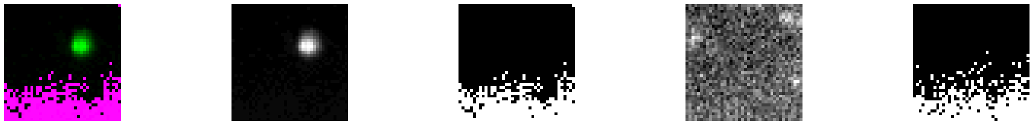				
25529	152	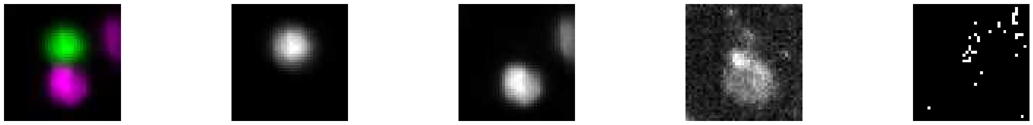				
25530	152	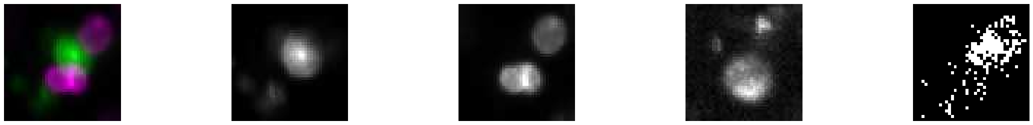				
25531	152	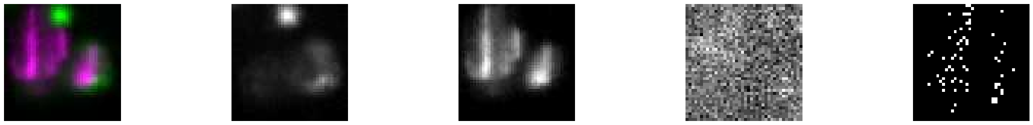				
25532	152	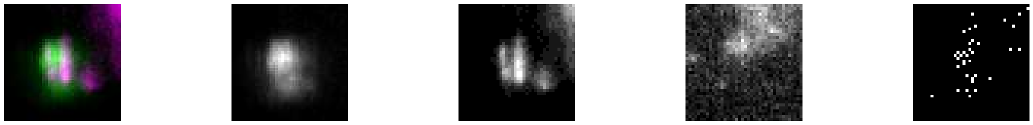				
25533	152	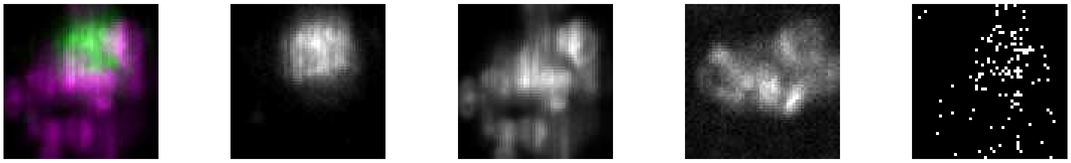				

Ereignis	Aufnahme	DAPI/CK-PE	CK-PE	DAPI	CD45-APC	HER-2/neu
25534	152	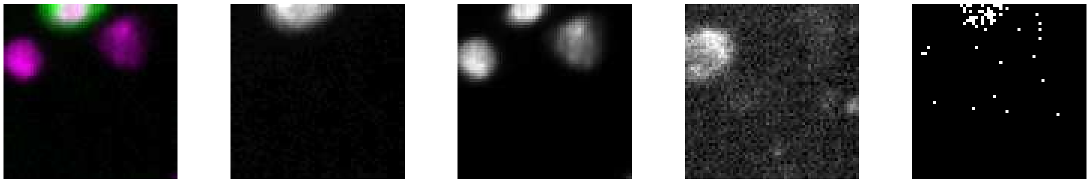				
25535	152	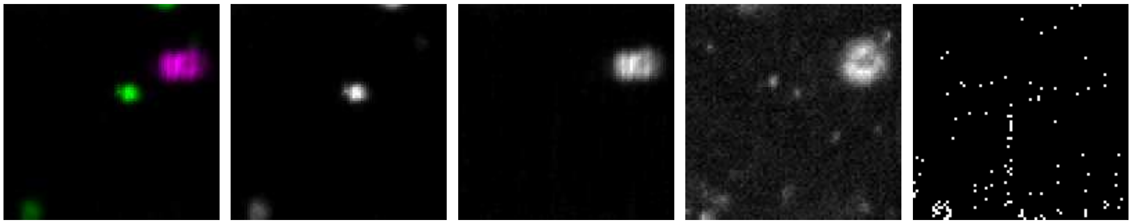				
25536	152	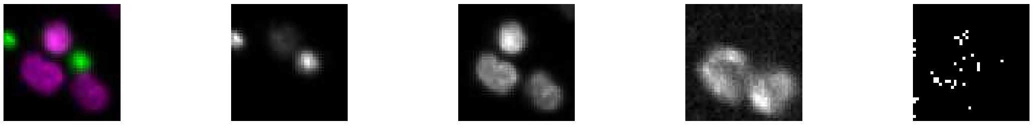				
25537	152	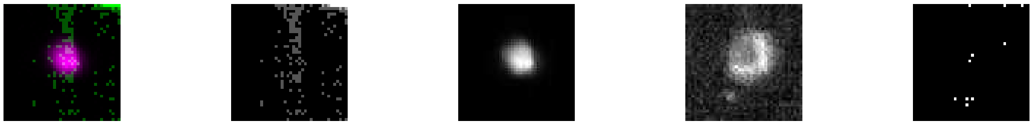				
25538	152	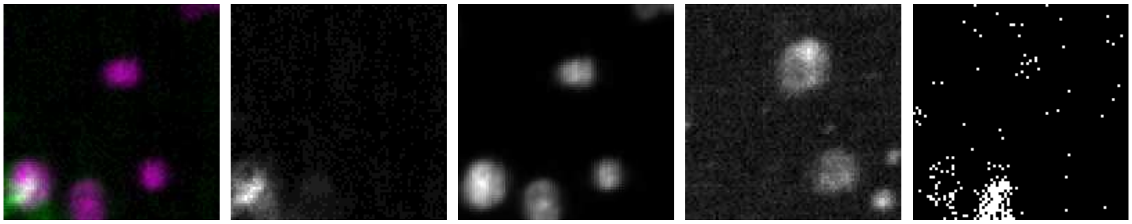				
25539	152	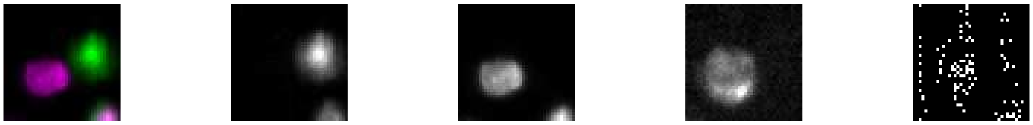				
25540	152	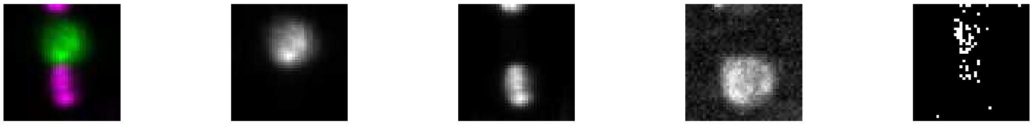				
25541	152	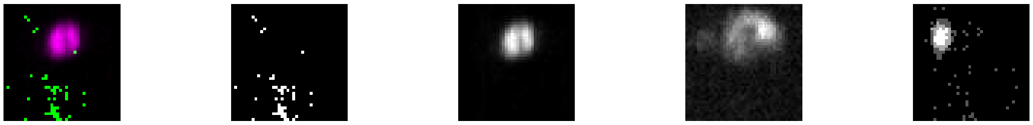				
25542	152	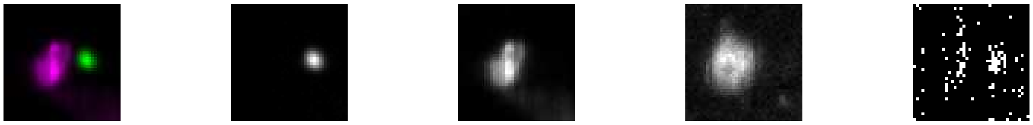				
25543	152	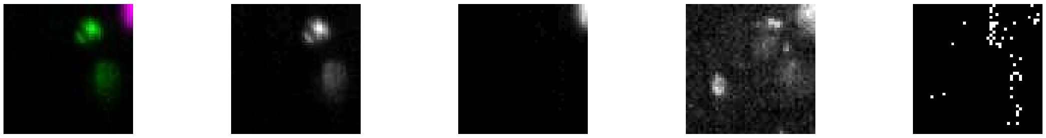				
25544	152	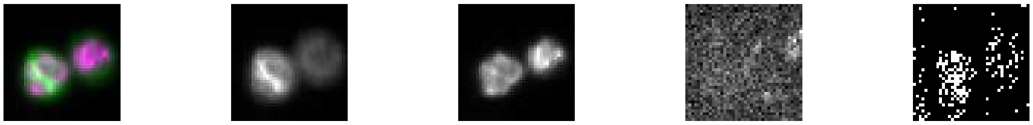				
25545	152	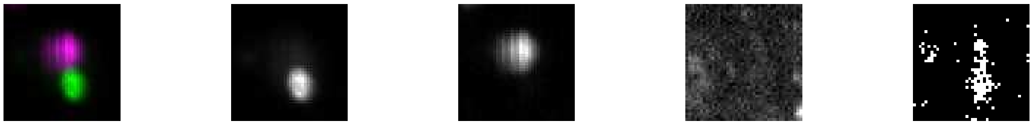				

Ereignis	Aufnahme	DAPI/CK-PE	CK-PE	DAPI	CD45-APC	HER-2/neu
25546	152	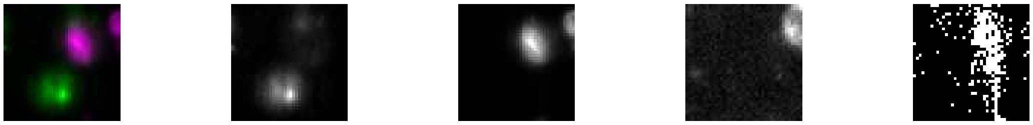				
25547	152	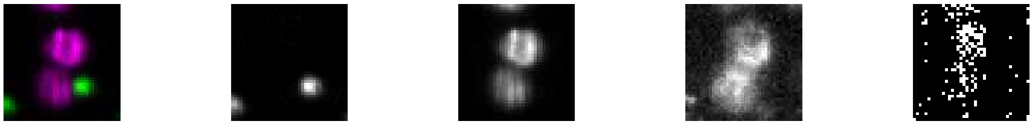				
25548	152	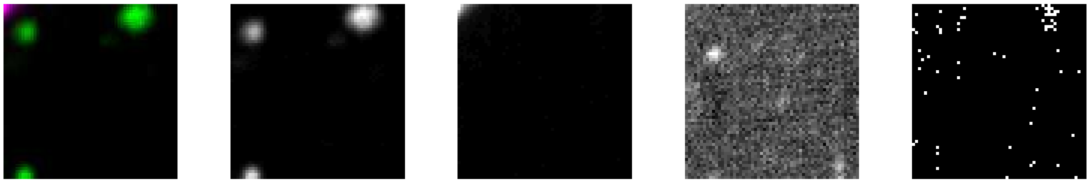				
25549	152	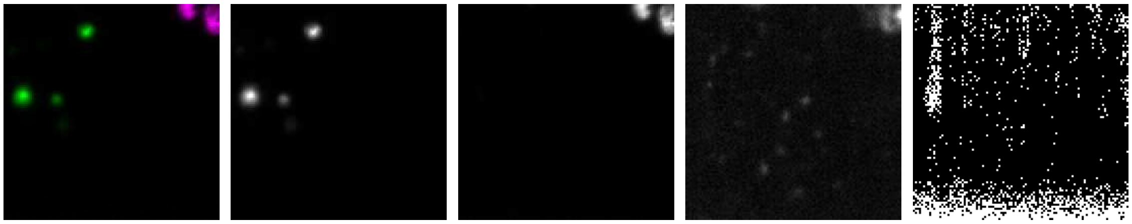				
25550	152	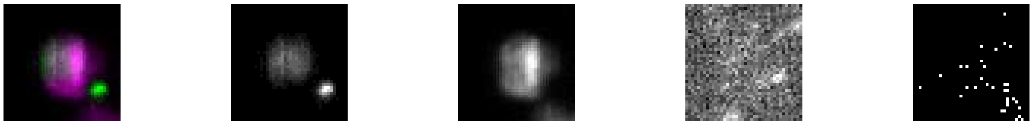				
25551	152	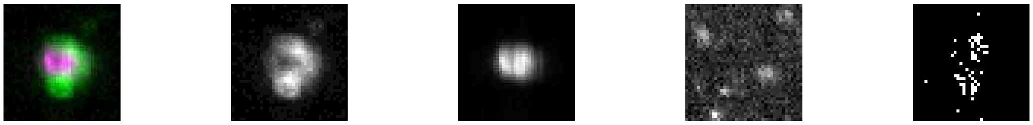				
25552	152	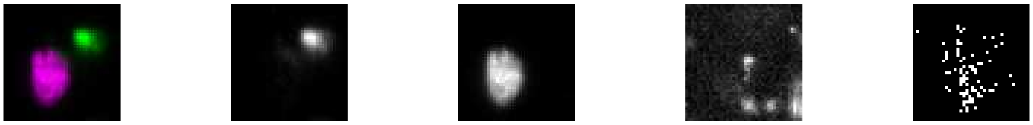				
25553	152	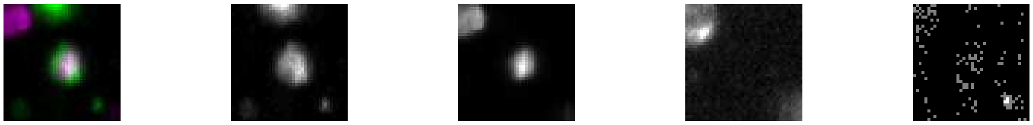				
25554	152	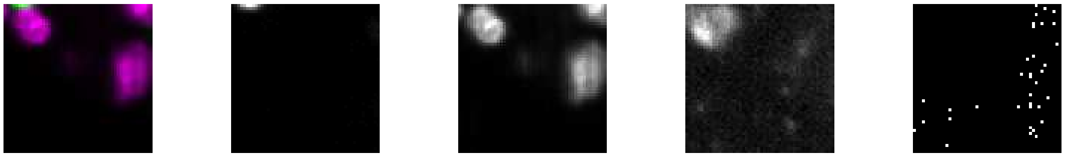				
25555	152	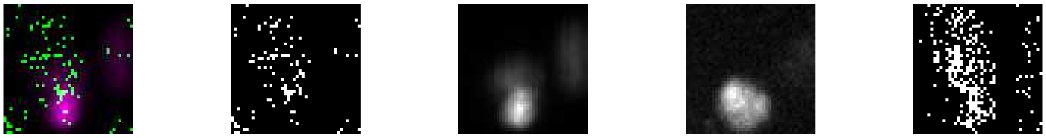				
25556	152	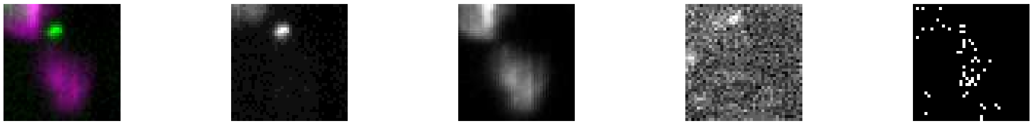				
25557	152	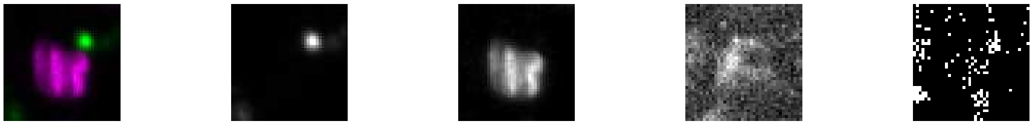				

Ereignis	Aufnahme	DAPI/CK-PE	CK-PE	DAPI	CD45-APC	HER-2/neu
25558	152	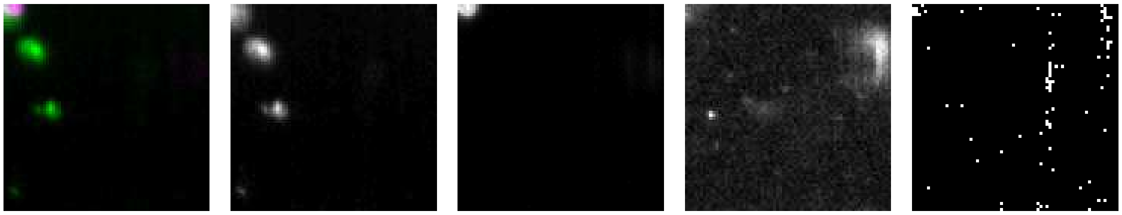				
25559	152	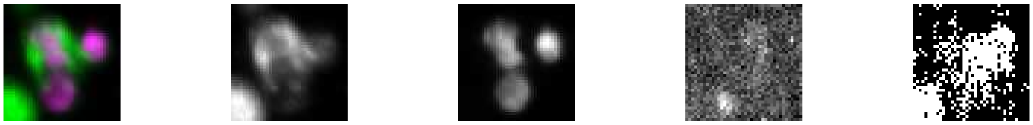				
25560	152	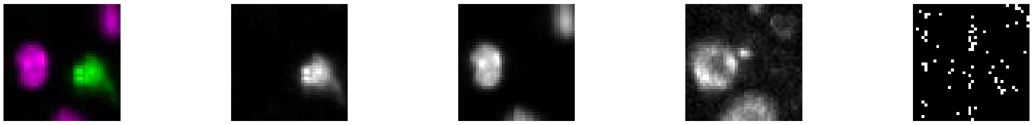				
25561	152	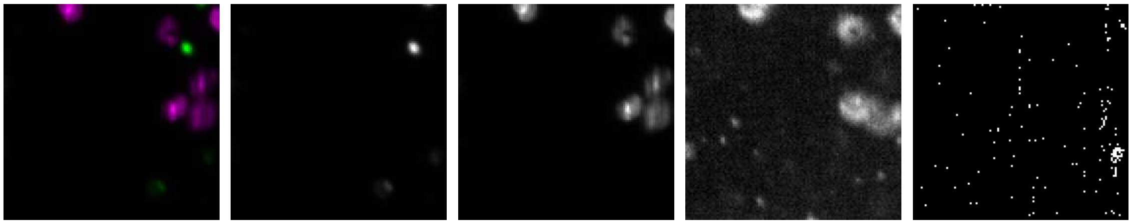				
25562	152	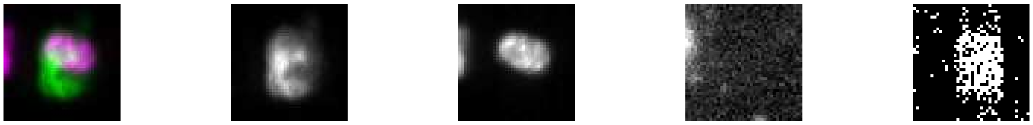				
25563	152	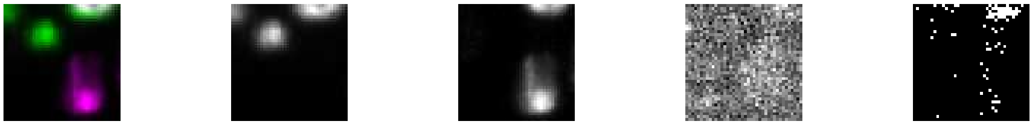				
25564	152	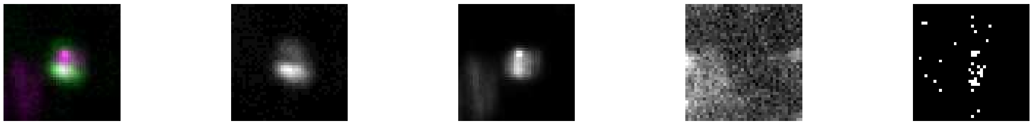				
25565	152	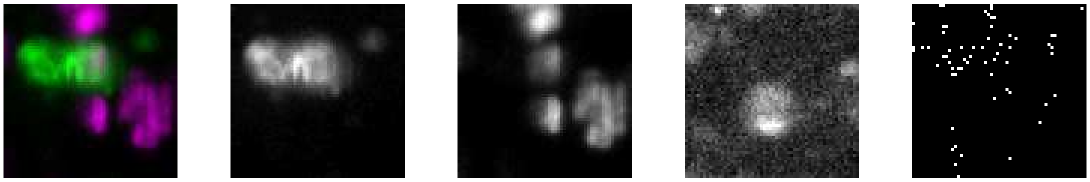				
25566	152	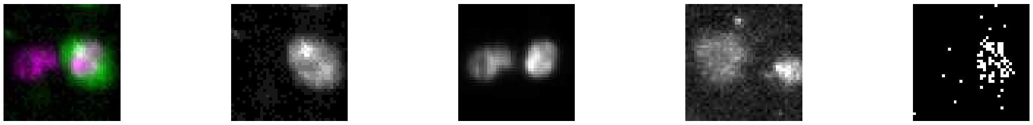				
25567	152	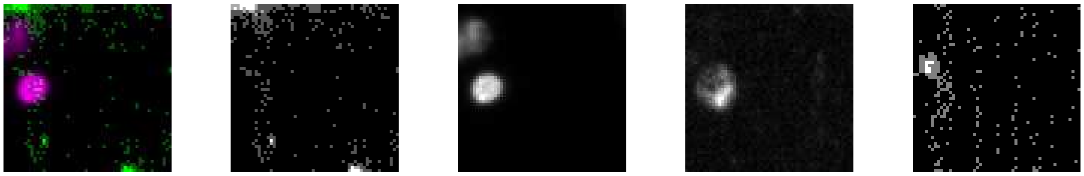				
25568	152	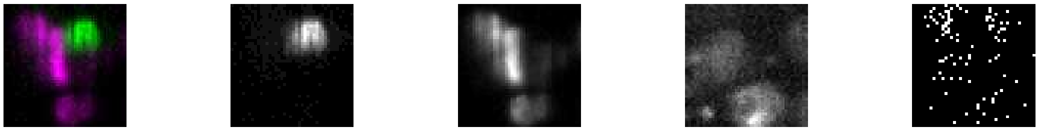				
25569	152	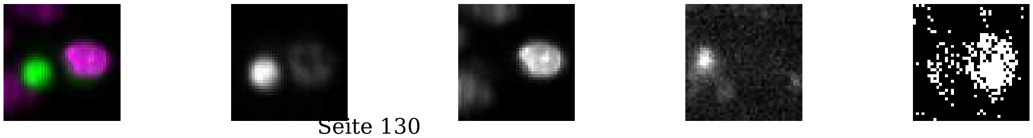				

Ereignis	Aufnahme	DAPI/CK-PE	CK-PE	DAPI	CD45-APC	HER-2/neu
25570	152	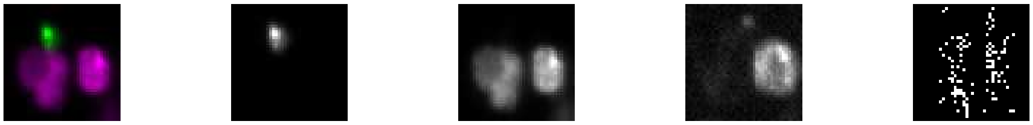				
25571	152	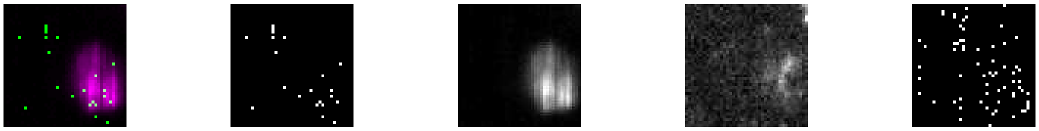				
25572	152	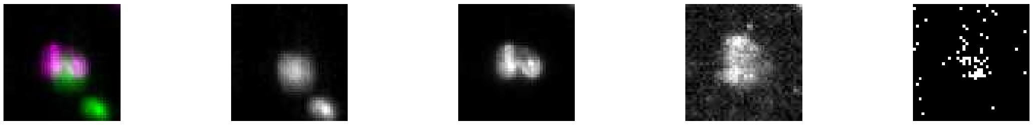				
25573	152	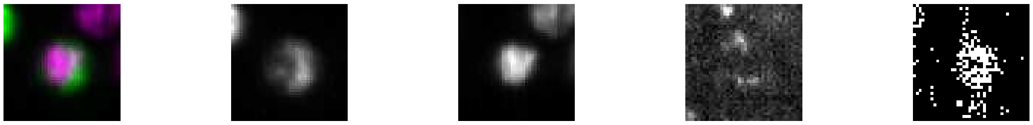				
25574	152	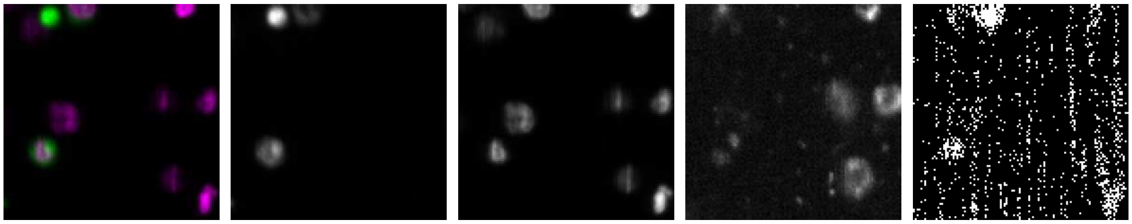				
25575	152	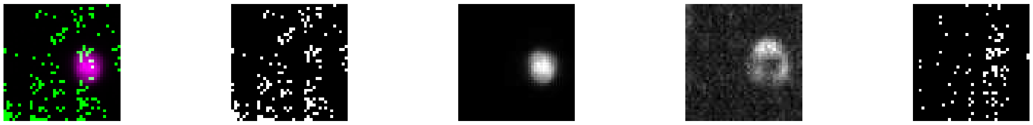				
25576	152	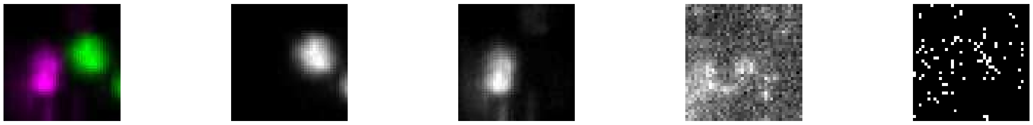				
25577	152	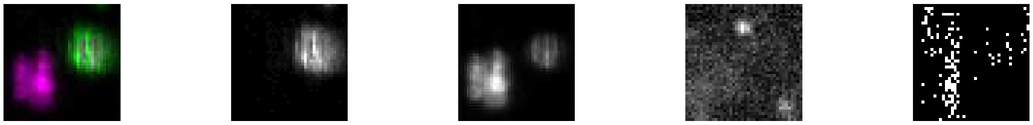				
25578	152	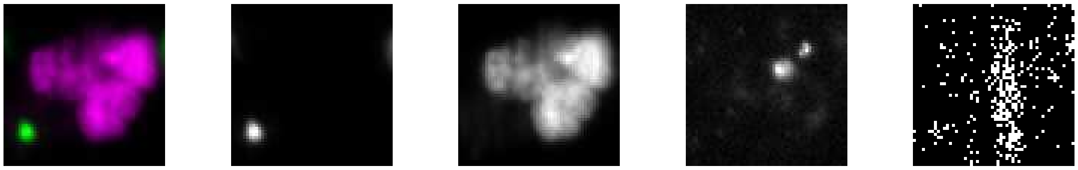				
25579	152	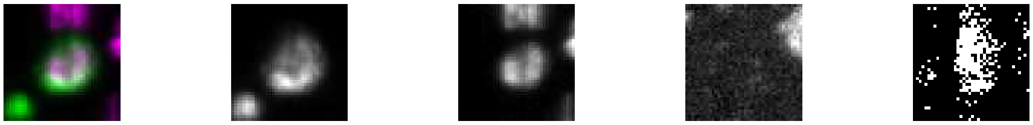				
25580	152	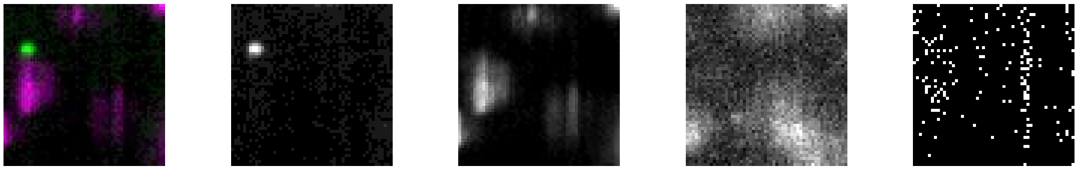				
25581	152	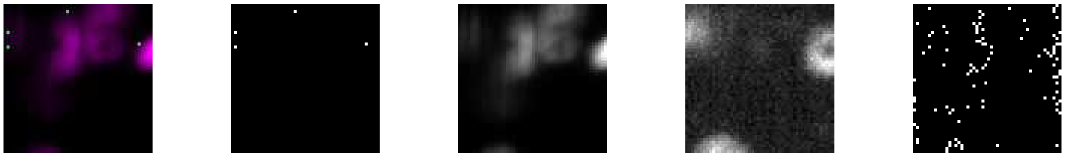				

Ereignis	Aufnahme	DAPI/CK-PE	CK-PE	DAPI	CD45-APC	HER-2/neu
25582	152	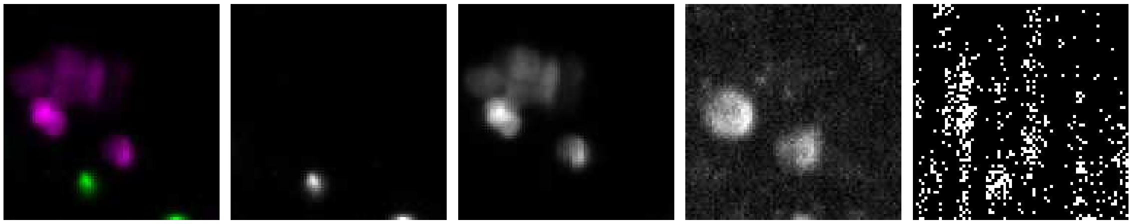				
25583	152	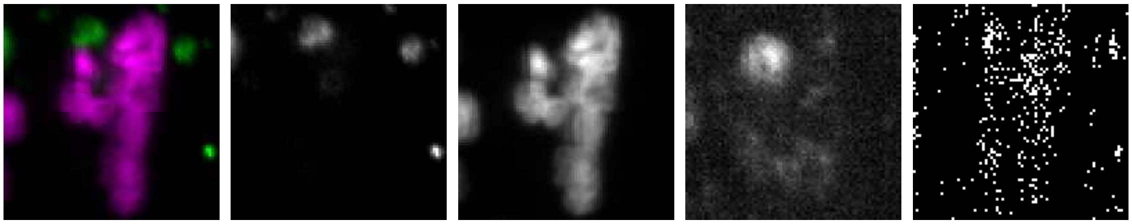				
25584	152	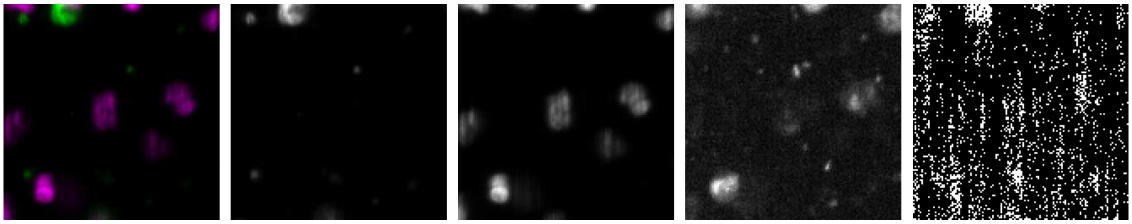				
25585	152	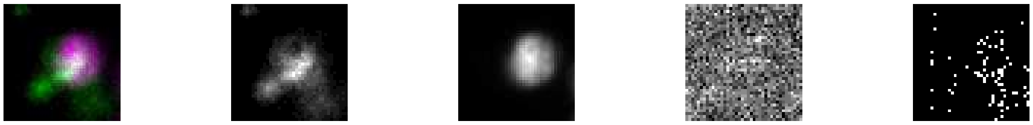				
25586	152	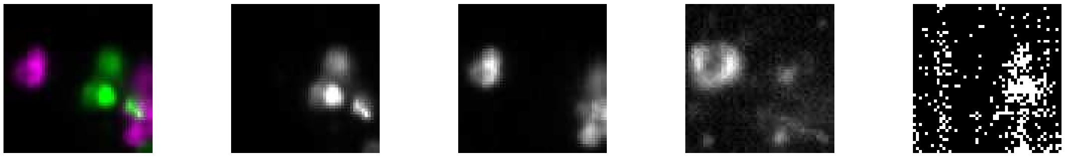				
25587	152	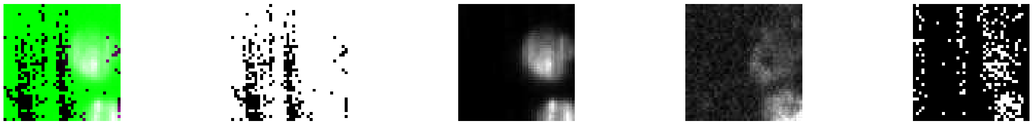				
25588	152	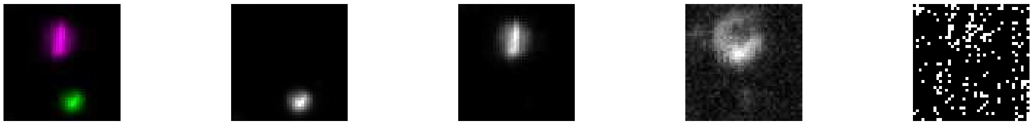				
25589	152	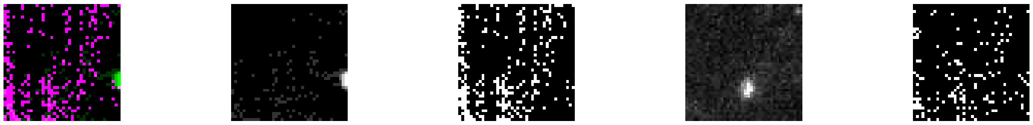				
25590	152	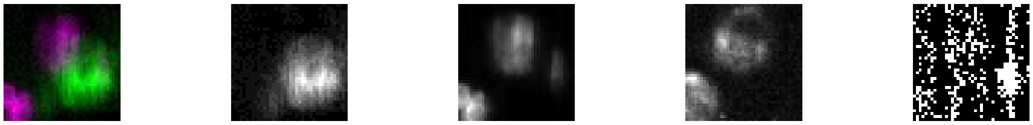				
25591	152	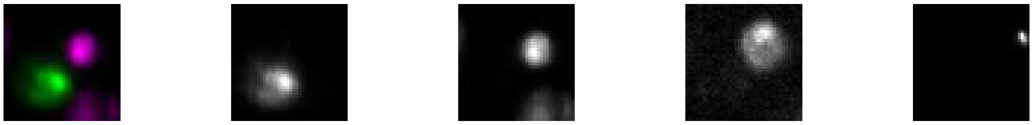				
25592	152	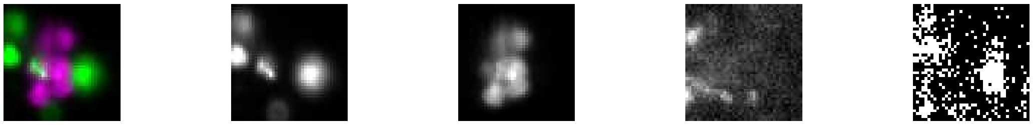				

Ereignis	Aufnahme	DAPI/CK-PE	CK-PE	DAPI	CD45-APC	HER-2/neu
25593	152	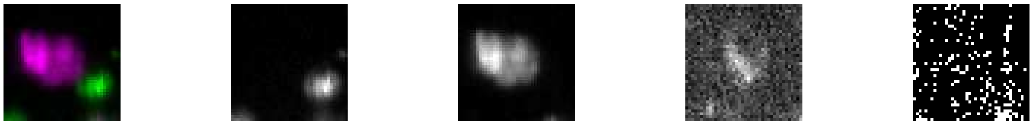				
25594	152	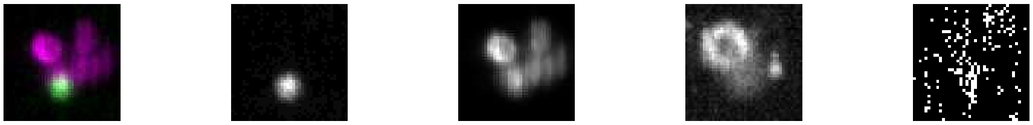				
25595	152	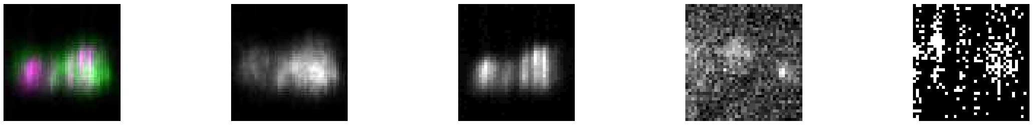				
25596	152	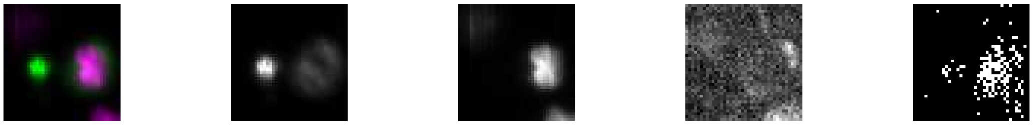				
25597	152	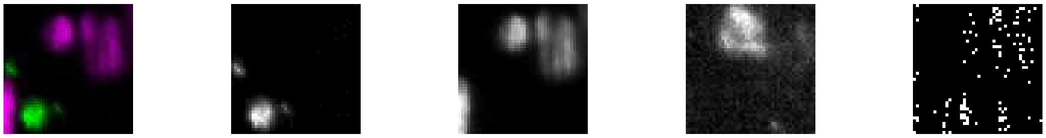				
25598	152	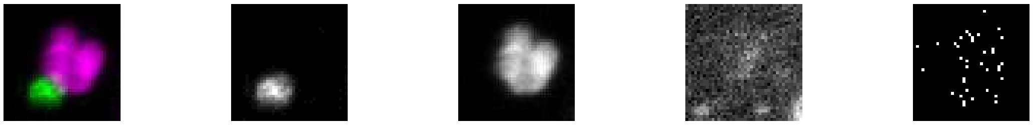				
25599	152	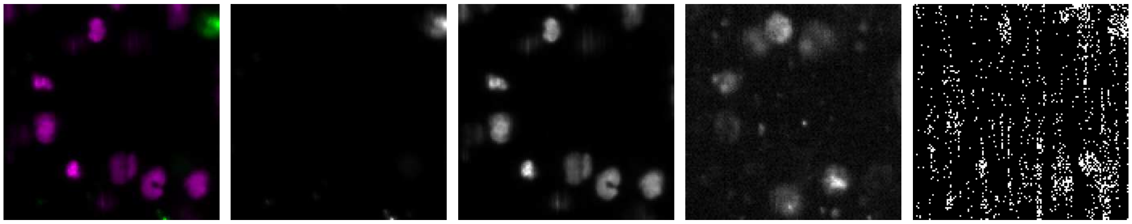				
25600	152	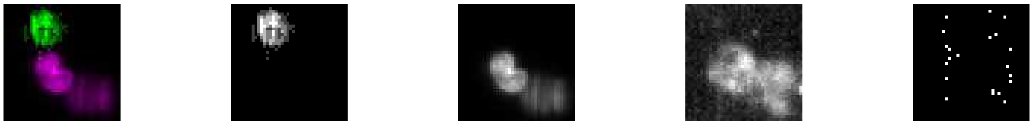				
25601	152	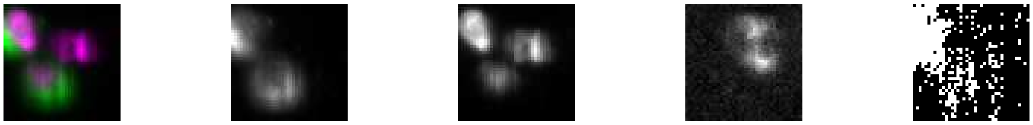				
25602	152	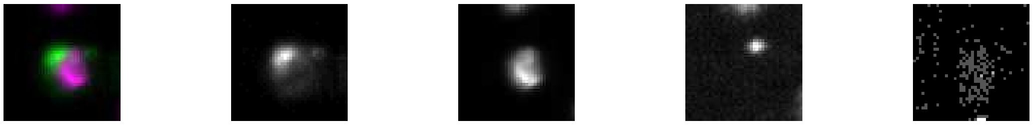				
25603	152	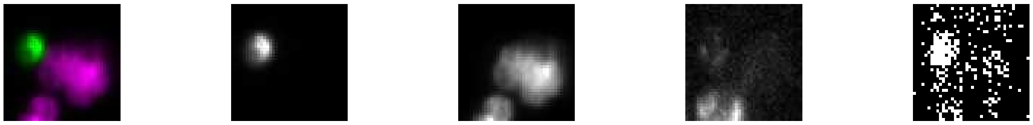				
25604	152	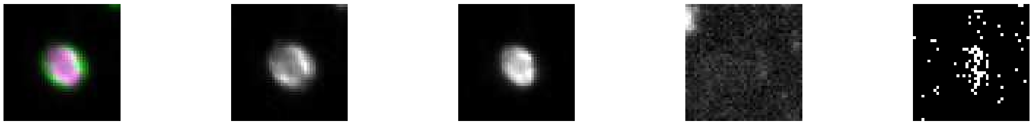				
25605	152	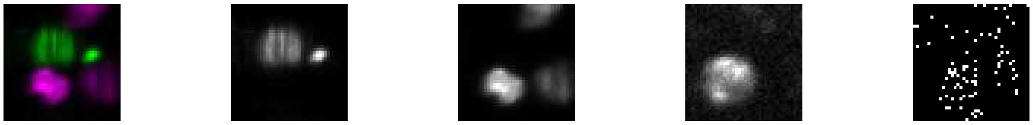				

Ereignis	Aufnahme	DAPI/CK-PE	CK-PE	DAPI	CD45-APC	HER-2/neu
25606	152	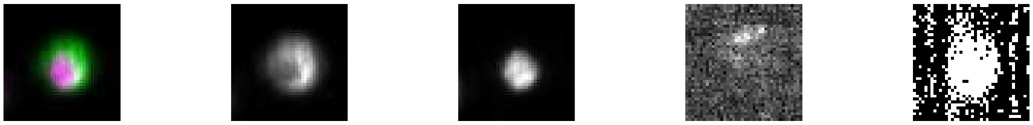				
25607	152	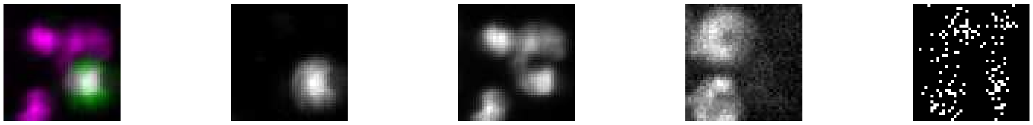				
25608	152	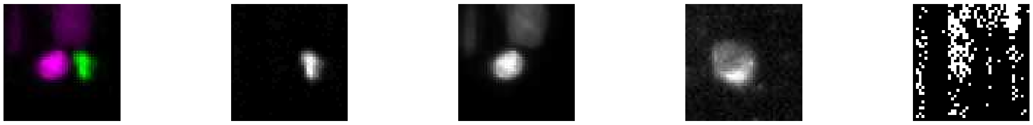				
25609	152	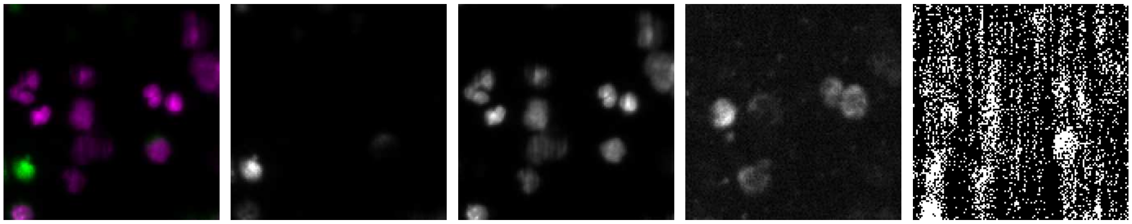				
25610	152	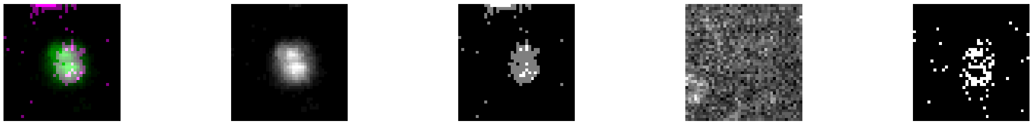				
25611	152	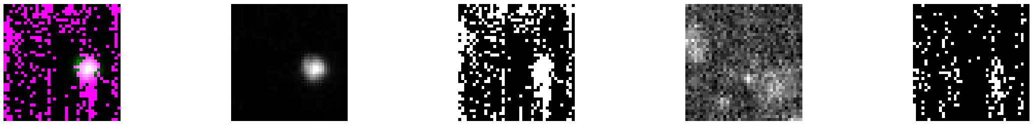				
25612	152	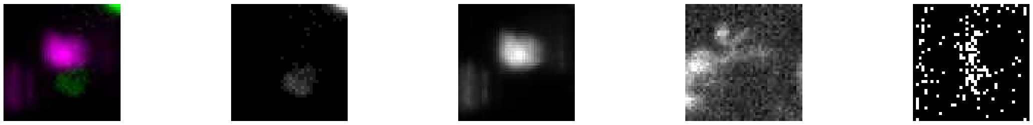				
25613	152	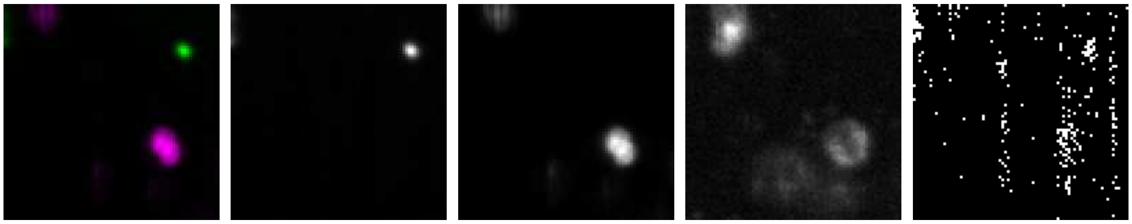				
25614	152	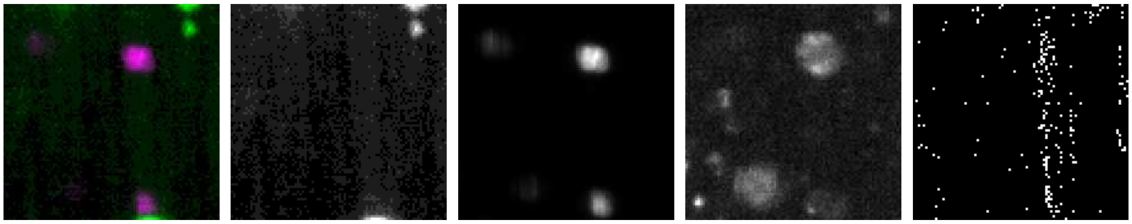				
25615	152	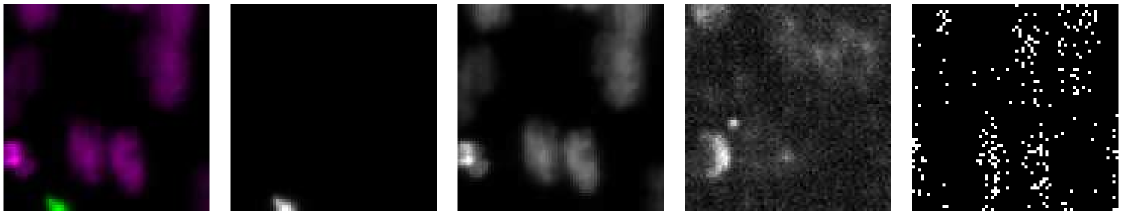				
25616	152	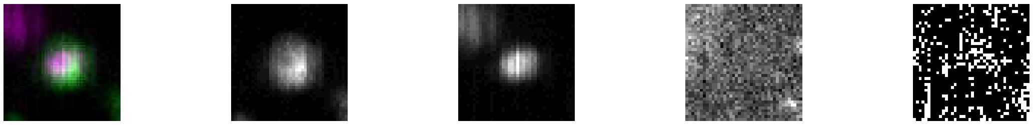				

Ereignis	Aufnahme	DAPI/CK-PE	CK-PE	DAPI	CD45-APC	HER-2/neu
25617	152	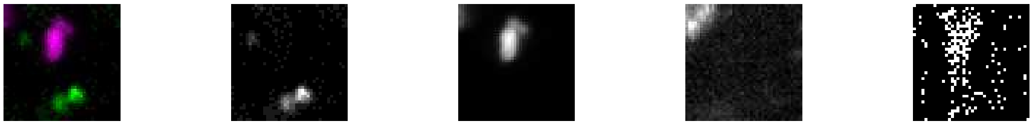				
25618	152	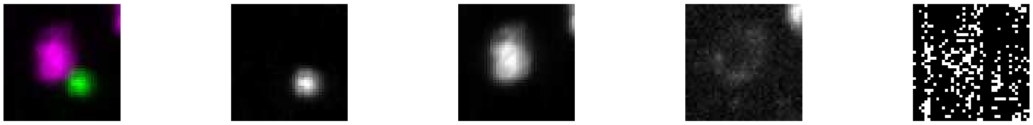				
25619	152	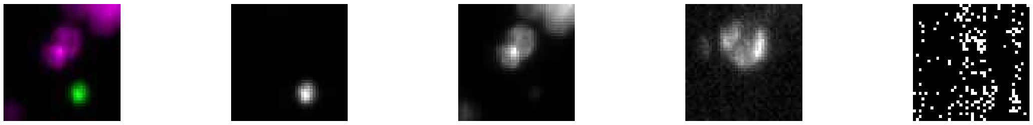				
25620	152	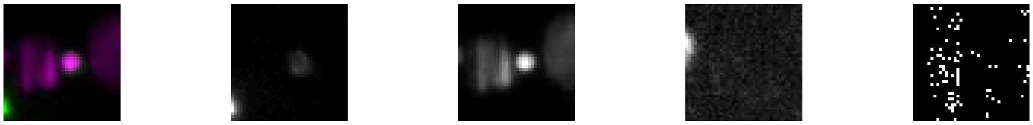				
25621	152	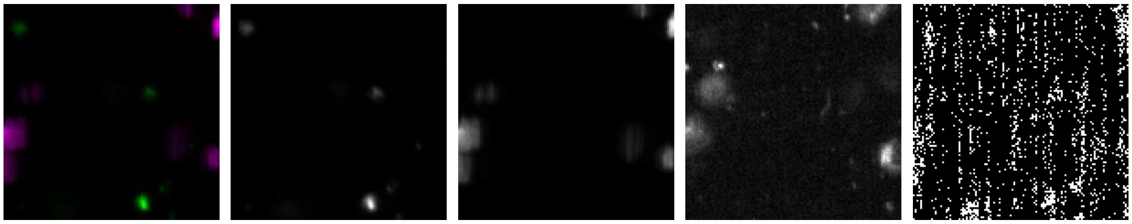				
25622	152	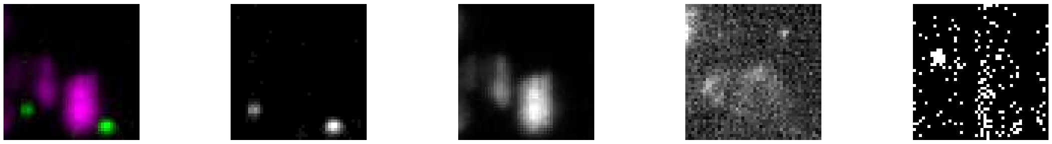				
25623	152	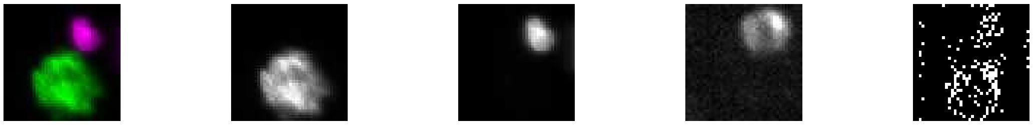				
25624	152	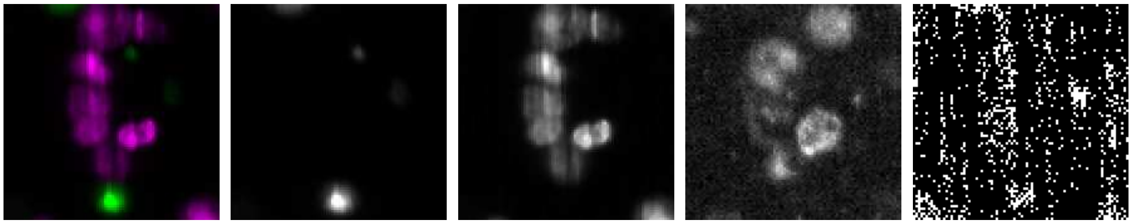				
25625	152	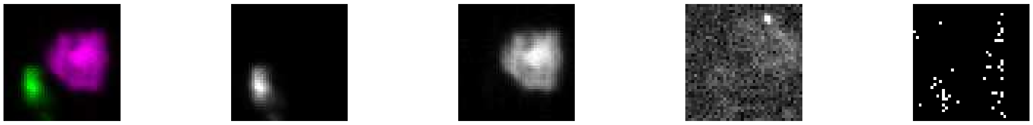				
25626	152	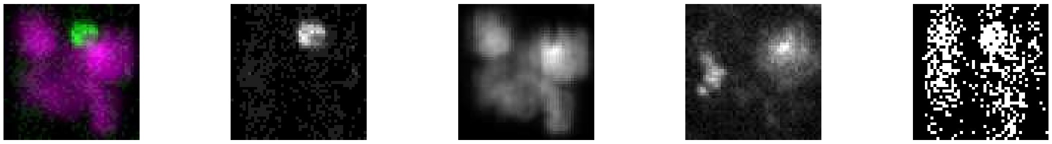				
25627	152	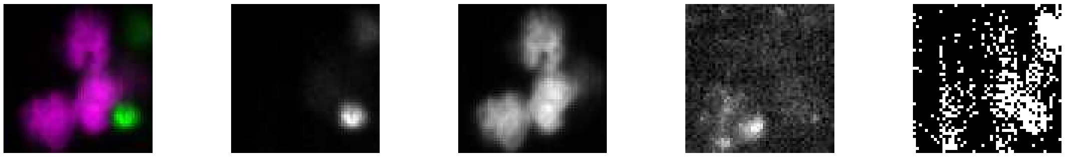				
25628	152	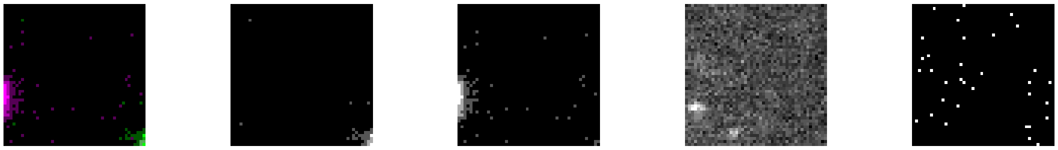				

Ereignis	Aufnahme	DAPI/CK-PE	CK-PE	DAPI	CD45-APC	HER-2/neu
25629	152	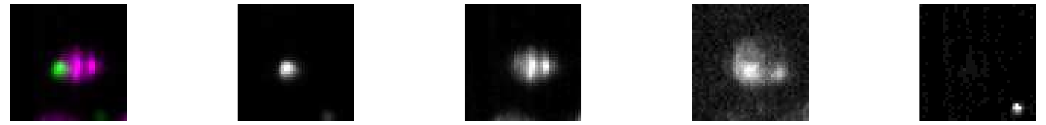				
25630	152	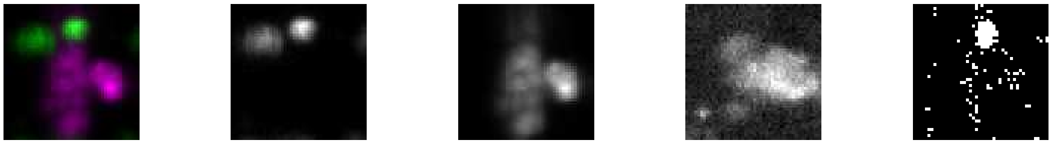				
25631	152	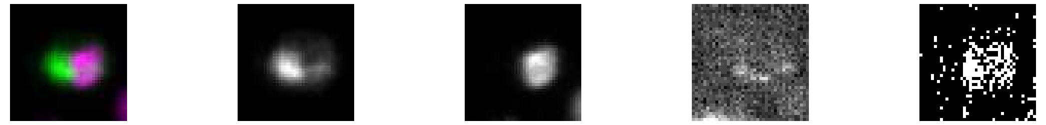				
25632	152	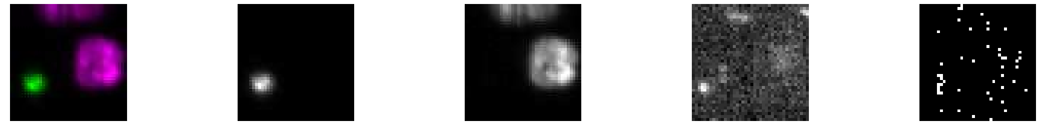				
25633	152	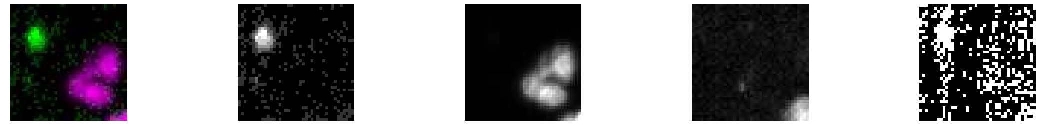				
25634	152	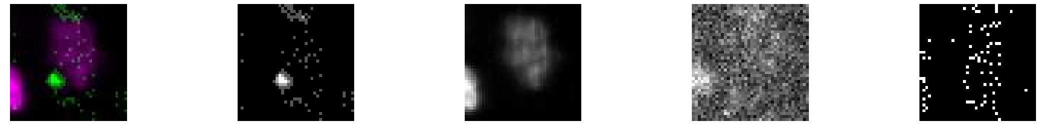				
25635	152	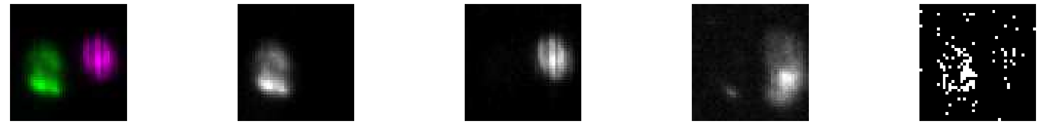				
25636	152	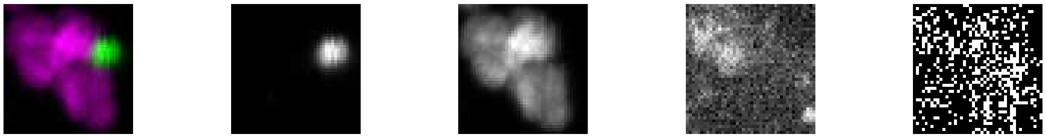				
25637	152	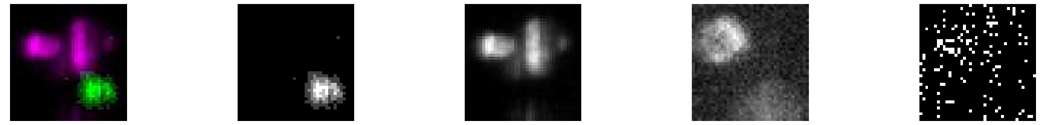				
25638	152	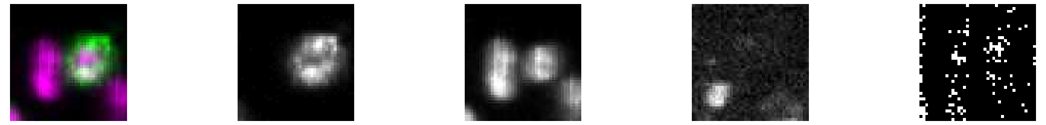				
25639	152	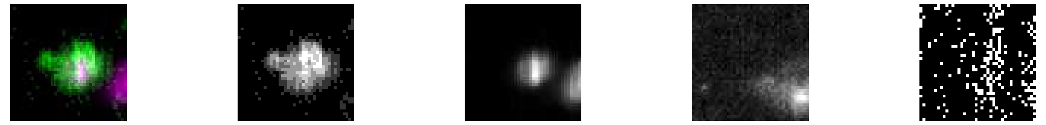				
25640	152	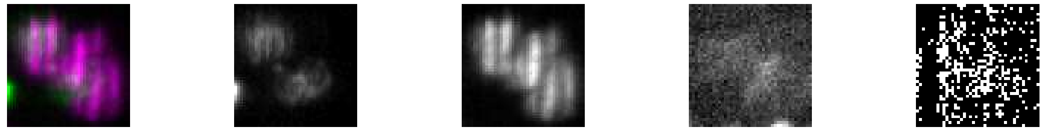				
25641	152	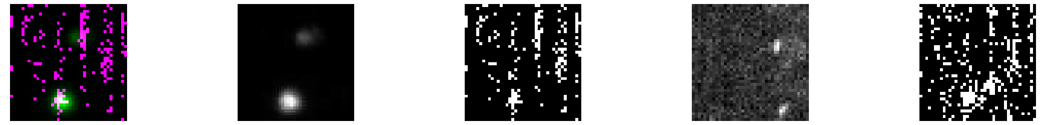				
25642	152	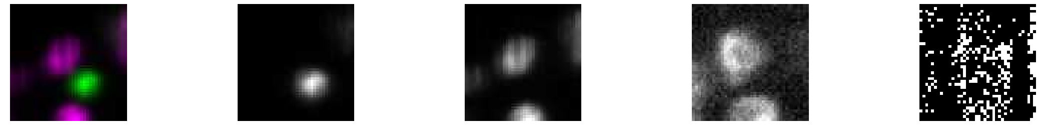				

Ereignis	Aufnahme	DAPI/CK-PE, CK-PE, DAPI, CD45-APC, HER-2/neu
25643	152	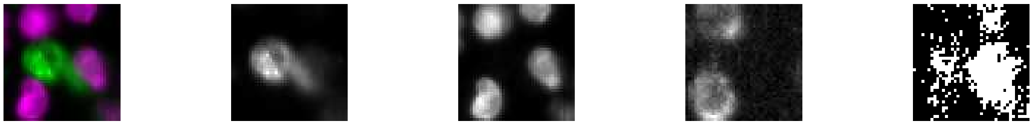
25644	152	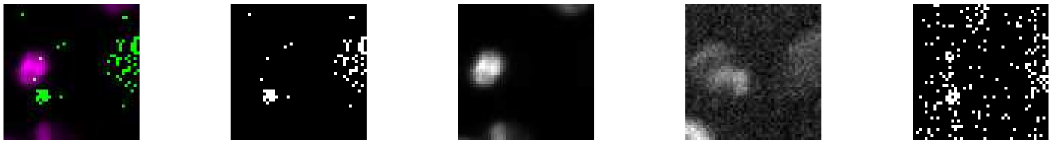
25645	152	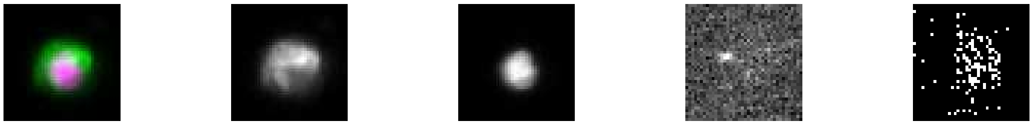
25646	152	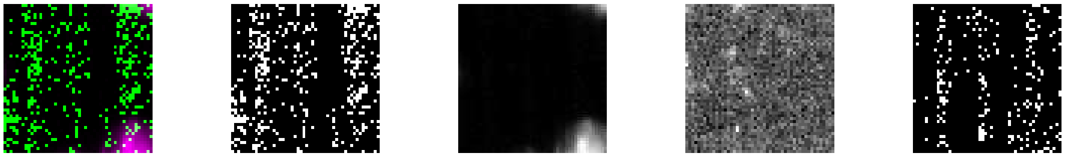
25647	152	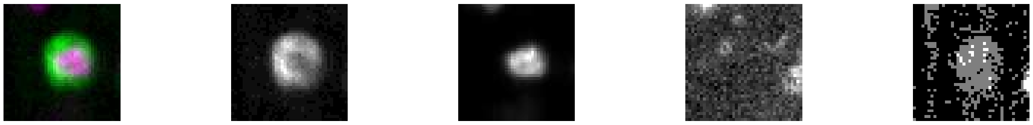
25648	152	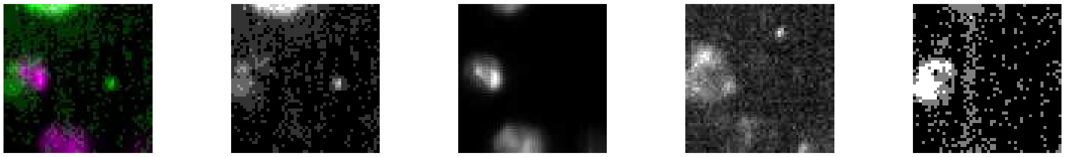
25649	152	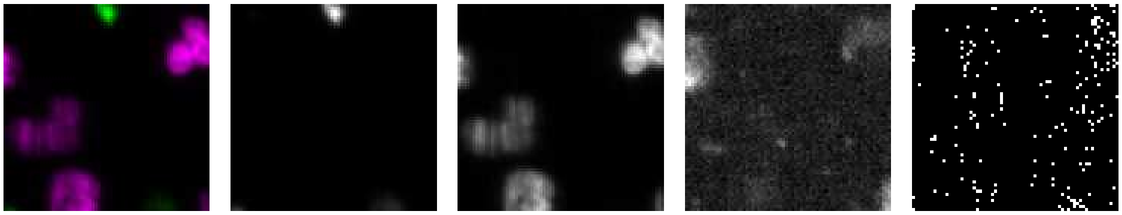
25650	152	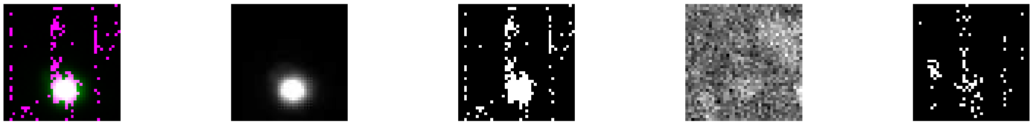
25651	152	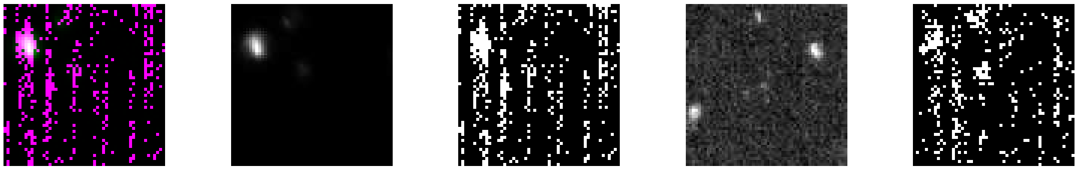
25652	152	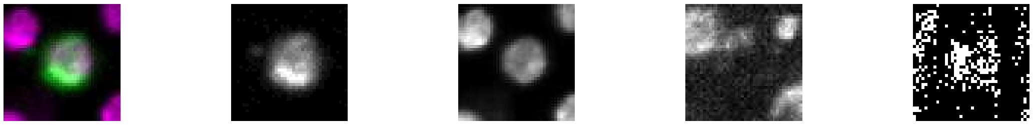
25653	152	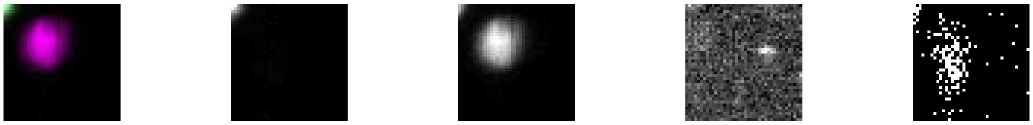
25654	152	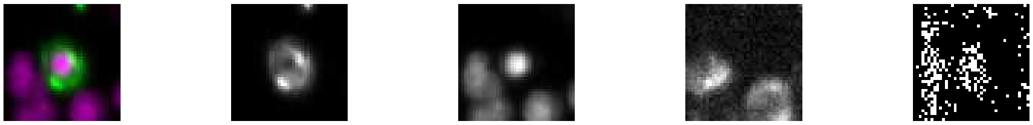

Ereignis	Aufnahme	DAPI/CK-PE	CK-PE	DAPI	CD45-APC	HER-2/neu
25655	152	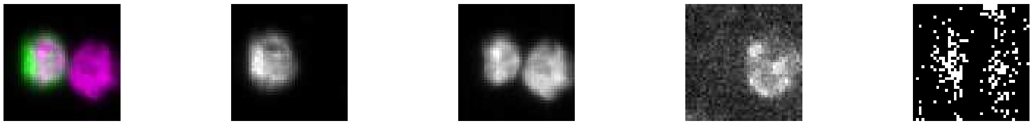				
25656	152	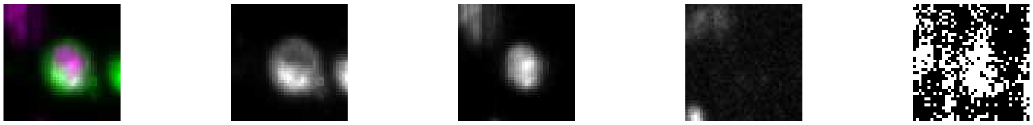				
25657	152	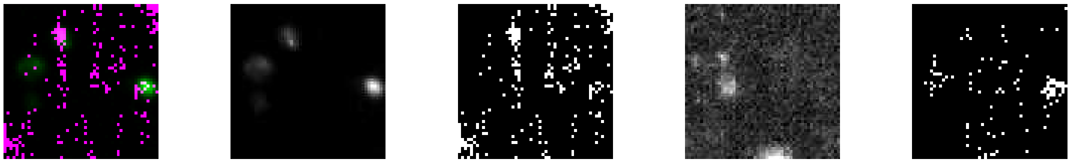				
25658	152	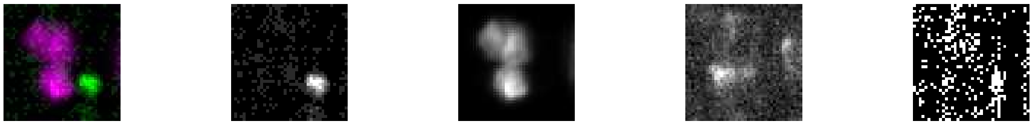				
25659	152	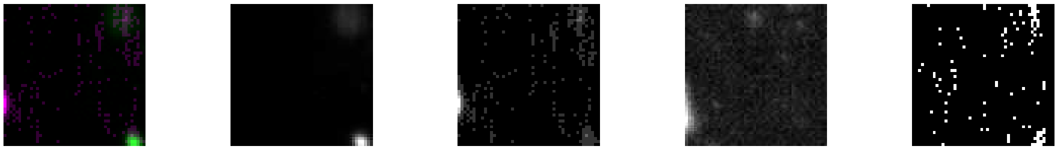				
25660	152	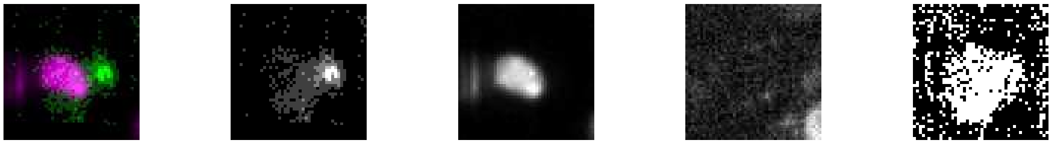				
25661	129	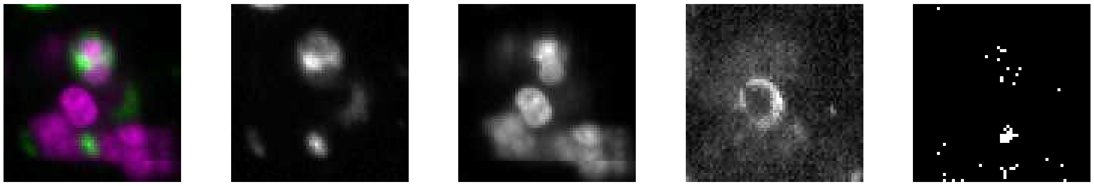				
25662	151	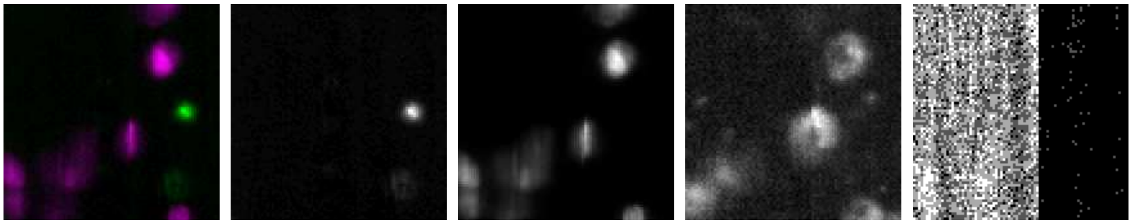				
25663	152	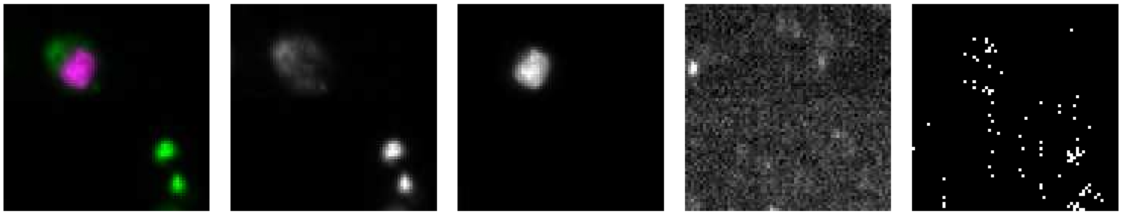				
25664	129	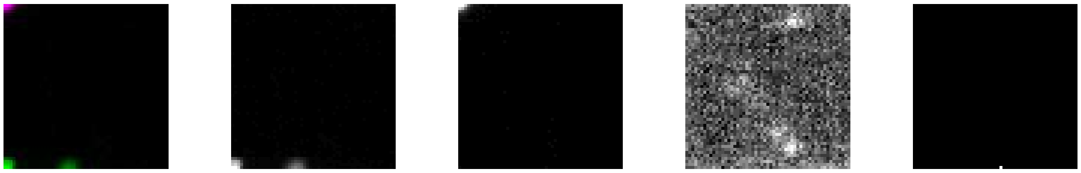				

Ereignis	Aufnahme	DAPI/CK-PE	CK-PE	DAPI	CD45-APC	HER-2/neu
25665	129	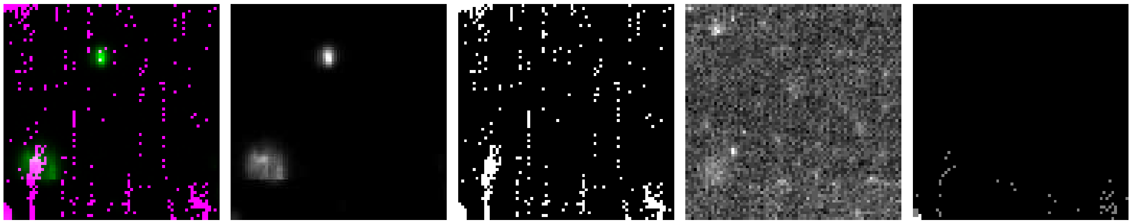				
25666	129	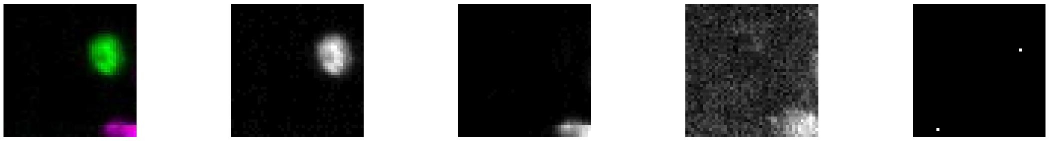				
25667	152	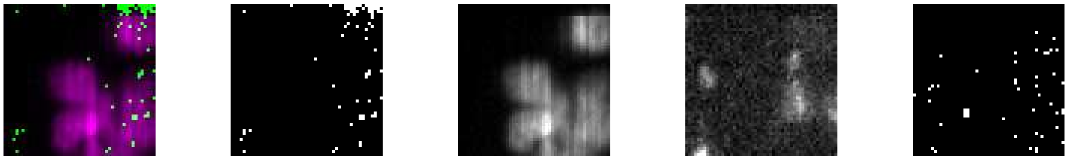				
25668	152	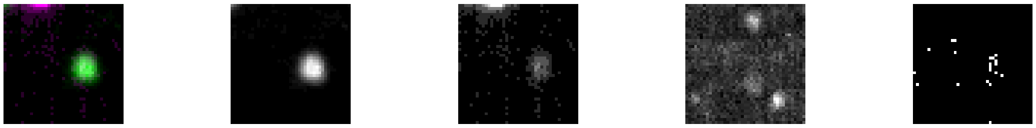				
25669	152	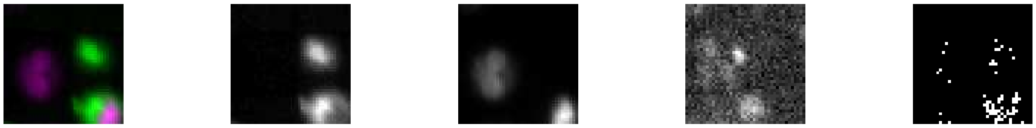				
25670	152	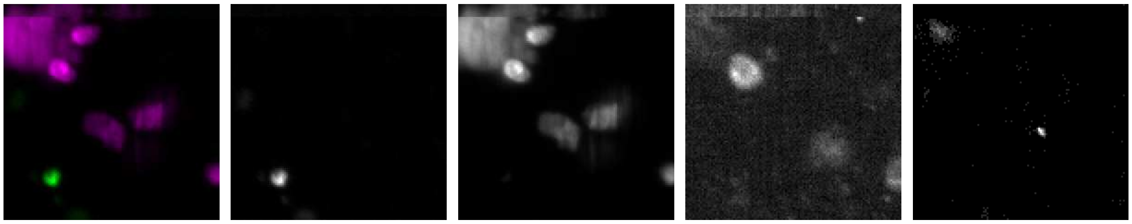				
25671	129	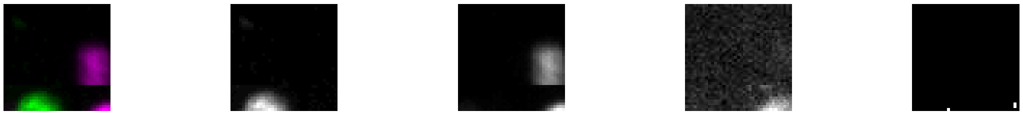				
25672	153	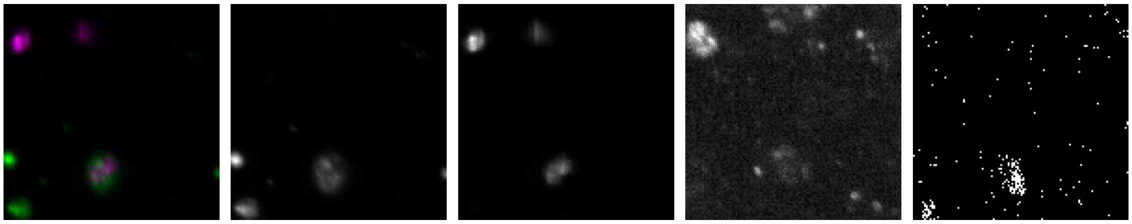				
25673	153	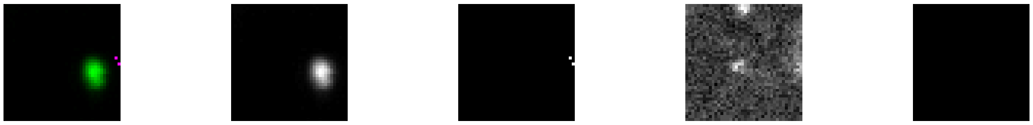				
25674	153	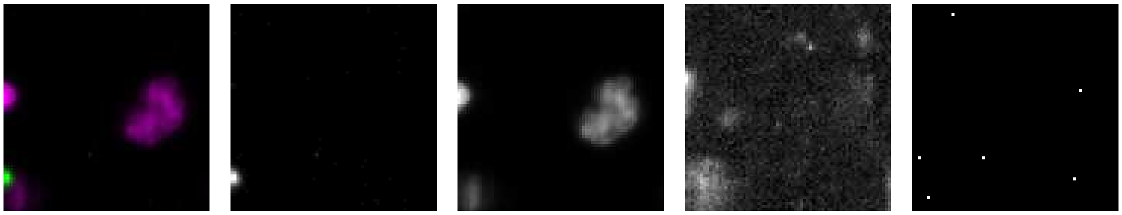				

Ereignis	Aufnahme	DAPI/CK-PE	CK-PE	DAPI	CD45-APC	HER-2/neu
25675	153	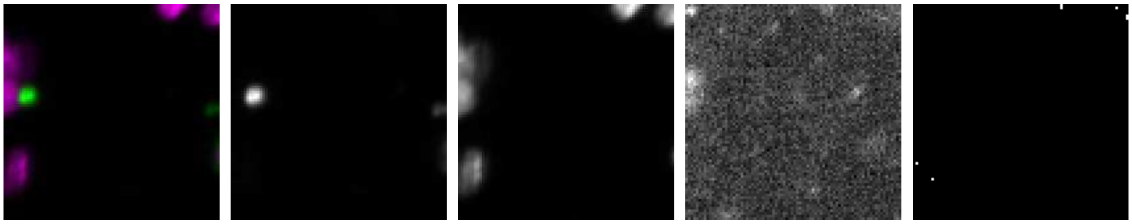				
25676	153	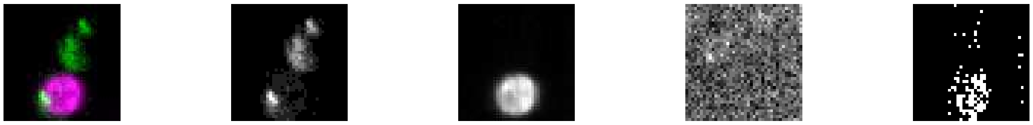				
25677	153	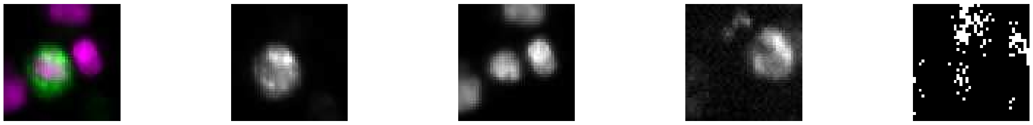				
25678	153	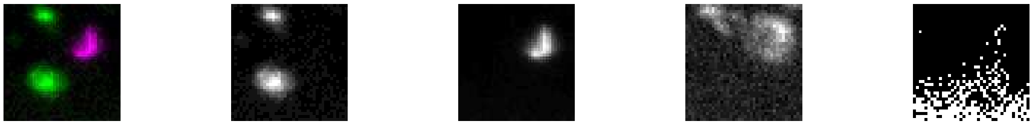				
25679	153	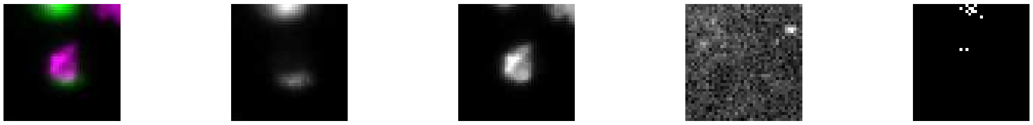				
25680	153	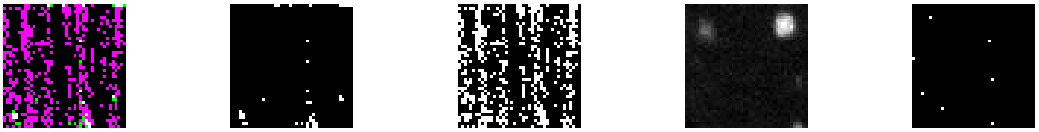				
25681	153	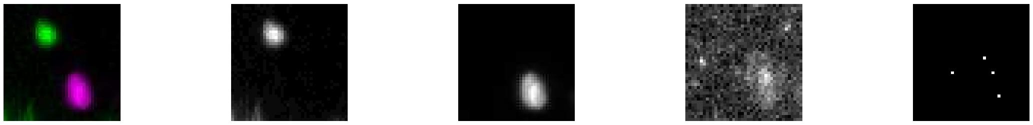				
25682	153	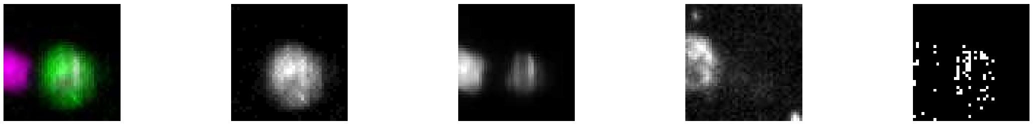				
25683	153	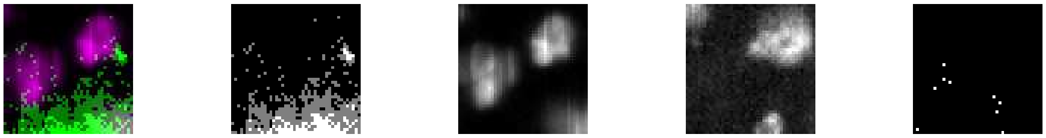				
25684	153	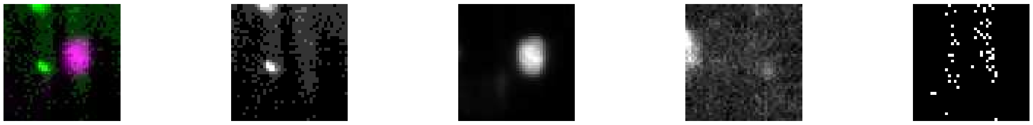				
25685	153	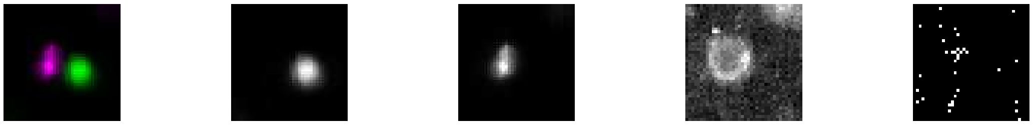				
25686	153	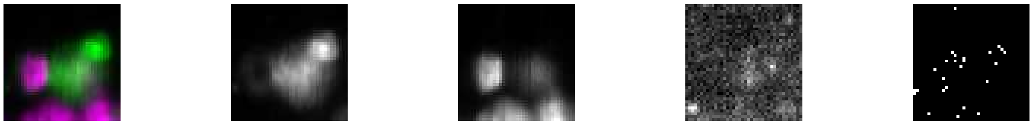				
25687	153	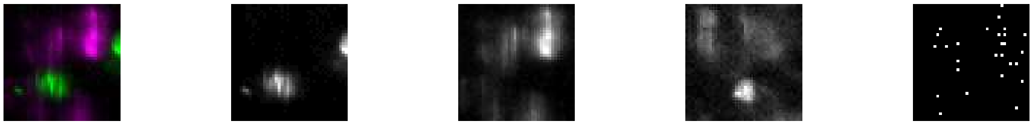				

Ereignis	Aufnahme	DAPI/CK-PE	CK-PE	DAPI	CD45-APC	HER-2/neu
25688	153	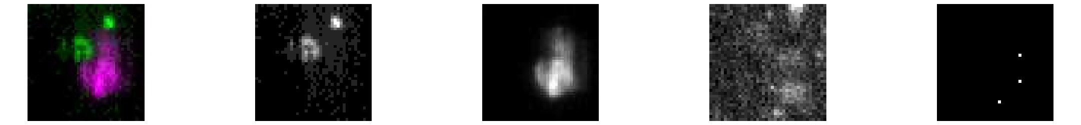				
25689	153	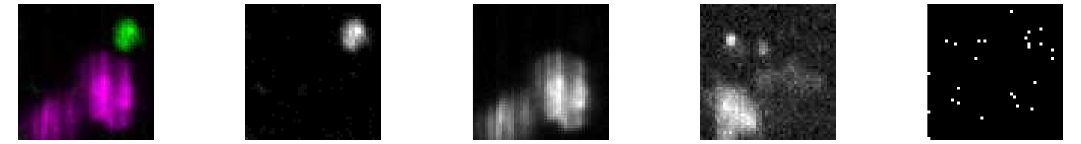				
25690	153	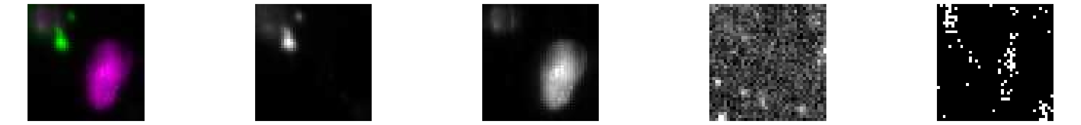				
25691	153	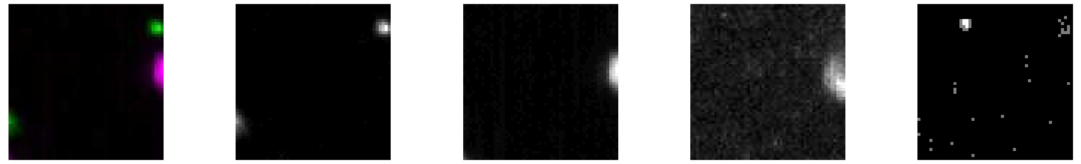				
25692	153	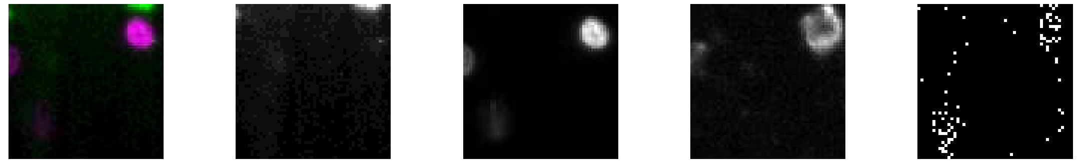				
25693	153	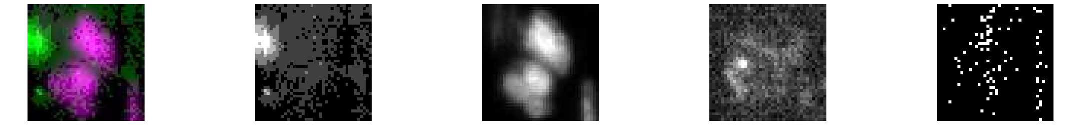				
25694	153	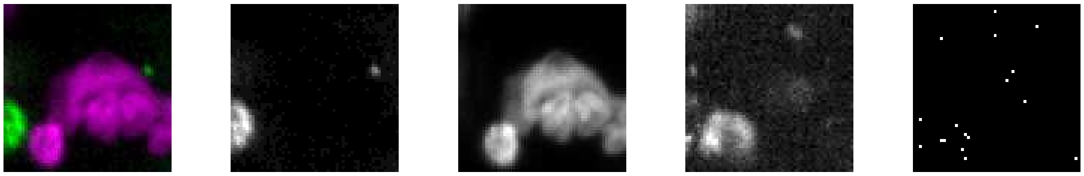				
25695	153	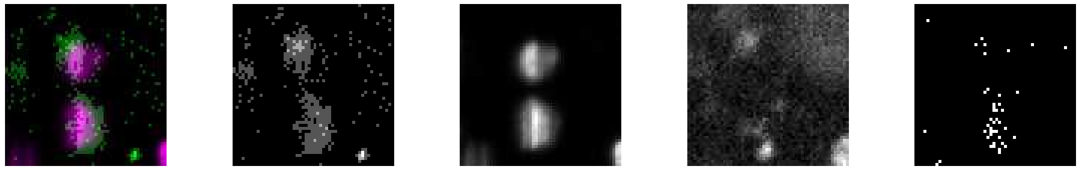				
25696	153	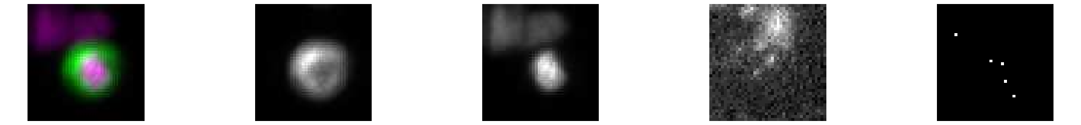				
25697	153	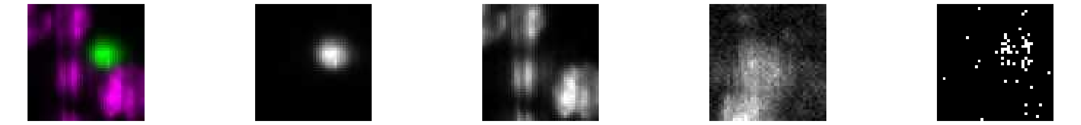				
25698	153	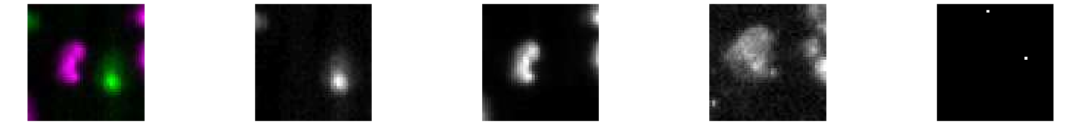				
25699	153	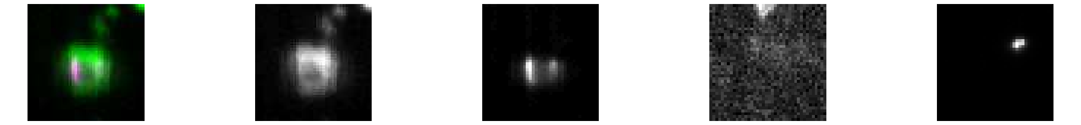				

Ereignis	Aufnahme	DAPI/CK-PE	CK-PE	DAPI	CD45-APC	HER-2/neu
25700	153	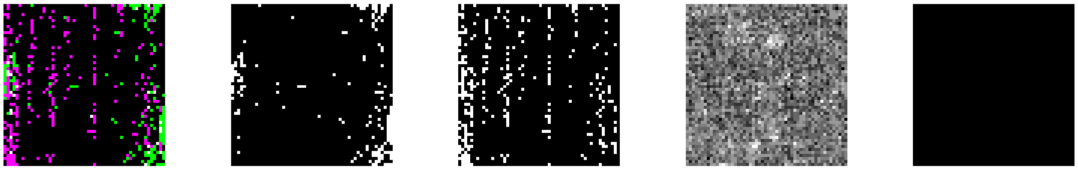				
25701	153	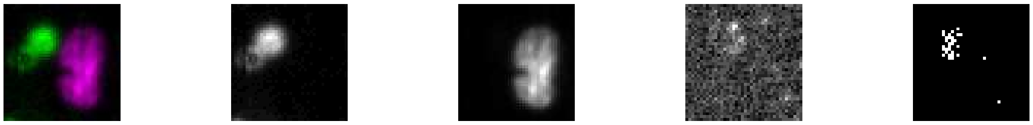				
25702	153	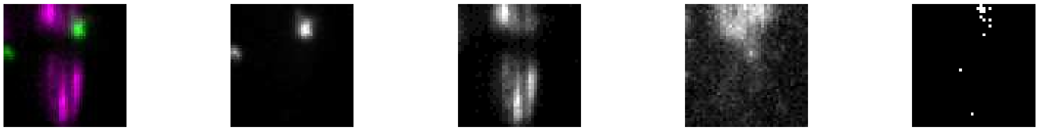				
25703	153	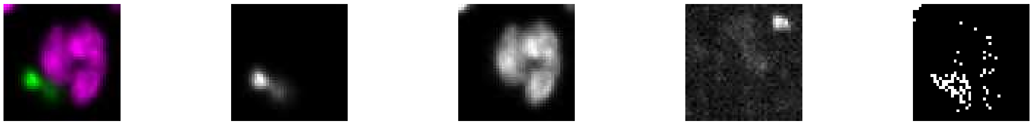				
25704	153	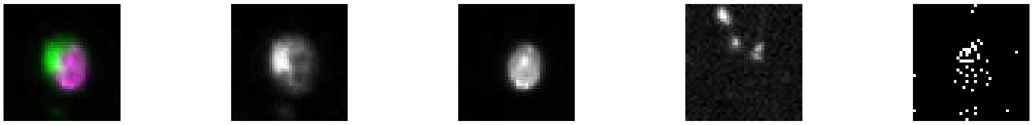				
25705	153	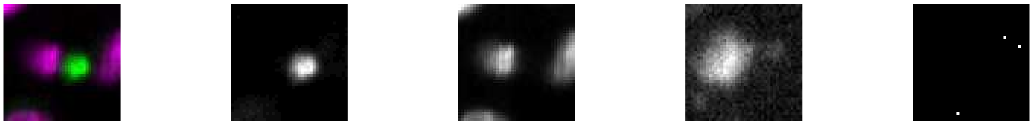				
25706	153	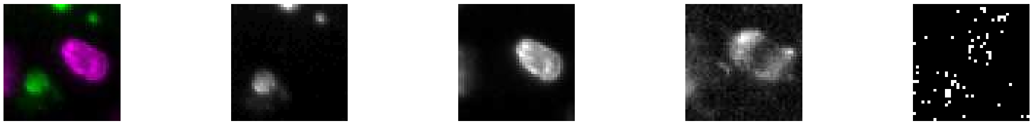				
25707	153	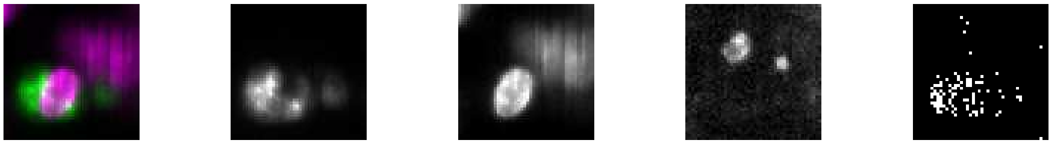				
25708	153	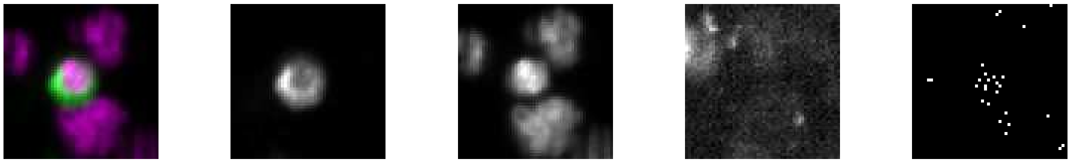				
25709	153	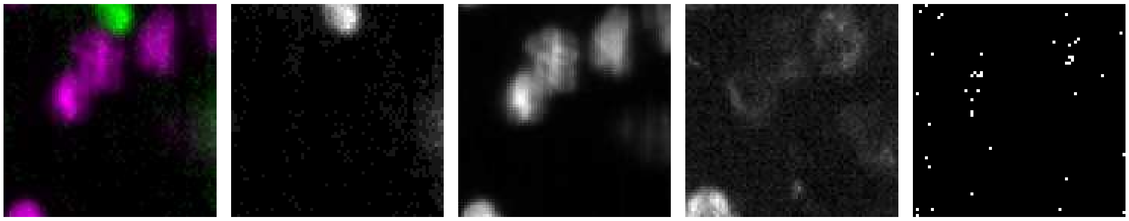				
25710	153	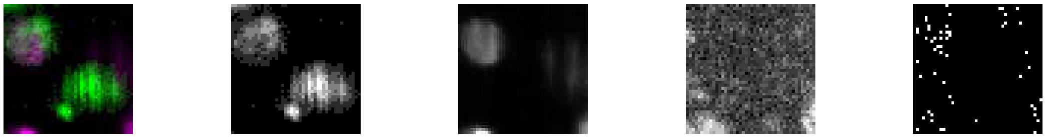				

Druckdatum: 02/06/2020 17:25

Proben-ID: Gyn14.208 **Kassetten-ID:** 01327106 **Patienten-ID:**

Ereignis	Aufnahme	DAPI/CK-PE	CK-PE	DAPI	CD45-APC	HER-2/neu
25711	153	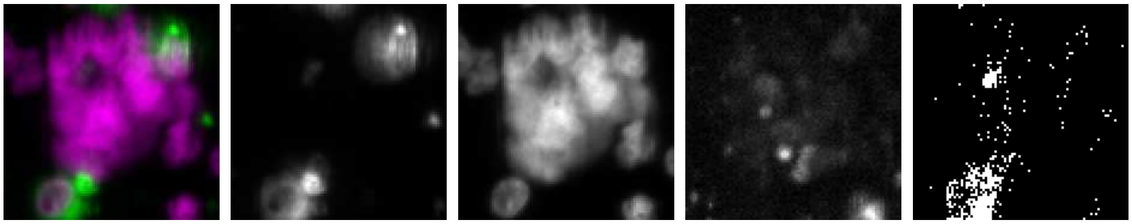				
25712	153	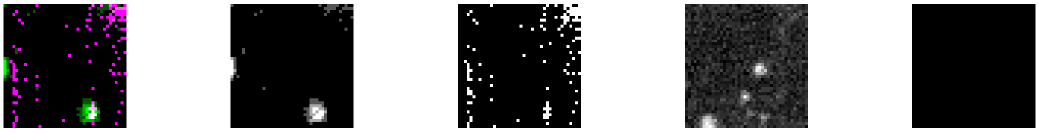				
25713	153	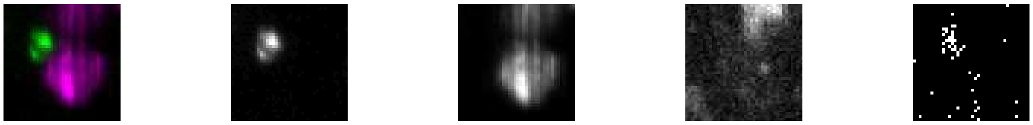				
25714	153	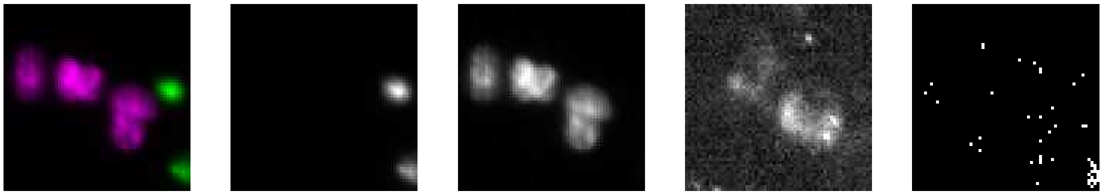				
25715	153	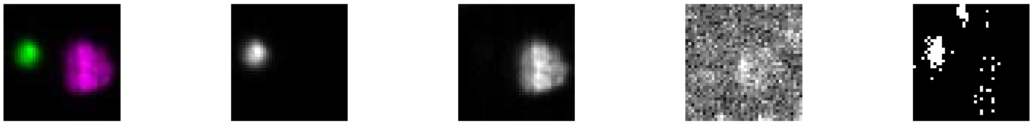				
25716	153	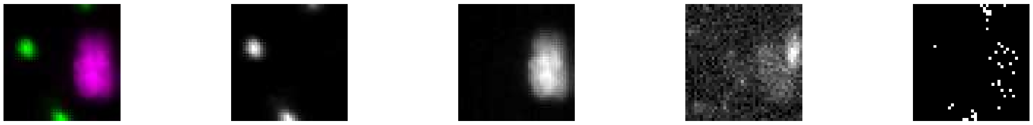				
25717	153	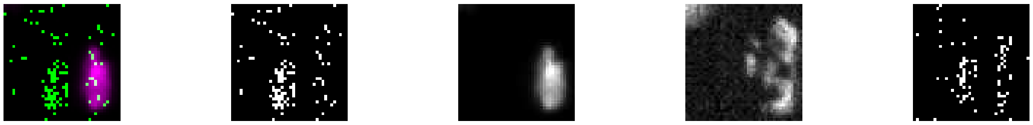				
25718	153	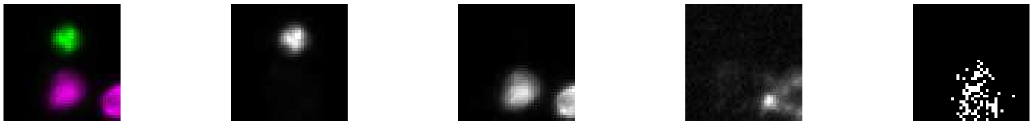				
25719	153	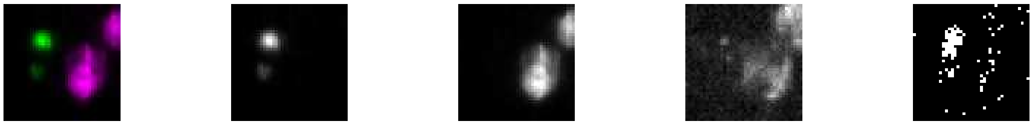				
25720	153	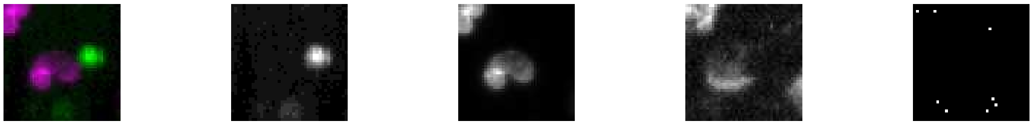				
25721	153	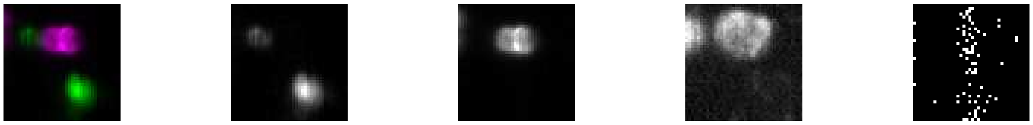				
25722	153	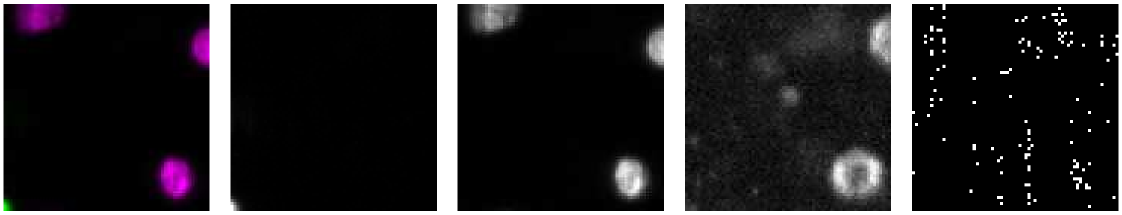				

Ereignis	Aufnahme	DAPI/CK-PE	CK-PE	DAPI	CD45-APC	HER-2/neu
25723	153	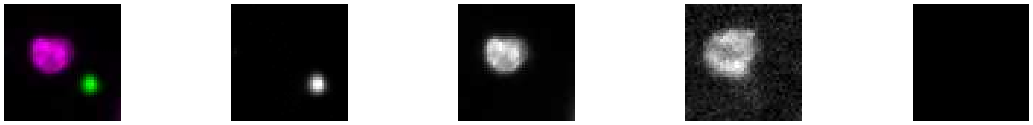				
25724	153	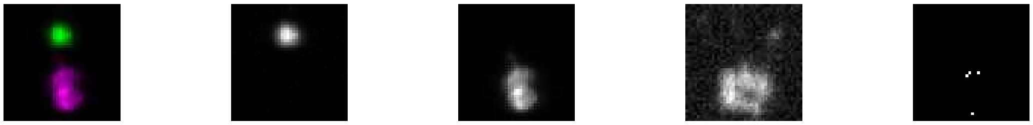				
25725	153	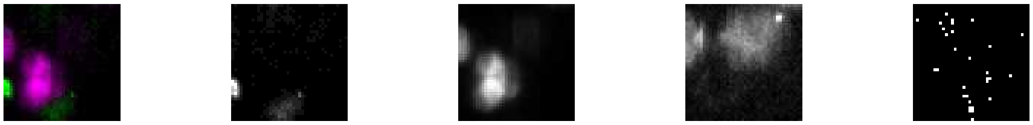				
25726	153	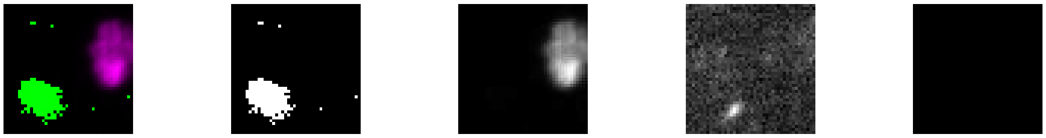				
25727	153	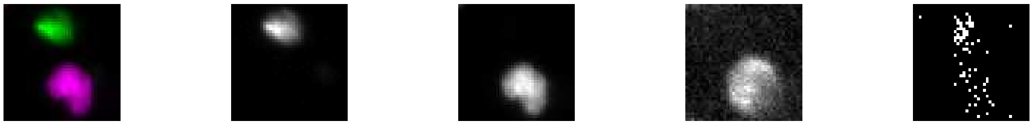				
25728	153	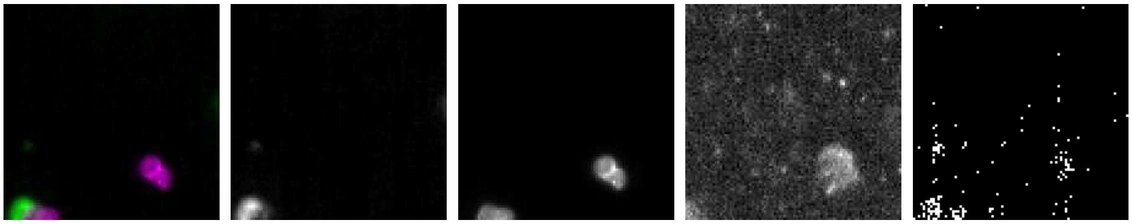				
25729	153	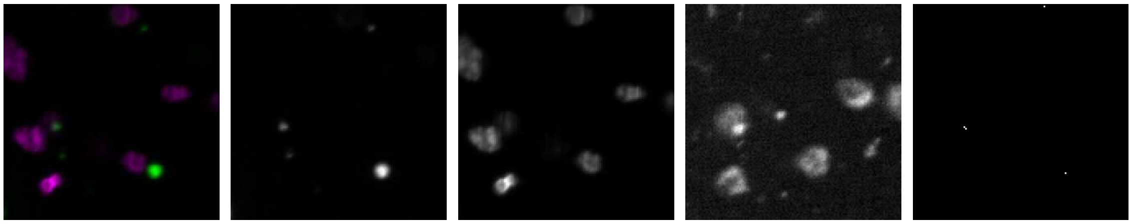				
25730	153	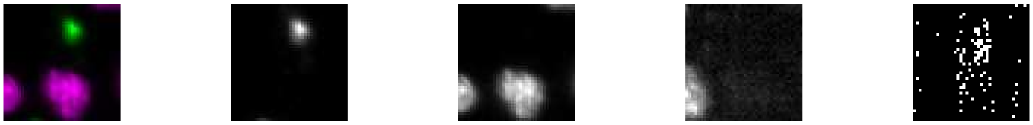				
25731	153	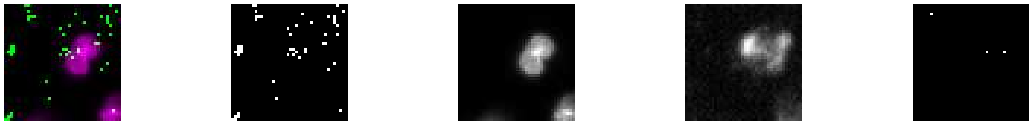				
25732	153	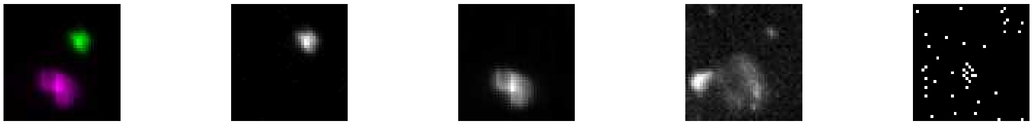				
25733	153	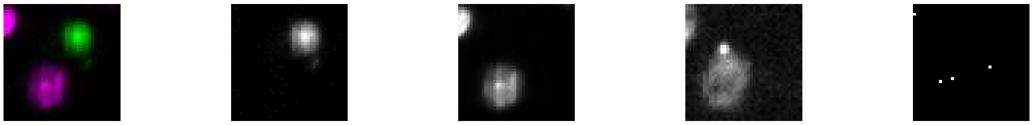				
25734	153	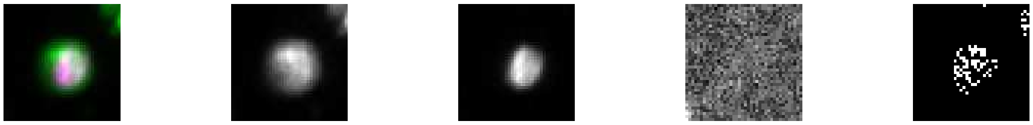				

Ereignis	Aufnahme	DAPI/CK-PE / CK-PE / DAPI / CD45-APC / HER-2/neu
25735	153	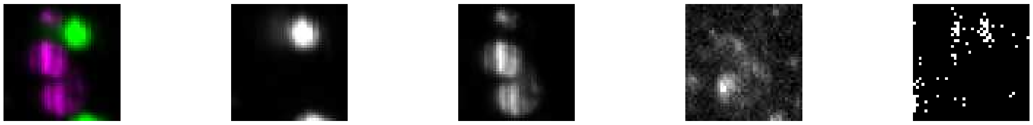
25736	153	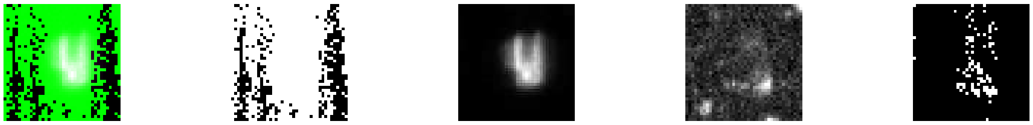
25737	153	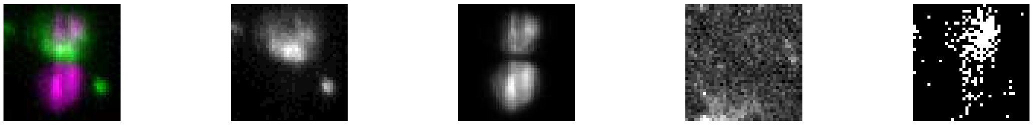
25738	153	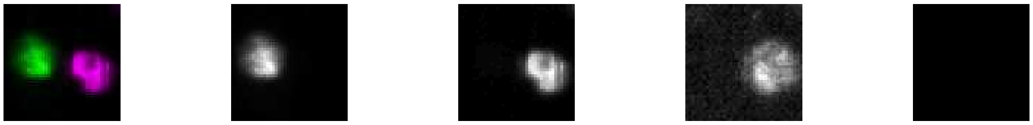
25739	153	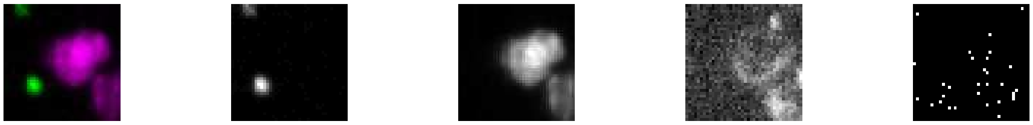
25740	153	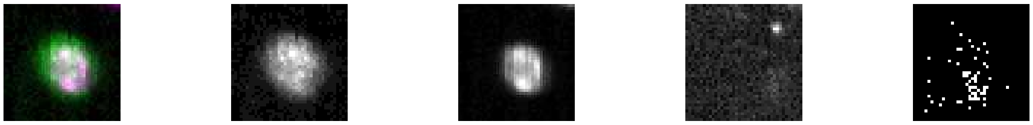
25741	153	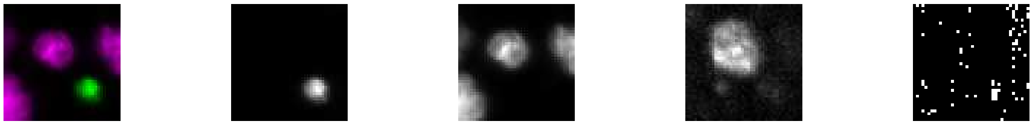
25742	153	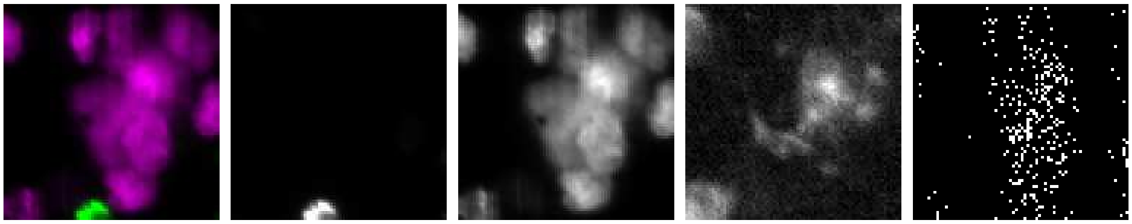
25743	153	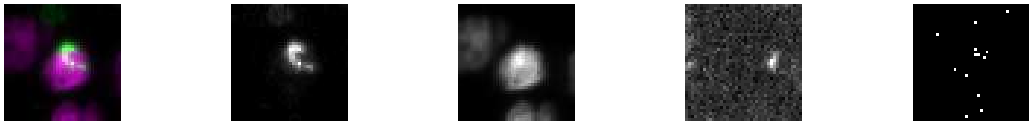
25744	153	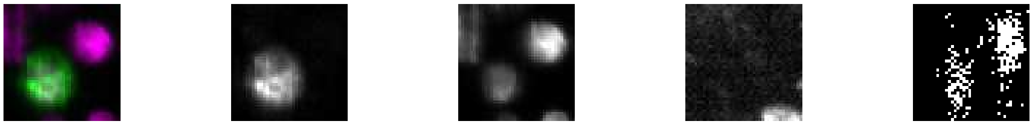
25745	153	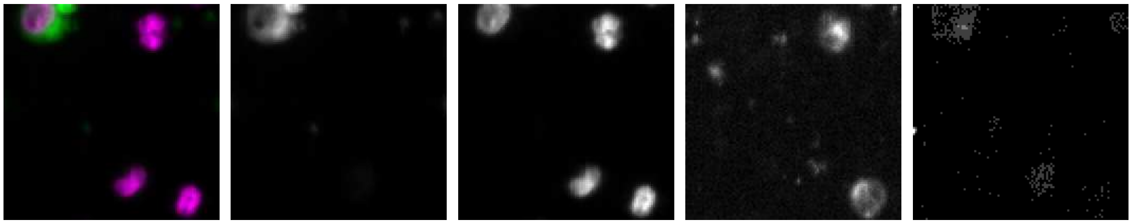
25746	153	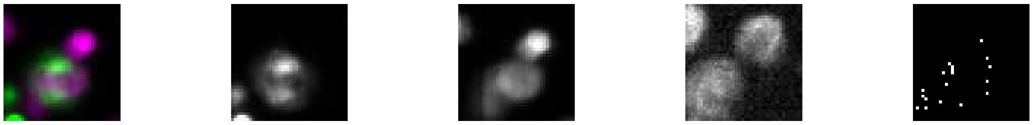

Ereignis	Aufnahme	DAPI/CK-PE	CK-PE	DAPI	CD45-APC	HER-2/neu
25747	153	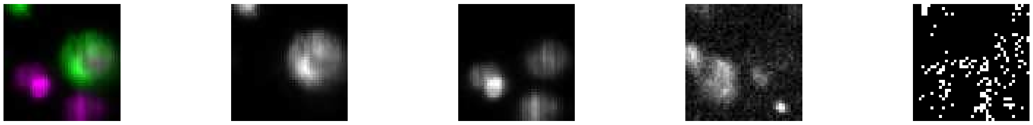				
25748	153	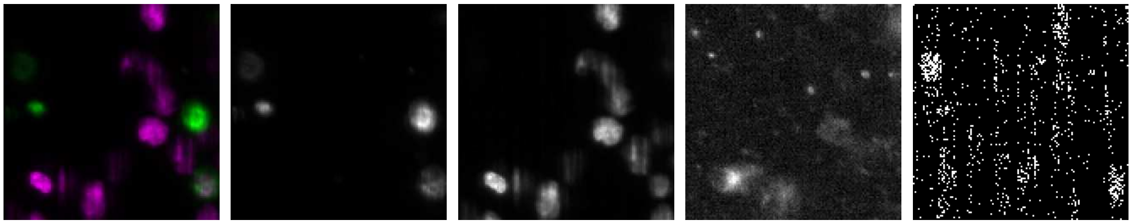				
25749	153	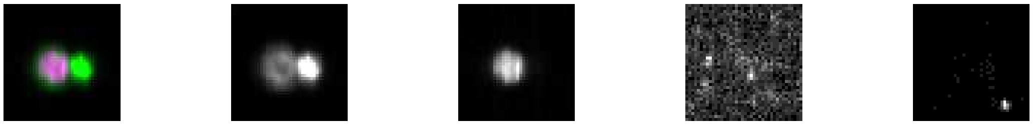				
25750	153	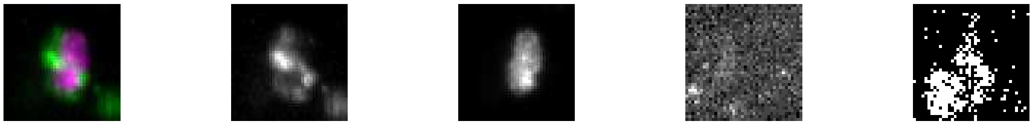				
25751	153	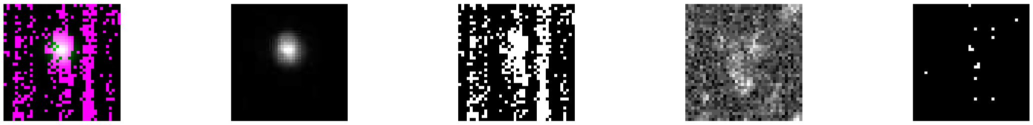				
25752	153	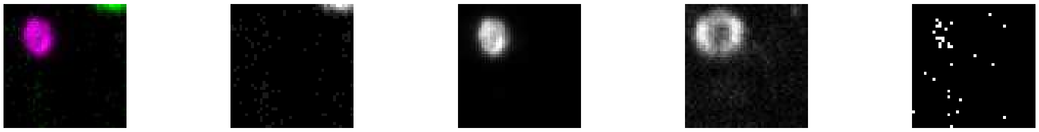				
25753	153	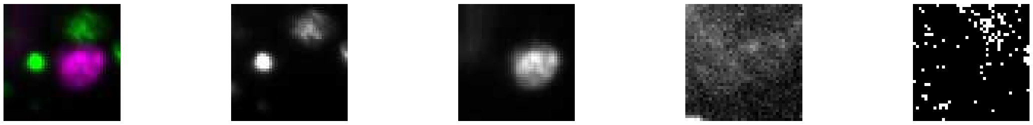				
25754	153	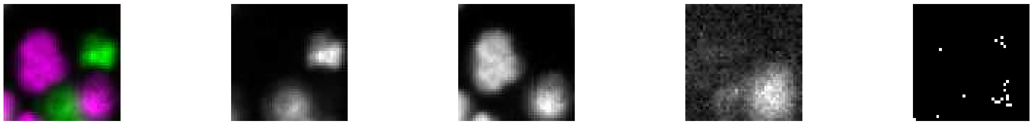				
25755	153	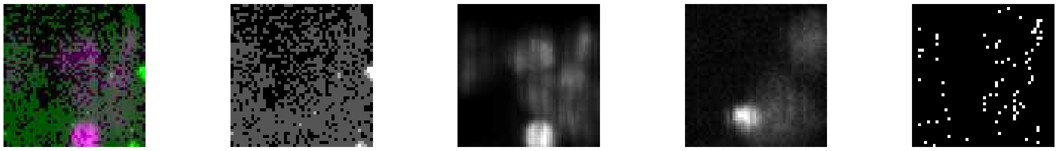				
25756	153	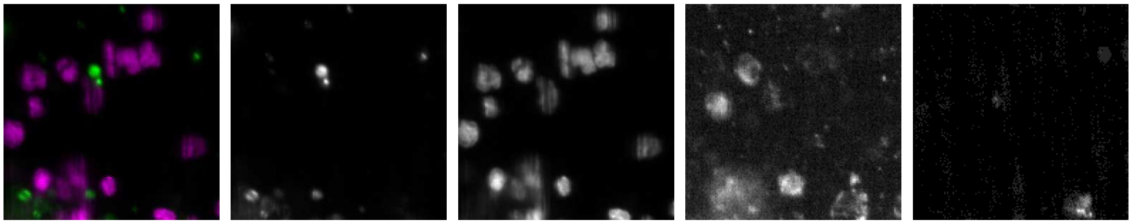				
25757	153	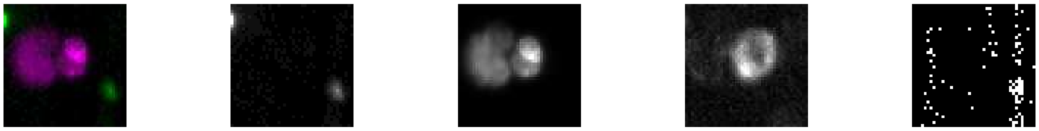				
25758	153	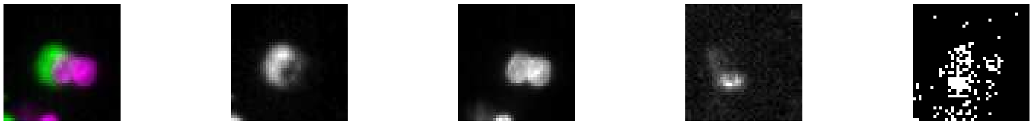				

Ereignis	Aufnahme	DAPI/CK-PE	CK-PE	DAPI	CD45-APC	HER-2/neu
25759	153	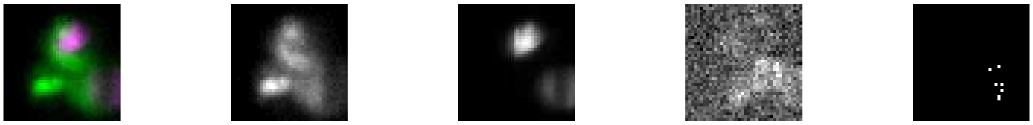				
25760	153	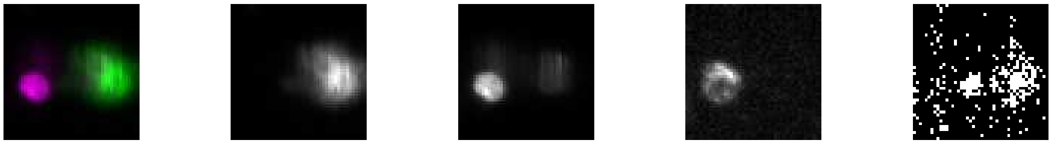				
25761	153	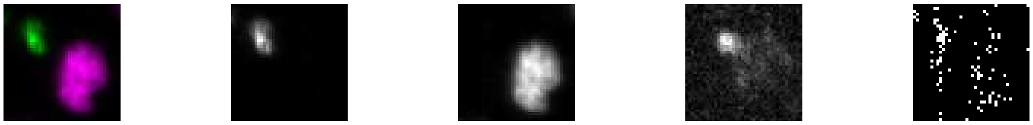				
25762	153	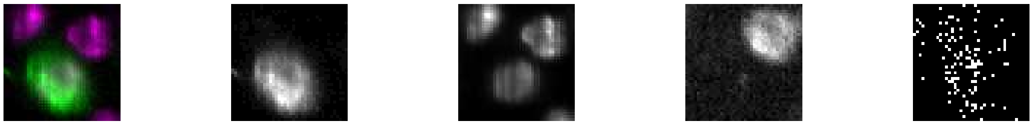				
25763	153	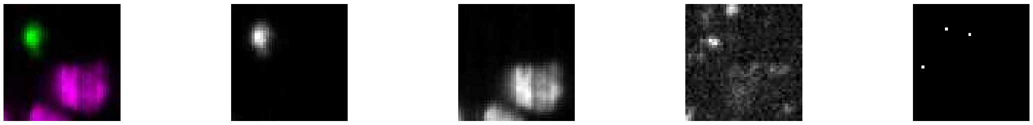				
25764	153	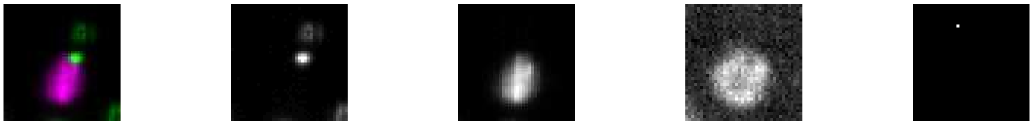				
25765	153	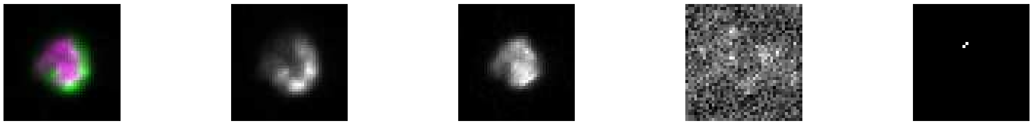				
25766	153	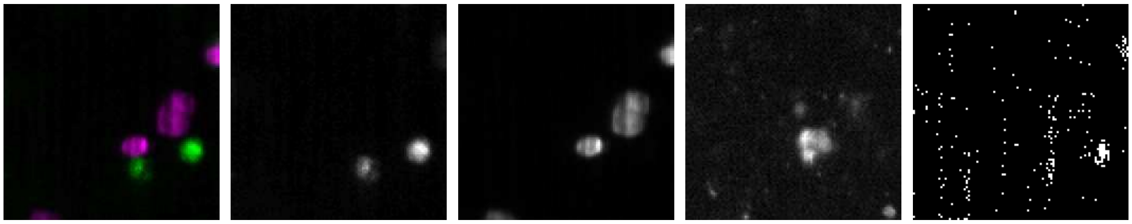				
25767	153	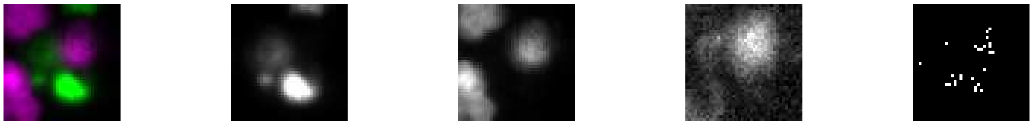				
25768	153	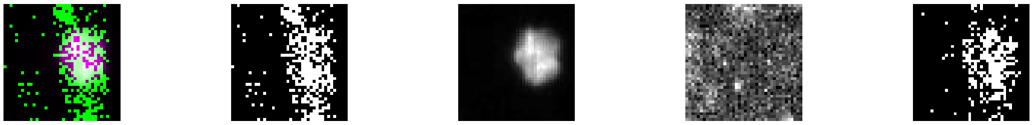				
25769	153	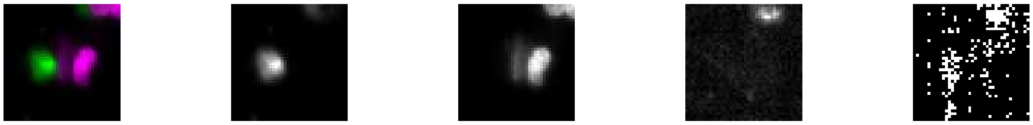				
25770	153	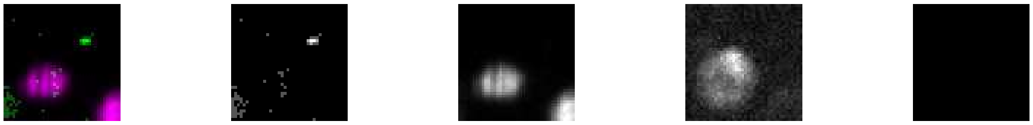				
25771	153	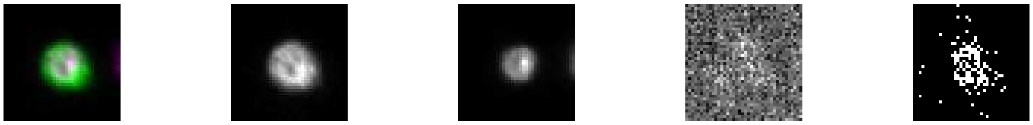				

Ereignis	Aufnahme	DAPI/CK-PE	CK-PE	DAPI	CD45-APC	HER-2/neu
25772	153	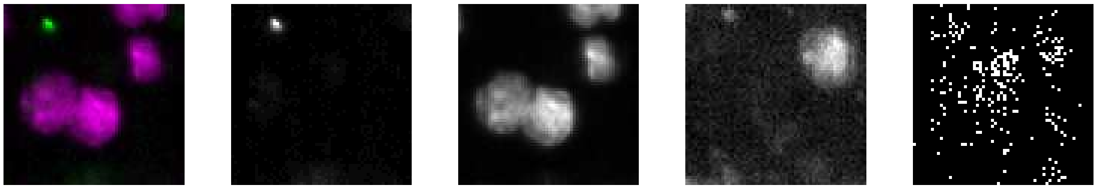				
25773	153	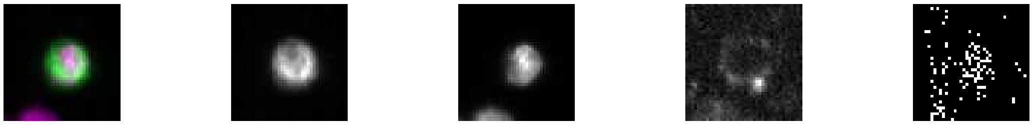				
25774	153	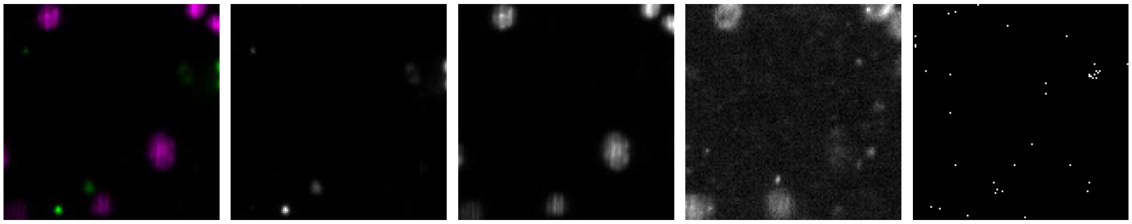				
25775	153	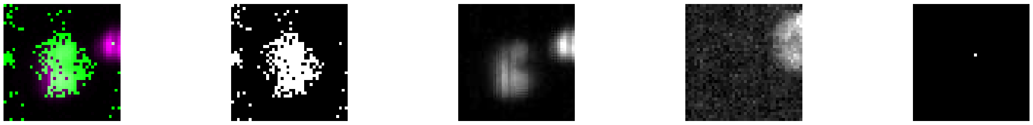				
25776	153	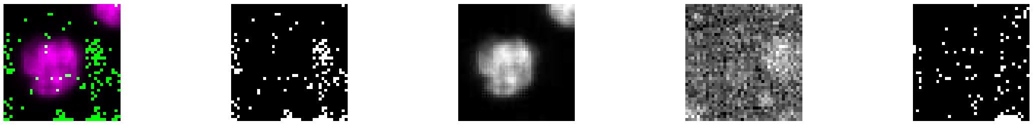				
25777	153	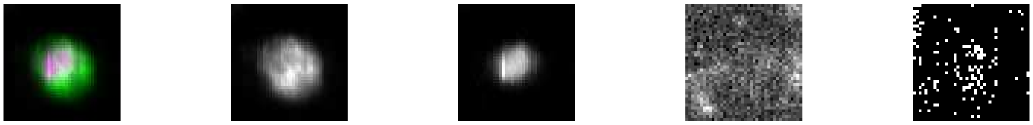				
25778	153	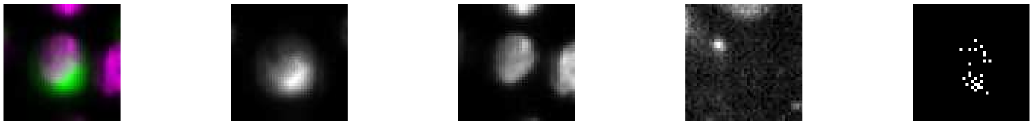				
25779	153	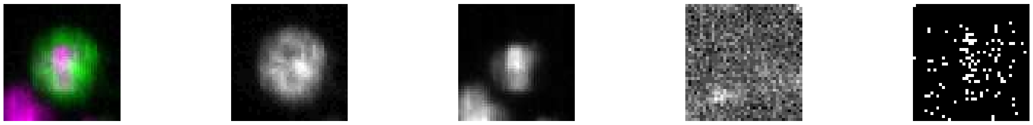				
25780	153	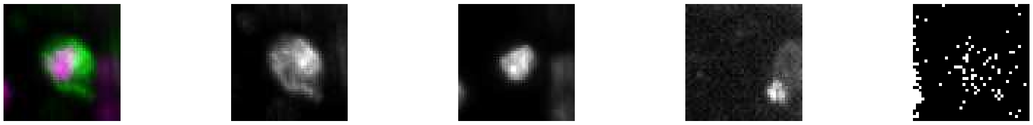				
25781	153	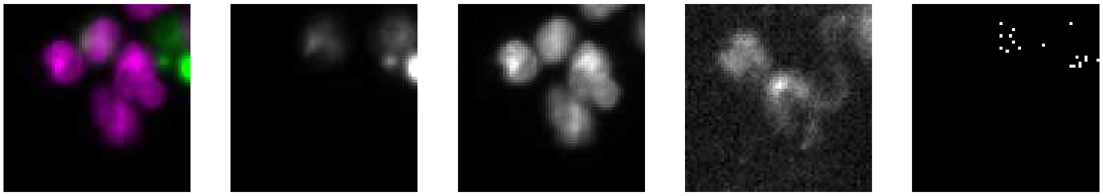				
25782	153	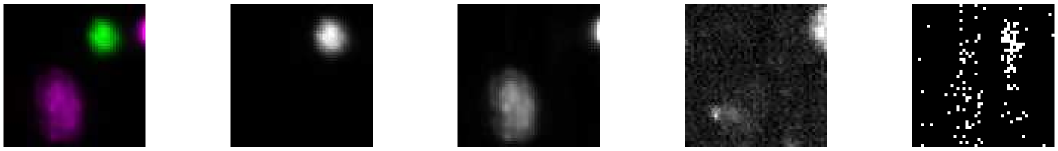				
25783	153	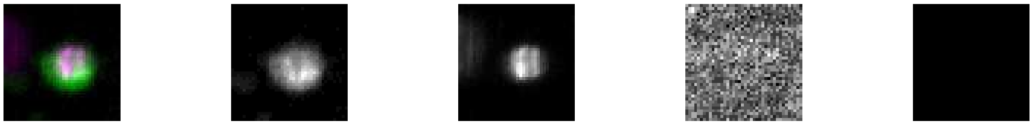				

Ereignis	Aufnahme	DAPI/CK-PE	CK-PE	DAPI	CD45-APC	HER-2/neu
25784	153	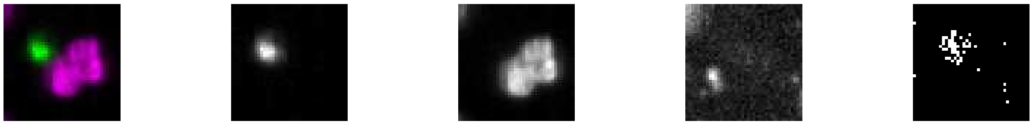				
25785	153	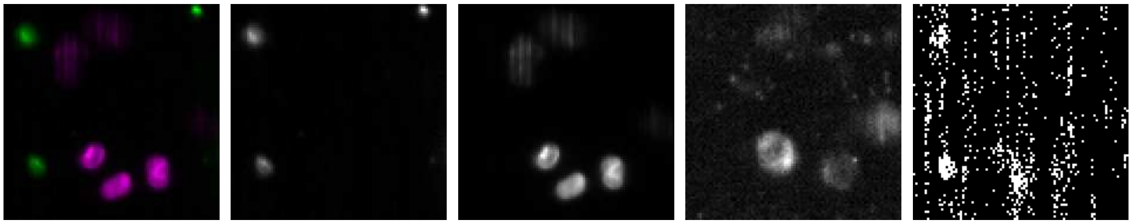				
25786	153	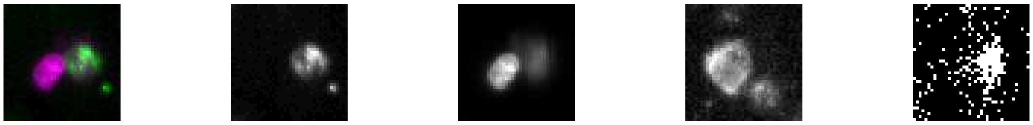				
25787	153	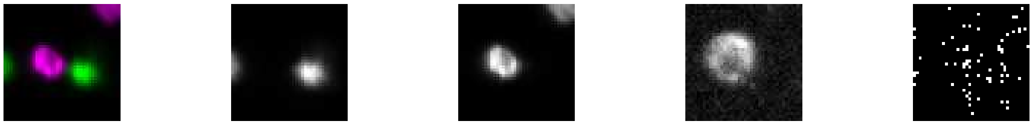				
25788	153	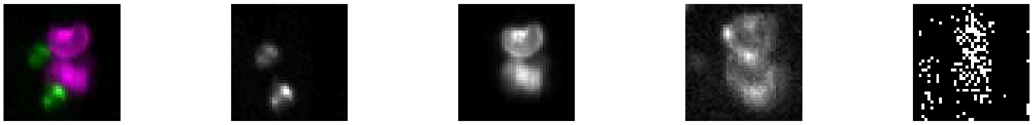				
25789	153	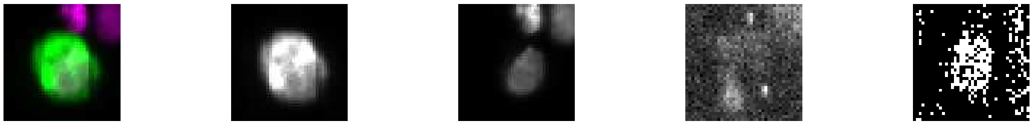				
25790	153	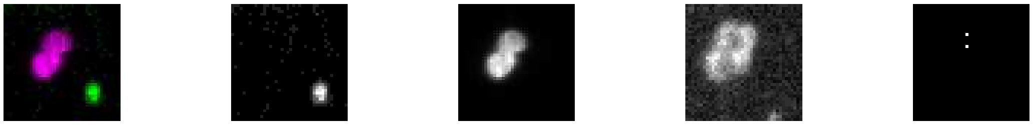				
25791	153	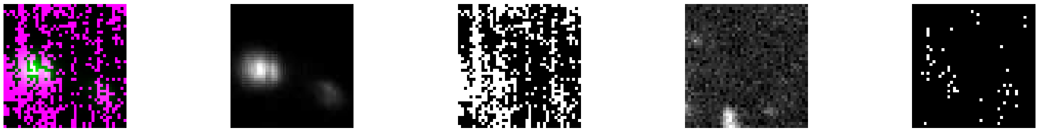				
25792	153	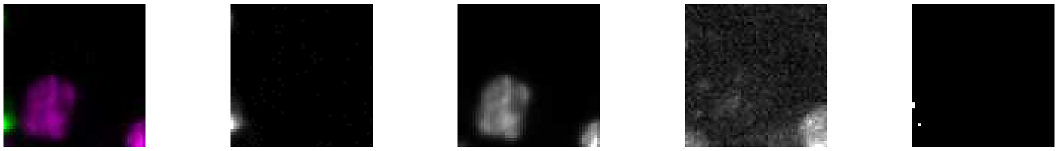				
25793	153	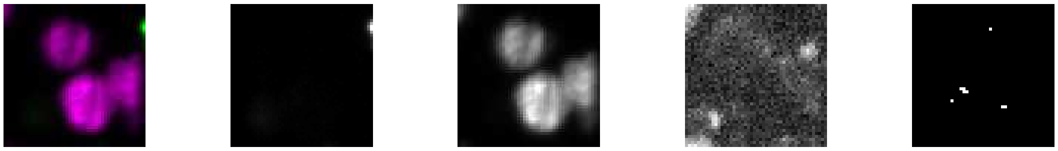				
25794	153	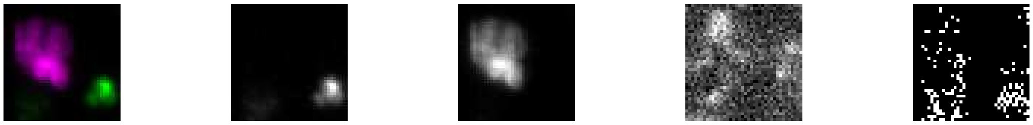				
25795	153	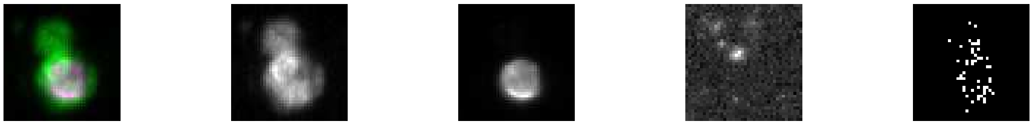				

Proben-ID: Gyn14.208 **Kassetten-ID:** 01327106 **Druckdatum:** 02/06/2020 17:25 **Patienten-ID:**

Ereignis	Aufnahme	DAPI/CK-PE	CK-PE	DAPI	CD45-APC	HER-2/neu
25796	153	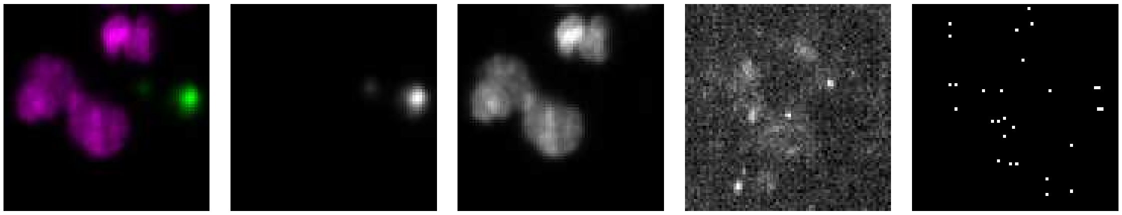				
25797	153	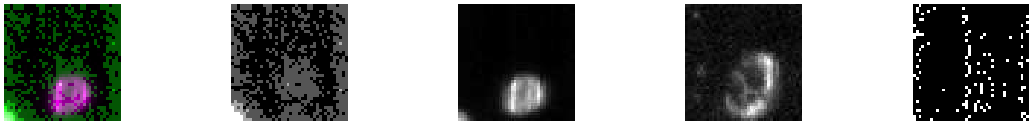				
25798	153	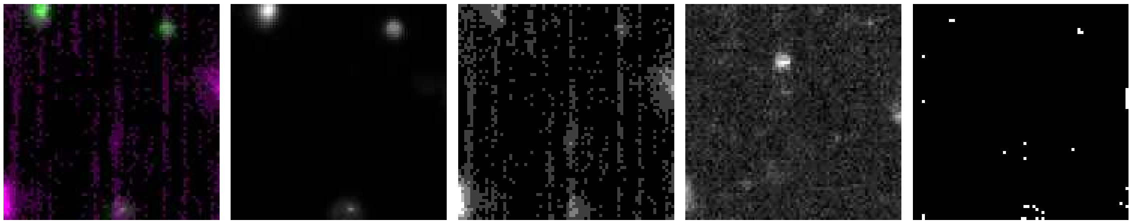				
25799	153	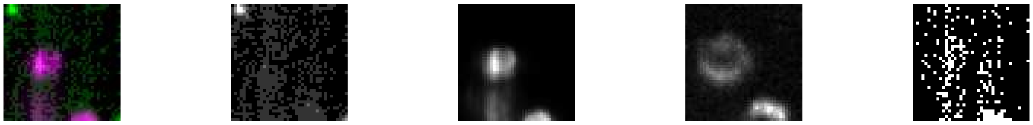				
25800	153	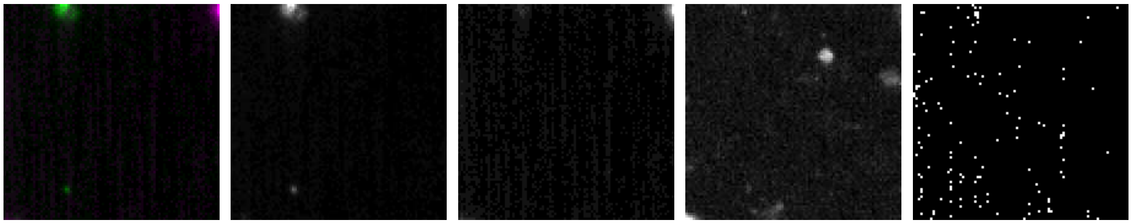				
25801	153	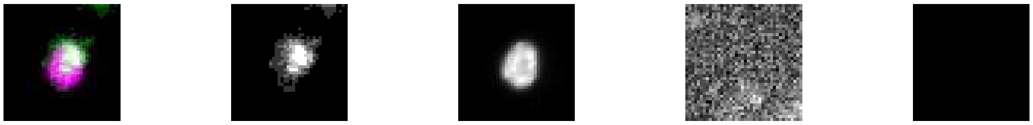				
25802	153	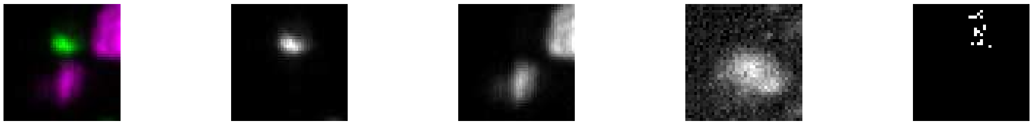				
25803	153	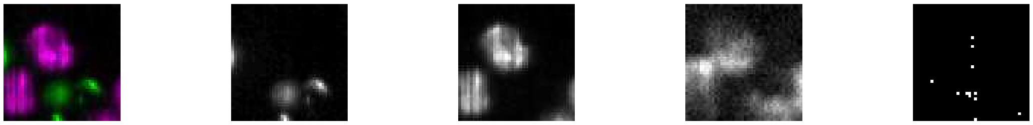				
25804	153	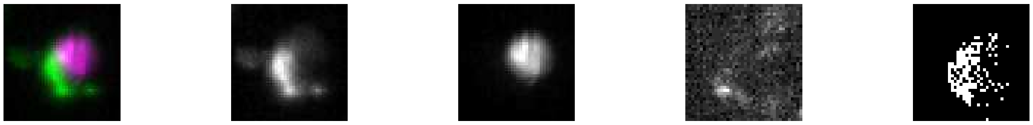				
25805	153	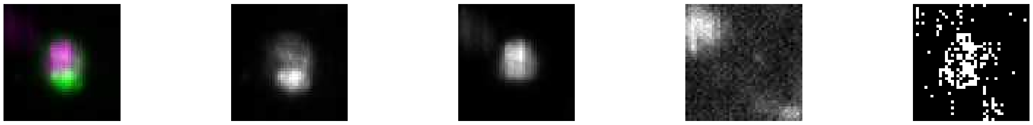				
25806	153	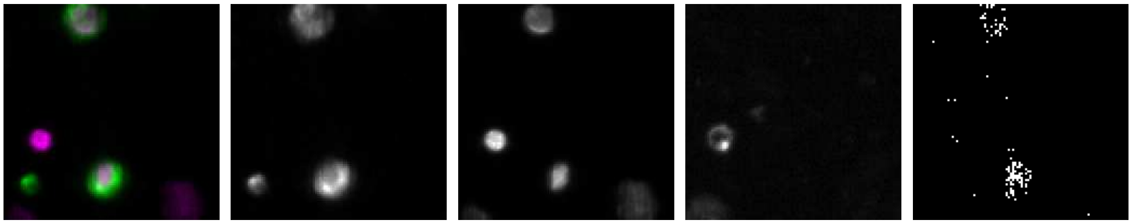				

Proben-ID: Gyn14.208 **Kassetten-ID:**01327106 **Patienten-ID:**

Ereignis	Aufnahme	DAPI/CK-PE	CK-PE	DAPI	CD45-APC	HER-2/neu
25807	153	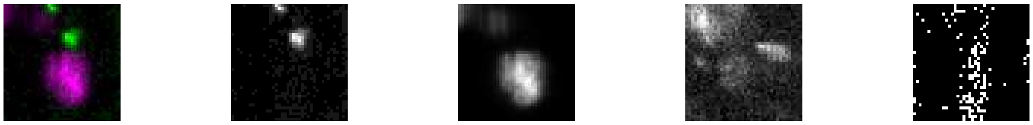				
25808	153	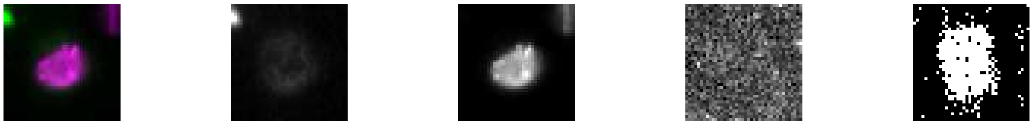				
25809	153	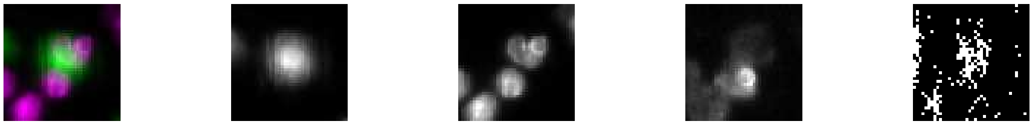				
25810	153	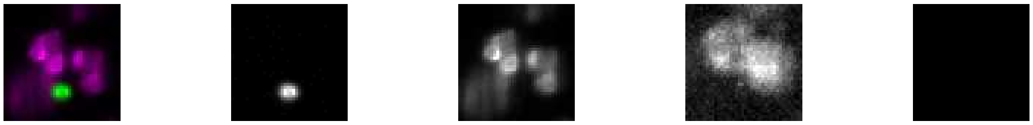				
25811	153	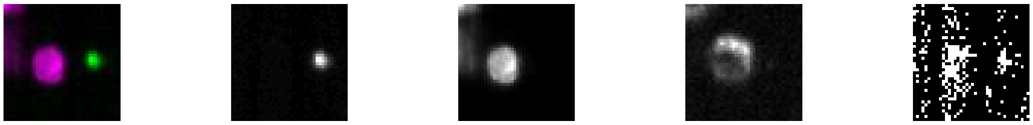				
25812	153	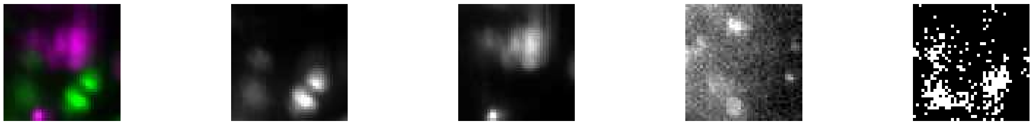				
25813	153	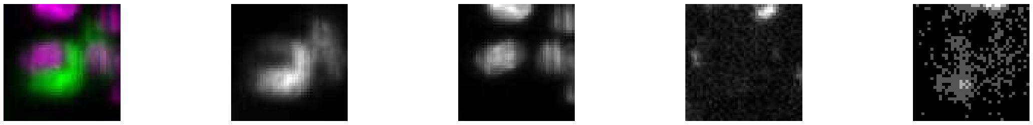				
25814	153	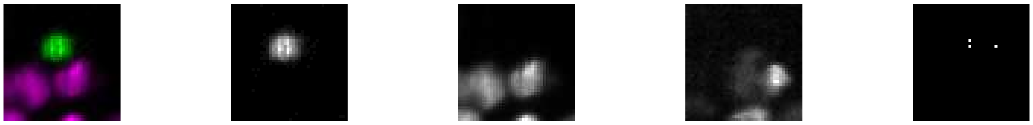				
25815	153	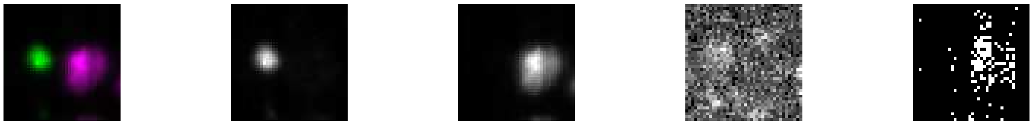				
25816	153	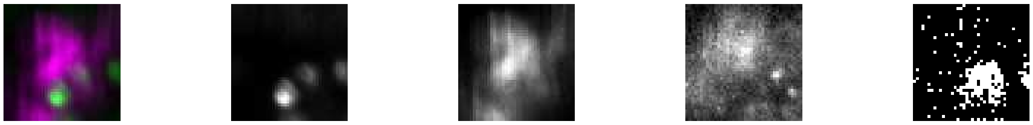				
25817	153	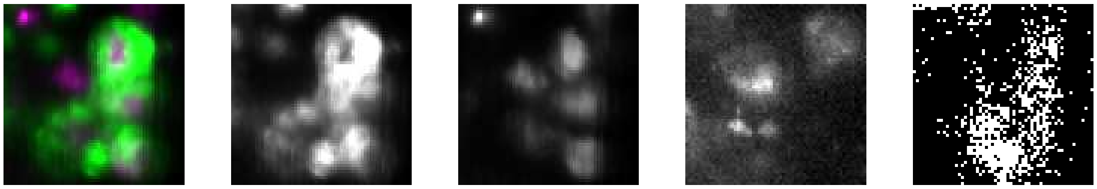				
25818	153	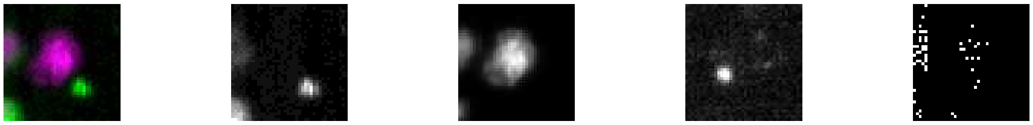				
25819	153	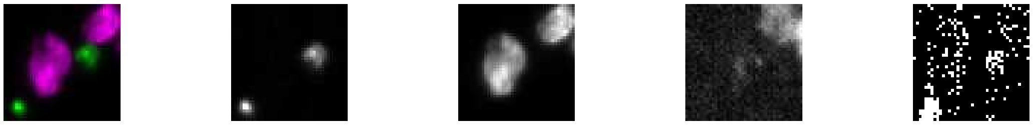				

Ereignis	Aufnahme	DAPI/CK-PE	CK-PE	DAPI	CD45-APC	HER-2/neu
25820	153	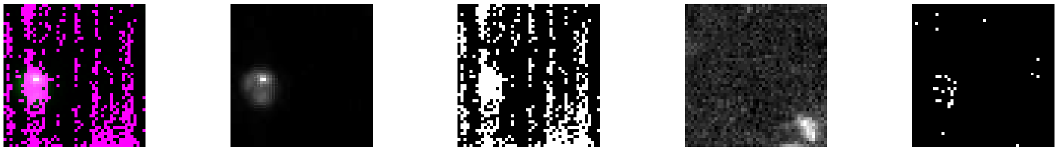				
25821	153	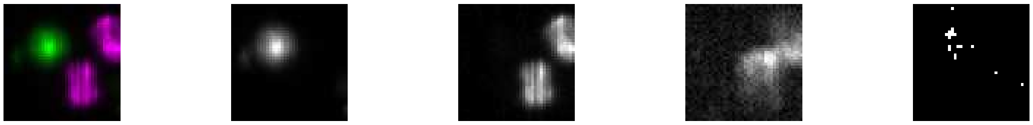				
25822	153	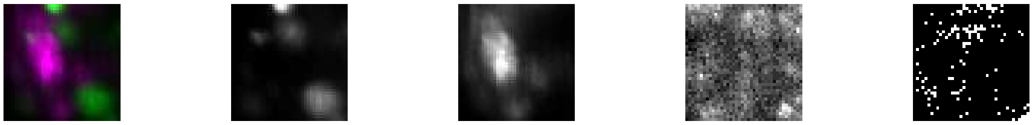				
25823	153	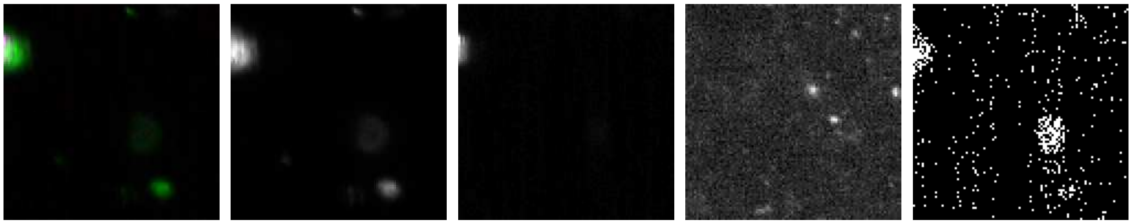				
25824	153	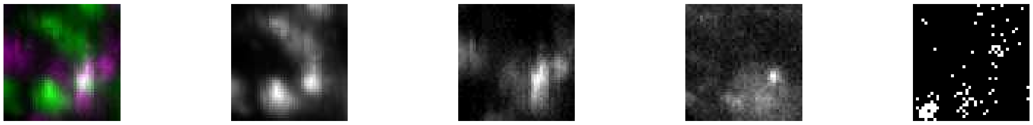				
25825	153	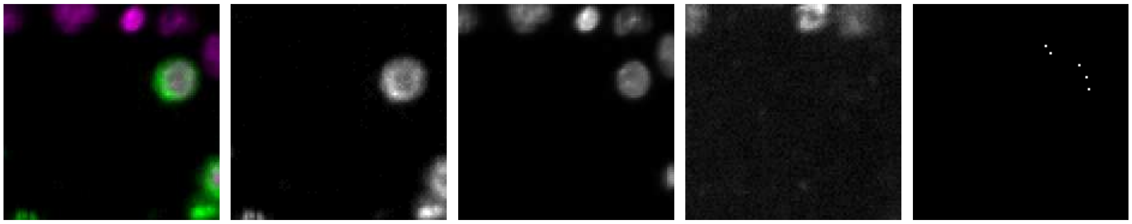				
25826	153	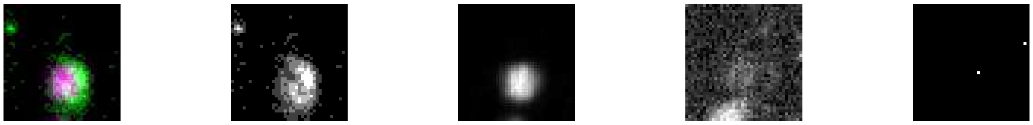				
25827	153	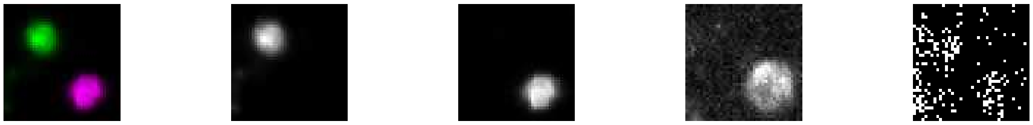				
25828	153	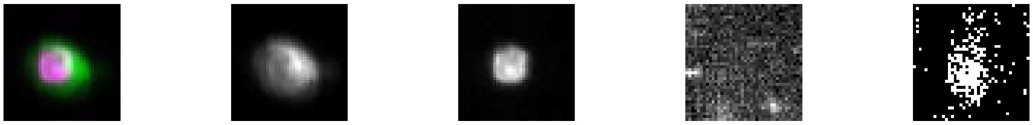				
25829	153	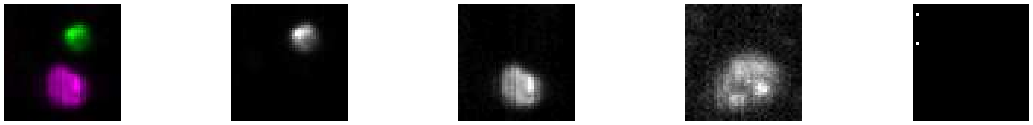				
25830	153	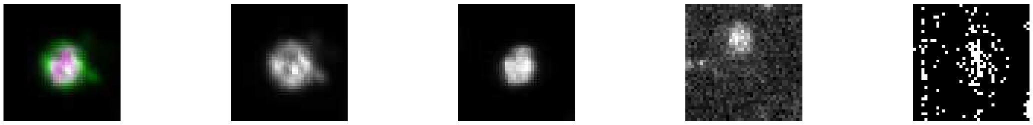				
25831	153	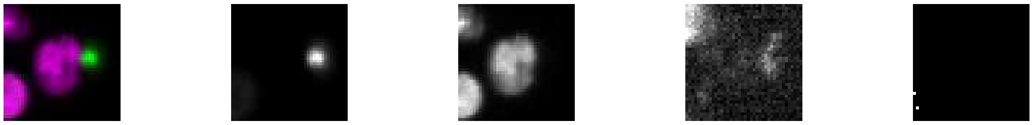				

Ereignis	Aufnahme	DAPI/CK-PE	CK-PE	DAPI	CD45-APC	HER-2/neu
25832	152	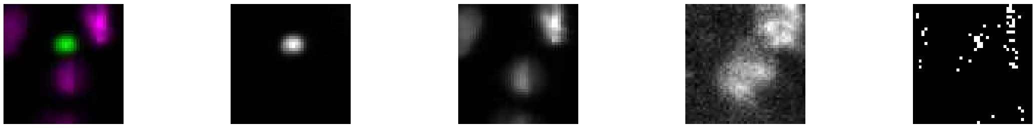				
25833	128	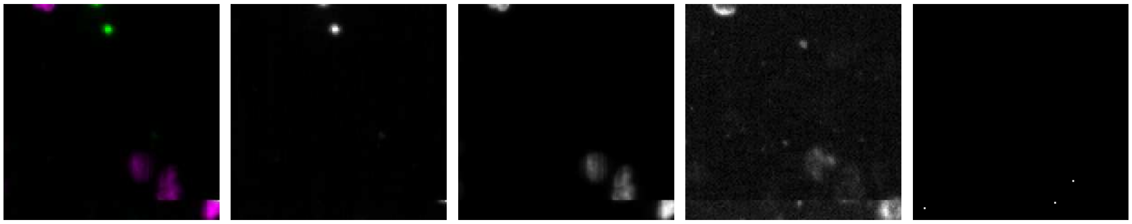				
25834	152	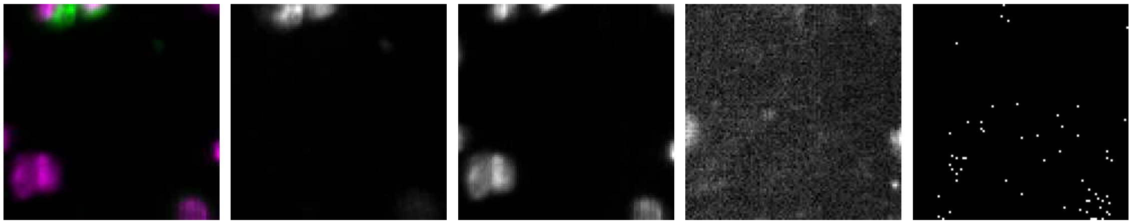				
25835	128	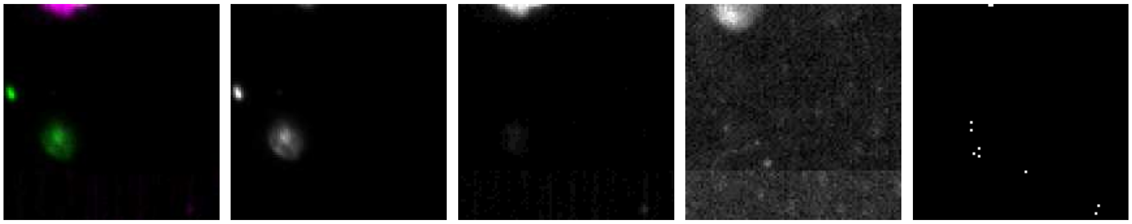				
25836	153	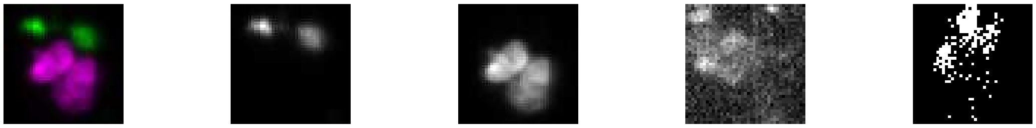				
25837	128	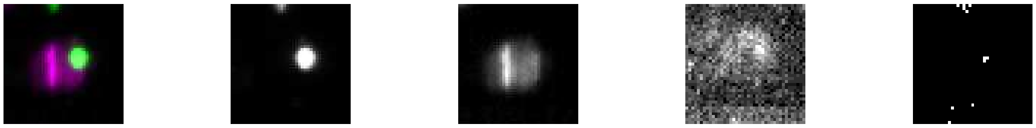				
25838	128	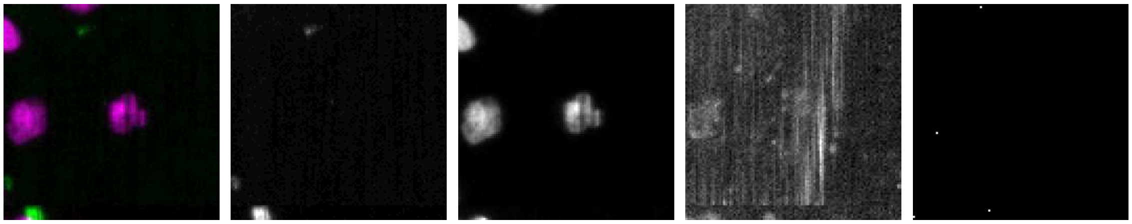				
25839	153	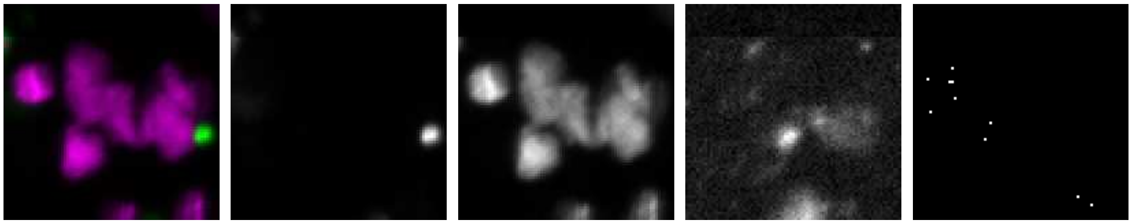				
25840	153	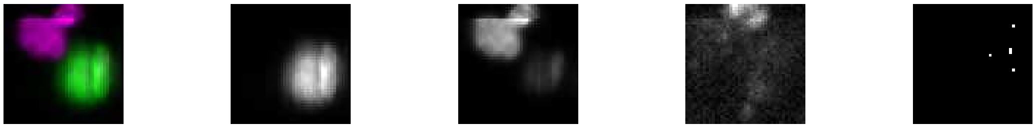				
25841	153	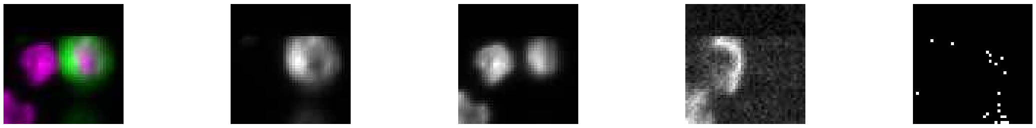				

Ereignis	Aufnahme	DAPI/CK-PE	CK-PE	DAPI	CD45-APC	HER-2/neu
25842	153	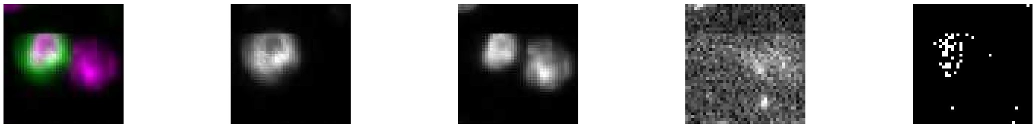				
25843	128	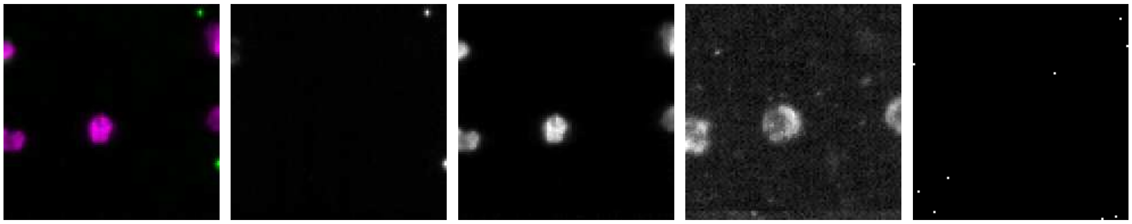				
25844	128	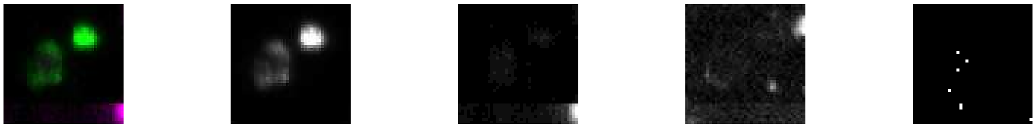				
25845	154	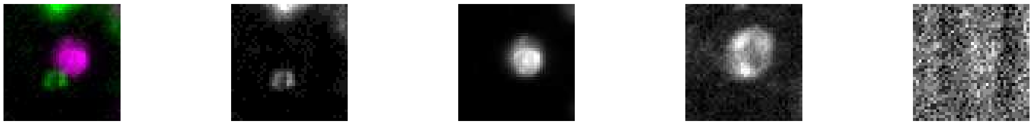				
25846	154	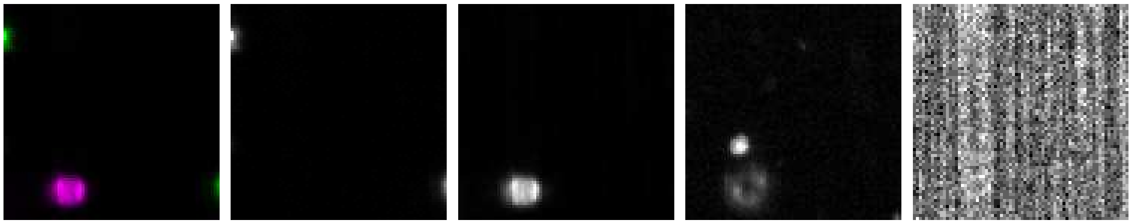				
25847	154	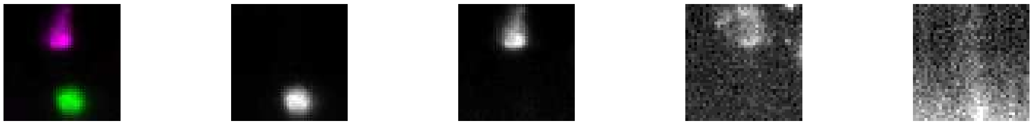				
25848	154	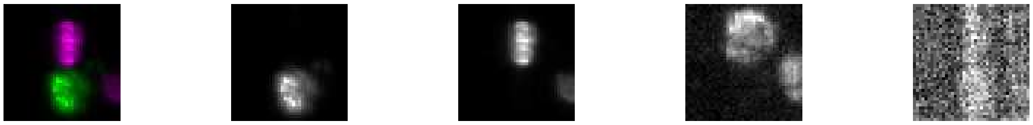				
25849	154	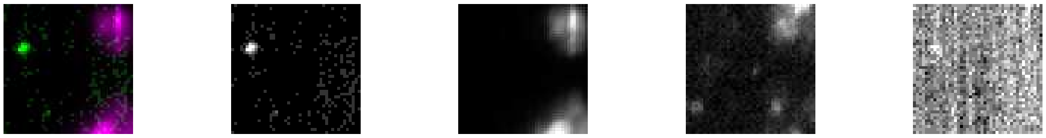				
25850	154	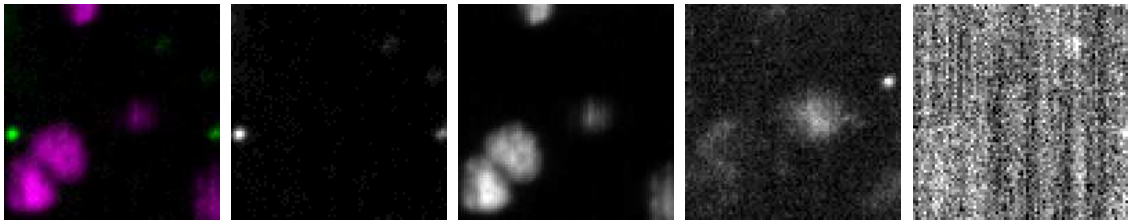				
25851	154	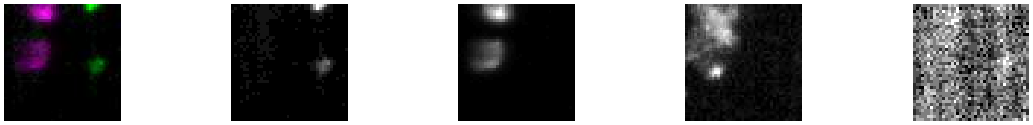				
25852	154	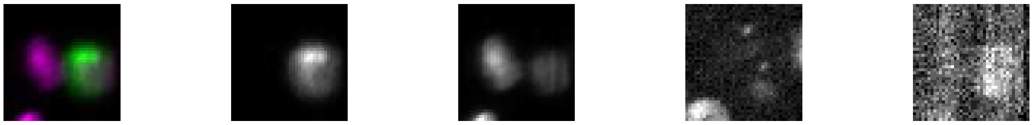				

Ereignis	Aufnahme	DAPI/CK-PE	CK-PE	DAPI	CD45-APC	HER-2/neu
25853	154	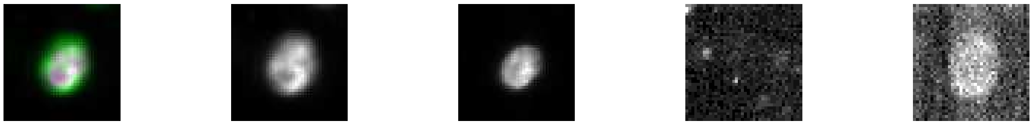				
25854	154	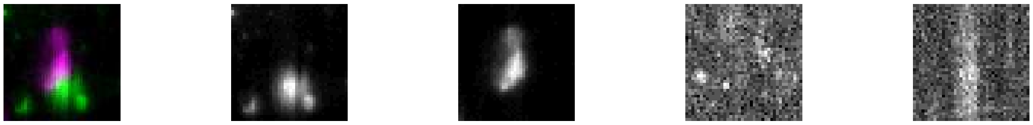				
25855	154	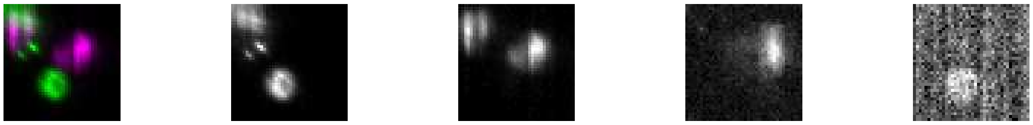				
25856	154	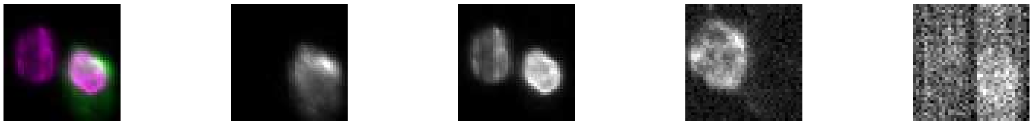				
25857	154	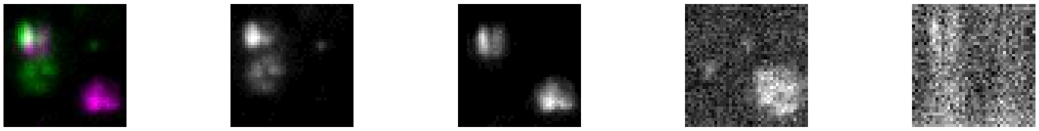				
25858	154	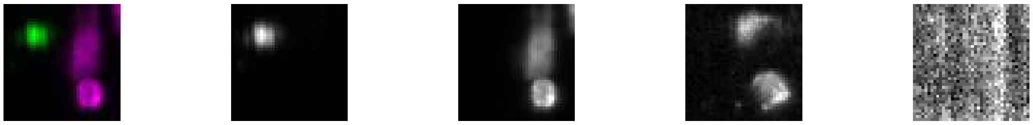				
25859	154	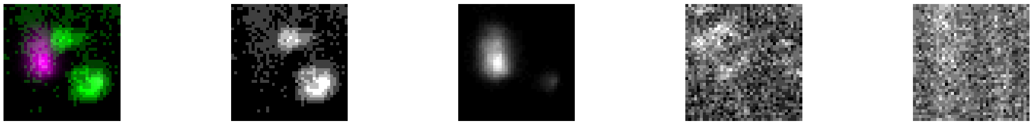				
25860	154	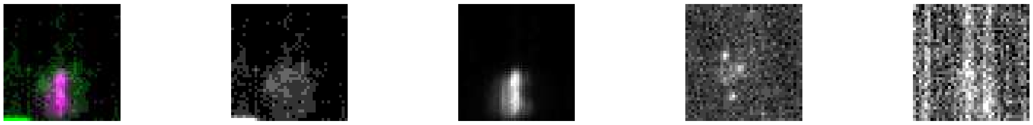				
25861	154	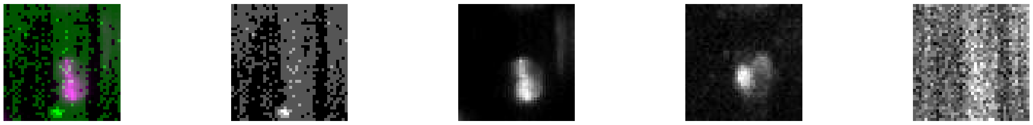				
25862	154	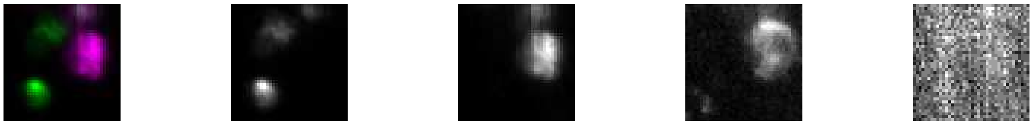				
25863	154	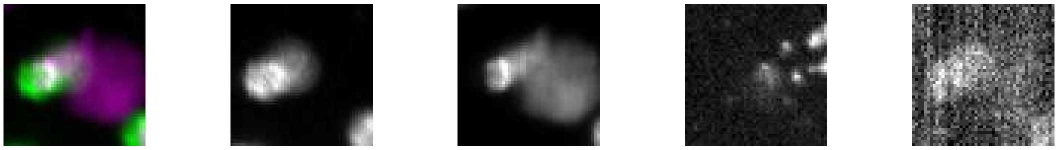				
25864	154	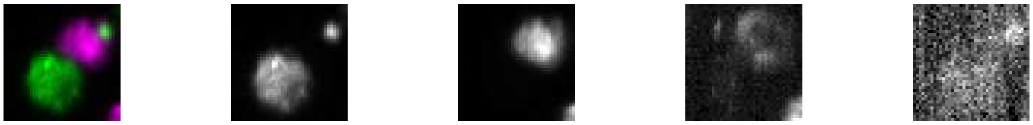				
25865	154	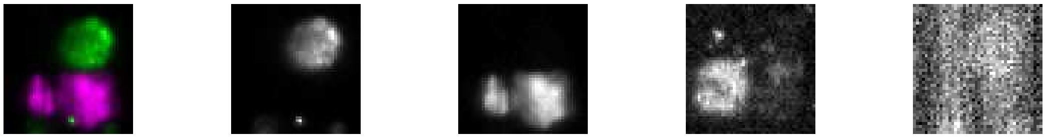				

Ereignis	Aufnahme	DAPI/CK-PE	CK-PE	DAPI	CD45-APC	HER-2/neu
25866	154	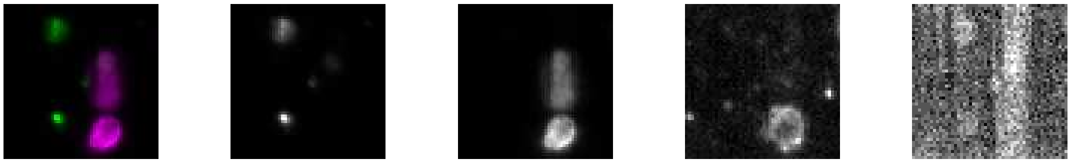				
25867	154	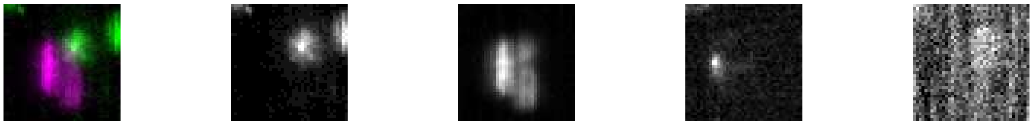				
25868	154	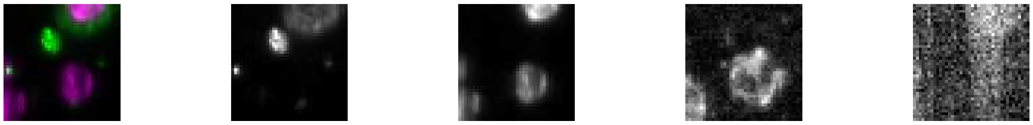				
25869	154	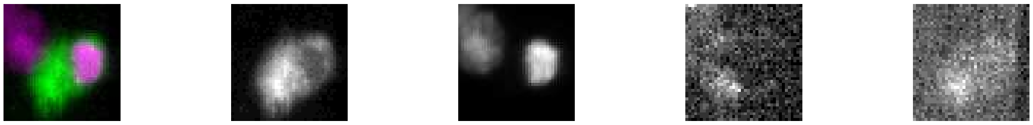				
25870	154	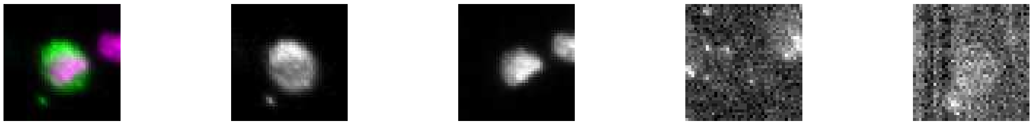				
25871	154	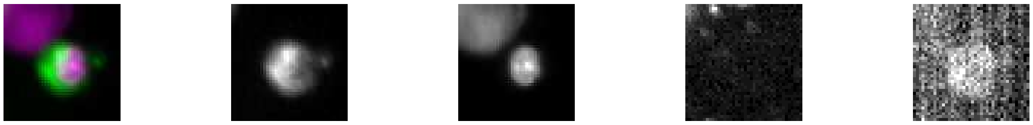				
25872	154	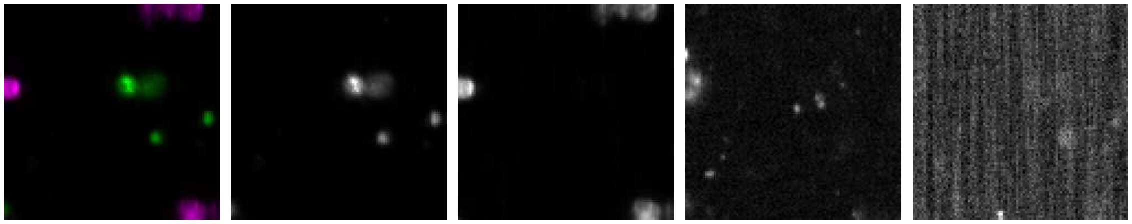				
25873	154	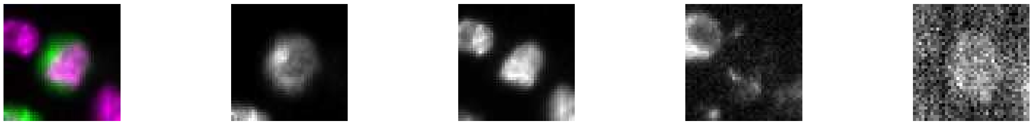				
25874	154	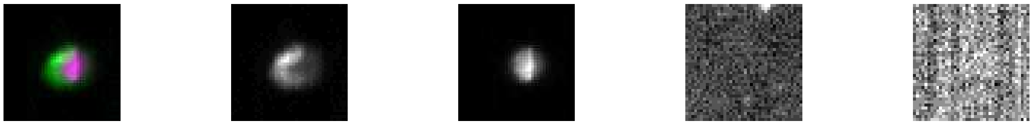				
25875	154	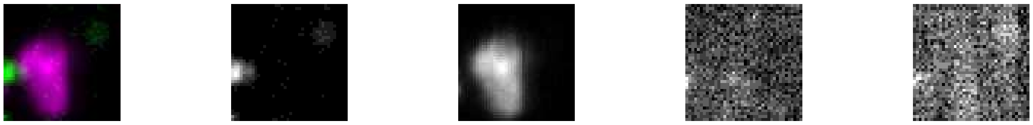				
25876	154	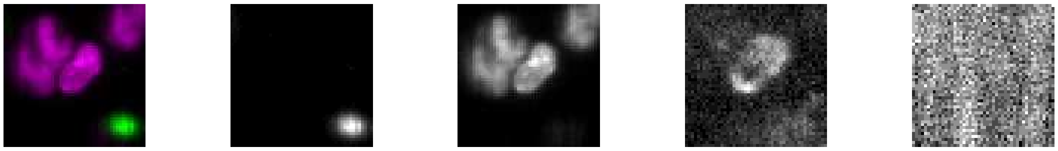				
25877	154	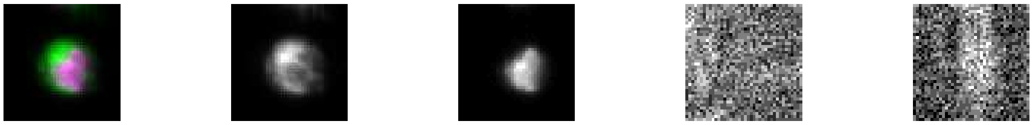				
25878	154	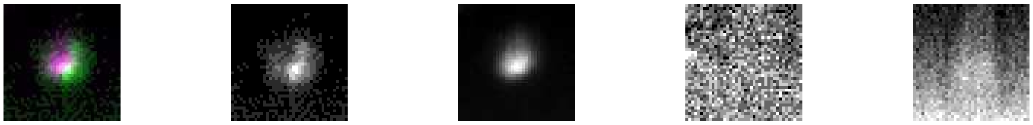				

Ereignis	Aufnahme	DAPI/CK-PE	CK-PE	DAPI	CD45-APC	HER-2/neu
25879	154	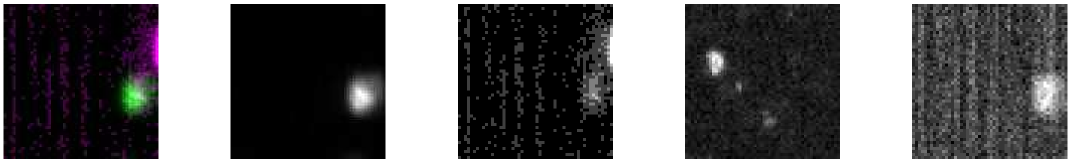				
25880	154	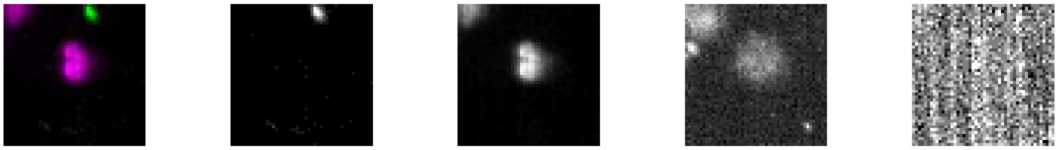				
25881	154	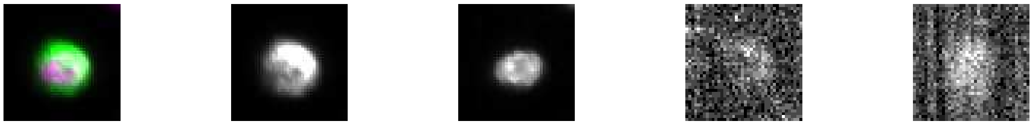				
25882	154	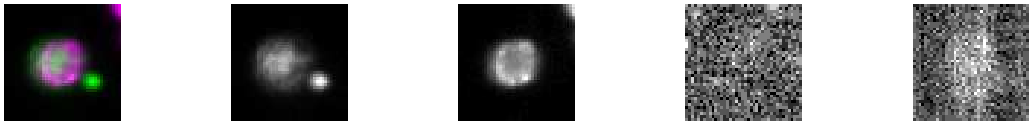				
25883	154	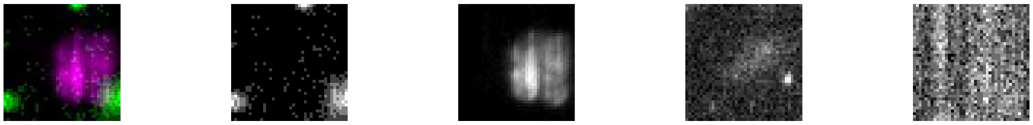				
25884	154	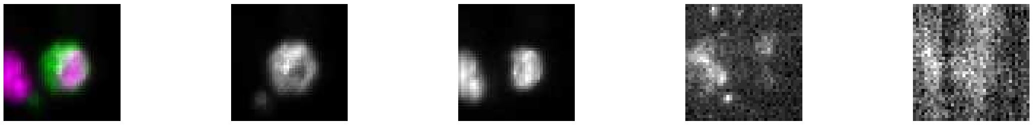				
25885	154	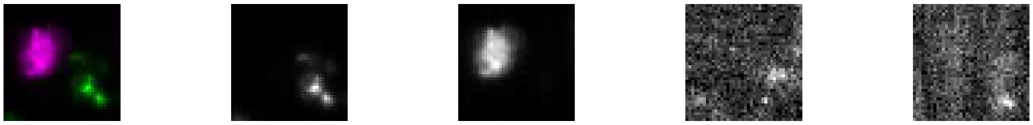				
25886	154	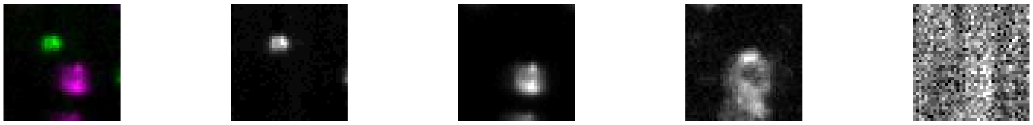				
25887	154	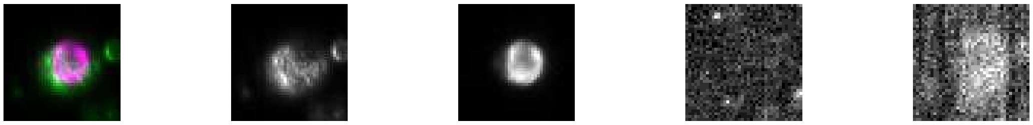				
25888	154	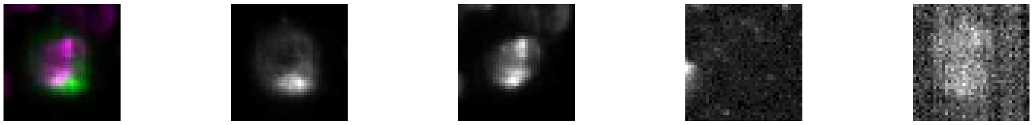				
25889	154	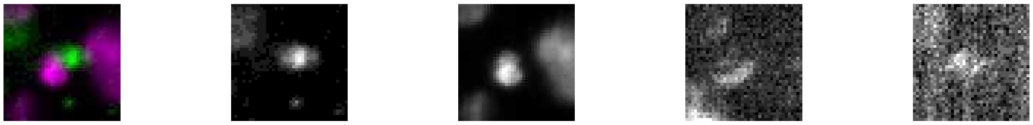				
25890	154	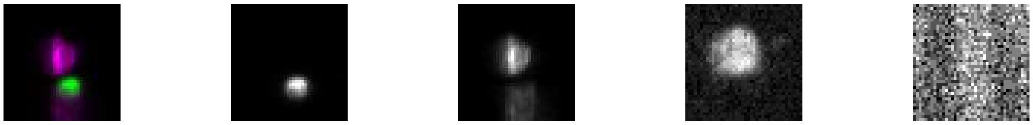				
25891	154	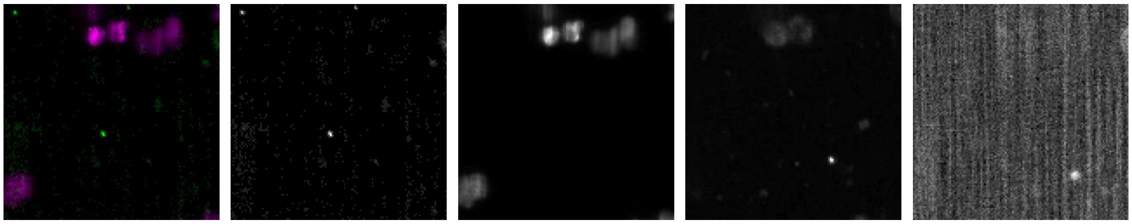				

Proben-ID: Gyn14.208 **Kassetten-ID:**01327106 **Patienten-ID:**

Ereignis	Aufnahme	DAPI/CK-PE	CK-PE	DAPI	CD45-APC	HER-2/neu
25892	154	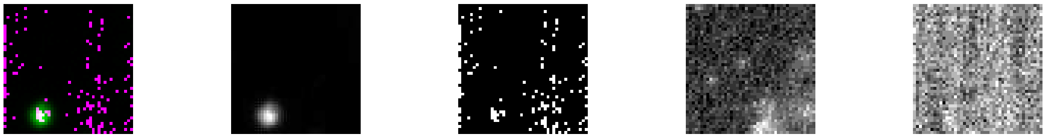				
25893	154	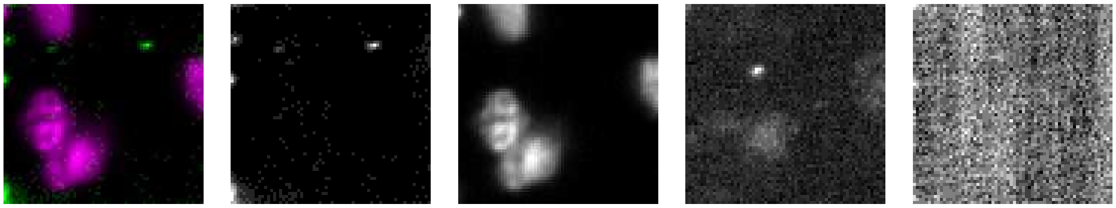				
25894	154	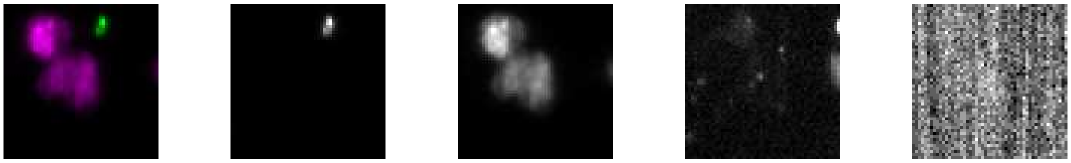				
25895	154	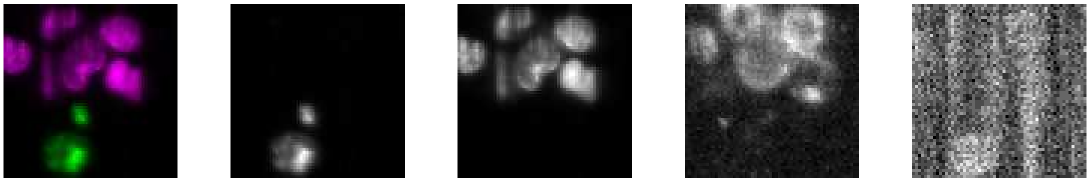				
25896	154	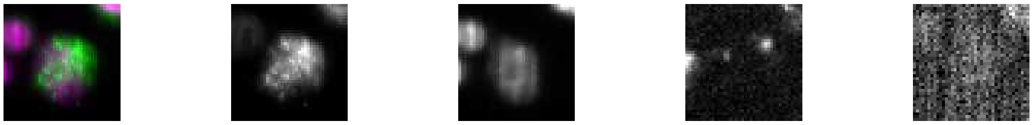				
25897	154	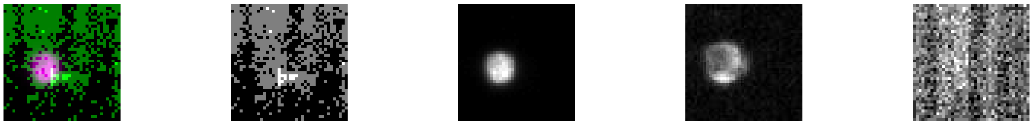				
25898	154	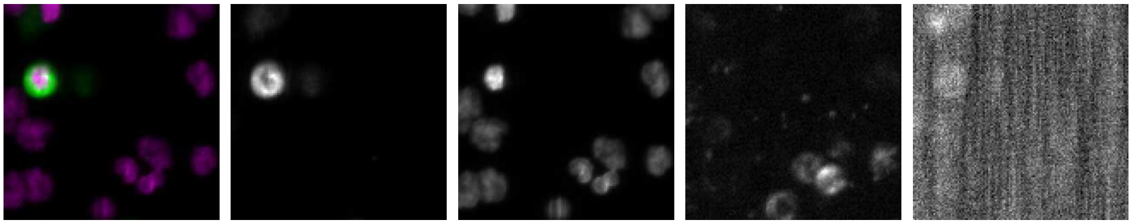				
25899	154	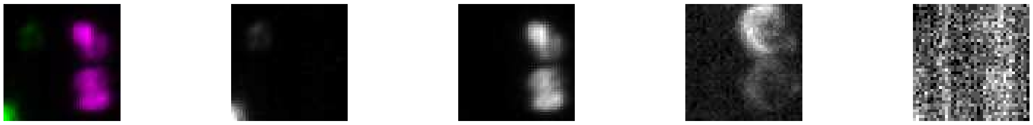				
25900	154	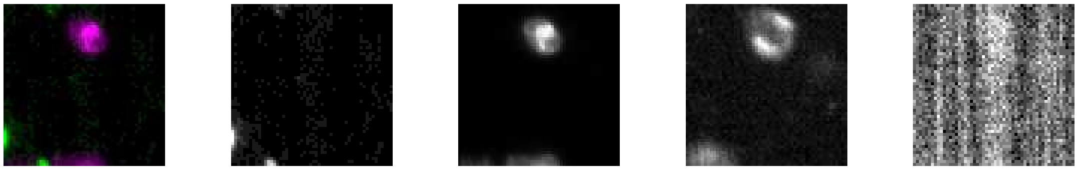				
25901	154	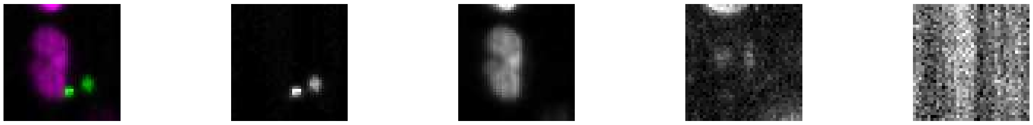				
25902	154	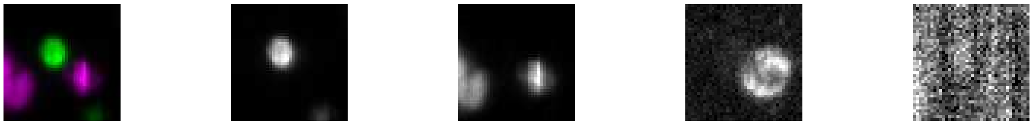				

Druckdatum:02/06/2020 17:25

Proben-ID: Gyn14.208 **Kassetten-ID:**01327106 **Patienten-ID:**

Ereignis	Aufnahme	DAPI/CK-PE	CK-PE	DAPI	CD45-APC	HER-2/neu
25903	154	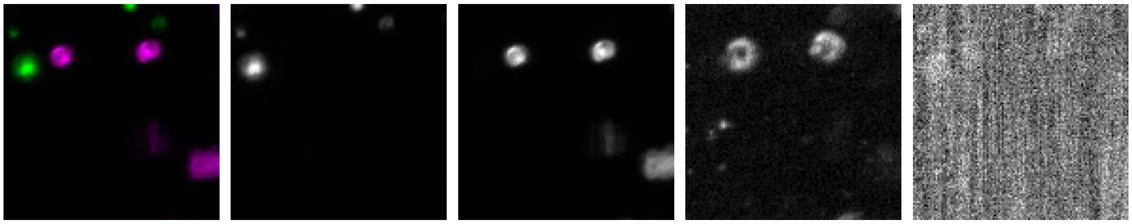				
25904	154	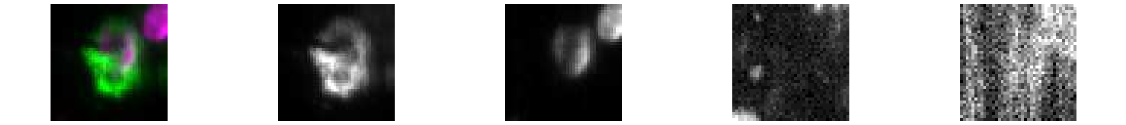				
25905	154	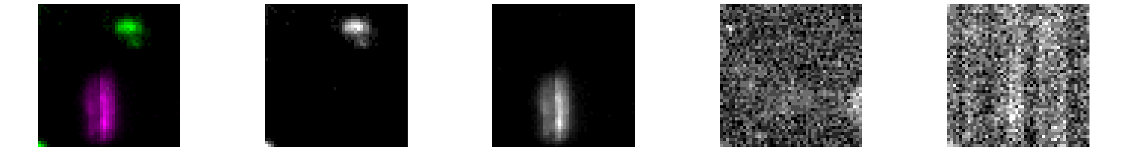				
25906	154	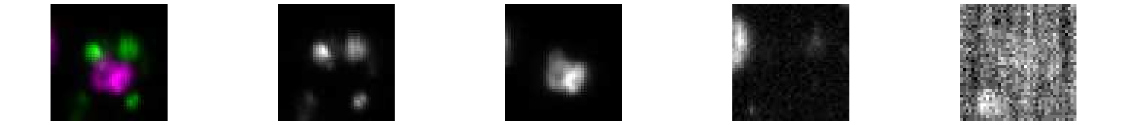				
25907	154	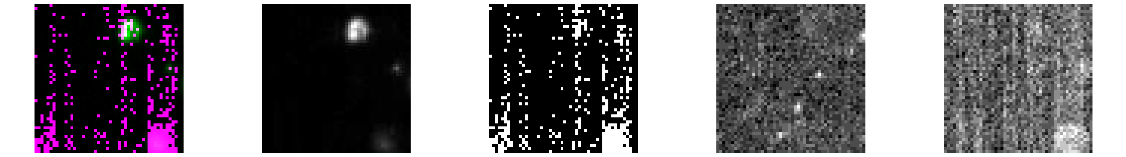				
25908	154	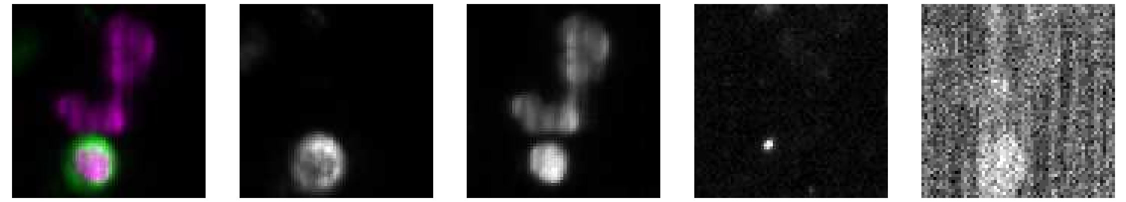				
25909	154	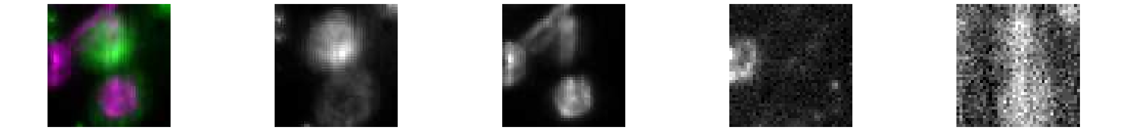				
25910	154	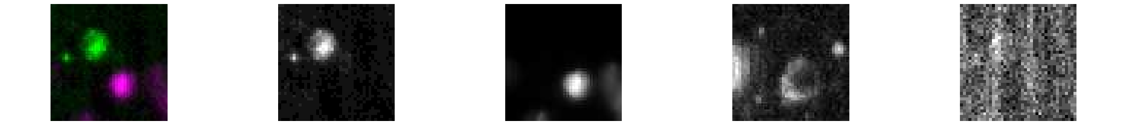				
25911	154	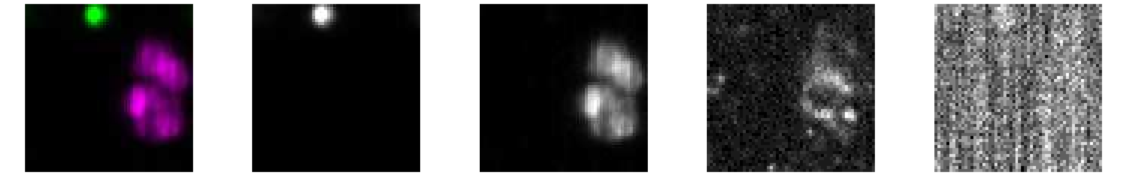				
25912	154	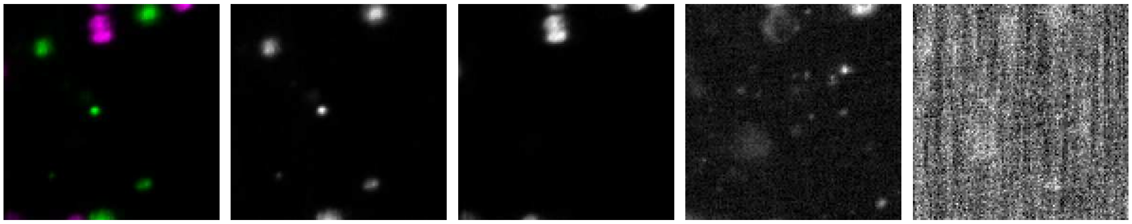				
25913	154	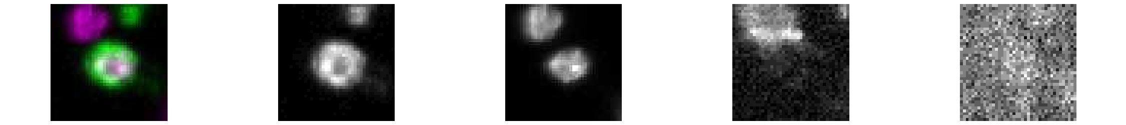				

Ereignis	Aufnahme	DAPI/CK-PE	CK-PE	DAPI	CD45-APC	HER-2/neu
25914	154	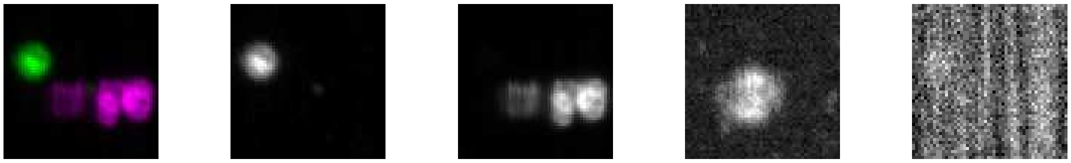				
25915	154	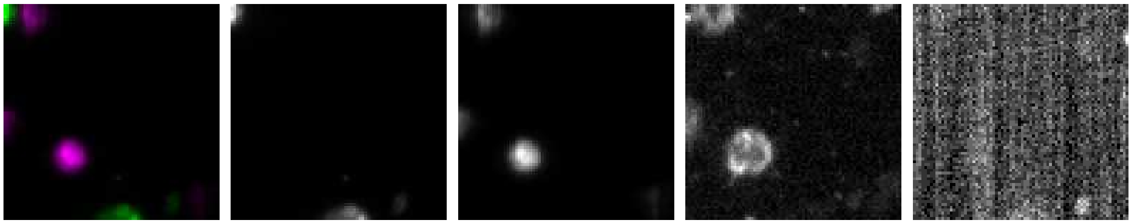				
25916	154	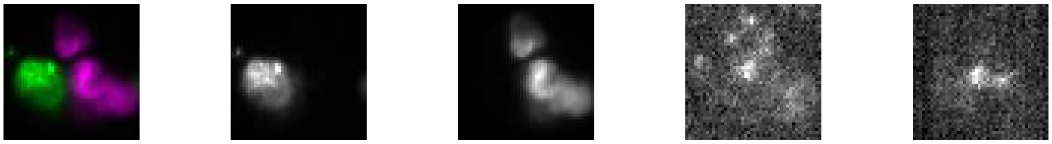				
25917	154	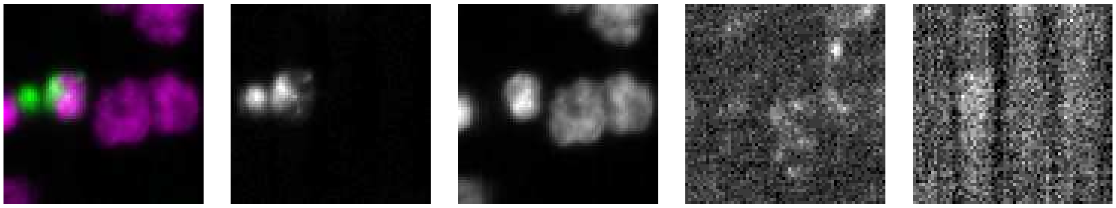				
25918	154	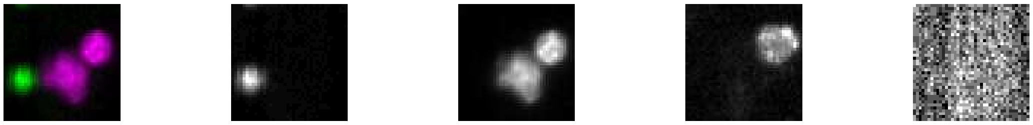				
25919	154	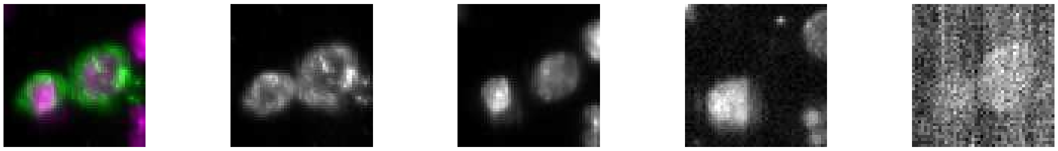				
25920	154	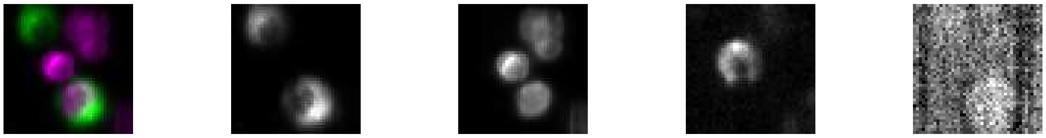				
25921	154	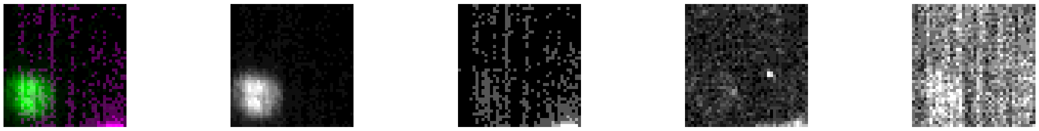				
25922	154	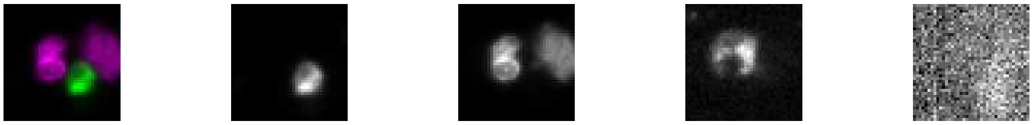				
25923	154	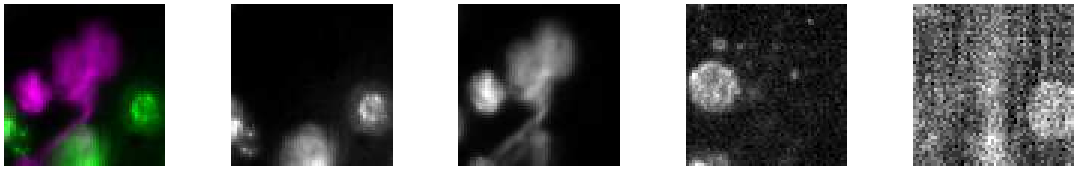				
25924	154	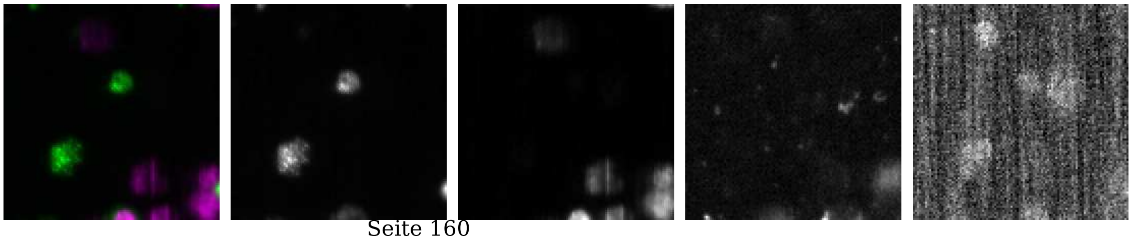				

Ereignis	Aufnahme	DAPI/CK-PE	CK-PE	DAPI	CD45-APC	HER-2/neu
25925	154	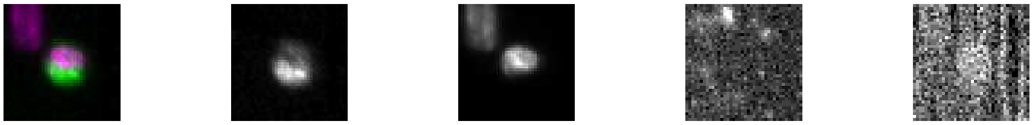				
25926	154	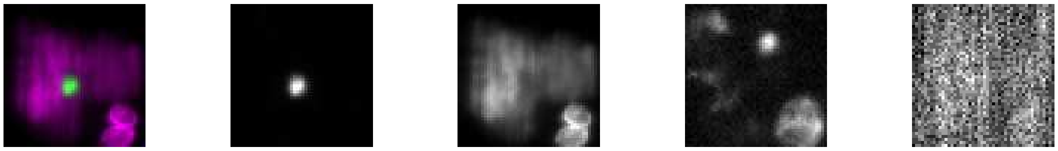				
25927	154	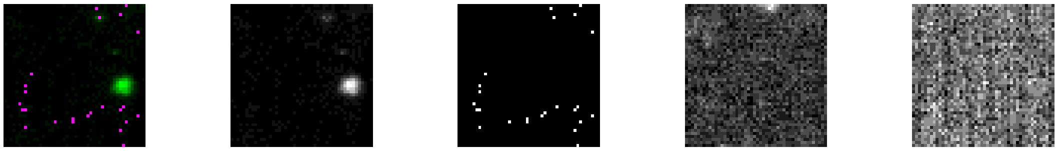				
25928	154	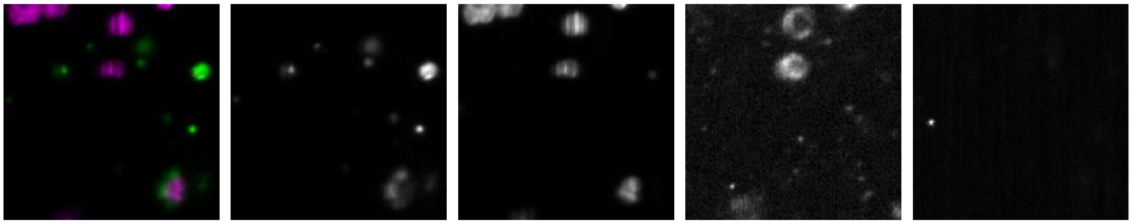				
25929	154	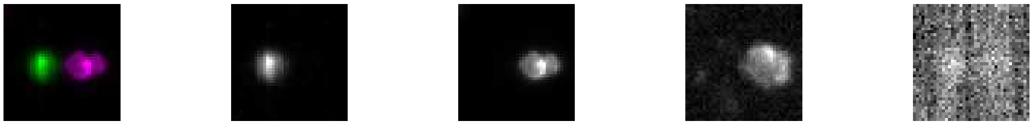				
25930	154	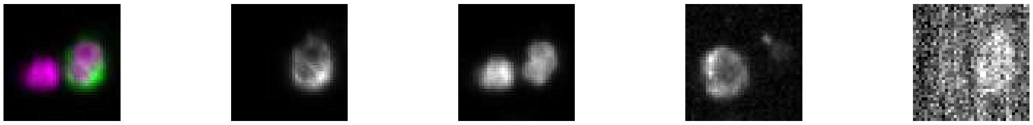				
25931	154	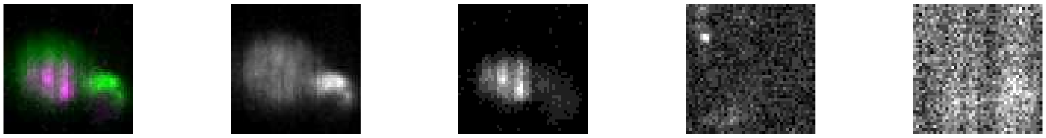				
25932	154	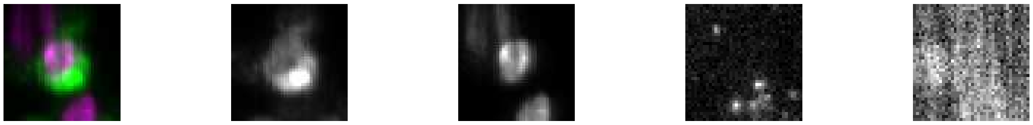				
25933	154	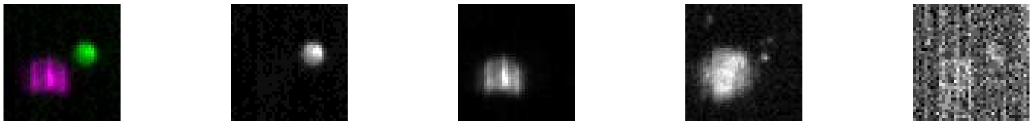				
25934	154	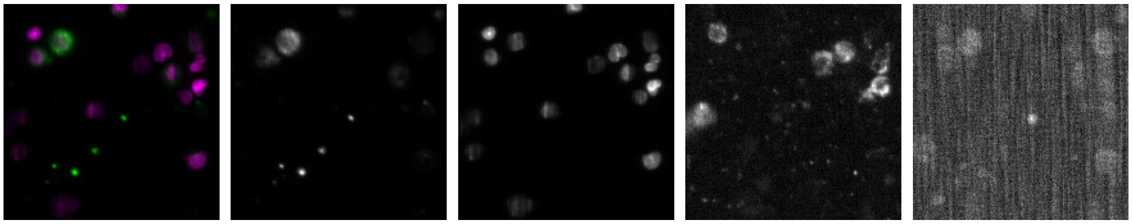				
25935	154	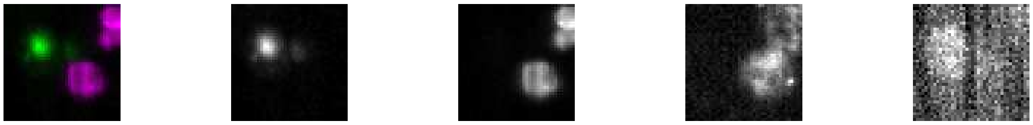				
25936	154	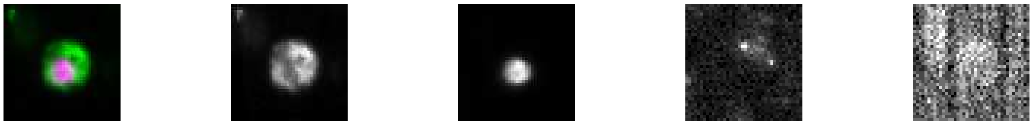				

Druckdatum:02/06/2020 17:25

Proben-ID: Gyn14.208 **Kassetten-ID:**01327106 **Patienten-ID:**

Ereignis	Aufnahme	DAPI/CK-PE	CK-PE	DAPI	CD45-APC	HER-2/neu
25937	154	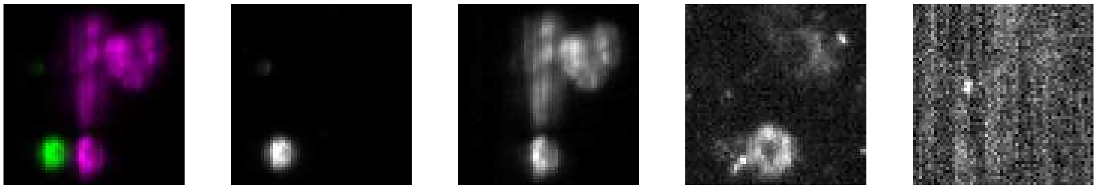				
25938	154	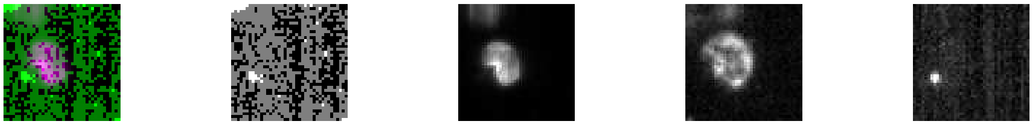				
25939	154	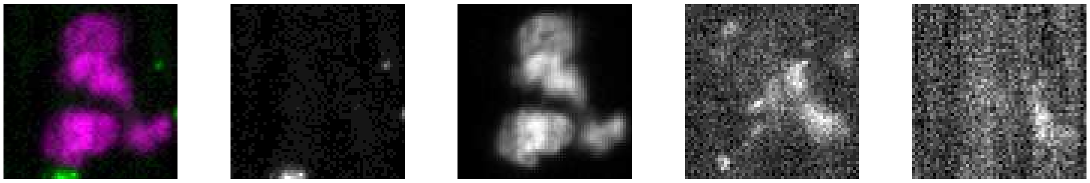				
25940	154	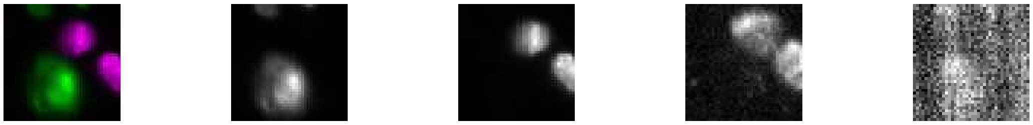				
25941	154	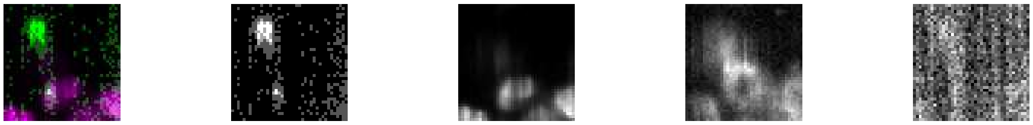				
25942	154	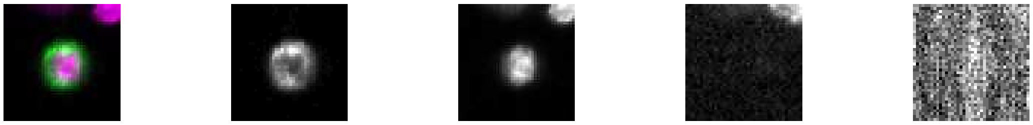				
25943	154	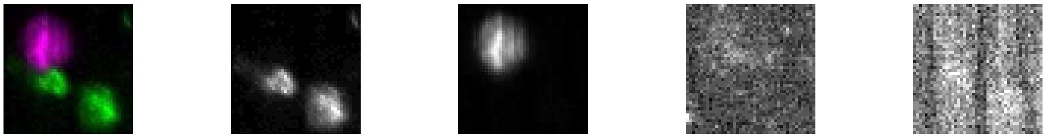				
25944	154	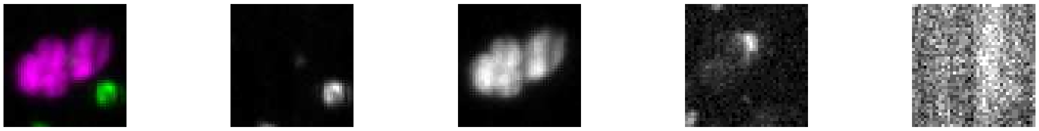				
25945	154	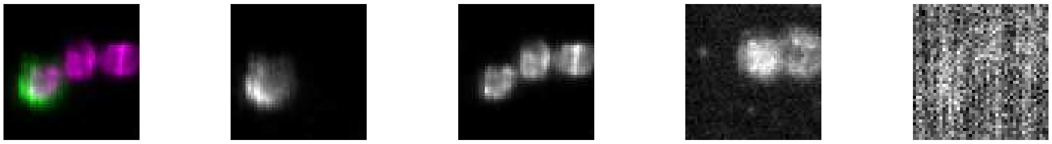				
25946	154	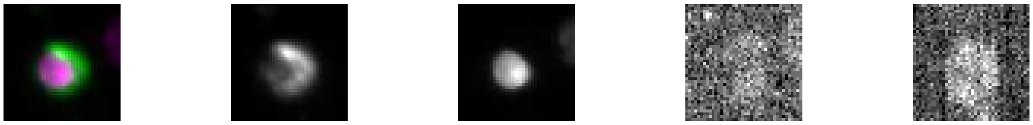				
25947	154	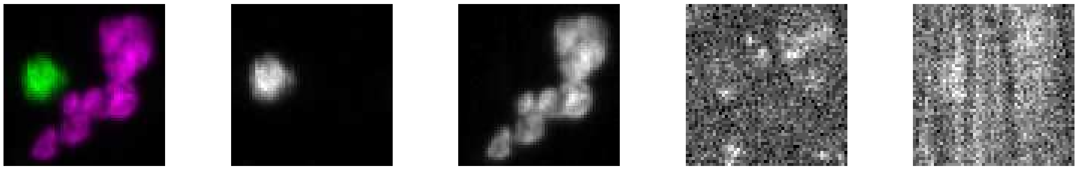				
25948	154	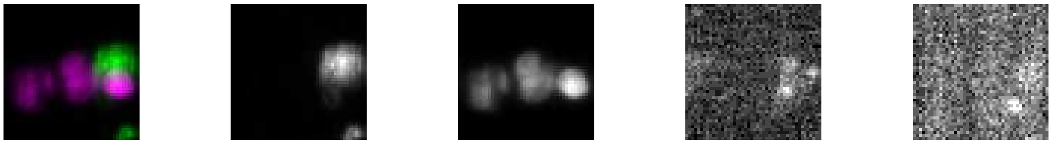				

Proben-ID: Gyn14.208 **Kassetten-ID:** 01327106 **Patienten-ID:**

Ereignis	Aufnahme	DAPI/CK-PE, CK-PE, DAPI, CD45-APC, HER-2/neu
25949	154	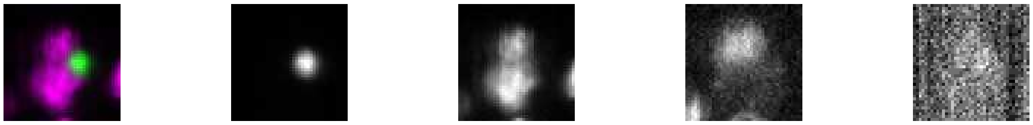
25950	154	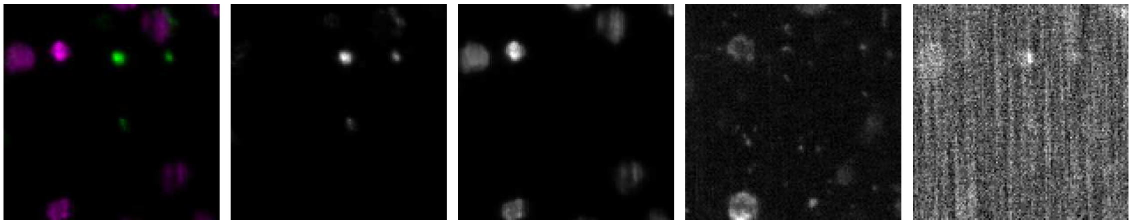
25951	154	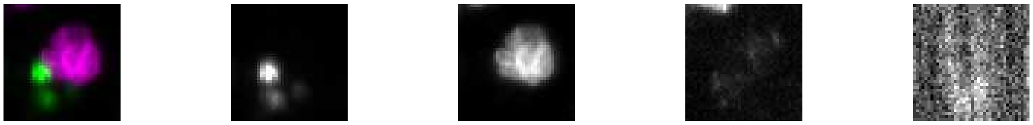
25952	154	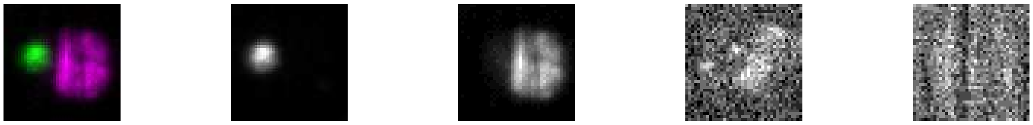
25953	154	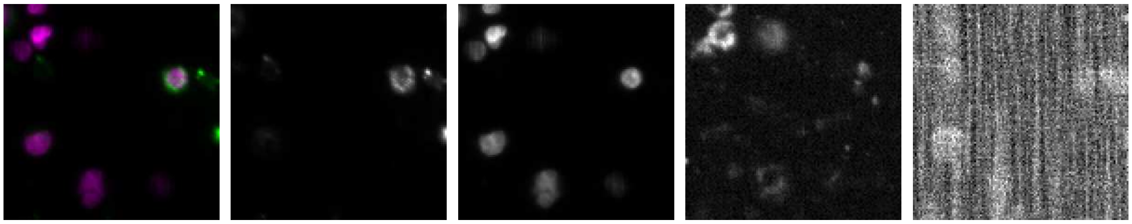
25954	154	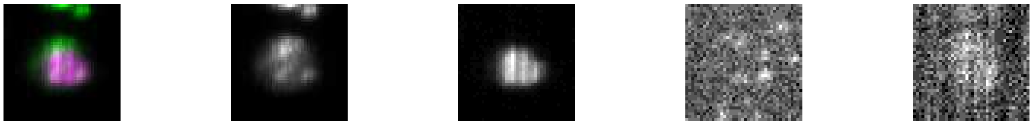
25955	154	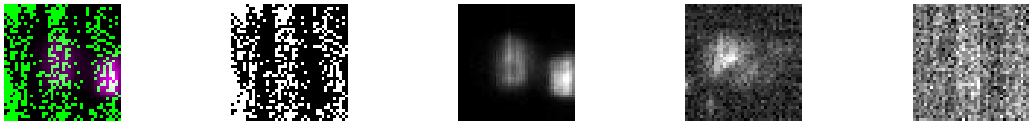
25956	154	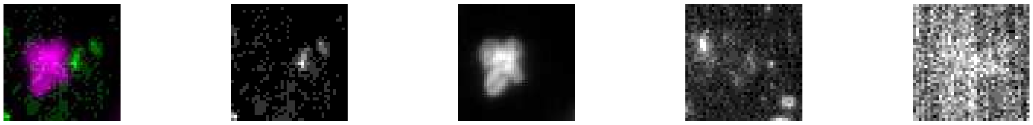
25957	154	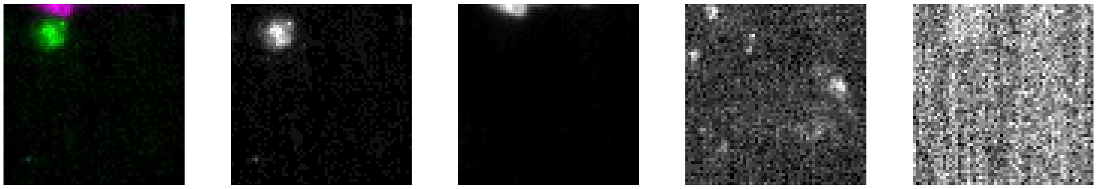
25958	154	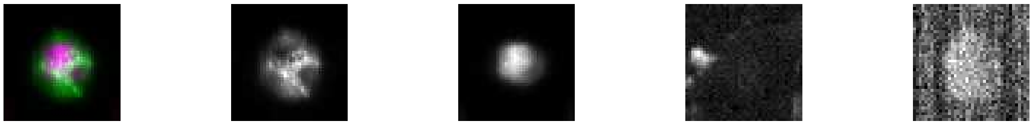
25959	154	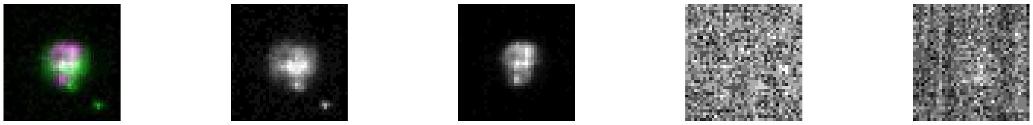

Ereignis	Aufnahme	DAPI/CK-PE	CK-PE	DAPI	CD45-APC	HER-2/neu
25960	154	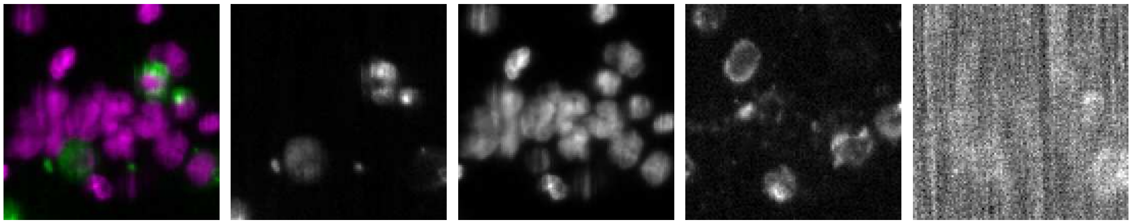				
25961	154	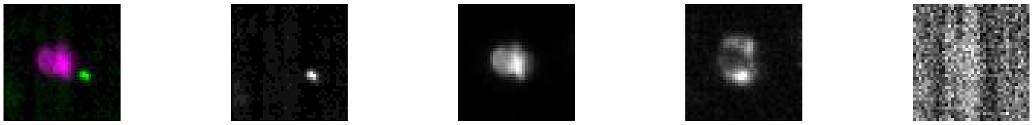				
25962	154	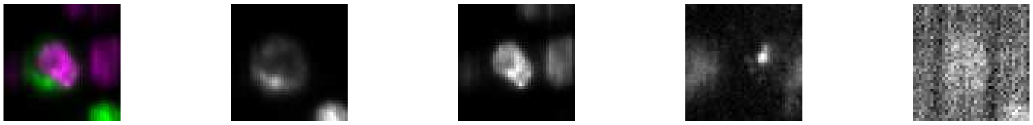				
25963	154	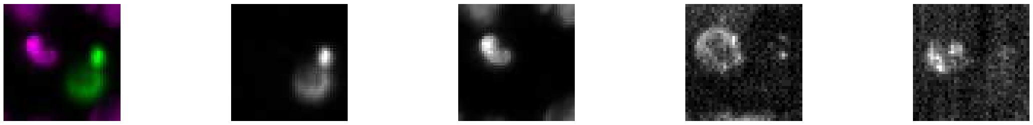				
25964	154	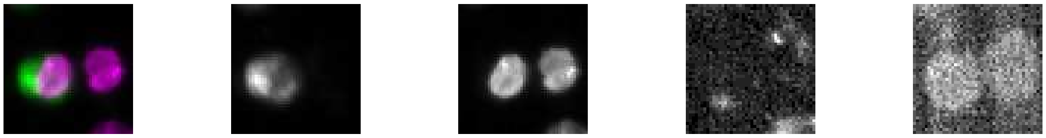				
25965	154	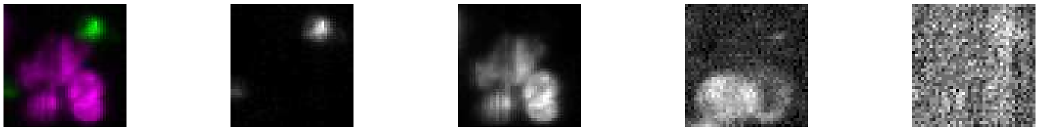				
25966	154	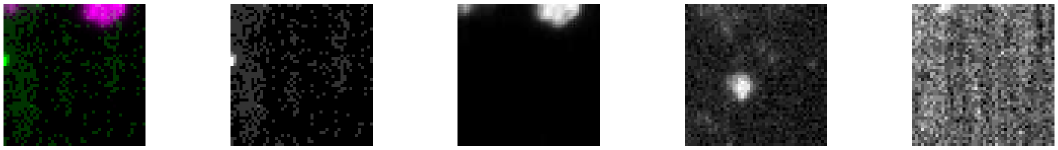				
25967	154	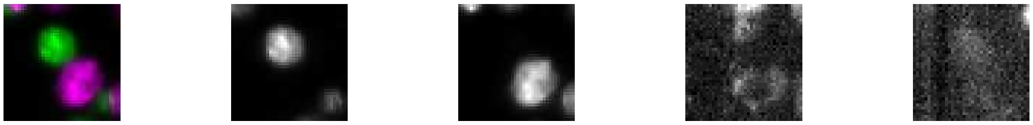				
25968	154	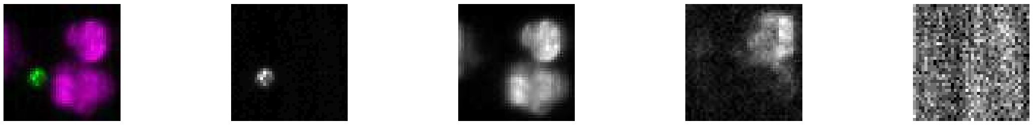				
25969	154	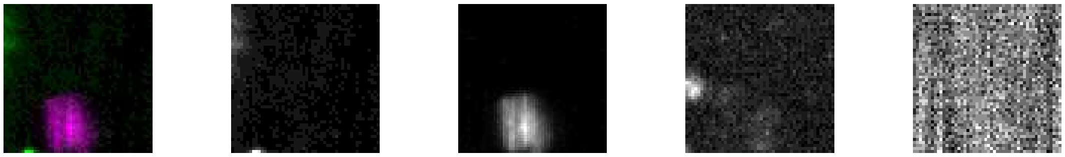				
25970	154	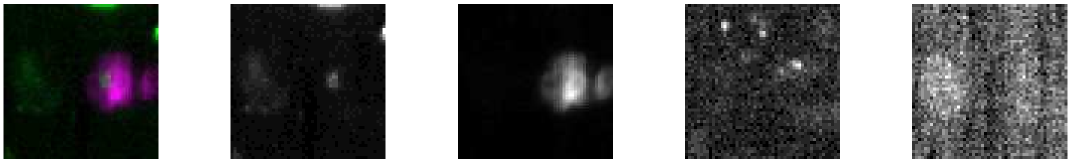				
25971	154	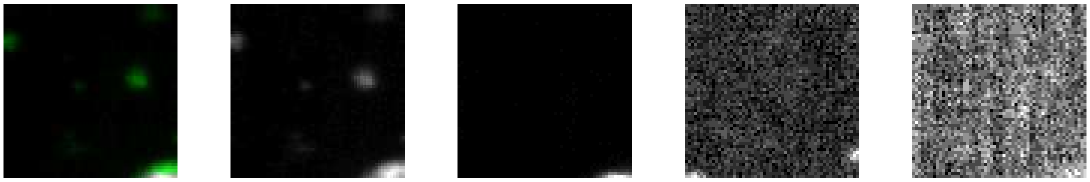				

Druckdatum:02/06/2020 17:25

Proben-ID: Gyn14.208 **Kassetten-ID:**01327106 **Patienten-ID:**

Ereignis	Aufnahme	DAPI/CK-PE	CK-PE	DAPI	CD45-APC	HER-2/neu
25972	154	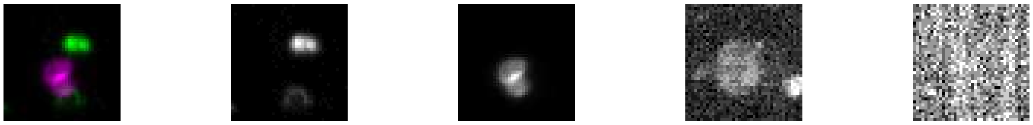				
25973	154	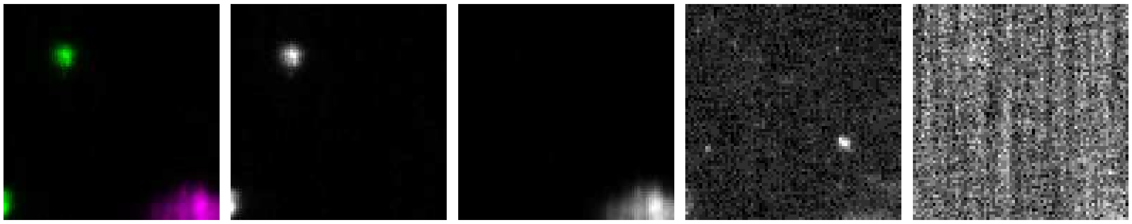				
25974	154	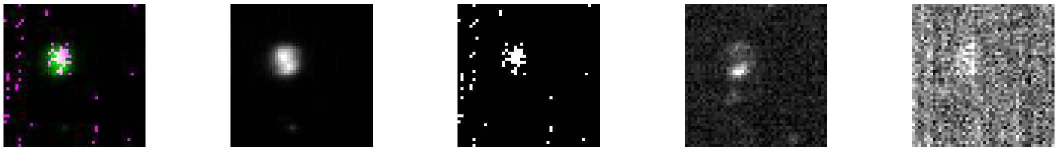				
25975	154	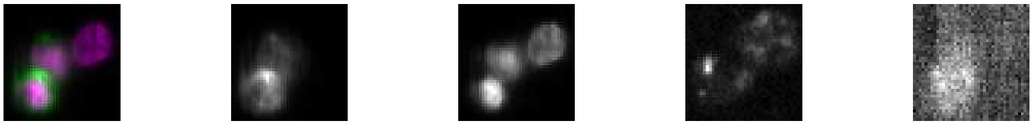				
25976	154	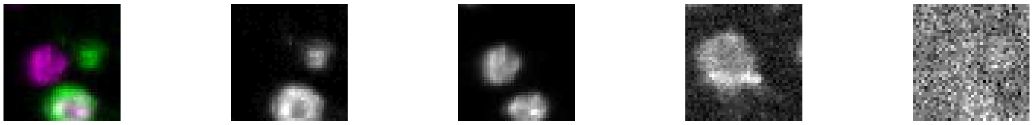				
25977	154	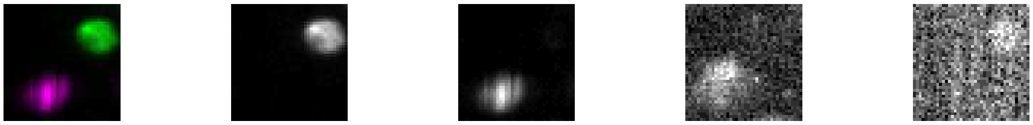				
25978	154	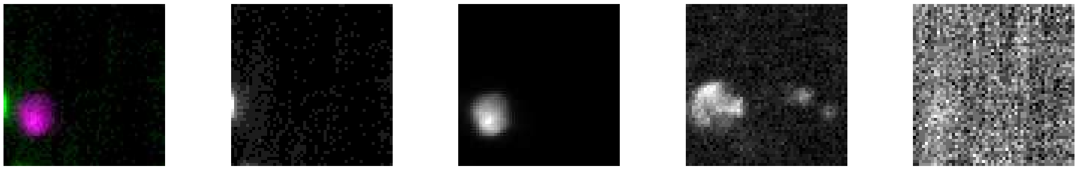				
25979	154	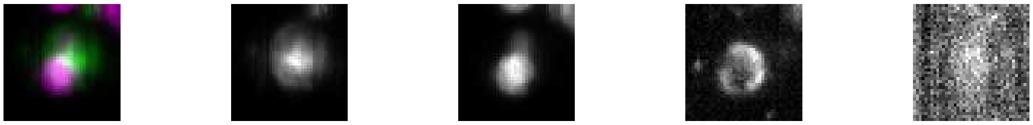				
25980	154	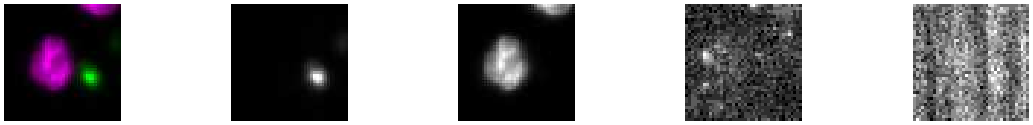				
25981	154	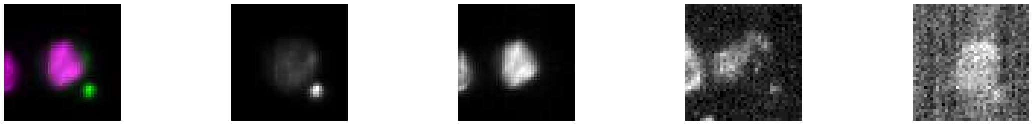				
25982	154	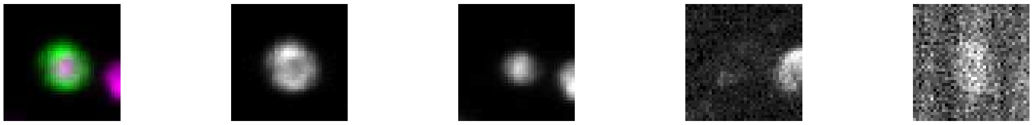				
25983	154	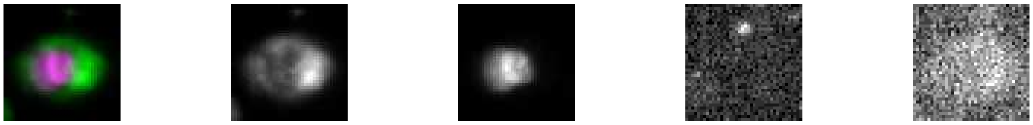				
25984	154	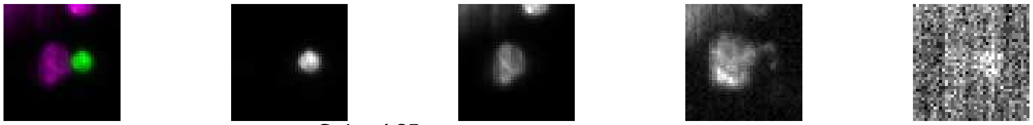				

Ereignis	Aufnahme	DAPI/CK-PE	CK-PE	DAPI	CD45-APC	HER-2/neu
25985	153	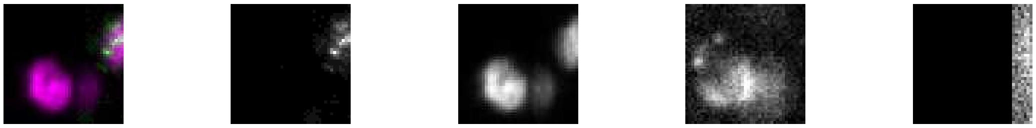				
25986	153	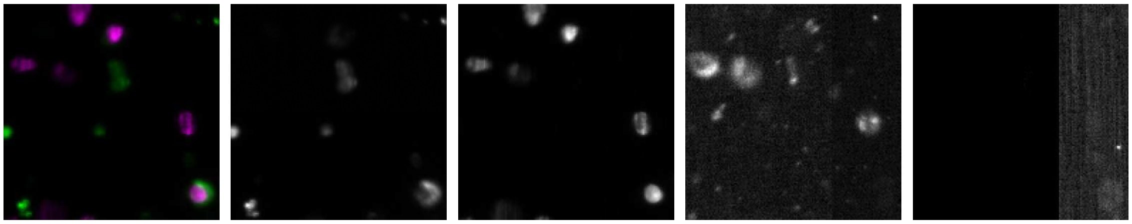				
25987	127	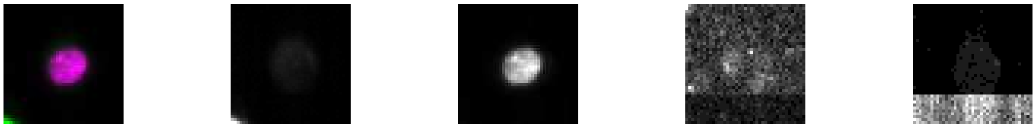				
25988	127	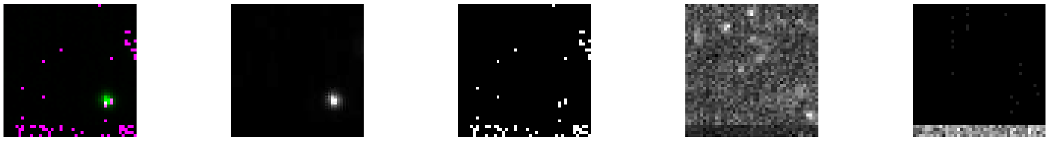				
25989	154	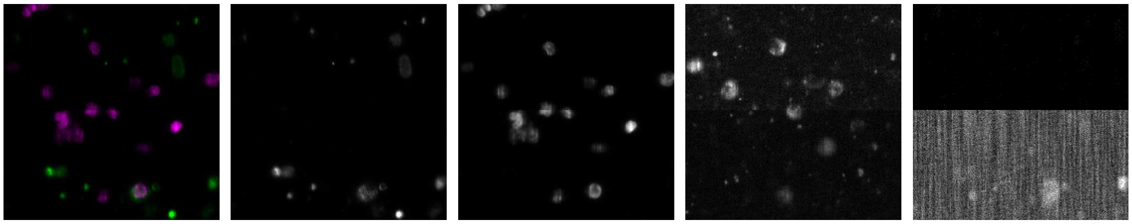				
25990	127	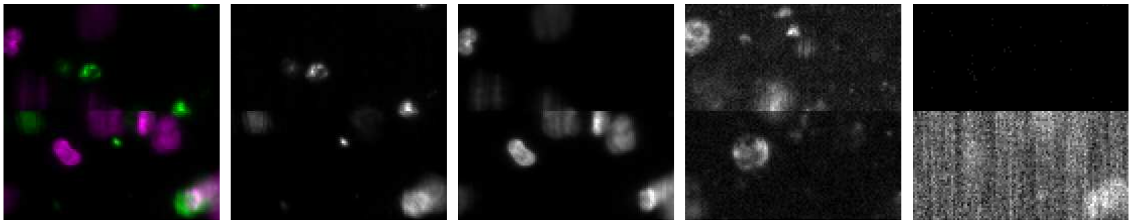				
25991	154	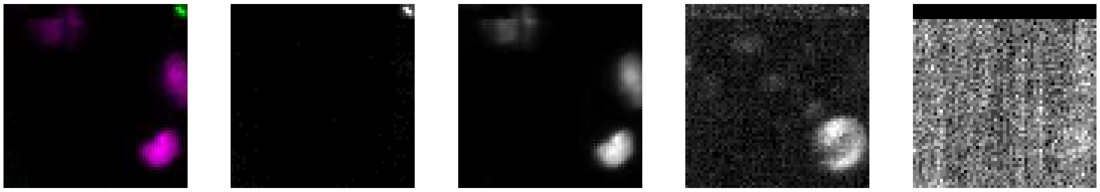				
25992	154	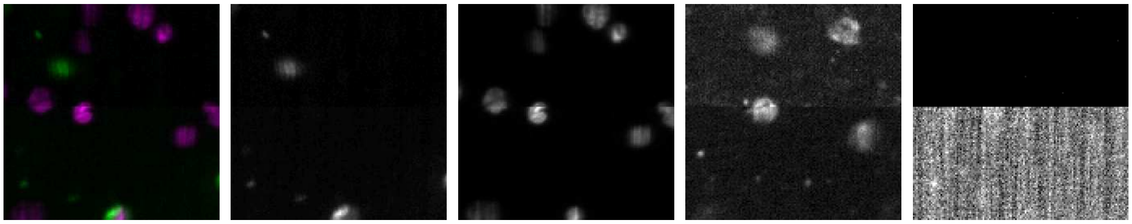				
25993	127	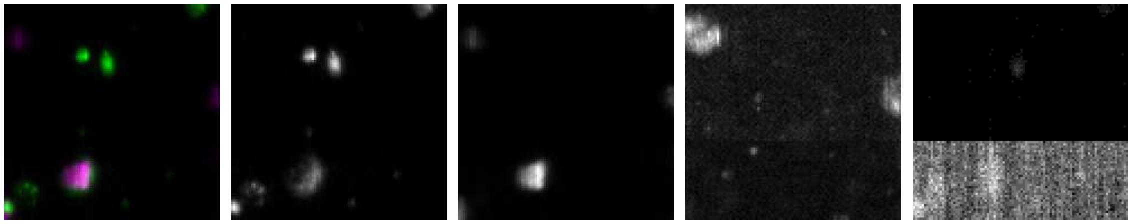				

Ereignis	Aufnahme	DAPI/CK-PE	CK-PE	DAPI	CD45-APC	HER-2/neu
25994	153	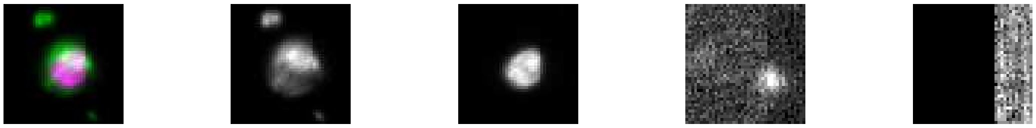				
25995	127	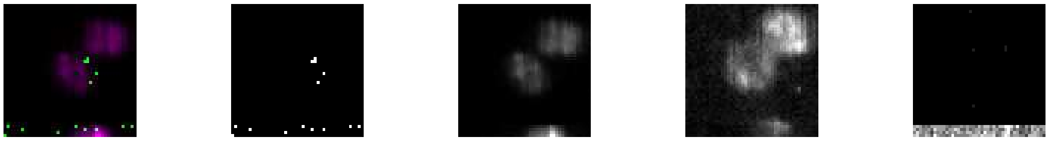				
25996	154	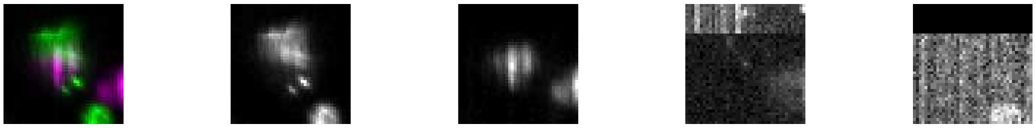				
25997	127	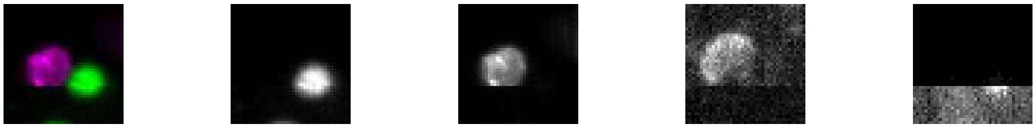				
25998	155	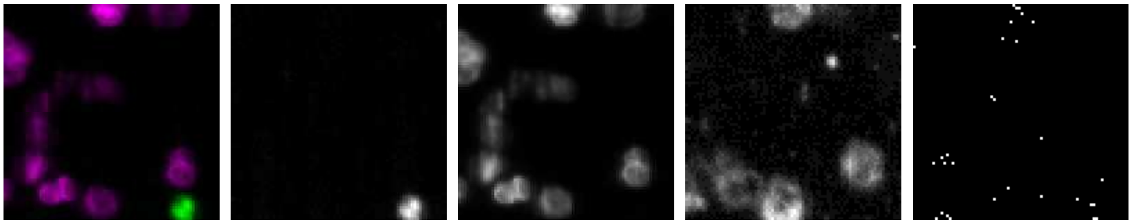				
25999	155	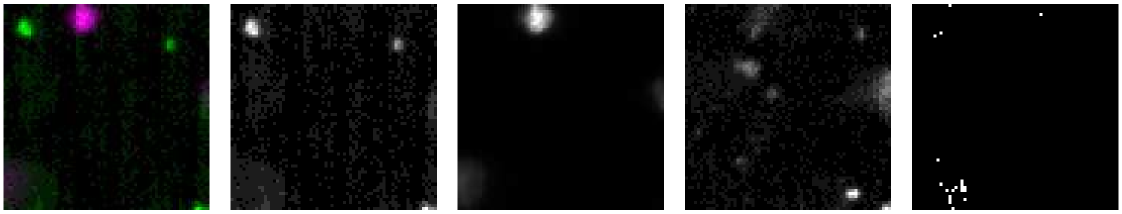				
26000	155	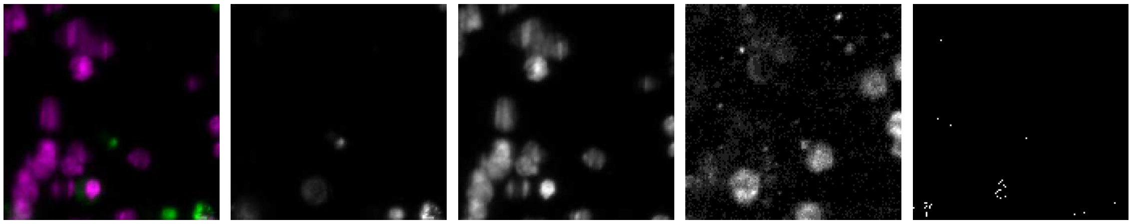				
